# Supplementary material for: Immunoinformatic identification of B cell and T cell epitopes in the SARS-CoV-2 proteome
Source: Sci Rep. 2020 Aug 25;10:14179. doi: 10.1038/s41598-020-70864-8 (PMC7447814; doi:10.1038/s41598-020-70864-8)
Supplement: Supplementary file 1 — Supplementary Information 1. [file 41598_2020_70864_MOESM1_ESM.pdf]

Immunoinformatic identification of B cell and T cell epitopes in the SARS-CoV-2 proteome

Stephen N. Crooke, Inna G. Ovsyannikova, Richard B. Kennedy, Gregory A. Poland*

Mayo Clinic Vaccine Research Group, Mayo Clinic, Rochester, MN USA

Correspondence:
Gregory A. Poland, M.D., Director – Vaccine Research Group, Mayo Clinic, Guggenheim Building 611C, 200 First Street SW, Rochester, MN 55905 USA

Phone: (507) 284-4968; E-mail: poland.gregory@mayo.edu

© 2020 Mayo Foundation for Medical Education and Research

```
CLUSTAL O(1.2.4) multiple sequence alignment


gb:MT020781|Organism:Severe      --------------------------------------------------TACCTTCCCA	10
gb:MT007544|Organism:Severe      --------------------------------------ATTAAAGGTTTATACCTTCCCA	22
gb:MN994467|Organism:Severe      --------------------------------------ATTAAAGGTTTATACCTTCCCA	22
gb:MT044257|Organism:Severe      --------------------------------------ATTAAAGGTTTATACCTTCCCA	22
gb:MT106054|Organism:Severe      --------------------------------------ATTAAAGGTTTATACCTTCCCA	22
gb:MT049951|Organism:Severe      --------------------------------------ATTAAAGGTTTATACCTTCCCA	22
gb:MN975262|Organism:Severe      --------------------------------------ATTAAAGGTTTATACCTTCCCA	22
gb:MT106052|Organism:Severe      --------------------------------------ATTAAAGGTTTATACCTTCCCA	22
gb:LC522975|Organism:Severe      -----------------------------------------AAAGGTTTATACCTTCCCA	19
gb:LC522973|Organism:Severe      -----------------------------------------AAAGGTTTATACCTTCCCA	19
gb:LC522974|Organism:Severe      -----------------------------------------AAAGGTTTATACCTTCCCA	19
gb:MN985325|Organism:Severe      --------------------------------------ATTAAAGGTTTATACCTTCCCA	22
gb:MT020881|Organism:Severe      --------------------------------------ATTAAAGGTTTATACCTTCCCA	22
gb:MT020880|Organism:Severe      --------------------------------------ATTAAAGGTTTATACCTTCCCA	22
gb:MT066175|Organism:Severe      --------------------------------------ATTAAAGGTTTATACCTTCCCA	22
gb:MN997409|Organism:Severe      --------------------------------------ATTAAAGGTTTATACCTTCCCA	22
gb:MN938384|Organism:Severe      ------------------------------------------------------------	0
gb:MT044258|Organism:Severe      --------------------------------------ATTAAAGGTTTATACCTTCCCA	22
gb:MT039890|Organism:Severe      --------------------------------------ATTAAAGGTTTATACCTTCCCA	22
gb:MN988713|Organism:Severe      --------------------------------------ATTAAAGGTTTATACCTTCCCA	22
gb:LC521925|Organism:Severe      -----------------------------------------AAAGGTTTATACCTTCCCA	19
gb:MT093571|Organism:Severe      --------------------------------------ATTAAAGGTTTATACCTTCCCA	22
gb:MT039887|Organism:Severe      --------------------------------------ATTAAAGGTTTATACCTTCCCA	22
gb:MT019530|Organism:Severe      --------------------------------------ATTAAAGGTTTATACCTTCCCA	22
gb:MT039888|Organism:Severe      --------------------------------------ATTAAAGGTTTATACCTTCCCA	22
gb:LC522972|Organism:Severe      -----------------------------------------AAAGGTTTATACCTTCCCA	19
gb:MT027063|Organism:Severe      --------------------------------------ATTAAAGGTTTATACCTTCCCA	22
gb:MT027062|Organism:Severe      --------------------------------------ATTAAAGGTTTATACCTTCCCA	22
gb:MT019529|Organism:Severe      --------------------------------------ATTAAAGGTTTATACCTTCCCA	22
gb:MN996529|Organism:Severe      --------------------------------------------------TACCTTCCCA	10
gb:MN996531|Organism:Severe      ---------------------------------------------------ACCTTCCCA	9
gb:MT066176|Organism:Severe      --------------------------------------ATTAAAGGTTTATACCTTCCCA	22
gb:MT027064|Organism:Severe      --------------------------------------ATTAAAGGTTTATACCTTCCCA	22
gb:MN994468|Organism:Severe      --------------------------------------ATTAAAGGTTTATACCTTCCCA	22
gb:MT072688|Organism:Severe      -----------------------------------------------------CTTCCCA	7
gb:MN996527|Organism:Severe      ------------------------------------------------------------	0
gb:MT093631|Organism:Severe      AGAGTGCTTATGAAAATTTTAATCAGCACGAAGTTCTACTTGCACCATTATACCTTCCCA	60
gb:MT106053|Organism:Severe      --------------------------------------ATTAAAGGTTTATACCTTCCCA	22
gb:MT019533|Organism:Severe      --------------------------------------ATTAAAGGTTTATACCTTCCCA	22
gb:MT019531|Organism:Severe      --------------------------------------ATTAAAGGTTTATACCTTCCCA	22
gb:MN996528|Organism:Severe      --------------------------------------ATTAAAGGTTTATACCTTCCCA	22
gb:MN996530|Organism:Severe      ----------------------------------------------------CCTTCCCA	8
gb:MN908947|Organism:Severe      --------------------------------------ATTAAAGGTTTATACCTTCCCA	22
gb:MT019532|Organism:Severe      --------------------------------------ATTAAAGGTTTATACCTTCCCA	22


gb:MT020781|Organism:Severe      GGTAACAAACCAACCAACTTTCGATCTCTTGTAGATCTGTTCTCTAAACGAACTTTAAAA	70
gb:MT007544|Organism:Severe      GGTAACAAACCAACCAACTTTCGATCTCTTGTAGATCTGTTCTCTAAACGAACTTTAAAA	82
gb:MN994467|Organism:Severe      GGTAACAAACCAACCAACTTTCGATCTCTTGTAGATCTGTTCTCTAAACGAACTTTAAAA	82
gb:MT044257|Organism:Severe      GGTAACAAACCAACCAACTTTCGATCTCTTGTAGATCTGTTCTCTAAACGAACTTTAAAA	82
gb:MT106054|Organism:Severe      GGTAACAAACCAACCAACTTTCGATCTCTTGTAGATCTGTTCTCTAAACGAACTTTAAAA	82
gb:MT049951|Organism:Severe      GGTAACAAACCAACCAACTTTCGATCTCTTGTAGATCTGTTCTCTAAACGAAATTTAAAA	82
gb:MN975262|Organism:Severe      GGTAACAAACCAACCAACTTTCGATCTCTTGTAGATCTGTTCTCTAAACGAACTTTAAAA	82
gb:MT106052|Organism:Severe      GGTAACAAACCAACCAACTTTCGATCTCTTGTAGATCTGTTCTCTAAACGAACTTTAAAA	82
gb:LC522975|Organism:Severe      GGTAACAAACCAACCAACTTTCGATCTCTTGTAGATCTGTTCTCTAAACGAACTTTAAAA	79
gb:LC522973|Organism:Severe      GGTAACAAACCAACCAACTTTCGATCTCTTGTAGATCTGTTCTCTAAACGAACTTTAAAA	79
gb:LC522974|Organism:Severe      GGTAACAAACCAACCAACTTTCGATCTCTTGTAGATCTGTTCTCTAAACGAACTTTAAAA	79
gb:MN985325|Organism:Severe      GGTAACAAACCAACCAACTTTCGATCTCTTGTAGATCTGTTCTCTAAACGAACTTTAAAA	82
gb:MT020881|Organism:Severe      GGTAACAAACCAACCAACTTTCGATCTCTTGTAGATCTGTTCTCTAAACGAACTTTAAAA	82
gb:MT020880|Organism:Severe      GGTAACAAACCAACCAACTTTCGATCTCTTGTAGATCTGTTCTCTAAACGAACTTTAAAA	82
gb:MT066175|Organism:Severe      GGTAACAAACCAACCAACTTTCGATCTCTTGTAGATCTGTTCTCTAAACGAACTTTAAAA	82
```

```
gb:MN997409|Organism:Severe      GGTAACAAACCAACCAACTTTCGATCTCTTGTAGATCTGTTCTCTAAACGAACTTTAAAA   82
gb:MN938384|Organism:Severe      ----------CAACCAACTTTCGATCTCTTGTAGATCTGTTCTCTAAACGAACTTTAAAA   50
gb:MT044258|Organism:Severe      GGTAACAAACCAACCAACTTTCGATCTCTTGTAGATCTGTTCTCTAAACGAACTTTAAAA   82
gb:MT039890|Organism:Severe      GGTAACAAACCAACCAACTTTCGATCTCTTGTAGATCTGTTCTCTAAACGAACTTTAAAA   82
gb:MN988713|Organism:Severe      GGTAACAAACCAACCAACTTTCGATCTCTTGTAGATCTGTTCTCTAAACGAACTTTAAAA   82
gb:LC521925|Organism:Severe      GGTAACAAACCAACCAACTTTCGATCTCTTGTAGATCTGTTCTCTAAACGAACTTTAAAA   79
gb:MT093571|Organism:Severe      GGTAACAAACCAACCAACTTTCGATCTCTTGTAGATCTGTTCTCTAAACGAACTTTAAAA   82
gb:MT039887|Organism:Severe      GGTAACAAACCAACCAACTTTCGATCTCTTGTAGATCTGTTCTCTAAACGAACTTTAAAA   82
gb:MT019530|Organism:Severe      GGTAACAAACCAACCAACTTTCGATCTCTTGTAGATCTGTTCTCTAAACGAACTTTAAAA   82
gb:MT039888|Organism:Severe      GGTAACAAACCAACCAACTTTCGATCTCTTGTAGATCTGTTCTCTAAACGAACTTTAAAA   82
gb:LC522972|Organism:Severe      GGTAACAAACCAACCAACTTTCGATCTCTTGTAGATCTGTTCTCTAAACGAACTTTAAAA   79
gb:MT027063|Organism:Severe      GGTAACAAACCAACCAACTTTCGATCTCTTGTAGATCTGTTCTCTAAACGAACTTTAAAA   82
gb:MT027062|Organism:Severe      GGTAACAAACCAACCAACTTTCGATCTCTTGTAGATCTGTTCTCTAAACGAACTTTAAAA   82
gb:MT019529|Organism:Severe      GGTAACAAACCAACCAACTTTCGATCTCTTGTAGATCTGTTCTCTAAACGAACTTTAAAA   82
gb:MN996529|Organism:Severe      GGTAACAAACCAACCAACTTTCGATCTCTTGTAGATCTGTTCTCTAAACGAACTTTAAAA   70
gb:MN996531|Organism:Severe      GGTAACAAACCAACCAACTTTCGATCTCTTGTAGATCTGTTCTCTAAACGAACTTTAAAA   69
gb:MT066176|Organism:Severe      GGTAACAAACCAACCAACTTTCGATCTCTTGTAGATCTGTTCTCTAAACGAACTTTAAAA   82
gb:MT027064|Organism:Severe      GGTAACAAACCAACCAACTTTCGATCTCTTGTAGATCTGTTCTCTAAACGAACTTTAAAA   82
gb:MN994468|Organism:Severe      GGTAACAAACCAACCAACTTTCGATCTCTTGTAGATCTGTTCTCTAAACGAACTTTAAAA   82
gb:MT072688|Organism:Severe      GGTAACAAACCAACCAACTTTCGATCTCTTGTAGATCTGTTCTCTAAACGAACTTTAAAA   67
gb:MN996527|Organism:Severe      -----------AACCAACTTTCGATCTCTTGTAGATCTGTTCTCTAAACGAACTTTAAAA   49
gb:MT093631|Organism:Severe      GGTAACAAACCAACCAACTTTCGATCTCTTGTAGATCTGTTCTCTAAACGAACTTTAAAA   120
gb:MT106053|Organism:Severe      GGTAACAAACCAACCAACTTTCGATCTCTTGTAGATCTGTTCTCTAAACGAACTTTAAAA   82
gb:MT019533|Organism:Severe      GGTAACAAACCAACCAACTTTCGATCTCTTGTAGATCTGTTCTCTAAACGAACTTTAAAA   82
gb:MT019531|Organism:Severe      GGTAACAAACCAACCAACTTTCGATCTCTTGTAGATCTGTTCTCTAAACGAACTTTAAAA   82
gb:MN996528|Organism:Severe      GGTAACAAACCAACCAACTTTCGATCTCTTGTAGATCTGTTCTCTAAACGAACTTTAAAA   82
gb:MN996530|Organism:Severe      GGTAACAAACCAACCAACTTTCGATCTCTTGTAGATCTGTTCTCTAAACGAACTTTAAAA   68
gb:MN908947|Organism:Severe      GGTAACAAACCAACCAACTTTCGATCTCTTGTAGATCTGTTCTCTAAACGAACTTTAAAA   82
gb:MT019532|Organism:Severe      GGTAACAAACCAACCAACTTTCGATCTCTTGTAGATCTGTTCTCTAAACGAACTTTAAAA   82
                                            ***************************************** *******

gb:MT020781|Organism:Severe      TCTGTGTGGCTGTCACTCGGCTGCATGCTTAGTGCACTCACGCAGTATAATTAATAACTA   130
gb:MT007544|Organism:Severe      TCTGTGTGGCTGTCACTCGGCTGCATGCTTAGTGCACTCACGCAGTATAATTAATAACTA   142
gb:MN994467|Organism:Severe      TCTGTGTGGCTGTCACTCGGCTGCATGCTTAGTGCACTCACGCAGTATAATTAATAACTA   142
gb:MT044257|Organism:Severe      TCTGTGTGGCTGTCACTCGGCTGCATGCTTAGTGCACTCACGCAGTATAATTAATAACTA   142
gb:MT106054|Organism:Severe      TCTGTGTGGCTGTCACTCGGCTGCATGCTTAGTGCACTCACGCAGTATAATTAATAACTA   142
gb:MT049951|Organism:Severe      TCTGTGTGGCTGTCACTCGGCTGCATGCTTAGTGCACTCACGCAGTATAATTAATAACTA   142
gb:MN975262|Organism:Severe      TCTGTGTGGCTGTCACTCGGCTGCATGCTTAGTGCACTCACGCAGTATAATTAATAACTA   142
gb:MT106052|Organism:Severe      TCTGTGTGGCTGTCACTCGGCTGCATGCTTAGTGCACTCACGCAGTATAATTAATAACTA   142
gb:LC522975|Organism:Severe      TCTGTGTGGCTGTCACTCGGCTGCATGCTTAGTGCACTCACGCAGTATAATTAATAACTA   139
gb:LC522973|Organism:Severe      TCTGTGTGGCTGTCACTCGGCTGCATGCTTAGTGCACTCACGCAGTATAATTAATAACTA   139
gb:LC522974|Organism:Severe      TCTGTGTGGCTGTCACTCGGCTGCATGCTTAGTGCACTCACGCAGTATAATTAATAACTA   139
gb:MN985325|Organism:Severe      TCTGTGTGGCTGTCACTCGGCTGCATGCTTAGTGCACTCACGCAGTATAATTAATAACTA   142
gb:MT020881|Organism:Severe      TCTGTGTGGCTGTCACTCGGCTGCATGCTTAGTGCACTCACGCAGTATAATTAATAACTA   142
gb:MT020880|Organism:Severe      TCTGTGTGGCTGTCACTCGGCTGCATGCTTAGTGCACTCACGCAGTATAATTAATAACTA   142
gb:MT066175|Organism:Severe      TCTGTGTGGCTGTCACTCGGCTGCATGCTTAGTGCACTCACGCAGTATAATTAATAACTA   142
gb:MN997409|Organism:Severe      TCTGTGTGGCTGTCACTCGGCTGCATGCTTAGTGCACTCACGCAGTATAATTAATAACTA   142
gb:MN938384|Organism:Severe      TCTGTGTGGCTGTCACTCGGCTGCATGCTTAGTGCACTCACGCAGTATAATTAATAACTA   110
gb:MT044258|Organism:Severe      TCTGTGTGGCTGTCACTCGGCTGCATGCTTAGTGCACTCACGCAGTATAATTAATAACTA   142
gb:MT039890|Organism:Severe      TCTGTGTGGCTGTCACTCGGCTGCATGCTTAGTGCACTCACGCAGTATAATTAATAACTA   142
gb:MN988713|Organism:Severe      TCTGTGTGGCTGTCACTCGGCTGCATGCTTAGTGCACTCACGCAGTATAATTAATAACTA   142
gb:LC521925|Organism:Severe      TCTGTGTGGCTGTCACTCGGCTGCATGCTTAGTGCACTCACGCAGTATAATTAATAACTA   139
gb:MT093571|Organism:Severe      TCTGTGTGGCTGTCACTCGGCTGCATGCTTAGTGCACTCACGCAGTATAATTAATAACTA   142
gb:MT039887|Organism:Severe      TCTGTGTGGCTGTCACTCGGCTGCATGCTTAGTGCACTCACGCAGTATAATTAATAACTA   142
gb:MT019530|Organism:Severe      TCTGTGTGGCTGTCACTCGGCAGCATGCCGAGTGCAGCCACACAGTATAATTAATAACTA   142
gb:MT039888|Organism:Severe      TCTGTGTGGCTGTCACTCGGCTGCATGCTTAGTGCACTCACGCAGTATAATTAATAACTA   142
gb:LC522972|Organism:Severe      TCTGTGTGGCTGTCACTCGGCTGCATGCTTAGTGCACTCACGCAGTATAATTAATAACTA   139
gb:MT027063|Organism:Severe      TCTGTGTGGCTGTCACTCGGCTGCATGCTTAGTGCACTCACGCAGTATAATTAATAACTA   142
gb:MT027062|Organism:Severe      TCTGTGTGGCTGTCACTCGGCTGCATGCTTAGTGCACTCACGCAGTATAATTAATAACTA   142
gb:MT019529|Organism:Severe      TCTGTGTGGCTGTCACTCGGCTGCATGCTTAGTGCACTCACGCAGTATAATTAATAACTA   142
gb:MN996529|Organism:Severe      TCTGTGTGGCTGTCACTCGGCTGCATGCTTAGTGCACTCACGCAGTATAATTAATAACTA   130
gb:MN996531|Organism:Severe      TCTGTGTGGCTGTCACTCGGCTGCATGCTTAGTGCACTCACGCAGTATAATTAATAACTA   129
gb:MT066176|Organism:Severe      TCTGTGTGGCTGTCACTCGGCTGCATGCTTAGTGCACTCACGCAGTATAATTAATAACTA   142
gb:MT027064|Organism:Severe      TCTGTGTGGCTGTCACTCGGCTGCATGCTTAGTGCACTCACGCAGTATAATTAATAACTA   142
gb:MN994468|Organism:Severe      TCTGTGTGGCTGTCACTCGGCTGCATGCTTAGTGCACTCACGCAGTATAATTAATAACTA   142
```

```
gb:MT072688|Organism:Severe      TCTGTGTGGCTGTCACTCGGCTGCATGCTTAGTGCACTCACGCAGTATAATTAATAACTA   127
gb:MN996527|Organism:Severe      TCTGTGTGGCTGTCACTCGGCTGCATGCTTAGTGCACTCACGCAGTATAATTAATAACTA   109
gb:MT093631|Organism:Severe      TCTGTGTGGCTGTCACTCGGCTGCATGCTTAGTGCACTCACGCAGTATAATTAATAACTA   180
gb:MT106053|Organism:Severe      TCTGTGTGGCTGTCACTCGGCTGCATGCTTAGTGCACTCACGCAGTATAATTAATAACTA   142
gb:MT019533|Organism:Severe      TCTGTGTGGCTGTCACTCGGCTGCATGCTTAGTGCACTCACGCAGTATAATTAATAACTA   142
gb:MT019531|Organism:Severe      TCTGTGTGGCTGTCACTCGGCTGCATGCTTAGTGCACTCACGCAGTATAATTAATAACTA   142
gb:MN996528|Organism:Severe      TCTGTGTGGCTGTCACTCGGCTGCATGCTTAGTGCACTCACGCAGTATAATTAATAACTA   142
gb:MN996530|Organism:Severe      TCTGTGTGGCTGTCACTCGGCTGCATGCTTAGTGCACTCACGCAGTATAATTAATAACTA   128
gb:MN908947|Organism:Severe      TCTGTGTGGCTGTCACTCGGCTGCATGCTTAGTGCACTCACGCAGTATAATTAATAACTA   142
gb:MT019532|Organism:Severe      TCTGTGTGGCTGTCACTCGGCTGCATGCTTAGTGCACTCACGCAGTATAATTAATAACTA   142
                                 ********************* ******  ******  *** ******************

gb:MT020781|Organism:Severe      ATTACTGTCGTTGACAGGACACGAGTAACTCGTCTATCTTCTGCAGGCTGCTTACGGTTT   190
gb:MT007544|Organism:Severe      ATTACTGTCGTTGACAGGACACGAGTAACTCGTCTATCTTCTGCAGGCTGCTTACGGTTT   202
gb:MN994467|Organism:Severe      ATTACTGTCGTTGACAGGACACGAGTAACTCGTCTATCTTCTGCAGGCTGCTTACGGTTT   202
gb:MT044257|Organism:Severe      ATTACTGTCGTTGACAGGACACGAGTAACTCGTCTATCTTCTGCAGGCTGCTTACGGTTT   202
gb:MT106054|Organism:Severe      ATTACTGTCGTTGACAGGACACGAGTAACTCGTCTATCTTCTGCAGGCTGCTTACGGTTT   202
gb:MT049951|Organism:Severe      ATTACTGTCGTTGACAGGACACGAGTAACTCGTCTATCTTCTGCAGGCTGCTTACGGTTT   202
gb:MN975262|Organism:Severe      ATTACTGTCGTTGACAGGACACGAGTAACTCGTCTATCTTCTGCAGGCTGCTTACGGTTT   202
gb:MT106052|Organism:Severe      ATTACTGTCGTTGACAGGACACGAGTAACTCGTCTATCTTCTGCAGGCTGCTTACGGTTT   202
gb:LC522975|Organism:Severe      ATTACTGTCGTTGACAGGACACGAGTAACTCGTCTATCTTCTGCAGGCTGCTTACGGTTT   199
gb:LC522973|Organism:Severe      ATTACTGTCGTTGACAGGACACGAGTAACTCGTCTATCTTCTGCAGGCTGCTTACGGTTT   199
gb:LC522974|Organism:Severe      ATTACTGTCGTTGACAGGACACGAGTAACTCGTCTATCTTCTGCAGGCTGCTTACGGTTT   199
gb:MN985325|Organism:Severe      ATTACTGTCGTTGACAGGACACGAGTAACTCGTCTATCTTCTGCAGGCTGCTTACGGTTT   202
gb:MT020881|Organism:Severe      ATTACTGTCGTTGACAGGACACGAGTAACTCGTCTATCTTCTGCAGGCTGCTTACGGTTT   202
gb:MT020880|Organism:Severe      ATTACTGTCGTTGACAGGACACGAGTAACTCGTCTATCTTCTGCAGGCTGCTTACGGTTT   202
gb:MT066175|Organism:Severe      ATTACTGTCGTTGACAGGACACGAGTAACTCGTCTATCTTCTGCAGGCTGCTTACGGTTT   202
gb:MN997409|Organism:Severe      ATTACTGTCGTTGACAGGACACGAGTAACTCGTCTATCTTCTGCAGGCTGCTTACGGTTT   202
gb:MN938384|Organism:Severe      ATTACTGTCGTTGACAGGACACGAGTAACTCGTCTATCTTCTGCAGGCTGCTTACGGTTT   170
gb:MT044258|Organism:Severe      ATTACTGTCGTTGACAGGACACGAGTAACTCGTCTATCTTCTGCAGGCTGCTTACGGTTT   202
gb:MT039890|Organism:Severe      ATTACTGTCGTTGACAGGACACGAGTAACTCGTCTATCTTCTGCAGGCTGCTTACGGTTT   202
gb:MN988713|Organism:Severe      ATTACTGTCGTTGACAGGACACGAGTAACTCGTCTATCTTCTGCAGGCTGCTTACGGTTT   202
gb:LC521925|Organism:Severe      ATTACTGTCGTTGACAGGACACGAGTAACTCGTCTATCTTCTGCAGGCTGCTTACGGTTT   199
gb:MT093571|Organism:Severe      ATTACTGTCGTTGACAGGACACGAGTAACTCGTCTATCTTCTGCAGGCTGCTTACGGTTT   202
gb:MT039887|Organism:Severe      ATTACTGTCGTTGACAGGACACGAGTAACTCGTCTATCTTCTGCAGGCTGCTTACGGTTT   202
gb:MT019530|Organism:Severe      ATTACTGTCGTTGACAGGACACGAGTAACTCGTCTATCTTCTGCAGGCTGCTTACGGTTT   202
gb:MT039888|Organism:Severe      ATTACTGTCGTTGACAGGACACGAGTAACTCGTCTATCTTCTGCAGGCTGCTTACGGTTT   202
gb:LC522972|Organism:Severe      ATTACTGTCGTTGACAGGACACGAGTAACTCGTCTATCTTCTGCAGGCTGCTTACGGTTT   199
gb:MT027063|Organism:Severe      ATTACTGTCGTTGACAGGACACGAGTAACTCGTCTATCTTCTGCAGGCTGCTTACGGTTT   202
gb:MT027062|Organism:Severe      ATTACTGTCGTTGACAGGACACGAGTAACTCGTCTATCTTCTGCAGGCTGCTTACGGTTT   202
gb:MT019529|Organism:Severe      ATTACTGTCGTTGACAGGACACGAGTAACTCGTCTATCTTCTGCAGGCTGCTTACGGTTT   202
gb:MN996529|Organism:Severe      ATTACTGTCGTTGACAGGACACGAGTAACTCGTCTATCTTCTGCAGGCTGCTTACGGTTT   190
gb:MN996531|Organism:Severe      ATTACTGTCGTTGACAGGACACGAGTAACTCGTCTATCTTCTGCAGGCTGCTTACGGTTT   189
gb:MT066176|Organism:Severe      ATTACTGTCGTTGACAGGACACGAGTAACTCGTCTATCTTCTGCAGGCTGCTTACGGTTT   202
gb:MT027064|Organism:Severe      ATTACTGTCGTTGACAGGACACGAGTAACTCGTCTATCTTCTGCAGGCTGCTTACGGTTT   202
gb:MN994468|Organism:Severe      ATTACTGTCGTTGACAGGACACGAGTAACTCGTCTATCTTCTGCAGGCTGCTTACGGTTT   202
gb:MT072688|Organism:Severe      ATTACTGTCGTTGACAGGACACGAGTAACTCGTCTATCTTCTGCAGGCTGCTTACGGTTT   187
gb:MN996527|Organism:Severe      ATTACTGTCGTTGACAGGACACGAGTAACTCGTCTATCTTCTGCAGGCTGCTTACGGTTT   169
gb:MT093631|Organism:Severe      ATTACTGTCGTTGACAGGACACGAGTAACTCGTCTATCTTCTGTAGGCTGCTTACGGTTT   240
gb:MT106053|Organism:Severe      ATTACTGTCGTTGACAGGACACGAGTAACTCGTCTATCTTCTGCAGGCTGCTTACGGTTT   202
gb:MT019533|Organism:Severe      ATTACTGTCGTTGACAGGACACGAGTAACTCGTCTATCTTCTGCAGGCTGCTTACGGTTT   202
gb:MT019531|Organism:Severe      ATTACTGTCGTTGACAGGACACGAGTAACTCGTCTATCTTCTGCAGGCTGCTTACGGTTT   202
gb:MN996528|Organism:Severe      ATTACTGTCGTTGACAGGACACGAGTAACTCGTCTATCTTCTGCAGGCTGCTTACGGTTT   202
gb:MN996530|Organism:Severe      ATTACTGTCGTTGACAGGACACGAGTAACTCGTCTATCTTCTGCAGGCTGCTTACGGTTT   188
gb:MN908947|Organism:Severe      ATTACTGTCGTTGACAGGACACGAGTAACTCGTCTATCTTCTGCAGGCTGCTTACGGTTT   202
gb:MT019532|Organism:Severe      ATTACTGTCGTTGACAGGACACGAGTAACTCGTCTATCTTCTGCAGGCTGCTTACGGTTT   202
                                 ******************************************* ****************

gb:MT020781|Organism:Severe      CGTCCGTGTTGCAGCCGATCATCAGCACATCTAGGTTTCGTCCGGGTGTGACCGAAAGGT   250
gb:MT007544|Organism:Severe      CGTCCGTGTTGCAGCCGATCATCAGCACATCTAGGTTTCGTCCGGGTGTGACCGAAAGGT   262
gb:MN994467|Organism:Severe      CGTCCGTGTTGCAGCCGATCATCAGCACATCTAGGTTTCGTCCGGGTGTGACCGAAAGGT   262
gb:MT044257|Organism:Severe      CGTCCGTGTTGCAGCCGATCATCAGCACATCTAGGTTTCGTCCGGGTGTGACCGAAAGGT   262
gb:MT106054|Organism:Severe      CGTCCGTGTTGCAGCCGATCATCAGCACATCTAGGTTTCGTCCGGGTGTGACCGAAAGGT   262
gb:MT049951|Organism:Severe      CGTCCGTGTTGCAGCCGATCATCAGCACATCTAGGTTTCGTCCGGGTGTGACCGAAAGGT   262
gb:MN975262|Organism:Severe      CGTCCGTGTTGCAGCCGATCATCAGCACATCTAGGTTTCGTCCGGGTGTGACCGAAAGGT   262
```

```
gb:MT106052|Organism:Severe      CGTCCGTGTTGCAGCCGATCATCAGCACATCTAGGTTTCGTCCGGGTGTGACCGAAAGGT	262
gb:LC522975|Organism:Severe      CGTCCGTGTTGCAGCCGATCATCAGCACATCTAGGTTTCGTCCGGGTGTGACCGAAAGGT	259
gb:LC522973|Organism:Severe      CGTCCGTGTTGCAGCCGATCATCAGCACATCTAGGTTTCGTCCGGGTGTGACCGAAAGGT	259
gb:LC522974|Organism:Severe      CGTCCGTGTTGCAGCCGATCATCAGCACATCTAGGTTTCGTCCGGGTGTGACCGAAAGGT	259
gb:MN985325|Organism:Severe      CGTCCGTGTTGCAGCCGATCATCAGCACATCTAGGTTTCGTCCGGGTGTGACCGAAAGGT	262
gb:MT020881|Organism:Severe      CGTCCGTGTTGCAGCCGATCATCAGCACATCTAGGTTTCGTCCGGGTGTGACCGAAAGGT	262
gb:MT020880|Organism:Severe      CGTCCGTGTTGCAGCCGATCATCAGCACATCTAGGTTTCGTCCGGGTGTGACCGAAAGGT	262
gb:MT066175|Organism:Severe      CGTCCGTGTTGCAGCCGATCATCAGCACATCTAGGTTTCGTCCGGGTGTGACCGAAAGGT	262
gb:MN997409|Organism:Severe      CGTCCGTGTTGCAGCCGATCATCAGCACATCTAGGTTTCGTCCGGGTGTGACCGAAAGGT	262
gb:MN938384|Organism:Severe      CGTCCGTGTTGCAGCCGATCATCAGCACATCTAGGTTTCGTCCGGGTGTGACCGAAAGGT	230
gb:MT044258|Organism:Severe      CGTCCGTGTTGCAGCCGATCATCAGCACATCTAGGTTTCGTCCGGGTGTGACCGAAAGGT	262
gb:MT039890|Organism:Severe      CGTCCGTGTTGCAGCCGATCATCAGCACATCTAGGTTTCGTCCGGGTGTGACCGAAAGGT	262
gb:MN988713|Organism:Severe      CGTCCGTGTTGCAGCCGATCATCAGCACATCTAGGTTTCGTCCGGGTGTGACCGAAAGGT	262
gb:LC521925|Organism:Severe      CGTCCGTGTTGCAGCCGATCATCAGCACATCTAGGTTTCGTCCGGGTGTGACCGAAAGGT	259
gb:MT093571|Organism:Severe      CGTCCGTGTTGCAGCCGATCATCAGCACATCTAGGTTTCGTCCGGGTGTGACCGAAAGGT	262
gb:MT039887|Organism:Severe      CGTCCGTGTTGCAGCCGATCATCAGCACATCTAGGTTTCGTCCGGGTGTGACCGAAAGGT	262
gb:MT019530|Organism:Severe      CGTCCGTGTTGCAGCCGATCATCAGCACATCTAGGTTTCGTCCGGGTGTGACCGAAAGGT	262
gb:MT039888|Organism:Severe      CGTCCGTGTTGCAGCCGATCATCAGCACATCTAGGTTTCGTCCGGGTGTGACCGAAAGGT	262
gb:LC522972|Organism:Severe      CGTCCGTGTTGCAGCCGATCATCAGCACATCTAGGTTTCGTCCGGGTGTGACCGAAAGGT	259
gb:MT027063|Organism:Severe      CGTCCGTGTTGCAGCCGATCATCAGCACATCTAGGTTTCGTCCGGGTGTGACCGAAAGGT	262
gb:MT027062|Organism:Severe      CGTCCGTGTTGCAGCCGATCATCAGCACATCTAGGTTTCGTCCGGGTGTGACCGAAAGGT	262
gb:MT019529|Organism:Severe      CGTCCGTGTTGCAGCCGATCATCAGCACATCTAGGTTTCGTCCGGGTGTGACCGAAAGGT	262
gb:MN996529|Organism:Severe      CGTCCGTGTTGCAGCCGATCATCAGCACATCTAGGTTTCGTCCGGGTGTGACCGAAAGGT	250
gb:MN996531|Organism:Severe      CGTCCGTGTTGCAGCCGATCATCAGCACATCTAGGTTTCGTCCGGGTGTGACCGAAAGGT	249
gb:MT066176|Organism:Severe      CGTCCGTGTTGCAGCCGATCATCAGCACATCTAGGTTTCGTCCGGGTGTGACCGAAAGGT	262
gb:MT027064|Organism:Severe      CGTCCGTGTTGCAGCCGATCATCAGCACATCTAGGTTTCGTCCGGGTGTGACCGAAAGGT	262
gb:MN994468|Organism:Severe      CGTCCGTGTTGCAGCCGATCATCAGCACATCTAGGTTTCGTCCGGGTGTGACCGAAAGGT	262
gb:MT072688|Organism:Severe      CGTCCGTGTTGCAGCCGATCATCAGCACATCTAGGTTTCGTCCGGGTGTGACCGAAAGGT	247
gb:MN996527|Organism:Severe      CGTCCGTGTTGCAGCCGATCATCAGCACATCTAGGTTTCGTCCGGGTGTGACCGAAAGGT	229
gb:MT093631|Organism:Severe      CGTCCGTGTTGCAGCCGATCATCAGCACATCTAGGTTTCGTCCGGGTGTGACCGAAAGGT	300
gb:MT106053|Organism:Severe      CGTCCGTGTTGCAGCCGATCATCAGCACATCTAGGTTTCGTCCGGGTGTGACCGAAAGGT	262
gb:MT019533|Organism:Severe      CGTCCGTGTTGCAGCCGATCATCAGCACATCTAGGTTTCGTCCGGGTGTGACCGAAAGGT	262
gb:MT019531|Organism:Severe      CGTCCGTGTTGCAGCCGATCATCAGCACATCTAGGTTTCGTCCGGGTGTGACCGAAAGGT	262
gb:MN996528|Organism:Severe      CGTCCGTGTTGCAGCCGATCATCAGCACATCTAGGTTTCGTCCGGGTGTGACCGAAAGGT	262
gb:MN996530|Organism:Severe      CGTCCGTGTTGCAGCCGATCATCAGCACATCTAGGTTTCGTCCGGGTGTGACCGAAAGGT	248
gb:MN908947|Organism:Severe      CGTCCGTGTTGCAGCCGATCATCAGCACATCTAGGTTTCGTCCGGGTGTGACCGAAAGGT	262
gb:MT019532|Organism:Severe      CGTCCGTGTTGCAGCCGATCATCAGCACATCTAGGTTTCGTCCGGGTGTGACCGAAAGGT	262
                                 ************************************************************

gb:MT020781|Organism:Severe      AAGATGGAGAGCCTTGTCCCTGGTTTCAACGAGAAAACACACGTCCAACTCAGTTTGCCT	310
gb:MT007544|Organism:Severe      AAGATGGAGAGCCTTGTCCCTGGTTTCAACGAGAAAACACACGTCCAACTCAGTTTGCCT	322
gb:MN994467|Organism:Severe      AAGATGGAGAGCCTTGTCCCTGGTTTCAACGAGAAAACACACGTCCAACTCAGTTTGCCT	322
gb:MT044257|Organism:Severe      AAGATGGAGAGCCTTGTCCCTGGTTTCAACGAGAAAACACACGTCCAACTCAGTTTGCCT	322
gb:MT106054|Organism:Severe      AAGATGGAGAGCCTTGTCCCTGGTTTCAACGAGAAAACACACGTCCAACTCAGTTTGCCT	322
gb:MT049951|Organism:Severe      AAGATGGAGAGCCTTGTCCCTGGTTTCAACGAGAAAACACACGTCCAACTCAGTTTGCCT	322
gb:MN975262|Organism:Severe      AAGATGGAGAGCCTTGTCCCTGGTTTCAACGAGAAAACACACGTCCAACTCAGTTTGCCT	322
gb:MT106052|Organism:Severe      AAGATGGAGAGCCTTGTCCCTGGTTTCAACGAGAAAACACACGTCCAACTCAGTTTGCCT	322
gb:LC522975|Organism:Severe      AAGATGGAGAGCCTTGTCCCTGGTTTCAACGAGAAAACACACGTCCAACTCAGTTTGCCT	319
gb:LC522973|Organism:Severe      AAGATGGAGAGCCTTGTCCCTGGTTTCAACGAGAAAACACACGTCCAACTCAGTTTGCCT	319
gb:LC522974|Organism:Severe      AAGATGGAGAGCCTTGTCCCTGGTTTCAACGAGAAAACACACGTCCAACTCAGTTTGCCT	319
gb:MN985325|Organism:Severe      AAGATGGAGAGCCTTGTCCCTGGTTTCAACGAGAAAACACACGTCCAACTCAGTTTGCCT	322
gb:MT020881|Organism:Severe      AAGATGGAGAGCCTTGTCCCTGGTTTCAACGAGAAAACACACGTCCAACTCAGTTTGCCT	322
gb:MT020880|Organism:Severe      AAGATGGAGAGCCTTGTCCCTGGTTTCAACGAGAAAACACACGTCCAACTCAGTTTGCCT	322
gb:MT066175|Organism:Severe      AAGATGGAGAGCCTTGTCCCTGGTTTCAACGAGAAAACACACGTCCAACTCAGTTTGCCT	322
gb:MN997409|Organism:Severe      AAGATGGAGAGCCTTGTCCCTGGTTTCAACGAGAAAACACACGTCCAACTCAGTTTGCCT	322
gb:MN938384|Organism:Severe      AAGATGGAGAGCCTTGTCCCTGGTTTCAACGAGAAAACACACGTCCAACTCAGTTTGCCT	290
gb:MT044258|Organism:Severe      AAGATGGAGAGCCTTGTCCCTGGTTTCAACGAGAAAACACACGTCCAACTCAGTTTGCCT	322
gb:MT039890|Organism:Severe      AAGATGGAGAGCCTTGTCCCTGGTTTCAACGAGAAAACACACGTCCAACTCAGTTTGCCT	322
gb:MN988713|Organism:Severe      AAGATGGAGAGCCTTGTCCCTGGTTTCAACGAGAAAACACACGTCCAACTCAGTTTGCCT	322
gb:LC521925|Organism:Severe      AAGATGGAGAGCCTTGTCCCTGGTTTCAACGAGAAAACACACGTCCAACTCAGTTTGCCT	319
gb:MT093571|Organism:Severe      AAGATGGAGAGCCTTGTCCCTGGTTTCAACGAGAAAACACACGTCCAACTCAGTTTGCCT	322
gb:MT039887|Organism:Severe      AAGATGGAGAGCCTTGTCCCTGGTTTCAACGAGAAAACACACGTCCAACTCAGTTTGCCT	322
gb:MT019530|Organism:Severe      AAGATGGAGAGCCTTGTCCCTGGTTTCAACGAGAAAACACACGTCCAACTCAGTTTGCCT	322
gb:MT039888|Organism:Severe      AAGATGGAGAGCCTTGTCCCTGGTTTCAACGAGAAAACACACGTCCAACTCAGTTTGCCT	322
gb:LC522972|Organism:Severe      AAGATGGAGAGCCTTGTCCCTGGTTTCAACGAGAAAACACACGTCCAACTCAGTTTGCCT	319
```

```
gb:MT027063|Organism:Severe      AAGATGGAGAGCCTTGTCCCTGGTTTCAACGAGAAAACACACGTCCAACTCAGTTTGCCT   322
gb:MT027062|Organism:Severe      AAGATGGAGAGCCTTGTCCCTGGTTTCAACGAGAAAACACACGTCCAACTCAGTTTGCCT   322
gb:MT019529|Organism:Severe      AAGATGGAGAGCCTTGTCCCTGGTTTCAACGAGAAAACACACGTCCAACTCAGTTTGCCT   322
gb:MN996529|Organism:Severe      AAGATGGAGAGCCTTGTCCCTGGTTTCAACGAGAAAACACACGTCCAACTCAGTTTGCCT   310
gb:MN996531|Organism:Severe      AAGATGGAGAGCCTTGTCCCTGGTTTCAACGAGAAAACACACGTCCAACTCAGTTTGCCT   309
gb:MT066176|Organism:Severe      AAGATGGAGAGCCTTGTCCCTGGTTTCAACGAGAAAACACACGTCCAACTCAGTTTGCCT   322
gb:MT027064|Organism:Severe      AAGATGGAGAGCCTTGTCCCTGGTTTCAACGAGAAAACACACGTCCAACTCAGTTTGCCT   322
gb:MN994468|Organism:Severe      AAGATGGAGAGCCTTGTCCCTGGTTTCAACGAGAAAACACACGTCCAACTCAGTTTGCCT   322
gb:MT072688|Organism:Severe      AAGATGGAGAGCCTTGTCCCTGGTTTCAACGAGAAAACACACGTCCAACTCAGTTTGCCT   307
gb:MN996527|Organism:Severe      AAGATGGAGAGCCTTGTCCCTGGTTTCAACGAGAAAACACACGTCCAACTCAGTTTGCCT   289
gb:MT093631|Organism:Severe      AAGATGGAGAGCCTTGTCCCTGGTTTCAACGAGAAAACACACGTCCAACTCAGTTTGCCT   360
gb:MT106053|Organism:Severe      AAGATGGAGAGCCTTGTCCCTGGTTTCAACGAGAAAACACACGTCCAACTCAGTTTGCCT   322
gb:MT019533|Organism:Severe      AAGATGGAGAGCCTTGTCCCTGGTTTCAACGAGAAAACACACGTCCAACTCAGTTTGCCT   322
gb:MT019531|Organism:Severe      AAGATGGAGAGCCTTGTCCCTGGTTTCAACGAGAAAACACACGTCCAACTCAGTTTGCCT   322
gb:MN996528|Organism:Severe      AAGATGGAGAGCCTTGTCCCTGGTTTCAACGAGAAAACACACGTCCAACTCAGTTTGCCT   322
gb:MN996530|Organism:Severe      AAGATGGAGAGCCTTGTCCCTGGTTTCAACGAGAAAACACACGTCCAACTCAGTTTGCCT   308
gb:MN908947|Organism:Severe      AAGATGGAGAGCCTTGTCCCTGGTTTCAACGAGAAAACACACGTCCAACTCAGTTTGCCT   322
gb:MT019532|Organism:Severe      AAGATGGAGAGCCTTGTCCCTGGTTTCAACGAGAAAACACACGTCCAACTCAGTTTGCCT   322
                                 ************************************************************

gb:MT020781|Organism:Severe      GTTTTACAGGTTCGCGACGTGCTCGTACGTGGCTTTGGAGACTCCGTGGAGGAGGTCTTA   370
gb:MT007544|Organism:Severe      GTTTTACAGGTTCGCGACGTGCTCGTACGTGGCTTTGGAGACTCCGTGGAGGAGGTCTTA   382
gb:MN994467|Organism:Severe      GTTTTACAGGTTCGCGACGTGCTCGTACGTGGCTTTGGAGACTCCGTGGAGGAGGTCTTA   382
gb:MT044257|Organism:Severe      GTTTTACAGGTTCGCGACGTGCTCGTACGTGGCTTTGGAGACTCCGTGGAGGAGGTCTTA   382
gb:MT106054|Organism:Severe      GTTTTACAGGTTCGCGACGTGCTCGTACGTGGCTTTGGAGACTCCGTGGAGGAGGTCTTA   382
gb:MT049951|Organism:Severe      GTTTTACAGGTTCGCGACGTGCTCGTACGTGGCTTTGGAGACTCCGTGGAGGAGGTCTTA   382
gb:MN975262|Organism:Severe      GTTTTACAGGTTCGCGACGTGCTCGTACGTGGCTTTGGAGACTCCGTGGAGGAGGTCTTA   382
gb:MT106052|Organism:Severe      GTTTTACAGGTTCGCGACGTGCTCGTACGTGGCTTTGGAGACTCCGTGGAGGAGGTCTTA   382
gb:LC522975|Organism:Severe      GTTTTACAGGTTCGCGACGTGCTCGTACGTGGCTTTGGAGACTCCGTGGAGGAGGTCTTA   379
gb:LC522973|Organism:Severe      GTTTTACAGGTTCGCGACGTGCTCGTACGTGGCTTTGGAGACTCCGTGGAGGAGGTCTTA   379
gb:LC522974|Organism:Severe      GTTTTACAGGTTCGCGACGTGCTCGTACGTGGCTTTGGAGACTCCGTGGAGGAGGTCTTA   379
gb:MN985325|Organism:Severe      GTTTTACAGGTTCGCGACGTGCTCGTACGTGGCTTTGGAGACTCCGTGGAGGAGGTCTTA   382
gb:MT020881|Organism:Severe      GTTTTACAGGTTCGCGACGTGCTCGTACGTGGCTTTGGAGACTCCGTGGAGGAGGTCTTA   382
gb:MT020880|Organism:Severe      GTTTTACAGGTTCGCGACGTGCTCGTACGTGGCTTTGGAGACTCCGTGGAGGAGGTCTTA   382
gb:MT066175|Organism:Severe      GTTTTACAGGTTCGCGACGTGCTCGTACGTGGCTTTGGAGACTCCGTGGAGGAGGTCTTA   382
gb:MN997409|Organism:Severe      GTTTTACAGGTTCGCGACGTGCTCGTACGTGGCTTTGGAGACTCCGTGGAGGAGGTCTTA   382
gb:MN938384|Organism:Severe      GTTTTACAGGTTCGCGACGTGCTCGTACGTGGCTTTGGAGACTCCGTGGAGGAGGTCTTA   350
gb:MT044258|Organism:Severe      GTTTTACAGGTTCGCGACGTGCTCGTACGTGGCTTTGGAGACTCCGTGGAGGAGGTCTTA   382
gb:MT039890|Organism:Severe      GTTTTACAGGTTCGCGACGTGCTCGTACGTGGCTTTGGAGACTCCGTGGAGGAGGTCTTA   382
gb:MN988713|Organism:Severe      GTTTTACAGGTTCGCGACGTGCTCGTACGTGGCTTTGGAGACTCCGTGGAGGAGGTCTTA   382
gb:LC521925|Organism:Severe      GTTTTACAGGTTCGCGACGTGCTCGTACGTGGCTTT------------------------   355
gb:MT093571|Organism:Severe      GTTTTACAGGTTCGCGACGTGCTCGTACGTGGCTTTGGAGACTCCGTGGAGGAGGTCTTA   382
gb:MT039887|Organism:Severe      GTTTTACAGGTTCGCGACGTGCTCGTACGTGGCTTTGGAGACTCCGTGGAGGAGGTCTTA   382
gb:MT019530|Organism:Severe      GTTTTACAGGTTCGCGACGTGCTCGTACGTGGCTTTGGAGACTCCGTGGAGGAGGTCTTA   382
gb:MT039888|Organism:Severe      GTTTTACAGGTTCGCGACGTGCTCGTACGTGGCTTTGGAGACTCCGTGGAGGAGGTCTTA   382
gb:LC522972|Organism:Severe      GTTTTACAGGTTCGCGACGTGCTCGTACGTGGCTTTGGAGACTCCGTGGAGGAGGTCTTA   379
gb:MT027063|Organism:Severe      GTTTTACAGGTTCGCGACGTGCTCGTACGTGGCTTTGGAGACTCCGTGGAGGAGGTCTTA   382
gb:MT027062|Organism:Severe      GTTTTACAGGTTCGCGACGTGCTCGTACGTGGCTTTGGAGACTCCGTGGAGGAGGTCTTA   382
gb:MT019529|Organism:Severe      GTTTTACAGGTTCGCGACGTGCTCGTACGTGGCTTTGGAGACTCCGTGGAGGAGGTCTTA   382
gb:MN996529|Organism:Severe      GTTTTACAGGTTCGCGACGTGCTCGTACGTGGCTTTGGAGACTCCGTGGAGGAGGTCTTA   370
gb:MN996531|Organism:Severe      GTTTTACAGGTTCGCGACGTGCTCGTACGTGGCTTTGGAGACTCCGTGGAGGAGGTCTTA   369
gb:MT066176|Organism:Severe      GTTTTACAGGTTCGCGACGTGCTCGTACGTGGCTTTGGAGACTCCGTGGAGGAGGTCTTA   382
gb:MT027064|Organism:Severe      GTTTTACAGGTTCGCGACGTGCTCGTACGTGGCTTTGGAGACTCCGTGGAGGAGGTCTTA   382
gb:MN994468|Organism:Severe      GTTTTACAGGTTCGCGACGTGCTCGTACGTGGCTTTGGAGACTCCGTGGAGGAGGTCTTA   382
gb:MT072688|Organism:Severe      GTTTTACAGGTTCGCGACGTGCTCGTACGTGGCTTTGGAGACTCCGTGGAGGAGGTCTTA   367
gb:MN996527|Organism:Severe      GTTTTACAGGTTCGCGACGTGCTCGTACGTGGCTTTGGAGACTCCGTGGAGGAGGTCTTA   349
gb:MT093631|Organism:Severe      GTTTTACAGGTTCGCGACGTGCTCGTACGTGGCTTTGGAGACTCCGTGGAGGAGGTCTTA   420
gb:MT106053|Organism:Severe      GTTTTACAGGTTCGCGACGTGCTCGTACGTGGCTTTGGAGACTCCGTGGAGGAGGTCTTA   382
gb:MT019533|Organism:Severe      GTTTTACAGGTTCGCGACGTGCTCGTACGTGGCTTTGGAGACTCCGTGGAGGAGGTCTTA   382
gb:MT019531|Organism:Severe      GTTTTACAGGTTCGCGACGTGCTCGTACGTGGCTTTGGAGACTCCGTGGAGGAGGTCTTA   382
gb:MN996528|Organism:Severe      GTTTTACAGGTTCGCGACGTGCTCGTACGTGGCTTTGGAGACTCCGTGGAGGAGGTCTTA   382
gb:MN996530|Organism:Severe      GTTTTACAGGTTCGCGACGTGCTCGTACGTGGCTTTGGAGACTCCGTGGAGGAGGTCTTA   368
gb:MN908947|Organism:Severe      GTTTTACAGGTTCGCGACGTGCTCGTACGTGGCTTTGGAGACTCCGTGGAGGAGGTCTTA   382
gb:MT019532|Organism:Severe      GTTTTACAGGTTCGCGACGTGCTCGTACGTGGCTTTGGAGACTCCGTGGAGGAGGTCTTA   382
                                 ************************************
```

```
gb:MT020781|Organism:Severe      TCAGAGGCACGTCAACATCTTAAAGATGGCACTTGTGGCTTAGTAGAAGTTGAAAAAGGC   430
gb:MT007544|Organism:Severe      TCAGAGGCACGTCAACATCTTAAAGATGGCACTTGTGGCTTAGTAGAAGTTGAAAAAGGC   442
gb:MN994467|Organism:Severe      TCAGAGGCACGTCAACATCTTAAAGATGGCACTTGTGGCTTAGTAGAAGTTGAAAAAGGC   442
gb:MT044257|Organism:Severe      TCAGAGGCACGTCAACATCTTAAAGATGGCACTTGTGGCTTAGTAGAAGTTGAAAAAGGC   442
gb:MT106054|Organism:Severe      TCAGAGGCACGTCAACATCTTAAAGATGGCACTTGTGGCTTAGTAGAAGTTGAAAAAGGC   442
gb:MT049951|Organism:Severe      TCAGAGGCACGTCAACATCTTAAAGATGGCACTTGTGGCTTAGTAGAAGTTGAAAAAGGC   442
gb:MN975262|Organism:Severe      TCAGAGGCACGTCAACATCTTAAAGATGGCACTTGTGGCTTAGTAGAAGTTGAAAAAGGC   442
gb:MT106052|Organism:Severe      TCAGAGGCACGTCAACATCTTAAAGATGGCACTTGTGGCTTAGTAGAAGTTGAAAAAGGC   442
gb:LC522975|Organism:Severe      TCAGAGGCACGTCAACATCTTAAAGATGGCACTTGTGGCTTAGTAGAAGTTGAAAAAGGC   439
gb:LC522973|Organism:Severe      TCAGAGGCACGTCAACATCTTAAAGATGGCACTTGTGGCTTAGTAGAAGTTGAAAAAGGC   439
gb:LC522974|Organism:Severe      TCAGAGGCACGTCAACATCTTAAAGATGGCACTTGTGGCTTAGTAGAAGTTGAAAAAGGC   439
gb:MN985325|Organism:Severe      TCAGAGGCACGTCAACATCTTAAAGATGGCACTTGTGGCTTAGTAGAAGTTGAAAAAGGC   442
gb:MT020881|Organism:Severe      TCAGAGGCACGTCAACATCTTAAAGATGGCACTTGTGGCTTAGTAGAAGTTGAAAAAGGC   442
gb:MT020880|Organism:Severe      TCAGAGGCACGTCAACATCTTAAAGATGGCACTTGTGGCTTAGTAGAAGTTGAAAAAGGC   442
gb:MT066175|Organism:Severe      TCAGAGGCACGTCAACATCTTAAAGATGGCACTTGTGGCTTAGTAGAAGTTGAAAAAGGC   442
gb:MN997409|Organism:Severe      TCAGAGGCACGTCAACATCTTAAAGATGGCACTTGTGGCTTAGTAGAAGTTGAAAAAGGC   442
gb:MN938384|Organism:Severe      TCAGAGGCACGTCAACATCTTAAAGATGGCACTTGTGGCTTAGTAGAAGTTGAAAAAGGC   410
gb:MT044258|Organism:Severe      TCAGAGGCACGTCAACATCTTAAAGATGGCACTTGTGGCTTAGTAGAAGTTGAAAAAGGC   442
gb:MT039890|Organism:Severe      TCAGAGGCACGTCAACATCTTAAAGATGGCACTTGTGGCTTAGTAGAAGTTGAAAAAGGC   442
gb:MN988713|Organism:Severe      TCAGAGGCACGTCAACATCTTAAAGATGGCACTTGTGGCTTAGTAGAAGTTGAAAAAGGC   442
gb:LC521925|Organism:Severe      TCAGAGGCACGTCAACATCTTAAAGATGGCACTTGTGGCTTAGTAGAAGTTGAAAAAGGC   415
gb:MT093571|Organism:Severe      TCAGAGGCACGTCAACATCTTAAAGATGGCACTTGTGGCTTAGTAGAAGTTGAAAAAGGC   442
gb:MT039887|Organism:Severe      TCAGAGGCACGTCAACATCTTAAAGATGGCACTTGTGGCTTAGTAGAAGTTGAAAAAGGC   442
gb:MT019530|Organism:Severe      TCAGAGGCACGTCAACATCTTAAAGATGGCACTTGTGGCTTAGTAGAAGTTGAAAAAGGC   442
gb:MT039888|Organism:Severe      TCAGAGGCACGTCAACATCTTAAAGATGGCACTTGTGGCTTAGTAGAAGTTGAAAAAGGC   442
gb:LC522972|Organism:Severe      TCAGAGGCACGTCAACATCTTAAAGATGGCACTTGTGGCTTAGTAGAAGTTGAAAAAGGC   439
gb:MT027063|Organism:Severe      TCAGAGGCACGTCAACATCTTAAAGATGGCACTTGTGGCTTAGTAGAAGTTGAAAAAGGC   442
gb:MT027062|Organism:Severe      TCAGAGGCACGTCAACATCTTAAAGATGGCACTTGTGGCTTAGTAGAAGTTGAAAAAGGC   442
gb:MT019529|Organism:Severe      TCAGAGGCACGTCAACATCTTAAAGATGGCACTTGTGGCTTAGTAGAAGTTGAAAAAGGC   442
gb:MN996529|Organism:Severe      TCAGAGGCACGTCAACATCTTAAAGATGGCACTTGTGGCTTAGTAGAAGTTGAAAAAGGC   430
gb:MN996531|Organism:Severe      TCAGAGGCACGTCAACATCTTAAAGATGGCACTTGTGGCTTAGTAGAAGTTGAAAAAGGC   429
gb:MT066176|Organism:Severe      TCAGAGGCACGTCAACATCTTAAAGATGGCACTTGTGGCTTAGTAGAAGTTGAAAAAGGC   442
gb:MT027064|Organism:Severe      TCAGAGGCACGTCAACATCTTAAAGATGGCACTTGTGGCTTAGTAGAAGTTGAAAAAGGC   442
gb:MN994468|Organism:Severe      TCAGAGGCACGTCAACATCTTAAAGATGGCACTTGTGGCTTAGTAGAAGTTGAAAAAGGC   442
gb:MT072688|Organism:Severe      TCAGAGGCACGTCAACATCTTAAAGATGGCACTTGTGGCTTAGTAGAAGTTGAAAAAGGC   427
gb:MN996527|Organism:Severe      TCAGAGGCACGTCAACATCTTAAAGATGGCACTTGTGGCTTAGTAGAAGTTGAAAAAGGC   409
gb:MT093631|Organism:Severe      TCAGAGGCACGTCAACATCTTAAAGATGGCACTTGTGGCTTAGTAGAAGTTGAAAAAGGC   480
gb:MT106053|Organism:Severe      TCAGAGGCACGTCAACATCTTAAAGATGGCACTTGTGGCTTAGTAGAAGTTGAAAAAGGC   442
gb:MT019533|Organism:Severe      TCAGAGGCACGTCAACATCTTAAAGATGGCACTTGTGGCTTAGTAGAAGTTGAAAAAGGC   442
gb:MT019531|Organism:Severe      TCAGAGGCACGTCAACATCTTAAAGATGGCACTTGTGGCTTAGTAGAAGTTGAAAAAGGC   442
gb:MN996528|Organism:Severe      TCAGAGGCACGTCAACATCTTAAAGATGGCACTTGTGGCTTAGTAGAAGTTGAAAAAGGC   442
gb:MN996530|Organism:Severe      TCAGAGGCACGTCAACATCTTAAAGATGGCACTTGTGGCTTAGTAGAAGTTGAAAAAGGC   428
gb:MN908947|Organism:Severe      TCAGAGGCACGTCAACATCTTAAAGATGGCACTTGTGGCTTAGTAGAAGTTGAAAAAGGC   442
gb:MT019532|Organism:Severe      TCAGAGGCACGTCAACATCTTAAAGATGGCACTTGTGGCTTAGTAGAAGTTGAAAAAGGC   442
                                 ************************************************************

gb:MT020781|Organism:Severe      GTTTTGCCTCAACTTGAACAGCCCTATGTGTTCATCAAACGTTCGGATGCTCGAACTGCA   490
gb:MT007544|Organism:Severe      GTTTTGCCTCAACTTGAACAGCCCTATGTGTTCATCAAACGTTCGGATGCTCGAACTGCA   502
gb:MN994467|Organism:Severe      GTTTTGCCTCAACTTGAACAGCCCTATGTGTTCATCAAACGTTCGGATGCTCGAACTGCA   502
gb:MT044257|Organism:Severe      GTTTTGCCTCAACTTGAACAGCCCTATGTGTTCATCAAACGTTCGGAAGCTCGAACTGCA   502
gb:MT106054|Organism:Severe      GTTTTGCCTCAACTTGAACAGCCCTATGTGTTCATCAAACGTTCGGATGCTCGAACTGCA   502
gb:MT049951|Organism:Severe      GTTTTGCCTCAACTTGAACAGCCCTATGTGTTCATCAAACGTTCGGATGCTCGAACTGCA   502
gb:MN975262|Organism:Severe      GTTTTGCCTCAACTTGAACAGCCCTATGTGTTCATCAAACGTTCGGATGCTCGAACTGCA   502
gb:MT106052|Organism:Severe      GTTTTGCCTCAACTTGAACAGCCCTATGTGTTCATCAAACGTTCGGATGCTCGAACTGCA   502
gb:LC522975|Organism:Severe      GTTTTGCCTCAACTTGAACAGCCCTATGTGTTCATCAAACGTTCGGATGCTCGAACTGCA   499
gb:LC522973|Organism:Severe      GTTTTGCCTCAACTTGAACAGCCCTATGTGTTCATCAAACGTTCGGATGCTCGAACTGCA   499
gb:LC522974|Organism:Severe      GTTTTGCCTCAACTTGAACAGCCCTATGTGTTCATCAAACGTTCGGATGCTCGAACTGCA   499
gb:MN985325|Organism:Severe      GTTTTGCCTCAACTTGAACAGCCCTATGTGTTCATCAAACGTTCGGATGCTCGAACTGCA   502
gb:MT020881|Organism:Severe      GTTTTGCCTCAACTTGAACAGCCCTATGTGTTCATCAAACGTTCGGATGCTCGAACTGCA   502
gb:MT020880|Organism:Severe      GTTTTGCCTCAACTTGAACAGCCCTATGTGTTCATCAAACGTTCGGATGCTCGAACTGCA   502
gb:MT066175|Organism:Severe      GTTTTGCCTCAACTTGAACAGCCCTATGTGTTCATCAAACGTTCGGATGCTCGAACTGCA   502
gb:MN997409|Organism:Severe      GTTTTGCCTCAACTTGAACAGCCCTATGTGTTCATCAAACGTTCGGATGCTCGAACTGCA   502
gb:MN938384|Organism:Severe      GTTTTGCCTCAACTTGAACAGCCCTATGTGTTCATCAAACGTTCGGATGCTCGAACTGCA   470
gb:MT044258|Organism:Severe      GTTTTGCCTCAACTTGAACAGCCCTATGTGTTCATCAAACGTTCGGATGCTCGAACTGCA   502
```

```
gb:MT039890|Organism:Severe      GTTTTGCCTCAACTTGAACAGCCCTATGTGTTCATCAAACGTTCGGATGCTCGAACTGCA   502
gb:MN988713|Organism:Severe      GTTTTGCCTCAACTTGAACAGCCCTATGTGTTCATCAAACGTTCGGAWGCTCGAACTGCA   502
gb:LC521925|Organism:Severe      GTTTTGCCTCAACTTGAACAGCCCTATGTGTTCATCAAACGTTCGGATGCTCGAACTGCA   475
gb:MT093571|Organism:Severe      GTTTTGCCTCAACTTGAACAGCCCTATGTGTTCATCAAACGTTCGGATGCTCGAACTGCA   502
gb:MT039887|Organism:Severe      GTTTTGCCTCAACTTGAACAGCCCTATGTGTTCATCAAACGTTCGGATGCTCGAACTGCA   502
gb:MT019530|Organism:Severe      GTTTTGCCTCAACTTGAACAGCCCTATGTGTTCATCAAACGTTCGGATGCTCGAACTGCA   502
gb:MT039888|Organism:Severe      GTTTTGCCTCAACTTGAACAGCCCTATGTGTTCATCAAACGTTCGGATGCTCGAACTGCA   502
gb:LC522972|Organism:Severe      GTTTTGCCTCAACTTGAACAGCCCTATGTGTTCATCAAACGTTCGGATGCTCGAACTGCA   499
gb:MT027063|Organism:Severe      GTTTTGCCTCAACTTGAACAGCCCTATGTGTTCATCAAACGTTCGGATGCTCGAACTGCA   502
gb:MT027062|Organism:Severe      GTTTTGCCTCAACTTGAACAGCCCTATGTGTTCATCAAACGTTCGGATGCTCGAACTGCA   502
gb:MT019529|Organism:Severe      GTTTTGCCTCAACTTGAACAGCCCTATGTGTTCATCAAACGTTCGGATGCTCGAACTGCA   502
gb:MN996529|Organism:Severe      GTTTTGCCTCAACTTGAACAGCCCTATGTGTTCATCAAACGTTCGGATGCTCGAACTGCA   490
gb:MN996531|Organism:Severe      GTTTTGCCTCAACTTGAACAGCCCTATGTGTTCATCAAACGTTCGGATGCTCGAACTGCA   489
gb:MT066176|Organism:Severe      GTTTTGCCTCAACTTGAACAGCCCTATGTGTTCATCAAACGTTCGGATGCTCGAACTGCA   502
gb:MT027064|Organism:Severe      GTTTTGCCTCAACTTGAACAGCCCTATGTGTTCATCAAACGTTCGGATGCTCGAACTGCA   502
gb:MN994468|Organism:Severe      GTTTTGCCTCAACTTGAACAGCCCTATGTGTTCATCAAACGTTCGGATGCTCGAACTGCA   502
gb:MT072688|Organism:Severe      GTTTTGCCTCAACTTGAACAGCCCTATGTGTTCATCAAACGTTCGGATGCTCGAACTGCA   487
gb:MN996527|Organism:Severe      GTTTTGCCTCAACTTGAACAGCCCTATGTGTTCATCAAACGTTCGGATGCTCGAACTGCA   469
gb:MT093631|Organism:Severe      GTTTTGCCTCAACTTGAACAGCCCTATGTGTTCATCAAACGTTCGGATGCTCGAACTGCA   540
gb:MT106053|Organism:Severe      GTTTTGCCTCAACTTGAACAGCCCTATGTGTTCATCAAACGTTCGGATGCTCGAACTGCA   502
gb:MT019533|Organism:Severe      GTTTTGCCTCAACTTGAACAGCCCTATGTGTTCATCAAACGTTCGGATGCTCGAACTGCA   502
gb:MT019531|Organism:Severe      GTTTTGCCTCAACTTGAACAGCCCTATGTGTTCATCAAACGTTCGGATGCTCGAACTGCA   502
gb:MN996528|Organism:Severe      GTTTTGCCTCAACTTGAACAGCCCTATGTGTTCATCAAACGTTCGGATGCTCGAACTGCA   502
gb:MN996530|Organism:Severe      GTTTTGCCTCAACTTGAACAGCCCTATGTGTTCATCAAACGTTCGGATGCTCGAACTGCA   488
gb:MN908947|Organism:Severe      GTTTTGCCTCAACTTGAACAGCCCTATGTGTTCATCAAACGTTCGGATGCTCGAACTGCA   502
gb:MT019532|Organism:Severe      GTTTTGCCTCAACTTGAACAGCCCTATGTGTTCATCAAACGTTCGGATGCTCGAACTGCA   502
                                 *********************************************** ************

gb:MT020781|Organism:Severe      CCTCATGGTCATGTTATGGTTGAGCTGGTAGCAGAACTCGAAGGCATTCAGTACGGTCGT   550
gb:MT007544|Organism:Severe      CCTCATGGTCATGTTATGGTTGAGCTGGTAGCAGAACTCGAAGGCATTCAGTACGGTCGT   562
gb:MN994467|Organism:Severe      CCTCATGGTCATGTTATGGTTGAGCTGGTAGCAGAACTCGAAGGCATTCAGTACGGTCGT   562
gb:MT044257|Organism:Severe      CCTCATGGTCATGTTATGGTTGAGCTGGTAGCAGAACTCGAAGGCATTCAGTACGGTCGT   562
gb:MT106054|Organism:Severe      CCTCATGGTCATGTTATGGTTGAGCTGGTAGCAGAACTCGAAGGCATTCAGTACGGTCGT   562
gb:MT049951|Organism:Severe      CCTCATGGTCATGTTATGGTTGAGCTGGTAGCAGAACTCGAAGGCATTCAGTACGGTCGT   562
gb:MN975262|Organism:Severe      CCTCATGGTCATGTTATGGTTGAGCTGGTAGCAGAACTCGAAGGCATTCAGTACGGTCGT   562
gb:MT106052|Organism:Severe      CCTCATGGTCATGTTATGGTTGAGCTGGTAGCAGAACTCGAAGGCATTCAGTACGGTCGT   562
gb:LC522975|Organism:Severe      CCTCATGGTCATGTTATGGTTGAGCTGGTAGCAGAACTCGAAGGCATTCAGTACGGTCGT   559
gb:LC522973|Organism:Severe      CCTCATGGTCATGTTATGGTTGAGCTGGTAGCAGAACTCGAAGGCATTCAGTACGGTCGT   559
gb:LC522974|Organism:Severe      CCTCATGGTCATGTTATGGTTGAGCTGGTAGCAGAACTCGAAGGCATTCAGTACGGTCGT   559
gb:MN985325|Organism:Severe      CCTCATGGTCATGTTATGGTTGAGCTGGTAGCAGAACTCGAAGGCATTCAGTACGGTCGT   562
gb:MT020881|Organism:Severe      CCTCATGGTCATGTTATGGTTGAGCTGGTAGCAGAACTCGAAGGCATTCAGTACGGTCGT   562
gb:MT020880|Organism:Severe      CCTCATGGTCATGTTATGGTTGAGCTGGTAGCAGAACTCGAAGGCATTCAGTACGGTCGT   562
gb:MT066175|Organism:Severe      CCTCATGGTCATGTTATGGTTGAGCTGGTAGCAGAACTCGAAGGCATTCAGTACGGTCGT   562
gb:MN997409|Organism:Severe      CCTCATGGTCATGTTATGGTTGAGCTGGTAGCAGAACTCGAAGGCATTCAGTACGGTCGT   562
gb:MN938384|Organism:Severe      CCTCATGGTCATGTTATGGTTGAGCTGGTAGCAGAACTCGAAGGCATTCAGTACGGTCGT   530
gb:MT044258|Organism:Severe      CCTCA---------------TGAGCTGGTAGCAGAACTCGAAGGCATTCAGTACGGTCGT   547
gb:MT039890|Organism:Severe      CCTCATGGTCATGTTATGGTTGAGCTGGTAGCAGAACTCGAAGGCATTCAGTACGGTCGT   562
gb:MN988713|Organism:Severe      CCTCATGGTCATGTTATGGTTGAGCTGGTAGCAGAACTCGAAGGCATTCAGTACGGTCGT   562
gb:LC521925|Organism:Severe      CCTCATGGTCATGTTATGGTTGAGCTGGTAGCAGAACTCGAAGGCATTCAGTACGGTCGT   535
gb:MT093571|Organism:Severe      CCTCATGGTCATGTTATGGTTGAGCTGGTAGCAGAACTCGAAGGCATTCAGTACGGTCGT   562
gb:MT039887|Organism:Severe      CCTCATGGTCATGTTATGGTTGAGCTGGTAGCAGAACTCGAAGGCATTCAGTACGGTCGT   562
gb:MT019530|Organism:Severe      CCTCATGGTCATGTTATGGTTGAGCTGGTAGCAGAACTCGAAGGCATTCAGTACGGTCGT   562
gb:MT039888|Organism:Severe      CCTCATGGTCATGTTATGGTTGAGCTGGTAGCAGAACTCGAAGGCATTCAGTACGGTCGT   562
gb:LC522972|Organism:Severe      CCTCATGGTCATGTTATGGTTGAGCTGGTAGCAGAACTCGAAGGCATTCAGTACGGTCGT   559
gb:MT027063|Organism:Severe      CCTCATGGTCATGTTATGGTTGAGCTGGTAGCAGAACTCGAAGGCATTCAGTACGGTCGT   562
gb:MT027062|Organism:Severe      CCTCATGGTCATGTTATGGTTGAGCTGGTAGCAGAACTCGAAGGCATTCAGTACGGTCGT   562
gb:MT019529|Organism:Severe      CCTCATGGTCATGTTATGGTTGAGCTGGTAGCAGAACTCGAAGGCATTCAGTACGGTCGT   562
gb:MN996529|Organism:Severe      CCTCATGGTCATGTTATGGTTGAGCTGGTAGCAGAACTCGAAGGCATTCAGTACGGTCGT   550
gb:MN996531|Organism:Severe      CCTCATGGTCATGTTATGGTTGAGCTGGTAGCAGAACTCGAAGGCATTCAGTACGGTCGT   549
gb:MT066176|Organism:Severe      CCTCATGGTCATGTTATGGTTGAGCTGGTAGCAGAACTCGAAGGCATTCAGTACGGTCGT   562
gb:MT027064|Organism:Severe      CCTCATGGTCATGTTATGGTTGAGCTGGTAGCAGAACTCGAAGGCATTCAGTACGGTCGT   562
gb:MN994468|Organism:Severe      CCTCATGGTCATGTTATGGTTGAGCTGGTAGCAGAACTCGAAGGCATTCAGTACGGTCGT   562
gb:MT072688|Organism:Severe      CCTCATGGTCATGTTATGGTTGAGCTGGTAGCAGAACTCGAAGGCATTCAGTACGGTCGT   547
gb:MN996527|Organism:Severe      CCTCATGGTCATGTTATGGTTGAGCTGGTAGCAGAACTCGAAGGCATTCAGTACGGTCGT   529
gb:MT093631|Organism:Severe      CCTCATGGTCATGTTATGGTTGAGCTGGTAGCAGAACTCGAAGGCATTCAGTACGGTCGT   600
```

```
gb:MT106053|Organism:Severe      CCTCATGGTCATGTTATGGTTGAGCTGGTAGCAGAACTCGAAGGCATTCAGTACGGTCGT   562
gb:MT019533|Organism:Severe      CCTCATGGTCATGTTATGGTTGAGCTGGTAGCAGAACTCGAAGGCATTCAGTACGGTCGT   562
gb:MT019531|Organism:Severe      CCTCATGGTCATGTTATGGTTGAGCTGGTAGCAGAACTCGAAGGCATTCAGTACGGTCGT   562
gb:MN996528|Organism:Severe      CCTCATGGTCATGTTATGGTTGAGCTGGTAGCAGAACTCGAAGGCATTCAGTACGGTCGT   562
gb:MN996530|Organism:Severe      CCTCATGGTCATGTTATGGTTGAGCTGGTAGCAGAACTCGAAGGCATTCAGTACGGTCGT   548
gb:MN908947|Organism:Severe      CCTCATGGTCATGTTATGGTTGAGCTGGTAGCAGAACTCGAAGGCATTCAGTACGGTCGT   562
gb:MT019532|Organism:Severe      CCTCATGGTCATGTTATGGTTGAGCTGGTAGCAGAACTCGAAGGCATTCAGTACGGTCGT   562
                                 *****               ****************************************

gb:MT020781|Organism:Severe      AGTGGTGAGACACTTGGTGTCCTTGTCCCTCATGTGGGCGAAATACCAGTGGCTTACCGC   610
gb:MT007544|Organism:Severe      AGTGGTGAGACACTTGGTGTCCTTGTCCCTCATGTGGGCGAAATACCAGTGGCTTACCGC   622
gb:MN994467|Organism:Severe      AGTGGTGAGACACTTGGTGTCCTTGTCCCTCATGTGGGCGAAATACCAGTGGCTTACCGC   622
gb:MT044257|Organism:Severe      AGTGGTGAGACACTTGGTGTCCTTGTCCCTCATGTGGGCGAAATACCAGTGGCTTACCGC   622
gb:MT106054|Organism:Severe      AGTGGTGAGACACTTGGTGTCCTTGTCCCTCATGTGGGCGAAATACCAGTGGCTTACCGC   622
gb:MT049951|Organism:Severe      AGTGGTGAGACACTTGGTGTCCTTGTCCCTCATGTGGGCGAAATACCAGTGGCTTACCGC   622
gb:MN975262|Organism:Severe      AGTGGTGAGACACTTGGTGTCCTTGTCCCTCATGTGGGCGAAATACCAGTGGCTTACCGC   622
gb:MT106052|Organism:Severe      AGTGGTGAGACACTTGGTGTCCTTGTCCCTCATGTGGGCGAAATACCAGTGGCTTACCGC   622
gb:LC522975|Organism:Severe      AGTGGTGAGACACTTGGTGTCCTTGTCCCTCATGTGGGCGAAATACCAGTGGCTTACCGC   619
gb:LC522973|Organism:Severe      AGTGGTGAGACACTTGGTGTCCTTGTCCCTCATGTGGGCGAAATACCAGTGGCTTACCGC   619
gb:LC522974|Organism:Severe      AGTGGTGAGACACTTGGTGTCCTTGTCCCTCATGTGGGCGAAATACCAGTGGCTTACCGC   619
gb:MN985325|Organism:Severe      AGTGGTGAGACACTTGGTGTCCTTGTCCCTCATGTGGGCGAAATACCAGTGGCTTACCGC   622
gb:MT020881|Organism:Severe      AGTGGTGAGACACTTGGTGTCCTTGTCCCTCATGTGGGCGAAATACCAGTGGCTTACCGC   622
gb:MT020880|Organism:Severe      AGTGGTGAGACACTTGGTGTCCTTGTCCCTCATGTGGGCGAAATACCAGTGGCTTACCGC   622
gb:MT066175|Organism:Severe      AGTGGTGAGACACTTGGTGTCCTTGTCCCTCATGTGGGCGAAATACCAGTGGCTTACCGC   622
gb:MN997409|Organism:Severe      AGTGGTGAGACACTTGGTGTCCTTGTCCCTCATGTGGGCGAAATACCAGTGGCTTACCGC   622
gb:MN938384|Organism:Severe      AGTGGTGAGACACTTGGTGTCCTTGTCCCTCATGTGGGCGAAATACCAGTGGCTTACCGC   590
gb:MT044258|Organism:Severe      AGTGGTGAGACACTTGGTGTCCTTGTCCCTCATGTGGGCGAAATACCAGTGGCTTACCGC   607
gb:MT039890|Organism:Severe      AGTGGTGAGACACTTGGTGTCCTTGTCCCTCATGTGGGCGAAATACCAGTGGCTTACCGC   622
gb:MN988713|Organism:Severe      AGTGGTGAGACACTTGGTGTCCTTGTCCCTCATGTGGGCGAAATACCAGTGGCTTACCGC   622
gb:LC521925|Organism:Severe      AGTGGTGAGACACTTGGTGTCCTTGTCCCTCATGTGGGCGAAATACCAGTGGCTTACCGC   595
gb:MT093571|Organism:Severe      AGTGGTGAGACACTTGGTGTCCTTGTCCCTCATGTGGGCGAAATACCAGTGGCTTACCGC   622
gb:MT039887|Organism:Severe      AGTGGTGAGACACTTGGTGTCCTTGTCCCTCATGTGGGCGAAATACCAGTGGCTTACCGC   622
gb:MT019530|Organism:Severe      AGTGGTGAGACACTTGGTGTCCTTGTCCCTCATGTGGGCGAAATACCAGTGGCTTACCGC   622
gb:MT039888|Organism:Severe      AGTGGTGAGACACTTGGTGTCCTTGTCCCTCATGTGGGCGAAATACCAGTGGCTTACCGC   622
gb:LC522972|Organism:Severe      AGTGGTGAGACACTTGGTGTCCTTGTCCCTCATGTGGGCGAAATACCAGTGGCTTACCGC   619
gb:MT027063|Organism:Severe      AGTGGTGAGACACTTGGTGTCCTTGTCCCTCATGTGGGCGAAATACCAGTGACTTACCGC   622
gb:MT027062|Organism:Severe      AGTGGTGAGACACTTGGTGTCCTTGTCCCTCATGTGGGCGAAATACCAGTGACTTACCGC   622
gb:MT019529|Organism:Severe      AGTGGTGAGACACTTGGTGTCCTTGTCCCTCATGTGGGCGAAATACCAGTGGCTTACCGC   622
gb:MN996529|Organism:Severe      AGTGGTGAGACACTTGGTGTCCTTGTCCCTCATGTGGGCGAAATACCAGTGGCTTACCGC   610
gb:MN996531|Organism:Severe      AGTGGTGAGACACTTGGTGTCCTTGTCCCTCATGTGGGCGAAATACCAGTGGCTTACCGC   609
gb:MT066176|Organism:Severe      AGTGGTGAGACACTTGGTGTCCTTGTCCCTCATGTGGGCGAAATACCAGTGGCTTACCGC   622
gb:MT027064|Organism:Severe      AGTGGTGAGACACTTGGTGTCCTTGTCCCTCATGTGGGCGAAATACCAGTGGCTTACCGC   622
gb:MN994468|Organism:Severe      AGTGGTGAGACACTTGGTGTCCTTGTCCCTCATGTGGGCGAAATACCAGTGGCTTACCGC   622
gb:MT072688|Organism:Severe      AGTGGTGAGACACTTGGTGTCCTTGTCCCTCATGTGGGCGAAATACCAGTGGCTTACCGC   607
gb:MN996527|Organism:Severe      AGTGGTGAGACACTTGGTGTCCTTGTCCCTCATGTGGGCGAAATACCAGTGGCTTACCGC   589
gb:MT093631|Organism:Severe      AGTGGTGAGACACTTGGTGTCCTTGTCCCTCATGTGGGCGAAATACCAGTGGCTTACCGC   660
gb:MT106053|Organism:Severe      AGTGGTGAGACACTTGGTGTCCTTGTCCCTCATGTGGGCGAAATACCAGTGGCTTACCGC   622
gb:MT019533|Organism:Severe      AGTGGTGAGACACTTGGTGTCCTTGTCCCTCATGTGGGCGAAATACCAGTGGCTTACCGC   622
gb:MT019531|Organism:Severe      AGTGGTGAGACACTTGGTGTCCTTGTCCCTCATGTGGGCGAAATACCAGTGGCTTACCGC   622
gb:MN996528|Organism:Severe      AGTGGTGAGACACTTGGTGTCCTTGTCCCTCATGTGGGCGAAATACCAGTGGCTTACCGC   622
gb:MN996530|Organism:Severe      AGTGGTGAGACACTTGGTGTCCTTGTCCCTCATGTGGGCGAAATACCAGTGGCTTACCGC   608
gb:MN908947|Organism:Severe      AGTGGTGAGACACTTGGTGTCCTTGTCCCTCATGTGGGCGAAATACCAGTGGCTTACCGC   622
gb:MT019532|Organism:Severe      AGTGGTGAGACACTTGGTGTCCTTGTCCCTCATGTGGGCGAAATACCAGTGGCTTACCGC   622
                                 *************************************************** ********

gb:MT020781|Organism:Severe      AAGGTTCTTCTTCGTAAGAACGGTAATAAAGGAGCTGGTGGCCATAGTTACGGCGCCGAT   670
gb:MT007544|Organism:Severe      AAGGTTCTTCTTCGTAAGAACGGTAATAAAGGAGCTGGTGGCCATAGTTACGGCGCCGAT   682
gb:MN994467|Organism:Severe      AAGGTTCTTCTTCGTAAGAACGGTAATAAAGGAGCTGGTGGCCATAGTTACGGCGCCGAT   682
gb:MT044257|Organism:Severe      AAGGTTCTTCTTCGTAAGAACGGTAATAAAGGAGCTGGTGGCCATAGTTACGGCGCCGAT   682
gb:MT106054|Organism:Severe      AAGGTTCTTCTTCGTAAGAACGGTAATAAAGGAGCTGGTGGCCATAGTTACGGCGCCGAT   682
gb:MT049951|Organism:Severe      AAGGTTCTTCTTCGTAAGAACGGTAATAAAGGAGCTGGTGGCCATAGTTACGGCGCCGAT   682
gb:MN975262|Organism:Severe      AAGGTTCTTCTTCGTAAGAACGGTAATAAAGGAGCTGGTGGCCATAGTTACGGCGCCGAT   682
gb:MT106052|Organism:Severe      AAGGTTCTTCTTCGTAAGAACGGTAATAAAGGAGCTGGTGGCCATAGTTACGGCGCCGAT   682
gb:LC522975|Organism:Severe      AAGGTTCTTCTTCGTAAGAACGGTAATAAAGGAGCTGGTGGCCATAGTTACGGCGCCGAT   679
gb:LC522973|Organism:Severe      AAGGTTCTTCTTCGTAAGAACGGTAATAAAGGAGCTGGTGGCCATAGTTACGGCGCCGAT   679
```

```
gb:LC522974|Organism:Severe      AAGGTTCTTCTTCGTAAGAACGGTAATAAAGGAGCTGGTGGCCATAGTTACGGCGCCGAT   679
gb:MN985325|Organism:Severe      AAGGTTCTTCTTCGTAAGAACGGTAATAAAGGAGCTGGTGGCCATAGTTACGGCGCCGAT   682
gb:MT020881|Organism:Severe      AAGGTTCTTCTTCGTAAGAACGGTAATAAAGGAGCTGGTGGCCATAGTTACGGCGCCGAT   682
gb:MT020880|Organism:Severe      AAGGTTCTTCTTCGTAAGAACGGTAATAAAGGAGCTGGTGGCCATAGTTACGGCGCCGAT   682
gb:MT066175|Organism:Severe      AAGGTTCTTCTTCGTAAGAACGGTAATAAAGGAGCTGGTGGCCATAGTTACGGCGCCGAT   682
gb:MN997409|Organism:Severe      AAGGTTCTTCTTCGTAAGAACGGTAATAAAGGAGCTGGTGGCCATAGTTACGGCGCCGAT   682
gb:MN938384|Organism:Severe      AAGGTTCTTCTTCGTAAGAACGGTAATAAAGGAGCTGGTGGCCATAGTTACGGCGCCGAT   650
gb:MT044258|Organism:Severe      AAGGTTCTTCTTCGTAAGAACGGTAATAAAGGAGCTGGTGGCCATAGTTACGGCGCCGAT   667
gb:MT039890|Organism:Severe      AAGGTTCTTCTTCGTAAGAACGGTAATAAAGGAGCTGGTGGCCATAGTTACGGCGCCGAT   682
gb:MN988713|Organism:Severe      AAGGTTCTTCTTCGTAAGAACGGTAATAAAGGAGCTGGTGGCCATAGTTACGGCGCCGAT   682
gb:LC521925|Organism:Severe      AAGGTTCTTCTTCGTAAGAACGGTAATAAAGGAGCTGGTGGCCATAGTTACGGCGCCGAT   655
gb:MT093571|Organism:Severe      AAGGTTCTTCTTCGTAAGAACGGTAATAAAGGAGCTGGTGGCCATAGTTACGGCGCCGAT   682
gb:MT039887|Organism:Severe      AAGGTTCTTCTTCGTAAGAACGGTAATAAAGGAGCTGGTGGCCATAGTTACGGCGCCGAT   682
gb:MT019530|Organism:Severe      AAGGTTCTTCTTCGTAAGAACGGTAATAAAGGAGCTGGTGGCCATAGTTACGGCGCCGAT   682
gb:MT039888|Organism:Severe      AAGGTTCTTCTTCGTAAGAACGGTAATAAAGGAGCTGGTGGCCATAGTTACGGCGCCGAT   682
gb:LC522972|Organism:Severe      AAGGTTCTTCTTCGTAAGAACGGTAATAAAGGAGCTGGTGGCCATAGTTACGGCGCCGAT   679
gb:MT027063|Organism:Severe      AAGGTTCTTCTTCGTAAGAACGGTAATAAAGGAGCTGGTGGCCATAGTTACGGCGCCGAT   682
gb:MT027062|Organism:Severe      AAGGTTCTTCTTCGTAAGAACGGTAATAAAGGAGCTGGTGGCCATAGTTACGGCGCCGAT   682
gb:MT019529|Organism:Severe      AAGGTTCTTCTTCGTAAGAACGGTAATAAAGGAGCTGGTGGCCATAGTTACGGCGCCGAT   682
gb:MN996529|Organism:Severe      AAGGTTCTTCTTCGTAAGAACGGTAATAAAGGAGCTGGTGGCCATAGTTACGGCGCCGAT   670
gb:MN996531|Organism:Severe      AAGGTTCTTCTTCGTAAGAACGGTAATAAAGGAGCTGGTGGCCATAGTTACGGCGCCGAT   669
gb:MT066176|Organism:Severe      AAGGTTCTTCTTCGTAAGAACGGTAATAAAGGAGCTGGTGGCCATAGTTACGGCGCCGAT   682
gb:MT027064|Organism:Severe      AAGGTTCTTCTTCGTAAGAACGGTAATAAAGGAGCTGGTGGCCATAGTTACGGCGCCGAT   682
gb:MN994468|Organism:Severe      AAGGTTCTTCTTCGTAAGAACGGTAATAAAGGAGCTGGTGGCCATAGTTACGGCGCCGAT   682
gb:MT072688|Organism:Severe      AAGGTTCTTCTTCGTAAGAACGGTAATAAAGGAGCTGGTGGCCATAGTTACGGCGCCGAT   667
gb:MN996527|Organism:Severe      AAGGTTCTTCTTCGTAAGAACGGTAATAAAGGAGCTGGTGGCCATAGTTACGGCGCCGAT   649
gb:MT093631|Organism:Severe      AAGGTTCTTCTTCGTAAGAACGGTAATAAAGGAGCTGGTGGCCATAGTTACGGCGCCGAT   720
gb:MT106053|Organism:Severe      AAGGTTCTTCTTCGTAAGAACGGTAATAAAGGAGCTGGTGGCCATAGTTACGGCGCCGAT   682
gb:MT019533|Organism:Severe      AAGGTTCTTCTTCGTAAGAACGGTAATAAAGGAGCTGGTGGCCATAGTTACGGCGCCGAT   682
gb:MT019531|Organism:Severe      AAGGTTCTTCTTCGTAAGAACGGTAATAAAGGAGCTGGTGGCCATAGTTACGGCGCCGAT   682
gb:MN996528|Organism:Severe      AAGGTTCTTCTTCGTAAGAACGGTAATAAAGGAGCTGGTGGCCATAGTTACGGCGCCGAT   682
gb:MN996530|Organism:Severe      AAGGTTCTTCTTCGTAAGAACGGTAATAAAGGAGCTGGTGGCCATAGTTACGGCGCCGAT   668
gb:MN908947|Organism:Severe      AAGGTTCTTCTTCGTAAGAACGGTAATAAAGGAGCTGGTGGCCATAGTTACGGCGCCGAT   682
gb:MT019532|Organism:Severe      AAGGTTCTTCTTCGTAAGAACGGTAATAAAGGAGCTGGTGGCCATAGTTACGGCGCCGAT   682
                                 ************************************************************

gb:MT020781|Organism:Severe      CTAAAGTCATTTGACTTAGGCGACGAGCTTGGCACTGATCCTTATGAAGATTTTCAAGAA   730
gb:MT007544|Organism:Severe      CTAAAGTCATTTGACTTAGGCGACGAGCTTGGCACTGATCCTTATGAAGATTTTCAAGAA   742
gb:MN994467|Organism:Severe      CTAAAGTCATTTGACTTAGGCGACGAGCTTGGCACTGATCCTTATGAAGATTTTCAAGAA   742
gb:MT044257|Organism:Severe      CTAAAGTCATTTGACTTAGGCGACGAGCTTGGCACTGATCCTTATGAAGATTTTCAAGAA   742
gb:MT106054|Organism:Severe      CTAAAGTCATTTGACTTAGGCGACGAGCTTGGCACTGATCCTTATGAAGATTTTCAAGAA   742
gb:MT049951|Organism:Severe      CTAAAGTCATTTGACTTAGGCGACGAGCTTGGCACTGATCCTTATGAAGATTTTCAAGAA   742
gb:MN975262|Organism:Severe      CTAAAGTCATTTGACTTAGGCGACGAGCTTGGCACTGATCCTTATGAAGATTTTCAAGAA   742
gb:MT106052|Organism:Severe      CTAAAGTCATTTGACTTAGGCGACGAGCTTGGCACTGATCCTTATGAAGATTTTCAAGAA   742
gb:LC522975|Organism:Severe      CTAAAGTCATTTGACTTAGGCGACGAGCTTGGCACTGATCCTTATGAAGATTTTCAAGAA   739
gb:LC522973|Organism:Severe      CTAAAGTCATTTGACTTAGGCGACGAGCTTGGCACTGATCCTTATGAAGATTTTCAAGAA   739
gb:LC522974|Organism:Severe      CTAAAGTCATTTGACTTAGGCGACGAGCTTGGCACTGATCCTTATGAAGATTTTCAAGAA   739
gb:MN985325|Organism:Severe      CTAAAGTCATTTGACTTAGGCGACGAGCTTGGCACTGATCCTTATGAAGATTTTCAAGAA   742
gb:MT020881|Organism:Severe      CTAAAGTCATTTGACTTAGGCGACGAGCTTGGCACTGATCCTTATGAAGATTTTCAAGAA   742
gb:MT020880|Organism:Severe      CTAAAGTCATTTGACTTAGGCGACGAGCTTGGCACTGATCCTTATGAAGATTTTCAAGAA   742
gb:MT066175|Organism:Severe      CTAAAGTCATTTGACTTAGGCGACGAGCTTGGCACTGATCCTTATGAAGATTTTCAAGAA   742
gb:MN997409|Organism:Severe      CTAAAGTCATTTGACTTAGGCGACGAGCTTGGCACTGATCCTTATGAAGATTTTCAAGAA   742
gb:MN938384|Organism:Severe      CTAAAGTCATTTGACTTAGGCGACGAGCTTGGCACTGATCCTTATGAAGATTTTCAAGAA   710
gb:MT044258|Organism:Severe      CTA---------GACTTAGGCGACGAGCTTGGCACTGATCCTTATGAAGATTTTCAAGAA   718
gb:MT039890|Organism:Severe      CTAAAGTCATTTGACTTAGGCGACGAGCTTGGCACTGATCCTTATGAAGATTTTCAAGAA   742
gb:MN988713|Organism:Severe      CTAAAGTCATTTGACTTAGGCGACGAGCTTGGCACTGATCCTTATGAAGATTTTCAAGAA   742
gb:LC521925|Organism:Severe      CTAAAGTCATTTGACTTAGGCGACGAGCTTGGCACTGATCCTTATGAAGATTTTCAAGAA   715
gb:MT093571|Organism:Severe      CTAAAGTCATTTGACTTAGGCGACGAGCTTGGCACTGATCCTTATGAAGATTTTCAAGAA   742
gb:MT039887|Organism:Severe      CTAAAGTCATTTGACTTAGGCGACGAGCTTGGCACTGATCCTTATGAAGATTTTCAAGAA   742
gb:MT019530|Organism:Severe      CTAAAGTCATTTGACTTAGGCGACGAGCTTGGCACTGATCCTTATGAAGATTTTCAAGAA   742
gb:MT039888|Organism:Severe      CTAAAGTCATTTGACTTAGGCGACGAGCTTGGCACTGATCCTTATGAAGATTTTCAAGAA   742
gb:LC522972|Organism:Severe      CTAAAGTCATTTGACTTAGGCGACGAGCTTGGCACTGATCCTTATGAAGATTTTCAAGAA   739
gb:MT027063|Organism:Severe      CTAAAGTCATTTGACTTAGGCGACGAGCTTGGCACTGATCCTTATGAAGATTTTCAAGAA   742
gb:MT027062|Organism:Severe      CTAAAGTCATTTGACTTAGGCGACGAGCTTGGCACTGATCCTTATGAAGATTTTCAAGAA   742
gb:MT019529|Organism:Severe      CTAAAGTCATTTGACTTAGGCGACGAGCTTGGCACTGATCCTTATGAAGATTTTCAAGAA   742
```

```
gb:MN996529|Organism:Severe      CTAAAGTCATTTGACTTAGGCGACGAGCTTGGCACTGATCCTTATGAAGATTTTCAAGAA   730
gb:MN996531|Organism:Severe      CTAAAGTCATTTGACTTAGGCGACGAGCTTGGCACTGATCCTTATGAAGATTTTCAAGAA   729
gb:MT066176|Organism:Severe      CTAAAGTCATTTGACTTAGGCGACGAGCTTGGCACTGATCCTTATGAAGATTTTCAAGAA   742
gb:MT027064|Organism:Severe      CTAAAGTCATTTGACTTAGGCGACGAGCTTGGCACTGATCCTTATGAAGATTTTCAAGAA   742
gb:MN994468|Organism:Severe      CTAAAGTCATTTGACTTAGGCGACGAGCTTGGCACTGATCCTTATGAAGATTTTCAAGAA   742
gb:MT072688|Organism:Severe      CTAAAGTCATTTGACTTAGGCGACGAGCTTGGCACTGATCCTTATGAAGATTTTCAAGAA   727
gb:MN996527|Organism:Severe      CTAAAGTCATTTGACTTAGGCGACGAGCTTGGCACTGATCCTTATGAAGATTTTCAAGAA   709
gb:MT093631|Organism:Severe      CTAAAGTCATTTGACTTAGGCGACGAGCTTGGCACTGATCCTTATGAAGATTTTCAAGAA   780
gb:MT106053|Organism:Severe      CTAAAGTCATTTGACTTAGGCGACGAGCTTGGCACTGATCCTTATGAAGATTTTCAAGAA   742
gb:MT019533|Organism:Severe      CTAAAGTCATTTGACTTAGGCGACGAGCTTGGCACTGATCCTTATGAAGATTTTCAAGAA   742
gb:MT019531|Organism:Severe      CTAAAGTCATTTGACTTAGGCGACGAGCTTGGCACTGATCCTTATGAAGATTTTCAAGAA   742
gb:MN996528|Organism:Severe      CTAAAGTCATTTGACTTAGGCGACGAGCTTGGCACTGATCCTTATGAAGATTTTCAAGAA   742
gb:MN996530|Organism:Severe      CTAAAGTCATTTGACTTAGGCGACGAGCTTGGCACTGATCCTTATGAAGATTTTCAAGAA   728
gb:MN908947|Organism:Severe      CTAAAGTCATTTGACTTAGGCGACGAGCTTGGCACTGATCCTTATGAAGATTTTCAAGAA   742
gb:MT019532|Organism:Severe      CTAAAGTCATTTGACTTAGGCGACGAGCTTGGCACTGATCCTTATGAAGATTTTCAAGAA   742
                                 ***         ************************************************

gb:MT020781|Organism:Severe      AACTGGAACACTAAACATAGCAGTGGTGTTACCCGTGAACTCATGCGTGAGCTTAACGGA   790
gb:MT007544|Organism:Severe      AACTGGAACACTAAACATAGCAGTGGTGTTACCCGTGAACTCATGCGTGAGCTTAACGGA   802
gb:MN994467|Organism:Severe      AACTGGAACACTAAACATAGCAGTGGTGTTACCCGTGAACTCATGCGTGAGCTTAACGGA   802
gb:MT044257|Organism:Severe      AACTGGAACACTAAACATAGCAGTGGTGTTACCCGTGAACTCATGCGTGAGCTTAACGGA   802
gb:MT106054|Organism:Severe      AACTGGAACACTAAACATAGCAGTGGTGTTACCCGTGAACTCATGCGTGAGCTTAACGGA   802
gb:MT049951|Organism:Severe      AACTGGAACACTAAACATAGCAGTGGTGTTACCCGTGAACTCATGCGTGAGCTTAACGGA   802
gb:MN975262|Organism:Severe      AACTGGAACACTAAACATAGCAGTGGTGTTACCCGTGAACTCATGCGTGAGCTTAACGGA   802
gb:MT106052|Organism:Severe      AACTGGAACACTAAACATAGCAGTGGTGTTACCCGTGAACTCATGCGTGAGCTTAACGGA   802
gb:LC522975|Organism:Severe      AACTGGAACACTAAACATAGCAGTGGTGTTACCCGTGAACTCATGCGTGAGCTTAACGGA   799
gb:LC522973|Organism:Severe      AACTGGAACACTAAACATAGCAGTGGTGTTACCCGTGAACTCATGCGTGAGCTTAACGGA   799
gb:LC522974|Organism:Severe      AACTGGAACACTAAACATAGCAGTGGTGTTACCCGTGAACTCATGCGTGAGCTTAACGGA   799
gb:MN985325|Organism:Severe      AACTGGAACACTAAACATAGCAGTGGTGTTACCCGTGAACTCATGCGTGAGCTTAACGGA   802
gb:MT020881|Organism:Severe      AACTGGAACACTAAACATAGCAGTGGTGTTACCCGTGAACTCATGCGTGAGCTTAACGGA   802
gb:MT020880|Organism:Severe      AACTGGAACACTAAACATAGCAGTGGTGTTACCCGTGAACTCATGCGTGAGCTTAACGGA   802
gb:MT066175|Organism:Severe      AACTGGAACACTAAACATAGCAGTGGTGTTACCCGTGAACTCATGCGTGAGCTTAACGGA   802
gb:MN997409|Organism:Severe      AACTGGAACACTAAACATAGCAGTGGTGTTACCCGTGAACTCATGCGTGAGCTTAACGGA   802
gb:MN938384|Organism:Severe      AACTGGAACACTAAACATAGCAGTGGTGTTACCCGTGAACTCATGCGTGAGCTTAACGGA   770
gb:MT044258|Organism:Severe      AACTGGAACACTAAACATAGCAGTGGTGTTACCCGTGAACTCATGCGTGAGCTTAACGGA   778
gb:MT039890|Organism:Severe      AACTGGAACACTAAACATAGCAGTGGTGTTACCCGTGAACTCATGCGTGAGCTTAACGGA   802
gb:MN988713|Organism:Severe      AACTGGAACACTAAACATAGCAGTGGTGTTACCCGTGAACTCATGCGTGAGCTTAACGGA   802
gb:LC521925|Organism:Severe      AACTGGAACACTAAACATAGCAGTGGTGTTACCCGTGAACTCATGCGTGAGCTTAACGGA   775
gb:MT093571|Organism:Severe      AACTGGAACACTAAACATAGCAGTGGTGTTACCCGTGAACTCATGCGTGAGCTTAACGGA   802
gb:MT039887|Organism:Severe      AACTGGAACACTAAACATAGCAGTGGTGTTACCCGTGAACTCATGCGTGAGCTTAACGGA   802
gb:MT019530|Organism:Severe      AACTGGAACACTAAACATAGCAGTGGTGTTACCCGTGAACTCATGCGTGAGCTTAACGGA   802
gb:MT039888|Organism:Severe      AACTGGAACACTAAACATAGCAGTGGTGTTACCCGTGAACTCATGCGTGAGCTTAACGGA   802
gb:LC522972|Organism:Severe      AACTGGAACACTAAACATAGCAGTGGTGTTACCCGTGAACTCATGCGTGAGCTTAACGGA   799
gb:MT027063|Organism:Severe      AACTGGAACACTAAACATAGCAGTGGTGTTACCCGTGAACTCATGCGTGAGCTTAACGGA   802
gb:MT027062|Organism:Severe      AACTGGAACACTAAACATAGCAGTGGTGTTACCCGTGAACTCATGCGTGAGCTTAACGGA   802
gb:MT019529|Organism:Severe      AACTGGAACACTAAACATAGCAGTGGTGTTACCCGTGAACTCATGCGTGAGCTTAACGGA   802
gb:MN996529|Organism:Severe      AACTGGAACACTAAACATAGCAGTGGTGTTACCCGTGAACTCATGCGTGAGCTTAACGGA   790
gb:MN996531|Organism:Severe      AACTGGAACACTAAACATAGCAGTGGTGTTACCCGTGAACTCATGCGTGAGCTTAACGGA   789
gb:MT066176|Organism:Severe      AACTGGAACACTAAACATAGCAGTGGTGTTACCCGTGAACTCATGCGTGAGCTTAACGGA   802
gb:MT027064|Organism:Severe      AACTGGAACACTAAACATAGCAGTGGTGTTACCCGTGAACTCATGCGTGAGCTTAACGGA   802
gb:MN994468|Organism:Severe      AACTGGAACACTAAACATAGCAGTGGTGTTACCCGTGAACTCATGCGTGAGCTTAACGGA   802
gb:MT072688|Organism:Severe      AACTGGAACACTAAACATAGCAGTGGTGTTACCCGTGAACTCATGCGTGAGCTTAACGGA   787
gb:MN996527|Organism:Severe      AACTGGAACACTAAACATAGCAGTGGTGTTACCCGTGAACTCATGCGTGAGCTTAACGGA   769
gb:MT093631|Organism:Severe      AACTGGAACACTAAACATAGCAGTGGTGTTACCCGTGAACTCATGCGTGAGCTTAACGGA   840
gb:MT106053|Organism:Severe      AACTGGAACACTAAACATAGCAGTGGTGTTACCCGTGAACTCATGCGTGAGCTTAACGGA   802
gb:MT019533|Organism:Severe      AACTGGAACACTAAACATAGCAGTGGTGTTACCCGTGAACTCATGCGTGAGCTTAACGGA   802
gb:MT019531|Organism:Severe      AACTGGAACACTAAACATAGCAGTGGTGTTACCCGTGAACTCATGCGTGAGCTTAACGGA   802
gb:MN996528|Organism:Severe      AACTGGAACACTAAACATAGCAGTGGTGTTACCCGTGAACTCATGCGTGAGCTTAACGGA   802
gb:MN996530|Organism:Severe      AACTGGAACACTAAACATAGCAGTGGTGTTACCCGTGAACTCATGCGTGAGCTTAACGGA   788
gb:MN908947|Organism:Severe      AACTGGAACACTAAACATAGCAGTGGTGTTACCCGTGAACTCATGCGTGAGCTTAACGGA   802
gb:MT019532|Organism:Severe      AACTGGAACACTAAACATAGCAGTGGTGTTACCCGTGAACTCATGCGTGAGCTTAACGGA   802
                                 ************************************************************

gb:MT020781|Organism:Severe      GGGGCATACACTCGCTATGTCGATAACAACTTCTGTGGCCCTGATGGCTACCCTCTTGAG   850
gb:MT007544|Organism:Severe      GGGGCATACACTCGCTATGTCGATAACAACTTCTGTGGCCCTGATGGCTACCCTCTTGAG   862
```

```
gb:MN994467|Organism:Severe      GGGGCATACACTCGCTATGTCGATAACAACTTCTGTGGCCCTGATGGCTACCCTCTTGAG    862
gb:MT044257|Organism:Severe      GGGGCATACACTCGCTATGTCGATAACAACTTCTGTGGCCCTGATGGCTACCCTCTTGAG    862
gb:MT106054|Organism:Severe      GGGGCATACACTCGCTATGTCGATAACAACTTCTGTGGCCCTGATGGCTACCCTCTTGAG    862
gb:MT049951|Organism:Severe      GGGGCATACACTCGCTATGTCGATAACAACTTCTGTGGCCCTGATGGCTACCCTCTTGAG    862
gb:MN975262|Organism:Severe      GGGGCATACACTCGCTATGTCGATAACAACTTCTGTGGCCCTGATGGCTACCCTCTTGAG    862
gb:MT106052|Organism:Severe      GGGGCATACACTCGCTATGTCGATAACAACTTCTGTGGCCCTGATGGCTACCCTCTTGAG    862
gb:LC522975|Organism:Severe      GGGGCATACACTCGCTATGTCGATAACAACTTCTGTGGCCCTGATGGCTACCCTCTTGAG    859
gb:LC522973|Organism:Severe      GGGGCATACACTCGCTATGTCGATAACAACTTCTGTGGCCCTGATGGCTACCCTCTTGAG    859
gb:LC522974|Organism:Severe      GGGGCATACACTCGCTATGTCGATAACAACTTCTGTGGCCCTGATGGCTACCCTCTTGAG    859
gb:MN985325|Organism:Severe      GGGGCATACACTCGCTATGTCGATAACAACTTCTGTGGCCCTGATGGCTACCCTCTTGAG    862
gb:MT020881|Organism:Severe      GGGGCATACACTCGCTATGTCGATAACAACTTCTGTGGCCCTGATGGCTACCCTCTTGAG    862
gb:MT020880|Organism:Severe      GGGGCATACACTCGCTATGTCGATAACAACTTCTGTGGCCCTGATGGCTACCCTCTTGAG    862
gb:MT066175|Organism:Severe      GGGGCATACACTCGCTATGTCGATAACAACTTCTGTGGCCCTGATGGCTACCCTCTTGAG    862
gb:MN997409|Organism:Severe      GGGGCATACACTCGCTATGTCGATAACAACTTCTGTGGCCCTGATGGCTACCCTCTTGAG    862
gb:MN938384|Organism:Severe      GGGGCATACACTCGCTATGTCGATAACAACTTCTGTGGCCCTGATGGCTACCCTCTTGAG    830
gb:MT044258|Organism:Severe      GGGGCATACACTCGCTATGTCGATAACAACTTCTGTGGCCCTGATGGCTACCCTCTTGAG    838
gb:MT039890|Organism:Severe      GGGGCATACACTCGCTATGTCGATAACAACTTCTGTGGCCCTGATGGCTACCCTCTTGAG    862
gb:MN988713|Organism:Severe      GGGGCATACACTCGCTATGTCGATAACAACTTCTGTGGCCCTGATGGCTACCCTCTTGAG    862
gb:LC521925|Organism:Severe      GGGGCATACACTCGCTATGTCGATAACAACTTCTGTGGCCCTGATGGCTACCCTCTTGAG    835
gb:MT093571|Organism:Severe      GGGGCATACACTCGCTATGTCGATAACAACTTCTGTGGCCCTGATGGCTACCCTCTTGAG    862
gb:MT039887|Organism:Severe      GGGGCATACACTCGCTATGTCGATAACAACTTCTGTGGCCCTGATGGCTACCCTCTTGAG    862
gb:MT019530|Organism:Severe      GGGGCATACACTCGCTATGTCGATAACAACTTCTGTGGCCCTGATGGCTACCCTCTTGAG    862
gb:MT039888|Organism:Severe      GGGGCATACACTCGCTATGTCGATAACAACTTCTGTGGCCCTGATGGCTACCCTCTTGAG    862
gb:LC522972|Organism:Severe      GGGGCATACACTCGCTATGTCGATAACAACTTCTGTGGCCCTGATGGCTACCCTCTTGAG    859
gb:MT027063|Organism:Severe      GGGGCATACACTCGCTATGTCGATAACAACTTCTGTGGCCCTGATGGCTACCCTCTTGAG    862
gb:MT027062|Organism:Severe      GGGGCATACACTCGCTATGTCGATAACAACTTCTGTGGCCCTGATGGCTACCCTCTTGAG    862
gb:MT019529|Organism:Severe      GGGGCATACACTCGCTATGTCGATAACAACTTCTGTGGCCCTGATGGCTACCCTCTTGAG    862
gb:MN996529|Organism:Severe      GGGGCATACACTCGCTATGTCGATAACAACTTCTGTGGCCCTGATGGCTACCCTCTTGAG    850
gb:MN996531|Organism:Severe      GGGGCATACACTCGCTATGTCGATAACAACTTCTGTGGCCCTGATGGCTACCCTCTTGAG    849
gb:MT066176|Organism:Severe      GGGGCATACACTCGCTATGTCGATAACAACTTCTGTGGCCCTGATGGCTACCCTCTTGAG    862
gb:MT027064|Organism:Severe      GGGGCATACACTCGCTATGTCGATAACAACTTCTGTGGCCCTGATGGCTACCCTCTTGAG    862
gb:MN994468|Organism:Severe      GGGGCATACACTCGCTATGTCGATAACAACTTCTGTGGCCCTGATGGCTACCCTCTTGAG    862
gb:MT072688|Organism:Severe      GGGGCATACACTCGCTATGTCGATAACAACTTCTGTGGCCCTGATGGCTACCCTCTTGAG    847
gb:MN996527|Organism:Severe      GGGGCATACACTCGCTATGTCGATAACAACTTCTGTGGCCCTGATGGCTACCCTCTTGAG    829
gb:MT093631|Organism:Severe      GGGGCATACACTCGCTATGTCGATAACAACTTCTGTGGCCCTGATGGCTACCCTCTTGAG    900
gb:MT106053|Organism:Severe      GGGGCATACACTCGCTATGTCGATAACAACTTCTGTGGCCCTGATGGCTACCCTCTTGAG    862
gb:MT019533|Organism:Severe      GGGGCATACACTCGCTATGTCGATAACAACTTCTGTGGCCCTGATGGCTACCCTCTTGAG    862
gb:MT019531|Organism:Severe      GGGGCATACACTCGCTATGTCGATAACAACTTCTGTGGCCCTGATGGCTACCCTCTTGAG    862
gb:MN996528|Organism:Severe      GGGGCATACACTCGCTATGTCGATAACAACTTCTGTGGCCCTGATGGCTACCCTCTTGAG    862
gb:MN996530|Organism:Severe      GGGGCATACACTCGCTATGTCGATAACAACTTCTGTGGCCCTGATGGCTACCCTCTTGAG    848
gb:MN908947|Organism:Severe      GGGGCATACACTCGCTATGTCGATAACAACTTCTGTGGCCCTGATGGCTACCCTCTTGAG    862
gb:MT019532|Organism:Severe      GGGGCATACACTCGCTATGTCGATAACAACTTCTGTGGCCCTGATGGCTACCCTCTTGAG    862
                                 ************************************************************

gb:MT020781|Organism:Severe      TGCATTAAAGACCTTCTAGCACGTGCTGGTAAAGCTTCATGCACTTTGTCCGAACAACTG    910
gb:MT007544|Organism:Severe      TGCATTAAAGACCTTCTAGCACGTGCTGGTAAAGCTTCATGCACTTTGTCCGAACAACTG    922
gb:MN994467|Organism:Severe      TGCATTAAAGACCTTCTAGCACGTGCTGGTAAAGCTTCATGCACTTTGTCCGAACAACTG    922
gb:MT044257|Organism:Severe      TGCATTAAAGACCTTCTAGCACGTGCTGGTAAAGCTTCATGCACTTTGTCCGAACAACTG    922
gb:MT106054|Organism:Severe      TGCATTAAAGACCTTCTAGCACGTGCTGGTAAAGCTTCATGCACTTTGTCCGAACAACTG    922
gb:MT049951|Organism:Severe      TGCATTAAAGACCTTCTAGCACGTGCTGGTAAAGCTTCATGCACTTTGTCCGAACAACTG    922
gb:MN975262|Organism:Severe      TGCATTAAAGACCTTCTAGCACGTGCTGGTAAAGCTTCATGCACTTTGTCCGAACAACTG    922
gb:MT106052|Organism:Severe      TGCATTAAAGACCTTCTAGCACGTGCTGGTAAAGCTTCATGCACTTTGTCCGAACAACTG    922
gb:LC522975|Organism:Severe      TGCATTAAAGACCTTCTAGCACGTGCTGGTAAAGCTTCATGCACTTTGTCCGAACAACTG    919
gb:LC522973|Organism:Severe      TGCATTAAAGACCTTCTAGCACGTGCTGGTAAAGCTTCATGCACTTTGTCCGAACAACTG    919
gb:LC522974|Organism:Severe      TGCATTAAAGACCTTCTAGCACGTGCTGGTAAAGCTTCATGCACTTTGTCCGAACAACTG    919
gb:MN985325|Organism:Severe      TGCATTAAAGACCTTCTAGCACGTGCTGGTAAAGCTTCATGCACTTTGTCCGAACAACTG    922
gb:MT020881|Organism:Severe      TGCATTAAAGACCTTCTAGCACGTGCTGGTAAAGCTTCATGCACTTTGTCCGAACAACTG    922
gb:MT020880|Organism:Severe      TGCATTAAAGACCTTCTAGCACGTGCTGGTAAAGCTTCATGCACTTTGTCCGAACAACTG    922
gb:MT066175|Organism:Severe      TGCATTAAAGACCTTCTAGCACGTGCTGGTAAAGCTTCATGCACTTTGTCCGAACAACTG    922
gb:MN997409|Organism:Severe      TGCATTAAAGACCTTCTAGCACGTGCTGGTAAAGCTTCATGCACTTTGTCCGAACAACTG    922
gb:MN938384|Organism:Severe      TGCATTAAAGACCTTCTAGCACGTGCTGGTAAAGCTTCATGCACTTTGTCCGAACAACTG    890
gb:MT044258|Organism:Severe      TGCATTAAAGACCTTCTAGCACGTGCTGGTAAAGCTTCATGCACTTTGTCCGAACAACTG    898
gb:MT039890|Organism:Severe      TGCATTAAAGACCTTCTAGCACGTGCTGGTAAAGCTTCATGCACTTTGTCCGAACAACTG    922
gb:MN988713|Organism:Severe      TGCATTAAAGACCTTCTAGCACGTGCTGGTAAAGCTTCATGCACTTTGTCCGAACAACTG    922
gb:LC521925|Organism:Severe      TGCATTAAAGACCTTCTAGCACGTGCTGGTAAAGCTTCATGCACTTTGTCCGAACAACTG    895
```

```
gb:MT093571|Organism:Severe      TGCATTAAAGACCTTCTAGCACGTGCTGGTAAAGCTTCATGCACTTTGTCCGAACAACTG   922
gb:MT039887|Organism:Severe      TGCATTAAAGACCTTCTAGCACGTGCTGGTAAAGCTTCATGCACTTTGTCCGAACAACTG   922
gb:MT019530|Organism:Severe      TGCATTAAAGACCTTCTAGCACGTGCTGGTAAAGCTTCATGCACTTTGTCCGAACAACTG   922
gb:MT039888|Organism:Severe      TGCATTAAAGACCTTCTAGCACGTGCTGGTAAAGCTTCATGCACTTTGTCCGAACAACTG   922
gb:LC522972|Organism:Severe      TGCATTAAAGACCTTCTAGCACGTGCTGGTAAAGCTTCATGCACTTTGTCCGAACAACTG   919
gb:MT027063|Organism:Severe      TGCATTAAAGACCTTCTAGCACGTGCTGGTAAAGCTTCATGCACTTTGTCCGAACAACTG   922
gb:MT027062|Organism:Severe      TGCATTAAAGACCTTCTAGCACGTGCTGGTAAAGCTTCATGCACTTTGTCCGAACAACTG   922
gb:MT019529|Organism:Severe      TGCATTAAAGACCTTCTAGCACGTGCTGGTAAAGCTTCATGCACTTTGTCCGAACAACTG   922
gb:MN996529|Organism:Severe      TGCATTAAAGACCTTCTAGCACGTGCTGGTAAAGCTTCATGCACTTTGTCCGAACAACTG   910
gb:MN996531|Organism:Severe      TGCATTAAAGACCTTCTAGCACGTGCTGGTAAAGCTTCATGCACTTTGTCCGAACAACTG   909
gb:MT066176|Organism:Severe      TGCATTAAAGACCTTCTAGCACGTGCTGGTAAAGCTTCATGCACTTTGTCCGAACAACTG   922
gb:MT027064|Organism:Severe      TGCATTAAAGACCTTCTAGCACGTGCTGGTAAAGCTTCATGCACTTTGTCCGAACAACTG   922
gb:MN994468|Organism:Severe      TGCATTAAAGACCTTCTAGCACGTGCTGGTAAAGCTTCATGCACTTTGTCCGAACAACTG   922
gb:MT072688|Organism:Severe      TGCATTAAAGACCTTCTAGCACGTGCTGGTAAAGCTTCATGCACTTTGTCCGAACAACTG   907
gb:MN996527|Organism:Severe      TGCATTAAAGACCTTCTAGCACGTGCTGGTAAAGCTTCATGCACTTTGTCCGAACAACTG   889
gb:MT093631|Organism:Severe      TGCATTAAAGACCTTCTAGCACGTGCTGGTAAAGCTTCATGCACTTTGTCCGAACAACTG   960
gb:MT106053|Organism:Severe      TGCATTAAAGACCTTCTAGCACGTGCTGGTAAAGCTTCATGCACTTTGTCCGAACAACTG   922
gb:MT019533|Organism:Severe      TGCATTAAAGACCTTCTAGCACGTGCTGGTAAAGCTTCATGCACTTTGTCCGAACAACTG   922
gb:MT019531|Organism:Severe      TGCATTAAAGACCTTCTAGCACGTGCTGGTAAAGCTTCATGCACTTTGTCCGAACAACTG   922
gb:MN996528|Organism:Severe      TGCATTAAAGACCTTCTAGCACGTGCTGGTAAAGCTTCATGCACTTTGTCCGAACAACTG   922
gb:MN996530|Organism:Severe      TGCATTAAAGACCTTCTAGCACGTGCTGGTAAAGCTTCATGCACTTTGTCCGAACAACTG   908
gb:MN908947|Organism:Severe      TGCATTAAAGACCTTCTAGCACGTGCTGGTAAAGCTTCATGCACTTTGTCCGAACAACTG   922
gb:MT019532|Organism:Severe      TGCATTAAAGACCTTCTAGCACGTGCTGGTAAAGCTTCATGCACTTTGTCCGAACAACTG   922
                                 ************************************************************

gb:MT020781|Organism:Severe      GACTTTATTGACACTAAGAGGGGTGTATACTGCTGCCGTGAACATGAGCATGAAATTGCT   970
gb:MT007544|Organism:Severe      GACTTTATTGACACTAAGAGGGGTGTATACTGCTGCCGTGAACATGAGCATGAAATTGCT   982
gb:MN994467|Organism:Severe      GACTTTATTGACACTAAGAGGGGTGTATACTGCTGCCGTGAACATGAGCATGAAATTGCT   982
gb:MT044257|Organism:Severe      GACTTTATTGACACTAAGAGGGGTGTATACTGCTGCCGTGAACATGAGCATGAAATTGCT   982
gb:MT106054|Organism:Severe      GACTTTATTGACACTAAGAGGGGTGTATACTGCTGCCGTGAACATGAGCATGAAATTGCT   982
gb:MT049951|Organism:Severe      GACTTTATTGACACTAAGAGGGGTGTATACTGCTGCCGTGAACATGAGCATGAAATTGCT   982
gb:MN975262|Organism:Severe      GACTTTATTGACACTAAGAGGGGTGTATACTGCTGCCGTGAACATGAGCATGAAATTGCT   982
gb:MT106052|Organism:Severe      GACTTTATTGACACTAAGAGGGGTGTATACTGCTGCCGTGAACATGAGCATGAAATTGCT   982
gb:LC522975|Organism:Severe      GACTTTATTGACACTAAGAGGGGTGTATACTGCTGCCGTGAACATGAGCATGAAATTGCT   979
gb:LC522973|Organism:Severe      GACTTTATTGACACTAAGAGGGGTGTATACTGCTGCCGTGAACATGAGCATGAAATTGCT   979
gb:LC522974|Organism:Severe      GACTTTATTGACACTAAGAGGGGTGTATACTGCTGCCGTGAACATGAGCATGAAATTGCT   979
gb:MN985325|Organism:Severe      GACTTTATTGACACTAAGAGGGGTGTATACTGCTGCCGTGAACATGAGCATGAAATTGCT   982
gb:MT020881|Organism:Severe      GACTTTATTGACACTAAGAGGGGTGTATACTGCTGCCGTGAACATGAGCATGAAATTGCT   982
gb:MT020880|Organism:Severe      GACTTTATTGACACTAAGAGGGGTGTATACTGCTGCCGTGAACATGAGCATGAAATTGCT   982
gb:MT066175|Organism:Severe      GACTTTATTGACACTAAGAGGGGTGTATACTGCTGCCGTGAACATGAGCATGAAATTGCT   982
gb:MN997409|Organism:Severe      GACTTTATTGACACTAAGAGGGGTGTATACTGCTGCCGTGAACATGAGCATGAAATTGCT   982
gb:MN938384|Organism:Severe      GACTTTATTGACACTAAGAGGGGTGTATACTGCTGCCGTGAACATGAGCATGAAATTGCT   950
gb:MT044258|Organism:Severe      GACTTTATTGACACTAAGAGGGGTGTATACTGCTGCCGTGAACATGAGCATGAAATTGCT   958
gb:MT039890|Organism:Severe      GACTTTATTGACACTAAGAGGGGTGTATACTGCTGCCGTGAACATGAGCATGAAATTGCT   982
gb:MN988713|Organism:Severe      GACTTTATTGACACTAAGAGGGGTGTATACTGCTGCCGTGAACATGAGCATGAAATTGCT   982
gb:LC521925|Organism:Severe      GACTTTATTGACACTAAGAGGGGTGTATACTGCTGCCGTGAACATGAGCATGAAATTGCT   955
gb:MT093571|Organism:Severe      GACTTTATTGACACTAAGAGGGGTGTATACTGCTGCCGTGAACATGAGCATGAAATTGCT   982
gb:MT039887|Organism:Severe      GACTTTATTGACACTAAGAGGGGTGTATACTGCTGCCGTGAACATGAGCATGAAATTGCT   982
gb:MT019530|Organism:Severe      GACTTTATTGACACTAAGAGGGGTGTATACTGCTGCCGTGAACATGAGCATGAAATTGCT   982
gb:MT039888|Organism:Severe      GACTTTATTGACACTAAGAGGGGTGTATACTGCTGCCGTGAACATGAGCATGAAATTGCT   982
gb:LC522972|Organism:Severe      GACTTTATTGACACTAAGAGGGGTGTATACTGCTGCCGTGAACATGAGCATGAAATTGCT   979
gb:MT027063|Organism:Severe      GACTTTATTGACACTAAGAGGGGTGTATACTGCTGCCGTGAACATGAGCATGAAATTGCT   982
gb:MT027062|Organism:Severe      GACTTTATTGACACTAAGAGGGGTGTATACTGCTGCCGTGAACATGAGCATGAAATTGCT   982
gb:MT019529|Organism:Severe      GACTTTATTGACACTAAGAGGGGTGTATACTGCTGCCGTGAACATGAGCATGAAATTGCT   982
gb:MN996529|Organism:Severe      GACTTTATTGACACTAAGAGGGGTGTATACTGCTGCCGTGAACATGAGCATGAAATTGCT   970
gb:MN996531|Organism:Severe      GACTTTATTGACACTAAGAGGGGTGTATACTGCTGCCGTGAACATGAGCATGAAATTGCT   969
gb:MT066176|Organism:Severe      GACTTTATTGACACTAAGAGGGGTGTATACTGCTGCCGTGAACATGAGCATGAAATTGCT   982
gb:MT027064|Organism:Severe      GACTTTATTGACACTAAGAGGGGTGTATACTGCTGCCGTGAACATGAGCATGAAATTGCT   982
gb:MN994468|Organism:Severe      GACTTTATTGACACTAAGAGGGGTGTATACTGCTGCCGTGAACATGAGCATGAAATTGCT   982
gb:MT072688|Organism:Severe      GACTTTATTGACACTAAGAGGGGTGTATACTGCTGCCGTGAACATGAGCATGAAATTGCT   967
gb:MN996527|Organism:Severe      GACTTTATTGACACTAAGAGGGGTGTATACTGCTGCCGTGAACATGAGCATGAAATTGCT   949
gb:MT093631|Organism:Severe      GACTTTATTGACACTAAGAGGGGTGTATACTGCTGCCGTGAACATGAGCATGAAATTGCT   1020
gb:MT106053|Organism:Severe      GACTTTATTGACACTAAGAGGGGTGTATACTGCTGCCGTGAACATGAGCATGAAATTGCT   982
gb:MT019533|Organism:Severe      GACTTTATTGACACTAAGAGGGGTGTATACTGCTGCCGTGAACATGAGCATGAAATTGCT   982
gb:MT019531|Organism:Severe      GACTTTATTGACACTAAGAGGGGTGTATACTGCTGCCGTGAACATGAGCATGAAATTGCT   982
```

gb:MN996528|Organism:Severe GACTTTATTGACACTAAGAGGGGTGTATACTGCTGCCGTGAACATGAGCATGAAATTGCT 982
gb:MN996530|Organism:Severe GACTTTATTGACACTAAGAGGGGTGTATACTGCTGCCGTGAACATGAGCATGAAATTGCT 968
gb:MN908947|Organism:Severe GACTTTATTGACACTAAGAGGGGTGTATACTGCTGCCGTGAACATGAGCATGAAATTGCT 982
gb:MT019532|Organism:Severe GACTTTATTGACACTAAGAGGGGTGTATACTGCTGCCGTGAACATGAGCATGAAATTGCT 982
****

gb:MT020781|Organism:Severe TGGTACACGGAACGTTCTGAAAAGAGCTATGAATTGCAGACACCTTTTGAAATTAAATTG 1030
gb:MT007544|Organism:Severe TGGTACACGGAACGTTCTGAAAAGAGCTATGAATTGCAGACACCTTTTGAAATTAAATTG 1042
gb:MN994467|Organism:Severe TGGTACACGGAACGTTCTGAAAAGAGCTATGAATTGCAGACACCTTTTGAAATTAAATTG 1042
gb:MT044257|Organism:Severe TGGTACACGGAACGTTCTGAAAAGAGCTATGAATTGCAGACACCTTTTGAAATTAAATTG 1042
gb:MT106054|Organism:Severe TGGTACACGGAACGTTCTGAAAAGAGCTATGAATTGCAGACACCTTTTGAAATTAAATTG 1042
gb:MT049951|Organism:Severe TGGTACACGGAACGTTCTGAAAAGAGCTATGAATTGCAGACACCTTTTGAAATTAAATTG 1042
gb:MN975262|Organism:Severe TGGTACACGGAACGTTCTGAAAAGAGCTATGAATTGCAGACACCTTTTGAAATTAAATTG 1042
gb:MT106052|Organism:Severe TGGTACACGGAACGTTCTGAAAAGAGCTATGAATTGCAGACACCTTTTGAAATTAAATTG 1042
gb:LC522975|Organism:Severe TGGTACACGGAACGTTCTGAAAAGAGCTATGAATTGCAGACACCTTTTGAAATTAAATTG 1039
gb:LC522973|Organism:Severe TGGTACACGGAACGTTCTGAAAAGAGCTATGAATTGCAGACACCTTTTGAAATTAAATTG 1039
gb:LC522974|Organism:Severe TGGTACACGGAACGTTCTGAAAAGAGCTATGAATTGCAGACACCTTTTGAAATTAAATTG 1039
gb:MN985325|Organism:Severe TGGTACACGGAACGTTCTGAAAAGAGCTATGAATTGCAGACACCTTTTGAAATTAAATTG 1042
gb:MT020881|Organism:Severe TGGTACACGGAACGTTCTGAAAAGAGCTATGAATTGCAGACACCTTTTGAAATTAAATTG 1042
gb:MT020880|Organism:Severe TGGTACACGGAACGTTCTGAAAAGAGCTATGAATTGCAGACACCTTTTGAAATTAAATTG 1042
gb:MT066175|Organism:Severe TGGTACACGGAACGTTCTGAAAAGAGCTATGAATTGCAGACACCTTTTGAAATTAAATTG 1042
gb:MN997409|Organism:Severe TGGTACACGGAACGTTCTGAAAAGAGCTATGAATTGCAGACACCTTTTGAAATTAAATTG 1042
gb:MN938384|Organism:Severe TGGTACACGGAACGTTCTGAAAAGAGCTATGAATTGCAGACACCTTTTGAAATTAAATTG 1010
gb:MT044258|Organism:Severe TGGTACACGGAACGTTCTGAAAAGAGCTATGAATTGCAGACACCTTTTGAAATTAAATTG 1018
gb:MT039890|Organism:Severe TGGTACACGGAACGTTCTGAAAAGAGCTATGAATTGCAGACACCTTTTGAAATTAAATTG 1042
gb:MN988713|Organism:Severe TGGTACACGGAACGTTCTGAAAAGAGCTATGAATTGCAGACACCTTTTGAAATTAAATTG 1042
gb:LC521925|Organism:Severe TGGTACACGGAACGTTCTGAAAAGAGCTATGAATTGCAGACACCTTTTGAAATTAAATTG 1015
gb:MT093571|Organism:Severe TGGTACACGGAACGTTCTGAAAAGAGCTATGAATTGCAGACACCTTTTGAAATTAAATTG 1042
gb:MT039887|Organism:Severe TGGTACACGGAACGTTCTGAAAAGAGCTATGAATTGCAGACACCTTTTGAAATTAAATTG 1042
gb:MT019530|Organism:Severe TGGTACACGGAACGTTCTGAAAAGAGCTATGAATTGCAGACACCTTTTGAAATTAAATTG 1042
gb:MT039888|Organism:Severe TGGTACACGGAACGTTCTGAAAAGAGCTATGAATTGCAGACACCTTTTGAAATTAAATTG 1042
gb:LC522972|Organism:Severe TGGTACACGGAACGTTCTGAAAAGAGCTATGAATTGCAGACACCTTTTGAAATTAAATTG 1039
gb:MT027063|Organism:Severe TGGTACACGGAACGTTCTGAAAAGAGCTATGAATTGCAGACACCTTTTGAAATTAAATTG 1042
gb:MT027062|Organism:Severe TGGTACACGGAACGTTCTGAAAAGAGCTATGAATTGCAGACACCTTTTGAAATTAAATTG 1042
gb:MT019529|Organism:Severe TGGTACACGGAACGTTCTGAAAAGAGCTATGAATTGCAGACACCTTTTGAAATTAAATTG 1042
gb:MN996529|Organism:Severe TGGTACACGGAACGTTCTGAAAAGAGCTATGAATTGCAGACACCTTTTGAAATTAAATTG 1030
gb:MN996531|Organism:Severe TGGTACACGGAACGTTCTGAAAAGAGCTATGAATTGCAGACACCTTTTGAAATTAAATTG 1029
gb:MT066176|Organism:Severe TGGTACACGGAACGTTCTGAAAAGAGCTATGAATTGCAGACACCTTTTGAAATTAAATTG 1042
gb:MT027064|Organism:Severe TGGTACACGGAACGTTCTGAAAAGAGCTATGAATTGCAGACACCTTTTGAAATTAAATTG 1042
gb:MN994468|Organism:Severe TGGTACACGGAACGTTCTGAAAAGAGCTATGAATTGCAGACACCTTTTGAAATTAAATTG 1042
gb:MT072688|Organism:Severe TGGTACACGGAACGTTCTGAAAAGAGCTATGAATTGCAGACACCTTTTGAAATTAAATTG 1027
gb:MN996527|Organism:Severe TGGTACACGGAACGTTCTGAAAAGAGCTATGAATTGCAGACACCTTTTGAAATTAAATTG 1009
gb:MT093631|Organism:Severe TGGTACACGGAACGTTCTGAAAAGAGCTATGAATTGCAGACACCTTTTGAAATTAAATTG 1080
gb:MT106053|Organism:Severe TGGTACACGGAACGTTCTGAAAAGAGCTATGAATTGCAGACACCTTTTGAAATTAAATTG 1042
gb:MT019533|Organism:Severe TGGTACACGGAACGTTCTGAAAAGAGCTATGAATTGCAGACACCTTTTGAAATTAAATTG 1042
gb:MT019531|Organism:Severe TGGTACACGGAACGTTCTGAAAAGAGCTATGAATTGCAGACACCTTTTGAAATTAAATTG 1042
gb:MN996528|Organism:Severe TGGTACACGGAACGTTCTGAAAAGAGCTATGAATTGCAGACACCTTTTGAAATTAAATTG 1042
gb:MN996530|Organism:Severe TGGTACACGGAACGTTCTGAAAAGAGCTATGAATTGCAGACACCTTTTGAAATTAAATTG 1028
gb:MN908947|Organism:Severe TGGTACACGGAACGTTCTGAAAAGAGCTATGAATTGCAGACACCTTTTGAAATTAAATTG 1042
gb:MT019532|Organism:Severe TGGTACACGGAACGTTCTGAAAAGAGCTATGAATTGCAGACACCTTTTGAAATTAAATTG 1042
****

gb:MT020781|Organism:Severe GCAAAGAAATTTGACACCTTCAATGGGGAATGTCCAAATTTTGTATTTCCCTTAAATTCC 1090
gb:MT007544|Organism:Severe GCAAAGAAATTTGACACCTTCAATGGGGAATGTCCAAATTTTGTATTTCCCTTAAATTCC 1102
gb:MN994467|Organism:Severe GCAAAGAAATTTGACACCTTCAATGGGGAATGTCCAAATTTTGTATTTCCCTTAAATTCC 1102
gb:MT044257|Organism:Severe GCAAAGAAATTTGACACCTTCAATGGGGAATGTCCAAATTTTGTATTTCCCTTAAATTCC 1102
gb:MT106054|Organism:Severe GCAAAGAAATTTGACACCTTCAATGGGGAATGTCCAAATTTTGTATTTCCCTTAAATTCC 1102
gb:MT049951|Organism:Severe GCAAAGAAATTTGACACCTTCAATGGGGAATGTCCAAATTTTGTATTTCCCTTAAATTCC 1102
gb:MN975262|Organism:Severe GCAAAGAAATTTGACACCTTCAATGGGGAATGTCCAAATTTTGTATTTCCCTTAAATTCC 1102
gb:MT106052|Organism:Severe GCAAAGAAATTTGACACCTTCAATGGGGAATGTCCAAATTTTGTATTTCCCTTAAATTCC 1102
gb:LC522975|Organism:Severe GCAAAGAAATTTGACACCTTCAATGGGGAATGTCCAAATTTTGTATTTCCCTTAAATTCC 1099
gb:LC522973|Organism:Severe GCAAAGAAATTTGACACCTTCAATGGGGAATGTCCAAATTTTGTATTTCCCTTAAATTCC 1099
gb:LC522974|Organism:Severe GCAAAGAAATTTGACACCTTCAATGGGGAATGTCCAAATTTTGTATTTCCCTTAAATTCC 1099
gb:MN985325|Organism:Severe GCAAAGAAATTTGACACCTTCAATGGGGAATGTCCAAATTTTGTATTTCCCTTAAATTCC 1102
gb:MT020881|Organism:Severe GCAAAGAAATTTGACACCTTCAATGGGGAATGTCCAAATTTTGTATTTCCCTTAAATTCC 1102

```
gb:MT020880|Organism:Severe      GCAAAGAAATTTGACACCTTCAATGGGGAATGTCCAAATTTTGTATTTCCCTTAAATTCC    1102
gb:MT066175|Organism:Severe      GCAAAGAAATTTGACACCTTCAATGGGGAATGTCCAAATTTTGTATTTCCCTTAAATTCC    1102
gb:MN997409|Organism:Severe      GCAAAGAAATTTGACACCTTCAATGGGGAATGTCCAAATTTTGTATTTCCCTTAAATTCC    1102
gb:MN938384|Organism:Severe      GCAAAGAAATTTGACACCTTCAATGGGGAATGTCCAAATTTTGTATTTCCCTTAAATTCC    1070
gb:MT044258|Organism:Severe      GCAAAGAAATTTGACACCTTCAATGGGGAATGTCCAAATTTTGTATTTCCCTTAAATTCC    1078
gb:MT039890|Organism:Severe      GCAAAGAAATTTGACACCTTCAATGGGGAATGTCCAAATTTTGTATTTCCCTTAAATTCC    1102
gb:MN988713|Organism:Severe      GCAAAGAAATTTGACACCTTCAATGGGGAATGTCCAAATTTTGTATTTCCCTTAAATTCC    1102
gb:LC521925|Organism:Severe      GCAAAGAAATTTGACACCTTCAATGGGGAATGTCCAAATTTTGTATTTCCCTTAAATTCC    1075
gb:MT093571|Organism:Severe      GCAAAGAAATTTGACACCTTCAATGGGGAATGTCCAAATTTTGTATTTCCCTTAAATTCC    1102
gb:MT039887|Organism:Severe      GCAAAGAAATTTGACACCTTCAATGGGGAATGTCCAAATTTTGTATTTCCCTTAAATTCC    1102
gb:MT019530|Organism:Severe      GCAAAGAAATTTGACACCTTCAATGGGGAATGTCCAAATTTTGTATTTCCCTTAAATTCC    1102
gb:MT039888|Organism:Severe      GCAAAGAAATTTGACACCTTCAATGGGGAATGTCCAAATTTTGTATTTCCCTTAAATTCC    1102
gb:LC522972|Organism:Severe      GCAAAGAAATTTGACACCTTCAATGGGGAATGTCCAAATTTTGTATTTCCCTTAAATTCC    1099
gb:MT027063|Organism:Severe      GCAAAGAAATTTGACACCTTCAATGGGGAATGTCCAAATTTTGTATTTCCCTTAAATTCC    1102
gb:MT027062|Organism:Severe      GCAAAGAAATTTGACACCTTCAATGGGGAATGTCCAAATTTTGTATTTCCCTTAAATTCC    1102
gb:MT019529|Organism:Severe      GCAAAGAAATTTGACACCTTCAATGGGGAATGTCCAAATTTTGTATTTCCCTTAAATTCC    1102
gb:MN996529|Organism:Severe      GCAAAGAAATTTGACACCTTCAATGGGGAATGTCCAAATTTTGTATTTCCCTTAAATTCC    1090
gb:MN996531|Organism:Severe      GCAAAGAAATTTGACACCTTCAATGGGGAATGTCCAAATTTTGTATTTCCCTTAAATTCC    1089
gb:MT066176|Organism:Severe      GCAAAGAAATTTGACACCTTCAATGGGGAATGTCCAAATTTTGTATTTCCCTTAAATTCC    1102
gb:MT027064|Organism:Severe      GCAAAGAAATTTGACACCTTCAATGGGGAATGTCCAAATTTTGTATTTCCCTTAAATTCC    1102
gb:MN994468|Organism:Severe      GCAAAGAAATTTGACACCTTCAATGGGGAATGTCCAAATTTTGTATTTCCCTTAAATTCC    1102
gb:MT072688|Organism:Severe      GCAAAGAAATTTGACACCTTCAATGGGGAATGTCCAAATTTTGTATTTCCCTTAAATTCC    1087
gb:MN996527|Organism:Severe      GCAAAGAAATTTGACACCTTCAATGGGGAATGTCCAAATTTTGTATTTCCCTTAAATTCC    1069
gb:MT093631|Organism:Severe      GCAAAGAAATTTGACACCTTCAATGGGGAATGTCCAAATTTTGTATTTCCCTTAAATTCC    1140
gb:MT106053|Organism:Severe      GCAAAGAAATTTGACACCTTCAATGGGGAATGTCCAAATTTTGTATTTCCCTTAAATTCC    1102
gb:MT019533|Organism:Severe      GCAAAGAAATTTGACACCTTCAATGGGGAATGTCCAAATTTTGTATTTCCCTTAAATTCC    1102
gb:MT019531|Organism:Severe      GCAAAGAAATTTGACACCTTCAATGGGGAATGTCCAAATTTTGTATTTCCCTTAAATTCC    1102
gb:MN996528|Organism:Severe      GCAAAGAAATTTGACACCTTCAATGGGGAATGTCCAAATTTTGTATTTCCCTTAAATTCC    1102
gb:MN996530|Organism:Severe      GCAAAGAAATTTGACACCTTCAATGGGGAATGTCCAAATTTTGTATTTCCCTTAAATTCC    1088
gb:MN908947|Organism:Severe      GCAAAGAAATTTGACACCTTCAATGGGGAATGTCCAAATTTTGTATTTCCCTTAAATTCC    1102
gb:MT019532|Organism:Severe      GCAAAGAAATTTGACACCTTCAATGGGGAATGTCCAAATTTTGTATTTCCCTTAAATTCC    1102
                                 ************************************************************

gb:MT020781|Organism:Severe      ATAATCAAGACTATTCAACCAAGGGTTGAAAAGAAAAAGCTTGATGGCTTTATGGGTAGA    1150
gb:MT007544|Organism:Severe      ATAATCAAGACTATTCAACCAAGGGTTGAAAAGAAAAAGCTTGATGGCTTTATGGGTAGA    1162
gb:MN994467|Organism:Severe      ATAATCAAGACTATTCAACCAAGGGTTGAAAAGAAAAAGCTTGATGGCTTTATGGGTAGA    1162
gb:MT044257|Organism:Severe      ATAATCAAGACTATTCAACCAAGGGTTGAAAAGAAAAAGCTTGATGGCTTTATGGGTAGA    1162
gb:MT106054|Organism:Severe      ATAATCAAGACTATTCAACCAAGGGTTGAAAAGAAAAAGCTTGATGGCTTTATGGGTAGA    1162
gb:MT049951|Organism:Severe      ATAATCAAGACTATTCAACCAAGGGTTGAAAAGAAAAAGCTTGATGGCTTTATGGGTAGA    1162
gb:MN975262|Organism:Severe      ATAATCAAGACTATTCAACCAAGGGTTGAAAAGAAAAAGCTTGATGGCTTTATGGGTAGA    1162
gb:MT106052|Organism:Severe      ATAATCAAGACTATTCAACCAAGGGTTGAAAAGAAAAAGCTTGATGGCTTTATGGGTAGA    1162
gb:LC522975|Organism:Severe      ATAATCAAGACTATTCAACCAAGGGTTGAAAAGAAAAAGCTTGATGGCTTTATGGGTAGA    1159
gb:LC522973|Organism:Severe      ATAATCAAGACTATTCAACCAAGGGTTGAAAAGAAAAAGCTTGATGGCTTTATGGGTAGA    1159
gb:LC522974|Organism:Severe      ATAATCAAGACTATTCAACCAAGGGTTGAAAAGAAAAAGCTTGATGGCTTTATGGGTAGA    1159
gb:MN985325|Organism:Severe      ATAATCAAGACTATTCAACCAAGGGTTGAAAAGAAAAAGCTTGATGGCTTTATGGGTAGA    1162
gb:MT020881|Organism:Severe      ATAATCAAGACTATTCAACCAAGGGTTGAAAAGAAAAAGCTTGATGGCTTTATGGGTAGA    1162
gb:MT020880|Organism:Severe      ATAATCAAGACTATTCAACCAAGGGTTGAAAAGAAAAAGCTTGATGGCTTTATGGGTAGA    1162
gb:MT066175|Organism:Severe      ATAATCAAGACTATTCAACCAAGGGTTGAAAAGAAAAAGCTTGATGGCTTTATGGGTAGA    1162
gb:MN997409|Organism:Severe      ATAATCAAGACTATTCAACCAAGGGTTGAAAAGAAAAAGCTTGATGGCTTTATGGGTAGA    1162
gb:MN938384|Organism:Severe      ATAATCAAGACTATTCAACCAAGGGTTGAAAAGAAAAAGCTTGATGGCTTTATGGGTAGA    1130
gb:MT044258|Organism:Severe      ATAATCAAGACTATTCAACCAAGGGTTGAAAAGAAAAAGCTTGATGGCTTTATGGGTAGA    1138
gb:MT039890|Organism:Severe      ATAATCAAGACTATTCAACCAAGGGTTGAAAAGAAAAAGCTTGATGGCTTTATGGGTAGA    1162
gb:MN988713|Organism:Severe      ATAATCAAGACTATTCAACCAAGGGTTGAAAAGAAAAAGCTTGATGGCTTTATGGGTAGA    1162
gb:LC521925|Organism:Severe      ATAATCAAGACTATTCAACCAAGGGTTGAAAAGAAAAAGCTTGATGGCTTTATGGGTAGA    1135
gb:MT093571|Organism:Severe      ATAATCAAGACTATTCAACCAAGGGTTGAAAAGAAAAAGCTTGATGGCTTTATGGGTAGA    1162
gb:MT039887|Organism:Severe      ATAATCAAGACTATTCAACCAAGGGTTGAAAAGAAAAAGCTTGATGGCTTTATGGGTAGA    1162
gb:MT019530|Organism:Severe      ATAATCAAGACTATTCAACCAAGGGTTGAAAAGAAAAAGCTTGATGGCTTTATGGGTAGA    1162
gb:MT039888|Organism:Severe      ATAATCAAGACTATTCAACCAAGGGTTGAAAAGAAAAAGCTTGATGGCTTTATGGGTAGA    1162
gb:LC522972|Organism:Severe      ATAATCAAGACTATTCAACCAAGGGTTGAAAAGAAAAAGCTTGATGGCTTTATGGGTAGA    1159
gb:MT027063|Organism:Severe      ATAATCAAGACTATTCAACCAAGGGTTGAAAAGAAAAAGCTTGATGGCTTTATGGGTAGA    1162
gb:MT027062|Organism:Severe      ATAATCAAGACTATTCAACCAAGGGTTGAAAAGAAAAAGCTTGATGGCTTTATGGGTAGA    1162
gb:MT019529|Organism:Severe      ATAATCAAGACTATTCAACCAAGGGTTGAAAAGAAAAAGCTTGATGGCTTTATGGGTAGA    1162
gb:MN996529|Organism:Severe      ATAATCAAGACTATTCAACCAAGGGTTGAAAAGAAAAAGCTTGATGGCTTTATGGGTAGA    1150
gb:MN996531|Organism:Severe      ATAATCAAGACTATTCAACCAAGGGTTGAAAAGAAAAAGCTTGATGGCTTTATGGGTAGA    1149
gb:MT066176|Organism:Severe      ATAATCAAGACTATTCAACCAAGGGTTGAAAAGAAAAAGCTTGATGGCTTTATGGGTAGA    1162
```

```
gb:MT027064|Organism:Severe      ATAATCAAGACTATTCAACCAAGGGTTGAAAAGAAAAAGCTTGATGGCTTTATGGGTAGA    1162
gb:MN994468|Organism:Severe      ATAATCAAGACTATTCAACCAAGGGTTGAAAAGAAAAAGCTTGATGGCTTTATGGGTAGA    1162
gb:MT072688|Organism:Severe      ATAATCAAGACTATTCAACCAAGGGTTGAAAAGAAAAAGCTTGATGGCTTTATGGGTAGA    1147
gb:MN996527|Organism:Severe      ATAATCAAGACTATTCAACCAAGGGTTGAAAAGAAAAAGCTTGATGGCTTTATGGGTAGA    1129
gb:MT093631|Organism:Severe      ATAATCAAGACTATTCAACCAAGGGTTGAAAAGAAAAAGCTTGATGGCTTTATGGGTAGA    1200
gb:MT106053|Organism:Severe      ATAATCAAGACTATTCAACCAAGGGTTGAAAAGAAAAAGCTTGATGGCTTTATGGGTAGA    1162
gb:MT019533|Organism:Severe      ATAATCAAGACTATTCAACCAAGGGTTGAAAAGAAAAAGCTTGATGGCTTTATGGGTAGA    1162
gb:MT019531|Organism:Severe      ATAATCAAGACTATTCAACCAAGGGTTGAAAAGAAAAAGCTTGATGGCTTTATGGGTAGA    1162
gb:MN996528|Organism:Severe      ATAATCAAGACTATTCAACCAAGGGTTGAAAAGAAAAAGCTTGATGGCTTTATGGGTAGA    1162
gb:MN996530|Organism:Severe      ATAATCAAGACTATTCAACCAAGGGTTGAAAAGAAAAAGCTTGATGGCTTTATGGGTAGA    1148
gb:MN908947|Organism:Severe      ATAATCAAGACTATTCAACCAAGGGTTGAAAAGAAAAAGCTTGATGGCTTTATGGGTAGA    1162
gb:MT019532|Organism:Severe      ATAATCAAGACTATTCAACCAAGGGTTGAAAAGAAAAAGCTTGATGGCTTTATGGGTAGA    1162
                                 ************************************************************

gb:MT020781|Organism:Severe      ATTCGATCTGTCTATCCAGTTGCGTCACCAAATGAATGCAACCAAATGTGCCTTTCAACT    1210
gb:MT007544|Organism:Severe      ATTCGATCTGTCTATCCAGTTGCGTCACCAAATGAATGCAACCAAATGTGCCTTTCAACT    1222
gb:MN994467|Organism:Severe      ATTCGATCTGTCTATCCAGTTGCGTCACCAAATGAATGCAACCAAATGTGCCTTTCAACT    1222
gb:MT044257|Organism:Severe      ATTCGATCTGTCTATCCAGTTGCGTCACCAAATGAATGCAACCAAATGTGCCTTTCAACT    1222
gb:MT106054|Organism:Severe      ATTCGATCTGTCTATCCAGTTGCGTCACCAAATGAATGCAACCAAATGTGCCTTTCAACT    1222
gb:MT049951|Organism:Severe      ATTCGATCTGTCTATCCAGTTGCGTCACCAAATGAATGCAACCAAATGTGCCTTTCAACT    1222
gb:MN975262|Organism:Severe      ATTCGATCTGTCTATCCAGTTGCGTCACCAAATGAATGCAACCAAATGTGCCTTTCAACT    1222
gb:MT106052|Organism:Severe      ATTCGATCTGTCTATCCAGTTGCGTCACCAAATGAATGCAACCAAATGTGCCTTTCAACT    1222
gb:LC522975|Organism:Severe      ATTCGATCTGTCTATCCAGTTGCGTCACCAAATGAATGCAACCAAATGTGCCTTTCAACT    1219
gb:LC522973|Organism:Severe      ATTCGATCTGTCTATCCAGTTGCGTCACCAAATGAATGCAACCAAATGTGCCTTTCAACT    1219
gb:LC522974|Organism:Severe      ATTCGATCTGTCTATCCAGTTGCGTCACCAAATGAATGCAACCAAATGTGCCTTTCAACT    1219
gb:MN985325|Organism:Severe      ATTCGATCTGTCTATCCAGTTGCGTCACCAAATGAATGCAACCAAATGTGCCTTTCAACT    1222
gb:MT020881|Organism:Severe      ATTCGATCTGTCTATCCAGTTGCGTCACCAAATGAATGCAACCAAATGTGCCTTTCAACT    1222
gb:MT020880|Organism:Severe      ATTCGATCTGTCTATCCAGTTGCGTCACCAAATGAATGCAACCAAATGTGCCTTTCAACT    1222
gb:MT066175|Organism:Severe      ATTCGATCTGTCTATCCAGTTGCGTCACCAAATGAATGCAACCAAATGTGCCTTTCAACT    1222
gb:MN997409|Organism:Severe      ATTCGATCTGTCTATCCAGTTGCGTCACCAAATGAATGCAACCAAATGTGCCTTTCAACT    1222
gb:MN938384|Organism:Severe      ATTCGATCTGTCTATCCAGTTGCGTCACCAAATGAATGCAACCAAATGTGCCTTTCAACT    1190
gb:MT044258|Organism:Severe      ATTCGATCTGTCTATCCAGTTGCGTCACCAAATGAATGCAACCAAATGTGCCTTTCAACT    1198
gb:MT039890|Organism:Severe      ATTCGATCTGTCTATCCAGTTGCGTCACCAAATGAATGCAACCAAATGTGCCTTTCAACT    1222
gb:MN988713|Organism:Severe      ATTCGATCTGTCTATCCAGTTGCGTCACCAAATGAATGCAACCAAATGTGCCTTTCAACT    1222
gb:LC521925|Organism:Severe      ATTCGATCTGTCTATCCAGTTGCGTCACCAAATGAATGCAACCAAATGTGCCTTTCAACT    1195
gb:MT093571|Organism:Severe      ATTCGATCTGTCTATCCAGTTGCGTCACCAAATGAATGCAACCAAATGTGCCTTTCAACT    1222
gb:MT039887|Organism:Severe      ATTCGATCTGTCTATCCAGTTGCGTCACCAAATGAATGCAACCAAATGTGCCTTTCAACT    1222
gb:MT019530|Organism:Severe      ATTCGATCTGTCTATCCAGTTGCGTCACCAAATGAATGCAACCAAATGTGCCTTTCAACT    1222
gb:MT039888|Organism:Severe      ATTCGATCTGTCTATCCAGTTGCGTCACCAAATGAATGCAACCAAATGTGCCTTTCAACT    1222
gb:LC522972|Organism:Severe      ATTCGATCTGTCTATCCAGTTGCGTCACCAAATGAATGCAACCAAATGTGCCTTTCAACT    1219
gb:MT027063|Organism:Severe      ATTCGATCTGTCTATCCAGTTGCGTCACCAAATGAATGCAACCAAATGTGCCTTTCAACT    1222
gb:MT027062|Organism:Severe      ATTCGATCTGTCTATCCAGTTGCGTCACCAAATGAATGCAACCAAATGTGCCTTTCAACT    1222
gb:MT019529|Organism:Severe      ATTCGATCTGTCTATCCAGTTGCGTCACCAAATGAATGCAACCAAATGTGCCTTTCAACT    1222
gb:MN996529|Organism:Severe      ATTCGATCTGTCTATCCAGTTGCGTCACCAAATGAATGCAACCAAATGTGCCTTTCAACT    1210
gb:MN996531|Organism:Severe      ATTCGATCTGTCTATCCAGTTGCGTCACCAAATGAATGCAACCAAATGTGCCTTTCAACT    1209
gb:MT066176|Organism:Severe      ATTCGATCTGTCTATCCAGTTGCGTCACCAAATGAATGCAACCAAATGTGCCTTTCAACT    1222
gb:MT027064|Organism:Severe      ATTCGATCTGTCTATCCAGTTGCGTCACCAAATGAATGCAACCAAATGTGCCTTTCAACT    1222
gb:MN994468|Organism:Severe      ATTCGATCTGTCTATCCAGTTGCGTCACCAAATGAATGCAACCAAATGTGCCTTTCAACT    1222
gb:MT072688|Organism:Severe      ATTCGATCTGTCTATCCAGTTGCGTCACCAAATGAATGCAACCAAATGTGCCTTTCAACT    1207
gb:MN996527|Organism:Severe      ATTCGATCTGTCTATCCAGTTGCGTCACCAAATGAATGCAACCAAATGTGCCTTTCAACT    1189
gb:MT093631|Organism:Severe      ATTCGATCTGTCTATCCAGTTGCGTCACCAAATGAATGCAACCAAATGTGCCTTTCAACT    1260
gb:MT106053|Organism:Severe      ATTCGATCTGTCTATCCAGTTGCGTCACCAAATGAATGCAACCAAATGTGCCTTTCAACT    1222
gb:MT019533|Organism:Severe      ATTCGATCTGTCTATCCAGTTGCGTCACCAAATGAATGCAACCAAATGTGCCTTTCAACT    1222
gb:MT019531|Organism:Severe      ATTCGATCTGTCTATCCAGTTGCGTCACCAAATGAATGCAACCAAATGTGCCTTTCAACT    1222
gb:MN996528|Organism:Severe      ATTCGATCTGTCTATCCAGTTGCGTCACCAAATGAATGCAACCAAATGTGCCTTTCAACT    1222
gb:MN996530|Organism:Severe      ATTCGATCTGTCTATCCAGTTGCGTCACCAAATGAATGCAACCAAATGTGCCTTTCAACT    1208
gb:MN908947|Organism:Severe      ATTCGATCTGTCTATCCAGTTGCGTCACCAAATGAATGCAACCAAATGTGCCTTTCAACT    1222
gb:MT019532|Organism:Severe      ATTCGATCTGTCTATCCAGTTGCGTCACCAAATGAATGCAACCAAATGTGCCTTTCAACT    1222
                                 ************************************************************

gb:MT020781|Organism:Severe      CTCATGAAGTGTGATCATTGTGGTGAAACTTCATGGCAGACGGGCGATTTTGTTAAAGCC    1270
gb:MT007544|Organism:Severe      CTCATGAAGTGTGATCATTGTGGTGAAACTTCATGGCAGACGGGCGATTTTGTTAAAGCC    1282
gb:MN994467|Organism:Severe      CTCATGAAGTGTGATCATTGTGGTGAAACTTCATGGCAGACGGGCGATTTTGTTAAAGCC    1282
gb:MT044257|Organism:Severe      CTCATGAAGTGTGATCATTGTGGTGAAACTTCATGGCAGACGGGCGATTTTGTTAAAGCC    1282
gb:MT106054|Organism:Severe      CTCATGAAGTGTGATCATTGTGGTGAAACTTCATGGCAGACGGGCGATTTTGTTAAAGCC    1282
```

```
gb:MT049951|Organism:Severe      CTCATGAAGTGTGATCATTGTGGTGAAACTTCATGGCAGACGGGCGATTTTGTTAAAGCC    1282
gb:MN975262|Organism:Severe      CTCATGAAGTGTGATCATTGTGGTGAAACTTCATGGCAGACGGGCGATTTTGTTAAAGCC    1282
gb:MT106052|Organism:Severe      CTCATGAAGTGTGATCATTGTGGTGAAACTTCATGGCAGACGGGCGATTTTGTTAAAGCC    1282
gb:LC522975|Organism:Severe      CTCATGAAGTGTGATCATTGTGGTGAAACTTCATGGCAGACGGGCGATTTTGTTAAAGCC    1279
gb:LC522973|Organism:Severe      CTCATGAAGTGTGATCATTGTGGTGAAACTTCATGGCAGACGGGCGATTTTGTTAAAGCC    1279
gb:LC522974|Organism:Severe      CTCATGAAGTGTGATCATTGTGGTGAAACTTCATGGCAGACGGGCGATTTTGTTAAAGCC    1279
gb:MN985325|Organism:Severe      CTCATGAAGTGTGATCATTGTGGTGAAACTTCATGGCAGACGGGCGATTTTGTTAAAGCC    1282
gb:MT020881|Organism:Severe      CTCATGAAGTGTGATCATTGTGGTGAAACTTCATGGCAGACGGGCGATTTTGTTAAAGCC    1282
gb:MT020880|Organism:Severe      CTCATGAAGTGTGATCATTGTGGTGAAACTTCATGGCAGACGGGCGATTTTGTTAAAGCC    1282
gb:MT066175|Organism:Severe      CTCATGAAGTGTGATCATTGTGGTGAAACTTCATGGCAGACGGGCGATTTTGTTAAAGCC    1282
gb:MN997409|Organism:Severe      CTCATGAAGTGTGATCATTGTGGTGAAACTTCATGGCAGACGGGCGATTTTGTTAAAGCC    1282
gb:MN938384|Organism:Severe      CTCATGAAGTGTGATCATTGTGGTGAAACTTCATGGCAGACGGGCGATTTTGTTAAAGCC    1250
gb:MT044258|Organism:Severe      CTCATGAAGTGTGATCATTGTGGTGAAACTTCATGGCAGACGGGCGATTTTGTTAAAGCC    1258
gb:MT039890|Organism:Severe      CTCATGAAGTGTGATCATTGTGGTGAAACTTCATGGCAGACGGGCGATTTTGTTAAAGCC    1282
gb:MN988713|Organism:Severe      CTCATGAAGTGTGATCATTGTGGTGAAACTTCATGGCAGACGGGCGATTTTGTTAAAGCC    1282
gb:LC521925|Organism:Severe      CTCATGAAGTGTGATCATTGTGGTGAAACTTCATGGCAGACGGGCGATTTTGTTAAAGCC    1255
gb:MT093571|Organism:Severe      CTCATGAAGTGTGATCATTGTGGTGAAACTTCATGGCAGACGGGCGATTTTGTTAAAGCC    1282
gb:MT039887|Organism:Severe      CTCATGAAGTGTGATCATTGTGGTGAAACTTCATGGCAGACGGGCGATTTTGTTAAAGCC    1282
gb:MT019530|Organism:Severe      CTCATGAAGTGTGATCATTGTGGTGAAACTTCATGGCAGACGGGCGATTTTGTTAAAGCC    1282
gb:MT039888|Organism:Severe      CTCATGAAGTGTGATCATTGTGGTGAAACTTCATGGCAGACGGGCGATTTTGTTAAAGCC    1282
gb:LC522972|Organism:Severe      CTCATGAAGTGTGATCATTGTGGTGAAACTTCATGGCAGACGGGCGATTTTGTTAAAGCC    1279
gb:MT027063|Organism:Severe      CTCATGAAGTGTGATCATTGTGGTGAAACTTCATGGCAGACGGGCGATTTTGTTAAAGCC    1282
gb:MT027062|Organism:Severe      CTCATGAAGTGTGATCATTGTGGTGAAACTTCATGGCAGACGGGCGATTTTGTTAAAGCC    1282
gb:MT019529|Organism:Severe      CTCATGAAGTGTGATCATTGTGGTGAAACTTCATGGCAGACGGGCGATTTTGTTAAAGCC    1282
gb:MN996529|Organism:Severe      CTCATGAAGTGTGATCATTGTGGTGAAACTTCATGGCAGACGGGCGATTTTGTTAAAGCC    1270
gb:MN996531|Organism:Severe      CTCATGAAGTGTGATCATTGTGGTGAAACTTCATGGCAGACGGGCGATTTTGTTAAAGCC    1269
gb:MT066176|Organism:Severe      CTCATGAAGTGTGATCATTGTGGTGAAACTTCATGGCAGACGGGCGATTTTGTTAAAGCC    1282
gb:MT027064|Organism:Severe      CTCATGAAGTGTGATCATTGTGGTGAAACTTCATGGCAGACGGGCGATTTTGTTAAAGCC    1282
gb:MN994468|Organism:Severe      CTCATGAAGTGTGATCATTGTGGTGAAACTTCATGGCAGACGGGCGATTTTGTTAAAGCC    1282
gb:MT072688|Organism:Severe      CTCATGAAGTGTGATCATTGTGGTGAAACTTCATGGCAGACGGGCGATTTTGTTAAAGCC    1267
gb:MN996527|Organism:Severe      CTCATGAAGTGTGATCATTGTGGTGAAACTTCATGGCAGACGGGCGATTTTGTTAAAGCC    1249
gb:MT093631|Organism:Severe      CTCATGAAGTGTGATCATTGTGGTGAAACTTCATGGCAGACGGGCGATTTTGTTAAAGCC    1320
gb:MT106053|Organism:Severe      CTCATGAAGTGTGATCATTGTGGTGAAACTTCATGGCAGACGGGCGATTTTGTTAAAGCC    1282
gb:MT019533|Organism:Severe      CTCATGAAGTGTGATCATTGTGGTGAAACTTCATGGCAGACGGGCGATTTTGTTAAAGCC    1282
gb:MT019531|Organism:Severe      CTCATGAAGTGTGATCATTGTGGTGAAACTTCATGGCAGACGGGCGATTTTGTTAAAGCC    1282
gb:MN996528|Organism:Severe      CTCATGAAGTGTGATCATTGTGGTGAAACTTCATGGCAGACGGGCGATTTTGTTAAAGCC    1282
gb:MN996530|Organism:Severe      CTCATGAAGTGTGATCATTGTGGTGAAACTTCATGGCAGACGGGCGATTTTGTTAAAGCC    1268
gb:MN908947|Organism:Severe      CTCATGAAGTGTGATCATTGTGGTGAAACTTCATGGCAGACGGGCGATTTTGTTAAAGCC    1282
gb:MT019532|Organism:Severe      CTCATGAAGTGTGATCATTGTGGTGAAACTTCATGGCAGACGGGCGATTTTGTTAAAGCC    1282
                                 ************************************************************

gb:MT020781|Organism:Severe      ACTTGCGAATTTTGTGGCACTGAGAATTTGACTAAAGAAGGTGCCACTACTTGTGGTTAC    1330
gb:MT007544|Organism:Severe      ACTTGCGAATTTTGTGGCACTGAGAATTTGACTAAAGAAGGTGCCACTACTTGTGGTTAC    1342
gb:MN994467|Organism:Severe      ACTTGCGAATTTTGTGGCACTGAGAATTTGACTAAAGAAGGTGCCACTACTTGTGGTTAC    1342
gb:MT044257|Organism:Severe      ACTTGCGAATTTTGTGGCACTGAGAATTTGACTAAAGAAGGTGCCACTACTTGTGGTTAC    1342
gb:MT106054|Organism:Severe      ACTTGCGAATTTTGTGGCACTGAGAATTTGACTAAAGAAGGTGCCACTACTTGTGGTTAC    1342
gb:MT049951|Organism:Severe      ACTTGCGAATTTTGTGGCACTGAGAATTTGACTAAAGAAGGTGCCACTACTTGTGGTTAC    1342
gb:MN975262|Organism:Severe      ACTTGCGAATTTTGTGGCACTGAGAATTTGACTAAAGAAGGTGCCACTACTTGTGGTTAC    1342
gb:MT106052|Organism:Severe      ACTTGCGAATTTTGTGGCACTGAGAATTTGACTAAAGAAGGTGCCACTACTTGTGGTTAC    1342
gb:LC522975|Organism:Severe      ACTTGCGAATTTTGTGGCACTGAGAATTTGACTAAAGAAGGTGCCACTACTTGTGGTTAC    1339
gb:LC522973|Organism:Severe      ACTTGCGAATTTTGTGGCACTGAGAATTTGACTAAAGAAGGTGCCACTACTTGTGGTTAC    1339
gb:LC522974|Organism:Severe      ACTTGCGAATTTTGTGGCACTGAGAATTTGACTAAAGAAGGTGCCACTACTTGTGGTTAC    1339
gb:MN985325|Organism:Severe      ACTTGCGAATTTTGTGGCACTGAGAATTTGACTAAAGAAGGTGCCACTACTTGTGGTTAC    1342
gb:MT020881|Organism:Severe      ACTTGCGAATTTTGTGGCACTGAGAATTTGACTAAAGAAGGTGCCACTACTTGTGGTTAC    1342
gb:MT020880|Organism:Severe      ACTTGCGAATTTTGTGGCACTGAGAATTTGACTAAAGAAGGTGCCACTACTTGTGGTTAC    1342
gb:MT066175|Organism:Severe      ACTTGCGAATTTTGTGGCACTGAGAATTTGACTAAAGAAGGTGCCACTACTTGTGGTTAC    1342
gb:MN997409|Organism:Severe      ACTTGCGAATTTTGTGGCACTGAGAATTTGACTAAAGAAGGTGCCACTACTTGTGGTTAC    1342
gb:MN938384|Organism:Severe      ACTTGCGAATTTTGTGGCACTGAGAATTTGACTAAAGAAGGTGCCACTACTTGTGGTTAC    1310
gb:MT044258|Organism:Severe      ACTTGCGAATTTTGTGGCACTGAGAATTTGACTAAAGAAGGTGCCACTACTTGTGGTTAC    1318
gb:MT039890|Organism:Severe      ACTTGCGAATTTTGTGGCACTGAGAATTTGACTAAAGAAGGTGCCACTACTTGTGGTTAC    1342
gb:MN988713|Organism:Severe      ACTTGCGAATTTTGTGGCACTGAGAATTTGACTAAAGAAGGTGCCACTACTTGTGGTTAC    1342
gb:LC521925|Organism:Severe      ACTTGCGAATTTTGTGGCACTGAGAATTTGACTAAAGAAGGTGCCACTACTTGTGGTTAC    1315
gb:MT093571|Organism:Severe      ACTTGCGAATTTTGTGGCACTGAGAATTTGACTAAAGAAGGTGCCACTACTTGTGGTTAC    1342
gb:MT039887|Organism:Severe      ACTTGCGAATTTTGTGGCACTGAGAATTTGACTAAAGAAGGTGCCACTACTTGTGGTTAC    1342
gb:MT019530|Organism:Severe      ACTTGCGAATTTTGTGGCACTGAGAATTTGACTAAAGAAGGTGCCACTACTTGTGGTTAC    1342
```

```
gb:MT039888|Organism:Severe      ACTTGCGAATTTTGTGGCACTGAGAATTTGACTAAAGAAGGTGCCACTACTTGTGGTTAC	1342
gb:LC522972|Organism:Severe      ACTTGCGAATTTTGTGGCACTGAGAATTTGACTAAAGAAGGTGCCACTACTTGTGGTTAC	1339
gb:MT027063|Organism:Severe      ACTTGCGAATTTTGTGGCACTGAGAATTTGACTAAAGAAGGTGCCACTACTTGTGGTTAC	1342
gb:MT027062|Organism:Severe      ACTTGCGAATTTTGTGGCACTGAGAATTTGACTAAAGAAGGTGCCACTACTTGTGGTTAC	1342
gb:MT019529|Organism:Severe      ACTTGCGAATTTTGTGGCACTGAGAATTTGACTAAAGAAGGTGCCACTACTTGTGGTTAC	1342
gb:MN996529|Organism:Severe      ACTTGCGAATTTTGTGGCACTGAGAATTTGACTAAAGAAGGTGCCACTACTTGTGGTTAC	1330
gb:MN996531|Organism:Severe      ACTTGCGAATTTTGTGGCACTGAGAATTTGACTAAAGAAGGTGCCACTACTTGTGGTTAC	1329
gb:MT066176|Organism:Severe      ACTTGCGAATTTTGTGGCACTGAGAATTTGACTAAAGAAGGTGCCACTACTTGTGGTTAC	1342
gb:MT027064|Organism:Severe      ACTTGCGAATTTTGTGGCACTGAGAATTTGACTAAAGAAGGTGCCACTACTTGTGGTTAC	1342
gb:MN994468|Organism:Severe      ACTTGCGAATTTTGTGGCACTGAGAATTTGACTAAAGAAGGTGCCACTACTTGTGGTTAC	1342
gb:MT072688|Organism:Severe      ACTTGCGAATTTTGTGGCACTGAGAATTTGACTAAAGAAGGTGCCACTACTTGTGGTTAC	1327
gb:MN996527|Organism:Severe      ACTTGCGAATTTTGTGGCACTGAGAATTTGACTAAAGAAGGTGCCACTACTTGTGGTTAC	1309
gb:MT093631|Organism:Severe      ACTTGCGAATTTTGTGGCACTGAGAATTTGACTAAAGAAGGTGCCACTACTTGTGGTTAC	1380
gb:MT106053|Organism:Severe      ACTTGCGAATTTTGTGGCACTGAGAATTTGACTAAAGAAGGTGCCACTACTTGTGGTTAC	1342
gb:MT019533|Organism:Severe      ACTTGCGAATTTTGTGGCACTGAGAATTTGACTAAAGAAGGTGCCACTACTTGTGGTTAC	1342
gb:MT019531|Organism:Severe      ACTTGCGAATTTTGTGGCACTGAGAATTTGACTAAAGAAGGTGCCACTACTTGTGGTTAC	1342
gb:MN996528|Organism:Severe      ACTTGCGAATTTTGTGGCACTGAGAATTTGACTAAAGAAGGTGCCACTACTTGTGGTTAC	1342
gb:MN996530|Organism:Severe      ACTTGCGAATTTTGTGGCACTGAGAATTTGACTAAAGAAGGTGCCACTACTTGTGGTTAC	1328
gb:MN908947|Organism:Severe      ACTTGCGAATTTTGTGGCACTGAGAATTTGACTAAAGAAGGTGCCACTACTTGTGGTTAC	1342
gb:MT019532|Organism:Severe      ACTTGCGAATTTTGTGGCACTGAGAATTTGACTAAAGAAGGTGCCACTACTTGTGGTTAC	1342
                                 ************************************************************

gb:MT020781|Organism:Severe      TTACCCCAAAATGCTGTTGTTAAAATTTATTGTCCAGCATGTCACAATTCAGAAGTAGGA	1390
gb:MT007544|Organism:Severe      TTACCCCAAAATGCTGTTGTTAAAATTTATTGTCCAGCATGTCACAATTCAGAAGTAGGA	1402
gb:MN994467|Organism:Severe      TTACCCCAAAATGCTGTTGTTAAAATTTATTGTCCAGCATGTCACAATTCAGAAGTAGGA	1402
gb:MT044257|Organism:Severe      TTACCCCAAAATGCTGTTGTTAAAATTTATTGTCCAGCATGTCACAATTCAGAAGTAGGA	1402
gb:MT106054|Organism:Severe      TTACCCCAAAATGCTGTTGTTAAAATTTATTGTCCAGCATGTCACAATTCAGAAGTAGGA	1402
gb:MT049951|Organism:Severe      TTACCCCAAAATGCTGTTGTTAAAATTTATTGTCCAGCATGTCACAATTCAGAAGTAGGA	1402
gb:MN975262|Organism:Severe      TTACCCCAAAATGCTGTTGTTAAAATTTATTGTCCAGCATGTCACAATTCAGAAGTAGGA	1402
gb:MT106052|Organism:Severe      TTACCCCAAAATGCTGTTGTTAAAATTTATTGTCCAGCATGTCACAATTCAGAAGTAGGA	1402
gb:LC522975|Organism:Severe      TTACCCCAAAATGCTGTTGTTAAAATTTATTGTCCAGCATGTCACAATTCAGAAGTAGGA	1399
gb:LC522973|Organism:Severe      TTACCCCAAAATGCTGTTGTTAAAATTTATTGTCCAGCATGTCACAATTCAGAAGTAGGA	1399
gb:LC522974|Organism:Severe      TTACCCCAAAATGCTGTTGTTAAAATTTATTGTCCAGCATGTCACAATTCAGAAGTAGGA	1399
gb:MN985325|Organism:Severe      TTACCCCAAAATGCTGTTGTTAAAATTTATTGTCCAGCATGTCACAATTCAGAAGTAGGA	1402
gb:MT020881|Organism:Severe      TTACCCCAAAATGCTGTTGTTAAAATTTATTGTCCAGCATGTCACAATTCAGAAGTAGGA	1402
gb:MT020880|Organism:Severe      TTACCCCAAAATGCTGTTGTTAAAATTTATTGTCCAGCATGTCACAATTCAGAAGTAGGA	1402
gb:MT066175|Organism:Severe      TTACCCCAAAATGCTGTTGTTAAAATTTATTGTCCAGCATGTCACAATTCAGAAGTAGGA	1402
gb:MN997409|Organism:Severe      TTACCCCAAAATGCTGTTGTTAAAATTTATTGTCCAGCATGTCACAATTCAGAAGTAGGA	1402
gb:MN938384|Organism:Severe      TTACCCCAAAATGCTGTTGTTAAAATTTATTGTCCAGCATGTCACAATTCAGAAGTAGGA	1370
gb:MT044258|Organism:Severe      TTACCCCAAAATGCTGTTGTTAAAATTTATTGTCCAGCATGTCACAATTCAGAAGTAGGA	1378
gb:MT039890|Organism:Severe      TTACCCCAAAATGCTGTTGTTAAAATTTATTGTCCAGCATGTCACAATTCAGAAGTAGGA	1402
gb:MN988713|Organism:Severe      TTACCCCAAAATGCTGTTGTTAAAATTTATTGTCCAGCATGTCACAATTCAGAAGTAGGA	1402
gb:LC521925|Organism:Severe      TTACCCCAAAATGCTGTTGTTAAAATTTATTGTCCAGCATGTCACAATTCAGAAGTAGGA	1375
gb:MT093571|Organism:Severe      TTACCCCAAAATGCTGTTGTTAAAATTTATTGTCCAGCATGTCACAATTCAGAAGTAGGA	1402
gb:MT039887|Organism:Severe      TTACCCCAAAATGCTGTTGTTAAAATTTATTGTCCAGCATGTCACAATTCAGAAGTAGGA	1402
gb:MT019530|Organism:Severe      TTACCCCAAAATGCTGTTGTTAAAATTTATTGTCCAGCATGTCACAATTCAGAAGTAGGA	1402
gb:MT039888|Organism:Severe      TTACCCCAAAATGCTGTTGTTAAAATTTATTGTCCAGCATGTCACAATTCAGAAGTAGGA	1402
gb:LC522972|Organism:Severe      TTACCCCAAAATGCTGTTGTTAAAATTTATTGTCCAGCATGTCACAATTCAGAAGTAGGA	1399
gb:MT027063|Organism:Severe      TTACCCCAAAATGCTGTTGTTAAAATTTATTGTCCAGCATGTCACAATTCAGAAGTAGGA	1402
gb:MT027062|Organism:Severe      TTACCCCAAAATGCTGTTGTTAAAATTTATTGTCCAGCATGTCACAATTCAGAAGTAGGA	1402
gb:MT019529|Organism:Severe      TTACCCCAAAATGCTGTTGTTAAAATTTATTGTCCAGCATGTCACAATTCAGAAGTAGGA	1402
gb:MN996529|Organism:Severe      TTACCCCAAAATGCTGTTGTTAAAATTTATTGTCCAGCATGTCACAATTCAGAAGTAGGA	1390
gb:MN996531|Organism:Severe      TTACCCCAAAATGCTGTTGTTAAAATTTATTGTCCAGCATGTCACAATTCAGAAGTAGGA	1389
gb:MT066176|Organism:Severe      TTACCCCAAAATGCTGTTGTTAAAATTTATTGTCCAGCATGTCACAATTCAGAAGTAGGA	1402
gb:MT027064|Organism:Severe      TTACCCCAAAATGCTGTTGTTAAAATTTATTGTCCAGCATGTCACAATTCAGAAGTAGGA	1402
gb:MN994468|Organism:Severe      TTACCCCAAAATGCTGTTGTTAAAATTTATTGTCCAGCATGTCACAATTCAGAAGTAGGA	1402
gb:MT072688|Organism:Severe      TTACCCCAAAATGCTGTTGTTAAAATTTATTGTCCAGCATGTCACAATTCAGAAGTAGGA	1387
gb:MN996527|Organism:Severe      TTACCCCAAAATGCTGTTGTTAAAATTTATTGTCCAGCATGTCACAATTCAGAAGTAGGA	1369
gb:MT093631|Organism:Severe      TTACCCCAAAATGCTGTTGTTAAAATTTATTGTCCAGCATGTCACAATTCAGAAGTAGGA	1440
gb:MT106053|Organism:Severe      TTACCCCAAAATGCTGTTGTTAAAATTTATTGTCCAGCATGTCACAATTCAGAAGTAGGA	1402
gb:MT019533|Organism:Severe      TTACCCCAAAATGCTGTTGTTAAAATTTATTGTCCAGCATGTCACAATTCAGAAGTAGGA	1402
gb:MT019531|Organism:Severe      TTACCCCAAAATGCTGTTGTTAAAATTTATTGTCCAGCATGTCACAATTCAGAAGTAGGA	1402
gb:MN996528|Organism:Severe      TTACCCCAAAATGCTGTTGTTAAAATTTATTGTCCAGCATGTCACAATTCAGAAGTAGGA	1402
gb:MN996530|Organism:Severe      TTACCCCAAAATGCTGTTGTTAAAATTTATTGTCCAGCATGTCACAATTCAGAAGTAGGA	1388
gb:MN908947|Organism:Severe      TTACCCCAAAATGCTGTTGTTAAAATTTATTGTCCAGCATGTCACAATTCAGAAGTAGGA	1402
```

```
gb:MT019532|Organism:Severe      TTACCCCAAAATGCTGTTGTTAAAATTTATTGTCCAGCATGTCACAATTCAGAAGTAGGA   1402
                                 ************************************************************

gb:MT020781|Organism:Severe      CCTGAGCATAGTCTTGCCGAATACCATAATGAATCTGGCTTGAAAACCATTCTTCGTAAG   1450
gb:MT007544|Organism:Severe      CCTGAGCATAGTCTTGCCGAATACCATAATGAATCTGGCTTGAAAACCATTCTTCGTAAG   1462
gb:MN994467|Organism:Severe      CCTGAGCATAGTCTTGCCGAATACCATAATGAATCTGGCTTGAAAACCATTCTTCGTAAG   1462
gb:MT044257|Organism:Severe      CCTGAGCATAGTCTTGCCGAATACCATAATGAATCTGGCTTGAAAACCATTCTTCGTAAG   1462
gb:MT106054|Organism:Severe      CCTGAGCATAGTCTTGCCGAATACCATAATGAATCTGGCTTGAAAACCATTCTTCGTAAG   1462
gb:MT049951|Organism:Severe      CCTGAGCATAGTCTTGCCGAATACCATAATGAATCTGGCTTGAAAACCATTCTTCGTAAG   1462
gb:MN975262|Organism:Severe      CCTGAGCATAGTCTTGCCGAATACCATAATGAATCTGGCTTGAAAACCATTCTTCGTAAG   1462
gb:MT106052|Organism:Severe      CCTGAGCATAGTCTTGCCGAATACCATAATGAATCTGGCTTGAAAACCATTCTTCGTAAG   1462
gb:LC522975|Organism:Severe      CCTGAGCATAGTCTTGCCGAATACCATAATGAATCTGGCTTGAAAACCATTCTTCGTAAG   1459
gb:LC522973|Organism:Severe      CCTGAGCATAGTCTTGCCGAATACCATAATGAATCTGGCTTGAAAACCATTCTTCGTAAG   1459
gb:LC522974|Organism:Severe      CCTGAGCATAGTCTTGCCGAATACCATAATGAATCTGGCTTGAAAACCATTCTTCGTAAG   1459
gb:MN985325|Organism:Severe      CCTGAGCATAGTCTTGCCGAATACCATAATGAATCTGGCTTGAAAACCATTCTTCGTAAG   1462
gb:MT020881|Organism:Severe      CCTGAGCATAGTCTTGCCGAATACCATAATGAATCTGGCTTGAAAACCATTCTTCGTAAG   1462
gb:MT020880|Organism:Severe      CCTGAGCATAGTCTTGCCGAATACCATAATGAATCTGGCTTGAAAACCATTCTTCGTAAG   1462
gb:MT066175|Organism:Severe      CCTGAGCATAGTCTTGCCGAATACCATAATGAATCTGGCTTGAAAACCATTCTTCGTAAG   1462
gb:MN997409|Organism:Severe      CCTGAGCATAGTCTTGCCGAATACCATAATGAATCTGGCTTGAAAACCATTCTTCGTAAG   1462
gb:MN938384|Organism:Severe      CCTGAGCATAGTCTTGCCGAATACCATAATGAATCTGGCTTGAAAACCATTCTTCGTAAG   1430
gb:MT044258|Organism:Severe      CCTGAGCATAGTCTTGCCGAATACCATAATGAATCTGGCTTGAAAACCATTCTTCGTAAG   1438
gb:MT039890|Organism:Severe      CCTGAGCATAGTCTTGCCGAATACCATAATGAATCTGGCTTGAAAACCATTCTTCGTAAG   1462
gb:MN988713|Organism:Severe      CCTGAGCATAGTCTTGCCGAATACCATAATGAATCTGGCTTGAAAACCATTCTTCGTAAG   1462
gb:LC521925|Organism:Severe      CCTGAGCATAGTCTTGCCGAATACCATAATGAATCTGGCTTGAAAACCATTCTTCGTAAG   1435
gb:MT093571|Organism:Severe      CCTGAGCATAGTCTTGCCGAATACCATAATGAATCTGGCTTGAAAACCATTCTTCGTAAG   1462
gb:MT039887|Organism:Severe      CCTGAGCATAGTCTTGCCGAATACCATAATGAATCTGGCTTGAAAACCATTCTTCGTAAG   1462
gb:MT019530|Organism:Severe      CCTGAGCATAGTCTTGCCGAATACCATAATGAATCTGGCTTGAAAACCATTCTTCGTAAG   1462
gb:MT039888|Organism:Severe      CCTGAGCATAGTCTTGCCGAATACCATAATGAATCTGGCTTGAAAACCATTCTTCGTAAG   1462
gb:LC522972|Organism:Severe      CCTGAGCATAGTCTTGCCGAATACCATAATGAATCTGGCTTGAAAACCATTCTTCGTAAG   1459
gb:MT027063|Organism:Severe      CCTGAGCATAGTCTTGCCGAATACCATAATGAATCTGGCTTGAAAACCATTCTTCGTAAG   1462
gb:MT027062|Organism:Severe      CCTGAGCATAGTCTTGCCGAATACCATAATGAATCTGGCTTGAAAACCATTCTTCGTAAG   1462
gb:MT019529|Organism:Severe      CCTGAGCATAGTCTTGCCGAATACCATAATGAATCTGGCTTGAAAACCATTCTTCGTAAG   1462
gb:MN996529|Organism:Severe      CCTGAGCATAGTCTTGCCGAATACCATAATGAATCTGGCTTGAAAACCATTCTTCGTAAG   1450
gb:MN996531|Organism:Severe      CCTGAGCATAGTCTTGCCGAATACCATAATGAATCTGGCTTGAAAACCATTCTTCGTAAG   1449
gb:MT066176|Organism:Severe      CCTGAGCATAGTCTTGCCGAATACCATAATGAATCTGGCTTGAAAACCATTCTTCGTAAG   1462
gb:MT027064|Organism:Severe      CCTGAGCATAGTCTTGCCGAATACCATAATGAATCTGGCTTGAAAACCATTCTTCGTAAG   1462
gb:MN994468|Organism:Severe      CCTGAGCATAGTCTTGCCGAATACCATAATGAATCTGGCTTGAAAACCATTCTTCGTAAG   1462
gb:MT072688|Organism:Severe      CCTGAGCATAGTCTTGCCGAATACCATAATGAATCTGGCTTGAAAACCATTCTTCGTAAG   1447
gb:MN996527|Organism:Severe      CCTGAGCATAGTCTTGCCGAATACCATAATGAATCTGGCTTGAAAACCATTCTTCGTAAG   1429
gb:MT093631|Organism:Severe      CCTGAGCATAGTCTTGCCGAATACCATAATGAATCTGGCTTGAAAACCATTCTTCGTAAG   1500
gb:MT106053|Organism:Severe      CCTGAGCATAGTCTTGCCGAATACCATAATGAATCTGGCTTGAAAACCATTCTTCGTAAG   1462
gb:MT019533|Organism:Severe      CCTGAGCATAGTCTTGCCGAATACCATAATGAATCTGGCTTGAAAACCATTCTTCGTAAG   1462
gb:MT019531|Organism:Severe      CCTGAGCATAGTCTTGCCGAATACCATAATGAATCTGGCTTGAAAACCATTCTTCGTAAG   1462
gb:MN996528|Organism:Severe      CCTGAGCATAGTCTTGCCGAATACCATAATGAATCTGGCTTGAAAACCATTCTTCGTAAG   1462
gb:MN996530|Organism:Severe      CCTGAGCATAGTCTTGCCGAATACCATAATGAATCTGGCTTGAAAACCATTCTTCGTAAG   1448
gb:MN908947|Organism:Severe      CCTGAGCATAGTCTTGCCGAATACCATAATGAATCTGGCTTGAAAACCATTCTTCGTAAG   1462
gb:MT019532|Organism:Severe      CCTGAGCATAGTCTTGCCGAATACCATAATGAATCTGGCTTGAAAACCATTCTTCGTAAG   1462
                                 ************************************************************

gb:MT020781|Organism:Severe      GGTGGTCGCACTATTGCCTTTGGAGGCTGTGTGTTCTCTTATGTTGGTTGCCATAACAAG   1510
gb:MT007544|Organism:Severe      GGTGGTCGCACTATTGCCTTTGGAGGCTGTGTGTTCTCTTATGTTGGTTGCCATAACAAG   1522
gb:MN994467|Organism:Severe      GGTGGTCGCACTATTGCCTTTGGAGGCTGTGTGTTCTCTTATGTTGGTTGCCATAACAAG   1522
gb:MT044257|Organism:Severe      GGTGGTCGCACTATTGCCTTTGGAGGCTGTGTGTTCTCTTATGTTGGTTGCCATAACAAG   1522
gb:MT106054|Organism:Severe      GGTGGTCGCACTATTGCCTTTGGAGGCTGTGTGTTCTCTTATGTTGGTTGCCATAACAAG   1522
gb:MT049951|Organism:Severe      GGTGGTCGCACTATTGCCTTTGGAGGCTGTGTGTTCTCTTATGTTGGTTGCCATAACAAG   1522
gb:MN975262|Organism:Severe      GGTGGTCGCACTATTGCCTTTGGAGGCTGTGTGTTCTCTTATGTTGGTTGCCATAACAAG   1522
gb:MT106052|Organism:Severe      GGTGGTCGCACTATTGCCTTTGGAGGCTGTGTGTTCTCTTATGTTGGTTGCCATAACAAG   1522
gb:LC522975|Organism:Severe      GGTGGTCGCACTATTGCCTTTGGAGGCTGTGTGTTCTCTTATGTTGGTTGCCATAACAAG   1519
gb:LC522973|Organism:Severe      GGTGGTCGCACTATTGCCTTTGGAGGCTGTGTGTTCTCTTATGTTGGTTGCCATAACAAG   1519
gb:LC522974|Organism:Severe      GGTGGTCGCACTATTGCCTTTGGAGGCTGTGTGTTCTCTTATGTTGGTTGCCATAACAAG   1519
gb:MN985325|Organism:Severe      GGTGGTCGCACTATTGCCTTTGGAGGCTGTGTGTTCTCTTATGTTGGTTGCCATAACAAG   1522
gb:MT020881|Organism:Severe      GGTGGTCGCACTATTGCCTTTGGAGGCTGTGTGTTCTCTTATGTTGGTTGCCATAACAAG   1522
gb:MT020880|Organism:Severe      GGTGGTCGCACTATTGCCTTTGGAGGCTGTGTGTTCTCTTATGTTGGTTGCCATAACAAG   1522
gb:MT066175|Organism:Severe      GGTGGTCGCACTATTGCCTTTGGAGGCTGTGTGTTCTCTTATGTTGGTTGCCATAACAAG   1522
gb:MN997409|Organism:Severe      GGTGGTCGCACTATTGCCTTTGGAGGCTGTGTGTTCTCTTATGTTGGTTGCCATAACAAG   1522
```

```
gb:MN938384|Organism:Severe      GGTGGTCGCACTATTGCCTTTGGAGGCTGTGTGTTCTCTTATGTTGGTTGCCATAACAAG   1490
gb:MT044258|Organism:Severe      GGTGGTCGCACTATTGCCTTTGGAGGCTGTGTGTTCTCTTATGTTGGTTGCCATAACAAG   1498
gb:MT039890|Organism:Severe      GGTGGTCGCACTATTGCCTTTGGAGGCTGTGTGTTCTCTTATGTTGGTTGCCATAACAAG   1522
gb:MN988713|Organism:Severe      GGTGGTCGCACTATTGCCTTTGGAGGCTGTGTGTTCTCTTATGTTGGTTGCCATAACAAG   1522
gb:LC521925|Organism:Severe      GGTGGTCGCACTATTGCCTTTGGAGGCTGTGTGTTCTCTTATGTTGGTTGCCATAACAAG   1495
gb:MT093571|Organism:Severe      GGTGGTCGCACTATTGCCTTTGGAGGCTGTGTGTTCTCTTATGTTGGTTGCCATAACAAG   1522
gb:MT039887|Organism:Severe      GGTGGTCGCACTATTGCCTTTGGAGGCTGTGTGTTCTCTTATGTTGGTTGCCATAACAAG   1522
gb:MT019530|Organism:Severe      GGTGGTCGCACTATTGCCTTTGGAGGCTGTGTGTTCTCTTATGTTGGTTGCCATAACAAG   1522
gb:MT039888|Organism:Severe      GGTGGTCGCACTATTGCCTTTGGAGGCTGTGTGTTCTCTTATGTTGGTTGCCATAACAAG   1522
gb:LC522972|Organism:Severe      GGTGGTCGCACTATTGCCTTTGGAGGCTGTGTGTTCTCTTATGTTGGTTGCCATAACAAG   1519
gb:MT027063|Organism:Severe      GGTGGTCGCACTATTGCCTTTGGAGGCTGTGTGTTCTCTTATGTTGGTTGCCATAACAAG   1522
gb:MT027062|Organism:Severe      GGTGGTCGCACTATTGCCTTTGGAGGCTGTGTGTTCTCTTATGTTGGTTGCCATAACAAG   1522
gb:MT019529|Organism:Severe      GGTGGTCGCACTATTGCCTTTGGAGGCTGTGTGTTCTCTTATGTTGGTTGCCATAACAAG   1522
gb:MN996529|Organism:Severe      GGTGGTCGCACTATTGCCTTTGGAGGCTGTGTGTTCTCTTATGTTGGTTGCCATAACAAG   1510
gb:MN996531|Organism:Severe      GGTGGTCGCACTATTGCCTTTGGAGGCTGTGTGTTCTCTTATGTTGGTTGCCATAACAAG   1509
gb:MT066176|Organism:Severe      GGTGGTCGCACTATTGCCTTTGGAGGCTGTGTGTTCTCTTATGTTGGTTGCCATAACAAG   1522
gb:MT027064|Organism:Severe      GGTGGTCGCACTATTGCCTTTGGAGGCTGTGTGTTCTCTTATGTTGGTTGCCATAACAAG   1522
gb:MN994468|Organism:Severe      GGTGGTCGCACTATTGCCTTTGGAGGCTGTGTGTTCTCTTATGTTGGTTGCCATAACAAG   1522
gb:MT072688|Organism:Severe      GGTGGTCGCACTATTGCCTTTGGAGGCTGTGTGTTCTCTTATGTTGGTTGCCATAACAAG   1507
gb:MN996527|Organism:Severe      GGTGGTCGCACTATTGCCTTTGGAGGCTGTGTGTTCTCTTATGTTGGTTGCCATAACAAG   1489
gb:MT093631|Organism:Severe      GGTGGTCGCACTATTGCCTTTGGAGGCTGTGTGTTCTCTTATGTTGGTTGCCATAACAAG   1560
gb:MT106053|Organism:Severe      GGTGGTCGCACTATTGCCTTTGGAGGCTGTGTGTTCTCTTATGTTGGTTGCCATAACAAG   1522
gb:MT019533|Organism:Severe      GGTGGTCGCACTATTGCCTTTGGAGGCTGTGTGTTCTCTTATGTTGGTTGCCATAACAAG   1522
gb:MT019531|Organism:Severe      GGTGGTCGCACTATTGCCTTTGGAGGCTGTGTGTTCTCTTATGTTGGTTGCCATAACAAG   1522
gb:MN996528|Organism:Severe      GGTGGTCGCACTATTGCCTTTGGAGGCTGTGTGTTCTCTTATGTTGGTTGCCATAACAAG   1522
gb:MN996530|Organism:Severe      GGTGGTCGCACTATTGCCTTTGGAGGCTGTGTGTTCTCTTATGTTGGTTGCCATAACAAG   1508
gb:MN908947|Organism:Severe      GGTGGTCGCACTATTGCCTTTGGAGGCTGTGTGTTCTCTTATGTTGGTTGCCATAACAAG   1522
gb:MT019532|Organism:Severe      GGTGGTCGCACTATTGCCTTTGGAGGCTGTGTGTTCTCTTATGTTGGTTGCCATAACAAG   1522
                                 ************************************************************

gb:MT020781|Organism:Severe      TGTGCCTATTGGGTTCCACGTGCTAGCGCTAACATAGGTTGTAACCATACAGGTGTTGTT   1570
gb:MT007544|Organism:Severe      TGTGCCTATTGGGTTCCACGTGCTAGCGCTAACATAGGTTGTAACCATACAGGTGTTGTT   1582
gb:MN994467|Organism:Severe      TGTGCCTATTGGGTTCCACGTGCTAACGCTAACATAGGTTGTAACCATACAGGTGTTGTT   1582
gb:MT044257|Organism:Severe      TGTGCCTATTGGGTTCCACGTGCTAGCGCTAACATAGGTTGTAACCATACAGGTGTTGTT   1582
gb:MT106054|Organism:Severe      TGTGCCTATTGGGTTCCACGTGCTAGCGCTAACATAGGTTGTAACCATACAGGTGTTGTT   1582
gb:MT049951|Organism:Severe      TGTGCCTATTGGGTTCCACGTGCTAGCGCTAACATAGGTTGTAACCATACAGGTGTTGTT   1582
gb:MN975262|Organism:Severe      TGTGCCTATTGGGTTCCACGTGCTAGCGCTAACATAGGTTGTAACCATACAGGTGTTGTT   1582
gb:MT106052|Organism:Severe      TGTGCCTATTGGGTTCCACGTGCTAGCGCTAACATAGGTTGTAACCATACAGGTGTTGTT   1582
gb:LC522975|Organism:Severe      TGTGCCTATTGGGTTCCACGTGCTAGCGCTAACATAGGTTGTAACCATACAGGTGTTGTT   1579
gb:LC522973|Organism:Severe      TGTGCCTATTGGGTTCCACGTGCTAGCGCTAACATAGGTTGTAACCATACAGGTGTTGTT   1579
gb:LC522974|Organism:Severe      TGTGCCTATTGGGTTCCACGTGCTAGCGCTAACATAGGTTGTAACCATACAGGTGTTGTT   1579
gb:MN985325|Organism:Severe      TGTGCCTATTGGGTTCCACGTGCTAGCGCTAACATAGGTTGTAACCATACAGGTGTTGTT   1582
gb:MT020881|Organism:Severe      TGTGCCTATTGGGTTCCACGTGCTAGCGCTAACATAGGTTGTAACCATACAGGTGTTGTT   1582
gb:MT020880|Organism:Severe      TGTGCCTATTGGGTTCCACGTGCTAGCGCTAACATAGGTTGTAACCATACAGGTGTTGTT   1582
gb:MT066175|Organism:Severe      TGTGCCTATTGGGTTCCACGTGCTAGCGCTAACATAGGTTGTAACCATACAGGTGTTGTT   1582
gb:MN997409|Organism:Severe      TGTGCCTATTGGGTTCCACGTGCTAGCGCTAACATAGGTTGTAACCATACAGGTGTTGTT   1582
gb:MN938384|Organism:Severe      TGTGCCTATTGGGTTCCACGTGCTAGCGCTAACATAGGTTGTAACCATACAGGTGTTGTT   1550
gb:MT044258|Organism:Severe      TGTGCCTATTGGGTTCCACGTGCTAGCGCTAACATAGGTTGTAACCATACAGGTGTTGTT   1558
gb:MT039890|Organism:Severe      TGTGCCTATTGGGTTCCACGTGCTAGCGCTAACATAGGTTGTAACCATACAGGTGTTGTT   1582
gb:MN988713|Organism:Severe      TGTGCCTATTGGGTTCCACGTGCTAGCGCTAACATAGGTTGTAACCATACAGGTGTTGTT   1582
gb:LC521925|Organism:Severe      TGTGCCTATTGGGTTCCACGTGCTAGCGCTAACATAGGTTGTAACCATACAGGTGTTGTT   1555
gb:MT093571|Organism:Severe      TGTGCCTATTGGGTTCCACGTGCTAGCGCTAACATAGGTTGTAACCATACAGGTGTTGTT   1582
gb:MT039887|Organism:Severe      TGTGCCTATTGGGTTCCACGTGCTAGCGCTAACATAGGTTGTAACCATACAGGTGTTGTT   1582
gb:MT019530|Organism:Severe      TGTGCCTATTGGGTTCCACGTGCTAGCGCTAACATAGGTTGTAACCATACAGGTGTTGTT   1582
gb:MT039888|Organism:Severe      TGTGCCTATTGGGTTCCACGTGCTAGCGCTAACATAGGTTGTAACCATACAGGTGTTGTT   1582
gb:LC522972|Organism:Severe      TGTGCCTATTGGGTTCCACGTGCTAGCGCTAACATAGGTTGTAACCATACAGGTGTTGTT   1579
gb:MT027063|Organism:Severe      TGTGCCTATTGGGTTCCACGTGCTAGCGCTAACATAGGTTGTAACCATACAGGTGTTGTT   1582
gb:MT027062|Organism:Severe      TGTGCCTATTGGGTTCCACGTGCTAGCGCTAACATAGGTTGTAACCATACAGGTGTTGTT   1582
gb:MT019529|Organism:Severe      TGTGCCTATTGGGTTCCACGTGCTAGCGCTAACATAGGTTGTAACCATACAGGTGTTGTT   1582
gb:MN996529|Organism:Severe      TGTGCCTATTGGGTTCCACGTGCTAGCGCTAACATAGGTTGTAACCATACAGGTGTTGTT   1570
gb:MN996531|Organism:Severe      TGTGCCTATTGGGTTCCACGTGCTAGCGCTAACATAGGTTGTAACCATACAGGTGTTGTT   1569
gb:MT066176|Organism:Severe      TGTGCCTATTGGGTTCCACGTGCTAGCGCTAACATAGGTTGTAACCATACAGGTGTTGTT   1582
gb:MT027064|Organism:Severe      TGTGCCTATTGGGTTCCACGTGCTAGCGCTAACATAGGTTGTAACCATACAGGTGTTGTT   1582
gb:MN994468|Organism:Severe      TGTGCCTATTGGGTTCCACGTGCTAGCGCTAACATAGGTTGTAACCATACAGGTGTTGTT   1582
gb:MT072688|Organism:Severe      TGTGCCTATTGGGTTCCACGTGCTAGCGCTAACATAGGTTGTAACCATACAGGTGTTGTT   1567
```

```
gb:MN996527|Organism:Severe      TGTGCCTATTGGGTTCCACGTGCTAGCGCTAACATAGGTTGTAACCATACAGGTGTTGTT   1549
gb:MT093631|Organism:Severe      TGTGCCTATTGGGTTCCACGTGCTAGCGCTAACATAGGTTGTAACCATACAGGTGTTGTT   1620
gb:MT106053|Organism:Severe      TGTGCCTATTGGGTTCCACGTGCTAGCGCTAACATAGGTTGTAACCATACAGGTGTTGTT   1582
gb:MT019533|Organism:Severe      TGTGCCTATTGGGTTCCACGTGCTAGCGCTAACATAGGTTGTAACCATACAGGTGTTGTT   1582
gb:MT019531|Organism:Severe      TGTGCCTATTGGGTTCCACGTGCTAGCGCTAACATAGGTTGTAACCATACAGGTGTTGTT   1582
gb:MN996528|Organism:Severe      TGTGCCTATTGGGTTCCACGTGCTAGCGCTAACATAGGTTGTAACCATACAGGTGTTGTT   1582
gb:MN996530|Organism:Severe      TGTGCCTATTGGGTTCCACGTGCTAGCGCTAACATAGGTTGTAACCATACAGGTGTTGTT   1568
gb:MN908947|Organism:Severe      TGTGCCTATTGGGTTCCACGTGCTAGCGCTAACATAGGTTGTAACCATACAGGTGTTGTT   1582
gb:MT019532|Organism:Severe      TGTGCCTATTGGGTTCCACGTGCTAGCGCTAACATAGGTTGTAACCATACAGGTGTTGTT   1582
                                 ************************* **********************************

gb:MT020781|Organism:Severe      GGAGAAGGTTCCGAAGGTCTTAATGACAACCTTCTTGAAATACTCCAAAAAGAGAAAGTC   1630
gb:MT007544|Organism:Severe      GGAGAAGGTTCCGAAGGTCTTAATGACAACCTTCTTGAAATACTCCAAAAAGAGAAAGTC   1642
gb:MN994467|Organism:Severe      GGAGAAGGTTCCGAAGGTCTTAATGACAACCTTCTTGAAATACTCCAAAAAGAGAAAGTC   1642
gb:MT044257|Organism:Severe      GGAGAAGGTTCCGAAGGTCTTAATGACAACCTTCTTGAAATACTCCAAAAAGAGAAAGTC   1642
gb:MT106054|Organism:Severe      GGAGAAGGTTCCGAAGGTCTTAATGACAACCTTCTTGAAATACTCCAAAAAGAGAAAGTC   1642
gb:MT049951|Organism:Severe      GGAGAAGGTTCCGAAGGTCTTAATGACAACCTTCTTGAAATACTCCAAAAAGAGAAAGTC   1642
gb:MN975262|Organism:Severe      GGAGAAGGTTCCGAAGGTCTTAATGACAACCTTCTTGAAATACTCCAAAAAGAGAAAGTC   1642
gb:MT106052|Organism:Severe      GGAGAAGGTTCCGAAGGTCTTAATGACAACCTTCTTGAAATACTCCAAAAAGAGAAAGTC   1642
gb:LC522975|Organism:Severe      GGAGAAGGTTCCGAAGGTCTTAATGACAACCTTCTTGAAATACTCCAAAAAGAGAAAGTC   1639
gb:LC522973|Organism:Severe      GGAGAAGGTTCCGAAGGTCTTAATGACAACCTTCTTGAAATACTCCAAAAAGAGAAAGTC   1639
gb:LC522974|Organism:Severe      GGAGAAGGTTCCGAAGGTCTTAATGACAACCTTCTTGAAATACTCCAAAAAGAGAAAGTC   1639
gb:MN985325|Organism:Severe      GGAGAAGGTTCCGAAGGTCTTAATGACAACCTTCTTGAAATACTCCAAAAAGAGAAAGTC   1642
gb:MT020881|Organism:Severe      GGAGAAGGTTCCGAAGGTCTTAATGACAACCTTCTTGAAATACTCCAAAAAGAGAAAGTC   1642
gb:MT020880|Organism:Severe      GGAGAAGGTTCCGAAGGTCTTAATGACAACCTTCTTGAAATACTCCAAAAAGAGAAAGTC   1642
gb:MT066175|Organism:Severe      GGAGAAGGTTCCGAAGGTCTTAATGACAACCTTCTTGAAATACTCCAAAAAGAGAAAGTC   1642
gb:MN997409|Organism:Severe      GGAGAAGGTTCCGAAGGTCTTAATGACAACCTTCTTGAAATACTCCAAAAAGAGAAAGTC   1642
gb:MN938384|Organism:Severe      GGAGAAGGTTCCGAAGGTCTTAATGACAACCTTCTTGAAATACTCCAAAAAGAGAAAGTC   1610
gb:MT044258|Organism:Severe      GGAGAAGGTTCCGAAGGTCTTAATGACAACCTTCTTGAAATACTCCAAAAAGAGAAAGTC   1618
gb:MT039890|Organism:Severe      GGAGAAGGTTCCGAAGGTCTTAATGACAACCTTCTTGAAATACTCCAAAAAGAGAAAGTC   1642
gb:MN988713|Organism:Severe      GGAGAAGGTTCCGAAGGTCTTAATGACAACCTTCTTGAAATACTCCAAAAAGAGAAAGTC   1642
gb:LC521925|Organism:Severe      GGAGAAGGTTCCGAAGGTCTTAATGACAACCTTCTTGAAATACTCCAAAAAGAGAAAGTC   1615
gb:MT093571|Organism:Severe      GGAGAAGGTTCCGAAGGTCTTAATGACAACCTTCTTGAAATACTCCAAAAAGAGAAAGTC   1642
gb:MT039887|Organism:Severe      GGAGAAGGTTCCGAAGGTCTTAATGACAACCTTCTTGAAATACTCCAAAAAGAGAAAGTC   1642
gb:MT019530|Organism:Severe      GGAGAAGGTTCCGAAGGTCTTAATGACAACCTTCTTGAAATACTCCAAAAAGAGAAAGTC   1642
gb:MT039888|Organism:Severe      GGAGAAGGTTCCGAAGGTCTTAATGACAACCTTCTTGAAATACTCCAAAAAGAGAAAGTC   1642
gb:LC522972|Organism:Severe      GGAGAAGGTTCCGAAGGTCTTAATGACAACCTTCTTGAAATACTCCAAAAAGAGAAAGTC   1639
gb:MT027063|Organism:Severe      GGAGAAGGTTCCGAAGGTCTTAATGACAACCTTCTTGAAATACTCCAAAAAGAGAAAGTC   1642
gb:MT027062|Organism:Severe      GGAGAAGGTTCCGAAGGTCTTAATGACAACCTTCTTGAAATACTCCAAAAAGAGAAAGTC   1642
gb:MT019529|Organism:Severe      GGAGAAGGTTCCGAAGGTCTTAATGACAACCTTCTTGAAATACTCCAAAAAGAGAAAGTC   1642
gb:MN996529|Organism:Severe      GGAGAAGGTTCCGAAGGTCTTAATGACAACCTTCTTGAAATACTCCAAAAAGAGAAAGTC   1630
gb:MN996531|Organism:Severe      GGAGAAGGTTCCGAAGGTCTTAATGACAACCTTCTTGAAATACTCCAAAAAGAGAAAGTC   1629
gb:MT066176|Organism:Severe      GGAGAAGGTTCCGAAGGTCTTAATGACAACCTTCTTGAAATACTCCAAAAAGAGAAAGTC   1642
gb:MT027064|Organism:Severe      GGAGAAGGTTCCGAAGGTCTTAATGACAACCTTCTTGAAATACTCCAAAAAGAGAAAGTC   1642
gb:MN994468|Organism:Severe      GGAGAAGGTTCCGAAGGTCTTAATGACAACCTTCTTGAAATACTCCAAAAAGAGAAAGTC   1642
gb:MT072688|Organism:Severe      GGAGAAGGTTCCGAAGGTCTTAATGACAACCTTCTTGAAATACTCCAAAAAGAGAAAGTC   1627
gb:MN996527|Organism:Severe      GGAGAAGGTTCCGAAGGTCTTAATGACAACCTTCTTGAAATACTCCAAAAAGAGAAAGTC   1609
gb:MT093631|Organism:Severe      GGAGAAGGTTCCGAAGGTCTTAATGACAACCTTCTTGAAATACTCCAAAAAGAGAAAGTC   1680
gb:MT106053|Organism:Severe      GGAGAAGGTTCCGAAGGTCTTAATGACAACCTTCTTGAAATACTCCAAAAAGAGAAAGTC   1642
gb:MT019533|Organism:Severe      GGAGAAGGTTCCGAAGGTCTTAATGACAACCTTCTTGAAATACTCCAAAAAGAGAAAGTC   1642
gb:MT019531|Organism:Severe      GGAGAAGGTTCCGAAGGTCTTAATGACAACCTTCTTGAAATACTCCAAAAAGAGAAAGTC   1642
gb:MN996528|Organism:Severe      GGAGAAGGTTCCGAAGGTCTTAATGACAACCTTCTTGAAATACTCCAAAAAGAGAAAGTC   1642
gb:MN996530|Organism:Severe      GGAGAAGGTTCCGAAGGTCTTAATGACAACCTTCTTGAAATACTCCAAAAAGAGAAAGTC   1628
gb:MN908947|Organism:Severe      GGAGAAGGTTCCGAAGGTCTTAATGACAACCTTCTTGAAATACTCCAAAAAGAGAAAGTC   1642
gb:MT019532|Organism:Severe      GGAGAAGGTTCCGAAGGTCTTAATGACAACCTTCTTGAAATACTCCAAAAAGAGAAAGTC   1642
                                 ************************************************************

gb:MT020781|Organism:Severe      AACATCAATATTGTTGGTGACTTTAAACTTAATGAAGAGATCGCCATTATTTTGGCATCT   1690
gb:MT007544|Organism:Severe      AACATCAATATTGTTGGTGACTTTAAACTTAATGAAGAGATCGCCATTATTTTGGCATCT   1702
gb:MN994467|Organism:Severe      AACATCAATATTGTTGGTGACTTTAAACTTAATGAAGAGATCGCCATTATTTTGGCATCT   1702
gb:MT044257|Organism:Severe      AACATCAATATTGTTGGTGACTTTAAACTTAATGAAGAGATCGCCATTATTTTGGCATCT   1702
gb:MT106054|Organism:Severe      AACATCAATATTGTTGGTGACTTTAAACTTAATGAAGAGATCGCCATTATTTTGGCATCT   1702
gb:MT049951|Organism:Severe      AACATCAATATTGTTGGTGACTTTAAACTTAATGAAGAGATCGCCATTATTTTGGCATCT   1702
gb:MN975262|Organism:Severe      AACATCAATATTGTTGGTGACTTTAAACTTAATGAAGAGATCGCCATTATTTTGGCATCT   1702
gb:MT106052|Organism:Severe      AACATCAATATTGTTGGTGACTTTAAACTTAATGAAGAGATCGCCATTATTTTGGCATCT   1702
```

```
gb:LC522975|Organism:Severe      AACATCAATATTGTTGGTGACTTTAAACTTAATGAAGAGATCGCCATTATTTTGGCATCT   1699
gb:LC522973|Organism:Severe      AACATCAATATTGTTGGTGACTTTAAACTTAATGAAGAGATCGCCATTATTTTGGCATCT   1699
gb:LC522974|Organism:Severe      AACATCAATATTGTTGGTGACTTTAAACTTAATGAAGAGATCGCCATTATTTTGGCATCT   1699
gb:MN985325|Organism:Severe      AACATCAATATTGTTGGTGACTTTAAACTTAATGAAGAGATCGCCATTATTTTGGCATCT   1702
gb:MT020881|Organism:Severe      AACATCAATATTGTTGGTGACTTTAAACTTAATGAAGAGATCGCCATTATTTTGGCATCT   1702
gb:MT020880|Organism:Severe      AACATCAATATTGTTGGTGACTTTAAACTTAATGAAGAGATCGCCATTATTTTGGCATCT   1702
gb:MT066175|Organism:Severe      AACATCAATATTGTTGGTGACTTTAAACTTAATGAAGAGATCGCCATTATTTTGGCATCT   1702
gb:MN997409|Organism:Severe      AACATCAATATTGTTGGTGACTTTAAACTTAATGAAGAGATCGCCATTATTTTGGCATCT   1702
gb:MN938384|Organism:Severe      AACATCAATATTGTTGGTGACTTTAAACTTAATGAAGAGATCGCCATTATTTTGGCATCT   1670
gb:MT044258|Organism:Severe      AACATCAATATTGTTGGTGACTTTAAACTTAATGAAGAGATCGCCATTATTTTGGCATCT   1678
gb:MT039890|Organism:Severe      AACATCAATATTGTTGGTGACTTTAAACTTAATGAAGAGATCGCCATTATTTTGGCATCT   1702
gb:MN988713|Organism:Severe      AACATCAATATTGTTGGTGACTTTAAACTTAATGAAGAGATCGCCATTATTTTGGCATCT   1702
gb:LC521925|Organism:Severe      AACATCAATATTGTTGGTGACTTTAAACTTAATGAAGAGATCGCCATTATTTTGGCATCT   1675
gb:MT093571|Organism:Severe      AACATCAATATTGTTGGTGACTTTAAACTTAATGAAGAGATCGCCATTATTTTGGCATCT   1702
gb:MT039887|Organism:Severe      AACATCAATATTGTTGGTGACTTTAAACTTAATGAAGAGATCGCCATTATTTTGGCATCT   1702
gb:MT019530|Organism:Severe      AACATCAATATTGTTGGTGACTTTAAACTTAATGAAGAGATCGCCATTATTTTGGCATCT   1702
gb:MT039888|Organism:Severe      AACATCAATATTGTTGGTGACTTTAAACTTAATGAAGAGATCGCCATTATTTTGGCATCT   1702
gb:LC522972|Organism:Severe      AACATCAATATTGTTGGTGACTTTAAACTTAATGAAGAGATCGCCATTATTTTGGCATCT   1699
gb:MT027063|Organism:Severe      AACATCAATATTGTTGGTGACTTTAAACTTAATGAAGAGATCGCCATTATTTTGGCATCT   1702
gb:MT027062|Organism:Severe      AACATCAATATTGTTGGTGACTTTAAACTTAATGAAGAGATCGCCATTATTTTGGCATCT   1702
gb:MT019529|Organism:Severe      AACATCAATATTGTTGGTGACTTTAAACTTAATGAAGAGATCGCCATTATTTTGGCATCT   1702
gb:MN996529|Organism:Severe      AACATCAATATTGTTGGTGACTTTAAACTTAATGAAGAGATCGCCATTATTTTGGCATCT   1690
gb:MN996531|Organism:Severe      AACATCAATATTGTTGGTGACTTTAAACTTAATGAAGAGATCGCCATTATTTTGGCATCT   1689
gb:MT066176|Organism:Severe      AACATCAATATTGTTGGTGACTTTAAACTTAATGAAGAGATCGCCATTATTTTGGCATCT   1702
gb:MT027064|Organism:Severe      AACATCAATATTGTTGGTGACTTTAAACTTAATGAAGAGATCGCCATTATTTTGGCATCT   1702
gb:MN994468|Organism:Severe      AACATCAATATTGTTGGTGACTTTAAACTTAATGAAGAGATCGCCATTATTTTGGCATCT   1702
gb:MT072688|Organism:Severe      AACATCAATATTGTTGGTGACTTTAAACTTAATGAAGAGATCGCCATTATTTTGGCATCT   1687
gb:MN996527|Organism:Severe      AACATCAATATTGTTGGTGACTTTAAACTTAATGAAGAGATCGCCATTATTTTGGCATCT   1669
gb:MT093631|Organism:Severe      AACATCAATATTGTTGGTGACTTTAAACTTAATGAAGAGATCGCCATTATTTTGGCATCT   1740
gb:MT106053|Organism:Severe      AACATCAATATTGTTGGTGACTTTAAACTTAATGAAGAGATCGCCATTATTTTGGCATCT   1702
gb:MT019533|Organism:Severe      AACATCAATATTGTTGGTGACTTTAAACTTAATGAAGAGATCGCCATTATTTTGGCATCT   1702
gb:MT019531|Organism:Severe      AACATCAATATTGTTGGTGACTTTAAACTTAATGAAGAGATCGCCATTATTTTGGCATCT   1702
gb:MN996528|Organism:Severe      AACATCAATATTGTTGGTGACTTTAAACTTAATGAAGAGATCGCCATTATTTTGGCATCT   1702
gb:MN996530|Organism:Severe      AACATCAATATTGTTGGTGACTTTAAACTTAATGAAGAGATCGCCATTATTTTGGCATCT   1688
gb:MN908947|Organism:Severe      AACATCAATATTGTTGGTGACTTTAAACTTAATGAAGAGATCGCCATTATTTTGGCATCT   1702
gb:MT019532|Organism:Severe      AACATCAATATTGTTGGTGACTTTAAACTTAATGAAGAGATCGCCATTATTTTGGCATCT   1702
                                 ************************************************************

gb:MT020781|Organism:Severe      TTTTCTGCTTCCACAAGTGCTTTTGTGGAAACTGTGAAAGGTTTGGATTATAAAGCATTC   1750
gb:MT007544|Organism:Severe      TTTTCTGCTTCCACAAGTGCTTTTGTGGAAACTGTGAAAGGTTTGGATTATAAAGCATTC   1762
gb:MN994467|Organism:Severe      TTTTCTGCTTCCACAAGTGCTTTTGTGGAAACTGTGAAAGGTTTGGATTATAAAGCATTC   1762
gb:MT044257|Organism:Severe      TTTTCTGCTTCCACAAGTGCTTTTGTGGAAACTGTGAAAGGTTTGGATTATAAAGCATTC   1762
gb:MT106054|Organism:Severe      TTTTCTGCTTCCACAAGTGCTTTTGTGGAAACTGTGAAAGGTTTGGATTATAAAGCATTC   1762
gb:MT049951|Organism:Severe      TTTTCTGCTTCCACAAGTGCTTTTGTGGAAACTGTGAAAGGTTTGGATTATAAAGCATTC   1762
gb:MN975262|Organism:Severe      TTTTCTGCTTCCACAAGTGCTTTTGTGGAAACTGTGAAAGGTTTGGATTATAAAGCATTC   1762
gb:MT106052|Organism:Severe      TTTTCTGCTTCCACAAGTGCTTTTGTGGAAACTGTGAAAGGTTTGGATTATAAAGCATTC   1762
gb:LC522975|Organism:Severe      TTTTCTGCTTCCACAAGTGCTTTTGTGGAAACTGTGAAAGGTTTGGATTATAAAGCATTC   1759
gb:LC522973|Organism:Severe      TTTTCTGCTTCCACAAGTGCTTTTGTGGAAACTGTGAAAGGTTTGGATTATAAAGCATTC   1759
gb:LC522974|Organism:Severe      TTTTCTGCTTCCACAAGTGCTTTTGTGGAAACTGTGAAAGGTTTGGATTATAAAGCATTC   1759
gb:MN985325|Organism:Severe      TTTTCTGCTTCCACAAGTGCTTTTGTGGAAACTGTGAAAGGTTTGGATTATAAAGCATTC   1762
gb:MT020881|Organism:Severe      TTTTCTGCTTCCACAAGTGCTTTTGTGGAAACTGTGAAAGGTTTGGATTATAAAGCATTC   1762
gb:MT020880|Organism:Severe      TTTTCTGCTTCCACAAGTGCTTTTGTGGAAACTGTGAAAGGTTTGGATTATAAAGCATTC   1762
gb:MT066175|Organism:Severe      TTTTCTGCTTCCACAAGTGCTTTTGTGGAAACTGTGAAAGGTTTGGATTATAAAGCATTC   1762
gb:MN997409|Organism:Severe      TTTTCTGCTTCCACAAGTGCTTTTGTGGAAACTGTGAAAGGTTTGGATTATAAAGCATTC   1762
gb:MN938384|Organism:Severe      TTTTCTGCTTCCACAAGTGCTTTTGTGGAAACTGTGAAAGGTTTGGATTATAAAGCATTC   1730
gb:MT044258|Organism:Severe      TTTTCTGCTTCCACAAGTGCTTTTGTGGAAACTGTGAAAGGTTTGGATTATAAAGCATTC   1738
gb:MT039890|Organism:Severe      TTTTCTGCTTCCACAAGTGCTTTTGTGGAAACTGTGAAAGGTTTGGATTATAAAGCATTC   1762
gb:MN988713|Organism:Severe      TTTTCTGCTTCCACAAGTGCTTTTGTGGAAACTGTGAAAGGTTTGGATTATAAAGCATTC   1762
gb:LC521925|Organism:Severe      TTTTCTGCTTCCACAAGTGCTTTTGTGGAAACTGTGAAAGGTTTGGATTATAAAGCATTC   1735
gb:MT093571|Organism:Severe      TTTTCTGCTTCCACAAGTGCTTTTGTGGAAACTGTGAAAGGTTTGGATTATAAAGCATTC   1762
gb:MT039887|Organism:Severe      TTTTCTGCTTCCACAAGTGCTTTTGTGGAAACTGTGAAAGGTTTGGATTATAAAGCATTC   1762
gb:MT019530|Organism:Severe      TTTTCTGCTTCCACAAGTGCTTTTGTGGAAACTGTGAAAGGTTTGGATTATAAAGCATTC   1762
gb:MT039888|Organism:Severe      TTTTCTGCTTCCACAAGTGCTTTTGTGGAAACTGTGAAAGGTTTGGATTATAAAGCATTC   1762
gb:LC522972|Organism:Severe      TTTTCTGCTTCCACAAGTGCTTTTGTGGAAACTGTGAAAGGTTTGGATTATAAAGCATTC   1759
gb:MT027063|Organism:Severe      TTTTCTGCTTCCACAAGTGCTTTTGTGGAAACTGTGAAAGGTTTGGATTATAAAGCATTC   1762
```

```
gb:MT027062|Organism:Severe      TTTTCTGCTTCCACAAGTGCTTTTGTGGAAACTGTGAAAGGTTTGGATTATAAAGCATTC    1762
gb:MT019529|Organism:Severe      TTTTCTGCTTCCACAAGTGCTTTTGTGGAAACTGTGAAAGGTTTGGATTATAAAGCATTC    1762
gb:MN996529|Organism:Severe      TTTTCTGCTTCCACAAGTGCTTTTGTGGAAACTGTGAAAGGTTTGGATTATAAAGCATTC    1750
gb:MN996531|Organism:Severe      TTTTCTGCTTCCACAAGTGCTTTTGTGGAAACTGTGAAAGGTTTGGATTATAAAGCATTC    1749
gb:MT066176|Organism:Severe      TTTTCTGCTTCCACAAGTGCTTTTGTGGAAACTGTGAAAGGTTTGGATTATAAAGCATTC    1762
gb:MT027064|Organism:Severe      TTTTCTGCTTCCACAAGTGCTTTTGTGGAAACTGTGAAAGGTTTGGATTATAAAGCATTC    1762
gb:MN994468|Organism:Severe      TTTTCTGCTTCCACAAGTGCTTTTGTGGAAACTGTGAAAGGTTTGGATTATAAAGCATTC    1762
gb:MT072688|Organism:Severe      TTTTCTGCTTCCACAAGTGCTTTTGTGGAAACTGTGAAAGGTTTGGATTATAAAGCATTC    1747
gb:MN996527|Organism:Severe      TTTTCTGCTTCCACAAGTGCTTTTGTGGAAACTGTGAAAGGTTTGGATTATAAAGCATTC    1729
gb:MT093631|Organism:Severe      TTTTCTGCTTCCACAAGTGCTTTTGTGGAAACTGTGAAAGGTTTGGATTATAAAGCATTC    1800
gb:MT106053|Organism:Severe      TTTTCTGCTTCCACAAGTGCTTTTGTGGAAACTGTGAAAGGTTTGGATTATAAAGCATTC    1762
gb:MT019533|Organism:Severe      TTTTCTGCTTCCACAAGTGCTTTTGTGGAAACTGTGAAAGGTTTGGATTATAAAGCATTC    1762
gb:MT019531|Organism:Severe      TTTTCTGCTTCCACAAGTGCTTTTGTGGAAACTGTGAAAGGTTTGGATTATAAAGCATTC    1762
gb:MN996528|Organism:Severe      TTTTCTGCTTCCACAAGTGCTTTTGTGGAAACTGTGAAAGGTTTGGATTATAAAGCATTC    1762
gb:MN996530|Organism:Severe      TTTTCTGCTTCCACAAGTGCTTTTGTGGAAACTGTGAAAGGTTTGGATTATAAAGCATTC    1748
gb:MN908947|Organism:Severe      TTTTCTGCTTCCACAAGTGCTTTTGTGGAAACTGTGAAAGGTTTGGATTATAAAGCATTC    1762
gb:MT019532|Organism:Severe      TTTTCTGCTTCCACAAGTGCTTTTGTGGAAACTGTGAAAGGTTTGGATTATAAAGCATTC    1762
                                 ************************************************************

gb:MT020781|Organism:Severe      AAACAAATTGTTGAATCCTGTGGTAATTTTAAAGTTACAAAAGGAAAAGCTAAAAAAGGT    1810
gb:MT007544|Organism:Severe      AAACAAATTGTTGAATCCTGTGGTAATTTTAAAGTTACAAAAGGAAAAGCTAAAAAAGGT    1822
gb:MN994467|Organism:Severe      AAACAAATTGTTGAATCCTGTGGTAATTTTAAAGTTACAAAAGGAAAAGCTAAAAAAGGT    1822
gb:MT044257|Organism:Severe      AAACAAATTGTTGAATCCTGTGGTAATTTTAAAGTTACAAAAGGAAAAGCTAAAAAAGGT    1822
gb:MT106054|Organism:Severe      AAACAAATTGTTGAATCCTGTGGTAATTTTAAAGTTACAAAAGGAAAAGCTAAAAAAGGT    1822
gb:MT049951|Organism:Severe      AAACAAATTGTTGAATCCTGTGGTAATTTTAAAGTTACAAAAGGAAAAGCTAAAAAAGGT    1822
gb:MN975262|Organism:Severe      AAACAAATTGTTGAATCCTGTGGTAATTTTAAAGTTACAAAAGGAAAAGCTAAAAAAGGT    1822
gb:MT106052|Organism:Severe      AAACAAATTGTTGAATCCTGTGGTAATTTTAAAGTTACAAAAGGAAAAGCTAAAAAAGGT    1822
gb:LC522975|Organism:Severe      AAACAAATTGTTGAATCCTGTGGTAATTTTAAAGTTACAAAAGGAAAAGCTAAAAAAGGT    1819
gb:LC522973|Organism:Severe      AAACAAATTGTTGAATCCTGTGGTAATTTTAAAGTTACAAAAGGAAAAGCTAAAAAAGGT    1819
gb:LC522974|Organism:Severe      AAACAAATTGTTGAATCCTGTGGTAATTTTAAAGTTACAAAAGGAAAAGCTAAAAAAGGT    1819
gb:MN985325|Organism:Severe      AAACAAATTGTTGAATCCTGTGGTAATTTTAAAGTTACAAAAGGAAAAGCTAAAAAAGGT    1822
gb:MT020881|Organism:Severe      AAACAAATTGTTGAATCCTGTGGTAATTTTAAAGTTACAAAAGGAAAAGCTAAAAAAGGT    1822
gb:MT020880|Organism:Severe      AAACAAATTGTTGAATCCTGTGGTAATTTTAAAGTTACAAAAGGAAAAGCTAAAAAAGGT    1822
gb:MT066175|Organism:Severe      AAACAAATTGTTGAATCCTGTGGTAATTTTAAAGTTACAAAAGGAAAAGCTAAAAAAGGT    1822
gb:MN997409|Organism:Severe      AAACAAATTGTTGAATCCTGTGGTAATTTTAAAGTTACAAAAGGAAAAGCTAAAAAAGGT    1822
gb:MN938384|Organism:Severe      AAACAAATTGTTGAATCCTGTGGTAATTTTAAAGTTACAAAAGGAAAAGCTAAAAAAGGT    1790
gb:MT044258|Organism:Severe      AAACAAATTGTTGAATCCTGTGGTAATTTTAAAGTTACAAAAGGAAAAGCTAAAAAAGGT    1798
gb:MT039890|Organism:Severe      AAACAAATTGTTGAATCCTGTGGTAATTTTAAAGTTACAAAAGGAAAAGCTAAAAAAGGT    1822
gb:MN988713|Organism:Severe      AAACAAATTGTTGAATCCTGTGGTAATTTTAAAGTTACAAAAGGAAAAGCTAAAAAAGGT    1822
gb:LC521925|Organism:Severe      AAACAAATTGTTGAATCCTGTGGTAATTTTAAAGTTACAAAAGGAAAAGCTAAAAAAGGT    1795
gb:MT093571|Organism:Severe      AAACAAATTGTTGAATCCTGTGGTAATTTTAAAGTTACAAAAGGAAAAGCTAAAAAAGGT    1822
gb:MT039887|Organism:Severe      AAACAAATTGTTGAATCCTGTGGTAATTTTAAAGTTACAAAAGGAAAAGCTAAAAAAGGT    1822
gb:MT019530|Organism:Severe      AAACAAATTGTTGAATCCTGTGGTAATTTTAAAGTTACAAAAGGAAAAGCTAAAAAAGGT    1822
gb:MT039888|Organism:Severe      AAACAAATTGTTGAATCCTGTGGTAATTTTAAAGTTACAAAAGGAAAAGCTAAAAAAGGT    1822
gb:LC522972|Organism:Severe      AAACAAATTGTTGAATCCTGTGGTAATTTTAAAGTTACAAAAGGAAAAGCTAAAAAAGGT    1819
gb:MT027063|Organism:Severe      AAACAAATTGTTGAATCCTGTGGTAATTTTAAAGTTACAAAAGGAAAAGCTAAAAAAGGT    1822
gb:MT027062|Organism:Severe      AAACAAATTGTTGAATCCTGTGGTAATTTTAAAGTTACAAAAGGAAAAGCTAAAAAAGGT    1822
gb:MT019529|Organism:Severe      AAACAAATTGTTGAATCCTGTGGTAATTTTAAAGTTACAAAAGGAAAAGCTAAAAAAGGT    1822
gb:MN996529|Organism:Severe      AAACAAATTGTTGAATCCTGTGGTAATTTTAAAGTTACAAAAGGAAAAGCTAAAAAAGGT    1810
gb:MN996531|Organism:Severe      AAACAAATTGTTGAATCCTGTGGTAATTTTAAAGTTACAAAAGGAAAAGCTAAAAAAGGT    1809
gb:MT066176|Organism:Severe      AAACAAATTGTTGAATCCTGTGGTAATTTTAAAGTTACAAAAGGAAAAGCTAAAAAAGGT    1822
gb:MT027064|Organism:Severe      AAACAAATTGTTGAATCCTGTGGTAATTTTAAAGTTACAAAAGGAAAAGCTAAAAAAGGT    1822
gb:MN994468|Organism:Severe      AAACAAATTGTTGAATCCTGTGGTAATTTTAAAGTTACAAAAGGAAAAGCTAAAAAAGGT    1822
gb:MT072688|Organism:Severe      AAACAAATTGTTGAATCCTGTGGTAATTTTAAAGTTACAAAAGGAAAAGCTAAAAAAGGT    1807
gb:MN996527|Organism:Severe      AAACAAATTGTTGAATCCTGTGGTAATTTTAAAGTTACAAAAGGAAAAGCTAAAAAAGGT    1789
gb:MT093631|Organism:Severe      AAACAAATTGTTGAATCCTGTGGTAATTTTAAAGTTACAAAAGGAAAAGCTAAAAAAGGT    1860
gb:MT106053|Organism:Severe      AAACAAATTGTTGAATCCTGTGGTAATTTTAAAGTTACAAAAGGAAAAGCTAAAAAAGGT    1822
gb:MT019533|Organism:Severe      AAACAAATTGTTGAATCCTGTGGTAATTTTAAAGTTACAAAAGGAAAAGCTAAAAAAGGT    1822
gb:MT019531|Organism:Severe      AAACAAATTGTTGAATCCTGTGGTAATTTTAAAGTTACAAAAGGAAAAGCTAAAAAAGGT    1822
gb:MN996528|Organism:Severe      AAACAAATTGTTGAATCCTGTGGTAATTTTAAAGTTACAAAAGGAAAAGCTAAAAAAGGT    1822
gb:MN996530|Organism:Severe      AAACAAATTGTTGAATCCTGTGGTAATTTTAAAGTTACAAAAGGAAAAGCTAAAAAAGGT    1808
gb:MN908947|Organism:Severe      AAACAAATTGTTGAATCCTGTGGTAATTTTAAAGTTACAAAAGGAAAAGCTAAAAAAGGT    1822
gb:MT019532|Organism:Severe      AAACAAATTGTTGAATCCTGTGGTAATTTTAAAGTTACAAAAGGAAAAGCTAAAAAAGGT    1822
                                 ************************************************************
```

```
gb:MT020781|Organism:Severe      GCCTGGAATATTGGTGAACAGAAATCAATACTGAGTCCTCTTTATGCATTTGCATCAGAG    1870
gb:MT007544|Organism:Severe      GCCTGGAATATTGGTGAACAGAAATCAATACTGAGTCCTCTTTATGCATTTGCATCAGAG    1882
gb:MN994467|Organism:Severe      GCCTGGAATATTGGTGAACAGAAATCAATACTGAGTCCTCTTTATGCATTTGCATCAGAG    1882
gb:MT044257|Organism:Severe      GCCTGGAATATTGGTGAACAGAAATCAATACTGAGTCCTCTTTATGCATTTGCATCAGAG    1882
gb:MT106054|Organism:Severe      GCCTGGAATATTGGTGAACAGAAATCAATACTGAGTCCTCTTTATGCATTTGCATCAGAG    1882
gb:MT049951|Organism:Severe      GCCTGGAATATTGGTGAACAGAAATCAATACTGAGTCCTCTTTATGCATTTGCATCAGAG    1882
gb:MN975262|Organism:Severe      GCCTGGAATATTGGTGAACAGAAATCAATACTGAGTCCTCTTTATGCATTTGCATCAGAG    1882
gb:MT106052|Organism:Severe      GCCTGGAATATTGGTGAACAGAAATCAATACTGAGTCCTCTTTATGCATTTGCATCAGAG    1882
gb:LC522975|Organism:Severe      GCCTGGAATATTGGTGAACAGAAATCAATACTGAGTCCTCTTTATGCATTTGCATCAGAG    1879
gb:LC522973|Organism:Severe      GCCTGGAATATTGGTGAACAGAAATCAATACTGAGTCCTCTTTATGCATTTGCATCAGAG    1879
gb:LC522974|Organism:Severe      GCCTGGAATATTGGTGAACAGAAATCAATACTGAGTCCTCTTTATGCATTTGCATCAGAG    1879
gb:MN985325|Organism:Severe      GCCTGGAATATTGGTGAACAGAAATCAATACTGAGTCCTCTTTATGCATTTGCATCAGAG    1882
gb:MT020881|Organism:Severe      GCCTGGAATATTGGTGAACAGAAATCAATACTGAGTCCTCTTTATGCATTTGCATCAGAG    1882
gb:MT020880|Organism:Severe      GCCTGGAATATTGGTGAACAGAAATCAATACTGAGTCCTCTTTATGCATTTGCATCAGAG    1882
gb:MT066175|Organism:Severe      GCCTGGAATATTGGTGAACAGAAATCAATACTGAGTCCTCTTTATGCATTTGCATCAGAG    1882
gb:MN997409|Organism:Severe      GCCTGGAATATTGGTGAACAGAAATCAATACTGAGTCCTCTTTATGCATTTGCATCAGAG    1882
gb:MN938384|Organism:Severe      GCCTGGAATATTGGTGAACAGAAATCAATACTGAGTCCTCTTTATGCATTTGCATCAGAG    1850
gb:MT044258|Organism:Severe      GCCTGGAATATTGGTGAACAGAAATCAATACTGAGTCCTCTTTATGCATTTGCATCAGAG    1858
gb:MT039890|Organism:Severe      GCCTGGAATATTGGTGAACAGAAATCAATACTGAGTCCTCTTTATGCATTTGCATCAGAG    1882
gb:MN988713|Organism:Severe      GCCTGGAATATTGGTGAACAGAAATCAATACTGAGTCCTCTTTATGCATTTGCATCAGAG    1882
gb:LC521925|Organism:Severe      GCCTGGAATATTGGTGAACAGAAATCAATACTGAGTCCTCTTTATGCATTTGCATCAGAG    1855
gb:MT093571|Organism:Severe      GCCTGGAATATTGGTGAACAGAAATCAATACTGAGTCCTCTTTATGCATTTGCATCAGAG    1882
gb:MT039887|Organism:Severe      GCCTGGAATATTGGTGAACAGAAATCAATACTGAGTCCTCTTTATGCATTTGCATCAGAG    1882
gb:MT019530|Organism:Severe      GCCTGGAATATTGGTGAACAGAAATCAATACTGAGTCCTCTTTATGCATTTGCATCAGAG    1882
gb:MT039888|Organism:Severe      GCCTGGAATATTGGTGAACAGAAATCAATACTGAGTCCTCTTTATGCATTTGCATCAGAG    1882
gb:LC522972|Organism:Severe      GCCTGGAATATTGGTGAACAGAAATCAATACTGAGTCCTCTTTATGCATTTGCATCAGAG    1879
gb:MT027063|Organism:Severe      GCCTGGAATATTGGTGAACAGAAATCAATACTGAGTCCTCTTTATGCATTTGCATCAGAG    1882
gb:MT027062|Organism:Severe      GCCTGGAATATTGGTGAACAGAAATCAATACTGAGTCCTCTTTATGCATTTGCATCAGAG    1882
gb:MT019529|Organism:Severe      GCCTGGAATATTGGTGAACAGAAATCAATACTGAGTCCTCTTTATGCATTTGCATCAGAG    1882
gb:MN996529|Organism:Severe      GCCTGGAATATTGGTGAACAGAAATCAATACTGAGTCCTCTTTATGCATTTGCATCAGAG    1870
gb:MN996531|Organism:Severe      GCCTGGAATATTGGTGAACAGAAATCAATACTGAGTCCTCTTTATGCATTTGCATCAGAG    1869
gb:MT066176|Organism:Severe      GCCTGGAATATTGGTGAACAGAAATCAATACTGAGTCCTCTTTATGCATTTGCATCAGAG    1882
gb:MT027064|Organism:Severe      GCCTGGAATATTGGTGAACAGAAATCAATACTGAGTCCTCTTTATGCATTTGCATCAGAG    1882
gb:MN994468|Organism:Severe      GCCTGGAATATTGGTGAACAGAAATCAATACTGAGTCCTCTTTATGCATTTGCATCAGAG    1882
gb:MT072688|Organism:Severe      GCCTGGAATATTGGTGAACAGAAATCAATACTGAGTCCTCTTTATGCATTTGCATCAGAG    1867
gb:MN996527|Organism:Severe      GCCTGGAATATTGGTGAACAGAAATCAATACTGAGTCCTCTTTATGCATTTGCATCAGAG    1849
gb:MT093631|Organism:Severe      GCCTGGAATATTGGTGAACAGAAATCAATACTGAGTCCTCTTTATGCATTTGCATCAGAG    1920
gb:MT106053|Organism:Severe      GCCTGGAATATTGGTGAACAGAAATCAATACTGAGTCCTCTTTATGCATTTGCATCAGAG    1882
gb:MT019533|Organism:Severe      GCCTGGAATATTGGTGAACAGAAATCAATACTGAGTCCTCTTTATGCATTTGCATCAGAG    1882
gb:MT019531|Organism:Severe      GCCTGGAATATTGGTGAACAGAAATCAATACTGAGTCCTCTTTATGCATTTGCATCAGAG    1882
gb:MN996528|Organism:Severe      GCCTGGAATATTGGTGAACAGAAATCAATACTGAGTCCTCTTTATGCATTTGCATCAGAG    1882
gb:MN996530|Organism:Severe      GCCTGGAATATTGGTGAACAGAAATCAATACTGAGTCCTCTTTATGCATTTGCATCAGAG    1868
gb:MN908947|Organism:Severe      GCCTGGAATATTGGTGAACAGAAATCAATACTGAGTCCTCTTTATGCATTTGCATCAGAG    1882
gb:MT019532|Organism:Severe      GCCTGGAATATTGGTGAACAGAAATCAATACTGAGTCCTCTTTATGCATTTGCATCAGAG    1882
                                 ************************************************************

gb:MT020781|Organism:Severe      GCTGCTCGTGTTGTACGATCAATTTTCTCCCGCACTCTTGAAACTGCTCAAAATTCTGTG    1930
gb:MT007544|Organism:Severe      GCTGCTCGTGTTGTACGATCAATTTTCTCCCGCACTCTTGAAACTGCTCAAAATTCTGTG    1942
gb:MN994467|Organism:Severe      GCTGCTCGTGTTGTACGATCAATTTTCTCCCGCACTCTTGAAACTGCTCAAAATTCTGTG    1942
gb:MT044257|Organism:Severe      GCTGCTCGTGTTGTACGATCAATTTTCTCCCGCACTCTTGAAACTGCTCAAAATTCTGTG    1942
gb:MT106054|Organism:Severe      GCTGCTCGTGTTGTACGATCAATTTTCTCCCGCACTCTTGAAACTGCTCAAAATTCTGTG    1942
gb:MT049951|Organism:Severe      GCTGCTCGTGTTGTACGATCAATTTTCTCCCGCACTCTTGAAACTGCTCAAAATTCTGTG    1942
gb:MN975262|Organism:Severe      GCTGCTCGTGTTGTACGATCAATTTTCTCCCGCACTCTTGAAACTGCTCAAAATTCTGTG    1942
gb:MT106052|Organism:Severe      GCTGCTCGTGTTGTACGATCAATTTTCTCCCGCACTCTTGAAACTGCTCAAAATTCTGTG    1942
gb:LC522975|Organism:Severe      GCTGCTCGTGTTGTACGATCAATTTTCTCCCGCACTCTTGAAACTGCTCAAAATTCTGTG    1939
gb:LC522973|Organism:Severe      GCTGCTCGTGTTGTACGATCAATTTTCTCCCGCACTCTTGAAACTGCTCAAAATTCTGTG    1939
gb:LC522974|Organism:Severe      GCTGCTCGTGTTGTACGATCAATTTTCTCCCGCACTCTTGAAACTGCTCAAAATTCTGTG    1939
gb:MN985325|Organism:Severe      GCTGCTCGTGTTGTACGATCAATTTTCTCCCGCACTCTTGAAACTGCTCAAAATTCTGTG    1942
gb:MT020881|Organism:Severe      GCTGCTCGTGTTGTACGATCAATTTTCTCCCGCACTCTTGAAACTGCTCAAAATTCTGTG    1942
gb:MT020880|Organism:Severe      GCTGCTCGTGTTGTACGATCAATTTTCTCCCGCACTCTTGAAACTGCTCAAAATTCTGTG    1942
gb:MT066175|Organism:Severe      GCTGCTCGTGTTGTACGATCAATTTTCTCCCGCACTCTTGAAACTGCTCAAAATTCTGTG    1942
gb:MN997409|Organism:Severe      GCTGCTCGTGTTGTACGATCAATTTTCTCCCGCACTCTTGAAACTGCTCAAAATTCTGTG    1942
gb:MN938384|Organism:Severe      GCTGCTCGTGTTGTACGATCAATTTTCTCCCGCACTCTTGAAACTGCTCAAAATTCTGTG    1910
gb:MT044258|Organism:Severe      GCTGCTCGTGTTGTACGATCAATTTTCTCCCGCACTCTTGAAACTGCTCAAAATTCTGTG    1918
gb:MT039890|Organism:Severe      GCTGCTCGTGTTGTACGATCAATTTTCTCCCGCACTCTTGAAACTGCTCAAAATTCTGTG    1942
```

```
gb:MN988713|Organism:Severe      GCTGCTCGTGTTGTACGATCAATTTTCTCCCGCACTCTTGAAACTGCTCAAAATTCTGTG   1942
gb:LC521925|Organism:Severe      GCTGCTCGTGTTGTACGATCAATTTTCTCTCGCACTCTTGAAACTGCTCAAAATTCTGTG   1915
gb:MT093571|Organism:Severe      GCTGCTCGTGTTGTACGATCAATTTTCTCCCGCACTCTTGAAACTGCTCAAAATTCTGTG   1942
gb:MT039887|Organism:Severe      GCTGCTCGTGTTGTACGATCAATTTTCTCCCGCACTCTTGAAACTGCTCAAAATTCTGTG   1942
gb:MT019530|Organism:Severe      GCTGCTCGTGTTGTACGATCAATTTTCTCCCGCACTCTTGAAACTGCTCAAAATTCTGTG   1942
gb:MT039888|Organism:Severe      GCTGCTCGTGTTGTACGATCAATTTTCTCCCGCACTCTTGAAACTGCTCAAAATTCTGTG   1942
gb:LC522972|Organism:Severe      GCTGCTCGTGTTGTACGATCAATTTTCTCCCGCACTCTTGAAACTGCTCAAAATTCTGTG   1939
gb:MT027063|Organism:Severe      GCTGCTCGTGTTGTACGATCAATTTTCTCCCGCACTCTTGAAACTGCTCAAAATTCTGTG   1942
gb:MT027062|Organism:Severe      GCTGCTCGTGTTGTACGATCAATTTTCTCCCGCACTCTTGAAACTGCTCAAAATTCTGTG   1942
gb:MT019529|Organism:Severe      GCTGCTCGTGTTGTACGATCAATTTTCTCCCGCACTCTTGAAACTGCTCAAAATTCTGTG   1942
gb:MN996529|Organism:Severe      GCTGCTCGTGTTGTACGATCAATTTTCTCCCGCACTCTTGAAACTGCTCAAAATTCTGTG   1930
gb:MN996531|Organism:Severe      GCTGCTCGTGTTGTACGATCAATTTTCTCCCGCACTCTTGAAACTGCTCAAAATTCTGTG   1929
gb:MT066176|Organism:Severe      GCTGCTCGTGTTGTACGATCAATTTTCTCCCGCACTCTTGAAACTGCTCAAAATTCTGTG   1942
gb:MT027064|Organism:Severe      GCTGCTCGTGTTGTACGATCAATTTTCTCCCGCACTCTTGAAACTGCTCAAAATTCTGTG   1942
gb:MN994468|Organism:Severe      GCTGCTCGTGTTGTACGATCAATTTTCTCCCGCACTCTTGAAACTGCTCAAAATTCTGTG   1942
gb:MT072688|Organism:Severe      GCTGCTCGTGTTGTACGATCAATTTTCTCCCGCACTCTTGAAACTGCTCAAAATTCTGTG   1927
gb:MN996527|Organism:Severe      GCTGCTCGTGTTGTACGATCAATTTTCTCCCGCACTCTTGAAACTGCTCAAAATTCTGTG   1909
gb:MT093631|Organism:Severe      GCTGCTCGTGTTGTACGATCAATTTTCTCCCGCACTCTTGAAACTGCTCAAAATTCTGTG   1980
gb:MT106053|Organism:Severe      GCTGCTCGTGTTGTACGATCAATTTTCTCCCGCACTCTTGAAACTGCTCAAAATTCTGTG   1942
gb:MT019533|Organism:Severe      GCTGCTCGTGTTGTACGATCAATTTTCTCCCGCACTCTTGAAACTGCTCAAAATTCTGTG   1942
gb:MT019531|Organism:Severe      GCTGCTCGTGTTGTACGATCAATTTTCTCCCGCACTCTTGAAACTGCTCAAAATTCTGTG   1942
gb:MN996528|Organism:Severe      GCTGCTCGTGTTGTACGATCAATTTTCTCCCGCACTCTTGAAACTGCTCAAAATTCTGTG   1942
gb:MN996530|Organism:Severe      GCTGCTCGTGTTGTACGATCAATTTTCTCCCGCACTCTTGAAACTGCTCAAAATTCTGTG   1928
gb:MN908947|Organism:Severe      GCTGCTCGTGTTGTACGATCAATTTTCTCCCGCACTCTTGAAACTGCTCAAAATTCTGTG   1942
gb:MT019532|Organism:Severe      GCTGCTCGTGTTGTACGATCAATTTTCTCCCGCACTCTTGAAACTGCTCAAAATTCTGTG   1942
                                 ***************************** ******************************

gb:MT020781|Organism:Severe      CGTGTTTTACAGAAGGCCGCTATAACAATACTAGATGGAATTTCACAGTATTCACTGAGA   1990
gb:MT007544|Organism:Severe      CGTGTTTTACAGAAGGCCGCTATAACAATACTAGATGGAATTTCACAGTATTCACTGAGA   2002
gb:MN994467|Organism:Severe      CGTGTTTTACAGAAGGCCGCTATAACAATACTAGATGGAATTTCACAGTATTCACTGAGA   2002
gb:MT044257|Organism:Severe      CGTGTTTTACAGAAGGCCGCTATAACAATACTAGATGGAATTTCACAGTATTCACTGAGA   2002
gb:MT106054|Organism:Severe      CGTGTTTTACAGAAGGCCGCTATAACAATACTAGATGGAATTTCACAGTATTCACTGAGA   2002
gb:MT049951|Organism:Severe      CGTGTTTTACAGAAGGCCGCTATAACAATACTAGATGGAATTTCACAGTATTCACTGAGA   2002
gb:MN975262|Organism:Severe      CGTGTTTTACAGAAGGCCGCTATAACAATACTAGATGGAATTTCACAGTATTCACTGAGA   2002
gb:MT106052|Organism:Severe      CGTGTTTTACAGAAGGCCGCTATAACAATACTAGATGGAATTTCACAGTATTCACTGAGA   2002
gb:LC522975|Organism:Severe      CGTGTTTTACAGAAGGCCGCTATAACAATACTAGATGGAATTTCACAGTATTCACTGAGA   1999
gb:LC522973|Organism:Severe      CGTGTTTTACAGAAGGCCGCTATAACAATACTAGATGGAATTTCACAGTATTCACTGAGA   1999
gb:LC522974|Organism:Severe      CGTGTTTTACAGAAGGCCGCTATAACAATACTAGATGGAATTTCACAGTATTCACTGAGA   1999
gb:MN985325|Organism:Severe      CGTGTTTTACAGAAGGCCGCTATAACAATACTAGATGGAATTTCACAGTATTCACTGAGA   2002
gb:MT020881|Organism:Severe      CGTGTTTTACAGAAGGCCGCTATAACAATACTAGATGGAATTTCACAGTATTCACTGAGA   2002
gb:MT020880|Organism:Severe      CGTGTTTTACAGAAGGCCGCTATAACAATACTAGATGGAATTTCACAGTATTCACTGAGA   2002
gb:MT066175|Organism:Severe      CGTGTTTTACAGAAGGCCGCTATAACAATACTAGATGGAATTTCACAGTATTCACTGAGA   2002
gb:MN997409|Organism:Severe      CGTGTTTTACAGAAGGCCGCTATAACAATACTAGATGGAATTTCACAGTATTCACTGAGA   2002
gb:MN938384|Organism:Severe      CGTGTTTTACAGAAGGCCGCTATAACAATACTAGATGGAATTTCACAGTATTCACTGAGA   1970
gb:MT044258|Organism:Severe      CGTGTTTTACAGAAGGCCGCTATAACAATACTAGATGGAATTTCACAGTATTCACTGAGA   1978
gb:MT039890|Organism:Severe      CGTGTTTTACAGAAGGCCGCTATAACAATACTAGATGGAATTTCACAGTATTCACTGAGA   2002
gb:MN988713|Organism:Severe      CGTGTTTTACAGAAGGCCGCTATAACAATACTAGATGGAATTTCACAGTATTCACTGAGA   2002
gb:LC521925|Organism:Severe      CGTGTTTTACAGAAGGCCGCTATAACAATACTAGATGGAATTTCACAGTATTCACTGAGA   1975
gb:MT093571|Organism:Severe      CGTGTTTTACAGAAGGCCGCTATAACAATACTAGATGGAATTTCACAGTATTCACTGAGA   2002
gb:MT039887|Organism:Severe      CGTGTTTTACAGAAGGCCGCTATAACAATACTAGATGGAATTTCACAGTATTCACTGAGA   2002
gb:MT019530|Organism:Severe      CGTGTTTTACAGAAGGCCGCTATAACAATACTAGATGGAATTTCACAGTATTCACTGAGA   2002
gb:MT039888|Organism:Severe      CGTGTTTTACAGAAGGCCGCTATAACAATACTAGATGGAATTTCACAGTATTCACTGAGA   2002
gb:LC522972|Organism:Severe      CGTGTTTTACAGAAGGCCGCTATAACAATACTAGATGGAATTTCACAGTATTCACTGAGA   1999
gb:MT027063|Organism:Severe      CGTGTTTTACAGAAGGCCGCTATAACAATACTAGATGGAATTTCACAGTATTCACTGAGA   2002
gb:MT027062|Organism:Severe      CGTGTTTTACAGAAGGCCGCTATAACAATACTAGATGGAATTTCACAGTATTCACTGAGA   2002
gb:MT019529|Organism:Severe      CGTGTTTTACAGAAGGCCGCTATAACAATACTAGATGGAATTTCACAGTATTCACTGAGA   2002
gb:MN996529|Organism:Severe      CGTGTTTTACAGAAGGCCGCTATAACAATACTAGATGGAATTTCACAGTATTCACTGAGA   1990
gb:MN996531|Organism:Severe      CGTGTTTTACAGAAGGCCGCTATAACAATACTAGATGGAATTTCACAGTATTCACTGAGA   1989
gb:MT066176|Organism:Severe      CGTGTTTTACAGAAGGCCGCTATAACAATACTAGATGGAATTTCACAGTATTCACTGAGA   2002
gb:MT027064|Organism:Severe      CGTGTTTTACAGAAGGCCGCTATAACAATACTAGATGGAATTTCACAGTATTCACTGAGA   2002
gb:MN994468|Organism:Severe      CGTGTTTTACAGAAGGCCGCTATAACAATACTAGATGGAATTTCACAGTATTCACTGAGA   2002
gb:MT072688|Organism:Severe      CGTGTTTTACAGAAGGCCGCTATAACAATACTAGATGGAATTTCACAGTATTCACTGAGA   1987
gb:MN996527|Organism:Severe      CGTGTTTTACAGAAGGCCGCTATAACAATACTAGATGGAATTTCACAGTATTCACTGAGA   1969
gb:MT093631|Organism:Severe      CGTGTTTTACAGAAGGCCGCTATAACAATACTAGATGGAATTTCACAGTATTCACTGAGA   2040
gb:MT106053|Organism:Severe      CGTGTTTTACAGAAGGCCGCTATAACAATACTAGATGGAATTTCACAGTATTCACTGAGA   2002
```

```
gb:MT019533|Organism:Severe      CGTGTTTTACAGAAGGCCGCTATAACAATACTAGATGGAATTTCACAGTATTCACTGAGA   2002
gb:MT019531|Organism:Severe      CGTGTTTTACAGAAGGCCGCTATAACAATACTAGATGGAATTTCACAGTATTCACTGAGA   2002
gb:MN996528|Organism:Severe      CGTGTTTTACAGAAGGCCGCTATAACAATACTAGATGGAATTTCACAGTATTCACTGAGA   2002
gb:MN996530|Organism:Severe      CGTGTTTTACAGAAGGCCGCTATAACAATACTAGATGGAATTTCACAGTATTCACTGAGA   1988
gb:MN908947|Organism:Severe      CGTGTTTTACAGAAGGCCGCTATAACAATACTAGATGGAATTTCACAGTATTCACTGAGA   2002
gb:MT019532|Organism:Severe      CGTGTTTTACAGAAGGCCGCTATAACAATACTAGATGGAATTTCACAGTATTCACTGAGA   2002
                                 ************************************************************

gb:MT020781|Organism:Severe      CTCATTGATGCTATGATGTTCACATCTGATTTGGCTACTAACAATCTAGTTGTAATGGCC   2050
gb:MT007544|Organism:Severe      CTCATTGATGCTATGATGTTCACATCTGATTTGGCTACTAACAATCTAGTTGTAATGGCC   2062
gb:MN994467|Organism:Severe      CTCATTGATGCTATGATGTTCACATCTGATTTGGCTACTAACAATCTAGTTGTAATGGCC   2062
gb:MT044257|Organism:Severe      CTCATTGATGCTATGATGTTCACATCTGATTTGGCTACTAACAATCTAGTTGTAATGGCC   2062
gb:MT106054|Organism:Severe      CTCATTGATGCTATGATGTTCACATCTGATTTGGCTACTAACAATCTAGTTGTAATGGCC   2062
gb:MT049951|Organism:Severe      CTCATTGATGCTATGATGTTCACATCTGATTTGGCTACTAACAATCTAGTTGTAATGGCC   2062
gb:MN975262|Organism:Severe      CTCATTGATGCTATGATGTTCACATCTGATTTGGCTACTAACAATCTAGTTGTAATGGCC   2062
gb:MT106052|Organism:Severe      CTCATTGATGCTATGATGTTCACATCTGATTTGGCTACTAACAATCTAGTTGTAATGGCC   2062
gb:LC522975|Organism:Severe      CTCATTGATGCTATGATGTTCACATCTGATTTGGCTACTAACAATCTAGTTGTAATGGCC   2059
gb:LC522973|Organism:Severe      CTCATTGATGCTATGATGTTCACATCTGATTTGGCTACTAACAATCTAGTTGTAATGGCC   2059
gb:LC522974|Organism:Severe      CTCATTGATGCTATGATGTTCACATCTGATTTGGCTACTAACAATCTAGTTGTAATGGCC   2059
gb:MN985325|Organism:Severe      CTCATTGATGCTATGATGTTCACATCTGATTTGGCTACTAACAATCTAGTTGTAATGGCC   2062
gb:MT020881|Organism:Severe      CTCATTGATGCTATGATGTTCACATCTGATTTGGCTACTAACAATCTAGTTGTAATGGCC   2062
gb:MT020880|Organism:Severe      CTCATTGATGCTATGATGTTCACATCTGATTTGGCTACTAACAATCTAGTTGTAATGGCC   2062
gb:MT066175|Organism:Severe      CTCATTGATGCTATGATGTTCACATCTGATTTGGCTACTAACAATCTAGTTGTAATGGCC   2062
gb:MN997409|Organism:Severe      CTCATTGATGCTATGATGTTCACATCTGATTTGGCTACTAACAATCTAGTTGTAATGGCC   2062
gb:MN938384|Organism:Severe      CTCATTGATGCTATGATGTTCACATCTGATTTGGCTACTAACAATCTAGTTGTAATGGCC   2030
gb:MT044258|Organism:Severe      CTCATTGATGCTATGATGTTCACATCTGATTTGGCTACTAACAATCTAGTTGTAATGGCC   2038
gb:MT039890|Organism:Severe      CTCATTGATGCTATGATGTTCACATCTGATTTGGCTACTAACAATCTAGTTGTAATGGCC   2062
gb:MN988713|Organism:Severe      CTCATTGATGCTATGATGTTCACATCTGATTTGGCTACTAACAATCTAGTTGTAATGGCC   2062
gb:LC521925|Organism:Severe      CTCATTGATGCTATGATGTTCACATCTGATTTGGCTACTAACAATCTAGTTGTAATGGCC   2035
gb:MT093571|Organism:Severe      CTCATTGATGCTATGATGTTCACATCTGATTTGGCTACTAACAATCTAGTTGTAATGGCC   2062
gb:MT039887|Organism:Severe      CTCATTGATGCTATGATGTTCACATCTGATTTGGCTACTAACAATCTAGTTGTAATGGCC   2062
gb:MT019530|Organism:Severe      CTCATTGATGCTATGATGTTCACATCTGATTTGGCTACTAACAATCTAGTTGTAATGGCC   2062
gb:MT039888|Organism:Severe      CTCATTGATGCTATGATGTTCACATCTGATTTGGCTACTAACAATCTAGTTGTAATGGCC   2062
gb:LC522972|Organism:Severe      CTCATTGATGCTATGATGTTCACATCTGATTTGGCTACTAACAATCTAGTTGTAATGGCC   2059
gb:MT027063|Organism:Severe      CTCATTGATGCTATGATGTTCACATCTGATTTGGCTACTAACAATCTAGTTGTAATGGCC   2062
gb:MT027062|Organism:Severe      CTCATTGATGCTATGATGTTCACATCTGATTTGGCTACTAACAATCTAGTTGTAATGGCC   2062
gb:MT019529|Organism:Severe      CTCATTGATGCTATGATGTTCACATCTGATTTGGCTACTAACAATCTAGTTGTAATGGCC   2062
gb:MN996529|Organism:Severe      CTCATTGATGCTATGATGTTCACATCTGATTTGGCTACTAACAATCTAGTTGTAATGGCC   2050
gb:MN996531|Organism:Severe      CTCATTGATGCTATGATGTTCACATCTGATTTGGCTACTAACAATCTAGTTGTAATGGCC   2049
gb:MT066176|Organism:Severe      CTCATTGATGCTATGATGTTCACATCTGATTTGGCTACTAACAATCTAGTTGTAATGGCC   2062
gb:MT027064|Organism:Severe      CTCATTGATGCTATGATGTTCACATCTGATTTGGCTACTAACAATCTAGTTGTAATGGCC   2062
gb:MN994468|Organism:Severe      CTCATTGATGCTATGATGTTCACATCTGATTTGGCTACTAACAATCTAGTTGTAATGGCC   2062
gb:MT072688|Organism:Severe      CTCATTGATGCTATGATGTTCACATCTGATTTGGCTACTAACAATCTAGTTGTAATGGCC   2047
gb:MN996527|Organism:Severe      CTCATTGATGCTATGATGTTCACATCTGATTTGGCTACTAACAATCTAGTTGTAATGGCC   2029
gb:MT093631|Organism:Severe      CTCATTGATGCTATGATGTTCACATCTGATTTGGCTACTAACAATCTAGTTGTAATGGCC   2100
gb:MT106053|Organism:Severe      CTCATTGATGCTATGATGTTCACATCTGATTTGGCTACTAACAATCTAGTTGTAATGGCC   2062
gb:MT019533|Organism:Severe      CTCATTGATGCTATGATGTTCACATCTGATTTGGCTACTAACAATCTAGTTGTAATGGCC   2062
gb:MT019531|Organism:Severe      CTCATTGATGCTATGATGTTCACATCTGATTTGGCTACTAACAATCTAGTTGTAATGGCC   2062
gb:MN996528|Organism:Severe      CTCATTGATGCTATGATGTTCACATCTGATTTGGCTACTAACAATCTAGTTGTAATGGCC   2062
gb:MN996530|Organism:Severe      CTCATTGATGCTATGATGTTCACATCTGATTTGGCTACTAACAATCTAGTTGTAATGGCC   2048
gb:MN908947|Organism:Severe      CTCATTGATGCTATGATGTTCACATCTGATTTGGCTACTAACAATCTAGTTGTAATGGCC   2062
gb:MT019532|Organism:Severe      CTCATTGATGCTATGATGTTCACATCTGATTTGGCTACTAACAATCTAGTTGTAATGGCC   2062
                                 ************************************************************

gb:MT020781|Organism:Severe      TACATTACAGGTGGTGTTGTTCAGTTGACTTCGCAGTGGCTAACTAACATCTTTGGCACT   2110
gb:MT007544|Organism:Severe      TACATTACAGGTGGTGTTGTTCAGTTGACTTCGCAGTGGCTAACTAACATCTTTGGCACT   2122
gb:MN994467|Organism:Severe      TACATTACAGGTGGTGTTGTTCAGTTGACTTCGCAGTGGCTAACTAACATCTTTGGCACT   2122
gb:MT044257|Organism:Severe      TACATTACAGGTGGTGTTGTTCAGTTGACTTCGCAGTGGCTAACTAACATCTTTGGCACT   2122
gb:MT106054|Organism:Severe      TACATTACAGGTGGTGTTGTTCAGTTGACTTCGCAGTGGCTAACTAACATCTTTGGCACT   2122
gb:MT049951|Organism:Severe      TACATTACAGGTGGTGTTGTTCAGTTGACTTCGCAGTGGCTAACTAACATCTTTGGCACT   2122
gb:MN975262|Organism:Severe      TACATTACAGGTGGTGTTGTTCAGTTGACTTCGCAGTGGCTAACTAACATCTTTGGCACT   2122
gb:MT106052|Organism:Severe      TACATTACAGGTGGTGTTGTTCAGTTGACTTCGCAGTGGCTAACTAACATCTTTGGCACT   2122
gb:LC522975|Organism:Severe      TACATTACAGGTGGTGTTGTTCAGTTGACTTCGCAGTGGCTAACTAACATCTTTGGCACT   2119
gb:LC522973|Organism:Severe      TACATTACAGGTGGTGTTGTTCAGTTGACTTCGCAGTGGCTAACTAACATCTTTGGCACT   2119
gb:LC522974|Organism:Severe      TACATTACAGGTGGTGTTGTTCAGTTGACTTCGCAGTGGCTAACTAACATCTTTGGCACT   2119
```

```
gb:MN985325|Organism:Severe      TACATTACAGGTGGTGTTGTTCAGTTGACTTCGCAGTGGCTAACTAACATCTTTGGCACT   2122
gb:MT020881|Organism:Severe      TACATTACAGGTGGTGTTGTTCAGTTGACTTCGCAGTGGCTAACTAACATCTTTGGCACT   2122
gb:MT020880|Organism:Severe      TACATTACAGGTGGTGTTGTTCAGTTGACTTCGCAGTGGCTAACTAACATCTTTGGCACT   2122
gb:MT066175|Organism:Severe      TACATTACAGGTGGTGTTGTTCAGTTGACTTCGCAGTGGCTAACTAACATCTTTGGCACT   2122
gb:MN997409|Organism:Severe      TACATTACAGGTGGTGTTGTTCAGTTGACTTCGCAGTGGCTAACTAACATCTTTGGCACT   2122
gb:MN938384|Organism:Severe      TACATTACAGGTGGTGTTGTTCAGTTGACTTCGCAGTGGCTAACTAACATCTTTGGCACT   2090
gb:MT044258|Organism:Severe      TACATTACAGGTGGTGTTGTTCAGTTGACTTCGCAGTGGCTAACTAACATCTTTGGCACT   2098
gb:MT039890|Organism:Severe      TACATTACAGGTGGTGTTGTTCAGTTGACTTCGCAGTGGCTAACTAACATCTTTGGCACT   2122
gb:MN988713|Organism:Severe      TACATTACAGGTGGTGTTGTTCAGTTGACTTCGCAGTGGCTAACTAACATCTTTGGCACT   2122
gb:LC521925|Organism:Severe      TACATTACAGGTGGTGTTGTTCAGTTGACTTCGCAGTGGCTAACTAACATCTTTGGCACT   2095
gb:MT093571|Organism:Severe      TACATTACAGGTGGTGTTGTTCAGTTGACTTCGCAGTGGCTAACTAACATCTTTGGCACT   2122
gb:MT039887|Organism:Severe      TACATTACAGGTGGTGTTGTTCAGTTGACTTCGCAGTGGCTAACTAACATCTTTGGCACT   2122
gb:MT019530|Organism:Severe      TACATTACAGGTGGTGTTGTTCAGTTGACTTCGCAGTGGCTAACTAACATCTTTGGCACT   2122
gb:MT039888|Organism:Severe      TACATTACAGGTGGTGTTGTTCAGTTGACTTCGCAGTGGCTAACTAACATCTTTGGCACT   2122
gb:LC522972|Organism:Severe      TACATTACAGGTGGTGTTGTTCAGTTGACTTCGCAGTGGCTAACTAACATCTTTGGCACT   2119
gb:MT027063|Organism:Severe      TACATTACAGGTGGTGTTGTTCAGTTGACTTCGCAGTGGCTAACTAACATCTTTGGCACT   2122
gb:MT027062|Organism:Severe      TACATTACAGGTGGTGTTGTTCAGTTGACTTCGCAGTGGCTAACTAACATCTTTGGCACT   2122
gb:MT019529|Organism:Severe      TACATTACAGGTGGTGTTGTTCAGTTGACTTCGCAGTGGCTAACTAACATCTTTGGCACT   2122
gb:MN996529|Organism:Severe      TACATTACAGGTGGTGTTGTTCAGTTGACTTCGCAGTGGCTAACTAACATCTTTGGCACT   2110
gb:MN996531|Organism:Severe      TACATTACAGGTGGTGTTGTTCAGTTGACTTCGCAGTGGCTAACTAACATCTTTGGCACT   2109
gb:MT066176|Organism:Severe      TACATTACAGGTGGTGTTGTTCAGTTGACTTCGCAGTGGCTAACTAACATCTTTGGCACT   2122
gb:MT027064|Organism:Severe      TACATTACAGGTGGTGTTGTTCAGTTGATTTCGCAGTGGCTAACTAACATCTTTGGCACT   2122
gb:MN994468|Organism:Severe      TACATTACAGGTGGTGTTGTTCAGTTGACTTCGCAGTGGCTAACTAACATCTTTGGCACT   2122
gb:MT072688|Organism:Severe      TACATTACAGGTGGTGTTGTTCAGTTGACTTCGCAGTGGCTAACTAACATCTTTGGCACT   2107
gb:MN996527|Organism:Severe      TACATTACAGGTGGTGTTGTTCAGTTGACTTCGCAGTGGCTAACTAACATCTTTGGCACT   2089
gb:MT093631|Organism:Severe      TACATTACAGGTGGTGTTGTTCAGTTGACTTCGCAGTGGCTAACTAACATCTTTGGCACT   2160
gb:MT106053|Organism:Severe      TACATTACAGGTGGTGTTGTTCAGTTGACTTCGCAGTGGCTAACTAACATCTTTGGCACT   2122
gb:MT019533|Organism:Severe      TACATTACAGGTGGTGTTGTTCAGTTGACTTCGCAGTGGCTAACTAACATCTTTGGCACT   2122
gb:MT019531|Organism:Severe      TACATTACAGGTGGTGTTGTTCAGTTGACTTCGCAGTGGCTAACTAACATCTTTGGCACT   2122
gb:MN996528|Organism:Severe      TACATTACAGGTGGTGTTGTTCAGTTGACTTCGCAGTGGCTAACTAACATCTTTGGCACT   2122
gb:MN996530|Organism:Severe      TACATTACAGGTGGTGTTGTTCAGTTGACTTCGCAGTGGCTAACTAACATCTTTGGCACT   2108
gb:MN908947|Organism:Severe      TACATTACAGGTGGTGTTGTTCAGTTGACTTCGCAGTGGCTAACTAACATCTTTGGCACT   2122
gb:MT019532|Organism:Severe      TACATTACAGGTGGTGTTGTTCAGTTGACTTCGCAGTGGCTAACTAACATCTTTGGCACT   2122
                                 **************************** *******************************

gb:MT020781|Organism:Severe      GTTTATGAAAAACTCAAACCCGTCCTTGATTGGCTTGAAGAGAAGTTTAAGGAAGGTGTA   2170
gb:MT007544|Organism:Severe      GTTTATGAAAAACTCAAACCCGTCCTTGATTGGCTTGAAGAGAAGTTTAAGGAAGGTGTA   2182
gb:MN994467|Organism:Severe      GTTTATGAAAAACTCAAACCCGTCCTTGATTGGCTTGAAGAGAAGTTTAAGGAAGGTGTA   2182
gb:MT044257|Organism:Severe      GTTTATGAAAAACTCAAACCCGTCCTTGATTGGCTTGAAGAGAAGTTTAAGGAAGGTGTA   2182
gb:MT106054|Organism:Severe      GTTTATGAAAAACTCAAACCCGTCCTTGATTGGCTTGAAGAGAAGTTTAAGGAAGGTGTA   2182
gb:MT049951|Organism:Severe      GTTTATGAAAAACTCAAACCCGTCCTTGATTGGCTTGAAGAGAAGTTTAAGGAAGGTGTA   2182
gb:MN975262|Organism:Severe      GTTTATGAAAAACTCAAACCCGTCCTTGATTGGCTTGAAGAGAAGTTTAAGGAAGGTGTA   2182
gb:MT106052|Organism:Severe      GTTTATGAAAAACTCAAACCCGTCCTTGATTGGCTTGAAGAGAAGTTTAAGGAAGGTGTA   2182
gb:LC522975|Organism:Severe      GTTTATGAAAAACTCAAACCCGTCCTTGATTGGCTTGAAGAGAAGTTTAAGGAAGGTGTA   2179
gb:LC522973|Organism:Severe      GTTTATGAAAAACTCAAACCCGTCCTTGATTGGCTTGAAGAGAAGTTTAAGGAAGGTGTA   2179
gb:LC522974|Organism:Severe      GTTTATGAAAAACTCAAACCCGTCCTTGATTGGCTTGAAGAGAAGTTTAAGGAAGGTGTA   2179
gb:MN985325|Organism:Severe      GTTTATGAAAAACTCAAACCCGTCCTTGATTGGCTTGAAGAGAAGTTTAAGGAAGGTGTA   2182
gb:MT020881|Organism:Severe      GTTTATGAAAAACTCAAACCCGTCCTTGATTGGCTTGAAGAGAAGTTTAAGGAAGGTGTA   2182
gb:MT020880|Organism:Severe      GTTTATGAAAAACTCAAACCCGTCCTTGATTGGCTTGAAGAGAAGTTTAAGGAAGGTGTA   2182
gb:MT066175|Organism:Severe      GTTTATGAAAAACTCAAACCCGTCCTTGATTGGCTTGAAGAGAAGTTTAAGGAAGGTGTA   2182
gb:MN997409|Organism:Severe      GTTTATGAAAAACTCAAACCCGTCCTTGATTGGCTTGAAGAGAAGTTTAAGGAAGGTGTA   2182
gb:MN938384|Organism:Severe      GTTTATGAAAAACTCAAACCCGTCCTTGATTGGCTTGAAGAGAAGTTTAAGGAAGGTGTA   2150
gb:MT044258|Organism:Severe      GTTTATGAAAAACTCAAACCCGTCCTTGATTGGCTTGAAGAGAAGTTTAAGGAAGGTGTA   2158
gb:MT039890|Organism:Severe      GTTTATGAAAAACTCAAACCCGTCCTTGATTGGCTTGAAGAGAAGTTTAAGGAAGGTGTA   2182
gb:MN988713|Organism:Severe      GTTTATGAAAAACTCAAACCCGTCCTTGATTGGCTTGAAGAGAAGTTTAAGGAAGGTGTA   2182
gb:LC521925|Organism:Severe      GTTTATGAAAAACTCAAACCCGTCCTTGATTGGCTTGAAGAGAAGTTTAAGGAAGGTGTA   2155
gb:MT093571|Organism:Severe      GTTTATGAAAAACTCAAACCCGTCCTTGATTGGCTTGAAGAGAAGTTTAAGGAAGGTGTA   2182
gb:MT039887|Organism:Severe      GTTTATGAAAAACTCAAACCCGTCCTTGATTGGCTTGAAGAGAAGTTTAAGGAAGGTGTA   2182
gb:MT019530|Organism:Severe      GTTTATGAAAAACTCAAACCCGTCCTTGATTGGCTTGAAGAGAAGTTTAAGGAAGGTGTA   2182
gb:MT039888|Organism:Severe      GTTTATGAAAAACTCAAACCCGTCCTTGATTGGCTTGAAGAGAAGTTTAAGGAAGGTGTA   2182
gb:LC522972|Organism:Severe      GTTTATGAAAAACTCAAACCCGTCCTTGATTGGCTTGAAGAGAAGTTTAAGGAAGGTGTA   2179
gb:MT027063|Organism:Severe      GTTTATGAAAAACTCAAACCCGTCCTTGATTGGCTTGAAGAGAAGTTTAAGGAAGGTGTA   2182
gb:MT027062|Organism:Severe      GTTTATGAAAAACTCAAACCCGTCCTTGATTGGCTTGAAGAGAAGTTTAAGGAAGGTGTA   2182
gb:MT019529|Organism:Severe      GTTTATGAAAAACTCAAACCCGTCCTTGATTGGCTTGAAGAGAAGTTTAAGGAAGGTGTA   2182
gb:MN996529|Organism:Severe      GTTTATGAAAAACTCAAACCCGTCCTTGATTGGCTTGAAGAGAAGTTTAAGGAAGGTGTA   2170
```

```
gb:MN996531|Organism:Severe      GTTTATGAAAAACTCAAACCCGTCCTTGATTGGCTTGAAGAGAAGTTTAAGGAAGGTGTA   2169
gb:MT066176|Organism:Severe      GTTTATGAAAAACTCAAACCCGTCCTTGATTGGCTTGAAGAGAAGTTTAAGGAAGGTGTA   2182
gb:MT027064|Organism:Severe      GTTTATGAAAAACTCAAACCCGTCCTTGATTGGCTTGAAGAGAAGTTTAAGGAAGGTGTA   2182
gb:MN994468|Organism:Severe      GTTTATGAAAAACTCAAACCCGTCCTTGATTGGCTTGAAGAGAAGTTTAAGGAAGGTGTA   2182
gb:MT072688|Organism:Severe      GTTTATGAAAAACTCAAACCCGTCCTTGATTGGCTTGAAGAGAAGTTTAAGGAAGGTGTA   2167
gb:MN996527|Organism:Severe      GTTTATGAAAAACTCAAACCCGTCCTTGATTGGCTTGAAGAGAAGTTTAAGGAAGGTGTA   2149
gb:MT093631|Organism:Severe      GTTTATGAAAAACTCAAACCCGTCCTTGATTGGCTTGAAGAGAAGTTTAAGGAAGGTGTA   2220
gb:MT106053|Organism:Severe      GTTTATGAAAAACTCAAACCCGTCCTTGATTGGCTTGAAGAGAAGTTTAAGGAAGGTGTA   2182
gb:MT019533|Organism:Severe      GTTTATGAAAAACTCAAACCCGTCCTTGATTGGCTTGAAGAGAAGTTTAAGGAAGGTGTA   2182
gb:MT019531|Organism:Severe      GTTTATGAAAAACTCAAACCCGTCCTTGATTGGCTTGAAGAGAAGTTTAAGGAAGGTGTA   2182
gb:MN996528|Organism:Severe      GTTTATGAAAAACTCAAACCCGTCCTTGATTGGCTTGAAGAGAAGTTTAAGGAAGGTGTA   2182
gb:MN996530|Organism:Severe      GTTTATGAAAAACTCAAACCCGTCCTTGATTGGCTTGAAGAGAAGTTTAAGGAAGGTGTA   2168
gb:MN908947|Organism:Severe      GTTTATGAAAAACTCAAACCCGTCCTTGATTGGCTTGAAGAGAAGTTTAAGGAAGGTGTA   2182
gb:MT019532|Organism:Severe      GTTTATGAAAAACTCAAACCCGTCCTTGATTGGCTTGAAGAGAAGTTTAAGGAAGGTGTA   2182
                                 ************************************************************

gb:MT020781|Organism:Severe      GAGTTTCTTAGAGACGGTTGGGAAATTGTTAAATTTATCTCAACCTGTGCTTGTGAAATT   2230
gb:MT007544|Organism:Severe      GAGTTTCTTAGAGACGGTTGGGAAATTGTTAAATTTATCTCAACCTGTGCTTGTGAAATT   2242
gb:MN994467|Organism:Severe      GAGTTTCTTAGAGACGGTTGGGAAATTGTTAAATTTATCTCAACCTGTGCTTGTGAAATT   2242
gb:MT044257|Organism:Severe      GAGTTTCTTAGAGACGGTTGGGAAATTGTTAAATTTATCTCAACCTGTGCTTGTGAAATT   2242
gb:MT106054|Organism:Severe      GAGTTTCTTAGAGACGGTTGGGAAATTGTTAAATTTATCTCAACCTGTGCTTGTGAAATT   2242
gb:MT049951|Organism:Severe      GAGTTTCTTAGAGACGGTTGGGAAATTGTTAAATTTATCTCAACCTGTGCTTGTGAAATT   2242
gb:MN975262|Organism:Severe      GAGTTTCTTAGAGACGGTTGGGAAATTGTTAAATTTATCTCAACCTGTGCTTGTGAAATT   2242
gb:MT106052|Organism:Severe      GAGTTTCTTAGAGACGGTTGGGAAATTGTTAAATTTATCTCAACCTGTGCTTGTGAAATT   2242
gb:LC522975|Organism:Severe      GAGTTTCTTAGAGACGGTTGGGAAATTGTTAAATTTATCTCAACCTGTGCTTGTGAAATT   2239
gb:LC522973|Organism:Severe      GAGTTTCTTAGAGACGGTTGGGAAATTGTTAAATTTATCTCAACCTGTGCTTGTGAAATT   2239
gb:LC522974|Organism:Severe      GAGTTTCTTAGAGACGGTTGGGAAATTGTTAAATTTATCTCAACCTGTGCTTGTGAAATT   2239
gb:MN985325|Organism:Severe      GAGTTTCTTAGAGACGGTTGGGAAATTGTTAAATTTATCTCAACCTGTGCTTGTGAAATT   2242
gb:MT020881|Organism:Severe      GAGTTTCTTAGAGACGGTTGGGAAATTGTTAAATTTATCTCAACCTGTGCTTGTGAAATT   2242
gb:MT020880|Organism:Severe      GAGTTTCTTAGAGACGGTTGGGAAATTGTTAAATTTATCTCAACCTGTGCTTGTGAAATT   2242
gb:MT066175|Organism:Severe      GAGTTTCTTAGAGACGGTTGGGAAATTGTTAAATTTATCTCAACCTGTGCTTGTGAAATT   2242
gb:MN997409|Organism:Severe      GAGTTTCTTAGAGACGGTTGGGAAATTGTTAAATTTATCTCAACCTGTGCTTGTGAAATT   2242
gb:MN938384|Organism:Severe      GAGTTTCTTAGAGACGGTTGGGAAATTGTTAAATTTATCTCAACCTGTGCTTGTGAAATT   2210
gb:MT044258|Organism:Severe      GAGTTTCTTAGAGACGGTTGGGAAATTGTTAAATTTATCTCAACCTGTGCTTGTGAAATT   2218
gb:MT039890|Organism:Severe      GAGTTTCTTAGAGACGGTTGGGAAATTGTTAAATTTATCTCAACCTGTGCTTGTGAAATT   2242
gb:MN988713|Organism:Severe      GAGTTTCTTAGAGACGGTTGGGAAATTGTTAAATTTATCTCAACCTGTGCTTGTGAAATT   2242
gb:LC521925|Organism:Severe      GAGTTTCTTAGAGACGGTTGGGAAATTGTTAAATTTATCTCAACCTGTGCTTGTGAAATT   2215
gb:MT093571|Organism:Severe      GAGTTTCTTAGAGACGGTTGGGAAATTGTTAAATTTATCTCAACCTGTGCTTGTGAAATT   2242
gb:MT039887|Organism:Severe      GAGTTTCTTAGAGACGGTTGGGAAATTGTTAAATTTATCTCAACCTGTGCTTGTGAAATT   2242
gb:MT019530|Organism:Severe      GAGTTTCTTAGAGACGGTTGGGAAATTGTTAAATTTATCTCAACCTGTGCTTGTGAAATT   2242
gb:MT039888|Organism:Severe      GAGTTTCTTAGAGACGGTTGGGAAATTGTTAAATTTATCTCAACCTGTGCTTGTGAAATT   2242
gb:LC522972|Organism:Severe      GAGTTTCTTAGAGACGGTTGGGAAATTGTTAAATTTATCTCAACCTGTGCTTGTGAAATT   2239
gb:MT027063|Organism:Severe      GAGTTTCTTAGAGACGGTTGGGAAATTGTTAAATTTATCTCAACCTGTGCTTGTGAAATT   2242
gb:MT027062|Organism:Severe      GAGTTTCTTAGAGACGGTTGGGAAATTGTTAAATTTATCTCAACCTGTGCTTGTGAAATT   2242
gb:MT019529|Organism:Severe      GAGTTTCTTAGAGACGGTTGGGAAATTGTTAAATTTATCTCAACCTGTGCTTGTGAAATT   2242
gb:MN996529|Organism:Severe      GAGTTTCTTAGAGACGGTTGGGAAATTGTTAAATTTATCTCAACCTGTGCTTGTGAAATT   2230
gb:MN996531|Organism:Severe      GAGTTTCTTAGAGACGGTTGGGAAATTGTTAAATTTATCTCAACCTGTGCTTGTGAAATT   2229
gb:MT066176|Organism:Severe      GAGTTTCTTAGAGACGGTTGGGAAATTGTTAAATTTATCTCAACCTGTGCTTGTGAAATT   2242
gb:MT027064|Organism:Severe      GAGTTTCTTAGAGACGGTTGGGAAATTGTTAAATTTATCTCAACCTGTGCTTGTGAAATT   2242
gb:MN994468|Organism:Severe      GAGTTTCTTAGAGACGGTTGGGAAATTGTTAAATTTATCTCAACCTGTGCTTGTGAAATT   2242
gb:MT072688|Organism:Severe      GAGTTTCTTAGAGACGGTTGGGAAATTGTTAAATTTATCTCAACCTGTGCTTGTGAAATT   2227
gb:MN996527|Organism:Severe      GAGTTTCTTAGAGACGGTTGGGAAATTGTTAAATTTATCTCAACCTGTGCTTGTGAAATT   2209
gb:MT093631|Organism:Severe      GAGTTTCTTAGAGACGGTTGGGAAATTGTTAAATTTATCTCAACCTGTGCTTGTGAAATT   2280
gb:MT106053|Organism:Severe      GAGTTTCTTAGAGACGGTTGGGAAATTGTTAAATTTATCTCAACCTGTGCTTGTGAAATT   2242
gb:MT019533|Organism:Severe      GAGTTTCTTAGAGACGGTTGGGAAATTGTTAAATTTATCTCAACCTGTGCTTGTGAAATT   2242
gb:MT019531|Organism:Severe      GAGTTTCTTAGAGACGGTTGGGAAATTGTTAAATTTATCTCAACCTGTGCTTGTGAAATT   2242
gb:MN996528|Organism:Severe      GAGTTTCTTAGAGACGGTTGGGAAATTGTTAAATTTATCTCAACCTGTGCTTGTGAAATT   2242
gb:MN996530|Organism:Severe      GAGTTTCTTAGAGACGGTTGGGAAATTGTTAAATTTATCTCAACCTGTGCTTGTGAAATT   2228
gb:MN908947|Organism:Severe      GAGTTTCTTAGAGACGGTTGGGAAATTGTTAAATTTATCTCAACCTGTGCTTGTGAAATT   2242
gb:MT019532|Organism:Severe      GAGTTTCTTAGAGACGGTTGGGAAATTGTTAAATTTATCTCAACCTGTGCTTGTGAAATT   2242
                                 ************************************************************

gb:MT020781|Organism:Severe      GTCGGTGGACAAATTGTCACCTGTGCAAAGGAAATTAAGGAGAGTGTTCAGACATTCTTT   2290
gb:MT007544|Organism:Severe      GTCGGTGGACAAATTGTCACCTGTGCAAAGGAAATTAAGGAGAGTGTTCAGACATTCTTT   2302
gb:MN994467|Organism:Severe      GTCGGTGGACAAATTGTCACCTGTGCAAAGGAAATTAAGGAGAGTGTTCAGACATTCTTT   2302
```

```
gb:MT044257|Organism:Severe      GTCGGTGGACAAATTGTCACCTGTGCAAAGGAAATTAAGGAGAGTGTTCAGACATTCTTT	2302
gb:MT106054|Organism:Severe      GTCGGTGGACAAATTGTCACCTGTGCAAAGGAAATTAAGGAGAGTGTTCAGACATTCTTT	2302
gb:MT049951|Organism:Severe      GTCGGTGGACAAATTGTCACCTGTGCAAAGGAAATTAAGGAGAGTGTTCAGACATTCTTT	2302
gb:MN975262|Organism:Severe      GTCGGTGGACAAATTGTCACCTGTGCAAAGGAAATTAAGGAGAGTGTTCAGACATTCTTT	2302
gb:MT106052|Organism:Severe      GTCGGTGGACAAATTGTCACCTGTGCAAAGGAAATTAAGGAGAGTGTTCAGACATTCTTT	2302
gb:LC522975|Organism:Severe      GTCGGTGGACAAATTGTCACCTGTGCAAAGGAAATTAAGGAGAGTGTTCAGACATTCTTT	2299
gb:LC522973|Organism:Severe      GTCGGTGGACAAATTGTCACCTGTGCAAAGGAAATTAAGGAGAGTGTTCAGACATTCTTT	2299
gb:LC522974|Organism:Severe      GTCGGTGGACAAATTGTCACCTGTGCAAAGGAAATTAAGGAGAGTGTTCAGACATTCTTT	2299
gb:MN985325|Organism:Severe      GTCGGTGGACAAATTGTCACCTGTGCAAAGGAAATTAAGGAGAGTGTTCAGACATTCTTT	2302
gb:MT020881|Organism:Severe      GTCGGTGGACAAATTGTCACCTGTGCAAAGGAAATTAAGGAGAGTGTTCAGACATTCTTT	2302
gb:MT020880|Organism:Severe      GTCGGTGGACAAATTGTCACCTGTGCAAAGGAAATTAAGGAGAGTGTTCAGACATTCTTT	2302
gb:MT066175|Organism:Severe      GTCGGTGGACAAATTGTCACCTGTGCAAAGGAAATTAAGGAGAGTGTTCAGACATTCTTT	2302
gb:MN997409|Organism:Severe      GTCGGTGGACAAATTGTCACCTGTGCAAAGGAAATTAAGGAGAGTGTTCAGACATTCTTT	2302
gb:MN938384|Organism:Severe      GTCGGTGGACAAATTGTCACCTGTGCAAAGGAAATTAAGGAGAGTGTTCAGACATTCTTT	2270
gb:MT044258|Organism:Severe      GTCGGTGGACAAATTGTCACCTGTGCAAAGGAAATTAAGGAGAGTGTTCAGACATTCTTT	2278
gb:MT039890|Organism:Severe      GTCGGTGGACAAATTGTCACCTGTGCAAAGGAAATTAAGGAGAGTGTTCAGACATTCTTT	2302
gb:MN988713|Organism:Severe      GTCGGTGGACAAATTGTCACCTGTGCAAAGGAAATTAAGGAGAGTGTTCAGACATTCTTT	2302
gb:LC521925|Organism:Severe      GTCGGTGGACAAATTGTCACCTGTGCAAAGGAAATTAAGGAGAGTGTTCAGACATTCTTT	2275
gb:MT093571|Organism:Severe      GTCGGTGGACAAATTGTCACCTGTGCAAAGGAAATTAAGGAGAGTGTTCAGACATTCTTT	2302
gb:MT039887|Organism:Severe      GTCGGTGGACAAATTGTCACCTGTGCAAAGGAAATTAAGGAGAGTGTTCAGACATTCTTT	2302
gb:MT019530|Organism:Severe      GTCGGTGGACAAATTGTCACCTGTGCAAAGGAAATTAAGGAGAGTGTTCAGACATTCTTT	2302
gb:MT039888|Organism:Severe      GTCGGTGGACAAATTGTCACCTGTGCAAAGGAAATTAAGGAGAGTGTTCAGACATTCTTT	2302
gb:LC522972|Organism:Severe      GTCGGTGGACAAATTGTCACCTGTGCAAAGGAAATTAAGGAGAGTGTTCAGACATTCTTT	2299
gb:MT027063|Organism:Severe      GTCGGTGGACAAATTGTCACCTGTGCAAAGGAAATTAAGGAGAGTGTTCAGACATTCTTT	2302
gb:MT027062|Organism:Severe      GTCGGTGGACAAATTGTCACCTGTGCAAAGGAAATTAAGGAGAGTGTTCAGACATTCTTT	2302
gb:MT019529|Organism:Severe      GTCGGTGGACAAATTGTCACCTGTGCAAAGGAAATTAAGGAGAGTGTTCAGACATTCTTT	2302
gb:MN996529|Organism:Severe      GTCGGTGGACAAATTGTCACCTGTGCAAAGGAAATTAAGGAGAGTGTTCAGACATTCTTT	2290
gb:MN996531|Organism:Severe      GTCGGTGGACAAATTGTCACCTGTGCAAAGGAAATTAAGGAGAGTGTTCAGACATTCTTT	2289
gb:MT066176|Organism:Severe      GTCGGTGGACAAATTGTCACCTGTGCAAAGGAAATTAAGGAGAGTGTTCAGACATTCTTT	2302
gb:MT027064|Organism:Severe      GTCGGTGGACAAATTGTCACCTGTGCAAAGGAAATTAAGGAGAGTGTTCAGACATTCTTT	2302
gb:MN994468|Organism:Severe      GTCGGTGGACAAATTGTCACCTGTGCAAAGGAAATTAAGGAGAGTGTTCAGACATTCTTT	2302
gb:MT072688|Organism:Severe      GTCGGTGGACAAATTGTCACCTGTGCAAAGGAAATTAAGGAGAGTGTTCAGACATTCTTT	2287
gb:MN996527|Organism:Severe      GTCGGTGGACAAATTGTCACCTGTGCAAAGGAAATTAAGGAGAGTGTTCAGACATTCTTT	2269
gb:MT093631|Organism:Severe      GTCGGTGGACAAATTGTCACCTGTGCAAAGGAAATTAAGGAGAGTGTTCAGACATTCTTT	2340
gb:MT106053|Organism:Severe      GTCGGTGGACAAATTGTCACCTGTGCAAAGGAAATTAAGGAGAGTGTTCAGACATTCTTT	2302
gb:MT019533|Organism:Severe      GTCGGTGGACAAATTGTCACCTGTGCAAAGGAAATTAAGGAGAGTGTTCAGACATTCTTT	2302
gb:MT019531|Organism:Severe      GTCGGTGGACAAATTGTCACCTGTGCAAAGGAAATTAAGGAGAGTGTTCAGACATTCTTT	2302
gb:MN996528|Organism:Severe      GTCGGTGGACAAATTGTCACCTGTGCAAAGGAAATTAAGGAGAGTGTTCAGACATTCTTT	2302
gb:MN996530|Organism:Severe      GTCGGTGGACAAATTGTCACCTGTGCAAAGGAAATTAAGGAGAGTGTTCAGACATTCTTT	2288
gb:MN908947|Organism:Severe      GTCGGTGGACAAATTGTCACCTGTGCAAAGGAAATTAAGGAGAGTGTTCAGACATTCTTT	2302
gb:MT019532|Organism:Severe      GTCGGTGGACAAATTGTCACCTGTGCAAAGGAAATTAAGGAGAGTGTTCAGACATTCTTT	2302
                                 ************************************************************

gb:MT020781|Organism:Severe      AAGCTTGTAAATAAATTTTTGGCTTTGTGTGCTGACTCTATCATTATTGGTGGAGCTAAA	2350
gb:MT007544|Organism:Severe      AAGCTTGTAAATAAATTTTTGGCTTTGTGTGCTGACTCTATCATTATTGGTGGAGCTAAA	2362
gb:MN994467|Organism:Severe      AAGCTTGTAAATAAATTTTTGGCTTTGTGTGCTGACTCTATCATTATTGGTGGAGCTAAA	2362
gb:MT044257|Organism:Severe      AAGCTTGTAAATAAATTTTTGGCTTTGTGTGCTGACTCTATCATTATTGGTGGAGCTAAA	2362
gb:MT106054|Organism:Severe      AAGCTTGTAAATAAATTTTTGGCTTTGTGTGCTGACTCTATCATTATTGGTGGAGCTAAA	2362
gb:MT049951|Organism:Severe      AAGCTTGTAAATAAATTTTTGGCTTTGTGTGCTGACTCTATCATTATTGGTGGAGCTAAA	2362
gb:MN975262|Organism:Severe      AAGCTTGTAAATAAATTTTTGGCTTTGTGTGCTGACTCTATCATTATTGGTGGAGCTAAA	2362
gb:MT106052|Organism:Severe      AAGCTTGTAAATAAATTTTTGGCTTTGTGTGCTGACTCTATCATTATTGGTGGAGCTAAA	2362
gb:LC522975|Organism:Severe      AAGCTTGTAAATAAATTTTTGGCTTTGTGTGCTGACTCTATCATTATTGGTGGAGCTAAA	2359
gb:LC522973|Organism:Severe      AAGCTTGTAAATAAATTTTTGGCTTTGTGTGCTGACTCTATCATTATTGGTGGAGCTAAA	2359
gb:LC522974|Organism:Severe      AAGCTTGTAAATAAATTTTTGGCTTTGTGTGCTGACTCTATCATTATTGGTGGAGCTAAA	2359
gb:MN985325|Organism:Severe      AAGCTTGTAAATAAATTTTTGGCTTTGTGTGCTGACTCTATCATTATTGGTGGAGCTAAA	2362
gb:MT020881|Organism:Severe      AAGCTTGTAAATAAATTTTTGGCTTTGTGTGCTGACTCTATCATTATTGGTGGAGCTAAA	2362
gb:MT020880|Organism:Severe      AAGCTTGTAAATAAATTTTTGGCTTTGTGTGCTGACTCTATCATTATTGGTGGAGCTAAA	2362
gb:MT066175|Organism:Severe      AAGCTTGTAAATAAATTTTTGGCTTTGTGTGCTGACTCTATCATTATTGGTGGAGCTAAA	2362
gb:MN997409|Organism:Severe      AAGCTTGTAAATAAATTTTTGGCTTTGTGTGCTGACTCTATCATTATTGGTGGAGCTAAA	2362
gb:MN938384|Organism:Severe      AAGCTTGTAAATAAATTTTTGGCTTTGTGTGCTGACTCTATCATTATTGGTGGAGCTAAA	2330
gb:MT044258|Organism:Severe      AAGCTTGTAAATAAATTTTTGGCTTTGTGTGCTGACTCTATCATTATTGGTGGAGCTAAA	2338
gb:MT039890|Organism:Severe      AAGCTTGTAAATAAATTTTTGGCTTTGTGTGCTGACTCTATCATTATTGGTGGAGCTAAA	2362
gb:MN988713|Organism:Severe      AAGCTTGTAAATAAATTTTTGGCTTTGTGTGCTGACTCTATCATTATTGGTGGAGCTAAA	2362
gb:LC521925|Organism:Severe      AAGCTTGTAAATAAATTTTTGGCTTTGTGTGCTGACTCTATCATTATTGGTGGAGCTAAA	2335
gb:MT093571|Organism:Severe      AAGCTTGTAAATAAATTTTTGGCTTTGTGTGCTGACTCTATCATTATTGGTGGAGCTAAA	2362
```

```
gb:MT039887|Organism:Severe      AAGCTTGTAAATAAATTTTTGGCTTTGTGTGCTGACTCTATCATTATTGGTGGAGCTAAA    2362
gb:MT019530|Organism:Severe      AAGCTTGTAAATAAATTTTTGGCTTTGTGTGCTGACTCTATCATTATTGGTGGAGCTAAA    2362
gb:MT039888|Organism:Severe      AAGCTTGTAAATAAATTTTTGGCTTTGTGTGCTGACTCTATCATTATTGGTGGAGCTAAA    2362
gb:LC522972|Organism:Severe      AAGCTTGTAAATAAATTTTTGGCTTTGTGTGCTGACTCTATCATTATTGGTGGAGCTAAA    2359
gb:MT027063|Organism:Severe      AAGCTTGTAAATAAATTTTTGGCTTTGTGTGCTGACTCTATCATTATTGGTGGAGCTAAA    2362
gb:MT027062|Organism:Severe      AAGCTTGTAAATAAATTTTTGGCTTTGTGTGCTGACTCTATCATTATTGGTGGAGCTAAA    2362
gb:MT019529|Organism:Severe      AAGCTTGTAAATAAATTTTTGGCTTTGTGTGCTGACTCTATCATTATTGGTGGAGCTAAA    2362
gb:MN996529|Organism:Severe      AAGCTTGTAAATAAATTTTTGGCTTTGTGTGCTGACTCTATCATTATTGGTGGAGCTAAA    2350
gb:MN996531|Organism:Severe      AAGCTTGTAAATAAATTTTTGGCTTTGTGTGCTGACTCTATCATTATTGGTGGAGCTAAA    2349
gb:MT066176|Organism:Severe      AAGCTTGTAAATAAATTTTTGGCTTTGTGTGCTGACTCTATCATTATTGGTGGAGCTAAA    2362
gb:MT027064|Organism:Severe      AAGCTTGTAAATAAATTTTTGGCTTTGTGTGCTGACTCTATCATTATTGGTGGAGCTAAA    2362
gb:MN994468|Organism:Severe      AAGCTTGTAAATAAATTTTTGGCTTTGTGTGCTGACTCTATCATTATTGGTGGAGCTAAA    2362
gb:MT072688|Organism:Severe      AAGCTTGTAAATAAATTTTTGGCTTTGTGTGCTGACTCTATCATTATTGGTGGAGCTAAA    2347
gb:MN996527|Organism:Severe      AAGCTTGTAAATAAATTTTTGGCTTTGTGTGCTGACTCTATCATTATTGGTGGAGCTAAA    2329
gb:MT093631|Organism:Severe      AAGCTTGTAAATAAATTTTTGGCTTTGTGTGCTGACTCTATCATTATTGGTGGAGCTAAA    2400
gb:MT106053|Organism:Severe      AAGCTTGTAAATAAATTTTTGGCTTTGTGTGCTGACTCTATCATTATTGGTGGAGCTAAA    2362
gb:MT019533|Organism:Severe      AAGCTTGTAAATAAATTTTTGGCTTTGTGTGCTGACTCTATCATTATTGGTGGAGCTAAA    2362
gb:MT019531|Organism:Severe      AAGCTTGTAAATAAATTTTTGGCTTTGTGTGCTGACTCTATCATTATTGGTGGAGCTAAA    2362
gb:MN996528|Organism:Severe      AAGCTTGTAAATAAATTTTTGGCTTTGTGTGCTGACTCTATCATTATTGGTGGAGCTAAA    2362
gb:MN996530|Organism:Severe      AAGCTTGTAAATAAATTTTTGGCTTTGTGTGCTGACTCTATCATTATTGGTGGAGCTAAA    2348
gb:MN908947|Organism:Severe      AAGCTTGTAAATAAATTTTTGGCTTTGTGTGCTGACTCTATCATTATTGGTGGAGCTAAA    2362
gb:MT019532|Organism:Severe      AAGCTTGTAAATAAATTTTTGGCTTTGTGTGCTGACTCTATCATTATTGGTGGAGCTAAA    2362
                                 ************************************************************

gb:MT020781|Organism:Severe      CTTAAAGCCTTGAATTTAGGTGAAACATTTGTCACGCACTCAAAGGGATTGTACAGAAAG    2410
gb:MT007544|Organism:Severe      CTTAAAGCCTTGAATTTAGGTGAAACATTTGTCACGCACTCAAAGGGATTGTACAGAAAG    2422
gb:MN994467|Organism:Severe      CTTAAAGCCTTGAATTTAGGTGAAACATTTGTCACGCACTCAAAGGGATTGTACAGAAAG    2422
gb:MT044257|Organism:Severe      CTTAAAGCCTTGAATTTAGGTGAAACATTTGTCACGCACTCAAAGGGATTGTACAGAAAG    2422
gb:MT106054|Organism:Severe      CTTAAAGCCTTGAATTTAGGTGAAACATTTGTCACGCACTCAAAGGGATTGTACAGAAAG    2422
gb:MT049951|Organism:Severe      CTTAAAGCCTTGAATTTAGGTGAAACATTTGTCACGCACTCAAAGGGATTGTACAGAAAG    2422
gb:MN975262|Organism:Severe      CTTAAAGCCTTGAATTTAGGTGAAACATTTGTCACGCACTCAAAGGGATTGTACAGAAAG    2422
gb:MT106052|Organism:Severe      CTTAAAGCCTTGAATTTAGGTGAAACATTTGTCACGCACTCAAAGGGATTGTACAGAAAG    2422
gb:LC522975|Organism:Severe      CTTAAAGCCTTGAATTTAGGTGAAACATTTGTCACGCACTCAAAGGGATTGTACAGAAAG    2419
gb:LC522973|Organism:Severe      CTTAAAGCCTTGAATTTAGGTGAAACATTTGTCACGCACTCAAAGGGATTGTACAGAAAG    2419
gb:LC522974|Organism:Severe      CTTAAAGCCTTGAATTTAGGTGAAACATTTGTCACGCACTCAAAGGGATTGTACAGAAAG    2419
gb:MN985325|Organism:Severe      CTTAAAGCCTTGAATTTAGGTGAAACATTTGTCACGCACTCAAAGGGATTGTACAGAAAG    2422
gb:MT020881|Organism:Severe      CTTAAAGCCTTGAATTTAGGTGAAACATTTGTCACGCACTCAAAGGGATTGTACAGAAAG    2422
gb:MT020880|Organism:Severe      CTTAAAGCCTTGAATTTAGGTGAAACATTTGTCACGCACTCAAAGGGATTGTACAGAAAG    2422
gb:MT066175|Organism:Severe      CTTAAAGCCTTGAATTTAGGTGAAACATTTGTCACGCACTCAAAGGGATTGTACAGAAAG    2422
gb:MN997409|Organism:Severe      CTTAAAGCCTTGAATTTAGGTGAAACATTTGTCACGCACTCAAAGGGATTGTACAGAAAG    2422
gb:MN938384|Organism:Severe      CTTAAAGCCTTGAATTTAGGTGAAACATTTGTCACGCACTCAAAGGGATTGTACAGAAAG    2390
gb:MT044258|Organism:Severe      CTTAAAGCCTTGAATTTAGGTGAAACATTTGTCACGCACTCAAAGGGATTGTACAGAAAG    2398
gb:MT039890|Organism:Severe      CTTAAAGCCTTGAATTTAGGTGAAACATTTGTCACGCACTCAAAGGGATTGTACAGAAAG    2422
gb:MN988713|Organism:Severe      CTTAAAGCCTTGAATTTAGGTGAAACATTTGTCACGCACTCAAAGGGATTGTACAGAAAG    2422
gb:LC521925|Organism:Severe      CTTAAAGCCTTGAATTTAGGTGAAACATTTGTCACGCACTCAAAGGGATTGTACAGAAAG    2395
gb:MT093571|Organism:Severe      CTTAAAGCCTTGAATTTAGGTGAAACATTTGTCACGCACTCAAAGGGATTGTACAGAAAG    2422
gb:MT039887|Organism:Severe      CTTAAAGCCTTGAATTTAGGTGAAACATTTGTCACGCACTCAAAGGGATTGTACAGAAAG    2422
gb:MT019530|Organism:Severe      CTTAAAGCCTTGAATTTAGGTGAAACATTTGTCACGCACTCAAAGGGATTGTACAGAAAG    2422
gb:MT039888|Organism:Severe      CTTAAAGCCTTGAATTTAGGTGAAACATTTGTCACGCACTCAAAGGGATTGTACAGAAAG    2422
gb:LC522972|Organism:Severe      CTTAAAGCCTTGAATTTAGGTGAAACATTTGTCACGCACTCAAAGGGATTGTACAGAAAG    2419
gb:MT027063|Organism:Severe      CTTAAAGCCTTGAATTTAGGTGAAACATTTGTCACGCACTCAAAGGGATTGTACAGAAAG    2422
gb:MT027062|Organism:Severe      CTTAAAGCCTTGAATTTAGGTGAAACATTTGTCACGCACTCAAAGGGATTGTACAGAAAG    2422
gb:MT019529|Organism:Severe      CTTAAAGCCTTGAATTTAGGTGAAACATTTGTCACGCACTCAAAGGGATTGTACAGAAAG    2422
gb:MN996529|Organism:Severe      CTTAAAGCCTTGAATTTAGGTGAAACATTTGTCACGCACTCAAAGGGATTGTACAGAAAG    2410
gb:MN996531|Organism:Severe      CTTAAAGCCTTGAATTTAGGTGAAACATTTGTCACGCACTCAAAGGGATTGTACAGAAAG    2409
gb:MT066176|Organism:Severe      CTTAAAGCCTTGAATTTAGGTGAAACATTTGTCACGCACTCAAAGGGATTGTACAGAAAG    2422
gb:MT027064|Organism:Severe      CTTAAAGCCTTGAATTTAGGTGAAACATTTGTCACGCACTCAAAGGGATTGTACAGAAAG    2422
gb:MN994468|Organism:Severe      CTTAAAGCCTTGAATTTAGGTGAAACATTTGTCACGCACTCAAAGGGATTGTACAGAAAG    2422
gb:MT072688|Organism:Severe      CTTAAAGCCTTGAATTTAGGTGAAACATTTGTCACGCACTCAAAGGGATTGTACAGAAAG    2407
gb:MN996527|Organism:Severe      CTTAAAGCCTTGAATTTAGGTGAAACATTTGTCACGCACTCAAAGGGATTGTACAGAAAG    2389
gb:MT093631|Organism:Severe      CTTAAAGCCTTGAATTTAGGTGAAACATTTGTCACGCACTCAAAGGGATTGTACAGAAAG    2460
gb:MT106053|Organism:Severe      CTTAAAGCCTTGAATTTAGGTGAAACATTTGTCACGCACTCAAAGGGATTGTACAGAAAG    2422
gb:MT019533|Organism:Severe      CTTAAAGCCTTGAATTTAGGTGAAACATTTGTCACGCACTCAAAGGGATTGTACAGAAAG    2422
gb:MT019531|Organism:Severe      CTTAAAGCCTTGAATTTAGGTGAAACATTTGTCACGCACTCAAAGGGATTGTACAGAAAG    2422
gb:MN996528|Organism:Severe      CTTAAAGCCTTGAATTTAGGTGAAACATTTGTCACGCACTCAAAGGGATTGTACAGAAAG    2422
```

```
gb:MN996530|Organism:Severe      CTTAAAGCCTTGAATTTAGGTGAAACATTTGTCACGCACTCAAAGGGATTGTACAGAAAG   2408
gb:MN908947|Organism:Severe      CTTAAAGCCTTGAATTTAGGTGAAACATTTGTCACGCACTCAAAGGGATTGTACAGAAAG   2422
gb:MT019532|Organism:Severe      CTTAAAGCCTTGAATTTAGGTGAAACATTTGTCACGCACTCAAAGGGATTGTACAGAAAG   2422
                                 ************************************************************

gb:MT020781|Organism:Severe      TGTGTTAAATCCAGAGAAGAAACTGGCCTACTCATGCCTCTAAAAGCCCCAAAAGAAATT   2470
gb:MT007544|Organism:Severe      TGTGTTAAATCCAGAGAAGAAACTGGCCTACTCATGCCTCTAAAAGCCCCAAAAGAAATT   2482
gb:MN994467|Organism:Severe      TGTGTTAAATCCAGAGAAGAAACTGGCCTACTCATGCCTCTAAAAGCCCCAAAAGAAATT   2482
gb:MT044257|Organism:Severe      TGTGTTAAATCCAGAGAAGAAACTGGCCTACTCATGCCTCTAAAAGCCCCAAAAGAAATT   2482
gb:MT106054|Organism:Severe      TGTGTTAAATCCAGAGAAGAAACTGGCCTACTCATGCCTCTAAAAGCCCCAAAAGAAATT   2482
gb:MT049951|Organism:Severe      TGTGTTAAATCCAGAGAAGAAACTGGCCTACTCATGCCTCTAAAAGCCCCAAAAGAAATT   2482
gb:MN975262|Organism:Severe      TGTGTTAAATCCAGAGAAGAAACTGGCCTACTCATGCCTCTAAAAGCCCCAAAAGAAATT   2482
gb:MT106052|Organism:Severe      TGTGTTAAATCCAGAGAAGAAACTGGCCTACTCATGCCTCTAAAAGCCCCAAAAGAAATT   2482
gb:LC522975|Organism:Severe      TGTGTTAAATCCAGAGAAGAAACTGGCCTACTCATGCCTCTAAAAGCCCCAAAAGAAATT   2479
gb:LC522973|Organism:Severe      TGTGTTAAATCCAGAGAAGAAACTGGCCTACTCATGCCTCTAAAAGCCCCAAAAGAAATT   2479
gb:LC522974|Organism:Severe      TGTGTTAAATCCAGAGAAGAAACTGGCCTACTCATGCCTCTAAAAGCCCCAAAAGAAATT   2479
gb:MN985325|Organism:Severe      TGTGTTAAATCCAGAGAAGAAACTGGCCTACTCATGCCTCTAAAAGCCCCAAAAGAAATT   2482
gb:MT020881|Organism:Severe      TGTGTTAAATCCAGAGAAGAAACTGGCCTACTCATGCCTCTAAAAGCCCCAAAAGAAATT   2482
gb:MT020880|Organism:Severe      TGTGTTAAATCCAGAGAAGAAACTGGCCTACTCATGCCTCTAAAAGCCCCAAAAGAAATT   2482
gb:MT066175|Organism:Severe      TGTGTTAAATCCAGAGAAGAAACTGGCCTACTCATGCCTCTAAAAGCCCCAAAAGAAATT   2482
gb:MN997409|Organism:Severe      TGTGTTAAATCCAGAGAAGAAACTGGCCTACTCATGCCTCTAAAAGCCCCAAAAGAAATT   2482
gb:MN938384|Organism:Severe      TGTGTTAAATCCAGAGAAGAAACTGGCCTACTCATGCCTCTAAAAGCCCCAAAAGAAATT   2450
gb:MT044258|Organism:Severe      TGTGTTAAATCCAGAGAAGAAACTGGCCTACTCATGCCTCTAAAAGCCCCAAAAGAAATT   2458
gb:MT039890|Organism:Severe      TGTGTTAAATCCAGAGAAGAAACTGGCCTACTCATGCCTCTAAAAGCCCCAAAAGAAATT   2482
gb:MN988713|Organism:Severe      TGTGTTAAATCCAGAGAAGAAACTGGCCTACTCATGCCTCTAAAAGCCCCAAAAGAAATT   2482
gb:LC521925|Organism:Severe      TGTGTTAAATCCAGAGAAGAAACTGGCCTACTCATGCCTCTAAAAGCCCCAAAAGAAATT   2455
gb:MT093571|Organism:Severe      TGTGTTAAATCCAGAGAAGAAACTGGCCTACTCATGCCTCTAAAAGCCCCAAAAGAAATT   2482
gb:MT039887|Organism:Severe      TGTGTTAAATCCAGAGAAGAAACTGGCCTACTCATGCCTCTAAAAGCCCCAAAAGAAATT   2482
gb:MT019530|Organism:Severe      TGTGTTAAATCCAGAGAAGAAACTGGCCTACTCATGCCTCTAAAAGCCCCAAAAGAAATT   2482
gb:MT039888|Organism:Severe      TGTGTTAAATCCAGAGAAGAAACTGGCCTACTCATGCCTCTAAAAGCCCCAAAAGAAATT   2482
gb:LC522972|Organism:Severe      TGTGTTAAATCCAGAGAAGAAACTGGCCTACTCATGCCTCTAAAAGCCCCAAAAGAAATT   2479
gb:MT027063|Organism:Severe      TGTGTTAAATCCAGAGAAGAAACTGGCCTACTCATGCCTCTAAAAGCCCCAAAAGAAATT   2482
gb:MT027062|Organism:Severe      TGTGTTAAATCCAGAGAAGAAACTGGCCTACTCATGCCTCTAAAAGCCCCAAAAGAAATT   2482
gb:MT019529|Organism:Severe      TGTGTTAAATCCAGAGAAGAAACTGGCCTACTCATGCCTCTAAAAGCCCCAAAAGAAATT   2482
gb:MN996529|Organism:Severe      TGTGTTAAATCCAGAGAAGAAACTGGCCTACTCATGCCTCTAAAAGCCCCAAAAGAAATT   2470
gb:MN996531|Organism:Severe      TGTGTTAAATCCAGAGAAGAAACTGGCCTACTCATGCCTCTAAAAGCCCCAAAAGAAATT   2469
gb:MT066176|Organism:Severe      TGTGTTAAATCCAGAGAAGAAACTGGCCTACTCATGCCTCTAAAAGCCCCAAAAGAAATT   2482
gb:MT027064|Organism:Severe      TGTGTTAAATCCAGAGAAGAAACTGGCCTACTCATGCCTCTAAAAGCCCCAAAAGAAATT   2482
gb:MN994468|Organism:Severe      TGTGTTAAATCCAGAGAAGAAACTGGCCTACTCATGCCTCTAAAAGCCCCAAAAGAAATT   2482
gb:MT072688|Organism:Severe      TGTGTTAAATCCAGAGAAGAAACTGGCCTACTCATGCCTCTAAAAGCCCCAAAAGAAATT   2467
gb:MN996527|Organism:Severe      TGTGTTAAATCCAGAGAAGAAACTGGCCTACTCATGCCTCTAAAAGCCCCAAAAGAAATT   2449
gb:MT093631|Organism:Severe      TGTGTTAAATCCAGAGAAGAAACTGGCCTACTCATGCCTCTAAAAGCCCCAAAAGAAATT   2520
gb:MT106053|Organism:Severe      TGTGTTAAATCCAGAGAAGAAACTGGCCTACTCATGCCTCTAAAAGCCCCAAAAGAAATT   2482
gb:MT019533|Organism:Severe      TGTGTTAAATCCAGAGAAGAAACTGGCCTACTCATGCCTCTAAAAGCCCCAAAAGAAATT   2482
gb:MT019531|Organism:Severe      TGTGTTAAATCCAGAGAAGAAACTGGCCTACTCATGCCTCTAAAAGCCCCAAAAGAAATT   2482
gb:MN996528|Organism:Severe      TGTGTTAAATCCAGAGAAGAAACTGGCCTACTCATGCCTCTAAAAGCCCCAAAAGAAATT   2482
gb:MN996530|Organism:Severe      TGTGTTAAATCCAGAGAAGAAACTGGCCTACTCATGCCTCTAAAAGCCCCAAAAGAAATT   2468
gb:MN908947|Organism:Severe      TGTGTTAAATCCAGAGAAGAAACTGGCCTACTCATGCCTCTAAAAGCCCCAAAAGAAATT   2482
gb:MT019532|Organism:Severe      TGTGTTAAATCCAGAGAAGAAACTGGCCTACTCATGCCTCTAAAAGCCCCAAAAGAAATT   2482
                                 ************************************************************

gb:MT020781|Organism:Severe      ATCTTCTTAGAGGGAGAAACACTTCCCACAGAAGTGTTAACAGAGGAAGTTGTCTTGAAA   2530
gb:MT007544|Organism:Severe      ATCTTCTTAGAGGGAGAAACACTTCCCACAGAAGTGTTAACAGAGGAAGTTGTCTTGAAA   2542
gb:MN994467|Organism:Severe      ATCTTCTTAGAGGGAGAAACACTTCCCACAGAAGTGTTAACAGAGGAAGTTGTCTTGAAA   2542
gb:MT044257|Organism:Severe      ATCTTCTTAGAGGGAGAAACACTTCCCACAGAAGTGTTAACAGAGGAAGTTGTCTTGAAA   2542
gb:MT106054|Organism:Severe      ATCTTCTTAGAGGGAGAAACACTTCCCACAGAAGTGTTAACAGAGGAAGTTGTCTTGAAA   2542
gb:MT049951|Organism:Severe      ATCTTCTTAGAGGGAGAAACACTTCCCACAGAAGTGTTAACAGAGGAAGTTGTCTTGAAA   2542
gb:MN975262|Organism:Severe      ATCTTCTTAGAGGGAGAAACACTTCCCACAGAAGTGTTAACAGAGGAAGTTGTCTTGAAA   2542
gb:MT106052|Organism:Severe      ATCTTCTTAGAGGGAGAAACACTTCCCACAGAAGTGTTAACAGAGGAAGTTGTCTTGAAA   2542
gb:LC522975|Organism:Severe      ATCTTCTTAGAGGGAGAAACACTTCCCACAGAAGTGTTAACAGAGGAAGTTGTCTTGAAA   2539
gb:LC522973|Organism:Severe      ATCTTCTTAGAGGGAGAAACACTTCCCACAGAAGTGTTAACAGAGGAAGTTGTCTTGAAA   2539
gb:LC522974|Organism:Severe      ATCTTCTTAGAGGGAGAAACACTTCCCACAGAAGTGTTAACAGAGGAAGTTGTCTTGAAA   2539
gb:MN985325|Organism:Severe      ATCTTCTTAGAGGGAGAAACACTTCCCACAGAAGTGTTAACAGAGGAAGTTGTCTTGAAA   2542
gb:MT020881|Organism:Severe      ATCTTCTTAGAGGGAGAAACACTTCCCACAGAAGTGTTAACAGAGGAAGTTGTCTTGAAA   2542
gb:MT020880|Organism:Severe      ATCTTCTTAGAGGGAGAAACACTTCCCACAGAAGTGTTAACAGAGGAAGTTGTCTTGAAA   2542
```

```
gb:MT066175|Organism:Severe      ATCTTCTTAGAGGGAGAAACACTTCCCACAGAAGTGTTAACAGAGGAAGTTGTCTTGAAA   2542
gb:MN997409|Organism:Severe      ATCTTCTTAGAGGGAGAAACACTTCCCACAGAAGTGTTAACAGAGGAAGTTGTCTTGAAA   2542
gb:MN938384|Organism:Severe      ATCTTCTTAGAGGGAGAAACACTTCCCACAGAAGTGTTAACAGAGGAAGTTGTCTTGAAA   2510
gb:MT044258|Organism:Severe      ATCTTCTTAGAGGGAGAAACACTTCCCACAGAAGTGTTAACAGAGGAAGTTGTCTTGAAA   2518
gb:MT039890|Organism:Severe      ATCTTCTTAGAGGGAGAAACACTTCCCACAGAAGTGTTAACAGAGGAAGTTGTCTTGAAA   2542
gb:MN988713|Organism:Severe      ATCTTCTTAGAGGGAGAAACACTTCCCACAGAAGTGTTAACAGAGGAAGTTGTCTTGAAA   2542
gb:LC521925|Organism:Severe      ATCTTCTTAGAGGGAGAAACACTTCCCACAGAAGTGTTAACAGAGGAAGTTGTCTTGAAA   2515
gb:MT093571|Organism:Severe      ATCTTCTTAGAGGGAGAAACACTTCCCACAGAAGTGTTAACAGAGGAAGTTGTCTTGAAA   2542
gb:MT039887|Organism:Severe      ATCTTCTTAGAGGGAGAAACACTTCCCACAGAAGTGTTAACAGAGGAAGTTGTCTTGAAA   2542
gb:MT019530|Organism:Severe      ATCTTCTTAGAGGGAGAAACACTTCCCACAGAAGTGTTAACAGAGGAAGTTGTCTTGAAA   2542
gb:MT039888|Organism:Severe      ATCTTCTTAGAGGGAGAAACACTTCCCACAGAAGTGTTAACAGAGGAAGTTGTCTTGAAA   2542
gb:LC522972|Organism:Severe      ATCTTCTTAGAGGGAGAAACACTTCCCACAGAAGTGTTAACAGAGGAAGTTGTCTTGAAA   2539
gb:MT027063|Organism:Severe      ATCTTCTTAGAGGGAGAAACACTTCCCACAGAAGTGTTAACAGAGGAAGTTGTCTTGAAA   2542
gb:MT027062|Organism:Severe      ATCTTCTTAGAGGGAGAAACACTTCCCACAGAAGTGTTAACAGAGGAAGTTGTCTTGAAA   2542
gb:MT019529|Organism:Severe      ATCTTCTTAGAGGGAGAAACACTTCCCACAGAAGTGTTAACAGAGGAAGTTGTCTTGAAA   2542
gb:MN996529|Organism:Severe      ATCTTCTTAGAGGGAGAAACACTTCCCACAGAAGTGTTAACAGAGGAAGTTGTCTTGAAA   2530
gb:MN996531|Organism:Severe      ATCTTCTTAGAGGGAGAAACACTTCCCACAGAAGTGTTAACAGAGGAAGTTGTCTTGAAA   2529
gb:MT066176|Organism:Severe      ATCTTCTTAGAGGGAGAAACACTTCCCACAGAAGTGTTAACAGAGGAAGTTGTCTTGAAA   2542
gb:MT027064|Organism:Severe      ATCTTCTTAGAGGGAGAAACACTTCCCACAGAAGTGTTAACAGAGGAAGTTGTCTTGAAA   2542
gb:MN994468|Organism:Severe      ATCTTCTTAGAGGGAGAAACACTTCCCACAGAAGTGTTAACAGAGGAAGTTGTCTTGAAA   2542
gb:MT072688|Organism:Severe      ATCTTCTTAGAGGGAGAAACACTTCCCACAGAAGTGTTAACAGAGGAAGTTGTCTTGAAA   2527
gb:MN996527|Organism:Severe      ATCTTCTTAGAGGGAGAAACACTTCCCACAGAAGTGTTAACAGAGGAAGTTGTCTTGAAA   2509
gb:MT093631|Organism:Severe      ATCTTCTTAGAGGGAGAAACACTTCCCACAGAAGTGTTAACAGAGGAAGTTGTCTTGAAA   2580
gb:MT106053|Organism:Severe      ATCTTCTTAGAGGGAGAAACACTTCCCACAGAAGTGTTAACAGAGGAAGTTGTCTTGAAA   2542
gb:MT019533|Organism:Severe      ATCTTCTTAGAGGGAGAAACACTTCCCACAGAAGTGTTAACAGAGGAAGTTGTCTTGAAA   2542
gb:MT019531|Organism:Severe      ATCTTCTTAGAGGGAGAAACACTTCCCACAGAAGTGTTAACAGAGGAAGTTGTCTTGAAA   2542
gb:MN996528|Organism:Severe      ATCTTCTTAGAGGGAGAAACACTTCCCACAGAAGTGTTAACAGAGGAAGTTGTCTTGAAA   2542
gb:MN996530|Organism:Severe      ATCTTCTTAGAGGGAGAAACACTTCCCACAGAAGTGTTAACAGAGGAAGTTGTCTTGAAA   2528
gb:MN908947|Organism:Severe      ATCTTCTTAGAGGGAGAAACACTTCCCACAGAAGTGTTAACAGAGGAAGTTGTCTTGAAA   2542
gb:MT019532|Organism:Severe      ATCTTCTTAGAGGGAGAAACACTTCCCACAGAAGTGTTAACAGAGGAAGTTGTCTTGAAA   2542
                                 ************************************************************

gb:MT020781|Organism:Severe      ACTGGTGATTTACAACCATTAGAACAACCTACTAGTGAAGCTGTTGAAGCTCCATTGGTT   2590
gb:MT007544|Organism:Severe      ACTGGTGATTTACAACCATTAGAACAACCTACTAGTGAAGCTGTTGAAGCTCCATTGGTT   2602
gb:MN994467|Organism:Severe      ACTGGTGATTTACAACCATTAGAACAACCTACTAGTGAAGCTGTTGAAGCTCCATTGGTT   2602
gb:MT044257|Organism:Severe      ACTGGTGATTTACAACCATTAGAACAACCTACTAGTGAAGCTGTTGAAGCTCCATTGGTT   2602
gb:MT106054|Organism:Severe      ACTGGTGATTTACAACCATTAGAACAACCTACTAGTGAAGCTGTTGAAGCTCCATTGGTT   2602
gb:MT049951|Organism:Severe      ACTGGTGATTTACAACCATTAGAACAACCTACTAGTGAAGCTGTTGAAGCTCCATTGGTT   2602
gb:MN975262|Organism:Severe      ACTGGTGATTTACAACCATTAGAACAACCTACTAGTGAAGCTGTTGAAGCTCCATTGGTT   2602
gb:MT106052|Organism:Severe      ACTGGTGATTTACAACCATTAGAACAACCTACTAGTGAAGCTGTTGAAGCTCCATTGGTT   2602
gb:LC522975|Organism:Severe      ACTGGTGATTTACAACCATTAGAACAACCTACTAGTGAAGCTGTTGAAGCTCCATTGGTT   2599
gb:LC522973|Organism:Severe      ACTGGTGATTTACAACCATTAGAACAACCTACTAGTGAAGCTGTTGAAGCTCCATTGGTT   2599
gb:LC522974|Organism:Severe      ACTGGTGATTTACAACCATTAGAACAACCTACTAGTGAAGCTGTTGAAGCTCCATTGGTT   2599
gb:MN985325|Organism:Severe      ACTGGTGATTTACAACCATTAGAACAACCTACTAGTGAAGCTGTTGAAGCTCCATTGGTT   2602
gb:MT020881|Organism:Severe      ACTGGTGATTTACAACCATTAGAACAACCTACTAGTGAAGCTGTTGAAGCTCCATTGGTT   2602
gb:MT020880|Organism:Severe      ACTGGTGATTTACAACCATTAGAACAACCTACTAGTGAAGCTGTTGAAGCTCCATTGGTT   2602
gb:MT066175|Organism:Severe      ACTGGTGATTTACAACCATTAGAACAACCTACTAGTGAAGCTGTTGAAGCTCCATTGGTT   2602
gb:MN997409|Organism:Severe      ACTGGTGATTTACAACCATTAGAACAACCTACTAGTGAAGCTGTTGAAGCTCCATTGGTT   2602
gb:MN938384|Organism:Severe      ACTGGTGATTTACAACCATTAGAACAACCTACTAGTGAAGCTGTTGAAGCTCCATTGGTT   2570
gb:MT044258|Organism:Severe      ACTGGTGATTTACAACCATTAGAACAACCTACTAGTGAAGCTGTTGAAGCTCCATTGGTT   2578
gb:MT039890|Organism:Severe      ACTGGTGATTTACAACCATTAGAACAACCTACTAGTGAAGCTGTTGAAGCTCCATTGGTT   2602
gb:MN988713|Organism:Severe      ACTGGTGATTTACAACCATTAGAACAACCTACTAGTGAAGCTGTTGAAGCTCCATTGGTT   2602
gb:LC521925|Organism:Severe      ACTGGTGATTTACAACCATTAGAACAACCTACTAGTGAAGCTGTTGAAGCTCCATTGGTT   2575
gb:MT093571|Organism:Severe      ACTGGTGATTTACAACCATTAGAACAACCTACTAGTGAAGCTGTTGAAGCTCCATTGGTT   2602
gb:MT039887|Organism:Severe      ACTGGTGATTTACAACCATTAGAACAACCTACTAGTGAAGCTGTTGAAGCTCCATTGGTT   2602
gb:MT019530|Organism:Severe      ACTGGTGATTTACAACCATTAGAACAACCTACTAGTGAAGCTGTTGAAGCTCCATTGGTT   2602
gb:MT039888|Organism:Severe      ACTGGTGATTTACAACCATTAGAACAACCTACTAGTGAAGCTGTTGAAGCTCCATTGGTT   2602
gb:LC522972|Organism:Severe      ACTGGTGATTTACAACCATTAGAACAACCTACTAGTGAAGCTGTTGAAGCTCCATTGGTT   2599
gb:MT027063|Organism:Severe      ACTGGTGATTTACAACCATTAGAACAACCTACTAGTGAAGCTGTTGAAGCTCCATTGGTT   2602
gb:MT027062|Organism:Severe      ACTGGTGATTTACAACCATTAGAACAACCTACTAGTGAAGCTGTTGAAGCTCCATTGGTT   2602
gb:MT019529|Organism:Severe      ACTGGTGATTTACAACCATTAGAACAACCTACTAGTGAAGCTGTTGAAGCTCCATTGGTT   2602
gb:MN996529|Organism:Severe      ACTGGTGATTTACAACCATTAGAACAACCTACTAGTGAAGCTGTTGAAGCTCCATTGGTT   2590
gb:MN996531|Organism:Severe      ACTGGTGATTTACAACCATTAGAACAACCTACTAGTGAAGCTGTTGAAGCTCCATTGGTT   2589
gb:MT066176|Organism:Severe      ACTGGTGATTTACAACCATTAGAACAACCTACTAGTGAAGCTGTTGAAGCTCCATTGGTT   2602
gb:MT027064|Organism:Severe      ACTGGTGATTTACAACCATTAGAACAACCTACTAGTGAAGCTGTTGAAGCTCCATTGGTT   2602
```

```
gb:MN994468|Organism:Severe      ACTGGTGATTTACAACCATTAGAACAACCTACTAGTGAAGCTGTTGAAGCTCCATTGGTT    2602
gb:MT072688|Organism:Severe      ACTGGTGATTTACAACCATTAGAACAACCTACTAGTGAAGCTGTTGAAGCTCCATTGGTT    2587
gb:MN996527|Organism:Severe      ACTGGTGATTTACAACCATTAGAACAACCTACTAGTGAAGCTGTTGAAGCTCCATTGGTT    2569
gb:MT093631|Organism:Severe      ACTGGTGATTTACAACCATTAGAACAACCTACTAGTGAAGCTGTTGAAGCTCCATTGGTT    2640
gb:MT106053|Organism:Severe      ACTGGTGATTTACAACCATTAGAACAACCTACTAGTGAAGCTGTTGAAGCTCCATTGGTT    2602
gb:MT019533|Organism:Severe      ACTGGTGATTTACAACCATTAGAACAACCTACTAGTGAAGCTGTTGAAGCTCCATTGGTT    2602
gb:MT019531|Organism:Severe      ACTGGTGATTTACAACCATTAGAACAACCTACTAGTGAAGCTGTTGAAGCTCCATTGGTT    2602
gb:MN996528|Organism:Severe      ACTGGTGATTTACAACCATTAGAACAACCTACTAGTGAAGCTGTTGAAGCTCCATTGGTT    2602
gb:MN996530|Organism:Severe      ACTGGTGATTTACAACCATTAGAACAACCTACTAGTGAAGCTGTTGAAGCTCCATTGGTT    2588
gb:MN908947|Organism:Severe      ACTGGTGATTTACAACCATTAGAACAACCTACTAGTGAAGCTGTTGAAGCTCCATTGGTT    2602
gb:MT019532|Organism:Severe      ACTGGTGATTTACAACCATTAGAACAACCTACTAGTGAAGCTGTTGAAGCTCCATTGGTT    2602
                                 ************************************************************

gb:MT020781|Organism:Severe      GGTACACCAGTTTGTATTAACGGGCTTATGTTGCTCGAAATCAAAGACACAGAAAAGTAC    2650
gb:MT007544|Organism:Severe      GGTACACCAGTTTGTATTAACGGGCTTATGTTGCTCGAAATCAAAGACACAGAAAAGTAC    2662
gb:MN994467|Organism:Severe      GGTACACCAGTTTGTATTAACGGGCTTATGTTGCTCGAAATCAAAGACACAGAAAAGTAC    2662
gb:MT044257|Organism:Severe      GGTACACCAGTTTGTATTAACGGGCTTATGTTGCTCGAAATCAAAGACACAGAAAAGTAC    2662
gb:MT106054|Organism:Severe      GGTACACCAGTTTGTATTAACGGGCTTATGTTGCTCGAAATCAAAGACACAGAAAAGTAC    2662
gb:MT049951|Organism:Severe      GGTACACCAGTTTGTATTAACGGGCTTATGTTGCTCGAAATCAAAGACACAGAAAAGTAC    2662
gb:MN975262|Organism:Severe      GGTACACCAGTTTGTATTAACGGGCTTATGTTGCTCGAAATCAAAGACACAGAAAAGTAC    2662
gb:MT106052|Organism:Severe      GGTACACCAGTTTGTATTAACGGGCTTATGTTGCTCGAAATCAAAGACACAGAAAAGTAC    2662
gb:LC522975|Organism:Severe      GGTACACCAGTTTGTATTAACGGGCTTATGTTGCTCGAAATCAAAGACACAGAAAAGTAT    2659
gb:LC522973|Organism:Severe      GGTACACCAGTTTGTATTAACGGGCTTATGTTGCTCGAAATCAAAGACACAGAAAAGTAT    2659
gb:LC522974|Organism:Severe      GGTACACCAGTTTGTATTAACGGGCTTATGTTGCTCGAAATCAAAGACACAGAAAAGTAT    2659
gb:MN985325|Organism:Severe      GGTACACCAGTTTGTATTAACGGGCTTATGTTGCTCGAAATCAAAGACACAGAAAAGTAC    2662
gb:MT020881|Organism:Severe      GGTACACCAGTTTGTATTAACGGGCTTATGTTGCTCGAAATCAAAGACACAGAAAAGTAC    2662
gb:MT020880|Organism:Severe      GGTACACCAGTTTGTATTAACGGGCTTATGTTGCTCGAAATCAAAGACACAGAAAAGTAC    2662
gb:MT066175|Organism:Severe      GGTACACCAGTTTGTATTAACGGGCTTATGTTGCTCGAAATCAAAGACACAGAAAAGTAC    2662
gb:MN997409|Organism:Severe      GGTACACCAGTTTGTATTAACGGGCTTATGTTGCTCGAAATCAAAGACACAGAAAAGTAC    2662
gb:MN938384|Organism:Severe      GGTACACCAGTTTGTATTAACGGGCTTATGTTGCTCGAAATCAAAGACACAGAAAAGTAC    2630
gb:MT044258|Organism:Severe      GGTACACCAGTTTGTATTAACGGGCTTATGTTGCTCGAAATCAAAGACACAGAAAAGTAC    2638
gb:MT039890|Organism:Severe      GGTACACCAGTTTGTATTAACGGGCTTATGTTGCTCGAAATCAAAGACACAGAAAAGTAC    2662
gb:MN988713|Organism:Severe      GGTACACCAGTTTGTATTAACGGGCTTATGTTGCTCGAAATCAAAGACACAGAAAAGTAC    2662
gb:LC521925|Organism:Severe      GGTACACCAGTTTGTATTAACGGGCTTATGTTGCTCGAAATCAAAGACACAGAAAAGTAC    2635
gb:MT093571|Organism:Severe      GGTACACCAGTTTGTATTAACGGGCTTATGTTGCTCGAAATCAAAGACACAGAAAAGTAC    2662
gb:MT039887|Organism:Severe      GGTACACCAGTTTGTATTAACGGGCTTATGTTGCTCGAAATCAAAGACACAGAAAAGTAC    2662
gb:MT019530|Organism:Severe      GGTACACCAGTTTGTATTAACGGGCTTATGTTGCTCGAAATCAAAGACACAGAAAAGTAC    2662
gb:MT039888|Organism:Severe      GGTACACCAGTTTGTATTAACGGGCTTATGTTGCTCGAAATCAAAGACACAGAAAAGTAC    2662
gb:LC522972|Organism:Severe      GGTACACCAGTTTGTATTAACGGGCTTATGTTGCTCGAAATCAAAGACACAGAAAAGTAC    2659
gb:MT027063|Organism:Severe      GGTACACCAGTTTGTATTAACGGGCTTATGTTGCTCGAAATCAAAGACACAGAAAAGTAC    2662
gb:MT027062|Organism:Severe      GGTACACCAGTTTGTATTAACGGGCTTATGTTGCTCGAAATCAAAGACACAGAAAAGTAC    2662
gb:MT019529|Organism:Severe      GGTACACCAGTTTGTATTAACGGGCTTATGTTGCTCGAAATCAAAGACACAGAAAAGTAC    2662
gb:MN996529|Organism:Severe      GGTACACCAGTTTGTATTAACGGGCTTATGTTGCTCGAAATCAAAGACACAGAAAAGTAC    2650
gb:MN996531|Organism:Severe      GGTACACCAGTTTGTATTAACGGGCTTATGTTGCTCGAAATCAAAGACACAGAAAAGTAC    2649
gb:MT066176|Organism:Severe      GGTACACCAGTTTGTATTAACGGGCTTATGTTGCTCGAAATCAAAGACACAGAAAAGTAC    2662
gb:MT027064|Organism:Severe      GGTACACCAGTTTGTATTAACGGGCTTATGTTGCTCGAAATCAAAGACACAGAAAAGTAC    2662
gb:MN994468|Organism:Severe      GGTACACCAGTTTGTATTAACGGGCTTATGTTGCTCGAAATCAAAGACACAGAAAAGTAC    2662
gb:MT072688|Organism:Severe      GGTACACCAGTTTGTATTAACGGGCTTATGTTGCTCGAAATCAAAGACACAGAAAAGTAC    2647
gb:MN996527|Organism:Severe      GGTACACCAGTTTGTATTAACGGGCTTATGTTGCTCGAAATCAAAGACACAGAAAAGTAC    2629
gb:MT093631|Organism:Severe      GGTACACCAGTTTGTATTAACGGGCTTATGTTGCTCGAAATCAAAGACACAGAAAAGTAC    2700
gb:MT106053|Organism:Severe      GGTACACCAGTTTGTATTAACGGGCTTATGTTGCTCGAAATCAAAGACACAGAAAAGTAC    2662
gb:MT019533|Organism:Severe      GGTACACCAGTTTGTATTAACGGGCTTATGTTGCTCGAAATCAAAGACACAGAAAAGTAC    2662
gb:MT019531|Organism:Severe      GGTACACCAGTTTGTATTAACGGGCTTATGTTGCTCGAAATCAAAGACACAGAAAAGTAC    2662
gb:MN996528|Organism:Severe      GGTACACCAGTTTGTATTAACGGGCTTATGTTGCTCGAAATCAAAGACACAGAAAAGTAC    2662
gb:MN996530|Organism:Severe      GGTACACCAGTTTGTATTAACGGGCTTATGTTGCTCGAAATCAAAGACACAGAAAAGTAC    2648
gb:MN908947|Organism:Severe      GGTACACCAGTTTGTATTAACGGGCTTATGTTGCTCGAAATCAAAGACACAGAAAAGTAC    2662
gb:MT019532|Organism:Severe      GGTACACCAGTTTGTATTAACGGGCTTATGTTGCTCGAAATCAAAGACACAGAAAAGTAC    2662
                                 ***********************************************************

gb:MT020781|Organism:Severe      TGTGCCCTTGCACCTAATATGATGGTAACAAACAATACCTTCACACTCAAAGGCGGTGCA    2710
gb:MT007544|Organism:Severe      TGTGCCCTTGCACCTAATATGATGGTAACAAACAATACCTTCACACTCAAAGGCGGTGCA    2722
gb:MN994467|Organism:Severe      TGTGCCCTTGCACCTAATATGATGGTAACAAACAATACCTTCACACTCAAAGGCGGTGCA    2722
gb:MT044257|Organism:Severe      TGTGCCCTTGCACCTAATATGATGGTAACAAACAATACCTTCACACTCAAAGGCGGTGCA    2722
gb:MT106054|Organism:Severe      TGTGCCCTTGCACCTAATATGATGGTAACAAACAATACCTTCACACTCAAAGGCGGTGCA    2722
gb:MT049951|Organism:Severe      TGTGCCCTTGCACCTAATATGATGGTAACAAACAATACCTTCACACTCAAAGGCGGTGCA    2722
```

```
gb:MN975262|Organism:Severe      TGTGCCCTTGCACCTAATATGATGGTAACAAACAATACCTTCACACTCAAAGGCGGTGCA    2722
gb:MT106052|Organism:Severe      TGTGCCCTTGCACCTAATATGATGGTAACAAACAATACCTTCACACTCAAAGGCGGTGCA    2722
gb:LC522975|Organism:Severe      TGTGCCCTTGCACCTAATATGATGGTAACAAACAATACCTTCACACTCAAAGGCGGTGCA    2719
gb:LC522973|Organism:Severe      TGTGCCCTTGCACCTAATATGATGGTAACAAACAATACCTTCACACTCAAAGGCGGTGCA    2719
gb:LC522974|Organism:Severe      TGTGCCCTTGCACCTAATATGATGGTAACAAACAATACCTTCACACTCAAAGGCGGTGCA    2719
gb:MN985325|Organism:Severe      TGTGCCCTTGCACCTAATATGATGGTAACAAACAATACCTTCACACTCAAAGGCGGTGCA    2722
gb:MT020881|Organism:Severe      TGTGCCCTTGCACCTAATATGATGGTAACAAACAATACCTTCACACTCAAAGGCGGTGCA    2722
gb:MT020880|Organism:Severe      TGTGCCCTTGCACCTAATATGATGGTAACAAACAATACCTTCACACTCAAAGGCGGTGCA    2722
gb:MT066175|Organism:Severe      TGTGCCCTTGCACCTAATATGATGGTAACAAACAATACCTTCACACTCAAAGGCGGTGCA    2722
gb:MN997409|Organism:Severe      TGTGCCCTTGCACCTAATATGATGGTAACAAACAATACCTTCACACTCAAAGGCGGTGCA    2722
gb:MN938384|Organism:Severe      TGTGCCCTTGCACCTAATATGATGGTAACAAACAATACCTTCACACTCAAAGGCGGTGCA    2690
gb:MT044258|Organism:Severe      TGTGCCCTTGCACCTAATATGATGGTAACAAACAATACCTTCACACTCAAAGGCGGTGCA    2698
gb:MT039890|Organism:Severe      TGTGCCCTTGCACCTAATATGATGGTAACAAACAATACCTTCACACTCAAAGGCGGTGCA    2722
gb:MN988713|Organism:Severe      TGTGCCCTTGCACCTAATATGATGGTAACAAACAATACCTTCACACTCAAAGGCGGTGCA    2722
gb:LC521925|Organism:Severe      TGTGCCCTTGCACCTAATATGATGGTAACAAACAATACCTTCACACTCAAAGGCGGTGCA    2695
gb:MT093571|Organism:Severe      TGTGCCCTTGCACCTAATATGATGGTAACAAACAATACCTTCACACTCAAAGGCAGTGCA    2722
gb:MT039887|Organism:Severe      TGTGCCCTTGCACCTAATATGATGGTAACAAACAATACCTTCACACTCAAAGGCGGTGCA    2722
gb:MT019530|Organism:Severe      TGTGCCCTTGCACCTAATATGATGGTAACAAACAATACCTTCACACTCAAAGGCGGTGCA    2722
gb:MT039888|Organism:Severe      TGTGCCCTTGCACCTAATATGATGGTAACAAACAATACCTTCACACTCAAAGGCGGTGCA    2722
gb:LC522972|Organism:Severe      TGTGCCCTTGCACCTAATATGATGGTAACAAACAATACCTTCACACTCAAAGGCGGTGCA    2719
gb:MT027063|Organism:Severe      TGTGCCCTTGCACCTAATATGATGGTAACAAACAATACCTTCACACTCAAAGGCGGTGCA    2722
gb:MT027062|Organism:Severe      TGTGCCCTTGCACCTAATATGATGGTAACAAACAATACCTTCACACTCAAAGGCGGTGCA    2722
gb:MT019529|Organism:Severe      TGTGCCCTTGCACCTAATATGATGGTAACAAACAATACCTTCACACTCAAAGGCGGTGCA    2722
gb:MN996529|Organism:Severe      TGTGCCCTTGCACCTAATATGATGGTAACAAACAATACCTTCACACTCAAAGGCGGTGCA    2710
gb:MN996531|Organism:Severe      TGTGCCCTTGCACCTAATATGATGGTAACAAACAATACCTTCACACTCAAAGGCGGTGCA    2709
gb:MT066176|Organism:Severe      TGTGCCCTTGCACCTAATATGATGGTAACAAACAATACCTTCACACTCAAAGGCGGTGCA    2722
gb:MT027064|Organism:Severe      TGTGCCCTTGCACCTAATATGATGGTAACAAACAATACCTTCACACTCAAAGGCGGTGCA    2722
gb:MN994468|Organism:Severe      TGTGCCCTTGCACCTAATATGATGGTAACAAACAATACCTTCACACTCAAAGGCGGTGCA    2722
gb:MT072688|Organism:Severe      TGTGCCCTTGCACCTAATATGATGGTAACAAACAATACCTTCACACTCAAAGGCGGTGCA    2707
gb:MN996527|Organism:Severe      TGTGCCCTTGCACCTAATATGATGGTAACAAACAATACCTTCACACTCAAAGGCGGTGCA    2689
gb:MT093631|Organism:Severe      TGTGCCCTTGCACCTAATATGATGGTAACAAACAATACCTTCACACTCAAAGGCGGTGCA    2760
gb:MT106053|Organism:Severe      TGTGCCCTTGCACCTAATATGATGGTAACAAACAATACCTTCACACTCAAAGGCGGTGCA    2722
gb:MT019533|Organism:Severe      TGTGCCCTTGCACCTAATATGATGGTAACAAACAATACCTTCACACTCAAAGGCGGTGCA    2722
gb:MT019531|Organism:Severe      TGTGCCCTTGCACCTAATATGATGGTAACAAACAATACCTTCACACTCAAAGGCGGTGCA    2722
gb:MN996528|Organism:Severe      TGTGCCCTTGCACCTAATATGATGGTAACAAACAATACCTTCACACTCAAAGGCGGTGCA    2722
gb:MN996530|Organism:Severe      TGTGCCCTTGCACCTAATATGATGGTAACAAACAATACCTTCACACTCAAAGGCGGTGCA    2708
gb:MN908947|Organism:Severe      TGTGCCCTTGCACCTAATATGATGGTAACAAACAATACCTTCACACTCAAAGGCGGTGCA    2722
gb:MT019532|Organism:Severe      TGTGCCCTTGCACCTAATATGATGGTAACAAACAATACCTTCACACTCAAAGGCGGTGCA    2722
                                 ****************************************************** *****

gb:MT020781|Organism:Severe      CCAACAAAGGTTACTTTTGGTGATGACACTGTGATAGAAGTGCAAGGTTACAAGAGTGTG    2770
gb:MT007544|Organism:Severe      CCAACAAAGGTTACTTTTGGTGATGACACTGTGATAGAAGTGCAAGGTTACAAGAGTGTG    2782
gb:MN994467|Organism:Severe      CCAACAAAGGTTACTTTTGGTGATGACACTGTGATAGAAGTGCAAGGTTACAAGAGTGTG    2782
gb:MT044257|Organism:Severe      CCAACAAAGGTTACTTTTGGTGATGACACTGTGATAGAAGTGCAAGGTTACAAGAGTGTG    2782
gb:MT106054|Organism:Severe      CCAACAAAGGTTACTTTTGGTGATGACACTGTGATAGAAGTGCAAGGTTACAAGAGTGTG    2782
gb:MT049951|Organism:Severe      CCAACAAAGGTTACTTTTGGTGATGACACTGTGATAGAAGTGCAAGGTTACAAGAGTGTG    2782
gb:MN975262|Organism:Severe      CCAACAAAGGTTACTTTTGGTGATGACACTGTGATAGAAGTGCAAGGTTACAAGAGTGTG    2782
gb:MT106052|Organism:Severe      CCAACAAAGGTTACTTTTGGTGATGACACTGTGATAGAAGTGCAAGGTTACAAGAGTGTG    2782
gb:LC522975|Organism:Severe      CCAACAAAGGTTACTTTTGGTGATGACACTGTGATAGAAGTGCAAGGTTACAAGAGTGTG    2779
gb:LC522973|Organism:Severe      CCAACAAAGGTTACTTTTGGTGATGACACTGTGATAGAAGTGCAAGGTTACAAGAGTGTG    2779
gb:LC522974|Organism:Severe      CCAACAAAGGTTACTTTTGGTGATGACACTGTGATAGAAGTGCAAGGTTACAAGAGTGTG    2779
gb:MN985325|Organism:Severe      CCAACAAAGGTTACTTTTGGTGATGACACTGTGATAGAAGTGCAAGGTTACAAGAGTGTG    2782
gb:MT020881|Organism:Severe      CCAACAAAGGTTACTTTTGGTGATGACACTGTGATAGAAGTGCAAGGTTACAAGAGTGTG    2782
gb:MT020880|Organism:Severe      CCAACAAAGGTTACTTTTGGTGATGACACTGTGATAGAAGTGCAAGGTTACAAGAGTGTG    2782
gb:MT066175|Organism:Severe      CCAACAAAGGTTACTTTTGGTGATGACACTGTGATAGAAGTGCAAGGTTACAAGAGTGTG    2782
gb:MN997409|Organism:Severe      CCAACAAAGGTTACTTTTGGTGATGACACTGTGATAGAAGTGCAAGGTTACAAGAGTGTG    2782
gb:MN938384|Organism:Severe      CCAACAAAGGTTACTTTTGGTGATGACACTGTGATAGAAGTGCAAGGTTACAAGAGTGTG    2750
gb:MT044258|Organism:Severe      CCAACAAAGGTTACTTTTGGTGATGACACTGTGATAGAAGTGCAAGGTTACAAGAGTGTG    2758
gb:MT039890|Organism:Severe      CCAACAAAGGTTACTTTTGGTGATGACACTGTGATAGAAGTGCAAGGTTACAAGAGTGTG    2782
gb:MN988713|Organism:Severe      CCAACAAAGGTTACTTTTGGTGATGACACTGTGATAGAAGTGCAAGGTTACAAGAGTGTG    2782
gb:LC521925|Organism:Severe      CCAACAAAGGTTACTTTTGGTGATGACACTGTGATAGAAGTGCAAGGTTACAAGAGTGTG    2755
gb:MT093571|Organism:Severe      CCAACAAAGGTTACTTTTGGTGATGACACTGTGATAGAAGTGCAAGGTTACAAGAGTGTG    2782
gb:MT039887|Organism:Severe      CCAACAAAGGTTACTTTTGGTGATGACACTGTGATAGAAGTGCAAGGTTACAAGAGTGTG    2782
gb:MT019530|Organism:Severe      CCAACAAAGGTTACTTTTGGTGATGACACTGTGATAGAAGTGCAAGGTTACAAGAGTGTG    2782
gb:MT039888|Organism:Severe      CCAACAAAGGTTACTTTTGGTGATGACACTGTGATAGAAGTGCAAGGTTACAAGAGTGTG    2782
```

```
gb:LC522972|Organism:Severe      CCAACAAAGGTTACTTTTGGTGATGACACTGTGATAGAAGTGCAAGGTTACAAGAGTGTG    2779
gb:MT027063|Organism:Severe      CCAACAAAGGTTACTTTTGGTGATGACACTGTGATAGAAGTGCAAGGTTACAAGAGTGTG    2782
gb:MT027062|Organism:Severe      CCAACAAAGGTTACTTTTGGTGATGACACTGTGATAGAAGTGCAAGGTTACAAGAGTGTG    2782
gb:MT019529|Organism:Severe      CCAACAAAGGTTACTTTTGGTGATGACACTGTGATAGAAGTGCAAGGTTACAAGAGTGTG    2782
gb:MN996529|Organism:Severe      CCAACAAAGGTTACTTTTGGTGATGACACTGTGATAGAAGTGCAAGGTTACAAGAGTGTG    2770
gb:MN996531|Organism:Severe      CCAACAAAGGTTACTTTTGGTGATGACACTGTGATAGAAGTGCAAGGTTACAAGAGTGTG    2769
gb:MT066176|Organism:Severe      CCAACAAAGGTTACTTTTGGTGATGACACTGTGATAGAAGTGCAAGGTTACAAGAGTGTG    2782
gb:MT027064|Organism:Severe      CCAACAAAGGTTACTTTTGGTGATGACACTGTGATAGAAGTGCAAGGTTACAAGAGTGTG    2782
gb:MN994468|Organism:Severe      CCAACAAAGGTTACTTTTGGTGATGACACTGTGATAGAAGTGCAAGGTTACAAGAGTGTG    2782
gb:MT072688|Organism:Severe      CCAACAAAGGTTACTTTTGGTGATGACACTGTGATAGAAGTGCAAGGTTACAAGAGTGTG    2767
gb:MN996527|Organism:Severe      CCAACAAAGGTTACTTTTGGTGATGACACTGTGATAGAAGTGCAAGGTTACAAGAGTGTG    2749
gb:MT093631|Organism:Severe      CCAACAAAGGTTACTTTTGGTGATGACACTGTGATAGAAGTGCAAGGTTACAAGAGTGTG    2820
gb:MT106053|Organism:Severe      CCAACAAAGGTTACTTTTGGTGATGACACTGTGATAGAAGTGCAAGGTTACAAGAGTGTG    2782
gb:MT019533|Organism:Severe      CCAACAAAGGTTACTTTTGGTGATGACACTGTGATAGAAGTGCAAGGTTACAAGAGTGTG    2782
gb:MT019531|Organism:Severe      CCAACAAAGGTTACTTTTGGTGATGACACTGTGATAGAAGTGCAAGGTTACAAGAGTGTG    2782
gb:MN996528|Organism:Severe      CCAACAAAGGTTACTTTTGGTGATGACACTGTGATAGAAGTGCAAGGTTACAAGAGTGTG    2782
gb:MN996530|Organism:Severe      CCAACAAAGGTTACTTTTGGTGATGACACTGTGATAGAAGTGCAAGGTTACAAGAGTGTG    2768
gb:MN908947|Organism:Severe      CCAACAAAGGTTACTTTTGGTGATGACACTGTGATAGAAGTGCAAGGTTACAAGAGTGTG    2782
gb:MT019532|Organism:Severe      CCAACAAAGGTTACTTTTGGTGATGACACTGTGATAGAAGTGCAAGGTTACAAGAGTGTG    2782
                                 ************************************************************

gb:MT020781|Organism:Severe      AATATCACTTTTGAACTTGATGAAAGGATTGATAAAGTACTTAATGAGAAGTGCTCTGCC    2830
gb:MT007544|Organism:Severe      AATATCACTTTTGAACTTGATGAAAGGATTGATAAAGTACTTAATGAGAAGTGCTCTGCC    2842
gb:MN994467|Organism:Severe      AATATCACTTTTGAACTTGATGAAAGGATTGATAAAGTACTTAATGAGAAGTGCTCTGCC    2842
gb:MT044257|Organism:Severe      AATATCACTTTTGAACTTGATGAAAGGATTGATAAAGTACTTAATGAGAAGTGCTCTGCC    2842
gb:MT106054|Organism:Severe      AATATCACTTTTGAACTTGATGAAAGGATTGATAAAGTACTTAATGAGAAGTGCTCTGCC    2842
gb:MT049951|Organism:Severe      AATATCACTTTTGAACTTGATGAAAGGATTGATAAAGTACTTAATGAGAAGTGCTCTGCC    2842
gb:MN975262|Organism:Severe      AATATCACTTTTGAACTTGATGAAAGGATTGATAAAGTACTTAATGAGAAGTGCTCTGCC    2842
gb:MT106052|Organism:Severe      AATATCACTTTTGAACTTGATGAAAGGATTGATAAAGTACTTAATGAGAAGTGCTCTGCC    2842
gb:LC522975|Organism:Severe      AATATCACTTTTGAACTTGATGAAAGGATTGATAAAGTACTTAATGAGAAGTGCTCTGCC    2839
gb:LC522973|Organism:Severe      AATATCACTTTTGAACTTGATGAAAGGATTGATAAAGTACTTAATGAGAAGTGCTCTGCC    2839
gb:LC522974|Organism:Severe      AATATCACTTTTGAACTTGATGAAAGGATTGATAAAGTACTTAATGAGAAGTGCTCTGCC    2839
gb:MN985325|Organism:Severe      AATATCACTTTTGAACTTGATGAAAGGATTGATAAAGTACTTAATGAGAAGTGCTCTGCC    2842
gb:MT020881|Organism:Severe      AATATCACTTTTGAACTTGATGAAAGGATTGATAAAGTACTTAATGAGAAGTGCTCTGCC    2842
gb:MT020880|Organism:Severe      AATATCACTTTTGAACTTGATGAAAGGATTGATAAAGTACTTAATGAGAAGTGCTCTGCC    2842
gb:MT066175|Organism:Severe      AATATCACTTTTGAACTTGATGAAAGGATTGATAAAGTACTTAATGAGAAGTGCTCTGCC    2842
gb:MN997409|Organism:Severe      AATATCACTTTTGAACTTGATGAAAGGATTGATAAAGTACTTAATGAGAAGTGCTCTGCC    2842
gb:MN938384|Organism:Severe      AATATCACTTTTGAACTTGATGAAAGGATTGATAAAGTACTTAATGAGAAGTGCTCTGCC    2810
gb:MT044258|Organism:Severe      AATATCACTTTTGAACTTGATGAAAGGATTGATAAAGTACTTAATGAGAAGTGCTCTGCC    2818
gb:MT039890|Organism:Severe      AATATCACTTTTGAACTTGATGAAAGGATTGATAAAGTACTTAATGAGAAGTGCTCTGCC    2842
gb:MN988713|Organism:Severe      AATATCACTTTTGAACTTGATGAAAGGATTGATAAAGTACTTAATGAGAAGTGCTCTGCC    2842
gb:LC521925|Organism:Severe      AATATCACTTTTGAACTTGATGAAAGGATTGATAAAGTACTTAATGAGAAGTGCTCTGCC    2815
gb:MT093571|Organism:Severe      AATATCACTTTTGAACTTGATGAAAGGATTGATAAAGTACTTAATGAGAAGTGCTCTGCC    2842
gb:MT039887|Organism:Severe      AATATCACTTTTGAACTTGATGAAAGGATTGATAAAGTACTTAATGAGAAGTGCTCTGCC    2842
gb:MT019530|Organism:Severe      AATATCACTTTTGAACTTGATGAAAGGATTGATAAAGTACTTAATGAGAAGTGCTCTGCC    2842
gb:MT039888|Organism:Severe      AATATCACTTTTGAACTTGATGAAAGGATTGATAAAGTACTTAATGAGAAGTGCTCTGCC    2842
gb:LC522972|Organism:Severe      AATATCACTTTTGAACTTGATGAAAGGATTGATAAAGTACTTAATGAGAAGTGCTCTGCC    2839
gb:MT027063|Organism:Severe      AATATCACTTTTGAACTTGATGAAAGGATTGATAAAGTACTTAATGAGAAGTGCTCTGCC    2842
gb:MT027062|Organism:Severe      AATATCACTTTTGAACTTGATGAAAGGATTGATAAAGTACTTAATGAGAAGTGCTCTGCC    2842
gb:MT019529|Organism:Severe      AATATCACTTTTGAACTTGATGAAAGGATTGATAAAGTACTTAATGAGAAGTGCTCTGCC    2842
gb:MN996529|Organism:Severe      AATATCACTTTTGAACTTGATGAAAGGATTGATAAAGTACTTAATGAGAAGTGCTCTGCC    2830
gb:MN996531|Organism:Severe      AATATCACTTTTGAACTTGATGAAAGGATTGATAAAGTACTTAATGAGAAGTGCTCTGCC    2829
gb:MT066176|Organism:Severe      AATATCACTTTTGAACTTGATGAAAGGATTGATAAAGTACTTAATGAGAAGTGCTCTGCC    2842
gb:MT027064|Organism:Severe      AATATCACTTTTGAACTTGATGAAAGGATTGATAAAGTACTTAATGAGAAGTGCTCTGCC    2842
gb:MN994468|Organism:Severe      AATATCACTTTTGAACTTGATGAAAGGATTGATAAAGTACTTAATGAGAAGTGCTCTGCC    2842
gb:MT072688|Organism:Severe      AATATCACTTTTGAACTTGATGAAAGGATTGATAAAGTACTTAATGAGAAGTGCTCTGCC    2827
gb:MN996527|Organism:Severe      AATATCACTTTTGAACTTGATGAAAGGATTGATAAAGTACTTAATGAGAAGTGCTCTGCC    2809
gb:MT093631|Organism:Severe      AATATCACTTTTGAACTTGATGAAAGGATTGATAAAGTACTTAATGAGAAGTGCTCTGCC    2880
gb:MT106053|Organism:Severe      AATATCACTTTTGAACTTGATGAAAGGATTGATAAAGTACTTAATGAGAAGTGCTCTGCC    2842
gb:MT019533|Organism:Severe      AATATCACTTTTGAACTTGATGAAAGGATTGATAAAGTACTTAATGAGAAGTGCTCTGCC    2842
gb:MT019531|Organism:Severe      AATATCACTTTTGAACTTGATGAAAGGATTGATAAAGTACTTAATGAGAAGTGCTCTGCC    2842
gb:MN996528|Organism:Severe      AATATCACTTTTGAACTTGATGAAAGGATTGATAAAGTACTTAATGAGAAGTGCTCTGCC    2842
gb:MN996530|Organism:Severe      AATATCACTTTTGAACTTGATGAAAGGATTGATAAAGTACTTAATGAGAAGTGCTCTGCC    2828
gb:MN908947|Organism:Severe      AATATCACTTTTGAACTTGATGAAAGGATTGATAAAGTACTTAATGAGAAGTGCTCTGCC    2842
gb:MT019532|Organism:Severe      AATATCACTTTTGAACTTGATGAAAGGATTGATAAAGTACTTAATGAGAAGTGCTCTGCC    2842
```

```
                                    ************************************************************

gb:MT020781|Organism:Severe         TATACAGTTGAACTCGGTACAGAAGTAAATGAGTTCGCCTGTGTTGTGGCAGATGCTGTC   2890
gb:MT007544|Organism:Severe         TATACAGTTGAACTCGGTACAGAAGTAAATGAGTTCGCCTGTGTTGTGGCAGATGCTGTC   2902
gb:MN994467|Organism:Severe         TATACAGTTGAACTCGGTACAGAAGTAAATGAGTTCGCCTGTGTTGTGGCAGATGCTGTC   2902
gb:MT044257|Organism:Severe         TATACAGTTGAACTCGGTACAGAAGTAAATGAGTTCGCCTGTGTTGTGGCAGATGCTGTC   2902
gb:MT106054|Organism:Severe         TATACAGTTGAACTCGGTACAGAAGTAAATGAGTTCGCCTGTGTTGTGGCAGATGCTGTC   2902
gb:MT049951|Organism:Severe         TATACAGTTGAACTCGGTACAGAAGTAAATGAGTTCGCCTGTGTTGTGGCAGATGCTGTC   2902
gb:MN975262|Organism:Severe         TATACAGTTGAACTCGGTACAGAAGTAAATGAGTTCGCCTGTGTTGTGGCAGATGCTGTC   2902
gb:MT106052|Organism:Severe         TATACAGTTGAACTCGGTACAGAAGTAAATGAGTTCGCCTGTGTTGTGGCAGATGCTGTC   2902
gb:LC522975|Organism:Severe         TATACAGTTGAACTCGGTACAGAAGTAAATGAGTTCGCCTGTGTTGTGGCAGATGCTGTC   2899
gb:LC522973|Organism:Severe         TATACAGTTGAACTCGGTACAGAAGTAAATGAGTTCGCCTGTGTTGTGGCAGATGCTGTC   2899
gb:LC522974|Organism:Severe         TATACAGTTGAACTCGGTACAGAAGTAAATGAGTTCGCCTGTGTTGTGGCAGATGCTGTC   2899
gb:MN985325|Organism:Severe         TATACAGTTGAACTCGGTACAGAAGTAAATGAGTTCGCCTGTGTTGTGGCAGATGCTGTC   2902
gb:MT020881|Organism:Severe         TATACAGTTGAACTCGGTACAGAAGTAAATGAGTTCGCCTGTGTTGTGGCAGATGCTGTC   2902
gb:MT020880|Organism:Severe         TATACAGTTGAACTCGGTACAGAAGTAAATGAGTTCGCCTGTGTTGTGGCAGATGCTGTC   2902
gb:MT066175|Organism:Severe         TATACAGTTGAACTCGGTACAGAAGTAAATGAGTTCGCCTGTGTTGTGGCAGATGCTGTC   2902
gb:MN997409|Organism:Severe         TATACAGTTGAACTCGGTACAGAAGTAAATGAGTTCGCCTGTGTTGTGGCAGATGCTGTC   2902
gb:MN938384|Organism:Severe         TATACAGTTGAACTCGGTACAGAAGTAAATGAGTTCGCCTGTGTTGTGGCAGATGCTGTC   2870
gb:MT044258|Organism:Severe         TATACAGTTGAACTCGGTACAGAAGTAAATGAGTTCGCCTGTGTTGTGGCAGATGCTGTC   2878
gb:MT039890|Organism:Severe         TATACAGTTGAACTCGGTACAGAAGTAAATGAGTTCGCCTGTGTTGTGGCAGATGCTGTC   2902
gb:MN988713|Organism:Severe         TATACAGTTGAACTCGGTACAGAAGTAAATGAGTTCGCCTGTGTTGTGGCAGATGCTGTC   2902
gb:LC521925|Organism:Severe         TATACAGTTGAACTCGGTACAGAAGTAAATGAGTTCGCCTGTGTTGTGGCAGATGCTGTC   2875
gb:MT093571|Organism:Severe         TATACAGTTGAACTCGGTACAGAAGTAAATGAGTTCGCCTGTGTTGTGGCAGATGCTGTC   2902
gb:MT039887|Organism:Severe         TATACAGTTGAACTCGGTACAGAAGTAAATGAGTTCGCCTGTGTTGTGGCAGATGCTGTC   2902
gb:MT019530|Organism:Severe         TATACAGTTGAACTCGGTACAGAAGTAAATGAGTTCGCCTGTGTTGTGGCAGATGCTGTC   2902
gb:MT039888|Organism:Severe         TATACAGTTGAACTCGGTACAGAAGTAAATGAGTTCGCCTGTGTTGTGGCAGATGCTGTC   2902
gb:LC522972|Organism:Severe         TATACAGTTGAACTCGGTACAGAAGTAAATGAGTTCGCCTGTGTTGTGGCAGATGCTGTC   2899
gb:MT027063|Organism:Severe         TATACAGTTGAACTCGGTACAGAAGTAAATGAGTTCGCCTGTGTTGTGGCAGATGCTGTC   2902
gb:MT027062|Organism:Severe         TATACAGTTGAACTCGGTACAGAAGTAAATGAGTTCGCCTGTGTTGTGGCAGATGCTGTC   2902
gb:MT019529|Organism:Severe         TATACAGTTGAACTCGGTACAGAAGTAAATGAGTTCGCCTGTGTTGTGGCAGATGCTGTC   2902
gb:MN996529|Organism:Severe         TATACAGTTGAACTCGGTACAGAAGTAAATGAGTTCGCCTGTGTTGTGGCAGATGCTGTC   2890
gb:MN996531|Organism:Severe         TATACAGTTGAACTCGGTACAGAAGTAAATGAGTTCGCCTGTGTTGTGGCAGATGCTGTC   2889
gb:MT066176|Organism:Severe         TATACAGTTGAACTCGGTACAGAAGTAAATGAGTTCGCCTGTGTTGTGGCAGATGCTGTC   2902
gb:MT027064|Organism:Severe         TATACAGTTGAACTCGGTACAGAAGTAAATGAGTTCGCCTGTGTTGTGGCAGATGCTGTC   2902
gb:MN994468|Organism:Severe         TATACAGTTGAACTCGGTACAGAAGTAAATGAGTTCGCCTGTGTTGTGGCAGATGCTGTC   2902
gb:MT072688|Organism:Severe         TATACAGTTGAACTCGGTACAGAAGTAAATGAGTTCGCCTGTGTTGTGGCAGATGCTGTC   2887
gb:MN996527|Organism:Severe         TATACAGTTGAACTCGGTACAGAAGTAAATGAGTTCGCCTGTGTTGTGGCAGATGCTGTC   2869
gb:MT093631|Organism:Severe         TATACAGTTGAACTCGGTACAGAAGTAAATGAGTTCGCCTGTGTTGTGGCAGATGCTGTC   2940
gb:MT106053|Organism:Severe         TATACAGTTGAACTCGGTACAGAAGTAAATGAGTTCGCCTGTGTTGTGGCAGATGCTGTC   2902
gb:MT019533|Organism:Severe         TATACAGTTGAACTCGGTACAGAAGTAAATGAGTTCGCCTGTGTTGTGGCAGATGCTGTC   2902
gb:MT019531|Organism:Severe         TATACAGTTGAACTCGGTACAGAAGTAAATGAGTTCGCCTGTGTTGTGGCAGATGCTGTC   2902
gb:MN996528|Organism:Severe         TATACAGTTGAACTCGGTACAGAAGTAAATGAGTTCGCCTGTGTTGTGGCAGATGCTGTC   2902
gb:MN996530|Organism:Severe         TATACAGTTGAACTCGGTACAGAAGTAAATGAGTTCGCCTGTGTTGTGGCAGATGCTGTC   2888
gb:MN908947|Organism:Severe         TATACAGTTGAACTCGGTACAGAAGTAAATGAGTTCGCCTGTGTTGTGGCAGATGCTGTC   2902
gb:MT019532|Organism:Severe         TATACAGTTGAACTCGGTACAGAAGTAAATGAGTTCGCCTGTGTTGTGGCAGATGCTGTC   2902
                                    ************************************************************

gb:MT020781|Organism:Severe         ATAAAAACTTTGCAACCAGTATCTGAATTACTTACACCACTGGGCATTGATTTAGATGAG   2950
gb:MT007544|Organism:Severe         ATAAAAACTTTGCAACCAGTATCTGAATTACTTACACCACTGGGCATTGATTTAGATGAG   2962
gb:MN994467|Organism:Severe         ATAAAAACTTTGCAACCAGTATCTGAATTACTTACACCACTGGGCATTGATTTAGATGAG   2962
gb:MT044257|Organism:Severe         ATAAAAACTTTGCAACCAGTATCTGAATTACTTACACCACTGGGCATTGATTTAGATGAG   2962
gb:MT106054|Organism:Severe         ATAAAAACTTTGCAACCAGTATCTGAATTACTTACACCACTGGGCATTGATTTAGATGAG   2962
gb:MT049951|Organism:Severe         ATAAAAACTTTGCAACCAGTATCTGAATTACTTACACCACTGGGCATTGATTTAGATGAG   2962
gb:MN975262|Organism:Severe         ATAAAAACTTTGCAACCAGTATCTGAATTACTTACACCACTGGGCATTGATTTAGATGAG   2962
gb:MT106052|Organism:Severe         ATAAAAACTTTGCAACCAGTATCTGAATTACTTACACCACTGGGCATTGATTTAGATGAG   2962
gb:LC522975|Organism:Severe         ATAAAAACTTTGCAACCAGTATCTGAATTACTTACACCACTGGGCATTGATTTAGATGAG   2959
gb:LC522973|Organism:Severe         ATAAAAACTTTGCAACCAGTATCTGAATTACTTACACCACTGGGCATTGATTTAGATGAG   2959
gb:LC522974|Organism:Severe         ATAAAAACTTTGCAACCAGTATCTGAATTACTTACACCACTGGGCATTGATTTAGATGAG   2959
gb:MN985325|Organism:Severe         ATAAAAACTTTGCAACCAGTATCTGAATTACTTACACCACTGGGCATTGATTTAGATGAG   2962
gb:MT020881|Organism:Severe         ATAAAAACTTTGCAACCAGTATCTGAATTACTTACACCACTGGGCATTGATTTAGATGAG   2962
gb:MT020880|Organism:Severe         ATAAAAACTTTGCAACCAGTATCTGAATTACTTACACCACTGGGCATTGATTTAGATGAG   2962
gb:MT066175|Organism:Severe         ATAAAAACTTTGCAACCAGTATCTGAATTACTTACACCACTGGGCATTGATTTAGATGAG   2962
gb:MN997409|Organism:Severe         ATAAAAACTTTGCAACCAGTATCTGAATTACTTACACCACTGGGCATTGATTTAGATGAG   2962
gb:MN938384|Organism:Severe         ATAAAAACTTTGCAACCAGTATCTGAATTACTTACACCACTGGGCATTGATTTAGATGAG   2930
```

```
gb:MT044258|Organism:Severe      ATAAAAACTTTGCAACCAGTATCTGAATTACTTACACCACTGGGCATTGATTTAGATGAG   2938
gb:MT039890|Organism:Severe      ATAAAAACTTTGCAACCAGTATCTGAATTACTTACACCACTGGGCATTGATTTAGATGAG   2962
gb:MN988713|Organism:Severe      ATAAAAACTTTGCAACCAGTATCTGAATTACTTACACCACTGGGCATTGATTTAGATGAG   2962
gb:LC521925|Organism:Severe      ATAAAAACTTTGCAACCAGTATCTGAATTACTTACACCACTGGGCATTGATTTAGATGAG   2935
gb:MT093571|Organism:Severe      ATAAAAACTTTGCAACCAGTATCTGAATTACTTACACCACTGGGCATTGATTTAGATGAG   2962
gb:MT039887|Organism:Severe      ATAAAAACTTTGCAACCAGTATCTGAATTACTTACACCACTGGGCATTGATTTAGATGAG   2962
gb:MT019530|Organism:Severe      ATAAAAACTTTGCAACCAGTATCTGAATTACTTACACCACTGGGCATTGATTTAGATGAG   2962
gb:MT039888|Organism:Severe      ATAAAAACTTTGCAACCAGTATCTGAATTACTTACACCACTGGGCATTGATTTAGATGAG   2962
gb:LC522972|Organism:Severe      ATAAAAACTTTGCAACCAGTATCTGAATTACTTACACCACTGGGCATTGATTTAGATGAG   2959
gb:MT027063|Organism:Severe      ATAAAAACTTTGCAACCAGTATCTGAATTACTTACACCACTGGGCATTGATTTAGATGAG   2962
gb:MT027062|Organism:Severe      ATAAAAACTTTGCAACCAGTATCTGAATTACTTACACCACTGGGCATTGATTTAGATGAG   2962
gb:MT019529|Organism:Severe      ATAAAAACTTTGCAACCAGTATCTGAATTACTTACACCACTGGGCATTGATTTAGATGAG   2962
gb:MN996529|Organism:Severe      ATAAAAACTTTGCAACCAGTATCTGAATTACTTACACCACTGGGCATTGATTTAGATGAG   2950
gb:MN996531|Organism:Severe      ATAAAAACTTTGCAACCAGTATCTGAATTACTTACACCACTGGGCATTGATTTAGATGAG   2949
gb:MT066176|Organism:Severe      ATAAAAACTTTGCAACCAGTATCTGAATTACTTACACCACTGGGCATTGATTTAGATGAG   2962
gb:MT027064|Organism:Severe      ATAAAAACTTTGCAACCAGTATCTGAATTACTTACACCACTGGGCATTGATTTAGATGAG   2962
gb:MN994468|Organism:Severe      ATAAAAACTTTGCAACCAGTATCTGAATTACTTACACCACTGGGCATTGATTTAGATGAG   2962
gb:MT072688|Organism:Severe      ATAAAAACTTTGCAACCAGTATCTGAATTACTTACACCACTGGGCATTGATTTAGATGAG   2947
gb:MN996527|Organism:Severe      ATAAAAACTTTGCAACCAGTATCTGAATTACTTACACCACTGGGCATTGATTTAGATGAG   2929
gb:MT093631|Organism:Severe      ATAAAAACTTTGCAACCAGTATCTGAATTACTTACACCACTGGGCATTGATTTAGATGAG   3000
gb:MT106053|Organism:Severe      ATAAAAACTTTGCAACCAGTATCTGAATTACTTACACCACTGGGCATTGATTTAGATGAG   2962
gb:MT019533|Organism:Severe      ATAAAAACTTTGCAACCAGTATCTGAATTACTTACACCACTGGGCATTGATTTAGATGAG   2962
gb:MT019531|Organism:Severe      ATAAAAACTTTGCAACCAGTATCTGAATTACTTACACCACTGGGCATTGATTTAGATGAG   2962
gb:MN996528|Organism:Severe      ATAAAAACTTTGCAACCAGTATCTGAATTACTTACACCACTGGGCATTGATTTAGATGAG   2962
gb:MN996530|Organism:Severe      ATAAAAACTTTGCAACCAGTATCTGAATTACTTACACCACTGGGCATTGATTTAGATGAG   2948
gb:MN908947|Organism:Severe      ATAAAAACTTTGCAACCAGTATCTGAATTACTTACACCACTGGGCATTGATTTAGATGAG   2962
gb:MT019532|Organism:Severe      ATAAAAACTTTGCAACCAGTATCTGAATTACTTACACCACTGGGCATTGATTTAGATGAG   2962
                                 ************************************************************

gb:MT020781|Organism:Severe      TGGAGTATGGCTACATACTACTTATTTGATGAGTCTGGTGAGTTTAAATTGGCTTCACAT   3010
gb:MT007544|Organism:Severe      TGGAGTATGGCTACATACTACTTATTTGATGAGTCTGGTGAGTTTAAATTGGCTTCACAT   3022
gb:MN994467|Organism:Severe      TGGAGTATGGCTACATACTACTTATTTGATGAGTCTGGTGAGTTTAAATTGGCTTCACAT   3022
gb:MT044257|Organism:Severe      TGGAGTATGGCTACATACTACTTATTTGATGAGTCTGGTGAGTTTAAATTGGCTTCACAT   3022
gb:MT106054|Organism:Severe      TGGAGTATGGCTACATACTACTTATTTGATGAGTCTGGTGAGTTTAAATTGGCTTCACAT   3022
gb:MT049951|Organism:Severe      TGGAGTATGGCTACATACTACTTATTTGATGAGTCTGGTGAGTTTAAATTGGCTTCACAT   3022
gb:MN975262|Organism:Severe      TGGAGTATGGCTACATACTACTTATTTGATGAGTCTGGTGAGTTTAAATTGGCTTCACAT   3022
gb:MT106052|Organism:Severe      TGGAGTATGGCTACATACTACTTATTTGATGAGTCTGGTGAGTTTAAATTGGCTTCACAT   3022
gb:LC522975|Organism:Severe      TGGAGTATGGCTACATACTACTTATTTGATGAGTCTGGTGAGTTTAAATTGGCTTCACAT   3019
gb:LC522973|Organism:Severe      TGGAGTATGGCTACATACTACTTATTTGATGAGTCTGGTGAGTTTAAATTGGCTTCACAT   3019
gb:LC522974|Organism:Severe      TGGAGTATGGCTACATACTACTTATTTGATGAGTCTGGTGAGTTTAAATTGGCTTCACAT   3019
gb:MN985325|Organism:Severe      TGGAGTATGGCTACATACTACTTATTTGATGAGTCTGGTGAGTTTAAATTGGCTTCACAT   3022
gb:MT020881|Organism:Severe      TGGAGTATGGCTACATACTACTTATTTGATGAGTCTGGTGAGTTTAAATTGGCTTCACAT   3022
gb:MT020880|Organism:Severe      TGGAGTATGGCTACATACTACTTATTTGATGAGTCTGGTGAGTTTAAATTGGCTTCACAT   3022
gb:MT066175|Organism:Severe      TGGAGTATGGCTACATACTACTTATTTGATGAGTCTGGTGAGTTTAAATTGGCTTCACAT   3022
gb:MN997409|Organism:Severe      TGGAGTATGGCTACATACTACTTATTTGATGAGTCTGGTGAGTTTAAATTGGCTTCACAT   3022
gb:MN938384|Organism:Severe      TGGAGTATGGCTACATACTACTTATTTGATGAGTCTGGTGAGTTTAAATTGGCTTCACAT   2990
gb:MT044258|Organism:Severe      TGGAGTATGGCTACATACTACTTATTTGATGAGTCTGGTGAGTTTAAATTGGCTTCACAT   2998
gb:MT039890|Organism:Severe      TGGAGTATTGCTACATACTACTTATTTGATGAGTCTGGTGAGTTTAAATTGGCTTCACAT   3022
gb:MN988713|Organism:Severe      TGGAGTATGGCTACATACTACTTATTTGATGAGTCTGGTGAGTTTAAATTGGCTTCACAT   3022
gb:LC521925|Organism:Severe      TGGAGTATGGCTACATACTACTTATTTGATGAGTCTGGTGAGTTTAAATTGGCTTCACAT   2995
gb:MT093571|Organism:Severe      TGGAGTATGGCTACATACTACTTATTTGATGAGTCTGGTGAGTTTAAATTGGCTTCACAT   3022
gb:MT039887|Organism:Severe      TGGAGTATGGCTACATACTACTTATTTGATGAGTCTGGTGAGTTTAAATTGGCTTCACAT   3022
gb:MT019530|Organism:Severe      TGGAGTATGGCTACATACTACTTATTTGATGAGTCTGGTGAGTTTAAATTGGCTTCACAT   3022
gb:MT039888|Organism:Severe      TGGAGTATGGCTACATACTACTTATTTGATGAGTCTGGTGAGTTTAAATTGGCTTCACAT   3022
gb:LC522972|Organism:Severe      TGGAGTATGGCTACATACTACTTATTTGATGAGTCTGGTGAGTTTAAATTGGCTTCACAT   3019
gb:MT027063|Organism:Severe      TGGAGTATGGCTACATACTACTTATTTGATGAGTCTGGTGAGTTTAAATTGGCTTCACAT   3022
gb:MT027062|Organism:Severe      TGGAGTATGGCTACATACTACTTATTTGATGAGTCTGGTGAGTTTAAATTGGCTTCACAT   3022
gb:MT019529|Organism:Severe      TGGAGTATGGCTACATACTACTTATTTGATGAGTCTGGTGAGTTTAAATTGGCTTCACAT   3022
gb:MN996529|Organism:Severe      TGGAGTATGGCTACATACTACTTATTTGATGAGTCTGGTGAGTTTAAATTGGCTTCACAT   3010
gb:MN996531|Organism:Severe      TGGAGTATGGCTACATACTACTTATTTGATGAGTCTGGTGAGTTTAAATTGGCTTCACAT   3009
gb:MT066176|Organism:Severe      TGGAGTATGGCTACATACTACTTATTTGATGAGTCTGGTGAGTTTAAATTGGCTTCACAT   3022
gb:MT027064|Organism:Severe      TGGAGTATGGCTACATACTACTTATTTGATGAGTCTGGTGAGTTTAAATTGGCTTCACAT   3022
gb:MN994468|Organism:Severe      TGGAGTATGGCTACATACTACTTATTTGATGAGTCTGGTGAGTTTAAATTGGCTTCACAT   3022
gb:MT072688|Organism:Severe      TGGAGTATGGCTACATACTACTTATTTGATGAGTCTGGTGAGTTTAAATTGGCTTCACAT   3007
gb:MN996527|Organism:Severe      TGGAGTATGGCTACATACTACTTATTTGATGAGTCTGGTGAGTTTAAATTGGCTTCACAT   2989
```

```
gb:MT093631|Organism:Severe      TGGAGTATGGCTACATACTACTTATTTGATGAGTCTGGTGAGTTTAAATTGGCTTCACAT   3060
gb:MT106053|Organism:Severe      TGGAGTATGGCTACATACTACTTATTTGATGAGTCTGGTGAGTTTAAATTGGCTTCACAT   3022
gb:MT019533|Organism:Severe      TGGAGTATGGCTACATACTACTTATTTGATGAGTCTGGTGAGTTTAAATTGGCTTCACAT   3022
gb:MT019531|Organism:Severe      TGGAGTATGGCTACATACTACTTATTTGATGAGTCTGGTGAGTTTAAATTGGCTTCACAT   3022
gb:MN996528|Organism:Severe      TGGAGTATGGCTACATACTACTTATTTGATGAGTCTGGTGAGTTTAAATTGGCTTCACAT   3022
gb:MN996530|Organism:Severe      TGGAGTATGGCTACATACTACTTATTTGATGAGTCTGGTGAGTTTAAATTGGCTTCACAT   3008
gb:MN908947|Organism:Severe      TGGAGTATGGCTACATACTACTTATTTGATGAGTCTGGTGAGTTTAAATTGGCTTCACAT   3022
gb:MT019532|Organism:Severe      TGGAGTATGGCTACATACTACTTATTTGATGAGTCTGGTGAGTTTAAATTGGCTTCACAT   3022
                                 ******** ***************************************************

gb:MT020781|Organism:Severe      ATGTATTGTTCTTTCTACCCTCCAGATGAGGATGAAGAAGAAGGTGATTGTGAAGAAGAA   3070
gb:MT007544|Organism:Severe      ATGTATTGTTCTTTCTACCCTCCAGATGAGGATGAAGAAGAAGGTGATTGTGAAGAAGAA   3082
gb:MN994467|Organism:Severe      ATGTATTGTTCTTTCTACCCTCCAGATGAGGATGAAGAAGAAGGTGATTGTGAAGAAGAA   3082
gb:MT044257|Organism:Severe      ATGTATTGTTCTTTCTACCCTCCAGATGAGGATGAAGAAGAAGGTGATTGTGAAGAAGAA   3082
gb:MT106054|Organism:Severe      ATGTATTGTTCTTTCTACCCTCCAGATGAGGATGAAGAAGAAGGTGATTGTGAAGAAGAA   3082
gb:MT049951|Organism:Severe      ATGTATTGTTCTTTCTACCCTCCAGATGAGGATGAAGAAGAAGGTGATTGTGAAGAAGAA   3082
gb:MN975262|Organism:Severe      ATGTATTGTTCTTTCTACCCTCCAGATGAGGATGAAGAAGAAGGTGATTGTGAAGAAGAA   3082
gb:MT106052|Organism:Severe      ATGTATTGTTCTTTCTACCCTCCAGATGAGGATGAAGAAGAAGGTGATTGTGAAGAAGAA   3082
gb:LC522975|Organism:Severe      ATGTATTGTTCTTTCTACCCTCCAGATGAGGATGAAGAAGAAGGTGATTGTGAAGAAGAA   3079
gb:LC522973|Organism:Severe      ATGTATTGTTCTTTCTACCCTCCAGATGAGGATGAAGAAGAAGGTGATTGTGAAGAAGAA   3079
gb:LC522974|Organism:Severe      ATGTATTGTTCTTTCTACCCTCCAGATGAGGATGAAGAAGAAGGTGATTGTGAAGAAGAA   3079
gb:MN985325|Organism:Severe      ATGTATTGTTCTTTCTACCCTCCAGATGAGGATGAAGAAGAAGGTGATTGTGAAGAAGAA   3082
gb:MT020881|Organism:Severe      ATGTATTGTTCTTTCTACCCTCCAGATGAGGATGAAGAAGAAGGTGATTGTGAAGAAGAA   3082
gb:MT020880|Organism:Severe      ATGTATTGTTCTTTCTACCCTCCAGATGAGGATGAAGAAGAAGGTGATTGTGAAGAAGAA   3082
gb:MT066175|Organism:Severe      ATGTATTGTTCTTTCTACCCTCCAGATGAGGATGAAGAAGAAGGTGATTGTGAAGAAGAA   3082
gb:MN997409|Organism:Severe      ATGTATTGTTCTTTCTACCCTCCAGATGAGGATGAAGAAGAAGGTGATTGTGAAGAAGAA   3082
gb:MN938384|Organism:Severe      ATGTATTGTTCTTTCTACCCTCCAGATGAGGATGAAGAAGAAGGTGATTGTGAAGAAGAA   3050
gb:MT044258|Organism:Severe      ATGTATTGTTCTTTCTACCCTCCAGATGAGGATGAAGAAGAAGGTGATTGTGAAGAAGAA   3058
gb:MT039890|Organism:Severe      ATGTATTGTTCTTTCTACCCTCCAGATGAGGATGAAGAAGAAGGTGATTGTGAAGAAGAA   3082
gb:MN988713|Organism:Severe      ATGTATTGTTCTTTCTACCCTCCAGATGAGGATGAAGAAGAAGGTGATTGTGAAGAAGAA   3082
gb:LC521925|Organism:Severe      ATGTATTGTTCTTTCTACCCTCCAGATGAGGATGAAGAAGAAGGTGATTGTGAAGAAGAA   3055
gb:MT093571|Organism:Severe      ATGTATTGTTCTTTCTACCCTCCAGATGAGGATGAAGAAGAAGGTGATTGTGAAGAAGAA   3082
gb:MT039887|Organism:Severe      ATGTATTGTTCTTTCTACCCTCCAGATGAGGATGAAGAAGAAGGTGATTGTGAAGAAGAA   3082
gb:MT019530|Organism:Severe      ATGTATTGTTCTTTCTACCCTCCAGATGAGGATGAAGAAGAAGGTGATTGTGAAGAAGAA   3082
gb:MT039888|Organism:Severe      ATGTATTGTTCTTTCTACCCTCCAGATGAGGATGAAGAAGAAGGTGATTGTGAAGAAGAA   3082
gb:LC522972|Organism:Severe      ATGTATTGTTCTTTCTACCCTCCAGATGAGGATGAAGAAGAAGGTGATTGTGAAGAAGAA   3079
gb:MT027063|Organism:Severe      ATGTATTGTTCTTTCTACCCTCCAGATGAGGATGAAGAAGAAGGTGATTGTGAAGAAGAA   3082
gb:MT027062|Organism:Severe      ATGTATTGTTCTTTCTACCCTCCAGATGAGGATGAAGAAGAAGGTGATTGTGAAGAAGAA   3082
gb:MT019529|Organism:Severe      ATGTATTGTTCTTTCTACCCTCCAGATGAGGATGAAGAAGAAGGTGATTGTGAAGAAGAA   3082
gb:MN996529|Organism:Severe      ATGTATTGTTCTTTCTACCCTCCAGATGAGGATGAAGAAGAAGGTGATTGTGAAGAAGAA   3070
gb:MN996531|Organism:Severe      ATGTATTGTTCTTTCTACCCTCCAGATGAGGATGAAGAAGAAGGTGATTGTGAAGAAGAA   3069
gb:MT066176|Organism:Severe      ATGTATTGTTCTTTCTACCCTCCAGATGAGGATGAAGAAGAAGGTGATTGTGAAGAAGAA   3082
gb:MT027064|Organism:Severe      ATGTATTGTTCTTTCTACCCTCCAGATGAGGATGAAGAAGAAGGTGATTGTGAAGAAGAA   3082
gb:MN994468|Organism:Severe      ATGTATTGTTCTTTCTACCCTCCAGATGAGGATGAAGAAGAAGGTGATTGTGAAGAAGAA   3082
gb:MT072688|Organism:Severe      ATGTATTGTTCTTTCTACCCTCCAGATGAGGATGAAGAAGAAGGTGATTGTGAAGAAGAA   3067
gb:MN996527|Organism:Severe      ATGTATTGTTCTTTCTACCCTCCAGATGAGGATGAAGAAGAAGGTGATTGTGAAGAAGAA   3049
gb:MT093631|Organism:Severe      ATGTATTGTTCTTTCTACCCTCCAGATGAGGATGAAGAAGAAGGTGATTGTGAAGAAGAA   3120
gb:MT106053|Organism:Severe      ATGTATTGTTCTTTCTACCCTCCAGATGAGGATGAAGAAGAAGGTGATTGTGAAGAAGAA   3082
gb:MT019533|Organism:Severe      ATGTATTGTTCTTTCTACCCTCCAGATGAGGATGAAGAAGAAGGTGATTGTGAAGAAGAA   3082
gb:MT019531|Organism:Severe      ATGTATTGTTCTTTCTACCCTCCAGATGAGGATGAAGAAGAAGGTGATTGTGAAGAAGAA   3082
gb:MN996528|Organism:Severe      ATGTATTGTTCTTTCTACCCTCCAGATGAGGATGAAGAAGAAGGTGATTGTGAAGAAGAA   3082
gb:MN996530|Organism:Severe      ATGTATTGTTCTTTCTACCCTCCAGATGAGGATGAAGAAGAAGGTGATTGTGAAGAAGAA   3068
gb:MN908947|Organism:Severe      ATGTATTGTTCTTTCTACCCTCCAGATGAGGATGAAGAAGAAGGTGATTGTGAAGAAGAA   3082
gb:MT019532|Organism:Severe      ATGTATTGTTCTTTCTACCCTCCAGATGAGGATGAAGAAGAAGGTGATTGTGAAGAAGAA   3082
                                 ************************************************************

gb:MT020781|Organism:Severe      GAGTTTGAGCCATCAACTCAATATGAGTATGGTACTGAAGATGATTACCAAGGTAAACCT   3130
gb:MT007544|Organism:Severe      GAGTTTGAGCCATCAACTCAATATGAGTATGGTACTGAAGATGATTACCAAGGTAAACCT   3142
gb:MN994467|Organism:Severe      GAGTTTGAGCCATCAACTCAATATGAGTATGGTACTGAAGATGATTACCAAGGTAAACCT   3142
gb:MT044257|Organism:Severe      GAGTTTGAGCCATCAACTCAATATGAGTATGGTACTGAAGATGATTACCAAGGTAAACCT   3142
gb:MT106054|Organism:Severe      GAGTTTGAGCCATCAACTCAATATGAGTATGGTACTGAAGATGATTACCAAGGTAAACCT   3142
gb:MT049951|Organism:Severe      GAGTTTGAGCCATCAACTCAATATGAGTATGGTACTGAAGATGATTACCAAGGTAAACCT   3142
gb:MN975262|Organism:Severe      GAGTTTGAGCCATCAACTCAATATGAGTATGGTACTGAAGATGATTACCAAGGTAAACCT   3142
gb:MT106052|Organism:Severe      GAGTTTGAGCCATCAACTCAATATGAGTATGGTACTGAAGATGATTACCAAGGTAAACCT   3142
gb:LC522975|Organism:Severe      GAGTTTGAGCCATCAACTCAATATGAGTATGGTACTGAAGATGATTACCAAGGTAAACCT   3139
```

```
gb:LC522973|Organism:Severe      GAGTTTGAGCCATCAACTCAATATGAGTATGGTACTGAAGATGATTACCAAGGTAAACCT   3139
gb:LC522974|Organism:Severe      GAGTTTGAGCCATCAACTCAATATGAGTATGGTACTGAAGATGATTACCAAGGTAAACCT   3139
gb:MN985325|Organism:Severe      GAGTTTGAGCCATCAACTCAATATGAGTATGGTACTGAAGATGATTACCAAGGTAAACCT   3142
gb:MT020881|Organism:Severe      GAGTTTGAGCCATCAACTCAATATGAGTATGGTACTGAAGATGATTACCAAGGTAAACCT   3142
gb:MT020880|Organism:Severe      GAGTTTGAGCCATCAACTCAATATGAGTATGGTACTGAAGATGATTACCAAGGTAAACCT   3142
gb:MT066175|Organism:Severe      GAGTTTGAGCCATCAACTCAATATGAGTATGGTACTGAAGATGATTACCAAGGTAAACCT   3142
gb:MN997409|Organism:Severe      GAGTTTGAGCCATCAACTCAATATGAGTATGGTACTGAAGATGATTACCAAGGTAAACCT   3142
gb:MN938384|Organism:Severe      GAGTTTGAGCCATCAACTCAATATGAGTATGGTACTGAAGATGATTACCAAGGTAAACCT   3110
gb:MT044258|Organism:Severe      GAGTTTGAGCCATCAACTCAATATGAGTATGGTACTGAAGATGATTACCAAGGTAAACCT   3118
gb:MT039890|Organism:Severe      GAGTTTGAGCCATCAACTCAATATGAGTATGGTACTGAAGATGATTACCAAGGTAAACCT   3142
gb:MN988713|Organism:Severe      GAGTTTGAGCCATCAACTCAATATGAGTATGGTACTGAAGATGATTACCAAGGTAAACCT   3142
gb:LC521925|Organism:Severe      GAGTTTGAGCCATCAACTCAATATGAGTATGGTACTGAAGATGATTACCAAGGTAAACCT   3115
gb:MT093571|Organism:Severe      GAGTTTGAGCCATCAACTCAATATGAGTATGGTACTGAAGATGATTACCAAGGTAAACCT   3142
gb:MT039887|Organism:Severe      GAGTTTGAGCCATCAACTCAATATGAGTATGGTACTGAAGATGATTACCAAGGTAAACCT   3142
gb:MT019530|Organism:Severe      GAGTTTGAGCCATCAACTCAATATGAGTATGGTACTGAAGATGATTACCAAGGTAAACCT   3142
gb:MT039888|Organism:Severe      GAGTTTGAGCCATCAACTCAATATGAGTATGGTACTGAAGATGATTACCAAGGTAAACCT   3142
gb:LC522972|Organism:Severe      GAGTTTGAGCCATCAACTCAATATGAGTATGGTACTGAAGATGATTACCAAGGTAAACCT   3139
gb:MT027063|Organism:Severe      GAGTTTGAGCCATCAACTCAATATGAGTATGGTACTGAAGATGATTACCAAGGTAAACCT   3142
gb:MT027062|Organism:Severe      GAGTTTGAGCCATCAACTCAATATGAGTATGGTACTGAAGATGATTACCAAGGTAAACCT   3142
gb:MT019529|Organism:Severe      GAGTTTGAGCCATCAACTCAATATGAGTATGGTACTGAAGATGATTACCAAGGTAAACCT   3142
gb:MN996529|Organism:Severe      GAGTTTGAGCCATCAACTCAATATGAGTATGGTACTGAAGATGATTACCAAGGTAAACCT   3130
gb:MN996531|Organism:Severe      GAGTTTGAGCCATCAACTCAATATGAGTATGGTACTGAAGATGATTACCAAGGTAAACCT   3129
gb:MT066176|Organism:Severe      GAGTTTGAGCCATCAACTCAATATGAGTATGGTACTGAAGATGATTACCAAGGTAAACCT   3142
gb:MT027064|Organism:Severe      GAGTTTGAGCCATCAACTCAATATGAGTATGGTACTGAAGATGATTACCAAGGTAAACCT   3142
gb:MN994468|Organism:Severe      GAGTTTGAGCCATCAACTCAATATGAGTATGGTACTGAAGATGATTACCAAGGTAAACCT   3142
gb:MT072688|Organism:Severe      GAGTTTGAGCCATCAACTCAATATGAGTATGGTACTGAAGATGATTACCAAGGTAAACCT   3127
gb:MN996527|Organism:Severe      GAGTTTGAGCCATCAACTCAATATGAGTATGGTACTGAAGATGATTACCAAGGTAAACCT   3109
gb:MT093631|Organism:Severe      GAGTTTGAGCCATCAACTCAATATGAGTATGGTACTGAAGATGATTACCAAGGTAAACCT   3180
gb:MT106053|Organism:Severe      GAGTTTGAGCCATCAACTCAATATGAGTATGGTACTGAAGATGATTACCAAGGTAAACCT   3142
gb:MT019533|Organism:Severe      GAGTTTGAGCCATCAACTCAATATGAGTATGGTACTGAAGATGATTACCAAGGTAAACCT   3142
gb:MT019531|Organism:Severe      GAGTTTGAGCCATCAACTCAATATGAGTATGGTACTGAAGATGATTACCAAGGTAAACCT   3142
gb:MN996528|Organism:Severe      GAGTTTGAGCCATCAACTCAATATGAGTATGGTACTGAAGATGATTACCAAGGTAAACCT   3142
gb:MN996530|Organism:Severe      GAGTTTGAGCCATCAACTCAATATGAGTATGGTACTGAAGATGATTACCAAGGTAAACCT   3128
gb:MN908947|Organism:Severe      GAGTTTGAGCCATCAACTCAATATGAGTATGGTACTGAAGATGATTACCAAGGTAAACCT   3142
gb:MT019532|Organism:Severe      GAGTTTGAGCCATCAACTCAATATGAGTATGGTACTGAAGATGATTACCAAGGTAAACCT   3142
                                 ************************************************************

gb:MT020781|Organism:Severe      TTGGAATTTGGTGCCACTTCTGCTGCTCTTCAACCTGAAGAAGAGCAAGAAGAAGATTGG   3190
gb:MT007544|Organism:Severe      TTGGAATTTGGTGCCACTTCTGCTGCTCTTCAACCTGAAGAAGAGCAAGAAGAAGATTGG   3202
gb:MN994467|Organism:Severe      TTGGAATTTGGTGCCACTTCTGCTGCTCTTCAACCTGAAGAAGAGCAAGAAGAAGATTGG   3202
gb:MT044257|Organism:Severe      TTGGAATTTGGTGCCACTTCTGCTGCTCTTCAACTTGAAGAAGAGCAAGAAGAAGATTGG   3202
gb:MT106054|Organism:Severe      TTGGAATTTGGTGCCACTTCTGCTGCTCTTCAACCTGAAGAAGAGCAAGAAGAAGATTGG   3202
gb:MT049951|Organism:Severe      TTGGAATTTGGTGCCACTTCTGCTGCTCTTCAACCTGAAGAAGAGCAAGAAGAAGATTGG   3202
gb:MN975262|Organism:Severe      TTGGAATTTGGTGCCACTTCTGCTGCTCTTCAACCTGAAGAAGAGCAAGAAGAAGATTGG   3202
gb:MT106052|Organism:Severe      TTGGAATTTGGTGCCACTTCTGCTGCTCTTCAACCTGAAGAAGAGCAAGAAGAAGATTGG   3202
gb:LC522975|Organism:Severe      TTGGAATTTGGTGCCACTTCTGCTGCTCTTCAACCTGAAGAAGAGCAAGAAGAAGATTGG   3199
gb:LC522973|Organism:Severe      TTGGAATTTGGTGCCACTTCTGCTGCTCTTCAACCTGAAGAAGAGCAAGAAGAAGATTGG   3199
gb:LC522974|Organism:Severe      TTGGAATTTGGTGCCACTTCTGCTGCTCTTCAACCTGAAGAAGAGCAAGAAGAAGATTGG   3199
gb:MN985325|Organism:Severe      TTGGAATTTGGTGCCACTTCTGCTGCTCTTCAACCTGAAGAAGAGCAAGAAGAAGATTGG   3202
gb:MT020881|Organism:Severe      TTGGAATTTGGTGCCACTTCTGCTGCTCTTCAACCTGAAGAAGAGCAAGAAGAAGATTGG   3202
gb:MT020880|Organism:Severe      TTGGAATTTGGTGCCACTTCTGCTGCTCTTCAACCTGAAGAAGAGCAAGAAGAAGATTGG   3202
gb:MT066175|Organism:Severe      TTGGAATTTGGTGCCACTTCTGCTGCTCTTCAACCTGAAGAAGAGCAAGAAGAAGATTGG   3202
gb:MN997409|Organism:Severe      TTGGAATTTGGTGCCACTTCTGCTGCTCTTCAACCTGAAGAAGAGCAAGAAGAAGATTGG   3202
gb:MN938384|Organism:Severe      TTGGAATTTGGTGCCACTTCTGCTGCTCTTCAACCTGAAGAAGAGCAAGAAGAAGATTGG   3170
gb:MT044258|Organism:Severe      TTGGAATTTGGTGCCACTTCTGCTGCTCTTCAACCTGAAGAAGAGCAAGAAGAAGATTGG   3178
gb:MT039890|Organism:Severe      TTGGAATTTGGTGCCACTTCTGCTGCTCTTCAACCTGAAGAAGAGCAAGAAGAAGATTGG   3202
gb:MN988713|Organism:Severe      TTGGAATTTGGTGCCACTTCTGCTGCTCTTCAACYTGAAGAAGAGCAAGAAGAAGATTGG   3202
gb:LC521925|Organism:Severe      TTGGAATTTGGTGCCACTTCTGCTGCTCTTCAACCTGAAGAAGAGCAAGAAGAAGATTGG   3175
gb:MT093571|Organism:Severe      TTGGAATTTGGTGCCACTTCTGCTGCTCTTCAACCTGAAGAAGAGCAAGAAGAAGATTGG   3202
gb:MT039887|Organism:Severe      TTGGAATTTGGTGCCACTTCTGCTGCTCTTCAACCTGAAGAAGAGCAAGAAGAAGATTGG   3202
gb:MT019530|Organism:Severe      TTGGAATTTGGTGCCACTTCTGCTGCTCTTCAACCTGAAGAAGAGCAAGAAGAAGATTGG   3202
gb:MT039888|Organism:Severe      TTGGAATTTGGTGCCACTTCTGCTGCTCTTCAACCTGAAGAAGAGCAAGAAGAAGATTGG   3202
gb:LC522972|Organism:Severe      TTGGAATTTGGTGCCACTTCTGCTGCTCTTCAACCTGAAGAAGAGCAAGAAGAAGATTGG   3199
gb:MT027063|Organism:Severe      TTGGAATTTGGTGCCACTTCTGCTGCTCTTCAACCTGAAGAAGAGCAAGAAGAAGATTGG   3202
gb:MT027062|Organism:Severe      TTGGAATTTGGTGCCACTTCTGCTGCTCTTCAACCTGAAGAAGAGCAAGAAGAAGATTGG   3202
```

```
gb:MT019529|Organism:Severe      TTGGAATTTGGTGCCACTTCTGCTGCTCTTCAACCTGAAGAAGAGCAAGAAGAAGATTGG    3202
gb:MN996529|Organism:Severe      TTGGAATTTGGTGCCACTTCTGCTGCTCTTCAACCTGAAGAAGAGCAAGAAGAAGATTGG    3190
gb:MN996531|Organism:Severe      TTGGAATTTGGTGCCACTTCTGCTGCTCTTCAACCTGAAGAAGAGCAAGAAGAAGATTGG    3189
gb:MT066176|Organism:Severe      TTGGAATTTGGTGCCACTTCTGCTGCTCTTCAACCTGAAGAAGAGCAAGAAGAAGATTGG    3202
gb:MT027064|Organism:Severe      TTGGAATTTGGTGCCACTTCTGCTGCTCTTCAACCTGAAGAAGAGCAAGAAGAAGATTGG    3202
gb:MN994468|Organism:Severe      TTGGAATTTGGTGCCACTTCTGCTGCTCTTCAACCTGAAGAAGAGCAAGAAGAAGATTGG    3202
gb:MT072688|Organism:Severe      TTGGAATTTGGTGCCACTTCTGCTGCTCTTCAACCTGAAGAAGAGCAAGAAGAAGATTGG    3187
gb:MN996527|Organism:Severe      TTGGAATTTGGTGCCACTTCTGCTGCTCTTCAACCTGAAGAAGAGCAAGAAGAAGATTGG    3169
gb:MT093631|Organism:Severe      TTGGAATTTGGTGCCACTTCTGCTGCTCTTCAACCTGAAGAAGAGCAAGAAGAAGATTGG    3240
gb:MT106053|Organism:Severe      TTGGAATTTGGTGCCACTTCTGCTGCTCTTCAACCTGAAGAAGAGCAAGAAGAAGATTGG    3202
gb:MT019533|Organism:Severe      TTGGAATTTGGTGCCACTTCTGCTGCTCTTCAACCTGAAGAAGAGCAAGAAGAAGATTGG    3202
gb:MT019531|Organism:Severe      TTGGAATTTGGTGCCACTTCTGCTGCTCTTCAACCTGAAGAAGAGCAAGAAGAAGATTGG    3202
gb:MN996528|Organism:Severe      TTGGAATTTGGTGCCACTTCTGCTGCTCTTCAACCTGAAGAAGAGCAAGAAGAAGATTGG    3202
gb:MN996530|Organism:Severe      TTGGAATTTGGTGCCACTTCTGCTGCTCTTCAACCTGAAGAAGAGCAAGAAGAAGATTGG    3188
gb:MN908947|Organism:Severe      TTGGAATTTGGTGCCACTTCTGCTGCTCTTCAACCTGAAGAAGAGCAAGAAGAAGATTGG    3202
gb:MT019532|Organism:Severe      TTGGAATTTGGTGCCACTTCTGCTGCTCTTCAACCTGAAGAAGAGCAAGAAGAAGATTGG    3202
                                 ********************************** *************************

gb:MT020781|Organism:Severe      TTAGATGATGATAGTCAACAAACTGTTGGTCAACAAGACGGCAGTGAGGACAATCAGACA    3250
gb:MT007544|Organism:Severe      TTAGATGATGATAGTCAACAAACTGTTGGTCAACAAGACGGCAGTGAGGACAATCAGACA    3262
gb:MN994467|Organism:Severe      TTAGATGATGATAGTCAACAAACTGTTGGTCAACAAGACGGCAGTGAGGACAATCAGACA    3262
gb:MT044257|Organism:Severe      TTAGATGATGATAGTCAACAAACTGTTGGTCAACAAGACGGCAGTGAGGACAATCAGACA    3262
gb:MT106054|Organism:Severe      TTAGATGATGATAGTCAACAAACTGTTGGTCAACAAGACGGCAGTGAGGACAATCAGACA    3262
gb:MT049951|Organism:Severe      TTAGATGATGATAGTCAACAAACTGTTGGTCAACAAGACGGCAGTGAGGACAATCAGACA    3262
gb:MN975262|Organism:Severe      TTAGATGATGATAGTCAACAAACTGTTGGTCAACAAGACGGCAGTGAGGACAATCAGACA    3262
gb:MT106052|Organism:Severe      TTAGATGATGATAGTCAACAAACTGTTGGTCAACAAGACGGCAGTGAGGACAATCAGACA    3262
gb:LC522975|Organism:Severe      TTAGATGATGATAGTCAACAAACTGTTGGTCAACAAGACGGCAGTGAGGACAATCAGACA    3259
gb:LC522973|Organism:Severe      TTAGATGATGATAGTCAACAAACTGTTGGTCAACAAGACGGCAGTGAGGACAATCAGACA    3259
gb:LC522974|Organism:Severe      TTAGATGATGATAGTCAACAAACTGTTGGTCAACAAGACGGCAGTGAGGACAATCAGACA    3259
gb:MN985325|Organism:Severe      TTAGATGATGATAGTCAACAAACTGTTGGTCAACAAGACGGCAGTGAGGACAATCAGACA    3262
gb:MT020881|Organism:Severe      TTAGATGATGATAGTCAACAAACTGTTGGTCAACAAGACGGCAGTGAGGACAATCAGACA    3262
gb:MT020880|Organism:Severe      TTAGATGATGATAGTCAACAAACTGTTGGTCAACAAGACGGCAGTGAGGACAATCAGACA    3262
gb:MT066175|Organism:Severe      TTAGATGATGATAGTCAACAAACTGTTGGTCAACAAGACGGCAGTGAGGACAATCAGACA    3262
gb:MN997409|Organism:Severe      TTAGATGATGATAGTCAACAAACTGTTGGTCAACAAGACGGCAGTGAGGACAATCAGACA    3262
gb:MN938384|Organism:Severe      TTAGATGATGATAGTCAACAAACTGTTGGTCAACAAGACGGCAGTGAGGACAATCAGACA    3230
gb:MT044258|Organism:Severe      TTAGATGATGATAGTCAACAAACTGTTGGTCAACAAGACGGCAGTGAGGACAATCAGACA    3238
gb:MT039890|Organism:Severe      TTAGATGATGATAGTCAACAAACTGTTGGTCAACAAGACGGCAGTGAGGACAATCAGACA    3262
gb:MN988713|Organism:Severe      TTAGATGATGATAGTCAACAAACTGTTGGTCAACAAGACGGCAGTGAGGACAATCAGACA    3262
gb:LC521925|Organism:Severe      TTAGATGATGATAGTCAACAAACTGTTGGTCAACAAGACGGCAGTGAGGACAATCAGACA    3235
gb:MT093571|Organism:Severe      TTAGATGATGATAGTCAACAAACTGTTGGTCAACAAGACGGCAGTGAGGACAATCAGACA    3262
gb:MT039887|Organism:Severe      TTAGATGATGATAGTCAACAAACTGTTGGTCAACAAGACGGCAGTGAGGACAATCAGACA    3262
gb:MT019530|Organism:Severe      TTAGATGATGATAGTCAACAAACTGTTGGTCAACAAGACGGCAGTGAGGACAATCAGACA    3262
gb:MT039888|Organism:Severe      TTAGATGATGATAGTCAACAAACTGTTGGTCAACAAGACGGCAGTGAGGACAATCAGACA    3262
gb:LC522972|Organism:Severe      TTAGATGATGATAGTCAACAAACTGTTGGTCAACAAGACGGCAGTGAGGACAATCAGACA    3259
gb:MT027063|Organism:Severe      TTAGATGATGATAGTCAACAAACTGTTGGTCAACAAGACGGCAGTGAGGACAATCAGACA    3262
gb:MT027062|Organism:Severe      TTAGATGATGATAGTCAACAAACTGTTGGTCAACAAGACGGCAGTGAGGACAATCAGACA    3262
gb:MT019529|Organism:Severe      TTAGATGATGATAGTCAACAAACTGTTGGTCAACAAGACGGCAGTGAGGACAATCAGACA    3262
gb:MN996529|Organism:Severe      TTAGATGATGATAGTCAACAAACTGTTGGTCAACAAGACGGCAGTGAGGACAATCAGACA    3250
gb:MN996531|Organism:Severe      TTAGATGATGATAGTCAACAAACTGTTGGTCAACAAGACGGCAGTGAGGACAATCAGACA    3249
gb:MT066176|Organism:Severe      TTAGATGATGATAGTCAACAAACTGTTGGTCAACAAGACGGCAGTGAGGACAATCAGACA    3262
gb:MT027064|Organism:Severe      TTAGATGATGATAGTCAACAAACTGTTGGTCAACAAGACGGCAGTGAGGACAATCAGACA    3262
gb:MN994468|Organism:Severe      TTAGATGATGATAGTCAACAAACTGTTGGTCAACAAGACGGCAGTGAGGACAATCAGACA    3262
gb:MT072688|Organism:Severe      TTAGATGATGATAGTCAACAAACTGTTGGTCAACAAGACGGCAGTGAGGACAATCAGACA    3247
gb:MN996527|Organism:Severe      TTAGATGATGATAGTCAACAAACTGTTGGTCAACAAGACGGCAGTGAGGACAATCAGACA    3229
gb:MT093631|Organism:Severe      TTAGATGATGATAGTCAACAAACTGTTGGTCAACAAGACGGCAGTGAGGACAATCAGACA    3300
gb:MT106053|Organism:Severe      TTAGATGATGATAGTCAACAAACTGTTGGTCAACAAGACGGCAGTGAGGACAATCAGACA    3262
gb:MT019533|Organism:Severe      TTAGATGATGATAGTCAACAAACTGTTGGTCAACAAGACGGCAGTGAGGACAATCAGACA    3262
gb:MT019531|Organism:Severe      TTAGATGATGATAGTCAACAAACTGTTGGTCAACAAGACGGCAGTGAGGACAATCAGACA    3262
gb:MN996528|Organism:Severe      TTAGATGATGATAGTCAACAAACTGTTGGTCAACAAGACGGCAGTGAGGACAATCAGACA    3262
gb:MN996530|Organism:Severe      TTAGATGATGATAGTCAACAAACTGTTGGTCAACAAGACGGCAGTGAGGACAATCAGACA    3248
gb:MN908947|Organism:Severe      TTAGATGATGATAGTCAACAAACTGTTGGTCAACAAGACGGCAGTGAGGACAATCAGACA    3262
gb:MT019532|Organism:Severe      TTAGATGATGATAGTCAACAAACTGTTGGTCAACAAGACGGCAGTGAGGACAATCAGACA    3262
                                 ************************************************************

gb:MT020781|Organism:Severe      ACTACTATTCAAACAATTGTTGAGGTTCAACCTCAATTAGAGATGGAACTTACACCAGTT    3310
```

```
gb:MT007544|Organism:Severe      ACTACTATTCAAACAATTGTTGAGGTTCAACCTCAATTAGAGATGGAACTTACACCAGTT   3322
gb:MN994467|Organism:Severe      ACTACTATTCAAACAATTGTTGAGGTTCAACCTCAATTAGAGATGGAACTTACACCAGTT   3322
gb:MT044257|Organism:Severe      ACTACTATTCAAACAATTGTTGAGGTTCAACCTCAATTAGAGATGGAACTTACACCAGTT   3322
gb:MT106054|Organism:Severe      ACTACTATTCAAACAATTGTTGAGGTTCAACCTCAATTAGAGATGGAACTTACACCAGTT   3322
gb:MT049951|Organism:Severe      ACTACTATTCAAACAATTGTTGAGGTTCAACCTCAATTAGAGATGGAACTTACACCAGTT   3322
gb:MN975262|Organism:Severe      ACTACTATTCAAACAATTGTTGAGGTTCAACCTCAATTAGAGATGGAACTTACACCAGTT   3322
gb:MT106052|Organism:Severe      ACTACTATTCAAACAATTGTTGAGGTTCAACCTCAATTAGAGATGGAACTTACACCAGTT   3322
gb:LC522975|Organism:Severe      ACTACTATTCAAACAATTGTTGAGGTTCAACCTCAATTAGAGATGGAACTTACACCAGTT   3319
gb:LC522973|Organism:Severe      ACTACTATTCAAACAATTGTTGAGGTTCAACCTCAATTAGAGATGGAACTTACACCAGTT   3319
gb:LC522974|Organism:Severe      ACTACTATTCAAACAATTGTTGAGGTTCAACCTCAATTAGAGATGGAACTTACACCAGTT   3319
gb:MN985325|Organism:Severe      ACTACTATTCAAACAATTGTTGAGGTTCAACCTCAATTAGAGATGGAACTTACACCAGTT   3322
gb:MT020881|Organism:Severe      ACTACTATTCAAACAATTGTTGAGGTTCAACCTCAATTAGAGATGGAACTTACACCAGTT   3322
gb:MT020880|Organism:Severe      ACTACTATTCAAACAATTGTTGAGGTTCAACCTCAATTAGAGATGGAACTTACACCAGTT   3322
gb:MT066175|Organism:Severe      ACTACTATTCAAACAATTGTTGAGGTTCAACCTCAATTAGAGATGGAACTTACACCAGTT   3322
gb:MN997409|Organism:Severe      ACTACTATTCAAACAATTGTTGAGGTTCAACCTCAATTAGAGATGGAACTTACACCAGTT   3322
gb:MN938384|Organism:Severe      ACTACTATTCAAACAATTGTTGAGGTTCAACCTCAATTAGAGATGGAACTTACACCAGTT   3290
gb:MT044258|Organism:Severe      ACTACTATTCAAACAATTGTTGAGGTTCAACCTCAATTAGAGATGGAACTTACACCAGTT   3298
gb:MT039890|Organism:Severe      ACTACTATTCAAACAATTGTTGAGGTTCAACCTCAATTAGAGATGGAACTTACACCAGTT   3322
gb:MN988713|Organism:Severe      ACTACTATTCAAACAATTGTTGAGGTTCAACCTCAATTAGAGATGGAACTTACACCAGTT   3322
gb:LC521925|Organism:Severe      ACTACTATTCAAACAATTGTTGAGGTTCAACCTCAATTAGAGATGGAACTTACACCAGTT   3295
gb:MT093571|Organism:Severe      ACTACTATTCAAACAATTGTTGAGGTTCAACCTCAATTAGAGATGGAACTTACACCAGTT   3322
gb:MT039887|Organism:Severe      ACTACTATTCAAACAATTGTTGAGGTTCAACCTCAATTAGAGATGGAACTTACACCAGTT   3322
gb:MT019530|Organism:Severe      ACTACTATTCAAACAATTGTTGAGGTTCAACCTCAATTAGAGATGGAACTTACACCAGTT   3322
gb:MT039888|Organism:Severe      ACTACTATTCAAACAATTGTTGAGGTTCAACCTCAATTAGAGATGGAACTTACACCAGTT   3322
gb:LC522972|Organism:Severe      ACTACTATTCAAACAATTGTTGAGGTTCAACCTCAATTAGAGATGGAACTTACACCAGTT   3319
gb:MT027063|Organism:Severe      ACTACTATTCAAACAATTGTTGAGGTTCAACCTCAATTAGAGATGGAACTTACACCAGTT   3322
gb:MT027062|Organism:Severe      ACTACTATTCAAACAATTGTTGAGGTTCAACCTCAATTAGAGATGGAACTTACACCAGTT   3322
gb:MT019529|Organism:Severe      ACTACTATTCAAACAATTGTTGAGGTTCAACCTCAATTAGAGATGGAACTTACACCAGTT   3322
gb:MN996529|Organism:Severe      ACTACTATTCAAACAATTGTTGAGGTTCAACCTCAATTAGAGATGGAACTTACACCAGTT   3310
gb:MN996531|Organism:Severe      ACTACTATTCAAACAATTGTTGAGGTTCAACCTCAATTAGAGATGGAACTTACACCAGTT   3309
gb:MT066176|Organism:Severe      ACTACTATTCAAACAATTGTTGAGGTTCAACCTCAATTAGAGATGGAACTTACACCAGTT   3322
gb:MT027064|Organism:Severe      ACTACTATTCAAACAATTGTTGAGGTTCAACCTCAATTAGAGATGGAACTTACACCAGTT   3322
gb:MN994468|Organism:Severe      ACTACTATTCAAACAATTGTTGAGGTTCAACCTCAATTAGAGATGGAACTTACACCAGTT   3322
gb:MT072688|Organism:Severe      ACTACTATTCAAACAATTGTTGAGGTTCAACCTCAATTAGAGATGGAACTTACACCAGTT   3307
gb:MN996527|Organism:Severe      ACTACTATTCAAACAATTGTTGAGGTTCAACCTCAATTAGAGATGGAACTTACACCAGTT   3289
gb:MT093631|Organism:Severe      ACTACTATTCAAACAATTGTTGAGGTTCAACCTCAATTAGAGATGGAACTTACACCAGTT   3360
gb:MT106053|Organism:Severe      ACTACTATTCAAACAATTGTTGAGGTTCAACCTCAATTAGAGATGGAACTTACACCAGTT   3322
gb:MT019533|Organism:Severe      ACTACTATTCAAACAATTGTTGAGGTTCAACCTCAATTAGAGATGGAACTTACACCAGTT   3322
gb:MT019531|Organism:Severe      ACTACTATTCAAACAATTGTTGAGGTTCAACCTCAATTAGAGATGGAACTTACACCAGTT   3322
gb:MN996528|Organism:Severe      ACTACTATTCAAACAATTGTTGAGGTTCAACCTCAATTAGAGATGGAACTTACACCAGTT   3322
gb:MN996530|Organism:Severe      ACTACTATTCAAACAATTGTTGAGGTTCAACCTCAATTAGAGATGGAACTTACACCAGTT   3308
gb:MN908947|Organism:Severe      ACTACTATTCAAACAATTGTTGAGGTTCAACCTCAATTAGAGATGGAACTTACACCAGTT   3322
gb:MT019532|Organism:Severe      ACTACTATTCAAACAATTGTTGAGGTTCAACCTCAATTAGAGATGGAACTTACACCAGTT   3322
                                 ************************************************************

gb:MT020781|Organism:Severe      GTTCAGACTATTGAAGTGAATAGTTTTAGTGGTTATTTAAAACTTACTGACAATGTATAC   3370
gb:MT007544|Organism:Severe      GTTCAGACTATTGAAGTGAATAGTTTTAGTGGTTATTTAAAACTTACTGACAATGTATAC   3382
gb:MN994467|Organism:Severe      GTTCAGACTATTGAAGTGAATAGTTTTAGTGGTTATTTAAAACTTACTGACAATGTATAC   3382
gb:MT044257|Organism:Severe      GTTCAGACTATTGAAGTGAATAGTTTTAGTGGTTATTTAAAACTTACTGACAATGTATAC   3382
gb:MT106054|Organism:Severe      GTTCAGACTATTGAAGTGAATAGTTTTAGTGGTTATTTAAAACTTACTGACAATGTATAC   3382
gb:MT049951|Organism:Severe      GTTCAGACTATTGAAGTGAATAGTTTTAGTGGTTATTTAAAACTTACTGACAATGTATAC   3382
gb:MN975262|Organism:Severe      GTTCAGACTATTGAAGTGAATAGTTTTAGTGGTTATTTAAAACTTACTGACAATGTATAC   3382
gb:MT106052|Organism:Severe      GTTCAGACTATTGAAGTGAATAGTTTTAGTGGTTATTTAAAACTTACTGACAATGTATAC   3382
gb:LC522975|Organism:Severe      GTTCAGACTATTGAAGTGAATAGTTTTAGTGGTTATTTAAAACTTACTGACAATGTATAC   3379
gb:LC522973|Organism:Severe      GTTCAGACTATTGAAGTGAATAGTTTTAGTGGTTATTTAAAACTTACTGACAATGTATAC   3379
gb:LC522974|Organism:Severe      GTTCAGACTATTGAAGTGAATAGTTTTAGTGGTTATTTAAAACTTACTGACAATGTATAC   3379
gb:MN985325|Organism:Severe      GTTCAGACTATTGAAGTGAATAGTTTTAGTGGTTATTTAAAACTTACTGACAATGTATAC   3382
gb:MT020881|Organism:Severe      GTTCAGACTATTGAAGTGAATAGTTTTAGTGGTTATTTAAAACTTACTGACAATGTATAC   3382
gb:MT020880|Organism:Severe      GTTCAGACTATTGAAGTGAATAGTTTTAGTGGTTATTTAAAACTTACTGACAATGTATAC   3382
gb:MT066175|Organism:Severe      GTTCAGACTATTGAAGTGAATAGTTTTAGTGGTTATTTAAAACTTACTGACAATGTATAC   3382
gb:MN997409|Organism:Severe      GTTCAGACTATTGAAGTGAATAGTTTTAGTGGTTATTTAAAACTTACTGACAATGTATAC   3382
gb:MN938384|Organism:Severe      GTTCAGACTATTGAAGTGAATAGTTTTAGTGGTTATTTAAAACTTACTGACAATGTATAC   3350
gb:MT044258|Organism:Severe      GTTCAGACTATTGAAGTGAATAGTTTTAGTGGTTATTTAAAACTTACTGACAATGTATAC   3358
gb:MT039890|Organism:Severe      GTTCAGACTATTGAAGTGAATAGTTTTAGTGGTTATTTAAAACTTACTGACAATGTATAC   3382
gb:MN988713|Organism:Severe      GTTCAGACTATTGAAGTGAATAGTTTTAGTGGTTATTTAAAACTTACTGACAATGTATAC   3382
```

```
gb:LC521925|Organism:Severe      GTTCAGACTATTGAAGTGAATAGTTTTAGTGGTTATTTAAAACTTACTGACAATGTATAC    3355
gb:MT093571|Organism:Severe      GTTCAGACTATTGAAGTGAATAGTTTTAGTGGTTATTTAAAACTTACTGACAATGTATAC    3382
gb:MT039887|Organism:Severe      GTTCAGACTATTGAAGTGAATAGTTTTAGTGGTTATTTAAAACTTACTGACAATGTATAC    3382
gb:MT019530|Organism:Severe      GTTCAGACTATTGAAGTGAATAGTTTTAGTGGTTATTTAAAACTTACTGACAATGTATAC    3382
gb:MT039888|Organism:Severe      GTTCAGACTATTGAAGTGAATAGTTTTAGTGGTTATTTAAAACTTACTGACAATGTATAC    3382
gb:LC522972|Organism:Severe      GTTCAGACTATTGAAGTGAATAGTTTTAGTGGTTATTTAAAACTTACTGACAATGTATAC    3379
gb:MT027063|Organism:Severe      GTTCAGACTATTGAAGTGAATAGTTTTAGTGGTTATTTAAAACTTACTGACAATGTATAC    3382
gb:MT027062|Organism:Severe      GTTCAGACTATTGAAGTGAATAGTTTTAGTGGTTATTTAAAACTTACTGACAATGTATAC    3382
gb:MT019529|Organism:Severe      GTTCAGACTATTGAAGTGAATAGTTTTAGTGGTTATTTAAAACTTACTGACAATGTATAC    3382
gb:MN996529|Organism:Severe      GTTCAGACTATTGAAGTGAATAGTTTTAGTGGTTATTTAAAACTTACTGACAATGTATAC    3370
gb:MN996531|Organism:Severe      GTTCAGACTATTGAAGTGAATAGTTTTAGTGGTTATTTAAAACTTACTGACAATGTATAC    3369
gb:MT066176|Organism:Severe      GTTCAGACTATTGAAGTGAATAGTTTTAGTGGTTATTTAAAACTTACTGACAATGTATAC    3382
gb:MT027064|Organism:Severe      GTTCAGACTATTGAAGTGAATAGTTTTAGTGGTTATTTAAAACTTACTGACAATGTATAC    3382
gb:MN994468|Organism:Severe      GTTCAGACTATTGAAGTGAATAGTTTTAGTGGTTATTTAAAACTTACTGACAATGTATAC    3382
gb:MT072688|Organism:Severe      GTTCAGACTATTGAAGTGAATAGTTTTAGTGGTTATTTAAAACTTACTGACAATGTATAC    3367
gb:MN996527|Organism:Severe      GTTCAGACTATTGAAGTGAATAGTTTTAGTGGTTATTTAAAACTTACTGACAATGTATAC    3349
gb:MT093631|Organism:Severe      GTTCAGACTATTGAAGTGAATAGTTTTAGTGGTTATTTAAAACTTACTGACAATGTATAC    3420
gb:MT106053|Organism:Severe      GTTCAGACTATTGAAGTGAATAGTTTTAGTGGTTATTTAAAACTTACTGACAATGTATAC    3382
gb:MT019533|Organism:Severe      GTTCAGACTATTGAAGTGAATAGTTTTAGTGGTTATTTAAAACTTACTGACAATGTATAC    3382
gb:MT019531|Organism:Severe      GTTCAGACTATTGAAGTGAATAGTTTTAGTGGTTATTTAAAACTTACTGACAATGTATAC    3382
gb:MN996528|Organism:Severe      GTTCAGACTATTGAAGTGAATAGTTTTAGTGGTTATTTAAAACTTACTGACAATGTATAC    3382
gb:MN996530|Organism:Severe      GTTCAGACTATTGAAGTGAATAGTTTTAGTGGTTATTTAAAACTTACTGACAATGTATAC    3368
gb:MN908947|Organism:Severe      GTTCAGACTATTGAAGTGAATAGTTTTAGTGGTTATTTAAAACTTACTGACAATGTATAC    3382
gb:MT019532|Organism:Severe      GTTCAGACTATTGAAGTGAATAGTTTTAGTGGTTATTTAAAACTTACTGACAATGTATAC    3382
                                 ************************************************************

gb:MT020781|Organism:Severe      ATTAAAAATGCAGACATTGTGGAAGAAGCTAAAAAGGTAAAACCAACAGTGGTTGTTAAT    3430
gb:MT007544|Organism:Severe      ATTAAAAATGCAGACATTGTGGAAGAAGCTAAAAAGGTAAAACCAACAGTGGTTGTTAAT    3442
gb:MN994467|Organism:Severe      ATTAAAAATGCAGACATTGTGGAAGAAGCTAAAAAGGTAAAACCAACAGTGGTTGTTAAT    3442
gb:MT044257|Organism:Severe      ATTAAAAATGCAGACATTGTGGAAGAAGCTAAAAAGGTAAAACCAACAGTGGTTGTTAAT    3442
gb:MT106054|Organism:Severe      ATTAAAAATGCAGACATTGTGGAAGAAGCTAAAAAGGTAAAACCAACAGTGGTTGTTAAT    3442
gb:MT049951|Organism:Severe      ATTAAAAATGCAGACATTGTGGAAGAAGCTAAAAAGGTAAAACCAACAGTGGTTGTTAAT    3442
gb:MN975262|Organism:Severe      ATTAAAAATGCAGACATTGTGGAAGAAGCTAAAAAGGTAAAACCAACAGTGGTTGTTAAT    3442
gb:MT106052|Organism:Severe      ATTAAAAATGCAGACATTGTGGAAGAAGCTAAAAAGGTAAAACCAACAGTGGTTGTTAAT    3442
gb:LC522975|Organism:Severe      ATTAAAAATGCAGACATTGTGGAAGAAGCTAAAAAGGTAAAACCAACAGTGGTTGTTAAT    3439
gb:LC522973|Organism:Severe      ATTAAAAATGCAGACATTGTGGAAGAAGCTAAAAAGGTAAAACCAACAGTGGTTGTTAAT    3439
gb:LC522974|Organism:Severe      ATTAAAAATGCAGACATTGTGGAAGAAGCTAAAAAGGTAAAACCAACAGTGGTTGTTAAT    3439
gb:MN985325|Organism:Severe      ATTAAAAATGCAGACATTGTGGAAGAAGCTAAAAAGGTAAAACCAACAGTGGTTGTTAAT    3442
gb:MT020881|Organism:Severe      ATTAAAAATGCAGACATTGTGGAAGAAGCTAAAAAGGTAAAACCAACAGTGGTTGTTAAT    3442
gb:MT020880|Organism:Severe      ATTAAAAATGCAGACATTGTGGAAGAAGCTAAAAAGGTAAAACCAACAGTGGTTGTTAAT    3442
gb:MT066175|Organism:Severe      ATTAAAAATGCAGACATTGTGGAAGAAGCTAAAAAGGTAAAACCAACAGTGGTTGTTAAT    3442
gb:MN997409|Organism:Severe      ATTAAAAATGCAGACATTGTGGAAGAAGCTAAAAAGGTAAAACCAACAGTGGTTGTTAAT    3442
gb:MN938384|Organism:Severe      ATTAAAAATGCAGACATTGTGGAAGAAGCTAAAAAGGTAAAACCAACAGTGGTTGTTAAT    3410
gb:MT044258|Organism:Severe      ATTAAAAATGCAGACATTGTGGAAGAAGCTAAAAAGGTAAAACCAACAGTGGTTGTTAAT    3418
gb:MT039890|Organism:Severe      ATTAAAAATGCAGACATTGTGGAAGAAGCTAAAAAGGTAAAACCAACAGTGGTTGTTAAT    3442
gb:MN988713|Organism:Severe      ATTAAAAATGCAGACATTGTGGAAGAAGCTAAAAAGGTAAAACCAACAGTGGTTGTTAAT    3442
gb:LC521925|Organism:Severe      ATTAAAAATGCAGACATTGTGGAAGAAGCTAAAAAGGTAAAACCAACAGTGGTTGTTAAT    3415
gb:MT093571|Organism:Severe      ATTAAAAATGCAGACATTGTGGAAGAAGCTAAAAAGGTAAAACCAACAGTGGTTGTTAAT    3442
gb:MT039887|Organism:Severe      ATTAAAAATGCAGACATTGTGGAAGAAGCTAAAAAGGTAAAACCAACAGTGGTTGTTAAT    3442
gb:MT019530|Organism:Severe      ATTAAAAATGCAGACATTGTGGAAGAAGCTAAAAAGGTAAAACCAACAGTGGTTGTTAAT    3442
gb:MT039888|Organism:Severe      ATTAAAAATGCAGACATTGTGGAAGAAGCTAAAAAGGTAAAACCAACAGTGGTTGTTAAT    3442
gb:LC522972|Organism:Severe      ATTAAAAATGCAGACATTGTGGAAGAAGCTAAAAAGGTAAAACCAACAGTGGTTGTTAAT    3439
gb:MT027063|Organism:Severe      ATTAAAAATGCAGACATTGTGGAAGAAGCTAAAAAGGTAAAACCAACAGTGGTTGTTAAT    3442
gb:MT027062|Organism:Severe      ATTAAAAATGCAGACATTGTGGAAGAAGCTAAAAAGGTAAAACCAACAGTGGTTGTTAAT    3442
gb:MT019529|Organism:Severe      ATTAAAAATGCAGACATTGTGGAAGAAGCTAAAAAGGTAAAACCAACAGTGGTTGTTAAT    3442
gb:MN996529|Organism:Severe      ATTAAAAATGCAGACATTGTGGAAGAAGCTAAAAAGGTAAAACCAACAGTGGTTGTTAAT    3430
gb:MN996531|Organism:Severe      ATTAAAAATGCAGACATTGTGGAAGAAGCTAAAAAGGTAAAACCAACAGTGGTTGTTAAT    3429
gb:MT066176|Organism:Severe      ATTAAAAATGCAGACATTGTGGAAGAAGCTAAAAAGGTAAAACCAACAGTGGTTGTTAAT    3442
gb:MT027064|Organism:Severe      ATTAAAAATGCAGACATTGTGGAAGAAGCTAAAAAGGTAAAACCAACAGTGGTTGTTAAT    3442
gb:MN994468|Organism:Severe      ATTAAAAATGCAGACATTGTGGAAGAAGCTAAAAAGGTAAAACCAACAGTGGTTGTTAAT    3442
gb:MT072688|Organism:Severe      ATTAAAAATGCAGACATTGTGGAAGAAGCTAAAAAGGTAAAACCAACAGTGGTTGTTAAT    3427
gb:MN996527|Organism:Severe      ATTAAAAATGCAGACATTGTGGAAGAAGCTAAAAAGGTAAAACCAACAGTGGTTGTTAAT    3409
gb:MT093631|Organism:Severe      ATTAAAAATGCAGACATTGTGGAAGAAGCTAAAAAGGTAAAACCAACAGTGGTTGTTAAT    3480
gb:MT106053|Organism:Severe      ATTAAAAATGCAGACATTGTGGAAGAAGCTAAAAAGGTAAAACCAACAGTGGTTGTTAAT    3442
gb:MT019533|Organism:Severe      ATTAAAAATGCAGACATTGTGGAAGAAGCTAAAAAGGTAAAACCAACAGTGGTTGTTAAT    3442
```

```
gb:MT019531|Organism:Severe      ATTAAAAATGCAGACATTGTGGAAGAAGCTAAAAAGGTAAAACCAACAGTGGTTGTTAAT   3442
gb:MN996528|Organism:Severe      ATTAAAAATGCAGACATTGTGGAAGAAGCTAAAAAGGTAAAACCAACAGTGGTTGTTAAT   3442
gb:MN996530|Organism:Severe      ATTAAAAATGCAGACATTGTGGAAGAAGCTAAAAAGGTAAAACCAACAGTGGTTGTTAAT   3428
gb:MN908947|Organism:Severe      ATTAAAAATGCAGACATTGTGGAAGAAGCTAAAAAGGTAAAACCAACAGTGGTTGTTAAT   3442
gb:MT019532|Organism:Severe      ATTAAAAATGCAGACATTGTGGAAGAAGCTAAAAAGGTAAAACCAACAGTGGTTGTTAAT   3442
                                 ************************************************************

gb:MT020781|Organism:Severe      GCAGCCAATGTTTACCTTAAACATGGAGGAGGTGTTGCAGGAGCCTTAAATAAGGCTACT   3490
gb:MT007544|Organism:Severe      GCAGCCAATGTTTACCTTAAACATGGAGGAGGTGTTGCAGGAGCCTTAAATAAGGCTACT   3502
gb:MN994467|Organism:Severe      GCAGCCAATGTTTACCTTAAACATGGAGGAGGTGTTGCAGGAGCCTTAAATAAGGCTACT   3502
gb:MT044257|Organism:Severe      GCAGCCAATGTTTACCTTAAACATGGAGGAGGTGTTGCAGGAGCCTTAAATAAGGCTACT   3502
gb:MT106054|Organism:Severe      GCAGCCAATGTTTACCTTAAACATGGAGGAGGTGTTGCAGGAGCCTTAAATAAGGCTACT   3502
gb:MT049951|Organism:Severe      GCAGCCAATGTTTACCTTAAACATGGAGGAGGTGTTGCAGGAGCCTTAAATAAGGCTACT   3502
gb:MN975262|Organism:Severe      GCAGCCAATGTTTACCTTAAACATGGAGGAGGTGTTGCAGGAGCCTTAAATAAGGCTACT   3502
gb:MT106052|Organism:Severe      GCAGCCAATGTTTACCTTAAACATGGAGGAGGTGTTGCAGGAGCCTTAAATAAGGCTACT   3502
gb:LC522975|Organism:Severe      GCAGCCAATGTTTACCTTAAACATGGAGGAGGTGTTGCAGGAGCCTTAAATAAGGCTACT   3499
gb:LC522973|Organism:Severe      GCAGCCAATGTTTACCTTAAACATGGAGGAGGTGTTGCAGGAGCCTTAAATAAGGCTACT   3499
gb:LC522974|Organism:Severe      GCAGCCAATGTTTACCTTAAACATGGAGGAGGTGTTGCAGGAGCCTTAAATAAGGCTACT   3499
gb:MN985325|Organism:Severe      GCAGCCAATGTTTACCTTAAACATGGAGGAGGTGTTGCAGGAGCCTTAAATAAGGCTACT   3502
gb:MT020881|Organism:Severe      GCAGCCAATGTTTACCTTAAACATGGAGGAGGTGTTGCAGGAGCCTTAAATAAGGCTACT   3502
gb:MT020880|Organism:Severe      GCAGCCAATGTTTACCTTAAACATGGAGGAGGTGTTGCAGGAGCCTTAAATAAGGCTACT   3502
gb:MT066175|Organism:Severe      GCAGCCAATGTTTACCTTAAACATGGAGGAGGTGTTGCAGGAGCCTTAAATAAGGCTACT   3502
gb:MN997409|Organism:Severe      GCAGCCAATGTTTACCTTAAACATGGAGGAGGTGTTGCAGGAGCCTTAAATAAGGCTACT   3502
gb:MN938384|Organism:Severe      GCAGCCAATGTTTACCTTAAACATGGAGGAGGTGTTGCAGGAGCCTTAAATAAGGCTACT   3470
gb:MT044258|Organism:Severe      GCAGCCAATGTTTACCTTAAACATGGAGGAGGTGTTGCAGGAGCCTTAAATAAGGCTACT   3478
gb:MT039890|Organism:Severe      GCAGCCAATGTTTACCTTAAACATGGAGGAGGTGTTGCAGGAGCCTTAAATAAGGCTACT   3502
gb:MN988713|Organism:Severe      GCAGCCAATGTTTACCTTAAACATGGAGGAGGTGTTGCAGGAGCCTTAAATAAGGCTACT   3502
gb:LC521925|Organism:Severe      GCAGCCAATGTTTACCTTAAACATGGAGGAGGTGTTGCAGGAGCCTTAAATAAGGCTACT   3475
gb:MT093571|Organism:Severe      GCAGCCAATGTTTACCTTAAACATGGAGGAGGTGTTGCAGGAGCCTTAAATAAGGCTACT   3502
gb:MT039887|Organism:Severe      GCAGCCAATGTTTACCTTAAACATGGAGGAGGTGTTGCAGGAGCCTTAAATAAGGCTACT   3502
gb:MT019530|Organism:Severe      GCAGCCAATGTTTACCTTAAACATGGAGGAGGTGTTGCAGGAGCCTTAAATAAGGCTACT   3502
gb:MT039888|Organism:Severe      GCAGCCAATGTTTACCTTAAACATGGAGGAGGTGTTGCAGGAGCCTTAAATAAGGCTACT   3502
gb:LC522972|Organism:Severe      GCAGCCAATGTTTACCTTAAACATGGAGGAGGTGTTGCAGGAGCCTTAAATAAGGCTACT   3499
gb:MT027063|Organism:Severe      GCAGCCAATGTTTACCTTAAACATGGAGGAGGTGTTGCAGGAGCCTTAAATAAGGCTACT   3502
gb:MT027062|Organism:Severe      GCAGCCAATGTTTACCTTAAACATGGAGGAGGTGTTGCAGGAGCCTTAAATAAGGCTACT   3502
gb:MT019529|Organism:Severe      GCAGCCAATGTTTACCTTAAACATGGAGGAGGTGTTGCAGGAGCCTTAAATAAGGCTACT   3502
gb:MN996529|Organism:Severe      GCAGCCAATGTTTACCTTAAACATGGAGGAGGTGTTGCAGGAGCCTTAAATAAGGCTACT   3490
gb:MN996531|Organism:Severe      GCAGCCAATGTTTACCTTAAACATGGAGGAGGTGTTGCAGGAGCCTTAAATAAGGCTACT   3489
gb:MT066176|Organism:Severe      GCAGCCAATGTTTACCTTAAACATGGAGGAGGTGTTGCAGGAGCCTTAAATAAGGCTACT   3502
gb:MT027064|Organism:Severe      GCAGCCAATGTTTACCTTAAACATGGAGGAGGTGTTGCAGGAGCCTTAAATAAGGCTACT   3502
gb:MN994468|Organism:Severe      GCAGCCAATGTTTACCTTAAACATGGAGGAGGTGTTGCAGGAGCCTTAAATAAGGCTACT   3502
gb:MT072688|Organism:Severe      GCAGCCAATGTTTACCTTAAACATGGAGGAGGTGTTGCAGGAGCCTTAAATAAGGCTACT   3487
gb:MN996527|Organism:Severe      GCAGCCAATGTTTACCTTAAACATGGAGGAGGTGTTGCAGGAGCCTTAAATAAGGCTACT   3469
gb:MT093631|Organism:Severe      GCAGCCAATGTTTACCTTAAACATGGAGGAGGTGTTGCAGGAGCCTTAAATAAGGCTACT   3540
gb:MT106053|Organism:Severe      GCAGCCAATGTTTACCTTAAACATGGAGGAGGTGTTGCAGGAGCCTTAAATAAGGCTACT   3502
gb:MT019533|Organism:Severe      GCAGCCAATGTTTACCTTAAACATGGAGGAGGTGTTGCAGGAGCCTTAAATAAGGCTACT   3502
gb:MT019531|Organism:Severe      GCAGCCAATGTTTACCTTAAACATGGAGGAGGTGTTGCAGGAGCCTTAAATAAGGCTACT   3502
gb:MN996528|Organism:Severe      GCAGCCAATGTTTACCTTAAACATGGAGGAGGTGTTGCAGGAGCCTTAAATAAGGCTACT   3502
gb:MN996530|Organism:Severe      GCAGCCAATGTTTACCTTAAACATGGAGGAGGTGTTGCAGGAGCCTTAAATAAGGCTACT   3488
gb:MN908947|Organism:Severe      GCAGCCAATGTTTACCTTAAACATGGAGGAGGTGTTGCAGGAGCCTTAAATAAGGCTACT   3502
gb:MT019532|Organism:Severe      GCAGCCAATGTTTACCTTAAACATGGAGGAGGTGTTGCAGGAGCCTTAAATAAGGCTACT   3502
                                 ************************************************************

gb:MT020781|Organism:Severe      AACAATGCCATGCAAGTTGAATCTGATGATTACATAGCTACTAATGGACCACTTAAAGTG   3550
gb:MT007544|Organism:Severe      AACAATGCCATGCAAGTTGAATCTGATGATTACATAGCTACTAATGGACCACTTAAAGTG   3562
gb:MN994467|Organism:Severe      AACAATGCCATGCAAGTTGAATCTGATGATTACATAGCTACTAATGGACCACTTAAAGTG   3562
gb:MT044257|Organism:Severe      AACAATGCCATGCAAGTTGAATCTGATGATTACATAGCTACTAATGGACCACTTAAAGTG   3562
gb:MT106054|Organism:Severe      AACAATGCCATGCAAGTTGAATCTGATGATTACATAGCTACTAATGGACCACTTAAAGTG   3562
gb:MT049951|Organism:Severe      AACAATGCCATGCAAGTTGAATCTGATGATTACATAGCTACTAATGGACCACTTAAAGTG   3562
gb:MN975262|Organism:Severe      AACAATGCCATGCAAGTTGAATCTGATGATTACATAGCTACTAATGGACCACTTAAAGTG   3562
gb:MT106052|Organism:Severe      AACAATGCCATGCAAGTTGAATCTGATGATTACATAGCTACTAATGGACCACTTAAAGTG   3562
gb:LC522975|Organism:Severe      AACAATGCCATGCAAGTTGAATCTGATGATTACATAGCTACTAATGGACCACTTAAAGTG   3559
gb:LC522973|Organism:Severe      AACAATGCCATGCAAGTTGAATCTGATGATTACATAGCTACTAATGGACCACTTAAAGTG   3559
gb:LC522974|Organism:Severe      AACAATGCCATGCAAGTTGAATCTGATGATTACATAGCTACTAATGGACCACTTAAAGTG   3559
gb:MN985325|Organism:Severe      AACAATGCCATGCAAGTTGAATCTGATGATTACATAGCTACTAATGGACCACTTAAAGTG   3562
```

```
gb:MT020881|Organism:Severe      AACAATGCCATGCAAGTTGAATCTGATGATTACATAGCTACTAATGGACCACTTAAAGTG   3562
gb:MT020880|Organism:Severe      AACAATGCCATGCAAGTTGAATCTGATGATTACATAGCTACTAATGGACCACTTAAAGTG   3562
gb:MT066175|Organism:Severe      AACAATGCCATGCAAGTTGAATCTGATGATTACATAGCTACTAATGGACCACTTAAAGTG   3562
gb:MN997409|Organism:Severe      AACAATGCCATGCAAGTTGAATCTGATGATTACATAGCTACTAATGGACCACTTAAAGTG   3562
gb:MN938384|Organism:Severe      AACAATGCCATGCAAGTTGAATCTGATGATTACATAGCTACTAATGGACCACTTAAAGTG   3530
gb:MT044258|Organism:Severe      AACAATGCCATGCAAGTTGAATCTGATGATTACATAGCTACTAATGGACCACTTAAAGTG   3538
gb:MT039890|Organism:Severe      AACAATGCCATGCAAGTTGAATCTGATGATTACATAGCTACTAATGGACCACTTAAAGTG   3562
gb:MN988713|Organism:Severe      AACAATGCCATGCAAGTTGAATCTGATGATTACATAGCTACTAATGGACCACTTAAAGTG   3562
gb:LC521925|Organism:Severe      AACAATGCCATGCAAGTTGAATCTGATGATTACATAGCTACTAATGGACCACTTAAAGTG   3535
gb:MT093571|Organism:Severe      AACAATGCCATGCAAGTTGAATCTGATGATTACATAGCTACTAATGGACCACTTAAAGTG   3562
gb:MT039887|Organism:Severe      AACAATGCCATGCAAGTTGAATCTGATGATTACATAGCTACTAATGGACCACTTAAAGTG   3562
gb:MT019530|Organism:Severe      AACAATGCCATGCAAGTTGAATCTGATGATTACATAGCTACTAATGGACCACTTAAAGTG   3562
gb:MT039888|Organism:Severe      AACAATGCCATGCAATTTGAATCTGATGATTACATAGCTACTAATGGACCACTTAAAGTG   3562
gb:LC522972|Organism:Severe      AACAATGCCATGCAAGTTGAATCTGATGATTACATAGCTACTAATGGACCACTTAAAGTG   3559
gb:MT027063|Organism:Severe      AACAATGCCATGCAAGTTGAATCTGATGATTACATAGCTACTAATGGACCACTTAAAGTG   3562
gb:MT027062|Organism:Severe      AACAATGCCATGCAAGTTGAATCTGATGATTACATAGCTACTAATGGACCACTTAAAGTG   3562
gb:MT019529|Organism:Severe      AACAATGCCATGCAAGTTGAATCTGATGATTACATAGCTACTAATGGACCACTTAAAGTG   3562
gb:MN996529|Organism:Severe      AACAATGCCATGCAAGTTGAATCTGATGATTACATAGCTACTAATGGACCACTTAAAGTG   3550
gb:MN996531|Organism:Severe      AACAATGCCATGCAAGTTGAATCTGATGATTACATAGCTACTAATGGACCACTTAAAGTG   3549
gb:MT066176|Organism:Severe      AACAATGCCATGCAAGTTGAATCTGATGATTACATAGCTACTAATGGACCACTTAAAGTG   3562
gb:MT027064|Organism:Severe      AACAATGCCATGCAAGTTGAATCTGATGATTACATAGCTACTAATGGACCACTTAAAGTG   3562
gb:MN994468|Organism:Severe      AACAATGCCATGCAAGTTGAATCTGATGATTACATAGCTACTAATGGACCACTTAAAGTG   3562
gb:MT072688|Organism:Severe      AACAATGCCATGCAAGTTGAATCTGATGATTACATAGCTACTAATGGACCACTTAAAGTG   3547
gb:MN996527|Organism:Severe      AACAATGCCATGCAAGTTGAATCTGATGATTACATAGCTACTAATGGACCACTTAAAGTG   3529
gb:MT093631|Organism:Severe      AACAATGCCATGCAAGTTGAATCTGATGATTACATAGCTACTAATGGACCACTTAAAGTG   3600
gb:MT106053|Organism:Severe      AACAATGCCATGCAAGTTGAATCTGATGATTACATAGCTACTAATGGACCACTTAAAGTG   3562
gb:MT019533|Organism:Severe      AACAATGCCATGCAAGTTGAATCTGATGATTACATAGCTACTAATGGACCACTTAAAGTG   3562
gb:MT019531|Organism:Severe      AACAATGCCATGCAAGTTGAATCTGATGATTACATAGCTACTAATGGACCACTTAAAGTG   3562
gb:MN996528|Organism:Severe      AACAATGCCATGCAAGTTGAATCTGATGATTACATAGCTACTAATGGACCACTTAAAGTG   3562
gb:MN996530|Organism:Severe      AACAATGCCATGCAAGTTGAATCTGATGATTACATAGCTACTAATGGACCACTTAAAGTG   3548
gb:MN908947|Organism:Severe      AACAATGCCATGCAAGTTGAATCTGATGATTACATAGCTACTAATGGACCACTTAAAGTG   3562
gb:MT019532|Organism:Severe      AACAATGCCATGCAAGTTGAATCTGATGATTACATAGCTACTAATGGACCACTTAAAGTG   3562
                                 *************** ********************************************

gb:MT020781|Organism:Severe      GGTGGTAGTTGTGTTTTAAGCGGACACAATCTTGCTAAACACTGTCTTCATGTTGTCGGC   3610
gb:MT007544|Organism:Severe      GGTGGTAGTTGTGTTTTAAGCGGACACAATCTTGCTAAACACTGTCTTCATGTTGTCGGC   3622
gb:MN994467|Organism:Severe      GGTGGTAGTTGTGTTTTAAGCGGACACAATCTTGCTAAACACTGTCTTCATGTTGTCGGC   3622
gb:MT044257|Organism:Severe      GGTGGTAGTTGTGTTTTAAGCGGACACAATCTTGCTAAACACTGTCTTCATGTTGTCGGC   3622
gb:MT106054|Organism:Severe      GGTGGTAGTTGTGTTTTAAGCGGACACAATCTTGCTAAACACTGTCTTCATGTTGTCGGC   3622
gb:MT049951|Organism:Severe      GGTGGTAGTTGTGTTTTAAGCGGACACAATCTTGCTAAACACTGTCTTCATGTTGTCGGC   3622
gb:MN975262|Organism:Severe      GGTGGTAGTTGTGTTTTAAGCGGACACAATCTTGCTAAACACTGTCTTCATGTTGTCGGC   3622
gb:MT106052|Organism:Severe      GGTGGTAGTTGTGTTTTAAGCGGACACAATCTTGCTAAACACTGTCTTCATGTTGTCGGC   3622
gb:LC522975|Organism:Severe      GGTGGTAGTTGTGTTTTAAGCGGACACAATCTTGCTAAACACTGTCTTCATGTTGTCGGC   3619
gb:LC522973|Organism:Severe      GGTGGTAGTTGTGTTTTAAGCGGACACAATCTTGCTAAACACTGTCTTCATGTTGTCGGC   3619
gb:LC522974|Organism:Severe      GGTGGTAGTTGTGTTTTAAGCGGACACAATCTTGCTAAACACTGTCTTCATGTTGTCGGC   3619
gb:MN985325|Organism:Severe      GGTGGTAGTTGTGTTTTAAGCGGACACAATCTTGCTAAACACTGTCTTCATGTTGTCGGC   3622
gb:MT020881|Organism:Severe      GGTGGTAGTTGTGTTTTAAGCGGACACAATCTTGCTAAACACTGTCTTCATGTTGTCGGC   3622
gb:MT020880|Organism:Severe      GGTGGTAGTTGTGTTTTAAGCGGACACAATCTTGCTAAACACTGTCTTCATGTTGTCGGC   3622
gb:MT066175|Organism:Severe      GGTGGTAGTTGTGTTTTAAGCGGACACAATCTTGCTAAACACTGTCTTCATGTTGTCGGC   3622
gb:MN997409|Organism:Severe      GGTGGTAGTTGTGTTTTAAGCGGACACAATCTTGCTAAACACTGTCTTCATGTTGTCGGC   3622
gb:MN938384|Organism:Severe      GGTGGTAGTTGTGTTTTAAGCGGACACAATCTTGCTAAACACTGTCTTCATGTTGTCGGC   3590
gb:MT044258|Organism:Severe      GGTGGTAGTTGTGTTTTAAGCGGACACAATCTTGCTAAACACTGTCTTCATGTTGTCGGC   3598
gb:MT039890|Organism:Severe      GGTGGTAGTTGTGTTTTAAGCGGACACAATCTTGCTAAACACTGTCTTCATGTTGTCGGC   3622
gb:MN988713|Organism:Severe      GGTGGTAGTTGTGTTTTAAGCGGACACAATCTTGCTAAACACTGTCTTCATGTTGTCGGC   3622
gb:LC521925|Organism:Severe      GGTGGTAGTTGTGTTTTAAGCGGACACAATCTTGCTAAACACTGTCTTCATGTTGTCGGC   3595
gb:MT093571|Organism:Severe      GGTGGTAGTTGTGTTTTAAGCGGACACAATCTTGCTAAACACTGTCTTCATGTTGTCGGC   3622
gb:MT039887|Organism:Severe      GGTGGTAGTTGTGTTTTAAGCGGACACAATCTTGCTAAACACTGTCTTCATGTTGTCGGC   3622
gb:MT019530|Organism:Severe      GGTGGTAGTTGTGTTTTAAGCGGACACAATCTTGCTAAACACTGTCTTCATGTTGTCGGC   3622
gb:MT039888|Organism:Severe      GGTGGTAGTTGTGTTTTAAGCGGACACAATCTTGCTAAACACTGTCTTCATGTTGTCGGC   3622
gb:LC522972|Organism:Severe      GGTGGTAGTTGTGTTTTAAGCGGACACAATCTTGCTAAACACTGTCTTCATGTTGTCGGC   3619
gb:MT027063|Organism:Severe      GGTGGTAGTTGTGTTTTAAGCGGACACAATCTTGCTAAACACTGTCTTCATGTTGTCGGC   3622
gb:MT027062|Organism:Severe      GGTGGTAGTTGTGTTTTAAGCGGACACAATCTTGCTAAACACTGTCTTCATGTTGTCGGC   3622
gb:MT019529|Organism:Severe      GGTGGTAGTTGTGTTTTAAGCGGACACAATCTTGCTAAACACTGTCTTCATGTTGTCGGC   3622
gb:MN996529|Organism:Severe      GGTGGTAGTTGTGTTTTAAGCGGACACAATCTTGCTAAACACTGTCTTCATGTTGTCGGC   3610
gb:MN996531|Organism:Severe      GGTGGTAGTTGTGTTTTAAGCGGACACAATCTTGCTAAACACTGTCTTCATGTTGTCGGC   3609
```

```
gb:MT066176|Organism:Severe      GGTGGTAGTTGTGTTTTAAGCGGACACAATCTTGCTAAACACTGTCTTCATGTTGTCGGC    3622
gb:MT027064|Organism:Severe      GGTGGTAGTTGTGTTTTAAGCGGACACAATCTTGCTAAACACTGTCTTCATGTTGTCGGC    3622
gb:MN994468|Organism:Severe      GGTGGTAGTTGTGTTTTAAGCGGACACAATCTTGCTAAACACTGTCTTCATGTTGTCGGC    3622
gb:MT072688|Organism:Severe      GGTGGTAGTTGTGTTTTAAGCGGACACAATCTTGCTAAACACTGTCTTCATGTTGTCGGC    3607
gb:MN996527|Organism:Severe      GGTGGTAGTTGTGTTTTAAGCGGACACAATCTTGCTAAACACTGTCTTCATGTTGTCGGC    3589
gb:MT093631|Organism:Severe      GGTGGTAGTTGTGTTTTAAGCGGACACAATCTTGCTAAACACTGTCTTCATGTTGTCGGC    3660
gb:MT106053|Organism:Severe      GGTGGTAGTTGTGTTTTAAGCGGACACAATCTTGCTAAACACTGTCTTCATGTTGTCGGC    3622
gb:MT019533|Organism:Severe      GGTGGTAGTTGTGTTTTAAGCGGACACAATCTTGCTAAACACTGTCTTCATGTTGTCGGC    3622
gb:MT019531|Organism:Severe      GGTGGTAGTTGTGTTTTAAGCGGACACAATCTTGCTAAACACTGTCTTCATGTTGTCGGC    3622
gb:MN996528|Organism:Severe      GGTGGTAGTTGTGTTTTAAGCGGACACAATCTTGCTAAACACTGTCTTCATGTTGTCGGC    3622
gb:MN996530|Organism:Severe      GGTGGTAGTTGTGTTTTAAGCGGACACAATCTTGCTAAACACTGTCTTCATGTTGTCGGC    3608
gb:MN908947|Organism:Severe      GGTGGTAGTTGTGTTTTAAGCGGACACAATCTTGCTAAACACTGTCTTCATGTTGTCGGC    3622
gb:MT019532|Organism:Severe      GGTGGTAGTTGTGTTTTAAGCGGACACAATCTTGCTAAACACTGTCTTCATGTTGTCGGC    3622
                                 ************************************************************

gb:MT020781|Organism:Severe      CCAAATGTTAACAAAGGTGAAGACATTCAACTTCTTAAGAGTGCTTATGAAAATTTTAAT    3670
gb:MT007544|Organism:Severe      CCAAATGTTAACAAAGGTGAAGACATTCAACTTCTTAAGAGTGCTTATGAAAATTTTAAT    3682
gb:MN994467|Organism:Severe      CCAAATGTTAACAAAGGTGAAGACATTCAACTTCTTAAGAGTGCTTATGAAAATTTTAAT    3682
gb:MT044257|Organism:Severe      CCAAATGTTAACAAAGGTGAAGACATTCAACTTCTTAAGAGTGCTTATGAAAATTTTAAT    3682
gb:MT106054|Organism:Severe      CCAAATGTTAACAAAGGTGAAGACATTCAACTTCTTAAGAGTGCTTATGAAAATTTTAAT    3682
gb:MT049951|Organism:Severe      CCAAATGTTAACAAAGGTGAAGACATTCAACTTCTTAAGAGTGCTTATGAAAATTTTAAT    3682
gb:MN975262|Organism:Severe      CCAAATGTTAACAAAGGTGAAGACATTCAACTTCTTAAGAGTGCTTATGAAAATTTTAAT    3682
gb:MT106052|Organism:Severe      CCAAATGTTAACAAAGGTGAAGACATTCAACTTCTTAAGAGTGCTTATGAAAATTTTAAT    3682
gb:LC522975|Organism:Severe      CCAAATGTTAACAAAGGTGAAGACATTCAACTTCTTAAGAGTGCTTATGAAAATTTTAAT    3679
gb:LC522973|Organism:Severe      CCAAATGTTAACAAAGGTGAAGACATTCAACTTCTTAAGAGTGCTTATGAAAATTTTAAT    3679
gb:LC522974|Organism:Severe      CCAAATGTTAACAAAGGTGAAGACATTCAACTTCTTAAGAGTGCTTATGAAAATTTTAAT    3679
gb:MN985325|Organism:Severe      CCAAATGTTAACAAAGGTGAAGACATTCAACTTCTTAAGAGTGCTTATGAAAATTTTAAT    3682
gb:MT020881|Organism:Severe      CCAAATGTTAACAAAGGTGAAGACATTCAACTTCTTAAGAGTGCTTATGAAAATTTTAAT    3682
gb:MT020880|Organism:Severe      CCAAATGTTAACAAAGGTGAAGACATTCAACTTCTTAAGAGTGCTTATGAAAATTTTAAT    3682
gb:MT066175|Organism:Severe      CCAAATGTTAACAAAGGTGAAGACATTCAACTTCTTAAGAGTGCTTATGAAAATTTTAAT    3682
gb:MN997409|Organism:Severe      CCAAATGTTAACAAAGGTGAAGACATTCAACTTCTTAAGAGTGCTTATGAAAATTTTAAT    3682
gb:MN938384|Organism:Severe      CCAAATGTTAACAAAGGTGAAGACATTCAACTTCTTAAGAGTGCTTATGAAAATTTTAAT    3650
gb:MT044258|Organism:Severe      CCAAATGTTAACAAAGGTGAAGACATTCAACTTCTTAAGAGTGCTTATGAAAATTTTAAT    3658
gb:MT039890|Organism:Severe      CCAAATGTTAACAAAGGTGAAGACATTCAACTTCTTAAGAGTGCTTATGAAAATTTTAAT    3682
gb:MN988713|Organism:Severe      CCAAATGTTAACAAAGGTGAAGACATTCAACTTCTTAAGAGTGCTTATGAAAATTTTAAT    3682
gb:LC521925|Organism:Severe      CCAAATGTTAACAAAGGTGAAGACATTCAACTTCTTAAGAGTGCTTATGAAAATTTTAAT    3655
gb:MT093571|Organism:Severe      CCAAATGTTAACAAAGGTGAAGACATTCAACTTCTTAAGAGTGCTTATGAAAATTTTAAT    3682
gb:MT039887|Organism:Severe      CCAAATGTTAACAAAGGTGAAGACATTCAACTTCTTAAGAGTGCTTATGAAAATTTTAAT    3682
gb:MT019530|Organism:Severe      CCAAATGTTAACAAAGGTGAAGACATTCAACTTCTTAAGAGTGCTTATGAAAATTTTAAT    3682
gb:MT039888|Organism:Severe      CCAAATGTTAACAAAGGTGAAGACATTCAACTTCTTAAGAGTGCTTATGAAAATTTTAAT    3682
gb:LC522972|Organism:Severe      CCAAATGTTAACAAAGGTGAAGACATTCAACTTCTTAAGAGTGCTTATGAAAATTTTAAT    3679
gb:MT027063|Organism:Severe      CCAAATGTTAACAAAGGTGAAGACATTCAACTTCTTAAGAGTGCTTATGAAAATTTTAAT    3682
gb:MT027062|Organism:Severe      CCAAATGTTAACAAAGGTGAAGACATTCAACTTCTTAAGAGTGCTTATGAAAATTTTAAT    3682
gb:MT019529|Organism:Severe      CCAAATGTTAACAAAGGTGAAGACATTCAACTTCTTAAGAGTGCTTATGAAAATTTTAAT    3682
gb:MN996529|Organism:Severe      CCAAATGTTAACAAAGGTGAAGACATTCAACTTCTTAAGAGTGCTTATGAAAATTTTAAT    3670
gb:MN996531|Organism:Severe      CCAAATGTTAACAAAGGTGAAGACATTCAACTTCTTAAGAGTGCTTATGAAAATTTTAAT    3669
gb:MT066176|Organism:Severe      CCAAATGTTAACAAAGGTGAAGACATTCAACTTCTTAAGAGTGCTTATGAAAATTTTAAT    3682
gb:MT027064|Organism:Severe      CCAAATGTTAACAAAGGTGAAGACATTCAACTTCTTAAGAGTGCTTATGAAAATTTTAAT    3682
gb:MN994468|Organism:Severe      CCAAATGTTAACAAAGGTGAAGACATTCAACTTCTTAAGAGTGCTTATGAAAATTTTAAT    3682
gb:MT072688|Organism:Severe      CCAAATGTTAACAAAGGTGAAGACATTCAACTTCTTAAGAGTGCTTATGAAAATTTTAAT    3667
gb:MN996527|Organism:Severe      CCAAATGTTAACAAAGGTGAAGACATTCAACTTCTTAAGAGTGCTTATGAAAATTTTAAT    3649
gb:MT093631|Organism:Severe      CCAAATGTTAACAAAGGTGAAGACATTCAACTTCTTAAGAGTGCTTATGAAAATTTTAAT    3720
gb:MT106053|Organism:Severe      CCAAATGTTAACAAAGGTGAAGACATTCAACTTCTTAAGAGTGCTTATGAAAATTTTAAT    3682
gb:MT019533|Organism:Severe      CCAAATGTTAACAAAGGTGAAGACATTCAACTTCTTAAGAGTGCTTATGAAAATTTTAAT    3682
gb:MT019531|Organism:Severe      CCAAATGTTAACAAAGGTGAAGACATTCAACTTCTTAAGAGTGCTTATGAAAATTTTAAT    3682
gb:MN996528|Organism:Severe      CCAAATGTTAACAAAGGTGAAGACATTCAACTTCTTAAGAGTGCTTATGAAAATTTTAAT    3682
gb:MN996530|Organism:Severe      CCAAATGTTAACAAAGGTGAAGACATTCAACTTCTTAAGAGTGCTTATGAAAATTTTAAT    3668
gb:MN908947|Organism:Severe      CCAAATGTTAACAAAGGTGAAGACATTCAACTTCTTAAGAGTGCTTATGAAAATTTTAAT    3682
gb:MT019532|Organism:Severe      CCAAATGTTAACAAAGGTGAAGACATTCAACTTCTTAAGAGTGCTTATGAAAATTTTAAT    3682
                                 ************************************************************

gb:MT020781|Organism:Severe      CAGCACGAAGTTCTACTTGCACCATTATTATCAGCTGGTATTTTTGGTGCTGACCCTATA    3730
gb:MT007544|Organism:Severe      CAGCACGAAGTTCTACTTGCACCATTATTATCAGCTGGTATTTTTGGTGCTGACCCTATA    3742
gb:MN994467|Organism:Severe      CAGCACGAAGTTCTACTTGCACCATTATTATCAGCTGGTATTTTTGGTGCTGACCCTATA    3742
gb:MT044257|Organism:Severe      CAGCACGAAGTTCTACTTGCACCATTATTATCAGCTGGTATTTTTGGTGCTGACCCTATA    3742
```

```
gb:MT106054|Organism:Severe      CAGCACGAAGTTCTACTTGCACCATTATTATCAGCTGGTATTTTTGGTGCTGACCCTATA   3742
gb:MT049951|Organism:Severe      CAGCACGAAGTTCTACTTGCACCATTATTATCAGCTGGTATTTTTGGTGCTGACCCTATA   3742
gb:MN975262|Organism:Severe      CAGCACGAAGTTCTACTTGCACCATTATTATCAGCTGGTATTTTTGGTGCTGACCCTATA   3742
gb:MT106052|Organism:Severe      CAGCACGAAGTTCTACTTGCACCATTATTATCAGCTGGTATTTTTGGTGCTGACCCTATA   3742
gb:LC522975|Organism:Severe      CAGCACGAAGTTCTACTTGCACCATTATTATCAGCTGGTATTTTTGGTGCTGACCCTATA   3739
gb:LC522973|Organism:Severe      CAGCACGAAGTTCTACTTGCACCATTATTATCAGCTGGTATTTTTGGTGCTGACCCTATA   3739
gb:LC522974|Organism:Severe      CAGCACGAAGTTCTACTTGCACCATTATTATCAGCTGGTATTTTTGGTGCTGACCCTATA   3739
gb:MN985325|Organism:Severe      CAGCACGAAGTTCTACTTGCACCATTATTATCAGCTGGTATTTTTGGTGCTGACCCTATA   3742
gb:MT020881|Organism:Severe      CAGCACGAAGTTCTACTTGCACCATTATTATCAGCTGGTATTTTTGGTGCTGACCCTATA   3742
gb:MT020880|Organism:Severe      CAGCACGAAGTTCTACTTGCACCATTATTATCAGCTGGTATTTTTGGTGCTGACCCTATA   3742
gb:MT066175|Organism:Severe      CAGCACGAAGTTCTACTTGCACCATTATTATCAGCTGGTATTTTTGGTGCTGACCCTATA   3742
gb:MN997409|Organism:Severe      CAGCACGAAGTTCTACTTGCACCATTATTATCAGCTGGTATTTTTGGTGCTGACCCTATA   3742
gb:MN938384|Organism:Severe      CAGCACGAAGTTCTACTTGCACCATTATTATCAGCTGGTATTTTTGGTGCTGACCCTATA   3710
gb:MT044258|Organism:Severe      CAGCACGAAGTTCTACTTGCACCATTATTATCAGCTGGTATTTTTGGTGCTGACCCTATA   3718
gb:MT039890|Organism:Severe      CAGCACGAAGTTCTACTTGCACCATTATTATCAGCTGGTATTTTTGGTGCTGACCCTATA   3742
gb:MN988713|Organism:Severe      CAGCACGAAGTTCTACTTGCACCATTATTATCAGCTGGTATTTTTGGTGCTGACCCTATA   3742
gb:LC521925|Organism:Severe      CAGCACGAAGTTCTACTTGCACCATTATTATCAGCTGGTATTTTTGGTGCTGACCCTATA   3715
gb:MT093571|Organism:Severe      CAGCACGAAGTTCTACTTGCACCATTATTATCAGCTGGTATTTTTGGTGCTGACCCTATA   3742
gb:MT039887|Organism:Severe      CAGCACGAAGTTCTACTTGCACCATTATTATCAGCTGGTATTTTTGGTGCTGACCCTATA   3742
gb:MT019530|Organism:Severe      CAGCACGAAGTTCTACTTGCACCATTATTATCAGCTGGTATTTTTGGTGCTGACCCTATA   3742
gb:MT039888|Organism:Severe      CAGCACGAAGTTCTACTTGCACCATTATTATCAGCTGGTATTTTTGGTGCTGACCCTATA   3742
gb:LC522972|Organism:Severe      CAGCACGAAGTTCTACTTGCACCATTATTATCAGCTGGTATTTTTGGTGCTGACCCTATA   3739
gb:MT027063|Organism:Severe      CAGCACGAAGTTCTACTTGCACCATTATTATCAGCTGGTATTTTTGGTGCTGACCCTATA   3742
gb:MT027062|Organism:Severe      CAGCACGAAGTTCTACTTGCACCATTATTATCAGCTGGTATTTTTGGTGCTGACCCTATA   3742
gb:MT019529|Organism:Severe      CAGCACGAAGTTCTACTTGCACCATTATTATCAGCTGGTATTTTTGGTGCTGACCCTATA   3742
gb:MN996529|Organism:Severe      CAGCACGAAGTTCTACTTGCACCATTATTATCAGCTGGTATTTTTGGTGCTGACCCTATA   3730
gb:MN996531|Organism:Severe      CAGCACGAAGTTCTACTTGCACCATTATTATCAGCTGGTATTTTTGGTGCTGACCCTATA   3729
gb:MT066176|Organism:Severe      CAGCACGAAGTTCTACTTGCACCATTATTATCAGCTGGTATTTTTGGTGCTGACCCTATA   3742
gb:MT027064|Organism:Severe      CAGCACGAAGTTCTACTTGCACCATTATTATCAGCTGGTATTTTTGGTGCTGACCCTATA   3742
gb:MN994468|Organism:Severe      CAGCACGAAGTTCTACTTGCACCATTATTATCAGCTGGTATTTTTGGTGCTGACCCTATA   3742
gb:MT072688|Organism:Severe      CAGCACGAAGTTCTACTTGCACCATTATTATCAGCTGGTATTTTTGGTGCTGACCCTATA   3727
gb:MN996527|Organism:Severe      CAGCACGAAGTTCTACTTGCACCATTATTATCAGCTGGTATTTTTGGTGCTGACCCTATA   3709
gb:MT093631|Organism:Severe      CAGCACGAAGTTCTACTTGCACCATTATTATCAGCTGGTATTTTTGGTGCTGACCCTATA   3780
gb:MT106053|Organism:Severe      CAGCACGAAGTTCTACTTGCACCATTATTATCAGCTGGTATTTTTGGTGCTGACCCTATA   3742
gb:MT019533|Organism:Severe      CAGCACGAAGTTCTACTTGCACCATTATTATCAGCTGGTATTTTTGGTGCTGACCCTATA   3742
gb:MT019531|Organism:Severe      CAGCACGAAGTTCTACTTGCACCATTATTATCAGCTGGTATTTTTGGTGCTGACCCTATA   3742
gb:MN996528|Organism:Severe      CAGCACGAAGTTCTACTTGCACCATTATTATCAGCTGGTATTTTTGGTGCTGACCCTATA   3742
gb:MN996530|Organism:Severe      CAGCACGAAGTTCTACTTGCACCATTATTATCAGCTGGTATTTTTGGTGCTGACCCTATA   3728
gb:MN908947|Organism:Severe      CAGCACGAAGTTCTACTTGCACCATTATTATCAGCTGGTATTTTTGGTGCTGACCCTATA   3742
gb:MT019532|Organism:Severe      CAGCACGAAGTTCTACTTGCACCATTATTATCAGCTGGTATTTTTGGTGCTGACCCTATA   3742
                                 ************************************************************

gb:MT020781|Organism:Severe      CATTCTTTAAGAGTTTGTGTAGATACTGTTCGCACAAATGTCTACTTAGCTGTCTTTGAT   3790
gb:MT007544|Organism:Severe      CATTCTTTAAGAGTTTGTGTAGATACTGTTCGCACAAATGTCTACTTAGCTGTCTTTGAT   3802
gb:MN994467|Organism:Severe      CATTCTTTAAGAGTTTGTGTAGATACTGTTCGCACAAATGTCTACTTAGCTGTCTTTGAT   3802
gb:MT044257|Organism:Severe      CATTCTTTAAGAGTTTGTGTAGATACTGTTCGCACAAATGTCTACTTAGCTGTCTTTGAT   3802
gb:MT106054|Organism:Severe      CATTCTTTAAGAGTTTGTGTAGATACTGTTCGCACAAATGTCTACTTAGCTGTCTTTGAT   3802
gb:MT049951|Organism:Severe      CATTCTTTAAGAGTTTGTGTAGATACTGTTCGCACAAATGTCTACTTAGCTGTCTTTGAT   3802
gb:MN975262|Organism:Severe      CATTCTTTAAGAGTTTGTGTAGATACTGTTCGCACAAATGTCTACTTAGCTGTCTTTGAT   3802
gb:MT106052|Organism:Severe      CATTCTTTAAGAGTTTGTGTAGATACTGTTCGCACAAATGTCTACTTAGCTGTCTTTGAT   3802
gb:LC522975|Organism:Severe      CATTCTTTAAGAGTTTGTGTAGATACTGTTCGCACAAATGTCTACTTAGCTGTCTTTGAT   3799
gb:LC522973|Organism:Severe      CATTCTTTAAGAGTTTGTGTAGATACTGTTCGCACAAATGTCTACTTAGTTGTCTTTGAT   3799
gb:LC522974|Organism:Severe      CATTCTTTAAGAGTTTGTGTAGATACTGTTCGCACAAATGTCTACTTAGCTGTCTTTGAT   3799
gb:MN985325|Organism:Severe      CATTCTTTAAGAGTTTGTGTAGATACTGTTCGCACAAATGTCTACTTAGCTGTCTTTGAT   3802
gb:MT020881|Organism:Severe      CATTCTTTAAGAGTTTGTGTAGATACTGTTCGCACAAATGTCTACTTAGCTGTCTTTGAT   3802
gb:MT020880|Organism:Severe      CATTCTTTAAGAGTTTGTGTAGATACTGTTCGCACAAATGTCTACTTAGCTGTCTTTGAT   3802
gb:MT066175|Organism:Severe      CATTCTTTAAGAGTTTGTGTAGATACTGTTCGCACAAATGTCTACTTAGCTGTCTTTGAT   3802
gb:MN997409|Organism:Severe      CATTCTTTAAGAGTTTGTGTAGATACTGTTCGCACAAATGTCTACTTAGCTGTCTTTGAT   3802
gb:MN938384|Organism:Severe      CATTCTTTAAGAGTTTGTGTAGATACTGTTCGCACAAATGTCTACTTAGCTGTCTTTGAT   3770
gb:MT044258|Organism:Severe      CATTCTTTAAGAGTTTGTGTAGATACTGTTCGCACAAATGTCTACTTAGCTGTCTTTGAT   3778
gb:MT039890|Organism:Severe      CATTCTTTAAGAGTTTGTGTAGATACTGTTCGCACAAATGTCTACTTAGCTGTCTTTGAT   3802
gb:MN988713|Organism:Severe      CATTCTTTAAGAGTTTGTGTAGATACTGTTCGCACAAATGTCTACTTAGCTGTCTTTGAT   3802
gb:LC521925|Organism:Severe      CATTCTTTAAGAGTTTGTGTAGATACTGTTCGCACAAATGTCTACTTAGCTGTCTTTGAT   3775
gb:MT093571|Organism:Severe      CATTCTTTAAGAGTTTGTGTAGATACTGTTCGCACAAATGTCTACTTAGCTGTCTTTGAT   3802
gb:MT039887|Organism:Severe      CATTCTTTAAGAGTTTGTGTAGATACTGTTCGCACAAATGTCTACTTAGCTGTCTTTGAT   3802
```

```
gb:MT019530|Organism:Severe      CATTCTTTAAGAGTTTGTGTAGATACTGTTCGCACAAATGTCTACTTAGCTGTCTTTGAT   3802
gb:MT039888|Organism:Severe      CATTCTTTAAGAGTTTGTGTAGATACTGTTCGCACAAATGTCTACTTAGCTGTCTTTGAT   3802
gb:LC522972|Organism:Severe      CATTCTTTAAGAGTTTGTGTAGATACTGTTCGCACAAATGTCTACTTAGCTGTCTTTGAT   3799
gb:MT027063|Organism:Severe      CATTCTTTAAGAGTTTGTGTAGATACTGTTCGCACAAATGTCTACTTAGCTGTCTTTGAT   3802
gb:MT027062|Organism:Severe      CATTCTTTAAGAGTTTGTGTAGATACTGTTCGCACAAATGTCTACTTAGCTGTCTTTGAT   3802
gb:MT019529|Organism:Severe      CATTCTTTAAGAGTTTGTGTAGATACTGTTCGCACGAATGTCTACTTAGCTGTCTTTGAT   3802
gb:MN996529|Organism:Severe      CATTCTTTAAGAGTTTGTGTAGATACTGTTCGCACAAATGTCTACTTAGCTGTCTTTGAT   3790
gb:MN996531|Organism:Severe      CATTCTTTAAGAGTTTGTGTAGATACTGTTCGCACAAATGTCTACTTAGCTGTCTTTGAT   3789
gb:MT066176|Organism:Severe      CATTCTTTAAGAGTTTGTGTAGATACTGTTCGCACAAATGTCTACTTAGCTGTCTTTGAT   3802
gb:MT027064|Organism:Severe      CATTCTTTAAGAGTTTGTGTAGATACTGTTCGCACAAATGTCTACTTAGCTGTCTTTGAT   3802
gb:MN994468|Organism:Severe      CATTCTTTAAGAGTTTGTGTAGATACTGTTCGCACAAATGTCTACTTAGCTGTCTTTGAT   3802
gb:MT072688|Organism:Severe      CATTCTTTAAGAGTTTGTGTAGATACTGTTCGCACAAATGTCTACTTAGCTGTCTTTGAT   3787
gb:MN996527|Organism:Severe      CATTCTTTAAGAGTTTGTGTAGATACTGTTCGCACAAATGTCTACTTAGCTGTCTTTGAT   3769
gb:MT093631|Organism:Severe      CATTCTTTAAGAGTTTGTGTAGATACTGTTCGCACAAATGTCTACTTAGCTGTCTTTGAT   3840
gb:MT106053|Organism:Severe      CATTCTTTAAGAGTTTGTGTAGATACTGTTCGCACAAATGTCTACTTAGCTGTCTTTGAT   3802
gb:MT019533|Organism:Severe      CATTCTTTAAGAGTTTGTGTAGATACTGTTCGCACAAATGTCTACTTAGCTGTCTTTGAT   3802
gb:MT019531|Organism:Severe      CATTCTTTAAGAGTTTGTGTAGATACTGTTCGCACAAATGTCTACTTAGCTGTCTTTGAT   3802
gb:MN996528|Organism:Severe      CATTCTTTAAGAGTTTGTGTAGATACTGTTCGCACAAATGTCTACTTAGCTGTCTTTGAT   3802
gb:MN996530|Organism:Severe      CATTCTTTAAGAGTTTGTGTAGATACTGTTCGCACAAATGTCTACTTAGCTGTCTTTGAT   3788
gb:MN908947|Organism:Severe      CATTCTTTAAGAGTTTGTGTAGATACTGTTCGCACAAATGTCTACTTAGCTGTCTTTGAT   3802
gb:MT019532|Organism:Severe      CATTCTTTAAGAGTTTGTGTAGATACTGTTCGCACAAATGTCTACTTAGCTGTCTTTGAT   3802
                                 *********************************** ************* **********

gb:MT020781|Organism:Severe      AAAAATCTCTATGACAAACTTGTTTCAAGCTTTTTGGAAATGAAGAGTGAAAAGCAAGTT   3850
gb:MT007544|Organism:Severe      AAAAATCTCTATGACAAACTTGTTTCAAGCTTTTTGGAAATGAAGAGTGAAAAGCAAGTT   3862
gb:MN994467|Organism:Severe      AAAAATCTCTATGACAAACTTGTTTCAAGCTTTTTGGAAATGAAGAGTGAAAAGCAAGTT   3862
gb:MT044257|Organism:Severe      AAAAATCTCTATGACAAACTTGTTTCAAGCTTTTTGGAAATGAAGAGTGAAAAGCAAGTT   3862
gb:MT106054|Organism:Severe      AAAAATCTCTATGACAAACTTGTTTCAAGCTTTTTGGAAATGAAGAGTGAAAAGCAAGTT   3862
gb:MT049951|Organism:Severe      AAAAATCTCTATGACAAACTTGTTTCAAGCTTTTTGGAAATGAAGAGTGAAAAGCAAGTT   3862
gb:MN975262|Organism:Severe      AAAAATCTCTATGACAAACTTGTTTCAAGCTTTTTGGAAATGAAGAGTGAAAAGCAAGTT   3862
gb:MT106052|Organism:Severe      AAAAATCTCTATGACAAACTTGTTTCAAGCTTTTTGGAAATGAAGAGTGAAAAGCAAGTT   3862
gb:LC522975|Organism:Severe      AAAAATCTCTATGACAAACTTGTTTCAAGCTTTTTGGAAATGAAGAGTGAAAAGCAAGTT   3859
gb:LC522973|Organism:Severe      AAAAATCTCTATGACAAACTTGTTTCAAGCTTTTTGGAAATGAAGAGTGAAAAGCAAGTT   3859
gb:LC522974|Organism:Severe      AAAAATCTCTATGACAAACTTGTTTCAAGCTTTTTGGAAATGAAGAGTGAAAAGCAAGTT   3859
gb:MN985325|Organism:Severe      AAAAATCTCTATGACAAACTTGTTTCAAGCTTTTTGGAAATGAAGAGTGAAAAGCAAGTT   3862
gb:MT020881|Organism:Severe      AAAAATCTCTATGACAAACTTGTTTCAAGCTTTTTGGAAATGAAGAGTGAAAAGCAAGTT   3862
gb:MT020880|Organism:Severe      AAAAATCTCTATGACAAACTTGTTTCAAGCTTTTTGGAAATGAAGAGTGAAAAGCAAGTT   3862
gb:MT066175|Organism:Severe      AAAAATCTCTATGACAAACTTGTTTCAAGCTTTTTGGAAATGAAGAGTGAAAAGCAAGTT   3862
gb:MN997409|Organism:Severe      AAAAATCTCTATGACAAACTTGTTTCAAGCTTTTTGGAAATGAAGAGTGAAAAGCAAGTT   3862
gb:MN938384|Organism:Severe      AAAAATCTCTATGACAAACTTGTTTCAAGCTTTTTGGAAATGAAGAGTGAAAAGCAAGTT   3830
gb:MT044258|Organism:Severe      AAAAATCTCTATGACAAACTTGTTTCAAGCTTTTTGGAAATGAAGAGTGAAAAGCAAGTT   3838
gb:MT039890|Organism:Severe      AAAAATCTCTATGACAAACTTGTTTCAAGCTTTTTGGAAATGAAGAGTGAAAAGCAAGTT   3862
gb:MN988713|Organism:Severe      AAAAATCTCTATGACAAACTTGTTTCAAGCTTTTTGGAAATGAAGAGTGAAAAGCAAGTT   3862
gb:LC521925|Organism:Severe      AAAAATCTCTATGACAAACTTGTTTCAAGCTTTTTGGAAATGAAGAGTGAAAAGCAAGTT   3835
gb:MT093571|Organism:Severe      AAAAATCTCTATGACAAACTTGTTTCAAGCTTTTTGGAAATGAAGAGTGAAAAGCAAGTT   3862
gb:MT039887|Organism:Severe      AAAAATCTCTATGACAAACTTGTTTCAAGCTTTTTGGAAATGAAGAGTGAAAAGCAAGTT   3862
gb:MT019530|Organism:Severe      AAAAATCTCTATGACAAACTTGTTTCAAGCTTTTTGGAAATGAAGAGTGAAAAGCAAGTT   3862
gb:MT039888|Organism:Severe      AAAAATCTCTATGACAAACTTGTTTCAAGCTTTTTGGAAATGAAGAGTGAAAAGCAAGTT   3862
gb:LC522972|Organism:Severe      AAAAATCTCTATGACAAACTTGTTTCAAGCTTTTTGGAAATGAAGAGTGAAAAGCAAGTT   3859
gb:MT027063|Organism:Severe      AAAAATCTCTATGACAAACTTGTTTCAAGCTTTTTGGAAATGAAGAGTGAAAAGCAAGTT   3862
gb:MT027062|Organism:Severe      AAAAATCTCTATGACAAACTTGTTTCAAGCTTTTTGGAAATGAAGAGTGAAAAGCAAGTT   3862
gb:MT019529|Organism:Severe      AAAAATCTCTATGACAAACTTGTTTCAAGCTTTTTGGAAATGAAGAGTGAAAAGCAAGTT   3862
gb:MN996529|Organism:Severe      AAAAATCTCTATGACAAACTTGTTTCAAGCTTTTTGGAAATGAAGAGTGAAAAGCAAGTT   3850
gb:MN996531|Organism:Severe      AAAAATCTCTATGACAAACTTGTTTCAAGCTTTTTGGAAATGAAGAGTGAAAAGCAAGTT   3849
gb:MT066176|Organism:Severe      AAAAATCTCTATGACAAACTTGTTTCAAGCTTTTTGGAAATGAAGAGTGAAAAGCAAGTT   3862
gb:MT027064|Organism:Severe      AAAAATCTCTATGACAAACTTGTTTCAAGCTTTTTGGAAATGAAGAGTGAAAAGCAAGTT   3862
gb:MN994468|Organism:Severe      AAAAATCTCTATGACAAACTTGTTTCAAGCTTTTTGGAAATGAAGAGTGAAAAGCAAGTT   3862
gb:MT072688|Organism:Severe      AAAAATCTCTATGACAAACTTGTTTCAAGCTTTTTGGAAATGAAGAGTGAAAAGCAAGTT   3847
gb:MN996527|Organism:Severe      AAAAATCTCTATGACAAACTTGTTTCAAGCTTTTTGGAAATGAAGAGTGAAAAGCAAGTT   3829
gb:MT093631|Organism:Severe      AAAAATCTCTATGACAAACTTGTTTCAAGCTTTTTGGAAATGAAGAGTGAAAAGCAAGTT   3900
gb:MT106053|Organism:Severe      AAAAATCTCTATGACAAACTTGTTTCAAGCTTTTTGGAAATGAAGAGTGAAAAGCAAGTT   3862
gb:MT019533|Organism:Severe      AAAAATCTCTATGACAAACTTGTTTCAAGCTTTTTGGAAATGAAGAGTGAAAAGCAAGTT   3862
gb:MT019531|Organism:Severe      AAAAATCTCTATGACAAACTTGTTTCAAGCTTTTTGGAAATGAAGAGTGAAAAGCAAGTT   3862
gb:MN996528|Organism:Severe      AAAAATCTCTATGACAAACTTGTTTCAAGCTTTTTGGAAATGAAGAGTGAAAAGCAAGTT   3862
gb:MN996530|Organism:Severe      AAAAATCTCTATGACAAACTTGTTTCAAGCTTTTTGGAAATGAAGAGTGAAAAGCAAGTT   3848
```

```
gb:MN908947|Organism:Severe      AAAAATCTCTATGACAAACTTGTTTCAAGCTTTTTGGAAATGAAGAGTGAAAAGCAAGTT    3862
gb:MT019532|Organism:Severe      AAAAATCTCTATGACAAACTTGTTTCAAGCTTTTTGGAAATGAAGAGTGAAAAGCAAGTT    3862
                                 ************************************************************

gb:MT020781|Organism:Severe      GAACAAAAGATCGCTGAGATTCCTAAAGAGGAAGTTAAGCCATTTATAACTGAAAGTAAA    3910
gb:MT007544|Organism:Severe      GAACAAAAGATCGCTGAGATTCCTAAAGAGGAAGTTAAGCCATTTATAACTGAAAGTAAA    3922
gb:MN994467|Organism:Severe      GAACAAAAGATCGCTGAGATTCCTAAAGAGGAAGTTAAGCCATTTATAACTGAAAGTAAA    3922
gb:MT044257|Organism:Severe      GAACAAAAGATCGCTGAGATTCCTAAAGAGGAAGTTAAGCCATTTATAACTGAAAGTAAA    3922
gb:MT106054|Organism:Severe      GAACAAAAGATCGCTGAGATTCCTAAAGAGGAAGTTAAGCCATTTATAACTGAAAGTAAA    3922
gb:MT049951|Organism:Severe      GAACAAAAGATCGCTGAGATTCCTAAAGAGGAAGTTAAGCCATTTATAACTGAAAGTAAA    3922
gb:MN975262|Organism:Severe      GAACAAAAGATCGCTGAGATTCCTAAAGAGGAAGTTAAGCCATTTATAACTGAAAGTAAA    3922
gb:MT106052|Organism:Severe      GAACAAAAGATCGCTGAGATTCCTAAAGAGGAAGTTAAGCCATTTATAACTGAAAGTAAA    3922
gb:LC522975|Organism:Severe      GAACAAAAGATCGCTGAGATTCCTAAAGAGGAAGTTAAGCCATTTATAACTGAAAGTAAA    3919
gb:LC522973|Organism:Severe      GAACAAAAGATCGCTGAGATTCCTAAAGAGGAAGTTAAGCCATTTATAACTGAAAGTAAA    3919
gb:LC522974|Organism:Severe      GAACAAAAGATCGCTGAGATTCCTAAAGAGGAAGTTAAGCCATTTATAACTGAAAGTAAA    3919
gb:MN985325|Organism:Severe      GAACAAAAGATCGCTGAGATTCCTAAAGAGGAAGTTAAGCCATTTATAACTGAAAGTAAA    3922
gb:MT020881|Organism:Severe      GAACAAAAGATCGCTGAGATTCCTAAAGAGGAAGTTAAGCCATTTATAACTGAAAGTAAA    3922
gb:MT020880|Organism:Severe      GAACAAAAGATCGCTGAGATTCCTAAAGAGGAAGTTAAGCCATTTATAACTGAAAGTAAA    3922
gb:MT066175|Organism:Severe      GAACAAAAGATCGCTGAGATTCCTAAAGAGGAAGTTAAGCCATTTATAACTGAAAGTAAA    3922
gb:MN997409|Organism:Severe      GAACAAAAGATCGCTGAGATTCCTAAAGAGGAAGTTAAGCCATTTATAACTGAAAGTAAA    3922
gb:MN938384|Organism:Severe      GAACAAAAGATCGCTGAGATTCCTAAAGAGGAAGTTAAGCCATTTATAACTGAAAGTAAA    3890
gb:MT044258|Organism:Severe      GAACAAAAGATCGCTGAGATTCCTAAAGAGGAAGTTAAGCCATTTATAACTGAAAGTAAA    3898
gb:MT039890|Organism:Severe      GAACAAAAGATCGCTGAGATTCCTAAAGAGGAAGTTAAGCCATTTATAACTGAAAGTAAA    3922
gb:MN988713|Organism:Severe      GAACAAAAGATCGCTGAGATTCCTAAAGAGGAAGTTAAGCCATTTATAACTGAAAGTAAA    3922
gb:LC521925|Organism:Severe      GAACAAAAGATCGCTGAGATTCCTAAAGAGGAAGTTAAGCCATTTATAACTGAAAGTAAA    3895
gb:MT093571|Organism:Severe      GAACAAAAGATCGCTGAGATTCCTAAAGAGGAAGTTAAGCCATTTATAACTGAAAGTAAA    3922
gb:MT039887|Organism:Severe      GAACAAAAGATCGCTGAGATTCCTAAAGAGGAAGTTAAGCCATTTATAACTGAAAGTAAA    3922
gb:MT019530|Organism:Severe      GAACAAAAGATCGCTGAGATTCCTAAAGAGGAAGTTAAGCCATTTATAACTGAAAGTAAA    3922
gb:MT039888|Organism:Severe      GAACAAAAGATCGCTGAGATTCCTAAAGAGGAAGTTAAGCCATTTATAACTGAAAGTAAA    3922
gb:LC522972|Organism:Severe      GAACAAAAGATCGCTGAGATTCCTAAAGAGGAAGTTAAGCCATTTATAACTGAAAGTAAA    3919
gb:MT027063|Organism:Severe      GAACAAAAGATCGCTGAGATTCCTAAAGAGGAAGTTAAGCCATTTATAACTGAAAGTAAA    3922
gb:MT027062|Organism:Severe      GAACAAAAGATCGCTGAGATTCCTAAAGAGGAAGTTAAGCCATTTATAACTGAAAGTAAA    3922
gb:MT019529|Organism:Severe      GAACAAAAGATCGCTGAGATTCCTAAAGAGGAAGTTAAGCCATTTATAACTGAAAGTAAA    3922
gb:MN996529|Organism:Severe      GAACAAAAGATCGCTGAGATTCCTAAAGAGGAAGTTAAGCCATTTATAACTGAAAGTAAA    3910
gb:MN996531|Organism:Severe      GAACAAAAGATCGCTGAGATTCCTAAAGAGGAAGTTAAGCCATTTATAACTGAAAGTAAA    3909
gb:MT066176|Organism:Severe      GAACAAAAGATCGCTGAGATTCCTAAAGAGGAAGTTAAGCCATTTATAACTGAAAGTAAA    3922
gb:MT027064|Organism:Severe      GAACAAAAGATCGCTGAGATTCCTAAAGAGGAAGTTAAGCCATTTATAACTGAAAGTAAA    3922
gb:MN994468|Organism:Severe      GAACAAAAGATCGCTGAGATTCCTAAAGAGGAAGTTAAGCCATTTATAACTGAAAGTAAA    3922
gb:MT072688|Organism:Severe      GAACAAAAGATCGCTGAGATTCCTAAAGAGGAAGTTAAGCCATTTATAACTGAAAGTAAA    3907
gb:MN996527|Organism:Severe      GAACAAAAGATCGCTGAGATTCCTAAAGAGGAAGTTAAGCCATTTATAACTGAAAGTAAA    3889
gb:MT093631|Organism:Severe      GAACAAAAGATCGCTGAGATTCCTAAAGAGGAAGTTAAGCCATTTATAACTGAAAGTAAA    3960
gb:MT106053|Organism:Severe      GAACAAAAGATCGCTGAGATTCCTAAAGAGGAAGTTAAGCCATTTATAACTGAAAGTAAA    3922
gb:MT019533|Organism:Severe      GAACAAAAGATCGCTGAGATTCCTAAAGAGGAAGTTAAGCCATTTATAACTGAAAGTAAA    3922
gb:MT019531|Organism:Severe      GAACAAAAGATCGCTGAGATTCCTAAAGAGGAAGTTAAGCCATTTATAACTGAAAGTAAA    3922
gb:MN996528|Organism:Severe      GAACAAAAGATCGCTGAGATTCCTAAAGAGGAAGTTAAGCCATTTATAACTGAAAGTAAA    3922
gb:MN996530|Organism:Severe      GAACAAAAGATCGCTGAGATTCCTAAAGAGGAAGTTAAGCCATTTATAACTGAAAGTAAA    3908
gb:MN908947|Organism:Severe      GAACAAAAGATCGCTGAGATTCCTAAAGAGGAAGTTAAGCCATTTATAACTGAAAGTAAA    3922
gb:MT019532|Organism:Severe      GAACAAAAGATCGCTGAGATTCCTAAAGAGGAAGTTAAGCCATTTATAACTGAAAGTAAA    3922
                                 ************************************************************

gb:MT020781|Organism:Severe      CCTTCAGTTGAACAGAGAAAACAAGATGATAAGAAAATCAAAGCTTGTGTTGAAGAAGTT    3970
gb:MT007544|Organism:Severe      CCTTCAGTTGAACAGAGAAAACAAGATGATAAGAAAATCAAAGCTTGTGTTGAAGAAGTT    3982
gb:MN994467|Organism:Severe      CCTTCAGTTGAACAGAGAAAACAAGATGATAAGAAAATCAAAGCTTGTGTTGAAGAAGTT    3982
gb:MT044257|Organism:Severe      CCTTCAGTTGAACAGAGAAAACAAGATGATAAGAAAATCAAAGCTTGTGTTGAAGAAGTT    3982
gb:MT106054|Organism:Severe      CCTTCAGTTGAACAGAGAAAACAAGATGATAAGAAAATCAAAGCTTGTGTTGAAGAAGTT    3982
gb:MT049951|Organism:Severe      CCTTCAGTTGAACAGAGAAAACAAGATGATAAGAAAATCAAAGCTTGTGTTGAAGAAGTT    3982
gb:MN975262|Organism:Severe      CCTTCAGTTGAACAGAGAAAACAAGATGATAAGAAAATCAAAGCTTGTGTTGAAGAAGTT    3982
gb:MT106052|Organism:Severe      CCTTCAGTTGAACAGAGAAAACAAGATGATAAGAAAATCAAAGCTTGTGTTGAAGAAGTT    3982
gb:LC522975|Organism:Severe      CCTTCAGTTGAACAGAGAAAACAAGATGATAAGAAAATCAAAGCTTGTGTTGAAGAAGTT    3979
gb:LC522973|Organism:Severe      CCTTCAGTTGAACAGAGAAAACAAGATGATAAGAAAATCAAAGCTTGTGTTGAAGAAGTT    3979
gb:LC522974|Organism:Severe      CCTTCAGTTGAACAGAGAAAACAAGATGATAAGAAAATCAAAGCTTGTGTTGAAGAAGTT    3979
gb:MN985325|Organism:Severe      CCTTCAGTTGAACAGAGAAAACAAGATGATAAGAAAATCAAAGCTTGTGTTGAAGAAGTT    3982
gb:MT020881|Organism:Severe      CCTTCAGTTGAACAGAGAAAACAAGATGATAAGAAAATCAAAGCTTGTGTTGAAGAAGTT    3982
gb:MT020880|Organism:Severe      CCTTCAGTTGAACAGAGAAAACAAGATGATAAGAAAATCAAAGCTTGTGTTGAAGAAGTT    3982
gb:MT066175|Organism:Severe      CCTTCAGTTGAACAGAGAAAACAAGATGATAAGAAAATCAAAGCTTGTGTTGAAGAAGTT    3982
```

```
gb:MN997409|Organism:Severe      CCTTCAGTTGAACAGAGAAAACAAGATGATAAGAAAATCAAAGCTTGTGTTGAAGAAGTT   3982
gb:MN938384|Organism:Severe      CCTTCAGTTGAACAGAGAAAACAAGATGATAAGAAAATCAAAGCTTGTGTTGAAGAAGTT   3950
gb:MT044258|Organism:Severe      CCTTCAGTTGAACAGAGAAAACAAGATGATAAGAAAATCAAAGCTTGTGTTGAAGAAGTT   3958
gb:MT039890|Organism:Severe      CCTTCAGTTGAACAGAGAAAACAAGATGATAAGAAAATCAAAGCTTGTGTTGAAGAAGTT   3982
gb:MN988713|Organism:Severe      CCTTCAGTTGAACAGAGAAAACAAGATGATAAGAAAATCAAAGCTTGTGTTGAAGAAGTT   3982
gb:LC521925|Organism:Severe      CCTTCAGTTGAACAGAGAAAACAAGATGATAAGAAAATCAAAGCTTGTGTTGAAGAAGTT   3955
gb:MT093571|Organism:Severe      CCTTCAGTTGAACAGAGAAAACAAGATGATAAGAAAATCAAAGCTTGTGTTGAAGAAGTT   3982
gb:MT039887|Organism:Severe      CCTTCAGTTGAACAGAGAAAACAAGATGATAAGAAAATCAAAGCTTGTGTTGAAGAAGTT   3982
gb:MT019530|Organism:Severe      CCTTCAGTTGAACAGAGAAAACAAGATGATAAGAAAATCAAAGCTTGTGTTGAAGAAGTT   3982
gb:MT039888|Organism:Severe      CCTTCAGTTGAACAGAGAAAACAAGATGATAAGAAAATCAAAGCTTGTGTTGAAGAAGTT   3982
gb:LC522972|Organism:Severe      CCTTCAGTTGAACAGAGAAAACAAGATGATAAGAAAATCAAAGCTTGTGTTGAAGAAGTT   3979
gb:MT027063|Organism:Severe      CCTTCAGTTGAACAGAGAAAACAAGATGATAAGAAAATCAAAGCTTGTGTTGAAGAAGTT   3982
gb:MT027062|Organism:Severe      CCTTCAGTTGAACAGAGAAAACAAGATGATAAGAAAATCAAAGCTTGTGTTGAAGAAGTT   3982
gb:MT019529|Organism:Severe      CCTTCAGTTGAACAGAGAAAACAAGATGATAAGAAAATCAAAGCTTGTGTTGAAGAAGTT   3982
gb:MN996529|Organism:Severe      CCTTCAGTTGAACAGAGAAAACAAGATGATAAGAAAATCAAAGCTTGTGTTGAAGAAGTT   3970
gb:MN996531|Organism:Severe      CCTTCAGTTGAACAGAGAAAACAAGATGATAAGAAAATCAAAGCTTGTGTTGAAGAAGTT   3969
gb:MT066176|Organism:Severe      CCTTCAGTTGAACAGAGAAAACAAGATGATAAGAAAATCAAAGCTTGTGTTGAAGAAGTT   3982
gb:MT027064|Organism:Severe      CCTTCAGTTGAACAGAGAAAACAAGATGATAAGAAAATCAAAGCTTGTGTTGAAGAAGTT   3982
gb:MN994468|Organism:Severe      CCTTCAGTTGAACAGAGAAAACAAGATGATAAGAAAATCAAAGCTTGTGTTGAAGAAGTT   3982
gb:MT072688|Organism:Severe      CCTTCAGTTGAACAGAGAAAACAAGATGATAAGAAAATCAAAGCTTGTGTTGAAGAAGTT   3967
gb:MN996527|Organism:Severe      CCTTCAGTTGAACAGAGAAAACAAGATGATAAGAAAATCAAAGCTTGTGTTGAAGAAGTT   3949
gb:MT093631|Organism:Severe      CCTTCAGTTGAACAGAGAAAACAAGATGATAAGAAAATCAAAGCTTGTGTTGAAGAAGTT   4020
gb:MT106053|Organism:Severe      CCTTCAGTTGAACAGAGAAAACAAGATGATAAGAAAATCAAAGCTTGTGTTGAAGAAGTT   3982
gb:MT019533|Organism:Severe      CCTTCAGTTGAACAGAGAAAACAAGATGATAAGAAAATCAAAGCTTGTGTTGAAGAAGTT   3982
gb:MT019531|Organism:Severe      CCTTCAGTTGAACAGAGAAAACAAGATGATAAGAAAATCAAAGCTTGTGTTGAAGAAGTT   3982
gb:MN996528|Organism:Severe      CCTTCAGTTGAACAGAGAAAACAAGATGATAAGAAAATCAAAGCTTGTGTTGAAGAAGTT   3982
gb:MN996530|Organism:Severe      CCTTCAGTTGAACAGAGAAAACAAGATGATAAGAAAATCAAAGCTTGTGTTGAAGAAGTT   3968
gb:MN908947|Organism:Severe      CCTTCAGTTGAACAGAGAAAACAAGATGATAAGAAAATCAAAGCTTGTGTTGAAGAAGTT   3982
gb:MT019532|Organism:Severe      CCTTCAGTTGAACAGAGAAAACAAGATGATAAGAAAATCAAAGCTTGTGTTGAAGAAGTT   3982
                                 ************************************************************

gb:MT020781|Organism:Severe      ACAACAACTCTGGAAGAAACTAAGTTCCTCACAGAAAACTTGTTACTTTATATTGACATT   4030
gb:MT007544|Organism:Severe      ACAACAACTCTGGAAGAAACTAAGTTCCTCACAGAAAACTTGTTACTTTATATTGACATT   4042
gb:MN994467|Organism:Severe      ACAACAACTCTGGAAGAAACTAAGTTCCTCACAGAAAACTTGTTACTTTATATTGACATT   4042
gb:MT044257|Organism:Severe      ACAACAACTCTGGAAGAAACTAAGTTCCTCACAGAAAACTTGTTACTTTATATTGACATT   4042
gb:MT106054|Organism:Severe      ACAACAACTCTGGAAGAAACTAAGTTCCTCACAGAAAACTTGTTACTTTATATTGACATT   4042
gb:MT049951|Organism:Severe      ACAACAACTCTGGAAGAAACTAAGTTCCTCACAGAAAACTTGTTACTTTATATTGACATT   4042
gb:MN975262|Organism:Severe      ACAACAACTCTGGAAGAAACTAAGTTCCTCACAGAAAACTTGTTACTTTATATTGACATT   4042
gb:MT106052|Organism:Severe      ACAACAACTCTGGAAGAAACTAAGTTCCTCACAGAAAACTTGTTACTTTATATTGACATT   4042
gb:LC522975|Organism:Severe      ACAACAACTCTGGAAGAAACTAAGTTCCTCACAGAAAACTTGTTACTTTATATTGACATT   4039
gb:LC522973|Organism:Severe      ACAACAACTCTGGAAGAAACTAAGTTCCTCACAGAAAACTTGTTACTTTATATTGACATT   4039
gb:LC522974|Organism:Severe      ACAACAACTCTGGAAGAAACTAAGTTCCTCACAGAAAACTTGTTACTTTATATTGACATT   4039
gb:MN985325|Organism:Severe      ACAACAACTCTGGAAGAAACTAAGTTCCTCACAGAAAACTTGTTACTTTATATTGACATT   4042
gb:MT020881|Organism:Severe      ACAACAACTCTGGAAGAAACTAAGTTCCTCACAGAAAACTTGTTACTTTATATTGACATT   4042
gb:MT020880|Organism:Severe      ACAACAACTCTGGAAGAAACTAAGTTCCTCACAGAAAACTTGTTACTTTATATTGACATT   4042
gb:MT066175|Organism:Severe      ACAACAACTCTGGAAGAAACTAAGTTCCTCACAGAAAACTTGTTACTTTATATTGACATT   4042
gb:MN997409|Organism:Severe      ACAACAACTCTGGAAGAAACTAAGTTCCTCACAGAAAACTTGTTACTTTATATTGACATT   4042
gb:MN938384|Organism:Severe      ACAACAACTCTGGAAGAAACTAAGTTCCTCACAGAAAACTTGTTACTTTATATTGACATT   4010
gb:MT044258|Organism:Severe      ACAACAACTCTGGAAGAAACTAAGTTCCTCACAGAAAACTTGTTACTTTATATTGACATT   4018
gb:MT039890|Organism:Severe      ACAACAACTCTGGAAGAAACTAAGTTCCTCACAGAAAACTTGTTACTTTATATTGACATT   4042
gb:MN988713|Organism:Severe      ACAACAACTCTGGAAGAAACTAAGTTCCTCACAGAAAACTTGTTACTTTATATTGACATT   4042
gb:LC521925|Organism:Severe      ACAACAACTCTGGAAGAAACTAAGTTCCTCACAGAAAACTTGTTACTTTATATTGACATT   4015
gb:MT093571|Organism:Severe      ACAACAACTCTGGAAGAAACTAAGTTCCTCACAGAAAACTTGTTACTTTATATTGACATT   4042
gb:MT039887|Organism:Severe      ACAACAACTCTGGAAGAAACTAAGTTCCTCACAGAAAACTTGTTACTTTATATTGACATT   4042
gb:MT019530|Organism:Severe      ACAACAACTCTGGAAGAAACTAAGTTCCTCACAGAAAACTTGTTACTTTATATTGACATT   4042
gb:MT039888|Organism:Severe      ACAACAACTCTGGAAGAAACTAAGTTCCTCACAGAAAACTTGTTACTTTATATTGACATT   4042
gb:LC522972|Organism:Severe      ACAACAACTCTGGAAGAAACTAAGTTCCTCACAGAAAACTTGTTACTTTATATTGACATT   4039
gb:MT027063|Organism:Severe      ACAACAACTCTGGAAGAAACTAAGTTCCTCACAGAAAACTTGTTACTTTATATTGACATT   4042
gb:MT027062|Organism:Severe      ACAACAACTCTGGAAGAAACTAAGTTCCTCACAGAAAACTTGTTACTTTATATTGACATT   4042
gb:MT019529|Organism:Severe      ACAACAACTCTGGAAGAAACTAAGTTCCTCACAGAAAACTTGTTACTTTATATTGACATT   4042
gb:MN996529|Organism:Severe      ACAACAACTCTGGAAGAAACTAAGTTCCTCACAGAAAACTTGTTACTTTATATTGACATT   4030
gb:MN996531|Organism:Severe      ACAACAACTCTGGAAGAAACTAAGTTCCTCACAGAAAACTTGTTACTTTATATTGACATT   4029
gb:MT066176|Organism:Severe      ACAACAACTCTGGAAGAAACTAAGTTCCTCACAGAAAACTTGTTACTTTATATTGACATT   4042
gb:MT027064|Organism:Severe      ACAACAACTCTGGAAGAAACTAAGTTCCTCACAGAAAACTTGTTACTTTATATTGACATT   4042
gb:MN994468|Organism:Severe      ACAACAACTCTGGAAGAAACTAAGTTCCTCACAGAAAACTTGTTACTTTATATTGACATT   4042
```

```
gb:MT072688|Organism:Severe      ACAACAACTCTGGAAGAAACTAAGTTCCTCACAGAAAACTTGTTACTTTATATTGACATT    4027
gb:MN996527|Organism:Severe      ACAACAACTCTGGAAGAAACTAAGTTCCTCACAGAAAACTTGTTACTTTATATTGACATT    4009
gb:MT093631|Organism:Severe      ACAACAACTCTGGAAGAAACTAAGTTCCTCACAGAAAACTTGTTACTTTATATTGACATT    4080
gb:MT106053|Organism:Severe      ACAACAACTCTGGAAGAAACTAAGTTCCTCACAGAAAACTTGTTACTTTATATTGACATT    4042
gb:MT019533|Organism:Severe      ACAACAACTCTGGAAGAAACTAAGTTCCTCACAGAAAACTTGTTACTTTATATTGACATT    4042
gb:MT019531|Organism:Severe      ACAACAACTCTGGAAGAAACTAAGTTCCTCACAGAAAACTTGTTACTTTATATTGACATT    4042
gb:MN996528|Organism:Severe      ACAACAACTCTGGAAGAAACTAAGTTCCTCACAGAAAACTTGTTACTTTATATTGACATT    4042
gb:MN996530|Organism:Severe      ACAACAACTCTGGAAGAAACTAAGTTCCTCACAGAAAACTTGTTACTTTATATTGACATT    4028
gb:MN908947|Organism:Severe      ACAACAACTCTGGAAGAAACTAAGTTCCTCACAGAAAACTTGTTACTTTATATTGACATT    4042
gb:MT019532|Organism:Severe      ACAACAACTCTGGAAGAAACTAAGTTCCTCACAGAAAACTTGTTACTTTATATTGACATT    4042
                                 ************************************************************

gb:MT020781|Organism:Severe      AATGGCAATCTTCATCCAGATTCTGCCACTCTTGTTAGTGACATTGACATCACTTTCTTA    4090
gb:MT007544|Organism:Severe      AATGGCAATCTTCATCCAGATTCTGCCACTCTTGTTAGTGACATTGACATCACTTTCTTA    4102
gb:MN994467|Organism:Severe      AATGGCAATCTTCATCCAGATTCTGCCACTCTTGTTAGTGACATTGACATCACTTTCTTA    4102
gb:MT044257|Organism:Severe      AATGGCAATCTTCATCCAGATTCTGCCACTCTTGTTAGTGACATTGACATCACTTTCTTA    4102
gb:MT106054|Organism:Severe      AATGGCAATCTTCATCCAGATTCTGCCACTCTTGTTAGTGACATTGACATCACTTTCTTA    4102
gb:MT049951|Organism:Severe      AATGGCAATCTTCATCCAGATTCTGCCACTCTTGTTAGTGACATTGACATCACTTTCTTA    4102
gb:MN975262|Organism:Severe      AATGGCAATCTTCATCCAGATTCTGCCACTCTTGTTAGTGACATTGACATCACTTTCTTA    4102
gb:MT106052|Organism:Severe      AATGGCAATCTTCATCCAGATTCTGCCACTCTTGTTAGTGACATTGACATCACTTTCTTA    4102
gb:LC522975|Organism:Severe      AATGGCAATCTTCATCCAGATTCTGCCACTCTTGTTAGTGACATTGACATCACTTTCTTA    4099
gb:LC522973|Organism:Severe      AATGGCAATCTTCATCCAGATTCTGCCACTCTTGTTAGTGACATTGACATCACTTTCTTA    4099
gb:LC522974|Organism:Severe      AATGGCAATCTTCATCCAGATTCTGCCACTCTTGTTAGTGACATTGACATCACTTTCTTA    4099
gb:MN985325|Organism:Severe      AATGGCAATCTTCATCCAGATTCTGCCACTCTTGTTAGTGACATTGACATCACTTTCTTA    4102
gb:MT020881|Organism:Severe      AATGGCAATCTTCATCCAGATTCTGCCACTCTTGTTAGTGACATTGACATCACTTTCTTA    4102
gb:MT020880|Organism:Severe      AATGGCAATCTTCATCCAGATTCTGCCACTCTTGTTAGTGACATTGACATCACTTTCTTA    4102
gb:MT066175|Organism:Severe      AATGGCAATCTTCATCCAGATTCTGCCACTCTTGTTAGTGACATTGACATCACTTTCTTA    4102
gb:MN997409|Organism:Severe      AATGGCAATCTTCATCCAGATTCTGCCACTCTTGTTAGTGACATTGACATCACTTTCTTA    4102
gb:MN938384|Organism:Severe      AATGGCAATCTTCATCCAGATTCTGCCACTCTTGTTAGTGACATTGACATCACTTTCTTA    4070
gb:MT044258|Organism:Severe      AATGGCAATCTTCATCCAGATTCTGCCACTCTTGTTAGTGACATTGACATCACTTTCTTA    4078
gb:MT039890|Organism:Severe      AATGGCAATCTTCATCCAGATTCTGCCACTCTTGTTAGTGACATTGACATCACTTTCTTA    4102
gb:MN988713|Organism:Severe      AATGGCAATCTTCATCCAGATTCTGCCACTCTTGTTAGTGACATTGACATCACTTTCTTA    4102
gb:LC521925|Organism:Severe      AATGGCAATCTTCATCCAGATTCTGCCACTCTTGTTAGTGACATTGACATCACTTTCTTA    4075
gb:MT093571|Organism:Severe      AATGGCAATCTTCATCCAGATTCTGCCACTCTTGTTAGTGACATTGACATCACTTTCTTA    4102
gb:MT039887|Organism:Severe      AATGGCAATCTTCATCCAGATTCTGCCACTCTTGTTAGTGACATTGACATCACTTTCTTA    4102
gb:MT019530|Organism:Severe      AATGGCAATCTTCATCCAGATTCTGCCACTCTTGTTAGTGACATTGACATCACTTTCTTA    4102
gb:MT039888|Organism:Severe      AATGGCAATCTTCATCCAGATTCTGCCACTCTTGTTAGTGACATTGACATCACTTTCTTA    4102
gb:LC522972|Organism:Severe      AATGGCAATCTTCATCCAGATTCTGCCACTCTTGTTAGTGACATTGACATCACTTTCTTA    4099
gb:MT027063|Organism:Severe      AATGGCAATCTTCATCCAGATTCTGCCACTCTTGTTAGTGACATTGACATCACTTTCTTA    4102
gb:MT027062|Organism:Severe      AATGGCAATCTTCATCCAGATTCTGCCACTCTTGTTAGTGACATTGACATCACTTTCTTA    4102
gb:MT019529|Organism:Severe      AATGGCAATCTTCATCCAGATTCTGCCACTCTTGTTAGTGACATTGACATCACTTTCTTA    4102
gb:MN996529|Organism:Severe      AATGGCAATCTTCATCCAGATTCTGCCACTCTTGTTAGTGACATTGACATCACTTTCTTA    4090
gb:MN996531|Organism:Severe      AATGGCAATCTTCATCCAGATTCTGCCACTCTTGTTAGTGACATTGACATCACTTTCTTA    4089
gb:MT066176|Organism:Severe      AATGGCAATCTTCATCCAGATTCTGCCACTCTTGTTAGTGACATTGACATCACTTTCTTA    4102
gb:MT027064|Organism:Severe      AATGGCAATCTTCATCCAGATTCTGCCACTCTTGTTAGTGACATTGACATCACTTTCTTA    4102
gb:MN994468|Organism:Severe      AATGGCAATCTTCATCCAGATTCTGCCACTCTTGTTAGTGACATTGACATCACTTTCTTA    4102
gb:MT072688|Organism:Severe      AATGGCAATCTTCATCCAGATTCTGCCACTCTTGTTAGTGACATTGACATCACTTTCTTA    4087
gb:MN996527|Organism:Severe      AATGGCAATCTTCATCCAGATTCTGCCACTCTTGTTAGTGACATTGACATCACTTTCTTA    4069
gb:MT093631|Organism:Severe      AATGGCAATCTTCATCCAGATTCTGCCACTCTTGTTAGTGACATTGACATCACTTTCTTA    4140
gb:MT106053|Organism:Severe      AATGGCAATCTTCATCCAGATTCTGCCACTCTTGTTAGTGACATTGACATCACTTTCTTA    4102
gb:MT019533|Organism:Severe      AATGGCAATCTTCATCCAGATTCTGCCACTCTTGTTAGTGACATTGACATCACTTTCTTA    4102
gb:MT019531|Organism:Severe      AATGGCAATCTTCATCCAGATTCTGCCACTCTTGTTAGTGACATTGACATCACTTTCTTA    4102
gb:MN996528|Organism:Severe      AATGGCAATCTTCATCCAGATTCTGCCACTCTTGTTAGTGACATTGACATCACTTTCTTA    4102
gb:MN996530|Organism:Severe      AATGGCAATCTTCATCCAGATTCTGCCACTCTTGTTAGTGACATTGACATCACTTTCTTA    4088
gb:MN908947|Organism:Severe      AATGGCAATCTTCATCCAGATTCTGCCACTCTTGTTAGTGACATTGACATCACTTTCTTA    4102
gb:MT019532|Organism:Severe      AATGGCAATCTTCATCCAGATTCTGCCACTCTTGTTAGTGACATTGACATCACTTTCTTA    4102
                                 ************************************************************

gb:MT020781|Organism:Severe      AAGAAAGATGCTCCATATATAGTGGGTGATGTTGTTCAAGAGGGTGTTTTAACTGCTGTG    4150
gb:MT007544|Organism:Severe      AAGAAAGATGCTCCATATATAGTGGGTGATGTTGTTCAAGAGGGTGTTTTAACTGCTGTG    4162
gb:MN994467|Organism:Severe      AAGAAAGATGCTCCATATATAGTGGGTGATGTTGTTCAAGAGGGTGTTTTAACTGCTGTG    4162
gb:MT044257|Organism:Severe      AAGAAAGATGCTCCATATATAGTGGGTGATGTTGTTCAAGAGGGTGTTTTAACTGCTGTG    4162
gb:MT106054|Organism:Severe      AAGAAAGATGCTCCATATATAGTGGGTGATGTTGTTCAAGAGGGTGTTTTAACTGCTGTG    4162
gb:MT049951|Organism:Severe      AAGAAAGATGCTCCATATATAGTGGGTGATGTTGTTCAAGAGGGTGTTTTAACTGCTGTG    4162
gb:MN975262|Organism:Severe      AAGAAAGATGCTCCATATATAGTGGGTGATGTTGTTCAAGAGGGTGTTTTAACTGCTGTG    4162
```

```
gb:MT106052|Organism:Severe      AAGAAAGATGCTCCATATATAGTGGGTGATGTTGTTCAAGAGGGTGTTTTAACTGCTGTG    4162
gb:LC522975|Organism:Severe      AAGAAAGATGCTCCATATATAGTGGGTGATGTTGTTCAAGAGGGTGTTTTAACTGCTGTG    4159
gb:LC522973|Organism:Severe      AAGAAAGATGCTCCATATATAGTGGGTGATGTTGTTCAAGAGGGTGTTTTAACTGCTGTG    4159
gb:LC522974|Organism:Severe      AAGAAAGATGCTCCATATATAGTGGGTGATGTTGTTCAAGAGGGTGTTTTAACTGCTGTG    4159
gb:MN985325|Organism:Severe      AAGAAAGATGCTCCATATATAGTGGGTGATGTTGTTCAAGAGGGTGTTTTAACTGCTGTG    4162
gb:MT020881|Organism:Severe      AAGAAAGATGCTCCATATATAGTGGGTGATGTTGTTCAAGAGGGTGTTTTAACTGCTGTG    4162
gb:MT020880|Organism:Severe      AAGAAAGATGCTCCATATATAGTGGGTGATGTTGTTCAAGAGGGTGTTTTAACTGCTGTG    4162
gb:MT066175|Organism:Severe      AAGAAAGATGCTCCATATATAGTGGGTGATGTTGTTCAAGAGGGTGTTTTAACTGCTGTG    4162
gb:MN997409|Organism:Severe      AAGAAAGATGCTCCATATATAGTGGGTGATGTTGTTCAAGAGGGTGTTTTAACTGCTGTG    4162
gb:MN938384|Organism:Severe      AAGAAAGATGCTCCATATATAGTGGGTGATGTTGTTCAAGAGGGTGTTTTAACTGCTGTG    4130
gb:MT044258|Organism:Severe      AAGAAAGATGCTCCATATATAGTGGGTGATGTTGTTCAAGAGGGTGTTTTAACTGCTGTG    4138
gb:MT039890|Organism:Severe      AAGAAAGATGCTCCATATATAGTGGGTGATGTTGTTCAAGAGGGTGTTTTAACTGCTGTG    4162
gb:MN988713|Organism:Severe      AAGAAAGATGCTCCATATATAGTGGGTGATGTTGTTCAAGAGGGTGTTTTAACTGCTGTG    4162
gb:LC521925|Organism:Severe      AAGAAAGATGCTCCATATATAGTGGGTGATGTTGTTCAAGAGGGTGTTTTAACTGCTGTG    4135
gb:MT093571|Organism:Severe      AAGAAAGATGCTCCATATATAGTGGGTGATGTTGTTCAAGAGGGTGTTTTAACTGCTGTG    4162
gb:MT039887|Organism:Severe      AAGAAAGATGCTCCATATATAGTGGGTGATGTTGTTCAAGAGGGTGTTTTAACTGCTGTG    4162
gb:MT019530|Organism:Severe      AAGAAAGATGCTCCATATATAGTGGGTGATGTTGTTCAAGAGGGTGTTTTAACTGCTGTG    4162
gb:MT039888|Organism:Severe      AAGAAAGATGCTCCATATATAGTGGGTGATGTTGTTCAAGAGGGTGTTTTAACTGCTGTG    4162
gb:LC522972|Organism:Severe      AAGAAAGATGCTCCATATATAGTGGGTGATGTTGTTCAAGAGGGTGTTTTAACTGCTGTG    4159
gb:MT027063|Organism:Severe      AAGAAAGATGCTCCATATATAGTGGGTGATGTTGTTCAAGAGGGTGTTTTAACTGCTGTG    4162
gb:MT027062|Organism:Severe      AAGAAAGATGCTCCATATATAGTGGGTGATGTTGTTCAAGAGGGTGTTTTAACTGCTGTG    4162
gb:MT019529|Organism:Severe      AAGAAAGATGCTCCATATATAGTGGGTGATGTTGTTCAAGAGGGTGTTTTAACTGCTGTG    4162
gb:MN996529|Organism:Severe      AAGAAAGATGCTCCATATATAGTGGGTGATGTTGTTCAAGAGGGTGTTTTAACTGCTGTG    4150
gb:MN996531|Organism:Severe      AAGAAAGATGCTCCATATATAGTGGGTGATGTTGTTCAAGAGGGTGTTTTAACTGCTGTG    4149
gb:MT066176|Organism:Severe      AAGAAAGATGCTCCATATATAGTGGGTGATGTTGTTCAAGAGGGTGTTTTAACTGCTGTG    4162
gb:MT027064|Organism:Severe      AAGAAAGATGCTCCATATATAGTGGGTGATGTTGTTCAAGAGGGTGTTTTAACTGCTGTG    4162
gb:MN994468|Organism:Severe      AAGAAAGATGCTCCATATATAGTGGGTGATGTTGTTCAAGAGGGTGTTTTAACTGCTGTG    4162
gb:MT072688|Organism:Severe      AAGAAAGATGCTCCATATATAGTGGGTGATGTTGTTCAAGAGGGTGTTTTAACTGCTGTG    4147
gb:MN996527|Organism:Severe      AAGAAAGATGCTCCATATATAGTGGGTGATGTTGTTCAAGAGGGTGTTTTAACTGCTGTG    4129
gb:MT093631|Organism:Severe      AAGAAAGATGCTCCATATATAGTGGGTGATGTTGTTCAAGAGGGTGTTTTAACTGCTGTG    4200
gb:MT106053|Organism:Severe      AAGAAAGATGCTCCATATATAGTGGGTGATGTTGTTCAAGAGGGTGTTTTAACTGCTGTG    4162
gb:MT019533|Organism:Severe      AAGAAAGATGCTCCATATATAGTGGGTGATGTTGTTCAAGAGGGTGTTTTAACTGCTGTG    4162
gb:MT019531|Organism:Severe      AAGAAAGATGCTCCATATATAGTGGGTGATGTTGTTCAAGAGGGTGTTTTAACTGCTGTG    4162
gb:MN996528|Organism:Severe      AAGAAAGATGCTCCATATATAGTGGGTGATGTTGTTCAAGAGGGTGTTTTAACTGCTGTG    4162
gb:MN996530|Organism:Severe      AAGAAAGATGCTCCATATATAGTGGGTGATGTTGTTCAAGAGGGTGTTTTAACTGCTGTG    4148
gb:MN908947|Organism:Severe      AAGAAAGATGCTCCATATATAGTGGGTGATGTTGTTCAAGAGGGTGTTTTAACTGCTGTG    4162
gb:MT019532|Organism:Severe      AAGAAAGATGCTCCATATATAGTGGGTGATGTTGTTCAAGAGGGTGTTTTAACTGCTGTG    4162
                                 ************************************************************

gb:MT020781|Organism:Severe      GTTATACCTACTAAAAAGGCTGGTGGCACTACTGAAATGCTAGCGAAAGCTTTGAGAAAA    4210
gb:MT007544|Organism:Severe      GTTATACCTACTAAAAAGGCTGGTGGCACTACTGAAATGCTAGCGAAAGCTTTGAGAAAA    4222
gb:MN994467|Organism:Severe      GTTATACCTACTAAAAAGGCTGGTGGCACTACTGAAATGCTAGCGAAAGCTTTGAGAAAA    4222
gb:MT044257|Organism:Severe      GTTATACCTACTAAAAAGGCTGGTGGCACTACTGAAATGCTAGCGAAAGCTTTGAGAAAA    4222
gb:MT106054|Organism:Severe      GTTATACCTACTAAAAAGGCTGGTGGCACTACTGAAATGCTAGCGAAAGCTTTGAGAAAA    4222
gb:MT049951|Organism:Severe      GTTATACCTACTAAAAAGGCTGGTGGCACTACTGAAATGCTAGCGAAAGCTTTGAGAAAA    4222
gb:MN975262|Organism:Severe      GTTATACCTACTAAAAAGGCTGGTGGCACTACTGAAATGCTAGCGAAAGCTTTGAGAAAA    4222
gb:MT106052|Organism:Severe      GTTATACCTACTAAAAAGGCTGGTGGCACTACTGAAATGCTAGCGAAAGCTTTGAGAAAA    4222
gb:LC522975|Organism:Severe      GTTATACCTACTAAAAAGGCTGGTGGCACTACTGAAATGCTAGCGAAAGCTTTGAGAAAA    4219
gb:LC522973|Organism:Severe      GTTATACCTACTAAAAAGGCTGGTGGCACTACTGAAATGCTAGCGAAAGCTTTGAGAAAA    4219
gb:LC522974|Organism:Severe      GTTATACCTACTAAAAAGGCTGGTGGCACTACTGAAATGCTAGCGAAAGCTTTGAGAAAA    4219
gb:MN985325|Organism:Severe      GTTATACCTACTAAAAAGGCTGGTGGCACTACTGAAATGCTAGCGAAAGCTTTGAGAAAA    4222
gb:MT020881|Organism:Severe      GTTATACCTACTAAAAAGGCTGGTGGCACTACTGAAATGCTAGCGAAAGCTTTGAGAAAA    4222
gb:MT020880|Organism:Severe      GTTATACCTACTAAAAAGGCTGGTGGCACTACTGAAATGCTAGCGAAAGCTTTGAGAAAA    4222
gb:MT066175|Organism:Severe      GTTATACCTACTAAAAAGGCTGGTGGCACTACTGAAATGCTAGCGAAAGCTTTGAGAAAA    4222
gb:MN997409|Organism:Severe      GTTATACCTACTAAAAAGGCTGGTGGCACTACTGAAATGCTAGCGAAAGCTTTGAGAAAA    4222
gb:MN938384|Organism:Severe      GTTATACCTACTAAAAAGGCTGGTGGCACTACTGAAATGCTAGCGAAAGCTTTGAGAAAA    4190
gb:MT044258|Organism:Severe      GTTATACCTACTAAAAAGGCTGGTGGCACTACTGAAATGCTAGCGAAAGCTTTGAGAAAA    4198
gb:MT039890|Organism:Severe      GTTATACCTACTAAAAAGGCTGGTGGCACTACTGAAATGCTAGCGAAAGCTTTGAGAAAA    4222
gb:MN988713|Organism:Severe      GTTATACCTACTAAAAAGGCTGGTGGCACTACTGAAATGCTAGCGAAAGCTTTGAGAAAA    4222
gb:LC521925|Organism:Severe      GTTATACCTACTAAAAAGGCTGGTGGCACTACTGAAATGCTAGCGAAAGCTTTGAGAAAA    4195
gb:MT093571|Organism:Severe      GTTATACCTACTAAAAAGGCTGGTGGCACTACTGAAATGCTAGCGAAAGCTTTGAGAAAA    4222
gb:MT039887|Organism:Severe      GTTATACCTACTAAAAAGGCTGGTGGCACTACTGAAATGCTAGCGAAAGCTTTGAGAAAA    4222
gb:MT019530|Organism:Severe      GTTATACCTACTAAAAAGGCTGGTGGCACTACTGAAATGCTAGCGAAAGCTTTGAGAAAA    4222
gb:MT039888|Organism:Severe      GTTATACCTACTAAAAAGGCTGGTGGCACTACTGAAATGCTAGCGAAAGCTTTGAGAAAA    4222
gb:LC522972|Organism:Severe      GTTATACCTACTAAAAAGGCTGGTGGCACTACTGAAATGCTAGCGAAAGCTTTGAGAAAA    4219
```

```
gb:MT027063|Organism:Severe      GTTATACCTACTAAAAAGGCTGGTGGCACTACTGAAATGCTAGCGAAAGCTTTGAGAAAA   4222
gb:MT027062|Organism:Severe      GTTATACCTACTAAAAAGGCTGGTGGCACTACTGAAATGCTAGCGAAAGCTTTGAGAAAA   4222
gb:MT019529|Organism:Severe      GTTATACCTACTAAAAAGGCTGGTGGCACTACTGAAATGCTAGCGAAAGCTTTGAGAAAA   4222
gb:MN996529|Organism:Severe      GTTATACCTACTAAAAAGGCTGGTGGCACTACTGAAATGCTAGCGAAAGCTTTGAGAAAA   4210
gb:MN996531|Organism:Severe      GTTATACCTACTAAAAAGGCTGGTGGCACTACTGAAATGCTAGCGAAAGCTTTGAGAAAA   4209
gb:MT066176|Organism:Severe      GTTATACCTACTAAAAAGGCTGGTGGCACTACTGAAATGCTAGCGAAAGCTTTGAGAAAA   4222
gb:MT027064|Organism:Severe      GTTATACCTACTAAAAAGGCTGGTGGCACTACTGAAATGCTAGCGAAAGCTTTGAGAAAA   4222
gb:MN994468|Organism:Severe      GTTATACCTACTAAAAAGGCTGGTGGCACTACTGAAATGCTAGCGAAAGCTTTGAGAAAA   4222
gb:MT072688|Organism:Severe      GTTATACCTACTAAAAAGGCTGGTGGCACTACTGAAATGCTAGCGAAAGCTTTGAGAAAA   4207
gb:MN996527|Organism:Severe      GTTATACCTACTAAAAAGGCTGGTGGCACTACTGAAATGCTAGCGAAAGCTTTGAGAAAA   4189
gb:MT093631|Organism:Severe      GTTATACCTACTAAAAAGGCTGGTGGCACTACTGAAATGCTAGCGAAAGCTTTGAGAAAA   4260
gb:MT106053|Organism:Severe      GTTATACCTACTAAAAAGGCTGGTGGCACTACTGAAATGCTAGCGAAAGCTTTGAGAAAA   4222
gb:MT019533|Organism:Severe      GTTATACCTACTAAAAAGGCTGGTGGCACTACTGAAATGCTAGCGAAAGCTTTGAGAAAA   4222
gb:MT019531|Organism:Severe      GTTATACCTACTAAAAAGGCTGGTGGCACTACTGAAATGCTAGCGAAAGCTTTGAGAAAA   4222
gb:MN996528|Organism:Severe      GTTATACCTACTAAAAAGGCTGGTGGCACTACTGAAATGCTAGCGAAAGCTTTGAGAAAA   4222
gb:MN996530|Organism:Severe      GTTATACCTACTAAAAAGGCTGGTGGCACTACTGAAATGCTAGCGAAAGCTTTGAGAAAA   4208
gb:MN908947|Organism:Severe      GTTATACCTACTAAAAAGGCTGGTGGCACTACTGAAATGCTAGCGAAAGCTTTGAGAAAA   4222
gb:MT019532|Organism:Severe      GTTATACCTACTAAAAAGGCTGGTGGCACTACTGAAATGCTAGCGAAAGCTTTGAGAAAA   4222
                                 ************************************************************

gb:MT020781|Organism:Severe      GTGCCAACAGACAATTATATAACCACTTACCCGGGTCAGGGTTTAAATGGTTACACTGTA   4270
gb:MT007544|Organism:Severe      GTGCCAACAGACAATTATATAACCACTTACCCGGGTCAGGGTTTAAATGGTTACACTGTA   4282
gb:MN994467|Organism:Severe      GTGCCAACAGACAATTATATAACCACTTACCCGGGTCAGGGTTTAAATGGTTACACTGTA   4282
gb:MT044257|Organism:Severe      GTGCCAACAGACAATTATATAACCACTTACCCGGGTCAGGGTTTAAATGGTTACACTGTA   4282
gb:MT106054|Organism:Severe      GTGCCAACAGACAATTATATAACCACTTACCCGGGTCAGGGTTTAAATGGTTACACTGTA   4282
gb:MT049951|Organism:Severe      GTGCCAACAGACAATTATATAACCACTTACCCGGGTCAGGGTTTAAATGGTTACACTGTA   4282
gb:MN975262|Organism:Severe      GTGCCAACAGACAATTATATAACCACTTACCCGGGTCAGGGTTTAAATGGTTACACTGTA   4282
gb:MT106052|Organism:Severe      GTGCCAACAGACAATTATATAACCACTTACCCGGGTCAGGGTTTAAATGGTTACACTGTA   4282
gb:LC522975|Organism:Severe      GTGCCAACAGACAATTATATAACCACTTACCCGGGTCAGGGTTTAAATGGTTACACTGTA   4279
gb:LC522973|Organism:Severe      GTGCCAACAGACAATTATATAACCACTTACCCGGGTCAGGGTTTAAATGGTTACACTGTA   4279
gb:LC522974|Organism:Severe      GTGCCAACAGACAATTATATAACCACTTACCCGGGTCAGGGTTTAAATGGTTACACTGTA   4279
gb:MN985325|Organism:Severe      GTGCCAACAGACAATTATATAACCACTTACCCGGGTCAGGGTTTAAATGGTTACACTGTA   4282
gb:MT020881|Organism:Severe      GTGCCAACAGACAATTATATAACCACTTACCCGGGTCAGGGTTTAAATGGTTACACTGTA   4282
gb:MT020880|Organism:Severe      GTGCCAACAGACAATTATATAACCACTTACCCGGGTCAGGGTTTAAATGGTTACACTGTA   4282
gb:MT066175|Organism:Severe      GTGCCAACAGACAATTATATAACCACTTACCCGGGTCAGGGTTTAAATGGTTACACTGTA   4282
gb:MN997409|Organism:Severe      GTGCCAACAGACAATTATATAACCACTTACCCGGGTCAGGGTTTAAATGGTTACACTGTA   4282
gb:MN938384|Organism:Severe      GTGCCAACAGACAATTATATAACCACTTACCCGGGTCAGGGTTTAAATGGTTACACTGTA   4250
gb:MT044258|Organism:Severe      GTGCCAACAGACAATTATATAACCACTTACCCGGGTCAGGGTTTAAATGGTTACACTGTA   4258
gb:MT039890|Organism:Severe      GTGCCAACAGACAATTATATAACCACTTACCCGGGTCAGGGTTTAAATGGTTACACTGTA   4282
gb:MN988713|Organism:Severe      GTGCCAACAGACAATTATATAACCACTTACCCGGGTCAGGGTTTAAATGGTTACACTGTA   4282
gb:LC521925|Organism:Severe      GTGCCAACAGACAATTATATAACCACTTACCCGGGTCAGGGTTTAAATGGTTACACTGTA   4255
gb:MT093571|Organism:Severe      GTGCCAACAGACAATTATATAACCACTTACCCGGGTCAGGGTTTAAATGGTTACACTGTA   4282
gb:MT039887|Organism:Severe      GTGCCAACAGACAATTATATAACCACTTACCCGGGTCAGGGTTTAAATGGTTACACTGTA   4282
gb:MT019530|Organism:Severe      GTGCCAACAGACAATTATATAACCACTTACCCGGGTCAGGGTTTAAATGGTTACACTGTA   4282
gb:MT039888|Organism:Severe      GTGCCAACAGACAATTATATAACCACTTACCCGGGTCAGGGTTTAAATGGTTACACTGTA   4282
gb:LC522972|Organism:Severe      GTGCCAACAGACAATTATATAACCACTTACCCGGGTCAGGGTTTAAATGGTTACACTGTA   4279
gb:MT027063|Organism:Severe      GTGCCAACAGACAATTATATAACCACTTACCCGGGTCAGGGTTTAAATGGTTACACTGTA   4282
gb:MT027062|Organism:Severe      GTGCCAACAGACAATTATATAACCACTTACCCGGGTCAGGGTTTAAATGGTTACACTGTA   4282
gb:MT019529|Organism:Severe      GTGCCAACAGACAATTATATAACCACTTACCCGGGTCAGGGTTTAAATGGTTACACTGTA   4282
gb:MN996529|Organism:Severe      GTGCCAACAGACAATTATATAACCACTTACCCGGGTCAGGGTTTAAATGGTTACACTGTA   4270
gb:MN996531|Organism:Severe      GTGCCAACAGACAATTATATAACCACTTACCCGGGTCAGGGTTTAAATGGTTACACTGTA   4269
gb:MT066176|Organism:Severe      GTGCCAACAGACAATTATATAACCACTTACCCGGGTCAGGGTTTAAATGGTTACACTGTA   4282
gb:MT027064|Organism:Severe      GTGCCAACAGACAATTATATAACCACTTACCCGGGTCAGGGTTTAAATGGTTACACTGTA   4282
gb:MN994468|Organism:Severe      GTGCCAACAGACAATTATATAACCACTTACCCGGGTCAGGGTTTAAATGGTTACACTGTA   4282
gb:MT072688|Organism:Severe      GTGCCAACAGACAATTATATAACCACTTACCCGGGTCAGGGTTTAAATGGTTACACTGTA   4267
gb:MN996527|Organism:Severe      GTGCCAACAGACAATTATATAACCACTTACCCGGGTCAGGGTTTAAATGGTTACACTGTA   4249
gb:MT093631|Organism:Severe      GTGCCAACAGACAATTATATAACCACTTACCCGGGTCAGGGTTTAAATGGTTACACTGTA   4320
gb:MT106053|Organism:Severe      GTGCCAACAGACAATTATATAACCACTTACCCGGGTCAGGGTTTAAATGGTTACACTGTA   4282
gb:MT019533|Organism:Severe      GTGCCAACAGACAATTATATAACCACTTACCCGGGTCAGGGTTTAAATGGTTACACTGTA   4282
gb:MT019531|Organism:Severe      GTGCCAACAGACAATTATATAACCACTTACCCGGGTCAGGGTTTAAATGGTTACACTGTA   4282
gb:MN996528|Organism:Severe      GTGCCAACAGACAATTATATAACCACTTACCCGGGTCAGGGTTTAAATGGTTACACTGTA   4282
gb:MN996530|Organism:Severe      GTGCCAACAGACAATTATATAACCACTTACCCGGGTCAGGGTTTAAATGGTTACACTGTA   4268
gb:MN908947|Organism:Severe      GTGCCAACAGACAATTATATAACCACTTACCCGGGTCAGGGTTTAAATGGTTACACTGTA   4282
gb:MT019532|Organism:Severe      GTGCCAACAGACAATTATATAACCACTTACCCGGGTCAGGGTTTAAATGGTTACACTGTA   4282
                                 ************************************************************
```

```
gb:MT020781|Organism:Severe      GAGGAGGCAAAGACAGTGCTTAAAAAGTGTAAAAGTGCCTTTTACATTCTACCATCTATT   4330
gb:MT007544|Organism:Severe      GAGGAGGCAAAGACAGTGCTTAAAAAGTGTAAAAGTGCCTTTTACATTCTACCATCTATT   4342
gb:MN994467|Organism:Severe      GAGGAGGCAAAGACAGTGCTTAAAAAGTGTAAAAGTGCCTTTTACATTCTACCATCTATT   4342
gb:MT044257|Organism:Severe      GAGGAGGCAAAGACAGTGCTTAAAAAGTGTAAAAGTGCCTTTTACATTCTACCATCTATT   4342
gb:MT106054|Organism:Severe      GAGGAGGCAAAGACAGTGCTTAAAAAGTGTAAAAGTGCCTTTTACATTCTACCATCTATT   4342
gb:MT049951|Organism:Severe      GAGGAGGCAAAGACAGTGCTTAAAAAGTGTAAAAGTGCCTTTTACATTCTACCATCTATT   4342
gb:MN975262|Organism:Severe      GAGGAGGCAAAGACAGTGCTTAAAAAGTGTAAAAGTGCCTTTTACATTCTACCATCTATT   4342
gb:MT106052|Organism:Severe      GAGGAGGCAAAGACAGTGCTTAAAAAGTGTAAAAGTGCCTTTTACATTCTACCATCTATT   4342
gb:LC522975|Organism:Severe      GAGGAGGCAAAGACAGTGCTTAAAAAGTGTAAAAGTGCCTTTTACATTCTACCATCTATT   4339
gb:LC522973|Organism:Severe      GAGGAGGCAAAGACAGTGCTTAAAAAGTGTAAAAGTGCCTTTTACATTCTACCATCTATT   4339
gb:LC522974|Organism:Severe      GAGGAGGCAAAGACAGTGCTTAAAAAGTGTAAAAGTGCCTTTTACATTCTACCATCTATT   4339
gb:MN985325|Organism:Severe      GAGGAGGCAAAGACAGTGCTTAAAAAGTGTAAAAGTGCCTTTTACATTCTACCATCTATT   4342
gb:MT020881|Organism:Severe      GAGGAGGCAAAGACAGTGCTTAAAAAGTGTAAAAGTGCCTTTTACATTCTACCATCTATT   4342
gb:MT020880|Organism:Severe      GAGGAGGCAAAGACAGTGCTTAAAAAGTGTAAAAGTGCCTTTTACATTCTACCATCTATT   4342
gb:MT066175|Organism:Severe      GAGGAGGCAAAGACAGTGCTTAAAAAGTGTAAAAGTGCCTTTTACATTCTACCATCTATT   4342
gb:MN997409|Organism:Severe      GAGGAGGCAAAGACAGTGCTTAAAAAGTGTAAAAGTGCCTTTTACATTCTACCATCTATT   4342
gb:MN938384|Organism:Severe      GAGGAGGCAAAGACAGTGCTTAAAAAGTGTAAAAGTGCCTTTTACATTCTACCATCTATT   4310
gb:MT044258|Organism:Severe      GAGGAGGCAAAGACAGTGCTTAAAAAGTGTAAAAGTGCCTTTTACATTCTACCATCTATT   4318
gb:MT039890|Organism:Severe      GAGGAGGCAAAGACAGTGCTTAAAAAGTGTAAAAGTGCCTTTTACATTCTACCATCTATT   4342
gb:MN988713|Organism:Severe      GAGGAGGCAAAGACAGTGCTTAAAAAGTGTAAAAGTGCCTTTTACATTCTACCATCTATT   4342
gb:LC521925|Organism:Severe      GAGGAGGCAAAGACAGTGCTTAAAAAGTGTAAAAGTGCCTTTTACATTCTACCATCTATT   4315
gb:MT093571|Organism:Severe      GAGGAGGCAAAGACAGTGCTTAAAAAGTGTAAAAGTGCCTTTTACATTCTACCATCTATT   4342
gb:MT039887|Organism:Severe      GAGGAGGCAAAGACAGTGCTTAAAAAGTGTAAAAGTGCCTTTTACATTCTACCATCTATT   4342
gb:MT019530|Organism:Severe      GAGGAGGCAAAGACAGTGCTTAAAAAGTGTAAAAGTGCCTTTTACATTCTACCATCTATT   4342
gb:MT039888|Organism:Severe      GAGGAGGCAAAGACAGTGCTTAAAAAGTGTAAAAGTGCCTTTTACATTCTACCATCTATT   4342
gb:LC522972|Organism:Severe      GAGGAGGCAAAGACAGTGCTTAAAAAGTGTAAAAGTGCCTTTTACATTCTACCATCTATT   4339
gb:MT027063|Organism:Severe      GAGGAGGCAAAGACAGTGCTTAAAAAGTGTAAAAGTGCCTTTTACATTCTACCATCTATT   4342
gb:MT027062|Organism:Severe      GAGGAGGCAAAGACAGTGCTTAAAAAGTGTAAAAGTGCCTTTTACATTCTACCATCTATT   4342
gb:MT019529|Organism:Severe      GAGGAGGCAAAGACAGTGCTTAAAAAGTGTAAAAGTGCCTTTTACATTCTACCATCTATT   4342
gb:MN996529|Organism:Severe      GAGGAGGCAAAGACAGTGCTTAAAAAGTGTAAAAGTGCCTTTTACATTCTACCATCTATT   4330
gb:MN996531|Organism:Severe      GAGGAGGCAAAGACAGTGCTTAAAAAGTGTAAAAGTGCCTTTTACATTCTACCATCTATT   4329
gb:MT066176|Organism:Severe      GAGGAGGCAAAGACAGTGCTTAAAAAGTGTAAAAGTGCCTTTTACATTCTACCATCTATT   4342
gb:MT027064|Organism:Severe      GAGGAGGCAAAGACAGTGCTTAAAAAGTGTAAAAGTGCCTTTTACATTCTACCATCTATT   4342
gb:MN994468|Organism:Severe      GAGGAGGCAAAGACAGTGCTTAAAAAGTGTAAAAGTGCCTTTTACATTCTACCATCTATT   4342
gb:MT072688|Organism:Severe      GAGGAGGCAAAGACAGTGCTTAAAAAGTGTAAAAGTGCCTTTTACATTCTACCATCTATT   4327
gb:MN996527|Organism:Severe      GAGGAGGCAAAGACAGTGCTTAAAAAGTGTAAAAGTGCCTTTTACATTCTACCATCTATT   4309
gb:MT093631|Organism:Severe      GAGGAGGCAAAGACAGTGCTTAAAAAGTGTAAAAGTGCCTTTTACATTCTACCATCTATT   4380
gb:MT106053|Organism:Severe      GAGGAGGCAAAGACAGTGCTTAAAAAGTGTAAAAGTGCCTTTTACATTCTACCATCTATT   4342
gb:MT019533|Organism:Severe      GAGGAGGCAAAGACAGTGCTTAAAAAGTGTAAAAGTGCCTTTTACATTCTACCATCTATT   4342
gb:MT019531|Organism:Severe      GAGGAGGCAAAGACAGTGCTTAAAAAGTGTAAAAGTGCCTTTTACATTCTACCATCTATT   4342
gb:MN996528|Organism:Severe      GAGGAGGCAAAGACAGTGCTTAAAAAGTGTAAAAGTGCCTTTTACATTCTACCATCTATT   4342
gb:MN996530|Organism:Severe      GAGGAGGCAAAGACAGTGCTTAAAAAGTGTAAAAGTGCCTTTTACATTCTACCATCTATT   4328
gb:MN908947|Organism:Severe      GAGGAGGCAAAGACAGTGCTTAAAAAGTGTAAAAGTGCCTTTTACATTCTACCATCTATT   4342
gb:MT019532|Organism:Severe      GAGGAGGCAAAGACAGTGCTTAAAAAGTGTAAAAGTGCCTTTTACATTCTACCATCTATT   4342
                                 ************************************************************

gb:MT020781|Organism:Severe      ATCTCTAATGAGAAGCAAGAAATTCTTGGAACTGTTTCTTGGAATTTGCGAGAAATGCTT   4390
gb:MT007544|Organism:Severe      ATCTCTAATGAGAAGCAAGAAATTCTTGGAACTGTTTCTTGGAATTTGCGAGAAATGCTT   4402
gb:MN994467|Organism:Severe      ATCTCTAATGAGAAGCAAGAAATTCTTGGAACTGTTTCTTGGAATTTGCGAGAAATGCTT   4402
gb:MT044257|Organism:Severe      ATCTCTAATGAGAAGCAAGAAATTCTTGGAACTGTTTCTTGGAATTTGCGAGAAATGCTT   4402
gb:MT106054|Organism:Severe      ATCTCTAATGAGAAGCAAGAAATTCTTGGAACTGTTTCTTGGAATTTGCGAGAAATGCTT   4402
gb:MT049951|Organism:Severe      ATCTCTAATGAGAAGCAAGAAATTCTTGGAACTGTTTCTTGGAATTTGCGAGAAATGCTT   4402
gb:MN975262|Organism:Severe      ATCTCTAATGAGAAGCAAGAAATTCTTGGAACTGTTTCTTGGAATTTGCGAGAAATGCTT   4402
gb:MT106052|Organism:Severe      ATCTCTAATGAGAAGCAAGAAATTCTTGGAACTGTTTCTTGGAATTTGCGAGAAATGCTT   4402
gb:LC522975|Organism:Severe      ATCTCTAATGAGAAGCAAGAAATTCTTGGAACTGTTTCTTGGAATTTGCGAGAAATGCTT   4399
gb:LC522973|Organism:Severe      ATCTCTAATGAGAAGCAAGAAATTCTTGGAACTGTTTCTTGGAATTTGCGAGAAATGCTT   4399
gb:LC522974|Organism:Severe      ATCTCTAATGAGAAGCAAGAAATTCTTGGAACTGTTTCTTGGAATTTGCGAGAAATGCTT   4399
gb:MN985325|Organism:Severe      ATCTCTAATGAGAAGCAAGAAATTCTTGGAACTGTTTCTTGGAATTTGCGAGAAATGCTT   4402
gb:MT020881|Organism:Severe      ATCTCTAATGAGAAGCAAGAAATTCTTGGAACTGTTTCTTGGAATTTGCGAGAAATGCTT   4402
gb:MT020880|Organism:Severe      ATCTCTAATGAGAAGCAAGAAATTCTTGGAACTGTTTCTTGGAATTTGCGAGAAATGCTT   4402
gb:MT066175|Organism:Severe      ATCTCTAATGAGAAGCAAGAAATTCTTGGAACTGTTTCTTGGAATTTGCGAGAAATGCTT   4402
gb:MN997409|Organism:Severe      ATCTCTAATGAGAAGCAAGAAATTCTTGGAACTGTTTCTTGGAATTTGCGAGAAATGCTT   4402
gb:MN938384|Organism:Severe      ATCTCTAATGAGAAGCAAGAAATTCTTGGAACTGTTTCTTGGAATTTGCGAGAAATGCTT   4370
gb:MT044258|Organism:Severe      ATCTCTAATGAGAAGCAAGAAATTCTTGGAACTGTTTCTTGGAATTTGCGAGAAATGCTT   4378
```

```
gb:MT039890|Organism:Severe      ATCTCTAATGAGAAGCAAGAAATTCTTGGAACTGTTTCTTGGAATTTGCGAGAAATGCTT   4402
gb:MN988713|Organism:Severe      ATCTCTAATGAGAAGCAAGAAATTCTTGGAACTGTTTCTTGGAATTTGCGAGAAATGCTT   4402
gb:LC521925|Organism:Severe      ATCTCTAATGAGAAGCAAGAAATTCTTGGAACTGTTTCTTGGAATTTGCGAGAAATGCTT   4375
gb:MT093571|Organism:Severe      ATCTCTAATGAGAAGCAAGAAATTCTTGGAACTGTTTCTTGGAATTTGCGAGAAATGCTT   4402
gb:MT039887|Organism:Severe      ATCTCTAATGAGAAGCAAGAAATTCTTGGAACTGTTTCTTGGAATTTGCGAGAAATGCTT   4402
gb:MT019530|Organism:Severe      ATCTCTAATGAGAAGCAAGAAATTCTTGGAACTGTTTCTTGGAATTTGCGAGAAATGCTT   4402
gb:MT039888|Organism:Severe      ATCTCTAATGAGAAGCAAGAAATTCTTGGAACTGTTTCTTGGAATTTGCGAGAAATGCTT   4402
gb:LC522972|Organism:Severe      ATCTCTAATGAGAAGCAAGAAATTCTTGGAACTGTTTCTTGGAATTTGCGAGAAATGCTT   4399
gb:MT027063|Organism:Severe      ATCTCTAATGAGAAGCAAGAAATTCTTGGAACTGTTTCTTGGAATTTGCGAGAAATGCTT   4402
gb:MT027062|Organism:Severe      ATCTCTAATGAGAAGCAAGAAATTCTTGGAACTGTTTCTTGGAATTTGCGAGAAATGCTT   4402
gb:MT019529|Organism:Severe      ATCTCTAATGAGAAGCAAGAAATTCTTGGAACTGTTTCTTGGAATTTGCGAGAAATGCTT   4402
gb:MN996529|Organism:Severe      ATCTCTAATGAGAAGCAAGAAATTCTTGGAACTGTTTCTTGGAATTTGCGAGAAATGCTT   4390
gb:MN996531|Organism:Severe      ATCTCTAATGAGAAGCAAGAAATTCTTGGAACTGTTTCTTGGAATTTGCGAGAAATGCTT   4389
gb:MT066176|Organism:Severe      ATCTCTAATGAGAAGCAAGAAATTCTTGGAACTGTTTCTTGGAATTTGCGAGAAATGCTT   4402
gb:MT027064|Organism:Severe      ATCTCTAATGAGAAGCAAGAAATTCTTGGAACTGTTTCTTGGAATTTGCGAGAAATGCTT   4402
gb:MN994468|Organism:Severe      ATCTCTAATGAGAAGCAAGAAATTCTTGGAACTGTTTCTTGGAATTTGCGAGAAATGCTT   4402
gb:MT072688|Organism:Severe      ATCTCTAATGAGAAGCAAGAAATTCTTGGAACTGTTTCTTGGAATTTGCGAGAAATGCTT   4387
gb:MN996527|Organism:Severe      ATCTCTAATGAGAAGCAAGAAATTCTTGGAACTGTTTCTTGGAATTTGCGAGAAATGCTT   4369
gb:MT093631|Organism:Severe      ATCTCTAATGAGAAGCAAGAAATTCTTGGAACTGTTTCTTGGAATTTGCGAGAAATGCTT   4440
gb:MT106053|Organism:Severe      ATCTCTAATGAGAAGCAAGAAATTCTTGGAACTGTTTCTTGGAATTTGCGAGAAATGCTT   4402
gb:MT019533|Organism:Severe      ATCTCTAATGAGAAGCAAGAAATTCTTGGAACTGTTTCTTGGAATTTGCGAGAAATGCTT   4402
gb:MT019531|Organism:Severe      ATCTCTAATGAGAAGCAAGAAATTCTTGGAACTGTTTCTTGGAATTTGCGAGAAATGCTT   4402
gb:MN996528|Organism:Severe      ATCTCTAATGAGAAGCAAGAAATTCTTGGAACTGTTTCTTGGAATTTGCGAGAAATGCTT   4402
gb:MN996530|Organism:Severe      ATCTCTAATGAGAAGCAAGAAATTCTTGGAACTGTTTCTTGGAATTTGCGAGAAATGCTT   4388
gb:MN908947|Organism:Severe      ATCTCTAATGAGAAGCAAGAAATTCTTGGAACTGTTTCTTGGAATTTGCGAGAAATGCTT   4402
gb:MT019532|Organism:Severe      ATCTCTAATGAGAAGCAAGAAATTCTTGGAACTGTTTCTTGGAATTTGCGAGAAATGCTT   4402
                                 ************************************************************

gb:MT020781|Organism:Severe      GCACATGCAGAAGAAACACGCAAATTAATGCCTGTCTGTGTGGAAACTAAAGCCATAGTT   4450
gb:MT007544|Organism:Severe      GCACATGCAGAAGAAACACGCAAATTAATGCCTGTCTGTGTGGAAACTAAAGCCATAGTT   4462
gb:MN994467|Organism:Severe      GCACATGCAGAAGAAACACGCAAATTAATGCCTGTCTGTGTGGAAACTAAAGCCATAGTT   4462
gb:MT044257|Organism:Severe      GCACATGCAGAAGAAACACGCAAATTAATGCCTGTCTGTGTGGAAACTAAAGCCATAGTT   4462
gb:MT106054|Organism:Severe      GCACATGCAGAAGAAACACGCAAATTAATGCCTGTCTGTGTGGAAACTAAAGCCATAGTT   4462
gb:MT049951|Organism:Severe      GCACATGCAGAAGAAACACGCAAATTAATGCCTGTCTGTGTGGAAACTAAAGCCATAGTT   4462
gb:MN975262|Organism:Severe      GCACATGCAGAAGAAACACGCAAATTAATGCCTGTCTGTGTGGAAACTAAAGCCATAGTT   4462
gb:MT106052|Organism:Severe      GCACATGCAGAAGAAACACGCAAATTAATGCCTGTCTGTGTGGAAACTAAAGCCATAGTT   4462
gb:LC522975|Organism:Severe      GCACATGCAGAAGAAACACGCAAATTAATGCCTGTCTGTGTGGAAACTAAAGCCATAGTT   4459
gb:LC522973|Organism:Severe      GCACATGCAGAAGAAACACGCAAATTAATGCCTGTCTGTGTGGAAACTAAAGCCATAGTT   4459
gb:LC522974|Organism:Severe      GCACATGCAGAAGAAACACGCAAATTAATGCCTGTCTGTGTGGAAACTAAAGCCATAGTT   4459
gb:MN985325|Organism:Severe      GCACATGCAGAAGAAACACGCAAATTAATGCCTGTCTGTGTGGAAACTAAAGCCATAGTT   4462
gb:MT020881|Organism:Severe      GCACATGCAGAAGAAACACGCAAATTAATGCCTGTCTGTGTGGAAACTAAAGCCATAGTT   4462
gb:MT020880|Organism:Severe      GCACATGCAGAAGAAACACGCAAATTAATGCCTGTCTGTGTGGAAACTAAAGCCATAGTT   4462
gb:MT066175|Organism:Severe      GCACATGCAGAAGAAACACGCAAATTAATGCCTGTCTGTGTGGAAACTAAAGCCATAGTT   4462
gb:MN997409|Organism:Severe      GCACATGCAGAAGAAACACGCAAATTAATGCCTGTCTGTGTGGAAACTAAAGCCATAGTT   4462
gb:MN938384|Organism:Severe      GCACATGCAGAAGAAACACGCAAATTAATGCCTGTCTGTGTGGAAACTAAAGCCATAGTT   4430
gb:MT044258|Organism:Severe      GCACATGCAGAAGAAACACGCAAATTAATGCCTGTCTGTGTGGAAACTAAAGCCATAGTT   4438
gb:MT039890|Organism:Severe      GCACATGCAGAAGAAACACGCAAATTAATGCCTGTCTGTGTGGAAACTAAAGCCATAGTT   4462
gb:MN988713|Organism:Severe      GCACATGCAGAAGAAACACGCAAATTAATGCCTGTCTGTGTGGAAACTAAAGCCATAGTT   4462
gb:LC521925|Organism:Severe      GCACATGCAGAAGAAACACGCAAATTAATGCCTGTCTGTGTGGAAACTAAAGCCATAGTT   4435
gb:MT093571|Organism:Severe      GCACATGCAGAAGAAACACGCAAATTAATGCCTGTCTGTGTGGAAACTAAAGCCATAGTT   4462
gb:MT039887|Organism:Severe      GCACATGCAGAAGAAACACGCAAATTAATGCCTGTCTGTGTGGAAACTAAAGCCATAGTT   4462
gb:MT019530|Organism:Severe      GCACATGCAGAAGAAACACGCAAATTAATGCCTGTCTGTGTGGAAACTAAAGCCATAGTT   4462
gb:MT039888|Organism:Severe      GCACATGCAGAAGAAACACGCAAATTAATGCCTGTCTGTGTGGAAACTAAAGCCATAGTT   4462
gb:LC522972|Organism:Severe      GCACATGCAGAAGAAACACGCAAATTAATGCCTGTCTGTGTGGAAACTAAAGCCATAGTT   4459
gb:MT027063|Organism:Severe      GCACATGCAGAAGAAACACGCAAATTAATGCCTGTCTGTGTGGAAACTAAAGCCATAGTT   4462
gb:MT027062|Organism:Severe      GCACATGCAGAAGAAACACGCAAATTAATGCCTGTCTGTGTGGAAACTAAAGCCATAGTT   4462
gb:MT019529|Organism:Severe      GCACATGCAGAAGAAACACGCAAATTAATGCCTGTCTGTGTGGAAACTAAAGCCATAGTT   4462
gb:MN996529|Organism:Severe      GCACATGCAGAAGAAACACGCAAATTAATGCCTGTCTGTGTGGAAACTAAAGCCATAGTT   4450
gb:MN996531|Organism:Severe      GCACATGCAGAAGAAACACGCAAATTAATGCCTGTCTGTGTGGAAACTAAAGCCATAGTT   4449
gb:MT066176|Organism:Severe      GCACATGCAGAAGAAACACGCAAATTAATGCCTGTCTGTGTGGAAACTAAAGCCATAGTT   4462
gb:MT027064|Organism:Severe      GCACATGCAGAAGAAACACGCAAATTAATGCCTGTCTGTGTGGAAACTAAAGCCATAGTT   4462
gb:MN994468|Organism:Severe      GCACATGCAGAAGAAACACGCAAATTAATGCCTGTCTGTGTGGAAACTAAAGCCATAGTT   4462
gb:MT072688|Organism:Severe      GCACATGCAGAAGAAACACGCAAATTAATGCCTGTCTGTGTGGAAACTAAAGCCATAGTT   4447
gb:MN996527|Organism:Severe      GCACATGCAGAAGAAACACGCAAATTAATGCCTGTCTGTGTGGAAACTAAAGCCATAGTT   4429
gb:MT093631|Organism:Severe      GCACATGCAGAAGAAACACGCAAATTAATGCCTGTCTGTGTGGAAACTAAAGCCATAGTT   4500
```

```
gb:MT106053|Organism:Severe      GCACATGCAGAAGAAACACGCAAATTAATGCCTGTCTGTGTGGAAACTAAAGCCATAGTT    4462
gb:MT019533|Organism:Severe      GCACATGCAGAAGAAACACGCAAATTAATGCCTGTCTGTGTGGAAACTAAAGCCATAGTT    4462
gb:MT019531|Organism:Severe      GCACATGCAGAAGAAACACGCAAATTAATGCCTGTCTGTGTGGAAACTAAAGCCATAGTT    4462
gb:MN996528|Organism:Severe      GCACATGCAGAAGAAACACGCAAATTAATGCCTGTCTGTGTGGAAACTAAAGCCATAGTT    4462
gb:MN996530|Organism:Severe      GCACATGCAGAAGAAACACGCAAATTAATGCCTGTCTGTGTGGAAACTAAAGCCATAGTT    4448
gb:MN908947|Organism:Severe      GCACATGCAGAAGAAACACGCAAATTAATGCCTGTCTGTGTGGAAACTAAAGCCATAGTT    4462
gb:MT019532|Organism:Severe      GCACATGCAGAAGAAACACGCAAATTAATGCCTGTCTGTGTGGAAACTAAAGCCATAGTT    4462
                                 ************************************************************

gb:MT020781|Organism:Severe      TCAACTATACAGCGTAAATATAAGGGTATTAAAATACAAGAGGGTGTGGTTGATTATGGT    4510
gb:MT007544|Organism:Severe      TCAACTATACAGCGTAAATATAAGGGTATTAAAATACAAGAGGGTGTGGTTGATTATGGT    4522
gb:MN994467|Organism:Severe      TCAACTATACAGCGTAAATATAAGGGTATTAAAATACAAGAGGGTGTGGTTGATTATGGT    4522
gb:MT044257|Organism:Severe      TCAACTATACAGCGTAAATATAAGGGTATTAAAATACAAGAGGGTGTGGTTGATTATGGT    4522
gb:MT106054|Organism:Severe      TCAACTATACAGCGTAAATATAAGGGTATTAAAATACAAGAGGGTGTGGTTGATTATGGT    4522
gb:MT049951|Organism:Severe      TCAACTATACAGCGTAAATATAAGGGTATTAAAATACAAGAGGGTGTGGTTGATTATGGT    4522
gb:MN975262|Organism:Severe      TCAACTATACAGCGTAAATATAAGGGTATTAAAATACAAGAGGGTGTGGTTGATTATGGT    4522
gb:MT106052|Organism:Severe      TCAACTATACAGCGTAAATATAAGGGTATTAAAATACAAGAGGGTGTGGTTGATTATGGT    4522
gb:LC522975|Organism:Severe      TCAACTATACAGCGTAAATATAAGGGTATTAAAATACAAGAGGGTGTGGTTGATTATGGT    4519
gb:LC522973|Organism:Severe      TCAACTATACAGCGTAAATATAAGGGTATTAAAATACAAGAGGGTGTGGTTGATTATGGT    4519
gb:LC522974|Organism:Severe      TCAACTATACAGCGTAAATATAAGGGTATTAAAATACAAGAGGGTGTGGTTGATTATGGT    4519
gb:MN985325|Organism:Severe      TCAACTATACAGCGTAAATATAAGGGTATTAAAATACAAGAGGGTGTGGTTGATTATGGT    4522
gb:MT020881|Organism:Severe      TCAACTATACAGCGTAAATATAAGGGTATTAAAATACAAGAGGGTGTGGTTGATTATGGT    4522
gb:MT020880|Organism:Severe      TCAACTATACAGCGTAAATATAAGGGTATTAAAATACAAGAGGGTGTGGTTGATTATGGT    4522
gb:MT066175|Organism:Severe      TCAACTATACAGCGTAAATATAAGGGTATTAAAATACAAGAGGGTGTGGTTGATTATGGT    4522
gb:MN997409|Organism:Severe      TCAACTATACAGCGTAAATATAAGGGTATTAAAATACAAGAGGGTGTGGTTGATTATGGT    4522
gb:MN938384|Organism:Severe      TCAACTATACAGCGTAAATATAAGGGTATTAAAATACAAGAGGGTGTGGTTGATTATGGT    4490
gb:MT044258|Organism:Severe      TCAACTATACAGCGTAAATATAAGGGTATTAAAATACAAGAGGGTGTGGTTGATTATGGT    4498
gb:MT039890|Organism:Severe      TCAACTATACAGCGTAAATATAAGGGTATTAAAATACAAGAGGGTGTGGTTGATTATGGT    4522
gb:MN988713|Organism:Severe      TCAACTATACAGCGTAAATATAAGGGTATTAAAATACAAGAGGGTGTGGTTGATTATGGT    4522
gb:LC521925|Organism:Severe      TCAACTATACAGCGTAAATATAAGGGTATTAAAATACAAGAGGGTGTGGTTGATTATGGT    4495
gb:MT093571|Organism:Severe      TCAACTATACAGCGTAAATATAAGGGTATTAAAATACAAGAGGGTGTGGTTGATTATGGT    4522
gb:MT039887|Organism:Severe      TCAACTATACAGCGTAAATATAAGGGTATTAAAATACAAGAGGGTGTGGTTGATTATGGT    4522
gb:MT019530|Organism:Severe      TCAACTATACAGCGTAAATATAAGGGTATTAAAATACAAGAGGGTGTGGTTGATTATGGT    4522
gb:MT039888|Organism:Severe      TCAACTATACAGCGTAAATATAAGGGTATTAAAATACAAGAGGGTGTGGTTGATTATGGT    4522
gb:LC522972|Organism:Severe      TCAACTATACAGCGTAAATATAAGGGTATTAAAATACAAGAGGGTGTGGTTGATTATGGT    4519
gb:MT027063|Organism:Severe      TCAACTATACAGCGTAAATATAAGGGTATTAAAATACAAGAGGGTGTGGTTGATTATGGT    4522
gb:MT027062|Organism:Severe      TCAACTATACAGCGTAAATATAAGGGTATTAAAATACAAGAGGGTGTGGTTGATTATGGT    4522
gb:MT019529|Organism:Severe      TCAACTATACAGCGTAAATATAAGGGTATTAAAATACAAGAGGGTGTGGTTGATTATGGT    4522
gb:MN996529|Organism:Severe      TCAACTATACAGCGTAAATATAAGGGTATTAAAATACAAGAGGGTGTGGTTGATTATGGT    4510
gb:MN996531|Organism:Severe      TCAACTATACAGCGTAAATATAAGGGTATTAAAATACAAGAGGGTGTGGTTGATTATGGT    4509
gb:MT066176|Organism:Severe      TCAACTATACAGCGTAAATATAAGGGTATTAAAATACAAGAGGGTGTGGTTGATTATGGT    4522
gb:MT027064|Organism:Severe      TCAACTATACAGCGTAAATATAAGGGTATTAAAATACAAGAGGGTGTGGTTGATTATGGT    4522
gb:MN994468|Organism:Severe      TCAACTATACAGCGTAAATATAAGGGTATTAAAATACAAGAGGGTGTGGTTGATTATGGT    4522
gb:MT072688|Organism:Severe      TCAACTATACAGCGTAAATATAAGGGTATTAAAATACAAGAGGGTGTGGTTGATTATGGT    4507
gb:MN996527|Organism:Severe      TCAACTATACAGCGTAAATATAAGGGTATTAAAATACAAGAGGGTGTGGTTGATTATGGT    4489
gb:MT093631|Organism:Severe      TCAACTATACAGCGTAAATATAAGGGTATTAAAATACAAGAGGGTGTGGTTGATTATGGT    4560
gb:MT106053|Organism:Severe      TCAACTATACAGCGTAAATATAAGGGTATTAAAATACAAGAGGGTGTGGTTGATTATGGT    4522
gb:MT019533|Organism:Severe      TCAACTATACAGCGTAAATATAAGGGTATTAAAATACAAGAGGGTGTGGTTGATTATGGT    4522
gb:MT019531|Organism:Severe      TCAACTATACAGCGTAAATATAAGGGTATTAAAATACAAGAGGGTGTGGTTGATTATGGT    4522
gb:MN996528|Organism:Severe      TCAACTATACAGCGTAAATATAAGGGTATTAAAATACAAGAGGGTGTGGTTGATTATGGT    4522
gb:MN996530|Organism:Severe      TCAACTATACAGCGTAAATATAAGGGTATTAAAATACAAGAGGGTGTGGTTGATTATGGT    4508
gb:MN908947|Organism:Severe      TCAACTATACAGCGTAAATATAAGGGTATTAAAATACAAGAGGGTGTGGTTGATTATGGT    4522
gb:MT019532|Organism:Severe      TCAACTATACAGCGTAAATATAAGGGTATTAAAATACAAGAGGGTGTGGTTGATTATGGT    4522
                                 ************************************************************

gb:MT020781|Organism:Severe      GCTAGATTTTACTTTTACACCAGTAAAACAACTGTAGCGTCACTTATCAACACACTTAAC    4570
gb:MT007544|Organism:Severe      GCTAGATTTTACTTTTACACCAGTAAAACAACTGTAGCGTCACTTATCAACACACTTAAC    4582
gb:MN994467|Organism:Severe      GCTAGATTTTACTTTTACACCAGTAAAACAACTGTAGCGTCACTTATCAACACACTTAAC    4582
gb:MT044257|Organism:Severe      GCTAGATTTTACTTTTACACCAGTAAAACAACTGTAGCGTCACTTATCAACACACTTAAC    4582
gb:MT106054|Organism:Severe      GCTAGATTTTACTTTTACACCAGTAAAACAACTGTAGCGTCACTTATCAACACACTTAAC    4582
gb:MT049951|Organism:Severe      GCTAGATTTTACTTTTACACCAGTAAAACAACTGTAGCGTCACTTATCAACACACTTAAC    4582
gb:MN975262|Organism:Severe      GCTAGATTTTACTTTTACACCAGTAAAACAACTGTAGCGTCACTTATCAACACACTTAAC    4582
gb:MT106052|Organism:Severe      GCTAGATTTTACTTTTACACCAGTAAAACAACTGTAGCGTCACTTATCAACACACTTAAC    4582
gb:LC522975|Organism:Severe      GCTAGATTTTACTTTTACACCAGTAAAACAACTGTAGCGTCACTTATCAACACACTTAAC    4579
gb:LC522973|Organism:Severe      GCTAGATTTTACTTTTACACCAGTAAAACAACTGTAGCGTCACTTATCAACACACTTAAC    4579
```

```
gb:LC522974|Organism:Severe      GCTAGATTTTACTTTTACACCAGTAAAACAACTGTAGCGTCACTTATCAACACACTTAAC   4579
gb:MN985325|Organism:Severe      GCTAGATTTTACTTTTACACCAGTAAAACAACTGTAGCGTCACTTATCAACACACTTAAC   4582
gb:MT020881|Organism:Severe      GCTAGATTTTACTTTTACACCAGTAAAACAACTGTAGCGTCACTTATCAACACACTTAAC   4582
gb:MT020880|Organism:Severe      GCTAGATTTTACTTTTACACCAGTAAAACAACTGTAGCGTCACTTATCAACACACTTAAC   4582
gb:MT066175|Organism:Severe      GCTAGATTTTACTTTTACACCAGTAAAACAACTGTAGCGTCACTTATCAACACACTTAAC   4582
gb:MN997409|Organism:Severe      GCTAGATTTTACTTTTACACCAGTAAAACAACTGTAGCGTCACTTATCAACACACTTAAC   4582
gb:MN938384|Organism:Severe      GCTAGATTTTACTTTTACACCAGTAAAACAACTGTAGCGTCACTTATCAACACACTTAAC   4550
gb:MT044258|Organism:Severe      GCTAGATTTTACTTTTACACCAGTAAAACAACTGTAGCGTCACTTATCAACACACTTAAC   4558
gb:MT039890|Organism:Severe      GCTAGATTTTACTTTTACACCAGTAAAACAACTGTAGCGTCACTTATCAACACACTTAAC   4582
gb:MN988713|Organism:Severe      GCTAGATTTTACTTTTACACCAGTAAAACAACTGTAGCGTCACTTATCAACACACTTAAC   4582
gb:LC521925|Organism:Severe      GCTAGATTTTACTTTTACACCAGTAAAACAACTGTAGCGTCACTTATCAACACACTTAAC   4555
gb:MT093571|Organism:Severe      GCTAGATTTTACTTTTACACCAGTAAAACAACTGTAGCGTCACTTATCAACACACTTAAC   4582
gb:MT039887|Organism:Severe      GCTAGATTTTACTTTTACACCAGTAAAACAACTGTAGCGTCACTTATCAACACACTTAAC   4582
gb:MT019530|Organism:Severe      GCTAGATTTTACTTTTACACCAGTAAAACAACTGTAGCGTCACTTATCAACACACTTAAC   4582
gb:MT039888|Organism:Severe      GCTAGATTTTACTTTTACACCAGTAAAACAACTGTAGCGTCACTTATCAACACACTTAAC   4582
gb:LC522972|Organism:Severe      GCTAGATTTTACTTTTACACCAGTAAAACAACTGTAGCGTCACTTATCAACACACTTAAC   4579
gb:MT027063|Organism:Severe      GCTAGATTTTACTTTTACACCAGTAAAACAACTGTAGCGTCACTTATCAACACACTTAAC   4582
gb:MT027062|Organism:Severe      GCTAGATTTTACTTTTACACCAGTAAAACAACTGTAGCGTCACTTATCAACACACTTAAC   4582
gb:MT019529|Organism:Severe      GCTAGATTTTACTTTTACACCAGTAAAACAACTGTAGCGTCACTTATCAACACACTTAAC   4582
gb:MN996529|Organism:Severe      GCTAGATTTTACTTTTACACCAGTAAAACAACTGTAGCGTCACTTATCAACACACTTAAC   4570
gb:MN996531|Organism:Severe      GCTAGATTTTACTTTTACACCAGTAAAACAACTGTAGCGTCACTTATCAACACACTTAAC   4569
gb:MT066176|Organism:Severe      GCTAGATTTTACTTTTACACCAGTAAAACAACTGTAGCGTCACTTATCAACACACTTAAC   4582
gb:MT027064|Organism:Severe      GCTAGATTTTACTTTTACACCAGTAAAACAACTGTAGCGTCACTTATCAACACACTTAAC   4582
gb:MN994468|Organism:Severe      GCTAGATTTTACTTTTACACCAGTAAAACAACTGTAGCGTCACTTATCAACACACTTAAC   4582
gb:MT072688|Organism:Severe      GCTAGATTTTACTTTTACACCAGTAAAACAACTGTAGCGTCACTTATCAACACACTTAAC   4567
gb:MN996527|Organism:Severe      GCTAGATTTTACTTTTACACCAGTAAAACAACTGTAGCGTCACTTATCAACACACTTAAC   4549
gb:MT093631|Organism:Severe      GCTAGATTTTACTTTTACACCAGTAAAACAACTGTAGCGTCACTTATCAACACACTTAAC   4620
gb:MT106053|Organism:Severe      GCTAGATTTTACTTTTACACCAGTAAAACAACTGTAGCGTCACTTATCAACACACTTAAC   4582
gb:MT019533|Organism:Severe      GCTAGATTTTACTTTTACACCAGTAAAACAACTGTAGCGTCACTTATCAACACACTTAAC   4582
gb:MT019531|Organism:Severe      GCTAGATTTTACTTTTACACCAGTAAAACAACTGTAGCGTCACTTATCAACACACTTAAC   4582
gb:MN996528|Organism:Severe      GCTAGATTTTACTTTTACACCAGTAAAACAACTGTAGCGTCACTTATCAACACACTTAAC   4582
gb:MN996530|Organism:Severe      GCTAGATTTTACTTTTACACCAGTAAAACAACTGTAGCGTCACTTATCAACACACTTAAC   4568
gb:MN908947|Organism:Severe      GCTAGATTTTACTTTTACACCAGTAAAACAACTGTAGCGTCACTTATCAACACACTTAAC   4582
gb:MT019532|Organism:Severe      GCTAGATTTTACTTTTACACCAGTAAAACAACTGTAGCGTCACTTATCAACACACTTAAC   4582
                                 ************************************************************

gb:MT020781|Organism:Severe      GATCTAAATGAAACTCTTGTTACAATGCCACTTGGCTATGTAACACATGGCTTAAATTTG   4630
gb:MT007544|Organism:Severe      GATCTAAATGAAACTCTTGTTACAATGCCACTTGGCTATGTAACACATGGCTTAAATTTG   4642
gb:MN994467|Organism:Severe      GATCTAAATGAAACTCTTGTTACAATGCCACTTGGCTATGTAACACATGGCTTAAATTTG   4642
gb:MT044257|Organism:Severe      GATCTAAATGAAACTCTTGTTACAATGCCACTTGGCTATGTAACACATGGCTTAAATTTG   4642
gb:MT106054|Organism:Severe      GATCTAAATGAAACTCTTGTTACAATGCCACTTGGCTATGTAACACATGGCTTAAATTTG   4642
gb:MT049951|Organism:Severe      GATCTAAATGAAACTCTTGTTACAATGCCACTTGGCTATGTAACACATGGCTTAAATTTG   4642
gb:MN975262|Organism:Severe      GATCTAAATGAAACTCTTGTTACAATGCCACTTGGCTATGTAACACATGGCTTAAATTTG   4642
gb:MT106052|Organism:Severe      GATCTAAATGAAACTCTTGTTACAATGCCACTTGGCTATGTAACACATGGCTTAAATTTG   4642
gb:LC522975|Organism:Severe      GATCTAAATGAAACTCTTGTTACAATGCCACTTGGCTATGTAACACATGGCTTAAATTTG   4639
gb:LC522973|Organism:Severe      GATCTAAATGAAACTCTTGTTACAATGCCACTTGGCTATGTAACACATGGCTTAAATTTG   4639
gb:LC522974|Organism:Severe      GATCTAAATGAAACTCTTGTTACAATGCCACTTGGCTATGTAACACATGGCTTAAATTTG   4639
gb:MN985325|Organism:Severe      GATCTAAATGAAACTCTTGTTACAATGCCACTTGGCTATGTAACACATGGCTTAAATTTG   4642
gb:MT020881|Organism:Severe      GATCTAAATGAAACTCTTGTTACAATGCCACTTGGCTATGTAACACATGGCTTAAATTTG   4642
gb:MT020880|Organism:Severe      GATCTAAATGAAACTCTTGTTACAATGCCACTTGGCTATGTAACACATGGCTTAAATTTG   4642
gb:MT066175|Organism:Severe      GATCTAAATGAAACTCTTGTTACAATGCCACTTGGCTATGTAACACATGGCTTAAATTTG   4642
gb:MN997409|Organism:Severe      GATCTAAATGAAACTCTTGTTACAATGCCACTTGGCTATGTAACACATGGCTTAAATTTG   4642
gb:MN938384|Organism:Severe      GATCTAAATGAAACTCTTGTTACAATGCCACTTGGCTATGTAACACATGGCTTAAATTTG   4610
gb:MT044258|Organism:Severe      GATCTAAATGAAACTCTTGTTACAATGCCACTTGGCTATGTAACACATGGCTTAAATTTG   4618
gb:MT039890|Organism:Severe      GATCTAAATGAAACTCTTGTTACAATGCCACTTGGCTATGTAACACATGGCTTAAATTTG   4642
gb:MN988713|Organism:Severe      GATCTAAATGAAACTCTTGTTACAATGCCACTTGGCTATGTAACACATGGCTTAAATTTG   4642
gb:LC521925|Organism:Severe      GATCTAAATGAAACTCTTGTTACAATGCCACTTGGCTATGTAACACATGGCTTAAATTTG   4615
gb:MT093571|Organism:Severe      GATCTAAATGAAACTCTTGTTACAATGCCACTTGGCTATGTAACACATGGCTTAAATTTG   4642
gb:MT039887|Organism:Severe      GATCTAAATGAAACTCTTGTTACAATGCCACTTGGCTATGTAACACATGGCTTAAATTTG   4642
gb:MT019530|Organism:Severe      GATCTAAATGAAACTCTTGTTACAATGCCACTTGGCTATGTAACACATGGCTTAAATTTG   4642
gb:MT039888|Organism:Severe      GATCTAAATGAAACTCTTGTTACAATGCCACTTGGCTATGTAACACATGGCTTAAATTTG   4642
gb:LC522972|Organism:Severe      GATCTAAATGAAACTCTTGTTACAATGCCACTTGGCTATGTAACACATGGCTTAAATTTG   4639
gb:MT027063|Organism:Severe      GATCTAAATGAAACTCTTGTTACAATGCCACTTGGCTATGTAACACATGGCTTAAATTTG   4642
gb:MT027062|Organism:Severe      GATCTAAATGAAACTCTTGTTACAATGCCACTTGGCTATGTAACACATGGCTTAAATTTG   4642
gb:MT019529|Organism:Severe      GATCTAAATGAAACTCTTGTTACAATGCCACTTGGCTATGTAACACATGGCTTAAATTTG   4642
```

```
gb:MN996529|Organism:Severe      GATCTAAATGAAACTCTTGTTACAATGCCACTTGGCTATGTAACACATGGCTTAAATTTG   4630
gb:MN996531|Organism:Severe      GATCTAAATGAAACTCTTGTTACAATGCCACTTGGCTATGTAACACATGGCTTAAATTTG   4629
gb:MT066176|Organism:Severe      GATCTAAATGAAACTCTTGTTACAATGCCACTTGGCTATGTAACACATGGCTTAAATTTG   4642
gb:MT027064|Organism:Severe      GATCTAAATGAAACTCTTGTTACAATGCCACTTGGCTATGTAACACATGGCTTAAATTTG   4642
gb:MN994468|Organism:Severe      GATCTAAATGAAACTCTTGTTACAATGCCACTTGGCTATGTAACACATGGCTTAAATTTG   4642
gb:MT072688|Organism:Severe      GATCTAAATGAAACTCTTGTTACAATGCCACTTGGCTATGTAACACATGGCTTAAATTTG   4627
gb:MN996527|Organism:Severe      GATCTAAATGAAACTCTTGTTACAATGCCACTTGGCTATGTAACACATGGCTTAAATTTG   4609
gb:MT093631|Organism:Severe      GATCTAAATGAAACTCTTGTTACAATGCCACTTGGCTATGTAACACATGGCTTAAATTTG   4680
gb:MT106053|Organism:Severe      GATCTAAATGAAACTCTTGTTACAATGCCACTTGGCTATGTAACACATGGCTTAAATTTG   4642
gb:MT019533|Organism:Severe      GATCTAAATGAAACTCTTGTTACAATGCCACTTGGCTATGTAACACATGGCTTAAATTTG   4642
gb:MT019531|Organism:Severe      GATCTAAATGAAACTCTTGTTACAATGCCACTTGGCTATGTAACACATGGCTTAAATTTG   4642
gb:MN996528|Organism:Severe      GATCTAAATGAAACTCTTGTTACAATGCCACTTGGCTATGTAACACATGGCTTAAATTTG   4642
gb:MN996530|Organism:Severe      GATCTAAATGAAACTCTTGTTACAATGCCACTTGGCTATGTAACACATGGCTTAAATTTG   4628
gb:MN908947|Organism:Severe      GATCTAAATGAAACTCTTGTTACAATGCCACTTGGCTATGTAACACATGGCTTAAATTTG   4642
gb:MT019532|Organism:Severe      GATCTAAATGAAACTCTTGTTACAATGCCACTTGGCTATGTAACACATGGCTTAAATTTG   4642
                                 ************************************************************

gb:MT020781|Organism:Severe      GAAGAAGCTGCTCGGTATATGAGATCTCTCAAAGTGCCAGCTACAGTTTCTGTTTCTTCA   4690
gb:MT007544|Organism:Severe      GAAGAAGCTGCTCGGTATATGAGATCTCTCAAAGTGCCAGCTACAGTTTCTGTTTCTTCA   4702
gb:MN994467|Organism:Severe      GAAGAAGCTGCTCGGTATATGAGATCTCTCAAAGTGCCAGCTACAGTTTCTGTTTCTTCA   4702
gb:MT044257|Organism:Severe      GAAGAAGCTGCTCGGTATATGAGATCTCTCAAAGTGCCAGCTACAGTTTCTGTTTCTTCA   4702
gb:MT106054|Organism:Severe      GAAGAAGCTGCTCGGTATATGAGATCTCTCAAAGTGCCAGCTACAGTTTCTGTTTCTTCA   4702
gb:MT049951|Organism:Severe      GAAGAAGCTGCTCGGTATATGAGATCTCTCAAAGTGCCAGCTACAGTTTCTGTTTCTTCA   4702
gb:MN975262|Organism:Severe      GAAGAAGCTGCTCGGTATATGAGATCTCTCAAAGTGCCAGCTACAGTTTCTGTTTCTTCA   4702
gb:MT106052|Organism:Severe      GAAGAAGCTGCTCGGTATATGAGATCTCTCAAAGTGCCAGCTACAGTTTCTGTTTCTTCA   4702
gb:LC522975|Organism:Severe      GAAGAAGCTGCTCGGTATATGAGATCTCTCAAAGTGCCAGCTACAGTTTCTGTTTCTTCA   4699
gb:LC522973|Organism:Severe      GAAGAAGCTGCTCGGTATATGAGATCTCTCAAAGTGCCAGCTACAGTTTCTGTTTCTTCA   4699
gb:LC522974|Organism:Severe      GAAGAAGCTGCTCGGTATATGAGATCTCTCAAAGTGCCAGCTACAGTTTCTGTTTCTTCA   4699
gb:MN985325|Organism:Severe      GAAGAAGCTGCTCGGTATATGAGATCTCTCAAAGTGCCAGCTACAGTTTCTGTTTCTTCA   4702
gb:MT020881|Organism:Severe      GAAGAAGCTGCTCGGTATATGAGATCTCTCAAAGTGCCAGCTACAGTTTCTGTTTCTTCA   4702
gb:MT020880|Organism:Severe      GAAGAAGCTGCTCGGTATATGAGATCTCTCAAAGTGCCAGCTACAGTTTCTGTTTCTTCA   4702
gb:MT066175|Organism:Severe      GAAGAAGCTGCTCGGTATATGAGATCTCTCAAAGTGCCAGCTACAGTTTCTGTTTCTTCA   4702
gb:MN997409|Organism:Severe      GAAGAAGCTGCTCGGTATATGAGATCTCTCAAAGTGCCAGCTACAGTTTCTGTTTCTTCA   4702
gb:MN938384|Organism:Severe      GAAGAAGCTGCTCGGTATATGAGATCTCTCAAAGTGCCAGCTACAGTTTCTGTTTCTTCA   4670
gb:MT044258|Organism:Severe      GAAGAAGCTGCTCGGTATATGAGATCTCTCAAAGTGCCAGCTACAGTTTCTGTTTCTTCA   4678
gb:MT039890|Organism:Severe      GAAGAAGCTGCTCGGTATATGAGATCTCTCAAAGTGCCAGCTACAGTTTCTGTTTCTTCA   4702
gb:MN988713|Organism:Severe      GAAGAAGCTGCTCGGTATATGAGATCTCTCAAAGTGCCAGCTACAGTTTCTGTTTCTTCA   4702
gb:LC521925|Organism:Severe      GAAGAAGCTGCTCGGTATATGAGATCTCTCAAAGTGCCAGCTACAGTTTCTGTTTCTTCA   4675
gb:MT093571|Organism:Severe      GAAGAAGCTGCTCGGTATATGAGATCTCTCAAAGTGCCAGCTACAGTTTCTGTTTCTTCA   4702
gb:MT039887|Organism:Severe      GAAGAAGCTGCTCGGTATATGAGATCTCTCAAAGTGCCAGCTACAGTTTCTGTTTCTTCA   4702
gb:MT019530|Organism:Severe      GAAGAAGCTGCTCGGTATATGAGATCTCTCAAAGTGCCAGCTACAGTTTCTGTTTCTTCA   4702
gb:MT039888|Organism:Severe      GAAGAAGCTGCTCGGTATATGAGATCTCTCAAAGTGCCAGCTACAGTTTCTGTTTCTTCA   4702
gb:LC522972|Organism:Severe      GAAGAAGCTGCTCGGTATATGAGATCTCTCAAAGTGCCAGCTACAGTTTCTGTTTCTTCA   4699
gb:MT027063|Organism:Severe      GAAGAAGCTGCTCGGTATATGAGATCTCTCAAAGTGCCAGCTACAGTTTCTGTTTCTTCA   4702
gb:MT027062|Organism:Severe      GAAGAAGCTGCTCGGTATATGAGATCTCTCAAAGTGCCAGCTACAGTTTCTGTTTCTTCA   4702
gb:MT019529|Organism:Severe      GAAGAAGCTGCTCGGTATATGAGATCTCTCAAAGTGCCAGCTACAGTTTCTGTTTCTTCA   4702
gb:MN996529|Organism:Severe      GAAGAAGCTGCTCGGTATATGAGATCTCTCAAAGTGCCAGCTACAGTTTCTGTTTCTTCA   4690
gb:MN996531|Organism:Severe      GAAGAAGCTGCTCGGTATATGAGATCTCTCAAAGTGCCAGCTACAGTTTCTGTTTCTTCA   4689
gb:MT066176|Organism:Severe      GAAGAAGCTGCTCGGTATATGAGATCTCTCAAAGTGCCAGCTACAGTTTCTGTTTCTTCA   4702
gb:MT027064|Organism:Severe      GAAGAAGCTGCTCGGTATATGAGATCTCTCAAAGTGCCAGCTACAGTTTCTGTTTCTTCA   4702
gb:MN994468|Organism:Severe      GAAGAAGCTGCTCGGTATATGAGATCTCTCAAAGTGCCAGCTACAGTTTCTGTTTCTTCA   4702
gb:MT072688|Organism:Severe      GAAGAAGCTGCTCGGTATATGAGATCTCTCAAAGTGCCAGCTACAGTTTCTGTTTCTTCA   4687
gb:MN996527|Organism:Severe      GAAGAAGCTGCTCGGTATATGAGATCTCTCAAAGTGCCAGCTACAGTTTCTGTTTCTTCA   4669
gb:MT093631|Organism:Severe      GAAGAAGCTGCTCGGTATATGAGATCTCTCAAAGTGCCAGCTACAGTTTCTGTTTCTTCA   4740
gb:MT106053|Organism:Severe      GAAGAAGCTGCTCGGTATATGAGATCTCTCAAAGTGCCAGCTACAGTTTCTGTTTCTTCA   4702
gb:MT019533|Organism:Severe      GAAGAAGCTGCTCGGTATATGAGATCTCTCAAAGTGCCAGCTACAGTTTCTGTTTCTTCA   4702
gb:MT019531|Organism:Severe      GAAGAAGCTGCTCGGTATATGAGATCTCTCAAAGTGCCAGCTACAGTTTCTGTTTCTTCA   4702
gb:MN996528|Organism:Severe      GAAGAAGCTGCTCGGTATATGAGATCTCTCAAAGTGCCAGCTACAGTTTCTGTTTCTTCA   4702
gb:MN996530|Organism:Severe      GAAGAAGCTGCTCGGTATATGAGATCTCTCAAAGTGCCAGCTACAGTTTCTGTTTCTTCA   4688
gb:MN908947|Organism:Severe      GAAGAAGCTGCTCGGTATATGAGATCTCTCAAAGTGCCAGCTACAGTTTCTGTTTCTTCA   4702
gb:MT019532|Organism:Severe      GAAGAAGCTGCTCGGTATATGAGATCTCTCAAAGTGCCAGCTACAGTTTCTGTTTCTTCA   4702
                                 ************************************************************

gb:MT020781|Organism:Severe      CCTGATGCTGTTACAGCGTATAATGGTTATCTTACTTCTTCTTCTAAAACACCTGAAGAA   4750
gb:MT007544|Organism:Severe      CCTGATGCTGTTACAGCGTATAATGGTTATCTTACTTCTTCTTCTAAAACACCTGAAGAA   4762
```

```
gb:MN994467|Organism:Severe      CCTGATGCTGTTACAGCGTATAATGGTTATCTTACTTCTTCTTCTAAAACACCTGAAGAA    4762
gb:MT044257|Organism:Severe      CCTGATGCTGTTACAGCGTATAATGGTTATCTTACTTCTTCTTCTAAAACACCTGAAGAA    4762
gb:MT106054|Organism:Severe      CCTGATGCTGTTACAGCGTATAATGGTTATCTTACTTCTTCTTCTAAAACACCTGAAGAA    4762
gb:MT049951|Organism:Severe      CCTGATGCTGTTACAGCGTATAATGGTTATCTTACTTCTTCTTCTAAAACACCTGAAGAA    4762
gb:MN975262|Organism:Severe      CCTGATGCTGTTACAGCGTATAATGGTTATCTTACTTCTTCTTCTAAAACACCTGAAGAA    4762
gb:MT106052|Organism:Severe      CCTGATGCTGTTACAGCGTATAATGGTTATCTTACTTCTTCTTCTAAAACACCTGAAGAA    4762
gb:LC522975|Organism:Severe      CCTGATGCTGTTACAGCGTATAATGGTTATCTTACTTCTTCTTCTAAAACACCTGAAGAA    4759
gb:LC522973|Organism:Severe      CCTGATGCTGTTACAGCGTATAATGGTTATCTTACTTCTTCTTCTAAAACACCTGAAGAA    4759
gb:LC522974|Organism:Severe      CCTGATGCTGTTACAGCGTATAATGGTTATCTTACTTCTTCTTCTAAAACACCTGAAGAA    4759
gb:MN985325|Organism:Severe      CCTGATGCTGTTACAGCGTATAATGGTTATCTTACTTCTTCTTCTAAAACACCTGAAGAA    4762
gb:MT020881|Organism:Severe      CCTGATGCTGTTACAGCGTATAATGGTTATCTTACTTCTTCTTCTAAAACACCTGAAGAA    4762
gb:MT020880|Organism:Severe      CCTGATGCTGTTACAGCGTATAATGGTTATCTTACTTCTTCTTCTAAAACACCTGAAGAA    4762
gb:MT066175|Organism:Severe      CCTGATGCTGTTACAGCGTATAATGGTTATCTTACTTCTTCTTCTAAAACACCTGAAGAA    4762
gb:MN997409|Organism:Severe      CCTGATGCTGTTACAGCGTATAATGGTTATCTTACTTCTTCTTCTAAAACACCTGAAGAA    4762
gb:MN938384|Organism:Severe      CCTGATGCTGTTACAGCGTATAATGGTTATCTTACTTCTTCTTCTAAAACACCTGAAGAA    4730
gb:MT044258|Organism:Severe      CCTGATGCTGTTACAGCGTATAATGGTTATCTTACTTCTTCTTCTAAAACACCTGAAGAA    4738
gb:MT039890|Organism:Severe      CCTGATGCTGTTACAGCGTATAATGGTTATCTTACTTCTTCTTCTAAAACACCTGAAGAA    4762
gb:MN988713|Organism:Severe      CCTGATGCTGTTACAGCGTATAATGGTTATCTTACTTCTTCTTCTAAAACACCTGAAGAA    4762
gb:LC521925|Organism:Severe      CCTGATGCTGTTACAGCGTATAATGGTTATCTTACTTCTTCTTCTAAAACACCTGAAGAA    4735
gb:MT093571|Organism:Severe      CCTGATGCTGTTACAGCGTATAATGGTTATCTTACTTCTTCTTCTAAAACACCTGAAGAA    4762
gb:MT039887|Organism:Severe      CCTGATGCTGTTACAGCGTATAATGGTTATCTTACTTCTTCTTCTAAAACACCTGAAGAA    4762
gb:MT019530|Organism:Severe      CCTGATGCTGTTACAGCGTATAATGGTTATCTTACTTCTTCTTCTAAAACACCTGAAGAA    4762
gb:MT039888|Organism:Severe      CCTGATGCTGTTACAGCGTATAATGGTTATCTTACTTCTTCTTCTAAAACACCTGAAGAA    4762
gb:LC522972|Organism:Severe      CCTGATGCTGTTACAGCGTATAATGGTTATCTTACTTCTTCTTCTAAAACACCTGAAGAA    4759
gb:MT027063|Organism:Severe      CCTGATGCTGTTACAGCGTATAATGGTTATCTTACTTCTTCTTCTAAAACACCTGAAGAA    4762
gb:MT027062|Organism:Severe      CCTGATGCTGTTACAGCGTATAATGGTTATCTTACTTCTTCTTCTAAAACACCTGAAGAA    4762
gb:MT019529|Organism:Severe      CCTGATGCTGTTACAGCGTATAATGGTTATCTTACTTCTTCTTCTAAAACACCTGAAGAA    4762
gb:MN996529|Organism:Severe      CCTGATGCTGTTACAGCGTATAATGGTTATCTTACTTCTTCTTCTAAAACACCTGAAGAA    4750
gb:MN996531|Organism:Severe      CCTGATGCTGTTACAGCGTATAATGGTTATCTTACTTCTTCTTCTAAAACACCTGAAGAA    4749
gb:MT066176|Organism:Severe      CCTGATGCTGTTACAGCGTATAATGGTTATCTTACTTCTTCTTCTAAAACACCTGAAGAA    4762
gb:MT027064|Organism:Severe      CCTGATGCTGTTACAGCGTATAATGGTTATCTTACTTCTTCTTCTAAAACACCTGAAGAA    4762
gb:MN994468|Organism:Severe      CCTGATGCTGTTACAGCGTATAATGGTTATCTTACTTCTTCTTCTAAAACACCTGAAGAA    4762
gb:MT072688|Organism:Severe      CCTGATGCTGTTACAGCGTATAATGGTTATCTTACTTCTTCTTCTAAAACACCTGAAGAA    4747
gb:MN996527|Organism:Severe      CCTGATGCTGTTACAGCGTATAATGGTTATCTTACTTCTTCTTCTAAAACACCTGAAGAA    4729
gb:MT093631|Organism:Severe      CCTGATGCTGTTACAGCGTATAATGGTTATCTTACTTCTTCTTCTAAAACACCTGAAGAA    4800
gb:MT106053|Organism:Severe      CCTGATGCTGTTACAGCGTATAATGGTTATCTTACTTCTTCTTCTAAAACACCTGAAGAA    4762
gb:MT019533|Organism:Severe      CCTGATGCTGTTACAGCGTATAATGGTTATCTTACTTCTTCTTCTAAAACACCTGAAGAA    4762
gb:MT019531|Organism:Severe      CCTGATGCTGTTACAGCGTATAATGGTTATCTTACTTCTTCTTCTAAAACACCTGAAGAA    4762
gb:MN996528|Organism:Severe      CCTGATGCTGTTACAGCGTATAATGGTTATCTTACTTCTTCTTCTAAAACACCTGAAGAA    4762
gb:MN996530|Organism:Severe      CCTGATGCTGTTACAGCGTATAATGGTTATCTTACTTCTTCTTCTAAAACACCTGAAGAA    4748
gb:MN908947|Organism:Severe      CCTGATGCTGTTACAGCGTATAATGGTTATCTTACTTCTTCTTCTAAAACACCTGAAGAA    4762
gb:MT019532|Organism:Severe      CCTGATGCTGTTACAGCGTATAATGGTTATCTTACTTCTTCTTCTAAAACACCTGAAGAA    4762
                                 ************************************************************

gb:MT020781|Organism:Severe      CATTTTATTGAAACCATCTCACTTGCTGGTTCCTATAAAGATTGGTCCTATTCTGGACAA    4810
gb:MT007544|Organism:Severe      CATTTTATTGAAACCATCTCACTTGCTGGTTCCTATAAAGATTGGTCCTATTCTGGACAA    4822
gb:MN994467|Organism:Severe      CATTTTATTGAAACCATCTCACTTGCTGGTTCCTATAAAGATTGGTCCTATTCTGGACAA    4822
gb:MT044257|Organism:Severe      CATTTTATTGAAACCATCTCACTTGCTGGTTCCTATAAAGATTGGTCCTATTCTGGACAA    4822
gb:MT106054|Organism:Severe      CATTTTATTGAAACCATCTCACTTGCTGGTTCCTATAAAGATTGGTCCTATTCTGGACAA    4822
gb:MT049951|Organism:Severe      CATTTTATTGAAACCATCTCACTTGCTGGTTCCTATAAAGATTGGTCCTATTCTGGACAA    4822
gb:MN975262|Organism:Severe      CATTTTATTGAAACCATCTCACTTGCTGGTTCCTATAAAGATTGGTCCTATTCTGGACAA    4822
gb:MT106052|Organism:Severe      CATTTTATTGAAACCATCTCACTTGCTGGTTCCTATAAAGATTGGTCCTATTCTGGACAA    4822
gb:LC522975|Organism:Severe      CATTTTATTGAAACCATCTCACTTGCTGGTTCCTATAAAGATTGGTCCTATTCTGGACAA    4819
gb:LC522973|Organism:Severe      CATTTTATTGAAACCATCTCACTTGCTGGTTCCTATAAAGATTGGTCCTATTCTGGACAA    4819
gb:LC522974|Organism:Severe      CATTTTATTGAAACCATCTCACTTGCTGGTTCCTATAAAGATTGGTCCTATTCTGGACAA    4819
gb:MN985325|Organism:Severe      CATTTTATTGAAACCATCTCACTTGCTGGTTCCTATAAAGATTGGTCCTATTCTGGACAA    4822
gb:MT020881|Organism:Severe      CATTTTATTGAAACCATCTCACTTGCTGGTTCCTATAAAGATTGGTCCTATTCTGGACAA    4822
gb:MT020880|Organism:Severe      CATTTTATTGAAACCATCTCACTTGCTGGTTCCTATAAAGATTGGTCCTATTCTGGACAA    4822
gb:MT066175|Organism:Severe      CATTTTATTGAAACCATCTCACTTGCTGGTTCCTATAAAGATTGGTCCTATTCTGGACAA    4822
gb:MN997409|Organism:Severe      CATTTTATTGAAACCATCTCACTTGCTGGTTCCTATAAAGATTGGTCCTATTCTGGACAA    4822
gb:MN938384|Organism:Severe      CATTTTATTGAAACCATCTCACTTGCTGGTTCCTATAAAGATTGGTCCTATTCTGGACAA    4790
gb:MT044258|Organism:Severe      CATTTTATTGAAACCATCTCACTTGCTGGTTCCTATAAAGATTGGTCCTATTCTGGACAA    4798
gb:MT039890|Organism:Severe      CATTTTATTGAAACCATCTCACTTGCTGGTTCCTATAAAGATTGGTCCTATTCTGGACAA    4822
gb:MN988713|Organism:Severe      CATTTTATTGAAACCATCTCACTTGCTGGTTCCTATAAAGATTGGTCCTATTCTGGACAA    4822
gb:LC521925|Organism:Severe      CATTTTATTGAAACCATCTCACTTGCTGGTTCCTATAAAGATTGGTCCTATTCTGGACAA    4795
```

```
gb:MT093571|Organism:Severe      CATTTTATTGAAACCATCTCACTTGCTGGTTCCTATAAAGATTGGTCCTATTCTGGACAA   4822
gb:MT039887|Organism:Severe      CATTTTATTGAAACCATCTCACTTGCTGGTTCCTATAAAGATTGGTCCTATTCTGGACAA   4822
gb:MT019530|Organism:Severe      CATTTTATTGAAACCATCTCACTTGCTGGTTCCTATAAAGATTGGTCCTATTCTGGACAA   4822
gb:MT039888|Organism:Severe      CATTTTATTGAAACCATCTCACTTGCTGGTTCCTATAAAGATTGGTCCTATTCTGGACAA   4822
gb:LC522972|Organism:Severe      CATTTTATTGAAACCATCTCACTTGCTGGTTCCTATAAAGATTGGTCCTATTCTGGACAA   4819
gb:MT027063|Organism:Severe      CATTTTATTGAAACCATCTCACTTGCTGGTTCCTATAAAGATTGGTCCTATTCTGGACAA   4822
gb:MT027062|Organism:Severe      CATTTTATTGAAACCATCTCACTTGCTGGTTCCTATAAAGATTGGTCCTATTCTGGACAA   4822
gb:MT019529|Organism:Severe      CATTTTATTGAAACCATCTCACTTGCTGGTTCCTATAAAGATTGGTCCTATTCTGGACAA   4822
gb:MN996529|Organism:Severe      CATTTTATTGAAACCATCTCACTTGCTGGTTCCTATAAAGATTGGTCCTATTCTGGACAA   4810
gb:MN996531|Organism:Severe      CATTTTATTGAAACCATCTCACTTGCTGGTTCCTATAAAGATTGGTCCTATTCTGGACAA   4809
gb:MT066176|Organism:Severe      CATTTTATTGAAACCATCTCACTTGCTGGTTCCTATAAAGATTGGTCCTATTCTGGACAA   4822
gb:MT027064|Organism:Severe      CATTTTATTGAAACCATCTCACTTGCTGGTTCCTATAAAGATTGGTCCTATTCTGGACAA   4822
gb:MN994468|Organism:Severe      CATTTTATTGAAACCATCTCACTTGCTGGTTCCTATAAAGATTGGTCCTATTCTGGACAA   4822
gb:MT072688|Organism:Severe      CATTTTATTGAAACCATCTCACTTGCTGGTTCCTATAAAGATTGGTCCTATTCTGGACAA   4807
gb:MN996527|Organism:Severe      CATTTTATTGAAACCATCTCACTTGCTGGTTCCTATAAAGATTGGTCCTATTCTGGACAA   4789
gb:MT093631|Organism:Severe      CATTTTATTGAAACCATCTCACTTGCTGGTTCCTATAAAGATTGGTCCTATTCTGGACAA   4860
gb:MT106053|Organism:Severe      CATTTTATTGAAACCATCTCACTTGCTGGTTCCTATAAAGATTGGTCCTATTCTGGACAA   4822
gb:MT019533|Organism:Severe      CATTTTATTGAAACCATCTCACTTGCTGGTTCCTATAAAGATTGGTCCTATTCTGGACAA   4822
gb:MT019531|Organism:Severe      CATTTTATTGAAACCATCTCACTTGCTGGTTCCTATAAAGATTGGTCCTATTCTGGACAA   4822
gb:MN996528|Organism:Severe      CATTTTATTGAAACCATCTCACTTGCTGGTTCCTATAAAGATTGGTCCTATTCTGGACAA   4822
gb:MN996530|Organism:Severe      CATTTTATTGAAACCATCTCACTTGCTGGTTCCTATAAAGATTGGTCCTATTCTGGACAA   4808
gb:MN908947|Organism:Severe      CATTTTATTGAAACCATCTCACTTGCTGGTTCCTATAAAGATTGGTCCTATTCTGGACAA   4822
gb:MT019532|Organism:Severe      CATTTTATTGAAACCATCTCACTTGCTGGTTCCTATAAAGATTGGTCCTATTCTGGACAA   4822
                                 ************************************************************

gb:MT020781|Organism:Severe      TCTACACAACTAGGTATAGAATTTCTTAAGAGAGGTGATAAAAGTGTATATTACACTAGT   4870
gb:MT007544|Organism:Severe      TCTACACAACTAGGTATAGAATTTCTTAAGAGAGGTGATAAAAGTGTATATTACACTAGT   4882
gb:MN994467|Organism:Severe      TCTACACAACTAGGTATAGAATTTCTTAAGAGAGGTGATAAAAGTGTATATTACACTAGT   4882
gb:MT044257|Organism:Severe      TCTACACAACTAGGTATAGAATTTCTTAAGAGAGGTGATAAAAGTGTATATTACACTAGT   4882
gb:MT106054|Organism:Severe      TCTACACAACTAGGTATAGAATTTCTTAAGAGAGGTGATAAAAGTGTATATTACACTAGT   4882
gb:MT049951|Organism:Severe      TCTACACAACTAGGTATAGAATTTCTTAAGAGAGGTGATAAAAGTGTATATTACACTAGT   4882
gb:MN975262|Organism:Severe      TCTACACAACTAGGTATAGAATTTCTTAAGAGAGGTGATAAAAGTGTATATTACACTAGT   4882
gb:MT106052|Organism:Severe      TCTACACAACTAGGTATAGAATTTCTTAAGAGAGGTGATAAAAGTGTATATTACACTAGT   4882
gb:LC522975|Organism:Severe      TCTACACAACTAGGTATAGAATTTCTTAAGAGAGGTGATAAAAGTGTATATTACACTAGT   4879
gb:LC522973|Organism:Severe      TCTACACAACTAGGTATAGAATTTCTTAAGAGAGGTGATAAAAGTGTATATTACACTAGT   4879
gb:LC522974|Organism:Severe      TCTACACAACTAGGTATAGAATTTCTTAAGAGAGGTGATAAAAGTGTATATTACACTAGT   4879
gb:MN985325|Organism:Severe      TCTACACAACTAGGTATAGAATTTCTTAAGAGAGGTGATAAAAGTGTATATTACACTAGT   4882
gb:MT020881|Organism:Severe      TCTACACAACTAGGTATAGAATTTCTTAAGAGAGGTGATAAAAGTGTATATTACACTAGT   4882
gb:MT020880|Organism:Severe      TCTACACAACTAGGTATAGAATTTCTTAAGAGAGGTGATAAAAGTGTATATTACACTAGT   4882
gb:MT066175|Organism:Severe      TCTACACAACTAGGTATAGAATTTCTTAAGAGAGGTGATAAAAGTGTATATTACACTAGT   4882
gb:MN997409|Organism:Severe      TCTACACAACTAGGTATAGAATTTCTTAAGAGAGGTGATAAAAGTGTATATTACACTAGT   4882
gb:MN938384|Organism:Severe      TCTACACAACTAGGTATAGAATTTCTTAAGAGAGGTGATAAAAGTGTATATTACACTAGT   4850
gb:MT044258|Organism:Severe      TCTACACAACTAGGTATAGAATTTCTTAAGAGAGGTGATAAAAGTGTATATTACACTAGT   4858
gb:MT039890|Organism:Severe      TCTACACAACTAGGTATAGAATTTCTTAAGAGAGGTGATAAAAGTGTATATTACACTAGT   4882
gb:MN988713|Organism:Severe      TCTACACAACTAGGTATAGAATTTCTTAAGAGAGGTGATAAAAGTGTATATTACACTAGT   4882
gb:LC521925|Organism:Severe      TCTACACAACTAGGTATAGAATTTCTTAAGAGAGGTGATAAAAGTGTATATTACACTAGT   4855
gb:MT093571|Organism:Severe      TCTACACAACTAGGTATAGAATTTCTTAAGAGAGGTGATAAAAGTGTATATTACACTAGT   4882
gb:MT039887|Organism:Severe      TCTACACAACTAGGTATAGAATTTCTTAAGAGAGGTGATAAAAGTGTATATTACACTAGT   4882
gb:MT019530|Organism:Severe      TCTACACAACTAGGTATAGAATTTCTTAAGAGAGGTGATAAAAGTGTATATTACACTAGT   4882
gb:MT039888|Organism:Severe      TCTACACAACTAGGTATAGAATTTCTTAAGAGAGGTGATAAAAGTGTATATTACACTAGT   4882
gb:LC522972|Organism:Severe      TCTACACAACTAGGTATAGAATTTCTTAAGAGAGGTGATAAAAGTGTATATTACACTAGT   4879
gb:MT027063|Organism:Severe      TCTACACAACTAGGTATAGAATTTCTTAAGAGAGGTGATAAAAGTGTATATTACACTAGT   4882
gb:MT027062|Organism:Severe      TCTACACAACTAGGTATAGAATTTCTTAAGAGAGGTGATAAAAGTGTATATTACACTAGT   4882
gb:MT019529|Organism:Severe      TCTACACAACTAGGTATAGAATTTCTTAAGAGAGGTGATAAAAGTGTATATTACACTAGT   4882
gb:MN996529|Organism:Severe      TCTACACAACTAGGTATAGAATTTCTTAAGAGAGGTGATAAAAGTGTATATTACACTAGT   4870
gb:MN996531|Organism:Severe      TCTACACAACTAGGTATAGAATTTCTTAAGAGAGGTGATAAAAGTGTATATTACACTAGT   4869
gb:MT066176|Organism:Severe      TCTACACAACTAGGTATAGAATTTCTTAAGAGAGGTGATAAAAGTGTATATTACACTAGT   4882
gb:MT027064|Organism:Severe      TCTACACAACTAGGTATAGAATTTCTTAAGAGAGGTGATAAAAGTGTATATTACACTAGT   4882
gb:MN994468|Organism:Severe      TCTACACAACTAGGTATAGAATTTCTTAAGAGAGGTGATAAAAGTGTATATTACACTAGT   4882
gb:MT072688|Organism:Severe      TCTACACAACTAGGTATAGAATTTCTTAAGAGAGGTGATAAAAGTGTATATTACACTAGT   4867
gb:MN996527|Organism:Severe      TCTACACAACTAGGTATAGAATTTCTTAAGAGAGGTGATAAAAGTGTATATTACACTAGT   4849
gb:MT093631|Organism:Severe      TCTACACAACTAGGTATAGAATTTCTTAAGAGAGGTGATAAAAGTGTATATTACACTAGT   4920
gb:MT106053|Organism:Severe      TCTACACAACTAGGTATAGAATTTCTTAAGAGAGGTGATAAAAGTGTATATTACACTAGT   4882
gb:MT019533|Organism:Severe      TCTACACAACTAGGTATAGAATTTCTTAAGAGAGGTGATAAAAGTGTATATTACACTAGT   4882
gb:MT019531|Organism:Severe      TCTACACAACTAGGTATAGAATTTCTTAAGAGAGGTGATAAAAGTGTATATTACACTAGT   4882
```

```
gb:MN996528|Organism:Severe      TCTACACAACTAGGTATAGAATTTCTTAAGAGAGGTGATAAAAGTGTATATTACACTAGT    4882
gb:MN996530|Organism:Severe      TCTACACAACTAGGTATAGAATTTCTTAAGAGAGGTGATAAAAGTGTATATTACACTAGT    4868
gb:MN908947|Organism:Severe      TCTACACAACTAGGTATAGAATTTCTTAAGAGAGGTGATAAAAGTGTATATTACACTAGT    4882
gb:MT019532|Organism:Severe      TCTACACAACTAGGTATAGAATTTCTTAAGAGAGGTGATAAAAGTGTATATTACACTAGT    4882
                                 ************************************************************

gb:MT020781|Organism:Severe      AATCCTACCACATTCCACCTAGATGGTGAAGTTATCACCTTTGACAATCTTAAGACACTT    4930
gb:MT007544|Organism:Severe      AATCCTACCACATTCCACCTAGATGGTGAAGTTATCACCTTTGACAATCTTAAGACACTT    4942
gb:MN994467|Organism:Severe      AATCCTACCACATTCCACCTAGATGGTGAAGTTATCACCTTTGACAATCTTAAGACACTT    4942
gb:MT044257|Organism:Severe      AATCCTACCACATTCCACCTAGATGGTGAAGTTATCACCTTTGACAATCTTAAGACACTT    4942
gb:MT106054|Organism:Severe      AATCCTACCACATTCCACCTAGATGGTGAAGTTATCACCTTTGACAATCTTAAGACACTT    4942
gb:MT049951|Organism:Severe      AATCCTACCACATTCCACCTAGATGGTGAAGTTATCACCTTTGACAATCTTAAGACACTT    4942
gb:MN975262|Organism:Severe      AATCCTACCACATTCCACCTAGATGGTGAAGTTATCACCTTTGACAATCTTAAGACACTT    4942
gb:MT106052|Organism:Severe      AATCCTACCACATTCCACCTAGATGGTGAAGTTATCACCTTTGACAATCTTAAGACACTT    4942
gb:LC522975|Organism:Severe      AATCCTACCACATTCCACCTAGATGGTGAAGTTATCACCTTTGACAATCTTAAGACACTT    4939
gb:LC522973|Organism:Severe      AATCCTACCACATTCCACCTAGATGGTGAAGTTATCACCTTTGACAATCTTAAGACACTT    4939
gb:LC522974|Organism:Severe      AATCCTACCACATTCCACCTAGATGGTGAAGTTATCACCTTTGACAATCTTAAGACACTT    4939
gb:MN985325|Organism:Severe      AATCCTACCACATTCCACCTAGATGGTGAAGTTATCACCTTTGACAATCTTAAGACACTT    4942
gb:MT020881|Organism:Severe      AATCCTACCACATTCCACCTAGATGGTGAAGTTATCACCTTTGACAATCTTAAGACACTT    4942
gb:MT020880|Organism:Severe      AATCCTACCACATTCCACCTAGATGGTGAAGTTATCACCTTTGACAATCTTAAGACACTT    4942
gb:MT066175|Organism:Severe      AATCCTACCACATTCCACCTAGATGGTGAAGTTATCACCTTTGACAATCTTAAGACACTT    4942
gb:MN997409|Organism:Severe      AATCCTACCACATTCCACCTAGATGGTGAAGTTATCACCTTTGACAATCTTAAGACACTT    4942
gb:MN938384|Organism:Severe      AATCCTACCACATTCCACCTAGATGGTGAAGTTATCACCTTTGACAATCTTAAGACACTT    4910
gb:MT044258|Organism:Severe      AATCCTACCACATTCCACCTAGATGGTGAAGTTATCACCTTTGACAATCTTAAGACACTT    4918
gb:MT039890|Organism:Severe      AATCCTACCACATTCCACCTAGATGGTGAAGTTATCACCTTTGACAATCTTAAGACACTT    4942
gb:MN988713|Organism:Severe      AATCCTACCACATTCCACCTAGATGGTGAAGTTATCACCTTTGACAATCTTAAGACACTT    4942
gb:LC521925|Organism:Severe      AATCCTACCACATTCCACCTAGATGGTGAAGTTATCACCTTTGACAATCTTAAGACACTT    4915
gb:MT093571|Organism:Severe      AATCCTACCACATTCCACCTAGATGGTGAAGTTATCACCTTTGACAATCTTAAGACACTT    4942
gb:MT039887|Organism:Severe      AATCCTACCACATTCCACCTAGATGGTGAAGTTATCACCTTTGACAATCTTAAGACACTT    4942
gb:MT019530|Organism:Severe      AATCCTACCACATTCCACCTAGATGGTGAAGTTATCACCTTTGACAATCTTAAGACACTT    4942
gb:MT039888|Organism:Severe      AATCCTACCACATTCCACCTAGATGGTGAAGTTATCACCTTTGACAATCTTAAGACACTT    4942
gb:LC522972|Organism:Severe      AATCCTACCACATTCCACCTAGATGGTGAAGTTATCACCTTTGACAATCTTAAGACACTT    4939
gb:MT027063|Organism:Severe      AATCCTACCACATTCCACCTAGATGGTGAAGTTATCACCTTTGACAATCTTAAGACACTT    4942
gb:MT027062|Organism:Severe      AATCCTACCACATTCCACCTAGATGGTGAAGTTATCACCTTTGACAATCTTAAGACACTT    4942
gb:MT019529|Organism:Severe      AATCCTACCACATTCCACCTAGATGGTGAAGTTATCACCTTTGACAATCTTAAGACACTT    4942
gb:MN996529|Organism:Severe      AATCCTACCACATTCCACCTAGATGGTGAAGTTATCACCTTTGACAATCTTAAGACACTT    4930
gb:MN996531|Organism:Severe      AATCCTACCACATTCCACCTAGATGGTGAAGTTATCACCTTTGACAATCTTAAGACACTT    4929
gb:MT066176|Organism:Severe      AATCCTACCACATTCCACCTAGATGGTGAAGTTATCACCTTTGACAATCTTAAGACACTT    4942
gb:MT027064|Organism:Severe      AATCCTACCACATTCCACCTAGATGGTGAAGTTATCACCTTTGACAATCTTAAGACACTT    4942
gb:MN994468|Organism:Severe      AATCCTACCACATTCCACCTAGATGGTGAAGTTATCACCTTTGACAATCTTAAGACACTT    4942
gb:MT072688|Organism:Severe      AATCCTACCACATTCCACCTAGATGGTGAAGTTATCACCTTTGACAATCTTAAGACACTT    4927
gb:MN996527|Organism:Severe      AATCCTACCACATTCCACCTAGATGGTGAAGTTATCACCTTTGACAATCTTAAGACACTT    4909
gb:MT093631|Organism:Severe      AATCCTACCACATTCCACCTAGATGGTGAAGTTATCACCTTTGACAATCTTAAGACACTT    4980
gb:MT106053|Organism:Severe      AATCCTACCACATTCCACCTAGATGGTGAAGTTATCACCTTTGACAATCTTAAGACACTT    4942
gb:MT019533|Organism:Severe      AATCCTACCACATTCCACCTAGATGGTGAAGTTATCACCTTTGACAATCTTAAGACACTT    4942
gb:MT019531|Organism:Severe      AATCCTACCACATTCCACCTAGATGGTGAAGTTATCACCTTTGACAATCTTAAGACACTT    4942
gb:MN996528|Organism:Severe      AATCCTACCACATTCCACCTAGATGGTGAAGTTATCACCTTTGACAATCTTAAGACACTT    4942
gb:MN996530|Organism:Severe      AATCCTACCACATTCCACCTAGATGGTGAAGTTATCACCTTTGACAATCTTAAGACACTT    4928
gb:MN908947|Organism:Severe      AATCCTACCACATTCCACCTAGATGGTGAAGTTATCACCTTTGACAATCTTAAGACACTT    4942
gb:MT019532|Organism:Severe      AATCCTACCACATTCCACCTAGATGGTGAAGTTATCACCTTTGACAATCTTAAGACACTT    4942
                                 ************************************************************

gb:MT020781|Organism:Severe      CTTTCTTTGAGAGAAGTGAGGACTATTAAGGTGTTTACAACAGTAGACAACATTAACCTC    4990
gb:MT007544|Organism:Severe      CTTTCTTTGAGAGAAGTGAGGACTATTAAGGTGTTTACAACAGTAGACAACATTAACCTC    5002
gb:MN994467|Organism:Severe      CTTTCTTTGAGAGAAGTGAGGACTATTAAGGTGTTTACAACAGTAGACAACATTAACCTC    5002
gb:MT044257|Organism:Severe      CTTTCTTTGAGAGAAGTGAGGACTATTAAGGTGTTTACAACAGTAGACAACATTAACCTC    5002
gb:MT106054|Organism:Severe      CTTTCTTTGAGAGAAGTGAGGACTATTAAGGTGTTTACAACAGTAGACAACATTAACCTC    5002
gb:MT049951|Organism:Severe      CTTTCTTTGAGAGAAGTGAGGACTATTAAGGTGTTTACAACAGTAGACAACATTAACCTC    5002
gb:MN975262|Organism:Severe      CTTTCTTTGAGAGAAGTGAGGACTATTAAGGTGTTTACAACAGTAGACAACATTAACCTC    5002
gb:MT106052|Organism:Severe      CTTTCTTTGAGAGAAGTGAGGACTATTAAGGTGTTTACAACAGTAGACAACATTAACCTC    5002
gb:LC522975|Organism:Severe      CTTTCTTTGAGAGAAGTGAGGACTATTAAGGTGTTTACAACAGTAGACAACATTAACCTC    4999
gb:LC522973|Organism:Severe      CTTTCTTTGAGAGAAGTGAGGACTATTAAGGTGTTTACAACAGTAGACAACATTAACCTC    4999
gb:LC522974|Organism:Severe      CTTTCTTTGAGAGAAGTGAGGACTATTAAGGTGTTTACAACAGTAGACAACATTAACCTC    4999
gb:MN985325|Organism:Severe      CTTTCTTTGAGAGAAGTGAGGACTATTAAGGTGTTTACAACAGTAGACAACATTAACCTC    5002
gb:MT020881|Organism:Severe      CTTTCTTTGAGAGAAGTGAGGACTATTAAGGTGTTTACAACAGTAGACAACATTAACCTC    5002
```

```
gb:MT020880|Organism:Severe      CTTTCTTTGAGAGAAGTGAGGACTATTAAGGTGTTTACAACAGTAGACAACATTAACCTC    5002
gb:MT066175|Organism:Severe      CTTTCTTTGAGAGAAGTGAGGACTATTAAGGTGTTTACAACAGTAGACAACATTAACCTC    5002
gb:MN997409|Organism:Severe      CTTTCTTTGAGAGAAGTGAGGACTATTAAGGTGTTTACAACAGTAGACAACATTAACCTC    5002
gb:MN938384|Organism:Severe      CTTTCTTTGAGAGAAGTGAGGACTATTAAGGTGTTTACAACAGTAGACAACATTAACCTC    4970
gb:MT044258|Organism:Severe      CTTTCTTTGAGAGAAGTGAGGACTATTAAGGTGTTTACAACAGTAGACAACATTAACCTC    4978
gb:MT039890|Organism:Severe      CTTTCTTTGAGAGAAGTGAGGACTATTAAGGTGTTTACAACAGTAGACAACATTAACCTC    5002
gb:MN988713|Organism:Severe      CTTTCTTTGAGAGAAGTGAGGACTATTAAGGTGTTTACAACAGTAGACAACATTAACCTC    5002
gb:LC521925|Organism:Severe      CTTTCTTTGAGAGAAGTGAGGACTATTAAGGTGTTTACAACAGTAGACAACATTAACCTC    4975
gb:MT093571|Organism:Severe      CTTTCTTTGAGAGAAGTGAGGACTATTAAGGTGTTTACAACAGTAGACAACATTAACCTC    5002
gb:MT039887|Organism:Severe      CTTTCTTTGAGAGAAGTGAGGACTATTAAGGTGTTTACAACAGTAGACAACATTAACCTC    5002
gb:MT019530|Organism:Severe      CTTTCTTTGAGAGAAGTGAGGACTATTAAGGTGTTTACAACAGTAGACAACATTAACCTC    5002
gb:MT039888|Organism:Severe      CTTTCTTTGAGAGAAGTGAGGACTATTAAGGTGTTTACAACAGTAGACAACATTAACCTC    5002
gb:LC522972|Organism:Severe      CTTTCTTTGAGAGAAGTGAGGACTATTAAGGTGTTTACAACAGTAGACAACATTAACCTC    4999
gb:MT027063|Organism:Severe      CTTTCTTTGAGAGAAGTGAGGACTATTAAGGTGTTTACAACAGTAGACAACATTAACCTC    5002
gb:MT027062|Organism:Severe      CTTTCTTTGAGAGAAGTGAGGACTATTAAGGTGTTTACAACAGTAGACAACATTAACCTC    5002
gb:MT019529|Organism:Severe      CTTTCTTTGAGAGAAGTGAGGACTATTAAGGTGTTTACAACAGTAGACAACATTAACCTC    5002
gb:MN996529|Organism:Severe      CTTTCTTTGAGAGAAGTGAGGACTATTAAGGTGTTTACAACAGTAGACAACATTAACCTC    4990
gb:MN996531|Organism:Severe      CTTTCTTTGAGAGAAGTGAGGACTATTAAGGTGTTTACAACAGTAGACAACATTAACCTC    4989
gb:MT066176|Organism:Severe      CTTTCTTTGAGAGAAGTGAGGACTATTAAGGTGTTTACAACAGTAGACAACATTAACCTC    5002
gb:MT027064|Organism:Severe      CTTTCTTTGAGAGAAGTGAGGACTATTAAGGTGTTTACAACAGTAGACAACATTAACCTC    5002
gb:MN994468|Organism:Severe      CTTTCTTTGAGAGAAGTGAGGACTATTAAGGTGTTTACAACAGTAGACAACATTAACCTC    5002
gb:MT072688|Organism:Severe      CTTTCTTTGAGAGAAGTGAGGACTATTAAGGTGTTTACAACAGTAGACAACATTAACCTC    4987
gb:MN996527|Organism:Severe      CTTTCTTTGAGAGAAGTGAGGACTATTAAGGTGTTTACAACAGTAGACAACATTAACCTC    4969
gb:MT093631|Organism:Severe      CTTTCTTTGAGAGAAGTGAGGACTATTAAGGTGTTTACAACAGTAGACAACATTAACCTC    5040
gb:MT106053|Organism:Severe      CTTTCTTTGAGAGAAGTGAGGACTATTAAGGTGTTTACAACAGTAGACAACATTAACCTC    5002
gb:MT019533|Organism:Severe      CTTTCTTTGAGAGAAGTGAGGACTATTAAGGTGTTTACAACAGTAGACAACATTAACCTC    5002
gb:MT019531|Organism:Severe      CTTTCTTTGAGAGAAGTGAGGACTATTAAGGTGTTTACAACAGTAGACAACATTAACCTC    5002
gb:MN996528|Organism:Severe      CTTTCTTTGAGAGAAGTGAGGACTATTAAGGTGTTTACAACAGTAGACAACATTAACCTC    5002
gb:MN996530|Organism:Severe      CTTTCTTTGAGAGAAGTGAGGACTATTAAGGTGTTTACAACAGTAGACAACATTAACCTC    4988
gb:MN908947|Organism:Severe      CTTTCTTTGAGAGAAGTGAGGACTATTAAGGTGTTTACAACAGTAGACAACATTAACCTC    5002
gb:MT019532|Organism:Severe      CTTTCTTTGAGAGAAGTGAGGACTATTAAGGTGTTTACAACAGTAGACAACATTAACCTC    5002
                                 ************************************************************

gb:MT020781|Organism:Severe      CACACGCAAGTTGTGGACATGTCAATGACATATGGACAACAGTTTGGTCCAACTTATTTG    5050
gb:MT007544|Organism:Severe      CACACGCAAGTTGTGGACATGTCAATGACATATGGACAACAGTTTGGTCCAACTTATTTG    5062
gb:MN994467|Organism:Severe      CACACGCAAGTTGTGGACATGTCAATGACATATGGACAACAGTTTGGTCCAACTTATTTG    5062
gb:MT044257|Organism:Severe      CACACGCAAGTTGTGGACATGTCAATGACATATGGACAACAGTTTGGTCCAACTTATTTG    5062
gb:MT106054|Organism:Severe      CACACGCAAGTTGTGGACATGTCAATGACATATGGACAACAGTTTGGTCCAACTTATTTG    5062
gb:MT049951|Organism:Severe      CACACGCAAGTTGTGGACATGTCAATGACATATGGACAACAGTTTGGTCCAACTTATTTG    5062
gb:MN975262|Organism:Severe      CACACGCAAGTTGTGGACATGTCAATGACATATGGACAACAGTTTGGTCCAACTTATTTG    5062
gb:MT106052|Organism:Severe      CACACGCAAGTTGTGGACATGTCAATGACATATGGACAACAGTTTGGTCCAACTTATTTG    5062
gb:LC522975|Organism:Severe      CACACGCAAGTTGTGGACATGTCAATGACATATGGACAACAGTTTGGTCCAACTTATTTG    5059
gb:LC522973|Organism:Severe      CACACGCAAGTTGTGGACATGTCAATGACATATGGACAACAGTTTGGTCCAACTTATTTG    5059
gb:LC522974|Organism:Severe      CACACGCAAGTTGTGGACATGTCAATGACATATGGACAACAGTTTGGTCCAACTTATTTG    5059
gb:MN985325|Organism:Severe      CACACGCAAGTTGTGGACATGTCAATGACATATGGACAACAGTTTGGTCCAACTTATTTG    5062
gb:MT020881|Organism:Severe      CACACGCAAGTTGTGGACATGTCAATGACATATGGACAACAGTTTGGTCCAACTTATTTG    5062
gb:MT020880|Organism:Severe      CACACGCAAGTTGTGGACATGTCAATGACATATGGACAACAGTTTGGTCCAACTTATTTG    5062
gb:MT066175|Organism:Severe      CACACGCAAGTTGTGGACATGTCAATGACATATGGACAACAGTTTGGTCCAACTTATTTG    5062
gb:MN997409|Organism:Severe      CACACGCAAGTTGTGGACATGTCAATGACATATGGACAACAGTTTGGTCCAACTTATTTG    5062
gb:MN938384|Organism:Severe      CACACGCAAGTTGTGGACATGTCAATGACATATGGACAACAGTTTGGTCCAACTTATTTG    5030
gb:MT044258|Organism:Severe      CACACGCAAGTTGTGGACATGTCAATGACATATGGACAACAGTTTGGTCCAACTTATTTG    5038
gb:MT039890|Organism:Severe      CACACGCAAGTTGTGGACATGTCAATGACATATGGACAACAGTTTGGTCCAACTTATTTG    5062
gb:MN988713|Organism:Severe      CACACGCAAGTTGTGGACATGTCAATGACATATGGACAACAGTTTGGTCCAACTTATTTG    5062
gb:LC521925|Organism:Severe      CACACGCAAGTTGTGGACATGTCAATGACATATGGACAACAGTTTGGTCCAACTTATTTG    5035
gb:MT093571|Organism:Severe      CACACGCAAGTTGTGGACATGTCAATGACATATGGACAACAGTTTGGTCCAACTTATTTG    5062
gb:MT039887|Organism:Severe      CACACGCAAGTTGTGGACATGTCAATGACATATGGACAACAGTTTGGTCCAACTTATTTG    5062
gb:MT019530|Organism:Severe      CACACGCAAGTTGTGGACATGTCAATGACATATGGACAACAGTTTGGTCCAACTTATTTG    5062
gb:MT039888|Organism:Severe      CACACGCAAGTTGTGGACATGTCAATGACATATGGACAACAGTTTGGTCCAACTTATTTG    5062
gb:LC522972|Organism:Severe      CACACGCAAGTTGTGGACATGTCAATGACATATGGACAACAGTTTGGTCCAACTTATTTG    5059
gb:MT027063|Organism:Severe      CACACGCAAGTTGTGGACATGTCAATGACATATGGACAACAGTTTGGTCCAACTTATTTG    5062
gb:MT027062|Organism:Severe      CACACGCAAGTTGTGGACATGTCAATGACATATGGACAACAGTTTGGTCCAACTTATTTG    5062
gb:MT019529|Organism:Severe      CACACGCAAGTTGTGGACATGTCAATGACATATGGACAACAGTTTGGTCCAACTTATTTG    5062
gb:MN996529|Organism:Severe      CACACGCAAGTTGTGGACATGTCAATGACATATGGACAACAGTTTGGTCCAACTTATTTG    5050
gb:MN996531|Organism:Severe      CACACGCAAGTTGTGGACATGTCAATGACATATGGACAACAGTTTGGTCCAACTTATTTG    5049
gb:MT066176|Organism:Severe      CACACGCAAGTTGTGGACATGTCAATGACATATGGACAACAGTTTGGTCCAACTTATTTG    5062
```

```
gb:MT027064|Organism:Severe      CACACGCAAGTTGTGGACATGTCAATGACATATGGACAACAGTTTGGTCCAACTTATTTG	5062
gb:MN994468|Organism:Severe      CACACGCAAGTTGTGGACATGTCAATGACATATGGACAACAGTTTGGTCCAACTTATTTG	5062
gb:MT072688|Organism:Severe      CACACGCAAGTTGTGGACATGTCAATGACATATGGACAACAGTTTGGTCCAACTTATTTG	5047
gb:MN996527|Organism:Severe      CACACGCAAGTTGTGGACATGTCAATGACATATGGACAACAGTTTGGTCCAACTTATTTG	5029
gb:MT093631|Organism:Severe      CACACGCAAGTTGTGGACATGTCAATGACATATGGACAACAGTTTGGTCCAACTTATTTG	5100
gb:MT106053|Organism:Severe      CACACGCAAGTTGTGGACATGTCAATGACATATGGACAACAGTTTGGTCCAACTTATTTG	5062
gb:MT019533|Organism:Severe      CACACGCAAGTTGTGGACATGTCAATGACATATGGACAACAGTTTGGTCCAACTTATTTG	5062
gb:MT019531|Organism:Severe      CACACGCAAGTTGTGGACATGTCAATGACATATGGACAACAGTTTGGTCCAACTTATTTG	5062
gb:MN996528|Organism:Severe      CACACGCAAGTTGTGGACATGTCAATGACATATGGACAACAGTTTGGTCCAACTTATTTG	5062
gb:MN996530|Organism:Severe      CACACGCAAGTTGTGGACATGTCAATGACATATGGACAACAGTTTGGTCCAACTTATTTG	5048
gb:MN908947|Organism:Severe      CACACGCAAGTTGTGGACATGTCAATGACATATGGACAACAGTTTGGTCCAACTTATTTG	5062
gb:MT019532|Organism:Severe      CACACGCAAGTTGTGGACATGTCAATGACATATGGACAACAGTTTGGTCCAACTTATTTG	5062
                                 ************************************************************

gb:MT020781|Organism:Severe      GATGGAGCTGATGTTACTAAAATAAAACCTCATAATTCACATGAAGGTAAAACATTTTAT	5110
gb:MT007544|Organism:Severe      GATGGAGCTGATGTTACTAAAATAAAACCTCATAATTCACATGAAGGTAAAACATTTTAT	5122
gb:MN994467|Organism:Severe      GATGGAGCTGATGTTACTAAAATAAAACCTCATAATTCACATGAAGGTAAAACATTTTAT	5122
gb:MT044257|Organism:Severe      GATGGAGCTGATGTTACTAAAATAAAACCTCATAATTCACATGAAGGTAAAACATTTTAT	5122
gb:MT106054|Organism:Severe      GATGGAGCTGATGTTACTAAAATAAAACCTCATAATTCACATGAAGGTAAAACATTTTAT	5122
gb:MT049951|Organism:Severe      GATGGAGCTGATGTTACTAAAATAAAACCTCATAATTCACATGAAGGTAAAACATTTTAT	5122
gb:MN975262|Organism:Severe      GATGGAGCTGATGTTACTAAAATAAAACCTCATAATTCACATGAAGGTAAAACATTTTAT	5122
gb:MT106052|Organism:Severe      GATGGAGCTGATGTTACTAAAATAAAACCTCATAATTCACATGAAGGTAAAACATTTTAT	5122
gb:LC522975|Organism:Severe      GATGGAGCTGATGTTACTAAAATAAAACCTCATAATTCACATGAAGGTAAAACATTTTAT	5119
gb:LC522973|Organism:Severe      GATGGAGCTGATGTTACTAAAATAAAACCTCATAATTCACATGAAGGTAAAACATTTTAT	5119
gb:LC522974|Organism:Severe      GATGGAGCTGATGTTACTAAAATAAAACCTCATAATTCACATGAAGGTAAAACATTTTAT	5119
gb:MN985325|Organism:Severe      GATGGAGCTGATGTTACTAAAATAAAACCTCATAATTCACATGAAGGTAAAACATTTTAT	5122
gb:MT020881|Organism:Severe      GATGGAGCTGATGTTACTAAAATAAAACCTCATAATTCACATGAAGGTAAAACATTTTAT	5122
gb:MT020880|Organism:Severe      GATGGAGCTGATGTTACTAAAATAAAACCTCATAATTCACATGAAGGTAAAACATTTTAT	5122
gb:MT066175|Organism:Severe      GATGGAGCTGATGTTACTAAAATAAAACCTCATAATTCACATGAAGGTAAAACATTTTAT	5122
gb:MN997409|Organism:Severe      GATGGAGCTGATGTTACTAAAATAAAACCTCATAATTCACATGAAGGTAAAACATTTTAT	5122
gb:MN938384|Organism:Severe      GATGGAGCTGATGTTACTAAAATAAAACCTCATAATTCACATGAAGGTAAAACATTTTAT	5090
gb:MT044258|Organism:Severe      GATGGAGCTGATGTTACTAAAATAAAACCTCATAATTCACATGAAGGTAAAACATTTTAT	5098
gb:MT039890|Organism:Severe      GATGGAGCTGATGTTACTAAAATAAAACCTCATAATTCACATGAAGGTAAAACATTTTAT	5122
gb:MN988713|Organism:Severe      GATGGAGCTGATGTTACTAAAATAAAACCTCATAATTCACATGAAGGTAAAACATTTTAT	5122
gb:LC521925|Organism:Severe      GATGGAGCTGATGTTACTAAAATAAAACCTCATAATTCACATGAAGGTAAAACATTTTAT	5095
gb:MT093571|Organism:Severe      GATGGAGCTGATGTTACTAAAATAAAACCTCATAATTCACATGAAGGTAAAACATTTTAT	5122
gb:MT039887|Organism:Severe      GATGGAGCTGATGTTACTAAAATAAAACCTCATAATTCACATGAAGGTAAAACATTTTAT	5122
gb:MT019530|Organism:Severe      GATGGAGCTGATGTTACTAAAATAAAACCTCATAATTCACATGAAGGTAAAACATTTTAT	5122
gb:MT039888|Organism:Severe      GATGGAGCTGATGTTACTAAAATAAAACCTCATAATTCACATGAAGGTAAAACATTTTAT	5122
gb:LC522972|Organism:Severe      GATGGAGCTGATGTTACTAAAATAAAACCTCATAATTCACATGAAGGTAAAACATTTTAT	5119
gb:MT027063|Organism:Severe      GATGGAGCTGATGTTACTAAAGTAAAACCTCATAATTCACATGAAGGTAAAACATTTTAT	5122
gb:MT027062|Organism:Severe      GATGGAGCTGATGTTACTAAAGTAAAACCTCATAATTCACATGAAGGTAAAACATTTTAT	5122
gb:MT019529|Organism:Severe      GATGGAGCTGATGTTACTAAAATAAAACCTCATAATTCACATGAAGGTAAAACATTTTAT	5122
gb:MN996529|Organism:Severe      GATGGAGCTGATGTTACTAAAATAAAACCTCATAATTCACATGAAGGTAAAACATTTTAT	5110
gb:MN996531|Organism:Severe      GATGGAGCTGATGTTACTAAAATAAAACCTCATAATTCACATGAAGGTAAAACATTTTAT	5109
gb:MT066176|Organism:Severe      GATGGAGCTGATGTTACTAAAATAAAACCTCATAATTCACATGAAGGTAAAACATTTTAT	5122
gb:MT027064|Organism:Severe      GATGGAGCTGATGTTACTAAAATAAAACCTCATAATTCACATGAAGGTAAAACATTTTAT	5122
gb:MN994468|Organism:Severe      GATGGAGCTGATGTTACTAAAATAAAACCTCATAATTCACATGAAGGTAAAACATTTTAT	5122
gb:MT072688|Organism:Severe      GATGGAGCTGATGTTACTAAAATAAAACCTCATAATTCACATGAAGGTAAAACATTTTAT	5107
gb:MN996527|Organism:Severe      GATGGAGCTGATGTTACTAAAATAAAACCTCATAATTCACATGAAGGTAAAACATTTTAT	5089
gb:MT093631|Organism:Severe      GATGGAGCTGATGTTACTAAAATAAAACCTCATAATTCACATGAAGGTAAAACATTTTAT	5160
gb:MT106053|Organism:Severe      GATGGAGCTGATGTTACTAAAATAAAACCTCATAATTCACATGAAGGTAAAACATTTTAT	5122
gb:MT019533|Organism:Severe      GATGGAGCTGATGTTACTAAAATAAAACCTCATAATTCACATGAAGGTAAAACATTTTAT	5122
gb:MT019531|Organism:Severe      GATGGAGCTGATGTTACTAAAATAAAACCTCATAATTCACATGAAGGTAAAACATTTTAT	5122
gb:MN996528|Organism:Severe      GATGGAGCTGATGTTACTAAAATAAAACCTCATAATTCACATGAAGGTAAAACATTTTAT	5122
gb:MN996530|Organism:Severe      GATGGAGCTGATGTTACTAAAATAAAACCTCATAATTCACATGAAGGTAAAACATTTTAT	5108
gb:MN908947|Organism:Severe      GATGGAGCTGATGTTACTAAAATAAAACCTCATAATTCACATGAAGGTAAAACATTTTAT	5122
gb:MT019532|Organism:Severe      GATGGAGCTGATGTTACTAAAATAAAACCTCATAATTCACATGAAGGTAAAACATTTTAT	5122
                                 ********************* **************************************

gb:MT020781|Organism:Severe      GTTTTACCTAATGATGACACTCTACGTGTTGAGGCTTTTGAGTACTACCACACAACTGAT	5170
gb:MT007544|Organism:Severe      GTTTTACCTAATGATGACACTCTACGTGTTGAGGCTTTTGAGTACTACCACACAACTGAT	5182
gb:MN994467|Organism:Severe      GTTTTACCTAATGATGACACTCTACGTGTTGAGGCTTTTGAGTACTACCACACAACTGAT	5182
gb:MT044257|Organism:Severe      GTTTTACCTAATGATGACACTCTACGTGTTGAGGCTTTTGAGTACTACCACACAACTGAT	5182
gb:MT106054|Organism:Severe      GTTTTACCTAATGATGACACTCTACGTGTTGAGGCTTTTGAGTACTACCACACAACTGAT	5182
```

```
gb:MT049951|Organism:Severe      GTTTTACCTAATGATGACACTCTACGTGTTGAGGCTTTTGAGTACTACCACACAACTGAT    5182
gb:MN975262|Organism:Severe      GTTTTACCTAATGATGACACTCTACGTGTTGAGGCTTTTGAGTACTACCACACAACTGAT    5182
gb:MT106052|Organism:Severe      GTTTTACCTAATGATGACACTCTACGTGTTGAGGCTTTTGAGTACTACCACACAACTGAT    5182
gb:LC522975|Organism:Severe      GTTTTACCTAATGATGACACTCTACGTGTTGAGGCTTTTGAGTACTACCACACAACTGAT    5179
gb:LC522973|Organism:Severe      GTTTTACCTAATGATGACACTCTACGTGTTGAGGCTTTTGAGTACTACCACACAACTGAT    5179
gb:LC522974|Organism:Severe      GTTTTACCTAATGATGACACTCTACGTGTTGAGGCTTTTGAGTACTACCACACAACTGAT    5179
gb:MN985325|Organism:Severe      GTTTTACCTAATGATGACACTCTACGTGTTGAGGCTTTTGAGTACTACCACACAACTGAT    5182
gb:MT020881|Organism:Severe      GTTTTACCTAATGATGACACTCTACGTGTTGAGGCTTTTGAGTACTACCACACAACTGAT    5182
gb:MT020880|Organism:Severe      GTTTTACCTAATGATGACACTCTACGTGTTGAGGCTTTTGAGTACTACCACACAACTGAT    5182
gb:MT066175|Organism:Severe      GTTTTACCTAATGATGACACTCTACGTGTTGAGGCTTTTGAGTACTACCACACAACTGAT    5182
gb:MN997409|Organism:Severe      GTTTTACCTAATGATGACACTCTACGTGTTGAGGCTTTTGAGTACTACCACACAACTGAT    5182
gb:MN938384|Organism:Severe      GTTTTACCTAATGATGACACTCTACGTGTTGAGGCTTTTGAGTACTACCACACAACTGAT    5150
gb:MT044258|Organism:Severe      GTTTTACCTAATGATGACACTCTACGTGTTGAGGCTTTTGAGTACTACCACACAACTGAT    5158
gb:MT039890|Organism:Severe      GTTTTACCTAATGATGACACTCTACGTGTTGAGGCTTTTGAGTACTACCACACAACTGAT    5182
gb:MN988713|Organism:Severe      GTTTTACCTAATGATGACACTCTACGTGTTGAGGCTTTTGAGTACTACCACACAACTGAT    5182
gb:LC521925|Organism:Severe      GTTTTACCTAATGATGACACTCTACGTGTTGAGGCTTTTGAGTACTACCACACAACTGAT    5155
gb:MT093571|Organism:Severe      GTTTTACCTAATGATGACACTCTACGTGTTGAGGCTTTTGAGTACTACCACACAACTGAT    5182
gb:MT039887|Organism:Severe      GTTTTACCTAATGATGACACTCTACGTGTTGAGGCTTTTGAGTACTACCACACAACTGAT    5182
gb:MT019530|Organism:Severe      GTTTTACCTAATGATGACACTCTACGTGTTGAGGCTTTTGAGTACTACCACACAACTGAT    5182
gb:MT039888|Organism:Severe      GTTTTACCTAATGATGACACTCTACGTGTTGAGGCTTTTGAGTACTACCACACAACTGAT    5182
gb:LC522972|Organism:Severe      GTTTTACCTAATGATGACACTCTACGTGTTGAGGCTTTTGAGTACTACCACACAACTGAT    5179
gb:MT027063|Organism:Severe      GTTTTACCTAATGATGACACTCTACGTGTTGAGGCTTTTGAGTACTACCACACAACTGAT    5182
gb:MT027062|Organism:Severe      GTTTTACCTAATGATGACACTCTACGTGTTGAGGCTTTTGAGTACTACCACACAACTGAT    5182
gb:MT019529|Organism:Severe      GTTTTACCTAATGATGACACTCTACGTGTTGAGGCTTTTGAGTACTACCACACAACTGAT    5182
gb:MN996529|Organism:Severe      GTTTTACCTAATGATGACACTCTACGTGTTGAGGCTTTTGAGTACTACCACACAACTGAT    5170
gb:MN996531|Organism:Severe      GTTTTACCTAATGATGACACTCTACGTGTTGAGGCTTTTGAGTACTACCACACAACTGAT    5169
gb:MT066176|Organism:Severe      GTTTTACCTAATGATGACACTCTACGTGTTGAGGCTTTTGAGTACTACCACACAACTGAT    5182
gb:MT027064|Organism:Severe      GTTTTACCTAATGATGACACTCTACGTGTTGAGGCTTTTGAGTACTACCACACAACTGAT    5182
gb:MN994468|Organism:Severe      GTTTTACCTAATGATGACACTCTACGTGTTGAGGCTTTTGAGTACTACCACACAACTGAT    5182
gb:MT072688|Organism:Severe      GTTTTACCTAATGATGACACTCTACGTGTTGAGGCTTTTGAGTACTACCACACAACTGAT    5167
gb:MN996527|Organism:Severe      GTTTTACCTAATGATGACACTCTACGTGTTGAGGCTTTTGAGTACTACCACACAACTGAT    5149
gb:MT093631|Organism:Severe      GTTTTACCTAATGATGACACTCTACGTGTTGAGGCTTTTGAGTACTACCACACAACTGAT    5220
gb:MT106053|Organism:Severe      GTTTTACCTAATGATGACACTCTACGTGTTGAGGCTTTTGAGTACTACCACACAACTGAT    5182
gb:MT019533|Organism:Severe      GTTTTACCTAATGATGACACTCTACGTGTTGAGGCTTTTGAGTACTACCACACAACTGAT    5182
gb:MT019531|Organism:Severe      GTTTTACCTAATGATGACACTCTACGTGTTGAGGCTTTTGAGTACTACCACACAACTGAT    5182
gb:MN996528|Organism:Severe      GTTTTACCTAATGATGACACTCTACGTGTTGAGGCTTTTGAGTACTACCACACAACTGAT    5182
gb:MN996530|Organism:Severe      GTTTTACCTAATGATGACACTCTACGTGTTGAGGCTTTTGAGTACTACCACACAACTGAT    5168
gb:MN908947|Organism:Severe      GTTTTACCTAATGATGACACTCTACGTGTTGAGGCTTTTGAGTACTACCACACAACTGAT    5182
gb:MT019532|Organism:Severe      GTTTTACCTAATGATGACACTCTACGTGTTGAGGCTTTTGAGTACTACCACACAACTGAT    5182
                                 ************************************************************

gb:MT020781|Organism:Severe      CCTAGTTTTCTGGGTAGGTACATGTCAGCATTAAATCACACTAAAAAGTGGAAATACCCA    5230
gb:MT007544|Organism:Severe      CCTAGTTTTCTGGGTAGGTACATGTCAGCATTAAATCACACTAAAAAGTGGAAATACCCA    5242
gb:MN994467|Organism:Severe      CCTAGTTTTCTGGGTAGGTACATGTCAGCATTAAATCACACTAAAAAGTGGAAATACCCA    5242
gb:MT044257|Organism:Severe      CCTAGTTTTCTGGGTAGGTACATGTCAGCATTAAATCACACTAAAAAGTGGAAATACCCA    5242
gb:MT106054|Organism:Severe      CCTAGTTTTCTGGGTAGGTACATGTCAGCATTAAATCACACTAAAAAGTGGAAATACCCA    5242
gb:MT049951|Organism:Severe      CCTAGTTTTCTGGGTAGGTACATGTCAGCATTAAATCACACTAAAAAGTGGAAATACCCA    5242
gb:MN975262|Organism:Severe      CCTAGTTTTCTGGGTAGGTACATGTCAGCATTAAATCACACTAAAAAGTGGAAATACCCA    5242
gb:MT106052|Organism:Severe      CCTAGTTTTCTGGGTAGGTACATGTCAGCATTAAATCACACTAAAAAGTGGAAATACCCA    5242
gb:LC522975|Organism:Severe      CCTAGTTTTCTGGGTAGGTACATGTCAGCATTAAATCACACTAAAAAGTGGAAATACCCA    5239
gb:LC522973|Organism:Severe      CCTAGTTTTCTGGGTAGGTACATGTCAGCATTAAATCACACTAAAAAGTGGAAATACCCA    5239
gb:LC522974|Organism:Severe      CCTAGTTTTCTGGGTAGGTACATGTCAGCATTAAATCACACTAAAAAGTGGAAATACCCA    5239
gb:MN985325|Organism:Severe      CCTAGTTTTCTGGGTAGGTACATGTCAGCATTAAATCACACTAAAAAGTGGAAATACCCA    5242
gb:MT020881|Organism:Severe      CCTAGTTTTCTGGGTAGGTACATGTCAGCATTAAATCACACTAAAAAGTGGAAATACCCA    5242
gb:MT020880|Organism:Severe      CCTAGTTTTCTGGGTAGGTACATGTCAGCATTAAATCACACTAAAAAGTGGAAATACCCA    5242
gb:MT066175|Organism:Severe      CCTAGTTTTCTGGGTAGGTACATGTCAGCATTAAATCACACTAAAAAGTGGAAATACCCA    5242
gb:MN997409|Organism:Severe      CCTAGTTTTCTGGGTAGGTACATGTCAGCATTAAATCACACTAAAAAGTGGAAATACCCA    5242
gb:MN938384|Organism:Severe      CCTAGTTTTCTGGGTAGGTACATGTCAGCATTAAATCACACTAAAAAGTGGAAATACCCA    5210
gb:MT044258|Organism:Severe      CCTAGTTTTCTGGGTAGGTACATGTCAGCATTAAATCACACTAAAAAGTGGAAATACCCA    5218
gb:MT039890|Organism:Severe      CCTAGTTTTCTGGGTAGGTACATGTCAGCATTAAATCACACTAAAAAGTGGAAATACCCA    5242
gb:MN988713|Organism:Severe      CCTAGTTTTCTGGGTAGGTACATGTCAGCATTAAATCACACTAAAAAGTGGAAATACCCA    5242
gb:LC521925|Organism:Severe      CCTAGTTTTCTGGGTAGGTACATGTCAGCATTAAATCACACTAAAAAGTGGAAATACCCA    5215
gb:MT093571|Organism:Severe      CCTAGTTTTCTGGGTAGGTACATGTCAGCATTAAATCACACTAAAAAGTGGAAATACCCA    5242
gb:MT039887|Organism:Severe      CCTAGTTTTCTGGGTAGGTACATGTCAGCATTAAATCACACTAAAAAGTGGAAATACCCA    5242
gb:MT019530|Organism:Severe      CCTAGTTTTCTGGGTAGGTACATGTCAGCATTAAATCACACTAAAAAGTGGAAATACCCA    5242
```

```
gb:MT039888|Organism:Severe      CCTAGTTTTCTGGGTAGGTACATGTCAGCATTAAATCACACTAAAAAGTGGAAATACCCA    5242
gb:LC522972|Organism:Severe      CCTAGTTTTCTGGGTAGGTACATGTCAGCATTAAATCACACTAAAAAGTGGAAATACCCA    5239
gb:MT027063|Organism:Severe      CCTAGTTTTCTGGGTAGGTACATGTCAGCATTAAATCACACTAAAAAGTGGAAATACCCA    5242
gb:MT027062|Organism:Severe      CCTAGTTTTCTGGGTAGGTACATGTCAGCATTAAATCACACTAAAAAGTGGAAATACCCA    5242
gb:MT019529|Organism:Severe      CCTAGTTTTCTGGGTAGGTACATGTCAGCATTAAATCACACTAAAAAGTGGAAATACCCA    5242
gb:MN996529|Organism:Severe      CCTAGTTTTCTGGGTAGGTACATGTCAGCATTAAATCACACTAAAAAGTGGAAATACCCA    5230
gb:MN996531|Organism:Severe      CCTAGTTTTCTGGGTAGGTACATGTCAGCATTAAATCACACTAAAAAGTGGAAATACCCA    5229
gb:MT066176|Organism:Severe      CCTAGTTTTCTGGGTAGGTACATGTCAGCATTAAATCACACTAAAAAGTGGAAATACCCA    5242
gb:MT027064|Organism:Severe      CCTAGTTTTCTGGGTAGGTACATGTCAGCATTAAATCACACTAAAAAGTGGAAATACCCA    5242
gb:MN994468|Organism:Severe      CCTAGTTTTCTGGGTAGGTACATGTCAGCATTAAATCACACTAAAAAGTGGAAATACCCA    5242
gb:MT072688|Organism:Severe      CCTAGTTTTCTGGGTAGGTACATGTCAGCATTAAATCACACTAAAAAGTGGAAATACCCA    5227
gb:MN996527|Organism:Severe      CCTAGTTTTCTGGGTAGGTACATGTCAGCATTAAATCACACTAAAAAGTGGAAATACCCA    5209
gb:MT093631|Organism:Severe      CCTAGTTTTCTGGGTAGGTACATGTCAGCATTAAATCACACTAAAAAGTGGAAATACCCA    5280
gb:MT106053|Organism:Severe      CCTAGTTTTCTGGGTAGGTACATGTCAGCATTAAATCACACTAAAAAGTGGAAATACCCA    5242
gb:MT019533|Organism:Severe      CCTAGTTTTCTGGGTAGGTACATGTCAGCATTAAATCACACTAAAAAGTGGAAATACCCA    5242
gb:MT019531|Organism:Severe      CCTAGTTTTCTGGGTAGGTACATGTCAGCATTAAATCACACTAAAAAGTGGAAATACCCA    5242
gb:MN996528|Organism:Severe      CCTAGTTTTCTGGGTAGGTACATGTCAGCATTAAATCACACTAAAAAGTGGAAATACCCA    5242
gb:MN996530|Organism:Severe      CCTAGTTTTCTGGGTAGGTACATGTCAGCATTAAATCACACTAAAAAGTGGAAATACCCA    5228
gb:MN908947|Organism:Severe      CCTAGTTTTCTGGGTAGGTACATGTCAGCATTAAATCACACTAAAAAGTGGAAATACCCA    5242
gb:MT019532|Organism:Severe      CCTAGTTTTCTGGGTAGGTACATGTCAGCATTAAATCACACTAAAAAGTGGAAATACCCA    5242
                                 ************************************************************

gb:MT020781|Organism:Severe      CAAGTTAATGGTTTAACTTCTATTAAATGGGCAGATAACAACTGTTATCTTGCCACTGCA    5290
gb:MT007544|Organism:Severe      CAAGTTAATGGTTTAACTTCTATTAAATGGGCAGATAACAACTGTTATCTTGCCACTGCA    5302
gb:MN994467|Organism:Severe      CAAGTTAATGGTTTAACTTCTATTAAATGGGCAGATAACAACTGTTATCTTGCCACTGCA    5302
gb:MT044257|Organism:Severe      CAAGTTAATGGTTTAACTTCTATTAAATGGGCAGATAACAACTGTTATCTTGCCACTGCA    5302
gb:MT106054|Organism:Severe      CAAGTTAATGGTTTAACTTCTATTAAATGGGCAGATAACAACTGTTATCTTGCCACTGCA    5302
gb:MT049951|Organism:Severe      CAAGTTAATGGTTTAACTTCTATTAAATGGGCAGATAACAACTGTTATCTTGCCACTGCA    5302
gb:MN975262|Organism:Severe      CAAGTTAATGGTTTAACTTCTATTAAATGGGCAGATAACAACTGTTATCTTGCCACTGCA    5302
gb:MT106052|Organism:Severe      CAAGTTAATGGTTTAACTTCTATTAAATGGGCAGATAACAACTGTTATCTTGCCACTGCA    5302
gb:LC522975|Organism:Severe      CAAGTTAATGGTTTAACTTCTATTAAATGGGCAGATAACAACTGTTATCTTGCCACTGCA    5299
gb:LC522973|Organism:Severe      CAAGTTAATGGTTTAACTTCTATTAAATGGGCAGATAACAACTGTTATCTTGCCACTGCA    5299
gb:LC522974|Organism:Severe      CAAGTTAATGGTTTAACTTCTATTAAATGGGCAGATAACAACTGTTATCTTGCCACTGCA    5299
gb:MN985325|Organism:Severe      CAAGTTAATGGTTTAACTTCTATTAAATGGGCAGATAACAACTGTTATCTTGCCACTGCA    5302
gb:MT020881|Organism:Severe      CAAGTTAATGGTTTAACTTCTATTAAATGGGCAGATAACAACTGTTATCTTGCCACTGCA    5302
gb:MT020880|Organism:Severe      CAAGTTAATGGTTTAACTTCTATTAAATGGGCAGATAACAACTGTTATCTTGCCACTGCA    5302
gb:MT066175|Organism:Severe      CAAGTTAATGGTTTAACTTCTATTAAATGGGCAGATAACAACTGTTATCTTGCCACTGCA    5302
gb:MN997409|Organism:Severe      CAAGTTAATGGTTTAACTTCTATTAAATGGGCAGATAACAACTGTTATCTTGCCACTGCA    5302
gb:MN938384|Organism:Severe      CAAGTTAATGGTTTAACTTCTATTAAATGGGCAGATAACAACTGTTATCTTGCCACTGCA    5270
gb:MT044258|Organism:Severe      CAAGTTAATGGTTTAACTTCTATTAAATGGGCAGATAACAACTGTTATCTTGCCACTGCA    5278
gb:MT039890|Organism:Severe      CAAGTTAATGGTTTAACTTCTATTAAATGGGCAGATAACAACTGTTATCTTGCCACTGCA    5302
gb:MN988713|Organism:Severe      CAAGTTAATGGTTTAACTTCTATTAAATGGGCAGATAACAACTGTTATCTTGCCACTGCA    5302
gb:LC521925|Organism:Severe      CAAGTTAATGGTTTAACTTCTATTAAATGGGCAGATAACAACTGTTATCTTGCCACTGCA    5275
gb:MT093571|Organism:Severe      CAAGTTAATGGTTTAACTTCTATTAAATGGGCAGATAACAACTGTTATCTTGCCACTGCA    5302
gb:MT039887|Organism:Severe      CAAGTTAATGGTTTAACTTCTATTAAATGGGCAGATAACAACTGTTATCTTGCCACTGCA    5302
gb:MT019530|Organism:Severe      CAAGTTAATGGTTTAACTTCTATTAAATGGGCAGATAACAACTGTTATCTTGCCACTGCA    5302
gb:MT039888|Organism:Severe      CAAGTTAATGGTTTAACTTCTATTAAATGGGCAGATAACAACTGTTATCTTGCCACTGCA    5302
gb:LC522972|Organism:Severe      CAAGTTAATGGTTTAACTTCTATTAAATGGGCAGATAACAACTGTTATCTTGCCACTGCA    5299
gb:MT027063|Organism:Severe      CAAGTTAATGGTTTAACTTCTATTAAATGGGCAGATAACAACTGTTATCTTGCCACTGCA    5302
gb:MT027062|Organism:Severe      CAAGTTAATGGTTTAACTTCTATTAAATGGGCAGATAACAACTGTTATCTTGCCACTGCA    5302
gb:MT019529|Organism:Severe      CAAGTTAATGGTTTAACTTCTATTAAATGGGCAGATAACAACTGTTATCTTGCCACTGCA    5302
gb:MN996529|Organism:Severe      CAAGTTAATGGTTTAACTTCTATTAAATGGGCAGATAACAACTGTTATCTTGCCACTGCA    5290
gb:MN996531|Organism:Severe      CAAGTTAATGGTTTAACTTCTATTAAATGGGCAGATAACAACTGTTATCTTGCCACTGCA    5289
gb:MT066176|Organism:Severe      CAAGTTAATGGTTTAACTTCTATTAAATGGGCAGATAACAACTGTTATCTTGCCACTGCA    5302
gb:MT027064|Organism:Severe      CAAGTTAATGGTTTAACTTCTATTAAATGGGCAGATAACAACTGTTATCTTGCCACTGCA    5302
gb:MN994468|Organism:Severe      CAAGTTAATGGTTTAACTTCTATTAAATGGGCAGATAACAACTGTTATCTTGCCACTGCA    5302
gb:MT072688|Organism:Severe      CAAGTTAATGGTTTAACTTCTATTAAATGGGCAGATAACAACTGTTATCTTGCCACTGCA    5287
gb:MN996527|Organism:Severe      CAAGTTAATGGTTTAACTTCTATTAAATGGGCAGATAACAACTGTTATCTTGCCACTGCA    5269
gb:MT093631|Organism:Severe      CAAGTTAATGGTTTAACTTCTATTAAATGGGCAGATAACAACTGTTATCTTGCCACTGCA    5340
gb:MT106053|Organism:Severe      CAAGTTAATGGTTTAACTTCTATTAAATGGGCAGATAACAACTGTTATCTTGCCACTGCA    5302
gb:MT019533|Organism:Severe      CAAGTTAATGGTTTAACTTCTATTAAATGGGCAGATAACAACTGTTATCTTGCCACTGCA    5302
gb:MT019531|Organism:Severe      CAAGTTAATGGTTTAACTTCTATTAAATGGGCAGATAACAACTGTTATCTTGCCACTGCA    5302
gb:MN996528|Organism:Severe      CAAGTTAATGGTTTAACTTCTATTAAATGGGCAGATAACAACTGTTATCTTGCCACTGCA    5302
gb:MN996530|Organism:Severe      CAAGTTAATGGTTTAACTTCTATTAAATGGGCAGATAACAACTGTTATCTTGCCACTGCA    5288
gb:MN908947|Organism:Severe      CAAGTTAATGGTTTAACTTCTATTAAATGGGCAGATAACAACTGTTATCTTGCCACTGCA    5302
```

```
gb:MT019532|Organism:Severe      CAAGTTAATGGTTTAACTTCTATTAAATGGGCAGATAACAACTGTTATCTTGCCACTGCA   5302
                                 ************************************************************

gb:MT020781|Organism:Severe      TTGTTAACACTCCAACAAATAGAGTTGAAGTTTAATCCACCTGCTCTACAAGATGCTTAT   5350
gb:MT007544|Organism:Severe      TTGTTAACACTCCAACAAATAGAGTTGAAGTTTAATCCACCTGCTCTACAAGATGCTTAT   5362
gb:MN994467|Organism:Severe      TTGTTAACACTCCAACAAATAGAGTTGAAGTTTAATCCACCTGCTCTACAAGATGCTTAT   5362
gb:MT044257|Organism:Severe      TTGTTAACACTCCAACAAATAGAGTTGAAGTTTAATCCACCTGCTCTACAAGATGCTTAT   5362
gb:MT106054|Organism:Severe      TTGTTAACACTCCAACAAATAGAGTTGAAGTTTAATCCACCTGCTCTACAAGATGCTTAT   5362
gb:MT049951|Organism:Severe      TTGTTAACACTCCAACAAATAGAGTTGAAGTTTAATCCACCTGCTCTACAAGATGCTTAT   5362
gb:MN975262|Organism:Severe      TTGTTAACACTCCAACAAATAGAGTTGAAGTTTAATCCACCTGCTCTACAAGATGCTTAT   5362
gb:MT106052|Organism:Severe      TTGTTAACACTCCAACAAATAGAGTTGAAGTTTAATCCACCTGCTCTACAAGATGCTTAT   5362
gb:LC522975|Organism:Severe      TTGTTAACACTCCAACAAATAGAGTTGAAGTTTAATCCACCTGCTCTACAAGATGCTTAT   5359
gb:LC522973|Organism:Severe      TTGTTAACACTCCAACAAATAGAGTTGAAGTTTAATCCACCTGCTCTACAAGATGCTTAT   5359
gb:LC522974|Organism:Severe      TTGTTAACACTCCAACAAATAGAGTTGAAGTTTAATCCACCTGCTCTACAAGATGCTTAT   5359
gb:MN985325|Organism:Severe      TTGTTAACACTCCAACAAATAGAGTTGAAGTTTAATCCACCTGCTCTACAAGATGCTTAT   5362
gb:MT020881|Organism:Severe      TTGTTAACACTCCAACAAATAGAGTTGAAGTTTAATCCACCTGCTCTACAAGATGCTTAT   5362
gb:MT020880|Organism:Severe      TTGTTAACACTCCAACAAATAGAGTTGAAGTTTAATCCACCTGCTCTACAAGATGCTTAT   5362
gb:MT066175|Organism:Severe      TTGTTAACACTCCAACAAATAGAGTTGAAGTTTAATCCACCTGCTCTACAAGATGCTTAT   5362
gb:MN997409|Organism:Severe      TTGTTAACACTCCAACAAATAGAGTTGAAGTTTAATCCACCTGCTCTACAAGATGCTTAT   5362
gb:MN938384|Organism:Severe      TTGTTAACACTCCAACAAATAGAGTTGAAGTTTAATCCACCTGCTCTACAAGATGCTTAT   5330
gb:MT044258|Organism:Severe      TTGTTAACACTCCAACAAATAGAGTTGAAGTTTAATCCACCTGCTCTACAAGATGCTTAT   5338
gb:MT039890|Organism:Severe      TTGTTAACACTCCAACAAATAGAGTTGAAGTTTAATCCACCTGCTCTACAAGATGCTTAT   5362
gb:MN988713|Organism:Severe      TTGTTAACACTCCAACAAATAGAGTTGAAGTTTAATCCACCTGCTCTACAAGATGCTTAT   5362
gb:LC521925|Organism:Severe      TTGTTAACACTCCAACAAATAGAGTTGAAGTTTAATCCACCTGCTCTACAAGATGCTTAT   5335
gb:MT093571|Organism:Severe      TTGTTAACACTCCAACAAATAGAGTTGAAGTTTAATCCACCTGCTCTACAAGATGCTTAT   5362
gb:MT039887|Organism:Severe      TTGTTAACACTCCAACAAATAGAGTTGAAGTTTAATCCACCTGCTCTACAAGATGCTTAT   5362
gb:MT019530|Organism:Severe      TTGTTAACACTCCAACAAATAGAGTTGAAGTTTAATCCACCTGCTCTACAAGATGCTTAT   5362
gb:MT039888|Organism:Severe      TTGTTAACACTCCAACAAATAGAGTTGAAGTTTAATCCACCTGCTCTACAAGATGCTTAT   5362
gb:LC522972|Organism:Severe      TTGTTAACACTCCAACAAATAGAGTTGAAGTTTAATCCACCTGCTCTACAAGATGCTTAT   5359
gb:MT027063|Organism:Severe      TTGTTAACACTCCAACAAATAGAGTTGAAGTTTAATCCACCTGCTCTACAAGATGCTTAT   5362
gb:MT027062|Organism:Severe      TTGTTAACACTCCAACAAATAGAGTTGAAGTTTAATCCACCTGCTCTACAAGATGCTTAT   5362
gb:MT019529|Organism:Severe      TTGTTAACACTCCAACAAATAGAGTTGAAGTTTAATCCACCTGCTCTACAAGATGCTTAT   5362
gb:MN996529|Organism:Severe      TTGTTAACACTCCAACAAATAGAGTTGAAGTTTAATCCACCTGCTCTACAAGATGCTTAT   5350
gb:MN996531|Organism:Severe      TTGTTAACACTCCAACAAATAGAGTTGAAGTTTAATCCACCTGCTCTACAAGATGCTTAT   5349
gb:MT066176|Organism:Severe      TTGTTAACACTCCAACAAATAGAGTTGAAGTTTAATCCACCTGCTCTACAAGATGCTTAT   5362
gb:MT027064|Organism:Severe      TTGTTAACACTCCAACAAATAGAGTTGAAGTTTAATCCACCTGCTCTACAAGATGCTTAT   5362
gb:MN994468|Organism:Severe      TTGTTAACACTCCAACAAATAGAGTTGAAGTTTAATCCACCTGCTCTACAAGATGCTTAT   5362
gb:MT072688|Organism:Severe      TTGTTAACACTCCAACAAATAGAGTTGAAGTTTAATCCACCTGCTCTACAAGATGCTTAT   5347
gb:MN996527|Organism:Severe      TTGTTAACACTCCAACAAATAGAGTTGAAGTTTAATCCACCTGCTCTACAAGATGCTTAT   5329
gb:MT093631|Organism:Severe      TTGTTAACACTCCAACAAATAGAGTTGAAGTTTAATCCACCTGCTCTACAAGATGCTTAT   5400
gb:MT106053|Organism:Severe      TTGTTAACACTCCAACAAATAGAGTTGAAGTTTAATCCACCTGCTCTACAAGATGCTTAT   5362
gb:MT019533|Organism:Severe      TTGTTAACACTCCAACAAATAGAGTTGAAGTTTAATCCACCTGCTCTACAAGATGCTTAT   5362
gb:MT019531|Organism:Severe      TTGTTAACACTCCAACAAATAGAGTTGAAGTTTAATCCACCTGCTCTACAAGATGCTTAT   5362
gb:MN996528|Organism:Severe      TTGTTAACACTCCAACAAATAGAGTTGAAGTTTAATCCACCTGCTCTACAAGATGCTTAT   5362
gb:MN996530|Organism:Severe      TTGTTAACACTCCAACAAATAGAGTTGAAGTTTAATCCACCTGCTCTACAAGATGCTTAT   5348
gb:MN908947|Organism:Severe      TTGTTAACACTCCAACAAATAGAGTTGAAGTTTAATCCACCTGCTCTACAAGATGCTTAT   5362
gb:MT019532|Organism:Severe      TTGTTAACACTCCAACAAATAGAGTTGAAGTTTAATCCACCTGCTCTACAAGATGCTTAT   5362
                                 ************************************************************

gb:MT020781|Organism:Severe      TACAGAGCAAGGGCTGGTGAAGCTGCTAACTTTTGTGCACTTATCTTAGCCTACTGTAAT   5410
gb:MT007544|Organism:Severe      TACAGAGCAAGGGCTGGTGAAGCTGCTAACTTTTGTGCACTTATCTTAGCCTACTGTAAT   5422
gb:MN994467|Organism:Severe      TACAGAGCAAGGGCTGGTGAAGCTGCTAACTTTTGTGCACTTATCTTAGCCTACTGTAAT   5422
gb:MT044257|Organism:Severe      TACAGAGCAAGGGCTGGTGAAGCTGCTAACTTTTGTGCACTTATCTTAGCCTACTGTAAT   5422
gb:MT106054|Organism:Severe      TACAGAGCAAGGGCTGGTGAAGCTGCTAACTTTTGTGCACTTATCTTAGCCTACTGTAAT   5422
gb:MT049951|Organism:Severe      TACAGAGCAAGGGCTGGTGAAGCTGCTAACTTTTGTGCACTTATCTTAGCCTACTGTAAT   5422
gb:MN975262|Organism:Severe      TACAGAGCAAGGGCTGGTGAAGCTGCTAACTTTTGTGCACTTATCTTAGCCTACTGTAAT   5422
gb:MT106052|Organism:Severe      TACAGAGCAAGGGCTGGTGAAGCTGCTAACTTTTGTGCACTTATCTTAGCCTACTGTAAT   5422
gb:LC522975|Organism:Severe      TACAGAGCAAGGGCTGGTGAAGCTGCTAACTTTTGTGCACTTATCTTAGCCTACTGTAAT   5419
gb:LC522973|Organism:Severe      TACAGAGCAAGGGCTGGTGAAGCTGCTAACTTTTGTGCACTTATCTTAGCCTACTGTAAT   5419
gb:LC522974|Organism:Severe      TACAGAGCAAGGGCTGGTGAAGCTGCTAACTTTTGTGCACTTATCTTAGCCTACTGTAAT   5419
gb:MN985325|Organism:Severe      TACAGAGCAAGGGCTGGTGAAGCTGCTAACTTTTGTGCACTTATCTTAGCCTACTGTAAT   5422
gb:MT020881|Organism:Severe      TACAGAGCAAGGGCTGGTGAAGCTGCTAACTTTTGTGCACTTATCTTAGCCTACTGTAAT   5422
gb:MT020880|Organism:Severe      TACAGAGCAAGGGCTGGTGAAGCTGCTAACTTTTGTGCACTTATCTTAGCCTACTGTAAT   5422
gb:MT066175|Organism:Severe      TACAGAGCAAGGGCTGGTGAAGCTGCTAACTTTTGTGCACTTATCTTAGCCTACTGTAAT   5422
gb:MN997409|Organism:Severe      TACAGAGCAAGGGCTGGTGAAGCTGCTAACTTTTGTGCACTTATCTTAGCCTACTGTAAT   5422
```

```
gb:MN938384|Organism:Severe      TACAGAGCAAGGGCTGGTGAAGCTGCTAACTTTTGTGCACTTATCTTAGCCTACTGTAAT   5390
gb:MT044258|Organism:Severe      TACAGAGCAAGGGCTGGTGAAGCTGCTAACTTTTGTGCACTTATCTTAGCCTACTGTAAT   5398
gb:MT039890|Organism:Severe      TACAGAGCAAGGGCTGGTGAAGCTGCTAACTTTTGTGCACTTATCTTAGCCTACTGTAAT   5422
gb:MN988713|Organism:Severe      TACAGAGCAAGGGCTGGTGAAGCTGCTAACTTTTGTGCACTTATCTTAGCCTACTGTAAT   5422
gb:LC521925|Organism:Severe      TACAGAGCAAGGGCTGGTGAAGCTGCTAACTTTTGTGCACTTATCTTAGCCTACTGTAAT   5395
gb:MT093571|Organism:Severe      TACAGAGCAAGGGCTGGTGAAGCTGCTAACTTTTGTGCACTTATCTTAGCCTACTGTAAT   5422
gb:MT039887|Organism:Severe      TACAGAGCAAGGGCTGGTGAAGCTGCTAACTTTTGTGCACTTATCTTAGCCTACTGTAAT   5422
gb:MT019530|Organism:Severe      TACAGAGCAAGGGCTGGTGAAGCTGCTAACTTTTGTGCACTTATCTTAGCCTACTGTAAT   5422
gb:MT039888|Organism:Severe      TACAGAGCAAGGGCTGGTGAAGCTGCTAACTTTTGTGCACTTATCTTAGCCTACTGTAAT   5422
gb:LC522972|Organism:Severe      TACAGAGCAAGGGCTGGTGAAGCTGCTAACTTTTGTGCACTTATCTTAGCCTACTGTAAT   5419
gb:MT027063|Organism:Severe      TACAGAGCAAGGGCTGGTGAAGCTGCTAACTTTTGTGCACTTATCTTAGCCTACTGTAAT   5422
gb:MT027062|Organism:Severe      TACAGAGCAAGGGCTGGTGAAGCTGCTAACTTTTGTGCACTTATCTTAGCCTACTGTAAT   5422
gb:MT019529|Organism:Severe      TACAGAGCAAGGGCTGGTGAAGCTGCTAACTTTTGTGCACTTATCTTAGCCTACTGTAAT   5422
gb:MN996529|Organism:Severe      TACAGAGCAAGGGCTGGTGAAGCTGCTAACTTTTGTGCACTTATCTTAGCCTACTGTAAT   5410
gb:MN996531|Organism:Severe      TACAGAGCAAGGGCTGGTGAAGCTGCTAACTTTTGTGCACTTATCTTAGCCTACTGTAAT   5409
gb:MT066176|Organism:Severe      TACAGAGCAAGGGCTGGTGAAGCTGCTAACTTTTGTGCACTTATCTTAGCCTACTGTAAT   5422
gb:MT027064|Organism:Severe      TACAGAGCAAGGGCTGGTGAAGCTGCTAACTTTTGTGCACTTATCTTAGCCTACTGTAAT   5422
gb:MN994468|Organism:Severe      TACAGAGCAAGGGCTGGTGAAGCTGCTAACTTTTGTGCACTTATCTTAGCCTACTGTAAT   5422
gb:MT072688|Organism:Severe      TACAGAGCAAGGGCTGGTGAAGCTGCTAACTTTTGTGCACTTATCTTAGCCTACTGTAAT   5407
gb:MN996527|Organism:Severe      TACAGAGCAAGGGCTGGTGAAGCTGCTAACTTTTGTGCACTTATCTTAGCCTACTGTAAT   5389
gb:MT093631|Organism:Severe      TACAGAGCAAGGGCTGGTGAAGCTGCTAACTTTTGTGCACTTATCTTAGCCTACTGTAAT   5460
gb:MT106053|Organism:Severe      TACAGAGCAAGGGCTGGTGAAGCTGCTAACTTTTGTGCACTTATCTTAGCCTACTGTAAT   5422
gb:MT019533|Organism:Severe      TACAGAGCAAGGGCTGGTGAAGCTGCTAACTTTTGTGCACTTATCTTAGCCTACTGTAAT   5422
gb:MT019531|Organism:Severe      TACAGAGCAAGGGCTGGTGAAGCTGCTAACTTTTGTGCACTTATCTTAGCCTACTGTAAT   5422
gb:MN996528|Organism:Severe      TACAGAGCAAGGGCTGGTGAAGCTGCTAACTTTTGTGCACTTATCTTAGCCTACTGTAAT   5422
gb:MN996530|Organism:Severe      TACAGAGCAAGGGCTGGTGAAGCTGCTAACTTTTGTGCACTTATCTTAGCCTACTGTAAT   5408
gb:MN908947|Organism:Severe      TACAGAGCAAGGGCTGGTGAAGCTGCTAACTTTTGTGCACTTATCTTAGCCTACTGTAAT   5422
gb:MT019532|Organism:Severe      TACAGAGCAAGGGCTGGTGAAGCTGCTAACTTTTGTGCACTTATCTTAGCCTACTGTAAT   5422
                                 ************************************************************

gb:MT020781|Organism:Severe      AAGACAGTAGGTGAGTTAGGTGATGTTAGAGAAACAATGAGTTACTTGTTTCAACATGCC   5470
gb:MT007544|Organism:Severe      AAGACAGTAGGTGAGTTAGGTGATGTTAGAGAAACAATGAGTTACTTGTTTCAACATGCC   5482
gb:MN994467|Organism:Severe      AAGACAGTAGGTGAGTTAGGTGATGTTAGAGAAACAATGAGTTACTTGTTTCAACATGCC   5482
gb:MT044257|Organism:Severe      AAGACAGTAGGTGAGTTAGGTGATGTTAGAGAAACAATGAGTTACTTGTTTCAACATGCC   5482
gb:MT106054|Organism:Severe      AAGACAGTAGGTGAGTTAGGTGATGTTAGAGAAACAATGAGTTACTTGTTTCAACATGCC   5482
gb:MT049951|Organism:Severe      AAGACAGTAGGTGAGTTAGGTGATGTTAGAGAAACAATGAGTTACTTGTTTCAACATGCC   5482
gb:MN975262|Organism:Severe      AAGACAGTAGGTGAGTTAGGTGATGTTAGAGAAACAATGAGTTACTTGTTTCAACATGCC   5482
gb:MT106052|Organism:Severe      AAGACAGTAGGTGAGTTAGGTGATGTTAGAGAAACAATGAGTTACTTGTTTCAACATGCC   5482
gb:LC522975|Organism:Severe      AAGACAGTAGGTGAGTTAGGTGATGTTAGAGAAACAATGAGTTACTTGTTTCAACATGCC   5479
gb:LC522973|Organism:Severe      AAGACAGTAGGTGAGTTAGGTGATGTTAGAGAAACAATGAGTTACTTGTTTCAACATGCC   5479
gb:LC522974|Organism:Severe      AAGACAGTAGGTGAGTTAGGTGATGTTAGAGAAACAATGAGTTACTTGTTTCAACATGCC   5479
gb:MN985325|Organism:Severe      AAGACAGTAGGTGAGTTAGGTGATGTTAGAGAAACAATGAGTTACTTGTTTCAACATGCC   5482
gb:MT020881|Organism:Severe      AAGACAGTAGGTGAGTTAGGTGATGTTAGAGAAACAATGAGTTACTTGTTTCAACATGCC   5482
gb:MT020880|Organism:Severe      AAGACAGTAGGTGAGTTAGGTGATGTTAGAGAAACAATGAGTTACTTGTTTCAACATGCC   5482
gb:MT066175|Organism:Severe      AAGACAGTAGGTGAGTTAGGTGATGTTAGAGAAACAATGAGTTACTTGTTTCAACATGCC   5482
gb:MN997409|Organism:Severe      AAGACAGTAGGTGAGTTAGGTGATGTTAGAGAAACAATGAGTTACTTGTTTCAACATGCC   5482
gb:MN938384|Organism:Severe      AAGACAGTAGGTGAGTTAGGTGATGTTAGAGAAACAATGAGTTACTTGTTTCAACATGCC   5450
gb:MT044258|Organism:Severe      AAGACAGTAGGTGAGTTAGGTGATGTTAGAGAAACAATGAGTTACTTGTTTCAACATGCC   5458
gb:MT039890|Organism:Severe      AAGACAGTAGGTGAGTTAGGTGATGTTAGAGAAACAATGAGTTACTTGTTTCAACATGCC   5482
gb:MN988713|Organism:Severe      AAGACAGTAGGTGAGTTAGGTGATGTTAGAGAAACAATGAGTTACTTGTTTCAACATGCC   5482
gb:LC521925|Organism:Severe      AAGACAGTAGGTGAGTTAGGTGATGTTAGAGAAACAATGAGTTACTTGTTTCAACATGCC   5455
gb:MT093571|Organism:Severe      AAGACAGTAGGTGAGTTAGGTGATGTTAGAGAAACAATGAGTTACTTGTTTCAACATGCC   5482
gb:MT039887|Organism:Severe      AAGACAGTAGGTGAGTTAGGTGATGTTAGAGAAACAATGAGTTACTTGTTTCAACATGCC   5482
gb:MT019530|Organism:Severe      AAGACAGTAGGTGAGTTAGGTGATGTTAGAGAAACAATGAGTTACTTGTTTCAACATGCC   5482
gb:MT039888|Organism:Severe      AAGACAGTAGGTGAGTTAGGTGATGTTAGAGAAACAATGAGTTACTTGTTTCAACATRCC   5482
gb:LC522972|Organism:Severe      AAGACAGTAGGTGAGTTAGGTGATGTTAGAGAAACAATGAGTTACTTGTTTCAACATGCC   5479
gb:MT027063|Organism:Severe      AAGACAGTAGGTGAGTTAGGTGATGTTAGAGAAACAATGAGTTACTTGTTTCAACATGCC   5482
gb:MT027062|Organism:Severe      AAGACAGTAGGTGAGTTAGGTGATGTTAGAGAAACAATGAGTTACTTGTTTCAACATGCC   5482
gb:MT019529|Organism:Severe      AAGACAGTAGGTGAGTTAGGTGATGTTAGAGAAACAATGAGTTACTTGTTTCAACATGCC   5482
gb:MN996529|Organism:Severe      AAGACAGTAGGTGAGTTAGGTGATGTTAGAGAAACAATGAGTTACTTGTTTCAACATGCC   5470
gb:MN996531|Organism:Severe      AAGACAGTAGGTGAGTTAGGTGATGTTAGAGAAACAATGAGTTACTTGTTTCAACATGCC   5469
gb:MT066176|Organism:Severe      AAGACAGTAGGTGAGTTAGGTGATGTTAGAGAAACAATGAGTTACTTGTTTCAACATGCC   5482
gb:MT027064|Organism:Severe      AAGACAGTAGGTGAGTTAGGTGATGTTAGAGAAACAATGAGTTACTTGTTTCAACATGCC   5482
gb:MN994468|Organism:Severe      AAGACAGTAGGTGAGTTAGGTGATGTTAGAGAAACAATGAGTTACTTGTTTCAACATGCC   5482
gb:MT072688|Organism:Severe      AAGACAGTAGGTGAGTTAGGTGATGTTAGAGAAACAATGAGTTACTTGTTTCAACATGCC   5467
```

```
gb:MN996527|Organism:Severe      AAGACAGTAGGTGAGTTAGGTGATGTTAGAGAAACAATGAGTTACTTGTTTCAACATGCC	5449
gb:MT093631|Organism:Severe      AAGACAGTAGGTGAGTTAGGTGATGTTAGAGAAACAATGAGTTACTTGTTTCAACATGCC	5520
gb:MT106053|Organism:Severe      AAGACAGTAGGTGAGTTAGGTGATGTTAGAGAAACAATGAGTTACTTGTTTCAACATGCC	5482
gb:MT019533|Organism:Severe      AAGACAGTAGGTGAGTTAGGTGATGTTAGAGAAACAATGAGTTACTTGTTTCAACATGCC	5482
gb:MT019531|Organism:Severe      AAGACAGTAGGTGAGTTAGGTGATGTTAGAGAAACAATGAGTTACTTGTTTCAACATGCC	5482
gb:MN996528|Organism:Severe      AAGACAGTAGGTGAGTTAGGTGATGTTAGAGAAACAATGAGTTACTTGTTTCAACATGCC	5482
gb:MN996530|Organism:Severe      AAGACAGTAGGTGAGTTAGGTGATGTTAGAGAAACAATGAGTTACTTGTTTCAACATGCC	5468
gb:MN908947|Organism:Severe      AAGACAGTAGGTGAGTTAGGTGATGTTAGAGAAACAATGAGTTACTTGTTTCAACATGCC	5482
gb:MT019532|Organism:Severe      AAGACAGTAGGTGAGTTAGGTGATGTTAGAGAAACAATGAGTTACTTGTTTCAACATGCC	5482
                                 ********************************************************* **

gb:MT020781|Organism:Severe      AATTTAGATTCTTGCAAAAGAGTCTTGAACGTGGTGTGTAAAACTTGTGGACAACAGCAG	5530
gb:MT007544|Organism:Severe      AATTTAGATTCTTGCAAAAGAGTCTTGAACGTGGTGTGTAAAACTTGTGGACAACAGCAG	5542
gb:MN994467|Organism:Severe      AATTTAGATTCTTGCAAAAGAGTCTTGAACGTGGTGTGTAAAACTTGTGGACAACAGCAG	5542
gb:MT044257|Organism:Severe      AATTTAGATTCTTGCAAAAGAGTCTTGAACGTGGTGTGTAAAACTTGTGGACAACAGCAG	5542
gb:MT106054|Organism:Severe      AATTTAGATTCTTGCAAAAGAGTCTTGAACGTGGTGTGTAAAACTTGTGGACAACAGCAG	5542
gb:MT049951|Organism:Severe      AATTTAGATTCTTGCAAAAGAGTCTTGAACGTGGTGTGTAAAACTTGTGGACAACAGCAG	5542
gb:MN975262|Organism:Severe      AATTTAGATTCTTGCAAAAGAGTCTTGAACGTGGTGTGTAAAACTTGTGGACAACAGCAG	5542
gb:MT106052|Organism:Severe      AATTTAGATTCTTGCAAAAGAGTCTTGAACGTGGTGTGTAAAACTTGTGGACAACAGCAG	5542
gb:LC522975|Organism:Severe      AATTTAGATTCTTGCAAAAGAGTCTTGAACGTGGTGTGTAAAACTTGTGGACAACAGCAG	5539
gb:LC522973|Organism:Severe      AATTTAGATTCTTGCAAAAGAGTCTTGAACGTGGTGTGTAAAACTTGTGGACAACAGCAG	5539
gb:LC522974|Organism:Severe      AATTTAGATTCTTGCAAAAGAGTCTTGAACGTGGTGTGTAAAACTTGTGGACAACAGCAG	5539
gb:MN985325|Organism:Severe      AATTTAGATTCTTGCAAAAGAGTCTTGAACGTGGTGTGTAAAACTTGTGGACAACAGCAG	5542
gb:MT020881|Organism:Severe      AATTTAGATTCTTGCAAAAGAGTCTTGAACGTGGTGTGTAAAACTTGTGGACAACAGCAG	5542
gb:MT020880|Organism:Severe      AATTTAGATTCTTGCAAAAGAGTCTTGAACGTGGTGTGTAAAACTTGTGGACAACAGCAG	5542
gb:MT066175|Organism:Severe      AATTTAGATTCTTGCAAAAGAGTCTTGAACGTGGTGTGTAAAACTTGTGGACAACAGCAG	5542
gb:MN997409|Organism:Severe      AATTTAGATTCTTGCAAAAGAGTCTTGAACGTGGTGTGTAAAACTTGTGGACAACAGCAG	5542
gb:MN938384|Organism:Severe      AATTTAGATTCTTGCAAAAGAGTCTTGAACGTGGTGTGTAAAACTTGTGGACAACAGCAG	5510
gb:MT044258|Organism:Severe      AATTTAGATTCTTGCAAAAGAGTCTTGAACGTGGTGTGTAAAACTTGTGGACAACAGCAG	5518
gb:MT039890|Organism:Severe      AATTTAGATTCTTGCAAAAGAGTCTTGAACGTGGTGTGTAAAACTTGTGGACAACAGCAG	5542
gb:MN988713|Organism:Severe      AATTTAGATTCTTGCAAAAGAGTCTTGAACGTGGTGTGTAAAACTTGTGGACAACAGCAG	5542
gb:LC521925|Organism:Severe      AATTTAGATTCTTGCAAAAGAGTCTTGAACGTGGTGTGTAAAACTTGTGGACAACAGCAG	5515
gb:MT093571|Organism:Severe      AATTTAGATTCTTGCAAAAGAGTCTTGAACGTGGTGTGTAAAACTTGTGGACAACAGCAG	5542
gb:MT039887|Organism:Severe      AATTTAGATTCTTGCAAAAGAGTCTTGAACGTGGTGTGTAAAACTTGTGGACAACAGCAG	5542
gb:MT019530|Organism:Severe      AATTTAGATTCTTGCAAAAGAGTCTTGAACGTGGTGTGTAAAACTTGTGGACAACAGCAG	5542
gb:MT039888|Organism:Severe      AATTTAGATTCTTGCAAAAGAGTCTTGAACGTGGTGTGTAAAACTTGTGGACAACAGCAG	5542
gb:LC522972|Organism:Severe      AATTTAGATTCTTGCAAAAGAGTCTTGAACGTGGTGTGTAAAACTTGTGGACAACAGCAG	5539
gb:MT027063|Organism:Severe      AATTTAGATTCTTGCAAAAGAGTCTTGAACGTGGTGTGTAAAACTTGTGGACAACAGCAG	5542
gb:MT027062|Organism:Severe      AATTTAGATTCTTGCAAAAGAGTCTTGAACGTGGTGTGTAAAACTTGTGGACAACAGCAG	5542
gb:MT019529|Organism:Severe      AATTTAGATTCTTGCAAAAGAGTCTTGAACGTGGTGTGTAAAACTTGTGGACAACAGCAG	5542
gb:MN996529|Organism:Severe      AATTTAGATTCTTGCAAAAGAGTCTTGAACGTGGTGTGTAAAACTTGTGGACAACAGCAG	5530
gb:MN996531|Organism:Severe      AATTTAGATTCTTGCAAAAGAGTCTTGAACGTGGTGTGTAAAACTTGTGGACAACAGCAG	5529
gb:MT066176|Organism:Severe      AATTTAGATTCTTGCAAAAGAGTCTTGAACGTGGTGTGTAAAACTTGTGGACAACAGCAG	5542
gb:MT027064|Organism:Severe      AATTTAGATTCTTGCAAAAGAGTCTTGAACGTGGTGTGTAAAACTTGTGGACAACAGCAG	5542
gb:MN994468|Organism:Severe      AATTTAGATTCTTGCAAAAGAGTCTTGAACGTGGTGTGTAAAACTTGTGGACAACAGCAG	5542
gb:MT072688|Organism:Severe      AATTTAGATTCTTGCAAAAGAGTCTTGAACGTGGTGTGTAAAACTTGTGGACAACAGCAG	5527
gb:MN996527|Organism:Severe      AATTTAGATTCTTGCAAAAGAGTCTTGAACGTGGTGTGTAAAACTTGTGGACAACAGCAG	5509
gb:MT093631|Organism:Severe      AATTTAGATTCTTGCAAAAGAGTCTTGAACGTGGTGTGTAAAACTTGTGGACAACAGCAG	5580
gb:MT106053|Organism:Severe      AATTTAGATTCTTGCAAAAGAGTCTTGAACGTGGTGTGTAAAACTTGTGGACAACAGCAG	5542
gb:MT019533|Organism:Severe      AATTTAGATTCTTGCAAAAGAGTCTTGAACGTGGTGTGTAAAACTTGTGGACAACAGCAG	5542
gb:MT019531|Organism:Severe      AATTTAGATTCTTGCAAAAGAGTCTTGAACGTGGTGTGTAAAACTTGTGGACAACAGCAG	5542
gb:MN996528|Organism:Severe      AATTTAGATTCTTGCAAAAGAGTCTTGAACGTGGTGTGTAAAACTTGTGGACAACAGCAG	5542
gb:MN996530|Organism:Severe      AATTTAGATTCTTGCAAAAGAGTCTTGAACGTGGTGTGTAAAACTTGTGGACAACAGCAG	5528
gb:MN908947|Organism:Severe      AATTTAGATTCTTGCAAAAGAGTCTTGAACGTGGTGTGTAAAACTTGTGGACAACAGCAG	5542
gb:MT019532|Organism:Severe      AATTTAGATTCTTGCAAAAGAGTCTTGAACGTGGTGTGTAAAACTTGTGGACAACAGCAG	5542
                                 ************************************************************

gb:MT020781|Organism:Severe      ACAACCCTTAAGGGTGTAGAAGCTGTTATGTACATGGGCACACTTTCTTATGAACAATTT	5590
gb:MT007544|Organism:Severe      ACAACCCTTAAGGGTGTAGAAGCTGTTATGTACATGGGCACACTTTCTTATGAACAATTT	5602
gb:MN994467|Organism:Severe      ACAACCCTTAAGGGTGTAGAAGCTGTTATGTACATGGGCACACTTTCTTATGAACAATTT	5602
gb:MT044257|Organism:Severe      ACAACCCTTAAGGGTGTAGAAGCTGTTATGTACATGGGCACACTTTCTTATGAACAATTT	5602
gb:MT106054|Organism:Severe      ACAACCCTTAAGGGTGTAGAAGCTGTTATGTACATGGGCACACTTTCTTATGAACAATTT	5602
gb:MT049951|Organism:Severe      ACAACCCTTAAGGGTGTAGAAGCTGTTATGTACATGGGCACACTTTCTTATGAACAATTT	5602
gb:MN975262|Organism:Severe      ACAACCCTTAAGGGTGTAGAAGCTGTTATGTACATGGGCACACTTTCTTATGAACAATTT	5602
gb:MT106052|Organism:Severe      ACAACCCTTAAGGGTGTAGAAGCTGTTATGTACATGGGCACACTTTCTTATGAACAATTT	5602
```

```
gb:LC522975|Organism:Severe      ACAACCCTTAAGGGTGTAGAAGCTGTTATGTACATGGGCACACTTTCTTATGAACAATTT   5599
gb:LC522973|Organism:Severe      ACAACCCTTAAGGGTGTAGAAGCTGTTATGTACATGGGCACACTTTCTTATGAACAATTT   5599
gb:LC522974|Organism:Severe      ACAACCCTTAAGGGTGTAGAAGCTGTTATGTACATGGGCACACTTTCTTATGAACAATTT   5599
gb:MN985325|Organism:Severe      ACAACCCTTAAGGGTGTAGAAGCTGTTATGTACATGGGCACACTTTCTTATGAACAATTT   5602
gb:MT020881|Organism:Severe      ACAACCCTTAAGGGTGTAGAAGCTGTTATGTACATGGGCACACTTTCTTATGAACAATTT   5602
gb:MT020880|Organism:Severe      ACAACCCTTAAGGGTGTAGAAGCTGTTATGTACATGGGCACACTTTCTTATGAACAATTT   5602
gb:MT066175|Organism:Severe      ACAACCCTTAAGGGTGTAGAAGCTGTTATGTACATGGGCACACTTTCTTATGAACAATTT   5602
gb:MN997409|Organism:Severe      ACAACCCTTAAGGGTGTAGAAGCTGTTATGTACATGGGCACACTTTCTTATGAACAATTT   5602
gb:MN938384|Organism:Severe      ACAACCCTTAAGGGTGTAGAAGCTGTTATGTACATGGGCACACTTTCTTATGAACAATTT   5570
gb:MT044258|Organism:Severe      ACAACCCTTAAGGGTGTAGAAGCTGTTATGTACATGGGCACACTTTCTTATGAACAATTT   5578
gb:MT039890|Organism:Severe      ACAACCCTTAAGGGTGTAGAAGCTGTTATGTACATGGGCACACTTTCTTATGAACAATTT   5602
gb:MN988713|Organism:Severe      ACAACCCTTAAGGGTGTAGAAGCTGTTATGTACATGGGCACACTTTCTTATGAACAATTT   5602
gb:LC521925|Organism:Severe      ACAACCCTTAAGGGTGTAGAAGCTGTTATGTACATGGGCACACTTTCTTATGAACAATTT   5575
gb:MT093571|Organism:Severe      ACAACCCTTAAGGGTGTAGAAGCTGTTATGTACATGGGCACACTTTCTTATGAACAATTT   5602
gb:MT039887|Organism:Severe      ACAACCCTTAAGGGTGTAGAAGCTGTTATGTACATGGGCACACTTTCTTATGAACAATTT   5602
gb:MT019530|Organism:Severe      ACAACCCTTAAGGGTGTAGAAGCTGTTATGTACATGGGCACACTTTCTTATGAACAATTT   5602
gb:MT039888|Organism:Severe      ACAACCCTTAAGGGTGTAGAAGCTGTTATGTACATGGGCACACTTTCTTATGAACAATTT   5602
gb:LC522972|Organism:Severe      ACAACCCTTAAGGGTGTAGAAGCTGTTATGTACATGGGCACACTTTCTTATGAACAATTT   5599
gb:MT027063|Organism:Severe      ACAACCCTTAAGGGTGTAGAAGCTGTTATGTACATGGGCACACTTTCTTATGAACAATTT   5602
gb:MT027062|Organism:Severe      ACAACCCTTAAGGGTGTAGAAGCTGTTATGTACATGGGCACACTTTCTTATGAACAATTT   5602
gb:MT019529|Organism:Severe      ACAACCCTTAAGGGTGTAGAAGCTGTTATGTACATGGGCACACTTTCTTATGAACAATTT   5602
gb:MN996529|Organism:Severe      ACAACCCTTAAGGGTGTAGAAGCTGTTATGTACATGGGCACACTTTCTTATGAACAATTT   5590
gb:MN996531|Organism:Severe      ACAACCCTTAAGGGTGTAGAAGCTGTTATGTACATGGGCACACTTTCTTATGAACAATTT   5589
gb:MT066176|Organism:Severe      ACAACCCTTAAGGGTGTAGAAGCTGTTATGTACATGGGCACACTTTCTTATGAACAATTT   5602
gb:MT027064|Organism:Severe      ACAACCCTTAAGGGTGTAGAAGCTGTTATGTACATGGGCACACTTTCTTATGAACAATTT   5602
gb:MN994468|Organism:Severe      ACAACCCTTAAGGGTGTAGAAGCTGTTATGTACATGGGCACACTTTCTTATGAACAATTT   5602
gb:MT072688|Organism:Severe      ACAACCCTTAAGGGTGTAGAAGCTGTTATGTACATGGGCACACTTTCTTATGAACAATTT   5587
gb:MN996527|Organism:Severe      ACAACCCTTAAGGGTGTAGAAGCTGTTATGTACATGGGCACACTTTCTTATGAACAATTT   5569
gb:MT093631|Organism:Severe      ACAACCCTTAAGGGTGTAGAAGCTGTTATGTACATGGGCACACTTTCTTATGAACAATTT   5640
gb:MT106053|Organism:Severe      ACAACCCTTAAGGGTGTAGAAGCTGTTATGTACATGGGCACACTTTCTTATGAACAATTT   5602
gb:MT019533|Organism:Severe      ACAACCCTTAAGGGTGTAGAAGCTGTTATGTACATGGGCACACTTTCTTATGAACAATTT   5602
gb:MT019531|Organism:Severe      ACAACCCTTAAGGGTGTAGAAGCTGTTATGTACATGGGCACACTTTCTTATGAACAATTT   5602
gb:MN996528|Organism:Severe      ACAACCCTTAAGGGTGTAGAAGCTGTTATGTACATGGGCACACTTTCTTATGAACAATTT   5602
gb:MN996530|Organism:Severe      ACAACCCTTAAGGGTGTAGAAGCTGTTATGTACATGGGCACACTTTCTTATGAACAATTT   5588
gb:MN908947|Organism:Severe      ACAACCCTTAAGGGTGTAGAAGCTGTTATGTACATGGGCACACTTTCTTATGAACAATTT   5602
gb:MT019532|Organism:Severe      ACAACCCTTAAGGGTGTAGAAGCTGTTATGTACATGGGCACACTTTCTTATGAACAATTT   5602
                                 ************************************************************

gb:MT020781|Organism:Severe      AAGAAAGGTGTTCAGATACCTTGTACGTGTGGTAAACAAGCTACAAAATATCTAGTACAA   5650
gb:MT007544|Organism:Severe      AAGAAAGGTGTTCAGATACCTTGTACGTGTGGTAAACAAGCTACAAAATATCTAGTACAA   5662
gb:MN994467|Organism:Severe      AAGAAAGGTGTTCAGATACCTTGTACGTGTGGTAAACAAGCTACAAAATATCTAGTACAA   5662
gb:MT044257|Organism:Severe      AAGAAAGGTGTTCAGATACCTTGTACGTGTGGTAAACAAGCTACAAAATATCTAGTACAA   5662
gb:MT106054|Organism:Severe      AAGAAAGGTGTTCAGATACCTTGTACGTGTGGTAAACAAGCTACAAAATATCTAGTACAA   5662
gb:MT049951|Organism:Severe      AAGAAAGGTGTTCAGATACCTTGTACGTGTGGTAAACAAGCTACAAAATATCTAGTACAA   5662
gb:MN975262|Organism:Severe      AAGAAAGGTGTTCAGATACCTTGTACGTGTGGTAAACAAGCTACAAAATATCTAGTACAA   5662
gb:MT106052|Organism:Severe      AAGAAAGGTGTTCAGATACCTTGTACGTGTGGTAAACAAGCTACAAAATATCTAGTACAA   5662
gb:LC522975|Organism:Severe      AAGAAAGGTGTTCAGATACCTTGTACGTGTGGTAAACAAGCTACAAAATATCTAGTACAA   5659
gb:LC522973|Organism:Severe      AAGAAAGGTGTTCAGATACCTTGTACGTGTGGTAAACAAGCTACAAAATATCTAGTACAA   5659
gb:LC522974|Organism:Severe      AAGAAAGGTGTTCAGATACCTTGTACGTGTGGTAAACAAGCTACAAAATATCTAGTACAA   5659
gb:MN985325|Organism:Severe      AAGAAAGGTGTTCAGATACCTTGTACGTGTGGTAAACAAGCTACAAAATATCTAGTACAA   5662
gb:MT020881|Organism:Severe      AAGAAAGGTGTTCAGATACCTTGTACGTGTGGTAAACAAGCTACAAAATATCTAGTACAA   5662
gb:MT020880|Organism:Severe      AAGAAAGGTGTTCAGATACCTTGTACGTGTGGTAAACAAGCTACAAAATATCTAGTACAA   5662
gb:MT066175|Organism:Severe      AAGAAAGGTGTTCAGATACCTTGTACGTGTGGTAAACAAGCTACAAAATATCTAGTACAA   5662
gb:MN997409|Organism:Severe      AAGAAAGGTGTTCAGATACCTTGTACGTGTGGTAAACAAGCTACAAAATATCTAGTACAA   5662
gb:MN938384|Organism:Severe      AAGAAAGGTGTTCAGATACCTTGTACGTGTGGTAAACAAGCTACAAAATATCTAGTACAA   5630
gb:MT044258|Organism:Severe      AAGAAAGGTGTTCAGATACCTTGTACGTGTGGTAAACAAGCTACAAAATATCTAGTACAA   5638
gb:MT039890|Organism:Severe      AAGAAAGGTGTTCAGATACCTTGTACGTGTGGTAAACAAGCTACAAAATATCTAGTACAA   5662
gb:MN988713|Organism:Severe      AAGAAAGGTGTTCAGATACCTTGTACGTGTGGTAAACAAGCTACAAAATATCTAGTACAA   5662
gb:LC521925|Organism:Severe      AAGAAAGGTGTTCAGATACCTTGTACGTGTGGTAAACAAGCTACAAAATATCTAGTACAA   5635
gb:MT093571|Organism:Severe      AAGAAAGGTGTTCAGATACCTTGTACGTGTGGTAAACAAGCTACAAAATATCTAGTACAA   5662
gb:MT039887|Organism:Severe      AAGAAAGGTGTTCAGATACCTTGTACGTGTGGTAAACAAGCTACAAAATATCTAGTACAA   5662
gb:MT019530|Organism:Severe      AAGAAAGGTGTTCAGATACCTTGTACGTGTGGTAAACAAGCTACAAAATATCTAGTACAA   5662
gb:MT039888|Organism:Severe      AAGAAAGGTGTTCAGATACCTTGTACGTGTGGTAAACAAGCTACAAAATATCTAGTACAA   5662
gb:LC522972|Organism:Severe      AAGAAAGGTGTTCAGATACCTTGTACGTGTGGTAAACAAGCTACAAAATATCTAGTACAA   5659
gb:MT027063|Organism:Severe      AAGAAAGGTGTTCAGATACCTTGTACGTGTGGTAAACAAGCTACAAAATATCTAGTACAA   5662
```

```
gb:MT027062|Organism:Severe      AAGAAAGGTGTTCAGATACCTTGTACGTGTGGTAAACAAGCTACAAAATATCTAGTACAA    5662
gb:MT019529|Organism:Severe      AAGAAAGGTGTTCAGATACCTTGTACGTGTGGTAAACAAGCTACAAAATATCTAGTACAA    5662
gb:MN996529|Organism:Severe      AAGAAAGGTGTTCAGATACCTTGTACGTGTGGTAAACAAGCTACAAAATATCTAGTACAA    5650
gb:MN996531|Organism:Severe      AAGAAAGGTGTTCAGATACCTTGTACGTGTGGTAAACAAGCTACAAAATATCTAGTACAA    5649
gb:MT066176|Organism:Severe      AAGAAAGGTGTTCAGATACCTTGTACGTGTGGTAAACAAGCTACAAAATATCTAGTACAA    5662
gb:MT027064|Organism:Severe      AAGAAAGGTGTTCAGATACCTTGTACGTGTGGTAAACAAGCTACAAAATATCTAGTACAA    5662
gb:MN994468|Organism:Severe      AAGAAAGGTGTTCAGATACCTTGTACGTGTGGTAAACAAGCTACAAAATATCTAGTACAA    5662
gb:MT072688|Organism:Severe      AAGAAAGGTGTTCAGATACCTTGTACGTGTGGTAAACAAGCTACAAAATATCTAGTACAA    5647
gb:MN996527|Organism:Severe      AAGAAAGGTGTTCAGATACCTTGTACGTGTGGTAAACAAGCTACAAAATATCTAGTACAA    5629
gb:MT093631|Organism:Severe      AAGAAAGGTGTTCAGATACCTTGTACGTGTGGTAAACAAGCTACAAAATATCTAGTACAA    5700
gb:MT106053|Organism:Severe      AAGAAAGGTGTTCAGATACCTTGTACGTGTGGTAAACAAGCTACAAAATATCTAGTACAA    5662
gb:MT019533|Organism:Severe      AAGAAAGGTGTTCAGATACCTTGTACGTGTGGTAAACAAGCTACAAAATATCTAGTACAA    5662
gb:MT019531|Organism:Severe      AAGAAAGGTGTTCAGATACCTTGTACGTGTGGTAAACAAGCTACAAAATATCTAGTACAA    5662
gb:MN996528|Organism:Severe      AAGAAAGGTGTTCAGATACCTTGTACGTGTGGTAAACAAGCTACAAAATATCTAGTACAA    5662
gb:MN996530|Organism:Severe      AAGAAAGGTGTTCAGATACCTTGTACGTGTGGTAAACAAGCTACAAAATATCTAGTACAA    5648
gb:MN908947|Organism:Severe      AAGAAAGGTGTTCAGATACCTTGTACGTGTGGTAAACAAGCTACAAAATATCTAGTACAA    5662
gb:MT019532|Organism:Severe      AAGAAAGGTGTTCAGATACCTTGTACGTGTGGTAAACAAGCTACAAAATATCTAGTACAA    5662
                                 ************************************************************

gb:MT020781|Organism:Severe      CAGGAGTCACCTTTTGTTATGATGTCAGCACCACCTGCTCAGTATGAACTTAAGCATGGT    5710
gb:MT007544|Organism:Severe      CAGGAGTCACCTTTTGTTATGATGTCAGCACCACCTGCTCAGTATGAACTTAAGCATGGT    5722
gb:MN994467|Organism:Severe      CAGGAGTCACCTTTTGTTATGATGTCAGCACCACCTGCTCAGTATGAACTTAAGCATGGT    5722
gb:MT044257|Organism:Severe      CAGGAGTCACCTTTTGTTATGATGTCAGCACCACCTGCTCAGTATGAACTTAAGCATGGT    5722
gb:MT106054|Organism:Severe      CAGGAGTCACCTTTTGTTATGATGTCAGCACCACCTGCTCAGTATGAACTTAAGCATGGT    5722
gb:MT049951|Organism:Severe      CAGGAGTCACCTTTTGTTATGATGTCAGCACCACCTGCTCAGTATGAACTTAAGCATGGT    5722
gb:MN975262|Organism:Severe      CAGGAGTCACCTTTTGTTATGATGTCAGCACCACCTGCTCAGTATGAACTTAAGCATGGT    5722
gb:MT106052|Organism:Severe      CAGGAGTCACCTTTTGTTATGATGTCAGCACCACCTGCTCAGTATGAACTTAAGCATGGT    5722
gb:LC522975|Organism:Severe      CAGGAGTCACCTTTTGTTATGATGTCAGCACCACCTGCTCAGTATGAACTTAAGCATGGT    5719
gb:LC522973|Organism:Severe      CAGGAGTCACCTTTTGTTATGATGTCAGCACCACCTGCTCAGTATGAACTTAAGCATGGT    5719
gb:LC522974|Organism:Severe      CAGGAGTCACCTTTTGTTATGATGTCAGCACCACCTGCTCAGTATGAACTTAAGCATGGT    5719
gb:MN985325|Organism:Severe      CAGGAGTCACCTTTTGTTATGATGTCAGCACCACCTGCTCAGTATGAACTTAAGCATGGT    5722
gb:MT020881|Organism:Severe      CAGGAGTCACCTTTTGTTATGATGTCAGCACCACCTGCTCAGTATGAACTTAAGCATGGT    5722
gb:MT020880|Organism:Severe      CAGGAGTCACCTTTTGTTATGATGTCAGCACCACCTGCTCAGTATGAACTTAAGCATGGT    5722
gb:MT066175|Organism:Severe      CAGGAGTCACCTTTTGTTATGATGTCAGCACCACCTGCTCAGTATGAACTTAAGCATGGT    5722
gb:MN997409|Organism:Severe      CAGGAGTCACCTTTTGTTATGATGTCAGCACCACCTGCTCAGTATGAACTTAAGCATGGT    5722
gb:MN938384|Organism:Severe      CAGGAGTCACCTTTTGTTATGATGTCAGCACCACCTGCTCAGTATGAACTTAAGCATGGT    5690
gb:MT044258|Organism:Severe      CAGGAGTCACCTTTTGTTATGATGTCAGCACCACCTGCTCAGTATGAACTTAAGCATGGT    5698
gb:MT039890|Organism:Severe      CAGGAGTCACCTTTTGTTATGATGTCAGCACCACCTGCTCAGTATGAACTTAAGCATGGT    5722
gb:MN988713|Organism:Severe      CAGGAGTCACCTTTTGTTATGATGTCAGCACCACCTGCTCAGTATGAACTTAAGCATGGT    5722
gb:LC521925|Organism:Severe      CAGGAGTCACCTTTTGTTATGATGTCAGCACCACCTGCTCAGTATGAACTTAAGCATGGT    5695
gb:MT093571|Organism:Severe      CAGGAGTCACCTTTTGTTATGATGTCAGCACCACCTGCTCAGTATGAACTTAAGCATGGT    5722
gb:MT039887|Organism:Severe      CAGGAGTCACCTTTTGTTATGATGTCAGCACCACCTGCTCAGTATGAACTTAAGCATGGT    5722
gb:MT019530|Organism:Severe      CAGGAGTCACCTTTTGTTATGATGTCAGCACCACCTGCTCAGTATGAACTTAAGCATGGT    5722
gb:MT039888|Organism:Severe      CAGGAGTCACCTTTTGTTATGATGTCAGCACCACCTGCTCAGTATGAACTTAAGCATGGT    5722
gb:LC522972|Organism:Severe      CAGGAGTCACCTTTTGTTATGATGTCAGCACCACCTGCTCAGTATGAACTTAAGCATGGT    5719
gb:MT027063|Organism:Severe      CAGGAGTCACCTTTTGTTATGATGTCAGCACCACCTGCTCAGTATGAACTTAAGCATGGT    5722
gb:MT027062|Organism:Severe      CAGGAGTCACCTTTTGTTATGATGTCAGCACCACCTGCTCAGTATGAACTTAAGCATGGT    5722
gb:MT019529|Organism:Severe      CAGGAGTCACCTTTTGTTATGATGTCAGCACCACCTGCTCAGTATGAACTTAAGCATGGT    5722
gb:MN996529|Organism:Severe      CAGGAGTCACCTTTTGTTATGATGTCAGCACCACCTGCTCAGTATGAACTTAAGCATGGT    5710
gb:MN996531|Organism:Severe      CAGGAGTCACCTTTTGTTATGATGTCAGCACCACCTGCTCAGTATGAACTTAAGCATGGT    5709
gb:MT066176|Organism:Severe      CAGGAGTCACCTTTTGTTATGATGTCAGCACCACCTGCTCAGTATGAACTTAAGCATGGT    5722
gb:MT027064|Organism:Severe      CAGGAGTCACCTTTTGTTATGATGTCAGCACCACCTGCTCAGTATGAACTTAAGCATGGT    5722
gb:MN994468|Organism:Severe      CAGGAGTCACCTTTTGTTATGATGTCAGCACCACCTGCTCAGTATGAACTTAAGCATGGT    5722
gb:MT072688|Organism:Severe      CAGGAGTCACCTTTTGTTATGATGTCAGCACCACCTGCTCAGTATGAACTTAAGCATGGT    5707
gb:MN996527|Organism:Severe      CAGGAGTCACCTTTTGTTATGATGTCAGCACCACCTGCTCAGTATGAACTTAAGCATGGT    5689
gb:MT093631|Organism:Severe      CAGGAGTCACCTTTTGTTATGATGTCAGCACCACCTGCTCAGTATGAACTTAAGCATGGT    5760
gb:MT106053|Organism:Severe      CAGGAGTCACCTTTTGTTATGATGTCAGCACCACCTGCTCAGTATGAACTTAAGCATGGT    5722
gb:MT019533|Organism:Severe      CAGGAGTCACCTTTTGTTATGATGTCAGCACCACCTGCTCAGTATGAACTTAAGCATGGT    5722
gb:MT019531|Organism:Severe      CAGGAGTCACCTTTTGTTATGATGTCAGCACCACCTGCTCAGTATGAACTTAAGCATGGT    5722
gb:MN996528|Organism:Severe      CAGGAGTCACCTTTTGTTATGATGTCAGCACCACCTGCTCAGTATGAACTTAAGCATGGT    5722
gb:MN996530|Organism:Severe      CAGGAGTCACCTTTTGTTATGATGTCAGCACCACCTGCTCAGTATGAACTTAAGCATGGT    5708
gb:MN908947|Organism:Severe      CAGGAGTCACCTTTTGTTATGATGTCAGCACCACCTGCTCAGTATGAACTTAAGCATGGT    5722
gb:MT019532|Organism:Severe      CAGGAGTCACCTTTTGTTATGATGTCAGCACCACCTGCTCAGTATGAACTTAAGCATGGT    5722
                                 ************************************************************
```

```
gb:MT020781|Organism:Severe      ACATTTACTTGTGCTAGTGAGTACACTGGTAATTACCAGTGTGGTCACTATAAACATATA   5770
gb:MT007544|Organism:Severe      ACATTTACTTGTGCTAGTGAGTACACTGGTAATTACCAGTGTGGTCACTATAAACATATA   5782
gb:MN994467|Organism:Severe      ACATTTACTTGTGCTAGTGAGTACACTGGTAATTACCAGTGTGGTCACTATAAACATATA   5782
gb:MT044257|Organism:Severe      ACATTTACTTGTGCTAGTGAGTACACTGGTAATTACCAGTGTGGTCACTATAAACATATA   5782
gb:MT106054|Organism:Severe      ACATTTACTTGTGCTAGTGAGTACACTGGTAATTACCAGTGTGGTCACTATAAACATATA   5782
gb:MT049951|Organism:Severe      ACATTTACTTGTGCTAGTGAGTACACTGGTAATTACCAGTGTGGTCACTATAAACATATA   5782
gb:MN975262|Organism:Severe      ACATTTACTTGTGCTAGTGAGTACACTGGTAATTACCAGTGTGGTCACTATAAACATATA   5782
gb:MT106052|Organism:Severe      ACATTTACTTGTGCTAGTGAGTACACTGGTAATTACCAGTGTGGTCACTATAAACATATA   5782
gb:LC522975|Organism:Severe      ACATTTACTTGTGCTAGTGAGTACACTGGTAATTACCAGTGTGGTCACTATAAACATATA   5779
gb:LC522973|Organism:Severe      ACATTTACTTGTGCTAGTGAGTACACTGGTAATTACCAGTGTGGTCACTATAAACATATA   5779
gb:LC522974|Organism:Severe      ACATTTACTTGTGCTAGTGAGTACACTGGTAATTACCAGTGTGGTCACTATAAACATATA   5779
gb:MN985325|Organism:Severe      ACATTTACTTGTGCTAGTGAGTACACTGGTAATTACCAGTGTGGTCACTATAAACATATA   5782
gb:MT020881|Organism:Severe      ACATTTACTTGTGCTAGTGAGTACACTGGTAATTACCAGTGTGGTCACTATAAACATATA   5782
gb:MT020880|Organism:Severe      ACATTTACTTGTGCTAGTGAGTACACTGGTAATTACCAGTGTGGTCACTATAAACATATA   5782
gb:MT066175|Organism:Severe      ACATTTACTTGTGCTAGTGAGTACACTGGTAATTACCAGTGTGGTCACTATAAACATATA   5782
gb:MN997409|Organism:Severe      ACATTTACTTGTGCTAGTGAGTACACTGGTAATTACCAGTGTGGTCACTATAAACATATA   5782
gb:MN938384|Organism:Severe      ACATTTACTTGTGCTAGTGAGTACACTGGTAATTACCAGTGTGGTCACTATAAACATATA   5750
gb:MT044258|Organism:Severe      ACATTTACTTGTGCTAGTGAGTACACTGGTAATTACCAGTGTGGTCACTATAAACATATA   5758
gb:MT039890|Organism:Severe      ACATTTACTTGTGCTAGTGAGTACACTGGTAATTACCAGTGTGGTCACTATAAACATATA   5782
gb:MN988713|Organism:Severe      ACATTTACTTGTGCTAGTGAGTACACTGGTAATTACCAGTGTGGTCACTATAAACATATA   5782
gb:LC521925|Organism:Severe      ACATTTACTTGTGCTAGTGAGTACACTGGTAATTACCAGTGTGGTCACTATAAACATATA   5755
gb:MT093571|Organism:Severe      ACATTTACTTGTGCTAGTGAGTACACTGGTAATTACCAGTGTGGTCACTATAAACATATA   5782
gb:MT039887|Organism:Severe      ACATTTACTTGTGCTAGTGAGTACACTGGTAATTACCAGTGTGGTCACTATAAACATATA   5782
gb:MT019530|Organism:Severe      ACATTTACTTGTGCTAGTGAGTACACTGGTAATTACCAGTGTGGTCACTATAAACATATA   5782
gb:MT039888|Organism:Severe      ACATTTACTTGTGCTAGTGAGTACACTGGTAATTACCAGTGTGGTCACTATAAACATATA   5782
gb:LC522972|Organism:Severe      ACATTTACTTGTGCTAGTGAGTACACTGGTAATTACCAGTGTGGTCACTATAAACATATA   5779
gb:MT027063|Organism:Severe      ACATTTACTTGTGCTAGTGAGTACACTGGTAATTACCAGTGTGGTCACTATAAACATATA   5782
gb:MT027062|Organism:Severe      ACATTTACTTGTGCTAGTGAGTACACTGGTAATTACCAGTGTGGTCACTATAAACATATA   5782
gb:MT019529|Organism:Severe      ACATTTACTTGTGCTAGTGAGTACACTGGTAATTACCAGTGTGGTCACTATAAACATATA   5782
gb:MN996529|Organism:Severe      ACATTTACTTGTGCTAGTGAGTACACTGGTAATTACCAGTGTGGTCACTATAAACATATA   5770
gb:MN996531|Organism:Severe      ACATTTACTTGTGCTAGTGAGTACACTGGTAATTACCAGTGTGGTCACTATAAACATATA   5769
gb:MT066176|Organism:Severe      ACATTTACTTGTGCTAGTGAGTACACTGGTAATTACCAGTGTGGTCACTATAAACATATA   5782
gb:MT027064|Organism:Severe      ACATTTACTTGTGCTAGTGAGTACACTGGTAATTACCAGTGTGGTCACTATAAACATATA   5782
gb:MN994468|Organism:Severe      ACATTTACTTGTGCTAGTGAGTACACTGGTAATTACCAGTGTGGTCACTATAAACATATA   5782
gb:MT072688|Organism:Severe      ACATTTACTTGTGCTAGTGAGTACACTGGTAATTACCAGTGTGGTCACTATAAACATATA   5767
gb:MN996527|Organism:Severe      ACATTTACTTGTGCTAGTGAGTACACTGGTAATTACCAGTGTGGTCACTATAAACATATA   5749
gb:MT093631|Organism:Severe      ACATTTACTTGTGCTAGTGAGTACACTGGTAATTACCAGTGTGGTCACTATAAACATATA   5820
gb:MT106053|Organism:Severe      ACATTTACTTGTGCTAGTGAGTACACTGGTAATTACCAGTGTGGTCACTATAAACATATA   5782
gb:MT019533|Organism:Severe      ACATTTACTTGTGCTAGTGAGTACACTGGTAATTACCAGTGTGGTCACTATAAACATATA   5782
gb:MT019531|Organism:Severe      ACATTTACTTGTGCTAGTGAGTACACTGGTAATTACCAGTGTGGTCACTATAAACATATA   5782
gb:MN996528|Organism:Severe      ACATTTACTTGTGCTAGTGAGTACACTGGTAATTACCAGTGTGGTCACTATAAACATATA   5782
gb:MN996530|Organism:Severe      ACATTTACTTGTGCTAGTGAGTACACTGGTAATTACCAGTGTGGTCACTATAAACATATA   5768
gb:MN908947|Organism:Severe      ACATTTACTTGTGCTAGTGAGTACACTGGTAATTACCAGTGTGGTCACTATAAACATATA   5782
gb:MT019532|Organism:Severe      ACATTTACTTGTGCTAGTGAGTACACTGGTAATTACCAGTGTGGTCACTATAAACATATA   5782
                                 ************************************************************

gb:MT020781|Organism:Severe      ACTTCTAAAGAAACTTTGTATTGCATAGACGGTGCTTTACTTACAAAGTCCTCAGAATAC   5830
gb:MT007544|Organism:Severe      ACTTCTAAAGAAACTTTGTATTGCATAGACGGTGCTTTACTTACAAAGTCCTCAGAATAC   5842
gb:MN994467|Organism:Severe      ACTTCTAAAGAAACTTTGTATTGCATAGACGGTGCTTTACTTACAAAGTCCTCAGAATAC   5842
gb:MT044257|Organism:Severe      ACTTCTAAAGAAACTTTGTATTGCATAGACGGTGCTTTACTTACAAAGTCCTCAGAATAC   5842
gb:MT106054|Organism:Severe      ACTTCTAAAGAAACTTTGTATTGCATAGACGGTGCTTTACTTACAAAGTCCTCAGAATAC   5842
gb:MT049951|Organism:Severe      ACTTCTAAAGAAACTTTGTATTGCATAGACGGTGCTTTACTTACAAAGTCCTCAGAATAC   5842
gb:MN975262|Organism:Severe      ACTTCTAAAGAAACTTTGTATTGCATAGACGGTGCTTTACTTACAAAGTCCTCAGAATAC   5842
gb:MT106052|Organism:Severe      ACTTCTAAAGAAACTTTGTATTGCATAGACGGTGCTTTACTTACAAAGTCCTCAGAATAC   5842
gb:LC522975|Organism:Severe      ACTTCTAAAGAAACTTTGTATTGCATAGACGGTGCTTTACTTACAAAGTCCTCAGAATAC   5839
gb:LC522973|Organism:Severe      ACTTCTAAAGAAACTTTGTATTGCATAGACGGTGCTTTACTTACAAAGTCCTCAGAATAC   5839
gb:LC522974|Organism:Severe      ACTTCTAAAGAAACTTTGTATTGCATAGACGGTGCTTTACTTACAAAGTCCTCAGAATAC   5839
gb:MN985325|Organism:Severe      ACTTCTAAAGAAACTTTGTATTGCATAGACGGTGCTTTACTTACAAAGTCCTCAGAATAC   5842
gb:MT020881|Organism:Severe      ACTTCTAAAGAAACTTTGTATTGCATAGACGGTGCTTTACTTACAAAGTCCTCAGAATAC   5842
gb:MT020880|Organism:Severe      ACTTCTAAAGAAACTTTGTATTGCATAGACGGTGCTTTACTTACAAAGTCCTCAGAATAC   5842
gb:MT066175|Organism:Severe      ACTTCTAAAGAAACTTTGTATTGCATAGACGGTGCTTTACTTACAAAGTCCTCAGAATAC   5842
gb:MN997409|Organism:Severe      ACTTCTAAAGAAACTTTGTATTGCATAGACGGTGCTTTACTTACAAAGTCCTCAGAATAC   5842
gb:MN938384|Organism:Severe      ACTTCTAAAGAAACTTTGTATTGCATAGACGGTGCTTTACTTACAAAGTCCTCAGAATAC   5810
gb:MT044258|Organism:Severe      ACTTCTAAAGAAACTTTGTATTGCATAGACGGTGCTTTACTTACAAAGTCCTCAGAATAC   5818
gb:MT039890|Organism:Severe      ACTTCTAAAGAAACTTTGTATTGCATAGACGGTGCTTTACTTACAAAGTCCTCAGAATAC   5842
```

```
gb:MN988713|Organism:Severe      ACTTCTAAAGAAACTTTGTATTGCATAGACGGTGCTTTACTTACAAAGTCCTCAGAATAC    5842
gb:LC521925|Organism:Severe      ACTTCTAAAGAAACTTTGTATTGCATAGACGGTGCTTTACTTACAAAGTCCTCAGAATAC    5815
gb:MT093571|Organism:Severe      ACTTCTAAAGAAACTTTGTATTGCATAGACGGTGCTTTACTTACAAAGTCCTCAGAATAC    5842
gb:MT039887|Organism:Severe      ACTTCTAAAGAAACTTTGTATTGCATAGACGGTGCTTTACTTACAAAGTCCTCAGAATAC    5842
gb:MT019530|Organism:Severe      ACTTCTAAAGAAACTTTGTATTGCATAGACGGTGCTTTACTTACAAAGTCCTCAGAATAC    5842
gb:MT039888|Organism:Severe      ACTTCTAAAGAAACTTTGTATTGCATAGACGGTGCTTTACTTACAAAGTCCTCAGAATAC    5842
gb:LC522972|Organism:Severe      ACTTCTAAAGAAACTTTGTATTGCATAGACGGTGCTTTACTTACAAAGTCCTCAGAATAC    5839
gb:MT027063|Organism:Severe      ACTTCTAAAGAAACTTTGTATTGCATAGACGGTGCTTTACTTACAAAGTCCTCAGAATAC    5842
gb:MT027062|Organism:Severe      ACTTCTAAAGAAACTTTGTATTGCATAGACGGTGCTTTACTTACAAAGTCCTCAGAATAC    5842
gb:MT019529|Organism:Severe      ACTTCTAAAGAAACTTTGTATTGCATAGACGGTGCTTTACTTACAAAGTCCTCAGAATAC    5842
gb:MN996529|Organism:Severe      ACTTCTAAAGAAACTTTGTATTGCATAGACGGTGCTTTACTTACAAAGTCCTCAGAATAC    5830
gb:MN996531|Organism:Severe      ACTTCTAAAGAAACTTTGTATTGCATAGACGGTGCTTTACTTACAAAGTCCTCAGAATAC    5829
gb:MT066176|Organism:Severe      ACTTCTAAAGAAACTTTGTATTGCATAGACGGTGCTTTACTTACAAAGTCCTCAGAATAC    5842
gb:MT027064|Organism:Severe      ACTTCTAAAGAAACTTTGTATTGCATAGACGGTGCTTTACTTACAAAGTCCTCAGAATAC    5842
gb:MN994468|Organism:Severe      ACTTCTAAAGAAACTTTGTATTGCATAGACGGTGCTTTACTTACAAAGTCCTCAGAATAC    5842
gb:MT072688|Organism:Severe      ACTTCTAAAGAAACTTTGTATTGCATAGACGGTGCTTTACTTACAAAGTCCTCAGAATAC    5827
gb:MN996527|Organism:Severe      ACTTCTAAAGAAACTTTGTATTGCATAGACGGTGCTTTACTTACAAAGTCCTCAGAATAC    5809
gb:MT093631|Organism:Severe      ACTTCTAAAGAAACTTTGTATTGCATAGACGGTGCTTTACTTACAAAGTCCTCAGAATAC    5880
gb:MT106053|Organism:Severe      ACTTCTAAAGAAACTTTGTATTGCATAGACGGTGCTTTACTTACAAAGTCCTCAGAATAC    5842
gb:MT019533|Organism:Severe      ACTTCTAAAGAAACTTTGTATTGCATAGACGGTGCTTTACTTACAAAGTCCTCAGAATAC    5842
gb:MT019531|Organism:Severe      ACTTCTAAAGAAACTTTGTATTGCATAGACGGTGCTTTACTTACAAAGTCCTCAGAATAC    5842
gb:MN996528|Organism:Severe      ACTTCTAAAGAAACTTTGTATTGCATAGACGGTGCTTTACTTACAAAGTCCTCAGAATAC    5842
gb:MN996530|Organism:Severe      ACTTCTAAAGAAACTTTGTATTGCATAGACGGTGCTTTACTTACAAAGTCCTCAGAATAC    5828
gb:MN908947|Organism:Severe      ACTTCTAAAGAAACTTTGTATTGCATAGACGGTGCTTTACTTACAAAGTCCTCAGAATAC    5842
gb:MT019532|Organism:Severe      ACTTCTAAAGAAACTTTGTATTGCATAGACGGTGCTTTACTTACAAAGTCCTCAGAATAC    5842
                                 ************************************************************

gb:MT020781|Organism:Severe      AAAGGTCCTATTACGGATGTTTTCTACAAAGAAAACAGTTACACAACAACCATAAAACCA    5890
gb:MT007544|Organism:Severe      AAAGGTCCTATTACGGATGTTTTCTACAAAGAAAACAGTTACACAACAACCATAAAACCA    5902
gb:MN994467|Organism:Severe      AAAGGTCCTATTACGGATGTTTTCTACAAAGAAAACAGTTACACAACAACCATAAAACCA    5902
gb:MT044257|Organism:Severe      AAAGGTCCTATTACGGATGTTTTCTACAAAGAAAACAGTTACACAACAACCATAAAACCA    5902
gb:MT106054|Organism:Severe      AAAGGTCCTATTACGGATGTTTTCTACAAAGAAAACAGTTACACAACAACCATAAAACCA    5902
gb:MT049951|Organism:Severe      AAAGGTCCTATTACGGATGTTTTCTACAAAGAAAACAGTTACACAACAACCATAAAACCA    5902
gb:MN975262|Organism:Severe      AAAGGTCCTATTACGGATGTTTTCTACAAAGAAAACAGTTACACAACAACCATAAAACCA    5902
gb:MT106052|Organism:Severe      AAAGGTCCTATTACGGATGTTTTCTACAAAGAAAACAGTTACACAACAACCATAAAACCA    5902
gb:LC522975|Organism:Severe      AAAGGTCCTATTACGGATGTTTTCTACAAAGAAAACAGTTACACAACAACCATAAAACCA    5899
gb:LC522973|Organism:Severe      AAAGGTCCTATTACGGATGTTTTCTACAAAGAAAACAGTTACACAACAACCATAAAACCA    5899
gb:LC522974|Organism:Severe      AAAGGTCCTATTACGGATGTTTTCTACAAAGAAAACAGTTACACAACAACCATAAAACCA    5899
gb:MN985325|Organism:Severe      AAAGGTCCTATTACGGATGTTTTCTACAAAGAAAACAGTTACACAACAACCATAAAACCA    5902
gb:MT020881|Organism:Severe      AAAGGTCCTATTACGGATGTTTTCTACAAAGAAAACAGTTACACAACAACCATAAAACCA    5902
gb:MT020880|Organism:Severe      AAAGGTCCTATTACGGATGTTTTCTACAAAGAAAACAGTTACACAACAACCATAAAACCA    5902
gb:MT066175|Organism:Severe      AAAGGTCCTATTACGGATGTTTTCTACAAAGAAAACAGTTACACAACAACCATAAAACCA    5902
gb:MN997409|Organism:Severe      AAAGGTCCTATTACGGATGTTTTCTACAAAGAAAACAGTTACACAACAACCATAAAACCA    5902
gb:MN938384|Organism:Severe      AAAGGTCCTATTACGGATGTTTTCTACAAAGAAAACAGTTACACAACAACCATAAAACCA    5870
gb:MT044258|Organism:Severe      AAAGGTCCTATTACGGATGTTTTCTACAAAGAAAACAGTTACACAACAACCATAAAACCA    5878
gb:MT039890|Organism:Severe      AAAGGTCCTATTACGGATGTTTTCTACAAAGAAAACAGTTACACAACAACCATAAAACCA    5902
gb:MN988713|Organism:Severe      AAAGGTCCTATTACGGATGTTTTCTACAAAGAAAACAGTTACACAACAACCATAAAACCA    5902
gb:LC521925|Organism:Severe      AAAGGTCCTATTACGGATGTTTTCTACAAAGAAAACAGTTACACAACAACCATAAAACCA    5875
gb:MT093571|Organism:Severe      AAAGGTCCTATTACGGATGTTTTCTACAAAGAAAACAGTTACACAACAACCATAAAACCA    5902
gb:MT039887|Organism:Severe      AAAGGTCCTATTACGGATGTTTTCTACAAAGAAAACAGTTACACAACAACCATAAAACCA    5902
gb:MT019530|Organism:Severe      AAAGGTCCTATTACGGATGTTTTCTACAAAGAAAACAGTTACACAACAACCATAAAACCA    5902
gb:MT039888|Organism:Severe      AAAGGTCCTATTACGGATGTTTTCTACAAAGAAAACAGTTACACAACAACCATAAAACCA    5902
gb:LC522972|Organism:Severe      AAAGGTCCTATTACGGATGTTTTCTACAAAGAAAACAGTTACACAACAACCATAAAACCA    5899
gb:MT027063|Organism:Severe      AAAGGTCCTATTACGGATGTTTTCTACAAAGAAAACAGTTACACAACAACCATAAAACCA    5902
gb:MT027062|Organism:Severe      AAAGGTCCTATTACGGATGTTTTCTACAAAGAAAACAGTTACACAACAACCATAAAACCA    5902
gb:MT019529|Organism:Severe      AAAGGTCCTATTACGGATGTTTTCTACAAAGAAAACAGTTACACAACAACCATAAAACCA    5902
gb:MN996529|Organism:Severe      AAAGGTCCTATTACGGATGTTTTCTACAAAGAAAACAGTTACACAACAACCATAAAACCA    5890
gb:MN996531|Organism:Severe      AAAGGTCCTATTACGGATGTTTTCTACAAAGAAAACAGTTACACAACAACCATAAAACCA    5889
gb:MT066176|Organism:Severe      AAAGGTCCTATTACGGATGTTTTCTACAAAGAAAACAGTTACACAACAACCATAAAACCA    5902
gb:MT027064|Organism:Severe      AAAGGTCCTATTACGGATGTTTTCTACAAAGAAAACAGTTACACAACAACCATAAAACCA    5902
gb:MN994468|Organism:Severe      AAAGGTCCTATTACGGATGTTTTCTACAAAGAAAACAGTTACACAACAACCATAAAACCA    5902
gb:MT072688|Organism:Severe      AAAGGTCCTATTACGGATGTTTTCTACAAAGAAAACAGTTACACAACAACCATAAAACCA    5887
gb:MN996527|Organism:Severe      AAAGGTCCTATTACGGATGTTTTCTACAAAGAAAACAGTTACACAACAACCATAAAACCA    5869
gb:MT093631|Organism:Severe      AAAGGTCCTATTACGGATGTTTTCTACAAAGAAAACAGTTACACAACAACCATAAAACCA    5940
gb:MT106053|Organism:Severe      AAAGGTCCTATTACGGATGTTTTCTACAAAGAAAACAGTTACACAACAACCATAAAACCA    5902
```

```
gb:MT019533|Organism:Severe      AAAGGTCCTATTACGGATGTTTTCTACAAAGAAAACAGTTACACAACAACCATAAAACCA   5902
gb:MT019531|Organism:Severe      AAAGGTCCTATTACGGATGTTTTCTACAAAGAAAACAGTTACACAACAACCATAAAACCA   5902
gb:MN996528|Organism:Severe      AAAGGTCCTATTACGGATGTTTTCTACAAAGAAAACAGTTACACAACAACCATAAAACCA   5902
gb:MN996530|Organism:Severe      AAAGGTCCTATTACGGATGTTTTCTACAAAGAAAACAGTTACACAACAACCATAAAACCA   5888
gb:MN908947|Organism:Severe      AAAGGTCCTATTACGGATGTTTTCTACAAAGAAAACAGTTACACAACAACCATAAAACCA   5902
gb:MT019532|Organism:Severe      AAAGGTCCTATTACGGATGTTTTCTACAAAGAAAACAGTTACACAACAACCATAAAACCA   5902
                                 ************************************************************

gb:MT020781|Organism:Severe      GTTACTTATAAATTGGATGGTGTTGTTTGTACAGAAATTGACCCTAAGTTGGACAATTAT   5950
gb:MT007544|Organism:Severe      GTTACTTATAAATTGGATGGTGTTGTTTGTACAGAAATTGACCCTAAGTTGGACAATTAT   5962
gb:MN994467|Organism:Severe      GTTACTTATAAATTGGATGGTGTTGTTTGTACAGAAATTGACCCTAAGTTGGACAATTAT   5962
gb:MT044257|Organism:Severe      GTTACTTATAAATTGGATGGTGTTGTTTGTACAGAAATTGACCCTAAGTTGGACAATTAT   5962
gb:MT106054|Organism:Severe      GTTACTTATAAATTGGATGGTGTTGTTTGTACAGAAATTGACCCTAAGTTGGACAATTAT   5962
gb:MT049951|Organism:Severe      GTTACTTATAAATTGGATGGTGTTGTTTGTACAGAAATTGACCCTAAGTTGGACAATTAT   5962
gb:MN975262|Organism:Severe      GTTACTTATAAATTGGATGGTGTTGTTTGTACAGAAATTGACCCTAAGTTGGACAATTAT   5962
gb:MT106052|Organism:Severe      GTTACTTATAAATTGGATGGTGTTGTTTGTACAGAAATTGACCCTAAGTTGGACAATTAT   5962
gb:LC522975|Organism:Severe      GTTACTTATAAATTGGATGGTGTTGTTTGTACAGAAATTGACCCTAAGTTGGACAATTAT   5959
gb:LC522973|Organism:Severe      GTTACTTATAAATTGGATGGTGTTGTTTGTACAGAAATTGACCCTAAGTTGGACAATTAT   5959
gb:LC522974|Organism:Severe      GTTACTTATAAATTGGATGGTGTTGTTTGTACAGAAATTGACCCTAAGTTGGACAATTAT   5959
gb:MN985325|Organism:Severe      GTTACTTATAAATTGGATGGTGTTGTTTGTACAGAAATTGACCCTAAGTTGGACAATTAT   5962
gb:MT020881|Organism:Severe      GTTACTTATAAATTGGATGGTGTTGTTTGTACAGAAATTGACCCTAAGTTGGACAATTAT   5962
gb:MT020880|Organism:Severe      GTTACTTATAAATTGGATGGTGTTGTTTGTACAGAAATTGACCCTAAGTTGGACAATTAT   5962
gb:MT066175|Organism:Severe      GTTACTTATAAATTGGATGGTGTTGTTTGTACAGAAATTGACCCTAAGTTGGACAATTAT   5962
gb:MN997409|Organism:Severe      GTTACTTATAAATTGGATGGTGTTGTTTGTACAGAAATTGACCCTAAGTTGGACAATTAT   5962
gb:MN938384|Organism:Severe      GTTACTTATAAATTGGATGGTGTTGTTTGTACAGAAATTGACCCTAAGTTGGACAATTAT   5930
gb:MT044258|Organism:Severe      GTTACTTATAAATTGGATGGTGTTGTTTGTACAGAAATTGACCCTAAGTTGGACAATTAT   5938
gb:MT039890|Organism:Severe      GTTACTTATAAATTGGATGGTGTTGTTTGTACAGAAATTGACCCTAAGTTGGACAATTAT   5962
gb:MN988713|Organism:Severe      GTTACTTATAAATTGGATGGTGTTGTTTGTACAGAAATTGACCCTAAGTTGGACAATTAT   5962
gb:LC521925|Organism:Severe      GTTACTTATAAATTGGATGGTGTTGTTTGTACAGAAATTGACCCTAAGTTGGACAATTAT   5935
gb:MT093571|Organism:Severe      GTTACTTATAAATTGGATGGTGTTGTTTGTACAGAAATTGACCCTAAGTTGGACAATTAT   5962
gb:MT039887|Organism:Severe      GTTACTTATAAATTGGATGGTGTTGTTTGTACAGAAATTGACCCTAAGTTGGACAATTAT   5962
gb:MT019530|Organism:Severe      GTTACTTATAAATTGGATGGTGTTGTTTGTACAGAAATTGACCCTAAGTTGGACAATTAT   5962
gb:MT039888|Organism:Severe      GTTACTTATAAATTGGATGGTGTTGTTTGTACAGAAATTGACCCTAAGTTGGACAATTAT   5962
gb:LC522972|Organism:Severe      GTTACTTATAAATTGGATGGTGTTGTTTGTACAGAAATTGACCCTAAGTTGGACAATTAT   5959
gb:MT027063|Organism:Severe      GTTACTTATAAATTGGATGGTGTTGTTTGTACAGAAATTGACCCTAAGTTGGACAATTAT   5962
gb:MT027062|Organism:Severe      GTTACTTATAAATTGGATGGTGTTGTTTGTACAGAAATTGACCCTAAGTTGGACAATTAT   5962
gb:MT019529|Organism:Severe      GTTACTTATAAATTGGATGGTGTTGTTTGTACAGAAATTGACCCTAAGTTGGACAATTAT   5962
gb:MN996529|Organism:Severe      GTTACTTATAAATTGGATGGTGTTGTTTGTACAGAAATTGACCCTAAGTTGGACAATTAT   5950
gb:MN996531|Organism:Severe      GTTACTTATAAATTGGATGGTGTTGTTTGTACAGAAATTGACCCTAAGTTGGACAATTAT   5949
gb:MT066176|Organism:Severe      GTTACTTATAAATTGGATGGTGTTGTTTGTACAGAAATTGACCCTAAGTTGGACAATTAT   5962
gb:MT027064|Organism:Severe      GTTACTTATAAATTGGATGGTGTTGTTTGTACAGAAATTGACCCTAAGTTGGACAATTAT   5962
gb:MN994468|Organism:Severe      GTTACTTATAAATTGGATGGTGTTGTTTGTACAGAAATTGACCCTAAGTTGGACAATTAT   5962
gb:MT072688|Organism:Severe      GTTACTTATAAATTGGATGGTGTTGTTTGTACAGAAATTGACCCTAAGTTGGACAATTAT   5947
gb:MN996527|Organism:Severe      GTTACTTATAAATTGGATGGTGTTGTTTGTACAGAAATTGACCCTAAGTTGGACAATTAT   5929
gb:MT093631|Organism:Severe      GTTACTTATAAATTGGATGGTGTTGTTTGTACAGAAATTGACCCTAAGTTGGACAATTAT   6000
gb:MT106053|Organism:Severe      GTTACTTATAAATTGGATGGTGTTGTTTGTACAGAAATTGACCCTAAGTTGGACAATTAT   5962
gb:MT019533|Organism:Severe      GTTACTTATAAATTGGATGGTGTTGTTTGTACAGAAATTGACCCTAAGTTGGACAATTAT   5962
gb:MT019531|Organism:Severe      GTTACTTATAAATTGGATGGTGTTGTTTGTACAGAAATTGACCCTAAGTTGGACAATTAT   5962
gb:MN996528|Organism:Severe      GTTACTTATAAATTGGATGGTGTTGTTTGTACAGAAATTGACCCTAAGTTGGACAATTAT   5962
gb:MN996530|Organism:Severe      GTTACTTATAAATTGGATGGTGTTGTTTGTACAGAAATTGACCCTAAGTTGGACAATTAT   5948
gb:MN908947|Organism:Severe      GTTACTTATAAATTGGATGGTGTTGTTTGTACAGAAATTGACCCTAAGTTGGACAATTAT   5962
gb:MT019532|Organism:Severe      GTTACTTATAAATTGGATGGTGTTGTTTGTACAGAAATTGACCCTAAGTTGGACAATTAT   5962
                                 ************************************************************

gb:MT020781|Organism:Severe      TATAAGAAAGACAATTCTTATTTCACAGAGCAACCAATTGATCTTGTACCAAACCAACCA   6010
gb:MT007544|Organism:Severe      TATAAGAAAGACAATTCTTATTTCACAGAGCAACCAATTGATCTTGTACCAAACCAACCA   6022
gb:MN994467|Organism:Severe      TATAAGAAAGACAATTCTTATTTCACAGAGCAACCAATTGATCTTGTACCAAACCAACCA   6022
gb:MT044257|Organism:Severe      TATAAGAAAGACAATTCTTATTTCACAGAGCAACCAATTGATCTTGTACCAAACCAACCA   6022
gb:MT106054|Organism:Severe      TATAAGAAAGACAATTCTTATTTCACAGAGCAACCAATTGATCTTGTACCAAACCAACCA   6022
gb:MT049951|Organism:Severe      TATAAGAAAGACAATTCTTATTTCACAGAGCAACCAATTGATCTTGTACCAAACCAACCA   6022
gb:MN975262|Organism:Severe      TATAAGAAAGACAATTCTTATTTCACAGAGCAACCAATTGATCTTGTACCAAACCAACCA   6022
gb:MT106052|Organism:Severe      TATAAGAAAGACAATTCTTATTTCACAGAGCAACCAATTGATCTTGTACCAAACCAACCA   6022
gb:LC522975|Organism:Severe      TATAAGAAAGACAATTCTTATTTCACAGAGCAACCAATTGATCTTGTACCAAACCAACCA   6019
gb:LC522973|Organism:Severe      TATAAGAAAGACAATTCTTATTTCACAGAGCAACCAATTGATCTTGTACCAAACCAACCA   6019
gb:LC522974|Organism:Severe      TATAAGAAAGACAATTCTTATTTCACAGAGCAACCAATTGATCTTGTACCAAACCAACCA   6019
```

```
gb:MN985325|Organism:Severe      TATAAGAAAGACAATTCTTATTTCACAGAGCAACCAATTGATCTTGTACCAAACCAACCA   6022
gb:MT020881|Organism:Severe      TATAAGAAAGACAATTCTTATTTCACAGAGCAACCAATTGATCTTGTACCAAACCAACCA   6022
gb:MT020880|Organism:Severe      TATAAGAAAGACAATTCTTATTTCACAGAGCAACCAATTGATCTTGTACCAAACCAACCA   6022
gb:MT066175|Organism:Severe      TATAAGAAAGACAATTCTTATTTCACAGAGCAACCAATTGATCTTGTACCAAACCAACCA   6022
gb:MN997409|Organism:Severe      TATAAGAAAGACAATTCTTATTTCACAGAGCAACCAATTGATCTTGTACCAAACCAACCA   6022
gb:MN938384|Organism:Severe      TATAAGAAAGACAATTCTTATTTCACAGAGCAACCAATTGATCTTGTACCAAACCAACCA   5990
gb:MT044258|Organism:Severe      TATAAGAAAGACAATTCTTATTTCACAGAGCAACCAATTGATCTTGTACCAAACCAACCA   5998
gb:MT039890|Organism:Severe      TATAAGAAAGACAATTCTTATTTCACAGAGCAACCAATTGATCTTGTACCAAACCAACCA   6022
gb:MN988713|Organism:Severe      TATAAGAAAGACAATTCTTATTTCACAGAGCAACCAATTGATCTTGTACCAAACCAACCA   6022
gb:LC521925|Organism:Severe      TATAAGAAAGACAATTCTTATTTCACAGAGCAACCAATTGATCTTGTACCAAACCAACCA   5995
gb:MT093571|Organism:Severe      TATAAGAAAGACAATTCTTATTTCACAGAGCAACCAATTGATCTTGTACCAAACCAACCA   6022
gb:MT039887|Organism:Severe      TATAAGAAAGACAATTCTTATTTCACAGAGCAACCAATTGATCTTGTACCAAACCAACCA   6022
gb:MT019530|Organism:Severe      TATAAGAAAGACAATTCTTATTTCACAGAGCAACCAATTGATCTTGTACCAAACCAACCA   6022
gb:MT039888|Organism:Severe      TATAAGAAAGACAATTCTTATTTCACAGAGCAACCAATTGATCTTGTACCAAACCAACCA   6022
gb:LC522972|Organism:Severe      TATAAGAAAGACAATTCTTATTTCACAGAGCAACCAATTGATCTTGTACCAAACCAACCA   6019
gb:MT027063|Organism:Severe      TATAAGAAAGACAATTCTTATTTCACAGAGCAACCAATTGATCTTGTACCAAACCAACCA   6022
gb:MT027062|Organism:Severe      TATAAGAAAGACAATTCTTATTTCACAGAGCAACCAATTGATCTTGTACCAAACCAACCA   6022
gb:MT019529|Organism:Severe      TATAAGAAAGACAATTCTTATTTCACAGAGCAACCAATTGATCTTGTACCAAACCAACCA   6022
gb:MN996529|Organism:Severe      TATAAGAAAGACAATTCTTATTTCACAGAGCAACCAATTGATCTTGTACCAAACCAACCA   6010
gb:MN996531|Organism:Severe      TATAAGAAAGACAATTCTTATTTCACAGAGCAACCAATTGATCTTGTACCAAACCAACCA   6009
gb:MT066176|Organism:Severe      TATAAGAAAGACAATTCTTATTTCACAGAGCAACCAATTGATCTTGTACCAAACCAACCA   6022
gb:MT027064|Organism:Severe      TATAAGAAAGACAATTCTTATTTCACAGAGCAACCAATTGATCTTGTACCAAACCAACCA   6022
gb:MN994468|Organism:Severe      TATAAGAAAGACAATTCTTATTTCACAGAGCAACCAATTGATCTTGTACCAAACCAACCA   6022
gb:MT072688|Organism:Severe      TATAAGAAAGACAATTCTTATTTCACAGAGCAACCAATTGATCTTGTACCAAACCAACCA   6007
gb:MN996527|Organism:Severe      TATAAGAAAGACAATTCTTATTTCACAGAGCAACCAATTGATCTTGTACCAAACCAACCA   5989
gb:MT093631|Organism:Severe      TATAAGAAAGACAATTCTTATTTCACAGAGCAACCAATTGATCTTGTACCAAACCAACCA   6060
gb:MT106053|Organism:Severe      TATAAGAAAGACAATTCTTATTTCACAGAGCAACCAATTGATCTTGTACCAAACCAACCA   6022
gb:MT019533|Organism:Severe      TATAAGAAAGACAATTCTTATTTCACAGAGCAACCAATTGATCTTGTACCAAACCAACCA   6022
gb:MT019531|Organism:Severe      TATAAGAAAGACAATTCTTATTTCACAGAGCAACCAATTGATCTTGTACCAAACCAACCA   6022
gb:MN996528|Organism:Severe      TATAAGAAAGACAATTCTTATTTCACAGAGCAACCAATTGATCTTGTACCAAACCAACCA   6022
gb:MN996530|Organism:Severe      TATAAGAAAGACAATTCTTATTTCACAGAGCAACCAATTGATCTTGTACCAAACCAACCA   6008
gb:MN908947|Organism:Severe      TATAAGAAAGACAATTCTTATTTCACAGAGCAACCAATTGATCTTGTACCAAACCAACCA   6022
gb:MT019532|Organism:Severe      TATAAGAAAGACAATTCTTATTTCACAGAGCAACCAATTGATCTTGTACCAAACCAACCA   6022
                                 ************************************************************

gb:MT020781|Organism:Severe      TATCCAAACGCAAGCTTCGATAATTTTAAGTTTGTATGTGATAATATCAAATTTGCTGAT   6070
gb:MT007544|Organism:Severe      TATCCAAACGCAAGCTTCGATAATTTTAAGTTTGTATGTGATAATATCAAATTTGCTGAT   6082
gb:MN994467|Organism:Severe      TATCCAAACGCAAGCTTCGATAATTTTAAGTTTGTATGTGATAATATCAAATTTGCTGAT   6082
gb:MT044257|Organism:Severe      TATCCAAACGCAAGCTTCGATAATTTTAAGTTTGTATGTGATAATATCAAATTTGCTGAT   6082
gb:MT106054|Organism:Severe      TATCCAAACGCAAGCTTCGATAATTTTAAGTTTGTATGTGATAATATCAAATTTGCTGAT   6082
gb:MT049951|Organism:Severe      TATCCAAACGCAAGCTTCGATAATTTTAAGTTTGTATGTGATAATATCAAATTTGCTGAT   6082
gb:MN975262|Organism:Severe      TATCCAAACGCAAGCTTCGATAATTTTAAGTTTGTATGTGATAATATCAAATTTGCTGAT   6082
gb:MT106052|Organism:Severe      TATCCAAACGCAAGCTTCGATAATTTTAAGTTTGTATGTGATAATATCAAATTTGCTGAT   6082
gb:LC522975|Organism:Severe      TATCCAAACGCAAGCTTCGATAATTTTAAGTTTGTATGTGATAATATCAAATTTGCTGAT   6079
gb:LC522973|Organism:Severe      TATCCAAACGCAAGCTTCGATAATTTTAAGTTTGTATGTGATAATATCAAATTTGCTGAT   6079
gb:LC522974|Organism:Severe      TATCCAAACGCAAGCTTCGATAATTTTAAGTTTGTATGTGATAATATCAAATTTGCTGAT   6079
gb:MN985325|Organism:Severe      TATCCAAACGCAAGCTTCGATAATTTTAAGTTTGTATGTGATAATATCAAATTTGCTGAT   6082
gb:MT020881|Organism:Severe      TATCCAAACGCAAGCTTCGATAATTTTAAGTTTGTATGTGATAATATCAAATTTGCTGAT   6082
gb:MT020880|Organism:Severe      TATCCAAACGCAAGCTTCGATAATTTTAAGTTTGTATGTGATAATATCAAATTTGCTGAT   6082
gb:MT066175|Organism:Severe      TATCCAAACGCAAGCTTCGATAATTTTAAGTTTGTATGTGATAATATCAAATTTGCTGAT   6082
gb:MN997409|Organism:Severe      TATCCAAACGCAAGCTTCGATAATTTTAAGTTTGTATGTGATAATATCAAATTTGCTGAT   6082
gb:MN938384|Organism:Severe      TATCCAAACGCAAGCTTCGATAATTTTAAGTTTGTATGTGATAATATCAAATTTGCTGAT   6050
gb:MT044258|Organism:Severe      TATCCAAACGCAAGCTTCGATAATTTTAAGTTTGTATGTGATAATATCAAATTTGCTGAT   6058
gb:MT039890|Organism:Severe      TATCCAAATGCAAGCTTCGATAATTTTAAGTTTGTATGTGATAATATCAAATTTGCTGAT   6082
gb:MN988713|Organism:Severe      TATCCAAACGCAAGCTTCGATAATTTTAAGTTTGTATGTGATAATATCAAATTTGCTGAT   6082
gb:LC521925|Organism:Severe      TATCCAAACGCAAGCTTCGATAATTTTAAGTTTGTATGTGATAATATCAAATTTGCTGAT   6055
gb:MT093571|Organism:Severe      TATCCAAACGCAAGCTTCGATAATTTTAAGTTTGTATGTGATAATATCAAATTTGCTGAT   6082
gb:MT039887|Organism:Severe      TATCCAAACGCAAGCTTCGATAATTTTAAGTTTGTATGTGATAATATCAAATTTGCTGAT   6082
gb:MT019530|Organism:Severe      TATCCAAACGCAAGCTTCGATAATTTTAAGTTTGTATGTGATAATATCAAATTTGCTGAT   6082
gb:MT039888|Organism:Severe      TATCCAAACGCAAGCTTCGATAATTTTAAGTTTGTATGTGATAATATCAAATTTGCTGAT   6082
gb:LC522972|Organism:Severe      TATCCAAACGCAAGCTTCGATAATTTTAAGTTTGTATGTGATAATATCAAATTTGCTGAT   6079
gb:MT027063|Organism:Severe      TATCCAAACGCAAGCTTCGATAATTTTAAGTTTGTATGTGATAATATCAAATTTGCTGAT   6082
gb:MT027062|Organism:Severe      TATCCAAACGCAAGCTTCGATAATTTTAAGTTTGTATGTGATAATATCAAATTTGCTGAT   6082
gb:MT019529|Organism:Severe      TATCCAAACGCAAGCTTCGATAATTTTAAGTTTGTATGTGATAATATCAAATTTGCTGAT   6082
gb:MN996529|Organism:Severe      TATCCAAACGCAAGCTTCGATAATTTTAAGTTTGTATGTGATAATATCAAATTTGCTGAT   6070
```

```
gb:MN996531|Organism:Severe      TATCCAAACGCAAGCTTCGATAATTTTAAGTTTGTATGTGATAATATCAAATTTGCTGAT   6069
gb:MT066176|Organism:Severe      TATCCAAACGCAAGCTTCGATAATTTTAAGTTTGTATGTGATAATATCAAATTTGCTGAT   6082
gb:MT027064|Organism:Severe      TATCCAAACGCAAGCTTCGATAATTTTAAGTTTGTATGTGATAATATCAAATTTGCTGAT   6082
gb:MN994468|Organism:Severe      TATCCAAACGCAAGCTTCGATAATTTTAAGTTTGTATGTGATAATATCAAATTTGCTGAT   6082
gb:MT072688|Organism:Severe      TATCCAAACGCAAGCTTCGATAATTTTAAGTTTGTATGTGATAATATCAAATTTGCTGAT   6067
gb:MN996527|Organism:Severe      TATCCAAACGCAAGCTTCGATAATTTTAAGTTTGTATGTGATAATATCAAATTTGCTGAT   6049
gb:MT093631|Organism:Severe      TATCCAAACGCAAGCTTCGATAATTTTAAGTTTGTATGTGATAATATCAAATTTGCTGAT   6120
gb:MT106053|Organism:Severe      TATCCAAACGCAAGCTTCGATAATTTTAAGTTTGTATGTGATAATATCAAATTTGCTGAT   6082
gb:MT019533|Organism:Severe      TATCCAAACGCAAGCTTCGATAATTTTAAGTTTGTATGTGATAATATCAAATTTGCTGAT   6082
gb:MT019531|Organism:Severe      TATCCAAACGCAAGCTTCGATAATTTTAAGTTTGTATGTGATAATATCAAATTTGCTGAT   6082
gb:MN996528|Organism:Severe      TATCCAAACGCAAGCTTCGATAATTTTAAGTTTGTATGTGATAATATCAAATTTGCTGAT   6082
gb:MN996530|Organism:Severe      TATCCAAACGCAAGCTTCGATAATTTTAAGTTTGTATGTGATAATATCAAATTTGCTGAT   6068
gb:MN908947|Organism:Severe      TATCCAAACGCAAGCTTCGATAATTTTAAGTTTGTATGTGATAATATCAAATTTGCTGAT   6082
gb:MT019532|Organism:Severe      TATCCAAACGCAAGCTTCGATAATTTTAAGTTTGTATGTGATAATATCAAATTTGCTGAT   6082
                                 ******** ***************************************************

gb:MT020781|Organism:Severe      GATTTAAACCAGTTAACTGGTTATAAGAAACCTGCTTCAAGAGAGCTTAAAGTTACATTT   6130
gb:MT007544|Organism:Severe      GATTTAAACCAGTTAACTGGTTATAAGAAACCTGCTTCAAGAGAGCTTAAAGTTACATTT   6142
gb:MN994467|Organism:Severe      GATTTAAACCAGTTAACTGGTTATAAGAAACCTGCTTCAAGAGAGCTTAAAGTTACATTT   6142
gb:MT044257|Organism:Severe      GATTTAAACCAGTTAACTGGTTATAAGAAACCTGCTTCAAGAGAGCTTAAAGTTACATTT   6142
gb:MT106054|Organism:Severe      GATTTAAACCAGTTAACTGGTTATAAGAAACCTGCTTCAAGAGAGCTTAAAGTTACATTT   6142
gb:MT049951|Organism:Severe      GATTTAAACCAGTTAACTGGTTATAAGAAACCTGCTTCAAGAGAGCTTAAAGTTACATTT   6142
gb:MN975262|Organism:Severe      GATTTAAACCAGTTAACTGGTTATAAGAAACCTGCTTCAAGAGAGCTTAAAGTTACATTT   6142
gb:MT106052|Organism:Severe      GATTTAAACCAGTTAACTGGTTATAAGAAACCTGCTTCAAGAGAGCTTAAAGTTACATTT   6142
gb:LC522975|Organism:Severe      GATTTAAACCAGTTAACTGGTTATAAGAAACCTGCTTCAAGAGAGCTTAAAGTTACATTT   6139
gb:LC522973|Organism:Severe      GATTTAAACCAGTTAACTGGTTATAAGAAACCTGCTTCAAGAGAGCTTAAAGTTACATTT   6139
gb:LC522974|Organism:Severe      GATTTAAACCAGTTAACTGGTTATAAGAAACCTGCTTCAAGAGAGCTTAAAGTTACATTT   6139
gb:MN985325|Organism:Severe      GATTTAAACCAGTTAACTGGTTATAAGAAACCTGCTTCAAGAGAGCTTAAAGTTACATTT   6142
gb:MT020881|Organism:Severe      GATTTAAACCAGTTAACTGGTTATAAGAAACCTGCTTCAAGAGAGCTTAAAGTTACATTT   6142
gb:MT020880|Organism:Severe      GATTTAAACCAGTTAACTGGTTATAAGAAACCTGCTTCAAGAGAGCTTAAAGTTACATTT   6142
gb:MT066175|Organism:Severe      GATTTAAACCAGTTAACTGGTTATAAGAAACCTGCTTCAAGAGAGCTTAAAGTTACATTT   6142
gb:MN997409|Organism:Severe      GATTTAAACCAGTTAACTGGTTATAAGAAACCTGCTTCAAGAGAGCTTAAAGTTACATTT   6142
gb:MN938384|Organism:Severe      GATTTAAACCAGTTAACTGGTTATAAGAAACCTGCTTCAAGAGAGCTTAAAGTTACATTT   6110
gb:MT044258|Organism:Severe      GATTTAAACCAGTTAACTGGTTATAAGAAACCTGCTTCAAGAGAGCTTAAAGTTACATTT   6118
gb:MT039890|Organism:Severe      GATTTAAACCAGTTAACTGGTTATAAGAAACCTGCTTCAAGAGAGCTTAAAGTTACATTT   6142
gb:MN988713|Organism:Severe      GATTTAAACCAGTTAACTGGTTATAAGAAACCTGCTTCAAGAGAGCTTAAAGTTACATTT   6142
gb:LC521925|Organism:Severe      GATTTAAACCAGTTAACTGGTTATAAGAAACCTGCTTCAAGAGAGCTTAAAGTTACATTT   6115
gb:MT093571|Organism:Severe      GATTTAAACCAGTTAACTGGTTATAAGAAACCTGCTTCAAGAGAGCTTAAAGTTACATTT   6142
gb:MT039887|Organism:Severe      GATTTAAACCAGTTAACTGGTTATAAGAAACCTGCTTCAAGAGAGCTTAAAGTTACATTT   6142
gb:MT019530|Organism:Severe      GATTTAAACCAGTTAACTGGTTATAAGAAACCTGCTTCAAGAGAGCTTAAAGTTACATTT   6142
gb:MT039888|Organism:Severe      GATTTAAACCAGTTAACTGGTTATAAGAAACCTGCTTCAAGAGAGCTTAAAGTTACATTT   6142
gb:LC522972|Organism:Severe      GATTTAAACCAGTTAACTGGTTATAAGAAACCTGCTTCAAGAGAGCTTAAAGTTACATTT   6139
gb:MT027063|Organism:Severe      GATTTAAACCAGTTAACTGGTTATAAGAAACCTGCTTCAAGAGAGCTTAAAGTTACATTT   6142
gb:MT027062|Organism:Severe      GATTTAAACCAGTTAACTGGTTATAAGAAACCTGCTTCAAGAGAGCTTAAAGTTACATTT   6142
gb:MT019529|Organism:Severe      GATTTAAACCAGTTAACTGGTTATAAGAAACCTGCTTCAAGAGAGCTTAAAGTTACATTT   6142
gb:MN996529|Organism:Severe      GATTTAAACCAGTTAACTGGTTATAAGAAACCTGCTTCAAGAGAGCTTAAAGTTACATTT   6130
gb:MN996531|Organism:Severe      GATTTAAACCAGTTAACTGGTTATAAGAAACCTGCTTCAAGAGAGCTTAAAGTTACATTT   6129
gb:MT066176|Organism:Severe      GATTTAAACCAGTTAACTGGTTATAAGAAACCTGCTTCAAGAGAGCTTAAAGTTACATTT   6142
gb:MT027064|Organism:Severe      GATTTAAACCAGTTAACTGGTTATAAGAAACCTGCTTCAAGAGAGCTTAAAGTTACATTT   6142
gb:MN994468|Organism:Severe      GATTTAAACCAGTTAACTGGTTATAAGAAACCTGCTTCAAGAGAGCTTAAAGTTACATTT   6142
gb:MT072688|Organism:Severe      GATTTAAACCAGTTAACTGGTTATAAGAAACCTGCTTCAAGAGAGCTTAAAGTTACATTT   6127
gb:MN996527|Organism:Severe      GATTTAAACCAGTTAACTGGTTATAAGAAACCTGCTTCAAGAGAGCTTAAAGTTACATTT   6109
gb:MT093631|Organism:Severe      GATTTAAACCAGTTAACTGGTTATAAGAAACCTGCTTCAAGAGAGCTTAAAGTTACATTT   6180
gb:MT106053|Organism:Severe      GATTTAAACCAGTTAACTGGTTATAAGAAACCTGCTTCAAGAGAGCTTAAAGTTACATTT   6142
gb:MT019533|Organism:Severe      GATTTAAACCAGTTAACTGGTTATAAGAAACCTGCTTCAAGAGAGCTTAAAGTTACATTT   6142
gb:MT019531|Organism:Severe      GATTTAAACCAGTTAACTGGTTATAAGAAACCTGCTTCAAGAGAGCTTAAAGTTACATTT   6142
gb:MN996528|Organism:Severe      GATTTAAACCAGTTAACTGGTTATAAGAAACCTGCTTCAAGAGAGCTTAAAGTTACATTT   6142
gb:MN996530|Organism:Severe      GATTTAAACCAGTTAACTGGTTATAAGAAACCTGCTTCAAGAGAGCTTAAAGTTACATTT   6128
gb:MN908947|Organism:Severe      GATTTAAACCAGTTAACTGGTTATAAGAAACCTGCTTCAAGAGAGCTTAAAGTTACATTT   6142
gb:MT019532|Organism:Severe      GATTTAAACCAGTTAACTGGTTATAAGAAACCTGCTTCAAGAGAGCTTAAAGTTACATTT   6142
                                 ************************************************************

gb:MT020781|Organism:Severe      TTCCCTGACTTAAATGGTGATGTGGTGGCTATTGATTATAAACACTACACACCCTCTTTT   6190
gb:MT007544|Organism:Severe      TTCCCTGACTTAAATGGTGATGTGGTGGCTATTGATTATAAACACTACACACCCTCTTTT   6202
gb:MN994467|Organism:Severe      TTCCCTGACTTAAATGGTGATGTGGTGGCTATTGATTATAAACACTACACACCCTCTTTT   6202
```

```
gb:MT044257|Organism:Severe      TTCCCTGACTTAAATGGTGATGTGGTGGCTATTGATTATAAACACTACACACCCTCTTTT   6202
gb:MT106054|Organism:Severe      TTCCCTGACTTAAATGGTGATGTGGTGGCTATTGATTATAAACACTACACACCCTCTTTT   6202
gb:MT049951|Organism:Severe      TTCCCTGACTTAAATGGTGATGTGGTGGCTATTGATTATAAACACTACACACCCTCTTTT   6202
gb:MN975262|Organism:Severe      TTCCCTGACTTAAATGGTGATGTGGTGGCTATTGATTATAAACACTACACACCCTCTTTT   6202
gb:MT106052|Organism:Severe      TTCCCTGACTTAAATGGTGATGTGGTGGCTATTGATTATAAACACTACACACCCTCTTTT   6202
gb:LC522975|Organism:Severe      TTCCCTGACTTAAATGGTGATGTGGTGGCTATTGATTATAAACACTACACACCCTCTTTT   6199
gb:LC522973|Organism:Severe      TTCCCTGACTTAAATGGTGATGTGGTGGCTATTGATTATAAACACTACACACCCTCTTTT   6199
gb:LC522974|Organism:Severe      TTCCCTGACTTAAATGGTGATGTGGTGGCTATTGATTATAAACACTACACACCCTCTTTT   6199
gb:MN985325|Organism:Severe      TTCCCTGACTTAAATGGTGATGTGGTGGCTATTGATTATAAACACTACACACCCTCTTTT   6202
gb:MT020881|Organism:Severe      TTCCCTGACTTAAATGGTGATGTGGTGGCTATTGATTATAAACACTACACACCCTCTTTT   6202
gb:MT020880|Organism:Severe      TTCCCTGACTTAAATGGTGATGTGGTGGCTATTGATTATAAACACTACACACCCTCTTTT   6202
gb:MT066175|Organism:Severe      TTCCCTGACTTAAATGGTGATGTGGTGGCTATTGATTATAAACACTACACACCCTCTTTT   6202
gb:MN997409|Organism:Severe      TTCCCTGACTTAAATGGTGATGTGGTGGCTATTGATTATAAACACTACACACCCTCTTTT   6202
gb:MN938384|Organism:Severe      TTCCCTGACTTAAATGGTGATGTGGTGGCTATTGATTATAAACACTACACACCCTCTTTT   6170
gb:MT044258|Organism:Severe      TTCCCTGACTTAAATGGTGATGTGGTGGCTATTGATTATAAACACTACACACCCTCTTTT   6178
gb:MT039890|Organism:Severe      TTCCCTGACTTAAATGGTGATGTGGTGGCTATTGATTATAAACACTACACACCCTCTTTT   6202
gb:MN988713|Organism:Severe      TTCCCTGACTTAAATGGTGATGTGGTGGCTATTGATTATAAACACTACACACCCTCTTTT   6202
gb:LC521925|Organism:Severe      TTCCCTGACTTAAATGGTGATGTGGTGGCTATTGATTATAAACACTACACACCCTCTTTT   6175
gb:MT093571|Organism:Severe      TTCCCTGACTTAAATGGTGATGTGGTGGCTATTGATTATAAACACTACACACCCTCTTTT   6202
gb:MT039887|Organism:Severe      TTCCCTGACTTAAATGGTGATGTGGTGGCTATTGATTATAAACACTACACACCCTCTTTT   6202
gb:MT019530|Organism:Severe      TTCCCTGACTTAAATGGTGATGTGGTGGCTATTGATTATAAACACTACACACCCTCTTTT   6202
gb:MT039888|Organism:Severe      TTCCCTGACTTAAATGGTGATGTGGTGGCTATTGATTATAAACACTACACACCCTCTTTT   6202
gb:LC522972|Organism:Severe      TTCCCTGACTTAAATGGTGATGTGGTGGCTATTGATTATAAACACTACACACCCTCTTTT   6199
gb:MT027063|Organism:Severe      TTCCCTGACTTAAATGGTGATGTGGTGGCTATTGATTATAAACACTACACACCCTCTTTT   6202
gb:MT027062|Organism:Severe      TTCCCTGACTTAAATGGTGATGTGGTGGCTATTGATTATAAACACTACACACCCTCTTTT   6202
gb:MT019529|Organism:Severe      TTCCCTGACTTAAATGGTGATGTGGTGGCTATTGATTATAAACACTACACACCCTCTTTT   6202
gb:MN996529|Organism:Severe      TTCCCTGACTTAAATGGTGATGTGGTGGCTATTGATTATAAACACTACACACCCTCTTTT   6190
gb:MN996531|Organism:Severe      TTCCCTGACTTAAATGGTGATGTGGTGGCTATTGATTATAAACACTACACACCCTCTTTT   6189
gb:MT066176|Organism:Severe      TTCCCTGACTTAAATGGTGATGTGGTGGCTATTGATTATAAACACTACACACCCTCTTTT   6202
gb:MT027064|Organism:Severe      TTCCCTGACTTAAATGGTGATGTGGTGGCTATTGATTATAAACACTACACACCCTCTTTT   6202
gb:MN994468|Organism:Severe      TTCCCTGACTTAAATGGTGATGTGGTGGCTATTGATTATAAACACTACACACCCTCTTTT   6202
gb:MT072688|Organism:Severe      TTCCCTGACTTAAATGGTGATGTGGTGGCTATTGATTATAAACACTACACACCCTCTTTT   6187
gb:MN996527|Organism:Severe      TTCCCTGACTTAAATGGTGATGTGGTGGCTATTGATTATAAACACTACACACCCTCTTTT   6169
gb:MT093631|Organism:Severe      TTCCCTGACTTAAATGGTGATGTGGTGGCTATTGATTATAAACACTACACACCCTCTTTT   6240
gb:MT106053|Organism:Severe      TTCCCTGACTTAAATGGTGATGTGGTGGCTATTGATTATAAACACTACACACCCTCTTTT   6202
gb:MT019533|Organism:Severe      TTCCCTGACTTAAATGGTGATGTGGTGGCTATTGATTATAAACACTACACACCCTCTTTT   6202
gb:MT019531|Organism:Severe      TTCCCTGACTTAAATGGTGATGTGGTGGCTATTGATTATAAACACTACACACCCTCTTTT   6202
gb:MN996528|Organism:Severe      TTCCCTGACTTAAATGGTGATGTGGTGGCTATTGATTATAAACACTACACACCCTCTTTT   6202
gb:MN996530|Organism:Severe      TTCCCTGACTTAAATGGTGATGTGGTGGCTATTGATTATAAACACTACACACCCTCTTTT   6188
gb:MN908947|Organism:Severe      TTCCCTGACTTAAATGGTGATGTGGTGGCTATTGATTATAAACACTACACACCCTCTTTT   6202
gb:MT019532|Organism:Severe      TTCCCTGACTTAAATGGTGATGTGGTGGCTATTGATTATAAACACTACACACCCTCTTTT   6202
                                 ************************************************************

gb:MT020781|Organism:Severe      AAGAAAGGAGCTAAATTGTTACATAAACCTATTGTTTGGCATGTTAACAATGCAACTAAT   6250
gb:MT007544|Organism:Severe      AAGAAAGGAGCTAAATTGTTACATAAACCTATTGTTTGGCATGTTAACAATGCAACTAAT   6262
gb:MN994467|Organism:Severe      AAGAAAGGAGCTAAATTGTTACATAAACCTATTGTTTGGCATGTTAACAATGCAACTAAT   6262
gb:MT044257|Organism:Severe      AAGAAAGGAGCTAAATTGTTACATAAACCTATTGTTTGGCATGTTAACAATGCAACTAAT   6262
gb:MT106054|Organism:Severe      AAGAAAGGAGCTAAATTGTTACATAAACCTATTGTTTGGCATGTTAACAATGCAACTAAT   6262
gb:MT049951|Organism:Severe      AAGAAAGGAGCTAAATTGTTACATAAACCTATTGTTTGGCATGTTAACAATGCAACTAAT   6262
gb:MN975262|Organism:Severe      AAGAAAGGAGCTAAATTGTTACATAAACCTATTGTTTGGCATGTTAACAATGCAACTAAT   6262
gb:MT106052|Organism:Severe      AAGAAAGGAGCTAAATTGTTACATAAACCTATTGTTTGGCATGTTAACAATGCAACTAAT   6262
gb:LC522975|Organism:Severe      AAGAAAGGAGCTAAATTGTTACATAAACCTATTGTTTGGCATGTTAACAATGCAACTAAT   6259
gb:LC522973|Organism:Severe      AAGAAAGGAGCTAAATTGTTACATAAACCTATTGTTTGGCATGTTAACAATGCAACTAAT   6259
gb:LC522974|Organism:Severe      AAGAAAGGAGCTAAATTGTTACATAAACCTATTGTTTGGCATGTTAACAATGCAACTAAT   6259
gb:MN985325|Organism:Severe      AAGAAAGGAGCTAAATTGTTACATAAACCTATTGTTTGGCATGTTAACAATGCAACTAAT   6262
gb:MT020881|Organism:Severe      AAGAAAGGAGCTAAATTGTTACATAAACCTATTGTTTGGCATGTTAACAATGCAACTAAT   6262
gb:MT020880|Organism:Severe      AAGAAAGGAGCTAAATTGTTACATAAACCTATTGTTTGGCATGTTAACAATGCAACTAAT   6262
gb:MT066175|Organism:Severe      AAGAAAGGAGCTAAATTGTTACATAAACCTATTGTTTGGCATGTTAACAATGCAACTAAT   6262
gb:MN997409|Organism:Severe      AAGAAAGGAGCTAAATTGTTACATAAACCTATTGTTTGGCATGTTAACAATGCAACTAAT   6262
gb:MN938384|Organism:Severe      AAGAAAGGAGCTAAATTGTTACATAAACCTATTGTTTGGCATGTTAACAATGCAACTAAT   6230
gb:MT044258|Organism:Severe      AAGAAAGGAGCTAAATTGTTACATAAACCTATTGTTTGGCATGTTAACAATGCAACTAAT   6238
gb:MT039890|Organism:Severe      AAGAAAGGAGCTAAATTGTTACATAAACCTATTGTTTGGCATGTTAACAATGCAACTAAT   6262
gb:MN988713|Organism:Severe      AAGAAAGGAGCTAAATTGTTACATAAACCTATTGTTTGGCATGTTAACAATGCAACTAAT   6262
gb:LC521925|Organism:Severe      AAGAAAGGAGCTAAATTGTTACATAAACCTATTGTTTGGCATGTTAACAATGCAACTAAT   6235
gb:MT093571|Organism:Severe      AAGAAAGGAGCTAAATTGTTACATAAACCTATTGTTTGGCATGTTAACAATGCAACTAAT   6262
```

```
gb:MT039887|Organism:Severe      AAGAAAGGAGCTAAATTGTTACATAAACCTATTGTTTGGCATGTTAACAATGCAACTAAT    6262
gb:MT019530|Organism:Severe      AAGAAAGGAGCTAAATTGTTACATAAACCTATTGTTTGGCATGTTAACAATGCAACTAAT    6262
gb:MT039888|Organism:Severe      AAGAAAGGAGCTAAATTGTTACATAAACCTATTGTTTGGCATGTTAACAATGCAACTAAT    6262
gb:LC522972|Organism:Severe      AAGAAAGGAGCTAAATTGTTACATAAACCTATTGTTTGGCATGTTAACAATGCAACTAAT    6259
gb:MT027063|Organism:Severe      AAGAAAGGAGCTAAATTGTTACATAAACCTATTGTTTGGCATGTTAACAATGCAACTAAT    6262
gb:MT027062|Organism:Severe      AAGAAAGGAGCTAAATTGTTACATAAACCTATTGTTTGGCATGTTAACAATGCAACTAAT    6262
gb:MT019529|Organism:Severe      AAGAAAGGAGCTAAATTGTTACATAAACCTATTGTTTGGCATGTTAACAATGCAACTAAT    6262
gb:MN996529|Organism:Severe      AAGAAAGGAGCTAAATTGTTACATAAACCTATTGTTTGGCATGTTAACAATGCAACTAAT    6250
gb:MN996531|Organism:Severe      AAGAAAGGAGCTAAATTGTTACATAAACCTATTGTTTGGCATGTTAACAATGCAACTAAT    6249
gb:MT066176|Organism:Severe      AAGAAAGGAGCTAAATTGTTACATAAACCTATTGTTTGGCATGTTAACAATGCAACTAAT    6262
gb:MT027064|Organism:Severe      AAGAAAGGAGCTAAATTGTTACATAAACCTATTGTTTGGCATGTTAACAATGCAACTAAT    6262
gb:MN994468|Organism:Severe      AAGAAAGGAGCTAAATTGTTACATAAACCTATTGTTTGGCATGTTAACAATGCAACTAAT    6262
gb:MT072688|Organism:Severe      AAGAAAGGAGCTAAATTGTTACATAAACCTATTGTTTGGCATGTTAACAATGCAACTAAT    6247
gb:MN996527|Organism:Severe      AAGAAAGGAGCTAAATTGTTACATAAACCTATTGTTTGGCATGTTAACAATGCAACTAAT    6229
gb:MT093631|Organism:Severe      AAGAAAGGAGCTAAATTGTTACATAAACCTATTGTTTGGCATGTTAACAATGCAACTAAT    6300
gb:MT106053|Organism:Severe      AAGAAAGGAGCTAAATTGTTACATAAACCTATTGTTTGGCATGTTAACAATGCAACTAAT    6262
gb:MT019533|Organism:Severe      AAGAAAGGAGCTAAATTGTTACATAAACCTATTGTTTGGCATGTTAACAATGCAACTAAT    6262
gb:MT019531|Organism:Severe      AAGAAAGGAGCTAAATTGTTACATAAACCTATTGTTTGGCATGTTAACAATGCAACTAAT    6262
gb:MN996528|Organism:Severe      AAGAAAGGAGCTAAATTGTTACATAAACCTATTGTTTGGCATGTTAACAATGCAACTAAT    6262
gb:MN996530|Organism:Severe      AAGAAAGGAGCTAAATTGTTACATAAACCTATTGTTTGGCATGTTAACAATGCAACTAAT    6248
gb:MN908947|Organism:Severe      AAGAAAGGAGCTAAATTGTTACATAAACCTATTGTTTGGCATGTTAACAATGCAACTAAT    6262
gb:MT019532|Organism:Severe      AAGAAAGGAGCTAAATTGTTACATAAACCTATTGTTTGGCATGTTAACAATGCAACTAAT    6262
                                 ************************************************************

gb:MT020781|Organism:Severe      AAAGCCACGTATAAACCAAATACCTGGTGTATACGTTGTCTTTGGAGCACAAAACCAGTT    6310
gb:MT007544|Organism:Severe      AAAGCCACGTATAAACCAAATACCTGGTGTATACGTTGTCTTTGGAGCACAAAACCAGTT    6322
gb:MN994467|Organism:Severe      AAAGCCACGTATAAACCAAATACCTGGTGTATACGTTGTCTTTGGAGCACAAAACCAGTT    6322
gb:MT044257|Organism:Severe      AAAGCCACGTATAAACCAAATACCTGGTGTATACGTTGTCTTTGGAGCACAAAACCAGTT    6322
gb:MT106054|Organism:Severe      AAAGCCACGTATAAACCAAATACCTGGTGTATACGTTGTCTTTGGAGCACAAAACCAGTT    6322
gb:MT049951|Organism:Severe      AAAGCCACGTATAAACCAAATACCTGGTGTATACGTTGTCTTTGGAGCACAAAACCAGTT    6322
gb:MN975262|Organism:Severe      AAAGCCACGTATAAACCAAATACCTGGTGTATACGTTGTCTTTGGAGCACAAAACCAGTT    6322
gb:MT106052|Organism:Severe      AAAGCCACGTATAAACCAAATACCTGGTGTATACGTTGTCTTTGGAGCACAAAACCAGTT    6322
gb:LC522975|Organism:Severe      AAAGCCACGTATAAACCAAATACCTGGTGTATACGTTGTCTTTGGAGCACAAAACCAGTT    6319
gb:LC522973|Organism:Severe      AAAGCCACGTATAAACCAAATACCTGGTGTATACGTTGTCTTTGGAGCACAAAACCAGTT    6319
gb:LC522974|Organism:Severe      AAAGCCACGTATAAACCAAATACCTGGTGTATACGTTGTCTTTGGAGCACAAAACCAGTT    6319
gb:MN985325|Organism:Severe      AAAGCCACGTATAAACCAAATACCTGGTGTATACGTTGTCTTTGGAGCACAAAACCAGTT    6322
gb:MT020881|Organism:Severe      AAAGCCACGTATAAACCAAATACCTGGTGTATACGTTGTCTTTGGAGCACAAAACCAGTT    6322
gb:MT020880|Organism:Severe      AAAGCCACGTATAAACCAAATACCTGGTGTATACGTTGTCTTTGGAGCACAAAACCAGTT    6322
gb:MT066175|Organism:Severe      AAAGCCACGTATAAACCAAATACCTGGTGTATACGTTGTCTTTGGAGCACAAAACCAGTT    6322
gb:MN997409|Organism:Severe      AAAGCCACGTATAAACCAAATACCTGGTGTATACGTTGTCTTTGGAGCACAAAACCAGTT    6322
gb:MN938384|Organism:Severe      AAAGCCACGTATAAACCAAATACCTGGTGTATACGTTGTCTTTGGAGCACAAAACCAGTT    6290
gb:MT044258|Organism:Severe      AAAGCCACGTATAAACCAAATACCTGGTGTATACGTTGTCTTTGGAGCACAAAACCAGTT    6298
gb:MT039890|Organism:Severe      AAAGCCACGTATAAACCAAATACCTGGTGTATACGTTGTCTTTGGAGCACAAAACCAGTT    6322
gb:MN988713|Organism:Severe      AAAGCCACGTATAAACCAAATACCTGGTGTATACGTTGTCTTTGGAGCACAAAACCAGTT    6322
gb:LC521925|Organism:Severe      AAAGCCACGTATAAACCAAATACCTGGTGTATACGTTGTCTTTGGAGCACAAAACCAGTT    6295
gb:MT093571|Organism:Severe      AAAGCCACGTATAAACCAAATACCTGGTGTATACGTTGTCTTTGGAGCACAAAACCAGTT    6322
gb:MT039887|Organism:Severe      AAAGCCACGTATAAACCAAATACCTGGTGTATACGTTGTCTTTGGAGCACAAAACCAGTT    6322
gb:MT019530|Organism:Severe      AAAGCCACGTATAAACCAAATACCTGGTGTATACGTTGTCTTTGGAGCACAAAACCAGTT    6322
gb:MT039888|Organism:Severe      AAAGCCACGTATAAACCAAATACCTGGTGTATACGTTGTCTTTGGAGCACAAAACCAGTT    6322
gb:LC522972|Organism:Severe      AAAGCCACGTATAAACCAAATACCTGGTGTATACGTTGTCTTTGGAGCACAAAACCAGTT    6319
gb:MT027063|Organism:Severe      AAAGCCACGTATAAACCAAATACCTGGTGTATACGTTGTCTTTGGAGCACAAAACCAGTT    6322
gb:MT027062|Organism:Severe      AAAGCCACGTATAAACCAAATACCTGGTGTATACGTTGTCTTTGGAGCACAAAACCAGTT    6322
gb:MT019529|Organism:Severe      AAAGCCACGTATAAACCAAATACCTGGTGTATACGTTGTCTTTGGAGCACAAAACCAGTT    6322
gb:MN996529|Organism:Severe      AAAGCCACGTATAAACCAAATACCTGGTGTATACGTTGTCTTTGGAGCACAAAACCAGTT    6310
gb:MN996531|Organism:Severe      AAAGCCACGTATAAACCAAATACCTGGTGTATACGTTGTCTTTGGAGCACAAAACCAGTT    6309
gb:MT066176|Organism:Severe      AAAGCCACGTATAAACCAAATACCTGGTGTATACGTTGTCTTTGGAGCACAAAACCAGTT    6322
gb:MT027064|Organism:Severe      AAAGCCACGTATAAACCAAATACCTGGTGTATACGTTGTCTTTGGAGCACAAAACCAGTT    6322
gb:MN994468|Organism:Severe      AAAGCCACGTATAAACCAAATACCTGGTGTATACGTTGTCTTTGGAGCACAAAACCAGTT    6322
gb:MT072688|Organism:Severe      AAAGCCACGTATAAACCAAATACCTGGTGTATACGTTGTCTTTGGAGCACAAAACCAGTT    6307
gb:MN996527|Organism:Severe      AAAGCCACGTATAAACCAAATACCTGGTGTATACGTTGTCTTTGGAGCACAAAACCAGTT    6289
gb:MT093631|Organism:Severe      AAAGCCACGTATAAACCAAATACCTGGTGTATACGTTGTCTTTGGAGCACAAAACCAGTT    6360
gb:MT106053|Organism:Severe      AAAGCCACGTATAAACCAAATACCTGGTGTATACGTTGTCTTTGGAGCACAAAACCAGTT    6322
gb:MT019533|Organism:Severe      AAAGCCACGTATAAACCAAATACCTGGTGTATACGTTGTCTTTGGAGCACAAAACCAGTT    6322
gb:MT019531|Organism:Severe      AAAGCCACGTATAAACCAAATACCTGGTGTATACGTTGTCTTTGGAGCACAAAACCAGTT    6322
gb:MN996528|Organism:Severe      AAAGCCACGTATAAACCAAATACCTGGTGTATACGTTGTCTTTGGAGCACAAAACCAGTT    6322
```

```
gb:MN996530|Organism:Severe      AAAGCCACGTATAAACCAAATACCTGGTGTATACGTTGTCTTTGGAGCACAAAACCAGTT   6308
gb:MN908947|Organism:Severe      AAAGCCACGTATAAACCAAATACCTGGTGTATACGTTGTCTTTGGAGCACAAAACCAGTT   6322
gb:MT019532|Organism:Severe      AAAGCCACGTATAAACCAAATACCTGGTGTATACGTTGTCTTTGGAGCACAAAACCAGTT   6322
                                 ************************************************************

gb:MT020781|Organism:Severe      GAAACATCAAATTCGTTTGATGTACTGAAGTCAGAGGACGCGCAGGGAATGGATAATCTT   6370
gb:MT007544|Organism:Severe      GAAACATCAAATTCGTTTGATGTACTGAAGTCAGAGGACGCGCAGGGAATGGATAATCTT   6382
gb:MN994467|Organism:Severe      GAAACATCAAATTCGTTTGATGTACTGAAGTCAGAGGACGCGCAGGGAATGGATAATCTT   6382
gb:MT044257|Organism:Severe      GAAACATCAAATTCGTTTGATGTACTGAAGTCAGAGGACGCGCAGGGAATGGATAATCTT   6382
gb:MT106054|Organism:Severe      GAAACATCAAATTCGTTTGATGTACTGAAGTCAGAGGACGCGCAGGGAATGGATAATCTT   6382
gb:MT049951|Organism:Severe      GAAACATCAAATTCGTTTGATGTACTGAAGTCAGAGGACGCGCAGGGAATGGATAATCTT   6382
gb:MN975262|Organism:Severe      GAAACATCAAATTCGTTTGATGTACTGAAGTCAGAGGACGCGCAGGGAATGGATAATCTT   6382
gb:MT106052|Organism:Severe      GAAACATCAAATTCGTTTGATGTACTGAAGTCAGAGGACGCGCAGGGAATGGATAATCTT   6382
gb:LC522975|Organism:Severe      GAAACATCAAATTCGTTTGATGTACTGAAGTCAGAGGACGCGCAGGGAATGGATAATCTT   6379
gb:LC522973|Organism:Severe      GAAACATCAAATTCGTTTGATGTACTGAAGTCAGAGGACGCGCAGGGAATGGATAATCTT   6379
gb:LC522974|Organism:Severe      GAAACATCAAATTCGTTTGATGTACTGAAGTCAGAGGACGCGCAGGGAATGGATAATCTT   6379
gb:MN985325|Organism:Severe      GAAACATCAAATTCGTTTGATGTACTGAAGTCAGAGGACGCGCAGGGAATGGATAATCTT   6382
gb:MT020881|Organism:Severe      GAAACATCAAATTCGTTTGATGTACTGAAGTCAGAGGACGCGCAGGGAATGGATAATCTT   6382
gb:MT020880|Organism:Severe      GAAACATCAAATTCGTTTGATGTACTGAAGTCAGAGGACGCGCAGGGAATGGATAATCTT   6382
gb:MT066175|Organism:Severe      GAAACATCAAATTCGTTTGATGTACTGAAGTCAGAGGACGCGCAGGGAATGGATAATCTT   6382
gb:MN997409|Organism:Severe      GAAACATCAAATTCGTTTGATGTACTGAAGTCAGAGGACGCGCAGGGAATGGATAATCTT   6382
gb:MN938384|Organism:Severe      GAAACATCAAATTCGTTTGATGTACTGAAGTCAGAGGACGCGCAGGGAATGGATAATCTT   6350
gb:MT044258|Organism:Severe      GAAACATCAAATTCGTTTGATGTACTGAAGTCAGAGGACGCGCAGGGAATGGATAATCTT   6358
gb:MT039890|Organism:Severe      GAAACATCAAATTCGTTTGATGTACTGAAGTCAGAGGACGCGCAGGGAATGGATAATCTT   6382
gb:MN988713|Organism:Severe      GAAACATCAAATTCGTTTGATGTACTGAAGTCAGAGGACGCGCAGGGAATGGATAATCTT   6382
gb:LC521925|Organism:Severe      GAAACATCAAATTCGTTTGATGTACTGAAGTCAGAGGACGCGCAGGGAATGGATAATCTT   6355
gb:MT093571|Organism:Severe      GAAACATCAAATTCGTTTGATGTACTGAAGTCAGAGGACGCGCAGGGAATGGATAATCTT   6382
gb:MT039887|Organism:Severe      GAAACATCAAATTCGTTTGATGTACTGAAGTCAGAGGACGCGCAGGGAATGGATAATCTT   6382
gb:MT019530|Organism:Severe      GAAACATCAAATTCGTTTGATGTACTGAAGTCAGAGGACGCGCAGGGAATGGATAATCTT   6382
gb:MT039888|Organism:Severe      GAAACATCAAATTCGTTTGATGTACTGAAGTCAGAGGACGCGCAGGGAATGGATAATCTT   6382
gb:LC522972|Organism:Severe      GAAACATCAAATTCGTTTGATGTACTGAAGTCAGAGGACGCGCAGGGAATGGATAATCTT   6379
gb:MT027063|Organism:Severe      GAAACATCAAATTCGTTTGATGTACTGAAGTCAGAGGACGCGCAGGGAATGGATAATCTT   6382
gb:MT027062|Organism:Severe      GAAACATCAAATTCGTTTGATGTACTGAAGTCAGAGGACGCGCAGGGAATGGATAATCTT   6382
gb:MT019529|Organism:Severe      GAAACATCAAATTCGTTTGATGTACTGAAGTCAGAGGACGCGCAGGGAATGGATAATCTT   6382
gb:MN996529|Organism:Severe      GAAACATCAAATTCGTTTGATGTACTGAAGTCAGAGGACGCGCAGGGAATGGATAATCTT   6370
gb:MN996531|Organism:Severe      GAAACATCAAATTCGTTTGATGTACTGAAGTCAGAGGACGCGCAGGGAATGGATAATCTT   6369
gb:MT066176|Organism:Severe      GAAACATCAAATTCGTTTGATGTACTGAAGTCAGAGGACGCGCAGGGAATGGATAATCTT   6382
gb:MT027064|Organism:Severe      GAAACATCAAATTCGTTTGATGTACTGAAGTCAGAGGACGCGCAGGGAATGGATAATCTT   6382
gb:MN994468|Organism:Severe      GAAACATCAAATTCGTTTGATGTACTGAAGTCAGAGGACGCGCAGGGAATGGATAATCTT   6382
gb:MT072688|Organism:Severe      GAAACATCAAATTCGTTTGATGTACTGAAGTCAGAGGACGCGCAGGGAATGGATAATCTT   6367
gb:MN996527|Organism:Severe      GAAACATCAAATTCGTTTGATGTACTGAAGTCAGAGGACGCGCAGGGAATGGATAATCTT   6349
gb:MT093631|Organism:Severe      GAAACATCAAATTCGTTTGATGTACTGAAGTCAGAGGACGCGCAGGGAATGGATAATCTT   6420
gb:MT106053|Organism:Severe      GAAACATCAAATTCGTTTGATGTACTGAAGTCAGAGGACGCGCAGGGAATGGATAATCTT   6382
gb:MT019533|Organism:Severe      GAAACATCAAATTCGTTTGATGTACTGAAGTCAGAGGACGCGCAGGGAATGGATAATCTT   6382
gb:MT019531|Organism:Severe      GAAACATCAAATTCGTTTGATGTACTGAAGTCAGAGGACGCGCAGGGAATGGATAATCTT   6382
gb:MN996528|Organism:Severe      GAAACATCAAATTCGTTTGATGTACTGAAGTCAGAGGACGCGCAGGGAATGGATAATCTT   6382
gb:MN996530|Organism:Severe      GAAACATCAAATTCGTTTGATGTACTGAAGTCAGAGGACGCGCAGGGAATGGATAATCTT   6368
gb:MN908947|Organism:Severe      GAAACATCAAATTCGTTTGATGTACTGAAGTCAGAGGACGCGCAGGGAATGGATAATCTT   6382
gb:MT019532|Organism:Severe      GAAACATCAAATTCGTTTGATGTACTGAAGTCAGAGGACGCGCAGGGAATGGATAATCTT   6382
                                 ************************************************************

gb:MT020781|Organism:Severe      GCCTGCGAAGATCTAAAACCAGTCTCTGAAGAAGTAGTGGAAAATCCTACCATACAGAAA   6430
gb:MT007544|Organism:Severe      GCCTGCGAAGATCTAAAACCAGTCTCTGAAGAAGTAGTGGAAAATCCTACCATACAGAAA   6442
gb:MN994467|Organism:Severe      GCCTGCGAAGATCTAAAACCAGTCTCTGAAGAAGTAGTGGAAAATCCTACCATACAGAAA   6442
gb:MT044257|Organism:Severe      GCCTGCGAAGATCTAAAACCAGTCTCTGAAGAAGTAGTGGAAAATCCTACCATACAGAAA   6442
gb:MT106054|Organism:Severe      GCCTGCGAAGATCTAAAACCAGTCTCTGAAGAAGTAGTGGAAAATCCTACCATACAGAAA   6442
gb:MT049951|Organism:Severe      GCCTGCGAAGATCTAAAACCAGTCTCTGAAGAAGTAGTGGAAAATCCTACCATACAGAAA   6442
gb:MN975262|Organism:Severe      GCCTGCGAAGATCTAAAACCAGTCTCTGAAGAAGTAGTGGAAAATCCTACCATACAGAAA   6442
gb:MT106052|Organism:Severe      GCCTGCGAAGATCTAAAACCAGTCTCTGAAGAAGTAGTGGAAAATCCTACCATACAGAAA   6442
gb:LC522975|Organism:Severe      GCCTGCGAAGATCTAAAACCAGTCTCTGAAGAAGTAGTGGAAAATCCTACCATACAGAAA   6439
gb:LC522973|Organism:Severe      GCCTGCGAAGATCTAAAACCAGTCTCTGAAGAAGTAGTGGAAAATCCTACCATACAGAAA   6439
gb:LC522974|Organism:Severe      GCCTGCGAAGATCTAAAACCAGTCTCTGAAGAAGTAGTGGAAAATCCTACCATACAGAAA   6439
gb:MN985325|Organism:Severe      GCCTGCGAAGATCTAAAACCAGTCTCTGAAGAAGTAGTGGAAAATCCTACCATACAGAAA   6442
gb:MT020881|Organism:Severe      GCCTGCGAAGATCTAAAACCAGTCTCTGAAGAAGTAGTGGAAAATCCTACCATACAGAAA   6442
gb:MT020880|Organism:Severe      GCCTGCGAAGATCTAAAACCAGTCTCTGAAGAAGTAGTGGAAAATCCTACCATACAGAAA   6442
```

```
gb:MT066175|Organism:Severe      GCCTGCGAAGATCTAAAACCAGTCTCTGAAGAAGTAGTGGAAAATCCTACCATACAGAAA   6442
gb:MN997409|Organism:Severe      GCCTGCGAAGATCTAAAACCAGTCTCTGAAGAAGTAGTGGAAAATCCTACCATACAGAAA   6442
gb:MN938384|Organism:Severe      GCCTGCGAAGATCTAAAACCAGTCTCTGAAGAAGTAGTGGAAAATCCTACCATACAGAAA   6410
gb:MT044258|Organism:Severe      GCCTGCGAAGATCTAAAACCAGTCTCTGAAGAAGTAGTGGAAAATCCTACCATACAGAAA   6418
gb:MT039890|Organism:Severe      GCCTGCGAAGATCTAAAACCAGTCTCTGAAGAAGTAGTGGAAAATCCTACCATACAGAAA   6442
gb:MN988713|Organism:Severe      GCCTGCGAAGATCTAAAACCAGTCTCTGAAGAAGTAGTGGAAAATCCTACCATACAGAAA   6442
gb:LC521925|Organism:Severe      GCCTGCGAAGATCTAAAACCAGTCTCTGAAGAAGTAGTGGAAAATCCTACCATACAGAAA   6415
gb:MT093571|Organism:Severe      GCCTGCGAAGATCTAAAACCAGTCTCTGAAGAAGTAGTGGAAAATCCTACCATACAGAAA   6442
gb:MT039887|Organism:Severe      GCCTGCGAAGATCTAAAACCAGTCTCTGAAGAAGTAGTGGAAAATCCTACCATACAGAAA   6442
gb:MT019530|Organism:Severe      GCCTGCGAAGATCTAAAACCAGTCTCTGAAGAAGTAGTGGAAAATCCTACCATACAGAAA   6442
gb:MT039888|Organism:Severe      GCCTGCGAAGATCTAAAACCAGTCTCTGAAGAAGTAGTGGAAAATCCTACCATACAGAAA   6442
gb:LC522972|Organism:Severe      GCCTGCGAAGATCTAAAACCAGTCTCTGAAGAAGTAGTGGAAAATCCTACCATACAGAAA   6439
gb:MT027063|Organism:Severe      GCCTGCGAAGATCTAAAACCAGTCTCTGAAGAAGTAGTGGAAAATCCTACCATACAGAAA   6442
gb:MT027062|Organism:Severe      GCCTGCGAAGATCTAAAACCAGTCTCTGAAGAAGTAGTGGAAAATCCTACCATACAGAAA   6442
gb:MT019529|Organism:Severe      GCCTGCGAAGATCTAAAACCAGTCTCTGAAGAAGTAGTGGAAAATCCTACCATACAGAAA   6442
gb:MN996529|Organism:Severe      GCCTGCGAAGATCTAAAACCAGTCTCTGAAGAAGTAGTGGAAAATCCTACCATACAGAAA   6430
gb:MN996531|Organism:Severe      GCCTGCGAAGATCTAAAACCAGTCTCTGAAGAAGTAGTGGAAAATCCTACCATACAGAAA   6429
gb:MT066176|Organism:Severe      GCCTGCGAAGATCTAAAACCAGTCTCTGAAGAAGTAGTGGAAAATCCTACCATACAGAAA   6442
gb:MT027064|Organism:Severe      GCCTGCGAAGATCTAAAACCAGTCTCTGAAGAAGTAGTGGAAAATCCTACCATACAGAAA   6442
gb:MN994468|Organism:Severe      GCCTGCGAAGATCTAAAACCAGTCTCTGAAGAAGTAGTGGAAAATCCTACCATACAGAAA   6442
gb:MT072688|Organism:Severe      GCCTGCGAAGATCTAAAACCAGTCTCTGAAGAAGTAGTGGAAAATCCTACCATACAGAAA   6427
gb:MN996527|Organism:Severe      GCCTGCGAAGATCTAAAACCAGTCTCTGAAGAAGTAGTGGAAAATCCTACCATACAGAAA   6409
gb:MT093631|Organism:Severe      GCCTGCGAAGATCTAAAACCAGTCTCTGAAGAAGTAGTGGAAAATCCTACCATACAGAAA   6480
gb:MT106053|Organism:Severe      GCCTGCGAAGATCTAAAACCAGTCTCTGAAGAAGTAGTGGAAAATCCTACCATACAGAAA   6442
gb:MT019533|Organism:Severe      GCCTGCGAAGATCTAAAACCAGTCTCTGAAGAAGTAGTGGAAAATCCTACCATACAGAAA   6442
gb:MT019531|Organism:Severe      GCCTGCGAAGATCTAAAACCAGTCTCTGAAGAAGTAGTGGAAAATCCTACCATACAGAAA   6442
gb:MN996528|Organism:Severe      GCCTGCGAAGATCTAAAACCAGTCTCTGAAGAAGTAGTGGAAAATCCTACCATACAGAAA   6442
gb:MN996530|Organism:Severe      GCCTGCGAAGATCTAAAACCAGTCTCTGAAGAAGTAGTGGAAAATCCTACCATACAGAAA   6428
gb:MN908947|Organism:Severe      GCCTGCGAAGATCTAAAACCAGTCTCTGAAGAAGTAGTGGAAAATCCTACCATACAGAAA   6442
gb:MT019532|Organism:Severe      GCCTGCGAAGATCTAAAACCAGTCTCTGAAGAAGTAGTGGAAAATCCTACCATACAGAAA   6442
                                 ************************************************************

gb:MT020781|Organism:Severe      GACGTTCTTGAGTGTAATGTGAAAACTACCGAAGTTGTAGGAGACATTATACTTAAACCA   6490
gb:MT007544|Organism:Severe      GACGTTCTTGAGTGTAATGTGAAAACTACCGAAGTTGTAGGAGACATTATACTTAAACCA   6502
gb:MN994467|Organism:Severe      GACGTTCTTGAGTGTAATGTGAAAACTACCGAAGTTGTAGGAGACATTATACTTAAACCA   6502
gb:MT044257|Organism:Severe      GACGTTCTTGAGTGTAATGTGAAAACTACCGAAGTTGTAGGAGACATTATACTTAAACCA   6502
gb:MT106054|Organism:Severe      GACGTTCTTGAGTGTAATGTGAAAACTACCGAAGTTGTAGGAGACATTATACTTAAACCA   6502
gb:MT049951|Organism:Severe      GACGTTCTTGAGTGTAATGTGAAAACTACCGAAGTTGTAGGAGACATTATACTTAAACCA   6502
gb:MN975262|Organism:Severe      GACGTTCTTGAGTGTAATGTGAAAACTACCGAAGTTGTAGGAGACATTATACTTAAACCA   6502
gb:MT106052|Organism:Severe      GACGTTCTTGAGTGTAATGTGAAAACTACCGAAGTTGTAGGAGACATTATACTTAAACCA   6502
gb:LC522975|Organism:Severe      GACGTTCTTGAGTGTAATGTGAAAACTACCGAAGTTGTAGGAGACATTATACTTAAACCA   6499
gb:LC522973|Organism:Severe      GACGTTCTTGAGTGTAATGTGAAAACTACCGAAGTTGTAGGAGACATTATACTTAAACCA   6499
gb:LC522974|Organism:Severe      GACGTTCTTGAGTGTAATGTGAAAACTACCGAAGTTGTAGGAGACATTATACTTAAACCA   6499
gb:MN985325|Organism:Severe      GACGTTCTTGAGTGTAATGTGAAAACTACCGAAGTTGTAGGAGACATTATACTTAAACCA   6502
gb:MT020881|Organism:Severe      GACGTTCTTGAGTGTAATGTGAAAACTACCGAAGTTGTAGGAGACATTATACTTAAACCA   6502
gb:MT020880|Organism:Severe      GACGTTCTTGAGTGTAATGTGAAAACTACCGAAGTTGTAGGAGACATTATACTTAAACCA   6502
gb:MT066175|Organism:Severe      GACGTTCTTGAGTGTAATGTGAAAACTACCGAAGTTGTAGGAGACATTATACTTAAACCA   6502
gb:MN997409|Organism:Severe      GACGTTCTTGAGTGTAATGTGAAAACTACCGAAGTTGTAGGAGACATTATACTTAAACCA   6502
gb:MN938384|Organism:Severe      GACGTTCTTGAGTGTAATGTGAAAACTACCGAAGTTGTAGGAGACATTATACTTAAACCA   6470
gb:MT044258|Organism:Severe      GACGTTCTTGAGTGTAATGTGAAAACTACCGAAGTTGTAGGAGACATTATACTTAAACCA   6478
gb:MT039890|Organism:Severe      GACGTTCTTGAGTGTAATGTGAAAACTACCGAAGTTGTAGGAGACATTATACTTAAACCA   6502
gb:MN988713|Organism:Severe      GACGTTCTTGAGTGTAATGTGAAAACTACCGAAGTTGTAGGAGACATTATACTTAAACCA   6502
gb:LC521925|Organism:Severe      GACGTTCTTGAGTGTAATGTGAAAACTACCGAAGTTGTAGGAGACATTATACTTAAACCA   6475
gb:MT093571|Organism:Severe      GACGTTCTTGAGTGTAATGTGAAAACTACCGAAGTTGTAGGAGACATTATACTTAAACCA   6502
gb:MT039887|Organism:Severe      GACGTTCTTGAGTGTAATGTGAAAACTACCGAAGTTGTAGGAGACATTATACTTAAACCA   6502
gb:MT019530|Organism:Severe      GACGTTCTTGAGTGTAATGTGAAAACTACCGAAGTTGTAGGAGACATTATACTTAAACCA   6502
gb:MT039888|Organism:Severe      GACGTTCTTGAGTGTAATGTGAAAACTACCGAAGTTGTAGGAGACATTATACTTAAACCA   6502
gb:LC522972|Organism:Severe      GACGTTCTTGAGTGTAATGTGAAAACTACCGAAGTTGTAGGAGACATTATACTTAAACCA   6499
gb:MT027063|Organism:Severe      GACGTTCTTGAGTGTAATGTGAAAACTACCGAAGTTGTAGGAGACATTATACTTAAACCA   6502
gb:MT027062|Organism:Severe      GACGTTCTTGAGTGTAATGTGAAAACTACCGAAGTTGTAGGAGACATTATACTTAAACCA   6502
gb:MT019529|Organism:Severe      GACGTTCTTGAGTGTAATGTGAAAACTACCGAAGTTGTAGGAGACATTATACTTAAACCA   6502
gb:MN996529|Organism:Severe      GACGTTCTTGAGTGTAATGTGAAAACTACCGAAGTTGTAGGAGACATTATACTTAAACCA   6490
gb:MN996531|Organism:Severe      GACGTTCTTGAGTGTAATGTGAAAACTACCGAAGTTGTAGGAGACATTATACTTAAACCA   6489
gb:MT066176|Organism:Severe      GACGTTCTTGAGTGTAATGTGAAAACTACCGAAGTTGTAGGAGACATTATACTTAAACCA   6502
gb:MT027064|Organism:Severe      GACGTTCTTGAGTGTAATGTGAAAACTACCGAAGTTGTAGGAGACATTATACTTAAACCA   6502
```

```
gb:MN994468|Organism:Severe      GACGTTCTTGAGTGTAATGTGAAAACTACCGAAGTTGTAGGAGACATTATACTTAAACCA   6502
gb:MT072688|Organism:Severe      GACGTTCTTGAGTGTAATGTGAAAACTACCGAAGTTGTAGGAGACATTATACTTAAACCA   6487
gb:MN996527|Organism:Severe      GACGTTCTTGAGTGTAATGTGAAAACTACCGAAGTTGTAGGAGACATTATACTTAAACCA   6469
gb:MT093631|Organism:Severe      GACGTTCTTGAGTGTAATGTGAAAACTACCGAAGTTGTAGGAGACATTATACTTAAACCA   6540
gb:MT106053|Organism:Severe      GACGTTCTTGAGTGTAATGTGAAAACTACCGAAGTTGTAGGAGACATTATACTTAAACCA   6502
gb:MT019533|Organism:Severe      GACGTTCTTGAGTGTAATGTGAAAACTACCGAAGTTGTAGGAGACATTATACTTAAACCA   6502
gb:MT019531|Organism:Severe      GACGTTCTTGAGTGTAATGTGAAAACTACCGAAGTTGTAGGAGACATTATACTTAAACCA   6502
gb:MN996528|Organism:Severe      GACGTTCTTGAGTGTAATGTGAAAACTACCGAAGTTGTAGGAGACATTATACTTAAACCA   6502
gb:MN996530|Organism:Severe      GACGTTCTTGAGTGTAATGTGAAAACTACCGAAGTTGTAGGAGACATTATACTTAAACCA   6488
gb:MN908947|Organism:Severe      GACGTTCTTGAGTGTAATGTGAAAACTACCGAAGTTGTAGGAGACATTATACTTAAACCA   6502
gb:MT019532|Organism:Severe      GACGTTCTTGAGTGTAATGTGAAAACTACCGAAGTTGTAGGAGACATTATACTTAAACCA   6502
                                 ************************************************************

gb:MT020781|Organism:Severe      GCAAATAATAGTTTAAAAATTACAGAAGAGGTTGGCCACACAGATCTAATGGCTGCTTAT   6550
gb:MT007544|Organism:Severe      GCAAATAATAGTTTAAAAATTACAGAAGAGGTTGGCCACACAGATCTAATGGCTGCTTAT   6562
gb:MN994467|Organism:Severe      GCAAATAATAGTTTAAAAATTACAGAAGAGGTTGGCCACACAGATCTAATGGCTGCTTAT   6562
gb:MT044257|Organism:Severe      GCAAATAATAGTTTAAAAATTACAGAAGAGGTTGGCCACACAGATCTAATGGCTGCTTAT   6562
gb:MT106054|Organism:Severe      GCAAATAATAGTTTAAAAATTACAGAAGAGGTTGGCCACACAGATCTAATGGCTGCTTAT   6562
gb:MT049951|Organism:Severe      GCAAATAATAGTTTAAAAATTACAGAAGAGGTTGGCCACACAGATCTAATGGCTGCTTAT   6562
gb:MN975262|Organism:Severe      GCAAATAATAGTTTAAAAATTACAGAAGAGGTTGGCCACACAGATCTAATGGCTGCTTAT   6562
gb:MT106052|Organism:Severe      GCAAATAATAGTTTAAAAATTACAGAAGAGGTTGGCCACACAGATCTAATGGCTGCTTAT   6562
gb:LC522975|Organism:Severe      GCAAATAATAGTTTAAAAATTACAGAAGAGGTTGGCCACACAGATCTAATGGCTGCTTAT   6559
gb:LC522973|Organism:Severe      GCAAATAATAGTTTAAAAATTACAGAAGAGGTTGGCCACACAGATCTAATGGCTGCTTAT   6559
gb:LC522974|Organism:Severe      GCAAATAATAGTTTAAAAATTACAGAAGAGGTTGGCCACACAGATCTAATGGCTGCTTAT   6559
gb:MN985325|Organism:Severe      GCAAATAATAGTTTAAAAATTACAGAAGAGGTTGGCCACACAGATCTAATGGCTGCTTAT   6562
gb:MT020881|Organism:Severe      GCAAATAATAGTTTAAAAATTACAGAAGAGGTTGGCCACACAGATCTAATGGCTGCTTAT   6562
gb:MT020880|Organism:Severe      GCAAATAATAGTTTAAAAATTACAGAAGAGGTTGGCCACACAGATCTAATGGCTGCTTAT   6562
gb:MT066175|Organism:Severe      GCAAATAATAGTTTAAAAATTACAGAAGAGGTTGGCCACACAGATCTAATGGCTGCTTAT   6562
gb:MN997409|Organism:Severe      GCAAATAATAGTTTAAAAATTACAGAAGAGGTTGGCCACACAGATCTAATGGCTGCTTAT   6562
gb:MN938384|Organism:Severe      GCAAATAATAGTTTAAAAATTACAGAAGAGGTTGGCCACACAGATCTAATGGCTGCTTAT   6530
gb:MT044258|Organism:Severe      GCAAATAATAGTTTAAAAATTACAGAAGAGGTTGGCCACACAGATCTAATGGCTGCTTAT   6538
gb:MT039890|Organism:Severe      GCAAATAATAGTTTAAAAATTACAGAAGAGGTTGGCCACACAGATCTAATGGCTGCTTAT   6562
gb:MN988713|Organism:Severe      GCAAATAATAGTTTAAAAATTACAGAAGAGGTTGGCCACACAGATCTAATGGCTGCTTAT   6562
gb:LC521925|Organism:Severe      GCAAATAATAGTTTAAAAATTACAGAAGAGGTTGGCCACACAGATCTAATGGCTGCTTAT   6535
gb:MT093571|Organism:Severe      GCAAATAATAGTTTAAAAATTACAGAAGAGGTTGGCCACACAGATCTAATGGCTGCTTAT   6562
gb:MT039887|Organism:Severe      GCAAATAATAGTTTAAAAATTACAGAAGAGGTTGGCCACACAGATCTAATGGCTGCTTAT   6562
gb:MT019530|Organism:Severe      GCAAATAATAGTTTAAAAATTACAGAAGAGGTTGGCCACACAGATCTAATGGCTGCTTAT   6562
gb:MT039888|Organism:Severe      GCAAATAATAGTTTAAAAATTACAGAAGAGGTTGGCCACACAGATCTAATGGCTGCTTAT   6562
gb:LC522972|Organism:Severe      GCAAATAATAGTTTAAAAATTACAGAAGAGGTTGGCCACACAGATCTAATGGCTGCTTAT   6559
gb:MT027063|Organism:Severe      GCAAATAATAGTTTAAAAATTACAGAAGAGGTTGGCCACACAGATCTAATGGCTGCTTAT   6562
gb:MT027062|Organism:Severe      GCAAATAATAGTTTAAAAATTACAGAAGAGGTTGGCCACACAGATCTAATGGCTGCTTAT   6562
gb:MT019529|Organism:Severe      GCAAATAATAGTTTAAAAATTACAGAAGAGGTTGGCCACACAGATCTAATGGCTGCTTAT   6562
gb:MN996529|Organism:Severe      GCAAATAATAGTTTAAAAATTACAGAAGAGGTTGGCCACACAGATCTAATGGCTGCTTAT   6550
gb:MN996531|Organism:Severe      GCAAATAATAGTTTAAAAATTACAGAAGAGGTTGGCCACACAGATCTAATGGCTGCTTAT   6549
gb:MT066176|Organism:Severe      GCAAATAATAGTTTAAAAATTACAGAAGAGGTTGGCCACACAGATCTAATGGCTGCTTAT   6562
gb:MT027064|Organism:Severe      GCAAATAATAGTTTAAAAATTACAGAAGAGGTTGGCCACACAGATCTAATGGCTGCTTAT   6562
gb:MN994468|Organism:Severe      GCAAATAATAGTTTAAAAATTACAGAAGAGGTTGGCCACACAGATCTAATGGCTGCTTAT   6562
gb:MT072688|Organism:Severe      GCAAATAATAGTTTAAAAATTACAGAAGAGGTTGGCCACACAGATCTAATGGCTGCTTAT   6547
gb:MN996527|Organism:Severe      GCAAATAATAGTTTAAAAATTACAGAAGAGGTTGGCCACACAGATCTAATGGCTGCTTAT   6529
gb:MT093631|Organism:Severe      GCAAATAATAGTTTAAAAATTACAGAAGAGGTTGGCCACACAGATCTAATGGCTGCTTAT   6600
gb:MT106053|Organism:Severe      GCAAATAATAGTTTAAAAATTACAGAAGAGGTTGGCCACACAGATCTAATGGCTGCTTAT   6562
gb:MT019533|Organism:Severe      GCAAATAATAGTTTAAAAATTACAGAAGAGGTTGGCCACACAGATCTAATGGCTGCTTAT   6562
gb:MT019531|Organism:Severe      GCAAATAATAGTTTAAAAATTACAGAAGAGGTTGGCCACACAGATCTAATGGCTGCTTAT   6562
gb:MN996528|Organism:Severe      GCAAATAATAGTTTAAAAATTACAGAAGAGGTTGGCCACACAGATCTAATGGCTGCTTAT   6562
gb:MN996530|Organism:Severe      GCAAATAATAGTTTAAAAATTACAGAAGAGGTTGGCCACACAGATCTAATGGCTGCTTAT   6548
gb:MN908947|Organism:Severe      GCAAATAATAGTTTAAAAATTACAGAAGAGGTTGGCCACACAGATCTAATGGCTGCTTAT   6562
gb:MT019532|Organism:Severe      GCAAATAATAGTTTAAAAATTACAGAAGAGGTTGGCCACACAGATCTAATGGCTGCTTAT   6562
                                 ************************************************************

gb:MT020781|Organism:Severe      GTAGACAATTCTAGTCTTACTATTAAGAAACCTAATGAATTATCTAGAGTATTAGGTTTG   6610
gb:MT007544|Organism:Severe      GTAGACAATTCTAGTCTTACTATTAAGAAACCTAATGAATTATCTAGAGTATTAGGTTTG   6622
gb:MN994467|Organism:Severe      GTAGACAATTCTAGTCTTACTATTAAGAAACCTAATGAATTATCTAGAGTATTAGGTTTG   6622
gb:MT044257|Organism:Severe      GTAGACAATTCTAGTCTTACTATTAAGAAACCTAATGAATTATCTAGAGTATTAGGTTTG   6622
gb:MT106054|Organism:Severe      GTAGACAATTCTAGTCTTACTATTAAGAAACCTAATGAATTATCTAGAGTATTAGGTTTG   6622
gb:MT049951|Organism:Severe      GTAGACAATTCTAGTCTTACTATTAAGAAACCTAATGAATTATCTAGAGTATTAGGTTTG   6622
```

```
gb:MN975262|Organism:Severe      GTAGACAATTCTAGTCTTACTATTAAGAAACCTAATGAATTATCTAGAGTATTAGGTTTG   6622
gb:MT106052|Organism:Severe      GTAGACAATTCTAGTCTTACTATTAAGAAACCTAATGAATTATCTAGAGTATTAGGTTTG   6622
gb:LC522975|Organism:Severe      GTAGACAATTCTAGTCTTACTATTAAGAAACCTAATGAATTATCTAGAGTATTAGGTTTG   6619
gb:LC522973|Organism:Severe      GTAGACAATTCTAGTCTTACTATTAAGAAACCTAATGAATTATCTAGAGTATTAGGTTTG   6619
gb:LC522974|Organism:Severe      GTAGACAATTCTAGTCTTACTATTAAGAAACCTAATGAATTATCTAGAGTATTAGGTTTG   6619
gb:MN985325|Organism:Severe      GTAGACAATTCTAGTCTTACTATTAAGAAACCTAATGAATTATCTAGAGTATTAGGTTTG   6622
gb:MT020881|Organism:Severe      GTAGACAATTCTAGTCTTACTATTAAGAAACCTAATGAATTATCTAGAGTATTAGGTTTG   6622
gb:MT020880|Organism:Severe      GTAGACAATTCTAGTCTTACTATTAAGAAACCTAATGAATTATCTAGAGTATTAGGTTTG   6622
gb:MT066175|Organism:Severe      GTAGACAATTCTAGTCTTACTATTAAGAAACCTAATGAATTATCTAGAGTATTAGGTTTG   6622
gb:MN997409|Organism:Severe      GTAGACAATTCTAGTCTTACTATTAAGAAACCTAATGAATTATCTAGAGTATTAGGTTTG   6622
gb:MN938384|Organism:Severe      GTAGACAATTCTAGTCTTACTATTAAGAAACCTAATGAATTATCTAGAGTATTAGGTTTG   6590
gb:MT044258|Organism:Severe      GTAGACAATTCTAGTCTTACTATTAAGAAACCTAATGAATTATCTAGAGTATTAGGTTTG   6598
gb:MT039890|Organism:Severe      GTAGACAATTCTAGTCTTACTATTAAGAAACCTAATGAATTATCTAGAGTATTAGGTTTG   6622
gb:MN988713|Organism:Severe      GTAGACAATTCTAGTCTTACTATTAAGAAACCTAATGAATTATCTAGAGTATTAGGTTTG   6622
gb:LC521925|Organism:Severe      GTAGACAATTCTAGTCTTACTATTAAGAAACCTAATGAATTATCTAGAGTATTAGGTTTG   6595
gb:MT093571|Organism:Severe      GTAGACAATTCTAGTCTTACTATTAAGAAACCTAATGAATTATCTAGAGTATTAGGTTTG   6622
gb:MT039887|Organism:Severe      GTAGACAATTCTAGTCTTACTATTAAGAAACCTAATGAATTATCTAGAGTATTAGGTTTG   6622
gb:MT019530|Organism:Severe      GTAGACAATTCTAGTCTTACTATTAAGAAACCTAATGAATTATCTAGAGTATTAGGTTTG   6622
gb:MT039888|Organism:Severe      GTAGACAATTCTAGTCTTACTATTAAGAAACCTAATGAATTATCTAGAGTATTAGGTTTG   6622
gb:LC522972|Organism:Severe      GTAGACAATTCTAGTCTTACTATTAAGAAACCTAATGAATTATCTAGAGTATTAGGTTTG   6619
gb:MT027063|Organism:Severe      GTAGACAATTCTAGTCTTACTATTAAGAAACCTAATGAATTATCTAGAGTATTAGGTTTG   6622
gb:MT027062|Organism:Severe      GTAGACAATTCTAGTCTTACTATTAAGAAACCTAATGAATTATCTAGAGTATTAGGTTTG   6622
gb:MT019529|Organism:Severe      GTAGACAATTCTAGTCTTACTATTAAGAAACCTAATGAATTATCTAGAGTATTAGGTTTG   6622
gb:MN996529|Organism:Severe      GTAGACAATTCTAGTCTTACTATTAAGAAACCTAATGAATTATCTAGAGTATTAGGTTTG   6610
gb:MN996531|Organism:Severe      GTAGACAATTCTAGTCTTACTATTAAGAAACCTAATGAATTATCTAGAGTATTAGGTTTG   6609
gb:MT066176|Organism:Severe      GTAGACAATTCTAGTCTTACTATTAAGAAACCTAATGAATTATCTAGAGTATTAGGTTTG   6622
gb:MT027064|Organism:Severe      GTAGACAATTCTAGTCTTACTATTAAGAAACCTAATGAATTATCTAGAGTATTAGGTTTG   6622
gb:MN994468|Organism:Severe      GTAGACAATTCTAGTCTTACTATTAAGAAACCTAATGAATTATCTAGAGTATTAGGTTTG   6622
gb:MT072688|Organism:Severe      GTAGACAATTCTAGTCTTACTATTAAGAAACCTAATGAATTATCTAGAGTATTAGGTTTG   6607
gb:MN996527|Organism:Severe      GTAGACAATTCTAGTCTTACTATTAAGAAACCTAATGAATTATCTAGAGTATTAGGTTTG   6589
gb:MT093631|Organism:Severe      GTAGACAATTCTAGTCTTACTATTAAGAAACCTAATGAATTATCTAGAGTATTAGGTTTG   6660
gb:MT106053|Organism:Severe      GTAGACAATTCTAGTCTTACTATTAAGAAACCTAATGAATTATCTAGAGTATTAGGTTTG   6622
gb:MT019533|Organism:Severe      GTAGACAATTCTAGTCTTACTATTAAGAAACCTAATGAATTATCTAGAGTATTAGGTTTG   6622
gb:MT019531|Organism:Severe      GTAGACAATTCTAGTCTTACTATTAAGAAACCTAATGAATTATCTAGAGTATTAGGTTTG   6622
gb:MN996528|Organism:Severe      GTAGACAATTCTAGTCTTACTATTAAGAAACCTAATGAATTATCTAGAGTATTAGGTTTG   6622
gb:MN996530|Organism:Severe      GTAGACAATTCTAGTCTTACTATTAAGAAACCTAATGAATTATCTAGAGTATTAGGTTTG   6608
gb:MN908947|Organism:Severe      GTAGACAATTCTAGTCTTACTATTAAGAAACCTAATGAATTATCTAGAGTATTAGGTTTG   6622
gb:MT019532|Organism:Severe      GTAGACAATTCTAGTCTTACTATTAAGAAACCTAATGAATTATCTAGAGTATTAGGTTTG   6622
                                 ************************************************************

gb:MT020781|Organism:Severe      AAAACCCTTGCTACTCATGGTTTAGCTGCTGTTAATAGTGTCCCTTGGGATACTATAGCT   6670
gb:MT007544|Organism:Severe      AAAACCCTTGCTACTCATGGTTTAGCTGCTGTTAATAGTGTCCCTTGGGATACTATAGCT   6682
gb:MN994467|Organism:Severe      AAAACCCTTGCTACTCATGGTTTAGCTGCTGTTAATAGTGTCCCTTGGGATACTATAGCT   6682
gb:MT044257|Organism:Severe      AAAACCCTTGCTACTCATGGTTTAGCTGCTGTTAATAGTGTCCCTTGGGATACTATAGCT   6682
gb:MT106054|Organism:Severe      AAAACCCTTGCTACTCATGGTTTAGCTGCTGTTAATAGTGTCCCTTGGGATACTATAGCT   6682
gb:MT049951|Organism:Severe      AAAACCCTTGCTACTCATGGTTTAGCTGCTGTTAATAGTGTCCCTTGGGATACTATAGCT   6682
gb:MN975262|Organism:Severe      AAAACCCTTGCTACTCATGGTTTAGCTGCTGTTAATAGTGTCCCTTGGGATACTATAGCT   6682
gb:MT106052|Organism:Severe      AAAACCCTTGCTACTCATGGTTTAGCTGCTGTTAATAGTGTCCCTTGGGATACTATAGCT   6682
gb:LC522975|Organism:Severe      AAAACCCTTGCTACTCATGGTTTAGCTGCTGTTAATAGTGTCCCTTGGGATACTATAGCT   6679
gb:LC522973|Organism:Severe      AAAACCCTTGCTACTCATGGTTTAGCTGCTGTTAATAGTGTCCCTTGGGATACTATAGCT   6679
gb:LC522974|Organism:Severe      AAAACCCTTGCTACTCATGGTTTAGCTGCTGTTAATAGTGTCCCTTGGGATACTATAGCT   6679
gb:MN985325|Organism:Severe      AAAACCCTTGCTACTCATGGTTTAGCTGCTGTTAATAGTGTCCCTTGGGATACTATAGCT   6682
gb:MT020881|Organism:Severe      AAAACCCTTGCTACTCATGGTTTAGCTGCTGTTAATAGTGTCCCTTGGGATACTATAGCT   6682
gb:MT020880|Organism:Severe      AAAACCCTTGCTACTCATGGTTTAGCTGCTGTTAATAGTGTCCCTTGGGATACTATAGCT   6682
gb:MT066175|Organism:Severe      AAAACCCTTGCTACTCATGGTTTAGCTGCTGTTAATAGTGTCCCTTGGGATACTATAGCT   6682
gb:MN997409|Organism:Severe      AAAACCCTTGCTACTCATGGTTTAGCTGCTGTTAATAGTGTCCCTTGGGATACTATAGCT   6682
gb:MN938384|Organism:Severe      AAAACCCTTGCTACTCATGGTTTAGCTGCTGTTAATAGTGTCCCTTGGGATACTATAGCT   6650
gb:MT044258|Organism:Severe      AAAACCCTTGCTACTCATGGTTTAGCTGCTGTTAATAGTGTCCCTTGGGATACTATAGCT   6658
gb:MT039890|Organism:Severe      AAAACCCTTGCTACTCATGGTTTAGCTGCTGTTAATAGTGTCCCTTGGGATACTATAGCT   6682
gb:MN988713|Organism:Severe      AAAACCCTTGCTACTCATGGTTTAGCTGCTGTTAATAGTGTCCCTTGGGATACTATAGCT   6682
gb:LC521925|Organism:Severe      AAAACCCTTGCTACTCATGGTTTAGCTGCTGTTAATAGTGTCCCTTGGGATACTATAGCT   6655
gb:MT093571|Organism:Severe      AAAACCCTTGCTACTCATGGTTTAGCTGCTGTTAATAGTGTCCCTTGGGATACTATAGCT   6682
gb:MT039887|Organism:Severe      AAAACCCTTGCTACTCATGGTTTAGCTGCTGTTAATAGTGTCCCTTGGGATACTATAGCT   6682
gb:MT019530|Organism:Severe      AAAACCCTTGCTACTCATGGTTTAGCTGCTGTTAATAGTGTCCCTTGGGATACTATAGCT   6682
gb:MT039888|Organism:Severe      AAAACCCTTGCTACTCATGGTTTAGCTGCTGTTAATAGTGTCCCTTGGGATACTATAGCT   6682
```

```
gb:LC522972|Organism:Severe      AAAACCCTTGCTACTCATGGTTTAGCTGCTGTTAATAGTGTCCCTTGGGATACTATAGCT   6679
gb:MT027063|Organism:Severe      AAAACCCTTGCTACTCATGGTTTAGCTGCTGTTAATAGTGTCCCTTGGGATACTATAGCT   6682
gb:MT027062|Organism:Severe      AAAACCCTTGCTACTCATGGTTTAGCTGCTGTTAATAGTGTCCCTTGGGATACTATAGCT   6682
gb:MT019529|Organism:Severe      AAAACCCTTGCTACTCATGGTTTAGCTGCTGTTAATAGTGTCCCTTGGGATACTATAGCT   6682
gb:MN996529|Organism:Severe      AAAACCCTTGCTACTCATGGTTTAGCTGCTGTTAATAGTGTCCCTTGGGATACTATAGCT   6670
gb:MN996531|Organism:Severe      AAAACCCTTGCTACTCATGGTTTAGCTGCTGTTAATAGTGTCCCTTGGGATACTATAGCT   6669
gb:MT066176|Organism:Severe      AAAACCCTTGCTACTCATGGTTTAGCTGCTGTTAATAGTGTCCCTTGGGATACTATAGCT   6682
gb:MT027064|Organism:Severe      AAAACCCTTGCTACTCATGGTTTAGCTGCTGTTAATAGTGTCCCTTGGGATACTATAGCT   6682
gb:MN994468|Organism:Severe      AAAACCCTTGCTACTCATGGTTTAGCTGCTGTTAATAGTGTCCCTTGGGATACTATAGCT   6682
gb:MT072688|Organism:Severe      AAAACCCTTGCTACTCATGGTTTAGCTGCTGTTAATAGTGTCCCTTGGGATACTATAGCT   6667
gb:MN996527|Organism:Severe      AAAACCCTTGCTACTCATGGTTTAGCTGCTGTTAATAGTGTCCCTTGGGATACTATAGCT   6649
gb:MT093631|Organism:Severe      AAAACCCTTGCTACTCATGGTTTAGCTGCTGTTAATAGTGTCCCTTGGGATACTATAGCT   6720
gb:MT106053|Organism:Severe      AAAACCCTTGCTACTCATGGTTTAGCTGCTGTTAATAGTGTCCCTTGGGATACTATAGCT   6682
gb:MT019533|Organism:Severe      AAAACCCTTGCTACTCATGGTTTAGCTGCTGTTAATAGTGTCCCTTGGGATACTATAGCT   6682
gb:MT019531|Organism:Severe      AAAACCCTTGCTACTCATGGTTTAGCTGCTGTTAATAGTGTCCCTTGGGATACTATAGCT   6682
gb:MN996528|Organism:Severe      AAAACCCTTGCTACTCATGGTTTAGCTGCTGTTAATAGTGTCCCTTGGGATACTATAGCT   6682
gb:MN996530|Organism:Severe      AAAACCCTTGCTACTCATGGTTTAGCTGCTGTTAATAGTGTCCCTTGGGATACTATAGCT   6668
gb:MN908947|Organism:Severe      AAAACCCTTGCTACTCATGGTTTAGCTGCTGTTAATAGTGTCCCTTGGGATACTATAGCT   6682
gb:MT019532|Organism:Severe      AAAACCCTTGCTACTCATGGTTTAGCTGCTGTTAATAGTGTCCCTTGGGATACTATAGCT   6682
                                 ************************************************************

gb:MT020781|Organism:Severe      AATTATGCTAAGCCTTTTCTTAACAAAGTTGTTAGTACAACTACTAACATAGTTACACGG   6730
gb:MT007544|Organism:Severe      AATTATGCTAAGCCTTTTCTTAACAAAGTTGTTAGTACAACTACTAACATAGTTACACGG   6742
gb:MN994467|Organism:Severe      AATTATGCTAAGCCTTTTCTTAACAAAGTTGTTAGTACAACTACTAACATAGTTACACGG   6742
gb:MT044257|Organism:Severe      AATTATGCTAAGCCTTTTCTTAACAAAGTTGTTAGTACAACTACTAACATAGTTACACGG   6742
gb:MT106054|Organism:Severe      AATTATGCTAAGCCTTTTCTTAACAAAGTTGTTAGTACAACTACTAACATAGTTACACGG   6742
gb:MT049951|Organism:Severe      AATTATGCTAAGCCTTTTCTTAACAAAGTTGTTAGTACAACTACTAACATAGTTACACGG   6742
gb:MN975262|Organism:Severe      AATTATGCTAAGCCTTTTCTTAACAAAGTTGTTAGTACAACTACTAACATAGTTACACGG   6742
gb:MT106052|Organism:Severe      AATTATGCTAAGCCTTTTCTTAACAAAGTTGTTAGTACAACTACTAACATAGTTACACGG   6742
gb:LC522975|Organism:Severe      AATTATGCTAAGCCTTTTCTTAACAAAGTTGTTAGTACAACTACTAACATAGTTACACGG   6739
gb:LC522973|Organism:Severe      AATTATGCTAAGCCTTTTCTTAACAAAGTTGTTAGTACAACTACTAACATAGTTACACGG   6739
gb:LC522974|Organism:Severe      AATTATGCTAAGCCTTTTCTTAACAAAGTTGTTAGTACAACTACTAACATAGTTACACGG   6739
gb:MN985325|Organism:Severe      AATTATGCTAAGCCTTTTCTTAACAAAGTTGTTAGTACAACTACTAACATAGTTACACGG   6742
gb:MT020881|Organism:Severe      AATTATGCTAAGCCTTTTCTTAACAAAGTTGTTAGTACAACTACTAACATAGTTACACGG   6742
gb:MT020880|Organism:Severe      AATTATGCTAAGCCTTTTCTTAACAAAGTTGTTAGTACAACTACTAACATAGTTACACGG   6742
gb:MT066175|Organism:Severe      AATTATGCTAAGCCTTTTCTTAACAAAGTTGTTAGTACAACTACTAACATAGTTACACGG   6742
gb:MN997409|Organism:Severe      AATTATGCTAAGCCTTTTCTTAACAAAGTTGTTAGTACAACTACTAACATAGTTACACGG   6742
gb:MN938384|Organism:Severe      AATTATGCTAAGCCTTTTCTTAACAAAGTTGTTAGTACAACTACTAACATAGTTACACGG   6710
gb:MT044258|Organism:Severe      AATTATGCTAAGCCTTTTCTTAACAAAGTTGTTAGTACAACTACTAACATAGTTACACGG   6718
gb:MT039890|Organism:Severe      AATTATGCTAAGCCTTTTCTTAACAAAGTTGTTAGTACAACTACTAACATAGTTACACGG   6742
gb:MN988713|Organism:Severe      AATTATGCTAAGCCTTTTCTTAACAAAGTTGTTAGTACAACTACTAACATAGTTACACGG   6742
gb:LC521925|Organism:Severe      AATTATGCTAAGCCTTTTCTTAACAAAGTTGTTAGTACAACTACTAACATAGTTACACGG   6715
gb:MT093571|Organism:Severe      AATTATGCTAAGCCTTTTCTTAACAAAGTTGTTAGTACAACTACTAACATAGTTACACGG   6742
gb:MT039887|Organism:Severe      AATTATGCTAAGCCTTTTCTTAACAAAGTTGTTAGTACAACTACTAACATAGTTACACGG   6742
gb:MT019530|Organism:Severe      AATTATGCTAAGCCTTTTCTTAACAAAGTTGTTAGTACAACTACTAACATAGTTACACGG   6742
gb:MT039888|Organism:Severe      AATTATGCTAAGCCTTTTCTTAACAAAGTTGTTAGTACAACTACTAACATAGTTACACGG   6742
gb:LC522972|Organism:Severe      AATTATGCTAAGCCTTTTCTTAACAAAGTTGTTAGTACAACTACTAACATAGTTACACGG   6739
gb:MT027063|Organism:Severe      AATTATGCTAAGCCTTTTCTTAACAAAGTTGTTAGTACAACTACTAACATAGTTACACGG   6742
gb:MT027062|Organism:Severe      AATTATGCTAAGCCTTTTCTTAACAAAGTTGTTAGTACAACTACTAACATAGTTACACGG   6742
gb:MT019529|Organism:Severe      AATTATGCTAAGCCTTTTCTTAACAAAGTTGTTAGTACAACTACTAACATAGTTACACGG   6742
gb:MN996529|Organism:Severe      AATTATGCTAAGCCTTTTCTTAACAAAGTTGTTAGTACAACTACTAACATAGTTACACGG   6730
gb:MN996531|Organism:Severe      AATTATGCTAAGCCTTTTCTTAACAAAGTTGTTAGTACAACTACTAACATAGTTACACGG   6729
gb:MT066176|Organism:Severe      AATTATGCTAAGCCTTTTCTTAACAAAGTTGTTAGTACAACTACTAACATAGTTACACGG   6742
gb:MT027064|Organism:Severe      AATTATGCTAAGCCTTTTCTTAACAAAGTTGTTAGTACAACTACTAACATAGTTACACGG   6742
gb:MN994468|Organism:Severe      AATTATGCTAAGCCTTTTCTTAACAAAGTTGTTAGTACAACTACTAACATAGTTACACGG   6742
gb:MT072688|Organism:Severe      AATTATGCTAAGCCTTTTCTTAACAAAGTTGTTAGTACAACTACTAACATAGTTACACGG   6727
gb:MN996527|Organism:Severe      AATTATGCTAAGCCTTTTCTTAACAAAGTTGTTAGTACAACTACTAACATAGTTACACGG   6709
gb:MT093631|Organism:Severe      AATTATGCTAAGCCTTTTCTTAACAAAGTTGTTAGTACAACTACTAACATAGTTACACGG   6780
gb:MT106053|Organism:Severe      AATTATGCTAAGCCTTTTCTTAACAAAGTTGTTAGTACAACTACTAACATAGTTACACGG   6742
gb:MT019533|Organism:Severe      AATTATGCTAAGCCTTTTCTTAACAAAGTTGTTAGTACAACTACTAACATAGTTACACGG   6742
gb:MT019531|Organism:Severe      AATTATGCTAAGCCTTTTCTTAACAAAGTTGTTAGTACAACTACTAACATAGTTACACGG   6742
gb:MN996528|Organism:Severe      AATTATGCTAAGCCTTTTCTTAACAAAGTTGTTAGTACAACTACTAACATAGTTACACGG   6742
gb:MN996530|Organism:Severe      AATTATGCTAAGCCTTTTCTTAACAAAGTTGTTAGTACAACTACTAACATAGTTACACGG   6728
gb:MN908947|Organism:Severe      AATTATGCTAAGCCTTTTCTTAACAAAGTTGTTAGTACAACTACTAACATAGTTACACGG   6742
gb:MT019532|Organism:Severe      AATTATGCTAAGCCTTTTCTTAACAAAGTTGTTAGTACAACTACTAACATAGTTACACGG   6742
```

```
                                  ************************************************************

gb:MT020781|Organism:Severe      TGTTTAAACCGTGTTTGTANNNNNNNNNNNNNNNNNNNNTTTACTTTATTGCTACAATTG   6790
gb:MT007544|Organism:Severe      TGTTTAAACCGTGTTTGTACTAATTATATGCCTTATTTCTTTACTTTATTGCTACAATTG   6802
gb:MN994467|Organism:Severe      TGTTTAAACCGTGTTTGTACTAATTATATGCCTTATTTCTTTACTTTATTGCTACAATTG   6802
gb:MT044257|Organism:Severe      TGTTTAAACCGTGTTTGTACTAATTATATGCCTTATTTCTTTACTTTATTGCTACAATTG   6802
gb:MT106054|Organism:Severe      TGTTTAAACCGTGTTTGTACTAATTATATGCCTTATTTCTTTACTTTATTGCTACAATTG   6802
gb:MT049951|Organism:Severe      TGTTTAAACCGTGTTTGTACTAATTATATGCCTTATTTCTTTACTTTATTGCTACAATTG   6802
gb:MN975262|Organism:Severe      TGTTTAAACCGTGTTTGTACTAATTATATGCCTTATTTCTTTACTTTATTGCTACAATTG   6802
gb:MT106052|Organism:Severe      TGTTTAAACCGTGTTTGTACTAATTATATGCCTTATTTCTTTACTTTATTGCTACAATTG   6802
gb:LC522975|Organism:Severe      TGTTTAAACCGTGTTTGTACTAATTATATGCCTTATTTCTTTACTTTATTGCTACAATTG   6799
gb:LC522973|Organism:Severe      TGTTTAAACCGTGTTTGTACTAATTATATGCCTTATTTCTTTACTTTATTGCTACAATTG   6799
gb:LC522974|Organism:Severe      TGTTTAAACCGTGTTTGTACTAATTATATGCCTTATTTCTTTACTTTATTGCTACAATTG   6799
gb:MN985325|Organism:Severe      TGTTTAAACCGTGTTTGTACTAATTATATGCCTTATTTCTTTACTTTATTGCTACAATTG   6802
gb:MT020881|Organism:Severe      TGTTTAAACCGTGTTTGTACTAATTATATGCCTTATTTCTTTACTTTATTGCTACAATTG   6802
gb:MT020880|Organism:Severe      TGTTTAAACCGTGTTTGTACTAATTATATGCCTTATTTCTTTACTTTATTGCTACAATTG   6802
gb:MT066175|Organism:Severe      TGTTTAAACCGTGTTTGTACTAATTATATGCCTTATTTCTTTACTTTATTGCTACAATTG   6802
gb:MN997409|Organism:Severe      TGTTTAAACCGTGTTTGTACTAATTATATGCCTTATTTCTTTACTTTATTGCTACAATTG   6802
gb:MN938384|Organism:Severe      TGTTTAAACCGTGTTTGTACTAATTATATGCCTTATTTCTTTACTTTATTGCTACAATTG   6770
gb:MT044258|Organism:Severe      TGTTTAAACCGTGTTTGTACTAATTATATGCCTTATTTCTTTACTTTATTGCTACAATTG   6778
gb:MT039890|Organism:Severe      TGTTTAAACCGTGTTTGTACTAATTATATGCCTTATTTCTTTACTTTATTGCTACAATTG   6802
gb:MN988713|Organism:Severe      TGTTTAAACCGTGTTTGTACTAATTATATGCCTTATTTCTTTACTTTATTGCTACAATTG   6802
gb:LC521925|Organism:Severe      TGTTTAAACCGTGTTTGTACTAATTATATGCCTTATTTCTTTACTTTATTGCTACAATTG   6775
gb:MT093571|Organism:Severe      TGTTTAAACCGTGTTTGTACTAATTATATGCCTTATTTCTTTACTTTATTGCTACAATTG   6802
gb:MT039887|Organism:Severe      TGTTTAAACCGTGTTTGTACTAATTATATGCCTTATTTCTTTACTTTATTGCTACAATTG   6802
gb:MT019530|Organism:Severe      TGTTTAAACCGTGTTTGTACTAATTATATGCCTTATTTCTTTACTTTATTGCTACAATTG   6802
gb:MT039888|Organism:Severe      TGTTTAAACCGTGTTTGTACTAATTATATGCCTTATTTCTTTACTTTATTGCTACAATTG   6802
gb:LC522972|Organism:Severe      TGTTTAAACCGTGTTTGTACTAATTATATGCCTTATTTCTTTACTTTATTGCTACAATTG   6799
gb:MT027063|Organism:Severe      TGTTTAAACCGTGTTTGTACTAATTATATGCCTTATTTCTTTACTTTATTGCTACAATTG   6802
gb:MT027062|Organism:Severe      TGTTTAAACCGTGTTTGTACTAATTATATGCCTTATTTCTTTACTTTATTGCTACAATTG   6802
gb:MT019529|Organism:Severe      TGTTTAAACCGTGTTTGTACTAATTATATGCCTTATTTCTTTACTTTATTGCTACAATTG   6802
gb:MN996529|Organism:Severe      TGTTTAAACCGTGTTTGTACTAATTATATGCCTTATTTCTTTACTTTATTGCTACAATTG   6790
gb:MN996531|Organism:Severe      TGTTTAAACCGTGTTTGTACTAATTATATGCCTTATTTCTTTACTTTATTGCTACAATTG   6789
gb:MT066176|Organism:Severe      TGTTTAAACCGTGTTTGTACTAATTATATGCCTTATTTCTTTACTTTATTGCTACAATTG   6802
gb:MT027064|Organism:Severe      TGTTTAAACCGTGTTTGTACTAATTATATGCCTTATTTCTTTACTTTATTGCTACAATTG   6802
gb:MN994468|Organism:Severe      TGTTTAAACCGTGTTTGTACTAATTATATGCCTTATTTCTTTACTTTATTGCTACAATTG   6802
gb:MT072688|Organism:Severe      TGTTTAAACCGTGTTTGTACTAATTATATGCCTTATTTCTTTACTTTATTGCTACAATTG   6787
gb:MN996527|Organism:Severe      TGTTTAAACCGTGTTTGTACTAATTATATGCCTTATTTCTTTACTTTATTGCTACAATTG   6769
gb:MT093631|Organism:Severe      TGTTTAAACCGTGTTTGTACTAATTATATGCCTTATTTCTTTACTTTATTGCTACAATTG   6840
gb:MT106053|Organism:Severe      TGTTTAAACCGTGTTTGTACTAATTATATGCCTTATTTCTTTACTTTATTGCTACAATTG   6802
gb:MT019533|Organism:Severe      TGTTTAAACCGTGTTTGTACTAATTATATGCCTTATTTCTTTACTTTATTGCTACAATTG   6802
gb:MT019531|Organism:Severe      TGTTTAAACCGTGTTTGTACTAATTATATGCCTTATTTCTTTACTTTATTGCTACAATTG   6802
gb:MN996528|Organism:Severe      TGTTTAAACCGTGTTTGTACTAATTATATGCCTTATTTCTTTACTTTATTGCTACAATTG   6802
gb:MN996530|Organism:Severe      TGTTTAAACCGTGTTTGTACTAATTATATGCCTTATTTCTTTACTTTATTGCTACAATTG   6788
gb:MN908947|Organism:Severe      TGTTTAAACCGTGTTTGTACTAATTATATGCCTTATTTCTTTACTTTATTGCTACAATTG   6802
gb:MT019532|Organism:Severe      TGTTTAAACCGTGTTTGTACTAATTATATGCCTTATTTCTTTACTTTATTGCTACAATTG   6802
                                  *******************                    *********************

gb:MT020781|Organism:Severe      TGTACTTTTACTAGAAGTACAAATTCTAGAATTAAAGCATCTATGCCGACTACTATAGCA   6850
gb:MT007544|Organism:Severe      TGTACTTTTACTAGAAGTACAAATTCTAGAATTAAAGCATCTATGCCGACTACTATAGCA   6862
gb:MN994467|Organism:Severe      TGTACTTTTACTAGAAGTACAAATTCTAGAATTAAAGCATCTATGCCGACTACTATAGCA   6862
gb:MT044257|Organism:Severe      TGTACTTTTACTAGAAGTACAAATTCTAGAATTAAAGCATCTATGCCGACTACTATAGCA   6862
gb:MT106054|Organism:Severe      TGTACTTTTACTAGAAGTACAAATTCTAGAATTAAAGCATCTATGCCGACTACTATAGCA   6862
gb:MT049951|Organism:Severe      TGTACTTTTACTAGAAGTACAAATTCTAGAATTAAAGCATCTATGCCGACTACTATAGCA   6862
gb:MN975262|Organism:Severe      TGTACTTTTACTAGAAGTACAAATTCTAGAATTAAAGCATCTATGCCGACTACTATAGCA   6862
gb:MT106052|Organism:Severe      TGTACTTTTACTAGAAGTACAAATTCTAGAATTAAAGCATCTATGCCGACTACTATAGCA   6862
gb:LC522975|Organism:Severe      TGTACTTTTACTAGAAGTACAAATTCTAGAATTAAAGCATCTATGCCGACTACTATAGCA   6859
gb:LC522973|Organism:Severe      TGTACTTTTACTAGAAGTACAAATTCTAGAATTAAAGCATCTATGCCGACTACTATAGCA   6859
gb:LC522974|Organism:Severe      TGTACTTTTACTAGAAGTACAAATTCTAGAATTAAAGCATCTATGCCGACTACTATAGCA   6859
gb:MN985325|Organism:Severe      TGTACTTTTACTAGAAGTACAAATTCTAGAATTAAAGCATCTATGCCGACTACTATAGCA   6862
gb:MT020881|Organism:Severe      TGTACTTTTACTAGAAGTACAAATTCTAGAATTAAAGCATCTATGCCGACTACTATAGCA   6862
gb:MT020880|Organism:Severe      TGTACTTTTACTAGAAGTACAAATTCTAGAATTAAAGCATCTATGCCGACTACTATAGCA   6862
gb:MT066175|Organism:Severe      TGTACTTTTACTAGAAGTACAAATTCTAGAATTAAAGCATCTATGCCGACTACTATAGCA   6862
gb:MN997409|Organism:Severe      TGTACTTTTACTAGAAGTACAAATTCTAGAATTAAAGCATCTATGCCGACTACTATAGCA   6862
gb:MN938384|Organism:Severe      TGTACTTTTACTAGAAGTACAAATTCTAGAATTAAAGCATCTATGCCGACTACTATAGCA   6830
```

```
gb:MT044258|Organism:Severe      TGTACTTTTACTAGAAGTACAAATTCTAGAATTAAAGCATCTATGCCGACTACTATAGCA   6838
gb:MT039890|Organism:Severe      TGTACTTTTACTAGAAGTACAAATTCTAGAATTAAAGCATCTATGCCGACTACTATAGCA   6862
gb:MN988713|Organism:Severe      TGTACTTTTACTAGAAGTACAAATTCTAGAATTAAAGCATCTATGCCGACTACTATAGCA   6862
gb:LC521925|Organism:Severe      TGTACTTTTACTAGAAGTACAAATTCTAGAATTAAAGCATCTATGCCGACTACTATAGCA   6835
gb:MT093571|Organism:Severe      TGTACTTTTACTAGAAGTACAAATTCTAGAATTAAAGCATCTATGCCGACTACTATAGCA   6862
gb:MT039887|Organism:Severe      TGTACTTTTACTAGAAGTACAAATTCTAGAATTAAAGCATCTATGCCGACTACTATAGCA   6862
gb:MT019530|Organism:Severe      TGTACTTTTACTAGAAGTACAAATTCTAGAATTAAAGCATCTATGCCGACTACTATAGCA   6862
gb:MT039888|Organism:Severe      TGTACTTTTACTAGAAGTACAAATTCTAGAATTAAAGCATCTATGCCGACTACTATAGCA   6862
gb:LC522972|Organism:Severe      TGTACTTTTACTAGAAGTACAAATTCTAGAATTAAAGCATCTATGCCGACTACTATAGCA   6859
gb:MT027063|Organism:Severe      TGTACTTTTACTAGAAGTACAAATTCTAGAATTAAAGCATCTATGCCGACTACTATAGCA   6862
gb:MT027062|Organism:Severe      TGTACTTTTACTAGAAGTACAAATTCTAGAATTAAAGCATCTATGCCGACTACTATAGCA   6862
gb:MT019529|Organism:Severe      TGTACTTTTACTAGAAGTACAAATTCTAGAATTAAAGCATCTATGCCGACTACTATAGCA   6862
gb:MN996529|Organism:Severe      TGTACTTTTACTAGAAGTACAAATTCTAGAATTAAAGCATCTATGCCGACTACTATAGCA   6850
gb:MN996531|Organism:Severe      TGTACTTTTACTAGAAGTACAAATTCTAGAATTAAAGCATCTATGCCGACTACTATAGCA   6849
gb:MT066176|Organism:Severe      TGTACTTTTACTAGAAGTACAAATTCTAGAATTAAAGCATCTATGCCGACTACTATAGCA   6862
gb:MT027064|Organism:Severe      TGTACTTTTACTAGAAGTACAAATTCTAGAATTAAAGCATCTATGCCGACTACTATAGCA   6862
gb:MN994468|Organism:Severe      TGTACTTTTACTAGAAGTACAAATTCTAGAATTAAAGCATCTATGCCGACTACTATAGCA   6862
gb:MT072688|Organism:Severe      TGTACTTTTACTAGAAGTACAAATTCTAGAATTAAAGCATCTATGCCGACTACTATAGCA   6847
gb:MN996527|Organism:Severe      TGTACTTTTACTAGAAGTACAAATTCTAGAATTAAAGCATCTATGCCGACTACTATAGCA   6829
gb:MT093631|Organism:Severe      TGTACTTTTACTAGAAGTACAAATTCTAGAATTAAAGCATCTATGCCGACTACTATAGCA   6900
gb:MT106053|Organism:Severe      TGTACTTTTACTAGAAGTACAAATTCTAGAATTAAAGCATCTATGCCGACTACTATAGCA   6862
gb:MT019533|Organism:Severe      TGTACTTTTACTAGAAGTACAAATTCTAGAATTAAAGCATCTATGCCGACTACTATAGCA   6862
gb:MT019531|Organism:Severe      TGTACTTTTACTAGAAGTACAAATTCTAGAATTAAAGCATCTATGCCGACTACTATAGCA   6862
gb:MN996528|Organism:Severe      TGTACTTTTACTAGAAGTACAAATTCTAGAATTAAAGCATCTATGCCGACTACTATAGCA   6862
gb:MN996530|Organism:Severe      TGTACTTTTACTAGAAGTACAAATTCTAGAATTAAAGCATCTATGCCGACTACTATAGCA   6848
gb:MN908947|Organism:Severe      TGTACTTTTACTAGAAGTACAAATTCTAGAATTAAAGCATCTATGCCGACTACTATAGCA   6862
gb:MT019532|Organism:Severe      TGTACTTTTACTAGAAGTACAAATTCTAGAATTAAAGCATCTATGCCGACTACTATAGCA   6862
                                 ************************************************************

gb:MT020781|Organism:Severe      AAGAATACTGTTAAGAGTGTCGGTAAATTTTGTCTAGAGGCTTCATTTAATTATTTGAAG   6910
gb:MT007544|Organism:Severe      AAGAATACTGTTAAGAGTGTCGGTAAATTTTGTCTAGAGGCTTCATTTAATTATTTGAAG   6922
gb:MN994467|Organism:Severe      AAGAATACTGTTAAGAGTGTCGGTAAATTTTGTCTAGAGGCTTCATTTAATTATTTGAAG   6922
gb:MT044257|Organism:Severe      AAGAATACTGTTAAGAGTGTCGGTAAATTTTGTCTAGAGGCTTCATTTAATTATTTGAAG   6922
gb:MT106054|Organism:Severe      AAGAATACTGTTAAGAGTGTCGGTAAATTTTGTCTAGAGGCTTCATTTAATTATTTGAAG   6922
gb:MT049951|Organism:Severe      AAGAATACTGTTAAGAGTGTCGGTAAATTTTGTCTAGAGGCTTCATTTAATTATTTGAAG   6922
gb:MN975262|Organism:Severe      AAGAATACTGTTAAGAGTGTCGGTAAATTTTGTCTAGAGGCTTCATTTAATTATTTGAAG   6922
gb:MT106052|Organism:Severe      AAGAATACTGTTAAGAGTGTCGGTAAATTTTGTCTAGAGGCTTCATTTAATTATTTGAAG   6922
gb:LC522975|Organism:Severe      AAGAATACTGTTAAGAGTGTCGGTAAATTTTGTCTAGAGGCTTCATTTAATTATTTGAAG   6919
gb:LC522973|Organism:Severe      AAGAATACTGTTAAGAGTGTCGGTAAATTTTGTCTAGAGGCTTCATTTAATTATTTGAAG   6919
gb:LC522974|Organism:Severe      AAGAATACTGTTAAGAGTGTCGGTAAATTTTGTCTAGAGGCTTCATTTAATTATTTGAAG   6919
gb:MN985325|Organism:Severe      AAGAATACTGTTAAGAGTGTCGGTAAATTTTGTCTAGAGGCTTCATTTAATTATTTGAAG   6922
gb:MT020881|Organism:Severe      AAGAATACTGTTAAGAGTGTCGGTAAATTTTGTCTAGAGGCTTCATTTAATTATTTGAAG   6922
gb:MT020880|Organism:Severe      AAGAATACTGTTAAGAGTGTCGGTAAATTTTGTCTAGAGGCTTCATTTAATTATTTGAAG   6922
gb:MT066175|Organism:Severe      AAGAATACTGTTAAGAGTGTCGGTAAATTTTGTCTAGAGGCTTCATTTAATTATTTGAAG   6922
gb:MN997409|Organism:Severe      AAGAATACTGTTAAGAGTGTCGGTAAATTTTGTCTAGAGGCTTCATTTAATTATTTGAAG   6922
gb:MN938384|Organism:Severe      AAGAATACTGTTAAGAGTGTCGGTAAATTTTGTCTAGAGGCTTCATTTAATTATTTGAAG   6890
gb:MT044258|Organism:Severe      AAGAATACTGTTAAGAGTGTCGGTAAATTTTGTCTAGAGGCTTCATTTAATTATTTGAAG   6898
gb:MT039890|Organism:Severe      AAGAATACTGTTAAGAGTGTCGGTAAATTTTGTCTAGAGGCTTCATTTAATTATTTGAAG   6922
gb:MN988713|Organism:Severe      AAGAATACTGTTAAGAGTGTCGGTAAATTTTGTCTAGAGGCTTCATTTAATTATTTGAAG   6922
gb:LC521925|Organism:Severe      AAGAATACTGTTAAGAGTGTCGGTAAATTTTGTCTAGAGGCTTCATTTAATTATTTGAAG   6895
gb:MT093571|Organism:Severe      AAGAATACTGTTAAGAGTGTCGGTAAATTTTGTCTAGAGGCTTCATTTAATTATTTGAAG   6922
gb:MT039887|Organism:Severe      AAGAATACTGTTAAGAGTGTCGGTAAATTTTGTCTAGAGGCTTCATTTAATTATTTGAAG   6922
gb:MT019530|Organism:Severe      AAGAATACTGTTAAGAGTGTCGGTAAATTTTGTCTAGAGGCTTCATTTAATTATTTGAAG   6922
gb:MT039888|Organism:Severe      AAGAATACTGTTAAGAGTGTCGGTAAATTTTGTCTAGAGGCTTCATTTAATTATTTGAAG   6922
gb:LC522972|Organism:Severe      AAGAATACTGTTAAGAGTGTCGGTAAATTTTGTCTAGAGGCTTCATTTAATTATTTGAAG   6919
gb:MT027063|Organism:Severe      AAGAATACTGTTAAGAGTGTCGGTAAATTTTGTCTAGAGGCTTCATTTAATTATTTGAAG   6922
gb:MT027062|Organism:Severe      AAGAATACTGTTAAGAGTGTCGGTAAATTTTGTCTAGAGGCTTCATTTAATTATTTGAAG   6922
gb:MT019529|Organism:Severe      AAGAATACTGTTAAGAGTGTCGGTAAATTTTGTCTAGAGGCTTCATTTAATTATTTGAAG   6922
gb:MN996529|Organism:Severe      AAGAATACTGTTAAGAGTGTCGGTAAATTTTGTCTAGAGGCTTCATTTAATTATTTGAAG   6910
gb:MN996531|Organism:Severe      AAGAATACTGTTAAGAGTGTCGGTAAATTTTGTCTAGAGGCTTCATTTAATTATTTGAAG   6909
gb:MT066176|Organism:Severe      AAGAATACTGTTAAGAGTGTCGGTAAATTTTGTCTAGAGGCTTCATTTAATTATTTGAAG   6922
gb:MT027064|Organism:Severe      AAGAATACTGTTAAGAGTGTCGGTAAATTTTGTCTAGAGGCTTCATTTAATTATTTGAAG   6922
gb:MN994468|Organism:Severe      AAGAATACTGTTAAGAGTGTCGGTAAATTTTGTCTAGAGGCTTCATTTAATTATTTGAAG   6922
gb:MT072688|Organism:Severe      AAGAATACTGTTAAGAGTGTCGGTAAATTTTGTCTAGAGGCTTCATTTAATTATTTGAAG   6907
gb:MN996527|Organism:Severe      AAGAATACTGTTAAGAGTGTCGGTAAATTTTGTCTAGAGGCTTCATTTAATTATTTGAAG   6889
```

```
gb:MT093631|Organism:Severe      AAGAATACTGTTAAGAGTGTCGGTAAATTTTGTCTAGAGGCTTCATTTAATTATTTGAAG   6960
gb:MT106053|Organism:Severe      AAGAATACTGTTAAGAGTGTCGGTAAATTTTGTCTAGAGGCTTCATTTAATTATTTGAAG   6922
gb:MT019533|Organism:Severe      AAGAATACTGTTAAGAGTGTCGGTAAATTTTGTCTAGAGGCTTCATTTAATTATTTGAAG   6922
gb:MT019531|Organism:Severe      AAGAATACTGTTAAGAGTGTCGGTAAATTTTGTCTAGAGGCTTCATTTAATTATTTGAAG   6922
gb:MN996528|Organism:Severe      AAGAATACTGTTAAGAGTGTCGGTAAATTTTGTCTAGAGGCTTCATTTAATTATTTGAAG   6922
gb:MN996530|Organism:Severe      AAGAATACTGTTAAGAGTGTCGGTAAATTTTGTCTAGAGGCTTCATTTAATTATTTGAAG   6908
gb:MN908947|Organism:Severe      AAGAATACTGTTAAGAGTGTCGGTAAATTTTGTCTAGAGGCTTCATTTAATTATTTGAAG   6922
gb:MT019532|Organism:Severe      AAGAATACTGTTAAGAGTGTCGGTAAATTTTGTCTAGAGGCTTCATTTAATTATTTGAAG   6922
                                 ************************************************************

gb:MT020781|Organism:Severe      TCACCTAATTTTTCTAAACTGATAAATATTATAATTTGGTTTTTACTATTAAGTGTTTGC   6970
gb:MT007544|Organism:Severe      TCACCTAATTTTTCTAAACTGATAAATATTATAATTTGGTTTTTACTATTAAGTGTTTGC   6982
gb:MN994467|Organism:Severe      TCACCTAATTTTTCTAAACTGATAAATATTATAATTTGGTTTTTACTATTAAGTGTTTGC   6982
gb:MT044257|Organism:Severe      TCACCTAATTTTTCTAAACTGATAAATATTATAATTTGGTTTTTACTATTAAGTGTTTGC   6982
gb:MT106054|Organism:Severe      TCACCTAATTTTTCTAAACTGATAAATATTATAATTTGGTTTTTACTATTAAGTGTTTGC   6982
gb:MT049951|Organism:Severe      TCACCTAATTTTTCTAAACTGATAAATATTATAATTTGGTTTTTACTATTAAGTGTTTGC   6982
gb:MN975262|Organism:Severe      TCACCTAATTTTTCTAAACTGATAAATATTATAATTTGGTTTTTACTATTAAGTGTTTGC   6982
gb:MT106052|Organism:Severe      TCACCTAATTTTTCTAAACTGATAAATATTATAATTTGGTTTTTACTATTAAGTGTTTGC   6982
gb:LC522975|Organism:Severe      TCACCTAATTTTTCTAAACTGATAAATATTATAATTTGGTTTTTACTATTAAGTGTTTGC   6979
gb:LC522973|Organism:Severe      TCACCTAATTTTTCTAAACTGATAAATATTATAATTTGGTTTTTACTATTAAGTGTTTGC   6979
gb:LC522974|Organism:Severe      TCACCTAATTTTTCTAAACTGATAAATATTATAATTTGGTTTTTACTATTAAGTGTTTGC   6979
gb:MN985325|Organism:Severe      TCACCTAATTTTTCTAAACTGATAAATATTATAATTTGGTTTTTACTATTAAGTGTTTGC   6982
gb:MT020881|Organism:Severe      TCACCTAATTTTTCTAAACTGATAAATATTATAATTTGGTTTTTACTATTAAGTGTTTGC   6982
gb:MT020880|Organism:Severe      TCACCTAATTTTTCTAAACTGATAAATATTATAATTTGGTTTTTACTATTAAGTGTTTGC   6982
gb:MT066175|Organism:Severe      TCACCTAATTTTTCTAAACTGATAAATATTATAATTTGGTTTTTACTATTAAGTGTTTGC   6982
gb:MN997409|Organism:Severe      TCACCTAATTTTTCTAAACTGATAAATATTATAATTTGGTTTTTACTATTAAGTGTTTGC   6982
gb:MN938384|Organism:Severe      TCACCTAATTTTTCTAAACTGATAAATATTATAATTTGGTTTTTACTATTAAGTGTTTGC   6950
gb:MT044258|Organism:Severe      TCACCTAATTTTTCTAAACTGATAAATATTATAATTTGGTTTTTACTATTAAGTGTTTGC   6958
gb:MT039890|Organism:Severe      TCACCTAATTTTTCTAAACTGATAAATATTATAATTTGGTTTTTACTATTAAGTGTTTGC   6982
gb:MN988713|Organism:Severe      TCACCTAATTTTTCTAAACTGATAAATATTATAATTTGGTTTTTACTATTAAGTGTTTGC   6982
gb:LC521925|Organism:Severe      TCACCTAATTTTTCTAAACTGATAAATATTATAATTTGGTTTTTACTATTAAGTGTTTGC   6955
gb:MT093571|Organism:Severe      TCACCTAATTTTTCTAAACTGATAAATATTATAATTTGGTTTTTACTATTAAGTGTTTGC   6982
gb:MT039887|Organism:Severe      TCACCTAATTTTTCTAAACTGATAAATATTATAATTTGGTTTTTACTATTAAGTGTTTGC   6982
gb:MT019530|Organism:Severe      TCACCTAATTTTTCTAAACTGATAAATATTATAATTTGGTTTTTACTATTAAGTGTTTGC   6982
gb:MT039888|Organism:Severe      TCACCTAATTTTTCTAAACTGATAAATATTATAATTTGGTTTTTACTATTAAGTGTTTGC   6982
gb:LC522972|Organism:Severe      TCACCTAATTTTTCTAAACTGATAAATATTATAATTTGGTTTTTACTATTAAGTGTTTGC   6979
gb:MT027063|Organism:Severe      TCACCTAATTTTTCTAAACTGATAAATATTATAATTTGGTTTTTACTATTAAGTGTTTGC   6982
gb:MT027062|Organism:Severe      TCACCTAATTTTTCTAAACTGATAAATATTATAATTTGGTTTTTACTATTAAGTGTTTGC   6982
gb:MT019529|Organism:Severe      TCACCTAATTTTTCTAAACTGATAAATATTATAATTTGGTTTTTACTATTAAGTGTTTGC   6982
gb:MN996529|Organism:Severe      TCACCTAATTTTTCTAAACTGATAAATATTATAATTTGGTTTTTACTATTAAGTGTTTGC   6970
gb:MN996531|Organism:Severe      TCACCTAATTTTTCTAAACTGATAAATATTATAATTTGGTTTTTACTATTAAGTGTTTGC   6969
gb:MT066176|Organism:Severe      TCACCTAATTTTTCTAAACTGATAAATATTATAATTTGGTTTTTACTATTAAGTGTTTGC   6982
gb:MT027064|Organism:Severe      TCACCTAATTTTTCTAAACTGATAAATATTATAATTTGGTTTTTACTATTAAGTGTTTGC   6982
gb:MN994468|Organism:Severe      TCACCTAATTTTTCTAAACTGATAAATATTATAATTTGGTTTTTACTATTAAGTGTTTGC   6982
gb:MT072688|Organism:Severe      TCACCTAATTTTTCTAAACTGATAAATATTATAATTTGGTTTTTACTATTAAGTGTTTGC   6967
gb:MN996527|Organism:Severe      TCACCTAATTTTTCTAAACTGATAAATATTATAATTTGGTTTTTACTATTAAGTGTTTGC   6949
gb:MT093631|Organism:Severe      TCACCTAATTTTTCTAAACTGATAAATATTATAATTTGGTTTTTACTATTAAGTGTTTGC   7020
gb:MT106053|Organism:Severe      TCACCTAATTTTTCTAAACTGATAAATATTATAATTTGGTTTTTACTATTAAGTGTTTGC   6982
gb:MT019533|Organism:Severe      TCACCTAATTTTTCTAAACTGATAAATATTATAATTTGGTTTTTACTATTAAGTGTTTGC   6982
gb:MT019531|Organism:Severe      TCACCTAATTTTTCTAAACTGATAAATATTATAATTTGGTTTTTACTATTAAGTGTTTGC   6982
gb:MN996528|Organism:Severe      TCACCTAATTTTTCTAAACTGATAAATATTATAATTTGGTTTTTACTATTAAGTGTTTGC   6982
gb:MN996530|Organism:Severe      TCACCTAATTTTTCTAAACTGATAAATATTATAATTTGGTTTTTACTATTAAGTGTTTGC   6968
gb:MN908947|Organism:Severe      TCACCTAATTTTTCTAAACTGATAAATATTATAATTTGGTTTTTACTATTAAGTGTTTGC   6982
gb:MT019532|Organism:Severe      TCACCTAATTTTTCTAAACTGATAAATATTATAATTTGGTTTTTACTATTAAGTGTTTGC   6982
                                 ************************************************************

gb:MT020781|Organism:Severe      CTAGGTTCTTTAATCTACTCAACCGCTGCTTTAGGTGTTTTAATGTCTAATTTAGGCATG   7030
gb:MT007544|Organism:Severe      CTAGGTTCTTTAATCTACTCAACCGCTGCTTTAGGTGTTTTAATGTCTAATTTAGGCATG   7042
gb:MN994467|Organism:Severe      CTAGGTTCTTTAATCTACTCAACCGCTGCTTTAGGTGTTTTAATGTCTAATTTAGGCATG   7042
gb:MT044257|Organism:Severe      CTAGGTTCTTTAATCTACTCAACCGCTGCTTTAGGTGTTTTAATGTCTAATTTAGGCATG   7042
gb:MT106054|Organism:Severe      CTAGGTTCTTTAATCTACTCAACCGCTGCTTTAGGTGTTTTAATGTCTAATTTAGGCATG   7042
gb:MT049951|Organism:Severe      CTAGGTTCTTTAATCTACTCAACCGCTGCTTTAGGTGTTTTAATGTCTAATTTAGGCATG   7042
gb:MN975262|Organism:Severe      CTAGGTTCTTTAATCTACTCAACCGCTGCTTTAGGTGTTTTAATGTCTAATTTAGGCATG   7042
gb:MT106052|Organism:Severe      CTAGGTTCTTTAATCTACTCAACCGCTGCTTTAGGTGTTTTAATGTCTAATTTAGGCATG   7042
gb:LC522975|Organism:Severe      CTAGGTTCTTTAATCTACTCAACCGCTGCTTTAGGTGTTTTAATGTCTAATTTAGGCATG   7039
```

```
gb:LC522973|Organism:Severe      CTAGGTTCTTTAATCTACTCAACCGCTGCTTTAGGTGTTTTAATGTCTAATTTAGGCATG   7039
gb:LC522974|Organism:Severe      CTAGGTTCTTTAATCTACTCAACCGCTGCTTTAGGTGTTTTAATGTCTAATTTAGGCATG   7039
gb:MN985325|Organism:Severe      CTAGGTTCTTTAATCTACTCAACCGCTGCTTTAGGTGTTTTAATGTCTAATTTAGGCATG   7042
gb:MT020881|Organism:Severe      CTAGGTTCTTTAATCTACTCAACCGCTGCTTTAGGTGTTTTAATGTCTAATTTAGGCATG   7042
gb:MT020880|Organism:Severe      CTAGGTTCTTTAATCTACTCAACCGCTGCTTTAGGTGTTTTAATGTCTAATTTAGGCATG   7042
gb:MT066175|Organism:Severe      CTAGGTTCTTTAATCTACTCAACCGCTGCTTTAGGTGTTTTAATGTCTAATTTAGGCATG   7042
gb:MN997409|Organism:Severe      CTAGGTTCTTTAATCTACTCAACCGCTGCTTTAGGTGTTTTAATGTCTAATTTAGGCATG   7042
gb:MN938384|Organism:Severe      CTAGGTTCTTTAATCTACTCAACCGCTGCTTTAGGTGTTTTAATGTCTAATTTAGGCATG   7010
gb:MT044258|Organism:Severe      CTAGGTTCTTTAATCTACTCAACCGCTGCTTTAGGTGTTTTAATGTCTAATTTAGGCATG   7018
gb:MT039890|Organism:Severe      CTAGGTTCTTTAATCTACTCAACCGCTGCTTTAGGTGTTTTAATGTCTAATTTAGGCATG   7042
gb:MN988713|Organism:Severe      CTAGGTTCTTTAATCTACTCAACCGCTGCTTTAGGTGTTTTAATGTCTAATTTAGGCATG   7042
gb:LC521925|Organism:Severe      CTAGGTTCTTTAATCTACTCAACCGCTGCTTTAGGTGTTTTAATGTCTAATTTAGGCATG   7015
gb:MT093571|Organism:Severe      CTAGGTTCTTTAATCTACTCAACCGCTGCTTTAGGTGTTTTAATGTCTAATTTAGGCATG   7042
gb:MT039887|Organism:Severe      CTAGGTTCTTTAATCTACTCAACCGCTGCTTTAGGTGTTTTAATGTCTAATTTAGGCATG   7042
gb:MT019530|Organism:Severe      CTAGGTTCTTTAATCTACTCAACCGCTGCTTTAGGTGTTTTAATGTCTAATTTAGGCATG   7042
gb:MT039888|Organism:Severe      CTAGGTTCTTTAATCTACTCAACCGCTGCTTTAGGTGTTTTAATGTCTAATTTAGGCATG   7042
gb:LC522972|Organism:Severe      CTAGGTTCTTTAATCTACTCAACCGCTGCTTTAGGTGTTTTAATGTCTAATTTAGGCATG   7039
gb:MT027063|Organism:Severe      CTAGGTTCTTTAATCTACTCAACCGCTGCTTTAGGTGTTTTAATGTCTAATTTAGGCATG   7042
gb:MT027062|Organism:Severe      CTAGGTTCTTTAATCTACTCAACCGCTGCTTTAGGTGTTTTAATGTCTAATTTAGGCATG   7042
gb:MT019529|Organism:Severe      CTAGGTTCTTTAATCTACTCAACCGCTGCTTTAGGTGTTTTAATGTCTAATTTAGGCATG   7042
gb:MN996529|Organism:Severe      CTAGGTTCTTTAATCTACTCAACCGCTGCTTTAAGTGTTTTAATGTCTAATTTAGGCATG   7030
gb:MN996531|Organism:Severe      CTAGGTTCTTTAATCTACTCAACCGCTGCTTTAGGTGTTTTAATGTCTAATTTAGGCATG   7029
gb:MT066176|Organism:Severe      CTAGGTTCTTTAATCTACTCAACCGCTGCTTTAGGTGTTTTAATGTCTAATTTAGGCATG   7042
gb:MT027064|Organism:Severe      CTAGGTTCTTTAATCTACTCAACCGCTGCTTTAGGTGTTTTAATGTCTAATTTAGGCATG   7042
gb:MN994468|Organism:Severe      CTAGGTTCTTTAATCTACTCAACCGCTGCTTTAGGTGTTTTAATGTCTAATTTAGGCATG   7042
gb:MT072688|Organism:Severe      CTAGGTTCTTTAATCTACTCAACCGCTGCTTTAGGTGTTTTAATGTCTAATTTAGGCATG   7027
gb:MN996527|Organism:Severe      CTAGGTTCTTTAATCTACTCAACCGCTGCTTTAGGTGTTTTAATGTCTAATTTAGGCATG   7009
gb:MT093631|Organism:Severe      CTAGGTTCTTTAATCTACTCAACCGCTGCTTTAGGTGTTTTAATGTCTAATTTAGGCATG   7080
gb:MT106053|Organism:Severe      CTAGGTTCTTTAATCTACTCAACCGCTGCTTTAGGTGTTTTAATGTCTAATTTAGGCATG   7042
gb:MT019533|Organism:Severe      CTAGGTTCTTTAATCTACTCAACCGCTGCTTTAGGTGTTTTAATGTCTAATTTAGGCATG   7042
gb:MT019531|Organism:Severe      CTAGGTTCTTTAACCTACTCAACCGCTGCTTTAGGTGTTTTAATGTCTAATTTAGGCATG   7042
gb:MN996528|Organism:Severe      CTAGGTTCTTTAATCTACTCAACCGCTGCTTTAGGTGTTTTAATGTCTAATTTAGGCATG   7042
gb:MN996530|Organism:Severe      CTAGGTTCTTTAATCTACTCAACCGCTGCTTTAGGTGTTTTAATGTCTAATTTAGGCATG   7028
gb:MN908947|Organism:Severe      CTAGGTTCTTTAATCTACTCAACCGCTGCTTTAGGTGTTTTAATGTCTAATTTAGGCATG   7042
gb:MT019532|Organism:Severe      CTAGGTTCTTTAATCTACTCAACCGCTGCTTTAGGTGTTTTAATGTCTAATTTAGGCATG   7042
                                 ************* ******************* **************************

gb:MT020781|Organism:Severe      CCTTCTTACTGTACTGGTTACAGAGAAGGCTATTTGAACTCTACTAATGTCACTATTGCA   7090
gb:MT007544|Organism:Severe      CCTTCTTACTGTACTGGTTACAGAGAAGGCTATTTGAACTCTACTAATGTCACTATTGCA   7102
gb:MN994467|Organism:Severe      CCTTCTTACTGTACTGGTTACAGAGAAGGCTATTTGAACTCTACTAATGTCACTATTGCA   7102
gb:MT044257|Organism:Severe      CCTTCTTACTGTACTGGTTACAGAGAAGGCTATTTGAACTCTACTAATGTCACTATTGCA   7102
gb:MT106054|Organism:Severe      CCTTCTTACTGTACTGGTTACAGAGAAGGCTATTTGAACTCTACTAATGTCACTATTGCA   7102
gb:MT049951|Organism:Severe      CCTTCTTACTGTACTGGTTACAGAGAAGGCTATTTGAACTCTACTAATGTCACTATTGCA   7102
gb:MN975262|Organism:Severe      CCTTCTTACTGTACTGGTTACAGAGAAGGCTATTTGAACTCTACTAATGTCACTATTGCA   7102
gb:MT106052|Organism:Severe      CCTTCTTACTGTACTGGTTACAGAGAAGGCTATTTGAACTCTACTAATGTCACTATTGCA   7102
gb:LC522975|Organism:Severe      CCTTCTTACTGTACTGGTTACAGAGAAGGCTATTTGAACTCTACTAATGTCACTATTGCA   7099
gb:LC522973|Organism:Severe      CCTTCTTACTGTACTGGTTACAGAGAAGGCTATTTGAACTCTACTAATGTCACTATTGCA   7099
gb:LC522974|Organism:Severe      CCTTCTTACTGTACTGGTTACAGAGAAGGCTATTTGAACTCTACTAATGTCACTATTGCA   7099
gb:MN985325|Organism:Severe      CCTTCTTACTGTACTGGTTACAGAGAAGGCTATTTGAACTCTACTAATGTCACTATTGCA   7102
gb:MT020881|Organism:Severe      CCTTCTTACTGTACTGGTTACAGAGAAGGCTATTTGAACTCTACTAATGTCACTATTGCA   7102
gb:MT020880|Organism:Severe      CCTTCTTACTGTACTGGTTACAGAGAAGGCTATTTGAACTCTACTAATGTCACTATTGCA   7102
gb:MT066175|Organism:Severe      CCTTCTTACTGTACTGGTTACAGAGAAGGCTATTTGAACTCTACTAATGTCACTATTGCA   7102
gb:MN997409|Organism:Severe      CCTTCTTACTGTACTGGTTACAGAGAAGGCTATTTGAACTCTACTAATGTCACTATTGCA   7102
gb:MN938384|Organism:Severe      CCTTCTTACTGTACTGGTTACAGAGAAGGCTATTTGAACTCTACTAATGTCACTATTGCA   7070
gb:MT044258|Organism:Severe      CCTTCTTACTGTACTGGTTACAGAGAAGGCTATTTGAACTCTACTAATGTCACTATTGCA   7078
gb:MT039890|Organism:Severe      CCTTCTTACTGTACTGGTTACAGAGAAGGCTATTTGAACTCTACTAATGTCACTATTGCA   7102
gb:MN988713|Organism:Severe      CCTTCTTACTGTACTGGTTACAGAGAAGGCTATTTGAACTCTACTAATGTCACTATTGCA   7102
gb:LC521925|Organism:Severe      CCTTCTTACTGTACTGGTTACAGAGAAGGCTATTTGAACTCTACTAATGTCACTATTGCA   7075
gb:MT093571|Organism:Severe      CCTTCTTACTGTACTGGTTACAGAGAAGGCTATTTGAACTCTACTAATGTCACTATTGCA   7102
gb:MT039887|Organism:Severe      CCTTCTTACTGTACTGGTTACAGAGAAGGCTATTTGAACTCTACTAATGTCACTATTGCA   7102
gb:MT019530|Organism:Severe      CCTTCTTACTGTACTGGTTACAGAGAAGGCTATTTGAACTCTACTAATGTCACTATTGCA   7102
gb:MT039888|Organism:Severe      CCTTCTTACTGTACTGGTTACAGAGAAGGCTATTTGAACTCTACTAATGTCACTATTGCA   7102
gb:LC522972|Organism:Severe      CCTTCTTACTGTACTGGTTACAGAGAAGGCTATTTGAACTCTACTAATGTCACTATTGCA   7099
gb:MT027063|Organism:Severe      CCTTCTTACTGTACTGGTTACAGAGAAGGCTATTTGAACTCTACTAATGTCACTATTGCA   7102
gb:MT027062|Organism:Severe      CCTTCTTACTGTACTGGTTACAGAGAAGGCTATTTGAACTCTACTAATGTCACTATTGCA   7102
```

```
gb:MT019529|Organism:Severe      CCTTCTTACTGTACTGGTTACAGAGAAGGCTATTTGAACTCTACTAATGTCACTATTGCA   7102
gb:MN996529|Organism:Severe      CCTTCTTACTGTACTGGTTACAGAGAAGGCTATTTGAACTCTACTAATGTCACTATTGCA   7090
gb:MN996531|Organism:Severe      CCTTCTTACTGTACTGGTTACAGAGAAGGCTATTTGAACTCTACTAATGTCACTATTGCA   7089
gb:MT066176|Organism:Severe      CCTTCTTACTGTACTGGTTACAGAGAAGGCTATTTGAACTCTACTAATGTCACTATTGCA   7102
gb:MT027064|Organism:Severe      CCTTCTTACTGTACTGGTTACAGAGAAGGCTATTTGAACTCTACTAATGTCACTATTGCA   7102
gb:MN994468|Organism:Severe      CCTTCTTACTGTACTGGTTACAGAGAAGGCTATTTGAACTCTACTAATGTCACTATTGCA   7102
gb:MT072688|Organism:Severe      CCTTCTTACTGTACTGGTTACAGAGAAGGCTATTTGAACTCTACTAATGTCACTATTGCA   7087
gb:MN996527|Organism:Severe      CCTTCTTACTGTACTGGTTACAGAGAAGGCTATTTGAACTCTACTAATGTCACTATTGCA   7069
gb:MT093631|Organism:Severe      CCTTCTTACTGTACTGGTTACAGAGAAGGCTATTTGAACTCTACTAATGTCACTATTGCA   7140
gb:MT106053|Organism:Severe      CCTTCTTACTGTACTGGTTACAGAGAAGGCTATTTGAACTCTACTAATGTCACTATTGCA   7102
gb:MT019533|Organism:Severe      CCTTCTTACTGTACTGGTTACAGAGAAGGCTATTTGAACTCTACTAATGTCACTATTGCA   7102
gb:MT019531|Organism:Severe      CCTTCTTACTGTACTGGTTACAGAGAAGGCTATTTGAACTCTACTAATGTCACTATTGCA   7102
gb:MN996528|Organism:Severe      CCTTCTTACTGTACTGGTTACAGAGAAGGCTATTTGAACTCTACTAATGTCACTATTGCA   7102
gb:MN996530|Organism:Severe      CCTTCTTACTGTACTGGTTACAGAGAAGGCTATTTGAACTCTACTAATGTCACTATTGCA   7088
gb:MN908947|Organism:Severe      CCTTCTTACTGTACTGGTTACAGAGAAGGCTATTTGAACTCTACTAATGTCACTATTGCA   7102
gb:MT019532|Organism:Severe      CCTTCTTACTGTACTGGTTACAGAGAAGGCTATTTGAACTCTACTAATGTCACTATTGCA   7102
                                 ************************************************************

gb:MT020781|Organism:Severe      ACCTACTGTACTGGTTCTATACCTTGTAGTGTTTGTCTTAGTGGTTTAGATTCTTTAGAC   7150
gb:MT007544|Organism:Severe      ACCTACTGTACTGGTTCTATACCTTGTAGTGTTTGTCTTAGTGGTTTAGATTCTTTAGAC   7162
gb:MN994467|Organism:Severe      ACCTACTGTACTGGTTCTATACCTTGTAGTGTTTGTCTTAGTGGTTTAGATTCTTTAGAC   7162
gb:MT044257|Organism:Severe      ACCTACTGTACTGGTTCTATACCTTGTAGTGTTTGTCTTAGTGGTTTAGATTCTTTAGAC   7162
gb:MT106054|Organism:Severe      ACCTACTGTACTGGTTCTATACCTTGTAGTGTTTGTCTTAGTGGTTTAGATTCTTTAGAC   7162
gb:MT049951|Organism:Severe      ACCTACTGTACTGGTTCTATACCTTGTAGTGTTTGTCTTAGTGGTTTAGATTCTTTAGAC   7162
gb:MN975262|Organism:Severe      ACCTACTGTACTGGTTCTATACCTTGTAGTGTTTGTCTTAGTGGTTTAGATTCTTTAGAC   7162
gb:MT106052|Organism:Severe      ACCTACTGTACTGGTTCTATACCTTGTAGTGTTTGTCTTAGTGGTTTAGATTCTTTAGAC   7162
gb:LC522975|Organism:Severe      ACCTACTGTACTGGTTCTATACCTTGTAGTGTTTGTCTTAGTGGTTTAGATTCTTTAGAC   7159
gb:LC522973|Organism:Severe      ACCTACTGTACTGGTTCTATACCTTGTAGTGTTTGTCTTAGTGGTTTAGATTCTTTAGAC   7159
gb:LC522974|Organism:Severe      ACCTACTGTACTGGTTCTATACCTTGTAGTGTTTGTCTTAGTGGTTTAGATTCTTTAGAC   7159
gb:MN985325|Organism:Severe      ACCTACTGTACTGGTTCTATACCTTGTAGTGTTTGTCTTAGTGGTTTAGATTCTTTAGAC   7162
gb:MT020881|Organism:Severe      ACCTACTGTACTGGTTCTATACCTTGTAGTGTTTGTCTTAGTGGTTTAGATTCTTTAGAC   7162
gb:MT020880|Organism:Severe      ACCTACTGTACTGGTTCTATACCTTGTAGTGTTTGTCTTAGTGGTTTAGATTCTTTAGAC   7162
gb:MT066175|Organism:Severe      ACCTACTGTACTGGTTCTATACCTTGTAGTGTTTGTCTTAGTGGTTTAGATTCTTTAGAC   7162
gb:MN997409|Organism:Severe      ACCTACTGTACTGGTTCTATACCTTGTAGTGTTTGTCTTAGTGGTTTAGATTCTTTAGAC   7162
gb:MN938384|Organism:Severe      ACCTACTGTACTGGTTCTATACCTTGTAGTGTTTGTCTTAGTGGTTTAGATTCTTTAGAC   7130
gb:MT044258|Organism:Severe      ACCTACTGTACTGGTTCTATACCTTGTAGTGTTTGTCTTAGTGGTTTAGATTCTTTAGAC   7138
gb:MT039890|Organism:Severe      ACCTACTGTACTGGTTCTATACCTTGTAGTGTTTGTCTTAGTGGTTTAGATTCTTTAGAC   7162
gb:MN988713|Organism:Severe      ACCTACTGTACTGGTTCTATACCTTGTAGTGTTTGTCTTAGTGGTTTAGATTCTTTAGAC   7162
gb:LC521925|Organism:Severe      ACCTACTGTACTGGTTCTATACCTTGTAGTGTTTGTCTTAGTGGTTTAGATTCTTTAGAC   7135
gb:MT093571|Organism:Severe      ACCTACTGTACTGGTTCTATACCTTGTAGTGTTTGTCTTAGTGGTTTAGATTCTTTAGAC   7162
gb:MT039887|Organism:Severe      ACCTACTGTACTGGTTCTATACCTTGTAGTGTTTGTCTTAGTGGTTTAGATTCTTTAGAC   7162
gb:MT019530|Organism:Severe      ACCTACTGTACTGGTTCTATACCTTGTAGTGTTTGTCTTAGTGGTTTAGATTCTTTAGAC   7162
gb:MT039888|Organism:Severe      ACCTACTGTACTGGTTCTATACCTTGTAGTGTTTGTCTTAGTGGTTTAGATTCTTTAGAC   7162
gb:LC522972|Organism:Severe      ACCTACTGTACTGGTTCTATACCTTGTAGTGTTTGTCTTAGTGGTTTAGATTCTTTAGAC   7159
gb:MT027063|Organism:Severe      ACCTACTGTACTGGTTCTATACCTTGTAGTGTTTGTCTTAGTGGTTTAGATTCTTTAGAC   7162
gb:MT027062|Organism:Severe      ACCTACTGTACTGGTTCTATACCTTGTAGTGTTTGTCTTAGTGGTTTAGATTCTTTAGAC   7162
gb:MT019529|Organism:Severe      ACCTACTGTACTGGTTCTATACCTTGTAGTGTTTGTCTTAGTGGTTTAGATTCTTTAGAC   7162
gb:MN996529|Organism:Severe      ACCTACTGTACTGGTTCTATACCTTGTAGTGTTTGTCTTAGTGGTTTAGATTCTTTAGAC   7150
gb:MN996531|Organism:Severe      ACCTACTGTACTGGTTCTATACCTTGTAGTGTTTGTCTTAGTGGTTTAGATTCTTTAGAC   7149
gb:MT066176|Organism:Severe      ACCTACTGTACTGGTTCTATACCTTGTAGTGTTTGTCTTAGTGGTTTAGATTCTTTAGAC   7162
gb:MT027064|Organism:Severe      ACCTACTGTACTGGTTCTATACCTTGTAGTGTTTGTCTTAGTGGTTTAGATTCTTTAGAC   7162
gb:MN994468|Organism:Severe      ACCTACTGTACTGGTTCTATACCTTGTAGTGTTTGTCTTAGTGGTTTAGATTCTTTAGAC   7162
gb:MT072688|Organism:Severe      ACCTACTGTACTGGTTCTATACCTTGTAGTGTTTGTCTTAGTGGTTTAGATTCTTTAGAC   7147
gb:MN996527|Organism:Severe      ACCTACTGTACTGGTTCTATACCTTGTAGTGTTTGTCTTAGTGGTTTAGATTCTTTAGAC   7129
gb:MT093631|Organism:Severe      ACCTACTGTACTGGTTCTATACCTTGTAGTGTTTGTCTTAGTGGTTTAGATTCTTTAGAC   7200
gb:MT106053|Organism:Severe      ACCTACTGTACTGGTTCTATACCTTGTAGTGTTTGTCTTAGTGGTTTAGATTCTTTAGAC   7162
gb:MT019533|Organism:Severe      ACCTACTGTACTGGTTCTATACCTTGTAGTGTTTGTCTTAGTGGTTTAGATTCTTTAGAC   7162
gb:MT019531|Organism:Severe      ACCTACTGTACTGGTTCTATACCTTGTAGTGTTTGTCTTAGTGGTTTAGATTCTTTAGAC   7162
gb:MN996528|Organism:Severe      ACCTACTGTACTGGTTCTATACCTTGTAGTGTTTGTCTTAGTGGTTTAGATTCTTTAGAC   7162
gb:MN996530|Organism:Severe      ACCTACTGTACTGGTTCTATACCTTGTAGTGTTTGTCTTAGTGGTTTAGATTCTTTAGAC   7148
gb:MN908947|Organism:Severe      ACCTACTGTACTGGTTCTATACCTTGTAGTGTTTGTCTTAGTGGTTTAGATTCTTTAGAC   7162
gb:MT019532|Organism:Severe      ACCTACTGTACTGGTTCTATACCTTGTAGTGTTTGTCTTAGTGGTTTAGATTCTTTAGAC   7162
                                 ************************************************************

gb:MT020781|Organism:Severe      ACCTATCCTTCTTTAGAAACTATACAAATTACCATTTCATCTTTTAAATGGGATTTAACT   7210
```

```
gb:MT007544|Organism:Severe      ACCTATCCTTCTTTAGAAACTATACAAATTACCATTTCATCTTTTAAATGGGATTTAACT   7222
gb:MN994467|Organism:Severe      ACCTATCCTTCTTTAGAAACTATACAAATTACCATTTCATCTTTTAAATGGGATTTAACT   7222
gb:MT044257|Organism:Severe      ACCTATCCTTCTTTAGAAACTATACAAATTACCATTTCATCTTTTAAATGGGATTTAACT   7222
gb:MT106054|Organism:Severe      ACCTATCCTTCTTTAGAAACTATACAAATTACCATTTCATCTTTTAAATGGGATTTAACT   7222
gb:MT049951|Organism:Severe      ACCTATCCTTCTTTAGAAACTATACAAATTACCATTTCATCTTTTAAATGGGATTTAACT   7222
gb:MN975262|Organism:Severe      ACCTATCCTTCTTTAGAAACTATACAAATTACCATTTCATCTTTTAAATGGGATTTAACT   7222
gb:MT106052|Organism:Severe      ACCTATCCTTCTTTAGAAACTATACAAATTACCATTTCATCTTTTAAATGGGATTTAACT   7222
gb:LC522975|Organism:Severe      ACCTATCCTTCTTTAGAAACTATACAAATTACCATTTCATCTTTTAAATGGGATTTAACT   7219
gb:LC522973|Organism:Severe      ACCTATCCTTCTTTAGAAACTATACAAATTACCATTTCATCTTTTAAATGGGATTTAACT   7219
gb:LC522974|Organism:Severe      ACCTATCCTTCTTTAGAAACTATACAAATTACCATTTCATCTTTTAAATGGGATTTAACT   7219
gb:MN985325|Organism:Severe      ACCTATCCTTCTTTAGAAACTATACAAATTACCATTTCATCTTTTAAATGGGATTTAACT   7222
gb:MT020881|Organism:Severe      ACCTATCCTTCTTTAGAAACTATACAAATTACCATTTCATCTTTTAAATGGGATTTAACT   7222
gb:MT020880|Organism:Severe      ACCTATCCTTCTTTAGAAACTATACAAATTACCATTTCATCTTTTAAATGGGATTTAACT   7222
gb:MT066175|Organism:Severe      ACCTATCCTTCTTTAGAAACTATACAAATTACCATTTCATCTTTTAAATGGGATTTAACT   7222
gb:MN997409|Organism:Severe      ACCTATCCTTCTTTAGAAACTATACAAATTACCATTTCATCTTTTAAATGGGATTTAACT   7222
gb:MN938384|Organism:Severe      ACCTATCCTTCTTTAGAAACTATACAAATTACCATTTCATCTTTTAAATGGGATTTAACT   7190
gb:MT044258|Organism:Severe      ACCTATCCTTCTTTAGAAACTATACAAATTACCATTTCATCTTTTAAATGGGATTTAACT   7198
gb:MT039890|Organism:Severe      ACCTATCCTTCTTTAGAAACTATACAAATTACCATTTCATCTTTTAAATGGGATTTAACT   7222
gb:MN988713|Organism:Severe      ACCTATCCTTCTTTAGAAACTATACAAATTACCATTTCATCTTTTAAATGGGATTTAACT   7222
gb:LC521925|Organism:Severe      ACCTATCCTTCTTTAGAAACTATACAAATTACCATTTCATCTTTTAAATGGGATTTAACT   7195
gb:MT093571|Organism:Severe      ACCTATCCTTCTTTAGAAACTATACAAATTACCATTTCATCTTTTAAATGGGATTTAACT   7222
gb:MT039887|Organism:Severe      ACCTATCCTTCTTTAGAAACTATACAAATTACCATTTCATCTTTTAAATGGGATTTAACT   7222
gb:MT019530|Organism:Severe      ACCTATCCTTCTTTAGAAACTATACAAATTACCATTTCATCTTTTAAATGGGATTTAACT   7222
gb:MT039888|Organism:Severe      ACCTATCCTTCTTTAGAAACTATACAAATTACCATTTCATCTTTTAAATGGGATTTAACT   7222
gb:LC522972|Organism:Severe      ACCTATCCTTCTTTAGAAACTATACAAATTACCATTTCATCTTTTAAATGGGATTTAACT   7219
gb:MT027063|Organism:Severe      ACCTATCCTTCTTTAGAAACTATACAAATTACCATTTCATCTTTTAAATGGGATTTAACT   7222
gb:MT027062|Organism:Severe      ACCTATCCTTCTTTAGAAACTATACAAATTACCATTTCATCTTTTAAATGGGATTTAACT   7222
gb:MT019529|Organism:Severe      ACCTATCCTTCTTTAGAAACTATACAAATTACCATTTCATCTTTTAAATGGGATTTAACT   7222
gb:MN996529|Organism:Severe      ACCTATCCTTCTTTAGAAACTATACAAATTACCATTTCATCTTTTAAATGGGATTTAACT   7210
gb:MN996531|Organism:Severe      ACCTATCCTTCTTTAGAAACTATACAAATTACCATTTCATCTTTTAAATGGGATTTAACT   7209
gb:MT066176|Organism:Severe      ACCTATCCTTCTTTAGAAACTATACAAATTACCATTTCATCTTTTAAATGGGATTTAACT   7222
gb:MT027064|Organism:Severe      ACCTATCCTTCTTTAGAAACTATACAAATTACCATTTCATCTTTTAAATGGGATTTAACT   7222
gb:MN994468|Organism:Severe      ACCTATCCTTCTTTAGAAACTATACAAATTACCATTTCATCTTTTAAATGGGATTTAACT   7222
gb:MT072688|Organism:Severe      ACCTATCCTTCTTTAGAAACTATACAAATTACCATTTCATCTTTTAAATGGGATTTAACT   7207
gb:MN996527|Organism:Severe      ACCTATCCTTCTTTAGAAACTATACAAATTACCATTTCATCTTTTAAATGGGATTTAACT   7189
gb:MT093631|Organism:Severe      ACCTATCCTTCTTTAGAAACTATACAAATTACCATTTCATCTTTTAAATGGGATTTAACT   7260
gb:MT106053|Organism:Severe      ACCTATCCTTCTTTAGAAACTATACAAATTACCATTTCATCTTTTAAATGGGATTTAACT   7222
gb:MT019533|Organism:Severe      ACCTATCCTTCTTTAGAAACTATACAAATTACCATTTCATCTTTTAAATGGGATTTAACT   7222
gb:MT019531|Organism:Severe      ACCTATCCTTCTTTAGAAACTATACAAATTACCATTTCATCTTTTAAATGGGATTTAACT   7222
gb:MN996528|Organism:Severe      ACCTATCCTTCTTTAGAAACTATACAAATTACCATTTCATCTTTTAAATGGGATTTAACT   7222
gb:MN996530|Organism:Severe      ACCTATCCTTCTTTAGAAACTATACAAATTACCATTTCATCTTTTAAATGGGATTTAACT   7208
gb:MN908947|Organism:Severe      ACCTATCCTTCTTTAGAAACTATACAAATTACCATTTCATCTTTTAAATGGGATTTAACT   7222
gb:MT019532|Organism:Severe      ACCTATCCTTCTTTAGAAACTATACAAATTACCATTTCATCTTTTAAATGGGATTTAACT   7222
                                 ************************************************************

gb:MT020781|Organism:Severe      GCTTTTGGCTTAGTTGCAGAGTGGTTTTTGGCATATATTCTTTTCACTAGGTTTTTCTAT   7270
gb:MT007544|Organism:Severe      GCTTTTGGCTTAGTTGCAGAGTGGTTTTTGGCATATATTCTTTTCACTAGGTTTTTCTAT   7282
gb:MN994467|Organism:Severe      GCTTTTGGCTTAGTTGCAGAGTGGTTTTTGGCATATATTCTTTTCACTAGGTTTTTCTAT   7282
gb:MT044257|Organism:Severe      GCTTTTGGCTTAGTTGCAGAGTGGTTTTTGGCATATATTCTTTTCACTAGGTTTTTCTAT   7282
gb:MT106054|Organism:Severe      GCTTTTGGCTTAGTTGCAGAGTGGTTTTTGGCATATATTCTTTTCACTAGGTTTTTCTAT   7282
gb:MT049951|Organism:Severe      GCTTTTGGCTTAGTTGCAGAGTGGTTTTTGGCATATATTCTTTTCACTAGGTTTTTCTAT   7282
gb:MN975262|Organism:Severe      GCTTTTGGCTTAGTTGCAGAGTGGTTTTTGGCATATATTCTTTTCACTAGGTTTTTCTAT   7282
gb:MT106052|Organism:Severe      GCTTTTGGCTTAGTTGCAGAGTGGTTTTTGGCATATATTCTTTTCACTAGGTTTTTCTAT   7282
gb:LC522975|Organism:Severe      GCTTTTGGCTTAGTTGCAGAGTGGTTTTTGGCATATATTCTTTTCACTAGGTTTTTCTAT   7279
gb:LC522973|Organism:Severe      GCTTTTGGCTTAGTTGCAGAGTGGTTTTTGGCATATATTCTTTTCACTAGGTTTTTCTAT   7279
gb:LC522974|Organism:Severe      GCTTTTGGCTTAGTTGCAGAGTGGTTTTTGGCATATATTCTTTTCACTAGGTTTTTCTAT   7279
gb:MN985325|Organism:Severe      GCTTTTGGCTTAGTTGCAGAGTGGTTTTTGGCATATATTCTTTTCACTAGGTTTTTCTAT   7282
gb:MT020881|Organism:Severe      GCTTTTGGCTTAGTTGCAGAGTGGTTTTTGGCATATATTCTTTTCACTAGGTTTTTCTAT   7282
gb:MT020880|Organism:Severe      GCTTTTGGCTTAGTTGCAGAGTGGTTTTTGGCATATATTCTTTTCACTAGGTTTTTCTAT   7282
gb:MT066175|Organism:Severe      GCTTTTGGCTTAGTTGCAGAGTGGTTTTTGGCATATATTCTTTTCACTAGGTTTTTCTAT   7282
gb:MN997409|Organism:Severe      GCTTTTGGCTTAGTTGCAGAGTGGTTTTTGGCATATATTCTTTTCACTAGGTTTTTCTAT   7282
gb:MN938384|Organism:Severe      GCTTTTGGCTTAGTTGCAGAGTGGTTTTTGGCATATATTCTTTTCACTAGGTTTTTCTAT   7250
gb:MT044258|Organism:Severe      GCTTTTGGCTTAGTTGCAGAGTGGTTTTTGGCATATATTCTTTTCACTAGGTTTTTCTAT   7258
gb:MT039890|Organism:Severe      GCTTTTGGCTTAGTTGCAGAGTGGTTTTTGGCATATATTCTTTTCACTAGGTTTTTCTAT   7282
gb:MN988713|Organism:Severe      GCTTTTGGCTTAGTTGCAGAGTGGTTTTTGGCATATATTCTTTTCACTAGGTTTTTCTAT   7282
```

```
gb:LC521925|Organism:Severe      GCTTTTGGCTTAGTTGCAGAGTGGTTTTTGGCATATATTCTTTTCACTAGGTTTTTCTAT   7255
gb:MT093571|Organism:Severe      GCTTTTGGCTTAGTTGCAGAGTGGTTTTTGGCATATATTCTTTTCACTAGGTTTTTCTAT   7282
gb:MT039887|Organism:Severe      GCTTTTGGCTTAGTTGCAGAGTGGTTTTTGGCATATATTCTTTTCACTAGGTTTTTCTAT   7282
gb:MT019530|Organism:Severe      GCTTTTGGCTTAGTTGCAGAGTGGTTTTTGGCATATATTCTTTTCACTAGGTTTTTCTAT   7282
gb:MT039888|Organism:Severe      GCTTTTGGCTTAGTTGCAGAGTGGTTTTTGGCATATATTCTTTTCACTAGGTTTTTCTAT   7282
gb:LC522972|Organism:Severe      GCTTTTGGCTTAGTTGCAGAGTGGTTTTTGGCATATATTCTTTTCACTAGGTTTTTCTAT   7279
gb:MT027063|Organism:Severe      GCTTTTGGCTTAGTTGCAGAGTGGTTTTTGGCATATATTCTTTTCACTAGGTTTTTCTAT   7282
gb:MT027062|Organism:Severe      GCTTTTGGCTTAGTTGCAGAGTGGTTTTTGGCATATATTCTTTTCACTAGGTTTTTCTAT   7282
gb:MT019529|Organism:Severe      GCTTTTGGCTTAGTTGCAGAGTGGTTTTTGGCATATATTCTTTTCACTAGGTTTTTCTAT   7282
gb:MN996529|Organism:Severe      GCTTTTGGCTTAGTTGCAGAGTGGTTTTTGGCATATATTCTTTTCACTAGGTTTTTCTAT   7270
gb:MN996531|Organism:Severe      GCTTTTGGCTTAGTTGCAGAGTGGTTTTTGGCATATATTCTTTTCACTAGGTTTTTCTAT   7269
gb:MT066176|Organism:Severe      GCTTTTGGCTTAGTTGCAGAGTGGTTTTTGGCATATATTCTTTTCACTAGGTTTTTCTAT   7282
gb:MT027064|Organism:Severe      GCTTTTGGCTTAGTTGCAGAGTGGTTTTTGGCATATATTCTTTTCACTAGGTTTTTCTAT   7282
gb:MN994468|Organism:Severe      GCTTTTGGCTTAGTTGCAGAGTGGTTTTTGGCATATATTCTTTTCACTAGGTTTTTCTAT   7282
gb:MT072688|Organism:Severe      GCTTTTGGCTTAGTTGCAGAGTGGTTTTTGGCATATATTCTTTTCACTAGGTTTTTCTAT   7267
gb:MN996527|Organism:Severe      GCTTTTGGCTTAGTTGCAGAGTGGTTTTTGGCATATATTCTTTTCACTAGGTTTTTCTAT   7249
gb:MT093631|Organism:Severe      GCTTTTGGCTTAGTTGCAGAGTGGTTTTTGGCATATATTCTTTTCACTAGGTTTTTCTAT   7320
gb:MT106053|Organism:Severe      GCTTTTGGCTTAGTTGCAGAGTGGTTTTTGGCATATATTCTTTTCACTAGGTTTTTCTAT   7282
gb:MT019533|Organism:Severe      GCTTTTGGCTTAGTTGCAGAGTGGTTTTTGGCATATATTCTTTTCACTAGGTTTTTCTAT   7282
gb:MT019531|Organism:Severe      GCTTTTGGCTTAGTTGCAGAGTGGTTTTTGGCATATATTCTTTTCACTAGGTTTTTCTAT   7282
gb:MN996528|Organism:Severe      GCTTTTGGCTTAGTTGCAGAGTGGTTTTTGGCATATATTCTTTTCACTAGGTTTTTCTAT   7282
gb:MN996530|Organism:Severe      GCTTTTGGCTTAGTTGCAGAGTGGTTTTTGGCATATATTCTTTTCACTAGGTTTTTCTAT   7268
gb:MN908947|Organism:Severe      GCTTTTGGCTTAGTTGCAGAGTGGTTTTTGGCATATATTCTTTTCACTAGGTTTTTCTAT   7282
gb:MT019532|Organism:Severe      GCTTTTGGCTTAGTTGCAGAGTGGTTTTTGGCATATATTCTTTTCACTAGGTTTTTCTAT   7282
                                 ************************************************************

gb:MT020781|Organism:Severe      GTACTTGGATTGGCTGCAATCATGCAATTGTTTTTCAGCTATTTTGCAGTACATTTTATT   7330
gb:MT007544|Organism:Severe      GTACTTGGATTGGCTGCAATCATGCAATTGTTTTTCAGCTATTTTGCAGTACATTTTATT   7342
gb:MN994467|Organism:Severe      GTACTTGGATTGGCTGCAATCATGCAATTGTTTTTCAGCTATTTTGCAGTACATTTTATT   7342
gb:MT044257|Organism:Severe      GTACTTGGATTGGCTGCAATCATGCAATTGTTTTTCAGCTATTTTGCAGTACATTTTATT   7342
gb:MT106054|Organism:Severe      GTACTTGGATTGGCTGCAATCATGCAATTGTTTTTCAGCTATTTTGCAGTACATTTTATT   7342
gb:MT049951|Organism:Severe      GTACTTGGATTGGCTGCAATCATGCAATTGTTTTTCAGCTATTTTGCAGTACATTTTATT   7342
gb:MN975262|Organism:Severe      GTACTTGGATTGGCTGCAATCATGCAATTGTTTTTCAGCTATTTTGCAGTACATTTTATT   7342
gb:MT106052|Organism:Severe      GTACTTGGATTGGCTGCAATCATGCAATTGTTTTTCAGCTATTTTGCAGTACATTTTATT   7342
gb:LC522975|Organism:Severe      GTACTTGGATTGGCTGCAATCATGCAATTGTTTTTCAGCTATTTTGCAGTACATTTTATT   7339
gb:LC522973|Organism:Severe      GTACTTGGATTGGCTGCAATCATGCAATTGTTTTTCAGCTATTTTGCAGTACATTTTATT   7339
gb:LC522974|Organism:Severe      GTACTTGGATTGGCTGCAATCATGCAATTGTTTTTCAGCTATTTTGCAGTACATTTTATT   7339
gb:MN985325|Organism:Severe      GTACTTGGATTGGCTGCAATCATGCAATTGTTTTTCAGCTATTTTGCAGTACATTTTATT   7342
gb:MT020881|Organism:Severe      GTACTTGGATTGGCTGCAATCATGCAATTGTTTTTCAGCTATTTTGCAGTACATTTTATT   7342
gb:MT020880|Organism:Severe      GTACTTGGATTGGCTGCAATCATGCAATTGTTTTTCAGCTATTTTGCAGTACATTTTATT   7342
gb:MT066175|Organism:Severe      GTACTTGGATTGGCTGCAATCATGCAATTGTTTTTCAGCTATTTTGCAGTACATTTTATT   7342
gb:MN997409|Organism:Severe      GTACTTGGATTGGCTGCAATCATGCAATTGTTTTTCAGCTATTTTGCAGTACATTTTATT   7342
gb:MN938384|Organism:Severe      GTACTTGGATTGGCTGCAATCATGCAATTGTTTTTCAGCTATTTTGCAGTACATTTTATT   7310
gb:MT044258|Organism:Severe      GTACTTGGATTGGCTGCAATCATGCAATTGTTTTTCAGCTATTTTGCAGTACATTTTATT   7318
gb:MT039890|Organism:Severe      GTACTTGGATTGGCTGCAATCATGCAATTGTTTTTCAGCTATTTTGCAGTACATTTTATT   7342
gb:MN988713|Organism:Severe      GTACTTGGATTGGCTGCAATCATGCAATTGTTTTTCAGCTATTTTGCAGTACATTTTATT   7342
gb:LC521925|Organism:Severe      GTACTTGGATTGGCTGCAATCATGCAATTGTTTTTCAGCTATTTTGCAGTACATTTTATT   7315
gb:MT093571|Organism:Severe      GTACTTGGATTGGCTGCAATCATGCAATTGTTTTTCAGCTATTTTGCAGTACATTTTATT   7342
gb:MT039887|Organism:Severe      GTACTTGGATTGGCTGCAATCATGCAATTGTTTTTCAGCTATTTTGCAGTACATTTTATT   7342
gb:MT019530|Organism:Severe      GTACTTGGATTGGCTGCAATCATGCAATTGTTTTTCAGCTATTTTGCAGTACATTTTATT   7342
gb:MT039888|Organism:Severe      GTACTTGGATTGGCTGCAATCATGCAATTGTTTTTCAGCTATTTTGCAGTACATTTTATT   7342
gb:LC522972|Organism:Severe      GTACTTGGATTGGCTGCAATCATGCAATTGTTTTTCAGCTATTTTGCAGTACATTTTATT   7339
gb:MT027063|Organism:Severe      GTACTTGGATTGGCTGCAATCATGCAATTGTTTTTCAGCTATTTTGCAGTACATTTTATT   7342
gb:MT027062|Organism:Severe      GTACTTGGATTGGCTGCAATCATGCAATTGTTTTTCAGCTATTTTGCAGTACATTTTATT   7342
gb:MT019529|Organism:Severe      GTACTTGGATTGGCTGCAATCATGCAATTGTTTTTCAGCTATTTTGCAGTACATTTTATT   7342
gb:MN996529|Organism:Severe      GTACTTGGATTGGCTGCAATCATGCAATTGTTTTTCAGCTATTTTGCAGTACATTTTATT   7330
gb:MN996531|Organism:Severe      GTACTTGGATTGGCTGCAATCATGCAATTGTTTTTCAGCTATTTTGCAGTACATTTTATT   7329
gb:MT066176|Organism:Severe      GTACTTGGATTGGCTGCAATCATGCAATTGTTTTTCAGCTATTTTGCAGTACATTTTATT   7342
gb:MT027064|Organism:Severe      GTACTTGGATTGGCTGCAATCATGCAATTGTTTTTCAGCTATTTTGCAGTACATTTTATT   7342
gb:MN994468|Organism:Severe      GTACTTGGATTGGCTGCAATCATGCAATTGTTTTTCAGCTATTTTGCAGTACATTTTATT   7342
gb:MT072688|Organism:Severe      GTACTTGGATTGGCTGCAATCATGCAATTGTTTTTCAGCTATTTTGCAGTACATTTTATT   7327
gb:MN996527|Organism:Severe      GTACTTGGATTGGCTGCAATCATGCAATTGTTTTTCAGCTATTTTGCAGTACATTTTATT   7309
gb:MT093631|Organism:Severe      GTACTTGGATTGGCTGCAATCATGCAATTGTTTTTCAGCTATTTTGCAGTACATTTTATT   7380
gb:MT106053|Organism:Severe      GTACTTGGATTGGCTGCAATCATGCAATTGTTTTTCAGCTATTTTGCAGTACATTTTATT   7342
gb:MT019533|Organism:Severe      GTACTTGGATTGGCTGCAATCATGCAATTGTTTTTCAGCTATTTTGCAGTACATTTTATT   7342
```

```
gb:MT019531|Organism:Severe      GTACTTGGATTGGCTGCAATCATGCAATTGTTTTTCAGCTATTTTGCAGTACATTTTATT   7342
gb:MN996528|Organism:Severe      GTACTTGGATTGGCTGCAATCATGCAATTGTTTTTCAGCTATTTTGCAGTACATTTTATT   7342
gb:MN996530|Organism:Severe      GTACTTGGATTGGCTGCAATCATGCAATTGTTTTTCAGCTATTTTGCAGTACATTTTATT   7328
gb:MN908947|Organism:Severe      GTACTTGGATTGGCTGCAATCATGCAATTGTTTTTCAGCTATTTTGCAGTACATTTTATT   7342
gb:MT019532|Organism:Severe      GTACTTGGATTGGCTGCAATCATGCAATTGTTTTTCAGCTATTTTGCAGTACATTTTATT   7342
                                 ************************************************************

gb:MT020781|Organism:Severe      AGTAATTCTTGGCTTATGTGGTTAATANNNNNNNNNNNNNNAAATGGCCCCGATTTCAGCT   7390
gb:MT007544|Organism:Severe      AGTAATTCTTGGCTTATGTGGTTAATAATTAATCTTGTACAAATGGCCCCGATTTCAGCT   7402
gb:MN994467|Organism:Severe      AGTAATTCTTGGCTTATGTGGTTAATAATTAATCTTGTACAAATGGCCCCGATTTCAGCT   7402
gb:MT044257|Organism:Severe      AGTAATTCTTGGCTTATGTGGTTAATAATTAATCTTGTACAAATGGCCCCGATTTCAGCT   7402
gb:MT106054|Organism:Severe      AGTAATTCTTGGCTTATGTGGTTAATAATTAATCTTGTACAAATGGCCCCGATTTCAGCT   7402
gb:MT049951|Organism:Severe      AGTAATTCTTGGCTTATGTGGTTAATAATTAATCTTGTACAAATGGCCCCGATTTCAGCT   7402
gb:MN975262|Organism:Severe      AGTAATTCTTGGCTTATGTGGTTAATAATTAATCTTGTACAAATGGCCCCGATTTCAGCT   7402
gb:MT106052|Organism:Severe      AGTAATTCTTGGCTTATGTGGTTAATAATTAATCTTGTACAAATGGCCCCGATTTCAGCT   7402
gb:LC522975|Organism:Severe      AGTAATTCTTGGCTTATGTGGTTAATAATTAATCTTGTACAAATGGCCCCGATTTCAGCT   7399
gb:LC522973|Organism:Severe      AGTAATTCTTGGCTTATGTGGTTAATAATTAATCTTGTACAAATGGCCCCGATTTCAGCT   7399
gb:LC522974|Organism:Severe      AGTAATTCTTGGCTTATGTGGTTAATAATTAATCTTGTACAAATGGCCCCGATTTCAGCT   7399
gb:MN985325|Organism:Severe      AGTAATTCTTGGCTTATGTGGTTAATAATTAATCTTGTACAAATGGCCCCGATTTCAGCT   7402
gb:MT020881|Organism:Severe      AGTAATTCTTGGCTTATGTGGTTAATAATTAATCTTGTACAAATGGCCCCGATTTCAGCT   7402
gb:MT020880|Organism:Severe      AGTAATTCTTGGCTTATGTGGTTAATAATTAATCTTGTACAAATGGCCCCGATTTCAGCT   7402
gb:MT066175|Organism:Severe      AGTAATTCTTGGCTTATGTGGTTAATAATTAATCTTGTACAAATGGCCCCGATTTCAGCT   7402
gb:MN997409|Organism:Severe      AGTAATTCTTGGCTTATGTGGTTAATAATTAATCTTGTACAAATGGCCCCGATTTCAGCT   7402
gb:MN938384|Organism:Severe      AGTAATTCTTGGCTTATGTGGTTAATAATTAATCTTGTACAAATGGCCCCGATTTCAGCT   7370
gb:MT044258|Organism:Severe      AGTAATTCTTGGCTTATGTGGTTAATAATTAATCTTGTACAAATGGCCCCGATTTCAGCT   7378
gb:MT039890|Organism:Severe      AGTAATTCTTGGCTTATGTGGTTAATAATTAATCTTGTACAAATGGCCCCGATTTCAGCT   7402
gb:MN988713|Organism:Severe      AGTAATTCTTGGCTTATGTGGTTAATAATTAATCTTGTACAAATGGCCCCGATTTCAGCT   7402
gb:LC521925|Organism:Severe      AGTAATTCTTGGCTTATGTGGTTAATAATTAATCTTGTACAAATGGCCCCGATTTCAGCT   7375
gb:MT093571|Organism:Severe      AGTAATTCTTGGCTTATGTGGTTAATAATTAATCTTGTACAAATGGCCCCGATTTCAGCT   7402
gb:MT039887|Organism:Severe      AGTAATTCTTGGCTTATGTGGTTAATAATTAATCTTGTACAAATGGCCCCGATTTCAGCT   7402
gb:MT019530|Organism:Severe      AGTAATTCTTGGCTTATGTGGTTAATAATTAATCTTGTACAAATGGCCCCGATTTCAGCT   7402
gb:MT039888|Organism:Severe      AGTAATTCTTGGCTTATGTGGTTAATAATTAATCTTGTACAAATGGCCCCGATTTCAGCT   7402
gb:LC522972|Organism:Severe      AGTAATTCTTGGCTTATGTGGTTAATAATTAATCTTGTACAAATGGCCCCGATTTCAGCT   7399
gb:MT027063|Organism:Severe      AGTAATTCTTGGCTTATGTGGTTAATAATTAATCTTGTACAAATGGCCCCGATTTCAGCT   7402
gb:MT027062|Organism:Severe      AGTAATTCTTGGCTTATGTGGTTAATAATTAATCTTGTACAAATGGCCCCGATTTCAGCT   7402
gb:MT019529|Organism:Severe      AGTAATTCTTGGCTTATGTGGTTAATAATTAATCTTGTACAAATGGCCCCGATTTCAGCT   7402
gb:MN996529|Organism:Severe      AGTAATTCTTGGCTTATGTGGTTAATAATTAATCTTGTACAAATGGCCCCGATTTCAGCT   7390
gb:MN996531|Organism:Severe      AGTAATTCTTGGCTTATGTGGTTAATAATTAATCTTGTACAAATGGCCCCGATTTCAGCT   7389
gb:MT066176|Organism:Severe      AGTAATTCTTGGCTTATGTGGTTAATAATTAATCTTGTACAAATGGCCCCGATTTCAGCT   7402
gb:MT027064|Organism:Severe      AGTAATTCTTGGCTTATGTGGTTAATAATTAATCTTGTACAAATGGCCCCGATTTCAGCT   7402
gb:MN994468|Organism:Severe      AGTAATTCTTGGCTTATGTGGTTAATAATTAATCTTGTACAAATGGCCCCGATTTCAGCT   7402
gb:MT072688|Organism:Severe      AGTAATTCTTGGCTTATGTGGTTAATAATTAATCTTGTACAAATGGCCCCGATTTCAGCT   7387
gb:MN996527|Organism:Severe      AGTAATTCTTGGCTTATGTGGTTAATAATTAATCTTGTACAAATGGCCCCGATTTCAGCT   7369
gb:MT093631|Organism:Severe      AGTAATTCTTGGCTTATGTGGTTAATAATTAATCTTGTACAAATGGCCCCGATTTCAGCT   7440
gb:MT106053|Organism:Severe      AGTAATTCTTGGCTTATGTGGTTAATAATTAATCTTGTACAAATGGCCCCGATTTCAGCT   7402
gb:MT019533|Organism:Severe      AGTAATTCTTGGCTTATGTGGTTAATAATTAATCTTGTACAAATGGCCCCGATTTCAGCT   7402
gb:MT019531|Organism:Severe      AGTAATTCTTGGCTTATGTGGTTAATAATTAATCTTGTACAAATGGCCCCGATTTCAGCT   7402
gb:MN996528|Organism:Severe      AGTAATTCTTGGCTTATGTGGTTAATAATTAATCTTGTACAAATGGCCCCGATTTCAGCT   7402
gb:MN996530|Organism:Severe      AGTAATTCTTGGCTTATGTGGTTAATAATTAATCTTGTACAAATGGCCCCGATTTCAGCT   7388
gb:MN908947|Organism:Severe      AGTAATTCTTGGCTTATGTGGTTAATAATTAATCTTGTACAAATGGCCCCGATTTCAGCT   7402
gb:MT019532|Organism:Severe      AGTAATTCTTGGCTTATGTGGTTAATAATTAATCTTGTACAAATGGCCCCGATTTCAGCT   7402
                                 ***************************              ********************

gb:MT020781|Organism:Severe      ATGGTTAGAATGTACATCTTCTTTGCATCATTTTATTATGTATGGAAAAGTTATGTGCAT   7450
gb:MT007544|Organism:Severe      ATGGTTAGAATGTACATCTTCTTTGCATCATTTTATTATGTATGGAAAAGTTATGTGCAT   7462
gb:MN994467|Organism:Severe      ATGGTTAGAATGTACATCTTCTTTGCATCATTTTATTATGTATGGAAAAGTTATGTGCAT   7462
gb:MT044257|Organism:Severe      ATGGTTAGAATGTACATCTTCTTTGCATCATTTTATTATGTATGGAAAAGTTATGTGCAT   7462
gb:MT106054|Organism:Severe      ATGGTTAGAATGTACATCTTCTTTGCATCATTTTATTATGTATGGAAAAGTTATGTGCAT   7462
gb:MT049951|Organism:Severe      ATGGTTAGAATGTACATCTTCTTTGCATCATTTTATTATGTATGGAAAAGTTATGTGCAT   7462
gb:MN975262|Organism:Severe      ATGGTTAGAATGTACATCTTCTTTGCATCATTTTATTATGTATGGAAAAGTTATGTGCAT   7462
gb:MT106052|Organism:Severe      ATGGTTAGAATGTACATCTTCTTTGCATCATTTTATTATGTATGGAAAAGTTATGTGCAT   7462
gb:LC522975|Organism:Severe      ATGGTTAGAATGTACATCTTCTTTGCATCATTTTATTATGTATGGAAAAGTTATGTGCAT   7459
gb:LC522973|Organism:Severe      ATGGTTAGAATGTACATCTTCTTTGCATCATTTTATTATGTATGGAAAAGTTATGTGCAT   7459
gb:LC522974|Organism:Severe      ATGGTTAGAATGTACATCTTCTTTGCATCATTTTATTATGTATGGAAAAGTTATGTGCAT   7459
gb:MN985325|Organism:Severe      ATGGTTAGAATGTACATCTTCTTTGCATCATTTTATTATGTATGGAAAAGTTATGTGCAT   7462
```

```
gb:MT020881|Organism:Severe      ATGGTTAGAATGTACATCTTCTTTGCATCATTTTATTATGTATGGAAAAGTTATGTGCAT    7462
gb:MT020880|Organism:Severe      ATGGTTAGAATGTACATCTTCTTTGCATCATTTTATTATGTATGGAAAAGTTATGTGCAT    7462
gb:MT066175|Organism:Severe      ATGGTTAGAATGTACATCTTCTTTGCATCATTTTATTATGTATGGAAAAGTTATGTGCAT    7462
gb:MN997409|Organism:Severe      ATGGTTAGAATGTACATCTTCTTTGCATCATTTTATTATGTATGGAAAAGTTATGTGCAT    7462
gb:MN938384|Organism:Severe      ATGGTTAGAATGTACATCTTCTTTGCATCATTTTATTATGTATGGAAAAGTTATGTGCAT    7430
gb:MT044258|Organism:Severe      ATGGTTAGAATGTACATCTTCTTTGCATCATTTTATTATGTATGGAAAAGTTATGTGCAT    7438
gb:MT039890|Organism:Severe      ATGGTTAGAATGTACATCTTCTTTGCATCATTTTATTATGTATGGAAAAGTTATGTGCAT    7462
gb:MN988713|Organism:Severe      ATGGTTAGAATGTACATCTTCTTTGCATCATTTTATTATGTATGGAAAAGTTATGTGCAT    7462
gb:LC521925|Organism:Severe      ATGGTTAGAATGTACATCTTCTTTGCATCATTTTATTATGTATGGAAAAGTTATGTGCAT    7435
gb:MT093571|Organism:Severe      ATGGTTAGAATGTACATCTTCTTTGCATCATTTTATTATGTATGGAAAAGTTATGTGCAT    7462
gb:MT039887|Organism:Severe      ATGGTTAGAATGTACATCTTCTTTGCATCATTTTATTATGTATGGAAAAGTTATGTGCAT    7462
gb:MT019530|Organism:Severe      ATGGTTAGAATGTACATCTTCTTTGCATCATTTTATTATGTATGGAAAAGTTATGTGCAT    7462
gb:MT039888|Organism:Severe      ATGGTTAGAATGTACATCTTCTTTGCATCATTTTATTATGTATGGAAAAGTTATGTGCAT    7462
gb:LC522972|Organism:Severe      ATGGTTAGAATGTACATCTTCTTTGCATCATTTTATTATGTATGGAAAAGTTATGTGCAT    7459
gb:MT027063|Organism:Severe      ATGGTTAGAATGTACATCTTCTTTGCATCATTTTATTATGTATGGAAAAGTTATGTGCAT    7462
gb:MT027062|Organism:Severe      ATGGTTAGAATGTACATCTTCTTTGCATCATTTTATTATGTATGGAAAAGTTATGTGCAT    7462
gb:MT019529|Organism:Severe      ATGGTTAGAATGTACATCTTCTTTGCATCATTTTATTATGTATGGAAAAGTTATGTGCAT    7462
gb:MN996529|Organism:Severe      ATGGTTAGAATGTACATCTTCTTTGCATCATTTTATTATGTATGGAAAAGTTATGTGCAT    7450
gb:MN996531|Organism:Severe      ATGGTTAGAATGTACATCTTCTTTGCATCATTTTATTATGTATGGAAAAGTTATGTGCAT    7449
gb:MT066176|Organism:Severe      ATGGTTAGAATGTACATCTTCTTTGCATCATTTTATTATGTATGGAAAAGTTATGTGCAT    7462
gb:MT027064|Organism:Severe      ATGGTTAGAATGTACATCTTCTTTGCATCATTTTATTATGTATGGAAAAGTTATGTGCAT    7462
gb:MN994468|Organism:Severe      ATGGTTAGAATGTACATCTTCTTTGCATCATTTTATTATGTATGGAAAAGTTATGTGCAT    7462
gb:MT072688|Organism:Severe      ATGGTTAGAATGTACATCTTCTTTGCATCATTTTATTATGTATGGAAAAGTTATGTGCAT    7447
gb:MN996527|Organism:Severe      ATGGTTAGAATGTACATCTTCTTTGCATCATTTTATTATGTATGGAAAAGTTATGTGCAT    7429
gb:MT093631|Organism:Severe      ATGGTTAGAATGTACATCTTCTTTGCATCATTTTATTATGTATGGAAAAGTTATGTGCAT    7500
gb:MT106053|Organism:Severe      ATGGTTAGAATGTACATCTTCTTTGCATCATTTTATTATGTATGGAAAAGTTATGTGCAT    7462
gb:MT019533|Organism:Severe      ATGGTTAGAATGTACATCTTCTTTGCATCATTTTATTATGTATGGAAAAGTTATGTGCAT    7462
gb:MT019531|Organism:Severe      ATGGTTAGAATGTACATCTTCTTTGCATCATTTTATTATGTATGGAAAAGTTATGTGCAT    7462
gb:MN996528|Organism:Severe      ATGGTTAGAATGTACATCTTCTTTGCATCATTTTATTATGTATGGAAAAGTTATGTGCAT    7462
gb:MN996530|Organism:Severe      ATGGTTAGAATGTACATCTTCTTTGCATCATTTTATTATGTATGGAAAAGTTATGTGCAT    7448
gb:MN908947|Organism:Severe      ATGGTTAGAATGTACATCTTCTTTGCATCATTTTATTATGTATGGAAAAGTTATGTGCAT    7462
gb:MT019532|Organism:Severe      ATGGTTAGAATGTACATCTTCTTTGCATCATTTTATTATGTATGGAAAAGTTATGTGCAT    7462
                                 ************************************************************

gb:MT020781|Organism:Severe      GTTGTAGACGGTTGTAATTCATCAACTTGTATGATGTGTTACAAACGTAATAGAGCAACA    7510
gb:MT007544|Organism:Severe      GTTGTAGACGGTTGTAATTCATCAACTTGTATGATGTGTTACAAACGTAATAGAGCAACA    7522
gb:MN994467|Organism:Severe      GTTGTAGACGGTTGTAATTCATCAACTTGTATGATGTGTTACAAACGTAATAGAGCAACA    7522
gb:MT044257|Organism:Severe      GTTGTAGACGGTTGTAATTCATCAACTTGTATGATGTGTTACAAACGTAATAGAGCAACA    7522
gb:MT106054|Organism:Severe      GTTGTAGACGGTTGTAATTCATCAACTTGTATGATGTGTTACAAACGTAATAGAGCAACA    7522
gb:MT049951|Organism:Severe      GTTGTAGACGGTTGTAATTCATCAACTTGTATGATGTGTTACAAACGTAATAGAGCAACA    7522
gb:MN975262|Organism:Severe      GTTGTAGACGGTTGTAATTCATCAACTTGTATGATGTGTTACAAACGTAATAGAGCAACA    7522
gb:MT106052|Organism:Severe      GTTGTAGACGGTTGTAATTCATCAACTTGTATGATGTGTTACAAACGTAATAGAGCAACA    7522
gb:LC522975|Organism:Severe      GTTGTAGACGGTTGTAATTCATCAACTTGTATGATGTGTTACAAACGTAATAGAGCAACA    7519
gb:LC522973|Organism:Severe      GTTGTAGACGGTTGTAATTCATCAACTTGTATGATGTGTTACAAACGTAATAGAGCAACA    7519
gb:LC522974|Organism:Severe      GTTGTAGACGGTTGTAATTCATCAACTTGTATGATGTGTTACAAACGTAATAGAGCAACA    7519
gb:MN985325|Organism:Severe      GTTGTAGACGGTTGTAATTCATCAACTTGTATGATGTGTTACAAACGTAATAGAGCAACA    7522
gb:MT020881|Organism:Severe      GTTGTAGACGGTTGTAATTCATCAACTTGTATGATGTGTTACAAACGTAATAGAGCAACA    7522
gb:MT020880|Organism:Severe      GTTGTAGACGGTTGTAATTCATCAACTTGTATGATGTGTTACAAACGTAATAGAGCAACA    7522
gb:MT066175|Organism:Severe      GTTGTAGACGGTTGTAATTCATCAACTTGTATGATGTGTTACAAACGTAATAGAGCAACA    7522
gb:MN997409|Organism:Severe      GTTGTAGACGGTTGTAATTCATCAACTTGTATGATGTGTTACAAACGTAATAGAGCAACA    7522
gb:MN938384|Organism:Severe      GTTGTAGACGGTTGTAATTCATCAACTTGTATGATGTGTTACAAACGTAATAGAGCAACA    7490
gb:MT044258|Organism:Severe      GTTGTAGACGGTTGTAATTCATCAACTTGTATGATGTGTTACAAACGTAATAGAGCAACA    7498
gb:MT039890|Organism:Severe      GTTGTAGACGGTTGTAATTCATCAACTTGTATGATGTGTTACAAACGTAATAGAGCAACA    7522
gb:MN988713|Organism:Severe      GTTGTAGACGGTTGTAATTCATCAACTTGTATGATGTGTTACAAACGTAATAGAGCAACA    7522
gb:LC521925|Organism:Severe      GTTGTAGACGGTTGTAATTCATCAACTTGTATGATGTGTTACAAACGTAATAGAGCAACA    7495
gb:MT093571|Organism:Severe      GTTGTAGACGGTTGTAATTCATCAACTTGTATGATGTGTTACAAACGTAATAGAGCAACA    7522
gb:MT039887|Organism:Severe      GTTGTAGACGGTTGTAATTCATCAACTTGTATGATGTGTTACAAACGTAATAGAGCAACA    7522
gb:MT019530|Organism:Severe      GTTGTAGACGGTTGTAATTCATCAACTTGTATGATGTGTTACAAACGTAATAGAGCAACA    7522
gb:MT039888|Organism:Severe      GTTGTAGACGGTTGTAATTCATCAACTTGTATGATGTGTTACAAACGTAATAGAGCAACA    7522
gb:LC522972|Organism:Severe      GTTGTAGACGGTTGTAATTCATCAACTTGTATGATGTGTTACAAACGTAATAGAGCAACA    7519
gb:MT027063|Organism:Severe      GTTGTAGACGGTTGTAATTCATCAACTTGTATGATGTGTTACAAACGTAATAGAGCAACA    7522
gb:MT027062|Organism:Severe      GTTGTAGACGGTTGTAATTCATCAACTTGTATGATGTGTTACAAACGTAATAGAGCAACA    7522
gb:MT019529|Organism:Severe      GTTGTAGACGGTTGTAATTCATCAACTTGTATGATGTGTTACAAACGTAATAGAGCAACA    7522
gb:MN996529|Organism:Severe      GTTGTAGACGGTTGTAATTCATCAACTTGTATGATGTGTTACAAACGTAATAGAGCAACA    7510
gb:MN996531|Organism:Severe      GTTGTAGACGGTTGTAATTCATCAACTTGTATGATGTGTTACAAACGTAATAGAGCAACA    7509
```

```
gb:MT066176|Organism:Severe      GTTGTAGACGGTTGTAATTCATCAACTTGTATGATGTGTTACAAACGTAATAGAGCAACA   7522
gb:MT027064|Organism:Severe      GTTGTAGACGGTTGTAATTCATCAACTTGTATGATGTGTTACAAACGTAATAGAGCAACA   7522
gb:MN994468|Organism:Severe      GTTGTAGACGGTTGTAATTCATCAACTTGTATGATGTGTTACAAACGTAATAGAGCAACA   7522
gb:MT072688|Organism:Severe      GTTGTAGACGGTTGTAATTCATCAACTTGTATGATGTGTTACAAACGTAATAGAGCAACA   7507
gb:MN996527|Organism:Severe      GTTGTAGACGGTTGTAATTCATCAACTTGTATGATGTGTTACAAACGTAATAGAGCAACA   7489
gb:MT093631|Organism:Severe      GTTGTAGACGGTTGTAATTCATCAACTTGTATGATGTGTTACAAACGTAATAGAGCAACA   7560
gb:MT106053|Organism:Severe      GTTGTAGACGGTTGTAATTCATCAACTTGTATGATGTGTTACAAACGTAATAGAGCAACA   7522
gb:MT019533|Organism:Severe      GTTGTAGACGGTTGTAATTCATCAACTTGTATGATGTGTTACAAACGTAATAGAGCAACA   7522
gb:MT019531|Organism:Severe      GTTGTAGACGGTTGTAATTCATCAACTTGTATGATGTGTTACAAACGTAATAGAGCAACA   7522
gb:MN996528|Organism:Severe      GTTGTAGACGGTTGTAATTCATCAACTTGTATGATGTGTTACAAACGTAATAGAGCAACA   7522
gb:MN996530|Organism:Severe      GTTGTAGACGGTTGTAATTCATCAACTTGTATGATGTGTTACAAACGTAATAGAGCAACA   7508
gb:MN908947|Organism:Severe      GTTGTAGACGGTTGTAATTCATCAACTTGTATGATGTGTTACAAACGTAATAGAGCAACA   7522
gb:MT019532|Organism:Severe      GTTGTAGACGGTTGTAATTCATCAACTTGTATGATGTGTTACAAACGTAATAGAGCAACA   7522
                                 ************************************************************

gb:MT020781|Organism:Severe      AGAGTCGAATGTACAACTATTGTTAATGGTGTTAGAAGGTCCTTTTATGTCTATGCTAAT   7570
gb:MT007544|Organism:Severe      AGAGTCGAATGTACAACTATTGTTAATGGTGTTAGAAGGTCCTTTTATGTCTATGCTAAT   7582
gb:MN994467|Organism:Severe      AGAGTCGAATGTACAACTATTGTTAATGGTGTTAGAAGGTCCTTTTATGTCTATGCTAAT   7582
gb:MT044257|Organism:Severe      AGAGTCGAATGTACAACTATTGTTAATGGTGTTAGAAGGTCCTTTTATGTCTATGCTAAT   7582
gb:MT106054|Organism:Severe      AGAGTCGAATGTACAACTATTGTTAATGGTGTTAGAAGGTCCTTTTATGTCTATGCTAAT   7582
gb:MT049951|Organism:Severe      AGAGTCGAATGTACAACTATTGTTAATGGTGTTAGAAGGTCCTTTTATGTCTATGCTAAT   7582
gb:MN975262|Organism:Severe      AGAGTCGAATGTACAACTATTGTTAATGGTGTTAGAAGGTCCTTTTATGTCTATGCTAAT   7582
gb:MT106052|Organism:Severe      AGAGTCGAATGTACAACTATTGTTAATGGTGTTAGAAGGTCCTTTTATGTCTATGCTAAT   7582
gb:LC522975|Organism:Severe      AGAGTCGAATGTACAACTATTGTTAATGGTGTTAGAAGGTCCTTTTATGTCTATGCTAAT   7579
gb:LC522973|Organism:Severe      AGAGTCGAATGTACAACTATTGTTAATGGTGTTAGAAGGTCCTTTTATGTCTATGCTAAT   7579
gb:LC522974|Organism:Severe      AGAGTCGAATGTACAACTATTGTTAATGGTGTTAGAAGGTCCTTTTATGTCTATGCTAAT   7579
gb:MN985325|Organism:Severe      AGAGTCGAATGTACAACTATTGTTAATGGTGTTAGAAGGTCCTTTTATGTCTATGCTAAT   7582
gb:MT020881|Organism:Severe      AGAGTCGAATGTACAACTATTGTTAATGGTGTTAGAAGGTCCTTTTATGTCTATGCTAAT   7582
gb:MT020880|Organism:Severe      AGAGTCGAATGTACAACTATTGTTAATGGTGTTAGAAGGTCCTTTTATGTCTATGCTAAT   7582
gb:MT066175|Organism:Severe      AGAGTCGAATGTACAACTATTGTTAATGGTGTTAGAAGGTCCTTTTATGTCTATGCTAAT   7582
gb:MN997409|Organism:Severe      AGAGTCGAATGTACAACTATTGTTAATGGTGTTAGAAGGTCCTTTTATGTCTATGCTAAT   7582
gb:MN938384|Organism:Severe      AGAGTCGAATGTACAACTATTGTTAATGGTGTTAGAAGGTCCTTTTATGTCTATGCTAAT   7550
gb:MT044258|Organism:Severe      AGAGTCGAATGTACAACTATTGTTAATGGTGTTAGAAGGTCCTTTTATGTCTATGCTAAT   7558
gb:MT039890|Organism:Severe      AGAGTCGAATGTACAACTATTGTTAATGGTGTTAGAAGGTCCTTTTATGTCTATGCTAAT   7582
gb:MN988713|Organism:Severe      AGAGTCGAATGTACAACTATTGTTAATGGTGTTAGAAGGTCCTTTTATGTCTATGCTAAT   7582
gb:LC521925|Organism:Severe      AGAGTCGAATGTACAACTATTGTTAATGGTGTTAGAAGGTCCTTTTATGTCTATGCTAAT   7555
gb:MT093571|Organism:Severe      AGAGTCGAATGTACAACTATTGTTAATGGTGTTAGAAGGTCCTTTTATGTCTATGCTAAT   7582
gb:MT039887|Organism:Severe      AGAGTCGAATGTACAACTATTGTTAATGGTGTTAGAAGGTCCTTTTATGTCTATGCTAAT   7582
gb:MT019530|Organism:Severe      AGAGTCGAATGTACAACTATTGTTAATGGTGTTAGAAGGTCCTTTTATGTCTATGCTAAT   7582
gb:MT039888|Organism:Severe      AGAGTCGAATGTACAACTATTGTTAATGGTGTTAGAAGGTCCTTTTATGTCTATGCTAAT   7582
gb:LC522972|Organism:Severe      AGAGTCGAATGTACAACTATTGTTAATGGTGTTAGAAGGTCCTTTTATGTCTATGCTAAT   7579
gb:MT027063|Organism:Severe      AGAGTCGAATGTACAACTATTGTTAATGGTGTTAGAAGGTCCTTTTATGTCTATGCTAAT   7582
gb:MT027062|Organism:Severe      AGAGTCGAATGTACAACTATTGTTAATGGTGTTAGAAGGTCCTTTTATGTCTATGCTAAT   7582
gb:MT019529|Organism:Severe      AGAGTCGAATGTACAACTATTGTTAATGGTGTTAGAAGGTCCTTTTATGTCTATGCTAAT   7582
gb:MN996529|Organism:Severe      AGAGTCGAATGTACAACTATTGTTAATGGTGTTAGAAGGTCCTTTTATGTCTATGCTAAT   7570
gb:MN996531|Organism:Severe      AGAGTCGAATGTACAACTATTGTTAATGGTGTTAGAAGGTCCTTTTATGTCTATGCTAAT   7569
gb:MT066176|Organism:Severe      AGAGTCGAATGTACAACTATTGTTAATGGTGTTAGAAGGTCCTTTTATGTCTATGCTAAT   7582
gb:MT027064|Organism:Severe      AGAGTCGAATGTACAACTATTGTTAATGGTGTTAGAAGGTCCTTTTATGTCTATGCTAAT   7582
gb:MN994468|Organism:Severe      AGAGTCGAATGTACAACTATTGTTAATGGTGTTAGAAGGTCCTTTTATGTCTATGCTAAT   7582
gb:MT072688|Organism:Severe      AGAGTCGAATGTACAACTATTGTTAATGGTGTTAGAAGGTCCTTTTATGTCTATGCTAAT   7567
gb:MN996527|Organism:Severe      AGAGTCGAATGTACAACTATTGTTAATGGTGTTAGAAGGTCCTTTTATGTCTATGCTAAT   7549
gb:MT093631|Organism:Severe      AGAGTCGAATGTACAACTATTGTTAATGGTGTTAGAAGGTCCTTTTATGTCTATGCTAAT   7620
gb:MT106053|Organism:Severe      AGAGTCGAATGTACAACTATTGTTAATGGTGTTAGAAGGTCCTTTTATGTCTATGCTAAT   7582
gb:MT019533|Organism:Severe      AGAGTCGAATGTACAACTATTGTTAATGGTGTTAGAAGGTCCTTTTATGTCTATGCTAAT   7582
gb:MT019531|Organism:Severe      AGAGTCGAATGTACAACTATTGTTAATGGTGTTAGAAGGTCCTTTTATGTCTATGCTAAT   7582
gb:MN996528|Organism:Severe      AGAGTCGAATGTACAACTATTGTTAATGGTGTTAGAAGGTCCTTTTATGTCTATGCTAAT   7582
gb:MN996530|Organism:Severe      AGAGTCGAATGTACAACTATTGTTAATGGTGTTAGAAGGTCCTTTTATGTCTATGCTAAT   7568
gb:MN908947|Organism:Severe      AGAGTCGAATGTACAACTATTGTTAATGGTGTTAGAAGGTCCTTTTATGTCTATGCTAAT   7582
gb:MT019532|Organism:Severe      AGAGTCGAATGTACAACTATTGTTAATGGTGTTAGAAGGTCCTTTTATGTCTATGCTAAT   7582
                                 ************************************************************

gb:MT020781|Organism:Severe      GGAGGTAAAGGCTTTTGCAAACTACACAATTGGAATTGTGTTAATTGTGATACATTCTGT   7630
gb:MT007544|Organism:Severe      GGAGGTAAAGGCTTTTGCAAACTACACAATTGGAATTGTGTTAATTGTGATACATTCTGT   7642
gb:MN994467|Organism:Severe      GGAGGTAAAGGCTTTTGCAAACTACACAATTGGAATTGTGTTAATTGTGATACATTCTGT   7642
gb:MT044257|Organism:Severe      GGAGGTAAAGGCTTTTGCAAACTACACAATTGGAATTGTGTTAATTGTGATACATTCTGT   7642
```

```
gb:MT106054|Organism:Severe      GGAGGTAAAGGCTTTTGCAAACTACACAATTGGAATTGTGTTAATTGTGATACATTCTGT    7642
gb:MT049951|Organism:Severe      GGAGGTAAAGGCTTTTGCAAACTACACAATTGGAATTGTGTTAATTGTGATACATTCTGT    7642
gb:MN975262|Organism:Severe      GGAGGTAAAGGCTTTTGCAAACTACACAATTGGAATTGTGTTAATTGTGATACATTCTGT    7642
gb:MT106052|Organism:Severe      GGAGGTAAAGGCTTTTGCAAACTACACAATTGGAATTGTGTTAATTGTGATACATTCTGT    7642
gb:LC522975|Organism:Severe      GGAGGTAAAGGCTTTTGCAAACTACACAATTGGAATTGTGTTAATTGTGATACATTCTGT    7639
gb:LC522973|Organism:Severe      GGAGGTAAAGGCTTTTGCAAACTACACAATTGGAATTGTGTTAATTGTGATACATTCTGT    7639
gb:LC522974|Organism:Severe      GGAGGTAAAGGCTTTTGCAAACTACACAATTGGAATTGTGTTAATTGTGATACATTCTGT    7639
gb:MN985325|Organism:Severe      GGAGGTAAAGGCTTTTGCAAACTACACAATTGGAATTGTGTTAATTGTGATACATTCTGT    7642
gb:MT020881|Organism:Severe      GGAGGTAAAGGCTTTTGCAAACTACACAATTGGAATTGTGTTAATTGTGATACATTCTGT    7642
gb:MT020880|Organism:Severe      GGAGGTAAAGGCTTTTGCAAACTACACAATTGGAATTGTGTTAATTGTGATACATTCTGT    7642
gb:MT066175|Organism:Severe      GGAGGTAAAGGCTTTTGCAAACTACACAATTGGAATTGTGTTAATTGTGATACATTCTGT    7642
gb:MN997409|Organism:Severe      GGAGGTAAAGGCTTTTGCAAACTACACAATTGGAATTGTGTTAATTGTGATACATTCTGT    7642
gb:MN938384|Organism:Severe      GGAGGTAAAGGCTTTTGCAAACTACACAATTGGAATTGTGTTAATTGTGATACATTCTGT    7610
gb:MT044258|Organism:Severe      GGAGGTAAAGGCTTTTGCAAACTACACAATTGGAATTGTGTTAATTGTGATACATTCTGT    7618
gb:MT039890|Organism:Severe      GGAGGTAAAGGCTTTTGCAAACTACACAATTGGAATTGTGTTAATTGTGATACATTCTGT    7642
gb:MN988713|Organism:Severe      GGAGGTAAAGGCTTTTGCAAACTACACAATTGGAATTGTGTTAATTGTGATACATTCTGT    7642
gb:LC521925|Organism:Severe      GGAGGTAAAGGCTTTTGCAAACTACACAATTGGAATTGTGTTAATTGTGATACATTCTGT    7615
gb:MT093571|Organism:Severe      GGAGGTAAAGGCTTTTGCAAACTACACAATTGGAATTGTGTTAATTGTGATACATTCTGT    7642
gb:MT039887|Organism:Severe      GGAGGTAAAGGCTTTTGCAAACTACACAATTGGAATTGTGTTAATTGTGATACATTCTGT    7642
gb:MT019530|Organism:Severe      GGAGGTAAAGGCTTTTGCAAACTACACAATTGGAATTGTGTTAATTGTGATACATTCTGT    7642
gb:MT039888|Organism:Severe      GGAGGTAAAGGCTTTTGCAAACTACACAATTGGAATTGTGTTAATTGTGATACATTCTGT    7642
gb:LC522972|Organism:Severe      GGAGGTAAAGGCTTTTGCAAACTACACAATTGGAATTGTGTTAATTGTGATACATTCTGT    7639
gb:MT027063|Organism:Severe      GGAGGTAAAGGCTTTTGCAAACTACACAATTGGAATTGTGTTAATTGTGATACATTCTGT    7642
gb:MT027062|Organism:Severe      GGAGGTAAAGGCTTTTGCAAACTACACAATTGGAATTGTGTTAATTGTGATACATTCTGT    7642
gb:MT019529|Organism:Severe      GGAGGTAAAGGCTTTTGCAAACTACACAATTGGAATTGTGTTAATTGTGATACATTCTGT    7642
gb:MN996529|Organism:Severe      GGAGGTAAAGGCTTTTGCAAACTACACAATTGGAATTGTGTTAATTGTGATACATTCTGT    7630
gb:MN996531|Organism:Severe      GGAGGTAAAGGCTTTTGCAAACTACACAATTGGAATTGTGTTAATTGTGATACATTCTGT    7629
gb:MT066176|Organism:Severe      GGAGGTAAAGGCTTTTGCAAACTACACAATTGGAATTGTGTTAATTGTGATACATTCTGT    7642
gb:MT027064|Organism:Severe      GGAGGTAAAGGCTTTTGCAAACTACACAATTGGAATTGTGTTAATTGTGATACATTCTGT    7642
gb:MN994468|Organism:Severe      GGAGGTAAAGGCTTTTGCAAACTACACAATTGGAATTGTGTTAATTGTGATACATTCTGT    7642
gb:MT072688|Organism:Severe      GGAGGTAAAGGCTTTTGCAAACTACACAATTGGAATTGTGTTAATTGTGATACATTCTGT    7627
gb:MN996527|Organism:Severe      GGAGGTAAAGGCTTTTGCAAACTACACAATTGGAATTGTGTTAATTGTGATACATTCTGT    7609
gb:MT093631|Organism:Severe      GGAGGTAAAGGCTTTTGCAAACTACACAATTGGAATTGTGTTAATTGTGATACATTCTGT    7680
gb:MT106053|Organism:Severe      GGAGGTAAAGGCTTTTGCAAACTACACAATTGGAATTGTGTTAATTGTGATACATTCTGT    7642
gb:MT019533|Organism:Severe      GGAGGTAAAGGCTTTTGCAAACTACACAATTGGAATTGTGTTAATTGTGATACATTCTGT    7642
gb:MT019531|Organism:Severe      GGAGGTAAAGGCTTTTGCAAACTACACAATTGGAATTGTGTTAATTGTGATACATTCTGT    7642
gb:MN996528|Organism:Severe      GGAGGTAAAGGCTTTTGCAAACTACACAATTGGAATTGTGTTAATTGTGATACATTCTGT    7642
gb:MN996530|Organism:Severe      GGAGGTAAAGGCTTTTGCAAACTACACAATTGGAATTGTGTTAATTGTGATACATTCTGT    7628
gb:MN908947|Organism:Severe      GGAGGTAAAGGCTTTTGCAAACTACACAATTGGAATTGTGTTAATTGTGATACATTCTGT    7642
gb:MT019532|Organism:Severe      GGAGGTAAAGGCTTTTGCAAACTACACAATTGGAATTGTGTTAATTGTGATACATTCTGT    7642
                                 ************************************************************

gb:MT020781|Organism:Severe      GCTGGTAGTACATTTATTAGTGATGAAGTTGCGAGAGACTTGTCACTACAGTTTAAAAGA    7690
gb:MT007544|Organism:Severe      GCTGGTAGTACATTTATTAGTGATGAAGTTGCGAGAGACTTGTCACTACAGTTTAAAAGA    7702
gb:MN994467|Organism:Severe      GCTGGTAGTACATTTATTAGTGATGAAGTTGCGAGAGACTTGTCACTACAGTTTAAAAGA    7702
gb:MT044257|Organism:Severe      GCTGGTAGTACATTTATTAGTGATGAAGTTGCGAGAGACTTGTCACTACAGTTTAAAAGA    7702
gb:MT106054|Organism:Severe      GCTGGTAGTACATTTATTAGTGATGAAGTTGCGAGAGACTTGTCACTACAGTTTAAAAGA    7702
gb:MT049951|Organism:Severe      GCTGGTAGTACATTTATTAGTGATGAAGTTGCGAGAGACTTGTCACTACAGTTTAAAAGA    7702
gb:MN975262|Organism:Severe      GCTGGTAGTACATTTATTAGTGATGAAGTTGCGAGAGACTTGTCACTACAGTTTAAAAGA    7702
gb:MT106052|Organism:Severe      GCTGGTAGTACATTTATTAGTGATGAAGTTGCGAGAGACTTGTCACTACAGTTTAAAAGA    7702
gb:LC522975|Organism:Severe      GCTGGTAGTACATTTATTAGTGATGAAGTTGCGAGAGACTTGTCACTACAGTTTAAAAGA    7699
gb:LC522973|Organism:Severe      GCTGGTAGTACATTTATTAGTGATGAAGTTGCGAGAGACTTGTCACTACAGTTTAAAAGA    7699
gb:LC522974|Organism:Severe      GCTGGTAGTACATTTATTAGTGATGAAGTTGCGAGAGACTTGTCACTACAGTTTAAAAGA    7699
gb:MN985325|Organism:Severe      GCTGGTAGTACATTTATTAGTGATGAAGTTGCGAGAGACTTGTCACTACAGTTTAAAAGA    7702
gb:MT020881|Organism:Severe      GCTGGTAGTACATTTATTAGTGATGAAGTTGCGAGAGACTTGTCACTACAGTTTAAAAGA    7702
gb:MT020880|Organism:Severe      GCTGGTAGTACATTTATTAGTGATGAAGTTGCGAGAGACTTGTCACTACAGTTTAAAAGA    7702
gb:MT066175|Organism:Severe      GCTGGTAGTACATTTATTAGTGATGAAGTTGCGAGAGACTTGTCACTACAGTTTAAAAGA    7702
gb:MN997409|Organism:Severe      GCTGGTAGTACATTTATTAGTGATGAAGTTGCGAGAGACTTGTCACTACAGTTTAAAAGA    7702
gb:MN938384|Organism:Severe      GCTGGTAGTACATTTATTAGTGATGAAGTTGCGAGAGACTTGTCACTACAGTTTAAAAGA    7670
gb:MT044258|Organism:Severe      GCTGGTAGTACATTTATTAGTGATGAAGTTGCGAGAGACTTGTCACTACAGTTTAAAAGA    7678
gb:MT039890|Organism:Severe      GCTGGTAGTACATTTATTAGTGATGAAGTTGCGAGAGACTTGTCACTACAGTTTAAAAGA    7702
gb:MN988713|Organism:Severe      GCTGGTAGTACATTTATTAGTGATGAAGTTGCGAGAGACTTGTCACTACAGTTTAAAAGA    7702
gb:LC521925|Organism:Severe      GCTGGTAGTACATTTATTAGTGATGAAGTTGCGAGAGACTTGTCACTACAGTTTAAAAGA    7675
gb:MT093571|Organism:Severe      GCTGGTAGTACATTTATTAGTGATGAAGTTGCGAGAGACTTGTCACTACAGTTTAAAAGA    7702
gb:MT039887|Organism:Severe      GCTGGTAGTACATTTATTAGTGATGAAGTTGCGAGAGACTTGTCACTACAGTTTAAAAGA    7702
```

```
gb:MT019530|Organism:Severe      GCTGGTAGTACATTTATTAGTGATGAAGTTGCGAGAGACTTGTCACTACAGTTTAAAAGA   7702
gb:MT039888|Organism:Severe      GCTGGTAGTACATTTATTAGTGATGAAGTTGCGAGAGACTTGTCACTACAGTTTAAAAGA   7702
gb:LC522972|Organism:Severe      GCTGGTAGTACATTTATTAGTGATGAAGTTGCGAGAGACTTGTCACTACAGTTTAAAAGA   7699
gb:MT027063|Organism:Severe      GCTGGTAGTACATTTATTAGTGATGAAGTTGCGAGAGACTTGTCACTACAGTTTAAAAGA   7702
gb:MT027062|Organism:Severe      GCTGGTAGTACATTTATTAGTGATGAAGTTGCGAGAGACTTGTCACTACAGTTTAAAAGA   7702
gb:MT019529|Organism:Severe      GCTGGTAGTACATTTATTAGTGATGAAGTTGCGAGAGACTTGTCACTACAGTTTAAAAGA   7702
gb:MN996529|Organism:Severe      GCTGGTAGTACATTTATTAGTGATGAAGTTGCGAGAGACTTGTCACTACAGTTTAAAAGA   7690
gb:MN996531|Organism:Severe      GCTGGTAGTACATTTATTAGTGATGAAGTTGCGAGAGACTTGTCACTACAGTTTAAAAGA   7689
gb:MT066176|Organism:Severe      GCTGGTAGTACATTTATTAGTGATGAAGTTGCGAGAGACTTGTCACTACAGTTTAAAAGA   7702
gb:MT027064|Organism:Severe      GCTGGTAGTACATTTATTAGTGATGAAGTTGCGAGAGACTTGTCACTACAGTTTAAAAGA   7702
gb:MN994468|Organism:Severe      GCTGGTAGTACATTTATTAGTGATGAAGTTGCGAGAGACTTGTCACTACAGTTTAAAAGA   7702
gb:MT072688|Organism:Severe      GCTGGTAGTACATTTATTAGTGATGAAGTTGCGAGAGACTTGTCACTACAGTTTAAAAGA   7687
gb:MN996527|Organism:Severe      GCTGGTAGTACATTTATTAGTGATGAAGTTGCGAGAGACTTGTCACTACAGTTTAAAAGA   7669
gb:MT093631|Organism:Severe      GCTGGTAGTACATTTATTAGTGATGAAGTTGCGAGAGACTTGTCACTACAGTTTAAAAGA   7740
gb:MT106053|Organism:Severe      GCTGGTAGTACATTTATTAGTGATGAAGTTGCGAGAGACTTGTCACTACAGTTTAAAAGA   7702
gb:MT019533|Organism:Severe      GCTGGTAGTACATTTATTAGTGATGAAGTTGCGAGAGACTTGTCACTACAGTTTAAAAGA   7702
gb:MT019531|Organism:Severe      GCTGGTAGTACATTTATTAGTGATGAAGTTGCGAGAGACTTGTCACTACAGTTTAAAAGA   7702
gb:MN996528|Organism:Severe      GCTGGTAGTACATTTATTAGTGATGAAGTTGCGAGAGACTTGTCACTACAGTTTAAAAGA   7702
gb:MN996530|Organism:Severe      GCTGGTAGTACATTTATTAGTGATGAAGTTGCGAGAGACTTGTCACTACAGTTTAAAAGA   7688
gb:MN908947|Organism:Severe      GCTGGTAGTACATTTATTAGTGATGAAGTTGCGAGAGACTTGTCACTACAGTTTAAAAGA   7702
gb:MT019532|Organism:Severe      GCTGGTAGTACATTTATTAGTGATGAAGTTGCGAGAGACTTGTCACTACAGTTTAAAAGA   7702
                                 ************************************************************

gb:MT020781|Organism:Severe      CCAATAAATCCTACTGACCAGTCTTCTTACATCGTTGATAGTGTTACAGTGAAGAATGGT   7750
gb:MT007544|Organism:Severe      CCAATAAATCCTACTGACCAGTCTTCTTACATCGTTGATAGTGTTACAGTGAAGAATGGT   7762
gb:MN994467|Organism:Severe      CCAATAAATCCTACTGACCAGTCTTCTTACATCGTTGATAGTGTTACAGTGAAGAATGGT   7762
gb:MT044257|Organism:Severe      CCAATAAATCCTACTGACCAGTCTTCTTACATCGTTGATAGTGTTACAGTGAAGAATGGT   7762
gb:MT106054|Organism:Severe      CCAATAAATCCTACTGACCAGTCTTCTTACATCGTTGATAGTGTTACAGTGAAGAATGGT   7762
gb:MT049951|Organism:Severe      CCAATAAATCCTACTGACCAGTCTTCTTACATCGTTGATAGTGTTACAGTGAAGAATGGT   7762
gb:MN975262|Organism:Severe      CCAATAAATCCTACTGACCAGTCTTCTTACATCGTTGATAGTGTTACAGTGAAGAATGGT   7762
gb:MT106052|Organism:Severe      CCAATAAATCCTACTGACCAGTCTTCTTACATCGTTGATAGTGTTACAGTGAAGAATGGT   7762
gb:LC522975|Organism:Severe      CCAATAAATCCTACTGACCAGTCTTCTTACATCGTTGATAGTGTTACAGTGAAGAATGGT   7759
gb:LC522973|Organism:Severe      CCAATAAATCCTACTGACCAGTCTTCTTACATCGTTGATAGTGTTACAGTGAAGAATGGT   7759
gb:LC522974|Organism:Severe      CCAATAAATCCTACTGACCAGTCTTCTTACATCGTTGATAGTGTTACAGTGAAGAATGGT   7759
gb:MN985325|Organism:Severe      CCAATAAATCCTACTGACCAGTCTTCTTACATCGTTGATAGTGTTACAGTGAAGAATGGT   7762
gb:MT020881|Organism:Severe      CCAATAAATCCTACTGACCAGTCTTCTTACATCGTTGATAGTGTTACAGTGAAGAATGGT   7762
gb:MT020880|Organism:Severe      CCAATAAATCCTACTGACCAGTCTTCTTACATCGTTGATAGTGTTACAGTGAAGAATGGT   7762
gb:MT066175|Organism:Severe      CCAATAAATCCTACTGACCAGTCTTCTTACATCGTTGATAGTGTTACAGTGAAGAATGGT   7762
gb:MN997409|Organism:Severe      CCAATAAATCCTACTGACCAGTCTTCTTACATCGTTGATAGTGTTACAGTGAAGAATGGT   7762
gb:MN938384|Organism:Severe      CCAATAAATCCTACTGACCAGTCTTCTTACATCGTTGATAGTGTTACAGTGAAGAATGGT   7730
gb:MT044258|Organism:Severe      CCAATAAATCCTACTGACCAGTCTTCTTACATCGTTGATAGTGTTACAGTGAAGAATGGT   7738
gb:MT039890|Organism:Severe      CCAATAAATCCTACTGACCAGTCTTCTTACATCGTTGATAGTGTTACAGTGAAGAATGGT   7762
gb:MN988713|Organism:Severe      CCAATAAATCCTACTGACCAGTCTTCTTACATCGTTGATAGTGTTACAGTGAAGAATGGT   7762
gb:LC521925|Organism:Severe      CCAATAAATCCTACTGACCAGTCTTCTTACATCGTTGATAGTGTTACAGTGAAGAATGGT   7735
gb:MT093571|Organism:Severe      CCAATAAATCCTACTGACCAGTCTTCTTACATCGTTGATAGTGTTACAGTGAAGAATGGT   7762
gb:MT039887|Organism:Severe      CCAATAAATCCTACTGACCAGTCTTCTTACATCGTTGATAGTGTTACAGTGAAGAATGGT   7762
gb:MT019530|Organism:Severe      CCAATAAATCCTACTGACCAGTCTTCTTACATCGTTGATAGTGTTACAGTGAAGAATGGT   7762
gb:MT039888|Organism:Severe      CCAATAAATCCTACTGACCAGTCTTCTTACATCGTTGATAGTGTTACAGTGAAGAATGGT   7762
gb:LC522972|Organism:Severe      CCAATAAATCCTACTGACCAGTCTTCTTACATCGTTGATAGTGTTACAGTGAAGAATGGT   7759
gb:MT027063|Organism:Severe      CCAATAAATCCTACTGACCAGTCTTCTTACATCGTTGATAGTGTTACAGTGAAGAATGGT   7762
gb:MT027062|Organism:Severe      CCAATAAATCCTACTGACCAGTCTTCTTACATCGTTGATAGTGTTACAGTGAAGAATGGT   7762
gb:MT019529|Organism:Severe      CCAATAAATCCTACTGACCAGTCTTCTTACATCGTTGATAGTGTTACAGTGAAGAATGGT   7762
gb:MN996529|Organism:Severe      CCAATAAATCCTACTGACCAGTCTTCTTACATCGTTGATAGTGTTACAGTGAAGAATGGT   7750
gb:MN996531|Organism:Severe      CCAATAAATCCTACTGACCAGTCTTCTTACATCGTTGATAGTGTTACAGTGAAGAATGGT   7749
gb:MT066176|Organism:Severe      CCAATAAATCCTACTGACCAGTCTTCTTACATCGTTGATAGTGTTACAGTGAAGAATGGT   7762
gb:MT027064|Organism:Severe      CCAATAAATCCTACTGACCAGTCTTCTTACATCGTTGATAGTGTTACAGTGAAGAATGGT   7762
gb:MN994468|Organism:Severe      CCAATAAATCCTACTGACCAGTCTTCTTACATCGTTGATAGTGTTACAGTGAAGAATGGT   7762
gb:MT072688|Organism:Severe      CCAATAAATCCTACTGACCAGTCTTCTTACATCGTTGATAGTGTTACAGTGAAGAATGGT   7747
gb:MN996527|Organism:Severe      CCAATAAATCCTACTGACCAGTCTTCTTACATCGTTGATAGTGTTACAGTGAAGAATGGT   7729
gb:MT093631|Organism:Severe      CCAATAAATCCTACTGACCAGTCTTCTTACATCGTTGATAGTGTTACAGTGAAGAATGGT   7800
gb:MT106053|Organism:Severe      CCAATAAATCCTACTGACCAGTCTTCTTACATCGTTGATAGTGTTACAGTGAAGAATGGT   7762
gb:MT019533|Organism:Severe      CCAATAAATCCTACTGACCAGTCTTCTTACATCGTTGATAGTGTTACAGTGAAGAATGGT   7762
gb:MT019531|Organism:Severe      CCAATAAATCCTACTGACCAGTCTTCTTACATCGTTGATAGTGTTACAGTGAAGAATGGT   7762
gb:MN996528|Organism:Severe      CCAATAAATCCTACTGACCAGTCTTCTTACATCGTTGATAGTGTTACAGTGAAGAATGGT   7762
gb:MN996530|Organism:Severe      CCAATAAATCCTACTGACCAGTCTTCTTACATCGTTGATAGTGTTACAGTGAAGAATGGT   7748
```

```
gb:MN908947|Organism:Severe      CCAATAAATCCTACTGACCAGTCTTCTTACATCGTTGATAGTGTTACAGTGAAGAATGGT    7762
gb:MT019532|Organism:Severe      CCAATAAATCCTACTGACCAGTCTTCTTACATCGTTGATAGTGTTACAGTGAAGAATGGT    7762
                                 ************************************************************

gb:MT020781|Organism:Severe      TCCATCCATCTTTACTTTGATAAAGCTGGTCAAAAGACTTATGAAAGACATTCTCTCTCT    7810
gb:MT007544|Organism:Severe      TCCATCCATCTTTACTTTGATAAAGCTGGTCAAAAGACTTATGAAAGACATTCTCTCTCT    7822
gb:MN994467|Organism:Severe      TCCATCCATCTTTACTTTGATAAAGCTGGTCAAAAGACTTATGAAAGACATTCTCTCTCT    7822
gb:MT044257|Organism:Severe      TCCATCCATCTTTACTTTGATAAAGCTGGTCAAAAGACTTATGAAAGACATTCTCTCTCT    7822
gb:MT106054|Organism:Severe      TCCATCCATCTTTACTTTGATAAAGCTGGTCAAAAGACTTATGAAAGACATTCTCTCTCT    7822
gb:MT049951|Organism:Severe      TCCATCCATCTTTACTTTGATAAAGCTGGTCAAAAGACTTATGAAAGACATTCTCTCTCT    7822
gb:MN975262|Organism:Severe      TCCATCCATCTTTACTTTGATAAAGCTGGTCAAAAGACTTATGAAAGACATTCTCTCTCT    7822
gb:MT106052|Organism:Severe      TCCATCCATCTTTACTTTGATAAAGCTGGTCAAAAGACTTATGAAAGACATTCTCTCTCT    7822
gb:LC522975|Organism:Severe      TCCATCCATCTTTACTTTGATAAAGCTGGTCAAAAGACTTATGAAAGACATTCTCTCTCT    7819
gb:LC522973|Organism:Severe      TCCATCCATCTTTACTTTGATAAAGCTGGTCAAAAGACTTATGAAAGACATTCTCTCTCT    7819
gb:LC522974|Organism:Severe      TCCATCCATCTTTACTTTGATAAAGCTGGTCAAAAGACTTATGAAAGACATTCTCTCTCT    7819
gb:MN985325|Organism:Severe      TCCATCCATCTTTACTTTGATAAAGCTGGTCAAAAGACTTATGAAAGACATTCTCTCTCT    7822
gb:MT020881|Organism:Severe      TCCATCCATCTTTACTTTGATAAAGCTGGTCAAAAGACTTATGAAAGACATTCTCTCTCT    7822
gb:MT020880|Organism:Severe      TCCATCCATCTTTACTTTGATAAAGCTGGTCAAAAGACTTATGAAAGACATTCTCTCTCT    7822
gb:MT066175|Organism:Severe      TCCATCCATCTTTACTTTGATAAAGCTGGTCAAAAGACTTATGAAAGACATTCTCTCTCT    7822
gb:MN997409|Organism:Severe      TCCATCCATCTTTACTTTGATAAAGCTGGTCAAAAGACTTATGAAAGACATTCTCTCTCT    7822
gb:MN938384|Organism:Severe      TCCATCCATCTTTACTTTGATAAAGCTGGTCAAAAGACTTATGAAAGACATTCTCTCTCT    7790
gb:MT044258|Organism:Severe      TCCATCCATCTTTACTTTGATAAAGCTGGTCAAAAGACTTATGAAAGACATTCTCTCTCT    7798
gb:MT039890|Organism:Severe      TCCATCCATCTTTACTTTGATAAAGCTGGTCAAAAGACTTATGAAAGACATTCTCTCTCT    7822
gb:MN988713|Organism:Severe      TCCATCCATCTTTACTTTGATAAAGCTGGTCAAAAGACTTATGAAAGACATTCTCTCTCT    7822
gb:LC521925|Organism:Severe      TCCATCCATCTTTACTTTGATAAAGCTGGTCAAAAGACTTATGAAAGACATTCTCTCTCT    7795
gb:MT093571|Organism:Severe      TCCATCCATCTTTACTTTGATAAAGCTGGTCAAAAGACTTATGAAAGACATTCTCTCTCT    7822
gb:MT039887|Organism:Severe      TCCATCCATCTTTACTTTGATAAAGCTGGTCAAAAGACTTATGAAAGACATTCTCTCTCT    7822
gb:MT019530|Organism:Severe      TCCATCCATCTTTACTTTGATAAAGCTGGTCAAAAGACTTATGAAAGACATTCTCTCTCT    7822
gb:MT039888|Organism:Severe      TCCATCCATCTTTACTTTGATAAAGCTGGTCAAAAGACTTATGAAAGACATTCTCTCTCT    7822
gb:LC522972|Organism:Severe      TCCATCCATCTTTACTTTGATAAAGCTGGTCAAAAGACTTATGAAAGACATTCTCTCTCT    7819
gb:MT027063|Organism:Severe      TCCATCCATCTTTACTTTGATAAAGCTGGTCAAAAGACTTATGAAAGACATTCTCTCTCT    7822
gb:MT027062|Organism:Severe      TCCATCCATCTTTACTTTGATAAAGCTGGTCAAAAGACTTATGAAAGACATTCTCTCTCT    7822
gb:MT019529|Organism:Severe      TCCATCCATCTTTACTTTGATAAAGCTGGTCAAAAGACTTATGAAAGACATTCTCTCTCT    7822
gb:MN996529|Organism:Severe      TCCATCCATCTTTACTTTGATAAAGCTGGTCAAAAGACTTATGAAAGACATTCTCTCTCT    7810
gb:MN996531|Organism:Severe      TCCATCCATCTTTACTTTGATAAAGCTGGTCAAAAGACTTATGAAAGACATTCTCTCTCT    7809
gb:MT066176|Organism:Severe      TCCATCCATCTTTACTTTGATAAAGCTGGTCAAAAGACTTATGAAAGACATTCTCTCTCT    7822
gb:MT027064|Organism:Severe      TCCATCCATCTTTACTTTGATAAAGCTGGTCAAAAGACTTATGAAAGACATTCTCTCTCT    7822
gb:MN994468|Organism:Severe      TCCATCCATCTTTACTTTGATAAAGCTGGTCAAAAGACTTATGAAAGACATTCTCTCTCT    7822
gb:MT072688|Organism:Severe      TCCATCCATCTTTACTTTGATAAAGCTGGTCAAAAGACTTATGAAAGACATTCTCTCTCT    7807
gb:MN996527|Organism:Severe      TCCATCCATCTTTACTTTGATAAAGCTGGTCAAAAGACTTATGAAAGACATTCTCTCTCT    7789
gb:MT093631|Organism:Severe      TCCATCCATCTTTACTTTGATAAAGCTGGTCAAAAGACTTATGAAAGACATTCTCTCTCT    7860
gb:MT106053|Organism:Severe      TCCATCCATCTTTACTTTGATAAAGCTGGTCAAAAGACTTATGAAAGACATTCTCTCTCT    7822
gb:MT019533|Organism:Severe      TCCATCCATCTTTACTTTGATAAAGCTGGTCAAAAGACTTATGAAAGACATTCTCTCTCT    7822
gb:MT019531|Organism:Severe      TCCATCCATCTTTACTTTGATAAAGCTGGTCAAAAGACTTATGAAAGACATTCTCTCTCT    7822
gb:MN996528|Organism:Severe      TCCATCCATCTTTACTTTGATAAAGCTGGTCAAAAGACTTATGAAAGACATTCTCTCTCT    7822
gb:MN996530|Organism:Severe      TCCATCCATCTTTACTTTGATAAAGCTGGTCAAAAGACTTATGAAAGACATTCTCTCTCT    7808
gb:MN908947|Organism:Severe      TCCATCCATCTTTACTTTGATAAAGCTGGTCAAAAGACTTATGAAAGACATTCTCTCTCT    7822
gb:MT019532|Organism:Severe      TCCATCCATCTTTACTTTGATAAAGCTGGTCAAAAGACTTATGAAAGACATTCTCTCTCT    7822
                                 ************************************************************

gb:MT020781|Organism:Severe      CATTTTGTTAACTTAGACAACCTGAGAGCTAATAACACTAAAGGTTCATTGCCTATTAAT    7870
gb:MT007544|Organism:Severe      CATTTTGTTAACTTAGACAACCTGAGAGCTAATAACACTAAAGGTTCATTGCCTATTAAT    7882
gb:MN994467|Organism:Severe      CATTTTGTTAACTTAGACAACCTGAGAGCTAATAACACTAAAGGTTCATTGCCTATTAAT    7882
gb:MT044257|Organism:Severe      CATTTTGTTAACTTAGACAACCTGAGAGCTAATAACACTAAAGGTTCATTGCCTATTAAT    7882
gb:MT106054|Organism:Severe      CATTTTGTTAACTTAGACAACCTGAGAGCTAATAACACTAAAGGTTCATTGCCTATTAAT    7882
gb:MT049951|Organism:Severe      CATTTTGTTAACTTAGACAACCTGAGAGCTAATAACACTAAAGGTTCATTGCCTATTAAT    7882
gb:MN975262|Organism:Severe      CATTTTGTTAACTTAGACAACCTGAGAGCTAATAACACTAAAGGTTCATTGCCTATTAAT    7882
gb:MT106052|Organism:Severe      CATTTTGTTAACTTAGACAACCTGAGAGCTAATAACACTAAAGGTTCATTGCCTATTAAT    7882
gb:LC522975|Organism:Severe      CATTTTGTTAACTTAGACAACCTGAGAGCTAATAACACTAAAGGTTCATTGCCTATTAAT    7879
gb:LC522973|Organism:Severe      CATTTTGTTAACTTAGACAACCTGAGAGCTAATAACACTAAAGGTTCATTGCCTATTAAT    7879
gb:LC522974|Organism:Severe      CATTTTGTTAACTTAGACAACCTGAGAGCTAATAACACTAAAGGTTCATTGCCTATTAAT    7879
gb:MN985325|Organism:Severe      CATTTTGTTAACTTAGACAACCTGAGAGCTAATAACACTAAAGGTTCATTGCCTATTAAT    7882
gb:MT020881|Organism:Severe      CATTTTGTTAACTTAGACAACCTGAGAGCTAATAACACTAAAGGTTCATTGCCTATTAAT    7882
gb:MT020880|Organism:Severe      CATTTTGTTAACTTAGACAACCTGAGAGCTAATAACACTAAAGGTTCATTGCCTATTAAT    7882
gb:MT066175|Organism:Severe      CATTTTGTTAACTTAGACAACCTGAGAGCTAATAACACTAAAGGTTCATTGCCTATTAAT    7882
```

```
gb:MN997409|Organism:Severe      CATTTTGTTAACTTAGACAACCTGAGAGCTAATAACACTAAAGGTTCATTGCCTATTAAT   7882
gb:MN938384|Organism:Severe      CATTTTGTTAACTTAGACAACCTGAGAGCTAATAACACTAAAGGTTCATTGCCTATTAAT   7850
gb:MT044258|Organism:Severe      CATTTTGTTAACTTAGACAACCTGAGAGCTAATAACACTAAAGGTTCATTGCCTATTAAT   7858
gb:MT039890|Organism:Severe      CATTTTGTTAACTTAGACAACCTGAGAGCTAATAACACTAAAGGTTCATTGCCTATTAAT   7882
gb:MN988713|Organism:Severe      CATTTTGTTAACTTAGACAACCTGAGAGCTAATAACACTAAAGGTTCATTGCCTATTAAT   7882
gb:LC521925|Organism:Severe      CATTTTGTTAACTTAGACAACCTGAGAGCTAATAACACTAAAGGTTCATTGCCTATTAAT   7855
gb:MT093571|Organism:Severe      CATTTTGTTAACTTAGACAACCTGAGAGCTAATAACACTAAAGGTTCATTGCCTATTAAT   7882
gb:MT039887|Organism:Severe      CATTTTGTTAACTTAGACAACCTGAGAGCTAATAACACTAAAGGTTCATTGCCTATTAAT   7882
gb:MT019530|Organism:Severe      CATTTTGTTAACTTAGACAACCTGAGAGCTAATAACACTAAAGGTTCATTGCCTATTAAT   7882
gb:MT039888|Organism:Severe      CATTTTGTTAACTTAGACAACCTGAGAGCTAATAACACTAAAGGTTCATTGCCTATTAAT   7882
gb:LC522972|Organism:Severe      CATTTTGTTAACTTAGACAACCTGAGAGCTAATAACACTAAAGGTTCATTGCCTATTAAT   7879
gb:MT027063|Organism:Severe      CATTTTGTTAACTTAGACAACCTGAGAGCTAATAACACTAAAGGTTCATTGCCTATTAAT   7882
gb:MT027062|Organism:Severe      CATTTTGTTAACTTAGACAACCTGAGAGCTAATAACACTAAAGGTTCATTGCCTATTAAT   7882
gb:MT019529|Organism:Severe      CATTTTGTTAACTTAGACAACCTGAGAGCTAATAACACTAAAGGTTCATTGCCTATTAAT   7882
gb:MN996529|Organism:Severe      CATTTTGTTAACTTAGACAACCTGAGAGCTAATAACACTAAAGGTTCATTGCCTATTAAT   7870
gb:MN996531|Organism:Severe      CATTTTGTTAACTTAGACAACCTGAGAGCTAATAACACTAAAGGTTCATTGCCTATTAAT   7869
gb:MT066176|Organism:Severe      CATTTTGTTAACTTAGACAACCTGAGAGCTAATAACACTAAAGGTTCATTGCCTATTAAT   7882
gb:MT027064|Organism:Severe      CATTTTGTTAACTTAGACAACCTGAGAGCTAATAACACTAAAGGTTCATTGCCTATTAAT   7882
gb:MN994468|Organism:Severe      CATTTTGTTAACTTAGACAACCTGAGAGCTAATAACACTAAAGGTTCATTGCCTATTAAT   7882
gb:MT072688|Organism:Severe      CATTTTGTTAACTTAGACAACCTGAGAGCTAATAACACTAAAGGTTCATTGCCTATTAAT   7867
gb:MN996527|Organism:Severe      CATTTTGTTAACTTAGACAACCTGAGAGCTAATAACACTAAAGGTTCATTGCCTATTAAT   7849
gb:MT093631|Organism:Severe      CATTTTGTTAACTTAGACAACCTGAGAGCTAATAACACTAAAGGTTCATTGCCTATTAAT   7920
gb:MT106053|Organism:Severe      CATTTTGTTAACTTAGACAACCTGAGAGCTAATAACACTAAAGGTTCATTGCCTATTAAT   7882
gb:MT019533|Organism:Severe      CATTTTGTTAACTTAGACAACCTGAGAGCTAATAACACTAAAGTTTCATTGCCTATTAAT   7882
gb:MT019531|Organism:Severe      CATTTTGTTAACTTAGACAACCTGAGAGCTAATAACACTAAAGGTTCATTGCCTATTAAT   7882
gb:MN996528|Organism:Severe      CATTTTGTTAACTTAGACAACCTGAGAGCTAATAACACTAAAGGTTCATTGCCTATTAAT   7882
gb:MN996530|Organism:Severe      CATTTTGTTAACTTAGACAACCTGAGAGCTAATAACACTAAAGGTTCATTGCCTATTAAT   7868
gb:MN908947|Organism:Severe      CATTTTGTTAACTTAGACAACCTGAGAGCTAATAACACTAAAGGTTCATTGCCTATTAAT   7882
gb:MT019532|Organism:Severe      CATTTTGTTAACTTAGACAACCTGAGAGCTAATAACACTAAAGGTTCATTGCCTATTAAT   7882
                                 ******************************************* ****************

gb:MT020781|Organism:Severe      GTTATAGTTTTTGATGGTAAATCAAAATGTGAAGAATCATCTGCAAAATCAGCGTCTGTT   7930
gb:MT007544|Organism:Severe      GTTATAGTTTTTGATGGTAAATCAAAATGTGAAGAATCATCTGCAAAATCAGCGTCTGTT   7942
gb:MN994467|Organism:Severe      GTTATAGTTTTTGATGGTAAATCAAAATGTGAAGAATCATCTGCAAAATCAGCGTCTGTT   7942
gb:MT044257|Organism:Severe      GTTATAGTTTTTGATGGTAAATCAAAATGTGAAGAATCATCTGCAAAATCAGCGTCTGTT   7942
gb:MT106054|Organism:Severe      GTTATAGTTTTTGATGGTAAATCAAAATGTGAAGAATCATCTGCAAAATCAGCGTCTGTT   7942
gb:MT049951|Organism:Severe      GTTATAGTTTTTGATGGTAAATCAAAATGTGAAGAATCATCTGCAAAATCAGCGTCTGTT   7942
gb:MN975262|Organism:Severe      GTTATAGTTTTTGATGGTAAATCAAAATGTGAAGAATCATCTGCAAAATCAGCGTCTGTT   7942
gb:MT106052|Organism:Severe      GTTATAGTTTTTGATGGTAAATCAAAATGTGAAGAATCATCTGCAAAATCAGCGTCTGTT   7942
gb:LC522975|Organism:Severe      GTTATAGTTTTTGATGGTAAATCAAAATGTGAAGAATCATCTGCAAAATCAGCGTCTGTT   7939
gb:LC522973|Organism:Severe      GTTATAGTTTTTGATGGTAAATCAAAATGTGAAGAATCATCTGCAAAATCAGCGTCTGTT   7939
gb:LC522974|Organism:Severe      GTTATAGTTTTTGATGGTAAATCAAAATGTGAAGAATCATCTGCAAAATCAGCGTCTGTT   7939
gb:MN985325|Organism:Severe      GTTATAGTTTTTGATGGTAAATCAAAATGTGAAGAATCATCTGCAAAATCAGCGTCTGTT   7942
gb:MT020881|Organism:Severe      GTTATAGTTTTTGATGGTAAATCAAAATGTGAAGAATCATCTGCAAAATCAGCGTCTGTT   7942
gb:MT020880|Organism:Severe      GTTATAGTTTTTGATGGTAAATCAAAATGTGAAGAATCATCTGCAAAATCAGCGTCTGTT   7942
gb:MT066175|Organism:Severe      GTTATAGTTTTTGATGGTAAATCAAAATGTGAAGAATCATCTGCAAAATCAGCGTCTGTT   7942
gb:MN997409|Organism:Severe      GTTATAGTTTTTGATGGTAAATCAAAATGTGAAGAATCATCTGCAAAATCAGCGTCTGTT   7942
gb:MN938384|Organism:Severe      GTTATAGTTTTTGATGGTAAATCAAAATGTGAAGAATCATCTGCAAAATCAGCGTCTGTT   7910
gb:MT044258|Organism:Severe      GTTATAGTTTTTGATGGTAAATCAAAATGTGAAGAATCATCTGCAAAATCAGCGTCTGTT   7918
gb:MT039890|Organism:Severe      GTTATAGTTTTTGATGGTAAATCAAAATGTGAAGAATCATCTGCAAAATCAGCGTCTGTT   7942
gb:MN988713|Organism:Severe      GTTATAGTTTTTGATGGTAAATCAAAATGTGAAGAATCATCTGCAAAATCAGCGTCTGTT   7942
gb:LC521925|Organism:Severe      GTTATAGTTTTTGATGGTAAATCAAAATGTGAAGAATCATCTGCAAAATCAGCGTCTGTT   7915
gb:MT093571|Organism:Severe      GTTATAGTTTTTGATGGTAAATCAAAATGTGAAGAATCATCTGCAAAATCAGCGTCTGTT   7942
gb:MT039887|Organism:Severe      GTTATAGTTTTTGATGGTAAATCAAAATGTGAAGAATCATCTGCAAAATCAGCGTCTGTT   7942
gb:MT019530|Organism:Severe      GTTATAGTTTTTGATGGTAAATCAAAATGTGAAGAATCATCTGCAAAATCAGCGTCTGTT   7942
gb:MT039888|Organism:Severe      GTTATAGTTTTTGATGGTAAATCAAAATGTGAAGAATCATCTGCAAAATCAGCGTCTGTT   7942
gb:LC522972|Organism:Severe      GTTATAGTTTTTGATGGTAAATCAAAATGTGAAGAATCATCTGCAAAATCAGCGTCTGTT   7939
gb:MT027063|Organism:Severe      GTTATAGTTTTTGATGGTAAATCAAAATGTGAAGAATCATCTGCAAAATCAGCGTCTGTT   7942
gb:MT027062|Organism:Severe      GTTATAGTTTTTGATGGTAAATCAAAATGTGAAGAATCATCTGCAAAATCAGCGTCTGTT   7942
gb:MT019529|Organism:Severe      GTTATAGTTTTTGATGGTAAATCAAAATGTGAAGAATCATCTGCAAAATCAGCGTCTGTT   7942
gb:MN996529|Organism:Severe      GTTATAGTTTTTGATGGTAAATCAAAATGTGAAGAATCATCTGCAAAATCAGCGTCTGTT   7930
gb:MN996531|Organism:Severe      GTTATAGTTTTTGATGGTAAATCAAAATGTGAAGAATCATCTGCAAAATCAGCGTCTGTT   7929
gb:MT066176|Organism:Severe      GTTATAGTTTTTGATGGTAAATCAAAATGTGAAGAATCATCTGCAAAATCAGCGTCTGTT   7942
gb:MT027064|Organism:Severe      GTTATAGTTTTTGATGGTAAATCAAAATGTGAAGAATCATCTGCAAAATCAGCGTCTGTT   7942
gb:MN994468|Organism:Severe      GTTATAGTTTTTGATGGTAAATCAAAATGTGAAGAATCATCTGCAAAATCAGCGTCTGTT   7942
```

```
gb:MT072688|Organism:Severe      GTTATAGTTTTTGATGGTAAATCAAAATGTGAAGAATCATCTGCAAAATCAGCGTCTGTT	7927
gb:MN996527|Organism:Severe      GTTATAGTTTTTGATGGTAAATCAAAATGTGAAGAATCATCTGCAAAATCAGCGTCTGTT	7909
gb:MT093631|Organism:Severe      GTTATAGTTTTTGATGGTAAATCAAAATGTGAAGAATCATCTGCAAAATCAGCGTCTGTT	7980
gb:MT106053|Organism:Severe      GTTATAGTTTTTGATGGTAAATCAAAATGTGAAGAATCATCTGCAAAATCAGCGTCTGTT	7942
gb:MT019533|Organism:Severe      GTTATAGTTTTTGATGGTAAATCAAAATGTGAAGAATCATCTGCAAAATCAGCGTCTGTT	7942
gb:MT019531|Organism:Severe      GTTATAGTTTTTGATGGTAAATCAAAATGTGAAGAATCATCTGCAAAATCAGCGTCTGTT	7942
gb:MN996528|Organism:Severe      GTTATAGTTTTTGATGGTAAATCAAAATGTGAAGAATCATCTGCAAAATCAGCGTCTGTT	7942
gb:MN996530|Organism:Severe      GTTATAGTTTTTGATGGTAAATCAAAATGTGAAGAATCATCTGCAAAATCAGCGTCTGTT	7928
gb:MN908947|Organism:Severe      GTTATAGTTTTTGATGGTAAATCAAAATGTGAAGAATCATCTGCAAAATCAGCGTCTGTT	7942
gb:MT019532|Organism:Severe      GTTATAGTTTTTGATGGTAAATCAAAATGTGAAGAATCATCTGCAAAATCAGCGTCTGTT	7942
                                 ************************************************************

gb:MT020781|Organism:Severe      TACTACAGTCAGCTTATGTGTCAACCTATACTGTTACTAGATCAGGCATTAGTGTCTGAT	7990
gb:MT007544|Organism:Severe      TACTACAGTCAGCTTATGTGTCAACCTATACTGTTACTAGATCAGGCATTAGTGTCTGAT	8002
gb:MN994467|Organism:Severe      TACTACAGTCAGCTTATGTGTCAACCTATACTGTTACTAGATCAGGCATTAGTGTCTGAT	8002
gb:MT044257|Organism:Severe      TACTACAGTCAGCTTATGTGTCAACCTATACTGTTACTAGATCAGGCATTAGTGTCTGAT	8002
gb:MT106054|Organism:Severe      TACTACAGTCAGCTTATGTGTCAACCTATACTGTTACTAGATCAGGCATTAGTGTCTGAT	8002
gb:MT049951|Organism:Severe      TACTACAGTCAGCTTATGTGTCAACCTATACTGTTACTAGATCAGGCATTAGTGTCTGAT	8002
gb:MN975262|Organism:Severe      TACTACAGTCAGCTTATGTGTCAACCTATACTGTTACTAGATCAGGCATTAGTGTCTGAT	8002
gb:MT106052|Organism:Severe      TACTACAGTCAGCTTATGTGTCAACCTATACTGTTACTAGATCAGGCATTAGTGTCTGAT	8002
gb:LC522975|Organism:Severe      TACTACAGTCAGCTTATGTGTCAACCTATACTGTTACTAGATCAGGCATTAGTGTCTGAT	7999
gb:LC522973|Organism:Severe      TACTACAGTCAGCTTATGTGTCAACCTATACTGTTACTAGATCAGGCATTAGTGTCTGAT	7999
gb:LC522974|Organism:Severe      TACTACAGTCAGCTTATGTGTCAACCTATACTGTTACTAGATCAGGCATTAGTGTCTGAT	7999
gb:MN985325|Organism:Severe      TACTACAGTCAGCTTATGTGTCAACCTATACTGTTACTAGATCAGGCATTAGTGTCTGAT	8002
gb:MT020881|Organism:Severe      TACTACAGTCAGCTTATGTGTCAACCTATACTGTTACTAGATCAGGCATTAGTGTCTGAT	8002
gb:MT020880|Organism:Severe      TACTACAGTCAGCTTATGTGTCAACCTATACTGTTACTAGATCAGGCATTAGTGTCTGAT	8002
gb:MT066175|Organism:Severe      TACTACAGTCAGCTTATGTGTCAACCTATACTGTTACTAGATCAGGCATTAGTGTCTGAT	8002
gb:MN997409|Organism:Severe      TACTACAGTCAGCTTATGTGTCAACCTATACTGTTACTAGATCAGGCATTAGTGTCTGAT	8002
gb:MN938384|Organism:Severe      TACTACAGTCAGCTTATGTGTCAACCTATACTGTTACTAGATCAGGCATTAGTGTCTGAT	7970
gb:MT044258|Organism:Severe      TACTACAGTCAGCTTATGTGTCAACCTATACTGTTACTAGATCAGGCATTAGTGTCTGAT	7978
gb:MT039890|Organism:Severe      TACTACAGTCAGCTTATGTGTCAACCTATACTGTTACTAGATCAGGCATTAGTGTCTGAT	8002
gb:MN988713|Organism:Severe      TACTACAGTCAGCTTATGTGTCAACCTATACTGTTACTAGATCAGGCATTAGTGTCTGAT	8002
gb:LC521925|Organism:Severe      TACTACAGTCAGCTTATGTGTCAACCTATACTGTTACTAGATCAGGCATTAGTGTCTGAT	7975
gb:MT093571|Organism:Severe      TACTACAGTCAGCTTATGTGTCAACCTATACTGTTACTAGATCAGGCATTAGTGTCTGAT	8002
gb:MT039887|Organism:Severe      TACTACAGTCAGCTTATGTGTCAACCTATACTGTTACTAGATCAGGCATTAGTGTCTGAT	8002
gb:MT019530|Organism:Severe      TACTACAGTCAGCTTATGTGTCAACCTATACTGTTACTAGATCAGGCATTAGTGTCTGAT	8002
gb:MT039888|Organism:Severe      TACTACAGTCAGCTTATGTGTCAACCTATACTGTTACTAGATCAGGCATTAGTGTCTGAT	8002
gb:LC522972|Organism:Severe      TACTACAGTCAGCTTATGTGTCAACCTATACTGTTACTAGATCAGGCATTAGTGTCTGAT	7999
gb:MT027063|Organism:Severe      TACTACAGTCAGCTTATGTGTCAACCTATACTGTTACTAGATCAGGCATTAGTGTCTGAT	8002
gb:MT027062|Organism:Severe      TACTACAGTCAGCTTATGTGTCAACCTATACTGTTACTAGATCAGGCATTAGTGTCTGAT	8002
gb:MT019529|Organism:Severe      TACTACAGTCAGCTTATGTGTCAACCTATACTGTTACTAGATCAGGCATTAGTGTCTGAT	8002
gb:MN996529|Organism:Severe      TACTACAGTCAGCTTATGTGTCAACCTATACTGTTACTAGATCAGGCATTAGTGTCTGAT	7990
gb:MN996531|Organism:Severe      TACTACAGTCAGCTTATGTGTCAACCTATACTGTTACTAGATCAGGCATTAGTGTCTGCT	7989
gb:MT066176|Organism:Severe      TACTACAGTCAGCTTATGTGTCAACCTATACTGTTACTAGATCAGGCATTAGTGTCTGAT	8002
gb:MT027064|Organism:Severe      TACTACAGTCAGCTTATGTGTCAACCTATACTGTTACTAGATCAGGCATTAGTGTCTGAT	8002
gb:MN994468|Organism:Severe      TACTACAGTCAGCTTATGTGTCAACCTATACTGTTACTAGATCAGGCATTAGTGTCTGAT	8002
gb:MT072688|Organism:Severe      TACTACAGTCAGCTTATGTGTCAACCTATACTGTTACTAGATCAGGCATTAGTGTCTGAT	7987
gb:MN996527|Organism:Severe      TACTACAGTCAGCTTATGTGTCAACCTATACTGTTACTAGATCAGGCATTAGTGTCTGAT	7969
gb:MT093631|Organism:Severe      TACTACAGTCAGCTTATGTGTCAACCTATACTGTTACTAGATCAGGCATTAGTGTCTGAT	8040
gb:MT106053|Organism:Severe      TACTACAGTCAGCTTATGTGTCAACCTATACTGTTACTAGATCAGGCATTAGTGTCTGAT	8002
gb:MT019533|Organism:Severe      TACTACAGTCAGCTTATGTGTCAACCTATACTGTTACTAGATCAGGCATTAGTGTCTGAT	8002
gb:MT019531|Organism:Severe      TACTACAGTCAGCTTATGTGTCAACCTATACTGTTACTAGATCAGGCATTAGTGTCTGAT	8002
gb:MN996528|Organism:Severe      TACTACAGTCAGCTTATGTGTCAACCTATACTGTTACTAGATCAGGCATTAGTGTCTGAT	8002
gb:MN996530|Organism:Severe      TACTACAGTCAGCTTATGTGTCAACCTATACTGTTACTAGATCAGGCATTAGTGTCTGAT	7988
gb:MN908947|Organism:Severe      TACTACAGTCAGCTTATGTGTCAACCTATACTGTTACTAGATCAGGCATTAGTGTCTGAT	8002
gb:MT019532|Organism:Severe      TACTACAGTCAGCTTATGTGTCAACCTATACTGTTACTAGATCAGGCATTAGTGTCTGAT	8002
                                 ********************************************************** *

gb:MT020781|Organism:Severe      GTTGGTGATAGTGCGGAAGTTGCAGTTAAAATGTTTGATGCTTACGTTAATACGTTTTCA	8050
gb:MT007544|Organism:Severe      GTTGGTGATAGTGCGGAAGTTGCAGTTAAAATGTTTGATGCTTACGTTAATACGTTTTCA	8062
gb:MN994467|Organism:Severe      GTTGGTGATAGTGCGGAAGTTGCAGTTAAAATGTTTGATGCTTACGTTAATACGTTTTCA	8062
gb:MT044257|Organism:Severe      GTTGGTGATAGTGCGGAAGTTGCAGTTAAAATGTTTGATGCTTACGTTAATACGTTTTCA	8062
gb:MT106054|Organism:Severe      GTTGGTGATAGTGCGGAAGTTGCAGTTAAAATGTTTGATGCTTACGTTAATACGTTTTCA	8062
gb:MT049951|Organism:Severe      GTTGGTGATAGTGCGGAAGTTGCAGTTAAAATGTTTGATGCTTACGTTAATACGTTTTCA	8062
gb:MN975262|Organism:Severe      GTTGGTGATAGTGCGGAAGTTGCAGTTAAAATGTTTGATGCTTACGTTAATACGTTTTCA	8062
```

```
gb:MT106052|Organism:Severe      GTTGGTGATAGTGCGGAAGTTGCAGTTAAAATGTTTGATGCTTACGTTAATACGTTTTCA   8062
gb:LC522975|Organism:Severe      GTTGGTGATAGTGCGGAAGTTGCAGTTAAAATGTTTGATGCTTACGTTAATACGTTTTCA   8059
gb:LC522973|Organism:Severe      GTTGGTGATAGTGCGGAAGTTGCAGTTAAAATGTTTGATGCTTACGTTAATACGTTTTCA   8059
gb:LC522974|Organism:Severe      GTTGGTGATAGTGCGGAAGTTGCAGTTAAAATGTTTGATGCTTACGTTAATACGTTTTCA   8059
gb:MN985325|Organism:Severe      GTTGGTGATAGTGCGGAAGTTGCAGTTAAAATGTTTGATGCTTACGTTAATACGTTTTCA   8062
gb:MT020881|Organism:Severe      GTTGGTGATAGTGCGGAAGTTGCAGTTAAAATGTTTGATGCTTACGTTAATACGTTTTCA   8062
gb:MT020880|Organism:Severe      GTTGGTGATAGTGCGGAAGTTGCAGTTAAAATGTTTGATGCTTACGTTAATACGTTTTCA   8062
gb:MT066175|Organism:Severe      GTTGGTGATAGTGCGGAAGTTGCAGTTAAAATGTTTGATGCTTACGTTAATACGTTTTCA   8062
gb:MN997409|Organism:Severe      GTTGGTGATAGTGCGGAAGTTGCAGTTAAAATGTTTGATGCTTACGTTAATACGTTTTCA   8062
gb:MN938384|Organism:Severe      GTTGGTGATAGTGCGGAAGTTGCAGTTAAAATGTTTGATGCTTACGTTAATACGTTTTCA   8030
gb:MT044258|Organism:Severe      GTTGGTGATAGTGCGGAAGTTGCAGTTAAAATGTTTGATGCTTACGTTAATACGTTTTCA   8038
gb:MT039890|Organism:Severe      GTTGGTGATAGTGCGGAAGTTGCAGTTAAAATGTTTGATGCTTACGTTAATACGTTTTCA   8062
gb:MN988713|Organism:Severe      GTTGGTGATAGTGCGGAAGTTGCAGTTAAAATGTTTGATGCTTACGTTAATACGTTTTCA   8062
gb:LC521925|Organism:Severe      GTTGGTGATAGTGCGGAAGTTGCAGTTAAAATGTTTGATGCTTACGTTAATACGTTTTCA   8035
gb:MT093571|Organism:Severe      GTTGGTGATAGTGCGGAAGTTGCAGTTAAAATGTTTGATGCTTACGTTAATACGTTTTCA   8062
gb:MT039887|Organism:Severe      GTTGGTGATAGTGCGGAAGTTGCAGTTAAAATGTTTGATGCTTACGTTAATACGTTTTCA   8062
gb:MT019530|Organism:Severe      GTTGGTGATAGTGCGGAAGTTGCAGTTAAAATGTTTGATGCTTACGTTAATACGTTTTCA   8062
gb:MT039888|Organism:Severe      GTTGGTGATAGTGCGGAAGTTGCAGTTAAAATGTTTGATGCTTACGTTAATACGTTTTCA   8062
gb:LC522972|Organism:Severe      GTTGGTGATAGTGCGGAAGTTGCAGTTAAAATGTTTGATGCTTACGTTAATACGTTTTCA   8059
gb:MT027063|Organism:Severe      GTTGGTGATAGTGCGGAAGTTGCAGTTAAAATGTTTGATGCTTACGTTAATACGTTTTCA   8062
gb:MT027062|Organism:Severe      GTTGGTGATAGTGCGGAAGTTGCAGTTAAAATGTTTGATGCTTACGTTAATACGTTTTCA   8062
gb:MT019529|Organism:Severe      GTTGGTGATAGTGCGGAAGTTGCAGTTAAAATGTTTGATGCTTACGTTAATACGTTTTCA   8062
gb:MN996529|Organism:Severe      GTTGGTGATAGTGCGGAAGTTGCAGTTAAAATGTTTGATGCTTACGTTAATACGTTTTCA   8050
gb:MN996531|Organism:Severe      GTTGGTGATAGTGCGGAAGTTGCAGTTAAAATGTTTGATGCTTACGTTAATACGTTTTCA   8049
gb:MT066176|Organism:Severe      GTTGGTGATAGTGCGGAAGTTGCAGTTAAAATGTTTGATGCTTACGTTAATACGTTTTCA   8062
gb:MT027064|Organism:Severe      GTTGGTGATAGTGCGGAAGTTGCAGTTAAAATGTTTGATGCTTACGTTAATACGTTTTCA   8062
gb:MN994468|Organism:Severe      GTTGGTGATAGTGCGGAAGTTGCAGTTAAAATGTTTGATGCTTACGTTAATACGTTTTCA   8062
gb:MT072688|Organism:Severe      GTTGGTGATAGTGCGGAAGTTGCAGTTAAAATGTTTGATGCTTACGTTAATACGTTTTCA   8047
gb:MN996527|Organism:Severe      GTTGGTGATAGTGCGGAAGTTGCAGTTAAAATGTTTGATGCTTACGTTAATACGTTTTCA   8029
gb:MT093631|Organism:Severe      GTTGGTGATAGTGCGGAAGTTGCAGTTAAAATGTTTGATGCTTACGTTAATACGTTTTCA   8100
gb:MT106053|Organism:Severe      GTTGGTGATAGTGCGGAAGTTGCAGTTAAAATGTTTGATGCTTACGTTAATACGTTTTCA   8062
gb:MT019533|Organism:Severe      GTTGGTGATAGTGCGGAAGTTGCAGTTAAAATGTTTGATGCTTACGTTAATACGTTTTCA   8062
gb:MT019531|Organism:Severe      GTTGGTGATAGTGCGGAAGTTGCAGTTAAAATGTTTGATGCTTACGTTAATACGTTTTCA   8062
gb:MN996528|Organism:Severe      GTTGGTGATAGTGCGGAAGTTGCAGTTAAAATGTTTGATGCTTACGTTAATACGTTTTCA   8062
gb:MN996530|Organism:Severe      GTTGGTGATAGTGCGGAAGTTGCAGTTAAAATGTTTGATGCTTACGTTAATACGTTTTCA   8048
gb:MN908947|Organism:Severe      GTTGGTGATAGTGCGGAAGTTGCAGTTAAAATGTTTGATGCTTACGTTAATACGTTTTCA   8062
gb:MT019532|Organism:Severe      GTTGGTGATAGTGCGGAAGTTGCAGTTAAAATGTTTGATGCTTACGTTAATACGTTTTCA   8062
                                 ************************************************************

gb:MT020781|Organism:Severe      TCAACTTTTAACGTACCAATGGAAAAACTCAAAACACTAGTTGCAACTGCAGAAGCTGAA   8110
gb:MT007544|Organism:Severe      TCAACTTTTAACGTACCAATGGAAAAACTCAAAACACTAGTTGCAACTGCAGAAGCTGAA   8122
gb:MN994467|Organism:Severe      TCAACTTTTAACGTACCAATGGAAAAACTCAAAACACTAGTTGCAACTGCAGAAGCTGAA   8122
gb:MT044257|Organism:Severe      TCAACTTTTAACGTACCAATGGAAAAACTCAAAACACTAGTTGCAACTGCAGAAGCTGAA   8122
gb:MT106054|Organism:Severe      TCAACTTTTAACGTACCAATGGAAAAACTCAAAACACTAGTTGCAACTGCAGAAGCTGAA   8122
gb:MT049951|Organism:Severe      TCAACTTTTAACGTACCAATGGAAAAACTCAAAACACTAGTTGCAACTGCAGAAGCTGAA   8122
gb:MN975262|Organism:Severe      TCAACTTTTAACGTACCAATGGAAAAACTCAAAACACTAGTTGCAACTGCAGAAGCTGAA   8122
gb:MT106052|Organism:Severe      TCAACTTTTAACGTACCAATGGAAAAACTCAAAACACTAGTTGCAACTGCAGAAGCTGAA   8122
gb:LC522975|Organism:Severe      TCAACTTTTAACGTACCAATGGAAAAACTCAAAACACTAGTTGCAACTGCAGAAGCTGAA   8119
gb:LC522973|Organism:Severe      TCAACTTTTAACGTACCAATGGAAAAACTCAAAACACTAGTTGCAACTGCAGAAGCTGAA   8119
gb:LC522974|Organism:Severe      TCAACTTTTAACGTACCAATGGAAAAACTCAAAACACTAGTTGCAACTGCAGAAGCTGAA   8119
gb:MN985325|Organism:Severe      TCAACTTTTAACGTACCAATGGAAAAACTCAAAACACTAGTTGCAACTGCAGAAGCTGAA   8122
gb:MT020881|Organism:Severe      TCAACTTTTAACGTACCAATGGAAAAACTCAAAACACTAGTTGCAACTGCAGAAGCTGAA   8122
gb:MT020880|Organism:Severe      TCAACTTTTAACGTACCAATGGAAAAACTCAAAACACTAGTTGCAACTGCAGAAGCTGAA   8122
gb:MT066175|Organism:Severe      TCAACTTTTAACGTACCAATGGAAAAACTCAAAACACTAGTTGCAACTGCAGAAGCTGAA   8122
gb:MN997409|Organism:Severe      TCAACTTTTAACGTACCAATGGAAAAACTCAAAACACTAGTTGCAACTGCAGAAGCTGAA   8122
gb:MN938384|Organism:Severe      TCAACTTTTAACGTACCAATGGAAAAACTCAAAACACTAGTTGCAACTGCAGAAGCTGAA   8090
gb:MT044258|Organism:Severe      TCAACTTTTAACGTACCAATGGAAAAACTCAAAACACTAGTTGCAACTGCAGAAGCTGAA   8098
gb:MT039890|Organism:Severe      TCAACTTTTAACGTACCAATGGAAAAACTCAAAACACTAGTTGCAACTGCAGAAGCTGAA   8122
gb:MN988713|Organism:Severe      TCAACTTTTAACGTACCAATGGAAAAACTCAAAACACTAGTTGCAACTGCAGAAGCTGAA   8122
gb:LC521925|Organism:Severe      TCAACTTTTAACGTACCAATGGAAAAACTCAAAACACTAGTTGCAACTGCAGAAGCTGAA   8095
gb:MT093571|Organism:Severe      TCAACTTTTAACGTACCAATGGAAAAACTCAAAACACTAGTTGCAACTGCAGAAGCTGAA   8122
gb:MT039887|Organism:Severe      TCAACTTTTAACGTACCAATGGAAAAACTCAAAACACTAGTTGCAACTGCAGAAGCTGAA   8122
gb:MT019530|Organism:Severe      TCAACTTTTAACGTACCAATGGAAAAACTCAAAACACTAGTTGCAACTGCAGAAGCTGAA   8122
gb:MT039888|Organism:Severe      TCAACTTTTAACGTACCAATGGAAAAACTCAAAACACTAGTTGCAACTGCAGAAGCTGAA   8122
gb:LC522972|Organism:Severe      TCAACTTTTAACGTACCAATGGAAAAACTCAAAACACTAGTTGCAACTGCAGAAGCTGAA   8119
```

```
gb:MT027063|Organism:Severe      TCAACTTTTAACGTACCAATGGAAAAACTCAAAACACTAGTTGCAACTGCAGAAGCTGAA   8122
gb:MT027062|Organism:Severe      TCAACTTTTAACGTACCAATGGAAAAACTCAAAACACTAGTTGCAACTGCAGAAGCTGAA   8122
gb:MT019529|Organism:Severe      TCAACTTTTAACGTACCAATGGAAAAACTCAAAACACTAGTTGCAACTGCAGAAGCTGAA   8122
gb:MN996529|Organism:Severe      TCAACTTTTAACGTACCAATGGAAAAACTCAAAACACTAGTTGCAACTGCAGAAGCTGAA   8110
gb:MN996531|Organism:Severe      TCAACTTTTAACGTACCAATGGAAAAACTCAAAACACTAGTTGCAACTGCAGAAGCTGAA   8109
gb:MT066176|Organism:Severe      TCAACTTTTAACGTACCAATGGAAAAACTCAAAACACTAGTTGCAACTGCAGAAGCTGAA   8122
gb:MT027064|Organism:Severe      TCAACTTTTAACGTACCAATGGAAAAACTCAAAACACTAGTTGCAACTGCAGAAGCTGAA   8122
gb:MN994468|Organism:Severe      TCAACTTTTAACGTACCAATGGAAAAACTCAAAACACTAGTTGCAACTGCAGAAGCTGAA   8122
gb:MT072688|Organism:Severe      TCAACTTTTAACGTACCAATGGAAAAACTCAAAACACTAGTTGCAACTGCAGAAGCTGAA   8107
gb:MN996527|Organism:Severe      TCAACTTTTAACGTACCAATGGAAAAACTCAAAACACTAGTTGCAACTGCAGAAGCTGAA   8089
gb:MT093631|Organism:Severe      TCAACTTTTAACGTACCAATGGAAAAACTCAAAACACTAGTTGCAACTGCAGAAGCTGAA   8160
gb:MT106053|Organism:Severe      TCAACTTTTAACGTACCAATGGAAAAACTCAAAACACTAGTTGCAACTGCAGAAGCTGAA   8122
gb:MT019533|Organism:Severe      TCAACTTTTAACGTACCAATGGAAAAACTCAAAACACTAGTTGCAACTGCAGAAGCTGAA   8122
gb:MT019531|Organism:Severe      TCAACTTTTAACGTACCAATGGAAAAACTCAAAACACTAGTTGCAACTGCAGAAGCTGAA   8122
gb:MN996528|Organism:Severe      TCAACTTTTAACGTACCAATGGAAAAACTCAAAACACTAGTTGCAACTGCAGAAGCTGAA   8122
gb:MN996530|Organism:Severe      TCAACTTTTAACGTACCAATGGAAAAACTCAAAACACTAGTTGCAACTGCAGAAGCTGAA   8108
gb:MN908947|Organism:Severe      TCAACTTTTAACGTACCAATGGAAAAACTCAAAACACTAGTTGCAACTGCAGAAGCTGAA   8122
gb:MT019532|Organism:Severe      TCAACTTTTAACGTACCAATGGAAAAACTCAAAACACTAGTTGCAACTGCAGAAGCTGAA   8122
                                 ************************************************************

gb:MT020781|Organism:Severe      CTTGCAAAGAATGTGTCCTTAGACAATGTCTTATCTACTTTTATTTCAGCAGCTCGGCAA   8170
gb:MT007544|Organism:Severe      CTTGCAAAGAATGTGTCCTTAGACAATGTCTTATCTACTTTTATTTCAGCAGCTCGGCAA   8182
gb:MN994467|Organism:Severe      CTTGCAAAGAATGTGTCCTTAGACAATGTCTTATCTACTTTTATTTCAGCAGCTCGGCAA   8182
gb:MT044257|Organism:Severe      CTTGCAAAGAATGTGTCCTTAGACAATGTCTTATCTACTTTTATTTCAGCAGCTCGGCAA   8182
gb:MT106054|Organism:Severe      CTTGCAAAGAATGTGTCCTTAGACAATGTCTTATCTACTTTTATTTCAGCAGCTCGGCAA   8182
gb:MT049951|Organism:Severe      CTTGCAAAGAATGTGTCCTTAGACAATGTCTTATCTACTTTTATTTCAGCAGCTCGGCAA   8182
gb:MN975262|Organism:Severe      CTTGCAAAGAATGTGTCCTTAGACAATGTCTTATCTACTTTTATTTCAGCAGCTCGGCAA   8182
gb:MT106052|Organism:Severe      CTTGCAAAGAATGTGTCCTTAGACAATGTCTTATCTACTTTTATTTCAGCAGCTCGGCAA   8182
gb:LC522975|Organism:Severe      CTTGCAAAGAATGTGTCCTTAGACAATGTCTTATCTACTTTTATTTCAGCAGCTCGGCAA   8179
gb:LC522973|Organism:Severe      CTTGCAAAGAATGTGTCCTTAGACAATGTCTTATCTACTTTTATTTCAGCAGCTCGGCAA   8179
gb:LC522974|Organism:Severe      CTTGCAAAGAATGTGTCCTTAGACAATGTCTTATCTACTTTTATTTCAGCAGCTCGGCAA   8179
gb:MN985325|Organism:Severe      CTTGCAAAGAATGTGTCCTTAGACAATGTCTTATCTACTTTTATTTCAGCAGCTCGGCAA   8182
gb:MT020881|Organism:Severe      CTTGCAAAGAATGTGTCCTTAGACAATGTCTTATCTACTTTTATTTCAGCAGCTCGGCAA   8182
gb:MT020880|Organism:Severe      CTTGCAAAGAATGTGTCCTTAGACAATGTCTTATCTACTTTTATTTCAGCAGCTCGGCAA   8182
gb:MT066175|Organism:Severe      CTTGCAAAGAATGTGTCCTTAGACAATGTCTTATCTACTTTTATTTCAGCAGCTCGGCAA   8182
gb:MN997409|Organism:Severe      CTTGCAAAGAATGTGTCCTTAGACAATGTCTTATCTACTTTTATTTCAGCAGCTCGGCAA   8182
gb:MN938384|Organism:Severe      CTTGCAAAGAATGTGTCCTTAGACAATGTCTTATCTACTTTTATTTCAGCAGCTCGGCAA   8150
gb:MT044258|Organism:Severe      CTTGCAAAGAATGTGTCCTTAGACAATGTCTTATCTACTTTTATTTCAGCAGCTCGGCAA   8158
gb:MT039890|Organism:Severe      CTTGCAAAGAATGTGTCCTTAGACAATGTCTTATCTACTTTTATTTCAGCAGCTCGGCAA   8182
gb:MN988713|Organism:Severe      CTTGCAAAGAATGTGTCCTTAGACAATGTCTTATCTACTTTTATTTCAGCAGCTCGGCAA   8182
gb:LC521925|Organism:Severe      CTTGCAAAGAATGTGTCCTTAGACAATGTCTTATCTACTTTTATTTCAGCAGCTCGGCAA   8155
gb:MT093571|Organism:Severe      CTTGCAAAGAATGTGTCCTTAGACAATGTCTTATCTACTTTTATTTCAGCAGCTCGGCAA   8182
gb:MT039887|Organism:Severe      CTTGCAAAGAATGTGTCCTTAGACAATGTCTTATCTACTTTTATTTCAGCAGCTCGGCAA   8182
gb:MT019530|Organism:Severe      CTTGCAAAGAATGTGTCCTTAGACAATGTCTTATCTACTTTTATTTCAGCAGCTCGGCAA   8182
gb:MT039888|Organism:Severe      CTTGCAAAGAATGTGTCCTTAGACAATGTCTTATCTACTTTTATTTCAGCAGCTCGGCAA   8182
gb:LC522972|Organism:Severe      CTTGCAAAGAATGTGTCCTTAGACAATGTCTTATCTACTTTTATTTCAGCAGCTCGGCAA   8179
gb:MT027063|Organism:Severe      CTTGCAAAGAATGTGTCCTTAGACAATGTCTTATCTACTTTTATTTCAGCAGCTCGGCAA   8182
gb:MT027062|Organism:Severe      CTTGCAAAGAATGTGTCCTTAGACAATGTCTTATCTACTTTTATTTCAGCAGCTCGGCAA   8182
gb:MT019529|Organism:Severe      CTTGCAAAGAATGTGTCCTTAGACAATGTCTTATCTACTTTTATTTCAGCAGCTCGGCAA   8182
gb:MN996529|Organism:Severe      CTTGCAAAGAATGTGTCCTTAGACAATGTCTTATCTACTTTTATTTCAGCAGCTCGGCAA   8170
gb:MN996531|Organism:Severe      CTTGCAAAGAATGTGTCCTTAGACAATGTCTTATCTACTTTTATTTCAGCAGCTCGGCAA   8169
gb:MT066176|Organism:Severe      CTTGCAAAGAATGTGTCCTTAGACAATGTCTTATCTACTTTTATTTCAGCAGCTCGGCAA   8182
gb:MT027064|Organism:Severe      CTTGCAAAGAATGTGTCCTTAGACAATGTCTTATCTACTTTTATTTCAGCAGCTCGGCAA   8182
gb:MN994468|Organism:Severe      CTTGCAAAGAATGTGTCCTTAGACAATGTCTTATCTACTTTTATTTCAGCAGCTCGGCAA   8182
gb:MT072688|Organism:Severe      CTTGCAAAGAATGTGTCCTTAGACAATGTCTTATCTACTTTTATTTCAGCAGCTCGGCAA   8167
gb:MN996527|Organism:Severe      CTTGCAAAGAATGTGTCCTTAGACAATGTCTTATCTACTTTTATTTCAGCAGCTCGGCAA   8149
gb:MT093631|Organism:Severe      CTTGCAAAGAATGTGTCCTTAGACAATGTCTTATCTACTTTTATTTCAGCAGCTCGGCAA   8220
gb:MT106053|Organism:Severe      CTTGCAAAGAATGTGTCCTTAGACAATGTCTTATCTACTTTTATTTCAGCAGCTCGGCAA   8182
gb:MT019533|Organism:Severe      CTTGCAAAGAATGTGTCCTTAGACAATGTCTTATCTACTTTTATTTCAGCAGCTCGGCAA   8182
gb:MT019531|Organism:Severe      CTTGCAAAGAATGTGTCCTTAGACAATGTCTTATCTACTTTTATTTCAGCAGCTCGGCAA   8182
gb:MN996528|Organism:Severe      CTTGCAAAGAATGTGTCCTTAGACAATGTCTTATCTACTTTTATTTCAGCAGCTCGGCAA   8182
gb:MN996530|Organism:Severe      CTTGCAAAGAATGTGTCCTTAGACAATGTCTTATCTACTTTTATTTCAGCAGCTCGGCAA   8168
gb:MN908947|Organism:Severe      CTTGCAAAGAATGTGTCCTTAGACAATGTCTTATCTACTTTTATTTCAGCAGCTCGGCAA   8182
gb:MT019532|Organism:Severe      CTTGCAAAGAATGTGTCCTTAGACAATGTCTTATCTACTTTTATTTCAGCAGCTCGGCAA   8182
                                 ************************************************************
```

```
gb:MT020781|Organism:Severe      GGGTTTGTTGATTCAGATGTAGAAACTAAAGATGTTGTTGAATGTCTTAAATTGTCACAT   8230
gb:MT007544|Organism:Severe      GGGTTTGTTGATTCAGATGTAGAAACTAAAGATGTTGTTGAATGTCTTAAATTGTCACAT   8242
gb:MN994467|Organism:Severe      GGGTTTGTTGATTCAGATGTAGAAACTAAAGATGTTGTTGAATGTCTTAAATTGTCACAT   8242
gb:MT044257|Organism:Severe      GGGTTTGTTGATTCAGATGTAGAAACTAAAGATGTTGTTGAATGTCTTAAATTGTCACAT   8242
gb:MT106054|Organism:Severe      GGGTTTGTTGATTCAGATGTAGAAACTAAAGATGTTGTTGAATGTCTTAAATTGTCACAT   8242
gb:MT049951|Organism:Severe      GGGTTTGTTGATTCAGATGTAGAAACTAAAGATGTTGTTGAATGTCTTAAATTGTCACAT   8242
gb:MN975262|Organism:Severe      GGGTTTGTTGATTCAGATGTAGAAACTAAAGATGTTGTTGAATGTCTTAAATTGTCACAT   8242
gb:MT106052|Organism:Severe      GGGTTTGTTGATTCAGATGTAGAAACTAAAGATGTTGTTGAATGTCTTAAATTGTCACAT   8242
gb:LC522975|Organism:Severe      GGGTTTGTTGATTCAGATGTAGAAACTAAAGATGTTGTTGAATGTCTTAAATTGTCACAT   8239
gb:LC522973|Organism:Severe      GGGTTTGTTGATTCAGATGTAGAAACTAAAGATGTTGTTGAATGTCTTAAATTGTCACAT   8239
gb:LC522974|Organism:Severe      GGGTTTGTTGATTCAGATGTAGAAACTAAAGATGTTGTTGAATGTCTTAAATTGTCACAT   8239
gb:MN985325|Organism:Severe      GGGTTTGTTGATTCAGATGTAGAAACTAAAGATGTTGTTGAATGTCTTAAATTGTCACAT   8242
gb:MT020881|Organism:Severe      GGGTTTGTTGATTCAGATGTAGAAACTAAAGATGTTGTTGAATGTCTTAAATTGTCACAT   8242
gb:MT020880|Organism:Severe      GGGTTTGTTGATTCAGATGTAGAAACTAAAGATGTTGTTGAATGTCTTAAATTGTCACAT   8242
gb:MT066175|Organism:Severe      GGGTTTGTTGATTCAGATGTAGAAACTAAAGATGTTGTTGAATGTCTTAAATTGTCACAT   8242
gb:MN997409|Organism:Severe      GGGTTTGTTGATTCAGATGTAGAAACTAAAGATGTTGTTGAATGTCTTAAATTGTCACAT   8242
gb:MN938384|Organism:Severe      GGGTTTGTTGATTCAGATGTAGAAACTAAAGATGTTGTTGAATGTCTTAAATTGTCACAT   8210
gb:MT044258|Organism:Severe      GGGTTTGTTGATTCAGATGTAGAAACTAAAGATGTTGTTGAATGTCTTAAATTGTCACAT   8218
gb:MT039890|Organism:Severe      GGGTTTGTTGATTCAGATGTAGAAACTAAAGATGTTGTTGAATGTCTTAAATTGTCACAT   8242
gb:MN988713|Organism:Severe      GGGTTTGTTGATTCAGATGTAGAAACTAAAGATGTTGTTGAATGTCTTAAATTGTCACAT   8242
gb:LC521925|Organism:Severe      GGGTTTGTTGATTCAGATGTAGAAACTAAAGATGTTGTTGAATGTCTTAAATTGTCACAT   8215
gb:MT093571|Organism:Severe      GGGTTTGTTGATTCAGATGTAGAAACTAAAGATGTTGTTGAATGTCTTAAATTGTCACAT   8242
gb:MT039887|Organism:Severe      GGGTTTGTTGATTCAGATGTAGAAACTAAAGATGTTGTTGAATGTCTTAAATTGTCACAT   8242
gb:MT019530|Organism:Severe      GGGTTTGTTGATTCAGATGTAGAAACTAAAGATGTTGTTGAATGTCTTAAATTGTCACAT   8242
gb:MT039888|Organism:Severe      GGGTTTGTTGATTCAGATGTAGAAACTAAAGATGTTGTTGAATGTCTTAAATTGTCACAT   8242
gb:LC522972|Organism:Severe      GGGTTTGTTGATTCAGATGTAGAAACTAAAGATGTTGTTGAATGTCTTAAATTGTCACAT   8239
gb:MT027063|Organism:Severe      GGGTTTGTTGATTCAGATGTAGAAACTAAAGATGTTGTTGAATGTCTTAAATTGTCACAT   8242
gb:MT027062|Organism:Severe      GGGTTTGTTGATTCAGATGTAGAAACTAAAGATGTTGTTGAATGTCTTAAATTGTCACAT   8242
gb:MT019529|Organism:Severe      GGGTTTGTTGATTCAGATGTAGAAACTAAAGATGTTGTTGAATGTCTTAAATTGTCACAT   8242
gb:MN996529|Organism:Severe      GGGTTTGTTGATTCAGATGTAGAAACTAAAGATGTTGTTGAATGTCTTAAATTGTCACAT   8230
gb:MN996531|Organism:Severe      GGGTTTGTTGATTCAGATGTAGAAACTAAAGATGTTGTTGAATGTCTTAAATTGTCACAT   8229
gb:MT066176|Organism:Severe      GGGTTTGTTGATTCAGATGTAGAAACTAAAGATGTTGTTGAATGTCTTAAATTGTCACAT   8242
gb:MT027064|Organism:Severe      GGGTTTGTTGATTCAGATGTAGAAACTAAAGATGTTGTTGAATGTCTTAAATTGTCACAT   8242
gb:MN994468|Organism:Severe      GGGTTTGTTGATTCAGATGTAGAAACTAAAGATGTTGTTGAATGTCTTAAATTGTCACAT   8242
gb:MT072688|Organism:Severe      GGGTTTGTTGATTCAGATGTAGAAACTAAAGATGTTGTTGAATGTCTTAAATTGTCACAT   8227
gb:MN996527|Organism:Severe      GGGTTTGTTGATTCAGATGTAGAAACTAAAGATGTTGTTGAATGTCTTAAATTGTCACAT   8209
gb:MT093631|Organism:Severe      GGGTTTGTTGATTCAGATGTAGAAACTAAAGATGTTGTTGAATGTCTTAAATTGTCACAT   8280
gb:MT106053|Organism:Severe      GGGTTTGTTGATTCAGATGTAGAAACTAAAGATGTTGTTGAATGTCTTAAATTGTCACAT   8242
gb:MT019533|Organism:Severe      GGGTTTGTTGATTCAGATGTAGAAACTAAAGATGTTGTTGAATGTCTTAAATTGTCACAT   8242
gb:MT019531|Organism:Severe      GGGTTTGTTGATTCAGATGTAGAAACTAAAGATGTTGTTGAATGTCTTAAATTGTCACAT   8242
gb:MN996528|Organism:Severe      GGGTTTGTTGATTCAGATGTAGAAACTAAAGATGTTGTTGAATGTCTTAAATTGTCACAT   8242
gb:MN996530|Organism:Severe      GGGTTTGTTGATTCAGATGTAGAAACTAAAGATGTTGTTGAATGTCTTAAATTGTCACAT   8228
gb:MN908947|Organism:Severe      GGGTTTGTTGATTCAGATGTAGAAACTAAAGATGTTGTTGAATGTCTTAAATTGTCACAT   8242
gb:MT019532|Organism:Severe      GGGTTTGTTGATTCAGATGTAGAAACTAAAGATGTTGTTGAATGTCTTAAATTGTCACAT   8242
                                 ************************************************************

gb:MT020781|Organism:Severe      CAATCTGACATAGAAGTTACTGGCGATAGTTGTAATAACTATATGCTCACCTATAACAAA   8290
gb:MT007544|Organism:Severe      CAATCTGACATAGAAGTTACTGGCGATAGTTGTAATAACTATATGCTCACCTATAACAAA   8302
gb:MN994467|Organism:Severe      CAATCTGACATAGAAGTTACTGGCGATAGTTGTAATAACTATATGCTCACCTATAACAAA   8302
gb:MT044257|Organism:Severe      CAATCTGACATAGAAGTTACTGGCGATAGTTGTAATAACTATATGCTCACCTATAACAAA   8302
gb:MT106054|Organism:Severe      CAATCTGACATAGAAGTTACTGGCGATAGTTGTAATAACTATATGCTCACCTATAACAAA   8302
gb:MT049951|Organism:Severe      CAATCTGACATAGAAGTTACTGGCGATAGTTGTAATAACTATATGCTCACCTATAACAAA   8302
gb:MN975262|Organism:Severe      CAATCTGACATAGAAGTTACTGGCGATAGTTGTAATAACTATATGCTCACCTATAACAAA   8302
gb:MT106052|Organism:Severe      CAATCTGACATAGAAGTTACTGGCGATAGTTGTAATAACTATATGCTCACCTATAACAAA   8302
gb:LC522975|Organism:Severe      CAATCTGACATAGAAGTTACTGGCGATAGTTGTAATAACTATATGCTCACCTATAACAAA   8299
gb:LC522973|Organism:Severe      CAATCTGACATAGAAGTTACTGGCGATAGTTGTAATAACTATATGCTCACCTATAACAAA   8299
gb:LC522974|Organism:Severe      CAATCTGACATAGAAGTTACTGGCGATAGTTGTAATAACTATATGCTCACCTATAACAAA   8299
gb:MN985325|Organism:Severe      CAATCTGACATAGAAGTTACTGGCGATAGTTGTAATAACTATATGCTCACCTATAACAAA   8302
gb:MT020881|Organism:Severe      CAATCTGACATAGAAGTTACTGGCGATAGTTGTAATAACTATATGCTCACCTATAACAAA   8302
gb:MT020880|Organism:Severe      CAATCTGACATAGAAGTTACTGGCGATAGTTGTAATAACTATATGCTCACCTATAACAAA   8302
gb:MT066175|Organism:Severe      CAATCTGACATAGAAGTTACTGGCGATAGTTGTAATAACTATATGCTCACCTATAACAAA   8302
gb:MN997409|Organism:Severe      CAATCTGACATAGAAGTTACTGGCGATAGTTGTAATAACTATATGCTCACCTATAACAAA   8302
gb:MN938384|Organism:Severe      CAATCTGACATAGAAGTTACTGGCGATAGTTGTAATAACTATATGCTCACCTATAACAAA   8270
gb:MT044258|Organism:Severe      CAATCTGACATAGAAGTTACTGGCGATAGTTGTAATAACTATATGCTCACCTATAACAAA   8278
```

```
gb:MT039890|Organism:Severe      CAATCTGACATAGAAGTTACTGGCGATAGTTGTAATAACTATATGCTCACCTATAACAAA   8302
gb:MN988713|Organism:Severe      CAATCTGACATAGAAGTTACTGGCGATAGTTGTAATAACTATATGCTCACCTATAACAAA   8302
gb:LC521925|Organism:Severe      CAATCTGACATAGAAGTTACTGGCGATAGTTGTAATAACTATATGCTCACCTATAACAAA   8275
gb:MT093571|Organism:Severe      CAATCTGACATAGAAGTTACTGGCGATAGTTGTAATAACTATATGCTCACCTATAACAAA   8302
gb:MT039887|Organism:Severe      CAATCTGACATAGAAGTTACTGGCGATAGTTGTAATAACTATATGCTCACCTATAACAAA   8302
gb:MT019530|Organism:Severe      CAATCTGACATAGAAGTTACTGGCGATAGTTGTAATAACTATATGCTCACCTATAACAAA   8302
gb:MT039888|Organism:Severe      CAATCTGACATAGAAGTTACTGGCGATAGTTGTAATAACTATATGCTCACCTATAACAAA   8302
gb:LC522972|Organism:Severe      CAATCTGACATAGAAGTTACTGGCGATAGTTGTAATAACTATATGCTCACCTATAACAAA   8299
gb:MT027063|Organism:Severe      CAATCTGACATAGAAGTTACTGGCGATAGTTGTAATAACTATATGCTCACCTATAACAAA   8302
gb:MT027062|Organism:Severe      CAATCTGACATAGAAGTTACTGGCGATAGTTGTAATAACTATATGCTCACCTATAACAAA   8302
gb:MT019529|Organism:Severe      CAATCTGACATAGAAGTTACTGGCGATAGTTGTAATAACTATATGCTCACCTATAACAAA   8302
gb:MN996529|Organism:Severe      CAATCTGACATAGAAGTTACTGGCGATAGTTGTAATAACTATATGCTCACCTATAACAAA   8290
gb:MN996531|Organism:Severe      CAATCTGACATAGAAGTTACTGGCGATAGTTGTAATAACTATATGCTCACCTATAACAAA   8289
gb:MT066176|Organism:Severe      CAATCTGACATAGAAGTTACTGGCGATAGTTGTAATAACTATATGCTCACCTATAACAAA   8302
gb:MT027064|Organism:Severe      CAATCTGACATAGAAGTTACTGGCGATAGTTGTAATAACTATATGCTCACCTATAACAAA   8302
gb:MN994468|Organism:Severe      CAATCTGACATAGAAGTTACTGGCGATAGTTGTAATAACTATATGCTCACCTATAACAAA   8302
gb:MT072688|Organism:Severe      CAATCTGACATAGAAGTTACTGGCGATAGTTGTAATAACTATATGCTCACCTATAACAAA   8287
gb:MN996527|Organism:Severe      CAATCTGACATAGAAGTTACTGGCGATAGTTGTAATAACTATATGCTCACCTATAACAAA   8269
gb:MT093631|Organism:Severe      CAATCTGACATAGAAGTTACTGGCGATAGTTGTAATAACTATATGCTCACCTATAACAAA   8340
gb:MT106053|Organism:Severe      CAATCTGACATAGAAGTTACTGGCGATAGTTGTAATAACTATATGCTCACCTATAACAAA   8302
gb:MT019533|Organism:Severe      CAATCTGACATAGAAGTTACTGGCGATAGTTGTAATAACTATATGCTCACCTATAACAAA   8302
gb:MT019531|Organism:Severe      CAATCTGACATAGAAGTTACTGGCGATAGTTGTAATAACTATATGCTCACCTATAACAAA   8302
gb:MN996528|Organism:Severe      CAATCTGACATAGAAGTTACTGGCGATAGTTGTAATAACTATATGCTCACCTATAACAAA   8302
gb:MN996530|Organism:Severe      CAATCTGACATAGAAGTTACTGGCGATAGTTGTAATAACTATATGCTCACCTATAACAAA   8288
gb:MN908947|Organism:Severe      CAATCTGACATAGAAGTTACTGGCGATAGTTGTAATAACTATATGCTCACCTATAACAAA   8302
gb:MT019532|Organism:Severe      CAATCTGACATAGAAGTTACTGGCGATAGTTGTAATAACTATATGCTCACCTATAACAAA   8302
                                 ************************************************************

gb:MT020781|Organism:Severe      GTTGAAAACATGACACCCCGTGACCTTGGTGCTTGTATTGACTGTAGTGCGCGTCATATT   8350
gb:MT007544|Organism:Severe      GTTGAAAACATGACACCCCGTGACCTTGGTGCTTGTATTGACTGTAGTGCGCGTCATATT   8362
gb:MN994467|Organism:Severe      GTTGAAAACATGACACCCCGTGACCTTGGTGCTTGTATTGACTGTAGTGCGCGTCATATT   8362
gb:MT044257|Organism:Severe      GTTGAAAACATGACACCCCGTGACCTTGGTGCTTGTATTGACTGTAGTGCGCGTCATATT   8362
gb:MT106054|Organism:Severe      GTTGAAAACATGACACCCCGTGACCTTGGTGCTTGTATTGACTGTAGTGCGCGTCATATT   8362
gb:MT049951|Organism:Severe      GTTGAAAACATGACACCCCGTGACCTTGGTGCTTGTATTGACTGTAGTGCGCGTCATATT   8362
gb:MN975262|Organism:Severe      GTTGAAAACATGACACCCCGTGACCTTGGTGCTTGTATTGACTGTAGTGCGCGTCATATT   8362
gb:MT106052|Organism:Severe      GTTGAAAACATGACACCCCGTGACCTTGGTGCTTGTATTGACTGTAGTGCGCGTCATATT   8362
gb:LC522975|Organism:Severe      GTTGAAAACATGACACCCCGTGACCTTGGTGCTTGTATTGACTGTAGTGCGCGTCATATT   8359
gb:LC522973|Organism:Severe      GTTGAAAACATGACACCCCGTGACCTTGGTGCTTGTATTGACTGTAGTGCGCGTCATATT   8359
gb:LC522974|Organism:Severe      GTTGAAAACATGACACCCCGTGACCTTGGTGCTTGTATTGACTGTAGTGCGCGTCATATT   8359
gb:MN985325|Organism:Severe      GTTGAAAACATGACACCCCGTGACCTTGGTGCTTGTATTGACTGTAGTGCGCGTCATATT   8362
gb:MT020881|Organism:Severe      GTTGAAAACATGACACCCCGTGACCTTGGTGCTTGTATTGACTGTAGTGCGCGTCATATT   8362
gb:MT020880|Organism:Severe      GTTGAAAACATGACACCCCGTGACCTTGGTGCTTGTATTGACTGTAGTGCGCGTCATATT   8362
gb:MT066175|Organism:Severe      GTTGAAAACATGACACCCCGTGACCTTGGTGCTTGTATTGACTGTAGTGCGCGTCATATT   8362
gb:MN997409|Organism:Severe      GTTGAAAACATGACACCCCGTGACCTTGGTGCTTGTATTGACTGTAGTGCGCGTCATATT   8362
gb:MN938384|Organism:Severe      GTTGAAAACATGACACCCCGTGACCTTGGTGCTTGTATTGACTGTAGTGCGCGTCATATT   8330
gb:MT044258|Organism:Severe      GTTGAAAACATGACACCCCGTGACCTTGGTGCTTGTATTGACTGTAGTGCGCGTCATATT   8338
gb:MT039890|Organism:Severe      GTTGAAAACATGACACCCCGTGACCTTGGTGCTTGTATTGACTGTAGTGCGCGTCATATT   8362
gb:MN988713|Organism:Severe      GTTGAAAACATGACACCCCGTGACCTTGGTGCTTGTATTGACTGTAGTGCGCGTCATATT   8362
gb:LC521925|Organism:Severe      GTTGAAAACATGACACCCCGTGACCTTGGTGCTTGTATTGACTGTAGTGCGCGTCATATT   8335
gb:MT093571|Organism:Severe      GTTGAAAACATGACACCCCGTGACCTTGGTGCTTGTATTGACTGTAGTGCGCGTCATATT   8362
gb:MT039887|Organism:Severe      GTTGAAAACATGACACCCCGTGACCTTGGTGCTTGTATTGACTGTAGTGCGCGTCATATT   8362
gb:MT019530|Organism:Severe      GTTGAAAACATGACACCCCGTGACCTTGGTGCTTGTATTGACTGTAGTGCGCGTCATATT   8362
gb:MT039888|Organism:Severe      GTTGAAAACATGACACCCCGTGACCTTGGTGCTTGTATTGACTGTAGTGCGCGTCATATT   8362
gb:LC522972|Organism:Severe      GTTGAAAACATGACACCCCGTGACCTTGGTGCTTGTATTGACTGTAGTGCGCGTCATATT   8359
gb:MT027063|Organism:Severe      GTTGAAAACATGACACCCCGTGACCTTGGTGCTTGTATTGACTGTAGTGCGCGTCATATT   8362
gb:MT027062|Organism:Severe      GTTGAAAACATGACACCCCGTGACCTTGGTGCTTGTATTGACTGTAGTGCGCGTCATATT   8362
gb:MT019529|Organism:Severe      GTTGAAAACATGACACCCCGTGACCTTGGTGCTTGTATTGACTGTAGTGCGCGTCATATT   8362
gb:MN996529|Organism:Severe      GTTGAAAACATGACACCCCGTGACCTTGGTGCTTGTATTGACTGTAGTGCGCGTCATATT   8350
gb:MN996531|Organism:Severe      GTTGAAAACATGACACCCCGTGACCTTGGTGCTTGTATTGACTGTAGTGCGCGTCATATT   8349
gb:MT066176|Organism:Severe      GTTGAAAACATGACACCCCGTGACCTTGGTGCTTGTATTGACTGTAGTGCGCGTCATATT   8362
gb:MT027064|Organism:Severe      GTTGAAAACATGACACCCCGTGACCTTGGTGCTTGTATTGACTGTAGTGCGCGTCATATT   8362
gb:MN994468|Organism:Severe      GTTGAAAACATGACACCCCGTGACCTTGGTGCTTGTATTGACTGTAGTGCGCGTCATATT   8362
gb:MT072688|Organism:Severe      GTTGAAAACATGACACCCCGTGACCTTGGTGCTTGTATTGACTGTAGTGCGCGTCATATT   8347
gb:MN996527|Organism:Severe      GTTGAAAACATGACACCCCGTGACCTTGGTGCTTGTATTGACTGTAGTGCGCGTCATATT   8329
gb:MT093631|Organism:Severe      GTTGAAAACATGACACCCCGTGACCTTGGTGCTTGTATTGACTGTAGTGCGCGTCATATT   8400
```

```
gb:MT106053|Organism:Severe      GTTGAAAACATGACACCCCGTGACCTTGGTGCTTGTATTGACTGTAGTGCGCGTCATATT   8362
gb:MT019533|Organism:Severe      GTTGAAAACATGACACCCCGTGACCTTGGTGCTTGTATTGACTGTAGTGCGCGTCATATT   8362
gb:MT019531|Organism:Severe      GTTGAAAACATGACACCCCGTGACCTTGGTGCTTGTATTGACTGTAGTGCGCGTCATATT   8362
gb:MN996528|Organism:Severe      GTTGAAAACATGACACCCCGTGACCTTGGTGCTTGTATTGACTGTAGTGCGCGTCATATT   8362
gb:MN996530|Organism:Severe      GTTGAAAACATGACACCCCGTGACCTTGGTGCTTGTATTGACTGTAGTGCGCGTCATATT   8348
gb:MN908947|Organism:Severe      GTTGAAAACATGACACCCCGTGACCTTGGTGCTTGTATTGACTGTAGTGCGCGTCATATT   8362
gb:MT019532|Organism:Severe      GTTGAAAACATGACACCCCGTGACCTTGGTGCTTGTATTGACTGTAGTGCGCGTCATATT   8362
                                 ************************************************************

gb:MT020781|Organism:Severe      AATGCGCAGGTAGCAAAAAGTCACAACATTGCTTTGATATGGAACGTTAAAGATTTCATG   8410
gb:MT007544|Organism:Severe      AATGCGCAGGTAGCAAAAAGTCACAACATTGCTTTGATATGGAACGTTAAAGATTTCATG   8422
gb:MN994467|Organism:Severe      AATGCGCAGGTAGCAAAAAGTCACAACATTGCTTTGATATGGAACGTTAAAGATTTCATG   8422
gb:MT044257|Organism:Severe      AATGCGCAGGTAGCAAAAAGTCACAACATTGCTTTGATATGGAACGTTAAAGATTTCATG   8422
gb:MT106054|Organism:Severe      AATGCGCAGGTAGCAAAAAGTCACAACATTGCTTTGATATGGAACGTTAAAGATTTCATG   8422
gb:MT049951|Organism:Severe      AATGCGCAGGTAGCAAAAAGTCACAACATTGCTTTGATATGGAACGTTAAAGATTTCATG   8422
gb:MN975262|Organism:Severe      AATGCGCAGGTAGCAAAAAGTCACAACATTGCTTTGATATGGAACGTTAAAGATTTCATG   8422
gb:MT106052|Organism:Severe      AATGCGCAGGTAGCAAAAAGTCACAACATTGCTTTGATATGGAACGTTAAAGATTTCATG   8422
gb:LC522975|Organism:Severe      AATGCGCAGGTAGCAAAAAGTCACAACATTGCTTTGATATGGAACGTTAAAGATTTCATG   8419
gb:LC522973|Organism:Severe      AATGCGCAGGTAGCAAAAAGTCACAACATTGCTTTGATATGGAACGTTAAAGATTTCATG   8419
gb:LC522974|Organism:Severe      AATGCGCAGGTAGCAAAAAGTCACAACATTGCTTTGATATGGAACGTTAAAGATTTCATG   8419
gb:MN985325|Organism:Severe      AATGCGCAGGTAGCAAAAAGTCACAACATTGCTTTGATATGGAACGTTAAAGATTTCATG   8422
gb:MT020881|Organism:Severe      AATGCGCAGGTAGCAAAAAGTCACAACATTGCTTTGATATGGAACGTTAAAGATTTCATG   8422
gb:MT020880|Organism:Severe      AATGCGCAGGTAGCAAAAAGTCACAACATTGCTTTGATATGGAACGTTAAAGATTTCATG   8422
gb:MT066175|Organism:Severe      AATGCGCAGGTAGCAAAAAGTCACAACATTGCTTTGATATGGAACGTTAAAGATTTCATG   8422
gb:MN997409|Organism:Severe      AATGCGCAGGTAGCAAAAAGTCACAACATTGCTTTGATATGGAACGTTAAAGATTTCATG   8422
gb:MN938384|Organism:Severe      AATGCGCAGGTAGCAAAAAGTCACAACATTGCTTTGATATGGAACGTTAAAGATTTCATG   8390
gb:MT044258|Organism:Severe      AATGCGCAGGTAGCAAAAAGTCACAACATTGCTTTGATATGGAACGTTAAAGATTTCATG   8398
gb:MT039890|Organism:Severe      AATGCGCAGGTAGCAAAAAGTCACAACATTGCTTTGATATGGAACGTTAAAGATTTCATG   8422
gb:MN988713|Organism:Severe      AATGCGCAGGTAGCAAAAAGTCACAACATTGCTTTGATATGGAACGTTAAAGATTTCATG   8422
gb:LC521925|Organism:Severe      AATGCGCAGGTAGCAAAAAGTCACAACATTGCTTTGATATGGAACGTTAAAGATTTCATG   8395
gb:MT093571|Organism:Severe      AATGCGCAGGTAGCAAAAAGTCACAACATTGCTTTGATATGGAACGTTAAAGATTTCATG   8422
gb:MT039887|Organism:Severe      AATGCGCAGGTAGCAAAAAGTCACAACATTGCTTTGATATGGAACGTTAAAGATTTCATG   8422
gb:MT019530|Organism:Severe      AATGCGCAGGTAGCAAAAAGTCACAACATTGCTTTGATATGGAACGTTAAAGATTTCATG   8422
gb:MT039888|Organism:Severe      AATGCGCAGGTAGCAAAAAGTCACAACATTGCTTTGATATGGAACGTTAAAGATTTCATG   8422
gb:LC522972|Organism:Severe      AATGCGCAGGTAGCAAAAAGTCACAACATTGCTTTGATATGGAACGTTAAAGATTTCATG   8419
gb:MT027063|Organism:Severe      AATGCGCAGGTAGCAAAAAGTCACAACATTGCTTTGATATGGAACGTTAAAGATTTCATG   8422
gb:MT027062|Organism:Severe      AATGCGCAGGTAGCAAAAAGTCACAACATTGCTTTGATATGGAACGTTAAAGATTTCATG   8422
gb:MT019529|Organism:Severe      AATGCGCAGGTAGCAAAAAGTCACAGCATTGCTTTGATATGGAACGTTAAAGATTTCATG   8422
gb:MN996529|Organism:Severe      AATGCGCAGGTAGCAAAAAGTCACAACATTGCTTTGATATGGAACGTTAAAGATTTCATG   8410
gb:MN996531|Organism:Severe      AATGCGCAGGTAGCAAAAAGTCACAACATTGCTTTGATATGGAACGTTAAAGATTTCATG   8409
gb:MT066176|Organism:Severe      AATGCGCAGGTAGCAAAAAGTCACAACATTGCTTTGATATGGAACGTTAAAGATTTCATG   8422
gb:MT027064|Organism:Severe      AATGCGCAGGTAGCAAAAAGTCACAACATTGCTTTGATATGGAACGTTAAAGATTTCATG   8422
gb:MN994468|Organism:Severe      AATGCGCAGGTAGCAAAAAGTCACAACATTGCTTTGATATGGAACGTTAAAGATTTCATG   8422
gb:MT072688|Organism:Severe      AATGCGCAGGTAGCAAAAAGTCACAACATTGCTTTGATATGGAACGTTAAAGATTTCATG   8407
gb:MN996527|Organism:Severe      AATGCGCAGGTAGCAAAAAGTCACAACATTGCTTTGATATGGAACGTTAAAGATTTCATG   8389
gb:MT093631|Organism:Severe      AATGCGCAGGTAGCAAAAAGTCACAACATTGCTTTGATATGGAACGTTAAAGATTTCATG   8460
gb:MT106053|Organism:Severe      AATGCGCAGGTAGCAAAAAGTCACAACATTGCTTTGATATGGAACGTTAAAGATTTCATG   8422
gb:MT019533|Organism:Severe      AATGCGCAGGTAGCAAAAAGTCACAACATTGCTTTGATATGGAACGTTAAAGATTTCATG   8422
gb:MT019531|Organism:Severe      AATGCGCAGGTAGCAAAAAGTCACAACATTGCTTTGATATGGAACGTTAAAGATTTCATG   8422
gb:MN996528|Organism:Severe      AATGCGCAGGTAGCAAAAAGTCACAACATTGCTTTGATATGGAACGTTAAAGATTTCATG   8422
gb:MN996530|Organism:Severe      AATGCGCAGGTAGCAAAAAGTCACAACATTGCTTTGATATGGAACGTTAAAGATTTCATG   8408
gb:MN908947|Organism:Severe      AATGCGCAGGTAGCAAAAAGTCACAACATTGCTTTGATATGGAACGTTAAAGATTTCATG   8422
gb:MT019532|Organism:Severe      AATGCGCAGGTAGCAAAAAGTCACAACATTGCTTTGATATGGAACGTTAAAGATTTCATG   8422
                                 ************************* **********************************

gb:MT020781|Organism:Severe      TCATTGTCTGAACAACTACGAAAACAAATACGTAGTGCTGCTAAAAAGAATAACTTACCT   8470
gb:MT007544|Organism:Severe      TCATTGTCTGAACAACTACGAAAACAAATACGTAGTGCTGCTAAAAAGAATAACTTACCT   8482
gb:MN994467|Organism:Severe      TCATTGTCTGAACAACTACGAAAACAAATACGTAGTGCTGCTAAAAAGAATAACTTACCT   8482
gb:MT044257|Organism:Severe      TCATTGTCTGAACAACTACGAAAACAAATACGTAGTGCTGCTAAAAAGAATAACTTACCT   8482
gb:MT106054|Organism:Severe      TCATTGTCTGAACAACTACGAAAACAAATACGTAGTGCTGCTAAAAAGAATAACTTACCT   8482
gb:MT049951|Organism:Severe      TCATTGTCTGAACAACTACGAAAACAAATACGTAGTGCTGCTAAAAAGAATAACTTACCT   8482
gb:MN975262|Organism:Severe      TCATTGTCTGAACAACTACGAAAACAAATACGTAGTGCTGCTAAAAAGAATAACTTACCT   8482
gb:MT106052|Organism:Severe      TCATTGTCTGAACAACTACGAAAACAAATACGTAGTGCTGCTAAAAAGAATAACTTACCT   8482
gb:LC522975|Organism:Severe      TCATTGTCTGAACAACTACGAAAACAAATACGTAGTGCTGCTAAAAAGAATAACTTACCT   8479
gb:LC522973|Organism:Severe      TCATTGTCTGAACAACTACGAAAACAAATACGTAGTGCTGCTAAAAAGAATAACTTACCT   8479
```

```
gb:LC522974|Organism:Severe      TCATTGTCTGAACAACTACGAAAACAAATACGTAGTGCTGCTAAAAAGAATAACTTACCT   8479
gb:MN985325|Organism:Severe      TCATTGTCTGAACAACTACGAAAACAAATACGTAGTGCTGCTAAAAAGAATAACTTACCT   8482
gb:MT020881|Organism:Severe      TCATTGTCTGAACAACTACGAAAACAAATACGTAGTGCTGCTAAAAAGAATAACTTACCT   8482
gb:MT020880|Organism:Severe      TCATTGTCTGAACAACTACGAAAACAAATACGTAGTGCTGCTAAAAAGAATAACTTACCT   8482
gb:MT066175|Organism:Severe      TCATTGTCTGAACAACTACGAAAACAAATACGTAGTGCTGCTAAAAAGAATAACTTACCT   8482
gb:MN997409|Organism:Severe      TCATTGTCTGAACAACTACGAAAACAAATACGTAGTGCTGCTAAAAAGAATAACTTACCT   8482
gb:MN938384|Organism:Severe      TCATTGTCTGAACAACTACGAAAACAAATACGTAGTGCTGCTAAAAAGAATAACTTACCT   8450
gb:MT044258|Organism:Severe      TCATTGTCTGAACAACTACGAAAACAAATACGTAGTGCTGCTAAAAAGAATAACTTACCT   8458
gb:MT039890|Organism:Severe      TCATTGTCTGAACAACTACGAAAACAAATACGTAGTGCTGCTAAAAAGAATAACTTACCT   8482
gb:MN988713|Organism:Severe      TCATTGTCTGAACAACTACGAAAACAAATACGTAGTGCTGCTAAAAAGAATAACTTACCT   8482
gb:LC521925|Organism:Severe      TCATTGTCTGAACAACTACGAAAACAAATACGTAGTGCTGCTAAAAAGAATAACTTACCT   8455
gb:MT093571|Organism:Severe      TCATTGTCTGAACAACTACGAAAACAAATACGTAGTGCTGCTAAAAAGAATAACTTACCT   8482
gb:MT039887|Organism:Severe      TCATTGTCTGAACAACTACGAAAACAAATACGTAGTGCTGCTAAAAAGAATAACTTACCT   8482
gb:MT019530|Organism:Severe      TCATTGTCTGAACAACTACGAAAACAAATACGTAGTGCTGCTAAAAAGAATAACTTACCT   8482
gb:MT039888|Organism:Severe      TCATTGTCTGAACAACTACGAAAACAAATACGTAGTGCTGCTAAAAAGAATAACTTACCT   8482
gb:LC522972|Organism:Severe      TCATTGTCTGAACAACTACGAAAACAAATACGTAGTGCTGCTAAAAAGAATAACTTACCT   8479
gb:MT027063|Organism:Severe      TCATTGTCTGAACAACTACGAAAACAAATACGTAGTGCTGCTAAAAAGAATAACTTACCT   8482
gb:MT027062|Organism:Severe      TCATTGTCTGAACAACTACGAAAACAAATACGTAGTGCTGCTAAAAAGAATAACTTACCT   8482
gb:MT019529|Organism:Severe      TCATTGTCTGAACAACTACGAAAACAAATACGTAGTGCTGCTAAAAAGAATAACTTACCT   8482
gb:MN996529|Organism:Severe      TCATTGTCTGAACAACTACGAAAACAAATACGTAGTGCTGCTAAAAAGAATAACTTACCT   8470
gb:MN996531|Organism:Severe      TCATTGTCTGAACAACTACGAAAACAAATACGTAGTGCTGCTAAAAAGAATAACTTACCT   8469
gb:MT066176|Organism:Severe      TCATTGTCTGAACAACTACGAAAACAAATACGTAGTGCTGCTAAAAAGAATAACTTACCT   8482
gb:MT027064|Organism:Severe      TCATTGTCTGAACAACTACGAAAACAAATACGTAGTGCTGCTAAAAAGAATAACTTACCT   8482
gb:MN994468|Organism:Severe      TCATTGTCTGAACAACTACGAAAACAAATACGTAGTGCTGCTAAAAAGAATAACTTACCT   8482
gb:MT072688|Organism:Severe      TCATTGTCTGAACAACTACGAAAACAAATACGTAGTGCTGCTAAAAAGAATAACTTACCT   8467
gb:MN996527|Organism:Severe      TCATTGTCTGAACAACTACGAAAACAAATACGTAGTGCTGCTAAAAAGAATAACTTACCT   8449
gb:MT093631|Organism:Severe      TCATTGTCTGAACAACTACGAAAACAAATACGTAGTGCTGCTAAAAAGAATAACTTACCT   8520
gb:MT106053|Organism:Severe      TCATTGTCTGAACAACTACGAAAACAAATACGTAGTGCTGCTAAAAAGAATAACTTACCT   8482
gb:MT019533|Organism:Severe      TCATTGTCTGAACAACTACGAAAACAAATACGTAGTGCTGCTAAAAAGAATAACTTACCT   8482
gb:MT019531|Organism:Severe      TCATTGTCTGAACAACTACGAAAACAAATACGTAGTGCTGCTAAAAAGAATAACTTACCT   8482
gb:MN996528|Organism:Severe      TCATTGTCTGAACAACTACGAAAACAAATACGTAGTGCTGCTAAAAAGAATAACTTACCT   8482
gb:MN996530|Organism:Severe      TCATTGTCTGAACAACTACGAAAACAAATACGTAGTGCTGCTAAAAAGAATAACTTACCT   8468
gb:MN908947|Organism:Severe      TCATTGTCTGAACAACTACGAAAACAAATACGTAGTGCTGCTAAAAAGAATAACTTACCT   8482
gb:MT019532|Organism:Severe      TCATTGTCTGAACAACTACGAAAACAAATACGTAGTGCTGCTAAAAAGAATAACTTACCT   8482
                                 ************************************************************

gb:MT020781|Organism:Severe      TTTAAGTTGACATGTGCAACTACTAGACAAGTTGTTAATGTTGTAACAACAAAGATAGCA   8530
gb:MT007544|Organism:Severe      TTTAAGTTGACATGTGCAACTACTAGACAAGTTGTTAATGTTGTAACAACAAAGATAGCA   8542
gb:MN994467|Organism:Severe      TTTAAGTTGACATGTGCAACTACTAGACAAGTTGTTAATGTTGTAACAACAAAGATAGCA   8542
gb:MT044257|Organism:Severe      TTTAAGTTGACATGTGCAACTACTAGACAAGTTGTTAATGTTGTAACAACAAAGATAGCA   8542
gb:MT106054|Organism:Severe      TTTAAGTTGACATGTGCAACTACTAGACAAGTTGTTAATGTTGTAACAACAAAGATAGCA   8542
gb:MT049951|Organism:Severe      TTTAAGTTGACATGTGCAACTACTAGACAAGTTGTTAATGTTGTAACAACAAAGATAGCA   8542
gb:MN975262|Organism:Severe      TTTAAGTTGACATGTGCAACTACTAGACAAGTTGTTAATGTTGTAACAACAAAGATAGCA   8542
gb:MT106052|Organism:Severe      TTTAAGTTGACATGTGCAACTACTAGACAAGTTGTTAATGTTGTAACAACAAAGATAGCA   8542
gb:LC522975|Organism:Severe      TTTAAGTTGACATGTGCAACTACTAGACAAGTTGTTAATGTTGTAACAACAAAGATAGCA   8539
gb:LC522973|Organism:Severe      TTTAAGTTGACATGTGCAACTACTAGACAAGTTGTTAATGTTGTAACAACAAAGATAGCA   8539
gb:LC522974|Organism:Severe      TTTAAGTTGACATGTGCAACTACTAGACAAGTTGTTAATGTTGTAACAACAAAGATAGCA   8539
gb:MN985325|Organism:Severe      TTTAAGTTGACATGTGCAACTACTAGACAAGTTGTTAATGTTGTAACAACAAAGATAGCA   8542
gb:MT020881|Organism:Severe      TTTAAGTTGACATGTGCAACTACTAGACAAGTTGTTAATGTTGTAACAACAAAGATAGCA   8542
gb:MT020880|Organism:Severe      TTTAAGTTGACATGTGCAACTACTAGACAAGTTGTTAATGTTGTAACAACAAAGATAGCA   8542
gb:MT066175|Organism:Severe      TTTAAGTTGACATGTGCAACTACTAGACAAGTTGTTAATGTTGTAACAACAAAGATAGCA   8542
gb:MN997409|Organism:Severe      TTTAAGTTGACATGTGCAACTACTAGACAAGTTGTTAATGTTGTAACAACAAAGATAGCA   8542
gb:MN938384|Organism:Severe      TTTAAGTTGACATGTGCAACTACTAGACAAGTTGTTAATGTTGTAACAACAAAGATAGCA   8510
gb:MT044258|Organism:Severe      TTTAAGTTGACATGTGCAACTACTAGACAAGTTGTTAATGTTGTAACAACAAAGATAGCA   8518
gb:MT039890|Organism:Severe      TTTAAGTTGACATGTGCAACTACTAGACAAGTTGTTAATGTTGTAACAACAAAGATAGCA   8542
gb:MN988713|Organism:Severe      TTTAAGTTGACATGTGCAACTACTAGACAAGTTGTTAATGTTGTAACAACAAAGATAGCA   8542
gb:LC521925|Organism:Severe      TTTAAGTTGACATGTGCAACTACTAGACAAGTTGTTAATGTTGTAACAACAAAGATAGCA   8515
gb:MT093571|Organism:Severe      TTTAAGTTGACATGTGCAACTACTAGACAAGTTGTTAATGTTGTAACAACAAAGATAGCA   8542
gb:MT039887|Organism:Severe      TTTAAGTTGACATGTGCAACTACTAGACAAGTTGTTAATGTTGTAACAACAAAGATAGCA   8542
gb:MT019530|Organism:Severe      TTTAAGTTGACATGTGCAACTACTAGACAAGTTGTTAATGTTGTAACAACAAAGATAGCA   8542
gb:MT039888|Organism:Severe      TTTAAGTTGACATGTGCAACTACTAGACAAGTTGTTAATGTTGTAACAACAAAGATAGCA   8542
gb:LC522972|Organism:Severe      TTTAAGTTGACATGTGCAACTACTAGACAAGTTGTTAATGTTGTAACAACAAAGATAGCA   8539
gb:MT027063|Organism:Severe      TTTAAGTTGACATGTGCAACTACTAGACAAGTTGTTAATGTTGTAACAACAAAGATAGCA   8542
gb:MT027062|Organism:Severe      TTTAAGTTGACATGTGCAACTACTAGACAAGTTGTTAATGTTGTAACAACAAAGATAGCA   8542
gb:MT019529|Organism:Severe      TTTAAGTTGACATGTGCAACTACTAGACAAGTTGTTAATGTTGTAACAACAAAGATAGCA   8542
```

```
gb:MN996529|Organism:Severe      TTTAAGTTGACATGTGCAACTACTAGACAAGTTGTTAATGTTGTAACAACAAAGATAGCA   8530
gb:MN996531|Organism:Severe      TTTAAGTTGACATGTGCAACTACTAGACAAGTTGTTAATGTTGTAACAACAAAGATAGCA   8529
gb:MT066176|Organism:Severe      TTTAAGTTGACATGTGCAACTACTAGACAAGTTGTTAATGTTGTAACAACAAAGATAGCA   8542
gb:MT027064|Organism:Severe      TTTAAGTTGACATGTGCAACTACTAGACAAGTTGTTAATGTTGTAACAACAAAGATAGCA   8542
gb:MN994468|Organism:Severe      TTTAAGTTGACATGTGCAACTACTAGACAAGTTGTTAATGTTGTAACAACAAAGATAGCA   8542
gb:MT072688|Organism:Severe      TTTAAGTTGACATGTGCAACTACTAGACAAGTTGTTAATGTTGTAACAACAAAGATAGCA   8527
gb:MN996527|Organism:Severe      TTTAAGTTGACATGTGCAACTACTAGACAAGTTGTTAATGTTGTAACAACAAAGATAGCA   8509
gb:MT093631|Organism:Severe      TTTAAGTTGACATGTGCAACTACTAGACAAGTTGTTAATGTTGTAACAACAAAGATAGCA   8580
gb:MT106053|Organism:Severe      TTTAAGTTGACATGTGCAACTACTAGACAAGTTGTTAATGTTGTAACAACAAAGATAGCA   8542
gb:MT019533|Organism:Severe      TTTAAGTTGACATGTGCAACTACTAGACAAGTTGTTAATGTTGTAACAACAAAGATAGCA   8542
gb:MT019531|Organism:Severe      TTTAAGTTGACATGTGCAACTACTAGACAAGTTGTTAATGTTGTAACAACAAAGATAGCA   8542
gb:MN996528|Organism:Severe      TTTAAGTTGACATGTGCAACTACTAGACAAGTTGTTAATGTTGTAACAACAAAGATAGCA   8542
gb:MN996530|Organism:Severe      TTTAAGTTGACATGTGCAACTACTAGACAAGTTGTTAATGTTGTAACAACAAAGATAGCA   8528
gb:MN908947|Organism:Severe      TTTAAGTTGACATGTGCAACTACTAGACAAGTTGTTAATGTTGTAACAACAAAGATAGCA   8542
gb:MT019532|Organism:Severe      TTTAAGTTGACATGTGCAACTACTAGACAAGTTGTTAATGTTGTAACAACAAAGATAGCA   8542
                                 ************************************************************

gb:MT020781|Organism:Severe      CTTAAGGGTGGTAAAATTGTTAATAATTGGTTGAAGCAGTTAATTAAAGTTACACTTGTG   8590
gb:MT007544|Organism:Severe      CTTAAGGGTGGTAAAATTGTTAATAATTGGTTGAAGCAGTTAATTAAAGTTACACTTGTG   8602
gb:MN994467|Organism:Severe      CTTAAGGGTGGTAAAATTGTTAATAATTGGTTGAAGCAGTTAATTAAAGTTACACTTGTG   8602
gb:MT044257|Organism:Severe      CTTAAGGGTGGTAAAATTGTTAATAATTGGTTGAAGCAGTTAATTAAAGTTACACTTGTG   8602
gb:MT106054|Organism:Severe      CTTAAGGGTGGTAAAATTGTTAATAATTGGTTGAAGCAGTTAATTAAAGTTACACTTGTG   8602
gb:MT049951|Organism:Severe      CTTAAGGGTGGTAAAATTGTTAATAATTGGTTGAAGCAGTTAATTAAAGTTACACTTGTG   8602
gb:MN975262|Organism:Severe      CTTAAGGGTGGTAAAATTGTTAATAATTGGTTGAAGCAGTTAATTAAAGTTACACTTGTG   8602
gb:MT106052|Organism:Severe      CTTAAGGGTGGTAAAATTGTTAATAATTGGTTGAAGCAGTTAATTAAAGTTACACTTGTG   8602
gb:LC522975|Organism:Severe      CTTAAGGGTGGTAAAATTGTTAATAATTGGTTGAAGCAGTTAATTAAAGTTACACTTGTG   8599
gb:LC522973|Organism:Severe      CTTAAGGGTGGTAAAATTGTTAATAATTGGTTGAAGCAGTTAATTAAAGTTACACTTGTG   8599
gb:LC522974|Organism:Severe      CTTAAGGGTGGTAAAATTGTTAATAATTGGTTGAAGCAGTTAATTAAAGTTACACTTGTG   8599
gb:MN985325|Organism:Severe      CTTAAGGGTGGTAAAATTGTTAATAATTGGTTGAAGCAGTTAATTAAAGTTACACTTGTG   8602
gb:MT020881|Organism:Severe      CTTAAGGGTGGTAAAATTGTTAATAATTGGTTGAAGCAGTTAATTAAAGTTACACTTGTG   8602
gb:MT020880|Organism:Severe      CTTAAGGGTGGTAAAATTGTTAATAATTGGTTGAAGCAGTTAATTAAAGTTACACTTGTG   8602
gb:MT066175|Organism:Severe      CTTAAGGGTGGTAAAATTGTTAATAATTGGTTGAAGCAGTTAATTAAAGTTACACTTGTG   8602
gb:MN997409|Organism:Severe      CTTAAGGGTGGTAAAATTGTTAATAATTGGTTGAAGCAGTTAATTAAAGTTACACTTGTG   8602
gb:MN938384|Organism:Severe      CTTAAGGGTGGTAAAATTGTTAATAATTGGTTGAAGCAGTTAATTAAAGTTACACTTGTG   8570
gb:MT044258|Organism:Severe      CTTAAGGGTGGTAAAATTGTTAATAATTGGTTGAAGCAGTTAATTAAAGTTACACTTGTG   8578
gb:MT039890|Organism:Severe      CTTAAGGGTGGTAAAATTGTTAATAATTGGTTGAAGCAGTTAATTAAAGTTACACTTGTG   8602
gb:MN988713|Organism:Severe      CTTAAGGGTGGTAAAATTGTTAATAATTGGTTGAAGCAGTTAATTAAAGTTACACTTGTG   8602
gb:LC521925|Organism:Severe      CTTAAGGGTGGTAAAATTGTTAATAATTGGTTGAAGCAGTTAATTAAAGTTACACTTGTG   8575
gb:MT093571|Organism:Severe      CTTAAGGGTGGTAAAATTGTTAATAATTGGTTGAAGCAGTTAATTAAAGTTACACTTGTG   8602
gb:MT039887|Organism:Severe      CTTAAGGGTGGTAAAATTGTTAATAATTGGTTGAAGCAGTTAATTAAAGTTACACTTGTG   8602
gb:MT019530|Organism:Severe      CTTAAGGGTGGTAAAATTGTTAATAATTGGTTGAAGCAGTTAATTAAAGTTACACTTGTG   8602
gb:MT039888|Organism:Severe      CTTAAGGGTGGTAAAATTGTTAATAATTGGTTGAAGCAGTTAATTAAAGTTACACTTGTG   8602
gb:LC522972|Organism:Severe      CTTAAGGGTGGTAAAATTGTTAATAATTGGTTGAAGCAGTTAATTAAAGTTACACTTGTG   8599
gb:MT027063|Organism:Severe      CTTAAGGGTGGTAAAATTGTTAATAATTGGTTGAAGCAGTTAATTAAAGTTACACTTGTG   8602
gb:MT027062|Organism:Severe      CTTAAGGGTGGTAAAATTGTTAATAATTGGTTGAAGCAGTTAATTAAAGTTACACTTGTG   8602
gb:MT019529|Organism:Severe      CTTAAGGGTGGTAAAATTGTTAATAATTGGTTGAAGCAGTTAATTAAAGTTACACTTGTG   8602
gb:MN996529|Organism:Severe      CTTAAGGGTGGTAAAATTGTTAATAATTGGTTGAAGCAGTTAATTAAAGTTACACTTGTG   8590
gb:MN996531|Organism:Severe      CTTAAGGGTGGTAAAATTGTTAATAATTGGTTGAAGCAGTTAATTAAAGTTACACTTGTG   8589
gb:MT066176|Organism:Severe      CTTAAGGGTGGTAAAATTGTTAATAATTGGTTGAAGCAGTTAATTAAAGTTACACTTGTG   8602
gb:MT027064|Organism:Severe      CTTAAGGGTGGTAAAATTGTTAATAATTGGTTGAAGCAGTTAATTAAAGTTACACTTGTG   8602
gb:MN994468|Organism:Severe      CTTAAGGGTGGTAAAATTGTTAATAATTGGTTGAAGCAGTTAATTAAAGTTACACTTGTG   8602
gb:MT072688|Organism:Severe      CTTAAGGGTGGTAAAATTGTTAATAATTGGTTGAAGCAGTTAATTAAAGTTACACTTGTG   8587
gb:MN996527|Organism:Severe      CTTAAGGGTGGTAAAATTGTTAATAATTGGTTGAAGCAGTTAATTAAAGTTACACTTGTG   8569
gb:MT093631|Organism:Severe      CTTAAGGGTGGTAAAATTGTTAATAATTGGTTGAAGCAGTTAATTAAAGTTACACTTGTG   8640
gb:MT106053|Organism:Severe      CTTAAGGGTGGTAAAATTGTTAATAATTGGTTGAAGCAGTTAATTAAAGTTACACTTGTG   8602
gb:MT019533|Organism:Severe      CTTAAGGGTGGTAAAATTGTTAATAATTGGTTGAAGCAGTTAATTAAAGTTACACTTGTG   8602
gb:MT019531|Organism:Severe      CTTAAGGGTGGTAAAATTGTTAATAATTGGTTGAAGCAGTTAATTAAAGTTACACTTGTG   8602
gb:MN996528|Organism:Severe      CTTAAGGGTGGTAAAATTGTTAATAATTGGTTGAAGCAGTTAATTAAAGTTACACTTGTG   8602
gb:MN996530|Organism:Severe      CTTAAGGGTGGTAAAATTGTTAATAATTGGTTGAAGCAGTTAATTAAAGTTACACTTGTG   8588
gb:MN908947|Organism:Severe      CTTAAGGGTGGTAAAATTGTTAATAATTGGTTGAAGCAGTTAATTAAAGTTACACTTGTG   8602
gb:MT019532|Organism:Severe      CTTAAGGGTGGTAAAATTGTTAATAATTGGTTGAAGCAGTTAATTAAAGTTACACTTGTG   8602
                                 ************************************************************

gb:MT020781|Organism:Severe      TTCCTTTTTGTTGCTGCTATTTTCTATTTAATAACACCTGTTCATGTCATGTCTAAACAT   8650
gb:MT007544|Organism:Severe      TTCCTTTTTGTTGCTGCTATTTTCTATTTAATAACACCTGTTCATGTCATGTCTAAACAT   8662
```

```
gb:MN994467|Organism:Severe      TTCCTTTTTGTTGCTGCTATTTTCTATTTAATAACACCTGTTCATGTCATGTCTAAACAT    8662
gb:MT044257|Organism:Severe      TTCCTTTTTGTTGCTGCTATTTTCTATTTAATAACACCTGTTCATGTCATGTCTAAACAT    8662
gb:MT106054|Organism:Severe      TTCCTTTTTGTTGCTGCTATTTTCTATTTAATAACACCTGTTCATGTCATGTCTAAACAT    8662
gb:MT049951|Organism:Severe      TTCCTTTTTGTTGCTGCTATTTTCTATTTAATAACACCTGTTCATGTCATGTCTAAACAT    8662
gb:MN975262|Organism:Severe      TTCCTTTTTGTTGCTGCTATTTTCTATTTAATAACACCTGTTCATGTCATGTCTAAACAT    8662
gb:MT106052|Organism:Severe      TTCCTTTTTGTTGCTGCTATTTTCTATTTAATAACACCTGTTCATGTCATGTCTAAACAT    8662
gb:LC522975|Organism:Severe      TTCCTTTTTGTTGCTGCTATTTTCTATTTAATAACACCTGTTCATGTCATGTCTAAACAT    8659
gb:LC522973|Organism:Severe      TTCCTTTTTGTTGCTGCTATTTTCTATTTAATAACACCTGTTCATGTCATGTCTAAACAT    8659
gb:LC522974|Organism:Severe      TTCCTTTTTGTTGCTGCTATTTTCTATTTAATAACACCTGTTCATGTCATGTCTAAACAT    8659
gb:MN985325|Organism:Severe      TTCCTTTTTGTTGCTGCTATTTTCTATTTAATAACACCTGTTCATGTCATGTCTAAACAT    8662
gb:MT020881|Organism:Severe      TTCCTTTTTGTTGCTGCTATTTTCTATTTAATAACACCTGTTCATGTCATGTCTAAACAT    8662
gb:MT020880|Organism:Severe      TTCCTTTTTGTTGCTGCTATTTTCTATTTAATAACACCTGTTCATGTCATGTCTAAACAT    8662
gb:MT066175|Organism:Severe      TTCCTTTTTGTTGCTGCTATTTTCTATTTAATAACACCTGTTCATGTCATGTCTAAACAT    8662
gb:MN997409|Organism:Severe      TTCCTTTTTGTTGCTGCTATTTTCTATTTAATAACACCTGTTCATGTCATGTCTAAACAT    8662
gb:MN938384|Organism:Severe      TTCCTTTTTGTTGCTGCTATTTTCTATTTAATAACACCTGTTCATGTCATGTCTAAACAT    8630
gb:MT044258|Organism:Severe      TTCCTTTTTGTTGCTGCTATTTTCTATTTAATAACACCTGTTCATGTCATGTCTAAACAT    8638
gb:MT039890|Organism:Severe      TTCCTTTTTGTTGCTGCTATTTTCTATTTAATAACACCTGTTCATGTCATGTCTAAACAT    8662
gb:MN988713|Organism:Severe      TTCCTTTTTGTTGCTGCTATTTTCTATTTAATAACACCTGTTCATGTCATGTCTAAACAT    8662
gb:LC521925|Organism:Severe      TTCCTTTTTGTTGCTGCTATTTTCTATTTAATAACACCTGTTCATGTCATGTCTAAACAT    8635
gb:MT093571|Organism:Severe      TTCCTTTTTGTTGCTGCTATTTTCTATTTAATAACACCTGTTCATGTCATGTCTAAACAT    8662
gb:MT039887|Organism:Severe      TTCCTTTTTGTTGCTGCTATTTTCTATTTAATAACACCTGTTCATGTCATGTCTAAACAT    8662
gb:MT019530|Organism:Severe      TTCCTTTTTGTTGCTGCTATTTTCTATTTAATAACACCTGTTCATGTCATGTCTAAACAT    8662
gb:MT039888|Organism:Severe      TTCCTTTTTGTTGCTGCTATTTTCTATTTAATAACACCTGTTCATGTCATGTCTAAACAT    8662
gb:LC522972|Organism:Severe      TTCCTTTTTGTTGCTGCTATTTTCTATTTAATAACACCTGTTCATGTCATGTCTAAACAT    8659
gb:MT027063|Organism:Severe      TTCCTTTTTGTTGCTGCTATTTTCTATTTAATAACACCTGTTCATGTCATGTCTAAACAT    8662
gb:MT027062|Organism:Severe      TTCCTTTTTGTTGCTGCTATTTTCTATTTAATAACACCTGTTCATGTCATGTCTAAACAT    8662
gb:MT019529|Organism:Severe      TTCCTTTTTGTTGCTGCTATTTTCTATTTAATAACACCTGTTCATGTCATGTCTAAACAT    8662
gb:MN996529|Organism:Severe      TTCCTTTTTGTTGCTGCTATTTTCTATTTAATAACACCTGTTCATGTCATGTCTAAACAT    8650
gb:MN996531|Organism:Severe      TTCCTTTTTGTTGCTGCTATTTTCTATTTAATAACACCTGTTCATGTCATGTCTAAACAT    8649
gb:MT066176|Organism:Severe      TTCCTTTTTGTTGCTGCTATTTTCTATTTAATAACACCTGTTCATGTCATGTCTAAACAT    8662
gb:MT027064|Organism:Severe      TTCCTTTTTGTTGCTGCTATTTTCTATTTAATAACACCTGTTCATGTCATGTCTAAACAT    8662
gb:MN994468|Organism:Severe      TTCCTTTTTGTTGCTGCTATTTTCTATTTAATAACACCTGTTCATGTCATGTCTAAACAT    8662
gb:MT072688|Organism:Severe      TTCCTTTTTGTTGCTGCTATTTTCTATTTAATAACACCTGTTCATGTCATGTCTAAACAT    8647
gb:MN996527|Organism:Severe      TTCCTTTTTGTTGCTGCTATTTTCTATTTAATAACACCTGTTCATGTCATGTCTAAACAT    8629
gb:MT093631|Organism:Severe      TTCCTTTTTGTTGCTGCTATTTTCTATTTAATAACACCTGTTCATGTCATGTCTAAACAT    8700
gb:MT106053|Organism:Severe      TTCCTTTTTGTTGCTGCTATTTTCTATTTAATAACACCTGTTCATGTCATGTCTAAACAT    8662
gb:MT019533|Organism:Severe      TTCCTTTTTGTTGCTGCTATTTTCTATTTAATAACACCTGTTCATGTCATGTCTAAACAT    8662
gb:MT019531|Organism:Severe      TTCCTTTTTGTTGCTGCTATTTTCTATTTAATAACACCTGTTCATGTCATGTCTAAACAT    8662
gb:MN996528|Organism:Severe      TTCCTTTTTGTTGCTGCTATTTTCTATTTAATAACACCTGTTCATGTCATGTCTAAACAT    8662
gb:MN996530|Organism:Severe      TTCCTTTTTGTTGCTGCTATTTTCTATTTAATAACACCTGTTCATGTCATGTCTAAACAT    8648
gb:MN908947|Organism:Severe      TTCCTTTTTGTTGCTGCTATTTTCTATTTAATAACACCTGTTCATGTCATGTCTAAACAT    8662
gb:MT019532|Organism:Severe      TTCCTTTTTGTTGCTGCTATTTTCTATTTAATAACACCTGTTCATGTCATGTCTAAACAT    8662
                                 ************************************************************

gb:MT020781|Organism:Severe      ACTGACTTTTCAAGTGAAATCATAGGATACAAGGCTATTGATGGTGGTGTCACTCGTGAC    8710
gb:MT007544|Organism:Severe      ACTGACTTTTCAAGTGAAATCATAGGATACAAGGCTATTGATGGTGGTGTCACTCGTGAC    8722
gb:MN994467|Organism:Severe      ACTGACTTTTCAAGTGAAATCATAGGATACAAGGCTATTGATGGTGGTGTCACTCGTGAC    8722
gb:MT044257|Organism:Severe      ACTGACTTTTCAAGTGAAATCATAGGATACAAGGCTATTGATGGTGGTGTCACTCGTGAC    8722
gb:MT106054|Organism:Severe      ACTGACTTTTCAAGTGAAATCATAGGATACAAGGCTATTGATGGTGGTGTCACTCGTGAC    8722
gb:MT049951|Organism:Severe      ACTGACTTTTCAAGTGAAATCATAGGATACAAGGCTATTGATGGTGGTGTCACTCGTGAC    8722
gb:MN975262|Organism:Severe      ACTGACTTTTCAAGTGAAATCATAGGATACAAGGCTATTGATGGTGGTGTCACTCGTGAC    8722
gb:MT106052|Organism:Severe      ACTGACTTTTCAAGTGAAATCATAGGATACAAGGCTATTGATGGTGGTGTCACTCGTGAC    8722
gb:LC522975|Organism:Severe      ACTGACTTTTCAAGTGAAATCATAGGATACAAGGCTATTGATGGTGGTGTCACTCGTGAC    8719
gb:LC522973|Organism:Severe      ACTGACTTTTCAAGTGAAATCATAGGATACAAGGCTATTGATGGTGGTGTCACTCGTGAC    8719
gb:LC522974|Organism:Severe      ACTGACTTTTCAAGTGAAATCATAGGATACAAGGCTATTGATGGTGGTGTCACTCGTGAC    8719
gb:MN985325|Organism:Severe      ACTGACTTTTCAAGTGAAATCATAGGATACAAGGCTATTGATGGTGGTGTCACTCGTGAC    8722
gb:MT020881|Organism:Severe      ACTGACTTTTCAAGTGAAATCATAGGATACAAGGCTATTGATGGTGGTGTCACTCGTGAC    8722
gb:MT020880|Organism:Severe      ACTGACTTTTCAAGTGAAATCATAGGATACAAGGCTATTGATGGTGGTGTCACTCGTGAC    8722
gb:MT066175|Organism:Severe      ACTGACTTTTCAAGTGAAATCATAGGATACAAGGCTATTGATGGTGGTGTCACTCGTGAC    8722
gb:MN997409|Organism:Severe      ACTGACTTTTCAAGTGAAATCATAGGATACAAGGCTATTGATGGTGGTGTCACTCGTGAC    8722
gb:MN938384|Organism:Severe      ACTGACTTTTCAAGTGAAATCATAGGATACAAGGCTATTGATGGTGGTGTCACTCGTGAC    8690
gb:MT044258|Organism:Severe      ACTGACTTTTCAAGTGAAATCATAGGATACAAGGCTATTGATGGTGGTGTCACTCGTGAC    8698
gb:MT039890|Organism:Severe      ACTGACTTTTCAAGTGAAATCATAGGATACAAGGCTATTGATGGTGGTGTCACTCGTGAC    8722
gb:MN988713|Organism:Severe      ACTGACTTTTCAAGTGAAATCATAGGATACAAGGCTATTGATGGTGGTGTCACTCGTGAC    8722
gb:LC521925|Organism:Severe      ACTGACTTTTCAAGTGAAATCATAGGATACAAGGCTATTGATGGTGGTGTCACTCGTGAC    8695
```

```
gb:MT093571|Organism:Severe      ACTGACTTTTCAAGTGAAATCATAGGATACAAGGCTATTGATGGTGGTGTCACTCGTGAC    8722
gb:MT039887|Organism:Severe      ACTGACTTTTCAAGTGAAATCATAGGATACAAGGCTATTGATGGTGGTGTCACTCGTGAC    8722
gb:MT019530|Organism:Severe      ACTGACTTTTCAAGTGAAATCATAGGATACAAGGCTATTGATGGTGGTGTCACTCGTGAC    8722
gb:MT039888|Organism:Severe      ACTGACTTTTCAAGTGAAATCATAGGATACAAGGCTATTGATGGTGGTGTCACTCGTGAC    8722
gb:LC522972|Organism:Severe      ACTGACTTTTCAAGTGAAATCATAGGATACAAGGCTATTGATGGTGGTGTCACTCGTGAC    8719
gb:MT027063|Organism:Severe      ACTGACTTTTCAAGTGAAATCATAGGATACAAGGCTATTGATGGTGGTGTCACTCGTGAC    8722
gb:MT027062|Organism:Severe      ACTGACTTTTCAAGTGAAATCATAGGATACAAGGCTATTGATGGTGGTGTCACTCGTGAC    8722
gb:MT019529|Organism:Severe      ACTGACTTTTCAAGTGAAATCATAGGATACAAGGCTATTGATGGTGGTGTCACTCGTGAC    8722
gb:MN996529|Organism:Severe      ACTGACTTTTCAAGTGAAATCATAGGATACAAGGCTATTGATGGTGGTGTCACTCGTGAC    8710
gb:MN996531|Organism:Severe      ACTGACTTTTCAAGTGAAATCATAGGATACAAGGCTATTGATGGTGGTGTCACTCGTGAC    8709
gb:MT066176|Organism:Severe      ACTGACTTTTCAAGTGAAATCATAGGATACAAGGCTATTGATGGTGGTGTCACTCGTGAC    8722
gb:MT027064|Organism:Severe      ACTGACTTTTCAAGTGAAATCATAGGATACAAGGCTATTGATGGTGGTGTCACTCGTGAC    8722
gb:MN994468|Organism:Severe      ACTGACTTTTCAAGTGAAATCATAGGATACAAGGCTATTGATGGTGGTGTCACTCGTGAC    8722
gb:MT072688|Organism:Severe      ACTGACTTTTCAAGTGAAATCATAGGATACAAGGCTATTGATGGTGGTGTCACTCGTGAC    8707
gb:MN996527|Organism:Severe      ACTGACTTTTCAAGTGAAATCATAGGATACAAGGCTATTGATGGTGGTGTCACTCGTGAC    8689
gb:MT093631|Organism:Severe      ACTGACTTTTCAAGTGAAATCATAGGATACAAGGCTATTGATGGTGGTGTCACTCGTGAC    8760
gb:MT106053|Organism:Severe      ACTGACTTTTCAAGTGAAATCATAGGATACAAGGCTATTGATGGTGGTGTCACTCGTGAC    8722
gb:MT019533|Organism:Severe      ACTGACTTTTCAAGTGAAATCATAGGATACAAGGCTATTGATGGTGGTGTCACTCGTGAC    8722
gb:MT019531|Organism:Severe      ACTGACTTTTCAAGTGAAATCATAGGATACAAGGCTATTGATGGTGGTGTCACTCGTGAC    8722
gb:MN996528|Organism:Severe      ACTGACTTTTCAAGTGAAATCATAGGATACAAGGCTATTGATGGTGGTGTCACTCGTGAC    8722
gb:MN996530|Organism:Severe      ACTGACTTTTCAAGTGAAATCATAGGATACAAGGCTATTGATGGTGGTGTCACTCGTGAC    8708
gb:MN908947|Organism:Severe      ACTGACTTTTCAAGTGAAATCATAGGATACAAGGCTATTGATGGTGGTGTCACTCGTGAC    8722
gb:MT019532|Organism:Severe      ACTGACTTTTCAAGTGAAATCATAGGATACAAGGCTATTGATGGTGGTGTCACTCGTGAC    8722
                                 ************************************************************

gb:MT020781|Organism:Severe      ATAGCATCTACAGATACTTGTTTTGCTAACAAACATGCTGATTTTGACACATGGTTTAGC    8770
gb:MT007544|Organism:Severe      ATAGCATCTACAGATACTTGTTTTGCTAACAAACATGCTGATTTTGACACATGGTTTAGC    8782
gb:MN994467|Organism:Severe      ATAGCATCTACAGATACTTGTTTTGCTAACAAACATGCTGATTTTGACACATGGTTTAGT    8782
gb:MT044257|Organism:Severe      ATAGCATCTACAGATACTTGTTTTGCTAACAAACATGCTGATTTTGACACATGGTTTAGT    8782
gb:MT106054|Organism:Severe      ATAGCATCTACAGATACTTGTTTTGCTAACAAACATGCTGATTTTGACACATGGTTTAGT    8782
gb:MT049951|Organism:Severe      ATAGCATCTACAGATACTTGTTTTGCTAACAAACATGCTGATTTTGACACATGGTTTAGT    8782
gb:MN975262|Organism:Severe      ATAGCATCTACAGATACTTGTTTTGCTAACAAACATGCTGATTTTGACACATGGTTTAGT    8782
gb:MT106052|Organism:Severe      ATAGCATCTACAGATACTTGTTTTGCTAACAAACATGCTGATTTTGACACATGGTTTAGT    8782
gb:LC522975|Organism:Severe      ATAGCATCTACAGATACTTGTTTTGCTAACAAACATGCTGATTTTGACACATGGTTTAGT    8779
gb:LC522973|Organism:Severe      ATAGCATCTACAGATACTTGTTTTGCTAACAAACATGCTGATTTTGACACATGGTTTAGT    8779
gb:LC522974|Organism:Severe      ATAGCATCTACAGATACTTGTTTTGCTAACAAACATGCTGATTTTGACACATGGTTTAGT    8779
gb:MN985325|Organism:Severe      ATAGCATCTACAGATACTTGTTTTGCTAACAAACATGCTGATTTTGACACATGGTTTAGT    8782
gb:MT020881|Organism:Severe      ATAGCATCTACAGATACTTGTTTTGCTAACAAACATGCTGATTTTGACACATGGTTTAGT    8782
gb:MT020880|Organism:Severe      ATAGCATCTACAGATACTTGTTTTGCTAACAAACATGCTGATTTTGACACATGGTTTAGT    8782
gb:MT066175|Organism:Severe      ATAGCATCTACAGATACTTGTTTTGCTAACAAACATGCTGATTTTGACACATGGTTTAGT    8782
gb:MN997409|Organism:Severe      ATAGCATCTACAGATACTTGTTTTGCTAACAAACATGCTGATTTTGACACATGGTTTAGT    8782
gb:MN938384|Organism:Severe      ATAGCATCTACAGATACTTGTTTTGCTAACAAACATGCTGATTTTGACACATGGTTTAGT    8750
gb:MT044258|Organism:Severe      ATAGCATCTACAGATACTTGTTTTGCTAACAAACATGCTGATTTTGACACATGGTTTAGC    8758
gb:MT039890|Organism:Severe      ATAGCATCTACAGATACTTGTTTTGCTAACAAACATGCTGATTTTGACACATGGTTTAGC    8782
gb:MN988713|Organism:Severe      ATAGCATCTACAGATACTTGTTTTGCTAACAAACATGCTGATTTTGACACATGGTTTAGY    8782
gb:LC521925|Organism:Severe      ATAGCATCTACAGATACTTGTTTTGCTAACAAACATGCTGATTTTGACACATGGTTTAGC    8755
gb:MT093571|Organism:Severe      ATAGCATCTACAGATACTTGTTTTGCTAACAAACATGCTGATTTTGACACATGGTTTAGC    8782
gb:MT039887|Organism:Severe      ATAGCATCTACAGATACTTGTTTTGCTAACAAACATGCTGATTTTGACACATGGTTTAGC    8782
gb:MT019530|Organism:Severe      ATAGCATCTACAGATACTTGTTTTGCTAACAAACATGCTGATTTTGACACATGGTTTAGC    8782
gb:MT039888|Organism:Severe      ATAGCATCTACAGATACTTGTTTTGCTAACAAACATGCTGATTTTGACACATGGTTTAGC    8782
gb:LC522972|Organism:Severe      ATAGCATCTACAGATACTTGTTTTGCTAACAAACATGCTGATTTTGACACATGGTTTAGC    8779
gb:MT027063|Organism:Severe      ATAGCATCTACAGATACTTGTTTTGCTAACAAACATGCTGATTTTGACACATGGTTTAGC    8782
gb:MT027062|Organism:Severe      ATAGCATCTACAGATACTTGTTTTGCTAACAAACATGCTGATTTTGACACATGGTTTAGC    8782
gb:MT019529|Organism:Severe      ATAGCATCTACAGATACTTGTTTTGCTAACAAACATGCTGATTTTGACACATGGTTTAGC    8782
gb:MN996529|Organism:Severe      ATAGCATCTACAGATACTTGTTTTGCTAACAAACATGCTGATTTTGACACATGGTTTAGC    8770
gb:MN996531|Organism:Severe      ATAGCATCTACAGATACTTGTTTTGCTAACAAACATGCTGATTTTGACACATGGTTTAGC    8769
gb:MT066176|Organism:Severe      ATAGCATCTACAGATACTTGTTTTGCTAACAAACATGCTGATTTTGACACATGGTTTAGC    8782
gb:MT027064|Organism:Severe      ATAGCATCTACAGATACTTGTTTTGCTAACAAACATGCTGATTTTGACACATGGTTTAGC    8782
gb:MN994468|Organism:Severe      ATAGCATCTACAGATACTTGTTTTGCTAACAAACATGCTGATTTTGACACATGGTTTAGC    8782
gb:MT072688|Organism:Severe      ATAGCATCTACAGATACTTGTTTTGCTAACAAACATGCTGATTTTGACACATGGTTTAGC    8767
gb:MN996527|Organism:Severe      ATAGCATCTACAGATACTTGTTTTGCTAACAAACATGCTGATTTTGACACATGGTTTAGC    8749
gb:MT093631|Organism:Severe      ATAGCATCTACAGATACTTGTTTTGCTAACAAACATGCTGATTTTGACACATGGTTTAGC    8820
gb:MT106053|Organism:Severe      ATAGCATCTACAGATACTTGTTTTGCTAACAAACATGCTGATTTTGACACATGGTTTAGC    8782
gb:MT019533|Organism:Severe      ATAGCATCTACAGATACTTGTTTTGCTAACAAACATGCTGATTTTGACACATGGTTTAGC    8782
gb:MT019531|Organism:Severe      ATAGCATCTACAGATACTTGTTTTGCTAACAAACATGCTGATTTTGACACATGGTTTAGC    8782
```

```
gb:MN996528|Organism:Severe      ATAGCATCTACAGATACTTGTTTTGCTAACAAACATGCTGATTTTGACACATGGTTTAGC   8782
gb:MN996530|Organism:Severe      ATAGCATCTACAGATACTTGTTTTGCTAACAAACATGCTGATTTTGACACATGGTTTAGC   8768
gb:MN908947|Organism:Severe      ATAGCATCTACAGATACTTGTTTTGCTAACAAACATGCTGATTTTGACACATGGTTTAGC   8782
gb:MT019532|Organism:Severe      ATAGCATCTACAGATACTTGTTTTGCTAACAAACATGCTGATTTTGACACATGGTTTAGC   8782
                                 **********************************************************

gb:MT020781|Organism:Severe      CAGCGTGGTGGTAGTTATACTAATGACAAAGCTTGCCCATTGATTGCTGCAGTCATAACA   8830
gb:MT007544|Organism:Severe      CAGCGTGGTGGTAGTTATACTAATGACAAAGCTTGCCCATTGATTGCTGCAGTCATAACA   8842
gb:MN994467|Organism:Severe      CAGCGTGGTGGTAGTTATACTAATGACAAAGCTTGCCCATTGATTGCTGCAGTCATAACA   8842
gb:MT044257|Organism:Severe      CAGCGTGGTGGTAGTTATACTAATGACAAAGCTTGCCCATTGATTGCTGCAGTCATAACA   8842
gb:MT106054|Organism:Severe      CAGCGTGGTGGTAGTTATACTAATGACAAAGCTTGCCCATTGATTGCTGCAGTCATAACA   8842
gb:MT049951|Organism:Severe      CAGCGTGGTGGTAGTTATACTAATGACAAAGCTTGCCCATTGATTGCTGCAGTCATAACA   8842
gb:MN975262|Organism:Severe      CAGCGTGGTGGTAGTTATACTAATGACAAAGCTTGCCCATTGATTGCTGCAGTCATAACA   8842
gb:MT106052|Organism:Severe      CAGCGTGGTGGTAGTTATACTAATGACAAAGCTTGCCCATTGATTGCTGCAGTCATAACA   8842
gb:LC522975|Organism:Severe      CAGCGTGGTGGTAGTTATACTAATGACAAAGCTTGCCCATTGATTGCTGCAGTCATAACA   8839
gb:LC522973|Organism:Severe      CAGCGTGGTGGTAGTTATACTAATGACAAAGCTTGCCCATTGATTGCTGCAGTCATAACA   8839
gb:LC522974|Organism:Severe      CAGCGTGGTGGTAGTTATACTAATGACAAAGCTTGCCCATTGATTGCTGCAGTCATAACA   8839
gb:MN985325|Organism:Severe      CAGCGTGGTGGTAGTTATACTAATGACAAAGCTTGCCCATTGATTGCTGCAGTCATAACA   8842
gb:MT020881|Organism:Severe      CAGCGTGGTGGTAGTTATACTAATGACAAAGCTTGCCCATTGATTGCTGCAGTCATAACA   8842
gb:MT020880|Organism:Severe      CAGCGTGGTGGTAGTTATACTAATGACAAAGCTTGCCCATTGATTGCTGCAGTCATAACA   8842
gb:MT066175|Organism:Severe      CAGCGTGGTGGTAGTTATACTAATGACAAAGCTTGCCCATTGATTGCTGCAGTCATAACA   8842
gb:MN997409|Organism:Severe      CAGCGTGGTGGTAGTTATACTAATGACAAAGCTTGCCCATTGATTGCTGCAGTCATAACA   8842
gb:MN938384|Organism:Severe      CAGCGTGGTGGTAGTTATACTAATGACAAAGCTTGCCCATTGATTGCTGCAGTCATAACA   8810
gb:MT044258|Organism:Severe      CAGCGTGGTGGTAGTTATACTAATGACAAAGCTTGCCCATTGATTGCTGCAGTCATAACA   8818
gb:MT039890|Organism:Severe      CAGCGTGGTGGTAGTTATACTAATGACAAAGCTTGCCCATTGATTGCTGCAGTCATAACA   8842
gb:MN988713|Organism:Severe      CAGCGTGGTGGTAGTTATACTAATGACAAAGCTTGCCCATTGATTGCTGCAGTCATAACA   8842
gb:LC521925|Organism:Severe      CAGCGTGGTGGTAGTTATACTAATGACAAAGCTTGCCCATTGATTGCTGCAGTCATAACA   8815
gb:MT093571|Organism:Severe      CAGCGTGGTGGTAGTTATACTAATGACAAAGCTTGCCCATTGATTGCTGCAGTCATAACA   8842
gb:MT039887|Organism:Severe      CAGCGTGGTGGTAGTTATACTAATGACAAAGCTTGCCCATTGATTGCTGCAGTCATAACA   8842
gb:MT019530|Organism:Severe      CAGCGTGGTGGTAGTTATACTAATGACAAAGCTTGCCCATTGATTGCTGCAGTCATAACA   8842
gb:MT039888|Organism:Severe      CAGCGTGGTGGTAGTTATACTAATGACAAAGCTTGCCCATTGATTGCTGCAGTCATAACA   8842
gb:LC522972|Organism:Severe      CAGCGTGGTGGTAGTTATACTAATGACAAAGCTTGCCCATTGATTGCTGCAGTCATAACA   8839
gb:MT027063|Organism:Severe      CAGCGTGGTGGTAGTTATACTAATGACAAAGCTTGCCCATTGATTGCTGCAGTCATAACA   8842
gb:MT027062|Organism:Severe      CAGCGTGGTGGTAGTTATACTAATGACAAAGCTTGCCCATTGATTGCTGCAGTCATAACA   8842
gb:MT019529|Organism:Severe      CAGCGTGGTGGTAGTTATACTAATGACAAAGCTTGCCCATTGATTGCTGCAGTCATAACA   8842
gb:MN996529|Organism:Severe      CAGCGTGGTGGTAGTTATACTAATGACAAAGCTTGCCCATTGATTGCTGCAGTCATAACA   8830
gb:MN996531|Organism:Severe      CAGCGTGGTGGTAGTTATACTAATGACAAAGCTTGCCCATTGATTGCTGCAGTCATAACA   8829
gb:MT066176|Organism:Severe      CAGCGTGGTGGTAGTTATACTAATGACAAAGCTTGCCCATTGATTGCTGCAGTCATAACA   8842
gb:MT027064|Organism:Severe      CAGCGTGGTGGTAGTTATACTAATGACAAAGCTTGCCCATTGATTGCTGCAGTCATAACA   8842
gb:MN994468|Organism:Severe      CAGCGTGGTGGTAGTTATACTAATGACAAAGCTTGCCCATTGATTGCTGCAGTCATAACA   8842
gb:MT072688|Organism:Severe      CAGCGTGGTGGTAGTTATACTAATGACAAAGCTTGCCCATTGATTGCTGCAGTCATAACA   8827
gb:MN996527|Organism:Severe      CAGCGTGGTGGTAGTTATACTAATGACAAAGCTTGCCCATTGATTGCTGCAGTCATAACA   8809
gb:MT093631|Organism:Severe      CAGCGTGGTGGTAGTTATACTAATGACAAAGCTTGCCCATTGATTGCTGCAGTCATAACA   8880
gb:MT106053|Organism:Severe      CAGCGTGGTGGTAGTTATACTAATGACAAAGCTTGCCCATTGATTGCTGCAGTCATAACA   8842
gb:MT019533|Organism:Severe      CAGCGTGGTGGTAGTTATACTAATGACAAAGCTTGCCCATTGATTGCTGCAGTCATAACA   8842
gb:MT019531|Organism:Severe      CAGCGTGGTGGTAGTTATACTAATGACAAAGCTTGCCCATTGATTGCTGCAGTCATAACA   8842
gb:MN996528|Organism:Severe      CAGCGTGGTGGTAGTTATACTAATGACAAAGCTTGCCCATTGATTGCTGCAGTCATAACA   8842
gb:MN996530|Organism:Severe      CAGCGTGGTGGTAGTTATACTAATGACAAAGCTTGCCCATTGATTGCTGCAGTCATAACA   8828
gb:MN908947|Organism:Severe      CAGCGTGGTGGTAGTTATACTAATGACAAAGCTTGCCCATTGATTGCTGCAGTCATAACA   8842
gb:MT019532|Organism:Severe      CAGCGTGGTGGTAGTTATACTAATGACAAAGCTTGCCCATTGATTGCTGCAGTCATAACA   8842
                                 ************************************************************

gb:MT020781|Organism:Severe      AGAGAAGTGGGTTTTGTCGTGCCTGGTTTGCCTGGCACGATATTACGCACAACTAATGGT   8890
gb:MT007544|Organism:Severe      AGAGAAGTGGGTTTTGTCGTGCCTGGTTTGCCTGGCACGATATTACGCACAACTAATGGT   8902
gb:MN994467|Organism:Severe      AGAGAAGTGGGTTTTGTCGTGCCTGGTTTGCCTGGCACGATATTACGCACAACTAATGGT   8902
gb:MT044257|Organism:Severe      AGAGAAGTGGGTTTTGTCGTGCCTGGTTTGCCTGGCACGATATTACGCACAACTAATGGT   8902
gb:MT106054|Organism:Severe      AGAGAAGTGGGTTTTGTCGTGCCTGGTTTGCCTGGCACGATATTACGCACAACTAATGGT   8902
gb:MT049951|Organism:Severe      AGAGAAGTGGGTTTTGTCGTGCCTGGTTTGCCTGGCACGATATTACGCACAACTAATGGT   8902
gb:MN975262|Organism:Severe      AGAGAAGTGGGTTTTGTCGTGCCTGGTTTGCCTGGCACGATATTACGCACAACTAATGGT   8902
gb:MT106052|Organism:Severe      AGAGAAGTGGGTTTTGTCGTGCCTGGTTTGCCTGGCACGATATTACGCACAACTAATGGT   8902
gb:LC522975|Organism:Severe      AGAGAAGTGGGTTTTGTCGTGCCTGGTTTGCCTGGCACGATATTACGCACAACTAATGGT   8899
gb:LC522973|Organism:Severe      AGAGAAGTGGGTTTTGTCGTGCCTGGTTTGCCTGGCACGATATTACGCACAACTAATGGT   8899
gb:LC522974|Organism:Severe      AGAGAAGTGGGTTTTGTCGTGCCTGGTTTGCCTGGCACGATATTACGCACAACTAATGGT   8899
gb:MN985325|Organism:Severe      AGAGAAGTGGGTTTTGTCGTGCCTGGTTTGCCTGGCACGATATTACGCACAACTAATGGT   8902
gb:MT020881|Organism:Severe      AGAGAAGTGGGTTTTGTCGTGCCTGGTTTGCCTGGCACGATATTACGCACAACTAATGGT   8902
```

```
gb:MT020880|Organism:Severe      AGAGAAGTGGGTTTTGTCGTGCCTGGTTTGCCTGGCACGATATTACGCACAACTAATGGT   8902
gb:MT066175|Organism:Severe      AGAGAAGTGGGTTTTGTCGTGCCTGGTTTGCCTGGCACGATATTACGCACAACTAATGGT   8902
gb:MN997409|Organism:Severe      AGAGAAGTGGGTTTTGTCGTGCCTGGTTTGCCTGGCACGATATTACGCACAACTAATGGT   8902
gb:MN938384|Organism:Severe      AGAGAAGTGGGTTTTGTCGTGCCTGGTTTGCCTGGCACGATATTACGCACAACTAATGGT   8870
gb:MT044258|Organism:Severe      AGAGAAGTGGGTTTTGTCGTGCCTGGTTTGCCTGGCACGATATTACGCACAACTAATGGT   8878
gb:MT039890|Organism:Severe      AGAGAAGTGGGTTTTGTCGTGCCTGGTTTGCCTGGCACGATATTACGCACAACTAATGGT   8902
gb:MN988713|Organism:Severe      AGAGAAGTGGGTTTTGTCGTGCCTGGTTTGCCTGGCACGATATTACGCACAACTAATGGT   8902
gb:LC521925|Organism:Severe      AGAGAAGTGGGTTTTGTCGTGCCTGGTTTGCCTGGCACGATATTACGCACAACTAATGGT   8875
gb:MT093571|Organism:Severe      AGAGAAGTGGGTTTTGTCGTGCCTGGTTTGCCTGGCACGATATTACGCACAACTAATGGT   8902
gb:MT039887|Organism:Severe      AGAGAAGTGGGTTTTGTCGTGCCTGGTTTGCCTGGCACGATATTACGCACAACTAATGGT   8902
gb:MT019530|Organism:Severe      AGAGAAGTGGGTTTTGTCGTGCCTGGTTTGCCTGGCACGATATTACGCACAACTAATGGT   8902
gb:MT039888|Organism:Severe      AGAGAAGTGGGTTTTGTCGTGCCTGGTTTGCCTGGCACGATATTACGCACAACTAATGGT   8902
gb:LC522972|Organism:Severe      AGAGAAGTGGGTTTTGTCGTGCCTGGTTTGCCTGGCACGATATTACGCACAACTAATGGT   8899
gb:MT027063|Organism:Severe      AGAGAAGTGGGTTTTGTCGTGCCTGGTTTGCCTGGCACGATATTACGCACAACTAATGGT   8902
gb:MT027062|Organism:Severe      AGAGAAGTGGGTTTTGTCGTGCCTGGTTTGCCTGGCACGATATTACGCACAACTAATGGT   8902
gb:MT019529|Organism:Severe      AGAGAAGTGGGTTTTGTCGTGCCTGGTTTGCCTGGCACGATATTACGCACAACTAATGGT   8902
gb:MN996529|Organism:Severe      AGAGAAGTGGGTTTTGTCGTGCCTGGTTTGCCTGGCACGATATTACGCACAACTAATGGT   8890
gb:MN996531|Organism:Severe      AGAGAAGTGGGTTTTGTCGTGCCTGGTTTGCCTGGCACGATATTACGCACAACTAATGGT   8889
gb:MT066176|Organism:Severe      AGAGAAGTGGGTTTTGTCGTGCCTGGTTTGCCTGGCACGATATTACGCACAACTAATGGT   8902
gb:MT027064|Organism:Severe      AGAGAAGTGGGTTTTGTCGTGCCTGGTTTGCCTGGCACGATATTACGCACAACTAATGGT   8902
gb:MN994468|Organism:Severe      AGAGAAGTGGGTTTTGTCGTGCCTGGTTTGCCTGGCACGATATTACGCACAACTAATGGT   8902
gb:MT072688|Organism:Severe      AGAGAAGTGGGTTTTGTCGTGCCTGGTTTGCCTGGCACGATATTACGCACAACTAATGGT   8887
gb:MN996527|Organism:Severe      AGAGAAGTGGGTTTTGTCGTGCCTGGTTTGCCTGGCACGATATTACGCACAACTAATGGT   8869
gb:MT093631|Organism:Severe      AGAGAAGTGGGTTTTGTCGTGCCTGGTTTGCCTGGCACGATATTACGCACAACTAATGGT   8940
gb:MT106053|Organism:Severe      AGAGAAGTGGGTTTTGTCGTGCCTGGTTTGCCTGGCACGATATTACGCACAACTAATGGT   8902
gb:MT019533|Organism:Severe      AGAGAAGTGGGTTTTGTCGTGCCTGGTTTGCCTGGCACGATATTACGCACAACTAATGGT   8902
gb:MT019531|Organism:Severe      AGAGAAGTGGGTTTTGTCGTGCCTGGTTTGCCTGGCACGATATTACGCACAACTAATGGT   8902
gb:MN996528|Organism:Severe      AGAGAAGTGGGTTTTGTCGTGCCTGGTTTGCCTGGCACGATATTACGCACAACTAATGGT   8902
gb:MN996530|Organism:Severe      AGAGAAGTGGGTTTTGTCGTGCCTGGTTTGCCTGGCACGATATTACGCACAACTAATGGT   8888
gb:MN908947|Organism:Severe      AGAGAAGTGGGTTTTGTCGTGCCTGGTTTGCCTGGCACGATATTACGCACAACTAATGGT   8902
gb:MT019532|Organism:Severe      AGAGAAGTGGGTTTTGTCGTGCCTGGTTTGCCTGGCACGATATTACGCACAACTAATGGT   8902
                                 ************************************************************

gb:MT020781|Organism:Severe      GACTTTTTGCATTTCTTACCTAGAGTTTTTAGTGCAGTTGGTAACATCTGTTACACACCA   8950
gb:MT007544|Organism:Severe      GACTTTTTGCATTTCTTACCTAGAGTTTTTAGTGCAGTTGGTAACATCTGTTACACACCA   8962
gb:MN994467|Organism:Severe      GACTTTTTGCATTTCTTACCTAGAGTTTTTAGTGCAGTTGGTAACATCTGTTACACACCA   8962
gb:MT044257|Organism:Severe      GACTTTTTGCATTTCTTACCTAGAGTTTTTAGTGCAGTTGGTAACATCTGTTACACACCA   8962
gb:MT106054|Organism:Severe      GACTTTTTGCATTTCTTACCTAGAGTTTTTAGTGCAGTTGGTAACATCTGTTACACACCA   8962
gb:MT049951|Organism:Severe      GACTTTTTGCATTTCTTACCTAGAGTTTTTAGTGCAGTTGGTAACATCTGTTACACACCA   8962
gb:MN975262|Organism:Severe      GACTTTTTGCATTTCTTACCTAGAGTTTTTAGTGCAGTTGGTAACATCTGTTACACACCA   8962
gb:MT106052|Organism:Severe      GACTTTTTGCATTTCTTACCTAGAGTTTTTAGTGCAGTTGGTAACATCTGTTACACACCA   8962
gb:LC522975|Organism:Severe      GACTTTTTGCATTTCTTACCTAGAGTTTTTAGTGCAGTTGGTAACATCTGTTACACACCA   8959
gb:LC522973|Organism:Severe      GACTTTTTGCATTTCTTACCTAGAGTTTTTAGTGCAGTTGGTAACATCTGTTACACACCA   8959
gb:LC522974|Organism:Severe      GACTTTTTGCATTTCTTACCTAGAGTTTTTAGTGCAGTTGGTAACATCTGTTACACACCA   8959
gb:MN985325|Organism:Severe      GACTTTTTGCATTTCTTACCTAGAGTTTTTAGTGCAGTTGGTAACATCTGTTACACACCA   8962
gb:MT020881|Organism:Severe      GACTTTTTGCATTTCTTACCTAGAGTTTTTAGTGCAGTTGGTAACATCTGTTACACACCA   8962
gb:MT020880|Organism:Severe      GACTTTTTGCATTTCTTACCTAGAGTTTTTAGTGCAGTTGGTAACATCTGTTACACACCA   8962
gb:MT066175|Organism:Severe      GACTTTTTGCATTTCTTACCTAGAGTTTTTAGTGCAGTTGGTAACATCTGTTACACACCA   8962
gb:MN997409|Organism:Severe      GACTTTTTGCATTTCTTACCTAGAGTTTTTAGTGCAGTTGGTAACATCTGTTACACACCA   8962
gb:MN938384|Organism:Severe      GACTTTTTGCATTTCTTACCTAGAGTTTTTAGTGCAGTTGGTAACATCTGTTACACACCA   8930
gb:MT044258|Organism:Severe      GACTTTTTGCATTTCTTACCTAGAGTTTTTAGTGCAGTTGGTAACATCTGTTACACACCA   8938
gb:MT039890|Organism:Severe      GACTTTTTGCATTTCTTACCTAGAGTTTTTAGTGCAGTTGGTAACATCTGTTACACACCA   8962
gb:MN988713|Organism:Severe      GACTTTTTGCATTTCTTACCTAGAGTTTTTAGTGCAGTTGGTAACATCTGTTACACACCA   8962
gb:LC521925|Organism:Severe      GACTTTTTGCATTTCTTACCTAGAGTTTTTAGTGCAGTTGGTAACATCTGTTACACACCA   8935
gb:MT093571|Organism:Severe      GACTTTTTGCATTTCTTACCTAGAGTTTTTAGTGCAGTTGGTAACATCTGTTACACACCA   8962
gb:MT039887|Organism:Severe      GACTTTTTGCATTTCTTACCTAGAGTTTTTAGTGCAGTTGGTAACATCTGTTACACACCA   8962
gb:MT019530|Organism:Severe      GACTTTTTGCATTTCTTACCTAGAGTTTTTAGTGCAGTTGGTAACATCTGTTACACACCA   8962
gb:MT039888|Organism:Severe      GACTTTTTGCATTTCTTACCTAGAGTTTTTAGTGCAGTTGGTAACATCTGTTACACACCA   8962
gb:LC522972|Organism:Severe      GACTTTTTGCATTTCTTACCTAGAGTTTTTAGTGCAGTTGGTAACATCTGTTACACACCA   8959
gb:MT027063|Organism:Severe      GACTTTTTGCATTTCTTACCTAGAGTTTTTAGTGCAGTTGGTAACATCTGTTACACACCA   8962
gb:MT027062|Organism:Severe      GACTTTTTGCATTTCTTACCTAGAGTTTTTAGTGCAGTTGGTAACATCTGTTACACACCA   8962
gb:MT019529|Organism:Severe      GACTTTTTGCATTTCTTACCTAGAGTTTTTAGTGCAGTTGGTAACATCTGTTACACACCA   8962
gb:MN996529|Organism:Severe      GACTTTTTGCATTTCTTACCTAGAGTTTTTAGTGCAGTTGGTAACATCTGTTACACACCA   8950
gb:MN996531|Organism:Severe      GACTTTTTGCATTTCTTACCTAGAGTTTTTAGTGCAGTTGGTAACATCTGTTACACACCA   8949
gb:MT066176|Organism:Severe      GACTTTTTGCATTTCTTACCTAGAGTTTTTAGTGCAGTTGGTAACATCTGTTACACACCA   8962
```

```
gb:MT027064|Organism:Severe      GACTTTTTGCATTTCTTACCTAGAGTTTTTAGTGCAGTTGGTAACATCTGTTACACACCA   8962
gb:MN994468|Organism:Severe      GACTTTTTGCATTTCTTACCTAGAGTTTTTAGTGCAGTTGGTAACATCTGTTACACACCA   8962
gb:MT072688|Organism:Severe      GACTTTTTGCATTTCTTACCTAGAGTTTTTAGTGCAGTTGGTAACATCTGTTACACACCA   8947
gb:MN996527|Organism:Severe      GACTTTTTGCATTTCTTACCTAGAGTTTTTAGTGCAGTTGGTAACATCTGTTACACACCA   8929
gb:MT093631|Organism:Severe      GACTTTTTGCATTTCTTACCTAGAGTTTTTAGTGCAGTTGGTAACATCTGTTACACACCA   9000
gb:MT106053|Organism:Severe      GACTTTTTGCATTTCTTACCTAGAGTTTTTAGTGCAGTTGGTAACATCTGTTACACACCA   8962
gb:MT019533|Organism:Severe      GACTTTTTGCATTTCTTACCTAGAGTTTTTAGTGCAGTTGGTAACATCTGTTACACACCA   8962
gb:MT019531|Organism:Severe      GACTTTTTGCATTTCTTACCTAGAGTTTTTAGTGCAGTTGGTAACATCTGTTACACACCA   8962
gb:MN996528|Organism:Severe      GACTTTTTGCATTTCTTACCTAGAGTTTTTAGTGCAGTTGGTAACATCTGTTACACACCA   8962
gb:MN996530|Organism:Severe      GACTTTTTGCATTTCTTACCTAGAGTTTTTAGTGCAGTTGGTAACATCTGTTACACACCA   8948
gb:MN908947|Organism:Severe      GACTTTTTGCATTTCTTACCTAGAGTTTTTAGTGCAGTTGGTAACATCTGTTACACACCA   8962
gb:MT019532|Organism:Severe      GACTTTTTGCATTTCTTACCTAGAGTTTTTAGTGCAGTTGGTAACATCTGTTACACACCA   8962
                                 ************************************************************

gb:MT020781|Organism:Severe      TCAAAACTTATAGAGTACACTGACTTTGCAACATCAGCTTGTGTTTTGGCTGCTGAATGT   9010
gb:MT007544|Organism:Severe      TCAAAACTTATAGAGTACACTGACTTTGCAACATCAGCTTGTGTTTTGGCTGCTGAATGT   9022
gb:MN994467|Organism:Severe      TCAAAACTTATAGAGTACACTGACTTTGCAACATCAGCTTGTGTTTTGGCTGCTGAATGT   9022
gb:MT044257|Organism:Severe      TCAAAACTTATAGAGTACACTGACTTTGCAACATCAGCTTGTGTTTTGGCTGCTGAATGT   9022
gb:MT106054|Organism:Severe      TCAAAACTTATAGAGTACACTGACTTTGCAACATCAGCTTGTGTTTTGGCTGCTGAATGT   9022
gb:MT049951|Organism:Severe      TCAAAACTTATAGAGTACACTGACTTTGCAACATCAGCTTGTGTTTTGGCTGCTGAATGT   9022
gb:MN975262|Organism:Severe      TCAAAACTTATAGAGTACACTGACTTTGCAACATCAGCTTGTGTTTTGGCTGCTGAATGT   9022
gb:MT106052|Organism:Severe      TCAAAACTTATAGAGTACACTGACTTTGCAACATCAGCTTGTGTTTTGGCTGCTGAATGT   9022
gb:LC522975|Organism:Severe      TCAAAACTTATAGAGTACACTGACTTTGCAACATCAGCTTGTGTTTTGGCTGCTGAATGT   9019
gb:LC522973|Organism:Severe      TCAAAACTTATAGAGTACACTGACTTTGCAACATCAGCTTGTGTTTTGGCTGCTGAATGT   9019
gb:LC522974|Organism:Severe      TCAAAACTTATAGAGTACACTGACTTTGCAACATCAGCTTGTGTTTTGGCTGCTGAATGT   9019
gb:MN985325|Organism:Severe      TCAAAACTTATAGAGTACACTGACTTTGCAACATCAGCTTGTGTTTTGGCTGCTGAATGT   9022
gb:MT020881|Organism:Severe      TCAAAACTTATAGAGTACACTGACTTTGCAACATCAGCTTGTGTTTTGGCTGCTGAATGT   9022
gb:MT020880|Organism:Severe      TCAAAACTTATAGAGTACACTGACTTTGCAACATCAGCTTGTGTTTTGGCTGCTGAATGT   9022
gb:MT066175|Organism:Severe      TCAAAACTTATAGAGTACACTGACTTTGCAACATCAGCTTGTGTTTTGGCTGCTGAATGT   9022
gb:MN997409|Organism:Severe      TCAAAACTTATAGAGTACACTGACTTTGCAACATCAGCTTGTGTTTTGGCTGCTGAATGT   9022
gb:MN938384|Organism:Severe      TCAAAACTTATAGAGTACACTGACTTTGCAACATCAGCTTGTGTTTTGGCTGCTGAATGT   8990
gb:MT044258|Organism:Severe      TCAAAACTTATAGAGTACACTGACTTTGCAACATCAGCTTGTGTTTTGGCTGCTGAATGT   8998
gb:MT039890|Organism:Severe      TCAAAACTTATAGAGTACACTGACTTTGCAACATCAGCTTGTGTTTTGGCTGCTGAATGT   9022
gb:MN988713|Organism:Severe      TCAAAACTTATAGAGTACACTGACTTTGCAACATCAGCTTGTGTTTTGGCTGCTGAATGT   9022
gb:LC521925|Organism:Severe      TCAAAACTTATAGAGTACACTGACTTTGCAACATCAGCTTGTGTTTTGGCTGCTGAATGT   8995
gb:MT093571|Organism:Severe      TCAAAACTTATAGAGTACACTGACTTTGCAACATCAGCTTGTGTTTTGGCTGCTGAATGT   9022
gb:MT039887|Organism:Severe      TCAAAACTTATAGAGTACACTGACTTTGCAACATCAGCTTGTGTTTTGGCTGCTGAATGT   9022
gb:MT019530|Organism:Severe      TCAAAACTTATAGAGTACACTGACTTTGCAACATCAGCTTGTGTTTTGGCTGCTGAATGT   9022
gb:MT039888|Organism:Severe      TCAAAACTTATAGAGTACACTGACTTTGCAACATCAGCTTGTGTTTTGGCTGCTGAATGT   9022
gb:LC522972|Organism:Severe      TCAAAACTTATAGAGTACACTGACTTTGCAACATCAGCTTGTGTTTTGGCTGCTGAATGT   9019
gb:MT027063|Organism:Severe      TCAAAACTTATAGAGTACACTGACTTTGCAACATCAGCTTGTGTTTTGGCTGCTGAATGT   9022
gb:MT027062|Organism:Severe      TCAAAACTTATAGAGTACACTGACTTTGCAACATCAGCTTGTGTTTTGGCTGCTGAATGT   9022
gb:MT019529|Organism:Severe      TCAAAACTTATAGAGTACACTGACATTGCAACATCAGCTTGTGTTTTGGCTGCTGAATGT   9022
gb:MN996529|Organism:Severe      TCAAAACTTATAGAGTACACTGACTTTGCAACATCAGCTTGTGTTTTGGCTGCTGAATGT   9010
gb:MN996531|Organism:Severe      TCAAAACTTATAGAGTACACTGACTTTGCAACATCAGCTTGTGTTTTGGCTGCTGAATGT   9009
gb:MT066176|Organism:Severe      TCAAAACTTATAGAGTACACTGACTTTGCAACATCAGCTTGTGTTTTGGCTGCTGAATGT   9022
gb:MT027064|Organism:Severe      TCAAAACTTATAGAGTACACTGACTTTGCAACATCAGCTTGTGTTTTGGCTGCTGAATGT   9022
gb:MN994468|Organism:Severe      TCAAAACTTATAGAGTACACTGACTTTGCAACATCAGCTTGTGTTTTGGCTGCTGAATGT   9022
gb:MT072688|Organism:Severe      TCAAAACTTATAGAGTACACTGACTTTGCAACATCAGCTTGTGTTTTGGCTGCTGAATGT   9007
gb:MN996527|Organism:Severe      TCAAAACTTATAGAGTACACTGACTTTGCAACATCAGCTTGTGTTTTGGCTGCTGAATGT   8989
gb:MT093631|Organism:Severe      TCAAAACTTATAGAGTACACTGACTTTGCAACATCAGCTTGTGTTTTGGCTGCTGAATGT   9060
gb:MT106053|Organism:Severe      TCAAAACTTATAGAGTACACTGACTTTGCAACATCAGCTTGTGTTTTGGCTGCTGAATGT   9022
gb:MT019533|Organism:Severe      TCAAAACTTATAGAGTACACTGACTTTGCAACATCAGCTTGTGTTTTGGCTGCTGAATGT   9022
gb:MT019531|Organism:Severe      TCAAAACTTATAGAGTACACTGACTTTGCAACATCAGCTTGTGTTTTGGCTGCTGAATGT   9022
gb:MN996528|Organism:Severe      TCAAAACTTATAGAGTACACTGACTTTGCAACATCAGCTTGTGTTTTGGCTGCTGAATGT   9022
gb:MN996530|Organism:Severe      TCAAAACTTATAGAGTACACTGACTTTGCAACATCAGCTTGTGTTTTGGCTGCTGAATGT   9008
gb:MN908947|Organism:Severe      TCAAAACTTATAGAGTACACTGACTTTGCAACATCAGCTTGTGTTTTGGCTGCTGAATGT   9022
gb:MT019532|Organism:Severe      TCAAAACTTATAGAGTACACTGACTTTGCAACATCAGCTTGTGTTTTGGCTGCTGAATGT   9022
                                 ************************ ***********************************

gb:MT020781|Organism:Severe      ACAATTTTTAAAGATGCTTCTGGTAAGCCAGTACCATATTGTTATGATACCAATGTACTA   9070
gb:MT007544|Organism:Severe      ACAATTTTTAAAGATGCTTCTGGTAAGCCAGTACCATATTGTTATGATACCAATGTACTA   9082
gb:MN994467|Organism:Severe      ACAATTTTTAAAGATGCTTCTGGTAAGCCAGTACCATATTGTTATGATACCAATGTACTA   9082
gb:MT044257|Organism:Severe      ACAATTTTTAAAGATGCTTCTGGTAAGCCAGTACCATATTGTTATGATACCAATGTACTA   9082
gb:MT106054|Organism:Severe      ACAATTTTTAAAGATGCTTCTGGTAAGCCAGTACCATATTGTTATGATACCAATGTACTA   9082
```

```
gb:MT049951|Organism:Severe      ACAATTTTTAAAGATGCTTCTGGTAAGCCAGTACCATATTGTTATGATACCAATGTACTA   9082
gb:MN975262|Organism:Severe      ACAATTTTTAAAGATGCTTCTGGTAAGCCAGTACCATATTGTTATGATACCAATGTACTA   9082
gb:MT106052|Organism:Severe      ACAATTTTTAAAGATGCTTCTGGTAAGCCAGTACCATATTGTTATGATACCAATGTACTA   9082
gb:LC522975|Organism:Severe      ACAATTTTTAAAGATGCTTCTGGTAAGCCAGTACCATATTGTTATGATACCAATGTACTA   9079
gb:LC522973|Organism:Severe      ACAATTTTTAAAGATGCTTCTGGTAAGCCAGTACCATATTGTTATGATACCAATGTACTA   9079
gb:LC522974|Organism:Severe      ACAATTTTTAAAGATGCTTCTGGTAAGCCAGTACCATATTGTTATGATACCAATGTACTA   9079
gb:MN985325|Organism:Severe      ACAATTTTTAAAGATGCTTCTGGTAAGCCAGTACCATATTGTTATGATACCAATGTACTA   9082
gb:MT020881|Organism:Severe      ACAATTTTTAAAGATGCTTCTGGTAAGCCAGTACCATATTGTTATGATACCAATGTACTA   9082
gb:MT020880|Organism:Severe      ACAATTTTTAAAGATGCTTCTGGTAAGCCAGTACCATATTGTTATGATACCAATGTACTA   9082
gb:MT066175|Organism:Severe      ACAATTTTTAAAGATGCTTCTGGTAAGCCAGTACCATATTGTTATGATACCAATGTACTA   9082
gb:MN997409|Organism:Severe      ACAATTTTTAAAGATGCTTCTGGTAAGCCAGTACCATATTGTTATGATACCAATGTACTA   9082
gb:MN938384|Organism:Severe      ACAATTTTTAAAGATGCTTCTGGTAAGCCAGTACCATATTGTTATGATACCAATGTACTA   9050
gb:MT044258|Organism:Severe      ACAATTTTTAAAGATGCTTCTGGTAAGCCAGTACCATATTGTTATGATACCAATGTACTA   9058
gb:MT039890|Organism:Severe      ACAATTTTTAAAGATGCTTCTGGTAAGCCAGTACCATATTGTTATGATACCAATGTACTA   9082
gb:MN988713|Organism:Severe      ACAATTTTTAAAGATGCTTCTGGTAAGCCAGTACCATATTGTTATGATACCAATGTACTA   9082
gb:LC521925|Organism:Severe      ACAATTTTTAAAGATGCTTCTGGTAAGCCAGTACCATATTGTTATGATACCAATGTACTA   9055
gb:MT093571|Organism:Severe      ACAATTTTTAAAGATGCTTCTGGTAAGCCAGTACCATATTGTTATGATACCAATGTACTA   9082
gb:MT039887|Organism:Severe      ACAATTTTTAAAGATGCTTCTGGTAAGCCAGTACCATATTGTTATGATACCAATGTACTA   9082
gb:MT019530|Organism:Severe      ACAATTTTTAAAGATGCTTCTGGTAAGCCAGTACCATATTGTTATGATACCAATGTACTA   9082
gb:MT039888|Organism:Severe      ACAATTTTTAAAGATGCTTCTGGTAAGCCAGTACCATATTGTTATGATACCAATGTACTA   9082
gb:LC522972|Organism:Severe      ACAATTTTTAAAGATGCTTCTGGTAAGCCAGTACCATATTGTTATGATACCAATGTACTA   9079
gb:MT027063|Organism:Severe      ACAATTTTTAAAGATGCTTCTGGTAAGCCAGTACCATATTGTTATGATACCAATGTACTA   9082
gb:MT027062|Organism:Severe      ACAATTTTTAAAGATGCTTCTGGTAAGCCAGTACCATATTGTTATGATACCAATGTACTA   9082
gb:MT019529|Organism:Severe      ACAATTTTTAAAGATGCTTCTGGTAAGCCAGTACCATATTGTTATGATACCAATGTACTA   9082
gb:MN996529|Organism:Severe      ACAATTTTTAAAGATGCTTCTGGTAAGCCAGTACCATATTGTTATGATACCAATGTACTA   9070
gb:MN996531|Organism:Severe      ACAATTTTTAAAGATGCTTCTGGTAAGCCAGTACCATATTGTTATGATACCAATGTACTA   9069
gb:MT066176|Organism:Severe      ACAATTTTTAAGGATGCTTCTGGTAAGCCAGTACCATATTGTTATGATACCAATGTACTA   9082
gb:MT027064|Organism:Severe      ACAATTTTTAAAGATGCTTCTGGTAAGCCAGTACCATATTGTTATGATACCAATGTACTA   9082
gb:MN994468|Organism:Severe      ACAATTTTTAAAGATGCTTCTGGTAAGCCAGTACCATATTGTTATGATACCAATGTACTA   9082
gb:MT072688|Organism:Severe      ACAATTTTTAAAGATGCTTCTGGTAAGCCAGTACCATATTGTTATGATACCAATGTACTA   9067
gb:MN996527|Organism:Severe      ACAATTTTTAAAGATGCTTCTGGTAAGCCAGTACCATATTGTTATGATACCAATGTACTA   9049
gb:MT093631|Organism:Severe      ACAATTTTTAAAGATGCTTCTGGTAAGCCAGTACCATATTGTTATGATACCAATGTACTA   9120
gb:MT106053|Organism:Severe      ACAATTTTTAAAGATGCTTCTGGTAAGCCAGTACCATATTGTTATGATACCAATGTACTA   9082
gb:MT019533|Organism:Severe      ACAATTTTTAAAGATGCTTCTGGTAAGCCAGTACCATATTGTTATGATACCAATGTACTA   9082
gb:MT019531|Organism:Severe      ACAATTTTTAAAGATGCTTCTGGTAAGCCAGTACCATATTGTTATGATACCAATGTACTA   9082
gb:MN996528|Organism:Severe      ACAATTTTTAAAGATGCTTCTGGTAAGCCAGTACCATATTGTTATGATACCAATGTACTA   9082
gb:MN996530|Organism:Severe      ACAATTTTTAAAGATGCTTCTGGTAAGCCAGTACCATATTGTTATGATACCAATGTACTA   9068
gb:MN908947|Organism:Severe      ACAATTTTTAAAGATGCTTCTGGTAAGCCAGTACCATATTGTTATGATACCAATGTACTA   9082
gb:MT019532|Organism:Severe      ACAATTTTTAAAGATGCTTCTGGTAAGCCAGTACCATATTGTTATGATACCAATGTACTA   9082
                                 *********** ************************************************

gb:MT020781|Organism:Severe      GAAGGTTCTGTTGCTTATGAAAGTTTACGCCCTGACACACGTTATGTGCTCATGGATGGC   9130
gb:MT007544|Organism:Severe      GAAGGTTCTGTTGCTTATGAAAGTTTACGCCCTGACACACGTTATGTGCTCATGGATGGC   9142
gb:MN994467|Organism:Severe      GAAGGTTCTGTTGCTTATGAAAGTTTACGCCCTGACACACGTTATGTGCTCATGGATGGC   9142
gb:MT044257|Organism:Severe      GAAGGTTCTGTTGCTTATGAAAGTTTACGCCCTGACACACGTTATGTGCTCATGGATGGC   9142
gb:MT106054|Organism:Severe      GAAGGTTCTGTTGCTTATGAAAGTTTACGCCCTGACACACGTTATGTGCTCATGGATGGC   9142
gb:MT049951|Organism:Severe      GAAGGTTCTGTTGCTTATGAAAGTTTACGCCCTGACACACGTTATGTGCTCATGGATGGC   9142
gb:MN975262|Organism:Severe      GAAGGTTCTGTTGCTTATGAAAGTTTACGCCCTGACACACGTTATGTGCTCATGGATGGC   9142
gb:MT106052|Organism:Severe      GAAGGTTCTGTTGCTTATGAAAGTTTACGCCCTGACACACGTTATGTGCTCATGGATGGC   9142
gb:LC522975|Organism:Severe      GAAGGTTCTGTTGCTTATGAAAGTTTACGCCCTGACACACGTTATGTGCTCATGGATGGC   9139
gb:LC522973|Organism:Severe      GAAGGTTCTGTTGCTTATGAAAGTTTACGCCCTGACACACGTTATGTGCTCATGGATGGC   9139
gb:LC522974|Organism:Severe      GAAGGTTCTGTTGCTTATGAAAGTTTACGCCCTGACACACGTTATGTGCTCATGGATGGC   9139
gb:MN985325|Organism:Severe      GAAGGTTCTGTTGCTTATGAAAGTTTACGCCCTGACACACGTTATGTGCTCATGGATGGC   9142
gb:MT020881|Organism:Severe      GAAGGTTCTGTTGCTTATGAAAGTTTACGCCCTGACACACGTTATGTGCTCATGGATGGC   9142
gb:MT020880|Organism:Severe      GAAGGTTCTGTTGCTTATGAAAGTTTACGCCCTGACACACGTTATGTGCTCATGGATGGC   9142
gb:MT066175|Organism:Severe      GAAGGTTCTGTTGCTTATGAAAGTTTACGCCCTGACACACGTTATGTGCTCATGGATGGC   9142
gb:MN997409|Organism:Severe      GAAGGTTCTGTTGCTTATGAAAGTTTACGCCCTGACACACGTTATGTGCTCATGGATGGC   9142
gb:MN938384|Organism:Severe      GAAGGTTCTGTTGCTTATGAAAGTTTACGCCCTGACACACGTTATGTGCTCATGGATGGC   9110
gb:MT044258|Organism:Severe      GAAGGTTCTGTTGCTTATGAAAGTTTACGCCCTGACACACGTTATGTGCTCATGGATGGC   9118
gb:MT039890|Organism:Severe      GAAGGTTCTGTTGCTTATGAAAGTTTACGCCCTGACACACGTTATGTGCTCATGGATGGC   9142
gb:MN988713|Organism:Severe      GAAGGTTCTGTTGCTTATGAAAGTTTACGCCCTGACACACGTTATGTGCTCATGGATGGC   9142
gb:LC521925|Organism:Severe      GAAGGTTCTGTTGCTTATGAAAGTTTACGCCCTGACACACGTTATGTGCTCATGGATGGC   9115
gb:MT093571|Organism:Severe      GAAGGTTCTGTTGCTTATGAAAGTTTACGCCCTGACACACGTTATGTGCTCATGGATGGC   9142
gb:MT039887|Organism:Severe      GAAGGTTCTGTTGCTTATGAAAGTTTACGCCCTGACACACGTTATGTGCTCATGGATGGC   9142
gb:MT019530|Organism:Severe      GAAGGTTCTGTTGCTTATGAAAGTTTACGCCCTGACACACGTTATGTGCTCATGGATGGC   9142
```

```
gb:MT039888|Organism:Severe      GAAGGTTCTGTTGCTTATGAAAGTTTACGCCCTGACACACGTTATGTGCTCATGGATGGC   9142
gb:LC522972|Organism:Severe      GAAGGTTCTGTTGCTTATGAAAGTTTACGCCCTGACACACGTTATGTGCTCATGGATGGC   9139
gb:MT027063|Organism:Severe      GAAGGTTCTGTTGCTTATGAAAGTTTACGCCCTGACACACGTTATGTGCTCATGGATGGC   9142
gb:MT027062|Organism:Severe      GAAGGTTCTGTTGCTTATGAAAGTTTACGCCCTGACACACGTTATGTGCTCATGGATGGC   9142
gb:MT019529|Organism:Severe      GAAGGTTCTGTTGCTTATGAAAGTTTACGCCCTGACACACGTTATGTGCTCATGGATGGC   9142
gb:MN996529|Organism:Severe      GAAGGTTCTGTTGCTTATGAAAGTTTACGCCCTGACACACGTTATGTGCTCATGGATGGC   9130
gb:MN996531|Organism:Severe      GAAGGTTCTGTTGCTTATGAAAGTTTACGCCCTGACACACGTTATGTGCTCATGGATGGC   9129
gb:MT066176|Organism:Severe      GAAGGTTCTGTTGCTTATGAAAGTTTACGCCCTGACACACGTTATGTGCTCATGGATGGC   9142
gb:MT027064|Organism:Severe      GAAGGTTCTGTTGCTTATGAAAGTTTACGCCCTGACACACGTTATGTGCTCATGGATGGC   9142
gb:MN994468|Organism:Severe      GAAGGTTCTGTTGCTTATGAAAGTTTACGCCCTGACACACGTTATGTGCTCATGGATGGC   9142
gb:MT072688|Organism:Severe      GAAGGTTCTGTTGCTTATGAAAGTTTACGCCCTGACACACGTTATGTGCTCATGGATGGC   9127
gb:MN996527|Organism:Severe      GAAGGTTCTGTTGCTTATGAAAGTTTACGCCCTGACACACGTTATGTGCTCATGGATGGC   9109
gb:MT093631|Organism:Severe      GAAGGTTCTGTTGCTTATGAAAGTTTACGCCCTGACACACGTTATGTGCTCATGGATGGC   9180
gb:MT106053|Organism:Severe      GAAGGTTCTGTTGCTTATGAAAGTTTACGCCCTGACACACGTTATGTGCTCATGGATGGC   9142
gb:MT019533|Organism:Severe      GAAGGTTCTGTTGCTTATGAAAGTTTACGCCCTGACACACGTTATGTGCTCATGGATGGC   9142
gb:MT019531|Organism:Severe      GAAGGTTCTGTTGCTTATGAAAGTTTACGCCCTGACACACGTTATGTGCTCATGGATGGC   9142
gb:MN996528|Organism:Severe      GAAGGTTCTGTTGCTTATGAAAGTTTACGCCCTGACACACGTTATGTGCTCATGGATGGC   9142
gb:MN996530|Organism:Severe      GAAGGTTCTGTTGCTTATGAAAGTTTACGCCCTGACACACGTTATGTGCTCATGGATGGC   9128
gb:MN908947|Organism:Severe      GAAGGTTCTGTTGCTTATGAAAGTTTACGCCCTGACACACGTTATGTGCTCATGGATGGC   9142
gb:MT019532|Organism:Severe      GAAGGTTCTGTTGCTTATGAAAGTTTACGCCCTGACACACGTTATGTGCTCATGGATGGC   9142
                                 ************************************************************

gb:MT020781|Organism:Severe      TCTATTATTCAATTTCCTAACACCTACCTTGAAGGTTCTGTTAGAGTGGTAACAACTTTT   9190
gb:MT007544|Organism:Severe      TCTATTATTCAATTTCCTAACACCTACCTTGAAGGTTCTGTTAGAGTGGTAACAACTTTT   9202
gb:MN994467|Organism:Severe      TCTATTATTCAATTTCCTAACACCTACCTTGAAGGTTCTGTTAGAGTGGTAACAACTTTT   9202
gb:MT044257|Organism:Severe      TCTATTATTCAATTTCCTAACACCTACCTTGAAGGTTCTGTTAGAGTGGTAACAACTTTT   9202
gb:MT106054|Organism:Severe      TCTATTATTCAATTTCCTAACACCTACCTTGAAGGTTCTGTTAGAGTGGTAACAACTTTT   9202
gb:MT049951|Organism:Severe      TCTATTATTCAATTTCCTAACACCTACCTTGAAGGTTCTGTTAGAGTGGTAACAACTTTT   9202
gb:MN975262|Organism:Severe      TCTATTATTCAATTTCCTAACACCTACCTTGAAGGTTCTGTTAGAGTGGTAACAACTTTT   9202
gb:MT106052|Organism:Severe      TCTATTATTCAATTTCCTAACACCTACCTTGAAGGTTCTGTTAGAGTGGTAACAACTTTT   9202
gb:LC522975|Organism:Severe      TCTATTATTCAATTTCCTAACACCTACCTTGAAGGTTCTGTTAGAGTGGTAACAACTTTT   9199
gb:LC522973|Organism:Severe      TCTATTATTCAATTTCCTAACACCTACCTTGAAGGTTCTGTTAGAGTGGTAACAACTTTT   9199
gb:LC522974|Organism:Severe      TCTATTATTCAATTTCCTAACACCTACCTTGAAGGTTCTGTTAGAGTGGTAACAACTTTT   9199
gb:MN985325|Organism:Severe      TCTATTATTCAATTTCCTAACACCTACCTTGAAGGTTCTGTTAGAGTGGTAACAACTTTT   9202
gb:MT020881|Organism:Severe      TCTATTATTCAATTTCCTAACACCTACCTTGAAGGTTCTGTTAGAGTGGTAACAACTTTT   9202
gb:MT020880|Organism:Severe      TCTATTATTCAATTTCCTAACACCTACCTTGAAGGTTCTGTTAGAGTGGTAACAACTTTT   9202
gb:MT066175|Organism:Severe      TCTATTATTCAATTTCCTAACACCTACCTTGAAGGTTCTGTTAGAGTGGTAACAACTTTT   9202
gb:MN997409|Organism:Severe      TCTATTATTCAATTTCCTAACACCTACCTTGAAGGTTCTGTTAGAGTGGTAACAACTTTT   9202
gb:MN938384|Organism:Severe      TCTATTATTCAATTTCCTAACACCTACCTTGAAGGTTCTGTTAGAGTGGTAACAACTTTT   9170
gb:MT044258|Organism:Severe      TCTATTATTCAATTTCCTAACACCTACCTTGAAGGTTCTGTTAGAGTGGTAACAACTTTT   9178
gb:MT039890|Organism:Severe      TCTATTATTCAATTTCCTAACACCTACCTTGAAGGTTCTGTTAGAGTGGTAACAACTTTT   9202
gb:MN988713|Organism:Severe      TCTATTATTCAATTTCCTAACACCTACCTTGAAGGTTCTGTTAGAGTGGTAACAACTTTT   9202
gb:LC521925|Organism:Severe      TCTATTATTCAATTTCCTAACACCTACCTTGAAGGTTCTGTTAGAGTGGTAACAACTTTT   9175
gb:MT093571|Organism:Severe      TCTATTATTCAATTTCCTAACACCTACCTTGAAGGTTCTGTTAGAGTGGTAACAACTTTT   9202
gb:MT039887|Organism:Severe      TCTATTATTCAATTTCCTAACACCTACCTTGAAGGTTCTGTTAGAGTGGTAACAACTTTT   9202
gb:MT019530|Organism:Severe      TCTATTATTCAATTTCCTAACACCTACCTTGAAGGTTCTGTTAGAGTGGTAACAACTTTT   9202
gb:MT039888|Organism:Severe      TCTATTATTCAATTTCCTAACACCTACCTTGAAGGTTCTGTTAGAGTGGTAACAACTTTT   9202
gb:LC522972|Organism:Severe      TCTATTATTCAATTTCCTAACACCTACCTTGAAGGTTCTGTTAGAGTGGTAACAACTTTT   9199
gb:MT027063|Organism:Severe      TCTATTATTCAATTTCCTAACACCTACCTTGAAGGTTCTGTTAGAGTGGTAACAACTTTT   9202
gb:MT027062|Organism:Severe      TCTATTATTCAATTTCCTAACACCTACCTTGAAGGTTCTGTTAGAGTGGTAACAACTTTT   9202
gb:MT019529|Organism:Severe      TCTATTATTCAATTTCCTAACACCTACCTTGAAGGTTCTGTTAGAGTGGTAACAACTTTT   9202
gb:MN996529|Organism:Severe      TCTATTATTCAATTTCCTAACACCTACCTTGAAGGTTCTGTTAGAGTGGTAACAACTTTT   9190
gb:MN996531|Organism:Severe      TCTATTATTCAATTTCCTAACACCTACCTTGAAGGTTCTGTTAGAGTGGTAACAACTTTT   9189
gb:MT066176|Organism:Severe      TCTATTATTCAATTTCCTAACACCTACCTTGAAGGTTCTGTTAGAGTGGTAACAACTTTT   9202
gb:MT027064|Organism:Severe      TCTATTATTCAATTTCCTAACACCTACCTTGAAGGTTCTGTTAGAGTGGTAACAACTTTT   9202
gb:MN994468|Organism:Severe      TCTATTATTCAATTTCCTAACACCTACCTTGAAGGTTCTGTTAGAGTGGTAACAACTTTT   9202
gb:MT072688|Organism:Severe      TCTATTATTCAATTTCCTAACACCTACCTTGAAGGTTCTGTTAGAGTGGTAACAACTTTT   9187
gb:MN996527|Organism:Severe      TCTATTATTCAATTTCCTAACACCTACCTTGAAGGTTCTGTTAGAGTGGTAACAACTTTT   9169
gb:MT093631|Organism:Severe      TCTATTATTCAATTTCCTAACACCTACCTTGAAGGTTCTGTTAGAGTGGTAACAACTTTT   9240
gb:MT106053|Organism:Severe      TCTATTATTCAATTTCCTAACACCTACCTTGAAGGTTCTGTTAGAGTGGTAACAACTTTT   9202
gb:MT019533|Organism:Severe      TCTATTATTCAATTTCCTAACACCTACCTTGAAGGTTCTGTTAGAGTGGTAACAACTTTT   9202
gb:MT019531|Organism:Severe      TCTATTATTCAATTTCCTAACACCTACCTTGAAGGTTCTGTTAGAGTGGTAACAACTTTT   9202
gb:MN996528|Organism:Severe      TCTATTATTCAATTTCCTAACACCTACCTTGAAGGTTCTGTTAGAGTGGTAACAACTTTT   9202
gb:MN996530|Organism:Severe      TCTATTATTCAATTTCCTAACACCTACCTTGAAGGTTCTGTTAGAGTGGTAACAACTTTT   9188
gb:MN908947|Organism:Severe      TCTATTATTCAATTTCCTAACACCTACCTTGAAGGTTCTGTTAGAGTGGTAACAACTTTT   9202
```

```
gb:MT019532|Organism:Severe      TCTATTATTCAATTTCCTAACACCTACCTTGAAGGTTCTGTTAGAGTGGTAACAACTTTT   9202
                                 ************************************************************

gb:MT020781|Organism:Severe      GATTCTGAGTACTGTAGGCACGGCACTTGTGAAAGATCAGAAGCTGGTGTTTGTGTATCT   9250
gb:MT007544|Organism:Severe      GATTCTGAGTACTGTAGGCACGGCACTTGTGAAAGATCAGAAGCTGGTGTTTGTGTATCT   9262
gb:MN994467|Organism:Severe      GATTCTGAGTACTGTAGGCACGGCACTTGTGAAAGATCAGAAGCTGGTGTTTGTGTATCT   9262
gb:MT044257|Organism:Severe      GATTCTGAGTACTGTAGGCACGGCACTTGTGAAAGATCAGAAGCTGGTGTTTGTGTATCT   9262
gb:MT106054|Organism:Severe      GATTCTGAGTACTGTAGGCACGGCACTTGTGAAAGATCAGAAGCTGGTGTTTGTGTATCT   9262
gb:MT049951|Organism:Severe      GATTCTGAGTACTGTAGGCACGGCACTTGTGAAAGATCAGAAGCTGGTGTTTGTGTATCT   9262
gb:MN975262|Organism:Severe      GATTCTGAGTACTGTAGGCACGGCACTTGTGAAAGATCAGAAGCTGGTGTTTGTGTATCT   9262
gb:MT106052|Organism:Severe      GATTCTGAGTACTGTAGGCACGGCACTTGTGAAAGATCAGAAGCTGGTGTTTGTGTATCT   9262
gb:LC522975|Organism:Severe      GATTCTGAGTACTGTAGGCACGGCACTTGTGAAAGATCAGAAGCTGGTGTTTGTGTATCT   9259
gb:LC522973|Organism:Severe      GATTCTGAGTACTGTAGGCACGGCACTTGTGAAAGATCAGAAGCTGGTGTTTGTGTATCT   9259
gb:LC522974|Organism:Severe      GATTCTGAGTACTGTAGGCACGGCACTTGTGAAAGATCAGAAGCTGGTGTTTGTGTATCT   9259
gb:MN985325|Organism:Severe      GATTCTGAGTACTGTAGGCACGGCACTTGTGAAAGATCAGAAGCTGGTGTTTGTGTATCT   9262
gb:MT020881|Organism:Severe      GATTCTGAGTACTGTAGGCACGGCACTTGTGAAAGATCAGAAGCTGGTGTTTGTGTATCT   9262
gb:MT020880|Organism:Severe      GATTCTGAGTACTGTAGGCACGGCACTTGTGAAAGATCAGAAGCTGGTGTTTGTGTATCT   9262
gb:MT066175|Organism:Severe      GATTCTGAGTACTGTAGGCACGGCACTTGTGAAAGATCAGAAGCTGGTGTTTGTGTATCT   9262
gb:MN997409|Organism:Severe      GATTCTGAGTACTGTAGGCACGGCACTTGTGAAAGATCAGAAGCTGGTGTTTGTGTATCT   9262
gb:MN938384|Organism:Severe      GATTCTGAGTACTGTAGGCACGGCACTTGTGAAAGATCAGAAGCTGGTGTTTGTGTATCT   9230
gb:MT044258|Organism:Severe      GATTCTGAGTACTGTAGGCACGGCACTTGTGAAAGATCAGAAGCTGGTGTTTGTGTATCT   9238
gb:MT039890|Organism:Severe      GATTCTGAGTACTGTAGGCACGGCACTTGTGAAAGATCAGAAGCTGGTGTTTGTGTATCT   9262
gb:MN988713|Organism:Severe      GATTCTGAGTACTGTAGGCACGGCACTTGTGAAAGATCAGAAGCTGGTGTTTGTGTATCT   9262
gb:LC521925|Organism:Severe      GATTCTGAGTACTGTAGGCACGGCACTTGTGAAAGATCAGAAGCTGGTGTTTGTGTATCT   9235
gb:MT093571|Organism:Severe      GATTCTGAGTACTGTAGGCACGGCACTTGTGAAAGATCAGAAGCTGGTGTTTGTGTATCT   9262
gb:MT039887|Organism:Severe      GATTCTGAGTACTGTAGGCACGGCACTTGTGAAAGATCAGAAGCTGGTGTTTGTGTATCT   9262
gb:MT019530|Organism:Severe      GATTCTGAGTACTGTAGGCACGGCACTTGTGAAAGATCAGAAGCTGGTGTTTGTGTATCT   9262
gb:MT039888|Organism:Severe      GATTCTGAGTACTGTAGGCACGGCACTTGTGAAAGATCAGAAGCTGGTGTTTGTGTATCT   9262
gb:LC522972|Organism:Severe      GATTCTGAGTACTGTAGGCACGGCACTTGTGAAAGATCAGAAGCTGGTGTTTGTGTATCT   9259
gb:MT027063|Organism:Severe      GATTCTGAGTACTGTAGGCACGGCACTTGTGAAAGATCAGAAGCTGGTGTTTGTGTATCT   9262
gb:MT027062|Organism:Severe      GATTCTGAGTACTGTAGGCACGGCACTTGTGAAAGATCAGAAGCTGGTGTTTGTGTATCT   9262
gb:MT019529|Organism:Severe      GATTCTGAGTACTGTAGGCACGGCACTTGTGAAAGATCAGAAGCTGGTGTTTGTGTATCT   9262
gb:MN996529|Organism:Severe      GATTCTGAGTACTGTAGGCACGGCACTTGTGAAAGATCAGAAGCTGGTGTTTGTGTATCT   9250
gb:MN996531|Organism:Severe      GATTCTGAGTACTGTAGGCACGGCACTTGTGAAAGATCAGAAGCTGGTGTTTGTGTATCT   9249
gb:MT066176|Organism:Severe      GATTCTGAGTACTGTAGGCACGGCACTTGTGAAAGATCAGAAGCTGGTGTTTGTGTATCT   9262
gb:MT027064|Organism:Severe      GATTCTGAGTACTGTAGGCACGGCACTTGTGAAAGATCAGAAGCTGGTGTTTGTGTATCT   9262
gb:MN994468|Organism:Severe      GATTCTGAGTACTGTAGGCACGGCACTTGTGAAAGATCAGAAGCTGGTGTTTGTGTATCT   9262
gb:MT072688|Organism:Severe      GATTCTGAGTACTGTAGGCACGGCACTTGTGAAAGATCAGAAGCTGGTGTTTGTGTATCT   9247
gb:MN996527|Organism:Severe      GATTCTGAGTACTGTAGGCACGGCACTTGTGAAAGATCAGAAGCTGGTGTTTGTGTATCT   9229
gb:MT093631|Organism:Severe      GATTCTGAGTACTGTAGGCACGGCACTTGTGAAAGATCAGAAGCTGGTGTTTGTGTATCT   9300
gb:MT106053|Organism:Severe      GATTCTGAGTACTGTAGGCACGGCACTTGTGAAAGATCAGAAGCTGGTGTTTGTGTATCT   9262
gb:MT019533|Organism:Severe      GATTCTGAGTACTGTAGGCACGGCACTTGTGAAAGATCAGAAGCTGGTGTTTGTGTATCT   9262
gb:MT019531|Organism:Severe      GATTCTGAGTACTGTAGGCACGGCACTTGTGAAAGATCAGAAGCTGGTGTTTGTGTATCT   9262
gb:MN996528|Organism:Severe      GATTCTGAGTACTGTAGGCACGGCACTTGTGAAAGATCAGAAGCTGGTGTTTGTGTATCT   9262
gb:MN996530|Organism:Severe      GATTCTGAGTACTGTAGGCACGGCACTTGTGAAAGATCAGAAGCTGGTGTTTGTGTATCT   9248
gb:MN908947|Organism:Severe      GATTCTGAGTACTGTAGGCACGGCACTTGTGAAAGATCAGAAGCTGGTGTTTGTGTATCT   9262
gb:MT019532|Organism:Severe      GATTCTGAGTACTGTAGGCACGGCACTTGTGAAAGATCAGAAGCTGGTGTTTGTGTATCT   9262
                                 ************************************************************

gb:MT020781|Organism:Severe      ACTAGTGGTAGATGGGTACTTAACAATGATTATTACAGATCTTTACCAGGAGTTTTCTGT   9310
gb:MT007544|Organism:Severe      ACTAGTGGTAGATGGGTACTTAACAATGATTATTACAGATCTTTACCAGGAGTTTTCTGT   9322
gb:MN994467|Organism:Severe      ACTAGTGGTAGATGGGTACTTAACAATGATTATTACAGATCTTTACCAGGAGTTTTCTGT   9322
gb:MT044257|Organism:Severe      ACTAGTGGTAGATGGGTACTTAACAATGATTATTACAGATCTTTACCAGGAGTTTTCTGT   9322
gb:MT106054|Organism:Severe      ACTAGTGGTAGATGGGTACTTAACAATGATTATTACAGATCTTTACCAGGAGTTTTCTGT   9322
gb:MT049951|Organism:Severe      ACTAGTGGTAGATGGGTACTTAACAATGATTATTACAGATCTTTACCAGGAGTTTTCTGT   9322
gb:MN975262|Organism:Severe      ACTAGTGGTAGATGGGTACTTAACAATGATTATTACAGATCTTTACCAGGAGTTTTCTGT   9322
gb:MT106052|Organism:Severe      ACTAGTGGTAGATGGGTACTTAACAATGATTATTACAGATCTTTACCAGGAGTTTTCTGT   9322
gb:LC522975|Organism:Severe      ACTAGTGGTAGATGGGTACTTAACAATGATTATTACAGATCTTTACCAGGAGTTTTCTGT   9319
gb:LC522973|Organism:Severe      ACTAGTGGTAGATGGGTACTTAACAATGATTATTACAGATCTTTACCAGGAGTTTTCTGT   9319
gb:LC522974|Organism:Severe      ACTAGTGGTAGATGGGTACTTAACAATGATTATTACAGATCTTTACCAGGAGTTTTCTGT   9319
gb:MN985325|Organism:Severe      ACTAGTGGTAGATGGGTACTTAACAATGATTATTACAGATCTTTACCAGGAGTTTTCTGT   9322
gb:MT020881|Organism:Severe      ACTAGTGGTAGATGGGTACTTAACAATGATTATTACAGATCTTTACCAGGAGTTTTCTGT   9322
gb:MT020880|Organism:Severe      ACTAGTGGTAGATGGGTACTTAACAATGATTATTACAGATCTTTACCAGGAGTTTTCTGT   9322
gb:MT066175|Organism:Severe      ACTAGTGGTAGATGGGTACTTAACAATGATTATTACAGATCTTTACCAGGAGTTTTCTGT   9322
gb:MN997409|Organism:Severe      ACTAGTGGTAGATGGGTACTTAACAATGATTATTACAGATCTTTACCAGGAGTTTTCTGT   9322
```

```
gb:MN938384|Organism:Severe      ACTAGTGGTAGATGGGTACTTAACAATGATTATTACAGATCTTTACCAGGAGTTTTCTGT   9290
gb:MT044258|Organism:Severe      ACTAGTGGTAGATGGGTACTTAACAATGATTATTACAGATCTTTACCAGGAGTTTTCTGT   9298
gb:MT039890|Organism:Severe      ACTAGTGGTAGATGGGTACTTAACAATGATTATTACAGATCTTTACCAGGAGTTTTCTGT   9322
gb:MN988713|Organism:Severe      ACTAGTGGTAGATGGGTACTTAACAATGATTATTACAGATCTTTACCAGGAGTTTTCTGT   9322
gb:LC521925|Organism:Severe      ACTAGTGGTAGATGGGTACTTAACAATGATTATTACAGATCTTTACCAGGAGTTTTCTGT   9295
gb:MT093571|Organism:Severe      ACTAGTGGTAGGTGGGTACTTAACAATGATTATTACAGATCTTTACCAGGAGTTTTCTGT   9322
gb:MT039887|Organism:Severe      ACTAGTGGTAGATGGGTACTTAACAATGATTATTACAGATCTTTACCAGGAGTTTTCTGT   9322
gb:MT019530|Organism:Severe      ACTAGTGGTAGATGGGTACTTAACAATGATTATTACAGATCTTTACCAGGAGTTTTCTGT   9322
gb:MT039888|Organism:Severe      ACTAGTGGTAGATGGGTACTTAACAATGATTATTACAGATCTTTACCAGGAGTTTTCTGT   9322
gb:LC522972|Organism:Severe      ACTAGTGGTAGATGGGTACTTAACAATGATTATTACAGATCTTTACCAGGAGTTTTCTGT   9319
gb:MT027063|Organism:Severe      ACTAGTGGTAGATGGGTACTTAACAATGATTATTACAGATCTTTACCAGGAGTTTTCTGT   9322
gb:MT027062|Organism:Severe      ACTAGTGGTAGATGGGTACTTAACAATGATTATTACAGATCTTTACCAGGAGTTTTCTGT   9322
gb:MT019529|Organism:Severe      ACTAGTGGTAGATGGGTACTTAACAATGATTATTACAGATCTTTACCAGGAGTTTTCTGT   9322
gb:MN996529|Organism:Severe      ACTAGTGGTAGATGGGTACTTAACAATGATTATTACAGATCTTTACCAGGAGTTTTCTGT   9310
gb:MN996531|Organism:Severe      ACTAGTGGTAGATGGGTACTTAACAATGATTATTACAGATCTTTACCAGGAGTTTTCTGT   9309
gb:MT066176|Organism:Severe      ACTAGTGGTAGATGGGTACTTAACAATGATTATTACAGATCTTTACCAGGAGTTTTCTGT   9322
gb:MT027064|Organism:Severe      ACTAGTGGTAGATGGGTACTTAACAATGATTATTACAGATCTTTACCAGGAGTTTTCTGT   9322
gb:MN994468|Organism:Severe      ACTAGTGGTAGATGGGTACTTAACAATGATTATTACAGATCTTTACCAGGAGTTTTCTGT   9322
gb:MT072688|Organism:Severe      ACTAGTGGTAGATGGGTACTTAACAATGATTATTACAGATCTTTACCAGGAGTTTTCTGT   9307
gb:MN996527|Organism:Severe      ACTAGTGGTAGATGGGTACTTAACAATGATTATTACAGATCTTTACCAGGAGTTTTCTGT   9289
gb:MT093631|Organism:Severe      ACTAGTGGTAGATGGGTACTTAACAATGATTATTACAGATCTTTACCAGGAGTTTTCTGT   9360
gb:MT106053|Organism:Severe      ACTAGTGGTAGATGGGTACTTAACAATGATTATTACAGATCTTTACCAGGAGTTTTCTGT   9322
gb:MT019533|Organism:Severe      ACTAGTGGTAGATGGGTACTTAACAATGATTATTACAGATCTTTACCAGGAGTTTTCTGT   9322
gb:MT019531|Organism:Severe      ACTAGTGGTAGATGGGTACTTAACAATGATTATTACAGATCTTTACCAGGAGTTTTCTGT   9322
gb:MN996528|Organism:Severe      ACTAGTGGTAGATGGGTACTTAACAATGATTATTACAGATCTTTACCAGGAGTTTTCTGT   9322
gb:MN996530|Organism:Severe      ACTAGTGGTAGATGGGTACTTAACAATGATTATTACAGATCTTTACCAGGAGTTTTCTGT   9308
gb:MN908947|Organism:Severe      ACTAGTGGTAGATGGGTACTTAACAATGATTATTACAGATCTTTACCAGGAGTTTTCTGT   9322
gb:MT019532|Organism:Severe      ACTAGTGGTAGATGGGTACTTAACAATGATTATTACAGATCTTTACCAGGAGTTTTCTGT   9322
                                 *********** ************************************************

gb:MT020781|Organism:Severe      GGTGTAGATGCTGTAAATTTACTTACTAATATGTTTACACCACTAATTCAACCTATTGGT   9370
gb:MT007544|Organism:Severe      GGTGTAGATGCTGTAAATTTACTTACTAATATGTTTACACCACTAATTCAACCTATTGGT   9382
gb:MN994467|Organism:Severe      GGTGTAGATGCTGTAAATTTACTTACTAATATGTTTACACCACTAATTCAACCTATTGGT   9382
gb:MT044257|Organism:Severe      GGTGTAGATGCTGTAAATTTACTTACTAATATGTTTACACCACTAATTCAACCTATTGGT   9382
gb:MT106054|Organism:Severe      GGTGTAGATGCTGTAAATTTACTTACTAATATGTTTACACCACTAATTCAACCTATTGGT   9382
gb:MT049951|Organism:Severe      GGTGTAGATGCTGTAAATTTACTTACTAATATGTTTACACCACTAATTCAACCTATTGGT   9382
gb:MN975262|Organism:Severe      GGTGTAGATGCTGTAAATTTACTTACTAATATGTTTACACCACTAATTCAACCTATTGGT   9382
gb:MT106052|Organism:Severe      GGTGTAGATGCTGTAAATTTACTTACTAATATGTTTACACCACTAATTCAACCTATTGGT   9382
gb:LC522975|Organism:Severe      GGTGTAGATGCTGTAAATTTACTTACTAATATGTTTACACCACTAATTCAACCTATTGGT   9379
gb:LC522973|Organism:Severe      GGTGTAGATGCTGTAAATTTACTTACTAATATGTTTACACCACTAATTCAACCTATTGGT   9379
gb:LC522974|Organism:Severe      GGTGTAGATGCTGTAAATTTACTTACTAATATGTTTACACCACTAATTCAACCTATTGGT   9379
gb:MN985325|Organism:Severe      GGTGTAGATGCTGTAAATTTACTTACTAATATGTTTACACCACTAATTCAACCTATTGGT   9382
gb:MT020881|Organism:Severe      GGTGTAGATGCTGTAAATTTACTTACTAATATGTTTACACCACTAATTCAACCTATTGGT   9382
gb:MT020880|Organism:Severe      GGTGTAGATGCTGTAAATTTACTTACTAATATGTTTACACCACTAATTCAACCTATTGGT   9382
gb:MT066175|Organism:Severe      GGTGTAGATGCTGTAAATTTACTTACTAATATGTTTACACCACTAATTCAACCTATTGGT   9382
gb:MN997409|Organism:Severe      GGTGTAGATGCTGTAAATTTACTTACTAATATGTTTACACCACTAATTCAACCTATTGGT   9382
gb:MN938384|Organism:Severe      GGTGTAGATGCTGTAAATTTACTTACTAATATGTTTACACCACTAATTCAACCTATTGGT   9350
gb:MT044258|Organism:Severe      GGTGTAGATGCTGTAAATTTACTTACTAATATGTTTACACCACTAATTCAACCTATTGGT   9358
gb:MT039890|Organism:Severe      GGTGTAGATGCTGTAAATTTACTTACTAATATGTTTACACCACTAATTCAACCTATTGGT   9382
gb:MN988713|Organism:Severe      GGTGTAGATGCTGTAAATTTACTTACTAATATGTTTACACCACTAATTCAACCTATTGGT   9382
gb:LC521925|Organism:Severe      GGTGTAGATGCTGTAAATTTACTTACTAATATGTTTACACCACTAATTCAACCTATTGGT   9355
gb:MT093571|Organism:Severe      GGTGTAGATGCTGTAAATTTACTTACTAATATGTTTACACCACTAATTCAACCTATTGGT   9382
gb:MT039887|Organism:Severe      GGTGTAGATGCTGTAAATTTACTTACTAATATGTTTACACCACTAATTCAACCTATTGGT   9382
gb:MT019530|Organism:Severe      GGTGTAGATGCTGTAAATTTACTTACTAATATGTTTACACCACTAATTCAACCTATTGGT   9382
gb:MT039888|Organism:Severe      GGTGTAGATGCTGTAAATTTACTTACTAATATGTTTACACCACTAATTCAACCTATTGGT   9382
gb:LC522972|Organism:Severe      GGTGTAGATGCTGTAAATTTACTTACTAATATGTTTACACCACTAATTCAACCTATTGGT   9379
gb:MT027063|Organism:Severe      GGTGTAGATGCTGTAAATTTACTTACTAATATGTTTACACCACTAATTCAACCTATTGGT   9382
gb:MT027062|Organism:Severe      GGTGTAGATGCTGTAAATTTACTTACTAATATGTTTACACCACTAATTCAACCTATTGGT   9382
gb:MT019529|Organism:Severe      GGTGTAGATGCTGTAAATTTACTTACTAATATGTTTACACCACTAATTCAACCTATTGGT   9382
gb:MN996529|Organism:Severe      GGTGTAGATGCTGTAAATTTACTTACTAATATGTTTACACCACTAATTCAACCTATTGGT   9370
gb:MN996531|Organism:Severe      GGTGTAGATGCTGTAAATTTACTTACTAATATGTTTACACCACTAATTCAACCTATTGGT   9369
gb:MT066176|Organism:Severe      GGTGTAGATGCTGTAAATTTACTTACTAATATGTTTACACCACTAATTCAACCTATTGGT   9382
gb:MT027064|Organism:Severe      GGTGTAGATGCTGTAAATTTACTTACTAATATGTTTACACCACTAATTCAACCTATTGGT   9382
gb:MN994468|Organism:Severe      GGTGTAGATGCTGTAAATTTACTTACTAATATGTTTACACCACTAATTCAACCTATTGGT   9382
gb:MT072688|Organism:Severe      GGTGTAGATGCTGTAAATTTACTTACTAATATGTTTACACCACTAATTCAACCTATTGGT   9367
```

```
gb:MN996527|Organism:Severe      GGTGTAGATGCTGTAAATTTACTTACTAATATGTTTACACCACTAATTCAACCTATTGGT   9349
gb:MT093631|Organism:Severe      GGTGTAGATGCTGTAAATTTACTTACTAATATGTTTACACCACTAATTCAACCTATTGGT   9420
gb:MT106053|Organism:Severe      GGTGTAGATGCTGTAAATTTACTTACTAATATGTTTACACCACTAATTCAACCTATTGGT   9382
gb:MT019533|Organism:Severe      GGTGTAGATGCTGTAAATTTACTTACTAATATGTTTACACCACTAATTCAACCTATTGGT   9382
gb:MT019531|Organism:Severe      GGTGTAGATGCTGTAAATTTACTTACTAATATGTTTACACCACTAATTCAACCTATTGGT   9382
gb:MN996528|Organism:Severe      GGTGTAGATGCTGTAAATTTACTTACTAATATGTTTACACCACTAATTCAACCTATTGGT   9382
gb:MN996530|Organism:Severe      GGTGTAGATGCTGTAAATTTACTTACTAATATGTTTACACCACTAATTCAACCTATTGGT   9368
gb:MN908947|Organism:Severe      GGTGTAGATGCTGTAAATTTACTTACTAATATGTTTACACCACTAATTCAACCTATTGGT   9382
gb:MT019532|Organism:Severe      GGTGTAGATGCTGTAAATTTACTTACTAATATGTTTACACCACTAATTCAACCTATTGGT   9382
                                 ************************************************************

gb:MT020781|Organism:Severe      GCTTTGGACATATCAGCATCTATAGTAGCTGGTGGTATTGTAGCTATCGTAGTAACATGC   9430
gb:MT007544|Organism:Severe      GCTTTGGACATATCAGCATCTATAGTAGCTGGTGGTATTGTAGCTATCGTAGTAACATGC   9442
gb:MN994467|Organism:Severe      GCTTTGGACATATCAGCATCTATAGTAGCTGGTGGTATTGTAGCTATCGTAGTAACATGC   9442
gb:MT044257|Organism:Severe      GCTTTGGACATATCAGCATCTATAGTAGCTGGTGGTATTGTAGCTATCGTAGTAACATGC   9442
gb:MT106054|Organism:Severe      GCTTTGGACATATCAGCATCTATAGTAGCTGGTGGTATTGTAGCTATCGTAGTAACATGC   9442
gb:MT049951|Organism:Severe      GCTTTGGACATATCAGCATCTATAGTAGCTGGTGGTATTGTAGCTATCGTAGTAACATGC   9442
gb:MN975262|Organism:Severe      GCTTTGGACATATCAGCATCTATAGTAGCTGGTGGTATTGTAGCTATCGTAGTAACATGC   9442
gb:MT106052|Organism:Severe      GCTTTGGACATATCAGCATCTATAGTAGCTGGTGGTATTGTAGCTATCGTAGTAACATGC   9442
gb:LC522975|Organism:Severe      GCTTTGGACATATCAGCATCTATAGTAGCTGGTGGTATTGTAGCTATCGTAGTAACATGC   9439
gb:LC522973|Organism:Severe      GCTTTGGACATATCAGCATCTATAGTAGCTGGTGGTATTGTAGCTATCGTAGTAACATGC   9439
gb:LC522974|Organism:Severe      GCTTTGGACATATCAGCATCTATAGTAGCTGGTGGTATTGTAGCTATCGTAGTAACATGC   9439
gb:MN985325|Organism:Severe      GCTTTGGACATATCAGCATCTATAGTAGCTGGTGGTATTGTAGCTATCGTAGTAACATGC   9442
gb:MT020881|Organism:Severe      GCTTTGGACATATCAGCATCTATAGTAGCTGGTGGTATTGTAGCTATCGTAGTAACATGC   9442
gb:MT020880|Organism:Severe      GCTTTGGACATATCAGCATCTATAGTAGCTGGTGGTATTGTAGCTATCGTAGTAACATGC   9442
gb:MT066175|Organism:Severe      GCTTTGGACATATCAGCATCTATAGTAGCTGGTGGTATTGTAGCTATCGTAGTAACATGC   9442
gb:MN997409|Organism:Severe      GCTTTGGACATATCAGCATCTATAGTAGCTGGTGGTATTGTAGCTATCGTAGTAACATGC   9442
gb:MN938384|Organism:Severe      GCTTTGGACATATCAGCATCTATAGTAGCTGGTGGTATTGTAGCTATCGTAGTAACATGC   9410
gb:MT044258|Organism:Severe      GCTTTGGACATATCAGCATCTATAGTAGCTGGTGGTATTGTAGCTATCGTAGTAACATGC   9418
gb:MT039890|Organism:Severe      GCTTTGGACATATCAGCATCTATAGTAGCTGGTGGTATTGTAGCTATCGTAGTAACATGC   9442
gb:MN988713|Organism:Severe      GCTTTGGACATATCAGCATCTATAGTAGCTGGTGGTATTGTAGCTATCGTAGTAACATGC   9442
gb:LC521925|Organism:Severe      GCTTTGGACATATCAGCATCTATAGTAGCTGGTGGTATTGTAGCTATCGTAGTAACATGC   9415
gb:MT093571|Organism:Severe      GCTTTGGACATATCAGCATCTATAGTAGCTGGTGGTATTGTAGCTATCGTAGTAACATGC   9442
gb:MT039887|Organism:Severe      GCTTTGGACATATCAGCATCTATAGTAGCTGGTGGTATTGTAGCTATCGTAGTAACATGC   9442
gb:MT019530|Organism:Severe      GCTTTGGACATATCAGCATCTATAGTAGCTGGTGGTATTGTAGCTATCGTAGTAACATGC   9442
gb:MT039888|Organism:Severe      GCTTTGGACATATCAGCATCTATAGTAGCTGGTGGTATTGTAGCTATCGTAGTAACATGC   9442
gb:LC522972|Organism:Severe      GCTTTGGACATATCAGCATCTATAGTAGCTGGTGGTATTGTAGCTATCGTAGTAACATGC   9439
gb:MT027063|Organism:Severe      GCTTTGGACATATCAGCATCTATAGTAGCTGGTGGTATTGTAGCTATCGTAGTAACATGC   9442
gb:MT027062|Organism:Severe      GCTTTGGACATATCAGCATCTATAGTAGCTGGTGGTATTGTAGCTATCGTAGTAACATGC   9442
gb:MT019529|Organism:Severe      GCTTTGGACATATCAGCATCTATAGTAGCTGGTGGTATTGTAGCTATCGTAGTAACATGC   9442
gb:MN996529|Organism:Severe      GCTTTGGACATATCAGCATCTATAGTAGCTGGTGGTATTGTAGCTATCGTAGTAACATGC   9430
gb:MN996531|Organism:Severe      GCTTTGGACATATCAGCATCTATAGTAGCTGGTGGTATTGTAGCTATCGTAGTAACATGC   9429
gb:MT066176|Organism:Severe      GCTTTGGACATATCAGCATCTATAGTAGCTGGTGGTATTGTAGCTATCGTAGTAACATGC   9442
gb:MT027064|Organism:Severe      GCTTTGGACATATCAGCATCTATAGTAGCTGGTGGTATTGTAGCTATCGTAGTAACATGC   9442
gb:MN994468|Organism:Severe      GCTTTGGACATATCAGCATCTATAGTAGCTGGTGGTATTGTAGCTATCGTAGTAACATGC   9442
gb:MT072688|Organism:Severe      GCTTTGGACATATCAGCATCTATAGTAGCTGGTGGTATTGTAGCTATCGTAGTAACATGC   9427
gb:MN996527|Organism:Severe      GCTTTGGACATATCAGCATCTATAGTAGCTGGTGGTATTGTAGCTATCGTAGTAACATGC   9409
gb:MT093631|Organism:Severe      GCTTTGGACATATCAGCATCTATAGTAGCTGGTGGTATTGTAGCTATCGTAGTAACATGC   9480
gb:MT106053|Organism:Severe      GCTTTGGACATATCAGCATCTATAGTAGCTGGTGGTATTGTAGCTATCGTAGTAACATGC   9442
gb:MT019533|Organism:Severe      GCTTTGGACATATCAGCATCTATAGTAGCTGGTGGTATTGTAGCTATCGTAGTAACATGC   9442
gb:MT019531|Organism:Severe      GCTTTGGACATATCAGCATCTATAGTAGCTGGTGGTATTGTAGCTATCGTAGTAACATGC   9442
gb:MN996528|Organism:Severe      GCTTTGGACATATCAGCATCTATAGTAGCTGGTGGTATTGTAGCTATCGTAGTAACATGC   9442
gb:MN996530|Organism:Severe      GCTTTGGACATATCAGCATCTATAGTAGCTGGTGGTATTGTAGCTATCGTAGTAACATGC   9428
gb:MN908947|Organism:Severe      GCTTTGGACATATCAGCATCTATAGTAGCTGGTGGTATTGTAGCTATCGTAGTAACATGC   9442
gb:MT019532|Organism:Severe      GCTTTGGACATATCAGCATCTATAGTAGCTGGTGGTATTGTAGCTATCGTAGTAACATGC   9442
                                 ************************************************************

gb:MT020781|Organism:Severe      CTTGCCTACTATTTTATGAGGTTTAGAAGAGCTTTTGGTGAATACAGTCATGTAGTTGCC   9490
gb:MT007544|Organism:Severe      CTTGCCTACTATTTTATGAGGTTTAGAAGAGCTTTTGGTGAATACAGTCATGTAGTTGCC   9502
gb:MN994467|Organism:Severe      CTTGCCTACTATTTTATGAGGTTTAGAAGAGCTTTTGGTGAATACAGTCATGTAGTTGCC   9502
gb:MT044257|Organism:Severe      CTTGCCTACTATTTTATGAGGTTTAGAAGAGCTTTTGGTGAATACAGTCATGTAGTTGCC   9502
gb:MT106054|Organism:Severe      CTTGCCTACTATTTTATGAGGTTTAGAAGAGCTTTTGGTGAATACAGTCATGTAGTTGCC   9502
gb:MT049951|Organism:Severe      CTTGCCTACTATTTTATGAGGTTTAGAAGAGCTTTTGGTGAATACAGTCATGTAGTTGCC   9502
gb:MN975262|Organism:Severe      CTTGCCTACTATTTTATGAGGTTTAGAAGAGCTTTTGGTGAATACAGTCATGTAGTTGCC   9502
gb:MT106052|Organism:Severe      CTTGCCTACTATTTTATGAGGTTTAGAAGAGCTTTTGGTGAATACAGTCATGTAGTTGCC   9502
```

```
gb:LC522975|Organism:Severe      CTTGCCTACTATTTTATGAGGTTTAGAAGAGCTTTTGGTGAATACAGTCATGTAGTTGCC   9499
gb:LC522973|Organism:Severe      CTTGCCTACTATTTTATGAGGTTTAGAAGAGCTTTTGGTGAATACAGTCATGTAGTTGCC   9499
gb:LC522974|Organism:Severe      CTTGCCTACTATTTTATGAGGTTTAGAAGAGCTTTTGGTGAATACAGTCATGTAGTTGCC   9499
gb:MN985325|Organism:Severe      CTTGCCTACTATTTTATGAGGTTTAGAAGAGCTTTTGGTGAATACAGTCATGTAGTTGCC   9502
gb:MT020881|Organism:Severe      CTTGCCTACTATTTTATGAGGTTTAGAAGAGCTTTTGGTGAATACAGTCATGTAGTTGCC   9502
gb:MT020880|Organism:Severe      CTTGCCTACTATTTTATGAGGTTTAGAAGAGCTTTTGGTGAATACAGTCATGTAGTTGCC   9502
gb:MT066175|Organism:Severe      CTTGCCTACTATTTTATGAGGTTTAGAAGAGCTTTTGGTGAATACAGTCATGTAGTTGCC   9502
gb:MN997409|Organism:Severe      CTTGCCTACTATTTTATGAGGTTTAGAAGAGCTTTTGGTGAATACAGTCATGTAGTTGCC   9502
gb:MN938384|Organism:Severe      CTTGCCTACTATTTTATGAGGTTTAGAAGAGCTTTTGGTGAATACAGTCATGTAGTTGCC   9470
gb:MT044258|Organism:Severe      CTTGCCTACTATTTTATGAGGTTTAGAAGAGCTTTTGGTGAATACAGTCATGTAGTTGCC   9478
gb:MT039890|Organism:Severe      CTTGCCTACTATTTTATGAGGTTTAGAAGAGCTTTTGGTGAATACAGTCATGTAGTTGCC   9502
gb:MN988713|Organism:Severe      CTTGCCTACTATTTTATGAGGTTTAGAAGAGCTTTTGGTGAATACAGTCATGTAGTTGCC   9502
gb:LC521925|Organism:Severe      CTTGCCTACTATTTTATGAGGTTTAGAAGAGCTTTTGGTGAATACAGTCATGTAGTTGCC   9475
gb:MT093571|Organism:Severe      CTTGCCTACTATTTTATGAGGTTTAGAAGAGCTTTTGGTGAATACAGTCATGTAGTTGCC   9502
gb:MT039887|Organism:Severe      CTTGCCTACTATTTTATGAGGTTTAGAAGAGCTTTTGGTGAATACAGTCATGTAGTTGCC   9502
gb:MT019530|Organism:Severe      CTTGCCTACTATTTTATGAGGTTTAGAAGAGCTTTTGGTGAATACAGTCATGTAGTTGCC   9502
gb:MT039888|Organism:Severe      CTTGCCTACTATTTTATGAGGTTTAGAAGAGCTTTTGGTGAATACAGTCATGTAGTTGCC   9502
gb:LC522972|Organism:Severe      CTTGCCTACTATTTTATGAGGTTTAGAAGAGCTTTTGGTGAATACAGTCATGTAGTTGCC   9499
gb:MT027063|Organism:Severe      CTTGCCTACTATTTTATGAGGTTTAGAAGAGCTTTTGGTGAATACAGTCATGTAGTTGCC   9502
gb:MT027062|Organism:Severe      CTTGCCTACTATTTTATGAGGTTTAGAAGAGCTTTTGGTGAATACAGTCATGTAGTTGCC   9502
gb:MT019529|Organism:Severe      CTTGCCTACTATTTTATGAGGTTTAGAAGAGCTTTTGGTGAATACAGTCATGTAGTTGCC   9502
gb:MN996529|Organism:Severe      CTTGCCTACTATTTTATGAGGTTTAGAAGAGCTTTTGGTGAATACAGTCATGTAGTTGCC   9490
gb:MN996531|Organism:Severe      CTTGCCTACTATTTTATGAGGTTTAGAAGAGCTTTTGGTGAATACAGTCATGTAGTTGCC   9489
gb:MT066176|Organism:Severe      CTTGCCTACTATTTTATGAGGTTTAGAAGAGCTTTTGGTGAATACAGTTATGTAGTTGCC   9502
gb:MT027064|Organism:Severe      CTTGCCTACTATTTTATGAGGTTTAGAAGAGCTTTTGGTGAATACAGTCATGTAGTTGCC   9502
gb:MN994468|Organism:Severe      CTTGCCTACTATTTTATGAGGTTTAGAAGAGCTTTTGGTGAATACAGTCATGTAGTTGCC   9502
gb:MT072688|Organism:Severe      CTTGCCTACTATTTTATGAGGTTTAGAAGAGCTTTTGGTGAATACAGTCATGTAGTTGCC   9487
gb:MN996527|Organism:Severe      CTTGCCTACTATTTTATGAGGTTTAGAAGAGCTTTTGGTGAATACAGTCATGTAGTTGCC   9469
gb:MT093631|Organism:Severe      CTTGCCTACTATTTTATGAGGTTTAGAAGAGCTTTTGGTGAATACAGTCATGTAGTTGCC   9540
gb:MT106053|Organism:Severe      CTTGCCTACTATTTTATGAGGTTTAGAAGAGCTTTTGGTGAATACAGTCATGTAGTTGCC   9502
gb:MT019533|Organism:Severe      CTTGCCTACTATTTTATGAGGTTTAGAAGAGCTTTTGGTGAATACAGTCATGTAGTTGCC   9502
gb:MT019531|Organism:Severe      CTTGCCTACTATTTTATGAGGTTTAGAAGAGCTTTTGGTGAATACAGTCATGTAGTTGCC   9502
gb:MN996528|Organism:Severe      CTTGCCTACTATTTTATGAGGTTTAGAAGAGCTTTTGGTGAATACAGTCATGTAGTTGCC   9502
gb:MN996530|Organism:Severe      CTTGCCTACTATTTTATGAGGTTTAGAAGAGCTTTTGGTGAATACAGTCATGTAGTTGCC   9488
gb:MN908947|Organism:Severe      CTTGCCTACTATTTTATGAGGTTTAGAAGAGCTTTTGGTGAATACAGTCATGTAGTTGCC   9502
gb:MT019532|Organism:Severe      CTTGCCTACTATTTTATGAGGTTTAGAAGAGCTTTTGGTGAATACAGTCATGTAGTTGCC   9502
                                 ************************************************ ***********

gb:MT020781|Organism:Severe      TTTAATACTTTACTATTCCTTATGTCATTCACTGTACTCTGTTTAACACCAGTTTACTCA   9550
gb:MT007544|Organism:Severe      TTTAATACTTTACTATTCCTTATGTCATTCACTGTACTCTGTTTAACACCAGTTTACTCA   9562
gb:MN994467|Organism:Severe      TTTAATACTTTACTATTCCTTATGTCATTCACTGTACTCTGTTTAACACCAGTTTACTCA   9562
gb:MT044257|Organism:Severe      TTTAATACTTTACTATTCCTTATGTCATTCACTGTACTCTGTTTAACACCAGTTTACTCA   9562
gb:MT106054|Organism:Severe      TTTAATACTTTACTATTCCTTATGTCATTCACTGTACTCTGTTTAACACCAGTTTACTCA   9562
gb:MT049951|Organism:Severe      TTTAATACTTTACTATTCCTTATGTCATTCACTGTACTCTGTTTAACACCAGTTTACTCA   9562
gb:MN975262|Organism:Severe      TTTAATACTTTACTATTCCTTATGTCATTCACTGTACTCTGTTTAACACCAGTTTACTTA   9562
gb:MT106052|Organism:Severe      TTTAATACTTTACTATTCCTTATGTCATTCACTGTACTCTGTTTAACACCAGTTTACTCA   9562
gb:LC522975|Organism:Severe      TTTAATACTTTACTATTCCTTATGTCATTCACTGTACTCTGTTTAACACCAGTTTACTCA   9559
gb:LC522973|Organism:Severe      TTTAATACTTTACTATTCCTTATGTCATTCACTGTACTCTGTTTAACACCAGTTTACTCA   9559
gb:LC522974|Organism:Severe      TTTAATACTTTACTATTCCTTATGTCATTCACTGTACTCTGTTTAACACCAGTTTACTCA   9559
gb:MN985325|Organism:Severe      TTTAATACTTTACTATTCCTTATGTCATTCACTGTACTCTGTTTAACACCAGTTTACTCA   9562
gb:MT020881|Organism:Severe      TTTAATACTTTACTATTCCTTATGTCATTCACTGTACTCTGTTTAACACCAGTTTACTCA   9562
gb:MT020880|Organism:Severe      TTTAATACTTTACTATTCCTTATGTCATTCACTGTACTCTGTTTAACACCAGTTTACTCA   9562
gb:MT066175|Organism:Severe      TTTAATACTTTACTATTCCTTATGTCATTCACTGTACTCTGTTTAACACCAGTTTACTCA   9562
gb:MN997409|Organism:Severe      TTTAATACTTTACTATTCCTTATGTCATTCACTGTACTCTGTTTAACACCAGTTTACTCA   9562
gb:MN938384|Organism:Severe      TTTAATACTTTACTATTCCTTATGTCATTCACTGTACTCTGTTTAACACCAGTTTACTCA   9530
gb:MT044258|Organism:Severe      TTTAATACTTTACTATTCCTTATGTCATTCACTGTACTCTGTTTAACACCAGTTTACTCA   9538
gb:MT039890|Organism:Severe      TTTAATACTTTACTATTCCTTATGTCATTCACTGTACTCTGTTTAACACCAGTTTACTCA   9562
gb:MN988713|Organism:Severe      TTTAATACTTTACTATTCCTTATGTCATTCACTGTACTCTGTTTAACACCAGTTTACTCA   9562
gb:LC521925|Organism:Severe      TTTAATACTTTACTATTCCTTATGTCATTCACTGTACTCTGTTTAACACCAGTTTACTCA   9535
gb:MT093571|Organism:Severe      TTTAATACTTTACTATTCCTTATGTCATTCACTGTACTCTGTTTAACACCAGTTTACTCA   9562
gb:MT039887|Organism:Severe      TTTAATACTTTACTATTCCTTATGTCATTCACTGTACTCTGTTTAACACCAGTTTACTCA   9562
gb:MT019530|Organism:Severe      TTTAATACTTTACTATTCCTTATGTCATTCACTGTACTCTGTTTAACACCAGTTTACTCA   9562
gb:MT039888|Organism:Severe      TTTAATACTTTACTATTCCTTATGTCATTCACTGTACTCTGTTTAACACCAGTTTACTCA   9562
gb:LC522972|Organism:Severe      TTTAATACTTTACTATTCCTTATGTCATTCACTGTACTCTGTTTAACACCAGTTTACTCA   9559
gb:MT027063|Organism:Severe      TTTAATACTTTACTATTCCTTATGTCATTCACTGTACTCTGTTTAACACCAGTTTACTCA   9562
```

```
gb:MT027062|Organism:Severe      TTTAATACTTTACTATTCCTTATGTCATTCACTGTACTCTGTTTAACACCAGTTTACTCA   9562
gb:MT019529|Organism:Severe      TTTAATACTTTACTATTCCTTATGTCATTCACTGTACTCTGTTTAACACCAGTTTACTCA   9562
gb:MN996529|Organism:Severe      TTTAATACTTTACTATTCCTTATGTCATTCACTGTACTCTGTTTAACACCAGTTTACTCA   9550
gb:MN996531|Organism:Severe      TTTAATACTTTACTATTCCTTATGTCATTCATTGTACTCTGTTTAACACCAGTTTACTCA   9549
gb:MT066176|Organism:Severe      TTTAATACTTTACTATTCCTTATGTCATTCACTGTACTCTGTTTAACACCAGTTTACTCA   9562
gb:MT027064|Organism:Severe      TTTAATACTTTACTATTCCTTATGTCATTCACTGTACTCTGTTTAACACCAGTTTACTCA   9562
gb:MN994468|Organism:Severe      TTTAATACTTTACTATTCCTTATGTCATTCACTGTACTCTGTTTAACACCAGTTTACTCA   9562
gb:MT072688|Organism:Severe      TTTAATACTTTACTATTCCTTATGTCATTCACTGTACTCTGTTTAACACCAGTTTACTCA   9547
gb:MN996527|Organism:Severe      TTTAATACTTTACTATTCCTTATGTCATTCACTGTACTCTGTTTAACACCAGTTTACTCA   9529
gb:MT093631|Organism:Severe      TTTAATACTTTACTATTCCTTATGTCATTCACTGTACTCTGTTTAACACCAGTTTACTCA   9600
gb:MT106053|Organism:Severe      TTTAATACTTTACTATTCCTTATGTCATTCACTGTACTCTGTTTAACACCAGTTTACTCA   9562
gb:MT019533|Organism:Severe      TTTAATACTTTACTATTCCTTATGTCATTCACTGTACTCTGTTTAACACCAGTTTACTCA   9562
gb:MT019531|Organism:Severe      TTTAATACTTTACTATTCCTTATGTCATTCACTGTACTCTGTTTAACACCAGTTTACTCA   9562
gb:MN996528|Organism:Severe      TTTAATACTTTACTATTCCTTATGTCATTCACTGTACTCTGTTTAACACCAGTTTACTCA   9562
gb:MN996530|Organism:Severe      TTTAATACTTTACTATTCCTTATGTCATTCACTGTACTCTGTTTAACACCAGTTTACTCA   9548
gb:MN908947|Organism:Severe      TTTAATACTTTACTATTCCTTATGTCATTCACTGTACTCTGTTTAACACCAGTTTACTCA   9562
gb:MT019532|Organism:Severe      TTTAATACTTTACTATTCCTTATGTCATTCACTGTACTCTGTTTAACACCAGTTTACTCA   9562
                                 ******************************* ************************** *

gb:MT020781|Organism:Severe      TTCTTACCTGGTGTTTATTCTGTTATTTACTTGTACTTGACATTTTATCTTACTAATGAT   9610
gb:MT007544|Organism:Severe      TTCTTACCTGGTGTTTATTCTGTTATTTACTTGTACTTGACATTTTATCTTACTAATGAT   9622
gb:MN994467|Organism:Severe      TTCTTACCTGGTGTTTATTCTGTTATTTACTTGTACTTGACATTTTATCTTACTAATGAT   9622
gb:MT044257|Organism:Severe      TTCTTACCTGGTGTTTATTCTGTTATTTACTTGTACTTGACATTTTATCTTACTAATGAT   9622
gb:MT106054|Organism:Severe      TTCTTACCTGGTGTTTATTCTGTTATTTACTTGTACTTGACATTTTATCTTACTAATGAT   9622
gb:MT049951|Organism:Severe      TTCTTACCTGGTGTTTATTCTGTTATTTACTTGTACTTGACATTTTATCTTACTAATGAT   9622
gb:MN975262|Organism:Severe      TTCTTACCTGGTGTTTATTCTGTTATTTACTTGTACTTGACATTTTATCTTACTAATGAT   9622
gb:MT106052|Organism:Severe      TTCTTACCTGGTGTTTATTCTGTTATTTACTTGTACTTGACATTTTATCTTACTAATGAT   9622
gb:LC522975|Organism:Severe      TTCTTACCTGGTGTTTATTCTGTTATTTACTTGTACTTGACATTTTATCTTACTAATGAT   9619
gb:LC522973|Organism:Severe      TTCTTACCTGGTGTTTATTCTGTTATTTACTTGTACTTGACATTTTATCTTACTAATGAT   9619
gb:LC522974|Organism:Severe      TTCTTACCTGGTGTTTATTCTGTTATTTACTTGTACTTGACATTTTATCTTACTAATGAT   9619
gb:MN985325|Organism:Severe      TTCTTACCTGGTGTTTATTCTGTTATTTACTTGTACTTGACATTTTATCTTACTAATGAT   9622
gb:MT020881|Organism:Severe      TTCTTACCTGGTGTTTATTCTGTTATTTACTTGTACTTGACATTTTATCTTACTAATGAT   9622
gb:MT020880|Organism:Severe      TTCTTACCTGGTGTTTATTCTGTTATTTACTTGTACTTGACATTTTATCTTACTAATGAT   9622
gb:MT066175|Organism:Severe      TTCTTACCTGGTGTTTATTCTGTTATTTACTTGTACTTGACATTTTATCTTACTAATGAT   9622
gb:MN997409|Organism:Severe      TTCTTACCTGGTGTTTATTCTGTTATTTACTTGTACTTGACATTTTATCTTACTAATGAT   9622
gb:MN938384|Organism:Severe      TTCTTACCTGGTGTTTATTCTGTTATTTACTTGTACTTGACATTTTATCTTACTAATGAT   9590
gb:MT044258|Organism:Severe      TTCTTACCTGGTGTTTATTCTGTTATTTACTTGTACTTGACATTTTATCTTACTAATGAT   9598
gb:MT039890|Organism:Severe      TTCTTACCTGGTGTTTATTCTGTTATTTACTTGTACTTGACATTTTATCTTACTAATGAT   9622
gb:MN988713|Organism:Severe      TTCTTACCTGGTGTTTATTCTGTTATTTACTTGTACTTGACATTTTATCTTACTAATGAT   9622
gb:LC521925|Organism:Severe      TTCTTACCTGGTGTTTATTCTGTTATTTACTTGTACTTGACATTTTATCTTACTAATGAT   9595
gb:MT093571|Organism:Severe      TTCTTACCTGGTGTTTATTCTGTTATTTACTTGTACTTGACATTTTATCTTACTAATGAT   9622
gb:MT039887|Organism:Severe      TTCTTACCTGGTGTTTATTCTGTTATTTACTTGTACTTGACATTTTATCTTACTAATGAT   9622
gb:MT019530|Organism:Severe      TTCTTACCTGGTGTTTATTCTGTTATTTACTTGTACTTGACATTTTATCTTACTAATGAT   9622
gb:MT039888|Organism:Severe      TTCTTACCTGGTGTTTATTCTGTTATTTACTTGTACTTGACATTTTATCTTACTAATGAT   9622
gb:LC522972|Organism:Severe      TTCTTACCTGGTGTTTATTCTGTTATTTACTTGTACTTGACATTTTATCTTACTAATGAT   9619
gb:MT027063|Organism:Severe      TTCTTACCTGGTGTTTATTCTGTTATTTACTTGTACTTGACATTTTATCTTACTAATGAT   9622
gb:MT027062|Organism:Severe      TTCTTACCTGGTGTTTATTCTGTTATTTACTTGTACTTGACATTTTATCTTACTAATGAT   9622
gb:MT019529|Organism:Severe      TTCTTACCTGGTGTTTATTCTGTTATTTACTTGTACTTGACATTTTATCTTACTAATGAT   9622
gb:MN996529|Organism:Severe      TTCTTACCTGGTGTTTATTCTGTTATTTACTTGTACTTGACATTTTATCTTACTAATGAT   9610
gb:MN996531|Organism:Severe      TTCTTACCTGGTGTTTATTCTGTTATTTACTTGTACTTGACATTTTATCTTACTAATGAT   9609
gb:MT066176|Organism:Severe      TTCTTACCTGGTGTTTATTCTGTTATTTACTTGTACTTGACATTTTATCTTACTAATGAT   9622
gb:MT027064|Organism:Severe      TTCTTACCTGGTGTTTATTCTGTTATTTACTTGTACTTGACATTTTATCTTACTAATGAT   9622
gb:MN994468|Organism:Severe      TTCTTACCTGGTGTTTATTCTGTTATTTACTTGTACTTGACATTTTATCTTACTAATGAT   9622
gb:MT072688|Organism:Severe      TTCTTACCTGGTGTTTATTCTGTTATTTACTTGTACTTGACATTTTATCTTACTAATGAT   9607
gb:MN996527|Organism:Severe      TTCTTACCTGGTGTTTATTCTGTTATTTACTTGTACTTGACATTTTATCTTACTAATGAT   9589
gb:MT093631|Organism:Severe      TTCTTACCTGGTGTTTATTCTGTTATTTACTTGTACTTGACATTTTATCTTACTAATGAT   9660
gb:MT106053|Organism:Severe      TTCTTACCTGGTGTTTATTCTGTTATTTACTTGTACTTGACATTTTATCTTACTAATGAT   9622
gb:MT019533|Organism:Severe      TTCTTACCTGGTGTTTATTCTGTTATTTACTTGTACTTGACATTTTATCTTACTAATGAT   9622
gb:MT019531|Organism:Severe      TTCTTACCTGGTGTTTATTCTGTTATTTACTTGTACTTGACATTTTATCTTACTAATGAT   9622
gb:MN996528|Organism:Severe      TTCTTACCTGGTGTTTATTCTGTTATTTACTTGTACTTGACATTTTATCTTACTAATGAT   9622
gb:MN996530|Organism:Severe      TTCTTACCTGGTGTTTATTCTGTTATTTACTTGTACTTGACATTTTATCTTACTAATGAT   9608
gb:MN908947|Organism:Severe      TTCTTACCTGGTGTTTATTCTGTTATTTACTTGTACTTGACATTTTATCTTACTAATGAT   9622
gb:MT019532|Organism:Severe      TTCTTACCTGGTGTTTATTCTGTTATTTACTTGTACTTGACATTTTATCTTACTAATGAT   9622
                                 ************************************************************
```

```
gb:MT020781|Organism:Severe      GTTTCTTTTTTAGCACATATTCAGTGGATGGTTATGTTCACACCTTTAGTACCTTTCTGG   9670
gb:MT007544|Organism:Severe      GTTTCTTTTTTAGCACATATTCAGTGGATGGTTATGTTCACACCTTTAGTACCTTTCTGG   9682
gb:MN994467|Organism:Severe      GTTTCTTTTTTAGCACATATTCAGTGGATGGTTATGTTCACACCTTTAGTACCTTTCTGG   9682
gb:MT044257|Organism:Severe      GTTTCTTTTTTAGCACATATTCAGTGGATGGTTATGTTCACACCTTTAGTACCTTTCTGG   9682
gb:MT106054|Organism:Severe      GTTTCTTTTTTAGCACATATTCAGTGGATGGTTATGTTCACACCTTTAGTACCTTTCTGG   9682
gb:MT049951|Organism:Severe      GTTTCTTTTTTAGCACATATTCAGTGGATGGTTATGTTCACACCTTTAGTACCTTTCTGG   9682
gb:MN975262|Organism:Severe      GTTTCTTTTTTAGCACATATTCAGTGGATGGTTATGTTCACACCTTTAGTACCTTTCTGG   9682
gb:MT106052|Organism:Severe      GTTTCTTTTTTAGCACATATTCAGTGGATGGTTATGTTCACACCTTTAGTACCTTTCTGG   9682
gb:LC522975|Organism:Severe      GTTTCTTTTTTAGCACATATTCAGTGGATGGTTATGTTCACACCTTTAGTACCTTTCTGG   9679
gb:LC522973|Organism:Severe      GTTTCTTTTTTAGCACATATTCAGTGGATGGTTATGTTCACACCTTTAGTACCTTTCTGG   9679
gb:LC522974|Organism:Severe      GTTTCTTTTTTAGCACATATTCAGTGGATGGTTATGTTCACACCTTTAGTACCTTTCTGG   9679
gb:MN985325|Organism:Severe      GTTTCTTTTTTAGCACATATTCAGTGGATGGTTATGTTCACACCTTTAGTACCTTTCTGG   9682
gb:MT020881|Organism:Severe      GTTTCTTTTTTAGCACATATTCAGTGGATGGTTATGTTCACACCTTTAGTACCTTTCTGG   9682
gb:MT020880|Organism:Severe      GTTTCTTTTTTAGCACATATTCAGTGGATGGTTATGTTCACACCTTTAGTACCTTTCTGG   9682
gb:MT066175|Organism:Severe      GTTTCTTTTTTAGCACATATTCAGTGGATGGTTATGTTCACACCTTTAGTACCTTTCTGG   9682
gb:MN997409|Organism:Severe      GTTTCTTTTTTAGCACATATTCAGTGGATGGTTATGTTCACACCTTTAGTACCTTTCTGG   9682
gb:MN938384|Organism:Severe      GTTTCTTTTTTAGCACATATTCAGTGGATGGTTATGTTCACACCTTTAGTACCTTTCTGG   9650
gb:MT044258|Organism:Severe      GTTTCTTTTTTAGCACATATTCAGTGGATGGTTATGTTCACACCTTTAGTACCTTTCTGG   9658
gb:MT039890|Organism:Severe      GTTTCTTTTTTAGCACATATTCAGTGGATGGTTATGTTCACACCTTTAGTACCTTTCTGG   9682
gb:MN988713|Organism:Severe      GTTTCTTTTTTAGCACATATTCAGTGGATGGTTATGTTCACACCTTTAGTACCTTTCTGG   9682
gb:LC521925|Organism:Severe      GTTTCTTTTTTAGCACATATTCAGTGGATGGTTATGTTCACACCTTTAGTACCTTTCTGG   9655
gb:MT093571|Organism:Severe      GTTTCTTTTTTAGCACATATTCAGTGGATGGTTATGTTCACACCTTTAGTACCTTTCTGG   9682
gb:MT039887|Organism:Severe      GTTTCTTTTTTAGCACATATTCAGTGGATGGTTATGTTCACACCTTTAGTACCTTTCTGG   9682
gb:MT019530|Organism:Severe      GTTTCTTTTTTAGCACATATTCAGTGGATGGTTATGTTCACACCTTTAGTACCTTTCTGG   9682
gb:MT039888|Organism:Severe      GTTTCTTTTTTAGCACATATTCAGTGGATGGTTATGTTCACACCTTTAGTACCTTTCTGG   9682
gb:LC522972|Organism:Severe      GTTTCTTTTTTAGCACATATTCAGTGGATGGTTATGTTCACACCTTTAGTACCTTTCTGG   9679
gb:MT027063|Organism:Severe      GTTTCTTTTTTAGCACATATTCAGTGGATGGTTATGTTCACACCTTTAGTACCTTTCTGG   9682
gb:MT027062|Organism:Severe      GTTTCTTTTTTAGCACATATTCAGTGGATGGTTATGTTCACACCTTTAGTACCTTTCTGG   9682
gb:MT019529|Organism:Severe      GTTTCTTTTTTAGCACATATTCAGTGGATGGTTATGTTCACACCTTTAGTACCTTTCTGG   9682
gb:MN996529|Organism:Severe      GTTTCTTTTTTAGCACATATTCAGTGGATGGTTATGTTCACACCTTTAGTACCTTTCTGG   9670
gb:MN996531|Organism:Severe      GTTTCTTTTTTAGCACATATTCAGTGGATGGTTATGTTCACACCTTTAGTACCTTTCTGG   9669
gb:MT066176|Organism:Severe      GTTTCTTTTTTAGCACATATTCAGTGGATGGTTATGTTCACACCTTTAGTACCTTTCTGG   9682
gb:MT027064|Organism:Severe      GTTTCTTTTTTAGCACATATTCAGTGGATGGTTATGTTCACACCTTTAGTACCTTTCTGG   9682
gb:MN994468|Organism:Severe      GTTTCTTTTTTAGCACATATTCAGTGGATGGTTATGTTCACACCTTTAGTACCTTTCTGG   9682
gb:MT072688|Organism:Severe      GTTTCTTTTTTAGCACATATTCAGTGGATGGTTATGTTCACACCTTTAGTACCTTTCTGG   9667
gb:MN996527|Organism:Severe      GTTTCTTTTTTAGCACATATTCAGTGGATGGTTATGTTCACACCTTTAGTACCTTTCTGG   9649
gb:MT093631|Organism:Severe      GTTTCTTTTTTAGCACATATTCAGTGGATGGTTATGTTCACACCTTTAGTACCTTTCTGG   9720
gb:MT106053|Organism:Severe      GTTTCTTTTTTAGCACATATTCAGTGGATGGTTATGTTCACACCTTTAGTACCTTTCTGG   9682
gb:MT019533|Organism:Severe      GTTTCTTTTTTAGCACATATTCAGTGGATGGTTATGTTCACACCTTTAGTACCTTTCTGG   9682
gb:MT019531|Organism:Severe      GTTTCTTTTTTAGCACATATTCAGTGGATGGTTATGTTCACACCTTTAGTACCTTTCTGG   9682
gb:MN996528|Organism:Severe      GTTTCTTTTTTAGCACATATTCAGTGGATGGTTATGTTCACACCTTTAGTACCTTTCTGG   9682
gb:MN996530|Organism:Severe      GTTTCTTTTTTAGCACATATTCAGTGGATGGTTATGTTCACACCTTTAGTACCTTTCTGG   9668
gb:MN908947|Organism:Severe      GTTTCTTTTTTAGCACATATTCAGTGGATGGTTATGTTCACACCTTTAGTACCTTTCTGG   9682
gb:MT019532|Organism:Severe      GTTTCTTTTTTAGCACATATTCAGTGGATGGTTATGTTCACACCTTTAGTACCTTTCTGG   9682
                                 ************************************************************

gb:MT020781|Organism:Severe      ATAACAATTGCTTATATCATTTGTATTTCCACAAAGCATTTCTATTGGTTCTTTAGTAAT   9730
gb:MT007544|Organism:Severe      ATAACAATTGCTTATATCATTTGTATTTCCACAAAGCATTTCTATTGGTTCTTTAGTAAT   9742
gb:MN994467|Organism:Severe      ATAACAATTGCTTATATCATTTGTATTTCCACAAAGCATTTCTATTGGTTCTTTAGTAAT   9742
gb:MT044257|Organism:Severe      ATAACAATTGCTTATATCATTTGTATTTCCACAAAGCATTTCTATTGGTTCTTTAGTAAT   9742
gb:MT106054|Organism:Severe      ATAACAATTGCTTATATCATTTGTATTTCCACAAAGCATTTCTATTGGTTCTTTAGTAAT   9742
gb:MT049951|Organism:Severe      ATAACAATTGCTTATATCATTTGTATTTCCACAAAGCATTTCTATTGGTTCTTTAGTAAT   9742
gb:MN975262|Organism:Severe      ATAACAATTGCTTATATCATTTGTATTTCCACAAAGCATTTCTATTGGTTCTTTAGTAAT   9742
gb:MT106052|Organism:Severe      ATAACAATTGCTTATATCATTTGTATTTCCACAAAGCATTTCTATTGGTTCTTTAGTAAT   9742
gb:LC522975|Organism:Severe      ATAACAATTGCTTATATCATTTGTATTTCCACAAAGCATTTCTATTGGTTCTTTAGTAAT   9739
gb:LC522973|Organism:Severe      ATAACAATTGCTTATATCATTTGTATTTCCACAAAGCATTTCTATTGGTTCTTTAGTAAT   9739
gb:LC522974|Organism:Severe      ATAACAATTGCTTATATCATTTGTATTTCCACAAAGCATTTCTATTGGTTCTTTAGTAAT   9739
gb:MN985325|Organism:Severe      ATAACAATTGCTTATATCATTTGTATTTCCACAAAGCATTTCTATTGGTTCTTTAGTAAT   9742
gb:MT020881|Organism:Severe      ATAACAATTGCTTATATCATTTGTATTTCCACAAAGCATTTCTATTGGTTCTTTAGTAAT   9742
gb:MT020880|Organism:Severe      ATAACAATTGCTTATATCATTTGTATTTCCACAAAGCATTTCTATTGGTTCTTTAGTAAT   9742
gb:MT066175|Organism:Severe      ATAACAATTGCTTATATCATTTGTATTTCCACAAAGCATTTCTATTGGTTCTTTAGTAAT   9742
gb:MN997409|Organism:Severe      ATAACAATTGCTTATATCATTTGTATTTCCACAAAGCATTTCTATTGGTTCTTTAGTAAT   9742
gb:MN938384|Organism:Severe      ATAACAATTGCTTATATCATTTGTATTTCCACAAAGCATTTCTATTGGTTCTTTAGTAAT   9710
gb:MT044258|Organism:Severe      ATAACAATTGCTTATATCATTTGTATTTCCACAAAGCATTTCTATTGGTTCTTTAGTAAT   9718
gb:MT039890|Organism:Severe      ATAACAATTGCTTATATCATTTGTATTTCCACAAAGCATTTCTATTGGTTCTTTAGTAAT   9742
```

```
gb:MN988713|Organism:Severe      ATAACAATTGCTTATATCATTTGTATTTCCACAAAGCATTTCTATTGGTTCTTTAGTAAT   9742
gb:LC521925|Organism:Severe      ATAACAATTGCTTATATCATTTGTATTTCCACAAAGCATTTCTATTGGTTCTTTAGTAAT   9715
gb:MT093571|Organism:Severe      ATAACAATTGCTTATATCATTTGTATTTCCACAAAGCATTTCTATTGGTTCTTTAGTAAT   9742
gb:MT039887|Organism:Severe      ATAACAATTGCTTATATCATTTGTATTTCCACAAAGCATTTCTATTGGTTCTTTAGTAAT   9742
gb:MT019530|Organism:Severe      ATAACAATTGCTTATATCATTTGTATTTCCACAAAGCATTTCTATTGGTTCTTTAGTAAT   9742
gb:MT039888|Organism:Severe      ATAACAATTGCTTATATCATTTGTATTTCCACAAAGCATTTCTATTGGTTCTTTAGTAAT   9742
gb:LC522972|Organism:Severe      ATAACAATTGCTTATATCATTTGTATTTCCACAAAGCATTTCTATTGGTTCTTTAGTAAT   9739
gb:MT027063|Organism:Severe      ATAACAATTGCTTATATCATTTGTATTTCCACAAAGCATTTCTATTGGTTCTTTAGTAAT   9742
gb:MT027062|Organism:Severe      ATAACAATTGCTTATATCATTTGTATTTCCACAAAGCATTTCTATTGGTTCTTTAGTAAT   9742
gb:MT019529|Organism:Severe      ATAACAATTGCTTATATCATTTGTATTTCCACAAAGCATTTCTATTGGTTCTTTAGTAAT   9742
gb:MN996529|Organism:Severe      ATAACAATTGCTTATATCATTTGTATTTCCACAAAGCATTTCTATTGGTTCTTTAGTAAT   9730
gb:MN996531|Organism:Severe      ATAACAATTGCTTATATCATTTGTATTTCCACAAAGCATTTCTATTGGTTCTTTAGTAAT   9729
gb:MT066176|Organism:Severe      ATAACAATTGCTTATATCATTTGTATTTCCACAAAGCATTTCTATTGGTTCTTTAGTAAT   9742
gb:MT027064|Organism:Severe      ATAACAATTGCTTATATCATTTGTATTTCCACAAAGCATTTCTATTGGTTCTTTAGTAAT   9742
gb:MN994468|Organism:Severe      ATAACAATTGCTTATATCATTTGTATTTCCACAAAGCATTTCTATTGGTTCTTTAGTAAT   9742
gb:MT072688|Organism:Severe      ATAACAATTGCTTATATCATTTGTATTTCCACAAAGCATTTCTATTGGTTCTTTAGTAAT   9727
gb:MN996527|Organism:Severe      ATAACAATTGCTTATATCATTTGTATTTCCACAAAGCATTTCTATTGGTTCTTTAGTAAT   9709
gb:MT093631|Organism:Severe      ATAACAATTGCTTATATCATTTGTATTTCCACAAAGCATTTCTATTGGTTCTTTAGTAAT   9780
gb:MT106053|Organism:Severe      ATAACAATTGCTTATATCATTTGTATTTCCACAAAGCATTTCTATTGGTTCTTTAGTAAT   9742
gb:MT019533|Organism:Severe      ATAACAATTGCTTATATCATTTGTATTTCCACAAAGCATTTCTATTGGTTCTTTAGTAAT   9742
gb:MT019531|Organism:Severe      ATAACAATTGCTTATATCATTTGTATTTCCACAAAGCATTTCTATTGGTTCTTTAGTAAT   9742
gb:MN996528|Organism:Severe      ATAACAATTGCTTATATCATTTGTATTTCCACAAAGCATTTCTATTGGTTCTTTAGTAAT   9742
gb:MN996530|Organism:Severe      ATAACAATTGCTTATATCATTTGTATTTCCACAAAGCATTTCTATTGGTTCTTTAGTAAT   9728
gb:MN908947|Organism:Severe      ATAACAATTGCTTATATCATTTGTATTTCCACAAAGCATTTCTATTGGTTCTTTAGTAAT   9742
gb:MT019532|Organism:Severe      ATAACAATTGCTTATATCATTTGTATTTCCACAAAGCATTTCTATTGGTTCTTTAGTAAT   9742
                                 ************************************************************

gb:MT020781|Organism:Severe      TACCTAAAGAGACGTGTAGTCTTTAATGGTGTTTCCTTTAGTACTTTTGAAGAAGCTGCG   9790
gb:MT007544|Organism:Severe      TACCTAAAGAGACGTGTAGTCTTTAATGGTGTTTCCTTTAGTACTTTTGAAGAAGCTGCG   9802
gb:MN994467|Organism:Severe      TACCTAAAGAGACGTGTAGTCTTTAATGGTGTTTCCTTTAGTACTTTTGAAGAAGCTGCG   9802
gb:MT044257|Organism:Severe      TACCTAAAGAGACGTGTAGTCTTTAATGGTGTTTCCTTTAGTACTTTTGAAGAAGCTGCG   9802
gb:MT106054|Organism:Severe      TACCTAAAGAGACGTGTAGTCTTTAATGGTGTTTCCTTTAGTACTTTTGAAGAAGCTGCG   9802
gb:MT049951|Organism:Severe      TACCTAAAGAGACGTGTAGTCTTTAATGGTGTTTCCTTTAGTACTTTTGAAGAAGCTGCG   9802
gb:MN975262|Organism:Severe      TACCTAAAGAGACGTGTAGTCTTTAATGGTGTTTCCTTTAGTACTTTTGAAGAAGCTGCG   9802
gb:MT106052|Organism:Severe      TACCTAAAGAGACGTGTAGTCTTTAATGGTGTTTCCTTTAGTACTTTTGAAGAAGCTGCG   9802
gb:LC522975|Organism:Severe      TACCTAAAGAGACGTGTAGTCTTTAATGGTGTTTCCTTTAGTACTTTTGAAGAAGCTGCG   9799
gb:LC522973|Organism:Severe      TACCTAAAGAGACGTGTAGTCTTTAATGGTGTTTCCTTTAGTACTTTTGAAGAAGCTGCG   9799
gb:LC522974|Organism:Severe      TACCTAAAGAGACGTGTAGTCTTTAATGGTGTTTCCTTTAGTACTTTTGAAGAAGCTGCG   9799
gb:MN985325|Organism:Severe      TACCTAAAGAGACGTGTAGTCTTTAATGGTGTTTCCTTTAGTACTTTTGAAGAAGCTGCG   9802
gb:MT020881|Organism:Severe      TACCTAAAGAGACGTGTAGTCTTTAATGGTGTTTCCTTTAGTACTTTTGAAGAAGCTGCG   9802
gb:MT020880|Organism:Severe      TACCTAAAGAGACGTGTAGTCTTTAATGGTGTTTCCTTTAGTACTTTTGAAGAAGCTGCG   9802
gb:MT066175|Organism:Severe      TACCTAAAGAGACGTGTAGTCTTTAATGGTGTTTCCTTTAGTACTTTTGAAGAAGCTGCG   9802
gb:MN997409|Organism:Severe      TACCTAAAGAGACGTGTAGTCTTTAATGGTGTTTCCTTTAGTACTTTTGAAGAAGCTGCG   9802
gb:MN938384|Organism:Severe      TACCTAAAGAGACGTGTAGTCTTTAATGGTGTTTCCTTTAGTACTTTTGAAGAAGCTGCG   9770
gb:MT044258|Organism:Severe      TACCTAAAGAGACGTGTAGTCTTTAATGGTGTTTCCTTTAGTACTTTTGAAGAAGCTGCG   9778
gb:MT039890|Organism:Severe      TACCTAAAGAGACGTGTAGTCTTTAATGGTGTTTCCTTTAGTACTTTTGAAGAAGCTGCG   9802
gb:MN988713|Organism:Severe      TACCTAAAGAGACGTGTAGTCTTTAATGGTGTTTCCTTTAGTACTTTTGAAGAAGCTGCG   9802
gb:LC521925|Organism:Severe      TACCTAAAGAGACGTGTAGTCTTTAATGGTGTTTCCTTTAGTACTTTTGAAGAAGCTGCG   9775
gb:MT093571|Organism:Severe      TACCTAAAGAGACGTGTAGTCTTTAATGGTGTTTCCTTTAGTACTTTTGAAGAAGCTGCG   9802
gb:MT039887|Organism:Severe      TACCTAAAGAGACGTGTAGTCTTTAATGGTGTTTCCTTTAGTACTTTTGAAGAAGCTGCG   9802
gb:MT019530|Organism:Severe      TACCTAAAGAGACGTGTAGTCTTTAATGGTGTTTCCTTTAGTACTTTTGAAGAAGCTGCG   9802
gb:MT039888|Organism:Severe      TACCTAAAGAGACGTGTAGTCTTTAATGGTGTTTCCTTTAGTACTTTTGAAGAAGCTGCG   9802
gb:LC522972|Organism:Severe      TACCTAAAGAGACGTGTAGTCTTTAATGGTGTTTCCTTTAGTACTTTTGAAGAAGCTGCG   9799
gb:MT027063|Organism:Severe      TACCTAAAGAGACGTGTAGTCTTTAATGGTGTTTCCTTTAGTACTTTTGAAGAAGCTGCG   9802
gb:MT027062|Organism:Severe      TACCTAAAGAGACGTGTAGTCTTTAATGGTGTTTCCTTTAGTACTTTTGAAGAAGCTGCG   9802
gb:MT019529|Organism:Severe      TACCTAAAGAGACGTGTAGTCTTTAATGGTGTTTCCTTTAGTACTTTTGAAGAAGCTGCG   9802
gb:MN996529|Organism:Severe      TACCTAAAGAGACGTGTAGTCTTTAATGGTGTTTCCTTTAGTACTTTTGAAGAAGCTGCG   9790
gb:MN996531|Organism:Severe      TACCTAAAGAGACGTGTAGTCTTTAATGGTGTTTCCTTTAGTACTTTTGAAGAAGCTGCG   9789
gb:MT066176|Organism:Severe      TACCTAAAGAGACGTGTAGTCTTTAATGGTGTTTCCTTTAGTACTTTTGAAGAAGCTGCG   9802
gb:MT027064|Organism:Severe      TACCTAAAGAGACGTGTAGTCTTTAATGGTGTTTCCTTTAGTACTTTTGAAGAAGCTGCG   9802
gb:MN994468|Organism:Severe      TACCTAAAGAGACGTGTAGTCTTTAATGGTGTTTCCTTTAGTACTTTTGAAGAAGCTGCG   9802
gb:MT072688|Organism:Severe      TACCTAAAGAGACGTGTAGTCTTTAATGGTGTTTCCTTTAGTACTTTTGAAGAAGCTGCG   9787
gb:MN996527|Organism:Severe      TACCTAAAGAGACGTGTAGTCTTTAATGGTGTTTCCTTTAGTACTTTTGAAGAAGCTGCG   9769
gb:MT093631|Organism:Severe      TACCTAAAGAGACGTGTAGTCTTTAATGGTGTTTCCTTTAGTACTTTTGAAGAAGCTGCG   9840
gb:MT106053|Organism:Severe      TACCTAAAGAGACGTGTAGTCTTTAATGGTGTTTCCTTTAGTACTTTTGAAGAAGCTGCG   9802
```

```
gb:MT019533|Organism:Severe      TACCTAAAGAGACGTGTAGTCTTTAATGGTGTTTCCTTTAGTACTTTTGAAGAAGCTGCG   9802
gb:MT019531|Organism:Severe      TACCTAAAGAGACGTGTAGTCTTTAATGGTGTTTCCTTTAGTACTTTTGAAGAAGCTGCG   9802
gb:MN996528|Organism:Severe      TACCTAAAGAGACGTGTAGTCTTTAATGGTGTTTCCTTTAGTACTTTTGAAGAAGCTGCG   9802
gb:MN996530|Organism:Severe      TACCTAAAGAGACGTGTAGTCTTTAATGGTGTTTCCTTTAGTACTTTTGAAGAAGCTGCG   9788
gb:MN908947|Organism:Severe      TACCTAAAGAGACGTGTAGTCTTTAATGGTGTTTCCTTTAGTACTTTTGAAGAAGCTGCG   9802
gb:MT019532|Organism:Severe      TACCTAAAGAGACGTGTAGTCTTTAATGGTGTTTCCTTTAGTACTTTTGAAGAAGCTGCG   9802
                                 ************************************************************

gb:MT020781|Organism:Severe      CTGTGCACCTTTTTGTTAAATAAAGAAATGTATCTAAAGTTGCGTAGTGATGTGCTATTA   9850
gb:MT007544|Organism:Severe      CTGTGCACCTTTTTGTTAAATAAAGAAATGTATCTAAAGTTGCGTAGTGATGTGCTATTA   9862
gb:MN994467|Organism:Severe      CTGTGCACCTTTTTGTTAAATAAAGAAATGTATCTAAAGTTGCGTAGTGATGTGCTATTA   9862
gb:MT044257|Organism:Severe      CTGTGCACCTTTTTGTTAAATAAAGAAATGTATCTAAAGTTGCGTAGTGATGTGCTATTA   9862
gb:MT106054|Organism:Severe      CTGTGCACCTTTTTGTTAAATAAAGAAATGTATCTAAAGTTGCGTAGTGATGTGCTATTA   9862
gb:MT049951|Organism:Severe      CTGTGCACCTTTTTGTTAAATAAAGAAATGTATCTAAAGTTGCGTAGTGATGTGCTATTA   9862
gb:MN975262|Organism:Severe      CTGTGCACCTTTTTGTTAAATAAAGAAATGTATCTAAAGTTGCGTAGTGATGTGCTATTA   9862
gb:MT106052|Organism:Severe      CTGTGCACCTTTTTGTTAAATAAAGAAATGTATCTAAAGTTGCGTAGTGATGTGCTATTA   9862
gb:LC522975|Organism:Severe      CTGTGCACCTTTTTGTTAAATAAAGAAATGTATCTAAAGTTGCGTAGTGATGTGCTATTA   9859
gb:LC522973|Organism:Severe      CTGTGCACCTTTTTGTTAAATAAAGAAATGTATCTAAAGTTGCGTAGTGATGTGCTATTA   9859
gb:LC522974|Organism:Severe      CTGTGCACCTTTTTGTTAAATAAAGAAATGTATCTAAAGTTGCGTAGTGATGTGCTATTA   9859
gb:MN985325|Organism:Severe      CTGTGCACCTTTTTGTTAAATAAAGAAATGTATCTAAAGTTGCGTAGTGATGTGCTATTA   9862
gb:MT020881|Organism:Severe      CTGTGCACCTTTTTGTTAAATAAAGAAATGTATCTAAAGTTGCGTAGTGATGTGCTATTA   9862
gb:MT020880|Organism:Severe      CTGTGCACCTTTTTGTTAAATAAAGAAATGTATCTAAAGTTGCGTAGTGATGTGCTATTA   9862
gb:MT066175|Organism:Severe      CTGTGCACCTTTTTGTTAAATAAAGAAATGTATCTAAAGTTGCGTAGTGATGTGCTATTA   9862
gb:MN997409|Organism:Severe      CTGTGCACCTTTTTGTTAAATAAAGAAATGTATCTAAAGTTGCGTAGTGATGTGCTATTA   9862
gb:MN938384|Organism:Severe      CTGTGCACCTTTTTGTTAAATAAAGAAATGTATCTAAAGTTGCGTAGTGATGTGCTATTA   9830
gb:MT044258|Organism:Severe      CTGTGCACCTTTTTGTTAAATAAAGAAATGTATCTAAAGTTGCGTAGTGATGTGCTATTA   9838
gb:MT039890|Organism:Severe      CTGTGCACCTTTTTGTTAAATAAAGAAATGTATCTAAAGTTGCGTAGTGATGTGCTATTA   9862
gb:MN988713|Organism:Severe      CTGTGCACCTTTTTGTTAAATAAAGAAATGTATCTAAAGTTGCGTAGTGATGTGCTATTA   9862
gb:LC521925|Organism:Severe      CTGTGCACCTTTTTGTTAAATAAAGAAATGTATCTAAAGTTGCGTAGTGATGTGCTATTA   9835
gb:MT093571|Organism:Severe      CTGTGCACCTTTTTGTTAAATAAAGAAATGTATCTAAAGTTGCGTAGTGATGTGCTATTA   9862
gb:MT039887|Organism:Severe      CTGTGCACCTTTTTGTTAAATAAAGAAATGTATCTAAAGTTGCGTAGTGATGTGCTATTA   9862
gb:MT019530|Organism:Severe      CTGTGCACCTTTTTGTTAAATAAAGAAATGTATCTAAAGTTGCGTAGTGATGTGCTATTA   9862
gb:MT039888|Organism:Severe      CTGTGCACCTTTTTGTTAAATAAAGAAATGTATCTAAAGTTGCGTAGTGATGTGCTATTA   9862
gb:LC522972|Organism:Severe      CTGTGCACCTTTTTGTTAAATAAAGAAATGTATCTAAAGTTGCGTAGTGATGTGCTATTA   9859
gb:MT027063|Organism:Severe      CTGTGCACCTTTTTGTTAAATAAAGAAATGTATCTAAAGTTGCGTAGTGATGTGCTATTA   9862
gb:MT027062|Organism:Severe      CTGTGCACCTTTTTGTTAAATAAAGAAATGTATCTAAAGTTGCGTAGTGATGTGCTATTA   9862
gb:MT019529|Organism:Severe      CTGTGCACCTTTTTGTTAAATAAAGAAATGTATCTAAAGTTGCGTAGTGATGTGCTATTA   9862
gb:MN996529|Organism:Severe      CTGTGCACCTTTTTGTTAAATAAAGAAATGTATCTAAAGTTGCGTAGTGATGTGCTATTA   9850
gb:MN996531|Organism:Severe      CTGTGCACCTTTTTGTTAAATAAAGAAATGTATCTAAAGTTGCGTAGTGATGTGCTATTA   9849
gb:MT066176|Organism:Severe      CTGTGCACCTTTTTGTTAAATAAAGAAATGTATCTAAAGTTGCGTAGTGATGTGCTATTA   9862
gb:MT027064|Organism:Severe      CTGTGCACCTTTTTGTTAAATAAAGAAATGTATCTAAAGTTGCGTAGTGATGTGCTATTA   9862
gb:MN994468|Organism:Severe      CTGTGCACCTTTTTGTTAAATAAAGAAATGTATCTAAAGTTGCGTAGTGATGTGCTATTA   9862
gb:MT072688|Organism:Severe      CTGTGCACCTTTTTGTTAAATAAAGAAATGTATCTAAAGTTGCGTAGTGATGTGCTATTA   9847
gb:MN996527|Organism:Severe      CTGTGCACCTTTTTGTTAAATAAAGAAATGTATCTAAAGTTGCGTAGTGATGTGCTATTA   9829
gb:MT093631|Organism:Severe      CTGTGCACCTTTTTGTTAAATAAAGAAATGTATCTAAAGTTGCGTAGTGATGTGCTATTA   9900
gb:MT106053|Organism:Severe      CTGTGCACCTTTTTGTTAAATAAAGAAATGTATCTAAAGTTGCGTAGTGATGTGCTATTA   9862
gb:MT019533|Organism:Severe      CTGTGCACCTTTTTGTTAAATAAAGAAATGTATCTAAAGTTGCGTAGTGATGTGCTATTA   9862
gb:MT019531|Organism:Severe      CTGTGCACCTTTTTGTTAAATAAAGAAATGTATCTAAAGTTGCGTAGTGATGTGCTATTA   9862
gb:MN996528|Organism:Severe      CTGTGCACCTTTTTGTTAAATAAAGAAATGTATCTAAAGTTGCGTAGTGATGTGCTATTA   9862
gb:MN996530|Organism:Severe      CTGTGCACCTTTTTGTTAAATAAAGAAATGTATCTAAAGTTGCGTAGTGATGTGCTATTA   9848
gb:MN908947|Organism:Severe      CTGTGCACCTTTTTGTTAAATAAAGAAATGTATCTAAAGTTGCGTAGTGATGTGCTATTA   9862
gb:MT019532|Organism:Severe      CTGTGCACCTTTTTGTTAAATAAAGAAATGTATCTAAAGTTGCGTAGTGATGTGCTATTA   9862
                                 ************************************************************

gb:MT020781|Organism:Severe      CCTCTTACGCAATATAATAGATACTTAGCTCTTTATAATAAGTACAAGTATTTTAGTGGA   9910
gb:MT007544|Organism:Severe      CCTCTTACGCAATATAATAGATACTTAGCTCTTTATAATAAGTACAAGTATTTTAGTGGA   9922
gb:MN994467|Organism:Severe      CCTCTTACGCAATATAATAGATACTTAGCTCTTTATAATAAGTACAAGTATTTTAGTGGA   9922
gb:MT044257|Organism:Severe      CCTCTTACGCAATATAATAGATACTTAGCTCTTTATAATAAGTACAAGTATTTTAGTGGA   9922
gb:MT106054|Organism:Severe      CCTCTTACGCAATATAATAGATACTTAGCTCTTTATAATAAGTACAAGTATTTTAGTGGA   9922
gb:MT049951|Organism:Severe      CCTCTTACGCAATATAATAGATACTTAGCTCTTTATAATAAGTACAAGTATTTTAGTGGA   9922
gb:MN975262|Organism:Severe      CCTCTTACGCAATATAATAGATACTTAGCTCTTTATAATAAGTACAAGTATTTTAGTGGA   9922
gb:MT106052|Organism:Severe      CCTCTTACGCAATATAATAGATACTTAGCTCTTTATAATAAGTACAAGTATTTTAGTGGA   9922
gb:LC522975|Organism:Severe      CCTCTTACGCAATATAATAGATACTTAGCTCTTTATAATAAGTACAAGTATTTTAGTGGA   9919
gb:LC522973|Organism:Severe      CCTCTTACGCAATATAATAGATACTTAGCTCTTTATAATAAGTACAAGTATTTTAGTGGA   9919
gb:LC522974|Organism:Severe      CCTCTTACGCAATATAATAGATACTTAGCTCTTTATAATAAGTACAAGTATTTTAGTGGA   9919
```

```
gb:MN985325|Organism:Severe      CCTCTTACGCAATATAATAGATACTTAGCTCTTTATAATAAGTACAAGTATTTTAGTGGA   9922
gb:MT020881|Organism:Severe      CCTCTTACGCAATATAATAGATACTTAGCTCTTTATAATAAGTACAAGTATTTTAGTGGA   9922
gb:MT020880|Organism:Severe      CCTCTTACGCAATATAATAGATACTTAGCTCTTTATAATAAGTACAAGTATTTTAGTGGA   9922
gb:MT066175|Organism:Severe      CCTCTTACGCAATATAATAGATACTTAGCTCTTTATAATAAGTACAAGTATTTTAGTGGA   9922
gb:MN997409|Organism:Severe      CCTCTTACGCAATATAATAGATACTTAGCTCTTTATAATAAGTACAAGTATTTTAGTGGA   9922
gb:MN938384|Organism:Severe      CCTCTTACGCAATATAATAGATACTTAGCTCTTTATAATAAGTACAAGTATTTTAGTGGA   9890
gb:MT044258|Organism:Severe      CCTCTTACGCAATATAATAGATACTTAGCTCTTTATAATAAGTACAAGTATTTTAGTGGA   9898
gb:MT039890|Organism:Severe      CCTCTTACGCAATATAATAGATACTTAGCTCTTTATAATAAGTACAAGTATTTTAGTGGA   9922
gb:MN988713|Organism:Severe      CCTCTTACGCAATATAATAGATACTTAGCTCTTTATAATAAGTACAAGTATTTTAGTGGA   9922
gb:LC521925|Organism:Severe      CCTCTTACGCAATATAATAGATACTTAGCTCTTTATAATAAGTACAAGTATTTTAGTGGA   9895
gb:MT093571|Organism:Severe      CCTCTTACGCAATATAATAGATACTTAGCTCTTTATAATAAGTACAAGTATTTTAGTGGA   9922
gb:MT039887|Organism:Severe      CCTCTTACGCAATATAATAGATACTTAGCTCTTTATAATAAGTACAAGTATTTTAGTGGA   9922
gb:MT019530|Organism:Severe      CCTCTTACGCAATATAATAGATACTTAGCTCTTTATAATAAGTACAAGTATTTTAGTGGA   9922
gb:MT039888|Organism:Severe      CCTCTTACGCAATATAATAGATACTTAGCTCTTTATAATAAGTACAAGTATTTTAGTGGA   9922
gb:LC522972|Organism:Severe      CCTCTTACGCAATATAATAGATACTTAGCTCTTTATAATAAGTACAAGTATTTTAGTGGA   9919
gb:MT027063|Organism:Severe      CCTCTTACGCAATATAATAGATACTTAGCTCTTTATAATAAGTACAAGTATTTTAGTGGA   9922
gb:MT027062|Organism:Severe      CCTCTTACGCAATATAATAGATACTTAGCTCTTTATAATAAGTACAAGTATTTTAGTGGA   9922
gb:MT019529|Organism:Severe      CCTCTTACGCAATATAATAGATACTTAGCTCTTTATAATAAGTACAAGTATTTTAGTGGA   9922
gb:MN996529|Organism:Severe      CCTCTTACGCAATATAATAGATACTTAGCTCTTTATAATAAGTACAAGTATTTTAGTGGA   9910
gb:MN996531|Organism:Severe      CCTCTTACGCAATATAATAGATACTTAGCTCTTTATAATAAGTACAAGTATTTTAGTGGA   9909
gb:MT066176|Organism:Severe      CCTCTTACGCAATATAATAGATACTTAGCTCTTTATAATAAGTACAAGTATTTTAGTGGA   9922
gb:MT027064|Organism:Severe      CCTCTTACGCAATATAATAGATACTTAGCTCTTTATAATAAGTACAAGTATTTTAGTGGA   9922
gb:MN994468|Organism:Severe      CCTCTTACGCAATATAATAGATACTTAGCTCTTTATAATAAGTACAAGTATTTTAGTGGA   9922
gb:MT072688|Organism:Severe      CCTCTTACGCAATATAATAGATACTTAGCTCTTTATAATAAGTACAAGTATTTTAGTGGA   9907
gb:MN996527|Organism:Severe      CCTCTTACGCAATATAATAGATACTTAGCTCTTTATAATAAGTACAAGTATTTTAGTGGA   9889
gb:MT093631|Organism:Severe      CCTCTTACGCAATATAATAGATACTTAGCTCTTTATAATAAGTACAAGTATTTTAGTGGA   9960
gb:MT106053|Organism:Severe      CCTCTTACGCAATATAATAGATACTTAGCTCTTTATAATAAGTACAAGTATTTTAGTGGA   9922
gb:MT019533|Organism:Severe      CCTCTTACGCAATATAATAGATACTTAGCTCTTTATAATAAGTACAAGTATTTTAGTGGA   9922
gb:MT019531|Organism:Severe      CCTCTTACGCAATATAATAGATACTTAGCTCTTTATAATAAGTACAAGTATTTTAGTGGA   9922
gb:MN996528|Organism:Severe      CCTCTTACGCAATATAATAGATACTTAGCTCTTTATAATAAGTACAAGTATTTTAGTGGA   9922
gb:MN996530|Organism:Severe      CCTCTTACGCAATATAATAGATACTTAGCTCTTTATAATAAGTACAAGTATTTTAGTGGA   9908
gb:MN908947|Organism:Severe      CCTCTTACGCAATATAATAGATACTTAGCTCTTTATAATAAGTACAAGTATTTTAGTGGA   9922
gb:MT019532|Organism:Severe      CCTCTTACGCAATATAATAGATACTTAGCTCTTTATAATAAGTACAAGTATTTTAGTGGA   9922
                                 ************************************************************

gb:MT020781|Organism:Severe      GCAATGGATACAACTAGCTACAGAGAAGCTGCTTGTTGTCATCTCGCAAAGGCTCTCAAT   9970
gb:MT007544|Organism:Severe      GCAATGGATACAACTAGCTACAGAGAAGCTGCTTGTTGTCATCTCGCAAAGGCTCTCAAT   9982
gb:MN994467|Organism:Severe      GCAATGGATACAACTAGCTACAGAGAAGCTGCTTGTTGTCATCTCGCAAAGGCTCTCAAT   9982
gb:MT044257|Organism:Severe      GCAATGGATACAACTAGCTACAGAGAAGCTGCTTGTTGTCATCTCGCAAAGGCTCTCAAT   9982
gb:MT106054|Organism:Severe      GCAATGGATACAACTAGCTACAGAGAAGCTGCTTGTTGTCATCTCGCAAAGGCTCTCAAT   9982
gb:MT049951|Organism:Severe      GCAATGGATACAACTAGCTACAGAGAAGCTGCTTGTTGTCATCTCGCAAAGGCTCTCAAT   9982
gb:MN975262|Organism:Severe      GCAATGGATACAACTAGCTACAGAGAAGCTGCTTGTTGTCATCTCGCAAAGGCTCTCAAT   9982
gb:MT106052|Organism:Severe      GCAATGGATACAACTAGCTACAGAGAAGCTGCTTGTTGTCATCTCGCAAAGGCTCTCAAT   9982
gb:LC522975|Organism:Severe      GCAATGGATACAACTAGCTACAGAGAAGCTGCTTGTTGTCATCTCGCAAAGGCTCTCAAT   9979
gb:LC522973|Organism:Severe      GCAATGGATACAACTAGCTACAGAGAAGCTGCTTGTTGTCATCTCGCAAAGGCTCTCAAT   9979
gb:LC522974|Organism:Severe      GCAATGGATACAACTAGCTACAGAGAAGCTGCTTGTTGTCATCTCGCAAAGGCTCTCAAT   9979
gb:MN985325|Organism:Severe      GCAATGGATACAACTAGCTACAGAGAAGCTGCTTGTTGTCATCTCGCAAAGGCTCTCAAT   9982
gb:MT020881|Organism:Severe      GCAATGGATACAACTAGCTACAGAGAAGCTGCTTGTTGTCATCTCGCAAAGGCTCTCAAT   9982
gb:MT020880|Organism:Severe      GCAATGGATACAACTAGCTACAGAGAAGCTGCTTGTTGTCATCTCGCAAAGGCTCTCAAT   9982
gb:MT066175|Organism:Severe      GCAATGGATACAACTAGCTACAGAGAAGCTGCTTGTTGTCATCTCGCAAAGGCTCTCAAT   9982
gb:MN997409|Organism:Severe      GCAATGGATACAACTAGCTACAGAGAAGCTGCTTGTTGTCATCTCGCAAAGGCTCTCAAT   9982
gb:MN938384|Organism:Severe      GCAATGGATACAACTAGCTACAGAGAAGCTGCTTGTTGTCATCTCGCAAAGGCTCTCAAT   9950
gb:MT044258|Organism:Severe      GCAATGGATACAACTAGCTACAGAGAAGCTGCTTGTTGTCATCTCGCAAAGGCTCTCAAT   9958
gb:MT039890|Organism:Severe      GCAATGGATACAACTAGCTACAGAGAAGCTGCTTGTTGTCATCTCGCAAAGGCTCTCAAT   9982
gb:MN988713|Organism:Severe      GCAATGGATACAACTAGCTACAGAGAAGCTGCTTGTTGTCATCTCGCAAAGGCTCTCAAT   9982
gb:LC521925|Organism:Severe      GCAATGGATACAACTAGCTACAGAGAAGCTGCTTGTTGTCATCTCGCAAAGGCTCTCAAT   9955
gb:MT093571|Organism:Severe      GCAATGGATACAACTAGCTACAGAGAAGCTGCTTGTTGTCATCTCGCAAAGGCTCTCAAT   9982
gb:MT039887|Organism:Severe      GCAATGGATACAACTAGCTACAGAGAAGCTGCTTGTTGTCATCTCGCAAAGGCTCTCAAT   9982
gb:MT019530|Organism:Severe      GCAATGGATACAACTAGCTACAGAGAAGCTGCTTGTTGTCATCTCGCAAAGGCTCTCAAT   9982
gb:MT039888|Organism:Severe      GCAATGGATACAACTAGCTACAGAGAAGCTGCTTGTTGTCATCTCGCAAAGGCTCTCAAT   9982
gb:LC522972|Organism:Severe      GCAATGGATACAACTAGCTACAGAGAAGCTGCTTGTTGTCATCTCGCAAAGGCTCTCAAT   9979
gb:MT027063|Organism:Severe      GCAATGGATACAACTAGCTACAGAGAAGCTGCTTGTTGTCATCTCGCAAAGGCTCTCAAT   9982
gb:MT027062|Organism:Severe      GCAATGGATACAACTAGCTACAGAGAAGCTGCTTGTTGTCATCTCGCAAAGGCTCTCAAT   9982
gb:MT019529|Organism:Severe      GCAATGGATACAACTAGCTACAGAGAAGCTGCTTGTTGTCATCTCGCAAAGGCTCTCAAT   9982
gb:MN996529|Organism:Severe      GCAATGGATACAACTAGCTACAGAGAAGCTGCTTGTTGTCATCTCGCAAAGGCTCTCAAT   9970
```

```
gb:MN996531|Organism:Severe      GCAATGGATACAACTAGCTACAGAGAAGCTGCTTGTTGTCATCTCGCAAAGGCTCTCAAT   9969
gb:MT066176|Organism:Severe      GCAATGGATACAACTAGCTACAGAGAAGCTGCTTGTTGTCATCTCGCAAAGGCTCTCAAT   9982
gb:MT027064|Organism:Severe      GCAATGGATACAACTAGCTACAGAGAAGCTGCTTGTTGTCATCTCGCAAAGGCTCTCAAT   9982
gb:MN994468|Organism:Severe      GCAATGGATACAACTAGCTACAGAGAAGCTGCTTGTTGTCATCTCGCAAAGGCTCTCAAT   9982
gb:MT072688|Organism:Severe      GCAATGGATACAACTAGCTACAGAGAAGCTGCTTGTTGTCATCTCGCAAAGGCTCTCAAT   9967
gb:MN996527|Organism:Severe      GCAATGGATACAACTAGCTACAGAGAAGCTGCTTGTTGTCATCTCGCAAAGGCTCTCAAT   9949
gb:MT093631|Organism:Severe      GCAATGGATACAACTAGCTACAGAGAAGCTGCTTGTTGTCATCTCGCAAAGGCTCTCAAT   10020
gb:MT106053|Organism:Severe      GCAATGGATACAACTAGCTACAGAGAAGCTGCTTGTTGTCATCTCGCAAAGGCTCTCAAT   9982
gb:MT019533|Organism:Severe      GCAATGGATACAACTAGCTACAGAGAAGCTGCTTGTTGTCATCTCGCAAAGGCTCTCAAT   9982
gb:MT019531|Organism:Severe      GCAATGGATACAACTAGCTACAGAGAAGCTGCTTGTTGTCATCTCGCAAAGGCTCTCAAT   9982
gb:MN996528|Organism:Severe      GCAATGGATACAACTAGCTACAGAGAAGCTGCTTGTTGTCATCTCGCAAAGGCTCTCAAT   9982
gb:MN996530|Organism:Severe      GCAATGGATACAACTAGCTACAGAGAAGCTGCTTGTTGTCATCTCGCAAAGGCTCTCAAT   9968
gb:MN908947|Organism:Severe      GCAATGGATACAACTAGCTACAGAGAAGCTGCTTGTTGTCATCTCGCAAAGGCTCTCAAT   9982
gb:MT019532|Organism:Severe      GCAATGGATACAACTAGCTACAGAGAAGCTGCTTGTTGTCATCTCGCAAAGGCTCTCAAT   9982
                                 ************************************************************

gb:MT020781|Organism:Severe      GACTTCAGTAACTCAGGTTCTGATGTTCTTTACCAACCACCACAAACCTCTATCACCTCA   10030
gb:MT007544|Organism:Severe      GACTTCAGTAACTCAGGTTCTGATGTTCTTTACCAACCACCACAAACCTCTATCACCTCA   10042
gb:MN994467|Organism:Severe      GACTTCAGTAACTCAGGTTCTGATGTTCTTTACCAACCACCACAAACCTCTATCACCTCA   10042
gb:MT044257|Organism:Severe      GACTTCAGTAACTCAGGTTCTGATGTTCTTTACCAACCACCACAAACCTCTATCACCTCA   10042
gb:MT106054|Organism:Severe      GACTTCAGTAACTCAGGTTCTGATGTTCTTTACCAACCACCACAAACCTCTATCACCTCA   10042
gb:MT049951|Organism:Severe      GACTTCAGTAACTCAGGTTCTGATGTTCTTTACCAACCACCACAAACCTCTATCACCTCA   10042
gb:MN975262|Organism:Severe      GACTTCAGTAACTCAGGTTCTGATGTTCTTTACCAACCACCACAAACCTCTATCACCTCA   10042
gb:MT106052|Organism:Severe      GACTTCAGTAACTCAGGTTCTGATGTTCTTTACCAACCACCACAAACCTCTATCACCTCA   10042
gb:LC522975|Organism:Severe      GACTTCAGTAACTCAGGTTCTGATGTTCTTTACCAACCACCACAAACCTCTATCACCTCA   10039
gb:LC522973|Organism:Severe      GACTTCAGTAACTCAGGTTCTGATGTTCTTTACCAACCACCACAAACCTCTATCACCTCA   10039
gb:LC522974|Organism:Severe      GACTTCAGTAACTCAGGTTCTGATGTTCTTTACCAACCACCACAAACCTCTATCACCTCA   10039
gb:MN985325|Organism:Severe      GACTTCAGTAACTCAGGTTCTGATGTTCTTTACCAACCACCACAAACCTCTATCACCTCA   10042
gb:MT020881|Organism:Severe      GACTTCAGTAACTCAGGTTCTGATGTTCTTTACCAACCACCACAAACCTCTATCACCTCA   10042
gb:MT020880|Organism:Severe      GACTTCAGTAACTCAGGTTCTGATGTTCTTTACCAACCACCACAAACCTCTATCACCTCA   10042
gb:MT066175|Organism:Severe      GACTTCAGTAACTCAGGTTCTGATGTTCTTTACCAACCACCACAAACCTCTATCACCTCA   10042
gb:MN997409|Organism:Severe      GACTTCAGTAACTCAGGTTCTGATGTTCTTTACCAACCACCACAAACCTCTATCACCTCA   10042
gb:MN938384|Organism:Severe      GACTTCAGTAACTCAGGTTCTGATGTTCTTTACCAACCACCACAAACCTCTATCACCTCA   10010
gb:MT044258|Organism:Severe      GACTTCAGTAACTCAGGTTCTGATGTTCTTTACCAACCACCACAAACCTCTATCACCTCA   10018
gb:MT039890|Organism:Severe      GACTTCAGTAACTCAGGTTCTGATGTTCTTTACCAACCACCACAAACCTCTATCACCTCA   10042
gb:MN988713|Organism:Severe      GACTTCAGTAACTCAGGTTCTGATGTTCTTTACCAACCACCACAAACCTCTATCACCTCA   10042
gb:LC521925|Organism:Severe      GACTTCAGTAACTCAGGTTCTGATGTTCTTTACCAACCACCACAAACCTCTATCACCTCA   10015
gb:MT093571|Organism:Severe      GACTTCAGTAACTCAGGTTCTGATGTTCTTTACCAACCACCACAAACCTCTATCACCTCA   10042
gb:MT039887|Organism:Severe      GACTTCAGTAACTCAGGTTCTGATGTTCTTTACCAACCACCACAAACCTCTATCACCTCA   10042
gb:MT019530|Organism:Severe      GACTTCAGTAACTCAGGTTCTGATGTTCTTTACCAACCACCACAAACCTCTATCACCTCA   10042
gb:MT039888|Organism:Severe      GACTTCAGTAACTCAGGTTCTGATGTTCTTTACCAACCACCACAAACCTCTATCACCTCA   10042
gb:LC522972|Organism:Severe      GACTTCAGTAACTCAGGTTCTGATGTTCTTTACCAACCACCACAAACCTCTATCACCTCA   10039
gb:MT027063|Organism:Severe      GACTTCAGTAACTCAGGTTCTGATGTTCTTTACCAACCACCACAAACCTCTATCACCTCA   10042
gb:MT027062|Organism:Severe      GACTTCAGTAACTCAGGTTCTGATGTTCTTTACCAACCACCACAAACCTCTATCACCTCA   10042
gb:MT019529|Organism:Severe      GACTTCAGTAACTCAGGTTCTGATGTTCTTTACCAACCACCACAAACCTCTATCACCTCA   10042
gb:MN996529|Organism:Severe      GACTTCAGTAACTCAGGTTCTGATGTTCTTTACCAACCACCACAAACCTCTATCACCTCA   10030
gb:MN996531|Organism:Severe      GACTTCAGTAACTCAGGTTCTGATGTTCTTTACCAACCACCACAAACCTCTATCACCTCA   10029
gb:MT066176|Organism:Severe      GACTTCAGTAACTCAGGTTCTGATGTTCTTTACCAACCACCACAAACCTCTATCACCTCA   10042
gb:MT027064|Organism:Severe      GACTTCAGTAACTCAGGTTCTGATGTTCTTTACCAACCACCACAAACCTCTATCACCTCA   10042
gb:MN994468|Organism:Severe      GACTTCAGTAACTCAGGTTCTGATGTTCTTTACCAACCACCACAAACCTCTATCACCTCA   10042
gb:MT072688|Organism:Severe      GACTTCAGTAACTCAGGTTCTGATGTTCTTTACCAACCACCACAAACCTCTATCACCTCA   10027
gb:MN996527|Organism:Severe      GACTTCAGTAACTCAGGTTCTGATGTTCTTTACCAACCACCACAAACCTCTATCACCTCA   10009
gb:MT093631|Organism:Severe      GACTTCAGTAACTCAGGTTCTGATGTTCTTTACCAACCACCACAAACCTCTATCACCTCA   10080
gb:MT106053|Organism:Severe      GACTTCAGTAACTCAGGTTCTGATGTTCTTTACCAACCACCACAAACCTCTATCACCTCA   10042
gb:MT019533|Organism:Severe      GACTTCAGTAACTCAGGTTCTGATGTTCTTTACCAACCACCACAAACCTCTATCACCTCA   10042
gb:MT019531|Organism:Severe      GACTTCAGTAACTCAGGTTCTGATGTTCTTTACCAACCACCACAAACCTCTATCACCTCA   10042
gb:MN996528|Organism:Severe      GACTTCAGTAACTCAGGTTCTGATGTTCTTTACCAACCACCACAAACCTCTATCACCTCA   10042
gb:MN996530|Organism:Severe      GACTTCAGTAACTCAGGTTCTGATGTTCTTTACCAACCACCACAAACCTCTATCACCTCA   10028
gb:MN908947|Organism:Severe      GACTTCAGTAACTCAGGTTCTGATGTTCTTTACCAACCACCACAAACCTCTATCACCTCA   10042
gb:MT019532|Organism:Severe      GACTTCAGTAACTCAGGTTCTGATGTTCTTTACCAACCACCACAAACCTCTATCACCTCA   10042
                                 ************************************************************

gb:MT020781|Organism:Severe      GCTGTTTTGCAGAGTGGTTTTAGAAAAATGGCATTCCCATCTGGTAAAGTTGAGGGTTGT   10090
gb:MT007544|Organism:Severe      GCTGTTTTGCAGAGTGGTTTTAGAAAAATGGCATTCCCATCTGGTAAAGTTGAGGGTTGT   10102
gb:MN994467|Organism:Severe      GCTGTTTTGCAGAGTGGTTTTAGAAAAATGGCATTCCCATCTGGTAAAGTTGAGGGTTGT   10102
```

```
gb:MT044257|Organism:Severe      GCTGTTTTGCAGAGTGGTTTTAGAAAAATGGCATTCCCATCTGGTAAAGTTGAGGGTTGT   10102
gb:MT106054|Organism:Severe      GCTGTTTTGCAGAGTGGTTTTAGAAAAATGGCATTCCCATCTGGTAAAGTTGAGGGTTGT   10102
gb:MT049951|Organism:Severe      GCTGTTTTGCAGAGTGGTTTTAGAAAAATGGCATTCCCATCTGGTAAAGTTGAGGGTTGT   10102
gb:MN975262|Organism:Severe      GCTGTTTTGCAGAGTGGTTTTAGAAAAATGGCATTCCCATCTGGTAAAGTTGAGGGTTGT   10102
gb:MT106052|Organism:Severe      GCTGTTTTGCAGAGTGGTTTTAGAAAAATGGCATTCCCATCTGGTAAAGTTGAGGGTTGT   10102
gb:LC522975|Organism:Severe      GCTGTTTTGCAGAGTGGTTTTAGAAAAATGGCATTCCCATCTGGTAAAGTTGAGGGTTGT   10099
gb:LC522973|Organism:Severe      GCTGTTTTGCAGAGTGGTTTTAGAAAAATGGCATTCCCATCTGGTAAAGTTGAGGGTTGT   10099
gb:LC522974|Organism:Severe      GCTGTTTTGCAGAGTGGTTTTAGAAAAATGGCATTCCCATCTGGTAAAGTTGAGGGTTGT   10099
gb:MN985325|Organism:Severe      GCTGTTTTGCAGAGTGGTTTTAGAAAAATGGCATTCCCATCTGGTAAAGTTGAGGGTTGT   10102
gb:MT020881|Organism:Severe      GCTGTTTTGCAGAGTGGTTTTAGAAAAATGGCATTCCCATCTGGTAAAGTTGAGGGTTGT   10102
gb:MT020880|Organism:Severe      GCTGTTTTGCAGAGTGGTTTTAGAAAAATGGCATTCCCATCTGGTAAAGTTGAGGGTTGT   10102
gb:MT066175|Organism:Severe      GCTGTTTTGCAGAGTGGTTTTAGAAAAATGGCATTCCCATCTGGTAAAGTTGAGGGTTGT   10102
gb:MN997409|Organism:Severe      GCTGTTTTGCAGAGTGGTTTTAGAAAAATGGCATTCCCATCTGGTAAAGTTGAGGGTTGT   10102
gb:MN938384|Organism:Severe      GCTGTTTTGCAGAGTGGTTTTAGAAAAATGGCATTCCCATCTGGTAAAGTTGAGGGTTGT   10070
gb:MT044258|Organism:Severe      GCTGTTTTGCAGAGTGGTTTTAGAAAAATGGCATTCCCATCTGGTAAAGTTGAGGGTTGT   10078
gb:MT039890|Organism:Severe      GCTGTTTTGCAGAGTGGTTTTAGAAAAATGGCATTCCCATCTGGTAAAGTTGAGGGTTGT   10102
gb:MN988713|Organism:Severe      GCTGTTTTGCAGAGTGGTTTTAGAAAAATGGCATTCCCATCTGGTAAAGTTGAGGGTTGT   10102
gb:LC521925|Organism:Severe      GCTGTTTTGCAGAGTGGTTTTAGAAAAATGGCATTCCCATCTGGTAAAGTTGAGGGTTGT   10075
gb:MT093571|Organism:Severe      GCTGTTTTGCAGAGTGGTTTTAGAAAAATGGCATTCCCATCTGGTAAAGTTGAGGGTTGT   10102
gb:MT039887|Organism:Severe      GCTGTTTTGCAGAGTGGTTTTAGAAAAATGGCATTCCCATCTGGTAAAGTTGAGGGTTGT   10102
gb:MT019530|Organism:Severe      GCTGTTTTGCAGAGTGGTTTTAGAAAAATGGCATTCCCATCTGGTAAAGTTGAGGGTTGT   10102
gb:MT039888|Organism:Severe      GCTGTTTTGCAGAGTGGTTTTAGAAAAATGGCATTCCCATCTGGTAAAGTTGAGGGTTGT   10102
gb:LC522972|Organism:Severe      GCTGTTTTGCAGAGTGGTTTTAGAAAAATGGCATTCCCATCTGGTAAAGTTGAGGGTTGT   10099
gb:MT027063|Organism:Severe      GCTGTTTTGCAGAGTGGTTTTAGAAAAATGGCATTCCCATCTGGTAAAGTTGAGGGTTGT   10102
gb:MT027062|Organism:Severe      GCTGTTTTGCAGAGTGGTTTTAGAAAAATGGCATTCCCATCTGGTAAAGTTGAGGGTTGT   10102
gb:MT019529|Organism:Severe      GCTGTTTTGCAGAGTGGTTTTAGAAAAATGGCATTCCCATCTGGTAAAGTTGAGGGTTGT   10102
gb:MN996529|Organism:Severe      GCTGTTTTGCAGAGTGGTTTTAGAAAAATGGCATTCCCATCTGGTAAAGTTGAGGGTTGT   10090
gb:MN996531|Organism:Severe      GCTGTTTTGCAGAGTGGTTTTAGAAAAATGGCATTCCCATCTGGTAAAGTTGAGGGTTGT   10089
gb:MT066176|Organism:Severe      GCTGTTTTGCAGAGTGGTTTTAGAAAAATGGCATTCCCATCTGGTAAAGTTGAGGGTTGT   10102
gb:MT027064|Organism:Severe      GCTGTTTTGCAGAGTGGTTTTAGAAAAATGGCATTCCCATCTGGTAAAGTTGAGGGTTGT   10102
gb:MN994468|Organism:Severe      GCTGTTTTGCAGAGTGGTTTTAGAAAAATGGCATTCCCATCTGGTAAAGTTGAGGGTTGT   10102
gb:MT072688|Organism:Severe      GCTGTTTTGCAGAGTGGTTTTAGAAAAATGGCATTCCCATCTGGTAAAGTTGAGGGTTGT   10087
gb:MN996527|Organism:Severe      GCTGTTTTGCAGAGTGGTTTTAGAAAAATGGCATTCCCATCTGGTAAAGTTGAGGGTTGT   10069
gb:MT093631|Organism:Severe      GCTGTTTTGCAGAGTGGTTTTAGAAAAATGGCATTCCCATCTGGTAAAGTTGAGGGTTGT   10140
gb:MT106053|Organism:Severe      GCTGTTTTGCAGAGTGGTTTTAGAAAAATGGCATTCCCATCTGGTAAAGTTGAGGGTTGT   10102
gb:MT019533|Organism:Severe      GCTGTTTTGCAGAGTGGTTTTAGAAAAATGGCATTCCCATCTGGTAAAGTTGAGGGTTGT   10102
gb:MT019531|Organism:Severe      GCTGTTTTGCAGAGTGGTTTTAGAAAAATGGCATTCCCATCTGGTAAAGTTGAGGGTTGT   10102
gb:MN996528|Organism:Severe      GCTGTTTTGCAGAGTGGTTTTAGAAAAATGGCATTCCCATCTGGTAAAGTTGAGGGTTGT   10102
gb:MN996530|Organism:Severe      GCTGTTTTGCAGAGTGGTTTTAGAAAAATGGCATTCCCATCTGGTAAAGTTGAGGGTTGT   10088
gb:MN908947|Organism:Severe      GCTGTTTTGCAGAGTGGTTTTAGAAAAATGGCATTCCCATCTGGTAAAGTTGAGGGTTGT   10102
gb:MT019532|Organism:Severe      GCTGTTTTGCAGAGTGGTTTTAGAAAAATGGCATTCCCATCTGGTAAAGTTGAGGGTTGT   10102
                                 ************************************************************

gb:MT020781|Organism:Severe      ATGGTACAAGTAACTTGTGGTACAACTACACTTAACGGTCTTTGGCTTGATGACGTAGTT   10150
gb:MT007544|Organism:Severe      ATGGTACAAGTAACTTGTGGTACAACTACACTTAACGGTCTTTGGCTTGATGACGTAGTT   10162
gb:MN994467|Organism:Severe      ATGGTACAAGTAACTTGTGGTACAACTACACTTAACGGTCTTTGGCTTGATGACGTAGTT   10162
gb:MT044257|Organism:Severe      ATGGTACAAGTAACTTGTGGTACAACTACACTTAACGGTCTTTGGCTTGATGACGTAGTT   10162
gb:MT106054|Organism:Severe      ATGGTACAAGTAACTTGTGGTACAACTACACTTAACGGTCTTTGGCTTGATGACGTAGTT   10162
gb:MT049951|Organism:Severe      ATGGTACAAGTAACTTGTGGTACAACTACACTTAACGGTCTTTGGCTTGATGACGTAGTT   10162
gb:MN975262|Organism:Severe      ATGGTACAAGTAACTTGTGGTACAACTACACTTAACGGTCTTTGGCTTGATGACGTAGTT   10162
gb:MT106052|Organism:Severe      ATGGTACAAGTAACTTGTGGTACAACTACACTTAACGGTCTTTGGCTTGATGACGTAGTT   10162
gb:LC522975|Organism:Severe      ATGGTACAAGTAACTTGTGGTACAACTACACTTAACGGTCTTTGGCTTGATGACGTAGTT   10159
gb:LC522973|Organism:Severe      ATGGTACAAGTAACTTGTGGTACAACTACACTTAACGGTCTTTGGCTTGATGACGTAGTT   10159
gb:LC522974|Organism:Severe      ATGGTACAAGTAACTTGTGGTACAACTACACTTAACGGTCTTTGGCTTGATGACGTAGTT   10159
gb:MN985325|Organism:Severe      ATGGTACAAGTAACTTGTGGTACAACTACACTTAACGGTCTTTGGCTTGATGACGTAGTT   10162
gb:MT020881|Organism:Severe      ATGGTACAAGTAACTTGTGGTACAACTACACTTAACGGTCTTTGGCTTGATGACGTAGTT   10162
gb:MT020880|Organism:Severe      ATGGTACAAGTAACTTGTGGTACAACTACACTTAACGGTCTTTGGCTTGATGACGTAGTT   10162
gb:MT066175|Organism:Severe      ATGGTACAAGTAACTTGTGGTACAACTACACTTAACGGTCTTTGGCTTGATGACGTAGTT   10162
gb:MN997409|Organism:Severe      ATGGTACAAGTAACTTGTGGTACAACTACACTTAACGGTCTTTGGCTTGATGACGTAGTT   10162
gb:MN938384|Organism:Severe      ATGGTACAAGTAACTTGTGGTACAACTACACTTAACGGTCTTTGGCTTGATGACGTAGTT   10130
gb:MT044258|Organism:Severe      ATGGTACAAGTAACTTGTGGTACAACTACACTTAACGGTCTTTGGCTTGATGACGTAGTT   10138
gb:MT039890|Organism:Severe      ATGGTACAAGTAACTTGTGGTACAACTACACTTAACGGTCTTTGGCTTGATGACGTAGTT   10162
gb:MN988713|Organism:Severe      ATGGTACAAGTAACTTGTGGTACAACTACACTTAACGGTCTTTGGCTTGATGACGTAGTT   10162
gb:LC521925|Organism:Severe      ATGGTACAAGTAACTTGTGGTACAACTACACTTAACGGTCTTTGGCTTGATGACGTAGTT   10135
gb:MT093571|Organism:Severe      ATGGTACAAGTAACTTGTGGTACAACTACACTTAACGGTCTTTGGCTTGATGACGTAGTT   10162
```

```
gb:MT039887|Organism:Severe      ATGGTACAAGTAACTTGTGGTACAACTACACTTAACGGTCTTTGGCTTGATGACGTAGTT   10162
gb:MT019530|Organism:Severe      ATGGTACAAGTAACTTGTGGTACAACTACACTTAACGGTCTTTGGCTTGATGACGTAGTT   10162
gb:MT039888|Organism:Severe      ATGGTACAAGTAACTTGTGGTACAACTACACTTAACGGTCTTTGGCTTGATGACGTAGTT   10162
gb:LC522972|Organism:Severe      ATGGTACAAGTAACTTGTGGTACAACTACACTTAACGGTCTTTGGCTTGATGACGTAGTT   10159
gb:MT027063|Organism:Severe      ATGGTACAAGTAACTTGTGGTACAACTACACTTAACGGTCTTTGGCTTGATGACGTAGTT   10162
gb:MT027062|Organism:Severe      ATGGTACAAGTAACTTGTGGTACAACTACACTTAACGGTCTTTGGCTTGATGACGTAGTT   10162
gb:MT019529|Organism:Severe      ATGGTACAAGTAACTTGTGGTACAACTACACTTAACGGTCTTTGGCTTGATGACGTAGTT   10162
gb:MN996529|Organism:Severe      ATGGTACAAGTAACTTGTGGTACAACTACACTTAACGGTCTTTGGCTTGATGACGTAGTT   10150
gb:MN996531|Organism:Severe      ATGGTACAAGTAACTTGTGGTACAACTACACTTAACGGTCTTTGGCTTGATGACGTAGTT   10149
gb:MT066176|Organism:Severe      ATGGTACAAGTAACTTGTGGTACAACTACACTTAACGGTCTTTGGCTTGATGACGTAGTT   10162
gb:MT027064|Organism:Severe      ATGGTACAAGTAACTTGTGGTACAACTACACTTAACGGTCTTTGGCTTGATGACGTAGTT   10162
gb:MN994468|Organism:Severe      ATGGTACAAGTAACTTGTGGTACAACTACACTTAACGGTCTTTGGCTTGATGACGTAGTT   10162
gb:MT072688|Organism:Severe      ATGGTACAAGTAACTTGTGGTACAACTACACTTAACGGTCTTTGGCTTGATGACGTAGTT   10147
gb:MN996527|Organism:Severe      ATGGTACAAGTAACTTGTGGTACAACTACACTTAACGGTCTTTGGCTTGATGACGTAGTT   10129
gb:MT093631|Organism:Severe      ATGGTACAAGTAACTTGTGGTACAACTACACTTAACGGTCTTTGGCTTGATGACGTAGTT   10200
gb:MT106053|Organism:Severe      ATGGTACAAGTAACTTGTGGTACAACTACACTTAACGGTCTTTGGCTTGATGACGTAGTT   10162
gb:MT019533|Organism:Severe      ATGGTACAAGTAACTTGTGGTACAACTACACTTAACGGTCTTTGGCTTGATGACGTAGTT   10162
gb:MT019531|Organism:Severe      ATGGTACAAGTAACTTGTGGTACAACTACACTTAACGGTCTTTGGCTTGATGACGTAGTT   10162
gb:MN996528|Organism:Severe      ATGGTACAAGTAACTTGTGGTACAACTACACTTAACGGTCTTTGGCTTGATGACGTAGTT   10162
gb:MN996530|Organism:Severe      ATGGTACAAGTAACTTGTGGTACAACTACACTTAACGGTCTTTGGCTTGATGACGTAGTT   10148
gb:MN908947|Organism:Severe      ATGGTACAAGTAACTTGTGGTACAACTACACTTAACGGTCTTTGGCTTGATGACGTAGTT   10162
gb:MT019532|Organism:Severe      ATGGTACAAGTAACTTGTGGTACAACTACACTTAACGGTCTTTGGCTTGATGACGTAGTT   10162
                                 ************************************************************

gb:MT020781|Organism:Severe      TACTGTCCAAGACATGTGATCTGCACCTCTGAAGACATGCTTAACCCTAATTATGAAGAT   10210
gb:MT007544|Organism:Severe      TACTGTCCAAGACATGTGATCTGCACCTCTGAAGACATGCTTAACCCTAATTATGAAGAT   10222
gb:MN994467|Organism:Severe      TACTGTCCAAGACATGTGATCTGCACCTCTGAAGACATGCTTAACCCTAATTATGAAGAT   10222
gb:MT044257|Organism:Severe      TACTGTCCAAGACATGTGATCTGCACCTCTGAAGACATGCTTAACCCTAATTATGAAGAT   10222
gb:MT106054|Organism:Severe      TACTGTCCAAGACATGTGATCTGCACCTCTGAAGACATGCTTAACCCTAATTATGAAGAT   10222
gb:MT049951|Organism:Severe      TACTGTCCAAGACATGTGATCTGCACCTCTGAAGACATGCTTAACCCTAATTATGAAGAT   10222
gb:MN975262|Organism:Severe      TACTGTCCAAGACATGTGATCTGCACCTCTGAAGACATGCTTAACCCTAATTATGAAGAT   10222
gb:MT106052|Organism:Severe      TACTGTCCAAGACATGTGATCTGCACCTCTGAAGACATGCTTAACCCTAATTATGAAGAT   10222
gb:LC522975|Organism:Severe      TACTGTCCAAGACATGTGATCTGCACCTCTGAAGACATGCTTAACCCTAATTATGAAGAT   10219
gb:LC522973|Organism:Severe      TACTGTCCAAGACATGTGATCTGCACCTCTGAAGACATGCTTAACCCTAATTATGAAGAT   10219
gb:LC522974|Organism:Severe      TACTGTCCAAGACATGTGATCTGCACCTCTGAAGACATGCTTAACCCTAATTATGAAGAT   10219
gb:MN985325|Organism:Severe      TACTGTCCAAGACATGTGATCTGCACCTCTGAAGACATGCTTAACCCTAATTATGAAGAT   10222
gb:MT020881|Organism:Severe      TACTGTCCAAGACATGTGATCTGCACCTCTGAAGACATGCTTAACCCTAATTATGAAGAT   10222
gb:MT020880|Organism:Severe      TACTGTCCAAGACATGTGATCTGCACCTCTGAAGACATGCTTAACCCTAATTATGAAGAT   10222
gb:MT066175|Organism:Severe      TACTGTCCAAGACATGTGATCTGCACCTCTGAAGACATGCTTAACCCTAATTATGAAGAT   10222
gb:MN997409|Organism:Severe      TACTGTCCAAGACATGTGATCTGCACCTCTGAAGACATGCTTAACCCTAATTATGAAGAT   10222
gb:MN938384|Organism:Severe      TACTGTCCAAGACATGTGATCTGCACCTCTGAAGACATGCTTAACCCTAATTATGAAGAT   10190
gb:MT044258|Organism:Severe      TACTGTCCAAGACATGTGATCTGCACCTCTGAAGACATGCTTAACCCTAATTATGAAGAT   10198
gb:MT039890|Organism:Severe      TACTGTCCAAGACATGTGATCTGCACCTCTGAAGACATGCTTAACCCTAATTATGAAGAT   10222
gb:MN988713|Organism:Severe      TACTGTCCAAGACATGTGATCTGCACCTCTGAAGACATGCTTAACCCTAATTATGAAGAT   10222
gb:LC521925|Organism:Severe      TACTGTCCAAGACATGTGATCTGCACCTCTGAAGACATGCTTAACCCTAATTATGAAGAT   10195
gb:MT093571|Organism:Severe      TACTGTCCAAGACATGTGATCTGCACCTCTGAAGACATGCTTAACCCTAATTATGAAGAT   10222
gb:MT039887|Organism:Severe      TACTGTCCAAGACATGTGATCTGCACCTCTGAAGACATGCTTAACCCTAATTATGAAGAT   10222
gb:MT019530|Organism:Severe      TACTGTCCAAGACATGTGATCTGCACCTCTGAAGACATGCTTAACCCTAATTATGAAGAT   10222
gb:MT039888|Organism:Severe      TACTGTCCAAGACATGTGATCTGCACCTCTGAAGACATGCTTAACCCTAATTATGAAGAT   10222
gb:LC522972|Organism:Severe      TACTGTCCAAGACATGTGATCTGCACCTCTGAAGACATGCTTAACCCTAATTATGAAGAT   10219
gb:MT027063|Organism:Severe      TACTGTCCAAGACATGTGATCTGCACCTCTGAAGACATGCTTAACCCTAATTATGAAGAT   10222
gb:MT027062|Organism:Severe      TACTGTCCAAGACATGTGATCTGCACCTCTGAAGACATGCTTAACCCTAATTATGAAGAT   10222
gb:MT019529|Organism:Severe      TACTGTCCAAGACATGTGATCTGCACCTCTGAAGACATGCTTAACCCTAATTATGAAGAT   10222
gb:MN996529|Organism:Severe      TACTGTCCAAGACATGTGATCTGCACCTCTGAAGACATGCTTAACCCTAATTATGAAGAT   10210
gb:MN996531|Organism:Severe      TACTGTCCAAGACATGTGATCTGCACCTCTGAAGACATGCTTAACCCTAATTATGAAGAT   10209
gb:MT066176|Organism:Severe      TACTGTCCAAGACATGTGATCTGCACCTCTGAAGACATGCTTAACCCTAATTATGAAGAT   10222
gb:MT027064|Organism:Severe      TACTGTCCAAGACATGTGATCTGCACCTCTGAAGACATGCTTAACCCTAATTATGAAGAT   10222
gb:MN994468|Organism:Severe      TACTGTCCAAGACATGTGATCTGCACCTCTGAAGACATGCTTAACCCTAATTATGAAGAT   10222
gb:MT072688|Organism:Severe      TACTGTCCAAGACATGTGATCTGCACCTCTGAAGACATGCTTAACCCTAATTATGAAGAT   10207
gb:MN996527|Organism:Severe      TACTGTCCAAGACATGTGATCTGCACCTCTGAAGACATGCTTAACCCTAATTATGAAGAT   10189
gb:MT093631|Organism:Severe      TACTGTCCAAGACATGTGATCTGCACCTCTGAAGACATGCTTAACCCTAATTATGAAGAT   10260
gb:MT106053|Organism:Severe      TACTGTCCAAGACATGTGATCTGCACCTCTGAAGACATGCTTAACCCTAATTATGAAGAT   10222
gb:MT019533|Organism:Severe      TACTGTCCAAGACATGTGATCTGCACCTCTGAAGACATGCTTAACCCTAATTATGAAGAT   10222
gb:MT019531|Organism:Severe      TACTGTCCAAGACATGTGATCTGCACCTCTGAAGACATGCTTAACCCTAATTATGAAGAT   10222
gb:MN996528|Organism:Severe      TACTGTCCAAGACATGTGATCTGCACCTCTGAAGACATGCTTAACCCTAATTATGAAGAT   10222
```

```
gb:MN996530|Organism:Severe      TACTGTCCAAGACATGTGATCTGCACCTCTGAAGACATGCTTAACCCTAATTATGAAGAT   10208
gb:MN908947|Organism:Severe      TACTGTCCAAGACATGTGATCTGCACCTCTGAAGACATGCTTAACCCTAATTATGAAGAT   10222
gb:MT019532|Organism:Severe      TACTGTCCAAGACATGTGATCTGCACCTCTGAAGACATGCTTAACCCTAATTATGAAGAT   10222
                                 ************************************************************

gb:MT020781|Organism:Severe      TTACTCATTCGTAAGTCTAATCATAATTTCTTGGTACAGGCTGGTAATGTTCAACTCAGG   10270
gb:MT007544|Organism:Severe      TTACTCATTCGTAAGTCTAATCATAATTTCTTGGTACAGGCTGGTAATGTTCAACTCAGG   10282
gb:MN994467|Organism:Severe      TTACTCATTCGTAAGTCTAATCATAATTTCTTGGTACAGGCTGGTAATGTTCAACTCAGG   10282
gb:MT044257|Organism:Severe      TTACTCATTCGTAAGTCTAATCATAATTTCTTGGTACAGGCTGGTAATGTTCAACTCAGG   10282
gb:MT106054|Organism:Severe      TTACTCATTCGTAAGTCTAATCATAATTTCTTGGTACAGGCTGGTAATGTTCAACTCAGG   10282
gb:MT049951|Organism:Severe      TTACTCATTCGTAAGTCTAATCATAATTTCTTGGTACAGGCTGGTAATGTTCAACTCAGG   10282
gb:MN975262|Organism:Severe      TTACTCATTCGTAAGTCTAATCATAATTTCTTGGTACAGGCTGGTAATGTTCAACTCAGG   10282
gb:MT106052|Organism:Severe      TTACTCATTCGTAAGTCTAATCATAATTTCTTGGTACAGGCTGGTAATGTTCAACTCAGG   10282
gb:LC522975|Organism:Severe      TTACTCATTCGTAAGTCTAATCATAATTTCTTGGTACAGGCTGGTAATGTTCAACTCAGG   10279
gb:LC522973|Organism:Severe      TTACTCATTCGTAAGTCTAATCATAATTTCTTGGTACAGGCTGGTAATGTTCAACTCAGG   10279
gb:LC522974|Organism:Severe      TTACTCATTCGTAAGTCTAATCATAATTTCTTGGTACAGGCTGGTAATGTTCAACTCAGG   10279
gb:MN985325|Organism:Severe      TTACTCATTCGTAAGTCTAATCATAATTTCTTGGTACAGGCTGGTAATGTTCAACTCAGG   10282
gb:MT020881|Organism:Severe      TTACTCATTCGTAAGTCTAATCATAATTTCTTGGTACAGGCTGGTAATGTTCAACTCAGG   10282
gb:MT020880|Organism:Severe      TTACTCATTCGTAAGTCTAATCATAATTTCTTGGTACAGGCTGGTAATGTTCAACTCAGG   10282
gb:MT066175|Organism:Severe      TTACTCATTCGTAAGTCTAATCATAATTTCTTGGTACAGGCTGGTAATGTTCAACTCAGG   10282
gb:MN997409|Organism:Severe      TTACTCATTCGTAAGTCTAATCATAATTTCTTGGTACAGGCTGGTAATGTTCAACTCAGG   10282
gb:MN938384|Organism:Severe      TTACTCATTCGTAAGTCTAATCATAATTTCTTGGTACAGGCTGGTAATGTTCAACTCAGG   10250
gb:MT044258|Organism:Severe      TTACTCATTCGTAAGTCTAATCATAATTTCTTGGTACAGGCTGGTAATGTTCAACTCAGG   10258
gb:MT039890|Organism:Severe      TTACTCATTCGTAAGTCTAATCATAATTTCTTGGTACAGGCTGGTAATGTTCAACTCAGG   10282
gb:MN988713|Organism:Severe      TTACTCATTCGTAAGTCTAATCATAATTTCTTGGTACAGGCTGGTAATGTTCAACTCAGG   10282
gb:LC521925|Organism:Severe      TTACTCATTCGTAAGTCTAATCATAATTTCTTGGTACAGGCTGGTAATGTTCAACTCAGG   10255
gb:MT093571|Organism:Severe      TTACTCATTCGTAAGTCTAATCATAATTTCTTGGTACAGGCTGGTAATGTTCAACTCAGG   10282
gb:MT039887|Organism:Severe      TTACTCATTCGTAAGTCTAATCATAATTTCTTGGTACAGGCTGGTAATGTTCAACTCAGG   10282
gb:MT019530|Organism:Severe      TTACTCATTCGTAAGTCTAATCATAATTTCTTGGTACAGGCTGGTAATGTTCAACTCAGG   10282
gb:MT039888|Organism:Severe      TTACTCATTCGTAAGTCTAATCATAATTTCTTGGTACAGGCTGGTAATGTTCAACTCAGG   10282
gb:LC522972|Organism:Severe      TTACTCATTCGTAAGTCTAATCATAATTTCTTGGTACAGGCTGGTAATGTTCAACTCAGG   10279
gb:MT027063|Organism:Severe      TTACTCATTCGTAAGTCTAATCATAATTTCTTGGTACAGGCTGGTAATGTTCAACTCAGG   10282
gb:MT027062|Organism:Severe      TTACTCATTCGTAAGTCTAATCATAATTTCTTGGTACAGGCTGGTAATGTTCAACTCAGG   10282
gb:MT019529|Organism:Severe      TTACTCATTCGTAAGTCTAATCATAATTTCTTGGTACAGGCTGGTAATGTTCAACTCAGG   10282
gb:MN996529|Organism:Severe      TTACTCATTCGTAAGTCTAATCATAATTTCTTGGTACAGGCTGGTAATGTTCAACTCAGG   10270
gb:MN996531|Organism:Severe      TTACTCATTCGTAAGTCTAATCATAATTTCTTGGTACAGGCTGGTAATGTTCAACTCAGG   10269
gb:MT066176|Organism:Severe      TTACTCATTCGTAAGTCTAATCATAATTTCTTGGTACAGGCTGGTAATGTTCAACTCAGG   10282
gb:MT027064|Organism:Severe      TTACTCATTCGTAAGTCTAATCATAATTTCTTGGTACAGGCTGGTAATGTTCAACTCAGG   10282
gb:MN994468|Organism:Severe      TTACTCATTCGTAAGTCTAATCATAATTTCTTGGTACAGGCTGGTAATGTTCAACTCAGG   10282
gb:MT072688|Organism:Severe      TTACTCATTCGTAAGTCTAATCATAATTTCTTGGTACAGGCTGGTAATGTTCAACTCAGG   10267
gb:MN996527|Organism:Severe      TTACTCATTCGTAAGTCTAATCATAATTTCTTGGTACAGGCTGGTAATGTTCAACTCAGG   10249
gb:MT093631|Organism:Severe      TTACTCATTCGTAAGTCTAATCATAATTTCTTGGTACAGGCTGGTAATGTTCAACTCAGG   10320
gb:MT106053|Organism:Severe      TTACTCATTCGTAAGTCTAATCATAATTTCTTGGTACAGGCTGGTAATGTTCAACTCAGG   10282
gb:MT019533|Organism:Severe      TTACTCATTCGTAAGTCTAATCATAATTTCTTGGTACAGGCTGGTAATGTTCAACTCAGG   10282
gb:MT019531|Organism:Severe      TTACTCATTCGTAAGTCTAATCATAATTTCTTGGTACAGGCTGGTAATGTTCAACTCAGG   10282
gb:MN996528|Organism:Severe      TTACTCATTCGTAAGTCTAATCATAATTTCTTGGTACAGGCTGGTAATGTTCAACTCAGG   10282
gb:MN996530|Organism:Severe      TTACTCATTCGTAAGTCTAATCATAATTTCTTGGTACAGGCTGGTAATGTTCAACTCAGG   10268
gb:MN908947|Organism:Severe      TTACTCATTCGTAAGTCTAATCATAATTTCTTGGTACAGGCTGGTAATGTTCAACTCAGG   10282
gb:MT019532|Organism:Severe      TTACTCATTCGTAAGTCTAATCATAATTTCTTGGTACAGGCTGGTAATGTTCAACTCAGG   10282
                                 ************************************************************

gb:MT020781|Organism:Severe      GTTATTGGACATTCTATGCAAAATTGTGTACTTAAGCTTAAGGTTGATACAGCCAATCCT   10330
gb:MT007544|Organism:Severe      GTTATTGGACATTCTATGCAAAATTGTGTACTTAAGCTTAAGGTTGATACAGCCAATCCT   10342
gb:MN994467|Organism:Severe      GTTATTGGACATTCTATGCAAAATTGTGTACTTAAGCTTAAGGTTGATACAGCCAATCCT   10342
gb:MT044257|Organism:Severe      GTTATTGGACATTCTATGCAAAATTGTGTACTTAAGCTTAAGGTTGATACAGCCAATCCT   10342
gb:MT106054|Organism:Severe      GTTATTGGACATTCTATGCAAAATTGTGTACTTAAGCTTAAGGTTGATACAGCCAATCCT   10342
gb:MT049951|Organism:Severe      GTTATTGGACATTCTATGCAAAATTGTGTACTTAAGCTTAAGGTTGATACAGCCAATCCT   10342
gb:MN975262|Organism:Severe      GTTATTGGACATTCTATGCAAAATTGTGTACTTAAGCTTAAGGTTGATACAGCCAATCCT   10342
gb:MT106052|Organism:Severe      GTTATTGGACATTCTATGCAAAATTGTGTACTTAAGCTTAAGGTTGATACAGCCAATCCT   10342
gb:LC522975|Organism:Severe      GTTATTGGACATTCTATGCAAAATTGTGTACTTAAGCTTAAGGTTGATACAGCCAATCCT   10339
gb:LC522973|Organism:Severe      GTTATTGGACATTCTATGCAAAATTGTGTACTTAAGCTTAAGGTTGATACAGCCAATCCT   10339
gb:LC522974|Organism:Severe      GTTATTGGACATTCTATGCAAAATTGTGTACTTAAGCTTAAGGTTGATACAGCCAATCCT   10339
gb:MN985325|Organism:Severe      GTTATTGGACATTCTATGCAAAATTGTGTACTTAAGCTTAAGGTTGATACAGCCAATCCT   10342
gb:MT020881|Organism:Severe      GTTATTGGACATTCTATGCAAAATTGTGTACTTAAGCTTAAGGTTGATACAGCCAATCCT   10342
gb:MT020880|Organism:Severe      GTTATTGGACATTCTATGCAAAATTGTGTACTTAAGCTTAAGGTTGATACAGCCAATCCT   10342
```

```
gb:MT066175|Organism:Severe      GTTATTGGACATTCTATGCAAAATTGTGTACTTAAGCTTAAGGTTGATACAGCCAATCCT   10342
gb:MN997409|Organism:Severe      GTTATTGGACATTCTATGCAAAATTGTGTACTTAAGCTTAAGGTTGATACAGCCAATCCT   10342
gb:MN938384|Organism:Severe      GTTATTGGACATTCTATGCAAAATTGTGTACTTAAGCTTAAGGTTGATACAGCCAATCCT   10310
gb:MT044258|Organism:Severe      GTTATTGGACATTCTATGCAAAATTGTGTACTTAAGCTTAAGGTTGATACAGCCAATCCT   10318
gb:MT039890|Organism:Severe      GTTATTGGACATTCTATGCAAAATTGTGTACTTAAGCTTAAGGTTGATACAGCCAATCCT   10342
gb:MN988713|Organism:Severe      GTTATTGGACATTCTATGCAAAATTGTGTACTTAAGCTTAAGGTTGATACAGCCAATCCT   10342
gb:LC521925|Organism:Severe      GTTATTGGACATTCTATGCAAAATTGTGTACTTAAGCTTAAGGTTGATACAGCCAATCCT   10315
gb:MT093571|Organism:Severe      GTTATTGGACATTCTATGCAAAATTGTGTACTTAAGCTTAAGGTTGATACAGCCAATCCT   10342
gb:MT039887|Organism:Severe      GTTATTGGACATTCTATGCAAAATTGTGTACTTAAGCTTAAGGTTGATACAGCCAATCCT   10342
gb:MT019530|Organism:Severe      GTTATTGGACATTCTATGCAAAATTGTGTACTTAAGCTTAAGGTTGATACAGCCAATCCT   10342
gb:MT039888|Organism:Severe      GTTATTGGACATTCTATGCAAAATTGTGTACTTAAGCTTAAGGTTGATACAGCCAATCCT   10342
gb:LC522972|Organism:Severe      GTTATTGGACATTCTATGCAAAATTGTGTACTTAAGCTTAAGGTTGATACAGCCAATCCT   10339
gb:MT027063|Organism:Severe      GTTATTGGACATTCTATGCAAAATTGTGTACTTAAGCTTAAGGTTGATACAGCCAATCCT   10342
gb:MT027062|Organism:Severe      GTTATTGGACATTCTATGCAAAATTGTGTACTTAAGCTTAAGGTTGATACAGCCAATCCT   10342
gb:MT019529|Organism:Severe      GTTATTGGACATTCTATGCAAAATTGTGTACTTAAGCTTAAGGTTGATACAGCCAATCCT   10342
gb:MN996529|Organism:Severe      GTTATTGGACATTCTATGCAAAATTGTGTACTTAAGCTTAAGGTTGATACAGCCAATCCT   10330
gb:MN996531|Organism:Severe      GTTATTGGACATTCTATGCAAAATTGTGTACTTAAGCTTAAGGTTGATACAGCCAATCCT   10329
gb:MT066176|Organism:Severe      GTTATTGGACATTCTATGCAAAATTGTGTACTTAAGCTTAAGGTTGATACAGCCAATCCT   10342
gb:MT027064|Organism:Severe      GTTATTGGACATTCTATGCAAAATTGTGTACTTAAGCTTAAGGTTGATACAGCCAATCCT   10342
gb:MN994468|Organism:Severe      GTTATTGGACATTCTATGCAAAATTGTGTACTTAAGCTTAAGGTTGATACAGCCAATCCT   10342
gb:MT072688|Organism:Severe      GTTATTGGACATTCTATGCAAAATTGTGTACTTAAGCTTAAGGTTGATACAGCCAATCCT   10327
gb:MN996527|Organism:Severe      GTTATTGGACATTCTATGCAAAATTGTGTACTTAAGCTTAAGGTTGATACAGCCAATCCT   10309
gb:MT093631|Organism:Severe      GTTATTGGACATTCTATGCAAAATTGTGTACTTAAGCTTAAGGTTGATACAGCCAATCCT   10380
gb:MT106053|Organism:Severe      GTTATTGGACATTCTATGCAAAATTGTGTACTTAAGCTTAAGGTTGATACAGCCAATCCT   10342
gb:MT019533|Organism:Severe      GTTATTGGACATTCTATGCAAAATTGTGTACTTAAGCTTAAGGTTGATACAGCCAATCCT   10342
gb:MT019531|Organism:Severe      GTTATTGGACATTCTATGCAAAATTGTGTACTTAAGCTTAAGGTTGATACAGCCAATCCT   10342
gb:MN996528|Organism:Severe      GTTATTGGACATTCTATGCAAAATTGTGTACTTAAGCTTAAGGTTGATACAGCCAATCCT   10342
gb:MN996530|Organism:Severe      GTTATTGGACATTCTATGCAAAATTGTGTACTTAAGCTTAAGGTTGATACAGCCAATCCT   10328
gb:MN908947|Organism:Severe      GTTATTGGACATTCTATGCAAAATTGTGTACTTAAGCTTAAGGTTGATACAGCCAATCCT   10342
gb:MT019532|Organism:Severe      GTTATTGGACATTCTATGCAAAATTGTGTACTTAAGCTTAAGGTTGATACAGCCAATCCT   10342
                                 ************************************************************

gb:MT020781|Organism:Severe      AAGACACCTAAGTATAAGTTTGTTCGCATTCAACCAGGACAGACTTTTTCAGTGTTAGCT   10390
gb:MT007544|Organism:Severe      AAGACACCTAAGTATAAGTTTGTTCGCATTCAACCAGGACAGACTTTTTCAGTGTTAGCT   10402
gb:MN994467|Organism:Severe      AAGACACCTAAGTATAAGTTTGTTCGCATTCAACCAGGACAGACTTTTTCAGTGTTAGCT   10402
gb:MT044257|Organism:Severe      AAGACACCTAAGTATAAGTTTGTTCGCATTCAACCAGGACAGACTTTTTCAGTGTTAGCT   10402
gb:MT106054|Organism:Severe      AAGACACCTAAGTATAAGTTTGTTCGCATTCAACCAGGACAGACTTTTTCAGTGTTAGCT   10402
gb:MT049951|Organism:Severe      AAGACACCTAAGTATAAGTTTGTTCGCATTCAACCAGGACAGACTTTTTCAGTGTTAGCT   10402
gb:MN975262|Organism:Severe      AAGACACCTAAGTATAAGTTTGTTCGCATTCAACCAGGACAGACTTTTTCAGTGTTAGCT   10402
gb:MT106052|Organism:Severe      AAGACACCTAAGTATAAGTTTGTTCGCATTCAACCAGGACAGACTTTTTCAGTGTTAGCT   10402
gb:LC522975|Organism:Severe      AAGACACCTAAGTATAAGTTTGTTCGCATTCAACCAGGACAGACTTTTTCAGTGTTAGCT   10399
gb:LC522973|Organism:Severe      AAGACACCTAAGTATAAGTTTGTTCGCATTCAACCAGGACAGACTTTTTCAGTGTTAGCT   10399
gb:LC522974|Organism:Severe      AAGACACCTAAGTATAAGTTTGTTCGCATTCAACCAGGACAGACTTTTTCAGTGTTAGCT   10399
gb:MN985325|Organism:Severe      AAGACACCTAAGTATAAGTTTGTTCGCATTCAACCAGGACAGACTTTTTCAGTGTTAGCT   10402
gb:MT020881|Organism:Severe      AAGACACCTAAGTATAAGTTTGTTCGCATTCAACCAGGACAGACTTTTTCAGTGTTAGCT   10402
gb:MT020880|Organism:Severe      AAGACACCTAAGTATAAGTTTGTTCGCATTCAACCAGGACAGACTTTTTCAGTGTTAGCT   10402
gb:MT066175|Organism:Severe      AAGACACCTAAGTATAAGTTTGTTCGCATTCAACCAGGACAGACTTTTTCAGTGTTAGCT   10402
gb:MN997409|Organism:Severe      AAGACACCTAAGTATAAGTTTGTTCGCATTCAACCAGGACAGACTTTTTCAGTGTTAGCT   10402
gb:MN938384|Organism:Severe      AAGACACCTAAGTATAAGTTTGTTCGCATTCAACCAGGACAGACTTTTTCAGTGTTAGCT   10370
gb:MT044258|Organism:Severe      AAGACACCTAAGTATAAGTTTGTTCGCATTCAACCAGGACAGACTTTTTCAGTGTTAGCT   10378
gb:MT039890|Organism:Severe      AAGACACCTAAGTATAAGTTTGTTCGCATTCAACCAGGACAGACTTTTTCAGTGTTAGCT   10402
gb:MN988713|Organism:Severe      AAGACACCTAAGTATAAGTTTGTTCGCATTCAACCAGGACAGACTTTTTCAGTGTTAGCT   10402
gb:LC521925|Organism:Severe      AAGACACCTAAGTATAAGTTTGTTCGCATTCAACCAGGACAGACTTTTTCAGTGTTAGCT   10375
gb:MT093571|Organism:Severe      AAGACACCTAAGTATAAGTTTGTTCGCATTCAACCAGGACAGACTTTTTCAGTGTTAGCT   10402
gb:MT039887|Organism:Severe      AAGACACCTAAGTATAAGTTTGTTCGCATTCAACCAGGACAGACTTTTTCAGTGTTAGCT   10402
gb:MT019530|Organism:Severe      AAGACACCTAAGTATAAGTTTGTTCGCATTCAACCAGGACAGACTTTTTCAGTGTTAGCT   10402
gb:MT039888|Organism:Severe      AAGACACCTAAGTATAAGTTTGTTCGCATTCAACCAGGACAGACTTTTTCAGTGTTAGCT   10402
gb:LC522972|Organism:Severe      AAGACACCTAAGTATAAGTTTGTTCGCATTCAACCAGGACAGACTTTTTCAGTGTTAGCT   10399
gb:MT027063|Organism:Severe      AAGACACCTAAGTATAAGTTTGTTCGCATTCAACCAGGACAGACTTTTTCAGTGTTAGCT   10402
gb:MT027062|Organism:Severe      AAGACACCTAAGTATAAGTTTGTTCGCATTCAACCAGGACAGACTTTTTCAGTGTTAGCT   10402
gb:MT019529|Organism:Severe      AAGACACCTAAGTATAAGTTTGTTCGCATTCAACCAGGACAGACTTTTTCAGTGTTAGCT   10402
gb:MN996529|Organism:Severe      AAGACACCTAAGTATAAGTTTGTTCGCATTCAACCAGGACAGACTTTTTCAGTGTTAGCT   10390
gb:MN996531|Organism:Severe      AAGACACCTAAGTATAAGTTTGTTCGCATTCAACCAGGACAGACTTTTTCAGTGTTAGCT   10389
gb:MT066176|Organism:Severe      AAGACACCTAAGTATAAGTTTGTTCGCATTCAACCAGGACAGACTTTTTCAGTGTTAGCT   10402
gb:MT027064|Organism:Severe      AAGACACCTAAGTATAAGTTTGTTCGCATTCAACCAGGACAGACTTTTTCAGTGTTAGCT   10402
```

```
gb:MN994468|Organism:Severe      AAGACACCTAAGTATAAGTTTGTTCGCATTCAACCAGGACAGACTTTTTCAGTGTTAGCT   10402
gb:MT072688|Organism:Severe      AAGACACCTAAGTATAAGTTTGTTCGCATTCAACCAGGACAGACTTTTTCAGTGTTAGCT   10387
gb:MN996527|Organism:Severe      AAGACACCTAAGTATAAGTTTGTTCGCATTCAACCAGGACAGACTTTTTCAGTGTTAGCT   10369
gb:MT093631|Organism:Severe      AAGACACCTAAGTATAAGTTTGTTCGCATTCAACCAGGACAGACTTTTTCAGTGTTAGCT   10440
gb:MT106053|Organism:Severe      AAGACACCTAAGTATAAGTTTGTTCGCATTCAACCAGGACAGACTTTTTCAGTGTTAGCT   10402
gb:MT019533|Organism:Severe      AAGACACCTAAGTATAAGTTTGTTCGCATTCAACCAGGACAGACTTTTTCAGTGTTAGCT   10402
gb:MT019531|Organism:Severe      AAGACACCTAAGTATAAGTTTGTTCGCATTCAACCAGGACAGACTTTTTCAGTGTTAGCT   10402
gb:MN996528|Organism:Severe      AAGACACCTAAGTATAAGTTTGTTCGCATTCAACCAGGACAGACTTTTTCAGTGTTAGCT   10402
gb:MN996530|Organism:Severe      AAGACACCTAAGTATAAGTTTGTTCGCATTCAACCAGGACAGACTTTTTCAGTGTTAGCT   10388
gb:MN908947|Organism:Severe      AAGACACCTAAGTATAAGTTTGTTCGCATTCAACCAGGACAGACTTTTTCAGTGTTAGCT   10402
gb:MT019532|Organism:Severe      AAGACACCTAAGTATAAGTTTGTTCGCATTCAACCAGGACAGACTTTTTCAGTGTTAGCT   10402
                                 ************************************************************

gb:MT020781|Organism:Severe      TGTTACAATGGTTCACCATCTGGTGTTTACCAATGTGCTATGAGGCCCAATTTCACTATT   10450
gb:MT007544|Organism:Severe      TGTTACAATGGTTCACCATCTGGTGTTTACCAATGTGCTATGAGGCCCAATTTCACTATT   10462
gb:MN994467|Organism:Severe      TGTTACAATGGTTCACCATCTGGTGTTTACCAATGTGCTATGAGGCCCAATTTCACTATT   10462
gb:MT044257|Organism:Severe      TGTTACAATGGTTCACCATCTGGTGTTTACCAATGTGCTATGAGGCCCAATTTCACTATT   10462
gb:MT106054|Organism:Severe      TGTTACAATGGTTCACCATCTGGTGTTTACCAATGTGCTATGAGGCCCAATTTCACTATT   10462
gb:MT049951|Organism:Severe      TGTTACAATGGTTCACCATCTGGTGTTTACCAATGTGCTATGAGGCCCAATTTCACTATT   10462
gb:MN975262|Organism:Severe      TGTTACAATGGTTCACCATCTGGTGTTTACCAATGTGCTATGAGGCCCAATTTCACTATT   10462
gb:MT106052|Organism:Severe      TGTTACAATGGTTCACCATCTGGTGTTTACCAATGTGCTATGAGGCCCAATTTCACTATT   10462
gb:LC522975|Organism:Severe      TGTTACAATGGTTCACCATCTGGTGTTTACCAATGTGCTATGAGGCCCAATTTCACTATT   10459
gb:LC522973|Organism:Severe      TGTTACAATGGTTCACCATCTGGTGTTTACCAATGTGCTATGAGGCCCAATTTCACTATT   10459
gb:LC522974|Organism:Severe      TGTTACAATGGTTCACCATCTGGTGTTTACCAATGTGCTATGAGGCCCAATTTCACTATT   10459
gb:MN985325|Organism:Severe      TGTTACAATGGTTCACCATCTGGTGTTTACCAATGTGCTATGAGGCCCAATTTCACTATT   10462
gb:MT020881|Organism:Severe      TGTTACAATGGTTCACCATCTGGTGTTTACCAATGTGCTATGAGGCCCAATTTCACTATT   10462
gb:MT020880|Organism:Severe      TGTTACAATGGTTCACCATCTGGTGTTTACCAATGTGCTATGAGGCCCAATTTCACTATT   10462
gb:MT066175|Organism:Severe      TGTTACAATGGTTCACCATCTGGTGTTTACCAATGTGCTATGAGGCCCAATTTCACTATT   10462
gb:MN997409|Organism:Severe      TGTTACAATGGTTCACCATCTGGTGTTTACCAATGTGCTATGAGGCCCAATTTCACTATT   10462
gb:MN938384|Organism:Severe      TGTTACAATGGTTCACCATCTGGTGTTTACCAATGTGCTATGAGGCCCAATTTCACTATT   10430
gb:MT044258|Organism:Severe      TGTTACAATGGTTCACCATCTGGTGTTTACCAATGTGCTATGAGGCCCAATTTCACTATT   10438
gb:MT039890|Organism:Severe      TGTTACAATGGTTCACCATCTGGTGTTTACCAATGTGCTATGAGGCCCAATTTCACTATT   10462
gb:MN988713|Organism:Severe      TGTTACAATGGTTCACCATCTGGTGTTTACCAATGTGCTATGAGGCCCAATTTCACTATT   10462
gb:LC521925|Organism:Severe      TGTTACAATGGTTCACCATCTGGTGTTTACCAATGTGCTATGAGGCCCAATTTCACTATT   10435
gb:MT093571|Organism:Severe      TGTTACAATGGTTCACCATCTGGTGTTTACCAATGTGCTATGAGGCCCAATTTCACTATT   10462
gb:MT039887|Organism:Severe      TGTTACAATGGTTCACCATCTGGTGTTTACCAATGTGCTATGAGGCCCAATTTCACTATT   10462
gb:MT019530|Organism:Severe      TGTTACAATGGTTCACCATCTGGTGTTTACCAATGTGCTATGAGGCCCAATTTCACTATT   10462
gb:MT039888|Organism:Severe      TGTTACAATGGTTCACCATCTGGTGTTTACCAATGTGCTATGAGGCCCAATTTCACTATT   10462
gb:LC522972|Organism:Severe      TGTTACAATGGTTCACCATCTGGTGTTTACCAATGTGCTATGAGGCCCAATTTCACTATT   10459
gb:MT027063|Organism:Severe      TGTTACAATGGTTCACCATCTGGTGTTTACCAATGTGCTATGAGGCCCAATTTCACTATT   10462
gb:MT027062|Organism:Severe      TGTTACAATGGTTCACCATCTGGTGTTTACCAATGTGCTATGAGGCCCAATTTCACTATT   10462
gb:MT019529|Organism:Severe      TGTTACAATGGTTCACCATCTGGTGTTTACCAATGTGCTATGAGGCCCAATTTCACTATT   10462
gb:MN996529|Organism:Severe      TGTTACAATGGTTCACCATCTGGTGTTTACCAATGTGCTATGAGGCCCAATTTCACTATT   10450
gb:MN996531|Organism:Severe      TGTTACAATGGTTCACCATCTGGTGTTTACCAATGTGCTATGAGGCCCAATTTCACTATT   10449
gb:MT066176|Organism:Severe      TGTTACAATGGTTCACCATCTGGTGTTTACCAATGTGCTATGAGGCCCAATTTCACTATT   10462
gb:MT027064|Organism:Severe      TGTTACAATGGTTCACCATCTGGTGTTTACCAATGTGCTATGAGGCCCAATTTCACTATT   10462
gb:MN994468|Organism:Severe      TGTTACAATGGTTCACCATCTGGTGTTTACCAATGTGCTATGAGGCCCAATTTCACTATT   10462
gb:MT072688|Organism:Severe      TGTTACAATGGTTCACCATCTGGTGTTTACCAATGTGCTATGAGGCCCAATTTCACTATT   10447
gb:MN996527|Organism:Severe      TGTTACAATGGTTCACCATCTGGTGTTTACCAATGTGCTATGAGGCCCAATTTCACTATT   10429
gb:MT093631|Organism:Severe      TGTTACAATGGTTCACCATCTGGTGTTTACCAATGTGCTATGAGGCCCAATTTCACTATT   10500
gb:MT106053|Organism:Severe      TGTTACAATGGTTCACCATCTGGTGTTTACCAATGTGCTATGAGGCCCAATTTCACTATT   10462
gb:MT019533|Organism:Severe      TGTTACAATGGTTCACCATCTGGTGTTTACCAATGTGCTATGAGGCCCAATTTCACTATT   10462
gb:MT019531|Organism:Severe      TGTTACAATGGTTCACCATCTGGTGTTTACCAATGTGCTATGAGGCCCAATTTCACTATT   10462
gb:MN996528|Organism:Severe      TGTTACAATGGTTCACCATCTGGTGTTTACCAATGTGCTATGAGGCCCAATTTCACTATT   10462
gb:MN996530|Organism:Severe      TGTTACAATGGTTCACCATCTGGTGTTTACCAATGTGCTATGAGGCCCAATTTCACTATT   10448
gb:MN908947|Organism:Severe      TGTTACAATGGTTCACCATCTGGTGTTTACCAATGTGCTATGAGGCCCAATTTCACTATT   10462
gb:MT019532|Organism:Severe      TGTTACAATGGTTCACCATCTGGTGTTTACCAATGTGCTATGAGGCCCAATTTCACTATT   10462
                                 ************************************************************

gb:MT020781|Organism:Severe      AAGGGTTCATTCCTTAATGGTTCATGTGGTAGTGTTGGTTTTAACATAGATTATGACTGT   10510
gb:MT007544|Organism:Severe      AAGGGTTCATTCCTTAATGGTTCATGTGGTAGTGTTGGTTTTAACATAGATTATGACTGT   10522
gb:MN994467|Organism:Severe      AAGGGTTCATTCCTTAATGGTTCATGTGGTAGTGTTGGTTTTAACATAGATTATGACTGT   10522
gb:MT044257|Organism:Severe      AAGGGTTCATTCCTTAATGGTTCATGTGGTAGTGTTGGTTTTAACATAGATTATGACTGT   10522
gb:MT106054|Organism:Severe      AAGGGTTCATTCCTTAATGGTTCATGTGGTAGTGTTGGTTTTAACATAGATTATGACTGT   10522
gb:MT049951|Organism:Severe      AAGGGTTCATTCCTTAATGGTTCATGTGGTAGTGTTGGTTTTAACATAGATTATGACTGT   10522
```

```
gb:MN975262|Organism:Severe      AAGGGTTCATTCCTTAATGGTTCATGTGGTAGTGTTGGTTTTAACATAGATTATGACTGT   10522
gb:MT106052|Organism:Severe      AAGGGTTCATTCCTTAATGGTTCATGTGGTAGTGTTGGTTTTAACATAGATTATGACTGT   10522
gb:LC522975|Organism:Severe      AAGGGTTCATTCCTTAATGGTTCATGTGGTAGTGTTGGTTTTAACATAGATTATGACTGT   10519
gb:LC522973|Organism:Severe      AAGGGTTCATTCCTTAATGGTTCATGTGGTAGTGTTGGTTTTAACATAGATTATGACTGT   10519
gb:LC522974|Organism:Severe      AAGGGTTCATTCCTTAATGGTTCATGTGGTAGTGTTGGTTTTAACATAGATTATGACTGT   10519
gb:MN985325|Organism:Severe      AAGGGTTCATTCCTTAATGGTTCATGTGGTAGTGTTGGTTTTAACATAGATTATGACTGT   10522
gb:MT020881|Organism:Severe      AAGGGTTCATTCCTTAATGGTTCATGTGGTAGTGTTGGTTTTAACATAGATTATGACTGT   10522
gb:MT020880|Organism:Severe      AAGGGTTCATTCCTTAATGGTTCATGTGGTAGTGTTGGTTTTAACATAGATTATGACTGT   10522
gb:MT066175|Organism:Severe      AAGGGTTCATTCCTTAATGGTTCATGTGGTAGTGTTGGTTTTAACATAGATTATGACTGT   10522
gb:MN997409|Organism:Severe      AAGGGTTCATTCCTTAATGGTTCATGTGGTAGTGTTGGTTTTAACATAGATTATGACTGT   10522
gb:MN938384|Organism:Severe      AAGGGTTCATTCCTTAATGGTTCATGTGGTAGTGTTGGTTTTAACATAGATTATGACTGT   10490
gb:MT044258|Organism:Severe      AAGGGTTCATTCCTTAATGGTTCATGTGGTAGTGTTGGTTTTAACATAGATTATGACTGT   10498
gb:MT039890|Organism:Severe      AAGGGTTCATTCCTTAATGGTTCATGTGGTAGTGTTGGTTTTAACATAGATTATGACTGT   10522
gb:MN988713|Organism:Severe      AAGGGTTCATTCCTTAATGGTTCATGTGGTAGTGTTGGTTTTAACATAGATTATGACTGT   10522
gb:LC521925|Organism:Severe      AAGGGTTCATTCCTTAATGGTTCATGTGGTAGTGTTGGTTTTAACATAGATTATGACTGT   10495
gb:MT093571|Organism:Severe      AAGGGTTCATTCCTTAATGGTTCATGTGGTAGTGTTGGTTTTAACATAGATTATGACTGT   10522
gb:MT039887|Organism:Severe      AAGGGTTCATTCCTTAATGGTTCATGTGGTAGTGTTGGTTTTAACATAGATTATGACTGT   10522
gb:MT019530|Organism:Severe      AAGGGTTCATTCCTTAATGGTTCATGTGGTAGTGTTGGTTTTAACATAGATTATGACTGT   10522
gb:MT039888|Organism:Severe      AAGGGTTCATTCCTTAATGGTTCATGTGGTAGTGTTGGTTTTAACATAGATTATGACTGT   10522
gb:LC522972|Organism:Severe      AAGGGTTCATTCCTTAATGGTTCATGTGGTAGTGTTGGTTTTAACATAGATTATGACTGT   10519
gb:MT027063|Organism:Severe      AAGGGTTCATTCCTTAATGGTTCATGTGGTAGTGTTGGTTTTAACATAGATTATGACTGT   10522
gb:MT027062|Organism:Severe      AAGGGTTCATTCCTTAATGGTTCATGTGGTAGTGTTGGTTTTAACATAGATTATGACTGT   10522
gb:MT019529|Organism:Severe      AAGGGTTCATTCCTTAATGGTTCATGTGGTAGTGTTGGTTTTAACATAGATTATGACTGT   10522
gb:MN996529|Organism:Severe      AAGGGTTCATTCCTTAATGGTTCATGTGGTAGTGTTGGTTTTAACATAGATTATGACTGT   10510
gb:MN996531|Organism:Severe      AAGGGTTCATTCCTTAATGGTTCATGTGGTAGTGTTGGTTTTAACATAGATTATGACTGT   10509
gb:MT066176|Organism:Severe      AAGGGTTCATTCCTTAATGGTTCATGTGGTAGTGTTGGTTTTAACATAGATTATGACTGT   10522
gb:MT027064|Organism:Severe      AAGGGTTCATTCCTTAATGGTTCATGTGGTAGTGTTGGTTTTAACATAGATTATGACTGT   10522
gb:MN994468|Organism:Severe      AAGGGTTCATTCCTTAATGGTTCATGTGGTAGTGTTGGTTTTAACATAGATTATGACTGT   10522
gb:MT072688|Organism:Severe      AAGGGTTCATTCCTTAATGGTTCATGTGGTAGTGTTGGTTTTAACATAGATTATGACTGT   10507
gb:MN996527|Organism:Severe      AAGGGTTCATTCCTTAATGGTTCATGTGGTAGTGTTGGTTTTAACATAGATTATGACTGT   10489
gb:MT093631|Organism:Severe      AAGGGTTCATTCCTTAATGGTTCATGTGGTAGTGTTGGTTTTAACATAGATTATGACTGT   10560
gb:MT106053|Organism:Severe      AAGGGTTCATTCCTTAATGGTTCATGTGGTAGTGTTGGTTTTAACATAGATTATGACTGT   10522
gb:MT019533|Organism:Severe      AAGGGTTCATTCCTTAATGGTTCATGTGGTAGTGTTGGTTTTAACATAGATTATGACTGT   10522
gb:MT019531|Organism:Severe      AAGGGTTCATTCCTTAATGGTTCATGTGGTAGTGTTGGTTTTAACATAGATTATGACTGT   10522
gb:MN996528|Organism:Severe      AAGGGTTCATTCCTTAATGGTTCATGTGGTAGTGTTGGTTTTAACATAGATTATGACTGT   10522
gb:MN996530|Organism:Severe      AAGGGTTCATTCCTTAATGGTTCATGTGGTAGTGTTGGTTTTAACATAGATTATGACTGT   10508
gb:MN908947|Organism:Severe      AAGGGTTCATTCCTTAATGGTTCATGTGGTAGTGTTGGTTTTAACATAGATTATGACTGT   10522
gb:MT019532|Organism:Severe      AAGGGTTCATTCCTTAATGGTTCATGTGGTAGTGTTGGTTTTAACATAGATTATGACTGT   10522
                                 ************************************************************

gb:MT020781|Organism:Severe      GTCTCTTTTTGTTACATGCACCATATGGAATTACCAACTGGAGTTCATGCTGGCACAGAC   10570
gb:MT007544|Organism:Severe      GTCTCTTTTTGTTACATGCACCATATGGAATTACCAACTGGAGTTCATGCTGGCACAGAC   10582
gb:MN994467|Organism:Severe      GTCTCTTTTTGTTACATGCACCATATGGAATTACCAACTGGAGTTCATGCTGGCACAGAC   10582
gb:MT044257|Organism:Severe      GTCTCTTTTTGTTACATGCACCATATGGAATTACCAACTGGAGTTCATGCTGGCACAGAC   10582
gb:MT106054|Organism:Severe      GTCTCTTTTTGTTACATGCACCATATGGAATTACCAACTGGAGTTCATGCTGGCACAGAC   10582
gb:MT049951|Organism:Severe      GTCTCTTTTTGTTACATGCACCATATGGAATTACCAACTGGAGTTCATGCTGGCACAGAC   10582
gb:MN975262|Organism:Severe      GTCTCTTTTTGTTACATGCACCATATGGAATTACCAACTGGAGTTCATGCTGGCACAGAC   10582
gb:MT106052|Organism:Severe      GTCTCTTTTTGTTACATGCACCATATGGAATTACCAACTGGAGTTCATGCTGGCACAGAC   10582
gb:LC522975|Organism:Severe      GTCTCTTTTTGTTACATGCACCATATGGAATTACCAACTGGAGTTCATGCTGGCACAGAC   10579
gb:LC522973|Organism:Severe      GTCTCTTTTTGTTACATGCACCATATGGAATTACCAACTGGAGTTCATGCTGGCACAGAC   10579
gb:LC522974|Organism:Severe      GTCTCTTTTTGTTACATGCACCATATGGAATTACCAACTGGAGTTCATGCTGGCACAGAC   10579
gb:MN985325|Organism:Severe      GTCTCTTTTTGTTACATGCACCATATGGAATTACCAACTGGAGTTCATGCTGGCACAGAC   10582
gb:MT020881|Organism:Severe      GTCTCTTTTTGTTACATGCACCATATGGAATTACCAACTGGAGTTCATGCTGGCACAGAC   10582
gb:MT020880|Organism:Severe      GTCTCTTTTTGTTACATGCACCATATGGAATTACCAACTGGAGTTCATGCTGGCACAGAC   10582
gb:MT066175|Organism:Severe      GTCTCTTTTTGTTACATGCACCATATGGAATTACCAACTGGAGTTCATGCTGGCACAGAC   10582
gb:MN997409|Organism:Severe      GTCTCTTTTTGTTACATGCACCATATGGAATTACCAACTGGAGTTCATGCTGGCACAGAC   10582
gb:MN938384|Organism:Severe      GTCTCTTTTTGTTACATGCACCATATGGAATTACCAACTGGAGTTCATGCTGGCACAGAC   10550
gb:MT044258|Organism:Severe      GTCTCTTTTTGTTACATGCACCATATGGAATTACCAACTGGAGTTCATGCTGGCACAGAC   10558
gb:MT039890|Organism:Severe      GTCTCTTTTTGTTACATGCACCATATGGAATTACCAACTGGAGTTCATGCTGGCACAGAC   10582
gb:MN988713|Organism:Severe      GTCTCTTTTTGTTACATGCACCATATGGAATTACCAACTGGAGTTCATGCTGGCACAGAC   10582
gb:LC521925|Organism:Severe      GTCTCTTTTTGTTACATGCACCATATGGAATTACCAACTGGAGTTCATGCTGGCACAGAC   10555
gb:MT093571|Organism:Severe      GTCTCTTTTTGTTACATGCACCATATGGAATTACCAACTGGAGTTCATGCTGGCACAGAC   10582
gb:MT039887|Organism:Severe      GTCTCTTTTTGTTACATGCACCATATGGAATTACCAACTGGAGTTCATGCTGGCACAGAC   10582
gb:MT019530|Organism:Severe      GTCTCTTTTTGTTACATGCACCATATGGAATTACCAACTGGAGTTCATGCTGGCACAGAC   10582
gb:MT039888|Organism:Severe      GTCTCTTTTTGTTACATGCACCATATGGAATTACCAACTGGAGTTCATGCTGGCACAGAC   10582
```

```
gb:LC522972|Organism:Severe      GTCTCTTTTTGTTACATGCACCATATGGAATTACCAACTGGAGTTCATGCTGGCACAGAC   10579
gb:MT027063|Organism:Severe      GTCTCTTTTTGTTACATGCACCATATGGAATTACCAACTGGAGTTCATGCTGGCACAGAC   10582
gb:MT027062|Organism:Severe      GTCTCTTTTTGTTACATGCACCATATGGAATTACCAACTGGAGTTCATGCTGGCACAGAC   10582
gb:MT019529|Organism:Severe      GTCTCTTTTTGTTACATGCACCATATGGAATTACCAACTGGAGTTCATGCTGGCACAGAC   10582
gb:MN996529|Organism:Severe      GTCTCTTTTTGTTACATGCACCATATGGAATTACCAACTGGAGTTCATGCTGGCACAGAC   10570
gb:MN996531|Organism:Severe      GTCTCTTTTTGTTACATGCACCATATGGAATTACCAACTGGAGTTCATGCTGGCACAGAC   10569
gb:MT066176|Organism:Severe      GTCTCTTTTTGTTACATGCACCATATGGAATTACCAACTGGAGTTCATGCTGGCACAGAC   10582
gb:MT027064|Organism:Severe      GTCTCTTTTTGTTACATGCACCATATGGAATTACCAACTGGAGTTCATGCTGGCACAGAC   10582
gb:MN994468|Organism:Severe      GTCTCTTTTTGTTACATGCACCATATGGAATTACCAACTGGAGTTCATGCTGGCACAGAC   10582
gb:MT072688|Organism:Severe      GTCTCTTTTTGTTACATGCACCATATGGAATTACCAACTGGAGTTCATGCTGGCACAGAC   10567
gb:MN996527|Organism:Severe      GTCTCTTTTTGTTACATGCACCATATGGAATTACCAACTGGAGTTCATGCTGGCACAGAC   10549
gb:MT093631|Organism:Severe      GTCTCTTTTTGTTACATGCACCATATGGAATTACCAACTGGAGTTCATGCTGGCACAGAC   10620
gb:MT106053|Organism:Severe      GTCTCTTTTTGTTACATGCACCATATGGAATTACCAACTGGAGTTCATGCTGGCACAGAC   10582
gb:MT019533|Organism:Severe      GTCTCTTTTTGTTACATGCACCATATGGAATTACCAACTGGAGTTCATGCTGGCACAGAC   10582
gb:MT019531|Organism:Severe      GTCTCTTTTTGTTACATGCACCATATGGAATTACCAACTGGAGTTCATGCTGGCACAGAC   10582
gb:MN996528|Organism:Severe      GTCTCTTTTTGTTACATGCACCATATGGAATTACCAACTGGAGTTCATGCTGGCACAGAC   10582
gb:MN996530|Organism:Severe      GTCTCTTTTTGTTACATGCACCATATGGAATTACCAACTGGAGTTCATGCTGGCACAGAC   10568
gb:MN908947|Organism:Severe      GTCTCTTTTTGTTACATGCACCATATGGAATTACCAACTGGAGTTCATGCTGGCACAGAC   10582
gb:MT019532|Organism:Severe      GTCTCTTTTTGTTACATGCACCATATGGAATTACCAACTGGAGTTCATGCTGGCACAGAC   10582
                                 ************************************************************

gb:MT020781|Organism:Severe      TTAGAAGGTAACTTTTATGGACCTTTTGTTGACAGGCAAACAGCACAAGCAGCTGGTACG   10630
gb:MT007544|Organism:Severe      TTAGAAGGTAACTTTTATGGACCTTTTGTTGACAGGCAAACAGCACAAGCAGCTGGTACG   10642
gb:MN994467|Organism:Severe      TTAGAAGGTAACTTTTATGGACCTTTTGTTGACAGGCAAACAGCACAAGCAGCTGGTACG   10642
gb:MT044257|Organism:Severe      TTAGAAGGTAACTTTTATGGACCTTTTGTTGACAGGCAAACAGCACAAGCAGCTGGTACG   10642
gb:MT106054|Organism:Severe      TTAGAAGGTAACTTTTATGGACCTTTTGTTGACAGGCAAACAGCACAAGCAGCTGGTACG   10642
gb:MT049951|Organism:Severe      TTAGAAGGTAACTTTTATGGACCTTTTGTTGACAGGCAAACAGCACAAGCAGCTGGTACG   10642
gb:MN975262|Organism:Severe      TTAGAAGGTAACTTTTATGGACCTTTTGTTGACAGGCAAACAGCACAAGCAGCTGGTACG   10642
gb:MT106052|Organism:Severe      TTAGAAGGTAACTTTTATGGACCTTTTGTTGACAGGCAAACAGCACAAGCAGCTGGTACG   10642
gb:LC522975|Organism:Severe      TTAGAAGGTAACTTTTATGGACCTTTTGTTGACAGGCAAACAGCACAAGCAGCTGGTACG   10639
gb:LC522973|Organism:Severe      TTAGAAGGTAACTTTTATGGACCTTTTGTTGACAGGCAAACAGCACAAGCAGCTGGTACG   10639
gb:LC522974|Organism:Severe      TTAGAAGGTAACTTTTATGGACCTTTTGTTGACAGGCAAACAGCACAAGCAGCTGGTACG   10639
gb:MN985325|Organism:Severe      TTAGAAGGTAACTTTTATGGACCTTTTGTTGACAGGCAAACAGCACAAGCAGCTGGTACG   10642
gb:MT020881|Organism:Severe      TTAGAAGGTAACTTTTATGGACCTTTTGTTGACAGGCAAACAGCACAAGCAGCTGGTACG   10642
gb:MT020880|Organism:Severe      TTAGAAGGTAACTTTTATGGACCTTTTGTTGACAGGCAAACAGCACAAGCAGCTGGTACG   10642
gb:MT066175|Organism:Severe      TTAGAAGGTAACTTTTATGGACCTTTTGTTGACAGGCAAACAGCACAAGCAGCTGGTACG   10642
gb:MN997409|Organism:Severe      TTAGAAGGTAACTTTTATGGACCTTTTGTTGACAGGCAAACAGCACAAGCAGCTGGTACG   10642
gb:MN938384|Organism:Severe      TTAGAAGGTAACTTTTATGGACCTTTTGTTGACAGGCAAACAGCACAAGCAGCTGGTACG   10610
gb:MT044258|Organism:Severe      TTAGAAGGTAACTTTTATGGACCTTTTGTTGACAGGCAAACAGCACAAGCAGCTGGTACG   10618
gb:MT039890|Organism:Severe      TTAGAAGGTAACTTTTATGGACCTTTTGTTGACAGGCAAACAGCACAAGCAGCTGGTACG   10642
gb:MN988713|Organism:Severe      TTAGAAGGTAACTTTTATGGACCTTTTGTTGACAGGCAAACAGCACAAGCAGCTGGTACG   10642
gb:LC521925|Organism:Severe      TTAGAAGGTAACTTTTATGGACCTTTTGTTGACAGGCAAACAGCACAAGCAGCTGGTACG   10615
gb:MT093571|Organism:Severe      TTAGAAGGTAACTTTTATGGACCTTTTGTTGACAGGCAAACAGCACAAGCAGCTGGTACG   10642
gb:MT039887|Organism:Severe      TTAGAAGGTAACTTTTATGGACCTTTTGTTGACAGGCAAACAGCACAAGCAGCTGGTACG   10642
gb:MT019530|Organism:Severe      TTAGAAGGTAACTTTTATGGACCTTTTGTTGACAGGCAAACAGCACAAGCAGCTGGTACG   10642
gb:MT039888|Organism:Severe      TTAGAAGGTAACTTTTATGGACCTTTTGTTGACAGGCAAACAGCACAAGCAGCTGGTACG   10642
gb:LC522972|Organism:Severe      TTAGAAGGTAACTTTTATGGACCTTTTGTTGACAGGCAAACAGCACAAGCAGCTGGTACG   10639
gb:MT027063|Organism:Severe      TTAGAAGGTAACTTTTATGGACCTTTTGTTGACAGGCAAACAGCACAAGCAGCTGGTACG   10642
gb:MT027062|Organism:Severe      TTAGAAGGTAACTTTTATGGACCTTTTGTTGACAGGCAAACAGCACAAGCAGCTGGTACG   10642
gb:MT019529|Organism:Severe      TTAGAAGGTAACTTTTATGGACCTTTTGTTGACAGGCAAACAGCACAAGCAGCTGGTACG   10642
gb:MN996529|Organism:Severe      TTAGAAGGTAACTTTTATGGACCTTTTGTTGACAGGCAAACAGCACAAGCAGCTGGTACG   10630
gb:MN996531|Organism:Severe      TTAGAAGGTAACTTTTATGGACCTTTTGTTGACAGGCAAACAGCACAAGCAGCTGGTACG   10629
gb:MT066176|Organism:Severe      TTAGAAGGTAACTTTTATGGACCTTTTGTTGACAGGCAAACAGCACAAGCAGCTGGTACG   10642
gb:MT027064|Organism:Severe      TTAGAAGGTAACTTTTATGGACCTTTTGTTGACAGGCAAACAGCACAAGCAGCTGGTACG   10642
gb:MN994468|Organism:Severe      TTAGAAGGTAACTTTTATGGACCTTTTGTTGACAGGCAAACAGCACAAGCAGCTGGTACG   10642
gb:MT072688|Organism:Severe      TTAGAAGGTAACTTTTATGGACCTTTTGTTGACAGGCAAACAGCACAAGCAGCTGGTACG   10627
gb:MN996527|Organism:Severe      TTAGAAGGTAACTTTTATGGACCTTTTGTTGACAGGCAAACAGCACAAGCAGCTGGTACG   10609
gb:MT093631|Organism:Severe      TTAGAAGGTAACTTTTATGGACCTTTTGTTGACAGGCAAACAGCACAAGCAGCTGGTACG   10680
gb:MT106053|Organism:Severe      TTAGAAGGTAACTTTTATGGACCTTTTGTTGACAGGCAAACAGCACAAGCAGCTGGTACG   10642
gb:MT019533|Organism:Severe      TTAGAAGGTAACTTTTATGGACCTTTTGTTGACAGGCAAACAGCACAAGCAGCTGGTACG   10642
gb:MT019531|Organism:Severe      TTAGAAGGTAACTTTTATGGACCTTTTGTTGACAGGCAAACAGCACAAGCAGCTGGTACG   10642
gb:MN996528|Organism:Severe      TTAGAAGGTAACTTTTATGGACCTTTTGTTGACAGGCAAACAGCACAAGCAGCTGGTACG   10642
gb:MN996530|Organism:Severe      TTAGAAGGTAACTTTTATGGACCTTTTGTTGACAGGCAAACAGCACAAGCAGCTGGTACG   10628
gb:MN908947|Organism:Severe      TTAGAAGGTAACTTTTATGGACCTTTTGTTGACAGGCAAACAGCACAAGCAGCTGGTACG   10642
gb:MT019532|Organism:Severe      TTAGAAGGTAACTTTTATGGACCTTTTGTTGACAGGCAAACAGCACAAGCAGCTGGTACG   10642
```

```
                                 ************************************************************

gb:MT020781|Organism:Severe      GACACAACTATTACAGTTAATGTTTTAGCTTGGTTGTACGCTGCTGTTATAAATGGAGAC   10690
gb:MT007544|Organism:Severe      GACACAACTATTACAGTTAATGTTTTAGCTTGGTTGTACGCTGCTGTTATAAATGGAGAC   10702
gb:MN994467|Organism:Severe      GACACAACTATTACAGTTAATGTTTTAGCTTGGTTGTACGCTGCTGTTATAAATGGAGAC   10702
gb:MT044257|Organism:Severe      GACACAACTATTACAGTTAATGTTTTAGCTTGGTTGTACGCTGCTGTTATAAATGGAGAC   10702
gb:MT106054|Organism:Severe      GACACAACTATTACAGTTAATGTTTTAGCTTGGTTGTACGCTGCTGTTATAAATGGAGAC   10702
gb:MT049951|Organism:Severe      GACACAACTATTACAGTTAATGTTTTAGCTTGGTTGTACGCTGCTGTTATAAATGGAGAC   10702
gb:MN975262|Organism:Severe      GACACAACTATTACAGTTAATGTTTTAGCTTGGTTGTACGCTGCTGTTATAAATGGAGAC   10702
gb:MT106052|Organism:Severe      GACACAACTATTACAGTTAATGTTTTAGCTTGGTTGTACGCTGCTGTTATAAATGGAGAC   10702
gb:LC522975|Organism:Severe      GACACAACTATTACAGTTAATGTTTTAGCTTGGTTGTACGCTGCTGTTATAAATGGAGAC   10699
gb:LC522973|Organism:Severe      GACACAACTATTACAGTTAATGTTTTAGCTTGGTTGTACGCTGCTGTTATAAATGGAGAC   10699
gb:LC522974|Organism:Severe      GACACAACTATTACAGTTAATGTTTTAGCTTGGTTGTACGCTGCTGTTATAAATGGAGAC   10699
gb:MN985325|Organism:Severe      GACACAACTATTACAGTTAATGTTTTAGCTTGGTTGTACGCTGCTGTTATAAATGGAGAC   10702
gb:MT020881|Organism:Severe      GACACAACTATTACAGTTAATGTTTTAGCTTGGTTGTACGCTGCTGTTATAAATGGAGAC   10702
gb:MT020880|Organism:Severe      GACACAACTATTACAGTTAATGTTTTAGCTTGGTTGTACGCTGCTGTTATAAATGGAGAC   10702
gb:MT066175|Organism:Severe      GACACAACTATTACAGTTAATGTTTTAGCTTGGTTGTACGCTGCTGTTATAAATGGAGAC   10702
gb:MN997409|Organism:Severe      GACACAACTATTACAGTTAATGTTTTAGCTTGGTTGTACGCTGCTGTTATAAATGGAGAC   10702
gb:MN938384|Organism:Severe      GACACAACTATTACAGTTAATGTTTTAGCTTGGTTGTACGCTGCTGTTATAAATGGAGAC   10670
gb:MT044258|Organism:Severe      GACACAACTATTACAGTTAATGTTTTAGCTTGGTTGTACGCTGCTGTTATAAATGGAGAC   10678
gb:MT039890|Organism:Severe      GACACAACTATTACAGTTAATGTTTTAGCTTGGTTGTACGCTGCTGTTATAAATGGAGAC   10702
gb:MN988713|Organism:Severe      GACACAACTATTACAGTTAATGTTTTAGCTTGGTTGTACGCTGCTGTTATAAATGGAGAC   10702
gb:LC521925|Organism:Severe      GACACAACTATTACAGTTAATGTTTTAGCTTGGTTGTACGCTGCTGTTATAAATGGAGAC   10675
gb:MT093571|Organism:Severe      GACACAACTATTACAGTTAATGTTTTAGCTTGGTTGTACGCTGCTGTTATAAATGGAGAC   10702
gb:MT039887|Organism:Severe      GACACAACTATTACAGTTAATGTTTTAGCTTGGTTGTACGCTGCTGTTATAAATGGAGAC   10702
gb:MT019530|Organism:Severe      GACACAACTATTACAGTTAATGTTTTAGCTTGGTTGTACGCTGCTGTTATAAATGGAGAC   10702
gb:MT039888|Organism:Severe      GACACAACTATTACAGTTAATGTTTTAGCTTGGTTGTACGCTGCTGTTATAAATGGAGAC   10702
gb:LC522972|Organism:Severe      GACACAACTATTACAGTTAATGTTTTAGCTTGGTTGTACGCTGCTGTTATAAATGGAGAC   10699
gb:MT027063|Organism:Severe      GACACAACTATTACAGTTAATGTTTTAGCTTGGTTGTACGCTGCTGTTATAAATGGAGAC   10702
gb:MT027062|Organism:Severe      GACACAACTATTACAGTTAATGTTTTAGCTTGGTTGTACGCTGCTGTTATAAATGGAGAC   10702
gb:MT019529|Organism:Severe      GACACAACTATTACAGTTAATGTTTTAGCTTGGTTGTACGCTGCTGTTATAAATGGAGAC   10702
gb:MN996529|Organism:Severe      GACACAACTATTACAGTTAATGTTTTAGCTTGGTTGTACGCTGCTGTTATAAATGGAGAC   10690
gb:MN996531|Organism:Severe      GACACAACTATTACAGTTAATGTTTTAGCTTGGTTGTACGCTGCTGTTATAAATGGAGAC   10689
gb:MT066176|Organism:Severe      GACACAACTATTACAGTTAATGTTTTAGCTTGGTTGTACGCTGCTGTTATAAATGGAGAC   10702
gb:MT027064|Organism:Severe      GACACAACTATTACAGTTAATGTTTTAGCTTGGTTGTACGCTGCTGTTATAAATGGAGAC   10702
gb:MN994468|Organism:Severe      GACACAACTATTACAGTTAATGTTTTAGCTTGGTTGTACGCTGCTGTTATAAATGGAGAC   10702
gb:MT072688|Organism:Severe      GACACAACTATTACAGTTAATGTTTTAGCTTGGTTGTACGCTGCTGTTATAAATGGAGAC   10687
gb:MN996527|Organism:Severe      GACACAACTATTACAGTTAATGTTTTAGCTTGGTTGTACGCTGCTGTTATAAATGGAGAC   10669
gb:MT093631|Organism:Severe      GACACAACTATTACAGTTAATGTTTTAGCTTGGTTGTACGCTGCTGTTATAAATGGAGAC   10740
gb:MT106053|Organism:Severe      GACACAACTATTACAGTTAATGTTTTAGCTTGGTTGTACGCTGCTGTTATAAATGGAGAC   10702
gb:MT019533|Organism:Severe      GACACAACTATTACAGTTAATGTTTTAGCTTGGTTGTACGCTGCTGTTATAAATGGAGAC   10702
gb:MT019531|Organism:Severe      GACACAACTATTACAGTTAATGTTTTAGCTTGGTTGTACGCTGCTGTTATAAATGGAGAC   10702
gb:MN996528|Organism:Severe      GACACAACTATTACAGTTAATGTTTTAGCTTGGTTGTACGCTGCTGTTATAAATGGAGAC   10702
gb:MN996530|Organism:Severe      GACACAACTATTACAGTTAATGTTTTAGCTTGGTTGTACGCTGCTGTTATAAATGGAGAC   10688
gb:MN908947|Organism:Severe      GACACAACTATTACAGTTAATGTTTTAGCTTGGTTGTACGCTGCTGTTATAAATGGAGAC   10702
gb:MT019532|Organism:Severe      GACACAACTATTACAGTTAATGTTTTAGCTTGGTTGTACGCTGCTGTTATAAATGGAGAC   10702
                                 ************************************************************

gb:MT020781|Organism:Severe      AGGTGGTTTCTCAATCGATTTACCACAACTCTTAATGACTTTAACCTTGTGGCTATGAAG   10750
gb:MT007544|Organism:Severe      AGGTGGTTTCTCAATCGATTTACCACAACTCTTAATGACTTTAACCTTGTGGCTATGAAG   10762
gb:MN994467|Organism:Severe      AGGTGGTTTCTCAATCGATTTACCACAACTCTTAATGACTTTAACCTTGTGGCTATGAAG   10762
gb:MT044257|Organism:Severe      AGGTGGTTTCTCAATCGATTTACCACAACTCTTAATGACTTTAACCTTGTGGCTATGAAG   10762
gb:MT106054|Organism:Severe      AGGTGGTTTCTCAATCGATTTACCACAACTCTTAATGACTTTAACCTTGTGGCTATGAAG   10762
gb:MT049951|Organism:Severe      AGGTGGTTTCTCAATCGATTTACCACAACTCTTAATGACTTTAACCTTGTGGCTATGAAG   10762
gb:MN975262|Organism:Severe      AGGTGGTTTCTCAATCGATTTACCACAACTCTTAATGACTTTAACCTTGTGGCTATGAAG   10762
gb:MT106052|Organism:Severe      AGGTGGTTTCTCAATCGATTTACCACAACTCTTAATGACTTTAACCTTGTGGCTATGAAG   10762
gb:LC522975|Organism:Severe      AGGTGGTTTCTCAATCGATTTACCACAACTCTTAATGACTTTAACCTTGTGGCTATGAAG   10759
gb:LC522973|Organism:Severe      AGGTGGTTTCTCAATCGATTTACCACAACTCTTAATGACTTTAACCTTGTGGCTATGAAG   10759
gb:LC522974|Organism:Severe      AGGTGGTTTCTCAATCGATTTACCACAACTCTTAATGACTTTAACCTTGTGGCTATGAAG   10759
gb:MN985325|Organism:Severe      AGGTGGTTTCTCAATCGATTTACCACAACTCTTAATGACTTTAACCTTGTGGCTATGAAG   10762
gb:MT020881|Organism:Severe      AGGTGGTTTCTCAATCGATTTACCACAACTCTTAATGACTTTAACCTTGTGGCTATGAAG   10762
gb:MT020880|Organism:Severe      AGGTGGTTTCTCAATCGATTTACCACAACTCTTAATGACTTTAACCTTGTGGCTATGAAG   10762
gb:MT066175|Organism:Severe      AGGTGGTTTCTCAATCGATTTACCACAACTCTTAATGACTTTAACCTTGTGGCTATGAAG   10762
gb:MN997409|Organism:Severe      AGGTGGTTTCTCAATCGATTTACCACAACTCTTAATGACTTTAACCTTGTGGCTATGAAG   10762
gb:MN938384|Organism:Severe      AGGTGGTTTCTCAATCGATTTACCACAACTCTTAATGACTTTAACCTTGTGGCTATGAAG   10730
```

```
gb:MT044258|Organism:Severe      AGGTGGTTTCTCAATCGATTTACCACAACTCTTAATGACTTTAACCTTGTGGCTATGAAG   10738
gb:MT039890|Organism:Severe      AGGTGGTTTCTCAATCGATTTACCACAACTCTTAATGACTTTAACCTTGTGGCTATGAAG   10762
gb:MN988713|Organism:Severe      AGGTGGTTTCTCAATCGATTTACCACAACTCTTAATGACTTTAACCTTGTGGCTATGAAG   10762
gb:LC521925|Organism:Severe      AGGTGGTTTCTCAATCGATTTACCACAACTCTTAATGACTTTAACCTTGTGGCTATGAAG   10735
gb:MT093571|Organism:Severe      AGGTGGTTTCTCAATCGATTTACCACAACTCTTAATGACTTTAACCTTGTGGCTATGAAG   10762
gb:MT039887|Organism:Severe      AGGTGGTTTCTCAATCGATTTACCACAACTCTTAATGACTTTAACCTTGTGGCTATGAAG   10762
gb:MT019530|Organism:Severe      AGGTGGTTTCTCAATCGATTTACCACAACTCTTAATGACTTTAACCTTGTGGCTATGAAG   10762
gb:MT039888|Organism:Severe      AGGTGGTTTCTCAATCGATTTACCACAACTCTTAATGACTTTAACCTTGTGGCTATGAAG   10762
gb:LC522972|Organism:Severe      AGGTGGTTTCTCAATCGATTTACCACAACTCTTAATGACTTTAACCTTGTGGCTATGAAG   10759
gb:MT027063|Organism:Severe      AGGTGGTTTCTCAATCGATTTACCACAACTCTTAATGACTTTAACCTTGTGGCTATGAAG   10762
gb:MT027062|Organism:Severe      AGGTGGTTTCTCAATCGATTTACCACAACTCTTAATGACTTTAACCTTGTGGCTATGAAG   10762
gb:MT019529|Organism:Severe      AGGTGGTTTCTCAATCGATTTACCACAACTCTTAATGACTTTAACCTTGTGGCTATGAAG   10762
gb:MN996529|Organism:Severe      AGGTGGTTTCTCAATCGATTTACCACAACTCTTAATGACTTTAACCTTGTGGCTATGAAG   10750
gb:MN996531|Organism:Severe      AGGTGGTTTCTCAATCGATTTACCACAACTCTTAATGACTTTAACCTTGTGGCTATGAAG   10749
gb:MT066176|Organism:Severe      AGGTGGTTTCTCAATCGATTTACCACAACTCTTAATGACTTTAACCTTGTGGCTATGAAG   10762
gb:MT027064|Organism:Severe      AGGTGGTTTCTCAATCGATTTACCACAACTCTTAATGACTTTAACCTTGTGGCTATGAAG   10762
gb:MN994468|Organism:Severe      AGGTGGTTTCTCAATCGATTTACCACAACTCTTAATGACTTTAACCTTGTGGCTATGAAG   10762
gb:MT072688|Organism:Severe      AGGTGGTTTCTCAATCGATTTACCACAACTCTTAATGACTTTAACCTTGTGGCTATGAAG   10747
gb:MN996527|Organism:Severe      AGGTGGTTTCTCAATCGATTTACCACAACTCTTAATGACTTTAACCTTGTGGCTATGAAG   10729
gb:MT093631|Organism:Severe      AGGTGGTTTCTCAATCGATTTACCACAACTCTTAATGACTTTAACCTTGTGGCTATGAAG   10800
gb:MT106053|Organism:Severe      AGGTGGTTTCTCAATCGATTTACCACAACTCTTAATGACTTTAACCTTGTGGCTATGAAG   10762
gb:MT019533|Organism:Severe      AGGTGGTTTCTCAATCGATTTACCACAACTCTTAATGACTTTAACCTTGTGGCTATGAAG   10762
gb:MT019531|Organism:Severe      AGGTGGTTTCTCAATCGATTTACCACAACTCTTAATGACTTTAACCTTGTGGCTATGAAG   10762
gb:MN996528|Organism:Severe      AGGTGGTTTCTCAATCGATTTACCACAACTCTTAATGACTTTAACCTTGTGGCTATGAAG   10762
gb:MN996530|Organism:Severe      AGGTGGTTTCTCAATCGATTTACCACAACTCTTAATGACTTTAACCTTGTGGCTATGAAG   10748
gb:MN908947|Organism:Severe      AGGTGGTTTCTCAATCGATTTACCACAACTCTTAATGACTTTAACCTTGTGGCTATGAAG   10762
gb:MT019532|Organism:Severe      AGGTGGTTTCTCAATCGATTTACCACAACTCTTAATGACTTTAACCTTGTGGCTATGAAG   10762
                                 ************************************************************

gb:MT020781|Organism:Severe      TACAATTATGAACCTCTAACACAAGACCATGTTGACATACTAGGACCTCTTTCTGCTCAA   10810
gb:MT007544|Organism:Severe      TACAATTATGAACCTCTAACACAAGACCATGTTGACATACTAGGACCTCTTTCTGCTCAA   10822
gb:MN994467|Organism:Severe      TACAATTATGAACCTCTAACACAAGACCATGTTGACATACTAGGACCTCTTTCTGCTCAA   10822
gb:MT044257|Organism:Severe      TACAATTATGAACCTCTAACACAAGACCATGTTGACATACTAGGACCTCTTTCTGCTCAA   10822
gb:MT106054|Organism:Severe      TACAATTATGAACCTCTAACACAAGACCATGTTGACATACTAGGACCTCTTTCTGCTCAA   10822
gb:MT049951|Organism:Severe      TACAATTATGAACCTCTAACACAAGACCATGTTGACATACTAGGACCTCTTTCTGCTCAA   10822
gb:MN975262|Organism:Severe      TACAATTATGAACCTCTAACACAAGACCATGTTGACATACTAGGACCTCTTTCTGCTCAA   10822
gb:MT106052|Organism:Severe      TACAATTATGAACCTCTAACACAAGACCATGTTGACATACTAGGACCTCTTTCTGCTCAA   10822
gb:LC522975|Organism:Severe      TACAATTATGAACCTCTAACACAAGACCATGTTGACATACTAGGACCTCTTTCTGCTCAA   10819
gb:LC522973|Organism:Severe      TACAATTATGAACCTCTAACACAAGACCATGTTGACATACTAGGACCTCTTTCTGCTCAA   10819
gb:LC522974|Organism:Severe      TACAATTATGAACCTCTAACACAAGACCATGTTGACATACTAGGACCTCTTTCTGCTCAA   10819
gb:MN985325|Organism:Severe      TACAATTATGAACCTCTAACACAAGACCATGTTGACATACTAGGACCTCTTTCTGCTCAA   10822
gb:MT020881|Organism:Severe      TACAATTATGAACCTCTAACACAAGACCATGTTGACATACTAGGACCTCTTTCTGCTCAA   10822
gb:MT020880|Organism:Severe      TACAATTATGAACCTCTAACACAAGACCATGTTGACATACTAGGACCTCTTTCTGCTCAA   10822
gb:MT066175|Organism:Severe      TACAATTATGAACCTCTAACACAAGACCATGTTGACATACTAGGACCTCTTTCTGCTCAA   10822
gb:MN997409|Organism:Severe      TACAATTATGAACCTCTAACACAAGACCATGTTGACATACTAGGACCTCTTTCTGCTCAA   10822
gb:MN938384|Organism:Severe      TACAATTATGAACCTCTAACACAAGACCATGTTGACATACTAGGACCTCTTTCTGCTCAA   10790
gb:MT044258|Organism:Severe      TACAATTATGAACCTCTAACACAAGACCATGTTGACATACTAGGACCTCTTTCTGCTCAA   10798
gb:MT039890|Organism:Severe      TACAATTATGAACCTCTAACACAAGACCATGTTGACATACTAGGACCTCTTTCTGCTCAA   10822
gb:MN988713|Organism:Severe      TACAATTATGAACCTCTAACACAAGACCATGTTGACATACTAGGACCTCTTTCTGCTCAA   10822
gb:LC521925|Organism:Severe      TACAATTATGAACCTCTAACACAAGACCATGTTGACATACTAGGACCTCTTTCTGCTCAA   10795
gb:MT093571|Organism:Severe      TACAATTATGAACCTCTAACACAAGACCATGTTGACATACTAGGACCTCTTTCTGCTCAA   10822
gb:MT039887|Organism:Severe      TACAATTATGAACCTCTAACACAAGACCATGTTGACATACTAGGACCTCTTTCTGCTCAA   10822
gb:MT019530|Organism:Severe      TACAATTATGAACCTCTAACACAAGACCATGTTGACATACTAGGACCTCTTTCTGCTCAA   10822
gb:MT039888|Organism:Severe      TACAATTATGAACCTCTAACACAAGACCATGTTGACATACTAGGACCTCTTTCTGCTCAA   10822
gb:LC522972|Organism:Severe      TACAATTATGAACCTCTAACACAAGACCATGTTGACATACTAGGACCTCTTTCTGCTCAA   10819
gb:MT027063|Organism:Severe      TACAATTATGAACCTCTAACACAAGACCATGTTGACATACTAGGACCTCTTTCTGCTCAA   10822
gb:MT027062|Organism:Severe      TACAATTATGAACCTCTAACACAAGACCATGTTGACATACTAGGACCTCTTTCTGCTCAA   10822
gb:MT019529|Organism:Severe      TACAATTATGAACCTCTAACACAAGACCATGTTGACATACTAGGACCTCTTTCTGCTCAA   10822
gb:MN996529|Organism:Severe      TACAATTATGAACCTCTAACACAAGACCATGTTGACATACTAGGACCTCTTTCTGCTCAA   10810
gb:MN996531|Organism:Severe      TACAATTATGAACCTCTAACACAAGACCATGTTGACATACTAGGACCTCTTTCTGCTCAA   10809
gb:MT066176|Organism:Severe      TACAATTATGAACCTCTAACACAAGACCATGTTGACATACTAGGACCTCTTTCTGCTCAA   10822
gb:MT027064|Organism:Severe      TACAATTATGAACCTCTAACACAAGACCATGTTGACATACTAGGACCTCTTTCTGCTCAA   10822
gb:MN994468|Organism:Severe      TACAATTATGAACCTCTAACACAAGACCATGTTGACATACTAGGACCTCTTTCTGCTCAA   10822
gb:MT072688|Organism:Severe      TACAATTATGAACCTCTAACACAAGACCATGTTGACATACTAGGACCTCTTTCTGCTCAA   10807
gb:MN996527|Organism:Severe      TACAATTATGAACCTCTAACACAAGACCATGTTGACATACTAGGACCTCTTTCTGCTCAA   10789
```

```
gb:MT093631|Organism:Severe      TACAATTATGAACCTCTAACACAAGACCATGTTGACATACTAGGACCTCTTTCTGCTCAA   10860
gb:MT106053|Organism:Severe      TACAATTATGAACCTCTAACACAAGACCATGTTGACATACTAGGACCTCTTTCTGCTCAA   10822
gb:MT019533|Organism:Severe      TACAATTATGAACCTCTAACACAAGACCATGTTGACATACTAGGACCTCTTTCTGCTCAA   10822
gb:MT019531|Organism:Severe      TACAATTATGAACCTCTAACACAAGACCATGTTGACATACTAGGACCTCTTTCTGCTCAA   10822
gb:MN996528|Organism:Severe      TACAATTATGAACCTCTAACACAAGACCATGTTGACATACTAGGACCTCTTTCTGCTCAA   10822
gb:MN996530|Organism:Severe      TACAATTATGAACCTCTAACACAAGACCATGTTGACATACTAGGACCTCTTTCTGCTCAA   10808
gb:MN908947|Organism:Severe      TACAATTATGAACCTCTAACACAAGACCATGTTGACATACTAGGACCTCTTTCTGCTCAA   10822
gb:MT019532|Organism:Severe      TACAATTATGAACCTCTAACACAAGACCATGTTGACATACTAGGACCTCTTTCTGCTCAA   10822
                                 ************************************************************

gb:MT020781|Organism:Severe      ACTGGAATTGCCGTTTTAGATATGTGTGCTTCATTAAAAGAATTACTGCAAAATGGTATG   10870
gb:MT007544|Organism:Severe      ACTGGAATTGCCGTTTTAGATATGTGTGCTTCATTAAAAGAATTACTGCAAAATGGTATG   10882
gb:MN994467|Organism:Severe      ACTGGAATTGCCGTTTTAGATATGTGTGCTTCATTAAAAGAATTACTGCAAAATGGTATG   10882
gb:MT044257|Organism:Severe      ACTGGAATTGCCGTTTTAGATATGTGTGCTTCATTAAAAGAATTACTGCAAAATGGTATG   10882
gb:MT106054|Organism:Severe      ACTGGAATTGCCGTTTTAGATATGTGTGCTTCATTAAAAGAATTACTGCAAAATGGTATG   10882
gb:MT049951|Organism:Severe      ACTGGAATTGCCGTTTTAGATATGTGTGCTTCATTAAAAGAATTACTGCAAAATGGTATG   10882
gb:MN975262|Organism:Severe      ACTGGAATTGCCGTTTTAGATATGTGTGCTTCATTAAAAGAATTACTGCAAAATGGTATG   10882
gb:MT106052|Organism:Severe      ACTGGAATTGCCGTTTTAGATATGTGTGCTTCATTAAAAGAATTACTGCAAAATGGTATG   10882
gb:LC522975|Organism:Severe      ACTGGAATTGCCGTTTTAGATATGTGTGCTTCATTAAAAGAATTACTGCAAAATGGTATG   10879
gb:LC522973|Organism:Severe      ACTGGAATTGCCGTTTTAGATATGTGTGCTTCATTAAAAGAATTACTGCAAAATGGTATG   10879
gb:LC522974|Organism:Severe      ACTGGAATTGCCGTTTTAGATATGTGTGCTTCATTAAAAGAATTACTGCAAAATGGTATG   10879
gb:MN985325|Organism:Severe      ACTGGAATTGCCGTTTTAGATATGTGTGCTTCATTAAAAGAATTACTGCAAAATGGTATG   10882
gb:MT020881|Organism:Severe      ACTGGAATTGCCGTTTTAGATATGTGTGCTTCATTAAAAGAATTACTGCAAAATGGTATG   10882
gb:MT020880|Organism:Severe      ACTGGAATTGCCGTTTTAGATATGTGTGCTTCATTAAAAGAATTACTGCAAAATGGTATG   10882
gb:MT066175|Organism:Severe      ACTGGAATTGCCGTTTTAGATATGTGTGCTTCATTAAAAGAATTACTGCAAAATGGTATG   10882
gb:MN997409|Organism:Severe      ACTGGAATTGCCGTTTTAGATATGTGTGCTTCATTAAAAGAATTACTGCAAAATGGTATG   10882
gb:MN938384|Organism:Severe      ACTGGAATTGCCGTTTTAGATATGTGTGCTTCATTAAAAGAATTACTGCAAAATGGTATG   10850
gb:MT044258|Organism:Severe      ACTGGAATTGCCGTTTTAGATATGTGTGCTTCATTAAAAGAATTACTGCAAAATGGTATG   10858
gb:MT039890|Organism:Severe      ACTGGAATTGCCGTTTTAGATATGTGTGCTTCATTAAAAGAATTACTGCAAAATGGTATG   10882
gb:MN988713|Organism:Severe      ACTGGAATTGCCGTTTTAGATATGTGTGCTTCATTAAAAGAATTACTGCAAAATGGTATG   10882
gb:LC521925|Organism:Severe      ACTGGAATTGCCGTTTTAGATATGTGTGCTTCATTAAAAGAATTACTGCAAAATGGTATG   10855
gb:MT093571|Organism:Severe      ACTGGAATTGCCGTTTTAGATATGTGTGCTTCATTAAAAGAATTACTGCAAAATGGTATG   10882
gb:MT039887|Organism:Severe      ACTGGAATTGCCGTTTTAGATATGTGTGCTTCATTAAAAGAATTACTGCAAAATGGTATG   10882
gb:MT019530|Organism:Severe      ACTGGAATTGCCGTTTTAGATATGTGTGCTTCATTAAAAGAATTACTGCAAAATGGTATG   10882
gb:MT039888|Organism:Severe      ACTGGAATTGCCGTTTTAGATATGTGTGCTTCATTAAAAGAATTACTGCAAAATGGTATG   10882
gb:LC522972|Organism:Severe      ACTGGAATTGCCGTTTTAGATATGTGTGCTTCATTAAAAGAATTACTGCAAAATGGTATG   10879
gb:MT027063|Organism:Severe      ACTGGAATTGCCGTTTTAGATATGTGTGCTTCATTAAAAGAATTACTGCAAAATGGTATG   10882
gb:MT027062|Organism:Severe      ACTGGAATTGCCGTTTTAGATATGTGTGCTTCATTAAAAGAATTACTGCAAAATGGTATG   10882
gb:MT019529|Organism:Severe      ACTGGAATTGCCGTTTTAGATATGTGTGCTTCATTAAAAGAATTACTGCAAAATGGTATG   10882
gb:MN996529|Organism:Severe      ACTGGAATTGCCGTTTTAGATATGTGTGCTTCATTAAAAGAATTACTGCAAAATGGTATG   10870
gb:MN996531|Organism:Severe      ACTGGAATTGCCGTTTTAGATATGTGTGCTTCATTAAAAGAATTACTGCAAAATGGTATG   10869
gb:MT066176|Organism:Severe      ACTGGAATTGCCGTTTTAGATATGTGTGCTTCATTAAAAGAATTACTGCAAAATGGTATG   10882
gb:MT027064|Organism:Severe      ACTGGAATTGCCGTTTTAGATATGTGTGCTTCATTAAAAGAATTACTGCAAAATGGTATG   10882
gb:MN994468|Organism:Severe      ACTGGAATTGCCGTTTTAGATATGTGTGCTTCATTAAAAGAATTACTGCAAAATGGTATG   10882
gb:MT072688|Organism:Severe      ACTGGAATTGCCGTTTTAGATATGTGTGCTTCATTAAAAGAATTACTGCAAAATGGTATG   10867
gb:MN996527|Organism:Severe      ACTGGAATTGCCGTTTTAGATATGTGTGCTTCATTAAAAGAATTACTGCAAAATGGTATG   10849
gb:MT093631|Organism:Severe      ACTGGAATTGCCGTTTTAGATATGTGTGCTTCATTAAAAGAATTACTGCAAAATGGTATG   10920
gb:MT106053|Organism:Severe      ACTGGAATTGCCGTTTTAGATATGTGTGCTTCATTAAAAGAATTACTGCAAAATGGTATG   10882
gb:MT019533|Organism:Severe      ACTGGAATTGCCGTTTTAGATATGTGTGCTTCATTAAAAGAATTACTGCAAAATGGTATG   10882
gb:MT019531|Organism:Severe      ACTGGAATTGCCGTTTTAGATATGTGTGCTTCATTAAAAGAATTACTGCAAAATGGTATG   10882
gb:MN996528|Organism:Severe      ACTGGAATTGCCGTTTTAGATATGTGTGCTTCATTAAAAGAATTACTGCAAAATGGTATG   10882
gb:MN996530|Organism:Severe      ACTGGAATTGCCGTTTTAGATATGTGTGCTTCATTAAAAGAATTACTGCAAAATGGTATG   10868
gb:MN908947|Organism:Severe      ACTGGAATTGCCGTTTTAGATATGTGTGCTTCATTAAAAGAATTACTGCAAAATGGTATG   10882
gb:MT019532|Organism:Severe      ACTGGAATTGCCGTTTTAGATATGTGTGCTTCATTAAAAGAATTACTGCAAAATGGTATG   10882
                                 ************************************************************

gb:MT020781|Organism:Severe      AATGGACGTACCATATTGGGTAGTGCTTTATTAGAAGATGAATTTACACCTTTTGATGTT   10930
gb:MT007544|Organism:Severe      AATGGACGTACCATATTGGGTAGTGCTTTATTAGAAGATGAATTTACACCTTTTGATGTT   10942
gb:MN994467|Organism:Severe      AATGGACGTACCATATTGGGTAGTGCTTTATTAGAAGATGAATTTACACCTTTTGATGTT   10942
gb:MT044257|Organism:Severe      AATGGACGTACCATATTGGGTAGTGCTTTATTAGAAGATGAATTTACACCTTTTGATGTT   10942
gb:MT106054|Organism:Severe      AATGGACGTACCATATTGGGTAGTGCTTTATTAGAAGATGAATTTACACCTTTTGATGTT   10942
gb:MT049951|Organism:Severe      AATGGACGTACCATATTGGGTAGTGCTTTATTAGAAGATGAATTTACACCTTTTGATGTT   10942
gb:MN975262|Organism:Severe      AATGGACGTACCATATTGGGTAGTGCTTTATTAGAAGATGAATTTACACCTTTTGATGTT   10942
gb:MT106052|Organism:Severe      AATGGACGTACCATATTGGGTAGTGCTTTATTAGAAGATGAATTTACACCTTTTGATGTT   10942
gb:LC522975|Organism:Severe      AATGGACGTACCATATTGGGTAGTGCTTTATTAGAAGATGAATTTACACCTTTTGATGTT   10939
```

```
gb:LC522973|Organism:Severe      AATGGACGTACCATATTGGGTAGTGCTTTATTAGAAGATGAATTTACACCTTTTGATGTT   10939
gb:LC522974|Organism:Severe      AATGGACGTACCATATTGGGTAGTGCTTTATTAGAAGATGAATTTACACCTTTTGATGTT   10939
gb:MN985325|Organism:Severe      AATGGACGTACCATATTGGGTAGTGCTTTATTAGAAGATGAATTTACACCTTTTGATGTT   10942
gb:MT020881|Organism:Severe      AATGGACGTACCATATTGGGTAGTGCTTTATTAGAAGATGAATTTACACCTTTTGATGTT   10942
gb:MT020880|Organism:Severe      AATGGACGTACCATATTGGGTAGTGCTTTATTAGAAGATGAATTTACACCTTTTGATGTT   10942
gb:MT066175|Organism:Severe      AATGGACGTACCATATTGGGTAGTGCTTTATTAGAAGATGAATTTACACCTTTTGATGTT   10942
gb:MN997409|Organism:Severe      AATGGACGTACCATATTGGGTAGTGCTTTATTAGAAGATGAATTTACACCTTTTGATGTT   10942
gb:MN938384|Organism:Severe      AATGGACGTACCATATTGGGTAGTGCTTTATTAGAAGATGAATTTACACCTTTTGATGTT   10910
gb:MT044258|Organism:Severe      AATGGACGTACCATATTGGGTAGTGCTTTATTAGAAGATGAATTTACACCTTTTGATGTT   10918
gb:MT039890|Organism:Severe      AATGGACGTACCATATTGGGTAGTGCTTTATTAGAAGATGAATTTACACCTTTTGATGTT   10942
gb:MN988713|Organism:Severe      AATGGACGTACCATATTGGGTAGTGCTTTATTAGAAGATGAATTTACACCTTTTGATGTT   10942
gb:LC521925|Organism:Severe      AATGGACGTACCATATTGGGTAGTGCTTTATTAGAAGATGAATTTACACCTTTTGATGTT   10915
gb:MT093571|Organism:Severe      AATGGACGTACCATATTGGGTAGTGCTTTATTAGAAGATGAATTTACACCTTTTGATGTT   10942
gb:MT039887|Organism:Severe      AATGGACGTACCATATTGGGTAGTGCTTTATTAGAAGATGAATTTACACCTTTTGATGTT   10942
gb:MT019530|Organism:Severe      AATGGACGTACCATATTGGGTAGTGCTTTATTAGAAGATGAATTTACACCTTTTGATGTT   10942
gb:MT039888|Organism:Severe      AATGGACGTACCATATTGGGTAGTGCTTTATTAGAAGATGAATTTACACCTTTTGATGTT   10942
gb:LC522972|Organism:Severe      AATGGACGTACCATATTGGGTAGTGCTTTATTAGAAGATGAATTTACACCTTTTGATGTT   10939
gb:MT027063|Organism:Severe      AATGGACGTACCATATTGGGTAGTGCTTTATTAGAAGATGAATTTACACCTTTTGATGTT   10942
gb:MT027062|Organism:Severe      AATGGACGTACCATATTGGGTAGTGCTTTATTAGAAGATGAATTTACACCTTTTGATGTT   10942
gb:MT019529|Organism:Severe      AATGGACGTACCATATTGGGTAGTGCTTTATTAGAAGATGAATTTACACCTTTTGATGTT   10942
gb:MN996529|Organism:Severe      AATGGACGTACCATATTGGGTAGTGCTTTATTAGAAGATGAATTTACACCTTTTGATGTT   10930
gb:MN996531|Organism:Severe      AATGGACGTACCATATTGGGTAGTGCTTTATTAGAAGATGAATTTACACCTTTTGATGTT   10929
gb:MT066176|Organism:Severe      AATGGACGTACCATATTGGGTAGTGCTTTATTAGAAGATGAATTTACACCTTTTGATGTT   10942
gb:MT027064|Organism:Severe      AATGGACGTACCATATTGGGTAGTGCTTTATTAGAAGATGAATTTACACCTTTTGATGTT   10942
gb:MN994468|Organism:Severe      AATGGACGTACCATATTGGGTAGTGCTTTATTAGAAGATGAATTTACACCTTTTGATGTT   10942
gb:MT072688|Organism:Severe      AATGGACGTACCATATTGGGTAGTGCTTTATTAGAAGATGAATTTACACCTTTTGATGTT   10927
gb:MN996527|Organism:Severe      AATGGACGTACCATATTGGGTAGTGCTTTATTAGAAGATGAATTTACACCTTTTGATGTT   10909
gb:MT093631|Organism:Severe      AATGGACGTACCATATTGGGTAGTGCTTTATTAGAAGATGAATTTACACCTTTTGATGTT   10980
gb:MT106053|Organism:Severe      AATGGACGTACCATATTGGGTAGTGCTTTATTAGAAGATGAATTTACACCTTTTGATGTT   10942
gb:MT019533|Organism:Severe      AATGGACGTACCATATTGGGTAGTGCTTTATTAGAAGATGAATTTACACCTTTTGATGTT   10942
gb:MT019531|Organism:Severe      AATGGACGTACCATATTGGGTAGTGCTTTATTAGAAGATGAATTTACACCTTTTGATGTT   10942
gb:MN996528|Organism:Severe      AATGGACGTACCATATTGGGTAGTGCTTTATTAGAAGATGAATTTACACCTTTTGATGTT   10942
gb:MN996530|Organism:Severe      AATGGACGTACCATATTGGGTAGTGCTTTATTAGAAGATGAATTTACACCTTTTGATGTT   10928
gb:MN908947|Organism:Severe      AATGGACGTACCATATTGGGTAGTGCTTTATTAGAAGATGAATTTACACCTTTTGATGTT   10942
gb:MT019532|Organism:Severe      AATGGACGTACCATATTGGGTAGTGCTTTATTAGAAGATGAATTTACACCTTTTGATGTT   10942
                                 ************************************************************

gb:MT020781|Organism:Severe      GTTAGACAATGCTCAGGTGTTACTTTCCAAAGTGCAGTGAAAAGAACAATCAAGGGTACA   10990
gb:MT007544|Organism:Severe      GTTAGACAATGCTCAGGTGTTACTTTCCAAAGTGCAGTGAAAAGAACAATCAAGGGTACA   11002
gb:MN994467|Organism:Severe      GTTAGACAATGCTCAGGTGTTACTTTCCAAAGTGCAGTGAAAAGAACAATCAAGGGTACA   11002
gb:MT044257|Organism:Severe      GTTAGACAATGCTCAGGTGTTACTTTCCAAAGTGCAGTGAAAAGAACAATCAAGGGTACA   11002
gb:MT106054|Organism:Severe      GTTAGACAATGCTCAGGTGTTACTTTCCAAAGTGCAGTGAAAAGAACAATCAAGGGTACA   11002
gb:MT049951|Organism:Severe      GTTAGACAATGCTCAGGTGTTACTTTCCAAAGTGCAGTGAAAAGAACAATCAAGGGTACA   11002
gb:MN975262|Organism:Severe      GTTAGACAATGCTCAGGTGTTACTTTCCAAAGTGCAGTGAAAAGAACAATCAAGGGTACA   11002
gb:MT106052|Organism:Severe      GTTAGACAATGCTCAGGTGTTACTTTCCAAAGTGCAGTGAAAAGAACAATCAAGGGTACA   11002
gb:LC522975|Organism:Severe      GTTAGACAATGCTCAGGTGTTACTTTCCAAAGTGCAGTGAAAAGAACAATCAAGGGTACA   10999
gb:LC522973|Organism:Severe      GTTAGACAATGCTCAGGTGTTACTTTCCAAAGTGCAGTGAAAAGAACAATCAAGGGTACA   10999
gb:LC522974|Organism:Severe      GTTAGACAATGCTCAGGTGTTACTTTCCAAAGTGCAGTGAAAAGAACAATCAAGGGTACA   10999
gb:MN985325|Organism:Severe      GTTAGACAATGCTCAGGTGTTACTTTCCAAAGTGCAGTGAAAAGAACAATCAAGGGTACA   11002
gb:MT020881|Organism:Severe      GTTAGACAATGCTCAGGTGTTACTTTCCAAAGTGCAGTGAAAAGAACAATCAAGGGTACA   11002
gb:MT020880|Organism:Severe      GTTAGACAATGCTCAGGTGTTACTTTCCAAAGTGCAGTGAAAAGAACAATCAAGGGTACA   11002
gb:MT066175|Organism:Severe      GTTAGACAATGCTCAGGTGTTACTTTCCAAAGTGCAGTGAAAAGAACAATCAAGGGTACA   11002
gb:MN997409|Organism:Severe      GTTAGACAATGCTCAGGTGTTACTTTCCAAAGTGCAGTGAAAAGAACAATCAAGGGTACA   11002
gb:MN938384|Organism:Severe      GTTAGACAATGCTCAGGTGTTACTTTCCAAAGTGCAGTGAAAAGAACAATCAAGGGTACA   10970
gb:MT044258|Organism:Severe      GTTAGACAATGCTCAGGTGTTACTTTCCAAAGTGCAGTGAAAAGAACAATCAAGGGTACA   10978
gb:MT039890|Organism:Severe      GTTAGACAATGCTCAGGTGTTACTTTCCAAAGTGCAGTGAAAAGAACAATCAAGGGTACA   11002
gb:MN988713|Organism:Severe      GTTAGACAATGCTCAGGTGTTACTTTCCAAAGTGCAGTGAAAAGAACAATCAAGGGTACA   11002
gb:LC521925|Organism:Severe      GTTAGACAATGCTCAGGTGTTACTTTCCAAAGTGCAGTGAAAAGAACAATCAAGGGTACA   10975
gb:MT093571|Organism:Severe      GTTAGACAATGCTCAGGTGTTACTTTCCAAAGTGCAGTGAAAAGAACAATCAAGGGTACA   11002
gb:MT039887|Organism:Severe      GTTAGACAATGCTCAGGTGTTACTTTCCAAAGTGCAGTGAAAAGAACAATCAAGGGTACA   11002
gb:MT019530|Organism:Severe      GTTAGACAATGCTCAGGTGTTACTTTCCAAAGTGCAGTGAAAAGAACAATCAAGGGTACA   11002
gb:MT039888|Organism:Severe      GTTAGACAATGCTCAGGTGTTACTTTCCAAAGTGCAGTGAAAAGAACAATCAAGGGTACA   11002
gb:LC522972|Organism:Severe      GTTAGACAATGCTCAGGTGTTACTTTCCAAAGTGCAGTGAAAAGAACAATCAAGGGTACA   10999
gb:MT027063|Organism:Severe      GTTAGACAATGCTCAGGTGTTACTTTCCAAAGTGCAGTGAAAAGAACAATCAAGGGTACA   11002
gb:MT027062|Organism:Severe      GTTAGACAATGCTCAGGTGTTACTTTCCAAAGTGCAGTGAAAAGAACAATCAAGGGTACA   11002
```

```
gb:MT019529|Organism:Severe      GTTAGACAATGCTCAGGTGTTACTTTCCAAAGTGCAGTGAAAAGAACAATCAAGGGTACA   11002
gb:MN996529|Organism:Severe      GTTAGACAATGCTCAGGTGTTACTTTCCAAAGTGCAGTGAAAAGAACAATCAAGGGTACA   10990
gb:MN996531|Organism:Severe      GTTAGACAATGCTCAGGTGTTACTTTCCAAAGTGCAGTGAAAAGAACAATCAAGGGTACA   10989
gb:MT066176|Organism:Severe      GTTAGACAATGCTCAGGTGTTACTTTCCAAAGTGCAGTGAAAAGAACAATCAAGGGTACA   11002
gb:MT027064|Organism:Severe      GTTAGACAATGCTCAGGTGTTACTTTCCAAAGTGCAGTGAAAAGAACAATCAAGGGTACA   11002
gb:MN994468|Organism:Severe      GTTAGACAATGCTCAGGTGTTACTTTCCAAAGTGCAGTGAAAAGAACAATCAAGGGTACA   11002
gb:MT072688|Organism:Severe      GTTAGACAATGCTCAGGTGTTACTTTCCAAAGTGCAGTGAAAAGAACAATCAAGGGTACA   10987
gb:MN996527|Organism:Severe      GTTAGACAATGCTCAGGTGTTACTTTCCAAAGTGCAGTGAAAAGAACAATCAAGGGTACA   10969
gb:MT093631|Organism:Severe      GTTAGACAATGCTCAGGTGTTACTTTCCAAAGTGCAGTGAAAAGAACAATCAAGGGTACA   11040
gb:MT106053|Organism:Severe      GTTAGACAATGCTCAGGTGTTACTTTCCAAAGTGCAGTGAAAAGAACAATCAAGGGTACA   11002
gb:MT019533|Organism:Severe      GTTAGACAATGCTCAGGTGTTACTTTCCAAAGTGCAGTGAAAAGAACAATCAAGGGTACA   11002
gb:MT019531|Organism:Severe      GTTAGACAATGCTCAGGTGTTACTTTCCAAAGTGCAGTGAAAAGAACAATCAAGGGTACA   11002
gb:MN996528|Organism:Severe      GTTAGACAATGCTCAGGTGTTACTTTCCAAAGTGCAGTGAAAAGAACAATCAAGGGTACA   11002
gb:MN996530|Organism:Severe      GTTAGACAATGCTCAGGTGTTACTTTCCAAAGTGCAGTGAAAAGAACAATCAAGGGTACA   10988
gb:MN908947|Organism:Severe      GTTAGACAATGCTCAGGTGTTACTTTCCAAAGTGCAGTGAAAAGAACAATCAAGGGTACA   11002
gb:MT019532|Organism:Severe      GTTAGACAATGCTCAGGTGTTACTTTCCAAAGTGCAGTGAAAAGAACAATCAAGGGTACA   11002
                                 ************************************************************

gb:MT020781|Organism:Severe      CACCACTGGTTGTTACTCACAATTTTGACTTCACTTTTAGTTTTAGTCCAGAGTACTCAA   11050
gb:MT007544|Organism:Severe      CACCACTGGTTGTTACTCACAATTTTGACTTCACTTTTAGTTTTAGTCCAGAGTACTCAA   11062
gb:MN994467|Organism:Severe      CACCACTGGTTGTTACTCACAATTTTGACTTCACTTTTAGTTTTAGTCCAGAGTACTCAA   11062
gb:MT044257|Organism:Severe      CACCACTGGTTGTTACTCACAATTTTGACTTCACTTTTAGTTTTAGTCCAGAGTACTCAA   11062
gb:MT106054|Organism:Severe      CACCACTGGTTGTTACTCACAATTTTGACTTCACTTTTAGTTTTAGTCCAGAGTACTCAA   11062
gb:MT049951|Organism:Severe      CACCACTGGTTGTTACTCACAATTTTGACTTCACTTTTAGTTTTAGTCCAGAGTACTCAA   11062
gb:MN975262|Organism:Severe      CACCACTGGTTGTTACTCACAATTTTGACTTCACTTTTAGTTTTAGTCCAGAGTACTCAA   11062
gb:MT106052|Organism:Severe      CACCACTGGTTGTTACTCACAATTTTGACTTCACTTTTAGTTTTAGTCCAGAGTACTCAA   11062
gb:LC522975|Organism:Severe      CACCACTGGTTGTTACTCACAATTTTGACTTCACTTTTAGTTTTAGTCCAGAGTACTCAA   11059
gb:LC522973|Organism:Severe      CACCACTGGTTGTTACTCACAATTTTGACTTCACTTTTAGTTTTAGTCCAGAGTACTCAA   11059
gb:LC522974|Organism:Severe      CACCACTGGTTGTTACTCACAATTTTGACTTCACTTTTAGTTTTAGTCCAGAGTACTCAA   11059
gb:MN985325|Organism:Severe      CACCACTGGTTGTTACTCACAATTTTGACTTCACTTTTAGTTTTAGTCCAGAGTACTCAA   11062
gb:MT020881|Organism:Severe      CACCACTGGTTGTTACTCACAATTTTGACTTCACTTTTAGTTTTAGTCCAGAGTACTCAA   11062
gb:MT020880|Organism:Severe      CACCACTGGTTGTTACTCACAATTTTGACTTCACTTTTAGTTTTAGTCCAGAGTACTCAA   11062
gb:MT066175|Organism:Severe      CACCACTGGTTGTTACTCACAATTTTGACTTCACTTTTAGTTTTAGTCCAGAGTACTCAA   11062
gb:MN997409|Organism:Severe      CACCACTGGTTGTTACTCACAATTTTGACTTCACTTTTAGTTTTAGTCCAGAGTACTCAA   11062
gb:MN938384|Organism:Severe      CACCACTGGTTGTTACTCACAATTTTGACTTCACTTTTAGTTTTAGTCCAGAGTACTCAA   11030
gb:MT044258|Organism:Severe      CACCACTGGTTGTTACTCACAATTTTGACTTCACTTTTAGTTTTAGTCCAGAGTACTCAA   11038
gb:MT039890|Organism:Severe      CACCACTGGTTGTTACTCACAATTTTGACTTCACTTTTAGTTTTAGTCCAGAGTACTCAA   11062
gb:MN988713|Organism:Severe      CACCACTGGTTGTTACTCACAATTTTGACTTCACTTTTAGTTTTAGTCCAGAGTACTCAA   11062
gb:LC521925|Organism:Severe      CACCACTGGTTGTTACTCACAATTTTGACTTCACTTTTAGTTTTAGTCCAGAGTACTCAA   11035
gb:MT093571|Organism:Severe      CACCACTGGTTGTTACTCACAATTTTGACTTCACTTTTAGTTTTAGTCCAGAGTACTCAA   11062
gb:MT039887|Organism:Severe      CACCACTGGTTGTTACTCACAATTTTGACTTCACTTTTAGTTTTAGTCCAGAGTACTCAA   11062
gb:MT019530|Organism:Severe      CACCACTGGTTGTTACTCACAATTTTGACTTCACTTTTAGTTTTAGTCCAGAGTACTCAA   11062
gb:MT039888|Organism:Severe      CACCACTGGTTGTTACTCACAATTTTGACTTCACTTTTAGTTTTAGTCCAGAGTACTCAA   11062
gb:LC522972|Organism:Severe      CACCACTGGTTGTTACTCACAATTTTGACTTCACTTTTAGTTTTAGTCCAGAGTACTCAA   11059
gb:MT027063|Organism:Severe      CACCACTGGTTGTTACTCACAATTTTGACTTCACTTTTAGTTTTAGTCCAGAGTACTCAA   11062
gb:MT027062|Organism:Severe      CACCACTGGTTGTTACTCACAATTTTGACTTCACTTTTAGTTTTAGTCCAGAGTACTCAA   11062
gb:MT019529|Organism:Severe      CACCACTGGTTGTTACTCACAATTTTGACTTCACTTTTAGTTTTAGTCCAGAGTACTCAA   11062
gb:MN996529|Organism:Severe      CACCACTGGTTGTTACTCACAATTTTGACTTCACTTTTAGTTTTAGTCCAGAGTACTCAA   11050
gb:MN996531|Organism:Severe      CACCACTGGTTGTTACTCACAATTTTGACTTCACTTTTAGTTTTAGTCCAGAGTACTCAA   11049
gb:MT066176|Organism:Severe      CACCACTGGTTGTTACTCACAATTTTGACTTCACTTTTAGTTTTAGTCCAGAGTACTCAA   11062
gb:MT027064|Organism:Severe      CACCACTGGTTGTTACTCACAATTTTGACTTCACTTTTAGTTTTAGTCCAGAGTACTCAA   11062
gb:MN994468|Organism:Severe      CACCACTGGTTGTTACTCACAATTTTGACTTCACTTTTAGTTTTAGTCCAGAGTACTCAA   11062
gb:MT072688|Organism:Severe      CACCACTGGTTGTTACTCACAATTTTGACTTCACTTTTAGTTTTAGTCCAGAGTACTCAA   11047
gb:MN996527|Organism:Severe      CACCACTGGTTGTTACTCACAATTTTGACTTCACTTTTAGTTTTAGTCCAGAGTACTCAA   11029
gb:MT093631|Organism:Severe      CACCACTGGTTGTTACTCACAATTTTGACTTCACTTTTAGTTTTAGTCCAGAGTACTCAA   11100
gb:MT106053|Organism:Severe      CACCACTGGTTGTTACTCACAATTTTGACTTCACTTTTAGTTTTAGTCCAGAGTACTCAA   11062
gb:MT019533|Organism:Severe      CACCACTGGTTGTTACTCACAATTTTGACTTCACTTTTAGTTTTAGTCCAGAGTACTCAA   11062
gb:MT019531|Organism:Severe      CACCACTGGTTGTTACTCACAATTTTGACTTCACTTTTAGTTTTAGTCCAGAGTACTCAA   11062
gb:MN996528|Organism:Severe      CACCACTGGTTGTTACTCACAATTTTGACTTCACTTTTAGTTTTAGTCCAGAGTACTCAA   11062
gb:MN996530|Organism:Severe      CACCACTGGTTGTTACTCACAATTTTGACTTCACTTTTAGTTTTAGTCCAGAGTACTCAA   11048
gb:MN908947|Organism:Severe      CACCACTGGTTGTTACTCACAATTTTGACTTCACTTTTAGTTTTAGTCCAGAGTACTCAA   11062
gb:MT019532|Organism:Severe      CACCACTGGTTGTTACTCACAATTTTGACTTCACTTTTAGTTTTAGTCCAGAGTACTCAA   11062
                                 ************************************************************

gb:MT020781|Organism:Severe      TGGTCTTTGTTCTTTTTTTTGTATGAAAATGCCTTTTTACCTTTTGCTATGGGTATTATT   11110
```

```
gb:MT007544|Organism:Severe      TGGTCTTTGTTCTTTTTTTTGTATGAAAATGCCTTTTTACCTTTTGCTATGGGTATTATT   11122
gb:MN994467|Organism:Severe      TGGTCTTTGTTCTTTTTTTTGTATGAAAATGCCTTTTTACCTTTTGCTATGGGTATTATT   11122
gb:MT044257|Organism:Severe      TGGTCTTTGTTCTTTTTTTTGTATGAAAATGCCTTTTTACCTTTTGCTATGGGTATTATT   11122
gb:MT106054|Organism:Severe      TGGTCTTTGTTCTTTTTTTTGTATGAAAATGCCTTTTTACCTTTTGCTATGGGTATTATT   11122
gb:MT049951|Organism:Severe      TGGTCTTTGTTCTTTTTTTTCTATGAAAATGCCTTTTTACCTTTTGCTATGGGTATTATT   11122
gb:MN975262|Organism:Severe      TGGTCTTTGTTCTTTTTTTTGTATGAAAATGCCTTTTTACCTTTTGCTATGGGTATTATT   11122
gb:MT106052|Organism:Severe      TGGTCTTTGTTCTTTTTTTTGTATGAAAATGCCTTTTTACCTTTTGCTATGGGTATTATT   11122
gb:LC522975|Organism:Severe      TGGTCTTTGTTCTTTTTTTTGTATGAAAATGCCTTTTTACCTTTTGCTATGGGTATTATT   11119
gb:LC522973|Organism:Severe      TGGTCTTTGTTCTTTTTTTTGTATGAAAATGCCTTTTTACCTTTTGCTATGGGTATTATT   11119
gb:LC522974|Organism:Severe      TGGTCTTTGTTCTTTTTTTTGTATGAAAATGCCTTTTTACCTTTTGCTATGGGTATTATT   11119
gb:MN985325|Organism:Severe      TGGTCTTTGTTCTTTTTTTTGTATGAAAATGCCTTTTTACCTTTTGCTATGGGTATTATT   11122
gb:MT020881|Organism:Severe      TGGTCTTTGTTCTTTTTTTTGTATGAAAATGCCTTTTTACCTTTTGCTATGGGTATTATT   11122
gb:MT020880|Organism:Severe      TGGTCTTTGTTCTTTTTTTTGTATGAAAATGCCTTTTTACCTTTTGCTATGGGTATTATT   11122
gb:MT066175|Organism:Severe      TGGTCTTTGTTCTTTTTTTTGTATGAAAATGCCTTTTTACCTTTTGCTATGGGTATTATT   11122
gb:MN997409|Organism:Severe      TGGTCTTTGTTCTTTTTTTTTTATGAAAATGCCTTTTTACCTTTTGCTATGGGTATTATT   11122
gb:MN938384|Organism:Severe      TGGTCTTTGTTCTTTTTTTTGTATGAAAATGCCTTTTTACCTTTTGCTATGGGTATTATT   11090
gb:MT044258|Organism:Severe      TGGTCTTTGTTCTTTTTTTTGTATGAAAATGCCTTTTTACCTTTTGCTATGGGTATTATT   11098
gb:MT039890|Organism:Severe      TGGTCTTTGTTCTTTTTTTTGTATGAAAATGCCTTTTTACCTTTTGCTATGGGTATTATT   11122
gb:MN988713|Organism:Severe      TGGTCTTTGTTCTTTTTTTTGTATGAAAATGCCTTTTTACCTTTTGCTATGGGTATTATT   11122
gb:LC521925|Organism:Severe      TGGTCTTTGTTCTTTTTTTTGTATGAAAATGCCTTTTTACCTTTTGCTATGGGTATTATT   11095
gb:MT093571|Organism:Severe      TGGTCTTTGTTCTTTTTTTTGTATGAAAATGCCTTTTTACCTTTTGCTATGGGTATTATT   11122
gb:MT039887|Organism:Severe      TGGTCTTTGTTCTTTTTTTTGTATGAAAATGCCTTTTTACCTTTTGCTATGGGTATTATT   11122
gb:MT019530|Organism:Severe      TGGTCTTTGTTCTTTTTTTTGTATGAAAATGCCTTTTTACCTTTTGCTATGGGTATTATT   11122
gb:MT039888|Organism:Severe      TGGTCTTTGTTCTTTTTTTTGTATGAAAATGCCTTTTTACCTTTTGCTATGGGTATTATT   11122
gb:LC522972|Organism:Severe      TGGTCTTTGTTCTTTTTTTTGTATGAAAATGCCTTTTTACCTTTTGCTATGGGTATTATT   11119
gb:MT027063|Organism:Severe      TGGTCTTTGTTCTTTTTTTTGTATGAAAATGCCTTTTTACCTTTTGCTATGGGTATTATT   11122
gb:MT027062|Organism:Severe      TGGTCTTTGTTCTTTTTTTTGTATGAAAATGCCTTTTTACCTTTTGCTATGGGTATTATT   11122
gb:MT019529|Organism:Severe      TGGTCTTTGTTCTTTTTTTTGTATGAAAATGCCTTTTTACCTTTTGCTATGGGTATTATT   11122
gb:MN996529|Organism:Severe      TGGTCTTTGTTCTTTTTTTTGTATGAAAATGCCTTTTTACCTTTTGCTATGGGTATTATT   11110
gb:MN996531|Organism:Severe      TGGTCTTTGTTCTTTTTTTTGTATGAAAATGCCTTTTTACCTTTTGCTATGGGTATTATT   11109
gb:MT066176|Organism:Severe      TGGTCTTTGTTCTTTTTTTTGTATGAAAATGCCTTTTTACCTTTTGCTATGGGTATTATT   11122
gb:MT027064|Organism:Severe      TGGTCTTTGTTCTTTTTTTTGTATGAAAATGCCTTTTTACCTTTTGCTATGGGTATTATT   11122
gb:MN994468|Organism:Severe      TGGTCTTTGTTCTTTTTTTTGTATGAAAATGCCTTTTTACCTTTTGCTATGGGTATTATT   11122
gb:MT072688|Organism:Severe      TGGTCTTTGTTCTTTTTTTTGTATGAAAATGCCTTTTTACCTTTTGCTATGGGTATTATT   11107
gb:MN996527|Organism:Severe      TGGTCTTTGTTCTTTTTTTTGTATGAAAATGCCTTTTTACCTTTTGCTATGGGTATTATT   11089
gb:MT093631|Organism:Severe      TGGTCTTTGTTCTTTTTTTTGTATGAAAATGCCTTTTTACCTTTTGCTATGGGTATTATT   11160
gb:MT106053|Organism:Severe      TGGTCTTTGTTCTTTTTTTTGTATGAAAATGCCTTTTTACCTTTTGCTATGGGTATTATT   11122
gb:MT019533|Organism:Severe      TGGTCTTTGTTCTTTTTTTTGTATGAAAATGCCTTTTTACCTTTTGCTATGGGTATTATT   11122
gb:MT019531|Organism:Severe      TGGTCTTTGTTCTTTTTTTTGTATGAAAATGCCTTTTTACCTTTTGCTATGGGTATTATT   11122
gb:MN996528|Organism:Severe      TGGTCTTTGTTCTTTTTTTTGTATGAAAATGCCTTTTTACCTTTTGCTATGGGTATTATT   11122
gb:MN996530|Organism:Severe      TGGTCTTTGTTCTTTTTTTTGTATGAAAATGCCTTTTTACCTTTTGCTATGGGTATTATT   11108
gb:MN908947|Organism:Severe      TGGTCTTTGTTCTTTTTTTTGTATGAAAATGCCTTTTTACCTTTTGCTATGGGTATTATT   11122
gb:MT019532|Organism:Severe      TGGTCTTTGTTCTTTTTTTTGTATGAAAATGCCTTTTTACCTTTTGCTATGGGTATTATT   11122
                                 ******************** ***************************************

gb:MT020781|Organism:Severe      GCTATGTCTGCTTTTGCAATGATGTTTGTCAAACATAAGCATGCATTTCTCTGTTTGTTT   11170
gb:MT007544|Organism:Severe      GCTATGTCTGCTTTTGCAATGATGTTTGTCAAACATAAGCATGCATTTCTCTGTTTGTTT   11182
gb:MN994467|Organism:Severe      GCTATGTCTGCTTTTGCAATGATGTTTGTCAAACATAAGCATGCATTTCTCTGTTTGTTT   11182
gb:MT044257|Organism:Severe      GCTATGTCTGCTTTTGCAATGATGTTTGTCAAACATAAGCATGCATTTCTCTGTTTGTTT   11182
gb:MT106054|Organism:Severe      GCTATGTCTGCTTTTGCAATGATGTTTGTCAAACATAAGCATGCATTTCTCTGTTTGTTT   11182
gb:MT049951|Organism:Severe      GCTATGTCTGCTTTTGCAATGATGTTTGTCAAACATAAGCATGCATTTCTCTGTTTGTTT   11182
gb:MN975262|Organism:Severe      GCTATGTCTGCTTTTGCAATGATGTTTGTCAAACATAAGCATGCATTTCTCTGTTTGTTT   11182
gb:MT106052|Organism:Severe      GCTATGTCTGCTTTTGCAATGATGTTTGTCAAACATAAGCATGCATTTCTCTGTTTGTTT   11182
gb:LC522975|Organism:Severe      GCTATGTCTGCTTTTGCAATGATGTTTGTCAAACATAAGCATGCATTTCTCTGTTTGTTT   11179
gb:LC522973|Organism:Severe      GCTATGTCTGCTTTTGCAATGATGTTTGTCAAACATAAGCATGCATTTCTCTGTTTGTTT   11179
gb:LC522974|Organism:Severe      GCTATGTCTGCTTTTGCAATGATGTTTGTCAAACATAAGCATGCATTTCTCTGTTTGTTT   11179
gb:MN985325|Organism:Severe      GCTATGTCTGCTTTTGCAATGATGTTTGTCAAACATAAGCATGCATTTCTCTGTTTGTTT   11182
gb:MT020881|Organism:Severe      GCTATGTCTGCTTTTGCAATGATGTTTGTCAAACATAAGCATGCATTTCTCTGTTTGTTT   11182
gb:MT020880|Organism:Severe      GCTATGTCTGCTTTTGCAATGATGTTTGTCAAACATAAGCATGCATTTCTCTGTTTGTTT   11182
gb:MT066175|Organism:Severe      GCTATGTCTGCTTTTGCAATGATGTTTGTCAAACATAAGCATGCATTTCTCTGTTTGTTT   11182
gb:MN997409|Organism:Severe      GCTATGTCTGCTTTTGCAATGATGTTTGTCAAACATAAGCATGCATTTCTCTGTTTGTTT   11182
gb:MN938384|Organism:Severe      GCTATGTCTGCTTTTGCAATGATGTTTGTCAAACATAAGCATGCATTTCTCTGTTTGTTT   11150
gb:MT044258|Organism:Severe      GCTATGTCTGCTTTTGCAATGATGTTTGTCAAACATAAGCATGCATTTCTCTGTTTGTTT   11158
gb:MT039890|Organism:Severe      GCTATGTCTGCTTTTGCAATGATGTTTGTCAAACATAAGCATGCATTTCTCTGTTTGTTT   11182
gb:MN988713|Organism:Severe      GCTATGTCTGCTTTTGCAATGATGTTTGTCAAACATAAGCATGCATTTCTCTGTTTGTTT   11182
```

```
gb:LC521925|Organism:Severe      GCTATGTCTGCTTTTGCAATGATGTTTGTCAAACATAAGCATGCATTTCTCTGTTTGTTT   11155
gb:MT093571|Organism:Severe      GCTATGTCTGCTTTTGCAATGATGTTTGTCAAACATAAGCATGCATTTCTCTGTTTGTTT   11182
gb:MT039887|Organism:Severe      GCTATGTCTGCTTTTGCAATGATGTTTGTCAAACATAAGCATGCATTTCTCTGTTTGTTT   11182
gb:MT019530|Organism:Severe      GCTATGTCTGCTTTTGCAATGATGTTTGTCAAACATAAGCATGCATTTCTCTGTTTGTTT   11182
gb:MT039888|Organism:Severe      GCTATGTCTGCTTTTGCAATGATGTTTGTCAAACATAAGCATGCATTTCTCTGTTTGTTT   11182
gb:LC522972|Organism:Severe      GCTATGTCTGCTTTTGCAATGATGTTTGTCAAACATAAGCATGCATTTCTCTGTTTGTTT   11179
gb:MT027063|Organism:Severe      GCTATGTCTGCTTTTGCAATGATGTTTGTCAAACATAAGCATGCATTTCTCTGTTTGTTT   11182
gb:MT027062|Organism:Severe      GCTATGTCTGCTTTTGCAATGATGTTTGTCAAACATAAGCATGCATTTCTCTGTTTGTTT   11182
gb:MT019529|Organism:Severe      GCTATGTCTGCTTTTGCAATGATGTTTGTCAAACATAAGCATGCATTTCTCTGTTTGTTT   11182
gb:MN996529|Organism:Severe      GCTATGTCTGCTTTTGCAATGATGTTTGTCAAACATAAGCATGCATTTCTCTGTTTGTTT   11170
gb:MN996531|Organism:Severe      GCTATGTCTGCTTTTGCAATGATGTTTGTCAAACATAAGCATGCATTTCTCTGTTTGTTT   11169
gb:MT066176|Organism:Severe      GCTATGTCTGCTTTTGCAATGATGTTTGTCAAACATAAGCATGCATTTCTCTGTTTGTTT   11182
gb:MT027064|Organism:Severe      GCTATGTCTGCTTTTGCAATGATGTTTGTCAAACATAAGCATGCATTTCTCTGTTTGTTT   11182
gb:MN994468|Organism:Severe      GCTATGTCTGCTTTTGCAATGATGTTTGTCAAACATAAGCATGCATTTCTCTGTTTGTTT   11182
gb:MT072688|Organism:Severe      GCTATGTCTGCTTTTGCAATGATGTTTGTCAAACATAAGCATGCATTTCTCTGTTTGTTT   11167
gb:MN996527|Organism:Severe      GCTATGTCTGCTTTTGCAATGATGTTTGTCAAACATAAGCATGCATTTCTCTGTTTGTTT   11149
gb:MT093631|Organism:Severe      GCTATGTCTGCTTTTGCAATGATGTTTGTCAAACATAAGCATGCATTTCTCTGTTTGTTT   11220
gb:MT106053|Organism:Severe      GCTATGTCTGCTTTTGCAATGATGTTTGTCAAACATAAGCATGCATTTCTCTGTTTGTTT   11182
gb:MT019533|Organism:Severe      GCTATGTCTGCTTTTGCAATGATGTTTGTCAAACATAAGCATGCATTTCTCTGTTTGTTT   11182
gb:MT019531|Organism:Severe      GCTATGTCTGCTTTTGCAATGATGTTTGTCAAACATAAGCATGCATTTCTCTGTTTGTTT   11182
gb:MN996528|Organism:Severe      GCTATGTCTGCTTTTGCAATGATGTTTGTCAAACATAAGCATGCATTTCTCTGTTTGTTT   11182
gb:MN996530|Organism:Severe      GCTATGTCTGCTTTTGCAATGATGTTTGTCAAACATAAGCATGCATTTCTCTGTTTGTTT   11168
gb:MN908947|Organism:Severe      GCTATGTCTGCTTTTGCAATGATGTTTGTCAAACATAAGCATGCATTTCTCTGTTTGTTT   11182
gb:MT019532|Organism:Severe      GCTATGTCTGCTTTTGCAATGATGTTTGTCAAACATAAGCATGCATTTCTCTGTTTGTTT   11182
                                 ************************************************************

gb:MT020781|Organism:Severe      TTGTTACCTTCTCTTGCCACTGTAGCTTATTTTAATATGGTCTATATGCCTGCTAGTTGG   11230
gb:MT007544|Organism:Severe      TTGTTACCTTCTCTTGCCACTGTAGCTTATTTTAATATGGTCTATATGCCTGCTAGTTGG   11242
gb:MN994467|Organism:Severe      TTGTTACCTTCTCTTGCCACTGTAGCTTATTTTAATATGGTCTATATGCCTGCTAGTTGG   11242
gb:MT044257|Organism:Severe      TTGTTACCTTCTCTTGCCACTGTAGCTTATTTTAATATGGTCTATATGCCTGCTAGTTGG   11242
gb:MT106054|Organism:Severe      TTGTTACCTTCTCTTGCCACTGTAGCTTATTTTAATATGGTCTATATGCCTGCTAGTTGG   11242
gb:MT049951|Organism:Severe      TTGTTACCTTCTCTTGCCACTGTAGCTTATTTTAATATGGTCTATATGCCTGCTAGTTGG   11242
gb:MN975262|Organism:Severe      TTGTTACCTTCTCTTGCCACTGTAGCTTATTTTAATATGGTCTATATGCCTGCTAGTTGG   11242
gb:MT106052|Organism:Severe      TTGTTACCTTCTCTTGCCACTGTAGCTTATTTTAATATGGTCTATATGCCTGCTAGTTGG   11242
gb:LC522975|Organism:Severe      TTGTTACCTTCTCTTGCCACTGTAGCTTATTTTAATATGGTCTATATGCCTGCTAGTTGG   11239
gb:LC522973|Organism:Severe      TTGTTACCTTCTCTTGCCACTGTAGCTTATTTTAATATGGTCTATATGCCTGCTAGTTGG   11239
gb:LC522974|Organism:Severe      TTGTTACCTTCTCTTGCCACTGTAGCTTATTTTAATATGGTCTATATGCCTGCTAGTTGG   11239
gb:MN985325|Organism:Severe      TTGTTACCTTCTCTTGCCACTGTAGCTTATTTTAATATGGTCTATATGCCTGCTAGTTGG   11242
gb:MT020881|Organism:Severe      TTGTTACCTTCTCTTGCCACTGTAGCTTATTTTAATATGGTCTATATGCCTGCTAGTTGG   11242
gb:MT020880|Organism:Severe      TTGTTACCTTCTCTTGCCACTGTAGCTTATTTTAATATGGTCTATATGCCTGCTAGTTGG   11242
gb:MT066175|Organism:Severe      TTGTTACCTTCTCTTGCCACTGTAGCTTATTTTAATATGGTCTATATGCCTGCTAGTTGG   11242
gb:MN997409|Organism:Severe      TTGTTACCTTCTCTTGCCACTGTAGCTTATTTTAATATGGTCTATATGCCTGCTAGTTGG   11242
gb:MN938384|Organism:Severe      TTGTTACCTTCTCTTGCCACTGTAGCTTATTTTAATATGGTCTATATGCCTGCTAGTTGG   11210
gb:MT044258|Organism:Severe      TTGTTACCTTCTCTTGCCACTGTAGCTTATTTTAATATGGTCTATATGCCTGCTAGTTGG   11218
gb:MT039890|Organism:Severe      TTGTTACCTTCTCTTGCCACTGTAGCTTATTTTAATATGGTCTATATGCCTGCTAGTTGG   11242
gb:MN988713|Organism:Severe      TTGTTACCTTCTCTTGCCACTGTAGCTTATTTTAATATGGTCTATATGCCTGCTAGTTGG   11242
gb:LC521925|Organism:Severe      TTGTTACCTTCTCTTGCCACTGTAGCTTATTTTAATATGGTCTATATGCCTGCTAGTTGG   11215
gb:MT093571|Organism:Severe      TTGTTACCTTCTCTTGCCACTGTAGCTTATTTTAATATGGTCTATATGCCTGCTAGTTGG   11242
gb:MT039887|Organism:Severe      TTGTTACCTTCTCTTGCCACTGTAGCTTATTTTAATATGGTCTATATGCCTGCTAGTTGG   11242
gb:MT019530|Organism:Severe      TTGTTACCTTCTCTTGCCACTGTAGCTTATTTTAATATGGTCTATATGCCTGCTAGTTGG   11242
gb:MT039888|Organism:Severe      TTGTTACCTTCTCTTGCCACTGTAGCTTATTTTAATATGGTCTATATGCCTGCTAGTTGG   11242
gb:LC522972|Organism:Severe      TTGTTACCTTCTCTTGCCACTGTAGCTTATTTTAATATGGTCTATATGCCTGCTAGTTGG   11239
gb:MT027063|Organism:Severe      TTGTTACCTTCTCTTGCCACTGTAGCTTATTTTAATATGGTCTATATGCCTGCTAGTTGG   11242
gb:MT027062|Organism:Severe      TTGTTACCTTCTCTTGCCACTGTAGCTTATTTTAATATGGTCTATATGCCTGCTAGTTGG   11242
gb:MT019529|Organism:Severe      TTGTTACCTTCTCTTGCCACTGTAGCTTATTTTAATATGGTCTATATGCCTGCTAGTTGG   11242
gb:MN996529|Organism:Severe      TTGTTACCTTCTCTTGCCACTGTAGCTTATTTTAATATGGTCTATATGCCTGCTAGTTGG   11230
gb:MN996531|Organism:Severe      TTGTTACCTTCTCTTGCCACTGTAGCTTATTTTAATATGGTCTATATGCCTGCTAGTTGG   11229
gb:MT066176|Organism:Severe      TTGTTACCTTCTCTTGCCACTGTAGCTTATTTTAATATGGTCTATATGCCTGCTAGTTGG   11242
gb:MT027064|Organism:Severe      TTGTTACCTTCTCTTGCCACTGTAGCTTATTTTAATATGGTCTATATGCCTGCTAGTTGG   11242
gb:MN994468|Organism:Severe      TTGTTACCTTCTCTTGCCACTGTAGCTTATTTTAATATGGTCTATATGCCTGCTAGTTGG   11242
gb:MT072688|Organism:Severe      TTGTTACCTTCTCTTGCCACTGTAGCTTATTTTAATATGGTCTATATGCCTGCTAGTTGG   11227
gb:MN996527|Organism:Severe      TTGTTACCTTCTCTTGCCACTGTAGCTTATTTTAATATGGTCTATATGCCTGCTAGTTGG   11209
gb:MT093631|Organism:Severe      TTGTTACCTTCTCTTGCCACTGTAGCTTATTTTAATATGGTCTATATGCCTGCTAGTTGG   11280
gb:MT106053|Organism:Severe      TTGTTACCTTCTCTTGCCACTGTAGCTTATTTTAATATGGTCTATATGCCTGCTAGTTGG   11242
gb:MT019533|Organism:Severe      TTGTTACCTTCTCTTGCCACTGTAGCTTATTTTAATATGGTCTATATGCCTGCTAGTTGG   11242
```

```
gb:MT019531|Organism:Severe      TTGTTACCTTCTCTTGCCACTGTAGCTTATTTTAATATGGTCTATATGCCTGCTAGTTGG   11242
gb:MN996528|Organism:Severe      TTGTTACCTTCTCTTGCCACTGTAGCTTATTTTAATATGGTCTATATGCCTGCTAGTTGG   11242
gb:MN996530|Organism:Severe      TTGTTACCTTCTCTTGCCACTGTAGCTTATTTTAATATGGTCTATATGCCTGCTAGTTGG   11228
gb:MN908947|Organism:Severe      TTGTTACCTTCTCTTGCCACTGTAGCTTATTTTAATATGGTCTATATGCCTGCTAGTTGG   11242
gb:MT019532|Organism:Severe      TTGTTACCTTCTCTTGCCACTGTAGCTTATTTTAATATGGTCTATATGCCTGCTAGTTGG   11242
                                 ************************************************************

gb:MT020781|Organism:Severe      GTGATGCGTATTATGACATGGTTGGATATGGTTGATACTAGTTTGTCTGGTTTTAAGCTA   11290
gb:MT007544|Organism:Severe      GTGATGCGTATTATGACATGGTTGGATATGGTTGATACTAGTTTGTCTGGTTTTAAGCTA   11302
gb:MN994467|Organism:Severe      GTGATGCGTATTATGACATGGTTGGATATGGTTGATACTAGTTTGTCTGGTTTTAAGCTA   11302
gb:MT044257|Organism:Severe      GTGATGCGTATTATGACATGGTTGGATATGGTTGATACTAGTTTGTCTGGTTTTAAGCTA   11302
gb:MT106054|Organism:Severe      GTGATGCGTATTATGACATGGTTGGATATGGTTGATACTAGTTTGTCTGGTTTTAAGCTA   11302
gb:MT049951|Organism:Severe      GTGATGCGTATTATGACATGGTTGGATATGGTTGATACTAGTTTGTCTGGTTTTAAGCTA   11302
gb:MN975262|Organism:Severe      GTGATGCGTATTATGACATGGTTGGATATGGTTGATACTAGTTTGTCTGGTTTTAAGCTA   11302
gb:MT106052|Organism:Severe      GTGATGCGTATTATGACATGGTTGGATATGGTTGATACTAGTTTGTCTGGTTTTAAGCTA   11302
gb:LC522975|Organism:Severe      GTGATGCGTATTATGACATGGTTGGATATGGTTGATACTAGTTTGTCTGGTTTTAAGCTA   11299
gb:LC522973|Organism:Severe      GTGATGCGTATTATGACATGGTTGGATATGGTTGATACTAGTTTGTCTGGTTTTAAGCTA   11299
gb:LC522974|Organism:Severe      GTGATGCGTATTATGACATGGTTGGATATGGTTGATACTAGTTTGTCTGGTTTTAAGCTA   11299
gb:MN985325|Organism:Severe      GTGATGCGTATTATGACATGGTTGGATATGGTTGATACTAGTTTGTCTGGTTTTAAGCTA   11302
gb:MT020881|Organism:Severe      GTGATGCGTATTATGACATGGTTGGATATGGTTGATACTAGTTTGTCTGGTTTTAAGCTA   11302
gb:MT020880|Organism:Severe      GTGATGCGTATTATGACATGGTTGGATATGGTTGATACTAGTTTGTCTGGTTTTAAGCTA   11302
gb:MT066175|Organism:Severe      GTGATGCGTATTATGACATGGTTGGATATGGTTGATACTAGTTTGTCTGGTTTTAAGCTA   11302
gb:MN997409|Organism:Severe      GTGATGCGTATTATGACATGGTTGGATATGGTTGATACTAGTTTGTCTGGTTTTAAGCTA   11302
gb:MN938384|Organism:Severe      GTGATGCGTATTATGACATGGTTGGATATGGTTGATACTAGTTTGTCTGGTTTTAAGCTA   11270
gb:MT044258|Organism:Severe      GTGATGCGTATTATGACATGGTTGGATATGGTTGATACTAGTTTGTCTGGTTTTAAGCTA   11278
gb:MT039890|Organism:Severe      GTGATGCGTATTATGACATGGTTGGATATGGTTGATACTAGTTTGTCTGGTTTTAAGCTA   11302
gb:MN988713|Organism:Severe      GTGATGCGTATTATGACATGGTTGGATATGGTTGATACTAGTTTGTCTGGTTTTAAGCTA   11302
gb:LC521925|Organism:Severe      GTGATGCGTATTATGACATGGTTGGATATGGTTGATACTAGTTTGTCTGGTTTTAAGCTA   11275
gb:MT093571|Organism:Severe      GTGATGCGTATTATGACATGGTTGGATATGGTTGATACTAGTTTGTCTGGTTTTAAGCTA   11302
gb:MT039887|Organism:Severe      GTGATGCGTATTATGACATGGTTGGATATGGTTGATACTAGTTTGTCTGGTTTTAAGCTA   11302
gb:MT019530|Organism:Severe      GTGATGCGTATTATGACATGGTTGGATATGGTTGATACTAGTTTGTCTGGTTTTAAGCTA   11302
gb:MT039888|Organism:Severe      GTGATGCGTATTATGACATGGTTGGATATGGTTGATACTAGTTTGTCTGGTTTTAAGCTA   11302
gb:LC522972|Organism:Severe      GTGATGCGTATTATGACATGGTTGGATATGGTTGATACTAGTTTGTCTGGTTTTAAGCTA   11299
gb:MT027063|Organism:Severe      GTGATGCGTATTATGACATGGTTGGATATGGTTGATACTAGTTTGTCTGGTTTTAAGCTA   11302
gb:MT027062|Organism:Severe      GTGATGCGTATTATGACATGGTTGGATATGGTTGATACTAGTTTGTCTGGTTTTAAGCTA   11302
gb:MT019529|Organism:Severe      GTGATGCGTATTATGACATGGTTGGATATGGTTGATACTAGTTTGTCTGGTTTTAAGCTA   11302
gb:MN996529|Organism:Severe      GTGATGCGTATTATGACATGGTTGGATATGGTTGATACTAGTTTGTCTGGTTTTAAGCTA   11290
gb:MN996531|Organism:Severe      GTGATGCGTATTATGACATGGTTGGATATGGTTGATACTAGTTTGTCTGGTTTTAAGCTA   11289
gb:MT066176|Organism:Severe      GTGATGCGTATTATGACATGGTTGGATATGGTTGATACTAGTTTGTCTGGTTTTAAGCTA   11302
gb:MT027064|Organism:Severe      GTGATGCGTATTATGACATGGTTGGATATGGTTGATACTAGTTTGTCTGGTTTTAAGCTA   11302
gb:MN994468|Organism:Severe      GTGATGCGTATTATGACATGGTTGGATATGGTTGATACTAGTTTGTCTGGTTTTAAGCTA   11302
gb:MT072688|Organism:Severe      GTGATGCGTATTATGACATGGTTGGATATGGTTGATACTAGTTTGTCTGGTTTTAAGCTA   11287
gb:MN996527|Organism:Severe      GTGATGCGTATTATGACATGGTTGGATATGGTTGATACTAGTTTGTCTGGTTTTAAGCTA   11269
gb:MT093631|Organism:Severe      GTGATGCGTATTATGACATGGTTGGATATGGTTGATACTAGTTTGTCTGGTTTTAAGCTA   11340
gb:MT106053|Organism:Severe      GTGATGCGTATTATGACATGGTTGGATATGGTTGATACTAGTTTGTCTGGTTTTAAGCTA   11302
gb:MT019533|Organism:Severe      GTGATGCGTATTATGACATGGTTGGATATGGTTGATACTAGTTTGTCTGGTTTTAAGCTA   11302
gb:MT019531|Organism:Severe      GTGATGCGTATTATGACATGGTTGGATATGGTTGATACTAGTTTGTCTGGTTTTAAGCTA   11302
gb:MN996528|Organism:Severe      GTGATGCGTATTATGACATGGTTGGATATGGTTGATACTAGTTTGTCTGGTTTTAAGCTA   11302
gb:MN996530|Organism:Severe      GTGATGCGTATTATGACATGGTTGGATATGGTTGATACTAGTTTGTCTGGTTTTAAGCTA   11288
gb:MN908947|Organism:Severe      GTGATGCGTATTATGACATGGTTGGATATGGTTGATACTAGTTTGTCTGGTTTTAAGCTA   11302
gb:MT019532|Organism:Severe      GTGATGCGTATTATGACATGGTTGGATATGGTTGATACTAGTTTGTCTGGTTTTAAGCTA   11302
                                 ************************************************************

gb:MT020781|Organism:Severe      AAAGACTGTGTTATGTATGCATCAGCTGTAGTGTTACTAATCCTTATGACAGCAAGAACT   11350
gb:MT007544|Organism:Severe      AAAGACTGTGTTATGTATGCATCAGCTGTAGTGTTACTAATCCTTATGACAGCAAGAACT   11362
gb:MN994467|Organism:Severe      AAAGACTGTGTTATGTATGCATCAGCTGTAGTGTTACTAATCCTTATGACAGCAAGAACT   11362
gb:MT044257|Organism:Severe      AAAGACTGTGTTATGTATGCATCAGCTGTAGTGTTACTAATCCTTATGACAGCAAGAACT   11362
gb:MT106054|Organism:Severe      AAAGACTGTGTTATGTATGCATCAGCTGTAGTGTTACTAATCCTTATGACAGCAAGAACT   11362
gb:MT049951|Organism:Severe      AAAGACTGTGTTATGTATGCATCAGCTGTAGTGTTACTAATCCTTATGACAGCAAGAACT   11362
gb:MN975262|Organism:Severe      AAAGACTGTGTTATGTATGCATCAGCTGTAGTGTTACTAATCCTTATGACAGCAAGAACT   11362
gb:MT106052|Organism:Severe      AAAGACTGTGTTATGTATGCATCAGCTGTAGTGTTACTAATCCTTATGACAGCAAGAACT   11362
gb:LC522975|Organism:Severe      AAAGACTGTGTTATGTATGCATCAGCTGTAGTGTTACTAATCCTTATGACAGCAAGAACT   11359
gb:LC522973|Organism:Severe      AAAGACTGTGTTATGTATGCATCAGCTGTAGTGTTACTAATCCTTATGACAGCAAGAACT   11359
gb:LC522974|Organism:Severe      AAAGACTGTGTTATGTATGCATCAGCTGTAGTGTTACTAATCCTTATGACAGCAAGAACT   11359
gb:MN985325|Organism:Severe      AAAGACTGTGTTATGTATGCATCAGCTGTAGTGTTACTAATCCTTATGACAGCAAGAACT   11362
```

```
gb:MT020881|Organism:Severe      AAAGACTGTGTTATGTATGCATCAGCTGTAGTGTTACTAATCCTTATGACAGCAAGAACT    11362
gb:MT020880|Organism:Severe      AAAGACTGTGTTATGTATGCATCAGCTGTAGTGTTACTAATCCTTATGACAGCAAGAACT    11362
gb:MT066175|Organism:Severe      AAAGACTGTGTTATGTATGCATCAGCTGTAGTGTTACTAATCCTTATGACAGCAAGAACT    11362
gb:MN997409|Organism:Severe      AAAGACTGTGTTATGTATGCATCAGCTGTAGTGTTACTAATCCTTATGACAGCAAGAACT    11362
gb:MN938384|Organism:Severe      AAAGACTGTGTTATGTATGCATCAGCTGTAGTGTTACTAATCCTTATGACAGCAAGAACT    11330
gb:MT044258|Organism:Severe      AAAGACTGTGTTATGTATGCATCAGCTGTAGTGTTACTAATCCTTATGACAGCAAGAACT    11338
gb:MT039890|Organism:Severe      AAAGACTGTGTTATGTATGCATCAGCTGTAGTGTTACTAATCCTTATGACAGCAAGAACT    11362
gb:MN988713|Organism:Severe      AAAGACTGTGTTATGTATGCATCAGCTGTAGTGTTACTAATCCTTATGACAGCAAGAACT    11362
gb:LC521925|Organism:Severe      AAAGACTGTGTTATGTATGCATCAGCTGTAGTGTTACTAATCCTTATGACAGCAAGAACT    11335
gb:MT093571|Organism:Severe      AAAGACTGTGTTATGTATGCATCAGCTGTAGTGTTACTAATCCTTATGACAGCAAGAACT    11362
gb:MT039887|Organism:Severe      AAAGACTGTGTTATGTATGCATCAGCTGTAGTGTTACTAATCCTTATGACAGCAAGAACT    11362
gb:MT019530|Organism:Severe      AAAGACTGTGTTATGTATGCATCAGCTGTAGTGTTACTAATCCTTATGACAGCAAGAACT    11362
gb:MT039888|Organism:Severe      AAAGACTGTGTTATGTATGCATCAGCTGTAGTGTTACTAATCCTTATGACAGCAAGAACT    11362
gb:LC522972|Organism:Severe      AAAGACTGTGTTATGTATGCATCAGCTGTAGTGTTACTAATCCTTATGACAGCAAGAACT    11359
gb:MT027063|Organism:Severe      AAAGACTGTGTTATGTATGCATCAGCTGTAGTGTTACTAATCCTTATGACAGCAAGAACT    11362
gb:MT027062|Organism:Severe      AAAGACTGTGTTATGTATGCATCAGCTGTAGTGTTACTAATCCTTATGACAGCAAGAACT    11362
gb:MT019529|Organism:Severe      AAAGACTGTGTTATGTATGCATCAGCTGTAGTGTTACTAATCCTTATGACAGCAAGAACT    11362
gb:MN996529|Organism:Severe      AAAGACTGTGTTATGTATGCATCAGCTGTAGTGTTACTAATCCTTATGACAGCAAGAACT    11350
gb:MN996531|Organism:Severe      AAAGACTGTGTTATGTATGCATCAGCTGTAGTGTTACTAATCCTTATGACAGCAAGAACT    11349
gb:MT066176|Organism:Severe      AAAGACTGTGTTATGTATGCATCAGCTGTAGTGTTACTAATCCTTATGACAGCAAGAACT    11362
gb:MT027064|Organism:Severe      AAAGACTGTGTTATGTATGCATCAGCTGTAGTGTTACTAATCCTTATGACAGCAAGAACT    11362
gb:MN994468|Organism:Severe      AAAGACTGTGTTATGTATGCATCAGCTGTAGTGTTACTAATCCTTATGACAGCAAGAACT    11362
gb:MT072688|Organism:Severe      AAAGACTGTGTTATGTATGCATCAGCTGTAGTGTTACTAATCCTTATGACAGCAAGAACT    11347
gb:MN996527|Organism:Severe      AAAGACTGTGTTATGTATGCATCAGCTGTAGTGTTACTAATCCTTATGACAGCAAGAACT    11329
gb:MT093631|Organism:Severe      AAAGACTGTGTTATGTATGCATCAGCTGTAGTGTTACTAATCCTTATGACAGCAAGAACT    11400
gb:MT106053|Organism:Severe      AAAGACTGTGTTATGTATGCATCAGCTGTAGTGTTACTAATCCTTATGACAGCAAGAACT    11362
gb:MT019533|Organism:Severe      AAAGACTGTGTTATGTATGCATCAGCTGTAGTGTTACTAATCCTTATGACAGCAAGAACT    11362
gb:MT019531|Organism:Severe      AAAGACTGTGTTATGTATGCATCAGCTGTAGTGTTACTAATCCTTATGACAGCAAGAACT    11362
gb:MN996528|Organism:Severe      AAAGACTGTGTTATGTATGCATCAGCTGTAGTGTTACTAATCCTTATGACAGCAAGAACT    11362
gb:MN996530|Organism:Severe      AAAGACTGTGTTATGTATGCATCAGCTGTAGTGTTACTAATCCTTATGACAGCAAGAACT    11348
gb:MN908947|Organism:Severe      AAAGACTGTGTTATGTATGCATCAGCTGTAGTGTTACTAATCCTTATGACAGCAAGAACT    11362
gb:MT019532|Organism:Severe      AAAGACTGTGTTATGTATGCATCAGCTGTAGTGTTACTAATCCTTATGACAGCAAGAACT    11362
                                 ************************************************************

gb:MT020781|Organism:Severe      GTGTATGATGATGGTGCTAGGAGAGTGTGGACACTTATGAATGTCTTGACACTCGTTTAT    11410
gb:MT007544|Organism:Severe      GTGTATGATGATGGTGCTAGGAGAGTGTGGACACTTATGAATGTCTTGACACTCGTTTAT    11422
gb:MN994467|Organism:Severe      GTGTATGATGATGGTGCTAGGAGAGTGTGGACACTTATGAATGTCTTGACACTCGTTTAT    11422
gb:MT044257|Organism:Severe      GTGTATGATGATGGTGCTAGGAGAGTGTGGACACTTATGAATGTCTTGACACTCGTTTAT    11422
gb:MT106054|Organism:Severe      GTGTATGATGATGGTGCTAGGAGAGTGTGGACACTTATGAATGTCTTGACACTCGTTTAT    11422
gb:MT049951|Organism:Severe      GTGTATGATGATGGTGCTAGGAGAGTGTGGACACTTATGAATGTCTTGACACTCGTTTAT    11422
gb:MN975262|Organism:Severe      GTGTATGATGATGGTGCTAGGAGAGTGTGGACACTTATGAATGTCTTGACACTCGTTTAT    11422
gb:MT106052|Organism:Severe      GTGTATGATGATGGTGCTAGGAGAGTGTGGACACTTATGAATGTCTTGACACTCGTTTAT    11422
gb:LC522975|Organism:Severe      GTGTATGATGATGGTGCTAGGAGAGTGTGGACACTTATGAATGTCTTGACACTCGTTTAT    11419
gb:LC522973|Organism:Severe      GTGTATGATGATGGTGCTAGGAGAGTGTGGACACTTATGAATGTCTTGACACTCGTTTAT    11419
gb:LC522974|Organism:Severe      GTGTATGATGATGGTGCTAGGAGAGTGTGGACACTTATGAATGTCTTGACACTCGTTTAT    11419
gb:MN985325|Organism:Severe      GTGTATGATGATGGTGCTAGGAGAGTGTGGACACTTATGAATGTCTTGACACTCGTTTAT    11422
gb:MT020881|Organism:Severe      GTGTATGATGATGGTGCTAGGAGAGTGTGGACACTTATGAATGTCTTGACACTCGTTTAT    11422
gb:MT020880|Organism:Severe      GTGTATGATGATGGTGCTAGGAGAGTGTGGACACTTATGAATGTCTTGACACTCGTTTAT    11422
gb:MT066175|Organism:Severe      GTGTATGATGATGGTGCTAGGAGAGTGTGGACACTTATGAATGTCTTGACACTCGTTTAT    11422
gb:MN997409|Organism:Severe      GTGTATGATGATGGTGCTAGGAGAGTGTGGACACTTATGAATGTCTTGACACTCGTTTAT    11422
gb:MN938384|Organism:Severe      GTGTATGATGATGGTGCTAGGAGAGTGTGGACACTTATGAATGTCTTGACACTCGTTTAT    11390
gb:MT044258|Organism:Severe      GTGTATGATGATGGTGCTAGGAGAGTGTGGACACTTATGAATGTCTTGACACTCGTTTAT    11398
gb:MT039890|Organism:Severe      GTGTATGATGATGGTGCTAGGAGAGTGTGGACACTTATGAATGTCTTGACACTCGTTTAT    11422
gb:MN988713|Organism:Severe      GTGTATGATGATGGTGCTAGGAGAGTGTGGACACTTATGAATGTCTTGACACTCGTTTAT    11422
gb:LC521925|Organism:Severe      GTGTATGATGATGGTGCTAGGAGAGTGTGGACACTTATGAATGTCTTGACACTCGTTTAT    11395
gb:MT093571|Organism:Severe      GTGTATGATGATGGTGCTAGGAGAGTGTGGACACTTATGAATGTCTTGACACTCGTTTAT    11422
gb:MT039887|Organism:Severe      GTGTATGATGATGGTGCTAGGAGAGTGTGGACACTTATGAATGTCTTGACACTCGTTTAT    11422
gb:MT019530|Organism:Severe      GTGTATGATGATGGTGCTAGGAGAGTGTGGACACTTATGAATGTCTTGACACTCGTTTAT    11422
gb:MT039888|Organism:Severe      GTGTATGATGATGGTGCTAGGAGAGTGTGGACACTTATGAATGTCTTGACACTCGTTTAT    11422
gb:LC522972|Organism:Severe      GTGTATGATGATGGTGCTAGGAGAGTGTGGACACTTATGAATGTCTTGACACTCGTTTAT    11419
gb:MT027063|Organism:Severe      GTGTATGATGATGGTGCTAGGAGAGTGTGGACACTTATGAATGTCTTGACACTCGTTTAT    11422
gb:MT027062|Organism:Severe      GTGTATGATGATGGTGCTAGGAGAGTGTGGACACTTATGAATGTCTTGACACTCGTTTAT    11422
gb:MT019529|Organism:Severe      GTGTATGATGATGGTGCTAGGAGAGTGTGGACACTTATGAATGTCTTGACACTCGTTTAT    11422
gb:MN996529|Organism:Severe      GTGTATGATGATGGTGCTAGGAGAGTGTGGACACTTATGAATGTCTTGACACTCGTTTAT    11410
gb:MN996531|Organism:Severe      GTGTATGATGATGGTGCTAGGAGAGTGTGGACACTTATGAATGTCTTGACACTCGTTTAT    11409
```

```
gb:MT066176|Organism:Severe      GTGTATGATGATGGTGCTAGGAGAGTGTGGACACTTATGAATGTCTTGACACTCGTTTAT   11422
gb:MT027064|Organism:Severe      GTGTATGATGATGGTGCTAGGAGAGTGTGGACACTTATGAATGTCTTGACACTCGTTTAT   11422
gb:MN994468|Organism:Severe      GTGTATGATGATGGTGCTAGGAGAGTGTGGACACTTATGAATGTCTTGACACTCGTTTAT   11422
gb:MT072688|Organism:Severe      GTGTATGATGATGGTGCTAGGAGAGTGTGGACACTTATGAATGTCTTGACACTCGTTTAT   11407
gb:MN996527|Organism:Severe      GTGTATGATGATGGTGCTAGGAGAGTGTGGACACTTATGAATGTCTTGACACTCGTTTAT   11389
gb:MT093631|Organism:Severe      GTGTATGATGATGGTGCTAGGAGAGTGTGGACACTTATGAATGTCTTGACACTCGTTTAT   11460
gb:MT106053|Organism:Severe      GTGTATGATGATGGTGCTAGGAGAGTGTGGACACTTATGAATGTCTTGACACTCGTTTAT   11422
gb:MT019533|Organism:Severe      GTGTATGATGATGGTGCTAGGAGAGTGTGGACACTTATGAATGTCTTGACACTCGTTTAT   11422
gb:MT019531|Organism:Severe      GTGTATGATGATGGTGCTAGGAGAGTGTGGACACTTATGAATGTCTTGACACTCGTTTAT   11422
gb:MN996528|Organism:Severe      GTGTATGATGATGGTGCTAGGAGAGTGTGGACACTTATGAATGTCTTGACACTCGTTTAT   11422
gb:MN996530|Organism:Severe      GTGTATGATGATGGTGCTAGGAGAGTGTGGACACTTATGAATGTCTTGACACTCGTTTAT   11408
gb:MN908947|Organism:Severe      GTGTATGATGATGGTGCTAGGAGAGTGTGGACACTTATGAATGTCTTGACACTCGTTTAT   11422
gb:MT019532|Organism:Severe      GTGTATGATGATGGTGCTAGGAGAGTGTGGACACTTATGAATGTCTTGACACTCGTTTAT   11422
                                 ************************************************************

gb:MT020781|Organism:Severe      AAAGTTTATTATGGTAATGCTTTAGATCAAGCCATTTCCATGTGGGCTCTTATAATCTCT   11470
gb:MT007544|Organism:Severe      AAAGTTTATTATGGTAATGCTTTAGATCAAGCCATTTCCATGTGGGCTCTTATAATCTCT   11482
gb:MN994467|Organism:Severe      AAAGTTTATTATGGTAATGCTTTAGATCAAGCCATTTCCATGTGGGCTCTTATAATCTCT   11482
gb:MT044257|Organism:Severe      AAAGTTTATTATGGTAATGCTTTAGATCAAGCCATTTCCATGTGGGCTCTTATAATCTCT   11482
gb:MT106054|Organism:Severe      AAAGTTTATTATGGTAATGCTTTAGATCAAGCCATTTCCATGTGGGCTCTTATAATCTCT   11482
gb:MT049951|Organism:Severe      AAAGTTTATTATGGTAATGCTTTAGATCAAGCCATTTCCATGTGGGCTCTTATAATCTCT   11482
gb:MN975262|Organism:Severe      AAAGTTTATTATGGTAATGCTTTAGATCAAGCCATTTCCATGTGGGCTCTTATAATCTCT   11482
gb:MT106052|Organism:Severe      AAAGTTTATTATGGTAATGCTTTAGATCAAGCCATTTCCATGTGGGCTCTTATAATCTCT   11482
gb:LC522975|Organism:Severe      AAAGTTTATTATGGTAATGCTTTAGATCAAGCCATTTCCATGTGGGCTCTTATAATCTCT   11479
gb:LC522973|Organism:Severe      AAAGTTTATTATGGTAATGCTTTAGATCAAGCCATTTCCATGTGGGCTCTTATAATCTCT   11479
gb:LC522974|Organism:Severe      AAAGTTTATTATGGTAATGCTTTAGATCAAGCCATTTCCATGTGGGCTCTTATAATCTCT   11479
gb:MN985325|Organism:Severe      AAAGTTTATTATGGTAATGCTTTAGATCAAGCCATTTCCATGTGGGCTCTTATAATCTCT   11482
gb:MT020881|Organism:Severe      AAAGTTTATTATGGTAATGCTTTAGATCAAGCCATTTCCATGTGGGCTCTTATAATCTCT   11482
gb:MT020880|Organism:Severe      AAAGTTTATTATGGTAATGCTTTAGATCAAGCCATTTCCATGTGGGCTCTTATAATCTCT   11482
gb:MT066175|Organism:Severe      AAAGTTTATTATGGTAATGCTTTAGATCAAGCCATTTCCATGTGGGCTCTTATAATCTCT   11482
gb:MN997409|Organism:Severe      AAAGTTTATTATGGTAATGCTTTAGATCAAGCCATTTCCATGTGGGCTCTTATAATCTCT   11482
gb:MN938384|Organism:Severe      AAAGTTTATTATGGTAATGCTTTAGATCAAGCCATTTCCATGTGGGCTCTTATAATCTCT   11450
gb:MT044258|Organism:Severe      AAAGTTTATTATGGTAATGCTTTAGATCAAGCCATTTCCATGTGGGCTCTTATAATCTCT   11458
gb:MT039890|Organism:Severe      AAAGTTTATTATGGTAATGCTTTAGATCAAGCCATTTCCATGTGGGCTCTTATAATCTCT   11482
gb:MN988713|Organism:Severe      AAAGTTTATTATGGTAATGCTTTAGATCAAGCCATTTCCATGTGGGCTCTTATAATCTCT   11482
gb:LC521925|Organism:Severe      AAAGTTTATTATGGTAATGCTTTAGATCAAGCCATTTCCATGTGGGCTCTTATAATCTCT   11455
gb:MT093571|Organism:Severe      AAAGTTTATTATGGTAATGCTTTAGATCAAGCCATTTCCATGTGGGCTCTTATAATCTCT   11482
gb:MT039887|Organism:Severe      AAAGTTTATTATGGTAATGCTTTAGATCAAGCCATTTCCATGTGGGCTCTTATAATCTCT   11482
gb:MT019530|Organism:Severe      AAAGTTTATTATGGTAATGCTTTAGATCAAGCCATTTCCATGTGGGCTCTTATAATCTCT   11482
gb:MT039888|Organism:Severe      AAAGTTTATTATGGTAATGCTTTAGATCAAGCCATTTCCATGTGGGCTCTTATAATCTCT   11482
gb:LC522972|Organism:Severe      AAAGTTTATTATGGTAATGCTTTAGATCAAGCCATTTCCATGTGGGCTCTTATAATCTCT   11479
gb:MT027063|Organism:Severe      AAAGTTTATTATGGTAATGCTTTAGATCAAGCCATTTCCATGTGGGCTCTTATAATCTCT   11482
gb:MT027062|Organism:Severe      AAAGTTTATTATGGTAATGCTTTAGATCAAGCCATTTCCATGTGGGCTCTTATAATCTCT   11482
gb:MT019529|Organism:Severe      AAAGTTTATTATGGTAATGCTTTAGATCAAGCCATTTCCATGTGGGCTCTTATAATCTCT   11482
gb:MN996529|Organism:Severe      AAAGTTTATTATGGTAATGCTTTAGATCAAGCCATTTCCATGTGGGCTCTTATAATCTCT   11470
gb:MN996531|Organism:Severe      AAAGTTTATTATGGTAATGCTTTAGATCAAGCCATTTCCATGTGGGCTCTTATAATCTCT   11469
gb:MT066176|Organism:Severe      AAAGTTTATTATGGTAATGCTTTAGATCAAGCCATTTCCATGTGGGCTCTTATAATCTCT   11482
gb:MT027064|Organism:Severe      AAAGTTTATTATGGTAATGCTTTAGATCAAGCCATTTCCATGTGGGCTCTTATAATCTCT   11482
gb:MN994468|Organism:Severe      AAAGTTTATTATGGTAATGCTTTAGATCAAGCCATTTCCATGTGGGCTCTTATAATCTCT   11482
gb:MT072688|Organism:Severe      AAAGTTTATTATGGTAATGCTTTAGATCAAGCCATTTCCATGTGGGCTCTTATAATCTCT   11467
gb:MN996527|Organism:Severe      AAAGTTTATTATGGTAATGCTTTAGATCAAGCCATTTCCATGTGGGCTCTTATAATCTCT   11449
gb:MT093631|Organism:Severe      AAAGTTTATTATGGTAATGCTTTAGATCAAGCCATTTCCATGTGGGCTCTTATAATCTCT   11520
gb:MT106053|Organism:Severe      AAAGTTTATTATGGTAATGCTTTAGATCAAGCCATTTCCATGTGGGCTCTTATAATCTCT   11482
gb:MT019533|Organism:Severe      AAAGTTTATTATGGTAATGCTTTAGATCAAGCCATTTCCATGTGGGCTCTTATAATCTCT   11482
gb:MT019531|Organism:Severe      AAAGTTTATTATGGTAATGCTTTAGATCAAGCCATTTCCATGTGGGCTCTTATAATCTCT   11482
gb:MN996528|Organism:Severe      AAAGTTTATTATGGTAATGCTTTAGATCAAGCCATTTCCATGTGGGCTCTTATAATCTCT   11482
gb:MN996530|Organism:Severe      AAAGTTTATTATGGTAATGCTTTAGATCAAGCCATTTCCATGTGGGCTCTTATAATCTCT   11468
gb:MN908947|Organism:Severe      AAAGTTTATTATGGTAATGCTTTAGATCAAGCCATTTCCATGTGGGCTCTTATAATCTCT   11482
gb:MT019532|Organism:Severe      AAAGTTTATTATGGTAATGCTTTAGATCAAGCCATTTCCATGTGGGCTCTTATAATCTCT   11482
                                 ************************************************************

gb:MT020781|Organism:Severe      GTTACTTCTAACTACTCAGGTGTAGTTACAACTGTCATGTTTTTGGCCAGAGGTATTGTT   11530
gb:MT007544|Organism:Severe      GTTACTTCTAACTACTCAGGTGTAGTTACAACTGTCATGTTTTTGGCCAGAGGTATTGTT   11542
gb:MN994467|Organism:Severe      GTTACTTCTAACTACTCAGGTGTAGTTACAACTGTCATGTTTTTGGCCAGAGGTATTGTT   11542
gb:MT044257|Organism:Severe      GTTACTTCTAACTACTCAGGTGTAGTTACAACTGTCATGTTTTTGGCCAGAGGTATTGTT   11542
```

```
gb:MT106054|Organism:Severe      GTTACTTCTAACTACTCAGGTGTAGTTACAACTGTCATGTTTTTGGCCAGAGGTATTGTT   11542
gb:MT049951|Organism:Severe      GTTACTTCTAACTACTCAGGTGTAGTTACAACTGTCATGTTTTTGGCCAGAGGTATTGTT   11542
gb:MN975262|Organism:Severe      GTTACTTCTAACTACTCAGGTGTAGTTACAACTGTCATGTTTTTGGCCAGAGGTATTGTT   11542
gb:MT106052|Organism:Severe      GTTACTTCTAACTACTCAGGTGTAGTTACAACTGTCATGTTTTTGGCCAGAGGTATTGTT   11542
gb:LC522975|Organism:Severe      GTTACTTCTAACTACTCAGGTGTAGTTACAACTGTCATGTTTTTGGCCAGAGGTATTGTT   11539
gb:LC522973|Organism:Severe      GTTACTTCTAACTACTCAGGTGTAGTTACAACTGTCATGTTTTTGGCCAGAGGTATTGTT   11539
gb:LC522974|Organism:Severe      GTTACTTCTAACTACTCAGGTGTAGTTACAACTGTCATGTTTTTGGCCAGAGGTATTGTT   11539
gb:MN985325|Organism:Severe      GTTACTTCTAACTACTCAGGTGTAGTTACAACTGTCATGTTTTTGGCCAGAGGTATTGTT   11542
gb:MT020881|Organism:Severe      GTTACTTCTAACTACTCAGGTGTAGTTACAACTGTCATGTTTTTGGCCAGAGGTATTGTT   11542
gb:MT020880|Organism:Severe      GTTACTTCTAACTACTCAGGTGTAGTTACAACTGTCATGTTTTTGGCCAGAGGTATTGTT   11542
gb:MT066175|Organism:Severe      GTTACTTCTAACTACTCAGGTGTAGTTACAACTGTCATGTTTTTGGCCAGAGGTATTGTT   11542
gb:MN997409|Organism:Severe      GTTACTTCTAACTACTCAGGTGTAGTTACAACTGTCATGTTTTTGGCCAGAGGTATTGTT   11542
gb:MN938384|Organism:Severe      GTTACTTCTAACTACTCAGGTGTAGTTACAACTGTCATGTTTTTGGCCAGAGGTATTGTT   11510
gb:MT044258|Organism:Severe      GTTACTTCTAACTACTCAGGTGTAGTTACAACTGTCATGTTTTTGGCCAGAGGTATTGTT   11518
gb:MT039890|Organism:Severe      GTTACTTCTAACTACTCAGGTGTAGTTACAACTGTCATGTTTTTGGCCAGAGGTATTGTT   11542
gb:MN988713|Organism:Severe      GTTACTTCTAACTACTCAGGTGTAGTTACAACTGTCATGTTTTTGGCCAGAGGTATTGTT   11542
gb:LC521925|Organism:Severe      GTTACTTCTAACTACTCAGGTGTAGTTACAACTGTCATGTTTTTGGCCAGAGGTATTGTT   11515
gb:MT093571|Organism:Severe      GTTACTTCTAACTACTCAGGTGTAGTTACAACTGTCATGTTTTTGGCCAGAGGTATTGTT   11542
gb:MT039887|Organism:Severe      GTTACTTCTAACTACTCAGGTGTAGTTACAACTGTCATGTTTTTGGCCAGAGGTATTGTT   11542
gb:MT019530|Organism:Severe      GTTACTTCTAACTACTCAGGTGTAGTTACAACTGTCATGTTTTTGGCCAGAGGTATTGTT   11542
gb:MT039888|Organism:Severe      GTTACTTCTAACTACTCAGGTGTAGTTACAACTGTCATGTTTTTGGCCAGAGGTATTGTT   11542
gb:LC522972|Organism:Severe      GTTACTTCTAACTACTCAGGTGTAGTTACAACTGTCATGTTTTTGGCCAGAGGTATTGTT   11539
gb:MT027063|Organism:Severe      GTTACTTCTAACTACTCAGGTGTAGTTACAACTGTCATGTTTTTGGCCAGAGGTATTGTT   11542
gb:MT027062|Organism:Severe      GTTACTTCTAACTACTCAGGTGTAGTTACAACTGTCATGTTTTTGGCCAGAGGTATTGTT   11542
gb:MT019529|Organism:Severe      GTTACTTCTAACTACTCAGGTGTAGTTACAACTGTCATGTTTTTGGCCAGAGGTATTGTT   11542
gb:MN996529|Organism:Severe      GTTACTTCTAACTACTCAGGTGTAGTTACAACTGTCATGTTTTTGGCCAGAGGTATTGTT   11530
gb:MN996531|Organism:Severe      GTTACTTCTAACTACTCAGGTGTAGTTACAACTGTCATGTTTTTGGCCAGAGGTATTGTT   11529
gb:MT066176|Organism:Severe      GTTACTTCTAACTACTCAGGTGTAGTTACAACTGTCATGTTTTTGGCCAGAGGTATTGTT   11542
gb:MT027064|Organism:Severe      GTTACTTCTAACTACTCAGGTGTAGTTACAACTGTCATGTTTTTGGCCAGAGGTATTGTT   11542
gb:MN994468|Organism:Severe      GTTACTTCTAACTACTCAGGTGTAGTTACAACTGTCATGTTTTTGGCCAGAGGTATTGTT   11542
gb:MT072688|Organism:Severe      GTTACTTCTAACTACTCAGGTGTAGTTACAACTGTCATGTTTTTGGCCAGAGGTATTGTT   11527
gb:MN996527|Organism:Severe      GTTACTTCTAACTACTCAGGTGTAGTTACAACTGTCATGTTTTTGGCCAGAGGTATTGTT   11509
gb:MT093631|Organism:Severe      GTTACTTCTAACTACTCAGGTGTAGTTACAACTGTCATGTTTTTGGCCAGAGGTATTGTT   11580
gb:MT106053|Organism:Severe      GTTACTTCTAACTACTCAGGTGTAGTTACAACTGTCATGTTTTTGGCCAGAGGTATTGTT   11542
gb:MT019533|Organism:Severe      GTTACTTCTAACTACTCAGGTGTAGTTACAACTGTCATGTTTTTGGCCAGAGGTATTGTT   11542
gb:MT019531|Organism:Severe      GTTACTTCTAACTACTCAGGTGTAGTTACAACTGTCATGTTTTTGGCCAGAGGTATTGTT   11542
gb:MN996528|Organism:Severe      GTTACTTCTAACTACTCAGGTGTAGTTACAACTGTCATGTTTTTGGCCAGAGGTATTGTT   11542
gb:MN996530|Organism:Severe      GTTACTTCTAACTACTCAGGTGTAGTTACAACTGTCATGTTTTTGGCCAGAGGTATTGTT   11528
gb:MN908947|Organism:Severe      GTTACTTCTAACTACTCAGGTGTAGTTACAACTGTCATGTTTTTGGCCAGAGGTATTGTT   11542
gb:MT019532|Organism:Severe      GTTACTTCTAACTACTCAGGTGTAGTTACAACTGTCATGTTTTTGGCCAGAGGTATTGTT   11542
                                 ************************************************************

gb:MT020781|Organism:Severe      TTTATGTGTGTTGAGTATTGCCCTATTTTCTTCATAACTGGTAATACACTTCAGTGTATA   11590
gb:MT007544|Organism:Severe      TTTATGTGTGTTGAGTATTGCCCTATTTTCTTCATAACTGGTAATACACTTCAGTGTATA   11602
gb:MN994467|Organism:Severe      TTTATGTGTGTTGAGTATTGCCCTATTTTCTTCATAACTGGTAATACACTTCAGTGTATA   11602
gb:MT044257|Organism:Severe      TTTATGTGTGTTGAGTATTGCCCTATTTTCTTCATAACTGGTAATACACTTCAGTGTATA   11602
gb:MT106054|Organism:Severe      TTTATGTGTGTTGAGTATTGCCCTATTTTCTTCATAACTGGTAATACACTTCAGTGTATA   11602
gb:MT049951|Organism:Severe      TTTATGTGTGTTGAGTATTGCCCTATTTTCTTCATAACTGGTAATACACTTCAGTGTATA   11602
gb:MN975262|Organism:Severe      TTTATGTGTGTTGAGTATTGCCCTATTTTCTTCATAACTGGTAATACACTTCAGTGTATA   11602
gb:MT106052|Organism:Severe      TTTATGTGTGTTGAGTATTGCCCTATTTTCTTCATAACTGGTAATACACTTCAGTGTATA   11602
gb:LC522975|Organism:Severe      TTTATGTGTGTTGAGTATTGCCCTATTTTCTTCATAACTGGTAATACACTTCAGTGTATA   11599
gb:LC522973|Organism:Severe      TTTATGTGTGTTGAGTATTGCCCTATTTTCTTCATAACTGGTAATACACTTCAGTGTATA   11599
gb:LC522974|Organism:Severe      TTTATGTGTGTTGAGTATTGCCCTATTTTCTTCATAACTGGTAATACACTTCAGTGTATA   11599
gb:MN985325|Organism:Severe      TTTATGTGTGTTGAGTATTGCCCTATTTTCTTCATAACTGGTAATACACTTCAGTGTATA   11602
gb:MT020881|Organism:Severe      TTTATGTGTGTTGAGTATTGCCCTATTTTCTTCATAACTGGTAATACACTTCAGTGTATA   11602
gb:MT020880|Organism:Severe      TTTATGTGTGTTGAGTATTGCCCTATTTTCTTCATAACTGGTAATACACTTCAGTGTATA   11602
gb:MT066175|Organism:Severe      TTTATGTGTGTTGAGTATTGCCCTATTTTCTTCATAACTGGTAATACACTTCAGTGTATA   11602
gb:MN997409|Organism:Severe      TTTATGTGTGTTGAGTATTGCCCTATTTTCTTCATAACTGGTAATACACTTCAGTGTATA   11602
gb:MN938384|Organism:Severe      TTTATGTGTGTTGAGTATTGCCCTATTTTCTTCATAACTGGTAATACACTTCAGTGTATA   11570
gb:MT044258|Organism:Severe      TTTATGTGTGTTGAGTATTGCCCTATTTTCTTCATAACTGGTAATACACTTCAGTGTATA   11578
gb:MT039890|Organism:Severe      TTTATGTGTGTTGAGTATTGCCCTATTTTCTTCATAACTGGTAATACACTTCAGTGTATA   11602
gb:MN988713|Organism:Severe      TTTATGTGTGTTGAGTATTGCCCTATTTTCTTCATAACTGGTAATACACTTCAGTGTATA   11602
gb:LC521925|Organism:Severe      TTTATGTGTGTTGAGTATTGCCCTATTTTCTTCATAACTGGTAATACACTTCAGTGTATA   11575
gb:MT093571|Organism:Severe      TTTATGTGTGTTGAGTATTGCCCTATTTTCTTCATAACTGGTAATACACTTCAGTGTATA   11602
gb:MT039887|Organism:Severe      TTTATGTGTGTTGAGTATTGCCCTATTTTCTTCATAACTGGTAATACACTTCAGTGTATA   11602
```

```
gb:MT019530|Organism:Severe      TTTATGTGTGTTGAGTATTGCCCTATTTTCTTCATAACTGGTAATACACTTCAGTGTATA   11602
gb:MT039888|Organism:Severe      TTTATGTGTGTTGAGTATTGCCCTATTTTCTTCATAACTGGTAATACACTTCAGTGTATA   11602
gb:LC522972|Organism:Severe      TTTATGTGTGTTGATTATTGCCCTATTTTCTTCATAACTGGTAATACACTTCAGTGTATA   11599
gb:MT027063|Organism:Severe      TTTATGTGTGTTGAGTATTGCCCTATTTTCTTCATAACTGGTAATACACTTCAGTGTATA   11602
gb:MT027062|Organism:Severe      TTTATGTGTGTTGAGTATTGCCCTATTTTCTTCATAACTGGTAATACACTTCAGTGTATA   11602
gb:MT019529|Organism:Severe      TTTATGTGTGTTGAGTATTGCCCTATTTTCTTCATAACTGGTAATACACTTCAGTGTATA   11602
gb:MN996529|Organism:Severe      TTTATGTGTGTTGAGTATTGCCCTATTTTCTTCATAACTGGTAATACACTTCAGTGTATA   11590
gb:MN996531|Organism:Severe      TTTATGTGTGTTGAGTATTGCCCTATTTTCTTCATAACTGGTAATACACTTCAGTGTATA   11589
gb:MT066176|Organism:Severe      TTTATGTGTGTTGAGTATTGCCCTATTTTCTTCATAACTGGTAATACACTTCAGTGTATA   11602
gb:MT027064|Organism:Severe      TTTATGTGTGTTGAGTATTGCCCTATTTTCTTCATAACTGGTAATACACTTCAGTGTATA   11602
gb:MN994468|Organism:Severe      TTTATGTGTGTTGAGTATTGCCCTATTTTCTTCATAACTGGTAATACACTTCAGTGTATA   11602
gb:MT072688|Organism:Severe      TTTATGTGTGTTGAGTATTGCCCTATTTTCTTCATAACTGGTAATACACTTCAGTGTATA   11587
gb:MN996527|Organism:Severe      TTTATGTGTGTTGAGTATTGCCCTATTTTCTTCATAACTGGTAATACACTTCAGTGTATA   11569
gb:MT093631|Organism:Severe      TTTATGTGTGTTGAGTATTGCCCTATTTTCTTCATAACTGGTAATACACTTCAGTGTATA   11640
gb:MT106053|Organism:Severe      TTTATGTGTGTTGAGTATTGCCCTATTTTCTTCATAACTGGTAATACACTTCAGTGTATA   11602
gb:MT019533|Organism:Severe      TTTATGTGTGTTGAGTATTGCCCTATTTTCTTCATAACTGGTAATACACTTCAGTGTATA   11602
gb:MT019531|Organism:Severe      TTTATGTGTGTTGAGTATTGCCCTATTTTCTTCATAACTGGTAATACACTTCAGTGTATA   11602
gb:MN996528|Organism:Severe      TTTATGTGTGTTGAGTATTGCCCTATTTTCTTCATAACTGGTAATACACTTCAGTGTATA   11602
gb:MN996530|Organism:Severe      TTTATGTGTGTTGAGTATTGCCCTATTTTCTTCATAACTGGTAATACACTTCAGTGTATA   11588
gb:MN908947|Organism:Severe      TTTATGTGTGTTGAGTATTGCCCTATTTTCTTCATAACTGGTAATACACTTCAGTGTATA   11602
gb:MT019532|Organism:Severe      TTTATGTGTGTTGAGTATTGCCCTATTTTCTTCATAACTGGTAATACACTTCAGTGTATA   11602
                                 ************** *********************************************

gb:MT020781|Organism:Severe      ATGCTAGTTTATTGTTTCTTAGGCTATTTTTGTACTTGTTACTTTGGCCTCTTTTGTTTA   11650
gb:MT007544|Organism:Severe      ATGCTAGTTTATTGTTTCTTAGGCTATTTTTGTACTTGTTACTTTGGCCTCTTTTGTTTA   11662
gb:MN994467|Organism:Severe      ATGCTAGTTTATTGTTTCTTAGGCTATTTTTGTACTTGTTACTTTGGCCTCTTTTGTTTA   11662
gb:MT044257|Organism:Severe      ATGCTAGTTTATTGTTTCTTAGGCTATTTTTGTACTTGTTACTTTGGCCTCTTTTGTTTA   11662
gb:MT106054|Organism:Severe      ATGCTAGTTTATTGTTTCTTAGGCTATTTTTGTACTTGTTACTTTGGCCTCTTTTGTTTA   11662
gb:MT049951|Organism:Severe      ATGCTAGTTTATTGTTTCTTAGGCTATTTTTGTACTTGTTACTTTGGCCTCTTTTGTTTA   11662
gb:MN975262|Organism:Severe      ATGCTAGTTTATTGTTTCTTAGGCTATTTTTGTACTTGTTACTTTGGCCTCTTTTGTTTA   11662
gb:MT106052|Organism:Severe      ATGCTAGTTTATTGTTTCTTAGGCTATTTTTGTACTTGTTACTTTGGCCTCTTTTGTTTA   11662
gb:LC522975|Organism:Severe      ATGCTAGTTTATTGTTTCTTAGGCTATTTTTGTACTTGTTACTTTGGCCTCTTTTGTTTA   11659
gb:LC522973|Organism:Severe      ATGCTAGTTTATTGTTTCTTAGGCTATTTTTGTACTTGTTACTTTGGCCTCTTTTGTTTA   11659
gb:LC522974|Organism:Severe      ATGCTAGTTTATTGTTTCTTAGGCTATTTTTGTACTTGTTACTTTGGCCTCTTTTGTTTA   11659
gb:MN985325|Organism:Severe      ATGCTAGTTTATTGTTTCTTAGGCTATTTTTGTACTTGTTACTTTGGCCTCTTTTGTTTA   11662
gb:MT020881|Organism:Severe      ATGCTAGTTTATTGTTTCTTAGGCTATTTTTGTACTTGTTACTTTGGCCTCTTTTGTTTA   11662
gb:MT020880|Organism:Severe      ATGCTAGTTTATTGTTTCTTAGGCTATTTTTGTACTTGTTACTTTGGCCTCTTTTGTTTA   11662
gb:MT066175|Organism:Severe      ATGCTAGTTTATTGTTTCTTAGGCTATTTTTGTACTTGTTACTTTGGCCTCTTTTGTTTA   11662
gb:MN997409|Organism:Severe      ATGCTAGTTTATTGTTTCTTAGGCTATTTTTGTACTTGTTACTTTGGCCTCTTTTGTTTA   11662
gb:MN938384|Organism:Severe      ATGCTAGTTTATTGTTTCTTAGGCTATTTTTGTACTTGTTACTTTGGCCTCTTTTGTTTA   11630
gb:MT044258|Organism:Severe      ATGCTAGTTTATTGTTTCTTAGGCTATTTTTGTACTTGTTACTTTGGCCTCTTTTGTTTA   11638
gb:MT039890|Organism:Severe      ATGCTAGTTTATTGTTTCTTAGGCTATTTTTGTACTTGTTACTTTGGCCTCTTTTGTTTA   11662
gb:MN988713|Organism:Severe      ATGCTAGTTTATTGTTTCTTAGGCTATTTTTGTACTTGTTACTTTGGCCTCTTTTGTTTA   11662
gb:LC521925|Organism:Severe      ATGCTAGTTTATTGTTTCTTAGGCTATTTTTGTACTTGTTACTTTGGCCTCTTTTGTTTA   11635
gb:MT093571|Organism:Severe      ATGCTAGTTTATTGTTTCTTAGGCTATTTTTGTACTTGTTACTTTGGCCTCTTTTGTTTA   11662
gb:MT039887|Organism:Severe      ATGCTAGTTTATTGTTTCTTAGGCTATTTTTGTACTTGTTACTTTGGCCTCTTTTGTTTA   11662
gb:MT019530|Organism:Severe      ATGCTAGTTTATTGTTTCTTAGGCTATTTTTGTACTTGTTACTTTGGCCTCTTTTGTTTA   11662
gb:MT039888|Organism:Severe      ATGCTAGTTTATTGTTTCTTAGGCTATTTTTGTACTTGTTACTTTGGCCTCTTTTGTTTA   11662
gb:LC522972|Organism:Severe      ATGCTAGTTTATTGTTTCTTAGGCTATTTTTGTACTTGTTACTTTGGCCTCTTTTGTTTA   11659
gb:MT027063|Organism:Severe      ATGCTAGTTTATTGTTTCTTAGGCTATTTTTGTACTTGTTACTTTGGCCTCTTTTGTTTA   11662
gb:MT027062|Organism:Severe      ATGCTAGTTTATTGTTTCTTAGGCTATTTTTGTACTTGTTACTTTGGCCTCTTTTGTTTA   11662
gb:MT019529|Organism:Severe      ATGCTAGTTTATTGTTTCTTAGGCTATTTTTGTACTTGTTACTTTGGCCTCTTTTGTTTA   11662
gb:MN996529|Organism:Severe      ATGCTAGTTTATTGTTTCTTAGGCTATTTTTGTACTTGTTACTTTGGCCTCTTTTGTTTA   11650
gb:MN996531|Organism:Severe      ATGCTAGTTTATTGTTTCTTAGGCTATTTTTGTACTTGTTACTTTGGCCTCTTTTGTTTA   11649
gb:MT066176|Organism:Severe      ATGCTAGTTTATTGTTTCTTAGGCTATTTTTGTACTTGTTACTTTGGCCTCTTTTGTTTA   11662
gb:MT027064|Organism:Severe      ATGCTAGTTTATTGTTTCTTAGGCTATTTTTGTACTTGTTACTTTGGCCTCTTTTGTTTA   11662
gb:MN994468|Organism:Severe      ATGCTAGTTTATTGTTTCTTAGGCTATTTTTGTACTTGTTACTTTGGCCTCTTTTGTTTA   11662
gb:MT072688|Organism:Severe      ATGCTAGTTTATTGTTTCTTAGGCTATTTTTGTACTTGTTACTTTGGCCTCTTTTGTTTA   11647
gb:MN996527|Organism:Severe      ATGCTAGTTTATTGTTTCTTAGGCTATTTTTGTACTTGTTACTTTGGCCTCTTTTGTTTA   11629
gb:MT093631|Organism:Severe      ATGCTAGTTTATTGTTTCTTAGGCTATTTTTGTACTTGTTACTTTGGCCTCTTTTGTTTA   11700
gb:MT106053|Organism:Severe      ATGCTAGTTTATTGTTTCTTAGGCTATTTTTGTACTTGTTACTTTGGCCTCTTTTGTTTA   11662
gb:MT019533|Organism:Severe      ATGCTAGTTTATTGTTTCTTAGGCTATTTTTGTACTTGTTACTTTGGCCTCTTTTGTTTA   11662
gb:MT019531|Organism:Severe      ATGCTAGTTTATTGTTTCTTAGGCTATTTTTGTACTTGTTACTTTGGCCTCTTTTGTTTA   11662
gb:MN996528|Organism:Severe      ATGCTAGTTTATTGTTTCTTAGGCTATTTTTGTACTTGTTACTTTGGCCTCTTTTGTTTA   11662
gb:MN996530|Organism:Severe      ATGCTAGTTTATTGTTTCTTAGGCTATTTTTGTACTTGTTACTTTGGCCTCTTTTGTTTA   11648
```

```
gb:MN908947|Organism:Severe      ATGCTAGTTTATTGTTTCTTAGGCTATTTTTGTACTTGTTACTTTGGCCTCTTTTGTTTA   11662
gb:MT019532|Organism:Severe      ATGCTAGTTTATTGTTTCTTAGGCTATTTTTGTACTTGTTACTTTGGCCTCTTTTGTTTA   11662
                                 ************************************************************

gb:MT020781|Organism:Severe      CTCAACCGCTACTTTAGACTGACTCTTGGTGTTTATGATTACTTAGTTTCTACACAGGAG   11710
gb:MT007544|Organism:Severe      CTCAACCGCTACTTTAGACTGACTCTTGGTGTTTATGATTACTTAGTTTCTACACAGGAG   11722
gb:MN994467|Organism:Severe      CTCAACCGCTACTTTAGACTGACTCTTGGTGTTTATGATTACTTAGTTTCTACACAGGAG   11722
gb:MT044257|Organism:Severe      CTCAACCGCTACTTTAGACTGACTCTTGGTGTTTATGATTACTTAGTTTCTACACAGGAG   11722
gb:MT106054|Organism:Severe      CTCAACCGCTACTTTAGACTGACTCTTGGTGTTTATGATTACTTAGTTTCTACACAGGAG   11722
gb:MT049951|Organism:Severe      CTCAACCGCTACTTTAGACTGACTCTTGGTGTTTATGATTACTTAGTTTCTACACAGGAG   11722
gb:MN975262|Organism:Severe      CTCAACCGCTACTTTAGACTGACTCTTGGTGTTTATGATTACTTAGTTTCTACACAGGAG   11722
gb:MT106052|Organism:Severe      CTCAACCGCTACTTTAGACTGACTCTTGGTGTTTATGATTACTTAGTTTCTACACAGGAG   11722
gb:LC522975|Organism:Severe      CTCAACCGCTACTTTAGACTGACTCTTGGTGTTTATGATTACTTAGTTTCTACACAGGAG   11719
gb:LC522973|Organism:Severe      CTCAACCGCTACTTTAGACTGACTCTTGGTGTTTATGATTACTTAGTTTCTACACAGGAG   11719
gb:LC522974|Organism:Severe      CTCAACCGCTACTTTAGACTGACTCTTGGTGTTTATGATTACTTAGTTTCTACACAGGAG   11719
gb:MN985325|Organism:Severe      CTCAACCGCTACTTTAGACTGACTCTTGGTGTTTATGATTACTTAGTTTCTACACAGGAG   11722
gb:MT020881|Organism:Severe      CTCAACCGCTACTTTAGACTGACTCTTGGTGTTTATGATTACTTAGTTTCTACACAGGAG   11722
gb:MT020880|Organism:Severe      CTCAACCGCTACTTTAGACTGACTCTTGGTGTTTATGATTACTTAGTTTCTACACAGGAG   11722
gb:MT066175|Organism:Severe      CTCAACCGCTACTTTAGACTGACTCTTGGTGTTTATGATTACTTAGTTTCTACACAGGAG   11722
gb:MN997409|Organism:Severe      CTCAACCGCTACTTTAGACTGACTCTTGGTGTTTATGATTACTTAGTTTCTACACAGGAG   11722
gb:MN938384|Organism:Severe      CTCAACCGCTACTTTAGACTGACTCTTGGTGTTTATGATTACTTAGTTTCTACACAGGAG   11690
gb:MT044258|Organism:Severe      CTCAACCGCTACTTTAGACTGACTCTTGGTGTTTATGATTACTTAGTTTCTACACAGGAG   11698
gb:MT039890|Organism:Severe      CTCAACCGCTACTTTAGACTGACTCTTGGTGTTTATGATTACTTAGTTTCTACACAGGAG   11722
gb:MN988713|Organism:Severe      CTCAACCGCTACTTTAGACTGACTCTTGGTGTTTATGATTACTTAGTTTCTACACAGGAG   11722
gb:LC521925|Organism:Severe      CTCAACCGCTACTTTAGACTGACTCTTGGTGTTTATGATTACTTAGTTTCTACACAGGAG   11695
gb:MT093571|Organism:Severe      CTCAACCGCTACTTTAGACTGACTCTTGGTGTTTATGATTACTTAGTTTCTACACAGGAG   11722
gb:MT039887|Organism:Severe      CTCAACCGCTACTTTAGACTGACTCTTGGTGTTTATGATTACTTAGTTTCTACACAGGAG   11722
gb:MT019530|Organism:Severe      CTCAACCGCTACTTTAGACTGACTCTTGGTGTTTATGATTACTTAGTTTCTACACAGGAG   11722
gb:MT039888|Organism:Severe      CTCAACCGCTACTTTAGACTGACTCTTGGTGTTTATGATTACTTAGTTTCTACACAGGAG   11722
gb:LC522972|Organism:Severe      CTCAACCGCTACTTTAGACTGACTCTTGGTGTTTATGATTACTTAGTTTCTACACAGGAG   11719
gb:MT027063|Organism:Severe      CTCAACCGCTACTTTAGACTGACTCTTGGTGTTTATGATTACTTAGTTTCTACACAGGAG   11722
gb:MT027062|Organism:Severe      CTCAACCGCTACTTTAGACTGACTCTTGGTGTTTATGATTACTTAGTTTCTACACAGGAG   11722
gb:MT019529|Organism:Severe      CTCAACCGCTACTTTAGACTGACTCTTGGTGTTTATGATTACTTAGTTTCTACACAGGAG   11722
gb:MN996529|Organism:Severe      CTCAACCGCTACTTTAGACTGACTCTTGGTGTTTATGATTACTTAGTTTCTACACAGGAG   11710
gb:MN996531|Organism:Severe      CTCAACCGCTACTTTAGACTGACTCTTGGTGTTTATGATTACTTAGTTTCTACACAGGAG   11709
gb:MT066176|Organism:Severe      CTCAACCGCTACTTTAGACTGACTCTTGGTGTTTATGATTACTTAGTTTCTACACAGGAG   11722
gb:MT027064|Organism:Severe      CTCAACCGCTACTTTAGACTGACTCTTGGTGTTTATGATTACTTAGTTTCTACACAGGAG   11722
gb:MN994468|Organism:Severe      CTCAACCGCTACTTTAGACTGACTCTTGGTGTTTATGATTACTTAGTTTCTACACAGGAG   11722
gb:MT072688|Organism:Severe      CTCAACCGCTACTTTAGACTGACTCTTGGTGTTTATGATTACTTAGTTTCTACACAGGAG   11707
gb:MN996527|Organism:Severe      CTCAACCGCTACTTTAGACTGACTCTTGGTGTTTATGATTACTTAGTTTCTACACAGGAG   11689
gb:MT093631|Organism:Severe      CTCAACCGCTACTTTAGACTGACTCTTGGTGTTTATGATTACTTAGTTTCTACACAGGAG   11760
gb:MT106053|Organism:Severe      CTCAACCGCTACTTTAGACTGACTCTTGGTGTTTATGATTACTTAGTTTCTACACAGGAG   11722
gb:MT019533|Organism:Severe      CTCAACCGCTACTTTAGACTGACTCTTGGTGTTTATGATTACTTAGTTTCTACACAGGAG   11722
gb:MT019531|Organism:Severe      CTCAACCGCTACTTTAGACTGACTCTTGGTGTTTATGATTACTTAGTTTCTACACAGGAG   11722
gb:MN996528|Organism:Severe      CTCAACCGCTACTTTAGACTGACTCTTGGTGTTTATGATTACTTAGTTTCTACACAGGAG   11722
gb:MN996530|Organism:Severe      CTCAACCGCTACTTTAGACTGACTCTTGGTGTTTATGATTACTTAGTTTCTACACAGGAG   11708
gb:MN908947|Organism:Severe      CTCAACCGCTACTTTAGACTGACTCTTGGTGTTTATGATTACTTAGTTTCTACACAGGAG   11722
gb:MT019532|Organism:Severe      CTCAACCGCTACTTTAGACTGACTCTTGGTGTTTATGATTACTTAGTTTCTACACAGGAG   11722
                                 ************************************************************

gb:MT020781|Organism:Severe      TTTAGATATATGAATTCACAGGGACTACTCCCACCCAAGAATAGCATAGATGCCTTCAAA   11770
gb:MT007544|Organism:Severe      TTTAGATATATGAATTCACAGGGACTACTCCCACCCAAGAATAGCATAGATGCCTTCAAA   11782
gb:MN994467|Organism:Severe      TTTAGATATATGAATTCACAGGGACTACTCCCACCCAAGAATAGCATAGATGCCTTCAAA   11782
gb:MT044257|Organism:Severe      TTTAGATATATGAATTCACAGGGACTACTCCCACCCAAGAATAGCATAGATGCCTTCAAA   11782
gb:MT106054|Organism:Severe      TTTAGATATATGAATTCACAGGGACTACTCCCACCCAAGAATAGCATAGATGCCTTCAAA   11782
gb:MT049951|Organism:Severe      TTTAGATATATGAATTCACAGGGACTACTCCCACCCAAGAATAGCATAGATGCCTTCAAA   11782
gb:MN975262|Organism:Severe      TTTAGATATATGAATTCACAGGGACTACTCCCACCCAAGAATAGCATAGATGCCTTCAAA   11782
gb:MT106052|Organism:Severe      TTTAGATATATGAATTCACAGGGACTACTCCCACCCAAGAATAGCATAGATGCCTTCAAA   11782
gb:LC522975|Organism:Severe      TTTAGATATATGAATTCACAGGGACTACTCCCACCCAAGAATAGCATAGATGCCTTCAAA   11779
gb:LC522973|Organism:Severe      TTTAGATATATGAATTCACAGGGACTACTCCCACCCAAGAATAGCATAGATGCCTTCAAA   11779
gb:LC522974|Organism:Severe      TTTAGATATATGAATTCACAGGGACTACTCCCACCCAAGAATAGCATAGATGCCTTCAAA   11779
gb:MN985325|Organism:Severe      TTTAGATATATGAATTCACAGGGACTACTCCCACCCAAGAATAGCATAGATGCCTTCAAA   11782
gb:MT020881|Organism:Severe      TTTAGATATATGAATTCACAGGGACTACTCCCACCCAAGAATAGCATAGATGCCTTCAAA   11782
gb:MT020880|Organism:Severe      TTTAGATATATGAATTCACAGGGACTACTCCCACCCAAGAATAGCATAGATGCCTTCAAA   11782
gb:MT066175|Organism:Severe      TTTAGATATATGAATTCACAGGGACTACTCCCACCCAAGAATAGCATAGATGCCTTCAAA   11782
```

```
gb:MN997409|Organism:Severe      TTTAGATATATGAATTCACAGGGACTACTCCCACCCAAGAATAGCATAGATGCCTTCAAA   11782
gb:MN938384|Organism:Severe      TTTAGATATATGAATTCACAGGGACTACTCCCACCCAAGAATAGCATAGATGCCTTCAAA   11750
gb:MT044258|Organism:Severe      TTTAGATATATGAATTCACAGGGACTACTCCCACCCAAGAATAGCATAGATGCCTTCAAA   11758
gb:MT039890|Organism:Severe      TTTAGATATATGAATTCACAGGGACTACTCCCACCCAAGAATAGCATAGATGCCTTCAAA   11782
gb:MN988713|Organism:Severe      TTTAGATATATGAATTCACAGGGACTACTCCCACCCAAGAATAGCATAGATGCCTTCAAA   11782
gb:LC521925|Organism:Severe      TTTAGATATATGAATTCACAGGGACTACTCCCACCCAAGAATAGCATAGATGCCTTCAAA   11755
gb:MT093571|Organism:Severe      TTTAGATATATGAATTCACAGGGACTACTCCCACCCAAGAATAGCATAGATGCCTTCAAA   11782
gb:MT039887|Organism:Severe      TTTAGATATATGAATTCACAGGGACTACTCCCACCCAAGAATAGCATAGATGCCTTCAAA   11782
gb:MT019530|Organism:Severe      TTTAGATATATGAATTCACAGGGACTACTCCCACCCAAGAATAGCATAGATGCCTTCAAA   11782
gb:MT039888|Organism:Severe      TTTAGATATATGAATTCACAGGGACTACTCCCACCCAAGAATAGCATAGATGCCTTCAAA   11782
gb:LC522972|Organism:Severe      TTTAGATATATGAATTCACAGGGACTACTCCCACCCAAGAATAGCATAGATGCCTTCAAA   11779
gb:MT027063|Organism:Severe      TTTAGATATATGAATTCACAGGGACTACTCCCACCCAAGAATAGCATAGATGCCTTCAAA   11782
gb:MT027062|Organism:Severe      TTTAGATATATGAATTCACAGGGACTACTCCCACCCAAGAATAGCATAGATGCCTTCAAA   11782
gb:MT019529|Organism:Severe      TTTAGATATATGAATTCACAGGGACTACTCCCACCCAAGAATAGCATAGATGCCTTCAAA   11782
gb:MN996529|Organism:Severe      TTTAGATATATGAATTCACAGGGACTACTCCCACCCAAGAATAGCATAGATGCCTTCAAA   11770
gb:MN996531|Organism:Severe      TTTAGATATATGAATTCACAGGGACTACTCCCACCCAAGAATAGCATAGATGCCTTCAAA   11769
gb:MT066176|Organism:Severe      TTTAGATATATGAATTCACAGGGACTACTCCCACCCAAGAATAGCATAGATGCCTTCAAA   11782
gb:MT027064|Organism:Severe      TTTAGATATATGAATTCACAGGGACTACTCCCACCCAAGAATAGCATAGATGCCTTCAAA   11782
gb:MN994468|Organism:Severe      TTTAGATATATGAATTCACAGGGACTACTCCCACCCAAGAATAGCATAGATGCCTTCAAA   11782
gb:MT072688|Organism:Severe      TTTAGATATATGAATTCACAGGGACTACTCCCACCCAAGAATAGCATAGATGCCTTCAAA   11767
gb:MN996527|Organism:Severe      TTTAGATATATGAATTCACAGGGACTACTCCCACCCAAGAATAGCATAGATGCCTTCAAA   11749
gb:MT093631|Organism:Severe      TTTAGATATATGAATTCACAGGGACTACTCCCACCCAAGAATAGCATAGATGCCTTCAAA   11820
gb:MT106053|Organism:Severe      TTTAGATATATGAATTCACAGGGACTACTCCCACCCAAGAATAGCATAGATGCCTTCAAA   11782
gb:MT019533|Organism:Severe      TTTAGATATATGAATTCACAGGGACTACTCCCACCCAAGAATAGCATAGATGCCTTCAAA   11782
gb:MT019531|Organism:Severe      TTTAGATATATGAATTCACAGGGACTACTCCCACCCAAGAATAGCATAGATGCCTTCAAA   11782
gb:MN996528|Organism:Severe      TTTAGATATATGAATTCACAGGGACTACTCCCACCCAAGAATAGCATAGATGCCTTCAAA   11782
gb:MN996530|Organism:Severe      TTTAGATATATGAATTCACAGGGACTACTCCCACCCAAGAATAGCATAGATGCCTTCAAA   11768
gb:MN908947|Organism:Severe      TTTAGATATATGAATTCACAGGGACTACTCCCACCCAAGAATAGCATAGATGCCTTCAAA   11782
gb:MT019532|Organism:Severe      TTTAGATATATGAATTCACAGGGACTACTCCCACCCAAGAATAGCATAGATGCCTTCAAA   11782
                                 ************************************************************

gb:MT020781|Organism:Severe      CTCAACATTAAATTGTTGGGTGTTGGTGGCAAACCTTGTATCAAAGTAGCCACTGTACAG   11830
gb:MT007544|Organism:Severe      CTCAACATTAAATTGTTGGGTGTTGGTGGCAAACCTTGTATCAAAGTAGCCACTGTACAG   11842
gb:MN994467|Organism:Severe      CTCAACATTAAATTGTTGGGTGTTGGTGGCAAACCTTGTATCAAAGTAGCCACTGTACAG   11842
gb:MT044257|Organism:Severe      CTCAACATTAAATTGTTGGGTGTTGGTGGCAAACCTTGTATCAAAGTAGCCACTGTACAG   11842
gb:MT106054|Organism:Severe      CTCAACATTAAATTGTTGGGTGTTGGTGGCAAACCTTGTATCAAAGTAGCCACTGTACAG   11842
gb:MT049951|Organism:Severe      CTCAACATTAAATTGTTGGGTGTTGGTGGCAAACCTTGTATCAAAGTAGCCACTGTACAG   11842
gb:MN975262|Organism:Severe      CTCAACATTAAATTGTTGGGTGTTGGTGGCAAACCTTGTATCAAAGTAGCCACTGTACAG   11842
gb:MT106052|Organism:Severe      CTCAACATTAAATTGTTGGGTGTTGGTGGCAAACCTTGTATCAAAGTAGCCACTGTACAG   11842
gb:LC522975|Organism:Severe      CTCAACATTAAATTGTTGGGTGTTGGTGGCAAACCTTGTATCAAAGTAGCCACTGTACAG   11839
gb:LC522973|Organism:Severe      CTCAACATTAAATTGTTGGGTGTTGGTGGCAAACCTTGTATCAAAGTAGCCACTGTACAG   11839
gb:LC522974|Organism:Severe      CTCAACATTAAATTGTTGGGTGTTGGTGGCAAACCTTGTATCAAAGTAGCCACTGTACAG   11839
gb:MN985325|Organism:Severe      CTCAACATTAAATTGTTGGGTGTTGGTGGCAAACCTTGTATCAAAGTAGCCACTGTACAG   11842
gb:MT020881|Organism:Severe      CTCAACATTAAATTGTTGGGTGTTGGTGGCAAACCTTGTATCAAAGTAGCCACTGTACAG   11842
gb:MT020880|Organism:Severe      CTCAACATTAAATTGTTGGGTGTTGGTGGCAAACCTTGTATCAAAGTAGCCACTGTACAG   11842
gb:MT066175|Organism:Severe      CTCAACATTAAATTGTTGGGTGTTGGTGGCAAACCTTGTATCAAAGTAGCCACTGTACAG   11842
gb:MN997409|Organism:Severe      CTCAACATTAAATTGTTGGGTGTTGGTGGCAAACCTTGTATCAAAGTAGCCACTGTACAG   11842
gb:MN938384|Organism:Severe      CTCAACATTAAATTGTTGGGTGTTGGTGGCAAACCTTGTATCAAAGTAGCCACTGTACAG   11810
gb:MT044258|Organism:Severe      CTCAACATTAAATTGTTGGGTGTTGGTGGCAAACCTTGTATCAAAGTAGCCACTGTACAG   11818
gb:MT039890|Organism:Severe      CTCAACATTAAATTGTTGGGTGTTGGTGGCAAACCTTGTATCAAAGTAGCCACTGTACAG   11842
gb:MN988713|Organism:Severe      CTCAACATTAAATTGTTGGGTGTTGGTGGCAAACCTTGTATCAAAGTAGCCACTGTACAG   11842
gb:LC521925|Organism:Severe      CTCAACATTAAATTGTTGGGTGTTGGTGGCAAACCTTGTATCAAAGTAGCCACTGTACAG   11815
gb:MT093571|Organism:Severe      CTCAACATTAAATTGTTGGGTGTTGGTGGCAAACCTTGTATCAAAGTAGCCACTGTACAG   11842
gb:MT039887|Organism:Severe      CTCAACATTAAATTGTTGGGTGTTGGTGGCAAACCTTGTATCAAAGTAGCCACTGTACAG   11842
gb:MT019530|Organism:Severe      CTCAACATTAAATTGTTGGGTGTTGGTGGCAAACCTTGTATCAAAGTAGCCACTGTACAG   11842
gb:MT039888|Organism:Severe      CTCAACATTAAATTGTTGGGTGTTGGTGGCAAACCTTGTATCAAAGTAGCCACTGTACAG   11842
gb:LC522972|Organism:Severe      CTCAACATTAAATTGTTGGGTGTTGGTGGCAAACCTTGTATCAAAGTAGCCACTGTACAG   11839
gb:MT027063|Organism:Severe      CTCAACATTAAATTGTTGGGTGTTGGTGGCAAACCTTGTATCAAAGTAGCCACTGTACAG   11842
gb:MT027062|Organism:Severe      CTCAACATTAAATTGTTGGGTGTTGGTGGCAAACCTTGTATCAAAGTAGCCACTGTACAG   11842
gb:MT019529|Organism:Severe      CTCAACATTAAATTGTTGGGTGTTGGTGGCAAACCTTGTATCAAAGTAGCCACTGTACAG   11842
gb:MN996529|Organism:Severe      CTCAACATTAAATTGTTGGGTGTTGGTGGCAAACCTTGTATCAAAGTAGCCACTGTACAG   11830
gb:MN996531|Organism:Severe      CTCAACATTAAATTGTTGGGTGTTGGTGGCAAACCTTGTATCAAAGTAGCCACTGTACAG   11829
gb:MT066176|Organism:Severe      CTCAACATTAAATTGTTGGGTGTTGGTGGCAAACCTTGTATCAAAGTAGCCACTGTACAG   11842
gb:MT027064|Organism:Severe      CTCAACATTAAATTGTTGGGTGTTGGTGGCAAACCTTGTATCAAAGTAGCCACTGTACAG   11842
gb:MN994468|Organism:Severe      CTCAACATTAAATTGTTGGGTGTTGGTGGCAAACCTTGTATCAAAGTAGCCACTGTACAG   11842
```

```
gb:MT072688|Organism:Severe      CTCAACATTAAATTGTTGGGTGTTGGTGGCAAACCTTGTATCAAAGTAGCCACTGTACAG   11827
gb:MN996527|Organism:Severe      CTCAACATTAAATTGTTGGGTGTTGGTGGCAAACCTTGTATCAAAGTAGCCACTGTACAG   11809
gb:MT093631|Organism:Severe      CTCAACATTAAATTGTTGGGTGTTGGTGGCAAACCTTGTATCAAAGTAGCCACTGTACAG   11880
gb:MT106053|Organism:Severe      CTCAACATTAAATTGTTGGGTGTTGGTGGCAAACCTTGTATCAAAGTAGCCACTGTACAG   11842
gb:MT019533|Organism:Severe      CTCAACATTAAATTGTTGGGTGTTGGTGGCAAACCTTGTATCAAAGTAGCCACTGTACAG   11842
gb:MT019531|Organism:Severe      CTCAACATTAAATTGTTGGGTGTTGGTGGCAAACCTTGTATCAAAGTAGCCACTGTACAG   11842
gb:MN996528|Organism:Severe      CTCAACATTAAATTGTTGGGTGTTGGTGGCAAACCTTGTATCAAAGTAGCCACTGTACAG   11842
gb:MN996530|Organism:Severe      CTCAACATTAAATTGTTGGGTGTTGGTGGCAAACCTTGTATCAAAGTAGCCACTGTACAG   11828
gb:MN908947|Organism:Severe      CTCAACATTAAATTGTTGGGTGTTGGTGGCAAACCTTGTATCAAAGTAGCCACTGTACAG   11842
gb:MT019532|Organism:Severe      CTCAACATTAAATTGTTGGGTGTTGGTGGCAAACCTTGTATCAAAGTAGCCACTGTACAG   11842
                                 ************************************************************

gb:MT020781|Organism:Severe      TCTAAAATGTCAGATGTAAAGTGCACATCAGTAGTCTTACTCTCAGTTTTGCAACAACTC   11890
gb:MT007544|Organism:Severe      TCTAAAATGTCAGATGTAAAGTGCACATCAGTAGTCTTACTCTCAGTTTTGCAACAACTC   11902
gb:MN994467|Organism:Severe      TCTAAAATGTCAGATGTAAAGTGCACATCAGTAGTCTTACTCTCAGTTTTGCAACAACTC   11902
gb:MT044257|Organism:Severe      TCTAAAATGTCAGATGTAAAGTGCACATCAGTAGTCTTACTCTCAGTTTTGCAACAACTC   11902
gb:MT106054|Organism:Severe      TCTAAAATGTCAGATGTAAAGTGCACATCAGTAGTCTTACTCTCAGTTTTGCAACAACTC   11902
gb:MT049951|Organism:Severe      TCTAAAATGTCAGATGTAAAGTGCACATCAGTAGTCTTACTCTCAGTTTTGCAACAACTC   11902
gb:MN975262|Organism:Severe      TCTAAAATGTCAGATGTAAAGTGCACATCAGTAGTCTTACTCTCAGTTTTGCAACAACTC   11902
gb:MT106052|Organism:Severe      TCTAAAATGTCAGATGTAAAGTGCACATCAGTAGTCTTACTCTCAGTTTTGCAACAACTC   11902
gb:LC522975|Organism:Severe      TCTAAAATGTCAGATGTAAAGTGCACATCAGTAGTCTTACTCTCAGTTTTGCAACAACTC   11899
gb:LC522973|Organism:Severe      TCTAAAATGTCAGATGTAAAGTGCACATCAGTAGTCTTACTCTCAGTTTTGCAACAACTC   11899
gb:LC522974|Organism:Severe      TCTAAAATGTCAGATGTAAAGTGCACATCAGTAGTCTTACTCTCAGTTTTGCAACAACTC   11899
gb:MN985325|Organism:Severe      TCTAAAATGTCAGATGTAAAGTGCACATCAGTAGTCTTACTCTCAGTTTTGCAACAACTC   11902
gb:MT020881|Organism:Severe      TCTAAAATGTCAGATGTAAAGTGCACATCAGTAGTCTTACTCTCAGTTTTGCAACAACTC   11902
gb:MT020880|Organism:Severe      TCTAAAATGTCAGATGTAAAGTGCACATCAGTAGTCTTACTCTCAGTTTTGCAACAACTC   11902
gb:MT066175|Organism:Severe      TCTAAAATGTCAGATGTAAAGTGCACATCAGTAGTCTTACTCTCAGTTTTGCAACAACTC   11902
gb:MN997409|Organism:Severe      TCTAAAATGTCAGATGTAAAGTGCACATCAGTAGTCTTACTCTCAGTTTTGCAACAACTC   11902
gb:MN938384|Organism:Severe      TCTAAAATGTCAGATGTAAAGTGCACATCAGTAGTCTTACTCTCAGTTTTGCAACAACTC   11870
gb:MT044258|Organism:Severe      TCTAAAATGTCAGATGTAAAGTGCACATCAGTAGTCTTACTCTCAGTTTTGCAACAACTC   11878
gb:MT039890|Organism:Severe      TCTAAAATGTCAGATGTAAAGTGCACATCAGTAGTCTTACTCTCAGTTTTGCAACAACTC   11902
gb:MN988713|Organism:Severe      TCTAAAATGTCAGATGTAAAGTGCACATCAGTAGTCTTACTCTCAGTTTTGCAACAACTC   11902
gb:LC521925|Organism:Severe      TCTAAAATGTCAGATGTAAAGTGCACATCAGTAGTCTTACTCTCAGTTTTGCAACAACTC   11875
gb:MT093571|Organism:Severe      TCTAAAATGTCAGATGTAAAGTGCACATCAGTAGTCTTACTCTCAGTTTTGCAACAACTC   11902
gb:MT039887|Organism:Severe      TCTAAAATGTCAGATGTAAAGTGCACATCAGTAGTCTTACTCTCAGTTTTGCAACAACTC   11902
gb:MT019530|Organism:Severe      TCTAAAATGTCAGATGTAAAGTGCACATCAGTAGTCTTACTCTCAGTTTTGCAACAACTC   11902
gb:MT039888|Organism:Severe      TCTAAAATGTCAGATGTAAAGTGCACATCAGTAGTCTTACTCTCAGTTTTGCAACAACTC   11902
gb:LC522972|Organism:Severe      TCTAAAATGTCAGATGTAAAGTGCACATCAGTAGTCTTACTCTCAGTTTTGCAACAACTC   11899
gb:MT027063|Organism:Severe      TCTAAAATGTCAGATGTAAAGTGCACATCAGTAGTCTTACTCTCAGTTTTGCAACAACTC   11902
gb:MT027062|Organism:Severe      TCTAAAATGTCAGATGTAAAGTGCACATCAGTAGTCTTACTCTCAGTTTTGCAACAACTC   11902
gb:MT019529|Organism:Severe      TCTAAAATGTCAGATGTAAAGTGCACATCAGTAGTCTTACTCTCAGTTTTGCAACAACTC   11902
gb:MN996529|Organism:Severe      TCTAAAATGTCAGATGTAAAGTGCACATCAGTAGTCTTACTCTCAGTTTTGCAACAACTC   11890
gb:MN996531|Organism:Severe      TCTAAAATGTCAGATGTAAAGTGCACATCAGTAGTCTTACTCTCAGTTTTGCAACAACTC   11889
gb:MT066176|Organism:Severe      TCTAAAATGTCAGATGTAAAGTGCACATCAGTAGTCTTACTCTCAGTTTTGCAACAACTC   11902
gb:MT027064|Organism:Severe      TCTAAAATGTCAGATGTAAAGTGCACATCAGTAGTCTTACTCTCAGTTTTGCAACAACTC   11902
gb:MN994468|Organism:Severe      TCTAAAATGTCAGATGTAAAGTGCACATCAGTAGTCTTACTCTCAGTTTTGCAACAACTC   11902
gb:MT072688|Organism:Severe      TCTAAAATGTCAGATGTAAAGTGCACATCAGTAGTCTTACTCTCAGTTTTGCAACAACTC   11887
gb:MN996527|Organism:Severe      TCTAAAATGTCAGATGTAAAGTGCACATCAGTAGTCTTACTCTCAGTTTTGCAACAACTC   11869
gb:MT093631|Organism:Severe      TCTAAAATGTCAGATGTAAAGTGCACATCAGTAGTCTTACTCTCAGTTTTGCAACAACTC   11940
gb:MT106053|Organism:Severe      TCTAAAATGTCAGATGTAAAGTGCACATCAGTAGTCTTACTCTCAGTTTTGCAACAACTC   11902
gb:MT019533|Organism:Severe      TCTAAAATGTCAGATGTAAAGTGCACATCAGTAGTCTTACTCTCAGTTTTGCAACAACTC   11902
gb:MT019531|Organism:Severe      TCTAAAATGTCAGATGTAAAGTGCACATCAGTAGTCTTACTCTCAGTTTTGCAACAACTC   11902
gb:MN996528|Organism:Severe      TCTAAAATGTCAGATGTAAAGTGCACATCAGTAGTCTTACTCTCAGTTTTGCAACAACTC   11902
gb:MN996530|Organism:Severe      TCTAAAATGTCAGATGTAAAGTGCACATCAGTAGTCTTACTCTCAGTTTTGCAACAACTC   11888
gb:MN908947|Organism:Severe      TCTAAAATGTCAGATGTAAAGTGCACATCAGTAGTCTTACTCTCAGTTTTGCAACAACTC   11902
gb:MT019532|Organism:Severe      TCTAAAATGTCAGATGTAAAGTGCACATCAGTAGTCTTACTCTCAGTTTTGCAACAACTC   11902
                                 ************************************************************

gb:MT020781|Organism:Severe      AGAGTAGAATCATCATCTAAATTGTGGGCTCAATGTGTCCAGTTACACAATGACATTCTC   11950
gb:MT007544|Organism:Severe      AGAGTAGAATCATCATCTAAATTGTGGGCTCAATGTGTCCAGTTACACAATGACATTCTC   11962
gb:MN994467|Organism:Severe      AGAGTAGAATCATCATCTAAATTGTGGGCTCAATGTGTCCAGTTACACAATGACATTCTC   11962
gb:MT044257|Organism:Severe      AGAGTAGAATCATCATCTAAATTGTGGGCTCAATGTGTCCAGTTACACAATGACATTCTC   11962
gb:MT106054|Organism:Severe      AGAGTAGAATCATCATCTAAATTGTGGGCTCAATGTGTCCAGTTACACAATGACATTCTC   11962
gb:MT049951|Organism:Severe      AGAGTAGAATCATCATCTAAATTGTGGGCTCAATGTGTCCAGTTACACAATGACATTCTC   11962
gb:MN975262|Organism:Severe      AGAGTAGAATCATCATCTAAATTGTGGGCTCAATGTGTCCAGTTACACAATGACATTCTC   11962
```

```
gb:MT106052|Organism:Severe      AGAGTAGAATCATCATCTAAATTGTGGGCTCAATGTGTCCAGTTACACAATGACATTCTC    11962
gb:LC522975|Organism:Severe      AGAGTAGAATCATCATCTAAATTGTGGGCTCAATGTGTCCAGTTACACAATGACATTCTC    11959
gb:LC522973|Organism:Severe      AGAGTAGAATCATCATCTAAATTGTGGGCTCAATGTGTCCAGTTACACAATGACATTCTC    11959
gb:LC522974|Organism:Severe      AGAGTAGAATCATCATCTAAATTGTGGGCTCAATGTGTCCAGTTACACAATGACATTCTC    11959
gb:MN985325|Organism:Severe      AGAGTAGAATCATCATCTAAATTGTGGGCTCAATGTGTCCAGTTACACAATGACATTCTC    11962
gb:MT020881|Organism:Severe      AGAGTAGAATCATCATCTAAATTGTGGGCTCAATGTGTCCAGTTACACAATGACATTCTC    11962
gb:MT020880|Organism:Severe      AGAGTAGAATCATCATCTAAATTGTGGGCTCAATGTGTCCAGTTACACAATGACATTCTC    11962
gb:MT066175|Organism:Severe      AGAGTAGAATCATCATCTAAATTGTGGGCTCAATGTGTCCAGTTACACAATGACATTCTC    11962
gb:MN997409|Organism:Severe      AGAGTAGAATCATCATCTAAATTGTGGGCTCAATGTGTCCAGTTACACAATGACATTCTC    11962
gb:MN938384|Organism:Severe      AGAGTAGAATCATCATCTAAATTGTGGGCTCAATGTGTCCAGTTACACAATGACATTCTC    11930
gb:MT044258|Organism:Severe      AGAGTAGAATCATCATCTAAATTGTGGGCTCAATGTGTCCAGTTACACAATGACATTCTC    11938
gb:MT039890|Organism:Severe      AGAGTAGAATCATCATCTAAATTGTGGGCTCAATGTGTCCAGTTACACAATGACATTCTC    11962
gb:MN988713|Organism:Severe      AGAGTAGAATCATCATCTAAATTGTGGGCTCAATGTGTCCAGTTACACAATGACATTCTC    11962
gb:LC521925|Organism:Severe      AGAGTAGAATCATCATCTAAATTGTGGGCTCAATGTGTCCAGTTACACAATGACATTCTC    11935
gb:MT093571|Organism:Severe      AGAGTAGAATCATCATCTAAATTGTGGGCTCAATGTGTCCAGTTACACAATGACATTCTC    11962
gb:MT039887|Organism:Severe      AGAGTAGAATCATCATCTAAATTGTGGGCTCAATGTGTCCAGTTACACAATGACATTCTC    11962
gb:MT019530|Organism:Severe      AGAGTAGAATCATCATCTAAATTGTGGGCTCAATGTGTCCAGTTACACAATGACATTCTC    11962
gb:MT039888|Organism:Severe      AGAGTAGAATCATCATCTAAATTGTGGGCTCAATGTGTCCAGTTACACAATGACATTCTC    11962
gb:LC522972|Organism:Severe      AGAGTAGAATCATCATCTAAATTGTGGGCTCAATGTGTCCAGTTACACAATGACATTCTC    11959
gb:MT027063|Organism:Severe      AGAGTAGAATCATCATCTAAATTGTGGGCTCAATGTGTCCAGTTACACAATGACATTCTC    11962
gb:MT027062|Organism:Severe      AGAGTAGAATCATCATCTAAATTGTGGGCTCAATGTGTCCAGTTACACAATGACATTCTC    11962
gb:MT019529|Organism:Severe      AGAGTAGAATCATCATCTAAATTGTGGGCTCAATGTGTCCAGTTACACAATGACATTCTC    11962
gb:MN996529|Organism:Severe      AGAGTAGAATCATCATCTAAATTGTGGGCTCAATGTGTCCAGTTACACAATGACATTCTC    11950
gb:MN996531|Organism:Severe      AGAGTAGAATCATCATCTAAATTGTGGGCTCAATGTGTCCAGTTACACAATGACATTCTC    11949
gb:MT066176|Organism:Severe      AGAGTAGAATCATCATCTAAATTGTGGGCTCAATGTGTCCAGTTACACAATGACATTCTC    11962
gb:MT027064|Organism:Severe      AGAGTAGAATCATCATCTAAATTGTGGGCTCAATGTGTCCAGTTACACAATGACATTCTC    11962
gb:MN994468|Organism:Severe      AGAGTAGAATCATCATCTAAATTGTGGGCTCAATGTGTCCAGTTACACAATGACATTCTC    11962
gb:MT072688|Organism:Severe      AGAGTAGAATCATCATCTAAATTGTGGGCTCAATGTGTCCAGTTACACAATGACATTCTC    11947
gb:MN996527|Organism:Severe      AGAGTAGAATCATCATCTAAATTGTGGGCTCAATGTGTCCAGTTACACAATGACATTCTC    11929
gb:MT093631|Organism:Severe      AGAGTAGAATCATCATCTAAATTGTGGGCTCAATGTGTCCAGTTACACAATGACATTCTC    12000
gb:MT106053|Organism:Severe      AGAGTAGAATCATCATCTAAATTGTGGGCTCAATGTGTCCAGTTACACAATGACATTCTC    11962
gb:MT019533|Organism:Severe      AGAGTAGAATCATCATCTAAATTGTGGGCTCAATGTGTCCAGTTACACAATGACATTCTC    11962
gb:MT019531|Organism:Severe      AGAGTAGAATCATCATCTAAATTGTGGGCTCAATGTGTCCAGTTACACAATGACATTCTC    11962
gb:MN996528|Organism:Severe      AGAGTAGAATCATCATCTAAATTGTGGGCTCAATGTGTCCAGTTACACAATGACATTCTC    11962
gb:MN996530|Organism:Severe      AGAGTAGAATCATCATCTAAATTGTGGGCTCAATGTGTCCAGTTACACAATGACATTCTC    11948
gb:MN908947|Organism:Severe      AGAGTAGAATCATCATCTAAATTGTGGGCTCAATGTGTCCAGTTACACAATGACATTCTC    11962
gb:MT019532|Organism:Severe      AGAGTAGAATCATCATCTAAATTGTGGGCTCAATGTGTCCAGTTACACAATGACATTCTC    11962
                                 ************************************************************

gb:MT020781|Organism:Severe      TTAGCTAAAGATACTACTGAAGCCTTTGAAAAAATGGTTTCACTACTTTCTGTTTTGCTT    12010
gb:MT007544|Organism:Severe      TTAGCTAAAGATACTACTGAAGCCTTTGAAAAAATGGTTTCACTACTTTCTGTTTTGCTT    12022
gb:MN994467|Organism:Severe      TTAGCTAAAGATACTACTGAAGCCTTTGAAAAAATGGTTTCACTACTTTCTGTTTTGCTT    12022
gb:MT044257|Organism:Severe      TTAGCTAAAGATACTACTGAAGCCTTTGAAAAAATGGTTTCACTACTTTCTGTTTTGCTT    12022
gb:MT106054|Organism:Severe      TTAGCTAAAGATACTACTGAAGCCTTTGAAAAAATGGTTTCACTACTTTCTGTTTTGCTT    12022
gb:MT049951|Organism:Severe      TTAGCTAAAGATACTACTGAAGCCTTTGAAAAAATGGTTTCACTACTTTCTGTTTTGCTT    12022
gb:MN975262|Organism:Severe      TTAGCTAAAGATACTACTGAAGCCTTTGAAAAAATGGTTTCACTACTTTCTGTTTTGCTT    12022
gb:MT106052|Organism:Severe      TTAGCTAAAGATACTACTGAAGCCTTTGAAAAAATGGTTTCACTACTTTCTGTTTTGCTT    12022
gb:LC522975|Organism:Severe      TTAGCTAAAGATACTACTGAAGCCTTTGAAAAAATGGTTTCACTACTTTCTGTTTTGCTT    12019
gb:LC522973|Organism:Severe      TTAGCTAAAGATACTACTGAAGCCTTTGAAAAAATGGTTTCACTACTTTCTGTTTTGCTT    12019
gb:LC522974|Organism:Severe      TTAGCTAAAGATACTACTGAAGCCTTTGAAAAAATGGTTTCACTACTTTCTGTTTTGCTT    12019
gb:MN985325|Organism:Severe      TTAGCTAAAGATACTACTGAAGCCTTTGAAAAAATGGTTTCACTACTTTCTGTTTTGCTT    12022
gb:MT020881|Organism:Severe      TTAGCTAAAGATACTACTGAAGCCTTTGAAAAAATGGTTTCACTACTTTCTGTTTTGCTT    12022
gb:MT020880|Organism:Severe      TTAGCTAAAGATACTACTGAAGCCTTTGAAAAAATGGTTTCACTACTTTCTGTTTTGCTT    12022
gb:MT066175|Organism:Severe      TTAGCTAAAGATACTACTGAAGCCTTTGAAAAAATGGTTTCACTACTTTCTGTTTTGCTT    12022
gb:MN997409|Organism:Severe      TTAGCTAAAGATACTACTGAAGCCTTTGAAAAAATGGTTTCACTACTTTCTGTTTTGCTT    12022
gb:MN938384|Organism:Severe      TTAGCTAAAGATACTACTGAAGCCTTTGAAAAAATGGTTTCACTACTTTCTGTTTTGCTT    11990
gb:MT044258|Organism:Severe      TTAGCTAAAGATACTACTGAAGCCTTTGAAAAAATGGTTTCACTACTTTCTGTTTTGCTT    11998
gb:MT039890|Organism:Severe      TTAGCTAAAGATACTACTGAAGCCTTTGAAAAAATGGTTTCACTACTTTCTGTTTTGCTT    12022
gb:MN988713|Organism:Severe      TTAGCTAAAGATACTACTGAAGCCTTTGAAAAAATGGTTTCACTACTTTCTGTTTTGCTT    12022
gb:LC521925|Organism:Severe      TTAGCTAAAGATACTACTGAAGCCTTTGAAAAAATGGTTTCACTACTTTCTGTTTTGCTT    11995
gb:MT093571|Organism:Severe      TTAGCTAAAGATACTACTGAAGCCTTTGAAAAAATGGTTTCACTACTTTCTGTTTTGCTT    12022
gb:MT039887|Organism:Severe      TTAGCTAAAGATACTACTGAAGCCTTTGAAAAAATGGTTTCACTACTTTCTGTTTTGCTT    12022
gb:MT019530|Organism:Severe      TTAGCTAAAGATACTACTGAAGCCTTTGAAAAAATGGTTTCACTACTTTCTGTTTTGCTT    12022
gb:MT039888|Organism:Severe      TTAGCTAAAGATACTACTGAAGCCTTTGAAAAAATGGTTTCACTACTTTCTGTTTTGCTT    12022
gb:LC522972|Organism:Severe      TTAGCTAAAGATACTACTGAAGCCTTTGAAAAAATGGTTTCACTACTTTCTGTTTTGCTT    12019
```

```
gb:MT027063|Organism:Severe      TTAGCTAAAGATACTACTGAAGCCTTTGAAAAAATGGTTTCACTACTTTCTGTTTTGCTT   12022
gb:MT027062|Organism:Severe      TTAGCTAAAGATACTACTGAAGCCTTTGAAAAAATGGTTTCACTACTTTCTGTTTTGCTT   12022
gb:MT019529|Organism:Severe      TTAGCTAAAGATACTACTGAAGCCTTTGAAAAAATGGTTTCACTACTTTCTGTTTTGCTT   12022
gb:MN996529|Organism:Severe      TTAGCTAAAGATACTACTGAAGCCTTTGAAAAAATGGTTTCACTACTTTCTGTTTTGCTT   12010
gb:MN996531|Organism:Severe      TTAGCTAAAGATACTACTGAAGCCTTTGAAAAAATGGTTTCACTACTTTCTGTTTTGCTT   12009
gb:MT066176|Organism:Severe      TTAGCTAAAGATACTACTGAAGCCTTTGAAAAAATGGTTTCACTACTTTCTGTTTTGCTT   12022
gb:MT027064|Organism:Severe      TTAGCTAAAGATACTACTGAAGCCTTTGAAAAAATGGTTTCACTACTTTCTGTTTTGCTT   12022
gb:MN994468|Organism:Severe      TTAGCTAAAGATACTACTGAAGCCTTTGAAAAAATGGTTTCACTACTTTCTGTTTTGCTT   12022
gb:MT072688|Organism:Severe      TTAGCTAAAGATACTACTGAAGCCTTTGAAAAAATGGTTTCACTACTTTCTGTTTTGCTT   12007
gb:MN996527|Organism:Severe      TTAGCTAAAGATACTACTGAAGCCTTTGAAAAAATGGTTTCACTACTTTCTGTTTTGCTT   11989
gb:MT093631|Organism:Severe      TTAGCTAAAGATACTACTGAAGCCTTTGAAAAAATGGTTTCACTACTTTCTGTTTTGCTT   12060
gb:MT106053|Organism:Severe      TTAGCTAAAGATACTACTGAAGCCTTTGAAAAAATGGTTTCACTACTTTCTGTTTTGCTT   12022
gb:MT019533|Organism:Severe      TTAGCTAAAGATACTACTGAAGCCTTTGAAAAAATGGTTTCACTACTTTCTGTTTTGCTT   12022
gb:MT019531|Organism:Severe      TTAGCTAAAGATACTACTGAAGCCTTTGAAAAAATGGTTTCACTACTTTCTGTTTTGCTT   12022
gb:MN996528|Organism:Severe      TTAGCTAAAGATACTACTGAAGCCTTTGAAAAAATGGTTTCACTACTTTCTGTTTTGCTT   12022
gb:MN996530|Organism:Severe      TTAGCTAAAGATACTACTGAAGCCTTTGAAAAAATGGTTTCACTACTTTCTGTTTTGCTT   12008
gb:MN908947|Organism:Severe      TTAGCTAAAGATACTACTGAAGCCTTTGAAAAAATGGTTTCACTACTTTCTGTTTTGCTT   12022
gb:MT019532|Organism:Severe      TTAGCTAAAGATACTACTGAAGCCTTTGAAAAAATGGTTTCACTACTTTCTGTTTTGCTT   12022
                                 ************************************************************

gb:MT020781|Organism:Severe      TCCATGCAGGGTGCTGTAGACATAAACAAGCTTTGTGAAGAAATGCTGGACAACAGGGCA   12070
gb:MT007544|Organism:Severe      TCCATGCAGGGTGCTGTAGACATAAACAAGCTTTGTGAAGAAATGCTGGACAACAGGGCA   12082
gb:MN994467|Organism:Severe      TCCATGCAGGGTGCTGTAGACATAAACAAGCTTTGTGAAGAAATGCTGGACAACAGGGCA   12082
gb:MT044257|Organism:Severe      TCCATGCAGGGTGCTGTAGACATAAACAAGCTTTGTGAAGAAATGCTGGACAACAGGGCA   12082
gb:MT106054|Organism:Severe      TCCATGCAGGGTGCTGTAGACATAAACAAGCTTTGTGAAGAAATGCTGGACAACAGGGCA   12082
gb:MT049951|Organism:Severe      TCCATGCAGGGTGCTGTAGACATAAACAAGCTTTGTGAAGAAATGCTGGACAACAGGGCA   12082
gb:MN975262|Organism:Severe      TCCATGCAGGGTGCTGTAGACATAAACAAGCTTTGTGAAGAAATGCTGGACAACAGGGCA   12082
gb:MT106052|Organism:Severe      TCCATGCAGGGTGCTGTAGACATAAACAAGCTTTGTGAAGAAATGCTGGACAACAGGGCA   12082
gb:LC522975|Organism:Severe      TCCATGCAGGGTGCTGTAGACATAAACAAGCTTTGTGAAGAAATGCTGGACAACAGGGCA   12079
gb:LC522973|Organism:Severe      TCCATGCAGGGTGCTGTAGACATAAACAAGCTTTGTGAAGAAATGCTGGACAACAGGGCA   12079
gb:LC522974|Organism:Severe      TCCATGCAGGGTGCTGTAGACATAAACAAGCTTTGTGAAGAAATGCTGGACAACAGGGCA   12079
gb:MN985325|Organism:Severe      TCCATGCAGGGTGCTGTAGACATAAACAAGCTTTGTGAAGAAATGCTGGACAACAGGGCA   12082
gb:MT020881|Organism:Severe      TCCATGCAGGGTGCTGTAGACATAAACAAGCTTTGTGAAGAAATGCTGGACAACAGGGCA   12082
gb:MT020880|Organism:Severe      TCCATGCAGGGTGCTGTAGACATAAACAAGCTTTGTGAAGAAATGCTGGACAACAGGGCA   12082
gb:MT066175|Organism:Severe      TCCATGCAGGGTGCTGTAGACATAAACAAGCTTTGTGAAGAAATGCTGGACAACAGGGCA   12082
gb:MN997409|Organism:Severe      TCCATGCAGGGTGCTGTAGACATAAACAAGCTTTGTGAAGAAATGCTGGACAACAGGGCA   12082
gb:MN938384|Organism:Severe      TCCATGCAGGGTGCTGTAGACATAAACAAGCTTTGTGAAGAAATGCTGGACAACAGGGCA   12050
gb:MT044258|Organism:Severe      TCCATGCAGGGTGCTGTAGACATAAACAAGCTTTGTGAAGAAATGCTGGACAACAGGGCA   12058
gb:MT039890|Organism:Severe      TCCATGCAGGGTGCTGTAGACATAAACAAGCTTTGTGAAGAAATGCTGGACAACAGGGCA   12082
gb:MN988713|Organism:Severe      TCCATGCAGGGTGCTGTAGACATAAACAAGCTTTGTGAAGAAATGCTGGACAACAGGGCA   12082
gb:LC521925|Organism:Severe      TCCATGCAGGGTGCTGTAGACATAAACAAGCTTTGTGAAGAAATGCTGGACAACAGGGCA   12055
gb:MT093571|Organism:Severe      TCCATGCAGGGTGCTGTAGACATAAACAAGCTTTGTGAAGAAATGCTGGACAACAGGGCA   12082
gb:MT039887|Organism:Severe      TCCATGCAGGGTGCTGTAGACATAAACAAGCTTTGTGAAGAAATGCTGGACAACAGGGCA   12082
gb:MT019530|Organism:Severe      TCCATGCAGGGTGCTGTAGACATAAACAAGCTTTGTGAAGAAATGCTGGACAACAGGGCA   12082
gb:MT039888|Organism:Severe      TCCATGCAGGGTGCTGTAGACATAAACAAGCTTTGTGAAGAAATGCTGGACAACAGGGCA   12082
gb:LC522972|Organism:Severe      TCCATGCAGGGTGCTGTAGACATAAACAAGCTTTGTGAAGAAATGCTGGACAACAGGGCA   12079
gb:MT027063|Organism:Severe      TCCATGCAGGGTGCTGTAGACATAAACAAGCTTTGTGAAGAAATGCTGGACAACAGGGCA   12082
gb:MT027062|Organism:Severe      TCCATGCAGGGTGCTGTAGACATAAACAAGCTTTGTGAAGAAATGCTGGACAACAGGGCA   12082
gb:MT019529|Organism:Severe      TCCATGCAGGGTGCTGTAGACATAAACAAGCTTTGTGAAGAAATGCTGGACAACAGGGCA   12082
gb:MN996529|Organism:Severe      TCCATGCAGGGTGCTGTAGACATAAACAAGCTTTGTGAAGAAATGCTGGACAACAGGGCA   12070
gb:MN996531|Organism:Severe      TCCATGCAGGGTGCTGTAGACATAAACAAGCTTTGTGAAGAAATGCTGGACAACAGGGCA   12069
gb:MT066176|Organism:Severe      TCCATGCAGGGTGCTGTAGACATAAACAAGCTTTGTGAAGAAATGCTGGACAACAGGGCA   12082
gb:MT027064|Organism:Severe      TCCATGCAGGGTGCTGTAGACATAAACAAGCTTTGTGAAGAAATGCTGGACAACAGGGCA   12082
gb:MN994468|Organism:Severe      TCCATGCAGGGTGCTGTAGACATAAACAAGCTTTGTGAAGAAATGCTGGACAACAGGGCA   12082
gb:MT072688|Organism:Severe      TCCATGCAGGGTGCTGTAGACATAAACAAGCTTTGTGAAGAAATGCTGGACAACAGGGCA   12067
gb:MN996527|Organism:Severe      TCCATGCAGGGTGCTGTAGACATAAACAAGCTTTGTGAAGAAATGCTGGACAACAGGGCA   12049
gb:MT093631|Organism:Severe      TCCATGCAGGGTGCTGTAGACATAAACAAGCTTTGTGAAGAAATGCTGGACAACAGGGCA   12120
gb:MT106053|Organism:Severe      TCCATGCAGGGTGCTGTAGACATAAACAAGCTTTGTGAAGAAATGCTGGACAACAGGGCA   12082
gb:MT019533|Organism:Severe      TCCATGCAGGGTGCTGTAGACATAAACAAGCTTTGTGAAGAAATGCTGGACAACAGGGCA   12082
gb:MT019531|Organism:Severe      TCCATGCAGGGTGCTGTAGACATAAACAAGCTTTGTGAAGAAATGCTGGACAACAGGGCA   12082
gb:MN996528|Organism:Severe      TCCATGCAGGGTGCTGTAGACATAAACAAGCTTTGTGAAGAAATGCTGGACAACAGGGCA   12082
gb:MN996530|Organism:Severe      TCCATGCAGGGTGCTGTAGACATAAACAAGCTTTGTGAAGAAATGCTGGACAACAGGGCA   12068
gb:MN908947|Organism:Severe      TCCATGCAGGGTGCTGTAGACATAAACAAGCTTTGTGAAGAAATGCTGGACAACAGGGCA   12082
gb:MT019532|Organism:Severe      TCCATGCAGGGTGCTGTAGACATAAACAAGCTTTGTGAAGAAATGCTGGACAACAGGGCA   12082
                                 ************************************************************
```

```
gb:MT020781|Organism:Severe      ACCTTACAAGCTATAGCCTCAGAGTTTAGTTCCCTTCCATCATATGCAGCTTTTGCTACT   12130
gb:MT007544|Organism:Severe      ACCTTACAAGCTATAGCCTCAGAGTTTAGTTCCCTTCCATCATATGCAGCTTTTGCTACT   12142
gb:MN994467|Organism:Severe      ACCTTACAAGCTATAGCCTCAGAGTTTAGTTCCCTTCCATCATATGCAGCTTTTGCTACT   12142
gb:MT044257|Organism:Severe      ACCTTACAAGCTATAGCCTCAGAGTTTAGTTCCCTTCCATCATATGCAGCTTTTGCTACT   12142
gb:MT106054|Organism:Severe      ACCTTACAAGCTATAGCCTCAGAGTTTAGTTCCCTTCCATCATATGCAGCTTTTGCTACT   12142
gb:MT049951|Organism:Severe      ACCTTACAAGCTATAGCCTCAGAGTTTAGTTCCCTTCCATCATATGCAGCTTTTGCTACT   12142
gb:MN975262|Organism:Severe      ACCTTACAAGCTATAGCCTCAGAGTTTAGTTCCCTTCCATCATATGCAGCTTTTGCTACT   12142
gb:MT106052|Organism:Severe      ACCTTACAAGCTATAGCCTCAGAGTTTAGTTCCCTTCCATCATATGCAGCTTTTGCTACT   12142
gb:LC522975|Organism:Severe      ACCTTACAAGCTATAGCCTCAGAGTTTAGTTCCCTTCCATCATATGCAGCTTTTGCTACT   12139
gb:LC522973|Organism:Severe      ACCTTACAAGCTATAGCCTCAGAGTTTAGTTCCCTTCCATCATATGCAGCTTTTGCTACT   12139
gb:LC522974|Organism:Severe      ACCTTACAAGCTATAGCCTCAGAGTTTAGTTCCCTTCCATCATATGCAGCTTTTGCTACT   12139
gb:MN985325|Organism:Severe      ACCTTACAAGCTATAGCCTCAGAGTTTAGTTCCCTTCCATCATATGCAGCTTTTGCTACT   12142
gb:MT020881|Organism:Severe      ACCTTACAAGCTATAGCCTCAGAGTTTAGTTCCCTTCCATCATATGCAGCTTTTGCTACT   12142
gb:MT020880|Organism:Severe      ACCTTACAAGCTATAGCCTCAGAGTTTAGTTCCCTTCCATCATATGCAGCTTTTGCTACT   12142
gb:MT066175|Organism:Severe      ACCTTACAAGCTATAGCCTCAGAGTTTAGTTCCCTTCCATCATATGCAGCTTTTGCTACT   12142
gb:MN997409|Organism:Severe      ACCTTACAAGCTATAGCCTCAGAGTTTAGTTCCCTTCCATCATATGCAGCTTTTGCTACT   12142
gb:MN938384|Organism:Severe      ACCTTACAAGCTATAGCCTCAGAGTTTAGTTCCCTTCCATCATATGCAGCTTTTGCTACT   12110
gb:MT044258|Organism:Severe      ACCTTACAAGCTATAGCCTCAGAGTTTAGTTCCCTTCCATCATATGCAGCTTTTGCTACT   12118
gb:MT039890|Organism:Severe      ACCTTACAAGCTATAGCCTCAGAGTTTAGTTCTCTTCCATCATATGCAGCTTTTGCTACT   12142
gb:MN988713|Organism:Severe      ACCTTACAAGCTATAGCCTCAGAGTTTAGTTCCCTTCCATCATATGCAGCTTTTGCTACT   12142
gb:LC521925|Organism:Severe      ACCTTACAAGCTATAGCCTCAGAGTTTAGTTCCCTTCCATCATATGCAGCTTTTGCTACT   12115
gb:MT093571|Organism:Severe      ACCTTACAAGCTATAGCCTCAGAGTTTAGTTCCCTTCCATCATATGCAGCTTTTGCTACT   12142
gb:MT039887|Organism:Severe      ACCTTACAAGCTATAGCCTCAGAGTTTAGTTCCCTTCCATCATATGCAGCTTTTGCTACT   12142
gb:MT019530|Organism:Severe      ACCTTACAAGCTATAGCCTCAGAGTTTAGTTCCCTTCCATCATATGCAGCTTTTGCTACT   12142
gb:MT039888|Organism:Severe      ACCTTACAAGCTATAGCCTCAGAGTTTAGTTCCCTTCCATCATATGCAGCTTTTGCTACT   12142
gb:LC522972|Organism:Severe      ACCTTACAAGCTATAGCCTCAGAGTTTAGTTCCCTTCCATCATATGCAGCTTTTGCTACT   12139
gb:MT027063|Organism:Severe      ACCTTACAAGCTATAGCCTCAGAGTTTAGTTCCCTTCCATCATATGCAGCTTTTGCTACT   12142
gb:MT027062|Organism:Severe      ACCTTACAAGCTATAGCCTCAGAGTTTAGTTCCCTTCCATCATATGCAGCTTTTGCTACT   12142
gb:MT019529|Organism:Severe      ACCTTACAAGCTATAGCCTCAGAGTTTAGTTCCCTTCCATCATATGCAGCTTTTGCTACT   12142
gb:MN996529|Organism:Severe      ACCTTACAAGCTATAGCCTCAGAGTTTAGTTCCCTTCCATCATATGCAGCTTTTGCTACT   12130
gb:MN996531|Organism:Severe      ACCTTACAAGCTATAGCCTCAGAGTTTAGTTCCCTTCCATCATATGCAGCTTTTGCTACT   12129
gb:MT066176|Organism:Severe      ACCTTACAAGCTATAGCCTCAGAGTTTAGTTCCCTTCCATCATATGCAGCTTTTGCTACT   12142
gb:MT027064|Organism:Severe      ACCTTACAAGCTATAGCCTCAGAGTTTAGTTCCCTTCCATCATATGCAGCTTTTGCTACT   12142
gb:MN994468|Organism:Severe      ACCTTACAAGCTATAGCCTCAGAGTTTAGTTCCCTTCCATCATATGCAGCTTTTGCTACT   12142
gb:MT072688|Organism:Severe      ACCTTACAAGCTATAGCCTCAGAGTTTAGTTCCCTTCCATCATATGCAGCTTTTGCTACT   12127
gb:MN996527|Organism:Severe      ACCTTACAAGCTATAGCCTCAGAGTTTAGTTCCCTTCCATCATATGCAGCTTTTGCTACT   12109
gb:MT093631|Organism:Severe      ACCTTACAAGCTATAGCCTCAGAGTTTAGTTCCCTTCCATCATATGCAGCTTTTGCTACT   12180
gb:MT106053|Organism:Severe      ACCTTACAAGCTATAGCCTCAGAGTTTAGTTCCCTTCCATCATATGCAGCTTTTGCTACT   12142
gb:MT019533|Organism:Severe      ACCTTACAAGCTATAGCCTCAGAGTTTAGTTCCCTTCCATCATATGCAGCTTTTGCTACT   12142
gb:MT019531|Organism:Severe      ACCTTACAAGCTATAGCCTCAGAGTTTAGTTCCCTTCCATCATATGCAGCTTTTGCTACT   12142
gb:MN996528|Organism:Severe      ACCTTACAAGCTATAGCCTCAGAGTTTAGTTCCCTTCCATCATATGCAGCTTTTGCTACT   12142
gb:MN996530|Organism:Severe      ACCTTACAAGCTATAGCCTCAGAGTTTAGTTCCCTTCCATCATATGCAGCTTTTGCTACT   12128
gb:MN908947|Organism:Severe      ACCTTACAAGCTATAGCCTCAGAGTTTAGTTCCCTTCCATCATATGCAGCTTTTGCTACT   12142
gb:MT019532|Organism:Severe      ACCTTACAAGCTATAGCCTCAGAGTTTAGTTCCCTTCCATCATATGCAGCTTTTGCTACT   12142
                                 ******************************** ***************************

gb:MT020781|Organism:Severe      GCTCAAGAAGCTTATGAGCAGGCTGTTGCTAATGGTGATTCTGAAGTTGTTCTTAAAAAG   12190
gb:MT007544|Organism:Severe      GCTCAAGAAGCTTATGAGCAGGCTGTTGCTAATGGTGATTCTGAAGTTGTTCTTAAAAAG   12202
gb:MN994467|Organism:Severe      GCTCAAGAAGCTTATGAGCAGGCTGTTGCTAATGGTGATTCTGAAGTTGTTCTTAAAAAG   12202
gb:MT044257|Organism:Severe      GCTCAAGAAGCTTATGAGCAGGCTGTTGCTAATGGTGATTCTGAAGTTGTTCTTAAAAAG   12202
gb:MT106054|Organism:Severe      GCTCAAGAAGCTTATGAGCAGGCTGTTGCTAATGGTGATTCTGAAGTTGTTCTTAAAAAG   12202
gb:MT049951|Organism:Severe      GCTCAAGAAGCTTATGAGCAGGCTGTTGCTAATGGTGATTCTGAAGTTGTTCTTAAAAAG   12202
gb:MN975262|Organism:Severe      GCTCAAGAAGCTTATGAGCAGGCTGTTGCTAATGGTGATTCTGAAGTTGTTCTTAAAAAG   12202
gb:MT106052|Organism:Severe      GCTCAAGAAGCTTATGAGCAGGCTGTTGCTAATGGTGATTCTGAAGTTGTTCTTAAAAAG   12202
gb:LC522975|Organism:Severe      GCTCAAGAAGCTTATGAGCAGGCTGTTGCTAATGGTGATTCTGAAGTTGTTCTTAAAAAG   12199
gb:LC522973|Organism:Severe      GCTCAAGAAGCTTATGAGCAGGCTGTTGCTAATGGTGATTCTGAAGTTGTTCTTAAAAAG   12199
gb:LC522974|Organism:Severe      GCTCAAGAAGCTTATGAGCAGGCTGTTGCTAATGGTGATTCTGAAGTTGTTCTTAAAAAG   12199
gb:MN985325|Organism:Severe      GCTCAAGAAGCTTATGAGCAGGCTGTTGCTAATGGTGATTCTGAAGTTGTTCTTAAAAAG   12202
gb:MT020881|Organism:Severe      GCTCAAGAAGCTTATGAGCAGGCTGTTGCTAATGGTGATTCTGAAGTTGTTCTTAAAAAG   12202
gb:MT020880|Organism:Severe      GCTCAAGAAGCTTATGAGCAGGCTGTTGCTAATGGTGATTCTGAAGTTGTTCTTAAAAAG   12202
gb:MT066175|Organism:Severe      GCTCAAGAAGCTTATGAGCAGGCTGTTGCTAATGGTGATTCTGAAGTTGTTCTTAAAAAG   12202
gb:MN997409|Organism:Severe      GCTCAAGAAGCTTATGAGCAGGCTGTTGCTAATGGTGATTCTGAAGTTGTTCTTAAAAAG   12202
gb:MN938384|Organism:Severe      GCTCAAGAAGCTTATGAGCAGGCTGTTGCTAATGGTGATTCTGAAGTTGTTCTTAAAAAG   12170
gb:MT044258|Organism:Severe      GCTCAAGAAGCTTATGAGCAGGCTGTTGCTAATGGTGATTCTGAAGTTGTTCTTAAAAAG   12178
```

```
gb:MT039890|Organism:Severe      GCTCAAGAAGCTTATGAGCAGGCTGTTGCTAATGGTGATTCTGAAGTTGTTCTTAAAAAG   12202
gb:MN988713|Organism:Severe      GCTCAAGAAGCTTATGAGCAGGCTGTTGCTAATGGTGATTCTGAAGTTGTTCTTAAAAAG   12202
gb:LC521925|Organism:Severe      GCTCAAGAAGCTTATGAGCAGGCTGTTGCTAATGGTGATTCTGAAGTTGTTCTTAAAAAG   12175
gb:MT093571|Organism:Severe      GCTCAAGAAGCTTATGAGCAGGCTGTTGCTAATGGTGATTCTGAAGTTGTTCTTAAAAAG   12202
gb:MT039887|Organism:Severe      GCTCAAGAAGCTTATGAGCAGGCTGTTGCTAATGGTGATTCTGAAGTTGTTCTTAAAAAG   12202
gb:MT019530|Organism:Severe      GCTCAAGAAGCTTATGAGCAGGCTGTTGCTAATGGTGATTCTGAAGTTGTTCTTAAAAAG   12202
gb:MT039888|Organism:Severe      GCTCAAGAAGCTTATGAGCAGGCTGTTGCTAATGGTGATTCTGAAGTTGTTCTTAAAAAG   12202
gb:LC522972|Organism:Severe      GCTCAAGAAGCTTATGAGCAGGCTGTTGCTAATGGTGATTCTGAAGTTGTTCTTAAAAAG   12199
gb:MT027063|Organism:Severe      GCTCAAGAAGCTTATGAGCAGGCTGTTGCTAATGGTGATTCTGAAGTTGTTCTTAAAAAG   12202
gb:MT027062|Organism:Severe      GCTCAAGAAGCTTATGAGCAGGCTGTTGCTAATGGTGATTCTGAAGTTGTTCTTAAAAAG   12202
gb:MT019529|Organism:Severe      GCTCAAGAAGCTTATGAGCAGGCTGTTGCTAATGGTGATTCTGAAGTTGTTCTTAAAAAG   12202
gb:MN996529|Organism:Severe      GCTCAAGAAGCTTATGAGCAGGCTGTTGCTAATGGTGATTCTGAAGTTGTTCTTAAAAAG   12190
gb:MN996531|Organism:Severe      GCTCAAGAAGCTTATGAGCAGGCTGTTGCTAATGGTGATTCTGAAGTTGTTCTTAAAAAG   12189
gb:MT066176|Organism:Severe      GCTCAAGAAGCTTATGAGCAGGCTGTTGCTAATGGTGATTCTGAAGTTGTTCTTAAAAAG   12202
gb:MT027064|Organism:Severe      GCTCAAGAAGCTTATGAGCAGGCTGTTGCTAATGGTGATTCTGAAGTTGTTCTTAAAAAG   12202
gb:MN994468|Organism:Severe      GCTCAAGAAGCTTATGAGCAGGCTGTTGCTAATGGTGATTCTGAAGTTGTTCTTAAAAAG   12202
gb:MT072688|Organism:Severe      GCTCAAGAAGCTTATGAGCAGGCTGTTGCTAATGGTGATTCTGAAGTTGTTCTTAAAAAG   12187
gb:MN996527|Organism:Severe      GCTCAAGAAGCTTATGAGCAGGCTGTTGCTAATGGTGATTCTGAAGTTGTTCTTAAAAAG   12169
gb:MT093631|Organism:Severe      GCTCAAGAAGCTTATGAGCAGGCTGTTGCTAATGGTGATTCTGAAGTTGTTCTTAAAAAG   12240
gb:MT106053|Organism:Severe      GCTCAAGAAGCTTATGAGCAGGCTGTTGCTAATGGTGATTCTGAAGTTGTTCTTAAAAAG   12202
gb:MT019533|Organism:Severe      GCTCAAGAAGCTTATGAGCAGGCTGTTGCTAATGGTGATTCTGAAGTTGTTCTTAAAAAG   12202
gb:MT019531|Organism:Severe      GCTCAAGAAGCTTATGAGCAGGCTGTTGCTAATGGTGATTCTGAAGTTGTTCTTAAAAAG   12202
gb:MN996528|Organism:Severe      GCTCAAGAAGCTTATGAGCAGGCTGTTGCTAATGGTGATTCTGAAGTTGTTCTTAAAAAG   12202
gb:MN996530|Organism:Severe      GCTCAAGAAGCTTATGAGCAGGCTGTTGCTAATGGTGATTCTGAAGTTGTTCTTAAAAAG   12188
gb:MN908947|Organism:Severe      GCTCAAGAAGCTTATGAGCAGGCTGTTGCTAATGGTGATTCTGAAGTTGTTCTTAAAAAG   12202
gb:MT019532|Organism:Severe      GCTCAAGAAGCTTATGAGCAGGCTGTTGCTAATGGTGATTCTGAAGTTGTTCTTAAAAAG   12202
                                 ************************************************************

gb:MT020781|Organism:Severe      TTGAAGAAGTCTTTGAATGTGGCTAAATCTGAATTTGACCGTGATGCAGCCATGCAACGT   12250
gb:MT007544|Organism:Severe      TTGAAGAAGTCTTTGAATGTGGCTAAATCTGAATTTGACCGTGATGCAGCCATGCAACGT   12262
gb:MN994467|Organism:Severe      TTGAAGAAGTCTTTGAATGTGGCTAAATCTGAATTTGACCGTGATGCAGCCATGCAACGT   12262
gb:MT044257|Organism:Severe      TTGAAGAAGTCTTTGAATGTGGCTAAATCTGAATTTGACCGTGATGCAGCCATGCAACGT   12262
gb:MT106054|Organism:Severe      TTGAAGAAGTCTTTGAATGTGGCTAAATCTGAATTTGACCGTGATGCAGCCATGCAACGT   12262
gb:MT049951|Organism:Severe      TTGAAGAAGTCTTTGAATGTGGCTAAATCTGAATTTGACCGTGATGCAGCCATGCAACGT   12262
gb:MN975262|Organism:Severe      TTGAAGAAGTCTTTGAATGTGGCTAAATCTGAATTTGACCGTGATGCAGCCATGCAACGT   12262
gb:MT106052|Organism:Severe      TTGAAGAAGTCTTTGAATGTGGCTAAATCTGAATTTGACCGTGATGCAGCCATGCAACGT   12262
gb:LC522975|Organism:Severe      TTGAAGAAGTCTTTGAATGTGGCTAAATCTGAATTTGACCGTGATGCAGCCATGCAACGT   12259
gb:LC522973|Organism:Severe      TTGAAGAAGTCTTTGAATGTGGCTAAATCTGAATTTGACCGTGATGCAGCCATGCAACGT   12259
gb:LC522974|Organism:Severe      TTGAAGAAGTCTTTGAATGTGGCTAAATCTGAATTTGACCGTGATGCAGCCATGCAACGT   12259
gb:MN985325|Organism:Severe      TTGAAGAAGTCTTTGAATGTGGCTAAATCTGAATTTGACCGTGATGCAGCCATGCAACGT   12262
gb:MT020881|Organism:Severe      TTGAAGAAGTCTTTGAATGTGGCTAAATCTGAATTTGACCGTGATGCAGCCATGCAACGT   12262
gb:MT020880|Organism:Severe      TTGAAGAAGTCTTTGAATGTGGCTAAATCTGAATTTGACCGTGATGCAGCCATGCAACGT   12262
gb:MT066175|Organism:Severe      TTGAAGAAGTCTTTGAATGTGGCTAAATCTGAATTTGACCGTGATGCAGCCATGCAACGT   12262
gb:MN997409|Organism:Severe      TTGAAGAAGTCTTTGAATGTGGCTAAATCTGAATTTGACCGTGATGCAGCCATGCAACGT   12262
gb:MN938384|Organism:Severe      TTGAAGAAGTCTTTGAATGTGGCTAAATCTGAATTTGACCGTGATGCAGCCATGCAACGT   12230
gb:MT044258|Organism:Severe      TTGAAGAAGTCTTTGAATGTGGCTAAATCTGAATTTGACCGTGATGCAGCCATGCAACGT   12238
gb:MT039890|Organism:Severe      TTGAAGAAGTCTTTGAATGTGGCTAAATCTGAATTTGACCGTGATGCAGCCATGCAACGT   12262
gb:MN988713|Organism:Severe      TTGAAGAAGTCTTTGAATGTGGCTAAATCTGAATTTGACCGTGATGCAGCCATGCAACGT   12262
gb:LC521925|Organism:Severe      TTGAAGAAGTCTTTGAATGTGGCTAAATCTGAATTTGACCGTGATGCAGCCATGCAACGT   12235
gb:MT093571|Organism:Severe      TTGAAGAAGTCTTTGAATGTGGCTAAATCTGAATTTGACCGTGATGCAGCCATGCAACGT   12262
gb:MT039887|Organism:Severe      TTGAAGAAGTCTTTGAATGTGGCTAAATCTGAATTTGACCGTGATGCAGCCATGCAACGT   12262
gb:MT019530|Organism:Severe      TTGAAGAAGTCTTTGAATGTGGCTAAATCTGAATTTGACCGTGATGCAGCCATGCAACGT   12262
gb:MT039888|Organism:Severe      TTGAAGAAGTCTTTGAATGTGGCTAAATCTGAATTTGACCGTGATGCAGCCATGCAACGT   12262
gb:LC522972|Organism:Severe      TTGAAGAAGTCTTTGAATGTGGCTAAATCTGAATTTGACCGTGATGCAGCCATGCAACGT   12259
gb:MT027063|Organism:Severe      TTGAAGAAGTCTTTGAATGTGGCTAAATCTGAATTTGACCGTGATGCAGCCATGCAACGT   12262
gb:MT027062|Organism:Severe      TTGAAGAAGTCTTTGAATGTGGCTAAATCTGAATTTGACCGTGATGCAGCCATGCAACGT   12262
gb:MT019529|Organism:Severe      TTGAAGAAGTCTTTGAATGTGGCTAAATCTGAATTTGACCGTGATGCAGCCATGCAACGT   12262
gb:MN996529|Organism:Severe      TTGAAGAAGTCTTTGAATGTGGCTAAATCTGAATTTGACCGTGATGCAGCCATGCAACGT   12250
gb:MN996531|Organism:Severe      TTGAAGAAGTCTTTGAATGTGGCTAAATCTGAATTTGACCGTGATGCAGCCATGCAACGT   12249
gb:MT066176|Organism:Severe      TTGAAGAAGTCTTTGAATGTGGCTAAATCTGAATTTGACCGTGATGCAGCCATGCAACGT   12262
gb:MT027064|Organism:Severe      TTGAAGAAGTCTTTGAATGTGGCTAAATCTGAATTTGACCGTGATGCAGCCATGCAACGT   12262
gb:MN994468|Organism:Severe      TTGAAGAAGTCTTTGAATGTGGCTAAATCTGAATTTGACCGTGATGCAGCCATGCAACGT   12262
gb:MT072688|Organism:Severe      TTGAAGAAGTCTTTGAATGTGGCTAAATCTGAATTTGACCGTGATGCAGCCATGCAACGT   12247
gb:MN996527|Organism:Severe      TTGAAGAAGTCTTTGAATGTGGCTAAATCTGAATTTGACCGTGATGCAGCCATGCAACGT   12229
gb:MT093631|Organism:Severe      TTGAAGAAGTCTTTGAATGTGGCTAAATCTGAATTTGACCGTGATGCAGCCATGCAACGT   12300
```

```
gb:MT106053|Organism:Severe      TTGAAGAAGTCTTTGAATGTGGCTAAATCTGAATTTGACCGTGATGCAGCCATGCAACGT    12262
gb:MT019533|Organism:Severe      TTGAAGAAGTCTTTGAATGTGGCTAAATCTGAATTTGACCGTGATGCAGCCATGCAACGT    12262
gb:MT019531|Organism:Severe      TTGAAGAAGTCTTTGAATGTGGCTAAATCTGAATTTGACCGTGATGCAGCCATGCAACGT    12262
gb:MN996528|Organism:Severe      TTGAAGAAGTCTTTGAATGTGGCTAAATCTGAATTTGACCGTGATGCAGCCATGCAACGT    12262
gb:MN996530|Organism:Severe      TTGAAGAAGTCTTTGAATGTGGCTAAATCTGAATTTGACCGTGATGCAGCCATGCAACGT    12248
gb:MN908947|Organism:Severe      TTGAAGAAGTCTTTGAATGTGGCTAAATCTGAATTTGACCGTGATGCAGCCATGCAACGT    12262
gb:MT019532|Organism:Severe      TTGAAGAAGTCTTTGAATGTGGCTAAATCTGAATTTGACCGTGATGCAGCCATGCAACGT    12262
                                 ************************************************************

gb:MT020781|Organism:Severe      AAGTTGGAAAAGATGGCTGATCAAGCTATGACCCAAATGTATAAACAGGCTAGATCTGAG    12310
gb:MT007544|Organism:Severe      AAGTTGGAAAAGATGGCTGATCAAGCTATGACCCAAATGTATAAACAGGCTAGATCTGAG    12322
gb:MN994467|Organism:Severe      AAGTTGGAAAAGATGGCTGATCAAGCTATGACCCAAATGTATAAACAGGCTAGATCTGAG    12322
gb:MT044257|Organism:Severe      AAGTTGGAAAAGATGGCTGATCAAGCTATGACCCAAATGTATAAACAGGCTAGATCTGAG    12322
gb:MT106054|Organism:Severe      AAGTTGGAAAAGATGGCTGATCAAGCTATGACCCAAATGTATAAACAGGCTAGATCTGAG    12322
gb:MT049951|Organism:Severe      AAGTTGGAAAAGATGGCTGATCAAGCTATGACCCAAATGTATAAACAGGCTAGATCTGAG    12322
gb:MN975262|Organism:Severe      AAGTTGGAAAAGATGGCTGATCAAGCTATGACCCAAATGTATAAACAGGCTAGATCTGAG    12322
gb:MT106052|Organism:Severe      AAGTTGGAAAAGATGGCTGATCAAGCTATGACCCAAATGTATAAACAGGCTAGATCTGAG    12322
gb:LC522975|Organism:Severe      AAGTTGGAAAAGATGGCTGATCAAGCTATGACCCAAATGTATAAACAGGCTAGATCTGAG    12319
gb:LC522973|Organism:Severe      AAGTTGGAAAAGATGGCTGATCAAGCTATGACCCAAATGTATAAACAGGCTAGATCTGAG    12319
gb:LC522974|Organism:Severe      AAGTTGGAAAAGATGGCTGATCAAGCTATGACCCAAATGTATAAACAGGCTAGATCTGAG    12319
gb:MN985325|Organism:Severe      AAGTTGGAAAAGATGGCTGATCAAGCTATGACCCAAATGTATAAACAGGCTAGATCTGAG    12322
gb:MT020881|Organism:Severe      AAGTTGGAAAAGATGGCTGATCAAGCTATGACCCAAATGTATAAACAGGCTAGATCTGAG    12322
gb:MT020880|Organism:Severe      AAGTTGGAAAAGATGGCTGATCAAGCTATGACCCAAATGTATAAACAGGCTAGATCTGAG    12322
gb:MT066175|Organism:Severe      AAGTTGGAAAAGATGGCTGATCAAGCTATGACCCAAATGTATAAACAGGCTAGATCTGAG    12322
gb:MN997409|Organism:Severe      AAGTTGGAAAAGATGGCTGATCAAGCTATGACCCAAATGTATAAACAGGCTAGATCTGAG    12322
gb:MN938384|Organism:Severe      AAGTTGGAAAAGATGGCTGATCAAGCTATGACCCAAATGTATAAACAGGCTAGATCTGAG    12290
gb:MT044258|Organism:Severe      AAGTTGGAAAAGATGGCTGATCAAGCTATGACCCAAATGTATAAACAGGCTAGATCTGAG    12298
gb:MT039890|Organism:Severe      AAGTTGGAAAAGATGGCTGATCAAGCTATGACCCAAATGTATAAACAGGCTAGATCTGAG    12322
gb:MN988713|Organism:Severe      AAGTTGGAAAAGATGGCTGATCAAGCTATGACCCAAATGTATAAACAGGCTAGATCTGAG    12322
gb:LC521925|Organism:Severe      AAGTTGGAAAAGATGGCTGATCAAGCTATGACCCAAATGTATAAACAGGCTAGATCTGAG    12295
gb:MT093571|Organism:Severe      AAGTTGGAAAAGATGGCTGATCAAGCTATGACCCAAATGTATAAACAGGCTAGATCTGAG    12322
gb:MT039887|Organism:Severe      AAGTTGGAAAAGATGGCTGATCAAGCTATGACCCAAATGTATAAACAGGCTAGATCTGAG    12322
gb:MT019530|Organism:Severe      AAGTTGGAAAAGATGGCTGATCAAGCTATGACCCAAATGTATAAACAGGCTAGATCTGAG    12322
gb:MT039888|Organism:Severe      AAGTTGGAAAAGATGGCTGATCAAGCTATGACCCAAATGTATAAACAGGCTAGATCTGAG    12322
gb:LC522972|Organism:Severe      AAGTTGGAAAAGATGGCTGATCAAGCTATGACCCAAATGTATAAACAGGCTAGATCTGAG    12319
gb:MT027063|Organism:Severe      AAGTTGGAAAAGATGGCTGATCAAGCTATGACCCAAATGTATAAACAGGCTAGATCTGAG    12322
gb:MT027062|Organism:Severe      AAGTTGGAAAAGATGGCTGATCAAGCTATGACCCAAATGTATAAACAGGCTAGATCTGAG    12322
gb:MT019529|Organism:Severe      AAGTTGGAAAAGATGGCTGATCAAGCTATGACCCAAATGTATAAACAGGCTAGATCTGAG    12322
gb:MN996529|Organism:Severe      AAGTTGGAAAAGATGGCTGATCAAGCTATGACCCAAATGTATAAACAGGCTAGATCTGAG    12310
gb:MN996531|Organism:Severe      AAGTTGGAAAAGATGGCTGATCAAGCTATGACCCAAATGTATAAACAGGCTAGATCTGAG    12309
gb:MT066176|Organism:Severe      AAGTTGGAAAAGATGGCTGATCAAGCTATGACCCAAATGTATAAACAGGCTAGATCTGAG    12322
gb:MT027064|Organism:Severe      AAGTTGGAAAAGATGGCTGATCAAGCTATGACCCAAATGTATAAACAGGCTAGATCTGAG    12322
gb:MN994468|Organism:Severe      AAGTTGGAAAAGATGGCTGATCAAGCTATGACCCAAATGTATAAACAGGCTAGATCTGAG    12322
gb:MT072688|Organism:Severe      AAGTTGGAAAAGATGGCTGATCAAGCTATGACCCAAATGTATAAACAGGCTAGATCTGAG    12307
gb:MN996527|Organism:Severe      AAGTTGGAAAAGATGGCTGATCAAGCTATGACCCAAATGTATAAACAGGCTAGATCTGAG    12289
gb:MT093631|Organism:Severe      AAGTTGGAAAAGATGGCTGATCAAGCTATGACCCAAATGTATAAACAGGCTAGATCTGAG    12360
gb:MT106053|Organism:Severe      AAGTTGGAAAAGATGGCTGATCAAGCTATGACCCAAATGTATAAACAGGCTAGATCTGAG    12322
gb:MT019533|Organism:Severe      AAGTTGGAAAAGATGGCTGATCAAGCTATGACCCAAATGTATAAACAGGCTAGATCTGAG    12322
gb:MT019531|Organism:Severe      AAGTTGGAAAAGATGGCTGATCAAGCTATGACCCAAATGTATAAACAGGCTAGATCTGAG    12322
gb:MN996528|Organism:Severe      AAGTTGGAAAAGATGGCTGATCAAGCTATGACCCAAATGTATAAACAGGCTAGATCTGAG    12322
gb:MN996530|Organism:Severe      AAGTTGGAAAAGATGGCTGATCAAGCTATGACCCAAATGTATAAACAGGCTAGATCTGAG    12308
gb:MN908947|Organism:Severe      AAGTTGGAAAAGATGGCTGATCAAGCTATGACCCAAATGTATAAACAGGCTAGATCTGAG    12322
gb:MT019532|Organism:Severe      AAGTTGGAAAAGATGGCTGATCAAGCTATGACCCAAATGTATAAACAGGCTAGATCTGAG    12322
                                 ************************************************************

gb:MT020781|Organism:Severe      GACAAGAGGGCAAAAGTTACTAGTGCTATGCAGACAATGCTTTTCACTATGCTTAGAAAG    12370
gb:MT007544|Organism:Severe      GACAAGAGGGCAAAAGTTACTAGTGCTATGCAGACAATGCTTTTCACTATGCTTAGAAAG    12382
gb:MN994467|Organism:Severe      GACAAGAGGGCAAAAGTTACTAGTGCTATGCAGACAATGCTTTTCACTATGCTTAGAAAG    12382
gb:MT044257|Organism:Severe      GACAAGAGGGCAAAAGTTACTAGTGCTATGCAGACAATGCTTTTCACTATGCTTAGAAAG    12382
gb:MT106054|Organism:Severe      GACAAGAGGGCAAAAGTTACTAGTGCTATGCAGACAATGCTTTTCACTATGCTTAGAAAG    12382
gb:MT049951|Organism:Severe      GACAAGAGGGCAAAAGTTACTAGTGCTATGCAGACAATGCTTTTCACTATGCTTAGAAAG    12382
gb:MN975262|Organism:Severe      GACAAGAGGGCAAAAGTTACTAGTGCTATGCAGACAATGCTTTTCACTATGCTTAGAAAG    12382
gb:MT106052|Organism:Severe      GACAAGAGGGCAAAAGTTACTAGTGCTATGCAGACAATGCTTTTCACTATGCTTAGAAAG    12382
gb:LC522975|Organism:Severe      GACAAGAGGGCAAAAGTTACTAGTGCTATGCAGACAATGCTTTTCACTATGCTTAGAAAG    12379
gb:LC522973|Organism:Severe      GACAAGAGGGCAAAAGTTACTAGTGCTATGCAGACAATGCTTTTCACTATGCTTAGAAAG    12379
```

```
gb:LC522974|Organism:Severe      GACAAGAGGGCAAAAGTTACTAGTGCTATGCAGACAATGCTTTTCACTATGCTTAGAAAG   12379
gb:MN985325|Organism:Severe      GACAAGAGGGCAAAAGTTACTAGTGCTATGCAGACAATGCTTTTCACTATGCTTAGAAAG   12382
gb:MT020881|Organism:Severe      GACAAGAGGGCAAAAGTTACTAGTGCTATGCAGACAATGCTTTTCACTATGCTTAGAAAG   12382
gb:MT020880|Organism:Severe      GACAAGAGGGCAAAAGTTACTAGTGCTATGCAGACAATGCTTTTCACTATGCTTAGAAAG   12382
gb:MT066175|Organism:Severe      GACAAGAGGGCAAAAGTTACTAGTGCTATGCAGACAATGCTTTTCACTATGCTTAGAAAG   12382
gb:MN997409|Organism:Severe      GACAAGAGGGCAAAAGTTACTAGTGCTATGCAGACAATGCTTTTCACTATGCTTAGAAAG   12382
gb:MN938384|Organism:Severe      GACAAGAGGGCAAAAGTTACTAGTGCTATGCAGACAATGCTTTTCACTATGCTTAGAAAG   12350
gb:MT044258|Organism:Severe      GACAAGAGGGCAAAAGTTACTAGTGCTATGCAGACAATGCTTTTCACTATGCTTAGAAAG   12358
gb:MT039890|Organism:Severe      GACAAGAGGGCAAAAGTTACTAGTGCTATGCAGACAATGCTTTTCACTATGCTTAGAAAG   12382
gb:MN988713|Organism:Severe      GACAAGAGGGCAAAAGTTACTAGTGCTATGCAGACAATGCTTTTCACTATGCTTAGAAAG   12382
gb:LC521925|Organism:Severe      GACAAGAGGGCAAAAGTTACTAGTGCTATGCAGACAATGCTTTTCACTATGCTTAGAAAG   12355
gb:MT093571|Organism:Severe      GACAAGAGGGCAAAAGTTACTAGTGCTATGCAGACAATGCTTTTCACTATGCTTAGAAAG   12382
gb:MT039887|Organism:Severe      GACAAGAGGGCAAAAGTTACTAGTGCTATGCAGACAATGCTTTTCACTATGCTTAGAAAG   12382
gb:MT019530|Organism:Severe      GACAAGAGGGCAAAAGTTACTAGTGCTATGCAGACAATGCTTTTCACTATGCTTAGAAAG   12382
gb:MT039888|Organism:Severe      GACAAGAGGGCAAAAGTTACTAGTGCTATGCAGACAATGCTTTTCACTATGCTTAGAAAG   12382
gb:LC522972|Organism:Severe      GACAAGAGGGCAAAAGTTACTAGTGCTATGCAGACAATGCTTTTCACTATGCTTAGAAAG   12379
gb:MT027063|Organism:Severe      GACAAGAGGGCAAAAGTTACTAGTGCTATGCAGACAATGCTTTTCACTATGCTTAGAAAG   12382
gb:MT027062|Organism:Severe      GACAAGAGGGCAAAAGTTACTAGTGCTATGCAGACAATGCTTTTCACTATGCTTAGAAAG   12382
gb:MT019529|Organism:Severe      GACAAGAGGGCAAAAGTTACTAGTGCTATGCAGACAATGCTTTTCACTATGCTTAGAAAG   12382
gb:MN996529|Organism:Severe      GACAAGAGGGCAAAAGTTACTAGTGCTATGCAGACAATGCTTTTCACTATGCTTAGAAAG   12370
gb:MN996531|Organism:Severe      GACAAGAGGGCAAAAGTTACTAGTGCTATGCAGACAATGCTTTTCACTATGCTTAGAAAG   12369
gb:MT066176|Organism:Severe      GACAAGAGGGCAAAAGTTACTAGTGCTATGCAGACAATGCTTTTCACTATGCTTAGAAAG   12382
gb:MT027064|Organism:Severe      GACAAGAGGGCAAAAGTTACTAGTGCTATGCAGACAATGCTTTTCACTATGCTTAGAAAG   12382
gb:MN994468|Organism:Severe      GACAAGAGGGCAAAAGTTACTAGTGCTATGCAGACAATGCTTTTCACTATGCTTAGAAAG   12382
gb:MT072688|Organism:Severe      GACAAGAGGGCAAAAGTTACTAGTGCTATGCAGACAATGCTTTTCACTATGCTTAGAAAG   12367
gb:MN996527|Organism:Severe      GACAAGAGGGCAAAAGTTACTAGTGCTATGCAGACAATGCTTTTCACTATGCTTAGAAAG   12349
gb:MT093631|Organism:Severe      GACAAGAGGGCAAAAGTTACTAGTGCTATGCAGACAATGCTTTTCACTATGCTTAGAAAG   12420
gb:MT106053|Organism:Severe      GACAAGAGGGCAAAAGTTACTAGTGCTATGCAGACAATGCTTTTCACTATGCTTAGAAAG   12382
gb:MT019533|Organism:Severe      GACAAGAGGGCAAAAGTTACTAGTGCTATGCAGACAATGCTTTTCACTATGCTTAGAAAG   12382
gb:MT019531|Organism:Severe      GACAAGAGGGCAAAAGTTACTAGTGCTATGCAGACAATGCTTTTCACTATGCTTAGAAAG   12382
gb:MN996528|Organism:Severe      GACAAGAGGGCAAAAGTTACTAGTGCTATGCAGACAATGCTTTTCACTATGCTTAGAAAG   12382
gb:MN996530|Organism:Severe      GACAAGAGGGCAAAAGTTACTAGTGCTATGCAGACAATGCTTTTCACTATGCTTAGAAAG   12368
gb:MN908947|Organism:Severe      GACAAGAGGGCAAAAGTTACTAGTGCTATGCAGACAATGCTTTTCACTATGCTTAGAAAG   12382
gb:MT019532|Organism:Severe      GACAAGAGGGCAAAAGTTACTAGTGCTATGCAGACAATGCTTTTCACTATGCTTAGAAAG   12382
                                 ************************************************************

gb:MT020781|Organism:Severe      TTGGATAATGATGCACTCAACAACATTATCAACAATGCAAGAGATGGTTGTGTTCCCTTG   12430
gb:MT007544|Organism:Severe      TTGGATAATGATGCACTCAACAACATTATCAACAATGCAAGAGATGGTTGTGTTCCCTTG   12442
gb:MN994467|Organism:Severe      TTGGATAATGATGCACTCAACAACATTATCAACAATGCAAGAGATGGTTGTGTTCCCTTG   12442
gb:MT044257|Organism:Severe      TTGGATAATGATGCACTCAACAACATTATCAACAATGCAAGAGATGGTTGTGTTCCCTTG   12442
gb:MT106054|Organism:Severe      TTGGATAATGATGCACTCAACAACATTATCAACAATGCAAGAGATGGTTGTGTTCCCTTG   12442
gb:MT049951|Organism:Severe      TTGGATAATGATGCACTCAACAACATTATCAACAATGCAAGAGATGGTTGTGTTCCCTTG   12442
gb:MN975262|Organism:Severe      TTGGATAATGATGCACTCAACAACATTATCAACAATGCAAGAGATGGTTGTGTTCCCTTG   12442
gb:MT106052|Organism:Severe      TTGGATAATGATGCACTCAACAACATTATCAACAATGCAAGAGATGGTTGTGTTCCCTTG   12442
gb:LC522975|Organism:Severe      TTGGATAATGATGCACTCAACAACATTATCAACAATGCAAGAGATGGTTGTGTTCCCTTG   12439
gb:LC522973|Organism:Severe      TTGGATAATGATGCACTCAACAACATTATCAACAATGCAAGAGATGGTTGTGTTCCCTTG   12439
gb:LC522974|Organism:Severe      TTGGATAATGATGCACTCAACAACATTATCAACAATGCAAGAGATGGTTGTGTTCCCTTG   12439
gb:MN985325|Organism:Severe      TTGGATAATGATGCACTCAACAACATTATCAACAATGCAAGAGATGGTTGTGTTCCCTTG   12442
gb:MT020881|Organism:Severe      TTGGATAATGATGCACTCAACAACATTATCAACAATGCAAGAGATGGTTGTGTTCCCTTG   12442
gb:MT020880|Organism:Severe      TTGGATAATGATGCACTCAACAACATTATCAACAATGCAAGAGATGGTTGTGTTCCCTTG   12442
gb:MT066175|Organism:Severe      TTGGATAATGATGCACTCAACAACATTATCAACAATGCAAGAGATGGTTGTGTTCCCTTG   12442
gb:MN997409|Organism:Severe      TTGGATAATGATGCACTCAACAACATTATCAACAATGCAAGAGATGGTTGTGTTCCCTTG   12442
gb:MN938384|Organism:Severe      TTGGATAATGATGCACTCAACAACATTATCAACAATGCAAGAGATGGTTGTGTTCCCTTG   12410
gb:MT044258|Organism:Severe      TTGGATAATGATGCACTCAACAACATTATCAACAATGCAAGAGATGGTTGTGTTCCCTTG   12418
gb:MT039890|Organism:Severe      TTGGATAATGATGCACTCAACAACATTATCAACAATGCAAGAGATGGTTGTGTTCCCTTG   12442
gb:MN988713|Organism:Severe      TTGGATAATGATGCACTCAACAACATTATCAACAATGCAAGAGATGGTTGTGTTCCCTTG   12442
gb:LC521925|Organism:Severe      TTGGATAATGATGCACTCAACAACATTATCAACAATGCAAGAGATGGTTGTGTTCCCTTG   12415
gb:MT093571|Organism:Severe      TTGGATAATGATGCACTCAACAACATTATCAACAATGCAAGAGATGGTTGTGTTCCCTTG   12442
gb:MT039887|Organism:Severe      TTGGATAATGATGCACTCAACAACATTATCAACAATGCAAGAGATGGTTGTGTTCCCTTG   12442
gb:MT019530|Organism:Severe      TTGGATAATGATGCACTCAACAACATTATCAACAATGCAAGAGATGGTTGTGTTCCCTTG   12442
gb:MT039888|Organism:Severe      TTGGATAATGATGCACTCAACAACATTATCAACAATGCAAGAGATGGTTGTGTTCCCTTG   12442
gb:LC522972|Organism:Severe      TTGGATAATGATGCACTCAACAACATTATCAACAATGCAAGAGATGGTTGTGTTCCCTTG   12439
gb:MT027063|Organism:Severe      TTGGATAATGATGCACTCAACAACATTATCAACAATGCAAGAGATGGTTGTGTTCCCTTG   12442
gb:MT027062|Organism:Severe      TTGGATAATGATGCACTCAACAACATTATCAACAATGCAAGAGATGGTTGTGTTCCCTTG   12442
gb:MT019529|Organism:Severe      TTGGATAATGATGCACTCAACAACATTATCAACAATGCAAGAGATGGTTGTGTTCCCTTG   12442
```

```
gb:MN996529|Organism:Severe      TTGGATAATGATGCACTCAACAACATTATCAACAATGCAAGAGATGGTTGTGTTCCCTTG   12430
gb:MN996531|Organism:Severe      TTGGATAATGATGCACTCAACAACATTATCAACAATGCAAGAGATGGTTGTGTTCCCTTG   12429
gb:MT066176|Organism:Severe      TTGGATAATGATGCACTCAACAACATTATCAACAATGCAAGAGATGGTTGTGTTCCCTTG   12442
gb:MT027064|Organism:Severe      TTGGATAATGATGCACTCAACAACATTATCAACAATGCAAGAGATGGTTGTGTTCCCTTG   12442
gb:MN994468|Organism:Severe      TTGGATAATGATGCACTCAACAACATTATCAACAATGCAAGAGATGGTTGTGTTCCCTTG   12442
gb:MT072688|Organism:Severe      TTGGATAATGATGCACTCAACAACATTATCAACAATGCAAGAGATGGTTGTGTTCCCTTG   12427
gb:MN996527|Organism:Severe      TTGGATAATGATGCACTCAACAACATTATCAACAATGCAAGAGATGGTTGTGTTCCCTTG   12409
gb:MT093631|Organism:Severe      TTGGATAATGATGCACTCAACAACATTATCAACAATGCAAGAGATGGTTGTGTTCCCTTG   12480
gb:MT106053|Organism:Severe      TTGGATAATGATGCACTCAACAACATTATCAACAATGCAAGAGATGGTTGTGTTCCCTTG   12442
gb:MT019533|Organism:Severe      TTGGATAATGATGCACTCAACAACATTATCAACAATGCAAGAGATGGTTGTGTTCCCTTG   12442
gb:MT019531|Organism:Severe      TTGGATAATGATGCACTCAACAACATTATCAACAATGCAAGAGATGGTTGTGTTCCCTTG   12442
gb:MN996528|Organism:Severe      TTGGATAATGATGCACTCAACAACATTATCAACAATGCAAGAGATGGTTGTGTTCCCTTG   12442
gb:MN996530|Organism:Severe      TTGGATAATGATGCACTCAACAACATTATCAACAATGCAAGAGATGGTTGTGTTCCCTTG   12428
gb:MN908947|Organism:Severe      TTGGATAATGATGCACTCAACAACATTATCAACAATGCAAGAGATGGTTGTGTTCCCTTG   12442
gb:MT019532|Organism:Severe      TTGGATAATGATGCACTCAACAACATTATCAACAATGCAAGAGATGGTTGTGTTCCCTTG   12442
                                 ************************************************************

gb:MT020781|Organism:Severe      AACATAATACCTCTTACAACAGCAGCCAAACTAATGGTTGTCATACCAGACTATAACACA   12490
gb:MT007544|Organism:Severe      AACATAATACCTCTTACAACAGCAGCCAAACTAATGGTTGTCATACCAGACTATAACACA   12502
gb:MN994467|Organism:Severe      AACATAATACCTCTTACAACAGCAGCCAAACTAATGGTTGTCATACCAGACTATAACACA   12502
gb:MT044257|Organism:Severe      AACATAATACCTCTTACAACAGCAGCCAAACTAATGGTTGTCATACCAGACTATAACACA   12502
gb:MT106054|Organism:Severe      AACATAATACCTCTTACAACAGCAGCCAAACTAATGGTTGTCATACCAGACTATAACACA   12502
gb:MT049951|Organism:Severe      AACATAATACCTCTTACAACAGCAGCCAAACTAATGGTTGTCATACCAGACTATAACACA   12502
gb:MN975262|Organism:Severe      AACATAATACCTCTTACAACAGCAGCCAAACTAATGGTTGTCATACCAGACTATAACACA   12502
gb:MT106052|Organism:Severe      AACATAATACCTCTTACAACAGCAGCCAAACTAATGGTTGTCATACCAGACTATAACACA   12502
gb:LC522975|Organism:Severe      AACATAATACCTCTTACAACAGCAGCCAAACTAATGGTTGTCATACCAGACTATAACACA   12499
gb:LC522973|Organism:Severe      AACATAATACCTCTTACAACAGCAGCCAAACTAATGGTTGTCATACCAGACTATAACACA   12499
gb:LC522974|Organism:Severe      AACATAATACCTCTTACAACAGCAGCCAAACTAATGGTTGTCATACCAGACTATAACACA   12499
gb:MN985325|Organism:Severe      AACATAATACCTCTTACAACAGCAGCCAAACTAATGGTTGTCATACCAGACTATAACACA   12502
gb:MT020881|Organism:Severe      AACATAATACCTCTTACAACAGCAGCCAAACTAATGGTTGTCATACCAGACTATAACACA   12502
gb:MT020880|Organism:Severe      AACATAATACCTCTTACAACAGCAGCCAAACTAATGGTTGTCATACCAGACTATAACACA   12502
gb:MT066175|Organism:Severe      AACATAATACCTCTTACAACAGCAGCCAAACTAATGGTTGTCATACCAGACTATAACACA   12502
gb:MN997409|Organism:Severe      AACATAATACCTCTTACAACAGCAGCCAAACTAATGGTTGTCATACCAGACTATAACACA   12502
gb:MN938384|Organism:Severe      AACATAATACCTCTTACAACAGCAGCCAAACTAATGGTTGTCATACCAGACTATAACACA   12470
gb:MT044258|Organism:Severe      AACATAATACCTCTTACAACAGCAGCCAAACTAATGGTTGTCATACCAGACTATAACACA   12478
gb:MT039890|Organism:Severe      AACATAATACCTCTTACAACAGCAGCCAAACTAATGGTTGTCATACCAGACTATAACACA   12502
gb:MN988713|Organism:Severe      AACATAATACCTCTTACAACAGCAGCCAAACTAATGGTTGTCATACCAGACTATAACACA   12502
gb:LC521925|Organism:Severe      AACATAATACCTCTTACAACAGCAGCCAAACTAATGGTTGTCATACCAGACTATAACACA   12475
gb:MT093571|Organism:Severe      AACATAATACCTCTTACAACAGCAGCCAAACTAATGGTTGTCATACCAGACTATAACACA   12502
gb:MT039887|Organism:Severe      AACATAATACCTCTTACAACAGCAGCCAAACTAATGGTTGTCATACCAGACTATAACACA   12502
gb:MT019530|Organism:Severe      AACATAATACCTCTTACAACAGCAGCCAAACTAATGGTTGTCATACCAGACTATAACACA   12502
gb:MT039888|Organism:Severe      AACATAATACCTCTTACAACAGCAGCCAAACTAATGGTTGTCATACCAGACTATAACACA   12502
gb:LC522972|Organism:Severe      AACATAATACCTCTTACAACAGCAGCCAAACTAATGGTTGTCATACCAGACTATAACACA   12499
gb:MT027063|Organism:Severe      AACATAATACCTCTTACAACAGCAGCCAAACTAATGGTTGTCATACCAGACTATAACACA   12502
gb:MT027062|Organism:Severe      AACATAATACCTCTTACAACAGCAGCCAAACTAATGGTTGTCATACCAGACTATAACACA   12502
gb:MT019529|Organism:Severe      AACATAATACCTCTTACAACAGCAGCCAAACTAATGGTTGTCATACCAGACTATAACACA   12502
gb:MN996529|Organism:Severe      AACATAATACCTCTTACAACAGCAGCCAAACTAATGGTTGTCATACCAGACTATAACACA   12490
gb:MN996531|Organism:Severe      AACATAATACCTCTTACAACAGCAGCCAAACTAATGGTTGTCATACCAGACTATAACACA   12489
gb:MT066176|Organism:Severe      AACATAATACCTCTTACAACAGCAGCCAAACTAATGGTTGTCATACCAGACTATAACACA   12502
gb:MT027064|Organism:Severe      AACATAATACCTCTTACAACAGCAGCCAAACTAATGGTTGTCATACCAGACTATAACACA   12502
gb:MN994468|Organism:Severe      AACATAATACCTCTTACAACAGCAGCCAAACTAATGGTTGTCATACCAGACTATAACACA   12502
gb:MT072688|Organism:Severe      AACATAATACCTCTTACAACAGCAGCCAAACTAATGGTTGTCATACCAGACTATAACACA   12487
gb:MN996527|Organism:Severe      AACATAATACCTCTTACAACAGCAGCCAAACTAATGGTTGTCATACCAGACTATAACACA   12469
gb:MT093631|Organism:Severe      AACATAATACCTCTTACAACAGCAGCCAAACTAATGGTTGTCATACCAGACTATAACACA   12540
gb:MT106053|Organism:Severe      AACATAATACCTCTTACAACAGCAGCCAAACTAATGGTTGTCATACCAGACTATAACACA   12502
gb:MT019533|Organism:Severe      AACATAATACCTCTTACAACAGCAGCCAAACTAATGGTTGTCATACCAGACTATAACACA   12502
gb:MT019531|Organism:Severe      AACATAATACCTCTTACAACAGCAGCCAAACTAATGGTTGTCATACCAGACTATAACACA   12502
gb:MN996528|Organism:Severe      AACATAATACCTCTTACAACAGCAGCCAAACTAATGGTTGTCATACCAGACTATAACACA   12502
gb:MN996530|Organism:Severe      AACATAATACCTCTTACAACAGCAGCCAAACTAATGGTTGTCATACCAGACTATAACACA   12488
gb:MN908947|Organism:Severe      AACATAATACCTCTTACAACAGCAGCCAAACTAATGGTTGTCATACCAGACTATAACACA   12502
gb:MT019532|Organism:Severe      AACATAATACCTCTTACAACAGCAGCCAAACTAATGGTTGTCATACCAGACTATAACACA   12502
                                 ************************************************************

gb:MT020781|Organism:Severe      TATAAAAATACGTGTGATGGTACAACATTTACTTATGCATCAGCATTGTGGGAAATCCAA   12550
gb:MT007544|Organism:Severe      TATAAAAATACGTGTGATGGTACAACATTTACTTATGCATCAGCATTGTGGGAAATCCAA   12562
```

```
gb:MN994467|Organism:Severe      TATAAAAATACGTGTGATGGTACAACATTTACTTATGCATCAGCATTGTGGGAAATCCAA   12562
gb:MT044257|Organism:Severe      TATAAAAATACGTGTGATGGTACAACATTTACTTATGCATCAGCATTGTGGGAAATCCAA   12562
gb:MT106054|Organism:Severe      TATAAAAATACGTGTGATGGTACAACATTTACTTATGCATCAGCATTGTGGGAAATCCAA   12562
gb:MT049951|Organism:Severe      TATAAAAATACGTGTGATGGTACAACATTTACTTATGCATCAGCATTGTGGGAAATCCAA   12562
gb:MN975262|Organism:Severe      TATAAAAATACGTGTGATGGTACAACATTTACTTATGCATCAGCATTGTGGGAAATCCAA   12562
gb:MT106052|Organism:Severe      TATAAAAATACGTGTGATGGTACAACATTTACTTATGCATCAGCATTGTGGGAAATCCAA   12562
gb:LC522975|Organism:Severe      TATAAAAATACGTGTGATGGTACAACATTTACTTATGCATCAGCATTGTGGGAAATCCAA   12559
gb:LC522973|Organism:Severe      TATAAAAATACGTGTGATGGTACAACATTTACTTATGCATCAGCATTGTGGGAAATCCAA   12559
gb:LC522974|Organism:Severe      TATAAAAATACGTGTGATGGTACAACATTTACTTATGCATCAGCATTGTGGGAAATCCAA   12559
gb:MN985325|Organism:Severe      TATAAAAATACGTGTGATGGTACAACATTTACTTATGCATCAGCATTGTGGGAAATCCAA   12562
gb:MT020881|Organism:Severe      TATAAAAATACGTGTGATGGTACAACATTTACTTATGCATCAGCATTGTGGGAAATCCAA   12562
gb:MT020880|Organism:Severe      TATAAAAATACGTGTGATGGTACAACATTTACTTATGCATCAGCATTGTGGGAAATCCAA   12562
gb:MT066175|Organism:Severe      TATAAAAATACGTGTGATGGTACAACATTTACTTATGCATCAGCATTGTGGGAAATCCAA   12562
gb:MN997409|Organism:Severe      TATAAAAATACGTGTGATGGTACAACATTTACTTATGCATCAGCATTGTGGGAAATCCAA   12562
gb:MN938384|Organism:Severe      TATAAAAATACGTGTGATGGTACAACATTTACTTATGCATCAGCATTGTGGGAAATCCAA   12530
gb:MT044258|Organism:Severe      TATAAAAATACGTGTGATGGTACAACATTTACTTATGCATCAGCATTGTGGGAAATCCAA   12538
gb:MT039890|Organism:Severe      TATAAAAATACGTGTGATGGTACAACATTTACTTATGCATCAGCATTGTGGGAAATCCAA   12562
gb:MN988713|Organism:Severe      TATAAAAATACGTGTGATGGTACAACATTTACTTATGCATCAGCATTGTGGGAAATCCAA   12562
gb:LC521925|Organism:Severe      TATAAAAATACGTGTGATGGTACAACATTTACTTATGCATCAGCATTGTGGGAAATCCAA   12535
gb:MT093571|Organism:Severe      TATAAAAATACGTGTGATGGTACAACATTTACTTATGCATCAGCATTGTGGGAAATCCAA   12562
gb:MT039887|Organism:Severe      TATAAAAATACGTGTGATGGTACAACATTTACTTATGCATCAGCATTGTGGGAAATCCAA   12562
gb:MT019530|Organism:Severe      TATAAAAATACGTGTGATGGTACAACATTTACTTATGCATCAGCATTGTGGGAAATCCAA   12562
gb:MT039888|Organism:Severe      TATAAAAATACGTGTGATGGTACAACATTTACTTATGCATCAGCATTGTGGGAAATCCAA   12562
gb:LC522972|Organism:Severe      TATAAAAATACGTGTGATGGTACAACATTTACTTATGCATCAGCATTGTGGGAAATCCAA   12559
gb:MT027063|Organism:Severe      TATAAAAATACGTGTGATGGTACAACATTTACTTATGCATCAGCATTGTGGGAAATCCAA   12562
gb:MT027062|Organism:Severe      TATAAAAATACGTGTGATGGTACAACATTTACTTATGCATCAGCATTGTGGGAAATCCAA   12562
gb:MT019529|Organism:Severe      TATAAAAATACGTGTGATGGTACAACATTTACTTATGCATCAGCATTGTGGGAAATCCAA   12562
gb:MN996529|Organism:Severe      TATAAAAATACGTGTGATGGTACAACATTTACTTATGCATCAGCATTGTGGGAAATCCAA   12550
gb:MN996531|Organism:Severe      TATAAAAATACGTGTGATGGTACAACATTTACTTATGCATCAGCATTGTGGGAAATCCAA   12549
gb:MT066176|Organism:Severe      TATAAAAATACGTGTGATGGTACAACATTTACTTATGCATCAGCATTGTGGGAAATCCAA   12562
gb:MT027064|Organism:Severe      TATAAAAATACGTGTGATGGTACAACATTTACTTATGCATCAGCATTGTGGGAAATCCAA   12562
gb:MN994468|Organism:Severe      TATAAAAATACGTGTGATGGTACAACATTTACTTATGCATCAGCATTGTGGGAAATCCAA   12562
gb:MT072688|Organism:Severe      TATAAAAATACGTGTGATGGTACAACATTTACTTATGCATCAGCATTGTGGGAAATCCAA   12547
gb:MN996527|Organism:Severe      TATAAAAATACGTGTGATGGTACAACATTTACTTATGCATCAGCATTGTGGGAAATCCAA   12529
gb:MT093631|Organism:Severe      TATAAAAATACGTGTGATGGTACAACATTTACTTATGCATCAGCATTGTGGGAAATCCAA   12600
gb:MT106053|Organism:Severe      TATAAAAATACGTGTGATGGTACAACATTTACTTATGCATCAGCATTGTGGGAAATCCAA   12562
gb:MT019533|Organism:Severe      TATAAAAATACGTGTGATGGTACAACATTTACTTATGCATCAGCATTGTGGGAAATCCAA   12562
gb:MT019531|Organism:Severe      TATAAAAATACGTGTGATGGTACAACATTTACTTATGCATCAGCATTGTGGGAAATCCAA   12562
gb:MN996528|Organism:Severe      TATAAAAATACGTGTGATGGTACAACATTTACTTATGCATCAGCATTGTGGGAAATCCAA   12562
gb:MN996530|Organism:Severe      TATAAAAATACGTGTGATGGTACAACATTTACTTATGCATCAGCATTGTGGGAAATCCAA   12548
gb:MN908947|Organism:Severe      TATAAAAATACGTGTGATGGTACAACATTTACTTATGCATCAGCATTGTGGGAAATCCAA   12562
gb:MT019532|Organism:Severe      TATAAAAATACGTGTGATGGTACAACATTTACTTATGCATCAGCATTGTGGGAAATCCAA   12562
                                 ************************************************************

gb:MT020781|Organism:Severe      CAGGTTGTAGATGCAGATAGTAAAATTGTTCAACTTAGTGAAATTAGTATGGACAATTCA   12610
gb:MT007544|Organism:Severe      CAGGTTGTAGATGCAGATAGTAAAATTGTTCAACTTAGTGAAATTAGTATGGACAATTCA   12622
gb:MN994467|Organism:Severe      CAGGTTGTAGATGCAGATAGTAAAATTGTTCAACTTAGTGAAATTAGTATGGACAATTCA   12622
gb:MT044257|Organism:Severe      CAGGTTGTAGATGCAGATAGTAAAATTGTTCAACTTAGTGAAATTAGTATGGACAATTCA   12622
gb:MT106054|Organism:Severe      CAGGTTGTAGATGCAGATAGTAAAATTGTTCAACTTAGTGAAATTAGTATGGACAATTCA   12622
gb:MT049951|Organism:Severe      CAGGTTGTAGATGCAGATAGTAAAATTGTTCAACTTAGTGAAATTAGTATGGACAATTCA   12622
gb:MN975262|Organism:Severe      CAGGTTGTAGATGCAGATAGTAAAATTGTTCAACTTAGTGAAATTAGTATGGACAATTCA   12622
gb:MT106052|Organism:Severe      CAGGTTGTAGATGCAGATAGTAAAATTGTTCAACTTAGTGAAATTAGTATGGACAATTCA   12622
gb:LC522975|Organism:Severe      CAGGTTGTAGATGCAGATAGTAAAATTGTTCAACTTAGTGAAATTAGTATGGACAATTCA   12619
gb:LC522973|Organism:Severe      CAGGTTGTAGATGCAGATAGTAAAATTGTTCAACTTAGTGAAATTAGTATGGACAATTCA   12619
gb:LC522974|Organism:Severe      CAGGTTGTAGATGCAGATAGTAAAATTGTTCAACTTAGTGAAATTAGTATGGACAATTCA   12619
gb:MN985325|Organism:Severe      CAGGTTGTAGATGCAGATAGTAAAATTGTTCAACTTAGTGAAATTAGTATGGACAATTCA   12622
gb:MT020881|Organism:Severe      CAGGTTGTAGATGCAGATAGTAAAATTGTTCAACTTAGTGAAATTAGTATGGACAATTCA   12622
gb:MT020880|Organism:Severe      CAGGTTGTAGATGCAGATAGTAAAATTGTTCAACTTAGTGAAATTAGTATGGACAATTCA   12622
gb:MT066175|Organism:Severe      CAGGTTGTAGATGCAGATAGTAAAATTGTTCAACTTAGTGAAATTAGTATGGACAATTCA   12622
gb:MN997409|Organism:Severe      CAGGTTGTAGATGCAGATAGTAAAATTGTTCAACTTAGTGAAATTAGTATGGACAATTCA   12622
gb:MN938384|Organism:Severe      CAGGTTGTAGATGCAGATAGTAAAATTGTTCAACTTAGTGAAATTAGTATGGACAATTCA   12590
gb:MT044258|Organism:Severe      CAGGTTGTAGATGCAGATAGTAAAATTGTTCAACTTAGTGAAATTAGTATGGACAATTCA   12598
gb:MT039890|Organism:Severe      CAGGTTGTAGATGCAGATAGTAAAATTGTTCAACTTAGTGAAATTAGTATGGACAATTCA   12622
gb:MN988713|Organism:Severe      CAGGTTGTAGATGCAGATAGTAAAATTGTTCAACTTAGTGAAATTAGTATGGACAATTCA   12622
gb:LC521925|Organism:Severe      CAGGTTGTAGATGCAGATAGTAAAATTGTTCAACTTAGTGAAATTAGTATGGACAATTCA   12595
```

```
gb:MT093571|Organism:Severe      CAGGTTGTAGATGCAGATAGTAAAATTGTTCAACTTAGTGAAATTAGTATGGACAATTCA    12622
gb:MT039887|Organism:Severe      CAGGTTGTAGATGCAGATAGTAAAATTGTTCAACTTAGTGAAATTAGTATGGACAATTCA    12622
gb:MT019530|Organism:Severe      CAGGTTGTAGATGCAGATAGTAAAATTGTTCAACTTAGTGAAATTAGTATGGACAATTCA    12622
gb:MT039888|Organism:Severe      CAGGTTGTAGATGCAGATAGTAAAATTGTTCAACTTAGTGAAATTAGTATGGACAATTCA    12622
gb:LC522972|Organism:Severe      CAGGTTGTAGATGCAGATAGTAAAATTGTTCAACTTAGTGAAATTAGTATGGACAATTCA    12619
gb:MT027063|Organism:Severe      CAGGTTGTAGATGCAGATAGTAAAATTGTTCAACTTAGTGAAATTAGTATGGACAATTCA    12622
gb:MT027062|Organism:Severe      CAGGTTGTAGATGCAGATAGTAAAATTGTTCAACTTAGTGAAATTAGTATGGACAATTCA    12622
gb:MT019529|Organism:Severe      CAGGTTGTAGATGCAGATAGTAAAATTGTTCAACTTAGTGAAATTAGTATGGACAATTCA    12622
gb:MN996529|Organism:Severe      CAGGTTGTAGATGCAGATAGTAAAATTGTTCAACTTAGTGAAATTAGTATGGACAATTCA    12610
gb:MN996531|Organism:Severe      CAGGTTGTAGATGCAGATAGTAAAATTGTTCAACTTAGTGAAATTAGTATGGACAATTCA    12609
gb:MT066176|Organism:Severe      CAGGTTGTAGATGCAGATAGTAAAATTGTTCAACTTAGTGAAATTAGTATGGACAATTCA    12622
gb:MT027064|Organism:Severe      CAGGTTGTAGATGCAGATAGTAAAATTGTTCAACTTAGTGAAATTAGTATGGACAATTCA    12622
gb:MN994468|Organism:Severe      CAGGTTGTAGATGCAGATAGTAAAATTGTTCAACTTAGTGAAATTAGTATGGACAATTCA    12622
gb:MT072688|Organism:Severe      CAGGTTGTAGATGCAGATAGTAAAATTGTTCAACTTAGTGAAATTAGTATGGACAATTCA    12607
gb:MN996527|Organism:Severe      CAGGTTGTAGATGCAGATAGTAAAATTGTTCAACTTAGTGAAATTAGTATGGACAATTCA    12589
gb:MT093631|Organism:Severe      CAGGTTGTAGATGCAGATAGTAAAATTGTTCAACTTAGTGAAATTAGTATGGACAATTCA    12660
gb:MT106053|Organism:Severe      CAGGTTGTAGATGCAGATAGTAAAATTGTTCAACTTAGTGAAATTAGTATGGACAATTCA    12622
gb:MT019533|Organism:Severe      CAGGTTGTAGATGCAGATAGTAAAATTGTTCAACTTAGTGAAATTAGTATGGACAATTCA    12622
gb:MT019531|Organism:Severe      CAGGTTGTAGATGCAGATAGTAAAATTGTTCAACTTAGTGAAATTAGTATGGACAATTCA    12622
gb:MN996528|Organism:Severe      CAGGTTGTAGATGCAGATAGTAAAATTGTTCAACTTAGTGAAATTAGTATGGACAATTCA    12622
gb:MN996530|Organism:Severe      CAGGTTGTAGATGCAGATAGTAAAATTGTTCAACTTAGTGAAATTAGTATGGACAATTCA    12608
gb:MN908947|Organism:Severe      CAGGTTGTAGATGCAGATAGTAAAATTGTTCAACTTAGTGAAATTAGTATGGACAATTCA    12622
gb:MT019532|Organism:Severe      CAGGTTGTAGATGCAGATAGTAAAATTGTTCAACTTAGTGAAATTAGTATGGACAATTCA    12622
                                 ************************************************************

gb:MT020781|Organism:Severe      CCTAATTTAGCATGGCCTCTTATTGTAACAGCTTTAAGGGCCAATTCTGCTGTCAAATTA    12670
gb:MT007544|Organism:Severe      CCTAATTTAGCATGGCCTCTTATTGTAACAGCTTTAAGGGCCAATTCTGCTGTCAAATTA    12682
gb:MN994467|Organism:Severe      CCTAATTTAGCATGGCCTCTTATTGTAACAGCTTTAAGGGCCAATTCTGCTGTCAAATTA    12682
gb:MT044257|Organism:Severe      CCTAATTTAGCATGGCCTCTTATTGTAACAGCTTTAAGGGCCAATTCTGCTGTCAAATTA    12682
gb:MT106054|Organism:Severe      CCTAATTTAGCATGGCCTCTTATTGTAACAGCTTTAAGGGCCAATTCTGCTGTCAAATTA    12682
gb:MT049951|Organism:Severe      CCTAATTTAGCATGGCCTCTTATTGTAACAGCTTTAAGGGCCAATTCTGCTGTCAAATTA    12682
gb:MN975262|Organism:Severe      CCTAATTTAGCATGGCCTCTTATTGTAACAGCTTTAAGGGCCAATTCTGCTGTCAAATTA    12682
gb:MT106052|Organism:Severe      CCTAATTTAGCATGGCCTCTTATTGTAACAGCTTTAAGGGCCAATTCTGCTGTCAAATTA    12682
gb:LC522975|Organism:Severe      CCTAATTTAGCATGGCCTCTTATTGTAACAGCTTTAAGGGCCAATTCTGCTGTCAAATTA    12679
gb:LC522973|Organism:Severe      CCTAATTTAGCATGGCCTCTTATTGTAACAGCTTTAAGGGCCAATTCTGCTGTCAAATTA    12679
gb:LC522974|Organism:Severe      CCTAATTTAGCATGGCCTCTTATTGTAACAGCTTTAAGGGCCAATTCTGCTGTCAAATTA    12679
gb:MN985325|Organism:Severe      CCTAATTTAGCATGGCCTCTTATTGTAACAGCTTTAAGGGCCAATTCTGCTGTCAAATTA    12682
gb:MT020881|Organism:Severe      CCTAATTTAGCATGGCCTCTTATTGTAACAGCTTTAAGGGCCAATTCTGCTGTCAAATTA    12682
gb:MT020880|Organism:Severe      CCTAATTTAGCATGGCCTCTTATTGTAACAGCTTTAAGGGCCAATTCTGCTGTCAAATTA    12682
gb:MT066175|Organism:Severe      CCTAATTTAGCATGGCCTCTTATTGTAACAGCTTTAAGGGCCAATTCTGCTGTCAAATTA    12682
gb:MN997409|Organism:Severe      CCTAATTTAGCATGGCCTCTTATTGTAACAGCTTTAAGGGCCAATTCTGCTGTCAAATTA    12682
gb:MN938384|Organism:Severe      CCTAATTTAGCATGGCCTCTTATTGTAACAGCTTTAAGGGCCAATTCTGCTGTCAAATTA    12650
gb:MT044258|Organism:Severe      CCTAATTTAGCATGGCCTCTTATTGTAACAGCTTTAAGGGCCAATTCTGCTGTCAAATTA    12658
gb:MT039890|Organism:Severe      CCTAATTTAGCATGGCCTCTTATTGTAACAGCTTTAAGGGCCAATTCTGCTGTCAAATTA    12682
gb:MN988713|Organism:Severe      CCTAATTTAGCATGGCCTCTTATTGTAACAGCTTTAAGGGCCAATTCTGCTGTCAAATTA    12682
gb:LC521925|Organism:Severe      CCTAATTTAGCATGGCCTCTTATTGTAACAGCTTTAAGGGCCAATTCTGCTGTCAAATTA    12655
gb:MT093571|Organism:Severe      CCTAATTTAGCATGGCCTCTTATTGTAACAGCTTTAAGGGCCAATTCTGCTGTCAAATTA    12682
gb:MT039887|Organism:Severe      CCTAATTTAGCATGGCCTCTTATTGTAACAGCTTTAAGGGCCAATTCTGCTGTCAAATTA    12682
gb:MT019530|Organism:Severe      CCTAATTTAGCATGGCCTCTTATTGTAACAGCTTTAAGGGCCAATTCTGCTGTCAAATTA    12682
gb:MT039888|Organism:Severe      CCTAATTTAGCATGGCCTCTTATTGTAACAGCTTTAAGGGCCAATTCTGCTGTCAAATTA    12682
gb:LC522972|Organism:Severe      CCTAATTTAGCATGGCCTCTTATTGTAACAGCTTTAAGGGCCAATTCTGCTGTCAAATTA    12679
gb:MT027063|Organism:Severe      CCTAATTTAGCATGGCCTCTTATTGTAACAGCTTTAAGGGCCAATTCTGCTGTCAAATTA    12682
gb:MT027062|Organism:Severe      CCTAATTTAGCATGGCCTCTTATTGTAACAGCTTTAAGGGCCAATTCTGCTGTCAAATTA    12682
gb:MT019529|Organism:Severe      CCTAATTTAGCATGGCCTCTTATTGTAACAGCTTTAAGGGCCAATTCTGCTGTCAAATTA    12682
gb:MN996529|Organism:Severe      CCTAATTTAGCATGGCCTCTTATTGTAACAGCTTTAAGGGCCAATTCTGCTGTCAAATTA    12670
gb:MN996531|Organism:Severe      CCTAATTTAGCATGGCCTCTTATTGTAACAGCTTTAAGGGCCAATTCTGCTGTCAAATTA    12669
gb:MT066176|Organism:Severe      CCTAATTTAGCATGGCCTCTTATTGTAACAGCTTTAAGGGCCAATTCTGCTGTCAAATTA    12682
gb:MT027064|Organism:Severe      CCTAATTTAGCATGGCCTCTTATTGTAACAGCTTTAAGGGCCAATTCTGCTGTCAAATTA    12682
gb:MN994468|Organism:Severe      CCTAATTTAGCATGGCCTCTTATTGTAACAGCTTTAAGGGCCAATTCTGCTGTCAAATTA    12682
gb:MT072688|Organism:Severe      CCTAATTTAGCATGGCCTCTTATTGTAACAGCTTTAAGGGCCAATTCTGCTGTCAAATTA    12667
gb:MN996527|Organism:Severe      CCTAATTTAGCATGGCCTCTTATTGTAACAGCTTTAAGGGCCAATTCTGCTGTCAAATTA    12649
gb:MT093631|Organism:Severe      CCTAATTTAGCATGGCCTCTTATTGTAACAGCTTTAAGGGCCAATTCTGCTGTCAAATTA    12720
gb:MT106053|Organism:Severe      CCTAATTTAGCATGGCCTCTTATTGTAACAGCTTTAAGGGCCAATTCTGCTGTCAAATTA    12682
gb:MT019533|Organism:Severe      CCTAATTTAGCATGGCCTCTTATTGTAACAGCTTTAAGGGCCAATTCTGCTGTCAAATTA    12682
gb:MT019531|Organism:Severe      CCTAATTTAGCATGGCCTCTTATTGTAACAGCTTTAAGGGCCAATTCTGCTGTCAAATTA    12682
```

```
gb:MN996528|Organism:Severe      CCTAATTTAGCATGGCCTCTTATTGTAACAGCTTTAAGGGCCAATTCTGCTGTCAAATTA    12682
gb:MN996530|Organism:Severe      CCTAATTTAGCATGGCCTCTTATTGTAACAGCTTTAAGGGCCAATTCTGCTGTCAAATTA    12668
gb:MN908947|Organism:Severe      CCTAATTTAGCATGGCCTCTTATTGTAACAGCTTTAAGGGCCAATTCTGCTGTCAAATTA    12682
gb:MT019532|Organism:Severe      CCTAATTTAGCATGGCCTCTTATTGTAACAGCTTTAAGGGCCAATTCTGCTGTCAAATTA    12682
                                 ************************************************************

gb:MT020781|Organism:Severe      CAGAATAATGAGCTTAGTCCTGTTGCACTACGACAGATGTCTTGTGCTGCCGGTACTACA    12730
gb:MT007544|Organism:Severe      CAGAATAATGAGCTTAGTCCTGTTGCACTACGACAGATGTCTTGTGCTGCCGGTACTACA    12742
gb:MN994467|Organism:Severe      CAGAATAATGAGCTTAGTCCTGTTGCACTACGACAGATGTCTTGTGCTGCCGGTACTACA    12742
gb:MT044257|Organism:Severe      CAGAATAATGAGCTTAGTCCTGTTGCACTACGACAGATGTCTTGTGCTGCCGGTACTACA    12742
gb:MT106054|Organism:Severe      CAGAATAATGAGCTTAGTCCTGTTGCACTACGACAGATGTCTTGTGCTGCCGGTACTACA    12742
gb:MT049951|Organism:Severe      CAGAATAATGAGCTTAGTCCTGTTGCACTACGACAGATGTCTTGTGCTGCCGGTACTACA    12742
gb:MN975262|Organism:Severe      CAGAATAATGAGCTTAGTCCTGTTGCACTACGACAGATGTCTTGTGCTGCCGGTACTACA    12742
gb:MT106052|Organism:Severe      CAGAATAATGAGCTTAGTCCTGTTGCACTACGACAGATGTCTTGTGCTGCCGGTACTACA    12742
gb:LC522975|Organism:Severe      CAGAATAATGAGCTTAGTCCTGTTGCACTACGACAGATGTCTTGTGCTGCCGGTACTACA    12739
gb:LC522973|Organism:Severe      CAGAATAATGAGCTTAGTCCTGTTGCACTACGACAGATGTCTTGTGCTGCCGGTACTACA    12739
gb:LC522974|Organism:Severe      CAGAATAATGAGCTTAGTCCTGTTGCACTACGACAGATGTCTTGTGCTGCCGGTACTACA    12739
gb:MN985325|Organism:Severe      CAGAATAATGAGCTTAGTCCTGTTGCACTACGACAGATGTCTTGTGCTGCCGGTACTACA    12742
gb:MT020881|Organism:Severe      CAGAATAATGAGCTTAGTCCTGTTGCACTACGACAGATGTCTTGTGCTGCCGGTACTACA    12742
gb:MT020880|Organism:Severe      CAGAATAATGAGCTTAGTCCTGTTGCACTACGACAGATGTCTTGTGCTGCCGGTACTACA    12742
gb:MT066175|Organism:Severe      CAGAATAATGAGCTTAGTCCTGTTGCACTACGACAGATGTCTTGTGCTGCCGGTACTACA    12742
gb:MN997409|Organism:Severe      CAGAATAATGAGCTTAGTCCTGTTGCACTACGACAGATGTCTTGTGCTGCCGGTACTACA    12742
gb:MN938384|Organism:Severe      CAGAATAATGAGCTTAGTCCTGTTGCACTACGACAGATGTCTTGTGCTGCCGGTACTACA    12710
gb:MT044258|Organism:Severe      CAGAATAATGAGCTTAGTCCTGTTGCACTACGACAGATGTCTTGTGCTGCCGGTACTACA    12718
gb:MT039890|Organism:Severe      CAGAATAATGAGCTTAGTCCTGTTGCACTACGACAGATGTCTTGTGCTGCCGGTACTACA    12742
gb:MN988713|Organism:Severe      CAGAATAATGAGCTTAGTCCTGTTGCACTACGACAGATGTCTTGTGCTGCCGGTACTACA    12742
gb:LC521925|Organism:Severe      CAGAATAATGAGCTTAGTCCTGTTGCACTACGACAGATGTCTTGTGCTGCCGGTACTACA    12715
gb:MT093571|Organism:Severe      CAGAATAATGAGCTTAGTCCTGTTGCACTACGACAGATGTCTTGTGCTGCCGGTACTACA    12742
gb:MT039887|Organism:Severe      CAGAATAATGAGCTTAGTCCTGTTGCACTACGACAGATGTCTTGTGCTGCCGGTACTACA    12742
gb:MT019530|Organism:Severe      CAGAATAATGAGCTTAGTCCTGTTGCACTACGACAGATGTCTTGTGCTGCCGGTACTACA    12742
gb:MT039888|Organism:Severe      CAGAATAATGAGCTTAGTCCTGTTGCACTACGACAGATGTCTTGTGCTGCCGGTACTACA    12742
gb:LC522972|Organism:Severe      CAGAATAATGAGCTTAGTCCTGTTGCACTACGACAGATGTCTTGTGCTGCCGGTACTACA    12739
gb:MT027063|Organism:Severe      CAGAATAATGAGCTTAGTCCTGTTGCACTACGACAGATGTCTTGTGCTGCCGGTACTACA    12742
gb:MT027062|Organism:Severe      CAGAATAATGAGCTTAGTCCTGTTGCACTACGACAGATGTCTTGTGCTGCCGGTACTACA    12742
gb:MT019529|Organism:Severe      CAGAATAATGAGCTTAGTCCTGTTGCACTACGACAGATGTCTTGTGCTGCCGGTACTACA    12742
gb:MN996529|Organism:Severe      CAGAATAATGAGCTTAGTCCTGTTGCACTACGACAGATGTCTTGTGCTGCCGGTACTACA    12730
gb:MN996531|Organism:Severe      CAGAATAATGAGCTTAGTCCTGTTGCACTACGACAGATGTCTTGTGCTGCCGGTACTACA    12729
gb:MT066176|Organism:Severe      CAGAATAATGAGCTTAGTCCTGTTGCACTACGACAGATGTCTTGTGCTGCCGGTACTACA    12742
gb:MT027064|Organism:Severe      CAGAATAATGAGCTTAGTCCTGTTGCACTACGACAGATGTCTTGTGCTGCCGGTACTACA    12742
gb:MN994468|Organism:Severe      CAGAATAATGAGCTTAGTCCTGTTGCACTACGACAGATGTCTTGTGCTGCCGGTACTACA    12742
gb:MT072688|Organism:Severe      CAGAATAATGAGCTTAGTCCTGTTGCACTACGACAGATGTCTTGTGCTGCCGGTACTACA    12727
gb:MN996527|Organism:Severe      CAGAATAATGAGCTTAGTCCTGTTGCACTACGACAGATGTCTTGTGCTGCCGGTACTACA    12709
gb:MT093631|Organism:Severe      CAGAATAATGAGCTTAGTCCTGTTGCACTACGACAGATGTCTTGTGCTGCCGGTACTACA    12780
gb:MT106053|Organism:Severe      CAGAATAATGAGCTTAGTCCTGTTGCACTACGACAGATGTCTTGTGCTGCCGGTACTACA    12742
gb:MT019533|Organism:Severe      CAGAATAATGAGCTTAGTCCTGTTGCACTACGACAGATGTCTTGTGCTGCCGGTACTACA    12742
gb:MT019531|Organism:Severe      CAGAATAATGAGCTTAGTCCTGTTGCACTACGACAGATGTCTTGTGCTGCCGGTACTACA    12742
gb:MN996528|Organism:Severe      CAGAATAATGAGCTTAGTCCTGTTGCACTACGACAGATGTCTTGTGCTGCCGGTACTACA    12742
gb:MN996530|Organism:Severe      CAGAATAATGAGCTTAGTCCTGTTGCACTACGACAGATGTCTTGTGCTGCCGGTACTACA    12728
gb:MN908947|Organism:Severe      CAGAATAATGAGCTTAGTCCTGTTGCACTACGACAGATGTCTTGTGCTGCCGGTACTACA    12742
gb:MT019532|Organism:Severe      CAGAATAATGAGCTTAGTCCTGTTGCACTACGACAGATGTCTTGTGCTGCCGGTACTACA    12742
                                 ************************************************************

gb:MT020781|Organism:Severe      CAAACTGCTTGCACTGATGACAATGCGTTAGCTTACTACAACACAACAAAGGGAGGTAGG    12790
gb:MT007544|Organism:Severe      CAAACTGCTTGCACTGATGACAATGCGTTAGCTTACTACAACACAACAAAGGGAGGTAGG    12802
gb:MN994467|Organism:Severe      CAAACTGCTTGCACTGATGACAATGCGTTAGCTTACTACAACACAACAAAGGGAGGTAGG    12802
gb:MT044257|Organism:Severe      CAAACTGCTTGCACTGATGACAATGCGTTAGCTTACTACAACACAACAAAGGGAGGTAGG    12802
gb:MT106054|Organism:Severe      CAAACTGCTTGCACTGATGACAATGCGTTAGCTTACTACAACACAACAAAGGGAGGTAGG    12802
gb:MT049951|Organism:Severe      CAAACTGCTTGCACTGATGACAATGCGTTAGCTTACTACAACACAACAAAGGGAGGTAGG    12802
gb:MN975262|Organism:Severe      CAAACTGCTTGCACTGATGACAATGCGTTAGCTTACTACAACACAACAAAGGGAGGTAGG    12802
gb:MT106052|Organism:Severe      CAAACTGCTTGCACTGATGACAATGCGTTAGCTTACTACAACACAACAAAGGGAGGTAGG    12802
gb:LC522975|Organism:Severe      CAAACTGCTTGCACTGATGACAATGCGTTAGCTTACTACAACACAACAAAGGGAGGTAGG    12799
gb:LC522973|Organism:Severe      CAAACTGCTTGCACTGATGACAATGCGTTAGCTTACTACAACACAACAAAGGGAGGTAGG    12799
gb:LC522974|Organism:Severe      CAAACTGCTTGCACTGATGACAATGCGTTAGCTTACTACAACACAACAAAGGGAGGTAGG    12799
gb:MN985325|Organism:Severe      CAAACTGCTTGCACTGATGACAATGCGTTAGCTTACTACAACACAACAAAGGGAGGTAGG    12802
gb:MT020881|Organism:Severe      CAAACTGCTTGCACTGATGACAATGCGTTAGCTTACTACAACACAACAAAGGGAGGTAGG    12802
```

```
gb:MT020880|Organism:Severe      CAAACTGCTTGCACTGATGACAATGCGTTAGCTTACTACAACACAACAAAGGGAGGTAGG   12802
gb:MT066175|Organism:Severe      CAAACTGCTTGCACTGATGACAATGCGTTAGCTTACTACAACACAACAAAGGGAGGTAGG   12802
gb:MN997409|Organism:Severe      CAAACTGCTTGCACTGATGACAATGCGTTAGCTTACTACAACACAACAAAGGGAGGTAGG   12802
gb:MN938384|Organism:Severe      CAAACTGCTTGCACTGATGACAATGCGTTAGCTTACTACAACACAACAAAGGGAGGTAGG   12770
gb:MT044258|Organism:Severe      CAAACTGCTTGCACTGATGACAATGCGTTAGCTTACTACAACACAACAAAGGGAGGTAGG   12778
gb:MT039890|Organism:Severe      CAAACTGCTTGCACTGATGACAATGCGTTAGCTTACTACAACACAACAAAGGGAGGTAGG   12802
gb:MN988713|Organism:Severe      CAAACTGCTTGCACTGATGACAATGCGTTAGCTTACTACAACACAACAAAGGGAGGTAGG   12802
gb:LC521925|Organism:Severe      CAAACTGCTTGCACTGATGACAATGCGTTAGCTTACTACAACACAACAAAGGGAGGTAGG   12775
gb:MT093571|Organism:Severe      CAAACTGCTTGCACTGATGACAATGCGTTAGCTTACTACAACACAACAAAGGGAGGTAGG   12802
gb:MT039887|Organism:Severe      CAAACTGCTTGCACTGATGACAATGCGTTAGCTTACTACAACACAACAAAGGGAGGTAGG   12802
gb:MT019530|Organism:Severe      CAAACTGCTTGCACTGATGACAATGCGTTAGCTTACTACAACACAACAAAGGGAGGTAGG   12802
gb:MT039888|Organism:Severe      CAAACTGCTTGCACTGATGACAATGCGTTAGCTTACTACAACACAACAAAGGGAGGTAGG   12802
gb:LC522972|Organism:Severe      CAAACTGCTTGCACTGATGACAATGCGTTAGCTTACTACAACACAACAAAGGGAGGTAGG   12799
gb:MT027063|Organism:Severe      CAAACTGCTTGCACTGATGACAATGCGTTAGCTTACTACAACACAACAAAGGGAGGTAGG   12802
gb:MT027062|Organism:Severe      CAAACTGCTTGCACTGATGACAATGCGTTAGCTTACTACAACACAACAAAGGGAGGTAGG   12802
gb:MT019529|Organism:Severe      CAAACTGCTTGCACTGATGACAATGCGTTAGCTTACTACAACACAACAAAGGGAGGTAGG   12802
gb:MN996529|Organism:Severe      CAAACTGCTTGCACTGATGACAATGCGTTAGCTTACTACAACACAACAAAGGGAGGTAGG   12790
gb:MN996531|Organism:Severe      CAAACTGCTTGCACTGATGACAATGCGTTAGCTTACTACAACACAACAAAGGGAGGTAGG   12789
gb:MT066176|Organism:Severe      CAAACTGCTTGCACTGATGACAATGCGTTAGCTTACTACAACACAACAAAGGGAGGTAGG   12802
gb:MT027064|Organism:Severe      CAAACTGCTTGCACTGATGACAATGCGTTAGCTTACTACAACACAACAAAGGGAGGTAGG   12802
gb:MN994468|Organism:Severe      CAAACTGCTTGCACTGATGACAATGCGTTAGCTTACTACAACACAACAAAGGGAGGTAGG   12802
gb:MT072688|Organism:Severe      CAAACTGCTTGCACTGATGACAATGCGTTAGCTTACTACAACACAACAAAGGGAGGTAGG   12787
gb:MN996527|Organism:Severe      CAAACTGCTTGCACTGATGACAATGCGTTAGCTTACTACAACACAACAAAGGGAGGTAGG   12769
gb:MT093631|Organism:Severe      CAAACTGCTTGCACTGATGACAATGCGTTAGCTTACTACAACACAACAAAGGGAGGTAGG   12840
gb:MT106053|Organism:Severe      CAAACTGCTTGCACTGATGACAATGCGTTAGCTTACTACAACACAACAAAGGGAGGTAGG   12802
gb:MT019533|Organism:Severe      CAAACTGCTTGCACTGATGACAATGCGTTAGCTTACTACAACACAACAAAGGGAGGTAGG   12802
gb:MT019531|Organism:Severe      CAAACTGCTTGCACTGATGACAATGCGTTAGCTTACTACAACACAACAAAGGGAGGTAGG   12802
gb:MN996528|Organism:Severe      CAAACTGCTTGCACTGATGACAATGCGTTAGCTTACTACAACACAACAAAGGGAGGTAGG   12802
gb:MN996530|Organism:Severe      CAAACTGCTTGCACTGATGACAATGCGTTAGCTTACTACAACACAACAAAGGGAGGTAGG   12788
gb:MN908947|Organism:Severe      CAAACTGCTTGCACTGATGACAATGCGTTAGCTTACTACAACACAACAAAGGGAGGTAGG   12802
gb:MT019532|Organism:Severe      CAAACTGCTTGCACTGATGACAATGCGTTAGCTTACTACAACACAACAAAGGGAGGTAGG   12802
                                 ************************************************************

gb:MT020781|Organism:Severe      TTTGTACTTGCACTGTTATCCGATTTACAGGATTTGAAATGGGCTAGATTCCCTAAGAGT   12850
gb:MT007544|Organism:Severe      TTTGTACTTGCACTGTTATCCGATTTACAGGATTTGAAATGGGCTAGATTCCCTAAGAGT   12862
gb:MN994467|Organism:Severe      TTTGTACTTGCACTGTTATCCGATTTACAGGATTTGAAATGGGCTAGATTCCCTAAGAGT   12862
gb:MT044257|Organism:Severe      TTTGTACTTGCACTGTTATCCGATTTACAGGATTTGAAATGGGCTAGATTCCCTAAGAGT   12862
gb:MT106054|Organism:Severe      TTTGTACTTGCACTGTTATCCGATTTACAGGATTTGAAATGGGCTAGATTCCCTAAGAGT   12862
gb:MT049951|Organism:Severe      TTTGTACTTGCACTGTTATCCGATTTACAGGATTTGAAATGGGCTAGATTCCCTAAGAGT   12862
gb:MN975262|Organism:Severe      TTTGTACTTGCACTGTTATCCGATTTACAGGATTTGAAATGGGCTAGATTCCCTAAGAGT   12862
gb:MT106052|Organism:Severe      TTTGTACTTGCACTGTTATCCGATTTACAGGATTTGAAATGGGCTAGATTCCCTAAGAGT   12862
gb:LC522975|Organism:Severe      TTTGTACTTGCACTGTTATCCGATTTACAGGATTTGAAATGGGCTAGATTCCCTAAGAGT   12859
gb:LC522973|Organism:Severe      TTTGTACTTGCACTGTTATCCGATTTACAGGATTTGAAATGGGCTAGATTCCCTAAGAGT   12859
gb:LC522974|Organism:Severe      TTTGTACTTGCACTGTTATCCGATTTACAGGATTTGAAATGGGCTAGATTCCCTAAGAGT   12859
gb:MN985325|Organism:Severe      TTTGTACTTGCACTGTTATCCGATTTACAGGATTTGAAATGGGCTAGATTCCCTAAGAGT   12862
gb:MT020881|Organism:Severe      TTTGTACTTGCACTGTTATCCGATTTACAGGATTTGAAATGGGCTAGATTCCCTAAGAGT   12862
gb:MT020880|Organism:Severe      TTTGTACTTGCACTGTTATCCGATTTACAGGATTTGAAATGGGCTAGATTCCCTAAGAGT   12862
gb:MT066175|Organism:Severe      TTTGTACTTGCACTGTTATCCGATTTACAGGATTTGAAATGGGCTAGATTCCCTAAGAGT   12862
gb:MN997409|Organism:Severe      TTTGTACTTGCACTGTTATCCGATTTACAGGATTTGAAATGGGCTAGATTCCCTAAGAGT   12862
gb:MN938384|Organism:Severe      TTTGTACTTGCACTGTTATCCGATTTACAGGATTTGAAATGGGCTAGATTCCCTAAGAGT   12830
gb:MT044258|Organism:Severe      TTTGTACTTGCACTGTTATCCGATTTACAGGATTTGAAATGGGCTAGATTCCCTAAGAGT   12838
gb:MT039890|Organism:Severe      TTTGTACTTGCACTGTTATCCGATTTACAGGATTTGAAATGGGCTAGATTCCCTAAGAGT   12862
gb:MN988713|Organism:Severe      TTTGTACTTGCACTGTTATCCGATTTACAGGATTTGAAATGGGCTAGATTCCCTAAGAGT   12862
gb:LC521925|Organism:Severe      TTTGTACTTGCACTGTTATCCGATTTACAGGATTTGAAATGGGCTAGATTCCCTAAGAGT   12835
gb:MT093571|Organism:Severe      TTTGTACTTGCACTGTTATCCGATTTACAGGATTTGAAATGGGCTAGATTCCCTAAGAGT   12862
gb:MT039887|Organism:Severe      TTTGTACTTGCACTGTTATCCGATTTACAGGATTTGAAATGGGCTAGATTCCCTAAGAGT   12862
gb:MT019530|Organism:Severe      TTTGTACTTGCACTGTTATCCGATTTACAGGATTTGAAATGGGCTAGATTCCCTAAGAGT   12862
gb:MT039888|Organism:Severe      TTTGTACTTGCACTGTTATCCGATTTACAGGATTTGAAATGGGCTAGATTCCCTAAGAGT   12862
gb:LC522972|Organism:Severe      TTTGTACTTGCACTGTTATCCGATTTACAGGATTTGAAATGGGCTAGATTCCCTAAGAGT   12859
gb:MT027063|Organism:Severe      TTTGTACTTGCACTGTTATCCGATTTACAGGATTTGAAATGGGCTAGATTCCCTAAGAGT   12862
gb:MT027062|Organism:Severe      TTTGTACTTGCACTGTTATCCGATTTACAGGATTTGAAATGGGCTAGATTCCCTAAGAGT   12862
gb:MT019529|Organism:Severe      TTTGTACTTGCACTGTTATCCGATTTACAGGATTTGAAATGGGCTAGATTCCCTAAGAGT   12862
gb:MN996529|Organism:Severe      TTTGTACTTGCACTGTTATCCGATTTACAGGATTTGAAATGGGCTAGATTCCCTAAGAGT   12850
gb:MN996531|Organism:Severe      TTTGTACTTGCACTGTTATCCGATTTACAGGATTTGAAATGGGCTAGATTCCCTAAGAGT   12849
gb:MT066176|Organism:Severe      TTTGTACTTGCACTGTTATCCGATTTACAGGATTTGAAATGGGCTAGATTCCCTAAGAGT   12862
```

```
gb:MT027064|Organism:Severe      TTTGTACTTGCACTGTTATCCGATTTACAGGATTTGAAATGGGCTAGATTCCCTAAGAGT   12862
gb:MN994468|Organism:Severe      TTTGTACTTGCACTGTTATCCGATTTACAGGATTTGAAATGGGCTAGATTCCCTAAGAGT   12862
gb:MT072688|Organism:Severe      TTTGTACTTGCACTGTTATCCGATTTACAGGATTTGAAATGGGCTAGATTCCCTAAGAGT   12847
gb:MN996527|Organism:Severe      TTTGTACTTGCACTGTTATCCGATTTACAGGATTTGAAATGGGCTAGATTCCCTAAGAGT   12829
gb:MT093631|Organism:Severe      TTTGTACTTGCACTGTTATCCGATTTACAGGATTTGAAATGGGCTAGATTCCCTAAGAGT   12900
gb:MT106053|Organism:Severe      TTTGTACTTGCACTGTTATCCGATTTACAGGATTTGAAATGGGCTAGATTCCCTAAGAGT   12862
gb:MT019533|Organism:Severe      TTTGTACTTGCACTGTTATCCGATTTACAGGATTTGAAATGGGCTAGATTCCCTAAGAGT   12862
gb:MT019531|Organism:Severe      TTTGTACTTGCACTGTTATCCGATTTACAGGATTTGAAATGGGCTAGATTCCCTAAGAGT   12862
gb:MN996528|Organism:Severe      TTTGTACTTGCACTGTTATCCGATTTACAGGATTTGAAATGGGCTAGATTCCCTAAGAGT   12862
gb:MN996530|Organism:Severe      TTTGTACTTGCACTGTTATCCGATTTACAGGATTTGAAATGGGCTAGATTCCCTAAGAGT   12848
gb:MN908947|Organism:Severe      TTTGTACTTGCACTGTTATCCGATTTACAGGATTTGAAATGGGCTAGATTCCCTAAGAGT   12862
gb:MT019532|Organism:Severe      TTTGTACTTGCACTGTTATCCGATTTACAGGATTTGAAATGGGCTAGATTCCCTAAGAGT   12862
                                 ************************************************************

gb:MT020781|Organism:Severe      GATGGAACTGGTACTATCTATACAGAACTGGAACCACCTTGTAGGTTTGTTACAGACACA   12910
gb:MT007544|Organism:Severe      GATGGAACTGGTACTATCTATACAGAACTGGAACCACCTTGTAGGTTTGTTACAGACACA   12922
gb:MN994467|Organism:Severe      GATGGAACTGGTACTATCTATACAGAACTGGAACCACCTTGTAGGTTTGTTACAGACACA   12922
gb:MT044257|Organism:Severe      GATGGAACTGGTACTATCTATACAGAACTGGAACCACCTTGTAGGTTTGTTACAGACACA   12922
gb:MT106054|Organism:Severe      GATGGAACTGGTACTATCTATACAGAACTGGAACCACCTTGTAGGTTTGTTACAGACACA   12922
gb:MT049951|Organism:Severe      GATGGAACTGGTACTATCTATACAGAACTGGAACCACCTTGTAGGTTTGTTACAGACACA   12922
gb:MN975262|Organism:Severe      GATGGAACTGGTACTATCTATACAGAACTGGAACCACCTTGTAGGTTTGTTACAGACACA   12922
gb:MT106052|Organism:Severe      GATGGAACTGGTACTATCTATACAGAACTGGAACCACCTTGTAGGTTTGTTACAGACACA   12922
gb:LC522975|Organism:Severe      GATGGAACTGGTACTATCTATACAGAACTGGAACCACCTTGTAGGTTTGTTACAGACACA   12919
gb:LC522973|Organism:Severe      GATGGAACTGGTACTATCTATACAGAACTGGAACCACCTTGTAGGTTTGTTACAGACACA   12919
gb:LC522974|Organism:Severe      GATGGAACTGGTACTATCTATACAGAACTGGAACCACCTTGTAGGTTTGTTACAGACACA   12919
gb:MN985325|Organism:Severe      GATGGAACTGGTACTATCTATACAGAACTGGAACCACCTTGTAGGTTTGTTACAGACACA   12922
gb:MT020881|Organism:Severe      GATGGAACTGGTACTATCTATACAGAACTGGAACCACCTTGTAGGTTTGTTACAGACACA   12922
gb:MT020880|Organism:Severe      GATGGAACTGGTACTATCTATACAGAACTGGAACCACCTTGTAGGTTTGTTACAGACACA   12922
gb:MT066175|Organism:Severe      GATGGAACTGGTACTATCTATACAGAACTGGAACCACCTTGTAGGTTTGTTACAGACACA   12922
gb:MN997409|Organism:Severe      GATGGAACTGGTACTATCTATACAGAACTGGAACCACCTTGTAGGTTTGTTACAGACACA   12922
gb:MN938384|Organism:Severe      GATGGAACTGGTACTATCTATACAGAACTGGAACCACCTTGTAGGTTTGTTACAGACACA   12890
gb:MT044258|Organism:Severe      GATGGAACTGGTACTATCTATACAGAACTGGAACCACCTTGTAGGTTTGTTACAGACACA   12898
gb:MT039890|Organism:Severe      GATGGAACTGGTACTATCTATACAGAACTGGAACCACCTTGTAGGTTTGTTACAGACACA   12922
gb:MN988713|Organism:Severe      GATGGAACTGGTACTATCTATACAGAACTGGAACCACCTTGTAGGTTTGTTACAGACACA   12922
gb:LC521925|Organism:Severe      GATGGAACTGGTACTATCTATACAGAACTGGAACCACCTTGTAGGTTTGTTACAGACACA   12895
gb:MT093571|Organism:Severe      GATGGAACTGGTACTATCTATACAGAACTGGAACCACCTTGTAGGTTTGTTACAGACACA   12922
gb:MT039887|Organism:Severe      GATGGAACTGGTACTATCTATACAGAACTGGAACCACCTTGTAGGTTTGTTACAGACACA   12922
gb:MT019530|Organism:Severe      GATGGAACTGGTACTATCTATACAGAACTGGAACCACCTTGTAGGTTTGTTACAGACACA   12922
gb:MT039888|Organism:Severe      GATGGAACTGGTACTATCTATACAGAACTGGAACCACCTTGTAGGTTTGTTACAGACACA   12922
gb:LC522972|Organism:Severe      GATGGAACTGGTACTATCTATACAGAACTGGAACCACCTTGTAGGTTTGTTACAGACACA   12919
gb:MT027063|Organism:Severe      GATGGAACTGGTACTATCTATACAGAACTGGAACCACCTTGTAGGTTTGTTACAGACACA   12922
gb:MT027062|Organism:Severe      GATGGAACTGGTACTATCTATACAGAACTGGAACCACCTTGTAGGTTTGTTACAGACACA   12922
gb:MT019529|Organism:Severe      GATGGAACTGGTACTATCTATACAGAACTGGAACCACCTTGTAGGTTTGTTACAGACACA   12922
gb:MN996529|Organism:Severe      GATGGAACTGGTACTATCTATACAGAACTGGAACCACCTTGTAGGTTTGTTACAGACACA   12910
gb:MN996531|Organism:Severe      GATGGAACTGGTACTATCTATACAGAACTGGAACCACCTTGTAGGTTTGTTACAGACACA   12909
gb:MT066176|Organism:Severe      GATGGAACTGGTACTATCTATACAGAACTGGAACCACCTTGTAGGTTTGTTACAGACACA   12922
gb:MT027064|Organism:Severe      GATGGAACTGGTACTATCTATACAGAACTGGAACCACCTTGTAGGTTTGTTACAGACACA   12922
gb:MN994468|Organism:Severe      GATGGAACTGGTACTATCTATACAGAACTGGAACCACCTTGTAGGTTTGTTACAGACACA   12922
gb:MT072688|Organism:Severe      GATGGAACTGGTACTATCTATACAGAACTGGAACCACCTTGTAGGTTTGTTACAGACACA   12907
gb:MN996527|Organism:Severe      GATGGAACTGGTACTATCTATACAGAACTGGAACCACCTTGTAGGTTTGTTACAGACACA   12889
gb:MT093631|Organism:Severe      GATGGAACTGGTACTATCTATACAGAACTGGAACCACCTTGTAGGTTTGTTACAGACACA   12960
gb:MT106053|Organism:Severe      GATGGAACTGGTACTATCTATACAGAACTGGAACCACCTTGTAGGTTTGTTACAGACACA   12922
gb:MT019533|Organism:Severe      GATGGAACTGGTACTATCTATACAGAACTGGAACCACCTTGTAGGTTTGTTACAGACACA   12922
gb:MT019531|Organism:Severe      GATGGAACTGGTACTATCTATACAGAACTGGAACCACCTTGTAGGTTTGTTACAGACACA   12922
gb:MN996528|Organism:Severe      GATGGAACTGGTACTATCTATACAGAACTGGAACCACCTTGTAGGTTTGTTACAGACACA   12922
gb:MN996530|Organism:Severe      GATGGAACTGGTACTATCTATACAGAACTGGAACCACCTTGTAGGTTTGTTACAGACACA   12908
gb:MN908947|Organism:Severe      GATGGAACTGGTACTATCTATACAGAACTGGAACCACCTTGTAGGTTTGTTACAGACACA   12922
gb:MT019532|Organism:Severe      GATGGAACTGGTACTATCTATACAGAACTGGAACCACCTTGTAGGTTTGTTACAGACACA   12922
                                 ************************************************************

gb:MT020781|Organism:Severe      CCTAAAGGTCCTAAAGTGAAGTATTTATACTTTATTAAAGGATTAAACAACCTAAATAGA   12970
gb:MT007544|Organism:Severe      CCTAAAGGTCCTAAAGTGAAGTATTTATACTTTATTAAAGGATTAAACAACCTAAATAGA   12982
gb:MN994467|Organism:Severe      CCTAAAGGTCCTAAAGTGAAGTATTTATACTTTATTAAAGGATTAAACAACCTAAATAGA   12982
gb:MT044257|Organism:Severe      CCTAAAGGTCCTAAAGTGAAGTATTTATACTTTATTAAAGGATTAAACAACCTAAATAGA   12982
gb:MT106054|Organism:Severe      CCTAAAGGTCCTAAAGTGAAGTATTTATACTTTATTAAAGGATTAAACAACCTAAATAGA   12982
```

```
gb:MT049951|Organism:Severe      CCTAAAGGTCCTAAAGTGAAGTATTTATACTTTATTAAAGGATTAAACAACCTAAATAGA   12982
gb:MN975262|Organism:Severe      CCTAAAGGTCCTAAAGTGAAGTATTTATACTTTATTAAAGGATTAAACAACCTAAATAGA   12982
gb:MT106052|Organism:Severe      CCTAAAGGTCCTAAAGTGAAGTATTTATACTTTATTAAAGGATTAAACAACCTAAATAGA   12982
gb:LC522975|Organism:Severe      CCTAAAGGTCCTAAAGTGAAGTATTTATACTTTATTAAAGGATTAAACAACCTAAATAGA   12979
gb:LC522973|Organism:Severe      CCTAAAGGTCCTAAAGTGAAGTATTTATACTTTATTAAAGGATTAAACAACCTAAATAGA   12979
gb:LC522974|Organism:Severe      CCTAAAGGTCCTAAAGTGAAGTATTTATACTTTATTAAAGGATTAAACAACCTAAATAGA   12979
gb:MN985325|Organism:Severe      CCTAAAGGTCCTAAAGTGAAGTATTTATACTTTATTAAAGGATTAAACAACCTAAATAGA   12982
gb:MT020881|Organism:Severe      CCTAAAGGTCCTAAAGTGAAGTATTTATACTTTATTAAAGGATTAAACAACCTAAATAGA   12982
gb:MT020880|Organism:Severe      CCTAAAGGTCCTAAAGTGAAGTATTTATACTTTATTAAAGGATTAAACAACCTAAATAGA   12982
gb:MT066175|Organism:Severe      CCTAAAGGTCCTAAAGTGAAGTATTTATACTTTATTAAAGGATTAAACAACCTAAATAGA   12982
gb:MN997409|Organism:Severe      CCTAAAGGTCCTAAAGTGAAGTATTTATACTTTATTAAAGGATTAAACAACCTAAATAGA   12982
gb:MN938384|Organism:Severe      CCTAAAGGTCCTAAAGTGAAGTATTTATACTTTATTAAAGGATTAAACAACCTAAATAGA   12950
gb:MT044258|Organism:Severe      CCTAAAGGTCCTAAAGTGAAGTATTTATACTTTATTAAAGGATTAAACAACCTAAATAGA   12958
gb:MT039890|Organism:Severe      CCTAAAGGTCCTAAAGTGAAGTATTTATACTTTATTAAAGGATTAAACAACCTAAATAGA   12982
gb:MN988713|Organism:Severe      CCTAAAGGTCCTAAAGTGAAGTATTTATACTTTATTAAAGGATTAAACAACCTAAATAGA   12982
gb:LC521925|Organism:Severe      CCTAAAGGTCCTAAAGTGAAGTATTTATACTTTATTAAAGGATTAAACAACCTAAATAGA   12955
gb:MT093571|Organism:Severe      CCTAAAGGTCCTAAAGTGAAGTATTTATACTTTATTAAAGGATTAAACAACCTAAATAGA   12982
gb:MT039887|Organism:Severe      CCTAAAGGTCCTAAAGTGAAGTATTTATACTTTATTAAAGGATTAAACAACCTAAATAGA   12982
gb:MT019530|Organism:Severe      CCTAAAGGTCCTAAAGTGAAGTATTTATACTTTATTAAAGGATTAAACAACCTAAATAGA   12982
gb:MT039888|Organism:Severe      CCTAAAGGTCCTAAAGTGAAGTATTTATACTTTATTAAAGGATTAAACAACCTAAATAGA   12982
gb:LC522972|Organism:Severe      CCTAAAGGTCCTAAAGTGAAGTATTTATACTTTATTAAAGGATTAAACAACCTAAATAGA   12979
gb:MT027063|Organism:Severe      CCTAAAGGTCCTAAAGTGAAGTATTTATACTTTATTAAAGGATTAAACAACCTAAATAGA   12982
gb:MT027062|Organism:Severe      CCTAAAGGTCCTAAAGTGAAGTATTTATACTTTATTAAAGGATTAAACAACCTAAATAGA   12982
gb:MT019529|Organism:Severe      CCTAAAGGTCCTAAAGTGAAGTATTTATACTTTATTAAAGGATTAAACAACCTAAATAGA   12982
gb:MN996529|Organism:Severe      CCTAAAGGTCCTAAAGTGAAGTATTTATACTTTATTAAAGGATTAAACAACCTAAATAGA   12970
gb:MN996531|Organism:Severe      CCTAAAGGTCCTAAAGTGAAGTATTTATACTTTATTAAAGGATTAAACAACCTAAATAGA   12969
gb:MT066176|Organism:Severe      CCTAAAGGTCCTAAAGTGAAGTATTTATACTTTATTAAAGGATTAAACAACCTAAATAGA   12982
gb:MT027064|Organism:Severe      CCTAAAGGTCCTAAAGTGAAGTATTTATACTTTATTAAAGGATTAAACAACCTAAATAGA   12982
gb:MN994468|Organism:Severe      CCTAAAGGTCCTAAAGTGAAGTATTTATACTTTATTAAAGGATTAAACAACCTAAATAGA   12982
gb:MT072688|Organism:Severe      CCTAAAGGTCCTAAAGTGAAGTATTTATACTTTATTAAAGGATTAAACAACCTAAATAGA   12967
gb:MN996527|Organism:Severe      CCTAAAGGTCCTAAAGTGAAGTATTTATACTTTATTAAAGGATTAAACAACCTAAATAGA   12949
gb:MT093631|Organism:Severe      CCTAAAGGTCCTAAAGTGAAGTATTTATACTTTATTAAAGGATTAAACAACCTAAATAGA   13020
gb:MT106053|Organism:Severe      CCTAAAGGTCCTAAAGTGAAGTATTTATACTTTATTAAAGGATTAAACAACCTAAATAGA   12982
gb:MT019533|Organism:Severe      CCTAAAGGTCCTAAAGTGAAGTATTTATACTTTATTAAAGGATTAAACAACCTAAATAGA   12982
gb:MT019531|Organism:Severe      CCTAAAGGTCCTAAAGTGAAGTATTTATACTTTATTAAAGGATTAAACAACCTAAATAGA   12982
gb:MN996528|Organism:Severe      CCTAAAGGTCCTAAAGTGAAGTATTTATACTTTATTAAAGGATTAAACAACCTAAATAGA   12982
gb:MN996530|Organism:Severe      CCTAAAGGTCCTAAAGTGAAGTATTTATACTTTATTAAAGGATTAAACAACCTAAATAGA   12968
gb:MN908947|Organism:Severe      CCTAAAGGTCCTAAAGTGAAGTATTTATACTTTATTAAAGGATTAAACAACCTAAATAGA   12982
gb:MT019532|Organism:Severe      CCTAAAGGTCCTAAAGTGAAGTATTTATACTTTATTAAAGGATTAAACAACCTAAATAGA   12982
                                 ************************************************************

gb:MT020781|Organism:Severe      GGTATGGTACTTGGTAGTTTAGCTGCCACAGTACGTCTACAAGCTGGTAATGCAACAGAA   13030
gb:MT007544|Organism:Severe      GGTATGGTACTTGGTAGTTTAGCTGCCACAGTACGTCTACAAGCTGGTAATGCAACAGAA   13042
gb:MN994467|Organism:Severe      GGTATGGTACTTGGTAGTTTAGCTGCCACAGTACGTCTACAAGCTGGTAATGCAACAGAA   13042
gb:MT044257|Organism:Severe      GGTATGGTACTTGGTAGTTTAGCTGCCACAGTACGTCTACAAGCTGGTAATGCAACAGAA   13042
gb:MT106054|Organism:Severe      GGTATGGTACTTGGTAGTTTAGCTGCCACAGTACGTCTACAAGCTGGTAATGCAACAGAA   13042
gb:MT049951|Organism:Severe      GGTATGGTACTTGGTAGTTTAGCTGCCACAGTACGTCTACAAGCTGGTAATGCAACAGAA   13042
gb:MN975262|Organism:Severe      GGTATGGTACTTGGTAGTTTAGCTGCCACAGTACGTCTACAAGCTGGTAATGCAACAGAA   13042
gb:MT106052|Organism:Severe      GGTATGGTACTTGGTAGTTTAGCTGCCACAGTACGTCTACAAGCTGGTAATGCAACAGAA   13042
gb:LC522975|Organism:Severe      GGTATGGTACTTGGTAGTTTAGCTGCCACAGTACGTCTACAAGCTGGTAATGCAACAGAA   13039
gb:LC522973|Organism:Severe      GGTATGGTACTTGGTAGTTTAGCTGCCACAGTACGTCTACAAGCTGGTAATGCAACAGAA   13039
gb:LC522974|Organism:Severe      GGTATGGTACTTGGTAGTTTAGCTGCCACAGTACGTCTACAAGCTGGTAATGCAACAGAA   13039
gb:MN985325|Organism:Severe      GGTATGGTACTTGGTAGTTTAGCTGCCACAGTACGTCTACAAGCTGGTAATGCAACAGAA   13042
gb:MT020881|Organism:Severe      GGTATGGTACTTGGTAGTTTAGCTGCCACAGTACGTCTACAAGCTGGTAATGCAACAGAA   13042
gb:MT020880|Organism:Severe      GGTATGGTACTTGGTAGTTTAGCTGCCACAGTACGTCTACAAGCTGGTAATGCAACAGAA   13042
gb:MT066175|Organism:Severe      GGTATGGTACTTGGTAGTTTAGCTGCCACAGTACGTCTACAAGCTGGTAATGCAACAGAA   13042
gb:MN997409|Organism:Severe      GGTATGGTACTTGGTAGTTTAGCTGCCACAGTACGTCTACAAGCTGGTAATGCAACAGAA   13042
gb:MN938384|Organism:Severe      GGTATGGTACTTGGTAGTTTAGCTGCCACAGTACGTCTACAAGCTGGTAATGCAACAGAA   13010
gb:MT044258|Organism:Severe      GGTATGGTACTTGGTAGTTTAGCTGCCACAGTACGTCTACAAGCTGGTAATGCAACAGAA   13018
gb:MT039890|Organism:Severe      GGTATGGTACTTGGTAGTTTAGCTGCCACAGTACGTCTACAAGCTGGTAATGCAACAGAA   13042
gb:MN988713|Organism:Severe      GGTATGGTACTTGGTAGTTTAGCTGCCACAGTACGTCTACAAGCTGGTAATGCAACAGAA   13042
gb:LC521925|Organism:Severe      GGTATGGTACTTGGTAGTTTAGCTGCCACAGTACGTCTACAAGCTGGTAATGCAACAGAA   13015
gb:MT093571|Organism:Severe      GGTATGGTACTTGGTAGTTTAGCTGCCACAGTACGTCTACAAGCTGGTAATGCAACAGAA   13042
gb:MT039887|Organism:Severe      GGTATGGTACTTGGTAGTTTAGCTGCCACAGTACGTCTACAAGCTGGTAATGCAACAGAA   13042
gb:MT019530|Organism:Severe      GGTATGGTACTTGGTAGTTTAGCTGCCACAGTACGTCTACAAGCTGGTAATGCAACAGAA   13042
```

```
gb:MT039888|Organism:Severe      GGTATGGTACTTGGTAGTTTAGCTGCCACAGTACGTCTACAAGCTGGTAATGCAACAGAA   13042
gb:LC522972|Organism:Severe      GGTATGGTACTTGGTAGTTTAGCTGCCACAGTACGTCTACAAGCTGGTAATGCAACAGAA   13039
gb:MT027063|Organism:Severe      GGTATGGTACTTGGTAGTTTAGCTGCCACAGTACGTCTACAAGCTGGTAATGCAACAGAA   13042
gb:MT027062|Organism:Severe      GGTATGGTACTTGGTAGTTTAGCTGCCACAGTACGTCTACAAGCTGGTAATGCAACAGAA   13042
gb:MT019529|Organism:Severe      GGTATGGTACTTGGTAGTTTAGCTGCCACAGTACGTCTACAAGCTGGTAATGCAACAGAA   13042
gb:MN996529|Organism:Severe      GGTATGGTACTTGGTAGTTTAGCTGCCACAGTACGTCTACAAGCTGGTAATGCAACAGAA   13030
gb:MN996531|Organism:Severe      GGTATGGTACTTGGTAGTTTAGCTGCCACAGTACGTCTACAAGCTGGTAATGCAACAGAA   13029
gb:MT066176|Organism:Severe      GGTATGGTACTTGGTAGTTTAGCTGCCACAGTACGTCTACAAGCTGGTAATGCAACAGAA   13042
gb:MT027064|Organism:Severe      GGTATGGTACTTGGTAGTTTAGCTGCCACAGTACGTCTACAAGCTGGTAATGCAACAGAA   13042
gb:MN994468|Organism:Severe      GGTATGGTACTTGGTAGTTTAGCTGCCACAGTACGTCTACAAGCTGGTAATGCAACAGAA   13042
gb:MT072688|Organism:Severe      GGTATGGTACTTGGTAGTTTAGCTGCCACAGTACGTCTACAAGCTGGTAATGCAACAGAA   13027
gb:MN996527|Organism:Severe      GGTATGGTACTTGGTAGTTTAGCTGCCACAGTACGTCTACAAGCTGGTAATGCAACAGAA   13009
gb:MT093631|Organism:Severe      GGTATGGTACTTGGTAGTTTAGCTGCCACAGTACGTCTACAAGCTGGTAATGCAACAGAA   13080
gb:MT106053|Organism:Severe      GGTATGGTACTTGGTAGTTTAGCTGCCACAGTACGTCTACAAGCTGGTAATGCAACAGAA   13042
gb:MT019533|Organism:Severe      GGTATGGTACTTGGTAGTTTAGCTGCCACAGTACGTCTACAAGCTGGTAATGCAACAGAA   13042
gb:MT019531|Organism:Severe      GGTATGGTACTTGGTAGTTTAGCTGCCACAGTACGTCTACAAGCTGGTAATGCAACAGAA   13042
gb:MN996528|Organism:Severe      GGTATGGTACTTGGTAGTTTAGCTGCCACAGTACGTCTACAAGCTGGTAATGCAACAGAA   13042
gb:MN996530|Organism:Severe      GGTATGGTACTTGGTAGTTTAGCTGCCACAGTACGTCTACAAGCTGGTAATGCAACAGAA   13028
gb:MN908947|Organism:Severe      GGTATGGTACTTGGTAGTTTAGCTGCCACAGTACGTCTACAAGCTGGTAATGCAACAGAA   13042
gb:MT019532|Organism:Severe      GGTATGGTACTTGGTAGTTTAGCTGCCACAGTACGTCTACAAGCTGGTAATGCAACAGAA   13042
                                 ************************************************************

gb:MT020781|Organism:Severe      GTGCCTGCCAATTCAACTGTATTATCTTTCTGTGCTTTTGCTGTAGATGCTGCTAAAGCT   13090
gb:MT007544|Organism:Severe      GTGCCTGCCAATTCAACTGTATTATCTTTCTGTGCTTTTGCTGTAGATGCTGCTAAAGCT   13102
gb:MN994467|Organism:Severe      GTGCCTGCCAATTCAACTGTATTATCTTTCTGTGCTTTTGCTGTAGATGCTGCTAAAGCT   13102
gb:MT044257|Organism:Severe      GTGCCTGCCAATTCAACTGTATTATCTTTCTGTGCTTTTGCTGTAGATGCTGCTAAAGCT   13102
gb:MT106054|Organism:Severe      GTGCCTGCCAATTCAACTGTATTATCTTTCTGTGCTTTTGCTGTAGATGCTGCTAAAGCT   13102
gb:MT049951|Organism:Severe      GTGCCTGCCAATTCAACTGTATTATCTTTCTGTGCTTTTGCTGTAGATGCTGCTAAAGCT   13102
gb:MN975262|Organism:Severe      GTGCCTGCCAATTCAACTGTATTATCTTTCTGTGCTTTTGCTGTAGATGCTGCTAAAGCT   13102
gb:MT106052|Organism:Severe      GTGCCTGCCAATTCAACTGTATTATCTTTCTGTGCTTTTGCTGTAGATGCTGCTAAAGCT   13102
gb:LC522975|Organism:Severe      GTGCCTGCCAATTCAACTGTATTATCTTTCTGTGCTTTTGCTGTAGATGCTGCTAAAGCT   13099
gb:LC522973|Organism:Severe      GTGCCTGCCAATTCAACTGTATTATCTTTCTGTGCTTTTGCTGTAGATGCTGCTAAAGCT   13099
gb:LC522974|Organism:Severe      GTGCCTGCCAATTCAACTGTATTATCTTTCTGTGCTTTTGCTGTAGATGCTGCTAAAGCT   13099
gb:MN985325|Organism:Severe      GTGCCTGCCAATTCAACTGTATTATCTTTCTGTGCTTTTGCTGTAGATGCTGCTAAAGCT   13102
gb:MT020881|Organism:Severe      GTGCCTGCCAATTCAACTGTATTATCTTTCTGTGCTTTTGCTGTAGATGCTGCTAAAGCT   13102
gb:MT020880|Organism:Severe      GTGCCTGCCAATTCAACTGTATTATCTTTCTGTGCTTTTGCTGTAGATGCTGCTAAAGCT   13102
gb:MT066175|Organism:Severe      GTGCCTGCCAATTCAACTGTATTATCTTTCTGTGCTTTTGCTGTAGATGCTGCTAAAGCT   13102
gb:MN997409|Organism:Severe      GTGCCTGCCAATTCAACTGTATTATCTTTCTGTGCTTTTGCTGTAGATGCTGCTAAAGCT   13102
gb:MN938384|Organism:Severe      GTGCCTGCCAATTCAACTGTATTATCTTTCTGTGCTTTTGCTGTAGATGCTGCTAAAGCT   13070
gb:MT044258|Organism:Severe      GTGCCTGCCAATTCAACTGTATTATCTTTCTGTGCTTTTGCTGTAGATGCTGCTAAAGCT   13078
gb:MT039890|Organism:Severe      GTGCCTGCCAATTCAACTGTATTATCTTTCTGTGCTTTTGCTGTAGATGCTGCTAAAGCT   13102
gb:MN988713|Organism:Severe      GTGCCTGCCAATTCAACTGTATTATCTTTCTGTGCTTTTGCTGTAGATGCTGCTAAAGCT   13102
gb:LC521925|Organism:Severe      GTGCCTGCCAATTCAACTGTATTATCTTTCTGTGCTTTTGCTGTAGATGCTGCTAAAGCT   13075
gb:MT093571|Organism:Severe      GTGCCTGCCAATTCAACTGTATTATCTTTCTGTGCTTTTGCTGTAGATGCTGCTAAAGCT   13102
gb:MT039887|Organism:Severe      GTGCCTGCCAATTCAACTGTATTATCTTTCTGTGCTTTTGCTGTAGATGCTGCTAAAGCT   13102
gb:MT019530|Organism:Severe      GTGCCTGCCAATTCAACTGTATTATCTTTCTGTGCTTTTGCTGTAGATGCTGCTAAAGCT   13102
gb:MT039888|Organism:Severe      GTGCCTGCCAATTCAACTGTATTATCTTTCTGTGCTTTTGCTGTAGATGCTGCTAAAGCT   13102
gb:LC522972|Organism:Severe      GTGCCTGCCAATTCAACTGTATTATCTTTCTGTGCTTTTGCTGTAGATGCTGCTAAAGCT   13099
gb:MT027063|Organism:Severe      GTGCCTGCCAATTCAACTGTATTATCTTTCTGTGCTTTTGCTGTAGATGCTGCTAAAGCT   13102
gb:MT027062|Organism:Severe      GTGCCTGCCAATTCAACTGTATTATCTTTCTGTGCTTTTGCTGTAGATGCTGCTAAAGCT   13102
gb:MT019529|Organism:Severe      GTGCCTGCCAATTCAACTGTATTATCTTTCTGTGCTTTTGCTGTAGATGCTGCTAAAGCT   13102
gb:MN996529|Organism:Severe      GTGCCTGCCAATTCAACTGTATTATCTTTCTGTGCTTTTGCTGTAGATGCTGCTAAAGCT   13090
gb:MN996531|Organism:Severe      GTGCCTGCCAATTCAACTGTATTATCTTTCTGTGCTTTTGCTGTAGATGCTGCTAAAGCT   13089
gb:MT066176|Organism:Severe      GTGCCTGCCAATTCAACTGTATTATCTTTCTGTGCTTTTGCTGTAGATGCTGCTAAAGCT   13102
gb:MT027064|Organism:Severe      GTGCCTGCCAATTCAACTGTATTATCTTTCTGTGCTTTTGCTGTAGATGCTGCTAAAGCT   13102
gb:MN994468|Organism:Severe      GTGCCTGCCAATTCAACTGTATTATCTTTCTGTGCTTTTGCTGTAGATGCTGCTAAAGCT   13102
gb:MT072688|Organism:Severe      GTGCCTGCCAATTCAACTGTATTATCTTTCTGTGCTTTTGCTGTAGATGCTGCTAAAGCT   13087
gb:MN996527|Organism:Severe      GTGCCTGCCAATTCAACTGTATTATCTTTCTGTGCTTTTGCTGTAGATGCTGCTAAAGCT   13069
gb:MT093631|Organism:Severe      GTGCCTGCCAATTCAACTGTATTATCTTTCTGTGCTTTTGCTGTAGATGCTGCTAAAGCT   13140
gb:MT106053|Organism:Severe      GTGCCTGCCAATTCAACTGTATTATCTTTCTGTGCTTTTGCTGTAGATGCTGCTAAAGCT   13102
gb:MT019533|Organism:Severe      GTGCCTGCCAATTCAACTGTATTATCTTTCTGTGCTTTTGCTGTAGATGCTGCTAAAGCT   13102
gb:MT019531|Organism:Severe      GTGCCTGCCAATTCAACTGTATTATCTTTCTGTGCTTTTGCTGTAGATGCTGCTAAAGCT   13102
gb:MN996528|Organism:Severe      GTGCCTGCCAATTCAACTGTATTATCTTTCTGTGCTTTTGCTGTAGATGCTGCTAAAGCT   13102
gb:MN996530|Organism:Severe      GTGCCTGCCAATTCAACTGTATTATCTTTCTGTGCTTTTGCTGTAGATGCTGCTAAAGCT   13088
gb:MN908947|Organism:Severe      GTGCCTGCCAATTCAACTGTATTATCTTTCTGTGCTTTTGCTGTAGATGCTGCTAAAGCT   13102
```

```
gb:MT019532|Organism:Severe      GTGCCTGCCAATTCAACTGTATTATCTTTCTGTGCTTTTGCTGTAGATGCTGCTAAAGCT   13102
                                 ************************************************************

gb:MT020781|Organism:Severe      TACAAAGATTATCTAGCTAGTGGGGGACAACCAATCACTAATTGTGTTAAGATGTTGTGT   13150
gb:MT007544|Organism:Severe      TACAAAGATTATCTAGCTAGTGGGGGACAACCAATCACTAATTGTGTTAAGATGTTGTGT   13162
gb:MN994467|Organism:Severe      TACAAAGATTATCTAGCTAGTGGGGGACAACCAATCACTAATTGTGTTAAGATGTTGTGT   13162
gb:MT044257|Organism:Severe      TACAAAGATTATCTAGCTAGTGGGGGACAACCAATCACTAATTGTGTTAAGATGTTGTGT   13162
gb:MT106054|Organism:Severe      TACAAAGATTATCTAGCTAGTGGGGGACAACCAATCACTAATTGTGTTAAGATGTTGTGT   13162
gb:MT049951|Organism:Severe      TACAAAGATTATCTAGCTAGTGGGGGACAACCAATCACTAATTGTGTTAAGATGTTGTGT   13162
gb:MN975262|Organism:Severe      TACAAAGATTATCTAGCTAGTGGGGGACAACCAATCACTAATTGTGTTAAGATGTTGTGT   13162
gb:MT106052|Organism:Severe      TACAAAGATTATCTAGCTAGTGGGGGACAACCAATCACTAATTGTGTTAAGATGTTGTGT   13162
gb:LC522975|Organism:Severe      TACAAAGATTATCTAGCTAGTGGGGGACAACCAATCACTAATTGTGTTAAGATGTTGTGT   13159
gb:LC522973|Organism:Severe      TACAAAGATTATCTAGCTAGTGGGGGACAACCAATCACTAATTGTGTTAAGATGTTGTGT   13159
gb:LC522974|Organism:Severe      TACAAAGATTATCTAGCTAGTGGGGGACAACCAATCACTAATTGTGTTAAGATGTTGTGT   13159
gb:MN985325|Organism:Severe      TACAAAGATTATCTAGCTAGTGGGGGACAACCAATCACTAATTGTGTTAAGATGTTGTGT   13162
gb:MT020881|Organism:Severe      TACAAAGATTATCTAGCTAGTGGGGGACAACCAATCACTAATTGTGTTAAGATGTTGTGT   13162
gb:MT020880|Organism:Severe      TACAAAGATTATCTAGCTAGTGGGGGACAACCAATCACTAATTGTGTTAAGATGTTGTGT   13162
gb:MT066175|Organism:Severe      TACAAAGATTATCTAGCTAGTGGGGGACAACCAATCACTAATTGTGTTAAGATGTTGTGT   13162
gb:MN997409|Organism:Severe      TACAAAGATTATCTAGCTAGTGGGGGACAACCAATCACTAATTGTGTTAAGATGTTGTGT   13162
gb:MN938384|Organism:Severe      TACAAAGATTATCTAGCTAGTGGGGGACAACCAATCACTAATTGTGTTAAGATGTTGTGT   13130
gb:MT044258|Organism:Severe      TACAAAGATTATCTAGCTAGTGGGGGACAACCAATCACTAATTGTGTTAAGATGTTGTGT   13138
gb:MT039890|Organism:Severe      TACAAAGATTATCTAGCTAGTGGGGGACAACCAATCACTAATTGTGTTAAGATGTTGTGT   13162
gb:MN988713|Organism:Severe      TACAAAGATTATCTAGCTAGTGGGGGACAACCAATCACTAATTGTGTTAAGATGTTGTGT   13162
gb:LC521925|Organism:Severe      TACAAAGATTATCTAGCTAGTGGGGGACAACCAATCACTAATTGTGTTAAGATGTTGTGT   13135
gb:MT093571|Organism:Severe      TACAAAGATTATCTAGCTAGTGGGGGACAACCAATCACTAATTGTGTTAAGATGTTGTGT   13162
gb:MT039887|Organism:Severe      TACAAAGATTATCTAGCTAGTGGGGGACAACCAATCACTAATTGTGTTAAGATGTTGTGT   13162
gb:MT019530|Organism:Severe      TACAAAGATTATCTAGCTAGTGGGGGACAACCAATCACTAATTGTGTTAAGATGTTGTGT   13162
gb:MT039888|Organism:Severe      TACAAAGATTATCTAGCTAGTGGGGGACAACCAATCACTAATTGTGTTAAGATGTTGTGT   13162
gb:LC522972|Organism:Severe      TACAAAGATTATCTAGCTAGTGGGGGACAACCAATCACTAATTGTGTTAAGATGTTGTGT   13159
gb:MT027063|Organism:Severe      TACAAAGATTATCTAGCTAGTGGGGGACAACCAATCACTAATTGTGTTAAGATGTTGTGT   13162
gb:MT027062|Organism:Severe      TACAAAGATTATCTAGCTAGTGGGGGACAACCAATCACTAATTGTGTTAAGATGTTGTGT   13162
gb:MT019529|Organism:Severe      TACAAAGATTATCTAGCTAGTGGGGGACAACCAATCACTAATTGTGTTAAGATGTTGTGT   13162
gb:MN996529|Organism:Severe      TACAAAGATTATCTAGCTAGTGGGGGACAACCAATCACTAATTGTGTTAAGATGTTGTGT   13150
gb:MN996531|Organism:Severe      TACAAAGATTATCTAGCTAGTGGGGGACAACCAATCACTAATTGTGTTAAGATGTTGTGT   13149
gb:MT066176|Organism:Severe      TACAAAGATTATCTAGCTAGTGGGGGACAACCAATCACTAATTGTGTTAAGATGTTGTGT   13162
gb:MT027064|Organism:Severe      TACAAAGATTATCTAGCTAGTGGGGGACAACCAATCACTAATTGTGTTAAGATGTTGTGT   13162
gb:MN994468|Organism:Severe      TACAAAGATTATCTAGCTAGTGGGGGACAACCAATCACTAATTGTGTTAAGATGTTGTGT   13162
gb:MT072688|Organism:Severe      TACAAAGATTATCTAGCTAGTGGGGGACAACCAATCACTAATTGTGTTAAGATGTTGTGT   13147
gb:MN996527|Organism:Severe      TACAAAGATTATCTAGCTAGTGGGGGACAACCAATCACTAATTGTGTTAAGATGTTGTGT   13129
gb:MT093631|Organism:Severe      TACAAAGATTATCTAGCTAGTGGGGGACAACCAATCACTAATTGTGTTAAGATGTTGTGT   13200
gb:MT106053|Organism:Severe      TACAAAGATTATCTAGCTAGTGGGGGACAACCAATCACTAATTGTGTTAAGATGTTGTGT   13162
gb:MT019533|Organism:Severe      TACAAAGATTATCTAGCTAGTGGGGGACAACCAATCACTAATTGTGTTAAGATGTTGTGT   13162
gb:MT019531|Organism:Severe      TACAAAGATTATCTAGCTAGTGGGGGACAACCAATCACTAATTGTGTTAAGATGTTGTGT   13162
gb:MN996528|Organism:Severe      TACAAAGATTATCTAGCTAGTGGGGGACAACCAATCACTAATTGTGTTAAGATGTTGTGT   13162
gb:MN996530|Organism:Severe      TACAAAGATTATCTAGCTAGTGGGGGACAACCAATCACTAATTGTGTTAAGATGTTGTGT   13148
gb:MN908947|Organism:Severe      TACAAAGATTATCTAGCTAGTGGGGGACAACCAATCACTAATTGTGTTAAGATGTTGTGT   13162
gb:MT019532|Organism:Severe      TACAAAGATTATCTAGCTAGTGGGGGACAACCAATCACTAATTGTGTTAAGATGTTGTGT   13162
                                 ************************************************************

gb:MT020781|Organism:Severe      ACACACACTGGTACTGGTCAGGCAATAACAGTTACACCGGAAGCCAATATGGATCAAGAA   13210
gb:MT007544|Organism:Severe      ACACACACTGGTACTGGTCAGGCAATAACAGTTACACCGGAAGCCAATATGGATCAAGAA   13222
gb:MN994467|Organism:Severe      ACACACACTGGTACTGGTCAGGCAATAACAGTTACACCGGAAGCCAATATGGATCAAGAA   13222
gb:MT044257|Organism:Severe      ACACACACTGGTACTGGTCAGGCAATAACAGTTACACCGGAAGCCAATATGGATCAAGAA   13222
gb:MT106054|Organism:Severe      ACACACACTGGTACTGGTCAGGCAATAACAGTTACACCGGAAGCCAATATGGATCAAGAA   13222
gb:MT049951|Organism:Severe      ACACACACTGGTACTGGTCAGGCAATAACAGTTACACCGGAAGCCAATATGGATCAAGAA   13222
gb:MN975262|Organism:Severe      ACACACACTGGTACTGGTCAGGCAATAACAGTTACACCGGAAGCCAATATGGATCAAGAA   13222
gb:MT106052|Organism:Severe      ACACACACTGGTACTGGTCAGGCAATAACAGTTACACCGGAAGCCAATATGGATCAAGAA   13222
gb:LC522975|Organism:Severe      ACACACACTGGTACTGGTCAGGCAATAACAGTTACACCGGAAGCCAATATGGATCAAGAA   13219
gb:LC522973|Organism:Severe      ACACACACTGGTACTGGTCAGGCAATAACAGTTACACCGGAAGCCAATATGGATCAAGAA   13219
gb:LC522974|Organism:Severe      ACACACACTGGTACTGGTCAGGCAATAACAGTTACACCGGAAGCCAATATGGATCAAGAA   13219
gb:MN985325|Organism:Severe      ACACACACTGGTACTGGTCAGGCAATAACAGTTACACCGGAAGCCAATATGGATCAAGAA   13222
gb:MT020881|Organism:Severe      ACACACACTGGTACTGGTCAGGCAATAACAGTTACACCGGAAGCCAATATGGATCAAGAA   13222
gb:MT020880|Organism:Severe      ACACACACTGGTACTGGTCAGGCAATAACAGTTACACCGGAAGCCAATATGGATCAAGAA   13222
gb:MT066175|Organism:Severe      ACACACACTGGTACTGGTCAGGCAATAACAGTTACACCGGAAGCCAATATGGATCAAGAA   13222
gb:MN997409|Organism:Severe      ACACACACTGGTACTGGTCAGGCAATAACAGTTACACCGGAAGCCAATATGGATCAAGAA   13222
```

```
gb:MN938384|Organism:Severe      ACACACACTGGTACTGGTCAGGCAATAACAGTTACACCGGAAGCCAATATGGATCAAGAA   13190
gb:MT044258|Organism:Severe      ACACACACTGGTACTGGTCAGGCAATAACAGTTACACCGGAAGCCAATATGGATCAAGAA   13198
gb:MT039890|Organism:Severe      ACACACACTGGTACTGGTCAGGCAATAACAGTTACACCGGAAGCCAATATGGATCAAGAA   13222
gb:MN988713|Organism:Severe      ACACACACTGGTACTGGTCAGGCAATAACAGTTACACCGGAAGCCAATATGGATCAAGAA   13222
gb:LC521925|Organism:Severe      ACACACACTGGTACTGGTCAGGCAATAACAGTTACACCGGAAGCCAATATGGATCAAGAA   13195
gb:MT093571|Organism:Severe      ACACACACTGGTACTGGTCAGGCAATAACAGTTACACCGGAAGCCAATATGGATCAAGAA   13222
gb:MT039887|Organism:Severe      ACACACACTGGTACTGGTCAGGCAATAACAGTTACACCGGAAGCCAATATGGATCAAGAA   13222
gb:MT019530|Organism:Severe      ACACACACTGGTACTGGTCAGGCAATAACAGTTACACCGGAAGCCAATATGGATCAAGAA   13222
gb:MT039888|Organism:Severe      ACACACACTGGTACTGGTCAGGCAATAACAGTTACACCGGAAGCCAATATGGATCAAGAA   13222
gb:LC522972|Organism:Severe      ACACACACTGGTACTGGTCAGGCAATAACAGTTACACCGGAAGCCAATATGGATCAAGAA   13219
gb:MT027063|Organism:Severe      ACACACACTGGTACTGGTCAGGCAATAACAGTTACACCGGAAGCCAATATGGATCAAGAA   13222
gb:MT027062|Organism:Severe      ACACACACTGGTACTGGTCAGGCAATAACAGTTACACCGGAAGCCAATATGGATCAAGAA   13222
gb:MT019529|Organism:Severe      ACACACACTGGTACTGGTCAGGCAATAACAGTTACACCGGAAGCCAATATGGATCAAGAA   13222
gb:MN996529|Organism:Severe      ACACACACTGGTACTGGTCAGGCAATAACAGTTACACCGGAAGCCAATATGGATCAAGAA   13210
gb:MN996531|Organism:Severe      ACACACACTGGTACTGGTCAGGCAATAACAGTTACACCGGAAGCCAATATGGATCAAGAA   13209
gb:MT066176|Organism:Severe      ACACACACTGGTACTGGTCAGGCAATAACAGTTACACCGGAAGCCAATATGGATCAAGAA   13222
gb:MT027064|Organism:Severe      ACACACACTGGTACTGGTCAGGCAATAACAGTTACACCGGAAGCCAATATGGATCAAGAA   13222
gb:MN994468|Organism:Severe      ACACACACTGGTACTGGTCAGGCAATAACAGTTACACCGGAAGCCAATATGGATCAAGAA   13222
gb:MT072688|Organism:Severe      ACACACACTGGTACTGGTCAGGCAATAACAGTTACACCGGAAGCCAATATGGATCAAGAA   13207
gb:MN996527|Organism:Severe      ACACACACTGGTACTGGTCAGGCAATAACAGTTACACCGGAAGCCAATATGGATCAAGAA   13189
gb:MT093631|Organism:Severe      ACACACACTGGTACTGGTCAGGCAATAACAGTTACACCGGAAGCCAATATGGATCAAGAA   13260
gb:MT106053|Organism:Severe      ACACACACTGGTACTGGTCAGGCAATAACAGTTACACCGGAAGCCAATATGGATCAAGAA   13222
gb:MT019533|Organism:Severe      ACACACACTGGTACTGGTCAGGCAATAACAGTTACACCGGAAGCCAATATGGATCAAGAA   13222
gb:MT019531|Organism:Severe      ACACACACTGGTACTGGTCAGGCAATAACAGTTACACCGGAAGCCAATATGGATCAAGAA   13222
gb:MN996528|Organism:Severe      ACACACACTGGTACTGGTCAGGCAATAACAGTTACACCGGAAGCCAATATGGATCAAGAA   13222
gb:MN996530|Organism:Severe      ACACACACTGGTACTGGTCAGGCAATAACAGTTACACCGGAAGCCAATATGGATCAAGAA   13208
gb:MN908947|Organism:Severe      ACACACACTGGTACTGGTCAGGCAATAACAGTTACACCGGAAGCCAATATGGATCAAGAA   13222
gb:MT019532|Organism:Severe      ACACACACTGGTACTGGTCAGGCAATAACAGTTACACCGGAAGCCAATATGGATCAAGAA   13222
                                 ************************************************************

gb:MT020781|Organism:Severe      TCCTTTGGTGGTGCATCGTGTTGTCTGTACTGCCGTTGCCACATAGATCATCCAAATCCT   13270
gb:MT007544|Organism:Severe      TCCTTTGGTGGTGCATCGTGTTGTCTGTACTGCCGTTGCCACATAGATCATCCAAATCCT   13282
gb:MN994467|Organism:Severe      TCCTTTGGTGGTGCATCGTGTTGTCTGTACTGCCGTTGCCACATAGATCATCCAAATCCT   13282
gb:MT044257|Organism:Severe      TCCTTTGGTGGTGCATCGTGTTGTCTGTACTGCCGTTGCCACATAGATCATCCAAATCCT   13282
gb:MT106054|Organism:Severe      TCCTTTGGTGGTGCATCGTGTTGTCTGTACTGCCGTTGCCACATAGATCATCCAAATCCT   13282
gb:MT049951|Organism:Severe      TCCTTTGGTGGTGCATCGTGTTGTCTGTACTGCCGTTGCCACATAGATCATCCAAATCCT   13282
gb:MN975262|Organism:Severe      TCCTTTGGTGGTGCATCGTGTTGTCTGTACTGCCGTTGCCACATAGATCATCCAAATCCT   13282
gb:MT106052|Organism:Severe      TCCTTTGGTGGTGCATCGTGTTGTCTGTACTGCCGTTGCCACATAGATCATCCAAATCCT   13282
gb:LC522975|Organism:Severe      TCCTTTGGTGGTGCATCGTGTTGTCTGTACTGCCGTTGCCACATAGATCATCCAAATCCT   13279
gb:LC522973|Organism:Severe      TCCTTTGGTGGTGCATCGTGTTGTCTGTACTGCCGTTGCCACATAGATCATCCAAATCCT   13279
gb:LC522974|Organism:Severe      TCCTTTGGTGGTGCATCGTGTTGTCTGTACTGCCGTTGCCACATAGATCATCCAAATCCT   13279
gb:MN985325|Organism:Severe      TCCTTTGGTGGTGCATCGTGTTGTCTGTACTGCCGTTGCCACATAGATCATCCAAATCCT   13282
gb:MT020881|Organism:Severe      TCCTTTGGTGGTGCATCGTGTTGTCTGTACTGCCGTTGCCACATAGATCATCCAAATCCT   13282
gb:MT020880|Organism:Severe      TCCTTTGGTGGTGCATCGTGTTGTCTGTACTGCCGTTGCCACATAGATCATCCAAATCCT   13282
gb:MT066175|Organism:Severe      TCCTTTGGTGGTGCATCGTGTTGTCTGTACTGCCGTTGCCACATAGATCATCCAAATCCT   13282
gb:MN997409|Organism:Severe      TCCTTTGGTGGTGCATCGTGTTGTCTGTACTGCCGTTGCCACATAGATCATCCAAATCCT   13282
gb:MN938384|Organism:Severe      TCCTTTGGTGGTGCATCGTGTTGTCTGTACTGCCGTTGCCACATAGATCATCCAAATCCT   13250
gb:MT044258|Organism:Severe      TCCTTTGGTGGTGCATCGTGTTGTCTGTACTGCCGTTGCCACATAGATCATCCAAATCCT   13258
gb:MT039890|Organism:Severe      TCCTTTGGTGGTGCATCGTGTTGTCTGTACTGCCGTTGCCACATAGATCATCCAAATCCT   13282
gb:MN988713|Organism:Severe      TCCTTTGGTGGTGCATCGTGTTGTCTGTACTGCCGTTGCCACATAGATCATCCAAATCCT   13282
gb:LC521925|Organism:Severe      TCCTTTGGTGGTGCATCGTGTTGTCTGTACTGCCGTTGCCACATAGATCATCCAAATCCT   13255
gb:MT093571|Organism:Severe      TCGCTTGGTGGTGCATCGTGTTGTCTGTACTGCCGTTGCCACATAGATCATCCAAATCCT   13282
gb:MT039887|Organism:Severe      TCCTTTGGTGGTGCATCGTGTTGTCTGTACTGCCGTTGCCACATAGATCATCCAAATCCT   13282
gb:MT019530|Organism:Severe      TCCTTTGGTGGTGCATCGTGTTGTCTGTACTGCCGTTGCCACATAGATCATCCAAATCCT   13282
gb:MT039888|Organism:Severe      TCCTTTGGTGGTGCATCGTGTTGTCTGTACTGCCGTTGCCACATAGATCATCCAAATCCT   13282
gb:LC522972|Organism:Severe      TCCTTTGGTGGTGCATCGTGTTGTCTGTACTGCCGTTGCCACATAGATCATCCAAATCCT   13279
gb:MT027063|Organism:Severe      TCCTTTGGTGGTGCATCGTGTTGTCTGTACTGCCGTTGCCACATAGATCATCCAAATCCT   13282
gb:MT027062|Organism:Severe      TCCTTTGGTGGTGCATCGTGTTGTCTGTACTGCCGTTGCCACATAGATCATCCAAATCCT   13282
gb:MT019529|Organism:Severe      TCCTTTGGTGGTGCATCGTGTTGTCTGTACTGCCGTTGCCACATAGATCATCCAAATCCT   13282
gb:MN996529|Organism:Severe      TCCTTTGGTGGTGCATCGTGTTGTCTGTACTGCCGTTGCCACATAGATCATCCAAATCCT   13270
gb:MN996531|Organism:Severe      TCCTTTGGTGGTGCATCGTGTTGTCTGTACTGCCGTTGCCACATAGATCATCCAAATCCT   13269
gb:MT066176|Organism:Severe      TCCTTTGGTGGTGCATCGTGTTGTCTGTACTGCCGTTGCCACATAGATCATCCAAATCCT   13282
gb:MT027064|Organism:Severe      TCCTTTGGTGGTGCATCGTGTTGTCTGTACTGCCGTTGCCACATAGATCATCCAAATCCT   13282
gb:MN994468|Organism:Severe      TCCTTTGGTGGTGCATCGTGTTGTCTGTACTGCCGTTGCCACATAGATCATCCAAATCCT   13282
gb:MT072688|Organism:Severe      TCCTTTGGTGGTGCATCGTGTTGTCTGTACTGCCGTTGCCACATAGATCATCCAAATCCT   13267
```

```
gb:MN996527|Organism:Severe      TCCTTTGGTGGTGCATCGTGTTGTCTGTACTGCCGTTGCCACATAGATCATCCAAATCCT   13249
gb:MT093631|Organism:Severe      TCCTTTGGTGGTGCATCGTGTTGTCTGTACTGCCGTTGCCACATAGATCATCCAAATCCT   13320
gb:MT106053|Organism:Severe      TCCTTTGGTGGTGCATCGTGTTGTCTGTACTGCCGTTGCCACATAGATCATCCAAATCCT   13282
gb:MT019533|Organism:Severe      TCCTTTGGTGGTGCATCGTGTTGTCTGTACTGCCGTTGCCACATAGATCATCCAAATCCT   13282
gb:MT019531|Organism:Severe      TCCTTTGGTGGTGCATCGTGTTGTCTGTACTGCCGTTGCCACATAGATCATCCAAATCCT   13282
gb:MN996528|Organism:Severe      TCCTTTGGTGGTGCATCGTGTTGTCTGTACTGCCGTTGCCACATAGATCATCCAAATCCT   13282
gb:MN996530|Organism:Severe      TCCTTTGGTGGTGCATCGTGTTGTCTGTACTGCCGTTGCCACATAGATCATCCAAATCCT   13268
gb:MN908947|Organism:Severe      TCCTTTGGTGGTGCATCGTGTTGTCTGTACTGCCGTTGCCACATAGATCATCCAAATCCT   13282
gb:MT019532|Organism:Severe      TCCTTTGGTGGTGCATCGTGTTGTCTGTACTGCCGTTGCCACATAGATCATCCAAATCCT   13282
                                 **  ********************************************************

gb:MT020781|Organism:Severe      AAAGGATTTTGTGACTTAAAAGGTAAGTATGTACAAATACCTACAACTTGTGCTAATGAC   13330
gb:MT007544|Organism:Severe      AAAGGATTTTGTGACTTAAAAGGTAAGTATGTACAAATACCTACAACTTGTGCTAATGAC   13342
gb:MN994467|Organism:Severe      AAAGGATTTTGTGACTTAAAAGGTAAGTATGTACAAATACCTACAACTTGTGCTAATGAC   13342
gb:MT044257|Organism:Severe      AAAGGATTTTGTGACTTAAAAGGTAAGTATGTACAAATACCTACAACTTGTGCTAATGAC   13342
gb:MT106054|Organism:Severe      AAAGGATTTTGTGACTTAAAAGGTAAGTATGTACAAATACCTACAACTTGTGCTAATGAC   13342
gb:MT049951|Organism:Severe      AAAGGATTTTGTGACTTAAAAGGTAAGTATGTACAAATACCTACAACTTGTGCTAATGAC   13342
gb:MN975262|Organism:Severe      AAAGGATTTTGTGACTTAAAAGGTAAGTATGTACAAATACCTACAACTTGTGCTAATGAC   13342
gb:MT106052|Organism:Severe      AAAGGATTTTGTGACTTAAAAGGTAAGTATGTACAAATACCTACAACTTGTGCTAATGAC   13342
gb:LC522975|Organism:Severe      AAAGGATTTTGTGACTTAAAAGGTAAGTATGTACAAATACCTACAACTTGTGCTAATGAC   13339
gb:LC522973|Organism:Severe      AAAGGATTTTGTGACTTAAAAGGTAAGTATGTACAAATACCTACAACTTGTGCTAATGAC   13339
gb:LC522974|Organism:Severe      AAAGGATTTTGTGACTTAAAAGGTAAGTATGTACAAATACCTACAACTTGTGCTAATGAC   13339
gb:MN985325|Organism:Severe      AAAGGATTTTGTGACTTAAAAGGTAAGTATGTACAAATACCTACAACTTGTGCTAATGAC   13342
gb:MT020881|Organism:Severe      AAAGGATTTTGTGACTTAAAAGGTAAGTATGTACAAATACCTACAACTTGTGCTAATGAC   13342
gb:MT020880|Organism:Severe      AAAGGATTTTGTGACTTAAAAGGTAAGTATGTACAAATACCTACAACTTGTGCTAATGAC   13342
gb:MT066175|Organism:Severe      AAAGGATTTTGTGACTTAAAAGGTAAGTATGTACAAATACCTACAACTTGTGCTAATGAC   13342
gb:MN997409|Organism:Severe      AAAGGATTTTGTGACTTAAAAGGTAAGTATGTACAAATACCTACAACTTGTGCTAATGAC   13342
gb:MN938384|Organism:Severe      AAAGGATTTTGTGACTTAAAAGGTAAGTATGTACAAATACCTACAACTTGTGCTAATGAC   13310
gb:MT044258|Organism:Severe      AAAGGATTTTGTGACTTAAAAGGTAAGTATGTACAAATACCTACAACTTGTGCTAATGAC   13318
gb:MT039890|Organism:Severe      AAAGGATTTTGTGACTTAAAAGGTAAGTATGTACAAATACCTACAACTTGTGCTAATGAC   13342
gb:MN988713|Organism:Severe      AAAGGATTTTGTGACTTAAAAGGTAAGTATGTACAAATACCTACAACTTGTGCTAATGAC   13342
gb:LC521925|Organism:Severe      AAAGGATTTTGTGACTTAAAAGGTAAGTATGTACAAATACCTACAACTTGTGCTAATGAC   13315
gb:MT093571|Organism:Severe      AAAGGATTTTGTGACTTAAAAGGTAAGTATGTACAAATACCTACAACTTGTGCTAATGAC   13342
gb:MT039887|Organism:Severe      AAAGGATTTTGTGACTTAAAAGGTAAGTATGTACAAATACCTACAACTTGTGCTAATGAC   13342
gb:MT019530|Organism:Severe      AAAGGATTTTGTGACTTAAAAGGTAAGTATGTACAAATACCTACAACTTGTGCTAATGAC   13342
gb:MT039888|Organism:Severe      AAAGGATTTTGTGACTTAAAAGGTAAGTATGTACAAATACCTACAACTTGTGCTAATGAC   13342
gb:LC522972|Organism:Severe      AAAGGATTTTGTGACTTAAAAGGTAAGTATGTACAAATACCTACAACTTGTGCTAATGAC   13339
gb:MT027063|Organism:Severe      AAAGGATTTTGTGACTTAAAAGGTAAGTATGTACAAATACCTACAACTTGTGCTAATGAC   13342
gb:MT027062|Organism:Severe      AAAGGATTTTGTGACTTAAAAGGTAAGTATGTACAAATACCTACAACTTGTGCTAATGAC   13342
gb:MT019529|Organism:Severe      AAAGGATTTTGTGACTTAAAAGGTAAGTATGTACAAATACCTACAACTTGTGCTAATGAC   13342
gb:MN996529|Organism:Severe      AAAGGATTTTGTGACTTAAAAGGTAAGTATGTACAAATACCTACAACTTGTGCTAATGAC   13330
gb:MN996531|Organism:Severe      AAAGGATTTTGTGACTTAAAAGGTAAGTATGTACAAATACCTACAACTTGTGCTAATGAC   13329
gb:MT066176|Organism:Severe      AAAGGATTTTGTGACTTAAAAGGTAAGTATGTACAAATACCTACAACTTGTGCTAATGAC   13342
gb:MT027064|Organism:Severe      AAAGGATTTTGTGACTTAAAAGGTAAGTATGTACAAATACCTACAACTTGTGCTAATGAC   13342
gb:MN994468|Organism:Severe      AAAGGATTTTGTGACTTAAAAGGTAAGTATGTACAAATACCTACAACTTGTGCTAATGAC   13342
gb:MT072688|Organism:Severe      AAAGGATTTTGTGACTTAAAAGGTAAGTATGTACAAATACCTACAACTTGTGCTAATGAC   13327
gb:MN996527|Organism:Severe      AAAGGATTTTGTGACTTAAAAGGTAAGTATGTACAAATACCTACAACTTGTGCTAATGAC   13309
gb:MT093631|Organism:Severe      AAAGGATTTTGTGACTTAAAAGGTAAGTATGTACAAATACCTACAACTTGTGCTAATGAC   13380
gb:MT106053|Organism:Severe      AAAGGATTTTGTGACTTAAAAGGTAAGTATGTACAAATACCTACAACTTGTGCTAATGAC   13342
gb:MT019533|Organism:Severe      AAAGGATTTTGTGACTTAAAAGGTAAGTATGTACAAATACCTACAACTTGTGCTAATGAC   13342
gb:MT019531|Organism:Severe      AAAGGATTTTGTGACTTAAAAGGTAAGTATGTACAAATACCTACAACTTGTGCTAATGAC   13342
gb:MN996528|Organism:Severe      AAAGGATTTTGTGACTTAAAAGGTAAGTATGTACAAATACCTACAACTTGTGCTAATGAC   13342
gb:MN996530|Organism:Severe      AAAGGATTTTGTGACTTAAAAGGTAAGTATGTACAAATACCTACAACTTGTGCTAATGAC   13328
gb:MN908947|Organism:Severe      AAAGGATTTTGTGACTTAAAAGGTAAGTATGTACAAATACCTACAACTTGTGCTAATGAC   13342
gb:MT019532|Organism:Severe      AAAGGATTTTGTGACTTAAAAGGTAAGTATGTACAAATACCTACAACTTGTGCTAATGAC   13342
                                 ************************************************************

gb:MT020781|Organism:Severe      CCTGTGGGTTTTACACTTAAAAACACAGTCTGTACCGTCTGCGGTATGTGGAAAGGTTAT   13390
gb:MT007544|Organism:Severe      CCTGTGGGTTTTACACTTAAAAACACAGTCTGTACCGTCTGCGGTATGTGGAAAGGTTAT   13402
gb:MN994467|Organism:Severe      CCTGTGGGTTTTACACTTAAAAACACAGTCTGTACCGTCTGCGGTATGTGGAAAGGTTAT   13402
gb:MT044257|Organism:Severe      CCTGTGGGTTTTACACTTAAAAACACAGTCTGTACCGTCTGCGGTATGTGGAAAGGTTAT   13402
gb:MT106054|Organism:Severe      CCTGTGGGTTTTACACTTAAAAACACAGTCTGTACCGTCTGCGGTATGTGGAAAGGTTAT   13402
gb:MT049951|Organism:Severe      CCTGTGGGTTTTACACTTAAAAACACAGTCTGTACCGTCTGCGGTATGTGGAAAGGTTAT   13402
gb:MN975262|Organism:Severe      CCTGTGGGTTTTACACTTAAAAACACAGTCTGTACCGTCTGCGGTATGTGGAAAGGTTAT   13402
gb:MT106052|Organism:Severe      CCTGTGGGTTTTACACTTAAAAACACAGTCTGTACCGTCTGCGGTATGTGGAAAGGTTAT   13402
```

```
gb:LC522975|Organism:Severe      CCTGTGGGTTTTACACTTAAAAACACAGTCTGTACCGTCTGCGGTATGTGGAAAGGTTAT   13399
gb:LC522973|Organism:Severe      CCTGTGGGTTTTACACTTAAAAACACAGTCTGTACCGTCTGCGGTATGTGGAAAGGTTAT   13399
gb:LC522974|Organism:Severe      CCTGTGGGTTTTACACTTAAAAACACAGTCTGTACCGTCTGCGGTATGTGGAAAGGTTAT   13399
gb:MN985325|Organism:Severe      CCTGTGGGTTTTACACTTAAAAACACAGTCTGTACCGTCTGCGGTATGTGGAAAGGTTAT   13402
gb:MT020881|Organism:Severe      CCTGTGGGTTTTACACTTAAAAACACAGTCTGTACCGTCTGCGGTATGTGGAAAGGTTAT   13402
gb:MT020880|Organism:Severe      CCTGTGGGTTTTACACTTAAAAACACAGTCTGTACCGTCTGCGGTATGTGGAAAGGTTAT   13402
gb:MT066175|Organism:Severe      CCTGTGGGTTTTACACTTAAAAACACAGTCTGTACCGTCTGCGGTATGTGGAAAGGTTAT   13402
gb:MN997409|Organism:Severe      CCTGTGGGTTTTACACTTAAAAACACAGTCTGTACCGTCTGCGGTATGTGGAAAGGTTAT   13402
gb:MN938384|Organism:Severe      CCTGTGGGTTTTACACTTAAAAACACAGTCTGTACCGTCTGCGGTATGTGGAAAGGTTAT   13370
gb:MT044258|Organism:Severe      CCTGTGGGTTTTACACTTAAAAACACAGTCTGTACCGTCTGCGGTATGTGGAAAGGTTAT   13378
gb:MT039890|Organism:Severe      CCTGTGGGTTTTACACTTAAAAACACAGTCTGTACCGTCTGCGGTATGTGGAAAGGTTAT   13402
gb:MN988713|Organism:Severe      CCTGTGGGTTTTACACTTAAAAACACAGTCTGTACCGTCTGCGGTATGTGGAAAGGTTAT   13402
gb:LC521925|Organism:Severe      CCTGTGGGTTTTACACTTAAAAACACAGTCTGTACCGTCTGCGGTATGTGGAAAGGTTAT   13375
gb:MT093571|Organism:Severe      CCTGTGGGTTTTACACTTAAAAACACAGTCTGTACCGTCTGCGGTATGTGGAAAGGTTAT   13402
gb:MT039887|Organism:Severe      CCTGTGGGTTTTACACTTAAAAACACAGTCTGTACCGTCTGCGGTATGTGGAAAGGTTAT   13402
gb:MT019530|Organism:Severe      CCTGTGGGTTTTACACTTAAAAACACAGTCTGTACCGTCTGCGGTATGTGGAAAGGTTAT   13402
gb:MT039888|Organism:Severe      CCTGTGGGTTTTACACTTAAAAACACAGTCTGTACCGTCTGCGGTATGTGGAAAGGTTAT   13402
gb:LC522972|Organism:Severe      CCTGTGGGTTTTACACTTAAAAACACAGTCTGTACCGTCTGCGGTATGTGGAAAGGTTAT   13399
gb:MT027063|Organism:Severe      CCTGTGGGTTTTACACTTAAAAACACAGTCTGTACCGTCTGCGGTATGTGGAAAGGTTAT   13402
gb:MT027062|Organism:Severe      CCTGTGGGTTTTACACTTAAAAACACAGTCTGTACCGTCTGCGGTATGTGGAAAGGTTAT   13402
gb:MT019529|Organism:Severe      CCTGTGGGTTTTACACTTAAAAACACAGTCTGTACCGTCTGCGGTATGTGGAAAGGTTAT   13402
gb:MN996529|Organism:Severe      CCTGTGGGTTTTACACTTAAAAACACAGTCTGTACCGTCTGCGGTATGTGGAAAGGTTAT   13390
gb:MN996531|Organism:Severe      CCTGTGGGTTTTACACTTAAAAACACAGTCTGTACCGTCTGCGGTATGTGGAAAGGTTAT   13389
gb:MT066176|Organism:Severe      CCTGTGGGTTTTACACTTAAAAACACAGTCTGTACCGTCTGCGGTATGTGGAAAGGTTAT   13402
gb:MT027064|Organism:Severe      CCTGTGGGTTTTACACTTAAAAACACAGTCTGTACCGTCTGCGGTATGTGGAAAGGTTAT   13402
gb:MN994468|Organism:Severe      CCTGTGGGTTTTACACTTAAAAACACAGTCTGTACCGTCTGCGGTATGTGGAAAGGTTAT   13402
gb:MT072688|Organism:Severe      CCTGTGGGTTTTACACTTAAAAACACAGTCTGTACCGTCTGCGGTATGTGGAAAGGTTAT   13387
gb:MN996527|Organism:Severe      CCTGTGGGTTTTACACTTAAAAACACAGTCTGTACCGTCTGCGGTATGTGGAAAGGTTAT   13369
gb:MT093631|Organism:Severe      CCTGTGGGTTTTACACTTAAAAACACAGTCTGTACCGTCTGCGGTATGTGGAAAGGTTAT   13440
gb:MT106053|Organism:Severe      CCTGTGGGTTTTACACTTAAAAACACAGTCTGTACCGTCTGCGGTATGTGGAAAGGTTAT   13402
gb:MT019533|Organism:Severe      CCTGTGGGTTTTACACTTAAAAACACAGTCTGTACCGTCTGCGGTATGTGGAAAGGTTAT   13402
gb:MT019531|Organism:Severe      CCTGTGGGTTTTACACTTAAAAACACAGTCTGTACCGTCTGCGGTATGTGGAAAGGTTAT   13402
gb:MN996528|Organism:Severe      CCTGTGGGTTTTACACTTAAAAACACAGTCTGTACCGTCTGCGGTATGTGGAAAGGTTAT   13402
gb:MN996530|Organism:Severe      CCTGTGGGTTTTACACTTAAAAACACAGTCTGTACCGTCTGCGGTATGTGGAAAGGTTAT   13388
gb:MN908947|Organism:Severe      CCTGTGGGTTTTACACTTAAAAACACAGTCTGTACCGTCTGCGGTATGTGGAAAGGTTAT   13402
gb:MT019532|Organism:Severe      CCTGTGGGTTTTACACTTAAAAACACAGTCTGTACCGTCTGCGGTATGTGGAAAGGTTAT   13402
                                 ************************************************************

gb:MT020781|Organism:Severe      GGCTGTAGTTGTGATCAACTCCGCGAACCCATGCTTCAGTCAGCTGATGCACAATCGTTT   13450
gb:MT007544|Organism:Severe      GGCTGTAGTTGTGATCAACTCCGCGAACCCATGCTTCAGTCAGCTGATGCACAATCGTTT   13462
gb:MN994467|Organism:Severe      GGCTGTAGTTGTGATCAACTCCGCGAACCCATGCTTCAGTCAGCTGATGCACAATCGTTT   13462
gb:MT044257|Organism:Severe      GGCTGTAGTTGTGATCAACTCCGCGAACCCATGCTTCAGTCAGCTGATGCACAATCGTTT   13462
gb:MT106054|Organism:Severe      GGCTGTAGTTGTGATCAACTCCGCGAACCCATGCTTCAGTCAGCTGATGCACAATCGTTT   13462
gb:MT049951|Organism:Severe      GGCTGTAGTTGTGATCAACTCCGCGAACCCATGCTTCAGTCAGCTGATGCACAATCGTTT   13462
gb:MN975262|Organism:Severe      GGCTGTAGTTGTGATCAACTCCGCGAACCCATGCTTCAGTCAGCTGATGCACAATCGTTT   13462
gb:MT106052|Organism:Severe      GGCTGTAGTTGTGATCAACTCCGCGAACCCATGCTTCAGTCAGCTGATGCACAATCGTTT   13462
gb:LC522975|Organism:Severe      GGCTGTAGTTGTGATCAACTCCGCGAACCCATGCTTCAGTCAGCTGATGCACAATCGTTT   13459
gb:LC522973|Organism:Severe      GGCTGTAGTTGTGATCAACTCCGCGAACCCATGCTTCAGTCAGCTGATGCACAATCGTTT   13459
gb:LC522974|Organism:Severe      GGCTGTAGTTGTGATCAACTCCGCGAACCCATGCTTCAGTCAGCTGATGCACAATCGTTT   13459
gb:MN985325|Organism:Severe      GGCTGTAGTTGTGATCAACTCCGCGAACCCATGCTTCAGTCAGCTGATGCACAATCGTTT   13462
gb:MT020881|Organism:Severe      GGCTGTAGTTGTGATCAACTCCGCGAACCCATGCTTCAGTCAGCTGATGCACAATCGTTT   13462
gb:MT020880|Organism:Severe      GGCTGTAGTTGTGATCAACTCCGCGAACCCATGCTTCAGTCAGCTGATGCACAATCGTTT   13462
gb:MT066175|Organism:Severe      GGCTGTAGTTGTGATCAACTCCGCGAACCCATGCTTCAGTCAGCTGATGCACAATCGTTT   13462
gb:MN997409|Organism:Severe      GGCTGTAGTTGTGATCAACTCCGCGAACCCATGCTTCAGTCAGCTGATGCACAATCGTTT   13462
gb:MN938384|Organism:Severe      GGCTGTAGTTGTGATCAACTCCGCGAACCCATGCTTCAGTCAGCTGATGCACAATCGTTT   13430
gb:MT044258|Organism:Severe      GGCTGTAGTTGTGATCAACTCCGCGAACCCATGCTTCAGTCAGCTGATGCACAATCGTTT   13438
gb:MT039890|Organism:Severe      GGCTGTAGTTGTGATCAACTCCGCGAACCCATGCTTCAGTCAGCTGATGCACAATCGTTT   13462
gb:MN988713|Organism:Severe      GGCTGTAGTTGTGATCAACTCCGCGAACCCATGCTTCAGTCAGCTGATGCACAATCGTTT   13462
gb:LC521925|Organism:Severe      GGCTGTAGTTGTGATCAACTCCGCGAACCCATGCTTCAGTCAGCTGATGCACAATCGTTT   13435
gb:MT093571|Organism:Severe      GGCTGTAGTTGTGATCAACTCCGCGAACCCATGCTTCAGTCAGCTGATGCACAATCGTTT   13462
gb:MT039887|Organism:Severe      GGCTGTAGTTGTGATCAACTCCGCGAACCCATGCTTCAGTCAGCTGATGCACAATCGTTT   13462
gb:MT019530|Organism:Severe      GGCTGTAGTTGTGATCAACTCCGCGAACCCATGCTTCAGTCAGCTGATGCACAATCGTTT   13462
gb:MT039888|Organism:Severe      GGCTGTAGTTGTGATCAACTCCGCGAACCCATGCTTCAGTCAGCTGATGCACAATCGTTT   13462
gb:LC522972|Organism:Severe      GGCTGTAGTTGTGATCAACTCCGCGAACCCATGCTTCAGTCAGCTGATGCACAATCGTTT   13459
gb:MT027063|Organism:Severe      GGCTGTAGTTGTGATCAACTCCGCGAACCCATGCTTCAGTCAGCTGATGCACAATCGTTT   13462
```

```
gb:MT027062|Organism:Severe      GGCTGTAGTTGTGATCAACTCCGCGAACCCATGCTTCAGTCAGCTGATGCACAATCGTTT   13462
gb:MT019529|Organism:Severe      GGCTGTAGTTGTGATCAACTCCGCGAACCCATGCTTCAGTCAGCTGATGCACAATCGTTT   13462
gb:MN996529|Organism:Severe      GGCTGTAGTTGTGATCAACTCCGCGAACCCATGCTTCAGTCAGCTGATGCACAATCGTTT   13450
gb:MN996531|Organism:Severe      GGCTGTAGTTGTGATCAACTCCGCGAACCCATGCTTCAGTCAGCTGATGCACAATCGTTT   13449
gb:MT066176|Organism:Severe      GGCTGTAGTTGTGATCAACTCCGCGAACCCATGCTTCAGTCAGCTGATGCACAATCGTTT   13462
gb:MT027064|Organism:Severe      GGCTGTAGTTGTGATCAACTCCGCGAACCCATGCTTCAGTCAGCTGATGCACAATCGTTT   13462
gb:MN994468|Organism:Severe      GGCTGTAGTTGTGATCAACTCCGCGAACCCATGCTTCAGTCAGCTGATGCACAATCGTTT   13462
gb:MT072688|Organism:Severe      GGCTGTAGTTGTGATCAACTCCGCGAACCCATGCTTCAGTCAGCTGATGCACAATCGTTT   13447
gb:MN996527|Organism:Severe      GGCTGTAGTTGTGATCAACTCCGCGAACCCATGCTTCAGTCAGCTGATGCACAATCGTTT   13429
gb:MT093631|Organism:Severe      GGCTGTAGTTGTGATCAACTCCGCGAACCCATGCTTCAGTCAGCTGATGCACAATCGTTT   13500
gb:MT106053|Organism:Severe      GGCTGTAGTTGTGATCAACTCCGCGAACCCATGCTTCAGTCAGCTGATGCACAATCGTTT   13462
gb:MT019533|Organism:Severe      GGCTGTAGTTGTGATCAACTCCGCGAACCCATGCTTCAGTCAGCTGATGCACAATCGTTT   13462
gb:MT019531|Organism:Severe      GGCTGTAGTTGTGATCAACTCCGCGAACCCATGCTTCAGTCAGCTGATGCACAATCGTTT   13462
gb:MN996528|Organism:Severe      GGCTGTAGTTGTGATCAACTCCGCGAACCCATGCTTCAGTCAGCTGATGCACAATCGTTT   13462
gb:MN996530|Organism:Severe      GGCTGTAGTTGTGATCAACTCCGCGAACCCATGCTTCAGTCAGCTGATGCACAATCGTTT   13448
gb:MN908947|Organism:Severe      GGCTGTAGTTGTGATCAACTCCGCGAACCCATGCTTCAGTCAGCTGATGCACAATCGTTT   13462
gb:MT019532|Organism:Severe      GGCTGTAGTTGTGATCAACTCCGCGAACCCATGCTTCAGTCAGCTGATGCACAATCGTTT   13462
                                 ************************************************************

gb:MT020781|Organism:Severe      TTAAACGGGTTTGCGGTGTAAGTGCAGCCCGTCTTACACCGTGCGGCACAGGCACTAGTA   13510
gb:MT007544|Organism:Severe      TTAAACGGGTTTGCGGTGTAAGTGCAGCCCGTCTTACACCGTGCGGCACAGGCACTAGTA   13522
gb:MN994467|Organism:Severe      TTAAACGGGTTTGCGGTGTAAGTGCAGCCCGTCTTACACCGTGCGGCACAGGCACTAGTA   13522
gb:MT044257|Organism:Severe      TTAAACGGGTTTGCGGTGTAAGTGCAGCCCGTCTTACACCGTGCGGCACAGGCACTAGTA   13522
gb:MT106054|Organism:Severe      TTAAACGGGTTTGCGGTGTAAGTGCAGCCCGTCTTACACCGTGCGGCACAGGCACTAGTA   13522
gb:MT049951|Organism:Severe      TTAAACGGGTTTGCGGTGTAAGTGCAGCCCGTCTTACACCGTGCGGCACAGGCACTAGTA   13522
gb:MN975262|Organism:Severe      TTAAACGGGTTTGCGGTGTAAGTGCAGCCCGTCTTACACCGTGCGGCACAGGCACTAGTA   13522
gb:MT106052|Organism:Severe      TTAAACGGGTTTGCGGTGTAAGTGCAGCCCGTCTTACACCGTGCGGCACAGGCACTAGTA   13522
gb:LC522975|Organism:Severe      TTAAACGGGTTTGCGGTGTAAGTGCAGCCCGTCTTACACCGTGCGGCACAGGCACTAGTA   13519
gb:LC522973|Organism:Severe      TTAAACGGGTTTGCGGTGTAAGTGCAGCCCGTCTTACACCGTGCGGCACAGGCACTAGTA   13519
gb:LC522974|Organism:Severe      TTAAACGGGTTTGCGGTGTAAGTGCAGCCCGTCTTACACCGTGCGGCACAGGCACTAGTA   13519
gb:MN985325|Organism:Severe      TTAAACGGGTTTGCGGTGTAAGTGCAGCCCGTCTTACACCGTGCGGCACAGGCACTAGTA   13522
gb:MT020881|Organism:Severe      TTAAACGGGTTTGCGGTGTAAGTGCAGCCCGTCTTACACCGTGCGGCACAGGCACTAGTA   13522
gb:MT020880|Organism:Severe      TTAAACGGGTTTGCGGTGTAAGTGCAGCCCGTCTTACACCGTGCGGCACAGGCACTAGTA   13522
gb:MT066175|Organism:Severe      TTAAACGGGTTTGCGGTGTAAGTGCAGCCCGTCTTACACCGTGCGGCACAGGCACTAGTA   13522
gb:MN997409|Organism:Severe      TTAAACGGGTTTGCGGTGTAAGTGCAGCCCGTCTTACACCGTGCGGCACAGGCACTAGTA   13522
gb:MN938384|Organism:Severe      TTAAACGGGTTTGCGGTGTAAGTGCAGCCCGTCTTACACCGTGCGGCACAGGCACTAGTA   13490
gb:MT044258|Organism:Severe      TTAAACGGGTTTGCGGTGTAAGTGCAGCCCGTCTTACACCGTGCGGCACAGGCACTAGTA   13498
gb:MT039890|Organism:Severe      TTAAACGGGTTTGCGGTGTAAGTGCAGCCCGTCTTACACCGTGCGGCACAGGCACTAGTA   13522
gb:MN988713|Organism:Severe      TTAAACGGGTTTGCGGTGTAAGTGCAGCCCGTCTTACACCGTGCGGCACAGGCACTAGTA   13522
gb:LC521925|Organism:Severe      TTAAACGGGTTTGCGGTGTAAGTGCAGCCCGTCTTACACCGTGCGGCACAGGCACTAGTA   13495
gb:MT093571|Organism:Severe      TTAAACGGGTTTGCGGTGTAAGTGCAGCCCGTCTTACACCGTGCGGCACAGGCACTAGTA   13522
gb:MT039887|Organism:Severe      TTAAACGGGTTTGCGGTGTAAGTGCAGCCCGTCTTACACCGTGCGGCACAGGCACTAGTA   13522
gb:MT019530|Organism:Severe      TTAAACGGGTTTGCGGTGTAAGTGCAGCCCGTCTTACACCGTGCGGCACAGGCACTAGTA   13522
gb:MT039888|Organism:Severe      TTAAACGGGTTTGCGGTGTAAGTGCAGCCCGTCTTACACCGTGCGGCACAGGCACTAGTA   13522
gb:LC522972|Organism:Severe      TTAAACGGGTTTGCGGTGTAAGTGCAGCCCGTCTTACACCGTGCGGCACAGGCACTAGTA   13519
gb:MT027063|Organism:Severe      TTAAACGGGTTTGCGGTGTAAGTGCAGCCCGTCTTACACCGTGCGGCACAGGCACTAGTA   13522
gb:MT027062|Organism:Severe      TTAAACGGGTTTGCGGTGTAAGTGCAGCCCGTCTTACACCGTGCGGCACAGGCACTAGTA   13522
gb:MT019529|Organism:Severe      TTAAACGGGTTTGCGGTGTAAGTGCAGCCCGTCTTACACCGTGCGGCACAGGCACTAGTA   13522
gb:MN996529|Organism:Severe      TTAAACGGGTTTGCGGTGTAAGTGCAGCCCGTCTTACACCGTGCGGCACAGGCACTAGTA   13510
gb:MN996531|Organism:Severe      TTAAACGGGTTTGCGGTGTAAGTGCAGCCCGTCTTACACCGTGCGGCACAGGCACTAGTA   13509
gb:MT066176|Organism:Severe      TTAAACGGGTTTGCGGTGTAAGTGCAGCCCGTCTTACACCGTGCGGCACAGGCACTAGTA   13522
gb:MT027064|Organism:Severe      TTAAACGGGTTTGCGGTGTAAGTGCAGCCCGTCTTACACCGTGCGGCACAGGCACTAGTA   13522
gb:MN994468|Organism:Severe      TTAAACGGGTTTGCGGTGTAAGTGCAGCCCGTCTTACACCGTGCGGCACAGGCACTAGTA   13522
gb:MT072688|Organism:Severe      TTAAACGGGTTTGCGGTGTAAGTGCAGCCCGTCTTACACCGTGCGGCACAGGCACTAGTA   13507
gb:MN996527|Organism:Severe      TTAAACGGGTTTGCGGTGTAAGTGCAGCCCGTCTTACACCGTGCGGCACAGGCACTAGTA   13489
gb:MT093631|Organism:Severe      TTAAACGGGTTTGCGGTGTAAGTGCAGCCCGTCTTACACCGTGCGGCACAGGCACTAGTA   13560
gb:MT106053|Organism:Severe      TTAAACGGGTTTGCGGTGTAAGTGCAGCCCGTCTTACACCGTGCGGCACAGGCACTAGTA   13522
gb:MT019533|Organism:Severe      TTAAACGGGTTTGCGGTGTAAGTGCAGCCCGTCTTACACCGTGCGGCACAGGCACTAGTA   13522
gb:MT019531|Organism:Severe      TTAAACGGGTTTGCGGTGTAAGTGCAGCCCGTCTTACACCGTGCGGCACAGGCACTAGTA   13522
gb:MN996528|Organism:Severe      TTAAACGGGTTTGCGGTGTAAGTGCAGCCCGTCTTACACCGTGCGGCACAGGCACTAGTA   13522
gb:MN996530|Organism:Severe      TTAAACGGGTTTGCGGTGTAAGTGCAGCCCGTCTTACACCGTGCGGCACAGGCACTAGTA   13508
gb:MN908947|Organism:Severe      TTAAACGGGTTTGCGGTGTAAGTGCAGCCCGTCTTACACCGTGCGGCACAGGCACTAGTA   13522
gb:MT019532|Organism:Severe      TTAAACGGGTTTGCGGTGTAAGTGCAGCCCGTCTTACACCGTGCGGCACAGGCACTAGTA   13522
                                 ************************************************************
```

```
gb:MT020781|Organism:Severe      CTGATGTCGTATACAGGGCTTTTGACATCTACAATGATAAAGTAGCTGGTTTTGCTAAAT   13570
gb:MT007544|Organism:Severe      CTGATGTCGTATACAGGGCTTTTGACATCTACAATGATAAAGTAGCTGGTTTTGCTAAAT   13582
gb:MN994467|Organism:Severe      CTGATGTCGTATACAGGGCTTTTGACATCTACAATGATAAAGTAGCTGGTTTTGCTAAAT   13582
gb:MT044257|Organism:Severe      CTGATGTCGTATACAGGGCTTTTGACATCTACAATGATAAAGTAGCTGGTTTTGCTAAAT   13582
gb:MT106054|Organism:Severe      CTGATGTCGTATACAGGGCTTTTGACATCTACAATGATAAAGTAGCTGGTTTTGCTAAAT   13582
gb:MT049951|Organism:Severe      CTGATGTCGTATACAGGGCTTTTGACATCTACAATGATAAAGTAGCTGGTTTTGCTAAAT   13582
gb:MN975262|Organism:Severe      CTGATGTCGTATACAGGGCTTTTGACATCTACAATGATAAAGTAGCTGGTTTTGCTAAAT   13582
gb:MT106052|Organism:Severe      CTGATGTCGTATACAGGGCTTTTGACATCTACAATGATAAAGTAGCTGGTTTTGCTAAAT   13582
gb:LC522975|Organism:Severe      CTGATGTCGTATACAGGGCTTTTGACATCTACAATGATAAAGTAGCTGGTTTTGCTAAAT   13579
gb:LC522973|Organism:Severe      CTGATGTCGTATACAGGGCTTTTGACATCTACAATGATAAAGTAGCTGGTTTTGCTAAAT   13579
gb:LC522974|Organism:Severe      CTGATGTCGTATACAGGGCTTTTGACATCTACAATGATAAAGTAGCTGGTTTTGCTAAAT   13579
gb:MN985325|Organism:Severe      CTGATGTCGTATACAGGGCTTTTGACATCTACAATGATAAAGTAGCTGGTTTTGCTAAAT   13582
gb:MT020881|Organism:Severe      CTGATGTCGTATACAGGGCTTTTGACATCTACAATGATAAAGTAGCTGGTTTTGCTAAAT   13582
gb:MT020880|Organism:Severe      CTGATGTCGTATACAGGGCTTTTGACATCTACAATGATAAAGTAGCTGGTTTTGCTAAAT   13582
gb:MT066175|Organism:Severe      CTGATGTCGTATACAGGGCTTTTGACATCTACAATGATAAAGTAGCTGGTTTTGCTAAAT   13582
gb:MN997409|Organism:Severe      CTGATGTCGTATACAGGGCTTTTGACATCTACAATGATAAAGTAGCTGGTTTTGCTAAAT   13582
gb:MN938384|Organism:Severe      CTGATGTCGTATACAGGGCTTTTGACATCTACAATGATAAAGTAGCTGGTTTTGCTAAAT   13550
gb:MT044258|Organism:Severe      CTGATGTCGTATACAGGGCTTTTGACATCTACAATGATAAAGTAGCTGGTTTTGCTAAAT   13558
gb:MT039890|Organism:Severe      CTGATGTCGTATACAGGGCTTTTGACATCTACAATGATAAAGTAGCTGGTTTTGCTAAAT   13582
gb:MN988713|Organism:Severe      CTGATGTCGTATACAGGGCTTTTGACATCTACAATGATAAAGTAGCTGGTTTTGCTAAAT   13582
gb:LC521925|Organism:Severe      CTGATGTCGTATACAGGGCTTTTGACATCTACAATGATAAAGTAGCTGGTTTTGCTAAAT   13555
gb:MT093571|Organism:Severe      CTGATGTCGTATACAGGGCTTTTGACATCTACAATGATAAAGTAGCTGGTTTTGCTAAAT   13582
gb:MT039887|Organism:Severe      CTGATGTCGTATACAGGGCTTTTGACATCTACAATGATAAAGTAGCTGGTTTTGCTAAAT   13582
gb:MT019530|Organism:Severe      CTGATGTCGTATACAGGGCTTTTGACATCTACAATGATAAAGTAGCTGGTTTTGCTAAAT   13582
gb:MT039888|Organism:Severe      CTGATGTCGTATACAGGGCTTTTGACATCTACAATGATAAAGTAGCTGGTTTTGCTAAAT   13582
gb:LC522972|Organism:Severe      CTGATGTCGTATACAGGGCTTTTGACATCTACAATGATAAAGTAGCTGGTTTTGCTAAAT   13579
gb:MT027063|Organism:Severe      CTGATGTCGTATACAGGGCTTTTGACATCTACAATGATAAAGTAGCTGGTTTTGCTAAAT   13582
gb:MT027062|Organism:Severe      CTGATGTCGTATACAGGGCTTTTGACATCTACAATGATAAAGTAGCTGGTTTTGCTAAAT   13582
gb:MT019529|Organism:Severe      CTGATGTCGTATACAGGGCTTTTGACATCTACAATGATAAAGTAGCTGGTTTTGCTAAAT   13582
gb:MN996529|Organism:Severe      CTGATGTCGTATACAGGGCTTTTGACATCTACAATGATAAAGTAGCTGGTTTTGCTAAAT   13570
gb:MN996531|Organism:Severe      CTGATGTCGTATACAGGGCTTTTGACATCTACAATGATAAAGTAGCTGGTTTTGCTAAAT   13569
gb:MT066176|Organism:Severe      CTGATGTCGTATACAGGGCTTTTGACATCTACAATGATAAAGTAGCTGGTTTTGCTAAAT   13582
gb:MT027064|Organism:Severe      CTGATGTCGTATACAGGGCTTTTGACATCTACAATGATAAAGTAGCTGGTTTTGCTAAAT   13582
gb:MN994468|Organism:Severe      CTGATGTCGTATACAGGGCTTTTGACATCTACAATGATAAAGTAGCTGGTTTTGCTAAAT   13582
gb:MT072688|Organism:Severe      CTGATGTCGTATACAGGGCTTTTGACATCTACAATGATAAAGTAGCTGGTTTTGCTAAAT   13567
gb:MN996527|Organism:Severe      CTGATGTCGTATACAGGGCTTTTGACATCTACAATGATAAAGTAGCTGGTTTTGCTAAAT   13549
gb:MT093631|Organism:Severe      CTGATGTCGTATACAGGGCTTTTGACATCTACAATGATAAAGTAGCTGGTTTTGCTAAAT   13620
gb:MT106053|Organism:Severe      CTGATGTCGTATACAGGGCTTTTGACATCTACAATGATAAAGTAGCTGGTTTTGCTAAAT   13582
gb:MT019533|Organism:Severe      CTGATGTCGTATACAGGGCTTTTGACATCTACAATGATAAAGTAGCTGGTTTTGCTAAAT   13582
gb:MT019531|Organism:Severe      CTGATGTCGTATACAGGGCTTTTGACATCTACAATGATAAAGTAGCTGGTTTTGCTAAAT   13582
gb:MN996528|Organism:Severe      CTGATGTCGTATACAGGGCTTTTGACATCTACAATGATAAAGTAGCTGGTTTTGCTAAAT   13582
gb:MN996530|Organism:Severe      CTGATGTCGTATACAGGGCTTTTGACATCTACAATGATAAAGTAGCTGGTTTTGCTAAAT   13568
gb:MN908947|Organism:Severe      CTGATGTCGTATACAGGGCTTTTGACATCTACAATGATAAAGTAGCTGGTTTTGCTAAAT   13582
gb:MT019532|Organism:Severe      CTGATGTCGTATACAGGGCTTTTGACATCTACAATGATAAAGTAGCTGGTTTTGCTAAAT   13582
                                 ************************************************************

gb:MT020781|Organism:Severe      TCCTAAAAACTAATTGTTGTCGCTTCCAAGAAAAGGACGAAGATGACAATTTAATTGATT   13630
gb:MT007544|Organism:Severe      TCCTAAAAACTAATTGTTGTCGCTTCCAAGAAAAGGACGAAGATGACAATTTAATTGATT   13642
gb:MN994467|Organism:Severe      TCCTAAAAACTAATTGTTGTCGCTTCCAAGAAAAGGACGAAGATGACAATTTAATTGATT   13642
gb:MT044257|Organism:Severe      TCCTAAAAACTAATTGTTGTCGCTTCCAAGAAAAGGACGAAGATGACAATTTAATTGATT   13642
gb:MT106054|Organism:Severe      TCCTAAAAACTAATTGTTGTCGCTTCCAAGAAAAGGACGAAGATGACAATTTAATTGATT   13642
gb:MT049951|Organism:Severe      TCCTAAAAACTAATTGTTGTCGCTTCCAAGAAAAGGACGAAGATGACAATTTAATTGATT   13642
gb:MN975262|Organism:Severe      TCCTAAAAACTAATTGTTGTCGCTTCCAAGAAAAGGACGAAGATGACAATTTAATTGATT   13642
gb:MT106052|Organism:Severe      TCCTAAAAACTAATTGTTGTCGCTTCCAAGAAAAGGACGAAGATGACAATTTAATTGATT   13642
gb:LC522975|Organism:Severe      TCCTAAAAACTAATTGTTGTCGCTTCCAAGAAAAGGACGAAGATGACAATTTAATTGATT   13639
gb:LC522973|Organism:Severe      TCCTAAAAACTAATTGTTGTCGCTTCCAAGAAAAGGACGAAGATGACAATTTAATTGATT   13639
gb:LC522974|Organism:Severe      TCCTAAAAACTAATTGTTGTCGCTTCCAAGAAAAGGACGAAGATGACAATTTAATTGATT   13639
gb:MN985325|Organism:Severe      TCCTAAAAACTAATTGTTGTCGCTTCCAAGAAAAGGACGAAGATGACAATTTAATTGATT   13642
gb:MT020881|Organism:Severe      TCCTAAAAACTAATTGTTGTCGCTTCCAAGAAAAGGACGAAGATGACAATTTAATTGATT   13642
gb:MT020880|Organism:Severe      TCCTAAAAACTAATTGTTGTCGCTTCCAAGAAAAGGACGAAGATGACAATTTAATTGATT   13642
gb:MT066175|Organism:Severe      TCCTAAAAACTAATTGTTGTCGCTTCCAAGAAAAGGACGAAGATGACAATTTAATTGATT   13642
gb:MN997409|Organism:Severe      TCCTAAAAACTAATTGTTGTCGCTTCCAAGAAAAGGACGAAGATGACAATTTAATTGATT   13642
gb:MN938384|Organism:Severe      TCCTAAAAACTAATTGTTGTCGCTTCCAAGAAAAGGACGAAGATGACAATTTAATTGATT   13610
gb:MT044258|Organism:Severe      TCCTAAAAACTAATTGTTGTCGCTTCCAAGAAAAGGACGAAGATGACAATTTAATTGATT   13618
gb:MT039890|Organism:Severe      TCCTAAAAACTAATTGTTGTCGCTTCCAAGAAAAGGACGAAGATGACAATTTAATTGATT   13642
```

```
gb:MN988713|Organism:Severe      TCCTAAAAACTAATTGTTGTCGCTTCCAAGAAAAGGACGAAGATGACAATTTAATTGATT   13642
gb:LC521925|Organism:Severe      TCCTAAAAACTAATTGTTGTCGCTTCCAAGAAAAGGACGAAGATGACAATTTAATTGATT   13615
gb:MT093571|Organism:Severe      TCCTAAAAACTAATTGTTGTCGCTTCCAAGAAAAGGACGAAGATGACAATTTAATTGATT   13642
gb:MT039887|Organism:Severe      TCCTAAAAACTAATTGTTGTCGCTTCCAAGAAAAGGACGAAGATGACAATTTAATTGATT   13642
gb:MT019530|Organism:Severe      TCCTAAAAACTAATTGTTGTCGCTTCCAAGAAAAGGACGAAGATGACAATTTAATTGATT   13642
gb:MT039888|Organism:Severe      TCCTAAAAACTAATTGTTGTCGCTTCCAAGAAAAGGACGAAGATGACAATTTAATTGATT   13642
gb:LC522972|Organism:Severe      TCCTAAAAACTAATTGTTGTCGCTTCCAAGAAAAGGACGAAGATGACAATTTAATTGATT   13639
gb:MT027063|Organism:Severe      TCCTAAAAACTAATTGTTGTCGCTTCCAAGAAAAGGACGAAGATGACAATTTAATTGATT   13642
gb:MT027062|Organism:Severe      TCCTAAAAACTAATTGTTGTCGCTTCCAAGAAAAGGACGAAGATGACAATTTAATTGATT   13642
gb:MT019529|Organism:Severe      TCCTAAAAACTAATTGTTGTCGCTTCCAAGAAAAGGACGAAGATGACAATTTAATTGATT   13642
gb:MN996529|Organism:Severe      TCCTAAAAACTAATTGTTGTCGCTTCCAAGAAAAGGACGAAGATGACAATTTAATTGATT   13630
gb:MN996531|Organism:Severe      TCCTAAAAACTAATTGTTGTCGCTTCCAAGAAAAGGACGAAGATGACAATTTAATTGATT   13629
gb:MT066176|Organism:Severe      TCCTAAAAACTAATTGTTGTCGCTTCCAAGAAAAGGACGAAGATGACAATTTAATTGATT   13642
gb:MT027064|Organism:Severe      TCCTAAAAACTAATTGTTGTCGCTTCCAAGAAAAGGACGAAGATGACAATTTAATTGATT   13642
gb:MN994468|Organism:Severe      TCCTAAAAACTAATTGTTGTCGCTTCCAAGAAAAGGACGAAGATGACAATTTAATTGATT   13642
gb:MT072688|Organism:Severe      TCCTAAAAACTAATTGTTGTCGCTTCCAAGAAAAGGACGAAGATGACAATTTAATTGATT   13627
gb:MN996527|Organism:Severe      TCCTAAAAACTAATTGTTGTCGCTTCCAAGAAAAGGACGAAGATGACAATTTAATTGATT   13609
gb:MT093631|Organism:Severe      TCCTAAAAACTAATTGTTGTCGCTTCCAAGAAAAGGACGAAGATGACAATTTAATTGATT   13680
gb:MT106053|Organism:Severe      TCCTAAAAACTAATTGTTGTCGCTTCCAAGAAAAGGACGAAGATGACAATTTAATTGATT   13642
gb:MT019533|Organism:Severe      TCCTAAAAACTAATTGTTGTCGCTTCCAAGAAAAGGACGAAGATGACAATTTAATTGATT   13642
gb:MT019531|Organism:Severe      TCCTAAAAACTAATTGTTGTCGCTTCCAAGAAAAGGACGAAGATGACAATTTAATTGATT   13642
gb:MN996528|Organism:Severe      TCCTAAAAACTAATTGTTGTCGCTTCCAAGAAAAGGACGAAGATGACAATTTAATTGATT   13642
gb:MN996530|Organism:Severe      TCCTAAAAACTAATTGTTGTCGCTTCCAAGAAAAGGACGAAGATGACAATTTAATTGATT   13628
gb:MN908947|Organism:Severe      TCCTAAAAACTAATTGTTGTCGCTTCCAAGAAAAGGACGAAGATGACAATTTAATTGATT   13642
gb:MT019532|Organism:Severe      TCCTAAAAACTAATTGTTGTCGCTTCCAAGAAAAGGACGAAGATGACAATTTAATTGATT   13642
                                 ************************************************************

gb:MT020781|Organism:Severe      CTTACTTTGTAGTTAAGAGACACACTTTCTCTAACTACCAACATGAAGAAACAATTTATA   13690
gb:MT007544|Organism:Severe      CTTACTTTGTAGTTAAGAGACACACTTTCTCTAACTACCAACATGAAGAAACAATTTATA   13702
gb:MN994467|Organism:Severe      CTTACTTTGTAGTTAAGAGACACACTTTCTCTAACTACCAACATGAAGAAACAATTTATA   13702
gb:MT044257|Organism:Severe      CTTACTTTGTAGTTAAGAGACACACTTTCTCTAACTACCAACATGAAGAAACAATTTATA   13702
gb:MT106054|Organism:Severe      CTTACTTTGTAGTTAAGAGACACACTTTCTCTAACTACCAACATGAAGAAACAATTTATA   13702
gb:MT049951|Organism:Severe      CTTACTTTGTAGTTAAGAGACACACTTTCTCTAACTACCAACATGAAGAAACAATTTATA   13702
gb:MN975262|Organism:Severe      CTTACTTTGTAGTTAAGAGACACACTTTCTCTAACTACCAACATGAAGAAACAATTTATA   13702
gb:MT106052|Organism:Severe      CTTACTTTGTAGTTAAGAGACACACTTTCTCTAACTACCAACATGAAGAAACAATTTATA   13702
gb:LC522975|Organism:Severe      CTTACTTTGTAGTTAAGAGACACACTTTCTCTAACTACCAACATGAAGAAACAATTTATA   13699
gb:LC522973|Organism:Severe      CTTACTTTGTAGTTAAGAGACACACTTTCTCTAACTACCAACATGAAGAAACAATTTATA   13699
gb:LC522974|Organism:Severe      CTTACTTTGTAGTTAAGAGACACACTTTCTCTAACTACCAACATGAAGAAACAATTTATA   13699
gb:MN985325|Organism:Severe      CTTACTTTGTAGTTAAGAGACACACTTTCTCTAACTACCAACATGAAGAAACAATTTATA   13702
gb:MT020881|Organism:Severe      CTTACTTTGTAGTTAAGAGACACACTTTCTCTAACTACCAACATGAAGAAACAATTTATA   13702
gb:MT020880|Organism:Severe      CTTACTTTGTAGTTAAGAGACACACTTTCTCTAACTACCAACATGAAGAAACAATTTATA   13702
gb:MT066175|Organism:Severe      CTTACTTTGTAGTTAAGAGACACACTTTCTCTAACTACCAACATGAAGAAACAATTTATA   13702
gb:MN997409|Organism:Severe      CTTACTTTGTAGTTAAGAGACACACTTTCTCTAACTACCAACATGAAGAAACAATTTATA   13702
gb:MN938384|Organism:Severe      CTTACTTTGTAGTTAAGAGACACACTTTCTCTAACTACCAACATGAAGAAACAATTTATA   13670
gb:MT044258|Organism:Severe      CTTACTTTGTAGTTAAGAGACACACTTTCTCTAACTACCAACATGAAGAAACAATTTATA   13678
gb:MT039890|Organism:Severe      CTTACTTTGTAGTTAAGAGACACACTTTCTCTAACTACCAACATGAAGAAACAATTTATA   13702
gb:MN988713|Organism:Severe      CTTACTTTGTAGTTAAGAGACACACTTTCTCTAACTACCAACATGAAGAAACAATTTATA   13702
gb:LC521925|Organism:Severe      CTTACTTTGTAGTTAAGAGACACACTTTCTCTAACTACCAACATGAAGAAACAATTTATA   13675
gb:MT093571|Organism:Severe      CTTACTTTGTAGTTAAGAGACACACTTTCTCTAACTACCAACATGAAGAAACAATTTATA   13702
gb:MT039887|Organism:Severe      CTTACTTTGTAGTTAAGAGACACACTTTCTCTAACTACCAACATGAAGAAACAATTTATA   13702
gb:MT019530|Organism:Severe      CTTACTTTGTAGTTAAGAGACACACTTTCTCTAACTACCAACATGAAGAAACAATTTATA   13702
gb:MT039888|Organism:Severe      CTTACTTTGTAGTTAAGAGACACACTTTCTCTAACTACCAACATGAAGAAACAATTTATA   13702
gb:LC522972|Organism:Severe      CTTACTTTGTAGTTAAGAGACACACTTTCTCTAACTACCAACATGAAGAAACAATTTATA   13699
gb:MT027063|Organism:Severe      CTTACTTTGTAGTTAAGAGACACACTTTCTCTAACTACCAACATGAAGAAACAATTTATA   13702
gb:MT027062|Organism:Severe      CTTACTTTGTAGTTAAGAGACACACTTTCTCTAACTACCAACATGAAGAAACAATTTATA   13702
gb:MT019529|Organism:Severe      CTTACTTTGTAGTTAAGAGACACACTTTCTCTAACTACCAACATGAAGAAACAATTTATA   13702
gb:MN996529|Organism:Severe      CTTACTTTGTAGTTAAGAGACACACTTTCTCTAACTACCAACATGAAGAAACAATTTATA   13690
gb:MN996531|Organism:Severe      CTTACTTTGTAGTTAAGAGACACACTTTCTCTAACTACCAACATGAAGAAACAATTTATA   13689
gb:MT066176|Organism:Severe      CTTACTTTGTAGTTAAGAGACACACTTTCTCTAACTACCAACATGAAGAAACAATTTATA   13702
gb:MT027064|Organism:Severe      CTTACTTTGTAGTTAAGAGACACACTTTCTCTAACTACCAACATGAAGAAACAATTTATA   13702
gb:MN994468|Organism:Severe      CTTACTTTGTAGTTAAGAGACACACTTTCTCTAACTACCAACATGAAGAAACAATTTATA   13702
gb:MT072688|Organism:Severe      CTTACTTTGTAGTTAAGAGACACACTTTCTCTAACTACCAACATGAAGAAACAATTTATA   13687
gb:MN996527|Organism:Severe      CTTACTTTGTAGTTAAGAGACACACTTTCTCTAACTACCAACATGAAGAAACAATTTATA   13669
gb:MT093631|Organism:Severe      CTTACTTTGTAGTTAAGAGACACACTTTCTCTAACTACCAACATGAAGAAACAATTTATA   13740
gb:MT106053|Organism:Severe      CTTACTTTGTAGTTAAGAGACACACTTTCTCTAACTACCAACATGAAGAAACAATTTATA   13702
```

```
gb:MT019533|Organism:Severe      CTTACTTTGTAGTTAAGAGACACACTTTCTCTAACTACCAACATGAAGAAACAATTTATA   13702
gb:MT019531|Organism:Severe      CTTACTTTGTAGTTAAGAGACACACTTTCTCTAACTACCAACATGAAGAAACAATTTATA   13702
gb:MN996528|Organism:Severe      CTTACTTTGTAGTTAAGAGACACACTTTCTCTAACTACCAACATGAAGAAACAATTTATA   13702
gb:MN996530|Organism:Severe      CTTACTTTGTAGTTAAGAGACACACTTTCTCTAACTACCAACATGAAGAAACAATTTATA   13688
gb:MN908947|Organism:Severe      CTTACTTTGTAGTTAAGAGACACACTTTCTCTAACTACCAACATGAAGAAACAATTTATA   13702
gb:MT019532|Organism:Severe      CTTACTTTGTAGTTAAGAGACACACTTTCTCTAACTACCAACATGAAGAAACAATTTATA   13702
                                 ************************************************************

gb:MT020781|Organism:Severe      ATTTACTTAAGGATTGTCCAGCTGTTGCTAAACATGACTTCTTTAAGTTTAGAATAGACG   13750
gb:MT007544|Organism:Severe      ATTTACTTAAGGATTGTCCAGCTGTTGCTAAACATGACTTCTTTAAGTTTAGAATAGACG   13762
gb:MN994467|Organism:Severe      ATTTACTTAAGGATTGTCCAGCTGTTGCTAAACATGACTTCTTTAAGTTTAGAATAGACG   13762
gb:MT044257|Organism:Severe      ATTTACTTAAGGATTGTCCAGCTGTTGCTAAACATGACTTCTTTAAGTTTAGAATAGACG   13762
gb:MT106054|Organism:Severe      ATTTACTTAAGGATTGTCCAGCTGTTGCTAAACATGACTTCTTTAAGTTTAGAATAGACG   13762
gb:MT049951|Organism:Severe      ATTTACTTAAGGATTGTCCAGCTGTTGCTAAACATGACTTCTTTAAGTTTAGAATAGACG   13762
gb:MN975262|Organism:Severe      ATTTACTTAAGGATTGTCCAGCTGTTGCTAAACATGACTTCTTTAAGTTTAGAATAGACG   13762
gb:MT106052|Organism:Severe      ATTTACTTAAGGATTGTCCAGCTGTTGCTAAACATGACTTCTTTAAGTTTAGAATAGACG   13762
gb:LC522975|Organism:Severe      ATTTACTTAAGGATTGTCCAGCTGTTGCTAAACATGACTTCTTTAAGTTTAGAATAGACG   13759
gb:LC522973|Organism:Severe      ATTTACTTAAGGATTGTCCAGCTGTTGCTAAACATGACTTCTTTAAGTTTAGAATAGACG   13759
gb:LC522974|Organism:Severe      ATTTACTTAAGGATTGTCCAGCTGTTGCTAAACATGACTTCTTTAAGTTTAGAATAGACG   13759
gb:MN985325|Organism:Severe      ATTTACTTAAGGATTGTCCAGCTGTTGCTAAACATGACTTCTTTAAGTTTAGAATAGACG   13762
gb:MT020881|Organism:Severe      ATTTACTTAAGGATTGTCCAGCTGTTGCTAAACATGACTTCTTTAAGTTTAGAATAGACG   13762
gb:MT020880|Organism:Severe      ATTTACTTAAGGATTGTCCAGCTGTTGCTAAACATGACTTCTTTAAGTTTAGAATAGACG   13762
gb:MT066175|Organism:Severe      ATTTACTTAAGGATTGTCCAGCTGTTGCTAAACATGACTTCTTTAAGTTTAGAATAGACG   13762
gb:MN997409|Organism:Severe      ATTTACTTAAGGATTGTCCAGCTGTTGCTAAACATGACTTCTTTAAGTTTAGAATAGACG   13762
gb:MN938384|Organism:Severe      ATTTACTTAAGGATTGTCCAGCTGTTGCTAAACATGACTTCTTTAAGTTTAGAATAGACG   13730
gb:MT044258|Organism:Severe      ATTTACTTAAGGATTGTCCAGCTGTTGCTAAACATGACTTCTTTAAGTTTAGAATAGACG   13738
gb:MT039890|Organism:Severe      ATTTACTTAAGGATTGTCCAGCTGTTGCTAAACATGACTTCTTTAAGTTTAGAATAGACG   13762
gb:MN988713|Organism:Severe      ATTTACTTAAGGATTGTCCAGCTGTTGCTAAACATGACTTCTTTAAGTTTAGAATAGACG   13762
gb:LC521925|Organism:Severe      ATTTACTTAAGGATTGTCCAGCTGTTGCTAAACATGACTTCTTTAAGTTTAGAATAGACG   13735
gb:MT093571|Organism:Severe      ATTTACTTAAGGATTGTCCAGCTGTTGCTAAACATGACTTCTTTAAGTTTAGAATAGACG   13762
gb:MT039887|Organism:Severe      ATTTACTTAAGGATTGTCCAGCTGTTGCTAAACATGACTTCTTTAAGTTTAGAATAGACG   13762
gb:MT019530|Organism:Severe      ATTTACTTAAGGATTGTCCAGCTGTTGCTAAACATGACTTCTTTAAGTTTAGAATAGACG   13762
gb:MT039888|Organism:Severe      ATTTACTTAAGGATTGTCCAGCTGTTGCTAAACATGACTTCTTTAAGTTTAGAATAGACG   13762
gb:LC522972|Organism:Severe      ATTTACTTAAGGATTGTCCAGCTGTTGCTAAACATGACTTCTTTAAGTTTAGAATAGACG   13759
gb:MT027063|Organism:Severe      ATTTACTTAAGGATTGTCCAGCTGTTGCTAAACATGACTTCTTTAAGTTTAGAATAGACG   13762
gb:MT027062|Organism:Severe      ATTTACTTAAGGATTGTCCAGCTGTTGCTAAACATGACTTCTTTAAGTTTAGAATAGACG   13762
gb:MT019529|Organism:Severe      ATTTACTTAAGGATTGTCCAGCTGTTGCTAAACATGACTTCTTTAAGTTTAGAATAGACG   13762
gb:MN996529|Organism:Severe      ATTTACTTAAGGATTGTCCAGCTGTTGCTAAACATGACTTCTTTAAGTTTAGAATAGACG   13750
gb:MN996531|Organism:Severe      ATTTACTTAAGGATTGTCCAGCTGTTGCTAAACATGACTTCTTTAAGTTTAGAATAGACG   13749
gb:MT066176|Organism:Severe      ATTTACTTAAGGATTGTCCAGCTGTTGCTAAACATGACTTCTTTAAGTTTAGAATAGACG   13762
gb:MT027064|Organism:Severe      ATTTACTTAAGGATTGTCCAGCTGTTGCTAAACATGACTTCTTTAAGTTTAGAATAGACG   13762
gb:MN994468|Organism:Severe      ATTTACTTAAGGATTGTCCAGCTGTTGCTAAACATGACTTCTTTAAGTTTAGAATAGACG   13762
gb:MT072688|Organism:Severe      ATTTACTTAAGGATTGTCCAGCTGTTGCTAAACATGACTTCTTTAAGTTTAGAATAGACG   13747
gb:MN996527|Organism:Severe      ATTTACTTAAGGATTGTCCAGCTGTTGCTAAACATGACTTCTTTAAGTTTAGAATAGACG   13729
gb:MT093631|Organism:Severe      ATTTACTTAAGGATTGTCCAGCTGTTGCTAAACATGACTTCTTTAAGTTTAGAATAGACG   13800
gb:MT106053|Organism:Severe      ATTTACTTAAGGATTGTCCAGCTGTTGCTAAACATGACTTCTTTAAGTTTAGAATAGACG   13762
gb:MT019533|Organism:Severe      ATTTACTTAAGGATTGTCCAGCTGTTGCTAAACATGACTTCTTTAAGTTTAGAATAGACG   13762
gb:MT019531|Organism:Severe      ATTTACTTAAGGATTGTCCAGCTGTTGCTAAACATGACTTCTTTAAGTTTAGAATAGACG   13762
gb:MN996528|Organism:Severe      ATTTACTTAAGGATTGTCCAGCTGTTGCTAAACATGACTTCTTTAAGTTTAGAATAGACG   13762
gb:MN996530|Organism:Severe      ATTTACTTAAGGATTGTCCAGCTGTTGCTAAACATGACTTCTTTAAGTTTAGAATAGACG   13748
gb:MN908947|Organism:Severe      ATTTACTTAAGGATTGTCCAGCTGTTGCTAAACATGACTTCTTTAAGTTTAGAATAGACG   13762
gb:MT019532|Organism:Severe      ATTTACTTAAGGATTGTCCAGCTGTTGCTAAACATGACTTCTTTAAGTTTAGAATAGACG   13762
                                 ************************************************************

gb:MT020781|Organism:Severe      GTGACATGGTACCACATATATCACGTCAACGTCTTACTAAATACACAATGGCAGACCTCG   13810
gb:MT007544|Organism:Severe      GTGACATGGTACCACATATATCACGTCAACGTCTTACTAAATACACAATGGCAGACCTCG   13822
gb:MN994467|Organism:Severe      GTGACATGGTACCACATATATCACGTCAACGTCTTACTAAATACACAATGGCAGACCTCG   13822
gb:MT044257|Organism:Severe      GTGACATGGTACCACATATATCACGTCAACGTCTTACTAAATACACAATGGCAGACCTCG   13822
gb:MT106054|Organism:Severe      GTGACATGGTACCACATATATCACGTCAACGTCTTACTAAATACACAATGGCAGACCTCG   13822
gb:MT049951|Organism:Severe      GTGACATGGTACCACATATATCACGTCAACGTCTTACTAAATACACAATGGCAGACCTCG   13822
gb:MN975262|Organism:Severe      GTGACATGGTACCACATATATCACGTCAACGTCTTACTAAATACACAATGGCAGACCTCG   13822
gb:MT106052|Organism:Severe      GTGACATGGTACCACATATATCACGTCAACGTCTTACTAAATACACAATGGCAGACCTCG   13822
gb:LC522975|Organism:Severe      GTGACATGGTACCACATATATCACGTCAACGTCTTACTAAATACACAATGGCAGACCTCG   13819
gb:LC522973|Organism:Severe      GTGACATGGTACCACATATATCACGTCAACGTCTTACTAAATACACAATGGCAGACCTCG   13819
gb:LC522974|Organism:Severe      GTGACATGGTACCACATATATCACGTCAACGTCTTACTAAATACACAATGGCAGACCTCG   13819
```

```
gb:MN985325|Organism:Severe      GTGACATGGTACCACATATATCACGTCAACGTCTTACTAAATACACAATGGCAGACCTCG   13822
gb:MT020881|Organism:Severe      GTGACATGGTACCACATATATCACGTCAACGTCTTACTAAATACACAATGGCAGACCTCG   13822
gb:MT020880|Organism:Severe      GTGACATGGTACCACATATATCACGTCAACGTCTTACTAAATACACAATGGCAGACCTCG   13822
gb:MT066175|Organism:Severe      GTGACATGGTACCACATATATCACGTCAACGTCTTACTAAATACACAATGGCAGACCTCG   13822
gb:MN997409|Organism:Severe      GTGACATGGTACCACATATATCACGTCAACGTCTTACTAAATACACAATGGCAGACCTCG   13822
gb:MN938384|Organism:Severe      GTGACATGGTACCACATATATCACGTCAACGTCTTACTAAATACACAATGGCAGACCTCG   13790
gb:MT044258|Organism:Severe      GTGACATGGTACCACATATATCACGTCAACGTCTTACTAAATACACAATGGCAGACCTCG   13798
gb:MT039890|Organism:Severe      GTGACATGGTACCACATATATCACGTCAACGTCTTACTAAATACACAATGGCAGACCTCG   13822
gb:MN988713|Organism:Severe      GTGACATGGTACCACATATATCACGTCAACGTCTTACTAAATACACAATGGCAGACCTCG   13822
gb:LC521925|Organism:Severe      GTGACATGGTACCACATATATCACGTCAACGTCTTACTAAATACACAATGGCAGACCTCG   13795
gb:MT093571|Organism:Severe      GTGACATGGTACCACATATATCACGTCAACGTCTTACTAAATACACAATGGCAGACCTCG   13822
gb:MT039887|Organism:Severe      GTGACATGGTACCACATATATCACGTCAACGTCTTACTAAATACACAATGGCAGACCTCG   13822
gb:MT019530|Organism:Severe      GTGACATGGTACCACATATATCACGTCAACGTCTTACTAAATACACAATGGCAGACCTCG   13822
gb:MT039888|Organism:Severe      GTGACATGGTACCACATATATCACGTCAACGTCTTACTAAATACACAATGGCAGACCTCG   13822
gb:LC522972|Organism:Severe      GTGACATGGTACCACATATATCACGTCAACGTCTTACTAAATACACAATGGCAGACCTCG   13819
gb:MT027063|Organism:Severe      GTGACATGGTACCACATATATCACGTCAACGTCTTACTAAATACACAATGGCAGACCTCG   13822
gb:MT027062|Organism:Severe      GTGACATGGTACCACATATATCACGTCAACGTCTTACTAAATACACAATGGCAGACCTCG   13822
gb:MT019529|Organism:Severe      GTGACATGGTACCACATATATCACGTCAACGTCTTACTAAATACACAATGGCAGACCTCG   13822
gb:MN996529|Organism:Severe      GTGACATGGTACCACATATATCACGTCAACGTCTTACTAAATACACAATGGCAGACCTCG   13810
gb:MN996531|Organism:Severe      GTGACATGGTACCACATATATCACGTCAACGTCTTACTAAATACACAATGGCAGACCTCG   13809
gb:MT066176|Organism:Severe      GTGACATGGTACCACATATATCACGTCAACGTCTTACTAAATACACAATGGCAGACCTCG   13822
gb:MT027064|Organism:Severe      GTGACATGGTACCACATATATCACGTCAACGTCTTACTAAATACACAATGGCAGACCTCG   13822
gb:MN994468|Organism:Severe      GTGACATGGTACCACATATATCACGTCAACGTCTTACTAAATACACAATGGCAGACCTCG   13822
gb:MT072688|Organism:Severe      GTGACATGGTACCACATATATCACGTCAACGTCTTACTAAATACACAATGGCAGACCTCG   13807
gb:MN996527|Organism:Severe      GTGACATGGTACCACATATATCACGTCAACGTCTTACTAAATACACAATGGCAGACCTCG   13789
gb:MT093631|Organism:Severe      GTGACATGGTACCACATATATCACGTCAACGTCTTACTAAATACACAATGGCAGACCTCG   13860
gb:MT106053|Organism:Severe      GTGACATGGTACCACATATATCACGTCAACGTCTTACTAAATACACAATGGCAGACCTCG   13822
gb:MT019533|Organism:Severe      GTGACATGGTACCACATATATCACGTCAACGTCTTACTAAATACACAATGGCAGACCTCG   13822
gb:MT019531|Organism:Severe      GTGACATGGTACCACATATATCACGTCAACGTCTTACTAAATACACAATGGCAGACCTCG   13822
gb:MN996528|Organism:Severe      GTGACATGGTACCACATATATCACGTCAACGTCTTACTAAATACACAATGGCAGACCTCG   13822
gb:MN996530|Organism:Severe      GTGACATGGTACCACATATATCACGTCAACGTCTTACTAAATACACAATGGCAGACCTCG   13808
gb:MN908947|Organism:Severe      GTGACATGGTACCACATATATCACGTCAACGTCTTACTAAATACACAATGGCAGACCTCG   13822
gb:MT019532|Organism:Severe      GTGACATGGTACCACATATATCACGTCAACGTCTTACTAAATACACAATGGCAGACCTCG   13822
                                 ************************************************************

gb:MT020781|Organism:Severe      TCTATGCTTTAAGGCATTTTGATGAAGGTAATTGTGACACATTAAAAGAAATACTTGTCA   13870
gb:MT007544|Organism:Severe      TCTATGCTTTAAGGCATTTTGATGAAGGTAATTGTGACACATTAAAAGAAATACTTGTCA   13882
gb:MN994467|Organism:Severe      TCTATGCTTTAAGGCATTTTGATGAAGGTAATTGTGACACATTAAAAGAAATACTTGTCA   13882
gb:MT044257|Organism:Severe      TCTATGCTTTAAGGCATTTTGATGAAGGTAATTGTGACACATTAAAAGAAATACTTGTCA   13882
gb:MT106054|Organism:Severe      TCTATGCTTTAAGGCATTTTGATGAAGGTAATTGTGACACATTAAAAGAAATACTTGTCA   13882
gb:MT049951|Organism:Severe      TCTATGCTTTAAGGCATTTTGATGAAGGTAATTGTGACACATTAAAAGAAATACTTGTCA   13882
gb:MN975262|Organism:Severe      TCTATGCTTTAAGGCATTTTGATGAAGGTAATTGTGACACATTAAAAGAAATACTTGTCA   13882
gb:MT106052|Organism:Severe      TCTATGCTTTAAGGCATTTTGATGAAGGTAATTGTGACACATTAAAAGAAATACTTGTCA   13882
gb:LC522975|Organism:Severe      TCTATGCTTTAAGGCATTTTGATGAAGGTAATTGTGACACATTAAAAGAAATACTTGTCA   13879
gb:LC522973|Organism:Severe      TCTATGCTTTAAGGCATTTTGATGAAGGTAATTGTGACACATTAAAAGAAATACTTGTCA   13879
gb:LC522974|Organism:Severe      TCTATGCTTTAAGGCATTTTGATGAAGGTAATTGTGACACATTAAAAGAAATACTTGTCA   13879
gb:MN985325|Organism:Severe      TCTATGCTTTAAGGCATTTTGATGAAGGTAATTGTGACACATTAAAAGAAATACTTGTCA   13882
gb:MT020881|Organism:Severe      TCTATGCTTTAAGGCATTTTGATGAAGGTAATTGTGACACATTAAAAGAAATACTTGTCA   13882
gb:MT020880|Organism:Severe      TCTATGCTTTAAGGCATTTTGATGAAGGTAATTGTGACACATTAAAAGAAATACTTGTCA   13882
gb:MT066175|Organism:Severe      TCTATGCTTTAAGGCATTTTGATGAAGGTAATTGTGACACATTAAAAGAAATACTTGTCA   13882
gb:MN997409|Organism:Severe      TCTATGCTTTAAGGCATTTTGATGAAGGTAATTGTGACACATTAAAAGAAATACTTGTCA   13882
gb:MN938384|Organism:Severe      TCTATGCTTTAAGGCATTTTGATGAAGGTAATTGTGACACATTAAAAGAAATACTTGTCA   13850
gb:MT044258|Organism:Severe      TCTATGCTTTAAGGCATTTTGATGAAGGTAATTGTGACACATTAAAAGAAATACTTGTCA   13858
gb:MT039890|Organism:Severe      TCTATGCTTTAAGGCATTTTGATGAAGGTAATTGTGACACATTAAAAGAAATACTTGTCA   13882
gb:MN988713|Organism:Severe      TCTATGCTTTAAGGCATTTTGATGAAGGTAATTGTGACACATTAAAAGAAATACTTGTCA   13882
gb:LC521925|Organism:Severe      TCTATGCTTTAAGGCATTTTGATGAAGGTAATTGTGACACATTAAAAGAAATACTTGTCA   13855
gb:MT093571|Organism:Severe      TCTATGCTTTAAGGCATTTTGATGAAGGTAATTGTGACACATTAAAAGAAATACTTGTCA   13882
gb:MT039887|Organism:Severe      TCTATGCTTTAAGGCATTTTGATGAAGGTAATTGTGACACATTAAAAGAAATACTTGTCA   13882
gb:MT019530|Organism:Severe      TCTATGCTTTAAGGCATTTTGATGAAGGTAATTGTGACACATTAAAAGAAATACTTGTCA   13882
gb:MT039888|Organism:Severe      TCTATGCTTTAAGGCATTTTGATGAAGGTAATTGTGACACATTAAAAGAAATACTTGTCA   13882
gb:LC522972|Organism:Severe      TCTATGCTTTAAGGCATTTTGATGAAGGTAATTGTGACACATTAAAAGAAATACTTGTCA   13879
gb:MT027063|Organism:Severe      TCTATGCTTTAAGGCATTTTGATGAAGGTAATTGTGACACATTAAAAGAAATACTTGTCA   13882
gb:MT027062|Organism:Severe      TCTATGCTTTAAGGCATTTTGATGAAGGTAATTGTGACACATTAAAAGAAATACTTGTCA   13882
gb:MT019529|Organism:Severe      TCTATGCTTTAAGGCATTTTGATGAAGGTAATTGTGACACATTAAAAGAAATACTTGTCA   13882
gb:MN996529|Organism:Severe      TCTATGCTTTAAGGCATTTTGATGAAGGTAATTGTGACACATTAAAAGAAATACTTGTCA   13870
```

```
gb:MN996531|Organism:Severe      TCTATGCTTTAAGGCATTTTGATGAAGGTAATTGTGACACATTAAAAGAAATACTTGTCA   13869
gb:MT066176|Organism:Severe      TCTATGCTTTAAGGCATTTTGATGAAGGTAATTGTGACACATTAAAAGAAATACTTGTCA   13882
gb:MT027064|Organism:Severe      TCTATGCTTTAAGGCATTTTGATGAAGGTAATTGTGACACATTAAAAGAAATACTTGTCA   13882
gb:MN994468|Organism:Severe      TCTATGCTTTAAGGCATTTTGATGAAGGTAATTGTGACACATTAAAAGAAATACTTGTCA   13882
gb:MT072688|Organism:Severe      TCTATGCTTTAAGGCATTTTGATGAAGGTAATTGTGACACATTAAAAGAAATACTTGTCA   13867
gb:MN996527|Organism:Severe      TCTATGCTTTAAGGCATTTTGATGAAGGTAATTGTGACACATTAAAAGAAATACTTGTCA   13849
gb:MT093631|Organism:Severe      TCTATGCTTTAAGGCATTTTGATGAAGGTAATTGTGACACATTAAAAGAAATACTTGTCA   13920
gb:MT106053|Organism:Severe      TCTATGCTTTAAGGCATTTTGATGAAGGTAATTGTGACACATTAAAAGAAATACTTGTCA   13882
gb:MT019533|Organism:Severe      TCTATGCTTTAAGGCATTTTGATGAAGGTAATTGTGACACATTAAAAGAAATACTTGTCA   13882
gb:MT019531|Organism:Severe      TCTATGCTTTAAGGCATTTTGATGAAGGTAATTGTGACACATTAAAAGAAATACTTGTCA   13882
gb:MN996528|Organism:Severe      TCTATGCTTTAAGGCATTTTGATGAAGGTAATTGTGACACATTAAAAGAAATACTTGTCA   13882
gb:MN996530|Organism:Severe      TCTATGCTTTAAGGCATTTTGATGAAGGTAATTGTGACACATTAAAAGAAATACTTGTCA   13868
gb:MN908947|Organism:Severe      TCTATGCTTTAAGGCATTTTGATGAAGGTAATTGTGACACATTAAAAGAAATACTTGTCA   13882
gb:MT019532|Organism:Severe      TCTATGCTTTAAGGCATTTTGATGAAGGTAATTGTGACACATTAAAAGAAATACTTGTCA   13882
                                 ************************************************************

gb:MT020781|Organism:Severe      CATACAATTGTTGTGATGATGATTATTTCAATAAAAAGGACTGGTATGATTTTGTAGAAA   13930
gb:MT007544|Organism:Severe      CATACAATTGTTGTGATGATGATTATTTCAATAAAAAGGACTGGTATGATTTTGTAGAAA   13942
gb:MN994467|Organism:Severe      CATACAATTGTTGTGATGATGATTATTTCAATAAAAAGGACTGGTATGATTTTGTAGAAA   13942
gb:MT044257|Organism:Severe      CATACAATTGTTGTGATGATGATTATTTCAATAAAAAGGACTGGTATGATTTTGTAGAAA   13942
gb:MT106054|Organism:Severe      CATACAATTGTTGTGATGATGATTATTTCAATAAAAAGGACTGGTATGATTTTGTAGAAA   13942
gb:MT049951|Organism:Severe      CATACAATTGTTGTGATGATGATTATTTCAATAAAAAGGACTGGTATGATTTTGTAGAAA   13942
gb:MN975262|Organism:Severe      CATACAATTGTTGTGATGATGATTATTTCAATAAAAAGGACTGGTATGATTTTGTAGAAA   13942
gb:MT106052|Organism:Severe      CATACAATTGTTGTGATGATGATTATTTCAATAAAAAGGACTGGTATGATTTTGTAGAAA   13942
gb:LC522975|Organism:Severe      CATACAATTGTTGTGATGATGATTATTTCAATAAAAAGGACTGGTATGATTTTGTAGAAA   13939
gb:LC522973|Organism:Severe      CATACAATTGTTGTGATGATGATTATTTCAATAAAAAGGACTGGTATGATTTTGTAGAAA   13939
gb:LC522974|Organism:Severe      CATACAATTGTTGTGATGATGATTATTTCAATAAAAAGGACTGGTATGATTTTGTAGAAA   13939
gb:MN985325|Organism:Severe      CATACAATTGTTGTGATGATGATTATTTCAATAAAAAGGACTGGTATGATTTTGTAGAAA   13942
gb:MT020881|Organism:Severe      CATACAATTGTTGTGATGATGATTATTTCAATAAAAAGGACTGGTATGATTTTGTAGAAA   13942
gb:MT020880|Organism:Severe      CATACAATTGTTGTGATGATGATTATTTCAATAAAAAGGACTGGTATGATTTTGTAGAAA   13942
gb:MT066175|Organism:Severe      CATACAATTGTTGTGATGATGATTATTTCAATAAAAAGGACTGGTATGATTTTGTAGAAA   13942
gb:MN997409|Organism:Severe      CATACAATTGTTGTGATGATGATTATTTCAATAAAAAGGACTGGTATGATTTTGTAGAAA   13942
gb:MN938384|Organism:Severe      CATACAATTGTTGTGATGATGATTATTTCAATAAAAAGGACTGGTATGATTTTGTAGAAA   13910
gb:MT044258|Organism:Severe      CATACAATTGTTGTGATGATGATTATTTCAATAAAAAGGACTGGTATGATTTTGTAGAAA   13918
gb:MT039890|Organism:Severe      CATACAATTGTTGTGATGATGATTATTTCAATAAAAAGGACTGGTATGATTTTGTAGAAA   13942
gb:MN988713|Organism:Severe      CATACAATTGTTGTGATGATGATTATTTCAATAAAAAGGACTGGTATGATTTTGTAGAAA   13942
gb:LC521925|Organism:Severe      CATACAATTGTTGTGATGATGATTATTTCAATAAAAAGGACTGGTATGATTTTGTAGAAA   13915
gb:MT093571|Organism:Severe      CATACAATTGTTGTGATGATGATTATTTCAATAAAAAGGACTGGTATGATTTTGTAGAAA   13942
gb:MT039887|Organism:Severe      CATACAATTGTTGTGATGATGATTATTTCAATAAAAAGGACTGGTATGATTTTGTAGAAA   13942
gb:MT019530|Organism:Severe      CATACAATTGTTGTGATGATGATTATTTCAATAAAAAGGACTGGTATGATTTTGTAGAAA   13942
gb:MT039888|Organism:Severe      CATACAATTGTTGTGATGATGATTATTTCAATAAAAAGGACTGGTATGATTTTGTAGAAA   13942
gb:LC522972|Organism:Severe      CATACAATTGTTGTGATGATGATTATTTCAATAAAAAGGACTGGTATGATTTTGTAGAAA   13939
gb:MT027063|Organism:Severe      CATACAATTGTTGTGATGATGATTATTTCAATAAAAAGGACTGGTATGATTTTGTAGAAA   13942
gb:MT027062|Organism:Severe      CATACAATTGTTGTGATGATGATTATTTCAATAAAAAGGACTGGTATGATTTTGTAGAAA   13942
gb:MT019529|Organism:Severe      CATACAATTGTTGTGATGATGATTATTTCAATAAAAAGGACTGGTATGATTTTGTAGAAA   13942
gb:MN996529|Organism:Severe      CATACAATTGTTGTGATGATGATTATTTCAATAAAAAGGACTGGTATGATTTTGTAGAAA   13930
gb:MN996531|Organism:Severe      CATACAATTGTTGTGATGATGATTATTTCAATAAAAAGGACTGGTATGATTTTGTAGAAA   13929
gb:MT066176|Organism:Severe      CATACAATTGTTGTGATGATGATTATTTCAATAAAAAGGACTGGTATGATTTTGTAGAAA   13942
gb:MT027064|Organism:Severe      CATACAATTGTTGTGATGATGATTATTTCAATAAAAAGGACTGGTATGATTTTGTAGAAA   13942
gb:MN994468|Organism:Severe      CATACAATTGTTGTGATGATGATTATTTCAATAAAAAGGACTGGTATGATTTTGTAGAAA   13942
gb:MT072688|Organism:Severe      CATACAATTGTTGTGATGATGATTATTTCAATAAAAAGGACTGGTATGATTTTGTAGAAA   13927
gb:MN996527|Organism:Severe      CATACAATTGTTGTGATGATGATTATTTCAATAAAAAGGACTGGTATGATTTTGTAGAAA   13909
gb:MT093631|Organism:Severe      CATACAATTGTTGTGATGATGATTATTTCAATAAAAAGGACTGGTATGATTTTGTAGAAA   13980
gb:MT106053|Organism:Severe      CATACAATTGTTGTGATGATGATTATTTCAATAAAAAGGACTGGTATGATTTTGTAGAAA   13942
gb:MT019533|Organism:Severe      CATACAATTGTTGTGATGATGATTATTTCAATAAAAAGGACTGGTATGATTTTGTAGAAA   13942
gb:MT019531|Organism:Severe      CATACAATTGTTGTGATGATGATTATTTCAATAAAAAGGACTGGTATGATTTTGTAGAAA   13942
gb:MN996528|Organism:Severe      CATACAATTGTTGTGATGATGATTATTTCAATAAAAAGGACTGGTATGATTTTGTAGAAA   13942
gb:MN996530|Organism:Severe      CATACAATTGTTGTGATGATGATTATTTCAATAAAAAGGACTGGTATGATTTTGTAGAAA   13928
gb:MN908947|Organism:Severe      CATACAATTGTTGTGATGATGATTATTTCAATAAAAAGGACTGGTATGATTTTGTAGAAA   13942
gb:MT019532|Organism:Severe      CATACAATTGTTGTGATGATGATTATTTCAATAAAAAGGACTGGTATGATTTTGTAGAAA   13942
                                 ************************************************************

gb:MT020781|Organism:Severe      ACCCAGATATATTACGCGTATACGCCAACTTAGGTGAACGTGTACGCCAAGCTTTGTTAA   13990
gb:MT007544|Organism:Severe      ACCCAGATATATTACGCGTATACGCCAACTTAGGTGAACGTGTACGCCAAGCTTTGTTAA   14002
gb:MN994467|Organism:Severe      ACCCAGATATATTACGCGTATACGCCAACTTAGGTGAACGTGTACGCCAAGCTTTGTTAA   14002
```

```
gb:MT044257|Organism:Severe      ACCCAGATATATTACGCGTATACGCCAACTTAGGTGAACGTGTACGCCAAGCTTTGTTAA   14002
gb:MT106054|Organism:Severe      ACCCAGATATATTACGCGTATACGCCAACTTAGGTGAACGTGTACGCCAAGCTTTGTTAA   14002
gb:MT049951|Organism:Severe      ACCCAGATATATTACGCGTATACGCCAACTTAGGTGAACGTGTACGCCAAGCTTTGTTAA   14002
gb:MN975262|Organism:Severe      ACCCAGATATATTACGCGTATACGCCAACTTAGGTGAACGTGTACGCCAAGCTTTGTTAA   14002
gb:MT106052|Organism:Severe      ACCCAGATATATTACGCGTATACGCCAACTTAGGTGAACGTGTACGCCAAGCTTTGTTAA   14002
gb:LC522975|Organism:Severe      ACCCAGATATATTACGCGTATACGCCAACTTAGGTGAACGTGTACGCCAAGCTTTGTTAA   13999
gb:LC522973|Organism:Severe      ACCCAGATATATTACGCGTATACGCCAACTTAGGTGAACGTGTACGCCAAGCTTTGTTAA   13999
gb:LC522974|Organism:Severe      ACCCAGATATATTACGCGTATACGCCAACTTAGGTGAACGTGTACGCCAAGCTTTGTTAA   13999
gb:MN985325|Organism:Severe      ACCCAGATATATTACGCGTATACGCCAACTTAGGTGAACGTGTACGCCAAGCTTTGTTAA   14002
gb:MT020881|Organism:Severe      ACCCAGATATATTACGCGTATACGCCAACTTAGGTGAACGTGTACGCCAAGCTTTGTTAA   14002
gb:MT020880|Organism:Severe      ACCCAGATATATTACGCGTATACGCCAACTTAGGTGAACGTGTACGCCAAGCTTTGTTAA   14002
gb:MT066175|Organism:Severe      ACCCAGATATATTACGCGTATACGCCAACTTAGGTGAACGTGTACGCCAAGCTTTGTTAA   14002
gb:MN997409|Organism:Severe      ACCCAGATATATTACGCGTATACGCCAACTTAGGTGAACGTGTACGCCAAGCTTTGTTAA   14002
gb:MN938384|Organism:Severe      ACCCAGATATATTACGCGTATACGCCAACTTAGGTGAACGTGTACGCCAAGCTTTGTTAA   13970
gb:MT044258|Organism:Severe      ACCCAGATATATTACGCGTATACGCCAACTTAGGTGAACGTGTACGCCAAGCTTTGTTAA   13978
gb:MT039890|Organism:Severe      ACCCAGATATATTACGCGTATACGCCAACTTAGGTGAACGTGTACGCCAAGCTTTGTTAA   14002
gb:MN988713|Organism:Severe      ACCCAGATATATTACGCGTATACGCCAACTTAGGTGAACGTGTACGCCAAGCTTTGTTAA   14002
gb:LC521925|Organism:Severe      ACCCAGATATATTACGCGTATACGCCAACTTAGGTGAACGTGTACGCCAAGCTTTGTTAA   13975
gb:MT093571|Organism:Severe      ACCCAGATATATTACGCGTATACGCCAACTTAGGTGAACGTGTACGCCAAGCTTTGTTAA   14002
gb:MT039887|Organism:Severe      ACCCAGATATATTACGCGTATACGCCAACTTAGGTGAACGTGTACGCCAAGCTTTGTTAA   14002
gb:MT019530|Organism:Severe      ACCCAGATATATTACGCGTATACGCCAACTTAGGTGAACGTGTACGCCAAGCTTTGTTAA   14002
gb:MT039888|Organism:Severe      ACCCAGATATATTACGCGTATACGCCAACTTAGGTGAACGTGTACGCCAAGCTTTGTTAA   14002
gb:LC522972|Organism:Severe      ACCCAGATATATTACGCGTATACGCCAACTTAGGTGAACGTGTACGCCAAGCTTTGTTAA   13999
gb:MT027063|Organism:Severe      ACCCAGATATATTACGCGTATACGCCAACTTAGGTGAACGTGTACGCCAAGCTTTGTTAA   14002
gb:MT027062|Organism:Severe      ACCCAGATATATTACGCGTATACGCCAACTTAGGTGAACGTGTACGCCAAGCTTTGTTAA   14002
gb:MT019529|Organism:Severe      ACCCAGATATATTACGCGTATACGCCAACTTAGGTGAACGTGTACGCCAAGCTTTGTTAA   14002
gb:MN996529|Organism:Severe      ACCCAGATATATTACGCGTATACGCCAACTTAGGTGAACGTGTACGCCAAGCTTTGTTAA   13990
gb:MN996531|Organism:Severe      ACCCAGATATATTACGCGTATACGCCAACTTAGGTGAACGTGTACGCCAAGCTTTGTTAA   13989
gb:MT066176|Organism:Severe      ACCCAGATATATTACGCGTATACGCCAACTTAGGTGAACGTGTACGCCAAGCTTTGTTAA   14002
gb:MT027064|Organism:Severe      ACCCAGATATATTACGCGTATACGCCAACTTAGGTGAACGTGTACGCCAAGCTTTGTTAA   14002
gb:MN994468|Organism:Severe      ACCCAGATATATTACGCGTATACGCCAACTTAGGTGAACGTGTACGCCAAGCTTTGTTAA   14002
gb:MT072688|Organism:Severe      ACCCAGATATATTACGCGTATACGCCAACTTAGGTGAACGTGTACGCCAAGCTTTGTTAA   13987
gb:MN996527|Organism:Severe      ACCCAGATATATTACGCGTATACGCCAACTTAGGTGAACGTGTACGCCAAGCTTTGTTAA   13969
gb:MT093631|Organism:Severe      ACCCAGATATATTACGCGTATACGCCAACTTAGGTGAACGTGTACGCCAAGCTTTGTTAA   14040
gb:MT106053|Organism:Severe      ACCCAGATATATTACGCGTATACGCCAACTTAGGTGAACGTGTACGCCAAGCTTTGTTAA   14002
gb:MT019533|Organism:Severe      ACCCAGATATATTACGCGTATACGCCAACTTAGGTGAACGTGTACGCCAAGCTTTGTTAA   14002
gb:MT019531|Organism:Severe      ACCCAGATATATTACGCGTATACGCCAACTTAGGTGAACGTGTACGCCAAGCTTTGTTAA   14002
gb:MN996528|Organism:Severe      ACCCAGATATATTACGCGTATACGCCAACTTAGGTGAACGTGTACGCCAAGCTTTGTTAA   14002
gb:MN996530|Organism:Severe      ACCCAGATATATTACGCGTATACGCCAACTTAGGTGAACGTGTACGCCAAGCTTTGTTAA   13988
gb:MN908947|Organism:Severe      ACCCAGATATATTACGCGTATACGCCAACTTAGGTGAACGTGTACGCCAAGCTTTGTTAA   14002
gb:MT019532|Organism:Severe      ACCCAGATATATTACGCGTATACGCCAACTTAGGTGAACGTGTACGCCAAGCTTTGTTAA   14002
                                 ************************************************************

gb:MT020781|Organism:Severe      AAACAGTACAATTCTGTGATGCCATGCGAAATGCTGGTATTGTTGGTGTACTGACATTAG   14050
gb:MT007544|Organism:Severe      AAACAGTACAATTCTGTGATGCCATGCGAAATGCTGGTATTGTTGGTGTACTGACATTAG   14062
gb:MN994467|Organism:Severe      AAACAGTACAATTCTGTGATGCCATGCGAAATGCTGGTATTGTTGGTGTACTGACATTAG   14062
gb:MT044257|Organism:Severe      AAACAGTACAATTCTGTGATGCCATGCGAAATGCTGGTATTGTTGGTGTACTGACATTAG   14062
gb:MT106054|Organism:Severe      AAACAGTACAATTCTGTGATGCCATGCGAAATGCTGGTATTGTTGGTGTACTGACATTAG   14062
gb:MT049951|Organism:Severe      AAACAGTACAATTCTGTGATGCCATGCGAAATGCTGGTATTGTTGGTGTACTGACATTAG   14062
gb:MN975262|Organism:Severe      AAACAGTACAATTCTGTGATGCCATGCGAAATGCTGGTATTGTTGGTGTACTGACATTAG   14062
gb:MT106052|Organism:Severe      AAACAGTACAATTCTGTGATGCCATGCGAAATGCTGGTATTGTTGGTGTACTGACATTAG   14062
gb:LC522975|Organism:Severe      AAACAGTACAATTCTGTGATGCCATGCGAAATGCTGGTATTGTTGGTGTACTGACATTAG   14059
gb:LC522973|Organism:Severe      AAACAGTACAATTCTGTGATGCCATGCGAAATGCTGGTATTGTTGGTGTACTGACATTAG   14059
gb:LC522974|Organism:Severe      AAACAGTACAATTCTGTGATGCCATGCGAAATGCTGGTATTGTTGGTGTACTGACATTAG   14059
gb:MN985325|Organism:Severe      AAACAGTACAATTCTGTGATGCCATGCGAAATGCTGGTATTGTTGGTGTACTGACATTAG   14062
gb:MT020881|Organism:Severe      AAACAGTACAATTCTGTGATGCCATGCGAAATGCTGGTATTGTTGGTGTACTGACATTAG   14062
gb:MT020880|Organism:Severe      AAACAGTACAATTCTGTGATGCCATGCGAAATGCTGGTATTGTTGGTGTACTGACATTAG   14062
gb:MT066175|Organism:Severe      AAACAGTACAATTCTGTGATGCCATGCGAAATGCTGGTATTGTTGGTGTACTGACATTAG   14062
gb:MN997409|Organism:Severe      AAACAGTACAATTCTGTGATGCCATGCGAAATGCTGGTATTGTTGGTGTACTGACATTAG   14062
gb:MN938384|Organism:Severe      AAACAGTACAATTCTGTGATGCCATGCGAAATGCTGGTATTGTTGGTGTACTGACATTAG   14030
gb:MT044258|Organism:Severe      AAACAGTACAATTCTGTGATGCCATGCGAAATGCTGGTATTGTTGGTGTACTGACATTAG   14038
gb:MT039890|Organism:Severe      AAACAGTACAATTCTGTGATGCCATGCGAAATGCTGGTATTGTTGGTGTACTGACATTAG   14062
gb:MN988713|Organism:Severe      AAACAGTACAATTCTGTGATGCCATGCGAAATGCTGGTATTGTTGGTGTACTGACATTAG   14062
gb:LC521925|Organism:Severe      AAACAGTACAATTCTGTGATGCCATGCGAAATGCTGGTATTGTTGGTGTACTGACATTAG   14035
gb:MT093571|Organism:Severe      AAACAGTACAATTCTGTGATGCCATGCGAAATGCTGGTATTGTTGGTGTACTGACATTAG   14062
```

```
gb:MT039887|Organism:Severe      AAACAGTACAATTCTGTGATGCCATGCGAAATGCTGGTATTGTTGGTGTACTGACATTAG    14062
gb:MT019530|Organism:Severe      AAACAGTACAATTCTGTGATGCCATGCGAAATGCTGGTATTGTTGGTGTACTGACATTAG    14062
gb:MT039888|Organism:Severe      AAACAGTACAATTCTGTGATGCCATGCGAAATGCTGGTATTGTTGGTGTACTGACATTAG    14062
gb:LC522972|Organism:Severe      AAACAGTACAATTCTGTGATGCCATGCGAAATGCTGGTATTGTTGGTGTACTGACATTAG    14059
gb:MT027063|Organism:Severe      AAACAGTACAATTCTGTGATGCCATGCGAAATGCTGGTATTGTTGGTGTACTGACATTAG    14062
gb:MT027062|Organism:Severe      AAACAGTACAATTCTGTGATGCCATGCGAAATGCTGGTATTGTTGGTGTACTGACATTAG    14062
gb:MT019529|Organism:Severe      AAACAGTACAATTCTGTGATGCCATGCGAAATGCTGGTATTGTTGGTGTACTGACATTAG    14062
gb:MN996529|Organism:Severe      AAACAGTACAATTCTGTGATGCCATGCGAAATGCTGGTATTGTTGGTGTACTGACATTAG    14050
gb:MN996531|Organism:Severe      AAACAGTACAATTCTGTGATGCCATGCGAAATGCTGGTATTGTTGGTGTACTGACATTAG    14049
gb:MT066176|Organism:Severe      AAACAGTACAATTCTGTGATGCCATGCGAAATGCTGGTATTGTTGGTGTACTGACATTAG    14062
gb:MT027064|Organism:Severe      AAACAGTACAATTCTGTGATGCCATGCGAAATGCTGGTATTGTTGGTGTACTGACATTAG    14062
gb:MN994468|Organism:Severe      AAACAGTACAATTCTGTGATGCCATGCGAAATGCTGGTATTGTTGGTGTACTGACATTAG    14062
gb:MT072688|Organism:Severe      AAACAGTACAATTCTGTGATGCCATGCGAAATGCTGGTATTGTTGGTGTACTGACATTAG    14047
gb:MN996527|Organism:Severe      AAACAGTACAATTCTGTGATGCCATGCGAAATGCTGGTATTGTTGGTGTACTGACATTAG    14029
gb:MT093631|Organism:Severe      AAACAGTACAATTCTGTGATGCCATGCGAAATGCTGGTATTGTTGGTGTACTGACATTAG    14100
gb:MT106053|Organism:Severe      AAACAGTACAATTCTGTGATGCCATGCGAAATGCTGGTATTGTTGGTGTACTGACATTAG    14062
gb:MT019533|Organism:Severe      AAACAGTACAATTCTGTGATGCCATGCGAAATGCTGGTATTGTTGGTGTACTGACATTAG    14062
gb:MT019531|Organism:Severe      AAACAGTACAATTCTGTGATGCCATGCGAAATGCTGGTATTGTTGGTGTACTGACATTAG    14062
gb:MN996528|Organism:Severe      AAACAGTACAATTCTGTGATGCCATGCGAAATGCTGGTATTGTTGGTGTACTGACATTAG    14062
gb:MN996530|Organism:Severe      AAACAGTACAATTCTGTGATGCCATGCGAAATGCTGGTATTGTTGGTGTACTGACATTAG    14048
gb:MN908947|Organism:Severe      AAACAGTACAATTCTGTGATGCCATGCGAAATGCTGGTATTGTTGGTGTACTGACATTAG    14062
gb:MT019532|Organism:Severe      AAACAGTACAATTCTGTGATGCCATGCGAAATGCTGGTATTGTTGGTGTACTGACATTAG    14062
                                 ************************************************************

gb:MT020781|Organism:Severe      ATAATCAAGATCTCAATGGTAACTGGTATGATTTCGGTGATTTCATACAAACCACGCCAG    14110
gb:MT007544|Organism:Severe      ATAATCAAGATCTCAATGGTAACTGGTATGATTTCGGTGATTTCATACAAACCACGCCAG    14122
gb:MN994467|Organism:Severe      ATAATCAAGATCTCAATGGTAACTGGTATGATTTCGGTGATTTCATACAAACCACGCCAG    14122
gb:MT044257|Organism:Severe      ATAATCAAGATCTCAATGGTAACTGGTATGATTTCGGTGATTTCATACAAACCACGCCAG    14122
gb:MT106054|Organism:Severe      ATAATCAAGATCTCAATGGTAACTGGTATGATTTCGGTGATTTCATACAAACCACGCCAG    14122
gb:MT049951|Organism:Severe      ATAATCAAGATCTCAATGGTAACTGGTATGATTTCGGTGATTTCATACAAACCACGCCAG    14122
gb:MN975262|Organism:Severe      ATAATCAAGATCTCAATGGTAACTGGTATGATTTCGGTGATTTCATACAAACCACGCCAG    14122
gb:MT106052|Organism:Severe      ATAATCAAGATCTCAATGGTAACTGGTATGATTTCGGTGATTTCATACAAACCACGCCAG    14122
gb:LC522975|Organism:Severe      ATAATCAAGATCTCAATGGTAACTGGTATGATTTCGGTGATTTCATACAAACCACGCCAG    14119
gb:LC522973|Organism:Severe      ATAATCAAGATCTCAATGGTAACTGGTATGATTTCGGTGATTTCATACAAACCACGCCAG    14119
gb:LC522974|Organism:Severe      ATAATCAAGATCTCAATGGTAACTGGTATGATTTCGGTGATTTCATACAAACCACGCCAG    14119
gb:MN985325|Organism:Severe      ATAATCAAGATCTCAATGGTAACTGGTATGATTTCGGTGATTTCATACAAACCACGCCAG    14122
gb:MT020881|Organism:Severe      ATAATCAAGATCTCAATGGTAACTGGTATGATTTCGGTGATTTCATACAAACCACGCCAG    14122
gb:MT020880|Organism:Severe      ATAATCAAGATCTCAATGGTAACTGGTATGATTTCGGTGATTTCATACAAACCACGCCAG    14122
gb:MT066175|Organism:Severe      ATAATCAAGATCTCAATGGTAACTGGTATGATTTCGGTGATTTCATACAAACCACGCCAG    14122
gb:MN997409|Organism:Severe      ATAATCAAGATCTCAATGGTAACTGGTATGATTTCGGTGATTTCATACAAACCACGCCAG    14122
gb:MN938384|Organism:Severe      ATAATCAAGATCTCAATGGTAACTGGTATGATTTCGGTGATTTCATACAAACCACGCCAG    14090
gb:MT044258|Organism:Severe      ATAATCAAGATCTCAATGGTAACTGGTATGATTTCGGTGATTTCATACAAACCACGCCAG    14098
gb:MT039890|Organism:Severe      ATAATCAAGATCTCAATGGTAACTGGTATGATTTCGGTGATTTCATACAAACCACGCCAG    14122
gb:MN988713|Organism:Severe      ATAATCAAGATCTCAATGGTAACTGGTATGATTTCGGTGATTTCATACAAACCACGCCAG    14122
gb:LC521925|Organism:Severe      ATAATCAAGATCTCAATGGTAACTGGTATGATTTCGGTGATTTCATACAAACCACGCCAG    14095
gb:MT093571|Organism:Severe      ATAATCAAGATCTCAATGGTAACTGGTATGATTTCGGTGATTTCATACAAACCACGCCAG    14122
gb:MT039887|Organism:Severe      ATAATCAAGATCTCAATGGTAACTGGTATGATTTCGGTGATTTCATACAAACCACGCCAG    14122
gb:MT019530|Organism:Severe      ATAATCAAGATCTCAATGGTAACTGGTATGATTTCGGTGATTTCATACAAACCACGCCAG    14122
gb:MT039888|Organism:Severe      ATAATCAAGATCTCAATGGTAACTGGTATGATTTCGGTGATTTCATACAAACCACGCCAG    14122
gb:LC522972|Organism:Severe      ATAATCAAGATCTCAATGGTAACTGGTATGATTTCGGTGATTTCATACAAACCACGCCAG    14119
gb:MT027063|Organism:Severe      ATAATCAAGATCTCAATGGTAACTGGTATGATTTCGGTGATTTCATACAAACCACGCCAG    14122
gb:MT027062|Organism:Severe      ATAATCAAGATCTCAATGGTAACTGGTATGATTTCGGTGATTTCATACAAACCACGCCAG    14122
gb:MT019529|Organism:Severe      ATAATCAAGATCTCAATGGTAACTGGTATGATTTCGGTGATTTCATACAAACCACGCCAG    14122
gb:MN996529|Organism:Severe      ATAATCAAGATCTCAATGGTAACTGGTATGATTTCGGTGATTTCATACAAACCACGCCAG    14110
gb:MN996531|Organism:Severe      ATAATCAAGATCTCAATGGTAACTGGTATGATTTCGGTGATTTCATACAAACCACGCCAG    14109
gb:MT066176|Organism:Severe      ATAATCAAGATCTCAATGGTAACTGGTATGATTTCGGTGATTTCATACAAACCACGCCAG    14122
gb:MT027064|Organism:Severe      ATAATCAAGATCTCAATGGTAACTGGTATGATTTCGGTGATTTCATACAAACCACGCCAG    14122
gb:MN994468|Organism:Severe      ATAATCAAGATCTCAATGGTAACTGGTATGATTTCGGTGATTTCATACAAACCACGCCAG    14122
gb:MT072688|Organism:Severe      ATAATCAAGATCTCAATGGTAACTGGTATGATTTCGGTGATTTCATACAAACCACGCCAG    14107
gb:MN996527|Organism:Severe      ATAATCAAGATCTCAATGGTAACTGGTATGATTTCGGTGATTTCATACAAACCACGCCAG    14089
gb:MT093631|Organism:Severe      ATAATCAAGATCTCAATGGTAACTGGTATGATTTCGGTGATTTCATACAAACCACGCCAG    14160
gb:MT106053|Organism:Severe      ATAATCAAGATCTCAATGGTAACTGGTATGATTTCGGTGATTTCATACAAACCACGCCAG    14122
gb:MT019533|Organism:Severe      ATAATCAAGATCTCAATGGTAACTGGTATGATTTCGGTGATTTCATACAAACCACGCCAG    14122
gb:MT019531|Organism:Severe      ATAATCAAGATCTCAATGGTAACTGGTATGATTTCGGTGATTTCATACAAACCACGCCAG    14122
gb:MN996528|Organism:Severe      ATAATCAAGATCTCAATGGTAACTGGTATGATTTCGGTGATTTCATACAAACCACGCCAG    14122
```

```
gb:MN996530|Organism:Severe      ATAATCAAGATCTCAATGGTAACTGGTATGATTTCGGTGATTTCATACAAACCACGCCAG    14108
gb:MN908947|Organism:Severe      ATAATCAAGATCTCAATGGTAACTGGTATGATTTCGGTGATTTCATACAAACCACGCCAG    14122
gb:MT019532|Organism:Severe      ATAATCAAGATCTCAATGGTAACTGGTATGATTTCGGTGATTTCATACAAACCACGCCAG    14122
                                 ************************************************************

gb:MT020781|Organism:Severe      GTAGTGGAGTTCCTGTTGTAGATTCTTATTATTCATTGTTAATGCCTATATTAACCTTGA    14170
gb:MT007544|Organism:Severe      GTAGTGGAGTTCCTGTTGTAGATTCTTATTATTCATTGTTAATGCCTATATTAACCTTGA    14182
gb:MN994467|Organism:Severe      GTAGTGGAGTTCCTGTTGTAGATTCTTATTATTCATTGTTAATGCCTATATTAACCTTGA    14182
gb:MT044257|Organism:Severe      GTAGTGGAGTTCCTGTTGTAGATTCTTATTATTCATTGTTAATGCCTATATTAACCTTGA    14182
gb:MT106054|Organism:Severe      GTAGTGGAGTTCCTGTTGTAGATTCTTATTATTCATTGTTAATGCCTATATTAACCTTGA    14182
gb:MT049951|Organism:Severe      GTAGTGGAGTTCCTGTTGTAGATTCTTATTATTCATTGTTAATGCCTATATTAACCTTGA    14182
gb:MN975262|Organism:Severe      GTAGTGGAGTTCCTGTTGTAGATTCTTATTATTCATTGTTAATGCCTATATTAACCTTGA    14182
gb:MT106052|Organism:Severe      GTAGTGGAGTTCCTGTTGTAGATTCTTATTATTCATTGTTAATGCCTATATTAACCTTGA    14182
gb:LC522975|Organism:Severe      GTAGTGGAGTTCCTGTTGTAGATTCTTATTATTCATTGTTAATGCCTATATTAACCTTGA    14179
gb:LC522973|Organism:Severe      GTAGTGGAGTTCCTGTTGTAGATTCTTATTATTCATTGTTAATGCCTATATTAACCTTGA    14179
gb:LC522974|Organism:Severe      GTAGTGGAGTTCCTGTTGTAGATTCTTATTATTCATTGTTAATGCCTATATTAACCTTGA    14179
gb:MN985325|Organism:Severe      GTAGTGGAGTTCCTGTTGTAGATTCTTATTATTCATTGTTAATGCCTATATTAACCTTGA    14182
gb:MT020881|Organism:Severe      GTAGTGGAGTTCCTGTTGTAGATTCTTATTATTCATTGTTAATGCCTATATTAACCTTGA    14182
gb:MT020880|Organism:Severe      GTAGTGGAGTTCCTGTTGTAGATTCTTATTATTCATTGTTAATGCCTATATTAACCTTGA    14182
gb:MT066175|Organism:Severe      GTAGTGGAGTTCCTGTTGTAGATTCTTATTATTCATTGTTAATGCCTATATTAACCTTGA    14182
gb:MN997409|Organism:Severe      GTAGTGGAGTTCCTGTTGTAGATTCTTATTATTCATTGTTAATGCCTATATTAACCTTGA    14182
gb:MN938384|Organism:Severe      GTAGTGGAGTTCCTGTTGTAGATTCTTATTATTCATTGTTAATGCCTATATTAACCTTGA    14150
gb:MT044258|Organism:Severe      GTAGTGGAGTTCCTGTTGTAGATTCTTATTATTCATTGTTAATGCCTATATTAACCTTGA    14158
gb:MT039890|Organism:Severe      GTAGTGGAGTTCCTGTTGTAGATTCTTATTATTCATTGTTAATGCCTATATTAACCTTGA    14182
gb:MN988713|Organism:Severe      GTAGTGGAGTTCCTGTTGTAGATTCTTATTATTCATTGTTAATGCCTATATTAACCTTGA    14182
gb:LC521925|Organism:Severe      GTAGTGGAGTTCCTGTTGTAGATTCTTATTATTCATTGTTAATGCCTATATTAACCTTGA    14155
gb:MT093571|Organism:Severe      GTAGTGGAGTTCCTGTTGTAGATTCTTATTATTCATTGTTAATGCCTATATTAACCTTGA    14182
gb:MT039887|Organism:Severe      GTAGTGGAGTTCCTGTTGTAGATTCTTATTATTCATTGTTAATGCCTATATTAACCTTGA    14182
gb:MT019530|Organism:Severe      GTAGTGGAGTTCCTGTTGTAGATTCTTATTATTCATTGTTAATGCCTATATTAACCTTGA    14182
gb:MT039888|Organism:Severe      GTAGTGGAGTTCCTGTTGTAGATTCTTATTATTCATTGTTAATGCCTATATTAACCTTGA    14182
gb:LC522972|Organism:Severe      GTAGTGGAGTTCCTGTTGTAGATTCTTATTATTCATTGTTAATGCCTATATTAACCTTGA    14179
gb:MT027063|Organism:Severe      GTAGTGGAGTTCCTGTTGTAGATTCTTATTATTCATTGTTAATGCCTATATTAACCTTGA    14182
gb:MT027062|Organism:Severe      GTAGTGGAGTTCCTGTTGTAGATTCTTATTATTCATTGTTAATGCCTATATTAACCTTGA    14182
gb:MT019529|Organism:Severe      GTAGTGGAGTTCCTGTTGTAGATTCTTATTATTCATTGTTAATGCCTATATTAACCTTGA    14182
gb:MN996529|Organism:Severe      GTAGTGGAGTTCCTGTTGTAGATTCTTATTATTCATTGTTAATGCCTATATTAACCTTGA    14170
gb:MN996531|Organism:Severe      GTAGTGGAGTTCCTGTTGTAGATTCTTATTATTCATTGTTAATGCCTATATTAACCTTGA    14169
gb:MT066176|Organism:Severe      GTAGTGGAGTTCCTGTTGTAGATTCTTATTATTCATTGTTAATGCCTATATTAACCTTGA    14182
gb:MT027064|Organism:Severe      GTAGTGGAGTTCCTGTTGTAGATTCTTATTATTCATTGTTAATGCCTATATTAACCTTGA    14182
gb:MN994468|Organism:Severe      GTAGTGGAGTTCCTGTTGTAGATTCTTATTATTCATTGTTAATGCCTATATTAACCTTGA    14182
gb:MT072688|Organism:Severe      GTAGTGGAGTTCCTGTTGTAGATTCTTATTATTCATTGTTAATGCCTATATTAACCTTGA    14167
gb:MN996527|Organism:Severe      GTAGTGGAGTTCCTGTTGTAGATTCTTATTATTCATTGTTAATGCCTATATTAACCTTGA    14149
gb:MT093631|Organism:Severe      GTAGTGGAGTTCCTGTTGTAGATTCTTATTATTCATTGTTAATGCCTATATTAACCTTGA    14220
gb:MT106053|Organism:Severe      GTAGTGGAGTTCCTGTTGTAGATTCTTATTATTCATTGTTAATGCCTATATTAACCTTGA    14182
gb:MT019533|Organism:Severe      GTAGTGGAGTTCCTGTTGTAGATTCTTATTATTCATTGTTAATGCCTATATTAACCTTGA    14182
gb:MT019531|Organism:Severe      GTAGTGGAGTTCCTGTTGTAGATTCTTATTATTCATTGTTAATGCCTATATTAACCTTGA    14182
gb:MN996528|Organism:Severe      GTAGTGGAGTTCCTGTTGTAGATTCTTATTATTCATTGTTAATGCCTATATTAACCTTGA    14182
gb:MN996530|Organism:Severe      GTAGTGGAGTTCCTGTTGTAGATTCTTATTATTCATTGTTAATGCCTATATTAACCTTGA    14168
gb:MN908947|Organism:Severe      GTAGTGGAGTTCCTGTTGTAGATTCTTATTATTCATTGTTAATGCCTATATTAACCTTGA    14182
gb:MT019532|Organism:Severe      GTAGTGGAGTTCCTGTTGTAGATTCTTATTATTCATTGTTAATGCCTATATTAACCTTGA    14182
                                 ************************************************************

gb:MT020781|Organism:Severe      CCAGGGCTTTAACTGCAGAGTCACATGTTGACACTGACTTAACAAAGCCTTACATTAAGT    14230
gb:MT007544|Organism:Severe      CCAGGGCTTTAACTGCAGAGTCACATGTTGACACTGACTTAACAAAGCCTTACATTAAGT    14242
gb:MN994467|Organism:Severe      CCAGGGCTTTAACTGCAGAGTCACATGTTGACACTGACTTAACAAAGCCTTACATTAAGT    14242
gb:MT044257|Organism:Severe      CCAGGGCTTTAACTGCAGAGTCACATGTTGACACTGACTTAACAAAGCCTTACATTAAGT    14242
gb:MT106054|Organism:Severe      CCAGGGCTTTAACTGCAGAGTCACATGTTGACACTGACTTAACAAAGCCTTACATTAAGT    14242
gb:MT049951|Organism:Severe      CCAGGGCTTTAACTGCAGAGTCACATGTTGACACTGACTTAACAAAGCCTTACATTAAGT    14242
gb:MN975262|Organism:Severe      CCAGGGCTTTAACTGCAGAGTCACATGTTGACACTGACTTAACAAAGCCTTACATTAAGT    14242
gb:MT106052|Organism:Severe      CCAGGGCTTTAACTGCAGAGTCACATGTTGACACTGACTTAACAAAGCCTTACATTAAGT    14242
gb:LC522975|Organism:Severe      CCAGGGCTTTAACTGCAGAGTCACATGTTGACACTGACTTAACAAAGCCTTACATTAAGT    14239
gb:LC522973|Organism:Severe      CCAGGGCTTTAACTGCAGAGTCACATGTTGACACTGACTTAACAAAGCCTTACATTAAGT    14239
gb:LC522974|Organism:Severe      CCAGGGCTTTAACTGCAGAGTCACATGTTGACACTGACTTAACAAAGCCTTACATTAAGT    14239
gb:MN985325|Organism:Severe      CCAGGGCTTTAACTGCAGAGTCACATGTTGACACTGACTTAACAAAGCCTTACATTAAGT    14242
gb:MT020881|Organism:Severe      CCAGGGCTTTAACTGCAGAGTCACATGTTGACACTGACTTAACAAAGCCTTACATTAAGT    14242
gb:MT020880|Organism:Severe      CCAGGGCTTTAACTGCAGAGTCACATGTTGACACTGACTTAACAAAGCCTTACATTAAGT    14242
```

```
gb:MT066175|Organism:Severe      CCAGGGCTTTAACTGCAGAGTCACATGTTGACACTGACTTAACAAAGCCTTACATTAAGT    14242
gb:MN997409|Organism:Severe      CCAGGGCTTTAACTGCAGAGTCACATGTTGACACTGACTTAACAAAGCCTTACATTAAGT    14242
gb:MN938384|Organism:Severe      CCAGGGCTTTAACTGCAGAGTCACATGTTGACACTGACTTAACAAAGCCTTACATTAAGT    14210
gb:MT044258|Organism:Severe      CCAGGGCTTTAACTGCAGAGTCACATGTTGACACTGACTTAACAAAGCCTTACATTAAGT    14218
gb:MT039890|Organism:Severe      CCAGGGCTTTAACTGCAGAGTCACATGTTGACACTGACTTAACAAAGCCTTACATTAAGT    14242
gb:MN988713|Organism:Severe      CCAGGGCTTTAACTGCAGAGTCACATGTTGACACTGACTTAACAAAGCCTTACATTAAGT    14242
gb:LC521925|Organism:Severe      CCAGGGCTTTAACTGCAGAGTCACATGTTGACACTGACTTAACAAAGCCTTACATTAAGT    14215
gb:MT093571|Organism:Severe      CCAGGGCTTTAACTGCAGAGTCACATGTTGACACTGACTTAACAAAGCCTTACATTAAGT    14242
gb:MT039887|Organism:Severe      CCAGGGCTTTAACTGCAGAGTCACATGTTGACACTGACTTAACAAAGCCTTACATTAAGT    14242
gb:MT019530|Organism:Severe      CCAGGGCTTTAACTGCAGAGTCACATGTTGACACTGACTTAACAAAGCCTTACATTAAGT    14242
gb:MT039888|Organism:Severe      CCAGGGCTTTAACTGCAGAGTCACATGTTGACACTGACTTAACAAAGCCTTACATTAAGT    14242
gb:LC522972|Organism:Severe      CCAGGGCTTTAACTGCAGAGTCACATGTTGACACTGACTTAACAAAGCCTTACATTAAGT    14239
gb:MT027063|Organism:Severe      CCAGGGCTTTAACTGCAGAGTCACATGTTGACACTGACTTAACAAAGCCTTACATTAAGT    14242
gb:MT027062|Organism:Severe      CCAGGGCTTTAACTGCAGAGTCACATGTTGACACTGACTTAACAAAGCCTTACATTAAGT    14242
gb:MT019529|Organism:Severe      CCAGGGCTTTAACTGCAGAGTCACATGTTGACACTGACTTAACAAAGCCTTACATTAAGT    14242
gb:MN996529|Organism:Severe      CCAGGGCTTTAACTGCAGAGTCACATGTTGACACTGACTTAACAAAGCCTTACATTAAGT    14230
gb:MN996531|Organism:Severe      CCAGGGCTTTAACTGCAGAGTCACATGTTGACACTGACTTAACAAAGCCTTACATTAAGT    14229
gb:MT066176|Organism:Severe      CCAGGGCTTTAACTGCAGAGTCACATGTTGACACTGACTTAACAAAGCCTTACATTAAGT    14242
gb:MT027064|Organism:Severe      CCAGGGCTTTAACTGCAGAGTCACATGTTGACACTGACTTAACAAAGCCTTACATTAAGT    14242
gb:MN994468|Organism:Severe      CCAGGGCTTTAACTGCAGAGTCACATGTTGACACTGACTTAACAAAGCCTTACATTAAGT    14242
gb:MT072688|Organism:Severe      CCAGGGCTTTAACTGCAGAGTCACATGTTGACACTGACTTAACAAAGCCTTACATTAAGT    14227
gb:MN996527|Organism:Severe      CCAGGGCTTTAACTGCAGAGTCACATGTTGACACTGACTTAACAAAGCCTTACATTAAGT    14209
gb:MT093631|Organism:Severe      CCAGGGCTTTAACTGCAGAGTCACATGTTGACACTGACTTAACAAAGCCTTACATTAAGT    14280
gb:MT106053|Organism:Severe      CCAGGGCTTTAACTGCAGAGTCACATGTTGACACTGACTTAACAAAGCCTTACATTAAGT    14242
gb:MT019533|Organism:Severe      CCAGGGCTTTAACTGCAGAGTCACATGTTGACACTGACTTAACAAAGCCTTACATTAAGT    14242
gb:MT019531|Organism:Severe      CCAGGGCTTTAACTGCAGAGTCACATGTTGACACTGACTTAACAAAGCCTTACATTAAGT    14242
gb:MN996528|Organism:Severe      CCAGGGCTTTAACTGCAGAGTCACATGTTGACACTGACTTAACAAAGCCTTACATTAAGT    14242
gb:MN996530|Organism:Severe      CCAGGGCTTTAACTGCAGAGTCACATGTTGACACTGACTTAACAAAGCCTTACATTAAGT    14228
gb:MN908947|Organism:Severe      CCAGGGCTTTAACTGCAGAGTCACATGTTGACACTGACTTAACAAAGCCTTACATTAAGT    14242
gb:MT019532|Organism:Severe      CCAGGGCTTTAACTGCAGAGTCACATGTTGACACTGACTTAACAAAGCCTTACATTAAGT    14242
                                 ************************************************************

gb:MT020781|Organism:Severe      GGGATTTGTTAAAATATGACTTCACGGAAGAGAGGTTAAAACTCTTTGACCGTTATTTTA    14290
gb:MT007544|Organism:Severe      GGGATTTGTTAAAATATGACTTCACGGAAGAGAGGTTAAAACTCTTTGACCGTTATTTTA    14302
gb:MN994467|Organism:Severe      GGGATTTGTTAAAATATGACTTCACGGAAGAGAGGTTAAAACTCTTTGACCGTTATTTTA    14302
gb:MT044257|Organism:Severe      GGGATTTGTTAAAATATGACTTCACGGAAGAGAGGTTAAAACTCTTTGACCGTTATTTTA    14302
gb:MT106054|Organism:Severe      GGGATTTGTTAAAATATGACTTCACGGAAGAGAGGTTAAAACTCTTTGACCGTTATTTTA    14302
gb:MT049951|Organism:Severe      GGGATTTGTTAAAATATGACTTCACGGAAGAGAGGTTAAAACTCTTTGACCGTTATTTTA    14302
gb:MN975262|Organism:Severe      GGGATTTGTTAAAATATGACTTCACGGAAGAGAGGTTAAAACTCTTTGACCGTTATTTTA    14302
gb:MT106052|Organism:Severe      GGGATTTGTTAAAATATGACTTCACGGAAGAGAGGTTAAAACTCTTTGACCGTTATTTTA    14302
gb:LC522975|Organism:Severe      GGGATTTGTTAAAATATGACTTCACGGAAGAGAGGTTAAAACTCTTTGACCGTTATTTTA    14299
gb:LC522973|Organism:Severe      GGGATTTGTTAAAATATGACTTCACGGAAGAGAGGTTAAAACTCTTTGACCGTTATTTTA    14299
gb:LC522974|Organism:Severe      GGGATTTGTTAAAATATGACTTCACGGAAGAGAGGTTAAAACTCTTTGACCGTTATTTTA    14299
gb:MN985325|Organism:Severe      GGGATTTGTTAAAATATGACTTCACGGAAGAGAGGTTAAAACTCTTTGACCGTTATTTTA    14302
gb:MT020881|Organism:Severe      GGGATTTGTTAAAATATGACTTCACGGAAGAGAGGTTAAAACTCTTTGACCGTTATTTTA    14302
gb:MT020880|Organism:Severe      GGGATTTGTTAAAATATGACTTCACGGAAGAGAGGTTAAAACTCTTTGACCGTTATTTTA    14302
gb:MT066175|Organism:Severe      GGGATTTGTTAAAATATGACTTCACGGAAGAGAGGTTAAAACTCTTTGACCGTTATTTTA    14302
gb:MN997409|Organism:Severe      GGGATTTGTTAAAATATGACTTCACGGAAGAGAGGTTAAAACTCTTTGACCGTTATTTTA    14302
gb:MN938384|Organism:Severe      GGGATTTGTTAAAATATGACTTCACGGAAGAGAGGTTAAAACTCTTTGACCGTTATTTTA    14270
gb:MT044258|Organism:Severe      GGGATTTGTTAAAATATGACTTCACGGAAGAGAGGTTAAAACTCTTTGACCGTTATTTTA    14278
gb:MT039890|Organism:Severe      GGGATTTGTTAAAATATGACTTCACGGAAGAGAGGTTAAAACTCTTTGACCGTTATTTTA    14302
gb:MN988713|Organism:Severe      GGGATTTGTTAAAATATGACTTCACGGAAGAGAGGTTAAAACTCTTTGACCGTTATTTTA    14302
gb:LC521925|Organism:Severe      GGGATTTGTTAAAATATGACTTCACGGAAGAGAGGTTAAAACTCTTTGACCGTTATTTTA    14275
gb:MT093571|Organism:Severe      GGGATTTGTTAAAATATGACTTCACGGAAGAGAGGTTAAAACTCTTTGACCGTTATTTTA    14302
gb:MT039887|Organism:Severe      GGGATTTGTTAAAATATGACTTCACGGAAGAGAGGTTAAAACTCTTTGACCGTTATTTTA    14302
gb:MT019530|Organism:Severe      GGGATTTGTTAAAATATGACTTCACGGAAGAGAGGTTAAAACTCTTTGACCGTTATTTTA    14302
gb:MT039888|Organism:Severe      GGGATTTGTTAAAATATGACTTCACGGAAGAGAGGTTAAAACTCTTTGACCGTTATTTTA    14302
gb:LC522972|Organism:Severe      GGGATTTGTTAAAATATGACTTCACGGAAGAGAGGTTAAAACTCTTTGACCGTTATTTTA    14299
gb:MT027063|Organism:Severe      GGGATTTGTTAAAATATGACTTCACGGAAGAGAGGTTAAAACTCTTTGACCGTTATTTTA    14302
gb:MT027062|Organism:Severe      GGGATTTGTTAAAATATGACTTCACGGAAGAGAGGTTAAAACTCTTTGACCGTTATTTTA    14302
gb:MT019529|Organism:Severe      GGGATTTGTTAAAATATGACTTCACGGAAGAGAGGTTAAAACTCTTTGACCGTTATTTTA    14302
gb:MN996529|Organism:Severe      GGGATTTGTTAAAATATGACTTCACGGAAGAGAGGTTAAAACTCTTTGACCGTTATTTTA    14290
gb:MN996531|Organism:Severe      GGGATTTGTTAAAATATGACTTCACGGAAGAGAGGTTAAAACTCTTTGACCGTTATTTTA    14289
gb:MT066176|Organism:Severe      GGGATTTGTTAAAATATGACTTCACGGAAGAGAGGTTAAAACTCTTTGACCGTTATTTTA    14302
gb:MT027064|Organism:Severe      GGGATTTGTTAAAATATGACTTCACGGAAGAGAGGTTAAAACTCTTTGACCGTTATTTTA    14302
```

```
gb:MN994468|Organism:Severe      GGGATTTGTTAAAATATGACTTCACGGAAGAGAGGTTAAAACTCTTTGACCGTTATTTTA   14302
gb:MT072688|Organism:Severe      GGGATTTGTTAAAATATGACTTCACGGAAGAGAGGTTAAAACTCTTTGACCGTTATTTTA   14287
gb:MN996527|Organism:Severe      GGGATTTGTTAAAATATGACTTCACGGAAGAGAGGTTAAAACTCTTTGACCGTTATTTTA   14269
gb:MT093631|Organism:Severe      GGGATTTGTTAAAATATGACTTCACGGAAGAGAGGTTAAAACTCTTTGACCGTTATTTTA   14340
gb:MT106053|Organism:Severe      GGGATTTGTTAAAATATGACTTCACGGAAGAGAGGTTAAAACTCTTTGACCGTTATTTTA   14302
gb:MT019533|Organism:Severe      GGGATTTGTTAAAATATGACTTCACGGAAGAGAGGTTAAAACTCTTTGACCGTTATTTTA   14302
gb:MT019531|Organism:Severe      GGGATTTGTTAAAATATGACTTCACGGAAGAGAGGTTAAAACTCTTTGACCGTTATTTTA   14302
gb:MN996528|Organism:Severe      GGGATTTGTTAAAATATGACTTCACGGAAGAGAGGTTAAAACTCTTTGACCGTTATTTTA   14302
gb:MN996530|Organism:Severe      GGGATTTGTTAAAATATGACTTCACGGAAGAGAGGTTAAAACTCTTTGACCGTTATTTTA   14288
gb:MN908947|Organism:Severe      GGGATTTGTTAAAATATGACTTCACGGAAGAGAGGTTAAAACTCTTTGACCGTTATTTTA   14302
gb:MT019532|Organism:Severe      GGGATTTGTTAAAATATGACTTCACGGAAGAGAGGTTAAAACTCTTTGACCGTTATTTTA   14302
                                 ************************************************************

gb:MT020781|Organism:Severe      AATATTGGGATCAGACATACCACCCAAATTGTGTTAACTGTTTGGATGACAGATGCATTC   14350
gb:MT007544|Organism:Severe      AATATTGGGATCAGACATACCACCCAAATTGTGTTAACTGTTTGGATGACAGATGCATTC   14362
gb:MN994467|Organism:Severe      AATATTGGGATCAGACATACCACCCAAATTGTGTTAACTGTTTGGATGACAGATGCATTC   14362
gb:MT044257|Organism:Severe      AATATTGGGATCAGACATACCACCCAAATTGTGTTAACTGTTTGGATGACAGATGCATTC   14362
gb:MT106054|Organism:Severe      AATATTGGGATCAGACATACCACCCAAATTGTGTTAACTGTTTGGATGACAGATGCATTC   14362
gb:MT049951|Organism:Severe      AATATTGGGATCAGACATACCACCCAAATTGTGTTAACTGTTTGGATGACAGATGCATTC   14362
gb:MN975262|Organism:Severe      AATATTGGGATCAGACATACCACCCAAATTGTGTTAACTGTTTGGATGACAGATGCATTC   14362
gb:MT106052|Organism:Severe      AATATTGGGATCAGACATACCACCCAAATTGTGTTAACTGTTTGGATGACAGATGCATTC   14362
gb:LC522975|Organism:Severe      AATATTGGGATCAGACATACCACCCAAATTGTGTTAACTGTTTGGATGACAGATGCATTC   14359
gb:LC522973|Organism:Severe      AATATTGGGATCAGACATACCACCCAAATTGTGTTAACTGTTTGGATGACAGATGCATTC   14359
gb:LC522974|Organism:Severe      AATATTGGGATCAGACATACCACCCAAATTGTGTTAACTGTTTGGATGACAGATGCATTC   14359
gb:MN985325|Organism:Severe      AATATTGGGATCAGACATACCACCCAAATTGTGTTAACTGTTTGGATGACAGATGCATTC   14362
gb:MT020881|Organism:Severe      AATATTGGGATCAGACATACCACCCAAATTGTGTTAACTGTTTGGATGACAGATGCATTC   14362
gb:MT020880|Organism:Severe      AATATTGGGATCAGACATACCACCCAAATTGTGTTAACTGTTTGGATGACAGATGCATTC   14362
gb:MT066175|Organism:Severe      AATATTGGGATCAGACATACCACCCAAATTGTGTTAACTGTTTGGATGACAGATGCATTC   14362
gb:MN997409|Organism:Severe      AATATTGGGATCAGACATACCACCCAAATTGTGTTAACTGTTTGGATGACAGATGCATTC   14362
gb:MN938384|Organism:Severe      AATATTGGGATCAGACATACCACCCAAATTGTGTTAACTGTTTGGATGACAGATGCATTC   14330
gb:MT044258|Organism:Severe      AATATTGGGATCAGACATACCACCCAAATTGTGTTAACTGTTTGGATGACAGATGCATTC   14338
gb:MT039890|Organism:Severe      AATATTGGGATCAGACATACCACCCAAATTGTGTTAACTGTTTGGATGACAGATGCATTC   14362
gb:MN988713|Organism:Severe      AATATTGGGATCAGACATACCACCCAAATTGTGTTAACTGTTTGGATGACAGATGCATTC   14362
gb:LC521925|Organism:Severe      AATATTGGGATCAGACATACCACCCAAATTGTGTTAACTGTTTGGATGACAGATGCATTC   14335
gb:MT093571|Organism:Severe      AATATTGGGATCAGACATACCACCCAAATTGTGTTAACTGTTTGGATGACAGATGCATTC   14362
gb:MT039887|Organism:Severe      AATATTGGGATCAGACATACCACCCAAATTGTGTTAACTGTTTGGATGACAGATGCATTC   14362
gb:MT019530|Organism:Severe      AATATTGGGATCAGACATACCACCCAAATTGTGTTAACTGTTTGGATGACAGATGCATTC   14362
gb:MT039888|Organism:Severe      AATATTGGGATCAGACATACCACCCAAATTGTGTTAACTGTTTGGATGACAGATGCATTC   14362
gb:LC522972|Organism:Severe      AATATTGGGATCAGACATACCACCCAAATTGTGTTAACTGTTTGGATGACAGATGCATTC   14359
gb:MT027063|Organism:Severe      AATATTGGGATCAGACATACCACCCAAATTGTGTTAACTGTTTGGATGACAGATGCATTC   14362
gb:MT027062|Organism:Severe      AATATTGGGATCAGACATACCACCCAAATTGTGTTAACTGTTTGGATGACAGATGCATTC   14362
gb:MT019529|Organism:Severe      AATATTGGGATCAGACATACCACCCAAATTGTGTTAACTGTTTGGATGACAGATGCATTC   14362
gb:MN996529|Organism:Severe      AATATTGGGATCAGACATACCACCCAAATTGTGTTAACTGTTTGGATGACAGATGCATTC   14350
gb:MN996531|Organism:Severe      AATATTGGGATCAGACATACCACCCAAATTGTGTTAACTGTTTGGATGACAGATGCATTC   14349
gb:MT066176|Organism:Severe      AATATTGGGATCAGACATACCACCCAAATTGTGTTAACTGTTTGGATGACAGATGCATTC   14362
gb:MT027064|Organism:Severe      AATATTGGGATCAGACATACCACCCAAATTGTGTTAACTGTTTGGATGACAGATGCATTC   14362
gb:MN994468|Organism:Severe      AATATTGGGATCAGACATACCACCCAAATTGTGTTAACTGTTTGGATGACAGATGCATTC   14362
gb:MT072688|Organism:Severe      AATATTGGGATCAGACATACCACCCAAATTGTGTTAACTGTTTGGATGACAGATGCATTC   14347
gb:MN996527|Organism:Severe      AATATTGGGATCAGACATACCACCCAAATTGTGTTAACTGTTTGGATGACAGATGCATTC   14329
gb:MT093631|Organism:Severe      AATATTGGGATCAGACATACCACCCAAATTGTGTTAACTGTTTGGATGACAGATGCATTC   14400
gb:MT106053|Organism:Severe      AATATTGGGATCAGACATACCACCCAAATTGTGTTAACTGTTTGGATGACAGATGCATTC   14362
gb:MT019533|Organism:Severe      AATATTGGGATCAGACATACCACCCAAATTGTGTTAACTGTTTGGATGACAGATGCATTC   14362
gb:MT019531|Organism:Severe      AATATTGGGATCAGACATACCACCCAAATTGTGTTAACTGTTTGGATGACAGATGCATTC   14362
gb:MN996528|Organism:Severe      AATATTGGGATCAGACATACCACCCAAATTGTGTTAACTGTTTGGATGACAGATGCATTC   14362
gb:MN996530|Organism:Severe      AATATTGGGATCAGACATACCACCCAAATTGTGTTAACTGTTTGGATGACAGATGCATTC   14348
gb:MN908947|Organism:Severe      AATATTGGGATCAGACATACCACCCAAATTGTGTTAACTGTTTGGATGACAGATGCATTC   14362
gb:MT019532|Organism:Severe      AATATTGGGATCAGACATACCACCCAAATTGTGTTAACTGTTTGGATGACAGATGCATTC   14362
                                 ************************************************************

gb:MT020781|Organism:Severe      TGCATTGTGCAAACTTTAATGTTTTATTCTCTACAGTGTTCCCACCTACAAGTTTTGGAC   14410
gb:MT007544|Organism:Severe      TGCATTGTGCAAACTTTAATGTTTTATTCTCTACAGTGTTCCCACCTACAAGTTTTGGAC   14422
gb:MN994467|Organism:Severe      TGCATTGTGCAAACTTTAATGTTTTATTCTCTACAGTGTTCCCACCTACAAGTTTTGGAC   14422
gb:MT044257|Organism:Severe      TGCATTGTGCAAACTTTAATGTTTTATTCTCTACAGTGTTCCCACCTACAAGTTTTGGAC   14422
gb:MT106054|Organism:Severe      TGCATTGTGCAAACTTTAATGTTTTATTCTCTACAGTGTTCCCACCTACAAGTTTTGGAC   14422
gb:MT049951|Organism:Severe      TGCATTGTGCAAACTTTAATGTTTTATTCTCTACAGTGTTCCCACCTACAAGTTTTGGAC   14422
```

```
gb:MN975262|Organism:Severe      TGCATTGTGCAAACTTTAATGTTTATTCTCTACAGTGTTCCCACCTACAAGTTTTGGAC    14422
gb:MT106052|Organism:Severe      TGCATTGTGCAAACTTTAATGTTTATTCTCTACAGTGTTCCCACCTACAAGTTTTGGAC    14422
gb:LC522975|Organism:Severe      TGCATTGTGCAAACTTTAATGTTTATTCTCTACAGTGTTCCCACCTACAAGTTTTGGAC    14419
gb:LC522973|Organism:Severe      TGCATTGTGCAAACTTTAATGTTTATTCTCTACAGTGTTCCCACCTACAAGTTTTGGAC    14419
gb:LC522974|Organism:Severe      TGCATTGTGCAAACTTTAATGTTTATTCTCTACAGTGTTCCCACCTACAAGTTTTGGAC    14419
gb:MN985325|Organism:Severe      TGCATTGTGCAAACTTTAATGTTTATTCTCTACAGTGTTCCCACCTACAAGTTTTGGAC    14422
gb:MT020881|Organism:Severe      TGCATTGTGCAAACTTTAATGTTTATTCTCTACAGTGTTCCCACCTACAAGTTTTGGAC    14422
gb:MT020880|Organism:Severe      TGCATTGTGCAAACTTTAATGTTTATTCTCTACAGTGTTCCCACCTACAAGTTTTGGAC    14422
gb:MT066175|Organism:Severe      TGCATTGTGCAAACTTTAATGTTTATTCTCTACAGTGTTCCCACCTACAAGTTTTGGAC    14422
gb:MN997409|Organism:Severe      TGCATTGTGCAAACTTTAATGTTTATTCTCTACAGTGTTCCCACCTACAAGTTTTGGAC    14422
gb:MN938384|Organism:Severe      TGCATTGTGCAAACTTTAATGTTTATTCTCTACAGTGTTCCCACCTACAAGTTTTGGAC    14390
gb:MT044258|Organism:Severe      TGCATTGTGCAAACTTTAATGTTTATTCTCTACAGTGTTCCCACCTACAAGTTTTGGAC    14398
gb:MT039890|Organism:Severe      TGCATTGTGCAAACTTTAATGTTTATTCTCTACAGTGTTCCCACCTACAAGTTTTGGAC    14422
gb:MN988713|Organism:Severe      TGCATTGTGCAAACTTTAATGTTTATTCTCTACAGTGTTCCCACCTACAAGTTTTGGAC    14422
gb:LC521925|Organism:Severe      TGCATTGTGCAAACTTTAATGTTTATTCTCTACAGTGTTCCCACCTACAAGTTTTGGAC    14395
gb:MT093571|Organism:Severe      TGCATTGTGCAAACTTTAATGTTTATTCTCTACAGTGTTCCCACCTACAAGTTTTGGAC    14422
gb:MT039887|Organism:Severe      TGCATTGTGCAAACTTTAATGTTTATTCTCTACAGTGTTCCCACCTACAAGTTTTGGAC    14422
gb:MT019530|Organism:Severe      TGCATTGTGCAAACTTTAATGTTTATTCTCTACAGTGTTCCCACCTACAAGTTTTGGAC    14422
gb:MT039888|Organism:Severe      TGCATTGTGCAAACTTTAATGTTTATTCTCTACAGTGTTCCCACCTACAAGTTTTGGAC    14422
gb:LC522972|Organism:Severe      TGCATTGTGCAAACTTTAATGTTTATTCTCTACAGTGTTCCCACCTACAAGTTTTGGAC    14419
gb:MT027063|Organism:Severe      TGCATTGTGCAAACTTTAATGTTTATTCTCTACAGTGTTCCCACCTACAAGTTTTGGAC    14422
gb:MT027062|Organism:Severe      TGCATTGTGCAAACTTTAATGTTTATTCTCTACAGTGTTCCCACCTACAAGTTTTGGAC    14422
gb:MT019529|Organism:Severe      TGCATTGTGCAAACTTTAATGTTTATTCTCTACAGTGTTCCCACCTACAAGTTTTGGAC    14422
gb:MN996529|Organism:Severe      TGCATTGTGCAAACTTTAATGTTTATTCTCTACAGTGTTCCCACCTACAAGTTTTGGAC    14410
gb:MN996531|Organism:Severe      TGCATTGTGCAAACTTTAATGTTTATTCTCTACAGTGTTCCCACCTACAAGTTTTGGAC    14409
gb:MT066176|Organism:Severe      TGCATTGTGCAAACTTTAATGTTTATTCTCTACAGTGTTCCCACCTACAAGTTTTGGAC    14422
gb:MT027064|Organism:Severe      TGCATTGTGCAAACTTTAATGTTTATTCTCTACAGTGTTCCCACCTACAAGTTTTGGAC    14422
gb:MN994468|Organism:Severe      TGCATTGTGCAAACTTTAATGTTTATTCTCTACAGTGTTCCCACCTACAAGTTTTGGAC    14422
gb:MT072688|Organism:Severe      TGCATTGTGCAAACTTTAATGTTTATTCTCTACAGTGTTCCCACCTACAAGTTTTGGAC    14407
gb:MN996527|Organism:Severe      TGCATTGTGCAAACTTTAATGTTTATTCTCTACAGTGTTCCCACCTACAAGTTTTGGAC    14389
gb:MT093631|Organism:Severe      TGCATTGTGCAAACTTTAATGTTTATTCTCTACAGTGTTCCCACCTACAAGTTTTGGAC    14460
gb:MT106053|Organism:Severe      TGCATTGTGCAAACTTTAATGTTTATTCTCTACAGTGTTCCCACCTACAAGTTTTGGAC    14422
gb:MT019533|Organism:Severe      TGCATTGTGCAAACTTTAATGTTTATTCTCTACAGTGTTCCCACCTACAAGTTTTGGAC    14422
gb:MT019531|Organism:Severe      TGCATTGTGCAAACTTTAATGTTTATTCTCTACAGTGTTCCCACCTACAAGTTTTGGAC    14422
gb:MN996528|Organism:Severe      TGCATTGTGCAAACTTTAATGTTTATTCTCTACAGTGTTCCCACCTACAAGTTTTGGAC    14422
gb:MN996530|Organism:Severe      TGCATTGTGCAAACTTTAATGTTTATTCTCTACAGTGTTCCCACCTACAAGTTTTGGAC    14408
gb:MN908947|Organism:Severe      TGCATTGTGCAAACTTTAATGTTTATTCTCTACAGTGTTCCCACCTACAAGTTTTGGAC    14422
gb:MT019532|Organism:Severe      TGCATTGTGCAAACTTTAATGTTTATTCTCTACAGTGTTCCCACCTACAAGTTTTGGAC    14422
                                 ************************************************************

gb:MT020781|Organism:Severe      CACTAGTGAGAAAAATATTTGTTGATGGTGTTCCATTTGTAGTTTCAACTGGATACCACT    14470
gb:MT007544|Organism:Severe      CACTAGTGAGAAAAATATTTGTTGATGGTGTTCCATTTGTAGTTTCAACTGGATACCACT    14482
gb:MN994467|Organism:Severe      CACTAGTGAGAAAAATATTTGTTGATGGTGTTCCATTTGTAGTTTCAACTGGATACCACT    14482
gb:MT044257|Organism:Severe      CACTAGTGAGAAAAATATTTGTTGATGGTGTTCCATTTGTAGTTTCAACTGGATACCACT    14482
gb:MT106054|Organism:Severe      CACTAGTGAGAAAAATATTTGTTGATGGTGTTCCATTTGTAGTTTCAACTGGATACCACT    14482
gb:MT049951|Organism:Severe      CACTAGTGAGAAAAATATTTGTTGATGGTGTTCCATTTGTAGTTTCAACTGGATACCACT    14482
gb:MN975262|Organism:Severe      CACTAGTGAGAAAAATATTTGTTGATGGTGTTCCATTTGTAGTTTCAACTGGATACCACT    14482
gb:MT106052|Organism:Severe      CACTAGTGAGAAAAATATTTGTTGATGGTGTTCCATTTGTAGTTTCAACTGGATACCACT    14482
gb:LC522975|Organism:Severe      CACTAGTGAGAAAAATATTTGTTGATGGTGTTCCATTTGTAGTTTCAACTGGATACCACT    14479
gb:LC522973|Organism:Severe      CACTAGTGAGAAAAATATTTGTTGATGGTGTTCCATTTGTAGTTTCAACTGGATACCACT    14479
gb:LC522974|Organism:Severe      CACTAGTGAGAAAAATATTTGTTGATGGTGTTCCATTTGTAGTTTCAACTGGATACCACT    14479
gb:MN985325|Organism:Severe      CACTAGTGAGAAAAATATTTGTTGATGGTGTTCCATTTGTAGTTTCAACTGGATACCACT    14482
gb:MT020881|Organism:Severe      CACTAGTGAGAAAAATATTTGTTGATGGTGTTCCATTTGTAGTTTCAACTGGATACCACT    14482
gb:MT020880|Organism:Severe      CACTAGTGAGAAAAATATTTGTTGATGGTGTTCCATTTGTAGTTTCAACTGGATACCACT    14482
gb:MT066175|Organism:Severe      CACTAGTGAGAAAAATATTTGTTGATGGTGTTCCATTTGTAGTTTCAACTGGATACCACT    14482
gb:MN997409|Organism:Severe      CACTAGTGAGAAAAATATTTGTTGATGGTGTTCCATTTGTAGTTTCAACTGGATACCACT    14482
gb:MN938384|Organism:Severe      CACTAGTGAGAAAAATATTTGTTGATGGTGTTCCATTTGTAGTTTCAACTGGATACCACT    14450
gb:MT044258|Organism:Severe      CACTAGTGAGAAAAATATTTGTTGATGGTGTTCCATTTGTAGTTTCAACTGGATACCACT    14458
gb:MT039890|Organism:Severe      CACTAGTGAGAAAAATATTTGTTGATGGTGTTCCATTTGTAGTTTCAACTGGATACCACT    14482
gb:MN988713|Organism:Severe      CACTAGTGAGAAAAATATTTGTTGATGGTGTTCCATTTGTAGTTTCAACTGGATACCACT    14482
gb:LC521925|Organism:Severe      CACTAGTGAGAAAAATATTTGTTGATGGTGTTCCATTTGTAGTTTCAACTGGATACCACT    14455
gb:MT093571|Organism:Severe      CACTAGTGAGAAAAATATTTGTTGATGGTGTTCCATTTGTAGTTTCAACTGGATACCACT    14482
gb:MT039887|Organism:Severe      CACTAGTGAGAAAAATATTTGTTGATGGTGTTCCATTTGTAGTTTCAACTGGATACCACT    14482
gb:MT019530|Organism:Severe      CACTAGTGAGAAAAATATTTGTTGATGGTGTTCCATTTGTAGTTTCAACTGGATACCACT    14482
gb:MT039888|Organism:Severe      CACTAGTGAGAAAAATATTTGTTGATGGTGTTCCATTTGTAGTTTCAACTGGATACCACT    14482
```

```
gb:LC522972|Organism:Severe      CACTAGTGAGAAAAATATTTGTTGATGGTGTTCCATTTGTAGTTTCAACTGGATACCACT   14479
gb:MT027063|Organism:Severe      CACTAGTGAGAAAAATATTTGTTGATGGTGTTCCATTTGTAGTTTCAACTGGATACCACT   14482
gb:MT027062|Organism:Severe      CACTAGTGAGAAAAATATTTGTTGATGGTGTTCCATTTGTAGTTTCAACTGGATACCACT   14482
gb:MT019529|Organism:Severe      CACTAGTGAGAAAAATATTTGTTGATGGTGTTCCATTTGTAGTTTCAACTGGATACCACT   14482
gb:MN996529|Organism:Severe      CACTAGTGAGAAAAATATTTGTTGATGGTGTTCCATTTGTAGTTTCAACTGGATACCACT   14470
gb:MN996531|Organism:Severe      CACTAGTGAGAAAAATATTTGTTGATGGTGTTCCATTTGTAGTTTCAACTGGATACCACT   14469
gb:MT066176|Organism:Severe      CACTAGTGAGAAAAATATTTGTTGATGGTGTTCCATTTGTAGTTTCAACTGGATACCACT   14482
gb:MT027064|Organism:Severe      CACTAGTGAGAAAAATATTTGTTGATGGTGTTCCATTTGTAGTTTCAACTGGATACCACT   14482
gb:MN994468|Organism:Severe      CACTAGTGAGAAAAATATTTGTTGATGGTGTTCCATTTGTAGTTTCAACTGGATACCACT   14482
gb:MT072688|Organism:Severe      CACTAGTGAGAAAAATATTTGTTGATGGTGTTCCATTTGTAGTTTCAACTGGATACCACT   14467
gb:MN996527|Organism:Severe      CACTAGTGAGAAAAATATTTGTTGATGGTGTTCCATTTGTAGTTTCAACTGGATACCACT   14449
gb:MT093631|Organism:Severe      CACTAGTGAGAAAAATATTTGTTGATGGTGTTCCATTTGTAGTTTCAACTGGATACCACT   14520
gb:MT106053|Organism:Severe      CACTAGTGAGAAAAATATTTGTTGATGGTGTTCCATTTGTAGTTTCAACTGGATACCACT   14482
gb:MT019533|Organism:Severe      CACTAGTGAGAAAAATATTTGTTGATGGTGTTCCATTTGTAGTTTCAACTGGATACCACT   14482
gb:MT019531|Organism:Severe      CACTAGTGAGAAAAATATTTGTTGATGGTGTTCCATTTGTAGTTTCAACTGGATACCACT   14482
gb:MN996528|Organism:Severe      CACTAGTGAGAAAAATATTTGTTGATGGTGTTCCATTTGTAGTTTCAACTGGATACCACT   14482
gb:MN996530|Organism:Severe      CACTAGTGAGAAAAATATTTGTTGATGGTGTTCCATTTGTAGTTTCAACTGGATACCACT   14468
gb:MN908947|Organism:Severe      CACTAGTGAGAAAAATATTTGTTGATGGTGTTCCATTTGTAGTTTCAACTGGATACCACT   14482
gb:MT019532|Organism:Severe      CACTAGTGAGAAAAATATTTGTTGATGGTGTTCCATTTGTAGTTTCAACTGGATACCACT   14482
                                 ************************************************************

gb:MT020781|Organism:Severe      TCAGAGAGCTAGGTGTTGTACATAATCAGGATGTAAACTTACATAGCTCTAGACTTAGTT   14530
gb:MT007544|Organism:Severe      TCAGAGAGCTAGGTGTTGTACATAATCAGGATGTAAACTTACATAGCTCTAGACTTAGTT   14542
gb:MN994467|Organism:Severe      TCAGAGAGCTAGGTGTTGTACATAATCAGGATGTAAACTTACATAGCTCTAGACTTAGTT   14542
gb:MT044257|Organism:Severe      TCAGAGAGCTAGGTGTTGTACATAATCAGGATGTAAACTTACATAGCTCTAGACTTAGTT   14542
gb:MT106054|Organism:Severe      TCAGAGAGCTAGGTGTTGTACATAATCAGGATGTAAACTTACATAGCTCTAGACTTAGTT   14542
gb:MT049951|Organism:Severe      TCAGAGAGCTAGGTGTTGTACATAATCAGGATGTAAACTTACATAGCTCTAGACTTAGTT   14542
gb:MN975262|Organism:Severe      TCAGAGAGCTAGGTGTTGTACATAATCAGGATGTAAACTTACATAGCTCTAGACTTAGTT   14542
gb:MT106052|Organism:Severe      TCAGAGAGCTAGGTGTTGTACATAATCAGGATGTAAACTTACATAGCTCTAGACTTAGTT   14542
gb:LC522975|Organism:Severe      TCAGAGAGCTAGGTGTTGTACATAATCAGGATGTAAACTTACATAGCTCTAGACTTAGTT   14539
gb:LC522973|Organism:Severe      TCAGAGAGCTAGGTGTTGTACATAATCAGGATGTAAACTTACATAGCTCTAGACTTAGTT   14539
gb:LC522974|Organism:Severe      TCAGAGAGCTAGGTGTTGTACATAATCAGGATGTAAACTTACATAGCTCTAGACTTAGTT   14539
gb:MN985325|Organism:Severe      TCAGAGAGCTAGGTGTTGTACATAATCAGGATGTAAACTTACATAGCTCTAGACTTAGTT   14542
gb:MT020881|Organism:Severe      TCAGAGAGCTAGGTGTTGTACATAATCAGGATGTAAACTTACATAGCTCTAGACTTAGTT   14542
gb:MT020880|Organism:Severe      TCAGAGAGCTAGGTGTTGTACATAATCAGGATGTAAACTTACATAGCTCTAGACTTAGTT   14542
gb:MT066175|Organism:Severe      TCAGAGAGCTAGGTGTTGTACATAATCAGGATGTAAACTTACATAGCTCTAGACTTAGTT   14542
gb:MN997409|Organism:Severe      TCAGAGAGCTAGGTGTTGTACATAATCAGGATGTAAACTTACATAGCTCTAGACTTAGTT   14542
gb:MN938384|Organism:Severe      TCAGAGAGCTAGGTGTTGTACATAATCAGGATGTAAACTTACATAGCTCTAGACTTAGTT   14510
gb:MT044258|Organism:Severe      TCAGAGAGCTAGGTGTTGTACATAATCAGGATGTAAACTTACATAGCTCTAGACTTAGTT   14518
gb:MT039890|Organism:Severe      TCAGAGAGCTAGGTGTTGTACATAATCAGGATGTAAACTTACATAGCTCTAGACTTAGTT   14542
gb:MN988713|Organism:Severe      TCAGAGAGCTAGGTGTTGTACATAATCAGGATGTAAACTTACATAGCTCTAGACTTAGTT   14542
gb:LC521925|Organism:Severe      TCAGAGAGCTAGGTGTTGTACATAATCAGGATGTAAACTTACATAGCTCTAGACTTAGTT   14515
gb:MT093571|Organism:Severe      TCAGAGAGCTAGGTGTTGTACATAATCAGGATGTAAACTTACATAGCTCTAGACTTAGTT   14542
gb:MT039887|Organism:Severe      TCAGAGAGCTAGGTGTTGTACATAATCAGGATGTAAACTTACATAGCTCTAGACTTAGTT   14542
gb:MT019530|Organism:Severe      TCAGAGAGCTAGGTGTTGTACATAATCAGGATGTAAACTTACATAGCTCTAGACTTAGTT   14542
gb:MT039888|Organism:Severe      TCAGAGAGCTAGGTGTTGTACATAATCAGGATGTAAACTTACATAGCTCTAGACTTAGTT   14542
gb:LC522972|Organism:Severe      TCAGAGAGCTAGGTGTTGTACATAATCAGGATGTAAACTTACATAGCTCTAGACTTAGTT   14539
gb:MT027063|Organism:Severe      TCAGAGAGCTAGGTGTTGTACATAATCAGGATGTAAACTTACATAGCTCTAGACTTAGTT   14542
gb:MT027062|Organism:Severe      TCAGAGAGCTAGGTGTTGTACATAATCAGGATGTAAACTTACATAGCTCTAGACTTAGTT   14542
gb:MT019529|Organism:Severe      TCAGAGAGCTAGGTGTTGTACATAATCAGGATGTAAACTTACATAGCTCTAGACTTAGTT   14542
gb:MN996529|Organism:Severe      TCAGAGAGCTAGGTGTTGTACATAATCAGGATGTAAACTTACATAGCTCTAGACTTAGTT   14530
gb:MN996531|Organism:Severe      TCAGAGAGCTAGGTGTTGTACATAATCAGGATGTAAACTTACATAGCTCTAGACTTAGTT   14529
gb:MT066176|Organism:Severe      TCAGAGAGCTAGGTGTTGTACATAATCAGGATGTAAACTTACATAGCTCTAGACTTAGTT   14542
gb:MT027064|Organism:Severe      TCAGAGAGCTAGGTGTTGTACATAATCAGGATGTAAACTTACATAGCTCTAGACTTAGTT   14542
gb:MN994468|Organism:Severe      TCAGAGAGCTAGGTGTTGTACATAATCAGGATGTAAACTTACATAGCTCTAGACTTAGTT   14542
gb:MT072688|Organism:Severe      TCAGAGAGCTAGGTGTTGTACATAATCAGGATGTAAACTTACATAGCTCTAGACTTAGTT   14527
gb:MN996527|Organism:Severe      TCAGAGAGCTAGGTGTTGTACATAATCAGGATGTAAACTTACATAGCTCTAGACTTAGTT   14509
gb:MT093631|Organism:Severe      TCAGAGAGCTAGGTGTTGTACATAATCAGGATGTAAACTTACATAGCTCTAGACTTAGTT   14580
gb:MT106053|Organism:Severe      TCAGAGAGCTAGGTGTTGTACATAATCAGGATGTAAACTTACATAGCTCTAGACTTAGTT   14542
gb:MT019533|Organism:Severe      TCAGAGAGCTAGGTGTTGTACATAATCAGGATGTAAACTTACATAGCTCTAGACTTAGTT   14542
gb:MT019531|Organism:Severe      TCAGAGAGCTAGGTGTTGTACATAATCAGGATGTAAACTTACATAGCTCTAGACTTAGTT   14542
gb:MN996528|Organism:Severe      TCAGAGAGCTAGGTGTTGTACATAATCAGGATGTAAACTTACATAGCTCTAGACTTAGTT   14542
gb:MN996530|Organism:Severe      TCAGAGAGCTAGGTGTTGTACATAATCAGGATGTAAACTTACATAGCTCTAGACTTAGTT   14528
gb:MN908947|Organism:Severe      TCAGAGAGCTAGGTGTTGTACATAATCAGGATGTAAACTTACATAGCTCTAGACTTAGTT   14542
gb:MT019532|Organism:Severe      TCAGAGAGCTAGGTGTTGTACATAATCAGGATGTAAACTTACATAGCTCTAGACTTAGTT   14542
```

```
                                  ************************************************************

gb:MT020781|Organism:Severe      TTAAGGAATTACTTGTGTATGCTGCTGACCCTGCTATGCACGCTGCTTCTGGTAATCTAT   14590
gb:MT007544|Organism:Severe      TTAAGGAATTACTTGTGTATGCTGCTGACCCTGCTATGCACGCTGCTTCTGGTAATCTAT   14602
gb:MN994467|Organism:Severe      TTAAGGAATTACTTGTGTATGCTGCTGACCCTGCTATGCACGCTGCTTCTGGTAATCTAT   14602
gb:MT044257|Organism:Severe      TTAAGGAATTACTTGTGTATGCTGCTGACCCTGCTATGCACGCTGCTTCTGGTAATCTAT   14602
gb:MT106054|Organism:Severe      TTAAGGAATTACTTGTGTATGCTGCTGACCCTGCTATGCACGCTGCTTCTGGTAATCTAT   14602
gb:MT049951|Organism:Severe      TTAAGGAATTACTTGTGTATGCTGCTGACCCTGCTATGCACGCTGCTTCTGGTAATCTAT   14602
gb:MN975262|Organism:Severe      TTAAGGAATTACTTGTGTATGCTGCTGACCCTGCTATGCACGCTGCTTCTGGTAATCTAT   14602
gb:MT106052|Organism:Severe      TTAAGGAATTACTTGTGTATGCTGCTGACCCTGCTATGCACGCTGCTTCTGGTAATCTAT   14602
gb:LC522975|Organism:Severe      TTAAGGAATTACTTGTGTATGCTGCTGACCCTGCTATGCACGCTGCTTCTGGTAATCTAT   14599
gb:LC522973|Organism:Severe      TTAAGGAATTACTTGTGTATGCTGCTGACCCTGCTATGCACGCTGCTTCTGGTAATCTAT   14599
gb:LC522974|Organism:Severe      TTAAGGAATTACTTGTGTATGCTGCTGACCCTGCTATGCACGCTGCTTCTGGTAATCTAT   14599
gb:MN985325|Organism:Severe      TTAAGGAATTACTTGTGTATGCTGCTGACCCTGCTATGCACGCTGCTTCTGGTAATCTAT   14602
gb:MT020881|Organism:Severe      TTAAGGAATTACTTGTGTATGCTGCTGACCCTGCTATGCACGCTGCTTCTGGTAATCTAT   14602
gb:MT020880|Organism:Severe      TTAAGGAATTACTTGTGTATGCTGCTGACCCTGCTATGCACGCTGCTTCTGGTAATCTAT   14602
gb:MT066175|Organism:Severe      TTAAGGAATTACTTGTGTATGCTGCTGACCCTGCTATGCACGCTGCTTCTGGTAATCTAT   14602
gb:MN997409|Organism:Severe      TTAAGGAATTACTTGTGTATGCTGCTGACCCTGCTATGCACGCTGCTTCTGGTAATCTAT   14602
gb:MN938384|Organism:Severe      TTAAGGAATTACTTGTGTATGCTGCTGACCCTGCTATGCACGCTGCTTCTGGTAATCTAT   14570
gb:MT044258|Organism:Severe      TTAAGGAATTACTTGTGTATGCTGCTGACCCTGCTATGCACGCTGCTTCTGGTAATCTAT   14578
gb:MT039890|Organism:Severe      TTAAGGAATTACTTGTGTATGCTGCTGACCCTGCTATGCACGCTGCTTCTGGTAATCTAT   14602
gb:MN988713|Organism:Severe      TTAAGGAATTACTTGTGTATGCTGCTGACCCTGCTATGCACGCTGCTTCTGGTAATCTAT   14602
gb:LC521925|Organism:Severe      TTAAGGAATTACTTGTGTATGCTGCTGACCCTGCTATGCACGCTGCTTCTGGTAATCTAT   14575
gb:MT093571|Organism:Severe      TTAAGGAATTACTTGTGTATGCTGCTGACCCTGCTATGCACGCTGCTTCTGGTAATCTAT   14602
gb:MT039887|Organism:Severe      TTAAGGAATTACTTGTGTATGCTGCTGACCCTGCTATGCACGCTGCTTCTGGTAATCTAT   14602
gb:MT019530|Organism:Severe      TTAAGGAATTACTTGTGTATGCTGCTGACCCTGCTATGCACGCTGCTTCTGGTAATCTAT   14602
gb:MT039888|Organism:Severe      TTAAGGAATTACTTGTGTATGCTGCTGACCCTGCTATGCACGCTGCTTCTGGTAATCTAT   14602
gb:LC522972|Organism:Severe      TTAAGGAATTACTTGTGTATGCTGCTGACCCTGCTATGCACGCTGCTTCTGGTAATCTAT   14599
gb:MT027063|Organism:Severe      TTAAGGAATTACTTGTGTATGCTGCTGACCCTGCTATGCACGCTGCTTCTGGTAATCTAT   14602
gb:MT027062|Organism:Severe      TTAAGGAATTACTTGTGTATGCTGCTGACCCTGCTATGCACGCTGCTTCTGGTAATCTAT   14602
gb:MT019529|Organism:Severe      TTAAGGAATTACTTGTGTATGCTGCTGACCCTGCTATGCACGCTGCTTCTGGTAATCTAT   14602
gb:MN996529|Organism:Severe      TTAAGGAATTACTTGTGTATGCTGCTGACCCTGCTATGCACGCTGCTTCTGGTAATCTAT   14590
gb:MN996531|Organism:Severe      TTAAGGAATTACTTGTGTATGCTGCTGACCCTGCTATGCACGCTGCTTCTGGTAATCTAT   14589
gb:MT066176|Organism:Severe      TTAAGGAATTACTTGTGTATGCTGCTGACCCTGCTATGCACGCTGCTTCTGGTAATCTAT   14602
gb:MT027064|Organism:Severe      TTAAGGAATTACTTGTGTATGCTGCTGACCCTGCTATGCACGCTGCTTCTGGTAATCTAT   14602
gb:MN994468|Organism:Severe      TTAAGGAATTACTTGTGTATGCTGCTGACCCTGCTATGCACGCTGCTTCTGGTAATCTAT   14602
gb:MT072688|Organism:Severe      TTAAGGAATTACTTGTGTATGCTGCTGACCCTGCTATGCACGCTGCTTCTGGTAATCTAT   14587
gb:MN996527|Organism:Severe      TTAAGGAATTACTTGTGTATGCTGCTGACCCTGCTATGCACGCTGCTTCTGGTAATCTAT   14569
gb:MT093631|Organism:Severe      TTAAGGAATTACTTGTGTATGCTGCTGACCCTGCTATGCACGCTGCTTCTGGTAATCTAT   14640
gb:MT106053|Organism:Severe      TTAAGGAATTACTTGTGTATGCTGCTGACCCTGCTATGCACGCTGCTTCTGGTAATCTAT   14602
gb:MT019533|Organism:Severe      TTAAGGAATTACTTGTGTATGCTGCTGACCCTGCTATGCACGCTGCTTCTGGTAATCTAT   14602
gb:MT019531|Organism:Severe      TTAAGGAATTACTTGTGTATGCTGCTGACCCTGCTATGCACGCTGCTTCTGGTAATCTAT   14602
gb:MN996528|Organism:Severe      TTAAGGAATTACTTGTGTATGCTGCTGACCCTGCTATGCACGCTGCTTCTGGTAATCTAT   14602
gb:MN996530|Organism:Severe      TTAAGGAATTACTTGTGTATGCTGCTGACCCTGCTATGCACGCTGCTTCTGGTAATCTAT   14588
gb:MN908947|Organism:Severe      TTAAGGAATTACTTGTGTATGCTGCTGACCCTGCTATGCACGCTGCTTCTGGTAATCTAT   14602
gb:MT019532|Organism:Severe      TTAAGGAATTACTTGTGTATGCTGCTGACCCTGCTATGCACGCTGCTTCTGGTAATCTAT   14602
                                  ************************************************************

gb:MT020781|Organism:Severe      TACTAGATAAACGCACTACGTGCTTTTCAGTAGCTGCACTTACTAACAATGTTGCTTTTC   14650
gb:MT007544|Organism:Severe      TACTAGATAAACGCACTACGTGCTTTTCAGTAGCTGCACTTACTAACAATGTTGCTTTTC   14662
gb:MN994467|Organism:Severe      TACTAGATAAACGCACTACGTGCTTTTCAGTAGCTGCACTTACTAACAATGTTGCTTTTC   14662
gb:MT044257|Organism:Severe      TACTAGATAAACGCACTACGTGCTTTTCAGTAGCTGCACTTACTAACAATGTTGCTTTTC   14662
gb:MT106054|Organism:Severe      TACTAGATAAACGCACTACGTGCTTTTCAGTAGCTGCACTTACTAACAATGTTGCTTTTC   14662
gb:MT049951|Organism:Severe      TACTAGATAAACGCACTACGTGCTTTTCAGTAGCTGCACTTACTAACAATGTTGCTTTTC   14662
gb:MN975262|Organism:Severe      TACTAGATAAACGCACTACGTGCTTTTCAGTAGCTGCACTTACTAACAATGTTGCTTTTC   14662
gb:MT106052|Organism:Severe      TACTAGATAAACGCACTACGTGCTTTTCAGTAGCTGCACTTACTAACAATGTTGCTTTTC   14662
gb:LC522975|Organism:Severe      TACTAGATAAACGCACTACGTGCTTTTCAGTAGCTGCACTTACTAACAATGTTGCTTTTC   14659
gb:LC522973|Organism:Severe      TACTAGATAAACGCACTACGTGCTTTTCAGTAGCTGCACTTACTAACAATGTTGCTTTTC   14659
gb:LC522974|Organism:Severe      TACTAGATAAACGCACTACGTGCTTTTCAGTAGCTGCACTTACTAACAATGTTGCTTTTC   14659
gb:MN985325|Organism:Severe      TACTAGATAAACGCACTACGTGCTTTTCAGTAGCTGCACTTACTAACAATGTTGCTTTTC   14662
gb:MT020881|Organism:Severe      TACTAGATAAACGCACTACGTGCTTTTCAGTAGCTGCACTTACTAACAATGTTGCTTTTC   14662
gb:MT020880|Organism:Severe      TACTAGATAAACGCACTACGTGCTTTTCAGTAGCTGCACTTACTAACAATGTTGCTTTTC   14662
gb:MT066175|Organism:Severe      TACTAGATAAACGCACTACGTGCTTTTCAGTAGCTGCACTTACTAACAATGTTGCTTTTC   14662
gb:MN997409|Organism:Severe      TACTAGATAAACGCACTACGTGCTTTTCAGTAGCTGCACTTACTAACAATGTTGCTTTTC   14662
gb:MN938384|Organism:Severe      TACTAGATAAACGCACTACGTGCTTTTCAGTAGCTGCACTTACTAACAATGTTGCTTTTC   14630
```

```
gb:MT044258|Organism:Severe      TACTAGATAAACGCACTACGTGCTTTTCAGTAGCTGCACTTACTAACAATGTTGCTTTTC   14638
gb:MT039890|Organism:Severe      TACTAGATAAACGCACTACGTGCTTTTCAGTAGCTGCACTTACTAACAATGTTGCTTTTC   14662
gb:MN988713|Organism:Severe      TACTAGATAAACGCACTACGTGCTTTTCAGTAGCTGCACTTACTAACAATGTTGCTTTTC   14662
gb:LC521925|Organism:Severe      TACTAGATAAACGCACTACGTGCTTTTCAGTAGCTGCACTTACTAACAATGTTGCTTTTC   14635
gb:MT093571|Organism:Severe      TACTAGATAAACGCACTACGTGCTTTTCAGTAGCTGCACTTACTAACAATGTTGCTTTTC   14662
gb:MT039887|Organism:Severe      TACTAGATAAACGCACTACGTGCTTTTCAGTAGCTGCACTTACTAACAATGTTGCTTTTC   14662
gb:MT019530|Organism:Severe      TACTAGATAAACGCACTACGTGCTTTTCAGTAGCTGCACTTACTAACAATGTTGCTTTTC   14662
gb:MT039888|Organism:Severe      TACTAGATAAACGCACTACGTGCTTTTCAGTAGCTGCACTTACTAACAATGTTGCTTTTC   14662
gb:LC522972|Organism:Severe      TACTAGATAAACGCACTACGTGCTTTTCAGTAGCTGCACTTACTAACAATGTTGCTTTTC   14659
gb:MT027063|Organism:Severe      TACTAGATAAACGCACTACGTGCTTTTCAGTAGCTGCACTTACTAACAATGTTGCTTTTC   14662
gb:MT027062|Organism:Severe      TACTAGATAAACGCACTACGTGCTTTTCAGTAGCTGCACTTACTAACAATGTTGCTTTTC   14662
gb:MT019529|Organism:Severe      TACTAGATAAACGCACTACGTGCTTTTCAGTAGCTGCACTTACTAACAATGTTGCTTTTC   14662
gb:MN996529|Organism:Severe      TACTAGATAAACGCACTACGTGCTTTTCAGTAGCTGCACTTACTAACAATGTTGCTTTTC   14650
gb:MN996531|Organism:Severe      TACTAGATAAACGCACTACGTGCTTTTCAGTAGCTGCACTTACTAACAATGTTGCTTTTC   14649
gb:MT066176|Organism:Severe      TACTAGATAAACGCACTACGTGCTTTTCAGTAGCTGCACTTACTAACAATGTTGCTTTTC   14662
gb:MT027064|Organism:Severe      TACTAGATAAACGCACTACGTGCTTTTCAGTAGCTGCACTTACTAACAATGTTGCTTTTC   14662
gb:MN994468|Organism:Severe      TACTAGATAAACGCACTACGTGCTTTTCAGTAGCTGCACTTACTAACAATGTTGCTTTTC   14662
gb:MT072688|Organism:Severe      TACTAGATAAACGCACTACGTGCTTTTCAGTAGCTGCACTTACTAACAATGTTGCTTTTC   14647
gb:MN996527|Organism:Severe      TACTAGATAAACGCACTACGTGCTTTTCAGTAGCTGCACTTACTAACAATGTTGCTTTTC   14629
gb:MT093631|Organism:Severe      TACTAGATAAACGCACTACGTGCTTTTCAGTAGCTGCACTTACTAACAATGTTGCTTTTC   14700
gb:MT106053|Organism:Severe      TACTAGATAAACGCACTACGTGCTTTTCAGTAGCTGCACTTACTAACAATGTTGCTTTTC   14662
gb:MT019533|Organism:Severe      TACTAGATAAACGCACTACGTGCTTTTCAGTAGCTGCACTTACTAACAATGTTGCTTTTC   14662
gb:MT019531|Organism:Severe      TACTAGATAAACGCACTACGTGCTTTTCAGTAGCTGCACTTACTAACAATGTTGCTTTTC   14662
gb:MN996528|Organism:Severe      TACTAGATAAACGCACTACGTGCTTTTCAGTAGCTGCACTTACTAACAATGTTGCTTTTC   14662
gb:MN996530|Organism:Severe      TACTAGATAAACGCACTACGTGCTTTTCAGTAGCTGCACTTACTAACAATGTTGCTTTTC   14648
gb:MN908947|Organism:Severe      TACTAGATAAACGCACTACGTGCTTTTCAGTAGCTGCACTTACTAACAATGTTGCTTTTC   14662
gb:MT019532|Organism:Severe      TACTAGATAAACGCACTACGTGCTTTTCAGTAGCTGCACTTACTAACAATGTTGCTTTTC   14662
                                 ************************************************************

gb:MT020781|Organism:Severe      AAACTGTCAAACCCGGTAATTTTAACAAAGACTTCTATGACTTTGCTGTGTCTAAGGGTT   14710
gb:MT007544|Organism:Severe      AAACTGTCAAACCCGGTAATTTTAACAAAGACTTCTATGACTTTGCTGTGTCTAAGGGTT   14722
gb:MN994467|Organism:Severe      AAACTGTCAAACCCGGTAATTTTAACAAAGACTTCTATGACTTTGCTGTGTCTAAGGGTT   14722
gb:MT044257|Organism:Severe      AAACTGTCAAACCCGGTAATTTTAACAAAGACTTCTATGACTTTGCTGTGTCTAAGGGTT   14722
gb:MT106054|Organism:Severe      AAACTGTCAAACCCGGTAATTTTAACAAAGACTTCTATGACTTTGCTGTGTCTAAGGGTT   14722
gb:MT049951|Organism:Severe      AAACTGTCAAACCCGGTAATTTTAACAAAGACTTCTATGACTTTGCTGTGTCTAAGGGTT   14722
gb:MN975262|Organism:Severe      AAACTGTCAAACCCGGTAATTTTAACAAAGACTTCTATGACTTTGCTGTGTCTAAGGGTT   14722
gb:MT106052|Organism:Severe      AAACTGTCAAACCCGGTAATTTTAACAAAGACTTCTATGACTTTGCTGTGTCTAAGGGTT   14722
gb:LC522975|Organism:Severe      AAACTGTCAAACCCGGTAATTTTAACAAAGACTTCTATGACTTTGCTGTGTCTAAGGGTT   14719
gb:LC522973|Organism:Severe      AAACTGTCAAACCCGGTAATTTTAACAAAGACTTCTATGACTTTGCTGTGTCTAAGGGTT   14719
gb:LC522974|Organism:Severe      AAACTGTCAAACCCGGTAATTTTAACAAAGACTTCTATGACTTTGCTGTGTCTAAGGGTT   14719
gb:MN985325|Organism:Severe      AAACTGTCAAACCCGGTAATTTTAACAAAGACTTCTATGACTTTGCTGTGTCTAAGGGTT   14722
gb:MT020881|Organism:Severe      AAACTGTCAAACCCGGTAATTTTAACAAAGACTTCTATGACTTTGCTGTGTCTAAGGGTT   14722
gb:MT020880|Organism:Severe      AAACTGTCAAACCCGGTAATTTTAACAAAGACTTCTATGACTTTGCTGTGTCTAAGGGTT   14722
gb:MT066175|Organism:Severe      AAACTGTCAAACCCGGTAATTTTAACAAAGACTTCTATGACTTTGCTGTGTCTAAGGGTT   14722
gb:MN997409|Organism:Severe      AAACTGTCAAACCCGGTAATTTTAACAAAGACTTCTATGACTTTGCTGTGTCTAAGGGTT   14722
gb:MN938384|Organism:Severe      AAACTGTCAAACCCGGTAATTTTAACAAAGACTTCTATGACTTTGCTGTGTCTAAGGGTT   14690
gb:MT044258|Organism:Severe      AAACTGTCAAACCCGGTAATTTTAACAAAGACTTCTATGACTTTGCTGTGTCTAAGGGTT   14698
gb:MT039890|Organism:Severe      AAACTGTCAAACCCGGTAATTTTAACAAAGACTTCTATGACTTTGCTGTGTCTAAGGGTT   14722
gb:MN988713|Organism:Severe      AAACTGTCAAACCCGGTAATTTTAACAAAGACTTCTATGACTTTGCTGTGTCTAAGGGTT   14722
gb:LC521925|Organism:Severe      AAACTGTCAAACCCGGTAATTTTAACAAAGACTTCTATGACTTTGCTGTGTCTAAGGGTT   14695
gb:MT093571|Organism:Severe      AAACTGTCAAACCCGGTAATTTTAACAAAGACTTCTATGACTTTGCTGTGTCTAAGGGTT   14722
gb:MT039887|Organism:Severe      AAACTGTCAAACCCGGTAATTTTAACAAAGACTTCTATGACTTTGCTGTGTCTAAGGGTT   14722
gb:MT019530|Organism:Severe      AAACTGTCAAACCCGGTAATTTTAACAAAGACTTCTATGACTTTGCTGTGTCTAAGGGTT   14722
gb:MT039888|Organism:Severe      AAACTGTCAAACCCGGTAATTTTAACAAAGACTTCTATGACTTTGCTGTGTCTAAGGGTT   14722
gb:LC522972|Organism:Severe      AAACTGTCAAACCCGGTAATTTTAACAAAGACTTCTATGACTTTGCTGTGTCTAAGGGTT   14719
gb:MT027063|Organism:Severe      AAACTGTCAAACCCGGTAATTTTAACAAAGACTTCTATGACTTTGCTGTGTCTAAGGGTT   14722
gb:MT027062|Organism:Severe      AAACTGTCAAACCCGGTAATTTTAACAAAGACTTCTATGACTTTGCTGTGTCTAAGGGTT   14722
gb:MT019529|Organism:Severe      AAACTGTCAAACCCGGTAATTTTAACAAAGACTTCTATGACTTTGCTGTGTCTAAGGGTT   14722
gb:MN996529|Organism:Severe      AAACTGTCAAACCCGGTAATTTTAACAAAGACTTCTATGACTTTGCTGTGTCTAAGGGTT   14710
gb:MN996531|Organism:Severe      AAACTGTCAAACCCGGTAATTTTAACAAAGACTTCTATGACTTTGCTGTGTCTAAGGGTT   14709
gb:MT066176|Organism:Severe      AAACTGTCAAACCCGGTAATTTTAACAAAGACTTCTATGACTTTGCTGTGTCTAAGGGTT   14722
gb:MT027064|Organism:Severe      AAACTGTCAAACCCGGTAATTTTAACAAAGACTTCTATGACTTTGCTGTGTCTAAGGGTT   14722
gb:MN994468|Organism:Severe      AAACTGTCAAACCCGGTAATTTTAACAAAGACTTCTATGACTTTGCTGTGTCTAAGGGTT   14722
gb:MT072688|Organism:Severe      AAACTGTCAAACCCGGTAATTTTAACAAAGACTTCTATGACTTTGCTGTGTCTAAGGGTT   14707
gb:MN996527|Organism:Severe      AAACTGTCAAACCCGGTAATTTTAACAAAGACTTCTATGACTTTGCTGTGTCTAAGGGTT   14689
```

```
gb:MT093631|Organism:Severe      AAACTGTCAAACCCGGTAATTTTAACAAAGACTTCTATGACTTTGCTGTGTCTAAGGGTT   14760
gb:MT106053|Organism:Severe      AAACTGTCAAACCCGGTAATTTTAACAAAGACTTCTATGACTTTGCTGTGTCTAAGGGTT   14722
gb:MT019533|Organism:Severe      AAACTGTCAAACCCGGTAATTTTAACAAAGACTTCTATGACTTTGCTGTGTCTAAGGGTT   14722
gb:MT019531|Organism:Severe      AAACTGTCAAACCCGGTAATTTTAACAAAGACTTCTATGACTTTGCTGTGTCTAAGGGTT   14722
gb:MN996528|Organism:Severe      AAACTGTCAAACCCGGTAATTTTAACAAAGACTTCTATGACTTTGCTGTGTCTAAGGGTT   14722
gb:MN996530|Organism:Severe      AAACTGTCAAACCCGGTAATTTTAACAAAGACTTCTATGACTTTGCTGTGTCTAAGGGTT   14708
gb:MN908947|Organism:Severe      AAACTGTCAAACCCGGTAATTTTAACAAAGACTTCTATGACTTTGCTGTGTCTAAGGGTT   14722
gb:MT019532|Organism:Severe      AAACTGTCAAACCCGGTAATTTTAACAAAGACTTCTATGACTTTGCTGTGTCTAAGGGTT   14722
                                 ************************************************************

gb:MT020781|Organism:Severe      TCTTTAAGGAAGGAAGTTCTGTTGAATTAAAACACTTCTTCTTTGCTCAGGATGGTAATG   14770
gb:MT007544|Organism:Severe      TCTTTAAGGAAGGAAGTTCTGTTGAATTAAAACACTTCTTCTTTGCTCAGGATGGTAATG   14782
gb:MN994467|Organism:Severe      TCTTTAAGGAAGGAAGTTCTGTTGAATTAAAACACTTCTTCTTTGCTCAGGATGGTAATG   14782
gb:MT044257|Organism:Severe      TCTTTAAGGAAGGAAGTTCTGTTGAATTAAAACACTTCTTCTTTGCTCAGGATGGTAATG   14782
gb:MT106054|Organism:Severe      TCTTTAAGGAAGGAAGTTCTGTTGAATTAAAACACTTCTTCTTTGCTCAGGATGGTAATG   14782
gb:MT049951|Organism:Severe      TCTTTAAGGAAGGAAGTTCTGTTGAATTAAAACACTTCTTCTTTGCTCAGGATGGTAATG   14782
gb:MN975262|Organism:Severe      TCTTTAAGGAAGGAAGTTCTGTTGAATTAAAACACTTCTTCTTTGCTCAGGATGGTAATG   14782
gb:MT106052|Organism:Severe      TCTTTAAGGAAGGAAGTTCTGTTGAATTAAAACACTTCTTCTTTGCTCAGGATGGTAATG   14782
gb:LC522975|Organism:Severe      TCTTTAAGGAAGGAAGTTCTGTTGAATTAAAACACTTCTTCTTTGCTCAGGATGGTAATG   14779
gb:LC522973|Organism:Severe      TCTTTAAGGAAGGAAGTTCTGTTGAATTAAAACACTTCTTCTTTGCTCAGGATGGTAATG   14779
gb:LC522974|Organism:Severe      TCTTTAAGGAAGGAAGTTCTGTTGAATTAAAACACTTCTTCTTTGCTCAGGATGGTAATG   14779
gb:MN985325|Organism:Severe      TCTTTAAGGAAGGAAGTTCTGTTGAATTAAAACACTTCTTCTTTGCTCAGGATGGTAATG   14782
gb:MT020881|Organism:Severe      TCTTTAAGGAAGGAAGTTCTGTTGAATTAAAACACTTCTTCTTTGCTCAGGATGGTAATG   14782
gb:MT020880|Organism:Severe      TCTTTAAGGAAGGAAGTTCTGTTGAATTAAAACACTTCTTCTTTGCTCAGGATGGTAATG   14782
gb:MT066175|Organism:Severe      TCTTTAAGGAAGGAAGTTCTGTTGAATTAAAACACTTCTTCTTTGCTCAGGATGGTAATG   14782
gb:MN997409|Organism:Severe      TCTTTAAGGAAGGAAGTTCTGTTGAATTAAAACACTTCTTCTTTGCTCAGGATGGTAATG   14782
gb:MN938384|Organism:Severe      TCTTTAAGGAAGGAAGTTCTGTTGAATTAAAACACTTCTTCTTTGCTCAGGATGGTAATG   14750
gb:MT044258|Organism:Severe      TCTTTAAGGAAGGAAGTTCTGTTGAATTAAAACACTTCTTCTTTGCTCAGGATGGTAATG   14758
gb:MT039890|Organism:Severe      TCTTTAAGGAAGGAAGTTCTGTTGAATTAAAACACTTCTTCTTTGCTCAGGATGGTAATG   14782
gb:MN988713|Organism:Severe      TCTTTAAGGAAGGAAGTTCTGTTGAATTAAAACACTTCTTCTTTGCTCAGGATGGTAATG   14782
gb:LC521925|Organism:Severe      TCTTTAAGGAAGGAAGTTCTGTTGAATTAAAACACTTCTTCTTTGCTCAGGATGGTAATG   14755
gb:MT093571|Organism:Severe      TCTTTAAGGAAGGAAGTTCTGTTGAATTAAAACACTTCTTCTTTGCTCAGGATGGTAATG   14782
gb:MT039887|Organism:Severe      TCTTTAAGGAAGGAAGTTCTGTTGAATTAAAACACTTCTTCTTTGCTCAGGATGGTAATG   14782
gb:MT019530|Organism:Severe      TCTTTAAGGAAGGAAGTTCTGTTGAATTAAAACACTTCTTCTTTGCTCAGGATGGTAATG   14782
gb:MT039888|Organism:Severe      TCTTTAAGGAAGGAAGTTCTGTTGAATTAAAACACTTCTTCTTTGCTCAGGATGGTAATG   14782
gb:LC522972|Organism:Severe      TCTTTAAGGAAGGAAGTTCTGTTGAATTAAAACACTTCTTCTTTGCTCAGGATGGTAATG   14779
gb:MT027063|Organism:Severe      TCTTTAAGGAAGGAAGTTCTGTTGAATTAAAACACTTCTTCTTTGCTCAGGATGGTAATG   14782
gb:MT027062|Organism:Severe      TCTTTAAGGAAGGAAGTTCTGTTGAATTAAAACACTTCTTCTTTGCTCAGGATGGTAATG   14782
gb:MT019529|Organism:Severe      TCTTTAAGGAAGGAAGTTCTGTTGAATTAAAACACTTCTTCTTTGCTCAGGATGGTAATG   14782
gb:MN996529|Organism:Severe      TCTTTAAGGAAGGAAGTTCTGTTGAATTAAAACACTTCTTCTTTGCTCAGGATGGTAATG   14770
gb:MN996531|Organism:Severe      TCTTTAAGGAAGGAAGTTCTGTTGAATTAAAACACTTCTTCTTTGCTCAGGATGGTAATG   14769
gb:MT066176|Organism:Severe      TCTTTAAGGAAGGAAGTTCTGTTGAATTAAAACACTTCTTCTTTGCTCAGGATGGTAATG   14782
gb:MT027064|Organism:Severe      TCTTTAAGGAAGGAAGTTCTGTTGAATTAAAACACTTCTTCTTTGCTCAGGATGGTAATG   14782
gb:MN994468|Organism:Severe      TCTTTAAGGAAGGAAGTTCTGTTGAATTAAAACACTTCTTCTTTGCTCAGGATGGTAATG   14782
gb:MT072688|Organism:Severe      TCTTTAAGGAAGGAAGTTCTGTTGAATTAAAACACTTCTTCTTTGCTCAGGATGGTAATG   14767
gb:MN996527|Organism:Severe      TCTTTAAGGAAGGAAGTTCTGTTGAATTAAAACACTTCTTCTTTGCTCAGGATGGTAATG   14749
gb:MT093631|Organism:Severe      TCTTTAAGGAAGGAAGTTCTGTTGAATTAAAACACTTCTTCTTTGCTCAGGATGGTAATG   14820
gb:MT106053|Organism:Severe      TCTTTAAGGAAGGAAGTTCTGTTGAATTAAAACACTTCTTCTTTGCTCAGGATGGTAATG   14782
gb:MT019533|Organism:Severe      TCTTTAAGGAAGGAAGTTCTGTTGAATTAAAACACTTCTTCTTTGCTCAGGATGGTAATG   14782
gb:MT019531|Organism:Severe      TCTTTAAGGAAGGAAGTTCTGTTGAATTAAAACACTTCTTCTTTGCTCAGGATGGTAATG   14782
gb:MN996528|Organism:Severe      TCTTTAAGGAAGGAAGTTCTGTTGAATTAAAACACTTCTTCTTTGCTCAGGATGGTAATG   14782
gb:MN996530|Organism:Severe      TCTTTAAGGAAGGAAGTTCTGTTGAATTAAAACACTTCTTCTTTGCTCAGGATGGTAATG   14768
gb:MN908947|Organism:Severe      TCTTTAAGGAAGGAAGTTCTGTTGAATTAAAACACTTCTTCTTTGCTCAGGATGGTAATG   14782
gb:MT019532|Organism:Severe      TCTTTAAGGAAGGAAGTTCTGTTGAATTAAAACACTTCTTCTTTGCTCAGGATGGTAATG   14782
                                 ************************************************************

gb:MT020781|Organism:Severe      CTGCTATCAGCGATTATGACTACTATCGTTATAATCTACCAACAATGTGTGATATCAGAC   14830
gb:MT007544|Organism:Severe      CTGCTATCAGCGATTATGACTACTATCGTTATAATCTACCAACAATGTGTGATATCAGAC   14842
gb:MN994467|Organism:Severe      CTGCTATCAGCGATTATGACTACTATCGTTATAATCTACCAACAATGTGTGATATCAGAC   14842
gb:MT044257|Organism:Severe      CTGCTATCAGCGATTATGACTACTATCGTTATAATCTACCAACAATGTGTGATATCAGAC   14842
gb:MT106054|Organism:Severe      CTGCTATCAGCGATTATGACTACTATCGTTATAATCTACCAACAATGTGTGATATCAGAC   14842
gb:MT049951|Organism:Severe      CTGCTATCAGCGATTATGACTACTATCGTTATAATCTACCAACAATGTGTGATATCAGAC   14842
gb:MN975262|Organism:Severe      CTGCTATCAGCGATTATGACTACTATCGTTATAATCTACCAACAATGTGTGATATCAGAC   14842
gb:MT106052|Organism:Severe      CTGCTATCAGCGATTATGACTACTATCGTTATAATCTACCAACAATGTGTGATATCAGAC   14842
gb:LC522975|Organism:Severe      CTGCTATCAGCGATTATGACTACTATCGTTATAATCTACCAACAATGTGTGATATCAGAC   14839
```

```
gb:LC522973|Organism:Severe      CTGCTATCAGCGATTATGACTACTATCGTTATAATCTACCAACAATGTGTGATATCAGAC   14839
gb:LC522974|Organism:Severe      CTGCTATCAGCGATTATGACTACTATCGTTATAATCTACCAACAATGTGTGATATCAGAC   14839
gb:MN985325|Organism:Severe      CTGCTATCAGCGATTATGACTACTATCGTTATAATCTACCAACAATGTGTGATATCAGAC   14842
gb:MT020881|Organism:Severe      CTGCTATCAGCGATTATGACTACTATCGTTATAATCTACCAACAATGTGTGATATCAGAC   14842
gb:MT020880|Organism:Severe      CTGCTATCAGCGATTATGACTACTATCGTTATAATCTACCAACAATGTGTGATATCAGAC   14842
gb:MT066175|Organism:Severe      CTGCTATCAGCGATTATGACTACTATCGTTATAATCTACCAACAATGTGTGATATCAGAC   14842
gb:MN997409|Organism:Severe      CTGCTATCAGCGATTATGACTACTATCGTTATAATCTACCAACAATGTGTGATATCAGAC   14842
gb:MN938384|Organism:Severe      CTGCTATCAGCGATTATGACTACTATCGTTATAATCTACCAACAATGTGTGATATCAGAC   14810
gb:MT044258|Organism:Severe      CTGCTATCAGCGATTATGACTACTATCGTTATAATCTACCAACAATGTGTGATATCAGAC   14818
gb:MT039890|Organism:Severe      CTGCTATCAGCGATTATGACTACTATCGTTATAATCTACCAACAATGTGTGATATCAGAC   14842
gb:MN988713|Organism:Severe      CTGCTATCAGCGATTATGACTACTATCGTTATAATCTACCAACAATGTGTGATATCAGAC   14842
gb:LC521925|Organism:Severe      CTGCTATCAGCGATTATGACTACTATCGTTATAATCTACCAACAATGTGTGATATCAGAC   14815
gb:MT093571|Organism:Severe      CTGCTATCAGCGATTATGACTACTATCGTTATAATCTACCAACAATGTGTGATATCAGAC   14842
gb:MT039887|Organism:Severe      CTGCTATCAGCGATTATGACTACTATCGTTATAATCTACCAACAATGTGTGATATCAGAC   14842
gb:MT019530|Organism:Severe      CTGCTATCAGCGATTATGACTACTATCGTTATAATCTACCAACAATGTGTGATATCAGAC   14842
gb:MT039888|Organism:Severe      CTGCTATCAGCGATTATGACTACTATCGTTATAATCTACCAACAATGTGTGATATCAGAC   14842
gb:LC522972|Organism:Severe      CTGCTATCAGCGATTATGACTACTATCGTTATAATCTACCAACAATGTGTGATATCAGAC   14839
gb:MT027063|Organism:Severe      CTGCTATCAGCGATTATGACTACTATCGTTATAATCTACCAACAATGTGTGATATCAGAC   14842
gb:MT027062|Organism:Severe      CTGCTATCAGCGATTATGACTACTATCGTTATAATCTACCAACAATGTGTGATATCAGAC   14842
gb:MT019529|Organism:Severe      CTGCTATCAGCGATTATGACTACTATCGTTATAATCTACCAACAATGTGTGATATCAGAC   14842
gb:MN996529|Organism:Severe      CTGCTATCAGCGATTATGACTACTATCGTTATAATCTACCAACAATGTGTGATATCAGAC   14830
gb:MN996531|Organism:Severe      CTGCTATCAGCGATTATGACTACTATCGTTATAATCTACCAACAATGTGTGATATCAGAC   14829
gb:MT066176|Organism:Severe      CTGCTATCAGCGATTATGACTACTATCGTTATAATCTACCAACAATGTGTGATATCAGAC   14842
gb:MT027064|Organism:Severe      CTGCTATCAGCGATTATGACTACTATCGTTATAATCTACCAACAATGTGTGATATCAGAC   14842
gb:MN994468|Organism:Severe      CTGCTATCAGCGATTATGACTACTATCGTTATAATCTACCAACAATGTGTGATATCAGAC   14842
gb:MT072688|Organism:Severe      CTGCTATCAGCGATTATGACTACTATCGTTATAATCTACCAACAATGTGTGATATCAGAC   14827
gb:MN996527|Organism:Severe      CTGCTATCAGCGATTATGACTACTATCGTTATAATCTACCAACAATGTGTGATATCAGAC   14809
gb:MT093631|Organism:Severe      CTGCTATCAGCGATTATGACTACTATCGTTATAATCTACCAACAATGTGTGATATCAGAC   14880
gb:MT106053|Organism:Severe      CTGCTATCAGCGATTATGACTACTATCGTTATAATCTACCAACAATGTGTGATATCAGAC   14842
gb:MT019533|Organism:Severe      CTGCTATCAGCGATTATGACTACTATCGTTATAATCTACCAACAATGTGTGATATCAGAC   14842
gb:MT019531|Organism:Severe      CTGCTATCAGCGATTATGACTACTATCGTTATAATCTACCAACAATGTGTGATATCAGAC   14842
gb:MN996528|Organism:Severe      CTGCTATCAGCGATTATGACTACTATCGTTATAATCTACCAACAATGTGTGATATCAGAC   14842
gb:MN996530|Organism:Severe      CTGCTATCAGCGATTATGACTACTATCGTTATAATCTACCAACAATGTGTGATATCAGAC   14828
gb:MN908947|Organism:Severe      CTGCTATCAGCGATTATGACTACTATCGTTATAATCTACCAACAATGTGTGATATCAGAC   14842
gb:MT019532|Organism:Severe      CTGCTATCAGCGATTATGACTACTATCGTTATAATCTACCAACAATGTGTGATATCAGAC   14842
                                 ************************************************************

gb:MT020781|Organism:Severe      AACTACTATTTGTAGTTGAAGTTGTTGATAAGTACTTTGATTGTTACGATGGTGGCTGTA   14890
gb:MT007544|Organism:Severe      AACTACTATTTGTAGTTGAAGTTGTTGATAAGTACTTTGATTGTTACGATGGTGGCTGTA   14902
gb:MN994467|Organism:Severe      AACTACTATTTGTAGTTGAAGTTGTTGATAAGTACTTTGATTGTTACGATGGTGGCTGTA   14902
gb:MT044257|Organism:Severe      AACTACTATTTGTAGTTGAAGTTGTTGATAAGTACTTTGATTGTTACGATGGTGGCTGTA   14902
gb:MT106054|Organism:Severe      AACTACTATTTGTAGTTGAAGTTGTTGATAAGTACTTTGATTGTTACGATGGTGGCTGTA   14902
gb:MT049951|Organism:Severe      AACTACTATTTGTAGTTGAAGTTGTTGATAAGTACTTTGATTGTTACGATGGTGGCTGTA   14902
gb:MN975262|Organism:Severe      AACTACTATTTGTAGTTGAAGTTGTTGATAAGTACTTTGATTGTTACGATGGTGGCTGTA   14902
gb:MT106052|Organism:Severe      AACTACTATTTGTAGTTGAAGTTGTTGATAAGTACTTTGATTGTTACGATGGTGGCTGTA   14902
gb:LC522975|Organism:Severe      AACTACTATTTGTAGTTGAAGTTGTTGATAAGTACTTTGATTGTTACGATGGTGGCTGTA   14899
gb:LC522973|Organism:Severe      AACTACTATTTGTAGTTGAAGTTGTTGATAAGTACTTTGATTGTTACGATGGTGGCTGTA   14899
gb:LC522974|Organism:Severe      AACTACTATTTGTAGTTGAAGTTGTTGATAAGTACTTTGATTGTTACGATGGTGGCTGTA   14899
gb:MN985325|Organism:Severe      AACTACTATTTGTAGTTGAAGTTGTTGATAAGTACTTTGATTGTTACGATGGTGGCTGTA   14902
gb:MT020881|Organism:Severe      AACTACTATTTGTAGTTGAAGTTGTTGATAAGTACTTTGATTGTTACGATGGTGGCTGTA   14902
gb:MT020880|Organism:Severe      AACTACTATTTGTAGTTGAAGTTGTTGATAAGTACTTTGATTGTTACGATGGTGGCTGTA   14902
gb:MT066175|Organism:Severe      AACTACTATTTGTAGTTGAAGTTGTTGATAAGTACTTTGATTGTTACGATGGTGGCTGTA   14902
gb:MN997409|Organism:Severe      AACTACTATTTGTAGTTGAAGTTGTTGATAAGTACTTTGATTGTTACGATGGTGGCTGTA   14902
gb:MN938384|Organism:Severe      AACTACTATTTGTAGTTGAAGTTGTTGATAAGTACTTTGATTGTTACGATGGTGGCTGTA   14870
gb:MT044258|Organism:Severe      AACTACTATTTGTAGTTGAAGTTGTTGATAAGTACTTTGATTGTTACGATGGTGGCTGTA   14878
gb:MT039890|Organism:Severe      AACTACTATTTGTAGTTGAAGTTGTTGATAAGTACTTTGATTGTTACGATGGTGGCTGTA   14902
gb:MN988713|Organism:Severe      AACTACTATTTGTAGTTGAAGTTGTTGATAAGTACTTTGATTGTTACGATGGTGGCTGTA   14902
gb:LC521925|Organism:Severe      AACTACTATTTGTAGTTGAAGTTGTTGATAAGTACTTTGATTGTTACGATGGTGGCTGTA   14875
gb:MT093571|Organism:Severe      AACTACTATTTGTAGTTGAAGTTGTTGATAAGTACTTTGATTGTTACGATGGTGGCTGTA   14902
gb:MT039887|Organism:Severe      AACTACTATTTGTAGTTGAAGTTGTTGATAAGTACTTTGATTGTTACGATGGTGGCTGTA   14902
gb:MT019530|Organism:Severe      AACTACTATTTGTAGTTGAAGTTGTTGATAAGTACTTTGATTGTTACGATGGTGGCTGTA   14902
gb:MT039888|Organism:Severe      AACTACTATTTGTAGTTGAAGTTGTTGATAAGTACTTTGATTGTTACGATGGTGGCTGTA   14902
gb:LC522972|Organism:Severe      AACTACTATTTGTAGTTGAAGTTGTTGATAAGTACTTTGATTGTTACGATGGTGGCTGTA   14899
gb:MT027063|Organism:Severe      AACTACTATTTGTAGTTGAAGTTGTTGATAAGTACTTTGATTGTTACGATGGTGGCTGTA   14902
gb:MT027062|Organism:Severe      AACTACTATTTGTAGTTGAAGTTGTTGATAAGTACTTTGATTGTTACGATGGTGGCTGTA   14902
```

```
gb:MT019529|Organism:Severe      AACTACTATTTGTAGTTGAAGTTGTTGATAAGTACTTTGATTGTTACGATGGTGGCTGTA   14902
gb:MN996529|Organism:Severe      AACTACTATTTGTAGTTGAAGTTGTTGATAAGTACTTTGATTGTTACGATGGTGGCTGTA   14890
gb:MN996531|Organism:Severe      AACTACTATTTGTAGTTGAAGTTGTTGATAAGTACTTTGATTGTTACGATGGTGGCTGTA   14889
gb:MT066176|Organism:Severe      AACTACTATTTGTAGTTGAAGTTGTTGATAAGTACTTTGATTGTTACGATGGTGGCTGTA   14902
gb:MT027064|Organism:Severe      AACTACTATTTGTAGTTGAAGTTGTTGATAAGTACTTTGATTGTTACGATGGTGGCTGTA   14902
gb:MN994468|Organism:Severe      AACTACTATTTGTAGTTGAAGTTGTTGATAAGTACTTTGATTGTTACGATGGTGGCTGTA   14902
gb:MT072688|Organism:Severe      AACTACTATTTGTAGTTGAAGTTGTTGATAAGTACTTTGATTGTTACGATGGTGGCTGTA   14887
gb:MN996527|Organism:Severe      AACTACTATTTGTAGTTGAAGTTGTTGATAAGTACTTTGATTGTTACGATGGTGGCTGTA   14869
gb:MT093631|Organism:Severe      AACTACTATTTGTAGTTGAAGTTGTTGATAAGTACTTTGATTGTTACGATGGTGGCTGTA   14940
gb:MT106053|Organism:Severe      AACTACTATTTGTAGTTGAAGTTGTTGATAAGTACTTTGATTGTTACGATGGTGGCTGTA   14902
gb:MT019533|Organism:Severe      AACTACTATTTGTAGTTGAAGTTGTTGATAAGTACTTTGATTGTTACGATGGTGGCTGTA   14902
gb:MT019531|Organism:Severe      AACTACTATTTGTAGTTGAAGTTGTTGATAAGTACTTTGATTGTTACGATGGTGGCTGTA   14902
gb:MN996528|Organism:Severe      AACTACTATTTGTAGTTGAAGTTGTTGATAAGTACTTTGATTGTTACGATGGTGGCTGTA   14902
gb:MN996530|Organism:Severe      AACTACTATTTGTAGTTGAAGTTGTTGATAAGTACTTTGATTGTTACGATGGTGGCTGTA   14888
gb:MN908947|Organism:Severe      AACTACTATTTGTAGTTGAAGTTGTTGATAAGTACTTTGATTGTTACGATGGTGGCTGTA   14902
gb:MT019532|Organism:Severe      AACTACTATTTGTAGTTGAAGTTGTTGATAAGTACTTTGATTGTTACGATGGTGGCTGTA   14902
                                 ************************************************************

gb:MT020781|Organism:Severe      TTAATGCTAACCAAGTCATCGTCAACAACCTAGACAAATCAGCTGGTTTTCCATTTAATA   14950
gb:MT007544|Organism:Severe      TTAATGCTAACCAAGTCATCGTCAACAACCTAGACAAATCAGCTGGTTTTCCATTTAATA   14962
gb:MN994467|Organism:Severe      TTAATGCTAACCAAGTCATCGTCAACAACCTAGACAAATCAGCTGGTTTTCCATTTAATA   14962
gb:MT044257|Organism:Severe      TTAATGCTAACCAAGTCATCGTCAACAACCTAGACAAATCAGCTGGTTTTCCATTTAATA   14962
gb:MT106054|Organism:Severe      TTAATGCTAACCAAGTCATCGTCAACAACCTAGACAAATCAGCTGGTTTTCCATTTAATA   14962
gb:MT049951|Organism:Severe      TTAATGCTAACCAAGTCATCGTCAACAACCTAGACAAATCAGCTGGTTTTCCATTTAATA   14962
gb:MN975262|Organism:Severe      TTAATGCTAACCAAGTCATCGTCAACAACCTAGACAAATCAGCTGGTTTTCCATTTAATA   14962
gb:MT106052|Organism:Severe      TTAATGCTAACCAAGTCATCGTCAACAACCTAGACAAATCAGCTGGTTTTCCATTTAATA   14962
gb:LC522975|Organism:Severe      TTAATGCTAACCAAGTCATCGTCAACAACCTAGACAAATCAGCTGGTTTTCCATTTAATA   14959
gb:LC522973|Organism:Severe      TTAATGCTAACCAAGTCATCGTCAACAACCTAGACAAATCAGCTGGTTTTCCATTTAATA   14959
gb:LC522974|Organism:Severe      TTAATGCTAACCAAGTCATCGTCAACAACCTAGACAAATCAGCTGGTTTTCCATTTAATA   14959
gb:MN985325|Organism:Severe      TTAATGCTAACCAAGTCATCGTCAACAACCTAGACAAATCAGCTGGTTTTCCATTTAATA   14962
gb:MT020881|Organism:Severe      TTAATGCTAACCAAGTCATCGTCAACAACCTAGACAAATCAGCTGGTTTTCCATTTAATA   14962
gb:MT020880|Organism:Severe      TTAATGCTAACCAAGTCATCGTCAACAACCTAGACAAATCAGCTGGTTTTCCATTTAATA   14962
gb:MT066175|Organism:Severe      TTAATGCTAACCAAGTCATCGTCAACAACCTAGACAAATCAGCTGGTTTTCCATTTAATA   14962
gb:MN997409|Organism:Severe      TTAATGCTAACCAAGTCATCGTCAACAACCTAGACAAATCAGCTGGTTTTCCATTTAATA   14962
gb:MN938384|Organism:Severe      TTAATGCTAACCAAGTCATCGTCAACAACCTAGACAAATCAGCTGGTTTTCCATTTAATA   14930
gb:MT044258|Organism:Severe      TTAATGCTAACCAAGTCATCGTCAACAACCTAGACAAATCAGCTGGTTTTCCATTTAATA   14938
gb:MT039890|Organism:Severe      TTAATGCTAACCAAGTCATCGTCAACAACCTAGACAAATCAGCTGGTTTTCCATTTAATA   14962
gb:MN988713|Organism:Severe      TTAATGCTAACCAAGTCATCGTCAACAACCTAGACAAATCAGCTGGTTTTCCATTTAATA   14962
gb:LC521925|Organism:Severe      TTAATGCTAACCAAGTCATCGTCAACAACCTAGACAAATCAGCTGGTTTTCCATTTAATA   14935
gb:MT093571|Organism:Severe      TTAATGCTAACCAAGTCATCGTCAACAACCTAGACAAATCAGCTGGTTTTCCATTTAATA   14962
gb:MT039887|Organism:Severe      TTAATGCTAACCAAGTCATCGTCAACAACCTAGACAAATCAGCTGGTTTTCCATTTAATA   14962
gb:MT019530|Organism:Severe      TTAATGCTAACCAAGTCATCGTCAACAACCTAGACAAATCAGCTGGTTTTCCATTTAATA   14962
gb:MT039888|Organism:Severe      TTAATGCTAACCAAGTCATCGTCAACAACCTAGACAAATCAGCTGGTTTTCCATTTAATA   14962
gb:LC522972|Organism:Severe      TTAATGCTAACCAAGTCATCGTCAACAACCTAGACAAATCAGCTGGTTTTCCATTTAATA   14959
gb:MT027063|Organism:Severe      TTAATGCTAACCAAGTCATCGTCAACAACCTAGACAAATCAGCTGGTTTTCCATTTAATA   14962
gb:MT027062|Organism:Severe      TTAATGCTAACCAAGTCATCGTCAACAACCTAGACAAATCAGCTGGTTTTCCATTTAATA   14962
gb:MT019529|Organism:Severe      TTAATGCTAACCAAGTCATCGTCAACAACCTAGACAAATCAGCTGGTTTTCCATTTAATA   14962
gb:MN996529|Organism:Severe      TTAATGCTAACCAAGTCATCGTCAACAACCTAGACAAATCAGCTGGTTTTCCATTTAATA   14950
gb:MN996531|Organism:Severe      TTAATGCTAACCAAGTCATCGTCAACAACCTAGACAAATCAGCTGGTTTTCCATTTAATA   14949
gb:MT066176|Organism:Severe      TTAATGCTAACCAAGTCATCGTCAACAACCTAGACAAATCAGCTGGTTTTCCATTTAATA   14962
gb:MT027064|Organism:Severe      TTAATGCTAACCAAGTCATCGTCAACAACCTAGACAAATCAGCTGGTTTTCCATTTAATA   14962
gb:MN994468|Organism:Severe      TTAATGCTAACCAAGTCATCGTCAACAACCTAGACAAATCAGCTGGTTTTCCATTTAATA   14962
gb:MT072688|Organism:Severe      TTAATGCTAACCAAGTCATCGTCAACAACCTAGACAAATCAGCTGGTTTTCCATTTAATA   14947
gb:MN996527|Organism:Severe      TTAATGCTAACCAAGTCATCGTCAACAACCTAGACAAATCAGCTGGTTTTCCATTTAATA   14929
gb:MT093631|Organism:Severe      TTAATGCTAACCAAGTCATCGTCAACAACCTAGACAAATCAGCTGGTTTTCCATTTAATA   15000
gb:MT106053|Organism:Severe      TTAATGCTAACCAAGTCATCGTCAACAACCTAGACAAATCAGCTGGTTTTCCATTTAATA   14962
gb:MT019533|Organism:Severe      TTAATGCTAACCAAGTCATCGTCAACAACCTAGACAAATCAGCTGGTTTTCCATTTAATA   14962
gb:MT019531|Organism:Severe      TTAATGCTAACCAAGTCATCGTCAACAACCTAGACAAATCAGCTGGTTTTCCATTTAATA   14962
gb:MN996528|Organism:Severe      TTAATGCTAACCAAGTCATCGTCAACAACCTAGACAAATCAGCTGGTTTTCCATTTAATA   14962
gb:MN996530|Organism:Severe      TTAATGCTAACCAAGTCATCGTCAACAACCTAGACAAATCAGCTGGTTTTCCATTTAATA   14948
gb:MN908947|Organism:Severe      TTAATGCTAACCAAGTCATCGTCAACAACCTAGACAAATCAGCTGGTTTTCCATTTAATA   14962
gb:MT019532|Organism:Severe      TTAATGCTAACCAAGTCATCGTCAACAACCTAGACAAATCAGCTGGTTTTCCATTTAATA   14962
                                 ************************************************************

gb:MT020781|Organism:Severe      AATGGGGTAAGGCTAGACTTTATTATGATTCAATGAGTTATGAGGATCAAGATGCACTTT   15010
```

```
gb:MT007544|Organism:Severe      AATGGGGTAAGGCTAGACTTTATTATGATTCAATGAGTTATGAGGATCAAGATGCACTTT   15022
gb:MN994467|Organism:Severe      AATGGGGTAAGGCTAGACTTTATTATGATTCAATGAGTTATGAGGATCAAGATGCACTTT   15022
gb:MT044257|Organism:Severe      AATGGGGTAAGGCTAGACTTTATTATGATTCAATGAGTTATGAGGATCAAGATGCACTTT   15022
gb:MT106054|Organism:Severe      AATGGGGTAAGGCTAGACTTTATTATGATTCAATGAGTTATGAGGATCAAGATGCACTTT   15022
gb:MT049951|Organism:Severe      AATGGGGTAAGGCTAGACTTTATTATGATTCAATGAGTTATGAGGATCAAGATGCACTTT   15022
gb:MN975262|Organism:Severe      AATGGGGTAAGGCTAGACTTTATTATGATTCAATGAGTTATGAGGATCAAGATGCACTTT   15022
gb:MT106052|Organism:Severe      AATGGGGTAAGGCTAGACTTTATTATGATTCAATGAGTTATGAGGATCAAGATGCACTTT   15022
gb:LC522975|Organism:Severe      AATGGGGTAAGGCTAGACTTTATTATGATTCAATGAGTTATGAGGATCAAGATGCACTTT   15019
gb:LC522973|Organism:Severe      AATGGGGTAAGGCTAGACTTTATTATGATTCAATGAGTTATGAGGATCAAGATGCACTTT   15019
gb:LC522974|Organism:Severe      AATGGGGTAAGGCTAGACTTTATTATGATTCAATGAGTTATGAGGATCAAGATGCACTTT   15019
gb:MN985325|Organism:Severe      AATGGGGTAAGGCTAGACTTTATTATGATTCAATGAGTTATGAGGATCAAGATGCACTTT   15022
gb:MT020881|Organism:Severe      AATGGGGTAAGGCTAGACTTTATTATGATTCAATGAGTTATGAGGATCAAGATGCACTTT   15022
gb:MT020880|Organism:Severe      AATGGGGTAAGGCTAGACTTTATTATGATTCAATGAGTTATGAGGATCAAGATGCACTTT   15022
gb:MT066175|Organism:Severe      AATGGGGTAAGGCTAGACTTTATTATGATTCAATGAGTTATGAGGATCAAGATGCACTTT   15022
gb:MN997409|Organism:Severe      AATGGGGTAAGGCTAGACTTTATTATGATTCAATGAGTTATGAGGATCAAGATGCACTTT   15022
gb:MN938384|Organism:Severe      AATGGGGTAAGGCTAGACTTTATTATGATTCAATGAGTTATGAGGATCAAGATGCACTTT   14990
gb:MT044258|Organism:Severe      AATGGGGTAAGGCTAGACTTTATTATGATTCAATGAGTTATGAGGATCAAGATGCACTTT   14998
gb:MT039890|Organism:Severe      AATGGGGTAAGGCTAGACTTTATTATGATTCAATGAGTTATGAGGATCAAGATGCACTTT   15022
gb:MN988713|Organism:Severe      AATGGGGTAAGGCTAGACTTTATTATGATTCAATGAGTTATGAGGATCAAGATGCACTTT   15022
gb:LC521925|Organism:Severe      AATGGGGTAAGGCTAGACTTTATTATGATTCAATGAGTTATGAGGATCAAGATGCACTTT   14995
gb:MT093571|Organism:Severe      AATGGGGTAAGGCTAGACTTTATTATGATTCAATGAGTTATGAGGATCAAGATGCACTTT   15022
gb:MT039887|Organism:Severe      AATGGGGTAAGGCTAGACTTTATTATGATTCAATGAGTTATGAGGATCAAGATGCACTTT   15022
gb:MT019530|Organism:Severe      AATGGGGTAAGGCTAGACTTTATTATGATTCAATGAGTTATGAGGATCAAGATGCACTTT   15022
gb:MT039888|Organism:Severe      AATGGGGTAAGGCTAGACTTTATTATGATTCAATGAGTTATGAGGATCAAGATGCACTTT   15022
gb:LC522972|Organism:Severe      AATGGGGTAAGGCTAGACTTTATTATGATTCAATGAGTTATGAGGATCAAGATGCACTTT   15019
gb:MT027063|Organism:Severe      AATGGGGTAAGGCTAGACTTTATTATGATTCAATGAGTTATGAGGATCAAGATGCACTTT   15022
gb:MT027062|Organism:Severe      AATGGGGTAAGGCTAGACTTTATTATGATTCAATGAGTTATGAGGATCAAGATGCACTTT   15022
gb:MT019529|Organism:Severe      AATGGGGTAAGGCTAGACTTTATTATGATTCAATGAGTTATGAGGATCAAGATGCACTTT   15022
gb:MN996529|Organism:Severe      AATGGGGTAAGGCTAGACTTTATTATGATTCAATGAGTTATGAGGATCAAGATGCACTTT   15010
gb:MN996531|Organism:Severe      AATGGGGTAAGGCTAGACTTTATTATGATTCAATGAGTTATGAGGATCAAGATGCACTTT   15009
gb:MT066176|Organism:Severe      AATGGGGTAAGGCTAGACTTTATTATGATTCAATGAGTTATGAGGATCAAGATGCACTTT   15022
gb:MT027064|Organism:Severe      AATGGGGTAAGGCTAGACTTTATTATGATTCAATGAGTTATGAGGATCAAGATGCACTTT   15022
gb:MN994468|Organism:Severe      AATGGGGTAAGGCTAGACTTTATTATGATTCAATGAGTTATGAGGATCAAGATGCACTTT   15022
gb:MT072688|Organism:Severe      AATGGGGTAAGGCTAGACTTTATTATGATTCAATGAGTTATGAGGATCAAGATGCACTTT   15007
gb:MN996527|Organism:Severe      AATGGGGTAAGGCTAGACTTTATTATGATTCAATGAGTTATGAGGATCAAGATGCACTTT   14989
gb:MT093631|Organism:Severe      AATGGGGTAAGGCTAGACTTTATTATGATTCAATGAGTTATGAGGATCAAGATGCACTTT   15060
gb:MT106053|Organism:Severe      AATGGGGTAAGGCTAGACTTTATTATGATTCAATGAGTTATGAGGATCAAGATGCACTTT   15022
gb:MT019533|Organism:Severe      AATGGGGTAAGGCTAGACTTTATTATGATTCAATGAGTTATGAGGATCAAGATGCACTTT   15022
gb:MT019531|Organism:Severe      AATGGGGTAAGGCTAGACTTTATTATGATTCAATGAGTTATGAGGATCAAGATGCACTTT   15022
gb:MN996528|Organism:Severe      AATGGGGTAAGGCTAGACTTTATTATGATTCAATGAGTTATGAGGATCAAGATGCACTTT   15022
gb:MN996530|Organism:Severe      AATGGGGTAAGGCTAGACTTTATTATGATTCAATGAGTTATGAGGATCAAGATGCACTTT   15008
gb:MN908947|Organism:Severe      AATGGGGTAAGGCTAGACTTTATTATGATTCAATGAGTTATGAGGATCAAGATGCACTTT   15022
gb:MT019532|Organism:Severe      AATGGGGTAAGGCTAGACTTTATTATGATTCAATGAGTTATGAGGATCAAGATGCACTTT   15022
                                 ************************************************************

gb:MT020781|Organism:Severe      TCGCATATACAAAACGTAATGTCATCCCTACTATAACTCAAATGAATCTTAAGTATGCCA   15070
gb:MT007544|Organism:Severe      TCGCATATACAAAACGTAATGTCATCCCTACTATAACTCAAATGAATCTTAAGTATGCCA   15082
gb:MN994467|Organism:Severe      TCGCATATACAAAACGTAATGTCATCCCTACTATAACTCAAATGAATCTTAAGTATGCCA   15082
gb:MT044257|Organism:Severe      TCGCATATACAAAACGTAATGTCATCCCTACTATAACTCAAATGAATCTTAAGTATGCCA   15082
gb:MT106054|Organism:Severe      TCGCATATACAAAACGTAATGTCATCCCTACTATAACTCAAATGAATCTTAAGTATGCCA   15082
gb:MT049951|Organism:Severe      TCGCATATACAAAACGTAATGTCATCCCTACTATAACTCAAATGAATCTTAAGTATGCCA   15082
gb:MN975262|Organism:Severe      TCGCATATACAAAACGTAATGTCATCCCTACTATAACTCAAATGAATCTTAAGTATGCCA   15082
gb:MT106052|Organism:Severe      TCGCATATACAAAACGTAATGTCATCCCTACTATAACTCAAATGAATCTTAAGTATGCCA   15082
gb:LC522975|Organism:Severe      TCGCATATACAAAACGTAATGTCATCCCTACTATAACTCAAATGAATCTTAAGTATGCCA   15079
gb:LC522973|Organism:Severe      TCGCATATACAAAACGTAATGTCATCCCTACTATAACTCAAATGAATCTTAAGTATGCCA   15079
gb:LC522974|Organism:Severe      TCGCATATACAAAACGTAATGTCATCCCTACTATAACTCAAATGAATCTTAAGTATGCCA   15079
gb:MN985325|Organism:Severe      TCGCATATACAAAACGTAATGTCATCCCTACTATAACTCAAATGAATCTTAAGTATGCCA   15082
gb:MT020881|Organism:Severe      TCGCATATACAAAACGTAATGTCATCCCTACTATAACTCAAATGAATCTTAAGTATGCCA   15082
gb:MT020880|Organism:Severe      TCGCATATACAAAACGTAATGTCATCCCTACTATAACTCAAATGAATCTTAAGTATGCCA   15082
gb:MT066175|Organism:Severe      TCGCATATACAAAACGTAATGTCATCCCTACTATAACTCAAATGAATCTTAAGTATGCCA   15082
gb:MN997409|Organism:Severe      TCGCATATACAAAACGTAATGTCATCCCTACTATAACTCAAATGAATCTTAAGTATGCCA   15082
gb:MN938384|Organism:Severe      TCGCATATACAAAACGTAATGTCATCCCTACTATAACTCAAATGAATCTTAAGTATGCCA   15050
gb:MT044258|Organism:Severe      TCGCATATACAAAACGTAATGTCATCCCTACTATAACTCAAATGAATCTTAAGTATGCCA   15058
gb:MT039890|Organism:Severe      TCGCATATACAAAACGTAATGTCATCCCTACTATAACTCAAATGAATCTTAAGTATGCCA   15082
gb:MN988713|Organism:Severe      TCGCATATACAAAACGTAATGTCATCCCTACTATAACTCAAATGAATCTTAAGTATGCCA   15082
```

```
gb:LC521925|Organism:Severe      TCGCATATACAAAACGTAATGTCATCCCTACTATAACTCAAATGAATCTTAAGTATGCCA   15055
gb:MT093571|Organism:Severe      TCGCATATACAAAACGTAATGTCATCCCTACTATAACTCAAATGAATCTTAAGTATGCCA   15082
gb:MT039887|Organism:Severe      TCGCATATACAAAACGTAATGTCATCCCTACTATAACTCAAATGAATCTTAAGTATGCCA   15082
gb:MT019530|Organism:Severe      TCGCATATACAAAACGTAATGTCATCCCTACTATAACTCAAATGAATCTTAAGTATGCCA   15082
gb:MT039888|Organism:Severe      TCGCATATACAAAACGTAATGTCATCCCTACTATAACTCAAATGAATCTTAAGTATGCCA   15082
gb:LC522972|Organism:Severe      TCGCATATACAAAACGTAATGTCATCCCTACTATAACTCAAATGAATCTTAAGTATGCCA   15079
gb:MT027063|Organism:Severe      TCGCATATACAAAACGTAATGTCATCCCTACTATAACTCAAATGAATCTTAAGTATGCCA   15082
gb:MT027062|Organism:Severe      TCGCATATACAAAACGTAATGTCATCCCTACTATAACTCAAATGAATCTTAAGTATGCCA   15082
gb:MT019529|Organism:Severe      TCGCATATACAAAACGTAATGTCATCCCTACTATAACTCAAATGAATCTTAAGTATGCCA   15082
gb:MN996529|Organism:Severe      TCGCATATACAAAACGTAATGTCATCCCTACTATAACTCAAATGAATCTTAAGTATGCCA   15070
gb:MN996531|Organism:Severe      TCGCATATACAAAACGTAATGTCATCCCTACTATAACTCAAATGAATCTTAAGTATGCCA   15069
gb:MT066176|Organism:Severe      TCGCATATACAAAACGTAATGTCATCCCTACTATAACTCAAATGAATCTTAAGTATGCCA   15082
gb:MT027064|Organism:Severe      TCGCATATACAAAACGTAATGTCATCCCTACTATAACTCAAATGAATCTTAAGTATGCCA   15082
gb:MN994468|Organism:Severe      TCGCATATACAAAACGTAATGTCATCCCTACTATAACTCAAATGAATCTTAAGTATGCCA   15082
gb:MT072688|Organism:Severe      TCGCATATACAAAACGTAATGTCATCCCTACTATAACTCAAATGAATCTTAAGTATGCCA   15067
gb:MN996527|Organism:Severe      TCGCATATACAAAACGTAATGTCATCCCTACTATAACTCAAATGAATCTTAAGTATGCCA   15049
gb:MT093631|Organism:Severe      TCGCATATACAAAACGTAATGTCATCCCTACTATAACTCAAATGAATCTTAAGTATGCCA   15120
gb:MT106053|Organism:Severe      TCGCATATACAAAACGTAATGTCATCCCTACTATAACTCAAATGAATCTTAAGTATGCCA   15082
gb:MT019533|Organism:Severe      TCGCATATACAAAACGTAATGTCATCCCTACTATAACTCAAATGAATCTTAAGTATGCCA   15082
gb:MT019531|Organism:Severe      TCGCATATACAAAACGTAATGTCATCCCTACTATAACTCAAATGAATCTTAAGTATGCCA   15082
gb:MN996528|Organism:Severe      TCGCATATACAAAACGTAATGTCATCCCTACTATAACTCAAATGAATCTTAAGTATGCCA   15082
gb:MN996530|Organism:Severe      TCGCATATACAAAACGTAATGTCATCCCTACTATAACTCAAATGAATCTTAAGTATGCCA   15068
gb:MN908947|Organism:Severe      TCGCATATACAAAACGTAATGTCATCCCTACTATAACTCAAATGAATCTTAAGTATGCCA   15082
gb:MT019532|Organism:Severe      TCGCATATACAAAACGTAATGTCATCCCTACTATAACTCAAATGAATCTTAAGTATGCCA   15082
                                 ************************************************************

gb:MT020781|Organism:Severe      TTAGTGCAAAGAATAGAGCTCGCACCGTAGCTGGTGTCTCTATCTGTAGTACTATGACCA   15130
gb:MT007544|Organism:Severe      TTAGTGCAAAGAATAGAGCTCGCACCGTAGCTGGTGTCTCTATCTGTAGTACTATGACCA   15142
gb:MN994467|Organism:Severe      TTAGTGCAAAGAATAGAGCTCGCACCGTAGCTGGTGTCTCTATCTGTAGTACTATGACCA   15142
gb:MT044257|Organism:Severe      TTAGTGCAAAGAATAGAGCTCGCACCGTAGCTGGTGTCTCTATCTGTAGTACTATGACCA   15142
gb:MT106054|Organism:Severe      TTAGTGCAAAGAATAGAGCTCGCACCGTAGCTGGTGTCTCTATCTGTAGTACTATGACCA   15142
gb:MT049951|Organism:Severe      TTAGTGCAAAGAATAGAGCTCGCACCGTAGCTGGTGTCTCTATCTGTAGTACTATGACCA   15142
gb:MN975262|Organism:Severe      TTAGTGCAAAGAATAGAGCTCGCACCGTAGCTGGTGTCTCTATCTGTAGTACTATGACCA   15142
gb:MT106052|Organism:Severe      TTAGTGCAAAGAATAGAGCTCGCACCGTAGCTGGTGTCTCTATCTGTAGTACTATGACCA   15142
gb:LC522975|Organism:Severe      TTAGTGCAAAGAATAGAGCTCGCACCGTAGCTGGTGTCTCTATCTGTAGTACTATGACCA   15139
gb:LC522973|Organism:Severe      TTAGTGCAAAGAATAGAGCTCGCACCGTAGCTGGTGTCTCTATCTGTAGTACTATGACCA   15139
gb:LC522974|Organism:Severe      TTAGTGCAAAGAATAGAGCTCGCACCGTAGCTGGTGTCTCTATCTGTAGTACTATGACCA   15139
gb:MN985325|Organism:Severe      TTAGTGCAAAGAATAGAGCTCGCACCGTAGCTGGTGTCTCTATCTGTAGTACTATGACCA   15142
gb:MT020881|Organism:Severe      TTAGTGCAAAGAATAGAGCTCGCACCGTAGCTGGTGTCTCTATCTGTAGTACTATGACCA   15142
gb:MT020880|Organism:Severe      TTAGTGCAAAGAATAGAGCTCGCACCGTAGCTGGTGTCTCTATCTGTAGTACTATGACCA   15142
gb:MT066175|Organism:Severe      TTAGTGCAAAGAATAGAGCTCGCACCGTAGCTGGTGTCTCTATCTGTAGTACTATGACCA   15142
gb:MN997409|Organism:Severe      TTAGTGCAAAGAATAGAGCTCGCACCGTAGCTGGTGTCTCTATCTGTAGTACTATGACCA   15142
gb:MN938384|Organism:Severe      TTAGTGCAAAGAATAGAGCTCGCACCGTAGCTGGTGTCTCTATCTGTAGTACTATGACCA   15110
gb:MT044258|Organism:Severe      TTAGTGCAAAGAATAGAGCTCGCACCGTAGCTGGTGTCTCTATCTGTAGTACTATGACCA   15118
gb:MT039890|Organism:Severe      TTAGTGCAAAGAATAGAGCTCGCACCGTAGCTGGTGTCTCTATCTGTAGTACTATGACCA   15142
gb:MN988713|Organism:Severe      TTAGTGCAAAGAATAGAGCTCGCACCGTAGCTGGTGTCTCTATCTGTAGTACTATGACCA   15142
gb:LC521925|Organism:Severe      TTAGTGCAAAGAATAGAGCTCGCACCGTAGCTGGTGTCTCTATCTGTAGTACTATGACCA   15115
gb:MT093571|Organism:Severe      TTAGTGCAAAGAATAGAGCTCGCACCGTAGCTGGTGTCTCTATCTGTAGTACTATGACCA   15142
gb:MT039887|Organism:Severe      TTAGTGCAAAGAATAGAGCTCGCACCGTAGCTGGTGTCTCTATCTGTAGTACTATGACCA   15142
gb:MT019530|Organism:Severe      TTAGTGCAAAGAATAGAGCTCGCACCGTAGCTGGTGTCTCTATCTGTAGTACTATGACCA   15142
gb:MT039888|Organism:Severe      TTAGTGCAAAGAATAGAGCTCGCACCGTAGCTGGTGTCTCTATCTGTAGTACTATGACCA   15142
gb:LC522972|Organism:Severe      TTAGTGCAAAGAATAGAGCTCGCACCGTAGCTGGTGTCTCTATCTGTAGTACTATGACCA   15139
gb:MT027063|Organism:Severe      TTAGTGCAAAGAATAGAGCTCGCACCGTAGCTGGTGTCTCTATCTGTAGTACTATGACCA   15142
gb:MT027062|Organism:Severe      TTAGTGCAAAGAATAGAGCTCGCACCGTAGCTGGTGTCTCTATCTGTAGTACTATGACCA   15142
gb:MT019529|Organism:Severe      TTAGTGCAAAGAATAGAGCTCGCACCGTAGCTGGTGTCTCTATCTGTAGTACTATGACCA   15142
gb:MN996529|Organism:Severe      TTAGTGCAAAGAATAGAGCTCGCACCGTAGCTGGTGTCTCTATCTGTAGTACTATGACCA   15130
gb:MN996531|Organism:Severe      TTAGTGCAAAGAATAGAGCTCGCACCGTAGCTGGTGTCTCTATCTGTAGTACTATGACCA   15129
gb:MT066176|Organism:Severe      TTAGTGCAAAGAATAGAGCTCGCACCGTAGCTGGTGTCTCTATCTGTAGTACTATGACCA   15142
gb:MT027064|Organism:Severe      TTAGTGCAAAGAATAGAGCTCGCACCGTAGCTGGTGTCTCTATCTGTAGTACTATGACCA   15142
gb:MN994468|Organism:Severe      TTAGTGCAAAGAATAGAGCTCGCACCGTAGCTGGTGTCTCTATCTGTAGTACTATGACCA   15142
gb:MT072688|Organism:Severe      TTAGTGCAAAGAATAGAGCTCGCACCGTAGCTGGTGTCTCTATCTGTAGTACTATGACCA   15127
gb:MN996527|Organism:Severe      TTAGTGCAAAGAATAGAGCTCGCACCGTAGCTGGTGTCTCTATCTGTAGTACTATGACCA   15109
gb:MT093631|Organism:Severe      TTAGTGCAAAGAATAGAGCTCGCACCGTAGCTGGTGTCTCTATCTGTAGTACTATGACCA   15180
gb:MT106053|Organism:Severe      TTAGTGCAAAGAATAGAGCTCGCACCGTAGCTGGTGTCTCTATCTGTAGTACTATGACCA   15142
gb:MT019533|Organism:Severe      TTAGTGCAAAGAATAGAGCTCGCACCGTAGCTGGTGTCTCTATCTGTAGTACTATGACCA   15142
```

```
gb:MT019531|Organism:Severe      TTAGTGCAAAGAATAGAGCTCGCACCGTAGCTGGTGTCTCTATCTGTAGTACTATGACCA   15142
gb:MN996528|Organism:Severe      TTAGTGCAAAGAATAGAGCTCGCACCGTAGCTGGTGTCTCTATCTGTAGTACTATGACCA   15142
gb:MN996530|Organism:Severe      TTAGTGCAAAGAATAGAGCTCGCACCGTAGCTGGTGTCTCTATCTGTAGTACTATGACCA   15128
gb:MN908947|Organism:Severe      TTAGTGCAAAGAATAGAGCTCGCACCGTAGCTGGTGTCTCTATCTGTAGTACTATGACCA   15142
gb:MT019532|Organism:Severe      TTAGTGCAAAGAATAGAGCTCGCACCGTAGCTGGTGTCTCTATCTGTAGTACTATGACCA   15142
                                 ************************************************************

gb:MT020781|Organism:Severe      ATAGACAGTTTCATCAAAAATTATTGAAATCAATAGCCGCCACTAGAGGAGCTACTGTAG   15190
gb:MT007544|Organism:Severe      ATAGACAGTTTCATCAAAAATTATTGAAATCAATAGCCGCCACTAGAGGAGCTACTGTAG   15202
gb:MN994467|Organism:Severe      ATAGACAGTTTCATCAAAAATTATTGAAATCAATAGCCGCCACTAGAGGAGCTACTGTAG   15202
gb:MT044257|Organism:Severe      ATAGACAGTTTCATCAAAAATTATTGAAATCAATAGCCGCCACTAGAGGAGCTACTGTAG   15202
gb:MT106054|Organism:Severe      ATAGACAGTTTCATCAAAAATTATTGAAATCAATAGCCGCCACTAGAGGAGCTACTGTAG   15202
gb:MT049951|Organism:Severe      ATAGACAGTTTCATCAAAAATTATTGAAATCAATAGCCGCCACTAGAGGAGCTACTGTAG   15202
gb:MN975262|Organism:Severe      ATAGACAGTTTCATCAAAAATTATTGAAATCAATAGCCGCCACTAGAGGAGCTACTGTAG   15202
gb:MT106052|Organism:Severe      ATAGACAGTTTCATCAAAAATTATTGAAATCAATAGCCGCCACTAGAGGAGCTACTGTAG   15202
gb:LC522975|Organism:Severe      ATAGACAGTTTCATCAAAAATTATTGAAATCAATAGCCGCCACTAGAGGAGCTACTGTAG   15199
gb:LC522973|Organism:Severe      ATAGACAGTTTCATCAAAAATTATTGAAATCAATAGCCGCCACTAGAGGAGCTACTGTAG   15199
gb:LC522974|Organism:Severe      ATAGACAGTTTCATCAAAAATTATTGAAATCAATAGCCGCCACTAGAGGAGCTACTGTAG   15199
gb:MN985325|Organism:Severe      ATAGACAGTTTCATCAAAAATTATTGAAATCAATAGCCGCCACTAGAGGAGCTACTGTAG   15202
gb:MT020881|Organism:Severe      ATAGACAGTTTCATCAAAAATTATTGAAATCAATAGCCGCCACTAGAGGAGCTACTGTAG   15202
gb:MT020880|Organism:Severe      ATAGACAGTTTCATCAAAAATTATTGAAATCAATAGCCGCCACTAGAGGAGCTACTGTAG   15202
gb:MT066175|Organism:Severe      ATAGACAGTTTCATCAAAAATTATTGAAATCAATAGCCGCCACTAGAGGAGCTACTGTAG   15202
gb:MN997409|Organism:Severe      ATAGACAGTTTCATCAAAAATTATTGAAATCAATAGCCGCCACTAGAGGAGCTACTGTAG   15202
gb:MN938384|Organism:Severe      ATAGACAGTTTCATCAAAAATTATTGAAATCAATAGCCGCCACTAGAGGAGCTACTGTAG   15170
gb:MT044258|Organism:Severe      ATAGACAGTTTCATCAAAAATTATTGAAATCAATAGCCGCCACTAGAGGAGCTACTGTAG   15178
gb:MT039890|Organism:Severe      ATAGACAGTTTCATCAAAAATTATTGAAATCAATAGCCGCCACTAGAGGAGCTACTGTAG   15202
gb:MN988713|Organism:Severe      ATAGACAGTTTCATCAAAAATTATTGAAATCAATAGCCGCCACTAGAGGAGCTACTGTAG   15202
gb:LC521925|Organism:Severe      ATAGACAGTTTCATCAAAAATTATTGAAATCAATAGCCGCCACTAGAGGAGCTACTGTAG   15175
gb:MT093571|Organism:Severe      ATAGACAGTTTCATCAAAAATTATTGAAATCAATAGCCGCCACTAGAGGAGCTACTGTAG   15202
gb:MT039887|Organism:Severe      ATAGACAGTTTCATCAAAAATTATTGAAATCAATAGCCGCCACTAGAGGAGCTACTGTAG   15202
gb:MT019530|Organism:Severe      ATAGACAGTTTCATCAAAAATTATTGAAATCAATAGCCGCCACTAGAGGAGCTACTGTAG   15202
gb:MT039888|Organism:Severe      ATAGACAGTTTCATCAAAAATTATTGAAATCAATAGCCGCCACTAGAGGAGCTACTGTAG   15202
gb:LC522972|Organism:Severe      ATAGACAGTTTCATCAAAAATTATTGAAATCAATAGCCGCCACTAGAGGAGCTACTGTAG   15199
gb:MT027063|Organism:Severe      ATAGACAGTTTCATCAAAAATTATTGAAATCAATAGCCGCCACTAGAGGAGCTACTGTAG   15202
gb:MT027062|Organism:Severe      ATAGACAGTTTCATCAAAAATTATTGAAATCAATAGCCGCCACTAGAGGAGCTACTGTAG   15202
gb:MT019529|Organism:Severe      ATAGACAGTTTCATCAAAAATTATTGAAATCAATAGCCGCCACTAGAGGAGCTACTGTAG   15202
gb:MN996529|Organism:Severe      ATAGACAGTTTCATCAAAAATTATTGAAATCAATAGCCGCCACTAGAGGAGCTACTGTAG   15190
gb:MN996531|Organism:Severe      ATAGACAGTTTCATCAAAAATTATTGAAATCAATAGCCGCCACTAGAGGAGCTACTGTAG   15189
gb:MT066176|Organism:Severe      ATAGACAGTTTCATCAAAAATTATTGAAATCAATAGCCGCCACTAGAGGAGCTACTGTAG   15202
gb:MT027064|Organism:Severe      ATAGACAGTTTCATCAAAAATTATTGAAATCAATAGCCGCCACTAGAGGAGCTACTGTAG   15202
gb:MN994468|Organism:Severe      ATAGACAGTTTCATCAAAAATTATTGAAATCAATAGCCGCCACTAGAGGAGCTACTGTAG   15202
gb:MT072688|Organism:Severe      ATAGACAGTTTCATCAAAAATTATTGAAATCAATAGCCGCCACTAGAGGAGCTACTGTAG   15187
gb:MN996527|Organism:Severe      ATAGACAGTTTCATCAAAAATTATTGAAATCAATAGCCGCCACTAGAGGAGCTACTGTAG   15169
gb:MT093631|Organism:Severe      ATAGACAGTTTCATCAAAAATTATTGAAATCAATAGCCGCCACTAGAGGAGCTACTGTAG   15240
gb:MT106053|Organism:Severe      ATAGACAGTTTCATCAAAAATTATTGAAATCAATAGCCGCCACTAGAGGAGCTACTGTAG   15202
gb:MT019533|Organism:Severe      ATAGACAGTTTCATCAAAAATTATTGAAATCAATAGCCGCCACTAGAGGAGCTACTGTAG   15202
gb:MT019531|Organism:Severe      ATAGACAGTTTCATCAAAAATTATTGAAATCAATAGCCGCCACTAGAGGAGCTACTGTAG   15202
gb:MN996528|Organism:Severe      ATAGACAGTTTCATCAAAAATTATTGAAATCAATAGCCGCCACTAGAGGAGCTACTGTAG   15202
gb:MN996530|Organism:Severe      ATAGACAGTTTCATCAAAAATTATTGAAATCAATAGCCGCCACTAGAGGAGCTACTGTAG   15188
gb:MN908947|Organism:Severe      ATAGACAGTTTCATCAAAAATTATTGAAATCAATAGCCGCCACTAGAGGAGCTACTGTAG   15202
gb:MT019532|Organism:Severe      ATAGACAGTTTCATCAAAAATTATTGAAATCAATAGCCGCCACTAGAGGAGCTACTGTAG   15202
                                 ************************************************************

gb:MT020781|Organism:Severe      TAATTGGAACAAGCAAATTCTATGGTGGTTGGCACAACATGTTAAAAACTGTTTATAGTG   15250
gb:MT007544|Organism:Severe      TAATTGGAACAAGCAAATTCTATGGTGGTTGGCACAACATGTTAAAAACTGTTTATAGTG   15262
gb:MN994467|Organism:Severe      TAATTGGAACAAGCAAATTCTATGGTGGTTGGCACAACATGTTAAAAACTGTTTATAGTG   15262
gb:MT044257|Organism:Severe      TAATTGGAACAAGCAAATTCTATGGTGGTTGGCACAACATGTTAAAAACTGTTTATAGTG   15262
gb:MT106054|Organism:Severe      TAATTGGAACAAGCAAATTCTATGGTGGTTGGCACAACATGTTAAAAACTGTTTATAGTG   15262
gb:MT049951|Organism:Severe      TAATTGGAACAAGCAAATTCTATGGTGGTTGGCACAACATGTTAAAAACTGTTTATAGTG   15262
gb:MN975262|Organism:Severe      TAATTGGAACAAGCAAATTCTATGGTGGTTGGCACAACATGTTAAAAACTGTTTATAGTG   15262
gb:MT106052|Organism:Severe      TAATTGGAACAAGCAAATTCTATGGTGGTTGGCACAACATGTTAAAAACTGTTTATAGTG   15262
gb:LC522975|Organism:Severe      TAATTGGAACAAGCAAATTCTATGGTGGTTGGCACAACATGTTAAAAACTGTTTATAGTG   15259
gb:LC522973|Organism:Severe      TAATTGGAACAAGCAAATTCTATGGTGGTTGGCACAACATGTTAAAAACTGTTTATAGTG   15259
gb:LC522974|Organism:Severe      TAATTGGAACAAGCAAATTCTATGGTGGTTGGCACAACATGTTAAAAACTGTTTATAGTG   15259
gb:MN985325|Organism:Severe      TAATTGGAACAAGCAAATTCTATGGTGGTTGGCACAACATGTTAAAAACTGTTTATAGTG   15262
```

```
gb:MT020881|Organism:Severe      TAATTGGAACAAGCAAATTCTATGGTGGTTGGCACAACATGTTAAAAACTGTTTATAGTG   15262
gb:MT020880|Organism:Severe      TAATTGGAACAAGCAAATTCTATGGTGGTTGGCACAACATGTTAAAAACTGTTTATAGTG   15262
gb:MT066175|Organism:Severe      TAATTGGAACAAGCAAATTCTATGGTGGTTGGCACAACATGTTAAAAACTGTTTATAGTG   15262
gb:MN997409|Organism:Severe      TAATTGGAACAAGCAAATTCTATGGTGGTTGGCACAACATGTTAAAAACTGTTTATAGTG   15262
gb:MN938384|Organism:Severe      TAATTGGAACAAGCAAATTCTATGGTGGTTGGCACAACATGTTAAAAACTGTTTATAGTG   15230
gb:MT044258|Organism:Severe      TAATTGGAACAAGCAAATTCTATGGTGGTTGGCACAACATGTTAAAAACTGTTTATAGTG   15238
gb:MT039890|Organism:Severe      TAATTGGAACAAGCAAATTCTATGGTGGTTGGCACAACATGTTAAAAACTGTTTATAGTG   15262
gb:MN988713|Organism:Severe      TAATTGGAACAAGCAAATTCTATGGTGGTTGGCACAACATGTTAAAAACTGTTTATAGTG   15262
gb:LC521925|Organism:Severe      TAATTGGAACAAGCAAATTCTATGGTGGTTGGCACAACATGTTAAAAACTGTTTATAGTG   15235
gb:MT093571|Organism:Severe      TAATTGGAACAAGCAAATTCTATGGTGGTTGGCACAACATGTTAAAAACTGTTTATAGTG   15262
gb:MT039887|Organism:Severe      TAATTGGAACAAGCAAATTCTATGGTGGTTGGCACAACATGTTAAAAACTGTTTATAGTG   15262
gb:MT019530|Organism:Severe      TAATTGGAACAAGCAAATTCTATGGTGGTTGGCACAACATGTTAAAAACTGTTTATAGTG   15262
gb:MT039888|Organism:Severe      TAATTGGAACAAGCAAATTCTATGGTGGTTGGCACAACATGTTAAAAACTGTTTATAGTG   15262
gb:LC522972|Organism:Severe      TAATTGGAACAAGCAAATTCTATGGTGGTTGGCACAACATGTTAAAAACTGTTTATAGTG   15259
gb:MT027063|Organism:Severe      TAATTGGAACAAGCAAATTCTATGGTGGTTGGCACAACATGTTAAAAACTGTTTATAGTG   15262
gb:MT027062|Organism:Severe      TAATTGGAACAAGCAAATTCTATGGTGGTTGGCACAACATGTTAAAAACTGTTTATAGTG   15262
gb:MT019529|Organism:Severe      TAATTGGAACAAGCAAATTCTATGGTGGTTGGCACAACATGTTAAAAACTGTTTATAGTG   15262
gb:MN996529|Organism:Severe      TAATTGGAACAAGCAAATTCTATGGTGGTTGGCACAACATGTTAAAAACTGTTTATAGTG   15250
gb:MN996531|Organism:Severe      TAATTGGAACAAGCAAATTCTATGGTGGTTGGCACAACATGTTAAAAACTGTTTATAGTG   15249
gb:MT066176|Organism:Severe      TAATTGGAACAAGCAAATTCTATGGTGGTTGGCACAACATGTTAAAAACTGTTTATAGTG   15262
gb:MT027064|Organism:Severe      TAATTGGAACAAGCAAATTCTATGGTGGTTGGCACAACATGTTAAAAACTGTTTATAGTG   15262
gb:MN994468|Organism:Severe      TAATTGGAACAAGCAAATTCTATGGTGGTTGGCACAACATGTTAAAAACTGTTTATAGTG   15262
gb:MT072688|Organism:Severe      TAATTGGAACAAGCAAATTCTATGGTGGTTGGCACAACATGTTAAAAACTGTTTATAGTG   15247
gb:MN996527|Organism:Severe      TAATTGGAACAAGCAAATTCTATGGTGGTTGGCACAACATGTTAAAAACTGTTTATAGTG   15229
gb:MT093631|Organism:Severe      TAATTGGAACAAGCAAATTCTATGGTGGTTGGCACAACATGTTAAAAACTGTTTATAGTG   15300
gb:MT106053|Organism:Severe      TAATTGGAACAAGCAAATTCTATGGTGGTTGGCACAACATGTTAAAAACTGTTTATAGTG   15262
gb:MT019533|Organism:Severe      TAATTGGAACAAGCAAATTCTATGGTGGTTGGCACAACATGTTAAAAACTGTTTATAGTG   15262
gb:MT019531|Organism:Severe      TAATTGGAACAAGCAAATTCTATGGTGGTTGGCACAACATGTTAAAAACTGTTTATAGTG   15262
gb:MN996528|Organism:Severe      TAATTGGAACAAGCAAATTCTATGGTGGTTGGCACAACATGTTAAAAACTGTTTATAGTG   15262
gb:MN996530|Organism:Severe      TAATTGGAACAAGCAAATTCTATGGTGGTTGGCACAACATGTTAAAAACTGTTTATAGTG   15248
gb:MN908947|Organism:Severe      TAATTGGAACAAGCAAATTCTATGGTGGTTGGCACAACATGTTAAAAACTGTTTATAGTG   15262
gb:MT019532|Organism:Severe      TAATTGGAACAAGCAAATTCTATGGTGGTTGGCACAACATGTTAAAAACTGTTTATAGTG   15262
                                 ************************************************************

gb:MT020781|Organism:Severe      ATGTAGAAAACCCTCACCTTATGGGTTGGGATTATCCTAAATGTGATAGAGCCATGCCTA   15310
gb:MT007544|Organism:Severe      ATGTAGAAAACCCTCACCTTATGGGTTGGGATTATCCTAAATGTGATAGAGCCATGCCTA   15322
gb:MN994467|Organism:Severe      ATGTAGAAAACCCTCACCTTATGGGTTGGGATTATCCTAAATGTGATAGAGCCATGCCTA   15322
gb:MT044257|Organism:Severe      ATGTAGAAAACCCTCACCTTATGGGTTGGGATTATCCTAAATGTGATAGAGCCATGCCTA   15322
gb:MT106054|Organism:Severe      ATGTAGAAAACCCTCACCTTATGGGTTGGGATTATCCTAAATGTGATAGAGCCATGCCTA   15322
gb:MT049951|Organism:Severe      ATGTAGAAAACCCTCACCTTATGGGTTGGGATTATCCTAAATGTGATAGAGCCATGCCTA   15322
gb:MN975262|Organism:Severe      ATGTAGAAAACCCTCACCTTATGGGTTGGGATTATCCTAAATGTGATAGAGCCATGCCTA   15322
gb:MT106052|Organism:Severe      ATGTAGAAAACCCTCACCTTATGGGTTGGGATTATCCTAAATGTGATAGAGCCATGCCTA   15322
gb:LC522975|Organism:Severe      ATGTAGAAAACCCTCACCTTATGGGTTGGGATTATCCTAAATGTGATAGAGCCATGCCTA   15319
gb:LC522973|Organism:Severe      ATGTAGAAAACCCTCACCTTATGGGTTGGGATTATCCTAAATGTGATAGAGCCATGCCTA   15319
gb:LC522974|Organism:Severe      ATGTAGAAAACCCTCACCTTATGGGTTGGGATTATCCTAAATGTGATAGAGCCATGCCTA   15319
gb:MN985325|Organism:Severe      ATGTAGAAAACCCTCACCTTATGGGTTGGGATTATCCTAAATGTGATAGAGCCATGCCTA   15322
gb:MT020881|Organism:Severe      ATGTAGAAAACCCTCACCTTATGGGTTGGGATTATCCTAAATGTGATAGAGCCATGCCTA   15322
gb:MT020880|Organism:Severe      ATGTAGAAAACCCTCACCTTATGGGTTGGGATTATCCTAAATGTGATAGAGCCATGCCTA   15322
gb:MT066175|Organism:Severe      ATGTAGAAAACCCTCACCTTATGGGTTGGGATTATCCTAAATGTGATAGAGCCATGCCTA   15322
gb:MN997409|Organism:Severe      ATGTAGAAAACCCTCACCTTATGGGTTGGGATTATCCTAAATGTGATAGAGCCATGCCTA   15322
gb:MN938384|Organism:Severe      ATGTAGAAAACCCTCACCTTATGGGTTGGGATTATCCTAAATGTGATAGAGCCATGCCTA   15290
gb:MT044258|Organism:Severe      ATGTAGAAAACCCTCACCTTATGGGTTGGGATTATCCTAAATGTGATAGAGCCATGCCTA   15298
gb:MT039890|Organism:Severe      ATGTAGAAAACCCTCACCTTATGGGTTGGGATTATCCTAAATGTGATAGAGCCATGCCTA   15322
gb:MN988713|Organism:Severe      ATGTAGAAAACCCTCACCTTATGGGTTGGGATTATCCTAAATGTGATAGAGCCATGCCTA   15322
gb:LC521925|Organism:Severe      ATGTAGAAAACCCTCACCTTATGGGTTGGGATTATCCTAAATGTGATAGAGCCATGCCTA   15295
gb:MT093571|Organism:Severe      ATGTAGAAAACCCTCACCTTATGGGTTGGGATTATCCTAAATGTGATAGAGCCATGCCTA   15322
gb:MT039887|Organism:Severe      ATGTAGAAAACCCTCACCTTATGGGTTGGGATTATCCTAAATGTGATAGAGCCATGCCTA   15322
gb:MT019530|Organism:Severe      ATGTAGAAAACCCTCACCTTATGGGTTGGGATTATCCTAAATGTGATAGAGCCATGCCTA   15322
gb:MT039888|Organism:Severe      ATGTAGAAAACCCTCACCTTATGGGTTGGGATTATCCTAAATGTGATAGAGCCATGCCTA   15322
gb:LC522972|Organism:Severe      ATGTAGAAAACCCTCACCTTATGGGTTGGGATTATCCTAAATGTGATAGAGCCATGCCTA   15319
gb:MT027063|Organism:Severe      ATGTAGAAAACCCTCACCTTATGGGTTGGGATTATCCTAAATGTGATAGAGCCATGCCTA   15322
gb:MT027062|Organism:Severe      ATGTAGAAAACCCTCACCTTATGGGTTGGGATTATCCTAAATGTGATAGAGCCATGCCTA   15322
gb:MT019529|Organism:Severe      ATGTAGAAAACCCTCACCTTATGGGTTGGGATTATCCTAAATGTGATAGAGCCATGCCTA   15322
gb:MN996529|Organism:Severe      ATGTAGAAAACCCTCACCTTATGGGTTGGGATTATCCTAAATGTGATAGAGCCATGCCTA   15310
gb:MN996531|Organism:Severe      ATGTAGAAAACCCTCACCTTATGGGTTGGGATTATCCTAAATGTGATAGAGCCATGCCTA   15309
```

```
gb:MT066176|Organism:Severe      ATGTAGAAAACCCTCACCTTATGGGTTGGGATTATCCTAAATGTGATAGAGCCATGCCTA   15322
gb:MT027064|Organism:Severe      ATGTAGAAAACCCTCACCTTATGGGTTGGGATTATCCTAAATGTGATAGAGCCATGCCTA   15322
gb:MN994468|Organism:Severe      ATGTAGAAAACCCTCACCTTATGGGTTGGGATTATCCTAAATGTGATAGAGCCATGCCTA   15322
gb:MT072688|Organism:Severe      ATGTAGAAAACCCTCACCTTATGGGTTGGGATTATCCTAAATGTGATAGAGCCATGCCTA   15307
gb:MN996527|Organism:Severe      ATGTAGAAAACCCTCACCTTATGGGTTGGGATTATCCTAAATGTGATAGAGCCATGCCTA   15289
gb:MT093631|Organism:Severe      ATGTAGAAAACCCTCACCTTATGGGTTGGGATTATCCTAAATGTGATAGAGCCATGCCTA   15360
gb:MT106053|Organism:Severe      ATGTAGAAAACCCTCACCTTATGGGTTGGGATTATCCTAAATGTGATAGAGCCATGCCTA   15322
gb:MT019533|Organism:Severe      ATGTAGAAAACCCTCACCTTATGGGTTGGGATTATCCTAAATGTGATAGAGCCATGCCTA   15322
gb:MT019531|Organism:Severe      ATGTAGAAAACCCTCACCTTATGGGTTGGGATTATCCTAAATGTGATAGAGCCATGCCTA   15322
gb:MN996528|Organism:Severe      ATGTAGAAAACCCTCACCTTATGGGTTGGGATTATCCTAAATGTGATAGAGCCATGCCTA   15322
gb:MN996530|Organism:Severe      ATGTAGAAAACCCTCACCTTATGGGTTGGGATTATCCTAAATGTGATAGAGCCATGCCTA   15308
gb:MN908947|Organism:Severe      ATGTAGAAAACCCTCACCTTATGGGTTGGGATTATCCTAAATGTGATAGAGCCATGCCTA   15322
gb:MT019532|Organism:Severe      ATGTAGAAAACCCTCACCTTATGGGTTGGGATTATCCTAAATGTGATAGAGCCATGCCTA   15322
                                 ************************************************************

gb:MT020781|Organism:Severe      ACATGCTTAGAATTATGGCCTCACTTGTTCTTGCTCGCAAACATACAACGTGTTGTAGCT   15370
gb:MT007544|Organism:Severe      ACATGCTTAGAATTATGGCCTCACTTGTTCTTGCTCGCAAACATACAACGTGTTGTAGCT   15382
gb:MN994467|Organism:Severe      ACATGCTTAGAATTATGGCCTCACTTGTTCTTGCTCGCAAACATACAACGTGTTGTAGCT   15382
gb:MT044257|Organism:Severe      ACATGCTTAGAATTATGGCCTCACTTGTTCTTGCTCGCAAACATACAACGTGTTGTAGCT   15382
gb:MT106054|Organism:Severe      ACATGCTTAGAATTATGGCCTCACTTGTTCTTGCTCGCAAACATACAACGTGTTGTAGCT   15382
gb:MT049951|Organism:Severe      ACATGCTTAGAATTATGGCCTCACTTGTTCTTGCTCGCAAACATACAACGTGTTGTAGCT   15382
gb:MN975262|Organism:Severe      ACATGCTTAGAATTATGGCCTCACTTGTTCTTGCTCGCAAACATACAACGTGTTGTAGCT   15382
gb:MT106052|Organism:Severe      ACATGCTTAGAATTATGGCCTCACTTGTTCTTGCTCGCAAACATACAACGTGTTGTAGCT   15382
gb:LC522975|Organism:Severe      ACATGCTTAGAATTATGGCCTCACTTGTTCTTGCTCGCAAACATACAACGTGTTGTAGCT   15379
gb:LC522973|Organism:Severe      ACATGCTTAGAATTATGGCCTCACTTGTTCTTGCTCGCAAACATACAACGTGTTGTAGCT   15379
gb:LC522974|Organism:Severe      ACATGCTTAGAATTATGGCCTCACTTGTTCTTGCTCGCAAACATACAACGTGTTGTAGCT   15379
gb:MN985325|Organism:Severe      ACATGCTTAGAATTATGGCCTCACTTGTTCTTGCTCGCAAACATACAACGTGTTGTAGCT   15382
gb:MT020881|Organism:Severe      ACATGCTTAGAATTATGGCCTCACTTGTTCTTGCTCGCAAACATACAACGTGTTGTAGCT   15382
gb:MT020880|Organism:Severe      ACATGCTTAGAATTATGGCCTCACTTGTTCTTGCTCGCAAACATACAACGTGTTGTAGCT   15382
gb:MT066175|Organism:Severe      ACATGCTTAGAATTATGGCCTCACTTGTTCTTGCTCGCAAACATACAACGTGTTGTAGCT   15382
gb:MN997409|Organism:Severe      ACATGCTTAGAATTATGGCCTCACTTGTTCTTGCTCGCAAACATACAACGTGTTGTAGCT   15382
gb:MN938384|Organism:Severe      ACATGCTTAGAATTATGGCCTCACTTGTTCTTGCTCGCAAACATACAACGTGTTGTAGCT   15350
gb:MT044258|Organism:Severe      ACATGCTTAGAATTATGGCCTCACTTGTTCTTGCTCGCAAACATACAACGTGTTGTAGCT   15358
gb:MT039890|Organism:Severe      ACATGCTTAGAATTATGGCCTCACTTGTTCTTGCTCGCAAACATACAACGTGTTGTAGCT   15382
gb:MN988713|Organism:Severe      ACATGCTTAGAATTATGGCCTCACTTGTTCTTGCTCGCAAACATACAACGTGTTGTAGCT   15382
gb:LC521925|Organism:Severe      ACATGCTTAGAATTATGGCCTCACTTGTTCTTGCTCGCAAACATACAACGTGTTGTAGCT   15355
gb:MT093571|Organism:Severe      ACATGCTTAGAATTATGGCCTCACTTGTTCTTGCTCGCAAACATACAACGTGTTGTAGCT   15382
gb:MT039887|Organism:Severe      ACATGCTTAGAATTATGGCCTCACTTGTTCTTGCTCGCAAACATACAACGTGTTGTAGCT   15382
gb:MT019530|Organism:Severe      ACATGCTTAGAATTATGGCCTCACTTGTTCTTGCTCGCAAACATACAACGTGTTGTAGCT   15382
gb:MT039888|Organism:Severe      ACATGCTTAGAATTATGGCCTCACTTGTTCTTGCTCGCAAACATACAACGTGTTGTAGCT   15382
gb:LC522972|Organism:Severe      ATATGCTTAGAATTATGGCCTCACTTGTTCTTGCTCGCAAACATACAACGTGTTGTAGCT   15379
gb:MT027063|Organism:Severe      ACATGCTTAGAATTATGGCCTCACTTGTTCTTGCTCGCAAACATACAACGTGTTGTAGCT   15382
gb:MT027062|Organism:Severe      ACATGCTTAGAATTATGGCCTCACTTGTTCTTGCTCGCAAACATACAACGTGTTGTAGCT   15382
gb:MT019529|Organism:Severe      ACATGCTTAGAATTATGGCCTCACTTGTTCTTGCTCGCAAACATACAACGTGTTGTAGCT   15382
gb:MN996529|Organism:Severe      ACATGCTTAGAATTATGGCCTCACTTGTTCTTGCTCGCAAACATACAACGTGTTGTAGCT   15370
gb:MN996531|Organism:Severe      ACATGCTTAGAATTATGGCCTCACTTGTTCTTGCTCGCAAACATACAACGTGTTGTAGCT   15369
gb:MT066176|Organism:Severe      ACATGCTTAGAATTATGGCCTCACTTGTTCTTGCTCGCAAACATACAACGTGTTGTAGCT   15382
gb:MT027064|Organism:Severe      ACATGCTTAGAATTATGGCCTCACTTGTTCTTGCTCGCAAACATACAACGTGTTGTAGCT   15382
gb:MN994468|Organism:Severe      ACATGCTTAGAATTATGGCCTCACTTGTTCTTGCTCGCAAACATACAACGTGTTGTAGCT   15382
gb:MT072688|Organism:Severe      ACATGCTTAGAATTATGGCCTCACTTGTTCTTGCTCGCAAACATACAACGTGTTGTAGCT   15367
gb:MN996527|Organism:Severe      ACATGCTTAGAATTATGGCCTCACTTGTTCTTGCTCGCAAACATACAACGTGTTGTAGCT   15349
gb:MT093631|Organism:Severe      ACATGCTTAGAATTATGGCCTCACTTGTTCTTGCTCGCAAACATACAACGTGTTGTAGCT   15420
gb:MT106053|Organism:Severe      ACATGCTTAGAATTATGGCCTCACTTGTTCTTGCTCGCAAACATACAACGTGTTGTAGCT   15382
gb:MT019533|Organism:Severe      ACATGCTTAGAATTATGGCCTCACTTGTTCTTGCTCGCAAACATACAACGTGTTGTAGCT   15382
gb:MT019531|Organism:Severe      ACATGCTTAGAATTATGGCCTCACTTGTTCTTGCTCGCAAACATACAACGTGTTGTAGCT   15382
gb:MN996528|Organism:Severe      ACATGCTTAGAATTATGGCCTCACTTGTTCTTGCTCGCAAACATACAACGTGTTGTAGCT   15382
gb:MN996530|Organism:Severe      ACATGCTTAGAATTATGGCCTCACTTGTTCTTGCTCGCAAACATACAACGTGTTGTAGCT   15368
gb:MN908947|Organism:Severe      ACATGCTTAGAATTATGGCCTCACTTGTTCTTGCTCGCAAACATACAACGTGTTGTAGCT   15382
gb:MT019532|Organism:Severe      ACATGCTTAGAATTATGGCCTCACTTGTTCTTGCTCGCAAACATACAACGTGTTGTAGCT   15382
                                 * **********************************************************

gb:MT020781|Organism:Severe      TGTCACACCGTTTCTATAGATTAGCTAATGAGTGTGCTCAAGTATTGAGTGAAATGGTCA   15430
gb:MT007544|Organism:Severe      TGTCACACCGTTTCTATAGATTAGCTAATGAGTGTGCTCAAGTATTGAGTGAAATGGTCA   15442
gb:MN994467|Organism:Severe      TGTCACACCGTTTCTATAGATTAGCTAATGAGTGTGCTCAAGTATTGAGTGAAATGGTCA   15442
gb:MT044257|Organism:Severe      TGTCACACCGTTTCTATAGATTAGCTAATGAGTGTGCTCAAGTATTGAGTGAAATGGTCA   15442
```

```
gb:MT106054|Organism:Severe      TGTCACACCGTTTCTATAGATTAGCTAATGAGTGTGCTCAAGTATTGAGTGAAATGGTCA   15442
gb:MT049951|Organism:Severe      TGTCACACCGTTTCTATAGATTAGCTAATGAGTGTGCTCAAGTATTGAGTGAAATGGTCA   15442
gb:MN975262|Organism:Severe      TGTCACACCGTTTCTATAGATTAGCTAATGAGTGTGCTCAAGTATTGAGTGAAATGGTCA   15442
gb:MT106052|Organism:Severe      TGTCACACCGTTTCTATAGATTAGCTAATGAGTGTGCTCAAGTATTGAGTGAAATGGTCA   15442
gb:LC522975|Organism:Severe      TGTCACACCGTTTCTATAGATTAGCTAATGAGTGTGCTCAAGTATTGAGTGAAATGGTCA   15439
gb:LC522973|Organism:Severe      TGTCACACCGTTTCTATAGATTAGCTAATGAGTGTGCTCAAGTATTGAGTGAAATGGTCA   15439
gb:LC522974|Organism:Severe      TGTCACACCGTTTCTATAGATTAGCTAATGAGTGTGCTCAAGTATTGAGTGAAATGGTCA   15439
gb:MN985325|Organism:Severe      TGTCACACCGTTTCTATAGATTAGCTAATGAGTGTGCTCAAGTATTGAGTGAAATGGTCA   15442
gb:MT020881|Organism:Severe      TGTCACACCGTTTCTATAGATTAGCTAATGAGTGTGCTCAAGTATTGAGTGAAATGGTCA   15442
gb:MT020880|Organism:Severe      TGTCACACCGTTTCTATAGATTAGCTAATGAGTGTGCTCAAGTATTGAGTGAAATGGTCA   15442
gb:MT066175|Organism:Severe      TGTCACACCGTTTCTATAGATTAGCTAATGAGTGTGCTCAAGTATTGAGTGAAATGGTCA   15442
gb:MN997409|Organism:Severe      TGTCACACCGTTTCTATAGATTAGCTAATGAGTGTGCTCAAGTATTGAGTGAAATGGTCA   15442
gb:MN938384|Organism:Severe      TGTCACACCGTTTCTATAGATTAGCTAATGAGTGTGCTCAAGTATTGAGTGAAATGGTCA   15410
gb:MT044258|Organism:Severe      TGTCACACCGTTTCTATAGATTAGCTAATGAGTGTGCTCAAGTATTGAGTGAAATGGTCA   15418
gb:MT039890|Organism:Severe      TGTCACACCGTTTCTATAGATTAGCTAATGAGTGTGCTCAAGTATTGAGTGAAATGGTCA   15442
gb:MN988713|Organism:Severe      TGTCACACCGTTTCTATAGATTAGCTAATGAGTGTGCTCAAGTATTGAGTGAAATGGTCA   15442
gb:LC521925|Organism:Severe      TGTCACACCGTTTCTATAGATTAGCTAATGAGTGTGCTCAAGTATTGAGTGAAATGGTCA   15415
gb:MT093571|Organism:Severe      TGTCACACCGTTTCTATAGATTAGCTAATGAGTGTGCTCAAGTATTGAGTGAAATGGTCA   15442
gb:MT039887|Organism:Severe      TGTCACACCGTTTCTATAGATTAGCTAATGAGTGTGCTCAAGTATTGAGTGAAATGGTCA   15442
gb:MT019530|Organism:Severe      TGTCACACCGTTTCTATAGATTAGCTAATGAGTGTGCTCAAGTATTGAGTGAAATGGTCA   15442
gb:MT039888|Organism:Severe      TGTCACACCGTTTCTATAGATTAGCTAATGAGTGTGCTCAAGTATTGAGTGAAATGGTCA   15442
gb:LC522972|Organism:Severe      TGTCACACCGTTTCTATAGATTAGCTAATGAGTGTGCTCAAGTATTGAGTGAAATGGTCA   15439
gb:MT027063|Organism:Severe      TGTCACACCGTTTCTATAGATTAGCTAATGAGTGTGCTCAAGTATTGAGTGAAATGGTCA   15442
gb:MT027062|Organism:Severe      TGTCACACCGTTTCTATAGATTAGCTAATGAGTGTGCTCAAGTATTGAGTGAAATGGTCA   15442
gb:MT019529|Organism:Severe      TGTCACACCGTTTCTATAGATTAGCTAATGAGTGTGCTCAAGTATTGAGTGAAATGGTCA   15442
gb:MN996529|Organism:Severe      TGTCACACCGTTTCTATAGATTAGCTAATGAGTGTGCTCAAGTATTGAGTGAAATGGTCA   15430
gb:MN996531|Organism:Severe      TGTCACACCGTTTCTATAGATTAGCTAATGAGTGTGCTCAAGTATTGAGTGAAATGGTCA   15429
gb:MT066176|Organism:Severe      TGTCACACCGTTTCTATAGATTAGCTAATGAGTGTGCTCAAGTATTGAGTGAAATGGTCA   15442
gb:MT027064|Organism:Severe      TGTCACACCGTTTCTATAGATTAGCTAATGAGTGTGCTCAAGTATTGAGTGAAATGGTCA   15442
gb:MN994468|Organism:Severe      TGTCACACCGTTTCTATAGATTAGCTAATGAGTGTGCTCAAGTATTGAGTGAAATGGTCA   15442
gb:MT072688|Organism:Severe      TGTCACACCGTTTCTATAGATTAGCTAATGAGTGTGCTCAAGTATTGAGTGAAATGGTCA   15427
gb:MN996527|Organism:Severe      TGTCACACCGTTTCTATAGATTAGCTAATGAGTGTGCTCAAGTATTGAGTGAAATGGTCA   15409
gb:MT093631|Organism:Severe      TGTCACACCGTTTCTATAGATTAGCTAATGAGTGTGCTCAAGTATTGAGTGAAATGGTCA   15480
gb:MT106053|Organism:Severe      TGTCACACCGTTTCTATAGATTAGCTAATGAGTGTGCTCAAGTATTGAGTGAAATGGTCA   15442
gb:MT019533|Organism:Severe      TGTCACACCGTTTCTATAGATTAGCTAATGAGTGTGCTCAAGTATTGAGTGAAATGGTCA   15442
gb:MT019531|Organism:Severe      TGTCACACCGTTTCTATAGATTAGCTAATGAGTGTGCTCAAGTATTGAGTGAAATGGTCA   15442
gb:MN996528|Organism:Severe      TGTCACACCGTTTCTATAGATTAGCTAATGAGTGTGCTCAAGTATTGAGTGAAATGGTCA   15442
gb:MN996530|Organism:Severe      TGTCACACCGTTTCTATAGATTAGCTAATGAGTGTGCTCAAGTATTGAGTGAAATGGTCA   15428
gb:MN908947|Organism:Severe      TGTCACACCGTTTCTATAGATTAGCTAATGAGTGTGCTCAAGTATTGAGTGAAATGGTCA   15442
gb:MT019532|Organism:Severe      TGTCACACCGTTTCTATAGATTAGCTAATGAGTGTGCTCAAGTATTGAGTGAAATGGTCA   15442
                                 ************************************************************

gb:MT020781|Organism:Severe      TGTGTGGCGGTTCACTATATGTTAAACCAGGTGGAACCTCATCAGGAGATGCCACAACTG   15490
gb:MT007544|Organism:Severe      TGTGTGGCGGTTCACTATATGTTAAACCAGGTGGAACCTCATCAGGAGATGCCACAACTG   15502
gb:MN994467|Organism:Severe      TGTGTGGCGGTTCACTATATGTTAAACCAGGTGGAACCTCATCAGGAGATGCCACAACTG   15502
gb:MT044257|Organism:Severe      TGTGTGGCGGTTCACTATATGTTAAACCAGGTGGAACCTCATCAGGAGATGCCACAACTG   15502
gb:MT106054|Organism:Severe      TGTGTGGCGGTTCACTATATGTTAAACCAGGTGGAACCTCATCAGGAGATGCCACAACTG   15502
gb:MT049951|Organism:Severe      TGTGTGGCGGTTCACTATATGTTAAACCAGGTGGAACCTCATCAGGAGATGCCACAACTG   15502
gb:MN975262|Organism:Severe      TGTGTGGCGGTTCACTATATGTTAAACCAGGTGGAACCTCATCAGGAGATGCCACAACTG   15502
gb:MT106052|Organism:Severe      TGTGTGGCGGTTCACTATATGTTAAACCAGGTGGAACCTCATCAGGAGATGCCACAACTG   15502
gb:LC522975|Organism:Severe      TGTGTGGCGGTTCACTATATGTTAAACCAGGTGGAACCTCATCAGGAGATGCCACAACTG   15499
gb:LC522973|Organism:Severe      TGTGTGGCGGTTCACTATATGTTAAACCAGGTGGAACCTCATCAGGAGATGCCACAACTG   15499
gb:LC522974|Organism:Severe      TGTGTGGCGGTTCACTATATGTTAAACCAGGTGGAACCTCATCAGGAGATGCCACAACTG   15499
gb:MN985325|Organism:Severe      TGTGTGGCGGTTCACTATATGTTAAACCAGGTGGAACCTCATCAGGAGATGCCACAACTG   15502
gb:MT020881|Organism:Severe      TGTGTGGCGGTTCACTATATGTTAAACCAGGTGGAACCTCATCAGGAGATGCCACAACTG   15502
gb:MT020880|Organism:Severe      TGTGTGGCGGTTCACTATATGTTAAACCAGGTGGAACCTCATCAGGAGATGCCACAACTG   15502
gb:MT066175|Organism:Severe      TGTGTGGCGGTTCACTATATGTTAAACCAGGTGGAACCTCATCAGGAGATGCCACAACTG   15502
gb:MN997409|Organism:Severe      TGTGTGGCGGTTCACTATATGTTAAACCAGGTGGAACCTCATCAGGAGATGCCACAACTG   15502
gb:MN938384|Organism:Severe      TGTGTGGCGGTTCACTATATGTTAAACCAGGTGGAACCTCATCAGGAGATGCCACAACTG   15470
gb:MT044258|Organism:Severe      TGTGTGGCGGTTCACTATATGTTAAACCAGGTGGAACCTCATCAGGAGATGCCACAACTG   15478
gb:MT039890|Organism:Severe      TGTGTGGCGGTTCACTATATGTTAAACCAGGTGGAACCTCATCAGGAGATGCCACAACTG   15502
gb:MN988713|Organism:Severe      TGTGTGGCGGTTCACTATATGTTAAACCAGGTGGAACCTCATCAGGAGATGCCACAACTG   15502
gb:LC521925|Organism:Severe      TGTGTGGCGGTTCACTATATGTTAAACCAGGTGGAACCTCATCAGGAGATGCCACAACTG   15475
gb:MT093571|Organism:Severe      TGTGTGGCGGTTCACTATATGTTAAACCAGGTGGAACCTCATCAGGAGATGCCACAACTG   15502
gb:MT039887|Organism:Severe      TGTGTGGCGGTTCACTATATGTTAAACCAGGTGGAACCTCATCAGGAGATGCCACAACTG   15502
```

```
gb:MT019530|Organism:Severe      TGTGTGGCGGTTCACTATATGTTAAACCAGGTGGAACCTCATCAGGAGATGCCACAACTG   15502
gb:MT039888|Organism:Severe      TGTGTGGCGGTTCACTATATGTTAAACCAGGTGGAACCTCATCAGGAGATGCCACAACTG   15502
gb:LC522972|Organism:Severe      TGTGTGGCGGTTCACTATATGTTAAACCAGGTGGAACCTCATCAGGAGATGCCACAACTG   15499
gb:MT027063|Organism:Severe      TGTGTGGCGGTTCACTATATGTTAAACCAGGTGGAACCTCATCAGGAGATGCCACAACTG   15502
gb:MT027062|Organism:Severe      TGTGTGGCGGTTCACTATATGTTAAACCAGGTGGAACCTCATCAGGAGATGCCACAACTG   15502
gb:MT019529|Organism:Severe      TGTGTGGCGGTTCACTATATGTTAAACCAGGTGGAACCTCATCAGGAGATGCCACAACTG   15502
gb:MN996529|Organism:Severe      TGTGTGGCGGTTCACTATATGTTAAACCAGGTGGAACCTCATCAGGAGATGCCACAACTG   15490
gb:MN996531|Organism:Severe      TGTGTGGCGGTTCACTATATGTTAAACCAGGTGGAACCTCATCAGGAGATGCCACAACTG   15489
gb:MT066176|Organism:Severe      TGTGTGGCGGTTCACTATATGTTAAACCAGGTGGAACCTCATCAGGAGATGCCACAACTG   15502
gb:MT027064|Organism:Severe      TGTGTGGCGGTTCACTATATGTTAAACCAGGTGGAACCTCATCAGGAGATGCCACAACTG   15502
gb:MN994468|Organism:Severe      TGTGTGGCGGTTCACTATATGTTAAACCAGGTGGAACCTCATCAGGAGATGCCACAACTG   15502
gb:MT072688|Organism:Severe      TGTGTGGCGGTTCACTATATGTTAAACCAGGTGGAACCTCATCAGGAGATGCCACAACTG   15487
gb:MN996527|Organism:Severe      TGTGTGGCGGTTCACTATATGTTAAACCAGGTGGAACCTCATCAGGAGATGCCACAACTG   15469
gb:MT093631|Organism:Severe      TGTGTGGCGGTTCACTATATGTTAAACCAGGTGGAACCTCATCAGGAGATGCCACAACTG   15540
gb:MT106053|Organism:Severe      TGTGTGGCGGTTCACTATATGTTAAACCAGGTGGAACCTCATCAGGAGATGCCACAACTG   15502
gb:MT019533|Organism:Severe      TGTGTGGCGGTTCACTATATGTTAAACCAGGTGGAACCTCATCAGGAGATGCCACAACTG   15502
gb:MT019531|Organism:Severe      TGTGTGGCGGTTCACTATATGTTAAACCAGGTGGAACCTCATCAGGAGATGCCACAACTG   15502
gb:MN996528|Organism:Severe      TGTGTGGCGGTTCACTATATGTTAAACCAGGTGGAACCTCATCAGGAGATGCCACAACTG   15502
gb:MN996530|Organism:Severe      TGTGTGGCGGTTCACTATATGTTAAACCAGGTGGAACCTCATCAGGAGATGCCACAACTG   15488
gb:MN908947|Organism:Severe      TGTGTGGCGGTTCACTATATGTTAAACCAGGTGGAACCTCATCAGGAGATGCCACAACTG   15502
gb:MT019532|Organism:Severe      TGTGTGGCGGTTCACTATATGTTAAACCAGGTGGAACCTCATCAGGAGATGCCACAACTG   15502
                                 ************************************************************

gb:MT020781|Organism:Severe      CTTATGCTAATAGTGTTTTTAACATTTGTCAAGCTGTCACGGCCAATGTTAATGCACTTT   15550
gb:MT007544|Organism:Severe      CTTATGCTAATAGTGTTTTTAACATTTGTCAAGCTGTCACGGCCAATGTTAATGCACTTT   15562
gb:MN994467|Organism:Severe      CTTATGCTAATAGTGTTTTTAACATTTGTCAAGCTGTCACGGCCAATGTTAATGCACTTT   15562
gb:MT044257|Organism:Severe      CTTATGCTAATAGTGTTTTTAACATTTGTCAAGCTGTCACGGCCAATGTTAATGCACTTT   15562
gb:MT106054|Organism:Severe      CTTATGCTAATAGTGTTTTTAACATTTGTCAAGCTGTCACGGCCAATGTTAATGCACTTT   15562
gb:MT049951|Organism:Severe      CTTATGCTAATAGTGTTTTTAACATTTGTCAAGCTGTCACGGCCAATGTTAATGCACTTT   15562
gb:MN975262|Organism:Severe      CTTATGCTAATAGTGTTTTTAACATTTGTCAAGCTGTCACGGCCAATGTTAATGCACTTT   15562
gb:MT106052|Organism:Severe      CTTATGCTAATAGTGTTTTTAACATTTGTCAAGCTGTCACGGCCAATGTTAATGCACTTT   15562
gb:LC522975|Organism:Severe      CTTATGCTAATAGTGTTTTTAACATTTGTCAAGCTGTCACGGCCAATGTTAATGCACTTT   15559
gb:LC522973|Organism:Severe      CTTATGCTAATAGTGTTTTTAACATTTGTCAAGCTGTCACGGCCAATGTTAATGCACTTT   15559
gb:LC522974|Organism:Severe      CTTATGCTAATAGTGTTTTTAACATTTGTCAAGCTGTCACGGCCAATGTTAATGCACTTT   15559
gb:MN985325|Organism:Severe      CTTATGCTAATAGTGTTTTTAACATTTGTCAAGCTGTCACGGCCAATGTTAATGCACTTT   15562
gb:MT020881|Organism:Severe      CTTATGCTAATAGTGTTTTTAACATTTGTCAAGCTGTCACGGCCAATGTTAATGCACTTT   15562
gb:MT020880|Organism:Severe      CTTATGCTAATAGTGTTTTTAACATTTGTCAAGCTGTCACGGCCAATGTTAATGCACTTT   15562
gb:MT066175|Organism:Severe      CTTATGCTAATAGTGTTTTTAACATTTGTCAAGCTGTCACGGCCAATGTTAATGCACTTT   15562
gb:MN997409|Organism:Severe      CTTATGCTAATAGTGTTTTTAACATTTGTCAAGCTGTCACGGCCAATGTTAATGCACTTT   15562
gb:MN938384|Organism:Severe      CTTATGCTAATAGTGTTTTTAACATTTGTCAAGCTGTCACGGCCAATGTTAATGCACTTT   15530
gb:MT044258|Organism:Severe      CTTATGCTAATAGTGTTTTTAACATTTGTCAAGCTGTCACGGCCAATGTTAATGCACTTT   15538
gb:MT039890|Organism:Severe      CTTATGCTAATAGTGTTTTTAACATTTGTCAAGCTGTCACGGCCAATGTTAATGCACTTT   15562
gb:MN988713|Organism:Severe      CTTATGCTAATAGTGTTTTTAACATTTGTCAAGCTGTCACGGCCAATGTTAATGCACTTT   15562
gb:LC521925|Organism:Severe      CTTATGCTAATAGTGTTTTTAACATTTGTCAAGCTGTCACGGCCAATGTTAATGCACTTT   15535
gb:MT093571|Organism:Severe      CTTATGCTAATAGTGTTTTTAACATTTGTCAAGCTGTCACGGCCAATGTTAATGCACTTT   15562
gb:MT039887|Organism:Severe      CTTATGCTAATAGTGTTTTTAACATTTGTCAAGCTGTCACGGCCAATGTTAATGCACTTT   15562
gb:MT019530|Organism:Severe      CTTATGCTAATAGTGTTTTTAACATTTGTCAAGCTGTCACGGCCAATGTTAATGCACTTT   15562
gb:MT039888|Organism:Severe      CTTATGCTAATAGTGTTTTTAACATTTGTCAAGCTGTCACGGCCAATGTTAATGCACTTT   15562
gb:LC522972|Organism:Severe      CTTATGCTAATAGTGTTTTTAACATTTGTCAAGCTGTCACGGCCAATGTTAATGCACTTT   15559
gb:MT027063|Organism:Severe      CTTATGCTAATAGTGTTTTTAACATTTGTCAAGCTGTCACGGCCAATGTTAATGCACTTT   15562
gb:MT027062|Organism:Severe      CTTATGCTAATAGTGTTTTTAACATTTGTCAAGCTGTCACGGCCAATGTTAATGCACTTT   15562
gb:MT019529|Organism:Severe      CTTATGCTAATAGTGTTTTTAACATTTGTCAAGCTGTCACGGCCAATGTTAATGCACTTT   15562
gb:MN996529|Organism:Severe      CTTATGCTAATAGTGTTTTTAACATTTGTCAAGCTGTCACGGCCAATGTTAATGCACTTT   15550
gb:MN996531|Organism:Severe      CTTATGCTAATAGTGTTTTTAACATTTGTCAAGCTGTCACGGCCAATGTTAATGCACTTT   15549
gb:MT066176|Organism:Severe      CTTATGCTAATAGTGTTTTTAACATTTGTCAAGCTGTCACGGCCAATGTTAATGCACTTT   15562
gb:MT027064|Organism:Severe      CTTATGCTAATAGTGTTTTTAACATTTGTCAAGCTGTCACGGCCAATGTTAATGCACTTT   15562
gb:MN994468|Organism:Severe      CTTATGCTAATAGTGTTTTTAACATTTGTCAAGCTGTCACGGCCAATGTTAATGCACTTT   15562
gb:MT072688|Organism:Severe      CTTATGCTAATAGTGTTTTTAACATTTGTCAAGCTGTCACGGCCAATGTTAATGCACTTT   15547
gb:MN996527|Organism:Severe      CTTATGCTAATAGTGTTTTTAACATTTGTCAAGCTGTCACGGCCAATGTTAATGCACTTT   15529
gb:MT093631|Organism:Severe      CTTATGCTAATAGTGTTTTTAACATTTGTCAAGCTGTCACGGCCAATGTTAATGCACTTT   15600
gb:MT106053|Organism:Severe      CTTATGCTAATAGTGTTTTTAACATTTGTCAAGCTGTCACGGCCAATGTTAATGCACTTT   15562
gb:MT019533|Organism:Severe      CTTATGCTAATAGTGTTTTTAACATTTGTCAAGCTGTCACGGCCAATGTTAATGCACTTT   15562
gb:MT019531|Organism:Severe      CTTATGCTAATAGTGTTTTTAACATTTGTCAAGCTGTCACGGCCAATGTTAATGCACTTT   15562
gb:MN996528|Organism:Severe      CTTATGCTAATAGTGTTTTTAACATTTGTCAAGCTGTCACGGCCAATGTTAATGCACTTT   15562
gb:MN996530|Organism:Severe      CTTATGCTAATAGTGTTTTTAACATTTGTCAAGCTGTCACGGCCAATGTTAATGCACTTT   15548
```

```
gb:MN908947|Organism:Severe      CTTATGCTAATAGTGTTTTTAACATTTGTCAAGCTGTCACGGCCAATGTTAATGCACTTT   15562
gb:MT019532|Organism:Severe      CTTATGCTAATAGTGTTTTTAACATTTGTCAAGCTGTCACGGCCAATGTTAATGCACTTT   15562
                                 ************************************************************

gb:MT020781|Organism:Severe      TATCTACTGATGGTAACAAAATTGCCGATAAGTATGTCCGCAATTTACAACACAGACTTT   15610
gb:MT007544|Organism:Severe      TATCTACTGATGGTAACAAAATTGCCGATAAGTATGTCCGCAATTTACAACACAGACTTT   15622
gb:MN994467|Organism:Severe      TATCTACTGATGGTAACAAAATTGCCGATAAGTATGTCCGCAATTTACAACACAGACTTT   15622
gb:MT044257|Organism:Severe      TATCTACTGATGGTAACAAAATTGCCGATAAGTATGTCCGCAATTTACAACACAGACTTT   15622
gb:MT106054|Organism:Severe      TATCTACTGATGGTAACAAAATTGCCGATAAGTATGTCCGCAATTTACAACACAGACTTT   15622
gb:MT049951|Organism:Severe      TATCTACTGATGGTAACAAAATTGCCGATAAGTATGTCCGCAATTTACAACACAGACTTT   15622
gb:MN975262|Organism:Severe      TATCTACTGATGGTAACAAAATTGCCGATAAGTATGTCCGCAATCTACAACACAGACTTT   15622
gb:MT106052|Organism:Severe      TATCTACTGATGGTAACAAAATTGCCGATAAGTATGTCCGCAATTTACAACACAGACTTT   15622
gb:LC522975|Organism:Severe      TATCTACTGATGGTAACAAAATTGCCGATAAGTATGTCCGCAATTTACAACACAGACTTT   15619
gb:LC522973|Organism:Severe      TATCTACTGATGGTAACAAAATTGCCGATAAGTATGTCCGCAATTTACAACACAGACTTT   15619
gb:LC522974|Organism:Severe      TATCTACTGATGGTAACAAAATTGCCGATAAGTATGTCCGCAATTTACAACACAGACTTT   15619
gb:MN985325|Organism:Severe      TATCTACTGATGGTAACAAAATTGCCGATAAGTATGTCCGCAATTTACAACACAGACTTT   15622
gb:MT020881|Organism:Severe      TATCTACTGATGGTAACAAAATTGCCGATAAGTATGTCCGCAATTTACAACACAGACTTT   15622
gb:MT020880|Organism:Severe      TATCTACTGATGGTAACAAAATTGCCGATAAGTATGTCCGCAATTTACAACACAGACTTT   15622
gb:MT066175|Organism:Severe      TATCTACTGATGGTAACAAAATTGCCGATAAGTATGTCCGCAATTTACAACACAGACTTT   15622
gb:MN997409|Organism:Severe      TATCTACTGATGGTAACAAAATTGCCGATAAGTATGTCCGCAATTTACAACACAGACTTT   15622
gb:MN938384|Organism:Severe      TATCTACTGATGGTAACAAAATTGCCGATAAGTATGTCCGCAATTTACAACACAGACTTT   15590
gb:MT044258|Organism:Severe      TATCTACTGATGGTAACAAAATTGCCGATAAGTATGTCCGCAATTTACAACACAGACTTT   15598
gb:MT039890|Organism:Severe      TATCTACTGATGGTAACAAAATTGCCGATAAGTACGTCCGCAATTTACAACACAGACTTT   15622
gb:MN988713|Organism:Severe      TATCTACTGATGGTAACAAAATTGCCGATAAGTATGTCCGCAATTTACAACACAGACTTT   15622
gb:LC521925|Organism:Severe      TATCTACTGATGGTAACAAAATTGCCGATAAGTATGTCCGCAATTTACAACACAGACTTT   15595
gb:MT093571|Organism:Severe      TATCTACTGATGGTAACAAAATTGCCGATAAGTATGTCCGCAATTTACAACACAGACTTT   15622
gb:MT039887|Organism:Severe      TATCTACTGATGGTAACAAAATTGCCGATAAGTATGTCCGCAATTTACAACACAGACTTT   15622
gb:MT019530|Organism:Severe      TATCTACTGATGGTAACAAAATTGCCGATAAGTATGTCCGCAATTTACAACACAGACTTT   15622
gb:MT039888|Organism:Severe      TATCTACTGATGGTAACAAAATTGCCGATAAGTATGTCCGCAATTTACAACACAGACTTT   15622
gb:LC522972|Organism:Severe      TATCTACTGATGGTAACAAAATTGCCGATAAGTATGTCCGCAATTTACAACACAGACTTT   15619
gb:MT027063|Organism:Severe      TATCTACTGATGGTAACAAAATTGCCGATAAGTATGTCCGCAATTTACAACACAGACTTT   15622
gb:MT027062|Organism:Severe      TATCTACTGATGGTAACAAAATTGCCGATAAGTATGTCCGCAATTTACAACACAGACTTT   15622
gb:MT019529|Organism:Severe      TATCTACTGATGGTAACAAAATTGCCGATAAGTATGTCCGCAATTTACAACACAGACTTT   15622
gb:MN996529|Organism:Severe      TATCTACTGATGGTAACAAAATTGCCGATAAGTATGTCCGCAATTTACAACACAGACTTT   15610
gb:MN996531|Organism:Severe      TATCTACTGATGGTAACAAAATTGCCGATAAGTATGTCCGCAATTTACAACACAGACTTT   15609
gb:MT066176|Organism:Severe      TATCTACTGATGGTAACAAAATTGCCGATAAGTATGTCCGCAATTTACAACACAGACTTT   15622
gb:MT027064|Organism:Severe      TATCTACTGATGGTAACAAAATTGCCGATAAGTATGTCCGCAATTTACAACACAGACTTT   15622
gb:MN994468|Organism:Severe      TATCTACTGATGGTAACAAAATTGCCGATAAGTATGTCCGCAATTTACAACACAGACTTT   15622
gb:MT072688|Organism:Severe      TATCTACTGATGGTAACAAAATTGCCGATAAGTATGTCCGCAATTTACAACACAGACTTT   15607
gb:MN996527|Organism:Severe      TATCTACTGATGGTAACAAAATTGCCGATAAGTATGTCCGCAATTTACAACACAGACTTT   15589
gb:MT093631|Organism:Severe      TATCTACTGATGGTAACAAAATTGCCGATAAGTATGTCCGCAATTTACAACACAGACTTT   15660
gb:MT106053|Organism:Severe      TATCTACTGATGGTAACAAAATTGCCGATAAGTATGTCCGCAATTTACAACACAGACTTT   15622
gb:MT019533|Organism:Severe      TATCTACTGATGGTAACAAAATTGCCGATAAGTATGTCCGCAATTTACAACACAGACTTT   15622
gb:MT019531|Organism:Severe      TATCTACTGATGGTAACAAAATTGCCGATAAGTATGTCCGCAATTTACAACACAGACTTT   15622
gb:MN996528|Organism:Severe      TATCTACTGATGGTAACAAAATTGCCGATAAGTATGTCCGCAATTTACAACACAGACTTT   15622
gb:MN996530|Organism:Severe      TATCTACTGATGGTAACAAAATTGCCGATAAGTATGTCCGCAATTTACAACACAGACTTT   15608
gb:MN908947|Organism:Severe      TATCTACTGATGGTAACAAAATTGCCGATAAGTATGTCCGCAATTTACAACACAGACTTT   15622
gb:MT019532|Organism:Severe      TATCTACTGATGGTAACAAAATTGCCGATAAGTATGTCCGCAATTTACAACACAGACTTT   15622
                                 ********************************** ******** **************

gb:MT020781|Organism:Severe      ATGAGTGTCTCTATAGAAATAGAGATGTTGACACAGACTTTGTGAATGAGTTTTACGCAT   15670
gb:MT007544|Organism:Severe      ATGAGTGTCTCTATAGAAATAGAGATGTTGACACAGACTTTGTGAATGAGTTTTACGCAT   15682
gb:MN994467|Organism:Severe      ATGAGTGTCTCTATAGAAATAGAGATGTTGACACAGACTTTGTGAATGAGTTTTACGCAT   15682
gb:MT044257|Organism:Severe      ATGAGTGTCTCTATAGAAATAGAGATGTTGACACAGACTTTGTGAATGAGTTTTACGCAT   15682
gb:MT106054|Organism:Severe      ATGAGTGTCTCTATAGAAATAGAGATGTTGACACAGACTTTGTGAATGAGTTTTACGCAT   15682
gb:MT049951|Organism:Severe      ATGAGTGTCTCTATAGAAATAGAGATGTTGACACAGACTTTGTGAATGAGTTTTACGCAT   15682
gb:MN975262|Organism:Severe      ATGAGTGTCTCTATAGAAATAGAGATGTTGACACAGACTTTGTGAATGAGTTTTACGCAT   15682
gb:MT106052|Organism:Severe      ATGAGTGTCTCTATAGAAATAGAGATGTTGACACAGACTTTGTGAATGAGTTTTACGCAT   15682
gb:LC522975|Organism:Severe      ATGAGTGTCTCTATAGAAATAGAGATGTTGACACAGACTTTGTGAATGAGTTTTACGCAT   15679
gb:LC522973|Organism:Severe      ATGAGTGTCTCTATAGAAATAGAGATGTTGACACAGACTTTGTGAATGAGTTTTACGCAT   15679
gb:LC522974|Organism:Severe      ATGAGTGTCTCTATAGAAATAGAGATGTTGACACAGACTTTGTGAATGAGTTTTACGCAT   15679
gb:MN985325|Organism:Severe      ATGAGTGTCTCTATAGAAATAGAGATGTTGACACAGACTTTGTGAATGAGTTTTACGCAT   15682
gb:MT020881|Organism:Severe      ATGAGTGTCTCTATAGAAATAGAGATGTTGACACAGACTTTGTGAATGAGTTTTACGCAT   15682
gb:MT020880|Organism:Severe      ATGAGTGTCTCTATAGAAATAGAGATGTTGACACAGACTTTGTGAATGAGTTTTACGCAT   15682
gb:MT066175|Organism:Severe      ATGAGTGTCTCTATAGAAATAGAGATGTTGACACAGACTTTGTGAATGAGTTTTACGCAT   15682
```

```
gb:MN997409|Organism:Severe      ATGAGTGTCTCTATAGAAATAGAGATGTTGACACAGACTTTGTGAATGAGTTTTACGCAT   15682
gb:MN938384|Organism:Severe      ATGAGTGTCTCTATAGAAATAGAGATGTTGACACAGACTTTGTGAATGAGTTTTACGCAT   15650
gb:MT044258|Organism:Severe      ATGAGTGTCTCTATAGAAATAGAGATGTTGACACAGACTTTGTGAATGAGTTTTACGCAT   15658
gb:MT039890|Organism:Severe      ATGAGTGTCTCTATAGAAATAGAGATGTTGACACAGACTTTGTGAATGAGTTTTACGCAT   15682
gb:MN988713|Organism:Severe      ATGAGTGTCTCTATAGAAATAGAGATGTTGACACAGACTTTGTGAATGAGTTTTACGCAT   15682
gb:LC521925|Organism:Severe      ATGAGTGTCTCTATAGAAATAGAGATGTTGACACAGACTTTGTGAATGAGTTTTACGCAT   15655
gb:MT093571|Organism:Severe      ATGAGTGTCTCTATAGAAATAGAGATGTTGACACAGACTTTGTGAATGAGTTTTACGCAT   15682
gb:MT039887|Organism:Severe      ATGAGTGTCTCTATAGAAATAGAGATGTTGACACAGACTTTGTGAATGAGTTTTACGCAT   15682
gb:MT019530|Organism:Severe      ATGAGTGTCTCTATAGAAATAGAGATGTTGACACAGACTTTGTGAATGAGTTTTACGCAT   15682
gb:MT039888|Organism:Severe      ATGAGTGTCTCTATAGAAATAGAGATGTTGACACAGACTTTGTGAATGAGTTTTACGCAT   15682
gb:LC522972|Organism:Severe      ATGAGTGTCTCTATAGAAATAGAGATGTTGACACAGACTTTGTGAATGAGTTTTACGCAT   15679
gb:MT027063|Organism:Severe      ATGAGTGTCTCTATAGAAATAGAGATGTTGACACAGACTTTGTGAATGAGTTTTACGCAT   15682
gb:MT027062|Organism:Severe      ATGAGTGTCTCTATAGAAATAGAGATGTTGACACAGACTTTGTGAATGAGTTTTACGCAT   15682
gb:MT019529|Organism:Severe      ATGAGTGTCTCTATAGAAATAGAGATGTTGACACAGACTTTGTGAATGAGTTTTACGCAT   15682
gb:MN996529|Organism:Severe      ATGAGTGTCTCTATAGAAATAGAGATGTTGACACAGACTTTGTGAATGAGTTTTACGCAT   15670
gb:MN996531|Organism:Severe      ATGAGTGTCTCTATAGAAATAGAGATGTTGACACAGACTTTGTGAATGAGTTTTACGCAT   15669
gb:MT066176|Organism:Severe      ATGAGTGTCTCTATAGAAATAGAGATGTTGACACAGACTTTGTGAATGAGTTTTACGCAT   15682
gb:MT027064|Organism:Severe      ATGAGTGTCTCTATAGAAATAGAGATGTTGACACAGACTTTGTGAATGAGTTTTACGCAT   15682
gb:MN994468|Organism:Severe      ATGAGTGTCTCTATAGAAATAGAGATGTTGACACAGACTTTGTGAATGAGTTTTACGCAT   15682
gb:MT072688|Organism:Severe      ATGAGTGTCTCTATAGAAATAGAGATGTTGACACAGACTTTGTGAATGAGTTTTACGCAT   15667
gb:MN996527|Organism:Severe      ATGAGTGTCTCTATAGAAATAGAGATGTTGACACAGACTTTGTGAATGAGTTTTACGCAT   15649
gb:MT093631|Organism:Severe      ATGAGTGTCTCTATAGAAATAGAGATGTTGACACAGACTTTGTGAATGAGTTTTACGCAT   15720
gb:MT106053|Organism:Severe      ATGAGTGTCTCTATAGAAATAGAGATGTTGACACAGACTTTGTGAATGAGTTTTACGCAT   15682
gb:MT019533|Organism:Severe      ATGAGTGTCTCTATAGAAATAGAGATGTTGACACAGACTTTGTGAATGAGTTTTACGCAT   15682
gb:MT019531|Organism:Severe      ATGAGTGTCTCTATAGAAATAGAGATGTTGACACAGACTTTGTGAATGAGTTTTACGCAT   15682
gb:MN996528|Organism:Severe      ATGAGTGTCTCTATAGAAATAGAGATGTTGACACAGACTTTGTGAATGAGTTTTACGCAT   15682
gb:MN996530|Organism:Severe      ATGAGTGTCTCTATAGAAATAGAGATGTTGACACAGACTTTGTGAATGAGTTTTACGCAT   15668
gb:MN908947|Organism:Severe      ATGAGTGTCTCTATAGAAATAGAGATGTTGACACAGACTTTGTGAATGAGTTTTACGCAT   15682
gb:MT019532|Organism:Severe      ATGAGTGTCTCTATAGAAATAGAGATGTTGACACAGACTTTGTGAATGAGTTTTACGCAT   15682
                                 ************************************************************

gb:MT020781|Organism:Severe      ATTTGCGTAAACATTTCTCAATGATGATACTCTCTGACGATGCTGTTGTGTGTTTCAATA   15730
gb:MT007544|Organism:Severe      ATTTGCGTAAACATTTCTCAATGATGATACTCTCTGACGATGCTGTTGTGTGTTTCAATA   15742
gb:MN994467|Organism:Severe      ATTTGCGTAAACATTTCTCAATGATGATACTCTCTGACGATGCTGTTGTGTGTTTCAATA   15742
gb:MT044257|Organism:Severe      ATTTGCGTAAACATTTCTCAATGATGATACTCTCTGACGATGCTGTTGTGTGTTTCAATA   15742
gb:MT106054|Organism:Severe      ATTTGCGTAAACATTTCTCAATGATGATACTCTCTGACGATGCTGTTGTGTGTTTCAATA   15742
gb:MT049951|Organism:Severe      ATTTGCGTAAACATTTCTCAATGATGATACTCTCTGACGATGCTGTTGTGTGTTTCAATA   15742
gb:MN975262|Organism:Severe      ATTTGCGTAAACATTTCTCAATGATGATACTCTCTGACGATGCTGTTGTGTGTTTCAATA   15742
gb:MT106052|Organism:Severe      ATTTGCGTAAACATTTCTCAATGATGATACTCTCTGACGATGCTGTTGTGTGTTTCAATA   15742
gb:LC522975|Organism:Severe      ATTTGCGTAAACATTTCTCAATGATGATACTCTCTGACGATGCTGTTGTGTGTTTCAATA   15739
gb:LC522973|Organism:Severe      ATTTGCGTAAACATTTCTCAATGATGATACTCTCTGACGATGCTGTTGTGTGTTTCAATA   15739
gb:LC522974|Organism:Severe      ATTTGCGTAAACATTTCTCAATGATGATACTCTCTGACGATGCTGTTGTGTGTTTCAATA   15739
gb:MN985325|Organism:Severe      ATTTGCGTAAACATTTCTCAATGATGATACTCTCTGACGATGCTGTTGTGTGTTTCAATA   15742
gb:MT020881|Organism:Severe      ATTTGCGTAAACATTTCTCAATGATGATACTCTCTGACGATGCTGTTGTGTGTTTCAATA   15742
gb:MT020880|Organism:Severe      ATTTGCGTAAACATTTCTCAATGATGATACTCTCTGACGATGCTGTTGTGTGTTTCAATA   15742
gb:MT066175|Organism:Severe      ATTTGCGTAAACATTTCTCAATGATGATACTCTCTGACGATGCTGTTGTGTGTTTCAATA   15742
gb:MN997409|Organism:Severe      ATTTGCGTAAACATTTCTCAATGATGATACTCTCTGACGATGCTGTTGTGTGTTTCAATA   15742
gb:MN938384|Organism:Severe      ATTTGCGTAAACATTTCTCAATGATGATACTCTCTGACGATGCTGTTGTGTGTTTCAATA   15710
gb:MT044258|Organism:Severe      ATTTGCGTAAACATTTCTCAATGATGATACTCTCTGACGATGCTGTTGTGTGTTTCAATA   15718
gb:MT039890|Organism:Severe      ATTTGCGTAAACATTTCTCAATGATGATACTCTCTGACGATGCTGTTGTGTGTTTCAATA   15742
gb:MN988713|Organism:Severe      ATTTGCGTAAACATTTCTCAATGATGATACTCTCTGACGATGCTGTTGTGTGTTTCAATA   15742
gb:LC521925|Organism:Severe      ATTTGCGTAAACATTTCTCAATGATGATACTCTCTGACGATGCTGTTGTGTGTTTCAATA   15715
gb:MT093571|Organism:Severe      ATTTGCGTAAACATTTCTCAATGATGATACTCTCTGACGATGCTGTTGTGTGTTTCAATA   15742
gb:MT039887|Organism:Severe      ATTTGCGTAAACATTTCTCAATGATGATACTCTCTGACGATGCTGTTGTGTGTTTCAATA   15742
gb:MT019530|Organism:Severe      ATTTGCGTAAACATTTCTCAATGATGATACTCTCTGACGATGCTGTTGTGTGTTTCAATA   15742
gb:MT039888|Organism:Severe      ATTTGCGTAAACATTTCTCAATGATGATACTCTCTGACGATGCTGTTGTGTGTTTCAATA   15742
gb:LC522972|Organism:Severe      ATTTGCGTAAACATTTCTCAATGATGATACTCTCTGACGATGCTGTTGTGTGTTTCAATA   15739
gb:MT027063|Organism:Severe      ATTTGCGTAAACATTTCTCAATGATGATACTCTCTGACGATGCTGTTGTGTGTTTCAATA   15742
gb:MT027062|Organism:Severe      ATTTGCGTAAACATTTCTCAATGATGATACTCTCTGACGATGCTGTTGTGTGTTTCAATA   15742
gb:MT019529|Organism:Severe      ATTTGCGTAAACATTTCTCAATGATGATACTCTCTGACGATGCTGTTGTGTGTTTCAATA   15742
gb:MN996529|Organism:Severe      ATTTGCGTAAACATTTCTCAATGATGATACTCTCTGACGATGCTGTTGTGTGTTTCAATA   15730
gb:MN996531|Organism:Severe      ATTTGCGTAAACATTTCTCAATGATGATACTCTCTGACGATGCTGTTGTGTGTTTCAATA   15729
gb:MT066176|Organism:Severe      ATTTGCGTAAACATTTCTCAATGATGATACTCTCTGACGATGCTGTTGTGTGTTTCAATA   15742
gb:MT027064|Organism:Severe      ATTTGCGTAAACATTTCTCAATGATGATACTCTCTGACGATGCTGTTGTGTGTTTCAATA   15742
gb:MN994468|Organism:Severe      ATTTGCGTAAACATTTCTCAATGATGATACTCTCTGACGATGCTGTTGTGTGTTTCAATA   15742
```

```
gb:MT072688|Organism:Severe      ATTTGCGTAAACATTTCTCAATGATGATACTCTCTGACGATGCTGTTGTGTGTTTCAATA   15727
gb:MN996527|Organism:Severe      ATTTGCGTAAACATTTCTCAATGATGATACTCTCTGACGATGCTGTTGTGTGTTTCAATA   15709
gb:MT093631|Organism:Severe      ATTTGCGTAAACATTTCTCAATGATGATACTCTCTGACGATGCTGTTGTGTGTTTCAATA   15780
gb:MT106053|Organism:Severe      ATTTGCGTAAACATTTCTCAATGATGATACTCTCTGACGATGCTGTTGTGTGTTTCAATA   15742
gb:MT019533|Organism:Severe      ATTTGCGTAAACATTTCTCAATGATGATACTCTCTGACGATGCTGTTGTGTGTTTCAATA   15742
gb:MT019531|Organism:Severe      ATTTGCGTAAACATTTCTCAATGATGATACTCTCTGACGATGCTGTTGTGTGTTTCAATA   15742
gb:MN996528|Organism:Severe      ATTTGCGTAAACATTTCTCAATGATGATACTCTCTGACGATGCTGTTGTGTGTTTCAATA   15742
gb:MN996530|Organism:Severe      ATTTGCGTAAACATTTCTCAATGATGATACTCTCTGACGATGCTGTTGTGTGTTTCAATA   15728
gb:MN908947|Organism:Severe      ATTTGCGTAAACATTTCTCAATGATGATACTCTCTGACGATGCTGTTGTGTGTTTCAATA   15742
gb:MT019532|Organism:Severe      ATTTGCGTAAACATTTCTCAATGATGATACTCTCTGACGATGCTGTTGTGTGTTTCAATA   15742
                                 ************************************************************

gb:MT020781|Organism:Severe      GCACTTATGCATCTCAAGGTCTAGTGGCTAGCATAAAGAACTTTAAGTCAGTTCTTTATT   15790
gb:MT007544|Organism:Severe      GCACTTATGCATCTCAAGGTCTAGTGGCTAGCATAAAGAACTTTAAGTCAGTTCTTTATT   15802
gb:MN994467|Organism:Severe      GCACTTATGCATCTCAAGGTCTAGTGGCTAGCATAAAGAACTTTAAGTCAGTTCTTTATT   15802
gb:MT044257|Organism:Severe      GCACTTATGCATCTCAAGGTCTAGTGGCTAGCATAAAGAACTTTAAGTCAGTTCTTTATT   15802
gb:MT106054|Organism:Severe      GCACTTATGCATCTCAAGGTCTAGTGGCTAGCATAAAGAACTTTAAGTCAGTTCTTTATT   15802
gb:MT049951|Organism:Severe      GCACTTATGCATCTCAAGGTCTAGTGGCTAGCATAAAGAACTTTAAGTCAGTTCTTTATT   15802
gb:MN975262|Organism:Severe      GCACTTATGCATCTCAAGGTCTAGTGGCTAGCATAAAGAACTTTAAGTCAGTTCTTTATT   15802
gb:MT106052|Organism:Severe      GCACTTATGCATCTCAAGGTCTAGTGGCTAGCATAAAGAACTTTAAGTCAGTTCTTTATT   15802
gb:LC522975|Organism:Severe      GCACTTATGCATCTCAAGGTCTAGTGGCTAGCATAAAGAACTTTAAGTCAGTTCTTTATT   15799
gb:LC522973|Organism:Severe      GCACTTATGCATCTCAAGGTCTAGTGGCTAGCATAAAGAACTTTAAGTCAGTTCTTTATT   15799
gb:LC522974|Organism:Severe      GCACTTATGCATCTCAAGGTCTAGTGGCTAGCATAAAGAACTTTAAGTCAGTTCTTTATT   15799
gb:MN985325|Organism:Severe      GCACTTATGCATCTCAAGGTCTAGTGGCTAGCATAAAGAACTTTAAGTCAGTTCTTTATT   15802
gb:MT020881|Organism:Severe      GCACTTATGCATCTCAAGGTCTAGTGGCTAGCATAAAGAACTTTAAGTCAGTTCTTTATT   15802
gb:MT020880|Organism:Severe      GCACTTATGCATCTCAAGGTCTAGTGGCTAGCATAAAGAACTTTAAGTCAGTTCTTTATT   15802
gb:MT066175|Organism:Severe      GCACTTATGCATCTCAAGGTCTAGTGGCTAGCATAAAGAACTTTAAGTCAGTTCTTTATT   15802
gb:MN997409|Organism:Severe      GCACTTATGCATCTCAAGGTCTAGTGGCTAGCATAAAGAACTTTAAGTCAGTTCTTTATT   15802
gb:MN938384|Organism:Severe      GCACTTATGCATCTCAAGGTCTAGTGGCTAGCATAAAGAACTTTAAGTCAGTTCTTTATT   15770
gb:MT044258|Organism:Severe      GCACTTATGCATCTCAAGGTCTAGTGGCTAGCATAAAGAACTTTAAGTCAGTTCTTTATT   15778
gb:MT039890|Organism:Severe      GCACTTATGCATCTCAAGGTCTAGTGGCTAGCATAAAGAACTTTAAGTCAGTTCTTTATT   15802
gb:MN988713|Organism:Severe      GCACTTATGCATCTCAAGGTCTAGTGGCTAGCATAAAGAACTTTAAGTCAGTTCTTTATT   15802
gb:LC521925|Organism:Severe      GCACTTATGCATCTCAAGGTCTAGTGGCTAGCATAAAGAACTTTAAGTCAGTTCTTTATT   15775
gb:MT093571|Organism:Severe      GCACTTATGCATCTCAAGGTCTAGTGGCTAGCATAAAGAACTTTAAGTCAGTTCTTTATT   15802
gb:MT039887|Organism:Severe      GCACTTATGCATCTCAAGGTCTAGTGGCTAGCATAAAGAACTTTAAGTCAGTTCTTTATT   15802
gb:MT019530|Organism:Severe      GCACTTATGCATCTCAAGGTCTAGTGGCTAGCATAAAGAACTTTAAGTCAGTTCTTTATT   15802
gb:MT039888|Organism:Severe      GCACTTATGCATCTCAAGGTCTAGTGGCTAGCATAAAGAACTTTAAGTCAGTTCTTTATT   15802
gb:LC522972|Organism:Severe      GCACTTATGCATCTCAAGGTCTAGTGGCTAGCATAAAGAACTTTAAGTCAGTTCTTTATT   15799
gb:MT027063|Organism:Severe      GCACTTATGCATCTCAAGGTCTAGTGGCTAGCATAAAGAACTTTAAGTCAGTTCTTTATT   15802
gb:MT027062|Organism:Severe      GCACTTATGCATCTCAAGGTCTAGTGGCTAGCATAAAGAACTTTAAGTCAGTTCTTTATT   15802
gb:MT019529|Organism:Severe      GCACTTATGCATCTCAAGGTCTAGTGGCTAGCATAAAGAACTTTAAGTCAGTTCTTTATT   15802
gb:MN996529|Organism:Severe      GCACTTATGCATCTCAAGGTCTAGTGGCTAGCATAAAGAACTTTAAGTCAGTTCTTTATT   15790
gb:MN996531|Organism:Severe      GCACTTATGCATCTCAAGGTCTAGTGGCTAGCATAAAGAACTTTAAGTCAGTTCTTTATT   15789
gb:MT066176|Organism:Severe      GCACTTATGCATCTCAAGGTCTAGTGGCTAGCATAAAGAACTTTAAGTCAGTTCTTTATT   15802
gb:MT027064|Organism:Severe      GCACTTATGCATCTCAAGGTCTAGTGGCTAGCATAAAGAACTTTAAGTCAGTTCTTTATT   15802
gb:MN994468|Organism:Severe      GCACTTATGCATCTCAAGGTCTAGTGGCTAGCATAAAGAACTTTAAGTCAGTTCTTTATT   15802
gb:MT072688|Organism:Severe      GCACTTATGCATCTCAAGGTCTAGTGGCTAGCATAAAGAACTTTAAGTCAGTTCTTTATT   15787
gb:MN996527|Organism:Severe      GCACTTATGCATCTCAAGGTCTAGTGGCTAGCATAAAGAACTTTAAGTCAGTTCTTTATT   15769
gb:MT093631|Organism:Severe      GCACTTATGCATCTCAAGGTCTAGTGGCTAGCATAAAGAACTTTAAGTCAGTTCTTTATT   15840
gb:MT106053|Organism:Severe      GCACTTATGCATCTCAAGGTCTAGTGGCTAGCATAAAGAACTTTAAGTCAGTTCTTTATT   15802
gb:MT019533|Organism:Severe      GCACTTATGCATCTCAAGGTCTAGTGGCTAGCATAAAGAACTTTAAGTCAGTTCTTTATT   15802
gb:MT019531|Organism:Severe      GCACTTATGCATCTCAAGGTCTAGTGGCTAGCATAAAGAACTTTAAGTCAGTTCTTTATT   15802
gb:MN996528|Organism:Severe      GCACTTATGCATCTCAAGGTCTAGTGGCTAGCATAAAGAACTTTAAGTCAGTTCTTTATT   15802
gb:MN996530|Organism:Severe      GCACTTATGCATCTCAAGGTCTAGTGGCTAGCATAAAGAACTTTAAGTCAGTTCTTTATT   15788
gb:MN908947|Organism:Severe      GCACTTATGCATCTCAAGGTCTAGTGGCTAGCATAAAGAACTTTAAGTCAGTTCTTTATT   15802
gb:MT019532|Organism:Severe      GCACTTATGCATCTCAAGGTCTAGTGGCTAGCATAAAGAACTTTAAGTCAGTTCTTTATT   15802
                                 ************************************************************

gb:MT020781|Organism:Severe      ATCAAAACAATGTTTTTATGTCTGAAGCAAAATGTTGGACTGAGACTGACCTTACTAAAG   15850
gb:MT007544|Organism:Severe      ATCAAAACAATGTTTTTATGTCTGAAGCAAAATGTTGGACTGAGACTGACCTTACTAAAG   15862
gb:MN994467|Organism:Severe      ATCAAAACAATGTTTTTATGTCTGAAGCAAAATGTTGGACTGAGACTGACCTTACTAAAG   15862
gb:MT044257|Organism:Severe      ATCAAAACAATGTTTTTATGTCTGAAGCAAAATGTTGGACTGAGACTGACCTTACTAAAG   15862
gb:MT106054|Organism:Severe      ATCAAAACAATGTTTTTATGTCTGAAGCAAAATGTTGGACTGAGACTGACCTTACTAAAG   15862
gb:MT049951|Organism:Severe      ATCAAAACAATGTTTTTATGTCTGAAGCAAAATGTTGGACTGAGACTGACCTTACTAAAG   15862
gb:MN975262|Organism:Severe      ATCAAAACAATGTTTTTATGTCTGAAGCAAAATGTTGGACTGAGACTGACCTTACTAAAG   15862
```

```
gb:MT106052|Organism:Severe      ATCAAAACAATGTTTTTATGTCTGAAGCAAAATGTTGGACTGAGACTGACCTTACTAAAG   15862
gb:LC522975|Organism:Severe      ATCAAAACAATGTTTTTATGTCTGAAGCAAAATGTTGGACTGAGACTGACCTTACTAAAG   15859
gb:LC522973|Organism:Severe      ATCAAAACAATGTTTTTATGTCTGAAGCAAAATGTTGGACTGAGACTGACCTTACTAAAG   15859
gb:LC522974|Organism:Severe      ATCAAAACAATGTTTTTATGTCTGAAGCAAAATGTTGGACTGAGACTGACCTTACTAAAG   15859
gb:MN985325|Organism:Severe      ATCAAAACAATGTTTTTATGTCTGAAGCAAAATGTTGGACTGAGACTGACCTTACTAAAG   15862
gb:MT020881|Organism:Severe      ATCAAAACAATGTTTTTATGTCTGAAGCAAAATGTTGGACTGAGACTGACCTTACTAAAG   15862
gb:MT020880|Organism:Severe      ATCAAAACAATGTTTTTATGTCTGAAGCAAAATGTTGGACTGAGACTGACCTTACTAAAG   15862
gb:MT066175|Organism:Severe      ATCAAAACAATGTTTTTATGTCTGAAGCAAAATGTTGGACTGAGACTGACCTTACTAAAG   15862
gb:MN997409|Organism:Severe      ATCAAAACAATGTTTTTATGTCTGAAGCAAAATGTTGGACTGAGACTGACCTTACTAAAG   15862
gb:MN938384|Organism:Severe      ATCAAAACAATGTTTTTATGTCTGAAGCAAAATGTTGGACTGAGACTGACCTTACTAAAG   15830
gb:MT044258|Organism:Severe      ATCAAAACAATGTTTTTATGTCTGAAGCAAAATGTTGGACTGAGACTGACCTTACTAAAG   15838
gb:MT039890|Organism:Severe      ATCAAAACAATGTTTTTATGTCTGAAGCAAAATGTTGGACTGAGACTGACCTTACTAAAG   15862
gb:MN988713|Organism:Severe      ATCAAAACAATGTTTTTATGTCTGAAGCAAAATGTTGGACTGAGACTGACCTTACTAAAG   15862
gb:LC521925|Organism:Severe      ATCAAAACAATGTTTTTATGTCTGAAGCAAAATGTTGGACTGAGACTGACCTTACTAAAG   15835
gb:MT093571|Organism:Severe      ATCAAAACAATGTTTTTATGTCTGAAGCAAAATGTTGGACTGAGACTGACCTTACTAAAG   15862
gb:MT039887|Organism:Severe      ATCAAAACAATGTTTTTATGTCTGAAGCAAAATGTTGGACTGAGACTGACCTTACTAAAG   15862
gb:MT019530|Organism:Severe      ATCAAAACAATGTTTTTATGTCTGAAGCAAAATGTTGGACTGAGACTGACCTTACTAAAG   15862
gb:MT039888|Organism:Severe      ATCAAAACAATGTTTTTATGTCTGAAGCAAAATGTTGGACTGAGACTGACCTTACTAAAG   15862
gb:LC522972|Organism:Severe      ATCAAAACAATGTTTTTATGTCTGAAGCAAAATGTTGGACTGAGACTGACCTTACTAAAG   15859
gb:MT027063|Organism:Severe      ATCAAAACAATGTTTTTATGTCTGAAGCAAAATGTTGGACTGAGACTGACCTTACTAAAG   15862
gb:MT027062|Organism:Severe      ATCAAAACAATGTTTTTATGTCTGAAGCAAAATGTTGGACTGAGACTGACCTTACTAAAG   15862
gb:MT019529|Organism:Severe      ATCAAAACAATGTTTTTATGTCTGAAGCAAAATGTTGGACTGAGACTGACCTTACTAAAG   15862
gb:MN996529|Organism:Severe      ATCAAAACAATGTTTTTATGTCTGAAGCAAAATGTTGGACTGAGACTGACCTTACTAAAG   15850
gb:MN996531|Organism:Severe      ATCAAAACAATGTTTTTATGTCTGAAGCAAAATGTTGGACTGAGACTGACCTTACTAAAG   15849
gb:MT066176|Organism:Severe      ATCAAAACAATGTTTTTATGTCTGAAGCAAAATGTTGGACTGAGACTGACCTTACTAAAG   15862
gb:MT027064|Organism:Severe      ATCAAAACAATGTTTTTATGTCTGAAGCAAAATGTTGGACTGAGACTGACCTTACTAAAG   15862
gb:MN994468|Organism:Severe      ATCAAAACAATGTTTTTATGTCTGAAGCAAAATGTTGGACTGAGACTGACCTTACTAAAG   15862
gb:MT072688|Organism:Severe      ATCAAAACAATGTTTTTATGTCTGAAGCAAAATGTTGGACTGAGACTGACCTTACTAAAG   15847
gb:MN996527|Organism:Severe      ATCAAAACAATGTTTTTATGTCTGAAGCAAAATGTTGGACTGAGACTGACCTTACTAAAG   15829
gb:MT093631|Organism:Severe      ATCAAAACAATGTTTTTATGTCTGAAGCAAAATGTTGGACTGAGACTGACCTTACTAAAG   15900
gb:MT106053|Organism:Severe      ATCAAAACAATGTTTTTATGTCTGAAGCAAAATGTTGGACTGAGACTGACCTTACTAAAG   15862
gb:MT019533|Organism:Severe      ATCAAAACAATGTTTTTATGTCTGAAGCAAAATGTTGGACTGAGACTGACCTTACTAAAG   15862
gb:MT019531|Organism:Severe      ATCAAAACAATGTTTTTATGTCTGAAGCAAAATGTTGGACTGAGACTGACCTTACTAAAG   15862
gb:MN996528|Organism:Severe      ATCAAAACAATGTTTTTATGTCTGAAGCAAAATGTTGGACTGAGACTGACCTTACTAAAG   15862
gb:MN996530|Organism:Severe      ATCAAAACAATGTTTTTATGTCTGAAGCAAAATGTTGGACTGAGACTGACCTTACTAAAG   15848
gb:MN908947|Organism:Severe      ATCAAAACAATGTTTTTATGTCTGAAGCAAAATGTTGGACTGAGACTGACCTTACTAAAG   15862
gb:MT019532|Organism:Severe      ATCAAAACAATGTTTTTATGTCTGAAGCAAAATGTTGGACTGAGACTGACCTTACTAAAG   15862
                                 ************************************************************

gb:MT020781|Organism:Severe      GACCTCATGAATTTTGCTCTCAACATACAATGCTAGTTAAACAGGGTGATGATTATGTGT   15910
gb:MT007544|Organism:Severe      GACCTCATGAATTTTGCTCTCAACATACAATGCTAGTTAAACAGGGTGATGATTATGTGT   15922
gb:MN994467|Organism:Severe      GACCTCATGAATTTTGCTCTCAACATACAATGCTAGTTAAACAGGGTGATGATTATGTGT   15922
gb:MT044257|Organism:Severe      GACCTCATGAATTTTGCTCTCAACATACAATGCTAGTTAAACAGGGTGATGATTATGTGT   15922
gb:MT106054|Organism:Severe      GACCTCATGAATTTTGCTCTCAACATACAATGCTAGTTAAACAGGGTGATGATTATGTGT   15922
gb:MT049951|Organism:Severe      GACCTCATGAATTTTGCTCTCAACATACAATGCTAGTTAAACAGGGTGATGATTATGTGT   15922
gb:MN975262|Organism:Severe      GACCTCATGAATTTTGCTCTCAACATACAATGCTAGTTAAACAGGGTGATGATTATGTGT   15922
gb:MT106052|Organism:Severe      GACCTCATGAATTTTGCTCTCAACATACAATGCTAGTTAAACAGGGTGATGATTATGTGT   15922
gb:LC522975|Organism:Severe      GACCTCATGAATTTTGCTCTCAACATACAATGCTAGTTAAACAGGGTGATGATTATGTGT   15919
gb:LC522973|Organism:Severe      GACCTCATGAATTTTGCTCTCAACATACAATGCTAGTTAAACAGGGTGATGATTATGTGT   15919
gb:LC522974|Organism:Severe      GACCTCATGAATTTTGCTCTCAACATACAATGCTAGTTAAACAGGGTGATGATTATGTGT   15919
gb:MN985325|Organism:Severe      GACCTCATGAATTTTGCTCTCAACATACAATGCTAGTTAAACAGGGTGATGATTATGTGT   15922
gb:MT020881|Organism:Severe      GACCTCATGAATTTTGCTCTCAACATACAATGCTAGTTAAACAGGGTGATGATTATGTGT   15922
gb:MT020880|Organism:Severe      GACCTCATGAATTTTGCTCTCAACATACAATGCTAGTTAAACAGGGTGATGATTATGTGT   15922
gb:MT066175|Organism:Severe      GACCTCATGAATTTTGCTCTCAACATACAATGCTAGTTAAACAGGGTGATGATTATGTGT   15922
gb:MN997409|Organism:Severe      GACCTCATGAATTTTGCTCTCAACATACAATGCTAGTTAAACAGGGTGATGATTATGTGT   15922
gb:MN938384|Organism:Severe      GACCTCATGAATTTTGCTCTCAACATACAATGCTAGTTAAACAGGGTGATGATTATGTGT   15890
gb:MT044258|Organism:Severe      GACCTCATGAATTTTGCTCTCAACATACAATGCTAGTTAAACAGGGTGATGATTATGTGT   15898
gb:MT039890|Organism:Severe      GACCTCATGAATTTTGCTCTCAACATACAATGCTAGTTAAACAGGGTGATGATTATGTGT   15922
gb:MN988713|Organism:Severe      GACCTCATGAATTTTGCTCTCAACATACAATGCTAGTTAAACAGGGTGATGATTATGTGT   15922
gb:LC521925|Organism:Severe      GACCTCATGAATTTTGCTCTCAACATACAATGCTAGTTAAACAGGGTGATGATTATGTGT   15895
gb:MT093571|Organism:Severe      GACCTCATGAATTTTGCTCTCAACATACAATGCTAGTTAAACAGGGTGATGATTATGTGT   15922
gb:MT039887|Organism:Severe      GACCTCATGAATTTTGCTCTCAACATACAATGCTAGTTAAACAGGGTGATGATTATGTGT   15922
gb:MT019530|Organism:Severe      GACCTCATGAATTTTGCTCTCAACATACAATGCTAGTTAAACAGGGTGATGATTATGTGT   15922
gb:MT039888|Organism:Severe      GACCTCATGAATTTTGCTCTCAACATACAATGCTAGTTAAACAGGGTGATGATTATGTGT   15922
gb:LC522972|Organism:Severe      GACCTCATGAATTTTGCTCTCAACATACAATGCTAGTTAAACAGGGTGATGATTATGTGT   15919
```

```
gb:MT027063|Organism:Severe      GACCTCATGAATTTTGCTCTCAACATACAATGCTAGTTAAACAGGGTGATGATTATGTGT   15922
gb:MT027062|Organism:Severe      GACCTCATGAATTTTGCTCTCAACATACAATGCTAGTTAAACAGGGTGATGATTATGTGT   15922
gb:MT019529|Organism:Severe      GACCTCATGAATTTTGCTCTCAACATACAATGCTAGTTAAACAGGGTGATGATTATGTGT   15922
gb:MN996529|Organism:Severe      GACCTCATGAATTTTGCTCTCAACATACAATGCTAGTTAAACAGGGTGATGATTATGTGT   15910
gb:MN996531|Organism:Severe      GACCTCATGAATTTTGCTCTCAACATACAATGCTAGTTAAACAGGGTGATGATTATGTGT   15909
gb:MT066176|Organism:Severe      GACCTCATGAATTTTGCTCTCAACATACAATGCTAGTTAAACAGGGTGATGATTATGTGT   15922
gb:MT027064|Organism:Severe      GACCTCATGAATTTTGCTCTCAACATACAATGCTAGTTAAACAGGGTGATGATTATGTGT   15922
gb:MN994468|Organism:Severe      GACCTCATGAATTTTGCTCTCAACATACAATGCTAGTTAAACAGGGTGATGATTATGTGT   15922
gb:MT072688|Organism:Severe      GACCTCATGAATTTTGCTCTCAACATACAATGCTAGTTAAACAGGGTGATGATTATGTGT   15907
gb:MN996527|Organism:Severe      GACCTCATGAATTTTGCTCTCAACATACAATGCTAGTTAAACAGGGTGATGATTATGTGT   15889
gb:MT093631|Organism:Severe      GACCTCATGAATTTTGCTCTCAACATACAATGCTAGTTAAACAGGGTGATGATTATGTGT   15960
gb:MT106053|Organism:Severe      GACCTCATGAATTTTGCTCTCAACATACAATGCTAGTTAAACAGGGTGATGATTATGTGT   15922
gb:MT019533|Organism:Severe      GACCTCATGAATTTTGCTCTCAACATACAATGCTAGTTAAACAGGGTGATGATTATGTGT   15922
gb:MT019531|Organism:Severe      GACCTCATGAATTTTGCTCTCAACATACAATGCTAGTTAAACAGGGTGATGATTATGTGT   15922
gb:MN996528|Organism:Severe      GACCTCATGAATTTTGCTCTCAACATACAATGCTAGTTAAACAGGGTGATGATTATGTGT   15922
gb:MN996530|Organism:Severe      GACCTCATGAATTTTGCTCTCAACATACAATGCTAGTTAAACAGGGTGATGATTATGTGT   15908
gb:MN908947|Organism:Severe      GACCTCATGAATTTTGCTCTCAACATACAATGCTAGTTAAACAGGGTGATGATTATGTGT   15922
gb:MT019532|Organism:Severe      GACCTCATGAATTTTGCTCTCAACATACAATGCTAGTTAAACAGGGTGATGATTATGTGT   15922
                                 ************************************************************

gb:MT020781|Organism:Severe      ACCTTCCTTACCCAGATCCATCAAGAATCCTAGGGGCCGGCTGTTTTGTAGATGATATCG   15970
gb:MT007544|Organism:Severe      ACCTTCCTTACCCAGATCCATCAAGAATCCTAGGGGCCGGCTGTTTTGTAGATGATATCG   15982
gb:MN994467|Organism:Severe      ACCTTCCTTACCCAGATCCATCAAGAATCCTAGGGGCCGGCTGTTTTGTAGATGATATCG   15982
gb:MT044257|Organism:Severe      ACCTTCCTTACCCAGATCCATCAAGAATCCTAGGGGCCGGCTGTTTTGTAGATGATATCG   15982
gb:MT106054|Organism:Severe      ACCTTCCTTACCCAGATCCATCAAGAATCCTAGGGGCCGGCTGTTTTGTAGATGATATCG   15982
gb:MT049951|Organism:Severe      ACCTTCCTTACCCAGATCCATCAAGAATCCTAGGGGCCGGCTGTTTTGTAGATGATATCG   15982
gb:MN975262|Organism:Severe      ACCTTCCTTACCCAGATCCATCAAGAATCCTAGGGGCCGGCTGTTTTGTAGATGATATCG   15982
gb:MT106052|Organism:Severe      ACCTTCCTTACCCAGATCCATCAAGAATCCTAGGGGCCGGCTGTTTTGTAGATGATATCG   15982
gb:LC522975|Organism:Severe      ACCTTCCTTACCCAGATCCATCAAGAATCCTAGGGGCCGGCTGTTTTGTAGATGATATCG   15979
gb:LC522973|Organism:Severe      ACCTTCCTTACCCAGATCCATCAAGAATCCTAGGGGCCGGCTGTTTTGTAGATGATATCG   15979
gb:LC522974|Organism:Severe      ACCTTCCTTACCCAGATCCATCAAGAATCCTAGGGGCCGGCTGTTTTGTAGATGATATCG   15979
gb:MN985325|Organism:Severe      ACCTTCCTTACCCAGATCCATCAAGAATCCTAGGGGCCGGCTGTTTTGTAGATGATATCG   15982
gb:MT020881|Organism:Severe      ACCTTCCTTACCCAGATCCATCAAGAATCCTAGGGGCCGGCTGTTTTGTAGATGATATCG   15982
gb:MT020880|Organism:Severe      ACCTTCCTTACCCAGATCCATCAAGAATCCTAGGGGCCGGCTGTTTTGTAGATGATATCG   15982
gb:MT066175|Organism:Severe      ACCTTCCTTACCCAGATCCATCAAGAATCCTAGGGGCCGGCTGTTTTGTAGATGATATCG   15982
gb:MN997409|Organism:Severe      ACCTTCCTTACCCAGATCCATCAAGAATCCTAGGGGCCGGCTGTTTTGTAGATGATATCG   15982
gb:MN938384|Organism:Severe      ACCTTCCTTACCCAGATCCATCAAGAATCCTAGGGGCCGGCTGTTTTGTAGATGATATCG   15950
gb:MT044258|Organism:Severe      ACCTTCCTTACCCAGATCCATCAAGAATCCTAGGGGCCGGCTGTTTTGTAGATGATATCG   15958
gb:MT039890|Organism:Severe      ACCTTCCTTACCCAGATCCATCAAGAATCCTAGGGGCCGGCTGTTTTGTAGATGATATCG   15982
gb:MN988713|Organism:Severe      ACCTTCCTTACCCAGATCCATCAAGAATCCTAGGGGCCGGCTGTTTTGTAGATGATATCG   15982
gb:LC521925|Organism:Severe      ACCTTCCTTACCCAGATCCATCAAGAATCCTAGGGGCCGGCTGTTTTGTAGATGATATCG   15955
gb:MT093571|Organism:Severe      ACCTTCCTTACCCAGATCCATCAAGAATCCTAGGGGCCGGCTGTTTTGTAGATGATATCG   15982
gb:MT039887|Organism:Severe      ACCTTCCTTACCCAGATCCATCAAGAATCCTAGGGGCCGGCTGTTTTGTAGATGATATCG   15982
gb:MT019530|Organism:Severe      ACCTTCCTTACCCAGATCCATCAAGAATCCTAGGGGCCGGCTGTTTTGTAGATGATATCG   15982
gb:MT039888|Organism:Severe      ACCTTCCTTACCCAGATCCATCAAGAATCCTAGGGGCCGGCTGTTTTGTAGATGATATCG   15982
gb:LC522972|Organism:Severe      ACCTTCCTTACCCAGATCCATCAAGAATCCTAGGGGCCGGCTGTTTTGTAGATGATATCG   15979
gb:MT027063|Organism:Severe      ACCTTCCTTACCCAGATCCATCAAGAATCCTAGGGGCCGGCTGTTTTGTAGATGATATCG   15982
gb:MT027062|Organism:Severe      ACCTTCCTTACCCAGATCCATCAAGAATCCTAGGGGCCGGCTGTTTTGTAGATGATATCG   15982
gb:MT019529|Organism:Severe      ACCTTCCTTACCCAGATCCATCAAGAATCCTAGGGGCCGGCTGTTTTGTAGATGATATCG   15982
gb:MN996529|Organism:Severe      ACCTTCCTTACCCAGATCCATCAAGAATCCTAGGGGCCGGCTGTTTTGTAGATGATATCG   15970
gb:MN996531|Organism:Severe      ACCTTCCTTACCCAGATCCATCAAGAATCCTAGGGGCCGGCTGTTTTGTAGATGATATCG   15969
gb:MT066176|Organism:Severe      ACCTTCCTTACCCAGATCCATCAAGAATCCTAGGGGCCGGCTGTTTTGTAGATGATATCG   15982
gb:MT027064|Organism:Severe      ACCTTCCTTACCCAGATCCATCAAGAATCCTAGGGGCCGGCTGTTTTGTAGATGATATCG   15982
gb:MN994468|Organism:Severe      ACCTTCCTTACCCAGATCCATCAAGAATCCTAGGGGCCGGCTGTTTTGTAGATGATATCG   15982
gb:MT072688|Organism:Severe      ACCTTCCTTACCCAGATCCATCAAGAATCCTAGGGGCCGGCTGTTTTGTAGATGATATCG   15967
gb:MN996527|Organism:Severe      ACCTTCCTTACCCAGATCCATCAAGAATCCTAGGGGCCGGCTGTTTTGTAGATGATATCG   15949
gb:MT093631|Organism:Severe      ACCTTCCTTACCCAGATCCATCAAGAATCCTAGGGGCCGGCTGTTTTGTAGATGATATCG   16020
gb:MT106053|Organism:Severe      ACCTTCCTTACCCAGATCCATCAAGAATCCTAGGGGCCGGCTGTTTTGTAGATGATATCG   15982
gb:MT019533|Organism:Severe      ACCTTCCTTACCCAGATCCATCAAGAATCCTAGGGGCCGGCTGTTTTGTAGATGATATCG   15982
gb:MT019531|Organism:Severe      ACCTTCCTTACCCAGATCCATCAAGAATCCTAGGGGCCGGCTGTTTTGTAGATGATATCG   15982
gb:MN996528|Organism:Severe      ACCTTCCTTACCCAGATCCATCAAGAATCCTAGGGGCCGGCTGTTTTGTAGATGATATCG   15982
gb:MN996530|Organism:Severe      ACCTTCCTTACCCAGATCCATCAAGAATCCTAGGGGCCGGCTGTTTTGTAGATGATATCG   15968
gb:MN908947|Organism:Severe      ACCTTCCTTACCCAGATCCATCAAGAATCCTAGGGGCCGGCTGTTTTGTAGATGATATCG   15982
gb:MT019532|Organism:Severe      ACCTTCCTTACCCAGATCCATCAAGAATCCTAGGGGCCGGCTGTTTTGTAGATGATATCG   15982
                                 ************************************************************
```

```
gb:MT020781|Organism:Severe      TAAAAACAGATGGTACACTTATGATTGAACGGTTCGTGTCTTTAGCTATAGATGCTTACC   16030
gb:MT007544|Organism:Severe      TAAAAACAGATGGTACACTTATGATTGAACGGTTCGTGTCTTTAGCTATAGATGCTTACC   16042
gb:MN994467|Organism:Severe      TAAAAACAGATGGTACACTTATGATTGAACGGTTCGTGTCTTTAGCTATAGATGCTTACC   16042
gb:MT044257|Organism:Severe      TAAAAACAGATGGTACACTTATGATTGAACGGTTCGTGTCTTTAGCTATAGATGCTTACC   16042
gb:MT106054|Organism:Severe      TAAAAACAGATGGTACACTTATGATTGAACGGTTCGTGTCTTTAGCTATAGATGCTTACC   16042
gb:MT049951|Organism:Severe      TAAAAACAGATGGTACACTTATGATTGAACGGTTCGTGTCTTTAGCTATAGATGCTTACC   16042
gb:MN975262|Organism:Severe      TAAAAACAGATGGTACACTTATGATTGAACGGTTCGTGTCTTTAGCTATAGATGCTTACC   16042
gb:MT106052|Organism:Severe      TAAAAACAGATGGTACACTTATGATTGAACGGTTCGTGTCTTTAGCTATAGATGCTTACC   16042
gb:LC522975|Organism:Severe      TAAAAACAGATGGTACACTTATGATTGAACGGTTCGTGTCTTTAGCTATAGATGCTTACC   16039
gb:LC522973|Organism:Severe      TAAAAACAGATGGTACACTTATGATTGAACGGTTCGTGTCTTTAGCTATAGATGCTTACC   16039
gb:LC522974|Organism:Severe      TAAAAACAGATGGTACACTTATGATTGAACGGTTCGTGTCTTTAGCTATAGATGCTTACC   16039
gb:MN985325|Organism:Severe      TAAAAACAGATGGTACACTTATGATTGAACGGTTCGTGTCTTTAGCTATAGATGCTTACC   16042
gb:MT020881|Organism:Severe      TAAAAACAGATGGTACACTTATGATTGAACGGTTCGTGTCTTTAGCTATAGATGCTTACC   16042
gb:MT020880|Organism:Severe      TAAAAACAGATGGTACACTTATGATTGAACGGTTCGTGTCTTTAGCTATAGATGCTTACC   16042
gb:MT066175|Organism:Severe      TAAAAACAGATGGTACACTTATGATTGAACGGTTCGTGTCTTTAGCTATAGATGCTTACC   16042
gb:MN997409|Organism:Severe      TAAAAACAGATGGTACACTTATGATTGAACGGTTCGTGTCTTTAGCTATAGATGCTTACC   16042
gb:MN938384|Organism:Severe      TAAAAACAGATGGTACACTTATGATTGAACGGTTCGTGTCTTTAGCTATAGATGCTTACC   16010
gb:MT044258|Organism:Severe      TAAAAACAGATGGTACACTTATGATTGAACGGTTCGTGTCTTTAGCTATAGATGCTTACC   16018
gb:MT039890|Organism:Severe      TAAAAACAGATGGTACACTTATGATTGAACGGTTCGTGTCTTTAGCTATAGATGCTTACC   16042
gb:MN988713|Organism:Severe      TAAAAACAGATGGTACACTTATGATTGAACGGTTCGTGTCTTTAGCTATAGATGCTTACC   16042
gb:LC521925|Organism:Severe      TAAAAACAGATGGTACACTTATGATTGAACGGTTCGTGTCTTTAGCTATAGATGCTTACC   16015
gb:MT093571|Organism:Severe      TAAAAACAGATGGTACACTTATGATTGAACGGTTCGTGTCTTTAGCTATAGATGCTTACC   16042
gb:MT039887|Organism:Severe      TAAAAACAGATGGTACACTTATGATTGAACGGTTCGTGTCTTTAGCTATAGATGCTTACC   16042
gb:MT019530|Organism:Severe      TAAAAACAGATGGTACACTTATGATTGAACGGTTCGTGTCTTTAGCTATAGATGCTTACC   16042
gb:MT039888|Organism:Severe      TAAAAACAGATGGTACACTTATGATTGAACGGTTCGTGTCTTTAGCTATAGATGCTTACC   16042
gb:LC522972|Organism:Severe      TAAAAACAGATGGTACACTTATGATTGAACGGTTCGTGTCTTTAGCTATAGATGCTTACC   16039
gb:MT027063|Organism:Severe      TAAAAACAGATGGTACACTTATGATTGAACGGTTCGTGTCTTTAGCTATAGATGCTTACC   16042
gb:MT027062|Organism:Severe      TAAAAACAGATGGTACACTTATGATTGAACGGTTCGTGTCTTTAGCTATAGATGCTTACC   16042
gb:MT019529|Organism:Severe      TAAAAACAGATGGTACACTTATGATTGAACGGTTCGTGTCTTTAGCTATAGATGCTTACC   16042
gb:MN996529|Organism:Severe      TAAAAACAGATGGTACACTTATGATTGAACGGTTCGTGTCTTTAGCTATAGATGCTTACC   16030
gb:MN996531|Organism:Severe      TAAAAACAGATGGTACACTTATGATTGAACGGTTCGTGTCTTTAGCTATAGATGCTTACC   16029
gb:MT066176|Organism:Severe      TAAAAACAGATGGTACACTTATGATTGAACGGTTCGTGTCTTTAGCTATAGATGCTTACC   16042
gb:MT027064|Organism:Severe      TAAAAACAGATGGTACACTTATGATTGAACGGTTCGTGTCTTTAGCTATAGATGCTTACC   16042
gb:MN994468|Organism:Severe      TAAAAACAGATGGTACACTTATGATTGAACGGTTCGTGTCTTTAGCTATAGATGCTTACC   16042
gb:MT072688|Organism:Severe      TAAAAACAGATGGTACACTTATGATTGAACGGTTCGTGTCTTTAGCTATAGATGCTTACC   16027
gb:MN996527|Organism:Severe      TAAAAACAGATGGTACACTTATGATTGAACGGTTCGTGTCTTTAGCTATAGATGCTTACC   16009
gb:MT093631|Organism:Severe      TAAAAACAGATGGTACACTTATGATTGAACGGTTCGTGTCTTTAGCTATAGATGCTTACC   16080
gb:MT106053|Organism:Severe      TAAAAACAGATGGTACACTTATGATTGAACGGTTCGTGTCTTTAGCTATAGATGCTTACC   16042
gb:MT019533|Organism:Severe      TAAAAACAGATGGTACACTTATGATTGAACGGTTCGTGTCTTTAGCTATAGATGCTTACC   16042
gb:MT019531|Organism:Severe      TAAAAACAGATGGTACACTTATGATTGAACGGTTCGTGTCTTTAGCTATAGATGCTTACC   16042
gb:MN996528|Organism:Severe      TAAAAACAGATGGTACACTTATGATTGAACGGTTCGTGTCTTTAGCTATAGATGCTTACC   16042
gb:MN996530|Organism:Severe      TAAAAACAGATGGTACACTTATGATTGAACGGTTCGTGTCTTTAGCTATAGATGCTTACC   16028
gb:MN908947|Organism:Severe      TAAAAACAGATGGTACACTTATGATTGAACGGTTCGTGTCTTTAGCTATAGATGCTTACC   16042
gb:MT019532|Organism:Severe      TAAAAACAGATGGTACACTTATGATTGAACGGTTCGTGTCTTTAGCTATAGATGCTTACC   16042
                                 ************************************************************

gb:MT020781|Organism:Severe      CACTTACTAAACATCCTAATCAGGAGTATGCTGATGTCTTTCATTTGTACTTACAATACA   16090
gb:MT007544|Organism:Severe      CACTTACTAAACATCCTAATCAGGAGTATGCTGATGTCTTTCATTTGTACTTACAATACA   16102
gb:MN994467|Organism:Severe      CACTTACTAAACATCCTAATCAGGAGTATGCTGATGTCTTTCATTTGTACTTACAATACA   16102
gb:MT044257|Organism:Severe      CACTTACTAAACATCCTAATCAGGAGTATGCTGATGTCTTTCATTTGTACTTACAATACA   16102
gb:MT106054|Organism:Severe      CACTTACTAAACATCCTAATCAGGAGTATGCTGATGTCTTTCATTTGTACTTACAATACA   16102
gb:MT049951|Organism:Severe      CACTTACTAAACATCCTAATCAGGAGTATGCTGATGTCTTTCATTTGTACTTACAATACA   16102
gb:MN975262|Organism:Severe      CACTTACTAAACATCCTAATCAGGAGTATGCTGATGTCTTTCATTTGTACTTACAATACA   16102
gb:MT106052|Organism:Severe      CACTTACTAAACATCCTAATCAGGAGTATGCTGATGTCTTTCATTTGTACTTACAATACA   16102
gb:LC522975|Organism:Severe      CACTTACTAAACATCCTAATCAGGAGTATGCTGATGTCTTTCATTTGTACTTACAATACA   16099
gb:LC522973|Organism:Severe      CACTTACTAAACATCCTAATCAGGAGTATGCTGATGTCTTTCATTTGTACTTACAATACA   16099
gb:LC522974|Organism:Severe      CACTTACTAAACATCCTAATCAGGAGTATGCTGATGTCTTTCATTTGTACTTACAATACA   16099
gb:MN985325|Organism:Severe      CACTTACTAAACATCCTAATCAGGAGTATGCTGATGTCTTTCATTTGTACTTACAATACA   16102
gb:MT020881|Organism:Severe      CACTTACTAAACATCCTAATCAGGAGTATGCTGATGTCTTTCATTTGTACTTACAATACA   16102
gb:MT020880|Organism:Severe      CACTTACTAAACATCCTAATCAGGAGTATGCTGATGTCTTTCATTTGTACTTACAATACA   16102
gb:MT066175|Organism:Severe      CACTTACTAAACATCCTAATCAGGAGTATGCTGATGTCTTTCATTTGTACTTACAATACA   16102
gb:MN997409|Organism:Severe      CACTTACTAAACATCCTAATCAGGAGTATGCTGATGTCTTTCATTTGTACTTACAATACA   16102
gb:MN938384|Organism:Severe      CACTTACTAAACATCCTAATCAGGAGTATGCTGATGTCTTTCATTTGTACTTACAATACA   16070
gb:MT044258|Organism:Severe      CACTTACTAAACATCCTAATCAGGAGTATGCTGATGTCTTTCATTTGTACTTACAATACA   16078
```

```
gb:MT039890|Organism:Severe      CACTTACTAAACATCCTAATCAGGAGTATGCTGATGTCTTTCATTTGTACTTACAATACA   16102
gb:MN988713|Organism:Severe      CACTTACTAAACATCCTAATCAGGAGTATGCTGATGTCTTTCATTTGTACTTACAATACA   16102
gb:LC521925|Organism:Severe      CACTTACTAAACATCCTAATCAGGAGTATGCTGATGTCTTTCATTTGTACTTACAATACA   16075
gb:MT093571|Organism:Severe      CACTTACTAAACATCCTAATCAGGAGTATGCTGATGTCTTTCATTTGTACTTACAATACA   16102
gb:MT039887|Organism:Severe      CACTTACTAAACATCCTAATCAGGAGTATGCTGATGTCTTTCATTTGTACTTACAATACA   16102
gb:MT019530|Organism:Severe      CACTTACTAAACATCCTAATCAGGAGTATGCTGATGTCTTTCATTTGTACTTACAATACA   16102
gb:MT039888|Organism:Severe      CACTTACTAAACATCCTAATCAGGAGTATGCTGATGTCTTTCATTTGTACTTACAATACA   16102
gb:LC522972|Organism:Severe      CACTTACTAAACATCCTAATCAGGAGTATGCTGATGTCTTTCATTTGTACTTACAATACA   16099
gb:MT027063|Organism:Severe      CACTTACTAAACATCCTAATCAGGAGTATGCTGATGTCTTTCATTTGTACTTACAATACA   16102
gb:MT027062|Organism:Severe      CACTTACTAAACATCCTAATCAGGAGTATGCTGATGTCTTTCATTTGTACTTACAATACA   16102
gb:MT019529|Organism:Severe      CACTTACTAAACATCCTAATCAGGAGTATGCTGATGTCTTTCATTTGTACTTACAATACA   16102
gb:MN996529|Organism:Severe      CACTTACTAAACATCCTAATCAGGAGTATGCTGATGTCTTTCATTTGTACTTACAATACA   16090
gb:MN996531|Organism:Severe      CACTTACTAAACATCCTAATCAGGAGTATGCTGATGTCTTTCATTTGTACTTACAATACA   16089
gb:MT066176|Organism:Severe      CACTTACTAAACATCCTAATCAGGAGTATGCTGATGTCTTTCATTTGTACTTACAATACA   16102
gb:MT027064|Organism:Severe      CACTTACTAAACATCCTAATCAGGAGTATGCTGATGTCTTTCATTTGTACTTACAATACA   16102
gb:MN994468|Organism:Severe      CACTTACTAAACATCCTAATCAGGAGTATGCTGATGTCTTTCATTTGTACTTACAATACA   16102
gb:MT072688|Organism:Severe      CACTTACTAAACATCCTAATCAGGAGTATGCTGATGTCTTTCATTTGTACTTACAATACA   16087
gb:MN996527|Organism:Severe      CACTTACTAAACATCCTAATCAGGAGTATGCTGATGTCTTTCATTTGTACTTACAATACA   16069
gb:MT093631|Organism:Severe      CACTTACTAAACATCCTAATCAGGAGTATGCTGATGTCTTTCATTTGTACTTACAATACA   16140
gb:MT106053|Organism:Severe      CACTTACTAAACATCCTAATCAGGAGTATGCTGATGTCTTTCATTTGTACTTACAATACA   16102
gb:MT019533|Organism:Severe      CACTTACTAAACATCCTAATCAGGAGTATGCTGATGTCTTTCATTTGTACTTACAATACA   16102
gb:MT019531|Organism:Severe      CACTTACTAAACATCCTAATCAGGAGTATGCTGATGTCTTTCATTTGTACTTACAATACA   16102
gb:MN996528|Organism:Severe      CACTTACTAAACATCCTAATCAGGAGTATGCTGATGTCTTTCATTTGTACTTACAATACA   16102
gb:MN996530|Organism:Severe      CACTTACTAAACATCCTAATCAGGAGTATGCTGATGTCTTTCATTTGTACTTACAATACA   16088
gb:MN908947|Organism:Severe      CACTTACTAAACATCCTAATCAGGAGTATGCTGATGTCTTTCATTTGTACTTACAATACA   16102
gb:MT019532|Organism:Severe      CACTTACTAAACATCCTAATCAGGAGTATGCTGATGTCTTTCATTTGTACTTACAATACA   16102
                                 ************************************************************

gb:MT020781|Organism:Severe      TAAGAAAGCTACATGATGAGTTAACAGGACACATGTTAGACATGTATTCTGTTATGCTTA   16150
gb:MT007544|Organism:Severe      TAAGAAAGCTACATGATGAGTTAACAGGACACATGTTAGACATGTATTCTGTTATGCTTA   16162
gb:MN994467|Organism:Severe      TAAGAAAGCTACATGATGAGTTAACAGGACACATGTTAGACATGTATTCTGTTATGCTTA   16162
gb:MT044257|Organism:Severe      TAAGAAAGCTACATGATGAGTTAACAGGACACATGTTAGACATGTATTCTGTTATGCTTA   16162
gb:MT106054|Organism:Severe      TAAGAAAGCTACATGATGAGTTAACAGGACACATGTTAGACATGTATTCTGTTATGCTTA   16162
gb:MT049951|Organism:Severe      TAAGAAAGCTACATGATGAGTTAACAGGACACATGTTAGACATGTATTCTGTTATGCTTA   16162
gb:MN975262|Organism:Severe      TAAGAAAGCTACATGATGAGTTAACAGGACACATGTTAGACATGTATTCTGTTATGCTTA   16162
gb:MT106052|Organism:Severe      TAAGAAAGCTACATGATGAGTTAACAGGACACATGTTAGACATGTATTCTGTTATGCTTA   16162
gb:LC522975|Organism:Severe      TAAGAAAGCTACATGATGAGTTAACAGGACACATGTTAGACATGTATTCTGTTATGCTTA   16159
gb:LC522973|Organism:Severe      TAAGAAAGCTACATGATGAGTTAACAGGACACATGTTAGACATGTATTCTGTTATGCTTA   16159
gb:LC522974|Organism:Severe      TAAGAAAGCTACATGATGAGTTAACAGGACACATGTTAGACATGTATTCTGTTATGCTTA   16159
gb:MN985325|Organism:Severe      TAAGAAAGCTACATGATGAGTTAACAGGACACATGTTAGACATGTATTCTGTTATGCTTA   16162
gb:MT020881|Organism:Severe      TAAGAAAGCTACATGATGAGTTAACAGGACACATGTTAGACATGTATTCTGTTATGCTTA   16162
gb:MT020880|Organism:Severe      TAAGAAAGCTACATGATGAGTTAACAGGACACATGTTAGACATGTATTCTGTTATGCTTA   16162
gb:MT066175|Organism:Severe      TAAGAAAGCTACATGATGAGTTAACAGGACACATGTTAGACATGTATTCTGTTATGCTTA   16162
gb:MN997409|Organism:Severe      TAAGAAAGCTACATGATGAGTTAACAGGACACATGTTAGACATGTATTCTGTTATGCTTA   16162
gb:MN938384|Organism:Severe      TAAGAAAGCTACATGATGAGTTAACAGGACACATGTTAGACATGTATTCTGTTATGCTTA   16130
gb:MT044258|Organism:Severe      TAAGAAAGCTACATGATGAGTTAACAGGACACATGTTAGACATGTATTCTGTTATGCTTA   16138
gb:MT039890|Organism:Severe      TAAGAAAGCTACATGATGAGTTAACAGGACACATGTTAGACATGTATTCTGTTATGCTTA   16162
gb:MN988713|Organism:Severe      TAAGAAAGCTACATGATGAGTTAACAGGACACATGTTAGACATGTATTCTGTTATGCTTA   16162
gb:LC521925|Organism:Severe      TAAGAAAGCTACATGATGAGTTAACAGGACACATGTTAGACATGTATTCTGTTATGCTTA   16135
gb:MT093571|Organism:Severe      TAAGAAAGCTACATGATGAGTTAACAGGACACATGTTAGACATGTATTCTGTTATGCTTA   16162
gb:MT039887|Organism:Severe      TAAGAAAGCTACATGATGAGTTAACAGGACACATGTTAGACATGTATTCTGTTATGCTTA   16162
gb:MT019530|Organism:Severe      TAAGAAAGCTACATGATGAGTTAACAGGACACATGTTAGACATGTATTCTGTTATGCTTA   16162
gb:MT039888|Organism:Severe      TAAGAAAGCTACATGATGAGTTAACAGGACACATGTTAGACATGTATTCTGTTATGCTTA   16162
gb:LC522972|Organism:Severe      TAAGAAAGCTACATGATGAGTTAACAGGACACATGTTAGACATGTATTCTGTTATGCTTA   16159
gb:MT027063|Organism:Severe      TAAGAAAGCTACATGATGAGTTAACAGGACACATGTTAGACATGTATTCTGTTATGCTTA   16162
gb:MT027062|Organism:Severe      TAAGAAAGCTACATGATGAGTTAACAGGACACATGTTAGACATGTATTCTGTTATGCTTA   16162
gb:MT019529|Organism:Severe      TAAGAAAGCTACATGATGAGTTAACAGGACACATGTTAGACATGTATTCTGTTATGCTTA   16162
gb:MN996529|Organism:Severe      TAAGAAAGCTACATGATGAGTTAACAGGACACATGTTAGACATGTATTCTGTTATGCTTA   16150
gb:MN996531|Organism:Severe      TAAGAAAGCTACATGATGAGTTAACAGGACACATGTTAGACATGTATTCTGTTATGCTTA   16149
gb:MT066176|Organism:Severe      TAAGAAAGCTACATGATGAGTTAACAGGACACATGTTAGACATGTATTCTGTTATGCTTA   16162
gb:MT027064|Organism:Severe      TAAGAAAGCTACATGATGAGTTAACAGGACACATGTTAGACATGTATTCTGTTATGCTTA   16162
gb:MN994468|Organism:Severe      TAAGAAAGCTACATGATGAGTTAACAGGACACATGTTAGACATGTATTCTGTTATGCTTA   16162
gb:MT072688|Organism:Severe      TAAGAAAGCTACATGATGAGTTAACAGGACACATGTTAGACATGTATTCTGTTATGCTTA   16147
gb:MN996527|Organism:Severe      TAAGAAAGCTACATGATGAGTTAACAGGACACATGTTAGACATGTATTCTGTTATGCTTA   16129
gb:MT093631|Organism:Severe      TAAGAAAGCTACATGATGAGTTAACAGGACACATGTTAGACATGTATTCTGTTATGCTTA   16200
```

```
gb:MT106053|Organism:Severe      TAAGAAAGCTACATGATGAGTTAACAGGACACATGTTAGACATGTATTCTGTTATGCTTA   16162
gb:MT019533|Organism:Severe      TAAGAAAGCTACATGATGAGTTAACAGGACACATGTTAGACATGTATTCTGTTATGCTTA   16162
gb:MT019531|Organism:Severe      TAAGAAAGCTACATGATGAGTTAACAGGACACATGTTAGACATGTATTCTGTTATGCTTA   16162
gb:MN996528|Organism:Severe      TAAGAAAGCTACATGATGAGTTAACAGGACACATGTTAGACATGTATTCTGTTATGCTTA   16162
gb:MN996530|Organism:Severe      TAAGAAAGCTACATGATGAGTTAACAGGACACATGTTAGACATGTATTCTGTTATGCTTA   16148
gb:MN908947|Organism:Severe      TAAGAAAGCTACATGATGAGTTAACAGGACACATGTTAGACATGTATTCTGTTATGCTTA   16162
gb:MT019532|Organism:Severe      TAAGAAAGCTACATGATGAGTTAACAGGACACATGTTAGACATGTATTCTGTTATGCTTA   16162
                                 ************************************************************

gb:MT020781|Organism:Severe      CTAATGATAACACTTCAAGGTATTGGGAACCTGAGTTTTATGAGGCTATGTACACACCGC   16210
gb:MT007544|Organism:Severe      CTAATGATAACACTTCAAGGTATTGGGAACCTGAGTTTTATGAGGCTATGTACACACCGC   16222
gb:MN994467|Organism:Severe      CTAATGATAACACTTCAAGGTATTGGGAACCTGAGTTTTATGAGGCTATGTACACACCGC   16222
gb:MT044257|Organism:Severe      CTAATGATAACACTTCAAGGTATTGGGAACCTGAGTTTTATGAGGCTATGTACACACCGC   16222
gb:MT106054|Organism:Severe      CTAATGATAACACTTCAAGGTATTGGGAACCTGAGTTTTATGAGGCTATGTACACACCGC   16222
gb:MT049951|Organism:Severe      CTAATGATAACACTTCAAGGTATTGGGAACCTGAGTTTTATGAGGCTATGTACACACCGC   16222
gb:MN975262|Organism:Severe      CTAATGATAACACTTCAAGGTATTGGGAACCTGAGTTTTATGAGGCTATGTACACACCGC   16222
gb:MT106052|Organism:Severe      CTAATGATAACACTTCAAGGTATTGGGAACCTGAGTTTTATGAGGCTATGTACACACCGC   16222
gb:LC522975|Organism:Severe      CTAATGATAACACTTCAAGGTATTGGGAACCTGAGTTTTATGAGGCTATGTACACACCGC   16219
gb:LC522973|Organism:Severe      CTAATGATAACACTTCAAGGTATTGGGAACCTGAGTTTTATGAGGCTATGTACACACCGC   16219
gb:LC522974|Organism:Severe      CTAATGATAACACTTCAAGGTATTGGGAACCTGAGTTTTATGAGGCTATGTACACACCGC   16219
gb:MN985325|Organism:Severe      CTAATGATAACACTTCAAGGTATTGGGAACCTGAGTTTTATGAGGCTATGTACACACCGC   16222
gb:MT020881|Organism:Severe      CTAATGATAACACTTCAAGGTATTGGGAACCTGAGTTTTATGAGGCTATGTACACACCGC   16222
gb:MT020880|Organism:Severe      CTAATGATAACACTTCAAGGTATTGGGAACCTGAGTTTTATGAGGCTATGTACACACCGC   16222
gb:MT066175|Organism:Severe      CTAATGATAACACTTCAAGGTATTGGGAACCTGAGTTTTATGAGGCTATGTACACACCGC   16222
gb:MN997409|Organism:Severe      CTAATGATAACACTTCAAGGTATTGGGAACCTGAGTTTTATGAGGCTATGTACACACCGC   16222
gb:MN938384|Organism:Severe      CTAATGATAACACTTCAAGGTATTGGGAACCTGAGTTTTATGAGGCTATGTACACACCGC   16190
gb:MT044258|Organism:Severe      CTAATGATAACACTTCAAGGTATTGGGAACCTGAGTTTTATGAGGCTATGTACACACCGC   16198
gb:MT039890|Organism:Severe      CTAATGATAACACTTCAAGGTATTGGGAACCTGAGTTTTATGAGGCTATGTACACACCGC   16222
gb:MN988713|Organism:Severe      CTAATGATAACACTTCAAGGTATTGGGAACCTGAGTTTTATGAGGCTATGTACACACCGC   16222
gb:LC521925|Organism:Severe      CTAATGATAACACTTCAAGGTATTGGGAACCTGAGTTTTATGAGGCTATGTACACACCGC   16195
gb:MT093571|Organism:Severe      CTAATGATAACACTTCAAGGTATTGGGAACCTGAGTTTTATGAGGCTATGTACACACCGC   16222
gb:MT039887|Organism:Severe      CTAATGATAACACTTCAAGGTATTGGGAACCTGAGTTTTATGAGGCTATGTACACACCGC   16222
gb:MT019530|Organism:Severe      CTAATGATAACACTTCAAGGTATTGGGAACCTGAGTTTTATGAGGCTATGTACACACCGC   16222
gb:MT039888|Organism:Severe      CTAATGATAACACTTCAAGGTATTGGGAACCTGAGTTTTATGAGGCTATGTACACACCGC   16222
gb:LC522972|Organism:Severe      CTAATGATAACACTTCAAGGTATTGGGAACCTGAGTTTTATGAGGCTATGTACACACCGC   16219
gb:MT027063|Organism:Severe      CTAATGATAACACTTCAAGGTATTGGGAACCTGAGTTTTATGAGGCTATGTACACACCGC   16222
gb:MT027062|Organism:Severe      CTAATGATAACACTTCAAGGTATTGGGAACCTGAGTTTTATGAGGCTATGTACACACCGC   16222
gb:MT019529|Organism:Severe      CTAATGATAACACTTCAAGGTATTGGGAACCTGAGTTTTATGAGGCTATGTACACACCGC   16222
gb:MN996529|Organism:Severe      CTAATGATAACACTTCAAGGTATTGGGAACCTGAGTTTTATGAGGCTATGTACACACCGC   16210
gb:MN996531|Organism:Severe      CTAATGATAACACTTCAAGGTATTGGGAACCTGAGTTTTATGAGGCTATGTACACACCGC   16209
gb:MT066176|Organism:Severe      CTAATGATAACACTTCAAGGTATTGGGAACCTGAGTTTTATGAGGCTATGTACACACCGC   16222
gb:MT027064|Organism:Severe      CTAATGATAACACTTCAAGGTATTGGGAACCTGAGTTTTATGAGGCTATGTACACACCGC   16222
gb:MN994468|Organism:Severe      CTAATGATAACACTTCAAGGTATTGGGAACCTGAGTTTTATGAGGCTATGTACACACCGC   16222
gb:MT072688|Organism:Severe      CTAATGATAACACTTCAAGGTATTGGGAACCTGAGTTTTATGAGGCTATGTACACACCGC   16207
gb:MN996527|Organism:Severe      CTAATGATAACACTTCAAGGTATTGGGAACCTGAGTTTTATGAGGCTATGTACACACCGC   16189
gb:MT093631|Organism:Severe      CTAATGATAACACTTCAAGGTATTGGGAACCTGAGTTTTATGAGGCTATGTACACACCGC   16260
gb:MT106053|Organism:Severe      CTAATGATAACACTTCAAGGTATTGGGAACCTGAGTTTTATGAGGCTATGTACACACCGC   16222
gb:MT019533|Organism:Severe      CTAATGATAACACTTCAAGGTATTGGGAACCTGAGTTTTATGAGGCTATGTACACACCGC   16222
gb:MT019531|Organism:Severe      CTAATGATAACACTTCAAGGTATTGGGAACCTGAGTTTTATGAGGCTATGTACACACCGC   16222
gb:MN996528|Organism:Severe      CTAATGATAACACTTCAAGGTATTGGGAACCTGAGTTTTATGAGGCTATGTACACACCGC   16222
gb:MN996530|Organism:Severe      CTAATGATAACACTTCAAGGTATTGGGAACCTGAGTTTTATGAGGCTATGTACACACCGC   16208
gb:MN908947|Organism:Severe      CTAATGATAACACTTCAAGGTATTGGGAACCTGAGTTTTATGAGGCTATGTACACACCGC   16222
gb:MT019532|Organism:Severe      CTAATGATAACACTTCAAGGTATTGGGAACCTGAGTTTTATGAGGCTATGTACACACCGC   16222
                                 ************************************************************

gb:MT020781|Organism:Severe      ATACAGTCTTACAGGCTGTTGGGGCTTGTGTTCTTTGCAATTCACAGACTTCATTAAGAT   16270
gb:MT007544|Organism:Severe      ATACAGTCTTACAGGCTGTTGGGGCTTGTGTTCTTTGCAATTCACAGACTTCATTAAGAT   16282
gb:MN994467|Organism:Severe      ATACAGTCTTACAGGCTGTTGGGGCTTGTGTTCTTTGCAATTCACAGACTTCATTAAGAT   16282
gb:MT044257|Organism:Severe      ATACAGTCTTACAGGCTGTTGGGGCTTGTGTTCTTTGCAATTCACAGACTTCATTAAGAT   16282
gb:MT106054|Organism:Severe      ATACAGTCTTACAGGCTGTTGGGGCTTGTGTTCTTTGCAATTCACAGACTTCATTAAGAT   16282
gb:MT049951|Organism:Severe      ATACAGTCTTACAGGCTGTTGGGGCTTGTGTTCTTTGCAATTCACAGACTTCATTAAGAT   16282
gb:MN975262|Organism:Severe      ATACAGTCTTACAGGCTGTTGGGGCTTGTGTTCTTTGCAATTCACAGACTTCATTAAGAT   16282
gb:MT106052|Organism:Severe      ATACAGTCTTACAGGCTGTTGGGGCTTGTGTTCTTTGCAATTCACAGACTTCATTAAGAT   16282
gb:LC522975|Organism:Severe      ATACAGTCTTACAGGCTGTTGGGGCTTGTGTTCTTTGCAATTCACAGACTTCATTAAGAT   16279
gb:LC522973|Organism:Severe      ATACAGTCTTACAGGCTGTTGGGGCTTGTGTTCTTTGCAATTCACAGACTTCATTAAGAT   16279
```

```
gb:LC522974|Organism:Severe      ATACAGTCTTACAGGCTGTTGGGGCTTGTGTTCTTTGCAATTCACAGACTTCATTAAGAT   16279
gb:MN985325|Organism:Severe      ATACAGTCTTACAGGCTGTTGGGGCTTGTGTTCTTTGCAATTCACAGACTTCATTAAGAT   16282
gb:MT020881|Organism:Severe      ATACAGTCTTACAGGCTGTTGGGGCTTGTGTTCTTTGCAATTCACAGACTTCATTAAGAT   16282
gb:MT020880|Organism:Severe      ATACAGTCTTACAGGCTGTTGGGGCTTGTGTTCTTTGCAATTCACAGACTTCATTAAGAT   16282
gb:MT066175|Organism:Severe      ATACAGTCTTACAGGCTGTTGGGGCTTGTGTTCTTTGCAATTCACAGACTTCATTAAGAT   16282
gb:MN997409|Organism:Severe      ATACAGTCTTACAGGCTGTTGGGGCTTGTGTTCTTTGCAATTCACAGACTTCATTAAGAT   16282
gb:MN938384|Organism:Severe      ATACAGTCTTACAGGCTGTTGGGGCTTGTGTTCTTTGCAATTCACAGACTTCATTAAGAT   16250
gb:MT044258|Organism:Severe      ATACAGTCTTACAGGCTGTTGGGGCTTGTGTTCTTTGCAATTCACAGACTTCATTAAGAT   16258
gb:MT039890|Organism:Severe      ATACAGTCTTACAGGCTGTTGGGGCTTGTGTTCTTTGCAATTCACAGACTTCATTAAGAT   16282
gb:MN988713|Organism:Severe      ATACAGTCTTACAGGCTGTTGGGGCTTGTGTTCTTTGCAATTCACAGACTTCATTAAGAT   16282
gb:LC521925|Organism:Severe      ATACAGTCTTACAGGCTGTTGGGGCTTGTGTTCTTTGCAATTCACAGACTTCATTAAGAT   16255
gb:MT093571|Organism:Severe      ATACAGTCTTACAGGCTGTTGGGGCTTGTGTTCTTTGCAATTCACAGACTTCATTAAGAT   16282
gb:MT039887|Organism:Severe      ATACAGTCTTACAGGCTGTTGGGGCTTGTGTTCTTTGCAATTCACAGACTTCATTAAGAT   16282
gb:MT019530|Organism:Severe      ATACAGTCTTACAGGCTGTTGGGGCTTGTGTTCTTTGCAATTCACAGACTTCATTAAGAT   16282
gb:MT039888|Organism:Severe      ATACAGTCTTACAGGCTGTTGGGGCTTGTGTTCTTTGCAATTCACAGACTTCATTAAGAT   16282
gb:LC522972|Organism:Severe      ATACAGTCTTACAGGCTGTTGGGGCTTGTGTTCTTTGCAATTCACAGACTTCATTAAGAT   16279
gb:MT027063|Organism:Severe      ATACAGTCTTACAGGCTGTTGGGGCTTGTGTTCTTTGCAATTCACAGACTTCATTAAGAT   16282
gb:MT027062|Organism:Severe      ATACAGTCTTACAGGCTGTTGGGGCTTGTGTTCTTTGCAATTCACAGACTTCATTAAGAT   16282
gb:MT019529|Organism:Severe      ATACAGTCTTACAGGCTGTTGGGGCTTGTGTTCTTTGCAATTCACAGACTTCATTAAGAT   16282
gb:MN996529|Organism:Severe      ATACAGTCTTACAGGCTGTTGGGGCTTGTGTTCTTTGCAATTCACAGACTTCATTAAGAT   16270
gb:MN996531|Organism:Severe      ATACAGTCTTACAGGCTGTTGGGGCTTGTGTTCTTTGCAATTCACAGACTTCATTAAGAT   16269
gb:MT066176|Organism:Severe      ATACAGTCTTACAGGCTGTTGGGGCTTGTGTTCTTTGCAATTCACAGACTTCATTAAGAT   16282
gb:MT027064|Organism:Severe      ATACAGTCTTACAGGCTGTTGGGGCTTGTGTTCTTTGCAATTCACAGACTTCATTAAGAT   16282
gb:MN994468|Organism:Severe      ATACAGTCTTACAGGCTGTTGGGGCTTGTGTTCTTTGCAATTCACAGACTTCATTAAGAT   16282
gb:MT072688|Organism:Severe      ATACAGTCTTACAGGCTGTTGGGGCTTGTGTTCTTTGCAATTCACAGACTTCATTAAGAT   16267
gb:MN996527|Organism:Severe      ATACAGTCTTACAGGCTGTTGGGGCTTGTGTTCTTTGCAATTCACAGACTTCATTAAGAT   16249
gb:MT093631|Organism:Severe      ATACAGTCTTACAGGCTGTTGGGGCTTGTGTTCTTTGCAATTCACAGACTTCATTAAGAT   16320
gb:MT106053|Organism:Severe      ATACAGTCTTACAGGCTGTTGGGGCTTGTGTTCTTTGCAATTCACAGACTTCATTAAGAT   16282
gb:MT019533|Organism:Severe      ATACAGTCTTACAGGCTGTTGGGGCTTGTGTTCTTTGCAATTCACAGACTTCATTAAGAT   16282
gb:MT019531|Organism:Severe      ATACAGTCTTACAGGCTGTTGGGGCTTGTGTTCTTTGCAATTCACAGACTTCATTAAGAT   16282
gb:MN996528|Organism:Severe      ATACAGTCTTACAGGCTGTTGGGGCTTGTGTTCTTTGCAATTCACAGACTTCATTAAGAT   16282
gb:MN996530|Organism:Severe      ATACAGTCTTACAGGCTGTTGGGGCTTGTGTTCTTTGCAATTCACAGACTTCATTAAGAT   16268
gb:MN908947|Organism:Severe      ATACAGTCTTACAGGCTGTTGGGGCTTGTGTTCTTTGCAATTCACAGACTTCATTAAGAT   16282
gb:MT019532|Organism:Severe      ATACAGTCTTACAGGCTGTTGGGGCTTGTGTTCTTTGCAATTCACAGACTTCATTAAGAT   16282
                                 ************************************************************

gb:MT020781|Organism:Severe      GTGGTGCTTGCATACGTAGACCATTCTTATGTTGTAAATGCTGTTACGACCATGTCATAT   16330
gb:MT007544|Organism:Severe      GTGGTGCTTGCATACGTAGACCATTCTTATGTTGTAAATGCTGTTACGACCATGTCATAT   16342
gb:MN994467|Organism:Severe      GTGGTGCTTGCATACGTAGACCATTCTTATGTTGTAAATGCTGTTACGACCATGTCATAT   16342
gb:MT044257|Organism:Severe      GTGGTGCTTGCATACGTAGACCATTCTTATGTTGTAAATGCTGTTACGACCATGTCATAT   16342
gb:MT106054|Organism:Severe      GTGGTGCTTGCATACGTAGACCATTCTTATGTTGTAAATGCTGTTACGACCATGTCATAT   16342
gb:MT049951|Organism:Severe      GTGGTGCTTGCATACGTAGACCATTCTTATGTTGTAAATGCTGTTACGACCATGTCATAT   16342
gb:MN975262|Organism:Severe      GTGGTGCTTGCATACGTAGACCATTCTTATGTTGTAAATGCTGTTACGACCATGTCATAT   16342
gb:MT106052|Organism:Severe      GTGGTGCTTGCATACGTAGACCATTCTTATGTTGTAAATGCTGTTACGACCATGTCATAT   16342
gb:LC522975|Organism:Severe      GTGGTGCTTGCATACGTAGACCATTCTTATGTTGTAAATGCTGTTACGACCATGTCATAT   16339
gb:LC522973|Organism:Severe      GTGGTGCTTGCATACGTAGACCATTCTTATGTTGTAAATGCTGTTACGACCATGTCATAT   16339
gb:LC522974|Organism:Severe      GTGGTGCTTGCATACGTAGACCATTCTTATGTTGTAAATGCTGTTACGACCATGTCATAT   16339
gb:MN985325|Organism:Severe      GTGGTGCTTGCATACGTAGACCATTCTTATGTTGTAAATGCTGTTACGACCATGTCATAT   16342
gb:MT020881|Organism:Severe      GTGGTGCTTGCATACGTAGACCATTCTTATGTTGTAAATGCTGTTACGACCATGTCATAT   16342
gb:MT020880|Organism:Severe      GTGGTGCTTGCATACGTAGACCATTCTTATGTTGTAAATGCTGTTACGACCATGTCATAT   16342
gb:MT066175|Organism:Severe      GTGGTGCTTGCATACGTAGACCATTCTTATGTTGTAAATGCTGTTACGACCATGTCATAT   16342
gb:MN997409|Organism:Severe      GTGGTGCTTGCATACGTAGACCATTCTTATGTTGTAAATGCTGTTACGACCATGTCATAT   16342
gb:MN938384|Organism:Severe      GTGGTGCTTGCATACGTAGACCATTCTTATGTTGTAAATGCTGTTACGACCATGTCATAT   16310
gb:MT044258|Organism:Severe      GTGGTGCTTGCATACGTAGACCATTCTTATGTTGTAAATGCTGTTACGACCATGTCATAT   16318
gb:MT039890|Organism:Severe      GTGGTGCTTGCATACGTAGACCATTCTTATGTTGTAAATGCTGTTACGACCATGTCATAT   16342
gb:MN988713|Organism:Severe      GTGGTGCTTGCATACGTAGACCATTCTTATGTTGTAAATGCTGTTACGACCATGTCATAT   16342
gb:LC521925|Organism:Severe      GTGGTGCTTGCATACGTAGACCATTCTTATGTTGTAAATGCTGTTACGACCATGTCATAT   16315
gb:MT093571|Organism:Severe      GTGGTGCTTGCATACGTAGACCATTCTTATGTTGTAAATGCTGTTACGACCATGTCATAT   16342
gb:MT039887|Organism:Severe      GTGGTGCTTGCATACGTAGACCATTCTTATGTTGTAAATGCTGTTACGACCATGTCATAT   16342
gb:MT019530|Organism:Severe      GTGGTGCTTGCATACGTAGACCATTCTTATGTTGTAAATGCTGTTACGACCATGTCATAT   16342
gb:MT039888|Organism:Severe      GTGGTGCTTGCATACGTAGACCATTCTTATGTTGTAAATGCTGTTACGACCATGTCATAT   16342
gb:LC522972|Organism:Severe      GTGGTGCTTGCATACGTAGACCATTCTTATGTTGTAAATGCTGTTACGACCATGTCATAT   16339
gb:MT027063|Organism:Severe      GTGGTGCTTGCATACGTAGACCATTCTTATGTTGTAAATGCTGTTACGACCATGTCATAT   16342
gb:MT027062|Organism:Severe      GTGGTGCTTGCATACGTAGACCATTCTTATGTTGTAAATGCTGTTACGACCATGTCATAT   16342
gb:MT019529|Organism:Severe      GTGGTGCTTGCATACGTAGACCATTCTTATGTTGTAAATGCTGTTACGACCATGTCATAT   16342
```

```
gb:MN996529|Organism:Severe      GTGGTGCTTGCATACGTAGACCATTCTTATGTTGTAAATGCTGTTACGACCATGTCATAT   16330
gb:MN996531|Organism:Severe      GTGGTGCTTGCATACGTAGACCATTCTTATGTTGTAAATGCTGTTACGACCATGTCATAT   16329
gb:MT066176|Organism:Severe      GTGGTGCTTGCATACGTAGACCATTCTTATGTTGTAAATGCTGTTACGACCATGTCATAT   16342
gb:MT027064|Organism:Severe      GTGGTGCTTGCATACGTAGACCATTCTTATGTTGTAAATGCTGTTACGACCATGTCATAT   16342
gb:MN994468|Organism:Severe      GTGGTGCTTGCATACGTAGACCATTCTTATGTTGTAAATGCTGTTACGACCATGTCATAT   16342
gb:MT072688|Organism:Severe      GTGGTGCTTGCATACGTAGACCATTCTTATGTTGTAAATGCTGTTACGACCATGTCATAT   16327
gb:MN996527|Organism:Severe      GTGGTGCTTGCATACGTAGACCATTCTTATGTTGTAAATGCTGTTACGACCATGTCATAT   16309
gb:MT093631|Organism:Severe      GTGGTGCTTGCATACGTAGACCATTCTTATGTTGTAAATGCTGTTACGACCATGTCATAT   16380
gb:MT106053|Organism:Severe      GTGGTGCTTGCATACGTAGACCATTCTTATGTTGTAAATGCTGTTACGACCATGTCATAT   16342
gb:MT019533|Organism:Severe      GTGGTGCTTGCATACGTAGACCATTCTTATGTTGTAAATGCTGTTACGACCATGTCATAT   16342
gb:MT019531|Organism:Severe      GTGGTGCTTGCATACGTAGACCATTCTTATGTTGTAAATGCTGTTACGACCATGTCATAT   16342
gb:MN996528|Organism:Severe      GTGGTGCTTGCATACGTAGACCATTCTTATGTTGTAAATGCTGTTACGACCATGTCATAT   16342
gb:MN996530|Organism:Severe      GTGGTGCTTGCATACGTAGACCATTCTTATGTTGTAAATGCTGTTACGACCATGTCATAT   16328
gb:MN908947|Organism:Severe      GTGGTGCTTGCATACGTAGACCATTCTTATGTTGTAAATGCTGTTACGACCATGTCATAT   16342
gb:MT019532|Organism:Severe      GTGGTGCTTGCATACGTAGACCATTCTTATGTTGTAAATGCTGTTACGACCATGTCATAT   16342
                                 ************************************************************

gb:MT020781|Organism:Severe      CAACATCACATAAATTAGTCTTGTCTGTTAATCCGTATGTTTGCAATGCTCCAGGTTGTG   16390
gb:MT007544|Organism:Severe      CAACATCACATAAATTAGTCTTGTCTGTTAATCCGTATGTTTGCAATGCTCCAGGTTGTG   16402
gb:MN994467|Organism:Severe      CAACATCACATAAATTAGTCTTGTCTGTTAATCCGTATGTTTGCAATGCTCCAGGTTGTG   16402
gb:MT044257|Organism:Severe      CAACATCACATAAATTAGTCTTGTCTGTTAATCCGTATGTTTGCAATGCTCCAGGTTGTG   16402
gb:MT106054|Organism:Severe      CAACATCACATAAATTAGTCTTGTCTGTTAATCCGTATGTTTGCAATGCTCCAGGTTGTG   16402
gb:MT049951|Organism:Severe      CAACATCACATAAATTAGTCTTGTCTGTTAATCCGTATGTTTGCAATGCTCCAGGTTGTG   16402
gb:MN975262|Organism:Severe      CAACATCACATAAATTAGTCTTGTCTGTTAATCCGTATGTTTGCAATGCTCCAGGTTGTG   16402
gb:MT106052|Organism:Severe      CAACATCACATAAATTAGTCTTGTCTGTTAATCCGTATGTTTGCAATGCTCCAGGTTGTG   16402
gb:LC522975|Organism:Severe      CAACATCACATAAATTAGTCTTGTCTGTTAATCCGTATGTTTGCAATGCTCCAGGTTGTG   16399
gb:LC522973|Organism:Severe      CAACATCACATAAATTAGTCTTGTCTGTTAATCCGTATGTTTGCAATGCTCCAGGTTGTG   16399
gb:LC522974|Organism:Severe      CAACATCACATAAATTAGTCTTGTCTGTTAATCCGTATGTTTGCAATGCTCCAGGTTGTG   16399
gb:MN985325|Organism:Severe      CAACATCACATAAATTAGTCTTGTCTGTTAATCCGTATGTTTGCAATGCTCCAGGTTGTG   16402
gb:MT020881|Organism:Severe      CAACATCACATAAATTAGTCTTGTCTGTTAATCCGTATGTTTGCAATGCTCCAGGTTGTG   16402
gb:MT020880|Organism:Severe      CAACATCACATAAATTAGTCTTGTCTGTTAATCCGTATGTTTGCAATGCTCCAGGTTGTG   16402
gb:MT066175|Organism:Severe      CAACATCACATAAATTAGTCTTGTCTGTTAATCCGTATGTTTGCAATGCTCCAGGTTGTG   16402
gb:MN997409|Organism:Severe      CAACATCACATAAATTAGTCTTGTCTGTTAATCCGTATGTTTGCAATGCTCCAGGTTGTG   16402
gb:MN938384|Organism:Severe      CAACATCACATAAATTAGTCTTGTCTGTTAATCCGTATGTTTGCAATGCTCCAGGTTGTG   16370
gb:MT044258|Organism:Severe      CAACATCACATAAATTAGTCTTGTCTGTTAATCCGTATGTTTGCAATGCTCCAGGTTGTG   16378
gb:MT039890|Organism:Severe      CAACATCACATAAATTAGTCTTGTCTGTTAATCCGTATGTTTGCAATGCTCCAGGTTGTG   16402
gb:MN988713|Organism:Severe      CAACATCACATAAATTAGTCTTGTCTGTTAATCCGTATGTTTGCAATGCTCCAGGTTGTG   16402
gb:LC521925|Organism:Severe      CAACATCACATAAATTAGTCTTGTCTGTTAATCCGTATGTTTGCAATGCTCCAGGTTGTG   16375
gb:MT093571|Organism:Severe      CAACATCACATAAATTAGTCTTGTCTGTTAATCCGTATGTTTGCAATGCTCCAGGTTGTG   16402
gb:MT039887|Organism:Severe      CAACATCACATAAATTAGTCTTGTCTGTTAATCCGTATGTTTGCAATGCTCCAGGTTGTG   16402
gb:MT019530|Organism:Severe      CAACATCACATAAATTAGTCTTGTCTGTTAATCCGTATGTTTGCAATGCTCCAGGTTGTG   16402
gb:MT039888|Organism:Severe      CAACATCACATAAATTAGTCTTGTCTGTTAATCCGTATGTTTGCAATGCTCCAGGTTGTG   16402
gb:LC522972|Organism:Severe      CAACATCACATAAATTAGTCTTGTCTGTTAATCCGTATGTTTGCAATGCTCCAGGTTGTG   16399
gb:MT027063|Organism:Severe      CAACATCACATAAATTAGTCTTGTCTGTTAATCCGTATGTTTGCAATGCTCCAGGTTGTG   16402
gb:MT027062|Organism:Severe      CAACATCACATAAATTAGTCTTGTCTGTTAATCCGTATGTTTGCAATGCTCCAGGTTGTG   16402
gb:MT019529|Organism:Severe      CAACATCACATAAATTAGTCTTGTCTGTTAATCCGTATGTTTGCAATGCTCCAGGTTGTG   16402
gb:MN996529|Organism:Severe      CAACATCACATAAATTAGTCTTGTCTGTTAATCCGTATGTTTGCAATGCTCCAGGTTGTG   16390
gb:MN996531|Organism:Severe      CAACATCACATAAATTAGTCTTGTCTGTTAATCCGTATGTTTGCAATGCTCCAGGTTGTG   16389
gb:MT066176|Organism:Severe      CAACATCACATAAATTAGTCTTGTCTGTTAATCCGTATGTTTGCAATGCTCCAGGTTGTG   16402
gb:MT027064|Organism:Severe      CAACATCACATAAATTAGTCTTGTCTGTTAATCCGTATGTTTGCAATGCTCCAGGTTGTG   16402
gb:MN994468|Organism:Severe      CAACATCACATAAATTAGTCTTGTCTGTTAATCCGTATGTTTGCAATGCTCCAGGTTGTG   16402
gb:MT072688|Organism:Severe      CAACATCACATAAATTAGTCTTGTCTGTTAATCCGTATGTTTGCAATGCTCCAGGTTGTG   16387
gb:MN996527|Organism:Severe      CAACATCACATAAATTAGTCTTGTCTGTTAATCCGTATGTTTGCAATGCTCCAGGTTGTG   16369
gb:MT093631|Organism:Severe      CAACATCACATAAATTAGTCTTGTCTGTTAATCCGTATGTTTGCAATGCTCCAGGTTGTG   16440
gb:MT106053|Organism:Severe      CAACATCACATAAATTAGTCTTGTCTGTTAATCCGTATGTTTGCAATGCTCCAGGTTGTG   16402
gb:MT019533|Organism:Severe      CAACATCACATAAATTAGTCTTGTCTGTTAATCCGTATGTTTGCAATGCTCCAGGTTGTG   16402
gb:MT019531|Organism:Severe      CAACATCACATAAATTAGTCTTGTCTGTTAATCCGTATGTTTGCAATGCTCCAGGTTGTG   16402
gb:MN996528|Organism:Severe      CAACATCACATAAATTAGTCTTGTCTGTTAATCCGTATGTTTGCAATGCTCCAGGTTGTG   16402
gb:MN996530|Organism:Severe      CAACATCACATAAATTAGTCTTGTCTGTTAATCCGTATGTTTGCAATGCTCCAGGTTGTG   16388
gb:MN908947|Organism:Severe      CAACATCACATAAATTAGTCTTGTCTGTTAATCCGTATGTTTGCAATGCTCCAGGTTGTG   16402
gb:MT019532|Organism:Severe      CAACATCACATAAATTAGTCTTGTCTGTTAATCCGTATGTTTGCAATGCTCCAGGTTGTG   16402
                                 ************************************************************

gb:MT020781|Organism:Severe      ATGTCACAGATGTGACTCAACTTTACTTAGGAGGTATGAGCTATTATTGTAAATCACATA   16450
gb:MT007544|Organism:Severe      ATGTCACAGATGTGACTCAACTTTACTTAGGAGGTATGAGCTATTATTGTAAATCACATA   16462
```

```
gb:MN994467|Organism:Severe      ATGTCACAGATGTGACTCAACTTTACTTAGGAGGTATGAGCTATTATTGTAAATCACATA   16462
gb:MT044257|Organism:Severe      ATGTCACAGATGTGACTCAACTTTACTTAGGAGGTATGAGCTATTATTGTAAATCACATA   16462
gb:MT106054|Organism:Severe      ATGTCACAGATGTGACTCAACTTTACTTAGGAGGTATGAGCTATTATTGTAAATCACATA   16462
gb:MT049951|Organism:Severe      ATGTCACAGATGTGACTCAACTTTACTTAGGAGGTATGAGCTATTATTGTAAATCACATA   16462
gb:MN975262|Organism:Severe      ATGTCACAGATGTGACTCAACTTTACTTAGGAGGTATGAGCTATTATTGTAAATCACATA   16462
gb:MT106052|Organism:Severe      ATGTCACAGATGTGACTCAACTTTACTTAGGAGGTATGAGCTATTATTGTAAATCACATA   16462
gb:LC522975|Organism:Severe      ATGTCACAGATGTGACTCAACTTTACTTAGGAGGTATGAGCTATTATTGTAAATCACATA   16459
gb:LC522973|Organism:Severe      ATGTCACAGATGTGACTCAACTTTACTTAGGAGGTATGAGCTATTATTGTAAATCACATA   16459
gb:LC522974|Organism:Severe      ATGTCACAGATGTGACTCAACTTTACTTAGGAGGTATGAGCTATTATTGTAAATCACATA   16459
gb:MN985325|Organism:Severe      ATGTCACAGATGTGACTCAACTTTACTTAGGAGGTATGAGCTATTATTGTAAATCACATA   16462
gb:MT020881|Organism:Severe      ATGTCACAGATGTGACTCAACTTTACTTAGGAGGTATGAGCTATTATTGTAAATCACATA   16462
gb:MT020880|Organism:Severe      ATGTCACAGATGTGACTCAACTTTACTTAGGAGGTATGAGCTATTATTGTAAATCACATA   16462
gb:MT066175|Organism:Severe      ATGTCACAGATGTGACTCAACTTTACTTAGGAGGTATGAGCTATTATTGTAAATCACATA   16462
gb:MN997409|Organism:Severe      ATGTCACAGATGTGACTCAACTTTACTTAGGAGGTATGAGCTATTATTGTAAATCACATA   16462
gb:MN938384|Organism:Severe      ATGTCACAGATGTGACTCAACTTTACTTAGGAGGTATGAGCTATTATTGTAAATCACATA   16430
gb:MT044258|Organism:Severe      ATGTCACAGATGTGACTCAACTTTACTTAGGAGGTATGAGCTATTATTGTAAATCACATA   16438
gb:MT039890|Organism:Severe      ATGTCACAGATGTGACTCAACTTTACTTAGGAGGTATGAGCTATTATTGTAAATCACATA   16462
gb:MN988713|Organism:Severe      ATGTCACAGATGTGACTCAACTTTACTTAGGAGGTATGAGCTATTATTGTAAATCACATA   16462
gb:LC521925|Organism:Severe      ATGTCACAGATGTGACTCAACTTTACTTAGGAGGTATGAGCTATTATTGTAAATCACATA   16435
gb:MT093571|Organism:Severe      ATGTCACAGATGTGACTCAACTTTACTTAGGAGGTATGAGCTATTATTGTAAATCACATA   16462
gb:MT039887|Organism:Severe      ATGTCACAGATGTGACTCAACTTTACTTAGGAGGTATGAGCTATTATTGTAAATCACATA   16462
gb:MT019530|Organism:Severe      ATGTCACAGATGTGACTCAACTTTACTTAGGAGGTATGAGCTATTATTGTAAATCACATA   16462
gb:MT039888|Organism:Severe      ATGTCACAGATGTGACTCAACTTTACTTAGGAGGTATGAGCTATTATTGTAAATCACATA   16462
gb:LC522972|Organism:Severe      ATGTCACAGATGTGACTCAACTTTACTTAGGAGGTATGAGCTATTATTGTAAATCACATA   16459
gb:MT027063|Organism:Severe      ATGTCACAGATGTGACTCAACTTTACTTAGGAGGTATGAGCTATTATTGTAAATCACATA   16462
gb:MT027062|Organism:Severe      ATGTCACAGATGTGACTCAACTTTACTTAGGAGGTATGAGCTATTATTGTAAATCACATA   16462
gb:MT019529|Organism:Severe      ATGTCACAGATGTGACTCAACTTTACTTAGGAGGTATGAGCTATTATTGTAAATCACATA   16462
gb:MN996529|Organism:Severe      ATGTCACAGATGTGACTCAACTTTACTTAGGAGGTATGAGCTATTATTGTAAATCACATA   16450
gb:MN996531|Organism:Severe      ATGTCACAGATGTGACTCAACTTTACTTAGGAGGTATGAGCTATTATTGTAAATCACATA   16449
gb:MT066176|Organism:Severe      ATGTCACAGATGTGACTCAACTTTACTTAGGAGGTATGAGCTATTATTGTAAATCACATA   16462
gb:MT027064|Organism:Severe      ATGTCACAGATGTGACTCAACTTTACTTAGGAGGTATGAGCTATTATTGTAAATCACATA   16462
gb:MN994468|Organism:Severe      ATGTCACAGATGTGACTCAACTTTACTTAGGAGGTATGAGCTATTATTGTAAATCACATA   16462
gb:MT072688|Organism:Severe      ATGTCACAGATGTGACTCAACTTTACTTAGGAGGTATGAGCTATTATTGTAAATCACATA   16447
gb:MN996527|Organism:Severe      ATGTCACAGATGTGACTCAACTTTACTTAGGAGGTATGAGCTATTATTGTAAATCACATA   16429
gb:MT093631|Organism:Severe      ATGTCACAGATGTGACTCAACTTTACTTAGGAGGTATGAGCTATTATTGTAAATCACATA   16500
gb:MT106053|Organism:Severe      ATGTCACAGATGTGACTCAACTTTACTTAGGAGGTATGAGCTATTATTGTAAATCACATA   16462
gb:MT019533|Organism:Severe      ATGTCACAGATGTGACTCAACTTTACTTAGGAGGTATGAGCTATTATTGTAAATCACATA   16462
gb:MT019531|Organism:Severe      ATGTCACAGATGTGACTCAACTTTACTTAGGAGGTATGAGCTATTATTGTAAATCACATA   16462
gb:MN996528|Organism:Severe      ATGTCACAGATGTGACTCAACTTTACTTAGGAGGTATGAGCTATTATTGTAAATCACATA   16462
gb:MN996530|Organism:Severe      ATGTCACAGATGTGACTCAACTTTACTTAGGAGGTATGAGCTATTATTGTAAATCACATA   16448
gb:MN908947|Organism:Severe      ATGTCACAGATGTGACTCAACTTTACTTAGGAGGTATGAGCTATTATTGTAAATCACATA   16462
gb:MT019532|Organism:Severe      ATGTCACAGATGTGACTCAACTTTACTTAGGAGGTATGAGCTATTATTGTAAATCACATA   16462
                                 ************************************************************

gb:MT020781|Organism:Severe      AACCACCCATTAGTTTTCCATTGTGTGCTAATGGACAAGTTTTTGGTTTATATAAAAATA   16510
gb:MT007544|Organism:Severe      AACCACCCATTAGTTTTCCATTGTGTGCTAATGGACAAGTTTTTGGTTTATATAAAAATA   16522
gb:MN994467|Organism:Severe      AACCACCCATTAGTTTTCCATTGTGTGCTAATGGACAAGTTTTTGGTTTATATAAAAATA   16522
gb:MT044257|Organism:Severe      AACCACCCATTAGTTTTCCATTGTGTGCTAATGGACAAGTTTTTGGTTTATATAAAAATA   16522
gb:MT106054|Organism:Severe      AACCACCCATTAGTTTTCCATTGTGTGCTAATGGACAAGTTTTTGGTTTATATAAAAATA   16522
gb:MT049951|Organism:Severe      AACCACCCATTAGTTTTCCATTGTGTGCTAATGGACAAGTTTTTGGTTTATATAAAAATA   16522
gb:MN975262|Organism:Severe      AACCACCCATTAGTTTTCCATTGTGTGCTAATGGACAAGTTTTTGGTTTATATAAAAATA   16522
gb:MT106052|Organism:Severe      AACCACCCATTAGTTTTCCATTGTGTGCTAATGGACAAGTTTTTGGTTTATATAAAAATA   16522
gb:LC522975|Organism:Severe      AACCACCCATTAGTTTTCCATTGTGTGCTAATGGACAAGTTTTTGGTTTATATAAAAATA   16519
gb:LC522973|Organism:Severe      AACCACCCATTAGTTTTCCATTGTGTGCTAATGGACAAGTTTTTGGTTTATATAAAAATA   16519
gb:LC522974|Organism:Severe      AACCACCCATTAGTTTTCCATTGTGTGCTAATGGACAAGTTTTTGGTTTATATAAAAATA   16519
gb:MN985325|Organism:Severe      AACCACCCATTAGTTTTCCATTGTGTGCTAATGGACAAGTTTTTGGTTTATATAAAAATA   16522
gb:MT020881|Organism:Severe      AACCACCCATTAGTTTTCCATTGTGTGCTAATGGACAAGTTTTTGGTTTATATAAAAATA   16522
gb:MT020880|Organism:Severe      AACCACCCATTAGTTTTCCATTGTGTGCTAATGGACAAGTTTTTGGTTTATATAAAAATA   16522
gb:MT066175|Organism:Severe      AACCACCCATTAGTTTTCCATTGTGTGCTAATGGACAAGTTTTTGGTTTATATAAAAATA   16522
gb:MN997409|Organism:Severe      AACCACCCATTAGTTTTCCATTGTGTGCTAATGGACAAGTTTTTGGTTTATATAAAAATA   16522
gb:MN938384|Organism:Severe      AACCACCCATTAGTTTTCCATTGTGTGCTAATGGACAAGTTTTTGGTTTATATAAAAATA   16490
gb:MT044258|Organism:Severe      AACCACCCATTAGTTTTCCATTGTGTGCTAATGGACAAGTTTTTGGTTTATATAAAAATA   16498
gb:MT039890|Organism:Severe      AACCACCCATTAGTTTTCCATTGTGTGCTAATGGACAAGTTTTTGGTTTATATAAAAATA   16522
gb:MN988713|Organism:Severe      AACCACCCATTAGTTTTCCATTGTGTGCTAATGGACAAGTTTTTGGTTTATATAAAAATA   16522
gb:LC521925|Organism:Severe      AACCACCCATTAGTTTTCCATTGTGTGCTAATGGACAAGTTTTTGGTTTATATAAAAATA   16495
```

```
gb:MT093571|Organism:Severe      AACCACCCATTAGTTTTCCATTGTGTGCTAATGGACAAGTTTTTGGTTTATATAAAAATA   16522
gb:MT039887|Organism:Severe      AACCACCCATTAGTTTTCCATTGTGTGCTAATGGACAAGTTTTTGGTTTATATAAAAATA   16522
gb:MT019530|Organism:Severe      AACCACCCATTAGTTTTCCATTGTGTGCTAATGGACAAGTTTTTGGTTTATATAAAAATA   16522
gb:MT039888|Organism:Severe      AACCACCCATTAGTTTTCCATTGTGTGCTAATGGACAAGTTTTTGGTTTATATAAAAATA   16522
gb:LC522972|Organism:Severe      AACCACCCATTAGTTTTCCATTGTGTGCTAATGGACAAGTTTTTGGTTTATATAAAAATA   16519
gb:MT027063|Organism:Severe      AACCACCCATTAGTTTTCCATTGTGTGCTAATGGACAAGTTTTTGGTTTATATAAAAATA   16522
gb:MT027062|Organism:Severe      AACCACCCATTAGTTTTCCATTGTGTGCTAATGGACAAGTTTTTGGTTTATATAAAAATA   16522
gb:MT019529|Organism:Severe      AACCACCCATTAGTTTTCCATTGTGTGCTAATGGACAAGTTTTTGGTTTATATAAAAATA   16522
gb:MN996529|Organism:Severe      AACCACCCATTAGTTTTCCATTGTGTGCTAATGGACAAGTTTTTGGTTTATATAAAAATA   16510
gb:MN996531|Organism:Severe      AACCACCCATTAGTTTTCCATTGTGTGCTAATGGACAAGTTTTTGGTTTATATAAAAATA   16509
gb:MT066176|Organism:Severe      AACCACCCATTAGTTTTCCATTGTGTGCTAATGGACAAGTTTTTGGTTTATATAAAAATA   16522
gb:MT027064|Organism:Severe      AACCACCCATTAGTTTTCCATTGTGTGCTAATGGACAAGTTTTTGGTTTATATAAAAATA   16522
gb:MN994468|Organism:Severe      AACCACCCATTAGTTTTCCATTGTGTGCTAATGGACAAGTTTTTGGTTTATATAAAAATA   16522
gb:MT072688|Organism:Severe      AACCACCCATTAGTTTTCCATTGTGTGCTAATGGACAAGTTTTTGGTTTATATAAAAATA   16507
gb:MN996527|Organism:Severe      AACCACCCATTAGTTTTCCATTGTGTGCTAATGGACAAGTTTTTGGTTTATATAAAAATA   16489
gb:MT093631|Organism:Severe      AACCACCCATTAGTTTTCCATTGTGTGCTAATGGACAAGTTTTTGGTTTATATAAAAATA   16560
gb:MT106053|Organism:Severe      AACCACCCATTAGTTTTCCATTGTGTGCTAATGGACAAGTTTTTGGTTTATATAAAAATA   16522
gb:MT019533|Organism:Severe      AACCACCCATTAGTTTTCCATTGTGTGCTAATGGACAAGTTTTTGGTTTATATAAAAATA   16522
gb:MT019531|Organism:Severe      AACCACCCATTAGTTTTCCATTGTGTGCTAATGGACAAGTTTTTGGTTTATATAAAAATA   16522
gb:MN996528|Organism:Severe      AACCACCCATTAGTTTTCCATTGTGTGCTAATGGACAAGTTTTTGGTTTATATAAAAATA   16522
gb:MN996530|Organism:Severe      AACCACCCATTAGTTTTCCATTGTGTGCTAATGGACAAGTTTTTGGTTTATATAAAAATA   16508
gb:MN908947|Organism:Severe      AACCACCCATTAGTTTTCCATTGTGTGCTAATGGACAAGTTTTTGGTTTATATAAAAATA   16522
gb:MT019532|Organism:Severe      AACCACCCATTAGTTTTCCATTGTGTGCTAATGGACAAGTTTTTGGTTTATATAAAAATA   16522
                                 ************************************************************

gb:MT020781|Organism:Severe      CATGTGTTGGTAGCGATAATGTTACTGACTTTAATGCAATTGCAACATGTGACTGGACAA   16570
gb:MT007544|Organism:Severe      CATGTGTTGGTAGCGATAATGTTACTGACTTTAATGCAATTGCAACATGTGACTGGACAA   16582
gb:MN994467|Organism:Severe      CATGTGTTGGTAGCGATAATGTTACTGACTTTAATGCAATTGCAACATGTGACTGGACAA   16582
gb:MT044257|Organism:Severe      CATGTGTTGGTAGCGATAATGTTACTGACTTTAATGCAATTGCAACATGTGACTGGACAA   16582
gb:MT106054|Organism:Severe      CATGTGTTGGTAGCGATAATGTTACTGACTTTAATGCAATTGCAACATGTGACTGGACAA   16582
gb:MT049951|Organism:Severe      CATGTGTTGGTAGCGATAATGTTACTGACTTTAATGCAATTGCAACATGTGACTGGACAA   16582
gb:MN975262|Organism:Severe      CATGTGTTGGTAGCGATAATGTTACTGACTTTAATGCAATTGCAACATGTGACTGGACAA   16582
gb:MT106052|Organism:Severe      CATGTGTTGGTAGCGATAATGTTACTGACTTTAATGCAATTGCAACATGTGACTGGACAA   16582
gb:LC522975|Organism:Severe      CATGTGTTGGTAGCGATAATGTTACTGACTTTAATGCAATTGCAACATGTGACTGGACAA   16579
gb:LC522973|Organism:Severe      CATGTGTTGGTAGCGATAATGTTACTGACTTTAATGCAATTGCAACATGTGACTGGACAA   16579
gb:LC522974|Organism:Severe      CATGTGTTGGTAGCGATAATGTTACTGACTTTAATGCAATTGCAACATGTGACTGGACAA   16579
gb:MN985325|Organism:Severe      CATGTGTTGGTAGCGATAATGTTACTGACTTTAATGCAATTGCAACATGTGACTGGACAA   16582
gb:MT020881|Organism:Severe      CATGTGTTGGTAGCGATAATGTTACTGACTTTAATGCAATTGCAACATGTGACTGGACAA   16582
gb:MT020880|Organism:Severe      CATGTGTTGGTAGCGATAATGTTACTGACTTTAATGCAATTGCAACATGTGACTGGACAA   16582
gb:MT066175|Organism:Severe      CATGTGTTGGTAGCGATAATGTTACTGACTTTAATGCAATTGCAACATGTGACTGGACAA   16582
gb:MN997409|Organism:Severe      CATGTGTTGGTAGCGATAATGTTACTGACTTTAATGCAATTGCAACATGTGACTGGACAA   16582
gb:MN938384|Organism:Severe      CATGTGTTGGTAGCGATAATGTTACTGACTTTAATGCAATTGCAACATGTGACTGGACAA   16550
gb:MT044258|Organism:Severe      CATGTGTTGGTAGCGATAATGTTACTGACTTTAATGCAATTGCAACATGTGACTGGACAA   16558
gb:MT039890|Organism:Severe      CATGTGTTGGTAGCGATAATGTTACTGACTTTAATGCAATTGCAACATGTGACTGGACAA   16582
gb:MN988713|Organism:Severe      CATGTGTTGGTAGCGATAATGTTACTGACTTTAATGCAATTGCAACATGTGACTGGACAA   16582
gb:LC521925|Organism:Severe      CATGTGTTGGTAGCGATAATGTTACTGACTTTAATGCAATTGCAACATGTGACTGGACAA   16555
gb:MT093571|Organism:Severe      CATGTGTTGGTAGCGATAATGTTACTGACTTTAATGCAATTGCAACATGTGACTGGACAA   16582
gb:MT039887|Organism:Severe      CATGTGTTGGTAGCGATAATGTTACTGACTTTAATGCAATTGCAACATGTGACTGGACAA   16582
gb:MT019530|Organism:Severe      CATGTGTTGGTAGCGATAATGTTACTGACTTTAATGCAATTGCAACATGTGACTGGACAA   16582
gb:MT039888|Organism:Severe      CATGTGTTGGTAGCGATAATGTTACTGACTTTAATGCAATTGCAACATGTGACTGGACAA   16582
gb:LC522972|Organism:Severe      CATGTGTTGGTAGCGATAATGTTACTGACTTTAATGCAATTGCAACATGTGACTGGACAA   16579
gb:MT027063|Organism:Severe      CATGTGTTGGTAGCGATAATGTTACTGACTTTAATGCAATTGCAACATGTGACTGGACAA   16582
gb:MT027062|Organism:Severe      CATGTGTTGGTAGCGATAATGTTACTGACTTTAATGCAATTGCAACATGTGACTGGACAA   16582
gb:MT019529|Organism:Severe      CATGTGTTGGTAGCGATAATGTTACTGACTTTAATGCAATTGCAACATGTGACTGGACAA   16582
gb:MN996529|Organism:Severe      CATGTGTTGGTAGCGATAATGTTACTGACTTTAATGCAATTGCAACATGTGACTGGACAA   16570
gb:MN996531|Organism:Severe      CATGTGTTGGTAGCGATAATGTTACTGACTTTAATGCAATTGCAACATGTGACTGGACAA   16569
gb:MT066176|Organism:Severe      CATGTGTTGGTAGCGATAATGTTACTGACTTTAATGCAATTGCAACATGTGACTGGACAA   16582
gb:MT027064|Organism:Severe      CATGTGTTGGTAGCGATAATGTTACTGACTTTAATGCAATTGCAACATGTGACTGGACAA   16582
gb:MN994468|Organism:Severe      CATGTGTTGGTAGCGATAATGTTACTGACTTTAATGCAATTGCAACATGTGACTGGACAA   16582
gb:MT072688|Organism:Severe      CATGTGTTGGTAGCGATAATGTTACTGACTTTAATGCAATTGCAACATGTGACTGGACAA   16567
gb:MN996527|Organism:Severe      CATGTGTTGGTAGCGATAATGTTACTGACTTTAATGCAATTGCAACATGTGACTGGACAA   16549
gb:MT093631|Organism:Severe      CATGTGTTGGTAGCGATAATGTTACTGACTTTAATGCAATTGCAACATGTGACTGGACAA   16620
gb:MT106053|Organism:Severe      CATGTGTTGGTAGCGATAATGTTACTGACTTTAATGCAATTGCAACATGTGACTGGACAA   16582
gb:MT019533|Organism:Severe      CATGTGTTGGTAGCGATAATGTTACTGACTTTAATGCAATTGCAACATGTGACTGGACAA   16582
gb:MT019531|Organism:Severe      CATGTGTTGGTAGCGATAATGTTACTGACTTTAATGCAATTGCAACATGTGACTGGACAA   16582
```

```
gb:MN996528|Organism:Severe      CATGTGTTGGTAGCGATAATGTTACTGACTTTAATGCAATTGCAACATGTGACTGGACAA    16582
gb:MN996530|Organism:Severe      CATGTGTTGGTAGCGATAATGTTACTGACTTTAATGCAATTGCAACATGTGACTGGACAA    16568
gb:MN908947|Organism:Severe      CATGTGTTGGTAGCGATAATGTTACTGACTTTAATGCAATTGCAACATGTGACTGGACAA    16582
gb:MT019532|Organism:Severe      CATGTGTTGGTAGCGATAATGTTACTGACTTTAATGCAATTGCAACATGTGACTGGACAA    16582
                                 ************************************************************

gb:MT020781|Organism:Severe      ATGCTGGTGATTACATTTTAGCTAACACCTGTACTGAAAGACTCAAGCTTTTTGCAGCAG    16630
gb:MT007544|Organism:Severe      ATGCTGGTGATTACATTTTAGCTAACACCTGTACTGAAAGACTCAAGCTTTTTGCAGCAG    16642
gb:MN994467|Organism:Severe      ATGCTGGTGATTACATTTTAGCTAACACCTGTACTGAAAGACTCAAGCTTTTTGCAGCAG    16642
gb:MT044257|Organism:Severe      ATGCTGGTGATTACATTTTAGCTAACACCTGTACTGAAAGACTCAAGCTTTTTGCAGCAG    16642
gb:MT106054|Organism:Severe      ATGCTGGTGATTACATTTTAGCTAACACCTGTACTGAAAGACTCAAGCTTTTTGCAGCAG    16642
gb:MT049951|Organism:Severe      ATGCTGGTGATTACATTTTAGCTAACACCTGTACTGAAAGACTCAAGCTTTTTGCAGCAG    16642
gb:MN975262|Organism:Severe      ATGCTGGTGATTACATTTTAGCTAACACCTGTACTGAAAGACTCAAGCTTTTTGCAGCAG    16642
gb:MT106052|Organism:Severe      ATGCTGGTGATTACATTTTAGCTAACACCTGTACTGAAAGACTCAAGCTTTTTGCAGCAG    16642
gb:LC522975|Organism:Severe      ATGCTGGTGATTACATTTTAGCTAACACCTGTACTGAAAGACTCAAGCTTTTTGCAGCAG    16639
gb:LC522973|Organism:Severe      ATGCTGGTGATTACATTTTAGCTAACACCTGTACTGAAAGACTCAAGCTTTTTGCAGCAG    16639
gb:LC522974|Organism:Severe      ATGCTGGTGATTACATTTTAGCTAACACCTGTACTGAAAGACTCAAGCTTTTTGCAGCAG    16639
gb:MN985325|Organism:Severe      ATGCTGGTGATTACATTTTAGCTAACACCTGTACTGAAAGACTCAAGCTTTTTGCAGCAG    16642
gb:MT020881|Organism:Severe      ATGCTGGTGATTACATTTTAGCTAACACCTGTACTGAAAGACTCAAGCTTTTTGCAGCAG    16642
gb:MT020880|Organism:Severe      ATGCTGGTGATTACATTTTAGCTAACACCTGTACTGAAAGACTCAAGCTTTTTGCAGCAG    16642
gb:MT066175|Organism:Severe      ATGCTGGTGATTACATTTTAGCTAACACCTGTACTGAAAGACTCAAGCTTTTTGCAGCAG    16642
gb:MN997409|Organism:Severe      ATGCTGGTGATTACATTTTAGCTAACACCTGTACTGAAAGACTCAAGCTTTTTGCAGCAG    16642
gb:MN938384|Organism:Severe      ATGCTGGTGATTACATTTTAGCTAACACCTGTACTGAAAGACTCAAGCTTTTTGCAGCAG    16610
gb:MT044258|Organism:Severe      ATGCTGGTGATTACATTTTAGCTAACACCTGTACTGAAAGACTCAAGCTTTTTGCAGCAG    16618
gb:MT039890|Organism:Severe      ATGCTGGTGATTACATTTTAGCTAACACCTGTACTGAAAGACTCAAGCTTTTTGCAGCAG    16642
gb:MN988713|Organism:Severe      ATGCTGGTGATTACATTTTAGCTAACACCTGTACTGAAAGACTCAAGCTTTTTGCAGCAG    16642
gb:LC521925|Organism:Severe      ATGCTGGTGATTACATTTTAGCTAACACCTGTACTGAAAGACTCAAGCTTTTTGCAGCAG    16615
gb:MT093571|Organism:Severe      ATGCTGGTGATTACATTTTAGCTAACACCTGTACTGAAAGACTCAAGCTTTTTGCAGCAG    16642
gb:MT039887|Organism:Severe      ATGCTGGTGATTACATTTTAGCTAACACCTGTACTGAAAGACTCAAGCTTTTTGCAGCAG    16642
gb:MT019530|Organism:Severe      ATGCTGGTGATTACATTTTAGCTAACACCTGTACTGAAAGACTCAAGCTTTTTGCAGCAG    16642
gb:MT039888|Organism:Severe      ATGCTGGTGATTACATTTTAGCTAACACCTGTACTGAAAGACTCAAGCTTTTTGCAGCAG    16642
gb:LC522972|Organism:Severe      ATGCTGGTGATTACATTTTAGCTAACACCTGTACTGAAAGACTCAAGCTTTTTGCAGCAG    16639
gb:MT027063|Organism:Severe      ATGCTGGTGATTACATTTTAGCTAACACCTGTACTGAAAGACTCAAGCTTTTTGCAGCAG    16642
gb:MT027062|Organism:Severe      ATGCTGGTGATTACATTTTAGCTAACACCTGTACTGAAAGACTCAAGCTTTTTGCAGCAG    16642
gb:MT019529|Organism:Severe      ATGCTGGTGATTACATTTTAGCTAACACCTGTACTGAAAGACTCAAGCTTTTTGCAGCAG    16642
gb:MN996529|Organism:Severe      ATGCTGGTGATTACATTTTAGCTAACACCTGTACTGAAAGACTCAAGCTTTTTGCAGCAG    16630
gb:MN996531|Organism:Severe      ATGCTGGTGATTACATTTTAGCTAACACCTGTACTGAAAGACTCAAGCTTTTTGCAGCAG    16629
gb:MT066176|Organism:Severe      ATGCTGGTGATTACATTTTAGCTAACACCTGTACTGAAAGACTCAAGCTTTTTGCAGCAG    16642
gb:MT027064|Organism:Severe      ATGCTGGTGATTACATTTTAGCTAACACCTGTACTGAAAGACTCAAGCTTTTTGCAGCAG    16642
gb:MN994468|Organism:Severe      ATGCTGGTGATTACATTTTAGCTAACACCTGTACTGAAAGACTCAAGCTTTTTGCAGCAG    16642
gb:MT072688|Organism:Severe      ATGCTGGTGATTACATTTTAGCTAACACCTGTACTGAAAGACTCAAGCTTTTTGCAGCAG    16627
gb:MN996527|Organism:Severe      ATGCTGGTGATTACATTTTAGCTAACACCTGTACTGAAAGACTCAAGCTTTTTGCAGCAG    16609
gb:MT093631|Organism:Severe      ATGCTGGTGATTACATTTTAGCTAACACCTGTACTGAAAGACTCAAGCTTTTTGCAGCAG    16680
gb:MT106053|Organism:Severe      ATGCTGGTGATTACATTTTAGCTAACACCTGTACTGAAAGACTCAAGCTTTTTGCAGCAG    16642
gb:MT019533|Organism:Severe      ATGCTGGTGATTACATTTTAGCTAACACCTGTACTGAAAGACTCAAGCTTTTTGCAGCAG    16642
gb:MT019531|Organism:Severe      ATGCTGGTGATTACATTTTAGCTAACACCTGTACTGAAAGACTCAAGCTTTTTGCAGCAG    16642
gb:MN996528|Organism:Severe      ATGCTGGTGATTACATTTTAGCTAACACCTGTACTGAAAGACTCAAGCTTTTTGCAGCAG    16642
gb:MN996530|Organism:Severe      ATGCTGGTGATTACATTTTAGCTAACACCTGTACTGAAAGACTCAAGCTTTTTGCAGCAG    16628
gb:MN908947|Organism:Severe      ATGCTGGTGATTACATTTTAGCTAACACCTGTACTGAAAGACTCAAGCTTTTTGCAGCAG    16642
gb:MT019532|Organism:Severe      ATGCTGGTGATTACATTTTAGCTAACACCTGTACTGAAAGACTCAAGCTTTTTGCAGCAG    16642
                                 ************************************************************

gb:MT020781|Organism:Severe      AAACGCTCAAAGCTACTGAGGAGACATTTAAACTGTCTTATGGTATTGCTACTGTACGTG    16690
gb:MT007544|Organism:Severe      AAACGCTCAAAGCTACTGAGGAGACATTTAAACTGTCTTATGGTATTGCTACTGTACGTG    16702
gb:MN994467|Organism:Severe      AAACGCTCAAAGCTACTGAGGAGACATTTAAACTGTCTTATGGTATTGCTACTGTACGTG    16702
gb:MT044257|Organism:Severe      AAACGCTCAAAGCTACTGAGGAGACATTTAAACTGTCTTATGGTATTGCTACTGTACGTG    16702
gb:MT106054|Organism:Severe      AAACGCTCAAAGCTACTGAGGAGACATTTAAACTGTCTTATGGTATTGCTACTGTACGTG    16702
gb:MT049951|Organism:Severe      AAACGCTCAAAGCTACTGAGGAGACATTTAAACTGTCTTATGGTATTGCTACTGTACGTG    16702
gb:MN975262|Organism:Severe      AAACGCTCAAAGCTACTGAGGAGACATTTAAACTGTCTTATGGTATTGCTACTGTACGTG    16702
gb:MT106052|Organism:Severe      AAACGCTCAAAGCTACTGAGGAGACATTTAAACTGTCTTATGGTATTGCTACTGTACGTG    16702
gb:LC522975|Organism:Severe      AAACGCTCAAAGCTACTGAGGAGACATTTAAACTGTCTTATGGTATTGCTACTGTACGTG    16699
gb:LC522973|Organism:Severe      AAACGCTCAAAGCTACTGAGGAGACATTTAAACTGTCTTATGGTATTGCTACTGTACGTG    16699
gb:LC522974|Organism:Severe      AAACGCTCAAAGCTACTGAGGAGACATTTAAACTGTCTTATGGTATTGCTACTGTACGTG    16699
gb:MN985325|Organism:Severe      AAACGCTCAAAGCTACTGAGGAGACATTTAAACTGTCTTATGGTATTGCTACTGTACGTG    16702
gb:MT020881|Organism:Severe      AAACGCTCAAAGCTACTGAGGAGACATTTAAACTGTCTTATGGTATTGCTACTGTACGTG    16702
```

```
gb:MT020880|Organism:Severe      AAACGCTCAAAGCTACTGAGGAGACATTTAAACTGTCTTATGGTATTGCTACTGTACGTG    16702
gb:MT066175|Organism:Severe      AAACGCTCAAAGCTACTGAGGAGACATTTAAACTGTCTTATGGTATTGCTACTGTACGTG    16702
gb:MN997409|Organism:Severe      AAACGCTCAAAGCTACTGAGGAGACATTTAAACTGTCTTATGGTATTGCTACTGTACGTG    16702
gb:MN938384|Organism:Severe      AAACGCTCAAAGCTACTGAGGAGACATTTAAACTGTCTTATGGTATTGCTACTGTACGTG    16670
gb:MT044258|Organism:Severe      AAACGCTCAAAGCTACTGAGGAGACATTTAAACTGTCTTATGGTATTGCTACTGTACGTG    16678
gb:MT039890|Organism:Severe      AAACGCTCAAAGCTACTGAGGAGACATTTAAACTGTCTTATGGTATTGCTACTGTACGTG    16702
gb:MN988713|Organism:Severe      AAACGCTCAAAGCTACTGAGGAGACATTTAAACTGTCTTATGGTATTGCTACTGTACGTG    16702
gb:LC521925|Organism:Severe      AAACGCTCAAAGCTACTGAGGAGACATTTAAACTGTCTTATGGTATTGCTACTGTACGTG    16675
gb:MT093571|Organism:Severe      AAACGCTCAAAGCTACTGAGGAGACATTTAAACTGTCTTATGGTATTGCTACTGTACGTG    16702
gb:MT039887|Organism:Severe      AAACGCTCAAAGCTACTGAGGAGACATTTAAACTGTCTTATGGTATTGCTACTGTACGTG    16702
gb:MT019530|Organism:Severe      AAACGCTCAAAGCTACTGAGGAGACATTTAAACTGTCTTATGGTATTGCTACTGTACGTG    16702
gb:MT039888|Organism:Severe      AAACGCTCAAAGCTACTGAGGAGACATTTAAACTGTCTTATGGTATTGCTACTGTACGTG    16702
gb:LC522972|Organism:Severe      AAACGCTCAAAGCTACTGAGGAGACATTTAAACTGTCTTATGGTATTGCTACTGTACGTG    16699
gb:MT027063|Organism:Severe      AAACGCTCAAAGCTACTGAGGAGACATTTAAACTGTCTTATGGTATTGCTACTGTACGTG    16702
gb:MT027062|Organism:Severe      AAACGCTCAAAGCTACTGAGGAGACATTTAAACTGTCTTATGGTATTGCTACTGTACGTG    16702
gb:MT019529|Organism:Severe      AAACGCTCAAAGCTACTGAGGAGACATTTAAACTGTCTTATGGTATTGCTACTGTACGTG    16702
gb:MN996529|Organism:Severe      AAACGCTCAAAGCTACTGAGGAGACATTTAAACTGTCTTATGGTATTGCTACTGTACGTG    16690
gb:MN996531|Organism:Severe      AAACGCTCAAAGCTACTGAGGAGACATTTAAACTGTCTTATGGTATTGCTACTGTACGTG    16689
gb:MT066176|Organism:Severe      AAACGCTCAAAGCTACTGAGGAGACATTTAAACTGTCTTATGGTATTGCTACTGTACGTG    16702
gb:MT027064|Organism:Severe      AAACGCTCAAAGCTACTGAGGAGACATTTAAACTGTCTTATGGTATTGCTACTGTACGTG    16702
gb:MN994468|Organism:Severe      AAACGCTCAAAGCTACTGAGGAGACATTTAAACTGTCTTATGGTATTGCTACTGTACGTG    16702
gb:MT072688|Organism:Severe      AAACGCTCAAAGCTACTGAGGAGACATTTAAACTGTCTTATGGTATTGCTACTGTACGTG    16687
gb:MN996527|Organism:Severe      AAACGCTCAAAGCTACTGAGGAGACATTTAAACTGTCTTATGGTATTGCTACTGTACGTG    16669
gb:MT093631|Organism:Severe      AAACGCTCAAAGCTACTGAGGAGACATTTAAACTGTCTTATGGTATTGCTACTGTACGTG    16740
gb:MT106053|Organism:Severe      AAACGCTCAAAGCTACTGAGGAGACATTTAAACTGTCTTATGGTATTGCTACTGTACGTG    16702
gb:MT019533|Organism:Severe      AAACGCTCAAAGCTACTGAGGAGACATTTAAACTGTCTTATGGTATTGCTACTGTACGTG    16702
gb:MT019531|Organism:Severe      AAACGCTCAAAGCTACTGAGGAGACATTTAAACTGTCTTATGGTATTGCTACTGTACGTG    16702
gb:MN996528|Organism:Severe      AAACGCTCAAAGCTACTGAGGAGACATTTAAACTGTCTTATGGTATTGCTACTGTACGTG    16702
gb:MN996530|Organism:Severe      AAACGCTCAAAGCTACTGAGGAGACATTTAAACTGTCTTATGGTATTGCTACTGTACGTG    16688
gb:MN908947|Organism:Severe      AAACGCTCAAAGCTACTGAGGAGACATTTAAACTGTCTTATGGTATTGCTACTGTACGTG    16702
gb:MT019532|Organism:Severe      AAACGCTCAAAGCTACTGAGGAGACATTTAAACTGTCTTATGGTATTGCTACTGTACGTG    16702
                                 ************************************************************

gb:MT020781|Organism:Severe      AAGTGCTGTCTGACAGAGAATTACATCTTTCATGGGAAGTTGGTAAACCTAGACCACCAC    16750
gb:MT007544|Organism:Severe      AAGTGCTGTCTGACAGAGAATTACATCTTTCATGGGAAGTTGGTAAACCTAGACCACCAC    16762
gb:MN994467|Organism:Severe      AAGTGCTGTCTGACAGAGAATTACATCTTTCATGGGAAGTTGGTAAACCTAGACCACCAC    16762
gb:MT044257|Organism:Severe      AAGTGCTGTCTGACAGAGAATTACATCTTTCATGGGAAGTTGGTAAACCTAGACCACCAC    16762
gb:MT106054|Organism:Severe      AAGTGCTGTCTGACAGAGAATTACATCTTTCATGGGAAGTTGGTAAACCTAGACCACCAC    16762
gb:MT049951|Organism:Severe      AAGTGCTGTCTGACAGAGAATTACATCTTTCATGGGAAGTTGGTAAACCTAGACCACCAC    16762
gb:MN975262|Organism:Severe      AAGTGCTGTCTGACAGAGAATTACATCTTTCATGGGAAGTTGGTAAACCTAGACCACCAC    16762
gb:MT106052|Organism:Severe      AAGTGCTGTCTGACAGAGAATTACATCTTTCATGGGAAGTTGGTAAACCTAGACCACCAC    16762
gb:LC522975|Organism:Severe      AAGTGCTGTCTGACAGAGAATTACATCTTTCATGGGAAGTTGGTAAACCTAGACCACCAC    16759
gb:LC522973|Organism:Severe      AAGTGCTGTCTGACAGAGAATTACATCTTTCATGGGAAGTTGGTAAACCTAGACCACCAC    16759
gb:LC522974|Organism:Severe      AAGTGCTGTCTGACAGAGAATTACATCTTTCATGGGAAGTTGGTAAACCTAGACCACCAC    16759
gb:MN985325|Organism:Severe      AAGTGCTGTCTGACAGAGAATTACATCTTTCATGGGAAGTTGGTAAACCTAGACCACCAC    16762
gb:MT020881|Organism:Severe      AAGTGCTGTCTGACAGAGAATTACATCTTTCATGGGAAGTTGGTAAACCTAGACCACCAC    16762
gb:MT020880|Organism:Severe      AAGTGCTGTCTGACAGAGAATTACATCTTTCATGGGAAGTTGGTAAACCTAGACCACCAC    16762
gb:MT066175|Organism:Severe      AAGTGCTGTCTGACAGAGAATTACATCTTTCATGGGAAGTTGGTAAACCTAGACCACCAC    16762
gb:MN997409|Organism:Severe      AAGTGCTGTCTGACAGAGAATTACATCTTTCATGGGAAGTTGGTAAACCTAGACCACCAC    16762
gb:MN938384|Organism:Severe      AAGTGCTGTCTGACAGAGAATTACATCTTTCATGGGAAGTTGGTAAACCTAGACCACCAC    16730
gb:MT044258|Organism:Severe      AAGTGCTGTCTGACAGAGAATTACATCTTTCATGGGAAGTTGGTAAACCTAGACCACCAC    16738
gb:MT039890|Organism:Severe      AAGTGCTGTCTGACAGAGAATTACATCTTTCATGGGAAGTTGGTAAACCTAGACCACCAC    16762
gb:MN988713|Organism:Severe      AAGTGCTGTCTGACAGAGAATTACATCTTTCATGGGAAGTTGGTAAACCTAGACCACCAC    16762
gb:LC521925|Organism:Severe      AAGTGCTGTCTGACAGAGAATTACATCTTTCATGGGAAGTTGGTAAACCTAGACCACCAC    16735
gb:MT093571|Organism:Severe      AAGTGCTGTCTGACAGAGAATTACATCTTTCATGGGAAGTTGGTAAACCTAGACCACCAC    16762
gb:MT039887|Organism:Severe      AAGTGCTGTCTGACAGAGAATTACATCTTTCATGGGAAGTTGGTAAACCTAGACCACCAC    16762
gb:MT019530|Organism:Severe      AAGTGCTGTCTGACAGAGAATTACATCTTTCATGGGAAGTTGGTAAACCTAGACCACCAC    16762
gb:MT039888|Organism:Severe      AAGTGCTGTCTGACAGAGAATTACATCTTTCATGGGAAGTTGGTAAACCTAGACCACCAC    16762
gb:LC522972|Organism:Severe      AAGTGCTGTCTGACAGAGAATTACATCTTTCATGGGAAGTTGGTAAACCTAGACCACCAC    16759
gb:MT027063|Organism:Severe      AAGTGCTGTCTGACAGAGAATTACATCTTTCATGGGAAGTTGGTAAACCTAGACCACCAC    16762
gb:MT027062|Organism:Severe      AAGTGCTGTCTGACAGAGAATTACATCTTTCATGGGAAGTTGGTAAACCTAGACCACCAC    16762
gb:MT019529|Organism:Severe      AAGTGCTGTCTGACAGAGAATTACATCTTTCATGGGAAGTTGGTAAACCTAGACCACCAC    16762
gb:MN996529|Organism:Severe      AAGTGCTGTCTGACAGAGAATTACATCTTTCATGGGAAGTTGGTAAACCTAGACCACCAC    16750
gb:MN996531|Organism:Severe      AAGTGCTGTCTGACAGAGAATTACATCTTTCATGGGAAGTTGGTAAACCTAGACCACCAC    16749
gb:MT066176|Organism:Severe      AAGTGCTGTCTGACAGAGAATTACATCTTTCATGGGAAGTTGGTAAACCTAGACCACCAC    16762
```

```
gb:MT027064|Organism:Severe      AAGTGCTGTCTGACAGAGAATTACATCTTTCATGGGAAGTTGGTAAACCTAGACCACCAC    16762
gb:MN994468|Organism:Severe      AAGTGCTGTCTGACAGAGAATTACATCTTTCATGGGAAGTTGGTAAACCTAGACCACCAC    16762
gb:MT072688|Organism:Severe      AAGTGCTGTCTGACAGAGAATTACATCTTTCATGGGAAGTTGGTAAACCTAGACCACCAC    16747
gb:MN996527|Organism:Severe      AAGTGCTGTCTGACAGAGAATTACATCTTTCATGGGAAGTTGGTAAACCTAGACCACCAC    16729
gb:MT093631|Organism:Severe      AAGTGCTGTCTGACAGAGAATTACATCTTTCATGGGAAGTTGGTAAACCTAGACCACCAC    16800
gb:MT106053|Organism:Severe      AAGTGCTGTCTGACAGAGAATTACATCTTTCATGGGAAGTTGGTAAACCTAGACCACCAC    16762
gb:MT019533|Organism:Severe      AAGTGCTGTCTGACAGAGAATTACATCTTTCATGGGAAGTTGGTAAACCTAGACCACCAC    16762
gb:MT019531|Organism:Severe      AAGTGCTGTCTGACAGAGAATTACATCTTTCATGGGAAGTTGGTAAACCTAGACCACCAC    16762
gb:MN996528|Organism:Severe      AAGTGCTGTCTGACAGAGAATTACATCTTTCATGGGAAGTTGGTAAACCTAGACCACCAC    16762
gb:MN996530|Organism:Severe      AAGTGCTGTCTGACAGAGAATTACATCTTTCATGGGAAGTTGGTAAACCTAGACCACCAC    16748
gb:MN908947|Organism:Severe      AAGTGCTGTCTGACAGAGAATTACATCTTTCATGGGAAGTTGGTAAACCTAGACCACCAC    16762
gb:MT019532|Organism:Severe      AAGTGCTGTCTGACAGAGAATTACATCTTTCATGGGAAGTTGGTAAACCTAGACCACCAC    16762
                                 ************************************************************

gb:MT020781|Organism:Severe      TTAACCGAAATTATGTCTTTACTGGTTATCGTGTAACTAAAAACAGTAAAGTACAAATAG    16810
gb:MT007544|Organism:Severe      TTAACCGAAATTATGTCTTTACTGGTTATCGTGTAACTAAAAACAGTAAAGTACAAATAG    16822
gb:MN994467|Organism:Severe      TTAACCGAAATTATGTCTTTACTGGTTATCGTGTAACTAAAAACAGTAAAGTACAAATAG    16822
gb:MT044257|Organism:Severe      TTAACCGAAATTATGTCTTTACTGGTTATCGTGTAACTAAAAACAGTAAAGTACAAATAG    16822
gb:MT106054|Organism:Severe      TTAACCGAAATTATGTCTTTACTGGTTATCGTGTAACTAAAAACAGTAAAGTACAAATAG    16822
gb:MT049951|Organism:Severe      TTAACCGAAATTATGTCTTTACTGGTTATCGTGTAACTAAAAACAGTAAAGTACAAATAG    16822
gb:MN975262|Organism:Severe      TTAACCGAAATTATGTCTTTACTGGTTATCGTGTAACTAAAAACAGTAAAGTACAAATAG    16822
gb:MT106052|Organism:Severe      TTAACCGAAATTATGTCTTTACTGGTTATCGTGTAACTAAAAACAGTAAAGTACAAATAG    16822
gb:LC522975|Organism:Severe      TTAACCGAAATTATGTCTTTACTGGTTATCGTGTAACTAAAAACAGTAAAGTACAAATAG    16819
gb:LC522973|Organism:Severe      TTAACCGAAATTATGTCTTTACTGGTTATCGTGTAACTAAAAACAGTAAAGTACAAATAG    16819
gb:LC522974|Organism:Severe      TTAACCGAAATTATGTCTTTACTGGTTATCGTGTAACTAAAAACAGTAAAGTACAAATAG    16819
gb:MN985325|Organism:Severe      TTAACCGAAATTATGTCTTTACTGGTTATCGTGTAACTAAAAACAGTAAAGTACAAATAG    16822
gb:MT020881|Organism:Severe      TTAACCGAAATTATGTCTTTACTGGTTATCGTGTAACTAAAAACAGTAAAGTACAAATAG    16822
gb:MT020880|Organism:Severe      TTAACCGAAATTATGTCTTTACTGGTTATCGTGTAACTAAAAACAGTAAAGTACAAATAG    16822
gb:MT066175|Organism:Severe      TTAACCGAAATTATGTCTTTACTGGTTATCGTGTAACTAAAAACAGTAAAGTACAAATAG    16822
gb:MN997409|Organism:Severe      TTAACCGAAATTATGTCTTTACTGGTTATCGTGTAACTAAAAACAGTAAAGTACAAATAG    16822
gb:MN938384|Organism:Severe      TTAACCGAAATTATGTCTTTACTGGTTATCGTGTAACTAAAAACAGTAAAGTACAAATAG    16790
gb:MT044258|Organism:Severe      TTAACCGAAATTATGTCTTTACTGGTTATCGTGTAACTAAAAACAGTAAAGTACAAATAG    16798
gb:MT039890|Organism:Severe      TTAACCGAAATTATGTCTTTACTGGTTATCGTGTAACTAAAAACAGTAAAGTACAAATAG    16822
gb:MN988713|Organism:Severe      TTAACCGAAATTATGTCTTTACTGGTTATCGTGTAACTAAAAACAGTAAAGTACAAATAG    16822
gb:LC521925|Organism:Severe      TTAACCGAAATTATGTCTTTACTGGTTATCGTGTAACTAAAAACAGTAAAGTACAAATAG    16795
gb:MT093571|Organism:Severe      TTAACCGAAATTATGTCTTTACTGGTTATCGTGTAACTAAAAACAGTAAAGTACAAATAG    16822
gb:MT039887|Organism:Severe      TTAACCGAAATTATGTCTTTACTGGTTATCGTGTAACTAAAAACAGTAAAGTACAAATAG    16822
gb:MT019530|Organism:Severe      TTAACCGAAATTATGTCTTTACTGGTTATCGTGTAACTAAAAACAGTAAAGTACAAATAG    16822
gb:MT039888|Organism:Severe      TTAACCGAAATTATGTCTTTACTGGTTATCGTGTAACTAAAAACAGTAAAGTACAAATAG    16822
gb:LC522972|Organism:Severe      TTAACCGAAATTATGTCTTTACTGGTTATCGTGTAACTAAAAACAGTAAAGTACAAATAG    16819
gb:MT027063|Organism:Severe      TTAACCGAAATTATGTCTTTACTGGTTATCGTGTAACTAAAAACAGTAAAGTACAAATAG    16822
gb:MT027062|Organism:Severe      TTAACCGAAATTATGTCTTTACTGGTTATCGTGTAACTAAAAACAGTAAAGTACAAATAG    16822
gb:MT019529|Organism:Severe      TTAACCGAAATTATGTCTTTACTGGTTATCGTGTAACTAAAAACAGTAAAGTACAAATAG    16822
gb:MN996529|Organism:Severe      TTAACCGAAATTATGTCTTTACTGGTTATCGTGTAACTAAAAACAGTAAAGTACAAATAG    16810
gb:MN996531|Organism:Severe      TTAACCGAAATTATGTCTTTACTGGTTATCGTGTAACTAAAAACAGTAAAGTACAAATAG    16809
gb:MT066176|Organism:Severe      TTAACCGAAATTATGTCTTTACTGGTTATCGTGTAACTAAAAACAGTAAAGTACAAATAG    16822
gb:MT027064|Organism:Severe      TTAACCGAAATTATGTCTTTACTGGTTATCGTGTAACTAAAAACAGTAAAGTACAAATAG    16822
gb:MN994468|Organism:Severe      TTAACCGAAATTATGTCTTTACTGGTTATCGTGTAACTAAAAACAGTAAAGTACAAATAG    16822
gb:MT072688|Organism:Severe      TTAACCGAAATTATGTCTTTACTGGTTATCGTGTAACTAAAAACAGTAAAGTACAAATAG    16807
gb:MN996527|Organism:Severe      TTAACCGAAATTATGTCTTTACTGGTTATCGTGTAACTAAAAACAGTAAAGTACAAATAG    16789
gb:MT093631|Organism:Severe      TTAACCGAAATTATGTCTTTACTGGTTATCGTGTAACTAAAAACAGTAAAGTACAAATAG    16860
gb:MT106053|Organism:Severe      TTAACCGAAATTATGTCTTTACTGGTTATCGTGTAACTAAAAACAGTAAAGTACAAATAG    16822
gb:MT019533|Organism:Severe      TTAACCGAAATTATGTCTTTACTGGTTATCGTGTAACTAAAAACAGTAAAGTACAAATAG    16822
gb:MT019531|Organism:Severe      TTAACCGAAATTATGTCTTTACTGGTTATCGTGTAACTAAAAACAGTAAAGTACAAATAG    16822
gb:MN996528|Organism:Severe      TTAACCGAAATTATGTCTTTACTGGTTATCGTGTAACTAAAAACAGTAAAGTACAAATAG    16822
gb:MN996530|Organism:Severe      TTAACCGAAATTATGTCTTTACTGGTTATCGTGTAACTAAAAACAGTAAAGTACAAATAG    16808
gb:MN908947|Organism:Severe      TTAACCGAAATTATGTCTTTACTGGTTATCGTGTAACTAAAAACAGTAAAGTACAAATAG    16822
gb:MT019532|Organism:Severe      TTAACCGAAATTATGTCTTTACTGGTTATCGTGTAACTAAAAACAGTAAAGTACAAATAG    16822
                                 ************************************************************

gb:MT020781|Organism:Severe      GAGAGTACACCTTTGAAAAAGGTGACTATGGTGATGCTGTTGTTTACCGAGGTACAACAA    16870
gb:MT007544|Organism:Severe      GAGAGTACACCTTTGAAAAAGGTGACTATGGTGATGCTGTTGTTTACCGAGGTACAACAA    16882
gb:MN994467|Organism:Severe      GAGAGTACACCTTTGAAAAAGGTGACTATGGTGATGCTGTTGTTTACCGAGGTACAACAA    16882
gb:MT044257|Organism:Severe      GAGAGTACACCTTTGAAAAAGGTGACTATGGTGATGCTGTTGTTTACCGAGGTACAACAA    16882
gb:MT106054|Organism:Severe      GAGAGTACACCTTTGAAAAAGGTGACTATGGTGATGCTGTTGTTTACCGAGGTACAACAA    16882
```

```
gb:MT049951|Organism:Severe      GAGAGTACACCTTTGAAAAAGGTGACTATGGTGATGCTGTTGTTTACCGAGGTACAACAA    16882
gb:MN975262|Organism:Severe      GAGAGTACACCTTTGAAAAAGGTGACTATGGTGATGCTGTTGTTTACCGAGGTACAACAA    16882
gb:MT106052|Organism:Severe      GAGAGTACACCTTTGAAAAAGGTGACTATGGTGATGCTGTTGTTTACCGAGGTACAACAA    16882
gb:LC522975|Organism:Severe      GAGAGTACACCTTTGAAAAAGGTGACTATGGTGATGCTGTTGTTTACCGAGGTACAACAA    16879
gb:LC522973|Organism:Severe      GAGAGTACACCTTTGAAAAAGGTGACTATGGTGATGCTGTTGTTTACCGAGGTACAACAA    16879
gb:LC522974|Organism:Severe      GAGAGTACACCTTTGAAAAAGGTGACTATGGTGATGCTGTTGTTTACCGAGGTACAACAA    16879
gb:MN985325|Organism:Severe      GAGAGTACACCTTTGAAAAAGGTGACTATGGTGATGCTGTTGTTTACCGAGGTACAACAA    16882
gb:MT020881|Organism:Severe      GAGAGTACACCTTTGAAAAAGGTGACTATGGTGATGCTGTTGTTTACCGAGGTACAACAA    16882
gb:MT020880|Organism:Severe      GAGAGTACACCTTTGAAAAAGGTGACTATGGTGATGCTGTTGTTTACCGAGGTACAACAA    16882
gb:MT066175|Organism:Severe      GAGAGTACACCTTTGAAAAAGGTGACTATGGTGATGCTGTTGTTTACCGAGGTACAACAA    16882
gb:MN997409|Organism:Severe      GAGAGTACACCTTTGAAAAAGGTGACTATGGTGATGCTGTTGTTTACCGAGGTACAACAA    16882
gb:MN938384|Organism:Severe      GAGAGTACACCTTTGAAAAAGGTGACTATGGTGATGCTGTTGTTTACCGAGGTACAACAA    16850
gb:MT044258|Organism:Severe      GAGAGTACACCTTTGAAAAAGGTGACTATGGTGATGCTGTTGTTTACCGAGGTACAACAA    16858
gb:MT039890|Organism:Severe      GAGAGTACACCTTTGAAAAAGGTGACTATGGTGATGCTGTTGTTTACCGAGGTACAACAA    16882
gb:MN988713|Organism:Severe      GAGAGTACACCTTTGAAAAAGGTGACTATGGTGATGCTGTTGTTTACCGAGGTACAACAA    16882
gb:LC521925|Organism:Severe      GAGAGTACACCTTTGAAAAAGGTGACTATGGTGATGCTGTTGTTTACCGAGGTACAACAA    16855
gb:MT093571|Organism:Severe      GAGAGTACACCTTTGAAAAAGGTGACTATGGTGATGCTGTTGTTTACCGAGGTACAACAA    16882
gb:MT039887|Organism:Severe      GAGAGTACACCTTTGAAAAAGGTGACTATGGTGATGCTGTTGTTTACCGAGGTACAACAA    16882
gb:MT019530|Organism:Severe      GAGAGTACACCTTTGAAAAAGGTGACTATGGTGATGCTGTTGTTTACCGAGGTACAACAA    16882
gb:MT039888|Organism:Severe      GAGAGTACACCTTTGAAAAAGGTGACTATGGTGATGCTGTTGTTTACCGAGGTACAACAA    16882
gb:LC522972|Organism:Severe      GAGAGTACACCTTTGAAAAAGGTGACTATGGTGATGCTGTTGTTTACCGAGGTACAACAA    16879
gb:MT027063|Organism:Severe      GAGAGTACACCTTTGAAAAAGGTGACTATGGTGATGCTGTTGTTTACCGAGGTACAACAA    16882
gb:MT027062|Organism:Severe      GAGAGTACACCTTTGAAAAAGGTGACTATGGTGATGCTGTTGTTTACCGAGGTACAACAA    16882
gb:MT019529|Organism:Severe      GAGAGTACACCTTTGAAAAAGGTGACTATGGTGATGCTGTTGTTTACCGAGGTACAACAA    16882
gb:MN996529|Organism:Severe      GAGAGTACACCTTTGAAAAAGGTGACTATGGTGATGCTGTTGTTTACCGAGGTACAACAA    16870
gb:MN996531|Organism:Severe      GAGAGTACACCTTTGAAAAAGGTGACTATGGTGATGCTGTTGTTTACCGAGGTACAACAA    16869
gb:MT066176|Organism:Severe      GAGAGTACACCTTTGAAAAAGGTGACTATGGTGATGCTGTTGTTTACCGAGGTACAACAA    16882
gb:MT027064|Organism:Severe      GAGAGTACACCTTTGAAAAAGGTGACTATGGTGATGCTGTTGTTTACCGAGGTACAACAA    16882
gb:MN994468|Organism:Severe      GAGAGTACACCTTTGAAAAAGGTGACTATGGTGATGCTGTTGTTTACCGAGGTACAACAA    16882
gb:MT072688|Organism:Severe      GAGAGTACACCTTTGAAAAAGGTGACTATGGTGATGCTGTTGTTTACCGAGGTACAACAA    16867
gb:MN996527|Organism:Severe      GAGAGTACACCTTTGAAAAAGGTGACTATGGTGATGCTGTTGTTTACCGAGGTACAACAA    16849
gb:MT093631|Organism:Severe      GAGAGTACACCTTTGAAAAAGGTGACTATGGTGATGCTGTTGTTTACCGAGGTACAACAA    16920
gb:MT106053|Organism:Severe      GAGAGTACACCTTTGAAAAAGGTGACTATGGTGATGCTGTTGTTTACCGAGGTACAACAA    16882
gb:MT019533|Organism:Severe      GAGAGTACACCTTTGAAAAAGGTGACTATGGTGATGCTGTTGTTTACCGAGGTACAACAA    16882
gb:MT019531|Organism:Severe      GAGAGTACACCTTTGAAAAAGGTGACTATGGTGATGCTGTTGTTTACCGAGGTACAACAA    16882
gb:MN996528|Organism:Severe      GAGAGTACACCTTTGAAAAAGGTGACTATGGTGATGCTGTTGTTTACCGAGGTACAACAA    16882
gb:MN996530|Organism:Severe      GAGAGTACACCTTTGAAAAAGGTGACTATGGTGATGCTGTTGTTTACCGAGGTACAACAA    16868
gb:MN908947|Organism:Severe      GAGAGTACACCTTTGAAAAAGGTGACTATGGTGATGCTGTTGTTTACCGAGGTACAACAA    16882
gb:MT019532|Organism:Severe      GAGAGTACACCTTTGAAAAAGGTGACTATGGTGATGCTGTTGTTTACCGAGGTACAACAA    16882
                                 ************************************************************

gb:MT020781|Organism:Severe      CTTACAAATTAAATGTTGGTGATTATTTTGTGCTGACATCACATACAGTAATGCCATTAA    16930
gb:MT007544|Organism:Severe      CTTACAAATTAAATGTTGGTGATTATTTTGTGCTGACATCACATACAGTAATGCCATTAA    16942
gb:MN994467|Organism:Severe      CTTACAAATTAAATGTTGGTGATTATTTTGTGCTGACATCACATACAGTAATGCCATTAA    16942
gb:MT044257|Organism:Severe      CTTACAAATTAAATGTTGGTGATTATTTTGTGCTGACATCACATACAGTAATGCCATTAA    16942
gb:MT106054|Organism:Severe      CTTACAAATTAAATGTTGGTGATTATTTTGTGCTGACATCACATACAGTAATGCCATTAA    16942
gb:MT049951|Organism:Severe      CTTACAAATTAAATGTTGGTGATTATTTTGTGCTGACATCACATACAGTAATGCCATTAA    16942
gb:MN975262|Organism:Severe      CTTACAAATTAAATGTTGGTGATTATTTTGTGCTGACATCACATACAGTAATGCCATTAA    16942
gb:MT106052|Organism:Severe      CTTACAAATTAAATGTTGGTGATTATTTTGTGCTGACATCACATACAGTAATGCCATTAA    16942
gb:LC522975|Organism:Severe      CTTACAAATTAAATGTTGGTGATTATTTTGTGCTGACATCACATACAGTAATGCCATTAA    16939
gb:LC522973|Organism:Severe      CTTACAAATTAAATGTTGGTGATTATTTTGTGCTGACATCACATACAGTAATGCCATTAA    16939
gb:LC522974|Organism:Severe      CTTACAAATTAAATGTTGGTGATTATTTTGTGCTGACATCACATACAGTAATGCCATTAA    16939
gb:MN985325|Organism:Severe      CTTACAAATTAAATGTTGGTGATTATTTTGTGCTGACATCACATACAGTAATGCCATTAA    16942
gb:MT020881|Organism:Severe      CTTACAAATTAAATGTTGGTGATTATTTTGTGCTGACATCACATACAGTAATGCCATTAA    16942
gb:MT020880|Organism:Severe      CTTACAAATTAAATGTTGGTGATTATTTTGTGCTGACATCACATACAGTAATGCCATTAA    16942
gb:MT066175|Organism:Severe      CTTACAAATTAAATGTTGGTGATTATTTTGTGCTGACATCACATACAGTAATGCCATTAA    16942
gb:MN997409|Organism:Severe      CTTACAAATTAAATGTTGGTGATTATTTTGTGCTGACATCACATACAGTAATGCCATTAA    16942
gb:MN938384|Organism:Severe      CTTACAAATTAAATGTTGGTGATTATTTTGTGCTGACATCACATACAGTAATGCCATTAA    16910
gb:MT044258|Organism:Severe      CTTACAAATTAAATGTTGGTGATTATTTTGTGCTGACATCACATACAGTAATGCCATTAA    16918
gb:MT039890|Organism:Severe      CTTACAAATTAAATGTTGGTGATTATTTTGTGCTGACATCACATACAGTAATGCCATTAA    16942
gb:MN988713|Organism:Severe      CTTACAAATTAAATGTTGGTGATTATTTTGTGCTGACATCACATACAGTAATGCCATTAA    16942
gb:LC521925|Organism:Severe      CTTACAAATTAAATGTTGGTGATTATTTTGTGCTGACATCACATACAGTAATGCCATTAA    16915
gb:MT093571|Organism:Severe      CTTACAAATTAAATGTTGGTGATTATTTTGTGCTGACATCACATACAGTAATGCCATTAA    16942
gb:MT039887|Organism:Severe      CTTACAAATTAAATGTTGGTGATTATTTTGTGCTGACATCACATACAGTAATGCCATTAA    16942
gb:MT019530|Organism:Severe      CTTACAAATTAAATGTTGGTGATTATTTTGTGCTGACATCACATACAGTAATGCCATTAA    16942
```

```
gb:MT039888|Organism:Severe      CTTACAAATTAAATGTTGGTGATTATTTTGTGCTGACATCACATACAGTAATGCCATTAA   16942
gb:LC522972|Organism:Severe      CTTACAAATTAAATGTTGGTGATTATTTTGTGCTGACATCACATACAGTAATGCCATTAA   16939
gb:MT027063|Organism:Severe      CTTACAAATTAAATGTTGGTGATTATTTTGTGCTGACATCACATACAGTAATGCCATTAA   16942
gb:MT027062|Organism:Severe      CTTACAAATTAAATGTTGGTGATTATTTTGTGCTGACATCACATACAGTAATGCCATTAA   16942
gb:MT019529|Organism:Severe      CTTACAAATTAAATGTTGGTGATTATTTTGTGCTGACATCACATACAGTAATGCCATTAA   16942
gb:MN996529|Organism:Severe      CTTACAAATTAAATGTTGGTGATTATTTTGTGCTGACATCACATACAGTAATGCCATTAA   16930
gb:MN996531|Organism:Severe      CTTACAAATTAAATGTTGGTGATTATTTTGTGCTGACATCACATACAGTAATGCCATTAA   16929
gb:MT066176|Organism:Severe      CTTACAAATTAAATGTTGGTGATTATTTTGTGCTGACATCACATACAGTAATGCCATTAA   16942
gb:MT027064|Organism:Severe      CTTACAAATTAAATGTTGGTGATTATTTTGTGCTGACATCACATACAGTAATGCCATTAA   16942
gb:MN994468|Organism:Severe      CTTACAAATTAAATGTTGGTGATTATTTTGTGCTGACATCACATACAGTAATGCCATTAA   16942
gb:MT072688|Organism:Severe      CTTACAAATTAAATGTTGGTGATTATTTTGTGCTGACATCACATACAGTAATGCCATTAA   16927
gb:MN996527|Organism:Severe      CTTACAAATTAAATGTTGGTGATTATTTTGTGCTGACATCACATACAGTAATGCCATTAA   16909
gb:MT093631|Organism:Severe      CTTACAAATTAAATGTTGGTGATTATTTTGTGCTGACATCACATACAGTAATGCCATTAA   16980
gb:MT106053|Organism:Severe      CTTACAAATTAAATGTTGGTGATTATTTTGTGCTGACATCACATACAGTAATGCCATTAA   16942
gb:MT019533|Organism:Severe      CTTACAAATTAAATGTTGGTGATTATTTTGTGCTGACATCACATACAGTAATGCCATTAA   16942
gb:MT019531|Organism:Severe      CTTACAAATTAAATGTTGGTGATTATTTTGTGCTGACATCACATACAGTAATGCCATTAA   16942
gb:MN996528|Organism:Severe      CTTACAAATTAAATGTTGGTGATTATTTTGTGCTGACATCACATACAGTAATGCCATTAA   16942
gb:MN996530|Organism:Severe      CTTACAAATTAAATGTTGGTGATTATTTTGTGCTGACATCACATACAGTAATGCCATTAA   16928
gb:MN908947|Organism:Severe      CTTACAAATTAAATGTTGGTGATTATTTTGTGCTGACATCACATACAGTAATGCCATTAA   16942
gb:MT019532|Organism:Severe      CTTACAAATTAAATGTTGGTGATTATTTTGTGCTGACATCACATACAGTAATGCCATTAA   16942
                                 ************************************************************

gb:MT020781|Organism:Severe      GTGCACCTACACTAGTGCCACAAGAGCACTATGTTAGAATTACTGGCTTATACCCAACAC   16990
gb:MT007544|Organism:Severe      GTGCACCTACACTAGTGCCACAAGAGCACTATGTTAGAATTACTGGCTTATACCCAACAC   17002
gb:MN994467|Organism:Severe      GTGCACCTACACTAGTGCCACAAGAGCACTATGTTAGAATTACTGGCTTATACCCAACAC   17002
gb:MT044257|Organism:Severe      GTGCACCTACACTAGTGCCACAAGAGCACTATGTTAGAATTACTGGCTTATACCCAACAC   17002
gb:MT106054|Organism:Severe      GTGCACCTACACTAGTGCCACAAGAGCACTATGTTAGAATTACTGGCTTATACCCAACAC   17002
gb:MT049951|Organism:Severe      GTGCACCTACACTAGTGCCACAAGAGCACTATGTTAGAATTACTGGCTTATACCCAACAC   17002
gb:MN975262|Organism:Severe      GTGCACCTACACTAGTGCCACAAGAGCACTATGTTAGAATTACTGGCTTATACCCAACAC   17002
gb:MT106052|Organism:Severe      GTGCACCTACACTAGTGCCACAAGAGCACTATGTTAGAATTACTGGCTTATACCCAACAC   17002
gb:LC522975|Organism:Severe      GTGCACCTACACTAGTGCCACAAGAGCACTATGTTAGAATTACTGGCTTATACCCAACAC   16999
gb:LC522973|Organism:Severe      GTGCACCTACACTAGTGCCACAAGAGCACTATGTTAGAATTACTGGCTTATACCCAACAC   16999
gb:LC522974|Organism:Severe      GTGCACCTACACTAGTGCCACAAGAGCACTATGTTAGAATTACTGGCTTATACCCAACAC   16999
gb:MN985325|Organism:Severe      GTGCACCTACACTAGTGCCACAAGAGCACTATGTTAGAATTACTGGCTTATACCCAACAC   17002
gb:MT020881|Organism:Severe      GTGCACCTACACTAGTGCCACAAGAGCACTATGTTAGAATTACTGGCTTATACCCAACAC   17002
gb:MT020880|Organism:Severe      GTGCACCTACACTAGTGCCACAAGAGCACTATGTTAGAATTACTGGCTTATACCCAACAC   17002
gb:MT066175|Organism:Severe      GTGCACCTACACTAGTGCCACAAGAGCACTATGTTAGAATTACTGGCTTATACCCAACAC   17002
gb:MN997409|Organism:Severe      GTGCACCTACACTAGTGCCACAAGAGCACTATGTTAGAATTACTGGCTTATACCCAACAC   17002
gb:MN938384|Organism:Severe      GTGCACCTACACTAGTGCCACAAGAGCACTATGTTAGAATTACTGGCTTATACCCAACAC   16970
gb:MT044258|Organism:Severe      GTGCACCTACACTAGTGCCACAAGAGCACTATGTTAGAATTACTGGCTTATACCCAACAC   16978
gb:MT039890|Organism:Severe      GTGCACCTACACTAGTGCCACAAGAGCACTATGTTAGAATTACTGGCTTATACCCAACAC   17002
gb:MN988713|Organism:Severe      GTGCACCTACACTAGTGCCACAAGAGCACTATGTTAGAATTACTGGCTTATACCCAACAC   17002
gb:LC521925|Organism:Severe      GTGCACCTACACTAGTGCCACAAGAGCACTATGTTAGAATTACTGGCTTATACCCAACAC   16975
gb:MT093571|Organism:Severe      GTGCACCTACACTAGTGCCACAAGAGCACTATGTTAGAATTACTGGCTTATACCCAACAC   17002
gb:MT039887|Organism:Severe      GTGCACCTACACTAGTGCCACAAGAGCACTATGTTAGAATTACTGGCTTATACCCAACAC   17002
gb:MT019530|Organism:Severe      GTGCACCTACACTAGTGCCACAAGAGCACTATGTTAGAATTACTGGCTTATACCCAACAC   17002
gb:MT039888|Organism:Severe      GTGCACCTACACTAGTGCCACAAGAGCACTATGTTAGAATTACTGGCTTATACCCAACAC   17002
gb:LC522972|Organism:Severe      GTGCACCTACACTAGTGCCACAAGAGCACTATGTTAGAATTACTGGCTTATACCCAACAC   16999
gb:MT027063|Organism:Severe      GTGCACCTACACTAGTGCCACAAGAGCACTATGTTAGAATTACTGGCTTATACCCAACAC   17002
gb:MT027062|Organism:Severe      GTGCACCTACACTAGTGCCACAAGAGCACTATGTTAGAATTACTGGCTTATACCCAACAC   17002
gb:MT019529|Organism:Severe      GTGCACCTACACTAGTGCCACAAGAGCACTATGTTAGAATTACTGGCTTATACCCAACAC   17002
gb:MN996529|Organism:Severe      GTGCACCTACACTAGTGCCACAAGAGCACTATGTTAGAATTACTGGCTTATACCCAACAC   16990
gb:MN996531|Organism:Severe      GTGCACCTACACTAGTGCCACAAGAGCACTATGTTAGAATTACTGGCTTATACCCAACAC   16989
gb:MT066176|Organism:Severe      GTGCACCTACACTAGTGCCACAAGAGCACTATGTTAGAATTACTGGCTTATACCCAACAC   17002
gb:MT027064|Organism:Severe      GTGCACCTACACTAGTGCCACAAGAGCACTATGTTAGAATTACTGGCTTATACCCAACAC   17002
gb:MN994468|Organism:Severe      GTGCACCTACACTAGTGCCACAAGAGCACTATGTTAGAATTACTGGCTTATACCCAATAC   17002
gb:MT072688|Organism:Severe      GTGCACCTACACTAGTGCCACAAGAGCACTATGTTAGAATTACTGGCTTATACCCAACAC   16987
gb:MN996527|Organism:Severe      GTGCACCTACACTAGTGCCACAAGAGCACTATGTTAGAATTACTGGCTTATACCCAACAC   16969
gb:MT093631|Organism:Severe      GTGCACCTACACTAGTGCCACAAGAGCACTATGTTAGAATTACTGGCTTATACCCAACAC   17040
gb:MT106053|Organism:Severe      GTGCACCTACACTAGTGCCACAAGAGCACTATGTTAGAATTACTGGCTTATACCCAACAC   17002
gb:MT019533|Organism:Severe      GTGCACCTACACTAGTGCCACAAGAGCACTATGTTAGAATTACTGGCTTATACCCAACAC   17002
gb:MT019531|Organism:Severe      GTGCACCTACACTAGTGCCACAAGAGCACTATGTTAGAATTACTGGCTTATACCCAACAC   17002
gb:MN996528|Organism:Severe      GTGCACCTACACTAGTGCCACAAGAGCACTATGTTAGAATTACTGGCTTATACCCAACAC   17002
gb:MN996530|Organism:Severe      GTGCACCTACACTAGTGCCACAAGAGCACTATGTTAGAATTACTGGCTTATACCCAACAC   16988
gb:MN908947|Organism:Severe      GTGCACCTACACTAGTGCCACAAGAGCACTATGTTAGAATTACTGGCTTATACCCAACAC   17002
```

```
gb:MT019532|Organism:Severe      GTGCACCTACACTAGTGCCACAAGAGCACTATGTTAGAATTACTGGCTTATACCCAACAC   17002
                                 ********************************************************* **

gb:MT020781|Organism:Severe      TCAATATCTCAGATGAGTTTTCTAGCAATGTTGCAAATTATCAAAAGGTTGGTATGCAAA   17050
gb:MT007544|Organism:Severe      TCAATATCTCAGATGAGTTTTCTAGCAATGTTGCAAATTATCAAAAGGTTGGTATGCAAA   17062
gb:MN994467|Organism:Severe      TCAATATCTCAGATGAGTTTTCTAGCAATGTTGCAAATTATCAAAAGGTTGGTATGCAAA   17062
gb:MT044257|Organism:Severe      TCAATATCTCAGATGAGTTTTCTAGCAATGTTGCAAATTATCAAAAGGTTGGTATGCAAA   17062
gb:MT106054|Organism:Severe      TCAATATCTCAGATGAGTTTTCTAGCAATGTTGCAAATTATCAAAAGGTTGGTATGCAAA   17062
gb:MT049951|Organism:Severe      TCAATATCTCAGATGAGTTTTCTAGCAATGTTGCAAATTATCAAAAGGTTGGTATGCAAA   17062
gb:MN975262|Organism:Severe      TCAATATCTCAGATGAGTTTTCTAGCAATGTTGCAAATTATCAAAAGGTTGGTATGCAAA   17062
gb:MT106052|Organism:Severe      TCAATATCTCAGATGAGTTTTCTAGCAATGTTGCAAATTATCAAAAGGTTGGTATGCAAA   17062
gb:LC522975|Organism:Severe      TCAATATCTCAGATGAGTTTTCTAGCAATGTTGCAAATTATCAAAAGGTTGGTATGCAAA   17059
gb:LC522973|Organism:Severe      TCAATATCTCAGATGAGTTTTCTAGCAATGTTGCAAATTATCAAAAGGTTGGTATGCAAA   17059
gb:LC522974|Organism:Severe      TCAATATCTCAGATGAGTTTTCTAGCAATGTTGCAAATTATCAAAAGGTTGGTATGCAAA   17059
gb:MN985325|Organism:Severe      TCAATATCTCAGATGAGTTTTCTAGCAATGTTGCAAATTATCAAAAGGTTGGTATGCAAA   17062
gb:MT020881|Organism:Severe      TCAATATCTCAGATGAGTTTTCTAGCAATGTTGCAAATTATCAAAAGGTTGGTATGCAAA   17062
gb:MT020880|Organism:Severe      TCAATATCTCAGATGAGTTTTCTAGCAATGTTGCAAATTATCAAAAGGTTGGTATGCAAA   17062
gb:MT066175|Organism:Severe      TCAATATCTCAGATGAGTTTTCTAGCAATGTTGCAAATTATCAAAAGGTTGGTATGCAAA   17062
gb:MN997409|Organism:Severe      TCAATATCTCAGATGAGTTTTCTAGCAATGTTGCAAATTATCAAAAGGTTGGTATGCAAA   17062
gb:MN938384|Organism:Severe      TCAATATCTCAGATGAGTTTTCTAGCAATGTTGCAAATTATCAAAAGGTTGGTATGCAAA   17030
gb:MT044258|Organism:Severe      TCAATATCTCAGATGAGTTTTCTAGCAATGTTGCAAATTATCAAAAGGTTGGTATGCAAA   17038
gb:MT039890|Organism:Severe      TCAATATCTCAGATGAGTTTTCTAGCAATGTTGCAAATTATCAAAAGGTTGGTATGCAAA   17062
gb:MN988713|Organism:Severe      TCAATATCTCAGATGAGTTTTCTAGCAATGTTGCAAATTATCAAAAGGTTGGTATGCAAA   17062
gb:LC521925|Organism:Severe      TCAATATCTCAGATGAGTTTTCTAGCAATGTTGCAAATTATCAAAAGGTTGGTATGCAAA   17035
gb:MT093571|Organism:Severe      TCAATATCTCAGATGAGTTTTCTAGCAATGTTGCAAATTATCAAAAGGTTGGTATGCAAA   17062
gb:MT039887|Organism:Severe      TCAATATCTCAGATGAGTTTTCTAGCAATGTTGCAAATTATCAAAAGGTTGGTATGCAAA   17062
gb:MT019530|Organism:Severe      TCAATATCTCAGATGAGTTTTCTAGCAATGTTGCAAATTATCAAAAGGTTGGTATGCAAA   17062
gb:MT039888|Organism:Severe      TCAATATCTCAGATGAGTTTTCTAGCAATGTTGCAAATTATCAAAAGGTTGGTATGCAAA   17062
gb:LC522972|Organism:Severe      TCAATATCTCAGATGAGTTTTCTAGCAATGTTGCAAATTATCAAAAGGTTGGTATGCAAA   17059
gb:MT027063|Organism:Severe      TCAATATCTCAGATGAGTTTTCTAGCAATGTTGCAAATTATCAAAAGGTTGGTATGCAAA   17062
gb:MT027062|Organism:Severe      TCAATATCTCAGATGAGTTTTCTAGCAATGTTGCAAATTATCAAAAGGTTGGTATGCAAA   17062
gb:MT019529|Organism:Severe      TCAATATCTCAGATGAGTTTTCTAGCAATGTTGCAAATTATCAAAAGGTTGGTATGCAAA   17062
gb:MN996529|Organism:Severe      TCAATATCTCAGATGAGTTTTCTAGCAATGTTGCAAATTATCAAAAGGTTGGTATGCAAA   17050
gb:MN996531|Organism:Severe      TCAATATCTCAGATGAGTTTTCTAGCAATGTTGCAAATTATCAAAAGGTTGGTATGCAAA   17049
gb:MT066176|Organism:Severe      TCAATATCTCAGATGAGTTTTCTAGCAATGTTGCAAATTATCAAAAGGTTGGTATGCAAA   17062
gb:MT027064|Organism:Severe      TCAATATCTCAGATGAGTTTTCTAGCAATGTTGCAAATTATCAAAAGGTTGGTATGCAAA   17062
gb:MN994468|Organism:Severe      TCAATATCTCAGATGAGTTTTCTAGCAATGTTGCAAATTATCAAAAGGTTGGTATGCAAA   17062
gb:MT072688|Organism:Severe      TCAATATCTCAGATGAGTTTTCTAGCAATGTTGCAAATTATCAAAAGGTTGGTATGCAAA   17047
gb:MN996527|Organism:Severe      TCAATATCTCAGATGAGTTTTCTAGCAATGTTGCAAATTATCAAAAGGTTGGTATGCAAA   17029
gb:MT093631|Organism:Severe      TCAATATCTCAGATGAGTTTTCTAGCAATGTTGCAAATTATCAAAAGGTTGGTATGCAAA   17100
gb:MT106053|Organism:Severe      TCAATATCTCAGATGAGTTTTCTAGCAATGTTGCAAATTATCAAAAGGTTGGTATGCAAA   17062
gb:MT019533|Organism:Severe      TCAATATCTCAGATGAGTTTTCTAGCAATGTTGCAAATTATCAAAAGGTTGGTATGCAAA   17062
gb:MT019531|Organism:Severe      TCAATATCTCAGATGAGTTTTCTAGCAATGTTGCAAATTATCAAAAGGTTGGTATGCAAA   17062
gb:MN996528|Organism:Severe      TCAATATCTCAGATGAGTTTTCTAGCAATGTTGCAAATTATCAAAAGGTTGGTATGCAAA   17062
gb:MN996530|Organism:Severe      TCAATATCTCAGATGAGTTTTCTAGCAATGTTGCAAATTATCAAAAGGTTGGTATGCAAA   17048
gb:MN908947|Organism:Severe      TCAATATCTCAGATGAGTTTTCTAGCAATGTTGCAAATTATCAAAAGGTTGGTATGCAAA   17062
gb:MT019532|Organism:Severe      TCAATATCTCAGATGAGTTTTCTAGCAATGTTGCAAATTATCAAAAGGTTGGTATGCAAA   17062
                                 ************************************************************

gb:MT020781|Organism:Severe      AGTATTCTACACTCCAGGGACCACCTGGTACTGGTAAGAGTCATTTTGCTATTGGCCTAG   17110
gb:MT007544|Organism:Severe      AGTATTCTACACTCCAGGGACCACCTGGTACTGGTAAGAGTCATTTTGCTATTGGCCTAG   17122
gb:MN994467|Organism:Severe      AGTATTCTACACTCCAGGGACCACCTGGTACTGGTAAGAGTCATTTTGCTATTGGCCTAG   17122
gb:MT044257|Organism:Severe      AGTATTCTACACTCCAGGGACCACCTGGTACTGGTAAGAGTCATTTTGCTATTGGCCTAG   17122
gb:MT106054|Organism:Severe      AGTATTCTACACTCCAGGGACCACCTGGTACTGGTAAGAGTCATTTTGCTATTGGCCTAG   17122
gb:MT049951|Organism:Severe      AGTATTCTACACTCCAGGGACCACCTGGTACTGGTAAGAGTCATTTTGCTATTGGCCTAG   17122
gb:MN975262|Organism:Severe      AGTATTCTACACTCCAGGGACCACCTGGTACTGGTAAGAGTCATTTTGCTATTGGCCTAG   17122
gb:MT106052|Organism:Severe      AGTATTCTACACTCCAGGGACCACCTGGTACTGGTAAGAGTCATTTTGCTATTGGCCTAG   17122
gb:LC522975|Organism:Severe      AGTATTCTACACTCCAGGGACCACCTGGTACTGGTAAGAGTCATTTTGCTATTGGCCTAG   17119
gb:LC522973|Organism:Severe      AGTATTCTACACTCCAGGGACCACCTGGTACTGGTAAGAGTCATTTTGCTATTGGCCTAG   17119
gb:LC522974|Organism:Severe      AGTATTCTACACTCCAGGGACCACCTGGTACTGGTAAGAGTCATTTTGCTATTGGCCTAG   17119
gb:MN985325|Organism:Severe      AGTATTCTACACTCCAGGGACCACCTGGTACTGGTAAGAGTCATTTTGCTATTGGCCTAG   17122
gb:MT020881|Organism:Severe      AGTATTCTACACTCCAGGGACCACCTGGTACTGGTAAGAGTCATTTTGCTATTGGCCTAG   17122
gb:MT020880|Organism:Severe      AGTATTCTACACTCCAGGGACCACCTGGTACTGGTAAGAGTCATTTTGCTATTGGCCTAG   17122
gb:MT066175|Organism:Severe      AGTATTCTACACTCCAGGGACCACCTGGTACTGGTAAGAGTCATTTTGCTATTGGCCTAG   17122
gb:MN997409|Organism:Severe      AGTATTCTACACTCCAGGGACCACCTGGTACTGGTAAGAGTCATTTTGCTATTGGCCTAG   17122
```

```
gb:MN938384|Organism:Severe      AGTATTCTACACTCCAGGGACCACCTGGTACTGGTAAGAGTCATTTTGCTATTGGCCTAG   17090
gb:MT044258|Organism:Severe      AGTATTCTACACTCCAGGGACCACCTGGTACTGGTAAGAGTCATTTTGCTATTGGCCTAG   17098
gb:MT039890|Organism:Severe      AGTATTCTACACTCCAGGGACCACCTGGTACTGGTAAGAGTCATTTTGCTATTGGCCTAG   17122
gb:MN988713|Organism:Severe      AGTATTCTACACTCCAGGGACCACCTGGTACTGGTAAGAGTCATTTTGCTATTGGCCTAG   17122
gb:LC521925|Organism:Severe      AGTATTCTACACTCCAGGGACCACCTGGTACTGGTAAGAGTCATTTTGCTATTGGCCTAG   17095
gb:MT093571|Organism:Severe      AGTATTCTACACTCCAGGGACCACCTGGTACTGGTAAGAGTCATTTTGCTATTGGCCTAG   17122
gb:MT039887|Organism:Severe      AGTATTCTACACTCCAGGGACCACCTGGTACTGGTAAGAGTCATTTTGCTATTGGCCTAG   17122
gb:MT019530|Organism:Severe      AGTATTCTACACTCCAGGGACCACCTGGTACTGGTAAGAGTCATTTTGCTATTGGCCTAG   17122
gb:MT039888|Organism:Severe      AGTATTCTACACTCCAGGGACCACCTGGTACTGGTAAGAGTCATTTTGCTATTGGCCTAG   17122
gb:LC522972|Organism:Severe      AGTATTCTACACTCCAGGGACCACCTGGTACTGGTAAGAGTCATTTTGCTATTGGCCTAG   17119
gb:MT027063|Organism:Severe      AGTATTCTACACTCCAGGGACCACCTGGTACTGGTAAGAGTCATTTTGCTATTGGCCTAG   17122
gb:MT027062|Organism:Severe      AGTATTCTACACTCCAGGGACCACCTGGTACTGGTAAGAGTCATTTTGCTATTGGCCTAG   17122
gb:MT019529|Organism:Severe      AGTATTCTACACTCCAGGGACCACCTGGTACTGGTAAGAGTCATTTTGCTATTGGCCTAG   17122
gb:MN996529|Organism:Severe      AGTATTCTACACTCCAGGGACCACCTGGTACTGGTAAGAGTCATTTTGCTATTGGCCTAG   17110
gb:MN996531|Organism:Severe      AGTATTCTACACTCCAGGGACCACCTGGTACTGGTAAGAGTCATTTTGCTATTGGCCTAG   17109
gb:MT066176|Organism:Severe      AGTATTCTACACTCCAGGGACCACCTGGTACTGGTAAGAGTCATTTTGCTATTGGCCTAG   17122
gb:MT027064|Organism:Severe      AGTATTCTACACTCCAGGGACCACCTGGTACTGGTAAGAGTCATTTTGCTATTGGCCTAG   17122
gb:MN994468|Organism:Severe      AGTATTCTACACTCCAGGGACCACCTGGTACTGGTAAGAGTCATTTTGCTATTGGCCTAG   17122
gb:MT072688|Organism:Severe      AGTATTCTACACTCCAGGGACCACCTGGTACTGGTAAGAGTCATTTTGCTATTGGCCTAG   17107
gb:MN996527|Organism:Severe      AGTATTCTACACTCCAGGGACCACCTGGTACTGGTAAGAGTCATTTTGCTATTGGCCTAG   17089
gb:MT093631|Organism:Severe      AGTATTCTACACTCCAGGGACCACCTGGTACTGGTAAGAGTCATTTTGCTATTGGCCTAG   17160
gb:MT106053|Organism:Severe      AGTATTCTACACTCCAGGGACCACCTGGTACTGGTAAGAGTCATTTTGCTATTGGCCTAG   17122
gb:MT019533|Organism:Severe      AGTATTCTACACTCCAGGGACCACCTGGTACTGGTAAGAGTCATTTTGCTATTGGCCTAG   17122
gb:MT019531|Organism:Severe      AGTATTCTACACTCCAGGGACCACCTGGTACTGGTAAGAGTCATTTTGCTATTGGCCTAG   17122
gb:MN996528|Organism:Severe      AGTATTCTACACTCCAGGGACCACCTGGTACTGGTAAGAGTCATTTTGCTATTGGCCTAG   17122
gb:MN996530|Organism:Severe      AGTATTCTACACTCCAGGGACCACCTGGTACTGGTAAGAGTCATTTTGCTATTGGCCTAG   17108
gb:MN908947|Organism:Severe      AGTATTCTACACTCCAGGGACCACCTGGTACTGGTAAGAGTCATTTTGCTATTGGCCTAG   17122
gb:MT019532|Organism:Severe      AGTATTCTACACTCCAGGGACCACCTGGTACTGGTAAGAGTCATTTTGCTATTGGCCTAG   17122
                                 ************************************************************

gb:MT020781|Organism:Severe      CTCTCTACTACCCTTCTGCTCGCATAGTGTATACAGCTTGCTCTCATGCCGCTGTTGATG   17170
gb:MT007544|Organism:Severe      CTCTCTACTACCCTTCTGCTCGCATAGTGTATACAGCTTGCTCTCATGCCGCTGTTGATG   17182
gb:MN994467|Organism:Severe      CTCTCTACTACCCTTCTGCTCGCATAGTGTATACAGCTTGCTCTCATGCCGCTGTTGATG   17182
gb:MT044257|Organism:Severe      CTCTCTACTACCCTTCTGCTCGCATAGTGTATACAGCTTGCTCTCATGCCGCTGTTGATG   17182
gb:MT106054|Organism:Severe      CTCTCTACTACCCTTCTGCTCGCATAGTGTATACAGCTTGCTCTCATGCCGCTGTTGATG   17182
gb:MT049951|Organism:Severe      CTCTCTACTACCCTTCTGCTCGCATAGTGTATACAGCTTGCTCTCATGCCGCTGTTGATG   17182
gb:MN975262|Organism:Severe      CTCTCTACTACCCTTCTGCTCGCATAGTGTATACAGCTTGCTCTCATGCCGCTGTTGATG   17182
gb:MT106052|Organism:Severe      CTCTCTACTACCCTTCTGCTCGCATAGTGTATACAGCTTGCTCTCATGCCGCTGTTGATG   17182
gb:LC522975|Organism:Severe      CTCTCTACTACCCTTCTGCTCGCATAGTGTATACAGCTTGCTCTCATGCCGCTGTTGATG   17179
gb:LC522973|Organism:Severe      CTCTCTACTACCCTTCTGCTCGCATAGTGTATACAGCTTGCTCTCATGCCGCTGTTGATG   17179
gb:LC522974|Organism:Severe      CTCTCTACTACCCTTCTGCTCGCATAGTGTATACAGCTTGCTCTCATGCCGCTGTTGATG   17179
gb:MN985325|Organism:Severe      CTCTCTACTACCCTTCTGCTCGCATAGTGTATACAGCTTGCTCTCATGCCGCTGTTGATG   17182
gb:MT020881|Organism:Severe      CTCTCTACTACCCTTCTGCTCGCATAGTGTATACAGCTTGCTCTCATGCCGCTGTTGATG   17182
gb:MT020880|Organism:Severe      CTCTCTACTACCCTTCTGCTCGCATAGTGTATACAGCTTGCTCTCATGCCGCTGTTGATG   17182
gb:MT066175|Organism:Severe      CTCTCTACTACCCTTCTGCTCGCATAGTGTATACAGCTTGCTCTCATGCCGCTGTTGATG   17182
gb:MN997409|Organism:Severe      CTCTCTACTACCCTTCTGCTCGCATAGTGTATACAGCTTGCTCTCATGCCGCTGTTGATG   17182
gb:MN938384|Organism:Severe      CTCTCTACTACCCTTCTGCTCGCATAGTGTATACAGCTTGCTCTCATGCCGCTGTTGATG   17150
gb:MT044258|Organism:Severe      CTCTCTACTACCCTTCTGCTCGCATAGTGTATACAGCTTGCTCTCATGCCGCTGTTGATG   17158
gb:MT039890|Organism:Severe      CTCTCTACTACCCTTCTGCTCGCATAGTGTATACAGCTTGCTCTCATGCCGCTGTTGATG   17182
gb:MN988713|Organism:Severe      CTCTCTACTACCCTTCTGCTCGCATAGTGTATACAGCTTGCTCTCATGCCGCTGTTGATG   17182
gb:LC521925|Organism:Severe      CTCTCTACTACCCTTCTGCTCGCATAGTGTATACAGCTTGCTCTCATGCCGCTGTTGATG   17155
gb:MT093571|Organism:Severe      CTCTCTACTACCCTTCTGCTCGCATAGTGTATACAGCTTGCTCTCATGCCGCTGTTGATG   17182
gb:MT039887|Organism:Severe      CTCTCTACTACCCTTCTGCTCGCATAGTGTATACAGCTTGCTCTCATGCCGCTGTTGATG   17182
gb:MT019530|Organism:Severe      CTCTCTACTACCCTTCTGCTCGCATAGTGTATACAGCTTGCTCTCATGCCGCTGTTGATG   17182
gb:MT039888|Organism:Severe      CTCTCTACTACCCTTCTGCTCGCATAGTGTATACAGCTTGCTCTCATGCCGCTGTTGATG   17182
gb:LC522972|Organism:Severe      CTCTCTACTACCCTTCTGCTCGCATAGTGTATACAGCTTGCTCTCATGCCGCTGTTGATG   17179
gb:MT027063|Organism:Severe      CTCTCTACTACCCTTCTGCTCGCATAGTGTATACAGCTTGCTCTCATGCCGCTGTTGATG   17182
gb:MT027062|Organism:Severe      CTCTCTACTACCCTTCTGCTCGCATAGTGTATACAGCTTGCTCTCATGCCGCTGTTGATG   17182
gb:MT019529|Organism:Severe      CTCTCTACTACCCTTCTGCTCGCATAGTGTATACAGCTTGCTCTCATGCCGCTGTTGATG   17182
gb:MN996529|Organism:Severe      CTCTCTACTACCCTTCTGCTCGCATAGTGTATACAGCTTGCTCTCATGCCGCTGTTGATG   17170
gb:MN996531|Organism:Severe      CTCTCTACTACCCTTCTGCTCGCATAGTGTATACAGCTTGCTCTCATGCCGCTGTTGATG   17169
gb:MT066176|Organism:Severe      CTCTCTACTACCCTTCTGCTCGCATAGTGTATACAGCTTGCTCTCATGCCGCTGTTGATG   17182
gb:MT027064|Organism:Severe      CTCTCTACTACCCTTCTGCTCGCATAGTGTATACAGCTTGCTCTCATGCCGCTGTTGATG   17182
gb:MN994468|Organism:Severe      CTCTCTACTACCCTTCTGCTCGCATAGTGTATACAGCTTGCTCTCATGCCGCTGTTGATG   17182
gb:MT072688|Organism:Severe      CTCTCTACTACCCTTCTGCTCGCATAGTGTATACAGCTTGCTCTCATGCCGCTGTTGATG   17167
```

```
gb:MN996527|Organism:Severe      CTCTCTACTACCCTTCTGCTCGCATAGTGTATACAGCTTGCTCTCATGCCGCTGTTGATG   17149
gb:MT093631|Organism:Severe      CTCTCTACTACCCTTCTGCTCGCATAGTGTATACAGCTTGCTCTCATGCCGCTGTTGATG   17220
gb:MT106053|Organism:Severe      CTCTCTACTACCCTTCTGCTCGCATAGTGTATACAGCTTGCTCTCATGCCGCTGTTGATG   17182
gb:MT019533|Organism:Severe      CTCTCTACTACCCTTCTGCTCGCATAGTGTATACAGCTTGCTCTCATGCCGCTGTTGATG   17182
gb:MT019531|Organism:Severe      CTCTCTACTACCCTTCTGCTCGCATAGTGTATACAGCTTGCTCTCATGCCGCTGTTGATG   17182
gb:MN996528|Organism:Severe      CTCTCTACTACCCTTCTGCTCGCATAGTGTATACAGCTTGCTCTCATGCCGCTGTTGATG   17182
gb:MN996530|Organism:Severe      CTCTCTACTACCCTTCTGCTCGCATAGTGTATACAGCTTGCTCTCATGCCGCTGTTGATG   17168
gb:MN908947|Organism:Severe      CTCTCTACTACCCTTCTGCTCGCATAGTGTATACAGCTTGCTCTCATGCCGCTGTTGATG   17182
gb:MT019532|Organism:Severe      CTCTCTACTACCCTTCTGCTCGCATAGTGTATACAGCTTGCTCTCATGCCGCTGTTGATG   17182
                                 ************************************************************

gb:MT020781|Organism:Severe      CACTATGTGAGAAGGCATTAAAATATTTGCCTATAGATAAATGTAGTAGAATTATACCTG   17230
gb:MT007544|Organism:Severe      CACTATGTGAGAAGGCATTAAAATATTTGCCTATAGATAAATGTAGTAGAATTATACCTG   17242
gb:MN994467|Organism:Severe      CACTATGTGAGAAGGCATTAAAATATTTGCCTATAGATAAATGTAGTAGAATTATACCTG   17242
gb:MT044257|Organism:Severe      CACTATGTGAGAAGGCATTAAAATATTTGCCTATAGATAAATGTAGTAGAATTATACCTG   17242
gb:MT106054|Organism:Severe      CACTATGTGAGAAGGCATTAAAATATTTGCCTATAGATAAATGTAGTAGAATTATACCTG   17242
gb:MT049951|Organism:Severe      CACTATGTGAGAAGGCATTAAAATATTTGCCTATAGATAAATGTAGTAGAATTATACCTG   17242
gb:MN975262|Organism:Severe      CACTATGTGAGAAGGCATTAAAATATTTGCCTATAGATAAATGTAGTAGAATTATACCTG   17242
gb:MT106052|Organism:Severe      CACTATGTGAGAAGGCATTAAAATATTTGCCTATAGATAAATGTAGTAGAATTATACCTG   17242
gb:LC522975|Organism:Severe      CACTATGTGAGAAGGCATTAAAATATTTGCCTATAGATAAATGTAGTAGAATTATACCTG   17239
gb:LC522973|Organism:Severe      CACTATGTGAGAAGGCATTAAAATATTTGCCTATAGATAAATGTAGTAGAATTATACCTG   17239
gb:LC522974|Organism:Severe      CACTATGTGAGAAGGCATTAAAATATTTGCCTATAGATAAATGTAGTAGAATTATACCTG   17239
gb:MN985325|Organism:Severe      CACTATGTGAGAAGGCATTAAAATATTTGCCTATAGATAAATGTAGTAGAATTATACCTG   17242
gb:MT020881|Organism:Severe      CACTATGTGAGAAGGCATTAAAATATTTGCCTATAGATAAATGTAGTAGAATTATACCTG   17242
gb:MT020880|Organism:Severe      CACTATGTGAGAAGGCATTAAAATATTTGCCTATAGATAAATGTAGTAGAATTATACCTG   17242
gb:MT066175|Organism:Severe      CACTATGTGAGAAGGCATTAAAATATTTGCCTATAGATAAATGTAGTAGAATTATACCTG   17242
gb:MN997409|Organism:Severe      CACTATGTGAGAAGGCATTAAAATATTTGCCTATAGATAAATGTAGTAGAATTATACCTG   17242
gb:MN938384|Organism:Severe      CACTATGTGAGAAGGCATTAAAATATTTGCCTATAGATAAATGTAGTAGAATTATACCTG   17210
gb:MT044258|Organism:Severe      CACTATGTGAGAAGGCATTAAAATATTTGCCTATAGATAAATGTAGTAGAATTATACCTG   17218
gb:MT039890|Organism:Severe      CACTATGTGAGAAGGCATTAAAATATTTGCCTATAGATAAATGTAGTAGAATTATACCTG   17242
gb:MN988713|Organism:Severe      CACTATGTGAGAAGGCATTAAAATATTTGCCTATAGATAAATGTAGTAGAATTATACCTG   17242
gb:LC521925|Organism:Severe      CACTATGTGAGAAGGCATTAAAATATTTGCCTATAGATAAATGTAGTAGAATTATACCTG   17215
gb:MT093571|Organism:Severe      CACTATGTGAGAAGGCATTAAAATATTTGCCTATAGATAAATGTAGTAGAATTATACCTG   17242
gb:MT039887|Organism:Severe      CACTATGTGAGAAGGCATTAAAATATTTGCCTATAGATAAATGTAGTAGAATTATACCTG   17242
gb:MT019530|Organism:Severe      CACTATGTGAGAAGGCATTAAAATATTTGCCTATAGATAAATGTAGTAGAATTATACCTG   17242
gb:MT039888|Organism:Severe      CACTATGTGAGAAGGCATTAAAATATTTGCCTATAGATAAATGTAGTAGAATTATACCTG   17242
gb:LC522972|Organism:Severe      CACTATGTGAGAAGGCATTAAAATATTTGCCTATAGATAAATGTAGTAGAATTATACCTG   17239
gb:MT027063|Organism:Severe      CACTATGTGAGAAGGCATTAAAATATTTGCCTATAGATAAATGTAGTAGAATTATACCTG   17242
gb:MT027062|Organism:Severe      CACTATGTGAGAAGGCATTAAAATATTTGCCTATAGATAAATGTAGTAGAATTATACCTG   17242
gb:MT019529|Organism:Severe      CACTATGTGAGAAGGCATTAAAATATTTGCCTATAGATAAATGTAGTAGAATTATACCTG   17242
gb:MN996529|Organism:Severe      CACTATGTGAGAAGGCATTAAAATATTTGCCTATAGATAAATGTAGTAGAATTATACCTG   17230
gb:MN996531|Organism:Severe      CACTATGTGAGAAGGCATTAAAATATTTGCCTATAGATAAATGTAGTAGAATTATACCTG   17229
gb:MT066176|Organism:Severe      CACTATGTGAGAAGGCATTAAAATATTTGCCTATAGATAAATGTAGTAGAATTATACCTG   17242
gb:MT027064|Organism:Severe      CACTATGTGAGAAGGCATTAAAATATTTGCCTATAGATAAATGTAGTAGAATTATACCTG   17242
gb:MN994468|Organism:Severe      CACTATGTGAGAAGGCATTAAAATATTTGCCTATAGATAAATGTAGTAGAATTATACCTG   17242
gb:MT072688|Organism:Severe      CACTATGTGAGAAGGCATTAAAATATTTGCCTATAGATAAATGTAGTAGAATTATACCTG   17227
gb:MN996527|Organism:Severe      CACTATGTGAGAAGGCATTAAAATATTTGCCTATAGATAAATGTAGTAGAATTATACCTG   17209
gb:MT093631|Organism:Severe      CACTATGTGAGAAGGCATTAAAATATTTGCCTATAGATAAATGTAGTAGAATTATACCTG   17280
gb:MT106053|Organism:Severe      CACTATGTGAGAAGGCATTAAAATATTTGCCTATAGATAAATGTAGTAGAATTATACCTG   17242
gb:MT019533|Organism:Severe      CACTATGTGAGAAGGCATTAAAATATTTGCCTATAGATAAATGTAGTAGAATTATACCTG   17242
gb:MT019531|Organism:Severe      CACTATGTGAGAAGGCATTAAAATATTTGCCTATAGATAAATGTAGTAGAATTATACCTG   17242
gb:MN996528|Organism:Severe      CACTATGTGAGAAGGCATTAAAATATTTGCCTATAGATAAATGTAGTAGAATTATACCTG   17242
gb:MN996530|Organism:Severe      CACTATGTGAGAAGGCATTAAAATATTTGCCTATAGATAAATGTAGTAGAATTATACCTG   17228
gb:MN908947|Organism:Severe      CACTATGTGAGAAGGCATTAAAATATTTGCCTATAGATAAATGTAGTAGAATTATACCTG   17242
gb:MT019532|Organism:Severe      CACTATGTGAGAAGGCATTAAAATATTTGCCTATAGATAAATGTAGTAGAATTATACCTG   17242
                                 ************************************************************

gb:MT020781|Organism:Severe      CACGTGCTCGTGTAGAGTGTTTTGATAAATTCAAAGTGAATTCAACATTAGAACAGTATG   17290
gb:MT007544|Organism:Severe      CACGTGCTCGTGTAGAGTGTTTTGATAAATTCAAAGTGAATTCAACATTAGAACAGTATG   17302
gb:MN994467|Organism:Severe      CACGTGCTCGTGTAGAGTGTTTTGATAAATTCAAAGTGAATTCAACATTAGAACAGTATG   17302
gb:MT044257|Organism:Severe      CACGTGCTCGTGTAGAGTGTTTTGATAAATTCAAAGTGAATTCAACATTAGAACAGTATG   17302
gb:MT106054|Organism:Severe      CACGTGCTCGTGTAGAGTGTTTTGATAAATTCAAAGTGAATTCAACATTAGAACAGTATG   17302
gb:MT049951|Organism:Severe      CACGTGCTCGTGTAGAGTGTTTTGATAAATTCAAAGTGAATTCAACATTAGAACAGTATG   17302
gb:MN975262|Organism:Severe      CACGTGCTCGTGTAGAGTGTTTTGATAAATTCAAAGTGAATTCAACATTAGAACAGTATG   17302
gb:MT106052|Organism:Severe      CACGTGCTCGTGTAGAGTGTTTTGATAAATTCAAAGTGAATTCAACATTAGAACAGTATG   17302
```

```
gb:LC522975|Organism:Severe      CACGTGCTCGTGTAGAGTGTTTTGATAAATTCAAAGTGAATTCAACATTAGAACAGTATG   17299
gb:LC522973|Organism:Severe      CACGTGCTCGTGTAGAGTGTTTTGATAAATTCAAAGTGAATTCAACATTAGAACAGTATG   17299
gb:LC522974|Organism:Severe      CACGTGCTCGTGTAGAGTGTTTTGATAAATTCAAAGTGAATTCAACATTAGAACAGTATG   17299
gb:MN985325|Organism:Severe      CACGTGCTCGTGTAGAGTGTTTTGATAAATTCAAAGTGAATTCAACATTAGAACAGTATG   17302
gb:MT020881|Organism:Severe      CACGTGCTCGTGTAGAGTGTTTTGATAAATTCAAAGTGAATTCAACATTAGAACAGTATG   17302
gb:MT020880|Organism:Severe      CACGTGCTCGTGTAGAGTGTTTTGATAAATTCAAAGTGAATTCAACATTAGAACAGTATG   17302
gb:MT066175|Organism:Severe      CACGTGCTCGTGTAGAGTGTTTTGATAAATTCAAAGTGAATTCAACATTAGAACAGTATG   17302
gb:MN997409|Organism:Severe      CACGTGCTCGTGTAGAGTGTTTTGATAAATTCAAAGTGAATTCAACATTAGAACAGTATG   17302
gb:MN938384|Organism:Severe      CACGTGCTCGTGTAGAGTGTTTTGATAAATTCAAAGTGAATTCAACATTAGAACAGTATG   17270
gb:MT044258|Organism:Severe      CACGTGCTCGTGTAGAGTGTTTTGATAAATTCAAAGTGAATTCAACATTAGAACAGTATG   17278
gb:MT039890|Organism:Severe      CACGTGCTCGTGTAGAGTGTTTTGATAAATTCAAAGTGAATTCAACATTAGAACAGTATG   17302
gb:MN988713|Organism:Severe      CACGTGCTCGTGTAGAGTGTTTTGATAAATTCAAAGTGAATTCAACATTAGAACAGTATG   17302
gb:LC521925|Organism:Severe      CACGTGCTCGTGTAGAGTGTTTTGATAAATTCAAAGTGAATTCAACATTAGAACAGTATG   17275
gb:MT093571|Organism:Severe      CACGTGCTCGTGTAGAGTGTTTTGATAAATTCAAAGTGAATTCAACATTAGAACAGTATG   17302
gb:MT039887|Organism:Severe      CACGTGCTCGTGTAGAGTGTTTTGATAAATTCAAAGTGAATTCAACATTAGAACAGTATG   17302
gb:MT019530|Organism:Severe      CACGTGCTCGTGTAGAGTGTTTTGATAAATTCAAAGTGAATTCAACATTAGAACAGTATG   17302
gb:MT039888|Organism:Severe      CACGTGCTCGTGTAGAGTGTTTTGATAAATTCAAAGTGAATTCAACATTAGAACAGTATG   17302
gb:LC522972|Organism:Severe      CACGTGCTCGTGTAGAGTGTTTTGATAAATTCAAAGTGAATTCAACATTAGAACAGTATG   17299
gb:MT027063|Organism:Severe      CACGTGCTCGTGTAGAGTGTTTTGATAAATTCAAAGTGAATTCAACATTAGAACAGTATG   17302
gb:MT027062|Organism:Severe      CACGTGCTCGTGTAGAGTGTTTTGATAAATTCAAAGTGAATTCAACATTAGAACAGTATG   17302
gb:MT019529|Organism:Severe      CACGTGCTCGTGTAGAGTGTTTTGATAAATTCAAAGTGAATTCAACATTAGAACAGTATG   17302
gb:MN996529|Organism:Severe      CACGTGCTCGTGTAGAGTGTTTTGATAAATTCAAAGTGAATTCAACATTAGAACAGTATG   17290
gb:MN996531|Organism:Severe      CACGTGCTCGTGTAGAGTGTTTTGATAAATTCAAAGTGAATTCAACATTAGAACAGTATG   17289
gb:MT066176|Organism:Severe      CACGTGCTCGTGTAGAGTGTTTTGATAAATTCAAAGTGAATTCAACATTAGAACAGTATG   17302
gb:MT027064|Organism:Severe      CACGTGCTCGTGTAGAGTGTTTTGATAAATTCAAAGTGAATTCAACATTAGAACAGTATG   17302
gb:MN994468|Organism:Severe      CACGTGCTCGTGTAGAGTGTTTTGATAAATTCAAAGTGAATTCAACATTAGAACAGTATG   17302
gb:MT072688|Organism:Severe      CACGTGCTCGTGTAGAGTGTTTTGATAAATTCAAAGTGAATTCAACATTAGAACAGTATG   17287
gb:MN996527|Organism:Severe      CACGTGCTCGTGTAGAGTGTTTTGATAAATTCAAAGTGAATTCAACATTAGAACAGTATG   17269
gb:MT093631|Organism:Severe      CACGTGCTCGTGTAGAGTGTTTTGATAAATTCAAAGTGAATTCAACATTAGAACAGTATG   17340
gb:MT106053|Organism:Severe      CACGTGCTCGTGTAGAGTGTTTTGATAAATTCAAAGTGAATTCAACATTAGAACAGTATG   17302
gb:MT019533|Organism:Severe      CACGTGCTCGTGTAGAGTGTTTTGATAAATTCAAAGTGAATTCAACATTAGAACAGTATG   17302
gb:MT019531|Organism:Severe      CACGTGCTCGTGTAGAGTGTTTTGATAAATTCAAAGTGAATTCAACATTAGAACAGTATG   17302
gb:MN996528|Organism:Severe      CACGTGCTCGTGTAGAGTGTTTTGATAAATTCAAAGTGAATTCAACATTAGAACAGTATG   17302
gb:MN996530|Organism:Severe      CACGTGCTCGTGTAGAGTGTTTTGATAAATTCAAAGTGAATTCAACATTAGAACAGTATG   17288
gb:MN908947|Organism:Severe      CACGTGCTCGTGTAGAGTGTTTTGATAAATTCAAAGTGAATTCAACATTAGAACAGTATG   17302
gb:MT019532|Organism:Severe      CACGTGCTCGTGTAGAGTGTTTTGATAAATTCAAAGTGAATTCAACATTAGAACAGTATG   17302
                                 ************************************************************

gb:MT020781|Organism:Severe      TCTTTTGTACTGTAAATGCATTGCCTGAGACGACAGCAGATATAGTTGTCTTTGATGAAA   17350
gb:MT007544|Organism:Severe      TCTTTTGTACTGTAAATGCATTGCCTGAGACGACAGCAGATATAGTTGTCTTTGATGAAA   17362
gb:MN994467|Organism:Severe      TCTTTTGTACTGTAAATGCATTGCCTGAGACGACAGCAGATATAGTTGTCTTTGATGAAA   17362
gb:MT044257|Organism:Severe      TCTTTTGTACTGTAAATGCATTGCCTGAGACGACAGCAGATATAGTTGTCTTTGATGAAA   17362
gb:MT106054|Organism:Severe      TCTTTTGTACTGTAAATGCATTGCCTGAGACGACAGCAGATATAGTTGTCTTTGATGAAA   17362
gb:MT049951|Organism:Severe      TCTTTTGTACTGTAAATGCATTGCCTGAGACGACAGCAGATATAGTTGTCTTTGATGAAA   17362
gb:MN975262|Organism:Severe      TCTTTTGTACTGTAAATGCATTGCCTGAGACGACAGCAGATATAGTTGTCTTTGATGAAA   17362
gb:MT106052|Organism:Severe      TCTTTTGTACTGTAAATGCATTGCCTGAGACGACAGCAGATATAGTTGTCTTTGATGAAA   17362
gb:LC522975|Organism:Severe      TCTTTTGTACTGTAAATGCATTGCCTGAGACGACAGCAGATATAGTTGTCTTTGATGAAA   17359
gb:LC522973|Organism:Severe      TCTTTTGTACTGTAAATGCATTGCCTGAGACGACAGCAGATATAGTTGTCTTTGATGAAA   17359
gb:LC522974|Organism:Severe      TCTTTTGTACTGTAAATGCATTGCCTGAGACGACAGCAGATATAGTTGTCTTTGATGAAA   17359
gb:MN985325|Organism:Severe      TCTTTTGTACTGTAAATGCATTGCCTGAGACGACAGCAGATATAGTTGTCTTTGATGAAA   17362
gb:MT020881|Organism:Severe      TCTTTTGTACTGTAAATGCATTGCCTGAGACGACAGCAGATATAGTTGTCTTTGATGAAA   17362
gb:MT020880|Organism:Severe      TCTTTTGTACTGTAAATGCATTGCCTGAGACGACAGCAGATATAGTTGTCTTTGATGAAA   17362
gb:MT066175|Organism:Severe      TCTTTTGTACTGTAAATGCATTGCCTGAGACGACAGCAGATATAGTTGTCTTTGATGAAA   17362
gb:MN997409|Organism:Severe      TCTTTTGTACTGTAAATGCATTGCCTGAGACGACAGCAGATATAGTTGTCTTTGATGAAA   17362
gb:MN938384|Organism:Severe      TCTTTTGTACTGTAAATGCATTGCCTGAGACGACAGCAGATATAGTTGTCTTTGATGAAA   17330
gb:MT044258|Organism:Severe      TCTTTTGTACTGTAAATGCATTGCCTGAGACGACAGCAGATATAGTTGTCTTTGATGAAA   17338
gb:MT039890|Organism:Severe      TCTTTTGTACTGTAAATGCATTGCCTGAGACGACAGCAGATATAGTTGTCTTTGATGAAA   17362
gb:MN988713|Organism:Severe      TCTTTTGTACTGTAAATGCATTGCCTGAGACGACAGCAGATATAGTTGTCTTTGATGAAA   17362
gb:LC521925|Organism:Severe      TCTTTTGTACTGTAAATGCATTGCCTGAGACGACAGCAGATATAGTTGTCTTTGATGAAA   17335
gb:MT093571|Organism:Severe      TCTTTTGTACTGTAAATGCATTGCCTGAGACGACAGCAGATATAGTTGTCTTTGATGAAA   17362
gb:MT039887|Organism:Severe      TCTTTTGTACTGTAAATGCATTGCCTGAGACGACAGCAGATATAGTTGTCTTTGATGAAA   17362
gb:MT019530|Organism:Severe      TCTTTTGTACTGTAAATGCATTGCCTGAGACGACAGCAGATATAGTTGTCTTTGATGAAA   17362
gb:MT039888|Organism:Severe      TCTTTTGTACTGTAAATGCATTGCCTGAGACGACAGCAGATATAGTTGTCTTTGATGAAA   17362
gb:LC522972|Organism:Severe      TCTTTTGTACTGTAAATGCATTGCCTGAGACGACAGCAGATATAGTTGTCTTTGATGAAA   17359
gb:MT027063|Organism:Severe      TCTTTTGTACTGTAAATGCATTGCCTGAGACGACAGCAGATATAGTTGTCTTTGATGAAA   17362
```

```
gb:MT027062|Organism:Severe      TCTTTTGTACTGTAAATGCATTGCCTGAGACGACAGCAGATATAGTTGTCTTTGATGAAA   17362
gb:MT019529|Organism:Severe      TCTTTTGTACTGTAAATGCATTGCCTGAGACGACAGCAGATATAGTTGTCTTTGATGAAA   17362
gb:MN996529|Organism:Severe      TCTTTTGTACTGTAAATGCATTGCCTGAGACGACAGCAGATATAGTTGTCTTTGATGAAA   17350
gb:MN996531|Organism:Severe      TCTTTTGTACTGTAAATGCATTGCCTGAGACGACAGCAGATATAGTTGTCTTTGATGAAA   17349
gb:MT066176|Organism:Severe      TCTTTTGTACTGTAAATGCATTGCCTGAGACGACAGCAGATATAGTTGTCTTTGATGAAA   17362
gb:MT027064|Organism:Severe      TCTTTTGTACTGTAAATGCATTGCCTGAGACGACAGCAGATATAGTTGTCTTTGATGAAA   17362
gb:MN994468|Organism:Severe      TCTTTTGTACTGTAAATGCATTGCCTGAGACGACAGCAGATATAGTTGTCTTTGATGAAA   17362
gb:MT072688|Organism:Severe      TCTTTTGTACTGTAAATGCATTGCCTGAGACGACAGCAGATATAGTTGTCTTTGATGAAA   17347
gb:MN996527|Organism:Severe      TCTTTTGTACTGTAAATGCATTGCCTGAGACGACAGCAGATATAGTTGTCTTTGATGAAA   17329
gb:MT093631|Organism:Severe      TCTTTTGTACTGTAAATGCATTGCCTGAGACGACAGCAGATATAGTTGTCTTTGATGAAA   17400
gb:MT106053|Organism:Severe      TCTTTTGTACTGTAAATGCATTGCCTGAGACGACAGCAGATATAGTTGTCTTTGATGAAA   17362
gb:MT019533|Organism:Severe      TCTTTTGTACTGTAAATGCATTGCCTGAGACGACAGCAGATATAGTTGTCTTTGATGAAA   17362
gb:MT019531|Organism:Severe      TCTTTTGTACTGTAAATGCATTGCCTGAGACGACAGCAGATATAGTTGTCTTTGATGAAA   17362
gb:MN996528|Organism:Severe      TCTTTTGTACTGTAAATGCATTGCCTGAGACGACAGCAGATATAGTTGTCTTTGATGAAA   17362
gb:MN996530|Organism:Severe      TCTTTTGTACTGTAAATGCATTGCCTGAGACGACAGCAGATATAGTTGTCTTTGATGAAA   17348
gb:MN908947|Organism:Severe      TCTTTTGTACTGTAAATGCATTGCCTGAGACGACAGCAGATATAGTTGTCTTTGATGAAA   17362
gb:MT019532|Organism:Severe      TCTTTTGTACTGTAAATGCATTGCCTGAGACGACAGCAGATATAGTTGTCTTTGATGAAA   17362
                                 ************************************************************

gb:MT020781|Organism:Severe      TTTCAATGGCCACAAATTATGATTTGAGTGTTGTCAATGCCAGATTACGTGCTAAGCACT   17410
gb:MT007544|Organism:Severe      TTTCAATGGCCACAAATTATGATTTGAGTGTTGTCAATGCCAGATTACGTGCTAAGCACT   17422
gb:MN994467|Organism:Severe      TTTCAATGGCCACAAATTATGATTTGAGTGTTGTCAATGCCAGATTACGTGCTAAGCACT   17422
gb:MT044257|Organism:Severe      TTTCAATGGCCACAAATTATGATTTGAGTGTTGTCAATGCCAGATTACGTGCTAAGCACT   17422
gb:MT106054|Organism:Severe      TTTCAATGGCCACAAATTATGATTTGAGTGTTGTCAATGCCAGATTACGTGCTAAGCACT   17422
gb:MT049951|Organism:Severe      TTTCAATGGCCACAAATTATGATTTGAGTGTTGTCAATGCCAGATTACGTGCTAAGCACT   17422
gb:MN975262|Organism:Severe      TTTCAATGGCCACAAATTATGATTTGAGTGTTGTCAATGCCAGATTACGTGCTAAGCACT   17422
gb:MT106052|Organism:Severe      TTTCAATGGCCACAAATTATGATTTGAGTGTTGTCAATGCCAGATTACGTGCTAAGCACT   17422
gb:LC522975|Organism:Severe      TTTCAATGGCCACAAATTATGATTTGAGTGTTGTCAATGCCAGATTACGTGCTAAGCACT   17419
gb:LC522973|Organism:Severe      TTTCAATGGCCACAAATTATGATTTGAGTGTTGTCAATGCCAGATTACGTGCTAAGCACT   17419
gb:LC522974|Organism:Severe      TTTCAATGGCCACAAATTATGATTTGAGTGTTGTCAATGCCAGATTACGTGCTAAGCACT   17419
gb:MN985325|Organism:Severe      TTTCAATGGCCACAAATTATGATTTGAGTGTTGTCAATGCCAGATTACGTGCTAAGCACT   17422
gb:MT020881|Organism:Severe      TTTCAATGGCCACAAATTATGATTTGAGTGTTGTCAATGCCAGATTACGTGCTAAGCACT   17422
gb:MT020880|Organism:Severe      TTTCAATGGCCACAAATTATGATTTGAGTGTTGTCAATGCCAGATTACGTGCTAAGCACT   17422
gb:MT066175|Organism:Severe      TTTCAATGGCCACAAATTATGATTTGAGTGTTGTCAATGCCAGATTACGTGCTAAGCACT   17422
gb:MN997409|Organism:Severe      TTTCAATGGCCACAAATTATGATTTGAGTGTTGTCAATGCCAGATTACGTGCTAAGCACT   17422
gb:MN938384|Organism:Severe      TTTCAATGGCCACAAATTATGATTTGAGTGTTGTCAATGCCAGATTACGTGCTAAGCACT   17390
gb:MT044258|Organism:Severe      TTTCAATGGCCACAAATTATGATTTGAGTGTTGTCAATGCCAGATTACGTGCTAAGCACT   17398
gb:MT039890|Organism:Severe      TTTCAATGGCCACAAATTATGATTTGAGTGTTGTCAATGCCAGATTACGTGCTAAGCACT   17422
gb:MN988713|Organism:Severe      TTTCAATGGCCACAAATTATGATTTGAGTGTTGTCAATGCCAGATTACGTGCTAAGCACT   17422
gb:LC521925|Organism:Severe      TTTCAATGGCCACAAATTATGATTTGAGTGTTGTCAATGCCAGATTACGTGCTAAGCACT   17395
gb:MT093571|Organism:Severe      TTTCAATGGCCACGAATTATGATTTGAGTGTTGTCAATGCCAGATTACGTGCTAAGCACT   17422
gb:MT039887|Organism:Severe      TTTCAATGGCTACAAATTATGATTTGAGTGTTGTCAATGCCAGATTACGTGCTAAGCACT   17422
gb:MT019530|Organism:Severe      TTTCAATGGCCACAAATTATGATTTGAGTGTTGTCAATGCCAGATTACGTGCTAAGCACT   17422
gb:MT039888|Organism:Severe      TTTCAATGGCCACAAATTATGATTTGAGTGTTGTCAATGCCAGATTACGTGCTAAGCACT   17422
gb:LC522972|Organism:Severe      TTTCAATGGCCACAAATTATGATTTGAGTGTTGTCAATGCCAGATTACGTGCTAAGCACT   17419
gb:MT027063|Organism:Severe      TTTCAATGGCCACAAATTATGATTTGAGTGTTGTCAATGCCAGATTACGTGCTAAGCACT   17422
gb:MT027062|Organism:Severe      TTTCAATGGCCACAAATTATGATTTGAGTGTTGTCAATGCCAGATTACGTGCTAAGCACT   17422
gb:MT019529|Organism:Severe      TTTCAATGGCCACAAATTATGATTTGAGTGTTGTCAATGCCAGATTACGTGCTAAGCACT   17422
gb:MN996529|Organism:Severe      TTTCAATGGCCACAAATTATGATTTGAGTGTTGTCAATGCCAGATTACGTGCTAAGCACT   17410
gb:MN996531|Organism:Severe      TTTCAATGGCCACAAATTATGATTTGAGTGTTGTCAATGCCAGATTACGTGCTAAGCACT   17409
gb:MT066176|Organism:Severe      TTTCAATGGCCACAAATTATGATTTGAGTGTTGTCAATGCCAGATTACGTGCTAAGCACT   17422
gb:MT027064|Organism:Severe      TTTCAATGGCCACAAATTATGATTTGAGTGTTGTCAATGCCAGATTACGTGCTAAGCACT   17422
gb:MN994468|Organism:Severe      TTTCAATGGCCACAAATTATGATTTGAGTGTTGTCAATGCCAGATTACGTGCTAAGCACT   17422
gb:MT072688|Organism:Severe      TTTCAATGGCCACAAATTATGATTTGAGTGTTGTCAATGCCAGATTACGTGCTAAGCACT   17407
gb:MN996527|Organism:Severe      TTTCAATGGCCACAAATTATGATTTGAGTGTTGTCAATGCCAGATTACGTGCTAAGCACT   17389
gb:MT093631|Organism:Severe      TTTCAATGGCCACAAATTATGATTTGAGTGTTGTCAATGCCAGATTACGTGCTAAGCACT   17460
gb:MT106053|Organism:Severe      TTTCAATGGCCACAAATTATGATTTGAGTGTTGTCAATGCCAGATTACGTGCTAAGCACT   17422
gb:MT019533|Organism:Severe      TTTCAATGGCCACAAATTATGATTTGAGTGTTGTCAATGCCAGATTACGTGCTAAGCACT   17422
gb:MT019531|Organism:Severe      TTTCAATGGCCACAAATTATGATTTGAGTGTTGTCAATGCCAGATTACGTGCTAAGCACT   17422
gb:MN996528|Organism:Severe      TTTCAATGGCCACAAATTATGATTTGAGTGTTGTCAATGCCAGATTACGTGCTAAGCACT   17422
gb:MN996530|Organism:Severe      TTTCAATGGCCACAAATTATGATTTGAGTGTTGTCAATGCCAGATTACGTGCTAAGCACT   17408
gb:MN908947|Organism:Severe      TTTCAATGGCCACAAATTATGATTTGAGTGTTGTCAATGCCAGATTACGTGCTAAGCACT   17422
gb:MT019532|Organism:Severe      TTTCAATGGCCACAAATTATGATTTGAGTGTTGTCAATGCCAGATTACGTGCTAAGCACT   17422
                                 ********** ** **********************************************
```

```
gb:MT020781|Organism:Severe      ATGTGTACATTGGCGACCCTGCTCAATTACCTGCACCACGCACATTGCTAACTAAGGGCA   17470
gb:MT007544|Organism:Severe      ATGTGTACATTGGCGACCCTGCTCAATTACCTGCACCACGCACATTGCTAACTAAGGGCA   17482
gb:MN994467|Organism:Severe      ATGTGTACATTGGCGACCCTGCTCAATTACCTGCACCACGCACATTGCTAACTAAGGGCA   17482
gb:MT044257|Organism:Severe      ATGTGTACATTGGCGACCCTGCTCAATTACCTGCACCACGCACATTGCTAACTAAGGGCA   17482
gb:MT106054|Organism:Severe      ATGTGTACATTGGCGACCCTGCTCAATTACCTGCACCACGCACATTGCTAACTAAGGGCA   17482
gb:MT049951|Organism:Severe      ATGTGTACATTGGCGACCCTGCTCAATTACCTGCACCACGCACATTGCTAACTAAGGGCA   17482
gb:MN975262|Organism:Severe      ATGTGTACATTGGCGACCCTGCTCAATTACCTGCACCACGCACATTGCTAACTAAGGGCA   17482
gb:MT106052|Organism:Severe      ATGTGTACATTGGCGACCCTGCTCAATTACCTGCACCACGCACATTGCTAACTAAGGGCA   17482
gb:LC522975|Organism:Severe      ATGTGTACATTGGCGACCCTGCTCAATTACCTGCACCACGCACATTGCTAACTAAGGGCA   17479
gb:LC522973|Organism:Severe      ATGTGTACATTGGCGACCCTGCTCAATTACCTGCACCACGCACATTGCTAACTAAGGGCA   17479
gb:LC522974|Organism:Severe      ATGTGTACATTGGCGACCCTGCTCAATTACCTGCACCACGCACATTGCTAACTAAGGGCA   17479
gb:MN985325|Organism:Severe      ATGTGTACATTGGCGACCCTGCTCAATTACCTGCACCACGCACATTGCTAACTAAGGGCA   17482
gb:MT020881|Organism:Severe      ATGTGTACATTGGCGACCCTGCTCAATTACCTGCACCACGCACATTGCTAACTAAGGGCA   17482
gb:MT020880|Organism:Severe      ATGTGTACATTGGCGACCCTGCTCAATTACCTGCACCACGCACATTGCTAACTAAGGGCA   17482
gb:MT066175|Organism:Severe      ATGTGTACATTGGCGACCCTGCTCAATTACCTGCACCACGCACATTGCTAACTAAGGGCA   17482
gb:MN997409|Organism:Severe      ATGTGTACATTGGCGACCCTGCTCAATTACCTGCACCACGCACATTGCTAACTAAGGGCA   17482
gb:MN938384|Organism:Severe      ATGTGTACATTGGCGACCCTGCTCAATTACCTGCACCACGCACATTGCTAACTAAGGGCA   17450
gb:MT044258|Organism:Severe      ATGTGTACATTGGCGACCCTGCTCAATTACCTGCACCACGCACATTGCTAACTAAGGGCA   17458
gb:MT039890|Organism:Severe      ATGTGTACATTGGCGACCCTGCTCAATTACCTGCACCACGCACATTGCTAACTAAGGGCA   17482
gb:MN988713|Organism:Severe      ATGTGTACATTGGCGACCCTGCTCAATTACCTGCACCACGCACATTGCTAACTAAGGGCA   17482
gb:LC521925|Organism:Severe      ATGTGTACATTGGCGACCCTGCTCAATTACCTGCACCACGCACATTGCTAACTAAGGGCA   17455
gb:MT093571|Organism:Severe      ATGTGTACATTGGCGACCCTGCTCAATTACCTGCACCACGCACATTGCTAACTAAGGGCA   17482
gb:MT039887|Organism:Severe      ATGTGTACATTGGCGACCCTGCTCAATTACCTGCACCACGCACATTGCTAACTAAGGGCA   17482
gb:MT019530|Organism:Severe      ATGTGTACATTGGCGACCCTGCTCAATTACCTGCACCACGCACATTGCTAACTAAGGGCA   17482
gb:MT039888|Organism:Severe      GTGTGTACATTGGCGACCCTGCTCAATTACCTGCACCACGCACATTGCTAACTAAGGGCA   17482
gb:LC522972|Organism:Severe      ATGTGTACATTGGCGACCCTGCTCAATTACCTGCACCACGCACATTGCTAACTAAGGGCA   17479
gb:MT027063|Organism:Severe      ATGTGTACATTGGCGACCCTGCTCAATTACCTGCACCACGCACATTGCTAACTAAGGGCA   17482
gb:MT027062|Organism:Severe      ATGTGTACATTGGCGACCCTGCTCAATTACCTGCACCACGCACATTGCTAACTAAGGGCA   17482
gb:MT019529|Organism:Severe      ATGTGTACATTGGCGACCCTGCTCAATTACCTGCACCACGCACATTGCTAACTAAGGGCA   17482
gb:MN996529|Organism:Severe      ATGTGTACATTGGCGACCCTGCTCAATTACCTGCACCACGCACATTGCTAACTAAGGGCA   17470
gb:MN996531|Organism:Severe      ATGTGTACATTGGCGACCCTGCTCAATTACCTGCACCACGCACATTGCTAACTAAGGGCA   17469
gb:MT066176|Organism:Severe      ATGTGTACATTGGCGACCCTGCTCAATTACCTGCACCACGCACATTGCTAACTAAGGGCA   17482
gb:MT027064|Organism:Severe      ATGTGTACATTGGCGACCCTGCTCAATTACCTGCACCACGCACATTGCTAACTAAGGGCA   17482
gb:MN994468|Organism:Severe      ATGTGTACATTGGCGACCCTGCTCAATTACCTGCACCACGCACATTGCTAACTAAGGGCA   17482
gb:MT072688|Organism:Severe      ATGTGTACATTGGCGACCCTGCTCAATTACCTGCACCACGCACATTGCTAACTAAGGGCA   17467
gb:MN996527|Organism:Severe      ATGTGTACATTGGCGACCCTGCTCAATTACCTGCACCACGCACATTGCTAACTAAGGGCA   17449
gb:MT093631|Organism:Severe      ATGTGTACATTGGCGACCCTGCTCAATTACCTGCACCACGCACATTGCTAACTAAGGGCA   17520
gb:MT106053|Organism:Severe      ATGTGTACATTGGCGACCCTGCTCAATTACCTGCACCACGCACATTGCTAACTAAGGGCA   17482
gb:MT019533|Organism:Severe      ATGTGTACATTGGCGACCCTGCTCAATTACCTGCACCACGCACATTGCTAACTAAGGGCA   17482
gb:MT019531|Organism:Severe      ATGTGTACATTGGCGACCCTGCTCAATTACCTGCACCACGCACATTGCTAACTAAGGGCA   17482
gb:MN996528|Organism:Severe      ATGTGTACATTGGCGACCCTGCTCAATTACCTGCACCACGCACATTGCTAACTAAGGGCA   17482
gb:MN996530|Organism:Severe      ATGTGTACATTGGCGACCCTGCTCAATTACCTGCACCACGCACATTGCTAACTAAGGGCA   17468
gb:MN908947|Organism:Severe      ATGTGTACATTGGCGACCCTGCTCAATTACCTGCACCACGCACATTGCTAACTAAGGGCA   17482
gb:MT019532|Organism:Severe      ATGTGTACATTGGCGACCCTGCTCAATTACCTGCACCACGCACATTGCTAACTAAGGGCA   17482
                                  ***********************************************************

gb:MT020781|Organism:Severe      CACTAGAACCAGAATATTTCAATTCAGTGTGTAGACTTATGAAAACTATAGGTCCAGACA   17530
gb:MT007544|Organism:Severe      CACTAGAACCAGAATATTTCAATTCAGTGTGTAGACTTATGAAAACTATAGGTCCAGACA   17542
gb:MN994467|Organism:Severe      CACTAGAACCAGAATATTTCAATTCAGTGTGTAGACTTATGAAAACTATAGGTCCAGACA   17542
gb:MT044257|Organism:Severe      CACTAGAACCAGAATATTTCAATTCAGTGTGTAGACTTATGAAAACTATAGGTCCAGACA   17542
gb:MT106054|Organism:Severe      CACTAGAACCAGAATATTTCAATTCAGTGTGTAGACTTATGAAAACTATAGGTCCAGACA   17542
gb:MT049951|Organism:Severe      CACTAGAACCAGAATATTTCAATTCAGTGTGTAGACTTATGAAAACTATAGGTCCAGACA   17542
gb:MN975262|Organism:Severe      CACTAGAACCAGAATATTTCAATTCAGTGTGTAGACTTATGAAAACTATAGGTCCAGACA   17542
gb:MT106052|Organism:Severe      CACTAGAACCAGAATATTTCAATTCAGTGTGTAGACTTATGAAAACTATAGGTCCAGACA   17542
gb:LC522975|Organism:Severe      CACTAGAACCAGAATATTTCAATTCAGTGTGTAGACTTATGAAAACTATAGGTCCAGACA   17539
gb:LC522973|Organism:Severe      CACTAGAACCAGAATATTTCAATTCAGTGTGTAGACTTATGAAAACTATAGGTCCAGACA   17539
gb:LC522974|Organism:Severe      CACTAGAACCAGAATATTTCAATTCAGTGTGTAGACTTATGAAAACTATAGGTCCAGACA   17539
gb:MN985325|Organism:Severe      CACTAGAACCAGAATATTTCAATTCAGTGTGTAGACTTATGAAAACTATAGGTCCAGACA   17542
gb:MT020881|Organism:Severe      CACTAGAACCAGAATATTTCAATTCAGTGTGTAGACTTATGAAAACTATAGGTCCAGACA   17542
gb:MT020880|Organism:Severe      CACTAGAACCAGAATATTTCAATTCAGTGTGTAGACTTATGAAAACTATAGGTCCAGACA   17542
gb:MT066175|Organism:Severe      CACTAGAACCAGAATATTTCAATTCAGTGTGTAGACTTATGAAAACTATAGGTCCAGACA   17542
gb:MN997409|Organism:Severe      CACTAGAACCAGAATATTTCAATTCAGTGTGTAGACTTATGAAAACTATAGGTCCAGACA   17542
gb:MN938384|Organism:Severe      CACTAGAACCAGAATATTTCAATTCAGTGTGTAGACTTATGAAAACTATAGGTCCAGACA   17510
gb:MT044258|Organism:Severe      CACTAGAACCAGAATATTTCAATTCAGTGTGTAGACTTATGAAAACTATAGGTCCAGACA   17518
gb:MT039890|Organism:Severe      CACTAGAACCAGAATATTTCAATTCAGTGTGTAGACTTATGAAAACTATAGGTCCAGACA   17542
```

```
gb:MN988713|Organism:Severe      CACTAGAACCAGAATATTTCAATTCAGTGTGTAGACTTATGAAAACTATAGGTCCAGACA   17542
gb:LC521925|Organism:Severe      CACTAGAACCAGAATATTTCAATTCAGTGTGTAGACTTATGAAAACTATAGGTCCAGACA   17515
gb:MT093571|Organism:Severe      CACTAGAACCAGAATATTTCAATTCAGTGTGTAGACTTATGAAAACTATAGGTCCAGACA   17542
gb:MT039887|Organism:Severe      CACTAGAACCAGAATATTTCAATTCAGTGTGTAGACTTATGAAAACTATAGGTCCAGACA   17542
gb:MT019530|Organism:Severe      CACTAGAACCAGAATATTTCAATTCAGTGTGTAGACTTATGAAAACTATAGGTCCAGACA   17542
gb:MT039888|Organism:Severe      CACTAGAACCAGAATATTTCAATTCAGTGTGTAGACTTATGAAAACTATAGGTCCAGACA   17542
gb:LC522972|Organism:Severe      CACTAGAACCAGAATATTTCAATTCAGTGTGTAGACTTATGAAAACTATAGGTCCAGACA   17539
gb:MT027063|Organism:Severe      CACTAGAACCAGAATATTTCAATTCAGTGTGTAGACTTATGAAAACTATAGGTCCAGACA   17542
gb:MT027062|Organism:Severe      CACTAGAACCAGAATATTTCAATTCAGTGTGTAGACTTATGAAAACTATAGGTCCAGACA   17542
gb:MT019529|Organism:Severe      CACTAGAACCAGAATATTTCAATTCAGTGTGTAGACTTATGAAAACTATAGGTCCAGACA   17542
gb:MN996529|Organism:Severe      CACTAGAACCAGAATATTTCAATTCAGTGTGTAGACTTATGAAAACTATAGGTCCAGACA   17530
gb:MN996531|Organism:Severe      CACTAGAACCAGAATATTTCAATTCAGTGTGTAGACTTATGAAAACTATAGGTCCAGACA   17529
gb:MT066176|Organism:Severe      CACTAGAACCAGAATATTTCAATTCAGTGTGTAGACTTATGAAAACTATAGGTCCAGACA   17542
gb:MT027064|Organism:Severe      CACTAGAACCAGAATATTTCAATTCAGTGTGTAGACTTATGAAAACTATAGGTCCAGACA   17542
gb:MN994468|Organism:Severe      CACTAGAACCAGAATATTTCAATTCAGTGTGTAGACTTATGAAAACTATAGGTCCAGACA   17542
gb:MT072688|Organism:Severe      CACTAGAACCAGAATATTTCAATTCAGTGTGTAGACTTATGAAAACTATAGGTCCAGACA   17527
gb:MN996527|Organism:Severe      CACTAGAACCAGAATATTTCAATTCAGTGTGTAGACTTATGAAAACTATAGGTCCAGACA   17509
gb:MT093631|Organism:Severe      CACTAGAACCAGAATATTTCAATTCAGTGTGTAGACTTATGAAAACTATAGGTCCAGACA   17580
gb:MT106053|Organism:Severe      CACTAGAACCAGAATATTTCAATTCAGTGTGTAGACTTATGAAAACTATAGGTCCAGACA   17542
gb:MT019533|Organism:Severe      CACTAGAACCAGAATATTTCAATTCAGTGTGTAGACTTATGAAAACTATAGGTCCAGACA   17542
gb:MT019531|Organism:Severe      CACTAGAACCAGAATATTTCAATTCAGTGTGTAGACTTATGAAAACTATAGGTCCAGACA   17542
gb:MN996528|Organism:Severe      CACTAGAACCAGAATATTTCAATTCAGTGTGTAGACTTATGAAAACTATAGGTCCAGACA   17542
gb:MN996530|Organism:Severe      CACTAGAACCAGAATATTTCAATTCAGTGTGTAGACTTATGAAAACTATAGGTCCAGACA   17528
gb:MN908947|Organism:Severe      CACTAGAACCAGAATATTTCAATTCAGTGTGTAGACTTATGAAAACTATAGGTCCAGACA   17542
gb:MT019532|Organism:Severe      CACTAGAACCAGAATATTTCAATTCAGTGTGTAGACTTATGAAAACTATAGGTCCAGACA   17542
                                 ************************************************************

gb:MT020781|Organism:Severe      TGTTCCTCGGAACTTGTCGGCGTTGTCCTGCTGAAATTGTTGACACTGTGAGTGCTTTGG   17590
gb:MT007544|Organism:Severe      TGTTCCTCGGAACTTGTCGGCGTTGTCCTGCTGAAATTGTTGACACTGTGAGTGCTTTGG   17602
gb:MN994467|Organism:Severe      TGTTCCTCGGAACTTGTCGGCGTTGTCCTGCTGAAATTGTTGACACTGTGAGTGCTTTGG   17602
gb:MT044257|Organism:Severe      TGTTCCTCGGAACTTGTCGGCGTTGTCCTGCTGAAATTGTTGACACTGTGAGTGCTTTGG   17602
gb:MT106054|Organism:Severe      TGTTCCTCGGAACTTGTCGGCGTTGTCCTGCTGAAATTGTTGACACTGTGAGTGCTTTGG   17602
gb:MT049951|Organism:Severe      TGTTCCTCGGAACTTGTCGGCGTTGTCCTGCTGAAATTGTTGACACTGTGAGTGCTTTGG   17602
gb:MN975262|Organism:Severe      TGTTCCTCGGAACTTGTCGGCGTTGTCCTGCTGAAATTGTTGACACTGTGAGTGCTTTGG   17602
gb:MT106052|Organism:Severe      TGTTCCTCGGAACTTGTCGGCGTTGTCCTGCTGAAATTGTTGACACTGTGAGTGCTTTGG   17602
gb:LC522975|Organism:Severe      TGTTCCTCGGAACTTGTCGGCGTTGTCCTGCTGAAATTGTTGACACTGTGAGTGCTTTGG   17599
gb:LC522973|Organism:Severe      TGTTCCTCGGAACTTGTCGGCGTTGTCCTGCTGAAATTGTTGACACTGTGAGTGCTTTGG   17599
gb:LC522974|Organism:Severe      TGTTCCTCGGAACTTGTCGGCGTTGTCCTGCTGAAATTGTTGACACTGTGAGTGCTTTGG   17599
gb:MN985325|Organism:Severe      TGTTCCTCGGAACTTGTCGGCGTTGTCCTGCTGAAATTGTTGACACTGTGAGTGCTTTGG   17602
gb:MT020881|Organism:Severe      TGTTCCTCGGAACTTGTCGGCGTTGTCCTGCTGAAATTGTTGACACTGTGAGTGCTTTGG   17602
gb:MT020880|Organism:Severe      TGTTCCTCGGAACTTGTCGGCGTTGTCCTGCTGAAATTGTTGACACTGTGAGTGCTTTGG   17602
gb:MT066175|Organism:Severe      TGTTCCTCGGAACTTGTCGGCGTTGTCCTGCTGAAATTGTTGACACTGTGAGTGCTTTGG   17602
gb:MN997409|Organism:Severe      TGTTCCTCGGAACTTGTCGGCGTTGTCCTGCTGAAATTGTTGACACTGTGAGTGCTTTGG   17602
gb:MN938384|Organism:Severe      TGTTCCTCGGAACTTGTCGGCGTTGTCCTGCTGAAATTGTTGACACTGTGAGTGCTTTGG   17570
gb:MT044258|Organism:Severe      TGTTCCTCGGAACTTGTCGGCGTTGTCCTGCTGAAATTGTTGACACTGTGAGTGCTTTGG   17578
gb:MT039890|Organism:Severe      TGTTCCTCGGAACTTGTCGGCGTTGTCCTGCTGAAATTGTTGACACTGTGAGTGCTTTGG   17602
gb:MN988713|Organism:Severe      TGTTCCTCGGAACTTGTCGGCGTTGTCCTGCTGAAATTGTTGACACTGTGAGTGCTTTGG   17602
gb:LC521925|Organism:Severe      TGTTCCTCGGAACTTGTCGGCGTTGTCCTGCTGAAATTGTTGACACTGTGAGTGCTTTGG   17575
gb:MT093571|Organism:Severe      TGTTCCTCGGAACTTGTCGGCGTTGTCCTGCTGAAATTGTTGACACTGTGAGTGCTTTGG   17602
gb:MT039887|Organism:Severe      TGTTCCTCGGAACTTGTCGGCGTTGTCCTGCTGAAATTGTTGACACTGTGAGTGCTTTGG   17602
gb:MT019530|Organism:Severe      TGTTCCTCGGAACTTGTCGGCGTTGTCCTGCTGAAATTGTTGACACTGTGAGTGCTTTGG   17602
gb:MT039888|Organism:Severe      TGTTCCTCGGAACTTGTCGGCGTTGTCCTGCTGAAATTGTTGACACTGTGAGTGCTTTGG   17602
gb:LC522972|Organism:Severe      TGTTCCTCGGAACTTGTCGGCGTTGTCCTGCTGAAATTGTTGACACTGTGAGTGCTTTGG   17599
gb:MT027063|Organism:Severe      TGTTCCTCGGAACTTGTCGGCGTTGTCCTGCTGAAATTGTTGACACTGTGAGTGCTTTGG   17602
gb:MT027062|Organism:Severe      TGTTCCTCGGAACTTGTCGGCGTTGTCCTGCTGAAATTGTTGACACTGTGAGTGCTTTGG   17602
gb:MT019529|Organism:Severe      TGTTCCTCGGAACTTGTCGGCGTTGTCCTGCTGAAATTGTTGACACTGTGAGTGCTTTGG   17602
gb:MN996529|Organism:Severe      TGTTCCTCGGAACTTGTCGGCGTTGTCCTGCTGAAATTGTTGACACTGTGAGTGCTTTGG   17590
gb:MN996531|Organism:Severe      TGTTCCTCGGAACTTGTCGGCGTTGTCCTGCTGAAATTGTTGACACTGTGAGTGCTTTGG   17589
gb:MT066176|Organism:Severe      TGTTCCTCGGAACTTGTCGGCGTTGTCCTGCTGAAATTGTTGACACTGTGAGTGCTTTGG   17602
gb:MT027064|Organism:Severe      TGTTCCTCGGAACTTGTCGGCGTTGTCCTGCTGAAATTGTTGACACTGTGAGTGCTTTGG   17602
gb:MN994468|Organism:Severe      TGTTCCTCGGAACTTGTCGGCGTTGTCCTGCTGAAATTGTTGACACTGTGAGTGCTTTGG   17602
gb:MT072688|Organism:Severe      TGTTCCTCGGAACTTGTCGGCGTTGTCCTGCTGAAATTGTTGACACTGTGAGTGCTTTGG   17587
gb:MN996527|Organism:Severe      TGTTCCTCGGAACTTGTCGGCGTTGTCCTGCTGAAATTGTTGACACTGTGAGTGCTTTGG   17569
gb:MT093631|Organism:Severe      TGTTCCTCGGAACTTGTCGGCGTTGTCCTGCTGAAATTGTTGACACTGTGAGTGCTTTGG   17640
gb:MT106053|Organism:Severe      TGTTCCTCGGAACTTGTCGGCGTTGTCCTGCTGAAATTGTTGACACTGTGAGTGCTTTGG   17602
```

```
gb:MT019533|Organism:Severe      TGTTCCTCGGAACTTGTCGGCGTTGTCCTGCTGAAATTGTTGACACTGTGAGTGCTTTGG   17602
gb:MT019531|Organism:Severe      TGTTCCTCGGAACTTGTCGGCGTTGTCCTGCTGAAATTGTTGACACTGTGAGTGCTTTGG   17602
gb:MN996528|Organism:Severe      TGTTCCTCGGAACTTGTCGGCGTTGTCCTGCTGAAATTGTTGACACTGTGAGTGCTTTGG   17602
gb:MN996530|Organism:Severe      TGTTCCTCGGAACTTGTCGGCGTTGTCCTGCTGAAATTGTTGACACTGTGAGTGCTTTGG   17588
gb:MN908947|Organism:Severe      TGTTCCTCGGAACTTGTCGGCGTTGTCCTGCTGAAATTGTTGACACTGTGAGTGCTTTGG   17602
gb:MT019532|Organism:Severe      TGTTCCTCGGAACTTGTCGGCGTTGTCCTGCTGAAATTGTTGACACTGTGAGTGCTTTGG   17602
                                 ************************************************************

gb:MT020781|Organism:Severe      TTTATGATAATAAGCTTAAAGCACATAAAGACAAATCAGCTCAATGCTTTAAAATGTTTT   17650
gb:MT007544|Organism:Severe      TTTATGATAATAAGCTTAAAGCACATAAAGACAAATCAGCTCAATGCTTTAAAATGTTTT   17662
gb:MN994467|Organism:Severe      TTTATGATAATAAGCTTAAAGCACATAAAGACAAATCAGCTCAATGCTTTAAAATGTTTT   17662
gb:MT044257|Organism:Severe      TTTATGATAATAAGCTTAAAGCACATAAAGACAAATCAGCTCAATGCTTTAAAATGTTTT   17662
gb:MT106054|Organism:Severe      TTTATGATAATAAGCTTAAAGCACATAAAGACAAATCAGCTCAATGCTTTAAAATGTTTT   17662
gb:MT049951|Organism:Severe      TTTATGATAATAAGCTTAAAGCACATAAAGACAAATCAGCTCAATGCTTTAAAATGTTTT   17662
gb:MN975262|Organism:Severe      TTTATGATAATAAGCTTAAAGCACATAAAGACAAATCAGCTCAATGCTTTAAAATGTTTT   17662
gb:MT106052|Organism:Severe      TTTATGATAATAAGCTTAAAGCACATAAAGACAAATCAGCTCAATGCTTTAAAATGTTTT   17662
gb:LC522975|Organism:Severe      TTTATGATAATAAGCTTAAAGCACATAAAGACAAATCAGCTCAATGCTTTAAAATGTTTT   17659
gb:LC522973|Organism:Severe      TTTATGATAATAAGCTTAAAGCACATAAAGACAAATCAGCTCAATGCTTTAAAATGTTTT   17659
gb:LC522974|Organism:Severe      TTTATGATAATAAGCTTAAAGCACATAAAGACAAATCAGCTCAATGCTTTAAAATGTTTT   17659
gb:MN985325|Organism:Severe      TTTATGATAATAAGCTTAAAGCACATAAAGACAAATCAGCTCAATGCTTTAAAATGTTTT   17662
gb:MT020881|Organism:Severe      TTTATGATAATAAGCTTAAAGCACATAAAGACAAATCAGCTCAATGCTTTAAAATGTTTT   17662
gb:MT020880|Organism:Severe      TTTATGATAATAAGCTTAAAGCACATAAAGACAAATCAGCTCAATGCTTTAAAATGTTTT   17662
gb:MT066175|Organism:Severe      TTTATGATAATAAGCTTAAAGCACATAAAGACAAATCAGCTCAATGCTTTAAAATGTTTT   17662
gb:MN997409|Organism:Severe      TTTATGATAATAAGCTTAAAGCACATAAAGACAAATCAGCTCAATGCTTTAAAATGTTTT   17662
gb:MN938384|Organism:Severe      TTTATGATAATAAGCTTAAAGCACATAAAGACAAATCAGCTCAATGCTTTAAAATGTTTT   17630
gb:MT044258|Organism:Severe      TTTATGATAATAAGCTTAAAGCACATAAAGACAAATCAGCTCAATGCTTTAAAATGTTTT   17638
gb:MT039890|Organism:Severe      TTTATGATAATAAGCTTAAAGCACATAAAGACAAATCAGCTCAATGCTTTAAAATGTTTT   17662
gb:MN988713|Organism:Severe      TTTATGATAATAAGCTTAAAGCACATAAAGACAAATCAGCTCAATGCTTTAAAATGTTTT   17662
gb:LC521925|Organism:Severe      TTTATGATAATAAGCTTAAAGCACATAAAGACAAATCAGCTCAATGCTTTAAAATGTTTT   17635
gb:MT093571|Organism:Severe      TTTATGATAATAAGCTTAAAGCACATAAAGACAAATCAGCTCAATGCTTTAAAATGTTTT   17662
gb:MT039887|Organism:Severe      TTTATGATAATAAGCTTAAAGCACATAAAGACAAATCAGCTCAATGCTTTAAAATGTTTT   17662
gb:MT019530|Organism:Severe      TTTATGATAATAAGCTTAAAGCACATAAAGACAAATCAGCTCAATGCTTTAAAATGTTTT   17662
gb:MT039888|Organism:Severe      TTTATGATAATAAGCTTAAAGCACATAAAGACAAATCAGCTCAATGCTTTAAAATGTTTT   17662
gb:LC522972|Organism:Severe      TTTATGATAATAAGCTTAAAGCACATAAAGACAAATCAGCTCAATGCTTTAAAATGTTTT   17659
gb:MT027063|Organism:Severe      TTTATGATAATAAGCTTAAAGCACATAAAGACAAATCAGCTCAATGCTTTAAAATGTTTT   17662
gb:MT027062|Organism:Severe      TTTATGATAATAAGCTTAAAGCACATAAAGACAAATCAGCTCAATGCTTTAAAATGTTTT   17662
gb:MT019529|Organism:Severe      TTTATGATAATAAGCTTAAAGCACATAAAGACAAATCAGCTCAATGCTTTAAAATGTTTT   17662
gb:MN996529|Organism:Severe      TTTATGATAATAAGCTTAAAGCACATAAAGACAAATCAGCTCAATGCTTTAAAATGTTTT   17650
gb:MN996531|Organism:Severe      TTTATGATAATAAGCTTAAAGCACATAAAGACAAATCAGCTCAATGCTTTAAAATGTTTT   17649
gb:MT066176|Organism:Severe      TTTATGATAATAAGCTTAAAGCACATAAAGACAAATCAGCTCAATGCTTTAAAATGTTTT   17662
gb:MT027064|Organism:Severe      TTTATGATAATAAGCTTAAAGCACATAAAGACAAATCAGCTCAATGCTTTAAAATGTTTT   17662
gb:MN994468|Organism:Severe      TTTATGATAATAAGCTTAAAGCACATAAAGACAAATCAGCTCAATGCTTTAAAATGTTTT   17662
gb:MT072688|Organism:Severe      TTTATGATAATAAGCTTAAAGCACATAAAGACAAATCAGCTCAATGCTTTAAAATGTTTT   17647
gb:MN996527|Organism:Severe      TTTATGATAATAAGCTTAAAGCACATAAAGACAAATCAGCTCAATGCTTTAAAATGTTTT   17629
gb:MT093631|Organism:Severe      TTTATGATAATAAGCTTAAAGCACATAAAGACAAATCAGCTCAATGCTTTAAAATGTTTT   17700
gb:MT106053|Organism:Severe      TTTATGATAATAAGCTTAAAGCACATAAAGACAAATCAGCTCAATGCTTTAAAATGTTTT   17662
gb:MT019533|Organism:Severe      TTTATGATAATAAGCTTAAAGCACATAAAGACAAATCAGCTCAATGCTTTAAAATGTTTT   17662
gb:MT019531|Organism:Severe      TTTATGATAATAAGCTTAAAGCACATAAAGACAAATCAGCTCAATGCTTTAAAATGTTTT   17662
gb:MN996528|Organism:Severe      TTTATGATAATAAGCTTAAAGCACATAAAGACAAATCAGCTCAATGCTTTAAAATGTTTT   17662
gb:MN996530|Organism:Severe      TTTATGATAATAAGCTTAAAGCACATAAAGACAAATCAGCTCAATGCTTTAAAATGTTTT   17648
gb:MN908947|Organism:Severe      TTTATGATAATAAGCTTAAAGCACATAAAGACAAATCAGCTCAATGCTTTAAAATGTTTT   17662
gb:MT019532|Organism:Severe      TTTATGATAATAAGCTTAAAGCACATAAAGACAAATCAGCTCAATGCTTTAAAATGTTTT   17662
                                 ************************************************************

gb:MT020781|Organism:Severe      ATAAGGGTGTTATCACGCATGATGTTTCATCTGCAATTAACAGGCCACAAATAGGCGTGG   17710
gb:MT007544|Organism:Severe      ATAAGGGTGTTATCACGCATGATGTTTCATCTGCAATTAACAGGCCACAAATAGGCGTGG   17722
gb:MN994467|Organism:Severe      ATAAGGGTGTTATCACGCATGATGTTTCATCTGCAATTAACAGGCCACAAATAGGCGTGG   17722
gb:MT044257|Organism:Severe      ATAAGGGTGTTATCACGCATGATGTTTCATCTGCAATTAACAGGCCACAAATAGGCGTGG   17722
gb:MT106054|Organism:Severe      ATAAGGGTGTTATCACGCATGATGTTTCATCTGCAATTAACAGGCCACAAATAGGCGTGG   17722
gb:MT049951|Organism:Severe      ATAAGGGTGTTATCACGCATGATGTTTCATCTGCAATTAACAGGCCACAAATAGGCGTGG   17722
gb:MN975262|Organism:Severe      ATAAGGGTGTTATCACGCATGATGTTTCATCTGCAATTAACAGGCCACAAATAGGCGTGG   17722
gb:MT106052|Organism:Severe      ATAAGGGTGTTATCACGCATGATGTTTCATCTGCAATTAACAGGCCACAAATAGGCGTGG   17722
gb:LC522975|Organism:Severe      ATAAGGGTGTTATCACGCATGATGTTTCATCTGCAATTAACAGGCCACAAATAGGCGTGG   17719
gb:LC522973|Organism:Severe      ATAAGGGTGTTATCACGCATGATGTTTCATCTGCAATTAACAGGCCACAAATAGGCGTGG   17719
gb:LC522974|Organism:Severe      ATAAGGGTGTTATCACGCATGATGTTTCATCTGCAATTAACAGGCCACAAATAGGCGTGG   17719
```

```
gb:MN985325|Organism:Severe      ATAAGGGTGTTATCACGCATGATGTTTCATCTGCAATTAACAGGCCACAAATAGGCGTGG    17722
gb:MT020881|Organism:Severe      ATAAGGGTGTTATCACGCATGATGTTTCATCTGCAATTAACAGGCCACAAATAGGCGTGG    17722
gb:MT020880|Organism:Severe      ATAAGGGTGTTATCACGCATGATGTTTCATCTGCAATTAACAGGCCACAAATAGGCGTGG    17722
gb:MT066175|Organism:Severe      ATAAGGGTGTTATCACGCATGATGTTTCATCTGCAATTAACAGGCCACAAATAGGCGTGG    17722
gb:MN997409|Organism:Severe      ATAAGGGTGTTATCACGCATGATGTTTCATCTGCAATTAACAGGCCACAAATAGGCGTGG    17722
gb:MN938384|Organism:Severe      ATAAGGGTGTTATCACGCATGATGTTTCATCTGCAATTAACAGGCCACAAATAGGCGTGG    17690
gb:MT044258|Organism:Severe      ATAAGGGTGTTATCACGCATGATGTTTCATCTGCAATTAACAGGCCACAAATAGGCGTGG    17698
gb:MT039890|Organism:Severe      ATAAGGGTGTTATCACGCATGATGTTTCATCTGCAATTAACAGGCCACAAATAGGCGTGG    17722
gb:MN988713|Organism:Severe      ATAAGGGTGTTATCACGCATGATGTTTCATCTGCAATTAACAGGCCACAAATAGGCGTGG    17722
gb:LC521925|Organism:Severe      ATAAGGGTGTTATCACGCATGATGTTTCATCTGCAATTAACAGGCCACAAATAGGCGTGG    17695
gb:MT093571|Organism:Severe      ATAAGGGTGTTATCACGCATGATGTTTCATCTGCAATTAACAGGCCACAAATAGGCGTGG    17722
gb:MT039887|Organism:Severe      ATAAGGGTGTTATCACGCATGATGTTTCATCTGCAATTAACAGGCCACAAATAGGCGTGG    17722
gb:MT019530|Organism:Severe      ATAAGGGTGTTATCACGCATGATGTTTCATCTGCAATTAACAGGCCACAAATAGGCGTGG    17722
gb:MT039888|Organism:Severe      ATAAGGGTGTTATCACGCATGATGTTTCATCTGCAATTAACAGGCCACAAATAGGCGTGG    17722
gb:LC522972|Organism:Severe      ATAAGGGTGTTATCACGCATGATGTTTCATCTGCAATTAACAGGCCACAAATAGGCGTGG    17719
gb:MT027063|Organism:Severe      ATAAGGGTGTTATCACGCATGATGTTTCATCTGCAATTAACAGGCCACAAATAGGCGTGG    17722
gb:MT027062|Organism:Severe      ATAAGGGTGTTATCACGCATGATGTTTCATCTGCAATTAACAGGCCACAAATAGGCGTGG    17722
gb:MT019529|Organism:Severe      ATAAGGGTGTTATCACGCATGATGTTTCATCTGCAATTAACAGGCCACAAATAGGCGTGG    17722
gb:MN996529|Organism:Severe      ATAAGGGTGTTATCACGCATGATGTTTCATCTGCAATTAACAGGCCACAAATAGGCGTGG    17710
gb:MN996531|Organism:Severe      ATAAGGGTGTTATCACGCATGATGTTTCATCTGCAATTAACAGGCCACAAATAGGCGTGG    17709
gb:MT066176|Organism:Severe      ATAAGGGTGTTATCACGCATGATGTTTCATCTGCAATTAACAGGCCACAAATAGGCGTGG    17722
gb:MT027064|Organism:Severe      ATAAGGGTGTTATCACGCATGATGTTTCATCTGCAATTAACAGGCCACAAATAGGCGTGG    17722
gb:MN994468|Organism:Severe      ATAAGGGTGTTATCACGCATGATGTTTCATCTGCAATTAACAGGCCACAAATAGGCGTGG    17722
gb:MT072688|Organism:Severe      ATAAGGGTGTTATCACGCATGATGTTTCATCTGCAATTAACAGGCCACAAATAGGCGTGG    17707
gb:MN996527|Organism:Severe      ATAAGGGTGTTATCACGCATGATGTTTCATCTGCAATTAACAGGCCACAAATAGGCGTGG    17689
gb:MT093631|Organism:Severe      ATAAGGGTGTTATCACGCATGATGTTTCATCTGCAATTAACAGGCCACAAATAGGCGTGG    17760
gb:MT106053|Organism:Severe      ATAAGGGTGTTATCACGCATGATGTTTCATCTGCAATTAACAGGCCACAAATAGGCGTGG    17722
gb:MT019533|Organism:Severe      ATAAGGGTGTTATCACGCATGATGTTTCATCTGCAATTAACAGGCCACAAATAGGCGTGG    17722
gb:MT019531|Organism:Severe      ATAAGGGTGTTATCACGCATGATGTTTCATCTGCAATTAACAGGCCACAAATAGGCGTGG    17722
gb:MN996528|Organism:Severe      ATAAGGGTGTTATCACGCATGATGTTTCATCTGCAATTAACAGGCCACAAATAGGCGTGG    17722
gb:MN996530|Organism:Severe      ATAAGGGTGTTATCACGCATGATGTTTCATCTGCAATTAACAGGCCACAAATAGGCGTGG    17708
gb:MN908947|Organism:Severe      ATAAGGGTGTTATCACGCATGATGTTTCATCTGCAATTAACAGGCCACAAATAGGCGTGG    17722
gb:MT019532|Organism:Severe      ATAAGGGTGTTATCACGCATGATGTTTCATCTGCAATTAACAGGCCACAAATAGGCGTGG    17722
                                 ************************************************************

gb:MT020781|Organism:Severe      TAAGAGAATTCCTTACACGTAACCCTGCTTGGAGAAAAGCTGTCTTTATTTCACCTTATA    17770
gb:MT007544|Organism:Severe      TAAGAGAATTCCTTACACGTAACCCTGCTTGGAGAAAAGCTGTCTTTATTTCACCTTATA    17782
gb:MN994467|Organism:Severe      TAAGAGAATTCCTTACACGTAACCCTGCTTGGAGAAAAGCTGTCTTTATTTCACCTTATA    17782
gb:MT044257|Organism:Severe      TAAGAGAATTCCTTACACGTAACCCTGCTTGGAGAAAAGCTGTCTTTATTTCACCTTATA    17782
gb:MT106054|Organism:Severe      TAAGAGAATTCCTTACACGTAACCCTGCTTGGAGAAAAGCTGTCTTTATTTCACCTTATA    17782
gb:MT049951|Organism:Severe      TAAGAGAATTCCTTACACGTAACCCTGCTTGGAGAAAAGCTGTCTTTATTTCACCTTATA    17782
gb:MN975262|Organism:Severe      TAAGAGAATTCCTTACACGTAACCCTGCTTGGAGAAAAGCTGTCTTTATTTCACCTTATA    17782
gb:MT106052|Organism:Severe      TAAGAGAATTCCTTACACGTAACCCTGCTTGGAGAAAAGCTGTCTTTATTTCACCTTATA    17782
gb:LC522975|Organism:Severe      TAAGAGAATTCCTTACACGTAACCCTGCTTGGAGAAAAGCTGTCTTTATTTCACCTTATA    17779
gb:LC522973|Organism:Severe      TAAGAGAATTCCTTACACGTAACCCTGCTTGGAGAAAAGCTGTCTTTATTTCACCTTATA    17779
gb:LC522974|Organism:Severe      TAAGAGAATTCCTTACACGTAACCCTGCTTGGAGAAAAGCTGTCTTTATTTCACCTTATA    17779
gb:MN985325|Organism:Severe      TAAGAGAATTCCTTACACGTAACCCTGCTTGGAGAAAAGCTGTCTTTATTTCACCTTATA    17782
gb:MT020881|Organism:Severe      TAAGAGAATTCCTTACACGTAACCCTGCTTGGAGAAAAGCTGTCTTTATTTCACCTTATA    17782
gb:MT020880|Organism:Severe      TAAGAGAATTCCTTACACGTAACCCTGCTTGGAGAAAAGCTGTCTTTATTTCACCTTATA    17782
gb:MT066175|Organism:Severe      TAAGAGAATTCCTTACACGTAACCCTGCTTGGAGAAAAGCTGTCTTTATTTCACCTTATA    17782
gb:MN997409|Organism:Severe      TAAGAGAATTCCTTACACGTAACCCTGCTTGGAGAAAAGCTGTCTTTATTTCACCTTATA    17782
gb:MN938384|Organism:Severe      TAAGAGAATTCCTTACACGTAACCCTGCTTGGAGAAAAGCTGTCTTTATTTCACCTTATA    17750
gb:MT044258|Organism:Severe      TAAGAGAATTCCTTACACGTAACCCTGCTTGGAGAAAAGCTGTCTTTATTTCACCTTATA    17758
gb:MT039890|Organism:Severe      TAAGAGAATTCCTTACACGTAACCCTGCTTGGAGAAAAGCTGTCTTTATTTCACCTTATA    17782
gb:MN988713|Organism:Severe      TAAGAGAATTCCTTACACGTAACCCTGCTTGGAGAAAAGCTGTCTTTATTTCACCTTATA    17782
gb:LC521925|Organism:Severe      TAAGAGAATTCCTTACACGTAACCCTGCTTGGAGAAAAGCTGTCTTTATTTCACCTTATA    17755
gb:MT093571|Organism:Severe      TAAGAGAATTCCTTACACGTAACCCTGCTTGGAGAAAAGCTGTCTTTATTTCACCTTATA    17782
gb:MT039887|Organism:Severe      TAAGAGAATTCCTTACACGTAACCCTGCTTGGAGAAAAGCTGTCTTTATTTCACCTTATA    17782
gb:MT019530|Organism:Severe      TAAGAGAATTCCTTACACGTAACCCTGCTTGGAGAAAAGCTGTCTTTATTTCACCTTATA    17782
gb:MT039888|Organism:Severe      TAAGAGAATTCCTTACACGTAACCCTGCTTGGAGAAAAGCTGTCTTTATTTCACCTTATA    17782
gb:LC522972|Organism:Severe      TAAGAGAATTCCTTACACGTAACCCTGCTTGGAGAAAAGCTGTCTTTATTTCACCTTATA    17779
gb:MT027063|Organism:Severe      TAAGAGAATTCCTTACACGTAACCCTGCTTGGAGAAAAGCTGTCTTTATTTCACCTTATA    17782
gb:MT027062|Organism:Severe      TAAGAGAATTCCTTACACGTAACCCTGCTTGGAGAAAAGCTGTCTTTATTTCACCTTATA    17782
gb:MT019529|Organism:Severe      TAAGAGAATTCCTTACACGTAACCCTGCTTGGAGAAAAGCTGTCTTTATTTCACCTTATA    17782
gb:MN996529|Organism:Severe      TAAGAGAATTCCTTACACGTAACCCTGCTTGGAGAAAAGCTGTCTTTATTTCACCTTATA    17770
```

```
gb:MN996531|Organism:Severe      TAAGAGAATTCCTTACACGTAACCCTGCTTGGAGAAAAGCTGTCTTTATTTCACCTTATA    17769
gb:MT066176|Organism:Severe      TAAGAGAATTCCTTACACGTAACCCTGCTTGGAGAAAAGCTGTCTTTATTTCACCTTATA    17782
gb:MT027064|Organism:Severe      TAAGAGAATTCCTTACACGTAACCCTGCTTGGAGAAAAGCTGTCTTTATTTCACCTTATA    17782
gb:MN994468|Organism:Severe      TAAGAGAATTCCTTACACGTAACCCTGCTTGGAGAAAAGCTGTCTTTATTTCACCTTATA    17782
gb:MT072688|Organism:Severe      TAAGAGAATTCCTTACACGTAACCCTGCTTGGAGAAAAGCTGTCTTTATTTCACCTTATA    17767
gb:MN996527|Organism:Severe      TAAGAGAATTCCTTACACGTAACCCTGCTTGGAGAAAAGCTGTCTTTATTTCACCTTATA    17749
gb:MT093631|Organism:Severe      TAAGAGAATTCCTTACACGTAACCCTGCTTGGAGAAAAGCTGTCTTTATTTCACCTTATA    17820
gb:MT106053|Organism:Severe      TAAGAGAATTCCTTACACGTAACCCTGCTTGGAGAAAAGCTGTCTTTATTTCACCTTATA    17782
gb:MT019533|Organism:Severe      TAAGAGAATTCCTTACACGTAACCCTGCTTGGAGAAAAGCTGTCTTTATTTCACCTTATA    17782
gb:MT019531|Organism:Severe      TAAGAGAATTCCTTACACGTAACCCTGCTTGGAGAAAAGCTGTCTTTATTTCACCTTATA    17782
gb:MN996528|Organism:Severe      TAAGAGAATTCCTTACACGTAACCCTGCTTGGAGAAAAGCTGTCTTTATTTCACCTTATA    17782
gb:MN996530|Organism:Severe      TAAGAGAATTCCTTACACGTAACCCTGCTTGGAGAAAAGCTGTCTTTATTTCACCTTATA    17768
gb:MN908947|Organism:Severe      TAAGAGAATTCCTTACACGTAACCCTGCTTGGAGAAAAGCTGTCTTTATTTCACCTTATA    17782
gb:MT019532|Organism:Severe      TAAGAGAATTCCTTACACGTAACCCTGCTTGGAGAAAAGCTGTCTTTATTTCACCTTATA    17782
                                 ************************************************************

gb:MT020781|Organism:Severe      ATTCACAGAATGCTGTAGCCTCAAAGATTTTGGGACTACCAACTCAAACTGTTGATTCAT    17830
gb:MT007544|Organism:Severe      ATTCACAGAATGCTGTAGCCTCAAAGATTTTGGGACTACCAACTCAAACTGTTGATTCAT    17842
gb:MN994467|Organism:Severe      ATTCACAGAATGCTGTAGCCTCAAAGATTTTGGGACTACCAACTCAAACTGTTGATTCAT    17842
gb:MT044257|Organism:Severe      ATTCACAGAATGCTGTAGCCTCAAAGATTTTGGGACTACCAACTCAAACTGTTGATTCAT    17842
gb:MT106054|Organism:Severe      ATTCACAGAATGCTGTAGCCTCAAAGATTTTGGGACTACCAACTCAAACTGTTGATTCAT    17842
gb:MT049951|Organism:Severe      ATTCACAGAATGCTGTAGCCTCAAAGATTTTGGGACTACCAACTCAAACTGTTGATTCAT    17842
gb:MN975262|Organism:Severe      ATTCACAGAATGCTGTAGCCTCAAAGATTTTGGGACTACCAACTCAAACTGTTGATTCAT    17842
gb:MT106052|Organism:Severe      ATTCACAGAATGCTGTAGCCTCAAAGATTTTGGGACTACCAACTCAAACTGTTGATTCAT    17842
gb:LC522975|Organism:Severe      ATTCACAGAATGCTGTAGCCTCAAAGATTTTGGGACTACCAACTCAAACTGTTGATTCAT    17839
gb:LC522973|Organism:Severe      ATTCACAGAATGCTGTAGCCTCAAAGATTTTGGGACTACCAACTCAAACTGTTGATTCAT    17839
gb:LC522974|Organism:Severe      ATTCACAGAATGCTGTAGCCTCAAAGATTTTGGGACTACCAACTCAAACTGTTGATTCAT    17839
gb:MN985325|Organism:Severe      ATTCACAGAATGCTGTAGCCTCAAAGATTTTGGGACTACCAACTCAAACTGTTGATTCAT    17842
gb:MT020881|Organism:Severe      ATTCACAGAATGCTGTAGCCTCAAAGATTTTGGGACTACCAACTCAAACTGTTGATTCAT    17842
gb:MT020880|Organism:Severe      ATTCACAGAATGCTGTAGCCTCAAAGATTTTGGGACTACCAACTCAAACTGTTGATTCAT    17842
gb:MT066175|Organism:Severe      ATTCACAGAATGCTGTAGCCTCAAAGATTTTGGGACTACCAACTCAAACTGTTGATTCAT    17842
gb:MN997409|Organism:Severe      ATTCACAGAATGCTGTAGCCTCAAAGATTTTGGGACTACCAACTCAAACTGTTGATTCAT    17842
gb:MN938384|Organism:Severe      ATTCACAGAATGCTGTAGCCTCAAAGATTTTGGGACTACCAACTCAAACTGTTGATTCAT    17810
gb:MT044258|Organism:Severe      ATTCACAGAATGCTGTAGCCTCAAAGATTTTGGGACTACCAACTCAAACTGTTGATTCAT    17818
gb:MT039890|Organism:Severe      ATTCACAGAATGCTGTAGCCTCAAAGATTTTGGGACTACCAACTCAAACTGTTGATTCAT    17842
gb:MN988713|Organism:Severe      ATTCACAGAATGCTGTAGCCTCAAAGATTTTGGGACTACCAACTCAAACTGTTGATTCAT    17842
gb:LC521925|Organism:Severe      ATTCACAGAATGCTGTAGCCTCAAAGATTTTGGGACTACCAACTCAAACTGTTGATTCAT    17815
gb:MT093571|Organism:Severe      ATTCACAGAATGCTGTAGCCTCAAAGATTTTGGGACTACCAACTCAAACTGTTGATTCAT    17842
gb:MT039887|Organism:Severe      ATTCACAGAATGCTGTAGCCTCAAAGATTTTGGGACTACCAACTCAAACTGTTGATTCAT    17842
gb:MT019530|Organism:Severe      ATTCACAGAATGCTGTAGCCTCAAAGATTTTGGGACTACCAACTCAAACTGTTGATTCAT    17842
gb:MT039888|Organism:Severe      ATTCACAGAATGCTGTAGCCTCAAAGATTTTGGGACTACCAACTCAAACTGTTGATTCAT    17842
gb:LC522972|Organism:Severe      ATTCACAGAATGCTGTAGCCTCAAAGATTTTGGGACTACCAACTCAAACTGTTGATTCAT    17839
gb:MT027063|Organism:Severe      ATTCACAGAATGCTGTAGCCTCAAAGATTTTGGGACTACCAACTCAAACTGTTGATTCAT    17842
gb:MT027062|Organism:Severe      ATTCACAGAATGCTGTAGCCTCAAAGATTTTGGGACTACCAACTCAAACTGTTGATTCAT    17842
gb:MT019529|Organism:Severe      ATTCACAGAATGCTGTAGCCTCAAAGATTTTGGGACTACCAACTCAAACTGTTGATTCAT    17842
gb:MN996529|Organism:Severe      ATTCACAGAATGCTGTAGCCTCAAAGATTTTGGGACTACCAACTCAAACTGTTGATTCAT    17830
gb:MN996531|Organism:Severe      ATTCACAGAATGCTGTAGCCTCAAAGATTTTGGGACTACCAACTCAAACTGTTGATTCAT    17829
gb:MT066176|Organism:Severe      ATTCACAGAATGCTGTAGCCTCAAAGATTTTGGGACTACCAACTCAAACTGTTGATTCAT    17842
gb:MT027064|Organism:Severe      ATTCACAGAATGCTGTAGCCTCAAAGATTTTGGGACTACCAACTCAAACTGTTGATTCAT    17842
gb:MN994468|Organism:Severe      ATTCACAGAATGCTGTAGCCTCAAAGATTTTGGGACTACCAACTCAAACTGTTGATTCAT    17842
gb:MT072688|Organism:Severe      ATTCACAGAATGCTGTAGCCTCAAAGATTTTGGGACTACCAACTCAAACTGTTGATTCAT    17827
gb:MN996527|Organism:Severe      ATTCACAGAATGCTGTAGCCTCAAAGATTTTGGGACTACCAACTCAAACTGTTGATTCAT    17809
gb:MT093631|Organism:Severe      ATTCACAGAATGCTGTAGCCTCAAAGATTTTGGGACTACCAACTCAAACTGTTGATTCAT    17880
gb:MT106053|Organism:Severe      ATTCACAGAATGCTGTAGCCTCAAAGATTTTGGGACTACCAACTCAAACTGTTGATTCAT    17842
gb:MT019533|Organism:Severe      ATTCACAGAATGCTGTAGCCTCAAAGATTTTGGGACTACCAACTCAAACTGTTGATTCAT    17842
gb:MT019531|Organism:Severe      ATTCACAGAATGCTGTAGCCTCAAAGATTTTGGGACTACCAACTCAAACTGTTGATTCAT    17842
gb:MN996528|Organism:Severe      ATTCACAGAATGCTGTAGCCTCAAAGATTTTGGGACTACCAACTCAAACTGTTGATTCAT    17842
gb:MN996530|Organism:Severe      ATTCACAGAATGCTGTAGCCTCAAAGATTTTGGGACTACCAACTCAAACTGTTGATTCAT    17828
gb:MN908947|Organism:Severe      ATTCACAGAATGCTGTAGCCTCAAAGATTTTGGGACTACCAACTCAAACTGTTGATTCAT    17842
gb:MT019532|Organism:Severe      ATTCACAGAATGCTGTAGCCTCAAAGATTTTGGGACTACCAACTCAAACTGTTGATTCAT    17842
                                 ************************************************************

gb:MT020781|Organism:Severe      CACAGGGCTCAGAATATGACTATGTCATATTCACTCAAACCACTGAAACAGCTCACTCTT    17890
gb:MT007544|Organism:Severe      CACAGGGCTCAGAATATGACTATGTCATATTCACTCAAACCACTGAAACAGCTCACTCTT    17902
gb:MN994467|Organism:Severe      CACAGGGCTCAGAATATGACTATGTCATATTCACTCAAACCACTGAAACAGCTCACTCTT    17902
```

```
gb:MT044257|Organism:Severe      CACAGGGCTCAGAATATGACTATGTCATATTCACTCAAACCACTGAAACAGCTCACTCTT   17902
gb:MT106054|Organism:Severe      CACAGGGCTCAGAATATGACTATGTCATATTCACTCAAACCACTGAAACAGCTCACTCTT   17902
gb:MT049951|Organism:Severe      CACAGGGCTCAGAATATGACTATGTCATATTCACTCAAACCACTGAAACAGCTCACTCTT   17902
gb:MN975262|Organism:Severe      CACAGGGCTCAGAATATGACTATGTCATATTCACTCAAACCACTGAAACAGCTCACTCTT   17902
gb:MT106052|Organism:Severe      CACAGGGCTCAGAATATGACTATGTCATATTCACTCAAACCACTGAAACAGCTCACTCTT   17902
gb:LC522975|Organism:Severe      CACAGGGCTCAGAATATGACTATGTCATATTCACTCAAACCACTGAAACAGCTCACTCTT   17899
gb:LC522973|Organism:Severe      CACAGGGCTCAGAATATGACTATGTCATATTCACTCAAACCACTGAAACAGCTCACTCTT   17899
gb:LC522974|Organism:Severe      CACAGGGCTCAGAATATGACTATGTCATATTCACTCAAACCACTGAAACAGCTCACTCTT   17899
gb:MN985325|Organism:Severe      CACAGGGCTCAGAATATGACTATGTCATATTCACTCAAACCACTGAAACAGCTCACTCTT   17902
gb:MT020881|Organism:Severe      CACAGGGCTCAGAATATGACTATGTCATATTCACTCAAACCACTGAAACAGCTCACTCTT   17902
gb:MT020880|Organism:Severe      CACAGGGCTCAGAATATGACTATGTCATATTCACTCAAACCACTGAAACAGCTCACTCTT   17902
gb:MT066175|Organism:Severe      CACAGGGCTCAGAATATGACTATGTCATATTCACTCAAACCACTGAAACAGCTCACTCTT   17902
gb:MN997409|Organism:Severe      CACAGGGCTCAGAATATGACTATGTCATATTCACTCAAACCACTGAAACAGCTCACTCTT   17902
gb:MN938384|Organism:Severe      CACAGGGCTCAGAATATGACTATGTCATATTCACTCAAACCACTGAAACAGCTCACTCTT   17870
gb:MT044258|Organism:Severe      CACAGGGCTCAGAATATGACTATGTCATATTCACTCAAACCACTGAAACAGCTCACTCTT   17878
gb:MT039890|Organism:Severe      CACAGGGCTCAGAATATGACTATGTCATATTCACTCAAACCACTGAAACAGCTCACTCTT   17902
gb:MN988713|Organism:Severe      CACAGGGCTCAGAATATGACTATGTCATATTCACTCAAACCACTGAAACAGCTCACTCTT   17902
gb:LC521925|Organism:Severe      CACAGGGCTCAGAATATGACTATGTCATATTCACTCAAACCACTGAAACAGCTCACTCTT   17875
gb:MT093571|Organism:Severe      CACAGGGCTCAGAATATGACTATGTCATATTCACTCAAACCACTGAAACAGCTCACTCTT   17902
gb:MT039887|Organism:Severe      CACAGGGCTCAGAATATGACTATGTCATATTCACTCAAACCACTGAAACAGCTCACTCTT   17902
gb:MT019530|Organism:Severe      CACAGGGCTCAGAATATGACTATGTCATATTCACTCAAACCACTGAAACAGCTCACTCTT   17902
gb:MT039888|Organism:Severe      CACAGGGCTCAGAATATGACTATGTCATATTCACTCAAACCACTGAAACAGCTCACTCTT   17902
gb:LC522972|Organism:Severe      CACAGGGCTCAGAATATGACTATGTCATATTCACTCAAACCACTGAAACAGCTCACTCTT   17899
gb:MT027063|Organism:Severe      CACAGGGCTCAGAATATGACTATGTCATATTCACTCAAACCACTGAAACAGCTCACTCTT   17902
gb:MT027062|Organism:Severe      CACAGGGCTCAGAATATGACTATGTCATATTCACTCAAACCACTGAAACAGCTCACTCTT   17902
gb:MT019529|Organism:Severe      CACAGGGCTCAGAATATGACTATGTCATATTCACTCAAACCACTGAAACAGCTCACTCTT   17902
gb:MN996529|Organism:Severe      CACAGGGCTCAGAATATGACTATGTCATATTCACTCAAACCACTGAAACAGCTCACTCTT   17890
gb:MN996531|Organism:Severe      CACAGGGCTCAGAATATGACTATGTCATATTCACTCAAACCACTGAAACAGCTCACTCTT   17889
gb:MT066176|Organism:Severe      CACAGGGCTCAGAATATGACTATGTCATATTCACTCAAACCACTGAAACAGCTCACTCTT   17902
gb:MT027064|Organism:Severe      CACAGGGCTCAGAATATGACTATGTCATATTCACTCAAACCACTGAAACAGCTCACTCTT   17902
gb:MN994468|Organism:Severe      CACAGGGCTCAGAATATGACTATGTCATATTCACTCAAACCACTGAAACAGCTCACTCTT   17902
gb:MT072688|Organism:Severe      CACAGGGCTCAGAATATGACTATGTCATATTCACTCAAACCACTGAAACAGCTCACTCTT   17887
gb:MN996527|Organism:Severe      CACAGGGCTCAGAATATGACTATGTCATATTCACTCAAACCACTGAAACAGCTCACTCTT   17869
gb:MT093631|Organism:Severe      CACAGGGCTCAGAATATGACTATGTCATATTCACTCAAACCACTGAAACAGCTCACTCTT   17940
gb:MT106053|Organism:Severe      CACAGGGCTCAGAATATGACTATGTCATATTCACTCAAACCACTGAAACAGCTCACTCTT   17902
gb:MT019533|Organism:Severe      CACAGGGCTCAGAATATGACTATGTCATATTCACTCAAACCACTGAAACAGCTCACTCTT   17902
gb:MT019531|Organism:Severe      CACAGGGCTCAGAATATGACTATGTCATATTCACTCAAACCACTGAAACAGCTCACTCTT   17902
gb:MN996528|Organism:Severe      CACAGGGCTCAGAATATGACTATGTCATATTCACTCAAACCACTGAAACAGCTCACTCTT   17902
gb:MN996530|Organism:Severe      CACAGGGCTCAGAATATGACTATGTCATATTCACTCAAACCACTGAAACAGCTCACTCTT   17888
gb:MN908947|Organism:Severe      CACAGGGCTCAGAATATGACTATGTCATATTCACTCAAACCACTGAAACAGCTCACTCTT   17902
gb:MT019532|Organism:Severe      CACAGGGCTCAGAATATGACTATGTCATATTCACTCAAACCACTGAAACAGCTCACTCTT   17902
                                 ************************************************************

gb:MT020781|Organism:Severe      GTAATGTAAACAGATTTAATGTTGCTATTACCAGAGCAAAAGTAGGCATACTTTGCATAA   17950
gb:MT007544|Organism:Severe      GTAATGTAAACAGATTTAATGTTGCTATTACCAGAGCAAAAGTAGGCATACTTTGCATAA   17962
gb:MN994467|Organism:Severe      GTAATGTAAACAGATTTAATGTTGCTATTACCAGAGCAAAAGTAGGCATACTTTGCATAA   17962
gb:MT044257|Organism:Severe      GTAATGTAAACAGATTTAATGTTGCTATTACCAGAGCAAAAGTAGGCATACTTTGCATAA   17962
gb:MT106054|Organism:Severe      GTAATGTAAACAGATTTAATGTTGCTATTACCAGAGCAAAAGTAGGCATACTTTGCATAA   17962
gb:MT049951|Organism:Severe      GTAATGTAAACAGATTTAATGTTGCTATTACCAGAGCAAAAGTAGGCATACTTTGCATAA   17962
gb:MN975262|Organism:Severe      GTAATGTAAACAGATTTAATGTTGCTATTACCAGAGCAAAAGTAGGCATACTTTGCATAA   17962
gb:MT106052|Organism:Severe      GTAATGTAAACAGATTTAATGTTGCTATTACCAGAGCAAAAGTAGGCATACTTTGCATAA   17962
gb:LC522975|Organism:Severe      GTAATGTAAACAGATTTAATGTTGCTATTACCAGAGCAAAAGTAGGCATACTTTGCATAA   17959
gb:LC522973|Organism:Severe      GTAATGTAAACAGATTTAATGTTGCTATTACCAGAGCAAAAGTAGGCATACTTTGCATAA   17959
gb:LC522974|Organism:Severe      GTAATGTAAACAGATTTAATGTTGCTATTACCAGAGCAAAAGTAGGCATACTTTGCATAA   17959
gb:MN985325|Organism:Severe      GTAATGTAAACAGATTTAATGTTGCTATTACCAGAGCAAAAGTAGGCATACTTTGCATAA   17962
gb:MT020881|Organism:Severe      GTAATGTAAACAGATTTAATGTTGCTATTACCAGAGCAAAAGTAGGCATACTTTGCATAA   17962
gb:MT020880|Organism:Severe      GTAATGTAAACAGATTTAATGTTGCTATTACCAGAGCAAAAGTAGGCATACTTTGCATAA   17962
gb:MT066175|Organism:Severe      GTAATGTAAACAGATTTAATGTTGCTATTACCAGAGCAAAAGTAGGCATACTTTGCATAA   17962
gb:MN997409|Organism:Severe      GTAATGTAAACAGATTTAATGTTGCTATTACCAGAGCAAAAGTAGGCATACTTTGCATAA   17962
gb:MN938384|Organism:Severe      GTAATGTAAACAGATTTAATGTTGCTATTACCAGAGCAAAAGTAGGCATACTTTGCATAA   17930
gb:MT044258|Organism:Severe      GTAATGTAAACAGATTTAATGTTGCTATTACCAGAGCAAAAGTAGGCATACTTTGCATAA   17938
gb:MT039890|Organism:Severe      GTAATGTAAACAGATTTAATGTTGCTATTACCAGAGCAAAAGTAGGCATACTTTGCATAA   17962
gb:MN988713|Organism:Severe      GTAATGTAAACAGATTTAATGTTGCTATTACCAGAGCAAAAGTAGGCATACTTTGCATAA   17962
gb:LC521925|Organism:Severe      GTAATGTAAACAGATTTAATGTTGCTATTACCAGAGCAAAAGTAGGCATACTTTGCATAA   17935
gb:MT093571|Organism:Severe      GTAATGTAAACAGATTTAATGTTGCTATTACCAGAGCAAAAGTAGGCATACTTTGCATAA   17962
```

```
gb:MT039887|Organism:Severe      GTAATGTAAACAGATTTAATGTTGCTATTACCAGAGCAAAAGTAGGCATACTTTGCATAA   17962
gb:MT019530|Organism:Severe      GTAATGTAAACAGATTTAATGTTGCTATTACCAGAGCAAAAGTAGGCATACTTTGCATAA   17962
gb:MT039888|Organism:Severe      GTAATGTAAACAGATTTAATGTTGCTATTACCAGAGCAAAAGTAGGCATACTTTGCATAA   17962
gb:LC522972|Organism:Severe      GTAATGTAAACAGATTTAATGTTGCTATTACCAGAGCAAAAGTAGGCATACTTTGCATAA   17959
gb:MT027063|Organism:Severe      GTAATGTAAACAGATTTAATGTTGCTATTACCAGAGCAAAAGTAGGCATACTTTGCATAA   17962
gb:MT027062|Organism:Severe      GTAATGTAAACAGATTTAATGTTGCTATTACCAGAGCAAAAGTAGGCATACTTTGCATAA   17962
gb:MT019529|Organism:Severe      GTAATGTAAACAGATTTAATGTTGCTATTACCAGAGCAAAAGTAGGCATACTTTGCATAA   17962
gb:MN996529|Organism:Severe      GTAATGTAAACAGATTTAATGTTGCTATTACCAGAGCAAAAGTAGGCATACTTTGCATAA   17950
gb:MN996531|Organism:Severe      GTAATGTAAACAGATTTAATGTTGCTATTACCAGAGCAAAAGTAGGCATACTTTGCATAA   17949
gb:MT066176|Organism:Severe      GTAATGTAAACAGATTTAATGTTGCTATTACCAGAGCAAAAGTAGGCATACTTTGCATAA   17962
gb:MT027064|Organism:Severe      GTAATGTAAACAGATTTAATGTTGCTATTACCAGAGCAAAAGTAGGCATACTTTGCATAA   17962
gb:MN994468|Organism:Severe      GTAATGTAAACAGATTTAATGTTGCTATTACCAGAGCAAAAGTAGGCATACTTTGCATAA   17962
gb:MT072688|Organism:Severe      GTAATGTAAACAGATTTAATGTTGCTATTACCAGAGCAAAAGTAGGCATACTTTGCATAA   17947
gb:MN996527|Organism:Severe      GTAATGTAAACAGATTTAATGTTGCTATTACCAGAGCAAAAGTAGGCATACTTTGCATAA   17929
gb:MT093631|Organism:Severe      GTAATGTAAACAGATTTAATGTTGCTATTACCAGAGCAAAAGTAGGCATACTTTGCATAA   18000
gb:MT106053|Organism:Severe      GTAATGTAAACAGATTTAATGTTGCTATTACCAGAGCAAAAGTAGGCATACTTTGCATAA   17962
gb:MT019533|Organism:Severe      GTAATGTAAACAGATTTAATGTTGCTATTACCAGAGCAAAAGTAGGCATACTTTGCATAA   17962
gb:MT019531|Organism:Severe      GTAATGTAAACAGATTTAATGTTGCTATTACCAGAGCAAAAGTAGGCATACTTTGCATAA   17962
gb:MN996528|Organism:Severe      GTAATGTAAACAGATTTAATGTTGCTATTACCAGAGCAAAAGTAGGCATACTTTGCATAA   17962
gb:MN996530|Organism:Severe      GTAATGTAAACAGATTTAATGTTGCTATTACCAGAGCAAAAGTAGGCATACTTTGCATAA   17948
gb:MN908947|Organism:Severe      GTAATGTAAACAGATTTAATGTTGCTATTACCAGAGCAAAAGTAGGCATACTTTGCATAA   17962
gb:MT019532|Organism:Severe      GTAATGTAAACAGATTTAATGTTGCTATTACCAGAGCAAAAGTAGGCATACTTTGCATAA   17962
                                 ************************************************************

gb:MT020781|Organism:Severe      TGTCTGATAGAGACCTTTATGACAAGTTGCAATTTACAAGTCTTGAAATTCCACGTAGGA   18010
gb:MT007544|Organism:Severe      TGTCTGATAGAGACCTTTATGACAAGTTGCAATTTACAAGTCTTGAAATTCCACGTAGGA   18022
gb:MN994467|Organism:Severe      TGTCTGATAGAGACCTTTATGACAAGTTGCAATTTACAAGTCTTGAAATTCCACGTAGGA   18022
gb:MT044257|Organism:Severe      TGTCTGATAGAGACCTTTATGACAAGTTGCAATTTACAAGTCTTGAAATTCCACGTAGGA   18022
gb:MT106054|Organism:Severe      TGTCTGATAGAGACCTTTATGACAAGTTGCAATTTACAAGTCTTGAAATTCCACGTAGGA   18022
gb:MT049951|Organism:Severe      TGTCTGATAGAGACCTTTATGACAAGTTGCAATTTACAAGTCTTGAAATTCCACGTAGGA   18022
gb:MN975262|Organism:Severe      TGTCTGATAGAGACCTTTATGACAAGTTGCAATTTACAAGTCTTGAAATTCCACGTAGGA   18022
gb:MT106052|Organism:Severe      TGTCTGATAGAGACCTTTATGACAAGTTGCAATTTACAAGTCTTGAAATTCCACGTAGGA   18022
gb:LC522975|Organism:Severe      TGTCTGATAGAGACCTTTATGACAAGTTGCAATTTACAAGTCTTGAAATTCCACGTAGGA   18019
gb:LC522973|Organism:Severe      TGTCTGATAGAGACCTTTATGACAAGTTGCAATTTACAAGTCTTGAAATTCCACGTAGGA   18019
gb:LC522974|Organism:Severe      TGTCTGATAGAGACCTTTATGACAAGTTGCAATTTACAAGTCTTGAAATTCCACGTAGGA   18019
gb:MN985325|Organism:Severe      TGTCTGATAGAGACCTTTATGACAAGTTGCAATTTACAAGTCTTGAAATTCCACGTAGGA   18022
gb:MT020881|Organism:Severe      TGTCTGATAGAGACCTTTATGACAAGTTGCAATTTACAAGTCTTGAAATTCCACGTAGGA   18022
gb:MT020880|Organism:Severe      TGTCTGATAGAGACCTTTATGACAAGTTGCAATTTACAAGTCTTGAAATTCCACGTAGGA   18022
gb:MT066175|Organism:Severe      TGTCTGATAGAGACCTTTATGACAAGTTGCAATTTACAAGTCTTGAAATTCCACGTAGGA   18022
gb:MN997409|Organism:Severe      TGTCTGATAGAGACCTTTATGACAAGTTGCAATTTACAAGTCTTGAAATTCCACGTAGGA   18022
gb:MN938384|Organism:Severe      TGTCTGATAGAGACCTTTATGACAAGTTGCAATTTACAAGTCTTGAAATTCCACGTAGGA   17990
gb:MT044258|Organism:Severe      TGTCTGATAGAGACCTTTATGACAAGTTGCAATTTACAAGTCTTGAAATTCCACGTAGGA   17998
gb:MT039890|Organism:Severe      TGTCTGATAGAGACCTTTATGACAAGTTGCAATTTACAAGTCTTGAAATTCCACGTAGGA   18022
gb:MN988713|Organism:Severe      TGTCTGATAGAGACCTTTATGACAAGTTGCAATTTACAAGTCTTGAAATTCCACGTAGGA   18022
gb:LC521925|Organism:Severe      TGTCTGATAGAGACCTTTATGACAAGTTGCAATTTACAAGTCTTGAAATTCCACGTAGGA   17995
gb:MT093571|Organism:Severe      TGTCTGATAGAGACCTTTATGACAAGTTGCAATTTACAAGTCTTGAAATTCCACGTAGGA   18022
gb:MT039887|Organism:Severe      TGTCTGATAGAGACCTTTATGACAAGTTGCAATTTACAAGTCTTGAAATTCCACGTAGGA   18022
gb:MT019530|Organism:Severe      TGTCTGATAGAGACCTTTATGACAAGTTGCAATTTACAAGTCTTGAAATTCCACGTAGGA   18022
gb:MT039888|Organism:Severe      TGTCTGATAGAGACCTTTATGACAAGTTGCAATTTACAAGTCTTGAAATTCCACGTAGGA   18022
gb:LC522972|Organism:Severe      TGTCTGATAGAGACCTTTATGACAAGTTGCAATTTACAAGTCTTGAAATTCCACGTAGGA   18019
gb:MT027063|Organism:Severe      TGTCTGATAGAGACCTTTATGACAAGTTGCAATTTACAAGTCTTGAAATTCCACGTAGGA   18022
gb:MT027062|Organism:Severe      TGTCTGATAGAGACCTTTATGACAAGTTGCAATTTACAAGTCTTGAAATTCCACGTAGGA   18022
gb:MT019529|Organism:Severe      TGTCTGATAGAGACCTTTATGACAAGTTGCAATTTACAAGTCTTGAAATTCCACGTAGGA   18022
gb:MN996529|Organism:Severe      TGTCTGATAGAGACCTTTATGACAAGTTGCAATTTACAAGTCTTGAAATTCCACGTAGGA   18010
gb:MN996531|Organism:Severe      TGTCTGATAGAGACCTTTATGACAAGTTGCAATTTACAAGTCTTGAAATTCCACGTAGGA   18009
gb:MT066176|Organism:Severe      TGTCTGATAGAGACCTTTATGACAAGTTGCAATTTACAAGTCTTGAAATTCCACGTAGGA   18022
gb:MT027064|Organism:Severe      TGTCTGATAGAGACCTTTATGACAAGTTGCAATTTACAAGTCTTGAAATTCCACGTAGGA   18022
gb:MN994468|Organism:Severe      TGTCTGATAGAGACCTTTATGACAAGTTGCAATTTACAAGTCTTGAAATTCCACGTAGGA   18022
gb:MT072688|Organism:Severe      TGTCTGATAGAGACCTTTATGACAAGTTGCAATTTACAAGTCTTGAAATTCCACGTAGGA   18007
gb:MN996527|Organism:Severe      TGTCTGATAGAGACCTTTATGACAAGTTGCAATTTACAAGTCTTGAAATTCCACGTAGGA   17989
gb:MT093631|Organism:Severe      TGTCTGATAGAGACCTTTATGACAAGTTGCAATTTACAAGTCTTGAAATTCCACGTAGGA   18060
gb:MT106053|Organism:Severe      TGTCTGATAGAGACCTTTATGACAAGTTGCAATTTACAAGTCTTGAAATTCCACGTAGGA   18022
gb:MT019533|Organism:Severe      TGTCTGATAGAGACCTTTATGACAAGTTGCAATTTACAAGTCTTGAAATTCCACGTAGGA   18022
gb:MT019531|Organism:Severe      TGTCTGATAGAGACCTTTATGACAAGTTGCAATTTACAAGTCTTGAAATTCCACGTAGGA   18022
gb:MN996528|Organism:Severe      TGTCTGATAGAGACCTTTATGACAAGTTGCAATTTACAAGTCTTGAAATTCCACGTAGGA   18022
```

```
gb:MN996530|Organism:Severe      TGTCTGATAGAGACCTTTATGACAAGTTGCAATTTACAAGTCTTGAAATTCCACGTAGGA    18008
gb:MN908947|Organism:Severe      TGTCTGATAGAGACCTTTATGACAAGTTGCAATTTACAAGTCTTGAAATTCCACGTAGGA    18022
gb:MT019532|Organism:Severe      TGTCTGATAGAGACCTTTATGACAAGTTGCAATTTACAAGTCTTGAAATTCCACGTAGGA    18022
                                 ************************************************************

gb:MT020781|Organism:Severe      ATGTGGCAACTTTACAAGCTGAAAATGTAACAGGACTCTTTAAAGATTGTAGTAAGGTAA    18070
gb:MT007544|Organism:Severe      ATGTGGCAACTTTACAAGCTGAAAATGTAACAGGACTCTTTAAAGATTGTAGTAAGGTAA    18082
gb:MN994467|Organism:Severe      ATGTGGCAACTTTACAAGCTGAAAATGTAACAGGACTCTTTAAAGATTGTAGTAAGGTAA    18082
gb:MT044257|Organism:Severe      ATGTGGCAACTTTACAAGCTGAAAATGTAACAGGACTCTTTAAAGATTGTAGTAAGGTAA    18082
gb:MT106054|Organism:Severe      ATGTGGCAACTTTACAAGCTGAAAATGTAACAGGACTCTTTAAAGATTGTAGTAAGGTAA    18082
gb:MT049951|Organism:Severe      ATGTGGCAACTTTACAAGCTGAAAATGTAACAGGACTCTTTAAAGATTGTAGTAAGGTAA    18082
gb:MN975262|Organism:Severe      ATGTGGCAACTTTACAAGCTGAAAATGTAACAGGACTCTTTAAAGATTGTAGTAAGGTAA    18082
gb:MT106052|Organism:Severe      ATGTGGCAACTTTACAAGCTGAAAATGTAACAGGACTCTTTAAAGATTGTAGTAAGGTAA    18082
gb:LC522975|Organism:Severe      ATGTGGCAACTTTACAAGCTGAAAATGTAACAGGACTCTTTAAAGATTGTAGTAAGGTAA    18079
gb:LC522973|Organism:Severe      ATGTGGCAACTTTACAAGCTGAAAATGTAACAGGACTCTTTAAAGATTGTAGTAAGGTAA    18079
gb:LC522974|Organism:Severe      ATGTGGCAACTTTACAAGCTGAAAATGTAACAGGACTCTTTAAAGATTGTAGTAAGGTAA    18079
gb:MN985325|Organism:Severe      ATGTGGCAACTTTACAAGCTGAAAATGTAACAGGACTTTTTAAAGATTGTAGTAAGGTAA    18082
gb:MT020881|Organism:Severe      ATGTGGCAACTTTACAAGCTGAAAATGTAACAGGACTTTTTAAAGATTGTAGTAAGGTAA    18082
gb:MT020880|Organism:Severe      ATGTGGCAACTTTACAAGCTGAAAATGTAACAGGACTTTTTAAAGATTGTAGTAAGGTAA    18082
gb:MT066175|Organism:Severe      ATGTGGCAACTTTACAAGCTGAAAATGTAACAGGACTCTTTAAAGATTGTAGTAAGGTAA    18082
gb:MN997409|Organism:Severe      ATGTGGCAACTTTACAAGCTGAAAATGTAACAGGACTCTTTAAAGATTGTAGTAAGGTAA    18082
gb:MN938384|Organism:Severe      ATGTGGCAACTTTACAAGCTGAAAATGTAACAGGACTCTTTAAAGATTGTAGTAAGGTAA    18050
gb:MT044258|Organism:Severe      ATGTGGCAACTTTACAAGCTGAAAATGTAACAGGACTCTTTAAAGATTGTAGTAAGGTAA    18058
gb:MT039890|Organism:Severe      ATGTGGCAACTTTACAAGCTGAAAATGTAACAGGACTCTTTAAAGATTGTAGTAAGGTAA    18082
gb:MN988713|Organism:Severe      ATGTGGCAACTTTACAAGCTGAAAATGTAACAGGACTCTTTAAAGATTGTAGTAAGGTAA    18082
gb:LC521925|Organism:Severe      ATGTGGCAACTTTACAAGCTGAAAATGTAACAGGACTCTTTAAAGATTGTAGTAAGGTAA    18055
gb:MT093571|Organism:Severe      ATGTGGCAACTTTACAAGCTGAAAATGTAACAGGACTCTTTAAAGATTGTAGTAAGGTAA    18082
gb:MT039887|Organism:Severe      ATGTGGCAACTTTACAAGCTGAAAATGTAACAGGACTCTTTAAAGATTGTAGTAAGGTAA    18082
gb:MT019530|Organism:Severe      ATGTGGCAACTTTACAAGCTGAAAATGTAACAGGACTCTTTAAAGATTGTAGTAAGGTAA    18082
gb:MT039888|Organism:Severe      ATGTGGCAACTTTACAAGCTGAAAATGTAACAGGACTCTTTAAAGATTGTAGTAAGGTAA    18082
gb:LC522972|Organism:Severe      ATGTGGCAACTTTACAAGCTGAAAATGTAACAGGACTCTTTAAAGATTGTAGTAAGGTAA    18079
gb:MT027063|Organism:Severe      ATGTGGCAACTTTACAAGCTGAAAATGTAACAGGACTCTTTAAAGATTGTAGTAAGGTAA    18082
gb:MT027062|Organism:Severe      ATGTGGCAACTTTACAAGCTGAAAATGTAACAGGACTCTTTAAAGATTGTAGTAAGGTAA    18082
gb:MT019529|Organism:Severe      ATGTGGCAACTTTACAAGCTGAAAATGTAACAGGACTCTTTAAAGATTGTAGTAAGGTAA    18082
gb:MN996529|Organism:Severe      ATGTGGCAACTTTACAAGCTGAAAATGTAACAGGACTCTTTAAAGATTGTAGTAAGGTAA    18070
gb:MN996531|Organism:Severe      ATGTGGCAACTTTACAAGCTGAAAATGTAACAGGACTCTTTAAAGATTGTAGTAAGGTAA    18069
gb:MT066176|Organism:Severe      ATGTGGCAACTTTACAAGCTGAAAATGTAACAGGACTCTTTAAAGATTGTAGTAAGGTAA    18082
gb:MT027064|Organism:Severe      ATGTGGCAACTTTACAAGCTGAAAATGTAACAGGACTCTTTAAAGATTGTAGTAAGGTAA    18082
gb:MN994468|Organism:Severe      ATGTGGCAACTTTACAAGCTGAAAATGTAACAGGACTCTTTAAAGATTGTAGTAAGGTAA    18082
gb:MT072688|Organism:Severe      ATGTGGCAACTTTACAAGCTGAAAATGTAACAGGACTCTTTAAAGATTGTAGTAAGGTAA    18067
gb:MN996527|Organism:Severe      ATGTGGCAACTTTACAAGCTGAAAATGTAACAGGACTCTTTAAAGATTGTAGTAAGGTAA    18049
gb:MT093631|Organism:Severe      ATGTGGCAACTTTACAAGCTGAAAATGTAACAGGACTCTTTAAAGATTGTAGTAAGGTAA    18120
gb:MT106053|Organism:Severe      ATGTGGCAACTTTACAAGCTGAAAATGTAACAGGACTCTTTAAAGATTGTAGTAAGGTAA    18082
gb:MT019533|Organism:Severe      ATGTGGCAACTTTACAAGCTGAAAATGTAACAGGACTCTTTAAAGATTGTAGTAAGGTAA    18082
gb:MT019531|Organism:Severe      ATGTGGCAACTTTACAAGCTGAAAATGTAACAGGACTCTTTAAAGATTGTAGTAAGGTAA    18082
gb:MN996528|Organism:Severe      ATGTGGCAACTTTACAAGCTGAAAATGTAACAGGACTCTTTAAAGATTGTAGTAAGGTAA    18082
gb:MN996530|Organism:Severe      ATGTGGCAACTTTACAAGCTGAAAATGTAACAGGACTCTTTAAAGATTGTAGTAAGGTAA    18068
gb:MN908947|Organism:Severe      ATGTGGCAACTTTACAAGCTGAAAATGTAACAGGACTCTTTAAAGATTGTAGTAAGGTAA    18082
gb:MT019532|Organism:Severe      ATGTGGCAACTTTACAAGCTGAAAATGTAACAGGACTCTTTAAAGATTGTAGTAAGGTAA    18082
                                 ************************************* **********************

gb:MT020781|Organism:Severe      TCACTGGGTTACATCCTACACAGGCACCTACACACCTCAGTGTTGACACTAAATTCAAAA    18130
gb:MT007544|Organism:Severe      TCACTGGGTTACATCCTACACAGGCACCTACACACCTCAGTGTTGACACTAAATTCAAAA    18142
gb:MN994467|Organism:Severe      TCACTGGGTTACATCCTACACAGGCACCTACACACCTCAGTGTTGACACTAAATTCAAAA    18142
gb:MT044257|Organism:Severe      TCACTGGGTTACATCCTACACAGGCACCTACACACCTCAGTGTTGACACTAAATTCAAAA    18142
gb:MT106054|Organism:Severe      TCACTGGGTTACATCCTACACAGGCACCTACACACCTCAGTGTTGACACTAAATTCAAAA    18142
gb:MT049951|Organism:Severe      TCACTGGGTTACATCCTACACAGGCACCTACACACCTCAGTGTTGACACTAAATTCAAAA    18142
gb:MN975262|Organism:Severe      TCACTGGGTTACATCCTACACAGGCACCTACACACCTCAGTGTTGACACTAAATTCAAAA    18142
gb:MT106052|Organism:Severe      TCACTGGGTTACATCCTACACAGGCACCTACACACCTCAGTGTTGACACTAAATTCAAAA    18142
gb:LC522975|Organism:Severe      TCACTGGGTTACATCCTACACAGGCACCTACACACCTCAGTGTTGACACTAAATTCAAAA    18139
gb:LC522973|Organism:Severe      TCACTGGGTTACATCCTACACAGGCACCTACACACCTCAGTGTTGACACTAAATTCAAAA    18139
gb:LC522974|Organism:Severe      TCACTGGGTTACATCCTACACAGGCACCTACACACCTCAGTGTTGACACTAAATTCAAAA    18139
gb:MN985325|Organism:Severe      TCACTGGGTTACATCCTACACAGGCACCTACACACCTCAGTGTTGACACTAAATTCAAAA    18142
gb:MT020881|Organism:Severe      TCACTGGGTTACATCCTACACAGGCACCTACACACCTCAGTGTTGACACTAAATTCAAAA    18142
gb:MT020880|Organism:Severe      TCACTGGGTTACATCCTACACAGGCACCTACACACCTCAGTGTTGACACTAAATTCAAAA    18142
```

```
gb:MT066175|Organism:Severe      TCACTGGGTTACATCCTACACAGGCACCTACACACCTCAGTGTTGACACTAAATTCAAAA   18142
gb:MN997409|Organism:Severe      TCACTGGGTTACATCCTACACAGGCACCTACACACCTCAGTGTTGACACTAAATTCAAAA   18142
gb:MN938384|Organism:Severe      TCACTGGGTTACATCCTACACAGGCACCTACACACCTCAGTGTTGACACTAAATTCAAAA   18110
gb:MT044258|Organism:Severe      TCACTGGGTTACATCCTACACAGGCACCTACACACCTCAGTGTTGACACTAAATTCAAAA   18118
gb:MT039890|Organism:Severe      TCACTGGGTTACATCCTACACAGGCACCTACACACCTCAGTGTTGACACTAAATTCAAAA   18142
gb:MN988713|Organism:Severe      TCACTGGGTTACATCCTACACAGGCACCTACACACCTCAGTGTTGACACTAAATTCAAAA   18142
gb:LC521925|Organism:Severe      TCACTGGGTTACATCCTACACAGGCACCTACACACCTCAGTGTTGACACTAAATTCAAAA   18115
gb:MT093571|Organism:Severe      TCACTGGGTTACATCCTACACAGGCACCTACACACCTCAGTGTTGACACTAAATTCAAAA   18142
gb:MT039887|Organism:Severe      TCACTGGGTTACATCCTACACAGGCACCTACACACCTCAGTGTTGACACTAAATTCAAAA   18142
gb:MT019530|Organism:Severe      TCACTGGGTTACATCCTACACAGGCACCTACACACCTCAGTGTTGACACTAAATTCAAAA   18142
gb:MT039888|Organism:Severe      TCACTGGGTTACATCCTACACAGGCACCTACACACCTCAGTGTTGACACTAAATTCAAAA   18142
gb:LC522972|Organism:Severe      TCACTGGGTTACATCCTACACAGGCACCTACACACCTCAGTGTTGACACTAAATTCAAAA   18139
gb:MT027063|Organism:Severe      TCACTGGGTTACATCCTACACAGGCACCTACACACCTCAGTGTTGACACTAAATTCAAAA   18142
gb:MT027062|Organism:Severe      TCACTGGGTTACATCCTACACAGGCACCTACACACCTCAGTGTTGACACTAAATTCAAAA   18142
gb:MT019529|Organism:Severe      TCACTGGGTTACATCCTACACAGGCACCTACACACCTCAGTGTTGACACTAAATTCAAAA   18142
gb:MN996529|Organism:Severe      TCACTGGGTTACATCCTACACAGGCACCTACACACCTCAGTGTTGACACTAAATTCAAAA   18130
gb:MN996531|Organism:Severe      TCACTGGGTTACATCCTACACAGGCACCTACACACCTCAGTGTTGACACTAAATTCAAAA   18129
gb:MT066176|Organism:Severe      TCACTGGGTTACATCCTACACAGGCACCTACACACCTCAGTGTTGACACTAAATTCAAAA   18142
gb:MT027064|Organism:Severe      TCACTGGGTTACATCCTACACAGGCACCTACACACCTCAGTGTTGACACTAAATTCAAAA   18142
gb:MN994468|Organism:Severe      TCACTGGGTTACATCCTACACAGGCACCTACACACCTCAGTGTTGACACTAAATTCAAAA   18142
gb:MT072688|Organism:Severe      TCACTGGGTTACATCCTACACAGGCACCTACACACCTCAGTGTTGACACTAAATTCAAAA   18127
gb:MN996527|Organism:Severe      TCACTGGGTTACATCCTACACAGGCACCTACACACCTCAGTGTTGACACTAAATTCAAAA   18109
gb:MT093631|Organism:Severe      TCACTGGGTTACATCCTACACAGGCACCTACACACCTCAGTGTTGACACTAAATTCAAAA   18180
gb:MT106053|Organism:Severe      TCACTGGGTTACATCCTACACAGGCACCTACACACCTCAGTGTTGACACTAAATTCAAAA   18142
gb:MT019533|Organism:Severe      TCACTGGGTTACATCCTACACAGGCACCTACACACCTCAGTGTTGACACTAAATTCAAAA   18142
gb:MT019531|Organism:Severe      TCACTGGGTTACATCCTACACAGGCACCTACACACCTCAGTGTTGACACTAAATTCAAAA   18142
gb:MN996528|Organism:Severe      TCACTGGGTTACATCCTACACAGGCACCTACACACCTCAGTGTTGACACTAAATTCAAAA   18142
gb:MN996530|Organism:Severe      TCACTGGGTTACATCCTACACAGGCACCTACACACCTCAGTGTTGACACTAAATTCAAAA   18128
gb:MN908947|Organism:Severe      TCACTGGGTTACATCCTACACAGGCACCTACACACCTCAGTGTTGACACTAAATTCAAAA   18142
gb:MT019532|Organism:Severe      TCACTGGGTTACATCCTACACAGGCACCTACACACCTCAGTGTTGACACTAAATTCAAAA   18142
                                 ************************************************************

gb:MT020781|Organism:Severe      CTGAAGGTTTATGTGTTGACATACCTGGCATACCTAAGGACATGACCTATAGAAGACTCA   18190
gb:MT007544|Organism:Severe      CTGAAGGTTTATGTGTTGACATACCTGGCATACCTAAGGACATGACCTATAGAAGACTCA   18202
gb:MN994467|Organism:Severe      CTGAAGGTTTATGTGTTGACATACCTGGCATACCTAAGGACATGACCTATAGAAGACTCA   18202
gb:MT044257|Organism:Severe      CTGAAGGTTTATGTGTTGACATACCTGGCATACCTAAGGACATGACCTATAGAAGACTCA   18202
gb:MT106054|Organism:Severe      CTGAAGGTTTATGTGTTGACATACCTGGCATACCTAAGGACATGACCTATAGAAGACTCA   18202
gb:MT049951|Organism:Severe      CTGAAGGTTTATGTGTTGACATACCTGGCATACCTAAGGACATGACCTATAGAAGACTCA   18202
gb:MN975262|Organism:Severe      CTGAAGGTTTATGTGTTGACATACCTGGCATACCTAAGGACATGACCTATAGAAGACTCA   18202
gb:MT106052|Organism:Severe      CTGAAGGTTTATGTGTTGACATACCTGGCATACCTAAGGACATGACCTATAGAAGACTCA   18202
gb:LC522975|Organism:Severe      CTGAAGGTTTATGTGTTGACATACCTGGCATACCTAAGGACATGACCTATAGAAGACTCA   18199
gb:LC522973|Organism:Severe      CTGAAGGTTTATGTGTTGACATACCTGGCATACCTAAGGACATGACCTATAGAAGACTCA   18199
gb:LC522974|Organism:Severe      CTGAAGGTTTATGTGTTGACATACCTGGCATACCTAAGGACATGACCTATAGAAGACTCA   18199
gb:MN985325|Organism:Severe      CTGAAGGTTTATGTGTTGACATACCTGGCATACCTAAGGACATGACCTATAGAAGACTCA   18202
gb:MT020881|Organism:Severe      CTGAAGGTTTATGTGTTGACATACCTGGCATACCTAAGGACATGACCTATAGAAGACTCA   18202
gb:MT020880|Organism:Severe      CTGAAGGTTTATGTGTTGACATACCTGGCATACCTAAGGACATGACCTATAGAAGACTCA   18202
gb:MT066175|Organism:Severe      CTGAAGGTTTATGTGTTGACATACCTGGCATACCTAAGGACATGACCTATAGAAGACTCA   18202
gb:MN997409|Organism:Severe      CTGAAGGTTTATGTGTTGACATACCTGGCATACCTAAGGACATGACCTATAGAAGACTCA   18202
gb:MN938384|Organism:Severe      CTGAAGGTTTATGTGTTGACATACCTGGCATACCTAAGGACATGACCTATAGAAGACTCA   18170
gb:MT044258|Organism:Severe      CTGAAGGTTTATGTGTTGACATACCTGGCATACCTAAGGACATGACCTATAGAAGACTCA   18178
gb:MT039890|Organism:Severe      CTGAAGGTTTATGTGTTGACATACCTGGCATACCTAAGGACATGACCTATAGAAGACTCA   18202
gb:MN988713|Organism:Severe      CTGAAGGTTTATGTGTTGACATACCTGGCATACCTAAGGACATGACCTATAGAAGACTCA   18202
gb:LC521925|Organism:Severe      CTGAAGGTTTATGTGTTGACATACCTGGCATACCTAAGGACATGACCTATAGAAGACTCA   18175
gb:MT093571|Organism:Severe      CTGAAGGTTTATGTGTTGACATACCTGGCATACCTAAGGACATGACCTATAGAAGACTCA   18202
gb:MT039887|Organism:Severe      CTGAAGGTTTATGTGTTGACATACCTGGCATACCTAAGGACATGACCTATAGAAGACTCA   18202
gb:MT019530|Organism:Severe      CTGAAGGTTTATGTGTTGACATACCTGGCATACCTAAGGACATGACCTATAGAAGACTCA   18202
gb:MT039888|Organism:Severe      CTGAAGGTTTATGTGTTGACATACCTGGCATACCTAAGGACATGACCTATAGAAGACTCA   18202
gb:LC522972|Organism:Severe      CTGAAGGTTTATGTGTTGACATACCTGGCATACCTAAGGACATGACCTATAGAAGACTCA   18199
gb:MT027063|Organism:Severe      CTGAAGGTTTATGTGTTGACATACCTGGCATACCTAAGGACATGACCTATAGAAGACTCA   18202
gb:MT027062|Organism:Severe      CTGAAGGTTTATGTGTTGACATACCTGGCATACCTAAGGACATGACCTATAGAAGACTCA   18202
gb:MT019529|Organism:Severe      CTGAAGGTTTATGTGTTGACATACCTGGCATACCTAAGGACATGACCTATAGAAGACTCA   18202
gb:MN996529|Organism:Severe      CTGAAGGTTTATGTGTTGACATACCTGGCATACCTAAGGACATGACCTATAGAAGACTCA   18190
gb:MN996531|Organism:Severe      CTGAAGGTTTATGTGTTGACATACCTGGCATACCTAAGGACATGACCTATAGAAGACTCA   18189
gb:MT066176|Organism:Severe      CTGAAGGTTTATGTGTTGACATACCTGGCATACCTAAGGACATGACCTATAGAAGACTCA   18202
gb:MT027064|Organism:Severe      CTGAAGGTTTATGTGTTGACATACCTGGCATACCTAAGGACATGACCTATAGAAGACTCA   18202
```

```
gb:MN994468|Organism:Severe      CTGAAGGTTTATGTGTTGACATACCTGGCATACCTAAGGACATGACCTATAGAAGACTCA   18202
gb:MT072688|Organism:Severe      CTGAAGGTTTATGTGTTGACATACCTGGCATACCTAAGGACATGACCTATAGAAGACTCA   18187
gb:MN996527|Organism:Severe      CTGAAGGTTTATGTGTTGACATACCTGGCATACCTAAGGACATGACCTATAGAAGACTCA   18169
gb:MT093631|Organism:Severe      CTGAAGGTTTATGTGTTGACATACCTGGCATACCTAAGGACATGACCTATAGAAGACTCA   18240
gb:MT106053|Organism:Severe      CTGAAGGTTTATGTGTTGACATACCTGGCATACCTAAGGACATGACCTATAGAAGACTCA   18202
gb:MT019533|Organism:Severe      CTGAAGGTTTATGTGTTGACATACCTGGCATACCTAAGGACATGACCTATAGAAGACTCA   18202
gb:MT019531|Organism:Severe      CTGAAGGTTTATGTGTTGACATACCTGGCATACCTAAGGACATGACCTATAGAAGACTCA   18202
gb:MN996528|Organism:Severe      CTGAAGGTTTATGTGTTGACATACCTGGCATACCTAAGGACATGACCTATAGAAGACTCA   18202
gb:MN996530|Organism:Severe      CTGAAGGTTTATGTGTTGACATACCTGGCATACCTAAGGACATGACCTATAGAAGACTCA   18188
gb:MN908947|Organism:Severe      CTGAAGGTTTATGTGTTGACATACCTGGCATACCTAAGGACATGACCTATAGAAGACTCA   18202
gb:MT019532|Organism:Severe      CTGAAGGTTTATGTGTTGACATACCTGGCATACCTAAGGACATGACCTATAGAAGACTCA   18202
                                 ************************************************************

gb:MT020781|Organism:Severe      TCTCTATGATGGGTTTTAAAATGAATTATCAAGTTAATGGTTACCCTAACATGTTTATCA   18250
gb:MT007544|Organism:Severe      TCTCTATGATGGGTTTTAAAATGAATTATCAAGTTAATGGTTACCCTAACATGTTTATCA   18262
gb:MN994467|Organism:Severe      TCTCTATGATGGGTTTTAAAATGAATTATCAAGTTAATGGTTACCCTAACATGTTTATCA   18262
gb:MT044257|Organism:Severe      TCTCTATGATGGGTTTTAAAATGAATTATCAAGTTAATGGTTACCCTAACATGTTTATCA   18262
gb:MT106054|Organism:Severe      TCTCTATGATGGGTTTTAAAATGAATTATCAAGTTAATGGTTACCCTAACATGTTTATCA   18262
gb:MT049951|Organism:Severe      TCTCTATGATGGGTTTTAAAATGAATTATCAAGTTAATGGTTACCCTAACATGTTTATCA   18262
gb:MN975262|Organism:Severe      TCTCTATGATGGGTTTTAAAATGAATTATCAAGTTAATGGTTACCCTAACATGTTTATCA   18262
gb:MT106052|Organism:Severe      TCTCTATGATGGGTTTTAAAATGAATTATCAAGTTAATGGTTACCCTAACATGTTTATCA   18262
gb:LC522975|Organism:Severe      TCTCTATGATGGGTTTTAAAATGAATTATCAAGTTAATGGTTACCCTAACATGTTTATCA   18259
gb:LC522973|Organism:Severe      TCTCTATGATGGGTTTTAAAATGAATTATCAAGTTAATGGTTACCCTAACATGTTTATCA   18259
gb:LC522974|Organism:Severe      TCTCTATGATGGGTTTTAAAATGAATTATCAAGTTAATGGTTACCCTAACATGTTTATCA   18259
gb:MN985325|Organism:Severe      TCTCTATGATGGGTTTTAAAATGAATTATCAAGTTAATGGTTACCCTAACATGTTTATCA   18262
gb:MT020881|Organism:Severe      TCTCTATGATGGGTTTTAAAATGAATTATCAAGTTAATGGTTACCCTAACATGTTTATCA   18262
gb:MT020880|Organism:Severe      TCTCTATGATGGGTTTTAAAATGAATTATCAAGTTAATGGTTACCCTAACATGTTTATCA   18262
gb:MT066175|Organism:Severe      TCTCTATGATGGGTTTTAAAATGAATTATCAAGTTAATGGTTACCCTAACATGTTTATCA   18262
gb:MN997409|Organism:Severe      TCTCTATGATGGGTTTTAAAATGAATTATCAAGTTAATGGTTACCCTAACATGTTTATCA   18262
gb:MN938384|Organism:Severe      TCTCTATGATGGGTTTTAAAATGAATTATCAAGTTAATGGTTACCCTAACATGTTTATCA   18230
gb:MT044258|Organism:Severe      TCTCTATGATGGGTTTTAAAATGAATTATCAAGTTAATGGTTACCCTAACATGTTTATCA   18238
gb:MT039890|Organism:Severe      TCTCTATGATGGGTTTTAAAATGAATTATCAAGTTAATGGTTACCCTAACATGTTTATCA   18262
gb:MN988713|Organism:Severe      TCTCTATGATGGGTTTTAAAATGAATTATCAAGTTAATGGTTACCCTAACATGTTTATCA   18262
gb:LC521925|Organism:Severe      TCTCTATGATGGGTTTTAAAATGAATTATCAAGTTAATGGTTACCCTAACATGTTTATCA   18235
gb:MT093571|Organism:Severe      TCTCTATGATGGGTTTTAAAATGAATTATCAAGTTAATGGTTACCCTAACATGTTTATCA   18262
gb:MT039887|Organism:Severe      TCTCTATGATGGGTTTTAAAATGAATTATCAAGTTAATGGTTACCCTAACATGTTTATCA   18262
gb:MT019530|Organism:Severe      TCTCTATGATGGGTTTTAAAATGAATTATCAAGTTAATGGTTACCCTAACATGTTTATCA   18262
gb:MT039888|Organism:Severe      TCTCTATGATGGGTTTTAAAATGAATTATCAAGTTAATGGTTACCCTAACATGTTTATCA   18262
gb:LC522972|Organism:Severe      TCTCTATGATGGGTTTTAAAATGAATTATCAAGTTAATGGTTACCCTAACATGTTTATCA   18259
gb:MT027063|Organism:Severe      TCTCTATGATGGGTTTTAAAATGAATTATCAAGTTAATGGTTACCCTAACATGTTTATCA   18262
gb:MT027062|Organism:Severe      TCTCTATGATGGGTTTTAAAATGAATTATCAAGTTAATGGTTACCCTAACATGTTTATCA   18262
gb:MT019529|Organism:Severe      TCTCTATGATGGGTTTTAAAATGAATTATCAAGTTAATGGTTACCCTAACATGTTTATCA   18262
gb:MN996529|Organism:Severe      TCTCTATGATGGGTTTTAAAATGAATTATCAAGTTAATGGTTACCCTAACATGTTTATCA   18250
gb:MN996531|Organism:Severe      TCTCTATGATGGGTTTTAAAATGAATTATCAAGTTAATGGTTACCCTAACATGTTTATCA   18249
gb:MT066176|Organism:Severe      TCTCTATGATGGGTTTTAAAATGAATTATCAAGTTAATGGTTACCCTAACATGTTTATCA   18262
gb:MT027064|Organism:Severe      TCTCTATGATGGGTTTTAAAATGAATTATCAAGTTAATGGTTACCCTAACATGTTTATCA   18262
gb:MN994468|Organism:Severe      TCTCTATGATGGGTTTTAAAATGAATTATCAAGTTAATGGTTACCCTAACATGTTTATCA   18262
gb:MT072688|Organism:Severe      TCTCTATGATGGGTTTTAAAATGAATTATCAAGTTAATGGTTACCCTAACATGTTTATCA   18247
gb:MN996527|Organism:Severe      TCTCTATGATGGGTTTTAAAATGAATTATCAAGTTAATGGTTACCCTAACATGTTTATCA   18229
gb:MT093631|Organism:Severe      TCTCTATGATGGGTTTTAAAATGAATTATCAAGTTAATGGTTACCCTAACATGTTTATCA   18300
gb:MT106053|Organism:Severe      TCTCTATGATGGGTTTTAAAATGAATTATCAAGTTAATGGTTACCCTAACATGTTTATCA   18262
gb:MT019533|Organism:Severe      TCTCTATGATGGGTTTTAAAATGAATTATCAAGTTAATGGTTACCCTAACATGTTTATCA   18262
gb:MT019531|Organism:Severe      TCTCTATGATGGGTTTTAAAATGAATTATCAAGTTAATGGTTACCCTAACATGTTTATCA   18262
gb:MN996528|Organism:Severe      TCTCTATGATGGGTTTTAAAATGAATTATCAAGTTAATGGTTACCCTAACATGTTTATCA   18262
gb:MN996530|Organism:Severe      TCTCTATGATGGGTTTTAAAATGAATTATCAAGTTAATGGTTACCCTAACATGTTTATCA   18248
gb:MN908947|Organism:Severe      TCTCTATGATGGGTTTTAAAATGAATTATCAAGTTAATGGTTACCCTAACATGTTTATCA   18262
gb:MT019532|Organism:Severe      TCTCTATGATGGGTTTTAAAATGAATTATCAAGTTAATGGTTACCCTAACATGTTTATCA   18262
                                 ************************************************************

gb:MT020781|Organism:Severe      CCCGCGAAGAAGCTATAAGACATGTACGTGCATGGATTGGCTTCGATGTCGAGGGGTGTC   18310
gb:MT007544|Organism:Severe      CCCGCGAAGAAGCTATAAGACATGTACGTGCATGGATTGGCTTCGATGTCGAGGGGTGTC   18322
gb:MN994467|Organism:Severe      CCCGCGAAGAAGCTATAAGACATGTACGTGCATGGATTGGCTTCGATGTCGAGGGGTGTC   18322
gb:MT044257|Organism:Severe      CCCGCGAAGAAGCTATAAGACATGTACGTGCATGGATTGGCTTCGATGTCGAGGGGTGTC   18322
gb:MT106054|Organism:Severe      CCCGCGAAGAAGCTATAAGACATGTACGTGCATGGATTGGCTTCGATGTCGAGGGGTGTC   18322
gb:MT049951|Organism:Severe      CCCGCGAAGAAGCTATAAGACATGTACGTGCATGGATTGGCTTCGATGTCGAGGGGTGTC   18322
```

```
gb:MN975262|Organism:Severe      CCCGCGAAGAAGCTATAAGACATGTACGTGCATGGATTGGCTTCGATGTCGAGGGGTGTC   18322
gb:MT106052|Organism:Severe      CCCGCGAAGAAGCTATAAGACATGTACGTGCATGGATTGGCTTCGATGTCGAGGGGTGTC   18322
gb:LC522975|Organism:Severe      CCCGCGAAGAAGCTATAAGACATGTACGTGCATGGATTGGCTTCGATGTCGAGGGGTGTC   18319
gb:LC522973|Organism:Severe      CCCGCGAAGAAGCTATAAGACATGTACGTGCATGGATTGGCTTCGATGTCGAGGGGTGTC   18319
gb:LC522974|Organism:Severe      CCCGCGAAGAAGCTATAAGACATGTACGTGCATGGATTGGCTTCGATGTCGAGGGGTGTC   18319
gb:MN985325|Organism:Severe      CCCGCGAAGAAGCTATAAGACATGTACGTGCATGGATTGGCTTCGATGTCGAGGGGTGTC   18322
gb:MT020881|Organism:Severe      CCCGCGAAGAAGCTATAAGACATGTACGTGCATGGATTGGCTTCGATGTCGAGGGGTGTC   18322
gb:MT020880|Organism:Severe      CCCGCGAAGAAGCTATAAGACATGTACGTGCATGGATTGGCTTCGATGTCGAGGGGTGTC   18322
gb:MT066175|Organism:Severe      CCCGCGAAGAAGCTATAAGACATGTACGTGCATGGATTGGCTTCGATGTCGAGGGGTGTC   18322
gb:MN997409|Organism:Severe      CCCGCGAAGAAGCTATAAGACATGTACGTGCATGGATTGGCTTCGATGTCGAGGGGTGTC   18322
gb:MN938384|Organism:Severe      CCCGCGAAGAAGCTATAAGACATGTACGTGCATGGATTGGCTTCGATGTCGAGGGGTGTC   18290
gb:MT044258|Organism:Severe      CCCGCGAAGAAGCTATAAGACATGTACGTGCATGGATTGGCTTCGATGTCGAGGGGTGTC   18298
gb:MT039890|Organism:Severe      CCCGCGAAGAAGCTATAAGACATGTACGTGCATGGATTGGCTTCGATGTCGAGGGGTGTC   18322
gb:MN988713|Organism:Severe      CCCGCGAAGAAGCTATAAGACATGTACGTGCATGGATTGGCTTCGATGTCGAGGGGTGTC   18322
gb:LC521925|Organism:Severe      CCCGCGAAGAAGCTATAAGACATGTACGTGCATGGATTGGCTTCGATGTCGAGGGGTGTC   18295
gb:MT093571|Organism:Severe      CCCGCGAAGAAGCTATAAGACATGTACGTGCATGGATTGGCTTCGATGTCGAGGGGTGTC   18322
gb:MT039887|Organism:Severe      CCCGCGAAGAAGCTATAAGACATGTACGTGCATGGATTGGCTTCGATGTCGAGGGGTGTC   18322
gb:MT019530|Organism:Severe      CCCGCGAAGAAGCTATAAGACATGTACGTGCATGGATTGGCTTCGATGTCGAGGGGTGTC   18322
gb:MT039888|Organism:Severe      CCCGCGAAGAAGCTATAAGACATGTACGTGCATGGATTGGCTTCGATGTCGAGGGGTGTC   18322
gb:LC522972|Organism:Severe      CCCGCGAAGAAGCTATAAGACATGTACGTGCATGGATTGGCTTCGATGTCGAGGGGTGTC   18319
gb:MT027063|Organism:Severe      CCCGCGAAGAAGCTATAAGACATGTACGTGCATGGATTGGCTTCGATGTCGAGGGGTGTC   18322
gb:MT027062|Organism:Severe      CCCGCGAAGAAGCTATAAGACATGTACGTGCATGGATTGGCTTCGATGTCGAGGGGTGTC   18322
gb:MT019529|Organism:Severe      CCCGCGAAGAAGCTATAAGACATGTACGTGCATGGATTGGCTTCGATGTCGAGGGGTGTC   18322
gb:MN996529|Organism:Severe      CCCGCGAAGAAGCTATAAGACATGTACGTGCATGGATTGGCTTCGATGTCGAGGGGTGTC   18310
gb:MN996531|Organism:Severe      CCCGCGAAGAAGCTATAAGACATGTACGTGCATGGATTGGCTTCGATGTCGAGGGGTGTC   18309
gb:MT066176|Organism:Severe      CCCGCGAAGAAGCTATAAGACATGTACGTGCATGGATTGGCTTCGATGTCGAGGGGTGTC   18322
gb:MT027064|Organism:Severe      CCCGCGAAGAAGCTATAAGACATGTACGTGCATGGATTGGCTTCGATGTCGAGGGGTGTC   18322
gb:MN994468|Organism:Severe      CCCGCGAAGAAGCTATAAGACATGTACGTGCATGGATTGGCTTCGATGTCGAGGGGTGTC   18322
gb:MT072688|Organism:Severe      CCCGCGAAGAAGCTATAAGACATGTACGTGCATGGATTGGCTTCGATGTCGAGGGGTGTC   18307
gb:MN996527|Organism:Severe      CCCGCGAAGAAGCTATAAGACATGTACGTGCATGGATTGGCTTCGATGTCGAGGGGTGTC   18289
gb:MT093631|Organism:Severe      CCCGCGAAGAAGCTATAAGACATGTACGTGCATGGATTGGCTTCGATGTCGAGGGGTGTC   18360
gb:MT106053|Organism:Severe      CCCGCGAAGAAGCTATAAGACATGTACGTGCATGGATTGGCTTCGATGTCGAGGGGTGTC   18322
gb:MT019533|Organism:Severe      CCCGCGAAGAAGCTATAAGACATGTACGTGCATGGATTGGCTTCGATGTCGAGGGGTGTC   18322
gb:MT019531|Organism:Severe      CCCGCGAAGAAGCTATAAGACATGTACGTGCATGGATTGGCTTCGATGTCGAGGGGTGTC   18322
gb:MN996528|Organism:Severe      CCCGCGAAGAAGCTATAAGACATGTACGTGCATGGATTGGCTTCGATGTCGAGGGGTGTC   18322
gb:MN996530|Organism:Severe      CCCGCGAAGAAGCTATAAGACATGTACGTGCATGGATTGGCTTCGATGTCGAGGGGTGTC   18308
gb:MN908947|Organism:Severe      CCCGCGAAGAAGCTATAAGACATGTACGTGCATGGATTGGCTTCGATGTCGAGGGGTGTC   18322
gb:MT019532|Organism:Severe      CCCGCGAAGAAGCTATAAGACATGTACGTGCATGGATTGGCTTCGATGTCGAGGGGTGTC   18322
                                 ************************************************************

gb:MT020781|Organism:Severe      ATGCTACTAGAGAAGCTGTTGGTACCAATTTACCTTTACAGCTAGGTTTTTCTACAGGTG   18370
gb:MT007544|Organism:Severe      ATGCTACTAGAGAAGCTGTTGGTACCAATTTACCTTTACAGCTAGGTTTTTCTACAGGTG   18382
gb:MN994467|Organism:Severe      ATGCTACTAGAGAAGCTGTTGGTACCAATTTACCTTTACAGCTAGGTTTTTCTACAGGTG   18382
gb:MT044257|Organism:Severe      ATGCTACTAGAGAAGCTGTTGGTACCAATTTACCTTTACAGCTAGGTTTTTCTACAGGTG   18382
gb:MT106054|Organism:Severe      ATGCTACTAGAGAAGCTGTTGGTACCAATTTACCTTTACAGCTAGGTTTTTCTACAGGTG   18382
gb:MT049951|Organism:Severe      ATGCTACTAGAGAAGCTGTTGGTACCAATTTACCTTTACAGCTAGGTTTTTCTACAGGTG   18382
gb:MN975262|Organism:Severe      ATGCTACTAGAGAAGCTGTTGGTACCAATTTACCTTTACAGCTAGGTTTTTCTACAGGTG   18382
gb:MT106052|Organism:Severe      ATGCTACTAGAGAAGCTGTTGGTACCAATTTACCTTTACAGCTAGGTTTTTCTACAGGTG   18382
gb:LC522975|Organism:Severe      ATGCTACTAGAGAAGCTGTTGGTACCAATTTACCTTTACAGCTAGGTTTTTCTACAGGTG   18379
gb:LC522973|Organism:Severe      ATGCTACTAGAGAAGCTGTTGGTACCAATTTACCTTTACAGCTAGGTTTTTCTACAGGTG   18379
gb:LC522974|Organism:Severe      ATGCTACTAGAGAAGCTGTTGGTACCAATTTACCTTTACAGCTAGGTTTTTCTACAGGTG   18379
gb:MN985325|Organism:Severe      ATGCTACTAGAGAAGCTGTTGGTACCAATTTACCTTTACAGCTAGGTTTTTCTACAGGTG   18382
gb:MT020881|Organism:Severe      ATGCTACTAGAGAAGCTGTTGGTACCAATTTACCTTTACAGCTAGGTTTTTCTACAGGTG   18382
gb:MT020880|Organism:Severe      ATGCTACTAGAGAAGCTGTTGGTACCAATTTACCTTTACAGCTAGGTTTTTCTACAGGTG   18382
gb:MT066175|Organism:Severe      ATGCTACTAGAGAAGCTGTTGGTACCAATTTACCTTTACAGCTAGGTTTTTCTACAGGTG   18382
gb:MN997409|Organism:Severe      ATGCTACTAGAGAAGCTGTTGGTACCAATTTACCTTTACAGCTAGGTTTTTCTACAGGTG   18382
gb:MN938384|Organism:Severe      ATGCTACTAGAGAAGCTGTTGGTACCAATTTACCTTTACAGCTAGGTTTTTCTACAGGTG   18350
gb:MT044258|Organism:Severe      ATGCTACTAGAGAAGCTGTTGGTACCAATTTACCTTTACAGCTAGGTTTTTCTACAGGTG   18358
gb:MT039890|Organism:Severe      ATGCTACTAGAGAAGCTGTTGGTACCAATTTACCTTTACAGCTAGGTTTTTCTACAGGTG   18382
gb:MN988713|Organism:Severe      ATGCTACTAGAGAAGCTGTTGGTACCAATTTACCTTTACAGCTAGGTTTTTCTACAGGTG   18382
gb:LC521925|Organism:Severe      ATGCTACTAGAGAAGCTGTTGGTACCAATTTACCTTTACAGCTAGGTTTTTCTACAGGTG   18355
gb:MT093571|Organism:Severe      ATGCTACTAGAGAAGCTGTTGGTACCAATTTACCTTTACAGCTAGGTTTTTCTACAGGTG   18382
gb:MT039887|Organism:Severe      ATGCTACTAGAGAAGCTGTTGGTACCAATTTACCTTTACAGCTAGGTTTTTCTACAGGTG   18382
gb:MT019530|Organism:Severe      ATGCTACTAGAGAAGCTGTTGGTACCAATTTACCTTTACAGCTAGGTTTTTCTACAGGTG   18382
gb:MT039888|Organism:Severe      ATGCTACTAGAGAAGCTGTTGGTACCAATTTACCTTTACAGCTAGGTTTTTCTACAGGTG   18382
```

```
gb:LC522972|Organism:Severe      ATGCTACTAGAGAAGCTGTTGGTACCAATTTACCTTTACAGCTAGGTTTTTCTACAGGTG   18379
gb:MT027063|Organism:Severe      ATGCTACTAGAGAAGCTGTTGGTACCAATTTACCTTTACAGCTAGGTTTTTCTACAGGTG   18382
gb:MT027062|Organism:Severe      ATGCTACTAGAGAAGCTGTTGGTACCAATTTACCTTTACAGCTAGGTTTTTCTACAGGTG   18382
gb:MT019529|Organism:Severe      ATGCTACTAGAGAAGCTGTTGGTACCAATTTACCTTTACAGCTAGGTTTTTCTACAGGTG   18382
gb:MN996529|Organism:Severe      ATGCTACTAGAGAAGCTGTTGGTACCAATTTACCTTTACAGCTAGGTTTTTCTACAGGTG   18370
gb:MN996531|Organism:Severe      ATGCTACTAGAGAAGCTGTTGGTACCAATTTACCTTTACAGCTAGGTTTTTCTACAGGTG   18369
gb:MT066176|Organism:Severe      ATGCTACTAGAGAAGCTGTTGGTACCAATTTACCTTTACAGCTAGGTTTTTCTACAGGTG   18382
gb:MT027064|Organism:Severe      ATGCTACTAGAGAAGCTGTTGGTACCAATTTACCTTTACAGCTAGGTTTTTCTACAGGTG   18382
gb:MN994468|Organism:Severe      ATGCTACTAGAGAAGCTGTTGGTACCAATTTACCTTTACAGCTAGGTTTTTCTACAGGTG   18382
gb:MT072688|Organism:Severe      ATGCTACTAGAGAAGCTGTTGGTACCAATTTACCTTTACAGCTAGGTTTTTCTACAGGTG   18367
gb:MN996527|Organism:Severe      ATGCTACTAGAGAAGCTGTTGGTACCAATTTACCTTTACAGCTAGGTTTTTCTACAGGTG   18349
gb:MT093631|Organism:Severe      ATGCTACTAGAGAAGCTGTTGGTACCAATTTACCTTTACAGCTAGGTTTTTCTACAGGTG   18420
gb:MT106053|Organism:Severe      ATGCTACTAGAGAAGCTGTTGGTACCAATTTACCTTTACAGCTAGGTTTTTCTACAGGTG   18382
gb:MT019533|Organism:Severe      ATGCTACTAGAGAAGCTGTTGGTACCAATTTACCTTTACAGCTAGGTTTTTCTACAGGTG   18382
gb:MT019531|Organism:Severe      ATGCTACTAGAGAAGCTGTTGGTACCAATTTACCTTTACAGCTAGGTTTTTCTACAGGTG   18382
gb:MN996528|Organism:Severe      ATGCTACTAGAGAAGCTGTTGGTACCAATTTACCTTTACAGCTAGGTTTTTCTACAGGTG   18382
gb:MN996530|Organism:Severe      ATGCTACTAGAGAAGCTGTTGGTACCAATTTACCTTTACAGCTAGGTTTTTCTACAGGTG   18368
gb:MN908947|Organism:Severe      ATGCTACTAGAGAAGCTGTTGGTACCAATTTACCTTTACAGCTAGGTTTTTCTACAGGTG   18382
gb:MT019532|Organism:Severe      ATGCTACTAGAGAAGCTGTTGGTACCAATTTACCTTTACAGCTAGGTTTTTCTACAGGTG   18382
                                 ************************************************************

gb:MT020781|Organism:Severe      TTAACCTAGTTGCTGTACCTACAGGTTATGTTGATACACCTAATAATACAGATTTTTCCA   18430
gb:MT007544|Organism:Severe      TTAACCTAGTTGCTGTACCTACAGGTTATGTTGATACACCTAATAATACAGATTTTTCCA   18442
gb:MN994467|Organism:Severe      TTAACCTAGTTGCTGTACCTACAGGTTATGTTGATACACCTAATAATACAGATTTTTCCA   18442
gb:MT044257|Organism:Severe      TTAACCTAGTTGCTGTACCTACAGGTTATGTTGATACACCTAATAATACAGATTTTTCCA   18442
gb:MT106054|Organism:Severe      TTAACCTAGTTGCTGTACCTACAGGTTATGTTGATACACCTAATAATACAGATTTTTCCA   18442
gb:MT049951|Organism:Severe      TTAACCTAGTTGCTGTACCTACAGGTTATGTTGATACACCTAATAATACAGATTTTTCCA   18442
gb:MN975262|Organism:Severe      TTAACCTAGTTGCTGTACCTACAGGTTATGTTGATACACCTAATAATACAGATTTTTCCA   18442
gb:MT106052|Organism:Severe      TTAACCTAGTTGCTGTACCTACAGGTTATGTTGATACACCTAATAATACAGATTTTTCCA   18442
gb:LC522975|Organism:Severe      TTAACCTAGTTGCTGTACCTACAGGTTATGTTGATACACCTAATAATACAGATTTTTCCA   18439
gb:LC522973|Organism:Severe      TTAACCTAGTTGCTGTACCTACAGGTTATGTTGATACACCTAATAATACAGATTTTTCCA   18439
gb:LC522974|Organism:Severe      TTAACCTAGTTGCTGTACCTACAGGTTATGTTGATACACCTAATAATACAGATTTTTCCA   18439
gb:MN985325|Organism:Severe      TTAACCTAGTTGCTGTACCTACAGGTTATGTTGATACACCTAATAATACAGATTTTTCCA   18442
gb:MT020881|Organism:Severe      TTAACCTAGTTGCTGTACCTACAGGTTATGTTGATACACCTAATAATACAGATTTTTCCA   18442
gb:MT020880|Organism:Severe      TTAACCTAGTTGCTGTACCTACAGGTTATGTTGATACACCTAATAATACAGATTTTTCCA   18442
gb:MT066175|Organism:Severe      TTAACCTAGTTGCTGTACCTACAGGTTATGTTGATACACCTAATAATACAGATTTTTCCA   18442
gb:MN997409|Organism:Severe      TTAACCTAGTTGCTGTACCTACAGGTTATGTTGATACACCTAATAATACAGATTTTTCCA   18442
gb:MN938384|Organism:Severe      TTAACCTAGTTGCTGTACCTACAGGTTATGTTGATACACCTAATAATACAGATTTTTCCA   18410
gb:MT044258|Organism:Severe      TTAACCTAGTTGCTGTACCTACAGGTTATGTTGATACACCTAATAATACAGATTTTTCCA   18418
gb:MT039890|Organism:Severe      TTAACCTAGTTGCTGTACCTACAGGTTATGTTGATACACCTAATAATACAGATTTTTCCA   18442
gb:MN988713|Organism:Severe      TTAACCTAGTTGCTGTACCTACAGGTTATGTTGATACACCTAATAATACAGATTTTTCCA   18442
gb:LC521925|Organism:Severe      TTAACCTAGTTGCTGTACCTACAGGTTATGTTGATACACCTAATAATACAGATTTTTCCA   18415
gb:MT093571|Organism:Severe      TTAACCTAGTTGCTGTACCTACAGGTTATGTTGATACACCTAATAATACAGATTTTTCCA   18442
gb:MT039887|Organism:Severe      TTAACCTAGTTGCTGTACCTACAGGTTATGTTGATACACCTAATAATACAGATTTTTCCA   18442
gb:MT019530|Organism:Severe      TTAACCTAGTTGCTGTACCTACAGGTTATGTTGATACACCTAATAATACAGATTTTTCCA   18442
gb:MT039888|Organism:Severe      TTAACCTAGTTGCTGTACCTACAGGTTATGTTGATACACCTAATAATACAGATTTTTCCA   18442
gb:LC522972|Organism:Severe      TTAACCTAGTTGCTGTACCTACAGGTTATGTTGATACACCTAATAATACAGATTTTTCCA   18439
gb:MT027063|Organism:Severe      TTAACCTAGTTGCTGTACCTACAGGTTATGTTGATACACCTAATAATACAGATTTTTCCA   18442
gb:MT027062|Organism:Severe      TTAACCTAGTTGCTGTACCTACAGGTTATGTTGATACACCTAATAATACAGATTTTTCCA   18442
gb:MT019529|Organism:Severe      TTAACCTAGTTGCTGTACCTACAGGTTATGTTGATACACCTAATAATACAGATTTTTCCA   18442
gb:MN996529|Organism:Severe      TTAACCTAGTTGCTGTACCTACAGGTTATGTTGATACACCTAATAATACAGATTTTTCCA   18430
gb:MN996531|Organism:Severe      TTAACCTAGTTGCTGTACCTACAGGTTATGTTGATACACCTAATAATACAGATTTTTCCA   18429
gb:MT066176|Organism:Severe      TTAACCTAGTTGCTGTACCTACAGGTTATGTTGATACACCTAATAATACAGATTTTTCCA   18442
gb:MT027064|Organism:Severe      TTAACCTAGTTGCTGTACCTACAGGTTATGTTGATACACCTAATAATACAGATTTTTCCA   18442
gb:MN994468|Organism:Severe      TTAACCTAGTTGCTGTACCTACAGGTTATGTTGATACACCTAATAATACAGATTTTTCCA   18442
gb:MT072688|Organism:Severe      TTAACCTAGTTGCTGTACCTACAGGTTATGTTGATACACCTAATAATACAGATTTTTCCA   18427
gb:MN996527|Organism:Severe      TTAACCTAGTTGCTGTACCTACAGGTTATGTTGATACACCTAATAATACAGATTTTTCCA   18409
gb:MT093631|Organism:Severe      TTAACCTAGTTGCTGTACCTACAGGTTATGTTGATACACCTAATAATACAGATTTTTCCA   18480
gb:MT106053|Organism:Severe      TTAACCTAGTTGCTGTACCTACAGGTTATGTTGATACACCTAATAATACAGATTTTTCCA   18442
gb:MT019533|Organism:Severe      TTAACCTAGTTGCTGTACCTACAGGTTATGTTGATACACCTAATAATACAGATTTTTCCA   18442
gb:MT019531|Organism:Severe      TTAACCTAGTTGCTGTACCTACAGGTTATGTTGATACACCTAATAATACAGATTTTTCCA   18442
gb:MN996528|Organism:Severe      TTAACCTAGTTGCTGTACCTACAGGTTATGTTGATACACCTAATAATACAGATTTTTCCA   18442
gb:MN996530|Organism:Severe      TTAACCTAGTTGCTGTACCTACAGGTTATGTTGATACACCTAATAATACAGATTTTTCCA   18428
gb:MN908947|Organism:Severe      TTAACCTAGTTGCTGTACCTACAGGTTATGTTGATACACCTAATAATACAGATTTTTCCA   18442
gb:MT019532|Organism:Severe      TTAACCTAGTTGCTGTACCTACAGGTTATGTTGATACACCTAATAATACAGATTTTTCCA   18442
```

```
                                     ************************************************************

gb:MT020781|Organism:Severe      GAGTTAGTGCTAAACCACCGCCTGGAGATCAATTTAAACACCTCATACCACTTATGTACA   18490
gb:MT007544|Organism:Severe      GAGTTAGTGCTAAACCACCGCCTGGAGATCAATTTAAACACCTCATACCACTTATGTACA   18502
gb:MN994467|Organism:Severe      GAGTTAGTGCTAAACCACCGCCTGGAGATCAATTTAAACACCTCATACCACTTATGTACA   18502
gb:MT044257|Organism:Severe      GAGTTAGTGCTAAACCACCGCCTGGAGATCAATTTAAACACCTCATACCACTTATGTACA   18502
gb:MT106054|Organism:Severe      GAGTTAGTGCTAAACCACCGCCTGGAGATCAATTTAAACACCTCATACCACTTATGTACA   18502
gb:MT049951|Organism:Severe      GAGTTAGTGCTAAACCACCGCCTGGAGATCAATTTAAACACCTCATACCACTTATGTACA   18502
gb:MN975262|Organism:Severe      GAGTTAGTGCTAAACCACCGCCTGGAGATCAATTTAAACACCTCATACCACTTATGTACA   18502
gb:MT106052|Organism:Severe      GAGTTAGTGCTAAACCACCGCCTGGAGATCAATTTAAACACCTCATACCACTTATGTACA   18502
gb:LC522975|Organism:Severe      GAGTTAGTGCTAAACCACCGCCTGGAGATCAATTTAAACACCTCATACCACTTATGTACA   18499
gb:LC522973|Organism:Severe      GAGTTAGTGCTAAACCACCGCCTGGAGATCAATTTAAACACCTCATACCACTTATGTACA   18499
gb:LC522974|Organism:Severe      GAGTTAGTGCTAAACCACCGCCTGGAGATCAATTTAAACACCTCATACCACTTATGTACA   18499
gb:MN985325|Organism:Severe      GAGTTAGTGCTAAACCACCGCCTGGAGATCAATTTAAACACCTCATACCACTTATGTACA   18502
gb:MT020881|Organism:Severe      GAGTTAGTGCTAAACCACCGCCTGGAGATCAATTTAAACACCTCATACCACTTATGTACA   18502
gb:MT020880|Organism:Severe      GAGTTAGTGCTAAACCACCGCCTGGAGATCAATTTAAACACCTCATACCACTTATGTACA   18502
gb:MT066175|Organism:Severe      GAGTTAGTGCTAAACCACCGCCTGGAGATCAATTTAAACACCTCATACCACTTATGTACA   18502
gb:MN997409|Organism:Severe      GAGTTAGTGCTAAACCACCGCCTGGAGATCAATTTAAACACCTCATACCACTTATGTACA   18502
gb:MN938384|Organism:Severe      GAGTTAGTGCTAAACCACCGCCTGGAGATCAATTTAAACACCTCATACCACTTATGTACA   18470
gb:MT044258|Organism:Severe      GAGTTAGTGCTAAACCACCGCCTGGAGATCAATTTAAACACCTCATACCACTTATGTACA   18478
gb:MT039890|Organism:Severe      GAGTTAGTGCTAAACCACCGCCTGGAGATCAATTTAAACACCTCATACCACTTATGTACA   18502
gb:MN988713|Organism:Severe      GAGTTAGTGCTAAACCACCGCCTGGAGATCAATTTAAACACCTCATACCACTTATGTACA   18502
gb:LC521925|Organism:Severe      GAGTTAGTGCTAAACCACCGCCTGGAGATCAATTTAAACACCTCATACCACTTATGTACA   18475
gb:MT093571|Organism:Severe      GAGTTAGTGCTAAACCACCGCCTGGAGATCAATTTAAACACCTCATACCACTTATGTACA   18502
gb:MT039887|Organism:Severe      GAGTTAGTGCTAAACCACCGCCTGGAGATCAATTTAAACACCTCATACCACTTATGTACA   18502
gb:MT019530|Organism:Severe      GAGTTAGTGCTAAACCACCGCCTGGAGATCAATTTAAACACCTCATACCACTTATGTACA   18502
gb:MT039888|Organism:Severe      GAGTTAGTGCTAAACCACCGCCTGGAGATCAATTTAAACACCTCATACCACTTATGTACA   18502
gb:LC522972|Organism:Severe      GAGTTAGTGCTAAACCACCGCCTGGAGATCAATTTAAACACCTCATACCACTTATGTACA   18499
gb:MT027063|Organism:Severe      GAGTTAGTGCTAAACCACCGCCTGGAGATCAATTTAAACACCTCATACCACTTATGTACA   18502
gb:MT027062|Organism:Severe      GAGTTAGTGCTAAACCACCGCCTGGAGATCAATTTAAACACCTCATACCACTTATGTACA   18502
gb:MT019529|Organism:Severe      GAGTTAGTGCTAAACCACCGCCTGGAGATCAATTTAAACACCTCATACCACTTATGTACA   18502
gb:MN996529|Organism:Severe      GAGTTAGTGCTAAACCACCGCCTGGAGATCAATTTAAACACCTCATACCACTTATGTACA   18490
gb:MN996531|Organism:Severe      GAGTTAGTGCTAAACCACCGCCTGGAGATCAATTTAAACACCTCATACCACTTATGTACA   18489
gb:MT066176|Organism:Severe      GAGTTAGTGCTAAACCACCGCCTGGAGATCAATTTAAACACCTCATACCACTTATGTACA   18502
gb:MT027064|Organism:Severe      GAGTTAGTGCTAAACCACCGCCTGGAGATCAATTTAAACACCTCATACCACTTATGTACA   18502
gb:MN994468|Organism:Severe      GAGTTAGTGCTAAACCACCGCCTGGAGATCAATTTAAACACCTCATACCACTTATGTACA   18502
gb:MT072688|Organism:Severe      GAGTTAGTGCTAAACCACCGCCTGGAGATCAATTTAAACACCTCATACCACTTATGTACA   18487
gb:MN996527|Organism:Severe      GAGTTAGTGCTAAACCACCGCCTGGAGATCAATTTAAACACCTCATACCACTTATGTACA   18469
gb:MT093631|Organism:Severe      GAGTTAGTGCTAAACCACCGCCTGGAGATCAATTTAAACACCTCATACCACTTATGTACA   18540
gb:MT106053|Organism:Severe      GAGTTAGTGCTAAACCACCGCCTGGAGATCAATTTAAACACCTCATACCACTTATGTACA   18502
gb:MT019533|Organism:Severe      GAGTTAGTGCTAAACCACCGCCTGGAGATCAATTTAAACACCTCATACCACTTATGTACA   18502
gb:MT019531|Organism:Severe      GAGTTAGTGCTAAACCACCGCCTGGAGATCAATTTAAACACCTCATACCACTTATGTACA   18502
gb:MN996528|Organism:Severe      GAGTTAGTGCTAAACCACCGCCTGGAGATCAATTTAAACACCTCATACCACTTATGTACA   18502
gb:MN996530|Organism:Severe      GAGTTAGTGCTAAACCACCGCCTGGAGATCAATTTAAACACCTCATACCACTTATGTACA   18488
gb:MN908947|Organism:Severe      GAGTTAGTGCTAAACCACCGCCTGGAGATCAATTTAAACACCTCATACCACTTATGTACA   18502
gb:MT019532|Organism:Severe      GAGTTAGTGCTAAACCACCGCCTGGAGATCAATTTAAACACCTCATACCACTTATGTACA   18502
                                 ************************************************************

gb:MT020781|Organism:Severe      AAGGACTTCCTTGGAATGTAGTGCGTATAAAGATTGTACAAATGTTAAGTGACACACTTA   18550
gb:MT007544|Organism:Severe      AAGGACTTCCTTGGAATGTAGTGCGTATAAAGATTGTACAAATGTTAAGTGACACACTTA   18562
gb:MN994467|Organism:Severe      AAGGACTTCCTTGGAATGTAGTGCGTATAAAGATTGTACAAATGTTAAGTGACACACTTA   18562
gb:MT044257|Organism:Severe      AAGGACTTCCTTGGAATGTAGTGCGTATAAAGATTGTACAAATGTTAAGTGACACACTTA   18562
gb:MT106054|Organism:Severe      AAGGACTTCCTTGGAATGTAGTGCGTATAAAGATTGTACAAATGTTAAGTGACACACTTA   18562
gb:MT049951|Organism:Severe      AAGGACTTCCTTGGAATGTAGTGCGTATAAAGATTGTACAAATGTTAAGTGACACACTTA   18562
gb:MN975262|Organism:Severe      AAGGACTTCCTTGGAATGTAGTGCGTATAAAGATTGTACAAATGTTAAGTGACACACTTA   18562
gb:MT106052|Organism:Severe      AAGGACTTCCTTGGAATGTAGTGCGTATAAAGATTGTACAAATGTTAAGTGACACACTTA   18562
gb:LC522975|Organism:Severe      AAGGACTTCCTTGGAATGTAGTGCGTATAAAGATTGTACAAATGTTAAGTGACACACTTA   18559
gb:LC522973|Organism:Severe      AAGGACTTCCTTGGAATGTAGTGCGTATAAAGATTGTACAAATGTTAAGTGACACACTTA   18559
gb:LC522974|Organism:Severe      AAGGACTTCCTTGGAATGTAGTGCGTATAAAGATTGTACAAATGTTAAGTGACACACTTA   18559
gb:MN985325|Organism:Severe      AAGGACTTCCTTGGAATGTAGTGCGTATAAAGATTGTACAAATGTTAAGTGACACACTTA   18562
gb:MT020881|Organism:Severe      AAGGACTTCCTTGGAATGTAGTGCGTATAAAGATTGTACAAATGTTAAGTGACACACTTA   18562
gb:MT020880|Organism:Severe      AAGGACTTCCTTGGAATGTAGTGCGTATAAAGATTGTACAAATGTTAAGTGACACACTTA   18562
gb:MT066175|Organism:Severe      AAGGACTTCCTTGGAATGTAGTGCGTATAAAGATTGTACAAATGTTAAGTGACACACTTA   18562
gb:MN997409|Organism:Severe      AAGGACTTCCTTGGAATGTAGTGCGTATAAAGATTGTACAAATGTTAAGTGACACACTTA   18562
gb:MN938384|Organism:Severe      AAGGACTTCCTTGGAATGTAGTGCGTATAAAGATTGTACAAATGTTAAGTGACACACTTA   18530
```

```
gb:MT044258|Organism:Severe      AAGGACTTCCTTGGAATGTAGTGCGTATAAAGATTGTACAAATGTTAAGTGACACACTTA    18538
gb:MT039890|Organism:Severe      AAGGACTTCCTTGGAATGTAGTGCGTATAAAGATTGTACAAATGTTAAGTGACACACTTA    18562
gb:MN988713|Organism:Severe      AAGGACTTCCTTGGAATGTAGTGCGTATAAAGATTGTACAAATGTTAAGTGACACACTTA    18562
gb:LC521925|Organism:Severe      AAGGACTTCTTTGGAATGTAGTGCGTATAAAGATTGTACAAATGTTAAGTGACACACTTA    18535
gb:MT093571|Organism:Severe      AAGGACTTCCTTGGAATGTAGTGCGTATAAAGATTGTACAAATGTTAAGTGACACACTTA    18562
gb:MT039887|Organism:Severe      AAGGACTTCCTTGGAATGTAGTGCGTATAAAGATTGTACAAATGTTAAGTGACACACTTA    18562
gb:MT019530|Organism:Severe      AAGGACTTCCTTGGAATGTAGTGCGTATAAAGATTGTACAAATGTTAAGTGACACACTTA    18562
gb:MT039888|Organism:Severe      AAGGACTTCCTTGGAATGTAGTGCGTATAAAGATTGTACAAATGTTAAGTGACACACTTA    18562
gb:LC522972|Organism:Severe      AAGGACTTCCTTGGAATGTAGTGCGTATAAAGATTGTACAAATGTTAAGTGACACACTTA    18559
gb:MT027063|Organism:Severe      AAGGACTTCCTTGGAATGTAGTGCGTATAAAGATTGTACAAATGTTAAGTGACACACTTA    18562
gb:MT027062|Organism:Severe      AAGGACTTCCTTGGAATGTAGTGCGTATAAAGATTGTACAAATGTTAAGTGACACACTTA    18562
gb:MT019529|Organism:Severe      AAGGACTTCCTTGGAATGTAGTGCGTATAAAGATTGTACAAATGTTAAGTGACACACTTA    18562
gb:MN996529|Organism:Severe      AAGGACTTCCTTGGAATGTAGTGCGTATAAAGATTGTACAAATGTTAAGTGACACACTTA    18550
gb:MN996531|Organism:Severe      AAGGACTTCCTTGGAATGTAGTGCGTATAAAGATTGTACAAATGTTAAGTGACACACTTA    18549
gb:MT066176|Organism:Severe      AAGGACTTCCTTGGAATGTAGTGCGTATAAAGATTGTACAAATGTTAAGTGACACACTTA    18562
gb:MT027064|Organism:Severe      AAGGACTTCCTTGGAATGTAGTGCGTATAAAGATTGTACAAATGTTAAGTGACACACTTA    18562
gb:MN994468|Organism:Severe      AAGGACTTCCTTGGAATGTAGTGCGTATAAAGATTGTACAAATGTTAAGTGACACACTTA    18562
gb:MT072688|Organism:Severe      AAGGACTTCCTTGGAATGTAGTGCGTATAAAGATTGTACAAATGTTAAGTGACACACTTA    18547
gb:MN996527|Organism:Severe      AAGGACTTCCTTGGAATGTAGTGCGTATAAAGATTGTACAAATGTTAAGTGACACACTTA    18529
gb:MT093631|Organism:Severe      AAGGACTTCCTTGGAATGTAGTGCGTATAAAGATTGTACAAATGTTAAGTGACACACTTA    18600
gb:MT106053|Organism:Severe      AAGGACTTCCTTGGAATGTAGTGCGTATAAAGATTGTACAAATGTTAAGTGACACACTTA    18562
gb:MT019533|Organism:Severe      AAGGACTTCCTTGGAATGTAGTGCGTATAAAGATTGTACAAATGTTAAGTGACACACTTA    18562
gb:MT019531|Organism:Severe      AAGGACTTCCTTGGAATGTAGTGCGTATAAAGATTGTACAAATGTTAAGTGACACACTTA    18562
gb:MN996528|Organism:Severe      AAGGACTTCCTTGGAATGTAGTGCGTATAAAGATTGTACAAATGTTAAGTGACACACTTA    18562
gb:MN996530|Organism:Severe      AAGGACTTCCTTGGAATGTAGTGCGTATAAAGATTGTACAAATGTTAAGTGACACACTTA    18548
gb:MN908947|Organism:Severe      AAGGACTTCCTTGGAATGTAGTGCGTATAAAGATTGTACAAATGTTAAGTGACACACTTA    18562
gb:MT019532|Organism:Severe      AAGGACTTCCTTGGAATGTAGTGCGTATAAAGATTGTACAAATGTTAAGTGACACACTTA    18562
                                 ********* **************************************************

gb:MT020781|Organism:Severe      AAAATCTCTCTGACAGAGTCGTATTTGTCTTATGGGCACATGGCTTTGAGTTGACATCTA    18610
gb:MT007544|Organism:Severe      AAAATCTCTCTGACAGAGTCGTATTTGTCTTATGGGCACATGGCTTTGAGTTGACATCTA    18622
gb:MN994467|Organism:Severe      AAAATCTCTCTGACAGAGTCGTATTTGTCTTATGGGCACATGGCTTTGAGTTGACATCTA    18622
gb:MT044257|Organism:Severe      AAAATCTCTCTGACAGAGTCGTATTTGTCTTATGGGCACATGGCTTTGAGTTGACATCTA    18622
gb:MT106054|Organism:Severe      AAAATCTCTCTGACAGAGTCGTATTTGTCTTATGGGCACACGGCTTTGAGTTGACATCTA    18622
gb:MT049951|Organism:Severe      AAAATCTCTCTGACAGAGTCGTATTTGTCTTATGGGCACATGGCTTTGAGTTGACATCTA    18622
gb:MN975262|Organism:Severe      AAAATCTCTCTGACAGAGTCGTATTTGTCTTATGGGCACATGGCTTTGAGTTGACATCTA    18622
gb:MT106052|Organism:Severe      AAAATCTCTCTGACAGAGTCGTATTTGTCTTATGGGCACATGGCTTTGAGTTGACATCTA    18622
gb:LC522975|Organism:Severe      AAAATCTCTCTGACAGAGTCGTATTTGTCTTATGGGCACATGGCTTTGAGTTGACATCTA    18619
gb:LC522973|Organism:Severe      AAAATCTCTCTGACAGAGTCGTATTTGTCTTATGGGCACATGGCTTTGAGTTGACATCTA    18619
gb:LC522974|Organism:Severe      AAAATCTCTCTGACAGAGTCGTATTTGTCTTATGGGCACATGGCTTTGAGTTGACATCTA    18619
gb:MN985325|Organism:Severe      AAAATCTCTCTGACAGAGTCGTATTTGTCTTATGGGCACATGGCTTTGAGTTGACATCTA    18622
gb:MT020881|Organism:Severe      AAAATCTCTCTGACAGAGTCGTATTTGTCTTATGGGCACATGGCTTTGAGTTGACATCTA    18622
gb:MT020880|Organism:Severe      AAAATCTCTCTGACAGAGTCGTATTTGTCTTATGGGCACATGGCTTTGAGTTGACATCTA    18622
gb:MT066175|Organism:Severe      AAAATCTCTCTGACAGAGTCGTATTTGTCTTATGGGCACATGGCTTTGAGTTGACATCTA    18622
gb:MN997409|Organism:Severe      AAAATCTCTCTGACAGAGTCGTATTTGTCTTATGGGCACATGGCTTTGAGTTGACATCTA    18622
gb:MN938384|Organism:Severe      AAAATCTCTCTGACAGAGTCGTATTTGTCTTATGGGCACATGGCTTTGAGTTGACATCTA    18590
gb:MT044258|Organism:Severe      AAAATCTCTCTGACAGAGTCGTATTTGTCTTATGGGCACATGGCTTTGAGTTGACATCTA    18598
gb:MT039890|Organism:Severe      AAAATCTCTCTGACAGAGTCGTATTTGTCTTATGGGCACATGGCTTTGAGTTGACATCTA    18622
gb:MN988713|Organism:Severe      AAAATCTCTCTGACAGAGTCGTATTTGTCTTATGGGCACATGGCTTTGAGTTGACATCTA    18622
gb:LC521925|Organism:Severe      AAAATCTCTCTGACAGAGTCGTATTTGTCTTATGGGCACATGGCTTTGAGTTGACATCTA    18595
gb:MT093571|Organism:Severe      AAAATCTCTCTGACAGAGTCGTATTTGTCTTATGGGCACATGGCTTTGAGTTGACATCTA    18622
gb:MT039887|Organism:Severe      AAAATCTCTCTGACAGAGTCGTATTTGTCTTATGGGCACATGGCTTTGAGTTGACATCTA    18622
gb:MT019530|Organism:Severe      AAAATCTCTCTGACAGAGTCGTATTTGTCTTATGGGCACATGGCTTTGAGTTGACATCTA    18622
gb:MT039888|Organism:Severe      AAAATCTCTCTGACAGAGTCGTATTTGTCTTATGGGCACATGGCTTTGAGTTGACATCTA    18622
gb:LC522972|Organism:Severe      AAAATCTCTCTGACAGAGTCGTATTTGTCTTATGGGCACATGGCTTTGAGTTGACATCTA    18619
gb:MT027063|Organism:Severe      AAAATCTCTCTGACAGAGTCGTATTTGTCTTATGGGCACATGGCTTTGAGTTGACATCTA    18622
gb:MT027062|Organism:Severe      AAAATCTCTCTGACAGAGTCGTATTTGTCTTATGGGCACATGGCTTTGAGTTGACATCTA    18622
gb:MT019529|Organism:Severe      AAAATCTCTCTGACAGAGTCGTATTTGTCTTATGGGCACATGGCTTTGAGTTGACATCTA    18622
gb:MN996529|Organism:Severe      AAAATCTCTCTGACAGAGTCGTATTTGTCTTATGGGCACATGGCTTTGAGTTGACATCTA    18610
gb:MN996531|Organism:Severe      AAAATCTCTCTGACAGAGTCGTATTTGTCTTATGGGCACATGGCTTTGAGTTGACATCTA    18609
gb:MT066176|Organism:Severe      AAAATCTCTCTGACAGAGTCGTATTTGTCTTATGGGCACATGGCTTTGAGTTGACATCTA    18622
gb:MT027064|Organism:Severe      AAAATCTCTCTGACAGAGTCGTATTTGTCTTATGGGCACATGGCTTTGAGTTGACATCTA    18622
gb:MN994468|Organism:Severe      AAAATCTCTCTGACAGAGTCGTATTTGTCTTATGGGCACATGGCTTTGAGTTGACATCTA    18622
gb:MT072688|Organism:Severe      AAAATCTCTCTGACAGAGTCGTATTTGTCTTATGGGCACATGGCTTTGAGTTGACATCTA    18607
gb:MN996527|Organism:Severe      AAAATCTCTCTGACAGAGTCGTATTTGTCTTATGGGCACATGGCTTTGAGTTGACATCTA    18589
```

```
gb:MT093631|Organism:Severe      AAAATCTCTCTGACAGAGTCGTATTTGTCTTATGGGCACATGGCTTTGAGTTGACATCTA   18660
gb:MT106053|Organism:Severe      AAAATCTCTCTGACAGAGTCGTATTTGTCTTATGGGCACATGGCTTTGAGTTGACATCTA   18622
gb:MT019533|Organism:Severe      AAAATCTCTCTGACAGAGTCGTATTTGTCTTATGGGCACATGGCTTTGAGTTGACATCTA   18622
gb:MT019531|Organism:Severe      AAAATCTCTCTGACAGAGTCGTATTTGTCTTATGGGCACATGGCTTTGAGTTGACATCTA   18622
gb:MN996528|Organism:Severe      AAAATCTCTCTGACAGAGTCGTATTTGTCTTATGGGCACATGGCTTTGAGTTGACATCTA   18622
gb:MN996530|Organism:Severe      AAAATCTCTCTGACAGAGTCGTATTTGTCTTATGGGCACATGGCTTTGAGTTGACATCTA   18608
gb:MN908947|Organism:Severe      AAAATCTCTCTGACAGAGTCGTATTTGTCTTATGGGCACATGGCTTTGAGTTGACATCTA   18622
gb:MT019532|Organism:Severe      AAAATCTCTCTGACAGAGTCGTATTTGTCTTATGGGCACATGGCTTTGAGTTGACATCTA   18622
                                 **************************************** *******************

gb:MT020781|Organism:Severe      TGAAGTATTTTGTGAAAATAGGACCTGAGCGCACCTGTTGTCTATGTGATAGACGTGCCA   18670
gb:MT007544|Organism:Severe      TGAAGTATTTTGTGAAAATAGGACCTGAGCGCACCTGTTGTCTATGTGATAGACGTGCCA   18682
gb:MN994467|Organism:Severe      TGAAGTATTTTGTGAAAATAGGACCTGAGCGCACCTGTTGTCTATGTGATAGACGTGCCA   18682
gb:MT044257|Organism:Severe      TGAAGTATTTTGTGAAAATAGGACCTGAGCGCACCTGTTGTCTATGTGATAGACGTGCCA   18682
gb:MT106054|Organism:Severe      TGAAGTATTTTGTGAAAATAGGACCTGAGCGCACCTGTTGTCTATGTGATAGACGTGCCA   18682
gb:MT049951|Organism:Severe      TGAAGTATTTTGTGAAAATAGGACCTGAGCGCACCTGTTGTCTATGTGATAGACGTGCCA   18682
gb:MN975262|Organism:Severe      TGAAGTATTTTGTGAAAATAGGACCTGAGCGCACCTGTTGTCTATGTGATAGACGTGCCA   18682
gb:MT106052|Organism:Severe      TGAAGTATTTTGTGAAAATAGGACCTGAGCGCACCTGTTGTCTATGTGATAGACGTGCCA   18682
gb:LC522975|Organism:Severe      TGAAGTATTTTGTGAAAATAGGACCTGAGCGCACCTGTTGTCTATGTGATAGACGTGCCA   18679
gb:LC522973|Organism:Severe      TGAAGTATTTTGTGAAAATAGGACCTGAGCGCACCTGTTGTCTATGTGATAGACGTGCCA   18679
gb:LC522974|Organism:Severe      TGAAGTATTTTGTGAAAATAGGACCTGAGCGCACCTGTTGTCTATGTGATAGACGTGCCA   18679
gb:MN985325|Organism:Severe      TGAAGTATTTTGTGAAAATAGGACCTGAGCGCACCTGTTGTCTATGTGATAGACGTGCCA   18682
gb:MT020881|Organism:Severe      TGAAGTATTTTGTGAAAATAGGACCTGAGCGCACCTGTTGTCTATGTGATAGACGTGCCA   18682
gb:MT020880|Organism:Severe      TGAAGTATTTTGTGAAAATAGGACCTGAGCGCACCTGTTGTCTATGTGATAGACGTGCCA   18682
gb:MT066175|Organism:Severe      TGAAGTATTTTGTGAAAATAGGACCTGAGCGCACCTGTTGTCTATGTGATAGACGTGCCA   18682
gb:MN997409|Organism:Severe      TGAAGTATTTTGTGAAAATAGGACCTGAGCGCACCTGTTGTCTATGTGATAGACGTGCCA   18682
gb:MN938384|Organism:Severe      TGAAGTATTTTGTGAAAATAGGACCTGAGCGCACCTGTTGTCTATGTGATAGACGTGCCA   18650
gb:MT044258|Organism:Severe      TGAAGTATTTTGTGAAAATAGGACCTGAGCGCACCTGTTGTCTATGTGATAGACGTGCCA   18658
gb:MT039890|Organism:Severe      TGAAGTATTTTGTGAAAATAGGACCTGAGCGCACCTGTTGTCTATGTGATAGACGTGCCA   18682
gb:MN988713|Organism:Severe      TGAAGTATTTTGTGAAAATAGGACCTGAGCGCACCTGTTGTCTATGTGATAGACGTGCCA   18682
gb:LC521925|Organism:Severe      TGAAGTATTTTGTGAAAATAGGACCTGAGCGCACCTGTTGTCTATGTGATAGACGTGCCA   18655
gb:MT093571|Organism:Severe      TGAAGTATTTTGTGAAAATAGGACCTGAGCGCACCTGTTGTCTATGTGATAGACGTGCCA   18682
gb:MT039887|Organism:Severe      TGAAGTATTTTGTGAAAATAGGACCTGAGCGCACCTGTTGTCTATGTGATAGACGTGCCA   18682
gb:MT019530|Organism:Severe      TGAAGTATTTTGTGAAAATAGGACCTGAGCGCACCTGTTGTCTATGTGATAGACGTGCCA   18682
gb:MT039888|Organism:Severe      TGAAGTATTTTGTGAAAATAGGACCTGAGCGCACCTGTTGTCTATGTGATAGACGTGCCA   18682
gb:LC522972|Organism:Severe      TGAAGTATTTTGTGAAAATAGGACCTGAGCGCACCTGTTGTCTATGTGATAGACGTGCCA   18679
gb:MT027063|Organism:Severe      TGAAGTATTTTGTGAAAATAGGACCTGAGCGCACCTGTTGTCTATGTGATAGACGTGCCA   18682
gb:MT027062|Organism:Severe      TGAAGTATTTTGTGAAAATAGGACCTGAGCGCACCTGTTGTCTATGTGATAGACGTGCCA   18682
gb:MT019529|Organism:Severe      TGAAGTATTTTGTGAAAATAGGACCTGAGCGCACCTGTTGTCTATGTGATAGACGTGCCA   18682
gb:MN996529|Organism:Severe      TGAAGTATTTTGTGAAAATAGGACCTGAGCGCACCTGTTGTCTATGTGATAGACGTGCCA   18670
gb:MN996531|Organism:Severe      TGAAGTATTTTGTGAAAATAGGACCTGAGCGCACCTGTTGTCTATGTGATAGACGTGCCA   18669
gb:MT066176|Organism:Severe      TGAAGTATTTTGTGAAAATAGGACCTGAGCGCACCTGTTGTCTATGTGATAGACGTGCCA   18682
gb:MT027064|Organism:Severe      TGAAGTATTTTGTGAAAATAGGACCTGAGCGCACCTGTTGTCTATGTGATAGACGTGCCA   18682
gb:MN994468|Organism:Severe      TGAAGTATTTTGTGAAAATAGGACCTGAGCGCACCTGTTGTCTATGTGATAGACGTGCCA   18682
gb:MT072688|Organism:Severe      TGAAGTATTTTGTGAAAATAGGACCTGAGCGCACCTGTTGTCTATGTGATAGACGTGCCA   18667
gb:MN996527|Organism:Severe      TGAAGTATTTTGTGAAAATAGGACCTGAGCGCACCTGTTGTCTATGTGATAGACGTGCCA   18649
gb:MT093631|Organism:Severe      TGAAGTATTTTGTGAAAATAGGACCTGAGCGCACCTGTTGTCTATGTGATAGACGTGCCA   18720
gb:MT106053|Organism:Severe      TGAAGTATTTTGTGAAAATAGGACCTGAGCGCACCTGTTGTCTATGTGATAGACGTGCCA   18682
gb:MT019533|Organism:Severe      TGAAGTATTTTGTGAAAATAGGACCTGAGCGCACCTGTTGTCTATGTGATAGACGTGCCA   18682
gb:MT019531|Organism:Severe      TGAAGTATTTTGTGAAAATAGGACCTGAGCGCACCTGTTGTCTATGTGATAGACGTGCCA   18682
gb:MN996528|Organism:Severe      TGAAGTATTTTGTGAAAATAGGACCTGAGCGCACCTGTTGTCTATGTGATAGACGTGCCA   18682
gb:MN996530|Organism:Severe      TGAAGTATTTTGTGAAAATAGGACCTGAGCGCACCTGTTGTCTATGTGATAGACGTGCCA   18668
gb:MN908947|Organism:Severe      TGAAGTATTTTGTGAAAATAGGACCTGAGCGCACCTGTTGTCTATGTGATAGACGTGCCA   18682
gb:MT019532|Organism:Severe      TGAAGTATTTTGTGAAAATAGGACCTGAGCGCACCTGTTGTCTATGTGATAGACGTGCCA   18682
                                 ************************************************************

gb:MT020781|Organism:Severe      CATGCTTTTCCACTGCTTCAGACACTTATGCCTGTTGGCATCATTCTATTGGATTTGATT   18730
gb:MT007544|Organism:Severe      CATGCTTTTCCACTGCTTCAGACACTTATGCCTGTTGGCATCATTCTATTGGATTTGATT   18742
gb:MN994467|Organism:Severe      CATGCTTTTCCACTGCTTCAGACACTTATGCCTGTTGGCATCATTCTATTGGATTTGATT   18742
gb:MT044257|Organism:Severe      CATGCTTTTCCACTGCTTCAGACACTTATGCCTGTTGGCATCATTCTATTGGATTTGATT   18742
gb:MT106054|Organism:Severe      CATGCTTTTCCACTGCTTCAGACACTTATGCCTGTTGGCATCATTCTATTGGATTTGATT   18742
gb:MT049951|Organism:Severe      CATGCTTTTCCACTGCTTCAGACACTTATGCCTGTTGGCATCATTCTATTGGATTTGATT   18742
gb:MN975262|Organism:Severe      CATGCTTTTCCACTGCTTCAGACACTTATGCCTGTTGGCATCATTCTATTGGATTTGATT   18742
gb:MT106052|Organism:Severe      CATGCTTTTCCACTGCTTCAGACACTTATGCCTGTTGGCATCATTCTATTGGATTTGATT   18742
gb:LC522975|Organism:Severe      CATGCTTTTCCACTGCTTCAGACACTTATGCCTGTTGGCATCATTCTATTGGATTTGATT   18739
```

```
gb:LC522973|Organism:Severe      CATGCTTTTCCACTGCTTCAGACACTTATGCCTGTTGGCATCATTCTATTGGATTTGATT   18739
gb:LC522974|Organism:Severe      CATGCTTTTCCACTGCTTCAGACACTTATGCCTGTTGGCATCATTCTATTGGATTTGATT   18739
gb:MN985325|Organism:Severe      CATGCTTTTCCACTGCTTCAGACACTTATGCCTGTTGGCATCATTCTATTGGATTTGATT   18742
gb:MT020881|Organism:Severe      CATGCTTTTCCACTGCTTCAGACACTTATGCCTGTTGGCATCATTCTATTGGATTTGATT   18742
gb:MT020880|Organism:Severe      CATGCTTTTCCACTGCTTCAGACACTTATGCCTGTTGGCATCATTCTATTGGATTTGATT   18742
gb:MT066175|Organism:Severe      CATGCTTTTCCACTGCTTCAGACACTTATGCCTGTTGGCATCATTCTATTGGATTTGATT   18742
gb:MN997409|Organism:Severe      CATGCTTTTCCACTGCTTCAGACACTTATGCCTGTTGGCATCATTCTATTGGATTTGATT   18742
gb:MN938384|Organism:Severe      CATGCTTTTCCACTGCTTCAGACACTTATGCCTGTTGGCATCATTCTATTGGATTTGATT   18710
gb:MT044258|Organism:Severe      CATGCTTTTCCACTGCTTCAGACACTTATGCCTGTTGGCATCATTCTATTGGATTTGATT   18718
gb:MT039890|Organism:Severe      CATGCTTTTCCACTGCTTCAGACACTTATGCCTGTTGGCATCATTCTATTGGATTTGATT   18742
gb:MN988713|Organism:Severe      CATGCTTTTCCACTGCTTCAGACACTTATGCCTGTTGGCATCATTCTATTGGATTTGATT   18742
gb:LC521925|Organism:Severe      CATGCTTTTCCACTGCTTCAGACACTTATGCCTGTTGGCATCATTCTATTGGATTTGATT   18715
gb:MT093571|Organism:Severe      CATGCTTTTCCACTGCTTCAGACACTTATGCCTGTTGGCATCATTCTATTGGATTTGATT   18742
gb:MT039887|Organism:Severe      CATGCTTTTCCACTGCTTCAGACACTTATGCCTGTTGGCATCATTCTATTGGATTTGATT   18742
gb:MT019530|Organism:Severe      CATGCTTTTCCACTGCTTCAGACACTTATGCCTGTTGGCATCATTCTATTGGATTTGATT   18742
gb:MT039888|Organism:Severe      CATGCTTTTCCACTGCTTCAGACACTTATGCCTGTTGGCATCATTCTATTGGATTTGATT   18742
gb:LC522972|Organism:Severe      CATGCTTTTCCACTGCTTCAGACACTTATGCCTGTTGGCATCATTCTATTGGATTTGATT   18739
gb:MT027063|Organism:Severe      CATGCTTTTCCACTGCTTCAGACACTTATGCCTGTTGGCATCATTCTATTGGATTTGATT   18742
gb:MT027062|Organism:Severe      CATGCTTTTCCACTGCTTCAGACACTTATGCCTGTTGGCATCATTCTATTGGATTTGATT   18742
gb:MT019529|Organism:Severe      CATGCTTTTCCACTGCTTCAGACACTTATGCCTGTTGGCATCATTCTATTGGATTTGATT   18742
gb:MN996529|Organism:Severe      CATGCTTTTCCACTGCTTCAGACACTTATGCCTGTTGGCATCATTCTATTGGATTTGATT   18730
gb:MN996531|Organism:Severe      CATGCTTTTCCACTGCTTCAGACACTTATGCCTGTTGGCATCATTCTATTGGATTTGATT   18729
gb:MT066176|Organism:Severe      CATGCTTTTCCACTGCTTCAGACACTTATGCCTGTTGGCATCATTCTATTGGATTTGATT   18742
gb:MT027064|Organism:Severe      CATGCTTTTCCACTGCTTCAGACACTTATGCCTGTTGGCATCATTCTATTGGATTTGATT   18742
gb:MN994468|Organism:Severe      CATGCTTTTCCACTGCTTCAGACACTTATGCCTGTTGGCATCATTCTATTGGATTTGATT   18742
gb:MT072688|Organism:Severe      CATGCTTTTCCACTGCTTCAGACACTTATGCCTGTTGGCATCATTCTATTGGATTTGATT   18727
gb:MN996527|Organism:Severe      CATGCTTTTCCACTGCTTCAGACACTTATGCCTGTTGGCATCATTCTATTGGATTTGATT   18709
gb:MT093631|Organism:Severe      CATGCTTTTCCACTGCTTCAGACACTTATGCCTGTTGGCATCATTCTATTGGATTTGATT   18780
gb:MT106053|Organism:Severe      CATGCTTTTCCACTGCTTCAGACACTTATGCCTGTTGGCATCATTCTATTGGATTTGATT   18742
gb:MT019533|Organism:Severe      CATGCTTTTCCACTGCTTCAGACACTTATGCCTGTTGGCATCATTCTATTGGATTTGATT   18742
gb:MT019531|Organism:Severe      CATGCTTTTCCACTGCTTCAGACACTTATGCCTGTTGGCATCATTCTATTGGATTTGATT   18742
gb:MN996528|Organism:Severe      CATGCTTTTCCACTGCTTCAGACACTTATGCCTGTTGGCATCATTCTATTGGATTTGATT   18742
gb:MN996530|Organism:Severe      CATGCTTTTCCACTGCTTCAGACACTTATGCCTGTTGGCATCATTCTATTGGATTTGATT   18728
gb:MN908947|Organism:Severe      CATGCTTTTCCACTGCTTCAGACACTTATGCCTGTTGGCATCATTCTATTGGATTTGATT   18742
gb:MT019532|Organism:Severe      CATGCTTTTCCACTGCTTCAGACACTTATGCCTGTTGGCATCATTCTATTGGATTTGATT   18742
                                 ************************************************************

gb:MT020781|Organism:Severe      ACGTCTATAATCCGTTTATGATTGATGTTCAACAATGGGGTTTTACAGGTAACCTACAAA   18790
gb:MT007544|Organism:Severe      ACGTCTATAATCCGTTTATGATTGATGTTCAACAATGGGGTTTTACAGGTAACCTACAAA   18802
gb:MN994467|Organism:Severe      ACGTCTATAATCCGTTTATGATTGATGTTCAACAATGGGGTTTTACAGGTAACCTACAAA   18802
gb:MT044257|Organism:Severe      ACGTCTATAATCCGTTTATGATTGATGTTCAACAATGGGGTTTTACAGGTAACCTACAAA   18802
gb:MT106054|Organism:Severe      ACGTCTATAATCCGTTTATGATTGATGTTCAACAATGGGGTTTTACAGGTAACCTACAAA   18802
gb:MT049951|Organism:Severe      ACGTCTATAATCCGTTTATGATTGATGTTCAACAATGGGGTTTTACAGGTAACCTACAAA   18802
gb:MN975262|Organism:Severe      ACGTCTATAATCCGTTTATGATTGATGTTCAACAATGGGGTTTTACAGGTAACCTACAAA   18802
gb:MT106052|Organism:Severe      ACGTCTATAATCCGTTTATGATTGATGTTCAACAATGGGGTTTTACAGGTAACCTACAAA   18802
gb:LC522975|Organism:Severe      ACGTCTATAATCCGTTTATGATTGATGTTCAACAATGGGGTTTTACAGGTAACCTACAAA   18799
gb:LC522973|Organism:Severe      ACGTCTATAATCCGTTTATGATTGATGTTCAACAATGGGGTTTTACAGGTAACCTACAAA   18799
gb:LC522974|Organism:Severe      ACGTCTATAATCCGTTTATGATTGATGTTCAACAATGGGGTTTTACAGGTAACCTACAAA   18799
gb:MN985325|Organism:Severe      ACGTCTATAATCCGTTTATGATTGATGTTCAACAATGGGGTTTTACAGGTAACCTACAAA   18802
gb:MT020881|Organism:Severe      ACGTCTATAATCCGTTTATGATTGATGTTCAACAATGGGGTTTTACAGGTAACCTACAAA   18802
gb:MT020880|Organism:Severe      ACGTCTATAATCCGTTTATGATTGATGTTCAACAATGGGGTTTTACAGGTAACCTACAAA   18802
gb:MT066175|Organism:Severe      ACGTCTATAATCCGTTTATGATTGATGTTCAACAATGGGGTTTTACAGGTAACCTACAAA   18802
gb:MN997409|Organism:Severe      ACGTCTATAATCCGTTTATGATTGATGTTCAACAATGGGGTTTTACAGGTAACCTACAAA   18802
gb:MN938384|Organism:Severe      ACGTCTATAATCCGTTTATGATTGATGTTCAACAATGGGGTTTTACAGGTAACCTACAAA   18770
gb:MT044258|Organism:Severe      ACGTCTATAATCCGTTTATGATTGATGTTCAACAATGGGGTTTTACAGGTAACCTACAAA   18778
gb:MT039890|Organism:Severe      ACGTCTATAATCCGTTTATGATTGATGTTCAACAATGGGGTTTTACAGGTAACCTACAAA   18802
gb:MN988713|Organism:Severe      ACGTCTATAATCCGTTTATGATTGATGTTCAACAATGGGGTTTTACAGGTAACCTACAAA   18802
gb:LC521925|Organism:Severe      ACGTCTATAATCCGTTTATGATTGATGTTCAACAATGGGGTTTTACAGGTAACCTACAAA   18775
gb:MT093571|Organism:Severe      ACGTCTATAATCCGTTTATGATTGATGTTCAACAATGGGGTTTTACAGGTAACCTACAAA   18802
gb:MT039887|Organism:Severe      ACGTCTATAATCCGTTTATGATTGATGTTCAACAATGGGGTTTTACAGGTAACCTACAAA   18802
gb:MT019530|Organism:Severe      ACGTCTATAATCCGTTTATGATTGATGTTCAACAATGGGGTTTTACAGGTAACCTACAAA   18802
gb:MT039888|Organism:Severe      ACGTCTATAATCCGTTTATGATTGATGTTCAACAATGGGGTTTTACAGGTAACCTACAAA   18802
gb:LC522972|Organism:Severe      ACGTCTATAATCCGTTTATGATTGATGTTCAACAATGGGGTTTTACAGGTAACCTACAAA   18799
gb:MT027063|Organism:Severe      ACGTCTATAATCCGTTTATGATTGATGTTCAACAATGGGGTTTTACAGGTAACCTACAAA   18802
gb:MT027062|Organism:Severe      ACGTCTATAATCCGTTTATGATTGATGTTCAACAATGGGGTTTTACAGGTAACCTACAAA   18802
```

```
gb:MT019529|Organism:Severe      ACGTCTATAATCCGTTTATGATTGATGTTCAACAATGGGGTTTTACAGGTAACCTACAAA    18802
gb:MN996529|Organism:Severe      ACGTCTATAATCCGTTTATGATTGATGTTCAACAATGGGGTTTTACAGGTAACCTACAAA    18790
gb:MN996531|Organism:Severe      ACGTCTATAATCCGTTTATGATTGATGTTCAACAATGGGGTTTTACAGGTAACCTACAAA    18789
gb:MT066176|Organism:Severe      ACGTCTATAATCCGTTTATGATTGATGTTCAACAATGGGGTTTTACAGGTAACCTACAAA    18802
gb:MT027064|Organism:Severe      ACGTCTATAATCCGTTTATGATTGATGTTCAACAATGGGGTTTTACAGGTAACCTACAAA    18802
gb:MN994468|Organism:Severe      ACGTCTATAATCCGTTTATGATTGATGTTCAACAATGGGGTTTTACAGGTAACCTACAAA    18802
gb:MT072688|Organism:Severe      ACGTCTATAATCCGTTTATGATTGATGTTCAACAATGGGGTTTTACAGGTAACCTACAAA    18787
gb:MN996527|Organism:Severe      ACGTCTATAATCCGTTTATGATTGATGTTCAACAATGGGGTTTTACAGGTAACCTACAAA    18769
gb:MT093631|Organism:Severe      ACGTCTATAATCCGTTTATGATTGATGTTCAACAATGGGGTTTTACAGGTAACCTACAAA    18840
gb:MT106053|Organism:Severe      ACGTCTATAATCCGTTTATGATTGATGTTCAACAATGGGGTTTTACAGGTAACCTACAAA    18802
gb:MT019533|Organism:Severe      ACGTCTATAATCCGTTTATGATTGATGTTCAACAATGGGGTTTTACAGGTAACCTACAAA    18802
gb:MT019531|Organism:Severe      ACGTCTATAATCCGTTTATGATTGATGTTCAACAATGGGGTTTTACAGGTAACCTACAAA    18802
gb:MN996528|Organism:Severe      ACGTCTATAATCCGTTTATGATTGATGTTCAACAATGGGGTTTTACAGGTAACCTACAAA    18802
gb:MN996530|Organism:Severe      ACGTCTATAATCCGTTTATGATTGATGTTCAACAATGGGGTTTTACAGGTAACCTACAAA    18788
gb:MN908947|Organism:Severe      ACGTCTATAATCCGTTTATGATTGATGTTCAACAATGGGGTTTTACAGGTAACCTACAAA    18802
gb:MT019532|Organism:Severe      ACGTCTATAATCCGTTTATGATTGATGTTCAACAATGGGGTTTTACAGGTAACCTACAAA    18802
                                 ************************************************************

gb:MT020781|Organism:Severe      GCAACCATGATCTGTATTGTCAAGTCCATGGTAATGCACATGTAGCTAGTTGTGATGCAA    18850
gb:MT007544|Organism:Severe      GCAACCATGATCTGTATTGTCAAGTCCATGGTAATGCACATGTAGCTAGTTGTGATGCAA    18862
gb:MN994467|Organism:Severe      GCAACCATGATCTGTATTGTCAAGTCCATGGTAATGCACATGTAGCTAGTTGTGATGCAA    18862
gb:MT044257|Organism:Severe      GCAACCATGATCTGTATTGTCAAGTCCATGGTAATGCACATGTAGCTAGTTGTGATGCAA    18862
gb:MT106054|Organism:Severe      GCAACCATGATCTGTATTGTCAAGTCCATGGTAATGCACATGTAGCTAGTTGTGATGCAA    18862
gb:MT049951|Organism:Severe      GCAACCATGATCTGTATTGTCAAGTCCATGGTAATGCACATGTAGCTAGTTGTGATGCAA    18862
gb:MN975262|Organism:Severe      GCAACCATGATCTGTATTGTCAAGTCCATGGTAATGCACATGTAGCTAGTTGTGATGCAA    18862
gb:MT106052|Organism:Severe      GCAACCATGATCTGTATTGTCAAGTCCATGGTAATGCACATGTAGCTAGTTGTGATGCAA    18862
gb:LC522975|Organism:Severe      GCAACCATGATCTGTATTGTCAAGTCCATGGTAATGCACATGTAGCTAGTTGTGATGCAA    18859
gb:LC522973|Organism:Severe      GCAACCATGATCTGTATTGTCAAGTCCATGGTAATGCACATGTAGCTAGTTGTGATGCAA    18859
gb:LC522974|Organism:Severe      GCAACCATGATCTGTATTGTCAAGTCCATGGTAATGCACATGTAGCTAGTTGTGATGCAA    18859
gb:MN985325|Organism:Severe      GCAACCATGATCTGTATTGTCAAGTCCATGGTAATGCACATGTAGCTAGTTGTGATGCAA    18862
gb:MT020881|Organism:Severe      GCAACCATGATCTGTATTGTCAAGTCCATGGTAATGCACATGTAGCTAGTTGTGATGCAA    18862
gb:MT020880|Organism:Severe      GCAACCATGATCTGTATTGTCAAGTCCATGGTAATGCACATGTAGCTAGTTGTGATGCAA    18862
gb:MT066175|Organism:Severe      GCAACCATGATCTGTATTGTCAAGTCCATGGTAATGCACATGTAGCTAGTTGTGATGCAA    18862
gb:MN997409|Organism:Severe      GCAACCATGATCTGTATTGTCAAGTCCATGGTAATGCACATGTAGCTAGTTGTGATGCAA    18862
gb:MN938384|Organism:Severe      GCAACCATGATCTGTATTGTCAAGTCCATGGTAATGCACATGTAGCTAGTTGTGATGCAA    18830
gb:MT044258|Organism:Severe      GCAACCATGATCTGTATTGTCAAGTCCATGGTAATGCACATGTAGCTAGTTGTGATGCAA    18838
gb:MT039890|Organism:Severe      GCAACCATGATCTGTATTGTCAAGTCCATGGTAATGCACATGTAGCTAGTTGTGATGCAA    18862
gb:MN988713|Organism:Severe      GCAACCATGATCTGTATTGTCAAGTCCATGGTAATGCACATGTAGCTAGTTGTGATGCAA    18862
gb:LC521925|Organism:Severe      GCAACCATGATCTGTATTGTCAAGTCCATGGTAATGCACATGTAGCTAGTTGTGATGCAA    18835
gb:MT093571|Organism:Severe      GCAACCATGATCTGTATTGTCAAGTCCATGGTAATGCACATGTAGCTAGTTGTGATGCAA    18862
gb:MT039887|Organism:Severe      GCAACCATGATCTGTATTGTCAAGTCCATGGTAATGCACATGTAGCTAGTTGTGATGCAA    18862
gb:MT019530|Organism:Severe      GCAACCATGATCTGTATTGTCAAGTCCATGGTAATGCACATGTAGCTAGTTGTGATGCAA    18862
gb:MT039888|Organism:Severe      GCAACCATGATCTGTATTGTCAAGTCCATGGTAATGCACATGTAGCTAGTTGTGATGCAA    18862
gb:LC522972|Organism:Severe      GCAACCATGATCTGTATTGTCAAGTCCATGGTAATGCACATGTAGCTAGTTGTGATGCAA    18859
gb:MT027063|Organism:Severe      GCAACCATGATCTGTATTGTCAAGTCCATGGTAATGCACATGTAGCTAGTTGTGATGCAA    18862
gb:MT027062|Organism:Severe      GCAACCATGATCTGTATTGTCAAGTCCATGGTAATGCACATGTAGCTAGTTGTGATGCAA    18862
gb:MT019529|Organism:Severe      GCAACCATGATCTGTATTGTCAAGTCCATGGTAATGCACATGTAGCTAGTTGTGATGCAA    18862
gb:MN996529|Organism:Severe      GCAACCATGATCTGTATTGTCAAGTCCATGGTAATGCACATGTAGCTAGTTGTGATGCAA    18850
gb:MN996531|Organism:Severe      GCAACCATGATCTGTATTGTCAAGTCCATGGTAATGCACATGTAGCTAGTTGTGATGCAA    18849
gb:MT066176|Organism:Severe      GCAACCATGATCTGTATTGTCAAGTCCATGGTAATGCACATGTAGCTAGTTGTGATGCAA    18862
gb:MT027064|Organism:Severe      GCAACCATGATCTGTATTGTCAAGTCCATGGTAATGCACATGTAGCTAGTTGTGATGCAA    18862
gb:MN994468|Organism:Severe      GCAACCATGATCTGTATTGTCAAGTCCATGGTAATGCACATGTAGCTAGTTGTGATGCAA    18862
gb:MT072688|Organism:Severe      GCAACCATGATCTGTATTGTCAAGTCCATGGTAATGCACATGTAGCTAGTTGTGATGCAA    18847
gb:MN996527|Organism:Severe      GCAACCATGATCTGTATTGTCAAGTCCATGGTAATGCACATGTAGCTAGTTGTGATGCAA    18829
gb:MT093631|Organism:Severe      GCAACCATGATCTGTATTGTCAAGTCCATGGTAATGCACATGTAGCTAGTTGTGATGCAA    18900
gb:MT106053|Organism:Severe      GCAACCATGATCTGTATTGTCAAGTCCATGGTAATGCACATGTAGCTAGTTGTGATGCAA    18862
gb:MT019533|Organism:Severe      GCAACCATGATCTGTATTGTCAAGTCCATGGTAATGCACATGTAGCTAGTTGTGATGCAA    18862
gb:MT019531|Organism:Severe      GCAACCATGATCTGTATTGTCAAGTCCATGGTAATGCACATGTAGCTAGTTGTGATGCAA    18862
gb:MN996528|Organism:Severe      GCAACCATGATCTGTATTGTCAAGTCCATGGTAATGCACATGTAGCTAGTTGTGATGCAA    18862
gb:MN996530|Organism:Severe      GCAACCATGATCTGTATTGTCAAGTCCATGGTAATGCACATGTAGCTAGTTGTGATGCAA    18848
gb:MN908947|Organism:Severe      GCAACCATGATCTGTATTGTCAAGTCCATGGTAATGCACATGTAGCTAGTTGTGATGCAA    18862
gb:MT019532|Organism:Severe      GCAACCATGATCTGTATTGTCAAGTCCATGGTAATGCACATGTAGCTAGTTGTGATGCAA    18862
                                 ************************************************************

gb:MT020781|Organism:Severe      TCATGACTAGGTGTCTAGCTGTCCACGAGTGCTTTGTTAAGCGTGTTGACTGGACTATTG    18910
```

```
gb:MT007544|Organism:Severe      TCATGACTAGGTGTCTAGCTGTCCACGAGTGCTTTGTTAAGCGTGTTGACTGGACTATTG   18922
gb:MN994467|Organism:Severe      TCATGACTAGGTGTCTAGCTGTCCACGAGTGCTTTGTTAAGCGTGTTGACTGGACTATTG   18922
gb:MT044257|Organism:Severe      TCATGACTAGGTGTCTAGCTGTCCACGAGTGCTTTGTTAAGCGTGTTGACTGGACTATTG   18922
gb:MT106054|Organism:Severe      TCATGACTAGGTGTCTAGCTGTCCACGAGTGCTTTGTTAAGCGTGTTGACTGGACTATTG   18922
gb:MT049951|Organism:Severe      TCATGACTAGGTGTCTAGCTGTCCACGAGTGCTTTGTTAAGCGTGTTGACTGGACTATTG   18922
gb:MN975262|Organism:Severe      TCATGACTAGGTGTCTAGCTGTCCACGAGTGCTTTGTTAAGCGTGTTGACTGGACTATTG   18922
gb:MT106052|Organism:Severe      TCATGACTAGGTGTCTAGCTGTCCACGAGTGCTTTGTTAAGCGTGTTGACTGGACTATTG   18922
gb:LC522975|Organism:Severe      TCATGACTAGGTGTCTAGCTGTCCACGAGTGCTTTGTTAAGCGTGTTGACTGGACTATTG   18919
gb:LC522973|Organism:Severe      TCATGACTAGGTGTCTAGCTGTCCACGAGTGCTTTGTTAAGCGTGTTGACTGGACTATTG   18919
gb:LC522974|Organism:Severe      TCATGACTAGGTGTCTAGCTGTCCACGAGTGCTTTGTTAAGCGTGTTGACTGGACTATTG   18919
gb:MN985325|Organism:Severe      TCATGACTAGGTGTCTAGCTGTCCACGAGTGCTTTGTTAAGCGTGTTGACTGGACTATTG   18922
gb:MT020881|Organism:Severe      TCATGACTAGGTGTCTAGCTGTCCACGAGTGCTTTGTTAAGCGTGTTGACTGGACTATTG   18922
gb:MT020880|Organism:Severe      TCATGACTAGGTGTCTAGCTGTCCACGAGTGCTTTGTTAAGCGTGTTGACTGGACTATTG   18922
gb:MT066175|Organism:Severe      TCATGACTAGGTGTCTAGCTGTCCACGAGTGCTTTGTTAAGCGTGTTGACTGGACTATTG   18922
gb:MN997409|Organism:Severe      TCATGACTAGGTGTCTAGCTGTCCACGAGTGCTTTGTTAAGCGTGTTGACTGGACTATTG   18922
gb:MN938384|Organism:Severe      TCATGACTAGGTGTCTAGCTGTCCACGAGTGCTTTGTTAAGCGTGTTGACTGGACTATTG   18890
gb:MT044258|Organism:Severe      TCATGACTAGGTGTCTAGCTGTCCACGAGTGCTTTGTTAAGCGTGTTGACTGGACTATTG   18898
gb:MT039890|Organism:Severe      TCATGACTAGGTGTCTAGCTGTCCACGAGTGCTTTGTTAAGCGTGTTGACTGGACTATTG   18922
gb:MN988713|Organism:Severe      TCATGACTAGGTGTCTAGCTGTCCACGAGTGCTTTGTTAAGCGTGTTGACTGGACTATTG   18922
gb:LC521925|Organism:Severe      TCATGACTAGGTGTCTAGCTGTCCACGAGTGCTTTGTTAAGCGTGTTGACTGGACTATTG   18895
gb:MT093571|Organism:Severe      TCATGACTAGGTGTCTAGCTGTCCACGAGTGCTTTGTTAAGCGTGTTGACTGGACTATTG   18922
gb:MT039887|Organism:Severe      TCATGACTAGGTGTCTAGCTGTCCACGAGTGCTTTGTTAAGCGTGTTGACTGGACTATTG   18922
gb:MT019530|Organism:Severe      TCATGACTAGGTGTCTAGCTGTCCACGAGTGCTTTGTTAAGCGTGTTGACTGGACTATTG   18922
gb:MT039888|Organism:Severe      TCATGACTAGGTGTCTAGCTGTCCACGAGTGCTTTGTTAAGCGTGTTGACTGGACTATTG   18922
gb:LC522972|Organism:Severe      TCATGACTAGGTGTCTAGCTGTCCACGAGTGCTTTGTTAAGCGTGTTGACTGGACTATTG   18919
gb:MT027063|Organism:Severe      TCATGACTAGGTGTCTAGCTGTCCACGAGTGCTTTGTTAAGCGTGTTGACTGGACTATTG   18922
gb:MT027062|Organism:Severe      TCATGACTAGGTGTCTAGCTGTCCACGAGTGCTTTGTTAAGCGTGTTGACTGGACTATTG   18922
gb:MT019529|Organism:Severe      TCATGACTAGGTGTCTAGCTGTCCACGAGTGCTTTGTTAAGCGTGTTGACTGGACTATTG   18922
gb:MN996529|Organism:Severe      TCATGACTAGGTGTCTAGCTGTCCACGAGTGCTTTGTTAAGCGTGTTGACTGGACTATTG   18910
gb:MN996531|Organism:Severe      TCATGACTAGGTGTCTAGCTGTCCACGAGTGCTTTGTTAAGCGTGTTGACTGGACTATTG   18909
gb:MT066176|Organism:Severe      TCATGACTAGGTGTCTAGCTGTCCACGAGTGCTTTGTTAAGCGTGTTGACTGGACTATTG   18922
gb:MT027064|Organism:Severe      TCATGACTAGGTGTCTAGCTGTCCACGAGTGCTTTGTTAAGCGTGTTGACTGGACTATTG   18922
gb:MN994468|Organism:Severe      TCATGACTAGGTGTCTAGCTGTCCACGAGTGCTTTGTTAAGCGTGTTGACTGGACTATTG   18922
gb:MT072688|Organism:Severe      TCATGACTAGGTGTCTAGCTGTCCACGAGTGCTTTGTTAAGCGTGTTGACTGGACTATTG   18907
gb:MN996527|Organism:Severe      TCATGACTAGGTGTCTAGCTGTCCACGAGTGCTTTGTTAAGCGTGTTGACTGGACTATTG   18889
gb:MT093631|Organism:Severe      TCATGACTAGGTGTCTAGCTGTCCACGAGTGCTTTGTTAAGCGTGTTGACTGGACTATTG   18960
gb:MT106053|Organism:Severe      TCATGACTAGGTGTCTAGCTGTCCACGAGTGCTTTGTTAAGCGTGTTGACTGGACTATTG   18922
gb:MT019533|Organism:Severe      TCATGACTAGGTGTCTAGCTGTCCACGAGTGCTTTGTTAAGCGTGTTGACTGGACTATTG   18922
gb:MT019531|Organism:Severe      TCATGACTAGGTGTCTAGCTGTCCACGAGTGCTTTGTTAAGCGTGTTGACTGGACTATTG   18922
gb:MN996528|Organism:Severe      TCATGACTAGGTGTCTAGCTGTCCACGAGTGCTTTGTTAAGCGTGTTGACTGGACTATTG   18922
gb:MN996530|Organism:Severe      TCATGACTAGGTGTCTAGCTGTCCACGAGTGCTTTGTTAAGCGTGTTGACTGGACTATTG   18908
gb:MN908947|Organism:Severe      TCATGACTAGGTGTCTAGCTGTCCACGAGTGCTTTGTTAAGCGTGTTGACTGGACTATTG   18922
gb:MT019532|Organism:Severe      TCATGACTAGGTGTCTAGCTGTCCACGAGTGCTTTGTTAAGCGTGTTGACTGGACTATTG   18922
                                 ************************************************************


gb:MT020781|Organism:Severe      AATATCCTATAATTGGTGATGAACTGAAGATTAATGCGGCTTGTAGAAAGGTTCAACACA   18970
gb:MT007544|Organism:Severe      AATATCCTATAATTGGTGATGAACTGAAGATTAATGCGGCTTGTAGAAAGGTTCAACACA   18982
gb:MN994467|Organism:Severe      AATATCCTATAATTGGTGATGAACTGAAGATTAATGCGGCTTGTAGAAAGGTTCAACACA   18982
gb:MT044257|Organism:Severe      AATATCCTATAATTGGTGATGAACTGAAGATTAATGCGGCTTGTAGAAAGGTTCAACACA   18982
gb:MT106054|Organism:Severe      AATATCCTATAATTGGTGATGAACTGAAGATTAATGCGGCTTGTAGAAAGGTACAACACA   18982
gb:MT049951|Organism:Severe      AATATCCTATAATTGGTGATGAACTGAAGATTAATGCGGCTTGTAGAAAGGTTCAACACA   18982
gb:MN975262|Organism:Severe      AATATCCTATAATTGGTGATGAACTGAAGATTAATGCGGCTTGTAGAAAGGTTCAACACA   18982
gb:MT106052|Organism:Severe      AATATCCTATAATTGGTGATGAACTGAAGATTAATGCGGCTTGTAGAAAGGTTCAACACA   18982
gb:LC522975|Organism:Severe      AATATCCTATAATTGGTGATGAACTGAAGATTAATGCGGCTTGTAGAAAGGTTCAACACA   18979
gb:LC522973|Organism:Severe      AATATCCTATAATTGGTGATGAACTGAAGATTAATGCGGCTTGTAGAAAGGTTCAACACA   18979
gb:LC522974|Organism:Severe      AATATCCTATAATTGGTGATGAACTGAAGATTAATGCGGCTTGTAGAAAGGTTCAACACA   18979
gb:MN985325|Organism:Severe      AATATCCTATAATTGGTGATGAACTGAAGATTAATGCGGCTTGTAGAAAGGTTCAACACA   18982
gb:MT020881|Organism:Severe      AATATCCTATAATTGGTGATGAACTGAAGATTAATGCGGCTTGTAGAAAGGTTCAACACA   18982
gb:MT020880|Organism:Severe      AATATCCTATAATTGGTGATGAACTGAAGATTAATGCGGCTTGTAGAAAGGTTCAACACA   18982
gb:MT066175|Organism:Severe      AATATCCTATAATTGGTGATGAACTGAAGATTAATGCGGCTTGTAGAAAGGTTCAACACA   18982
gb:MN997409|Organism:Severe      AATATCCTATAATTGGTGATGAACTGAAGATTAATGCGGCTTGTAGAAAGGTTCAACACA   18982
gb:MN938384|Organism:Severe      AATATCCTATAATTGGTGATGAACTGAAGATTAATGCGGCTTGTAGAAAGGTTCAACACA   18950
gb:MT044258|Organism:Severe      AATATCCTATAATTGGTGATGAACTGAAGATTAATGCGGCTTGTAGAAAGGTTCAACACA   18958
gb:MT039890|Organism:Severe      AATATCCTATAATTGGTGATGAACTGAAGATTAATGCGGCTTGTAGAAAGGTTCAACACA   18982
gb:MN988713|Organism:Severe      AATATCCTATAATTGGTGATGAACTGAAGATTAATGCGGCTTGTAGAAAGGTTCAACACA   18982
```

```
gb:LC521925|Organism:Severe      AATATCCTATAATTGGTGATGAACTGAAGATTAATGCGGCTTGTAGAAAGGTTCAACACA    18955
gb:MT093571|Organism:Severe      AATATCCTATAATTGGTGATGAACTGAAGATTAATGCGGCTTGTAGAAAGGTTCAACACA    18982
gb:MT039887|Organism:Severe      AATATCCTATAATTGGTGATGAACTGAAGATTAATGCGGCTTGTAGAAAGGTTCAACACA    18982
gb:MT019530|Organism:Severe      AATATCCTATAATTGGTGATGAACTGAAGATTAATGCGGCTTGTAGAAAGGTTCAACACA    18982
gb:MT039888|Organism:Severe      AATATCCTATAATTGGTGATGAACTGAAGATTAATGCGGCTTGTAGAAAGGTTCAACACA    18982
gb:LC522972|Organism:Severe      AATATCCTATAATTGGTGATGAACTGAAGATTAATGCGGCTTGTAGAAAGGTTCAACACA    18979
gb:MT027063|Organism:Severe      AATATCCTATAATTGGTGATGAACTGAAGATTAATGCGGCTTGTAGAAAGGTTCAACACA    18982
gb:MT027062|Organism:Severe      AATATCCTATAATTGGTGATGAACTGAAGATTAATGCGGCTTGTAGAAAGGTTCAACACA    18982
gb:MT019529|Organism:Severe      AATATCCTATAATTGGTGATGAACTGAAGATTAATGCGGCTTGTAGAAAGGTTCAACACA    18982
gb:MN996529|Organism:Severe      AATATCCTATAATTGGTGATGAACTGAAGATTAATGCGGCTTGTAGAAAGGTTCAACACA    18970
gb:MN996531|Organism:Severe      AATATCCTATAATTGGTGATGAACTGAAGATTAATGCGGCTTGTAGAAAGGTTCAACACA    18969
gb:MT066176|Organism:Severe      AATATCCTATAATTGGTGATGAACTGAAGATTAATGCGGCTTGTAGAAAGGTTCAACACA    18982
gb:MT027064|Organism:Severe      AATATCCTATAATTGGTGATGAACTGAAGATTAATGCGGCTTGTAGAAAGGTTCAACACA    18982
gb:MN994468|Organism:Severe      AATATCCTATAATTGGTGATGAACTGAAGATTAATGCGGCTTGTAGAAAGGTTCAACACA    18982
gb:MT072688|Organism:Severe      AATATCCTATAATTGGTGATGAACTGAAGATTAATGCGGCTTGTAGAAAGGTTCAACACA    18967
gb:MN996527|Organism:Severe      AATATCCTATAATTGGTGATGAACTGAAGATTAATGCGGCTTGTAGAAAGGTTCAACACA    18949
gb:MT093631|Organism:Severe      AATATCCTATAATTGGTGATGAACTGAAGATTAATGCGGCTTGTAGAAAGGTTCAACACA    19020
gb:MT106053|Organism:Severe      AATATCCTATAATTGGTGATGAACTGAAGATTAATGCGGCTTGTAGAAAGGTTCAACACA    18982
gb:MT019533|Organism:Severe      AATATCCTATAATTGGTGATGAACTGAAGATTAATGCGGCTTGTAGAAAGGTTCAACACA    18982
gb:MT019531|Organism:Severe      AATATCCTATAATTGGTGATGAACTGAAGATTAATGCGGCTTGTAGAAAGGTTCAACACA    18982
gb:MN996528|Organism:Severe      AATATCCTATAATTGGTGATGAACTGAAGATTAATGCGGCTTGTAGAAAGGTTCAACACA    18982
gb:MN996530|Organism:Severe      AATATCCTATAATTGGTGATGAACTGAAGATTAATGCGGCTTGTAGAAAGGTTCAACACA    18968
gb:MN908947|Organism:Severe      AATATCCTATAATTGGTGATGAACTGAAGATTAATGCGGCTTGTAGAAAGGTTCAACACA    18982
gb:MT019532|Organism:Severe      AATATCCTATAATTGGTGATGAACTGAAGATTAATGCGGCTTGTAGAAAGGTTCAACACA    18982
                                 **************************************************** *******

gb:MT020781|Organism:Severe      TGGTTGTTAAAGCTGCATTATTAGCAGACAAATTCCCAGTTCTTCACGACATTGGTAACC    19030
gb:MT007544|Organism:Severe      TGGTTGTTAAAGCTGCATTATTAGCAGACAAATTCCCAGTTCTTCACGACATTGGTAACC    19042
gb:MN994467|Organism:Severe      TGGTTGTTAAAGCTGCATTATTAGCAGACAAATTCCCAGTTCTTCACGACATTGGTAACC    19042
gb:MT044257|Organism:Severe      TGGTTGTTAAAGCTGCATTATTAGCAGACAAATTCCCAGTTCTTCACGACATTGGTAACC    19042
gb:MT106054|Organism:Severe      TGGTTGTTAAAGCTGCATTATTAGCAGACAAATTCCCAGTTCTTCACGACATTGGTAACC    19042
gb:MT049951|Organism:Severe      TGGTTGTTAAAGCTGCATTATTAGCAGACAAATTCCCAGTTCTTCACGACATTGGTAACC    19042
gb:MN975262|Organism:Severe      TGGTTGTTAAAGCTGCATTATTAGCAGACAAATTCCCAGTTCTTCACGACATTGGTAACC    19042
gb:MT106052|Organism:Severe      TGGTTGTTAAAGCTGCATTATTAGCAGACAAATTCCCAGTTCTTCACGACATTGGTAACC    19042
gb:LC522975|Organism:Severe      TGGTTGTTAAAGCTGCATTATTAGCAGACAAATTCCCAGTTCTTCACGACATTGGTAACC    19039
gb:LC522973|Organism:Severe      TGGTTGTTAAAGCTGCATTATTAGCAGACAAATTCCCAGTTCTTCACGACATTGGTAACC    19039
gb:LC522974|Organism:Severe      TGGTTGTTAAAGCTGCATTATTAGCAGACAAATTCCCAGTTCTTCACGACATTGGTAACC    19039
gb:MN985325|Organism:Severe      TGGTTGTTAAAGCTGCATTATTAGCAGACAAATTCCCAGTTCTTCACGACATTGGTAACC    19042
gb:MT020881|Organism:Severe      TGGTTGTTAAAGCTGCATTATTAGCAGACAAATTCCCAGTTCTTCACGACATTGGTAACC    19042
gb:MT020880|Organism:Severe      TGGTTGTTAAAGCTGCATTATTAGCAGACAAATTCCCAGTTCTTCACGACATTGGTAACC    19042
gb:MT066175|Organism:Severe      TGGTTGTTAAAGCTGCATTATTAGCAGACAAATTCCCAGTTCTTCACGACATTGGTAACC    19042
gb:MN997409|Organism:Severe      TGGTTGTTAAAGCTGCATTATTAGCAGACAAATTCCCAGTTCTTCACGACATTGGTAACC    19042
gb:MN938384|Organism:Severe      TGGTTGTTAAAGCTGCATTATTAGCAGACAAATTCCCAGTTCTTCACGACATTGGTAACC    19010
gb:MT044258|Organism:Severe      TGGTTGTTAAAGCTGCATTATTAGCAGACAAATTCCCAGTTCTTCACGACATTGGTAACC    19018
gb:MT039890|Organism:Severe      TGGTTGTTAAAGCTGCATTATTAGCAGACAAATTCCCAGTTCTTCACGACATTGGTAACC    19042
gb:MN988713|Organism:Severe      TGGTTGTTAAAGCTGCATTATTAGCAGACAAATTCCCAGTTCTTCACGACATTGGTAACC    19042
gb:LC521925|Organism:Severe      TGGTTGTTAAAGCTGCATTATTAGCAGACAAATTCCCAGTTCTTCACGACATTGGTAACC    19015
gb:MT093571|Organism:Severe      TGGTTGTTAAAGCTGCATTATTAGCAGACAAATTCCCAGTTCTTCACGACATTGGTAACC    19042
gb:MT039887|Organism:Severe      TGGTTGTTAAAGCTGCATTATTAGCAGACAAATTCCCAGTTCTTCACGACATTGGTAACC    19042
gb:MT019530|Organism:Severe      TGGTTGTTAAAGCTGCATTATTAGCAGACAAATTCCCAGTTCTTCACGACATTGGTAACC    19042
gb:MT039888|Organism:Severe      TGGTTGTTAAAGCTGCATTATTAGCAGACAAATTCCCAGTTCTTCACGACATTGGTAACC    19042
gb:LC522972|Organism:Severe      TGGTTGTTAAAGCTGCATTATTAGCAGACAAATTCCCAGTTCTTCACGACATTGGTAACC    19039
gb:MT027063|Organism:Severe      TGGTTGTTAAAGCTGCATTATTAGCAGACAAATTCCCAGTTCTTCACGACATTGGTAACC    19042
gb:MT027062|Organism:Severe      TGGTTGTTAAAGCTGCATTATTAGCAGACAAATTCCCAGTTCTTCACGACATTGGTAACC    19042
gb:MT019529|Organism:Severe      TGGTTGTTAAAGCTGCATTATTAGCAGACAAATTCCCAGTTCTTCACGACATTGGTAACC    19042
gb:MN996529|Organism:Severe      TGGTTGTTAAAGCTGCATTATTAGCAGACAAATTCCCAGTTCTTCACGACATTGGTAACC    19030
gb:MN996531|Organism:Severe      TGGTTGTTAAAGCTGCATTATTAGCAGACAAATTCCCAGTTCTTCACGACATTGGTAACC    19029
gb:MT066176|Organism:Severe      TGGTTGTTAAAGCTGCATTATTAGCAGACAAATTCCCAGTTCTTCACGACATTGGTAACC    19042
gb:MT027064|Organism:Severe      TGGTTGTTAAAGCTGCATTATTAGCAGACAAATTCCCAGTTCTTCACGACATTGGTAACC    19042
gb:MN994468|Organism:Severe      TGGTTGTTAAAGCTGCATTATTAGCAGACAAATTCCCAGTTCTTCACGACATTGGTAACC    19042
gb:MT072688|Organism:Severe      TGGTTGTTAAAGCTGCATTATTAGCAGACAAATTCCCAGTTCTTCACGACATTGGTAACC    19027
gb:MN996527|Organism:Severe      TGGTTGTTAAAGCTGCATTATTAGCAGACAAATTCCCAGTTCTTCACGACATTGGTAACC    19009
gb:MT093631|Organism:Severe      TGGTTGTTAAAGCTGCATTATTAGCAGACAAATTCCCAGTTCTTCACGACATTGGTAACC    19080
gb:MT106053|Organism:Severe      TGGTTGTTAAAGCTGCATTATTAGCAGACAAATTCCCAGTTCTTCACGACATTGGTAACC    19042
gb:MT019533|Organism:Severe      TGGTTGTTAAAGCTGCATTATTAGCAGACAAATTCCCAGTTCTTCACGACATTGGTAACC    19042
```

```
gb:MT019531|Organism:Severe      TGGTTGTTAAAGCTGCATTATTAGCAGACAAATTCCCAGTTCTTCACGACATTGGTAACC	19042
gb:MN996528|Organism:Severe      TGGTTGTTAAAGCTGCATTATTAGCAGACAAATTCCCAGTTCTTCACGACATTGGTAACC	19042
gb:MN996530|Organism:Severe      TGGTTGTTAAAGCTGCATTATTAGCAGACAAATTCCCAGTTCTTCACGACATTGGTAACC	19028
gb:MN908947|Organism:Severe      TGGTTGTTAAAGCTGCATTATTAGCAGACAAATTCCCAGTTCTTCACGACATTGGTAACC	19042
gb:MT019532|Organism:Severe      TGGTTGTTAAAGCTGCATTATTAGCAGACAAATTCCCAGTTCTTCACGACATTGGTAACC	19042
                                 ************************************************************

gb:MT020781|Organism:Severe      CTAAAGCTATTAAGTGTGTACCTCAAGCTGATGTAGAATGGAAGTTCTATGATGCACAGC	19090
gb:MT007544|Organism:Severe      CTAAAGCTATTAAGTGTGTACCCCAAGCTGATGTAGAATGGAAGTTCTATGATGCACAGC	19102
gb:MN994467|Organism:Severe      CTAAAGCTATTAAGTGTGTACCTCAAGCTGATGTAGAATGGAAGTTCTATGATGCACAGC	19102
gb:MT044257|Organism:Severe      CTAAAGCTATTAAGTGTGTACCTCAAGCTGATGTAGAATGGAAGTTCTATGATGCACAGC	19102
gb:MT106054|Organism:Severe      CTAAAGCTATTAAGTGTGTACCTCAAGCTGATGTAGAATGGAAGTTCTATGATGCACAGC	19102
gb:MT049951|Organism:Severe      CTAAAGCTATTAAGTGTGTACCTCAAGCTGATGTAGAATGGAAGTTCTATGATGCACAGC	19102
gb:MN975262|Organism:Severe      CTAAAGCTATTAAGTGTGTACCTCAAGCTGATGTAGAATGGAAGTTCTATGATGCACAGC	19102
gb:MT106052|Organism:Severe      CTAAAGCTATTAAGTGTGTACCTCAAGCTGATGTAGAATGGAAGTTCTATGATGCACAGC	19102
gb:LC522975|Organism:Severe      CTAAAGCTATTAAGTGTGTACCTCAAGCTGATGTAGAATGGAAGTTCTATGATGCACAGC	19099
gb:LC522973|Organism:Severe      CTAAAGCTATTAAGTGTGTACCTCAAGCTGATGTAGAATGGAAGTTCTATGATGCACAGC	19099
gb:LC522974|Organism:Severe      CTAAAGCTATTAAGTGTGTACCTCAAGCTGATGTAGAATGGAAGTTCTATGATGCACAGC	19099
gb:MN985325|Organism:Severe      CTAAAGCTATTAAGTGTGTACCTCAAGCTGATGTAGAATGGAAGTTCTATGATGCACAGC	19102
gb:MT020881|Organism:Severe      CTAAAGCTATTAAGTGTGTACCTCAAGCTGATGTAGAATGGAAGTTCTATGATGCACAGC	19102
gb:MT020880|Organism:Severe      CTAAAGCTATTAAGTGTGTACCTCAAGCTGATGTAGAATGGAAGTTCTATGATGCACAGC	19102
gb:MT066175|Organism:Severe      CTAAAGCTATTAAGTGTGTACCTCAAGCTGATGTAGAATGGAAGTTCTATGATGCACAGC	19102
gb:MN997409|Organism:Severe      CTAAAGCTATTAAGTGTGTACCTCAAGCTGATGTAGAATGGAAGTTCTATGATGCACAGC	19102
gb:MN938384|Organism:Severe      CTAAAGCTATTAAGTGTGTACCTCAAGCTGATGTAGAATGGAAGTTCTATGATGCACAGC	19070
gb:MT044258|Organism:Severe      CTAAAGCTATTAAGTGTGTACCTCAAGCTGATGTAGAATGGAAGTTCTATGATGCACAGC	19078
gb:MT039890|Organism:Severe      CTAAAGCTATTAAGTGTGTACCTCAAGCTGATGTAGAATGGAAGTTCTATGATGCACAGC	19102
gb:MN988713|Organism:Severe      CTAAAGCTATTAAGTGTGTACCTCAAGCTGATGTAGAATGGAAGTTCTATGATGCACAGC	19102
gb:LC521925|Organism:Severe      CTAAAGCTATTAAGTGTGTACCTCAAGCTGATGTAGAATGGAAGTTCTATGATGCACAGC	19075
gb:MT093571|Organism:Severe      CTAAAGCTATTAAGTGTGTACCTCAAGCTGATGTAGAATGGAAGTTCTATGATGCACAGC	19102
gb:MT039887|Organism:Severe      CTAAAGCTATTAAGTGTGTACCTCAAGCTGATGTAGAATGGAAGTTCTATGATGCACAGC	19102
gb:MT019530|Organism:Severe      CTAAAGCTATTAAGTGTGTACCTCAAGCTGATGTAGAATGGAAGTTCTATGATGCACAGC	19102
gb:MT039888|Organism:Severe      CTAAAGCTATTAAGTGTGTACCTCAAGCTGATGTAGAATGGAAGTTCTATGATGCACAGC	19102
gb:LC522972|Organism:Severe      CTAAAGCTATTAAGTGTGTACCTCAAGCTGATGTAGAATGGAAGTTCTATGATGCACAGC	19099
gb:MT027063|Organism:Severe      CTAAAGCTATTAAGTGTGTACCTCAAGCTGATGTAGAATGGAAGTTCTATGATGCACAGC	19102
gb:MT027062|Organism:Severe      CTAAAGCTATTAAGTGTGTACCTCAAGCTGATGTAGAATGGAAGTTCTATGATGCACAGC	19102
gb:MT019529|Organism:Severe      CTAAAGCTATTAAGTGTGTACCTCAAGCTGATGTAGAATGGAAGTTCTATGATGCACAGC	19102
gb:MN996529|Organism:Severe      CTAAAGCTATTAAGTGTGTACCTCAAGCTGATGTAGAATGGAAGTTCTATGATGCACAGC	19090
gb:MN996531|Organism:Severe      CTAAAGCTATTAAGTGTGTACCTCAAGCTGATGTAGAATGGAAGTTCTATGATGCACAGC	19089
gb:MT066176|Organism:Severe      CTAAAGCTATTAAGTGTGTACCTCAAGCTGATGTAGAATGGAAGTTCTATGATGCACAGC	19102
gb:MT027064|Organism:Severe      CTAAAGCTATTAAGTGTGTACCTCAAGCTGATGTAGAATGGAAGTTCTATGATGCACAGC	19102
gb:MN994468|Organism:Severe      CTAAAGCTATTAAGTGTGTACCTCAAGCTGATGTAGAATGGAAGTTCTATGATGCACAGC	19102
gb:MT072688|Organism:Severe      CTAAAGCTATTAAGTGTGTACCTCAAGCTGATGTAGAATGGAAGTTCTATGATGCACAGC	19087
gb:MN996527|Organism:Severe      CTAAAGCTATTAAGTGTGTACCTCAAGCTGATGTAGAATGGAAGTTCTATGATGCACAGC	19069
gb:MT093631|Organism:Severe      CTAAAGCTATTAAGTGTGTACCTCAAGCTGATGTAGAATGGAAGTTCTATGATGCACAGC	19140
gb:MT106053|Organism:Severe      CTAAAGCTATTAAGTGTGTACCTCAAGCTGATGTAGAATGGAAGTTCTATGATGCACAGC	19102
gb:MT019533|Organism:Severe      CTAAAGCTATTAAGTGTGTACCTCAAGCTGATGTAGAATGGAAGTTCTATGATGCACAGC	19102
gb:MT019531|Organism:Severe      CTAAAGCTATTAAGTGTGTACCTCAAGCTGATGTAGAATGGAAGTTCTATGATGCACAGC	19102
gb:MN996528|Organism:Severe      CTAAAGCTATTAAGTGTGTACCTCAAGCTGATGTAGAATGGAAGTTCTATGATGCACAGC	19102
gb:MN996530|Organism:Severe      CTAAAGCTATTAAGTGTGTACCTCAAGCTGATGTAGAATGGAAGTTCTATGATGCACAGC	19088
gb:MN908947|Organism:Severe      CTAAAGCTATTAAGTGTGTACCTCAAGCTGATGTAGAATGGAAGTTCTATGATGCACAGC	19102
gb:MT019532|Organism:Severe      CTAAAGCTATTAAGTGTGTACCTCAAGCTGATGTAGAATGGAAGTTCTATGATGCACAGC	19102
                                 ********************** *************************************

gb:MT020781|Organism:Severe      CTTGTAGTGACAAAGCTTATAAAATAGAAGAATTATTCTATTCTTATGCCACACATTCTG	19150
gb:MT007544|Organism:Severe      CTTGTAGTGACAAAGCTTATAAAATAGAAGAATTATTCTATTCTTATGCCACACATTCTG	19162
gb:MN994467|Organism:Severe      CTTGTAGTGACAAAGCTTATAAAATAGAAGAATTATTCTATTCTTATGCCACACATTCTG	19162
gb:MT044257|Organism:Severe      CTTGTAGTGACAAAGCTTATAAAATAGAAGAATTATTCTATTCTTATGCCACACATTCTG	19162
gb:MT106054|Organism:Severe      CTTGTAGTGACAAAGCTTATAAAATAGAAGAATTATTCTATTCTTATGCCACACATTCTG	19162
gb:MT049951|Organism:Severe      CTTGTAGTGACAAAGCTTATAAAATAGAAGAATTATTCTATTCTTATGCCACACATTCTG	19162
gb:MN975262|Organism:Severe      CTTGTAGTGACAAAGCTTATAAAATAGAAGAATTATTCTATTCTTATGCCACACATTCTG	19162
gb:MT106052|Organism:Severe      CTTGTAGTGACAAAGCTTATAAAATAGAAGAATTATTCTATTCTTATGCCACACATTCTG	19162
gb:LC522975|Organism:Severe      CTTGTAGTGACAAAGCTTATAAAATAGAAGAATTATTCTATTCTTATGCCACACATTCTG	19159
gb:LC522973|Organism:Severe      CTTGTAGTGACAAAGCTTATAAAATAGAAGAATTATTCTATTCTTATGCCACACATTCTG	19159
gb:LC522974|Organism:Severe      CTTGTAGTGACAAAGCTTATAAAATAGAAGAATTATTCTATTCTTATGCCACACATTCTG	19159
gb:MN985325|Organism:Severe      CTTGTAGTGACAAAGCTTATAAAATAGAAGAATTATTCTATTCTTATGCCACACATTCTG	19162
```

```
gb:MT020881|Organism:Severe      CTTGTAGTGACAAAGCTTATAAAATAGAAGAATTATTCTATTCTTATGCCACACATTCTG   19162
gb:MT020880|Organism:Severe      CTTGTAGTGACAAAGCTTATAAAATAGAAGAATTATTCTATTCTTATGCCACACATTCTG   19162
gb:MT066175|Organism:Severe      CTTGTAGTGACAAAGCTTATAAAATAGAAGAATTATTCTATTCTTATGCCACACATTCTG   19162
gb:MN997409|Organism:Severe      CTTGTAGTGACAAAGCTTATAAAATAGAAGAATTATTCTATTCTTATGCCACACATTCTG   19162
gb:MN938384|Organism:Severe      CTTGTAGTGACAAAGCTTATAAAATAGAAGAATTATTCTATTCTTATGCCACACATTCTG   19130
gb:MT044258|Organism:Severe      CTTGTAGTGACAAAGCTTATAAAATAGAAGAATTATTCTATTCTTATGCCACACATTCTG   19138
gb:MT039890|Organism:Severe      CTTGTAGTGACAAAGCTTATAAAATAGAAGAATTATTCTATTCTTATGCCACACATTCTG   19162
gb:MN988713|Organism:Severe      CTTGTAGTGACAAAGCTTATAAAATAGAAGAATTATTCTATTCTTATGCCACACATTCTG   19162
gb:LC521925|Organism:Severe      CTTGTAGTGACAAAGCTTATAAAATAGAAGAATTATTCTATTCTTATGCCACACATTCTG   19135
gb:MT093571|Organism:Severe      CTTGTAGTGACAAAGCTTATAAAATAGAAGAATTATTCTATTCTTATGCCACACATTCTG   19162
gb:MT039887|Organism:Severe      CTTGTAGTGACAAAGCTTATAAAATAGAAGAATTATTCTATTCTTATGCCACACATTCTG   19162
gb:MT019530|Organism:Severe      CTTGTAGTGACAAAGCTTATAAAATAGAAGAATTATTCTATTCTTATGCCACACATTCTG   19162
gb:MT039888|Organism:Severe      CTTGTAGTGACAAAGCTTATAAAATAGAAGAATTATTCTATTCTTATGCCACACATTCTG   19162
gb:LC522972|Organism:Severe      CTTGTAGTGACAAAGCTTATAAAATAGAAGAATTATTCTATTCTTATGCCACACATTCTG   19159
gb:MT027063|Organism:Severe      CTTGTAGTGACAAAGCTTATAAAATAGAAGAATTATTCTATTCTTATGCCACACATTCTG   19162
gb:MT027062|Organism:Severe      CTTGTAGTGACAAAGCTTATAAAATAGAAGAATTATTCTATTCTTATGCCACACATTCTG   19162
gb:MT019529|Organism:Severe      CTTGTAGTGACAAAGCTTATAAAATAGAAGAATTATTCTATTCTTATGCCACACATTCTG   19162
gb:MN996529|Organism:Severe      CTTGTAGTGACAAAGCTTATAAAATAGAAGAATTATTCTATTCTTATGCCACACATTCTG   19150
gb:MN996531|Organism:Severe      CTTGTAGTGACAAAGCTTATAAAATAGAAGAATTATTCTATTCTTATGCCACACATTCTG   19149
gb:MT066176|Organism:Severe      CTTGTAGTGACAAAGCTTATAAAATAGAAGAATTATTCTATTCTTATGCCACACATTCTG   19162
gb:MT027064|Organism:Severe      CTTGTAGTGACAAAGCTTATAAAATAGAAGAATTATTCTATTCTTATGCCACACATTCTG   19162
gb:MN994468|Organism:Severe      CTTGTAGTGACAAAGCTTATAAAATAGAAGAATTATTCTATTCTTATGCCACACATTCTG   19162
gb:MT072688|Organism:Severe      CTTGTAGTGACAAAGCTTATAAAATAGAAGAATTATTCTATTCTTATGCCACACATTCTG   19147
gb:MN996527|Organism:Severe      CTTGTAGTGACAAAGCTTATAAAATAGAAGAATTATTCTATTCTTATGCCACACATTCTG   19129
gb:MT093631|Organism:Severe      CTTGTAGTGACAAAGCTTATAAAATAGAAGAATTATTCTATTCTTATGCCACACATTCTG   19200
gb:MT106053|Organism:Severe      CTTGTAGTGACAAAGCTTATAAAATAGAAGAATTATTCTATTCTTATGCCACACATTCTG   19162
gb:MT019533|Organism:Severe      CTTGTAGTGACAAAGCTTATAAAATAGAAGAATTATTCTATTCTTATGCCACACATTCTG   19162
gb:MT019531|Organism:Severe      CTTGTAGTGACAAAGCTTATAAAATAGAAGAATTATTCTATTCTTATGCCACACATTCTG   19162
gb:MN996528|Organism:Severe      CTTGTAGTGACAAAGCTTATAAAATAGAAGAATTATTCTATTCTTATGCCACACATTCTG   19162
gb:MN996530|Organism:Severe      CTTGTAGTGACAAAGCTTATAAAATAGAAGAATTATTCTATTCTTATGCCACACATTCTG   19148
gb:MN908947|Organism:Severe      CTTGTAGTGACAAAGCTTATAAAATAGAAGAATTATTCTATTCTTATGCCACACATTCTG   19162
gb:MT019532|Organism:Severe      CTTGTAGTGACAAAGCTTATAAAATAGAAGAATTATTCTATTCTTATGCCACACATTCTG   19162
                                 ************************************************************

gb:MT020781|Organism:Severe      ACAAATTCACAGATGGTGTATGCCTATTTTGGAATTGCAATGTCGATAGATATCCTGCTA   19210
gb:MT007544|Organism:Severe      ACAAATTCACAGATGGTGTATGCCTATTTTGGAATTGCAATGTCGATAGATATCCTGCTA   19222
gb:MN994467|Organism:Severe      ACAAATTCACAGATGGTGTATGCCTATTTTGGAATTGCAATGTCGATAGATATCCTGCTA   19222
gb:MT044257|Organism:Severe      ACAAATTCACAGATGGTGTATGCCTATTTTGGAATTGCAATGTCGATAGATATCCTGCTA   19222
gb:MT106054|Organism:Severe      ACAAATTCACAGCTGGTGTATGCCTATTTTGGAATTGCAATGTCGATAGATATCCTGCTA   19222
gb:MT049951|Organism:Severe      ACAAATTCACAGATGGTGTATGCCTATTTTGGAATTGCAATGTCGATAGATATCCTGCTA   19222
gb:MN975262|Organism:Severe      ACAAATTCACAGATGGTGTATGCCTATTTTGGAATTGCAATGTCGATAGATATCCTGCTA   19222
gb:MT106052|Organism:Severe      ACAAATTCACAGATGGTGTATGCCTATTTTGGAATTGCAATGTCGATAGATATCCTGCTA   19222
gb:LC522975|Organism:Severe      ACAAATTCACAGATGGTGTATGCCTATTTTGGAATTGCAATGTCGATAGATATCCTGCTA   19219
gb:LC522973|Organism:Severe      ACAAATTCACAGATGGTGTATGCCTATTTTGGAATTGCAATGTCGATAGATATCCTGCTA   19219
gb:LC522974|Organism:Severe      ACAAATTCACAGATGGTGTATGCCTATTTTGGAATTGCAATGTCGATAGATATCCTGCTA   19219
gb:MN985325|Organism:Severe      ACAAATTCACAGATGGTGTATGCCTATTTTGGAATTGCAATGTCGATAGATATCCTGCTA   19222
gb:MT020881|Organism:Severe      ACAAATTCACAGATGGTGTATGCCTATTTTGGAATTGCAATGTCGATAGATATCCTGCTA   19222
gb:MT020880|Organism:Severe      ACAAATTCACAGATGGTGTATGCCTATTTTGGAATTGCAATGTCGATAGATATCCTGCTA   19222
gb:MT066175|Organism:Severe      ACAAATTCACAGATGGTGTATGCCTATTTTGGAATTGCAATGTCGATAGATATCCTGCTA   19222
gb:MN997409|Organism:Severe      ACAAATTCACAGATGGTGTATGCCTATTTTGGAATTGCAATGTCGATAGATATCCTGCTA   19222
gb:MN938384|Organism:Severe      ACAAATTCACAGATGGTGTATGCCTATTTTGGAATTGCAATGTCGATAGATATCCTGCTA   19190
gb:MT044258|Organism:Severe      ACAAATTCACAGATGGTGTATGCCTATTTTGGAATTGCAATGTCGATAGATATCCTGCTA   19198
gb:MT039890|Organism:Severe      ACAAATTCACAGATGGTGTATGCCTATTTTGGAATTGCAATGTCGATAGATATCCTGCTA   19222
gb:MN988713|Organism:Severe      ACAAATTCACAGATGGTGTATGCCTATTTTGGAATTGCAATGTCGATAGATATCCTGCTA   19222
gb:LC521925|Organism:Severe      ACAAATTCACAGATGGTGTATGCCTATTTTGGAATTGCAATGTCGATAGATATCCTGCTA   19195
gb:MT093571|Organism:Severe      ACAAATTCACAGATGGTGTATGCCTATTTTGGAATTGCAATGTCGATAGATATCCTGCTA   19222
gb:MT039887|Organism:Severe      ACAAATTCACAGATGGTGTATGCCTATTTTGGAATTGCAATGTCGATAGATATCCTGCTA   19222
gb:MT019530|Organism:Severe      ACAAATTCACAGATGGTGTATGCCTATTTTGGAATTGCAATGTCGATAGATATCCTGCTA   19222
gb:MT039888|Organism:Severe      ACAAATTCACAGATGGTGTATGCCTATTTTGGAATTGCAATGTCGATAGATATCCTGCTA   19222
gb:LC522972|Organism:Severe      ACAAATTCACAGATGGTGTATGCCTATTTTGGAATTGCAATGTCGATAGATATCCTGCTA   19219
gb:MT027063|Organism:Severe      ACAAATTCACAGATGGTGTATGCCTATTTTGGAATTGCAATGTCGATAGATATCCTGCTA   19222
gb:MT027062|Organism:Severe      ACAAATTCACAGATGGTGTATGCCTATTTTGGAATTGCAATGTCGATAGATATCCTGCTA   19222
gb:MT019529|Organism:Severe      ACAAATTCACAGATGGTGTATGCCTATTTTGGAATTGCAATGTCGATAGATATCCTGCTA   19222
gb:MN996529|Organism:Severe      ACAAATTCACAGATGGTGTATGCCTATTTTGGAATTGCAATGTCGATAGATATCCTGCTA   19210
gb:MN996531|Organism:Severe      ACAAATTCACAGATGGTGTATGCCTATTTTGGAATTGCAATGTCGATAGATATCCTGCTA   19209
```

```
gb:MT066176|Organism:Severe      ACAAATTCACAGATGGTGTATGCCTATTTTGGAATTGCAATGTCGATAGATATCCTGCTA   19222
gb:MT027064|Organism:Severe      ACAAATTCACAGATGGTGTATGCCTATTTTGGAATTGCAATGTCGATAGATATCCTGCTA   19222
gb:MN994468|Organism:Severe      ACAAATTCACAGATGGTGTATGCCTATTTTGGAATTGCAATGTCGATAGATATCCTGCTA   19222
gb:MT072688|Organism:Severe      ACAAATTCACAGATGGTGTATGCCTATTTTGGAATTGCAATGTCGATAGATATCCTGCTA   19207
gb:MN996527|Organism:Severe      ACAAATTCACAGATGGTGTATGCCTATTTTGGAATTGCAATGTCGATAGATATCCTGCTA   19189
gb:MT093631|Organism:Severe      ACAAATTCACAGATGGTGTATGCCTATTTTGGAATTGCAATGTCGATAGATATCCTGCTA   19260
gb:MT106053|Organism:Severe      ACAAATTCACAGATGGTGTATGCCTATTTTGGAATTGCAATGTCGATAGATATCCTGCTA   19222
gb:MT019533|Organism:Severe      ACAAATTCACAGATGGTGTATGCCTATTTTGGAATTGCAATGTCGATAGATATCCTGCTA   19222
gb:MT019531|Organism:Severe      ACAAATTCACAGATGGTGTATGCCTATTTTGGAATTGCAATGTCGATAGATATCCTGCTA   19222
gb:MN996528|Organism:Severe      ACAAATTCACAGATGGTGTATGCCTATTTTGGAATTGCAATGTCGATAGATATCCTGCTA   19222
gb:MN996530|Organism:Severe      ACAAATTCACAGATGGTGTATGCCTATTTTGGAATTGCAATGTCGATAGATATCCTGCTA   19208
gb:MN908947|Organism:Severe      ACAAATTCACAGATGGTGTATGCCTATTTTGGAATTGCAATGTCGATAGATATCCTGCTA   19222
gb:MT019532|Organism:Severe      ACAAATTCACAGATGGTGTATGCCTATTTTGGAATTGCAATGTCGATAGATATCCTGCTA   19222
                                 *********** ************************************************

gb:MT020781|Organism:Severe      ATTCCATTGTTTGTAGATTTGACACTAGAGTGCTATCTAACCTTAACTTGCCTGGTTGTG   19270
gb:MT007544|Organism:Severe      ATTCCATTGTTTGTAGATTTGACACTAGAGTGCTATCTAACCTTAACTTGCCTGGTTGTG   19282
gb:MN994467|Organism:Severe      ATTCCATTGTTTGTAGATTTGACACTAGAGTGCTATCTAACCTTAACTTGCCTGGTTGTG   19282
gb:MT044257|Organism:Severe      ATTCCATTGTTTGTAGATTTGACACTAGAGTGCTATCTAACCTTAACTTGCCTGGTTGTG   19282
gb:MT106054|Organism:Severe      ATTCCATTGTTTGTAGATTTGACACTAGAGTGCTATCTAACCTTAACTTGCCTGGTTGTG   19282
gb:MT049951|Organism:Severe      ATTCCATTGTTTGTAGATTTGACACTAGAGTGCTATCTAACCTTAACTTGCCTGGTTGTG   19282
gb:MN975262|Organism:Severe      ATTCCATTGTTTGTAGATTTGACACTAGAGTGCTATCTAACCTTAACTTGCCTGGTTGTG   19282
gb:MT106052|Organism:Severe      ATTCCATTGTTTGTAGATTTGACACTAGAGTGCTATCTAACCTTAACTTGCCTGGTTGTG   19282
gb:LC522975|Organism:Severe      ATTCCATTGTTTGTAGATTTGACACTAGAGTGCTATCTAACCTTAACTTGCCTGGTTGTG   19279
gb:LC522973|Organism:Severe      ATTCCATTGTTTGTAGATTTGACACTAGAGTGCTATCTAACCTTAACTTGCCTGGTTGTG   19279
gb:LC522974|Organism:Severe      ATTCCATTGTTTGTAGATTTGACACTAGAGTGCTATCTAACCTTAACTTGCCTGGTTGTG   19279
gb:MN985325|Organism:Severe      ATTCCATTGTTTGTAGATTTGACACTAGAGTGCTATCTAACCTTAACTTGCCTGGTTGTG   19282
gb:MT020881|Organism:Severe      ATTCCATTGTTTGTAGATTTGACACTAGAGTGCTATCTAACCTTAACTTGCCTGGTTGTG   19282
gb:MT020880|Organism:Severe      ATTCCATTGTTTGTAGATTTGACACTAGAGTGCTATCTAACCTTAACTTGCCTGGTTGTG   19282
gb:MT066175|Organism:Severe      ATTCCATTGTTTGTAGATTTGACACTAGAGTGCTATCTAACCTTAACTTGCCTGGTTGTG   19282
gb:MN997409|Organism:Severe      ATTCCATTGTTTGTAGATTTGACACTAGAGTGCTATCTAACCTTAACTTGCCTGGTTGTG   19282
gb:MN938384|Organism:Severe      ATTCCATTGTTTGTAGATTTGACACTAGAGTGCTATCTAACCTTAACTTGCCTGGTTGTG   19250
gb:MT044258|Organism:Severe      ATTCCATTGTTTGTAGATTTGACACTAGAGTGCTATCTAACCTTAACTTGCCTGGTTGTG   19258
gb:MT039890|Organism:Severe      ATTCCATTGTTTGTAGATTTGACACTAGAGTGCTATCTAACCTTAACTTGCCTGGTTGTG   19282
gb:MN988713|Organism:Severe      ATTCCATTGTTTGTAGATTTGACACTAGAGTGCTATCTAACCTTAACTTGCCTGGTTGTG   19282
gb:LC521925|Organism:Severe      ATTCCATTGTTTGTAGATTTGACACTAGAGTGCTATCTAACCTTAACTTGCCTGGTTGTG   19255
gb:MT093571|Organism:Severe      ATTCCATTGTTTGTAGATTTGACACTAGAGTGCTATCTAACCTTAACTTGCCTGGTTGTG   19282
gb:MT039887|Organism:Severe      ATTCCATTGTTTGTAGATTTGACACTAGAGTGCTATCTAACCTTAACTTGCCTGGTTGTG   19282
gb:MT019530|Organism:Severe      ATTCCATTGTTTGTAGATTTGACACTAGAGTGCTATCTAACCTTAACTTGCCTGGTTGTG   19282
gb:MT039888|Organism:Severe      ATTCCATTGTTTGTAGATTTGACACTAGAGTGCTATCTAACCTTAACTTGCCTGGTTGTG   19282
gb:LC522972|Organism:Severe      ATTCCATTGTTTGTAGATTTGACACTAGAGTGCTATCTAACCTTAACTTGCCTGGTTGTG   19279
gb:MT027063|Organism:Severe      ATTCCATTGTTTGTAGATTTGACACTAGAGTGCTATCTAACCTTAACTTGCCTGGTTGTG   19282
gb:MT027062|Organism:Severe      ATTCCATTGTTTGTAGATTTGACACTAGAGTGCTATCTAACCTTAACTTGCCTGGTTGTG   19282
gb:MT019529|Organism:Severe      ATTCCATTGTTTGTAGATTTGACACTAGAGTGCTATCTAACCTTAACTTGCCTGGTTGTG   19282
gb:MN996529|Organism:Severe      ATTCCATTGTTTGTAGATTTGACACTAGAGTGCTATCTAACCTTAACTTGCCTGGTTGTG   19270
gb:MN996531|Organism:Severe      ATTCCATTGTTTGTAGATTTGACACTAGAGTGCTATCTAACCTTAACTTGCCTGGTTGTG   19269
gb:MT066176|Organism:Severe      ATTCCATTGTTTGTAGATTTGACACTAGAGTGCTATCTAACCTTAACTTGCCTGGTTGTG   19282
gb:MT027064|Organism:Severe      ATTCCATTGTTTGTAGATTTGACACTAGAGTGCTATCTAACCTTAACTTGCCTGGTTGTG   19282
gb:MN994468|Organism:Severe      ATTCCATTGTTTGTAGATTTGACACTAGAGTGCTATCTAACCTTAACTTGCCTGGTTGTG   19282
gb:MT072688|Organism:Severe      ATTCCATTGTTTGTAGATTTGACACTAGAGTGCTATCTAACCTTAACTTGCCTGGTTGTG   19267
gb:MN996527|Organism:Severe      ATTCCATTGTTTGTAGATTTGACACTAGAGTGCTATCTAACCTTAACTTGCCTGGTTGTG   19249
gb:MT093631|Organism:Severe      ATTCCATTGTTTGTAGATTTGACACTAGAGTGCTATCTAACCTTAACTTGCCTGGTTGTG   19320
gb:MT106053|Organism:Severe      ATTCCATTGTTTGTAGATTTGACACTAGAGTGCTATCTAACCTTAACTTGCCTGGTTGTG   19282
gb:MT019533|Organism:Severe      ATTCCATTGTTTGTAGATTTGACACTAGAGTGCTATCTAACCTTAACTTGCCTGGTTGTG   19282
gb:MT019531|Organism:Severe      ATTCCATTGTTTGTAGATTTGACACTAGAGTGCTATCTAACCTTAACTTGCCTGGTTGTG   19282
gb:MN996528|Organism:Severe      ATTCCATTGTTTGTAGATTTGACACTAGAGTGCTATCTAACCTTAACTTGCCTGGTTGTG   19282
gb:MN996530|Organism:Severe      ATTCCATTGTTTGTAGATTTGACACTAGAGTGCTATCTAACCTTAACTTGCCTGGTTGTG   19268
gb:MN908947|Organism:Severe      ATTCCATTGTTTGTAGATTTGACACTAGAGTGCTATCTAACCTTAACTTGCCTGGTTGTG   19282
gb:MT019532|Organism:Severe      ATTCCATTGTTTGTAGATTTGACACTAGAGTGCTATCTAACCTTAACTTGCCTGGTTGTG   19282
                                 ************************************************************

gb:MT020781|Organism:Severe      ATGGTGGCAGTTTGTATGTAAATAAACATGCATTCCACACACCAGCTTTTGATAAAAGTG   19330
gb:MT007544|Organism:Severe      ATGGTGGCAGTTTGTATGTAAATAAACATGCATTCCACACACCAGCTTTTGATAAAAGTG   19342
gb:MN994467|Organism:Severe      ATGGTGGCAGTTTGTATGTAAATAAACATGCATTCCACACACCAGCTTTTGATAAAAGTG   19342
gb:MT044257|Organism:Severe      ATGGTGGCAGTTTGTATGTAAATAAACATGCATTCCACACACCAGCTTTTGATAAAAGTG   19342
```

```
gb:MT106054|Organism:Severe      ATGGTGGCAGTTTGTATGTAAATAAACATGCATTCCACACACCAGCTTTTGATAAAAGTG    19342
gb:MT049951|Organism:Severe      ATGGTGGCAGTTTGTATGTAAATAAACATGCATTCCACACACCAGCTTTTGATAAAAGTG    19342
gb:MN975262|Organism:Severe      ATGGTGGCAGTTTGTATGTAAATAAACATGCATTCCACACACCAGCTTTTGATAAAAGTG    19342
gb:MT106052|Organism:Severe      ATGGTGGCAGTTTGTATGTAAATAAACATGCATTCCACACACCAGCTTTTGATAAAAGTG    19342
gb:LC522975|Organism:Severe      ATGGTGGCAGTTTGTATGTAAATAAACATGCATTCCACACACCAGCTTTTGATAAAAGTG    19339
gb:LC522973|Organism:Severe      ATGGTGGCAGTTTGTATGTAAATAAACATGCATTCCACACACCAGCTTTTGATAAAAGTG    19339
gb:LC522974|Organism:Severe      ATGGTGGCAGTTTGTATGTAAATAAACATGCATTCCACACACCAGCTTTTGATAAAAGTG    19339
gb:MN985325|Organism:Severe      ATGGTGGCAGTTTGTATGTAAATAAACATGCATTCCACACACCAGCTTTTGATAAAAGTG    19342
gb:MT020881|Organism:Severe      ATGGTGGCAGTTTGTATGTAAATAAACATGCATTCCACACACCAGCTTTTGATAAAAGTG    19342
gb:MT020880|Organism:Severe      ATGGTGGCAGTTTGTATGTAAATAAACATGCATTCCACACACCAGCTTTTGATAAAAGTG    19342
gb:MT066175|Organism:Severe      ATGGTGGCAGTTTGTATGTAAATAAACATGCATTCCACACACCAGCTTTTGATAAAAGTG    19342
gb:MN997409|Organism:Severe      ATGGTGGCAGTTTGTATGTAAATAAACATGCATTCCACACACCAGCTTTTGATAAAAGTG    19342
gb:MN938384|Organism:Severe      ATGGTGGCAGTTTGTATGTAAATAAACATGCATTCCACACACCAGCTTTTGATAAAAGTG    19310
gb:MT044258|Organism:Severe      ATGGTGGCAGTTTGTATGTAAATAAACATGCATTCCACACACCAGCTTTTGATAAAAGTG    19318
gb:MT039890|Organism:Severe      ATGGTGGCAGTTTGTATGTAAATAAACATGCATTCCACACACCAGCTTTTGATAAAAGTG    19342
gb:MN988713|Organism:Severe      ATGGTGGCAGTTTGTATGTAAATAAACATGCATTCCACACACCAGCTTTTGATAAAAGTG    19342
gb:LC521925|Organism:Severe      ATGGTGGCAGTTTGTATGTAAATAAACATGCATTCCACACACCAGCTTTTGATAAAAGTG    19315
gb:MT093571|Organism:Severe      ATGGTGGCAGTTTGTATGTAAATAAACATGCATTCCACACACCAGCTTTTGATAAAAGTG    19342
gb:MT039887|Organism:Severe      ATGGTGGCAGTTTGTATGTAAATAAACATGCATTCCACACACCAGCTTTTGATAAAAGTG    19342
gb:MT019530|Organism:Severe      ATGGTGGCAGTTTGTATGTAAATAAACATGCATTCCACACACCAGCTTTTGATAAAAGTG    19342
gb:MT039888|Organism:Severe      ATGGTGGCAGTTTGTATGTAAATAAACATGCATTCCACACACCAGCTTTTGATAAAAGTG    19342
gb:LC522972|Organism:Severe      ATGGTGGCAGTTTGTATGTAAATAAACATGCATTCCACACACCAGCTTTTGATAAAAGTG    19339
gb:MT027063|Organism:Severe      ATGGTGGCAGTTTGTATGTAAATAAACATGCATTCCACACACCAGCTTTTGATAAAAGTG    19342
gb:MT027062|Organism:Severe      ATGGTGGCAGTTTGTATGTAAATAAACATGCATTCCACACACCAGCTTTTGATAAAAGTG    19342
gb:MT019529|Organism:Severe      ATGGTGGCAGTTTGTATGTAAATAAACATGCATTCCACACACCAGCTTTTGATAAAAGTG    19342
gb:MN996529|Organism:Severe      ATGGTGGCAGTTTGTATGTAAATAAACATGCATTCCACACACCAGCTTTTGATAAAAGTG    19330
gb:MN996531|Organism:Severe      ATGGTGGCAGTTTGTATGTAAATAAACATGCATTCCACACACCAGCTTTTGATAAAAGTG    19329
gb:MT066176|Organism:Severe      ATGGTGGCAGTTTGTATGTAAATAAACATGCATTCCACACACCAGCTTTTGATAAAAGTG    19342
gb:MT027064|Organism:Severe      ATGGTGGCAGTTTGTATGTAAATAAACATGCATTCCACACACCAGCTTTTGATAAAAGTG    19342
gb:MN994468|Organism:Severe      ATGGTGGCAGTTTGTATGTAAATAAACATGCATTCCACACACCAGCTTTTGATAAAAGTG    19342
gb:MT072688|Organism:Severe      ATGGTGGCAGTTTGTATGTAAATAAACATGCATTCCACACACCAGCTTTTGATAAAAGTG    19327
gb:MN996527|Organism:Severe      ATGGTGGCAGTTTGTATGTAAATAAACATGCATTCCACACACCAGCTTTTGATAAAAGTG    19309
gb:MT093631|Organism:Severe      ATGGTGGCAGTTTGTATGTAAATAAACATGCATTCCACACACCAGCTTTTGATAAAAGTG    19380
gb:MT106053|Organism:Severe      ATGGTGGCAGTTTGTATGTAAATAAACATGCATTCCACACACCAGCTTTTGATAAAAGTG    19342
gb:MT019533|Organism:Severe      ATGGTGGCAGTTTGTATGTAAATAAACATGCATTCCACACACCAGCTTTTGATAAAAGTG    19342
gb:MT019531|Organism:Severe      ATGGTGGCAGTTTGTATGTAAATAAACATGCATTCCACACACCAGCTTTTGATAAAAGTG    19342
gb:MN996528|Organism:Severe      ATGGTGGCAGTTTGTATGTAAATAAACATGCATTCCACACACCAGCTTTTGATAAAAGTG    19342
gb:MN996530|Organism:Severe      ATGGTGGCAGTTTGTATGTAAATAAACATGCATTCCACACACCAGCTTTTGATAAAAGTG    19328
gb:MN908947|Organism:Severe      ATGGTGGCAGTTTGTATGTAAATAAACATGCATTCCACACACCAGCTTTTGATAAAAGTG    19342
gb:MT019532|Organism:Severe      ATGGTGGCAGTTTGTATGTAAATAAACATGCATTCCACACACCAGCTTTTGATAAAAGTG    19342
                                 ************************************************************

gb:MT020781|Organism:Severe      CTTTTGTTAATTTAAAACAATTACCATTTTTCTATTACTCTGACAGTCCATGTGAGTCTC    19390
gb:MT007544|Organism:Severe      CTTTTGTTAATTTAAAACAATTACCATTTTTCTATTACTCTGACAGTCCATGTGAGTCTC    19402
gb:MN994467|Organism:Severe      CTTTTGTTAATTTAAAACAATTACCATTTTTCTATTACTCTGACAGTCCATGTGAGTCTC    19402
gb:MT044257|Organism:Severe      CTTTTGTTAATTTAAAACAATTACCATTTTTCTATTACTCTGACAGTCCATGTGAGTCTC    19402
gb:MT106054|Organism:Severe      CTTTTGTTAATTTAAAACAATTACCATTTTTCTATTACTCTGACAGTCCATGTGAGTCTC    19402
gb:MT049951|Organism:Severe      CTTTTGTTAATTTAAAACAATTACCATTTTTCTATTACTCTGACAGTCCATGTGAGTCTC    19402
gb:MN975262|Organism:Severe      CTTTTGTTAATTTAAAACAATTACCATTTTTCTATTACTCTGACAGTCCATGTGAGTCTC    19402
gb:MT106052|Organism:Severe      CTTTTGTTAATTTAAAACAATTACCATTTTTCTATTACTCTGACAGTCCATGTGAGTCTC    19402
gb:LC522975|Organism:Severe      CTTTTGTTAATTTAAAACAATTACCATTTTTCTATTACTCTGACAGTCCATGTGAGTCTC    19399
gb:LC522973|Organism:Severe      CTTTTGTTAATTTAAAACAATTACCATTTTTCTATTACTCTGACAGTCCATGTGAGTCTC    19399
gb:LC522974|Organism:Severe      CTTTTGTTAATTTAAAACAATTACCATTTTTCTATTACTCTGACAGTCCATGTGAGTCTC    19399
gb:MN985325|Organism:Severe      CTTTTGTTAATTTAAAACAATTACCATTTTTCTATTACTCTGACAGTCCATGTGAGTCTC    19402
gb:MT020881|Organism:Severe      CTTTTGTTAATTTAAAACAATTACCATTTTTCTATTACTCTGACAGTCCATGTGAGTCTC    19402
gb:MT020880|Organism:Severe      CTTTTGTTAATTTAAAACAATTACCATTTTTCTATTACTCTGACAGTCCATGTGAGTCTC    19402
gb:MT066175|Organism:Severe      CTTTTGTTAATTTAAAACAATTACCATTTTTCTATTACTCTGACAGTCCATGTGAGTCTC    19402
gb:MN997409|Organism:Severe      CTTTTGTTAATTTAAAACAATTACCATTTTTCTATTACTCTGACAGTCCATGTGAGTCTC    19402
gb:MN938384|Organism:Severe      CTTTTGTTAATTTAAAACAATTACCATTTTTCTATTACTCTGACAGTCCATGTGAGTCTC    19370
gb:MT044258|Organism:Severe      CTTTTGTTAATTTAAAACAATTACCATTTTTCTATTACTCTGACAGTCCATGTGAGTCTC    19378
gb:MT039890|Organism:Severe      CTTTTGTTAATTTAAAACAATTACCATTTTTCTATTACTCTGACAGTCCATGTGAGTCTC    19402
gb:MN988713|Organism:Severe      CTTTTGTTAATTTAAAACAATTACCATTTTTCTATTACTCTGACAGTCCATGTGAGTCTC    19402
gb:LC521925|Organism:Severe      CTTTTGTTAATTTAAAACAATTACCATTTTTCTATTACTCTGACAGTCCATGTGAGTCTC    19375
gb:MT093571|Organism:Severe      CTTTTGTTAATTTAAAACAATTACCATTTTTCTATTACTCTGACAGTCCATGTGAGTCTC    19402
gb:MT039887|Organism:Severe      CTTTTGTTAATTTAAAACAATTACCATTTTTCTATTACTCTGACAGTCCATGTGAGTCTC    19402
```

```
gb:MT019530|Organism:Severe      CTTTTGTTAATTTAAAACAATTACCATTTTTCTATTACTCTGACAGTCCATGTGAGTCTC   19402
gb:MT039888|Organism:Severe      CTTTTGTTAATTTAAAACAATTACCATTTTTCTATTACTCTGACAGTCCATGTGAGTCTC   19402
gb:LC522972|Organism:Severe      CTTTTGTTAATTTAAAACAATTACCATTTTTCTATTACTCTGACAGTCCATGTGAGTCTC   19399
gb:MT027063|Organism:Severe      CTTTTGTTAATTTAAAACAATTACCATTTTTCTATTACTCTGACAGTCCATGTGAGTCTC   19402
gb:MT027062|Organism:Severe      CTTTTGTTAATTTAAAACAATTACCATTTTTCTATTACTCTGACAGTCCATGTGAGTCTC   19402
gb:MT019529|Organism:Severe      CTTTTGTTAATTTAAAACAATTACCATTTTTCTATTACTCTGACAGTCCATGTGAGTCTC   19402
gb:MN996529|Organism:Severe      CTTTTGTTAATTTAAAACAATTACCATTTTTCTATTACTCTGACAGTCCATGTGAGTCTC   19390
gb:MN996531|Organism:Severe      CTTTTGTTAATTTAAAACAATTACCATTTTTCTATTACTCTGACAGTCCATGTGAGTCTC   19389
gb:MT066176|Organism:Severe      CTTTTGTTAATTTAAAACAATTACCATTTTTCTATTACTCTGACAGTCCATGTGAGTCTC   19402
gb:MT027064|Organism:Severe      CTTTTGTTAATTTAAAACAATTACCATTTTTCTATTACTCTGACAGTCCATGTGAGTCTC   19402
gb:MN994468|Organism:Severe      CTTTTGTTAATTTAAAACAATTACCATTTTTCTATTACTCTGACAGTCCATGTGAGTCTC   19402
gb:MT072688|Organism:Severe      CTTTTGTTAATTTAAAACAATTACCATTTTTCTATTACTCTGACAGTCCATGTGAGTCTC   19387
gb:MN996527|Organism:Severe      CTTTTGTTAATTTAAAACAATTACCATTTTTCTATTACTCTGACAGTCCATGTGAGTCTC   19369
gb:MT093631|Organism:Severe      CTTTTGTTAATTTAAAACAATTACCATTTTTCTATTACTCTGACAGTCCATGTGAGTCTC   19440
gb:MT106053|Organism:Severe      CTTTTGTTAATTTAAAACAATTACCATTTTTCTATTACTCTGACAGTCCATGTGAGTCTC   19402
gb:MT019533|Organism:Severe      CTTTTGTTAATTTAAAACAATTACCATTTTTCTATTACTCTGACAGTCCATGTGAGTCTC   19402
gb:MT019531|Organism:Severe      CTTTTGTTAATTTAAAACAATTACCATTTTTCTATTACTCTGACAGTCCATGTGAGTCTC   19402
gb:MN996528|Organism:Severe      CTTTTGTTAATTTAAAACAATTACCATTTTTCTATTACTCTGACAGTCCATGTGAGTCTC   19402
gb:MN996530|Organism:Severe      CTTTTGTTAATTTAAAACAATTACCATTTTTCTATTACTCTGACAGTCCATGTGAGTCTC   19388
gb:MN908947|Organism:Severe      CTTTTGTTAATTTAAAACAATTACCATTTTTCTATTACTCTGACAGTCCATGTGAGTCTC   19402
gb:MT019532|Organism:Severe      CTTTTGTTAATTTAAAACAATTACCATTTTTCTATTACTCTGACAGTCCATGTGAGTCTC   19402
                                 ************************************************************

gb:MT020781|Organism:Severe      ATGGAAAACAAGTAGTGTCAGATATAGATTATGTACCACTAAAGTCTGCTACGTGTATAA   19450
gb:MT007544|Organism:Severe      ATGGAAAACAAGTAGTGTCAGATATAGATTATGTACCACTAAAGTCTGCTACGTGTATAA   19462
gb:MN994467|Organism:Severe      ATGGAAAACAAGTAGTGTCAGATATAGATTATGTACCACTAAAGTCTGCTACGTGTATAA   19462
gb:MT044257|Organism:Severe      ATGGAAAACAAGTAGTGTCAGATATAGATTATGTACCACTAAAGTCTGCTACGTGTATAA   19462
gb:MT106054|Organism:Severe      ATGGAAAACAAGTAGTGTCAGATATAGATTATGTACCACTAAAGTCTGCTACGTGTATAA   19462
gb:MT049951|Organism:Severe      ATGGAAAACAAGTAGTGTCAGATATAGATTATGTACCACTAAAGTCTGCTACGTGTATAA   19462
gb:MN975262|Organism:Severe      ATGGAAAACAAGTAGTGTCAGATATAGATTATGTACCACTAAAGTCTGCTACGTGTATAA   19462
gb:MT106052|Organism:Severe      ATGGAAAACAAGTAGTGTCAGATATAGATTATGTACCACTAAAGTCTGCTACGTGTATAA   19462
gb:LC522975|Organism:Severe      ATGGAAAACAAGTAGTGTCAGATATAGATTATGTACCACTAAAGTCTGCTACGTGTATAA   19459
gb:LC522973|Organism:Severe      ATGGAAAACAAGTAGTGTCAGATATAGATTATGTACCACTAAAGTCTGCTACGTGTATAA   19459
gb:LC522974|Organism:Severe      ATGGAAAACAAGTAGTGTCAGATATAGATTATGTACCACTAAAGTCTGCTACGTGTATAA   19459
gb:MN985325|Organism:Severe      ATGGAAAACAAGTAGTGTCAGATATAGATTATGTACCACTAAAGTCTGCTACGTGTATAA   19462
gb:MT020881|Organism:Severe      ATGGAAAACAAGTAGTGTCAGATATAGATTATGTACCACTAAAGTCTGCTACGTGTATAA   19462
gb:MT020880|Organism:Severe      ATGGAAAACAAGTAGTGTCAGATATAGATTATGTACCACTAAAGTCTGCTACGTGTATAA   19462
gb:MT066175|Organism:Severe      ATGGAAAACAAGTAGTGTCAGATATAGATTATGTACCACTAAAGTCTGCTACGTGTATAA   19462
gb:MN997409|Organism:Severe      ATGGAAAACAAGTAGTGTCAGATATAGATTATGTACCACTAAAGTCTGCTACGTGTATAA   19462
gb:MN938384|Organism:Severe      ATGGAAAACAAGTAGTGTCAGATATAGATTATGTACCACTAAAGTCTGCTACGTGTATAA   19430
gb:MT044258|Organism:Severe      ATGGAAAACAAGTAGTGTCAGATATAGATTATGTACCACTAAAGTCTGCTACGTGTATAA   19438
gb:MT039890|Organism:Severe      ATGGAAAACAAGTAGTGTCAGATATAGATTATGTACCACTAAAGTCTGCTACGTGTATAA   19462
gb:MN988713|Organism:Severe      ATGGAAAACAAGTAGTGTCAGATATAGATTATGTACCACTAAAGTCTGCTACGTGTATAA   19462
gb:LC521925|Organism:Severe      ATGGAAAACAAGTAGTGTCAGATATAGATTATGTACCACTAAAGTCTGCTACGTGTATAA   19435
gb:MT093571|Organism:Severe      ATGGAAAACAAGTAGTGTCAGATATAGATTATGTACCACTAAAGTCTGCTACGTGTATAA   19462
gb:MT039887|Organism:Severe      ATGGAAAACAAGTAGTGTCAGATATAGATTATGTACCACTAAAGTCTGCTACGTGTATAA   19462
gb:MT019530|Organism:Severe      ATGGAAAACAAGTAGTGTCAGATATAGATTATGTACCACTAAAGTCTGCTACGTGTATAA   19462
gb:MT039888|Organism:Severe      ATGGAAAACAAGTAGTGTCAGATATAGATTATGTACCACTAAAGTCTGCTACGTGTATAA   19462
gb:LC522972|Organism:Severe      ATGGAAAACAAGTAGTGTCAGATATAGATTATGTACCACTAAAGTCTGCTACGTGTATAA   19459
gb:MT027063|Organism:Severe      ATGGAAAACAAGTAGTGTCAGATATAGATTATGTACCACTAAAGTCTGCTACGTGTATAA   19462
gb:MT027062|Organism:Severe      ATGGAAAACAAGTAGTGTCAGATATAGATTATGTACCACTAAAGTCTGCTACGTGTATAA   19462
gb:MT019529|Organism:Severe      ATGGAAAACAAGTAGTGTCAGATATAGATTATGTACCACTAAAGTCTGCTACGTGTATAA   19462
gb:MN996529|Organism:Severe      ATGGAAAACAAGTAGTGTCAGATATAGATTATGTACCACTAAAGTCTGCTACGTGTATAA   19450
gb:MN996531|Organism:Severe      ATGGAAAACAAGTAGTGTCAGATATAGATTATGTACCACTAAAGTCTGCTACGTGTATAA   19449
gb:MT066176|Organism:Severe      ATGGAAAACAAGTAGTGTCAGATATAGATTATGTACCACTAAAGTCTGCTACGTGTATAA   19462
gb:MT027064|Organism:Severe      ATGGAAAACAAGTAGTGTCAGATATAGATTATGTACCACTAAAGTCTGCTACGTGTATAA   19462
gb:MN994468|Organism:Severe      ATGGAAAACAAGTAGTGTCAGATATAGATTATGTACCACTAAAGTCTGCTACGTGTATAA   19462
gb:MT072688|Organism:Severe      ATGGAAAACAAGTAGTGTCAGATATAGATTATGTACCACTAAAGTCTGCTACGTGTATAA   19447
gb:MN996527|Organism:Severe      ATGGAAAACAAGTAGTGTCAGATATAGATTATGTACCACTAAAGTCTGCTACGTGTATAA   19429
gb:MT093631|Organism:Severe      ATGGAAAACAAGTAGTGTCAGATATAGATTATGTACCACTAAAGTCTGCTACGTGTATAA   19500
gb:MT106053|Organism:Severe      ATGGAAAACAAGTAGTGTCAGATATAGATTATGTACCACTAAAGTCTGCTACGTGTATAA   19462
gb:MT019533|Organism:Severe      ATGGAAAACAAGTAGTGTCAGATATAGATTATGTACCACTAAAGTCTGCTACGTGTATAA   19462
gb:MT019531|Organism:Severe      ATGGAAAACAAGTAGTGTCAGATATAGATTATGTACCACTAAAGTCTGCTACGTGTATAA   19462
gb:MN996528|Organism:Severe      ATGGAAAACAAGTAGTGTCAGATATAGATTATGTACCACTAAAGTCTGCTACGTGTATAA   19462
gb:MN996530|Organism:Severe      ATGGAAAACAAGTAGTGTCAGATATAGATTATGTACCACTAAAGTCTGCTACGTGTATAA   19448
```

```
gb:MN908947|Organism:Severe      ATGGAAAACAAGTAGTGTCAGATATAGATTATGTACCACTAAAGTCTGCTACGTGTATAA   19462
gb:MT019532|Organism:Severe      ATGGAAAACAAGTAGTGTCAGATATAGATTATGTACCACTAAAGTCTGCTACGTGTATAA   19462
                                 ************************************************************

gb:MT020781|Organism:Severe      CACGTTGCAATTTAGGTGGTGCTGTCTGTAGACATCATGCTAATGAGTACAGATTGTATC   19510
gb:MT007544|Organism:Severe      CACGTTGCAATTTAGGTGGTGCTGTCTGTAGACATCATGCTAATGAGTACAGATTGTATC   19522
gb:MN994467|Organism:Severe      CACGTTGCAATTTAGGTGGTGCTGTCTGTAGACATCATGCTAATGAGTACAGATTGTATC   19522
gb:MT044257|Organism:Severe      CACGTTGCAATTTAGGTGGTGCTGTCTGTAGACATCATGCTAATGAGTACAGATTGTATC   19522
gb:MT106054|Organism:Severe      CACGTTGCAATTTAGGTGGTGCTGTCTGTAGACATCATGCTAATGAGTACAGATTGTATC   19522
gb:MT049951|Organism:Severe      CACGTTGCAATTTAGGTGGTGCTGTCTGTAGACATCATGCTAATGAGTACAGATTGTATC   19522
gb:MN975262|Organism:Severe      CACGTTGCAATTTAGGTGGTGCTGTCTGTAGACATCATGCTAATGAGTACAGATTGTATC   19522
gb:MT106052|Organism:Severe      CACGTTGCAATTTAGGTGGTGCTGTCTGTAGACATCATGCTAATGAGTACAGATTGTATC   19522
gb:LC522975|Organism:Severe      CACGTTGCAATTTAGGTGGTGCTGTCTGTAGACATCATGCTAATGAGTACAGATTGTATC   19519
gb:LC522973|Organism:Severe      CACGTTGCAATTTAGGTGGTGCTGTCTGTAGACATCATGCTAATGAGTACAGATTGTATC   19519
gb:LC522974|Organism:Severe      CACGTTGCAATTTAGGTGGTGCTGTCTGTAGACATCATGCTAATGAGTACAGATTGTATC   19519
gb:MN985325|Organism:Severe      CACGTTGCAATTTAGGTGGTGCTGTCTGTAGACATCATGCTAATGAGTACAGATTGTATC   19522
gb:MT020881|Organism:Severe      CACGTTGCAATTTAGGTGGTGCTGTCTGTAGACATCATGCTAATGAGTACAGATTGTATC   19522
gb:MT020880|Organism:Severe      CACGTTGCAATTTAGGTGGTGCTGTCTGTAGACATCATGCTAATGAGTACAGATTGTATC   19522
gb:MT066175|Organism:Severe      CACGTTGCAATTTAGGTGGTGCTGTCTGTAGACATCATGCTAATGAGTACAGATTGTATC   19522
gb:MN997409|Organism:Severe      CACGTTGCAATTTAGGTGGTGCTGTCTGTAGACATCATGCTAATGAGTACAGATTGTATC   19522
gb:MN938384|Organism:Severe      CACGTTGCAATTTAGGTGGTGCTGTCTGTAGACATCATGCTAATGAGTACAGATTGTATC   19490
gb:MT044258|Organism:Severe      CACGTTGCAATTTAGGTGGTGCTGTCTGTAGACATCATGCTAATGAGTACAGATTGTATC   19498
gb:MT039890|Organism:Severe      CACGTTGCAATTTAGGTGGTGCTGTCTGTAGACATCATGCTAATGAGTACAGATTGTATC   19522
gb:MN988713|Organism:Severe      CACGTTGCAATTTAGGTGGTGCTGTCTGTAGACATCATGCTAATGAGTACAGATTGTATC   19522
gb:LC521925|Organism:Severe      CACGTTGCAATTTAGGTGGTGCTGTCTGTAGACATCATGCTAATGAGTACAGATTGTATC   19495
gb:MT093571|Organism:Severe      CACGTTGCAATTTAGGTGGTGCTGTCTGTAGACATCATGCTAATGAGTACAGATTGTATC   19522
gb:MT039887|Organism:Severe      CACGTTGCAATTTAGGTGGTGCTGTCTGTAGACATCATGCTAATGAGTACAGATTGTATC   19522
gb:MT019530|Organism:Severe      CACGTTGCAATTTAGGTGGTGCTGTCTGTAGACATCATGCTAATGAGTACAGATTGTATC   19522
gb:MT039888|Organism:Severe      CACGTTGCAATTTAGGTGGTGCTGTCTGTAGACATCATGCTAATGAGTACAGATTGTATC   19522
gb:LC522972|Organism:Severe      CACGTTGCAATTTAGGTGGTGCTGTCTGTAGACATCATGCTAATGAGTACAGATTGTATC   19519
gb:MT027063|Organism:Severe      CACGTTGCAATTTAGGTGGTGCTGTCTGTAGACATCATGCTAATGAGTACAGATTGTATC   19522
gb:MT027062|Organism:Severe      CACGTTGCAATTTAGGTGGTGCTGTCTGTAGACATCATGCTAATGAGTACAGATTGTATC   19522
gb:MT019529|Organism:Severe      CACGTTGCAATTTAGGTGGTGCTGTCTGTAGACATCATGCTAATGAGTACAGATTGTATC   19522
gb:MN996529|Organism:Severe      CACGTTGCAATTTAGGTGGTGCTGTCTGTAGACATCATGCTAATGAGTACAGATTGTATC   19510
gb:MN996531|Organism:Severe      CACGTTGCAATTTAGGTGGTGCTGTCTGTAGACATCATGCTAATGAGTACAGATTGTATC   19509
gb:MT066176|Organism:Severe      CACGTTGCAATTTAGGTGGTGCTGTCTGTAGACATCATGCTAATGAGTACAGATTGTATC   19522
gb:MT027064|Organism:Severe      CACGTTGCAATTTAGGTGGTGCTGTCTGTAGACATCATGCTAATGAGTACAGATTGTATC   19522
gb:MN994468|Organism:Severe      CACGTTGCAATTTAGGTGGTGCTGTCTGTAGACATCATGCTAATGAGTACAGATTGTATC   19522
gb:MT072688|Organism:Severe      CACGTTGCAATTTAGGTGGTGCTGTCTGTAGACATCATGCTAATGAGTACAGATTGTATC   19507
gb:MN996527|Organism:Severe      CACGTTGCAATTTAGGTGGTGCTGTCTGTAGACATCATGCTAATGAGTACAGATTGTATC   19489
gb:MT093631|Organism:Severe      CACGTTGCAATTTAGGTGGTGCTGTCTGTAGACATCATGCTAATGAGTACAGATTGTATC   19560
gb:MT106053|Organism:Severe      CACGTTGCAATTTAGGTGGTGCTGTCTGTAGACATCATGCTAATGAGTACAGATTGTATC   19522
gb:MT019533|Organism:Severe      CACGTTGCAATTTAGGTGGTGCTGTCTGTAGACATCATGCTAATGAGTACAGATTGTATC   19522
gb:MT019531|Organism:Severe      CACGTTGCAATTTAGGTGGTGCTGTCTGTAGACATCATGCTAATGAGTACAGATTGTATC   19522
gb:MN996528|Organism:Severe      CACGTTGCAATTTAGGTGGTGCTGTCTGTAGACATCATGCTAATGAGTACAGATTGTATC   19522
gb:MN996530|Organism:Severe      CACGTTGCAATTTAGGTGGTGCTGTCTGTAGACATCATGCTAATGAGTACAGATTGTATC   19508
gb:MN908947|Organism:Severe      CACGTTGCAATTTAGGTGGTGCTGTCTGTAGACATCATGCTAATGAGTACAGATTGTATC   19522
gb:MT019532|Organism:Severe      CACGTTGCAATTTAGGTGGTGCTGTCTGTAGACATCATGCTAATGAGTACAGATTGTATC   19522
                                 ************************************************************

gb:MT020781|Organism:Severe      TCGATGCTTATAACATGATGATCTCAGCTGGCTTTAGCTTGTGGGTTTACAAACAATTTG   19570
gb:MT007544|Organism:Severe      TCGATGCTTATAACATGATGATCTCAGCTGGCTTTAGCTTGTGGGTTTACAAACAATTTG   19582
gb:MN994467|Organism:Severe      TCGATGCTTATAACATGATGATCTCAGCTGGCTTTAGCTTGTGGGTTTACAAACAATTTG   19582
gb:MT044257|Organism:Severe      TCGATGCTTATAACATGATGATCTCAGCTGGCTTTAGCTTGTGGGTTTACAAACAATTTG   19582
gb:MT106054|Organism:Severe      TCGATGCTTATAACATGATGATCTCAGCTGGCTTTAGCTTGTGGGTTTACAAACAATTTG   19582
gb:MT049951|Organism:Severe      TCGATGCTTATAACATGATGATCTCAGCTGGCTTTAGCTTGTGGGTTTACAAACAATTTG   19582
gb:MN975262|Organism:Severe      TCGATGCTTATAACATGATGATCTCAGCTGGCTTTAGCTTGTGGGTTTACAAACAATTTG   19582
gb:MT106052|Organism:Severe      TCGATGCTTATAACATGATGATCTCAGCTGGCTTTAGCTTGTGGGTTTACAAACAATTTG   19582
gb:LC522975|Organism:Severe      TCGATGCTTATAACATGATGATCTCAGCTGGCTTTAGCTTGTGGGTTTACAAACAATTTG   19579
gb:LC522973|Organism:Severe      TCGATGCTTATAACATGATGATCTCAGCTGGCTTTAGCTTGTGGGTTTACAAACAATTTG   19579
gb:LC522974|Organism:Severe      TCGATGCTTATAACATGATGATCTCAGCTGGCTTTAGCTTGTGGGTTTACAAACAATTTG   19579
gb:MN985325|Organism:Severe      TCGATGCTTATAACATGATGATCTCAGCTGGCTTTAGCTTGTGGGTTTACAAACAATTTG   19582
gb:MT020881|Organism:Severe      TCGATGCTTATAACATGATGATCTCAGCTGGCTTTAGCTTGTGGGTTTACAAACAATTTG   19582
gb:MT020880|Organism:Severe      TCGATGCTTATAACATGATGATCTCAGCTGGCTTTAGCTTGTGGGTTTACAAACAATTTG   19582
gb:MT066175|Organism:Severe      TCGATGCTTATAACATGATGATCTCAGCTGGCTTTAGCTTGTGGGTTTACAAACAATTTG   19582
```

```
gb:MN997409|Organism:Severe      TCGATGCTTATAACATGATGATCTCAGCTGGCTTTAGCTTGTGGGTTTACAAACAATTTG   19582
gb:MN938384|Organism:Severe      TCGATGCTTATAACATGATGATCTCAGCTGGCTTTAGCTTGTGGGTTTACAAACAATTTG   19550
gb:MT044258|Organism:Severe      TCGATGCTTATAACATGATGATCTCAGCTGGCTTTAGCTTGTGGGTTTACAAACAATTTG   19558
gb:MT039890|Organism:Severe      TCGATGCTTATAACATGATGATCTCAGCTGGCTTTAGCTTGTGGGTTTACAAACAATTTG   19582
gb:MN988713|Organism:Severe      TCGATGCTTATAACATGATGATCTCAGCTGGCTTTAGCTTGTGGGTTTACAAACAATTTG   19582
gb:LC521925|Organism:Severe      TCGATGCTTATAACATGATGATCTCAGCTGGCTTTAGCTTGTGGGTTTACAAACAATTTG   19555
gb:MT093571|Organism:Severe      TCGATGCTTATAACATGATGATCTCAGCTGGCTTTAGCTTGTGGGTTTACAAACAATTTG   19582
gb:MT039887|Organism:Severe      TCGATGCTTATAACATGATGATCTCAGCTGGCTTTAGCTTGTGGGTTTACAAACAATTTG   19582
gb:MT019530|Organism:Severe      TCGATGCTTATAACATGATGATCTCAGCTGGCTTTAGCTTGTGGGTTTACAAACAATTTG   19582
gb:MT039888|Organism:Severe      TCGATGCTTATAACATGATGATCTCAGCTGGCTTTAGCTTGTGGGTTTACAAACAATTTG   19582
gb:LC522972|Organism:Severe      TCGATGCTTATAACATGATGATCTCAGCTGGCTTTAGCTTGTGGGTTTACAAACAATTTG   19579
gb:MT027063|Organism:Severe      TCGATGCTTATAACATGATGATCTCAGCTGGCTTTAGCTTGTGGGTTTACAAACAATTTG   19582
gb:MT027062|Organism:Severe      TCGATGCTTATAACATGATGATCTCAGCTGGCTTTAGCTTGTGGGTTTACAAACAATTTG   19582
gb:MT019529|Organism:Severe      TCGATGCTTATAACATGATGATCTCAGCTGGCTTTAGCTTGTGGGTTTACAAACAATTTG   19582
gb:MN996529|Organism:Severe      TCGATGCTTATAACATGATGATCTCAGCTGGCTTTAGCTTGTGGGTTTACAAACAATTTG   19570
gb:MN996531|Organism:Severe      TCGATGCTTATAACATGATGATCTCAGCTGGCTTTAGCTTGTGGGTTTACAAACAATTTG   19569
gb:MT066176|Organism:Severe      TCGATGCTTATAACATGATGATCTCAGCTGGCTTTAGCTTGTGGGTTTACAAACAATTTG   19582
gb:MT027064|Organism:Severe      TCGATGCTTATAACATGATGATCTCAGCTGGCTTTAGCTTGTGGGTTTACAAACAATTTG   19582
gb:MN994468|Organism:Severe      TCGATGCTTATAACATGATGATCTCAGCTGGCTTTAGCTTGTGGGTTTACAAACAATTTG   19582
gb:MT072688|Organism:Severe      TCGATGCTTATAACATGATGATCTCAGCTGGCTTTAGCTTGTGGGTTTACAAACAATTTG   19567
gb:MN996527|Organism:Severe      TCGATGCTTATAACATGATGATCTCAGCTGGCTTTAGCTTGTGGGTTTACAAACAATTTG   19549
gb:MT093631|Organism:Severe      TCGATGCTTATAACATGATGATCTCAGCTGGCTTTAGCTTGTGGGTTTACAAACAATTTG   19620
gb:MT106053|Organism:Severe      TCGATGCTTATAACATGATGATCTCAGCTGGCTTTAGCTTGTGGGTTTACAAACAATTTG   19582
gb:MT019533|Organism:Severe      TCGATGCTTATAACATGATGATCTCAGCTGGCTTTAGCTTGTGGGTTTACAAACAATTTG   19582
gb:MT019531|Organism:Severe      TCGATGCTTATAACATGATGATCTCAGCTGGCTTTAGCTTGTGGGTTTACAAACAATTTG   19582
gb:MN996528|Organism:Severe      TCGATGCTTATAACATGATGATCTCAGCTGGCTTTAGCTTGTGGGTTTACAAACAATTTG   19582
gb:MN996530|Organism:Severe      TCGATGCTTATAACATGATGATCTCAGCTGGCTTTAGCTTGTGGGTTTACAAACAATTTG   19568
gb:MN908947|Organism:Severe      TCGATGCTTATAACATGATGATCTCAGCTGGCTTTAGCTTGTGGGTTTACAAACAATTTG   19582
gb:MT019532|Organism:Severe      TCGATGCTTATAACATGATGATCTCAGCTGGCTTTAGCTTGTGGGTTTACAAACAATTTG   19582
                                 ************************************************************

gb:MT020781|Organism:Severe      ATACTTATAACCTCTGGAACACTTTTACAAGACTTCAGAGTTTAGAAAATGTGGCTTTTA   19630
gb:MT007544|Organism:Severe      ATACTTATAACCTCTGGAACACTTTTACAAGACTTCAGAGTTTAGAAAATGTGGCTTTTA   19642
gb:MN994467|Organism:Severe      ATACTTATAACCTCTGGAACACTTTTACAAGACTTCAGAGTTTAGAAAATGTGGCTTTTA   19642
gb:MT044257|Organism:Severe      ATACTTATAACCTCTGGAACACTTTTACAAGACTTCAGAGTTTAGAAAATGTGGCTTTTA   19642
gb:MT106054|Organism:Severe      ATACTTATAACCTCTGGAACACTTTTACAAGACTTCAGAGTTTAGAAAATGTGGCTTTTA   19642
gb:MT049951|Organism:Severe      ATACTTATAACCTCTGGAACACTTTTACAAGACTTCAGAGTTTAGAAAATGTGGCTTTTA   19642
gb:MN975262|Organism:Severe      ATACTTATAACCTCTGGAACACTTTTACAAGACTTCAGAGTTTAGAAAATGTGGCTTTTA   19642
gb:MT106052|Organism:Severe      ATACTTATAACCTCTGGAACACTTTTACAAGACTTCAGAGTTTAGAAAATGTGGCTTTTA   19642
gb:LC522975|Organism:Severe      ATACTTATAACCTCTGGAACACTTTTACAAGACTTCAGAGTTTAGAAAATGTGGCTTTTA   19639
gb:LC522973|Organism:Severe      ATACTTATAACCTCTGGAACACTTTTACAAGACTTCAGAGTTTAGAAAATGTGGCTTTTA   19639
gb:LC522974|Organism:Severe      ATACTTATAACCTCTGGAACACTTTTACAAGACTTCAGAGTTTAGAAAATGTGGCTTTTA   19639
gb:MN985325|Organism:Severe      ATACTTATAACCTCTGGAACACTTTTACAAGACTTCAGAGTTTAGAAAATGTGGCTTTTA   19642
gb:MT020881|Organism:Severe      ATACTTATAACCTCTGGAACACTTTTACAAGACTTCAGAGTTTAGAAAATGTGGCTTTTA   19642
gb:MT020880|Organism:Severe      ATACTTATAACCTCTGGAACACTTTTACAAGACTTCAGAGTTTAGAAAATGTGGCTTTTA   19642
gb:MT066175|Organism:Severe      ATACTTATAACCTCTGGAACACTTTTACAAGACTTCAGAGTTTAGAAAATGTGGCTTTTA   19642
gb:MN997409|Organism:Severe      ATACTTATAACCTCTGGAACACTTTTACAAGACTTCAGAGTTTAGAAAATGTGGCTTTTA   19642
gb:MN938384|Organism:Severe      ATACTTATAACCTCTGGAACACTTTTACAAGACTTCAGAGTTTAGAAAATGTGGCTTTTA   19610
gb:MT044258|Organism:Severe      ATACTTATAACCTCTGGAACACTTTTACAAGACTTCAGAGTTTAGAAAATGTGGCTTTTA   19618
gb:MT039890|Organism:Severe      ATACTTATAACCTCTGGAACACTTTTACAAGACTTCAGAGTTTAGAAAATGTGGCTTTTA   19642
gb:MN988713|Organism:Severe      ATACTTATAACCTCTGGAACACTTTTACAAGACTTCAGAGTTTAGAAAATGTGGCTTTTA   19642
gb:LC521925|Organism:Severe      ATACTTATAACCTCTGGAACACTTTTACAAGACTTCAGAGTTTAGAAAATGTGGCTTTTA   19615
gb:MT093571|Organism:Severe      ATACTTATAACCTCTGGAACACTTTTACAAGACTTCAGAGTTTAGAAAATGTGGCTTTTA   19642
gb:MT039887|Organism:Severe      ATACTTATAACCTCTGGAACACTTTTACAAGACTTCAGAGTTTAGAAAATGTGGCTTTTA   19642
gb:MT019530|Organism:Severe      ATACTTATAACCTCTGGAACACTTTTACAAGACTTCAGAGTTTAGAAAATGTGGCTTTTA   19642
gb:MT039888|Organism:Severe      ATACTTATAACCTCTGGAACACTTTTACAAGACTTCAGAGTTTAGAAAATGTGGCTTTTA   19642
gb:LC522972|Organism:Severe      ATACTTATAACCTCTGGAACACTTTTACAAGACTTCAGAGTTTAGAAAATGTGGCTTTTA   19639
gb:MT027063|Organism:Severe      ATACTTATAACCTCTGGAACACTTTTACAAGACTTCAGAGTTTAGAAAATGTGGCTTTTA   19642
gb:MT027062|Organism:Severe      ATACTTATAACCTCTGGAACACTTTTACAAGACTTCAGAGTTTAGAAAATGTGGCTTTTA   19642
gb:MT019529|Organism:Severe      ATACTTATAACCTCTGGAACACTTTTACAAGACTTCAGAGTTTAGAAAATGTGGCTTTTA   19642
gb:MN996529|Organism:Severe      ATACTTATAACCTCTGGAACACTTTTACAAGACTTCAGAGTTTAGAAAATGTGGCTTTTA   19630
gb:MN996531|Organism:Severe      ATACTTATAACCTCTGGAACACTTTTACAAGACTTCAGAGTTTAGAAAATGTGGCTTTTA   19629
gb:MT066176|Organism:Severe      ATACTTATAACCTCTGGAACACTTTTACAAGACTTCAGAGTTTAGAAAATGTGGCTTTTA   19642
gb:MT027064|Organism:Severe      ATACTTATAACCTCTGGAACACTTTTACAAGACTTCAGAGTTTAGAAAATGTGGCTTTTA   19642
gb:MN994468|Organism:Severe      ATACTTATAACCTCTGGAACACTTTTACAAGACTTCAGAGTTTAGAAAATGTGGCTTTTA   19642
```

```
gb:MT072688|Organism:Severe      ATACTTATAACCTCTGGAACACTTTTACAAGACTTCAGAGTTTAGAAAATGTGGCTTTTA   19627
gb:MN996527|Organism:Severe      ATACTTATAACCTCTGGAACACTTTTACAAGACTTCAGAGTTTAGAAAATGTGGCTTTTA   19609
gb:MT093631|Organism:Severe      ATACTTATAACCTCTGGAACACTTTTACAAGACTTCAGAGTTTAGAAAATGTGGCTTTTA   19680
gb:MT106053|Organism:Severe      ATACTTATAACCTCTGGAACACTTTTACAAGACTTCAGAGTTTAGAAAATGTGGCTTTTA   19642
gb:MT019533|Organism:Severe      ATACTTATAACCTCTGGAACACTTTTACAAGACTTCAGAGTTTAGAAAATGTGGCTTTTA   19642
gb:MT019531|Organism:Severe      ATACTTATAACCTCTGGAACACTTTTACAAGACTTCAGAGTTTAGAAAATGTGGCTTTTA   19642
gb:MN996528|Organism:Severe      ATACTTATAACCTCTGGAACACTTTTACAAGACTTCAGAGTTTAGAAAATGTGGCTTTTA   19642
gb:MN996530|Organism:Severe      ATACTTATAACCTCTGGAACACTTTTACAAGACTTCAGAGTTTAGAAAATGTGGCTTTTA   19628
gb:MN908947|Organism:Severe      ATACTTATAACCTCTGGAACACTTTTACAAGACTTCAGAGTTTAGAAAATGTGGCTTTTA   19642
gb:MT019532|Organism:Severe      ATACTTATAACCTCTGGAACACTTTTACAAGACTTCAGAGTTTAGAAAATGTGGCTTTTA   19642
                                 ************************************************************

gb:MT020781|Organism:Severe      ATGTTGTAAATAAGGGACACTTTGATGGACAACAGGGTGAAGTACCAGTTTCTATCATTA   19690
gb:MT007544|Organism:Severe      ATGTTGTAAATAAGGGACACTTTGATGGACAACAGGGTGAAGTACCAGTTTCTATCATTA   19702
gb:MN994467|Organism:Severe      ATGTTGTAAATAAGGGACACTTTGATGGACAACAGGGTGAAGTACCAGTTTCTATCATTA   19702
gb:MT044257|Organism:Severe      ATGTTGTAAATAAGGGACACTTTGATGGACAACAGGGTGAAGTACCAGTTTCTATCATTA   19702
gb:MT106054|Organism:Severe      ATGTTGTAAATAAGGGACACTTTGATGGACAACAGGGTGAAGTACCAGTTTCTATCATTA   19702
gb:MT049951|Organism:Severe      ATGTTGTAAATAAGGGACACTTTGATGGACAACAGGGTGAAGTACCAGTTTCTATCATTA   19702
gb:MN975262|Organism:Severe      ATGTTGTAAATAAGGGACACTTTGATGGACAACAGGGTGAAGTACCAGTTTCTATCATTA   19702
gb:MT106052|Organism:Severe      ATGTTGTAAATAAGGGACACTTTGATGGACAACAGGGTGAAGTACCAGTTTCTATCATTA   19702
gb:LC522975|Organism:Severe      ATGTTGTAAATAAGGGACACTTTGATGGACAACAGGGTGAAGTACCAGTTTCTATCATTA   19699
gb:LC522973|Organism:Severe      ATGTTGTAAATAAGGGACACTTTGATGGACAACAGGGTGAAGTACCAGTTTCTATCATTA   19699
gb:LC522974|Organism:Severe      ATGTTGTAAATAAGGGACACTTTGATGGACAACAGGGTGAAGTACCAGTTTCTATCATTA   19699
gb:MN985325|Organism:Severe      ATGTTGTAAATAAGGGACACTTTGATGGACAACAGGGTGAAGTACCAGTTTCTATCATTA   19702
gb:MT020881|Organism:Severe      ATGTTGTAAATAAGGGACACTTTGATGGACAACAGGGTGAAGTACCAGTTTCTATCATTA   19702
gb:MT020880|Organism:Severe      ATGTTGTAAATAAGGGACACTTTGATGGACAACAGGGTGAAGTACCAGTTTCTATCATTA   19702
gb:MT066175|Organism:Severe      ATGTTGTAAATAAGGGACACTTTGATGGACAACAGGGTGAAGTACCAGTTTCTATCATTA   19702
gb:MN997409|Organism:Severe      ATGTTGTAAATAAGGGACACTTTGATGGACAACAGGGTGAAGTACCAGTTTCTATCATTA   19702
gb:MN938384|Organism:Severe      ATGTTGTAAATAAGGGACACTTTGATGGACAACAGGGTGAAGTACCAGTTTCTATCATTA   19670
gb:MT044258|Organism:Severe      ATGTTGTAAATAAGGGACACTTTGATGGACAACAGGGTGAAGTACCAGTTTCTATCATTA   19678
gb:MT039890|Organism:Severe      ATGTTGTAAATAAGGGACACTTTGATGGACAACAGGGTGAAGTACCAGTTTCTATCATTA   19702
gb:MN988713|Organism:Severe      ATGTTGTAAATAAGGGACACTTTGATGGACAACAGGGTGAAGTACCAGTTTCTATCATTA   19702
gb:LC521925|Organism:Severe      ATGTTGTAAATAAGGGACACTTTGATGGACAACAGGGTGAAGTACCAGTTTCTATCATTA   19675
gb:MT093571|Organism:Severe      ATGTTGTAAATAAGGGACACTTTGATGGACAACAGGGTGAAGTACCAGTTTCTATCATTA   19702
gb:MT039887|Organism:Severe      ATGTTGTAAATAAGGGACACTTTGATGGACAACAGGGTGAAGTACCAGTTTCTATCATTA   19702
gb:MT019530|Organism:Severe      ATGTTGTAAATAAGGGACACTTTGATGGACAACAGGGTGAAGTACCAGTTTCTATCATTA   19702
gb:MT039888|Organism:Severe      ATGTTGTAAATAAGGGACACTTTGATGGACAACAGGGTGAAGTACCAGTTTCTATCATTA   19702
gb:LC522972|Organism:Severe      ATGTTGTAAATAAGGGACACTTTGATGGACAACAGGGTGAAGTACCAGTTTCTATCATTA   19699
gb:MT027063|Organism:Severe      ATGTTGTAAATAAGGGACACTTTGATGGACAACAGGGTGAAGTACCAGTTTCTATCATTA   19702
gb:MT027062|Organism:Severe      ATGTTGTAAATAAGGGACACTTTGATGGACAACAGGGTGAAGTACCAGTTTCTATCATTA   19702
gb:MT019529|Organism:Severe      ATGTTGTAAATAAGGGACACTTTGATGGACAACAGGGTGAAGTACCAGTTTCTATCATTA   19702
gb:MN996529|Organism:Severe      ATGTTGTAAATAAGGGACACTTTGATGGACAACAGGGTGAAGTACCAGTTTCTATCATTA   19690
gb:MN996531|Organism:Severe      ATGTTGTAAATAAGGGACACTTTGATGGACAACAGGGTGAAGTACCAGTTTCTATCATTA   19689
gb:MT066176|Organism:Severe      ATGTTGTAAATAAGGGACACTTTGATGGACAACAGGGTGAAGTACCAGTTTCTATCATTA   19702
gb:MT027064|Organism:Severe      ATGTTGTAAATAAGGGACACTTTGATGGACAACAGGGTGAAGTACCAGTTTCTATCATTA   19702
gb:MN994468|Organism:Severe      ATGTTGTAAATAAGGGACACTTTGATGGACAACAGGGTGAAGTACCAGTTTCTATCATTA   19702
gb:MT072688|Organism:Severe      ATGTTGTAAATAAGGGACACTTTGATGGACAACAGGGTGAAGTACCAGTTTCTATCATTA   19687
gb:MN996527|Organism:Severe      ATGTTGTAAATAAGGGACACTTTGATGGACAACAGGGTGAAGTACCAGTTTCTATCATTA   19669
gb:MT093631|Organism:Severe      ATGTTGTAAATAAGGGACACTTTGATGGACAACAGGGTGAAGTACCAGTTTCTATCATTA   19740
gb:MT106053|Organism:Severe      ATGTTGTAAATAAGGGACACTTTGATGGACAACAGGGTGAAGTACCAGTTTCTATCATTA   19702
gb:MT019533|Organism:Severe      ATGTTGTAAATAAGGGACACTTTGATGGACAACAGGGTGAAGTACCAGTTTCTATCATTA   19702
gb:MT019531|Organism:Severe      ATGTTGTAAATAAGGGACACTTTGATGGACAACAGGGTGAAGTACCAGTTTCTATCATTA   19702
gb:MN996528|Organism:Severe      ATGTTGTAAATAAGGGACACTTTGATGGACAACAGGGTGAAGTACCAGTTTCTATCATTA   19702
gb:MN996530|Organism:Severe      ATGTTGTAAATAAGGGACACTTTGATGGACAACAGGGTGAAGTACCAGTTTCTATCATTA   19688
gb:MN908947|Organism:Severe      ATGTTGTAAATAAGGGACACTTTGATGGACAACAGGGTGAAGTACCAGTTTCTATCATTA   19702
gb:MT019532|Organism:Severe      ATGTTGTAAATAAGGGACACTTTGATGGACAACAGGGTGAAGTACCAGTTTCTATCATTA   19702
                                 ************************************************************

gb:MT020781|Organism:Severe      ATAACACTGTTTACACAAAAGTTGATGGTGTTGATGTAGAATTGTTTGAAAATAAAACAA   19750
gb:MT007544|Organism:Severe      ATAACACTGTTTACACAAAAGTTGATGGTGTTGATGTAGAATTGTTTGAAAATAAAACAA   19762
gb:MN994467|Organism:Severe      ATAACACTGTTTACACAAAAGTTGATGGTGTTGATGTAGAATTGTTTGAAAATAAAACAA   19762
gb:MT044257|Organism:Severe      ATAACACTGTTTACACAAAAGTTGATGGTGTTGATGTAGAATTGTTTGAAAATAAAACAA   19762
gb:MT106054|Organism:Severe      ATAACACTGTTTACACAAAAGTTGATGGTGTTGATGTAGAATTGTTTGAAAATAAAACAA   19762
gb:MT049951|Organism:Severe      ATAACACTGTTTACACAAAAGTTGATGGTGTTGATGTAGAATTGTTTGAAAATAAAACAA   19762
gb:MN975262|Organism:Severe      ATAACACTGTTTACACAAAAGTTGATGGTGTTGATGTAGAATTGTTTGAAAATAAAACAA   19762
```

```
gb:MT106052|Organism:Severe      ATAACACTGTTTACACAAAAGTTGATGGTGTTGATGTAGAATTGTTTGAAAATAAAACAA    19762
gb:LC522975|Organism:Severe      ATAACACTGTTTACACAAAAGTTGATGGTGTTGATGTAGAATTGTTTGAAAATAAAACAA    19759
gb:LC522973|Organism:Severe      ATAACACTGTTTACACAAAAGTTGATGGTGTTGATGTAGAATTGTTTGAAAATAAAACAA    19759
gb:LC522974|Organism:Severe      ATAACACTGTTTACACAAAAGTTGATGGTGTTGATGTAGAATTGTTTGAAAATAAAACAA    19759
gb:MN985325|Organism:Severe      ATAACACTGTTTACACAAAAGTTGATGGTGTTGATGTAGAATTGTTTGAAAATAAAACAA    19762
gb:MT020881|Organism:Severe      ATAACACTGTTTACACAAAAGTTGATGGTGTTGATGTAGAATTGTTTGAAAATAAAACAA    19762
gb:MT020880|Organism:Severe      ATAACACTGTTTACACAAAAGTTGATGGTGTTGATGTAGAATTGTTTGAAAATAAAACAA    19762
gb:MT066175|Organism:Severe      ATAACACTGTTTACACAAAAGTTGATGGTGTTGATGTAGAATTGTTTGAAAATAAAACAA    19762
gb:MN997409|Organism:Severe      ATAACACTGTTTACACAAAAGTTGATGGTGTTGATGTAGAATTGTTTGAAAATAAAACAA    19762
gb:MN938384|Organism:Severe      ATAACACTGTTTACACAAAAGTTGATGGTGTTGATGTAGAATTGTTTGAAAATAAAACAA    19730
gb:MT044258|Organism:Severe      ATAACACTGTTTACACAAAAGTTGATGGTGTTGATGTAGAATTGTTTGAAAATAAAACAA    19738
gb:MT039890|Organism:Severe      ATAACACTGTTTACACAAAAGTTGATGGTGTTGATGTAGAATTGTTTGAAAATAAAACAA    19762
gb:MN988713|Organism:Severe      ATAACACTGTTTACACAAAAGTTGATGGTGTTGATGTAGAATTGTTTGAAAATAAAACAA    19762
gb:LC521925|Organism:Severe      ATAACACTGTTTACACAAAAGTTGATGGTGTTGATGTAGAATTGTTTGAAAATAAAACAA    19735
gb:MT093571|Organism:Severe      ATAACACTGTTTACACAAAAGTTGATGGTGTTGATGTAGAATTGTTTGAAAATAAAACAA    19762
gb:MT039887|Organism:Severe      ATAACACTGTTTACACAAAAGTTGATGGTGTTGATGTAGAATTGTTTGAAAATAAAACAA    19762
gb:MT019530|Organism:Severe      ATAACACTGTTTACACAAAAGTTGATGGTGTTGATGTAGAATTGTTTGAAAATAAAACAA    19762
gb:MT039888|Organism:Severe      ATAACACTGTTTACACAAAAGTTGATGGTGTTGATGTAGAATTGTTTGAAAATAAAACAA    19762
gb:LC522972|Organism:Severe      ATAACACTGTTTACACAAAAGTTGATGGTGTTGATGTAGAATTGTTTGAAAATAAAACAA    19759
gb:MT027063|Organism:Severe      ATAACACTGTTTACACAAAAGTTGATGGTGTTGATGTAGAATTGTTTGAAAATAAAACAA    19762
gb:MT027062|Organism:Severe      ATAACACTGTTTACACAAAAGTTGATGGTGTTGATGTAGAATTGTTTGAAAATAAAACAA    19762
gb:MT019529|Organism:Severe      ATAACACTGTTTACACAAAAGTTGATGGTGTTGATGTAGAATTGTTTGAAAATAAAACAA    19762
gb:MN996529|Organism:Severe      ATAACACTGTTTACACAAAAGTTGATGGTGTTGATGTAGAATTGTTTGAAAATAAAACAA    19750
gb:MN996531|Organism:Severe      ATAACACTGTTTACACAAAAGTTGATGGTGTTGATGTAGAATTGTTTGAAAATAAAACAA    19749
gb:MT066176|Organism:Severe      ATAACACTGTTTACACAAAAGTTGATGGTGTTGATGTAGAATTGTTTGAAAATAAAACAA    19762
gb:MT027064|Organism:Severe      ATAACACTGTTTACACAAAAGTTGATGGTGTTGATGTAGAATTGTTTGAAAATAAAACAA    19762
gb:MN994468|Organism:Severe      ATAACACTGTTTACACAAAAGTTGATGGTGTTGATGTAGAATTGTTTGAAAATAAAACAA    19762
gb:MT072688|Organism:Severe      ATAACACTGTTTACACAAAAGTTGATGGTGTTGATGTAGAATTGTTTGAAAATAAAACAA    19747
gb:MN996527|Organism:Severe      ATAACACTGTTTACACAAAAGTTGATGGTGTTGATGTAGAATTGTTTGAAAATAAAACAA    19729
gb:MT093631|Organism:Severe      ATAACACTGTTTACACAAAAGTTGATGGTGTTGATGTAGAATTGTTTGAAAATAAAACAA    19800
gb:MT106053|Organism:Severe      ATAACACTGTTTACACAAAAGTTGATGGTGTTGATGTAGAATTGTTTGAAAATAAAACAA    19762
gb:MT019533|Organism:Severe      ATAACACTGTTTACACAAAAGTTGATGGTGTTGATGTAGAATTGTTTGAAAATAAAACAA    19762
gb:MT019531|Organism:Severe      ATAACACTGTTTACACAAAAGTTGATGGTGTTGATGTAGAATTGTTTGAAAATAAAACAA    19762
gb:MN996528|Organism:Severe      ATAACACTGTTTACACAAAAGTTGATGGTGTTGATGTAGAATTGTTTGAAAATAAAACAA    19762
gb:MN996530|Organism:Severe      ATAACACTGTTTACACAAAAGTTGATGGTGTTGATGTAGAATTGTTTGAAAATAAAACAA    19748
gb:MN908947|Organism:Severe      ATAACACTGTTTACACAAAAGTTGATGGTGTTGATGTAGAATTGTTTGAAAATAAAACAA    19762
gb:MT019532|Organism:Severe      ATAACACTGTTTACACAAAAGTTGATGGTGTTGATGTAGAATTGTTTGAAAATAAAACAA    19762
                                 ************************************************************

gb:MT020781|Organism:Severe      CATTACCTGTTAATGTAGCATTTGAGCTTTGGGCTAAGCGCAACATTAAACCAGTACCAG    19810
gb:MT007544|Organism:Severe      CATTACCTGTTAATGTAGCATTTGAGCTTTGGGCTAAGCGCAACATTAAACCAGTACCAG    19822
gb:MN994467|Organism:Severe      CATTACCTGTTAATGTAGCATTTGAGCTTTGGGCTAAGCGCAACATTAAACCAGTACCAG    19822
gb:MT044257|Organism:Severe      CATTACCTGTTAATGTAGCATTTGAGCTTTGGGCTAAGCGCAACATTAAACCAGTACCAG    19822
gb:MT106054|Organism:Severe      CATTACCTGTTAATGTAGCATTTGAGCTTTGGGCTAAGCGCAACATTAAACCAGTACCAG    19822
gb:MT049951|Organism:Severe      CATTACCTGTTAATGTAGCATTTGAGCTTTGGGCTAAGCGCAACATTAAACCAGTACCAG    19822
gb:MN975262|Organism:Severe      CATTACCTGTTAATGTAGCATTTGAGCTTTGGGCTAAGCGCAACATTAAACCAGTACCAG    19822
gb:MT106052|Organism:Severe      CATTACCTGTTAATGTAGCATTTGAGCTTTGGGCTAAGCGCAACATTAAACCAGTACCAG    19822
gb:LC522975|Organism:Severe      CATTACCTGTTAATGTAGCATTTGAGCTTTGGGCTAAGCGCAACATTAAACCAGTACCAG    19819
gb:LC522973|Organism:Severe      CATTACCTGTTAATGTAGCATTTGAGCTTTGGGCTAAGCGCAACATTAAACCAGTACCAG    19819
gb:LC522974|Organism:Severe      CATTACCTGTTAATGTAGCATTTGAGCTTTGGGCTAAGCGCAACATTAAACCAGTACCAG    19819
gb:MN985325|Organism:Severe      CATTACCTGTTAATGTAGCATTTGAGCTTTGGGCTAAGCGCAACATTAAACCAGTACCAG    19822
gb:MT020881|Organism:Severe      CATTACCTGTTAATGTAGCATTTGAGCTTTGGGCTAAGCGCAACATTAAACCAGTACCAG    19822
gb:MT020880|Organism:Severe      CATTACCTGTTAATGTAGCATTTGAGCTTTGGGCTAAGCGCAACATTAAACCAGTACCAG    19822
gb:MT066175|Organism:Severe      CATTACCTGTTAATGTAGCATTTGAGCTTTGGGCTAAGCGCAACATTAAACCAGTACCAG    19822
gb:MN997409|Organism:Severe      CATTACCTGTTAATGTAGCATTTGAGCTTTGGGCTAAGCGCAACATTAAACCAGTACCAG    19822
gb:MN938384|Organism:Severe      CATTACCTGTTAATGTAGCATTTGAGCTTTGGGCTAAGCGCAACATTAAACCAGTACCAG    19790
gb:MT044258|Organism:Severe      CATTACCTGTTAATGTAGCATTTGAGCTTTGGGCTAAGCGCAACATTAAACCAGTACCAG    19798
gb:MT039890|Organism:Severe      CATTACCTGTTAATGTAGCATTTGAGCTTTGGGCTAAGCGCAACATTAAACCAGTACCAG    19822
gb:MN988713|Organism:Severe      CATTACCTGTTAATGTAGCATTTGAGCTTTGGGCTAAGCGCAACATTAAACCAGTACCAG    19822
gb:LC521925|Organism:Severe      CATTACCTGTTAATGTAGCATTTGAGCTTTGGGCTAAGCGCAACATTAAACCAGTACCAG    19795
gb:MT093571|Organism:Severe      CATTACCTGTTAATGTAGCATTTGAGCTTTGGGCTAAGCGCAACATTAAACCAGTACCAG    19822
gb:MT039887|Organism:Severe      CATTACCTGTTAATGTAGCATTTGAGCTTTGGGCTAAGCGCAACATTAAACCAGTACCAG    19822
gb:MT019530|Organism:Severe      CATTACCTGTTAATGTAGCATTTGAGCTTTGGGCTAAGCGCAACATTAAACCAGTACCAG    19822
gb:MT039888|Organism:Severe      CATTACCTGTTAATGTAGCATTTGAGCTTTGGGCTAAGCGCAACATTAAACCAGTACCAG    19822
gb:LC522972|Organism:Severe      CATTACCTGTTAATGTAGCATTTGAGCTTTGGGCTAAGCGCAACATTAAACCAGTACCAG    19819
```

```
gb:MT027063|Organism:Severe      CATTACCTGTTAATGTAGCATTTGAGCTTTGGGCTAAGCGCAACATTAAACCAGTACCAG   19822
gb:MT027062|Organism:Severe      CATTACCTGTTAATGTAGCATTTGAGCTTTGGGCTAAGCGCAACATTAAACCAGTACCAG   19822
gb:MT019529|Organism:Severe      CATTACCTGTTAATGTAGCATTTGAGCTTTGGGCTAAGCGCAACATTAAACCAGTACCAG   19822
gb:MN996529|Organism:Severe      CATTACCTGTTAATGTAGCATTTGAGCTTTGGGCTAAGCGCAACATTAAACCAGTACCAG   19810
gb:MN996531|Organism:Severe      CATTACCTGTTAATGTAGCATTTGAGCTTTGGGCTAAGCGCAACATTAAACCAGTACCAG   19809
gb:MT066176|Organism:Severe      CATTACCTGTTAATGTAGCATTTGAGCTTTGGGCTAAGCGCAACATTAAACCAGTACCAG   19822
gb:MT027064|Organism:Severe      CATTACCTGTTAATGTAGCATTTGAGCTTTGGGCTAAGCGCAACATTAAACCAGTACCAG   19822
gb:MN994468|Organism:Severe      CATTACCTGTTAATGTAGCATTTGAGCTTTGGGCTAAGCGCAACATTAAACCAGTACCAG   19822
gb:MT072688|Organism:Severe      CATTACCTGTTAATGTAGCATTTGAGCTTTGGGCTAAGCGCAACATTAAACCAGTACCAG   19807
gb:MN996527|Organism:Severe      CATTACCTGTTAATGTAGCATTTGAGCTTTGGGCTAAGCGCAACATTAAACCAGTACCAG   19789
gb:MT093631|Organism:Severe      CATTACCTGTTAATGTAGCATTTGAGCTTTGGGCTAAGCGCAACATTAAACCAGTACCAG   19860
gb:MT106053|Organism:Severe      CATTACCTGTTAATGTAGCATTTGAGCTTTGGGCTAAGCGCAACATTAAACCAGTACCAG   19822
gb:MT019533|Organism:Severe      CATTACCTGTTAATGTAGCATTTGAGCTTTGGGCTAAGCGCAACATTAAACCAGTACCAG   19822
gb:MT019531|Organism:Severe      CATTACCTGTTAATGTAGCATTTGAGCTTTGGGCTAAGCGCAACATTAAACCAGTACCAG   19822
gb:MN996528|Organism:Severe      CATTACCTGTTAATGTAGCATTTGAGCTTTGGGCTAAGCGCAACATTAAACCAGTACCAG   19822
gb:MN996530|Organism:Severe      CATTACCTGTTAATGTAGCATTTGAGCTTTGGGCTAAGCGCAACATTAAACCAGTACCAG   19808
gb:MN908947|Organism:Severe      CATTACCTGTTAATGTAGCATTTGAGCTTTGGGCTAAGCGCAACATTAAACCAGTACCAG   19822
gb:MT019532|Organism:Severe      CATTACCTGTTAATGTAGCATTTGAGCTTTGGGCTAAGCGCAACATTAAACCAGTACCAG   19822
                                 ************************************************************

gb:MT020781|Organism:Severe      AGGTGAAAATACTCAATAATTTGGGTGTGGACATTGCTGCTAATACTGTGATCTGGGACT   19870
gb:MT007544|Organism:Severe      AGGTGAAAATACTCAATAATTTGGGTGTGGACATTGCTGCTAATACTGTGATCTGGGACT   19882
gb:MN994467|Organism:Severe      AGGTGAAAATACTCAATAATTTGGGTGTGGACATTGCTGCTAATACTGTGATCTGGGACT   19882
gb:MT044257|Organism:Severe      AGGTGAAAATACTCAATAATTTGGGTGTGGACATTGCTGCTAATACTGTGATCTGGGACT   19882
gb:MT106054|Organism:Severe      AGGTGAAAATACTCAATAATTTGGGTGTGGACATTGCTGCTAATACTGTGATCTGGGACT   19882
gb:MT049951|Organism:Severe      AGGTGAAAATACTCAATAATTTGGGTGTGGACATTGCTGCTAATACTGTGATCTGGGACT   19882
gb:MN975262|Organism:Severe      AGGTGAAAATACTCAATAATTTGGGTGTGGACATTGCTGCTAATACTGTGATCTGGGACT   19882
gb:MT106052|Organism:Severe      AGGTGAAAATACTCAATAATTTGGGTGTGGACATTGCTGCTAATACTGTGATCTGGGACT   19882
gb:LC522975|Organism:Severe      AGGTGAAAATACTCAATAATTTGGGTGTGGACATTGCTGCTAATACTGTGATCTGGGACT   19879
gb:LC522973|Organism:Severe      AGGTGAAAATACTCAATAATTTGGGTGTGGACATTGCTGCTAATACTGTGATCTGGGACT   19879
gb:LC522974|Organism:Severe      AGGTGAAAATACTCAATAATTTGGGTGTGGACATTGCTGCTAATACTGTGATCTGGGACT   19879
gb:MN985325|Organism:Severe      AGGTGAAAATACTCAATAATTTGGGTGTGGACATTGCTGCTAATACTGTGATCTGGGACT   19882
gb:MT020881|Organism:Severe      AGGTGAAAATACTCAATAATTTGGGTGTGGACATTGCTGCTAATACTGTGATCTGGGACT   19882
gb:MT020880|Organism:Severe      AGGTGAAAATACTCAATAATTTGGGTGTGGACATTGCTGCTAATACTGTGATCTGGGACT   19882
gb:MT066175|Organism:Severe      AGGTGAAAATACTCAATAATTTGGGTGTGGACATTGCTGCTAATACTGTGATCTGGGACT   19882
gb:MN997409|Organism:Severe      AGGTGAAAATACTCAATAATTTGGGTGTGGACATTGCTGCTAATACTGTGATCTGGGACT   19882
gb:MN938384|Organism:Severe      AGGTGAAAATACTCAATAATTTGGGTGTGGACATTGCTGCTAATACTGTGATCTGGGACT   19850
gb:MT044258|Organism:Severe      AGGTGAAAATACTCAATAATTTGGGTGTGGACATTGCTGCTAATACTGTGATCTGGGACT   19858
gb:MT039890|Organism:Severe      AGGTGAAAATACTCAATAATTTGGGTGTGGACATTGCTGCTAATACTGTGATCTGGGACT   19882
gb:MN988713|Organism:Severe      AGGTGAAAATACTCAATAATTTGGGTGTGGACATTGCTGCTAATACTGTGATCTGGGACT   19882
gb:LC521925|Organism:Severe      AGGTGAAAATACTCAATAATTTGGGTGTGGACATTGCTGCTAATACTGTGATCTGGGACT   19855
gb:MT093571|Organism:Severe      AGGTGAAAATACTCAATAATTTGGGTGTGGACATTGCTGCTAATACTGTGATCTGGGACT   19882
gb:MT039887|Organism:Severe      AGGTGAAAATACTCAATAATTTGGGTGTGGACATTGCTGCTAATACTGTGATCTGGGACT   19882
gb:MT019530|Organism:Severe      AGGTGAAAATACTCAATAATTTGGGTGTGGACATTGCTGCTAATACTGTGATCTGGGACT   19882
gb:MT039888|Organism:Severe      AGGTGAAAATACTCAATAATTTGGGTGTGGACATTGCTGCTAATACTGTGATCTGGGACT   19882
gb:LC522972|Organism:Severe      AGGTGAAAATACTCAATAATTTGGGTGTGGACATTGCTGCTAATACTGTGATCTGGGACT   19879
gb:MT027063|Organism:Severe      AGGTGAAAATACTCAATAATTTGGGTGTGGACATTGCTGCTAATACTGTGATCTGGGACT   19882
gb:MT027062|Organism:Severe      AGGTGAAAATACTCAATAATTTGGGTGTGGACATTGCTGCTAATACTGTGATCTGGGACT   19882
gb:MT019529|Organism:Severe      AGGTGAAAATACTCAATAATTTGGGTGTGGACATTGCTGCTAATACTGTGATCTGGGACT   19882
gb:MN996529|Organism:Severe      AGGTGAAAATACTCAATAATTTGGGTGTGGACATTGCTGCTAATACTGTGATCTGGGACT   19870
gb:MN996531|Organism:Severe      AGGTGAAAATACTCAATAATTTGGGTGTGGACATTGCTGCTAATACTGTGATCTGGGACT   19869
gb:MT066176|Organism:Severe      AGGTGAAAATACTCAATAATTTGGGTGTGGACATTGCTGCTAATACTGTGATCTGGGACT   19882
gb:MT027064|Organism:Severe      AGGTGAAAATACTCAATAATTTGGGTGTGGACATTGCTGCTAATACTGTGATCTGGGACT   19882
gb:MN994468|Organism:Severe      AGGTGAAAATACTCAATAATTTGGGTGTGGACATTGCTGCTAATACTGTGATCTGGGACT   19882
gb:MT072688|Organism:Severe      AGGTGAAAATACTCAATAATTTGGGTGTGGACATTGCTGCTAATACTGTGATCTGGGACT   19867
gb:MN996527|Organism:Severe      AGGTGAAAATACTCAATAATTTGGGTGTGGACATTGCTGCTAATACTGTGATCTGGGACT   19849
gb:MT093631|Organism:Severe      AGGTGAAAATACTCAATAATTTGGGTGTGGACATTGCTGCTAATACTGTGATCTGGGACT   19920
gb:MT106053|Organism:Severe      AGGTGAAAATACTCAATAATTTGGGTGTGGACATTGCTGCTAATACTGTGATCTGGGACT   19882
gb:MT019533|Organism:Severe      AGGTGAAAATACTCAATAATTTGGGTGTGGACATTGCTGCTAATACTGTGATCTGGGACT   19882
gb:MT019531|Organism:Severe      AGGTGAAAATACTCAATAATTTGGGTGTGGACATTGCTGCTAATACTGTGATCTGGGACT   19882
gb:MN996528|Organism:Severe      AGGTGAAAATACTCAATAATTTGGGTGTGGACATTGCTGCTAATACTGTGATCTGGGACT   19882
gb:MN996530|Organism:Severe      AGGTGAAAATACTCAATAATTTGGGTGTGGACATTGCTGCTAATACTGTGATCTGGGACT   19868
gb:MN908947|Organism:Severe      AGGTGAAAATACTCAATAATTTGGGTGTGGACATTGCTGCTAATACTGTGATCTGGGACT   19882
gb:MT019532|Organism:Severe      AGGTGAAAATACTCAATAATTTGGGTGTGGACATTGCTGCTAATACTGTGATCTGGGACT   19882
                                 ************************************************************
```

```
gb:MT020781|Organism:Severe      ACAAAAGAGATGCTCCAGCACATATATCTACTATTGGTGTTTGTTCTATGACTGACATAG   19930
gb:MT007544|Organism:Severe      ACAAAAGAGATGCTCCAGCACATATATCTACTATTGGTGTTTGTTCTATGACTGACATAG   19942
gb:MN994467|Organism:Severe      ACAAAAGAGATGCTCCAGCACATATATCTACTATTGGTGTTTGTTCTATGACTGACATAG   19942
gb:MT044257|Organism:Severe      ACAAAAGAGATGCTCCAGCACATATATCTACTATTGGTGTTTGTTCTATGACTGACATAG   19942
gb:MT106054|Organism:Severe      ACAAAAGAGATGCTCCAGCACATATATCTACTATTGGTGTTTGTTCTATGACTGACATAG   19942
gb:MT049951|Organism:Severe      ACAAAAGAGATGCTCCAGCACATATATCTACTATTGGTGTTTGTTCTATGACTGACATAG   19942
gb:MN975262|Organism:Severe      ACAAAAGAGATGCTCCAGCACATATATCTACTATTGGTGTTTGTTCTATGACTGACATAG   19942
gb:MT106052|Organism:Severe      ACAAAAGAGATGCTCCAGCACATATATCTACTATTGGTGTTTGTTCTATGACTGACATAG   19942
gb:LC522975|Organism:Severe      ACAAAAGAGATGCTCCAGCACATATATCTACTATTGGTGTTTGTTCTATGACTGACATAG   19939
gb:LC522973|Organism:Severe      ACAAAAGAGATGCTCCAGCACATATATCTACTATTGGTGTTTGTTCTATGACTGACATAG   19939
gb:LC522974|Organism:Severe      ACAAAAGAGATGCTCCAGCACATATATCTACTATTGGTGTTTGTTCTATGACTGACATAG   19939
gb:MN985325|Organism:Severe      ACAAAAGAGATGCTCCAGCACATATATCTACTATTGGTGTTTGTTCTATGACTGACATAG   19942
gb:MT020881|Organism:Severe      ACAAAAGAGATGCTCCAGCACATATATCTACTATTGGTGTTTGTTCTATGACTGACATAG   19942
gb:MT020880|Organism:Severe      ACAAAAGAGATGCTCCAGCACATATATCTACTATTGGTGTTTGTTCTATGACTGACATAG   19942
gb:MT066175|Organism:Severe      ACAAAAGAGATGCTCCAGCACATATATCTACTATTGGTGTTTGTTCTATGACTGACATAG   19942
gb:MN997409|Organism:Severe      ACAAAAGAGATGCTCCAGCACATATATCTACTATTGGTGTTTGTTCTATGACTGACATAG   19942
gb:MN938384|Organism:Severe      ACAAAAGAGATGCTCCAGCACATATATCTACTATTGGTGTTTGTTCTATGACTGACATAG   19910
gb:MT044258|Organism:Severe      ACAAAAGAGATGCTCCAGCACATATATCTACTATTGGTGTTTGTTCTATGACTGACATAG   19918
gb:MT039890|Organism:Severe      ACAAAAGAGATGCTCCAGCACATATATCTACTATTGGTGTTTGTTCTATGACTGACATAG   19942
gb:MN988713|Organism:Severe      ACAAAAGAGATGCTCCAGCACATATATCTACTATTGGTGTTTGTTCTATGACTGACATAG   19942
gb:LC521925|Organism:Severe      ACAAAAGAGATGCTCCAGCACATATATCTACTATTGGTGTTTGTTCTATGACTGACATAG   19915
gb:MT093571|Organism:Severe      ACAAAAGAGATGCTCCAGCACATATATCTACTATTGGTGTTTGTTCTATGACTGACATAG   19942
gb:MT039887|Organism:Severe      ACAAAAGAGATGCTCCAGCACATATATCTACTATTGGTGTTTGTTCTATGACTGACATAG   19942
gb:MT019530|Organism:Severe      ACAAAAGAGATGCTCCAGCACATATATCTACTATTGGTGTTTGTTCTATGACTGACATAG   19942
gb:MT039888|Organism:Severe      ACAAAAGAGATGCTCCAGCACATATATCTACTATTGGTGTTTGTTCTATGACTGACATAG   19942
gb:LC522972|Organism:Severe      ACAAAAGAGATGCTCCAGCACATATATCTACTATTGGTGTTTGTTCTATGACTGACATAG   19939
gb:MT027063|Organism:Severe      ACAAAAGAGATGCTCCAGCACATATATCTACTATTGGTGTTTGTTCTATGACTGACATAG   19942
gb:MT027062|Organism:Severe      ACAAAAGAGATGCTCCAGCACATATATCTACTATTGGTGTTTGTTCTATGACTGACATAG   19942
gb:MT019529|Organism:Severe      ACAAAAGAGATGCTCCAGCACATATATCTACTATTGGTGTTTGTTCTATGACTGACATAG   19942
gb:MN996529|Organism:Severe      ACAAAAGAGATGCTCCAGCACATATATCTACTATTGGTGTTTGTTCTATGACTGACATAG   19930
gb:MN996531|Organism:Severe      ACAAAAGAGATGCTCCAGCACATATATCTACTATTGGTGTTTGTTCTATGACTGACATAG   19929
gb:MT066176|Organism:Severe      ACAAAAGAGATGCTCCAGCACATATATCTACTATTGGTGTTTGTTCTATGACTGACATAG   19942
gb:MT027064|Organism:Severe      ACAAAAGAGATGCTCCAGCACATATATCTACTATTGGTGTTTGTTCTATGACTGACATAG   19942
gb:MN994468|Organism:Severe      ACAAAAGAGATGCTCCAGCACATATATCTACTATTGGTGTTTGTTCTATGACTGACATAG   19942
gb:MT072688|Organism:Severe      ACAAAAGAGATGCTCCAGCACATATATCTACTATTGGTGTTTGTTCTATGACTGACATAG   19927
gb:MN996527|Organism:Severe      ACAAAAGAGATGCTCCAGCACATATATCTACTATTGGTGTTTGTTCTATGACTGACATAG   19909
gb:MT093631|Organism:Severe      ACAAAAGAGATGCTCCAGCACATATATCTACTATTGGTGTTTGTTCTATGACTGACATAG   19980
gb:MT106053|Organism:Severe      ACAAAAGAGATGCTCCAGCACATATATCTACTATTGGTGTTTGTTCTATGACTGACATAG   19942
gb:MT019533|Organism:Severe      ACAAAAGAGATGCTCCAGCACATATATCTACTATTGGTGTTTGTTCTATGACTGACATAG   19942
gb:MT019531|Organism:Severe      ACAAAAGAGATGCTCCAGCACATATATCTACTATTGGTGTTTGTTCTATGACTGACATAG   19942
gb:MN996528|Organism:Severe      ACAAAAGAGATGCTCCAGCACATATATCTACTATTGGTGTTTGTTCTATGACTGACATAG   19942
gb:MN996530|Organism:Severe      ACAAAAGAGATGCTCCAGCACATATATCTACTATTGGTGTTTGTTCTATGACTGACATAG   19928
gb:MN908947|Organism:Severe      ACAAAAGAGATGCTCCAGCACATATATCTACTATTGGTGTTTGTTCTATGACTGACATAG   19942
gb:MT019532|Organism:Severe      ACAAAAGAGATGCTCCAGCACATATATCTACTATTGGTGTTTGTTCTATGACTGACATAG   19942
                                 ************************************************************

gb:MT020781|Organism:Severe      CCAAGAAACCAACTGAAACGATTTGTGCACCACTCACTGTCTTTTTTGATGGTAGAGTTG   19990
gb:MT007544|Organism:Severe      CCAAGAAACCAACTGAAACGATTTGTGCACCACTCACTGTCTTTTTTGATGGTAGAGTTG   20002
gb:MN994467|Organism:Severe      CCAAGAAACCAACTGAAACGATTTGTGCACCACTCACTGTCTTTTTTGATGGTAGAGTTG   20002
gb:MT044257|Organism:Severe      CCAAGAAACCAACTGAAACGATTTGTGCACCACTCACTGTCTTTTTTGATGGTAGAGTTG   20002
gb:MT106054|Organism:Severe      CCAAGAAACCAACTGAAACGATTTGTGCACCACTCACTGTCTTTTTTGATGGTAGAGTTG   20002
gb:MT049951|Organism:Severe      CCAAGAAACCAACTGAAACGATTTGTGCACCACTCACTGTCTTTTTTGATGGTAGAGTTG   20002
gb:MN975262|Organism:Severe      CCAAGAAACCAACTGAAACGATTTGTGCACCACTCACTGTCTTTTTTGATGGTAGAGTTG   20002
gb:MT106052|Organism:Severe      CCAAGAAACCAACTGAAACGATTTGTGCACCACTCACTGTCTTTTTTGATGGTAGAGTTG   20002
gb:LC522975|Organism:Severe      CCAAGAAACCAACTGAAACGATTTGTGCACCACTCACTGTCTTTTTTGATGGTAGAGTTG   19999
gb:LC522973|Organism:Severe      CCAAGAAACCAACTGAAACGATTTGTGCACCACTCACTGTCTTTTTTGATGGTAGAGTTG   19999
gb:LC522974|Organism:Severe      CCAAGAAACCAACTGAAACGATTTGTGCACCACTCACTGTCTTTTTTGATGGTAGAGTTG   19999
gb:MN985325|Organism:Severe      CCAAGAAACCAACTGAAACGATTTGTGCACCACTCACTGTCTTTTTTGATGGTAGAGTTG   20002
gb:MT020881|Organism:Severe      CCAAGAAACCAACTGAAACGATTTGTGCACCACTCACTGTCTTTTTTGATGGTAGAGTTG   20002
gb:MT020880|Organism:Severe      CCAAGAAACCAACTGAAACGATTTGTGCACCACTCACTGTCTTTTTTGATGGTAGAGTTG   20002
gb:MT066175|Organism:Severe      CCAAGAAACCAACTGAAACGATTTGTGCACCACTCACTGTCTTTTTTGATGGTAGAGTTG   20002
gb:MN997409|Organism:Severe      CCAAGAAACCAACTGAAACGATTTGTGCACCACTCACTGTCTTTTTTGATGGTAGAGTTG   20002
gb:MN938384|Organism:Severe      CCAAGAAACCAACTGAAACGATTTGTGCACCACTCACTGTCTTTTTTGATGGTAGAGTTG   19970
gb:MT044258|Organism:Severe      CCAAGAAACCAACTGAAACGATTTGTGCACCACTCACTGTCTTTTTTGATGGTAGAGTTG   19978
```

```
gb:MT039890|Organism:Severe      CCAAGAAACCAACTGAAACGATTTGTGCACCACTCACTGTCTTTTTTGATGGTAGAGTTG   20002
gb:MN988713|Organism:Severe      CCAAGAAACCAACTGAAACGATTTGTGCACCACTCACTGTCTTTTTTGATGGTAGAGTTG   20002
gb:LC521925|Organism:Severe      CCAAGAAACCAACTGAAACGATTTGTGCACCACTCACTGTCTTTTTTGATGGTAGAGTTG   19975
gb:MT093571|Organism:Severe      CCAAGAAACCAACTGAAACGATTTGTGCACCACTCACTGTCTTTTTTGATGGTAGAGTTG   20002
gb:MT039887|Organism:Severe      CCAAGAAACCAACTGAAACGATTTGTGCACCACTCACTGTCTTTTTTGATGGTAGAGTTG   20002
gb:MT019530|Organism:Severe      CCAAGAAACCAACTGAAACGATTTGTGCACCACTCACTGTCTTTTTTGATGGTAGAGTTG   20002
gb:MT039888|Organism:Severe      CCAAGAAACCAACTGAAACGATTTGTGCACCACTCACTGTCTTTTTTGATGGTAGAGTTG   20002
gb:LC522972|Organism:Severe      CCAAGAAACCAACTGAAACGATTTGTGCACCACTCACTGTCTTTTTTGATGGTAGAGTTG   19999
gb:MT027063|Organism:Severe      CCAAGAAACCAACTGAAACGATTTGTGCACCACTCACTGTCTTTTTTGATGGTAGAGTTG   20002
gb:MT027062|Organism:Severe      CCAAGAAACCAACTGAAACGATTTGTGCACCACTCACTGTCTTTTTTGATGGTAGAGTTG   20002
gb:MT019529|Organism:Severe      CCAAGAAACCAACTGAAACGATTTGTGCACCACTCACTGTCTTTTTTGATGGTAGAGTTG   20002
gb:MN996529|Organism:Severe      CCAAGAAACCAACTGAAACGATTTGTGCACCACTCACTGTCTTTTTTGATGGTAGAGTTG   19990
gb:MN996531|Organism:Severe      CCAAGAAACCAACTGAAACGATTTGTGCACCACTCACTGTCTTTTTTGATGGTAGAGTTG   19989
gb:MT066176|Organism:Severe      CCAAGAAACCAACTGAAACGATTTGTGCACCACTCACTGTCTTTTTTGATGGTAGAGTTG   20002
gb:MT027064|Organism:Severe      CCAAGAAACCAACTGAAACGATTTGTGCACCACTCACTGTCTTTTTTGATGGTAGAGTTG   20002
gb:MN994468|Organism:Severe      CCAAGAAACCAACTGAAACGATTTGTGCACCACTCACTGTCTTTTTTGATGGTAGAGTTG   20002
gb:MT072688|Organism:Severe      CCAAGAAACCAACTGAAACGATTTGTGCACCACTCACTGTCTTTTTTGATGGTAGAGTTG   19987
gb:MN996527|Organism:Severe      CCAAGAAACCAACTGAAACGATTTGTGCACCACTCACTGTCTTTTTTGATGGTAGAGTTG   19969
gb:MT093631|Organism:Severe      CCAAGAAACCAACTGAAACGATTTGTGCACCACTCACTGTCTTTTTTGATGGTAGAGTTG   20040
gb:MT106053|Organism:Severe      CCAAGAAACCAACTGAAACGATTTGTGCACCACTCACTGTCTTTTTTGATGGTAGAGTTG   20002
gb:MT019533|Organism:Severe      CCAAGAAACCAACTGAAACGATTTGTGCACCACTCACTGTCTTTTTTGATGGTAGAGTTG   20002
gb:MT019531|Organism:Severe      CCAAGAAACCAACTGAAACGATTTGTGCACCACTCACTGTCTTTTTTGATGGTAGAGTTG   20002
gb:MN996528|Organism:Severe      CCAAGAAACCAACTGAAACGATTTGTGCACCACTCACTGTCTTTTTTGATGGTAGAGTTG   20002
gb:MN996530|Organism:Severe      CCAAGAAACCAACTGAAACGATTTGTGCACCACTCACTGTCTTTTTTGATGGTAGAGTTG   19988
gb:MN908947|Organism:Severe      CCAAGAAACCAACTGAAACGATTTGTGCACCACTCACTGTCTTTTTTGATGGTAGAGTTG   20002
gb:MT019532|Organism:Severe      CCAAGAAACCAACTGAAACGATTTGTGCACCACTCACTGTCTTTTTTGATGGTAGAGTTG   20002
                                 ************************************************************

gb:MT020781|Organism:Severe      ATGGTCAAGTAGACTTATTTAGAAATGCCCGTAATGGTGTTCTTATTACAGAAGGTAGTG   20050
gb:MT007544|Organism:Severe      ATGGTCAAGTAGACTTATTTAGAAATGCCCGTAATGGTGTTCTTATTACAGAAGGTAGTG   20062
gb:MN994467|Organism:Severe      ATGGTCAAGTAGACTTATTTAGAAATGCCCGTAATGGTGTTCTTATTACAGAAGGTAGTG   20062
gb:MT044257|Organism:Severe      ATGGTCAAGTAGACTTATTTAGAAATGCCCGTAATGGTGTTCTTATTACAGAAGGTAGTG   20062
gb:MT106054|Organism:Severe      ATGGTCAAGTAGACTTATTTAGAAATGCCCGTAATGGTGTTCTTATTACAGAAGGTAGTG   20062
gb:MT049951|Organism:Severe      ATGGTCAAGTAGACTTATTTAGAAATGCCCGTAATGGTGTTCTTATTACAGAAGGTAGTG   20062
gb:MN975262|Organism:Severe      ATGGTCAAGTAGACTTATTTAGAAATGCCCGTAATGGTGTTCTTATTACAGAAGGTAGTG   20062
gb:MT106052|Organism:Severe      ATGGTCAAGTAGACTTATTTAGAAATGCCCGTAATGGTGTTCTTATTACAGAAGGTAGTG   20062
gb:LC522975|Organism:Severe      ATGGTCAAGTAGACTTATTTAGAAATGCCCGTAATGGTGTTCTTATTACAGAAGGTAGTG   20059
gb:LC522973|Organism:Severe      ATGGTCAAGTAGACTTATTTAGAAATGCCCGTAATGGTGTTCTTATTACAGAAGGTAGTG   20059
gb:LC522974|Organism:Severe      ATGGTCAAGTAGACTTATTTAGAAATGCCCGTAATGGTGTTCTTATTACAGAAGGTAGTG   20059
gb:MN985325|Organism:Severe      ATGGTCAAGTAGACTTATTTAGAAATGCCCGTAATGGTGTTCTTATTACAGAAGGTAGTG   20062
gb:MT020881|Organism:Severe      ATGGTCAAGTAGACTTATTTAGAAATGCCCGTAATGGTGTTCTTATTACAGAAGGTAGTG   20062
gb:MT020880|Organism:Severe      ATGGTCAAGTAGACTTATTTAGAAATGCCCGTAATGGTGTTCTTATTACAGAAGGTAGTG   20062
gb:MT066175|Organism:Severe      ATGGTCAAGTAGACTTATTTAGAAATGCCCGTAATGGTGTTCTTATTACAGAAGGTAGTG   20062
gb:MN997409|Organism:Severe      ATGGTCAAGTAGACTTATTTAGAAATGCCCGTAATGGTGTTCTTATTACAGAAGGTAGTG   20062
gb:MN938384|Organism:Severe      ATGGTCAAGTAGACTTATTTAGAAATGCCCGTAATGGTGTTCTTATTACAGAAGGTAGTG   20030
gb:MT044258|Organism:Severe      ATGGTCAAGTAGACTTATTTAGAAATGCCCGTAATGGTGTTCTTATTACAGAAGGTAGTG   20038
gb:MT039890|Organism:Severe      ATGGTCAAGTAGACTTATTTAGAAATGCCCGTAATGGTGTTCTTATTACAGAAGGTAGTG   20062
gb:MN988713|Organism:Severe      ATGGTCAAGTAGACTTATTTAGAAATGCCCGTAATGGTGTTCTTATTACAGAAGGTAGTG   20062
gb:LC521925|Organism:Severe      ATGGTCAAGTAGACTTATTTAGAAATGCCCGTAATGGTGTTCTTATTACAGAAGGTAGTG   20035
gb:MT093571|Organism:Severe      ATGGTCAAGTAGACTTATTTAGAAATGCCCGTAATGGTGTTCTTATTACAGAAGGTAGTG   20062
gb:MT039887|Organism:Severe      ATGGTCAAGTAGACTTATTTAGAAATGCCCGTAATGGTGTTCTTATTACAGAAGGTAGTG   20062
gb:MT019530|Organism:Severe      ATGGTCAAGTAGACTTATTTAGAAATGCCCGTAATGGTGTTCTTATTACAGAAGGTAGTG   20062
gb:MT039888|Organism:Severe      ATGGTCAAGTAGACTTATTTAGAAATGCCCGTAATGGTGTTCTTATTACAGAAGGTAGTG   20062
gb:LC522972|Organism:Severe      ATGGTCAAGTAGACTTATTTAGAAATGCCCGTAATGGTGTTCTTATTACAGAAGGTAGTG   20059
gb:MT027063|Organism:Severe      ATGGTCAAGTAGACTTATTTAGAAATGCCCGTAATGGTGTTCTTATTACAGAAGGTAGTG   20062
gb:MT027062|Organism:Severe      ATGGTCAAGTAGACTTATTTAGAAATGCCCGTAATGGTGTTCTTATTACAGAAGGTAGTG   20062
gb:MT019529|Organism:Severe      ATGGTCAAGTAGACTTATTTAGAAATGCCCGTAATGGTGTTCTTATTACAGAAGGTAGTG   20062
gb:MN996529|Organism:Severe      ATGGTCAAGTAGACTTATTTAGAAATGCCCGTAATGGTGTTCTTATTACAGAAGGTAGTG   20050
gb:MN996531|Organism:Severe      ATGGTCAAGTAGACTTATTTAGAAATGCCCGTAATGGTGTTCTTATTACAGAAGGTAGTG   20049
gb:MT066176|Organism:Severe      ATGGTCAAGTAGACTTATTTAGAAATGCCCGTAATGGTGTTCTTATTACAGAAGGTAGTG   20062
gb:MT027064|Organism:Severe      ATGGTCAAGTAGACTTATTTAGAAATGCCCGTAATGGTGTTCTTATTACAGAAGGTAGTG   20062
gb:MN994468|Organism:Severe      ATGGTCAAGTAGACTTATTTAGAAATGCCCGTAATGGTGTTCTTATTACAGAAGGTAGTG   20062
gb:MT072688|Organism:Severe      ATGGTCAAGTAGACTTATTTAGAAATGCCCGTAATGGTGTTCTTATTACAGAAGGTAGTG   20047
gb:MN996527|Organism:Severe      ATGGTCAAGTAGACTTATTTAGAAATGCCCGTAATGGTGTTCTTATTACAGAAGGTAGTG   20029
gb:MT093631|Organism:Severe      ATGGTCAAGTAGACTTATTTAGAAATGCCCGTAATGGTGTTCTTATTACAGAAGGTAGTG   20100
```

```
gb:MT106053|Organism:Severe      ATGGTCAAGTAGACTTATTTAGAAATGCCCGTAATGGTGTTCTTATTACAGAAGGTAGTG   20062
gb:MT019533|Organism:Severe      ATGGTCAAGTAGACTTATTTAGAAATGCCCGTAATGGTGTTCTTATTACAGAAGGTAGTG   20062
gb:MT019531|Organism:Severe      ATGGTCAAGTAGACTTATTTAGAAATGCCCGTAATGGTGTTCTTATTACAGAAGGTAGTG   20062
gb:MN996528|Organism:Severe      ATGGTCAAGTAGACTTATTTAGAAATGCCCGTAATGGTGTTCTTATTACAGAAGGTAGTG   20062
gb:MN996530|Organism:Severe      ATGGTCAAGTAGACTTATTTAGAAATGCCCGTAATGGTGTTCTTATTACAGAAGGTAGTG   20048
gb:MN908947|Organism:Severe      ATGGTCAAGTAGACTTATTTAGAAATGCCCGTAATGGTGTTCTTATTACAGAAGGTAGTG   20062
gb:MT019532|Organism:Severe      ATGGTCAAGTAGACTTATTTAGAAATGCCCGTAATGGTGTTCTTATTACAGAAGGTAGTG   20062
                                 ************************************************************

gb:MT020781|Organism:Severe      TTAAAGGTTTACAACCATCTGTAGGTCCCAAACAAGCTAGTCTTAATGGAGTCACATTAA   20110
gb:MT007544|Organism:Severe      TTAAAGGTTTACAACCATCTGTAGGTCCCAAACAAGCTAGTCTTAATGGAGTCACATTAA   20122
gb:MN994467|Organism:Severe      TTAAAGGTTTACAACCATCTGTAGGTCCCAAACAAGCTAGTCTTAATGGAGTCACATTAA   20122
gb:MT044257|Organism:Severe      TTAAAGGTTTACAACCATCTGTAGGTCCCAAACAAGCTAGTCTTAATGGAGTCACATTAA   20122
gb:MT106054|Organism:Severe      TTAAAGGTTTACAACCATCTGTAGGTCCCAAACAAGCTAGTCTTAATGGAGTCACATTAA   20122
gb:MT049951|Organism:Severe      TTAAAGGTTTACAACCATCTGTAGGTCCCAAACAAGCTAGTCTTAATGGAGTCACATTAA   20122
gb:MN975262|Organism:Severe      TTAAAGGTTTACAACCATCTGTAGGTCCCAAACAAGCTAGTCTTAATGGAGTCACATTAA   20122
gb:MT106052|Organism:Severe      TTAAAGGTTTACAACCATCTGTAGGTCCCAAACAAGCTAGTCTTAATGGAGTCACATTAA   20122
gb:LC522975|Organism:Severe      TTAAAGGTTTACAACCATCTGTAGGTCCCAAACAAGCTAGTCTTAATGGAGTCACATTAA   20119
gb:LC522973|Organism:Severe      TTAAAGGTTTACAACCATCTGTAGGTCCCAAACAAGCTAGTCTTAATGGAGTCACATTAA   20119
gb:LC522974|Organism:Severe      TTAAAGGTTTACAACCATCTGTAGGTCCCAAACAAGCTAGTCTTAATGGAGTCACATTAA   20119
gb:MN985325|Organism:Severe      TTAAAGGTTTACAACCATCTGTAGGTCCCAAACAAGCTAGTCTTAATGGAGTCACATTAA   20122
gb:MT020881|Organism:Severe      TTAAAGGTTTACAACCATCTGTAGGTCCCAAACAAGCTAGTCTTAATGGAGTCACATTAA   20122
gb:MT020880|Organism:Severe      TTAAAGGTTTACAACCATCTGTAGGTCCCAAACAAGCTAGTCTTAATGGAGTCACATTAA   20122
gb:MT066175|Organism:Severe      TTAAAGGTTTACAACCATCTGTAGGTCCCAAACAAGCTAGTCTTAATGGAGTCACATTAA   20122
gb:MN997409|Organism:Severe      TTAAAGGTTTACAACCATCTGTAGGTCCCAAACAAGCTAGTCTTAATGGAGTCACATTAA   20122
gb:MN938384|Organism:Severe      TTAAAGGTTTACAACCATCTGTAGGTCCCAAACAAGCTAGTCTTAATGGAGTCACATTAA   20090
gb:MT044258|Organism:Severe      TTAAAGGTTTACAACCATCTGTAGGTCCCAAACAAGCTAGTCTTAATGGAGTCACATTAA   20098
gb:MT039890|Organism:Severe      TTAAAGGTTTACAACCATCTGTAGGTCCCAAACAAGCTAGTCTTAATGGAGTCACATTAA   20122
gb:MN988713|Organism:Severe      TTAAAGGTTTACAACCATCTGTAGGTCCCAAACAAGCTAGTCTTAATGGAGTCACATTAA   20122
gb:LC521925|Organism:Severe      TTAAAGGTTTACAACCATCTGTAGGTCCCAAACAAGCTAGTCTTAATGGAGTCACATTAA   20095
gb:MT093571|Organism:Severe      TTAAAGGTTTACAACCATCTGTAGGTCCCAAACAAGCTAGTCTTAATGGAGTCACATTAA   20122
gb:MT039887|Organism:Severe      TTAAAGGTTTACAACCATCTGTAGGTCCCAAACAAGCTAGTCTTAATGGAGTCACATTAA   20122
gb:MT019530|Organism:Severe      TTAAAGGTTTACAACCATCTGTAGGTCCCAAACAAGCTAGTCTTAATGGAGTCACATTAA   20122
gb:MT039888|Organism:Severe      TTAAAGGTTTACAACCATCTGTAGGTCCCAAACAAGCTAGTCTTAATGGAGTCACATTAA   20122
gb:LC522972|Organism:Severe      TTAAAGGTTTACAACCATCTGTAGGTCCCAAACAAGCTAGTCTTAATGGAGTCACATTAA   20119
gb:MT027063|Organism:Severe      TTAAAGGTTTACAACCATCTGTAGGTCCCAAACAAGCTAGTCTTAATGGAGTCACATTAA   20122
gb:MT027062|Organism:Severe      TTAAAGGTTTACAACCATCTGTAGGTCCCAAACAAGCTAGTCTTAATGGAGTCACATTAA   20122
gb:MT019529|Organism:Severe      TTAAAGGTTTACAACCATCTGTAGGTCCCAAACAAGCTAGTCTTAATGGAGTCACATTAA   20122
gb:MN996529|Organism:Severe      TTAAAGGTTTACAACCATCTGTAGGTCCCAAACAAGCTAGTCTTAATGGAGTCACATTAA   20110
gb:MN996531|Organism:Severe      TTAAAGGTTTACAACCATCTGTAGGTCCCAAACAAGCTAGTCTTAATGGAGTCACATTAA   20109
gb:MT066176|Organism:Severe      TTAAAGGTTTACAACCATCTGTAGGTCCCAAACAAGCTAGTCTTAATGGAGTCACATTAA   20122
gb:MT027064|Organism:Severe      TTAAAGGTTTACAACCATCTGTAGGTCCCAAACAAGCTAGTCTTAATGGAGTCACATTAA   20122
gb:MN994468|Organism:Severe      TTAAAGGTTTACAACCATCTGTAGGTCCCAAACAAGCTAGTCTTAATGGAGTCACATTAA   20122
gb:MT072688|Organism:Severe      TTAAAGGTTTACAACCATCTGTAGGTCCCAAACAAGCTAGTCTTAATGGAGTCACATTAA   20107
gb:MN996527|Organism:Severe      TTAAAGGTTTACAACCATCTGTAGGTCCCAAACAAGCTAGTCTTAATGGAGTCACATTAA   20089
gb:MT093631|Organism:Severe      TTAAAGGTTTACAACCATCTGTAGGTCCCAAACAAGCTAGTCTTAATGGAGTCACATTAA   20160
gb:MT106053|Organism:Severe      TTAAAGGTTTACAACCATCTGTAGGTCCCAAACAAGCTAGTCTTAATGGAGTCACATTAA   20122
gb:MT019533|Organism:Severe      TTAAAGGTTTACAACCATCTGTAGGTCCCAAACAAGCTAGTCTTAATGGAGTCACATTAA   20122
gb:MT019531|Organism:Severe      TTAAAGGTTTACAACCATCTGTAGGTCCCAAACAAGCTAGTCTTAATGGAGTCACATTAA   20122
gb:MN996528|Organism:Severe      TTAAAGGTTTACAACCATCTGTAGGTCCCAAACAAGCTAGTCTTAATGGAGTCACATTAA   20122
gb:MN996530|Organism:Severe      TTAAAGGTTTACAACCATCTGTAGGTCCCAAACAAGCTAGTCTTAATGGAGTCACATTAA   20108
gb:MN908947|Organism:Severe      TTAAAGGTTTACAACCATCTGTAGGTCCCAAACAAGCTAGTCTTAATGGAGTCACATTAA   20122
gb:MT019532|Organism:Severe      TTAAAGGTTTACAACCATCTGTAGGTCCCAAACAAGCTAGTCTTAATGGAGTCACATTAA   20122
                                 ************************************************************

gb:MT020781|Organism:Severe      TTGGAGAAGCCGTAAAAACACAGTTCAATTATTATAAGAAAGTTGATGGTGTTGTCCAAC   20170
gb:MT007544|Organism:Severe      TTGGAGAAGCCGTAAAAACACAGTTCAATTATTATAAGAAAGTTGATGGTGTTGTCCAAC   20182
gb:MN994467|Organism:Severe      TTGGAGAAGCCGTAAAAACACAGTTCAATTATTATAAGAAAGTTGATGGTGTTGTCCAAC   20182
gb:MT044257|Organism:Severe      TTGGAGAAGCCGTAAAAACACAGTTCAATTATTATAAGAAAGTTGATGGTGTTGTCCAAC   20182
gb:MT106054|Organism:Severe      TTGGAGAAGCCGTAAAAACACAGTTCAATTATTATAAGAAAGTTGATGGTGTTGTCCAAC   20182
gb:MT049951|Organism:Severe      TTGGAGAAGCCGTAAAAACACAGTTCAATTATTATAAGAAAGTTGATGGTGTTGTCCAAC   20182
gb:MN975262|Organism:Severe      TTGGAGAAGCCGTAAAAACACAGTTCAATTATTATAAGAAAGTTGATGGTGTTGTCCAAC   20182
gb:MT106052|Organism:Severe      TTGGAGAAGCCGTAAAAACACAGTTCAATTATTATAAGAAAGTTGATGGTGTTGTCCAAC   20182
gb:LC522975|Organism:Severe      TTGGAGAAGCCGTAAAAACACAGTTCAATTATTATAAGAAAGTTGATGGTGTTGTCCAAC   20179
gb:LC522973|Organism:Severe      TTGGAGAAGCCGTAAAAACACAGTTCAATTATTATAAGAAAGTTGATGGTGTTGTCCAAC   20179
```

```
gb:LC522974|Organism:Severe      TTGGAGAAGCCGTAAAAACACAGTTCAATTATTATAAGAAAGTTGATGGTGTTGTCCAAC   20179
gb:MN985325|Organism:Severe      TTGGAGAAGCCGTAAAAACACAGTTCAATTATTATAAGAAAGTTGATGGTGTTGTCCAAC   20182
gb:MT020881|Organism:Severe      TTGGAGAAGCCGTAAAAACACAGTTCAATTATTATAAGAAAGTTGATGGTGTTGTCCAAC   20182
gb:MT020880|Organism:Severe      TTGGAGAAGCCGTAAAAACACAGTTCAATTATTATAAGAAAGTTGATGGTGTTGTCCAAC   20182
gb:MT066175|Organism:Severe      TTGGAGAAGCCGTAAAAACACAGTTCAATTATTATAAGAAAGTTGATGGTGTTGTCCAAC   20182
gb:MN997409|Organism:Severe      TTGGAGAAGCCGTAAAAACACAGTTCAATTATTATAAGAAAGTTGATGGTGTTGTCCAAC   20182
gb:MN938384|Organism:Severe      TTGGAGAAGCCGTAAAAACACAGTTCAATTATTATAAGAAAGTTGATGGTGTTGTCCAAC   20150
gb:MT044258|Organism:Severe      TTGGAGAAGCCGTAAAAACACAGTTCAATTATTATAAGAAAGTTGATGGTGTTGTCCAAC   20158
gb:MT039890|Organism:Severe      TTGGAGAAGCCGTAAAAACACAGTTCAATTATTATAAGAAAGTTGATGGTGTTGTCCAAC   20182
gb:MN988713|Organism:Severe      TTGGAGAAGCCGTAAAAACACAGTTCAATTATTATAAGAAAGTTGATGGTGTTGTCCAAC   20182
gb:LC521925|Organism:Severe      TTGGAGAAGCCGTAAAAACACAGTTCAATTATTATAAGAAAGTTGATGGTGTTGTCCAAC   20155
gb:MT093571|Organism:Severe      TTGGAGAAGCCGTAAAAACACAGTTCAATTATTATAAGAAAGTTGATGGTGTTGTCCAAC   20182
gb:MT039887|Organism:Severe      TTGGAGAAGCCGTAAAAACACAGTTCAATTATTATAAGAAAGTTGATGGTGTTGTCCAAC   20182
gb:MT019530|Organism:Severe      TTGGAGAAGCCGTAAAAACACAGTTCAATTATTATAAGAAAGTTGATGGTGTTGTCCAAC   20182
gb:MT039888|Organism:Severe      TTGGAGAAGCCGTAAAAACACAGTTCAATTATTATAAGAAAGTTGATGGTGTTGTCCAAC   20182
gb:LC522972|Organism:Severe      TTGGAGAAGCCGTAAAAACACAGTTCAATTATTATAAGAAAGTTGATGGTGTTGTCCAAC   20179
gb:MT027063|Organism:Severe      TTGGAGAAGCCGTAAAAACACAGTTCAATTATTATAAGAAAGTTGATGGTGTTGTCCAAC   20182
gb:MT027062|Organism:Severe      TTGGAGAAGCCGTAAAAACACAGTTCAATTATTATAAGAAAGTTGATGGTGTTGTCCAAC   20182
gb:MT019529|Organism:Severe      TTGGAGAAGCCGTAAAAACACAGTTCAATTATTATAAGAAAGTTGATGGTGTTGTCCAAC   20182
gb:MN996529|Organism:Severe      TTGGAGAAGCCGTAAAAACACAGTTCAATTATTATAAGAAAGTTGATGGTGTTGTCCAAC   20170
gb:MN996531|Organism:Severe      TTGGAGAAGCCGTAAAAACACAGTTCAATTATTATAAGAAAGTTGATGGTGTTGTCCAAC   20169
gb:MT066176|Organism:Severe      TTGGAGAAGCCGTAAAAACACAGTTCAATTATTATAAGAAAGTTGATGGTGTTGTCCAAC   20182
gb:MT027064|Organism:Severe      TTGGAGAAGCCGTAAAAACACAGTTCAATTATTATAAGAAAGTTGATGGTGTTGTCCAAC   20182
gb:MN994468|Organism:Severe      TTGGAGAAGCCGTAAAAACACAGTTCAATTATTATAAGAAAGTTGATGGTGTTGTCCAAC   20182
gb:MT072688|Organism:Severe      TTGGAGAAGCCGTAAAAACACAGTTCAATTATTATAAGAAAGTTGATGGTGTTGTCCAAC   20167
gb:MN996527|Organism:Severe      TTGGAGAAGCCGTAAAAACACAGTTCAATTATTATAAGAAAGTTGATGGTGTTGTCCAAC   20149
gb:MT093631|Organism:Severe      TTGGAGAAGCCGTAAAAACACAGTTCAATTATTATAAGAAAGTTGATGGTGTTGTCCAAC   20220
gb:MT106053|Organism:Severe      TTGGAGAAGCCGTAAAAACACAGTTCAATTATTATAAGAAAGTTGATGGTGTTGTCCAAC   20182
gb:MT019533|Organism:Severe      TTGGAGAAGCCGTAAAAACACAGTTCAATTATTATAAGAAAGTTGATGGTGTTGTCCAAC   20182
gb:MT019531|Organism:Severe      TTGGAGAAGCCGTAAAAACACAGTTCAATTATTATAAGAAAGTTGATGGTGTTGTCCAAC   20182
gb:MN996528|Organism:Severe      TTGGAGAAGCCGTAAAAACACAGTTCAATTATTATAAGAAAGTTGATGGTGTTGTCCAAC   20182
gb:MN996530|Organism:Severe      TTGGAGAAGCCGTAAAAACACAGTTCAATTATTATAAGAAAGTTGATGGTGTTGTCCAAC   20168
gb:MN908947|Organism:Severe      TTGGAGAAGCCGTAAAAACACAGTTCAATTATTATAAGAAAGTTGATGGTGTTGTCCAAC   20182
gb:MT019532|Organism:Severe      TTGGAGAAGCCGTAAAAACACAGTTCAATTATTATAAGAAAGTTGATGGTGTTGTCCAAC   20182
                                 ************************************************************

gb:MT020781|Organism:Severe      AATTACCTGAAACTTACTTTACTCAGAGTAGAAATTTACAAGAATTTAAACCCAGGAGTC   20230
gb:MT007544|Organism:Severe      AATTACCTGAAACTTACTTTACTCAGAGTAGAAATTTACAAGAATTTAAACCCAGGAGTC   20242
gb:MN994467|Organism:Severe      AATTACCTGAAACTTACTTTACTCAGAGTAGAAATTTACAAGAATTTAAACCCAGGAGTC   20242
gb:MT044257|Organism:Severe      AATTACCTGAAACTTACTTTACTCAGAGTAGAAATTTACAAGAATTTAAACCCAGGAGTC   20242
gb:MT106054|Organism:Severe      AATTACCTGAAACTTACTTTACTCAGAGTAGAAATTTACAAGAATTTAAACCCAGGAGTC   20242
gb:MT049951|Organism:Severe      AATTACCTGAAACTTACTTTACTCAGAGTAGAAATTTACAAGAATTTAAACCCAGGAGTC   20242
gb:MN975262|Organism:Severe      AATTACCTGAAACTTACTTTACTCAGAGTAGAAATTTACAAGAATTTAAACCCAGGAGTC   20242
gb:MT106052|Organism:Severe      AATTACCTGAAACTTACTTTACTCAGAGTAGAAATTTACAAGAATTTAAACCCAGGAGTC   20242
gb:LC522975|Organism:Severe      AATTACCTGAAACTTACTTTACTCAGAGTAGAAATTTACAAGAATTTAAACCCAGGAGTC   20239
gb:LC522973|Organism:Severe      AATTACCTGAAACTTACTTTACTCAGAGTAGAAATTTACAAGAATTTAAACCCAGGAGTC   20239
gb:LC522974|Organism:Severe      AATTACCTGAAACTTACTTTACTCAGAGTAGAAATTTACAAGAATTTAAACCCAGGAGTC   20239
gb:MN985325|Organism:Severe      AATTACCTGAAACTTACTTTACTCAGAGTAGAAATTTACAAGAATTTAAACCCAGGAGTC   20242
gb:MT020881|Organism:Severe      AATTACCTGAAACTTACTTTACTCAGAGTAGAAATTTACAAGAATTTAAACCCAGGAGTC   20242
gb:MT020880|Organism:Severe      AATTACCTGAAACTTACTTTACTCAGAGTAGAAATTTACAAGAATTTAAACCCAGGAGTC   20242
gb:MT066175|Organism:Severe      AATTACCTGAAACTTACTTTACTCAGAGTAGAAATTTACAAGAATTTAAACCCAGGAGTC   20242
gb:MN997409|Organism:Severe      AATTACCTGAAACTTACTTTACTCAGAGTAGAAATTTACAAGAATTTAAACCCAGGAGTC   20242
gb:MN938384|Organism:Severe      AATTACCTGAAACTTACTTTACTCAGAGTAGAAATTTACAAGAATTTAAACCCAGGAGTC   20210
gb:MT044258|Organism:Severe      AATTACCTGAAACTTACTTTACTCAGAGTAGAAATTTACAAGAATTTAAACCCAGGAGTC   20218
gb:MT039890|Organism:Severe      AATTACCTGAAACTTACTTTACTCAGAGTAGAAATTTACAAGAATTTAAACCCAGGAGTC   20242
gb:MN988713|Organism:Severe      AATTACCTGAAACTTACTTTACTCAGAGTAGAAATTTACAAGAATTTAAACCCAGGAGTC   20242
gb:LC521925|Organism:Severe      AATTACCTGAAACTTACTTTACTCAGAGTAGAAATTTACAAGAATTTAAACCCAGGAGTC   20215
gb:MT093571|Organism:Severe      AATTACCTGAAACTTACTTTACTCAGAGTAGAAATTTACAAGAATTTAAACCCAGGAGTC   20242
gb:MT039887|Organism:Severe      AATTACCTGAAACTTACTTTACTCAGAGTAGAAATTTACAAGAATTTAAACCCAGGAGTC   20242
gb:MT019530|Organism:Severe      AATTACCTGAAACTTACTTTACTCAGAGTAGAAATTTACAAGAATTTAAACCCAGGAGTC   20242
gb:MT039888|Organism:Severe      AATTACCTGAAACTTACTTTACTCAGAGTAGAAATTTACAAGAATTTAAACCCAGGAGTC   20242
gb:LC522972|Organism:Severe      AATTACCTGAAACTTACTTTACTCAGAGTAGAAATTTACAAGAATTTAAACCCAGGAGTC   20239
gb:MT027063|Organism:Severe      AATTACCTGAAACTTACTTTACTCAGAGTAGAAATTTACAAGAATTTAAACCCAGGAGTC   20242
gb:MT027062|Organism:Severe      AATTACCTGAAACTTACTTTACTCAGAGTAGAAATTTACAAGAATTTAAACCCAGGAGTC   20242
gb:MT019529|Organism:Severe      AATTACCTGAAACTTACTTTACTCAGAGTAGAAATTTACAAGAATTTAAACCCAGGAGTC   20242
```

```
gb:MN996529|Organism:Severe      AATTACCTGAAACTTACTTTACTCAGAGTAGAAATTTACAAGAATTTAAACCCAGGAGTC   20230
gb:MN996531|Organism:Severe      AATTACCTGAAACTTACTTTACTCAGAGTAGAAATTTACAAGAATTTAAACCCAGGAGTC   20229
gb:MT066176|Organism:Severe      AATTACCTGAAACTTACTTTACTCAGAGTAGAAATTTACAAGAATTTAAACCCAGGAGTC   20242
gb:MT027064|Organism:Severe      AATTACCTGAAACTTACTTTACTCAGAGTAGAAATTTACAAGAATTTAAACCCAGGAGTC   20242
gb:MN994468|Organism:Severe      AATTACCTGAAACTTACTTTACTCAGAGTAGAAATTTACAAGAATTTAAACCCAGGAGTC   20242
gb:MT072688|Organism:Severe      AATTACCTGAAACTTACTTTACTCAGAGTAGAAATTTACAAGAATTTAAACCCAGGAGTC   20227
gb:MN996527|Organism:Severe      AATTACCTGAAACTTACTTTACTCAGAGTAGAAATTTACAAGAATTTAAACCCAGGAGTC   20209
gb:MT093631|Organism:Severe      AATTACCTGAAACTTACTTTACTCAGAGTAGAAATTTACAAGAATTTAAACCCAGGAGTC   20280
gb:MT106053|Organism:Severe      AATTACCTGAAACTTACTTTACTCAGAGTAGAAATTTACAAGAATTTAAACCCAGGAGTC   20242
gb:MT019533|Organism:Severe      AATTACCTGAAACTTACTTTACTCAGAGTAGAAATTTACAAGAATTTAAACCCAGGAGTC   20242
gb:MT019531|Organism:Severe      AATTACCTGAAACTTACTTTACTCAGAGTAGAAATTTACAAGAATTTAAACCCAGGAGTC   20242
gb:MN996528|Organism:Severe      AATTACCTGAAACTTACTTTACTCAGAGTAGAAATTTACAAGAATTTAAACCCAGGAGTC   20242
gb:MN996530|Organism:Severe      AATTACCTGAAACTTACTTTACTCAGAGTAGAAATTTACAAGAATTTAAACCCAGGAGTC   20228
gb:MN908947|Organism:Severe      AATTACCTGAAACTTACTTTACTCAGAGTAGAAATTTACAAGAATTTAAACCCAGGAGTC   20242
gb:MT019532|Organism:Severe      AATTACCTGAAACTTACTTTACTCAGAGTAGAAATTTACAAGAATTTAAACCCAGGAGTC   20242
                                 ************************************************************

gb:MT020781|Organism:Severe      AAATGGAAATTGATTTCTTAGAATTAGCTATGGATGAATTCATTGAACGGTATAAATTAG   20290
gb:MT007544|Organism:Severe      AAATGGAAATTGATTTCTTAGAATTAGCTATGGATGAATTCATTGAACGGTATAAATTAG   20302
gb:MN994467|Organism:Severe      AAATGGAAATTGATTTCTTAGAATTAGCTATGGATGAATTCATTGAACGGTATAAATTAG   20302
gb:MT044257|Organism:Severe      AAATGGAAATTGATTTCTTAGAATTAGCTATGGATGAATTCATTGAACGGTATAAATTAG   20302
gb:MT106054|Organism:Severe      AAATGGAAATTGATTTCTTAGAATTAGCTATGGATGAATTCATTGAACGGTATAAATTAG   20302
gb:MT049951|Organism:Severe      AAATGGAAATTGATTTCTTAGAATTAGCTATGGATGAATTCATTGAACGGTATAAATTAG   20302
gb:MN975262|Organism:Severe      AAATGGAAATTGATTTCTTAGAATTAGCTATGGATGAATTCATTGAACGGTATAAATTAG   20302
gb:MT106052|Organism:Severe      AAATGGAAATTGATTTCTTAGAATTAGCTATGGATGAATTCATTGAACGGTATAAATTAG   20302
gb:LC522975|Organism:Severe      AAATGGAAATTGATTTCTTAGAATTAGCTATGGATGAATTCATTGAACGGTATAAATTAG   20299
gb:LC522973|Organism:Severe      AAATGGAAATTGATTTCTTAGAATTAGCTATGGATGAATTCATTGAACGGTATAAATTAG   20299
gb:LC522974|Organism:Severe      AAATGGAAATTGATTTCTTAGAATTAGCTATGGATGAATTCATTGAACGGTATAAATTAG   20299
gb:MN985325|Organism:Severe      AAATGGAAATTGATTTCTTAGAATTAGCTATGGATGAATTCATTGAACGGTATAAATTAG   20302
gb:MT020881|Organism:Severe      AAATGGAAATTGATTTCTTAGAATTAGCTATGGATGAATTCATTGAACGGTATAAATTAG   20302
gb:MT020880|Organism:Severe      AAATGGAAATTGATTTCTTAGAATTAGCTATGGATGAATTCATTGAACGGTATAAATTAG   20302
gb:MT066175|Organism:Severe      AAATGGAAATTGATTTCTTAGAATTAGCTATGGATGAATTCATTGAACGGTATAAATTAG   20302
gb:MN997409|Organism:Severe      AAATGGAAATTGATTTCTTAGAATTAGCTATGGATGAATTCATTGAACGGTATAAATTAG   20302
gb:MN938384|Organism:Severe      AAATGGAAATTGATTTCTTAGAATTAGCTATGGATGAATTCATTGAACGGTATAAATTAG   20270
gb:MT044258|Organism:Severe      AAATGGAAATTGATTTCTTAGAATTAGCTATGGATGAATTCATTGAACGGTATAAATTAG   20278
gb:MT039890|Organism:Severe      AAATGGAAATTGATTTCTTAGAATTAGCTATGGATGAATTCATTGAACGGTATAAATTAG   20302
gb:MN988713|Organism:Severe      AAATGGAAATTGATTTCTTAGAATTAGCTATGGATGAATTCATTGAACGGTATAAATTAG   20302
gb:LC521925|Organism:Severe      AAATGGAAATTGATTTCTTAGAATTAGCTATGGATGAATTCATTGAACGGTATAAATTAG   20275
gb:MT093571|Organism:Severe      AAATGGAAATTGATTTCTTAGAATTAGCTATGGATGAATTCATTGAACGGTATAAATTAG   20302
gb:MT039887|Organism:Severe      AAATGGAAATTGATTTCTTAGAATTAGCTATGGATGAATTCATTGAACGGTATAA---AG   20299
gb:MT019530|Organism:Severe      AAATGGAAATTGATTTCTTAGAATTAGCTATGGATGAATTCATTGAACGGTATAAATTAG   20302
gb:MT039888|Organism:Severe      AAATGGAAATTGATTTCTTAGAATTAGCTATGGATGAATTCATTGAACGGTATAAATTAG   20302
gb:LC522972|Organism:Severe      AAATGGAAATTGATTTCTTAGAATTAGCTATGGATGAATTCATTGAACGGTATAAATTAG   20299
gb:MT027063|Organism:Severe      AAATGGAAATTGATTTCTTAGAATTAGCTATGGATGAATTCATTGAACGGTATAAATTAG   20302
gb:MT027062|Organism:Severe      AAATGGAAATTGATTTCTTAGAATTAGCTATGGATGAATTCATTGAACGGTATAAATTAG   20302
gb:MT019529|Organism:Severe      AAATGGAAATTGATTTCTTAGAATTAGCTATGGATGAATTCATTGAACGGTATAAATTAG   20302
gb:MN996529|Organism:Severe      AAATGGAAATTGATTTCTTAGAATTAGCTATGGATGAATTCATTGAACGGTATAAATTAG   20290
gb:MN996531|Organism:Severe      AAATGGAAATTGATTTCTTAGAATTAGCTATGGATGAATTCATTGAACGGTATAAATTAG   20289
gb:MT066176|Organism:Severe      AAATGGAAATTGATTTCTTAGAATTAGCTATGGATGAATTCATTGAACGGTATAAATTAG   20302
gb:MT027064|Organism:Severe      AAATGGAAATTGATTTCTTAGAATTAGCTATGGATGAATTCATTGAACGGTATAAATTAG   20302
gb:MN994468|Organism:Severe      AAATGGAAATTGATTTCTTAGAATTAGCTATGGATGAATTCATTGAACGGTATAAATTAG   20302
gb:MT072688|Organism:Severe      AAATGGAAATTGATTTCTTAGAATTAGCTATGGATGAATTCATTGAACGGTATAAATTAG   20287
gb:MN996527|Organism:Severe      AAATGGAAATTGATTTCTTAGAATTAGCTATGGATGAATTCATTGAACGGTATAAATTAG   20269
gb:MT093631|Organism:Severe      AAATGGAAATTGATTTCTTAGAATTAGCTATGGATGAATTCATTGAACGGTATAAATTAG   20340
gb:MT106053|Organism:Severe      AAATGGAAATTGATTTCTTAGAATTAGCTATGGATGAATTCATTGAACGGTATAAATTAG   20302
gb:MT019533|Organism:Severe      AAATGGAAATTGATTTCTTAGAATTAGCTATGGATGAATTCATTGAACGGTATAAATTAG   20302
gb:MT019531|Organism:Severe      AAATGGAAATTGATTTCTTAGAATTAGCTATGGATGAATTCATTGAACGGTATAAATTAG   20302
gb:MN996528|Organism:Severe      AAATGGAAATTGATTTCTTAGAATTAGCTATGGATGAATTCATTGAACGGTATAAATTAG   20302
gb:MN996530|Organism:Severe      AAATGGAAATTGATTTCTTAGAATTAGCTATGGATGAATTCATTGAACGGTATAAATTAG   20288
gb:MN908947|Organism:Severe      AAATGGAAATTGATTTCTTAGAATTAGCTATGGATGAATTCATTGAACGGTATAAATTAG   20302
gb:MT019532|Organism:Severe      AAATGGAAATTGATTTCTTAGAATTAGCTATGGATGAATTCATTGAACGGTATAAATTAG   20302
                                 *******************************************************   **

gb:MT020781|Organism:Severe      AAGGCTATGCCTTCGAACATATCGTTTATGGAGATTTAGTCATAGTCAGTTAGGTGGTT   20350
gb:MT007544|Organism:Severe      AAGGCTATGCCTTCGAACATATCGTTTATGGAGATTTAGTCATAGTCAGTTAGGTGGTT   20362
```

```
gb:MN994467|Organism:Severe      AAGGCTATGCCTTCGAACATATCGTTTATGGAGATTTTAGTCATAGTCAGTTAGGTGGTT   20362
gb:MT044257|Organism:Severe      AAGGCTATGCCTTCGAACATATCGTTTATGGAGATTTTAGTCATAGTCAGTTAGGTGGTT   20362
gb:MT106054|Organism:Severe      AAGGCTATGCCTTCGAACATATCGTTTATGGAGATTTTAGTCATAGTCAGTTAGGTGGTT   20362
gb:MT049951|Organism:Severe      AAGGCTATGCCTTCGAACATATCGTTTATGGAGATTTTAGTCATAGTCAGTTAGGTGGTT   20362
gb:MN975262|Organism:Severe      AAGGCTATGCCTTCGAACATATCGTTTATGGAGATTTTAGTCATAGTCAGTTAGGTGGTT   20362
gb:MT106052|Organism:Severe      AAGGCTATGCCTTCGAACATATCGTTTATGGAGATTTTAGTCATAGTCAGTTAGGTGGTT   20362
gb:LC522975|Organism:Severe      AAGGCTATGCCTTCGAACATATCGTTTATGGAGATTTTAGTCATAGTCAGTTAGGTGGTT   20359
gb:LC522973|Organism:Severe      AAGGCTATGCCTTCGAACATATCGTTTATGGAGATTTTAGTCATAGTCAGTTAGGTGGTT   20359
gb:LC522974|Organism:Severe      AAGGCTATGCCTTCGAACATATCGTTTATGGAGATTTTAGTCATAGTCAGTTAGGTGGTT   20359
gb:MN985325|Organism:Severe      AAGGCTATGCCTTCGAACATATCGTTTATGGAGATTTTAGTCATAGTCAGTTAGGTGGTT   20362
gb:MT020881|Organism:Severe      AAGGCTATGCCTTCGAACATATCGTTTATGGAGATTTTAGTCATAGTCAGTTAGGTGGTT   20362
gb:MT020880|Organism:Severe      AAGGCTATGCCTTCGAACATATCGTTTATGGAGATTTTAGTCATAGTCAGTTAGGTGGTT   20362
gb:MT066175|Organism:Severe      AAGGCTATGCCTTCGAACATATCGTTTATGGAGATTTTAGTCATAGTCAGTTAGGTGGTT   20362
gb:MN997409|Organism:Severe      AAGGCTATGCCTTCGAACATATCGTTTATGGAGATTTTAGTCATAGTCAGTTAGGTGGTT   20362
gb:MN938384|Organism:Severe      AAGGCTATGCCTTCGAACATATCGTTTATGGAGATTTTAGTCATAGTCAGTTAGGTGGTT   20330
gb:MT044258|Organism:Severe      AAGGCTATGCCTTCGAACATATCGTTTATGGAGATTTTAGTCATAGTCAGTTAGGTGGTT   20338
gb:MT039890|Organism:Severe      AAGGCTATGCCTTCGAACATATCGTTTATGGAGATTTTAGTCATAGTCAGTTAGGTGGTT   20362
gb:MN988713|Organism:Severe      AAGGCTATGCCTTCGAACATATCGTTTATGGAGATTTTAGTCATAGTCAGTTAGGTGGTT   20362
gb:LC521925|Organism:Severe      AAGGCTATGCCTTCGAACATATCGTTTATGGAGATTTTAGTCATAGTCAGTTAGGTGGTT   20335
gb:MT093571|Organism:Severe      AAGGCTATGCCTTCGAACATATCGTTTATGGAGATTTTAGTCATAGTCAGTTAGGTGGTT   20362
gb:MT039887|Organism:Severe      AAGGCTATGCCTTCGAACATATCGTTTATGGAGATTTTAGTCATAGTCAGTTAGGTGGTT   20359
gb:MT019530|Organism:Severe      AAGGCTATGCCTTCGAACATATCGTTTATGGAGATTTTAGTCATAGTCAGTTAGGTGGTT   20362
gb:MT039888|Organism:Severe      AAGGCTATGCCTTCGAACATATCGTTTATGGAGATTTTAGTCATAGTCAGTTAGGTGGTT   20362
gb:LC522972|Organism:Severe      AAGGCTATGCCTTCGAACATATCGTTTATGGAGATTTTAGTCATAGTCAGTTAGGTGGTT   20359
gb:MT027063|Organism:Severe      AAGGCTATGCCTTCGAACATATCGTTTATGGAGATTTTAGTCATAGTCAGTTAGGTGGTT   20362
gb:MT027062|Organism:Severe      AAGGCTATGCCTTCGAACATATCGTTTATGGAGATTTTAGTCATAGTCAGTTAGGTGGTT   20362
gb:MT019529|Organism:Severe      AAGGCTATGCCTTCGAACATATCGTTTATGGAGATTTTAGTCATAGTCAGTTAGGTGGTT   20362
gb:MN996529|Organism:Severe      AAGGCTATGCCTTCGAACATATCGTTTATGGAGATTTTAGTCATAGTCAGTTAGGTGGTT   20350
gb:MN996531|Organism:Severe      AAGGCTATGCCTTCGAACATATCGTTTATGGAGATTTTAGTCATAGTCAGTTAGGTGGTT   20349
gb:MT066176|Organism:Severe      AAGGCTATGCCTTCGAACATATCGTTTATGGAGATTTTAGTCATAGTCAGTTAGGTGGTT   20362
gb:MT027064|Organism:Severe      AAGGCTATGCCTTCGAACATATCGTTTATGGAGATTTTAGTCATAGTCAGTTAGGTGGTT   20362
gb:MN994468|Organism:Severe      AAGGCTATGCCTTCGAACATATCGTTTATGGAGATTTTAGTCATAGTCAGTTAGGTGGTT   20362
gb:MT072688|Organism:Severe      AAGGCTATGCCTTCGAACATATCGTTTATGGAGATTTTAGTCATAGTCAGTTAGGTGGTT   20347
gb:MN996527|Organism:Severe      AAGGCTATGCCTTCGAACATATCGTTTATGGAGATTTTAGTCATAGTCAGTTAGGTGGTT   20329
gb:MT093631|Organism:Severe      AAGGCTATGCCTTCGAACATATCGTTTATGGAGATTTTAGTCATAGTCAGTTAGGTGGTT   20400
gb:MT106053|Organism:Severe      AAGGCTATGCCTTCGAACATATCGTTTATGGAGATTTTAGTCATAGTCAGTTAGGTGGTT   20362
gb:MT019533|Organism:Severe      AAGGCTATGCCTTCGAACATATCGTTTATGGAGATTTTAGTCATAGTCAGTTAGGTGGTT   20362
gb:MT019531|Organism:Severe      AAGGCTATGCCTTCGAACATATCGTTTATGGAGATTTTAGTCATAGTCAGTTAGGTGGTT   20362
gb:MN996528|Organism:Severe      AAGGCTATGCCTTCGAACATATCGTTTATGGAGATTTTAGTCATAGTCAGTTAGGTGGTT   20362
gb:MN996530|Organism:Severe      AAGGCTATGCCTTCGAACATATCGTTTATGGAGATTTTAGTCATAGTCAGTTAGGTGGTT   20348
gb:MN908947|Organism:Severe      AAGGCTATGCCTTCGAACATATCGTTTATGGAGATTTTAGTCATAGTCAGTTAGGTGGTT   20362
gb:MT019532|Organism:Severe      AAGGCTATGCCTTCGAACATATCGTTTATGGAGATTTTAGTCATAGTCAGTTAGGTGGTT   20362
                                 ************************************************************

gb:MT020781|Organism:Severe      TACATCTACTGATTGGACTAGCTAAACGTTTTAAGGAATCACCTTTTGAATTAGAAGATT   20410
gb:MT007544|Organism:Severe      TACATCTACTGATTGGACTAGCTAAACGTTTTAAGGAATCACCTTTTGAATTAGAAGATT   20422
gb:MN994467|Organism:Severe      TACATCTACTGATTGGACTAGCTAAACGTTTTAAGGAATCACCTTTTGAATTAGAAGATT   20422
gb:MT044257|Organism:Severe      TACATCTACTGATTGGACTAGCTAAACGTTTTAAGGAATCACCTTTTGAATTAGAAGATT   20422
gb:MT106054|Organism:Severe      TACATCTACTGATTGGACTAGCTAAACGTTTTAAGGAATCACCTTTTGAATTAGAAGATT   20422
gb:MT049951|Organism:Severe      TACATCTACTGATTGGACTAGCTAAACGTTTTAAGGAATCACCTTTTGAATTAGAAGATT   20422
gb:MN975262|Organism:Severe      TACATCTACTGATTGGACTAGCTAAACGTTTTAAGGAATCACCTTTTGAATTAGAAGATT   20422
gb:MT106052|Organism:Severe      TACATCTACTGATTGGACTAGCTAAACGTTTTAAGGAATCACCTTTTGAATTAGAAGATT   20422
gb:LC522975|Organism:Severe      TACATCTACTGATTGGACTAGCTAAACGTTTTAAGGAATCACCTTTTGAATTAGAAGATT   20419
gb:LC522973|Organism:Severe      TACATCTACTGATTGGACTAGCTAAACGTTTTAAGGAATCACCTTTTGAATTAGAAGATT   20419
gb:LC522974|Organism:Severe      TACATCTACTGATTGGACTAGCTAAACGTTTTAAGGAATCACCTTTTGAATTAGAAGATT   20419
gb:MN985325|Organism:Severe      TACATCTACTGATTGGACTAGCTAAACGTTTTAAGGAATCACCTTTTGAATTAGAAGATT   20422
gb:MT020881|Organism:Severe      TACATCTACTGATTGGACTAGCTAAACGTTTTAAGGAATCACCTTTTGAATTAGAAGATT   20422
gb:MT020880|Organism:Severe      TACATCTACTGATTGGACTAGCTAAACGTTTTAAGGAATCACCTTTTGAATTAGAAGATT   20422
gb:MT066175|Organism:Severe      TACATCTACTGATTGGACTAGCTAAACGTTTTAAGGAATCACCTTTTGAATTAGAAGATT   20422
gb:MN997409|Organism:Severe      TACATCTACTGATTGGACTAGCTAAACGTTTTAAGGAATCACCTTTTGAATTAGAAGATT   20422
gb:MN938384|Organism:Severe      TACATCTACTGATTGGACTAGCTAAACGTTTTAAGGAATCACCTTTTGAATTAGAAGATT   20390
gb:MT044258|Organism:Severe      TACATCTACTGATTGGACTAGCTAAACGTTTTAAGGAATCACCTTTTGAATTAGAAGATT   20398
gb:MT039890|Organism:Severe      TACATCTACTGATTGGACTAGCTAAACGTTTTAAGGAATCACCTTTTGAATTAGAAGATT   20422
gb:MN988713|Organism:Severe      TACATCTACTGATTGGACTAGCTAAACGTTTTAAGGAATCACCTTTTGAATTAGAAGATT   20422
gb:LC521925|Organism:Severe      TACATCTACTGATTGGACTAGCTAAACGTTTTAAGGAATCACCTTTTGAATTAGAAGATT   20395
```

```
gb:MT093571|Organism:Severe      TACATCTACTGATTGGACTAGCTAAACGTTTTAAGGAATCACCTTTTGAATTAGAAGATT   20422
gb:MT039887|Organism:Severe      TACATCTACTGATTGGACTAGCTAAACGTTTTAAGGAATCACCTTTTGAATTAGAAGATT   20419
gb:MT019530|Organism:Severe      TACATCTACTGATTGGACTAGCTAAACGTTTTAAGGAATCACCTTTTGAATTAGAAGATT   20422
gb:MT039888|Organism:Severe      TACATCTACTGATTGGACTAGCTAAACGTTTTAAGGAATCACCTTTTGAATTAGAAGATT   20422
gb:LC522972|Organism:Severe      TACATCTACTGATTGGACTAGCTAAACGTTTTAAGGAATCACCTTTTGAATTAGAAGATT   20419
gb:MT027063|Organism:Severe      TACATCTACTGATTGGACTAGCTAAACGTTTTAAGGAATCACCTTTTGAATTAGAAGATT   20422
gb:MT027062|Organism:Severe      TACATCTACTGATTGGACTAGCTAAACGTTTTAAGGAATCACCTTTTGAATTAGAAGATT   20422
gb:MT019529|Organism:Severe      TACATCTACTGATTGGACTAGCTAAACGTTTTAAGGAATCACCTTTTGAATTAGAAGATT   20422
gb:MN996529|Organism:Severe      TACATCTACTGATTGGACTAGCTAAACGTTTTAAGGAATCACCTTTTGAATTAGAAGATT   20410
gb:MN996531|Organism:Severe      TACATCTACTGATTGGACTAGCTAAACGTTTTAAGGAATCACCTTTTGAATTAGAAGATT   20409
gb:MT066176|Organism:Severe      TACATCTACTGATTGGACTAGCTAAACGTTTTAAGGAATCACCTTTTGAATTAGAAGATT   20422
gb:MT027064|Organism:Severe      TACATCTACTGATTGGACTAGCTAAACGTTTTAAGGAATCACCTTTTGAATTAGAAGATT   20422
gb:MN994468|Organism:Severe      TACATCTACTGATTGGACTAGCTAAACGTTTTAAGGAATCACCTTTTGAATTAGAAGATT   20422
gb:MT072688|Organism:Severe      TACATCTACTGATTGGACTAGCTAAACGTTTTAAGGAATCACCTTTTGAATTAGAAGATT   20407
gb:MN996527|Organism:Severe      TACATCTACTGATTGGACTAGCTAAACGTTTTAAGGAATCACCTTTTGAATTAGAAGATT   20389
gb:MT093631|Organism:Severe      TACATCTACTGATTGGACTAGCTAAACGTTTTAAGGAATCACCTTTTGAATTAGAAGATT   20460
gb:MT106053|Organism:Severe      TACATCTACTGATTGGACTAGCTAAACGTTTTAAGGAATCACCTTTTGAATTAGAAGATT   20422
gb:MT019533|Organism:Severe      TACATCTACTGATTGGACTAGCTAAACGTTTTAAGGAATCACCTTTTGAATTAGAAGATT   20422
gb:MT019531|Organism:Severe      TACATCTACTGATTGGACTAGCTAAACGTTTTAAGGAATCACCTTTTGAATTAGAAGATT   20422
gb:MN996528|Organism:Severe      TACATCTACTGATTGGACTAGCTAAACGTTTTAAGGAATCACCTTTTGAATTAGAAGATT   20422
gb:MN996530|Organism:Severe      TACATCTACTGATTGGACTAGCTAAACGTTTTAAGGAATCACCTTTTGAATTAGAAGATT   20408
gb:MN908947|Organism:Severe      TACATCTACTGATTGGACTAGCTAAACGTTTTAAGGAATCACCTTTTGAATTAGAAGATT   20422
gb:MT019532|Organism:Severe      TACATCTACTGATTGGACTAGCTAAACGTTTTAAGGAATCACCTTTTGAATTAGAAGATT   20422
                                 ************************************************************

gb:MT020781|Organism:Severe      TTATTCCTATGGACAGTACAGTTAAAAACTATTTCATAACAGATGCGCAAACAGGTTCAT   20470
gb:MT007544|Organism:Severe      TTATTCCTATGGACAGTACAGTTAAAAACTATTTCATAACAGATGCGCAAACAGGTTCAT   20482
gb:MN994467|Organism:Severe      TTATTCCTATGGACAGTACAGTTAAAAACTATTTCATAACAGATGCGCAAACAGGTTCAT   20482
gb:MT044257|Organism:Severe      TTATTCCTATGGACAGTACAGTTAAAAACTATTTCATAACAGATGCGCAAACAGGTTCAT   20482
gb:MT106054|Organism:Severe      TTATTCCTATGGACAGTACAGTTAAAAACTATTTCATAACAGATGCGCAAACAGGTTCAT   20482
gb:MT049951|Organism:Severe      TTATTCCTATGGACAGTACAGTTAAAAACTATTTCATAACAGATGCGCAAACAGGTTCAT   20482
gb:MN975262|Organism:Severe      TTATTCCTATGGACAGTACAGTTAAAAACTATTTCATAACAGATGCGCAAACAGGTTCAT   20482
gb:MT106052|Organism:Severe      TTATTCCTATGGACAGTACAGTTAAAAACTATTTCATAACAGATGCGCAAACAGGTTCAT   20482
gb:LC522975|Organism:Severe      TTATTCCTATGGACAGTACAGTTAAAAACTATTTCATAACAGATGCGCAAACAGGTTCAT   20479
gb:LC522973|Organism:Severe      TTATTCCTATGGACAGTACAGTTAAAAACTATTTCATAACAGATGCGCAAACAGGTTCAT   20479
gb:LC522974|Organism:Severe      TTATTCCTATGGACAGTACAGTTAAAAACTATTTCATAACAGATGCGCAAACAGGTTCAT   20479
gb:MN985325|Organism:Severe      TTATTCCTATGGACAGTACAGTTAAAAACTATTTCATAACAGATGCGCAAACAGGTTCAT   20482
gb:MT020881|Organism:Severe      TTATTCCTATGGACAGTACAGTTAAAAACTATTTCATAACAGATGCGCAAACAGGTTCAT   20482
gb:MT020880|Organism:Severe      TTATTCCTATGGACAGTACAGTTAAAAACTATTTCATAACAGATGCGCAAACAGGTTCAT   20482
gb:MT066175|Organism:Severe      TTATTCCTATGGACAGTACAGTTAAAAACTATTTCATAACAGATGCGCAAACAGGTTCAT   20482
gb:MN997409|Organism:Severe      TTATTCCTATGGACAGTACAGTTAAAAACTATTTCATAACAGATGCGCAAACAGGTTCAT   20482
gb:MN938384|Organism:Severe      TTATTCCTATGGACAGTACAGTTAAAAACTATTTCATAACAGATGCGCAAACAGGTTCAT   20450
gb:MT044258|Organism:Severe      TTATTCCTATGGACAGTACAGTTAAAAACTATTTCATAACAGATGCGCAAACAGGTTCAT   20458
gb:MT039890|Organism:Severe      TTATTCCTATGGACAGTACAGTTAAAAACTATTTCATAACAGATGCGCAAACAGGTTCAT   20482
gb:MN988713|Organism:Severe      TTATTCCTATGGACAGTACAGTTAAAAACTATTTCATAACAGATGCGCAAACAGGTTCAT   20482
gb:LC521925|Organism:Severe      TTATTCCTATGGACAGTACAGTTAAAAACTATTTCATAACAGATGCGCAAACAGGTTCAT   20455
gb:MT093571|Organism:Severe      TTATTCCTATGGACAGTACAGTTAAAAACTATTTCATAACAGATGCGCAAACAGGTTCAT   20482
gb:MT039887|Organism:Severe      TTATTCCTATGGACAGTACAGTTAAAAACTATTTCATAACAGATGCGCAAACAGGTTCAT   20479
gb:MT019530|Organism:Severe      TTATTCCTATGGACAGTACAGTTAAAAACTATTTCATAACAGATGCGCAAACAGGTTCAT   20482
gb:MT039888|Organism:Severe      TTATTCCTATGGACAGTACAGTTAAAAACTATTTCATAACAGATGCGCAAACAGGTTCAT   20482
gb:LC522972|Organism:Severe      TTATTCCTATGGACAGTACAGTTAAAAACTATTTCATAACAGATGCGCAAACAGGTTCAT   20479
gb:MT027063|Organism:Severe      TTATTCCTATGGACAGTACAGTTAAAAACTATTTCATAACAGATGCGCAAACAGGTTCAT   20482
gb:MT027062|Organism:Severe      TTATTCCTATGGACAGTACAGTTAAAAACTATTTCATAACAGATGCGCAAACAGGTTCAT   20482
gb:MT019529|Organism:Severe      TTATTCCTATGGACAGTACAGTTAAAAACTATTTCATAACAGATGCGCAAACAGGTTCAT   20482
gb:MN996529|Organism:Severe      TTATTCCTATGGACAGTACAGTTAAAAACTATTTCATAACAGATGCGCAAACAGGTTCAT   20470
gb:MN996531|Organism:Severe      TTATTCCTATGGACAGTACAGTTAAAAACTATTTCATAACAGATGCGCAAACAGGTTCAT   20469
gb:MT066176|Organism:Severe      TTATTCCTATGGACAGTACAGTTAAAAACTATTTCATAACAGATGCGCAAACAGGTTCAT   20482
gb:MT027064|Organism:Severe      TTATTCCTATGGACAGTACAGTTAAAAACTATTTCATAACAGATGCGCAAACAGGTTCAT   20482
gb:MN994468|Organism:Severe      TTATTCCTATGGACAGTACAGTTAAAAACTATTTCATAACAGATGCGCAAACAGGTTCAT   20482
gb:MT072688|Organism:Severe      TTATTCCTATGGACAGTACAGTTAAAAACTATTTCATAACAGATGCGCAAACAGGTTCAT   20467
gb:MN996527|Organism:Severe      TTATTCCTATGGACAGTACAGTTAAAAACTATTTCATAACAGATGCGCAAACAGGTTCAT   20449
gb:MT093631|Organism:Severe      TTATTCCTATGGACAGTACAGTTAAAAACTATTTCATAACAGATGCGCAAACAGGTTCAT   20520
gb:MT106053|Organism:Severe      TTATTCCTATGGACAGTACAGTTAAAAACTATTTCATAACAGATGCGCAAACAGGTTCAT   20482
gb:MT019533|Organism:Severe      TTATTCCTATGGACAGTACAGTTAAAAACTATTTCATAACAGATGCGCAAACAGGTTCAT   20482
gb:MT019531|Organism:Severe      TTATTCCTATGGACAGTACAGTTAAAAACTATTTCATAACAGATGCGCAAACAGGTTCAT   20482
```

```
gb:MN996528|Organism:Severe      TTATTCCTATGGACAGTACAGTTAAAAACTATTTCATAACAGATGCGCAAACAGGTTCAT   20482
gb:MN996530|Organism:Severe      TTATTCCTATGGACAGTACAGTTAAAAACTATTTCATAACAGATGCGCAAACAGGTTCAT   20468
gb:MN908947|Organism:Severe      TTATTCCTATGGACAGTACAGTTAAAAACTATTTCATAACAGATGCGCAAACAGGTTCAT   20482
gb:MT019532|Organism:Severe      TTATTCCTATGGACAGTACAGTTAAAAACTATTTCATAACAGATGCGCAAACAGGTTCAT   20482
                                 ************************************************************

gb:MT020781|Organism:Severe      CTAAGTGTGTGTGTTCTGTTATTGATTTATTACTTGATGATTTTGTTGAAATAATAAAAT   20530
gb:MT007544|Organism:Severe      CTAAGTGTGTGTGTTCTGTTATTGATTTATTACTTGATGATTTTGTTGAAATAATAAAAT   20542
gb:MN994467|Organism:Severe      CTAAGTGTGTGTGTTCTGTTATTGATTTATTACTTGATGATTTTGTTGAAATAATAAAAT   20542
gb:MT044257|Organism:Severe      CTAAGTGTGTGTGTTCTGTTATTGATTTATTACTTGATGATTTTGTTGAAATAATAAAAT   20542
gb:MT106054|Organism:Severe      CTAAGTGTGTGTGTTCTGTTATTGATTTATTACTTGATGATTTTGTTGAAATAATAAAAT   20542
gb:MT049951|Organism:Severe      CTAAGTGTGTGTGTTCTGTTATTGATTTATTACTTGATGATTTTGTTGAAATAATAAAAT   20542
gb:MN975262|Organism:Severe      CTAAGTGTGTGTGTTCTGTTATTGATTTATTACTTGATGATTTTGTTGAAATAATAAAAT   20542
gb:MT106052|Organism:Severe      CTAAGTGTGTGTGTTCTGTTATTGATTTATTACTTGATGATTTTGTTGAAATAATAAAAT   20542
gb:LC522975|Organism:Severe      CTAAGTGTGTGTGTTCTGTTATTGATTTATTACTTGATGATTTTGTTGAAATAATAAAAT   20539
gb:LC522973|Organism:Severe      CTAAGTGTGTGTGTTCTGTTATTGATTTATTACTTGATGATTTTGTTGAAATAATAAAAT   20539
gb:LC522974|Organism:Severe      CTAAGTGTGTGTGTTCTGTTATTGATTTATTACTTGATGATTTTGTTGAAATAATAAAAT   20539
gb:MN985325|Organism:Severe      CTAAGTGTGTGTGTTCTGTTATTGATTTATTACTTGATGATTTTGTTGAAATAATAAAAT   20542
gb:MT020881|Organism:Severe      CTAAGTGTGTGTGTTCTGTTATTGATTTATTACTTGATGATTTTGTTGAAATAATAAAAT   20542
gb:MT020880|Organism:Severe      CTAAGTGTGTGTGTTCTGTTATTGATTTATTACTTGATGATTTTGTTGAAATAATAAAAT   20542
gb:MT066175|Organism:Severe      CTAAGTGTGTGTGTTCTGTTATTGATTTATTACTTGATGATTTTGTTGAAATAATAAAAT   20542
gb:MN997409|Organism:Severe      CTAAGTGTGTGTGTTCTGTTATTGATTTATTACTTGATGATTTTGTTGAAATAATAAAAT   20542
gb:MN938384|Organism:Severe      CTAAGTGTGTGTGTTCTGTTATTGATTTATTACTTGATGATTTTGTTGAAATAATAAAAT   20510
gb:MT044258|Organism:Severe      CTAAGTGTGTGTGTTCTGTTATTGATTTATTACTTGATGATTTTGTTGAAATAATAAAAT   20518
gb:MT039890|Organism:Severe      CTAAGTGTGTGTGTTCTGTTATTGATTTATTACTTGATGATTTTGTTGAAATAATAAAAT   20542
gb:MN988713|Organism:Severe      CTAAGTGTGTGTGTTCTGTTATTGATTTATTACTTGATGATTTTGTTGAAATAATAAAAT   20542
gb:LC521925|Organism:Severe      CTAAGTGTGTGTGTTCTGTTATTGATTTATTACTTGATGATTTTGTTGAAATAATAAAAT   20515
gb:MT093571|Organism:Severe      CTAAGTGTGTGTGTTCTGTTATTGATTTATTACTTGATGATTTTGTTGAAATAATAAAAT   20542
gb:MT039887|Organism:Severe      CTAAGTGTGTGTGTTCTGTTATTGATTTATTACTTGATGATTTTGTTGAAATAATAAAAT   20539
gb:MT019530|Organism:Severe      CTAAGTGTGTGTGTTCTGTTATTGATTTATTACTTGATGATTTTGTTGAAATAATAAAAT   20542
gb:MT039888|Organism:Severe      CTAAGTGTGTGTGTTCTGTTATTGATTTATTACTTGATGATTTTGTTGAAATAATAAAAT   20542
gb:LC522972|Organism:Severe      CTAAGTGTGTGTGTTCTGTTATTGATTTATTACTTGATGATTTTGTTGAAATAATAAAAT   20539
gb:MT027063|Organism:Severe      CTAAGTGTGTGTGTTCTGTTATTGATTTATTACTTGATGATTTTGTTGAAATAATAAAAT   20542
gb:MT027062|Organism:Severe      CTAAGTGTGTGTGTTCTGTTATTGATTTATTACTTGATGATTTTGTTGAAATAATAAAAT   20542
gb:MT019529|Organism:Severe      CTAAGTGTGTGTGTTCTGTTATTGATTTATTACTTGATGATTTTGTTGAAATAATAAAAT   20542
gb:MN996529|Organism:Severe      CTAAGTGTGTGTGTTCTGTTATTGATTTATTACTTGATGATTTTGTTGAAATAATAAAAT   20530
gb:MN996531|Organism:Severe      CTAAGTGTGTGTGTTCTGTTATTGATTTATTACTTGATGATTTTGTTGAAATAATAAAAT   20529
gb:MT066176|Organism:Severe      CTAAGTGTGTGTGTTCTGTTATTGATTTATTACTTGATGATTTTGTTGAAATAATAAAAT   20542
gb:MT027064|Organism:Severe      CTAAGTGTGTGTGTTCTGTTATTGATTTATTACTTGATGATTTTGTTGAAATAATAAAAT   20542
gb:MN994468|Organism:Severe      CTAAGTGTGTGTGTTCTGTTATTGATTTATTACTTGATGATTTTGTTGAAATAATAAAAT   20542
gb:MT072688|Organism:Severe      CTAAGTGTGTGTGTTCTGTTATTGATTTATTACTTGATGATTTTGTTGAAATAATAAAAT   20527
gb:MN996527|Organism:Severe      CTAAGTGTGTGTGTTCTGTTATTGATTTATTACTTGATGATTTTGTTGAAATAATAAAAT   20509
gb:MT093631|Organism:Severe      CTAAGTGTGTGTGTTCTGTTATTGATTTATTACTTGATGATTTTGTTGAAATAATAAAAT   20580
gb:MT106053|Organism:Severe      CTAAGTGTGTGTGTTCTGTTATTGATTTATTACTTGATGATTTTGTTGAAATAATAAAAT   20542
gb:MT019533|Organism:Severe      CTAAGTGTGTGTGTTCTGTTATTGATTTATTACTTGATGATTTTGTTGAAATAATAAAAT   20542
gb:MT019531|Organism:Severe      CTAAGTGTGTGTGTTCTGTTATTGATTTATTACTTGATGATTTTGTTGAAATAATAAAAT   20542
gb:MN996528|Organism:Severe      CTAAGTGTGTGTGTTCTGTTATTGATTTATTACTTGATGATTTTGTTGAAATAATAAAAT   20542
gb:MN996530|Organism:Severe      CTAAGTGTGTGTGTTCTGTTATTGATTTATTACTTGATGATTTTGTTGAAATAATAAAAT   20528
gb:MN908947|Organism:Severe      CTAAGTGTGTGTGTTCTGTTATTGATTTATTACTTGATGATTTTGTTGAAATAATAAAAT   20542
gb:MT019532|Organism:Severe      CTAAGTGTGTGTGTTCTGTTATTGATTTATTACTTGATGATTTTGTTGAAATAATAAAAT   20542
                                 ************************************************************

gb:MT020781|Organism:Severe      CCCAAGATTTATCTGTAGTTTCTAAGGTTGTCAAAGTGACTATTGACTATACAGAAATTT   20590
gb:MT007544|Organism:Severe      CCCAAGATTTATCTGTAGTTTCTAAGGTTGTCAAAGTGACTATTGACTATACAGAAATTT   20602
gb:MN994467|Organism:Severe      CCCAAGATTTATCTGTAGTTTCTAAGGTTGTCAAAGTGACTATTGACTATACAGAAATTT   20602
gb:MT044257|Organism:Severe      CCCAAGATTTATCTGTAGTTTCTAAGGTTGTCAAAGTGACTATTGACTATACAGAAATTT   20602
gb:MT106054|Organism:Severe      CCCAAGATTTATCTGTAGTTTCTAAGGTTGTCAAAGTGACTATTGACTATACAGAAATTT   20602
gb:MT049951|Organism:Severe      CCCAAGATTTATCTGTAGTTTCTAAGGTTGTCAAAGTGACTATTGACTATACAGAAATTT   20602
gb:MN975262|Organism:Severe      CCCAAGATTTATCTGTAGTTTCTAAGGTTGTCAAAGTGACTATTGACTATACAGAAATTT   20602
gb:MT106052|Organism:Severe      CCCAAGATTTATCTGTAGTTTCTAAGGTTGTCAAAGTGACTATTGACTATACAGAAATTT   20602
gb:LC522975|Organism:Severe      CCCAAGATTTATCTGTAGTTTCTAAGGTTGTCAAAGTGACTATTGACTATACAGAAATTT   20599
gb:LC522973|Organism:Severe      CCCAAGATTTATCTGTAGTTTCTAAGGTTGTCAAAGTGACTATTGACTATACAGAAATTT   20599
gb:LC522974|Organism:Severe      CCCAAGATTTATCTGTAGTTTCTAAGGTTGTCAAAGTGACTATTGACTATACAGAAATTT   20599
gb:MN985325|Organism:Severe      CCCAAGATTTATCTGTAGTTTCTAAGGTTGTCAAAGTGACTATTGACTATACAGAAATTT   20602
gb:MT020881|Organism:Severe      CCCAAGATTTATCTGTAGTTTCTAAGGTTGTCAAAGTGACTATTGACTATACAGAAATTT   20602
```

```
gb:MT020880|Organism:Severe      CCCAAGATTTATCTGTAGTTTCTAAGGTTGTCAAAGTGACTATTGACTATACAGAAATTT   20602
gb:MT066175|Organism:Severe      CCCAAGATTTATCTGTAGTTTCTAAGGTTGTCAAAGTGACTATTGACTATACAGAAATTT   20602
gb:MN997409|Organism:Severe      CCCAAGATTTATCTGTAGTTTCTAAGGTTGTCAAAGTGACTATTGACTATACAGAAATTT   20602
gb:MN938384|Organism:Severe      CCCAAGATTTATCTGTAGTTTCTAAGGTTGTCAAAGTGACTATTGACTATACAGAAATTT   20570
gb:MT044258|Organism:Severe      CCCAAGATTTATCTGTAGTTTCTAAGGTTGTCAAAGTGACTATTGACTATACAGAAATTT   20578
gb:MT039890|Organism:Severe      CCCAAGATTTATCTGTAGTTTCTAAGGTTGTCAAAGTGACTATTGACTATACAGAAATTT   20602
gb:MN988713|Organism:Severe      CCCAAGATTTATCTGTAGTTTCTAAGGTTGTCAAAGTGACTATTGACTATACAGAAATTT   20602
gb:LC521925|Organism:Severe      CCCAAGATTTATCTGTAGTTTCTAAGGTTGTCAAAGTGACTATTGACTATACAGAAATTT   20575
gb:MT093571|Organism:Severe      CCCAAGATTTATCTGTAGTTTCTAAGGTTGTCAAAGTGACTATTGACTATACAGAAATTT   20602
gb:MT039887|Organism:Severe      CCCAAGATTTATCTGTAGTTTCTAAGGTTGTCAAAGTGACTATTGACTATACAGAAATTT   20599
gb:MT019530|Organism:Severe      CCCAAGATTTATCTGTAGTTTCTAAGGTTGTCAAAGTGACTATTGACTATACAGAAATTT   20602
gb:MT039888|Organism:Severe      CCCAAGATTTATCTGTAGTTTCTAAGGTTGTCAAAGTGACTATTGACTATACAGAAATTT   20602
gb:LC522972|Organism:Severe      CCCAAGATTTATCTGTAGTTTCTAAGGTTGTCAAAGTGACTATTGACTATACAGAAATTT   20599
gb:MT027063|Organism:Severe      CCCAAGATTTATCTGTAGTTTCTAAGGTTGTCAAAGTGACTATTGACTATACAGAAATTT   20602
gb:MT027062|Organism:Severe      CCCAAGATTTATCTGTAGTTTCTAAGGTTGTCAAAGTGACTATTGACTATACAGAAATTT   20602
gb:MT019529|Organism:Severe      CCCAAGATTTATCTGTAGTTTCTAAGGTTGTCAAAGTGACTATTGACTATACAGAAATTT   20602
gb:MN996529|Organism:Severe      CCCAAGATTTATCTGTAGTTTCTAAGGTTGTCAAAGTGACTATTGACTATACAGAAATTT   20590
gb:MN996531|Organism:Severe      CCCAAGATTTATCTGTAGTTTCTAAGGTTGTCAAAGTGACTATTGACTATACAGAAATTT   20589
gb:MT066176|Organism:Severe      CCCAAGATTTATCTGTAGTTTCTAAGGTTGTCAAAGTGACTATTGACTATACAGAAATTT   20602
gb:MT027064|Organism:Severe      CCCAAGATTTATCTGTAGTTTCTAAGGTTGTCAAAGTGACTATTGACTATACAGAAATTT   20602
gb:MN994468|Organism:Severe      CCCAAGATTTATCTGTAGTTTCTAAGGTTGTCAAAGTGACTATTGACTATACAGAAATTT   20602
gb:MT072688|Organism:Severe      CCCAAGATTTATCTGTAGTTTCTAAGGTTGTCAAAGTGACTATTGACTATACAGAAATTT   20587
gb:MN996527|Organism:Severe      CCCAAGATTTATCTGTAGTTTCTAAGGTTGTCAAAGTGACTATTGACTATACAGAAATTT   20569
gb:MT093631|Organism:Severe      CCCAAGATTTATCTGTAGTTTCTAAGGTTGTCAAAGTGACTATTGACTATACAGAAATTT   20640
gb:MT106053|Organism:Severe      CCCAAGATTTATCTGTAGTTTCTAAGGTTGTCAAAGTGACTATTGACTATACAGAAATTT   20602
gb:MT019533|Organism:Severe      CCCAAGATTTATCTGTAGTTTCTAAGGTTGTCAAAGTGACTATTGACTATACAGAAATTT   20602
gb:MT019531|Organism:Severe      CCCAAGATTTATCTGTAGTTTCTAAGGTTGTCAAAGTGACTATTGACTATACAGAAATTT   20602
gb:MN996528|Organism:Severe      CCCAAGATTTATCTGTAGTTTCTAAGGTTGTCAAAGTGACTATTGACTATACAGAAATTT   20602
gb:MN996530|Organism:Severe      CCCAAGATTTATCTGTAGTTTCTAAGGTTGTCAAAGTGACTATTGACTATACAGAAATTT   20588
gb:MN908947|Organism:Severe      CCCAAGATTTATCTGTAGTTTCTAAGGTTGTCAAAGTGACTATTGACTATACAGAAATTT   20602
gb:MT019532|Organism:Severe      CCCAAGATTTATCTGTAGTTTCTAAGGTTGTCAAAGTGACTATTGACTATACAGAAATTT   20602
                                 ************************************************************

gb:MT020781|Organism:Severe      CATTTATGCTTTGGTGTAAAGATGGCCATGTAGAAACATTTTACCCAAAATTACAATCTA   20650
gb:MT007544|Organism:Severe      CATTTATGCTTTGGTGTAAAGATGGCCATGTAGAAACATTTTACCCAAAATTACAATCTA   20662
gb:MN994467|Organism:Severe      CATTTATGCTTTGGTGTAAAGATGGCCATGTAGAAACATTTTACCCAAAATTACAATCTA   20662
gb:MT044257|Organism:Severe      CATTTATGCTTTGGTGTAAAGATGGCCATGTAGAAACATTTTACCCAAAATTACAATCTA   20662
gb:MT106054|Organism:Severe      CATTTATGCTTTGGTGTAAAGATGGCCATGTAGAAACATTTTACCCAAAATTACAATCTA   20662
gb:MT049951|Organism:Severe      CATTTATGCTTTGGTGTAAAGATGGCCATGTAGAAACATTTTACCCAAAATTACAATCTA   20662
gb:MN975262|Organism:Severe      CATTTATGCTTTGGTGTAAAGATGGCCATGTAGAAACATTTTACCCAAAATTACAATCTA   20662
gb:MT106052|Organism:Severe      CATTTATGCTTTGGTGTAAAGATGGCCATGTAGAAACATTTTACCCAAAATTACAATCTA   20662
gb:LC522975|Organism:Severe      CATTTATGCTTTGGTGTAAAGATGGCCATGTAGAAACATTTTACCCAAAATTACAATCTA   20659
gb:LC522973|Organism:Severe      CATTTATGCTTTGGTGTAAAGATGGCCATGTAGAAACATTTTACCCAAAATTACAATCTA   20659
gb:LC522974|Organism:Severe      CATTTATGCTTTGGTGTAAAGATGGCCATGTAGAAACATTTTACCCAAAATTACAATCTA   20659
gb:MN985325|Organism:Severe      CATTTATGCTTTGGTGTAAAGATGGCCATGTAGAAACATTTTACCCAAAATTACAATCTA   20662
gb:MT020881|Organism:Severe      CATTTATGCTTTGGTGTAAAGATGGCCATGTAGAAACATTTTACCCAAAATTACAATCTA   20662
gb:MT020880|Organism:Severe      CATTTATGCTTTGGTGTAAAGATGGCCATGTAGAAACATTTTACCCAAAATTACAATCTA   20662
gb:MT066175|Organism:Severe      CATTTATGCTTTGGTGTAAAGATGGCCATGTAGAAACATTTTACCCAAAATTACAATCTA   20662
gb:MN997409|Organism:Severe      CATTTATGCTTTGGTGTAAAGATGGCCATGTAGAAACATTTTACCCAAAATTACAATCTA   20662
gb:MN938384|Organism:Severe      CATTTATGCTTTGGTGTAAAGATGGCCATGTAGAAACATTTTACCCAAAATTACAATCTA   20630
gb:MT044258|Organism:Severe      CATTTATGCTTTGGTGTAAAGATGGCCATGTAGAAACATTTTACCCAAAATTACAATCTA   20638
gb:MT039890|Organism:Severe      CATTTATGCTTTGGTGTAAAGATGGCCATGTAGAAACATTTTACCCAAAATTACAATCTA   20662
gb:MN988713|Organism:Severe      CATTTATGCTTTGGTGTAAAGATGGCCATGTAGAAACATTTTACCCAAAATTACAATCTA   20662
gb:LC521925|Organism:Severe      CATTTATGCTTTGGTGTAAAGATGGCCATGTAGAAACATTTTACCCAAAATTACAATCTA   20635
gb:MT093571|Organism:Severe      CATTTATGCTTTGGTGTAAAGATGGCCATGTAGAAACATTTTACCCAAAATTACAATCTA   20662
gb:MT039887|Organism:Severe      CATTTATGCTTTGGTGTAAAGATGGCCATGTAGAAACATTTTACCCAAAATTACAATCTA   20659
gb:MT019530|Organism:Severe      CATTTATGCTTTGGTGTAAAGATGGCCATGTAGAAACATTTTACCCAAAATTACAATCTA   20662
gb:MT039888|Organism:Severe      CATTTATGCTTTGGTGTAAAGATGGCCATGTAGAAACATTTTACCCAAAATTACAATCTA   20662
gb:LC522972|Organism:Severe      CATTTATGCTTTGGTGTAAAGATGGCCATGTAGAAACATTTTACCCAAAATTACAATCTA   20659
gb:MT027063|Organism:Severe      CATTTATGCTTTGGTGTAAAGATGGCCATGTAGAAACATTTTACCCAAAATTACAATCTA   20662
gb:MT027062|Organism:Severe      CATTTATGCTTTGGTGTAAAGATGGCCATGTAGAAACATTTTACCCAAAATTACAATCTA   20662
gb:MT019529|Organism:Severe      CATTTATGCTTTGGTGTAAAGATGGCCATGTAGAAACATTTTACCCAAAATTACAATCTA   20662
gb:MN996529|Organism:Severe      CATTTATGCTTTGGTGTAAAGATGGCCATGTAGAAACATTTTACCCAAAATTACAATCTA   20650
gb:MN996531|Organism:Severe      CATTTATGCTTTGGTGTAAAGATGGCCATGTAGAAACATTTTACCCAAAATTACAATCTA   20649
gb:MT066176|Organism:Severe      CATTTATGCTTTGGTGTAAAGATGGCCATGTAGAAACATTTTACCCAAAATTACAATCTA   20662
```

```
gb:MT027064|Organism:Severe      CATTTATGCTTTGGTGTAAAGATGGCCATGTAGAAACATTTTACCCAAAATTACAATCTA   20662
gb:MN994468|Organism:Severe      CATTTATGCTTTGGTGTAAAGATGGCCATGTAGAAACATTTTACCCAAAATTACAATCTA   20662
gb:MT072688|Organism:Severe      CATTTATGCTTTGGTGTAAAGATGGCCATGTAGAAACATTTTACCCAAAATTACAATCTA   20647
gb:MN996527|Organism:Severe      CATTTATGCTTTGGTGTAAAGATGGCCATGTAGAAACATTTTACCCAAAATTACAATCTA   20629
gb:MT093631|Organism:Severe      CATTTATGCTTTGGTGTAAAGATGGCCATGTAGAAACATTTTACCCAAAATTACAATCTA   20700
gb:MT106053|Organism:Severe      CATTTATGCTTTGGTGTAAAGATGGCCATGTAGAAACATTTTACCCAAAATTACAATCTA   20662
gb:MT019533|Organism:Severe      CATTTATGCTTTGGTGTAAAGATGGCCATGTAGAAACATTTTACCCAAAATTACAATCTA   20662
gb:MT019531|Organism:Severe      CATTTATGCTTTGGTGTAAAGATGGCCATGTAGAAACATTTTACCCAAAATTACAATCTA   20662
gb:MN996528|Organism:Severe      CATTTATGCTTTGGTGTAAAGATGGCCATGTAGAAACATTTTACCCAAAATTACAATCTA   20662
gb:MN996530|Organism:Severe      CATTTATGCTTTGGTGTAAAGATGGCCATGTAGAAACATTTTACCCAAAATTACAATCTA   20648
gb:MN908947|Organism:Severe      CATTTATGCTTTGGTGTAAAGATGGCCATGTAGAAACATTTTACCCAAAATTACAATCTA   20662
gb:MT019532|Organism:Severe      CATTTATGCTTTGGTGTAAAGATGGCCATGTAGAAACATTTTACCCAAAATTACAATCTA   20662
                                 ************************************************************

gb:MT020781|Organism:Severe      GTCAAGCGTGGCAACCGGGTGTTGCTATGCCTAATCTTTACAAAATGCAAAGAATGCTAT   20710
gb:MT007544|Organism:Severe      GTCAAGCGTGGCAACCGGGTGTTGCTATGCCTAATCTTTACAAAATGCAAAGAATGCTAT   20722
gb:MN994467|Organism:Severe      GTCAAGCGTGGCAACCGGGTGTTGCTATGCCTAATCTTTACAAAATGCAAAGAATGCTAT   20722
gb:MT044257|Organism:Severe      GTCAAGCGTGGCAACCGGGTGTTGCTATGCCTAATCTTTACAAAATGCAAAGAATGCTAT   20722
gb:MT106054|Organism:Severe      GTCAAGCGTGGCAACCGGGTGTTGCTATGCCTAATCTTTACAAAATGCAAAGAATGCTAT   20722
gb:MT049951|Organism:Severe      GTCAAGCGTGGCAACCGGGTGTTGCTATGCCTAATCTTTACAAAATGCAAAGAATGCTAT   20722
gb:MN975262|Organism:Severe      GTCAAGCGTGGCAACCGGGTGTTGCTATGCCTAATCTTTACAAAATGCAAAGAATGCTAT   20722
gb:MT106052|Organism:Severe      GTCAAGCGTGGCAACCGGGTGTTGCTATGCCTAATCTTTACAAAATGCAAAGAATGCTAT   20722
gb:LC522975|Organism:Severe      GTCAAGCGTGGCAACCGGGTGTTGCTATGCCTAATCTTTACAAAATGCAAAGAATGCTAT   20719
gb:LC522973|Organism:Severe      GTCAAGCGTGGCAACCGGGTGTTGCTATGCCTAATCTTTACAAAATGCAAAGAATGCTAT   20719
gb:LC522974|Organism:Severe      GTCAAGCGTGGCAACCGGGTGTTGCTATGCCTAATCTTTACAAAATGCAAAGAATGCTAT   20719
gb:MN985325|Organism:Severe      GTCAAGCGTGGCAACCGGGTGTTGCTATGCCTAATCTTTACAAAATGCAAAGAATGCTAT   20722
gb:MT020881|Organism:Severe      GTCAAGCGTGGCAACCGGGTGTTGCTATGCCTAATCTTTACAAAATGCAAAGAATGCTAT   20722
gb:MT020880|Organism:Severe      GTCAAGCGTGGCAACCGGGTGTTGCTATGCCTAATCTTTACAAAATGCAAAGAATGCTAT   20722
gb:MT066175|Organism:Severe      GTCAAGCGTGGCAACCGGGTGTTGCTATGCCTAATCTTTACAAAATGCAAAGAATGCTAT   20722
gb:MN997409|Organism:Severe      GTCAAGCGTGGCAACCGGGTGTTGCTATGCCTAATCTTTACAAAATGCAAAGAATGCTAT   20722
gb:MN938384|Organism:Severe      GTCAAGCGTGGCAACCGGGTGTTGCTATGCCTAATCTTTACAAAATGCAAAGAATGCTAT   20690
gb:MT044258|Organism:Severe      GTCAAGCGTGGCAACCGGGTGTTGCTATGCCTAATCTTTACAAAATGCAAAGAATGCTAT   20698
gb:MT039890|Organism:Severe      GTCAAGCGTGGCAACCGGGTGTTGCTATGCCTAATCTTTACAAAATGCAAAGAATGCTAT   20722
gb:MN988713|Organism:Severe      GTCAAGCGTGGCAACCGGGTGTTGCTATGCCTAATCTTTACAAAATGCAAAGAATGCTAT   20722
gb:LC521925|Organism:Severe      GTCAAGCGTGGCAACCGGGTGTTGCTATGCCTAATCTTTACAAAATGCAAAGAATGCTAT   20695
gb:MT093571|Organism:Severe      GTCAAGCGTGGCAACCGGGTGTTGCTATGCCTAATCTTTACAAAATGCAAAGAATGCTAT   20722
gb:MT039887|Organism:Severe      GTCAAGCGTGGCAACCGGGTGTTGCTATGCCTAATCTTTACAAAATGCAAAGAATGCTAT   20719
gb:MT019530|Organism:Severe      GTCAAGCGTGGCAACCGGGTGTTGCTATGCCTAATCTTTACAAAATGCAAAGAATGCTAT   20722
gb:MT039888|Organism:Severe      GTCAAGCGTGGCAACCGGGTGTTGCTATGCCTAATCTTTACAAAATGCAAAGAATGCTAT   20722
gb:LC522972|Organism:Severe      GTCAAGCGTGGCAACCGGGTGTTGCTATGCCTAATCTTTACAAAATGCAAAGAATGCTAT   20719
gb:MT027063|Organism:Severe      GTCAAGCGTGGCAACCGGGTGTTGCTATGCCTAATCTTTACAAAATGCAAAGAATGCTAT   20722
gb:MT027062|Organism:Severe      GTCAAGCGTGGCAACCGGGTGTTGCTATGCCTAATCTTTACAAAATGCAAAGAATGCTAT   20722
gb:MT019529|Organism:Severe      GTCAAGCGTGGCAACCGGGTGTTGCTATGCCTAATCTTTACAAAATGCAAAGAATGCTAT   20722
gb:MN996529|Organism:Severe      GTCAAGCGTGGCAACCGGGTGTTGCTATGCCTAATCTTTACAAAATGCAAAGAATGCTAT   20710
gb:MN996531|Organism:Severe      GTCAAGCGTGGCAACCGGGTGTTGCTATGCCTAATCTTTACAAAATGCAAAGAATGCTAT   20709
gb:MT066176|Organism:Severe      GTCAAGCGTGGCAACCGGGTGTTGCTATGCCTAATCTTTACAAAATGCAAAGAATGCTAT   20722
gb:MT027064|Organism:Severe      GTCAAGCGTGGCAACCGGGTGTTGCTATGCCTAATCTTTACAAAATGCAAAGAATGCTAT   20722
gb:MN994468|Organism:Severe      GTCAAGCGTGGCAACCGGGTGTTGCTATGCCTAATCTTTACAAAATGCAAAGAATGCTAT   20722
gb:MT072688|Organism:Severe      GTCAAGCGTGGCAACCGGGTGTTGCTATGCCTAATCTTTACAAAATGCAAAGAATGCTAT   20707
gb:MN996527|Organism:Severe      GTCAAGCGTGGCAACCGGGTGTTGCTATGCCTAATCTTTACAAAATGCAAAGAATGCTAT   20689
gb:MT093631|Organism:Severe      GTCAAGCGTGGCAACCGGGTGTTGCTATGCCTAATCTTTACAAAATGCAAAGAATGCTAT   20760
gb:MT106053|Organism:Severe      GTCAAGCGTGGCAACCGGGTGTTGCTATGCCTAATCTTTACAAAATGCAAAGAATGCTAT   20722
gb:MT019533|Organism:Severe      GTCAAGCGTGGCAACCGGGTGTTGCTATGCCTAATCTTTACAAAATGCAAAGAATGCTAT   20722
gb:MT019531|Organism:Severe      GTCAAGCGTGGCAACCGGGTGTTGCTATGCCTAATCTTTACAAAATGCAAAGAATGCTAT   20722
gb:MN996528|Organism:Severe      GTCAAGCGTGGCAACCGGGTGTTGCTATGCCTAATCTTTACAAAATGCAAAGAATGCTAT   20722
gb:MN996530|Organism:Severe      GTCAAGCGTGGCAACCGGGTGTTGCTATGCCTAATCTTTACAAAATGCAAAGAATGCTAT   20708
gb:MN908947|Organism:Severe      GTCAAGCGTGGCAACCGGGTGTTGCTATGCCTAATCTTTACAAAATGCAAAGAATGCTAT   20722
gb:MT019532|Organism:Severe      GTCAAGCGTGGCAACCGGGTGTTGCTATGCCTAATCTTTACAAAATGCAAAGAATGCTAT   20722
                                 ************************************************************

gb:MT020781|Organism:Severe      TAGAAAAGTGTGACCTTCAAAATTATGGTGATAGTGCAACATTACCTAAAGGCATAATGA   20770
gb:MT007544|Organism:Severe      TAGAAAAGTGTGACCTTCAAAATTATGGTGATAGTGCAACATTACCTAAAGGCATAATGA   20782
gb:MN994467|Organism:Severe      TAGAAAAGTGTGACCTTCAAAATTATGGTGATAGTGCAACATTACCTAAAGGCATAATGA   20782
gb:MT044257|Organism:Severe      TAGAAAAGTGTGACCTTCAAAATTATGGTGATAGTGCAACATTACCTAAAGGCATAATGA   20782
gb:MT106054|Organism:Severe      TAGAAAAGTGTGACCTTCAAAATTATGGTGATAGTGCAACATTACCTAAAGGCATAATGA   20782
```

```
gb:MT049951|Organism:Severe      TAGAAAAGTGTGACCTTCAAAATTATGGTGATAGTGCAACATTACCTAAAGGCATAATGA   20782
gb:MN975262|Organism:Severe      TAGAAAAGTGTGACCTTCAAAATTATGGTGATAGTGCAACATTACCTAAAGGCATAATGA   20782
gb:MT106052|Organism:Severe      TAGAAAAGTGTGACCTTCAAAATTATGGTGATAGTGCAACATTACCTAAAGGCATAATGA   20782
gb:LC522975|Organism:Severe      TAGAAAAGTGTGACCTTCAAAATTATGGTGATAGTGCAACATTACCTAAAGGCATAATGA   20779
gb:LC522973|Organism:Severe      TAGAAAAGTGTGACCTTCAAAATTATGGTGATAGTGCAACATTACCTAAAGGCATAATGA   20779
gb:LC522974|Organism:Severe      TAGAAAAGTGTGACCTTCAAAATTATGGTGATAGTGCAACATTACCTAAAGGCATAATGA   20779
gb:MN985325|Organism:Severe      TAGAAAAGTGTGACCTTCAAAATTATGGTGATAGTGCAACATTACCTAAAGGCATAATGA   20782
gb:MT020881|Organism:Severe      TAGAAAAGTGTGACCTTCAAAATTATGGTGATAGTGCAACATTACCTAAAGGCATAATGA   20782
gb:MT020880|Organism:Severe      TAGAAAAGTGTGACCTTCAAAATTATGGTGATAGTGCAACATTACCTAAAGGCATAATGA   20782
gb:MT066175|Organism:Severe      TAGAAAAGTGTGACCTTCAAAATTATGGTGATAGTGCAACATTACCTAAAGGCATAATGA   20782
gb:MN997409|Organism:Severe      TAGAAAAGTGTGACCTTCAAAATTATGGTGATAGTGCAACATTACCTAAAGGCATAATGA   20782
gb:MN938384|Organism:Severe      TAGAAAAGTGTGACCTTCAAAATTATGGTGATAGTGCAACATTACCTAAAGGCATAATGA   20750
gb:MT044258|Organism:Severe      TAGAAAAGTGTGACCTTCAAAATTATGGTGATAGTGCAACATTACCTAAAGGCATAATGA   20758
gb:MT039890|Organism:Severe      TAGAAAAGTGTGACCTTCAAAATTATGGTGATAGTGCAACATTACCTAAAGGCATAATGA   20782
gb:MN988713|Organism:Severe      TAGAAAAGTGTGACCTTCAAAATTATGGTGATAGTGCAACATTACCTAAAGGCATAATGA   20782
gb:LC521925|Organism:Severe      TAGAAAAGTGTGACCTTCAAAATTATGGTGATAGTGCAACATTACCTAAAGGCATAATGA   20755
gb:MT093571|Organism:Severe      TAGAAAAGTGTGACCTTCAAAATTATGGTGATAGTGCAACATTACCTAAAGGCATAATGA   20782
gb:MT039887|Organism:Severe      TAGAAAAGTGTGACCTTCAAAATTATGGTGATAGTGCAACATTACCTAAAGGCATAATGA   20779
gb:MT019530|Organism:Severe      TAGAAAAGTGTGACCTTCAAAATTATGGTGATAGTGCAACATTACCTAAAGGCATAATGA   20782
gb:MT039888|Organism:Severe      TAGAAAAGTGTGACCTTCAAAATTATGGTGATAGTGCAACATTACCTAAAGGCATAATGA   20782
gb:LC522972|Organism:Severe      TAGAAAAGTGTGACCTTCAAAATTATGGTGATAGTGCAACATTACCTAAAGGCATAATGA   20779
gb:MT027063|Organism:Severe      TAGAAAAGTGTGACCTTCAAAATTATGGTGATAGTGCAACATTACCTAAAGGCATAATGA   20782
gb:MT027062|Organism:Severe      TAGAAAAGTGTGACCTTCAAAATTATGGTGATAGTGCAACATTACCTAAAGGCATAATGA   20782
gb:MT019529|Organism:Severe      TAGAAAAGTGTGACCTTCAAAATTATGGTGATAGTGCAACATTACCTAAAGGCATAATGA   20782
gb:MN996529|Organism:Severe      TAGAAAAGTGTGACCTTCAAAATTATGGTGATAGTGCAACATTACCTAAAGGCATAATGA   20770
gb:MN996531|Organism:Severe      TAGAAAAGTGTGACCTTCAAAATTATGGTGATAGTGCAACATTACCTAAAGGCATAATGA   20769
gb:MT066176|Organism:Severe      TAGAAAAGTGTGACCTTCAAAATTATGGTGATAGTGCAACATTACCTAAAGGCATAATGA   20782
gb:MT027064|Organism:Severe      TAGAAAAGTGTGACCTTCAAAATTATGGTGATAGTGCAACATTACCTAAAGGCATAATGA   20782
gb:MN994468|Organism:Severe      TAGAAAAGTGTGACCTTCAAAATTATGGTGATAGTGCAACATTACCTAAAGGCATAATGA   20782
gb:MT072688|Organism:Severe      TAGAAAAGTGTGACCTTCAAAATTATGGTGATAGTGCAACATTACCTAAAGGCATAATGA   20767
gb:MN996527|Organism:Severe      TAGAAAAGTGTGACCTTCAAAATTATGGTGATAGTGCAACATTACCTAAAGGCATAATGA   20749
gb:MT093631|Organism:Severe      TAGAAAAGTGTGACCTTCAAAATTATGGTGATAGTGCAACATTACCTAAAGGCATAATGA   20820
gb:MT106053|Organism:Severe      TAGAAAAGTGTGACCTTCAAAATTATGGTGATAGTGCAACATTACCTAAAGGCATAATGA   20782
gb:MT019533|Organism:Severe      TAGAAAAGTGTGACCTTCAAAATTATGGTGATAGTGCAACATTACCTAAAGGCATAATGA   20782
gb:MT019531|Organism:Severe      TAGAAAAGTGTGACCTTCAAAATTATGGTGATAGTGCAACATTACCTAAAGGCATAATGA   20782
gb:MN996528|Organism:Severe      TAGAAAAGTGTGACCTTCAAAATTATGGTGATAGTGCAACATTACCTAAAGGCATAATGA   20782
gb:MN996530|Organism:Severe      TAGAAAAGTGTGACCTTCAAAATTATGGTGATAGTGCAACATTACCTAAAGGCATAATGA   20768
gb:MN908947|Organism:Severe      TAGAAAAGTGTGACCTTCAAAATTATGGTGATAGTGCAACATTACCTAAAGGCATAATGA   20782
gb:MT019532|Organism:Severe      TAGAAAAGTGTGACCTTCAAAATTATGGTGATAGTGCAACATTACCTAAAGGCATAATGA   20782
                                 ************************************************************

gb:MT020781|Organism:Severe      TGAATGTCGCAAAATATACTCAACTGTGTCAATATTTAAACACATTAACATTAGCTGTAC   20830
gb:MT007544|Organism:Severe      TGAATGTCGCAAAATATACTCAACTGTGTCAATATTTAAACACATTAACATTAGCTGTAC   20842
gb:MN994467|Organism:Severe      TGAATGTCGCAAAATATACTCAACTGTGTCAATATTTAAACACATTAACATTAGCTGTAC   20842
gb:MT044257|Organism:Severe      TGAATGTCGCAAAATATACTCAACTGTGTCAATATTTAAACACATTAACATTAGCTGTAC   20842
gb:MT106054|Organism:Severe      TGAATGTCGCAAAATATACTCAACTGTGTCAATATTTAAACACATTAACATTAGCTGTAC   20842
gb:MT049951|Organism:Severe      TGAATGTCGCAAAATATACTCAACTGTGTCAATATTTAAACACATTAACATTAGCTGTAC   20842
gb:MN975262|Organism:Severe      TGAATGTCGCAAAATATACTCAACTGTGTCAATATTTAAACACATTAACATTAGCTGTAC   20842
gb:MT106052|Organism:Severe      TGAATGTCGCAAAATATACTCAACTGTGTCAATATTTAAACACATTAACATTAGCTGTAC   20842
gb:LC522975|Organism:Severe      TGAATGTCGCAAAATATACTCAACTGTGTCAATATTTAAACACATTAACATTAGCTGTAC   20839
gb:LC522973|Organism:Severe      TGAATGTCGCAAAATATACTCAACTGTGTCAATATTTAAACACATTAACATTAGCTGTAC   20839
gb:LC522974|Organism:Severe      TGAATGTCGCAAAATATACTCAACTGTGTCAATATTTAAACACATTAACATTAGCTGTAC   20839
gb:MN985325|Organism:Severe      TGAATGTCGCAAAATATACTCAACTGTGTCAATATTTAAACACATTAACATTAGCTGTAC   20842
gb:MT020881|Organism:Severe      TGAATGTCGCAAAATATACTCAACTGTGTCAATATTTAAACACATTAACATTAGCTGTAC   20842
gb:MT020880|Organism:Severe      TGAATGTCGCAAAATATACTCAACTGTGTCAATATTTAAACACATTAACATTAGCTGTAC   20842
gb:MT066175|Organism:Severe      TGAATGTCGCAAAATATACTCAACTGTGTCAATATTTAAACACATTAACATTAGCTGTAC   20842
gb:MN997409|Organism:Severe      TGAATGTCGCAAAATATACTCAACTGTGTCAATATTTAAACACATTAACATTAGCTGTAC   20842
gb:MN938384|Organism:Severe      TGAATGTCGCAAAATATACTCAACTGTGTCAATATTTAAACACATTAACATTAGCTGTAC   20810
gb:MT044258|Organism:Severe      TGAATGTCGCAAAATATACTCAACTGTGTCAATATTTAAACACATTAACATTAGCTGTAC   20818
gb:MT039890|Organism:Severe      TGAATGTCGCAAAATATACTCAACTGTGTCAATATTTAAACACATTAACATTAGCTGTAC   20842
gb:MN988713|Organism:Severe      TGAATGTCGCAAAATATACTCAACTGTGTCAATATTTAAACACATTAACATTAGCTGTAC   20842
gb:LC521925|Organism:Severe      TGAATGTCGCAAAATATACTCAACTGTGTCAATATTTAAACACATTAACATTAGCTGTAC   20815
gb:MT093571|Organism:Severe      TGAATGTCGCAAAATATACTCAACTGTGTCAATATTTAAACACATTAACATTAGCTGTAC   20842
gb:MT039887|Organism:Severe      TGAATGTCGCAAAATATACTCAACTGTGTCAATATTTAAACACATTAACATTAGCTGTAC   20839
gb:MT019530|Organism:Severe      TGAATGTCGCAAAATATACTCAACTGTGTCAATATTTAAACACATTAACATTAGCTGTAC   20842
```

```
gb:MT039888|Organism:Severe      TGAATGTCGCAAAATATACTCAACTGTGTCAATATTTAAACACATTAACATTAGCTGTAC   20842
gb:LC522972|Organism:Severe      TGAATGTCGCAAAATATACTCAACTGTGTCAATATTTAAACACATTAACATTAGCTGTAC   20839
gb:MT027063|Organism:Severe      TGAATGTCGCAAAATATACTCAACTGTGTCAATATTTAAACACATTAACATTAGCTGTAC   20842
gb:MT027062|Organism:Severe      TGAATGTCGCAAAATATACTCAACTGTGTCAATATTTAAACACATTAACATTAGCTGTAC   20842
gb:MT019529|Organism:Severe      TGAATGTCGCAAAATATACTCAACTGTGTCAATATTTAAACACATTAACATTAGCTGTAC   20842
gb:MN996529|Organism:Severe      TGAATGTCGCAAAATATACTCAACTGTGTCAATATTTAAACACATTAACATTAGCTGTAC   20830
gb:MN996531|Organism:Severe      TGAATGTCGCAAAATATACTCAACTGTGTCAATATTTAAACACATTAACATTAGCTGTAC   20829
gb:MT066176|Organism:Severe      TGAATGTCGCAAAATATACTCAACTGTGTCAATATTTAAACACATTAACATTAGCTGTAC   20842
gb:MT027064|Organism:Severe      TGAATGTCGCAAAATATACTCAACTGTGTCAATATTTAAACACATTAACATTAGCTGTAC   20842
gb:MN994468|Organism:Severe      TGAATGTCGCAAAATATACTCAACTGTGTCAATATTTAAACACATTAACATTAGCTGTAC   20842
gb:MT072688|Organism:Severe      TGAATGTCGCAAAATATACTCAACTGTGTCAATATTTAAACACATTAACATTAGCTGTAC   20827
gb:MN996527|Organism:Severe      TGAATGTCGCAAAATATACTCAACTGTGTCAATATTTAAACACATTAACATTAGCTGTAC   20809
gb:MT093631|Organism:Severe      TGAATGTCGCAAAATATACTCAACTGTGTCAATATTTAAACACATTAACATTAGCTGTAC   20880
gb:MT106053|Organism:Severe      TGAATGTCGCAAAATATACTCAACTGTGTCAATATTTAAACACATTAACATTAGCTGTAC   20842
gb:MT019533|Organism:Severe      TGAATGTCGCAAAATATACTCAACTGTGTCAATATTTAAACACATTAACATTAGCTGTAC   20842
gb:MT019531|Organism:Severe      TGAATGTCGCAAAATATACTCAACTGTGTCAATATTTAAACACATTAACATTAGCTGTAC   20842
gb:MN996528|Organism:Severe      TGAATGTCGCAAAATATACTCAACTGTGTCAATATTTAAACACATTAACATTAGCTGTAC   20842
gb:MN996530|Organism:Severe      TGAATGTCGCAAAATATACTCAACTGTGTCAATATTTAAACACATTAACATTAGCTGTAC   20828
gb:MN908947|Organism:Severe      TGAATGTCGCAAAATATACTCAACTGTGTCAATATTTAAACACATTAACATTAGCTGTAC   20842
gb:MT019532|Organism:Severe      TGAATGTCGCAAAATATACTCAACTGTGTCAATATTTAAACACATTAACATTAGCTGTAC   20842
                                 ************************************************************

gb:MT020781|Organism:Severe      CCTATAATATGAGAGTTATACATTTTGGTGCTGGTTCTGATAAAGGAGTTGCACCAGGTA   20890
gb:MT007544|Organism:Severe      CCTATAATATGAGAGTTATACATTTTGGTGCTGGTTCTGATAAAGGAGTTGCACCAGGTA   20902
gb:MN994467|Organism:Severe      CCTATAATATGAGAGTTATACATTTTGGTGCTGGTTCTGATAAAGGAGTTGCACCAGGTA   20902
gb:MT044257|Organism:Severe      CCTATAATATGAGAGTTATACATTTTGGTGCTGGTTCTGATAAAGGAGTTGCACCAGGTA   20902
gb:MT106054|Organism:Severe      CCTATAATATGAGAGTTATACATTTTGGTGCTGGTTCTGATAAAGGAGTTGCACCAGGTA   20902
gb:MT049951|Organism:Severe      CCTATAATATGAGAGTTATACATTTTGGTGCTGGTTCTGATAAAGGAGTTGCACCAGGTA   20902
gb:MN975262|Organism:Severe      CCTATAATATGAGAGTTATACATTTTGGTGCTGGTTCTGATAAAGGAGTTGCACCAGGTA   20902
gb:MT106052|Organism:Severe      CCTATAATATGAGAGTTATACATTTTGGTGCTGGTTCTGATAAAGGAGTTGCACCAGGTA   20902
gb:LC522975|Organism:Severe      CCTATAATATGAGAGTTATACATTTTGGTGCTGGTTCTGATAAAGGAGTTGCACCAGGTA   20899
gb:LC522973|Organism:Severe      CCTATAATATGAGAGTTATACATTTTGGTGCTGGTTCTGATAAAGGAGTTGCACCAGGTA   20899
gb:LC522974|Organism:Severe      CCTATAATATGAGAGTTATACATTTTGGTGCTGGTTCTGATAAAGGAGTTGCACCAGGTA   20899
gb:MN985325|Organism:Severe      CCTATAATATGAGAGTTATACATTTTGGTGCTGGTTCTGATAAAGGAGTTGCACCAGGTA   20902
gb:MT020881|Organism:Severe      CCTATAATATGAGAGTTATACATTTTGGTGCTGGTTCTGATAAAGGAGTTGCACCAGGTA   20902
gb:MT020880|Organism:Severe      CCTATAATATGAGAGTTATACATTTTGGTGCTGGTTCTGATAAAGGAGTTGCACCAGGTA   20902
gb:MT066175|Organism:Severe      CCTATAATATGAGAGTTATACATTTTGGTGCTGGTTCTGATAAAGGAGTTGCACCAGGTA   20902
gb:MN997409|Organism:Severe      CCTATAATATGAGAGTTATACATTTTGGTGCTGGTTCTGATAAAGGAGTTGCACCAGGTA   20902
gb:MN938384|Organism:Severe      CCTATAATATGAGAGTTATACATTTTGGTGCTGGTTCTGATAAAGGAGTTGCACCAGGTA   20870
gb:MT044258|Organism:Severe      CCTATAATATGAGAGTTATACATTTTGGTGCTGGTTCTGATAAAGGAGTTGCACCAGGTA   20878
gb:MT039890|Organism:Severe      CCTATAATATGAGAGTTATACATTTTGGTGCTGGTTCTGATAAAGGAGTTGCACCAGGTA   20902
gb:MN988713|Organism:Severe      CCTATAATATGAGAGTTATACATTTTGGTGCTGGTTCTGATAAAGGAGTTGCACCAGGTA   20902
gb:LC521925|Organism:Severe      CCTATAATATGAGAGTTATACATTTTGGTGCTGGTTCTGATAAAGGAGTTGCACCAGGTA   20875
gb:MT093571|Organism:Severe      CCTATAATATGAGAGTTATACATTTTGGTGCTGGTTCTGATAAAGGAGTTGCACCAGGTA   20902
gb:MT039887|Organism:Severe      CCTATAATATGAGAGTTATACATTTTGGTGCTGGTTCTGATAAAGGAGTTGCACCAGGTA   20899
gb:MT019530|Organism:Severe      CCTATAATATGAGAGTTATACATTTTGGTGCTGGTTCTGATAAAGGAGTTGCACCAGGTA   20902
gb:MT039888|Organism:Severe      CCTATAATATGAGAGTTATACATTTTGGTGCTGGTTCTGATAAAGGAGTTGCACCAGGTA   20902
gb:LC522972|Organism:Severe      CCTATAATATGAGAGTTATACATTTTGGTGCTGGTTCTGATAAAGGAGTTGCACCAGGTA   20899
gb:MT027063|Organism:Severe      CCTATAATATGAGAGTTATACATTTTGGTGCTGGTTCTGATAAAGGAGTTGCACCAGGTA   20902
gb:MT027062|Organism:Severe      CCTATAATATGAGAGTTATACATTTTGGTGCTGGTTCTGATAAAGGAGTTGCACCAGGTA   20902
gb:MT019529|Organism:Severe      CCTATAATATGAGAGTTATACATTTTGGTGCTGGTTCTGATAAAGGAGTTGCACCAGGTA   20902
gb:MN996529|Organism:Severe      CCTATAATATGAGAGTTATACATTTTGGTGCTGGTTCTGATAAAGGAGTTGCACCAGGTA   20890
gb:MN996531|Organism:Severe      CCTATAATATGAGAGTTATACATTTTGGTGCTGGTTCTGATAAAGGAGTTGCACCAGGTA   20889
gb:MT066176|Organism:Severe      CCTATAATATGAGAGTTATACATTTTGGTGCTGGTTCTGATAAAGGAGTTGCACCAGGTA   20902
gb:MT027064|Organism:Severe      CCTATAATATGAGAGTTATACATTTTGGTGCTGGTTCTGATAAAGGAGTTGCACCAGGTA   20902
gb:MN994468|Organism:Severe      CCTATAATATGAGAGTTATACATTTTGGTGCTGGTTCTGATAAAGGAGTTGCACCAGGTA   20902
gb:MT072688|Organism:Severe      CCTATAATATGAGAGTTATACATTTTGGTGCTGGTTCTGATAAAGGAGTTGCACCAGGTA   20887
gb:MN996527|Organism:Severe      CCTATAATATGAGAGTTATACATTTTGGTGCTGGTTCTGATAAAGGAGTTGCACCAGGTA   20869
gb:MT093631|Organism:Severe      CCTATAATATGAGAGTTATACATTTTGGTGCTGGTTCTGATAAAGGAGTTGCACCAGGTA   20940
gb:MT106053|Organism:Severe      CCTATAATATGAGAGTTATACATTTTGGTGCTGGTTCTGATAAAGGAGTTGCACCAGGTA   20902
gb:MT019533|Organism:Severe      CCTATAATATGAGAGTTATACATTTTGGTGCTGGTTCTGATAAAGGAGTTGCACCAGGTA   20902
gb:MT019531|Organism:Severe      CCTATAATATGAGAGTTATACATTTTGGTGCTGGTTCTGATAAAGGAGTTGCACCAGGTA   20902
gb:MN996528|Organism:Severe      CCTATAATATGAGAGTTATACATTTTGGTGCTGGTTCTGATAAAGGAGTTGCACCAGGTA   20902
gb:MN996530|Organism:Severe      CCTATAATATGAGAGTTATACATTTTGGTGCTGGTTCTGATAAAGGAGTTGCACCAGGTA   20888
gb:MN908947|Organism:Severe      CCTATAATATGAGAGTTATACATTTTGGTGCTGGTTCTGATAAAGGAGTTGCACCAGGTA   20902
```

```
gb:MT019532|Organism:Severe      CCTATAATATGAGAGTTATACATTTTGGTGCTGGTTCTGATAAAGGAGTTGCACCAGGTA   20902
                                 ************************************************************

gb:MT020781|Organism:Severe      CAGCTGTTTTAAGACAGTGGTTGCCTACGGGTACGCTGCTTGTCGATTCAGATCTTAATG   20950
gb:MT007544|Organism:Severe      CAGCTGTTTTAAGACAGTGGTTGCCTACGGGTACGCTGCTTGTCGATTCAGATCTTAATG   20962
gb:MN994467|Organism:Severe      CAGCTGTTTTAAGACAGTGGTTGCCTACGGGTACGCTGCTTGTCGATTCAGATCTTAATG   20962
gb:MT044257|Organism:Severe      CAGCTGTTTTAAGACAGTGGTTGCCTACGGGTACGCTGCTTGTCGATTCAGATCTTAATG   20962
gb:MT106054|Organism:Severe      CAGCTGTTTTAAGACAGTGGTTGCCTACGGGTACGCTGCTTGTCGATTCAGATCTTAATG   20962
gb:MT049951|Organism:Severe      CAGCTGTTTTAAGACAGTGGTTGCCTACGGGTACGCTGCTTGTCGATTCAGATCTTAATG   20962
gb:MN975262|Organism:Severe      CAGCTGTTTTAAGACAGTGGTTGCCTACGGGTACGCTGCTTGTCGATTCAGATCTTAATG   20962
gb:MT106052|Organism:Severe      CAGCTGTTTTAAGACAGTGGTTGCCTACGGGTACGCTGCTTGTCGATTCAGATCTTAATG   20962
gb:LC522975|Organism:Severe      CAGCTGTTTTAAGACAGTGGTTGCCTACGGGTACGCTGCTTGTCGATTCAGATCTTAATG   20959
gb:LC522973|Organism:Severe      CAGCTGTTTTAAGACAGTGGTTGCCTACGGGTACGCTGCTTGTCGATTCAGATCTTAATG   20959
gb:LC522974|Organism:Severe      CAGCTGTTTTAAGACAGTGGTTGCCTACGGGTACGCTGCTTGTCGATTCAGATCTTAATG   20959
gb:MN985325|Organism:Severe      CAGCTGTTTTAAGACAGTGGTTGCCTACGGGTACGCTGCTTGTCGATTCAGATCTTAATG   20962
gb:MT020881|Organism:Severe      CAGCTGTTTTAAGACAGTGGTTGCCTACGGGTACGCTGCTTGTCGATTCAGATCTTAATG   20962
gb:MT020880|Organism:Severe      CAGCTGTTTTAAGACAGTGGTTGCCTACGGGTACGCTGCTTGTCGATTCAGATCTTAATG   20962
gb:MT066175|Organism:Severe      CAGCTGTTTTAAGACAGTGGTTGCCTACGGGTACGCTGCTTGTCGATTCAGATCTTAATG   20962
gb:MN997409|Organism:Severe      CAGCTGTTTTAAGACAGTGGTTGCCTACGGGTACGCTGCTTGTCGATTCAGATCTTAATG   20962
gb:MN938384|Organism:Severe      CAGCTGTTTTAAGACAGTGGTTGCCTACGGGTACGCTGCTTGTCGATTCAGATCTTAATG   20930
gb:MT044258|Organism:Severe      CAGCTGTTTTAAGACAGTGGTTGCCTACGGGTACGCTGCTTGTCGATTCAGATCTTAATG   20938
gb:MT039890|Organism:Severe      CAGCTGTTTTAAGACAGTGGTTGCCTACGGGTATGCTGCTTGTCGATTCAGATCTTAATG   20962
gb:MN988713|Organism:Severe      CAGCTGTTTTAAGACAGTGGTTGCCTACGGGTACGCTGCTTGTCGATTCAGATCTTAATG   20962
gb:LC521925|Organism:Severe      CAGCTGTTTTAAGACAGTGGTTGCCTACGGGTACGCTGCTTGTCGATTCAGATCTTAATG   20935
gb:MT093571|Organism:Severe      CAGCTGTTTTAAGACAGTGGTTGCCTACGGGTACGCTGCTTGTCGATTCAGATCTTAATG   20962
gb:MT039887|Organism:Severe      CAGCTGTTTTAAGACAGTGGTTGCCTACGGGTACGCTGCTTGTCGATTCAGATCTTAATG   20959
gb:MT019530|Organism:Severe      CAGCTGTTTTAAGACAGTGGTTGCCTACGGGTACGCTGCTTGTCGATTCAGATCTTAATG   20962
gb:MT039888|Organism:Severe      CAGCTGTTTTAAGACAGTGGTTGCCTACGGGTACGCTGCTTGTCGATTCAGATCTTAATG   20962
gb:LC522972|Organism:Severe      CAGCTGTTTTAAGACAGTGGTTGCCTACGGGTACGCTGCTTGTCGATTCAGATCTTAATG   20959
gb:MT027063|Organism:Severe      CAGCTGTTTTAAGACAGTGGTTGCCTACGGGTACGCTGCTTGTCGATTCAGATCTTAATG   20962
gb:MT027062|Organism:Severe      CAGCTGTTTTAAGACAGTGGTTGCCTACGGGTACGCTGCTTGTCGATTCAGATCTTAATG   20962
gb:MT019529|Organism:Severe      CAGCTGTTTTAAGACAGTGGTTGCCTACGGGTACGCTGCTTGTCGATTCAGATCTTAATG   20962
gb:MN996529|Organism:Severe      CAGCTGTTTTAAGACAGTGGTTGCCTACGGGTACGCTGCTTGTCGATTCAGATCTTAATG   20950
gb:MN996531|Organism:Severe      CAGCTGTTTTAAGACAGTGGTTGCCTACGGGTACGCTGCTTGTCGATTCAGATCTTAATG   20949
gb:MT066176|Organism:Severe      CAGCTGTTTTAAGACAGTGGTTGCCTACGGGTACGCTGCTTGTCGATTCAGATCTTAATG   20962
gb:MT027064|Organism:Severe      CAGCTGTTTTAAGACAGTGGTTGCCTACGGGTACGCTGCTTGTCGATTCAGATCTTAATG   20962
gb:MN994468|Organism:Severe      CAGCTGTTTTAAGACAGTGGTTGCCTACGGGTACGCTGCTTGTCGATTCAGATCTTAATG   20962
gb:MT072688|Organism:Severe      CAGCTGTTTTAAGACAGTGGTTGCCTACGGGTACGCTGCTTGTCGATTCAGATCTTAATG   20947
gb:MN996527|Organism:Severe      CAGCTGTTTTAAGACAGTGGTTGCCTACGGGTACGCTGCTTGTCGATTCAGATCTTAATG   20929
gb:MT093631|Organism:Severe      CAGCTGTTTTAAGACAGTGGTTGCCTACGGGTACGCTGCTTGTCGATTCAGATCTTAATG   21000
gb:MT106053|Organism:Severe      CAGCTGTTTTAAGACAGTGGTTGCCTACGGGTACGCTGCTTGTCGATTCAGATCTTAATG   20962
gb:MT019533|Organism:Severe      CAGCTGTTTTAAGACAGTGGTTGCCTACGGGTACGCTGCTTGTCGATTCAGATCTTAATG   20962
gb:MT019531|Organism:Severe      CAGCTGTTTTAAGACAGTGGTTGCCTACGGGTACGCTGCTTGTCGATTCAGATCTTAATG   20962
gb:MN996528|Organism:Severe      CAGCTGTTTTAAGACAGTGGTTGCCTACGGGTACGCTGCTTGTCGATTCAGATCTTAATG   20962
gb:MN996530|Organism:Severe      CAGCTGTTTTAAGACAGTGGTTGCCTACGGGTACGCTGCTTGTCGATTCAGATCTTAATG   20948
gb:MN908947|Organism:Severe      CAGCTGTTTTAAGACAGTGGTTGCCTACGGGTACGCTGCTTGTCGATTCAGATCTTAATG   20962
gb:MT019532|Organism:Severe      CAGCTGTTTTAAGACAGTGGTTGCCTACGGGTACGCTGCTTGTCGATTCAGATCTTAATG   20962
                                 ********************************* **************************

gb:MT020781|Organism:Severe      ACTTTGTCTCTGATGCAGATTCAACTTTGATTGGTGATTGTGCAACTGTACATACAGCTA   21010
gb:MT007544|Organism:Severe      ACTTTGTCTCTGATGCAGATTCAACTTTGATTGGTGATTGTGCAACTGTACATACAGCTA   21022
gb:MN994467|Organism:Severe      ACTTTGTCTCTGATGCAGATTCAACTTTGATTGGTGATTGTGCAACTGTACATACAGCTA   21022
gb:MT044257|Organism:Severe      ACTTTGTCTCTGATGCAGATTCAACTTTGATTGGTGATTGTGCAACTGTACATACAGCTA   21022
gb:MT106054|Organism:Severe      ACTTTGTCTCTGATGCAGATTCAACTTTGATTGGTGATTGTGCAACTGTACATACAGCTA   21022
gb:MT049951|Organism:Severe      ACTTTGTCTCTGATGCAGATTCAACTTTGATTGGTGATTGTGCAACTGTACATACAGCTA   21022
gb:MN975262|Organism:Severe      ACTTTGTCTCTGATGCAGATTCAACTTTGATTGGTGATTGTGCAACTGTACATACAGCTA   21022
gb:MT106052|Organism:Severe      ACTTTGTCTCTGATGCAGATTCAACTTTGATTGGTGATTGTGCAACTGTACATACAGCTA   21022
gb:LC522975|Organism:Severe      ACTTTGTCTCTGATGCAGATTCAACTTTGATTGGTGATTGTGCAACTGTACATACAGCTA   21019
gb:LC522973|Organism:Severe      ACTTTGTCTCTGATGCAGATTCAACTTTGATTGGTGATTGTGCAACTGTACATACAGCTA   21019
gb:LC522974|Organism:Severe      ACTTTGTCTCTGATGCAGATTCAACTTTGATTGGTGATTGTGCAACTGTACATACAGCTA   21019
gb:MN985325|Organism:Severe      ACTTTGTCTCTGATGCAGATTCAACTTTGATTGGTGATTGTGCAACTGTACATACAGCTA   21022
gb:MT020881|Organism:Severe      ACTTTGTCTCTGATGCAGATTCAACTTTGATTGGTGATTGTGCAACTGTACATACAGCTA   21022
gb:MT020880|Organism:Severe      ACTTTGTCTCTGATGCAGATTCAACTTTGATTGGTGATTGTGCAACTGTACATACAGCTA   21022
gb:MT066175|Organism:Severe      ACTTTGTCTCTGATGCAGATTCAACTTTGATTGGTGATTGTGCAACTGTACATACAGCTA   21022
gb:MN997409|Organism:Severe      ACTTTGTCTCTGATGCAGATTCAACTTTGATTGGTGATTGTGCAACTGTACATACAGCTA   21022
```

```
gb:MN938384|Organism:Severe      ACTTTGTCTCTGATGCAGATTCAACTTTGATTGGTGATTGTGCAACTGTACATACAGCTA   20990
gb:MT044258|Organism:Severe      ACTTTGTCTCTGATGCAGATTCAACTTTGATTGGTGATTGTGCAACTGTACATACAGCTA   20998
gb:MT039890|Organism:Severe      ACTTTGTCTCTGATGCAGATTCAACTTTGATTGGTGATTGTGCAACTGTACATACAGCTA   21022
gb:MN988713|Organism:Severe      ACTTTGTCTCTGATGCAGATTCAACTTTGATTGGTGATTGTGCAACTGTACATACAGCTA   21022
gb:LC521925|Organism:Severe      ACTTTGTCTCTGATGCAGATTCAACTTTGATTGGTGATTGTGCAACTGTACATACAGCTA   20995
gb:MT093571|Organism:Severe      ACTTTGTCTCTGATGCAGATTCAACTTTGATTGGTGATTGTGCAACTGTACATACAGCTA   21022
gb:MT039887|Organism:Severe      ACTTTGTCTCTGATGCAGATTCAACTTTGATTGGTGATTGTGCAACTGTACATACAGCTA   21019
gb:MT019530|Organism:Severe      ACTTTGTCTCTGATGCAGATTCAACTTTGATTGGTGATTGTGCAACTGTACATACAGCTA   21022
gb:MT039888|Organism:Severe      ACTTTGTCTCTGATGCAGATTCAACTTTGATTGGTGATTGTGCAACTGTACATACAGCTA   21022
gb:LC522972|Organism:Severe      ACTTTGTCTCTGATGCAGATTCAACTTTGATTGGTGATTGTGCAACTGTACATACAGCTA   21019
gb:MT027063|Organism:Severe      ACTTTGTCTCTGATGCAGATTCAACTTTGATTGGTGATTGTGCAACTGTACATACAGCTA   21022
gb:MT027062|Organism:Severe      ACTTTGTCTCTGATGCAGATTCAACTTTGATTGGTGATTGTGCAACTGTACATACAGCTA   21022
gb:MT019529|Organism:Severe      ACTTTGTCTCTGATGCAGATTCAACTTTGATTGGTGATTGTGCAACTGTACATACAGCTA   21022
gb:MN996529|Organism:Severe      ACTTTGTCTCTGATGCAGATTCAACTTTGATTGGTGATTGTGCAACTGTACATACAGCTA   21010
gb:MN996531|Organism:Severe      ACTTTGTCTCTGATGCAGATTCAACTTTGATTGGTGATTGTGCAACTGTACATACAGCTA   21009
gb:MT066176|Organism:Severe      ACTTTGTCTCTGATGCAGATTCAACTTTGATTGGTGATTGTGCAACTGTACATACAGCTA   21022
gb:MT027064|Organism:Severe      ACTTTGTCTCTGATGCAGATTCAACTTTGATTGGTGATTGTGCAACTGTACATACAGCTA   21022
gb:MN994468|Organism:Severe      ACTTTGTCTCTGATGCAGATTCAACTTTGATTGGTGATTGTGCAACTGTACATACAGCTA   21022
gb:MT072688|Organism:Severe      ACTTTGTCTCTGATGCAGATTCAACTTTGATTGGTGATTGTGCAACTGTACATACAGCTA   21007
gb:MN996527|Organism:Severe      ACTTTGTCTCTGATGCAGATTCAACTTTGATTGGTGATTGTGCAACTGTACATACAGCTA   20989
gb:MT093631|Organism:Severe      ACTTTGTCTCTGATGCAGATTCAACTTTGATTGGTGATTGTGCAACTGTACATACAGCTA   21060
gb:MT106053|Organism:Severe      ACTTTGTCTCTGATGCAGATTCAACTTTGATTGGTGATTGTGCAACTGTACATACAGCTA   21022
gb:MT019533|Organism:Severe      ACTTTGTCTCTGATGCAGATTCAACTTTGATTGGTGATTGTGCAACTGTACATACAGCTA   21022
gb:MT019531|Organism:Severe      ACTTTGTCTCTGATGCAGATTCAACTTTGATTGGTGATTGTGCAACTGTACATACAGCTA   21022
gb:MN996528|Organism:Severe      ACTTTGTCTCTGATGCAGATTCAACTTTGATTGGTGATTGTGCAACTGTACATACAGCTA   21022
gb:MN996530|Organism:Severe      ACTTTGTCTCTGATGCAGATTCAACTTTGATTGGTGATTGTGCAACTGTACATACAGCTA   21008
gb:MN908947|Organism:Severe      ACTTTGTCTCTGATGCAGATTCAACTTTGATTGGTGATTGTGCAACTGTACATACAGCTA   21022
gb:MT019532|Organism:Severe      ACTTTGTCTCTGATGCAGATTCAACTTTGATTGGTGATTGTGCAACTGTACATACAGCTA   21022
                                 ************************************************************

gb:MT020781|Organism:Severe      ATAAATGGGATCTCATTATTAGTGATATGTACGACCCTAAGACTAAAAATGTTACAAAAG   21070
gb:MT007544|Organism:Severe      ATAAATGGGATCTCATTATTAGTGATATGTACGACCCTAAGACTAAAAATGTTACAAAAG   21082
gb:MN994467|Organism:Severe      ATAAATGGGATCTCATTATTAGTGATATGTACGACCCTAAGACTAAAAATGTTACAAAAG   21082
gb:MT044257|Organism:Severe      ATAAATGGGATCTCATTATTAGTGATATGTACGACCCTAAGACTAAAAATGTTACAAAAG   21082
gb:MT106054|Organism:Severe      ATAAATGGGATCTCATTATTAGTGATATGTACGACCCTAAGACTAAAAATGTTACAAAAG   21082
gb:MT049951|Organism:Severe      ATAAATGGGATCTCATTATTAGTGATATGTACGACCCTAAGACTAAAAATGTTACAAAAG   21082
gb:MN975262|Organism:Severe      ATAAATGGGATCTCATTATTAGTGATATGTACGACCCTAAGACTAAAAATGTTACAAAAG   21082
gb:MT106052|Organism:Severe      ATAAATGGGATCTCATTATTAGTGATATGTACGACCCTAAGACTAAAAATGTTACAAAAG   21082
gb:LC522975|Organism:Severe      ATAAATGGGATCTCATTATTAGTGATATGTACGACCCTAAGACTAAAAATGTTACAAAAG   21079
gb:LC522973|Organism:Severe      ATAAATGGGATCTCATTATTAGTGATATGTACGACCCTAAGACTAAAAATGTTACAAAAG   21079
gb:LC522974|Organism:Severe      ATAAATGGGATCTCATTATTAGTGATATGTACGACCCTAAGACTAAAAATGTTACAAAAG   21079
gb:MN985325|Organism:Severe      ATAAATGGGATCTCATTATTAGTGATATGTACGACCCTAAGACTAAAAATGTTACAAAAG   21082
gb:MT020881|Organism:Severe      ATAAATGGGATCTCATTATTAGTGATATGTACGACCCTAAGACTAAAAATGTTACAAAAG   21082
gb:MT020880|Organism:Severe      ATAAATGGGATCTCATTATTAGTGATATGTACGACCCTAAGACTAAAAATGTTACAAAAG   21082
gb:MT066175|Organism:Severe      ATAAATGGGATCTCATTATTAGTGATATGTACGACCCTAAGACTAAAAATGTTACAAAAG   21082
gb:MN997409|Organism:Severe      ATAAATGGGATCTCATTATTAGTGATATGTACGACCCTAAGACTAAAAATGTTACAAAAG   21082
gb:MN938384|Organism:Severe      ATAAATGGGATCTCATTATTAGTGATATGTACGACCCTAAGACTAAAAATGTTACAAAAG   21050
gb:MT044258|Organism:Severe      ATAAATGGGATCTCATTATTAGTGATATGTACGACCCTAAGACTAAAAATGTTACAAAAG   21058
gb:MT039890|Organism:Severe      ATAAATGGGATCTCATTATTAGTGATATGTACGACCCTAAGACTAAAAATGTTACAAAAG   21082
gb:MN988713|Organism:Severe      ATAAATGGGATCTCATTATTAGTGATATGTACGACCCTAAGACTAAAAATGTTACAAAAG   21082
gb:LC521925|Organism:Severe      ATAAATGGGATCTCATTATTAGTGATATGTACGACCCTAAGACTAAAAATGTTACAAAAG   21055
gb:MT093571|Organism:Severe      ATAAATGGGATCTCATTATTAGTGATATGTACGACCCTAAGACTAAAAATGTTACAAAAG   21082
gb:MT039887|Organism:Severe      ATAAATGGGATCTCATTATTAGTGATATGTACGACCCTAAGACTAAAAATGTTACAAAAG   21079
gb:MT019530|Organism:Severe      ATAAATGGGATCTCATTATTAGTGATATGTACGACCCTAAGACTAAAAATGTTACAAAAG   21082
gb:MT039888|Organism:Severe      ATAAATGGGATCTCATTATTAGTGATATGTACGACCCTAAGACTAAAAATGTTACAAAAG   21082
gb:LC522972|Organism:Severe      ATAAATGGGATCTCATTATTAGTGATATGTACGACCCTAAGACTAAAAATGTTACAAAAG   21079
gb:MT027063|Organism:Severe      ATAAATGGGATCTCATTATTAGTGATATGTACGACCCTAAGACTAAAAATGTTACAAAAG   21082
gb:MT027062|Organism:Severe      ATAAATGGGATCTCATTATTAGTGATATGTACGACCCTAAGACTAAAAATGTTACAAAAG   21082
gb:MT019529|Organism:Severe      ATAAATGGGATCTCATTATTAGTGATATGTACGACCCTAAGACTAAAAATGTTACAAAAG   21082
gb:MN996529|Organism:Severe      ATAAATGGGATCTCATTATTAGTGATATGTACGACCCTAAGACTAAAAATGTTACAAAAG   21070
gb:MN996531|Organism:Severe      ATAAATGGGATCTCATTATTAGTGATATGTACGACCCTAAGACTAAAAATGTTACAAAAG   21069
gb:MT066176|Organism:Severe      ATAAATGGGATCTCATTATTAGTGATATGTACGACCCTAAGACTAAAAATGTTACAAAAG   21082
gb:MT027064|Organism:Severe      ATAAATGGGATCTCATTATTAGTGATATGTACGACCCTAAGACTAAAAATGTTACAAAAG   21082
gb:MN994468|Organism:Severe      ATAAATGGGATCTCATTATTAGTGATATGTACGACCCTAAGACTAAAAATGTTACAAAAG   21082
gb:MT072688|Organism:Severe      ATAAATGGGATCTCATTATTAGTGATATGTACGACCCTAAGACTAAAAATGTTACAAAAG   21067
```

```
gb:MN996527|Organism:Severe      ATAAATGGGATCTCATTATTAGTGATATGTACGACCCTAAGACTAAAAATGTTACAAAAG   21049
gb:MT093631|Organism:Severe      ATAAATGGGATCTCATTATTAGTGATATGTACGACCCTAAGACTAAAAATGTTACAAAAG   21120
gb:MT106053|Organism:Severe      ATAAATGGGATCTCATTATTAGTGATATGTACGACCCTAAGACTAAAAATGTTACAAAAG   21082
gb:MT019533|Organism:Severe      ATAAATGGGATCTCATTATTAGTGATATGTACGACCCTAAGACTAAAAATGTTACAAAAG   21082
gb:MT019531|Organism:Severe      ATAAATGGGATCTCATTATTAGTGATATGTACGACCCTAAGACTAAAAATGTTACAAAAG   21082
gb:MN996528|Organism:Severe      ATAAATGGGATCTCATTATTAGTGATATGTACGACCCTAAGACTAAAAATGTTACAAAAG   21082
gb:MN996530|Organism:Severe      ATAAATGGGATCTCATTATTAGTGATATGTACGACCCTAAGACTAAAAATGTTACAAAAG   21068
gb:MN908947|Organism:Severe      ATAAATGGGATCTCATTATTAGTGATATGTACGACCCTAAGACTAAAAATGTTACAAAAG   21082
gb:MT019532|Organism:Severe      ATAAATGGGATCTCATTATTAGTGATATGTACGACCCTAAGACTAAAAATGTTACAAAAG   21082
                                 ************************************************************

gb:MT020781|Organism:Severe      AAAATGACTCTAAAGAGGGTTTTTTCACTTACATTTGTGGGTTTATACAACAAAAGCTAG   21130
gb:MT007544|Organism:Severe      AAAATGACTCTAAAGAGGGTTTTTTCACTTACATTTGTGGGTTTATACAACAAAAGCTAG   21142
gb:MN994467|Organism:Severe      AAAATGACTCTAAAGAGGGTTTTTTCACTTACATTTGTGGGTTTATACAACAAAAGCTAG   21142
gb:MT044257|Organism:Severe      AAAATGACTCTAAAGAGGGTTTTTTCACTTACATTTGTGGGTTTATACAACAAAAGCTAG   21142
gb:MT106054|Organism:Severe      AAAATGACTCTAAAGAGGGTTTTTTCACTTACATTTGTGGGTTTATACAACAAAAGCTAG   21142
gb:MT049951|Organism:Severe      AAAATGACTCTAAAGAGGGTTTTTTCACTTACATTTGTGGGTTTATACAACAAAAGCTAG   21142
gb:MN975262|Organism:Severe      AAAATGACTCTAAAGAGGGTTTTTTCACTTACATTTGTGGGTTTATACAACAAAAGCTAG   21142
gb:MT106052|Organism:Severe      AAAATGACTCTAAAGAGGGTTTTTTCACTTACATTTGTGGGTTTATACAACAAAAGCTAG   21142
gb:LC522975|Organism:Severe      AAAATGACTCTAAAGAGGGTTTTTTCACTTACATTTGTGGGTTTATACAACAAAAGCTAG   21139
gb:LC522973|Organism:Severe      AAAATGACTCTAAAGAGGGTTTTTTCACTTACATTTGTGGGTTTATACAACAAAAGCTAG   21139
gb:LC522974|Organism:Severe      AAAATGACTCTAAAGAGGGTTTTTTCACTTACATTTGTGGGTTTATACAACAAAAGCTAG   21139
gb:MN985325|Organism:Severe      AAAATGACTCTAAAGAGGGTTTTTTCACTTACATTTGTGGGTTTATACAACAAAAGCTAG   21142
gb:MT020881|Organism:Severe      AAAATGACTCTAAAGAGGGTTTTTTCACTTACATTTGTGGGTTTATACAACAAAAGCTAG   21142
gb:MT020880|Organism:Severe      AAAATGACTCTAAAGAGGGTTTTTTCACTTACATTTGTGGGTTTATACAACAAAAGCTAG   21142
gb:MT066175|Organism:Severe      AAAATGACTCTAAAGAGGGTTTTTTCACTTACATTTGTGGGTTTATACAACAAAAGCTAG   21142
gb:MN997409|Organism:Severe      AAAATGACTCTAAAGAGGGTTTTTTCACTTACATTTGTGGGTTTATACAACAAAAGCTAG   21142
gb:MN938384|Organism:Severe      AAAATGACTCTAAAGAGGGTTTTTTCACTTACATTTGTGGGTTTATACAACAAAAGCTAG   21110
gb:MT044258|Organism:Severe      AAAATGACTCTAAAGAGGGTTTTTTCACTTACATTTGTGGGTTTATACAACAAAAGCTAG   21118
gb:MT039890|Organism:Severe      AAAATGACTCTAAAGAGGGTTTTTTCACTTACATTTGTGGGTTTATACAACAAAAGCTAG   21142
gb:MN988713|Organism:Severe      AAAATGACTCTAAAGAGGGTTTTTTCACTTACATTTGTGGGTTTATACAACAAAAGCTAG   21142
gb:LC521925|Organism:Severe      AAAATGACTCTAAAGAGGGTTTTTTCACTTACATTTGTGGGTTTATACAACAAAAGCTAG   21115
gb:MT093571|Organism:Severe      AAAATGACTCTAAAGAGGGTTTTTTCACTTACATTTGTGGGTTTATACAACAAAAGCTAG   21142
gb:MT039887|Organism:Severe      AAAATGACTCTAAAGAGGGTTTTTTCACTTACATTTGTGGGTTTATACAACAAAAGCTAG   21139
gb:MT019530|Organism:Severe      AAAATGACTCTAAAGAGGGTTTTTTCACTTACATTTGTGGGTTTATACAACAAAAGCTAG   21142
gb:MT039888|Organism:Severe      AAAATGACTCTAAAGAGGGTTTTTTCACTTACATTTGTGGGTTTATACAACAAAAGCTAG   21142
gb:LC522972|Organism:Severe      AAAATGACTCTAAAGAGGGTTTTTTCACTTACATTTGTGGGTTTATACAACAAAAGCTAG   21139
gb:MT027063|Organism:Severe      AAAATGACTCTAAAGAGGGTTTTTTCACTTACATTTGTGGGTTTATACAACAAAAGCTAG   21142
gb:MT027062|Organism:Severe      AAAATGACTCTAAAGAGGGTTTTTTCACTTACATTTGTGGGTTTATACAACAAAAGCTAG   21142
gb:MT019529|Organism:Severe      AAAATGACTCTAAAGAGGGTTTTTTCACTTACATTTGTGGGTTTATACAACAAAAGCTAG   21142
gb:MN996529|Organism:Severe      AAAATGACTCTAAAGAGGGTTTTTTCACTTACATTTGTGGGTTTATACAACAAAGGCTAG   21130
gb:MN996531|Organism:Severe      AAAATGACTCTAAAGAGGGTTTTTTCACTTACATTTGTGGGTTTATACAACAAAAGCTAG   21129
gb:MT066176|Organism:Severe      AAAATGACTCTAAAGAGGGTTTTTTCACTTACATTTGTGGGTTTATACAACAAAAGCTAG   21142
gb:MT027064|Organism:Severe      AAAATGACTCTAAAGAGGGTTTTTTCACTTACATTTGTGGGTTTATACAACAAAAGCTAG   21142
gb:MN994468|Organism:Severe      AAAATGACTCTAAAGAGGGTTTTTTCACTTACATTTGTGGGTTTATACAACAAAAGCTAG   21142
gb:MT072688|Organism:Severe      AAAATGACTCTAAAGAGGGTTTTTTCACTTACATTTGTGGGTTTATACAACAAAAGCTAG   21127
gb:MN996527|Organism:Severe      AAAATGACTCTAAAGAGGGTTTTTTCACTTACATTTGTGGGTTTATACAACAAAAGCTAG   21109
gb:MT093631|Organism:Severe      AAAATGACTCTAAAGAGGGTTTTTTCACTTACATTTGTGGGTTTATACAACAAAAGCTAG   21180
gb:MT106053|Organism:Severe      AAAATGACTCTAAAGAGGGTTTTTTCACTTACATTTGTGGGTTTATACAACAAAAGCTAG   21142
gb:MT019533|Organism:Severe      AAAATGACTCTAAAGAGGGTTTTTTCACTTACATTTGTGGGTTTATACAACAAAAGCTAG   21142
gb:MT019531|Organism:Severe      AAAATGACTCTAAAGAGGGTTTTTTCACTTACATTTGTGGGTTTATACAACAAAAGCTAG   21142
gb:MN996528|Organism:Severe      AAAATGACTCTAAAGAGGGTTTTTTCACTTACATTTGTGGGTTTATACAACAAAAGCTAG   21142
gb:MN996530|Organism:Severe      AAAATGACTCTAAAGAGGGTTTTTTCACTTACATTTGTGGGTTTATACAACAAAAGCTAG   21128
gb:MN908947|Organism:Severe      AAAATGACTCTAAAGAGGGTTTTTTCACTTACATTTGTGGGTTTATACAACAAAAGCTAG   21142
gb:MT019532|Organism:Severe      AAAATGACTCTAAAGAGGGTTTTTTCACTTACATTTGTGGGTTTATACAACAAAAGCTAG   21142
                                 ****************************************************** *****

gb:MT020781|Organism:Severe      CTCTTGGAGGTTCCGTGGCTATAAAGATAACAGAACATTCTTGGAATGCTGATCTTTATA   21190
gb:MT007544|Organism:Severe      CTCTTGGAGGTTCCGTGGCTATAAAGATAACAGAACATTCTTGGAATGCTGATCTTTATA   21202
gb:MN994467|Organism:Severe      CTCTTGGAGGTTCCGTGGCTATAAAGATAACAGAACATTCTTGGAATGCTGATCTTTATA   21202
gb:MT044257|Organism:Severe      CTCTTGGAGGTTCCGTGGCTATAAAGATAACAGAACATTCTTGGAATGCTGATCTTTATA   21202
gb:MT106054|Organism:Severe      CTCTTGGAGGTTCCGTGGCTATAAAGATAACAGAACATTCTTGGAATGCTGATCTTTATA   21202
gb:MT049951|Organism:Severe      CTCTTGGAGGTTCCGTGGCTATAAAGATAACAGAACATTCTTGGAATGCTGATCTTTATA   21202
gb:MN975262|Organism:Severe      CTCTTGGAGGTTCCGTGGCTATAAAGATAACAGAACATTCTTGGAATGCTGATCTTTATA   21202
gb:MT106052|Organism:Severe      CTCTTGGAGGTTCCGTGGCTATAAAGATAACAGAACATTCTTGGAATGCTGATCTTTATA   21202
```

```
gb:LC522975|Organism:Severe      CTCTTGGAGGTTCCGTGGCTATAAAGATAACAGAACATTCTTGGAATGCTGATCTTTATA   21199
gb:LC522973|Organism:Severe      CTCTTGGAGGTTCCGTGGCTATAAAGATAACAGAACATTCTTGGAATGCTGATCTTTATA   21199
gb:LC522974|Organism:Severe      CTCTTGGAGGTTCCGTGGCTATAAAGATAACAGAACATTCTTGGAATGCTGATCTTTATA   21199
gb:MN985325|Organism:Severe      CTCTTGGAGGTTCCGTGGCTATAAAGATAACAGAACATTCTTGGAATGCTGATCTTTATA   21202
gb:MT020881|Organism:Severe      CTCTTGGAGGTTCCGTGGCTATAAAGATAACAGAACATTCTTGGAATGCTGATCTTTATA   21202
gb:MT020880|Organism:Severe      CTCTTGGAGGTTCCGTGGCTATAAAGATAACAGAACATTCTTGGAATGCTGATCTTTATA   21202
gb:MT066175|Organism:Severe      CTCTTGGAGGTTCCGTGGCTATAAAGATAACAGAACATTCTTGGAATGCTGATCTTTATA   21202
gb:MN997409|Organism:Severe      CTCTTGGAGGTTCCGTGGCTATAAAGATAACAGAACATTCTTGGAATGCTGATCTTTATA   21202
gb:MN938384|Organism:Severe      CTCTTGGAGGTTCCGTGGCTATAAAGATAACAGAACATTCTTGGAATGCTGATCTTTATA   21170
gb:MT044258|Organism:Severe      CTCTTGGAGGTTCCGTGGCTATAAAGATAACAGAACATTCTTGGAATGCTGATCTTTATA   21178
gb:MT039890|Organism:Severe      CTCTTGGAGGTTCCGTGGCTATAAAGATAACAGAACATTCTTGGAATGCTGATCTTTATA   21202
gb:MN988713|Organism:Severe      CTCTTGGAGGTTCCGTGGCTATAAAGATAACAGAACATTCTTGGAATGCTGATCTTTATA   21202
gb:LC521925|Organism:Severe      CTCTTGGAGGTTCCGTGGCTATAAAGATAACAGAACATTCTTGGAATGCTGATCTTTATA   21175
gb:MT093571|Organism:Severe      CTCTTGGAGGTTCCGTGGCTATAAAGATAACAGAACATTCTTGGAATGCTGATCTTTATA   21202
gb:MT039887|Organism:Severe      CTCTTGGAGGTTCCGTGGCTATAAAGATAACAGAACATTCTTGGAATGCTGATCTTTATA   21199
gb:MT019530|Organism:Severe      CTCTTGGAGGTTCCGTGGCTATAAAGATAACAGAACATTCTTGGAATGCTGATCTTTATA   21202
gb:MT039888|Organism:Severe      CTCTTGGAGGTTCCGTGGCTATAAAGATAACAGAACATTCTTGGAATGCTGATCTTTATA   21202
gb:LC522972|Organism:Severe      CTCTTGGAGGTTCCGTGGCTATAAAGATAACAGAACATTCTTGGAATGCTGATCTTTATA   21199
gb:MT027063|Organism:Severe      CTCTTGGAGGTTCCGTGGCTATAAAGATAACAGAACATTCTTGGAATGCTGATCTTTATA   21202
gb:MT027062|Organism:Severe      CTCTTGGAGGTTCCGTGGCTATAAAGATAACAGAACATTCTTGGAATGCTGATCTTTATA   21202
gb:MT019529|Organism:Severe      CTCTTGGAGGTTCCGTGGCTATAAAGATAACAGAACATTCTTGGAATGCTGATCTTTATA   21202
gb:MN996529|Organism:Severe      CTCTTGGAGGTTCCGTGGCTATAAAGATAACAGAACATTCTTGGAATGCTGATCTTTATA   21190
gb:MN996531|Organism:Severe      CTCTTGGAGGTTCCGTGGCTATAAAGATAACAGAACATTCTTGGAATGCTGATCTTTATA   21189
gb:MT066176|Organism:Severe      CTCTTGGAGGTTCCGTGGCTATAAAGATAACAGAACATTCTTGGAATGCTGATCTTTATA   21202
gb:MT027064|Organism:Severe      CTCTTGGAGGTTCCGTGGCTATAAAGATAACAGAACATTCTTGGAATGCTGATCTTTATA   21202
gb:MN994468|Organism:Severe      CTCTTGGAGGTTCCGTGGCTATAAAGATAACAGAACATTCTTGGAATGCTGATCTTTATA   21202
gb:MT072688|Organism:Severe      CTCTTGGAGGTTCCGTGGCTATAAAGATAACAGAACATTCTTGGAATGCTGATCTTTATA   21187
gb:MN996527|Organism:Severe      CTCTTGGAGGTTCCGTGGCTATAAAGATAACAGAACATTCTTGGAATGCTGATCTTTATA   21169
gb:MT093631|Organism:Severe      CTCTTGGAGGTTCCGTGGCTATAAAGATAACAGAACATTCTTGGAATGCTGATCTTTATA   21240
gb:MT106053|Organism:Severe      CTCTTGGAGGTTCCGTGGCTATAAAGATAACAGAACATTCTTGGAATGCTGATCTTTATA   21202
gb:MT019533|Organism:Severe      CTCTTGGAGGTTCCGTGGCTATAAAGATAACAGAACATTCTTGGAATGCTGATCTTTATA   21202
gb:MT019531|Organism:Severe      CTCTTGGAGGTTCCGTGGCTATAAAGATAACAGAACATTCTTGGAATGCTGATCTTTATA   21202
gb:MN996528|Organism:Severe      CTCTTGGAGGTTCCGTGGCTATAAAGATAACAGAACATTCTTGGAATGCTGATCTTTATA   21202
gb:MN996530|Organism:Severe      CTCTTGGAGGTTCCGTGGCTATAAAGATAACAGAACATTCTTGGAATGCTGATCTTTATA   21188
gb:MN908947|Organism:Severe      CTCTTGGAGGTTCCGTGGCTATAAAGATAACAGAACATTCTTGGAATGCTGATCTTTATA   21202
gb:MT019532|Organism:Severe      CTCTTGGAGGTTCCGTGGCTATAAAGATAACAGAACATTCTTGGAATGCTGATCTTTATA   21202
                                 ************************************************************

gb:MT020781|Organism:Severe      AGCTCATGGGACACTTCGCATGGTGGACAGCCTTTGTTACTAATGTGAATGCGTCATCAT   21250
gb:MT007544|Organism:Severe      AGCTCATGGGACACTTCGCATGGTGGACAGCCTTTGTTACTAATGTGAATGCGTCATCAT   21262
gb:MN994467|Organism:Severe      AGCTCATGGGACACTTCGCATGGTGGACAGCCTTTGTTACTAATGTGAATGCGTCATCAT   21262
gb:MT044257|Organism:Severe      AGCTCATGGGACACTTCGCATGGTGGACAGCCTTTGTTACTAATGTGAATGCGTCATCAT   21262
gb:MT106054|Organism:Severe      AGCTCATGGGACACTTCGCATGGTGGACAGCCTTTGTTACTAATGTGAATGCGTCATCAT   21262
gb:MT049951|Organism:Severe      AGCTCATGGGACACTTCGCATGGTGGACAGCCTTTGTTACTAATGTGAATGCGTCATCAT   21262
gb:MN975262|Organism:Severe      AGCTCATGGGACACTTCGCATGGTGGACAGCCTTTGTTACTAATGTGAATGCGTCATCAT   21262
gb:MT106052|Organism:Severe      AGCTCATGGGACACTTCGCATGGTGGACAGCCTTTGTTACTAATGTGAATGCGTCATCAT   21262
gb:LC522975|Organism:Severe      AGCTCATGGGACACTTCGCATGGTGGACAGCCTTTGTTACTAATGTGAATGCGTCATCAT   21259
gb:LC522973|Organism:Severe      AGCTCATGGGACACTTCGCATGGTGGACAGCCTTTGTTACTAATGTGAATGCGTCATCAT   21259
gb:LC522974|Organism:Severe      AGCTCATGGGACACTTCGCATGGTGGACAGCCTTTGTTACTAATGTGAATGCGTCATCAT   21259
gb:MN985325|Organism:Severe      AGCTCATGGGACACTTCGCATGGTGGACAGCCTTTGTTACTAATGTGAATGCGTCATCAT   21262
gb:MT020881|Organism:Severe      AGCTCATGGGACACTTCGCATGGTGGACAGCCTTTGTTACTAATGTGAATGCGTCATCAT   21262
gb:MT020880|Organism:Severe      AGCTCATGGGACACTTCGCATGGTGGACAGCCTTTGTTACTAATGTGAATGCGTCATCAT   21262
gb:MT066175|Organism:Severe      AGCTCATGGGACACTTCGCATGGTGGACAGCCTTTGTTACTAATGTGAATGCGTCATCAT   21262
gb:MN997409|Organism:Severe      AGCTCATGGGACACTTCGCATGGTGGACAGCCTTTGTTACTAATGTGAATGCGTCATCAT   21262
gb:MN938384|Organism:Severe      AGCTCATGGGACACTTCGCATGGTGGACAGCCTTTGTTACTAATGTGAATGCGTCATCAT   21230
gb:MT044258|Organism:Severe      AGCTCATGGGACACTTCGCATGGTGGACAGCCTTTGTTACTAATGTGAATGCGTCATCAT   21238
gb:MT039890|Organism:Severe      AGCTCATGGGACACTTCGCATGGTGGACAGCCTTTGTTACTAATGTGAATGCGTCATCAT   21262
gb:MN988713|Organism:Severe      AGCTCATGGGACACTTCGCATGGTGGACAGCCTTTGTTACTAATGTGAATGCGTCATCAT   21262
gb:LC521925|Organism:Severe      AGCTCATGGGACACTTCGCATGGTGGACAGCCTTTGTTACTAATGTGAATGCGTCATCAT   21235
gb:MT093571|Organism:Severe      AGCTCATGGGACACTTCGCATGGTGGACAGCCTTTGTTACTAATGTGAATGCGTCATCAT   21262
gb:MT039887|Organism:Severe      AGCTCATGGGACACTTCGCATGGTGGACAGCCTTTGTTACTAATGTGAATGCGTCATCAT   21259
gb:MT019530|Organism:Severe      AGCTCATGGGACACTTCGCATGGTGGACAGCCTTTGTTACTAATGTGAATGCGTCATCAT   21262
gb:MT039888|Organism:Severe      AGCTCATGGGACACTTCGCATGGTGGACAGCCTTTGTTACTAATGTGAATGCGTCATCAT   21262
gb:LC522972|Organism:Severe      AGCTCATGGGACACTTCGCATGGTGGACAGCCTTTGTTACTAATGTGAATGCGTCATCAT   21259
gb:MT027063|Organism:Severe      AGCTCATGGGACACTTCGCATGGTGGACAGCCTTTGTTACTAATGTGAATGCGTCATCAT   21262
```

```
gb:MT027062|Organism:Severe      AGCTCATGGGACACTTCGCATGGTGGACAGCCTTTGTTACTAATGTGAATGCGTCATCAT   21262
gb:MT019529|Organism:Severe      AGCTCATGGGACACTTCGCATGGTGGACAGCCTTTGTTACTAATGTGAATGCGTCATCAT   21262
gb:MN996529|Organism:Severe      AGCTCATGGGACACTTCGCATGGTGGACAGCCTTTGTTACTAATGTGAATGCGTCATCAT   21250
gb:MN996531|Organism:Severe      AGCTCATGGGACACTTCGCATGGTGGACAGCCTTTGTTACTAATGTGAATGCGTCATCAT   21249
gb:MT066176|Organism:Severe      AGCTCATGGGACACTTCGCATGGTGGACAGCCTTTGTTACTAATGTGAATGCGTCATCAT   21262
gb:MT027064|Organism:Severe      AGCTCATGGGACACTTCGCATGGTGGACAGCCTTTGTTACTAATGTGAATGCGTCATCAT   21262
gb:MN994468|Organism:Severe      AGCTCATGGGACACTTCGCATGGTGGACAGCCTTTGTTACTAATGTGAATGCGTCATCAT   21262
gb:MT072688|Organism:Severe      AGCTCATGGGACACTTCGCATGGTGGACAGCCTTTGTTACTAATGTGAATGCGTCATCAT   21247
gb:MN996527|Organism:Severe      AGCTCATGGGACACTTCGCATGGTGGACAGCCTTTGTTACTAATGTGAATGCGTCATCAT   21229
gb:MT093631|Organism:Severe      AGCTCATGGGACACTTCGCATGGTGGACAGCCTTTGTTACTAATGTGAATGCGTCATCAT   21300
gb:MT106053|Organism:Severe      AGCTCATGGGACACTTCGCATGGTGGACAGCCTTTGTTACTAATGTGAATGCGTCATCAT   21262
gb:MT019533|Organism:Severe      AGCTCATGGGACACTTCGCATGGTGGACAGCCTTTGTTACTAATGTGAATGCGTCATCAT   21262
gb:MT019531|Organism:Severe      AGCTCATGGGACACTTCGCATGGTGGACAGCCTTTGTTACTAATGTGAATGCGTCATCAT   21262
gb:MN996528|Organism:Severe      AGCTCATGGGACACTTCGCATGGTGGACAGCCTTTGTTACTAATGTGAATGCGTCATCAT   21262
gb:MN996530|Organism:Severe      AGCTCATGGGACACTTCGCATGGTGGACAGCCTTTGTTACTAATGTGAATGCGTCATCAT   21248
gb:MN908947|Organism:Severe      AGCTCATGGGACACTTCGCATGGTGGACAGCCTTTGTTACTAATGTGAATGCGTCATCAT   21262
gb:MT019532|Organism:Severe      AGCTCATGGGACACTTCGCATGGTGGACAGCCTTTGTTACTAATGTGAATGCGTCATCAT   21262
                                 ************************************************************

gb:MT020781|Organism:Severe      CTGAAGCATTTTTAATTGGATGTAATTATCTTGGCAAACCACGCGAACAAATAGATGGTT   21310
gb:MT007544|Organism:Severe      CTGAAGCATTTTTAATTGGATGTAATTATCTTGGCAAACCACGCGAACAAATAGATGGTT   21322
gb:MN994467|Organism:Severe      CTGAAGCATTTTTAATTGGATGTAATTATCTTGGCAAACCACGCGAACAAATAGATGGTT   21322
gb:MT044257|Organism:Severe      CTGAAGCATTTTTAATTGGATGTAATTATCTTGGCAAACCACGCGAACAAATAGATGGTT   21322
gb:MT106054|Organism:Severe      CTGAAGCATTTTTAATTGGATGTAATTATCTTGGCAAACCACGCGAACAAATAGATGGTT   21322
gb:MT049951|Organism:Severe      CTGAAGCATTTTTAATTGGATGTAATTATCTTGGCAAACCACGCGAACAAATAGATGGTT   21322
gb:MN975262|Organism:Severe      CTGAAGCATTTTTAATTGGATGTAATTATCTTGGCAAACCACGCGAACAAATAGATGGTT   21322
gb:MT106052|Organism:Severe      CTGAAGCATTTTTAATTGGATGTAATTATCTTGGCAAACCACGCGAACAAATAGATGGTT   21322
gb:LC522975|Organism:Severe      CTGAAGCATTTTTAATTGGATGTAATTATCTTGGCAAACCACGCGAACAAATAGATGGTT   21319
gb:LC522973|Organism:Severe      CTGAAGCATTTTTAATTGGATGTAATTATCTTGGCAAACCACGCGAACAAATAGATGGTT   21319
gb:LC522974|Organism:Severe      CTGAAGCATTTTTAATTGGATGTAATTATCTTGGCAAACCACGCGAACAAATAGATGGTT   21319
gb:MN985325|Organism:Severe      CTGAAGCATTTTTAATTGGATGTAATTATCTTGGCAAACCACGCGAACAAATAGATGGTT   21322
gb:MT020881|Organism:Severe      CTGAAGCATTTTTAATTGGATGTAATTATCTTGGCAAACCACGCGAACAAATAGATGGTT   21322
gb:MT020880|Organism:Severe      CTGAAGCATTTTTAATTGGATGTAATTATCTTGGCAAACCACGCGAACAAATAGATGGTT   21322
gb:MT066175|Organism:Severe      CTGAAGCATTTTTAATTGGATGTAATTATCTTGGCAAACCACGCGAACAAATAGATGGTT   21322
gb:MN997409|Organism:Severe      CTGAAGCATTTTTAATTGGATGTAATTATCTTGGCAAACCACGCGAACAAATAGATGGTT   21322
gb:MN938384|Organism:Severe      CTGAAGCATTTTTAATTGGATGTAATTATCTTGGCAAACCACGCGAACAAATAGATGGTT   21290
gb:MT044258|Organism:Severe      CTGAAGCATTTTTAATTGGATGTAATTATCTTGGCAAACCACGCGAACAAATAGATGGTT   21298
gb:MT039890|Organism:Severe      CTGAAGCATTTTTAATTGGATGTAATTATCTTGGCAAACCACGCGAACAAATAGATGGTT   21322
gb:MN988713|Organism:Severe      CTGAAGCATTTTTAATTGGATGTAATTATCTTGGCAAACCACGCGAACAAATAGATGGTT   21322
gb:LC521925|Organism:Severe      CTGAAGCATTTTTAATTGGATGTAATTATCTTGGCAAACCACGCGAACAAATAGATGGTT   21295
gb:MT093571|Organism:Severe      CTGAAGCATTTTTAATTGGATGTAATTATCTTGGCAAACCACGCGAACAAATAGATGGTT   21322
gb:MT039887|Organism:Severe      CTGAAGCATTTTTAATTGGATGTAATTATCTTGGCAAACCACGCGAACAAATAGATGGTT   21319
gb:MT019530|Organism:Severe      CTGAAGCATTTTTAATTGGATGTAATTATCTTGGCAAACCACGCGAACAAATAGATGGTT   21322
gb:MT039888|Organism:Severe      CTGAAGCATTTTTAATTGGATGTAATTATCTTGGCAAACCACGCGAACAAATAGATGGTT   21322
gb:LC522972|Organism:Severe      CTGAAGCATTTTTAATTGGATGTAATTATCTTGGCAAACCACGCGAACAAATAGATGGTT   21319
gb:MT027063|Organism:Severe      CTGAAGCATTTTTAATTGGATGTAATTATCTTGGCAAACCACGCGAACAAATAGATGGTT   21322
gb:MT027062|Organism:Severe      CTGAAGCATTTTTAATTGGATGTAATTATCTTGGCAAACCACGCGAACAAATAGATGGTT   21322
gb:MT019529|Organism:Severe      CTGAAGCATTTTTAATTGGATGTAATTATCTTGGCAAACCACGCGAACAAATAGATGGTT   21322
gb:MN996529|Organism:Severe      CTGAAGCATTTTTAATTGGATGTAATTATCTTGGCAAACCACGCGAACAAATAGATGGTT   21310
gb:MN996531|Organism:Severe      CTGAAGCATTTTTAATTGGATGTAATTATCTTGGCAAACCACGCGAACAAATAGATGGTT   21309
gb:MT066176|Organism:Severe      CTGAAGCATTTTTAATTGGATGTAATTATCTTGGCAAACCACGCGAACAAATAGATGGTT   21322
gb:MT027064|Organism:Severe      CTGAAGCATTTTTAATTGGATGTAATTATCTTGGCAAACCACGCGAACAAATAGATGGTT   21322
gb:MN994468|Organism:Severe      CTGAAGCATTTTTAATTGGATGTAATTATCTTGGCAAACCACGCGAACAAATAGATGGTT   21322
gb:MT072688|Organism:Severe      CTGAAGCATTTTTAATTGGATGTAATTATCTTGGCAAACCACGCGAACAAATAGATGGTT   21307
gb:MN996527|Organism:Severe      CTGAAGCATTTTTAATTGGATGTAATTATCTTGGCAAACCACGCGAACAAATAAATGGTT   21289
gb:MT093631|Organism:Severe      CTGAAGCATTTTTAATTGGATGTAATTATCTTGGCAAACCACGCGAACAAATAGATGGTT   21360
gb:MT106053|Organism:Severe      CTGAAGCATTTTTAATTGGATGTAATTATCTTGGCAAACCACGCGAACAAATAGATGGTT   21322
gb:MT019533|Organism:Severe      CTGAAGCATTTTTAATTGGATGTAATTATCTTGGCAAACCACGCGAACAAATAGATGGTT   21322
gb:MT019531|Organism:Severe      CTGAAGCATTTTTAATTGGATGTAATTATCTTGGCAAACCACGCGAACAAATAGATGGTT   21322
gb:MN996528|Organism:Severe      CTGAAGCATTTTTAATTGGATGTAATTATCTTGGCAAACCACGCGAACAAATAGATGGTT   21322
gb:MN996530|Organism:Severe      CTGAAGCATTTTTAATTGGATGTAATTATCTTGGCAAACCACGCGAACAAATAGATGGTT   21308
gb:MN908947|Organism:Severe      CTGAAGCATTTTTAATTGGATGTAATTATCTTGGCAAACCACGCGAACAAATAGATGGTT   21322
gb:MT019532|Organism:Severe      CTGAAGCATTTTTAATTGGATGTAATTATCTTGGCAAACCACGCGAACAAATAGATGGTT   21322
                                 ***************************************************** ******
```

```
gb:MT020781|Organism:Severe      ATGTCATGCATGCAAATTACATATTTTGGAGGAATACAAATCCAATTCAGTTGTCTTCCT   21370
gb:MT007544|Organism:Severe      ATGTCATGCATGCAAATTACATATTTTGGAGGAATACAAATCCAATTCAGTTGTCTTCCT   21382
gb:MN994467|Organism:Severe      ATGTCATGCATGCAAATTACATATTTTGGAGGAATACAAATCCAATTCAGTTGTCTTCCT   21382
gb:MT044257|Organism:Severe      ATGTCATGCATGCAAATTACATATTTTGGAGGAATACAAATCCAATTCAGTTGTCTTCCT   21382
gb:MT106054|Organism:Severe      ATGTCATGCATGCAAATTACATATTTTGGAGGAATACAAATCCAATTCAGTTGTCTTCCT   21382
gb:MT049951|Organism:Severe      ATGTCATGCATGCAAATTACATATTTTGGAGGAATACAAATCCAATTCAGTTGTCTTCCT   21382
gb:MN975262|Organism:Severe      ATGTCATGCATGCAAATTACATATTTTGGAGGAATACAAATCCAATTCAGTTGTCTTCCT   21382
gb:MT106052|Organism:Severe      ATGTCATGCATGCAAATTACATATTTTGGAGGAATACAAATCCAATTCAGTTGTCTTCCT   21382
gb:LC522975|Organism:Severe      ATGTCATGCATGCAAATTACATATTTTGGAGGAATACAAATCCAATTCAGTTGTCTTCCT   21379
gb:LC522973|Organism:Severe      ATGTCATGCATGCAAATTACATATTTTGGAGGAATACAAATCCAATTCAGTTGTCTTCCT   21379
gb:LC522974|Organism:Severe      ATGTCATGCATGCAAATTACATATTTTGGAGGAATACAAATCCAATTCAGTTGTCTTCCT   21379
gb:MN985325|Organism:Severe      ATGTCATGCATGCAAATTACATATTTTGGAGGAATACAAATCCAATTCAGTTGTCTTCCT   21382
gb:MT020881|Organism:Severe      ATGTCATGCATGCAAATTACATATTTTGGAGGAATACAAATCCAATTCAGTTGTCTTCCT   21382
gb:MT020880|Organism:Severe      ATGTCATGCATGCAAATTACATATTTTGGAGGAATACAAATCCAATTCAGTTGTCTTCCT   21382
gb:MT066175|Organism:Severe      ATGTCATGCATGCAAATTACATATTTTGGAGGAATACAAATCCAATTCAGTTGTCTTCCT   21382
gb:MN997409|Organism:Severe      ATGTCATGCATGCAAATTACATATTTTGGAGGAATACAAATCCAATTCAGTTGTCTTCCT   21382
gb:MN938384|Organism:Severe      ATGTCATGCATGCAAATTACATATTTTGGAGGAATACAAATCCAATTCAGTTGTCTTCCT   21350
gb:MT044258|Organism:Severe      ATGTCATGCATGCAAATTACATATTTTGGAGGAATACAAATCCAATTCAGTTGTCTTCCT   21358
gb:MT039890|Organism:Severe      ATGTCATGCATGCAAATTACATATTTTGGAGGAATACAAATCCAATTCAGTTGTCTTCCT   21382
gb:MN988713|Organism:Severe      ATGTCATGCATGCAAATTACATATTTTGGAGGAATACAAATCCAATTCAGTTGTCTTCCT   21382
gb:LC521925|Organism:Severe      ATGTCATGCATGCAAATTACATATTTTGGAGGAATACAAATCCAATTCAGTTGTCTTCCT   21355
gb:MT093571|Organism:Severe      ATGTCATGCATGCAAATTACATATTTTGGAGGAATACAAATCCAATTCAGTTGTCTTCCT   21382
gb:MT039887|Organism:Severe      ATGTCATGCATGCAAATTACATATTTTGGAGGAATACAAATCCAATTCAGTTGTCTTCCT   21379
gb:MT019530|Organism:Severe      ATGTCATGCATGCAAATTACATATTTTGGAGGAATACAAATCCAATTCAGTTGTCTTCCT   21382
gb:MT039888|Organism:Severe      ATGTCATGCATGCAAATTACATATTTTGGAGGAATACAAATCCAATTCAGTTGTCTTCCT   21382
gb:LC522972|Organism:Severe      ATGTCATGCATGCAAATTACATATTTTGGAGGAATACAAATCCAATTCAGTTGTCTTCCT   21379
gb:MT027063|Organism:Severe      ATGTCATGCATGCAAATTACATATTTTGGAGGAATACAAATCCAATTCAGTTGTCTTCCT   21382
gb:MT027062|Organism:Severe      ATGTCATGCATGCAAATTACATATTTTGGAGGAATACAAATCCAATTCAGTTGTCTTCCT   21382
gb:MT019529|Organism:Severe      ATGTCATGCATGCAAATTACATATTTTGGAGGAATACAAATCCAATTCAGTTGTCTTCCT   21382
gb:MN996529|Organism:Severe      ATGTCATGCATGCAAATTACATATTTTGGAGGAATACAAATCCAATTCAGTTGTCTTCCT   21370
gb:MN996531|Organism:Severe      ATGTCATGCATGCAAATTACATATTTTGGAGGAATACAAATCCAATTCAGTTGTCTTCCT   21369
gb:MT066176|Organism:Severe      ATGTCATGCATGCAAATTACATATTTTGGAGGAATACAAATCCAATTCAGTTGTCTTCCT   21382
gb:MT027064|Organism:Severe      ATGTCATGCATGCAAATTACATATTTTGGAGGAATACAAATCCAATTCAGTTGTCTTCCT   21382
gb:MN994468|Organism:Severe      ATGTCATGCATGCAAATTACATATTTTGGAGGAATACAAATCCAATTCAGTTGTCTTCCT   21382
gb:MT072688|Organism:Severe      ATGTCATGCATGCAAATTACATATTTTGGAGGAATACAAATCCAATTCAGTTGTCTTCCT   21367
gb:MN996527|Organism:Severe      ATGTCATGCATGCAAATTACATATTTTGGAGGAATACAAATCCAATTCAGTTGTCTTCCT   21349
gb:MT093631|Organism:Severe      ATGTCATGCATGCAAATTACATATTTTGGAGGAATACAAATCCAATTCAGTTGTCTTCCT   21420
gb:MT106053|Organism:Severe      ATGTCATGCATGCAAATTACATATTTTGGAGGAATACAAATCCAATTCAGTTGTCTTCCT   21382
gb:MT019533|Organism:Severe      ATGTCATGCATGCAAATTACATATTTTGGAGGAATACAAATCCAATTCAGTTGTCTTCCT   21382
gb:MT019531|Organism:Severe      ATGTCATGCATGCAAATTACATATTTTGGAGGAATACAAATCCAATTCAGTTGTCTTCCT   21382
gb:MN996528|Organism:Severe      ATGTCATGCATGCAAATTACATATTTTGGAGGAATACAAATCCAATTCAGTTGTCTTCCT   21382
gb:MN996530|Organism:Severe      ATGTCATGCATGCAAATTACATATTTTGGAGGAATACAAATCCAATTCAGTTGTCTTCCT   21368
gb:MN908947|Organism:Severe      ATGTCATGCATGCAAATTACATATTTTGGAGGAATACAAATCCAATTCAGTTGTCTTCCT   21382
gb:MT019532|Organism:Severe      ATGTCATGCATGCAAATTACATATTTTGGAGGAATACAAATCCAATTCAGTTGTCTTCCT   21382
                                 ************************************************************

gb:MT020781|Organism:Severe      ATTCTTTATTTGACATGAGTAAATTTCCCCTTAAATTAAGGGGTACTGCTGTTATGTCTT   21430
gb:MT007544|Organism:Severe      ATTCTTTATTTGACATGAGTAAATTTCCCCTTAAATTAAGGGGTACTGCTGTTATGTCTT   21442
gb:MN994467|Organism:Severe      ATTCTTTATTTGACATGAGTAAATTTCCCCTTAAATTAAGGGGTACTGCTGTTATGTCTT   21442
gb:MT044257|Organism:Severe      ATTCTTTATTTGACATGAGTAAATTTCCCCTTAAATTAAGGGGTACTGCTGTTATGTCTT   21442
gb:MT106054|Organism:Severe      ATTCTTTATTTGACATGAGTAAATTTCCCCTTAAATTAAGGGGTACTGCTGTTATGTCTT   21442
gb:MT049951|Organism:Severe      ATTCTTTATTTGACATGAGTAAATTTCCCCTTAAATTAAGGGGTACTGCTGTTATGTCTT   21442
gb:MN975262|Organism:Severe      ATTCTTTATTTGACATGAGTAAATTTCCCCTTAAATTAAGGGGTACTGCTGTTATGTCTT   21442
gb:MT106052|Organism:Severe      ATTCTTTATTTGACATGAGTAAATTTCCCCTTAAATTAAGGGGTACTGCTGTTATGTCTT   21442
gb:LC522975|Organism:Severe      ATTCTTTATTTGACATGAGTAAATTTCCCCTTAAATTAAGGGGTACTGCTGTTATGTCTT   21439
gb:LC522973|Organism:Severe      ATTCTTTATTTGACATGAGTAAATTTCCCCTTAAATTAAGGGGTACTGCTGTTATGTCTT   21439
gb:LC522974|Organism:Severe      ATTCTTTATTTGACATGAGTAAATTTCCCCTTAAATTAAGGGGTACTGCTGTTATGTCTT   21439
gb:MN985325|Organism:Severe      ATTCTTTATTTGACATGAGTAAATTTCCCCTTAAATTAAGGGGTACTGCTGTTATGTCTT   21442
gb:MT020881|Organism:Severe      ATTCTTTATTTGACATGAGTAAATTTCCCCTTAAATTAAGGGGTACTGCTGTTATGTCTT   21442
gb:MT020880|Organism:Severe      ATTCTTTATTTGACATGAGTAAATTTCCCCTTAAATTAAGGGGTACTGCTGTTATGTCTT   21442
gb:MT066175|Organism:Severe      ATTCTTTATTTGACATGAGTAAATTTCCCCTTAAATTAAGGGGTACTGCTGTTATGTCTT   21442
gb:MN997409|Organism:Severe      ATTCTTTATTTGACATGAGTAAATTTCCCCTTAAATTAAGGGGTACTGCTGTTATGTCTT   21442
gb:MN938384|Organism:Severe      ATTCTTTATTTGACATGAGTAAATTTCCCCTTAAATTAAGGGGTACTGCTGTTATGTCTT   21410
gb:MT044258|Organism:Severe      ATTCTTTATTTGACATGAGTAAATTTCCCCTTAAATTAAGGGGTACTGCTGTTATGTCTT   21418
gb:MT039890|Organism:Severe      ATTCTTTATTTGACATGAGTAAATTTCCCCTTAAATTAAGGGGTACTGCTGTTATGTCTT   21442
```

```
gb:MN988713|Organism:Severe      ATTCTTTATTTGACATGAGTAAATTTCCCCTTAAATTAAGGGGTACTGCTGTTATGTCTT   21442
gb:LC521925|Organism:Severe      ATTCTTTATTTGACATGAGTAAATTTCCCCTTAAATTAAGGGGTACTGCTGTTATGTCTT   21415
gb:MT093571|Organism:Severe      ATTCTTTATTTGACATGAGTAAATTTCCCCTTAAATTAAGGGGTACTGCTGTTATGTCTT   21442
gb:MT039887|Organism:Severe      ATTCTTTATTTGACATGAGTAAATTTCCCCTTAAATTAAGGGGTACTGCTGTTATGTCTT   21439
gb:MT019530|Organism:Severe      ATTCTTTATTTGACATGAGTAAATTTCCCCTTAAATTAAGGGGTACTGCTGTTATGTCTT   21442
gb:MT039888|Organism:Severe      ATTCTTTATTTGACATGAGTAAATTTCCCCTTAAATTAAGGGGTACTGCTGTTATGTCTT   21442
gb:LC522972|Organism:Severe      ATTCTTTATTTGACATGAGTAAATTTCCCCTTAAATTAAGGGGTACTGCTGTTATGTCTT   21439
gb:MT027063|Organism:Severe      ATTCTTTATTTGACATGAGTAAATTTCCCCTTAAATTAAGGGGTACTGCTGTTATGTCTT   21442
gb:MT027062|Organism:Severe      ATTCTTTATTTGACATGAGTAAATTTCCCCTTAAATTAAGGGGTACTGCTGTTATGTCTT   21442
gb:MT019529|Organism:Severe      ATTCTTTATTTGACATGAGTAAATTTCCCCTTAAATTAAGGGGTACTGCTGTTATGTCTT   21442
gb:MN996529|Organism:Severe      ATTCTTTATTTGACATGAGTAAATTTCCCCTTAAATTAAGGGGTACTGCTGTTATGTCTT   21430
gb:MN996531|Organism:Severe      ATTCTTTATTTGACATGAGTAAATTTCCCCTTAAATTAAGGGGTACTGCTGTTATGTCTT   21429
gb:MT066176|Organism:Severe      ATTCTTTATTTGACATGAGTAAATTTCCCCTTAAATTAAGGGGTACTGCTGTTATGTCTT   21442
gb:MT027064|Organism:Severe      ATTCTTTATTTGACATGAGTAAATTTCCCCTTAAATTAAGGGGTACTGCTGTTATGTCTT   21442
gb:MN994468|Organism:Severe      ATTCTTTATTTGACATGAGTAAATTTCCCCTTAAATTAAGGGGTACTGCTGTTATGTCTT   21442
gb:MT072688|Organism:Severe      ATTCTTTATTTGACATGAGTAAATTTCCCCTTAAATTAAGGGGTACTGCTGTTATGTCTT   21427
gb:MN996527|Organism:Severe      ATTCTTTATTTGACATGAGTAAATTTCCCCTTAAATTAAGGGGTACTGCTGTTATGTCTT   21409
gb:MT093631|Organism:Severe      ATTCTTTATTTGACATGAGTAAATTTCCCCTTAAATTAAGGGGTACTGCTGTTATGTCTT   21480
gb:MT106053|Organism:Severe      ATTCTTTATTTGACATGAGTAAATTTCCCCTTAAATTAAGGGGTACTGCTGTTATGTCTT   21442
gb:MT019533|Organism:Severe      ATTCTTTATTTGACATGAGTAAATTTCCCCTTAAATTAAGGGGTACTGCTGTTATGTCTT   21442
gb:MT019531|Organism:Severe      ATTCTTTATTTGACATGAGTAAATTTCCCCTTAAATTAAGGGGTACTGCTGTTATGTCTT   21442
gb:MN996528|Organism:Severe      ATTCTTTATTTGACATGAGTAAATTTCCCCTTAAATTAAGGGGTACTGCTGTTATGTCTT   21442
gb:MN996530|Organism:Severe      ATTCTTTATTTGACATGAGTAAATTTCCCCTTAAATTAAGGGGTACTGCTGTTATGTCTT   21428
gb:MN908947|Organism:Severe      ATTCTTTATTTGACATGAGTAAATTTCCCCTTAAATTAAGGGGTACTGCTGTTATGTCTT   21442
gb:MT019532|Organism:Severe      ATTCTTTATTTGACATGAGTAAATTTCCCCTTAAATTAAGGGGTACTGCTGTTATGTCTT   21442
                                 ************************************************************

gb:MT020781|Organism:Severe      TAAAAGAAGGTCAAATCAATGATATGATTTTATCTCTTCTTAGTAAAGGTAGACTTATAA   21490
gb:MT007544|Organism:Severe      TAAAAGAAGGTCAAATCAATGATATGATTTTATCTCTTCTTAGTAAAGGTAGACTTATAA   21502
gb:MN994467|Organism:Severe      TAAAAGAAGGTCAAATCAATGATATGATTTTATCTCTTCTTAGTAAAGGTAGACTTATAA   21502
gb:MT044257|Organism:Severe      TAAAAGAAGGTCAAATCAATGATATGATTTTATCTCTTCTTAGTAAAGGTAGACTTATAA   21502
gb:MT106054|Organism:Severe      TAAAAGAAGGTCAAATCAATGATATGATTTTATCTCTTCTTAGTAAAGGTAGACTTATAA   21502
gb:MT049951|Organism:Severe      TAAAAGAAGGTCAAATCAATGATATGATTTTATCTCTTCTTAGTAAAGGTAGACTTATAA   21502
gb:MN975262|Organism:Severe      TAAAAGAAGGTCAAATCAATGATATGATTTTATCTCTTCTTAGTAAAGGTAGACTTATAA   21502
gb:MT106052|Organism:Severe      TAAAAGAAGGTCAAATCAATGATATGATTTTATCTCTTCTTAGTAAAGGTAGACTTATAA   21502
gb:LC522975|Organism:Severe      TAAAAGAAGGTCAAATCAATGATATGATTTTATCTCTTCTTAGTAAAGGTAGACTTATAA   21499
gb:LC522973|Organism:Severe      TAAAAGAAGGTCAAATCAATGATATGATTTTATCTCTTCTTAGTAAAGGTAGACTTATAA   21499
gb:LC522974|Organism:Severe      TAAAAGAAGGTCAAATCAATGATATGATTTTATCTCTTCTTAGTAAAGGTAGACTTATAA   21499
gb:MN985325|Organism:Severe      TAAAAGAAGGTCAAATCAATGATATGATTTTATCTCTTCTTAGTAAAGGTAGACTTATAA   21502
gb:MT020881|Organism:Severe      TAAAAGAAGGTCAAATCAATGATATGATTTTATCTCTTCTTAGTAAAGGTAGACTTATAA   21502
gb:MT020880|Organism:Severe      TAAAAGAAGGTCAAATCAATGATATGATTTTATCTCTTCTTAGTAAAGGTAGACTTATAA   21502
gb:MT066175|Organism:Severe      TAAAAGAAGGTCAAATCAATGATATGATTTTATCTCTTCTTAGTAAAGGTAGACTTATAA   21502
gb:MN997409|Organism:Severe      TAAAAGAAGGTCAAATCAATGATATGATTTTATCTCTTCTTAGTAAAGGTAGACTTATAA   21502
gb:MN938384|Organism:Severe      TAAAAGAAGGTCAAATCAATGATATGATTTTATCTCTTCTTAGTAAAGGTAGACTTATAA   21470
gb:MT044258|Organism:Severe      TAAAAGAAGGTCAAATCAATGATATGATTTTATCTCTTCTTAGTAAAGGTAGACTTATAA   21478
gb:MT039890|Organism:Severe      TAAAAGAAGGTCAAATCAATGATATGATTTTATCTCTTCTTAGTAAAGGTAGACTTATAA   21502
gb:MN988713|Organism:Severe      TAAAAGAAGGTCAAATCAATGATATGATTTTATCTCTTCTTAGTAAAGGTAGACTTATAA   21502
gb:LC521925|Organism:Severe      TAAAAGAAGGTCAAATCAATGATATGATTTTATCTCTTCTTAGTAAAGGTAGACTTATAA   21475
gb:MT093571|Organism:Severe      TAAAAGAAGGTCAAATCAATGATATGATTTTATCTCTTCTTAGTAAAGGTAGACTTATAA   21502
gb:MT039887|Organism:Severe      TAAAAGAAGGTCAAATCAATGATATGATTTTATCTCTTCTTAGTAAAGGTAGACTTATAA   21499
gb:MT019530|Organism:Severe      TAAAAGAAGGTCAAATCAATGATATGATTTTATCTCTTCTTAGTAAAGGTAGACTTATAA   21502
gb:MT039888|Organism:Severe      TAAAAGAAGGTCAAATCAATGATATGATTTTATCTCTTCTTAGTAAAGGTAGACTTATAA   21502
gb:LC522972|Organism:Severe      TAAAAGAAGGTCAAATCAATGATATGATTTTATCTCTTCTTAGTAAAGGTAGACTTATAA   21499
gb:MT027063|Organism:Severe      TAAAAGAAGGTCAAATCAATGATATGATTTTATCTCTTCTTAGTAAAGGTAGACTTATAA   21502
gb:MT027062|Organism:Severe      TAAAAGAAGGTCAAATCAATGATATGATTTTATCTCTTCTTAGTAAAGGTAGACTTATAA   21502
gb:MT019529|Organism:Severe      TAAAAGAAGGTCAAATCAATGATATGATTTTATCTCTTCTTAGTAAAGGTAGACTTATAA   21502
gb:MN996529|Organism:Severe      TAAAAGAAGGTCAAATCAATGATATGATTTTATCTCTTCTTAGTAAAGGTAGACTTATAA   21490
gb:MN996531|Organism:Severe      TAAAAGAAGGTCAAATCAATGATATGATTTTATCTCTTCTTAGTAAAGGTAGACTTATAA   21489
gb:MT066176|Organism:Severe      TAAAAGAAGGTCAAATCAATGATATGATTTTATCTCTTCTTAGTAAAGGTAGACTTATAA   21502
gb:MT027064|Organism:Severe      TAAAAGAAGGTCAAATCAATGATATGATTTTATCTCTTCTTAGTAAAGGTAGACTTATAA   21502
gb:MN994468|Organism:Severe      TAAAAGAAGGTCAAATCAATGATATGATTTTATCTCTTCTTAGTAAAGGTAGACTTATAA   21502
gb:MT072688|Organism:Severe      TAAAAGAAGGTCAAATCAATGATATGATTTTATCTCTTCTTAGTAAAGGTAGACTTATAA   21487
gb:MN996527|Organism:Severe      TAAAAGAAGGTCAAATCAATGATATGATTTTATCTCTTCTTAGTAAAGGTAGACTTATAA   21469
gb:MT093631|Organism:Severe      TAAAAGAAGGTCAAATCAATGATATGATTTTATCTCTTCTTAGTAAAGGTAGACTTATAA   21540
gb:MT106053|Organism:Severe      TAAAAGAAGGTCAAATCAATGATATGATTTTATCTCTTCTTAGTAAAGGTAGACTTATAA   21502
```

```
gb:MT019533|Organism:Severe      TAAAAGAAGGTCAAATCAATGATATGATTTTATCTCTTCTTAGTAAAGGTAGACTTATAA   21502
gb:MT019531|Organism:Severe      TAAAAGAAGGTCAAATCAATGATATGATTTTATCTCTTCTTAGTAAAGGTAGACTTATAA   21502
gb:MN996528|Organism:Severe      TAAAAGAAGGTCAAATCAATGATATGATTTTATCTCTTCTTAGTAAAGGTAGACTTATAA   21502
gb:MN996530|Organism:Severe      TAAAAGAAGGTCAAATCAATGATATGATTTTATCTCTTCTTAGTAAAGGTAGACTTATAA   21488
gb:MN908947|Organism:Severe      TAAAAGAAGGTCAAATCAATGATATGATTTTATCTCTTCTTAGTAAAGGTAGACTTATAA   21502
gb:MT019532|Organism:Severe      TAAAAGAAGGTCAAATCAATGATATGATTTTATCTCTTCTTAGTAAAGGTAGACTTATAA   21502
                                 ************************************************************

gb:MT020781|Organism:Severe      TTAGAGAAAACAACAGAGTTGTTATTTCTAGTGATGTTCTTGTTAACAACTAAACGAACA   21550
gb:MT007544|Organism:Severe      TTAGAGAAAACAACAGAGTTGTTATTTCTAGTGATGTTCTTGTTAACAACTAAACGAACA   21562
gb:MN994467|Organism:Severe      TTAGAGAAAACAACAGAGTTGTTATTTCTAGTGATGTTCTTGTTAACAACTAAACGAACA   21562
gb:MT044257|Organism:Severe      TTAGAGAAAACAACAGAGTTGTTATTTCTAGTGATGTTCTTGTTAACAACTAAACGAACA   21562
gb:MT106054|Organism:Severe      TTAGAGAAAACAACAGAGTTGTTATTTCTAGTGATGTTCTTGTTAACAACTAAACGAACA   21562
gb:MT049951|Organism:Severe      TTAGAGAAAACAACAGAGTTGTTATTTCTAGTGATGTTCTTGTTAACAACTAAACGAACA   21562
gb:MN975262|Organism:Severe      TTAGAGAAAACAACAGAGTTGTTATTTCTAGTGATGTTCTTGTTAACAACTAAACGAACA   21562
gb:MT106052|Organism:Severe      TTAGAGAAAACAACAGAGTTGTTATTTCTAGTGATGTTCTTGTTAACAACTAAACGAACA   21562
gb:LC522975|Organism:Severe      TTAGAGAAAACAACAGAGTTGTTATTTCTAGTGATGTTCTTGTTAACAACTAAACGAACA   21559
gb:LC522973|Organism:Severe      TTAGAGAAAACAACAGAGTTGTTATTTCTAGTGATGTTCTTGTTAACAACTAAACGAACA   21559
gb:LC522974|Organism:Severe      TTAGAGAAAACAACAGAGTTGTTATTTCTAGTGATGTTCTTGTTAACAACTAAACGAACA   21559
gb:MN985325|Organism:Severe      TTAGAGAAAACAACAGAGTTGTTATTTCTAGTGATGTTCTTGTTAACAACTAAACGAACA   21562
gb:MT020881|Organism:Severe      TTAGAGAAAACAACAGAGTTGTTATTTCTAGTGATGTTCTTGTTAACAACTAAACGAACA   21562
gb:MT020880|Organism:Severe      TTAGAGAAAACAACAGAGTTGTTATTTCTAGTGATGTTCTTGTTAACAACTAAACGAACA   21562
gb:MT066175|Organism:Severe      TTAGAGAAAACAACAGAGTTGTTATTTCTAGTGATGTTCTTGTTAACAACTAAACGAACA   21562
gb:MN997409|Organism:Severe      TTAGAGAAAACAACAGAGTTGTTATTTCTAGTGATGTTCTTGTTAACAACTAAACGAACA   21562
gb:MN938384|Organism:Severe      TTAGAGAAAACAACAGAGTTGTTATTTCTAGTGATGTTCTTGTTAACAACTAAACGAACA   21530
gb:MT044258|Organism:Severe      TTAGAGAAAACAACAGAGTTGTTATTTCTAGTGATGTTCTTGTTAACAACTAAACGAACA   21538
gb:MT039890|Organism:Severe      TTAGAGAAAACAACAGAGTTGTTATTTCTAGTGATGTTCTTGTTAACAACTAAACGAACA   21562
gb:MN988713|Organism:Severe      TTAGAGAAAACAACAGAGTTGTTATTTCTAGTGATGTTCTTGTTAACAACTAAACGAACA   21562
gb:LC521925|Organism:Severe      TTAGAGAAAACAACAGAGTTGTTATTTCTAGTGATGTTCTTGTTAACAACTAAACGAACA   21535
gb:MT093571|Organism:Severe      TTAGAGAAAACAACAGAGTTGTTATTTCTAGTGATGTTCTTGTTAACAACTAAACGAACA   21562
gb:MT039887|Organism:Severe      TTAGAGAAAACAACAGAGTTGTTATTTCTAGTGATGTTCTTGTTAACAACTAAACGAACA   21559
gb:MT019530|Organism:Severe      TTAGAGAAAACAACAGAGTTGTTATTTCTAGTGATGTTCTTGTTAACAACTAAACGAACA   21562
gb:MT039888|Organism:Severe      TTAGAGAAAACAACAGAGTTGTTATTTCTAGTGATGTTCTTGTTAACAACTAAACGAACA   21562
gb:LC522972|Organism:Severe      TTAGAGAAAACAACAGAGTTGTTATTTCTAGTGATGTTCTTGTTAACAACTAAACGAACA   21559
gb:MT027063|Organism:Severe      TTAGAGAAAACAACAGAGTTGTTATTTCTAGTGATGTTCTTGTTAACAACTAAACGAACA   21562
gb:MT027062|Organism:Severe      TTAGAGAAAACAACAGAGTTGTTATTTCTAGTGATGTTCTTGTTAACAACTAAACGAACA   21562
gb:MT019529|Organism:Severe      TTAGAGAAAACAACAGAGTTGTTATTTCTAGTGATGTTCTTGTTAACAACTAAACGAACA   21562
gb:MN996529|Organism:Severe      TTAGAGAAAACAACAGAGTTGTTATTTCTAGTGATGTTCTTGTTAACAACTAAACGAACA   21550
gb:MN996531|Organism:Severe      TTAGAGAAAACAACAGAGTTGTTATTTCTAGTGATGTTCTTGTTAACAACTAAACGAACA   21549
gb:MT066176|Organism:Severe      TTAGAGAAAACAACAGAGTTGTTATTTCTAGTGATGTTCTTGTTAACAACTAAACGAACA   21562
gb:MT027064|Organism:Severe      TTAGAGAAAACAACAGAGTTGTTATTTCTAGTGATGTTCTTGTTAACAACTAAACGAACA   21562
gb:MN994468|Organism:Severe      TTAGAGAAAACAACAGAGTTGTTATTTCTAGTGATGTTCTTGTTAACAACTAAACGAACA   21562
gb:MT072688|Organism:Severe      TTAGAGAAAACAACAGAGTTGTTATTTCTAGTGATGTTCTTGTTAACAACTAAACGAACA   21547
gb:MN996527|Organism:Severe      TTAGAGAAAACAACAGAGTTGTTATTTCTAGTGATGTTCTTGTTAACAACTAAACGAACA   21529
gb:MT093631|Organism:Severe      TTAGAGAAAACAACAGAGTTGTTATTTCTAGTGATGTTCTTGTTAACAACTAAACGAACA   21600
gb:MT106053|Organism:Severe      TTAGAGAAAACAACAGAGTTGTTATTTCTAGTGATGTTCTTGTTAACAACTAAACGAACA   21562
gb:MT019533|Organism:Severe      TTAGAGAAAACAACAGAGTTGTTATTTCTAGTGATGTTCTTGTTAACAACTAAACGAACA   21562
gb:MT019531|Organism:Severe      TTAGAGAAAACAACAGAGTTGTTATTTCTAGTGATGTTCTTGTTAACAACTAAACGAACA   21562
gb:MN996528|Organism:Severe      TTAGAGAAAACAACAGAGTTGTTATTTCTAGTGATGTTCTTGTTAACAACTAAACGAACA   21562
gb:MN996530|Organism:Severe      TTAGAGAAAACAACAGAGTTGTTATTTCTAGTGATGTTCTTGTTAACAACTAAACGAACA   21548
gb:MN908947|Organism:Severe      TTAGAGAAAACAACAGAGTTGTTATTTCTAGTGATGTTCTTGTTAACAACTAAACGAACA   21562
gb:MT019532|Organism:Severe      TTAGAGAAAACAACAGAGTTGTTATTTCTAGTGATGTTCTTGTTAACAACTAAACGAACA   21562
                                 ************************************************************

gb:MT020781|Organism:Severe      ATGTTTGTTTTTCTTGTTTTATTGCCACTAGTCTCTAGTCAGTGTGTTAATCTTACAACC   21610
gb:MT007544|Organism:Severe      ATGTTTGTTTTTCTTGTTTTATTGCCACTAGTCTCTAGTCAGTGTGTTAATCTTACAACC   21622
gb:MN994467|Organism:Severe      ATGTTTGTTTTTCTTGTTTTATTGCCACTAGTCTCTAGTCAGTGTGTTAATCTTACAACC   21622
gb:MT044257|Organism:Severe      ATGTTTGTTTTTCTTGTTTTATTGCCACTAGTCTCTAGTCAGTGTGTTAATCTTACAACC   21622
gb:MT106054|Organism:Severe      ATGTTTGTTTTTCTTGTTTTATTGCCACTAGTCTCTAGTCAGTGTGTTAATCTTACAACC   21622
gb:MT049951|Organism:Severe      ATGTTTGTTTTTCTTGTTTTATTGCCACTAGTCTCTAGTCAGTGTGTTAATCTTACAACC   21622
gb:MN975262|Organism:Severe      ATGTTTGTTTTTCTTGTTTTATTGCCACTAGTCTCTAGTCAGTGTGTTAATCTTACAACC   21622
gb:MT106052|Organism:Severe      ATGTTTGTTTTTCTTGTTTTATTGCCACTAGTCTCTAGTCAGTGTGTTAATCTTACAACC   21622
gb:LC522975|Organism:Severe      ATGTTTGTTTTTCTTGTTTTATTGCCACTAGTCTCTAGTCAGTGTGTTAATCTTACAACC   21619
gb:LC522973|Organism:Severe      ATGTTTGTTTTTCTTGTTTTATTGCCACTAGTCTCTAGTCAGTGTGTTAATCTTACAACC   21619
gb:LC522974|Organism:Severe      ATGTTTGTTTTTCTTGTTTTATTGCCACTAGTCTCTAGTCAGTGTGTTAATCTTACAACC   21619
```

```
gb:MN985325|Organism:Severe      ATGTTTGTTTTTCTTGTTTTATTGCCACTAGTCTCTAGTCAGTGTGTTAATCTTACAACC   21622
gb:MT020881|Organism:Severe      ATGTTTGTTTTTCTTGTTTTATTGCCACTAGTCTCTAGTCAGTGTGTTAATCTTACAACC   21622
gb:MT020880|Organism:Severe      ATGTTTGTTTTTCTTGTTTTATTGCCACTAGTCTCTAGTCAGTGTGTTAATCTTACAACC   21622
gb:MT066175|Organism:Severe      ATGTTTGTTTTTCTTGTTTTATTGCCACTAGTCTCTAGTCAGTGTGTTAATCTTACAACC   21622
gb:MN997409|Organism:Severe      ATGTTTGTTTTTCTTGTTTTATTGCCACTAGTCTCTAGTCAGTGTGTTAATCTTACAACC   21622
gb:MN938384|Organism:Severe      ATGTTTGTTTTTCTTGTTTTATTGCCACTAGTCTCTAGTCAGTGTGTTAATCTTACAACC   21590
gb:MT044258|Organism:Severe      ATGTTTGTTTTTCTTGTTTTATTGCCACTAGTCTCTAGTCAGTGTGTTAATCTTACAACC   21598
gb:MT039890|Organism:Severe      ATGTTTGTTTTTCTTGTTTTATTGCCACTAGTCTCTAGTCAGTGTGTTAATCTTACAACC   21622
gb:MN988713|Organism:Severe      ATGTTTGTTTTTCTTGTTTTATTGCCACTAGTCTCTAGTCAGTGTGTTAATCTTACAACC   21622
gb:LC521925|Organism:Severe      ATGTTTGTTTTTCTTGTTTTATTGCCACTAGTCTCTAGTCAGTGTGTTAATCTTACAACC   21595
gb:MT093571|Organism:Severe      ATGTTTGTTTTTCTTGTTTTATTGCCACTAGTCTCTAGTCAGTGTGTTAATCTTACAACC   21622
gb:MT039887|Organism:Severe      ATGTTTGTTTTTCTTGTTTTATTGCCACTAGTCTCTAGTCAGTGTGTTAATCTTACAACC   21619
gb:MT019530|Organism:Severe      ATGTTTGTTTTTCTTGTTTTATTGCCACTAGTCTCTAGTCAGTGTGTTAATCTTACAACC   21622
gb:MT039888|Organism:Severe      ATGTTTGTTTTTCTTGTTTTATTGCCACTAGTCTCTAGTCAGTGTGTTAATCTTACAACC   21622
gb:LC522972|Organism:Severe      ATGTTTGTTTTTCTTGTTTTATTGCCACTAGTCTCTAGTCAGTGTGTTAATCTTACAACC   21619
gb:MT027063|Organism:Severe      ATGTTTGTTTTTCTTGTTTTATTGCCACTAGTCTCTAGTCAGTGTGTTAATCTTACAACC   21622
gb:MT027062|Organism:Severe      ATGTTTGTTTTTCTTGTTTTATTGCCACTAGTCTCTAGTCAGTGTGTTAATCTTACAACC   21622
gb:MT019529|Organism:Severe      ATGTTTGTTTTTCTTGTTTTATTGCCACTAGTCTCTAGTCAGTGTGTTAATCTTACAACC   21622
gb:MN996529|Organism:Severe      ATGTTTGTTTTTCTTGTTTTATTGCCACTAGTCTCTAGTCAGTGTGTTAATCTTACAACC   21610
gb:MN996531|Organism:Severe      ATGTTTGTTTTTCTTGTTTTATTGCCACTAGTCTCTAGTCAGTGTGTTAATCTTACAACC   21609
gb:MT066176|Organism:Severe      ATGTTTGTTTTTCTTGTTTTATTGCCACTAGTCTCTAGTCAGTGTGTTAATCTTACAACC   21622
gb:MT027064|Organism:Severe      ATGTTTGTTTTTCTTGTTTTATTGCCACTAGTCTCTAGTCAGTGTGTTAATCTTACAACC   21622
gb:MN994468|Organism:Severe      ATGTTTGTTTTTCTTGTTTTATTGCCACTAGTCTCTAGTCAGTGTGTTAATCTTACAACC   21622
gb:MT072688|Organism:Severe      ATGTTTGTTTTTCTTGTTTTATTGCCACTAGTCTCTAGTCAGTGTGTTAATCTTACAACC   21607
gb:MN996527|Organism:Severe      ATGTTTGTTTTTCTTGTTTTATTGCCACTAGTCTCTAGTCAGTGTGTTAATCTTACAACC   21589
gb:MT093631|Organism:Severe      ATGTTTGTTTTTCTTGTTTTATTGCCACTAGTCTCTAGTCAGTGTGTTAATCTTACAACC   21660
gb:MT106053|Organism:Severe      ATGTTTGTTTTTCTTGTTTTATTGCCACTAGTCTCTAGTCAGTGTGTTAATCTTACAACC   21622
gb:MT019533|Organism:Severe      ATGTTTGTTTTTCTTGTTTTATTGCCACTAGTCTCTAGTCAGTGTGTTAATCTTACAACC   21622
gb:MT019531|Organism:Severe      ATGTTTGTTTTTCTTGTTTTATTGCCACTAGTCTCTAGTCAGTGTGTTAATCTTACAACC   21622
gb:MN996528|Organism:Severe      ATGTTTGTTTTTCTTGTTTTATTGCCACTAGTCTCTAGTCAGTGTGTTAATCTTACAACC   21622
gb:MN996530|Organism:Severe      ATGTTTGTTTTTCTTGTTTTATTGCCACTAGTCTCTAGTCAGTGTGTTAATCTTACAACC   21608
gb:MN908947|Organism:Severe      ATGTTTGTTTTTCTTGTTTTATTGCCACTAGTCTCTAGTCAGTGTGTTAATCTTACAACC   21622
gb:MT019532|Organism:Severe      ATGTTTGTTTTTCTTGTTTTATTGCCACTAGTCTCTAGTCAGTGTGTTAATCTTACAACC   21622
                                 ************************************************************

gb:MT020781|Organism:Severe      AGAANNNNNNNNNNNNNNNNNNNNNNNNNNNNNNNNNNNNNNNNNNNNNNNNNNNNNNNN   21670
gb:MT007544|Organism:Severe      AGAACTCAATTACCCCCTGCATACACTAATTCTTTCACACGTGGTGTTTATTACCCTGAC   21682
gb:MN994467|Organism:Severe      AGAACTCAATTACCCCCTGCATACACTAATTCTTTCACACGTGGTGTTTATTACCCTGAC   21682
gb:MT044257|Organism:Severe      AGAACTCAATTACCCCCTGCATACACTAATTCTTTCACACGTGGTGTTTATTACCCTGAC   21682
gb:MT106054|Organism:Severe      AGAACTCAATTACCCCCTGCATACACTAATTCTTTCACACGTGGTGTTTATTACCCTGAC   21682
gb:MT049951|Organism:Severe      AGAACTCAATTACCCCCTGCAAACACTAATTCTTTCACACGTGGTGTTTATTACCCTGAC   21682
gb:MN975262|Organism:Severe      AGAACTCAATTACCCCCTGCATACACTAATTCTTTCACACGTGGTGTTTATTACCCTGAC   21682
gb:MT106052|Organism:Severe      AGAACTCAATTACCCCCTGCATACACTAATTCTTTCACACGTGGTGTTTATTACCCTGAC   21682
gb:LC522975|Organism:Severe      AGAACTCAATTACCCCCTGCATACACTAATTCTTTCACACGTGGTGTTTATTACCCTGAC   21679
gb:LC522973|Organism:Severe      AGAACTCAATTACCCCCTGCATACACTAATTCTTTCACACGTGGTGTTTATTACCCTGAC   21679
gb:LC522974|Organism:Severe      AGAACTCAATTACCCCCTGCATACACTAATTCTTTCACACGTGGTGTTTATTACCCTGAC   21679
gb:MN985325|Organism:Severe      AGAACTCAATTACCCCCTGCATACACTAATTCTTTCACACGTGGTGTTTATTACCCTGAC   21682
gb:MT020881|Organism:Severe      AGAACTCAATTACCCCCTGCATACACTAATTCTTTCACACGTGGTGTTTATTACCCTGAC   21682
gb:MT020880|Organism:Severe      AGAACTCAATTACCCCCTGCATACACTAATTCTTTCACACGTGGTGTTTATTACCCTGAC   21682
gb:MT066175|Organism:Severe      AGAACTCAATTACCCCCTGCATACACTAATTCTTTCACACGTGGTGTTTATTACCCTGAC   21682
gb:MN997409|Organism:Severe      AGAACTCAATTACCCCCTGCATACACTAATTCTTTCACACGTGGTGTTTATTACCCTGAC   21682
gb:MN938384|Organism:Severe      AGAACTCAATTACCCCCTGCATACACTAATTCTTTCACACGTGGTGTTTATTACCCTGAC   21650
gb:MT044258|Organism:Severe      AGAACTCAATTACCCCCTGCATACACTAATTCTTTCACACGTGGTGTTTATTACCCTGAC   21658
gb:MT039890|Organism:Severe      AGAACTCAATTACCCCCTGCATACACTAATTCTTTCACACGTGGTGTTTATTACCCTGAC   21682
gb:MN988713|Organism:Severe      AGAACTCAATTACCCCCTGCATACACTAATTCTTTCACACGTGGTGTTTATTACCCTGAC   21682
gb:LC521925|Organism:Severe      AGAACTCAATTACCCCCTGCATACACTAATTCTTTCACACGTGGTGTTTATTACCCTGAC   21655
gb:MT093571|Organism:Severe      AGAACTCAATTACCCCCTGCATACACTAATTCTTTCACACGTGGTGTTTATTACCCTGAC   21682
gb:MT039887|Organism:Severe      AGAACTCAATTACCCCCTGCATACACTAATTCTTTCACACGTGGTGTTTATTACCCTGAC   21679
gb:MT019530|Organism:Severe      AGAACTCAATTACCCCCTGCATACACTAATTCTTTCACACGTGGTGTTTATTACCCTGAC   21682
gb:MT039888|Organism:Severe      AGAACTCAATTACCCCCTGCATACACTAATTCTTTCACACGTGGTGTTTATTACCCTGAC   21682
gb:LC522972|Organism:Severe      AGAACTCAATTACCCCCTGCATACACTAATTCTTTCACACGTGGTGTTTATTACCCTGAC   21679
gb:MT027063|Organism:Severe      AGAACTCAATTACCCCCTGCATACACTAATTCTTTCACACGTGGTGTTTATTACCCTGAC   21682
gb:MT027062|Organism:Severe      AGAACTCAATTACCCCCTGCATACACTAATTCTTTCACACGTGGTGTTTATTACCCTGAC   21682
gb:MT019529|Organism:Severe      AGAACTCAATTACCCCCTGCATACACTAATTCTTTCACACGTGGTGTTTATTACCCTGAC   21682
gb:MN996529|Organism:Severe      AGAACTCAATTACCCCCTGCATACACTAATTCTTTCACACGTGGTGTTTATTACCCTGAC   21670
```

```
gb:MN996531|Organism:Severe      AGAACTCAATTACCCCCTGCATACACTAATTCTTTCACACGTGGTGTTTATTACCCTGAC   21669
gb:MT066176|Organism:Severe      AGAACTCAATTACCCCCTGCATACACTAATTCTTTCACACGTGGTGTTTATTACCCTGAC   21682
gb:MT027064|Organism:Severe      AGAACTCAATTACCCCCTGCATACACTAATTCTTTCACACGTGGTGTTTATTACCCTGAC   21682
gb:MN994468|Organism:Severe      AGAACTCAATTACCCCCTGCATACACTAATTCTTTCACACGTGGTGTTTATTACCCTGAC   21682
gb:MT072688|Organism:Severe      AGAACTCAATTACCCCCTGCATACACTAATTCTTTCACACGTGGTGTTTATTACCCTGAC   21667
gb:MN996527|Organism:Severe      AGAACTCAATTACCCCCTGCATACACTAATTCTTTCACACGTGGTGTTTATTACCCTGAC   21649
gb:MT093631|Organism:Severe      AGAACTCAATTACCCCCTGCATACACTAATTCTTTCACACGTGGTGTTTATTACCCTGAC   21720
gb:MT106053|Organism:Severe      AGAACTCAATTACCCCCTGCATACACTAATTCTTTCACACGTGGTGTTTATTACCCTGAC   21682
gb:MT019533|Organism:Severe      AGAACTCAATTACCCCCTGCATACACTAATTCTTTCACACGTGGTGTTTATTACCCTGAC   21682
gb:MT019531|Organism:Severe      AGAACTCAATTACCCCCTGCATACACTAATTCTTTCACACGTGGTGTTTATTACCCTGAC   21682
gb:MN996528|Organism:Severe      AGAACTCAATTACCCCCTGCATACACTAATTCTTTCACACGTGGTGTTTATTACCCTGAC   21682
gb:MN996530|Organism:Severe      AGAACTCAATTACCCCCTGCATACACTAATTCTTTCACACGTGGTGTTTATTACCCTGAC   21668
gb:MN908947|Organism:Severe      AGAACTCAATTACCCCCTGCATACACTAATTCTTTCACACGTGGTGTTTATTACCCTGAC   21682
gb:MT019532|Organism:Severe      AGAACTCAATTACCCCCTGCATACACTAATTCTTTCACACGTGGTGTTTATTACCCTGAC   21682
                                 ****

gb:MT020781|Organism:Severe      NNNNNNNNNNNNNNNNNNNNNNNNNATTCAACTCAGGACTTGTTCTTACCTTTCTTTTCC   21730
gb:MT007544|Organism:Severe      AAAGTTTTCAGATCCTCAGTTTTACATTCAACTCAGGACTTGTTCTTACCTTTCTTTTCC   21742
gb:MN994467|Organism:Severe      AAAGTTTTCAGATCCTCAGTTTTACATTCAACTCAGGACTTGTTCTTACCTTTCTTTTCC   21742
gb:MT044257|Organism:Severe      AAAGTTTTCAGATCCTCAGTTTTACATTCAACTCAGGACTTGTTCTTACCTTTCTTTTCC   21742
gb:MT106054|Organism:Severe      AAAGTTTTCAGATCCTCAGTTTTACATTCAACTCAGGACTTGTTCTTACCTTTCTTTTCC   21742
gb:MT049951|Organism:Severe      AAAGTTTTCAGATCCTCAGTTTTACATTCAACTCAGGACTTGTTCTTACCTTTCTTTTCC   21742
gb:MN975262|Organism:Severe      AAAGTTTTCAGATCCTCAGTTTTACATTCAACTCAGGACTTGTTCTTACCTTTCTTTTCC   21742
gb:MT106052|Organism:Severe      AAAGTTTTCAGATCCTCAGTTTTACATTCAACTCAGGACTTGTTCTTACCTTTCTTTTCC   21742
gb:LC522975|Organism:Severe      AAAGTTTTCAGATCCTCAGTTTTACATTCAACTCAGGACTTGTTCTTACCTTTCTTTTCC   21739
gb:LC522973|Organism:Severe      AAAGTTTTCAGATCCTCAGTTTTACATTCAACTCAGGACTTGTTCTTACCTTTCTTTTCC   21739
gb:LC522974|Organism:Severe      AAAGTTTTCAGATCCTCAGTTTTACATTCAACTCAGGACTTGTTCTTACCTTTCTTTTCC   21739
gb:MN985325|Organism:Severe      AAAGTTTTCAGATCCTCAGTTTTACATTCAACTCAGGACTTGTTCTTACCTTTCTTTTCC   21742
gb:MT020881|Organism:Severe      AAAGTTTTCAGATCCTCAGTTTTACATTCAACTCAGGACTTGTTCTTACCTTTCTTTTCC   21742
gb:MT020880|Organism:Severe      AAAGTTTTCAGATCCTCAGTTTTACATTCAACTCAGGACTTGTTCTTACCTTTCTTTTCC   21742
gb:MT066175|Organism:Severe      AAAGTTTTCAGATCCTCAGTTTTACATTCAACTCAGGACTTGTTCTTACCTTTCTTTTCC   21742
gb:MN997409|Organism:Severe      AAAGTTTTCAGATCCTCAGTTTTACATTCAACTCAGGACTTGTTCTTACCTTTCTTTTCC   21742
gb:MN938384|Organism:Severe      AAAGTTTTCAGATCCTCAGTTTTACATTCAACTCAGGACTTGTTCTTACCTTTCTTTTCC   21710
gb:MT044258|Organism:Severe      AAAGTTTTCAGATCCTCAGTTTTACATTCAACTCAGGACTTGTTCTTACCTTTCTTTTCC   21718
gb:MT039890|Organism:Severe      AAAGTTTTCAGATCCTCAGTTTTACATTCAACTCAGGACTTGTTCTTACCTTTCTTTTCC   21742
gb:MN988713|Organism:Severe      AAAGTTTTCAGATCCTCAGTTTTACATTCAACTCAGGACTTGTTCTTACCTTTCTTTTCC   21742
gb:LC521925|Organism:Severe      AAAGTTTTCAGATCCTCAGTTTTACATTCAACTCAGGACTTGTTCTTACCTTTCTTTTCC   21715
gb:MT093571|Organism:Severe      AAAGTTTTCAGATCCTCAGTTTTACATTCAACTCAGGACTTGTTCTTACCTTTCTTTTCC   21742
gb:MT039887|Organism:Severe      AAAGTTTTCAGATCCTCAGTTTTACATTCAACTCAGGACTTGTTCTTACCTTTCTTTTCC   21739
gb:MT019530|Organism:Severe      AAAGTTTTCAGATCCTCAGTTTTACATTCAACTCAGGACTTGTTCTTACCTTTCTTTTCC   21742
gb:MT039888|Organism:Severe      AAAGTTTTCAGATCCTCAGTTTTACATTCAACTCAGGACTTGTTCTTACCTTTCTTTTCC   21742
gb:LC522972|Organism:Severe      AAAGTTTTCAGATCCTCAGTTTTACATTCAACTCAGGACTTGTTCTTACCTTTCTTTTCC   21739
gb:MT027063|Organism:Severe      AAAGTTTTCAGATCCTCAGTTTTACATTCAACTCAGGACTTGTTCTTACCTTTCTTTTCC   21742
gb:MT027062|Organism:Severe      AAAGTTTTCAGATCCTCAGTTTTACATTCAACTCAGGACTTGTTCTTACCTTTCTTTTCC   21742
gb:MT019529|Organism:Severe      AAAGTTTTCAGATCCTCAGTTTTACATTCAACTCAGGACTTGTTCTTACCTTTCTTTTCC   21742
gb:MN996529|Organism:Severe      AAAGTTTTCAGATCCTCAGTTTTACATTCAACTCAGGACTTGTTCTTACCTTTCTTTTCC   21730
gb:MN996531|Organism:Severe      AAAGTTTTCAGATCCTCAGTTTTACATTCAACTCAGGACTTGTTCTTACCTTTCTTTTCC   21729
gb:MT066176|Organism:Severe      AAAGTTTTCAGATCCTCAGTTTTACATTCAACTCAGGACTTGTTCTTACCTTTCTTTTCC   21742
gb:MT027064|Organism:Severe      AAAGTTTTCAGATCCTCAGTTTTATATTCAACTCAGGACTTGTTCTTACCTTTCTTTTCC   21742
gb:MN994468|Organism:Severe      AAAGTTTTCAGATCCTCAGTTTTACATTCAACTCAGGACTTGTTCTTACCTTTCTTTTCC   21742
gb:MT072688|Organism:Severe      AAAGTTTTCAGATCCTCAGTTTTACATTCAACTCAGGACTTGTTCTTACCTTTCTTTTCC   21727
gb:MN996527|Organism:Severe      AAAGTTTTCAGATCCTCAGTTTTACATTCAACTCAGGACTTGTTCTTACCTTTCTTTTCC   21709
gb:MT093631|Organism:Severe      AAAGTTTTCAGATCCTCAGTTTTACATTCAACTCAGGACTTGTTCTTACCTTTCTTTTCC   21780
gb:MT106053|Organism:Severe      AAAGTTTTCAGATCCTCAGTTTTACATTCAACTCAGGACTTGTTCTTACCTTTCTTTTCC   21742
gb:MT019533|Organism:Severe      AAAGTTTTCAGATCCTCAGTTTTACATTCAACTCAGGACTTGTTCTTACCTTTCTTTTCC   21742
gb:MT019531|Organism:Severe      AAAGTTTTCAGATCCTCAGTTTTACATTCAACTCAGGACTTGTTCTTACCTTTCTTTTCC   21742
gb:MN996528|Organism:Severe      AAAGTTTTCAGATCCTCAGTTTTACATTCAACTCAGGACTTGTTCTTACCTTTCTTTTCC   21742
gb:MN996530|Organism:Severe      AAAGTTTTCAGATCCTCAGTTTTACATTCAACTCAGGACTTGTTCTTACCTTTCTTTTCC   21728
gb:MN908947|Organism:Severe      AAAGTTTTCAGATCCTCAGTTTTACATTCAACTCAGGACTTGTTCTTACCTTTCTTTTCC   21742
gb:MT019532|Organism:Severe      AAAGTTTTCAGATCCTCAGTTTTACATTCAACTCAGGACTTGTTCTTACCTTTCTTTTCC   21742
                                                          ***********************************

gb:MT020781|Organism:Severe      AATGTTACTTGGTTCCATGCTATACATGTCTCTGGGACCAATGGTACTAAGAGGTTTGAT   21790
gb:MT007544|Organism:Severe      AATGTTACTTGGTTCCATGCTATACATGTCTCTGGGACCAATGGTACTAAGAGGTTTGAT   21802
gb:MN994467|Organism:Severe      AATGTTACTTGGTTCCATGCTATACATGTCTCTGGGACCAATGGTACTAAGAGGTTTGAT   21802
```

```
gb:MT044257|Organism:Severe      AATGTTACTTGGTTCCATGCTATACATGTCTCTGGGACCAATGGTACTAAGAGGTTTGAT   21802
gb:MT106054|Organism:Severe      AATGTTACTTGGTTCCATGCTATACATGTCTCTGGGACCAATGGTACTAAGAGGTTTGAT   21802
gb:MT049951|Organism:Severe      AATGTTACTTGGTTCCATGCTATACATGTCTCTGGGACCAATGGTACTAAGAGGTTTGAT   21802
gb:MN975262|Organism:Severe      AATGTTACTTGGTTCCATGCTATACATGTCTCTGGGACCAATGGTACTAAGAGGTTTGAT   21802
gb:MT106052|Organism:Severe      AATGTTACTTGGTTCCATGCTATACATGTCTCTGGGACCAATGGTACTAAGAGGTTTGAT   21802
gb:LC522975|Organism:Severe      AATGTTACTTGGTTCCATGCTATACATGTCTCTGGGACCAATGGTACTAAGAGGTTTGAT   21799
gb:LC522973|Organism:Severe      AATGTTACTTGGTTCCATGCTATACATGTCTCTGGGACCAATGGTACTAAGAGGTTTGAT   21799
gb:LC522974|Organism:Severe      AATGTTACTTGGTTCCATGCTATACATGTCTCTGGGACCAATGGTACTAAGAGGTTTGAT   21799
gb:MN985325|Organism:Severe      AATGTTACTTGGTTCCATGCTATACATGTCTCTGGGACCAATGGTACTAAGAGGTTTGAT   21802
gb:MT020881|Organism:Severe      AATGTTACTTGGTTCCATGCTATACATGTCTCTGGGACCAATGGTACTAAGAGGTTTGAT   21802
gb:MT020880|Organism:Severe      AATGTTACTTGGTTCCATGCTATACATGTCTCTGGGACCAATGGTACTAAGAGGTTTGAT   21802
gb:MT066175|Organism:Severe      AATGTTACTTGGTTCCATGCTATACATGTCTCTGGGACCAATGGTACTAAGAGGTTTGAT   21802
gb:MN997409|Organism:Severe      AATGTTACTTGGTTCCATGCTATACATGTCTCTGGGACCAATGGTACTAAGAGGTTTGAT   21802
gb:MN938384|Organism:Severe      AATGTTACTTGGTTCCATGCTATACATGTCTCTGGGACCAATGGTACTAAGAGGTTTGAT   21770
gb:MT044258|Organism:Severe      AATGTTACTTGGTTCCATGCTATACATGTCTCTGGGACCAATGGTACTAAGAGGTTTGAT   21778
gb:MT039890|Organism:Severe      AATGTTACTTGGTTCCATGCTATACATGTCTCTGGGACCAATGGTACTAAGAGGTTTGAT   21802
gb:MN988713|Organism:Severe      AATGTTACTTGGTTCCATGCTATACATGTCTCTGGGACCAATGGTACTAAGAGGTTTGAT   21802
gb:LC521925|Organism:Severe      AATGTTACTTGGTTCCATGCTATACATGTCTCTGGGACCAATGGTACTAAGAGGTTTGAT   21775
gb:MT093571|Organism:Severe      AATGTTACTTGGTTCCATGCTATACATGTCTCTGGGACCAATGGTACTAAGAGGTTTGAT   21802
gb:MT039887|Organism:Severe      AATGTTACTTGGTTCCATGCTATACATGTCTCTGGGACCAATGGTACTAAGAGGTTTGAT   21799
gb:MT019530|Organism:Severe      AATGTTACTTGGTTCCATGCTATACATGTCTCTGGGACCAATGGTACTAAGAGGTTTGAT   21802
gb:MT039888|Organism:Severe      AATGTTACTTGGTTCCATGCTATACATGTCTCTGGGACCAATGGTACTAAGAGGTTTGAT   21802
gb:LC522972|Organism:Severe      AATGTTACTTGGTTCCATGCTATACATGTCTCTGGGACCAATGGTACTAAGAGGTTTGAT   21799
gb:MT027063|Organism:Severe      AATGTTACTTGGTTCCATGCTATACATGTCTCTGGGACCAATGGTACTAAGAGGTTTGAT   21802
gb:MT027062|Organism:Severe      AATGTTACTTGGTTCCATGCTATACATGTCTCTGGGACCAATGGTACTAAGAGGTTTGAT   21802
gb:MT019529|Organism:Severe      AATGTTACTTGGTTCCATGCTATACATGTCTCTGGGACCAATGGTACTAAGAGGTTTGAT   21802
gb:MN996529|Organism:Severe      AATGTTACTTGGTTCCATGCTATACATGTCTCTGGGACCAATGGTACTAAGAGGTTTGAT   21790
gb:MN996531|Organism:Severe      AATGTTACTTGGTTCCATGCTATACATGTCTCTGGGACCAATGGTACTAAGAGGTTTGAT   21789
gb:MT066176|Organism:Severe      AATGTTACTTGGTTCCATGCTATACATGTCTCTGGGACCAATGGTACTAAGAGGTTTGAT   21802
gb:MT027064|Organism:Severe      AATGTTACTTGGTTCCATGCTATACATGTCTCTGGGACCAATGGTACTAAGAGGTTTGAT   21802
gb:MN994468|Organism:Severe      AATGTTACTTGGTTCCATGCTATACATGTCTCTGGGACCAATGGTACTAAGAGGTTTGAT   21802
gb:MT072688|Organism:Severe      AATGTTACTTGGTTCCATGCTATACATGTCTCTGGGACCAATGGTACTAAGAGGTTTGAT   21787
gb:MN996527|Organism:Severe      AATGTTACTTGGTTCCATGCTATACATGTCTCTGGGACCAATGGTACTAAGAGGTTTGAT   21769
gb:MT093631|Organism:Severe      AATGTTACTTGGTTCCATGCTATACATGTCTCTGGGACCAATGGTACTAAGAGGTTTGAT   21840
gb:MT106053|Organism:Severe      AATGTTACTTGGTTCCATGCTATACATGTCTCTGGGACCAATGGTACTAAGAGGTTTGAT   21802
gb:MT019533|Organism:Severe      AATGTTACTTGGTTCCATGCTATACATGTCTCTGGGACCAATGGTACTAAGAGGTTTGAT   21802
gb:MT019531|Organism:Severe      AATGTTACTTGGTTCCATGCTATACATGTCTCTGGGACCAATGGTACTAAGAGGTTTGAT   21802
gb:MN996528|Organism:Severe      AATGTTACTTGGTTCCATGCTATACATGTCTCTGGGACCAATGGTACTAAGAGGTTTGAT   21802
gb:MN996530|Organism:Severe      AATGTTACTTGGTTCCATGCTATACATGTCTCTGGGACCAATGGTACTAAGAGGTTTGAT   21788
gb:MN908947|Organism:Severe      AATGTTACTTGGTTCCATGCTATACATGTCTCTGGGACCAATGGTACTAAGAGGTTTGAT   21802
gb:MT019532|Organism:Severe      AATGTTACTTGGTTCCATGCTATACATGTCTCTGGGACCAATGGTACTAAGAGGTTTGAT   21802
                                 ************************************************************

gb:MT020781|Organism:Severe      AACCCTGTCCTACCATTTAATGATGGTGTTTATTTTGCTTCCACTGAGAAGTCTAACATA   21850
gb:MT007544|Organism:Severe      AACCCTGTCCTACCATTTAATGATGGTGTTTATTTTGCTTCCACTGAGAAGTCTAACATA   21862
gb:MN994467|Organism:Severe      AACCCTGTCCTACCATTTAATGATGGTGTTTATTTTGCTTCCACTGAGAAGTCTAACATA   21862
gb:MT044257|Organism:Severe      AACCCTGTCCTACCATTTAATGATGGTGTTTATTTTGCTTCCACTGAGAAGTCTAACATA   21862
gb:MT106054|Organism:Severe      AACCCTGTCCTACCATTTAATGATGGTGTTTATTTTGCTTCCACTGAGAAGTCTAACATA   21862
gb:MT049951|Organism:Severe      AACCCTGTCCTACCATTTAATGATGGTGTTTATTTTGCTTCCACTGAGAAGTCTAACATA   21862
gb:MN975262|Organism:Severe      AACCCTGTCCTACCATTTAATGATGGTGTTTATTTTGCTTCCACTGAGAAGTCTAACATA   21862
gb:MT106052|Organism:Severe      AACCCTGTCCTACCATTTAATGATGGTGTTTATTTTGCTTCCACTGAGAAGTCTAACATA   21862
gb:LC522975|Organism:Severe      AACCCTGTCCTACCATTTAATGATGGTGTTTATTTTGCTTCCACTGAGAAGTCTAACATA   21859
gb:LC522973|Organism:Severe      AACCCTGTCCTACCATTTAATGATGGTGTTTATTTTGCTTCCACTGAGAAGTCTAACATA   21859
gb:LC522974|Organism:Severe      AACCCTGTCCTACCATTTAATGATGGTGTTTATTTTGCTTCCACTGAGAAGTCTAACATA   21859
gb:MN985325|Organism:Severe      AACCCTGTCCTACCATTTAATGATGGTGTTTATTTTGCTTCCACTGAGAAGTCTAACATA   21862
gb:MT020881|Organism:Severe      AACCCTGTCCTACCATTTAATGATGGTGTTTATTTTGCTTCCACTGAGAAGTCTAACATA   21862
gb:MT020880|Organism:Severe      AACCCTGTCCTACCATTTAATGATGGTGTTTATTTTGCTTCCACTGAGAAGTCTAACATA   21862
gb:MT066175|Organism:Severe      AACCCTGTCCTACCATTTAATGATGGTGTTTATTTTGCTTCCACTGAGAAGTCTAACATA   21862
gb:MN997409|Organism:Severe      AACCCTGTCCTACCATTTAATGATGGTGTTTATTTTGCTTCCACTGAGAAGTCTAACATA   21862
gb:MN938384|Organism:Severe      AACCCTGTCCTACCATTTAATGATGGTGTTTATTTTGCTTCCACTGAGAAGTCTAACATA   21830
gb:MT044258|Organism:Severe      AACCCTGTCCTACCATTTAATGATGGTGTTTATTTTGCTTCCACTGAGAAGTCTAACATA   21838
gb:MT039890|Organism:Severe      AACCCTGTCCTACCATTTAATGATGGTGTTTATTTTGCTTCCACTGAGAAGTCTAACATA   21862
gb:MN988713|Organism:Severe      AACCCTGTCCTACCATTTAATGATGGTGTTTATTTTGCTTCCACTGAGAAGTCTAACATA   21862
gb:LC521925|Organism:Severe      AACCCTGTCCTACCATTTAATGATGGTGTTTATTTTGCTTCCACTGAGAAGTCTAACATA   21835
gb:MT093571|Organism:Severe      AACCCTGTCCTACCATTTAATGATGGTGTTTATTTTGCTTCCACTGAGAAGTCTAACATA   21862
```

```
gb:MT039887|Organism:Severe      AACCCTGTCCTACCATTTAATGATGGTGTTTATTTTGCTTCCACTGAGAAGTCTAACATA   21859
gb:MT019530|Organism:Severe      AACCCTGTCCTACCATTTAATGATGGTGTTTATTTTGCTTCCACTGAGAAGTCTAACATA   21862
gb:MT039888|Organism:Severe      AACCCTGTCCTACCATTTAATGATGGTGTTTATTTTGCTTCCACTGAGAAGTCTAACATA   21862
gb:LC522972|Organism:Severe      AACCCTGTCCTACCATTTAATGATGGTGTTTATTTTGCTTCCACTGAGAAGTCTAACATA   21859
gb:MT027063|Organism:Severe      AACCCTGTCCTACCATTTAATGATGGTGTTTATTTTGCTTCCACTGAGAAGTCTAACATA   21862
gb:MT027062|Organism:Severe      AACCCTGTCCTACCATTTAATGATGGTGTTTATTTTGCTTCCACTGAGAAGTCTAACATA   21862
gb:MT019529|Organism:Severe      AACCCTGTCCTACCATTTAATGATGGTGTTTATTTTGCTTCCACTGAGAAGTCTAACATA   21862
gb:MN996529|Organism:Severe      AACCCTGTCCTACCATTTAATGATGGTGTTTATTTTGCTTCCACTGAGAAGTCTAACATA   21850
gb:MN996531|Organism:Severe      AACCCTGTCCTACCATTTAATGATGGTGTTTATTTTGCTTCCACTGAGAAGTCTAACATA   21849
gb:MT066176|Organism:Severe      AACCCTGTCCTACCATTTAATGATGGTGTTTATTTTGCTTCCACTGAGAAGTCTAACATA   21862
gb:MT027064|Organism:Severe      AACCCTGTCCTACCATTTAATGATGGTGTTTATTTTGCTTCCACTGAGAAGTCTAACATA   21862
gb:MN994468|Organism:Severe      AACCCTGTCCTACCATTTAATGATGGTGTTTATTTTGCTTCCACTGAGAAGTCTAACATA   21862
gb:MT072688|Organism:Severe      AACCCTGTCCTACCATTTAATGATGGTGTTTATTTTGCTTCCACTGAGAAGTCTAACATA   21847
gb:MN996527|Organism:Severe      AACCCTGTCCTACCATTTAATGATGGTGTTTATTTTGCTTCCACTGAGAAGTCTAACATA   21829
gb:MT093631|Organism:Severe      AACCCTGTCCTACCATTTAATGATGGTGTTTATTTTGCTTCCACTGAGAAGTCTAACATA   21900
gb:MT106053|Organism:Severe      AACCCTGTCCTACCATTTAATGATGGTGTTTATTTTGCTTCCACTGAGAAGTCTAACATA   21862
gb:MT019533|Organism:Severe      AACCCTGTCCTACCATTTAATGATGGTGTTTATTTTGCTTCCACTGAGAAGTCTAACATA   21862
gb:MT019531|Organism:Severe      AACCCTGTCCTACCATTTAATGATGGTGTTTATTTTGCTTCCACTGAGAAGTCTAACATA   21862
gb:MN996528|Organism:Severe      AACCCTGTCCTACCATTTAATGATGGTGTTTATTTTGCTTCCACTGAGAAGTCTAACATA   21862
gb:MN996530|Organism:Severe      AACCCTGTCCTACCATTTAATGATGGTGTTTATTTTGCTTCCACTGAGAAGTCTAACATA   21848
gb:MN908947|Organism:Severe      AACCCTGTCCTACCATTTAATGATGGTGTTTATTTTGCTTCCACTGAGAAGTCTAACATA   21862
gb:MT019532|Organism:Severe      AACCCTGTCCTACCATTTAATGATGGTGTTTATTTTGCTTCCACTGAGAAGTCTAACATA   21862
                                 ************************************************************

gb:MT020781|Organism:Severe      ATAAGAGGCTGGATTTTTGGTACTACTTTAGATTCGAAGACCCAGTCCCTACTTATTGTT   21910
gb:MT007544|Organism:Severe      ATAAGAGGCTGGATTTTTGGTACTACTTTAGATTCGAAGACCCAGTCCCTACTTATTGTT   21922
gb:MN994467|Organism:Severe      ATAAGAGGCTGGATTTTTGGTACTACTTTAGATTCGAAGACCCAGTCCCTACTTATTGTT   21922
gb:MT044257|Organism:Severe      ATAAGAGGCTGGATTTTTGGTACTACTTTAGATTCGAAGACCCAGTCCCTACTTATTGTT   21922
gb:MT106054|Organism:Severe      ATAAGAGGCTGGATTTTTGGTACTACTTTAGATTCGAAGACCCAGTCCCTACTTATTGTT   21922
gb:MT049951|Organism:Severe      ATAAGAGGCTGGATTTTTGGTACTACTTTAGATTCGAAGACCCAGTCCCTACTTATTGTT   21922
gb:MN975262|Organism:Severe      ATAAGAGGCTGGATTTTTGGTACTACTTTAGATTCGAAGACCCAGTCCCTACTTATTGTT   21922
gb:MT106052|Organism:Severe      ATAAGAGGCTGGATTTTTGGTACTACTTTAGATTCGAAGACCCAGTCCCTACTTATTGTT   21922
gb:LC522975|Organism:Severe      ATAAGAGGCTGGATTTTTGGTACTACTTTAGATTCGAAGACCCAGTCCCTACTTATTGTT   21919
gb:LC522973|Organism:Severe      ATAAGAGGCTGGATTTTTGGTACTACTTTAGATTCGAAGACCCAGTCCCTACTTATTGTT   21919
gb:LC522974|Organism:Severe      ATAAGAGGCTGGATTTTTGGTACTACTTTAGATTCGAAGACCCAGTCCCTACTTATTGTT   21919
gb:MN985325|Organism:Severe      ATAAGAGGCTGGATTTTTGGTACTACTTTAGATTCGAAGACCCAGTCCCTACTTATTGTT   21922
gb:MT020881|Organism:Severe      ATAAGAGGCTGGATTTTTGGTACTACTTTAGATTCGAAGACCCAGTCCCTACTTATTGTT   21922
gb:MT020880|Organism:Severe      ATAAGAGGCTGGATTTTTGGTACTACTTTAGATTCGAAGACCCAGTCCCTACTTATTGTT   21922
gb:MT066175|Organism:Severe      ATAAGAGGCTGGATTTTTGGTACTACTTTAGATTCGAAGACCCAGTCCCTACTTATTGTT   21922
gb:MN997409|Organism:Severe      ATAAGAGGCTGGATTTTTGGTACTACTTTAGATTCGAAGACCCAGTCCCTACTTATTGTT   21922
gb:MN938384|Organism:Severe      ATAAGAGGCTGGATTTTTGGTACTACTTTAGATTCGAAGACCCAGTCCCTACTTATTGTT   21890
gb:MT044258|Organism:Severe      ATAAGAGGCTGGATTTTTGGTACTACTTTAGATTCGAAGACCCAGTCCCTACTTATTGTT   21898
gb:MT039890|Organism:Severe      ATAAGAGGCTGGATTTTTGGTACTACTTTAGATTCGAAGACCCAGTCCCTACTTATTGTT   21922
gb:MN988713|Organism:Severe      ATAAGAGGCTGGATTTTTGGTACTACTTTAGATTCGAAGACCCAGTCCCTACTTATTGTT   21922
gb:LC521925|Organism:Severe      ATAAGAGGCTGGATTTTTGGTACTACTTTAGATTCGAAGACCCAGTCCCTACTTATTGTT   21895
gb:MT093571|Organism:Severe      ATAAGAGGCTGGATTTTTGGTACTACTTTAGATTCGAAGACCCAGTCCCTACTTATTGTT   21922
gb:MT039887|Organism:Severe      ATAAGAGGCTGGATTTTTGGTACTACTTTAGATTCGAAGACCCAGTCCCTACTTATTGTT   21919
gb:MT019530|Organism:Severe      ATAAGAGGCTGGATTTTTGGTACTACTTTAGATTCGAAGACCCAGTCCCTACTTATTGTT   21922
gb:MT039888|Organism:Severe      ATAAGAGGCTGGATTTTTGGTACTACTTTAGATTCGAAGACCCAGTCCCTACTTATTGTT   21922
gb:LC522972|Organism:Severe      ATAAGAGGCTGGATTTTTGGTACTACTTTAGATTCGAAGACCCAGTCCCTACTTATTGTT   21919
gb:MT027063|Organism:Severe      ATAAGAGGCTGGATTTTTGGTACTACTTTAGATTCGAAGACCCAGTCCCTACTTATTGTT   21922
gb:MT027062|Organism:Severe      ATAAGAGGCTGGATTTTTGGTACTACTTTAGATTCGAAGACCCAGTCCCTACTTATTGTT   21922
gb:MT019529|Organism:Severe      ATAAGAGGCTGGATTTTTGGTACTACTTTAGATTCGAAGACCCAGTCCCTACTTATTGTT   21922
gb:MN996529|Organism:Severe      ATAAGAGGCTGGATTTTTGGTACTACTTTAGATTCGAAGACCCAGTCCCTACTTATTGTT   21910
gb:MN996531|Organism:Severe      ATAAGAGGCTGGATTTTTGGTACTACTTTAGATTCGAAGACCCAGTCCCTACTTATTGTT   21909
gb:MT066176|Organism:Severe      ATAAGAGGCTGGATTTTTGGTACTACTTTAGATTCGAAGACCCAGTCCCTACTTATTGTT   21922
gb:MT027064|Organism:Severe      ATAAGAGGCTGGATTTTTGGTACTACTTTAGATTCGAAGACCCAGTCCCTACTTATTGTT   21922
gb:MN994468|Organism:Severe      ATAAGAGGCTGGATTTTTGGTACTACTTTAGATTCGAAGACCCAGTCCCTACTTATTGTT   21922
gb:MT072688|Organism:Severe      ATAAGAGGCTGGATTTTTGGTACTACTTTAGATTCGAAGACCCAGTCCCTACTTATTGTT   21907
gb:MN996527|Organism:Severe      ATAAGAGGCTGGATTTTTGGTACTACTTTAGATTCGAAGACCCAGTCCCTACTTATTGTT   21889
gb:MT093631|Organism:Severe      ATAAGAGGCTGGATTTTTGGTACTACTTTAGATTCGAAGACCCAGTCCCTACTTATTGTT   21960
gb:MT106053|Organism:Severe      ATAAGAGGCTGGATTTTTGGTACTACTTTAGATTCGAAGACCCAGTCCCTACTTATTGTT   21922
gb:MT019533|Organism:Severe      ATAAGAGGCTGGATTTTTGGTACTACTTTAGATTCGAAGACCCAGTCCCTACTTATTGTT   21922
gb:MT019531|Organism:Severe      ATAAGAGGCTGGATTTTTGGTACTACTTTAGATTCGAAGACCCAGTCCCTACTTATTGTT   21922
gb:MN996528|Organism:Severe      ATAAGAGGCTGGATTTTTGGTACTACTTTAGATTCGAAGACCCAGTCCCTACTTATTGTT   21922
```

```
gb:MN996530|Organism:Severe      ATAAGAGGCTGGATTTTTGGTACTACTTTAGATTCGAAGACCCAGTCCCTACTTATTGTT    21908
gb:MN908947|Organism:Severe      ATAAGAGGCTGGATTTTTGGTACTACTTTAGATTCGAAGACCCAGTCCCTACTTATTGTT    21922
gb:MT019532|Organism:Severe      ATAAGAGGCTGGATTTTTGGTACTACTTTAGATTCGAAGACCCAGTCCCTACTTATTGTT    21922
                                 ************************************************************

gb:MT020781|Organism:Severe      AATAACGCTACTAATGTTGTTATTAAAGTCTGTGAATTTCAATTTTGTAATGATCCATTT    21970
gb:MT007544|Organism:Severe      AATAACGCTACTAATGTTGTTATTAAAGTCTGTGAATTTCAATTTTGTAATGATCCATTT    21982
gb:MN994467|Organism:Severe      AATAACGCTACTAATGTTGTTATTAAAGTCTGTGAATTTCAATTTTGTAATGATCCATTT    21982
gb:MT044257|Organism:Severe      AATAACGCTACTAATGTTGTTATTAAAGTCTGTGAATTTCAATTTTGTAATGATCCATTT    21982
gb:MT106054|Organism:Severe      AATAACGCTACTAATGTTGTTATTAAAGTCTGTGAATTTCAATTTTGTAATGATCCATTT    21982
gb:MT049951|Organism:Severe      AATAACGCTACTAATGTTGTTATTAAAGTCTGTGAATTTCAATTTTGTAATGATCCATTT    21982
gb:MN975262|Organism:Severe      AATAACGCTACTAATGTTGTTATTAAAGTCTGTGAATTTCAATTTTGTAATGATCCATTT    21982
gb:MT106052|Organism:Severe      AATAACGCTACTAATGTTGTTATTAAAGTCTGTGAATTTCAATTTTGTAATGATCCATTT    21982
gb:LC522975|Organism:Severe      AATAACGCTACTAATGTTGTTATTAAAGTCTGTGAATTTCAATTTTGTAATGATCCATTT    21979
gb:LC522973|Organism:Severe      AATAACGCTACTAATGTTGTTATTAAAGTCTGTGAATTTCAATTTTGTAATGATCCATTT    21979
gb:LC522974|Organism:Severe      AATAACGCTACTAATGTTGTTATTAAAGTCTGTGAATTTCAATTTTGTAATGATCCATTT    21979
gb:MN985325|Organism:Severe      AATAACGCTACTAATGTTGTTATTAAAGTCTGTGAATTTCAATTTTGTAATGATCCATTT    21982
gb:MT020881|Organism:Severe      AATAACGCTACTAATGTTGTTATTAAAGTCTGTGAATTTCAATTTTGTAATGATCCATTT    21982
gb:MT020880|Organism:Severe      AATAACGCTACTAATGTTGTTATTAAAGTCTGTGAATTTCAATTTTGTAATGATCCATTT    21982
gb:MT066175|Organism:Severe      AATAACGCTACTAATGTTGTTATTAAAGTCTGTGAATTTCAATTTTGTAATGATCCATTT    21982
gb:MN997409|Organism:Severe      AATAACGCTACTAATGTTGTTATTAAAGTCTGTGAATTTCAATTTTGTAATGATCCATTT    21982
gb:MN938384|Organism:Severe      AATAACGCTACTAATGTTGTTATTAAAGTCTGTGAATTTCAATTTTGTAATGATCCATTT    21950
gb:MT044258|Organism:Severe      AATAACGCTACTAATGTTGTTATTAAAGTCTGTGAATTTCAATTTTGTAATGATCCATTT    21958
gb:MT039890|Organism:Severe      AATAACGCTACTAATGTTGTTATTAAAGTCTGTGAATTTCAATTTTGTAATGATCCATTT    21982
gb:MN988713|Organism:Severe      AATAACGCTACTAATGTTGTTATTAAAGTCTGTGAATTTCAATTTTGTAATGATCCATTT    21982
gb:LC521925|Organism:Severe      AATAACGCTACTAATGTTGTTATTAAAGTCTGTGAATTTCAATTTTGTAATGATCCATTT    21955
gb:MT093571|Organism:Severe      AATAACGCTACTAATGTTGTTATTAAAGTCTGTGAATTTCAATTTTGTAATGATCCATTT    21982
gb:MT039887|Organism:Severe      AATAACGCTACTAATGTTGTTATTAAAGTCTGTGAATTTCAATTTTGTAATGATCCATTT    21979
gb:MT019530|Organism:Severe      AATAACGCTACTAATGTTGTTATTAAAGTCTGTGAATTTCAATTTTGTAATGATCCATTT    21982
gb:MT039888|Organism:Severe      AATAACGCTACTAATGTTGTTATTAAAGTCTGTGAATTTCAATTTTGTAATGATCCATTT    21982
gb:LC522972|Organism:Severe      AATAACGCTACTAATGTTGTTATTAAAGTCTGTGAATTTCAATTTTGTAATGATCCATTT    21979
gb:MT027063|Organism:Severe      AATAACGCTACTAATGTTGTTATTAAAGTCTGTGAATTTCAATTTTGTAATGATCCATTT    21982
gb:MT027062|Organism:Severe      AATAACGCTACTAATGTTGTTATTAAAGTCTGTGAATTTCAATTTTGTAATGATCCATTT    21982
gb:MT019529|Organism:Severe      AATAACGCTACTAATGTTGTTATTAAAGTCTGTGAATTTCAATTTTGTAATGATCCATTT    21982
gb:MN996529|Organism:Severe      AATAACGCTACTAATGTTGTTATTAAAGTCTGTGAATTTCAATTTTGTAATGATCCATTT    21970
gb:MN996531|Organism:Severe      AATAACGCTACTAATGTTGTTATTAAAGTCTGTGAATTTCAATTTTGTAATGATCCATTT    21969
gb:MT066176|Organism:Severe      AATAACGCTACTAATGTTGTTATTAAAGTCTGTGAATTTCAATTTTGTAATGATCCATTT    21982
gb:MT027064|Organism:Severe      AATAACGCTACTAATGTTGTTATTAAAGTCTGTGAATTTCAATTTTGTAATGATCCATTT    21982
gb:MN994468|Organism:Severe      AATAACGCTACTAATGTTGTTATTAAAGTCTGTGAATTTCAATTTTGTAATGATCCATTT    21982
gb:MT072688|Organism:Severe      AATAACGCTACTAATGTTGTTATTAAAGTCTGTGAATTTCAATTTTGTAATGATCCATTT    21967
gb:MN996527|Organism:Severe      AATAACGCTACTAATGTTGTTATTAAAGTCTGTGAATTTCAATTTTGTAATGATCCATTT    21949
gb:MT093631|Organism:Severe      AATAACGCTACTAATGTTGTTATTAAAGTCTGTGAATTTCAATTTTGTAATGATCCATTT    22020
gb:MT106053|Organism:Severe      AATAACGCTACTAATGTTGTTATTAAAGTCTGTGAATTTCAATTTTGTAATGATCCATTT    21982
gb:MT019533|Organism:Severe      AATAACGCTACTAATGTTGTTATTAAAGTCTGTGAATTTCAATTTTGTAATGATCCATTT    21982
gb:MT019531|Organism:Severe      AATAACGCTACTAATGTTGTTATTAAAGTCTGTGAATTTCAATTTTGTAATGATCCATTT    21982
gb:MN996528|Organism:Severe      AATAACGCTACTAATGTTGTTATTAAAGTCTGTGAATTTCAATTTTGTAATGATCCATTT    21982
gb:MN996530|Organism:Severe      AATAACGCTACTAATGTTGTTATTAAAGTCTGTGAATTTCAATTTTGTAATGATCCATTT    21968
gb:MN908947|Organism:Severe      AATAACGCTACTAATGTTGTTATTAAAGTCTGTGAATTTCAATTTTGTAATGATCCATTT    21982
gb:MT019532|Organism:Severe      AATAACGCTACTAATGTTGTTATTAAAGTCTGTGAATTTCAATTTTGTAATGATCCATTT    21982
                                 ************************************************************

gb:MT020781|Organism:Severe      TTGGGTGTTTATTACCACAAAAACAACAAAAGTTGGATGGAAAGTGAGTTCAGAGTTTAT    22030
gb:MT007544|Organism:Severe      TTGGGTGTTTATTACCACAAAAACAACAAAAGTTGGATGGAAAGTGAGTTCAGAGTTTAT    22042
gb:MN994467|Organism:Severe      TTGGGTGTTTATTACCACAAAAACAACAAAAGTTGGATGGAAAGTGAGTTCAGAGTTTAT    22042
gb:MT044257|Organism:Severe      TTGGGTGTTTATTACCACAAAAACAACAAAAGTTGGATGGAAAGTGAGTTCAGAGTTTAT    22042
gb:MT106054|Organism:Severe      TTGGGTGTTTATTACCACAAAAACAACAAAAGTTGGATGGAAAGTGAGTTCAGAGTTTAT    22042
gb:MT049951|Organism:Severe      TTGGGTGTTTATTACCACAAAAACAACAAAAGTTGGATGGAAAGTGAGTTCAGAGTTTAT    22042
gb:MN975262|Organism:Severe      TTGGGTGTTTATTACCACAAAAACAACAAAAGTTGGATGGAAAGTGAGTTCAGAGTTTAT    22042
gb:MT106052|Organism:Severe      TTGGGTGTTTATTACCACAAAAACAACAAAAGTTGGATGGAAAGTGAGTTCAGAGTTTAT    22042
gb:LC522975|Organism:Severe      TTGGGTGTTTATTACCACAAAAACAACAAAAGTTGGATGGAAAGTGAGTTCAGAGTTTAT    22039
gb:LC522973|Organism:Severe      TTGGGTGTTTATTACCACAAAAACAACAAAAGTTGGATGGAAAGTGAGTTCAGAGTTTAT    22039
gb:LC522974|Organism:Severe      TTGGGTGTTTATTACCACAAAAACAACAAAAGTTGGATGGAAAGTGAGTTCAGAGTTTAT    22039
gb:MN985325|Organism:Severe      TTGGGTGTTTATTACCACAAAAACAACAAAAGTTGGATGGAAAGTGAGTTCAGAGTTTAT    22042
gb:MT020881|Organism:Severe      TTGGGTGTTTATTACCACAAAAACAACAAAAGTTGGATGGAAAGTGAGTTCAGAGTTTAT    22042
gb:MT020880|Organism:Severe      TTGGGTGTTTATTACCACAAAAACAACAAAAGTTGGATGGAAAGTGAGTTCAGAGTTTAT    22042
```

```
gb:MT066175|Organism:Severe      TTGGGTGTTTATTACCACAAAAACAACAAAAGTTGGATGGAAAGTGAGTTCAGAGTTTAT   22042
gb:MN997409|Organism:Severe      TTGGGTGTTTATTACCACAAAAACAACAAAAGTTGGATGGAAAGTGAGTTCAGAGTTTAT   22042
gb:MN938384|Organism:Severe      TTGGGTGTTTATTACCACAAAAACAACAAAAGTTGGATGGAAAGTGAGTTCAGAGTTTAT   22010
gb:MT044258|Organism:Severe      TTGGGTGTTTATTACCACAAAAACAACAAAAGTTGGATGGAAAGTGAGTTCAGAGTTTAT   22018
gb:MT039890|Organism:Severe      TTGGGTGTTTATTACCACAAAAACAACAAAAGTTGGATGGAAAGTGAGTTCAGAGTTTAT   22042
gb:MN988713|Organism:Severe      TTGGGTGTTTATTACCACAAAAACAACAAAAGTTGGATGGAAAGTGAGTTCAGAGTTTAT   22042
gb:LC521925|Organism:Severe      TTGGGTGTTTATTACCACAAAAACAACAAAAGTTGGATGGAAAGTGAGTTCAGAGTTTAT   22015
gb:MT093571|Organism:Severe      TTGGGTGTTTATTACCACAAAAACAACAAAAGTTGGATGGAAAGTGAGTTCAGAGTTTAT   22042
gb:MT039887|Organism:Severe      TTGGGTGTTTATTACCACAAAAACAACAAAAGTTGGATGGAAAGTGAGTTCAGAGTTTAT   22039
gb:MT019530|Organism:Severe      TTGGGTGTTTATTACCACAAAAACAACAAAAGTTGGATGGAAAGTGAGTTCAGAGTTTAT   22042
gb:MT039888|Organism:Severe      TTGGGTGTTTATTACCACAAAAACAACAAAAGTTGGATGGAAAGTGAGTTCAGAGTTTAT   22042
gb:LC522972|Organism:Severe      TTGGGTGTTTATTACCACAAAAACAACAAAAGTTGGATGGAAAGTGAGTTCAGAGTTTAT   22039
gb:MT027063|Organism:Severe      TTGGGTGTTTATTACCACAAAAACAACAAAAGTTGGATGGAAAGTGAGTTCAGAGTTTAT   22042
gb:MT027062|Organism:Severe      TTGGGTGTTTATTACCACAAAAACAACAAAAGTTGGATGGAAAGTGAGTTCAGAGTTTAT   22042
gb:MT019529|Organism:Severe      TTGGGTGTTTATTACCACAAAAACAACAAAAGTTGGATGGAAAGTGAGTTCAGAGTTTAT   22042
gb:MN996529|Organism:Severe      TTGGGTGTTTATTACCACAAAAACAACAAAAGTTGGATGGAAAGTGAGTTCAGAGTTTAT   22030
gb:MN996531|Organism:Severe      TTGGGTGTTTATTACCACAAAAACAACAAAAGTTGGATGGAAAGTGAGTTCAGAGTTTAT   22029
gb:MT066176|Organism:Severe      TTGGGTGTTTATTACCACAAAAACAACAAAAGTTGGATGGAAAGTGAGTTCAGAGTTTAT   22042
gb:MT027064|Organism:Severe      TTGGGTGTTTATTACCACAAAAACAACAAAAGTTGGATGGAAAGTGAGTTCAGAGTTTAT   22042
gb:MN994468|Organism:Severe      TTGGGTGTTTATTACCACAAAAACAACAAAAGTTGGATGGAAAGTGAGTTCAGAGTTTAT   22042
gb:MT072688|Organism:Severe      TTGGGTGTTTATTACCACAAAAACAACAAAAGTTGGATGGAAAGTGAGTTCAGAGTTTAT   22027
gb:MN996527|Organism:Severe      TTGGGTGTTTATTACCACAAAAACAACAAAAGTTGGATGGAAAGTGAGTTCAGAGTTTAT   22009
gb:MT093631|Organism:Severe      TTGGGTGTTTATTACCACAAAAACAACAAAAGTTGGATGGAAAGTGAGTTCAGAGTTTAT   22080
gb:MT106053|Organism:Severe      TTGGGTGTTTATTACCACAAAAACAACAAAAGTTGGATGGAAAGTGAGTTCAGAGTTTAT   22042
gb:MT019533|Organism:Severe      TTGGGTGTTTATTACCACAAAAACAACAAAAGTTGGATGGAAAGTGAGTTCAGAGTTTAT   22042
gb:MT019531|Organism:Severe      TTGGGTGTTTATTACCACAAAAACAACAAAAGTTGGATGGAAAGTGAGTTCAGAGTTTAT   22042
gb:MN996528|Organism:Severe      TTGGGTGTTTATTACCACAAAAACAACAAAAGTTGGATGGAAAGTGAGTTCAGAGTTTAT   22042
gb:MN996530|Organism:Severe      TTGGGTGTTTATTACCACAAAAACAACAAAAGTTGGATGGAAAGTGAGTTCAGAGTTTAT   22028
gb:MN908947|Organism:Severe      TTGGGTGTTTATTACCACAAAAACAACAAAAGTTGGATGGAAAGTGAGTTCAGAGTTTAT   22042
gb:MT019532|Organism:Severe      TTGGGTGTTTATTACCACAAAAACAACAAAAGTTGGATGGAAAGTGAGTTCAGAGTTTAT   22042
                                 ************************************************************

gb:MT020781|Organism:Severe      TCTAGTGCGAATAATTGCACTTTTGAATATGTCTCTCAGCCTTTTCTTATGGACCTTGAA   22090
gb:MT007544|Organism:Severe      TCTAGTGCGAATAATTGCACTTTTGAATATGTCTCTCAGCCTTTTCTTATGGACCTTGAA   22102
gb:MN994467|Organism:Severe      TCTAGTGCGAATAATTGCACTTTTGAATATGTCTCTCAGCCTTTTCTTATGGACCTTGAA   22102
gb:MT044257|Organism:Severe      TCTAGTGCGAATAATTGCACTTTTGAATATGTCTCTCAGCCTTTTCTTATGGACCTTGAA   22102
gb:MT106054|Organism:Severe      TCTAGTGCGAATAATTGCACTTTTGAATATGTCTCTCAGCCTTTTCTTATGGACCTTGAA   22102
gb:MT049951|Organism:Severe      TCTAGTGCGAATAATTGCACTTTTGAATATGTCTCTCAGCCTTTTCTTATGGACCTTGAA   22102
gb:MN975262|Organism:Severe      TCTAGTGCGAATAATTGCACTTTTGAATATGTCTCTCAGCCTTTTCTTATGGACCTTGAA   22102
gb:MT106052|Organism:Severe      TCTAGTGCGAATAATTGCACTTTTGAATATGTCTCTCAGCCTTTTCTTATGGACCTTGAA   22102
gb:LC522975|Organism:Severe      TCTAGTGCGAATAATTGCACTTTTGAATATGTCTCTCAGCCTTTTCTTATGGACCTTGAA   22099
gb:LC522973|Organism:Severe      TCTAGTGCGAATAATTGCACTTTTGAATATGTCTCTCAGCCTTTTCTTATGGACCTTGAA   22099
gb:LC522974|Organism:Severe      TCTAGTGCGAATAATTGCACTTTTGAATATGTCTCTCAGCCTTTTCTTATGGACCTTGAA   22099
gb:MN985325|Organism:Severe      TCTAGTGCGAATAATTGCACTTTTGAATATGTCTCTCAGCCTTTTCTTATGGACCTTGAA   22102
gb:MT020881|Organism:Severe      TCTAGTGCGAATAATTGCACTTTTGAATATGTCTCTCAGCCTTTTCTTATGGACCTTGAA   22102
gb:MT020880|Organism:Severe      TCTAGTGCGAATAATTGCACTTTTGAATATGTCTCTCAGCCTTTTCTTATGGACCTTGAA   22102
gb:MT066175|Organism:Severe      TCTAGTGCGAATAATTGCACTTTTGAATATGTCTCTCAGCCTTTTCTTATGGACCTTGAA   22102
gb:MN997409|Organism:Severe      TCTAGTGCGAATAATTGCACTTTTGAATATGTCTCTCAGCCTTTTCTTATGGACCTTGAA   22102
gb:MN938384|Organism:Severe      TCTAGTGCGAATAATTGCACTTTTGAATATGTCTCTCAGCCTTTTCTTATGGACCTTGAA   22070
gb:MT044258|Organism:Severe      TCTAGTGCGAATAATTGCACTTTTGAATATGTCTCTCAGCCTTTTCTTATGGACCTTGAA   22078
gb:MT039890|Organism:Severe      TCTAGTGCGAATAATTGCACTTTTGAATATGTCTCTCAGCCTTTTCTTATGGACCTTGAA   22102
gb:MN988713|Organism:Severe      TCTAGTGCGAATAATTGCACTTTTGAATATGTCTCTCAGCCTTTTCTTATGGACCTTGAA   22102
gb:LC521925|Organism:Severe      TCTAGTGCGAATAATTGCACTTTTGAATATGTCTCTCAGCCTTTTCTTATGGACCTTGAA   22075
gb:MT093571|Organism:Severe      TCTAGTGCGAATAATTGCACTTTTGAATATGTCTCTCAGCCTTTTCTTATGGACCTTGAA   22102
gb:MT039887|Organism:Severe      TCTAGTGCGAATAATTGCACTTTTGAATATGTCTCTCAGCCTTTTCTTATGGACCTTGAA   22099
gb:MT019530|Organism:Severe      TCTAGTGCGAATAATTGCACTTTTGAATATGTCTCTCAGCCTTTTCTTATGGACCTTGAA   22102
gb:MT039888|Organism:Severe      TCTAGTGCGAATAATTGCACTTTTGAATATGTCTCTCAGCCTTTTCTTATGGACCTTGAA   22102
gb:LC522972|Organism:Severe      TCTAGTGCGAATAATTGCACTTTTGAATATGTCTCTCAGCCTTTTCTTATGGACCTTGAA   22099
gb:MT027063|Organism:Severe      TCTAGTGCGAATAATTGCACTTTTGAATATGTCTCTCAGCCTTTTCTTATGGACCTTGAA   22102
gb:MT027062|Organism:Severe      TCTAGTGCGAATAATTGCACTTTTGAATATGTCTCTCAGCCTTTTCTTATGGACCTTGAA   22102
gb:MT019529|Organism:Severe      TCTAGTGCGAATAATTGCACTTTTGAATATGTCTCTCAGCCTTTTCTTATGGACCTTGAA   22102
gb:MN996529|Organism:Severe      TCTAGTGCGAATAATTGCACTTTTGAATATGTCTCTCAGCCTTTTCTTATGGACCTTGAA   22090
gb:MN996531|Organism:Severe      TCTAGTGCGAATAATTGCACTTTTGAATATGTCTCTCAGCCTTTTCTTATGGACCTTGAA   22089
gb:MT066176|Organism:Severe      TCTAGTGCGAATAATTGCACTTTTGAATATGTCTCTCAGCCTTTTCTTATGGACCTTGAA   22102
gb:MT027064|Organism:Severe      TCTAGTGCGAATAATTGCACTTTTGAATATGTCTCTCAGCCTTTTCTTATGGACCTTGAA   22102
```

```
gb:MN994468|Organism:Severe      TCTAGTGCGAATAATTGCACTTTTGAATATGTCTCTCAGCCTTTTCTTATGGACCTTGAA   22102
gb:MT072688|Organism:Severe      TCTAGTGCGAATAATTGCACTTTTGAATATGTCTCTCAGCCTTTTCTTATGGACCTTGAA   22087
gb:MN996527|Organism:Severe      TCTAGTGCGAATAATTGCACTTTTGAATATGTCTCTCAGCCTTTTCTTATGGACCTTGAA   22069
gb:MT093631|Organism:Severe      TCTAGTGCGAATAATTGCACTTTTGAATATGTCTCTCAGCCTTTTCTTATGGACCTTGAA   22140
gb:MT106053|Organism:Severe      TCTAGTGCGAATAATTGCACTTTTGAATATGTCTCTCAGCCTTTTCTTATGGACCTTGAA   22102
gb:MT019533|Organism:Severe      TCTAGTGCGAATAATTGCACTTTTGAATATGTCTCTCAGCCTTTTCTTATGGACCTTGAA   22102
gb:MT019531|Organism:Severe      TCTAGTGCGAATAATTGCACTTTTGAATATGTCTCTCAGCCTTTTCTTATGGACCTTGAA   22102
gb:MN996528|Organism:Severe      TCTAGTGCGAATAATTGCACTTTTGAATATGTCTCTCAGCCTTTTCTTATGGACCTTGAA   22102
gb:MN996530|Organism:Severe      TCTAGTGCGAATAATTGCACTTTTGAATATGTCTCTCAGCCTTTTCTTATGGACCTTGAA   22088
gb:MN908947|Organism:Severe      TCTAGTGCGAATAATTGCACTTTTGAATATGTCTCTCAGCCTTTTCTTATGGACCTTGAA   22102
gb:MT019532|Organism:Severe      TCTAGTGCGAATAATTGCACTTTTGAATATGTCTCTCAGCCTTTTCTTATGGACCTTGAA   22102
                                 ************************************************************

gb:MT020781|Organism:Severe      GGAAAACAGGGTAATTTCAAAAATCTTAGGGAATTTGTGTTTAAGAATATTGATGGTTAT   22150
gb:MT007544|Organism:Severe      GGAAAACAGGGTAATTTCAAAAATCTTAGGGAATTTGTGTTTAAGAATATTGATGGTTAT   22162
gb:MN994467|Organism:Severe      GGAAAACAGGGTAATTTCAAAAATCTTAGGGAATTTGTGTTTAAGAATATTGATGGTTAT   22162
gb:MT044257|Organism:Severe      GGAAAACAGGGTAATTTCAAAAATCTTAGGGAATTTGTGTTTAAGAATATTGATGGTTAT   22162
gb:MT106054|Organism:Severe      GGAAAACAGGGTAATTTCAAAAATCTTAGGGAATTTGTGTTTAAGAATATTGATGGTTAT   22162
gb:MT049951|Organism:Severe      GGAAAACAGGGTAATTTCAAAAATCTTAGGGAATTTGTGTTTAAGAATATTGATGGTTAT   22162
gb:MN975262|Organism:Severe      GGAAAACAGGGTAATTTCAAAAATCTTAGGGAATTTGTGTTTAAGAATATTGATGGTTAT   22162
gb:MT106052|Organism:Severe      GGAAAACAGGGTAATTTCAAAAATCTTAGGGAATTTGTGTTTAAGAATATTGATGGTTAT   22162
gb:LC522975|Organism:Severe      GGAAAACAGGGTAATTTCAAAAATCTTAGGGAATTTGTGTTTAAGAATATTGATGGTTAT   22159
gb:LC522973|Organism:Severe      GGAAAACAGGGTAATTTCAAAAATCTTAGGGAATTTGTGTTTAAGAATATTGATGGTTAT   22159
gb:LC522974|Organism:Severe      GGAAAACAGGGTAATTTCAAAAATCTTAGGGAATTTGTGTTTAAGAATATTGATGGTTAT   22159
gb:MN985325|Organism:Severe      GGAAAACAGGGTAATTTCAAAAATCTTAGGGAATTTGTGTTTAAGAATATTGATGGTTAT   22162
gb:MT020881|Organism:Severe      GGAAAACAGGGTAATTTCAAAAATCTTAGGGAATTTGTGTTTAAGAATATTGATGGTTAT   22162
gb:MT020880|Organism:Severe      GGAAAACAGGGTAATTTCAAAAATCTTAGGGAATTTGTGTTTAAGAATATTGATGGTTAT   22162
gb:MT066175|Organism:Severe      GGAAAACAGGGTAATTTCAAAAATCTTAGGGAATTTGTGTTTAAGAATATTGATGGTTAT   22162
gb:MN997409|Organism:Severe      GGAAAACAGGGTAATTTCAAAAATCTTAGGGAATTTGTGTTTAAGAATATTGATGGTTAT   22162
gb:MN938384|Organism:Severe      GGAAAACAGGGTAATTTCAAAAATCTTAGGGAATTTGTGTTTAAGAATATTGATGGTTAT   22130
gb:MT044258|Organism:Severe      GGAAAACAGGGTAATTTCAAAAATCTTAGGGAATTTGTGTTTAAGAATATTGATGGTTAT   22138
gb:MT039890|Organism:Severe      GGAAAACAGGGTAATTTCAAAAATCTTAGGGAATTTGTGTTTAAGAATATTGATGGTTAT   22162
gb:MN988713|Organism:Severe      GGAAAACAGGGTAATTTCAAAAATCTTAGGGAATTTGTGTTTAAGAATATTGATGGTTAT   22162
gb:LC521925|Organism:Severe      GGAAAACAGGGTAATTTCAAAAATCTTAGGGAATTTGTGTTTAAGAATATTGATGGTTAT   22135
gb:MT093571|Organism:Severe      GGAAAACAGGGTAATTTCAAAAATCTTAGGGAATTTGTGTTTAAGAATATTGATGGTTAT   22162
gb:MT039887|Organism:Severe      GGAAAACAGGGTAATTTCAAAAATCTTAGGGAATTTGTGTTTAAGAATATTGATGGTTAT   22159
gb:MT019530|Organism:Severe      GGAAAACAGGGTAATTTCAAAAATCTTAGGGAATTTGTGTTTAAGAATATTGATGGTTAT   22162
gb:MT039888|Organism:Severe      GGAAAACAGGGTAATTTCAAAAATCTTAGGGAATTTGTGTTTAAGAATATTGATGGTTAT   22162
gb:LC522972|Organism:Severe      GGAAAACAGGGTAATTTCAAAAATCTTAGGGAATTTGTGTTTAAGAATATTGATGGTTAT   22159
gb:MT027063|Organism:Severe      GGAAAACAGGGTAATTTCAAAAATCTTAGGGAATTTGTGTTTAAGAATATTGATGGTTAT   22162
gb:MT027062|Organism:Severe      GGAAAACAGGGTAATTTCAAAAATCTTAGGGAATTTGTGTTTAAGAATATTGATGGTTAT   22162
gb:MT019529|Organism:Severe      GGAAAACAGGGTAATTTCAAAAATCTTAGGGAATTTGTGTTTAAGAATATTGATGGTTAT   22162
gb:MN996529|Organism:Severe      GGAAAACAGGGTAATTTCAAAAATCTTAGGGAATTTGTGTTTAAGAATATTGATGGTTAT   22150
gb:MN996531|Organism:Severe      GGAAAACAGGGTAATTTCAAAAATCTTAGGGAATTTGTGTTTAAGAATATTGATGGTTAT   22149
gb:MT066176|Organism:Severe      GGAAAACAGGGTAATTTCAAAAATCTTAGGGAATTTGTGTTTAAGAATATTGATGGTTAT   22162
gb:MT027064|Organism:Severe      GGAAAACAGGGTAATTTCAAAAATCTTAGGGAATTTGTGTTTAAGAATATTGATGGTTAT   22162
gb:MN994468|Organism:Severe      GGAAAACAGGGTAATTTCAAAAATCTTAGGGAATTTGTGTTTAAGAATATTGATGGTTAT   22162
gb:MT072688|Organism:Severe      GGAAAACAGGGTAATTTCAAAAATCTTAGGGAATTTGTGTTTAAGAATATTGATGGTTAT   22147
gb:MN996527|Organism:Severe      GGAAAACAGGGTAATTTCAAAAATCTTAGGGAATTTGTGTTTAAGAATATTGATGGTTAT   22129
gb:MT093631|Organism:Severe      GGAAAACAGGGTAATTTCAAAAATCTTAGGGAATTTGTGTTTAAGAATATTGATGGTTAT   22200
gb:MT106053|Organism:Severe      GGAAAACAGGGTAATTTCAAAAATCTTAGGGAATTTGTGTTTAAGAATATTGATGGTTAT   22162
gb:MT019533|Organism:Severe      GGAAAACAGGGTAATTTCAAAAATCTTAGGGAATTTGTGTTTAAGAATATTGATGGTTAT   22162
gb:MT019531|Organism:Severe      GGAAAACAGGGTAATTTCAAAAATCTTAGGGAATTTGTGTTTAAGAATATTGATGGTTAT   22162
gb:MN996528|Organism:Severe      GGAAAACAGGGTAATTTCAAAAATCTTAGGGAATTTGTGTTTAAGAATATTGATGGTTAT   22162
gb:MN996530|Organism:Severe      GGAAAACAGGGTAATTTCAAAAATCTTAGGGAATTTGTGTTTAAGAATATTGATGGTTAT   22148
gb:MN908947|Organism:Severe      GGAAAACAGGGTAATTTCAAAAATCTTAGGGAATTTGTGTTTAAGAATATTGATGGTTAT   22162
gb:MT019532|Organism:Severe      GGAAAACAGGGTAATTTCAAAAATCTTAGGGAATTTGTGTTTAAGAATATTGATGGTTAT   22162
                                 ************************************************************

gb:MT020781|Organism:Severe      TTTAAAATATATTCTAAGCACACGCCTATTAATTTAGTGCGTGATCTCCCTCAGGGTTTT   22210
gb:MT007544|Organism:Severe      TTTAAAATATATTCTAAGCACACGCCTATTAATTTAGTGCGTGATCTCCCTCAGGGTTTT   22222
gb:MN994467|Organism:Severe      TTTAAAATATATTCTAAGCACACGCCTATTAATTTAGTGCGTGATCTCCCTCAGGGTTTT   22222
gb:MT044257|Organism:Severe      TTTAAAATATATTCTAAGCACACGCCTATTAATTTAGTGCGTGATCTCCCTCAGGGTTTT   22222
gb:MT106054|Organism:Severe      TTTAAAATATATTCTAAGCACACGCCTATTAATTTAGTGCGTGATCTCCCTCAGGGTTTT   22222
gb:MT049951|Organism:Severe      TTTAAAATATATTCTAAGCACACGCCTATTAATTTAGTGCGTGATCTCCCTCAGGGTTTT   22222
```

```
gb:MN975262|Organism:Severe      TTTAAAATATATTCTAAGCACACGCCTATTAATTTAGTGCGTGATCTCCCTCAGGGTTTT   22222
gb:MT106052|Organism:Severe      TTTAAAATATATTCTAAGCACACGCCTATTAATTTAGTGCGTGATCTCCCTCAGGGTTTT   22222
gb:LC522975|Organism:Severe      TTTAAAATATATTCTAAGCACACGCCTATTAATTTAGTGCGTGATCTCCCTCAGGGTTTT   22219
gb:LC522973|Organism:Severe      TTTAAAATATATTCTAAGCACACGCCTATTAATTTAGTGCGTGATCTCCCTCAGGGTTTT   22219
gb:LC522974|Organism:Severe      TTTAAAATATATTCTAAGCACACGCCTATTAATTTAGTGCGTGATCTCCCTCAGGGTTTT   22219
gb:MN985325|Organism:Severe      TTTAAAATATATTCTAAGCACACGCCTATTAATTTAGTGCGTGATCTCCCTCAGGGTTTT   22222
gb:MT020881|Organism:Severe      TTTAAAATATATTCTAAGCACACGCCTATTAATTTAGTGCGTGATCTCCCTCAGGGTTTT   22222
gb:MT020880|Organism:Severe      TTTAAAATATATTCTAAGCACACGCCTATTAATTTAGTGCGTGATCTCCCTCAGGGTTTT   22222
gb:MT066175|Organism:Severe      TTTAAAATATATTCTAAGCACACGCCTATTAATTTAGTGCGTGATCTCCCTCAGGGTTTT   22222
gb:MN997409|Organism:Severe      TTTAAAATATATTCTAAGCACACGCCTATTAATTTAGTGCGTGATCTCCCTCAGGGTTTT   22222
gb:MN938384|Organism:Severe      TTTAAAATATATTCTAAGCACACGCCTATTAATTTAGTGCGTGATCTCCCTCAGGGTTTT   22190
gb:MT044258|Organism:Severe      TTTAAAATATATTCTAAGCACACGCCTATTAATTTAGTGCGTGATCTCCCTCAGGGTTTT   22198
gb:MT039890|Organism:Severe      TTTAAAATATATTCTAAGCACACGCCTATTAATTTAGTGCGTGATCTCCCTCAGGGTTTT   22222
gb:MN988713|Organism:Severe      TTTAAAATATATTCTAAGCACACGCCTATTAATTTAGTGCGTGATCTCCCTCAGGGTTTT   22222
gb:LC521925|Organism:Severe      TTTAAAATATATTCTAAGCACACGCCTATTAATTTAGTGCGTGATCTCCCTCAGGGTTTT   22195
gb:MT093571|Organism:Severe      TTTAAAATATATTCTAAGCACACGCCTATTAATTTAGTGCGTGATCTCCCTCAGGGTTTT   22222
gb:MT039887|Organism:Severe      TTTAAAATATATTCTAAGCACACGCCTATTAATTTAGTGCGTGATCTCCCTCAGGGTTTT   22219
gb:MT019530|Organism:Severe      TTTAAAATATATTCTAAGCACACGCCTATTAATTTAGTGCGTGATCTCCCTCAGGGTTTT   22222
gb:MT039888|Organism:Severe      TTTAAAATATATTCTAAGCACACGCCTATTAATTTAGTGCGTGATCTCCCTCAGGGTTTT   22222
gb:LC522972|Organism:Severe      TTTAAAATATATTCTAAGCACACGCCTATTAATTTAGTGCGTGATCTCCCTCAGGGTTTT   22219
gb:MT027063|Organism:Severe      TTTAAAATATATTCTAAGCACACGCCTATTAATTTAGTGCGTGATCTCCCTCAGGGTTTT   22222
gb:MT027062|Organism:Severe      TTTAAAATATATTCTAAGCACACGCCTATTAATTTAGTGCGTGATCTCCCTCAGGGTTTT   22222
gb:MT019529|Organism:Severe      TTTAAAATATATTCTAAGCACACGCCTATTAATTTAGTGCGTGATCTCCCTCAGGGTTTT   22222
gb:MN996529|Organism:Severe      TTTAAAATATATTCTAAGCACACGCCTATTAATTTAGTGCGTGATCTCCCTCAGGGTTTT   22210
gb:MN996531|Organism:Severe      TTTAAAATATATTCTAAGCACACGCCTATTAATTTAGTGCGTGATCTCCCTCAGGGTTTT   22209
gb:MT066176|Organism:Severe      TTTAAAATATATTCTAAGCACACGCCTATTAATTTAGTGCGTGATCTCCCTCAGGGTTTT   22222
gb:MT027064|Organism:Severe      TTTAAAATATATTCTAAGCACACGCCTATTAATTTAGTGCGTGATCTCCCTCAGGGTTTT   22222
gb:MN994468|Organism:Severe      TTTAAAATATATTCTAAGCACACGCCTATTAATTTAGTGCGTGATCTCCCTCAGGGTTTT   22222
gb:MT072688|Organism:Severe      TTTAAAATATATTCTAAGCACACGCCTATTAATTTAGTGCGTGATCTCCCTCAGGGTTTT   22207
gb:MN996527|Organism:Severe      TTTAAAATATATTCTAAGCACACGCCTATTAATTTAGTGCGTGATCTCCCTCAGGGTTTT   22189
gb:MT093631|Organism:Severe      TTTAAAATATATTCTAAGCACACGCCTATTAATTTAGTGCGTGATCTCCCTCAGGGTTTT   22260
gb:MT106053|Organism:Severe      TTTAAAATATATTCTAAGCACACGCCTATTAATTTAGTGCGTGATCTCCCTCAGGGTTTT   22222
gb:MT019533|Organism:Severe      TTTAAAATATATTCTAAGCACACGCCTATTAATTTAGTGCGTGATCTCCCTCAGGGTTTT   22222
gb:MT019531|Organism:Severe      TTTAAAATATATTCTAAGCACACGCCTATTAATTTAGTGCGTGATCTCCCTCAGGGTTTT   22222
gb:MN996528|Organism:Severe      TTTAAAATATATTCTAAGCACACGCCTATTAATTTAGTGCGTGATCTCCCTCAGGGTTTT   22222
gb:MN996530|Organism:Severe      TTTAAAATATATTCTAAGCACACGCCTATTAATTTAGTGCGTGATCTCCCTCAGGGTTTT   22208
gb:MN908947|Organism:Severe      TTTAAAATATATTCTAAGCACACGCCTATTAATTTAGTGCGTGATCTCCCTCAGGGTTTT   22222
gb:MT019532|Organism:Severe      TTTAAAATATATTCTAAGCACACGCCTATTAATTTAGTGCGTGATCTCCCTCAGGGTTTT   22222
                                 ************************************************************

gb:MT020781|Organism:Severe      TCGGCTTTAGAACCATTGGTAGATTTGCCAATAGGTATTAACATCACTAGGTTTCAAACT   22270
gb:MT007544|Organism:Severe      TCGGCTTTAGAACCATTGGTAGATTTGCCAATAGGTATTAACATCACTAGGTTTCAAACT   22282
gb:MN994467|Organism:Severe      TCGGCTTTAGAACCATTGGTAGATTTGCCAATAGGTATTAACATCACTAGGTTTCAAACT   22282
gb:MT044257|Organism:Severe      TCGGCTTTAGAACCATTGGTAGATTTGCCAATAGGTATTAACATCACTAGGTTTCAAACT   22282
gb:MT106054|Organism:Severe      TCGGCTTTAGAACCATTGGTAGATTTGCCAATAGGTATTAACATCACTAGGTTTCAAACT   22282
gb:MT049951|Organism:Severe      TCGGCTTTAGAACCATTGGTAGATTTGCCAATAGGTATTAACATCACTAGGTTTCAAACT   22282
gb:MN975262|Organism:Severe      TCGGCTTTAGAACCATTGGTAGATTTGCCAATAGGTATTAACATCACTAGGTTTCAAACT   22282
gb:MT106052|Organism:Severe      TCGGCTTTAGAACCATTGGTAGATTTGCCAATAGGTATTAACATCACTAGGTTTCAAACT   22282
gb:LC522975|Organism:Severe      TCGGCTTTAGAACCATTGGTAGATTTGCCAATAGGTATTAACATCACTAGGTTTCAAACT   22279
gb:LC522973|Organism:Severe      TCGGCTTTAGAACCATTGGTAGATTTGCCAATAGGTATTAACATCACTAGGTTTCAAACT   22279
gb:LC522974|Organism:Severe      TCGGCTTTAGAACCATTGGTAGATTTGCCAATAGGTATTAACATCACTAGGTTTCAAACT   22279
gb:MN985325|Organism:Severe      TCGGCTTTAGAACCATTGGTAGATTTGCCAATAGGTATTAACATCACTAGGTTTCAAACT   22282
gb:MT020881|Organism:Severe      TCGGCTTTAGAACCATTGGTAGATTTGCCAATAGGTATTAACATCACTAGGTTTCAAACT   22282
gb:MT020880|Organism:Severe      TCGGCTTTAGAACCATTGGTAGATTTGCCAATAGGTATTAACATCACTAGGTTTCAAACT   22282
gb:MT066175|Organism:Severe      TCGGCTTTAGAACCATTGGTAGATTTGCCAATAGGTATTAACATCACTAGGTTTCAAACT   22282
gb:MN997409|Organism:Severe      TCGGCTTTAGAACCATTGGTAGATTTGCCAATAGGTATTAACATCACTAGGTTTCAAACT   22282
gb:MN938384|Organism:Severe      TCGGCTTTAGAACCATTGGTAGATTTGCCAATAGGTATTAACATCACTAGGTTTCAAACT   22250
gb:MT044258|Organism:Severe      TCGGCTTTAGAACCATTGGTAGATTTGCCAATAGGTATTAACATCACTAGGTTTCAAACT   22258
gb:MT039890|Organism:Severe      TGGGCTTTAGAACCATTGGTAGATTTGCCAATAGGTATTAACATCACTAGGTTTCAAACT   22282
gb:MN988713|Organism:Severe      TCGGCTTTAGAACCATTGGTAGATTTGCCAATAGGTATTAACATCACTAGGTTTCAAACT   22282
gb:LC521925|Organism:Severe      TCGGCTTTAGAACCATTGGTAGATTTGCCAATAGGTATTAACATCACTAGGTTTCAAACT   22255
gb:MT093571|Organism:Severe      TCGGCTTTAGAACCATTGGTAGATTTGCCAATAGGTATTAACATCACTAGGTTTCAAACT   22282
gb:MT039887|Organism:Severe      TCGGCTTTAGAACCATTGGTAGATTTGCCAATAGGTATTAACATCACTAGGTTTCAAACT   22279
gb:MT019530|Organism:Severe      TCGGCTTTAGAACCATTGGTAGATTTGCCAATAGGTATTAACATCACTAGGTTTCAAACT   22282
gb:MT039888|Organism:Severe      TCGGCTTTAGAACCATTGGTAGATTTGCCAATAGGTATTAACATCACTAGGTTTCAAACT   22282
```

```
gb:LC522972|Organism:Severe      TCGGCTTTAGAACCATTGGTAGATTTGCCAATAGGTATTAACATCACTAGGTTTCAAACT   22279
gb:MT027063|Organism:Severe      TCGGCTTTAGAACCATTGGTAGATTTGCCAATAGGTATTAACATCACTAGGTTTCAAACT   22282
gb:MT027062|Organism:Severe      TCGGCTTTAGAACCATTGGTAGATTTGCCAATAGGTATTAACATCACTAGGTTTCAAACT   22282
gb:MT019529|Organism:Severe      TCGGCTTTAGAACCATTGGTAGATTTGCCAATAGGTATTAACATCACTAGGTTTCAAACT   22282
gb:MN996529|Organism:Severe      TCGGCTTTAGAACCATTGGTAGATTTGCCAATAGGTATTAACATCACTAGGTTTCAAACT   22270
gb:MN996531|Organism:Severe      TCGGCTTTAGAACCATTGGTAGATTTGCCAATAGGTATTAACATCACTAGGTTTCAAACT   22269
gb:MT066176|Organism:Severe      TCGGCTTTAGAACCATTGGTAGATTTGCCAATAGGTATTAACATCACTAGGTTTCAAACT   22282
gb:MT027064|Organism:Severe      TCGGCTTTAGAACCATTGGTAGATTTGCCAATAGGTATTAACATCACTAGGTTTCAAACT   22282
gb:MN994468|Organism:Severe      TCGGCTTTAGAACCATTGGTAGATTTGCCAATAGGTATTAACATCACTAGGTTTCAAACT   22282
gb:MT072688|Organism:Severe      TCGGCTTTAGAACCATTGGTAGATTTGCCAATAGGTATTAACATCACTAGGTTTCAAACT   22267
gb:MN996527|Organism:Severe      TCGGCTTTAGAACCATTGGTAGATTTGCCAATAGGTATTAACATCACTAGGTTTCAAACT   22249
gb:MT093631|Organism:Severe      TCGGCTTTAGAACCATTGGTAGATTTGCCAATAGGTATTAACATCACTAGGTTTCAAACT   22320
gb:MT106053|Organism:Severe      TCGGCTTTAGAACCATTGGTAGATTTGCCAATAGGTATTAACATCACTAGGTTTCAAACT   22282
gb:MT019533|Organism:Severe      TCGGCTTTAGAACCATTGGTAGATTTGCCAATAGGTATTAACATCACTAGGTTTCAAACT   22282
gb:MT019531|Organism:Severe      TCGGCTTTAGAACCATTGGTAGATTTGCCAATAGGTATTAACATCACTAGGTTTCAAACT   22282
gb:MN996528|Organism:Severe      TCGGCTTTAGAACCATTGGTAGATTTGCCAATAGGTATTAACATCACTAGGTTTCAAACT   22282
gb:MN996530|Organism:Severe      TCGGCTTTAGAACCATTGGTAGATTTGCCAATAGGTATTAACATCACTAGGTTTCAAACT   22268
gb:MN908947|Organism:Severe      TCGGCTTTAGAACCATTGGTAGATTTGCCAATAGGTATTAACATCACTAGGTTTCAAACT   22282
gb:MT019532|Organism:Severe      TCGGCTTTAGAACCATTGGTAGATTTGCCAATAGGTATTAACATCACTAGGTTTCAAACT   22282
                                 * **********************************************************

gb:MT020781|Organism:Severe      TTACTTGCTTTACATAGAAGTTATTTGACTCCTGGTGATTCTTCTTCAGGTTGGACAGCT   22330
gb:MT007544|Organism:Severe      TTACTTGCTTTACATAGAAGGTATTTGACTCCTGGTGATTCTTCTTCAGGTTGGACAGCT   22342
gb:MN994467|Organism:Severe      TTACTTGCTTTACATAGAAGTTATTTGACTCCTGGTGATTCTTCTTCAGGTTGGACAGCT   22342
gb:MT044257|Organism:Severe      TTACTTGCTTTACATAGAAGTTATTTGACTCCTGGTGATTCTTCTTCAGGTTGGACAGCT   22342
gb:MT106054|Organism:Severe      TTACTTGCTTTACATAGAAGTTATTTGACTCCTGGTGATTCTTCTTCAGGTTGGACAGCT   22342
gb:MT049951|Organism:Severe      TTACTTGCTTTACATAGAAGTTATTTGACTCCTGGTGATTCTTCTTCAGGTTGGACAGCT   22342
gb:MN975262|Organism:Severe      TTACTTGCTTTACATAGAAGTTATTTGACTCCTGGTGATTCTTCTTCAGGTTGGACAGCT   22342
gb:MT106052|Organism:Severe      TTACTTGCTTTACATAGAAGTTATTTGACTCCTGGTGATTCTTCTTCAGGTTGGACAGCT   22342
gb:LC522975|Organism:Severe      TTACTTGCTTTACATAGAAGTTATTTGACTCCTGGTGATTCTTCTTCAGGTTGGACAGCT   22339
gb:LC522973|Organism:Severe      TTACTTGCTTTACATAGAAGTTATTTGACTCCTGGTGATTCTTCTTCAGGTTGGACAGCT   22339
gb:LC522974|Organism:Severe      TTACTTGCTTTACATAGAAGTTATTTGACTCCTGGTGATTCTTCTTCAGGTTGGACAGCT   22339
gb:MN985325|Organism:Severe      TTACTTGCTTTACATAGAAGTTATTTGACTCCTGGTGATTCTTCTTCAGGTTGGACAGCT   22342
gb:MT020881|Organism:Severe      TTACTTGCTTTACATAGAAGTTATTTGACTCCTGGTGATTCTTCTTCAGGTTGGACAGCT   22342
gb:MT020880|Organism:Severe      TTACTTGCTTTACATAGAAGTTATTTGACTCCTGGTGATTCTTCTTCAGGTTGGACAGCT   22342
gb:MT066175|Organism:Severe      TTACTTGCTTTACATAGAAGTTATTTGACTCCTGGTGATTCTTCTTCAGGTTGGACAGCT   22342
gb:MN997409|Organism:Severe      TTACTTGCTTTACATAGAAGTTATTTGACTCCTGGTGATTCTTCTTCAGGTTGGACAGCT   22342
gb:MN938384|Organism:Severe      TTACTTGCTTTACATAGAAGTTATTTGACTCCTGGTGATTCTTCTTCAGGTTGGACAGCT   22310
gb:MT044258|Organism:Severe      TTACTTGCTTTACATAGAAGTTATTTGACTCCTGGTGATTCTTCTTCAGGTTGGACAGCT   22318
gb:MT039890|Organism:Severe      TTACTTGCTTTACATAGAAGTTATTTGACTCCTGGTGATTCTTCTTCAGGTTGGACAGCT   22342
gb:MN988713|Organism:Severe      TTACTTGCTTTACATAGAAGTTATTTGACTCCTGGTGATTCTTCTTCAGGTTGGACAGCT   22342
gb:LC521925|Organism:Severe      TTACTTGCTTTACATAGAAGTTATTTGACTCCTGGTGATTCTTCTTCAGGTTGGACAGCT   22315
gb:MT093571|Organism:Severe      TTACTTGCTTTACATAGAAGTTATTTGACTCCTGGTGATTCTTCTTCAGGTTGGACAGCT   22342
gb:MT039887|Organism:Severe      TTACTTGCTTTACATAGAAGTTATTTGACTCCTGGTGATTCTTCTTCAGGTTGGACAGCT   22339
gb:MT019530|Organism:Severe      TTACTTGCTTTACATAGAAGTTATTTGACTCCTGGTGATTCTTCTTCAGGTTGGACAGCT   22342
gb:MT039888|Organism:Severe      TTACTTGCTTTACATAGAAGTTATTTGACTCCTGGTGATTCTTCTTCAGGTTGGACAGCT   22342
gb:LC522972|Organism:Severe      TTACTTGCTTTACATAGAAGTTATTTGACTCCTGGTGATTCTTCTTCAGGTTGGACAGCT   22339
gb:MT027063|Organism:Severe      TTACTTGCTTTACATAGAAGTTATTTGACTCCTGGTGATTCTTCTTCAGGTTGGACAGCT   22342
gb:MT027062|Organism:Severe      TTACTTGCTTTACATAGAAGTTATTTGACTCCTGGTGATTCTTCTTCAGGTTGGACAGCT   22342
gb:MT019529|Organism:Severe      TTACTTGCTTTACATAGAAGTTATTTGACTCCTGGTGATTCTTCTTCAGGTTGGACAGCT   22342
gb:MN996529|Organism:Severe      TTACTTGCTTTACATAGAAGTTATTTGACTCCTGGTGATTCTTCTTCAGGTTGGACAGCT   22330
gb:MN996531|Organism:Severe      TTACTTGCTTTACATAGAAGTTATTTGACTCCTGGTGATTCTTCTTCAGGTTGGACAGCT   22329
gb:MT066176|Organism:Severe      TTACTTGCTTTACATAGAAGTTATTTGACTCCTGGTGATTCTTCTTCAGGTTGGACAGCT   22342
gb:MT027064|Organism:Severe      TTACTTGCTTTACATAGAAGTTATTTGACTCCTGGTGATTCTTCTTCAGGTTGGACAGCT   22342
gb:MN994468|Organism:Severe      TTACTTGCTTTACATAGAAGTTATTTGACTCCTGGTGATTCTTCTTCAGGTTGGACAGCT   22342
gb:MT072688|Organism:Severe      TTACTTGCTTTACATAGAAGTTATTTGACTCCTGGTGATTCTTCTTCAGGTTGGACAGCT   22327
gb:MN996527|Organism:Severe      TTACTTGCTTTACATAGAAGTTATTTGACTCCTGGTGATTCTTCTTCAGGTTGGACAGCT   22309
gb:MT093631|Organism:Severe      TTACTTGCTTTACATAGAAGTTATTTGACTCCTGGTGATTCTTCTTCAGGTTGGACAGCT   22380
gb:MT106053|Organism:Severe      TTACTTGCTTTACATAGAAGTTATTTGACTCCTGGTGATTCTTCTTCAGGTTGGACAGCT   22342
gb:MT019533|Organism:Severe      TTACTTGCTTTACATAGAAGTTATTTGACTCCTGGTGATTCTTCTTCAGGTTGGACAGCT   22342
gb:MT019531|Organism:Severe      TTACTTGCTTTACATAGAAGTTATTTGACTCCTGGTGATTCTTCTTCAGGTTGGACAGCT   22342
gb:MN996528|Organism:Severe      TTACTTGCTTTACATAGAAGTTATTTGACTCCTGGTGATTCTTCTTCAGGTTGGACAGCT   22342
gb:MN996530|Organism:Severe      TTACTTGCTTTACATAGAAGTTATTTGACTCCTGGTGATTCTTCTTCAGGTTGGACAGCT   22328
gb:MN908947|Organism:Severe      TTACTTGCTTTACATAGAAGTTATTTGACTCCTGGTGATTCTTCTTCAGGTTGGACAGCT   22342
gb:MT019532|Organism:Severe      TTACTTGCTTTACATAGAAGTTATTTGACTCCTGGTGATTCTTCTTCAGGTTGGACAGCT   22342
```

```
                                  ******************** ***************************************

gb:MT020781|Organism:Severe      GGTGCTGCAGCTTATTATGTGGGTTATCTTCAACCTAGGACTTTTCTATTAAAATATAAT   22390
gb:MT007544|Organism:Severe      GGTGCTGCAGCTTATTATGTGGGTTATCTTCAACCTAGGACTTTTCTATTAAAATATAAT   22402
gb:MN994467|Organism:Severe      GGTGCTGCAGCTTATTATGTGGGTTATCTTCAACCTAGGACTTTTCTATTAAAATATAAT   22402
gb:MT044257|Organism:Severe      GGTGCTGCAGCTTATTATGTGGGTTATCTTCAACCTAGGACTTTTCTATTAAAATATAAT   22402
gb:MT106054|Organism:Severe      GGTGCTGCAGCTTATTATGTGGGTTATCTTCAACCTAGGACTTTTCTATTAAAATATAAT   22402
gb:MT049951|Organism:Severe      GGTGCTGCAGCTTATTATGTGGGTTATCTTCAACCTAGGACTTTTCTATTAAAATATAAT   22402
gb:MN975262|Organism:Severe      GGTGCTGCAGCTTATTATGTGGGTTATCTTCAACCTAGGACTTTTCTATTAAAATATAAT   22402
gb:MT106052|Organism:Severe      GGTGCTGCAGCTTATTATGTGGGTTATCTTCAACCTAGGACTTTTCTATTAAAATATAAT   22402
gb:LC522975|Organism:Severe      GGTGCTGCAGCTTATTATGTGGGTTATCTTCAACCTAGGACTTTTCTATTAAAATATAAT   22399
gb:LC522973|Organism:Severe      GGTGCTGCAGCTTATTATGTGGGTTATCTTCAACCTAGGACTTTTCTATTAAAATATAAT   22399
gb:LC522974|Organism:Severe      GGTGCTGCAGCTTATTATGTGGGTTATCTTCAACCTAGGACTTTTCTATTAAAATATAAT   22399
gb:MN985325|Organism:Severe      GGTGCTGCAGCTTATTATGTGGGTTATCTTCAACCTAGGACTTTTCTATTAAAATATAAT   22402
gb:MT020881|Organism:Severe      GGTGCTGCAGCTTATTATGTGGGTTATCTTCAACCTAGGACTTTTCTATTAAAATATAAT   22402
gb:MT020880|Organism:Severe      GGTGCTGCAGCTTATTATGTGGGTTATCTTCAACCTAGGACTTTTCTATTAAAATATAAT   22402
gb:MT066175|Organism:Severe      GGTGCTGCAGCTTATTATGTGGGTTATCTTCAACCTAGGACTTTTCTATTAAAATATAAT   22402
gb:MN997409|Organism:Severe      GGTGCTGCAGCTTATTATGTGGGTTATCTTCAACCTAGGACTTTTCTATTAAAATATAAT   22402
gb:MN938384|Organism:Severe      GGTGCTGCAGCTTATTATGTGGGTTATCTTCAACCTAGGACTTTTCTATTAAAATATAAT   22370
gb:MT044258|Organism:Severe      GGTGCTGCAGCTTATTATGTGGGTTATCTTCAACCTAGGACTTTTCTATTAAAATATAAT   22378
gb:MT039890|Organism:Severe      GGTGCTGCAGCTTATTATGTGGGTTATCTTCAACCTAGGACTTTTCTATTAAAATATAAT   22402
gb:MN988713|Organism:Severe      GGTGCTGCAGCTTATTATGTGGGTTATCTTCAACCTAGGACTTTTCTATTAAAATATAAT   22402
gb:LC521925|Organism:Severe      GGTGCTGCAGCTTATTATGTGGGTTATCTTCAACCTAGGACTTTTCTATTAAAATATAAT   22375
gb:MT093571|Organism:Severe      GGTGCTGCAGCTTATTATGTGGGTTATCTTCAACCTAGGACTTTTCTATTAAAATATAAT   22402
gb:MT039887|Organism:Severe      GGTGCTGCAGCTTATTATGTGGGTTATCTTCAACCTAGGACTTTTCTATTAAAATATAAT   22399
gb:MT019530|Organism:Severe      GGTGCTGCAGCTTATTATGTGGGTTATCTTCAACCTAGGACTTTTCTATTAAAATATAAT   22402
gb:MT039888|Organism:Severe      GGTGCTGCAGCTTATTATGTGGGTTATCTTCAACCTAGGACTTTTCTATTAAAATATAAT   22402
gb:LC522972|Organism:Severe      GGTGCTGCAGCTTATTATGTGGGTTATCTTCAACCTAGGACTTTTCTATTAAAATATAAT   22399
gb:MT027063|Organism:Severe      GGTGCTGCAGCTTATTATGTGGGTTATCTTCAACCTAGGACTTTTCTATTAAAATATAAT   22402
gb:MT027062|Organism:Severe      GGTGCTGCAGCTTATTATGTGGGTTATCTTCAACCTAGGACTTTTCTATTAAAATATAAT   22402
gb:MT019529|Organism:Severe      GGTGCTGCAGCTTATTATGTGGGTTATCTTCAACCTAGGACTTTTCTATTAAAATATAAT   22402
gb:MN996529|Organism:Severe      GGTGCTGCAGCTTATTATGTGGGTTATCTTCAACCTAGGACTTTTCTATTAAAATATAAT   22390
gb:MN996531|Organism:Severe      GGTGCTGCAGCTTATTATGTGGGTTATCTTCAACCTAGGACTTTTCTATTAAAATATAAT   22389
gb:MT066176|Organism:Severe      GGTGCTGCAGCTTATTATGTGGGTTATCTTCAACCTAGGACTTTTCTATTAAAATATAAT   22402
gb:MT027064|Organism:Severe      GGTGCTGCAGCTTATTATGTGGGTTATCTTCAACCTAGGACTTTTCTATTAAAATATAAT   22402
gb:MN994468|Organism:Severe      GGTGCTGCAGCTTATTATGTGGGTTATCTTCAACCTAGGACTTTTCTATTAAAATATAAT   22402
gb:MT072688|Organism:Severe      GGTGCTGCAGCTTATTATGTGGGTTATCTTCAACCTAGGACTTTTCTATTAAAATATAAT   22387
gb:MN996527|Organism:Severe      GGTGCTGCAGCTTATTATGTGGGTTATCTTCAACCTAGGACTTTTCTATTAAAATATAAT   22369
gb:MT093631|Organism:Severe      GGTGCTGCAGCTTATTATGTGGGTTATCTTCAACCTAGGACTTTTCTATTAAAATATAAT   22440
gb:MT106053|Organism:Severe      GGTGCTGCAGCTTATTATGTGGGTTATCTTCAACCTAGGACTTTTCTATTAAAATATAAT   22402
gb:MT019533|Organism:Severe      GGTGCTGCAGCTTATTATGTGGGTTATCTTCAACCTAGGACTTTTCTATTAAAATATAAT   22402
gb:MT019531|Organism:Severe      GGTGCTGCAGCTTATTATGTGGGTTATCTTCAACCTAGGACTTTTCTATTAAAATATAAT   22402
gb:MN996528|Organism:Severe      GGTGCTGCAGCTTATTATGTGGGTTATCTTCAACCTAGGACTTTTCTATTAAAATATAAT   22402
gb:MN996530|Organism:Severe      GGTGCTGCAGCTTATTATGTGGGTTATCTTCAACCTAGGACTTTTCTATTAAAATATAAT   22388
gb:MN908947|Organism:Severe      GGTGCTGCAGCTTATTATGTGGGTTATCTTCAACCTAGGACTTTTCTATTAAAATATAAT   22402
gb:MT019532|Organism:Severe      GGTGCTGCAGCTTATTATGTGGGTTATCTTCAACCTAGGACTTTTCTATTAAAATATAAT   22402
                                  ************************************************************

gb:MT020781|Organism:Severe      GAAAATGGAACCATTACAGATGCTGTAGACTGTGCACTTGACCCTCTCTCAGAAACAAAG   22450
gb:MT007544|Organism:Severe      GAAAATGGAACCATTACAGATGCTGTAGACTGTGCACTTGACCCTCTCTCAGAAACAAAG   22462
gb:MN994467|Organism:Severe      GAAAATGGAACCATTACAGATGCTGTAGACTGTGCACTTGACCCTCTCTCAGAAACAAAG   22462
gb:MT044257|Organism:Severe      GAAAATGGAACCATTACAGATGCTGTAGACTGTGCACTTGACCCTCTCTCAGAAACAAAG   22462
gb:MT106054|Organism:Severe      GAAAATGGAACCATTACAGATGCTGTAGACTGTGCACTTGACCCTCTCTCAGAAACAAAG   22462
gb:MT049951|Organism:Severe      GAAAATGGAACCATTACAGATGCTGTAGACTGTGCACTTGACCCTCTCTCAGAAACAAAG   22462
gb:MN975262|Organism:Severe      GAAAATGGAACCATTACAGATGCTGTAGACTGTGCACTTGACCCTCTCTCAGAAACAAAG   22462
gb:MT106052|Organism:Severe      GAAAATGGAACCATTACAGATGCTGTAGACTGTGCACTTGACCCTCTCTCAGAAACAAAG   22462
gb:LC522975|Organism:Severe      GAAAATGGAACCATTACAGATGCTGTAGACTGTGCACTTGACCCTCTCTCAGAAACAAAG   22459
gb:LC522973|Organism:Severe      GAAAATGGAACCATTACAGATGCTGTAGACTGTGCACTTGACCCTCTCTCAGAAACAAAG   22459
gb:LC522974|Organism:Severe      GAAAATGGAACCATTACAGATGCTGTAGACTGTGCACTTGACCCTCTCTCAGAAACAAAG   22459
gb:MN985325|Organism:Severe      GAAAATGGAACCATTACAGATGCTGTAGACTGTGCACTTGACCCTCTCTCAGAAACAAAG   22462
gb:MT020881|Organism:Severe      GAAAATGGAACCATTACAGATGCTGTAGACTGTGCACTTGACCCTCTCTCAGAAACAAAG   22462
gb:MT020880|Organism:Severe      GAAAATGGAACCATTACAGATGCTGTAGACTGTGCACTTGACCCTCTCTCAGAAACAAAG   22462
gb:MT066175|Organism:Severe      GAAAATGGAACCATTACAGATGCTGTAGACTGTGCACTTGACCCTCTCTCAGAAACAAAG   22462
gb:MN997409|Organism:Severe      GAAAATGGAACCATTACAGATGCTGTAGACTGTGCACTTGACCCTCTCTCAGAAACAAAG   22462
gb:MN938384|Organism:Severe      GAAAATGGAACCATTACAGATGCTGTAGACTGTGCACTTGACCCTCTCTCAGAAACAAAG   22430
```

```
gb:MT044258|Organism:Severe      GAAAATGGAACCATTACAGATGCTGTAGACTGTGCACTTGACCCTCTCTCAGAAACAAAG   22438
gb:MT039890|Organism:Severe      GAAAATGGAACCATTACAGATGCTGTAGACTGTGCACTTGACCCTCTCTCAGAAACAAAG   22462
gb:MN988713|Organism:Severe      GAAAATGGAACCATTACAGATGCTGTAGACTGTGCACTTGACCCTCTCTCAGAAACAAAG   22462
gb:LC521925|Organism:Severe      GAAAATGGAACCATTACAGATGCTGTAGACTGTGCACTTGACCCTCTCTCAGAAACAAAG   22435
gb:MT093571|Organism:Severe      GAAAATGGAACCATTACAGATGCTGTAGACTGTGCACTTGACCCTCTCTCAGAAACAAAG   22462
gb:MT039887|Organism:Severe      GAAAATGGAACCATTACAGATGCTGTAGACTGTGCACTTGACCCTCTCTCAGAAACAAAG   22459
gb:MT019530|Organism:Severe      GAAAATGGAACCATTACAGATGCTGTAGACTGTGCACTTGACCCTCTCTCAGAAACAAAG   22462
gb:MT039888|Organism:Severe      GAAAATGGAACCATTACAGATGCTGTAGACTGTGCACTTGACCCTCTCTCAGAAACAAAG   22462
gb:LC522972|Organism:Severe      GAAAATGGAACCATTACAGATGCTGTAGACTGTGCACTTGACCCTCTCTCAGAAACAAAG   22459
gb:MT027063|Organism:Severe      GAAAATGGAACCATTACAGATGCTGTAGACTGTGCACTTGACCCTCTCTCAGAAACAAAG   22462
gb:MT027062|Organism:Severe      GAAAATGGAACCATTACAGATGCTGTAGACTGTGCACTTGACCCTCTCTCAGAAACAAAG   22462
gb:MT019529|Organism:Severe      GAAAATGGAACCATTACAGATGCTGTAGACTGTGCACTTGACCCTCTCTCAGAAACAAAG   22462
gb:MN996529|Organism:Severe      GAAAATGGAACCATTACAGATGCTGTAGACTGTGCACTTGACCCTCTCTCAGAAACAAAG   22450
gb:MN996531|Organism:Severe      GAAAATGGAACCATTACAGATGCTGTAGACTGTGCACTTGACCCTCTCTCAGAAACAAAG   22449
gb:MT066176|Organism:Severe      GAAAATGGAACCATTACAGATGCTGTAGACTGTGCACTTGACCCTCTCTCAGAAACAAAG   22462
gb:MT027064|Organism:Severe      GAAAATGGAACCATTACAGATGCTGTAGACTGTGCACTTGACCCTCTCTCAGAAACAAAG   22462
gb:MN994468|Organism:Severe      GAAAATGGAACCATTACAGATGCTGTAGACTGTGCACTTGACCCTCTCTCAGAAACAAAG   22462
gb:MT072688|Organism:Severe      GAAAATGGAACCATTACAGATGCTGTAGACTGTGCACTTGACCCTCTCTCAGAAACAAAG   22447
gb:MN996527|Organism:Severe      GAAAATGGAACCATTACAGATGCTGTAGACTGTGCACTTGACCCTCTCTCAGAAACAAAG   22429
gb:MT093631|Organism:Severe      GAAAATGGAACCATTACAGATGCTGTAGACTGTGCACTTGACCCTCTCTCAGAAACAAAG   22500
gb:MT106053|Organism:Severe      GAAAATGGAACCATTACAGATGCTGTAGACTGTGCACTTGACCCTCTCTCAGAAACAAAG   22462
gb:MT019533|Organism:Severe      GAAAATGGAACCATTACAGATGCTGTAGACTGTGCACTTGACCCTCTCTCAGAAACAAAG   22462
gb:MT019531|Organism:Severe      GAAAATGGAACCATTACAGATGCTGTAGACTGTGCACTTGACCCTCTCTCAGAAACAAAG   22462
gb:MN996528|Organism:Severe      GAAAATGGAACCATTACAGATGCTGTAGACTGTGCACTTGACCCTCTCTCAGAAACAAAG   22462
gb:MN996530|Organism:Severe      GAAAATGGAACCATTACAGATGCTGTAGACTGTGCACTTGACCCTCTCTCAGAAACAAAG   22448
gb:MN908947|Organism:Severe      GAAAATGGAACCATTACAGATGCTGTAGACTGTGCACTTGACCCTCTCTCAGAAACAAAG   22462
gb:MT019532|Organism:Severe      GAAAATGGAACCATTACAGATGCTGTAGACTGTGCACTTGACCCTCTCTCAGAAACAAAG   22462
                                 ************************************************************

gb:MT020781|Organism:Severe      TGTACGTTGAAATCCTTCACTGTAGAAAAAGGAATCTATCAAACTTCTAACTTTAGAGTC   22510
gb:MT007544|Organism:Severe      TGTACGTTGAAATCCTTCACTGTAGAAAAAGGAATCTATCAAACTTCTAACTTTAGAGTC   22522
gb:MN994467|Organism:Severe      TGTACGTTGAAATCCTTCACTGTAGAAAAAGGAATCTATCAAACTTCTAACTTTAGAGTC   22522
gb:MT044257|Organism:Severe      TGTACGTTGAAATCCTTCACTGTAGAAAAAGGAATCTATCAAACTTCTAACTTTAGAGTC   22522
gb:MT106054|Organism:Severe      TGTACGTTGAAATCCTTCACTGTAGAAAAAGGAATCTATCAAACTTCTAACTTTAGAGTC   22522
gb:MT049951|Organism:Severe      TGTACGTTGAAATCCTTCACTGTAGAAAAAGGAATCTATCAAACTTCTAACTTTAGAGTC   22522
gb:MN975262|Organism:Severe      TGTACGTTGAAATCCTTCACTGTAGAAAAAGGAATCTATCAAACTTCTAACTTTAGAGTC   22522
gb:MT106052|Organism:Severe      TGTACGTTGAAATCCTTCACTGTAGAAAAAGGAATCTATCAAACTTCTAACTTTAGAGTC   22522
gb:LC522975|Organism:Severe      TGTACGTTGAAATCCTTCACTGTAGAAAAAGGAATCTATCAAACTTCTAACTTTAGAGTC   22519
gb:LC522973|Organism:Severe      TGTACGTTGAAATCCTTCACTGTAGAAAAAGGAATCTATCAAACTTCTAACTTTAGAGTC   22519
gb:LC522974|Organism:Severe      TGTACGTTGAAATCCTTCACTGTAGAAAAAGGAATCTATCAAACTTCTAACTTTAGAGTC   22519
gb:MN985325|Organism:Severe      TGTACGTTGAAATCCTTCACTGTAGAAAAAGGAATCTATCAAACTTCTAACTTTAGAGTC   22522
gb:MT020881|Organism:Severe      TGTACGTTGAAATCCTTCACTGTAGAAAAAGGAATCTATCAAACTTCTAACTTTAGAGTC   22522
gb:MT020880|Organism:Severe      TGTACGTTGAAATCCTTCACTGTAGAAAAAGGAATCTATCAAACTTCTAACTTTAGAGTC   22522
gb:MT066175|Organism:Severe      TGTACGTTGAAATCCTTCACTGTAGAAAAAGGAATCTATCAAACTTCTAACTTTAGAGTC   22522
gb:MN997409|Organism:Severe      TGTACGTTGAAATCCTTCACTGTAGAAAAAGGAATCTATCAAACTTCTAACTTTAGAGTC   22522
gb:MN938384|Organism:Severe      TGTACGTTGAAATCCTTCACTGTAGAAAAAGGAATCTATCAAACTTCTAACTTTAGAGTC   22490
gb:MT044258|Organism:Severe      TGTACGTTGAAATCCTTCACTGTAGAAAAAGGAATCTATCAAACTTCTAACTTTAGAGTC   22498
gb:MT039890|Organism:Severe      TGTACGTTGAAATCCTTCACTGTAGAAAAAGGAATCTATCAAACTTCTAACTTTAGAGTC   22522
gb:MN988713|Organism:Severe      TGTACGTTGAAATCCTTCACTGTAGAAAAAGGAATCTATCAAACTTCTAACTTTAGAGTC   22522
gb:LC521925|Organism:Severe      TGTACGTTGAAATCCTTCACTGTAGAAAAAGGAATCTATCAAACTTCTAACTTTAGAGTC   22495
gb:MT093571|Organism:Severe      TGTACGTTGAAATCCTTCACTGTAGAAAAAGGAATCTATCAAACTTCTAACTTTAGAGTC   22522
gb:MT039887|Organism:Severe      TGTACGTTGAAATCCTTCACTGTAGAAAAAGGAATCTATCAAACTTCTAACTTTAGAGTC   22519
gb:MT019530|Organism:Severe      TGTACGTTGAAATCCTTCACTGTAGAAAAAGGAATCTATCAAACTTCTAACTTTAGAGTC   22522
gb:MT039888|Organism:Severe      TGTACGTTGAAATCCTTCACTGTAGAAAAAGGAATCTATCAAACTTCTAACTTTAGAGTC   22522
gb:LC522972|Organism:Severe      TGTACGTTGAAATCCTTCACTGTAGAAAAAGGAATCTATCAAACTTCTAACTTTAGAGTC   22519
gb:MT027063|Organism:Severe      TGTACGTTGAAATCCTTCACTGTAGAAAAAGGAATCTATCAAACTTCTAACTTTAGAGTC   22522
gb:MT027062|Organism:Severe      TGTACGTTGAAATCCTTCACTGTAGAAAAAGGAATCTATCAAACTTCTAACTTTAGAGTC   22522
gb:MT019529|Organism:Severe      TGTACGTTGAAATCCTTCACTGTAGAAAAAGGAATCTATCAAACTTCTAACTTTAGAGTC   22522
gb:MN996529|Organism:Severe      TGTACGTTGAAATCCTTCACTGTAGAAAAAGGAATCTATCAAACTTCTAACTTTAGAGTC   22510
gb:MN996531|Organism:Severe      TGTACGTTGAAATCCTTCACTGTAGAAAAAGGAATCTATCAAACTTCTAACTTTAGAGTC   22509
gb:MT066176|Organism:Severe      TGTACGTTGAAATCCTTCACTGTAGAAAAAGGAATCTATCAAACTTCTAACTTTAGAGTC   22522
gb:MT027064|Organism:Severe      TGTACGTTGAAATCCTTCACTGTAGAAAAAGGAATCTATCAAACTTCTAACTTTAGAGTC   22522
gb:MN994468|Organism:Severe      TGTACGTTGAAATCCTTCACTGTAGAAAAAGGAATCTATCAAACTTCTAACTTTAGAGTC   22522
gb:MT072688|Organism:Severe      TGTACGTTGAAATCCTTCACTGTAGAAAAAGGAATCTATCAAACTTCTAACTTTAGAGTC   22507
gb:MN996527|Organism:Severe      TGTACGTTGAAATCCTTCACTGTAGAAAAAGGAATCTATCAAACTTCTAACTTTAGAGTC   22489
```

```
gb:MT093631|Organism:Severe      TGTACGTTGAAATCCTTCACTGTAGAAAAAGGAATCTATCAAACTTCTAACTTTAGAGTC   22560
gb:MT106053|Organism:Severe      TGTACGTTGAAATCCTTCACTGTAGAAAAAGGAATCTATCAAACTTCTAACTTTAGAGTC   22522
gb:MT019533|Organism:Severe      TGTACGTTGAAATCCTTCACTGTAGAAAAAGGAATCTATCAAACTTCTAACTTTAGAGTC   22522
gb:MT019531|Organism:Severe      TGTACGTTGAAATCCTTCACTGTAGAAAAAGGAATCTATCAAACTTCTAACTTTAGAGTC   22522
gb:MN996528|Organism:Severe      TGTACGTTGAAATCCTTCACTGTAGAAAAAGGAATCTATCAAACTTCTAACTTTAGAGTC   22522
gb:MN996530|Organism:Severe      TGTACGTTGAAATCCTTCACTGTAGAAAAAGGAATCTATCAAACTTCTAACTTTAGAGTC   22508
gb:MN908947|Organism:Severe      TGTACGTTGAAATCCTTCACTGTAGAAAAAGGAATCTATCAAACTTCTAACTTTAGAGTC   22522
gb:MT019532|Organism:Severe      TGTACGTTGAAATCCTTCACTGTAGAAAAAGGAATCTATCAAACTTCTAACTTTAGAGTC   22522
                                 ************************************************************

gb:MT020781|Organism:Severe      CAACCAACAGAATCTATTGTTAGATTTCCTAATATTACAAACTTGTGCCCTTTTGGTGAA   22570
gb:MT007544|Organism:Severe      CAACCAACAGAATCTATTGTTAGATTTCCTAATATTACAAACTTGTGCCCTTTTGGTGAA   22582
gb:MN994467|Organism:Severe      CAACCAACAGAATCTATTGTTAGATTTCCTAATATTACAAACTTGTGCCCTTTTGGTGAA   22582
gb:MT044257|Organism:Severe      CAACCAACAGAATCTATTGTTAGATTTCCTAATATTACAAACTTGTGCCCTTTTGGTGAA   22582
gb:MT106054|Organism:Severe      CAACCAACAGAATCTATTGTTAGATTTCCTAATATTACAAACTTGTGCCCTTTTGGTGAA   22582
gb:MT049951|Organism:Severe      CAACCAACAGAATCTATTGTTAGATTTCCTAATATTACAAACTTGTGCCCTTTTGGTGAA   22582
gb:MN975262|Organism:Severe      CAACCAACAGAATCTATTGTTAGATTTCCTAATATTACAAACTTGTGCCCTTTTGGTGAA   22582
gb:MT106052|Organism:Severe      CAACCAACAGAATCTATTGTTAGATTTCCTAATATTACAAACTTGTGCCCTTTTGGTGAA   22582
gb:LC522975|Organism:Severe      CAACCAACAGAATCTATTGTTAGATTTCCTAATATTACAAACTTGTGCCCTTTTGGTGAA   22579
gb:LC522973|Organism:Severe      CAACCAACAGAATCTATTGTTAGATTTCCTAATATTACAAACTTGTGCCCTTTTGGTGAA   22579
gb:LC522974|Organism:Severe      CAACCAACAGAATCTATTGTTAGATTTCCTAATATTACAAACTTGTGCCCTTTTGGTGAA   22579
gb:MN985325|Organism:Severe      CAACCAACAGAATCTATTGTTAGATTTCCTAATATTACAAACTTGTGCCCTTTTGGTGAA   22582
gb:MT020881|Organism:Severe      CAACCAACAGAATCTATTGTTAGATTTCCTAATATTACAAACTTGTGCCCTTTTGGTGAA   22582
gb:MT020880|Organism:Severe      CAACCAACAGAATCTATTGTTAGATTTCCTAATATTACAAACTTGTGCCCTTTTGGTGAA   22582
gb:MT066175|Organism:Severe      CAACCAACAGAATCTATTGTTAGATTTCCTAATATTACAAACTTGTGCCCTTTTGGTGAA   22582
gb:MN997409|Organism:Severe      CAACCAACAGAATCTATTGTTAGATTTCCTAATATTACAAACTTGTGCCCTTTTGGTGAA   22582
gb:MN938384|Organism:Severe      CAACCAACAGAATCTATTGTTAGATTTCCTAATATTACAAACTTGTGCCCTTTTGGTGAA   22550
gb:MT044258|Organism:Severe      CAACCAACAGAATCTATTGTTAGATTTCCTAATATTACAAACTTGTGCCCTTTTGGTGAA   22558
gb:MT039890|Organism:Severe      CAACCAACAGAATCTATTGTTAGATTTCCTAATATTACAAACTTGTGCCCTTTTGGTGAA   22582
gb:MN988713|Organism:Severe      CAACCAACAGAATCTATTGTTAGATTTCCTAATATTACAAACTTGTGCCCTTTTGGTGAA   22582
gb:LC521925|Organism:Severe      CAACCAACAGAATCTATTGTTAGATTTCCTAATATTACAAACTTGTGCCCTTTTGGTGAA   22555
gb:MT093571|Organism:Severe      CAACCAACAGAATCTATTGTTAGATTTCCTAATATTACAAACTTGTGCCCTTTTGGTGAA   22582
gb:MT039887|Organism:Severe      CAACCAACAGAATCTATTGTTAGATTTCCTAATATTACAAACTTGTGCCCTTTTGGTGAA   22579
gb:MT019530|Organism:Severe      CAACCAACAGAATCTATTGTTAGATTTCCTAATATTACAAACTTGTGCCCTTTTGGTGAA   22582
gb:MT039888|Organism:Severe      CAACCAACAGAATCTATTGTTAGATTTCCTAATATTACAAACTTGTGCCCTTTTGGTGAA   22582
gb:LC522972|Organism:Severe      CAACCAACAGAATCTATTGTTAGATTTCCTAATATTACAAACTTGTGCCCTTTTGGTGAA   22579
gb:MT027063|Organism:Severe      CAACCAACAGAATCTATTGTTAGATTTCCTAATATTACAAACTTGTGCCCTTTTGGTGAA   22582
gb:MT027062|Organism:Severe      CAACCAACAGAATCTATTGTTAGATTTCCTAATATTACAAACTTGTGCCCTTTTGGTGAA   22582
gb:MT019529|Organism:Severe      CAACCAACAGAATCTATTGTTAGATTTCCTAATATTACAAACTTGTGCCCTTTTGGTGAA   22582
gb:MN996529|Organism:Severe      CAACCAACAGAATCTATTGTTAGATTTCCTAATATTACAAACTTGTGCCCTTTTGGTGAA   22570
gb:MN996531|Organism:Severe      CAACCAACAGAATCTATTGTTAGATTTCCTAATATTACAAACTTGTGCCCTTTTGGTGAA   22569
gb:MT066176|Organism:Severe      CAACCAACAGAATCTATTGTTAGATTTCCTAATATTACAAACTTGTGCCCTTTTGGTGAA   22582
gb:MT027064|Organism:Severe      CAACCAACAGAATCTATTGTTAGATTTCCTAATATTACAAACTTGTGCCCTTTTGGTGAA   22582
gb:MN994468|Organism:Severe      CAACCAACAGAATCTATTGTTAGATTTCCTAATATTACAAACTTGTGCCCTTTTGGTGAA   22582
gb:MT072688|Organism:Severe      CAACCAACAGAATCTATTGTTAGATTTCCTAATATTACAAACTTGTGCCCTTTTGGTGAA   22567
gb:MN996527|Organism:Severe      CAACCAACAGAATCTATTGTTAGATTTCCTAATATTACAAACTTGTGCCCTTTTGGTGAA   22549
gb:MT093631|Organism:Severe      CAACCAACAGAATCTATTGTTAGATTTCCTAATATTACAAACTTGTGCCCTTTTGGTGAA   22620
gb:MT106053|Organism:Severe      CAACCAACAGAATCTATTGTTAGATTTCCTAATATTACAAACTTGTGCCCTTTTGGTGAA   22582
gb:MT019533|Organism:Severe      CAACCAACAGAATCTATTGTTAGATTTCCTAATATTACAAACTTGTGCCCTTTTGGTGAA   22582
gb:MT019531|Organism:Severe      CAACCAACAGAATCTATTGTTAGATTTCCTAATATTACAAACTTGTGCCCTTTTGGTGAA   22582
gb:MN996528|Organism:Severe      CAACCAACAGAATCTATTGTTAGATTTCCTAATATTACAAACTTGTGCCCTTTTGGTGAA   22582
gb:MN996530|Organism:Severe      CAACCAACAGAATCTATTGTTAGATTTCCTAATATTACAAACTTGTGCCCTTTTGGTGAA   22568
gb:MN908947|Organism:Severe      CAACCAACAGAATCTATTGTTAGATTTCCTAATATTACAAACTTGTGCCCTTTTGGTGAA   22582
gb:MT019532|Organism:Severe      CAACCAACAGAATCTATTGTTAGATTTCCTAATATTACAAACTTGTGCCCTTTTGGTGAA   22582
                                 ************************************************************

gb:MT020781|Organism:Severe      GTTTTTAACGCCACCAGATTTGCATCTGTTTATGCTTGGAACAGGAAGAGAATCAGCAAC   22630
gb:MT007544|Organism:Severe      GTTTTTAACGCCACCAGATTTGCATCTGTTTATGCTTGGAACAGGAAGAGAATCAGCAAC   22642
gb:MN994467|Organism:Severe      GTTTTTAACGCCACCAGATTTGCATCTGTTTATGCTTGGAACAGGAAGAGAATCAGCAAC   22642
gb:MT044257|Organism:Severe      GTTTTTAACGCCACCAGATTTGCATCTGTTTATGCTTGGAACAGGAAGAGAATCAGCAAC   22642
gb:MT106054|Organism:Severe      GTTTTTAACGCCACCAGATTTGCATCTGTTTATGCTTGGAACAGGAAGAGAATCAGCAAC   22642
gb:MT049951|Organism:Severe      GTTTTTAACGCCACCAGATTTGCATCTGTTTATGCTTGGAACAGGAAGAGAATCAGCAAC   22642
gb:MN975262|Organism:Severe      GTTTTTAACGCCACCAGATTTGCATCTGTTTATGCTTGGAACAGGAAGAGAATCAGCAAC   22642
gb:MT106052|Organism:Severe      GTTTTTAACGCCACCAGATTTGCATCTGTTTATGCTTGGAACAGGAAGAGAATCAGCAAC   22642
gb:LC522975|Organism:Severe      GTTTTTAACGCCACCAGATTTGCATCTGTTTATGCTTGGAACAGGAAGAGAATCAGCAAC   22639
```

```
gb:LC522973|Organism:Severe      GTTTTTAACGCCACCAGATTTGCATCTGTTTATGCTTGGAACAGGAAGAGAATCAGCAAC   22639
gb:LC522974|Organism:Severe      GTTTTTAACGCCACCAGATTTGCATCTGTTTATGCTTGGAACAGGAAGAGAATCAGCAAC   22639
gb:MN985325|Organism:Severe      GTTTTTAACGCCACCAGATTTGCATCTGTTTATGCTTGGAACAGGAAGAGAATCAGCAAC   22642
gb:MT020881|Organism:Severe      GTTTTTAACGCCACCAGATTTGCATCTGTTTATGCTTGGAACAGGAAGAGAATCAGCAAC   22642
gb:MT020880|Organism:Severe      GTTTTTAACGCCACCAGATTTGCATCTGTTTATGCTTGGAACAGGAAGAGAATCAGCAAC   22642
gb:MT066175|Organism:Severe      GTTTTTAACGCCACCAGATTTGCATCTGTTTATGCTTGGAACAGGAAGAGAATCAGCAAC   22642
gb:MN997409|Organism:Severe      GTTTTTAACGCCACCAGATTTGCATCTGTTTATGCTTGGAACAGGAAGAGAATCAGCAAC   22642
gb:MN938384|Organism:Severe      GTTTTTAACGCCACCAGATTTGCATCTGTTTATGCTTGGAACAGGAAGAGAATCAGCAAC   22610
gb:MT044258|Organism:Severe      GTTTTTAACGCCACCAGATTTGCATCTGTTTATGCTTGGAACAGGAAGAGAATCAGCAAC   22618
gb:MT039890|Organism:Severe      GTTTTTAACGCCACCAGATTTGCATCTGTTTATGCTTGGAACAGGAAGAGAATCAGCAAC   22642
gb:MN988713|Organism:Severe      GTTTTTAACGCCACCAGATTTGCATCTGTTTATGCTTGGAACAGGAAGAGAATCAGCAAC   22642
gb:LC521925|Organism:Severe      GTTTTTAACGCCACCAGATTTGCATCTGTTTATGCTTGGAACAGGAAGAGAATCAGCAAC   22615
gb:MT093571|Organism:Severe      GTTTTTAACGCCACCAGATTTGCATCTGTTTATGCTTGGAACAGGAAGAGAATCAGCAAC   22642
gb:MT039887|Organism:Severe      GTTTTTAACGCCACCAGATTTGCATCTGTTTATGCTTGGAACAGGAAGAGAATCAGCAAC   22639
gb:MT019530|Organism:Severe      GTTTTTAACGCCACCAGATTTGCATCTGTTTATGCTTGGAACAGGAAGAGAATCAGCAAC   22642
gb:MT039888|Organism:Severe      GTTTTTAACGCCACCAGATTTGCATCTGTTTATGCTTGGAACAGGAAGAGAATCAGCAAC   22642
gb:LC522972|Organism:Severe      GTTTTTAACGCCACCAGATTTGCATCTGTTTATGCTTGGAACAGGAAGAGAATCAGCAAC   22639
gb:MT027063|Organism:Severe      GTTTTTAACGCCACCAGATTTGCATCTGTTTATGCTTGGAACAGGAAGAGAATCAGCAAC   22642
gb:MT027062|Organism:Severe      GTTTTTAACGCCACCAGATTTGCATCTGTTTATGCTTGGAACAGGAAGAGAATCAGCAAC   22642
gb:MT019529|Organism:Severe      GTTTTTAACGCCACCAGATTTGCATCTGTTTATGCTTGGAACAGGAAGAGAATCAGCAAC   22642
gb:MN996529|Organism:Severe      GTTTTTAACGCCACCAGATTTGCATCTGTTTATGCTTGGAACAGGAAGAGAATCAGCAAC   22630
gb:MN996531|Organism:Severe      GTTTTTAACGCCACCAGATTTGCATCTGTTTATGCTTGGAACAGGAAGAGAATCAGCAAC   22629
gb:MT066176|Organism:Severe      GTTTTTAACGCCACCAGATTTGCATCTGTTTATGCTTGGAACAGGAAGAGAATCAGCAAC   22642
gb:MT027064|Organism:Severe      GTTTTTAACGCCACCAGATTTGCATCTGTTTATGCTTGGAACAGGAAGAGAATCAGCAAC   22642
gb:MN994468|Organism:Severe      GTTTTTAACGCCACCAGATTTGCATCTGTTTATGCTTGGAACAGGAAGAGAATCAGCAAC   22642
gb:MT072688|Organism:Severe      GTTTTTAACGCCACCAGATTTGCATCTGTTTATGCTTGGAACAGGAAGAGAATCAGCAAC   22627
gb:MN996527|Organism:Severe      GTTTTTAACGCCACCAGATTTGCATCTGTTTATGCTTGGAACAGGAAGAGAATCAGCAAC   22609
gb:MT093631|Organism:Severe      GTTTTTAACGCCACCAGATTTGCATCTGTTTATGCTTGGAACAGGAAGAGAATCAGCAAC   22680
gb:MT106053|Organism:Severe      GTTTTTAACGCCACCAGATTTGCATCTGTTTATGCTTGGAACAGGAAGAGAATCAGCAAC   22642
gb:MT019533|Organism:Severe      GTTTTTAACGCCACCAGATTTGCATCTGTTTATGCTTGGAACAGGAAGAGAATCAGCAAC   22642
gb:MT019531|Organism:Severe      GTTTTTAACGCCACCAGATTTGCATCTGTTTATGCTTGGAACAGGAAGAGAATCAGCAAC   22642
gb:MN996528|Organism:Severe      GTTTTTAACGCCACCAGATTTGCATCTGTTTATGCTTGGAACAGGAAGAGAATCAGCAAC   22642
gb:MN996530|Organism:Severe      GTTTTTAACGCCACCAGATTTGCATCTGTTTATGCTTGGAACAGGAAGAGAATCAGCAAC   22628
gb:MN908947|Organism:Severe      GTTTTTAACGCCACCAGATTTGCATCTGTTTATGCTTGGAACAGGAAGAGAATCAGCAAC   22642
gb:MT019532|Organism:Severe      GTTTTTAACGCCACCAGATTTGCATCTGTTTATGCTTGGAACAGGAAGAGAATCAGCAAC   22642
                                 ************************************************************

gb:MT020781|Organism:Severe      TGTGTTGCTGATTATTCTGTCCTATATAATTCCGCATCATTTTCCACTTTTAAGTGTTAT   22690
gb:MT007544|Organism:Severe      TGTGTTGCTGATTATTCTGTCCTATATAATTCCGCATCATTTTCCACTTTTAAGTGTTAT   22702
gb:MN994467|Organism:Severe      TGTGTTGCTGATTATTCTGTCCTATATAATTCCGCATCATTTTCCACTTTTAAGTGTTAT   22702
gb:MT044257|Organism:Severe      TGTGTTGCTGATTATTCTGTCCTATATAATTCCGCATCATTTTCCACTTTTAAGTGTTAT   22702
gb:MT106054|Organism:Severe      TGTGTTGCTGATTATTCTGTCCTATATAATTCCGCATCATTTTCCACTTTTAAGTGTTAT   22702
gb:MT049951|Organism:Severe      TGTGTTGCTGATTATTCTGTCCTATATAATTCCGCATCATTTTCCACTTTTAAGTGTTAT   22702
gb:MN975262|Organism:Severe      TGTGTTGCTGATTATTCTGTCCTATATAATTCCGCATCATTTTCCACTTTTAAGTGTTAT   22702
gb:MT106052|Organism:Severe      TGTGTTGCTGATTATTCTGTCCTATATAATTCCGCATCATTTTCCACTTTTAAGTGTTAT   22702
gb:LC522975|Organism:Severe      TGTGTTGCTGATTATTCTGTCCTATATAATTCCGCATCATTTTCCACTTTTAAGTGTTAT   22699
gb:LC522973|Organism:Severe      TGTGTTGCTGATTATTCTGTCCTATATAATTCCGCATCATTTTCCACTTTTAAGTGTTAT   22699
gb:LC522974|Organism:Severe      TGTGTTGCTGATTATTCTGTCCTATATAATTCCGCATCATTTTCCACTTTTAAGTGTTAT   22699
gb:MN985325|Organism:Severe      TGTGTTGCTGATTATTCTGTCCTATATAATTCCGCATCATTTTCCACTTTTAAGTGTTAT   22702
gb:MT020881|Organism:Severe      TGTGTTGCTGATTATTCTGTCCTATATAATTCCGCATCATTTTCCACTTTTAAGTGTTAT   22702
gb:MT020880|Organism:Severe      TGTGTTGCTGATTATTCTGTCCTATATAATTCCGCATCATTTTCCACTTTTAAGTGTTAT   22702
gb:MT066175|Organism:Severe      TGTGTTGCTGATTATTCTGTCCTATATAATTCCGCATCATTTTCCACTTTTAAGTGTTAT   22702
gb:MN997409|Organism:Severe      TGTGTTGCTGATTATTCTGTCCTATATAATTCCGCATCATTTTCCACTTTTAAGTGTTAT   22702
gb:MN938384|Organism:Severe      TGTGTTGCTGATTATTCTGTCCTATATAATTCCGCATCATTTTCCACTTTTAAGTGTTAT   22670
gb:MT044258|Organism:Severe      TGTGTTGCTGATTATTCTGTCCTATATAATTCCGCATCATTTTCCACTTTTAAGTGTTAT   22678
gb:MT039890|Organism:Severe      TGTGTTGCTGATTATTCTGTCCTATATAATTCCGCATCATTTTCCACTTTTAAGTGTTAT   22702
gb:MN988713|Organism:Severe      TGTGTTGCTGATTATTCTGTCCTATATAATTCCGCATCATTTTCCACTTTTAAGTGTTAT   22702
gb:LC521925|Organism:Severe      TGTGTTGCTGATTATTCTGTCCTATATAATTCCGCATCATTTTCCACTTTTAAGTGTTAT   22675
gb:MT093571|Organism:Severe      TGTGTTGCTGATTATTCTGTCCTATATAATTCCGCATCATTTTCCACTTTTAAGTGTTAT   22702
gb:MT039887|Organism:Severe      TGTGTTGCTGATTATTCTGTCCTATATAATTCCGCATCATTTTCCACTTTTAAGTGTTAT   22699
gb:MT019530|Organism:Severe      TGTGTTGCTGATTATTCTGTCCTATATAATTCCGCATCATTTTCCACTTTTAAGTGTTAT   22702
gb:MT039888|Organism:Severe      TGTGTTGCTGATTATTCTGTCCTATATAATTCCGCATCATTTTCCACTTTTAAGTGTTAT   22702
gb:LC522972|Organism:Severe      TGTGTTGCTGATTATTCTGTCCTATATAATTCCGCATCATTTTCCACTTTTAAGTGTTAT   22699
gb:MT027063|Organism:Severe      TGTGTTGCTGATTATTCTGTCCTATATAATTCCGCATCATTTTCCACTTTTAAGTGTTAT   22702
gb:MT027062|Organism:Severe      TGTGTTGCTGATTATTCTGTCCTATATAATTCCGCATCATTTTCCACTTTTAAGTGTTAT   22702
```

```
gb:MT019529|Organism:Severe      TGTGTTGCTGATTATTCTGTCCTATATAATTCCGCATCATTTTCCACTTTTAAGTGTTAT    22702
gb:MN996529|Organism:Severe      TGTGTTGCTGATTATTCTGTCCTATATAATTCCGCATCATTTTCCACTTTTAAGTGTTAT    22690
gb:MN996531|Organism:Severe      TGTGTTGCTGATTATTCTGTCCTATATAATTCCGCATCATTTTCCACTTTTAAGTGTTAT    22689
gb:MT066176|Organism:Severe      TGTGTTGCTGATTATTCTGTCCTATATAATTCCGCATCATTTTCCACTTTTAAGTGTTAT    22702
gb:MT027064|Organism:Severe      TGTGTTGCTGATTATTCTGTCCTATATAATTCCGCATCATTTTCCACTTTTAAGTGTTAT    22702
gb:MN994468|Organism:Severe      TGTGTTGCTGATTATTCTGTCCTATATAATTCCGCATCATTTTCCACTTTTAAGTGTTAT    22702
gb:MT072688|Organism:Severe      TGTGTTGCTGATTATTCTGTCCTATATAATTCCGCATCATTTTCCACTTTTAAGTGTTAT    22687
gb:MN996527|Organism:Severe      TGTGTTGCTGATTATTCTGTCCTATATAATTCCGCATCATTTTCCACTTTTAAGTGTTAT    22669
gb:MT093631|Organism:Severe      TGTGTTGCTGATTATTCTGTCCTATATAATTCCGCATCATTTTCCACTTTTAAGTGTTAT    22740
gb:MT106053|Organism:Severe      TGTGTTGCTGATTATTCTGTCCTATATAATTCCGCATCATTTTCCACTTTTAAGTGTTAT    22702
gb:MT019533|Organism:Severe      TGTGTTGCTGATTATTCTGTCCTATATAATTCCGCATCATTTTCCACTTTTAAGTGTTAT    22702
gb:MT019531|Organism:Severe      TGTGTTGCTGATTATTCTGTCCTATATAATTCCGCATCATTTTCCACTTTTAAGTGTTAT    22702
gb:MN996528|Organism:Severe      TGTGTTGCTGATTATTCTGTCCTATATAATTCCGCATCATTTTCCACTTTTAAGTGTTAT    22702
gb:MN996530|Organism:Severe      TGTGTTGCTGATTATTCTGTCCTATATAATTCCGCATCATTTTCCACTTTTAAGTGTTAT    22688
gb:MN908947|Organism:Severe      TGTGTTGCTGATTATTCTGTCCTATATAATTCCGCATCATTTTCCACTTTTAAGTGTTAT    22702
gb:MT019532|Organism:Severe      TGTGTTGCTGATTATTCTGTCCTATATAATTCCGCATCATTTTCCACTTTTAAGTGTTAT    22702
                                 ************************************************************

gb:MT020781|Organism:Severe      GGAGTGTCTCCTACTAAATTAAATGATCTCTGCTTTACTAATGTCTATGCAGATTCATTT    22750
gb:MT007544|Organism:Severe      GGAGTGTCTCCTACTAAATTAAATGATCTCTGCTTTACTAATGTCTATGCAGATTCATTT    22762
gb:MN994467|Organism:Severe      GGAGTGTCTCCTACTAAATTAAATGATCTCTGCTTTACTAATGTCTATGCAGATTCATTT    22762
gb:MT044257|Organism:Severe      GGAGTGTCTCCTACTAAATTAAATGATCTCTGCTTTACTAATGTCTATGCAGATTCATTT    22762
gb:MT106054|Organism:Severe      GGAGTGTCTCCTACTAAATTAAATGATCTCTGCTTTACTAATGTCTATGCAGATTCATTT    22762
gb:MT049951|Organism:Severe      GGAGTGTCTCCTACTAAATTAAATGATCTCTGCTTTACTAATGTCTATGCAGATTCATTT    22762
gb:MN975262|Organism:Severe      GGAGTGTCTCCTACTAAATTAAATGATCTCTGCTTTACTAATGTCTATGCAGATTCATTT    22762
gb:MT106052|Organism:Severe      GGAGTGTCTCCTACTAAATTAAATGATCTCTGCTTTACTAATGTCTATGCAGATTCATTT    22762
gb:LC522975|Organism:Severe      GGAGTGTCTCCTACTAAATTAAATGATCTCTGCTTTACTAATGTCTATGCAGATTCATTT    22759
gb:LC522973|Organism:Severe      GGAGTGTCTCCTACTAAATTAAATGATCTCTGCTTTACTAATGTCTATGCAGATTCATTT    22759
gb:LC522974|Organism:Severe      GGAGTGTCTCCTACTAAATTAAATGATCTCTGCTTTACTAATGTCTATGCAGATTCATTT    22759
gb:MN985325|Organism:Severe      GGAGTGTCTCCTACTAAATTAAATGATCTCTGCTTTACTAATGTCTATGCAGATTCATTT    22762
gb:MT020881|Organism:Severe      GGAGTGTCTCCTACTAAATTAAATGATCTCTGCTTTACTAATGTCTATGCAGATTCATTT    22762
gb:MT020880|Organism:Severe      GGAGTGTCTCCTACTAAATTAAATGATCTCTGCTTTACTAATGTCTATGCAGATTCATTT    22762
gb:MT066175|Organism:Severe      GGAGTGTCTCCTACTAAATTAAATGATCTCTGCTTTACTAATGTCTATGCAGATTCATTT    22762
gb:MN997409|Organism:Severe      GGAGTGTCTCCTACTAAATTAAATGATCTCTGCTTTACTAATGTCTATGCAGATTCATTT    22762
gb:MN938384|Organism:Severe      GGAGTGTCTCCTACTAAATTAAATGATCTCTGCTTTACTAATGTCTATGCAGATTCATTT    22730
gb:MT044258|Organism:Severe      GGAGTGTCTCCTACTAAATTAAATGATCTCTGCTTTACTAATGTCTATGCAGATTCATTT    22738
gb:MT039890|Organism:Severe      GGAGTGTCTCCTACTAAATTAAATGATCTCTGCTTTACTAATGTCTATGCAGATTCATTT    22762
gb:MN988713|Organism:Severe      GGAGTGTCTCCTACTAAATTAAATGATCTCTGCTTTACTAATGTCTATGCAGATTCATTT    22762
gb:LC521925|Organism:Severe      GGAGTGTCTCCTACTAAATTAAATGATCTCTGCTTTACTAATGTCTATGCAGATTCATTT    22735
gb:MT093571|Organism:Severe      GGAGTGTCTCCTACTAAATTAAATGATCTCTGCTTTACTAATGTCTATGCAGATTCATTT    22762
gb:MT039887|Organism:Severe      GGAGTGTCTCCTACTAAATTAAATGATCTCTGCTTTACTAATGTCTATGCAGATTCATTT    22759
gb:MT019530|Organism:Severe      GGAGTGTCTCCTACTAAATTAAATGATCTCTGCTTTACTAATGTCTATGCAGATTCATTT    22762
gb:MT039888|Organism:Severe      GGAGTGTCTCCTACTAAATTAAATGATCTCTGCTTTACTAATGTCTATGCAGATTCATTT    22762
gb:LC522972|Organism:Severe      GGAGTGTCTCCTACTAAATTAAATGATCTCTGCTTTACTAATGTCTATGCAGATTCATTT    22759
gb:MT027063|Organism:Severe      GGAGTGTCTCCTACTAAATTAAATGATCTCTGCTTTACTAATGTCTATGCAGATTCATTT    22762
gb:MT027062|Organism:Severe      GGAGTGTCTCCTACTAAATTAAATGATCTCTGCTTTACTAATGTCTATGCAGATTCATTT    22762
gb:MT019529|Organism:Severe      GGAGTGTCTCCTACTAAATTAAATGATCTCTGCTTTACTAATGTCTATGCAGATTCATTT    22762
gb:MN996529|Organism:Severe      GGAGTGTCTCCTACTAAATTAAATGATCTCTGCTTTACTAATGTCTATGCAGATTCATTT    22750
gb:MN996531|Organism:Severe      GGAGTGTCTCCTACTAAATTAAATGATCTCTGCTTTACTAATGTCTATGCAGATTCATTT    22749
gb:MT066176|Organism:Severe      GGAGTGTCTCCTACTAAATTAAATGATCTCTGCTTTACTAATGTCTATGCAGATTCATTT    22762
gb:MT027064|Organism:Severe      GGAGTGTCTCCTACTAAATTAAATGATCTCTGCTTTACTAATGTCTATGCAGATTCATTT    22762
gb:MN994468|Organism:Severe      GGAGTGTCTCCTACTAAATTAAATGATCTCTGCTTTACTAATGTCTATGCAGATTCATTT    22762
gb:MT072688|Organism:Severe      GGAGTGTCTCCTACTAAATTAAATGATCTCTGCTTTACTAATGTCTATGCAGATTCATTT    22747
gb:MN996527|Organism:Severe      GGAGTGTCTCCTACTAAATTAAATGATCTCTGCTTTACTAATGTCTATGCAGATTCATTT    22729
gb:MT093631|Organism:Severe      GGAGTGTCTCCTACTAAATTAAATGATCTCTGCTTTACTAATGTCTATGCAGATTCATTT    22800
gb:MT106053|Organism:Severe      GGAGTGTCTCCTACTAAATTAAATGATCTCTGCTTTACTAATGTCTATGCAGATTCATTT    22762
gb:MT019533|Organism:Severe      GGAGTGTCTCCTACTAAATTAAATGATCTCTGCTTTACTAATGTCTATGCAGATTCATTT    22762
gb:MT019531|Organism:Severe      GGAGTGTCTCCTACTAAATTAAATGATCTCTGCTTTACTAATGTCTATGCAGATTCATTT    22762
gb:MN996528|Organism:Severe      GGAGTGTCTCCTACTAAATTAAATGATCTCTGCTTTACTAATGTCTATGCAGATTCATTT    22762
gb:MN996530|Organism:Severe      GGAGTGTCTCCTACTAAATTAAATGATCTCTGCTTTACTAATGTCTATGCAGATTCATTT    22748
gb:MN908947|Organism:Severe      GGAGTGTCTCCTACTAAATTAAATGATCTCTGCTTTACTAATGTCTATGCAGATTCATTT    22762
gb:MT019532|Organism:Severe      GGAGTGTCTCCTACTAAATTAAATGATCTCTGCTTTACTAATGTCTATGCAGATTCATTT    22762
                                 ************************************************************

gb:MT020781|Organism:Severe      GTAATTAGAGGTGATGAAGTCAGACAAATCGCTCCAGGGCAAACTGGAAAGATTGCTGAT    22810
```

```
gb:MT007544|Organism:Severe      GTAATTAGAGGTGATGAAGTCAGACAAATCGCTCCAGGGCAAACTGGAAAGATTGCTGAT    22822
gb:MN994467|Organism:Severe      GTAATTAGAGGTGATGAAGTCAGACAAATCGCTCCAGGGCAAACTGGAAAGATTGCTGAT    22822
gb:MT044257|Organism:Severe      GTAATTAGAGGTGATGAAGTCAGACAAATCGCTCCAGGGCAAACTGGAAAGATTGCTGAT    22822
gb:MT106054|Organism:Severe      GTAATTAGAGGTGATGAAGTCAGACAAATCGCTCCAGGGCAAACTGGAAAGATTGCTGAT    22822
gb:MT049951|Organism:Severe      GTAATTAGAGGTGATGAAGTCAGACAAATCGCTCCAGGGCAAACTGGAAAGATTGCTGAT    22822
gb:MN975262|Organism:Severe      GTAATTAGAGGTGATGAAGTCAGACAAATCGCTCCAGGGCAAACTGGAAAGATTGCTGAT    22822
gb:MT106052|Organism:Severe      GTAATTAGAGGTGATGAAGTCAGACAAATCGCTCCAGGGCAAACTGGAAAGATTGCTGAT    22822
gb:LC522975|Organism:Severe      GTAATTAGAGGTGATGAAGTCAGACAAATCGCTCCAGGGCAAACTGGAAAGATTGCTGAT    22819
gb:LC522973|Organism:Severe      GTAATTAGAGGTGATGAAGTCAGACAAATCGCTCCAGGGCAAACTGGAAAGATTGCTGAT    22819
gb:LC522974|Organism:Severe      GTAATTAGAGGTGATGAAGTCAGACAAATCGCTCCAGGGCAAACTGGAAAGATTGCTGAT    22819
gb:MN985325|Organism:Severe      GTAATTAGAGGTGATGAAGTCAGACAAATCGCTCCAGGGCAAACTGGAAAGATTGCTGAT    22822
gb:MT020881|Organism:Severe      GTAATTAGAGGTGATGAAGTCAGACAAATCGCTCCAGGGCAAACTGGAAAGATTGCTGAT    22822
gb:MT020880|Organism:Severe      GTAATTAGAGGTGATGAAGTCAGACAAATCGCTCCAGGGCAAACTGGAAAGATTGCTGAT    22822
gb:MT066175|Organism:Severe      GTAATTAGAGGTGATGAAGTCAGACAAATCGCTCCAGGGCAAACTGGAAAGATTGCTGAT    22822
gb:MN997409|Organism:Severe      GTAATTAGAGGTGATGAAGTCAGACAAATCGCTCCAGGGCAAACTGGAAAGATTGCTGAT    22822
gb:MN938384|Organism:Severe      GTAATTAGAGGTGATGAAGTCAGACAAATCGCTCCAGGGCAAACTGGAAAGATTGCTGAT    22790
gb:MT044258|Organism:Severe      GTAATTAGAGGTGATGAAGTCAGACAAATCGCTCCAGGGCAAACTGGAAAGATTGCTGAT    22798
gb:MT039890|Organism:Severe      GTAATTAGAGGTGATGAAGTCAGACAAATCGCTCCAGGGCAAACTGGAAAGATTGCTGAT    22822
gb:MN988713|Organism:Severe      GTAATTAGAGGTGATGAAGTCAGACAAATCGCTCCAGGGCAAACTGGAAAGATTGCTGAT    22822
gb:LC521925|Organism:Severe      GTAATTAGAGGTGATGAAGTCAGACAAATCGCTCCAGGGCAAACTGGAAAGATTGCTGAT    22795
gb:MT093571|Organism:Severe      GTAATTAGAGGTGATGAAGTCAGACAAATCGCTCCAGGGCAAACTGGAAAGATTGCTGAT    22822
gb:MT039887|Organism:Severe      GTAATTAGAGGTGATGAAGTCAGACAAATCGCTCCAGGGCAAACTGGAAAGATTGCTGAT    22819
gb:MT019530|Organism:Severe      GTAATTAGAGGTGATGAAGTCAGACAAATCGCTCCAGGGCAAACTGGAAAGATTGCTGAT    22822
gb:MT039888|Organism:Severe      GTAATTAGAGGTGATGAAGTCAGACAAATCGCTCCAGGGCAAACTGGAAAGATTGCTGAT    22822
gb:LC522972|Organism:Severe      GTAATTAGAGGTGATGAAGTCAGACAAATCGCTCCAGGGCAAACTGGAAAGATTGCTGAT    22819
gb:MT027063|Organism:Severe      GTAATTAGAGGTGATGAAGTCAGACAAATCGCTCCAGGGCAAACTGGAAAGATTGCTGAT    22822
gb:MT027062|Organism:Severe      GTAATTAGAGGTGATGAAGTCAGACAAATCGCTCCAGGGCAAACTGGAAAGATTGCTGAT    22822
gb:MT019529|Organism:Severe      GTAATTAGAGGTGATGAAGTCAGACAAATCGCTCCAGGGCAAACTGGAAAGATTGCTGAT    22822
gb:MN996529|Organism:Severe      GTAATTAGAGGTGATGAAGTCAGACAAATCGCTCCAGGGCAAACTGGAAAGATTGCTGAT    22810
gb:MN996531|Organism:Severe      GTAATTAGAGGTGATGAAGTCAGACAAATCGCTCCAGGGCAAACTGGAAAGATTGCTGAT    22809
gb:MT066176|Organism:Severe      GTAATTAGAGGTGATGAAGTCAGACAAATCGCTCCAGGGCAAACTGGAAAGATTGCTGAT    22822
gb:MT027064|Organism:Severe      GTAATTAGAGGTGATGAAGTCAGACAAATCGCTCCAGGGCAAACTGGAAAGATTGCTGAT    22822
gb:MN994468|Organism:Severe      GTAATTAGAGGTGATGAAGTCAGACAAATCGCTCCAGGGCAAACTGGAAAGATTGCTGAT    22822
gb:MT072688|Organism:Severe      GTAATTAGAGGTGATGAAGTCAGACAAATCGCTCCAGGGCAAACTGGAAAGATTGCTGAT    22807
gb:MN996527|Organism:Severe      GTAATTAGAGGTGATGAAGTCAGACAAATCGCTCCAGGGCAAACTGGAAAGATTGCTGAT    22789
gb:MT093631|Organism:Severe      GTAATTAGAGGTGATGAAGTCAGACAAATCGCTCCAGGGCAAACTGGAAAGATTGCTGAT    22860
gb:MT106053|Organism:Severe      GTAATTAGAGGTGATGAAGTCAGACAAATCGCTCCAGGGCAAACTGGAAAGATTGCTGAT    22822
gb:MT019533|Organism:Severe      GTAATTAGAGGTGATGAAGTCAGACAAATCGCTCCAGGGCAAACTGGAAAGATTGCTGAT    22822
gb:MT019531|Organism:Severe      GTAATTAGAGGTGATGAAGTCAGACAAATCGCTCCAGGGCAAACTGGAAAGATTGCTGAT    22822
gb:MN996528|Organism:Severe      GTAATTAGAGGTGATGAAGTCAGACAAATCGCTCCAGGGCAAACTGGAAAGATTGCTGAT    22822
gb:MN996530|Organism:Severe      GTAATTAGAGGTGATGAAGTCAGACAAATCGCTCCAGGGCAAACTGGAAAGATTGCTGAT    22808
gb:MN908947|Organism:Severe      GTAATTAGAGGTGATGAAGTCAGACAAATCGCTCCAGGGCAAACTGGAAAGATTGCTGAT    22822
gb:MT019532|Organism:Severe      GTAATTAGAGGTGATGAAGTCAGACAAATCGCTCCAGGGCAAACTGGAAAGATTGCTGAT    22822
                                 ************************************************************

gb:MT020781|Organism:Severe      TATAATTATAAATTACCAGATGATTTTACAGGCTGCGTTATAGCTTGGAATTCTAACAAT    22870
gb:MT007544|Organism:Severe      TATAATTATAAATTACCAGATGATTTTACAGGCTGCGTTATAGCTTGGAATTCTAACAAT    22882
gb:MN994467|Organism:Severe      TATAATTATAAATTACCAGATGATTTTACAGGCTGCGTTATAGCTTGGAATTCTAACAAT    22882
gb:MT044257|Organism:Severe      TATAATTATAAATTACCAGATGATTTTACAGGCTGCGTTATAGCTTGGAATTCTAACAAT    22882
gb:MT106054|Organism:Severe      TATAATTATAAATTACCAGATGATTTTACAGGCTGCGTTATAGCTTGGAATTCTAACAAT    22882
gb:MT049951|Organism:Severe      TATAATTATAAATTACCAGATGATTTTACAGGCTGCGTTATAGCTTGGAATTCTAACAAT    22882
gb:MN975262|Organism:Severe      TATAATTATAAATTACCAGATGATTTTACAGGCTGCGTTATAGCTTGGAATTCTAACAAT    22882
gb:MT106052|Organism:Severe      TATAATTATAAATTACCAGATGATTTTACAGGCTGCGTTATAGCTTGGAATTCTAACAAT    22882
gb:LC522975|Organism:Severe      TATAATTATAAATTACCAGATGATTTTACAGGCTGCGTTATAGCTTGGAATTCTAACAAT    22879
gb:LC522973|Organism:Severe      TATAATTATAAATTACCAGATGATTTTACAGGCTGCGTTATAGCTTGGAATTCTAACAAT    22879
gb:LC522974|Organism:Severe      TATAATTATAAATTACCAGATGATTTTACAGGCTGCGTTATAGCTTGGAATTCTAACAAT    22879
gb:MN985325|Organism:Severe      TATAATTATAAATTACCAGATGATTTTACAGGCTGCGTTATAGCTTGGAATTCTAACAAT    22882
gb:MT020881|Organism:Severe      TATAATTATAAATTACCAGATGATTTTACAGGCTGCGTTATAGCTTGGAATTCTAACAAT    22882
gb:MT020880|Organism:Severe      TATAATTATAAATTACCAGATGATTTTACAGGCTGCGTTATAGCTTGGAATTCTAACAAT    22882
gb:MT066175|Organism:Severe      TATAATTATAAATTACCAGATGATTTTACAGGCTGCGTTATAGCTTGGAATTCTAACAAT    22882
gb:MN997409|Organism:Severe      TATAATTATAAATTACCAGATGATTTTACAGGCTGCGTTATAGCTTGGAATTCTAACAAT    22882
gb:MN938384|Organism:Severe      TATAATTATAAATTACCAGATGATTTTACAGGCTGCGTTATAGCTTGGAATTCTAACAAT    22850
gb:MT044258|Organism:Severe      TATAATTATAAATTACCAGATGATTTTACAGGCTGCGTTATAGCTTGGAATTCTAACAAT    22858
gb:MT039890|Organism:Severe      TATAATTATAAATTACCAGATGATTTTACAGGCTGCGTTATAGCTTGGAATTCTAACAAT    22882
gb:MN988713|Organism:Severe      TATAATTATAAATTACCAGATGATTTTACAGGCTGCGTTATAGCTTGGAATTCTAACAAT    22882
```

```
gb:LC521925|Organism:Severe      TATAATTATAAATTACCAGATGATTTTACAGGCTGCGTTATAGCTTGGAATTCTAACAAT   22855
gb:MT093571|Organism:Severe      TATAATTATAAATTACCAGATGATTTTACAGGCTGCGTTATAGCTTGGAATTCTAACAAT   22882
gb:MT039887|Organism:Severe      TATAATTATAAATTACCAGATGATTTTACAGGCTGCGTTATAGCTTGGAATTCTAACAAT   22879
gb:MT019530|Organism:Severe      TATAATTATAAATTACCAGATGATTTTACAGGCTGCGTTATAGCTTGGAATTCTAACAAT   22882
gb:MT039888|Organism:Severe      TATAATTATAAATTACCAGATGATTTTACAGGCTGCGTTATAGCTTGGAATTCTAACAAT   22882
gb:LC522972|Organism:Severe      TATAATTATAAATTACCAGATGATTTTACAGGCTGCGTTATAGCTTGGAATTCTAACAAT   22879
gb:MT027063|Organism:Severe      TATAATTATAAATTACCAGATGATTTTACAGGCTGCGTTATAGCTTGGAATTCTAACAAT   22882
gb:MT027062|Organism:Severe      TATAATTATAAATTACCAGATGATTTTACAGGCTGCGTTATAGCTTGGAATTCTAACAAT   22882
gb:MT019529|Organism:Severe      TATAATTATAAATTACCAGATGATTTTACAGGCTGCGTTATAGCTTGGAATTCTAACAAT   22882
gb:MN996529|Organism:Severe      TATAATTATAAATTACCAGATGATTTTACAGGCTGCGTTATAGCTTGGAATTCTAACAAT   22870
gb:MN996531|Organism:Severe      TATAATTATAAATTACCAGATGATTTTACAGGCTGCGTTATAGCTTGGAATTCTAACAAT   22869
gb:MT066176|Organism:Severe      TATAATTATAAATTACCAGATGATTTTACAGGCTGCGTTATAGCTTGGAATTCTAACAAT   22882
gb:MT027064|Organism:Severe      TATAATTATAAATTACCAGATGATTTTACAGGCTGCGTTATAGCTTGGAATTCTAACAAT   22882
gb:MN994468|Organism:Severe      TATAATTATAAATTACCAGATGATTTTACAGGCTGCGTTATAGCTTGGAATTCTAACAAT   22882
gb:MT072688|Organism:Severe      TATAATTATAAATTACCAGATGATTTTACAGGCTGCGTTATAGCTTGGAATTCTAACAAT   22867
gb:MN996527|Organism:Severe      TATAATTATAAATTACCAGATGATTTTACAGGCTGCGTTATAGCTTGGAATTCTAACAAT   22849
gb:MT093631|Organism:Severe      TATAATTATAAATTACCAGATGATTTTACAGGCTGCGTTATAGCTTGGAATTCTAACAAT   22920
gb:MT106053|Organism:Severe      TATAATTATAAATTACCAGATGATTTTACAGGCTGCGTTATAGCTTGGAATTCTAACAAT   22882
gb:MT019533|Organism:Severe      TATAATTATAAATTACCAGATGATTTTACAGGCTGCGTTATAGCTTGGAATTCTAACAAT   22882
gb:MT019531|Organism:Severe      TATAATTATAAATTACCAGATGATTTTACAGGCTGCGTTATAGCTTGGAATTCTAACAAT   22882
gb:MN996528|Organism:Severe      TATAATTATAAATTACCAGATGATTTTACAGGCTGCGTTATAGCTTGGAATTCTAACAAT   22882
gb:MN996530|Organism:Severe      TATAATTATAAATTACCAGATGATTTTACAGGCTGCGTTATAGCTTGGAATTCTAACAAT   22868
gb:MN908947|Organism:Severe      TATAATTATAAATTACCAGATGATTTTACAGGCTGCGTTATAGCTTGGAATTCTAACAAT   22882
gb:MT019532|Organism:Severe      TATAATTATAAATTACCAGATGATTTTACAGGCTGCGTTATAGCTTGGAATTCTAACAAT   22882
                                 ************************************************************

gb:MT020781|Organism:Severe      CTTGATTCTAAGGTTGGTGGTAATTATAATTACCTGTATAGATTGTTTAGGAAGTCTAAT   22930
gb:MT007544|Organism:Severe      CTTGATTCTAAGGTTGGTGGTAATTATAATTACCTGTATAGATTGTTTAGGAAGTCTAAT   22942
gb:MN994467|Organism:Severe      CTTGATTCTAAGGTTGGTGGTAATTATAATTACCTGTATAGATTGTTTAGGAAGTCTAAT   22942
gb:MT044257|Organism:Severe      CTTGATTCTAAGGTTGGTGGTAATTATAATTACCTGTATAGATTGTTTAGGAAGTCTAAT   22942
gb:MT106054|Organism:Severe      CTTGATTCTAAGGTTGGTGGTAATTATAATTACCTGTATAGATTGTTTAGGAAGTCTAAT   22942
gb:MT049951|Organism:Severe      CTTGATTCTAAGGTTGGTGGTAATTATAATTACCTGTATAGATTGTTTAGGAAGTCTAAT   22942
gb:MN975262|Organism:Severe      CTTGATTCTAAGGTTGGTGGTAATTATAATTACCTGTATAGATTGTTTAGGAAGTCTAAT   22942
gb:MT106052|Organism:Severe      CTTGATTCTAAGGTTGGTGGTAATTATAATTACCTGTATAGATTGTTTAGGAAGTCTAAT   22942
gb:LC522975|Organism:Severe      CTTGATTCTAAGGTTGGTGGTAATTATAATTACCTGTATAGATTGTTTAGGAAGTCTAAT   22939
gb:LC522973|Organism:Severe      CTTGATTCTAAGGTTGGTGGTAATTATAATTACCTGTATAGATTGTTTAGGAAGTCTAAT   22939
gb:LC522974|Organism:Severe      CTTGATTCTAAGGTTGGTGGTAATTATAATTACCTGTATAGATTGTTTAGGAAGTCTAAT   22939
gb:MN985325|Organism:Severe      CTTGATTCTAAGGTTGGTGGTAATTATAATTACCTGTATAGATTGTTTAGGAAGTCTAAT   22942
gb:MT020881|Organism:Severe      CTTGATTCTAAGGTTGGTGGTAATTATAATTACCTGTATAGATTGTTTAGGAAGTCTAAT   22942
gb:MT020880|Organism:Severe      CTTGATTCTAAGGTTGGTGGTAATTATAATTACCTGTATAGATTGTTTAGGAAGTCTAAT   22942
gb:MT066175|Organism:Severe      CTTGATTCTAAGGTTGGTGGTAATTATAATTACCTGTATAGATTGTTTAGGAAGTCTAAT   22942
gb:MN997409|Organism:Severe      CTTGATTCTAAGGTTGGTGGTAATTATAATTACCTGTATAGATTGTTTAGGAAGTCTAAT   22942
gb:MN938384|Organism:Severe      CTTGATTCTAAGGTTGGTGGTAATTATAATTACCTGTATAGATTGTTTAGGAAGTCTAAT   22910
gb:MT044258|Organism:Severe      CTTGATTCTAAGGTTGGTGGTAATTATAATTACCTGTATAGATTGTTTAGGAAGTCTAAT   22918
gb:MT039890|Organism:Severe      CTTGATTCTAAGGTTGGTGGTAATTATAATTACCTGTATAGATTGTTTAGGAAGTCTAAT   22942
gb:MN988713|Organism:Severe      CTTGATTCTAAGGTTGGTGGTAATTATAATTACCTGTATAGATTGTTTAGGAAGTCTAAT   22942
gb:LC521925|Organism:Severe      CTTGATTCTAAGGTTGGTGGTAATTATAATTACCTGTATAGATTGTTTAGGAAGTCTAAT   22915
gb:MT093571|Organism:Severe      CTTGATTCTAAGGTTGGTGGTAATTATAATTACCTGTATAGATTGTTTAGGAAGTCTAAT   22942
gb:MT039887|Organism:Severe      CTTGATTCTAAGGTTGGTGGTAATTATAATTACCTGTATAGATTGTTTAGGAAGTCTAAT   22939
gb:MT019530|Organism:Severe      CTTGATTCTAAGGTTGGTGGTAATTATAATTACCTGTATAGATTGTTTAGGAAGTCTAAT   22942
gb:MT039888|Organism:Severe      CTTGATTCTAAGGTTGGTGGTAATTATAATTACCTGTATAGATTGTTTAGGAAGTCTAAT   22942
gb:LC522972|Organism:Severe      CTTGATTCTAAGGTTGGTGGTAATTATAATTACCTGTATAGATTGTTTAGGAAGTCTAAT   22939
gb:MT027063|Organism:Severe      CTTGATTCTAAGGTTGGTGGTAATTATAATTACCTGTATAGATTGTTTAGGAAGTCTAAT   22942
gb:MT027062|Organism:Severe      CTTGATTCTAAGGTTGGTGGTAATTATAATTACCTGTATAGATTGTTTAGGAAGTCTAAT   22942
gb:MT019529|Organism:Severe      CTTGATTCTAAGGTTGGTGGTAATTATAATTACCTGTATAGATTGTTTAGGAAGTCTAAT   22942
gb:MN996529|Organism:Severe      CTTGATTCTAAGGTTGGTGGTAATTATAATTACCTGTATAGATTGTTTAGGAAGTCTAAT   22930
gb:MN996531|Organism:Severe      CTTGATTCTAAGGTTGGTGGTAATTATAATTACCTGTATAGATTGTTTAGGAAGTCTAAT   22929
gb:MT066176|Organism:Severe      CTTGATTCTAAGGTTGGTGGTAATTATAATTACCTGTATAGATTGTTTAGGAAGTCTAAT   22942
gb:MT027064|Organism:Severe      CTTGATTCTAAGGTTGGTGGTAATTATAATTACCTGTATAGATTGTTTAGGAAGTCTAAT   22942
gb:MN994468|Organism:Severe      CTTGATTCTAAGGTTGGTGGTAATTATAATTACCTGTATAGATTGTTTAGGAAGTCTAAT   22942
gb:MT072688|Organism:Severe      CTTGATTCTAAGGTTGGTGGTAATTATAATTACCTGTATAGATTGTTTAGGAAGTCTAAT   22927
gb:MN996527|Organism:Severe      CTTGATTCTAAGGTTGGTGGTAATTATAATTACCTGTATAGATTGTTTAGGAAGTCTAAT   22909
gb:MT093631|Organism:Severe      CTTGATTCTAAGGTTGGTGGTAATTATAATTACCTGTATAGATTGTTTAGGAAGTCTAAT   22980
gb:MT106053|Organism:Severe      CTTGATTCTAAGGTTGGTGGTAATTATAATTACCTGTATAGATTGTTTAGGAAGTCTAAT   22942
gb:MT019533|Organism:Severe      CTTGATTCTAAGGTTGGTGGTAATTATAATTACCTGTATAGATTGTTTAGGAAGTCTAAT   22942
```

```
gb:MT019531|Organism:Severe      CTTGATTCTAAGGTTGGTGGTAATTATAATTACCTGTATAGATTGTTTAGGAAGTCTAAT   22942
gb:MN996528|Organism:Severe      CTTGATTCTAAGGTTGGTGGTAATTATAATTACCTGTATAGATTGTTTAGGAAGTCTAAT   22942
gb:MN996530|Organism:Severe      CTTGATTCTAAGGTTGGTGGTAATTATAATTACCTGTATAGATTGTTTAGGAAGTCTAAT   22928
gb:MN908947|Organism:Severe      CTTGATTCTAAGGTTGGTGGTAATTATAATTACCTGTATAGATTGTTTAGGAAGTCTAAT   22942
gb:MT019532|Organism:Severe      CTTGATTCTAAGGTTGGTGGTAATTATAATTACCTGTATAGATTGTTTAGGAAGTCTAAT   22942
                                 ************************************************************

gb:MT020781|Organism:Severe      CTCAAACCTTTTGAGAGAGATATTTCAACTGAAATCTATCAGGCCGGTAGCACACCTTGT   22990
gb:MT007544|Organism:Severe      CTCAAACCTTTTGAGAGAGATATTTCAACTGAAATCTATCAGGCCGGTAGCACACCTTGT   23002
gb:MN994467|Organism:Severe      CTCAAACCTTTTGAGAGAGATATTTCAACTGAAATCTATCAGGCCGGTAGCACACCTTGT   23002
gb:MT044257|Organism:Severe      CTCAAACCTTTTGAGAGAGATATTTCAACTGAAATCTATCAGGCCGGTAGCACACCTTGT   23002
gb:MT106054|Organism:Severe      CTCAAACCTTTTGAGAGAGATATTTCAACTGAAATCTATCAGGCCGGTAGCACACCTTGT   23002
gb:MT049951|Organism:Severe      CTCAAACCTTTTGAGAGAGATATTTCAACTGAAATCTATCAGGCCGGTAGCACACCTTGT   23002
gb:MN975262|Organism:Severe      CTCAAACCTTTTGAGAGAGATATTTCAACTGAAATCTATCAGGCCGGTAGCACACCTTGT   23002
gb:MT106052|Organism:Severe      CTCAAACCTTTTGAGAGAGATATTTCAACTGAAATCTATCAGGCCGGTAGCACACCTTGT   23002
gb:LC522975|Organism:Severe      CTCAAACCTTTTGAGAGAGATATTTCAACTGAAATCTATCAGGCCGGTAGCACACCTTGT   22999
gb:LC522973|Organism:Severe      CTCAAACCTTTTGAGAGAGATATTTCAACTGAAATCTATCAGGCCGGTAGCACACCTTGT   22999
gb:LC522974|Organism:Severe      CTCAAACCTTTTGAGAGAGATATTTCAACTGAAATCTATCAGGCCGGTAGCACACCTTGT   22999
gb:MN985325|Organism:Severe      CTCAAACCTTTTGAGAGAGATATTTCAACTGAAATCTATCAGGCCGGTAGCACACCTTGT   23002
gb:MT020881|Organism:Severe      CTCAAACCTTTTGAGAGAGATATTTCAACTGAAATCTATCAGGCCGGTAGCACACCTTGT   23002
gb:MT020880|Organism:Severe      CTCAAACCTTTTGAGAGAGATATTTCAACTGAAATCTATCAGGCCGGTAGCACACCTTGT   23002
gb:MT066175|Organism:Severe      CTCAAACCTTTTGAGAGAGATATTTCAACTGAAATCTATCAGGCCGGTAGCACACCTTGT   23002
gb:MN997409|Organism:Severe      CTCAAACCTTTTGAGAGAGATATTTCAACTGAAATCTATCAGGCCGGTAGCACACCTTGT   23002
gb:MN938384|Organism:Severe      CTCAAACCTTTTGAGAGAGATATTTCAACTGAAATCTATCAGGCCGGTAGCACACCTTGT   22970
gb:MT044258|Organism:Severe      CTCAAACCTTTTGAGAGAGATATTTCAACTGAAATCTATCAGGCCGGTAGCACACCTTGT   22978
gb:MT039890|Organism:Severe      CTCAAACCTTTTGAGAGAGATATTTCAACTGAAATCTATCAGGCCGGTAGCACACCTTGT   23002
gb:MN988713|Organism:Severe      CTCAAACCTTTTGAGAGAGATATTTCAACTGAAATCTATCAGGCCGGTAGCACACCTTGT   23002
gb:LC521925|Organism:Severe      CTCAAACCTTTTGAGAGAGATATTTCAACTGAAATCTATCAGGCCGGTAGCACACCTTGT   22975
gb:MT093571|Organism:Severe      CTCAAACCTTTTGAGAGAGATATTTCAACTGAAATCTATCAGGCCGGTAGCACACCTTGT   23002
gb:MT039887|Organism:Severe      CTCAAACCTTTTGAGAGAGATATTTCAACTGAAATCTATCAGGCCGGTAGCACACCTTGT   22999
gb:MT019530|Organism:Severe      CTCAAACCTTTTGAGAGAGATATTTCAACTGAAATCTATCAGGCCGGTAGCACACCTTGT   23002
gb:MT039888|Organism:Severe      CTCAAACCTTTTGAGAGAGATATTTCAACTGAAATCTATCAGGCCGGTAGCACACCTTGT   23002
gb:LC522972|Organism:Severe      CTCAAACCTTTTGAGAGAGATATTTCAACTGAAATCTATCAGGCCGGTAGCACACCTTGT   22999
gb:MT027063|Organism:Severe      CTCAAACCTTTTGAGAGAGATATTTCAACTGAAATCTATCAGGCCGGTAGCACACCTTGT   23002
gb:MT027062|Organism:Severe      CTCAAACCTTTTGAGAGAGATATTTCAACTGAAATCTATCAGGCCGGTAGCACACCTTGT   23002
gb:MT019529|Organism:Severe      CTCAAACCTTTTGAGAGAGATATTTCAACTGAAATCTATCAGGCCGGTAGCACACCTTGT   23002
gb:MN996529|Organism:Severe      CTCAAACCTTTTGAGAGAGATATTTCAACTGAAATCTATCAGGCCGGTAGCACACCTTGT   22990
gb:MN996531|Organism:Severe      CTCAAACCTTTTGAGAGAGATATTTCAACTGAAATCTATCAGGCCGGTAGCACACCTTGT   22989
gb:MT066176|Organism:Severe      CTCAAACCTTTTGAGAGAGATATTTCAACTGAAATCTATCAGGCCGGTAGCACACCTTGT   23002
gb:MT027064|Organism:Severe      CTCAAACCTTTTGAGAGAGATATTTCAACTGAAATCTATCAGGCCGGTAGCACACCTTGT   23002
gb:MN994468|Organism:Severe      CTCAAACCTTTTGAGAGAGATATTTCAACTGAAATCTATCAGGCCGGTAGCACACCTTGT   23002
gb:MT072688|Organism:Severe      CTCAAACCTTTTGAGAGAGATATTTCAACTGAAATCTATCAGGCCGGTAGCACACCTTGT   22987
gb:MN996527|Organism:Severe      CTCAAACCTTTTGAGAGAGATATTTCAACTGAAATCTATCAGGCCGGTAGCACACCTTGT   22969
gb:MT093631|Organism:Severe      CTCAAACCTTTTGAGAGAGATATTTCAACTGAAATCTATCAGGCCGGTAGCACACCTTGT   23040
gb:MT106053|Organism:Severe      CTCAAACCTTTTGAGAGAGATATTTCAACTGAAATCTATCAGGCCGGTAGCACACCTTGT   23002
gb:MT019533|Organism:Severe      CTCAAACCTTTTGAGAGAGATATTTCAACTGAAATCTATCAGGCCGGTAGCACACCTTGT   23002
gb:MT019531|Organism:Severe      CTCAAACCTTTTGAGAGAGATATTTCAACTGAAATCTATCAGGCCGGTAGCACACCTTGT   23002
gb:MN996528|Organism:Severe      CTCAAACCTTTTGAGAGAGATATTTCAACTGAAATCTATCAGGCCGGTAGCACACCTTGT   23002
gb:MN996530|Organism:Severe      CTCAAACCTTTTGAGAGAGATATTTCAACTGAAATCTATCAGGCCGGTAGCACACCTTGT   22988
gb:MN908947|Organism:Severe      CTCAAACCTTTTGAGAGAGATATTTCAACTGAAATCTATCAGGCCGGTAGCACACCTTGT   23002
gb:MT019532|Organism:Severe      CTCAAACCTTTTGAGAGAGATATTTCAACTGAAATCTATCAGGCCGGTAGCACACCTTGT   23002
                                 ************************************************************

gb:MT020781|Organism:Severe      AATGGTGTTGAAGGTTTTAATTGTTACTTTCCTTTACAATCATATGGTTTCCAACCCACT   23050
gb:MT007544|Organism:Severe      AATGGTGTTGAAGGTTTTAATTGTTACTTTCCTTTACAATCATATGGTTTCCAACCCACT   23062
gb:MN994467|Organism:Severe      AATGGTGTTGAAGGTTTTAATTGTTACTTTCCTTTACAATCATATGGTTTCCAACCCACT   23062
gb:MT044257|Organism:Severe      AATGGTGTTGAAGGTTTTAATTGTTACTTTCCTTTACAATCATATGGTTTCCAACCCACT   23062
gb:MT106054|Organism:Severe      AATGGTGTTGAAGGTTTTAATTGTTACTTTCCTTTACAATCATATGGTTTCCAACCCACT   23062
gb:MT049951|Organism:Severe      AATGGTGTTGAAGGTTTTAATTGTTACTTTCCTTTACAATCATATGGTTTCCAACCCACT   23062
gb:MN975262|Organism:Severe      AATGGTGTTGAAGGTTTTAATTGTTACTTTCCTTTACAATCATATGGTTTCCAACCCACT   23062
gb:MT106052|Organism:Severe      AATGGTGTTGAAGGTTTTAATTGTTACTTTCCTTTACAATCATATGGTTTCCAACCCACT   23062
gb:LC522975|Organism:Severe      AATGGTGTTGAAGGTTTTAATTGTTACTTTCCTTTACAATCATATGGTTTCCAACCCACT   23059
gb:LC522973|Organism:Severe      AATGGTGTTGAAGGTTTTAATTGTTACTTTCCTTTACAATCATATGGTTTCCAACCCACT   23059
gb:LC522974|Organism:Severe      AATGGTGTTGAAGGTTTTAATTGTTACTTTCCTTTACAATCATATGGTTTCCAACCCACT   23059
gb:MN985325|Organism:Severe      AATGGTGTTGAAGGTTTTAATTGTTACTTTCCTTTACAATCATATGGTTTCCAACCCACT   23062
```

```
gb:MT020881|Organism:Severe      AATGGTGTTGAAGGTTTTAATTGTTACTTTCCTTTACAATCATATGGTTTCCAACCCACT   23062
gb:MT020880|Organism:Severe      AATGGTGTTGAAGGTTTTAATTGTTACTTTCCTTTACAATCATATGGTTTCCAACCCACT   23062
gb:MT066175|Organism:Severe      AATGGTGTTGAAGGTTTTAATTGTTACTTTCCTTTACAATCATATGGTTTCCAACCCACT   23062
gb:MN997409|Organism:Severe      AATGGTGTTGAAGGTTTTAATTGTTACTTTCCTTTACAATCATATGGTTTCCAACCCACT   23062
gb:MN938384|Organism:Severe      AATGGTGTTGAAGGTTTTAATTGTTACTTTCCTTTACAATCATATGGTTTCCAACCCACT   23030
gb:MT044258|Organism:Severe      AATGGTGTTGAAGGTTTTAATTGTTACTTTCCTTTACAATCATATGGTTTCCAACCCACT   23038
gb:MT039890|Organism:Severe      AATGGTGTTGAAGGTTTTAATTGTTACTTTCCTTTACAATCATATGGTTTCCAACCCACT   23062
gb:MN988713|Organism:Severe      AATGGTGTTGAAGGTTTTAATTGTTACTTTCCTTTACAATCATATGGTTTCCAACCCACT   23062
gb:LC521925|Organism:Severe      AATGGTGTTGAAGGTTTTAATTGTTACTTTCCTTTACAATCATATGGTTTCCAACCCACT   23035
gb:MT093571|Organism:Severe      AATGGTGTTGAAGGTTTTAATTGTTACTTTCCTTTACAATCATATGGTTTCCAACCCACT   23062
gb:MT039887|Organism:Severe      AATGGTGTTGAAGGTTTTAATTGTTACTTTCCTTTACAATCATATGGTTTCCAACCCACT   23059
gb:MT019530|Organism:Severe      AATGGTGTTGAAGGTTTTAATTGTTACTTTCCTTTACAATCATATGGTTTCCAACCCACT   23062
gb:MT039888|Organism:Severe      AATGGTGTTGAAGGTTTTAATTGTTACTTTCCTTTACAATCATATGGTTTCCAACCCACT   23062
gb:LC522972|Organism:Severe      AATGGTGTTGAAGGTTTTAATTGTTACTTTCCTTTACAATCATATGGTTTCCAACCCACT   23059
gb:MT027063|Organism:Severe      AATGGTGTTGAAGGTTTTAATTGTTACTTTCCTTTACAATCATATGGTTTCCAACCCACT   23062
gb:MT027062|Organism:Severe      AATGGTGTTGAAGGTTTTAATTGTTACTTTCCTTTACAATCATATGGTTTCCAACCCACT   23062
gb:MT019529|Organism:Severe      AATGGTGTTGAAGGTTTTAATTGTTACTTTCCTTTACAATCATATGGTTTCCAACCCACT   23062
gb:MN996529|Organism:Severe      AATGGTGTTGAAGGTTTTAATTGTTACTTTCCTTTACAATCATATGGTTTCCAACCCACT   23050
gb:MN996531|Organism:Severe      AATGGTGTTGAAGGTTTTAATTGTTACTTTCCTTTACAATCATATGGTTTCCAACCCACT   23049
gb:MT066176|Organism:Severe      AATGGTGTTGAAGGTTTTAATTGTTACTTTCCTTTACAATCATATGGTTTCCAACCCACT   23062
gb:MT027064|Organism:Severe      AATGGTGTTGAAGGTTTTAATTGTTACTTTCCTTTACAATCATATGGTTTCCAACCCACT   23062
gb:MN994468|Organism:Severe      AATGGTGTTGAAGGTTTTAATTGTTACTTTCCTTTACAATCATATGGTTTCCAACCCACT   23062
gb:MT072688|Organism:Severe      AATGGTGTTGAAGGTTTTAATTGTTACTTTCCTTTACAATCATATGGTTTCCAACCCACT   23047
gb:MN996527|Organism:Severe      AATGGTGTTGAAGGTTTTAATTGTTACTTTCCTTTACAATCATATGGTTTCCAACCCACT   23029
gb:MT093631|Organism:Severe      AATGGTGTTGAAGGTTTTAATTGTTACTTTCCTTTACAATCATATGGTTTCCAACCCACT   23100
gb:MT106053|Organism:Severe      AATGGTGTTGAAGGTTTTAATTGTTACTTTCCTTTACAATCATATGGTTTCCAACCCACT   23062
gb:MT019533|Organism:Severe      AATGGTGTTGAAGGTTTTAATTGTTACTTTCCTTTACAATCATATGGTTTCCAACCCACT   23062
gb:MT019531|Organism:Severe      AATGGTGTTGAAGGTTTTAATTGTTACTTTCCTTTACAATCATATGGTTTCCAACCCACT   23062
gb:MN996528|Organism:Severe      AATGGTGTTGAAGGTTTTAATTGTTACTTTCCTTTACAATCATATGGTTTCCAACCCACT   23062
gb:MN996530|Organism:Severe      AATGGTGTTGAAGGTTTTAATTGTTACTTTCCTTTACAATCATATGGTTTCCAACCCACT   23048
gb:MN908947|Organism:Severe      AATGGTGTTGAAGGTTTTAATTGTTACTTTCCTTTACAATCATATGGTTTCCAACCCACT   23062
gb:MT019532|Organism:Severe      AATGGTGTTGAAGGTTTTAATTGTTACTTTCCTTTACAATCATATGGTTTCCAACCCACT   23062
                                 ************************************************************

gb:MT020781|Organism:Severe      AATGGTGTTGGTTACCAACCATACAGAGTAGTAGTACTTTCTTTTGAACTTCTACATGCA   23110
gb:MT007544|Organism:Severe      AATGGTGTTGGTTACCAACCATACAGAGTAGTAGTACTTTCTTTTGAACTTCTACATGCA   23122
gb:MN994467|Organism:Severe      AATGGTGTTGGTTACCAACCATACAGAGTAGTAGTACTTTCTTTTGAACTTCTACATGCA   23122
gb:MT044257|Organism:Severe      AATGGTGTTGGTTACCAACCATACAGAGTAGTAGTACTTTCTTTTGAACTTCTACATGCA   23122
gb:MT106054|Organism:Severe      AATGGTGTTGGTTACCAACCATACAGAGTAGTAGTACTTTCTTTTGAACTTCTACATGCA   23122
gb:MT049951|Organism:Severe      AATGGTGTTGGTTACCAACCATACAGAGTAGTAGTACTTTCTTTTGAACTTCTACATGCA   23122
gb:MN975262|Organism:Severe      AATGGTGTTGGTTACCAACCATACAGAGTAGTAGTACTTTCTTTTGAACTTCTACATGCA   23122
gb:MT106052|Organism:Severe      AATGGTGTTGGTTACCAACCATACAGAGTAGTAGTACTTTCTTTTGAACTTCTACATGCA   23122
gb:LC522975|Organism:Severe      AATGGTGTTGGTTACCAACCATACAGAGTAGTAGTACTTTCTTTTGAACTTCTACATGCA   23119
gb:LC522973|Organism:Severe      AATGGTGTTGGTTACCAACCATACAGAGTAGTAGTACTTTCTTTTGAACTTCTACATGCA   23119
gb:LC522974|Organism:Severe      AATGGTGTTGGTTACCAACCATACAGAGTAGTAGTACTTTCTTTTGAACTTCTACATGCA   23119
gb:MN985325|Organism:Severe      AATGGTGTTGGTTACCAACCATACAGAGTAGTAGTACTTTCTTTTGAACTTCTACATGCA   23122
gb:MT020881|Organism:Severe      AATGGTGTTGGTTACCAACCATACAGAGTAGTAGTACTTTCTTTTGAACTTCTACATGCA   23122
gb:MT020880|Organism:Severe      AATGGTGTTGGTTACCAACCATACAGAGTAGTAGTACTTTCTTTTGAACTTCTACATGCA   23122
gb:MT066175|Organism:Severe      AATGGTGTTGGTTACCAACCATACAGAGTAGTAGTACTTTCTTTTGAACTTCTACATGCA   23122
gb:MN997409|Organism:Severe      AATGGTGTTGGTTACCAACCATACAGAGTAGTAGTACTTTCTTTTGAACTTCTACATGCA   23122
gb:MN938384|Organism:Severe      AATGGTGTTGGTTACCAACCATACAGAGTAGTAGTACTTTCTTTTGAACTTCTACATGCA   23090
gb:MT044258|Organism:Severe      AATGGTGTTGGTTACCAACCATACAGAGTAGTAGTACTTTCTTTTGAACTTCTACATGCA   23098
gb:MT039890|Organism:Severe      AATGGTGTTGGTTACCAACCATACAGAGTAGTAGTACTTTCTTTTGAACTTCTACATGCA   23122
gb:MN988713|Organism:Severe      AATGGTGTTGGTTACCAACCATACAGAGTAGTAGTACTTTCTTTTGAACTTCTACATGCA   23122
gb:LC521925|Organism:Severe      AATGGTGTTGGTTACCAACCATACAGAGTAGTAGTACTTTCTTTTGAACTTCTACATGCA   23095
gb:MT093571|Organism:Severe      AATGGTGTTGGTTACCAACCATACAGAGTAGTAGTACTTTCTTTTGAACTTCTACATGCA   23122
gb:MT039887|Organism:Severe      AATGGTGTTGGTTACCAACCATACAGAGTAGTAGTACTTTCTTTTGAACTTCTACATGCA   23119
gb:MT019530|Organism:Severe      AATGGTGTTGGTTACCAACCATACAGAGTAGTAGTACTTTCTTTTGAACTTCTACATGCA   23122
gb:MT039888|Organism:Severe      AATGGTGTTGGTTACCAACCATACAGAGTAGTAGTACTTTCTTTTGAACTTCTACATGCA   23122
gb:LC522972|Organism:Severe      AATGGTGTTGGTTACCAACCATACAGAGTAGTAGTACTTTCTTTTGAACTTCTACATGCA   23119
gb:MT027063|Organism:Severe      AATGGTGTTGGTTACCAACCATACAGAGTAGTAGTACTTTCTTTTGAACTTCTACATGCA   23122
gb:MT027062|Organism:Severe      AATGGTGTTGGTTACCAACCATACAGAGTAGTAGTACTTTCTTTTGAACTTCTACATGCA   23122
gb:MT019529|Organism:Severe      AATGGTGTTGGTTACCAACCATACAGAGTAGTAGTACTTTCTTTTGAACTTCTACATGCA   23122
gb:MN996529|Organism:Severe      AATGGTGTTGGTTACCAACCATACAGAGTAGTAGTACTTTCTTTTGAACTTCTACATGCA   23110
gb:MN996531|Organism:Severe      AATGGTGTTGGTTACCAACCATACAGAGTAGTAGTACTTTCTTTTGAACTTCTACATGCA   23109
```

```
gb:MT066176|Organism:Severe      AATGGTGTTGGTTACCAACCATACAGAGTAGTAGTACTTTCTTTTGAACTTCTACATGCA   23122
gb:MT027064|Organism:Severe      AATGGTGTTGGTTACCAACCATACAGAGTAGTAGTACTTTCTTTTGAACTTCTACATGCA   23122
gb:MN994468|Organism:Severe      AATGGTGTTGGTTACCAACCATACAGAGTAGTAGTACTTTCTTTTGAACTTCTACATGCA   23122
gb:MT072688|Organism:Severe      AATGGTGTTGGTTACCAACCATACAGAGTAGTAGTACTTTCTTTTGAACTTCTACATGCA   23107
gb:MN996527|Organism:Severe      AATGGTGTTGGTTACCAACCATACAGAGTAGTAGTACTTTCTTTTGAACTTCTACATGCA   23089
gb:MT093631|Organism:Severe      AATGGTGTTGGTTACCAACCATACAGAGTAGTAGTACTTTCTTTTGAACTTCTACATGCA   23160
gb:MT106053|Organism:Severe      AATGGTGTTGGTTACCAACCATACAGAGTAGTAGTACTTTCTTTTGAACTTCTACATGCA   23122
gb:MT019533|Organism:Severe      AATGGTGTTGGTTACCAACCATACAGAGTAGTAGTACTTTCTTTTGAACTTCTACATGCA   23122
gb:MT019531|Organism:Severe      AATGGTGTTGGTTACCAACCATACAGAGTAGTAGTACTTTCTTTTGAACTTCTACATGCA   23122
gb:MN996528|Organism:Severe      AATGGTGTTGGTTACCAACCATACAGAGTAGTAGTACTTTCTTTTGAACTTCTACATGCA   23122
gb:MN996530|Organism:Severe      AATGGTGTTGGTTACCAACCATACAGAGTAGTAGTACTTTCTTTTGAACTTCTACATGCA   23108
gb:MN908947|Organism:Severe      AATGGTGTTGGTTACCAACCATACAGAGTAGTAGTACTTTCTTTTGAACTTCTACATGCA   23122
gb:MT019532|Organism:Severe      AATGGTGTTGGTTACCAACCATACAGAGTAGTAGTACTTTCTTTTGAACTTCTACATGCA   23122
                                 ************************************************************

gb:MT020781|Organism:Severe      CCAGCAACTGTTTGTGGACCTAAAAAGTCTACTAATTTGGTTAAAAACAAATGTGTCAAT   23170
gb:MT007544|Organism:Severe      CCAGCAACTGTTTGTGGACCTAAAAAGTCTACTAATTTGGTTAAAAACAAATGTGTCAAT   23182
gb:MN994467|Organism:Severe      CCAGCAACTGTTTGTGGACCTAAAAAGTCTACTAATTTGGTTAAAAACAAATGTGTCAAT   23182
gb:MT044257|Organism:Severe      CCAGCAACTGTTTGTGGACCTAAAAAGTCTACTAATTTGGTTAAAAACAAATGTGTCAAT   23182
gb:MT106054|Organism:Severe      CCAGCAACTGTTTGTGGACCTAAAAAGTCTACTAATTTGGTTAAAAACAAATGTGTCAAT   23182
gb:MT049951|Organism:Severe      CCAGCAACTGTTTGTGGACCTAAAAAGTCTACTAATTTGGTTAAAAACAAATGTGTCAAT   23182
gb:MN975262|Organism:Severe      CCAGCAACTGTTTGTGGACCTAAAAAGTCTACTAATTTGGTTAAAAACAAATGTGTCAAT   23182
gb:MT106052|Organism:Severe      CCAGCAACTGTTTGTGGACCTAAAAAGTCTACTAATTTGGTTAAAAACAAATGTGTCAAT   23182
gb:LC522975|Organism:Severe      CCAGCAACTGTTTGTGGACCTAAAAAGTCTACTAATTTGGTTAAAAACAAATGTGTCAAT   23179
gb:LC522973|Organism:Severe      CCAGCAACTGTTTGTGGACCTAAAAAGTCTACTAATTTGGTTAAAAACAAATGTGTCAAT   23179
gb:LC522974|Organism:Severe      CCAGCAACTGTTTGTGGACCTAAAAAGTCTACTAATTTGGTTAAAAACAAATGTGTCAAT   23179
gb:MN985325|Organism:Severe      CCAGCAACTGTTTGTGGACCTAAAAAGTCTACTAATTTGGTTAAAAACAAATGTGTCAAT   23182
gb:MT020881|Organism:Severe      CCAGCAACTGTTTGTGGACCTAAAAAGTCTACTAATTTGGTTAAAAACAAATGTGTCAAT   23182
gb:MT020880|Organism:Severe      CCAGCAACTGTTTGTGGACCTAAAAAGTCTACTAATTTGGTTAAAAACAAATGTGTCAAT   23182
gb:MT066175|Organism:Severe      CCAGCAACTGTTTGTGGACCTAAAAAGTCTACTAATTTGGTTAAAAACAAATGTGTCAAT   23182
gb:MN997409|Organism:Severe      CCAGCAACTGTTTGTGGACCTAAAAAGTCTACTAATTTGGTTAAAAACAAATGTGTCAAT   23182
gb:MN938384|Organism:Severe      CCAGCAACTGTTTGTGGACCTAAAAAGTCTACTAATTTGGTTAAAAACAAATGTGTCAAT   23150
gb:MT044258|Organism:Severe      CCAGCAACTGTTTGTGGACCTAAAAAGTCTACTAATTTGGTTAAAAACAAATGTGTCAAT   23158
gb:MT039890|Organism:Severe      CCAGCAACTGTTTGTGGACCTAAAAAGTCTACTAATTTGGTTAAAAACAAATGTGTCAAT   23182
gb:MN988713|Organism:Severe      CCAGCAACTGTTTGTGGACCTAAAAAGTCTACTAATTTGGTTAAAAACAAATGTGTCAAT   23182
gb:LC521925|Organism:Severe      CCAGCAACTGTTTGTGGACCTAAAAAGTCTACTAATTTGGTTAAAAACAAATGTGTCAAT   23155
gb:MT093571|Organism:Severe      CCAGCAACTGTTTGTGGACCTAAAAAGTCTACTAATTTGGTTAAAAACAAATGTGTCAAT   23182
gb:MT039887|Organism:Severe      CCAGCAACTGTTTGTGGACCTAAAAAGTCTACTAATTTGGTTAAAAACAAATGTGTCAAT   23179
gb:MT019530|Organism:Severe      CCAGCAACTGTTTGTGGACCTAAAAAGTCTACTAATTTGGTTAAAAACAAATGTGTCAAT   23182
gb:MT039888|Organism:Severe      CCAGCAACTGTTTGTGGACCTAAAAAGTCTACTAATTTGGTTAAAAACAAATGTGTCAAT   23182
gb:LC522972|Organism:Severe      CCAGCAACTGTTTGTGGACCTAAAAAGTCTACTAATTTGGTTAAAAACAAATGTGTCAAT   23179
gb:MT027063|Organism:Severe      CCAGCAACTGTTTGTGGACCTAAAAAGTCTACTAATTTGGTTAAAAACAAATGTGTCAAT   23182
gb:MT027062|Organism:Severe      CCAGCAACTGTTTGTGGACCTAAAAAGTCTACTAATTTGGTTAAAAACAAATGTGTCAAT   23182
gb:MT019529|Organism:Severe      CCAGCAACTGTTTGTGGACCTAAAAAGTCTACTAATTTGGTTAAAAACAAATGTGTCAAT   23182
gb:MN996529|Organism:Severe      CCAGCAACTGTTTGTGGACCTAAAAAGTCTACTAATTTGGTTAAAAACAAATGTGTCAAT   23170
gb:MN996531|Organism:Severe      CCAGCAACTGTTTGTGGACCTAAAAAGTCTACTAATTTGGTTAAAAACAAATGTGTCAAT   23169
gb:MT066176|Organism:Severe      CCAGCAACTGTTTGTGGACCTAAAAAGTCTACTAATTTGGTTAAAAACAAATGTGTCAAT   23182
gb:MT027064|Organism:Severe      CCAGCAACTGTTTGTGGACCTAAAAAGTCTACTAATTTGGTTAAAAACAAATGTGTCAAT   23182
gb:MN994468|Organism:Severe      CCAGCAACTGTTTGTGGACCTAAAAAGTCTACTAATTTGGTTAAAAACAAATGTGTCAAT   23182
gb:MT072688|Organism:Severe      CCAGCAACTGTTTGTGGACCTAAAAAGTCTACTAATTTGGTTAAAAACAAATGTGTCAAT   23167
gb:MN996527|Organism:Severe      CCAGCAACTGTTTGTGGACCTAAAAAGTCTACTAATTTGGTTAAAAACAAATGTGTCAAT   23149
gb:MT093631|Organism:Severe      CCAGCAACTGTTTGTGGACCTAAAAAGTCTACTAATTTGGTTAAAAACAAATGTGTCAAT   23220
gb:MT106053|Organism:Severe      CCAGCAACTGTTTGTGGACCTAAAAAGTCTACTAATTTGGTTAAAAACAAATGTGTCAAT   23182
gb:MT019533|Organism:Severe      CCAGCAACTGTTTGTGGACCTAAAAAGTCTACTAATTTGGTTAAAAACAAATGTGTCAAT   23182
gb:MT019531|Organism:Severe      CCAGCAACTGTTTGTGGACCTAAAAAGTCTACTAATTTGGTTAAAAACAAATGTGTCAAT   23182
gb:MN996528|Organism:Severe      CCAGCAACTGTTTGTGGACCTAAAAAGTCTACTAATTTGGTTAAAAACAAATGTGTCAAT   23182
gb:MN996530|Organism:Severe      CCAGCAACTGTTTGTGGACCTAAAAAGTCTACTAATTTGGTTAAAAACAAATGTGTCAAT   23168
gb:MN908947|Organism:Severe      CCAGCAACTGTTTGTGGACCTAAAAAGTCTACTAATTTGGTTAAAAACAAATGTGTCAAT   23182
gb:MT019532|Organism:Severe      CCAGCAACTGTTTGTGGACCTAAAAAGTCTACTAATTTGGTTAAAAACAAATGTGTCAAT   23182
                                 ************************************************************

gb:MT020781|Organism:Severe      TTCAACTTCAATGGTTTAACAGGCACAGGTGTTCTTACTGAGTCTAACAAAAAGTTTCTG   23230
gb:MT007544|Organism:Severe      TTCAACTTCAATGGTTTAACAGGCACAGGTGTTCTTACTGAGTCTAACAAAAAGTTTCTG   23242
gb:MN994467|Organism:Severe      TTCAACTTCAATGGTTTAACAGGCACAGGTGTTCTTACTGAGTCTAACAAAAAGTTTCTG   23242
gb:MT044257|Organism:Severe      TTCAACTTCAATGGTTTAACAGGCACAGGTGTTCTTACTGAGTCTAACAAAAAGTTTCTG   23242
```

```
gb:MT106054|Organism:Severe      TTCAACTTCAATGGTTTAACAGGCACAGGTGTTCTTACTGAGTCTAACAAAAAGTTTCTG   23242
gb:MT049951|Organism:Severe      TTCAACTTCAATGGTTTAACAGGCACAGGTGTTCTTACTGAGTCTAACAAAAAGTTTCTG   23242
gb:MN975262|Organism:Severe      TTCAACTTCAATGGTTTAACAGGCACAGGTGTTCTTACTGAGTCTAACAAAAAGTTTCTG   23242
gb:MT106052|Organism:Severe      TTCAACTTCAATGGTTTAACAGGCACAGGTGTTCTTACTGAGTCTAACAAAAAGTTTCTG   23242
gb:LC522975|Organism:Severe      TTCAACTTCAATGGTTTAACAGGCACAGGTGTTCTTACTGAGTCTAACAAAAAGTTTCTG   23239
gb:LC522973|Organism:Severe      TTCAACTTCAATGGTTTAACAGGCACAGGTGTTCTTACTGAGTCTAACAAAAAGTTTCTG   23239
gb:LC522974|Organism:Severe      TTCAACTTCAATGGTTTAACAGGCACAGGTGTTCTTACTGAGTCTAACAAAAAGTTTCTG   23239
gb:MN985325|Organism:Severe      TTCAACTTCAATGGTTTAACAGGCACAGGTGTTCTTACTGAGTCTAACAAAAAGTTTCTG   23242
gb:MT020881|Organism:Severe      TTCAACTTCAATGGTTTAACAGGCACAGGTGTTCTTACTGAGTCTAACAAAAAGTTTCTG   23242
gb:MT020880|Organism:Severe      TTCAACTTCAATGGTTTAACAGGCACAGGTGTTCTTACTGAGTCTAACAAAAAGTTTCTG   23242
gb:MT066175|Organism:Severe      TTCAACTTCAATGGTTTAACAGGCACAGGTGTTCTTACTGAGTCTAACAAAAAGTTTCTG   23242
gb:MN997409|Organism:Severe      TTCAACTTCAATGGTTTAACAGGCACAGGTGTTCTTACTGAGTCTAACAAAAAGTTTCTG   23242
gb:MN938384|Organism:Severe      TTCAACTTCAATGGTTTAACAGGCACAGGTGTTCTTACTGAGTCTAACAAAAAGTTTCTG   23210
gb:MT044258|Organism:Severe      TTCAACTTCAATGGTTTAACAGGCACAGGTGTTCTTACTGAGTCTAACAAAAAGTTTCTG   23218
gb:MT039890|Organism:Severe      TTCAACTTCAATGGTTTAACAGGCACAGGTGTTCTTACTGAGTCTAACAAAAAGTTTCTG   23242
gb:MN988713|Organism:Severe      TTCAACTTCAATGGTTTAACAGGCACAGGTGTTCTTACTGAGTCTAACAAAAAGTTTCTG   23242
gb:LC521925|Organism:Severe      TTCAACTTCAATGGTTTAACAGGCACAGGTGTTCTTACTGAGTCTAACAAAAAGTTTCTG   23215
gb:MT093571|Organism:Severe      TTCAACTTCAATGGTTTAACAGGCACAGGTGTTCTTACTGAGTCTAACAAAAAGTTTCTG   23242
gb:MT039887|Organism:Severe      TTCAACTTCAATGGTTTAACAGGCACAGGTGTTCTTACTGAGTCTAACAAAAAGTTTCTG   23239
gb:MT019530|Organism:Severe      TTCAACTTCAATGGTTTAACAGGCACAGGTGTTCTTACTGAGTCTAACAAAAAGTTTCTG   23242
gb:MT039888|Organism:Severe      TTCAACTTCAATGGTTTAACAGGCACAGGTGTTCTTACTGAGTCTAACAAAAAGTTTCTG   23242
gb:LC522972|Organism:Severe      TTCAACTTCAATGGTTTAACAGGCACAGGTGTTCTTACTGAGTCTAACAAAAAGTTTCTG   23239
gb:MT027063|Organism:Severe      TTCAACTTCAATGGTTTAACAGGCACAGGTGTTCTTACTGAGTCTAACAAAAAGTTTCTG   23242
gb:MT027062|Organism:Severe      TTCAACTTCAATGGTTTAACAGGCACAGGTGTTCTTACTGAGTCTAACAAAAAGTTTCTG   23242
gb:MT019529|Organism:Severe      TTCAACTTCAATGGTTTAACAGGCACAGGTGTTCTTACTGAGTCTAACAAAAAGTTTCTG   23242
gb:MN996529|Organism:Severe      TTCAACTTCAATGGTTTAACAGGCACAGGTGTTCTTACTGAGTCTAACAAAAAGTTTCTG   23230
gb:MN996531|Organism:Severe      TTCAACTTCAATGGTTTAACAGGCACAGGTGTTCTTACTGAGTCTAACAAAAAGTTTCTG   23229
gb:MT066176|Organism:Severe      TTCAACTTCAATGGTTTAACAGGCACAGGTGTTCTTACTGAGTCTAACAAAAAGTTTCTG   23242
gb:MT027064|Organism:Severe      TTCAACTTCAATGGTTTAACAGGCACAGGTGTTCTTACTGAGTCTAACAAAAAGTTTCTG   23242
gb:MN994468|Organism:Severe      TTCAACTTCAATGGTTTAACAGGCACAGGTGTTCTTACTGAGTCTAACAAAAAGTTTCTG   23242
gb:MT072688|Organism:Severe      TTCAACTTCAATGGTTTAACAGGCACAGGTGTTCTTACTGAGTCTAACAAAAAGTTTCTG   23227
gb:MN996527|Organism:Severe      TTCAACTTCAATGGTTTAACAGGCACAGGTGTTCTTACTGAGTCTAACAAAAAGTTTCTG   23209
gb:MT093631|Organism:Severe      TTCAACTTCAATGGTTTAACAGGCACAGGTGTTCTTACTGAGTCTAACAAAAAGTTTCTG   23280
gb:MT106053|Organism:Severe      TTCAACTTCAATGGTTTAACAGGCACAGGTGTTCTTACTGAGTCTAACAAAAAGTTTCTG   23242
gb:MT019533|Organism:Severe      TTCAACTTCAATGGTTTAACAGGCACAGGTGTTCTTACTGAGTCTAACAAAAAGTTTCTG   23242
gb:MT019531|Organism:Severe      TTCAACTTCAATGGTTTAACAGGCACAGGTGTTCTTACTGAGTCTAACAAAAAGTTTCTG   23242
gb:MN996528|Organism:Severe      TTCAACTTCAATGGTTTAACAGGCACAGGTGTTCTTACTGAGTCTAACAAAAAGTTTCTG   23242
gb:MN996530|Organism:Severe      TTCAACTTCAATGGTTTAACAGGCACAGGTGTTCTTACTGAGTCTAACAAAAAGTTTCTG   23228
gb:MN908947|Organism:Severe      TTCAACTTCAATGGTTTAACAGGCACAGGTGTTCTTACTGAGTCTAACAAAAAGTTTCTG   23242
gb:MT019532|Organism:Severe      TTCAACTTCAATGGTTTAACAGGCACAGGTGTTCTTACTGAGTCTAACAAAAAGTTTCTG   23242
                                 ************************************************************

gb:MT020781|Organism:Severe      CCTTTCCAACAATTTGGCAGAGACATTGCTGACACTACTGATGCTGTCCGTGATCCACAG   23290
gb:MT007544|Organism:Severe      CCTTTCCAACAATTTGGCAGAGACATTGCTGACACTACTGATGCTGTCCGTGATCCACAG   23302
gb:MN994467|Organism:Severe      CCTTTCCAACAATTTGGCAGAGACATTGCTGACACTACTGATGCTGTCCGTGATCCACAG   23302
gb:MT044257|Organism:Severe      CCTTTCCAACAATTTGGCAGAGACATTGCTGACACTACTGATGCTGTCCGTGATCCACAG   23302
gb:MT106054|Organism:Severe      CCTTTCCAACAATTTGGCAGAGACATTGCTGACACTACTGATGCTGTCCGTGATCCACAG   23302
gb:MT049951|Organism:Severe      CCTTTCCAACAATTTGGCAGAGACATTGCTGACACTACTGATGCTGTCCGTGATCCACAG   23302
gb:MN975262|Organism:Severe      CCTTTCCAACAATTTGGCAGAGACATTGCTGACACTACTGATGCTGTCCGTGATCCACAG   23302
gb:MT106052|Organism:Severe      CCTTTCCAACAATTTGGCAGAGACATTGCTGACACTACTGATGCTGTCCGTGATCCACAG   23302
gb:LC522975|Organism:Severe      CCTTTCCAACAATTTGGCAGAGACATTGCTGACACTACTGATGCTGTCCGTGATCCACAG   23299
gb:LC522973|Organism:Severe      CCTTTCCAACAATTTGGCAGAGACATTGCTGACACTACTGATGCTGTCCGTGATCCACAG   23299
gb:LC522974|Organism:Severe      CCTTTCCAACAATTTGGCAGAGACATTGCTGACACTACTGATGCTGTCCGTGATCCACAG   23299
gb:MN985325|Organism:Severe      CCTTTCCAACAATTTGGCAGAGACATTGCTGACACTACTGATGCTGTCCGTGATCCACAG   23302
gb:MT020881|Organism:Severe      CCTTTCCAACAATTTGGCAGAGACATTGCTGACACTACTGATGCTGTCCGTGATCCACAG   23302
gb:MT020880|Organism:Severe      CCTTTCCAACAATTTGGCAGAGACATTGCTGACACTACTGATGCTGTCCGTGATCCACAG   23302
gb:MT066175|Organism:Severe      CCTTTCCAACAATTTGGCAGAGACATTGCTGACACTACTGATGCTGTCCGTGATCCACAG   23302
gb:MN997409|Organism:Severe      CCTTTCCAACAATTTGGCAGAGACATTGCTGACACTACTGATGCTGTCCGTGATCCACAG   23302
gb:MN938384|Organism:Severe      CCTTTCCAACAATTTGGCAGAGACATTGCTGACACTACTGATGCTGTCCGTGATCCACAG   23270
gb:MT044258|Organism:Severe      CCTTTCCAACAATTTGGCAGAGACATTGCTGACACTACTGATGCTGTCCGTGATCCACAG   23278
gb:MT039890|Organism:Severe      CCTTTCCAACAATTTGGCAGAGACATTGCTGACACTACTGATGCTGTCCGTGATCCACAG   23302
gb:MN988713|Organism:Severe      CCTTTCCAACAATTTGGCAGAGACATTGCTGACACTACTGATGCTGTCCGTGATCCACAG   23302
gb:LC521925|Organism:Severe      CCTTTCCAACAATTTGGCAGAGACATTGCTGACACTACTGATGCTGTCCGTGATCCACAG   23275
gb:MT093571|Organism:Severe      CCTTTCCAACAATTTGGCAGAGACATTGCTGACACTACTGATGCTGTCCGTGATCCACAG   23302
gb:MT039887|Organism:Severe      CCTTTCCAACAATTTGGCAGAGACATTGCTGACACTACTGATGCTGTCCGTGATCCACAG   23299
```

```
gb:MT019530|Organism:Severe      CCTTTCCAACAATTTGGCAGAGACATTGCTGACACTACTGATGCTGTCCGTGATCCACAG    23302
gb:MT039888|Organism:Severe      CCTTTCCAACAATTTGGCAGAGACATTGCTGACACTACTGATGCTGTCCGTGATCCACAG    23302
gb:LC522972|Organism:Severe      CCTTTCCAACAATTTGGCAGAGACATTGCTGACACTACTGATGCTGTCCGTGATCCACAG    23299
gb:MT027063|Organism:Severe      CCTTTCCAACAATTTGGCAGAGACATTGCTGACACTACTGATGCTGTCCGTGATCCACAG    23302
gb:MT027062|Organism:Severe      CCTTTCCAACAATTTGGCAGAGACATTGCTGACACTACTGATGCTGTCCGTGATCCACAG    23302
gb:MT019529|Organism:Severe      CCTTTCCAACAATTTGGCAGAGACATTGCTGACACTACTGATGCTGTCCGTGATCCACAG    23302
gb:MN996529|Organism:Severe      CCTTTCCAACAATTTGGCAGAGACATTGCTGACACTACTGATGCTGTCCGTGATCCACAG    23290
gb:MN996531|Organism:Severe      CCTTTCCAACAATTTGGCAGAGACATTGCTGACACTACTGATGCTGTCCGTGATCCACAG    23289
gb:MT066176|Organism:Severe      CCTTTCCAACAATTTGGCAGAGACATTGCTGACACTACTGATGCTGTCCGTGATCCACAG    23302
gb:MT027064|Organism:Severe      CCTTTCCAACAATTTGGCAGAGACATTGCTGACACTACTGATGCTGTCCGTGATCCACAG    23302
gb:MN994468|Organism:Severe      CCTTTCCAACAATTTGGCAGAGACATTGCTGACACTACTGATGCTGTCCGTGATCCACAG    23302
gb:MT072688|Organism:Severe      CCTTTCCAACAATTTGGCAGAGACATTGCTGACACTACTGATGCTGTCCGTGATCCACAG    23287
gb:MN996527|Organism:Severe      CCTTTCCAACAATTTGGCAGAGACATTGCTGACACTACTGATGCTGTCCGTGATCCACAG    23269
gb:MT093631|Organism:Severe      CCTTTCCAACAATTTGGCAGAGACATTGCTGACACTACTGATGCTGTCCGTGATCCACAG    23340
gb:MT106053|Organism:Severe      CCTTTCCAACAATTTGGCAGAGACATTGCTGACACTACTGATGCTGTCCGTGATCCACAG    23302
gb:MT019533|Organism:Severe      CCTTTCCAACAATTTGGCAGAGACATTGCTGACACTACTGATGCTGTCCGTGATCCACAG    23302
gb:MT019531|Organism:Severe      CCTTTCCAACAATTTGGCAGAGACATTGCTGACACTACTGATGCTGTCCGTGATCCACAG    23302
gb:MN996528|Organism:Severe      CCTTTCCAACAATTTGGCAGAGACATTGCTGACACTACTGATGCTGTCCGTGATCCACAG    23302
gb:MN996530|Organism:Severe      CCTTTCCAACAATTTGGCAGAGACATTGCTGACACTACTGATGCTGTCCGTGATCCACAG    23288
gb:MN908947|Organism:Severe      CCTTTCCAACAATTTGGCAGAGACATTGCTGACACTACTGATGCTGTCCGTGATCCACAG    23302
gb:MT019532|Organism:Severe      CCTTTCCAACAATTTGGCAGAGACATTGCTGACACTACTGATGCTGTCCGTGATCCACAG    23302
                                 ************************************************************

gb:MT020781|Organism:Severe      ACACTTGAGATTCTTGACATTACACCATGTTCTTTTGGTGGTGTCAGTGTTATAACACCA    23350
gb:MT007544|Organism:Severe      ACACTTGAGATTCTTGACATTACACCATGTTCTTTTGGTGGTGTCAGTGTTATAACACCA    23362
gb:MN994467|Organism:Severe      ACACTTGAGATTCTTGACATTACACCATGTTCTTTTGGTGGTGTCAGTGTTATAACACCA    23362
gb:MT044257|Organism:Severe      ACACTTGAGATTCTTGACATTACACCATGTTCTTTTGGTGGTGTCAGTGTTATAACACCA    23362
gb:MT106054|Organism:Severe      ACACTTGAGATTCTTGACATTACACCATGTTCTTTTGGTGGTGTCAGTGTTATAACACCA    23362
gb:MT049951|Organism:Severe      ACACTTGAGATTCTTGACATTACACCATGTTCTTTTGGTGGTGTCAGTGTTATAACACCA    23362
gb:MN975262|Organism:Severe      ACACTTGAGATTCTTGACATTACACCATGTTCTTTTGGTGGTGTCAGTGTTATAACACCA    23362
gb:MT106052|Organism:Severe      ACACTTGAGATTCTTGACATTACACCATGTTCTTTTGGTGGTGTCAGTGTTATAACACCA    23362
gb:LC522975|Organism:Severe      ACACTTGAGATTCTTGACATTACACCATGTTCTTTTGGTGGTGTCAGTGTTATAACACCA    23359
gb:LC522973|Organism:Severe      ACACTTGAGATTCTTGACATTACACCATGTTCTTTTGGTGGTGTCAGTGTTATAACACCA    23359
gb:LC522974|Organism:Severe      ACACTTGAGATTCTTGACATTACACCATGTTCTTTTGGTGGTGTCAGTGTTATAACACCA    23359
gb:MN985325|Organism:Severe      ACACTTGAGATTCTTGACATTACACCATGTTCTTTTGGTGGTGTCAGTGTTATAACACCA    23362
gb:MT020881|Organism:Severe      ACACTTGAGATTCTTGACATTACACCATGTTCTTTTGGTGGTGTCAGTGTTATAACACCA    23362
gb:MT020880|Organism:Severe      ACACTTGAGATTCTTGACATTACACCATGTTCTTTTGGTGGTGTCAGTGTTATAACACCA    23362
gb:MT066175|Organism:Severe      ACACTTGAGATTCTTGACATTACACCATGTTCTTTTGGTGGTGTCAGTGTTATAACACCA    23362
gb:MN997409|Organism:Severe      ACACTTGAGATTCTTGACATTACACCATGTTCTTTTGGTGGTGTCAGTGTTATAACACCA    23362
gb:MN938384|Organism:Severe      ACACTTGAGATTCTTGACATTACACCATGTTCTTTTGGTGGTGTCAGTGTTATAACACCA    23330
gb:MT044258|Organism:Severe      ACACTTGAGATTCTTGACATTACACCATGTTCTTTTGGTGGTGTCAGTGTTATAACACCA    23338
gb:MT039890|Organism:Severe      ACACTTGAGATTCTTGACATTACACCATGTTCTTTTGGTGGTGTCAGTGTTATAACACCA    23362
gb:MN988713|Organism:Severe      ACACTTGAGATTCTTGACATTACACCATGTTCTTTTGGTGGTGTCAGTGTTATAACACCA    23362
gb:LC521925|Organism:Severe      ACACTTGAGATTCTTGACATTACACCATGTTCTTTTGGTGGTGTCAGTGTTATAACACCA    23335
gb:MT093571|Organism:Severe      ACACTTGAGATTCTTGACATTACACCATGTTCTTTTGGTGGTGTCAGTGTTATAACACCA    23362
gb:MT039887|Organism:Severe      ACACTTGAGATTCTTGACATTACACCATGTTCTTTTGGTGGTGTCAGTGTTATAACACCA    23359
gb:MT019530|Organism:Severe      ACACTTGAGATTCTTGACATTACACCATGTTCTTTTGGTGGTGTCAGTGTTATAACACCA    23362
gb:MT039888|Organism:Severe      ACACTTGAGATTCTTGACATTACACCATGTTCTTTTGGTGGTGTCAGTGTTATAACACCA    23362
gb:LC522972|Organism:Severe      ACACTTGAGATTCTTGACATTACACCATGTTCTTTTGGTGGTGTCAGTGTTATAACACCA    23359
gb:MT027063|Organism:Severe      ACACTTGAGATTCTTGACATTACACCATGTTCTTTTGGTGGTGTCAGTGTTATAACACCA    23362
gb:MT027062|Organism:Severe      ACACTTGAGATTCTTGACATTACACCATGTTCTTTTGGTGGTGTCAGTGTTATAACACCA    23362
gb:MT019529|Organism:Severe      ACACTTGAGATTCTTGACATTACACCATGTTCTTTTGGTGGTGTCAGTGTTATAACACCA    23362
gb:MN996529|Organism:Severe      ACACTTGAGATTCTTGACATTACACCATGTTCTTTTGGTGGTGTCAGTGTTATAACACCA    23350
gb:MN996531|Organism:Severe      ACACTTGAGATTCTTGACATTACACCATGTTCTTTTGGTGGTGTCAGTGTTATAACACCA    23349
gb:MT066176|Organism:Severe      ACACTTGAGATTCTTGACATTACACCATGTTCTTTTGGTGGTGTCAGTGTTATAACACCA    23362
gb:MT027064|Organism:Severe      ACACTTGAGATTCTTGACATTACACCATGTTCTTTTGGTGGTGTCAGTGTTATAACACCA    23362
gb:MN994468|Organism:Severe      ACACTTGAGATTCTTGACATTACACCATGTTCTTTTGGTGGTGTCAGTGTTATAACACCA    23362
gb:MT072688|Organism:Severe      ACACTTGAGATTCTTGACATTACACCATGTTCTTTTGGTGGTGTCAGTGTTATAACACCA    23347
gb:MN996527|Organism:Severe      ACACTTGAGATTCTTGACATTACACCATGTTCTTTTGGTGGTGTCAGTGTTATAACACCA    23329
gb:MT093631|Organism:Severe      ACACTTGAGATTCTTGACATTACACCATGTTCTTTTGGTGGTGTCAGTGTTATAACACCA    23400
gb:MT106053|Organism:Severe      ACACTTGAGATTCTTGACATTACACCATGTTCTTTTGGTGGTGTCAGTGTTATAACACCA    23362
gb:MT019533|Organism:Severe      ACACTTGAGATTCTTGACATTACACCATGTTCTTTTGGTGGTGTCAGTGTTATAACACCA    23362
gb:MT019531|Organism:Severe      ACACTTGAGATTCTTGACATTACACCATGTTCTTTTGGTGGTGTCAGTGTTATAACACCA    23362
gb:MN996528|Organism:Severe      ACACTTGAGATTCTTGACATTACACCATGTTCTTTTGGTGGTGTCAGTGTTATAACACCA    23362
gb:MN996530|Organism:Severe      ACACTTGAGATTCTTGACATTACACCATGTTCTTTTGGTGGTGTCAGTGTTATAACACCA    23348
```

```
gb:MN908947|Organism:Severe      ACACTTGAGATTCTTGACATTACACCATGTTCTTTTGGTGGTGTCAGTGTTATAACACCA   23362
gb:MT019532|Organism:Severe      ACACTTGAGATTCTTGACATTACACCATGTTCTTTTGGTGGTGTCAGTGTTATAACACCA   23362
                                 ************************************************************

gb:MT020781|Organism:Severe      GGAACAAATACTTCTAACCAGGTTGCTGTTCTTTATCAGGATGTTAACTGCACAGAAGTC   23410
gb:MT007544|Organism:Severe      GGAACAAATACTTCTAACCAGGTTGCTGTTCTTTATCAGGATGTTAACTGCACAGAAGTC   23422
gb:MN994467|Organism:Severe      GGAACAAATACTTCTAACCAGGTTGCTGTTCTTTATCAGGATGTTAACTGCACAGAAGTC   23422
gb:MT044257|Organism:Severe      GGAACAAATACTTCTAACCAGGTTGCTGTTCTTTATCAGGATGTTAACTGCACAGAAGTC   23422
gb:MT106054|Organism:Severe      GGAACAAATACTTCTAACCAGGTTGCTGTTCTTTATCAGGATGTTAACTGCACAGAAGTC   23422
gb:MT049951|Organism:Severe      GGAACAAATACTTCTAACCAGGTTGCTGTTCTTTATCAGGATGTTAACTGCACAGAAGTC   23422
gb:MN975262|Organism:Severe      GGAACAAATACTTCTAACCAGGTTGCTGTTCTTTATCAGGATGTTAACTGCACAGAAGTC   23422
gb:MT106052|Organism:Severe      GGAACAAATACTTCTAACCAGGTTGCTGTTCTTTATCAGGATGTTAACTGCACAGAAGTC   23422
gb:LC522975|Organism:Severe      GGAACAAATACTTCTAACCAGGTTGCTGTTCTTTATCAGGATGTTAACTGCACAGAAGTC   23419
gb:LC522973|Organism:Severe      GGAACAAATACTTCTAACCAGGTTGCTGTTCTTTATCAGGATGTTAACTGCACAGAAGTC   23419
gb:LC522974|Organism:Severe      GGAACAAATACTTCTAACCAGGTTGCTGTTCTTTATCAGGATGTTAACTGCACAGAAGTC   23419
gb:MN985325|Organism:Severe      GGAACAAATACTTCTAACCAGGTTGCTGTTCTTTATCAGGATGTTAACTGCACAGAAGTC   23422
gb:MT020881|Organism:Severe      GGAACAAATACTTCTAACCAGGTTGCTGTTCTTTATCAGGATGTTAACTGCACAGAAGTC   23422
gb:MT020880|Organism:Severe      GGAACAAATACTTCTAACCAGGTTGCTGTTCTTTATCAGGATGTTAACTGCACAGAAGTC   23422
gb:MT066175|Organism:Severe      GGAACAAATACTTCTAACCAGGTTGCTGTTCTTTATCAGGATGTTAACTGCACAGAAGTC   23422
gb:MN997409|Organism:Severe      GGAACAAATACTTCTAACCAGGTTGCTGTTCTTTATCAGGATGTTAACTGCACAGAAGTC   23422
gb:MN938384|Organism:Severe      GGAACAAATACTTCTAACCAGGTTGCTGTTCTTTATCAGGATGTTAACTGCACAGAAGTC   23390
gb:MT044258|Organism:Severe      GGAACAAATACTTCTAACCAGGTTGCTGTTCTTTATCAGGATGTTAACTGCACAGAAGTC   23398
gb:MT039890|Organism:Severe      GGAACAAATACTTCTAACCAGGTTGCTGTTCTTTATCAGGATGTTAACTGCACAGAAGTC   23422
gb:MN988713|Organism:Severe      GGAACAAATACTTCTAACCAGGTTGCTGTTCTTTATCAGGATGTTAACTGCACAGAAGTC   23422
gb:LC521925|Organism:Severe      GGAACAAATACTTCTAACCAGGTTGCTGTTCTTTATCAGGATGTTAACTGCACAGAAGTC   23395
gb:MT093571|Organism:Severe      GGAACAAATACTTCTAACCAGGTTGCTGTTCTTTATCAGGATGTTAACTGCACAGAAGTC   23422
gb:MT039887|Organism:Severe      GGAACAAATACTTCTAACCAGGTTGCTGTTCTTTATCAGGATGTTAACTGCACAGAAGTC   23419
gb:MT019530|Organism:Severe      GGAACAAATACTTCTAACCAGGTTGCTGTTCTTTATCAGGATGTTAACTGCACAGAAGTC   23422
gb:MT039888|Organism:Severe      GGAACAAATACTTCTAACCAGGTTGCTGTTCTTTATCAGGATGTTAACTGCACAGAAGTC   23422
gb:LC522972|Organism:Severe      GGAACAAATACTTCTAACCAGGTTGCTGTTCTTTATCAGGATGTTAACTGCACAGAAGTC   23419
gb:MT027063|Organism:Severe      GGAACAAATACTTCTAACCAGGTTGCTGTTCTTTATCAGGATGTTAACTGCACAGAAGTC   23422
gb:MT027062|Organism:Severe      GGAACAAATACTTCTAACCAGGTTGCTGTTCTTTATCAGGATGTTAACTGCACAGAAGTC   23422
gb:MT019529|Organism:Severe      GGAACAAATACTTCTAACCAGGTTGCTGTTCTTTATCAGGATGTTAACTGCACAGAAGTC   23422
gb:MN996529|Organism:Severe      GGAACAAATACTTCTAACCAGGTTGCTGTTCTTTATCAGGATGTTAACTGCACAGAAGTC   23410
gb:MN996531|Organism:Severe      GGAACAAATACTTCTAACCAGGTTGCTGTTCTTTATCAGGATGTTAACTGCACAGAAGTC   23409
gb:MT066176|Organism:Severe      GGAACAAATACTTCTAACCAGGTTGCTGTTCTTTATCAGGATGTTAACTGCACAGAAGTC   23422
gb:MT027064|Organism:Severe      GGAACAAATACTTCTAACCAGGTTGCTGTTCTTTATCAGGATGTTAACTGCACAGAAGTC   23422
gb:MN994468|Organism:Severe      GGAACAAATACTTCTAACCAGGTTGCTGTTCTTTATCAGGATGTTAACTGCACAGAAGTC   23422
gb:MT072688|Organism:Severe      GGAACAAATACTTCTAACCAGGTTGCTGTTCTTTATCAGGATGTTAACTGCACAGAAGTC   23407
gb:MN996527|Organism:Severe      GGAACAAATACTTCTAACCAGGTTGCTGTTCTTTATCAGGATGTTAACTGCACAGAAGTC   23389
gb:MT093631|Organism:Severe      GGAACAAATACTTCTAACCAGGTTGCTGTTCTTTATCAGGATGTTAACTGCACAGAAGTC   23460
gb:MT106053|Organism:Severe      GGAACAAATACTTCTAACCAGGTTGCTGTTCTTTATCAGGATGTTAACTGCACAGAAGTC   23422
gb:MT019533|Organism:Severe      GGAACAAATACTTCTAACCAGGTTGCTGTTCTTTATCAGGATGTTAACTGCACAGAAGTC   23422
gb:MT019531|Organism:Severe      GGAACAAATACTTCTAACCAGGTTGCTGTTCTTTATCAGGATGTTAACTGCACAGAAGTC   23422
gb:MN996528|Organism:Severe      GGAACAAATACTTCTAACCAGGTTGCTGTTCTTTATCAGGATGTTAACTGCACAGAAGTC   23422
gb:MN996530|Organism:Severe      GGAACAAATACTTCTAACCAGGTTGCTGTTCTTTATCAGGATGTTAACTGCACAGAAGTC   23408
gb:MN908947|Organism:Severe      GGAACAAATACTTCTAACCAGGTTGCTGTTCTTTATCAGGATGTTAACTGCACAGAAGTC   23422
gb:MT019532|Organism:Severe      GGAACAAATACTTCTAACCAGGTTGCTGTTCTTTATCAGGATGTTAACTGCACAGAAGTC   23422
                                 ************************************************************

gb:MT020781|Organism:Severe      CCTGTTGCTATTCATGCAGATCAACTTACTCCTACTTGGCGTGTTTATTCTACAGGTTCT   23470
gb:MT007544|Organism:Severe      CCTGTTGCTATTCATGCAGATCAACTTACTCCTACTTGGCGTGTTTATTCTACAGGTTCT   23482
gb:MN994467|Organism:Severe      CCTGTTGCTATTCATGCAGATCAACTTACTCCTACTTGGCGTGTTTATTCTACAGGTTCT   23482
gb:MT044257|Organism:Severe      CCTGTTGCTATTCATGCAGATCAACTTACTCCTACTTGGCGTGTTTATTCTACAGGTTCT   23482
gb:MT106054|Organism:Severe      CCTGTTGCTATTCATGCAGATCAACTTACTCCTACTTGGCGTGTTTATTCTACAGGTTCT   23482
gb:MT049951|Organism:Severe      CCTGTTGCTATTCATGCAGATCAACTTACTCCTACTTGGCGTGTTTATTCTACAGGTTCT   23482
gb:MN975262|Organism:Severe      CCTGTTGCTATTCATGCAGATCAACTTACTCCTACTTGGCGTGTTTATTCTACAGGTTCT   23482
gb:MT106052|Organism:Severe      CCTGTTGCTATTCATGCAGATCAACTTACTCCTACTTGGCGTGTTTATTCTACAGGTTCT   23482
gb:LC522975|Organism:Severe      CCTGTTGCTATTCATGCAGATCAACTTACTCCTACTTGGCGTGTTTATTCTACAGGTTCT   23479
gb:LC522973|Organism:Severe      CCTGTTGCTATTCATGCAGATCAACTTACTCCTACTTGGCGTGTTTATTCTACAGGTTCT   23479
gb:LC522974|Organism:Severe      CCTGTTGCTATTCATGCAGATCAACTTACTCCTACTTGGCGTGTTTATTCTACAGGTTCT   23479
gb:MN985325|Organism:Severe      CCTGTTGCTATTCATGCAGATCAACTTACTCCTACTTGGCGTGTTTATTCTACAGGTTCT   23482
gb:MT020881|Organism:Severe      CCTGTTGCTATTCATGCAGATCAACTTACTCCTACTTGGCGTGTTTATTCTACAGGTTCT   23482
gb:MT020880|Organism:Severe      CCTGTTGCTATTCATGCAGATCAACTTACTCCTACTTGGCGTGTTTATTCTACAGGTTCT   23482
gb:MT066175|Organism:Severe      CCTGTTGCTATTCATGCAGATCAACTTACTCCTACTTGGCGTGTTTATTCTACAGGTTCT   23482
```

```
gb:MN997409|Organism:Severe      CCTGTTGCTATTCATGCAGATCAACTTACTCCTACTTGGCGTGTTTATTCTACAGGTTCT   23482
gb:MN938384|Organism:Severe      CCTGTTGCTATTCATGCAGATCAACTTACTCCTACTTGGCGTGTTTATTCTACAGGTTCT   23450
gb:MT044258|Organism:Severe      CCTGTTGCTATTCATGCAGATCAACTTACTCCTACTTGGCGTGTTTATTCTACAGGTTCT   23458
gb:MT039890|Organism:Severe      CCTGTTGCTATTCATGCAGATCAACTTACTCCTACTTGGCGTGTTTATTCTACAGGTTCT   23482
gb:MN988713|Organism:Severe      CCTGTTGCTATTCATGCAGATCAACTTACTCCTACTTGGCGTGTTTATTCTACAGGTTCT   23482
gb:LC521925|Organism:Severe      CCTGTTGCTATTCATGCAGATCAACTTACTCCTACTTGGCGTGTTTATTCTACAGGTTCT   23455
gb:MT093571|Organism:Severe      CCTGTTGCTATTCATGCAGATCAACTTACTCCTACTTGGCGTGTTTATTCTACAGGTTCT   23482
gb:MT039887|Organism:Severe      CCTGTTGCTATTCATGCAGATCAACTTACTCCTACTTGGCGTGTTTATTCTACAGGTTCT   23479
gb:MT019530|Organism:Severe      CCTGTTGCTATTCATGCAGATCAACTTACTCCTACTTGGCGTGTTTATTCTACAGGTTCT   23482
gb:MT039888|Organism:Severe      CCTGTTGCTATTCATGCAGATCAACTTACTCCTACTTGGCGTGTTTATTCTACAGGTTCT   23482
gb:LC522972|Organism:Severe      CCTGTTGCTATTCATGCAGATCAACTTACTCCTACTTGGCGTGTTTATTCTACAGGTTCT   23479
gb:MT027063|Organism:Severe      CCTGTTGCTATTCATGCAGATCAACTTACTCCTACTTGGCGTGTTTATTCTACAGGTTCT   23482
gb:MT027062|Organism:Severe      CCTGTTGCTATTCATGCAGATCAACTTACTCCTACTTGGCGTGTTTATTCTACAGGTTCT   23482
gb:MT019529|Organism:Severe      CCTGTTGCTATTCATGCAGATCAACTTACTCCTACTTGGCGTGTTTATTCTACAGGTTCT   23482
gb:MN996529|Organism:Severe      CCTGTTGCTATTCATGCAGATCAACTTACTCCTACTTGGCGTGTTTATTCTACAGGTTCT   23470
gb:MN996531|Organism:Severe      CCTGTTGCTATTCATGCAGATCAACTTACTCCTACTTGGCGTGTTTATTCTACAGGTTCT   23469
gb:MT066176|Organism:Severe      CCTGTTGCTATTCATGCAGATCAACTTACTCCTACTTGGCGTGTTTATTCTACAGGTTCT   23482
gb:MT027064|Organism:Severe      CCTGTTGCTATTCATGCAGATCAACTTACTCCTACTTGGCGTGTTTATTCTACAGGTTCT   23482
gb:MN994468|Organism:Severe      CCTGTTGCTATTCATGCAGATCAACTTACTCCTACTTGGCGTGTTTATTCTACAGGTTCT   23482
gb:MT072688|Organism:Severe      CCTGTTGCTATTCATGCAGATCAACTTACTCCTACTTGGCGTGTTTATTCTACAGGTTCT   23467
gb:MN996527|Organism:Severe      CCTGTTGCTATTCATGCAGATCAACTTACTCCTACTTGGCGTGTTTATTCTACAGGTTCT   23449
gb:MT093631|Organism:Severe      CCTGTTGCTATTCATGCAGATCAACTTACTCCTACTTGGCGTGTTTATTCTACAGGTTCT   23520
gb:MT106053|Organism:Severe      CCTGTTGCTATTCATGCAGATCAACTTACTCCTACTTGGCGTGTTTATTCTACAGGTTCT   23482
gb:MT019533|Organism:Severe      CCTGTTGCTATTCATGCAGATCAACTTACTCCTACTTGGCGTGTTTATTCTACAGGTTCT   23482
gb:MT019531|Organism:Severe      CCTGTTGCTATTCATGCAGATCAACTTACTCCTACTTGGCGTGTTTATTCTACAGGTTCT   23482
gb:MN996528|Organism:Severe      CCTGTTGCTATTCATGCAGATCAACTTACTCCTACTTGGCGTGTTTATTCTACAGGTTCT   23482
gb:MN996530|Organism:Severe      CCTGTTGCTATTCATGCAGATCAACTTACTCCTACTTGGCGTGTTTATTCTACAGGTTCT   23468
gb:MN908947|Organism:Severe      CCTGTTGCTATTCATGCAGATCAACTTACTCCTACTTGGCGTGTTTATTCTACAGGTTCT   23482
gb:MT019532|Organism:Severe      CCTGTTGCTATTCATGCAGATCAACTTACTCCTACTTGGCGTGTTTATTCTACAGGTTCT   23482
                                 ************************************************************

gb:MT020781|Organism:Severe      AATGTTTTTCAAACACGTGCAGGCTGTTTAATAGGGGCTGAACATGTCAACAACTCATAT   23530
gb:MT007544|Organism:Severe      AATGTTTTTCAAACACGTGCAGGCTGTTTAATAGGGGCTGAACATGTCAACAACTCATAT   23542
gb:MN994467|Organism:Severe      AATGTTTTTCAAACACGTGCAGGCTGTTTAATAGGGGCTGAACATGTCAACAACTCATAT   23542
gb:MT044257|Organism:Severe      AATGTTTTTCAAACACGTGCAGGCTGTTTAATAGGGGCTGAACATGTCAACAACTCATAT   23542
gb:MT106054|Organism:Severe      AATGTTTTTCAAACACGTGCAGGCTGTTTAATAGGGGCTGAACATGTCAACAACTCATAT   23542
gb:MT049951|Organism:Severe      AATGTTTTTCAAACACGTGCAGGCTGTTTAATAGGGGCTGAACATGTCAACAACTCATAT   23542
gb:MN975262|Organism:Severe      AATGTTTTTCAAACACGTGCAGGCTGTTTAATAGGGGCTGAACATGTCAACAACTCATAT   23542
gb:MT106052|Organism:Severe      AATGTTTTTCAAACACGTGCAGGCTGTTTAATAGGGGCTGAACATGTCAACAACTCATAT   23542
gb:LC522975|Organism:Severe      AATGTTTTTCAAACACGTGCAGGCTGTTTAATAGGGGCTGAACATGTCAACAACTCATAT   23539
gb:LC522973|Organism:Severe      AATGTTTTTCAAACACGTGCAGGCTGTTTAATAGGGGCTGAACATGTCAACAACTCATAT   23539
gb:LC522974|Organism:Severe      AATGTTTTTCAAACACGTGCAGGCTGTTTAATAGGGGCTGAACATGTCAACAACTCATAT   23539
gb:MN985325|Organism:Severe      AATGTTTTTCAAACACGTGCAGGCTGTTTAATAGGGGCTGAACATGTCAACAACTCATAT   23542
gb:MT020881|Organism:Severe      AATGTTTTTCAAACACGTGCAGGCTGTTTAATAGGGGCTGAACATGTCAACAACTCATAT   23542
gb:MT020880|Organism:Severe      AATGTTTTTCAAACACGTGCAGGCTGTTTAATAGGGGCTGAACATGTCAACAACTCATAT   23542
gb:MT066175|Organism:Severe      AATGTTTTTCAAACACGTGCAGGCTGTTTAATAGGGGCTGAACATGTCAACAACTCATAT   23542
gb:MN997409|Organism:Severe      AATGTTTTTCAAACACGTGCAGGCTGTTTAATAGGGGCTGAACATGTCAACAACTCATAT   23542
gb:MN938384|Organism:Severe      AATGTTTTTCAAACACGTGCAGGCTGTTTAATAGGGGCTGAACATGTCAACAACTCATAT   23510
gb:MT044258|Organism:Severe      AATGTTTTTCAAACACGTGCAGGCTGTTTAATAGGGGCTGAACATGTCAACAACTCATAT   23518
gb:MT039890|Organism:Severe      AATGTTTTTCAAACACGTGCAGGCTGTTTAATAGGGGCTGAACATGTCAACAACTCATAT   23542
gb:MN988713|Organism:Severe      AATGTTTTTCAAACACGTGCAGGCTGTTTAATAGGGGCTGAACATGTCAACAACTCATAT   23542
gb:LC521925|Organism:Severe      AATGTTTTTCAAACACGTGCAGGCTGTTTAATAGGGGCTGAACATGTCAACAACTCATAT   23515
gb:MT093571|Organism:Severe      AATGTTTTTCAAACACGTGCAGGCTGTTTAATAGGGGCTGAACATGTCAACAACTCATAT   23542
gb:MT039887|Organism:Severe      AATGTTTTTCAAACACGTGCAGGCTGTTTAATAGGGGCTGAACATGTCAACAACTCATAT   23539
gb:MT019530|Organism:Severe      AATGTTTTTCAAACACGTGCAGGCTGTTTAATAGGGGCTGAACATGTCAACAACTCATAT   23542
gb:MT039888|Organism:Severe      AATGTTTTTCAAACACGTGCAGGCTGTTTAATAGGGGCTGAACATGTCAACAACTCATAT   23542
gb:LC522972|Organism:Severe      AATGTTTTTCAAACACGTGCAGGCTGTTTAATAGGGGCTGAACATGTCAACAACTCATAT   23539
gb:MT027063|Organism:Severe      AATGTTTTTCAAACACGTGCAGGCTGTTTAATAGGGGCTGAACATGTCAACAACTCATAT   23542
gb:MT027062|Organism:Severe      AATGTTTTTCAAACACGTGCAGGCTGTTTAATAGGGGCTGAACATGTCAACAACTCATAT   23542
gb:MT019529|Organism:Severe      AATGTTTTTCAAACACGTGCAGGCTGTTTAATAGGGGCTGAACATGTCAACAACTCATAT   23542
gb:MN996529|Organism:Severe      AATGTTTTTCAAACACGTGCAGGCTGTTTAATAGGGGCTGAACATGTCAACAACTCATAT   23530
gb:MN996531|Organism:Severe      AATGTTTTTCAAACACGTGCAGGCTGTTTAATAGGGGCTGAACATGTCAACAACTCATAT   23529
gb:MT066176|Organism:Severe      AATGTTTTTCAAACACGTGCAGGCTGTTTAATAGGGGCTGAACATGTCAACAACTCATAT   23542
gb:MT027064|Organism:Severe      AATGTTTTTCAAACACGTGCAGGCTGTTTAATAGGGGCTGAACATGTCAACAACTCATAT   23542
gb:MN994468|Organism:Severe      AATGTTTTTCAAACACGTGCAGGCTGTTTAATAGGGGCTGAACATGTCAACAACTCATAT   23542
```

```
gb:MT072688|Organism:Severe      AATGTTTTTCAAACACGTGCAGGCTGTTTAATAGGGGCTGAACATGTCAACAACTCATAT   23527
gb:MN996527|Organism:Severe      AATGTTTTTCAAACACGTGCAGGCTGTTTAATAGGGGCTGAACATGTCAACAACTCATAT   23509
gb:MT093631|Organism:Severe      AATGTTTTTCAAACACGTGCAGGCTGTTTAATAGGGGCTGAACATGTCAACAACTCATAT   23580
gb:MT106053|Organism:Severe      AATGTTTTTCAAACACGTGCAGGCTGTTTAATAGGGGCTGAACATGTCAACAACTCATAT   23542
gb:MT019533|Organism:Severe      AATGTTTTTCAAACACGTGCAGGCTGTTTAATAGGGGCTGAACATGTCAACAACTCATAT   23542
gb:MT019531|Organism:Severe      AATGTTTTTCAAACACGTGCAGGCTGTTTAATAGGGGCTGAACATGTCAACAACTCATAT   23542
gb:MN996528|Organism:Severe      AATGTTTTTCAAACACGTGCAGGCTGTTTAATAGGGGCTGAACATGTCAACAACTCATAT   23542
gb:MN996530|Organism:Severe      AATGTTTTTCAAACACGTGCAGGCTGTTTAATAGGGGCTGAACATGTCAACAACTCATAT   23528
gb:MN908947|Organism:Severe      AATGTTTTTCAAACACGTGCAGGCTGTTTAATAGGGGCTGAACATGTCAACAACTCATAT   23542
gb:MT019532|Organism:Severe      AATGTTTTTCAAACACGTGCAGGCTGTTTAATAGGGGCTGAACATGTCAACAACTCATAT   23542
                                 ************************************************************

gb:MT020781|Organism:Severe      GAGTGTGACATACCCATTGGTGCAGGTATATGCGCTAGTTATCAGACTCAGACTAATTCT   23590
gb:MT007544|Organism:Severe      GAGTGTGACATACCCATTGGTGCAGGTATATGCGCTAGTTATCAGACTCAGACTAATTCT   23602
gb:MN994467|Organism:Severe      GAGTGTGACATACCCATTGGTGCAGGTATATGCGCTAGTTATCAGACTCAGACTAATTCT   23602
gb:MT044257|Organism:Severe      GAGTGTGACATACCCATTGGTGCAGGTATATGCGCTAGTTATCAGACTCAGACTAATTCT   23602
gb:MT106054|Organism:Severe      GAGTGTGACATACCCATTGGTGCAGGTATATGCGCTAGTTATCAGACTCAGACTAATTCT   23602
gb:MT049951|Organism:Severe      GAGTGTGACATACCCATTGGTGCAGGTATATGCGCTAGTTATCAGACTCAGACTAATTCT   23602
gb:MN975262|Organism:Severe      GAGTGTGACATACCCATTGGTGCAGGTATATGCGCTAGTTATCAGACTCAGACTAATTCT   23602
gb:MT106052|Organism:Severe      GAGTGTGACATACCCATTGGTGCAGGTATATGCGCTAGTTATCAGACTCAGACTAATTCT   23602
gb:LC522975|Organism:Severe      GAGTGTGACATACCCATTGGTGCAGGTATATGCGCTAGTTATCAGACTCAGACTAATTCT   23599
gb:LC522973|Organism:Severe      GAGTGTGACATACCCATTGGTGCAGGTATATGCGCTAGTTATCAGACTCAGACTAATTCT   23599
gb:LC522974|Organism:Severe      GAGTGTGACATACCCATTGGTGCAGGTATATGCGCTAGTTATCAGACTCAGACTAATTCT   23599
gb:MN985325|Organism:Severe      GAGTGTGACATACCCATTGGTGCAGGTATATGCGCTAGTTATCAGACTCAGACTAATTCT   23602
gb:MT020881|Organism:Severe      GAGTGTGACATACCCATTGGTGCAGGTATATGCGCTAGTTATCAGACTCAGACTAATTCT   23602
gb:MT020880|Organism:Severe      GAGTGTGACATACCCATTGGTGCAGGTATATGCGCTAGTTATCAGACTCAGACTAATTCT   23602
gb:MT066175|Organism:Severe      GAGTGTGACATACCCATTGGTGCAGGTATATGCGCTAGTTATCAGACTCAGACTAATTCT   23602
gb:MN997409|Organism:Severe      GAGTGTGACATACCCATTGGTGCAGGTATATGCGCTAGTTATCAGACTCAGACTAATTCT   23602
gb:MN938384|Organism:Severe      GAGTGTGACATACCCATTGGTGCAGGTATATGCGCTAGTTATCAGACTCAGACTAATTCT   23570
gb:MT044258|Organism:Severe      GAGTGTGACATACCCATTGGTGCAGGTATATGCGCTAGTTATCAGACTCAGACTAATTCT   23578
gb:MT039890|Organism:Severe      GAGTGTGACATACCCATTGGTGCAGGTATATGCGCTAGTTATCAGACTCAGACTAATTCT   23602
gb:MN988713|Organism:Severe      GAGTGTGACATACCCATTGGTGCAGGTATATGCGCTAGTTATCAGACTCAGACTAATTCT   23602
gb:LC521925|Organism:Severe      GAGTGTGACATACCCATTGGTGCAGGTATATGCGCTAGTTATCAGACTCAGACTAATTCT   23575
gb:MT093571|Organism:Severe      GAGTGTGACATACCCATTGGTGCAGGTATATGCGCTAGTTATCAGACTCAGACTAATTCT   23602
gb:MT039887|Organism:Severe      GAGTGTGACATACCCATTGGTGCAGGTATATGCGCTAGTTATCAGACTCAGACTAATTCT   23599
gb:MT019530|Organism:Severe      GAGTGTGACATACCCATTGGTGCAGGTATATGCGCTAGTTATCAGACTCAGACTAATTCT   23602
gb:MT039888|Organism:Severe      GAGTGTGACATACCCATTGGTGCAGGTATATGCGCTAGTTATCAGACTCAGACTAATTCT   23602
gb:LC522972|Organism:Severe      GAGTGTGACATACCCATTGGTGCAGGTATATGCGCTAGTTATCAGACTCAGACTAATTCT   23599
gb:MT027063|Organism:Severe      GAGTGTGACATACCCATTGGTGCAGGTATATGCGCTAGTTATCAGACTCAGACTAATTCT   23602
gb:MT027062|Organism:Severe      GAGTGTGACATACCCATTGGTGCAGGTATATGCGCTAGTTATCAGACTCAGACTAATTCT   23602
gb:MT019529|Organism:Severe      GAGTGTGACATACCCATTGGTGCAGGTATATGCGCTAGTTATCAGACTCAGACTAATTCT   23602
gb:MN996529|Organism:Severe      GAGTGTGACATACCCATTGGTGCAGGTATATGCGCTAGTTATCAGACTCAGACTAATTCT   23590
gb:MN996531|Organism:Severe      GAGTGTGACATACCCATTGGTGCAGGTATATGCGCTAGTTATCAGACTCAGACTAATTCT   23589
gb:MT066176|Organism:Severe      GAGTGTGACATACCCATTGGTGCAGGTATATGCGCTAGTTATCAGACTCAGACTAATTCT   23602
gb:MT027064|Organism:Severe      GAGTGTGACATACCCATTGGTGCAGGTATATGCGCTAGTTATCAGACTCAGACTAATTCT   23602
gb:MN994468|Organism:Severe      GAGTGTGACATACCCATTGGTGCAGGTATATGCGCTAGTTATCAGACTCAGACTAATTCT   23602
gb:MT072688|Organism:Severe      GAGTGTGACATACCCATTGGTGCAGGTATATGCGCTAGTTATCAGACTCAGACTAATTCT   23587
gb:MN996527|Organism:Severe      GAGTGTGACATACCCATTGGTGCAGGTATATGCGCTAGTTATCAGACTCAGACTAATTCT   23569
gb:MT093631|Organism:Severe      GAGTGTGACATACCCATTGGTGCAGGTATATGCGCTAGTTATCAGACTCAGACTAATTCT   23640
gb:MT106053|Organism:Severe      GAGTGTGACATACCCATTGGTGCAGGTATATGCGCTAGTTATCAGACTCAGACTAATTCT   23602
gb:MT019533|Organism:Severe      GAGTGTGACATACCCATTGGTGCAGGTATATGCGCTAGTTATCAGACTCAGACTAATTCT   23602
gb:MT019531|Organism:Severe      GAGTGTGACATACCCATTGGTGCAGGTATATGCGCTAGTTATCAGACTCAGACTAATTCT   23602
gb:MN996528|Organism:Severe      GAGTGTGACATACCCATTGGTGCAGGTATATGCGCTAGTTATCAGACTCAGACTAATTCT   23602
gb:MN996530|Organism:Severe      GAGTGTGACATACCCATTGGTGCAGGTATATGCGCTAGTTATCAGACTCAGACTAATTCT   23588
gb:MN908947|Organism:Severe      GAGTGTGACATACCCATTGGTGCAGGTATATGCGCTAGTTATCAGACTCAGACTAATTCT   23602
gb:MT019532|Organism:Severe      GAGTGTGACATACCCATTGGTGCAGGTATATGCGCTAGTTATCAGACTCAGACTAATTCT   23602
                                 ************************************************************

gb:MT020781|Organism:Severe      CCTCGGCGGGCACGTAGTGTAGCTAGTCAATCCATCATTGCCTACACTATGTCACTTGGT   23650
gb:MT007544|Organism:Severe      CCTCGGCGGGCACGTAGTGTAGCTAGTCAATCCATCATTGCCTACACTATGTCACTTGGT   23662
gb:MN994467|Organism:Severe      CCTCGGCGGGCACGTAGTGTAGCTAGTCAATCCATCATTGCCTACACTATGTCACTTGGT   23662
gb:MT044257|Organism:Severe      CCTCGGCGGGCACGTAGTGTAGCTAGTCAATCCATCATTGCCTACACTATGTCACTTGGT   23662
gb:MT106054|Organism:Severe      CCTCGGCGGGCACGTAGTGTAGCTAGTCAATCCATCATTGCCTACACTATGTCACTTGGT   23662
gb:MT049951|Organism:Severe      CCTCGGCGGGCACGTAGTGTAGCTAGTCAATCCATCATTGCCTACACTATGTCACTTGGT   23662
gb:MN975262|Organism:Severe      CCTCGGCGGGCACGTAGTGTAGCTAGTCAATCCATCATTGCCTACACTATGTCACTTGGT   23662
```

```
gb:MT106052|Organism:Severe      CCTCGGCGGGCACGTAGTGTAGCTAGTCAATCCATCATTGCCTACACTATGTCACTTGGT    23662
gb:LC522975|Organism:Severe      CCTCGGCGGGCACGTAGTGTAGCTAGTCAATCCATCATTGCCTACACTATGTCACTTGGT    23659
gb:LC522973|Organism:Severe      CCTCGGCGGGCACGTAGTGTAGCTAGTCAATCCATCATTGCCTACACTATGTCACTTGGT    23659
gb:LC522974|Organism:Severe      CCTCGGCGGGCACGTAGTGTAGCTAGTCAATCCATCATTGCCTACACTATGTCACTTGGT    23659
gb:MN985325|Organism:Severe      CCTCGGCGGGCACGTAGTGTAGCTAGTCAATCCATCATTGCCTACACTATGTCACTTGGT    23662
gb:MT020881|Organism:Severe      CCTCGGCGGGCACGTAGTGTAGCTAGTCAATCCATCATTGCCTACACTATGTCACTTGGT    23662
gb:MT020880|Organism:Severe      CCTCGGCGGGCACGTAGTGTAGCTAGTCAATCCATCATTGCCTACACTATGTCACTTGGT    23662
gb:MT066175|Organism:Severe      CCTCGGCGGGCACGTAGTGTAGCTAGTCAATCCATCATTGCCTACACTATGTCACTTGGT    23662
gb:MN997409|Organism:Severe      CCTCGGCGGGCACGTAGTGTAGCTAGTCAATCCATCATTGCCTACACTATGTCACTTGGT    23662
gb:MN938384|Organism:Severe      CCTCGGCGGGCACGTAGTGTAGCTAGTCAATCCATCATTGCCTACACTATGTCACTTGGT    23630
gb:MT044258|Organism:Severe      CCTCGGCGGGCACGTAGTGTAGCTAGTCAATCCATCATTGCCTACACTATGTCACTTGGT    23638
gb:MT039890|Organism:Severe      CCTCGGCGGGCACGTAGTGTAGCTAGTCAATCCATCATTGCCTACACTATGTCACTTGGT    23662
gb:MN988713|Organism:Severe      CCTCGGCGGGCACGTAGTGTAGCTAGTCAATCCATCATTGCCTACACTATGTCACTTGGT    23662
gb:LC521925|Organism:Severe      CCTCGGCGGGCACGTAGTGTAGCTAGTCAATCCATCATTGCCTACACTATGTCACTTGGT    23635
gb:MT093571|Organism:Severe      CCTCGGCGGGCACGTAGTGTAGCTAGTCAATCCATCATTGCCTACACTATGTCACTTGGT    23662
gb:MT039887|Organism:Severe      CCTCGGCGGGCACGTAGTGTAGCTAGTCAATCCATCATTGCCTACACTATGTCACTTGGT    23659
gb:MT019530|Organism:Severe      CCTCGGCGGGCACGTAGTGTAGCTAGTCAATCCATCATTGCCTACACTATGTCACTTGGT    23662
gb:MT039888|Organism:Severe      CCTCGGCGGGCACGTAGTGTAGCTAGTCAATCCATCATTGCCTACACTATGTCACTTGGT    23662
gb:LC522972|Organism:Severe      CCTCGGCGGGCACGTAGTGTAGCTAGTCAATCCATCATTGCCTACACTATGTCACTTGGT    23659
gb:MT027063|Organism:Severe      CCTCGGCGGGCACGTAGTGTAGCTAGTCAATCCATCATTGCCTACACTATGTCACTTGGT    23662
gb:MT027062|Organism:Severe      CCTCGGCGGGCACGTAGTGTAGCTAGTCAATCCATCATTGCCTACACTATGTCACTTGGT    23662
gb:MT019529|Organism:Severe      CCTCGGCGGGCACGTAGTGTAGCTAGTCAATCCATCATTGCCTACACTATGTCACTTGGT    23662
gb:MN996529|Organism:Severe      CCTCGGCGGGCACGTAGTGTAGCTAGTCAATCCATCATTGCCTACACTATGTCACTTGGT    23650
gb:MN996531|Organism:Severe      CCTCGGCGGGCACGTAGTGTAGCTAGTCAATCCATCATTGCCTACACTATGTCACTTGGT    23649
gb:MT066176|Organism:Severe      CCTCGGCGGGCACGTAGTGTAGCTAGTCAATCCATCATTGCCTACACTATGTCACTTGGT    23662
gb:MT027064|Organism:Severe      CCTCGGCGGGCACGTAGTGTAGCTAGTCAATCCATCATTGCCTACACTATGTCACTTGGT    23662
gb:MN994468|Organism:Severe      CCTCGGCGGGCACGTAGTGTAGCTAGTCAATCCATCATTGCCTACACTATGTCACTTGGT    23662
gb:MT072688|Organism:Severe      CCTCGGCGGGCACGTAGTGTAGCTAGTCAATCCATCATTGCCTACACTATGTCACTTGGT    23647
gb:MN996527|Organism:Severe      CCTCGGCGGGCACGTAGTGTAGCTAGTCAATCCATCATTGCCTACACTATGTCACTTGGT    23629
gb:MT093631|Organism:Severe      CCTCGGCGGGCACGTAGTGTAGCTAGTCAATCCATCATTGCCTACACTATGTCACTTGGT    23700
gb:MT106053|Organism:Severe      CCTCGGCGGGCACGTAGTGTAGCTAGTCAATCCATCATTGCCTACACTATGTCACTTGGT    23662
gb:MT019533|Organism:Severe      CCTCGGCGGGCACGTAGTGTAGCTAGTCAATCCATCATTGCCTACACTATGTCACTTGGT    23662
gb:MT019531|Organism:Severe      CCTCGGCGGGCACGTAGTGTAGCTAGTCAATCCATCATTGCCTACACTATGTCACTTGGT    23662
gb:MN996528|Organism:Severe      CCTCGGCGGGCACGTAGTGTAGCTAGTCAATCCATCATTGCCTACACTATGTCACTTGGT    23662
gb:MN996530|Organism:Severe      CCTCGGCGGGCACGTAGTGTAGCTAGTCAATCCATCATTGCCTACACTATGTCACTTGGT    23648
gb:MN908947|Organism:Severe      CCTCGGCGGGCACGTAGTGTAGCTAGTCAATCCATCATTGCCTACACTATGTCACTTGGT    23662
gb:MT019532|Organism:Severe      CCTCGGCGGGCACGTAGTGTAGCTAGTCAATCCATCATTGCCTACACTATGTCACTTGGT    23662
                                 ************************************************************

gb:MT020781|Organism:Severe      GCAGAAAATTCAGTTGCTTACTCTAATAACTCTATTGCCATACCCACAAATTTTACTATT    23710
gb:MT007544|Organism:Severe      GCAGAAAATTCAGTTGCTTACTCTAATAACTCTATTGCCATACCCACAAATTTTACTATT    23722
gb:MN994467|Organism:Severe      GCAGAAAATTCAGTTGCTTACTCTAATAACTCTATTGCCATACCCACAAATTTTACTATT    23722
gb:MT044257|Organism:Severe      GCAGAAAATTCAGTTGCTTACTCTAATAACTCTATTGCCATACCCACAAATTTTACTATT    23722
gb:MT106054|Organism:Severe      GCAGAAAATTCAGTTGCTTACTCTAATAACTCTATTGCCATACCCACAAATTTTACTATT    23722
gb:MT049951|Organism:Severe      GCAGAAAATTCAGTTGCTTACTCTAATAACTCTATTGCCATACCCACAAATTTTACTATT    23722
gb:MN975262|Organism:Severe      GCAGAAAATTCAGTTGCTTACTCTAATAACTCTATTGCCATACCCACAAATTTTACTATT    23722
gb:MT106052|Organism:Severe      GCAGAAAATTCAGTTGCTTACTCTAATAACTCTATTGCCATACCCACAAATTTTACTATT    23722
gb:LC522975|Organism:Severe      GCAGAAAATTCAGTTGCTTACTCTAATAACTCTATTGCCATACCCACAAATTTTACTATT    23719
gb:LC522973|Organism:Severe      GCAGAAAATTCAGTTGCTTACTCTAATAACTCTATTGCCATACCCACAAATTTTACTATT    23719
gb:LC522974|Organism:Severe      GCAGAAAATTCAGTTGCTTACTCTAATAACTCTATTGCCATACCCACAAATTTTACTATT    23719
gb:MN985325|Organism:Severe      GCAGAAAATTCAGTTGCTTACTCTAATAACTCTATTGCCATACCCACAAATTTTACTATT    23722
gb:MT020881|Organism:Severe      GCAGAAAATTCAGTTGCTTACTCTAATAACTCTATTGCCATACCCACAAATTTTACTATT    23722
gb:MT020880|Organism:Severe      GCAGAAAATTCAGTTGCTTACTCTAATAACTCTATTGCCATACCCACAAATTTTACTATT    23722
gb:MT066175|Organism:Severe      GCAGAAAATTCAGTTGCTTACTCTAATAACTCTATTGCCATACCCACAAATTTTACTATT    23722
gb:MN997409|Organism:Severe      GCAGAAAATTCAGTTGCTTACTCTAATAACTCTATTGCCATACCCACAAATTTTACTATT    23722
gb:MN938384|Organism:Severe      GCAGAAAATTCAGTTGCTTACTCTAATAACTCTATTGCCATACCCACAAATTTTACTATT    23690
gb:MT044258|Organism:Severe      GCAGAAAATTCAGTTGCTTACTCTAATAACTCTATTGCCATACCCACAAATTTTACTATT    23698
gb:MT039890|Organism:Severe      GCAGAAAATTCAGTTGCTTACTCTAATAACTCTATTGCCATACCCACAAATTTTACTATT    23722
gb:MN988713|Organism:Severe      GCAGAAAATTCAGTTGCTTACTCTAATAACTCTATTGCCATACCCACAAATTTTACTATT    23722
gb:LC521925|Organism:Severe      GCAGAAAATTCAGTTGCTTACTCTAATAACTCTATTGCCATACCCACAAATTTTACTATT    23695
gb:MT093571|Organism:Severe      GCAGAAAATTCAGTTGCTTACTCTAATAACTCTATTGCCATACCCACAAATTTTACTATT    23722
gb:MT039887|Organism:Severe      GCAGAAAATTCAGTTGCTTACTCTAATAACTCTATTGCCATACCCACAAATTTTACTATT    23719
gb:MT019530|Organism:Severe      GCAGAAAATTCAGTTGCTTACTCTAATAACTCTATTGCCATACCCACAAATTTTACTATT    23722
gb:MT039888|Organism:Severe      GCAGAAAATTCAGTTGCTTACTCTAATAACTCTATTGCCATACCCACAAATTTTACTATT    23722
gb:LC522972|Organism:Severe      GCAGAAAATTCAGTTGCTTACTCTAATAACTCTATTGCCATACCCACAAATTTTACTATT    23719
```

```
gb:MT027063|Organism:Severe      GCAGAAAATTCAGTTGCTTACTCTAATAACTCTATTGCCATACCCACAAATTTTACTATT    23722
gb:MT027062|Organism:Severe      GCAGAAAATTCAGTTGCTTACTCTAATAACTCTATTGCCATACCCACAAATTTTACTATT    23722
gb:MT019529|Organism:Severe      GCAGAAAATTCAGTTGCTTACTCTAATAACTCTATTGCCATACCCACAAATTTTACTATT    23722
gb:MN996529|Organism:Severe      GCAGAAAATTCAGTTGCTTACTCTAATAACTCTATTGCCATACCCACAAATTTTACTATT    23710
gb:MN996531|Organism:Severe      GCAGAAAATTCAGTTGCTTACTCTAATAACTCTATTGCCATACCCACAAATTTTACTATT    23709
gb:MT066176|Organism:Severe      GCAGAAAATTCAGTTGCTTACTCTAATAACTCTATTGCCATACCCACAAATTTTACTATT    23722
gb:MT027064|Organism:Severe      GCAGAAAATTCAGTTGCTTACTCTAATAACTCTATTGCCATACCCACAAATTTTACTATT    23722
gb:MN994468|Organism:Severe      GCAGAAAATTCAGTTGCTTACTCTAATAACTCTATTGCCATACCCACAAATTTTACTATT    23722
gb:MT072688|Organism:Severe      GCAGAAAATTCAGTTGCTTACTCTAATAACTCTATTGCCATACCCACAAATTTTACTATT    23707
gb:MN996527|Organism:Severe      GCAGAAAATTCAGTTGCTTACTCTAATAACTCTATTGCCATACCCACAAATTTTACTATT    23689
gb:MT093631|Organism:Severe      GCAGAAAATTCAGTTGCTTACTCTAATAACTCTATTGCCATACCCACAAATTTTACTATT    23760
gb:MT106053|Organism:Severe      GCAGAAAATTCAGTTGCTTACTCTAATAACTCTATTGCCATACCCACAAATTTTACTATT    23722
gb:MT019533|Organism:Severe      GCAGAAAATTCAGTTGCTTACTCTAATAACTCTATTGCCATACCCACAAATTTTACTATT    23722
gb:MT019531|Organism:Severe      GCAGAAAATTCAGTTGCTTACTCTAATAACTCTATTGCCATACCCACAAATTTTACTATT    23722
gb:MN996528|Organism:Severe      GCAGAAAATTCAGTTGCTTACTCTAATAACTCTATTGCCATACCCACAAATTTTACTATT    23722
gb:MN996530|Organism:Severe      GCAGAAAATTCAGTTGCTTACTCTAATAACTCTATTGCCATACCCACAAATTTTACTATT    23708
gb:MN908947|Organism:Severe      GCAGAAAATTCAGTTGCTTACTCTAATAACTCTATTGCCATACCCACAAATTTTACTATT    23722
gb:MT019532|Organism:Severe      GCAGAAAATTCAGTTGCTTACTCTAATAACTCTATTGCCATACCCACAAATTTTACTATT    23722
                                 ************************************************************

gb:MT020781|Organism:Severe      AGTGTTACCACAGAAATTCTACCAGTGTCTATGACCAAGACATCAGTAGATTGTACAATG    23770
gb:MT007544|Organism:Severe      AGTGTTACCACAGAAATTCTACCAGTGTCTATGACCAAGACATCAGTAGATTGTACAATG    23782
gb:MN994467|Organism:Severe      AGTGTTACCACAGAAATTCTACCAGTGTCTATGACCAAGACATCAGTAGATTGTACAATG    23782
gb:MT044257|Organism:Severe      AGTGTTACCACAGAAATTCTACCAGTGTCTATGACCAAGACATCAGTAGATTGTACAATG    23782
gb:MT106054|Organism:Severe      AGTGTTACCACAGAAATTCTACCAGTGTCTATGACCAAGACATCAGTAGATTGTACAATG    23782
gb:MT049951|Organism:Severe      AGTGTTACCACAGAAATTCTACCAGTGTCTATGACCAAGACATCAGTAGATTGTACAATG    23782
gb:MN975262|Organism:Severe      AGTGTTACCACAGAAATTCTACCAGTGTCTATGACCAAGACATCAGTAGATTGTACAATG    23782
gb:MT106052|Organism:Severe      AGTGTTACCACAGAAATTCTACCAGTGTCTATGACCAAGACATCAGTAGATTGTACAATG    23782
gb:LC522975|Organism:Severe      AGTGTTACCACAGAAATTCTACCAGTGTCTATGACCAAGACATCAGTAGATTGTACAATG    23779
gb:LC522973|Organism:Severe      AGTGTTACCACAGAAATTCTACCAGTGTCTATGACCAAGACATCAGTAGATTGTACAATG    23779
gb:LC522974|Organism:Severe      AGTGTTACCACAGAAATTCTACCAGTGTCTATGACCAAGACATCAGTAGATTGTACAATG    23779
gb:MN985325|Organism:Severe      AGTGTTACCACAGAAATTCTACCAGTGTCTATGACCAAGACATCAGTAGATTGTACAATG    23782
gb:MT020881|Organism:Severe      AGTGTTACCACAGAAATTCTACCAGTGTCTATGACCAAGACATCAGTAGATTGTACAATG    23782
gb:MT020880|Organism:Severe      AGTGTTACCACAGAAATTCTACCAGTGTCTATGACCAAGACATCAGTAGATTGTACAATG    23782
gb:MT066175|Organism:Severe      AGTGTTACCACAGAAATTCTACCAGTGTCTATGACCAAGACATCAGTAGATTGTACAATG    23782
gb:MN997409|Organism:Severe      AGTGTTACCACAGAAATTCTACCAGTGTCTATGACCAAGACATCAGTAGATTGTACAATG    23782
gb:MN938384|Organism:Severe      AGTGTTACCACAGAAATTCTACCAGTGTCTATGACCAAGACATCAGTAGATTGTACAATG    23750
gb:MT044258|Organism:Severe      AGTGTTACCACAGAAATTCTACCAGTGTCTATGACCAAGACATCAGTAGATTGTACAATG    23758
gb:MT039890|Organism:Severe      AGTGTTACCACAGAAATTCTACCAGTGTCTATGACCAAGACATCAGTAGATTGTACAATG    23782
gb:MN988713|Organism:Severe      AGTGTTACCACAGAAATTCTACCAGTGTCTATGACCAAGACATCAGTAGATTGTACAATG    23782
gb:LC521925|Organism:Severe      AGTGTTACCACAGAAATTCTACCAGTGTCTATGACCAAGACATCAGTAGATTGTACAATG    23755
gb:MT093571|Organism:Severe      AGTGTTACCACAGAAATTCTACCAGTGTCTATGACCAAGACATCAGTAGATTGTACAATG    23782
gb:MT039887|Organism:Severe      AGTGTTACCACAGAAATTCTACCAGTGTCTATGACCAAGACATCAGTAGATTGTACAATG    23779
gb:MT019530|Organism:Severe      AGTGTTACCACAGAAATTCTACCAGTGTCTATGACCAAGACATCAGTAGATTGTACAATG    23782
gb:MT039888|Organism:Severe      AGTGTTACCACAGAAATTCTACCAGTGTCTATGACCAAGACATCAGTAGATTGTACAATG    23782
gb:LC522972|Organism:Severe      AGTGTTACCACAGAAATTCTACCAGTGTCTATGACCAAGACATCAGTAGATTGTACAATG    23779
gb:MT027063|Organism:Severe      AGTGTTACCACAGAAATTCTACCAGTGTCTATGACCAAGACATCAGTAGATTGTACAATG    23782
gb:MT027062|Organism:Severe      AGTGTTACCACAGAAATTCTACCAGTGTCTATGACCAAGACATCAGTAGATTGTACAATG    23782
gb:MT019529|Organism:Severe      AGTGTTACCACAGAAATTCTACCAGTGTCTATGACCAAGACATCAGTAGATTGTACAATG    23782
gb:MN996529|Organism:Severe      AGTGTTACCACAGAAATTCTACCAGTGTCTATGACCAAGACATCAGTAGATTGTACAATG    23770
gb:MN996531|Organism:Severe      AGTGTTACCACAGAAATTCTACCAGTGTCTATGACCAAGACATCAGTAGATTGTACAATG    23769
gb:MT066176|Organism:Severe      AGTGTTACCACAGAAATTCTACCAGTGTCTATGACCAAGACATCAGTAGATTGTACAATG    23782
gb:MT027064|Organism:Severe      AGTGTTACCACAGAAATTCTACCAGTGTCTATGACCAAGACATCAGTAGATTGTACAATG    23782
gb:MN994468|Organism:Severe      AGTGTTACCACAGAAATTCTACCAGTGTCTATGACCAAGACATCAGTAGATTGTACAATG    23782
gb:MT072688|Organism:Severe      AGTGTTACCACAGAAATTCTACCAGTGTCTATGACCAAGACATCAGTAGATTGTACAATG    23767
gb:MN996527|Organism:Severe      AGTGTTACCACAGAAATTCTACCAGTGTCTATGACCAAGACATCAGTAGATTGTACAATG    23749
gb:MT093631|Organism:Severe      AGTGTTACCACAGAAATTCTACCAGTGTCTATGACCAAGACATCAGTAGATTGTACAATG    23820
gb:MT106053|Organism:Severe      AGTGTTACCACAGAAATTCTACCAGTGTCTATGACCAAGACATCAGTAGATTGTACAATG    23782
gb:MT019533|Organism:Severe      AGTGTTACCACAGAAATTCTACCAGTGTCTATGACCAAGACATCAGTAGATTGTACAATG    23782
gb:MT019531|Organism:Severe      AGTGTTACCACAGAAATTCTACCAGTGTCTATGACCAAGACATCAGTAGATTGTACAATG    23782
gb:MN996528|Organism:Severe      AGTGTTACCACAGAAATTCTACCAGTGTCTATGACCAAGACATCAGTAGATTGTACAATG    23782
gb:MN996530|Organism:Severe      AGTGTTACCACAGAAATTCTACCAGTGTCTATGACCAAGACATCAGTAGATTGTACAATG    23768
gb:MN908947|Organism:Severe      AGTGTTACCACAGAAATTCTACCAGTGTCTATGACCAAGACATCAGTAGATTGTACAATG    23782
gb:MT019532|Organism:Severe      AGTGTTACCACAGAAATTCTACCAGTGTCTATGACCAAGACATCAGTAGATTGTACAATG    23782
                                 ************************************************************
```

```
gb:MT020781|Organism:Severe      TACATTTGTGGTGATTCAACTGAATGCAGCAATCTTTTGTTGCAATATGGCAGTTTTTGT   23830
gb:MT007544|Organism:Severe      TACATTTGTGGTGATTCAACTGAATGCAGCAATCTTTTGTTGCAATATGGCAGTTTTTGT   23842
gb:MN994467|Organism:Severe      TACATTTGTGGTGATTCAACTGAATGCAGCAATCTTTTGTTGCAATATGGCAGTTTTTGT   23842
gb:MT044257|Organism:Severe      TACATTTGTGGTGATTCAACTGAATGCAGCAATCTTTTGTTGCAATATGGCAGTTTTTGT   23842
gb:MT106054|Organism:Severe      TACATTTGTGGTGATTCAACTGAATGCAGCAATCTTTTGTTGCAATATGGCAGTTTTTGT   23842
gb:MT049951|Organism:Severe      TACATTTGTGGTGATTCAACTGAATGCAGCAATCTTTTGTTGCAATATGGCAGTTTTTGT   23842
gb:MN975262|Organism:Severe      TACATTTGTGGTGATTCAACTGAATGCAGCAATCTTTTGTTGCAATATGGCAGTTTTTGT   23842
gb:MT106052|Organism:Severe      TACATTTGTGGTGATTCAACTGAATGCAGCAATCTTTTGTTGCAATATGGCAGTTTTTGT   23842
gb:LC522975|Organism:Severe      TACATTTGTGGTGATTCAACTGAATGCAGCAATCTTTTGTTGCAATATGGCAGTTTTTGT   23839
gb:LC522973|Organism:Severe      TACATTTGTGGTGATTCAACTGAATGCAGCAATCTTTTGTTGCAATATGGCAGTTTTTGT   23839
gb:LC522974|Organism:Severe      TACATTTGTGGTGATTCAACTGAATGCAGCAATCTTTTGTTGCAATATGGCAGTTTTTGT   23839
gb:MN985325|Organism:Severe      TACATTTGTGGTGATTCAACTGAATGCAGCAATCTTTTGTTGCAATATGGCAGTTTTTGT   23842
gb:MT020881|Organism:Severe      TACATTTGTGGTGATTCAACTGAATGCAGCAATCTTTTGTTGCAATATGGCAGTTTTTGT   23842
gb:MT020880|Organism:Severe      TACATTTGTGGTGATTCAACTGAATGCAGCAATCTTTTGTTGCAATATGGCAGTTTTTGT   23842
gb:MT066175|Organism:Severe      TACATTTGTGGTGATTCAACTGAATGCAGCAATCTTTTGTTGCAATATGGCAGTTTTTGT   23842
gb:MN997409|Organism:Severe      TACATTTGTGGTGATTCAACTGAATGCAGCAATCTTTTGTTGCAATATGGCAGTTTTTGT   23842
gb:MN938384|Organism:Severe      TACATTTGTGGTGATTCAACTGAATGCAGCAATCTTTTGTTGCAATATGGCAGTTTTTGT   23810
gb:MT044258|Organism:Severe      TACATTTGTGGTGATTCAACTGAATGCAGCAATCTTTTGTTGCAATATGGCAGTTTTTGT   23818
gb:MT039890|Organism:Severe      TACATTTGTGGTGATTCAACTGAATGCAGCAATCTTTTGTTGCAATATGGCAGTTTTTGT   23842
gb:MN988713|Organism:Severe      TACATTTGTGGTGATTCAACTGAATGCAGCAATCTTTTGTTGCAATATGGCAGTTTTTGT   23842
gb:LC521925|Organism:Severe      TACATTTGTGGTGATTCAACTGAATGCAGCAATCTTTTGTTGCAATATGGCAGTTTTTGT   23815
gb:MT093571|Organism:Severe      TACATTTGTGGTGATTCAACTGAATGCAGCAATCTTTTGTTGCAATATGGCAGTTTTTGT   23842
gb:MT039887|Organism:Severe      TACATTTGTGGTGATTCAACTGAATGCAGCAATCTTTTGTTGCAATATGGCAGTTTTTGT   23839
gb:MT019530|Organism:Severe      TACATTTGTGGTGATTCAACTGAATGCAGCAATCTTTTGTTGCAATATGGCAGTTTTTGT   23842
gb:MT039888|Organism:Severe      TACATTTGTGGTGATTCAACTGAATGCAGCAATCTTTTGTTGCAATATGGCAGTTTTTGT   23842
gb:LC522972|Organism:Severe      TACATTTGTGGTGATTCAACTGAATGCAGCAATCTTTTGTTGCAATATGGCAGTTTTTGT   23839
gb:MT027063|Organism:Severe      TACATTTGTGGTGATTCAACTGAATGCAGCAATCTTTTGTTGCAATATGGCAGTTTTTGT   23842
gb:MT027062|Organism:Severe      TACATTTGTGGTGATTCAACTGAATGCAGCAATCTTTTGTTGCAATATGGCAGTTTTTGT   23842
gb:MT019529|Organism:Severe      TACATTTGTGGTGATTCAACTGAATGCAGCAATCTTTTGTTGCAATATGGCAGTTTTTGT   23842
gb:MN996529|Organism:Severe      TACATTTGTGGTGATTCAACTGAATGCAGCAATCTTTTGTTGCAATATGGCAGTTTTTGT   23830
gb:MN996531|Organism:Severe      TACATTTGTGGTGATTCAACTGAATGCAGCAATCTTTTGTTGCAATATGGCAGTTTTTGT   23829
gb:MT066176|Organism:Severe      TACATTTGTGGTGATTCAACTGAATGCAGCAATCTTTTGTTGCAATATGGCAGTTTTTGT   23842
gb:MT027064|Organism:Severe      TACATTTGTGGTGATTCAACTGAATGCAGCAATCTTTTGTTGCAATATGGCAGTTTTTGT   23842
gb:MN994468|Organism:Severe      TACATTTGTGGTGATTCAACTGAATGCAGCAATCTTTTGTTGCAATATGGCAGTTTTTGT   23842
gb:MT072688|Organism:Severe      TACATTTGTGGTGATTCAACTGAATGCAGCAATCTTTTGTTGCAATATGGCAGTTTTTGT   23827
gb:MN996527|Organism:Severe      TACATTTGTGGTGATTCAACTGAATGCAGCAATCTTTTGTTGCAATATGGCAGTTTTTGT   23809
gb:MT093631|Organism:Severe      TACATTTGTGGTGATTCAACTGAATGCAGCAATCTTTTGTTGCAATATGGCAGTTTTTGT   23880
gb:MT106053|Organism:Severe      TACATTTGTGGTGATTCAACTGAATGCAGCAATCTTTTGTTGCAATATGGCAGTTTTTGT   23842
gb:MT019533|Organism:Severe      TACATTTGTGGTGATTCAACTGAATGCAGCAATCTTTTGTTGCAATATGGCAGTTTTTGT   23842
gb:MT019531|Organism:Severe      TACATTTGTGGTGATTCAACTGAATGCAGCAATCTTTTGTTGCAATATGGCAGTTTTTGT   23842
gb:MN996528|Organism:Severe      TACATTTGTGGTGATTCAACTGAATGCAGCAATCTTTTGTTGCAATATGGCAGTTTTTGT   23842
gb:MN996530|Organism:Severe      TACATTTGTGGTGATTCAACTGAATGCAGCAATCTTTTGTTGCAATATGGCAGTTTTTGT   23828
gb:MN908947|Organism:Severe      TACATTTGTGGTGATTCAACTGAATGCAGCAATCTTTTGTTGCAATATGGCAGTTTTTGT   23842
gb:MT019532|Organism:Severe      TACATTTGTGGTGATTCAACTGAATGCAGCAATCTTTTGTTGCAATATGGCAGTTTTTGT   23842
                                 ************************************************************

gb:MT020781|Organism:Severe      ACACAATTAAACCGTGCTTTAACTGGAATAGCTGTTGAACAAGACAAAAACACCCAAGAA   23890
gb:MT007544|Organism:Severe      ACACAATTAAACCGTGCTTTAACTGGAATAGCTGTTGAACAAGACAAAAACACCCAAGAA   23902
gb:MN994467|Organism:Severe      ACACAATTAAACCGTGCTTTAACTGGAATAGCTGTTGAACAAGACAAAAACACCCAAGAA   23902
gb:MT044257|Organism:Severe      ACACAATTAAACCGTGCTTTAACTGGAATAGCTGTTGAACAAGACAAAAACACCCAAGAA   23902
gb:MT106054|Organism:Severe      ACACAATTAAACCGTGCTTTAACTGGAATAGCTGTTGAACAAGACAAAAACACCCAAGAA   23902
gb:MT049951|Organism:Severe      ACACAATTAAACCGTGCTTTAACTGGAATAGCTGTTGAACAAGACAAAAACACCCAAGAA   23902
gb:MN975262|Organism:Severe      ACACAATTAAACCGTGCTTTAACTGGAATAGCTGTTGAACAAGACAAAAACACCCAAGAA   23902
gb:MT106052|Organism:Severe      ACACAATTAAACCGTGCTTTAACTGGAATAGCTGTTGAACAAGACAAAAACACCCAAGAA   23902
gb:LC522975|Organism:Severe      ACACAATTAAACCGTGCTTTAACTGGAATAGCTGTTGAACAAGACAAAAACACCCAAGAA   23899
gb:LC522973|Organism:Severe      ACACAATTAAACCGTGCTTTAACTGGAATAGCTGTTGAACAAGACAAAAACACCCAAGAA   23899
gb:LC522974|Organism:Severe      ACACAATTAAACCGTGCTTTAACTGGAATAGCTGTTGAACAAGACAAAAACACCCAAGAA   23899
gb:MN985325|Organism:Severe      ACACAATTAAACCGTGCTTTAACTGGAATAGCTGTTGAACAAGACAAAAACACCCAAGAA   23902
gb:MT020881|Organism:Severe      ACACAATTAAACCGTGCTTTAACTGGAATAGCTGTTGAACAAGACAAAAACACCCAAGAA   23902
gb:MT020880|Organism:Severe      ACACAATTAAACCGTGCTTTAACTGGAATAGCTGTTGAACAAGACAAAAACACCCAAGAA   23902
gb:MT066175|Organism:Severe      ACACAATTAAACCGTGCTTTAACTGGAATAGCTGTTGAACAAGACAAAAACACCCAAGAA   23902
gb:MN997409|Organism:Severe      ACACAATTAAACCGTGCTTTAACTGGAATAGCTGTTGAACAAGACAAAAACACCCAAGAA   23902
gb:MN938384|Organism:Severe      ACACAATTAAACCGTGCTTTAACTGGAATAGCTGTTGAACAAGACAAAAACACCCAAGAA   23870
gb:MT044258|Organism:Severe      ACACAATTAAACCGTGCTTTAACTGGAATAGCTGTTGAACAAGACAAAAACACCCAAGAA   23878
```

```
gb:MT039890|Organism:Severe      ACACAATTAAACCGTGCTTTAACTGGAATAGCTGTTGAACAAGACAAAAACACCCAAGAA   23902
gb:MN988713|Organism:Severe      ACACAATTAAACCGTGCTTTAACTGGAATAGCTGTTGAACAAGACAAAAACACCCAAGAA   23902
gb:LC521925|Organism:Severe      ACACAATTAAACCGTGCTTTAACTGGAATAGCTGTTGAACAAGACAAAAACACCCAAGAA   23875
gb:MT093571|Organism:Severe      ACACAATTAAACCGTGCTTTAACTGGAATAGCTGTTGAACAAGACAAAAACACCCAAGAA   23902
gb:MT039887|Organism:Severe      ACACAATTAAACCGTGCTTTAACTGGAATAGCTGTTGAACAAGACAAAAACACCCAAGAA   23899
gb:MT019530|Organism:Severe      ACACAATTAAACCGTGCTTTAACTGGAATAGCTGTTGAACAAGACAAAAACACCCAAGAA   23902
gb:MT039888|Organism:Severe      ACACAATTAAACCGTGCTTTAACTGGAATAGCTGTTGAACAAGACAAAAACACCCAAGAA   23902
gb:LC522972|Organism:Severe      ACACAATTAAACCGTGCTTTAACTGGAATAGCTGTTGAACAAGACAAAAACACCCAAGAA   23899
gb:MT027063|Organism:Severe      ACACAATTAAACCGTGCTTTAACTGGAATAGCTGTTGAACAAGACAAAAACACCCAAGAA   23902
gb:MT027062|Organism:Severe      ACACAATTAAACCGTGCTTTAACTGGAATAGCTGTTGAACAAGACAAAAACACCCAAGAA   23902
gb:MT019529|Organism:Severe      ACACAATTAAACCGTGCTTTAACTGGAATAGCTGTTGAACAAGACAAAAACACCCAAGAA   23902
gb:MN996529|Organism:Severe      ACACAATTAAACCGTGCTTTAACTGGAATAGCTGTTGAACAAGACAAAAACACCCAAGAA   23890
gb:MN996531|Organism:Severe      ACACAATTAAACCGTGCTTTAACTGGAATAGCTGTTGAACAAGACAAAAACACCCAAGAA   23889
gb:MT066176|Organism:Severe      ACACAATTAAACCGTGCTTTAACTGGAATAGCTGTTGAACAAGACAAAAACACCCAAGAA   23902
gb:MT027064|Organism:Severe      ACACAATTAAACCGTGCTTTAACTGGAATAGCTGTTGAACAAGACAAAAACACCCAAGAA   23902
gb:MN994468|Organism:Severe      ACACAATTAAACCGTGCTTTAACTGGAATAGCTGTTGAACAAGACAAAAACACCCAAGAA   23902
gb:MT072688|Organism:Severe      ACACAATTAAACCGTGCTTTAACTGGAATAGCTGTTGAACAAGACAAAAACACCCAAGAA   23887
gb:MN996527|Organism:Severe      ACACAATTAAACCGTGCTTTAACTGGAATAGCTGTTGAACAAGACAAAAACACCCAAGAA   23869
gb:MT093631|Organism:Severe      ACACAATTAAACCGTGCTTTAACTGGAATAGCTGTTGAACAAGACAAAAACACCCAAGAA   23940
gb:MT106053|Organism:Severe      ACACAATTAAACCGTGCTTTAACTGGAATAGCTGTTGAACAAGACAAAAACACCCAAGAA   23902
gb:MT019533|Organism:Severe      ACACAATTAAACCGTGCTTTAACTGGAATAGCTGTTGAACAAGACAAAAACACCCAAGAA   23902
gb:MT019531|Organism:Severe      ACACAATTAAACCGTGCTTTAACTGGAATAGCTGTTGAACAAGACAAAAACACCCAAGAA   23902
gb:MN996528|Organism:Severe      ACACAATTAAACCGTGCTTTAACTGGAATAGCTGTTGAACAAGACAAAAACACCCAAGAA   23902
gb:MN996530|Organism:Severe      ACACAATTAAACCGTGCTTTAACTGGAATAGCTGTTGAACAAGACAAAAACACCCAAGAA   23888
gb:MN908947|Organism:Severe      ACACAATTAAACCGTGCTTTAACTGGAATAGCTGTTGAACAAGACAAAAACACCCAAGAA   23902
gb:MT019532|Organism:Severe      ACACAATTAAACCGTGCTTTAACTGGAATAGCTGTTGAACAAGACAAAAACACCCAAGAA   23902
                                 ************************************************************

gb:MT020781|Organism:Severe      GTTTTTGCACAAGTCAAACAAATTTACAAAACACCACCAATTAAAGATTTTGGTGGTTTT   23950
gb:MT007544|Organism:Severe      GTTTTTGCACAAGTCAAACAAATTTACAAAACACCACCAATTAAAGATTTTGGTGGTTTT   23962
gb:MN994467|Organism:Severe      GTTTTTGCACAAGTCAAACAAATTTACAAAACACCACCAATTAAAGATTTTGGTGGTTTT   23962
gb:MT044257|Organism:Severe      GTTTTTGCACAAGTCAAACAAATTTACAAAACACCACCAATTAAAGATTTTGGTGGTTTT   23962
gb:MT106054|Organism:Severe      GTTTTTGCACAAGTCAAACAAATTTACAAAACACCACCAATTAAAGATTTTGGTGGTTTT   23962
gb:MT049951|Organism:Severe      GTTTTTGCACAAGTCAAACAAATTTACAAAACACCACCAATTAAAGATTTTGGTGGTTTT   23962
gb:MN975262|Organism:Severe      GTTTTTGCACAAGTCAAACAAATTTACAAAACACCACCAATTAAAGATTTTGGTGGTTTT   23962
gb:MT106052|Organism:Severe      GTTTTTGCACAAGTCAAACAAATTTACAAAACACCACCAATTAAAGATTTTGGTGGTTTT   23962
gb:LC522975|Organism:Severe      GTTTTTGCACAAGTCAAACAAATTTACAAAACACCACCAATTAAAGATTTTGGTGGTTTT   23959
gb:LC522973|Organism:Severe      GTTTTTGCACAAGTCAAACAAATTTACAAAACACCACCAATTAAAGATTTTGGTGGTTTT   23959
gb:LC522974|Organism:Severe      GTTTTTGCACAAGTCAAACAAATTTACAAAACACCACCAATTAAAGATTTTGGTGGTTTT   23959
gb:MN985325|Organism:Severe      GTTTTTGCACAAGTCAAACAAATTTACAAAACACCACCAATTAAAGATTTTGGTGGTTTT   23962
gb:MT020881|Organism:Severe      GTTTTTGCACAAGTCAAACAAATTTACAAAACACCACCAATTAAAGATTTTGGTGGTTTT   23962
gb:MT020880|Organism:Severe      GTTTTTGCACAAGTCAAACAAATTTACAAAACACCACCAATTAAAGATTTTGGTGGTTTT   23962
gb:MT066175|Organism:Severe      GTTTTTGCACAAGTCAAACAAATTTACAAAACACCACCAATTAAAGATTTTGGTGGTTTT   23962
gb:MN997409|Organism:Severe      GTTTTTGCACAAGTCAAACAAATTTACAAAACACCACCAATTAAAGATTTTGGTGGTTTT   23962
gb:MN938384|Organism:Severe      GTTTTTGCACAAGTCAAACAAATTTACAAAACACCACCAATTAAAGATTTTGGTGGTTTT   23930
gb:MT044258|Organism:Severe      GTTTTTGCACAAGTCAAACAAATTTACAAAACACCACCAATTAAAGATTTTGGTGGTTTT   23938
gb:MT039890|Organism:Severe      GTTTTTGCACAAGTCAAACAAATTTACAAAACACCACCAATTAAAGATTTTGGTGGTTTT   23962
gb:MN988713|Organism:Severe      GTTTTTGCACAAGTCAAACAAATTTACAAAACACCACCAATTAAAGATTTTGGTGGTTTT   23962
gb:LC521925|Organism:Severe      GTTTTTGCACAAGTCAAACAAATTTACAAAACACCACCAATTAAAGATTTTGGTGGTTTT   23935
gb:MT093571|Organism:Severe      GTTTTTGCACAAGTCAAACAAATTTACAAAACACCACCAATTAAAGATTGTGGTGGTTTT   23962
gb:MT039887|Organism:Severe      GTTTTTGCACAAGTCAAACAAATTTACAAAACACCACCAATTAAAGATTTTGGTGGTTTT   23959
gb:MT019530|Organism:Severe      GTTTTTGCACAAGTCAAACAAATTTACAAAACACCACCAATTAAAGATTTTGGTGGTTTT   23962
gb:MT039888|Organism:Severe      GTTTTTGCACAAGTCAAACAAATTTACAAAACACCACCAATTAAAGATTTTGGTGGTTTT   23962
gb:LC522972|Organism:Severe      GTTTTTGCACAAGTCAAACAAATTTACAAAACACCACCAATTAAAGATTTTGGTGGTTTT   23959
gb:MT027063|Organism:Severe      GTTTTTGCACAAGTCAAACAAATTTACAAAACACCACCAATTAAAGATTTTGGTGGTTTT   23962
gb:MT027062|Organism:Severe      GTTTTTGCACAAGTCAAACAAATTTACAAAACACCACCAATTAAAGATTTTGGTGGTTTT   23962
gb:MT019529|Organism:Severe      GTTTTTGCACAAGTCAAACAAATTTACAAAACACCACCAATTAAAGATTTTGGTGGTTTT   23962
gb:MN996529|Organism:Severe      GTTTTTGCACAAGTCAAACAAATTTACAAAACACCACCAATTAAAGATTTTGGTGGTTTT   23950
gb:MN996531|Organism:Severe      GTTTTTGCACAAGTCAAACAAATTTACAAAACACCACCAATTAAAGATTTTGGTGGTTTT   23949
gb:MT066176|Organism:Severe      GTTTTTGCACAAGTCAAACAAATTTACAAAACACCACCAATTAAAGATTTTGGTGGTTTT   23962
gb:MT027064|Organism:Severe      GTTTTTGCACAAGTCAAACAAATTTACAAAACACCACCAATTAAAGATTTTGGTGGTTTT   23962
gb:MN994468|Organism:Severe      GTTTTTGCACAAGTCAAACAAATTTACAAAACACCACCAATTAAAGATTTTGGTGGTTTT   23962
gb:MT072688|Organism:Severe      GTTTTTGCACAAGTCAAACAAATTTACAAAACACCACCAATTAAAGATTTTGGTGGTTTT   23947
gb:MN996527|Organism:Severe      GTTTTTGCACAAGTCAAACAAATTTACAAAACACCACCAATTAAAGATTTTGGTGGTTTT   23929
gb:MT093631|Organism:Severe      GTTTTTGCACAAGTCAAACAAATTTACAAAACACCACCAATTAAAGATTTTGGTGGTTTT   24000
```

```
gb:MT106053|Organism:Severe      GTTTTTGCACAAGTCAAACAAATTTACAAAACACCACCAATTAAAGATTTTGGTGGTTTT   23962
gb:MT019533|Organism:Severe      GTTTTTGCACAAGTCAAACAAATTTACAAAACACCACCAATTAAAGATTTTGGTGGTTTT   23962
gb:MT019531|Organism:Severe      GTTTTTGCACAAGTCAAACAAATTTACAAAACACCACCAATTAAAGATTTTGGTGGTTTT   23962
gb:MN996528|Organism:Severe      GTTTTTGCACAAGTCAAACAAATTTACAAAACACCACCAATTAAAGATTTTGGTGGTTTT   23962
gb:MN996530|Organism:Severe      GTTTTTGCACAAGTCAAACAAATTTACAAAACACCACCAATTAAAGATTTTGGTGGTTTT   23948
gb:MN908947|Organism:Severe      GTTTTTGCACAAGTCAAACAAATTTACAAAACACCACCAATTAAAGATTTTGGTGGTTTT   23962
gb:MT019532|Organism:Severe      GTTTTTGCACAAGTCAAACAAATTTACAAAACACCACCAATTAAAGATTTTGGTGGTTTT   23962
                                 ************************************************* **********

gb:MT020781|Organism:Severe      AATTTTTCACAAATATTACCAGATCCATCAAAACCAAGCAAGAGGTCATTTATTGAAGAT   24010
gb:MT007544|Organism:Severe      AATTTTTCACAAATATTACCAGATCCATCAAAACCAAGCAAGAGGTCATTTATTGAAGAT   24022
gb:MN994467|Organism:Severe      AATTTTTCACAAATATTACCAGATCCATCAAAACCAAGCAAGAGGTCATTTATTGAAGAT   24022
gb:MT044257|Organism:Severe      AATTTTTCACAAATATTACCAGATCCATCAAAACCAAGCAAGAGGTCATTTATTGAAGAT   24022
gb:MT106054|Organism:Severe      AATTTTTCACAAATATTACCAGATCCATCAAAACCAAGCAAGAGGTCATTTATTGAAGAT   24022
gb:MT049951|Organism:Severe      AATTTTTCACAAATATTACCAGATCCATCAAAACCAAGCAAGAGGTCATTTATTGAAGAT   24022
gb:MN975262|Organism:Severe      AATTTTTCACAAATATTACCAGATCCATCAAAACCAAGCAAGAGGTCATTTATTGAAGAT   24022
gb:MT106052|Organism:Severe      AATTTTTCACAAATATTACCAGATCCATCAAAACCAAGCAAGAGGTCATTTATTGAAGAT   24022
gb:LC522975|Organism:Severe      AATTTTTCACAAATATTACCAGATCCATCAAAACCAAGCAAGAGGTCATTTATTGAAGAT   24019
gb:LC522973|Organism:Severe      AATTTTTCACAAATATTACCAGATCCATCAAAACCAAGCAAGAGGTCATTTATTGAAGAT   24019
gb:LC522974|Organism:Severe      AATTTTTCACAAATATTACCAGATCCATCAAAACCAAGCAAGAGGTCATTTATTGAAGAT   24019
gb:MN985325|Organism:Severe      AATTTTTCACAAATATTACCAGATCCATCAAAACCAAGCAAGAGGTCATTTATTGAAGAT   24022
gb:MT020881|Organism:Severe      AATTTTTCACAAATATTACCAGATCCATCAAAACCAAGCAAGAGGTCATTTATTGAAGAT   24022
gb:MT020880|Organism:Severe      AATTTTTCACAAATATTACCAGATCCATCAAAACCAAGCAAGAGGTCATTTATTGAAGAT   24022
gb:MT066175|Organism:Severe      AATTTTTCACAAATATTACCAGATCCATCAAAACCAAGCAAGAGGTCATTTATTGAAGAT   24022
gb:MN997409|Organism:Severe      AATTTTTCACAAATATTACCAGATCCATCAAAACCAAGCAAGAGGTCATTTATTGAAGAT   24022
gb:MN938384|Organism:Severe      AATTTTTCACAAATATTACCAGATCCATCAAAACCAAGCAAGAGGTCATTTATTGAAGAT   23990
gb:MT044258|Organism:Severe      AATTTTTCACAAATATTACCAGATCCATCAAAACCAAGCAAGAGGTCATTTATTGAAGAT   23998
gb:MT039890|Organism:Severe      AATTTTTCACAAATATTACCAGATCCATCAAAACCAAGCAAGAGGTCATTTATTGAAGAT   24022
gb:MN988713|Organism:Severe      AATTTTTCACAAATATTACCAGATCCATCAAAACCAAGCAAGAGGTCATTTATTGAAGAT   24022
gb:LC521925|Organism:Severe      AATTTTTCACAAATATTACCAGATCCATCAAAACCAAGCAAGAGGTCATTTATTGAAGAT   23995
gb:MT093571|Organism:Severe      AATTTTTCACAAATATTACCAGATCCATCAAAACCAAGCAAGAGGTCATTTATTGAAGAT   24022
gb:MT039887|Organism:Severe      AATTTTTCACAAATATTACCAGATCCATCAAAACCAAGCAAGAGGTCATTTATTGAAGAT   24019
gb:MT019530|Organism:Severe      AATTTTTCACAAATATTACCAGATCCATCAAAACCAAGCAAGAGGTCATTTATTGAAGAT   24022
gb:MT039888|Organism:Severe      AATTTTTCACAAATATTACCAGATCCATCAAAACCAAGCAAGAGGTCATTTATTGAAGAT   24022
gb:LC522972|Organism:Severe      AATTTTTCACAAATATTACCAGATCCATCAAAACCAAGCAAGAGGTCATTTATTGAAGAT   24019
gb:MT027063|Organism:Severe      AATTTTTCACAAATATTACCAGATCCATCAAAACCAAGCAAGAGGTCATTTATTGAAGAT   24022
gb:MT027062|Organism:Severe      AATTTTTCACAAATATTACCAGATCCATCAAAACCAAGCAAGAGGTCATTTATTGAAGAT   24022
gb:MT019529|Organism:Severe      AATTTTTCACAAATATTACCAGATCCATCAAAACCAAGCAAGAGGTCATTTATTGAAGAT   24022
gb:MN996529|Organism:Severe      AATTTTTCACAAATATTACCAGATCCATCAAAACCAAGCAAGAGGTCATTTATTGAAGAT   24010
gb:MN996531|Organism:Severe      AATTTTTCACAAATATTACCAGATCCATCAAAACCAAGCAAGAGGTCATTTATTGAAGAT   24009
gb:MT066176|Organism:Severe      AATTTTTCACAAATATTACCAGATCCATCAAAACCAAGCAAGAGGTCATTTATTGAAGAT   24022
gb:MT027064|Organism:Severe      AATTTTTCACAAATATTACCAGATCCATCAAAACCAAGCAAGAGGTCATTTATTGAAGAT   24022
gb:MN994468|Organism:Severe      AATTTTTCACAAATATTACCAGATCCATCAAAACCAAGCAAGAGGTCATTTATTGAAGAT   24022
gb:MT072688|Organism:Severe      AATTTTTCACAAATATTACCAGATCCATCAAAACCAAGCAAGAGGTCATTTATTGAAGAT   24007
gb:MN996527|Organism:Severe      AATTTTTCACAAATATTACCAGATCCATCAAAACCAAGCAAGAGGTCATTTATTGAAGAT   23989
gb:MT093631|Organism:Severe      AATTTTTCACAAATATTACCAGATCCATCAAAACCAAGCAAGAGGTCATTTATTGAAGAT   24060
gb:MT106053|Organism:Severe      AATTTTTCACAAATATTACCAGATCCATCAAAACCAAGCAAGAGGTCATTTATTGAAGAT   24022
gb:MT019533|Organism:Severe      AATTTTTCACAAATATTACCAGATCCATCAAAACCAAGCAAGAGGTCATTTATTGAAGAT   24022
gb:MT019531|Organism:Severe      AATTTTTCACAAATATTACCAGATCCATCAAAACCAAGCAAGAGGTCATTTATTGAAGAT   24022
gb:MN996528|Organism:Severe      AATTTTTCACAAATATTACCAGATCCATCAAAACCAAGCAAGAGGTCATTTATTGAAGAT   24022
gb:MN996530|Organism:Severe      AATTTTTCACAAATATTACCAGATCCATCAAAACCAAGCAAGAGGTCATTTATTGAAGAT   24008
gb:MN908947|Organism:Severe      AATTTTTCACAAATATTACCAGATCCATCAAAACCAAGCAAGAGGTCATTTATTGAAGAT   24022
gb:MT019532|Organism:Severe      AATTTTTCACAAATATTACCAGATCCATCAAAACCAAGCAAGAGGTCATTTATTGAAGAT   24022
                                 ************************************************************

gb:MT020781|Organism:Severe      CTACTTTTCAACAAAGTGACACTTGCAGATGCTGGCTTCATCAAACAATATGGTGATTGC   24070
gb:MT007544|Organism:Severe      CTACTTTTCAACAAAGTGACACTTGCAGATGCTGGCTTCATCAAACAATATGGTGATTGC   24082
gb:MN994467|Organism:Severe      CTACTTTTCAATAAAGTGACACTTGCAGATGCTGGCTTCATCAAACAATATGGTGATTGC   24082
gb:MT044257|Organism:Severe      CTACTTTTCAATAAAGTGACACTTGCAGATGCTGGCTTCATCAAACAATATGGTGATTGC   24082
gb:MT106054|Organism:Severe      CTACTTTTCAACAAAGTGACACTTGCAGATGCTGGCTTCATCAAACAATATGGTGATTGC   24082
gb:MT049951|Organism:Severe      CTACTTTTCAACAAAGTGACACTTGCAGATGCTGGCTTCATCAAACAATATGGTGATTGC   24082
gb:MN975262|Organism:Severe      CTACTTTTCAACAAAGTGACACTTGCAGATGCTGGCTTCATCAAACAATATGGTGATTGC   24082
gb:MT106052|Organism:Severe      CTACTTTTCAACAAAGTGACACTTGCAGATGCTGGCTTCATCAAACAATATGGTGATTGC   24082
gb:LC522975|Organism:Severe      CTACTTTTCAACAAAGTGACACTTGCAGATGCTGGCTTCATCAAACAATATGGTGATTGC   24079
gb:LC522973|Organism:Severe      CTACTTTTCAACAAAGTGACACTTGCAGATGCTGGCTTCATCAAACAATATGGTGATTGC   24079
```

```
gb:LC522974|Organism:Severe      CTACTTTTCAACAAAGTGACACTTGCAGATGCTGGCTTCATCAAACAATATGGTGATTGC   24079
gb:MN985325|Organism:Severe      CTACTTTTCAACAAAGTGACACTTGCAGATGCTGGCTTCATCAAACAATATGGTGATTGC   24082
gb:MT020881|Organism:Severe      CTACTTTTCAACAAAGTGACACTTGCAGATGCTGGCTTCATCAAACAATATGGTGATTGC   24082
gb:MT020880|Organism:Severe      CTACTTTTCAACAAAGTGACACTTGCAGATGCTGGCTTCATCAAACAATATGGTGATTGC   24082
gb:MT066175|Organism:Severe      CTACTTTTCAACAAAGTGACACTTGCAGATGCTGGCTTCATCAAACAATATGGTGATTGC   24082
gb:MN997409|Organism:Severe      CTACTTTTCAACAAAGTGACACTTGCAGATGCTGGCTTCATCAAACAATATGGTGATTGC   24082
gb:MN938384|Organism:Severe      CTACTTTTCAACAAAGTGACACTTGCAGATGCTGGCTTCATCAAACAATATGGTGATTGC   24050
gb:MT044258|Organism:Severe      CTACTTTTCAACAAAGTGACACTTGCAGATGCTGGCTTCATCAAACAATATGGTGATTGC   24058
gb:MT039890|Organism:Severe      CTACTTTTCAACAAAGTGACACTTGCAGATGCTGGCTTCATCAAACAATATGGTGATTGC   24082
gb:MN988713|Organism:Severe      CTACTTTTCAAYAAAGTGACACTTGCAGATGCTGGCTTCATCAAACAATATGGTGATTGC   24082
gb:LC521925|Organism:Severe      CTACTTTTCAACAAAGTGACACTTGCAGATGCTGGCTTCATCAAACAATATGGTGATTGC   24055
gb:MT093571|Organism:Severe      CTACTTTTCAACAAAGTGACACTTGCAGATGCTGGCTTCATCAAACAATATGGTGATTGC   24082
gb:MT039887|Organism:Severe      CTACTTTTCAACAAAGTGACACTTGCAGATGCTGGCTTCATCAAACAATATGGTGATTGC   24079
gb:MT019530|Organism:Severe      CTACTTTTCAACAAAGTGACACTTGCAGATGCTGGCTTCATCAAACAATATGGTGATTGC   24082
gb:MT039888|Organism:Severe      CTACTTTTCAATAAAGTGACACTTGCAGATGCTGGCTTCATCAAACAATATGGTGATTGC   24082
gb:LC522972|Organism:Severe      CTACTTTTCAACAAAGTGACACTTGCAGATGCTGGCTTCATCAAACAATATGGTGATTGC   24079
gb:MT027063|Organism:Severe      CTACTTTTCAACAAAGTGACACTTGCAGATGCTGGCTTCATCAAACAATATGGTGATTGC   24082
gb:MT027062|Organism:Severe      CTACTTTTCAACAAAGTGACACTTGCAGATGCTGGCTTCATCAAACAATATGGTGATTGC   24082
gb:MT019529|Organism:Severe      CTACTTTTCAACAAAGTGACACTTGCAGATGCTGGCTTCATCAAACAATATGGTGATTGC   24082
gb:MN996529|Organism:Severe      CTACTTTTCAACAAAGTGACACTTGCAGATGCTGGCTTCATCAAACAATATGGTGATTGC   24070
gb:MN996531|Organism:Severe      CTACTTTTCAACAAAGTGACACTTGCAGATGCTGGCTTCATCAAACAATATGGTGATTGC   24069
gb:MT066176|Organism:Severe      CTACTTTTCAACAAAGTGACACTTGCAGATGCTGGCTTCATCAAACAATATGGTGATTGC   24082
gb:MT027064|Organism:Severe      CTACTTTTCAACAAAGTGACACTTGCAGATGCTGGCTTCATCAAACAATATGGTGATTGC   24082
gb:MN994468|Organism:Severe      CTACTTTTCAACAAAGTGACACTTGCAGATGCTGGCTTCATCAAACAATATGGTGATTGC   24082
gb:MT072688|Organism:Severe      CTACTTTTCAATAAAGTGACACTTGCAGATGCTGGCTTCATCAAACAATATGGTGATTGC   24067
gb:MN996527|Organism:Severe      CTACTTTTCAACAAAGTGACACTTGCAGATGCTGGCTTCATCAAACAATATGGTGATTGC   24049
gb:MT093631|Organism:Severe      CTACTTTTCAACAAAGTGACACTTGCAGATGCTGGCTTCATCAAACAATATGGTGATTGC   24120
gb:MT106053|Organism:Severe      CTACTTTTCAACAAAGTGACACTTGCAGATGCTGGCTTCATCAAACAATATGGTGATTGC   24082
gb:MT019533|Organism:Severe      CTACTTTTCAACAAAGTGACACTTGCAGATGCTGGCTTCATCAAACAATATGGTGATTGC   24082
gb:MT019531|Organism:Severe      CTACTTTTCAACAAAGTGACACTTGCAGATGCTGGCTTCATCAAACAATATGGTGATTGC   24082
gb:MN996528|Organism:Severe      CTACTTTTCAACAAAGTGACACTTGCAGATGCTGGCTTCATCAAACAATATGGTGATTGC   24082
gb:MN996530|Organism:Severe      CTACTTTTCAACAAAGTGACACTTGCAGATGCTGGCTTCATCAAACAATATGGTGATTGC   24068
gb:MN908947|Organism:Severe      CTACTTTTCAACAAAGTGACACTTGCAGATGCTGGCTTCATCAAACAATATGGTGATTGC   24082
gb:MT019532|Organism:Severe      CTACTTTTCAACAAAGTGACACTTGCAGATGCTGGCTTCATCAAACAATATGGTGATTGC   24082
                                 *********** ************************************************

gb:MT020781|Organism:Severe      CTTGGTGATATTGCTGCTAGAGACCTCATTTGTGCACAAAAGTTTAACGGCCTTACTGTT   24130
gb:MT007544|Organism:Severe      CTTGGTGATATTGCTGCTAGAGACCTCATTTGTGCACAAAAGTTTAACGGCCTTACTGTT   24142
gb:MN994467|Organism:Severe      CTTGGTGATATTGCTGCTAGAGACCTCATTTGTGCACAAAAGTTTAACGGCCTTACTGTT   24142
gb:MT044257|Organism:Severe      CTTGGTGATATTGCTGCTAGAGACCTCATTTGTGCACAAAAGTTTAACGGCCTTACTGTT   24142
gb:MT106054|Organism:Severe      CTTGGTGATATTGCTGCTAGAGACCTCATTTGTGCACAAAAGTTTAACGGCCTTACTGTT   24142
gb:MT049951|Organism:Severe      CTTGGTGATATTGCTGCTAGAGACCTCATTTGTGCACAAAAGTTTAACGGCCTTACTGTT   24142
gb:MN975262|Organism:Severe      CTTGGTGATATTGCTGCTAGAGACCTCATTTGTGCACAAAAGTTTAACGGCCTTACTGTT   24142
gb:MT106052|Organism:Severe      CTTGGTGATATTGCTGCTAGAGACCTCATTTGTGCACAAAAGTTTAACGGCCTTACTGTT   24142
gb:LC522975|Organism:Severe      CTTGGTGATATTGCTGCTAGAGACCTCATTTGTGCACAAAAGTTTAACGGCCTTACTGTT   24139
gb:LC522973|Organism:Severe      CTTGGTGATATTGCTGCTAGAGACCTCATTTGTGCACAAAAGTTTAACGGCCTTACTGTT   24139
gb:LC522974|Organism:Severe      CTTGGTGATATTGCTGCTAGAGACCTCATTTGTGCACAAAAGTTTAACGGCCTTACTGTT   24139
gb:MN985325|Organism:Severe      CTTGGTGATATTGCTGCTAGAGACCTCATTTGTGCACAAAAGTTTAACGGCCTTACTGTT   24142
gb:MT020881|Organism:Severe      CTTGGTGATATTGCTGCTAGAGACCTCATTTGTGCACAAAAGTTTAACGGCCTTACTGTT   24142
gb:MT020880|Organism:Severe      CTTGGTGATATTGCTGCTAGAGACCTCATTTGTGCACAAAAGTTTAACGGCCTTACTGTT   24142
gb:MT066175|Organism:Severe      CTTGGTGATATTGCTGCTAGAGACCTCATTTGTGCACAAAAGTTTAACGGCCTTACTGTT   24142
gb:MN997409|Organism:Severe      CTTGGTGATATTGCTGCTAGAGACCTCATTTGTGCACAAAAGTTTAACGGCCTTACTGTT   24142
gb:MN938384|Organism:Severe      CTTGGTGATATTGCTGCTAGAGACCTCATTTGTGCACAAAAGTTTAACGGCCTTACTGTT   24110
gb:MT044258|Organism:Severe      CTTGGTGATATTGCTGCTAGAGACCTCATTTGTGCACAAAAGTTTAACGGCCTTACTGTT   24118
gb:MT039890|Organism:Severe      CTTGGTGATATTGCTGCTAGAGACCTCATTTGTGCACAAAAGTTTAACGGCCTTACTGTT   24142
gb:MN988713|Organism:Severe      CTTGGTGATATTGCTGCTAGAGACCTCATTTGTGCACAAAAGTTTAACGGCCTTACTGTT   24142
gb:LC521925|Organism:Severe      CTTGGTGATATTGCTGCTAGAGACCTCATTTGTGCACAAAAGTTTAACGGCCTTACTGTT   24115
gb:MT093571|Organism:Severe      CTTGGTGATATTGCTGCTAGAGACCTCATTTGTGCACAAAAGTTTAACGGCCTTACTGTT   24142
gb:MT039887|Organism:Severe      CTTGGTGATATTGCTGCTAGAGACCTCATTTGTGCACAAAAGTTTAACGGCCTTACTGTT   24139
gb:MT019530|Organism:Severe      CTTGGTGATATTGCTGCTAGAGACCTCATTTGTGCACAAAAGTTTAACGGCCTTACTGTT   24142
gb:MT039888|Organism:Severe      CTTGGTGATATTGCTGCTAGAGACCTCATTTGTGCACAAAAGTTTAACGGCCTTACTGTT   24142
gb:LC522972|Organism:Severe      CTTGGTGATATTGCTGCTAGAGACCTCATTTGTGCACAAAAGTTTAACGGCCTTACTGTT   24139
gb:MT027063|Organism:Severe      CTTGGTGATATTGCTGCTAGAGACCTCATTTGTGCACAAAAGTTTAACGGCCTTACTGTT   24142
gb:MT027062|Organism:Severe      CTTGGTGATATTGCTGCTAGAGACCTCATTTGTGCACAAAAGTTTAACGGCCTTACTGTT   24142
gb:MT019529|Organism:Severe      CTTGGTGATATTGCTGCTAGAGACCTCATTTGTGCACAAAAGTTTAACGGCCTTACTGTT   24142
```

```
gb:MN996529|Organism:Severe      CTTGGTGATATTGCTGCTAGAGACCTCATTTGTGCACAAAAGTTTAACGGCCTTACTGTT   24130
gb:MN996531|Organism:Severe      CTTGGTGATATTGCTGCTAGAGACCTCATTTGTGCACAAAAGTTTAACGGCCTTACTGTT   24129
gb:MT066176|Organism:Severe      CTTGGTGATATTGCTGCTAGAGACCTCATTTGTGCACAAAAGTTTAACGGCCTTACTGTT   24142
gb:MT027064|Organism:Severe      CTTGGTGATATTGCTGCTAGAGACCTCATTTGTGCACAAAAGTTTAACGGCCTTACTGTT   24142
gb:MN994468|Organism:Severe      CTTGGTGATATTGCTGCTAGAGACCTCATTTGTGCACAAAAGTTTAACGGCCTTACTGTT   24142
gb:MT072688|Organism:Severe      CTTGGTGATATTGCTGCTAGAGACCTCATTTGTGCACAAAAGTTTAACGGCCTTACTGTT   24127
gb:MN996527|Organism:Severe      CTTGGTGATATTGCTGCTAGAGACCTCATTTGTGCACAAAAGTTTAACGGCCTTACTGTT   24109
gb:MT093631|Organism:Severe      CTTGGTGATATTGCTGCTAGAGACCTCATTTGTGCACAAAAGTTTAACGGCCTTACTGTT   24180
gb:MT106053|Organism:Severe      CTTGGTGATATTGCTGCTAGAGACCTCATTTGTGCACAAAAGTTTAACGGCCTTACTGTT   24142
gb:MT019533|Organism:Severe      CTTGGTGATATTGCTGCTAGAGACCTCATTTGTGCACAAAAGTTTAACGGCCTTACTGTT   24142
gb:MT019531|Organism:Severe      CTTGGTGATATTGCTGCTAGAGACCTCATTTGTGCACAAAAGTTTAACGGCCTTACTGTT   24142
gb:MN996528|Organism:Severe      CTTGGTGATATTGCTGCTAGAGACCTCATTTGTGCACAAAAGTTTAACGGCCTTACTGTT   24142
gb:MN996530|Organism:Severe      CTTGGTGATATTGCTGCTAGAGACCTCATTTGTGCACAAAAGTTTAACGGCCTTACTGTT   24128
gb:MN908947|Organism:Severe      CTTGGTGATATTGCTGCTAGAGACCTCATTTGTGCACAAAAGTTTAACGGCCTTACTGTT   24142
gb:MT019532|Organism:Severe      CTTGGTGATATTGCTGCTAGAGACCTCATTTGTGCACAAAAGTTTAACGGCCTTACTGTT   24142
                                 ************************************************************

gb:MT020781|Organism:Severe      TTGCCACCTTTGCTCACAGATGAAATGATTGCTCAATACACTTCTGCACTGTTAGCGGGT   24190
gb:MT007544|Organism:Severe      TTGCCACCTTTGCTCACAGATGAAATGATTGCTCAATACACTTCTGCACTGTTAGCGGGT   24202
gb:MN994467|Organism:Severe      TTGCCACCTTTGCTCACAGATGAAATGATTGCTCAATACACTTCTGCACTGTTAGCGGGT   24202
gb:MT044257|Organism:Severe      TTGCCACCTTTGCTCACAGATGAAATGATTGCTCAATACACTTCTGCACTGTTAGCGGGT   24202
gb:MT106054|Organism:Severe      TTGCCACCTTTGCTCACAGATGAAATGATTGCTCAATACACTTCTGCACTGTTAGCGGGT   24202
gb:MT049951|Organism:Severe      TTGCCACCTTTGCTCACAGATGAAATGATTGCTCAATACACTTCTGCACTGTTAGCGGGT   24202
gb:MN975262|Organism:Severe      TTGCCACCTTTGCTCACAGATGAAATGATTGCTCAATACACTTCTGCACTGTTAGCGGGT   24202
gb:MT106052|Organism:Severe      TTGCCACCTTTGCTCACAGATGAAATGATTGCTCAATACACTTCTGCACTGTTAGCGGGT   24202
gb:LC522975|Organism:Severe      TTGCCACCTTTGCTCACAGATGAAATGATTGCTCAATACACTTCTGCACTGTTAGCGGGT   24199
gb:LC522973|Organism:Severe      TTGCCACCTTTGCTCACAGATGAAATGATTGCTCAATACACTTCTGCACTGTTAGCGGGT   24199
gb:LC522974|Organism:Severe      TTGCCACCTTTGCTCACAGATGAAATGATTGCTCAATACACTTCTGCACTGTTAGCGGGT   24199
gb:MN985325|Organism:Severe      TTGCCACCTTTGCTCACAGATGAAATGATTGCTCAATACACTTCTGCACTGTTAGCGGGT   24202
gb:MT020881|Organism:Severe      TTGCCACCTTTGCTCACAGATGAAATGATTGCTCAATACACTTCTGCACTGTTAGCGGGT   24202
gb:MT020880|Organism:Severe      TTGCCACCTTTGCTCACAGATGAAATGATTGCTCAATACACTTCTGCACTGTTAGCGGGT   24202
gb:MT066175|Organism:Severe      TTGCCACCTTTGCTCACAGATGAAATGATTGCTCAATACACTTCTGCACTGTTAGCGGGT   24202
gb:MN997409|Organism:Severe      TTGCCACCTTTGCTCACAGATGAAATGATTGCTCAATACACTTCTGCACTGTTAGCGGGT   24202
gb:MN938384|Organism:Severe      TTGCCACCTTTGCTCACAGATGAAATGATTGCTCAATACACTTCTGCACTGTTAGCGGGT   24170
gb:MT044258|Organism:Severe      TTGCCACCTTTGCTCACAGATGAAATGATTGCTCAATACACTTCTGCACTGTTAGCGGGT   24178
gb:MT039890|Organism:Severe      TTGCCACCTTTGCTCACAGATGAAATGATTGCTCAATACACTTCTGCACTGTTAGCGGGT   24202
gb:MN988713|Organism:Severe      TTGCCACCTTTGCTCACAGATGAAATGATTGCTCAATACACTTCTGCACTGTTAGCGGGT   24202
gb:LC521925|Organism:Severe      TTGCCACCTTTGCTCACAGATGAAATGATTGCTCAATACACTTCTGCACTGTTAGCGGGT   24175
gb:MT093571|Organism:Severe      TTGCCACCTTTGCTCACAGATGAAATGATTGCTCAATACACTTCTGCACTGTTAGCGGGT   24202
gb:MT039887|Organism:Severe      TTGCCACCTTTGCTCACAGATGAAATGATTGCTCAATACACTTCTGCACTGTTAGCGGGT   24199
gb:MT019530|Organism:Severe      TTGCCACCTTTGCTCACAGATGAAATGATTGCTCAATACACTTCTGCACTGTTAGCGGGT   24202
gb:MT039888|Organism:Severe      TTGCCACCTTTGCTCACAGATGAAATGATTGCTCAATACACTTCTGCACTGTTAGCGGGT   24202
gb:LC522972|Organism:Severe      TTGCCACCTTTGCTCACAGATGAAATGATTGCTCAATACACTTCTGCACTGTTAGCGGGT   24199
gb:MT027063|Organism:Severe      TTGCCACCTTTGCTCACAGATGAAATGATTGCTCAATACACTTCTGCACTGTTAGCGGGT   24202
gb:MT027062|Organism:Severe      TTGCCACCTTTGCTCACAGATGAAATGATTGCTCAATACACTTCTGCACTGTTAGCGGGT   24202
gb:MT019529|Organism:Severe      TTGCCACCTTTGCTCACAGATGAAATGATTGCTCAATACACTTCTGCACTGTTAGCGGGT   24202
gb:MN996529|Organism:Severe      TTGCCACCTTTGCTCACAGATGAAATGATTGCTCAATACACTTCTGCACTGTTAGCGGGT   24190
gb:MN996531|Organism:Severe      TTGCCACCTTTGCTCACAGATGAAATGATTGCTCAATACACTTCTGCACTGTTAGCGGGT   24189
gb:MT066176|Organism:Severe      TTGCCACCTTTGCTCACAGATGAAATGATTGCTCAATACACTTCTGCACTGTTAGCGGGT   24202
gb:MT027064|Organism:Severe      TTGCCACCTTTGCTCACAGATGAAATGATTGCTCAATACACTTCTGCACTGTTAGCGGGT   24202
gb:MN994468|Organism:Severe      TTGCCACCTTTGCTCACAGATGAAATGATTGCTCAATACACTTCTGCACTGTTAGCGGGT   24202
gb:MT072688|Organism:Severe      TTGCCACCTTTGCTCACAGATGAAATGATTGCTCAATACACTTCTGCACTGTTAGCGGGT   24187
gb:MN996527|Organism:Severe      TTGCCACCTTTGCTCACAGATGAAATGATTGCTCAATACACTTCTGCACTGTTAGCGGGT   24169
gb:MT093631|Organism:Severe      TTGCCACCTTTGCTCACAGATGAAATGATTGCTCAATACACTTCTGCACTGTTAGCGGGT   24240
gb:MT106053|Organism:Severe      TTGCCACCTTTGCTCACAGATGAAATGATTGCTCAATACACTTCTGCACTGTTAGCGGGT   24202
gb:MT019533|Organism:Severe      TTGCCACCTTTGCTCACAGATGAAATGATTGCTCAATACACTTCTGCACTGTTAGCGGGT   24202
gb:MT019531|Organism:Severe      TTGCCACCTTTGCTCACAGATGAAATGATTGCTCAATACACTTCTGCACTGTTAGCGGGT   24202
gb:MN996528|Organism:Severe      TTGCCACCTTTGCTCACAGATGAAATGATTGCTCAATACACTTCTGCACTGTTAGCGGGT   24202
gb:MN996530|Organism:Severe      TTGCCACCTTTGCTCACAGATGAAATGATTGCTCAATACACTTCTGCACTGTTAGCGGGT   24188
gb:MN908947|Organism:Severe      TTGCCACCTTTGCTCACAGATGAAATGATTGCTCAATACACTTCTGCACTGTTAGCGGGT   24202
gb:MT019532|Organism:Severe      TTGCCACCTTTGCTCACAGATGAAATGATTGCTCAATACACTTCTGCACTGTTAGCGGGT   24202
                                 ************************************************************

gb:MT020781|Organism:Severe      ACAATCACTTCTGGTTGGACCTTTGGTGCAGGTGCTGCATTACAAATACCATTTGCTATG   24250
gb:MT007544|Organism:Severe      ACAATCACTTCTGGTTGGACCTTTGGTGCAGGTGCTGCATTACAAATACCATTTGCTATG   24262
```

```
gb:MN994467|Organism:Severe      ACAATCACTTCTGGTTGGACCTTTGGTGCAGGTGCTGCATTACAAATACCATTTGCTATG   24262
gb:MT044257|Organism:Severe      ACAATCACTTCTGGTTGGACCTTTGGTGCAGGTGCTGCATTACAAATACCATTTGCTATG   24262
gb:MT106054|Organism:Severe      ACAATCACTTCTGGTTGGACCTTTGGTGCAGGTGCTGCATTACAAATACCATTTGCTATG   24262
gb:MT049951|Organism:Severe      ACAATCACTTCTGGTTGGACCTTTGGTGCAGGTGCTGCATTACAAATACCATTTGCTATG   24262
gb:MN975262|Organism:Severe      ACAATCACTTCTGGTTGGACCTTTGGTGCAGGTGCTGCATTACAAATACCATTTGCTATG   24262
gb:MT106052|Organism:Severe      ACAATCACTTCTGGTTGGACCTTTGGTGCAGGTGCTGCATTACAAATACCATTTGCTATG   24262
gb:LC522975|Organism:Severe      ACAATCACTTCTGGTTGGACCTTTGGTGCAGGTGCTGCATTACAAATACCATTTGCTATG   24259
gb:LC522973|Organism:Severe      ACAATCACTTCTGGTTGGACCTTTGGTGCAGGTGCTGCATTACAAATACCATTTGCTATG   24259
gb:LC522974|Organism:Severe      ACAATCACTTCTGGTTGGACCTTTGGTGCAGGTGCTGCATTACAAATACCATTTGCTATG   24259
gb:MN985325|Organism:Severe      ACAATCACTTCTGGTTGGACCTTTGGTGCAGGTGCTGCATTACAAATACCATTTGCTATG   24262
gb:MT020881|Organism:Severe      ACAATCACTTCTGGTTGGACCTTTGGTGCAGGTGCTGCATTACAAATACCATTTGCTATG   24262
gb:MT020880|Organism:Severe      ACAATCACTTCTGGTTGGACCTTTGGTGCAGGTGCTGCATTACAAATACCATTTGCTATG   24262
gb:MT066175|Organism:Severe      ACAATCACTTCTGGTTGGACCTTTGGTGCAGGTGCTGCATTACAAATACCATTTGCTATG   24262
gb:MN997409|Organism:Severe      ACAATCACTTCTGGTTGGACCTTTGGTGCAGGTGCTGCATTACAAATACCATTTGCTATG   24262
gb:MN938384|Organism:Severe      ACAATCACTTCTGGTTGGACCTTTGGTGCAGGTGCTGCATTACAAATACCATTTGCTATG   24230
gb:MT044258|Organism:Severe      ACAATCACTTCTGGTTGGACCTTTGGTGCAGGTGCTGCATTACAAATACCATTTGCTATG   24238
gb:MT039890|Organism:Severe      ACAATCACTTCTGGTTGGACCTTTGGTGCAGGTGCTGCATTACAAATACCATTTGCTATG   24262
gb:MN988713|Organism:Severe      ACAATCACTTCTGGTTGGACCTTTGGTGCAGGTGCTGCATTACAAATACCATTTGCTATG   24262
gb:LC521925|Organism:Severe      ACAATCACTTCTGGTTGGACCTTTGGTGCAGGTGCTGCATTACAAATACCATTTGCTATG   24235
gb:MT093571|Organism:Severe      ACAATCACTTCTGGTTGGACCTTTGGTGCAGGTGCTGCATTACAAATACCATTTGCTATG   24262
gb:MT039887|Organism:Severe      ACAATCACTTCTGGTTGGACCTTTGGTGCAGGTGCTGCATTACAAATACCATTTGCTATG   24259
gb:MT019530|Organism:Severe      ACAATCACTTCTGGTTGGACCTTTGGTGCAGGTGCTGCATTACAAATACCATTTGCTATG   24262
gb:MT039888|Organism:Severe      ACAATCACTTCTGGTTGGACCTTTGGTGCAGGTGCTGCATTACAAATACCATTTGCTATG   24262
gb:LC522972|Organism:Severe      ACAATCACTTCTGGTTGGACCTTTGGTGCAGGTGCTGCATTACAAATACCATTTGCTATG   24259
gb:MT027063|Organism:Severe      ACAATCACTTCTGGTTGGACCTTTGGTGCAGGTGCTGCATTACAAATACCATTTGCTATG   24262
gb:MT027062|Organism:Severe      ACAATCACTTCTGGTTGGACCTTTGGTGCAGGTGCTGCATTACAAATACCATTTGCTATG   24262
gb:MT019529|Organism:Severe      ACAATCACTTCTGGTTGGACCTTTGGTGCAGGTGCTGCATTACAAATACCATTTGCTATG   24262
gb:MN996529|Organism:Severe      ACAATCACTTCTGGTTGGACCTTTGGTGCAGGTGCTGCATTACAAATACCATTTGCTATG   24250
gb:MN996531|Organism:Severe      ACAATCACTTCTGGTTGGACCTTTGGTGCAGGTGCTGCATTACAAATACCATTTGCTATG   24249
gb:MT066176|Organism:Severe      ACAATCACTTCTGGTTGGACCTTTGGTGCAGGTGCTGCATTACAAATACCATTTGCTATG   24262
gb:MT027064|Organism:Severe      ACAATCACTTCTGGTTGGACCTTTGGTGCAGGTGCTGCATTACAAATACCATTTGCTATG   24262
gb:MN994468|Organism:Severe      ACAATCACTTCTGGTTGGACCTTTGGTGCAGGTGCTGCATTACAAATACCATTTGCTATG   24262
gb:MT072688|Organism:Severe      ACAATCACTTCTGGTTGGACCTTTGGTGCAGGTGCTGCATTACAAATACCATTTGCTATG   24247
gb:MN996527|Organism:Severe      ACAATCACTTCTGGTTGGACCTTTGGTGCAGGTGCTGCATTACAAATACCATTTGCTATG   24229
gb:MT093631|Organism:Severe      ACAATCACTTCTGGTTGGACCTTTGGTGCAGGTGCTGCATTACAAATACCATTTGCTATG   24300
gb:MT106053|Organism:Severe      ACAATCACTTCTGGTTGGACCTTTGGTGCAGGTGCTGCATTACAAATACCATTTGCTATG   24262
gb:MT019533|Organism:Severe      ACAATCACTTCTGGTTGGACCTTTGGTGCAGGTGCTGCATTACAAATACCATTTGCTATG   24262
gb:MT019531|Organism:Severe      ACAATCACTTCTGGTTGGACCTTTGGTGCAGGTGCTGCATTACAAATACCATTTGCTATG   24262
gb:MN996528|Organism:Severe      ACAATCACTTCTGGTTGGACCTTTGGTGCAGGTGCTGCATTACAAATACCATTTGCTATG   24262
gb:MN996530|Organism:Severe      ACAATCACTTCTGGTTGGACCTTTGGTGCAGGTGCTGCATTACAAATACCATTTGCTATG   24248
gb:MN908947|Organism:Severe      ACAATCACTTCTGGTTGGACCTTTGGTGCAGGTGCTGCATTACAAATACCATTTGCTATG   24262
gb:MT019532|Organism:Severe      ACAATCACTTCTGGTTGGACCTTTGGTGCAGGTGCTGCATTACAAATACCATTTGCTATG   24262
                                 ************************************************************

gb:MT020781|Organism:Severe      CAAATGGCTTATAGGTTTAATGGTATTGGAGTTACACAGAATGTTCTCTATGAGAACCAA   24310
gb:MT007544|Organism:Severe      CAAATGGCTTATAGGTTTAATGGTATTGGAGTTACACAGAATGTTCTCTATGAGAACCAA   24322
gb:MN994467|Organism:Severe      CAAATGGCTTATAGGTTTAATGGTATTGGAGTTACACAGAATGTTCTCTATGAGAACCAA   24322
gb:MT044257|Organism:Severe      CAAATGGCTTATAGGTTTAATGGTATTGGAGTTACACAGAATGTTCTCTATGAGAACCAA   24322
gb:MT106054|Organism:Severe      CAAATGGCTTATAGGTTTAATGGTATTGGAGTTACACAGAATGTTCTCTATGAGAACCAA   24322
gb:MT049951|Organism:Severe      CAAATGGCTTATAGGTTTAATGGTATTGGAGTTACACAGAATGTTCTCTATGAGAACCAA   24322
gb:MN975262|Organism:Severe      CAAATGGCTTATAGGTTTAATGGTATTGGAGTTACACAGAATGTTCTCTATGAGAACCAA   24322
gb:MT106052|Organism:Severe      CAAATGGCTTATAGGTTTAATGGTATTGGAGTTACACAGAATGTTCTCTATGAGAACCAA   24322
gb:LC522975|Organism:Severe      CAAATGGCTTATAGGTTTAATGGTATTGGAGTTACACAGAATGTTCTCTATGAGAACCAA   24319
gb:LC522973|Organism:Severe      CAAATGGCTTATAGGTTTAATGGTATTGGAGTTACACAGAATGTTCTCTATGAGAACCAA   24319
gb:LC522974|Organism:Severe      CAAATGGCTTATAGGTTTAATGGTATTGGAGTTACACAGAATGTTCTCTATGAGAACCAA   24319
gb:MN985325|Organism:Severe      CAAATGGCTTATAGGTTTAATGGTATTGGAGTTACACAGAATGTTCTCTATGAGAACCAA   24322
gb:MT020881|Organism:Severe      CAAATGGCTTATAGGTTTAATGGTATTGGAGTTACACAGAATGTTCTCTATGAGAACCAA   24322
gb:MT020880|Organism:Severe      CAAATGGCTTATAGGTTTAATGGTATTGGAGTTACACAGAATGTTCTCTATGAGAACCAA   24322
gb:MT066175|Organism:Severe      CAAATGGCTTATAGGTTTAATGGTATTGGAGTTACACAGAATGTTCTCTATGAGAACCAA   24322
gb:MN997409|Organism:Severe      CAAATGGCTTATAGGTTTAATGGTATTGGAGTTACACAGAATGTTCTCTATGAGAACCAA   24322
gb:MN938384|Organism:Severe      CAAATGGCTTATAGGTTTAATGGTATTGGAGTTACACAGAATGTTCTCTATGAGAACCAA   24290
gb:MT044258|Organism:Severe      CAAATGGCTTATAGGTTTAATGGTATTGGAGTTACACAGAATGTTCTCTATGAGAACCAA   24298
gb:MT039890|Organism:Severe      CAAATGGCTTATAGGTTTAATGGTATTGGAGTTACACAGAATGTTCTCTATGAGAACCAA   24322
gb:MN988713|Organism:Severe      CAAATGGCTTATAGGTTTAATGGTATTGGAGTTACACAGAATGTTCTCTATGAGAACCAA   24322
gb:LC521925|Organism:Severe      CAAATGGCTTATAGGTTTAATGGTATTGGAGTTACACAGAATGTTCTCTATGAGAACCAA   24295
```

```
gb:MT093571|Organism:Severe      CAAATGGCTTATAGGTTTAATGGTATTGGAGTTACACAGAATGTTCTCTATGAGAACCAA   24322
gb:MT039887|Organism:Severe      CAAATGGCTTATAGGTTTAATGGTATTGGAGTTACACAGAATGTTCTCTATGAGAACCAA   24319
gb:MT019530|Organism:Severe      CAAATGGCTTATAGGTTTAATGGTATTGGAGTTACACAGAATGTTCTCTATGAGAACCAA   24322
gb:MT039888|Organism:Severe      CAAATGGCTTATAGGTTTAATGGTATTGGAGTTACACAGAATGTTCTCTATGAGAACCAA   24322
gb:LC522972|Organism:Severe      CAAATGGCTTATAGGTTTAATGGTATTGGAGTTACACAGAATGTTCTCTATGAGAACCAA   24319
gb:MT027063|Organism:Severe      CAAATGGCTTATAGGTTTAATGGTATTGGAGTTACACAGAATGTTCTCTATGAGAACCAA   24322
gb:MT027062|Organism:Severe      CAAATGGCTTATAGGTTTAATGGTATTGGAGTTACACAGAATGTTCTCTATGAGAACCAA   24322
gb:MT019529|Organism:Severe      CAAATGGCTTATAGGTTTAATGGTATTGGAGTTACACAGAATGTTCTCTATGAGAACCAA   24322
gb:MN996529|Organism:Severe      CAAATGGCTTATAGGTTTAATGGTATTGGAGTTACACAGAATGTTCTCTATGAGAACCAA   24310
gb:MN996531|Organism:Severe      CAAATGGCTTATAGGTTTAATGGTATTGGAGTTACACAGAATGTTCTCTATGAGAACCAA   24309
gb:MT066176|Organism:Severe      CAAATGGCTTATAGGTTTAATGGTATTGGAGTTACACAGAATGTTCTCTATGAGAACCAA   24322
gb:MT027064|Organism:Severe      CAAATGGCTTATAGGTTTAATGGTATTGGAGTTACACAGAATGTTCTCTATGAGAACCAA   24322
gb:MN994468|Organism:Severe      CAAATGGCTTATAGGTTTAATGGTATTGGAGTTACACAGAATGTTCTCTATGAGAACCAA   24322
gb:MT072688|Organism:Severe      CAAATGGCTTATAGGTTTAATGGTATTGGAGTTACACAGAATGTTCTCTATGAGAACCAA   24307
gb:MN996527|Organism:Severe      CAAATGGCTTATAGGTTTAATGGTATTGGAGTTACACAGAATGTTCTCTATGAGAACCAA   24289
gb:MT093631|Organism:Severe      CAAATGGCTTATAGGTTTAATGGTATTGGAGTTACACAGAATGTTCTCTATGAGAACCAA   24360
gb:MT106053|Organism:Severe      CAAATGGCTTATAGGTTTAATGGTATTGGAGTTACACAGAATGTTCTCTATGAGAACCAA   24322
gb:MT019533|Organism:Severe      CAAATGGCTTATAGGTTTAATGGTATTGGAGTTACACAGAATGTTCTCTATGAGAACCAA   24322
gb:MT019531|Organism:Severe      CAAATGGCTTATAGGTTTAATGGTATTGGAGTTACACAGAATGTTCTCTATGAGAACCAA   24322
gb:MN996528|Organism:Severe      CAAATGGCTTATAGGTTTAATGGTATTGGAGTTACACAGAATGTTCTCTATGAGAACCAA   24322
gb:MN996530|Organism:Severe      CAAATGGCTTATAGGTTTAATGGTATTGGAGTTACACAGAATGTTCTCTATGAGAACCAA   24308
gb:MN908947|Organism:Severe      CAAATGGCTTATAGGTTTAATGGTATTGGAGTTACACAGAATGTTCTCTATGAGAACCAA   24322
gb:MT019532|Organism:Severe      CAAATGGCTTATAGGTTTAATGGTATTGGAGTTACACAGAATGTTCTCTATGAGAACCAA   24322
                                 ************************************************************

gb:MT020781|Organism:Severe      AAATTGATTGCCAACCAATTTAATAGTGCTATTGGCAAAATTCAAGACTCACTTTCTTCC   24370
gb:MT007544|Organism:Severe      AAATTGATTGCCAACCAATTTAATAGTGCTATTGGCAAAATTCAAGACTCACTTTCTTCC   24382
gb:MN994467|Organism:Severe      AAATTGATTGCCAACCAATTTAATAGTGCTATTGGCAAAATTCAAGACTCACTTTCTTCC   24382
gb:MT044257|Organism:Severe      AAATTGATTGCCAACCAATTTAATAGTGCTATTGGCAAAATTCAAGACTCACTTTCTTCC   24382
gb:MT106054|Organism:Severe      AAATTGATTGCCAACCAATTTAATAGTGCTATTGGCAAAATTCAAGACTCACTTTCTTCC   24382
gb:MT049951|Organism:Severe      AAATTGATTGCCAACCAATTTAATAGTGCTATTGGCAAAATTCAAGACTCACTTTCTTCC   24382
gb:MN975262|Organism:Severe      AAATTGATTGCCAACCAATTTAATAGTGCTATTGGCAAAATTCAAGACTCACTTTCTTCC   24382
gb:MT106052|Organism:Severe      AAATTGATTGCCAACCAATTTAATAGTGCTATTGGCAAAATTCAAGACTCACTTTCTTCC   24382
gb:LC522975|Organism:Severe      AAATTGATTGCCAACCAATTTAATAGTGCTATTGGCAAAATTCAAGACTCACTTTCTTCC   24379
gb:LC522973|Organism:Severe      AAATTGATTGCCAACCAATTTAATAGTGCTATTGGCAAAATTCAAGACTCACTTTCTTCC   24379
gb:LC522974|Organism:Severe      AAATTGATTGCCAACCAATTTAATAGTGCTATTGGCAAAATTCAAGACTCACTTTCTTCC   24379
gb:MN985325|Organism:Severe      AAATTGATTGCCAACCAATTTAATAGTGCTATTGGCAAAATTCAAGACTCACTTTCTTCC   24382
gb:MT020881|Organism:Severe      AAATTGATTGCCAACCAATTTAATAGTGCTATTGGCAAAATTCAAGACTCACTTTCTTCC   24382
gb:MT020880|Organism:Severe      AAATTGATTGCCAACCAATTTAATAGTGCTATTGGCAAAATTCAAGACTCACTTTCTTCC   24382
gb:MT066175|Organism:Severe      AAATTGATTGCCAACCAATTTAATAGTGCTATTGGCAAAATTCAAGACTCACTTTCTTCC   24382
gb:MN997409|Organism:Severe      AAATTGATTGCCAACCAATTTAATAGTGCTATTGGCAAAATTCAAGACTCACTTTCTTCC   24382
gb:MN938384|Organism:Severe      AAATTGATTGCCAACCAATTTAATAGTGCTATTGGCAAAATTCAAGACTCACTTTCTTCC   24350
gb:MT044258|Organism:Severe      AAATTGATTGCCAACCAATTTAATAGTGCTATTGGCAAAATTCAAGACTCACTTTCTTCC   24358
gb:MT039890|Organism:Severe      AAATTGATTGCCAACCAATTTAATAGTGCTATTGGCAAAATTCAAGACTCACTTTCTTCC   24382
gb:MN988713|Organism:Severe      AAATTGATTGCCAACCAATTTAATAGTGCTATTGGCAAAATTCAAGACTCACTTTCTTCC   24382
gb:LC521925|Organism:Severe      AAATTGATTGCCAACCAATTTAATAGTGCTATTGGCAAAATTCAAGACTCACTTTCTTCC   24355
gb:MT093571|Organism:Severe      AAATTGATTGCCAACCAATTTAATAGTGCTATTGGCAAAATTCAAGACTCACTTTCTTCC   24382
gb:MT039887|Organism:Severe      AAATTGATTGCCAACCAATTTAATAGTGCTATTGGCAAAATTCAAGACTCACTTTCTTCC   24379
gb:MT019530|Organism:Severe      AAATTGATTGCCAACCAATTTAATAGTGCTATTGGCAAAATTCAAGACTCACTTTCTTCC   24382
gb:MT039888|Organism:Severe      AAATTGATTGCCAACCAATTTAATAGTGCTATTGGCAAAATTCAAGACTCACTTTCTTCC   24382
gb:LC522972|Organism:Severe      AAATTGATTGCCAACCAATTTAATAGTGCTATTGGCAAAATTCAAGACTCACTTTCTTCC   24379
gb:MT027063|Organism:Severe      AAATTGATTGCCAACCAATTTAATAGTGCTATTGGCAAAATTCAAGACTCACTTTCTTCC   24382
gb:MT027062|Organism:Severe      AAATTGATTGCCAACCAATTTAATAGTGCTATTGGCAAAATTCAAGACTCACTTTCTTCC   24382
gb:MT019529|Organism:Severe      AAATTGATTGCCAACCAATTTAATAGTGCTATTGGCAAAATTCAAGACTCACTTTCTTCC   24382
gb:MN996529|Organism:Severe      AAATTGATTGCCAACCAATTTAATAGTGCTATTGGCAAAATTCAAGACTCACTTTCTTCC   24370
gb:MN996531|Organism:Severe      AAATTGATTGCCAACCAATTTAATAGTGCTATTGGCAAAATTCAAGACTCACTTTCTTCC   24369
gb:MT066176|Organism:Severe      AAATTGATTGCCAACCAATTTAATAGTGCTATTGGCAAAATTCAAGACTCACTTTCTTCC   24382
gb:MT027064|Organism:Severe      AAATTGATTGCCAACCAATTTAATAGTGCTATTGGCAAAATTCAAGACTCACTTTCTTCC   24382
gb:MN994468|Organism:Severe      AAATTGATTGCCAACCAATTTAATAGTGCTATTGGCAAAATTCAAGACTCACTTTCTTCC   24382
gb:MT072688|Organism:Severe      AAATTGATTGCCAACCAATTTAATAGTGCTATTGGCAAAATTCAAGACTCACTTTCTTCC   24367
gb:MN996527|Organism:Severe      AAGTTGATTGCCAACCAATTTAATAGTGCTATTGGCAAAATTCAAGACTCACTTTCTTCC   24349
gb:MT093631|Organism:Severe      AAATTGATTGCCAACCAATTTAATAGTGCTATTGGCAAAATTCAAGACTCACTTTCTTCC   24420
gb:MT106053|Organism:Severe      AAGTTGATTGCCAACCAATTTAATAGTGCTATTGGCAAAATTCAAGACTCACTTTCTTCC   24382
gb:MT019533|Organism:Severe      AAATTGATTGCCAACCAATTTAATAGTGCTATTGGCAAAATTCAAGACTCACTTTCTTCC   24382
gb:MT019531|Organism:Severe      AAATTGATTGCCAACCAATTTAATAGTGCTATTGGCAAAATTCAAGACTCACTTTCTTCC   24382
```

```
gb:MN996528|Organism:Severe      AAATTGATTGCCAACCAATTTAATAGTGCTATTGGCAAAATTCAAGACTCACTTTCTTCC   24382
gb:MN996530|Organism:Severe      AAATTGATTGCCAACCAATTTAATAGTGCTATTGGCAAAATTCAAGACTCACTTTCTTCC   24368
gb:MN908947|Organism:Severe      AAATTGATTGCCAACCAATTTAATAGTGCTATTGGCAAAATTCAAGACTCACTTTCTTCC   24382
gb:MT019532|Organism:Severe      AAATTGATTGCCAACCAATTTAATAGTGCTATTGGCAAAATTCAAGACTCACTTTCTTCC   24382
                                 ** *********************************************************

gb:MT020781|Organism:Severe      ACAGCAAGTGCACTTGGAAAACTTCAAGATGTGGTCAACCAAAATGCACAAGCTTTAAAC   24430
gb:MT007544|Organism:Severe      ACAGCAAGTGCACTTGGAAAACTTCAAGATGTGGTCAACCAAAATGCACAAGCTTTAAAC   24442
gb:MN994467|Organism:Severe      ACAGCAAGTGCACTTGGAAAACTTCAAGATGTGGTCAACCAAAATGCACAAGCTTTAAAC   24442
gb:MT044257|Organism:Severe      ACAGCAAGTGCACTTGGAAAACTTCAAGATGTGGTCAACCAAAATGCACAAGCTTTAAAC   24442
gb:MT106054|Organism:Severe      ACAGCAAGTGCACTTGGAAAACTTCAAGATGTGGTCAACCAAAATGCACAAGCTTTAAAC   24442
gb:MT049951|Organism:Severe      ACAGCAAGTGCACTTGGAAAACTTCAAGATGTGGTCAACCAAAATGCACAAGCTTTAAAC   24442
gb:MN975262|Organism:Severe      ACAGCAAGTGCACTTGGAAAACTTCAAGATGTGGTCAACCAAAATGCACAAGCTTTAAAC   24442
gb:MT106052|Organism:Severe      ACAGCAAGTGCACTTGGAAAACTTCAAGATGTGGTCAACCAAAATGCACAAGCTTTAAAC   24442
gb:LC522975|Organism:Severe      ACAGCAAGTGCACTTGGAAAACTTCAAGATGTGGTCAACCAAAATGCACAAGCTTTAAAC   24439
gb:LC522973|Organism:Severe      ACAGCAAGTGCACTTGGAAAACTTCAAGATGTGGTCAACCAAAATGCACAAGCTTTAAAC   24439
gb:LC522974|Organism:Severe      ACAGCAAGTGCACTTGGAAAACTTCAAGATGTGGTCAACCAAAATGCACAAGCTTTAAAC   24439
gb:MN985325|Organism:Severe      ACAGCAAGTGCACTTGGAAAACTTCAAGATGTGGTCAACCAAAATGCACAAGCTTTAAAC   24442
gb:MT020881|Organism:Severe      ACAGCAAGTGCACTTGGAAAACTTCAAGATGTGGTCAACCAAAATGCACAAGCTTTAAAC   24442
gb:MT020880|Organism:Severe      ACAGCAAGTGCACTTGGAAAACTTCAAGATGTGGTCAACCAAAATGCACAAGCTTTAAAC   24442
gb:MT066175|Organism:Severe      ACAGCAAGTGCACTTGGAAAACTTCAAGATGTGGTCAACCAAAATGCACAAGCTTTAAAC   24442
gb:MN997409|Organism:Severe      ACAGCAAGTGCACTTGGAAAACTTCAAGATGTGGTCAACCAAAATGCACAAGCTTTAAAC   24442
gb:MN938384|Organism:Severe      ACAGCAAGTGCACTTGGAAAACTTCAAGATGTGGTCAACCAAAATGCACAAGCTTTAAAC   24410
gb:MT044258|Organism:Severe      ACAGCAAGTGCACTTGGAAAACTTCAAGATGTGGTCAACCAAAATGCACAAGCTTTAAAC   24418
gb:MT039890|Organism:Severe      ACAGCAAGTGCACTTGGAAAACTTCAAGATGTGGTCAACCAAAATGCACAAGCTTTAAAC   24442
gb:MN988713|Organism:Severe      ACAGCAAGTGCACTTGGAAAACTTCAAGATGTGGTCAACCAAAATGCACAAGCTTTAAAC   24442
gb:LC521925|Organism:Severe      ACAGCAAGTGCACTTGGAAAACTTCAAGATGTGGTCAACCAAAATGCACAAGCTTTAAAC   24415
gb:MT093571|Organism:Severe      ACAGCAAGTGCACTTGGAAAACTTCAAGATGTGGTCAACCAAAATGCACAAGCTTTAAAC   24442
gb:MT039887|Organism:Severe      ACAGCAAGTGCACTTGGAAAACTTCAAGATGTGGTCAACCAAAATGCACAAGCTTTAAAC   24439
gb:MT019530|Organism:Severe      ACAGCAAGTGCACTTGGAAAACTTCAAGATGTGGTCAACCAAAATGCACAAGCTTTAAAC   24442
gb:MT039888|Organism:Severe      ACAGCAAGTGCACTTGGAAAACTTCAAGATGTGGTCAACCAAAATGCACAAGCTTTAAAC   24442
gb:LC522972|Organism:Severe      ACAGCAAGTGCACTTGGAAAACTTCAAGATGTGGTCAACCAAAATGCACAAGCTTTAAAC   24439
gb:MT027063|Organism:Severe      ACAGCAAGTGCACTTGGAAAACTTCAAGATGTGGTCAACCAAAATGCACAAGCTTTAAAC   24442
gb:MT027062|Organism:Severe      ACAGCAAGTGCACTTGGAAAACTTCAAGATGTGGTCAACCAAAATGCACAAGCTTTAAAC   24442
gb:MT019529|Organism:Severe      ACAGCAAGTGCACTTGGAAAACTTCAAGATGTGGTCAACCAAAATGCACAAGCTTTAAAC   24442
gb:MN996529|Organism:Severe      ACAGCAAGTGCACTTGGAAAACTTCAAGATGTGGTCAACCAAAATGCACAAGCTTTAAAC   24430
gb:MN996531|Organism:Severe      ACAGCAAGTGCACTTGGAAAACTTCAAGATGTGGTCAACCAAAATGCACAAGCTTTAAAC   24429
gb:MT066176|Organism:Severe      ACAGCAAGTGCACTTGGAAAACTTCAAGATGTGGTCAACCAAAATGCACAAGCTTTAAAC   24442
gb:MT027064|Organism:Severe      ACAGCAAGTGCACTTGGAAAACTTCAAGATGTGGTCAACCAAAATGCACAAGCTTTAAAC   24442
gb:MN994468|Organism:Severe      ACAGCAAGTGCACTTGGAAAACTTCAAGATGTGGTCAACCAAAATGCACAAGCTTTAAAC   24442
gb:MT072688|Organism:Severe      ACAGCAAGTGCACTTGGAAAACTTCAAGATGTGGTCAACCAAAATGCACAAGCTTTAAAC   24427
gb:MN996527|Organism:Severe      ACAGCAAGTGCACTTGGAAAACTTCAAGATGTGGTCAACCAAAATGCACAAGCTTTAAAC   24409
gb:MT093631|Organism:Severe      ACAGCAAGTGCACTTGGAAAACTTCAAGATGTGGTCAACCAAAATGCACAAGCTTTAAAC   24480
gb:MT106053|Organism:Severe      ACAGCAAGTGCACTTGGAAAACTTCAAGATGTGGTCAACCAAAATGCACAAGCTTTAAAC   24442
gb:MT019533|Organism:Severe      ACAGCAAGTGCACTTGGAAAACTTCAAGATGTGGTCAACCAAAATGCACAAGCTTTAAAC   24442
gb:MT019531|Organism:Severe      ACAGCAAGTGCACTTGGAAAACTTCAAGATGTGGTCAACCAAAATGCACAAGCTTTAAAC   24442
gb:MN996528|Organism:Severe      ACAGCAAGTGCACTTGGAAAACTTCAAGATGTGGTCAACCAAAATGCACAAGCTTTAAAC   24442
gb:MN996530|Organism:Severe      ACAGCAAGTGCACTTGGAAAACTTCAAGATGTGGTCAACCAAAATGCACAAGCTTTAAAC   24428
gb:MN908947|Organism:Severe      ACAGCAAGTGCACTTGGAAAACTTCAAGATGTGGTCAACCAAAATGCACAAGCTTTAAAC   24442
gb:MT019532|Organism:Severe      ACAGCAAGTGCACTTGGAAAACTTCAAGATGTGGTCAACCAAAATGCACAAGCTTTAAAC   24442
                                 ************************************************************

gb:MT020781|Organism:Severe      ACGCTTGTTAAACAACTTAGCTCCAATTTTGGTGCAATTTCAAGTGTTTTAAATGATATC   24490
gb:MT007544|Organism:Severe      ACGCTTGTTAAACAACTTAGCTCCAATTTTGGTGCAATTTCAAGTGTTTTAAATGATATC   24502
gb:MN994467|Organism:Severe      ACGCTTGTTAAACAACTTAGCTCCAATTTTGGTGCAATTTCAAGTGTTTTAAATGATATC   24502
gb:MT044257|Organism:Severe      ACGCTTGTTAAACAACTTAGCTCCAATTTTGGTGCAATTTCAAGTGTTTTAAATGATATC   24502
gb:MT106054|Organism:Severe      ACGCTTGTTAAACAACTTAGCTCCAATTTTGGTGCAATTTCAAGTGTTTTAAATGATATC   24502
gb:MT049951|Organism:Severe      ACGCTTGTTAAACAACTTAGCTCCAATTTTGGTGCAATTTCAAGTGTTTTAAATGATATC   24502
gb:MN975262|Organism:Severe      ACGCTTGTTAAACAACTTAGCTCCAATTTTGGTGCAATTTCAAGTGTTTTAAATGATATC   24502
gb:MT106052|Organism:Severe      ACGCTTGTTAAACAACTTAGCTCCAATTTTGGTGCAATTTCAAGTGTTTTAAATGATATC   24502
gb:LC522975|Organism:Severe      ACGCTTGTTAAACAACTTAGCTCCAATTTTGGTGCAATTTCAAGTGTTTTAAATGATATC   24499
gb:LC522973|Organism:Severe      ACGCTTGTTAAACAACTTAGCTCCAATTTTGGTGCAATTTCAAGTGTTTTAAATGATATC   24499
gb:LC522974|Organism:Severe      ACGCTTGTTAAACAACTTAGCTCCAATTTTGGTGCAATTTCAAGTGTTTTAAATGATATC   24499
gb:MN985325|Organism:Severe      ACGCTTGTTAAACAACTTAGCTCCAATTTTGGTGCAATTTCAAGTGTTTTAAATGATATC   24502
gb:MT020881|Organism:Severe      ACGCTTGTTAAACAACTTAGCTCCAATTTTGGTGCAATTTCAAGTGTTTTAAATGATATC   24502
```

```
gb:MT020880|Organism:Severe      ACGCTTGTTAAACAACTTAGCTCCAATTTTGGTGCAATTTCAAGTGTTTTAAATGATATC   24502
gb:MT066175|Organism:Severe      ACGCTTGTTAAACAACTTAGCTCCAATTTTGGTGCAATTTCAAGTGTTTTAAATGATATC   24502
gb:MN997409|Organism:Severe      ACGCTTGTTAAACAACTTAGCTCCAATTTTGGTGCAATTTCAAGTGTTTTAAATGATATC   24502
gb:MN938384|Organism:Severe      ACGCTTGTTAAACAACTTAGCTCCAATTTTGGTGCAATTTCAAGTGTTTTAAATGATATC   24470
gb:MT044258|Organism:Severe      ACGCTTGTTAAACAACTTAGCTCCAATTTTGGTGCAATTTCAAGTGTTTTAAATGATATC   24478
gb:MT039890|Organism:Severe      ACGCTTGTTAAACAACTTAGCTCCAATTTTGGTGCAATTTCAAGTGTTTTAAATGATATC   24502
gb:MN988713|Organism:Severe      ACGCTTGTTAAACAACTTAGCTCCAATTTTGGTGCAATTTCAAGTGTTTTAAATGATATC   24502
gb:LC521925|Organism:Severe      ACGCTTGTTAAACAACTTAGCTCCAATTTTGGTGCAATTTCAAGTGTTTTAAATGATATC   24475
gb:MT093571|Organism:Severe      ACGCTTGTTAAACAACTTAGCTCCAATTTTGGTGCAATTTCAAGTGTTTTAAATGATATC   24502
gb:MT039887|Organism:Severe      ACGCTTGTTAAACAACTTAGCTCCAATTTTGGTGCAATTTCAAGTGTTTTAAATGATATC   24499
gb:MT019530|Organism:Severe      ACGCTTGTTAAACAACTTAGCTCCAATTTTGGTGCAATTTCAAGTGTTTTAAATGATATC   24502
gb:MT039888|Organism:Severe      ACGCTTGTTAAACAACTTAGCTCCAATTTTGGTGCAATTTCAAGTGTTTTAAATGATATC   24502
gb:LC522972|Organism:Severe      ACGCTTGTTAAACAACTTAGCTCCAATTTTGGTGCAATTTCAAGTGTTTTAAATGATATC   24499
gb:MT027063|Organism:Severe      ACGCTTGTTAAACAACTTAGCTCCAATTTTGGTGCAATTTCAAGTGTTTTAAATGATATC   24502
gb:MT027062|Organism:Severe      ACGCTTGTTAAACAACTTAGCTCCAATTTTGGTGCAATTTCAAGTGTTTTAAATGATATC   24502
gb:MT019529|Organism:Severe      ACGCTTGTTAAACAACTTAGCTCCAATTTTGGTGCAATTTCAAGTGTTTTAAATGATATC   24502
gb:MN996529|Organism:Severe      ACGCTTGTTAAACAACTTAGCTCCAATTTTGGTGCAATTTCAAGTGTTTTAAATGATATC   24490
gb:MN996531|Organism:Severe      ACGCTTGTTAAACAACTTAGCTCCAATTTTGGTGCAATTTCAAGTGTTTTAAATGATATC   24489
gb:MT066176|Organism:Severe      ACGCTTGTTAAACAACTTAGCTCCAATTTTGGTGCAATTTCAAGTGTTTTAAATGATATC   24502
gb:MT027064|Organism:Severe      ACGCTTGTTAAACAACTTAGCTCCAATTTTGGTGCAATTTCAAGTGTTTTAAATGATATC   24502
gb:MN994468|Organism:Severe      ACGCTTGTTAAACAACTTAGCTCCAATTTTGGTGCAATTTCAAGTGTTTTAAATGATATC   24502
gb:MT072688|Organism:Severe      ACGCTTGTTAAACAACTTAGCTCCAATTTTGGTGCAATTTCAAGTGTTTTAAATGATATC   24487
gb:MN996527|Organism:Severe      ACGCTTGTTAAACAACTTAGCTCCAATTTTGGTGCAATTTCAAGTGTTTTAAATGATATC   24469
gb:MT093631|Organism:Severe      ACGCTTGTTAAACAACTTAGCTCCAATTTTGGTGCAATTTCAAGTGTTTTAAATGATATC   24540
gb:MT106053|Organism:Severe      ACGCTTGTTAAACAACTTAGCTCCAATTTTGGTGCAATTTCAAGTGTTTTAAATGATATC   24502
gb:MT019533|Organism:Severe      ACGCTTGTTAAACAACTTAGCTCCAATTTTGGTGCAATTTCAAGTGTTTTAAATGATATC   24502
gb:MT019531|Organism:Severe      ACGCTTGTTAAACAACTTAGCTCCAATTTTGGTGCAATTTCAAGTGTTTTAAATGATATC   24502
gb:MN996528|Organism:Severe      ACGCTTGTTAAACAACTTAGCTCCAATTTTGGTGCAATTTCAAGTGTTTTAAATGATATC   24502
gb:MN996530|Organism:Severe      ACGCTTGTTAAACAACTTAGCTCCAATTTTGGTGCAATTTCAAGTGTTTTAAATGATATC   24488
gb:MN908947|Organism:Severe      ACGCTTGTTAAACAACTTAGCTCCAATTTTGGTGCAATTTCAAGTGTTTTAAATGATATC   24502
gb:MT019532|Organism:Severe      ACGCTTGTTAAACAACTTAGCTCCAATTTTGGTGCAATTTCAAGTGTTTTAAATGATATC   24502
                                 ************************************************************

gb:MT020781|Organism:Severe      CTTTCACGTCTTGACAAAGTTGAGGCTGAAGTGCAAATTGATAGGTTGATCACAGGCAGA   24550
gb:MT007544|Organism:Severe      CTTTCACGTCTTGACAAAGTTGAGGCTGAAGTGCAAATTGATAGGTTGATCACAGGCAGA   24562
gb:MN994467|Organism:Severe      CTTTCACGTCTTGACAAAGTTGAGGCTGAAGTGCAAATTGATAGGTTGATCACAGGCAGA   24562
gb:MT044257|Organism:Severe      CTTTCACGTCTTGACAAAGTTGAGGCTGAAGTGCAAATTGATAGGTTGATCACAGGCAGA   24562
gb:MT106054|Organism:Severe      CTTTCACGTCTTGACAAAGTTGAGGCTGAAGTGCAAATTGATAGGTTGATCACAGGCAGA   24562
gb:MT049951|Organism:Severe      CTTTCACGTCTTGACAAAGTTGAGGCTGAAGTGCAAATTGATAGGTTGATCACAGGCAGA   24562
gb:MN975262|Organism:Severe      CTTTCACGTCTTGACAAAGTTGAGGCTGAAGTGCAAATTGATAGGTTGATCACAGGCAGA   24562
gb:MT106052|Organism:Severe      CTTTCACGTCTTGACAAAGTTGAGGCTGAAGTGCAAATTGATAGGTTGATCACAGGCAGA   24562
gb:LC522975|Organism:Severe      CTTTCACGTCTTGACAAAGTTGAGGCTGAAGTGCAAATTGATAGGTTGATCACAGGCAGA   24559
gb:LC522973|Organism:Severe      CTTTCACGTCTTGACAAAGTTGAGGCTGAAGTGCAAATTGATAGGTTGATCACAGGCAGA   24559
gb:LC522974|Organism:Severe      CTTTCACGTCTTGACAAAGTTGAGGCTGAAGTGCAAATTGATAGGTTGATCACAGGCAGA   24559
gb:MN985325|Organism:Severe      CTTTCACGTCTTGACAAAGTTGAGGCTGAAGTGCAAATTGATAGGTTGATCACAGGCAGA   24562
gb:MT020881|Organism:Severe      CTTTCACGTCTTGACAAAGTTGAGGCTGAAGTGCAAATTGATAGGTTGATCACAGGCAGA   24562
gb:MT020880|Organism:Severe      CTTTCACGTCTTGACAAAGTTGAGGCTGAAGTGCAAATTGATAGGTTGATCACAGGCAGA   24562
gb:MT066175|Organism:Severe      CTTTCACGTCTTGACAAAGTTGAGGCTGAAGTGCAAATTGATAGGTTGATCACAGGCAGA   24562
gb:MN997409|Organism:Severe      CTTTCACGTCTTGACAAAGTTGAGGCTGAAGTGCAAATTGATAGGTTGATCACAGGCAGA   24562
gb:MN938384|Organism:Severe      CTTTCACGTCTTGACAAAGTTGAGGCTGAAGTGCAAATTGATAGGTTGATCACAGGCAGA   24530
gb:MT044258|Organism:Severe      CTTTCACGTCTTGACAAAGTTGAGGCTGAAGTGCAAATTGATAGGTTGATCACAGGCAGA   24538
gb:MT039890|Organism:Severe      CTTTCACGTCTTGACAAAGTTGAGGCTGAAGTGCAAATTGATAGGTTGATCACAGGCAGA   24562
gb:MN988713|Organism:Severe      CTTTCACGTCTTGACAAAGTTGAGGCTGAAGTGCAAATTGATAGGTTGATCACAGGCAGA   24562
gb:LC521925|Organism:Severe      CTTTCACGTCTTGACAAAGTTGAGGCTGAAGTGCAAATTGATAGGTTGATCACAGGCAGA   24535
gb:MT093571|Organism:Severe      CTTTCACGTCTTGACAAAGTTGAGGCTGAAGTGCAAATTGATAGGTTGATCACAGGCAGA   24562
gb:MT039887|Organism:Severe      CTTTCACGTCTTGACAAAGTTGAGGCTGAAGTGCAAATTGATAGGTTGATCACAGGCAGA   24559
gb:MT019530|Organism:Severe      CTTTCACGTCTTGACAAAGTTGAGGCTGAAGTGCAAATTGATAGGTTGATCACAGGCAGA   24562
gb:MT039888|Organism:Severe      CTTTCACGTCTTGACAAAGTTGAGGCTGAAGTGCAAATTGATAGGTTGATCACAGGCAGA   24562
gb:LC522972|Organism:Severe      CTTTCACGTCTTGACAAAGTTGAGGCTGAAGTGCAAATTGATAGGTTGATCACAGGCAGA   24559
gb:MT027063|Organism:Severe      CTTTCACGTCTTGACAAAGTTGAGGCTGAAGTGCAAATTGATAGGTTGATCACAGGCAGA   24562
gb:MT027062|Organism:Severe      CTTTCACGTCTTGACAAAGTTGAGGCTGAAGTGCAAATTGATAGGTTGATCACAGGCAGA   24562
gb:MT019529|Organism:Severe      CTTTCACGTCTTGACAAAGTTGAGGCTGAAGTGCAAATTGATAGGTTGATCACAGGCAGA   24562
gb:MN996529|Organism:Severe      CTTTCACGTCTTGACAAAGTTGAGGCTGAAGTGCAAATTGATAGGTTGATCACAGGCAGA   24550
gb:MN996531|Organism:Severe      CTTTCACGTCTTGACAAAGTTGAGGCTGAAGTGCAAATTGATAGGTTGATCACAGGCAGA   24549
gb:MT066176|Organism:Severe      CTTTCACGTCTTGACAAAGTTGAGGCTGAAGTGCAAATTGATAGGTTGATCACAGGCAGA   24562
```

```
gb:MT027064|Organism:Severe      CTTTCACGTCTTGACAAAGTTGAGGCTGAAGTGCAAATTGATAGGTTGATCACAGGCAGA    24562
gb:MN994468|Organism:Severe      CTTTCACGTCTTGACAAAGTTGAGGCTGAAGTGCAAATTGATAGGTTGATCACAGGCAGA    24562
gb:MT072688|Organism:Severe      CTTTCACGTCTTGACAAAGTTGAGGCTGAAGTGCAAATTGATAGGTTGATCACAGGCAGA    24547
gb:MN996527|Organism:Severe      CTTTCACGTCTTGACAAAGTTGAGGCTGAAGTGCAAATTGATAGGTTGATCACAGGCAGA    24529
gb:MT093631|Organism:Severe      CTTTCACGTCTTGACAAAGTTGAGGCTGAAGTGCAAATTGATAGGTTGATCACAGGCAGA    24600
gb:MT106053|Organism:Severe      CTTTCACGTCTTGACAAAGTTGAGGCTGAAGTGCAAATTGATAGGTTGATCACAGGCAGA    24562
gb:MT019533|Organism:Severe      CTTTCACGTCTTGACAAAGTTGAGGCTGAAGTGCAAATTGATAGGTTGATCACAGGCAGA    24562
gb:MT019531|Organism:Severe      CTTTCACGTCTTGACAAAGTTGAGGCTGAAGTGCAAATTGATAGGTTGATCACAGGCAGA    24562
gb:MN996528|Organism:Severe      CTTTCACGTCTTGACAAAGTTGAGGCTGAAGTGCAAATTGATAGGTTGATCACAGGCAGA    24562
gb:MN996530|Organism:Severe      CTTTCACGTCTTGACAAAGTTGAGGCTGAAGTGCAAATTGATAGGTTGATCACAGGCAGA    24548
gb:MN908947|Organism:Severe      CTTTCACGTCTTGACAAAGTTGAGGCTGAAGTGCAAATTGATAGGTTGATCACAGGCAGA    24562
gb:MT019532|Organism:Severe      CTTTCACGTCTTGACAAAGTTGAGGCTGAAGTGCAAATTGATAGGTTGATCACAGGCAGA    24562
                                 ************************************************************

gb:MT020781|Organism:Severe      CTTCAAAGTTTGCAGACATATGTGACTCAACAATTAATTAGAGCTGCAGAAATCAGAGCT    24610
gb:MT007544|Organism:Severe      CTTCAAAGTTTGCAGACATATGTGACTCAACAATTAATTAGAGCTGCAGAAATCAGAGCT    24622
gb:MN994467|Organism:Severe      CTTCAAAGTTTGCAGACATATGTGACTCAACAATTAATTAGAGCTGCAGAAATCAGAGCT    24622
gb:MT044257|Organism:Severe      CTTCAAAGTTTGCAGACATATGTGACTCAACAATTAATTAGAGCTGCAGAAATCAGAGCT    24622
gb:MT106054|Organism:Severe      CTTCAAAGTTTGCAGACATATGTGACTCAACAATTAATTAGAGCTGCAGAAATCAGAGCT    24622
gb:MT049951|Organism:Severe      CTTCAAAGTTTGCAGACATATGTGACTCAACAATTAATTAGAGCTGCAGAAATCAGAGCT    24622
gb:MN975262|Organism:Severe      CTTCAAAGTTTGCAGACATATGTGACTCAACAATTAATTAGAGCTGCAGAAATCAGAGCT    24622
gb:MT106052|Organism:Severe      CTTCAAAGTTTGCAGACATATGTGACTCAACAATTAATTAGAGCTGCAGAAATCAGAGCT    24622
gb:LC522975|Organism:Severe      CTTCAAAGTTTGCAGACATATGTGACTCAACAATTAATTAGAGCTGCAGAAATCAGAGCT    24619
gb:LC522973|Organism:Severe      CTTCAAAGTTTGCAGACATATGTGACTCAACAATTAATTAGAGCTGCAGAAATCAGAGCT    24619
gb:LC522974|Organism:Severe      CTTCAAAGTTTGCAGACATATGTGACTCAACAATTAATTAGAGCTGCAGAAATCAGAGCT    24619
gb:MN985325|Organism:Severe      CTTCAAAGTTTGCAGACATATGTGACTCAACAATTAATTAGAGCTGCAGAAATCAGAGCT    24622
gb:MT020881|Organism:Severe      CTTCAAAGTTTGCAGACATATGTGACTCAACAATTAATTAGAGCTGCAGAAATCAGAGCT    24622
gb:MT020880|Organism:Severe      CTTCAAAGTTTGCAGACATATGTGACTCAACAATTAATTAGAGCTGCAGAAATCAGAGCT    24622
gb:MT066175|Organism:Severe      CTTCAAAGTTTGCAGACATATGTGACTCAACAATTAATTAGAGCTGCAGAAATCAGAGCT    24622
gb:MN997409|Organism:Severe      CTTCAAAGTTTGCAGACATATGTGACTCAACAATTAATTAGAGCTGCAGAAATCAGAGCT    24622
gb:MN938384|Organism:Severe      CTTCAAAGTTTGCAGACATATGTGACTCAACAATTAATTAGAGCTGCAGAAATCAGAGCT    24590
gb:MT044258|Organism:Severe      CTTCAAAGTTTGCAGACATATGTGACTCAACAATTAATTAGAGCTGCAGAAATCAGAGCT    24598
gb:MT039890|Organism:Severe      CTTCAAAGTTTGCAGACATATGTGACTCAACAATTAATTAGAGCTGCAGAAATCAGAGCT    24622
gb:MN988713|Organism:Severe      CTTCAAAGTTTGCAGACATATGTGACTCAACAATTAATTAGAGCTGCAGAAATCAGAGCT    24622
gb:LC521925|Organism:Severe      CTTCAAAGTTTGCAGACATATGTGACTCAACAATTAATTAGAGCTGCAGAAATCAGAGCT    24595
gb:MT093571|Organism:Severe      CTTCAAAGTTTGCAGACATATGTGACTCAACAATTAATTAGAGCTGCAGAAATCAGAGCT    24622
gb:MT039887|Organism:Severe      CTTCAAAGTTTGCAGACATATGTGACTCAACAATTAATTAGAGCTGCAGAAATCAGAGCT    24619
gb:MT019530|Organism:Severe      CTTCAAAGTTTGCAGACATATGTGACTCAACAATTAATTAGAGCTGCAGAAATCAGAGCT    24622
gb:MT039888|Organism:Severe      CTTCAAAGTTTGCAGACATATGTGACTCAACAATTAATTAGAGCTGCAGAAATCAGAGCT    24622
gb:LC522972|Organism:Severe      CTTCAAAGTTTGCAGACATATGTGACTCAACAATTAATTAGAGCTGCAGAAATCAGAGCT    24619
gb:MT027063|Organism:Severe      CTTCAAAGTTTGCAGACATATGTGACTCAACAATTAATTAGAGCTGCAGAAATCAGAGCT    24622
gb:MT027062|Organism:Severe      CTTCAAAGTTTGCAGACATATGTGACTCAACAATTAATTAGAGCTGCAGAAATCAGAGCT    24622
gb:MT019529|Organism:Severe      CTTCAAAGTTTGCAGACATATGTGACTCAACAATTAATTAGAGCTGCAGAAATCAGAGCT    24622
gb:MN996529|Organism:Severe      CTTCAAAGTTTGCAGACATATGTGACTCAACAATTAATTAGAGCTGCAGAAATCAGAGCT    24610
gb:MN996531|Organism:Severe      CTTCAAAGTTTGCAGACATATGTGACTCAACAATTAATTAGAGCTGCAGAAATCAGAGCT    24609
gb:MT066176|Organism:Severe      CTTCAAAGTTTGCAGACATATGTGACTCAACAATTAATTAGAGCTGCAGAAATCAGAGCT    24622
gb:MT027064|Organism:Severe      CTTCAAAGTTTGCAGACATATGTGACTCAACAATTAATTAGAGCTGCAGAAATCAGAGCT    24622
gb:MN994468|Organism:Severe      CTTCAAAGTTTGCAGACATATGTGACTCAACAATTAATTAGAGCTGCAGAAATCAGAGCT    24622
gb:MT072688|Organism:Severe      CTTCAAAGTTTGCAGACATATGTGACTCAACAATTAATTAGAGCTGCAGAAATCAGAGCT    24607
gb:MN996527|Organism:Severe      CTTCAAAGTTTGCAGACATATGTGACTCAACAATTAATTAGAGCTGCAGAAATCAGAGCT    24589
gb:MT093631|Organism:Severe      CTTCAAAGTTTGCAGACATATGTGACTCAACAATTAATTAGAGCTGCAGAAATCAGAGCT    24660
gb:MT106053|Organism:Severe      CTTCAAAGTTTGCAGACATATGTGACTCAACAATTAATTAGAGCTGCAGAAATCAGAGCT    24622
gb:MT019533|Organism:Severe      CTTCAAAGTTTGCAGACATATGTGACTCAACAATTAATTAGAGCTGCAGAAATCAGAGCT    24622
gb:MT019531|Organism:Severe      CTTCAAAGTTTGCAGACATATGTGACTCAACAATTAATTAGAGCTGCAGAAATCAGAGCT    24622
gb:MN996528|Organism:Severe      CTTCAAAGTTTGCAGACATATGTGACTCAACAATTAATTAGAGCTGCAGAAATCAGAGCT    24622
gb:MN996530|Organism:Severe      CTTCAAAGTTTGCAGACATATGTGACTCAACAATTAATTAGAGCTGCAGAAATCAGAGCT    24608
gb:MN908947|Organism:Severe      CTTCAAAGTTTGCAGACATATGTGACTCAACAATTAATTAGAGCTGCAGAAATCAGAGCT    24622
gb:MT019532|Organism:Severe      CTTCAAAGTTTGCAGACATATGTGACTCAACAATTAATTAGAGCTGCAGAAATCAGAGCT    24622
                                 ************************************************************

gb:MT020781|Organism:Severe      TCTGCTAATCTTGCTGCTACTAAAATGTCAGAGTGTGTACTTGGACAATCAAAAAGAGTT    24670
gb:MT007544|Organism:Severe      TCTGCTAATCTTGCTGCTACTAAAATGTCAGAGTGTGTACTTGGACAATCAAAAAGAGTT    24682
gb:MN994467|Organism:Severe      TCTGCTAATCTTGCTGCTACTAAAATGTCAGAGTGTGTACTTGGACAATCAAAAAGAGTT    24682
gb:MT044257|Organism:Severe      TCTGCTAATCTTGCTGCTACTAAAATGTCAGAGTGTGTACTTGGACAATCAAAAAGAGTT    24682
gb:MT106054|Organism:Severe      TCTGCTAATCTTGCTGCTACTAAAATGTCAGAGTGTGTACTTGGACAATCAAAAAGAGTT    24682
```

```
gb:MT049951|Organism:Severe      TCTGCTAATCTTGCTGCTACTAAAATGTCAGAGTGTGTACTTGGACAATCAAAAAGAGTT    24682
gb:MN975262|Organism:Severe      TCTGCTAATCTTGCTGCTACTAAAATGTCAGAGTGTGTACTTGGACAATCAAAAAGAGTT    24682
gb:MT106052|Organism:Severe      TCTGCTAATCTTGCTGCTACTAAAATGTCAGAGTGTGTACTTGGACAATCAAAAAGAGTT    24682
gb:LC522975|Organism:Severe      TCTGCTAATCTTGCTGCTACTAAAATGTCAGAGTGTGTACTTGGACAATCAAAAAGAGTT    24679
gb:LC522973|Organism:Severe      TCTGCTAATCTTGCTGCTACTAAAATGTCAGAGTGTGTACTTGGACAATCAAAAAGAGTT    24679
gb:LC522974|Organism:Severe      TCTGCTAATCTTGCTGCTACTAAAATGTCAGAGTGTGTACTTGGACAATCAAAAAGAGTT    24679
gb:MN985325|Organism:Severe      TCTGCTAATCTTGCTGCTACTAAAATGTCAGAGTGTGTACTTGGACAATCAAAAAGAGTT    24682
gb:MT020881|Organism:Severe      TCTGCTAATCTTGCTGCTACTAAAATGTCAGAGTGTGTACTTGGACAATCAAAAAGAGTT    24682
gb:MT020880|Organism:Severe      TCTGCTAATCTTGCTGCTACTAAAATGTCAGAGTGTGTACTTGGACAATCAAAAAGAGTT    24682
gb:MT066175|Organism:Severe      TCTGCTAATCTTGCTGCTACTAAAATGTCAGAGTGTGTACTTGGACAATCAAAAAGAGTT    24682
gb:MN997409|Organism:Severe      TCTGCTAATCTTGCTGCTACTAAAATGTCAGAGTGTGTACTTGGACAATCAAAAAGAGTT    24682
gb:MN938384|Organism:Severe      TCTGCTAATCTTGCTGCTACTAAAATGTCAGAGTGTGTACTTGGACAATCAAAAAGAGTT    24650
gb:MT044258|Organism:Severe      TCTGCTAATCTTGCTGCTACTAAAATGTCAGAGTGTGTACTTGGACAATCAAAAAGAGTT    24658
gb:MT039890|Organism:Severe      TCTGCTAATCTTGCTGCTACTAAAATGTCAGAGTGTGTACTTGGACAATCAAAAAGAGTT    24682
gb:MN988713|Organism:Severe      TCTGCTAATCTTGCTGCTACTAAAATGTCAGAGTGTGTACTTGGACAATCAAAAAGAGTT    24682
gb:LC521925|Organism:Severe      TCTGCTAATCTTGCTGCTACTAAAATGTCAGAGTGTGTACTTGGACAATCAAAAAGAGTT    24655
gb:MT093571|Organism:Severe      TCTGCTAATCTTGCTGCTACTAAAATGTCAGAGTGTGTACTTGGACAATCAAAAAGAGTT    24682
gb:MT039887|Organism:Severe      TCTGCTAATCTTGCTGCTACTAAAATGTCAGAGTGTGTACTTGGACAATCAAAAAGAGTT    24679
gb:MT019530|Organism:Severe      TCTGCTAATCTTGCTGCTACTAAAATGTCAGAGTGTGTACTTGGACAATCAAAAAGAGTT    24682
gb:MT039888|Organism:Severe      TCTGCTAATCTTGCTGCTACTAAAATGTCAGAGTGTGTACTTGGACAATCAAAAAGAGTT    24682
gb:LC522972|Organism:Severe      TCTGCTAATCTTGCTGCTACTAAAATGTCAGAGTGTGTACTTGGACAATCAAAAAGAGTT    24679
gb:MT027063|Organism:Severe      TCTGCTAATCTTGCTGCTACTAAAATGTCAGAGTGTGTACTTGGACAATCAAAAAGAGTT    24682
gb:MT027062|Organism:Severe      TCTGCTAATCTTGCTGCTACTAAAATGTCAGAGTGTGTACTTGGACAATCAAAAAGAGTT    24682
gb:MT019529|Organism:Severe      TCTGCTAATCTTGCTGCTACTAAAATGTCAGAGTGTGTACTTGGACAATCAAAAAGAGTT    24682
gb:MN996529|Organism:Severe      TCTGCTAATCTTGCTGCTACTAAAATGTCAGAGTGTGTACTTGGACAATCAAAAAGAGTT    24670
gb:MN996531|Organism:Severe      TCTGCTAATCTTGCTGCTACTAAAATGTCAGAGTGTGTACTTGGACAATCAAAAAGAGTT    24669
gb:MT066176|Organism:Severe      TCTGCTAATCTTGCTGCTACTAAAATGTCAGAGTGTGTACTTGGACAATCAAAAAGAGTT    24682
gb:MT027064|Organism:Severe      TCTGCTAATCTTGCTGCTACTAAAATGTCAGAGTGTGTACTTGGACAATCAAAAAGAGTT    24682
gb:MN994468|Organism:Severe      TCTGCTAATCTTGCTGCTACTAAAATGTCAGAGTGTGTACTTGGACAATCAAAAAGAGTT    24682
gb:MT072688|Organism:Severe      TCTGCTAATCTTGCTGCTACTAAAATGTCAGAGTGTGTACTTGGACAATCAAAAAGAGTT    24667
gb:MN996527|Organism:Severe      TCTGCTAATCTTGCTGCTACTAAAATGTCAGAGTGTGTACTTGGACAATCAAAAAGAGTT    24649
gb:MT093631|Organism:Severe      TCTGCTAATCTTGCTGCTACTAAAATGTCAGAGTGTGTACTTGGACAATCAAAAAGAGTT    24720
gb:MT106053|Organism:Severe      TCTGCTAATCTTGCTGCTACTAAAATGTCAGAGTGTGTACTTGGACAATCAAAAAGAGTT    24682
gb:MT019533|Organism:Severe      TCTGCTAATCTTGCTGCTACTAAAATGTCAGAGTGTGTACTTGGACAATCAAAAAGAGTT    24682
gb:MT019531|Organism:Severe      TCTGCTAATCTTGCTGCTACTAAAATGTCAGAGTGTGTACTTGGACAATCAAAAAGAGTT    24682
gb:MN996528|Organism:Severe      TCTGCTAATCTTGCTGCTACTAAAATGTCAGAGTGTGTACTTGGACAATCAAAAAGAGTT    24682
gb:MN996530|Organism:Severe      TCTGCTAATCTTGCTGCTACTAAAATGTCAGAGTGTGTACTTGGACAATCAAAAAGAGTT    24668
gb:MN908947|Organism:Severe      TCTGCTAATCTTGCTGCTACTAAAATGTCAGAGTGTGTACTTGGACAATCAAAAAGAGTT    24682
gb:MT019532|Organism:Severe      TCTGCTAATCTTGCTGCTACTAAAATGTCAGAGTGTGTACTTGGACAATCAAAAAGAGTT    24682
                                 ************************************************************

gb:MT020781|Organism:Severe      GATTTTTGTGGAAAGGGCTATCATCTTATGTCCTTCCCTCAGTCAGCACCTCATGGTGTA    24730
gb:MT007544|Organism:Severe      GATTTTTGTGGAAAGGGCTATCATCTTATGTCCTTCCCTCAGTCAGCACCTCATGGTGTA    24742
gb:MN994467|Organism:Severe      GATTTTTGTGGAAAGGGCTATCATCTTATGTCCTTCCCTCAGTCAGCACCTCATGGTGTA    24742
gb:MT044257|Organism:Severe      GATTTTTGTGGAAAGGGCTATCATCTTATGTCCTTCCCTCAGTCAGCACCTCATGGTGTA    24742
gb:MT106054|Organism:Severe      GATTTTTGTGGAAAGGGCTATCATCTTATGTCCTTCCCTCAGTCAGCACCTCATGGTGTA    24742
gb:MT049951|Organism:Severe      GATTTTTGTGGAAAGGGCTATCATCTTATGTCCTTCCCTCAGTCAGCACCTCATGGTGTA    24742
gb:MN975262|Organism:Severe      GATTTTTGTGGAAAGGGCTATCATCTTATGTCCTTCCCTCAGTCAGCACCTCATGGTGTA    24742
gb:MT106052|Organism:Severe      GATTTTTGTGGAAAGGGCTATCATCTTATGTCCTTCCCTCAGTCAGCACCTCATGGTGTA    24742
gb:LC522975|Organism:Severe      GATTTTTGTGGAAAGGGCTATCATCTTATGTCCTTCCCTCAGTCAGCACCTCATGGTGTA    24739
gb:LC522973|Organism:Severe      GATTTTTGTGGAAAGGGCTATCATCTTATGTCCTTCCCTCAGTCAGCACCTCATGGTGTA    24739
gb:LC522974|Organism:Severe      GATTTTTGTGGAAAGGGCTATCATCTTATGTCCTTCCCTCAGTCAGCACCTCATGGTGTA    24739
gb:MN985325|Organism:Severe      GATTTTTGTGGAAAGGGCTATCATCTTATGTCCTTCCCTCAGTCAGCACCTCATGGTGTA    24742
gb:MT020881|Organism:Severe      GATTTTTGTGGAAAGGGCTATCATCTTATGTCCTTCCCTCAGTCAGCACCTCATGGTGTA    24742
gb:MT020880|Organism:Severe      GATTTTTGTGGAAAGGGCTATCATCTTATGTCCTTCCCTCAGTCAGCACCTCATGGTGTA    24742
gb:MT066175|Organism:Severe      GATTTTTGTGGAAAGGGCTATCATCTTATGTCCTTCCCTCAGTCAGCACCTCATGGTGTA    24742
gb:MN997409|Organism:Severe      GATTTTTGTGGAAAGGGCTATCATCTTATGTCCTTCCCTCAGTCAGCACCTCATGGTGTA    24742
gb:MN938384|Organism:Severe      GATTTTTGTGGAAAGGGCTATCATCTTATGTCCTTCCCTCAGTCAGCACCTCATGGTGTA    24710
gb:MT044258|Organism:Severe      GATTTTTGTGGAAAGGGCTATCATCTTATGTCCTTCCCTCAGTCAGCACCTCATGGTGTA    24718
gb:MT039890|Organism:Severe      GATTTTTGTGGAAAGGGCTATCATCTTATGTCCTTCCCTCAGTCAGCACCTCATGGTGTA    24742
gb:MN988713|Organism:Severe      GATTTTTGTGGAAAGGGCTATCATCTTATGTCCTTCCCTCAGTCAGCACCTCATGGTGTA    24742
gb:LC521925|Organism:Severe      GATTTTTGTGGAAAGGGCTATCATCTTATGTCCTTCCCTCAGTCAGCACCTCATGGTGTA    24715
gb:MT093571|Organism:Severe      GATTTTTGTGGAAAGGGCTATCATCTTATGTCCTTCCCTCAGTCAGCACCTCATGGTGTA    24742
gb:MT039887|Organism:Severe      GATTTTTGTGGAAAGGGCTATCATCTTATGTCCTTCCCTCAGTCAGCACCTCATGGTGTA    24739
gb:MT019530|Organism:Severe      GATTTTTGTGGAAAGGGCTATCATCTTATGTCCTTCCCTCAGTCAGCACCTCATGGTGTA    24742
```

```
gb:MT039888|Organism:Severe      GATTTTGTGGAAAGGGCTATCATCTTATGTCCTTCCCTCAGTCAGCACCTCATGGTGTA   24742
gb:LC522972|Organism:Severe      GATTTTGTGGAAAGGGCTATCATCTTATGTCCTTCCCTCAGTCAGCACCTCATGGTGTA   24739
gb:MT027063|Organism:Severe      GATTTTGTGGAAAGGGCTATCATCTTATGTCCTTCCCTCAGTCAGCACCTCATGGTGTA   24742
gb:MT027062|Organism:Severe      GATTTTGTGGAAAGGGCTATCATCTTATGTCCTTCCCTCAGTCAGCACCTCATGGTGTA   24742
gb:MT019529|Organism:Severe      GATTTTGTGGAAAGGGCTATCATCTTATGTCCTTCCCTCAGTCAGCACCTCATGGTGTA   24742
gb:MN996529|Organism:Severe      GATTTTGTGGAAAGGGCTATCATCTTATGTCCTTCCCTCAGTCAGCACCTCATGGTGTA   24730
gb:MN996531|Organism:Severe      GATTTTGTGGAAAGGGCTATCATCTTATGTCCTTCCCTCAGTCAGCACCTCATGGTGTA   24729
gb:MT066176|Organism:Severe      GATTTTGTGGAAAGGGCTATCATCTTATGTCCTTCCCTCAGTCAGCACCTCATGGTGTA   24742
gb:MT027064|Organism:Severe      GATTTTGTGGAAAGGGCTATCATCTTATGTCCTTCCCTCAGTCAGCACCTCATGGTGTA   24742
gb:MN994468|Organism:Severe      GATTTTGTGGAAAGGGCTATCATCTTATGTCCTTCCCTCAGTCAGCACCTCATGGTGTA   24742
gb:MT072688|Organism:Severe      GATTTTGTGGAAAGGGCTATCATCTTATGTCCTTCCCTCAGTCAGCACCTCATGGTGTA   24727
gb:MN996527|Organism:Severe      GATTTTGTGGAAAGGGCTATCATCTTATGTCCTTCCCTCAGTCAGCACCTCATGGTGTA   24709
gb:MT093631|Organism:Severe      GATTTTGTGGAAAGGGCTATCATCTTATGTCCTTCCCTCAGTCAGCACCTCATGGTGTA   24780
gb:MT106053|Organism:Severe      GATTTTGTGGAAAGGGCTATCATCTTATGTCCTTCCCTCAGTCAGCACCTCATGGTGTA   24742
gb:MT019533|Organism:Severe      GATTTTGTGGAAAGGGCTATCATCTTATGTCCTTCCCTCAGTCAGCACCTCATGGTGTA   24742
gb:MT019531|Organism:Severe      GATTTTGTGGAAAGGGCTATCATCTTATGTCCTTCCCTCAGTCAGCACCTCATGGTGTA   24742
gb:MN996528|Organism:Severe      GATTTTGTGGAAAGGGCTATCATCTTATGTCCTTCCCTCAGTCAGCACCTCATGGTGTA   24742
gb:MN996530|Organism:Severe      GATTTTGTGGAAAGGGCTATCATCTTATGTCCTTCCCTCAGTCAGCACCTCATGGTGTA   24728
gb:MN908947|Organism:Severe      GATTTTGTGGAAAGGGCTATCATCTTATGTCCTTCCCTCAGTCAGCACCTCATGGTGTA   24742
gb:MT019532|Organism:Severe      GATTTTGTGGAAAGGGCTATCATCTTATGTCCTTCCCTCAGTCAGCACCTCATGGTGTA   24742
                                 ************************************************************

gb:MT020781|Organism:Severe      GTCTTCTTGCATGTGACTTATGTCCCTGCACAAGAAAAGAACTTCACAACTGCTCCTGCC   24790
gb:MT007544|Organism:Severe      GTCTTCTTGCATGTGACTTATGTCCCTGCACAAGAAAAGAACTTCACAACTGCTCCTGCC   24802
gb:MN994467|Organism:Severe      GTCTTCTTGCATGTGACTTATGTCCCTGCACAAGAAAAGAACTTCACAACTGCTCCTGCC   24802
gb:MT044257|Organism:Severe      GTCTTCTTGCATGTGACTTATGTCCCTGCACAAGAAAAGAACTTCACAACTGCTCCTGCC   24802
gb:MT106054|Organism:Severe      GTCTTCTTGCATGTGACTTATGTCCCTGCACAAGAAAAGAACTTCACAACTGCTCCTGCC   24802
gb:MT049951|Organism:Severe      GTCTTCTTGCATGTGACTTATGTCCCTGCACAAGAAAAGAACTTCACAACTGCTCCTGCC   24802
gb:MN975262|Organism:Severe      GTCTTCTTGCATGTGACTTATGTCCCTGCACAAGAAAAGAACTTCACAACTGCTCCTGCC   24802
gb:MT106052|Organism:Severe      GTCTTCTTGCATGTGACTTATGTCCCTGCACAAGAAAAGAACTTCACAACTGCTCCTGCC   24802
gb:LC522975|Organism:Severe      GTCTTCTTGCATGTGACTTATGTCCCTGCACAAGAAAAGAACTTCACAACTGCTCCTGCC   24799
gb:LC522973|Organism:Severe      GTCTTCTTGCATGTGACTTATGTCCCTGCACAAGAAAAGAACTTCACAACTGCTCCTGCC   24799
gb:LC522974|Organism:Severe      GTCTTCTTGCATGTGACTTATGTCCCTGCACAAGAAAAGAACTTCACAACTGCTCCTGCC   24799
gb:MN985325|Organism:Severe      GTCTTCTTGCATGTGACTTATGTCCCTGCACAAGAAAAGAACTTCACAACTGCTCCTGCC   24802
gb:MT020881|Organism:Severe      GTCTTCTTGCATGTGACTTATGTCCCTGCACAAGAAAAGAACTTCACAACTGCTCCTGCC   24802
gb:MT020880|Organism:Severe      GTCTTCTTGCATGTGACTTATGTCCCTGCACAAGAAAAGAACTTCACAACTGCTCCTGCC   24802
gb:MT066175|Organism:Severe      GTCTTCTTGCATGTGACTTATGTCCCTGCACAAGAAAAGAACTTCACAACTGCTCCTGCC   24802
gb:MN997409|Organism:Severe      GTCTTCTTGCATGTGACTTATGTCCCTGCACAAGAAAAGAACTTCACAACTGCTCCTGCC   24802
gb:MN938384|Organism:Severe      GTCTTCTTGCATGTGACTTATGTCCCTGCACAAGAAAAGAACTTCACAACTGCTCCTGCC   24770
gb:MT044258|Organism:Severe      GTCTTCTTGCATGTGACTTATGTCCCTGCACAAGAAAAGAACTTCACAACTGCTCCTGCC   24778
gb:MT039890|Organism:Severe      GTCTTCTTGCATGTGACTTATGTCCCTGCACAAGAAAAGAACTTCACAACTGCTCCTGCC   24802
gb:MN988713|Organism:Severe      GTCTTCTTGCATGTGACTTATGTCCCTGCACAAGAAAAGAACTTCACAACTGCTCCTGCC   24802
gb:LC521925|Organism:Severe      GTCTTCTTGCATGTGACTTATGTCCCTGCACAAGAAAAGAACTTCACAACTGCTCCTGCC   24775
gb:MT093571|Organism:Severe      GTCTTCTTGCATGTGACTTATGTCCCTGCACAAGAAAAGAACTTCACAACTGCTCCTGCC   24802
gb:MT039887|Organism:Severe      GTCTTCTTGCATGTGACTTATGTCCCTGCACAAGAAAAGAACTTCACAACTGCTCCTGCC   24799
gb:MT019530|Organism:Severe      GTCTTCTTGCATGTGACTTATGTCCCTGCACAAGAAAAGAACTTCACAACTGCTCCTGCC   24802
gb:MT039888|Organism:Severe      GTCTTCTTGCATGTGACTTATGTCCCTGCACAAGAAAAGAACTTCACAACTGCTCCTGCC   24802
gb:LC522972|Organism:Severe      GTCTTCTTGCATGTGACTTATGTCCCTGCACAAGAAAAGAACTTCACAACTGCTCCTGCC   24799
gb:MT027063|Organism:Severe      GTCTTCTTGCATGTGACTTATGTCCCTGCACAAGAAAAGAACTTCACAACTGCTCCTGCC   24802
gb:MT027062|Organism:Severe      GTCTTCTTGCATGTGACTTATGTCCCTGCACAAGAAAAGAACTTCACAACTGCTCCTGCC   24802
gb:MT019529|Organism:Severe      GTCTTCTTGCATGTGACTTATGTCCCTGCACAAGAAAAGAACTTCACAACTGCTCCTGCC   24802
gb:MN996529|Organism:Severe      GTCTTCTTGCATGTGACTTATGTCCCTGCACAAGAAAAGAACTTCACAACTGCTCCTGCC   24790
gb:MN996531|Organism:Severe      GTCTTCTTGCATGTGACTTATGTCCCTGCACAAGAAAAGAACTTCACAACTGCTCCTGCC   24789
gb:MT066176|Organism:Severe      GTCTTCTTGCATGTGACTTATGTCCCTGCACAAGAAAAGAACTTCACAACTGCTCCTGCC   24802
gb:MT027064|Organism:Severe      GTCTTCTTGCATGTGACTTATGTCCCTGCACAAGAAAAGAACTTCACAACTGCTCCTGCC   24802
gb:MN994468|Organism:Severe      GTCTTCTTGCATGTGACTTATGTCCCTGCACAAGAAAAGAACTTCACAACTGCTCCTGCC   24802
gb:MT072688|Organism:Severe      GTCTTCTTGCATGTGACTTATGTCCCTGCACAAGAAAAGAACTTCACAACTGCTCCTGCC   24787
gb:MN996527|Organism:Severe      GTCTTCTTGCATGTGACTTATGTCCCTGCACAAGAAAAGAACTTCACAACTGCTCCTGCC   24769
gb:MT093631|Organism:Severe      GTCTTCTTGCATGTGACTTATGTCCCTGCACAAGAAAAGAACTTCACAACTGCTCCTGCC   24840
gb:MT106053|Organism:Severe      GTCTTCTTGCATGTGACTTATGTCCCTGCACAAGAAAAGAACTTCACAACTGCTCCTGCC   24802
gb:MT019533|Organism:Severe      GTCTTCTTGCATGTGACTTATGTCCCTGCACAAGAAAAGAACTTCACAACTGCTCCTGCC   24802
gb:MT019531|Organism:Severe      GTCTTCTTGCATGTGACTTATGTCCCTGCACAAGAAAAGAACTTCACAACTGCTCCTGCC   24802
gb:MN996528|Organism:Severe      GTCTTCTTGCATGTGACTTATGTCCCTGCACAAGAAAAGAACTTCACAACTGCTCCTGCC   24802
gb:MN996530|Organism:Severe      GTCTTCTTGCATGTGACTTATGTCCCTGCACAAGAAAAGAACTTCACAACTGCTCCTGCC   24788
gb:MN908947|Organism:Severe      GTCTTCTTGCATGTGACTTATGTCCCTGCACAAGAAAAGAACTTCACAACTGCTCCTGCC   24802
```

```
gb:MT019532|Organism:Severe      GTCTTCTTGCATGTGACTTATGTCCCTGCACAAGAAAAGAACTTCACAACTGCTCCTGCC    24802
                                 ************************************************************

gb:MT020781|Organism:Severe      ATTTGTCATGATGGAAAAGCACACTTTCCTCGTGAAGGTGTCTTTGTTTCAAATGGCACA    24850
gb:MT007544|Organism:Severe      ATTTGTCATGATGGAAAAGCACACTTTCCTCGTGAAGGTGTCTTTGTTTCAAATGGCACA    24862
gb:MN994467|Organism:Severe      ATTTGTCATGATGGAAAAGCACACTTTCCTCGTGAAGGTGTCTTTGTTTCAAATGGCACA    24862
gb:MT044257|Organism:Severe      ATTTGTCATGATGGAAAAGCACACTTTCCTCGTGAAGGTGTCTTTGTTTCAAATGGCACA    24862
gb:MT106054|Organism:Severe      ATTTGTCATGATGGAAAAGCACACTTTCCTCGTGAAGGTGTCTTTGTTTCAAATGGCACA    24862
gb:MT049951|Organism:Severe      ATTTGTCATGATGGAAAAGCACACTTTCCTCGTGAAGGTGTCTTTGTTTCAAATGGCACA    24862
gb:MN975262|Organism:Severe      ATTTGTCATGATGGAAAAGCACACTTTCCTCGTGAAGGTGTCTTTGTTTCAAATGGCACA    24862
gb:MT106052|Organism:Severe      ATTTGTCATGATGGAAAAGCACACTTTCCTCGTGAAGGTGTCTTTGTTTCAAATGGCACA    24862
gb:LC522975|Organism:Severe      ATTTGTCATGATGGAAAAGCACACTTTCCTCGTGAAGGTGTCTTTGTTTCAAATGGCACA    24859
gb:LC522973|Organism:Severe      ATTTGTCATGATGGAAAAGCACACTTTCCTCGTGAAGGTGTCTTTGTTTCAAATGGCACA    24859
gb:LC522974|Organism:Severe      ATTTGTCATGATGGAAAAGCACACTTTCCTCGTGAAGGTGTCTTTGTTTCAAATGGCACA    24859
gb:MN985325|Organism:Severe      ATTTGTCATGATGGAAAAGCACACTTTCCTCGTGAAGGTGTCTTTGTTTCAAATGGCACA    24862
gb:MT020881|Organism:Severe      ATTTGTCATGATGGAAAAGCACACTTTCCTCGTGAAGGTGTCTTTGTTTCAAATGGCACA    24862
gb:MT020880|Organism:Severe      ATTTGTCATGATGGAAAAGCACACTTTCCTCGTGAAGGTGTCTTTGTTTCAAATGGCACA    24862
gb:MT066175|Organism:Severe      ATTTGTCATGATGGAAAAGCACACTTTCCTCGTGAAGGTGTCTTTGTTTCAAATGGCACA    24862
gb:MN997409|Organism:Severe      ATTTGTCATGATGGAAAAGCACACTTTCCTCGTGAAGGTGTCTTTGTTTCAAATGGCACA    24862
gb:MN938384|Organism:Severe      ATTTGTCATGATGGAAAAGCACACTTTCCTCGTGAAGGTGTCTTTGTTTCAAATGGCACA    24830
gb:MT044258|Organism:Severe      ATTTGTCATGATGGAAAAGCACACTTTCCTCGTGAAGGTGTCTTTGTTTCAAATGGCACA    24838
gb:MT039890|Organism:Severe      ATTTGTCATGATGGAAAAGCACACTTTCCTCGTGAAGGTGTCTTTGTTTCAAATGGCACA    24862
gb:MN988713|Organism:Severe      ATTTGTCATGATGGAAAAGCACACTTTCCTCGTGAAGGTGTCTTTGTTTCAAATGGCACA    24862
gb:LC521925|Organism:Severe      ATTTGTCATGATGGAAAAGCACACTTTCCTCGTGAAGGTGTCTTTGTTTCAAATGGCACA    24835
gb:MT093571|Organism:Severe      ATTTGTCATGATGGAAAAGCACACTTTCCTCGTGAAGGTGTCTTTGTTTCAAATGGCACA    24862
gb:MT039887|Organism:Severe      ATTTGTCATGATGGAAAAGCACACTTTCCTCGTGAAGGTGTCTTTGTTTCAAATGGCACA    24859
gb:MT019530|Organism:Severe      ATTTGTCATGATGGAAAAGCACACTTTCCTCGTGAAGGTGTCTTTGTTTCAAATGGCACA    24862
gb:MT039888|Organism:Severe      ATTTGTCATGATGGAAAAGCACACTTTCCTCGTGAAGGTGTCTTTGTTTCAAATGGCACA    24862
gb:LC522972|Organism:Severe      ATTTGTCATGATGGAAAAGCACACTTTCCTCGTGAAGGTGTCTTTGTTTCAAATGGCACA    24859
gb:MT027063|Organism:Severe      ATTTGTCATGATGGAAAAGCACACTTTCCTCGTGAAGGTGTCTTTGTTTCAAATGGCACA    24862
gb:MT027062|Organism:Severe      ATTTGTCATGATGGAAAAGCACACTTTCCTCGTGAAGGTGTCTTTGTTTCAAATGGCACA    24862
gb:MT019529|Organism:Severe      ATTTGTCATGATGGAAAAGCACACTTTCCTCGTGAAGGTGTCTTTGTTTCAAATGGCACA    24862
gb:MN996529|Organism:Severe      ATTTGTCATGATGGAAAAGCACACTTTCCTCGTGAAGGTGTCTTTGTTTCAAATGGCACA    24850
gb:MN996531|Organism:Severe      ATTTGTCATGATGGAAAAGCACACTTTCCTCGTGAAGGTGTCTTTGTTTCAAATGGCACA    24849
gb:MT066176|Organism:Severe      ATTTGTCATGATGGAAAAGCACACTTTCCTCGTGAAGGTGTCTTTGTTTCAAATGGCACA    24862
gb:MT027064|Organism:Severe      ATTTGTCATGATGGAAAAGCACACTTTCCTCGTGAAGGTGTCTTTGTTTCAAATGGCACA    24862
gb:MN994468|Organism:Severe      ATTTGTCATGATGGAAAAGCACACTTTCCTCGTGAAGGTGTCTTTGTTTCAAATGGCACA    24862
gb:MT072688|Organism:Severe      ATTTGTCATGATGGAAAAGCACACTTTCCTCGTGAAGGTGTCTTTGTTTCAAATGGCACA    24847
gb:MN996527|Organism:Severe      ATTTGTCATGATGGAAAAGCACACTTTCCTCGTGAAGGTGTCTTTGTTTCAAATGGCACA    24829
gb:MT093631|Organism:Severe      ATTTGTCATGATGGAAAAGCACACTTTCCTCGTGAAGGTGTCTTTGTTTCAAATGGCACA    24900
gb:MT106053|Organism:Severe      ATTTGTCATGATGGAAAAGCACACTTTCCTCGTGAAGGTGTCTTTGTTTCAAATGGCACA    24862
gb:MT019533|Organism:Severe      ATTTGTCATGATGGAAAAGCACACTTTCCTCGTGAAGGTGTCTTTGTTTCAAATGGCACA    24862
gb:MT019531|Organism:Severe      ATTTGTCATGATGGAAAAGCACACTTTCCTCGTGAAGGTGTCTTTGTTTCAAATGGCACA    24862
gb:MN996528|Organism:Severe      ATTTGTCATGATGGAAAAGCACACTTTCCTCGTGAAGGTGTCTTTGTTTCAAATGGCACA    24862
gb:MN996530|Organism:Severe      ATTTGTCATGATGGAAAAGCACACTTTCCTCGTGAAGGTGTCTTTGTTTCAAATGGCACA    24848
gb:MN908947|Organism:Severe      ATTTGTCATGATGGAAAAGCACACTTTCCTCGTGAAGGTGTCTTTGTTTCAAATGGCACA    24862
gb:MT019532|Organism:Severe      ATTTGTCATGATGGAAAAGCACACTTTCCTCGTGAAGGTGTCTTTGTTTCAAATGGCACA    24862
                                 ************************************************************

gb:MT020781|Organism:Severe      CACTGGTTTGTAACACAAAGGAATTTTTATGAACCACAAATCATTACTACAGACAACACA    24910
gb:MT007544|Organism:Severe      CACTGGTTTGTAACACAAAGGAATTTTTATGAACCACAAATCATTACTACAGACAACACA    24922
gb:MN994467|Organism:Severe      CACTGGTTTGTAACACAAAGGAATTTTTATGAACCACAAATCATTACTACAGACAACACA    24922
gb:MT044257|Organism:Severe      CACTGGTTTGTAACACAAAGGAATTTTTATGAACCACAAATCATTACTACAGACAACACA    24922
gb:MT106054|Organism:Severe      CACTGGTTTGTAACACAAAGGAATTTTTATGAACCACAAATCATTACTACAGACAACACA    24922
gb:MT049951|Organism:Severe      CACTGGTTTGTAACACAAAGGAATTTTTATGAACCACAAATCATTACTACAGACAACACA    24922
gb:MN975262|Organism:Severe      CACTGGTTTGTAACACAAAGGAATTTTTATGAACCACAAATCATTACTACAGACAACACA    24922
gb:MT106052|Organism:Severe      CACTGGTTTGTAACACAAAGGAATTTTTATGAACCACAAATCATTACTACAGACAACACA    24922
gb:LC522975|Organism:Severe      CACTGGTTTGTAACACAAAGGAATTTTTATGAACCACAAATCATTACTACAGACAACACA    24919
gb:LC522973|Organism:Severe      CACTGGTTTGTAACACAAAGGAATTTTTATGAACCACAAATCATTACTACAGACAACACA    24919
gb:LC522974|Organism:Severe      CACTGGTTTGTAACACAAAGGAATTTTTATGAACCACAAATCATTACTACAGACAACACA    24919
gb:MN985325|Organism:Severe      CACTGGTTTGTAACACAAAGGAATTTTTATGAACCACAAATCATTACTACAGACAACACA    24922
gb:MT020881|Organism:Severe      CACTGGTTTGTAACACAAAGGAATTTTTATGAACCACAAATCATTACTACAGACAACACA    24922
gb:MT020880|Organism:Severe      CACTGGTTTGTAACACAAAGGAATTTTTATGAACCACAAATCATTACTACAGACAACACA    24922
gb:MT066175|Organism:Severe      CACTGGTTTGTAACACAAAGGAATTTTTATGAACCACAAATCATTACTACAGACAACACA    24922
gb:MN997409|Organism:Severe      CACTGGTTTGTAACACAAAGGAATTTTTATGAACCACAAATCATTACTACAGACAACACA    24922
```

```
gb:MN938384|Organism:Severe      CACTGGTTTGTAACACAAAGGAATTTTTATGAACCACAAATCATTACTACAGACAACACA   24890
gb:MT044258|Organism:Severe      CACTGGTTTGTAACACAAAGGAATTTTTATGAACCACAAATCATTACTACAGACAACACA   24898
gb:MT039890|Organism:Severe      CACTGGTTTGTAACACAAAGGAATTTTTATGAACCACAAATCATTACTACAGACAACACA   24922
gb:MN988713|Organism:Severe      CACTGGTTTGTAACACAAAGGAATTTTTATGAACCACAAATCATTACTACAGACAACACA   24922
gb:LC521925|Organism:Severe      CACTGGTTTGTAACACAAAGGAATTTTTATGAACCACAAATCATTACTACAGACAACACA   24895
gb:MT093571|Organism:Severe      CACTGGTTTGTAACACAAAGGAATTTTTATGAACCACAAATCATTACTACAGACAACACA   24922
gb:MT039887|Organism:Severe      CACTGGTTTGTAACACAAAGGAATTTTTATGAACCACAAATCATTACTACAGACAACACA   24919
gb:MT019530|Organism:Severe      CACTGGTTTGTAACACAAAGGAATTTTTATGAACCACAAATCATTACTACAGACAACACA   24922
gb:MT039888|Organism:Severe      CACTGGTTTGTAACACAAAGGAATTTTTATGAACCACAAATCATTACTACAGACAACACA   24922
gb:LC522972|Organism:Severe      CACTGGTTTGTAACACAAAGGAATTTTTATGAACCACAAATCATTACTACAGACAACACA   24919
gb:MT027063|Organism:Severe      CACTGGTTTGTAACACAAAGGAATTTTTATGAACCACAAATCATTACTACAGACAACACA   24922
gb:MT027062|Organism:Severe      CACTGGTTTGTAACACAAAGGAATTTTTATGAACCACAAATCATTACTACAGACAACACA   24922
gb:MT019529|Organism:Severe      CACTGGTTTGTAACACAAAGGAATTTTTATGAACCACAAATCATTACTACAGACAACACA   24922
gb:MN996529|Organism:Severe      CACTGGTTTGTAACACAAAGGAATTTTTATGAACCACAAATCATTACTACAGACAACACA   24910
gb:MN996531|Organism:Severe      CACTGGTTTGTAACACAAAGGAATTTTTATGAACCACAAATCATTACTACAGACAACACA   24909
gb:MT066176|Organism:Severe      CACTGGTTTGTAACACAAAGGAATTTTTATGAACCACAAATCATTACTACAGACAACACA   24922
gb:MT027064|Organism:Severe      CACTGGTTTGTAACACAAAGGAATTTTTATGAACCACAAATCATTACTACAGACAACACA   24922
gb:MN994468|Organism:Severe      CACTGGTTTGTAACACAAAGGAATTTTTATGAACCACAAATCATTACTACAGACAACACA   24922
gb:MT072688|Organism:Severe      CACTGGTTTGTAACACAAAGGAATTTTTATGAACCACAAATCATTACTACAGACAACACA   24907
gb:MN996527|Organism:Severe      CACTGGTTTGTAACACAAAGGAATTTTTATGAACCACAAATCATTACTACAGACAACACA   24889
gb:MT093631|Organism:Severe      CACTGGTTTGTAACACAAAGGAATTTTTATGAACCACAAATCATTACTACAGACAACACA   24960
gb:MT106053|Organism:Severe      CACTGGTTTGTAACACAAAGGAATTTTTATGAACCACAAATCATTACTACAGACAACACA   24922
gb:MT019533|Organism:Severe      CACTGGTTTGTAACACAAAGGAATTTTTATGAACCACAAATCATTACTACAGACAACACA   24922
gb:MT019531|Organism:Severe      CACTGGTTTGTAACACAAAGGAATTTTTATGAACCACAAATCATTACTACAGACAACACA   24922
gb:MN996528|Organism:Severe      CACTGGTTTGTAACACAAAGGAATTTTTATGAACCACAAATCATTACTACAGACAACACA   24922
gb:MN996530|Organism:Severe      CACTGGTTTGTAACACAAAGGAATTTTTATGAACCACAAATCATTACTACAGACAACACA   24908
gb:MN908947|Organism:Severe      CACTGGTTTGTAACACAAAGGAATTTTTATGAACCACAAATCATTACTACAGACAACACA   24922
gb:MT019532|Organism:Severe      CACTGGTTTGTAACACAAAGGAATTTTTATGAACCACAAATCATTACTACAGACAACACA   24922
                                 ************************************************************

gb:MT020781|Organism:Severe      TTTGTGTCTGGTAACTGTGATGTTGTAATAGGAATTGTCAACAACACAGTTTATGATCCT   24970
gb:MT007544|Organism:Severe      TTTGTGTCTGGTAACTGTGATGTTGTAATAGGAATTGTCAACAACACAGTTTATGATCCT   24982
gb:MN994467|Organism:Severe      TTTGTGTCTGGTAACTGTGATGTTGTAATAGGAATTGTCAACAACACAGTTTATGATCCT   24982
gb:MT044257|Organism:Severe      TTTGTGTCTGGTAACTGTGATGTTGTAATAGGAATTGTCAACAACACAGTTTATGATCCT   24982
gb:MT106054|Organism:Severe      TTTGTGTCTGGTAACTGTGATGTTGTAATAGGAATTGTCAACAACACAGTTTATGATCCT   24982
gb:MT049951|Organism:Severe      TTTGTGTCTGGTAACTGTGATGTTGTAATAGGAATTGTCAACAACACAGTTTATGATCCT   24982
gb:MN975262|Organism:Severe      TTTGTGTCTGGTAACTGTGATGTTGTAATAGGAATTGTCAACAACACAGTTTATGATCCT   24982
gb:MT106052|Organism:Severe      TTTGTGTCTGGTAACTGTGATGTTGTAATAGGAATTGTCAACAACACAGTTTATGATCCT   24982
gb:LC522975|Organism:Severe      TTTGTGTCTGGTAACTGTGATGTTGTAATAGGAATTGTCAACAACACAGTTTATGATCCT   24979
gb:LC522973|Organism:Severe      TTTGTGTCTGGTAACTGTGATGTTGTAATAGGAATTGTCAACAACACAGTTTATGATCCT   24979
gb:LC522974|Organism:Severe      TTTGTGTCTGGTAACTGTGATGTTGTAATAGGAATTGTCAACAACACAGTTTATGATCCT   24979
gb:MN985325|Organism:Severe      TTTGTGTCTGGTAACTGTGATGTTGTAATAGGAATTGTCAACAACACAGTTTATGATCCT   24982
gb:MT020881|Organism:Severe      TTTGTGTCTGGTAACTGTGATGTTGTAATAGGAATTGTCAACAACACAGTTTATGATCCT   24982
gb:MT020880|Organism:Severe      TTTGTGTCTGGTAACTGTGATGTTGTAATAGGAATTGTCAACAACACAGTTTATGATCCT   24982
gb:MT066175|Organism:Severe      TTTGTGTCTGGTAACTGTGATGTTGTAATAGGAATTGTCAACAACACAGTTTATGATCCT   24982
gb:MN997409|Organism:Severe      TTTGTGTCTGGTAACTGTGATGTTGTAATAGGAATTGTCAACAACACAGTTTATGATCCT   24982
gb:MN938384|Organism:Severe      TTTGTGTCTGGTAACTGTGATGTTGTAATAGGAATTGTCAACAACACAGTTTATGATCCT   24950
gb:MT044258|Organism:Severe      TTTGTGTCTGGTAACTGTGATGTTGTAATAGGAATTGTCAACAACACAGTTTATGATCCT   24958
gb:MT039890|Organism:Severe      TTTGTGTCTGGTAACTGTGATGTTGTAATAGGAATTGTCAACAACACAGTTTATGATCCT   24982
gb:MN988713|Organism:Severe      TTTGTGTCTGGTAACTGTGATGTTGTAATAGGAATTGTCAACAACACAGTTTATGATCCT   24982
gb:LC521925|Organism:Severe      TTTGTGTCTGGTAACTGTGATGTTGTAATAGGAATTGTCAACAACACAGTTTATGATCCT   24955
gb:MT093571|Organism:Severe      TTTGTGTCTGGTAACTGTGATGTTGTAATAGGAATTGTCAACAACACAGTTTATGATCCT   24982
gb:MT039887|Organism:Severe      TTTGTGTCTGGTAACTGTGATGTTGTAATAGGAATTGTCAACAACACAGTTTATGATCCT   24979
gb:MT019530|Organism:Severe      TTTGTGTCTGGTAACTGTGATGTTGTAATAGGAATTGTCAACAACACAGTTTATGATCCT   24982
gb:MT039888|Organism:Severe      TTTGTGTCTGGTAACTGTGATGTTGTAATAGGAATTGTCAACAACACAGTTTATGATCCT   24982
gb:LC522972|Organism:Severe      TTTGTGTCTGGTAACTGTGATGTTGTAATAGGAATTGTCAACAACACAGTTTATGATCCT   24979
gb:MT027063|Organism:Severe      TTTGTGTCTGGTAACTGTGATGTTGTAATAGGAATTGTCAACAACACAGTTTATGATCCT   24982
gb:MT027062|Organism:Severe      TTTGTGTCTGGTAACTGTGATGTTGTAATAGGAATTGTCAACAACACAGTTTATGATCCT   24982
gb:MT019529|Organism:Severe      TTTGTGTCTGGTAACTGTGATGTTGTAATAGGAATTGTCAACAACACAGTTTATGATCCT   24982
gb:MN996529|Organism:Severe      TTTGTGTCTGGTAACTGTGATGTTGTAATAGGAATTGTCAACAACACAGTTTATGATCCT   24970
gb:MN996531|Organism:Severe      TTTGTGTCTGGTAACTGTGATGTTGTAATAGGAATTGTCAACAACACAGTTTATGATCCT   24969
gb:MT066176|Organism:Severe      TTTGTGTCTGGTAACTGTGATGTTGTAATAGGAATTGTCAACAACACAGTTTATGATCCT   24982
gb:MT027064|Organism:Severe      TTTGTGTCTGGTAACTGTGATGTTGTAATAGGAATTGTCAACAACACAGTTTATGATCCT   24982
gb:MN994468|Organism:Severe      TTTGTGTCTGGTAACTGTGATGTTGTAATAGGAATTGTCAACAACACAGTTTATGATCCT   24982
gb:MT072688|Organism:Severe      TTTGTGTCTGGTAACTGTGATGTTGTAATAGGAATTGTCAACAACACAGTTTATGATCCT   24967
```

```
gb:MN996527|Organism:Severe      TTTGTGTCTGGTAACTGTGATGTTGTAATAGGAATTGTCAACAACACAGTTTATGATCCT   24949
gb:MT093631|Organism:Severe      TTTGTGTCTGGTAACTGTGATGTTGTAATAGGAATTGTCAACAACACAGTTTATGATCCT   25020
gb:MT106053|Organism:Severe      TTTGTGTCTGGTAACTGTGATGTTGTAATAGGAATTGTCAACAACACAGTTTATGATCCT   24982
gb:MT019533|Organism:Severe      TTTGTGTCTGGTAACTGTGATGTTGTAATAGGAATTGTCAACAACACAGTTTATGATCCT   24982
gb:MT019531|Organism:Severe      TTTGTGTCTGGTAACTGTGATGTTGTAATAGGAATTGTCAACAACACAGTTTATGATCCT   24982
gb:MN996528|Organism:Severe      TTTGTGTCTGGTAACTGTGATGTTGTAATAGGAATTGTCAACAACACAGTTTATGATCCT   24982
gb:MN996530|Organism:Severe      TTTGTGTCTGGTAACTGTGATGTTGTAATAGGAATTGTCAACAACACAGTTTATGATCCT   24968
gb:MN908947|Organism:Severe      TTTGTGTCTGGTAACTGTGATGTTGTAATAGGAATTGTCAACAACACAGTTTATGATCCT   24982
gb:MT019532|Organism:Severe      TTTGTGTCTGGTAACTGTGATGTTGTAATAGGAATTGTCAACAACACAGTTTATGATCCT   24982
                                 ************************************************************

gb:MT020781|Organism:Severe      TTGCAACCTGAATTAGACTCATTCAAGGAGGAGTTAGATAAATATTTTAAGAATCATACA   25030
gb:MT007544|Organism:Severe      TTGCAACCTGAATTAGACTCATTCAAGGAGGAGTTAGATAAATATTTTAAGAATCATACA   25042
gb:MN994467|Organism:Severe      TTGCAACCTGAATTAGACTCATTCAAGGAGGAGTTAGATAAATATTTTAAGAATCATACA   25042
gb:MT044257|Organism:Severe      TTGCAACCTGAATTAGACTCATTCAAGGAGGAGTTAGATAAATATTTTAAGAATCATACA   25042
gb:MT106054|Organism:Severe      TTGCAACCTGAATTAGACTCATTCAAGGAGGAGTTAGATAAATATTTTAAGAATCATACA   25042
gb:MT049951|Organism:Severe      TTGCAACCTGAATTAGACTCATTCAAGGAGGAGTTAGATAAATATTTTAAGAATCATACA   25042
gb:MN975262|Organism:Severe      TTGCAACCTGAATTAGACTCATTCAAGGAGGAGTTAGATAAATATTTTAAGAATCATACA   25042
gb:MT106052|Organism:Severe      TTGCAACCTGAATTAGACTCATTCAAGGAGGAGTTAGATAAATATTTTAAGAATCATACA   25042
gb:LC522975|Organism:Severe      TTGCAACCTGAATTAGACTCATTCAAGGAGGAGTTAGATAAATATTTTAAGAATCATACA   25039
gb:LC522973|Organism:Severe      TTGCAACCTGAATTAGACTCATTCAAGGAGGAGTTAGATAAATATTTTAAGAATCATACA   25039
gb:LC522974|Organism:Severe      TTGCAACCTGAATTAGACTCATTCAAGGAGGAGTTAGATAAATATTTTAAGAATCATACA   25039
gb:MN985325|Organism:Severe      TTGCAACCTGAATTAGACTCATTCAAGGAGGAGTTAGATAAATATTTTAAGAATCATACA   25042
gb:MT020881|Organism:Severe      TTGCAACCTGAATTAGACTCATTCAAGGAGGAGTTAGATAAATATTTTAAGAATCATACA   25042
gb:MT020880|Organism:Severe      TTGCAACCTGAATTAGACTCATTCAAGGAGGAGTTAGATAAATATTTTAAGAATCATACA   25042
gb:MT066175|Organism:Severe      TTGCAACCTGAATTAGACTCATTCAAGGAGGAGTTAGATAAATATTTTAAGAATCATACA   25042
gb:MN997409|Organism:Severe      TTGCAACCTGAATTAGACTCATTCAAGGAGGAGTTAGATAAATATTTTAAGAATCATACA   25042
gb:MN938384|Organism:Severe      TTGCAACCTGAATTAGACTCATTCAAGGAGGAGTTAGATAAATATTTTAAGAATCATACA   25010
gb:MT044258|Organism:Severe      TTGCAACCTGAATTAGACTCATTCAAGGAGGAGTTAGATAAATATTTTAAGAATCATACA   25018
gb:MT039890|Organism:Severe      TTGCAACCTGAATTAGACTCATTCAAGGAGGAGTTAGATAAATATTTTAAGAATCATACA   25042
gb:MN988713|Organism:Severe      TTGCAACCTGAATTAGACTCATTCAAGGAGGAGTTAGATAAATATTTTAAGAATCATACA   25042
gb:LC521925|Organism:Severe      TTGCAACCTGAATTAGACTCATTCAAGGAGGAGTTAGATAAATATTTTAAGAATCATACA   25015
gb:MT093571|Organism:Severe      TTGCAACCTGAATTAGACTCATTCAAGGAGGAGTTAGATAAATATTTTAAGAATCATACA   25042
gb:MT039887|Organism:Severe      TTGCAACCTGAATTAGACTCATTCAAGGAGGAGTTAGATAAATATTTTAAGAATCATACA   25039
gb:MT019530|Organism:Severe      TTGCAACCTGAATTAGACTCATTCAAGGAGGAGTTAGATAAATATTTTAAGAATCATACA   25042
gb:MT039888|Organism:Severe      TTGCAACCTGAATTAGACTCATTCAAGGAGGAGTTAGATAAATATTTTAAGAATCATACA   25042
gb:LC522972|Organism:Severe      TTGCAACCTGAATTAGACTCATTCAAGGAGGAGTTAGATAAATATTTTAAGAATCATACA   25039
gb:MT027063|Organism:Severe      TTGCAACCTGAATTAGACTCATTCAAGGAGGAGTTAGATAAATATTTTAAGAATCATACA   25042
gb:MT027062|Organism:Severe      TTGCAACCTGAATTAGACTCATTCAAGGAGGAGTTAGATAAATATTTTAAGAATCATACA   25042
gb:MT019529|Organism:Severe      TTGCAACCTGAATTAGACTCATTCAAGGAGGAGTTAGATAAATATTTTAAGAATCATACA   25042
gb:MN996529|Organism:Severe      TTGCAACCTGAATTAGACTCATTCAAGGAGGAGTTAGATAAATATTTTAAGAATCATACA   25030
gb:MN996531|Organism:Severe      TTGCAACCTGAATTAGACTCATTCAAGGAGGAGTTAGATAAATATTTTAAGAATCATACA   25029
gb:MT066176|Organism:Severe      TTGCAACCTGAATTAGACTCATTCAAGGAGGAGTTAGATAAATATTTTAAGAATCATACA   25042
gb:MT027064|Organism:Severe      TTGCAACCTGAATTAGACTCATTCAAGGAGGAGTTAGATAAATATTTTAAGAATCATACA   25042
gb:MN994468|Organism:Severe      TTGCAACCTGAATTAGACTCATTCAAGGAGGAGTTAGATAAATATTTTAAGAATCATACA   25042
gb:MT072688|Organism:Severe      TTGCAACCTGAATTAGACTCATTCAAGGAGGAGTTAGATAAATATTTTAAGAATCATACA   25027
gb:MN996527|Organism:Severe      TTGCAACCTGAATTAGACTCATTCAAGGAGGAGTTAGATAAATATTTTAAGAATCATACA   25009
gb:MT093631|Organism:Severe      TTGCAACCTGAATTAGACTCATTCAAGGAGGAGTTAGATAAATATTTTAAGAATCATACA   25080
gb:MT106053|Organism:Severe      TTGCAACCTGAATTAGACTCATTCAAGGAGGAGTTAGATAAATATTTTAAGAATCATACA   25042
gb:MT019533|Organism:Severe      TTGCAACCTGAATTAGACTCATTCAAGGAGGAGTTAGATAAATATTTTAAGAATCATACA   25042
gb:MT019531|Organism:Severe      TTGCAACCTGAATTAGACTCATTCAAGGAGGAGTTAGATAAATATTTTAAGAATCATACA   25042
gb:MN996528|Organism:Severe      TTGCAACCTGAATTAGACTCATTCAAGGAGGAGTTAGATAAATATTTTAAGAATCATACA   25042
gb:MN996530|Organism:Severe      TTGCAACCTGAATTAGACTCATTCAAGGAGGAGTTAGATAAATATTTTAAGAATCATACA   25028
gb:MN908947|Organism:Severe      TTGCAACCTGAATTAGACTCATTCAAGGAGGAGTTAGATAAATATTTTAAGAATCATACA   25042
gb:MT019532|Organism:Severe      TTGCAACCTGAATTAGACTCATTCAAGGAGGAGTTAGATAAATATTTTAAGAATCATACA   25042
                                 ************************************************************

gb:MT020781|Organism:Severe      TCACCAGATGTTGATTTAGGTGACATCTCTGGCATTAATGCTTCAGTTGTAAACATTCAA   25090
gb:MT007544|Organism:Severe      TCACCAGATGTTGATTTAGGTGACATCTCTGGCATTAATGCTTCAGTTGTAAACATTCAA   25102
gb:MN994467|Organism:Severe      TCACCAGATGTTGATTTAGGTGACATCTCTGGCATTAATGCTTCAGTTGTAAACATTCAA   25102
gb:MT044257|Organism:Severe      TCACCAGATGTTGATTTAGGTGACATCTCTGGCATTAATGCTTCAGTTGTAAACATTCAA   25102
gb:MT106054|Organism:Severe      TCACCAGATGTTGATTTAGGTGACATCTCTGGCATTAATGCTTCAGTTGTAAACATTCAA   25102
gb:MT049951|Organism:Severe      TCACCAGATGTTGATTTAGGTGACATCTCTGGCATTAATGCTTCAGTTGTAAACATTCAA   25102
gb:MN975262|Organism:Severe      TCACCAGATGTTGATTTAGGTGACATCTCTGGCATTAATGCTTCAGTTGTAAACATTCAA   25102
gb:MT106052|Organism:Severe      TCACCAGATGTTGATTTAGGTGACATCTCTGGCATTAATGCTTCAGTTGTAAACATTCAA   25102
```

```
gb:LC522975|Organism:Severe      TCACCAGATGTTGATTTAGGTGACATCTCTGGCATTAATGCTTCAGTTGTAAACATTCAA   25099
gb:LC522973|Organism:Severe      TCACCAGATGTTGATTTAGGTGACATCTCTGGCATTAATGCTTCAGTTGTAAACATTCAA   25099
gb:LC522974|Organism:Severe      TCACCAGATGTTGATTTAGGTGACATCTCTGGCATTAATGCTTCAGTTGTAAACATTCAA   25099
gb:MN985325|Organism:Severe      TCACCAGATGTTGATTTAGGTGACATCTCTGGCATTAATGCTTCAGTTGTAAACATTCAA   25102
gb:MT020881|Organism:Severe      TCACCAGATGTTGATTTAGGTGACATCTCTGGCATTAATGCTTCAGTTGTAAACATTCAA   25102
gb:MT020880|Organism:Severe      TCACCAGATGTTGATTTAGGTGACATCTCTGGCATTAATGCTTCAGTTGTAAACATTCAA   25102
gb:MT066175|Organism:Severe      TCACCAGATGTTGATTTAGGTGACATCTCTGGCATTAATGCTTCAGTTGTAAACATTCAA   25102
gb:MN997409|Organism:Severe      TCACCAGATGTTGATTTAGGTGACATCTCTGGCATTAATGCTTCAGTTGTAAACATTCAA   25102
gb:MN938384|Organism:Severe      TCACCAGATGTTGATTTAGGTGACATCTCTGGCATTAATGCTTCAGTTGTAAACATTCAA   25070
gb:MT044258|Organism:Severe      TCACCAGATGTTGATTTAGGTGACATCTCTGGCATTAATGCTTCAGTTGTAAACATTCAA   25078
gb:MT039890|Organism:Severe      TCACCAGATGTTGATTTAGGTGACATCTCTGGCATTAATGCTTCAGTTGTAAACATTCAA   25102
gb:MN988713|Organism:Severe      TCACCAGATGTTGATTTAGGTGACATCTCTGGCATTAATGCTTCAGTTGTAAACATTCAA   25102
gb:LC521925|Organism:Severe      TCACCAGATGTTGATTTAGGTGACATCTCTGGCATTAATGCTTCAGTTGTAAACATTCAA   25075
gb:MT093571|Organism:Severe      TCACCAGATGTTGATTTAGGTGACATCTCTGGCATTAATGCTTCAGTTGTAAACATTCAA   25102
gb:MT039887|Organism:Severe      TCACCAGATGTTGATTTAGGTGACATCTCTGGCATTAATGCTTCAGTTGTAAACATTCAA   25099
gb:MT019530|Organism:Severe      TCACCAGATGTTGATTTAGGTGACATCTCTGGCATTAATGCTTCAGTTGTAAACATTCAA   25102
gb:MT039888|Organism:Severe      TCACCAGATGTTGATTTAGGTGACATCTCTGGCATTAATGCTTCAGTTGTAAACATTCAA   25102
gb:LC522972|Organism:Severe      TCACCAGATGTTGATTTAGGTGACATCTCTGGCATTAATGCTTCAGTTGTAAACATTCAA   25099
gb:MT027063|Organism:Severe      TCACCAGATGTTGATTTAGGTGACATCTCTGGCATTAATGCTTCAGTTGTAAACATTCAA   25102
gb:MT027062|Organism:Severe      TCACCAGATGTTGATTTAGGTGACATCTCTGGCATTAATGCTTCAGTTGTAAACATTCAA   25102
gb:MT019529|Organism:Severe      TCACCAGATGTTGATTTAGGTGACATCTCTGGCATTAATGCTTCAGTTGTAAACATTCAA   25102
gb:MN996529|Organism:Severe      TCACCAGATGTTGATTTAGGTGACATCTCTGGCATTAATGCTTCAGTTGTAAACATTCAA   25090
gb:MN996531|Organism:Severe      TCACCAGATGTTGATTTAGGTGACATCTCTGGCATTAATGCTTCAGTTGTAAACATTCAA   25089
gb:MT066176|Organism:Severe      TCACCAGATGTTGATTTAGGTGACATCTCTGGCATTAATGCTTCAGTTGTAAACATTCAA   25102
gb:MT027064|Organism:Severe      TCACCAGATGTTGATTTAGGTGACATCTCTGGCATTAATGCTTCAGTTGTAAACATTCAA   25102
gb:MN994468|Organism:Severe      TCACCAGATGTTGATTTAGGTGACATCTCTGGCATTAATGCTTCAGTTGTAAACATTCAA   25102
gb:MT072688|Organism:Severe      TCACCAGATGTTGATTTAGGTGACATCTCTGGCATTAATGCTTCAGTTGTAAACATTCAA   25087
gb:MN996527|Organism:Severe      TCACCAGATGTTGATTTAGGTGACATCTCTGGCATTAATGCTTCAGTTGTAAACATTCAA   25069
gb:MT093631|Organism:Severe      TCACCAGATGTTGATTTAGGTGACATCTCTGGCATTAATGCTTCAGTTGTAAACATTCAA   25140
gb:MT106053|Organism:Severe      TCACCAGATGTTGATTTAGGTGACATCTCTGGCATTAATGCTTCAGTTGTAAACATTCAA   25102
gb:MT019533|Organism:Severe      TCACCAGATGTTGATTTAGGTGACATCTCTGGCATTAATGCTTCAGTTGTAAACATTCAA   25102
gb:MT019531|Organism:Severe      TCACCAGATGTTGATTTAGGTGACATCTCTGGCATTAATGCTTCAGTTGTAAACATTCAA   25102
gb:MN996528|Organism:Severe      TCACCAGATGTTGATTTAGGTGACATCTCTGGCATTAATGCTTCAGTTGTAAACATTCAA   25102
gb:MN996530|Organism:Severe      TCACCAGATGTTGATTTAGGTGACATCTCTGGCATTAATGCTTCAGTTGTAAACATTCAA   25088
gb:MN908947|Organism:Severe      TCACCAGATGTTGATTTAGGTGACATCTCTGGCATTAATGCTTCAGTTGTAAACATTCAA   25102
gb:MT019532|Organism:Severe      TCACCAGATGTTGATTTAGGTGACATCTCTGGCATTAATGCTTCAGTTGTAAACATTCAA   25102
                                 ************************************************************

gb:MT020781|Organism:Severe      AAAGAAATTGACCGCCTCAATGAGGTTGCCAAGAATTTAAATGAATCTCTCATCGATCTC   25150
gb:MT007544|Organism:Severe      AAAGAAATTGACCGCCTCAATGAGGTTGCCAAGAATTTAAATGAATCTCTCATCGATCTC   25162
gb:MN994467|Organism:Severe      AAAGAAATTGACCGCCTCAATGAGGTTGCCAAGAATTTAAATGAATCTCTCATCGATCTC   25162
gb:MT044257|Organism:Severe      AAAGAAATTGACCGCCTCAATGAGGTTGCCAAGAATTTAAATGAATCTCTCATCGATCTC   25162
gb:MT106054|Organism:Severe      AAAGAAATTGACCGCCTCAATGAGGTTGCCAAGAATTTAAATGAATCTCTCATCGATCTC   25162
gb:MT049951|Organism:Severe      AAAGAAATTGACCGCCTCAATGAGGTTGCCAAGAATTTAAATGAATCTCTCATCGATCTC   25162
gb:MN975262|Organism:Severe      AAAGAAATTGACCGCCTCAATGAGGTTGCCAAGAATTTAAATGAATCTCTCATCGATCTC   25162
gb:MT106052|Organism:Severe      AAAGAAATTGACCGCCTCAATGAGGTTGCCAAGAATTTAAATGAATCTCTCATCGATCTC   25162
gb:LC522975|Organism:Severe      AAAGAAATTGACCGCCTCAATGAGGTTGCCAAGAATTTAAATGAATCTCTCATCGATCTC   25159
gb:LC522973|Organism:Severe      AAAGAAATTGACCGCCTCAATGAGGTTGCCAAGAATTTAAATGAATCTCTCATCGATCTC   25159
gb:LC522974|Organism:Severe      AAAGAAATTGACCGCCTCAATGAGGTTGCCAAGAATTTAAATGAATCTCTCATCGATCTC   25159
gb:MN985325|Organism:Severe      AAAGAAATTGACCGCCTCAATGAGGTTGCCAAGAATTTAAATGAATCTCTCATCGATCTC   25162
gb:MT020881|Organism:Severe      AAAGAAATTGACCGCCTCAATGAGGTTGCCAAGAATTTAAATGAATCTCTCATCGATCTC   25162
gb:MT020880|Organism:Severe      AAAGAAATTGACCGCCTCAATGAGGTTGCCAAGAATTTAAATGAATCTCTCATCGATCTC   25162
gb:MT066175|Organism:Severe      AAAGAAATTGACCGCCTCAATGAGGTTGCCAAGAATTTAAATGAATCTCTCATCGATCTC   25162
gb:MN997409|Organism:Severe      AAAGAAATTGACCGCCTCAATGAGGTTGCCAAGAATTTAAATGAATCTCTCATCGATCTC   25162
gb:MN938384|Organism:Severe      AAAGAAATTGACCGCCTCAATGAGGTTGCCAAGAATTTAAATGAATCTCTCATCGATCTC   25130
gb:MT044258|Organism:Severe      AAAGAAATTGACCGCCTCAATGAGGTTGCCAAGAATTTAAATGAATCTCTCATCGATCTC   25138
gb:MT039890|Organism:Severe      AAAGAAATTGACCGCCTCAATGAGGTTGCCAAGAATTTAAATGAATCTCTCATCGATCTC   25162
gb:MN988713|Organism:Severe      AAAGAAATTGACCGCCTCAATGAGGTTGCCAAGAATTTAAATGAATCTCTCATCGATCTC   25162
gb:LC521925|Organism:Severe      AAAGAAATTGACCGCCTCAATGAGGTTGCCAAGAATTTAAATGAATCTCTCATCGATCTC   25135
gb:MT093571|Organism:Severe      AAAGAAATTGACCGCCTCAATGAGGTTGCCAAGAATTTAAATGAATCTCTCATCGATCTC   25162
gb:MT039887|Organism:Severe      AAAGAAATTGACCGCCTCAATGAGGTTGCCAAGAATTTAAATGAATCTCTCATCGATCTC   25159
gb:MT019530|Organism:Severe      AAAGAAATTGACCGCCTCAATGAGGTTGCCAAGAATTTAAATGAATCTCTCATCGATCTC   25162
gb:MT039888|Organism:Severe      AAAGAAATTGACCGCCTCAATGAGGTTGCCAAGAATTTAAATGAATCTCTCATCGATCTC   25162
gb:LC522972|Organism:Severe      AAAGAAATTGACCGCCTCAATGAGGTTGCCAAGAATTTAAATGAATCTCTCATCGATCTC   25159
gb:MT027063|Organism:Severe      AAAGAAATTGACCGCCTCAATGAGGTTGCCAAGAATTTAAATGAATCTCTCATCGATCTC   25162
```

```
gb:MT027062|Organism:Severe      AAAGAAATTGACCGCCTCAATGAGGTTGCCAAGAATTTAAATGAATCTCTCATCGATCTC   25162
gb:MT019529|Organism:Severe      AAAGAAATTGACCGCCTCAATGAGGTTGCCAAGAATTTAAATGAATCTCTCATCGATCTC   25162
gb:MN996529|Organism:Severe      AAAGAAATTGACCGCCTCAATGAGGTTGCCAAGAATTTAAATGAATCTCTCATCGATCTC   25150
gb:MN996531|Organism:Severe      AAAGAAATTGACCGCCTCAATGAGGTTGCCAAGAATTTAAATGAATCTCTCATCGATCTC   25149
gb:MT066176|Organism:Severe      AAAGAAATTGACCGCCTCAATGAGGTTGCCAAGAATTTAAATGAATCTCTCATCGATCTC   25162
gb:MT027064|Organism:Severe      AAAGAAATTGACCGCCTCAATGAGGTTGCCAAGAATTTAAATGAATCTCTCATCGATCTC   25162
gb:MN994468|Organism:Severe      AAAGAAATTGACCGCCTCAATGAGGTTGCCAAGAATTTAAATGAATCTCTCATCGATCTC   25162
gb:MT072688|Organism:Severe      AAAGAAATTGACCGCCTCAATGAGGTTGCCAAGAATTTAAATGAATCTCTCATCGATCTC   25147
gb:MN996527|Organism:Severe      AAAGAAATTGACCGCCTCAATGAGGTTGCCAAGAATTTAAATGAATCTCTCATCGATCTC   25129
gb:MT093631|Organism:Severe      AAAGAAATTGACCGCCTCAATGAGGTTGCCAAGAATTTAAATGAATCTCTCATCGATCTC   25200
gb:MT106053|Organism:Severe      AAAGAAATTGACCGCCTCAATGAGGTTGCCAAGAATTTAAATGAATCTCTCATCGATCTC   25162
gb:MT019533|Organism:Severe      AAAGAAATTGACCGCCTCAATGAGGTTGCCAAGAATTTAAATGAATCTCTCATCGATCTC   25162
gb:MT019531|Organism:Severe      AAAGAAATTGACCGCCTCAATGAGGTTGCCAAGAATTTAAATGAATCTCTCATCGATCTC   25162
gb:MN996528|Organism:Severe      AAAGAAATTGACCGCCTCAATGAGGTTGCCAAGAATTTAAATGAATCTCTCATCGATCTC   25162
gb:MN996530|Organism:Severe      AAAGAAATTGACCGCCTCAATGAGGTTGCCAAGAATTTAAATGAATCTCTCATCGATCTC   25148
gb:MN908947|Organism:Severe      AAAGAAATTGACCGCCTCAATGAGGTTGCCAAGAATTTAAATGAATCTCTCATCGATCTC   25162
gb:MT019532|Organism:Severe      AAAGAAATTGACCGCCTCAATGAGGTTGCCAAGAATTTAAATGAATCTCTCATCGATCTC   25162
                                 ************************************************************

gb:MT020781|Organism:Severe      CAAGAACTTGGAAAGTATGAGCAGTATATAAAATGGCCATGGTACATTTGGCTAGGTTTT   25210
gb:MT007544|Organism:Severe      CAAGAACTTGGAAAGTATGAGCAGTATATAAAATGGCCATGGTACATTTGGCTAGGTTTT   25222
gb:MN994467|Organism:Severe      CAAGAACTTGGAAAGTATGAGCAGTATATAAAATGGCCATGGTACATTTGGCTAGGTTTT   25222
gb:MT044257|Organism:Severe      CAAGAACTTGGAAAGTATGAGCAGTATATAAAATGGCCATGGTACATTTGGCTAGGTTTT   25222
gb:MT106054|Organism:Severe      CAAGAACTTGGAAAGTATGAGCAGTATATAAAATGGCCATGGTACATTTGGCTAGGTTTT   25222
gb:MT049951|Organism:Severe      CAAGAACTTGGAAAGTATGAGCAGTATATAAAATGGCCATGGTACATTTGGCTAGGTTTT   25222
gb:MN975262|Organism:Severe      CAAGAACTTGGAAAGTATGAGCAGTATATAAAATGGCCATGGTACATTTGGCTAGGTTTT   25222
gb:MT106052|Organism:Severe      CAAGAACTTGGAAAGTATGAGCAGTATATAAAATGGCCATGGTACATTTGGCTAGGTTTT   25222
gb:LC522975|Organism:Severe      CAAGAACTTGGAAAGTATGAGCAGTATATAAAATGGCCATGGTACATTTGGCTAGGTTTT   25219
gb:LC522973|Organism:Severe      CAAGAACTTGGAAAGTATGAGCAGTATATAAAATGGCCATGGTACATTTGGCTAGGTTTT   25219
gb:LC522974|Organism:Severe      CAAGAACTTGGAAAGTATGAGCAGTATATAAAATGGCCATGGTACATTTGGCTAGGTTTT   25219
gb:MN985325|Organism:Severe      CAAGAACTTGGAAAGTATGAGCAGTATATAAAATGGCCATGGTACATTTGGCTAGGTTTT   25222
gb:MT020881|Organism:Severe      CAAGAACTTGGAAAGTATGAGCAGTATATAAAATGGCCATGGTACATTTGGCTAGGTTTT   25222
gb:MT020880|Organism:Severe      CAAGAACTTGGAAAGTATGAGCAGTATATAAAATGGCCATGGTACATTTGGCTAGGTTTT   25222
gb:MT066175|Organism:Severe      CAAGAACTTGGAAAGTATGAGCAGTATATAAAATGGCCATGGTACATTTGGCTAGGTTTT   25222
gb:MN997409|Organism:Severe      CAAGAACTTGGAAAGTATGAGCAGTATATAAAATGGCCATGGTACATTTGGCTAGGTTTT   25222
gb:MN938384|Organism:Severe      CAAGAACTTGGAAAGTATGAGCAGTATATAAAATGGCCATGGTACATTTGGCTAGGTTTT   25190
gb:MT044258|Organism:Severe      CAAGAACTTGGAAAGTATGAGCAGTATATAAAATGGCCATGGTACATTTGGCTAGGTTTT   25198
gb:MT039890|Organism:Severe      CAAGAACTTGGAAAGTATGAGCAGTATATAAAATGGCCATGGTACATTTGGCTAGGTTTT   25222
gb:MN988713|Organism:Severe      CAAGAACTTGGAAAGTATGAGCAGTATATAAAATGGCCATGGTACATTTGGCTAGGTTTT   25222
gb:LC521925|Organism:Severe      CAAGAACTTGGAAAGTATGAGCAGTATATAAAATGGCCATGGTACATTTGGCTAGGTTTT   25195
gb:MT093571|Organism:Severe      CAAGAACTTGGAAAGTATGAGCAGTATATAAAATGGCCATGGTACATTTGGCTAGGTTTT   25222
gb:MT039887|Organism:Severe      CAAGAACTTGGAAAGTATGAGCAGTATATAAAATGGCCATGGTACATTTGGCTAGGTTTT   25219
gb:MT019530|Organism:Severe      CAAGAACTTGGAAAGTATGAGCAGTATATAAAATGGCCATGGTACATTTGGCTAGGTTTT   25222
gb:MT039888|Organism:Severe      CAAGAACTTGGAAAGTATGAGCAGTATATAAAATGGCCATGGTACATTTGGCTAGGTTTT   25222
gb:LC522972|Organism:Severe      CAAGAACTTGGAAAGTATGAGCAGTATATAAAATGGCCATGGTACATTTGGCTAGGTTTT   25219
gb:MT027063|Organism:Severe      CAAGAACTTGGAAAGTATGAGCAGTATATAAAATGGCCATGGTACATTTGGCTAGGTTTT   25222
gb:MT027062|Organism:Severe      CAAGAACTTGGAAAGTATGAGCAGTATATAAAATGGCCATGGTACATTTGGCTAGGTTTT   25222
gb:MT019529|Organism:Severe      CAAGAACTTGGAAAGTATGAGCAGTATATAAAATGGCCATGGTACATTTGGCTAGGTTTT   25222
gb:MN996529|Organism:Severe      CAAGAACTTGGAAAGTATGAGCAGTATATAAAATGGCCATGGTACATTTGGCTAGGTTTT   25210
gb:MN996531|Organism:Severe      CAAGAACTTGGAAAGTATGAGCAGTATATAAAATGGCCATGGTACATTTGGCTAGGTTTT   25209
gb:MT066176|Organism:Severe      CAAGAACTTGGAAAGTATGAGCAGTATATAAAATGGCCATGGTACATTTGGCTAGGTTTT   25222
gb:MT027064|Organism:Severe      CAAGAACTTGGAAAGTATGAGCAGTATATAAAATGGCCATGGTACATTTGGCTAGGTTTT   25222
gb:MN994468|Organism:Severe      CAAGAACTTGGAAAGTATGAGCAGTATATAAAATGGCCATGGTACATTTGGCTAGGTTTT   25222
gb:MT072688|Organism:Severe      CAAGAACTTGGAAAGTATGAGCAGTATATAAAATGGCCATGGTACATTTGGCTAGGTTTT   25207
gb:MN996527|Organism:Severe      CAAGAACTTGGAAAGTATGAGCAGTATATAAAATGGCCATGGTACATTTGGCTAGGTTTT   25189
gb:MT093631|Organism:Severe      CAAGAACTTGGAAAGTATGAGCAGTATATAAAATGGCCATGGTACATTTGGCTAGGTTTT   25260
gb:MT106053|Organism:Severe      CAAGAACTTGGAAAGTATGAGCAGTATATAAAATGGCCATGGTACATTTGGCTAGGTTTT   25222
gb:MT019533|Organism:Severe      CAAGAACTTGGAAAGTATGAGCAGTATATAAAATGGCCATGGTACATTTGGCTAGGTTTT   25222
gb:MT019531|Organism:Severe      CAAGAACTTGGAAAGTATGAGCAGTATATAAAATGGCCATGGTACATTTGGCTAGGTTTT   25222
gb:MN996528|Organism:Severe      CAAGAACTTGGAAAGTATGAGCAGTATATAAAATGGCCATGGTACATTTGGCTAGGTTTT   25222
gb:MN996530|Organism:Severe      CAAGAACTTGGAAAGTATGAGCAGTATATAAAATGGCCATGGTACATTTGGCTAGGTTTT   25208
gb:MN908947|Organism:Severe      CAAGAACTTGGAAAGTATGAGCAGTATATAAAATGGCCATGGTACATTTGGCTAGGTTTT   25222
gb:MT019532|Organism:Severe      CAAGAACTTGGAAAGTATGAGCAGTATATAAAATGGCCATGGTACATTTGGCTAGGTTTT   25222
                                 ************************************************************
```

```
gb:MT020781|Organism:Severe      ATAGCTGGCTTGATTGCCATAGTAATGGTGACAATTATGCTTTGCTGTATGACCAGTTGC   25270
gb:MT007544|Organism:Severe      ATAGCTGGCTTGATTGCCATAGTAATGGTGACAATTATGCTTTGCTGTATGACCAGTTGC   25282
gb:MN994467|Organism:Severe      ATAGCTGGCTTGATTGCCATAGTAATGGTGACAATTATGCTTTGCTGTATGACCAGTTGC   25282
gb:MT044257|Organism:Severe      ATAGCTGGCTTGATTGCCATAGTAATGGTGACAATTATGCTTTGCTGTATGACCAGTTGC   25282
gb:MT106054|Organism:Severe      ATAGCTGGCTTGATTGCCATAGTAATGGTGACAATTATGCTTTGCTGTATGACCAGTTGC   25282
gb:MT049951|Organism:Severe      ATAGCTGGCTTGATTGCCATAGTAATGGTGACAATTATGCTTTGCTGTATGACCAGTTGC   25282
gb:MN975262|Organism:Severe      ATAGCTGGCTTGATTGCCATAGTAATGGTGACAATTATGCTTTGCTGTATGACCAGTTGC   25282
gb:MT106052|Organism:Severe      ATAGCTGGCTTGATTGCCATAGTAATGGTGACAATTATGCTTTGCTGTATGACCAGTTGC   25282
gb:LC522975|Organism:Severe      ATAGCTGGCTTGATTGCCATAGTAATGGTGACAATTATGCTTTGCTGTATGACCAGTTGC   25279
gb:LC522973|Organism:Severe      ATAGCTGGCTTGATTGCCATAGTAATGGTGACAATTATGCTTTGCTGTATGACCAGTTGC   25279
gb:LC522974|Organism:Severe      ATAGCTGGCTTGATTGCCATAGTAATGGTGACAATTATGCTTTGCTGTATGACCAGTTGC   25279
gb:MN985325|Organism:Severe      ATAGCTGGCTTGATTGCCATAGTAATGGTGACAATTATGCTTTGCTGTATGACCAGTTGC   25282
gb:MT020881|Organism:Severe      ATAGCTGGCTTGATTGCCATAGTAATGGTGACAATTATGCTTTGCTGTATGACCAGTTGC   25282
gb:MT020880|Organism:Severe      ATAGCTGGCTTGATTGCCATAGTAATGGTGACAATTATGCTTTGCTGTATGACCAGTTGC   25282
gb:MT066175|Organism:Severe      ATAGCTGGCTTGATTGCCATAGTAATGGTGACAATTATGCTTTGCTGTATGACCAGTTGC   25282
gb:MN997409|Organism:Severe      ATAGCTGGCTTGATTGCCATAGTAATGGTGACAATTATGCTTTGCTGTATGACCAGTTGC   25282
gb:MN938384|Organism:Severe      ATAGCTGGCTTGATTGCCATAGTAATGGTGACAATTATGCTTTGCTGTATGACCAGTTGC   25250
gb:MT044258|Organism:Severe      ATAGCTGGCTTGATTGCCATAGTAATGGTGACAATTATGCTTTGCTGTATGACCAGTTGC   25258
gb:MT039890|Organism:Severe      ATAGCTGGCTTGATTGCCATAGTAATGGTGACAATTATGCTTTGCTGTATGACCAGTTGC   25282
gb:MN988713|Organism:Severe      ATAGCTGGCTTGATTGCCATAGTAATGGTGACAATTATGCTTTGCTGTATGACCAGTTGC   25282
gb:LC521925|Organism:Severe      ATAGCTGGCTTGATTGCCATAGTAATGGTGACAATTATGCTTTGCTGTATGACCAGTTGC   25255
gb:MT093571|Organism:Severe      ATAGCTGGCTTGATTGCCATAGTAATGGTGACAATTATGCTTTGCTGTATGACCAGTTGC   25282
gb:MT039887|Organism:Severe      ATAGCTGGCTTGATTGCCATAGTAATGGTGACAATTATGCTTTGCTGTATGACCAGTTGC   25279
gb:MT019530|Organism:Severe      ATAGCTGGCTTGATTGCCATAGTAATGGTGACAATTATGCTTTGCTGTATGACCAGTTGC   25282
gb:MT039888|Organism:Severe      ATAGCTGGCTTGATTGCCATAGTAATGGTGACAATTATGCTTTGCTGTATGACCAGTTGC   25282
gb:LC522972|Organism:Severe      ATAGCTGGCTTGATTGCCATAGTAATGGTGACAATTATGCTTTGCTGTATGACCAGTTGC   25279
gb:MT027063|Organism:Severe      ATAGCTGGCTTGATTGCCATAGTAATGGTGACAATTATGCTTTGCTGTATGACCAGTTGC   25282
gb:MT027062|Organism:Severe      ATAGCTGGCTTGATTGCCATAGTAATGGTGACAATTATGCTTTGCTGTATGACCAGTTGC   25282
gb:MT019529|Organism:Severe      ATAGCTGGCTTGATTGCCATAGTAATGGTGACAATTATGCTTTGCTGTATGACCAGTTGC   25282
gb:MN996529|Organism:Severe      ATAGCTGGCTTGATTGCCATAGTAATGGTGACAATTATGCTTTGCTGTATGACCAGTTGC   25270
gb:MN996531|Organism:Severe      ATAGCTGGCTTGATTGCCATAGTAATGGTGACAATTATGCTTTGCTGTATGACCAGTTGC   25269
gb:MT066176|Organism:Severe      ATAGCTGGCTTGATTGCCATAGTAATGGTGACAATTATGCTTTGCTGTATGACCAGTTGC   25282
gb:MT027064|Organism:Severe      ATAGCTGGCTTGATTGCCATAGTAATGGTGACAATTATGCTTTGCTGTATGACCAGTTGC   25282
gb:MN994468|Organism:Severe      ATAGCTGGCTTGATTGCCATAGTAATGGTGACAATTATGCTTTGCTGTATGACCAGTTGC   25282
gb:MT072688|Organism:Severe      ATAGCTGGCTTGATTGCCATAGTAATGGTGACAATTATGCTTTGCTGTATGACCAGTTGC   25267
gb:MN996527|Organism:Severe      ATAGCTGGCTTGATTGCCATAGTAATGGTGACAATTATGCTTTGCTGTATGACCAGTTGC   25249
gb:MT093631|Organism:Severe      ATAGCTGGCTTGATTGCCATAGTAATGGTGACAATTATGCTTTGCTGTATGACCAGTTGC   25320
gb:MT106053|Organism:Severe      ATAGCTGGCTTGATTGCCATAGTAATGGTGACAATTATGCTTTGCTGTATGACCAGTTGC   25282
gb:MT019533|Organism:Severe      ATAGCTGGCTTGATTGCCATAGTAATGGTGACAATTATGCTTTGCTGTATGACCAGTTGC   25282
gb:MT019531|Organism:Severe      ATAGCTGGCTTGATTGCCATAGTAATGGTGACAATTATGCTTTGCTGTATGACCAGTTGC   25282
gb:MN996528|Organism:Severe      ATAGCTGGCTTGATTGCCATAGTAATGGTGACAATTATGCTTTGCTGTATGACCAGTTGC   25282
gb:MN996530|Organism:Severe      ATAGCTGGCTTGATTGCCATAGTAATGGTGACAATTATGCTTTGCTGTATGACCAGTTGC   25268
gb:MN908947|Organism:Severe      ATAGCTGGCTTGATTGCCATAGTAATGGTGACAATTATGCTTTGCTGTATGACCAGTTGC   25282
gb:MT019532|Organism:Severe      ATAGCTGGCTTGATTGCCATAGTAATGGTGACAATTATGCTTTGCTGTATGACCAGTTGC   25282
                                 ************************************************************

gb:MT020781|Organism:Severe      TGTAGTTGTCTCAAGGGCTGTTGTTCTTGTGGATCCTGCTGCAAATTTGATGAAGACGAC   25330
gb:MT007544|Organism:Severe      TGTAGTTGTCTCAAGGGCTGTTGTTCTTGTGGATCCTGCTGCAAATTTGATGAAGACGAC   25342
gb:MN994467|Organism:Severe      TGTAGTTGTCTCAAGGGCTGTTGTTCTTGTGGATCCTGCTGCAAATTTGATGAAGACGAC   25342
gb:MT044257|Organism:Severe      TGTAGTTGTCTCAAGGGCTGTTGTTCTTGTGGATCCTGCTGCAAATTTGATGAAGACGAC   25342
gb:MT106054|Organism:Severe      TGTAGTTGTCTCAAGGGCTGTTGTTCTTGTGGATCCTGCTGCAAATTTGATGAAGACGAC   25342
gb:MT049951|Organism:Severe      TGTAGTTGTCTCAAGGGCTGTTGTTCTTGTGGATCCTGCTGCAAATTTGATGAAGACGAC   25342
gb:MN975262|Organism:Severe      TGTAGTTGTCTCAAGGGCTGTTGTTCTTGTGGATCCTGCTGCAAATTTGATGAAGACGAC   25342
gb:MT106052|Organism:Severe      TGTAGTTGTCTCAAGGGCTGTTGTTCTTGTGGATCCTGCTGCAAATTTGATGAAGACGAC   25342
gb:LC522975|Organism:Severe      TGTAGTTGTCTCAAGGGCTGTTGTTCTTGTGGATCCTGCTGCAAATTTGATGAAGACGAC   25339
gb:LC522973|Organism:Severe      TGTAGTTGTCTCAAGGGCTGTTGTTCTTGTGGATCCTGCTGCAAATTTGATGAAGACGAC   25339
gb:LC522974|Organism:Severe      TGTAGTTGTCTCAAGGGCTGTTGTTCTTGTGGATCCTGCTGCAAATTTGATGAAGACGAC   25339
gb:MN985325|Organism:Severe      TGTAGTTGTCTCAAGGGCTGTTGTTCTTGTGGATCCTGCTGCAAATTTGATGAAGACGAC   25342
gb:MT020881|Organism:Severe      TGTAGTTGTCTCAAGGGCTGTTGTTCTTGTGGATCCTGCTGCAAATTTGATGAAGACGAC   25342
gb:MT020880|Organism:Severe      TGTAGTTGTCTCAAGGGCTGTTGTTCTTGTGGATCCTGCTGCAAATTTGATGAAGACGAC   25342
gb:MT066175|Organism:Severe      TGTAGTTGTCTCAAGGGCTGTTGTTCTTGTGGATCCTGCTGCAAATTTGATGAAGACGAC   25342
gb:MN997409|Organism:Severe      TGTAGTTGTCTCAAGGGCTGTTGTTCTTGTGGATCCTGCTGCAAATTTGATGAAGACGAC   25342
gb:MN938384|Organism:Severe      TGTAGTTGTCTCAAGGGCTGTTGTTCTTGTGGATCCTGCTGCAAATTTGATGAAGACGAC   25310
gb:MT044258|Organism:Severe      TGTAGTTGTCTCAAGGGCTGTTGTTCTTGTGGATCCTGCTGCAAATTTGATGAAGACGAC   25318
gb:MT039890|Organism:Severe      TGTAGTTGTCTCAAGGGCTGTTGTTCTTGTGGATCCTGCTGCAAATTTGATGAAGACGAC   25342
```

```
gb:MN988713|Organism:Severe      TGTAGTTGTCTCAAGGGCTGTTGTTCTTGTGGATCCTGCTGCAAATTTGATGAAGACGAC   25342
gb:LC521925|Organism:Severe      TGTAGTTGTCTCAAGGGCTGTTGTTCTTGTGGATCCTGCTGCAAATTTGATGAAGACGAC   25315
gb:MT093571|Organism:Severe      TGTAGTTGTCTCAAGGGCTGTTGTTCTTGTGGATCCTGCTGCAAATTTGATGAAGACGAC   25342
gb:MT039887|Organism:Severe      TGTAGTTGTCTCAAGGGCTGTTGTTCTTGTGGATCCTGCTGCAAATTTGATGAAGACGAC   25339
gb:MT019530|Organism:Severe      TGTAGTTGTCTCAAGGGCTGTTGTTCTTGTGGATCCTGCTGCAAATTTGATGAAGACGAC   25342
gb:MT039888|Organism:Severe      TGTAGTTGTCTCAAGGGCTGTTGTTCTTGTGGATCCTGCTGCAAATTTGATGAAGACGAC   25342
gb:LC522972|Organism:Severe      TGTAGTTGTCTCAAGGGCTGTTGTTCTTGTGGATCCTGCTGCAAATTTGATGAAGACGAC   25339
gb:MT027063|Organism:Severe      TGTAGTTGTCTCAAGGGCTGTTGTTCTTGTGGATCCTGCTGCAAATTTGATGAAGACGAC   25342
gb:MT027062|Organism:Severe      TGTAGTTGTCTCAAGGGCTGTTGTTCTTGTGGATCCTGCTGCAAATTTGATGAAGACGAC   25342
gb:MT019529|Organism:Severe      TGTAGTTGTCTCAAGGGCTGTTGTTCTTGTGGATCCTGCTGCAAATTTGATGAAGACGAC   25342
gb:MN996529|Organism:Severe      TGTAGTTGTCTCAAGGGCTGTTGTTCTTGTGGATCCTGCTGCAAATTTGATGAAGACGAC   25330
gb:MN996531|Organism:Severe      TGTAGTTGTCTCAAGGGCTGTTGTTCTTGTGGATCCTGCTGCAAATTTGATGAAGACGAC   25329
gb:MT066176|Organism:Severe      TGTAGTTGTCTCAAGGGCTGTTGTTCTTGTGGATCCTGCTGCAAATTTGATGAAGACGAC   25342
gb:MT027064|Organism:Severe      TGTAGTTGTCTCAAGGGCTGTTGTTCTTGTGGATCCTGCTGCAAATTTGATGAAGACGAC   25342
gb:MN994468|Organism:Severe      TGTAGTTGTCTCAAGGGCTGTTGTTCTTGTGGATCCTGCTGCAAATTTGATGAAGACGAC   25342
gb:MT072688|Organism:Severe      TGTAGTTGTCTCAAGGGCTGTTGTTCTTGTGGATCCTGCTGCAAATTTGATGAAGACGAC   25327
gb:MN996527|Organism:Severe      TGTAGTTGTCTCAAGGGCTGTTGTTCTTGTGGATCCTGCTGCAAATTTGATGAAGACGAC   25309
gb:MT093631|Organism:Severe      TGTAGTTGTCTCAAGGGCTGTTGTTCTTGTGGATCCTGCTGCAAATTTGATGAAGACGAC   25380
gb:MT106053|Organism:Severe      TGTAGTTGTCTCAAGGGCTGTTGTTCTTGTGGATCCTGCTGCAAATTTGATGAAGACGAC   25342
gb:MT019533|Organism:Severe      TGTAGTTGTCTCAAGGGCTGTTGTTCTTGTGGATCCTGCTGCAAATTTGATGAAGACGAC   25342
gb:MT019531|Organism:Severe      TGTAGTTGTCTCAAGGGCTGTTGTTCTTGTGGATCCTGCTGCAAATTTGATGAAGACGAC   25342
gb:MN996528|Organism:Severe      TGTAGTTGTCTCAAGGGCTGTTGTTCTTGTGGATCCTGCTGCAAATTTGATGAAGACGAC   25342
gb:MN996530|Organism:Severe      TGTAGTTGTCTCAAGGGCTGTTGTTCTTGTGGATCCTGCTGCAAATTTGATGAAGACGAC   25328
gb:MN908947|Organism:Severe      TGTAGTTGTCTCAAGGGCTGTTGTTCTTGTGGATCCTGCTGCAAATTTGATGAAGACGAC   25342
gb:MT019532|Organism:Severe      TGTAGTTGTCTCAAGGGCTGTTGTTCTTGTGGATCCTGCTGCAAATTTGATGAAGACGAC   25342
                                 ************************************************************

gb:MT020781|Organism:Severe      TCTGAGCCAGTGCTCAAAGGAGTCAAATTACATTACACATAAACGAACTTATGGATTTGT   25390
gb:MT007544|Organism:Severe      TCTGAGCCAGTGCTCAAAGGAGTCAAATTACATTACACATAAACGAACTTATGGATTTGT   25402
gb:MN994467|Organism:Severe      TCTGAGCCAGTGCTCAAAGGAGTCAAATTACATTACACATAAACGAACTTATGGATTTGT   25402
gb:MT044257|Organism:Severe      TCTGAGCCAGTGCTCAAAGGAGTCAAATTACATTACACATAAACGAACTTATGGATTTGT   25402
gb:MT106054|Organism:Severe      TCTGAGCCAGTGCTCAAAGGAGTCAAATTACATTACACATAAACGAACTTATGGATTTGT   25402
gb:MT049951|Organism:Severe      TCTGAGCCAGTGCTCAAAGGAGTCAAATTACATTACACATAAACGAACTTATGGATTTGT   25402
gb:MN975262|Organism:Severe      TCTGAGCCAGTGCTCAAAGGAGTCAAATTACATTACACATAAACGAACTTATGGATTTGT   25402
gb:MT106052|Organism:Severe      TCTGAGCCAGTGCTCAAAGGAGTCAAATTACATTACACATAAACGAACTTATGGATTTGT   25402
gb:LC522975|Organism:Severe      TCTGAGCCAGTGCTCAAAGGAGTCAAATTACATTACACATAAACGAACTTATGGATTTGT   25399
gb:LC522973|Organism:Severe      TCTGAGCCAGTGCTCAAAGGAGTCAAATTACATTACACATAAACGAACTTATGGATTTGT   25399
gb:LC522974|Organism:Severe      TCTGAGCCAGTGCTCAAAGGAGTCAAATTACATTACACATAAACGAACTTATGGATTTGT   25399
gb:MN985325|Organism:Severe      TCTGAGCCAGTGCTCAAAGGAGTCAAATTACATTACACATAAACGAACTTATGGATTTGT   25402
gb:MT020881|Organism:Severe      TCTGAGCCAGTGCTCAAAGGAGTCAAATTACATTACACATAAACGAACTTATGGATTTGT   25402
gb:MT020880|Organism:Severe      TCTGAGCCAGTGCTCAAAGGAGTCAAATTACATTACACATAAACGAACTTATGGATTTGT   25402
gb:MT066175|Organism:Severe      TCTGAGCCAGTGCTCAAAGGAGTCAAATTACATTACACATAAACGAACTTATGGATTTGT   25402
gb:MN997409|Organism:Severe      TCTGAGCCAGTGCTCAAAGGAGTCAAATTACATTACACATAAACGAACTTATGGATTTGT   25402
gb:MN938384|Organism:Severe      TCTGAGCCAGTGCTCAAAGGAGTCAAATTACATTACACATAAACGAACTTATGGATTTGT   25370
gb:MT044258|Organism:Severe      TCTGAGCCAGTGCTCAAAGGAGTCAAATTACATTACACATAAACGAACTTATGGATTTGT   25378
gb:MT039890|Organism:Severe      TCTGAGCCAGTGCTCAAAGGAGTCAAATTACATTACACATAAACGAACTTATGGATTTGT   25402
gb:MN988713|Organism:Severe      TCTGAGCCAGTGCTCAAAGGAGTCAAATTACATTACACATAAACGAACTTATGGATTTGT   25402
gb:LC521925|Organism:Severe      TCTGAGCCAGTGCTCAAAGGAGTCAAATTACATTACACATAAACGAACTTATGGATTTGT   25375
gb:MT093571|Organism:Severe      TCTGAGCCAGTGCTCAAAGGAGTCAAATTACATTACACATAAACGAACTTATGGATTTGT   25402
gb:MT039887|Organism:Severe      TCTGAGCCAGTGCTCAAAGGAGTCAAATTACATTACACATAAACGAACTTATGGATTTGT   25399
gb:MT019530|Organism:Severe      TCTGAGCCAGTGCTCAAAGGAGTCAAATTACATTACACATAAACGAACTTATGGATTTGT   25402
gb:MT039888|Organism:Severe      TCTGAGCCAGTGCTCAAAGGAGTCAAATTACATTACACATAAACGAACTTATGGATTTGT   25402
gb:LC522972|Organism:Severe      TCTGAGCCAGTGCTCAAAGGAGTCAAATTACATTACACATAAACGAACTTATGGATTTGT   25399
gb:MT027063|Organism:Severe      TCTGAGCCAGTGCTCAAAGGAGTCAAATTACATTACACATAAACGAACTTATGGATTTGT   25402
gb:MT027062|Organism:Severe      TCTGAGCCAGTGCTCAAAGGAGTCAAATTACATTACACATAAACGAACTTATGGATTTGT   25402
gb:MT019529|Organism:Severe      TCTGAGCCAGTGCTCAAAGGAGTCAAATTACATTACACATAAACGAACTTATGGATTTGT   25402
gb:MN996529|Organism:Severe      TCTGAGCCAGTGCTCAAAGGAGTCAAATTACATTACACATAAACGAACTTATGGATTTGT   25390
gb:MN996531|Organism:Severe      TCTGAGCCAGTGCTCAAAGGAGTCAAATTACATTACACATAAACGAACTTATGGATTTGT   25389
gb:MT066176|Organism:Severe      TCTGAGCCAGTGCTCAAAGGAGTCAAATTACATTACACATAAACGAACTTATGGATTTGT   25402
gb:MT027064|Organism:Severe      TCTGAGCCAGTGCTCAAAGGAGTCAAATTACATTACACATAAACGAACTTATGGATTTGT   25402
gb:MN994468|Organism:Severe      TCTGAGCCAGTGCTCAAAGGAGTCAAATTACATTACACATAAACGAACTTATGGATTTGT   25402
gb:MT072688|Organism:Severe      TCTGAGCCAGTGCTCAAAGGAGTCAAATTACATTACACATAAACGAACTTATGGATTTGT   25387
gb:MN996527|Organism:Severe      TCTGAGCCAGTGCTCAAAGGAGTCAAATTACATTACACATAAACGAACTTATGGATTTGT   25369
gb:MT093631|Organism:Severe      TCTGAGCCAGTGCTCAAAGGAGTCAAATTACATTACACATAAACGAACTTATGGATTTGT   25440
gb:MT106053|Organism:Severe      TCTGAGCCAGTGCTCAAAGGAGTCAAATTACATTACACATAAACGAACTTATGGATTTGT   25402
```

```
gb:MT019533|Organism:Severe      TCTGAGCCAGTGCTCAAAGGAGTCAAATTACATTACACATAAACGAACTTATGGATTTGT   25402
gb:MT019531|Organism:Severe      TCTGAGCCAGTGCTCAAAGGAGTCAAATTACATTACACATAAACGAACTTATGGATTTGT   25402
gb:MN996528|Organism:Severe      TCTGAGCCAGTGCTCAAAGGAGTCAAATTACATTACACATAAACGAACTTATGGATTTGT   25402
gb:MN996530|Organism:Severe      TCTGAGCCAGTGCTCAAAGGAGTCAAATTACATTACACATAAACGAACTTATGGATTTGT   25388
gb:MN908947|Organism:Severe      TCTGAGCCAGTGCTCAAAGGAGTCAAATTACATTACACATAAACGAACTTATGGATTTGT   25402
gb:MT019532|Organism:Severe      TCTGAGCCAGTGCTCAAAGGAGTCAAATTACATTACACATAAACGAACTTATGGATTTGT   25402
                                 ************************************************************

gb:MT020781|Organism:Severe      TTATGAGAATCTTCACAATTGGAACTGTAACTTTGAAGCAAGGTGAAATCAAGGATGCTA   25450
gb:MT007544|Organism:Severe      TTATGAGAATCTTCACAATTGGAACTGTAACTTTGAAGCAAGGTGAAATCAAGGATGCTA   25462
gb:MN994467|Organism:Severe      TTATGAGAATCTTCACAATTGGAACTGTAACTTTGAAGCAAGGTGAAATCAAGGATGCTA   25462
gb:MT044257|Organism:Severe      TTATGAGAATCTTCACAATTGGAACTGTAACTTTGAAGCAAGGTGAAATCAAGGATGCTA   25462
gb:MT106054|Organism:Severe      TTATGAGAATCTTCACAATTGGAACTGTAACTTTGAAGCAAGGTGAAATCAAGGATGCTA   25462
gb:MT049951|Organism:Severe      TTATGAGAATCTTCACAATTGGAACTGTAACTTTGAAGCAAGGTGAAATCAAGGATGCTA   25462
gb:MN975262|Organism:Severe      TTATGAGAATCTTCACAATTGGAACTGTAACTTTGAAGCAAGGTGAAATCAAGGATGCTA   25462
gb:MT106052|Organism:Severe      TTATGAGAATCTTCACAATTGGAACTGTAACTTTGAAGCAAGGTGAAATCAAGGATGCTA   25462
gb:LC522975|Organism:Severe      TTATGAGAATCTTCACAATTGGAACTGTAACTTTGAAGCAAGGTGAAATCAAGGATGCTA   25459
gb:LC522973|Organism:Severe      TTATGAGAATCTTCACAATTGGAACTGTAACTTTGAAGCAAGGTGAAATCAAGGATGCTA   25459
gb:LC522974|Organism:Severe      TTATGAGAATCTTCACAATTGGAACTGTAACTTTGAAGCAAGGTGAAATCAAGGATGCTA   25459
gb:MN985325|Organism:Severe      TTATGAGAATCTTCACAATTGGAACTGTAACTTTGAAGCAAGGTGAAATCAAGGATGCTA   25462
gb:MT020881|Organism:Severe      TTATGAGAATCTTCACAATTGGAACTGTAACTTTGAAGCAAGGTGAAATCAAGGATGCTA   25462
gb:MT020880|Organism:Severe      TTATGAGAATCTTCACAATTGGAACTGTAACTTTGAAGCAAGGTGAAATCAAGGATGCTA   25462
gb:MT066175|Organism:Severe      TTATGAGAATCTTCACAATTGGAACTGTAACTTTGAAGCAAGGTGAAATCAAGGATGCTA   25462
gb:MN997409|Organism:Severe      TTATGAGAATCTTCACAATTGGAACTGTAACTTTGAAGCAAGGTGAAATCAAGGATGCTA   25462
gb:MN938384|Organism:Severe      TTATGAGAATCTTCACAATTGGAACTGTAACTTTGAAGCAAGGTGAAATCAAGGATGCTA   25430
gb:MT044258|Organism:Severe      TTATGAGAATCTTCACAATTGGAACTGTAACTTTGAAGCAAGGTGAAATCAAGGATGCTA   25438
gb:MT039890|Organism:Severe      TTATGAGAATCTTCACAATTGGAACTGTAACTTTGAAGCAAGGTGAAATCAAGGATGCTA   25462
gb:MN988713|Organism:Severe      TTATGAGAATCTTCACAATTGGAACTGTAACTTTGAAGCAAGGTGAAATCAAGGATGCTA   25462
gb:LC521925|Organism:Severe      TTATGAGAATCTTCACAATTGGAACTGTAACTTTGAAGCAAGGTGAAATCAAGGATGCTA   25435
gb:MT093571|Organism:Severe      TTATGAGAATCTTCACAATTGGAACTGTAACTTTGAAGCAAGGTGAAATCAAGGATGCTA   25462
gb:MT039887|Organism:Severe      TTATGAGAATCTTCACAATTGGAACTGTAACTTTGAAGCAAGGTGAAATCAAGGATGCTA   25459
gb:MT019530|Organism:Severe      TTATGAGAATCTTCACAATTGGAACTGTAACTTTGAAGCAAGGTGAAATCAAGGATGCTA   25462
gb:MT039888|Organism:Severe      TTATGAGAATCTTCACAATTGGAACTGTAACTTTGAAGCAAGGTGAAATCAAGGATGCTA   25462
gb:LC522972|Organism:Severe      TTATGAGAATCTTCACAATTGGAACTGTAACTTTGAAGCAAGGTGAAATCAAGGATGCTA   25459
gb:MT027063|Organism:Severe      TTATGAGAATCTTCACAATTGGAACTGTAACTTTGAAGCAAGGTGAAATCAAGGATGCTA   25462
gb:MT027062|Organism:Severe      TTATGAGAATCTTCACAATTGGAACTGTAACTTTGAAGCAAGGTGAAATCAAGGATGCTA   25462
gb:MT019529|Organism:Severe      TTATGAGAATCTTCACAATTGGAACTGTAACTTTGAAGCAAGGTGAAATCAAGGATGCTA   25462
gb:MN996529|Organism:Severe      TTATGAGAATCTTCACAATTGGAACTGTAACTTTGAAGCAAGGTGAAATCAAGGATGCTA   25450
gb:MN996531|Organism:Severe      TTATGAGAATCTTCACAATTGGAACTGTAACTTTGAAGCAAGGTGAAATCAAGGATGCTA   25449
gb:MT066176|Organism:Severe      TTATGAGAATCTTCACAATTGGAACTGTAACTTTGAAGCAAGGTGAAATCAAGGATGCTA   25462
gb:MT027064|Organism:Severe      TTATGAGAATCTTCACAATTGGAACTGTAACTTTGAAGCAAGGTGAAATCAAGGATGCTA   25462
gb:MN994468|Organism:Severe      TTATGAGAATCTTCACAATTGGAACTGTAACTTTGAAGCAAGGTGAAATCAAGGATGCTA   25462
gb:MT072688|Organism:Severe      TTATGAGAATCTTCACAATTGGAACTGTAACTTTGAAGCAAGGTGAAATCAAGGATGCTA   25447
gb:MN996527|Organism:Severe      TTATGAGAATCTTCACAATTGGAACTGTAACTTTGAAGCAAGGTGAAATCAAGGATGCTA   25429
gb:MT093631|Organism:Severe      TTATGAGAATCTTCACAATTGGAACTGTAACTTTGAAGCAAGGTGAAATCAAGGATGCTA   25500
gb:MT106053|Organism:Severe      TTATGAGAATCTTCACAATTGGAACTGTAACTTTGAAGCAAGGTGAAATCAAGGATGCTA   25462
gb:MT019533|Organism:Severe      TTATGAGAATCTTCACAATTGGAACTGTAACTTTGAAGCAAGGTGAAATCAAGGATGCTA   25462
gb:MT019531|Organism:Severe      TTATGAGAATCTTCACAATTGGAACTGTAACTTTGAAGCAAGGTGAAATCAAGGATGCTA   25462
gb:MN996528|Organism:Severe      TTATGAGAATCTTCACAATTGGAACTGTAACTTTGAAGCAAGGTGAAATCAAGGATGCTA   25462
gb:MN996530|Organism:Severe      TTATGAGAATCTTCACAATTGGAACTGTAACTTTGAAGCAAGGTGAAATCAAGGATGCTA   25448
gb:MN908947|Organism:Severe      TTATGAGAATCTTCACAATTGGAACTGTAACTTTGAAGCAAGGTGAAATCAAGGATGCTA   25462
gb:MT019532|Organism:Severe      TTATGAGAATCTTCACAATTGGAACTGTAACTTTGAAGCAAGGTGAAATCAAGGATGCTA   25462
                                 ************************************************************

gb:MT020781|Organism:Severe      CTCCTTCAGATTTTGTTCGCGCTACTGCAACGATACCGATACAAGCCTCACTCCCTTTCG   25510
gb:MT007544|Organism:Severe      CTCCTTCAGATTTTGTTCGCGCTACTGCAACGATACCGATACAAGCCTCACTCCCTTTCG   25522
gb:MN994467|Organism:Severe      CTCCTTCAGATTTTGTTCGCGCTACTGCAACGATACCGATACAAGCCTCACTCCCTTTCG   25522
gb:MT044257|Organism:Severe      CTCCTTCAGATTTTGTTCGCGCTACTGCAACGATACCGATACAAGCCTCACTCCCTTTCG   25522
gb:MT106054|Organism:Severe      CTCCTTCAGATTTTGTTCGCGCTACTGCAACGATACCGATACAAGCCTCACTCCCTTTCG   25522
gb:MT049951|Organism:Severe      CTCCTTCAGATTTTGTTCGCGCTACTGCAACGATACCGATACAAGCCTCACTCCCTTTCG   25522
gb:MN975262|Organism:Severe      CTCCTTCAGATTTTGTTCGCGCTACTGCAACGATACCGATACAAGCCTCACTCCCTTTCG   25522
gb:MT106052|Organism:Severe      CTCCTTCAGATTTTGTTCGCGCTACTGCAACGATACCGATACAAGCCTCACTCCCTTTCG   25522
gb:LC522975|Organism:Severe      CTCCTTCAGATTTTGTTCGCGCTACTGCAACGATACCGATACAAGCCTCACTCCCTTTCG   25519
gb:LC522973|Organism:Severe      CTCCTTCAGATTTTGTTCGCGCTACTGCAACGATACCGATACAAGCCTCACTCCCTTTCG   25519
gb:LC522974|Organism:Severe      CTCCTTCAGATTTTGTTCGCGCTACTGCAACGATACCGATACAAGCCTCACTCCCTTTCG   25519
```

```
gb:MN985325|Organism:Severe      CTCCTTCAGATTTTGTTCGCGCTACTGCAACGATACCGATACAAGCCTCACTCCCTTTCG   25522
gb:MT020881|Organism:Severe      CTCCTTCAGATTTTGTTCGCGCTACTGCAACGATACCGATACAAGCCTCACTCCCTTTCG   25522
gb:MT020880|Organism:Severe      CTCCTTCAGATTTTGTTCGCGCTACTGCAACGATACCGATACAAGCCTCACTCCCTTTCG   25522
gb:MT066175|Organism:Severe      CTCCTTCAGATTTTGTTCGCGCTACTGCAACGATACCGATACAAGCCTCACTCCCTTTCG   25522
gb:MN997409|Organism:Severe      CTCCTTCAGATTTTGTTCGCGCTACTGCAACGATACCGATACAAGCCTCACTCCCTTTCG   25522
gb:MN938384|Organism:Severe      CTCCTTCAGATTTTGTTCGCGCTACTGCAACGATACCGATACAAGCCTCACTCCCTTTCG   25490
gb:MT044258|Organism:Severe      CTCCTTCAGATTTTGTTCGCGCTACTGCAACGATACCGATACAAGCCTCACTCCCTTTCG   25498
gb:MT039890|Organism:Severe      CTCCTTCAGATTTTGTTCGCGCTACTGCAACGATACCGATACAAGCCTCACTCCCTTTCG   25522
gb:MN988713|Organism:Severe      CTCCTTCAGATTTTGTTCGCGCTACTGCAACGATACCGATACAAGCCTCACTCCCTTTCG   25522
gb:LC521925|Organism:Severe      CTCCTTCAGATTTTGTTCGCGCTACTGCAACGATACCGATACAAGCCTCACTCCCTTTCG   25495
gb:MT093571|Organism:Severe      CTCCTTCAGATTTTGTTCGCGCTACTGCAACGATACCGATACAAGCCTCACTCCCTTTCG   25522
gb:MT039887|Organism:Severe      CTCCTTCAGATTTTGTTCGCGCTACTGCAACGATACCGATACAAGCCTCACTCCCTTTCG   25519
gb:MT019530|Organism:Severe      CTCCTTCAGATTTTGTTCGCGCTACTGCAACGATACCGATACAAGCCTCACTCCCTTTCG   25522
gb:MT039888|Organism:Severe      CTCCTTCAGATTTTGTTCGCGCTACTGCAACGATACCGATACAAGCCTCACTCCCTTTCG   25522
gb:LC522972|Organism:Severe      CTCCTTCAGATTTTGTTCGCGCTACTGCAACGATACCGATACAAGCCTCACTCCCTTTCG   25519
gb:MT027063|Organism:Severe      CTCCTTCAGATTTTGTTCGCGCTACTGCAACGATACCGATACAAGCCTCACTCCCTTTCG   25522
gb:MT027062|Organism:Severe      CTCCTTCAGATTTTGTTCGCGCTACTGCAACGATACCGATACAAGCCTCACTCCCTTTCG   25522
gb:MT019529|Organism:Severe      CTCCTTCAGATTTTGTTCGCGCTACTGCAACGATACCGATACAAGCCTCACTCCCTTTCG   25522
gb:MN996529|Organism:Severe      CTCCTTCAGATTTTGTTCGCGCTACTGCAACGATACCGATACAAGCCTCACTCCCTTTCG   25510
gb:MN996531|Organism:Severe      CTCCTTCAGATTTTGTTCGCGCTACTGCAACGATACCGATACAAGCCTCACTCCCTTTCG   25509
gb:MT066176|Organism:Severe      CTCCTTCAGATTTTGTTCGCGCTACTGCAACGATACCGATACAAGCCTCACTCCCTTTCG   25522
gb:MT027064|Organism:Severe      CTCCTTCAGATTTTGTTCGCGCTACTGCAACGATACCGATACAAGCCTCACTCCCTTTCG   25522
gb:MN994468|Organism:Severe      CTCCTTCAGATTTTGTTCGCGCTACTGCAACGATACCGATACAAGCCTCACTCCCTTTCG   25522
gb:MT072688|Organism:Severe      CTCCTTCAGATTTTGTTCGCGCTACTGCAACGATACCGATACAAGCCTCACTCCCTTTCG   25507
gb:MN996527|Organism:Severe      CTCCTTCAGATTTTGTTCGCGCTACTGCAACGATACCGATACAAGCCTCACTCCCTTTCG   25489
gb:MT093631|Organism:Severe      CTCCTTCAGATTTTGTTCGCGCTACTGCAACGATACCGATACAAGCCTCACTCCCTTTCG   25560
gb:MT106053|Organism:Severe      CTCCTTCAGATTTTGTTCGCGCTACTGCAACGATACCGATACAAGCCTCACTCCCTTTCG   25522
gb:MT019533|Organism:Severe      CTCCTTCAGATTTTGTTCGCGCTACTGCAACGATACCGATACAAGCCTCACTCCCTTTCG   25522
gb:MT019531|Organism:Severe      CTCCTTCAGATTTTGTTCGCGCTACTGCAACGATACCGATACAAGCCTCACTCCCTTTCG   25522
gb:MN996528|Organism:Severe      CTCCTTCAGATTTTGTTCGCGCTACTGCAACGATACCGATACAAGCCTCACTCCCTTTCG   25522
gb:MN996530|Organism:Severe      CTCCTTCAGATTTTGTTCGCGCTACTGCAACGATACCGATACAAGCCTCACTCCCTTTCG   25508
gb:MN908947|Organism:Severe      CTCCTTCAGATTTTGTTCGCGCTACTGCAACGATACCGATACAAGCCTCACTCCCTTTCG   25522
gb:MT019532|Organism:Severe      CTCCTTCAGATTTTGTTCGCGCTACTGCAACGATACCGATACAAGCCTCACTCCCTTTCG   25522
                                 ************************************************************

gb:MT020781|Organism:Severe      GATGGCTTATTGTTGGCGTTGCACTTCTTGCTGTTTTTCAGAGCGCTTCCAAAATCATAA   25570
gb:MT007544|Organism:Severe      GATGGCTTATTGTTGGCGTTGCACTTCTTGCTGTTTTTCAGAGCGCTTCCAAAATCATAA   25582
gb:MN994467|Organism:Severe      GATGGCTTATTGTTGGCGTTGCACTTCTTGCTGTTTTTCAGAGCGCTTCCAAAATCATAA   25582
gb:MT044257|Organism:Severe      GATGGCTTATTGTTGGCGTTGCACTTCTTGCTGTTTTTCAGAGCGCTTCCAAAATCATAA   25582
gb:MT106054|Organism:Severe      GATGGCTTATTGTTGGCGTTGCACTTCTTGCTGTTTTTCAGAGCGCTTCCAAAATCATAA   25582
gb:MT049951|Organism:Severe      GATGGCTTATTGTTGGCGTTGCACTTCTTGCTGTTTTTCAGAGCGCTTCCAAAATCATAA   25582
gb:MN975262|Organism:Severe      GATGGCTTATTGTTGGCGTTGCACTTCTTGCTGTTTTTCAGAGCGCTTCCAAAATCATAA   25582
gb:MT106052|Organism:Severe      GATGGCTTATTGTTGGCGTTGCACTTCTTGCTGTTTTTCAGAGCGCTTCCAAAATCATAA   25582
gb:LC522975|Organism:Severe      GATGGCTTATTGTTGGCGTTGCACTTCTTGCTGTTTTTCAGAGCGCTTCCAAAATCATAA   25579
gb:LC522973|Organism:Severe      GATGGCTTATTGTTGGCGTTGCACTTCTTGCTGTTTTTCAGAGCGCTTCCAAAATCATAA   25579
gb:LC522974|Organism:Severe      GATGGCTTATTGTTGGCGTTGCACTTCTTGCTGTTTTTCAGAGCGCTTCCAAAATCATAA   25579
gb:MN985325|Organism:Severe      GATGGCTTATTGTTGGCGTTGCACTTCTTGCTGTTTTTCAGAGCGCTTCCAAAATCATAA   25582
gb:MT020881|Organism:Severe      GATGGCTTATTGTTGGCGTTGCACTTCTTGCTGTTTTTCAGAGCGCTTCCAAAATCATAA   25582
gb:MT020880|Organism:Severe      GATGGCTTATTGTTGGCGTTGCACTTCTTGCTGTTTTTCAGAGCGCTTCCAAAATCATAA   25582
gb:MT066175|Organism:Severe      GATGGCTTATTGTTGGCGTTGCACTTCTTGCTGTTTTTCAGAGCGCTTCCAAAATCATAA   25582
gb:MN997409|Organism:Severe      GATGGCTTATTGTTGGCGTTGCACTTCTTGCTGTTTTTCAGAGCGCTTCCAAAATCATAA   25582
gb:MN938384|Organism:Severe      GATGGCTTATTGTTGGCGTTGCACTTCTTGCTGTTTTTCAGAGCGCTTCCAAAATCATAA   25550
gb:MT044258|Organism:Severe      GATGGCTTATTGTTGGCGTTGCACTTCTTGCTGTTTTTCAGAGCGCTTCCAAAATCATAA   25558
gb:MT039890|Organism:Severe      GATGGCTTATTGTTGGCGTTGCACTTCTTGCTGTTTTTCAGAGCGCTTCCAAAATCATAA   25582
gb:MN988713|Organism:Severe      GATGGCTTATTGTTGGCGTTGCACTTCTTGCTGTTTTTCAGAGCGCTTCCAAAATCATAA   25582
gb:LC521925|Organism:Severe      GATGGCTTATTGTTGGCGTTGCACTTCTTGCTGTTTTTCAGAGCGCTTCCAAAATCATAA   25555
gb:MT093571|Organism:Severe      GATGGCTTATTGTTGGCGTTGCACTTCTTGCTGTTTTTCAGAGCGCTTCCAAAATCATAA   25582
gb:MT039887|Organism:Severe      GATGGCTTATTGTTGGCGTTGCACTTCTTGCTGTTTTTCAGAGCGCTTCCAAAATCATAA   25579
gb:MT019530|Organism:Severe      GATGGCTTATTGTTGGCGTTGCACTTCTTGCTGTTTTTCAGAGCGCTTCCAAAATCATAA   25582
gb:MT039888|Organism:Severe      GATGGCTTATTGTTGGCGTTGCACTTCTTGCTGTTTTTCAGAGCGCTTCCAAAATCATAA   25582
gb:LC522972|Organism:Severe      GATGGCTTATTGTTGGCGTTGCACTTCTTGCTGTTTTTCAGAGCGCTTCCAAAATCATAA   25579
gb:MT027063|Organism:Severe      GATGGCTTATTGTTGGCGTTGCACTTCTTGCTGTTTTTCAGAGCGCTTCCAAAATCATAA   25582
gb:MT027062|Organism:Severe      GATGGCTTATTGTTGGCGTTGCACTTCTTGCTGTTTTTCAGAGCGCTTCCAAAATCATAA   25582
gb:MT019529|Organism:Severe      GATGGCTTATTGTTGGCGTTGCACTTCTTGCTGTTTTTCAGAGCGCTTCCAAAATCATAA   25582
gb:MN996529|Organism:Severe      GATGGCTTATTGTTGGCGTTGCACTTCTTGCTGTTTTTCAGAGCGCTTCCAAAATCATAA   25570
```

```
gb:MN996531|Organism:Severe      GATGGCTTATTGTTGGCGTTGCACTTCTTGCTGTTTTTCAGAGCGCTTCCAAAATCATAA    25569
gb:MT066176|Organism:Severe      GATGGCTTATTGTTGGCGTTGCACTTCTTGCTGTTTTTCAGAGCGCTTCCAAAATCATAA    25582
gb:MT027064|Organism:Severe      GATGGCTTATTGTTGGCGTTGCACTTCTTGCTGTTTTTCAGAGCGCTTCCAAAATCATAA    25582
gb:MN994468|Organism:Severe      GATGGCTTATTGTTGGCGTTGCACTTCTTGCTGTTTTTCAGAGCGCTTCCAAAATCATAA    25582
gb:MT072688|Organism:Severe      GATGGCTTATTGTTGGCGTTGCACTTCTTGCTGTTTTTCAGAGCGCTTCCAAAATCATAA    25567
gb:MN996527|Organism:Severe      GATGGCTTATTGTTGGCGTTGCACTTCTTGCTGTTTTTCAGAGCGCTTCCAAAATCATAA    25549
gb:MT093631|Organism:Severe      GATGGCTTATTGTTGGCGTTGCACTTCTTGCTGTTTTTCAGAGCGCTTCCAAAATCATAA    25620
gb:MT106053|Organism:Severe      GATGGCTTATTGTTGGCGTTGCACTTCTTGCTGTTTTTCAGAGCGCTTCCAAAATCATAA    25582
gb:MT019533|Organism:Severe      GATGGCTTATTGTTGGCGTTGCACTTCTTGCTGTTTTTCAGAGCGCTTCCAAAATCATAA    25582
gb:MT019531|Organism:Severe      GATGGCTTATTGTTGGCGTTGCACTTCTTGCTGTTTTTCAGAGCGCTTCCAAAATCATAA    25582
gb:MN996528|Organism:Severe      GATGGCTTATTGTTGGCGTTGCACTTCTTGCTGTTTTTCAGAGCGCTTCCAAAATCATAA    25582
gb:MN996530|Organism:Severe      GATGGCTTATTGTTGGCGTTGCACTTCTTGCTGTTTTTCAGAGCGCTTCCAAAATCATAA    25568
gb:MN908947|Organism:Severe      GATGGCTTATTGTTGGCGTTGCACTTCTTGCTGTTTTTCAGAGCGCTTCCAAAATCATAA    25582
gb:MT019532|Organism:Severe      GATGGCTTATTGTTGGCGTTGCACTTCTTGCTGTTTTTCAGAGCGCTTCCAAAATCATAA    25582
                                 ************************************************************

gb:MT020781|Organism:Severe      CCCTCAAAAAGAGATGGCAACTAGCACTCTCCAAGGGTGTTCACTTTGTTTGCAACTTGC    25630
gb:MT007544|Organism:Severe      CCCTCAAAAAGAGATGGCAACTAGCACTCTCCAAGGGTGTTCACTTTGTTTGCAACTTGC    25642
gb:MN994467|Organism:Severe      CCCTCAAAAAGAGATGGCAACTAGCACTCTCCAAGGGTGTTCACTTTGTTTGCAACTTGC    25642
gb:MT044257|Organism:Severe      CCCTCAAAAAGAGATGGCAACTAGCACTCTCCAAGGGTGTTCACTTTGTTTGCAACTTGC    25642
gb:MT106054|Organism:Severe      CCCTCAAAAAGAGATGGCAACTAGCACTCTCCAAGGGTGTTCACTTTGTTTGCAACTTGC    25642
gb:MT049951|Organism:Severe      CCCTCAAAAAGAGATGGCAACTAGCACTCTCCAAGGGTGTTCACTTTGTTTGCAACTTGC    25642
gb:MN975262|Organism:Severe      CCCTCAAAAAGAGATGGCAACTAGCACTCTCCAAGGGTGTTCACTTTGTTTGCAACTTGC    25642
gb:MT106052|Organism:Severe      CCCTCAAAAAGAGATGGCAACTAGCACTCTCCAAGGGTGTTCACTTTGTTTGCAACTTGC    25642
gb:LC522975|Organism:Severe      CCCTCAAAAAGAGATGGCAACTAGCACTCTCCAAGGGTGTTCACTTTGTTTGCAACTTGC    25639
gb:LC522973|Organism:Severe      CCCTCAAAAAGAGATGGCAACTAGCACTCTCCAAGGGTGTTCACTTTGTTTGCAACTTGC    25639
gb:LC522974|Organism:Severe      CCCTCAAAAAGAGATGGCAACTAGCACTCTCCAAGGGTGTTCACTTTGTTTGCAACTTGC    25639
gb:MN985325|Organism:Severe      CCCTCAAAAAGAGATGGCAACTAGCACTCTCCAAGGGTGTTCACTTTGTTTGCAACTTGC    25642
gb:MT020881|Organism:Severe      CCCTCAAAAAGAGATGGCAACTAGCACTCTCCAAGGGTGTTCACTTTGTTTGCAACTTGC    25642
gb:MT020880|Organism:Severe      CCCTCAAAAAGAGATGGCAACTAGCACTCTCCAAGGGTGTTCACTTTGTTTGCAACTTGC    25642
gb:MT066175|Organism:Severe      CCCTCAAAAAGAGATGGCAACTAGCACTCTCCAAGGGTGTTCACTTTGTTTGCAACTTGC    25642
gb:MN997409|Organism:Severe      CCCTCAAAAAGAGATGGCAACTAGCACTCTCCAAGGGTGTTCACTTTGTTTGCAACTTGC    25642
gb:MN938384|Organism:Severe      CCCTCAAAAAGAGATGGCAACTAGCACTCTCCAAGGGTGTTCACTTTGTTTGCAACTTGC    25610
gb:MT044258|Organism:Severe      CCCTCAAAAAGAGATGGCAACTAGCACTCTCCAAGGGTGTTCACTTTGTTTGCAACTTGC    25618
gb:MT039890|Organism:Severe      CCCTCAAAAAGAGATGGCAACTAGCACTCTCCAAGGGTGTTCACTTTGTTTGCAACTTGC    25642
gb:MN988713|Organism:Severe      CCCTCAAAAAGAGATGGCAACTAGCACTCTCCAAGGGTGTTCACTTTGTTTGCAACTTGC    25642
gb:LC521925|Organism:Severe      CCCTCAAAAAGAGATGGCAACTAGCACTCTCCAAGGGTGTTCACTTTGTTTGCAACTTGC    25615
gb:MT093571|Organism:Severe      CCCTCAAAAAGAGATGGCAACTAGCACTCTCCAAGGGTGTTCACTTTGTTTGCAACTTGC    25642
gb:MT039887|Organism:Severe      CCCTCAAAAAGAGATGGCAACTAGCACTCTCCAAGGGTGTTCACTTTGTTTGCAACTTGC    25639
gb:MT019530|Organism:Severe      CCCTCAAAAAGAGATGGCAACTAGCACTCTCCAAGGGTGTTCACTTTGTTTGCAACTTGC    25642
gb:MT039888|Organism:Severe      CCCTCAAAAAGAGATGGCAACTAGCACTCTCCAAGGGTGTTCACTTTGTTTGCAACTTGC    25642
gb:LC522972|Organism:Severe      CCCTCAAAAAGAGATGGCAACTAGCACTCTCCAAGGGTGTTCACTTTGTTTGCAACTTGC    25639
gb:MT027063|Organism:Severe      CCCTCAAAAAGAGATGGCAACTAGCACTCTCCAAGGGTGTTCACTTTGTTTGCAACTTGC    25642
gb:MT027062|Organism:Severe      CCCTCAAAAAGAGATGGCAACTAGCACTCTCCAAGGGTGTTCACTTTGTTTGCAACTTGC    25642
gb:MT019529|Organism:Severe      CCCTCAAAAAGAGATGGCAACTAGCACTCTCCAAGGGTGTTCACTTTGTTTGCAACTTGC    25642
gb:MN996529|Organism:Severe      CCCTCAAAAAGAGATGGCAACTAGCACTCTCCAAGGGTGTTCACTTTGTTTGCAACTTGC    25630
gb:MN996531|Organism:Severe      CCCTCAAAAAGAGATGGCAACTAGCACTCTCCAAGGGTGTTCACTTTGTTTGCAACTTGC    25629
gb:MT066176|Organism:Severe      CCCTCAAAAAGAGATGGCAACTAGCACTCTCCAAGGGTGTTCACTTTGTTTGCAACTTGC    25642
gb:MT027064|Organism:Severe      CCCTCAAAAAGAGATGGCAACTAGCACTCTCCAAGGGTGTTCACTTTGTTTGCAACTTGC    25642
gb:MN994468|Organism:Severe      CCCTCAAAAAGAGATGGCAACTAGCACTCTCCAAGGGTGTTCACTTTGTTTGCAACTTGC    25642
gb:MT072688|Organism:Severe      CCCTCAAAAAGAGATGGCAACTAGCACTCTCCAAGGGTGTTCACTTTGTTTGCAACTTGC    25627
gb:MN996527|Organism:Severe      CCCTCAAAAAGAGATGGCAACTAGCACTCTCCAAGGGTGTTCACTTTGTTTGCAACTTGC    25609
gb:MT093631|Organism:Severe      CCCTCAAAAAGAGATGGCAACTAGCACTCTCCAAGGGTGTTCACTTTGTTTGCAACTTGC    25680
gb:MT106053|Organism:Severe      CCCTCAAAAAGAGATGGCAACTAGCACTCTCCAAGGGTGTTCACTTTGTTTGCAACTTGC    25642
gb:MT019533|Organism:Severe      CCCTCAAAAAGAGATGGCAACTAGCACTCTCCAAGGGTGTTCACTTTGTTTGCAACTTGC    25642
gb:MT019531|Organism:Severe      CCCTCAAAAAGAGATGGCAACTAGCACTCTCCAAGGGTGTTCACTTTGTTTGCAACTTGC    25642
gb:MN996528|Organism:Severe      CCCTCAAAAAGAGATGGCAACTAGCACTCTCCAAGGGTGTTCACTTTGTTTGCAACTTGC    25642
gb:MN996530|Organism:Severe      CCCTCAAAAAGAGATGGCAACTAGCACTCTCCAAGGGTGTTCACTTTGTTTGCAACTTGC    25628
gb:MN908947|Organism:Severe      CCCTCAAAAAGAGATGGCAACTAGCACTCTCCAAGGGTGTTCACTTTGTTTGCAACTTGC    25642
gb:MT019532|Organism:Severe      CCCTCAAAAAGAGATGGCAACTAGCACTCTCCAAGGGTGTTCACTTTGTTTGCAACTTGC    25642
                                 ************************************************************

gb:MT020781|Organism:Severe      TGTTGTTGTTTGTAACAGTTTACTCACACCTTTTGCTCGTTGCTGCTGGCCTTGAAGCCC    25690
gb:MT007544|Organism:Severe      TGTTGTTGTTTGTAACAGTTTACTCACACCTTTTGCTCGTTGCTGCTGGCCTTGAAGCCC    25702
gb:MN994467|Organism:Severe      TGTTGTTGTTTGTAACAGTTTACTCACACCTTTTGCTCGTTGCTGCTGGCCTTGAAGCCC    25702
```

```
gb:MT044257|Organism:Severe      TGTTGTTGTTTGTAACAGTTTACTCACACCTTTTGCTCGTTGCTGCTGGCCTTGAAGCCC   25702
gb:MT106054|Organism:Severe      TGTTGTTGTTTGTAACAGTTTACTCACACCTTTTGCTCGTTGCTGCTGGCCTTGAAGCCC   25702
gb:MT049951|Organism:Severe      TGTTGTTGTTTGTAACAGTTTACTCACACCTTTTGCTCGTTGCTGCTGGCCTTGAAGCCC   25702
gb:MN975262|Organism:Severe      TGTTGTTGTTTGTAACAGTTTACTCACACCTTTTGCTCGTTGCTGCTGGCCTTGAAGCCC   25702
gb:MT106052|Organism:Severe      TGTTGTTGTTTGTAACAGTTTACTCACACCTTTTGCTCGTTGCTGCTGGCCTTGAAGCCC   25702
gb:LC522975|Organism:Severe      TGTTGTTGTTTGTAACAGTTTACTCACACCTTTTGCTCGTTGCTGCTGGCCTTGAAGCCC   25699
gb:LC522973|Organism:Severe      TGTTGTTGTTTGTAACAGTTTACTCACACCTTTTGCTCGTTGCTGCTGGCCTTGAAGCCC   25699
gb:LC522974|Organism:Severe      TGTTGTTGTTTGTAACAGTTTACTCACACCTTTTGCTCGTTGCTGCTGGCCTTGAAGCCC   25699
gb:MN985325|Organism:Severe      TGTTGTTGTTTGTAACAGTTTACTCACACCTTTTGCTCGTTGCTGCTGGCCTTGAAGCCC   25702
gb:MT020881|Organism:Severe      TGTTGTTGTTTGTAACAGTTTACTCACACCTTTTGCTCGTTGCTGCTGGCCTTGAAGCCC   25702
gb:MT020880|Organism:Severe      TGTTGTTGTTTGTAACAGTTTACTCACACCTTTTGCTCGTTGCTGCTGGCCTTGAAGCCC   25702
gb:MT066175|Organism:Severe      TGTTGTTGTTTGTAACAGTTTACTCACACCTTTTGCTCGTTGCTGCTGGCCTTGAAGCCC   25702
gb:MN997409|Organism:Severe      TGTTGTTGTTTGTAACAGTTTACTCACACCTTTTGCTCGTTGCTGCTGGCCTTGAAGCCC   25702
gb:MN938384|Organism:Severe      TGTTGTTGTTTGTAACAGTTTACTCACACCTTTTGCTCGTTGCTGCTGGCCTTGAAGCCC   25670
gb:MT044258|Organism:Severe      TGTTGTTGTTTGTAACAGTTTACTCACACCTTTTGCTCGTTGCTGCTGGCCTTGAAGCCC   25678
gb:MT039890|Organism:Severe      TGTTGTTGTTTGTAACAGTTTACTCACACCTTTTGCTCGTTGCTGCTGGCCTTGAAGCCC   25702
gb:MN988713|Organism:Severe      TGTTGTTGTTTGTAACAGTTTACTCACACCTTTTGCTCGTTGCTGCTGGCCTTGAAGCCC   25702
gb:LC521925|Organism:Severe      TGTTGTTGTTTGTAACAGTTTACTCACACCTTTTGCTCGTTGCTGCTGGCCTTGAAGCCC   25675
gb:MT093571|Organism:Severe      TGTTGTTGTTTGTAACAGTTTACTCACACCTTTTGCTCGTTGCTGCTGGCCTTGAAGCCC   25702
gb:MT039887|Organism:Severe      TGTTGTTGTTTGTAACAGTTTACTCACACCTTTTGCTCGTTGCTGCTGGCCTTGAAGCCC   25699
gb:MT019530|Organism:Severe      TGTTGTTGTTTGTAACAGTTTACTCACACCTTTTGCTCGTTGCTGCTGGCCTTGAAGCCC   25702
gb:MT039888|Organism:Severe      TGTTGTTGTTTGTAACAGTTTACTCACACCTTTTGCTCGTTGCTGCTGGCCTTGAAGCCC   25702
gb:LC522972|Organism:Severe      TGTTGTTGTTTGTAACAGTTTACTCACACCTTTTGCTCGTTGCTGCTGGCCTTGAAGCCC   25699
gb:MT027063|Organism:Severe      TGTTGTTGTTTGTAACAGTTTACTCACACCTTTTGCTCGTTGCTGCTGGCCTTGAAGCCC   25702
gb:MT027062|Organism:Severe      TGTTGTTGTTTGTAACAGTTTACTCACACCTTTTGCTCGTTGCTGCTGGCCTTGAAGCCC   25702
gb:MT019529|Organism:Severe      TGTTGTTGTTTGTAACAGTTTACTCACACCTTTTGCTCGTTGCTGCTGGCCTTGAAGCCC   25702
gb:MN996529|Organism:Severe      TGTTGTTGTTTGTAACAGTTTACTCACACCTTTTGCTCGTTGCTGCTGGCCTTGAAGCCC   25690
gb:MN996531|Organism:Severe      TGTTGTTGTTTGTAACAGTTTACTCACACCTTTTGCTCGTTGCTGCTGGCCTTGAAGCCC   25689
gb:MT066176|Organism:Severe      TGTTGTTGTTTGTAACAGTTTACTCACACCTTTTGCTCGTTGCTGCTGGCCTTGAAGCCC   25702
gb:MT027064|Organism:Severe      TGTTGTTGTTTGTAACAGTTTACTCACACCTTTTGCTCGTTGCTGCTGGCCTTGAAGCCC   25702
gb:MN994468|Organism:Severe      TGTTGTTGTTTGTAACAGTTTACTCACACCTTTTGCTCGTTGCTGCTGGCCTTGAAGCCC   25702
gb:MT072688|Organism:Severe      TGTTGTTGTTTGTAACAGTTTACTCACACCTTTTGCTCGTTGCTGCTGGCCTTGAAGCCC   25687
gb:MN996527|Organism:Severe      TGTTGTTGTTTGTAACAGTTTACTCACACCTTTTGCTCGTTGCTGCTGGCCTTGAAGCCC   25669
gb:MT093631|Organism:Severe      TGTTGTTGTTTGTAACAGTTTACTCACACCTTTTGCTCGTTGCTGCTGGCCTTGAAGCCC   25740
gb:MT106053|Organism:Severe      TGTTGTTGTTTGTAACAGTTTACTCACACCTTTTGCTCGTTGCTGCTGGCCTTGAAGCCC   25702
gb:MT019533|Organism:Severe      TGTTGTTGTTTGTAACAGTTTACTCACACCTTTTGCTCGTTGCTGCTGGCCTTGAAGCCC   25702
gb:MT019531|Organism:Severe      TGTTGTTGTTTGTAACAGTTTACTCACACCTTTTGCTCGTTGCTGCTGGCCTTGAAGCCC   25702
gb:MN996528|Organism:Severe      TGTTGTTGTTTGTAACAGTTTACTCACACCTTTTGCTCGTTGCTGCTGGCCTTGAAGCCC   25702
gb:MN996530|Organism:Severe      TGTTGTTGTTTGTAACAGTTTACTCACACCTTTTGCTCGTTGCTGCTGGCCTTGAAGCCC   25688
gb:MN908947|Organism:Severe      TGTTGTTGTTTGTAACAGTTTACTCACACCTTTTGCTCGTTGCTGCTGGCCTTGAAGCCC   25702
gb:MT019532|Organism:Severe      TGTTGTTGTTTGTAACAGTTTACTCACACCTTTTGCTCGTTGCTGCTGGCCTTGAAGCCC   25702
                                 ************************************************************

gb:MT020781|Organism:Severe      CTTTTCTCTATCTTTATGCTTTAGTCTACTTCTTGCAGAGTATAAACTTTGTAAGAATAA   25750
gb:MT007544|Organism:Severe      CTTTTCTCTATCTTTATGCTTTAGTCTACTTCTTGCAGAGTATAAACTTTGTAAGAATAA   25762
gb:MN994467|Organism:Severe      CTTTTCTCTATCTTTATGCTTTAGTCTACTTCTTGCAGAGTATAAACTTTGTAAGAATAA   25762
gb:MT044257|Organism:Severe      CTTTTCTCTATCTTTATGCTTTAGTCTACTTCTTGCAGAGTATAAACTTTGTAAGAATAA   25762
gb:MT106054|Organism:Severe      CTTTTCTCTATCTTTATGCTTTAGTCTACTTCTTGCAGAGTATAAACTTTGTAAGAATAA   25762
gb:MT049951|Organism:Severe      CTTTTCTCTATCTTTATGCTTTAGTCTACTTCTTGCAGAGTATAAACTTTGTAAGAATAA   25762
gb:MN975262|Organism:Severe      CTTTTCTCTATCTTTATGCTTTAGTCTACTTCTTGCAGAGTATAAACTTTGTAAGAATAA   25762
gb:MT106052|Organism:Severe      CTTTTCTCTATCTTTATGCTTTAGTCTACTTCTTGCAGAGTATAAACTTTGTAAGAATAA   25762
gb:LC522975|Organism:Severe      CTTTTCTCTATCTTTATGCTTTAGTCTACTTCTTGCAGAGTATAAACTTTGTAAGAATAA   25759
gb:LC522973|Organism:Severe      CTTTTCTCTATCTTTATGCTTTAGTCTACTTCTTGCAGAGTATAAACTTTGTAAGAATAA   25759
gb:LC522974|Organism:Severe      CTTTTCTCTATCTTTATGCTTTAGTCTACTTCTTGCAGAGTATAAACTTTGTAAGAATAA   25759
gb:MN985325|Organism:Severe      CTTTTCTCTATCTTTATGCTTTAGTCTACTTCTTGCAGAGTATAAACTTTGTAAGAATAA   25762
gb:MT020881|Organism:Severe      CTTTTCTCTATCTTTATGCTTTAGTCTACTTCTTGCAGAGTATAAACTTTGTAAGAATAA   25762
gb:MT020880|Organism:Severe      CTTTTCTCTATCTTTATGCTTTAGTCTACTTCTTGCAGAGTATAAACTTTGTAAGAATAA   25762
gb:MT066175|Organism:Severe      CTTTTCTCTATCTTTATGCTTTAGTCTACTTCTTGCAGAGTATAAACTTTGTAAGAATAA   25762
gb:MN997409|Organism:Severe      CTTTTCTCTATCTTTATGCTTTAGTCTACTTCTTGCAGAGTATAAACTTTGTAAGAATAA   25762
gb:MN938384|Organism:Severe      CTTTTCTCTATCTTTATGCTTTAGTCTACTTCTTGCAGAGTATAAACTTTGTAAGAATAA   25730
gb:MT044258|Organism:Severe      CTTTTCTCTATCTTTATGCTTTAGTCTACTTCTTGCAGAGTATAAACTTTGTAAGAATAA   25738
gb:MT039890|Organism:Severe      CTTTTCTCTATCTTTATGCTTTAGTCTACTTCTTGCAGAGTATAAACTTTGTAAGAATAA   25762
gb:MN988713|Organism:Severe      CTTTTCTCTATCTTTATGCTTTAGTCTACTTCTTGCAGAGTATAAACTTTGTAAGAATAA   25762
gb:LC521925|Organism:Severe      CTTTTCTCTATCTTTATGCTTTAGTCTACTTCTTGCAGAGTATAAACTTTGTAAGAATAA   25735
gb:MT093571|Organism:Severe      CTTTTCTCTATCTTTATGCTTTAGTCTACTTCTTGCAGAGTATAAACTTTGTAAGAATAA   25762
```

```
gb:MT039887|Organism:Severe      CTTTTCTCTATCTTTATGCTTTAGTCTACTTCTTGCAGAGTATAAACTTTGTAAGAATAA   25759
gb:MT019530|Organism:Severe      CTTTTCTCTATCTTTATGCTTTAGTCTACTTCTTGCAGAGTATAAACTTTGTAAGAATAA   25762
gb:MT039888|Organism:Severe      CTTTTCTCTATCTTTATGCTTTAGTCTACTTCTTGCAGAGTATAAACTTTGTAAGAATAA   25762
gb:LC522972|Organism:Severe      CTTTTCTCTATCTTTATGCTTTAGTCTACTTCTTGCAGAGTATAAACTTTGTAAGAATAA   25759
gb:MT027063|Organism:Severe      CTTTTCTCTATCTTTATGCTTTAGTCTACTTCTTGCAGAGTATAAACTTTGTAAGAATAA   25762
gb:MT027062|Organism:Severe      CTTTTCTCTATCTTTATGCTTTAGTCTACTTCTTGCAGAGTATAAACTTTGTAAGAATAA   25762
gb:MT019529|Organism:Severe      CTTTTCTCTATCTTTATGCTTTAGTCTACTTCTTGCAGAGTATAAACTTTGTAAGAATAA   25762
gb:MN996529|Organism:Severe      CTTTTCTCTATCTTTATGCTTTAGTCTACTTCTTGCAGAGTATAAACTTTGTAAGAATAA   25750
gb:MN996531|Organism:Severe      CTTTTCTCTATCTTTATGCTTTAGTCTACTTCTTGCAGAGTATAAACTTTGTAAGAATAA   25749
gb:MT066176|Organism:Severe      CTTTTCTCTATCTTTATGCTTTAGTCTACTTCTTGCAGAGTATAAACTTTGTAAGAATAA   25762
gb:MT027064|Organism:Severe      CTTTTCTCTATCTTTATGCTTTAGTCTACTTCTTGCAGAGTATAAACTTTGTAAGAATAA   25762
gb:MN994468|Organism:Severe      CTTTTCTCTATCTTTATGCTTTAGTCTACTTCTTGCAGAGTATAAACTTTGTAAGAATAA   25762
gb:MT072688|Organism:Severe      CTTTTCTCTATCTTTATGCTTTAGTCTACTTCTTGCAGAGTATAAACTTTGTAAGAATAA   25747
gb:MN996527|Organism:Severe      CTTTTCTCTATCTTTATGCTTTAGTCTACTTCTTGCAGAGTATAAACTTTGTAAGAATAA   25729
gb:MT093631|Organism:Severe      CTTTTCTCTATCTTTATGCTTTAGTCTACTTCTTGCAGAGTATAAACTTTGTAAGAATAA   25800
gb:MT106053|Organism:Severe      CTTTTCTCTATCTTTATGCTTTAGTCTACTTCTTGCAGAGTATAAACTTTGTAAGAATAA   25762
gb:MT019533|Organism:Severe      CTTTTCTCTATCTTTATGCTTTAGTCTACTTCTTGCAGAGTATAAACTTTGTAAGAATAA   25762
gb:MT019531|Organism:Severe      CTTTTCTCTATCTTTATGCTTTAGTCTACTTCTTGCAGAGTATAAACTTTGTAAGAATAA   25762
gb:MN996528|Organism:Severe      CTTTTCTCTATCTTTATGCTTTAGTCTACTTCTTGCAGAGTATAAACTTTGTAAGAATAA   25762
gb:MN996530|Organism:Severe      CTTTTCTCTATCTTTATGCTTTAGTCTACTTCTTGCAGAGTATAAACTTTGTAAGAATAA   25748
gb:MN908947|Organism:Severe      CTTTTCTCTATCTTTATGCTTTAGTCTACTTCTTGCAGAGTATAAACTTTGTAAGAATAA   25762
gb:MT019532|Organism:Severe      CTTTTCTCTATCTTTATGCTTTAGTCTACTTCTTGCAGAGTATAAACTTTGTAAGAATAA   25762
                                 ************************************************************

gb:MT020781|Organism:Severe      TAATGAGGCTTTGGCTTTGCTGGAAATGCCGTTCCAAAAACCCANNNNNNNNNNNNNNNN   25810
gb:MT007544|Organism:Severe      TAATGAGGCTTTGGCTTTGCTGGAAATGCCGTTCCAAAAACCCATTACTTTATGATGCCA   25822
gb:MN994467|Organism:Severe      TAATGAGGCTTTGGCTTTGCTGGAAATGCCGTTCCAAAAACCCATTACTTTATGATGCCA   25822
gb:MT044257|Organism:Severe      TAATGAGGCTTTGGCTTTGCTGGAAATGCCGTTCCAAAAACCCATTACTTTATGATGCCA   25822
gb:MT106054|Organism:Severe      TAATGAGGCTTTGGCTTTGCTGGAAATGCCGTTCCAAAAACCCATTACTTTATGATGCCA   25822
gb:MT049951|Organism:Severe      TAATGAGGCTTTGGCTTTGCTGGAAATGCCGTTCCAAAAACCCATTACTTTATGATGCCA   25822
gb:MN975262|Organism:Severe      TAATGAGGCTTTGGCTTTGCTGGAAATGCCGTTCCAAAAACCCATTACTTTATGATGCCA   25822
gb:MT106052|Organism:Severe      TAATGAGGCTTTGGCTTTGCTGGAAATGCCGTTCCAAAAACCCATTACTTTATGATGCCA   25822
gb:LC522975|Organism:Severe      TAATGAGGCTTTGGCTTTGCTGGAAATGCCGTTCCAAAAACCCATTACTTTATGATGCCA   25819
gb:LC522973|Organism:Severe      TAATGAGGCTTTGGCTTTGCTGGAAATGCCGTTCCAAAAACCCATTACTTTATGATGCCA   25819
gb:LC522974|Organism:Severe      TAATGAGGCTTTGGCTTTGCTGGAAATGCCGTTCCAAAAACCCATTACTTTATGATGCCA   25819
gb:MN985325|Organism:Severe      TAATGAGGCTTTGGCTTTGCTGGAAATGCCGTTCCAAAAACCCATTACTTTATGATGCCA   25822
gb:MT020881|Organism:Severe      TAATGAGGCTTTGGCTTTGCTGGAAATGCCGTTCCAAAAACCCATTACTTTATGATGCCA   25822
gb:MT020880|Organism:Severe      TAATGAGGCTTTGGCTTTGCTGGAAATGCCGTTCCAAAAACCCATTACTTTATGATGCCA   25822
gb:MT066175|Organism:Severe      TAATGAGGCTTTGGCTTTGCTGGAAATGCCGTTCCAAAAACCCATTACTTTATGATGCCA   25822
gb:MN997409|Organism:Severe      TAATGAGGCTTTGGCTTTGCTGGAAATGCCGTTCCAAAAACCCATTACTTTATGATGCCA   25822
gb:MN938384|Organism:Severe      TAATGAGGCTTTGGCTTTGCTGGAAATGCCGTTCCAAAAACCCATTACTTTATGATGCCA   25790
gb:MT044258|Organism:Severe      TAATGAGGCTTTGGCTTTGCTGGAAATGCCGTTCCAAAAACCCATTACTTTATGATGCCA   25798
gb:MT039890|Organism:Severe      TAATGAGGCTTTTGCTTTGCTGGAAATGCCGTTCCAAAAACCCATTACTTTATGATGCCA   25822
gb:MN988713|Organism:Severe      TAATGAGGCTTTGGCTTTGCTGGAAATGCCGTTCCAAAAACCCATTACTTTATGATGCCA   25822
gb:LC521925|Organism:Severe      TAATGAGGCTTTGGCTTTGCTGGAAATGCCGTTCCAAAAACCCATTACTTTATGATGCCA   25795
gb:MT093571|Organism:Severe      TAATGAGGCTTTGGCTTTGCTGGAAATGCCGTTCCAAAAACCCATTACTTTATGATGCCA   25822
gb:MT039887|Organism:Severe      TAATGAGGCTTTGGCTTTGCTGGAAATGCCGTTCCAAAAACCCATTACTTTATGATGCCA   25819
gb:MT019530|Organism:Severe      TAATGAGGCTTTGGCTTTGCTGGAAATGCCGTTCCAAAAACCCATTACTTTATGATGCCA   25822
gb:MT039888|Organism:Severe      TAATGAGGCTTTGGCTTTGCTGGAAATGCCGTTCCAAAAACCCATTACTTTATGATGCCA   25822
gb:LC522972|Organism:Severe      TAATGAGGCTTTGGCTTTGCTGGAAATGCCGTTCCAAAAACCCATTAGTTTATGATGCCA   25819
gb:MT027063|Organism:Severe      TAATGAGGCTTTGGCTTTGCTGGAAATGCCGTTCCAAAAACCCATTACTTTATGATGCCA   25822
gb:MT027062|Organism:Severe      TAATGAGGCTTTGGCTTTGCTGGAAATGCCGTTCCAAAAACCCATTACTTTATGATGCCA   25822
gb:MT019529|Organism:Severe      TAATGAGGCTTTGGCTTTGCTGGAAATGCCGTTCCAAAAACCCATTACTTTATGATGCCA   25822
gb:MN996529|Organism:Severe      TAATGAGGCTTTGGCTTTGCTGGAAATGCCGTTCCAAAAACCCATTACTTTATGATGCCA   25810
gb:MN996531|Organism:Severe      TAATGAGGCTTTGGCTTTGCTGGAAATGCCGTTCCAAAAACCCATTACTTTATGATGCCA   25809
gb:MT066176|Organism:Severe      TAATGAGGCTTTGGCTTTGCTGGAAATGCCGTTCCAAAAACCCATTACTTTATGATGCCA   25822
gb:MT027064|Organism:Severe      TAATGAGGCTTTGGCTTTGCTGGAAATGCCGTTCCAAAAACCCATTACTTTATGATGCCA   25822
gb:MN994468|Organism:Severe      TAATGAGGCTTTGGCTTTGCTGGAAATGCCGTTCCAAAAACCCATTACTTTATGATGCCA   25822
gb:MT072688|Organism:Severe      TAATGAGGCTTTGGCTTTGCTGGAAATGCCGTTCCAAAAACCCATTACTTTATGATGCCA   25807
gb:MN996527|Organism:Severe      TAATGAGGCTTTGGCTTTGCTGGAAATGCCGTTCCAAAAACCCATTACTTTATGATGCCA   25789
gb:MT093631|Organism:Severe      TAATGAGGCTTTGGCTTTGCTGGAAATGCCGTTCCAAAAACCCATTACTTTATGATGCCA   25860
gb:MT106053|Organism:Severe      TAATGAGGCTTTGGCTTTGCTGGAAATGCCGTTCCAAAAACCCATTACTTTATGATGCCA   25822
gb:MT019533|Organism:Severe      TAATGAGGCTTTGGCTTTGCTGGAAATGCCGTTCCAAAAACCCATTACTTTATGATGCCA   25822
gb:MT019531|Organism:Severe      TAATGAGGCTTTGGCTTTGCTGGAAATGCCGTTCCAAAAACCCATTACTTTATGATGCCA   25822
gb:MN996528|Organism:Severe      TAATGAGGCTTTGGCTTTGCTGGAAATGCCGTTCCAAAAACCCATTACTTTATGATGCCA   25822
```

```
gb:MN996530|Organism:Severe      TAATGAGGCTTTGGCTTTGCTGGAAATGCCGTTCCAAAAACCCATTACTTTATGATGCCA	25808
gb:MN908947|Organism:Severe      TAATGAGGCTTTGGCTTTGCTGGAAATGCCGTTCCAAAAACCCATTACTTTATGATGCCA	25822
gb:MT019532|Organism:Severe      TAATGAGGCTTTGGCTTTGCTGGAAATGCCGTTCCAAAAACCCATTACTTTATGATGCCA	25822
                                 ************ *******************************                

gb:MT020781|Organism:Severe      NNNNNNNNNNNNNNNNNNNNNNNNNNNNNNNNNACTATTGTATACCTTACAATAGTGTAA	25870
gb:MT007544|Organism:Severe      ACTATTTTCTTTGCTGGCATACTAATTGTTACGACTATTGTATACCTTACAATAGTGTAA	25882
gb:MN994467|Organism:Severe      ACTATTTTCTTTGCTGGCATACTAATTGTTACGACTATTGTATACCTTACAATAGTGTAA	25882
gb:MT044257|Organism:Severe      ACTATTTTCTTTGCTGGCATACTAATTGTTACGACTATTGTATACCTTACAATAGTGTAA	25882
gb:MT106054|Organism:Severe      ACTATTTTCTTTGCTGGCATACTAATTGTTACGACTATTGTATACCTTACAATAGTGTAA	25882
gb:MT049951|Organism:Severe      ACTATTTTCTTTGCTGGCATACTAATTGTTACGACTATTGTATACCTTACAATAGTGTAA	25882
gb:MN975262|Organism:Severe      ACTATTTTCTTTGCTGGCATACTAATTGTTACGACTATTGTATACCTTACAATAGTGTAA	25882
gb:MT106052|Organism:Severe      ACTATTTTCTTTGCTGGCATACTAATTGTTACGACTATTGTATACCTTACAATAGTGTAA	25882
gb:LC522975|Organism:Severe      ACTATTTTCTTTGCTGGCATACTAATTGTTACGACTATTGTATACCTTACAATAGTGTAA	25879
gb:LC522973|Organism:Severe      ACTATTTTCTTTGCTGGCATACTAATTGTTACGACTATTGTATACCTTACAATAGTGTAA	25879
gb:LC522974|Organism:Severe      ACTATTTTCTTTGCTGGCATACTAATTGTTACGACTATTGTATACCTTACAATAGTGTAA	25879
gb:MN985325|Organism:Severe      ACTATTTTCTTTGCTGGCATACTAATTGTTACGACTATTGTATACCTTACAATAGTGTAA	25882
gb:MT020881|Organism:Severe      ACTATTTTCTTTGCTGGCATACTAATTGTTACGACTATTGTATACCTTACAATAGTGTAA	25882
gb:MT020880|Organism:Severe      ACTATTTTCTTTGCTGGCATACTAATTGTTACGACTATTGTATACCTTACAATAGTGTAA	25882
gb:MT066175|Organism:Severe      ACTATTTTCTTTGCTGGCATACTAATTGTTACGACTATTGTATACCTTACAATAGTGTAA	25882
gb:MN997409|Organism:Severe      ACTATTTTCTTTGCTGGCATACTAATTGTTACGACTATTGTATACCTTACAATAGTGTAA	25882
gb:MN938384|Organism:Severe      ACTATTTTCTTTGCTGGCATACTAATTGTTACGACTATTGTATACCTTACAATAGTGTAA	25850
gb:MT044258|Organism:Severe      ACTATTTTCTTTGCTGGCATACTAATTGTTACGACTATTGTATACCTTACAATAGTGTAA	25858
gb:MT039890|Organism:Severe      ACTATTTTCTTTGCTGGCATACTAATTGTTACGACTATTGTATACCTTACAATAGTGTAA	25882
gb:MN988713|Organism:Severe      ACTATTTTCTTTGCTGGCATACTAATTGTTACGACTATTGTATACCTTACAATAGTGTAA	25882
gb:LC521925|Organism:Severe      ACTATTTTCTTTGCTGGCATACTAATTGTTACGACTATTGTATACCTTACAATAGTGTAA	25855
gb:MT093571|Organism:Severe      ACTATTTTCTTTGCTGGCATACTAATTGTTACGACTATTGTATACCTTACAATAGTGTAA	25882
gb:MT039887|Organism:Severe      ACTATTTTCTTTGCTGGCATACTAATTGTTACGACTATTGTATACCTTACAATAGTGTAA	25879
gb:MT019530|Organism:Severe      ACTATTTTCTTTGCTGGCATACTAATTGTTACGACTATTGTATACCTTACAATAGTGTAA	25882
gb:MT039888|Organism:Severe      ACTATTTTCTTTGCTGGCATACTAATTGTTACGACTATTGTATACCTTACAATAGTGTAA	25882
gb:LC522972|Organism:Severe      ACTATTTTCTTTGCTGGCATACTAATTGTTACGACTATTGTATACCTTACAATAGTGTAA	25879
gb:MT027063|Organism:Severe      ACTATTTTCTTTGCTGGCATACTAATTGTTACGACTATTGTATACCTTACAATAGTGTAA	25882
gb:MT027062|Organism:Severe      ACTATTTTCTTTGCTGGCATACTAATTGTTACGACTATTGTATACCTTACAATAGTGTAA	25882
gb:MT019529|Organism:Severe      ACTATTTTCTTTGCTGGCATACTAATTGTTACGACTATTGTATACCTTACAATAGTGTAA	25882
gb:MN996529|Organism:Severe      ACTATTTTCTTTGCTGGCATACTAATTGTTACGACTATTGTATACCTTACAATAGTGTAA	25870
gb:MN996531|Organism:Severe      ACTATTTTCTTTGCTGGCATACTAATTGTTACGACTATTGTATACCTTACAATAGTGTAA	25869
gb:MT066176|Organism:Severe      ACTATTTTCTTTGCTGGCATACTAATTGTTACGACTATTGTATACCTTACAATAGTGTAA	25882
gb:MT027064|Organism:Severe      ACTATTTTCTTTGCTGGCATACTAATTGTTACGACTATTGTATACCTTACAATAGTGTAA	25882
gb:MN994468|Organism:Severe      ACTATTTTCTTTGCTGGCATACTAATTGTTACGACTATTGTATACCTTACAATAGTGTAA	25882
gb:MT072688|Organism:Severe      ACTATTTTCTTTGCTGGCATACTAATTGTTACGACTATTGTATACCTTACAATAGTGTAA	25867
gb:MN996527|Organism:Severe      ACTATTTTCTTTGCTGGCATACTAATTGTTACGACTATTGTATACCTTACAATAGTGTAA	25849
gb:MT093631|Organism:Severe      ACTATTTTCTTTGCTGGCATACTAATTGTTACGACTATTGTATACCTTACAATAGTGTAA	25920
gb:MT106053|Organism:Severe      ACTATTTTCTTTGCTGGCATACTAATTGTTACGACTATTGTATACCTTACAATAGTGTAA	25882
gb:MT019533|Organism:Severe      ACTATTTTCTTTGCTGGCATACTAATTGTTACGACTATTGTATACCTTACAATAGTGTAA	25882
gb:MT019531|Organism:Severe      ACTATTTTCTTTGCTGGCATACTAATTGTTACGACTATTGTATACCTTACAATAGTGTAA	25882
gb:MN996528|Organism:Severe      ACTATTTTCTTTGCTGGCATACTAATTGTTACGACTATTGTATACCTTACAATAGTGTAA	25882
gb:MN996530|Organism:Severe      ACTATTTTCTTTGCTGGCATACTAATTGTTACGACTATTGTATACCTTACAATAGTGTAA	25868
gb:MN908947|Organism:Severe      ACTATTTTCTTTGCTGGCATACTAATTGTTACGACTATTGTATACCTTACAATAGTGTAA	25882
gb:MT019532|Organism:Severe      ACTATTTTCTTTGCTGGCATACTAATTGTTACGACTATTGTATACCTTACAATAGTGTAA	25882
                                                                  ***************************

gb:MT020781|Organism:Severe      CTTCTTCAATTGTCATTACTTCAGGTGATGGCACAACAAGTCCTATTTCTGAACATGACT	25930
gb:MT007544|Organism:Severe      CTTCTTCAATTGTCATTACTTCAGGTGATGGCACAACAAGTCCTATTTCTGAACATGACT	25942
gb:MN994467|Organism:Severe      CTTCTTCAATTGTCATTACTTCAGGTGATGGCACAACAAGTCCTATTTCTGAACATGACT	25942
gb:MT044257|Organism:Severe      CTTCTTCAATTGTCATTACTTCAGGTGATGGCACAACAAGTCCTATTTCTGAACATGACT	25942
gb:MT106054|Organism:Severe      CTTCTTCAATTGTCATTACTTCAGGTGATGGCACAACAAGTCCTATTTCTGAACATGACT	25942
gb:MT049951|Organism:Severe      CTTCTTCAATTGTCATTACTTCAGGTGATGGCACAACAAGTCCTATTTCTGAACATGACT	25942
gb:MN975262|Organism:Severe      CTTCTTCAATTGTCATTACTTCAGGTGATGGCACAACAAGTCCTATTTCTGAACATGACT	25942
gb:MT106052|Organism:Severe      CTTCTTCAATTGTCATTACTTCAGGTGATGGCACAACAAGTCCTATTTCTGAACATGACT	25942
gb:LC522975|Organism:Severe      CTTCTTCAATTGTCATTACTTCAGGTGATGGCACAACAAGTCCTATTTCTGAACATGACT	25939
gb:LC522973|Organism:Severe      CTTCTTCAATTGTCATTACTTCAGGTGATGGCACAACAAGTCCTATTTCTGAACATGACT	25939
gb:LC522974|Organism:Severe      CTTCTTCAATTGTCATTACTTCAGGTGATGGCACAACAAGTCCTATTTCTGAACATGACT	25939
gb:MN985325|Organism:Severe      CTTCTTCAATTGTCATTACTTCAGGTGATGGCACAACAAGTCCTATTTCTGAACATGACT	25942
gb:MT020881|Organism:Severe      CTTCTTCAATTGTCATTACTTCAGGTGATGGCACAACAAGTCCTATTTCTGAACATGACT	25942
gb:MT020880|Organism:Severe      CTTCTTCAATTGTCATTACTTCAGGTGATGGCACAACAAGTCCTATTTCTGAACATGACT	25942
```

```
gb:MT066175|Organism:Severe      CTTCTTCAATTGTCATTACTTCAGGTGATGGCACAACAAGTCCTATTTCTGAACATGACT   25942
gb:MN997409|Organism:Severe      CTTCTTCAATTGTCATTACTTCAGGTGATGGCACAACAAGTCCTATTTCTGAACATGACT   25942
gb:MN938384|Organism:Severe      CTTCTTCAATTGTCATTACTTCAGGTGATGGCACAACAAGTCCTATTTCTGAACATGACT   25910
gb:MT044258|Organism:Severe      CTTCTTCAATTGTCATTACTTCAGGTGATGGCACAACAAGTCCTATTTCTGAACATGACT   25918
gb:MT039890|Organism:Severe      CTTCTTCAATTGTCATTACTTCAGGTGATGGCACAACAAGTCCTATTTCTGAACATGACT   25942
gb:MN988713|Organism:Severe      CTTCTTCAATTGTCATTACTTCAGGTGATGGCACAACAAGTCCTATTTCTGAACATGACT   25942
gb:LC521925|Organism:Severe      CTTCTTCAATTGTCATTACTTCAGGTGATGGCACAACAAGTCCTATTTCTGAACATGACT   25915
gb:MT093571|Organism:Severe      CTTCTTCAATTGTCATTACTTCAGGTGATGGCACAACAAGTCCTATTTCTGAACATGACT   25942
gb:MT039887|Organism:Severe      CTTCTTCAATTGTCATTACTTCAGGTGATGGCACAACAAGTCCTATTTCTGAACATGACT   25939
gb:MT019530|Organism:Severe      CTTCTTCAATTGTCATTACTTCAGGTGATGGCACAACAAGTCCTATTTCTGAACATGACT   25942
gb:MT039888|Organism:Severe      CTTCTTCAATTGTCATTACTTCAGGTGATGGCACAACAAGTCCTATTTCTGAACATGACT   25942
gb:LC522972|Organism:Severe      CTTCTTCAATTGTCATTACTTCAGGTGATGGCACAACAAGTCCTATTTCTGAACATGACT   25939
gb:MT027063|Organism:Severe      CTTCTTCAATTGTCATTACTTCAGGTGATGGCACAACAAGTCCTATTTCTGAACATGACT   25942
gb:MT027062|Organism:Severe      CTTCTTCAATTGTCATTACTTCAGGTGATGGCACAACAAGTCCTATTTCTGAACATGACT   25942
gb:MT019529|Organism:Severe      CTTCTTCAATTGTCATTACTTCAGGTGATGGCACAACAAGTCCTATTTCTGAACATGACT   25942
gb:MN996529|Organism:Severe      CTTCTTCAATTGTCATTACTTCAGGTGATGGCACAACAAGTCCTATTTCTGAACATGACT   25930
gb:MN996531|Organism:Severe      CTTCTTCAATTGTCATTACTTCAGGTGATGGCACAACAAGTCCTATTTCTGAACATGACT   25929
gb:MT066176|Organism:Severe      CTTCTTCAATTGTCATTACTTCAGGTGATGGCACAACAAGTCCTATTTCTGAACATGACT   25942
gb:MT027064|Organism:Severe      CTTCTTCAATTGTCATTACTTCAGGTGATGGCACAACAAGTCCTATTTCTGAACATGACT   25942
gb:MN994468|Organism:Severe      CTTCTTCAATTGTCATTACTTCAGGTGATGGCACAACAAGTCCTATTTCTGAACATGACT   25942
gb:MT072688|Organism:Severe      CTTCTTCAATTGTCATTACTTCAGGTGATGGCACAACAAGTCCTATTTCTGAACATGACT   25927
gb:MN996527|Organism:Severe      CTTCTTCAATTGTCATTACTTCAGGTGATGGCACAACAAGTCCTATTTCTGAACATGACT   25909
gb:MT093631|Organism:Severe      CTTCTTCAATTGTCATTACTTCAGGTGATGGCACAACAAGTCCTATTTCTGAACATGACT   25980
gb:MT106053|Organism:Severe      CTTCTTCAATTGTCATTACTTCAGGTGATGGCACAACAAGTCCTATTTCTGAACATGACT   25942
gb:MT019533|Organism:Severe      CTTCTTCAATTGTCATTACTTCAGGTGATGGCACAACAAGTCCTATTTCTGAACATGACT   25942
gb:MT019531|Organism:Severe      CTTCTTCAATTGTCATTACTTCAGGTGATGGCACAACAAGTCCTATTTCTGAACATGACT   25942
gb:MN996528|Organism:Severe      CTTCTTCAATTGTCATTACTTCAGGTGATGGCACAACAAGTCCTATTTCTGAACATGACT   25942
gb:MN996530|Organism:Severe      CTTCTTCAATTGTCATTACTTCAGGTGATGGCACAACAAGTCCTATTTCTGAACATGACT   25928
gb:MN908947|Organism:Severe      CTTCTTCAATTGTCATTACTTCAGGTGATGGCACAACAAGTCCTATTTCTGAACATGACT   25942
gb:MT019532|Organism:Severe      CTTCTTCAATTGTCATTACTTCAGGTGATGGCACAACAAGTCCTATTTCTGAACATGACT   25942
                                 ************************************************************

gb:MT020781|Organism:Severe      ACCAGATTGGTGGTTATACTGAAAAATGGGAATCTGGAGTAAAAGACTGTGTTGTATTAC   25990
gb:MT007544|Organism:Severe      ACCAGATTGGTGGTTATACTGAAAAATGGGAATCTGGAGTAAAAGACTGTGTTGTATTAC   26002
gb:MN994467|Organism:Severe      ACCAGATTGGTGGTTATACTGAAAAATGGGAATCTGGAGTAAAAGACTGTGTTGTATTAC   26002
gb:MT044257|Organism:Severe      ACCAGATTGGTGGTTATACTGAAAAATGGGAATCTGGAGTAAAAGACTGTGTTGTATTAC   26002
gb:MT106054|Organism:Severe      ACCAGATTGGTGGTTATACTGAAAAATGGGAATCTGGAGTAAAAGACTGTGTTGTATTAC   26002
gb:MT049951|Organism:Severe      ACCAGATTGGTGGTTATACTGAAAAATGGGAATCTGGAGTAAAAGACTGTGTTGTATTAC   26002
gb:MN975262|Organism:Severe      ACCAGATTGGTGGTTATACTGAAAAATGGGAATCTGGAGTAAAAGACTGTGTTGTATTAC   26002
gb:MT106052|Organism:Severe      ACCAGATTGGTGGTTATACTGAAAAATGGGAATCTGGAGTAAAAGACTGTGTTGTATTAC   26002
gb:LC522975|Organism:Severe      ACCAGATTGGTGGTTATACTGAAAAATGGGAATCTGGAGTAAAAGACTGTGTTGTATTAC   25999
gb:LC522973|Organism:Severe      ACCAGATTGGTGGTTATACTGAAAAATGGGAATCTGGAGTAAAAGACTGTGTTGTATTAC   25999
gb:LC522974|Organism:Severe      ACCAGATTGGTGGTTATACTGAAAAATGGGAATCTGGAGTAAAAGACTGTGTTGTATTAC   25999
gb:MN985325|Organism:Severe      ACCAGATTGGTGGTTATACTGAAAAATGGGAATCTGGAGTAAAAGACTGTGTTGTATTAC   26002
gb:MT020881|Organism:Severe      ACCAGATTGGTGGTTATACTGAAAAATGGGAATCTGGAGTAAAAGACTGTGTTGTATTAC   26002
gb:MT020880|Organism:Severe      ACCAGATTGGTGGTTATACTGAAAAATGGGAATCTGGAGTAAAAGACTGTGTTGTATTAC   26002
gb:MT066175|Organism:Severe      ACCAGATTGGTGGTTATACTGAAAAATGGGAATCTGGAGTAAAAGACTGTGTTGTATTAC   26002
gb:MN997409|Organism:Severe      ACCAGATTGGTGGTTATACTGAAAAATGGGAATCTGGAGTAAAAGACTGTGTTGTATTAC   26002
gb:MN938384|Organism:Severe      ACCAGATTGGTGGTTATACTGAAAAATGGGAATCTGGAGTAAAAGACTGTGTTGTATTAC   25970
gb:MT044258|Organism:Severe      ACCAGATTGGTGGTTATACTGAAAAATGGGAATCTGGAGTAAAAGACTGTGTTGTATTAC   25978
gb:MT039890|Organism:Severe      ACCAGATTGGTGGTTATACTGAAAAATGGGAATCTGGAGTAAAAGACTGTGTTGTATTAC   26002
gb:MN988713|Organism:Severe      ACCAGATTGGTGGTTATACTGAAAAATGGGAATCTGGAGTAAAAGACTGTGTTGTATTAC   26002
gb:LC521925|Organism:Severe      ACCAGATTGGTGGTTATACTGAAAAATGGGAATCTGGAGTAAAAGACTGTGTTGTATTAC   25975
gb:MT093571|Organism:Severe      ACCAGATTGGTGGTTATACTGAAAAATGGGAATCTGGAGTAAAAGACTGTGTTGTATTAC   26002
gb:MT039887|Organism:Severe      ACCAGATTGGTGGTTATACTGAAAAATGGGAATCTGGAGTAAAAGACTGTGTTGTATTAC   25999
gb:MT019530|Organism:Severe      ACCAGATTGGTGGTTATACTGAAAAATGGGAATCTGGAGTAAAAGACTGTGTTGTATTAC   26002
gb:MT039888|Organism:Severe      ACCAGATTGGTGGTTATACTGAAAAATGGGAATCTGGAGTAAAAGACTGTGTTGTATTAC   26002
gb:LC522972|Organism:Severe      ACCAGATTGGTGGTTATACTGAAAAATGGGAATCTGGAGTAAAAGACTGTGTTGTATTAC   25999
gb:MT027063|Organism:Severe      ACCAGATTGGTGGTTATACTGAAAAATGGGAATCTGGAGTAAAAGACTGTGTTGTATTAC   26002
gb:MT027062|Organism:Severe      ACCAGATTGGTGGTTATACTGAAAAATGGGAATCTGGAGTAAAAGACTGTGTTGTATTAC   26002
gb:MT019529|Organism:Severe      ACCAGATTGGTGGTTATACTGAAAAATGGGAATCTGGAGTAAAAGACTGTGTTGTATTAC   26002
gb:MN996529|Organism:Severe      ACCAGATTGGTGGTTATACTGAAAAATGGGAATCTGGAGTAAAAGACTGTGTTGTATTAC   25990
gb:MN996531|Organism:Severe      ACCAGATTGGTGGTTATACTGAAAAATGGGAATCTGGAGTAAAAGACTGTGTTGTATTAC   25989
gb:MT066176|Organism:Severe      ACCAGATTGGTGGTTATACTGAAAAATGGGAATCTGGAGTAAAAGACTGTGTTGTATTAC   26002
gb:MT027064|Organism:Severe      ACCAGATTGGTGGTTATACTGAAAAATGGGAATCTGGAGTAAAAGACTGTGTTGTATTAC   26002
```

```
gb:MN994468|Organism:Severe      ACCAGATTGGTGGTTATACTGAAAAATGGGAATCTGGAGTAAAAGACTGTGTTGTATTAC    26002
gb:MT072688|Organism:Severe      ACCAGATTGGTGGTTATACTGAAAAATGGGAATCTGGAGTAAAAGACTGTGTTGTATTAC    25987
gb:MN996527|Organism:Severe      ACCAGATTGGTGGTTATACTGAAAAATGGGAATCTGGAGTAAAAGACTGTGTTGTATTAC    25969
gb:MT093631|Organism:Severe      ACCAGATTGGTGGTTATACTGAAAAATGGGAATCTGGAGTAAAAGACTGTGTTGTATTAC    26040
gb:MT106053|Organism:Severe      ACCAGATTGGTGGTTATACTGAAAAATGGGAATCTGGAGTAAAAGACTGTGTTGTATTAC    26002
gb:MT019533|Organism:Severe      ACCAGATTGGTGGTTATACTGAAAAATGGGAATCTGGAGTAAAAGACTGTGTTGTATTAC    26002
gb:MT019531|Organism:Severe      ACCAGATTGGTGGTTATACTGAAAAATGGGAATCTGGAGTAAAAGACTGTGTTGTATTAC    26002
gb:MN996528|Organism:Severe      ACCAGATTGGTGGTTATACTGAAAAATGGGAATCTGGAGTAAAAGACTGTGTTGTATTAC    26002
gb:MN996530|Organism:Severe      ACCAGATTGGTGGTTATACTGAAAAATGGGAATCTGGAGTAAAAGACTGTGTTGTATTAC    25988
gb:MN908947|Organism:Severe      ACCAGATTGGTGGTTATACTGAAAAATGGGAATCTGGAGTAAAAGACTGTGTTGTATTAC    26002
gb:MT019532|Organism:Severe      ACCAGATTGGTGGTTATACTGAAAAATGGGAATCTGGAGTAAAAGACTGTGTTGTATTAC    26002
                                 ************************************************************

gb:MT020781|Organism:Severe      ACAGTTACTTCACTTCAGACTATTACCAGCTGTACTCAACTCAATTGAGTACAGACACTG    26050
gb:MT007544|Organism:Severe      ACAGTTACTTCACTTCAGACTATTACCAGCTGTACTCAACTCAATTGAGTACAGACACTG    26062
gb:MN994467|Organism:Severe      ACAGTTACTTCACTTCAGACTATTACCAGCTGTACTCAACTCAATTGAGTACAGACACTG    26062
gb:MT044257|Organism:Severe      ACAGTTACTTCACTTCAGACTATTACCAGCTGTACTCAACTCAATTGAGTACAGACACTG    26062
gb:MT106054|Organism:Severe      ACAGTTACTTCACTTCAGACTATTACCAGCTGTACTCAACTCAATTGAGTACAGACACTG    26062
gb:MT049951|Organism:Severe      ACAGTTACTTCACTTCAGACTATTACCAGCTGTACTCAACTCAATTGAGTACAGACACTG    26062
gb:MN975262|Organism:Severe      ACAGTTACTTCACTTCAGACTATTACCAGCTGTACTCAACTCAATTGAGTACAGACACTG    26062
gb:MT106052|Organism:Severe      ACAGTTACTTCACTTCAGACTATTACCAGCTGTACTCAACTCAATTGAGTACAGACACTG    26062
gb:LC522975|Organism:Severe      ACAGTTACTTCACTTCAGACTATTACCAGCTGTACTCAACTCAATTGAGTACAGACACTG    26059
gb:LC522973|Organism:Severe      ACAGTTACTTCACTTCAGACTATTACCAGCTGTACTCAACTCAATTGAGTACAGACACTG    26059
gb:LC522974|Organism:Severe      ACAGTTACTTCACTTCAGACTATTACCAGCTGTACTCAACTCAATTGAGTACAGACACTG    26059
gb:MN985325|Organism:Severe      ACAGTTACTTCACTTCAGACTATTACCAGCTGTACTCAACTCAATTGAGTACAGACACTG    26062
gb:MT020881|Organism:Severe      ACAGTTACTTCACTTCAGACTATTACCAGCTGTACTCAACTCAATTGAGTACAGACACTG    26062
gb:MT020880|Organism:Severe      ACAGTTACTTCACTTCAGACTATTACCAGCTGTACTCAACTCAATTGAGTACAGACACTG    26062
gb:MT066175|Organism:Severe      ACAGTTACTTCACTTCAGACTATTACCAGCTGTACTCAACTCAATTGAGTACAGACACTG    26062
gb:MN997409|Organism:Severe      ACAGTTACTTCACTTCAGACTATTACCAGCTGTACTCAACTCAATTGAGTACAGACACTG    26062
gb:MN938384|Organism:Severe      ACAGTTACTTCACTTCAGACTATTACCAGCTGTACTCAACTCAATTGAGTACAGACACTG    26030
gb:MT044258|Organism:Severe      ACAGTTACTTCACTTCAGACTATTACCAGCTGTACTCAACTCAATTGAGTACAGACACTG    26038
gb:MT039890|Organism:Severe      ACAGTTACTTCACTTCAGACTATTACCAGCTGTACTCAACTCAATTGAGTACAGACACTG    26062
gb:MN988713|Organism:Severe      ACAGTTACTTCACTTCAGACTATTACCAGCTGTACTCAACTCAATTGAGTACAGACACTG    26062
gb:LC521925|Organism:Severe      ACAGTTACTTCACTTCAGACTATTACCAGCTGTACTCAACTCAATTGAGTACAGACACTG    26035
gb:MT093571|Organism:Severe      ACAGTTACTTCACTTCAGACTATTACCAGCTGTACTCAACTCAATTGAGTACAGACACTG    26062
gb:MT039887|Organism:Severe      ACAGTTACTTCACTTCAGACTATTACCAGCTGTACTCAACTCAATTGAGTACAGACACTG    26059
gb:MT019530|Organism:Severe      ACAGTTACTTCACTTCAGACTATTACCAGCTGTACTCAACTCAATTGAGTACAGACACTG    26062
gb:MT039888|Organism:Severe      ACAGTTACTTCACTTCAGACTATTACCAGCTGTACTCAACTCAATTGAGTACAGACACTG    26062
gb:LC522972|Organism:Severe      ACAGTTACTTCACTTCAGACTATTACCAGCTGTACTCAACTCAATTGAGTACAGACACTG    26059
gb:MT027063|Organism:Severe      ACAGTTACTTCACTTCAGACTATTACCAGCTGTACTCAACTCAATTGAGTACAGACACTG    26062
gb:MT027062|Organism:Severe      ACAGTTACTTCACTTCAGACTATTACCAGCTGTACTCAACTCAATTGAGTACAGACACTG    26062
gb:MT019529|Organism:Severe      ACAGTTACTTCACTTCAGACTATTACCAGCTGTACTCAACTCAATTGAGTACAGACACTG    26062
gb:MN996529|Organism:Severe      ACAGTTACTTCACTTCAGACTATTACCAGCTGTACTCAACTCAATTGAGTACAGACACTG    26050
gb:MN996531|Organism:Severe      ACAGTTACTTCACTTCAGACTATTACCAGCTGTACTCAACTCAATTGAGTACAGACACTG    26049
gb:MT066176|Organism:Severe      ACAGTTACTTCACTTCAGACTATTACCAGCTGTACTCAACTCAATTGAGTACAGACACTG    26062
gb:MT027064|Organism:Severe      ACAGTTACTTCACTTCAGACTATTACCAGCTGTACTCAACTCAATTGAGTACAGACACTG    26062
gb:MN994468|Organism:Severe      ACAGTTACTTCACTTCAGACTATTACCAGCTGTACTCAACTCAATTGAGTACAGACACTG    26062
gb:MT072688|Organism:Severe      ACAGTTACTTCACTTCAGACTATTACCAGCTGTACTCAACTCAATTGAGTACAGACACTG    26047
gb:MN996527|Organism:Severe      ACAGTTACTTCACTTCAGACTATTACCAGCTGTACTCAACTCAATTGAGTACAGACACTG    26029
gb:MT093631|Organism:Severe      ACAGTTACTTCACTTCAGACTATTACCAGCTGTACTCAACTCAATTGAGTACAGACACTG    26100
gb:MT106053|Organism:Severe      ACAGTTACTTCACTTCAGACTATTACCAGCTGTACTCAACTCAATTGAGTACAGACACTG    26062
gb:MT019533|Organism:Severe      ACAGTTACTTCACTTCAGACTATTACCAGCTGTACTCAACTCAATTGAGTACAGACACTG    26062
gb:MT019531|Organism:Severe      ACAGTTACTTCACTTCAGACTATTACCAGCTGTACTCAACTCAATTGAGTACAGACACTG    26062
gb:MN996528|Organism:Severe      ACAGTTACTTCACTTCAGACTATTACCAGCTGTACTCAACTCAATTGAGTACAGACACTG    26062
gb:MN996530|Organism:Severe      ACAGTTACTTCACTTCAGACTATTACCAGCTGTACTCAACTCAATTGAGTACAGACACTG    26048
gb:MN908947|Organism:Severe      ACAGTTACTTCACTTCAGACTATTACCAGCTGTACTCAACTCAATTGAGTACAGACACTG    26062
gb:MT019532|Organism:Severe      ACAGTTACTTCACTTCAGACTATTACCAGCTGTACTCAACTCAATTGAGTACAGACACTG    26062
                                 ************************************************************

gb:MT020781|Organism:Severe      GTGTTGAACATGTTACCTTCTTCATCTACAATAAAATTGTTGATGAGCCTGAAGAACATG    26110
gb:MT007544|Organism:Severe      GTGTTGAACATGTTACCTTCTTCATCTACAATAAAATTGTTGATGAGCCTGAAGAACATG    26122
gb:MN994467|Organism:Severe      GTGTTGAACATGTTACCTTCTTCATCTACAATAAAATTGTTGATGAGCCTGAAGAACATG    26122
gb:MT044257|Organism:Severe      GTGTTGAACATGTTACCTTCTTCATCTACAATAAAATTGTTGATGAGCCTGAAGAACATG    26122
gb:MT106054|Organism:Severe      GTGTTGAACATGTTACCTTCTTCATCTACAATAAAATTGTTGATGAGCCTGAAGAACATG    26122
gb:MT049951|Organism:Severe      GTGTTGAACATGTTACCTTCTTCATCTACAATAAAATTGTTGATGAGCCTGAAGAACATG    26122
```

```
gb:MN975262|Organism:Severe      GTGTTGAACATGTTACCTTCTTCATCTACAATAAAATTGTTGATGAGCCTGAAGAACATG    26122
gb:MT106052|Organism:Severe      GTGTTGAACATGTTACCTTCTTCATCTACAATAAAATTGTTGATGAGCCTGAAGAACATG    26122
gb:LC522975|Organism:Severe      GTGTTGAACATGTTACCTTCTTCATCTACAATAAAATTGTTGATGAGCCTGAAGAACATG    26119
gb:LC522973|Organism:Severe      GTGTTGAACATGTTACCTTCTTCATCTACAATAAAATTGTTGATGAGCCTGAAGAACATG    26119
gb:LC522974|Organism:Severe      GTGTTGAACATGTTACCTTCTTCATCTACAATAAAATTGTTGATGAGCCTGAAGAACATG    26119
gb:MN985325|Organism:Severe      GTGTTGAACATGTTACCTTCTTCATCTACAATAAAATTGTTGATGAGCCTGAAGAACATG    26122
gb:MT020881|Organism:Severe      GTGTTGAACATGTTACCTTCTTCATCTACAATAAAATTGTTGATGAGCCTGAAGAACATG    26122
gb:MT020880|Organism:Severe      GTGTTGAACATGTTACCTTCTTCATCTACAATAAAATTGTTGATGAGCCTGAAGAACATG    26122
gb:MT066175|Organism:Severe      GTGTTGAACATGTTACCTTCTTCATCTACAATAAAATTGTTGATGAGCCTGAAGAACATG    26122
gb:MN997409|Organism:Severe      GTGTTGAACATGTTACCTTCTTCATCTACAATAAAATTGTTGATGAGCCTGAAGAACATG    26122
gb:MN938384|Organism:Severe      GTGTTGAACATGTTACCTTCTTCATCTACAATAAAATTGTTGATGAGCCTGAAGAACATG    26090
gb:MT044258|Organism:Severe      GTGTTGAACATGTTACCTTCTTCATCTACAATAAAATTGTTGATGAGCCTGAAGAACATG    26098
gb:MT039890|Organism:Severe      GTGTTGAACATGTTACCTTCTTCATCTACAATAAAATTGTTGATGAGCCTGAAGAACATG    26122
gb:MN988713|Organism:Severe      GTGTTGAACATGTTACCTTCTTCATCTACAATAAAATTGTTGATGAGCCTGAAGAACATG    26122
gb:LC521925|Organism:Severe      GTGTTGAACATGTTACCTTCTTCATCTACAATAAAATTGTTGATGAGCCTGAAGAACATG    26095
gb:MT093571|Organism:Severe      GTGTTGAACATGTTACCTTCTTCATCTACAATAAAATTGTTGATGAGCCTGAAGAACATG    26122
gb:MT039887|Organism:Severe      GTGTTGAACATGTTACCTTCTTCATCTACAATAAAATTGTTGATGAGCCTGAAGAACATG    26119
gb:MT019530|Organism:Severe      GTGTTGAACATGTTACCTTCTTCATCTACAATAAAATTGTTGATGAGCCTGAAGAACATG    26122
gb:MT039888|Organism:Severe      GTGTTGAACATGTTACCTTCTTCATCTACAATAAAATTGTTGATGAGCCTGAAGAACATG    26122
gb:LC522972|Organism:Severe      GTGTTGAACATGTTACCTTCTTCATCTACAATAAAATTGTTGATGAGCCTGAAGAACATG    26119
gb:MT027063|Organism:Severe      GTGTTGAACATGTTACCTTCTTCATCTACAATAAAATTGTTGATGAGCCTGAAGAACATG    26122
gb:MT027062|Organism:Severe      GTGTTGAACATGTTACCTTCTTCATCTACAATAAAATTGTTGATGAGCCTGAAGAACATG    26122
gb:MT019529|Organism:Severe      GTGTTGAACATGTTACCTTCTTCATCTACAATAAAATTGTTGATGAGCCTGAAGAACATG    26122
gb:MN996529|Organism:Severe      GTGTTGAACATGTTACCTTCTTCATCTACAATAAAATTGTTGATGAGCCTGAAGAACATG    26110
gb:MN996531|Organism:Severe      GTGTTGAACATGTTACCTTCTTCATCTACAATAAAATTGTTGATGAGCCTGAAGAACATG    26109
gb:MT066176|Organism:Severe      GTGTTGAACATGTTACCTTCTTCATCTACAATAAAATTGTTGATGAGCCTGAAGAACATG    26122
gb:MT027064|Organism:Severe      GTGTTGAACATGTTACCTTCTTCATCTACAATAAAATTGTTGATGAGCCTGAAGAACATG    26122
gb:MN994468|Organism:Severe      GTGTTGAACATGTTACCTTCTTCATCTACAATAAAATTGTTGATGAGCCTGAAGAACATG    26122
gb:MT072688|Organism:Severe      GTGTTGAACATGTTACCTTCTTCATCTACAATAAAATTGTTGATGAGCCTGAAGAACATG    26107
gb:MN996527|Organism:Severe      GTGTTGAACATGTTACCTTCTTCATCTACAATAAAATTGTTGATGAGCCTGAAGAACATG    26089
gb:MT093631|Organism:Severe      GTGTTGAACATGTTACCTTCTTCATCTACAATAAAATTGTTGATGAGCCTGAAGAACATG    26160
gb:MT106053|Organism:Severe      GTGTTGAACATGTTACCTTCTTCATCTACAATAAAATTGTTGATGAGCCTGAAGAACATG    26122
gb:MT019533|Organism:Severe      GTGTTGAACATGTTACCTTCTTCATCTACAATAAAATTGTTGATGAGCCTGAAGAACATG    26122
gb:MT019531|Organism:Severe      GTGTTGAACATGTTACCTTCTTCATCTACAATAAAATTGTTGATGAGCCTGAAGAACATG    26122
gb:MN996528|Organism:Severe      GTGTTGAACATGTTACCTTCTTCATCTACAATAAAATTGTTGATGAGCCTGAAGAACATG    26122
gb:MN996530|Organism:Severe      GTGTTGAACATGTTACCTTCTTCATCTACAATAAAATTGTTGATGAGCCTGAAGAACATG    26108
gb:MN908947|Organism:Severe      GTGTTGAACATGTTACCTTCTTCATCTACAATAAAATTGTTGATGAGCCTGAAGAACATG    26122
gb:MT019532|Organism:Severe      GTGTTGAACATGTTACCTTCTTCATCTACAATAAAATTGTTGATGAGCCTGAAGAACATG    26122
                                 ************************************************************

gb:MT020781|Organism:Severe      TCCAAATTCACACAATCGACGGTTCATCCGGAGTTGTTAATCCAGTAATGGAACCAATTT    26170
gb:MT007544|Organism:Severe      TCCAAATTCACACAATCGACGTTTCATCCGGAGTTGTTAATCCAGTAATGGAACCAATTT    26182
gb:MN994467|Organism:Severe      TCCAAATTCACACAATCGACGGTTCATCCGGAGTTGTTAATCCAGTAATGGAACCAATTT    26182
gb:MT044257|Organism:Severe      TCCAAATTCACACAATCGACGGTTCATCCGGAGTTGTTAATCCAGTAATGGAACCAATTT    26182
gb:MT106054|Organism:Severe      TCCAAATTCACACAATCGACGGTTCATCCGGAGTTGTTAATCCAGTAATGGAACCAATTT    26182
gb:MT049951|Organism:Severe      TCCAAATTCACACAATCGACGGTTCATCCGGAGTTGTTAATCCAGTAATGGAACCAATTT    26182
gb:MN975262|Organism:Severe      TCCAAATTCACACAATCGACGGTTCATCCGGAGTTGTTAATCCAGTAATGGAACCAATTT    26182
gb:MT106052|Organism:Severe      TCCAAATTCACACAATCGACGGTTCATCCGGAGTTGTTAATCCAGTAATGGAACCAATTT    26182
gb:LC522975|Organism:Severe      TCCAAATTCACACAATCGACGGTTCATCCGGAGTTGTTAATCCAGTAATGGAACCAATTT    26179
gb:LC522973|Organism:Severe      TCCAAATTCACACAATCGACGGTTCATCCGGAGTTGTTAATCCAGTAATGGAACCAATTT    26179
gb:LC522974|Organism:Severe      TCCAAATTCACACAATCGACGGTTCATCCGGAGTTGTTAATCCAGTAATGGAACCAATTT    26179
gb:MN985325|Organism:Severe      TCCAAATTCACACAATCGACGGTTCATCCGGAGTTGTTAATCCAGTAATGGAACCAATTT    26182
gb:MT020881|Organism:Severe      TCCAAATTCACACAATCGACGGTTCATCCGGAGTTGTTAATCCAGTAATGGAACCAATTT    26182
gb:MT020880|Organism:Severe      TCCAAATTCACACAATCGACGGTTCATCCGGAGTTGTTAATCCAGTAATGGAACCAATTT    26182
gb:MT066175|Organism:Severe      TCCAAATTCACACAATCGACGGTTCATCCGGAGTTGTTAATCCAGTAATGGAACCAATTT    26182
gb:MN997409|Organism:Severe      TCCAAATTCACACAATCGACGGTTCATCCGGAGTTGTTAATCCAGTAATGGAACCAATTT    26182
gb:MN938384|Organism:Severe      TCCAAATTCACACAATCGACGGTTCATCCGGAGTTGTTAATCCAGTAATGGAACCAATTT    26150
gb:MT044258|Organism:Severe      TCCAAATTCACACAATCGACGGTTCATCCGGAGTTGTTAATCCAGTAATGGAACCAATTT    26158
gb:MT039890|Organism:Severe      TCCAAATTCACACAATCGACGTTTCATCCGGAGTTGTTAATCCAGTAATGGAACCAATTT    26182
gb:MN988713|Organism:Severe      TCCAAATTCACACAATCGACGGTTCATCCGGAGTTGTTAATCCAGTAATGGAACCAATTT    26182
gb:LC521925|Organism:Severe      TCCAAATTCACACAATCGACGGTTCATCCGGAGTTGTTAATCCAGTAATGGAACCAATTT    26155
gb:MT093571|Organism:Severe      TCCAAATTCACACAATCGACGTTTCATCCGGAGTTGTTAATCCAGTAATGGAACCAATTT    26182
gb:MT039887|Organism:Severe      TCCAAATTCACACAATCGACGGTTCATCCGGAGTTGTTAATCCAGTAATGGAACCAATTT    26179
gb:MT019530|Organism:Severe      TCCAAATTCACACAATCGACGGTTCATCCGGAGTTGTTAATCCAGTAATGGAACCAATTT    26182
gb:MT039888|Organism:Severe      TCCAAATTCACACAATCGACGGTTCATCCGGAGTTGTTAATCCAGTAATGGAACCAATTT    26182
```

```
gb:LC522972|Organism:Severe      TCCAAATTCACACAATCGACGGTTCATCCGGAGTTGTTAATCCAGTAATGGAACCAATTT   26179
gb:MT027063|Organism:Severe      TCCAAATTCACACAATCGACGGTTCATCCGGAGTTGTTAATCCAGTAATGGAACCAATTT   26182
gb:MT027062|Organism:Severe      TCCAAATTCACACAATCGACGGTTCATCCGGAGTTGTTAATCCAGTAATGGAACCAATTT   26182
gb:MT019529|Organism:Severe      TCCAAATTCACACAATCGACGGTTCATCCGGAGTTGTTAATCCAGTAATGGAACCAATTT   26182
gb:MN996529|Organism:Severe      TCCAAATTCACACAATCGACGGTTCATCCGGAGTTGTTAATCCAGTAATGGAACCAATTT   26170
gb:MN996531|Organism:Severe      TCCAAATTCACACAATCGACGGTTCATCCGGAGTTGTTAATCCAGTAATGGAACCAATTT   26169
gb:MT066176|Organism:Severe      TCCAAATTCACACAATCGACGGTTCATCCGGAGTTGTTAATCCAGTAATGGAACCAATTT   26182
gb:MT027064|Organism:Severe      TCCAAATTCACACAATCGACGGTTCATCCGGAGTTGTTAATCCAGTAATGGAACCAATTT   26182
gb:MN994468|Organism:Severe      TCCAAATTCACACAATCGACGTTTCATCCGGAGTTGTTAATCCAGTAATGGAACCAATTT   26182
gb:MT072688|Organism:Severe      TCCAAATTCACACAATCGACGGTTCATCCGGAGTTGTTAATCCAGTAATGGAACCAATTT   26167
gb:MN996527|Organism:Severe      TCCAAATTCACACAATCGACGGTTCATCCGGAGTTGTTAATCCAGTAATGGAACCAATTT   26149
gb:MT093631|Organism:Severe      TCCAAATTCACACAATCGACGGTTCATCCGGAGTTGTTAATCCAGTAATGGAACCAATTT   26220
gb:MT106053|Organism:Severe      TCCAAATTCACACAATCGACGGTTCATCCGGAGTTGTTAATCCAGTAATGGAACCAATTT   26182
gb:MT019533|Organism:Severe      TCCAAATTCACACAATCGACGGTTCATCCGGAGTTGTTAATCCAGTAATGGAACCAATTT   26182
gb:MT019531|Organism:Severe      TCCAAATTCACACAATCGACGGTTCATCCGGAGTTGTTAATCCAGTAATGGAACCAATTT   26182
gb:MN996528|Organism:Severe      TCCAAATTCACACAATCGACGGTTCATCCGGAGTTGTTAATCCAGTAATGGAACCAATTT   26182
gb:MN996530|Organism:Severe      TCCAAATTCACACAATCGACGGTTCATCCGGAGTTGTTAATCCAGTAATGGAACCAATTT   26168
gb:MN908947|Organism:Severe      TCCAAATTCACACAATCGACGGTTCATCCGGAGTTGTTAATCCAGTAATGGAACCAATTT   26182
```
[truncated: 287,159 more chars]
