# Supplementary material for: Immunoinformatic identification of B cell and T cell epitopes in the SARS-CoV-2 proteome
Source: Sci Rep. 2020 Aug 25;10:14179. doi: 10.1038/s41598-020-70864-8 (PMC7447814; doi:10.1038/s41598-020-70864-8)
Supplement: Supplementary file 2 — Supplementary Information 2. [file 41598_2020_70864_MOESM2_ESM.pdf]

Immunoinformatic identification of B cell and T cell epitopes in the SARS-CoV-2 proteome

Stephen N. Crooke, Inna G. Ovsyannikova, Richard B. Kennedy, Gregory A. Poland*

Mayo Clinic Vaccine Research Group, Mayo Clinic, Rochester, MN USA

Correspondence:
Gregory A. Poland, M.D., Director – Vaccine Research Group, Mayo Clinic, Guggenheim Building 611C, 200 First Street SW, Rochester, MN 55905 USA

Phone: (507) 284-4968; E-mail: poland.gregory@mayo.edu

© 2020 Mayo Foundation for Medical Education and Research

Supplementary Information for

Immunoinformatic identification of B cell and T cell epitopes in the SARS-CoV-2 proteome

Stephen N. Crooke, Inna G. Ovsyannikova, Richard B. Kennedy, Gregory A. Poland

Corresponding author: Gregory Poland, M.D.
Email: poland.gregory@mayo.edu

This PDF file includes:

Figures S1 to S2
Tables S1 to S2

Fig. S1 (separate file). Sequence alignments.

Fig. S2. NetCTL prediction scores by individual HLA class I supertype.

E glycoprotein

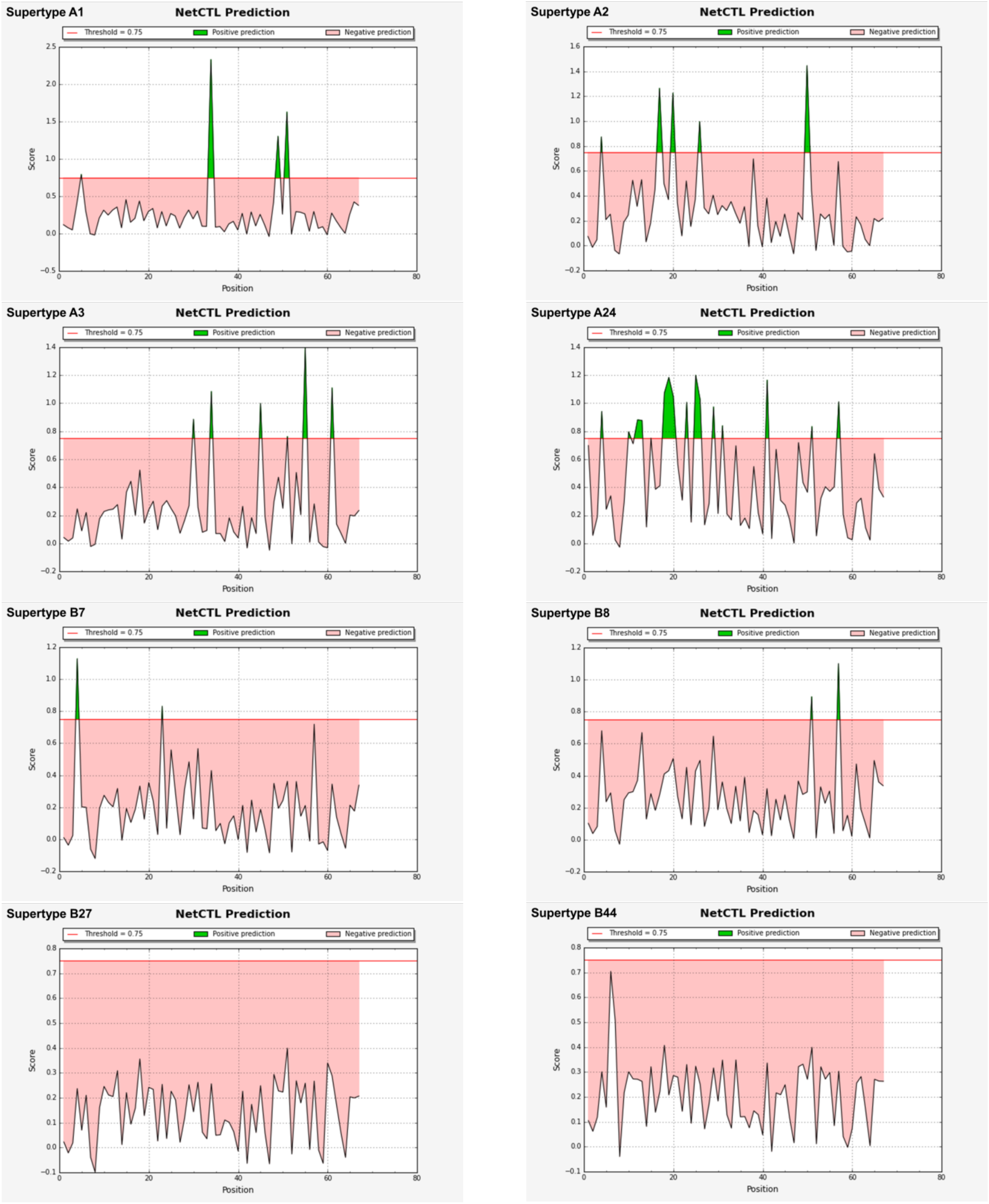

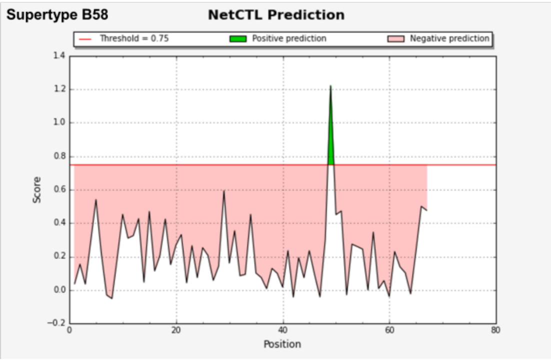

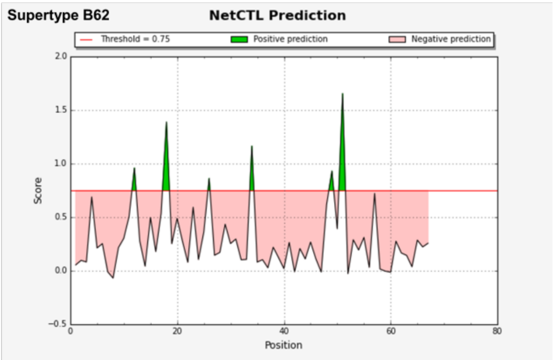

M glycoprotein

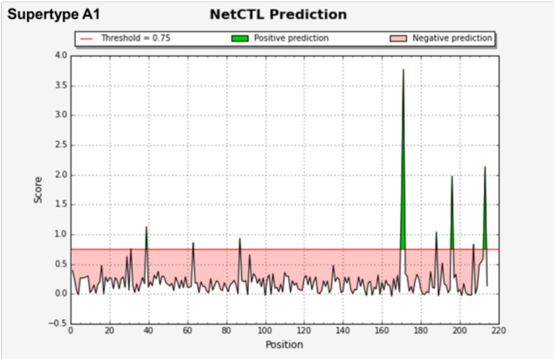

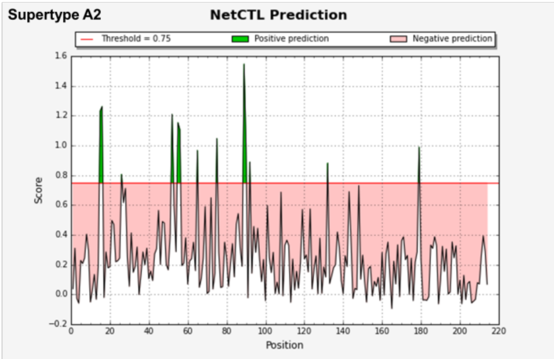

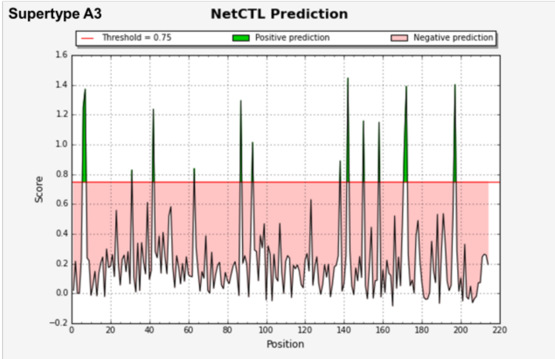

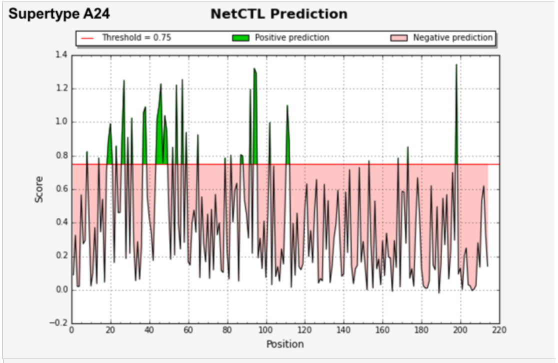

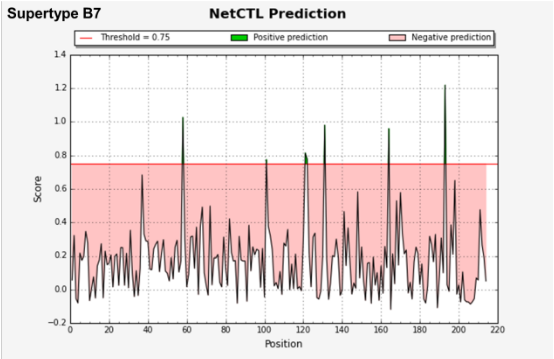

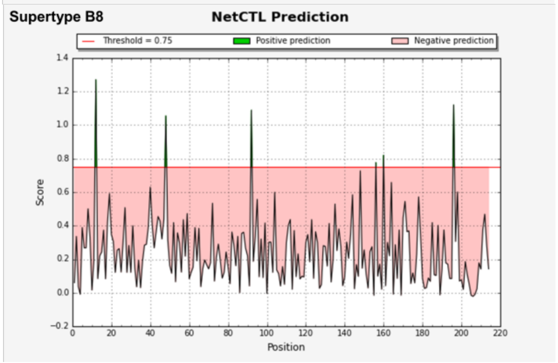

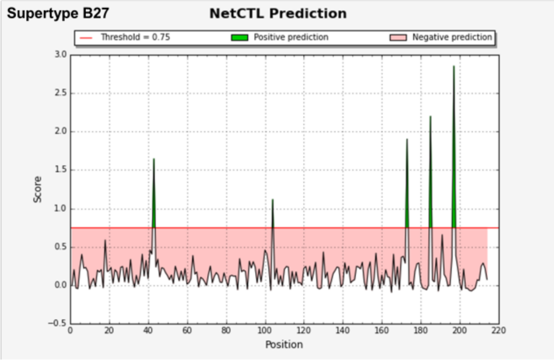

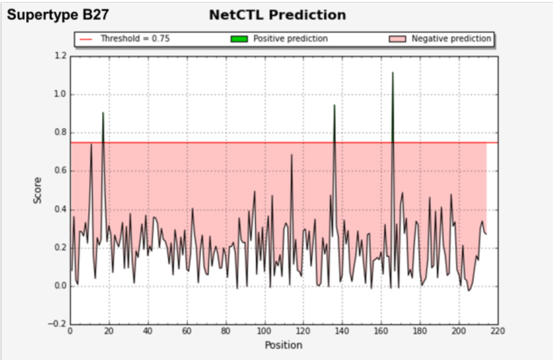

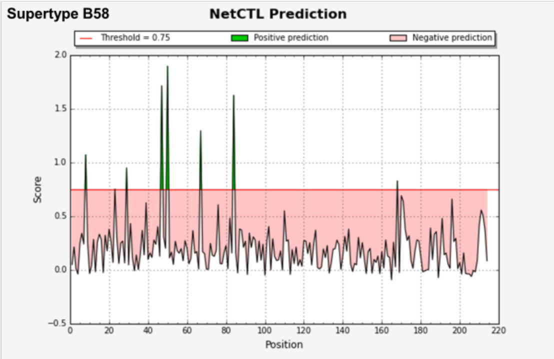

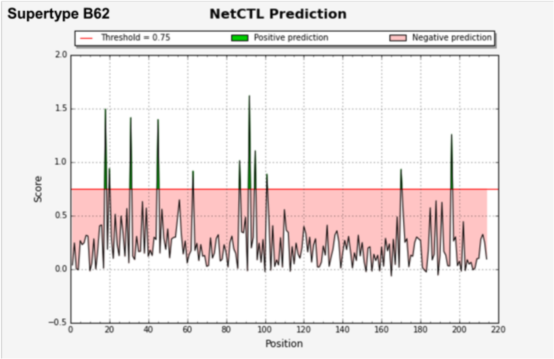

N phosphoprotein

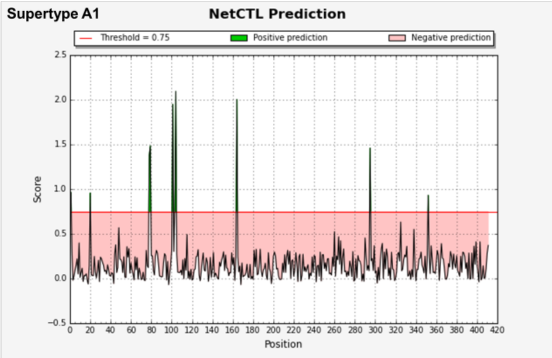

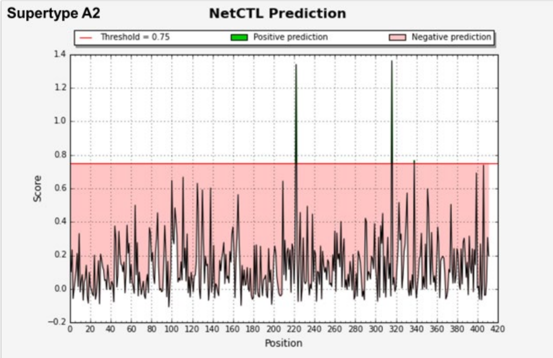

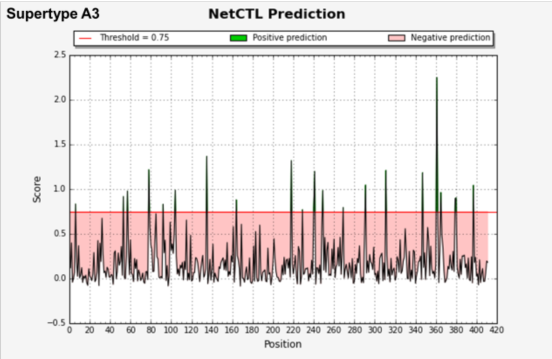

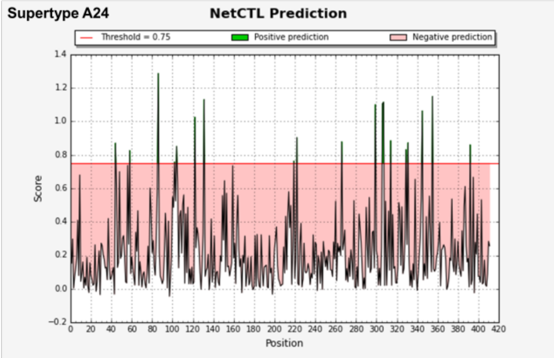

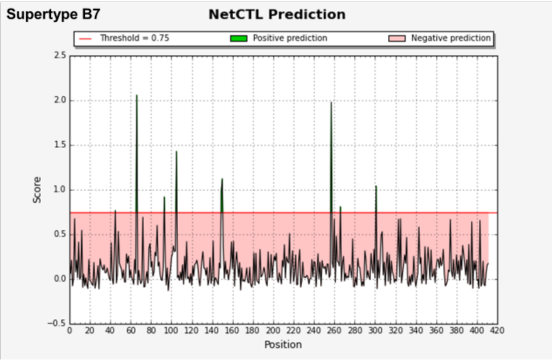

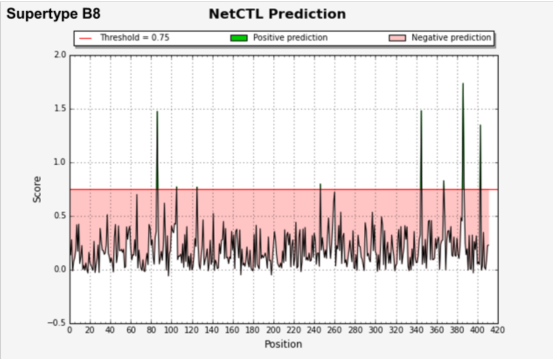

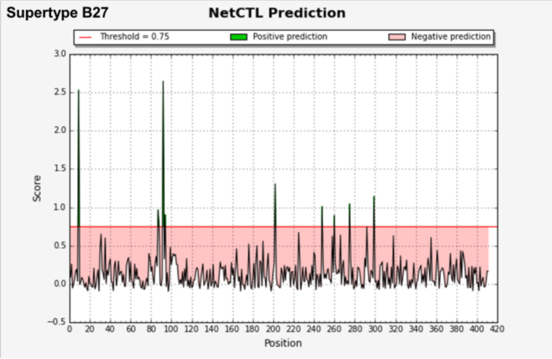

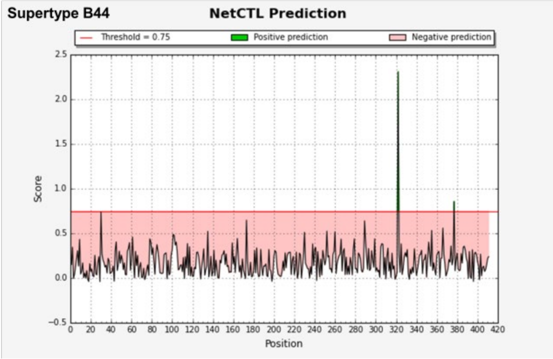

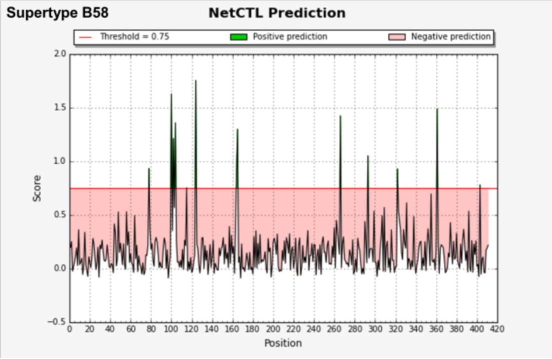

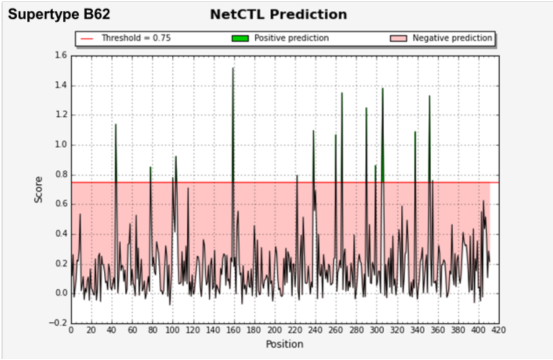

ORF3a protein

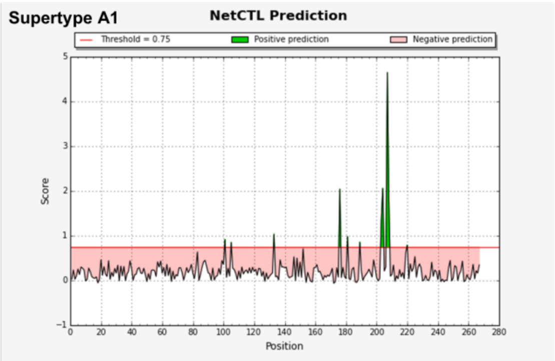

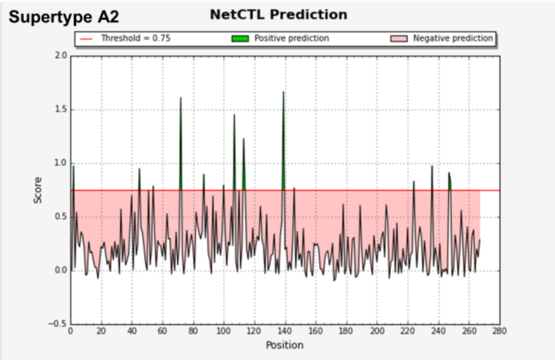

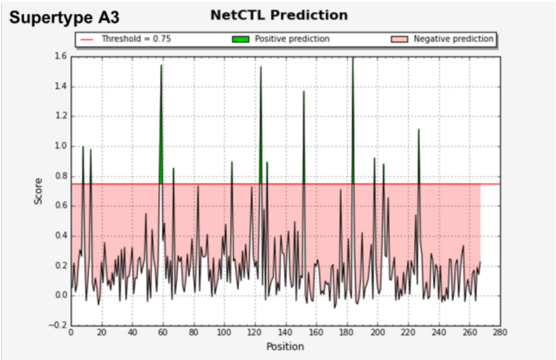
Supertype A3
NetCTL Prediction
Threshold = 0.75
Positive prediction
Negative prediction
Score
Position

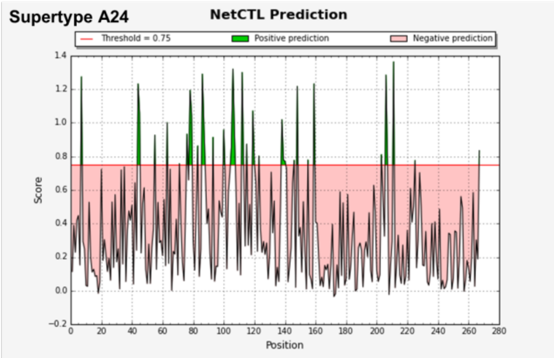
Supertype A24
NetCTL Prediction
Threshold = 0.75
Positive prediction
Negative prediction
Score
Position

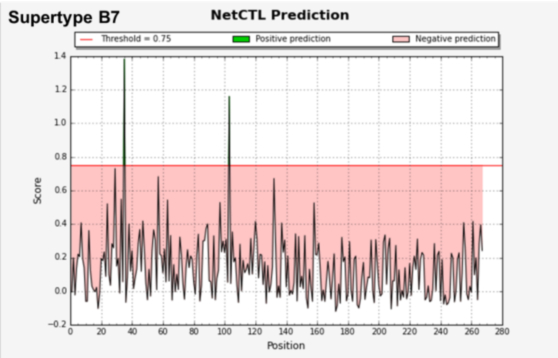
Supertype B7
NetCTL Prediction
Threshold = 0.75
Positive prediction
Negative prediction
Score
Position

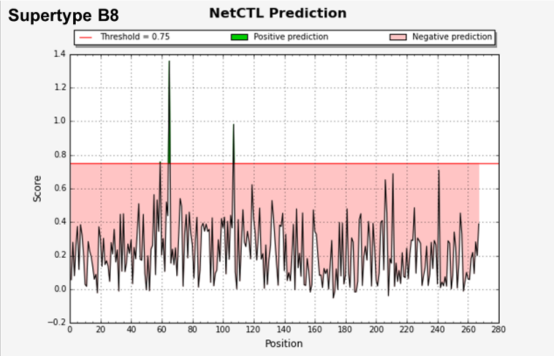
Supertype B8
NetCTL Prediction
Threshold = 0.75
Positive prediction
Negative prediction
Score
Position

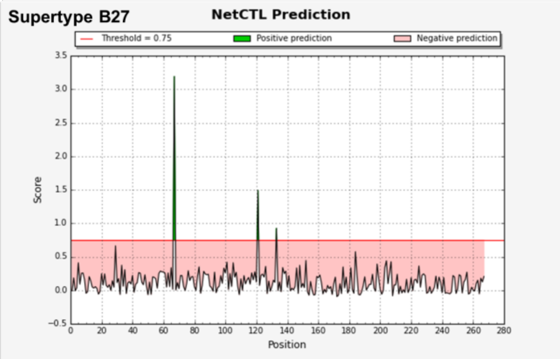
Supertype B27
NetCTL Prediction
Threshold = 0.75
Positive prediction
Negative prediction
Score
Position

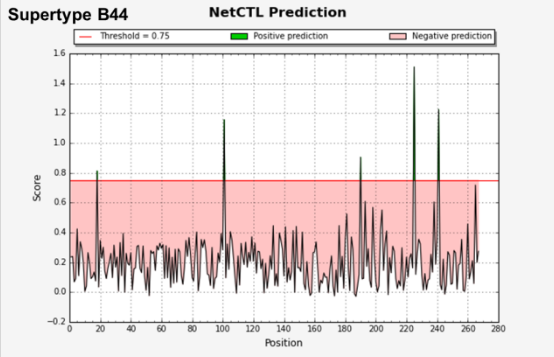
Supertype B44
NetCTL Prediction
Threshold = 0.75
Positive prediction
Negative prediction
Score
Position

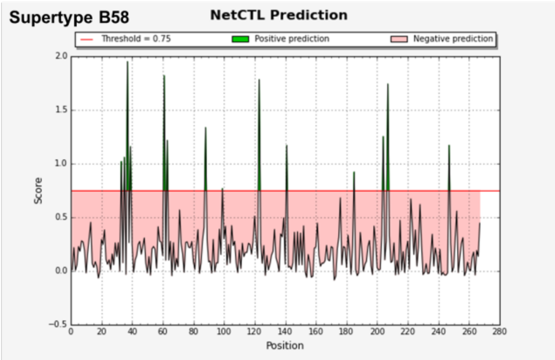
Supertype B58
NetCTL Prediction
Threshold = 0.75
Positive prediction
Negative prediction
Score
Position

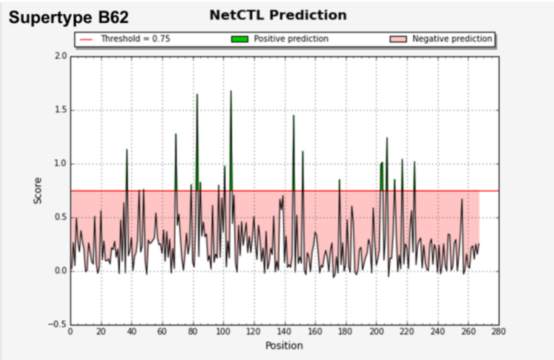
Supertype B62
NetCTL Prediction
Threshold = 0.75
Positive prediction
Negative prediction
Score
Position

ORF6 protein

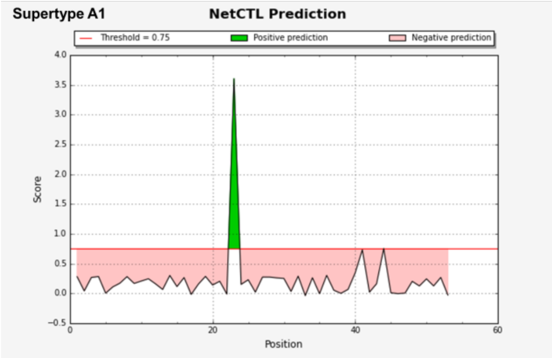

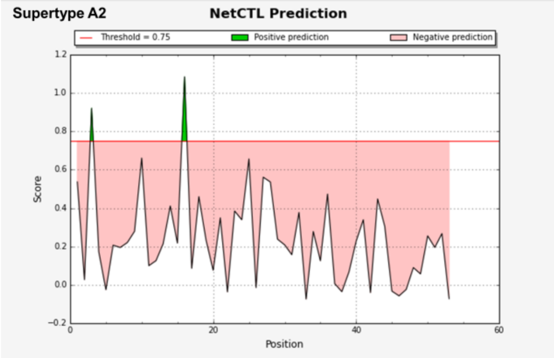

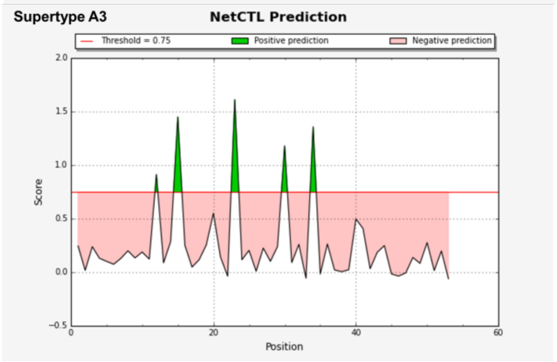

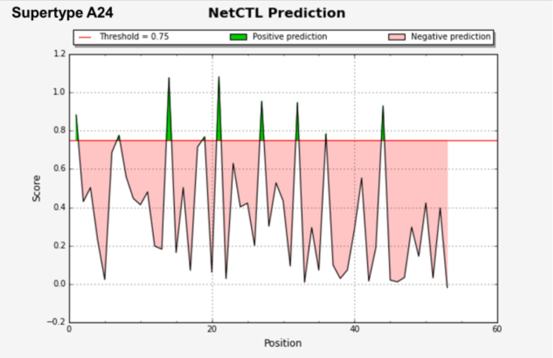

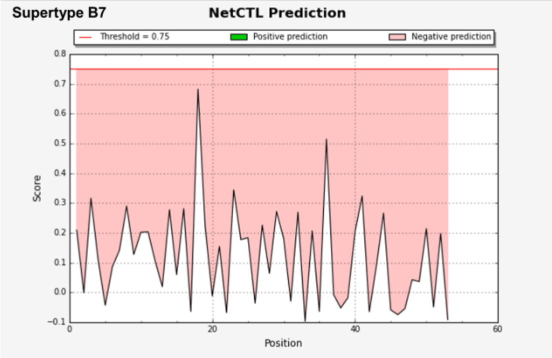

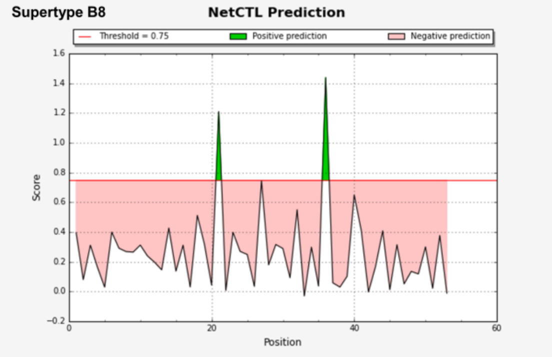

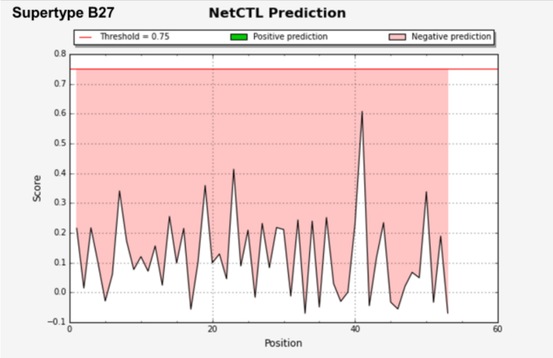

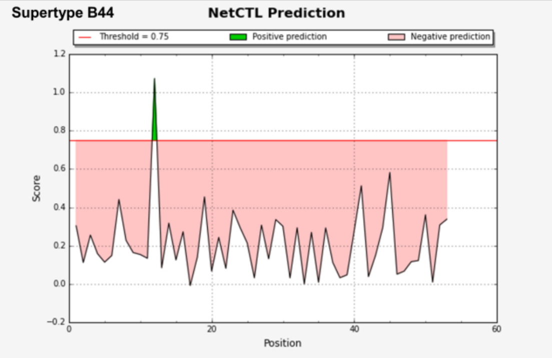

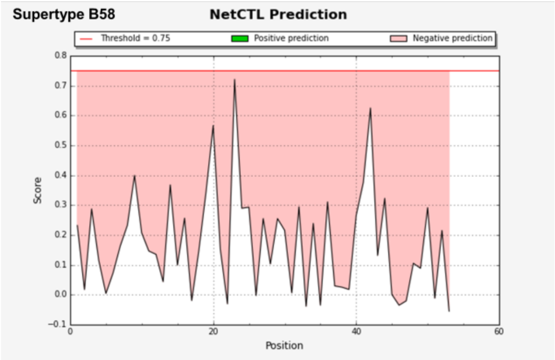

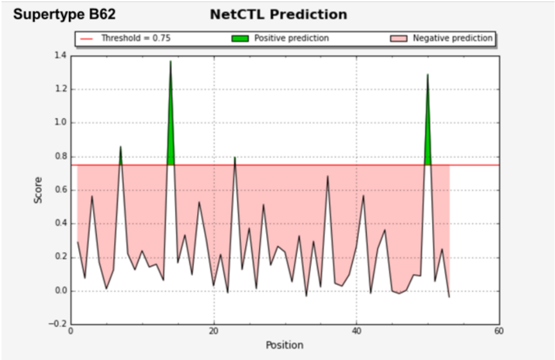

ORF7 protein

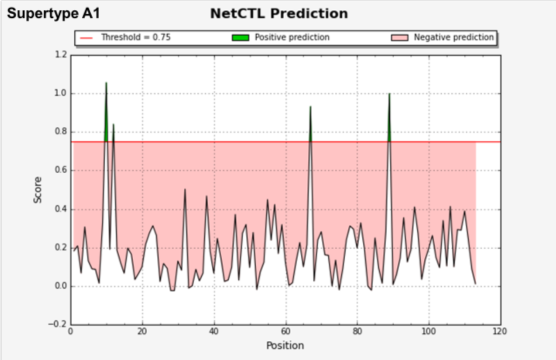

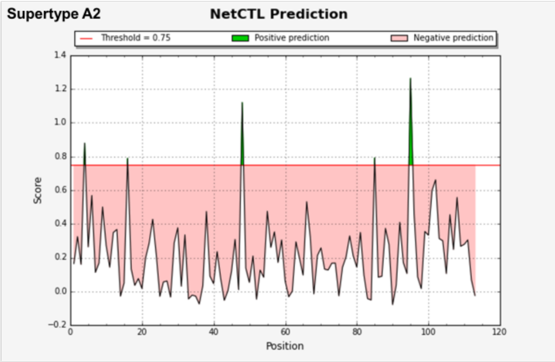

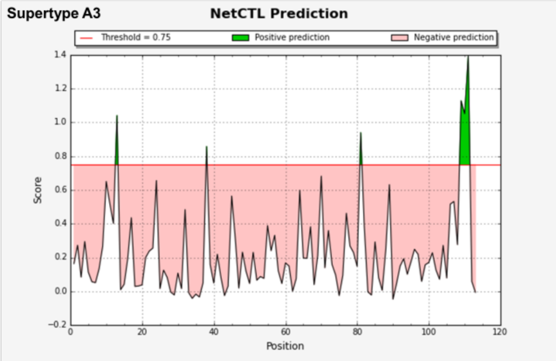

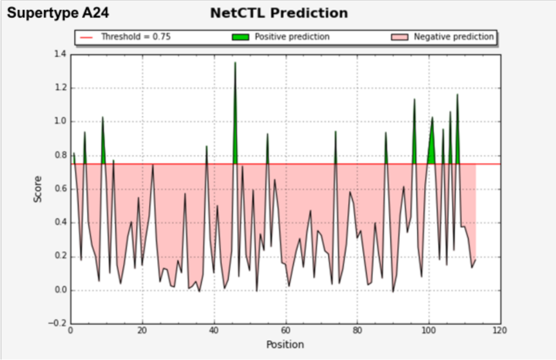

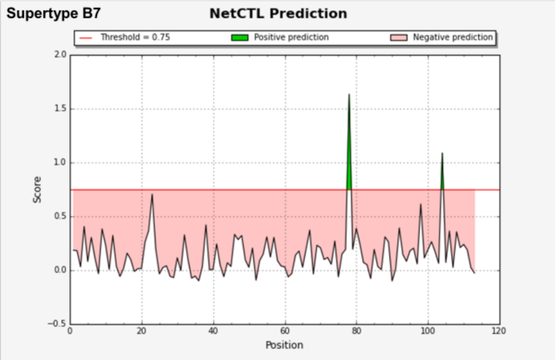

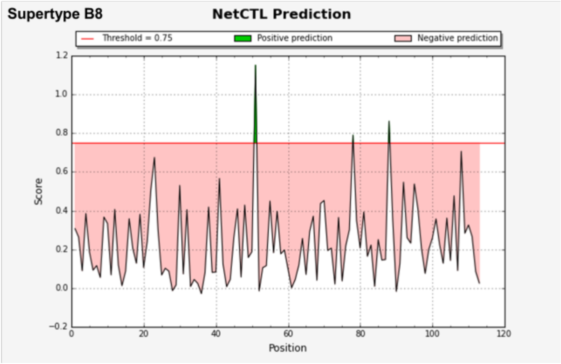

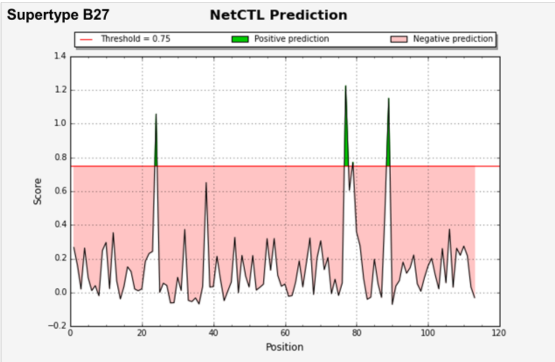

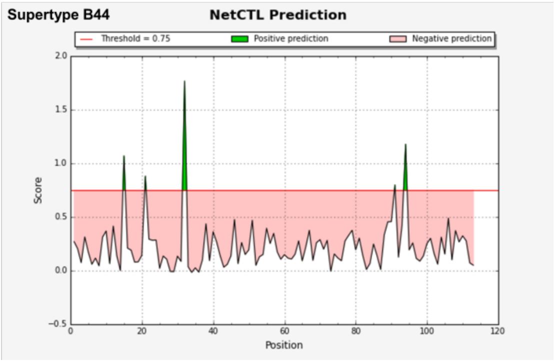

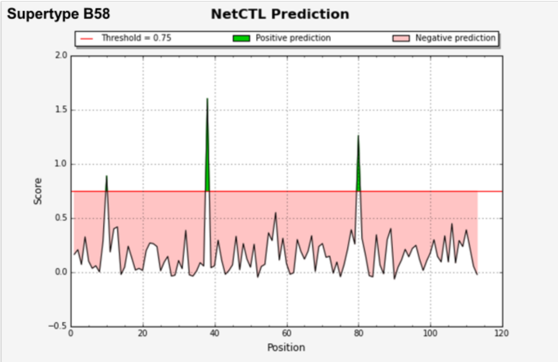

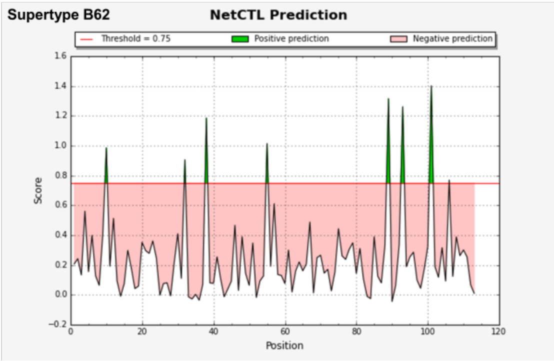

ORF8 protein

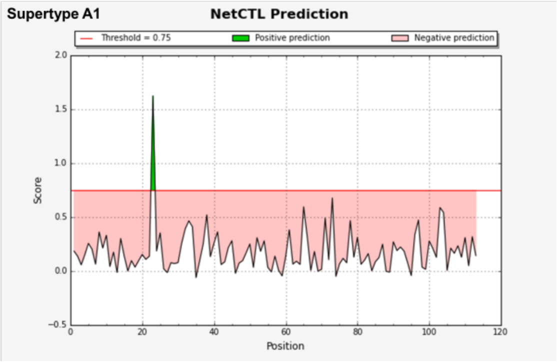

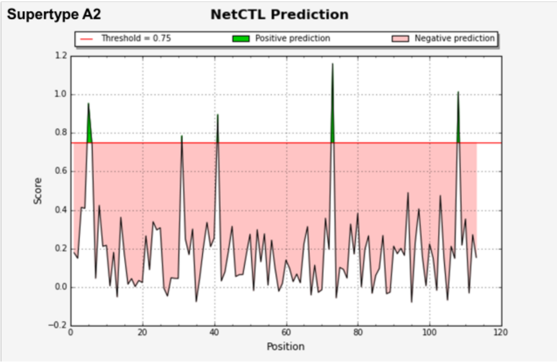

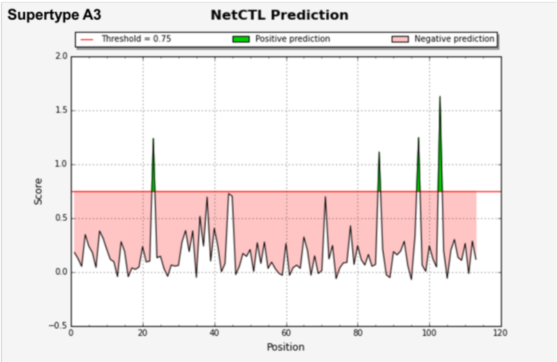

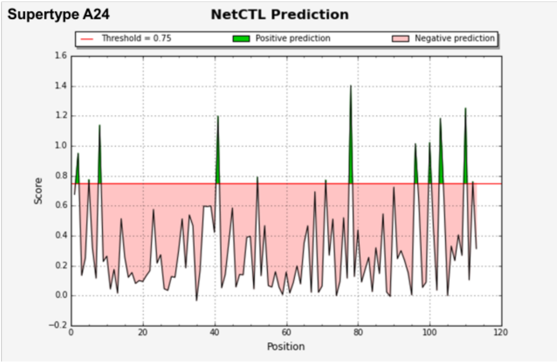

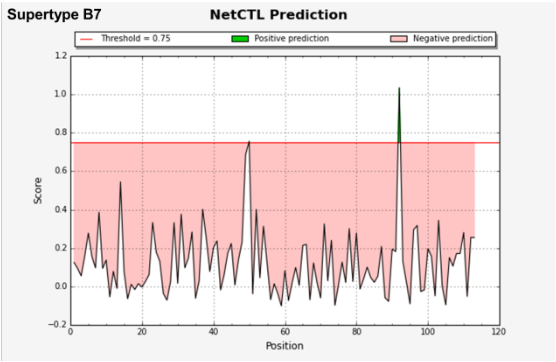
Supertype B7
NetCTL Prediction
Threshold = 0.75
Positive prediction
Negative prediction
Score
Position

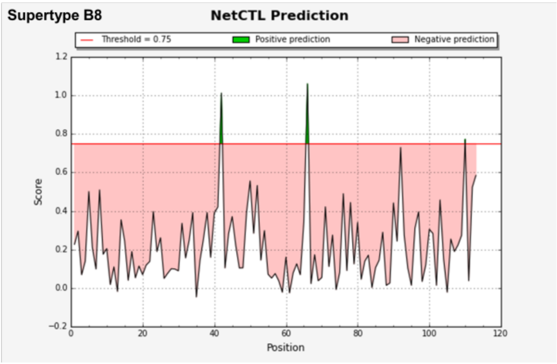
Supertype B8
NetCTL Prediction
Threshold = 0.75
Positive prediction
Negative prediction
Score
Position

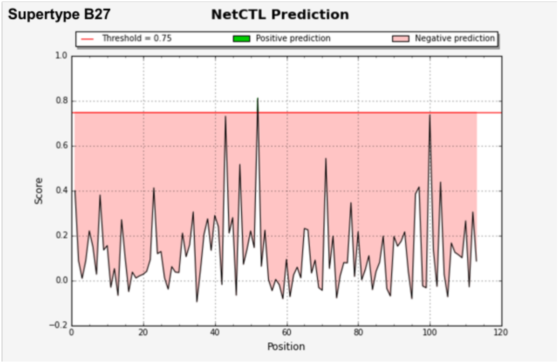
Supertype B27
NetCTL Prediction
Threshold = 0.75
Positive prediction
Negative prediction
Score
Position

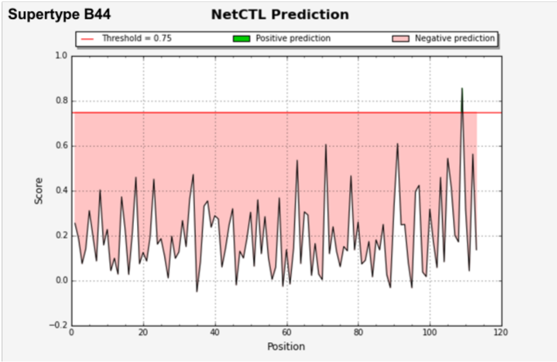
Supertype B44
NetCTL Prediction
Threshold = 0.75
Positive prediction
Negative prediction
Score
Position

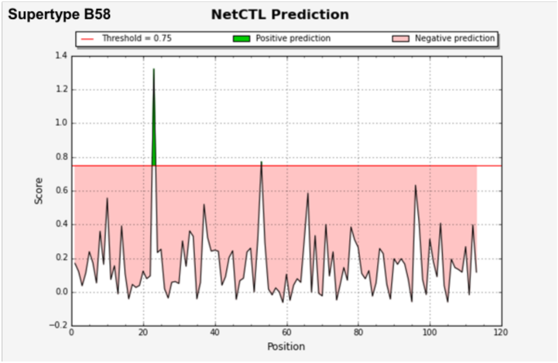
Supertype B58
NetCTL Prediction
Threshold = 0.75
Positive prediction
Negative prediction
Score
Position

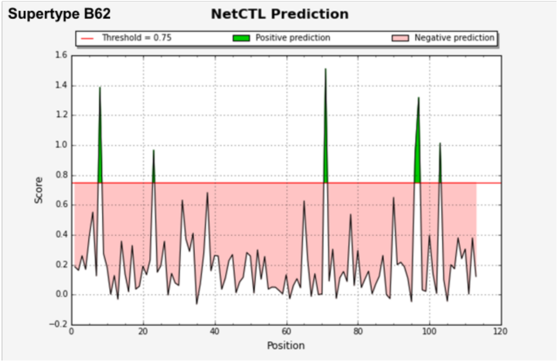
Supertype B62
NetCTL Prediction
Threshold = 0.75
Positive prediction
Negative prediction
Score
Position

ORF10

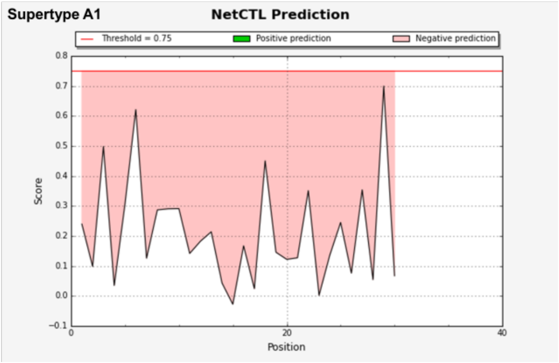
Supertype A1
NetCTL Prediction
Threshold = 0.75
Positive prediction
Negative prediction
Score
Position

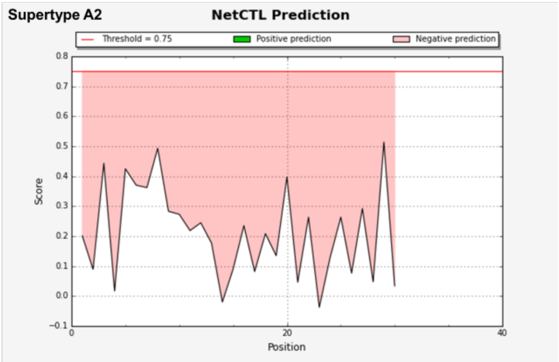
Supertype A2
NetCTL Prediction
Threshold = 0.75
Positive prediction
Negative prediction
Score
Position

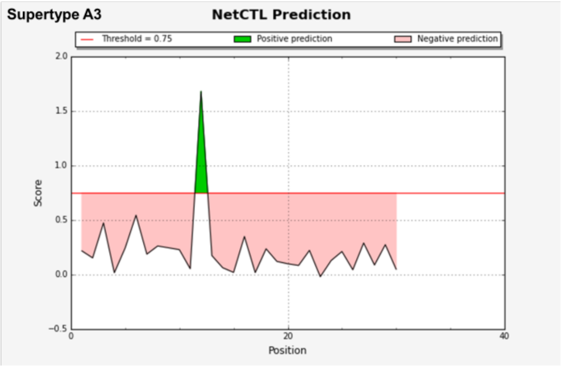
Supertype A3
NetCTL Prediction
Threshold = 0.75
Positive prediction
Negative prediction
Score
Position

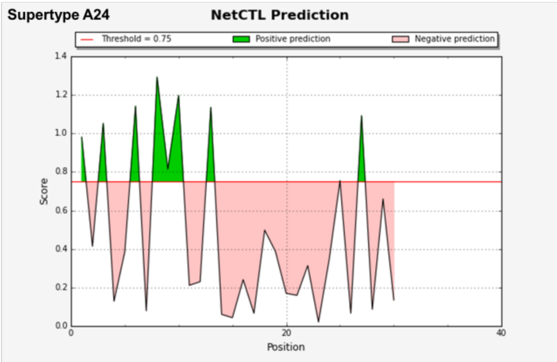
Supertype A24
NetCTL Prediction
Threshold = 0.75
Positive prediction
Negative prediction
Score
Position

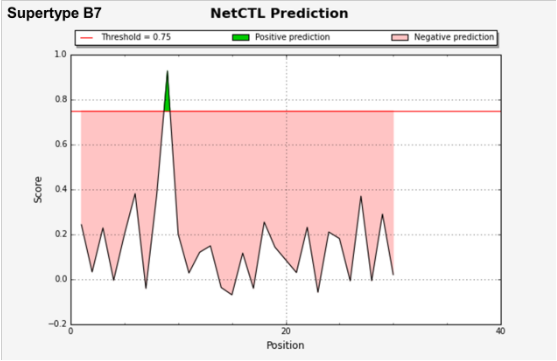
Supertype B7
NetCTL Prediction
Threshold = 0.75
Positive prediction
Negative prediction
Score
Position

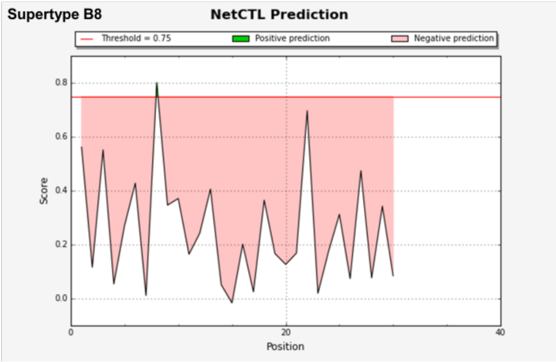
Supertype B8
NetCTL Prediction
Threshold = 0.75
Positive prediction
Negative prediction
Score
Position

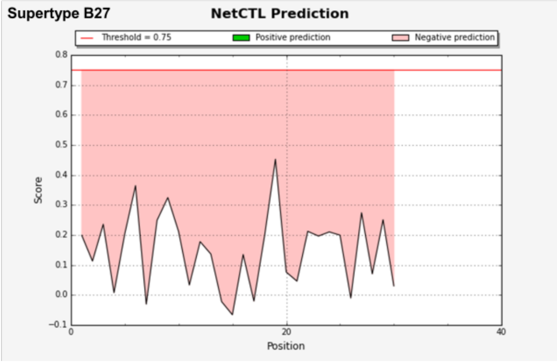
Supertype B27
NetCTL Prediction
Threshold = 0.75
Positive prediction
Negative prediction
Score
Position

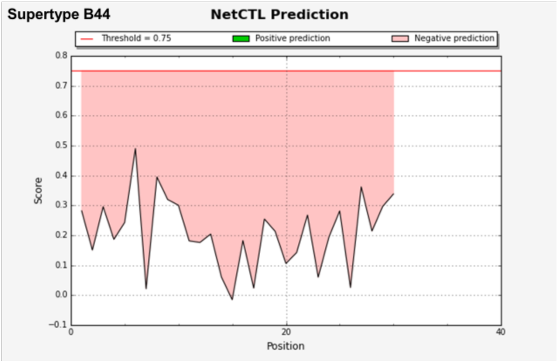
Supertype B44
NetCTL Prediction
Threshold = 0.75
Positive prediction
Negative prediction
Score
Position

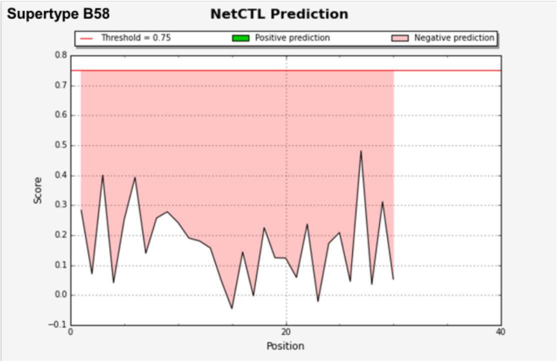
Supertype B58
NetCTL Prediction
Threshold = 0.75
Positive prediction
Negative prediction
Score
Position

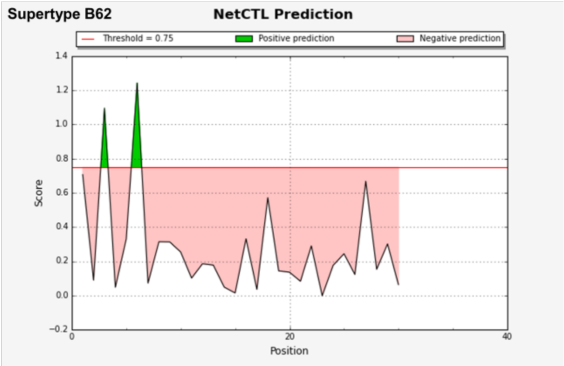
Supertype B62
NetCTL Prediction
Threshold = 0.75
Positive prediction
Negative prediction
Score
Position

S glycoprotein

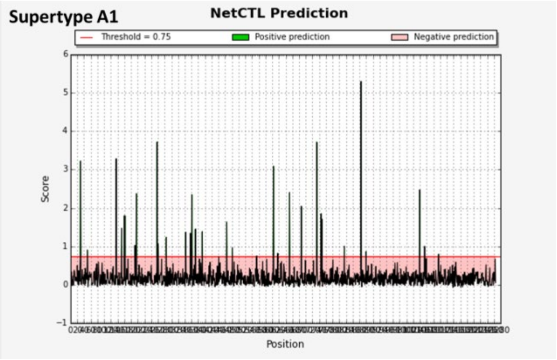

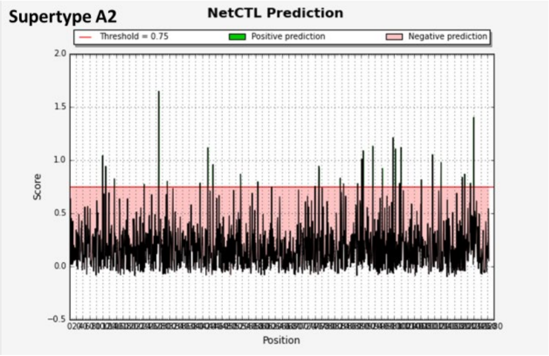

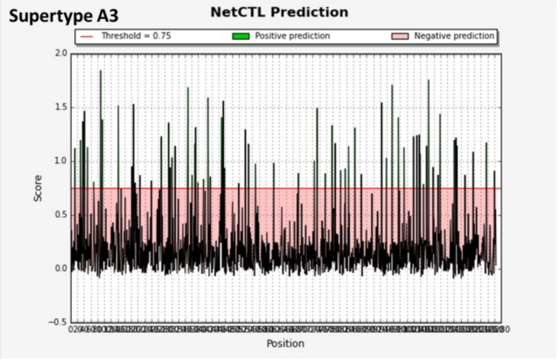

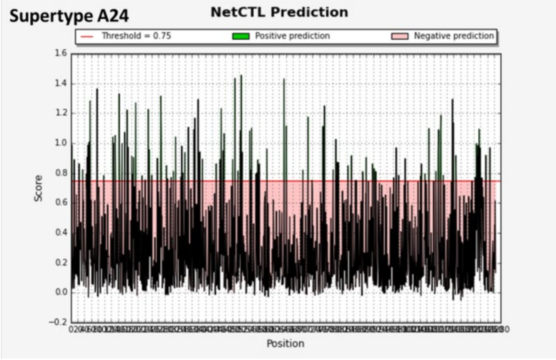

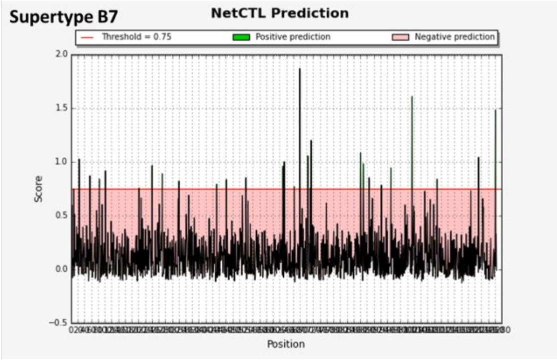

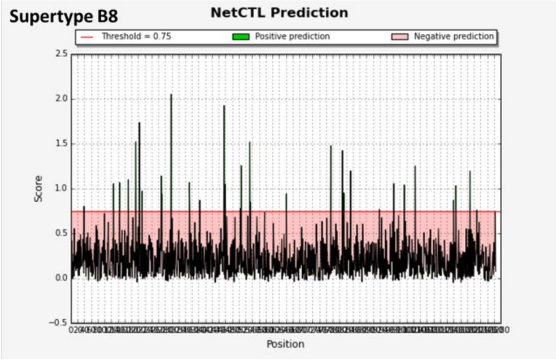

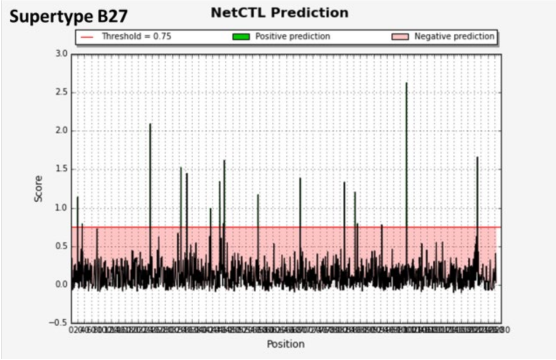

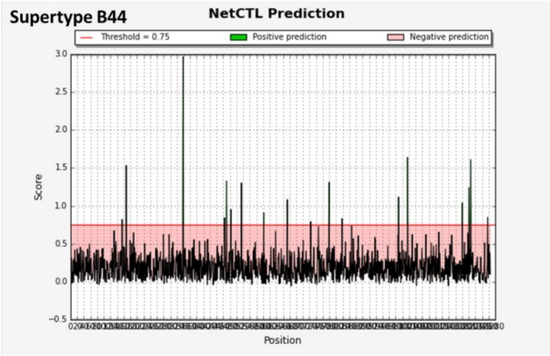

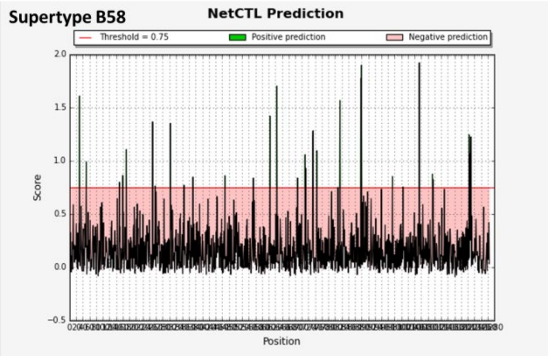

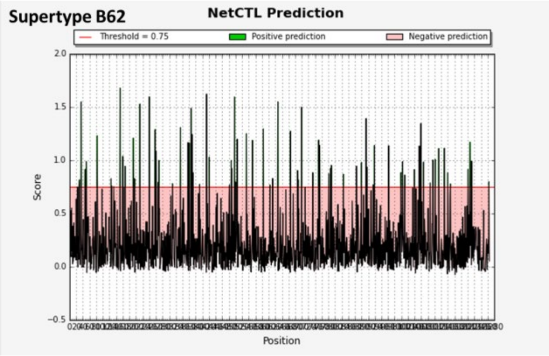

ORF1ab polyprotein

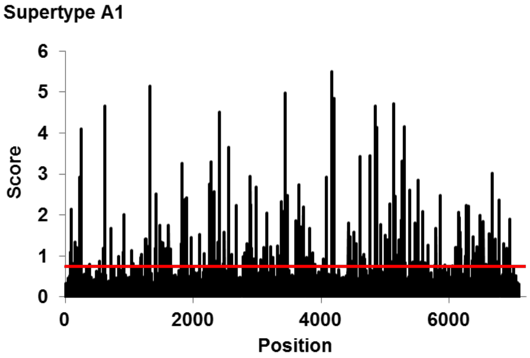

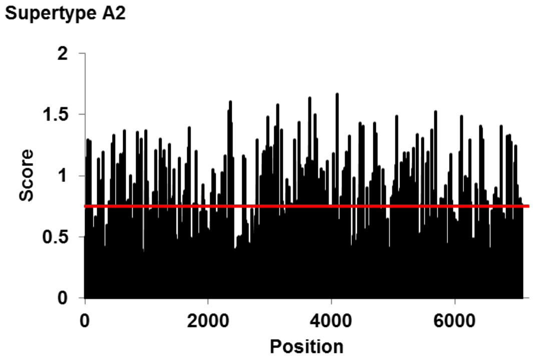

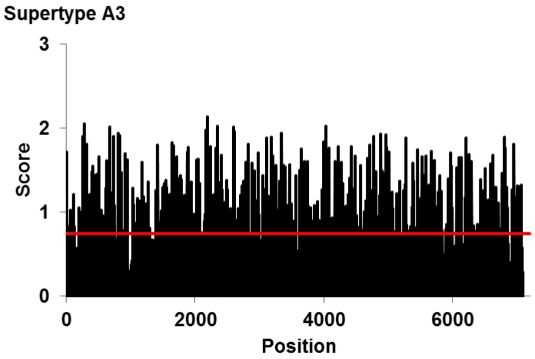

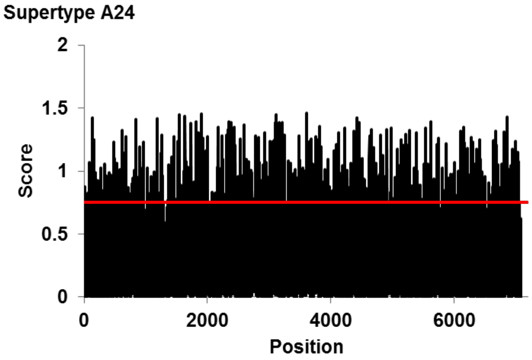

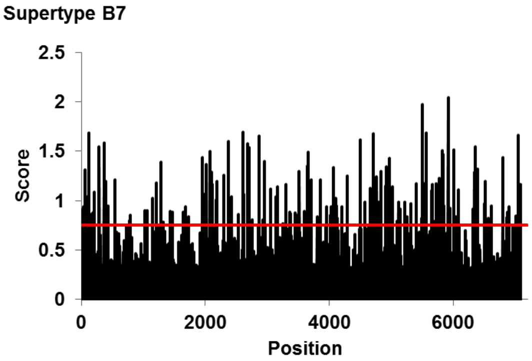

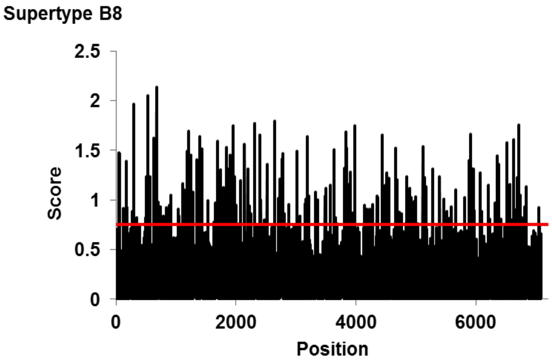

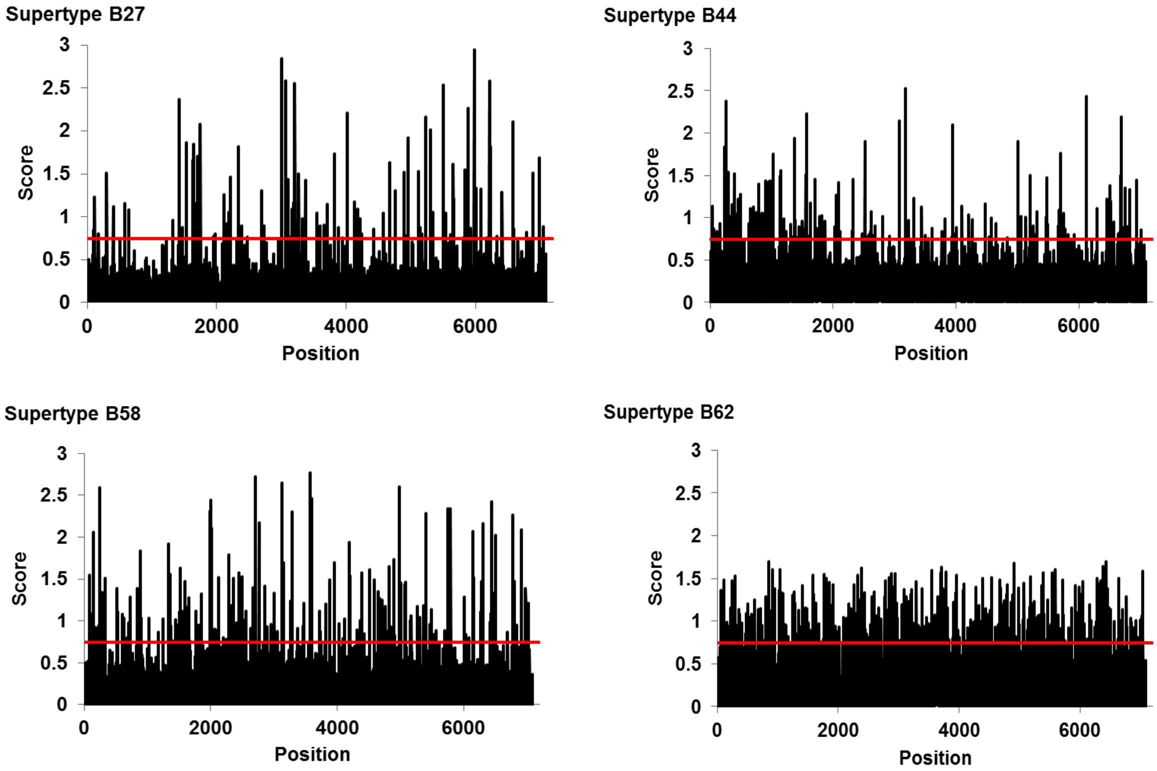

Plots illustrating the NetCTL score for each sequential peptide across the entire amino acid sequence for each SARS-CoV-2 protein. Individual panels represent the score trace for each major HLA class I supertype as indicated. The threshold for positive predictions was set at 0.75.

Figure S3. Global population coverage of top SARS-CoV-2 peptides.

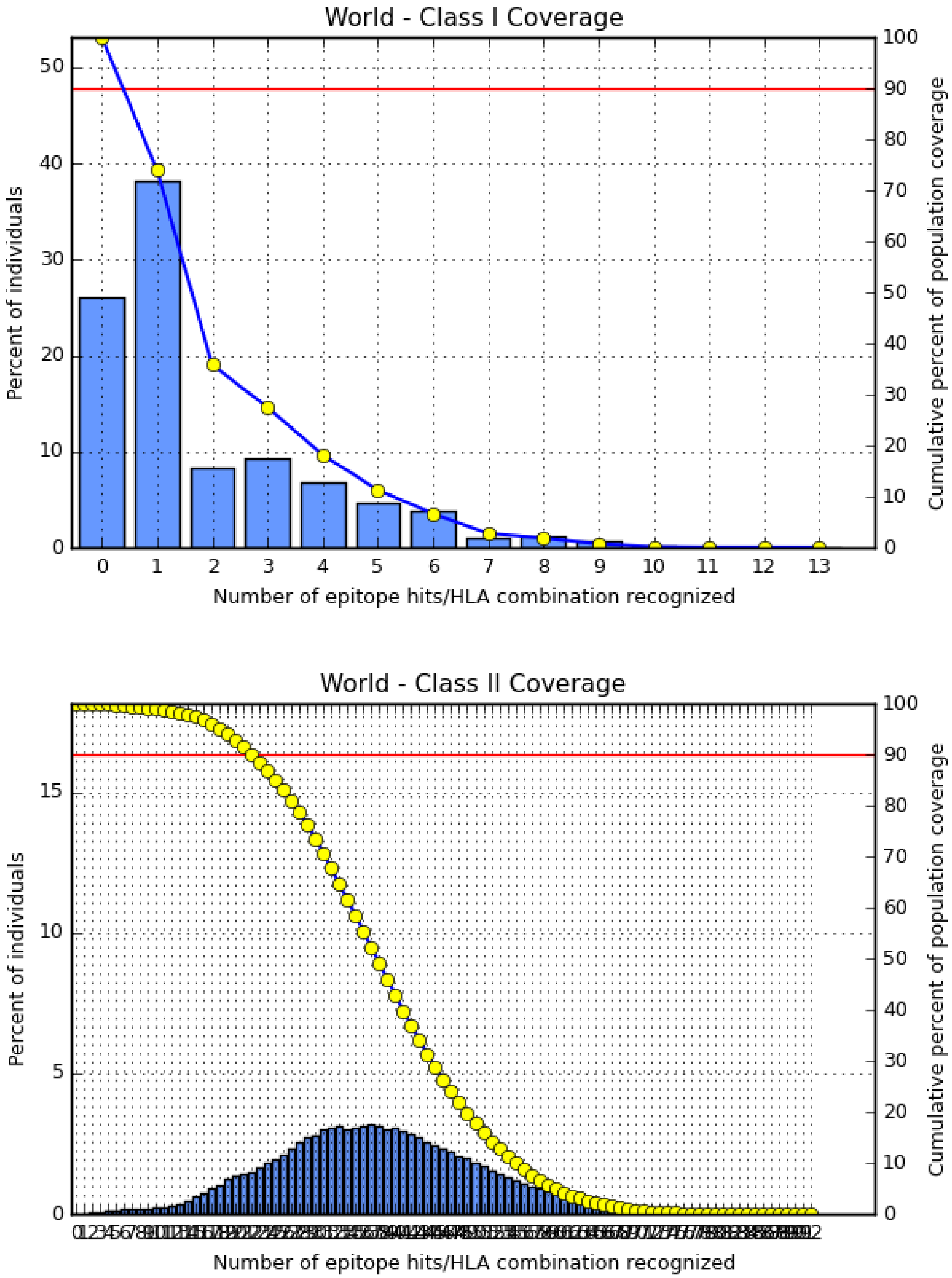

Table S1. Mutations identified across SARS-CoV-2 genomes.

Genome ID	Coding Mutation	Viral Protein[a]	Mutation Effect
MT039890	T26354A	E protein	L37H
MN994467/MT044257	T26729C	M protein	Silent
LC522972	C29300T	N protein	P344S
MN994467	A28792T	N protein	Silent
MT039888/MT027063/MT027062	C28853T	N protein	S194L
MT106052	G28878A	N protein	S202N
Several	C29094T	N protein	Silent
LC521925	DelG331-A355	ORF1ab	Deletion $G_{31}DSVEEVL_{38}$
LC521925	C1885T	ORF1ab	Silent
LC521925	C18483T	ORF1ab	P6083L
LC522972	G11554T	ORF1ab	E3765D
LC522972	C15321T	ORF1ab	Silent
LC522973	C3789T	ORF1ab	A1176V
MN975262	C9561T	ORF1ab	S3099L
MN975262	T15607C	ORF1ab	Silent
MN985325/MT020881/MT020880	C18060T	ORF1ab	Silent
MN994467	G1548A	ORF1ab	S428N
MN994468	C17000T	ORF1ab	T5579I
MN996527	G21283A	ORF1ab	D7018N
MN996529	G7004A	ORF1ab	G2251S
MN996529	A21125G	ORF1ab	K6958R
MN996531	A7988C	ORF1ab	D2579A
MN996531	C9520T	ORF1ab	T3090I
MN997409/MT049951	G11083T/C	ORF1ab	L3606F
MT007544	T19065C	ORF1ab	Silent
MT019529	A3778G	ORF1ab	Silent
MT019529	A8388G	ORF1ab	N2757S
MT019529	T8987A	ORF1ab	F2908I
MT019531	T6996C	ORF1ab	I2244T
MT019533	G7866T	ORF1ab	G2523V
MT027062/MT027063	G614A	ORF1ab	A117T
MT027063/MT027062	A5084G	ORF1ab	I1607V
MT027064	C2091T	ORF1ab	T609I
MT039887	C17373T	ORF1ab	Silent
MT039887	DelA20295-T20297	ORF1ab	L6678del
MT039888	G3518T	ORF1ab	V1085F
MT039888	G5480R (AorG)	ORF1ab	A1739X
MT039888	A17423G	ORF1ab	Y5720C
MT039890	G2971T	ORF1ab	M902I
MT039890	C6031T	ORF1ab	Silent
MT039890	C12115T	ORF1ab	Silent
MT039890	T15597C	ORF1ab	Silent
MT039890	C20936T	ORF1ab	T6891M
MT044257	T490A	ORF1ab	D75E

MT044257	C3177T	ORF1ab	P971L
MT044258	DelT508-T522	ORF1ab	Deletion $G_{82}HVMV_{86}$
MT044258	DelA671-T679	ORF1ab	Deletion $K_{141}SF_{143}$
MT066176	A9034G	ORF1ab	Silent
MT066176	C9491T	ORF1ab	H3076Y
MT093571	G2717A	ORF1ab	Silent
MT093571	A9274G	ORF1ab	Silent
MT093571	C13225G/T13226C	ORF1ab	F4321L
MT093571	A17376G	ORF1ab	Silent
MT106054	T18603C	ORF1ab	Silent
MT106054	T18975A	ORF1ab	Silent
MT106054	A19175C	ORF1ab	D6304A
Several	C8782T	ORF1ab	Silent
MT039890	G25775T	ORF3a	W128L
Several	G26144T	ORF3a	G251V
MN994467/MT044257	G28077C	ORF8	V12L
Several	T28144C	ORF8	L36S
MN994467/MT044257/MT039888	C24034T	Spike	Silent
MN996527/MT106053	A24292G	Spike	Silent
MT007544	T22303G	Spike	S247R
MT039890	C22224G	Spike	S221W
MT093571	T23952G	Spike	F797C
LC522975	G29701T	UTR	Unknown
MT007544	DelC29739-G29749	UTR	Unknown
MT019530	T104A	UTR	Unknown
MT019530	T111C	UTR	Unknown
MT019530	T112G	UTR	Unknown
MT019530	C119G	UTR	Unknown
MT019530	T120C	UTR	Unknown
MT019530	G124A	UTR	Unknown
MT049951	C75A	UTR	Unknown
MT093631	C224T	UTR	Unknown
MT106052	G29742A	UTR	Unknown

[a]UTR = untranslated region

Table S2. **Partially refined list of HLA class I epitopes from SARS-CoV-2 proteome.**

**Protein**	**Peptide**	**Predicted Alleles**	**Predicted Binding Affinity (nM)**
E	LTALRLCAY	HLA-A*01:01, HLA-B*15:01	139.4, 60.2
M	ATSRTLSYY	HLA-A*01:01, HLA-A*03:01, HLA-B*15:01, HLA-B*58:01	62.4, 184.4, 138.8, 238.6
M	RLFARTRSM	HLA-B*07:02, HLA-B*08:01, HLA-B*15:01	90.7, 27.6, 31.6
M	YANRNRFLY	HLA-A*01:01, HLA-B*15:01, HLA-B*58:01	119.2, 245.3, 65.7
M	YFIASFRLF	HLA-A*24:02, HLA-B*15:01	7.9, 415.6
N	FPRGQGVPI	HLA-B*07:02, HLA-B*08:01	4.7, 368.3

N	KAYNVTQAF	HLA-B*15:01, HLA-B*58:01	18.9, 17.7
N	KPRQKRTAT	HLA-B*07:02, HLA-B*08:01	18.8, 432.6
N	LLLDRLNQL	HLA-A*02:01, HLA-B*08:01	11.3, 136.8
N	LSPRWYFYY	HLA-A*01:01, HLA-B*58:01	76.9, 430.6
N	SPRWYFYYL	HLA-B*07:02, HLA-B*08:01	15.3, 42.1
S	FAMQMAYRF	HLA-A*24:02, HLA-B*15:01, HLA-B*58:01	142.9, 123.9, 23.4
S	MIAQYTSAL	HLA-A*02:01, HLA-B*07:02, HLA-B*08:01, HLA-B*15:01	82.1, 63.6, 80.4, 71.1
S	SIIAYTMSL	HLA-A*02:01, HLA-B*08:01, HLA-B*15:01	16.9, 239.2, 202.9
S	WTAGAAAYY	HLA-A*01:01, HLA-B*15:01, HLA-B*58:01	31.1, 65.7, 281.5
S	YLQPRTFLL	HLA-A*02:01, HLA-A*24:02, HLA-B*08:01	4.1, 201.3, 23.9
S	YRLFRKSNL	HLA-B*08:01, HLA-B*27:05	202.3, 68.3
S	YSSANNCTF	HLA-B*15:01, HLA-B*58:01	88.0, 33.1
ORF1ab	AMSAFAMMF	HLA-A*24:02, HLA-B*15:01	294.5, 19.1
ORF1ab	ASHMYCSFY	HLA-A*01:01, HLA-B*15:01	328.9, 86.2
ORF1ab	CTNYMPYFF	HLA-A*01:01, HLA-B*58:01	397.9, 30.1
ORF1ab	FLGRYMSAL	HLA-A*02:01, HLA-B*08:01	9.8, 55.1
ORF1ab	FLKTNCCRF	HLA-B*08:01, HLA-B*15:01	300.4, 51.4
ORF1ab	FLLNKEMYL	HLA-A*02:01, HLA-B*08:01	2.6, 91.4
ORF1ab	FLNRFTTTL	HLA-A*02:01, HLA-B*08:01	5.4, 83.7
ORF1ab	FTDGVCLFW	HLA-A*01:01, HLA-B*58:01	384.2, 28.6
ORF1ab	FVNLKQLPF	HLA-B*08:01, HLA-B*15:01	241.3, 68.6
ORF1ab	HSIGFDYVY	HLA-A*01:01, HLA-B*58:01	102.9, 26.2
ORF1ab	ILFTRFFYV	HLA-A*02:01, HLA-B*08:01	2.2, 130.8
ORF1ab	IMQLFFSYF	HLA-A*24:02, HLA-B*15:01	211.4, 85.8
ORF1ab	IPRRNVATL	HLA-B*07:02, HLA-B*08:01	5.3, 101.8
ORF1ab	IQLSSYSLF	HLA-A*24:02, HLA-B*15:01	50.2, 11.9
ORF1ab	ISTKHFYWF	HLA-A*24:02, HLA-B*58:01	172.1, 44.2
ORF1ab	KLAKKFDTF	HLA-A*24:02, HLA-B*15:01	195.7, 58.6
ORF1ab	KLMGHFAWW	HLA-A*24:02, HLA-B*58:01	294.3, 49.9
ORF1ab	KLVNKFLAL	HLA-A*02:01, HLA-B*08:01, HLA-B*15:01	30.4, 73.8, 236.5
ORF1ab	KMNYQVNGY	HLA-A*03:01, HLA-B*15:01	132.3, 49.1
ORF1ab	KSYELQTPF	HLA-B*15:01, HLA-B*58:01	28.5, 12.3
ORF1ab	LASHMYCSF	HLA-B*15:01, HLA-B*58:01	48.1, 66.1
ORF1ab	LLADKFPVL	HLA-A*02:01, HLA-B*08:01	11.1, 106.3
ORF1ab	LLTNMFTPL	HLA-A*02:01, HLA-B*08:01, HLA-B*15:01	27.2, 230.1, 200.1
ORF1ab	LMIERFVSL	HLA-A*02:01, HLA-B*08:01, HLA-B*15:01	6.8, 6.6, 43.2
ORF1ab	LMNVLTLVY	HLA-A*01:01, HLA-A*03:01, HLA-B*15:01	238.7, 115.2, 11.6
ORF1ab	LSFKELLVY	HLA-A*01:01, HLA-B*15:01, HLA-B*58:01	371.8, 42.6, 35.7
ORF1ab	LTNIFGTVY	HLA-A*01:01, HLA-B*15:01, HLA-B*58:01	64.6, 73.7, 479.9
ORF1ab	LVAEWFLAY	HLA-A*01:01, HLA-A*03:01,	191.6, 378.8, 32.1

		HLA-B*15:01	
ORF1ab	MFTPLVPFW	HLA-A*24:02, HLA-B*58:01	141.6, 80.6
ORF1ab	MMISAGFSL	HLA-A*02:01, HLA-B*08:01, HLA-B*15:01	6.9, 367.6, 16.2
ORF1ab	MMSAPPAQY	HLA-A*01:01, HLA-B*15:01, HLA-B*58:01	157.3, 14.4, 447.6
ORF1ab	MPYFFTLLL	HLA-B*07:02, HLA-B*08:01	103.1, 271.0
ORF1ab	MSMTYGQQF	HLA-B*15:01, HLA-B*58:01	28.1, 11.4
ORF1ab	MSNLGMPSY	HLA-A*01:01, HLA-B*15:01, HLA-B*58:01	184.2, 74.1, 87.6
ORF1ab	MVMCGGSLY	HLA-A*03:01, HLA-B*15:01	143.8, 14.1
ORF1ab	NPAWRKAVF	HLA-B*07:02, HLA-B*08:01	23.5, 158.4
ORF1ab	QLYLGGMSY	HLA-A*03:01, HLA-B*15:01	68.1, 25.2
ORF1ab	RLYYDSMSY	HLA-A*03:01, HLA-B*15:01	10.9, 7.9
ORF1ab	RMYIFFASF	HLA-A*24:02, HLA-B*15:01	196.9, 13.9
ORF1ab	RQFHQKLLK	HLA-A*03:01, HLA-B*27:05	16.9, 104.5
ORF1ab	RTAPHGHVM	HLA-B*07:02, HLA-B*15:01, HLA-B*58:01	56.4, 62.2, 189.1
ORF1ab	RTNVYLAVF	HLA-B*15:01, HLA-B*58:01	36.4, 24.4
ORF1ab	SMMGFKMNY	HLA-A*03:01, HLA-B*15:01	20.3, 23.4
ORF1ab	SSAKSASVY	HLA-A*01:01, HLA-B*15:01	444.5, 48.4
ORF1ab	SSLPSYAAF	HLA-B*15:01, HLA-B*58:01	49.2, 101.1
ORF1ab	STNVTIATY	HLA-A*01:01, HLA-B*15:01, HLA-B*58:01	241.1, 81.9, 294.5
ORF1ab	TQYNRYLAL	HLA-B*08:01, HLA-B*15:01	39.5, 73.2
ORF1ab	TVAYFNMVY	HLA-A*01:01, HLA-B*15:01	456.8, 57.8
ORF1ab	VAKSHNIAL	HLA-B*07:02, HLA-B*08:01	102.0, 98.0
ORF1ab	VMHANYIFW	HLA-A*24:02, HLA-B*58:01	188.0, 69.6
ORF1ab	VMYMGTLSY	HLA-A*03:01, HLA-B*15:01	9.2, 5.3
ORF1ab	VPMEKLKTL	HLA-B*07:02, HLA-B*08:01	17.3, 90.8
ORF1ab	VQSTQWSLF	HLA-A*24:02, HLA-B*15:01	258.7, 31.3
ORF1ab	VVYRGTTTY	HLA-A*03:01, HLA-B*15:01	70.9, 23.3
ORF1ab	WSMATYYLF	HLA-A*24:02, HLA-B*15:01, HLA-B*58:01	15.6, 42.7, 8.6
ORF1ab	YFMRFRRAF	HLA-A*24:02, HLA-B*08:01	124.5, 91.6
ORF1ab	YIFFASFYY	HLA-A*01:01, HLA-A*03:01	89.8, 195.5
ORF1ab	YLDAYNMMI	HLA-A*01:01, HLA-A*02:01	236.2, 2.9
ORF1ab	YLITPVHVM	HLA-A*02:01, HLA-B*15:01	26.5, 49.2
ORF1ab	YLRKHFSMM	HLA-B*07:02, HLA-B*08:01, HLA-B*15:01	168.3, 5.2, 37.8
ORF1ab	YMPYFFTLL	HLA-A*02:01, HLA-A*24:02	11.2, 177.5
ORF1ab	YTEISFMLW	HLA-A*01:01, HLA-B*58:01	305.4, 16.9
ORF1ab	YVMHANYIF	HLA-A*24:02, HLA-B*15:01, HLA-B*58:01	79.2, 28.8, 151.4
ORF3a	APFLYLYAL	HLA-B*07:02, HLA-B*08:01	50.1, 156.7
ORF7	ITLATCELY	HLA-A*01:01, HLA-B*58:01	285.7, 84.4

Table S3. Partially refined list of HLA class II epitopes from SARS-CoV-2 proteome.

Protein	Peptide	Predicted Alleles	Predicted Binding Affinity (nM)
E	FYVYSRVKNLNSSRV	HLA-DRB1*04:01, HLA-DRB1*04:05, HLA-DRB1*08:02, HLA-DRB1*11:01, HLA-DRB3*02:02, HLA-DRB5*01:01	50.1, 69.7, 148.1, 47.2, 130.8, 67.6
E	SFYVYSRVKNLNSSR	HLA-DRB1*11:01, HLA-DRB3*02:02, HLA-DRB5*01:01	42.3, 164.0, 62.1
E	LVTLAILTALRLCAY	HLA-DRB1*11:01, HLA-DRB1*12:01, HLA-DRB1*15:01, HLA-DRB4*01:01	82.2, 121.5, 75.5, 97.4
M	TLSYYKLGASQRVAG	HLA-DRB1*01:01, HLA-DRB5*01:01	7.3, 16.7
M	RTLSYYKLGASQRVA	HLA-DRB1*01:01, HLA-DRB1*04:01, HLA-DRB1*04:05, HLA-DRB1*07:01, HLA-DRB1*09:01	7.5, 71.3, 118.8, 31.5, 25.6
M	ASFRLFARTRSMWSF	HLA-DRB1*01:01, HLA-DRB1*07:01, HLA-DRB1*08:02, HLA-DRB1*09:01, HLA-DRB1*11:01, HLA-DRB5*01:01, HLA-DPA1*02:01/DPB1*05:01, HLA-DPA1*02:01/DPB1*14:01	19.2, 30.9, 53.5, 49.9, 12.2, 16.3, 256.2, 387.3
M	IASFRLFARTRSMWS	HLA-DRB1*01:01, HLA-DRB1*04:05	23.9, 77.8
M	LLQFAYANRNRFLYI	HLA-DRB1*03:01, HLA-DRB1*07:01, HLA-DRB1*08:02, HLA-DRB1*11:01, HLA-DRB1*13:02, HLA-DRB3*02:02, HLA-DRB5*01:01	179.8, 58.2, 225.6, 36.2, 27.8, 46.6, 26.3
M	SFRLFARTRSMWSFN	HLA-DRB1*04:01, HLA-DRB1*04:05	47.5, 53.7
M	ETNILLNVPLHGTIL	HLA-DRB1*08:02, HLA-DRB1*13:02, HLA-DRB3*02:02	228.4, 24.3, 66.5
M	LSYFIASFRLFARTR	HLA-DRB1*11:01, HLA-DRB1*15:01, HLA-DPA1*02:01/DPB1*01:01,HLA-DPA1*03:01/DPB1*04:02, HLA-DPA1*02:01/DPB1*05:01	27.4, 36.3, 75.5, 94.8, 112.8
M	ILRGHLRIAGHHLGR	HLA-DRB1*11:01, HLA-DRB4*01:01, HLA-DRB5*01:01	37.6, 87.1, 34.2
M	MWLSYFIASFRLFAR	HLA-DRB1*15:01, HLA-DPA1*02:01/DPB1*01:01,HLA-DPA1*01:03/DPB1*02:01, HLA-DPA1*01:03/DPB1*04:01, HLA-DPA1*03:01/DPB1*04:02, HLA-DPA1*02:01/DPB1*05:01	31.8, 63.4, 29.8, 42.5, 79.9, 133.8

M	YRINWITGGIAIAMA	HLA-DQA1*05:01/DQB1*03:01, HLA-DQA1*01:02/DQB1*06:02	20.2, 56.4
M	GLMWLSYFIASFRLF	HLA-DQA1*01:01/DQB1*05:01, HLA-DPA1*01:03/DPB1*02:01, HLA-DPA1*01:03/DPB1*04:01	141.4, 38.2, 54.4
M	SYFIASFRLFARTRS	HLA-DPA1*01:03/DPB1*02:01, HLA-DPA1*01:03/DPB1*04:01	52.6, 73.7
N	QIAQFAPSASAFFGM	HLA-DRB1*07:01, HLA-DRB1*09:01, HLA-DQA1*05:01/DQB1*03:01	32.2, 30.1, 57.8
N	QIGYYRRATRRIRGG	HLA-DRB1*08:02, HLA-DRB1*11:01	111.2, 14.9
N	QQTVTLLPAADLDDF	HLA-DQA1*05:01/DQB1*02:01, HLA-DQA1*04:01/DQB1*04:02	188.6, 453.6
S	AAEIRASANLAATKM	HLA-DRB1*08:02, HLA-DRB1*13:02, HLA-DRB3*02:02, HLA-DQA1*01:02/DQB1*06:02, HLA-DPA1*02:01/DPB1*14:01	101.3, 23.0, 52.7, 141.5, 327.4
S	ALQIPFAMQMAYRFN	HLA-DRB1*09:01, HLA-DRB1*12:01, HLA-DRB1*15:01	52.9, 159.5, 50.3
S	AQKFNGLTVLPPLLT	HLA-DRB1*04:01, HLA-DRB1*04:05	63.1, 46.9
S	DLFLPFFSNVTWFHA	HLA-DRB1*04:01, HLA-DRB1*04:05, HLA-DPA1*01:03/DPB1*02:01, HLA-DPA1*01:03/DPB1*04:01	74.8, 60.5, 64.7, 100.0
S	DSSSGWTAGAAAYYV	HLA-DQA1*05:01/DQB1*03:01, HLA-DQA1*04:01/DQB1*04:02	22.9, 455.2
S	FGEVFNATRFASVYA	HLA-DRB1*07:01, HLA-DPA1*02:01/DPB1*01:01, HLA-DPA1*01:03/DPB1*02:01, HLA-DPA1*01:03/DPB1*04:01, HLA-DPA1*03:01/DPB1*04:02, HLA-DPA1*02:01/DPB1*05:01, HLA-DPA1*02:01/DPB1*14:01	25.5, 35.9, 19.6, 24.6, 55.0, 160.5, 278.3
S	GINITRFQTLLALHR	HLA-DRB1*04:01, HLA-DRB1*04:05, HLA-DRB1*12:01, HLA-DRB1*15:01, HLA-DPA1*02:01/DPB1*01:01, HLA-DPA1*03:01/DPB1*04:02, HLA-DPA1*02:01/DPB1*14:01	64.3, 45.1, 145.1, 27.6, 106.0, 167.6, 433.6
S	GNYNYLYRLFRKSNL	HLA-DRB1*15:01, HLA-DRB5*01:01	57.5, 31.5
S	GWTFGAGAALQIPFA	HLA-DRB1*01:01, HLA-DRB1*09:01, HLA-DQA1*05:01/DQB1*03:01	16.2, 24.8, 20.8
S	IIAYTMSLGAENSVA	HLA-DRB1*01:01, HLA-DRB1*04:01, HLA-DRB1*09:01, HLA-DQA1*01:02/DQB1*06:02	8.9, 55.0, 24.1, 171.3
S	ITRFQTLLALHRSYL	HLA-DRB1*01:01, HLA-DRB1*12:01, HLA-DRB1*15:01, HLA-DRB5*01:01, HLA-DPA1*02:01/DPB1*05:01, HLA-DPA1*02:01/DPB1*14:01	10.7, 108.3, 34.9, 10.1, 156.2, 318.0
S	ITSGWTFGAGAALQI	HLA-DRB1*09:01, HLA-	36.2, 24.4

		DQA1*05:01/DQB1*03:01	
S	KRSFIEDLLFNKVTL	HLA-DPA1*02:01/DPB1*01:01, HLA-DPA1*01:03/DPB1*02:01, HLA-DPA1*01:03/DPB1*04:01, HLA-DPA1*03:01/DPB1*04:02, HLA-DPA1*02:01/DPB1*05:01	51.5, 36.1, 50.3, 68.5, 158.4
S	LFLPFFSNVTWFHAI	HLA-DRB1*04:05, HLA-DPA1*01:03/DPB1*02:01, HLA-DPA1*01:03/DPB1*04:01	62.9, 53.2, 83.1
S	LQIPFAMQMAYRFNG	HLA-DRB4*01:01, HLA-DRB5*01:01	68.8, 20.9
S	NCTFEYVSQPFLMDL	HLA-DPA1*02:01/DPB1*01:01, HLA-DPA1*01:03/DPB1*02:01, HLA-DPA1*01:03/DPB1*04:01, HLA-DPA1*03:01/DPB1*04:02	53.3, 30.9, 41.3, 70.1
S	NITRFQTLLALHRSY	HLA-DRB1*04:01, HLA-DRB1*04:05, HLA-DRB1*08:02, HLA-DRB4*01:01	57.3, 41.9, 125.1, 54.1
S	NYNYLYRLFRKSNLK	HLA-DRB1*08:02, HLA-DRB1*11:01, HLA-DRB1*15:01, HLA-DRB5*01:01, HLA-DPA1*02:01/DPB1*05:01	148.6, 16.6, 52.9, 168.8
S	PINLVRDLPQGFSAL	HLA-DRB1*03:01, HLA-DRB3*01:01	51.7, 56.8
S	PYRVVVLSFELLHAP	HLA-DPA1*02:01/DPB1*01:01, HLA-DPA1*01:03/DPB1*02:01, HLA-DPA1*01:03/DPB1*04:01, HLA-DPA1*03:01/DPB1*04:02	79.6, 53.3, 77.1, 92.9
S	QDLFLPFFSNVTWFH	HLA-DRB1*04:01, HLA-DRB1*15:01	88.4, 44.6
S	QPYRVVVLSFELLHA	HLA-DPA1*02:01/DPB1*01:01, HLA-DPA1*01:03/DPB1*02:01, HLA-DPA1*01:03/DPB1*04:01, HLA-DPA1*03:01/DPB1*04:02, HLA-DPA1*02:01/DPB1*05:01	73.2, 50.2, 71.4, 90.1, 211.1
S	QQLIRAAEIRASANL	HLA-DRB1*08:02, HLA-DRB4*01:01, HLA-DQA1*01:02/DQB1*06:02, HLA-DPA1*02:01/DPB1*14:01	76.1, 42.9, 53.9, 229.4
S	QSLLIVNNATNVVIK	HLA-DRB1*04:01, HLA-DRB1*13:02, HLA-DRB3*02:02	77.2, 5.9, 13.1
S	REGVFVSNGTHWFVT	HLA-DRB1*13:02, HLA-DRB3*02:02	21.4, 25.8
S	RSFIEDLLFNKVTLA	HLA-DPA1*01:03/DPB1*02:01, HLA-DPA1*01:03/DPB1*04:01	45.4, 63.1
S	SIIAYTMSLGAENSV	HLA-DRB1*04:05, HLA-DRB1*07:01	68.4, 35.7
S	TQLNRALTGIAVEQD	HLA-DQA1*03:01/DQB1*03:02, HLA-DQA1*04:01/DQB1*04:02	445.8, 373.3
S	TRFQTLLALHRSYLT	HLA-DRB1*11:01, HLA-DRB1*12:01	20.9, 117.1
S	TSNFRVQPTESIVRF	HLA-DRB1*04:01, HLA-DRB1*04:05	52.4, 83.9
S	VFNATRFASVYAWNR	HLA-DRB1*09:01, HLA-DQA1*01:02/DQB1*06:02	48.7, 141.4

S	VNFNFNGLTGTGVLT	HLA-DRB1*01:01, HLA-DRB1*09:01	14.5, 47.2
S	YQPYRVVVLSFELLH	HLA-DPA1*02:01/DPB1*01:01, HLA-DPA1*01:03/DPB1*04:01, HLA-DPA1*03:01/DPB1*04:02, HLA-DPA1*02:01/DPB1*05:01	102.2, 93.0, 127.5, 299.3
ORF1ab	AAIFYLITPVHVMSK	HLA-DRB1*01:01, HLA-DRB1*04:01, HLA-DRB1*04:05, HLA-DRB1*07:01, HLA-DRB1*08:02, HLA-DRB1*09:01, HLA-DRB1*15:01	9.9, 77.9, 59.6, 21.3, 116.2, 49.2, 49.4
ORF1ab	AARYMRSLKVPATVS	HLA-DRB1*01:01, HLA-DRB1*04:01, HLA-DRB1*04:05, HLA-DRB1*08:02, HLA-DRB1*11:01, HLA-DRB4*01:01, HLA-DPA1*02:01/DPB1*14:01	10.9, 64.7, 82.9, 54.4, 23.9, 59.1, 249.3
ORF1ab	ACRKVQHMVVKAALL	HLA-DRB4*01:01, HLA-DPA1*02:01/DPB1*14:01	77.5, 486.3
ORF1ab	AFGLVAEWFLAYILF	HLA-DPA1*01:03/DPB1*02:01, HLA-DPA1*01:03/DPB1*04:01	40.2, 66.6
ORF1ab	AHIQWMVMFTPLVPF	HLA-DQA1*01:01/DQB1*05:01, HLA-DPA1*03:01/DPB1*04:02	285.1, 141.4
ORF1ab	AIILASFSASTSAFV	HLA-DRB1*01:01, HLA-DRB1*04:01, HLA-DRB1*04:05, HLA-DQA1*05:01/DQB1*03:01, HLA-DQA1*01:02/DQB1*06:02	13.8, 76.6, 87.5, 49.3, 152.2
ORF1ab	AKRFKESPFELEDFI	HLA-DPA1*02:01/DPB1*01:01, HLA-DPA1*03:01/DPB1*04:02	73.2, 146.7
ORF1ab	ALDISASIVAGGIVA	HLA-DQA1*05:01/DQB1*03:01, HLA-DQA1*01:02/DQB1*06:02	32.9, 73.9
ORF1ab	AMMFTSDLATNNLVV	HLA-DRB1*04:01, HLA-DRB3*01:01	77.5, 63.1
ORF1ab	ANYIFWRNTNPIQLS	HLA-DRB1*04:05, HLA-DRB1*07:01, HLA-DRB1*13:02	89.9, 35.2, 13.5
ORF1ab	ARVVRSIFSRTLETA	HLA-DRB1*15:01, HLA-DPA1*02:01/DPB1*05:01	55.9, 282.2
ORF1ab	ARYMRSLKVPATVSV	HLA-DRB1*04:01, HLA-DRB1*04:05, HLA-DRB1*08:02	64.7, 87.3, 54.4
ORF1ab	ASFNYLKSPNFSKLI	HLA-DRB1*01:01, HLA-DRB1*04:01, HLA-DRB1*07:01, HLA-DRB5*01:01	12.1, 66.5, 36.4, 28.8
ORF1ab	AVNLLTNMFTPLIQP	HLA-DPA1*01:03/DPB1*02:01, HLA-DPA1*01:03/DPB1*04:01, HLA-DPA1*03:01/DPB1*04:02	55.8, 95.0, 142.2
ORF1ab	AVVLLILMTARTVYD	HLA-DRB1*01:01, HLA-DRB1*08:02, HLA-DRB4*01:01	14.5, 140.1, 77.3
ORF1ab	CTNYMPYFFTLLLQL	HLA-DPA1*02:01/DPB1*01:01, HLA-DPA1*01:03/DPB1*02:01, HLA-DPA1*01:03/DPB1*04:01, HLA-DPA1*03:01/DPB1*04:02	108.2, 40.8, 54.1, 112.4
ORF1ab	DMILSLLSKGRLIIR	HLA-DRB1*12:01, HLA-DRB1*15:01, HLA-DRB4*01:01	85.4, 38.9, 52.9
ORF1ab	EAARYMRSLKVPATV	HLA-DRB1*01:01, HLA-DRB1*15:01, HLA-DRB4*01:01, HLA-DRB5*01:01, HLA-	11.5, 50.4, 64.5, 31.0, 288.8

		DPA1*02:01/DPB1*14:01	
ORF1ab	EASFNYLKSPNFSKL	HLA-DRB1*04:05, HLA-DRB1*09:01, HLA-DRB5*01:01	69.6, 47.8, 33.6
ORF1ab	EKLKTLVATAEAELA	HLA-DQA1*05:01/DQB1*02:01, HLA-DQA1*03:01/DQB1*03:02, HLA-DQA1*04:01/DQB1*04:02, HLA-DPA1*02:01/DPB1*14:01	117.7, 309.6, 233.5, 476.4
ORF1ab	ELLVYAADPAMHAAS	HLA-DRB1*01:01, HLA-DRB1*03:01, HLA-DRB1*04:01, HLA-DRB1*04:05, HLA-DRB3*01:01, HLA-DRB3*02:02, HLA-DQA1*05:01/DQB1*02:01	14.9, 99.3, 37.3, 83.0, 29.6, 66.6, 240.7
ORF1ab	EMYLKLRSDVLLPLT	HLA-DRB3*01:01, HLA-DPA1*02:01/DPB1*01:01, HLA-DPA1*03:01/DPB1*04:02	56.9, 93.5, 148.9
ORF1ab	ERLKLFAAETLKATE	HLA-DRB1*01:01, HLA-DRB1*04:01, HLA-DRB1*04:05, HLA-DPA1*03:01/DPB1*04:02, HLA-DPA1*02:01/DPB1*05:01	11.9, 47.4, 61.4, 164.7, 160.2
ORF1ab	ERYKLEGYAFEHIVY	HLA-DPA1*02:01/DPB1*01:01, HLA-DPA1*01:03/DPB1*02:01, HLA-DPA1*01:03/DPB1*04:01	101.7, 61.4, 83.1
ORF1ab	EWFLAYILFTRFFYV	HLA-DPA1*02:01/DPB1*01:01, HLA-DPA1*01:03/DPB1*02:01, HLA-DPA1*01:03/DPB1*04:01, HLA-DPA1*03:01/DPB1*04:02, HLA-DPA1*02:01/DPB1*05:01	79.6, 26.3, 33.1, 73.4, 287.7
ORF1ab	EYSHVVAFNTLLFLM	HLA-DPA1*01:03/DPB1*02:01, HLA-DPA1*01:03/DPB1*04:01, HLA-DPA1*03:01/DPB1*04:02	59.2, 98.5, 147.3
ORF1ab	FAMGIIAMSAFAMMF	HLA-DRB1*12:01, HLA-DRB1*15:01, HLA-DQA1*05:01/DQB1*03:01	141.0, 38.6, 60.1
ORF1ab	FGEYSHVVAFNTLLF	HLA-DRB1*04:05, HLA-DRB1*07:01, HLA-DRB1*09:01	85.9, 29.7, 48.3
ORF1ab	FKWDLTAFGLVAEWF	HLA-DQA1*05:01/DQB1*02:01, HLA-DQA1*03:01/DQB1*03:02, HLA-DQA1*04:01/DQB1*04:02	178.3, 425.3, 349.3
ORF1ab	FLELAMDEFIERYKL	HLA-DRB1*03:01, HLA-DPA1*02:01/DPB1*01:01, HLA-DPA1*03:01/DPB1*04:02, HLA-DPA1*02:01/DPB1*05:01	98.0, 86.9, 147.1, 288.8
ORF1ab	FYAYLRKHFSMMILS	HLA-DRB1*07:01, HLA-DRB1*09:01, HLA-DRB1*15:01, HLA-DRB4*01:01, HLA-DPA1*02:01/DPB1*05:01, HLA-DPA1*02:01/DPB1*14:01	27.8, 49.7, 24.7, 87.6, 202.6, 420.7
ORF1ab	GDQFKHLIPLMYKGL	HLA-DRB1*04:05, HLA-DRB1*12:01, HLA-DRB5*01:01	80.6, 157.9, 31.4
ORF1ab	GDYFVLTSHTVMPLS	HLA-DRB1*01:01, HLA-DRB1*04:01, HLA-DRB1*04:05, HLA-DRB1*07:01, HLA-DRB1*09:01	10.0, 77.4, 83.9, 19.0, 42.9
ORF1ab	HIQWMVMFTPLVPFW	HLA-DQA1*01:01/DQB1*05:01, HLA-DPA1*02:01/DPB1*01:01,	293.1, 116.3, 84.6, 135.4

		HLA-DPA1*01:03/DPB1*04:01, HLA-DPA1*03:01/DPB1*04:02	
ORF1ab	HQKLLKSIAATRGAT	HLA-DRB1*01:01, HLA-DRB1*08:02, HLA-DRB5*01:01	9.9, 55.9, 16.7
ORF1ab	IASEFSSLPSYAAFA	HLA-DRB1*04:01, HLA-DRB1*04:05, HLA-DRB1*09:01	40.8, 47.9, 36.8
ORF1ab	IDFLELAMDEFIERY	HLA-DQA1*01:01/DQB1*05:01, HLA-DPA1*02:01/DPB1*01:01, HLA-DPA1*03:01/DPB1*04:02	114.7, 83.4, 151.0
ORF1ab	IINLVQMAPISAMVR	HLA-DRB1*01:01, HLA-DRB1*08:02, HLA-DRB4*01:01	12.8, 118.8, 54.7
ORF1ab	IKNFKSVLYYQNNVF	HLA-DPA1*02:01/DPB1*01:01, HLA-DPA1*01:03/DPB1*04:01, HLA-DPA1*02:01/DPB1*05:01	103.0, 83.0, 251.4
ORF1ab	INLVQMAPISAMVRM	HLA-DRB1*12:01, HLA-DRB4*01:01, HLA-DQA1*01:02/DQB1*06:02, HLA-DPA1*02:01/DPB1*14:01	176.9, 57.1, 116.5, 398.6
ORF1ab	IVFMCVEYCPIFFIT	HLA-DPA1*02:01/DPB1*01:01, HLA-DPA1*01:03/DPB1*02:01, HLA-DPA1*01:03/DPB1*04:01, HLA-DPA1*03:01/DPB1*04:02	116.2, 53.9, 70.9, 144.9
ORF1ab	IVTALRANSAVKLQN	HLA-DRB1*08:02, HLA-DRB1*13:02, HLA-DRB3*02:02, HLA-DPA1*02:01/DPB1*14:01	115.9, 9.4, 19.5, 408.7
ORF1ab	KEIKESVQTFFKLVN	HLA-DPA1*02:01/DPB1*01:01, HLA-DPA1*01:03/DPB1*02:01, HLA-DPA1*01:03/DPB1*04:01	107.1, 64.3, 94.0
ORF1ab	KEMYLKLRSDVLLPL	HLA-DRB1*01:01, HLA-DRB1*04:05, HLA-DPA1*02:01/DPB1*05:01	11.0, 68.0, 285.6
ORF1ab	KGRLIIRENNRVVIS	HLA-DRB1*12:01, HLA-DRB1*13:02, HLA-DRB1*15:01, HLA-DRB4*01:01	170.9, 9.5, 48.2, 58.8
ORF1ab	KHFYWFFSNYLKRRV	HLA-DRB1*15:01, HLA-DPA1*02:01/DPB1*01:01, HLA-DPA1*01:03/DPB1*02:01, HLA-DPA1*01:03/DPB1*04:01, HLA-DPA1*03:01/DPB1*04:02, HLA-DPA1*02:01/DPB1*05:01	38.8, 57.3, 27.1, 38.3, 88.8, 115.3
ORF1ab	KSAFYILPSIISNEK	HLA-DRB1*01:01, HLA-DRB1*04:01, HLA-DRB1*04:05, HLA-DRB1*08:02	9.3, 49.3, 47.5, 96.3
ORF1ab	KTPKYKFVRIQPGQT	HLA-DRB1*04:01, HLA-DRB1*04:05, HLA-DRB1*08:02	77.4, 90.3, 112.3
ORF1ab	KYKFVRIQPGQTFSV	HLA-DRB1*01:01, HLA-DRB1*07:01, HLA-DRB1*08:02, HLA-DRB1*09:01, HLA-DRB1*11:01, HLA-DRB1*15:01, HLA-DRB4*01:01, HLA-DRB5*01:01, HLA-DPA1*02:01/DPB1*14:01	7.2, 38.8, 67.3, 31.4, 30.6, 51.5, 58.3, 21.5, 421.1
ORF1ab	KYLYFIKGLNNLNRG	HLA-DRB1*08:02, HLA-DRB1*11:01, HLA-DRB1*15:01, HLA-DRB5*01:01	139.3, 38.1, 40.6, 32.8

ORF1ab	LASFSASTSAFVETV	HLA-DRB1*07:01, HLA-DRB1*09:01, HLA-DRB3*02:02, HLA-DQA1*05:01/DQB1*03:01, HLA-DQA1*03:01/DQB1*03:02, HLA-DQA1*04:01/DQB1*04:02, HLA-DQA1*01:02/DQB1*06:02	13.0, 20.8, 84.8, 62.8, 418.9, 302.9, 147.1
ORF1ab	LCTFLLNKEMYLKLR	HLA-DRB3*02:02, HLA-DPA1*02:01/DPB1*01:01, HLA-DPA1*01:03/DPB1*02:01, HLA-DPA1*01:03/DPB1*04:01, HLA-DPA1*03:01/DPB1*04:02	43.0, 96.4, 63.1, 92.6, 130.6
ORF1ab	LEETKFLTENLLLYI	HLA-DPA1*02:01/DPB1*01:01, HLA-DPA1*01:03/DPB1*02:01, HLA-DPA1*01:03/DPB1*04:01, HLA-DPA1*03:01/DPB1*04:02, HLA-DPA1*02:01/DPB1*05:01	36.9, 27.9, 37.3, 41.1, 145.1
ORF1ab	LFFFLYENAFLPFAM	HLA-DRB1*04:05, HLA-DQA1*05:01/DQB1*02:01, HLA-DQA1*01:01/DQB1*05:01, HLA-DPA1*02:01/DPB1*01:01, HLA-DPA1*01:03/DPB1*02:01, HLA-DPA1*01:03/DPB1*04:01, HLA-DPA1*03:01/DPB1*04:02, HLA-DPA1*02:01/DPB1*05:01	72.4, 188.7, 136.1, 31.2, 14.9, 21.5, 37.1, 155.7
ORF1ab	LGSLIYSTAALGVLM	HLA-DRB1*01:01, HLA-DRB1*07:01, HLA-DRB1*09:01, HLA-DQA1*01:02/DQB1*06:02	14.0, 18.3, 36.1, 156.5
ORF1ab	LIVTALRANSAVKLQ	HLA-DRB1*01:01, HLA-DRB1*07:01, HLA-DRB4*01:01, HLA-DQA1*01:02/DQB1*06:02, HLA-DPA1*02:01/DPB1*14:01	8.8, 39.2, 78.6, 142.5, 368.3
ORF1ab	LKHFFFAQDGNAAIS	HLA-DRB1*01:01, HLA-DRB1*04:01, HLA-DRB1*04:05, HLA-DRB3*01:01, HLA-DRB3*02:02	12.2, 33.6, 66.2, 32.8, 67.0
ORF1ab	LLKSIAATRGATVVI	HLA-DRB1*01:01, HLA-DRB1*07:01, HLA-DRB1*09:01, HLA-DRB1*13:02, HLA-DQA1*01:02/DQB1*06:02, HLA-DPA1*02:01/DPB1*14:01	10.9, 14.9, 26.5, 25.2, 161.4, 346.8
ORF1ab	LNSIIKTIQPRVEKK	HLA-DRB1*08:02, HLA-DRB1*12:01, HLA-DRB4*01:01	143.2, 170.3, 52.0
ORF1ab	LPSYAAFATAQEAYE	HLA-DQA1*05:01/DQB1*02:01, HLA-DQA1*03:01/DQB1*03:02, HLA-DQA1*04:01/DQB1*04:02	163.1, 306.4, 191.8
ORF1ab	LSRVLGLKTLATHGL	HLA-DRB1*08:02, HLA-DRB1*12:01, HLA-DRB4*01:01	101.6, 184.9, 81.0
ORF1ab	LTFYLTNDVSFLAHI	HLA-DRB1*03:01, HLA-DRB1*04:05, HLA-DRB3*01:01, HLA-DRB3*02:02	89.2, 70.5, 30.5, 40.4
ORF1ab	LVNKFLALCADSIII	HLA-DRB1*01:01, HLA-DRB1*04:01, HLA-DRB1*04:05, HLA-DRB1*07:01, HLA-DRB1*09:01, HLA-DRB1*15:01, HLA-DQA1*05:01/DQB1*02:01,	7.4, 56.7, 44.8, 29.9, 33.8, 59.3, 158.9, 234.9

		HLA-DQA1*01:01/DQB1*05:01	
ORF1ab	LVRKIFVDGVPFVVS	HLA-DRB1*03:01, HLA-DRB1*13:02, HLA-DRB3*01:01	46.5, 25.6, 22.8
ORF1ab	LYAFASEAARVVRSI	HLA-DRB1*07:01, HLA-DQA1*05:01/DQB1*03:01, HLA-DPA1*02:01/DPB1*14:01	28.5, 31.7, 313.2
ORF1ab	MIERFVSLAIDAYPL	HLA-DRB1*04:05, HLA-DQA1*05:01/DQB1*02:01, HLA-DQA1*04:01/DQB1*04:02, HLA-DPA1*02:01/DPB1*14:01	83.0, 96.3, 428.4, 392.1
ORF1ab	MLRIMASLVLARKHT	HLA-DRB1*11:01, HLA-DRB1*12:01, HLA-DRB1*15:01, HLA-DRB5*01:01	34.2, 97.2, 33.9, 23.7
ORF1ab	MPNMLRIMASLVLAR	HLA-DRB1*08:02, HLA-DRB4*01:01, HLA-DQA1*01:02/DQB1*06:02	98.1, 38.6, 93.7
ORF1ab	MQLFFSYFAVHFISN	HLA-DQA1*01:01/DQB1*05:01, HLA-DPA1*01:03/DPB1*02:01, HLA-DPA1*01:03/DPB1*04:01, HLA-DPA1*03:01/DPB1*04:02	232.8, 30.3, 45.4, 103.5
ORF1ab	MTYRRLISMMGFKMN	HLA-DRB1*01:01, HLA-DRB1*04:01, HLA-DRB1*04:05, HLA-DRB1*08:02, HLA-DRB1*09:01, HLA-DRB1*11:01, HLA-DRB5*01:01, HLA-DPA1*02:01/DPB1*14:01	6.4, 26.7, 27.7, 60.9, 42.4, 12.2, 13.5, 294.7
ORF1ab	NEYRLYLDAYNMMIS	HLA-DRB1*04:05, HLA-DRB3*01:01, HLA-DQA1*01:01/DQB1*05:01	85.5, 44.4, 160.5
ORF1ab	NFNVLFSTVFPPTSF	HLA-DPA1*02:01/DPB1*01:01, HLA-DPA1*01:03/DPB1*02:01, HLA-DPA1*01:03/DPB1*04:01, HLA-DPA1*03:01/DPB1*04:02	88.8, 34.5, 51.0, 127.9
ORF1ab	NGDFLHFLPRVFSAV	HLA-DRB1*11:01, HLA-DRB1*12:01, HLA-DRB1*15:01	30.2, 198.0, 50.0
ORF1ab	NLPFKLTCATTRQVV	HLA-DRB1*07:01, HLA-DRB1*09:01, HLA-DRB5*01:01	35.9, 58.6, 23.9
ORF1ab	NLYDKLVSSFLEMKS	HLA-DPA1*01:03/DPB1*02:01, HLA-DPA1*01:03/DPB1*04:01, HLA-DPA1*03:01/DPB1*04:02, HLA-DPA1*02:01/DPB1*05:01	36.7, 45.5, 64.9, 140.4
ORF1ab	NMLRIMASLVLARKH	HLA-DRB1*03:01, HLA-DQA1*01:02/DQB1*06:02, HLA-DPA1*02:01/DPB1*05:01, HLA-DPA1*02:01/DPB1*14:01	80.3, 164.2, 190.9, 210.1
ORF1ab	NNCYLATALLTLQQI	HLA-DPA1*02:01/DPB1*01:01, HLA-DPA1*01:03/DPB1*02:01, HLA-DPA1*01:03/DPB1*04:01, HLA-DPA1*03:01/DPB1*04:02	111.3, 58.7, 79.7, 112.8
ORF1ab	PASRELKVTFFPDLN	HLA-DPA1*02:01/DPB1*01:01, HLA-DPA1*01:03/DPB1*02:01, HLA-DPA1*01:03/DPB1*04:01, HLA-DPA1*03:01/DPB1*04:02	76.9, 48.9, 64.3, 149.5
ORF1ab	PFAMGIIAMSAFAMM	HLA-DRB1*01:01, HLA-DRB1*09:01, HLA-	12.3, 57.6, 45.6

		DQA1*05:01/DQB1*03:01	
ORF1ab	PNMLRIMASLVLARK	HLA-DRB1*01:01, HLA-DRB1*07:01, HLA-DRB1*08:02, HLA-DRB1*09:01, HLA-DRB1*12:01, HLA-DRB1*15:01, HLA-DRB4*01:01	8.9, 30.9, 82.4, 56.9, 69.9, 22.2, 34.2
ORF1ab	QESPFVMMSAPPAQY	HLA-DRB1*01:01, HLA-DRB1*04:05, HLA-DRB3*02:02	5.0, 36.2, 53.3
ORF1ab	QGLVASIKNFKSVLY	HLA-DRB1*04:01, HLA-DRB1*04:05, HLA-DRB1*08:02, HLA-DRB1*11:01, HLA-DRB1*12:01, HLA-DRB4*01:01, HLA-DRB5*01:01	18.3, 62.8, 86.5, 37.7, 115.4, 86.6, 24.5
ORF1ab	QKLLKSIAATRGATV	HLA-DRB1*01:01, HLA-DRB1*08:02, HLA-DRB4*01:01, HLA-DPA1*02:01/DPB1*14:01	9.1, 55.2, 73.1, 198.6
ORF1ab	QLCQYLNTLTLAVPY	HLA-DRB1*01:01, HLA-DRB1*04:01, HLA-DRB1*04:05, HLA-DRB1*07:01	16.1, 66.8, 65.9, 35.8
ORF1ab	QMEIDFLELAMDEFI	HLA-DQA1*05:01/DQB1*02:01, HLA-DQA1*03:01/DQB1*03:02, HLA-DQA1*01:01/DQB1*05:01	57.2, 450.7, 125.8
ORF1ab	QMNLKYAISAKNRAR	HLA-DRB1*01:01, HLA-DRB1*04:01, HLA-DRB1*08:02, HLA-DRB1*09:01, HLA-DRB1*11:01, HLA-DRB3*02:02, HLA-DPA1*02:01/DPB1*14:01	14.9, 56.9, 49.1, 45.2, 22.1, 84.9, 158.3
ORF1ab	QQKLALGGSVAIKIT	HLA-DRB1*01:01, HLA-DRB1*07:01, HLA-DRB1*09:01, HLA-DQA1*05:01/DQB1*03:01	12.6, 23.4, 32.3, 42.9
ORF1ab	QTFFKLVNKFLALCA	HLA-DRB1*08:02, HLA-DRB1*12:01, HLA-DRB1*15:01, HLA-DPA1*02:01/DPB1*01:01, HLA-DPA1*01:03/DPB1*02:01	133.7, 197.3, 48.8, 81.5, 53.4
ORF1ab	QWLTNIFGTVYEKLK	HLA-DPA1*02:01/DPB1*01:01, HLA-DPA1*01:03/DPB1*02:01, HLA-DPA1*01:03/DPB1*04:01, HLA-DPA1*03:01/DPB1*04:02	60.0, 36.2, 50.9, 97.1
ORF1ab	QYNRYLALYNKYKYF	HLA-DRB1*15:01, HLA-DRB5*01:01, HLA-DPA1*02:01/DPB1*05:01	50.0, 27.1, 242.9
ORF1ab	RFKESPFELEDFIPM	HLA-DPA1*02:01/DPB1*01:01, HLA-DPA1*01:03/DPB1*02:01, HLA-DPA1*01:03/DPB1*04:01, HLA-DPA1*03:01/DPB1*04:02	74.0, 65.9, 81.9, 130.6
ORF1ab	RFYFYTSKTTVASLI	HLA-DRB1*01:01, HLA-DRB1*04:01, HLA-DRB1*04:05, HLA-DRB1*07:01, HLA-DRB1*08:02, HLA-DRB1*09:01, HLA-DRB3*02:02, HLA-DPA1*02:01/DPB1*14:01	10.1, 44.5, 75.7, 17.5, 98.6, 30.5, 46.1, 466.8
ORF1ab	RIKIVQMLSDTLKNL	HLA-DRB1*04:01, HLA-DRB1*04:05, HLA-DRB1*15:01, HLA-DRB4*01:01	78.1, 58.8, 55.3, 43.4
ORF1ab	RLKLFAAETLKATEE	HLA-DRB1*08:02, HLA-	100.9, 58.6, 109.5

		DRB1*15:01, HLA-DPA1*02:01/DPB1*01:01	
ORF1ab	RRLISMMGFKMNYQV	HLA-DRB1*08:02, HLA-DRB1*11:01, HLA-DPA1*02:01/DPB1*05:01	134.2, 30.5, 251.6
ORF1ab	SAFAMMFVKHKHAFL	HLA-DRB1*08:02, HLA-DRB1*11:01, HLA-DRB1*15:01, HLA-DRB4*01:01, HLA-DRB5*01:01	110.4, 18.3, 50.9, 79.2, 15.1
ORF1ab	SFLAHIQWMVMFTPL	HLA-DPA1*02:01/DPB1*01:01, HLA-DPA1*01:03/DPB1*02:01, HLA-DPA1*01:03/DPB1*04:01, HLA-DPA1*03:01/DPB1*04:02	103.9, 47.8, 70.7, 140.6
ORF1ab	SHKLVLSVNPYVCNA	HLA-DRB1*04:01, HLA-DRB1*04:05, HLA-DRB1*08:02, HLA-DRB1*13:02, HLA-DRB1*15:01, HLA-DRB3*02:02	49.7, 72.9, 138.5, 11.4, 41.4, 32.5
ORF1ab	SHRFYRLANECAQVL	HLA-DRB1*01:01, HLA-DRB1*04:01, HLA-DRB1*04:05	14.3, 50.4, 56.9
ORF1ab	SIGFDYVYNPFMIDV	HLA-DPA1*02:01/DPB1*01:01, HLA-DPA1*01:03/DPB1*02:01, HLA-DPA1*01:03/DPB1*04:01, HLA-DPA1*03:01/DPB1*04:02	108.9, 47.1, 81.9, 137.6
ORF1ab	SLLMPILTLTRALTA	HLA-DRB1*11:01, HLA-DRB1*12:01, HLA-DRB4*01:01	37.3, 122.2, 59.9
ORF1ab	SPFVMMSAPPAQYEL	HLA-DRB1*08:02, HLA-DRB1*09:01, HLA-DQA1*05:01/DQB1*03:01	58.3, 33.8, 65.6
ORF1ab	SPLYAFASEAARVVR	HLA-DRB1*09:01, HLA-DQA1*01:02/DQB1*06:02, HLA-DPA1*02:01/DPB1*14:01	28.9, 173.6, 397.7
ORF1ab	SVQTFFKLVNKFLAL	HLA-DRB1*11:01, HLA-DPA1*02:01/DPB1*01:01, HLA-DPA1*01:03/DPB1*04:01, HLA-DPA1*03:01/DPB1*04:02, HLA-DPA1*02:01/DPB1*05:01	23.5, 76.9, 70.7, 111.5, 113.3
ORF1ab	TCLAYYFMRFRRAFG	HLA-DRB1*08:02, HLA-DRB1*11:01, HLA-DRB1*15:01	143.9, 16.4, 44.7
ORF1ab	TDFVNEFYAYLRKHF	HLA-DPA1*02:01/DPB1*01:01, HLA-DPA1*01:03/DPB1*02:01, HLA-DPA1*01:03/DPB1*04:01, HLA-DPA1*03:01/DPB1*04:02, HLA-DPA1*02:01/DPB1*05:01	90.9, 46.9, 67.2, 144.4, 239.2
ORF1ab	TEETFKLSYGIATVR	HLA-DRB1*01:01, HLA-DRB1*07:01, HLA-DRB1*09:01	8.7, 21.8, 25.9
ORF1ab	TEKYCALAPNMMVTN	HLA-DRB1*01:01, HLA-DRB1*04:01, HLA-DRB1*04:05, HLA-DRB1*09:01	8.5, 55.1, 71.1, 27.8
ORF1ab	TERLKLFAAETLKAT	HLA-DRB1*09:01, HLA-DRB1*15:01, HLA-DPA1*02:01/DPB1*01:01, HLA-DPA1*02:01/DPB1*05:01, HLA-DPA1*02:01/DPB1*14:01	51.9, 46.6, 99.4, 161.5, 214.9
ORF1ab	TEVNEFACVVADAVI	HLA-DQA1*05:01/DQB1*02:01, HLA-DQA1*03:01/DQB1*03:02,	136.8, 424.7, 351.1

		HLA-DQA1*04:01/DQB1*04:02	
ORF1ab	TNSRIKASMPTTIAK	HLA-DRB1*07:01, HLA-DRB1*09:01, HLA-DRB1*13:02	38.1, 51.7, 20.6
ORF1ab	TRYVLMDGSIIQFPN	HLA-DRB1*01:01, HLA-DRB1*03:01, HLA-DRB1*04:01, HLA-DRB1*04:05	10.7, 46.6, 58.2, 85.6
ORF1ab	TSAMQTMLFTMLRKL	HLA-DPA1*02:01/DPB1*01:01, HLA-DPA1*01:03/DPB1*04:01, HLA-DPA1*03:01/DPB1*04:02, HLA-DPA1*02:01/DPB1*05:01	98.6, 87.4, 113.4, 162.8
ORF1ab	TYRRLISMMGFKMNY	HLA-DRB1*12:01, HLA-DRB1*15:01, HLA-DRB4*01:01, HLA-DRB5*01:01, HLA-DPA1*02:01/DPB1*05:01	66.9, 15.3, 34.3, 13.9, 166.5
ORF1ab	VFTGYRVTKNSKVQI	HLA-DRB1*07:01, HLA-DRB1*09:01, HLA-DRB3*02:02, HLA-DRB5*01:01	19.1, 50.7, 53.1, 31.4
ORF1ab	VLSFCAFAVDAAKAY	HLA-DQA1*05:01/DQB1*02:01, HLA-DQA1*04:01/DQB1*04:02, HLA-DQA1*01:02/DQB1*06:02	203.9, 360.2, 143.4
ORF1ab	VLVQSTQWSLFFFLY	HLA-DPA1*02:01/DPB1*01:01, HLA-DPA1*01:03/DPB1*02:01, HLA-DPA1*01:03/DPB1*04:01, HLA-DPA1*03:01/DPB1*04:02	77.0, 35.3, 42.3, 93.1
ORF1ab	VNRFNVAITRAKVGI	HLA-DRB1*08:02, HLA-DRB1*11:01, HLA-DRB5*01:01, HLA-DPA1*02:01/DPB1*14:01	78.4, 31.7, 12.1, 389.7
ORF1ab	VNTFSSTFNVPMEKL	HLA-DRB1*04:01, HLA-DRB1*04:05, HLA-DRB1*07:01	77.6, 84.5, 38.7
ORF1ab	VQSTQWSLFFFLYEN	HLA-DPA1*02:01/DPB1*01:01, HLA-DPA1*01:03/DPB1*02:01, HLA-DPA1*03:01/DPB1*04:02	107.1, 49.9, 129.8
ORF1ab	VTCLAYYFMRFRRAF	HLA-DPA1*02:01/DPB1*01:01, HLA-DPA1*01:03/DPB1*02:01, HLA-DPA1*01:03/DPB1*04:01, HLA-DPA1*03:01/DPB1*04:02, HLA-DPA1*02:01/DPB1*05:01	94.2, 49.1, 62.2, 121.3, 132.7
ORF1ab	VVDSYYSLLMPILTL	HLA-DPA1*02:01/DPB1*01:01, HLA-DPA1*01:03/DPB1*02:01, HLA-DPA1*01:03/DPB1*04:01, HLA-DPA1*03:01/DPB1*04:02	102.1, 58.0, 84.6, 114.6
ORF1ab	VVLKKLKKSLNVAKS	HLA-DRB1*08:02, HLA-DRB4*01:01, HLA-DRB5*01:01	112.7, 76.3, 31.4
ORF1ab	WLIINLVQMAPISAM	HLA-DRB1*12:01, HLA-DRB4*01:01, HLA-DQA1*01:02/DQB1*06:02	130.6, 65.9, 139.6
ORF1ab	YFAVHFISNSWLMWL	HLA-DRB1*07:01, HLA-DPA1*02:01/DPB1*01:01, HLA-DPA1*01:03/DPB1*02:01, HLA-DPA1*01:03/DPB1*04:01	36.2, 84.0, 33.4, 55.3
ORF1ab	YFMRFRRAFGEYSHV	HLA-DRB1*04:01, HLA-DRB1*04:05, HLA-DRB1*15:01	53.9, 50.7, 53.5
ORF1ab	YFNMVYMPASWVMRI	HLA-DRB1*01:01, HLA-DRB1*04:05, HLA-DRB1*07:01, HLA-DRB1*09:01, HLA-	8.3, 80.2, 38.2, 37.4, 184.5, 30.1

		DRB1*12:01, HLA-DRB1*15:01	
ORF1ab	YLYFIKGLNNLNRGM	HLA-DRB1*01:01, HLA-DRB1*04:01, HLA-DRB1*04:05, HLA-DRB1*11:01, HLA-DRB3*02:02	9.7, 27.4, 32.3, 44.4, 70.3
ORF3	KKRWQLALSKGVHFV	HLA-DRB1*01:01, HLA-DRB1*07:01, HLA-DRB1*08:02, HLA-DRB1*09:01, HLA-DRB1*11:01, HLA-DRB1*12:01, HLA-DRB1*13:02, HLA-DRB1*15:01, HLA-DRB4*01:01, HLA-DRB5*01:01	9.2, 11.6, 200.3, 17.9, 43.1, 119.6, 30.0, 34.2, 79.8, 18.4
ORF3	SDFVRATATIPIQAS	HLA-DRB1*01:01, HLA-DRB1*07:01, HLA-DRB1*08:02, HLA-DRB1*09:01, HLA-DRB1*13:02	12.5, 16.0, 88.5, 27.8, 27.6
ORF3	PSDFVRATATIPIQA	HLA-DRB1*04:01, HLA-DRB1*04:05, HLA-DRB1*08:02	54.4, 90.0, 100.9
ORF3	YCIPYNSVTSSIVIT	HLA-DRB1*07:01, HLA-DRB1*09:01	24.3, 44.7
ORF3	ASKIITLKKRWQLAL	HLA-DRB1*08:02, HLA-DRB1*11:01	121.0, 20.5
ORF3	FVRATATIPIQASLP	HLA-DRB1*09:01, HLA-DQA1*01:02/DQB1*06:02, HLA-DPA1*02:01/DPB1*14:01	45.7, 47.8, 196.9
ORF3	VFQSASKIITLKKRW	HLA-DRB1*11:01,HLA-DRB5*01:01	32.2, 26.3
ORF3	YFLQSINFVRIIMRL	HLA-DRB1*12:01, HLA-DRB1*15:01, HLA-DRB4*01:01, HLA-DPA1*02:01/DPB1*05:01	124.4, 52.2, 81.9, 175.5
ORF3	VRATATIPIQASLPF	HLA-DQA1*04:01/DQB1*04:02, HLA-DQA1*01:02/DQB1*06:02	492.8, 51.5
ORF3	VYFLQSINFVRIIMR	HLA-DPA1*02:01/DPB1*01:01, HLA-DPA1*03:01/DPB1*04:02	58.7, 54.9
ORF3	VLHSYFTSDYYQLYS	HLA-DPA1*02:01/DPB1*01:01, HLA-DPA1*01:03/DPB1*02:01, HLA-DPA1*01:03/DPB1*04:01, HLA-DPA1*03:01/DPB1*04:02	65.6, 33.1, 40.3, 93.4
ORF3	LVYFLQSINFVRIIM	HLA-DPA1*01:03/DPB1*02:01, HLA-DPA1*01:03/DPB1*04:01	24.8, 32.9
ORF6	HLVDFQVTIAEILLI	HLA-DRB1*07:01, HLA-DQA1*05:01/DQB1*02:01	36.7, 198.5
ORF6	YIINLIIKNLSKSLT	HLA-DRB1*08:02, HLA-DRB1*11:01, HLA-DRB1*12:01, HLA-DRB1*13:02, HLA-DRB3*02:02, HLA-DRB4*01:01	113.5, 32.4, 135.4, 15.4, 43.6, 68.3
ORF6	IINLIIKNLSKSLTE	HLA-DRB1*08:02, HLA-DRB1*11:01	118.6, 31.7
ORF6	EILLIIMRTFKVSIW	HLA-DRB1*08:02, HLA-DRB1*12:01, HLA-DRB1*15:01, HLA-DRB4*01:01	130.2, 90.4, 20.2, 62.6
ORF6	DYIINLIIKNLSKSL	HLA-DRB1*11:01,HLA-DRB1*12:01, HLA-DRB3*02:02, HLA-DRB4*01:01	41.9, 166.1, 42.9, 77.1
ORF6	FKVSIWNLDYIINLI	HLA-DRB3*01:01, HLA-	66.1, 90.1

		DQA1*01:01/DQB1*05:01	
ORF6	MFHLVDFQVTIAEIL	HLA-DQA1*05:01/DQB1*02:01, HLA-DQA1*01:01/DQB1*05:01, HLA-DPA1*02:01/DPB1*01:01, HLA-DPA1*01:03/DPB1*04:01	192.0, 292.1, 108.3, 100.7
ORF6	MRTFKVSIWNLDYII	HLA-DPA1*02:01/DPB1*01:01, HLA-DPA1*01:03/DPB1*02:01, HLA-DPA1*01:03/DPB1*04:01, HLA-DPA1*03:01/DPB1*04:02, HLA-DPA1*02:01/DPB1*05:01	79.0, 44.3, 60.7, 110.1, 304.0
ORF7	VKHVYQLRARSVSPK	HLA-DRB1*01:01, HLA-DRB1*08:02, HLA-DRB1*11:01, HLA-DRB4*01:01	14.3, 150.6, 38.3, 86.6
ORF7	GVKHVYQLRARSVSP	HLA-DRB1*11:01, HLA-DRB4*01:01	39.6, 82.1
ORF7	NKFALTCFSTQFAFA	HLA-DPA1*02:01/DPB1*01:01, HLA-DPA1*01:03/DPB1*02:01, HLA-DPA1*01:03/DPB1*04:01, HLA-DPA1*03:01/DPB1*04:02, HLA-DPA1*02:01/DPB1*05:01	50.9, 29.1, 35.9, 80.2, 273.4
ORF7	EEVQELYSPIFLIVA	HLA-DPA1*02:01/DPB1*01:01, HLA-DPA1*01:03/DPB1*02:01	59.7, 30.7
ORF7	FALTCFSTQFAFACP	HLA-DPA1*01:03/DPB1*02:01, HLA-DPA1*01:03/DPB1*04:01	61.9, 76.1
ORF7	EVQELYSPIFLIVAA	HLA-DPA1*01:03/DPB1*04:01, HLA-DPA1*03:01/DPB1*04:02	44.2, 74.6
ORF8	SKWYIRVGARKSAPL	HLA-DRB1*01:01, HLA-DRB1*08:02, HLA-DRB1*09:01, HLA-DRB1*11:01, HLA-DRB5*01:01	13.7, 87.8, 50.7, 15.3, 8.8
ORF8	LVVRCSFYEDFLEYH	HLA-DQA1*01:01/DQB1*05:01, HLA-DPA1*02:01/DPB1*01:01, HLA-DPA1*01:03/DPB1*04:01, HLA-DPA1*03:01/DPB1*04:02	177.0, 87.4, 89.3, 166.8
ORF10	FAFPFTIYSLLLCRM	HLA-DPA1*02:01/DPB1*01:01, HLA-DPA1*01:03/DPB1*02:01, HLA-DPA1*01:03/DPB1*04:01, HLA-DPA1*03:01/DPB1*04:02	97.3, 37.2, 56.9, 104.8
ORF10	INVFAFPFTIYSLLL	HLA-DPA1*02:01/DPB1*01:01, HLA-DPA1*01:03/DPB1*02:01, HLA-DPA1*01:03/DPB1*04:01, HLA-DPA1*03:01/DPB1*04:02	102.1, 36.8, 57.3, 126.7
ORF10	YINVFAFPFTIYSLL	HLA-DPA1*01:03/DPB1*02:01, HLA-DPA1*03:01/DPB1*04:02	42.9, 142.3

Table S4. Allergenicity and toxicity for top predicted SARS-CoV-2 T cell epitopes.

**Peptide**	**Viral Protein**	**ToxinPred Score**	**AllerCatPro Result**
FAMQMAYRF	S	-1.23 (Non-toxin)	No hits
LSFKELLVY	ORF1ab	-1.24 (Non-toxin)	No hits
MMISAGFSL	ORF1ab	-1.13 (Non-toxin)	No hits
MSNLGMPSY	ORF1ab	-1.36 (Non-toxin)	No hits

STNVTIATY	ORF1ab	-0.92 (Non-toxin)	No hits
ASFRLFARTRSMWSF	M	-0.99 (Non-toxin)	No hits
LLQFAYANRNRFLYI	M	-0.27 (Non-toxin)	No hits
AAEIRASANLAATKM	S	-1.00 (Non-toxin)	No hits
ALQIPFAMQMAYRFN	S	-1.16 (Non-toxin)	No hits
PYRVVVLSFELLHAP	S	-1.50 (Non-toxin)	No hits
QPYRVVVLSFELLHA	S	-1.65 (Non-toxin)	No hits
YQPYRVVVLSFELLH	S	-1.53 (Non-toxin)	No hits
ANYIFWRNTNPIQLS	ORF1ab	-0.98 (Non-toxin)	No hits
FKWDLTAFGLVAEWF	ORF1ab	-1.11 (Non-toxin)	No hits
HIQWMVMFTPLVPFW	ORF1ab	-1.26 (Non-toxin)	No hits
IINLVQMAPISAMVR	ORF1ab	-0.93 (Non-toxin)	No hits
INLVQMAPISAMVRM	ORF1ab	-1.17 (Non-toxin)	No hits
IVFMCVEYCPIFFIT	ORF1ab	-0.04 (Non-toxin)	No hits
IVTALRANSAVKLQN	ORF1ab	-0.68 (Non-toxin)	No hits
KGRLIIRENNRVVIS	ORF1ab	-0.62 (Non-toxin)	No hits
KSAFYILPSIISNEK	ORF1ab	-1.50 (Non-toxin)	No hits
LIVTALRANSAVKLQ	ORF1ab	-0.76 (Non-toxin)	No hits
NLPFKLTCATTRQVV	ORF1ab	-0.37 (Non-toxin)	No hits
PASRELKVTFFPDLN	ORF1ab	-1.11 (Non-toxin)	No hits
PFAMGIIAMSAFAMM	ORF1ab	-1.03 (Non-toxin)	No hits
QMNLKYAISAKNRAR	ORF1ab	-0.93 (Non-toxin)	No hits
QQKLALGGSVAIKIT	ORF1ab	-1.35 (Non-toxin)	No hits
RFKESPFELEDFIPM	ORF1ab	-1.04 (Non-toxin)	No hits
SAFAMMFVKHKHAFL	ORF1ab	-0.99 (Non-toxin)	No hits
SFLAHIQWMVMFTPL	ORF1ab	-1.00 (Non-toxin)	No hits
SIGFDYVYNPFMIDV	ORF1ab	-0.90 (Non-toxin)	No hits
TEETFKLSYGIATVR	ORF1ab	-1.05 (Non-toxin)	No hits
VLVQSTQWSLFFFLY	ORF1ab	-0.68 (Non-toxin)	No hits
VQSTQWSLFFFLYEN	ORF1ab	-0.69 (Non-toxin)	No hits
WLIINLVQMAPISAM	ORF1ab	-1.03 (Non-toxin)	No hits
YFNMVYMPASWVMRI	ORF1ab	-1.93 (Non-toxin)	No hits
KKRWQLALSKGVHFV	ORF3	-1.49 (Non-toxin)	No hits
MFHLVDFQVTIAEIL	ORF6	-1.01 (Non-toxin)	No hits
VKHVYQLRARSVSPK	ORF7	-0.84 (Non-toxin)	No hits
NKFALTCFSTQFAFA	ORF7	-1.01 (Non-toxin)	No hits
SKWYIRVGARKSAPL	ORF8	-0.87 (Non-toxin)	No hits

Table S5. B cell epitopes identified from SARS-CoV-2 structural proteins.

**Linear B cell Epitopes***			
**Peptide**	**Residues**	**Viral Protein**	**Bepipred Score**[a]
MSDNGPQNQRNAPRITFGGPSDST GSNQNGERSGARSKQRRPQGLPN	1 – 51	N	1.53

NTAS			
QHGKEDLKFPRGQGVPINTNSSPD DQIG	58 – 85	N	1.09
RIRGGDGKMKDL	93 – 104	N	0.844
TGPEAGLPYGANK	115 – 127	N	1.19
GALNTPKDHIGTRNPANN	137 – 154	N	1.09
GTTLPKGFYAEGSRGGSQASSRSS SRSRNSSRNSTPGSSRGTSPARMA GNGGD	164 – 216	N	1.40
SKMSGKGQQQQGQTVTKKSAAEA SKKPRQKRTATKAYN	232 – 269	N	1.18
AFGRRGPEQTQGNFG	273 – 287	N	1.34
TDYKHW	296 – 301	N	0.759
QFAPS	306 – 310	N	0.579
EVTPSGTWL	323 – 331	N	0.678
KLDDKDPNFK	338 – 347	N	1.05
KTFPPTEPKKDKKKKADETQALPQ RQKKQQ	361 – 390	N	1.45
QQSMSSADSTQA	408 – 419	N	1.06
MADSNGTI	2 – 9	M	0.61
QRVAGDSGFA	186 – 195	M	0.783
LNTDHSSSSD	207 – 216	M	0.976
RTQLPPAYTNS	21 – 31	S – S1 domain	0.881
SGTNGTKRFDN	71 – 81	S – S1 domain	0.885
GKQGNF	181 – 186	S – S1 domain	0.637
LTPGDSSSGWTAG	249 – 261	S – S1 domain	1.36
NGTITD	282 – 287	S – S1 domain	0.804
FRVQPTE	318 – 324	S – S1 domain	0.561
**VRQIAPGQTGKIAD**	407 – 420	S – S1 domain	0.928
YKLPDD	423 – 428	S – S1 domain	0.602
N**NLDSKVGG**	439 – 447	S – S1 domain	0.770
YQAGSTPCNGV	473 – 483	S – S1 domain	0.985
Y**GFQPTNGVGYQ**	495 – 506	S – S1 domain	1.01
TVCGPKKSTN	523 – 532	S – S1 domain	0.867
R**DIADTT**DAVRDPQ	567 – 580	S – S1 domain	0.959
VITPGTNTSN	597 – 606	S – S1 domain	1.11
RVYST	634 – 638	S – S1 domain	0.483
QTQTNSPRRARSV	675 – 687	S – S2 domain	1.148
VEQDKNTQE	772 – 780	S – S2 domain	0.888
IYKT**PPIKD**F	788 – 797	S – S2 domain	0.751
ILPDPSKPSKRS	805 – 816	S – S2 domain	1.29
DSLSST	936 – 941	S – S2 domain	0.618
QSAPH	1054 – 1058	S – S2 domain	0.84
PAQEKNFTT	1069 – 1077	S – S2 domain	0.84
KAHFP	1086 – 1090	S – S2 domain	0.665

**VYDPLQPELDSF**	1137 – 1148	S – S2 domain	0.747
KNHTSPDVDLG	1157 – 1167	S – S2 domain	0.918
FDEDDSEPVL	1256 – 1265	S – S2 domain	1.09
**Discontinuous B cell Epitopes***			
**Peptide**[#]	**Residues**	**Viral Protein**	**DiscoTope Score**[a]
DKVF	40 – 43	S – S1 domain	-5.12
TPINLVR	208 – 214	S – S1 domain	-6.36
**DEVRQ**	405 – 409	S – S1 domain	-7.83
**PGQTGKIADY**	412 – 421	S – S1 domain	-5.79
**KLPDD**	424 – 428	S – S1 domain	-6.19
**NLDS---YN**	440 – 443; 449 – 450	S – S1 domain	-6.62
**QPTNGVG**	498 – 504	S – S1 domain	-6.33
NKKFLPFQQ	556 – 564	S – S1 domain	-5.09
**RDIADT**	567 – 572	S – S1 domain	-5.69
AENSVAYSN	701 – 709	S – S2 domain	-6.10
**PPIKD**	792 – 796	S – S2 domain	-3.28
DPSK	808 – 811	S- S2 domain	-2.61
SRLD---E	982 – 985; 988	S – S2 domain	-7.01
NGTH	1098 – 1101	S – S2 domain	-6.36
EPQI	1111 – 1114	S – S2 domain	-6.46
**YDPLQPELD**	1138 – 1146	S – S2 domain	-4.12

*Bold type indicates residues with overlapping sequences in both linear and discontinuous epitope analysis (see Table 2 in main text).
[a]Reported scores represent the average calculated across all amino acids.
[#]Gaps in represent residues in the amino acid sequence that were not resolved in the S protein crystal structure (PDB ID: 6VSB) and excluded from DiscoTope score predictions.
